# Natural product fragment combination to performance-diverse pseudo-natural products

**DOI:** 10.1038/s41467-021-22174-4

**Published:** 2021-03-25

**Authors:** Michael Grigalunas, Annina Burhop, Sarah Zinken, Axel Pahl, José-Manuel Gally, Niklas Wild, Yannik Mantel, Sonja Sievers, Daniel J. Foley, Rebecca Scheel, Carsten Strohmann, Andrey P. Antonchick, Herbert Waldmann

**Affiliations:** 1 Department of Chemical Biology, Max Planck Institute of Molecular Physiology, Dortmund, Germany; 2 Technical University Dortmund, Faculty of Chemistry and Chemical Biology, Dortmund, Germany; 3 Compound Management and Screening Center, Dortmund, Germany; 4 Technical University Dortmund, Faculty of Chemistry and Inorganic Chemistry, Dortmund, Germany; 5 Present Address: College of Science, University of Canterbury, Canterbury, New Zealand; 6 Present Address: College of Science and Technology, Nottingham Trent University, Nottingham, UK

**Keywords:** Chemical libraries, Chemical synthesis

## Abstract

Natural product structure and fragment-based compound development inspire pseudo-natural product design through different combinations of a given natural product fragment set to compound classes expected to be chemically and biologically diverse. We describe the synthetic combination of the fragment-sized natural products quinine, quinidine, sinomenine, and griseofulvin with chromanone or indole-containing fragments to provide a 244-member pseudo-natural product collection. Cheminformatic analyses reveal that the resulting eight pseudo-natural product classes are chemically diverse and share both drug- and natural product-like properties. Unbiased biological evaluation by cell painting demonstrates that bioactivity of pseudo-natural products, guiding natural products, and fragments differ and that combination of different fragments dominates establishment of unique bioactivity. Identification of phenotypic fragment dominance enables design of compound classes with correctly predicted bioactivity. The results demonstrate that fusion of natural product fragments in different combinations and arrangements can provide chemically and biologically diverse pseudo-natural product classes for wider exploration of biologically relevant chemical space.

## Abstract (web summary)

Natural products inspire the development of pseudo-natural products through combinations of fragments of compound classes that are chemically and biologically distinct. Here, the authors report a library of 244 pseudo-natural products, evaluate them in the cell painting essays and identify the phenotypic role of individual fragments.

## Introduction

The design and synthesis of pseudo-natural products (PNPs) through the combination of natural product (NP) fragments is a recently proposed principle for the discovery of bioactive compounds^1–6^. The concept aims to synthetically combine fragments of NPs that are biosynthetically unrelated and have different bioactivity to produce scaffolds that resemble NPs but are not accessible through existing biosynthetic pathways. This combination of NP-inspired and fragment-based compound development promises to cover wider areas of chemical and biological space than explored by the guiding NPs. Furthermore, different combinations and arrangements of a given set of NP-fragments are expected to yield chemically diverse PNP classes with diverse bioactivity profiles and targets.

Since the PNP frameworks are new, they can have unknown or unexpected biological targets and modes of action. Therefore, their bioactivity will best be evaluated in unbiased target-agnostic cell-based assays covering a wide range of biological processes^7^. However, often these assays examine only a fraction of cellular features, leaving the majority of phenotypic perturbations unexplored. As an alternative that potentially can overcome this shortcoming, morphological profiling has emerged as an unbiased method for the identification and characterization of bioactive compounds in a broader biological context^8,9^. The “cell painting assay” (CPA) is a morphological profiling assay that evaluates phenotypic changes in cells upon treatment with compound and condenses them into characteristic “fingerprints”^10,11^. Comparison to reference compounds with known bioactivities can give insight into the mode of action and/or biological target(s). Cell painting has been employed to differentiate bioactivity profiles based on structural variances and chemical properties^12,13^, to identify small molecule targets^6^, to identify mode of action even when the target is not a protein^14^, and to delineate qualitative structure-activity relationships^4^.

In light of this proven ability to characterize small molecule bioactivity in a broader cellular context, we hypothesized that the CPA would enable biological characterization and differentiation of structurally related PNP classes that will be obtained through different combinations of a given set of NP-fragments.

**Fig. 1:**
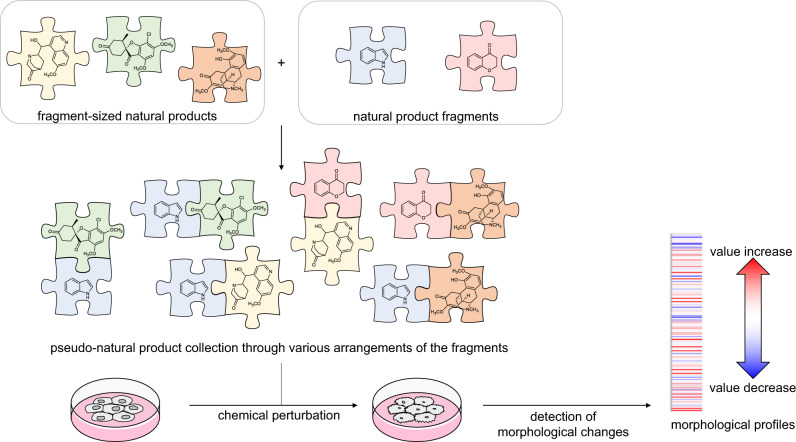
Overview of the design, combination, and biological evaluation of the pseudo-natural product collection. Fragment-sized natural products were combined with natural product fragments to generate a collection of 244 pseudo-natural products. The compounds were biologically evaluated by the cell painting assay and compared.

Here we describe the design, synthesis, and cheminformatic analysis of a PNP collection obtained by combination of four readily accessible, fragment-sized NPs^15–18^ and several smaller fragments representing two different NP classes, namely indoles and chromanones (Fig. 1). The synthesis yielded a total of 244 PNPs representing eight chemically diverse PNP classes that differ in structural features and/or the pairwise combination of the common fragment set. Analysis of the PNP classes in the cell painting assay (CPA) to determine whether they are also biologically diverse was facilitated by principal component analysis (PCA) and further verified with a cross-similarity evaluation. The bioactivity of the PNP classes differs from the profiles recorded for the guiding NPs. Based on the analysis, compounds with correctly predicted phenotypic behaviors were synthesized.

Our results demonstrate that de novo combination of a given set of NP fragments in different arrangements can successfully yield NP-inspired PNP classes with related structure covering wider chemical space, and with different bioactivity profiles, i.e. also covering wider biological space.

## Results

### Design and synthesis of a pseudo-natural product collection

**Fig. 2:**
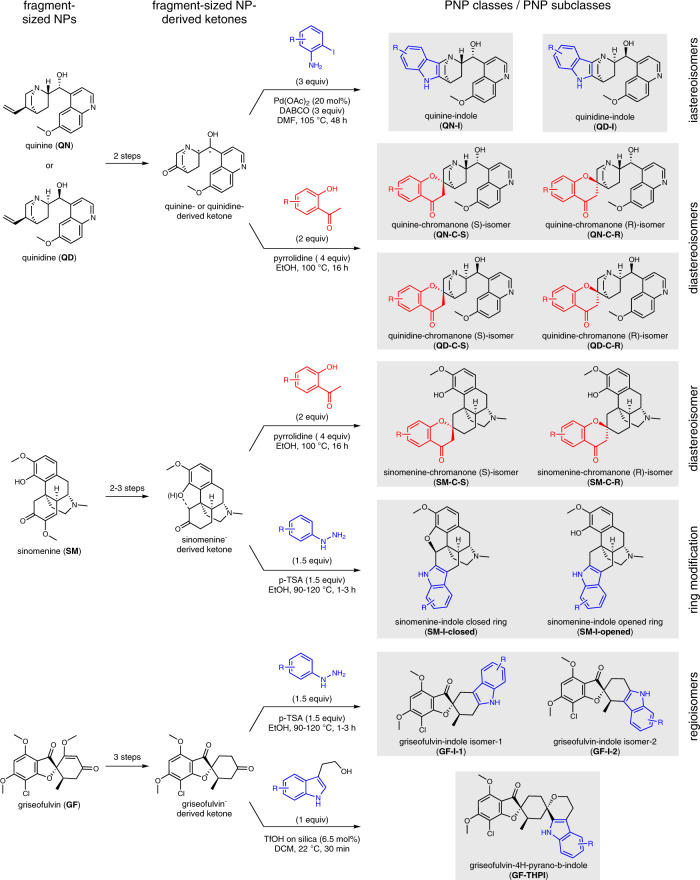
Overview of the synthesized pseudo-natural product classes and pseudo-natural product subclasses. Pseudo-natural product (PNP) classes are within the gray boxes. For syntheses of fragment-sized NP ketones, see Supplementary Fig. 1. Blue indicates an indole fragment or indole fragment precursor. Red indicates a chromanone fragment or chromanone fragment precursor. DABCO = 1,4-diazabicyclo(2.2.2.)octane; DMF = dimethylformamide; EtOH = ethanol; p-TSA = paratoluenesulfonic acid; TfOH = triflic acid; DCM = dichloromethane.

The PNP compound library was designed based on the combination of complex NPs, which themselves are fragment-sized, to smaller, more truncated NP fragments (Fig. 2). The NPs used in this study have fragment-like structures, considering that the typically employed empirical ‘rule of three’^19^ may not be entirely valid for NPs^20^. Thus, NPs with an Alog P < 3.5, a molecular weight of 120–350 Da, ≤ 3 hydrogen bond donors, ≤ 6 hydrogen bond acceptors and ≤ 6 rotatable bonds are considered ‘fragment-like’^15^. The cinchona alkaloids quinine and quinidine as well as the NPs sinomenine and griseofulvin meet these criteria and were employed as combination partners with an indole or chromanone fragment. The stereoisomeric cinchona alkaloids quinine (**QN**) and quinidine (**QD**) show several bioactivities including antimalarial and antiarrhythmic effects^21^. Sinomenine (**SM**) is a morphine derivative that has immunosuppressive activity and is used as an analgesic against rheumatism and arthritis^22^. The antimycotic agent griseofulvin (**GF**) is a known tubulin binder that inhibits mitosis^23,24^. All four NPs are readily available through commercial sources and can be functionalized to have suitably reactive ketone handles for the installation of a second fragment within a few synthetic steps (Supplementary Fig. 1).

Indoles and chromanones were used as smaller combination partners due to their prevalence in bioactive NPs and drugs^25,26^ and their facile access via robust synthetic methods in which ketones are well established substrates. The Fischer indole synthesis^27^ and a Pd-catalyzed annulation employing 2-halo anilines^28^ were used to access edge-fused indole PNPs (Fig. 2, **QN-I**, **QD-I**, **SM-I**, **GF-I**). In addition, an oxa-Pictet-Spengler reaction was used as a spirocycle-generating method to combine the indole-derived fragment 4*H*-pyrano-indole and a griseofulvin derivative (**GF-THPI**)^29^. The spirocyclic installation of chromanone fragments to ketone-containing NP derivatives was achieved via the Kabbe condensation by employing 2-hydroxyacetophenones (**QN-C**, **QD-C**, **SM-C**)^30^. The reactions discussed above used substrates and catalysts that were either commercially available or readily synthetically accessible. Furthermore, these methods proved to be robust in combining NP fragments in one step to afford a PNP collection that incorporates high structural complexity and diverse functionalities.

In all, 244 PNPs were synthesized and divided into eight ‘PNP classes’ based on fragment combinations (Fig. 2, individual PNP classes are within the gray boxes). The compounds were further classified into 13 ‘PNP subclasses’ based on structural features including diastereomeric-, ring modified- and regioisomeric variants. Compounds derived from the Kabbe condensation provided two chromanone-fused PNPs that are diastereomers at the spirocyclic point of fragment connection (**QN-C-S** and **QN-C-R**, **QD-C-S** and **QD-C-R**, and **SM-C-S** and **SM-C-R**). Additionally, the quinine- and quinidine-based compounds are diastereomers due to their two naturally inverted stereocenters (**QN-I** and **QD-I**, **QN-C-S** and **QD-C-S**, and **QN-C-R** and **QD-C-R**). Reacting different sinomenine-based starting materials in the Fischer indole reaction yielded ring modified derivatives with either a fused heptacyclic (**SM-I-closed**) or a fragmented hexacyclic (**SM-I-opened**) ring system. Regioisomers with edge fusion patterns were obtained when a griseofulvin-derived substrate was employed in a Fischer indole reaction (**GF-I-1** and **GF-I-2**). An oxa-Pictet-Spengler reaction provided access to a similar griseofulvin-indole fragment combination but with a different spirocyclic fusion pattern (**GF-THPI**).

### Cheminformatic analysis of the pseudo-natural product collection

To give insight into the structural and physiochemical properties of the PNP library, cheminformatic methods were employed. Tanimoto similarity of the Morgan fingerprints (ECFC4, count fingerprint, radius 2), as implemented in the RDKit^31^, revealed a high chemical similarity within the 13 subclasses (0.75 median similarity, Supplementary Fig. 2a, b) whereas inter-subclass comparisons have significantly lower chemical similarities (0.26 median similarity, Supplementary Fig. 2a, c). These results suggest that different combinations of a small set of NP fragments can lead to a chemically diverse library with homogeneous subclasses. Similar conclusions were obtained when different fingerprints of a different design were compared (ECFP6, bit fingerprint of length 1024, radius 3, see Supplementary Fig. 3).

**Fig. 3:**
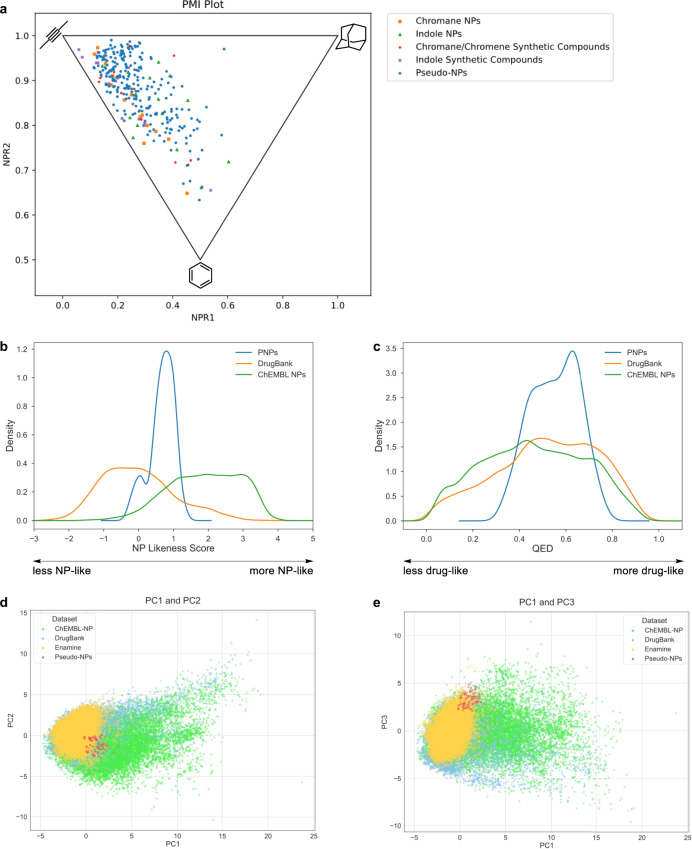
Cheminformatic analyses of the pseudo-natural product collection. **a** Principal moments of inertia (PMI) plot of the shape of the pseudo-natural product (PNP) collection (244 compounds, blue circles), chromane NPs (15 compounds, orange squares), indole NPs (15 compounds, green triangles), chromane/chromene synthetic compounds (15 compounds, red crosses), and indole synthetic compounds (15 compounds, purple xes). The corners of the triangle within the plot indicate a rod-like shape (top left), disk-like shape (bottom middle), and sphere-like shape (top right). Source data is provided in the repository and in the Supplementary Information. **b** NP-likeness score of the PNP collection (244 compounds, blue), compounds from the DrugBank (7472 compounds, orange), and NPs from ChEMBL (30655 compounds, green). **c** Quantitative estimation of drug-likeness (QED) of the PNP collection (244 compounds, blue), compounds from the DrugBank (7472 compounds, orange), and NPs from ChEMBL (30655 compounds, green). **d**, **e** Principal component analysis (PCA) plots (**d** PC1 and PC2; **e** PC1 and PC3) of 17 molecular descriptors (number of heavy atoms, molecular weight, number of rings, number of aromatic rings, number of aliphatic rings, number of hydrogen bond donors, number of hydrogen bond acceptors, SLogP, total polar surface area, number of rotatable bonds, number of oxygen atoms, number of nitrogen atoms, number of halogen atoms, number of bridgehead atoms, fraction sp^3^, number of violations of Lipinski’s rules, number of violations of Veber’s rules) of NPs from ChEMBL (30655 compounds, green), compounds from the DrugBank (7472 compounds, blue), compounds from the Enamine database (49968 compounds, yellow), and compounds from the PNP collection (244 compounds, red) (Expl. Var.: PC1 (37.0%), PC2 (15.7%), PC3 (11.9%)). A plot of PC2 and PC3 can be found at Supplementary Fig. 10. PC = principal component. Source data are provided in the online repository.

Characterization of molecular shape by a principal moments of inertia (PMI) analysis^32^ revealed that the PNP collection has a wide distribution of PMI values (Fig. 3a). The PNPs were compared to four reference sets comprised of chromane-containing NPs, indole-containing NPs, chromane/chromene-containing synthetic compounds, and indole-containing synthetic compounds. While the PNPs overlapped with all reference sets, a high density of PNPs were shifted away from the rod/disk-like axis where synthetic reference compounds and chromane-containing NPs lie (for individual plots, see Supplementary Fig. 4). This suggests many of the PNPs share the shape of indole-containing NPs that are more abundant in three-dimensional character than the other references^33,34^.

Substructure searches in the Dictionary of Natural Products (DNP) of fragment combinations found in the PNP library indicated that the fragment combinations are not found in NPs (see Supplementary Fig. 5). Furthermore, substructure searches in the DNP and COCONUT databases revealed that trimmed and truncated PNP scaffolds are not found in nature, and only severely truncated PNP scaffolds showed resemblance to NPs (Supplementary Fig. 6).

Atom connectivity was evaluated by a NP-likeness score^35^ and compared to compounds in the DrugBank^36^, which characterizes marketed and experimental drugs, as well as NPs in the ChEMBL database^37^. The PNP collection is mostly represented in the area where drugs and NPs intersect, indicating that some NP-identity is conserved while also providing further support that the fragment combinations may not occur in nature (Fig. 3b and Supplementary Fig. 7a). Similarly to previous cheminformatic studies^1^, compounds that are more nitrogen-rich, i.e. **QN-I** and **QD-I** ( ≥ 3 nitrogens), are less NP-like while oxygen-rich compounds are more NP-like (for NP-likeness scores of individual compound classes, see Supplementary Fig. 7b). Using NP-Scout^38^, the probability of the PNPs to be NPs was calculated and was found to lie between the DrugBank and ChEMBL NP reference sets (Supplementary Fig. 8a–d).

Overall, the collection has favorable properties for drug discovery based on the Quantitative Estimation of Drug-likeness^39^ (QED), with the PNPs corresponding to the area of highest density of Drugbank compounds (Fig. 3c; for QED of individual compound classes, see Supplementary Fig. 9). ADME-Tox predictions of the PNP collection were calculated and can be found in Supplementary Tables 1–2^40–42^. Additionally, 17 molecular descriptors were evaluated in a principal component analysis (PCA), comparing Drugbank, ChEMBL NPs and a random selection of the Enamine Advanced Screening Collection with the PNP set (see Supplementary Information for details). All three reference sets overlapped but are not superimposed, with Drugbank and Enamine being closest together (Fig. 3d and Supplementary Fig. 10). The PNPs are located at the interface of the reference sets.

### Biological evaluation of the pseudo-natural product collection in the cell painting assay

To investigate the bioactivity of the synthesized PNPs, they were subjected to the morphology-based cell painting assay. This assay monitors phenotypic changes in cells by employing six fluorescent dyes that selectively stain various compartments^10,11^. Imaging via multi-channel fluorescent microscopy extracts information about the changes of the cell’s morphology represented in 579 highly reproducible features to generate a characteristic profile^4^. These profiles were analyzed by various methods to allow for facile evaluation and comparison of the PNP collection.

For the analysis we focused in particular on the questions whether (i) the PNPs are bioactive and (ii) induce different morphological changes than the guiding NPs. We sought to (iii) analyze the phenotypic impact of structural differences such as fusion patterns and regio- or diastereomeric fragment combination. Finally, we asked (iv) how different combinations of a set of common fragments would influence the phenotypic profiles and whether (v) the information obtained from the CPA analysis could be employed to design compounds with predicted phenotypic trends.

To quantify the intensity of phenotypic activity, the number of significantly changed features in a profile relative to a DMSO control were calculated to provide an induction percentage (see Supplementary Information for more details). Similarities of phenotypic profiles could be calculated from the correlation distances between two profiles and is represented as a biosimilarity percentage. Comparisons with a biosimilarity ≥ 75% are considered biosimilar whereas < 75% are biologically dissimilar. Cross-similarity analysis followed by the calculation of a median biosimilarity percentage (MBP) allowed for the comparison of several compounds or entire compound classes and can be regarded as a quantitative representation of the entire comparison. As a tool for visualizing differences and similarities, principal component analysis (PCA) was used to condense the phenotypic profiles of several compounds into three-dimensional plots. Significant phenotypic differences between compound classes can be identified through PCA when clusters are formed. Conversely, compound classes that do not form clusters in PCA, i.e. scatter plot, are considered either phenotypically similar or have broad intra-class similarities. Through comparison of both MBP and PCA, compound classes can be determined to be similar or dissimilar.

Significant morphological changes relative to the DMSO control (i.e., high induction values) were observed for all PNP subclasses, indicating bioactivity (see Supplementary Fig. 11). Overall, 84% of the compounds were active, i.e., > 5% induction, with a median induction of 17% at 10 µM concentrations.

Initial PCA with a wide induction range (5–70%) resulted in a large correlation of the first principal component to the induction value and is representative of the number of changed cellular features rather than phenotypic changes (see Supplementary Information for details). Reducing the induction range resulted in the components becoming independent of the induction value. While each comparison varies, the best results were obtained with an induction range between 15–25% with overall induction values between 10–40%. Further concentrations (1, 3, 30, 50 µM) were employed when needed to broadly cover the suitable areas of induction and enable the adherence of small induction windows for analysis. For this analysis, compounds having an induction value between 20–40% were compared unless otherwise noted (118 compounds).

To evaluate the phenotypic character of the entire library, MBPs were calculated from a cross-similarity analysis of all PNP subclasses (Supplementary Figure 12). Within the subclasses, a moderately high 77% MBP was observed while interclass phenotypic similarities were significantly lower at 61%. These biosimilarity trends parallel the chemical trends mentioned above in that the library is phenotypically diverse with homogenous subclasses.

**Fig. 4:**
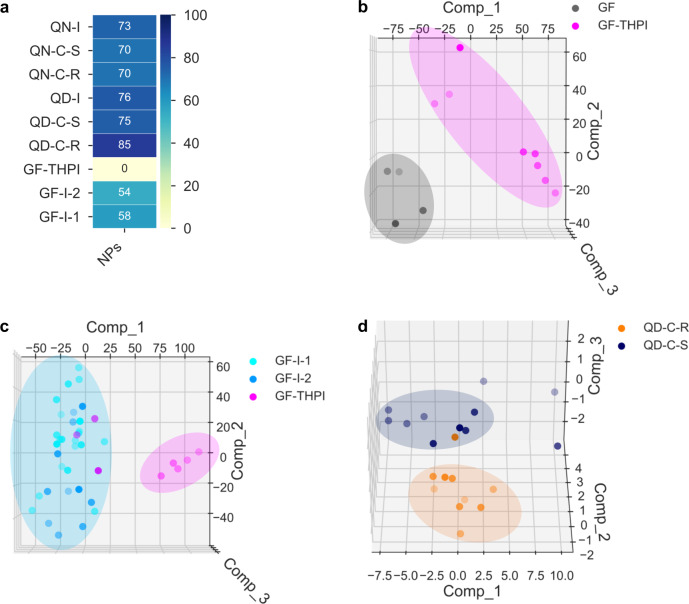
Comparisons of pseudo-natural products to their parent natural product, different fusion patterns, and diastereomers. **a** Median biosimilarity percentages for compound sub-classes vs. CPA-active fragment-sized NP from which the PNP was derived (i.e., quinine, quinidine, or griseofulvin); the different colors indicate high (dark blue) to low (yellow) MBP values. QN-I = quinine-indoles; QN-C-S = quinine-chromanones (S)-isomer; QN-C-R = quinine-chromanones (R)-isomer; QD-I = quinidine-indoles; QN-C-S = quinidine-chromanones (S)-isomer; QN-C-R = quinidine-chromanones (R)-isomer; GF-THPI = griseofulvin-tetrahydropyranoindoles; GF-I-2 = griseofulvin-indoles isomer-2; GF-I-1 = griseofulvin-indoles isomer-1; NPs = natural products. **b** Dimension reduction analysis (PCA) of cell painting assay (CPA) data for the NP griseofulvin and a griseofulvin-derived PNP (**GF** (griseofulvin) vs **GF-THPI** (griseofulvin-tetrahydropyranoindoles), Expl. Var.: PC1 (45.3%), PC2 (36.7%), PC3 (10.2%)). **c** PCA of griseofulvin-indoles with a different fusion pattern (**GF-I** (griseofulvin-indoles) vs **GF-THPI** (griseofulvin-tetrahydropyranoindoles), Expl. Var.: PC1 (39.9%), PC2 (25.8%), PC3 (9.4%)), **d**, PCA for diastereomers with one different stereogenic center (**QD-C-R** (quinidine-chromanones (R)-isomer) vs **QD-C-S** (quinidine-chromanones (S)-isomer), Expl. Var.: PC1 (62.0%), PC2 (10.8%), PC3 (6.1%)). PC = principal component; Comp = component. Source data are provided as a Source Data file or in the online repository.

To analyze whether the combination of NP fragments leads to different biological variances than the individual fragments, the profiles of the fragment-sized-NPs, indole fragments, and chromanone fragments were compared to the PNPs from which they were derived. Three of the fragment-sized NPs were active in the CPA, and the three quinidine-derived sub-classes were at or above the 75% median biosimilarity threshold (Fig. 4a). As a representative PCA, griseofulvin was compared to **GF-THPI** in which significant distance between compound class clusters were observed, indicating significant phenotypic differences (Fig. 4b). The fourth fragment-sized-NP, sinomenine, is inactive at up to 50 µM, whereas the sinomenine-derived PNPs **SM-C** and **SM-I** have high median induction values at 10 µM (68 and 53%, respectively), indicating substantial phenotypic differences. Overall, 10/13 compound sub-classes had different profiles than the NP from which they were derived. Inadequate morphological activity was observed for most small indole- and chromanone-containing compounds (MW < 300), while compounds that did have suitable induction values had significant morphological differences than the related PNPs. In general, the phenotypic profiles of the PNPs seem to be due to the combination of fragments and not the individual fragments themselves^4^.

We briefly investigated the influence of structural features on the phenotypic profiles. The **GF-I** and **GF-THPI** classes contain similar fragments but differ in connection by edge and spirocyclic fusion patterns, respectively. Both the **G-I-1** and **G-I-2** subclasses have low MBPs when compared to **GF-THPI** (1 and 9%, respectively) and occupy different space in a PCA (Fig. 4c) which signifies a phenotypic impact based on fusion pattern. **GF-I-1** and **GF-I-2** are regioisomers but do not form defined clusters in a PCA. For several cases, diastereomeric features did not result in significant phenotypic differences; however, PCA was able to distinguish **QD-C-S** and **QD-C-R** (Fig. 4d) despite an MBP of 74%. Last, comparison of **SM-I-closed** and the ring-fragmented derivative **SM-I-opened** had a high MBP (81%), suggesting ring fragmentation did not significantly affect the phenotypic profile. Overall, variation of structural features in the library provided some phenotypic diversity but not in all examples.

The principle of PNPs is based on the combination of unrelated fragments to access compounds that differ in bioactivity from the individual fragments. To address whether different combinations lead to different bioactivities, compounds containing either an indole or chromanone fused to the four different NP-derivatives were analyzed. From the analysis, the common fragments within the PNPs can be classified as non-dominating or dominating. If PNPs containing the same fragment have class-based clustering in PCA and/or a low MBP ( < 75%), the fragment is considered non-dominating, whereas compounds that do not cluster in PCA and have a high MBP are dominating.

**Fig. 5:**
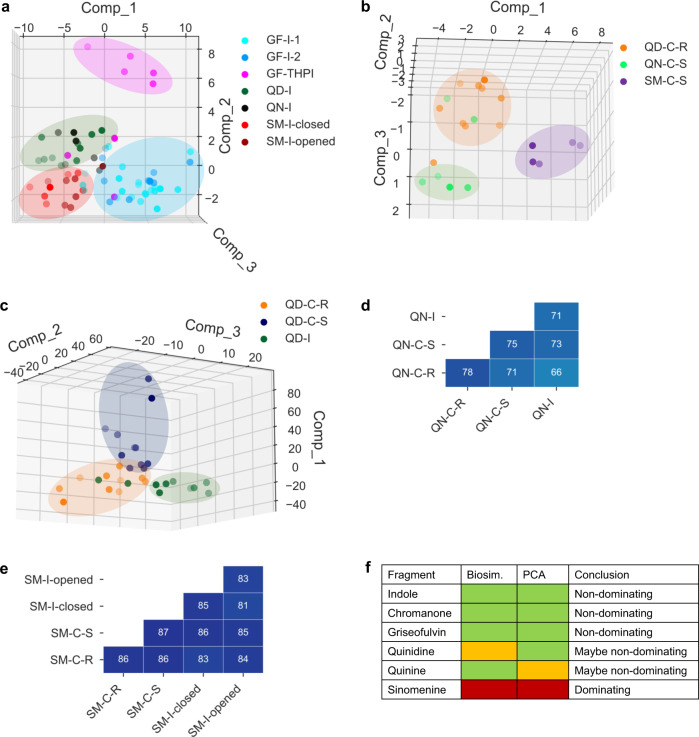
Comparison of PNP compound classes with one conserved fragment. **a** PCA of indole-containing PNPs: **GF-I-1** (griseofulvin-indoles isomer 1, light blue), **GF-I-2** (griseofulvin-indoles isomer 2, blue), **GF-THIP** (Griseofulvin-tetrahydropyranoindoles, pink), **QD-I** (quinidine-indoles, green, 15-40% induction), **QN-I** (quinine-indoles, black, 15-40% induction), **SM-I-closed** (sinomenine-indoles ring closed, red), and **SM-I-opened** (sinomenine-indoles ring opened, dark red). Expl. Var.: PC1 (55.1%), PC2 (13.0%), PC3 (8.6%). **b** PCA of chromanone-containing PNPs: **QD-C-R** (quinidine-chromanones (R)-isomer, orange), **QN-C-S** (quinine-chromanones (S)-isomer, light green), and **SM-C-S** (sinomenine-chromanones (S)-isomer, purple). Expl. Var.: PC1 (52.4%), PC2 (10.6%), PC3 (8.2%). **c** PCA of quinidine-containing PNPs: **QD-C-R** (quinidine-chromanones (R)- isomer, orange), **QD-C-S** (quinidine-chromanones (S)-isomer, dark blue) and **QD-I** (quinidine-indoles, green, 15-40% induction). Expl. Var.: PC1 (45.2%), PC2 (19.6%), PC3 (7.6%). **d** Biosimilarity table for quinine-derived PNPs (same color scale as Fig. 4a). **e** Biosimilarity table for sinomenine-derived PNPs (same color scale as Fig. 4a). **f** Summary table of classifications. Non-dominating: green, maybe non-dominating: yellow, dominating: red. PC = principal component; Comp = component; Biosim. = biosimilarity; PCA = principal component analysis. Source data are provided as a Source Data file or in the online repository.

PCA of indole-containing PNPs resulted in distinguishable clusters based on compound class (Fig. 5a). Furthermore, the low MBP between indole-derived subclasses (45%) signifies distinct phenotypic changes and suggests that the indole fragment is non-dominating. Three chromanone-containing subclasses (**QD-C-R**, **QN-C-S**, and **SM-C-S**) provided three clusters in PCA (Fig. 5b) but had an MBP close to the 75% cutoff (74%). Despite the high MBP, the two clusters from the PCA indicate different phenotypes and suggest that the chromanone fragment may be non-dominating.

The two compound classes derived from griseofulvin (**GF-I** and **GF-THPI**) both contain the indole fragment. Despite only varying in fusion pattern, clustering in PCA (Fig. 4c) and a significantly different MBP indicates that griseofulvin is a non-dominating fragment and further verifies that the indole fragment is non-dominating. PCA of quinidine-containing compounds provided three clusters (Fig. 5c) but are at the biosimilarity cutoff (MBP = 75%). Conversely, compounds derived from quinine did not form clear clusters in PCA (Supplementary Figure 13a) but did have a value below the MBP threshold (71%, Fig. 5d). Since both quinidine- and quinine-derived PNPs have phenotypic differences based on different fragment combinations in either PCA or MBP calculations, we concluded that both fragments may be non-dominating.

Analysis of sinomenine-derived compounds led to an unspecific distribution in the PCA plot in which no phenotypic differences could be distinguished. Regardless of fragment combination or structural variation, i.e. diastereomers or ring fragmentation, the sinomenine-containing subclasses do not cluster in PCA (Supplementary Fig. 13b) and all have high MBPs relative to each other (86%, Fig. 5e). Therefore, the sinomenine-based PNPs’ phenotypic profiles are likely dictated by a dominating sinomenine fragment.

Based on the insights gained from the CPA analysis, we hypothesized that our classification of non-dominating and dominating fragments could guide the design of compounds to expand the biological diversity of the collection (for a summary of classifications, see Fig. 5f). In the examples with the combination of non-dominating fragments, it is likely that the bioactivity profiles of theses PNPs are neither a representation of either individual fragment (Fig. 3a) nor the addition of both individual fragments’ profiles^4^. Instead, the unique profile may be due to the combination of fragments. It is therefore possible that other combinations of non-dominating fragments may lead to biological profiles that differ from the current collection. On the other hand, a different combination of a dominating and a non-dominating fragment is expected to provide a profile that is already represented by the dominating fragment in other combinations. By identifying dominating fragments, design of compounds with redundant profiles may be avoided.

**Fig. 6:**
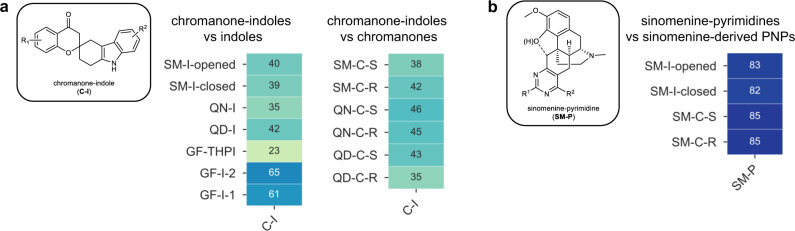
Biosimilarity comparisons for predicted compound classes. **a** Chromanone-indoles (**C-I**) compared to indole-containing subclasses and chromanone-containing subclasses (same color scale as Fig. 4a). **b** Sinomenine-pyrimidines (**SM-P**, 20–53% induction) compared to sinomenine-containing subclasses (20–40% induction) (same color scale as Fig. 4a). For references to subclass abbreviations and structures, see Fig. 2. Source data are provided in the online repository.

The first predictive compound class combines the chromanone and indole fragments to give chromanone-indoles (**C-I**). It is hypothesized that **C-I** will have a unique phenotype since both NP fragments were proposed to have a non-dominating effect on the phenotypic profile. A small collection of 31 **C-I** was synthesized (Supplementary Fig. 14a), subjected to the CPA, and compared to the previous described compounds containing either an indole or a chromanone fragment. As predicted, the profiles of the **C-I** class showed low similarity to all indole- (average MBP = 44%) and chromanone- (average MBP = 42%) containing classes (Fig. 6a). PCA shows a clear separation between **C-I** and chromanone-containing classes and clustering when compared to the other indole-containing classes (Supplementary Fig. 14b, c). These analyses agree with our prediction and support the hypothesis that chromanone and indole are non-dominating fragments.

Since sinomenine showed a dominating effect on the phenotypic profile, it was hypothesized that combining it with a fragment other than indole or chromanone would still give a PNP with a similar phenotypic response. To test our hypothesis, sinomenine was combined with different pyrimidines to give sinomenine-pyrimidines (**SM-P**) (Supplementary Fig. 15a). As indicated by high MBP values (average MBP = 84%; Fig. 6b) as well as by PCA (Supplementary Fig. 15b), the SM-P class has a similar profile to other sinomenine-containing PNPs and is indicative of the dominating nature of the sinomenine fragment.

Since it was possible to generate compound classes that show expected phenotypic behaviors based on certain fragment combinations, there is an indication of a predictive character of the CPA. Other compound classes that share a conserved fragment may be synthesized and subjected to a similar CPA analysis to further validate this approach as a method to design compound classes with diverse bioactivity profiles.

## Discussion

Herein, we have described the design, synthesis, and chemoinformatic analysis of eight PNP classes. The library was designed to have different fragment combinations and structural variances through the fusion of fragment-sized NPs to biosynthetically unrelated chromanone or indole-containing fragments. Combination of the fragments was achieved through multibond-forming reactions employing NPs with suitably reactive ketone handles as substrates to afford a compound collection. Cheminformatic analyses indicated that the PNP classes are chemically diverse to each other while the overall library has connectivity patterns that resemble both NPs and synthetic drugs. Furthermore, the collection has structural features that occupy a wide variety of shapes and favorable drug-like properties.

The cell painting assay was employed as an unbiased method to biologically evaluate the compound collection and to answer the five previously asked questions (vide supra). Analysis of the CPA data revealed that (i) the PNP classes are bioactive and, in most cases, (ii) differ from the morphological profiles of the NPs and NP fragments from which they are derived. We showed that while (iii) structural variations can influence the phenotypic profiles, the (iv) combination of different fragments is the most important aspect that leads to the diverse profiles in this compound set. Finally, (v) the CPA was able to identify the phenotypic role of individual fragments within the PNPs and categorize these as either phenotypically dominating fragments or suitable combination partners for different bioactivity. This information was used to design compound classes that were correctly predicted to have either unique or similar biological profiles relative to the compound set.

These results demonstrate that the fusion of a small set of NP fragments that differ in combination and structural features can provide many chemically diverse PNP-based compound classes with diverse bioactivity profiles. We anticipate that the principle of PNPs in conjunction with the CPA and related morphological profiling assays will continue to guide the design of compound collections that will have significant value towards the exploration of biologically relevant chemical space.

## Methods

### Synthetic methods

#### Synthesis of **QN-I** and **QD-I** classes

The quinine- or quinidine-derived ketone (1.0 equiv), a 2-iodoaniline derivative (3.0 equiv), DABCO (3.0 equiv) and Pd(OAc)_2_ (0.1 equiv) were added to a reaction vial equipped with a screw-cap lid^6^. The reactants were dissolved in anhydrous DMF (1.0 ml / 0.32 mmol) and degassed under a stream of Argon for 10 min then heated to 105 °C. The reaction progress was monitored every 24 h by HPLC-MS. In case of incomplete conversion, the reaction mixture was cooled to rt, and additional Pd(OAc)_2_ (0.1 equiv) was added. The resulting solution was degassed under a steam of argon for 10 min and returned to the heating block for additional 24 h. Following complete conversion of starting ketone (or after a maximum 72 h reaction time), the reaction mixture was cooled to rt, diluted with MeOH (5 ml) and passed through a syringe filter. After removal of the solvents in vacuo the crude products were purified by silica chromatography and/or by mass-directed preparative HPLC.

#### Synthesis of **QN-C**, **QD-C**, and **SM-C** classes

To an oven-dried pressure vessel equipped with a stir bar that was cooled under an atm of Ar was added a natural product ketone derivative (1 equiv). Then, anhydrous EtOH (1 ml/0.33 mmol) was added followed by a 2-hydroxyacetophenone derivative (2 equiv) and pyrrolidine (4 equiv). The pressure vessel was flushed with Ar for 5 min then sealed with the proper cap. The reaction was heated to 100 °C for 16 h. After cooling to room temperature, the reaction was diluted with DCM, transferred to round-bottomed flask, and concentrated. Purification could be achieved by silica chromatography and/or by mass-directed preparative HPLC to afford pure isomers. All of the reactions produced two diastereomers; however, after purification, sometimes only one of the diastereomers could be obtained in pure form.

#### Synthesis of **SM-I** and **GF-I** classes

To an oven-dried microwave vial under an atmosphere of argon was added a natural product ketone derivative (1 equiv), a phenylhydrazine HCl salt derivative (1.5 equiv), and paratolylsulfonic acid (pTSA) (1.5 equiv). Then, anhydrous EtOH (1.5 ml/0.13 mmol) was added, and the vial was flushed with argon again. A microwave cap was placed on the vial, and the reaction was heated to 90-130 °C for 1–3 h in a microwave. After the reaction had cooled to room temperature, it was diluted with DCM, transferred to a sept funnel, and saturated aqueous NaHCO_3_ was added. The aqueous layer was extracted with DCM (3 x 10 ml). The combined organic layers were dried and concentrated. The crude mixture was purified by silica chromatography and/or by mass-directed preparative HPLC. For **GF-I**, two regioisomers were formed and, in most cases, could be separated by silica chromatography.

#### Synthesis of **GF-THPI** class

To a 5 ml vial was added an ethanol-derived indole (1 equiv), DCM (1 ml / 0.1 mmol), the griseofulvin-derived ketone (1.2 equiv), and TfOH-SiO_2_ (0.065 equiv), successively. The reaction was stirred for 30 min at 22 °C. The reaction was filtered, concentrated, and purified by silica chromatography to afford a single diastereomer. NMRs of the crude reaction mixture indicated that only a single diastereomer was present for each reaction.

Syntheses of starting materials and detailed characterization data can be found in the Supplementary Information.

### Cheminformatic analyses

All details pertaining to the cheminformatic analyses can be found in the Supplementary Information or in the github repository^43^,10.5281/zenodo.4529728.

### Cell painting assay

Initially, 5 µl U2OS medium were added to each well of a 384-well plate (PerkinElmer CellCarrier-384 Ultra). Subsequently, U2OS cell were seeded with a density of 1600 cells per well in 20 µl medium. The plate was incubated for 10 min at the ambient temperature, followed by an additional 4 h incubation (37 °C, 5% CO_2_). Compound treatment was performed with the Echo 520 acoustic dispenser (Labcyte) at final concentrations of 10 µM, 3 µM or 1 µM. Incubation with compound was performed for 20 h (37 °C, 5% CO_2_). Subsequently, mitochondria were stained with Mito Tracker Deep Red (Thermo Fisher Scientific, Cat. No. M22426). The Mito Tracker Deep Red stock solution (1 mM) was diluted to a final concentration of 100 nM in prewarmed medium. The medium was removed from the plate leaving 10 µl residual volume and 25 µl of the Mito Tracker solution were added to each well. The plate was incubated for 30 min in darkness (37 °C, 5% CO_2_). To fix the cells 7 µl of 18.5 % formaldehyde in PBS were added, resulting in a final formaldehyde concentration of 3.7 %. Subsequently, the plate was incubated for another 20 min in darkness (RT) and washed three times with 70 µl of PBS. (Biotek Washer Elx405). Cells were permeabilized by addition of 25 µl 0.1% Triton X-100 to each well, followed by 15 min incubation (RT) in darkness. The cells were washed three times with PBS leaving a final volume of 10 µl. To each well 25 µl of a staining solution were added, which contains 1% BSA, 5 µl/ml Phalloidin (Alexa594 conjugate, Thermo Fisher Scientific, A12381), 25 µg/ml Concanavalin A (Alexa488 conjugate, Thermo Fisher Scientific, Cat. No. C11252), 5 µg/ml Hoechst 33342 (Sigma, Cat. No. B2261-25mg), 1.5 µg/ml WGA-Alexa594 conjugate (Thermo Fisher Scientific, Cat. No. W11262) and 1.5 µM SYTO 14 solution (Thermo Fisher Scientific, Cat. No. S7576). The plate is incubated for 30 min (RT) in darkness and washed three times with 70 µl PBS. After the final washing step, the PBS was not aspirated. The plates were sealed and centrifuged for 1 min at 500 rpm.

The plates were prepared in triplicates with shifted layouts to reduce plate effects and imaged using a Micro XL High-Content Screening System (Molecular Devices) in 5 channels (DAPI: Ex350-400/ Em410-480; FITC: Ex470-500/ Em510-540; Spectrum Gold: Ex520-545/ Em560-585; TxRed: Ex535-585/ Em600-650; Cy5: Ex605-650/ Em670-715) with 9 sites per well and 20x magnification (binning 2).

Detailed descriptions of processing and analysis of the cell painting assay data can be found in the Supplementary Information.

### Reporting summary

Further information on research design is available in the Nature Research Reporting Summary linked to this article.

## Supplementary information

Supplementary Information

Reporting Summary

## Data Availability

The data sets generated during and/or analyzed during the current study are available in the manuscript, in the Supplementary Information, or the github repository^43^, 10.5281/zenodo.4529728. Source data are provided with this paper. The crystallographic data for the structure of **GF-THPI-7** has been published as supplementary publication number 2047701 in the Cambridge Crystallographic Data Centre. Source data are provided with this paper.

## Source data

Source Data

## Code availability

The computer code used to analyze the data is available in the github repository, Grigalunas, M., Burhop, A., Zinken, S., Pahl, A., Gally, J-M., Wild, N., Mantel, Y., Sievers, S., Foley, D. J., Scheel, R., Strohmann, C., Antonchick, A. P., Waldmann & H., Natural product fragment combination to performance-diverse pseudo-natural products, 2021_grigalunas_burhop_zinken, 10.5281/zenodo.4529728 (2021).
