# Supplementary material for: Natural product fragment combination to performance-diverse pseudo-natural products
Source: Nat Commun. 2021 Mar 25;12:1883. doi: 10.1038/s41467-021-22174-4 (PMC7994817; doi:10.1038/s41467-021-22174-4)
Supplement: Supplementary file 2 — Reporting Summary [file 41467_2021_22174_MOESM2_ESM.pdf]

nature research

Corresponding author(s): Herbert Waldmann

Last updated by author(s): Feb 22, 2021

Reporting Summary

Nature Research wishes to improve the reproducibility of the work that we publish. This form provides structure for consistency and transparency in reporting. For further information on Nature Research policies, see our Editorial Policies and the Editorial Policy Checklist.

Statistics

For all statistical analyses, confirm that the following items are present in the figure legend, table legend, main text, or Methods section.

n/a	Confirmed	
☐	☒	The exact sample size (*n*) for each experimental group/condition, given as a discrete number and unit of measurement
☒	☐	A statement on whether measurements were taken from distinct samples or whether the same sample was measured repeatedly
☒	☐	The statistical test(s) used AND whether they are one- or two-sided<br>*Only common tests should be described solely by name; describe more complex techniques in the Methods section.*
☒	☐	A description of all covariates tested
☒	☐	A description of any assumptions or corrections, such as tests of normality and adjustment for multiple comparisons
☒	☐	A full description of the statistical parameters including central tendency (e.g. means) or other basic estimates (e.g. regression coefficient) AND variation (e.g. standard deviation) or associated estimates of uncertainty (e.g. confidence intervals)
☒	☐	For null hypothesis testing, the test statistic (e.g. *F*, *t*, *r*) with confidence intervals, effect sizes, degrees of freedom and *P* value noted<br>*Give P values as exact values whenever suitable.*
☒	☐	For Bayesian analysis, information on the choice of priors and Markov chain Monte Carlo settings
☒	☐	For hierarchical and complex designs, identification of the appropriate level for tests and full reporting of outcomes
☒	☐	Estimates of effect sizes (e.g. Cohen's *d*, Pearson's *r*), indicating how they were calculated

Our web collection on statistics for biologists contains articles on many of the points above.

Software and code

Policy information about availability of computer code

Data collection

* Natural products from ChEMBL 26
  * Two versions of ChEMBL 26 were downloaded from the FTP server [http://ftp.ebi.ac.uk/pub/databases/chembl/ChEMBLdb/releases/chembl_26/] on 25-Mar-2020:
    * chembl_26_postgresql.tar.gz
    * chembl_26.sdf.gz
* Approved and Experimental Drugs
  * The approved and experimental data sets from DrugBank were used [v 5.1.5, https://www.drugbank.com, downloaded on 25-Mar-2020].
* Enamine Advanced Screening Collection
  * The full Advanced Screening Collection was obtained from the Enamine site [https://enamine.net/hit-finding/compound-collections/screening-collection/advanced-collection), downloaded on 07-Dec-2020]

The reference data sets themselves are not included in this manuscript.
All processing steps performed on the data sets are described in the SI and the code is included in the Github repository (see Data Availability statement).

Data analysis	The data analysis was performed using the following Open Source software:<br>Python (https://www.python.org; v 3.8.6), the cheminformatics toolkit RDKit (RDKit: Open-Source Cheminformatics Software, v2020.03.6, http://www.rdkit.org/, last accessed 17-Dec-2020), the Scientific Python package (https://scipy.org/; v1.5.2), scikit-learn (https://scikit-learn.org; v 0.23.2) and KNIME (https://www.knime.com; v 4.2.3).<br>The data analysis was performed in a conda environment (https://www.anaconda.com/) using JupyterLab notebooks (https://jupyter.org/). A detailed list of the employed software packages as well as instructions for the reproduction of the environment can be found in the Github repository (see Data Availability statement).

For manuscripts utilizing custom algorithms or software that are central to the research but not yet described in published literature, software must be made available to editors and reviewers. We strongly encourage code deposition in a community repository (e.g. GitHub). See the Nature Research guidelines for submitting code & software for further information.

Data

Policy information about availability of data

All manuscripts must include a data availability statement. This statement should provide the following information, where applicable:

- Accession codes, unique identifiers, or web links for publicly available datasets
- A list of figures that have associated raw data
- A description of any restrictions on data availability

The data sets generated during and/or analyzed during the current study are available in the manuscript, in the Supplementary Information, or the github repository, http://dx.doi.org/10.5281/zenodo.4529728. Source data are provided with this paper. The crystallographic data for the structure of GF-THPI-7 has been published as supplementary publication number 2047701 in the Cambridge Crystallographic Data Centre.

Field-specific reporting

Please select the one below that is the best fit for your research. If you are not sure, read the appropriate sections before making your selection.

- [x] Life sciences
- [ ] Behavioural & social sciences
- [ ] Ecological, evolutionary & environmental sciences

For a reference copy of the document with all sections, see nature.com/documents/nr-reporting-summary-flat.pdf

Life sciences study design

All studies must disclose on these points even when the disclosure is negative.

Sample size	Does not apply to our study
Data exclusions	Does not apply to our study
Replication	The cell painting assay was run in triplicate.
Randomization	Does not apply to our study
Blinding	Does not apply to our study

Reporting for specific materials, systems and methods

We require information from authors about some types of materials, experimental systems and methods used in many studies. Here, indicate whether each material, system or method listed is relevant to your study. If you are not sure if a list item applies to your research, read the appropriate section before selecting a response.

Materials & experimental systems

n/a	Involved in the study	
x		Antibodies
	x	Eukaryotic cell lines
x		Palaeontology and archaeology
x		Animals and other organisms
x		Human research participants
x		Clinical data
x		Dual use research of concern

Methods

n/a	Involved in the study	
x		ChIP-seq
x		Flow cytometry
x		MRI-based neuroimaging

Eukaryotic cell lines

Policy information about cell lines

Cell line source(s)	U2OS
Authentication	The cell line was bought from ATCC at the start of the project in 2018
Mycoplasma contamination	Tests for mycoplasma contamination were performed every 6 months using the MycoAlert (Mycoplasma Detection Kit, Lonza, LT07-218) according to manufacturer's instructions.
Commonly misidentified lines (See ICLAC register)	None
