# Supplementary material for: Natural product fragment combination to performance-diverse pseudo-natural products
Source: Nat Commun. 2021 Mar 25;12:1883. doi: 10.1038/s41467-021-22174-4 (PMC7994817; doi:10.1038/s41467-021-22174-4)
Supplement: Supplementary file 1 — Supplementary Information [file 41467_2021_22174_MOESM1_ESM.pdf]

Supplementary Information

Natural Product Fragment Combination to Performance-Diverse Pseudo-Natural Products

Michael Grigalunas, Annina Burhop, Sarah Zinken, Axel Pahl, José-Manuel Gally, Niklas Wild, Yannik Mantel, Sonja Sievers, Daniel J. Foley, Rebecca Scheel, Carsten Strohmann, Andrey P. Antonchick, and Herbert Waldmann*

Table of Contents

Supplementary Materials 4
Supplementary Figures 4
Supplementary Tables 26
Compound Collection 28
Supplementary Methods 41
Cheminformatics Analysis 41
Data Preparation 41
NP Likeness and QED Scores 45
Principal Component Analysis of 17 Molecular Descriptors 46
Chemical Similarities 50
Biological Similarities 50
Principal Moment of Inertia Details 51
Cell Painting Assay Experimental and Analyses 55
Synthetic Experimental Details 59
Synthesis of quinine- and quinidine-based ketones: 60
General procedure for quinine- and quinidine-based indoles 61
General procedure for quinine-based chromanones 62
Compound data for quinine-based chromanones 63
General procedure for quinidine-based chromanones 93
Compound data for quinine-based chromanones 94
Synthesis of sinomenine-based ketones 117
General procedure for sinomenine-based chromanones 119
Compound data for sinomenine-based chromanones 120
General procedure for ring closed sinomenine-based indoles 138
Compound data for ring closed sinomenine-based indoles 139
General procedure for ring opened sinomenine-based indoles 157
Compound data for ring opened sinomenine-based indoles 158
Synthesis of the griseofulvin-based ketone 171
General procedures for griseofulvin-derived indoles 172
Compound data for griseofulvin-derived indoles 173
General procedure for the synthesis of 3-ethanolic indoles and compound data 205
General procedure for griseofulvin-based 4H-pyranoindoles and compound data 208
General procedure for chromanone-based ketones 215

Compound data for chromanone-based ketones 216
General procedure for chromanone-based indoles 218
Compound data for chromanone-based indoles 219
Synthesis and compound data of sinomenine-based pyrimadines 235
Representative NOESY correlations for the determination of diastereomers 239
X-ray analysis of compound GF-THPI-7: 245
HPLC Spectra 247
NMR Spectra 526
Supplementary References 1078

Supplementary Materials

Supplementary Figures

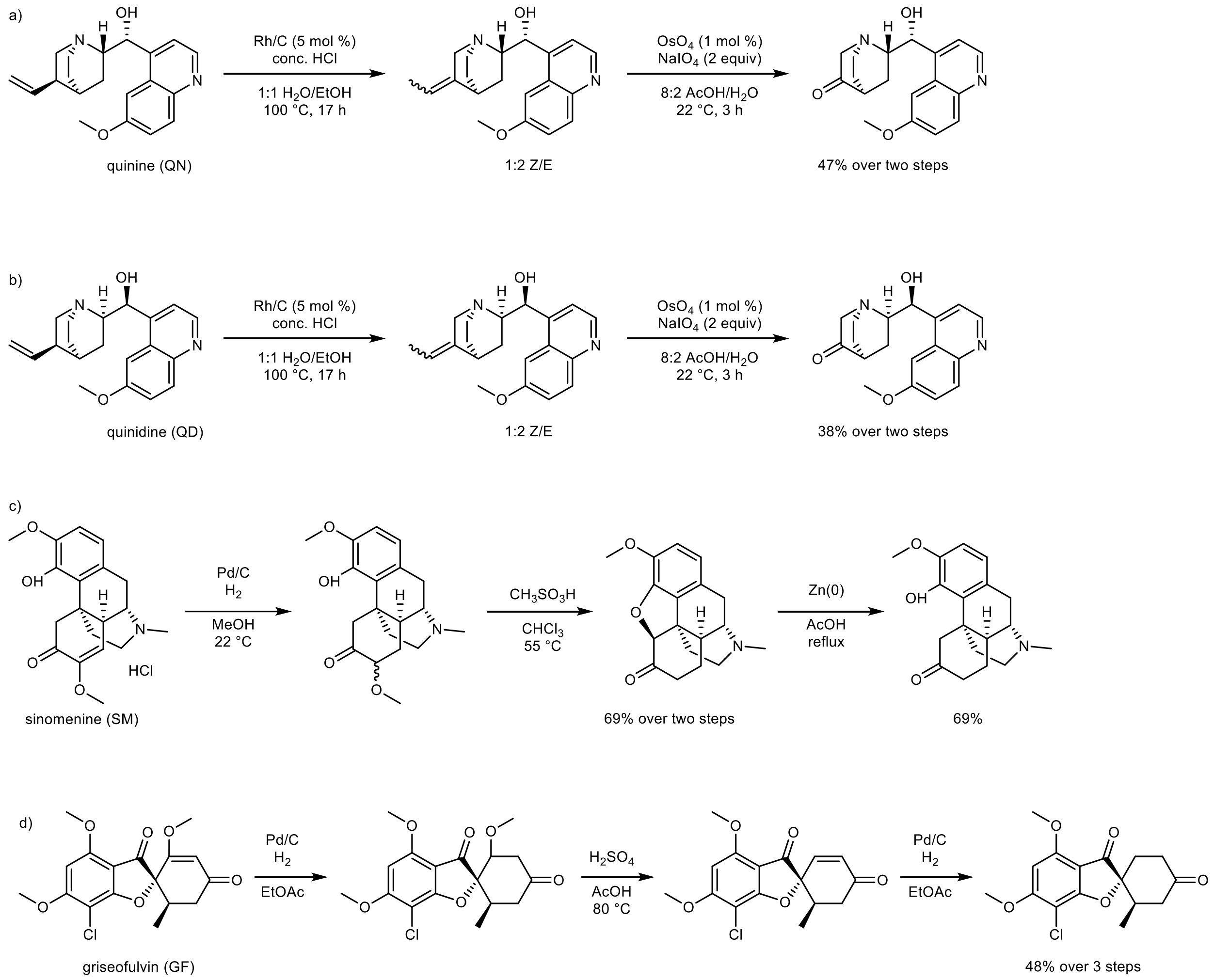

Supplementary Figure 1: **Synthesis of natural product ketone substrates.** Synthesis of a) quinine-[1], b) quinidine-[1], c) sinomenine-, and d) griseofulvin-derived ketones from the parent natural products.

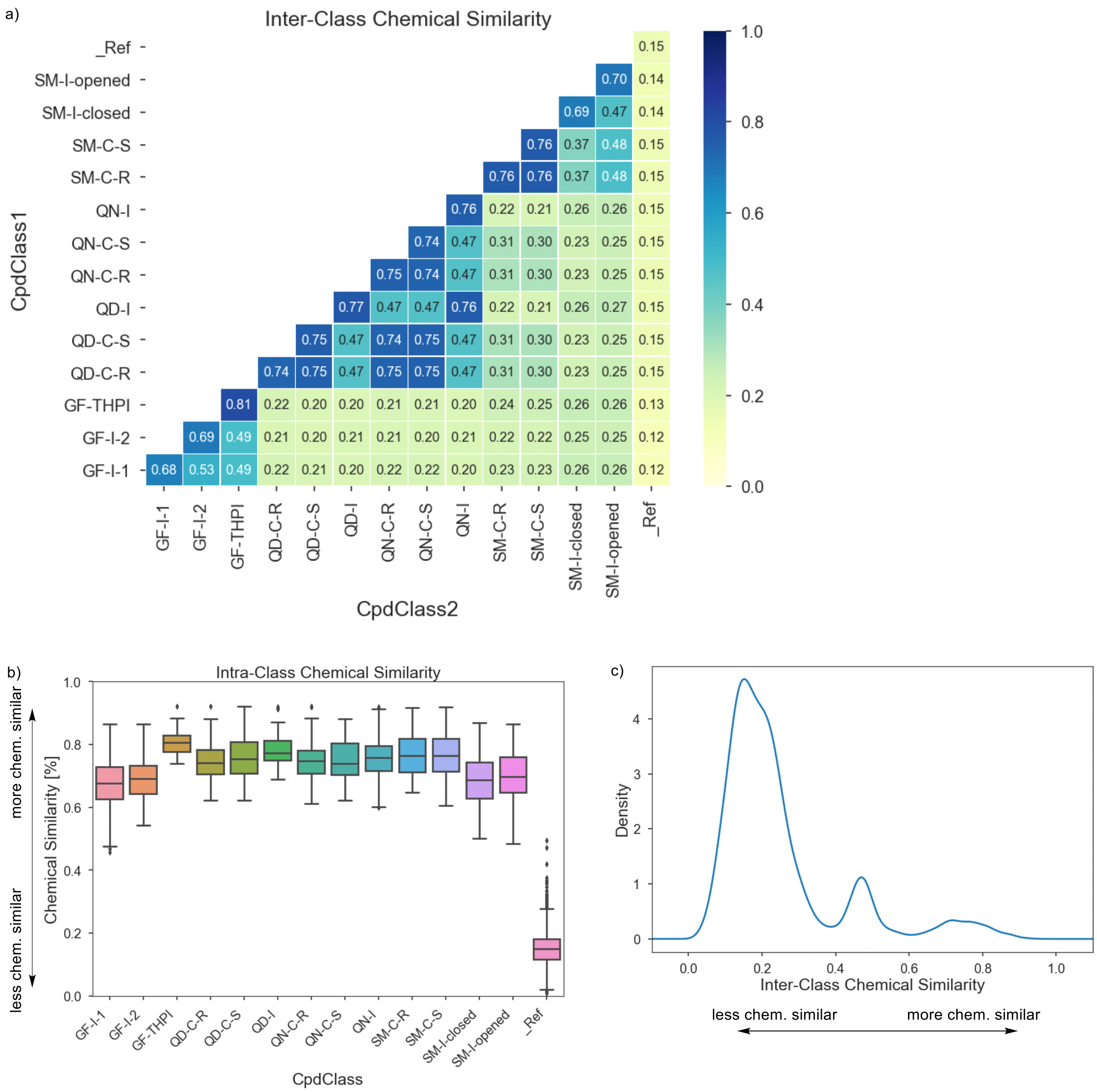

Supplementary Figure 2: **Tanimoto similarities of the Morgan fingerprints (ECFC4, count fingerprint, radius 2) of the pseudo-natural product library.** Tanimoto similarities of the Morgan fingerprints (ECFC4, count fingerprint, radius 2) of a) sub-class cross-comparisons (similarity color scale can be seen on the right), b) intra-subclass comparisons (box plot is following Tukey's definition with outliers[2]), and c) inter-subclass comparisons. "_Ref" is a random selection of 100 compounds from the Enamine Advanced Screening Collection (see the Chemical Similarities sections for details). Source data are provided in the online repository.

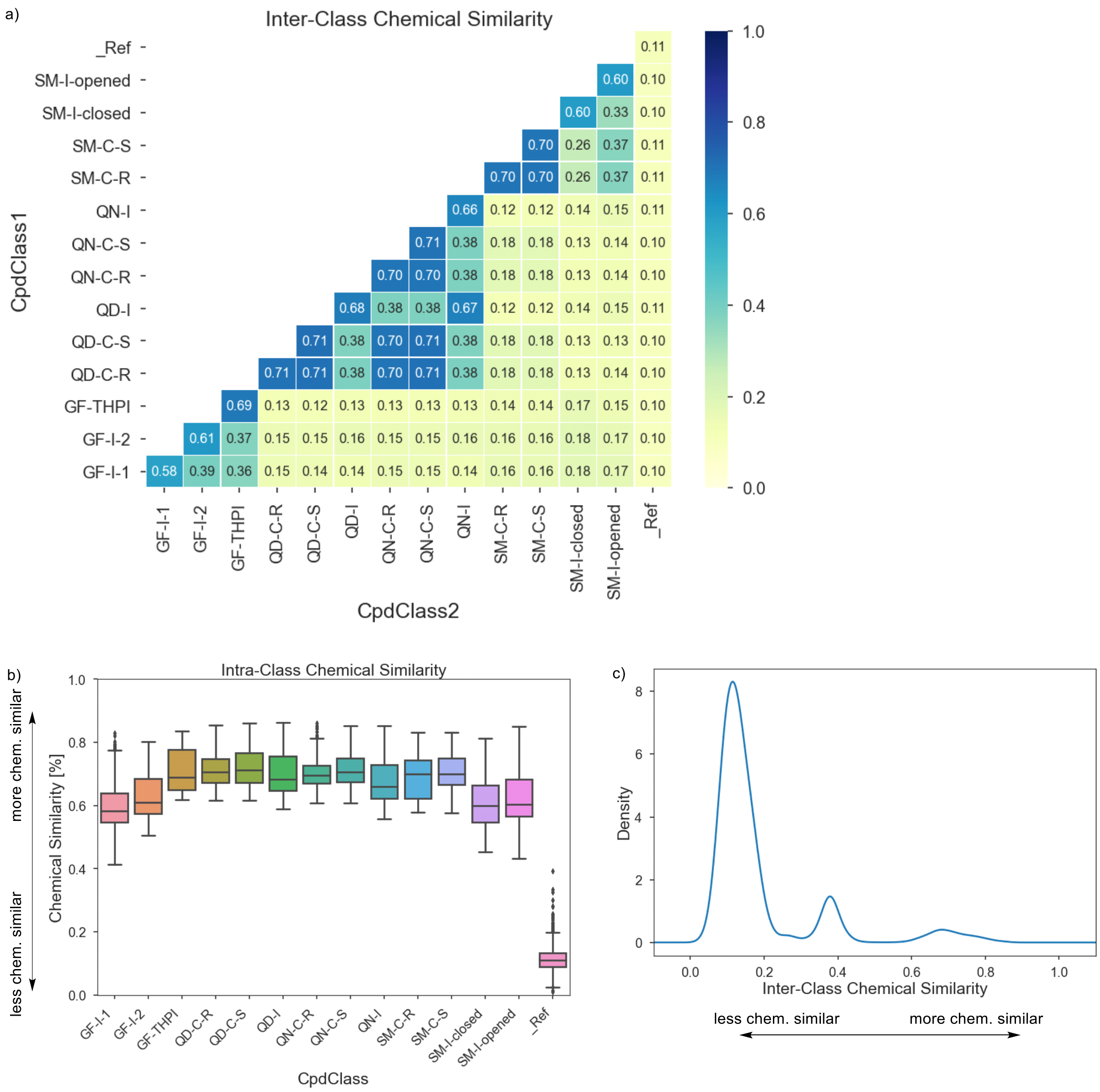

Supplementary Figure 3: **Tanimoto similarities of the Morgan (ECFP6, bit fingerprint of length 1024, radius 3) of the pseudo-natural product library.** Tanimoto similarities of the Morgan fingerprints (ECFP6, bit fingerprint of length 1024, radius 3) of a) sub-class cross-comparisons (similarity color scale can be seen on the right), b) intra-subclass comparisons (box plot is following Tukey's definition with outliers[2]), and c) inter-subclass comparisons. "_Ref" is a random selection of 100 compounds from the Enamine Advanced Screening Collection (see the Chemical Similarities sections for details). Source data are provided in the online repository.

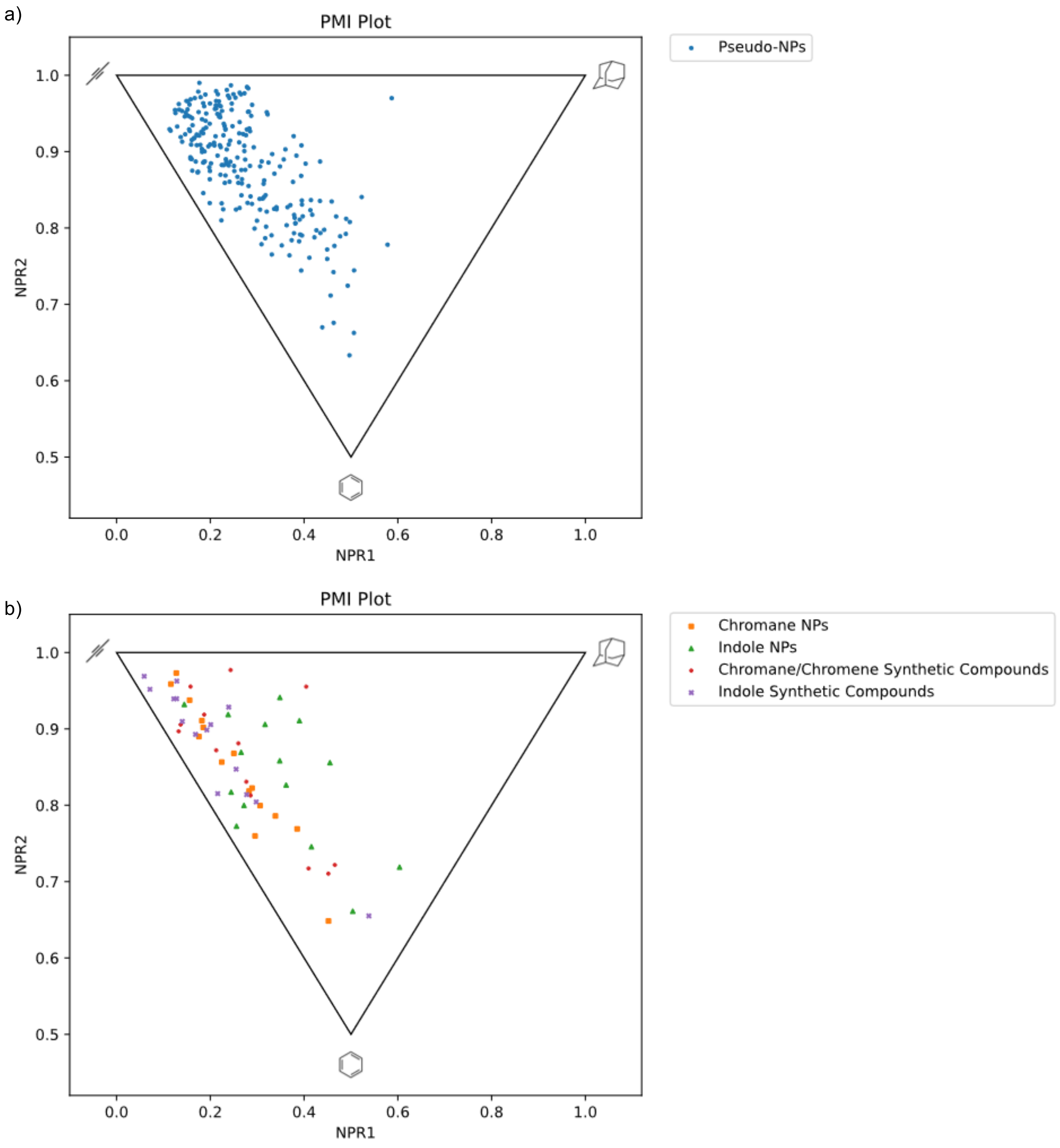

Supplementary Figure 4: **Principal moments of inertia plot of the PNP collection and reference compounds.** a) PMI plot of the pseudo-NP compound collection (244 compounds, blue circles). b) PMI plot of reference compounds comprised of chromane NPs (15 compounds, orange squares), indole NPs (15 compounds, green triangles), chromane/chromene synthetic compounds (15 compounds, red crosses), and indole synthetic compounds (15 compounds, purple xes). The corners of the triangle within the plot indicate a rod-like shape (top left), disk-like shape (bottom middle), and sphere-like shape (top right). Source data are provided in the online repository.

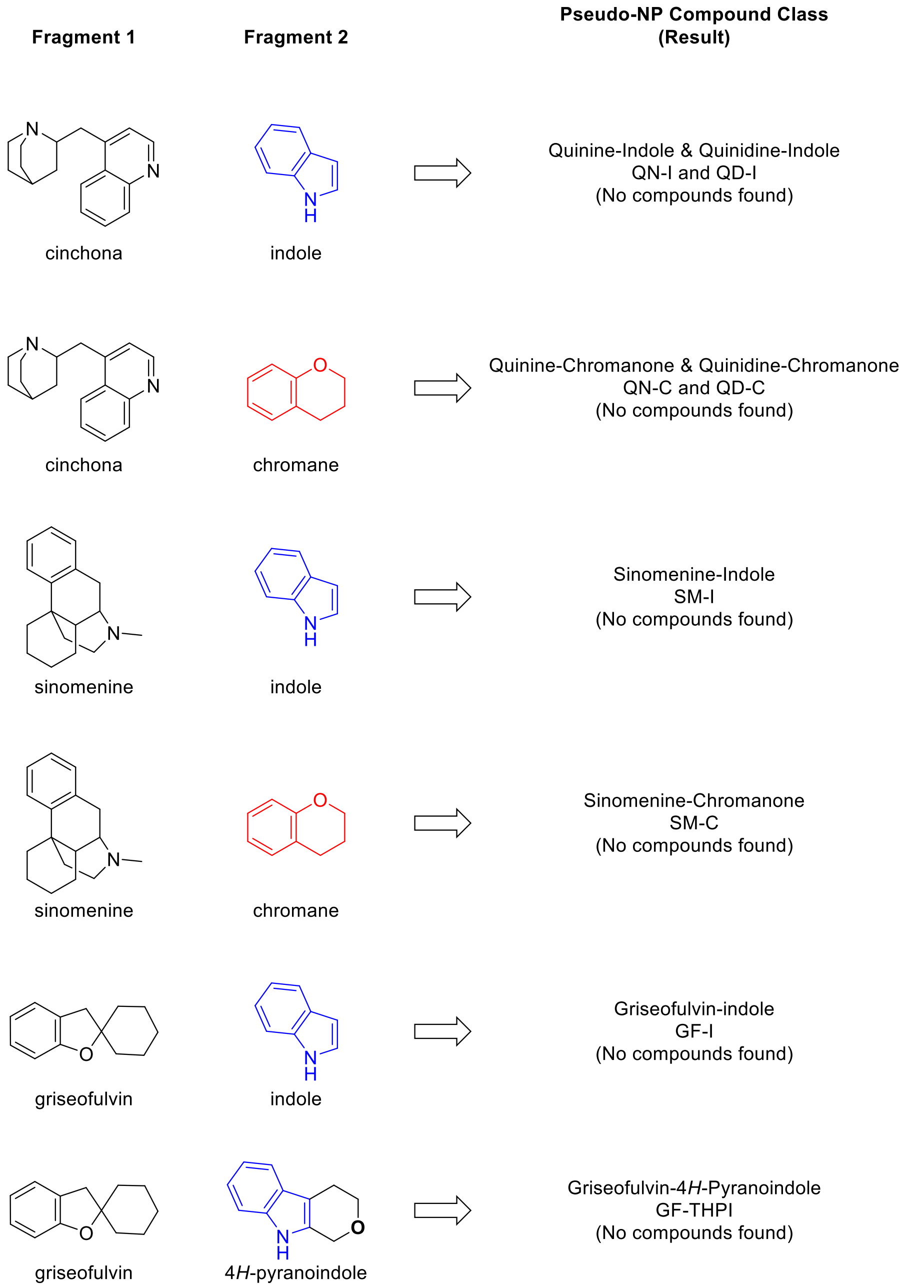

Supplementary Figure 5: **Fragment combination searches within the Dictionary of Natural Products.** These fragment combinations were investigated via a substructure search in the Dictionary of Natural Products with tautomers included (http://dnp.chemnetbase.com, accessed 10/12/2020).

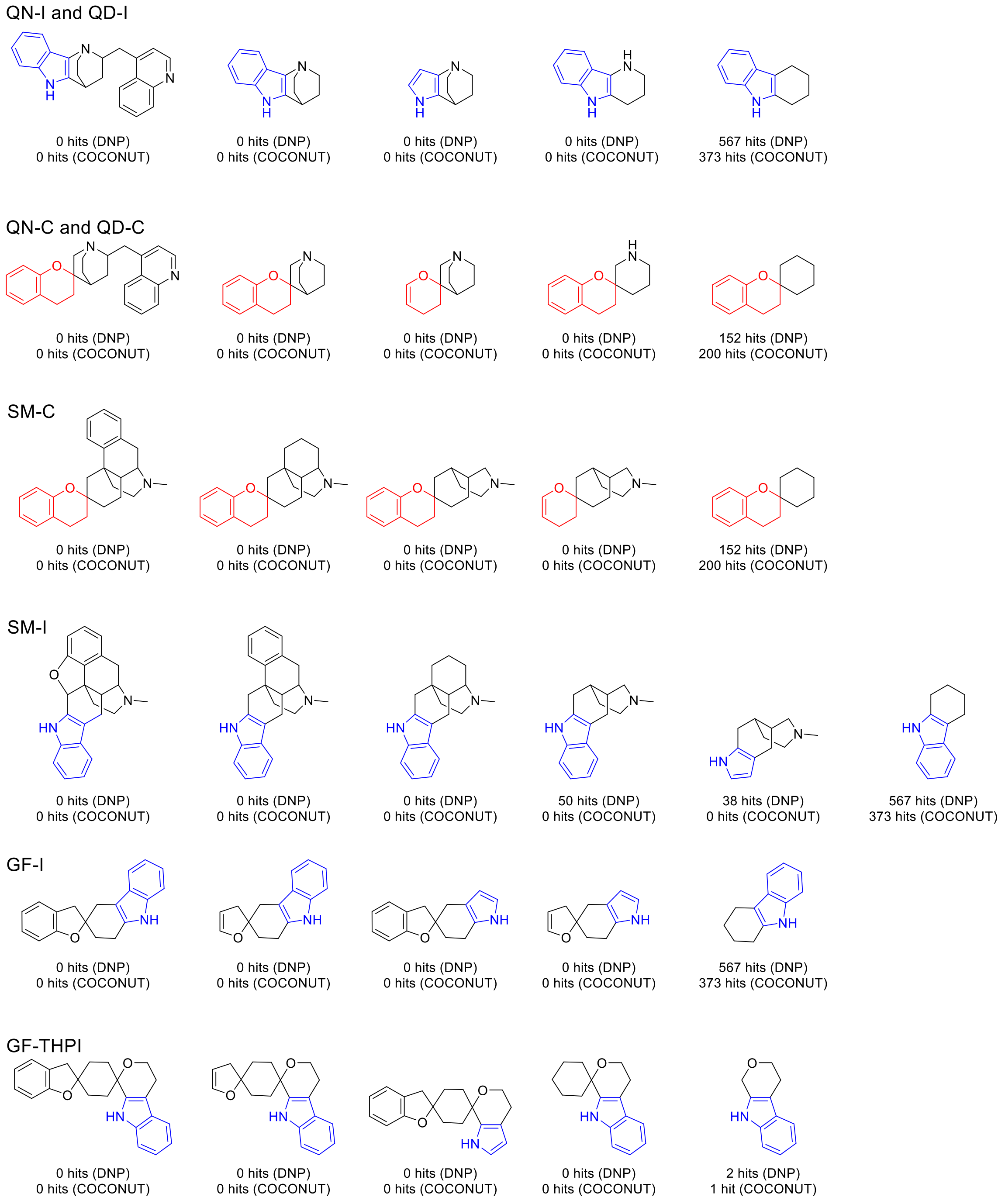

Supplementary Figure 6: **Substructure searches within the Dictionary of Natural Products and the COCONUT database.** These substructures were investigated in the Dictionary of Natural Products (http://dnp.chemnetbase.com, accessed 10/12/2020) with tautomers included. A second substructure search was conducted in the Collection of Open Natural Products (COCONUT) database (http://coconut.naturalproducts.net, accessed 10/12/2020) using the default substructure search (Ullmann algorithm). For the COCONUT database, each tautomer was manually entered and searched. The number of hits reflects the combination of all tautomers found.

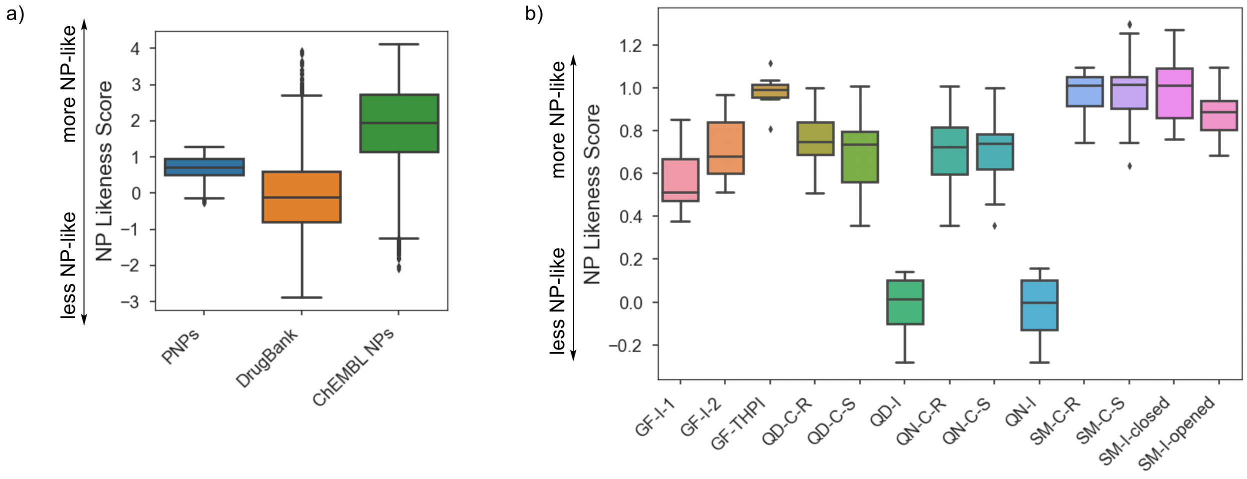

Supplementary Figure 7: **Box plots for NP-likeness scores.** Box plots of NP-likeness score of a) the PNP library relative to compounds in the DrugBank and NPs in ChEMBL as well as b) individual sub classes of the PNP library. Source data are provided in the online repository. Box plots are following Tukey's definition with outliers.[2]

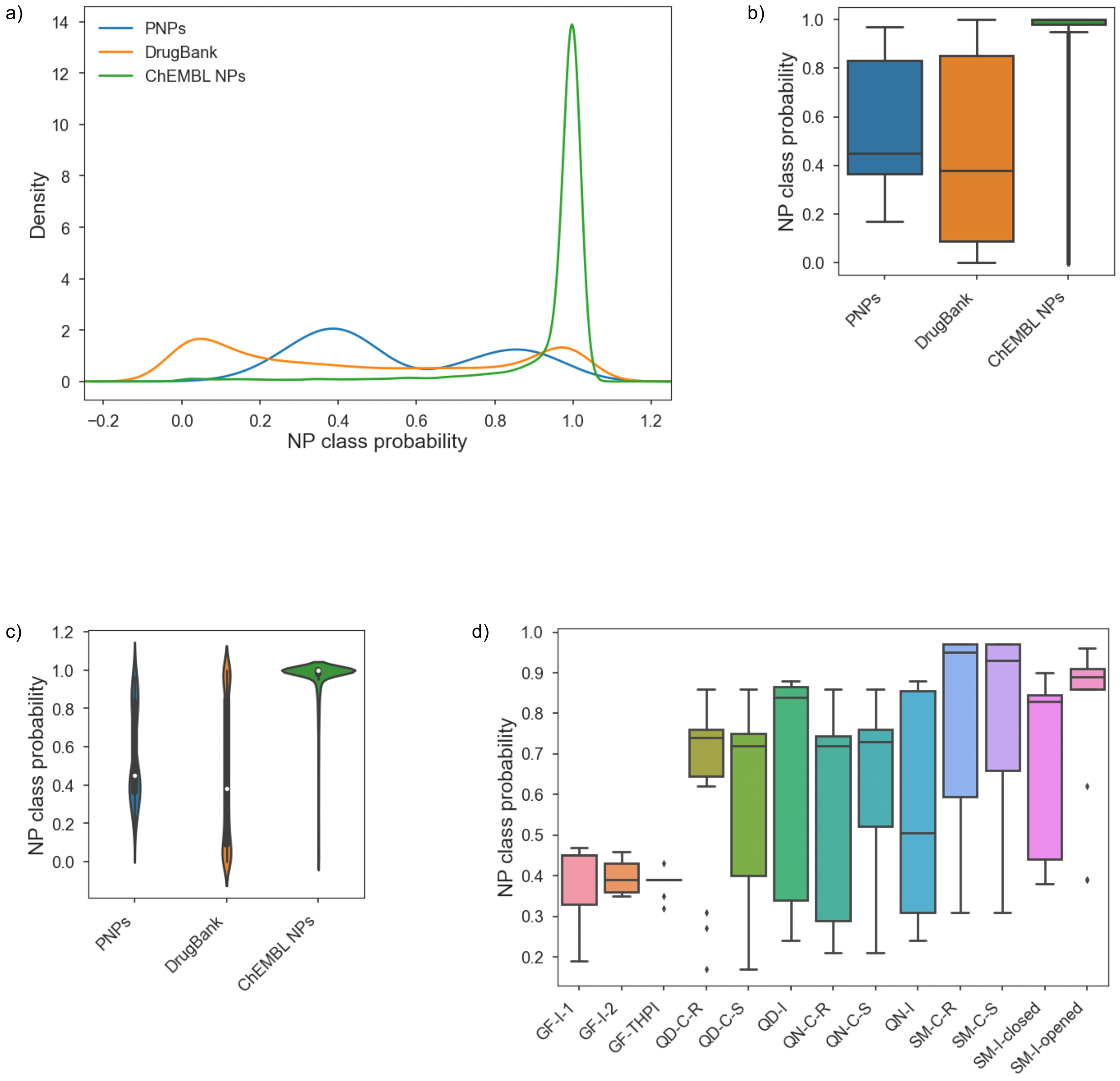

Supplementary Figure 8: **Prediction of natural product probability.** a) Density plot (PNPs are blue, DrugBank compounds are orange, ChEMBL NPs are green), b) box plot (PNPs are blue, DrugBank compounds are orange, ChEMBL NPs are green), and c) violin plot of NP class probability of the PNPs to the compounds in the DrugBank and NPs in ChEMBL (PNPs are blue, DrugBank compounds are orange, ChEMBL NPs are green). d) Box plot of NP class probability of PNP subclasses. Source data are provided in the online repository. Box plots are following Tukey's definition with outliers.[2]

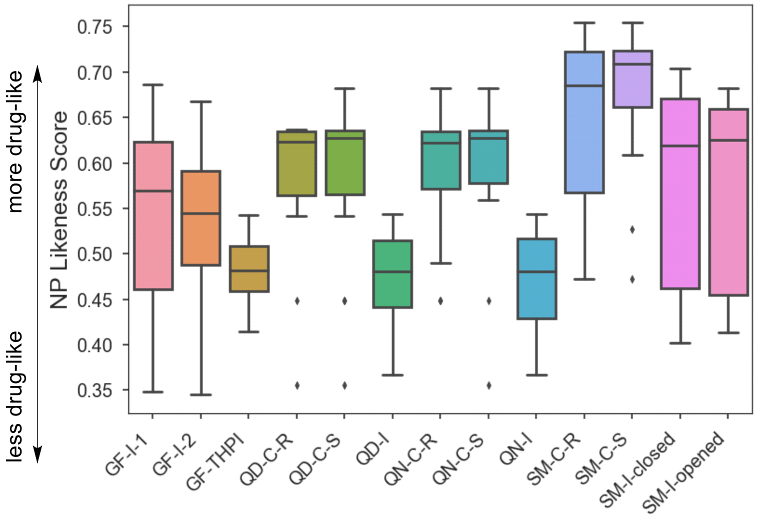

Supplementary Figure 9: **QED of the PNP library showing individual compound classes.** A drug-likeness score was generated for the PNP library. Measurements are between 0 (not drug-like) and 1 (drug-like). Source data are provided in the online repository. The box plot is following Tukey's definition with outliers.[2]

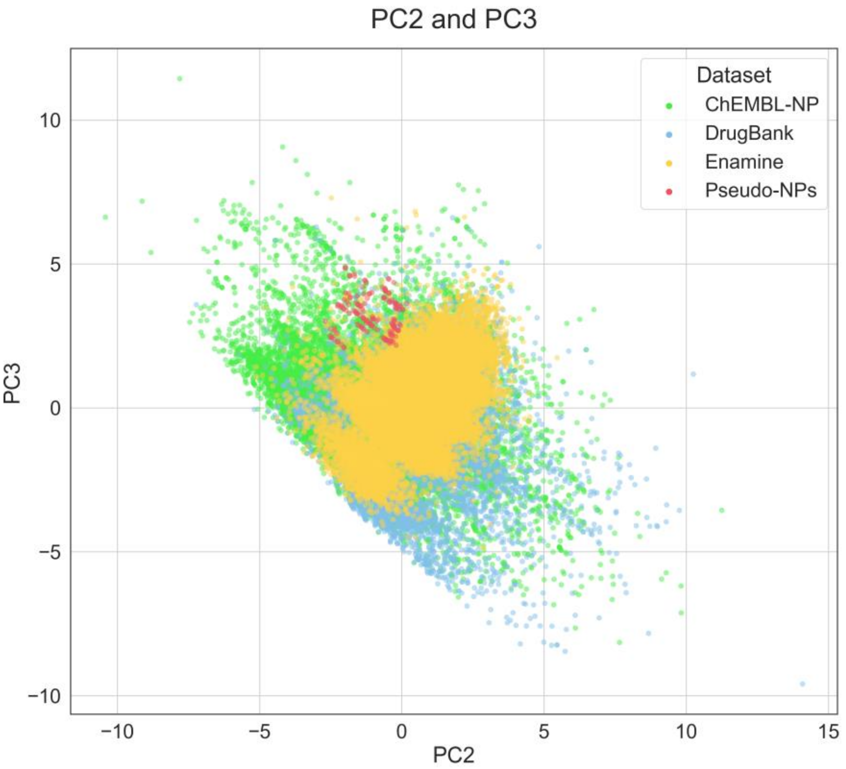

Supplementary Figure 10: **PCA plot of PC2 and PC3 of 17 molecular descriptors for the PNP collection and reference sets.** (PC2 and PC3) of 17 molecular descriptors (number of heavy atoms, molecular weight, number of rings, number of aromatic rings, number of aliphatic rings, number of hydrogen bond donors, number of hydrogen bond acceptors, SLogP, total polar surface area, number of rotatable bonds, number of oxygen atoms, number of nitrogen atoms, number of halogen atoms, number of bridgehead atoms, fraction sp^3, number of violations of Lipinski's rules, number of violations of Verber's rules) of NPs from ChEMBL (30655 compounds, green), compounds from the DrugBank (7472 compounds, blue), compounds from the Enamine database (49968 compounds, yellow), and compounds from the PNP collection (244 compounds, red) (Expl. Var.: PC2 (15.7%), PC3 (11.9%)). Source data are provided in the online repository.

a)

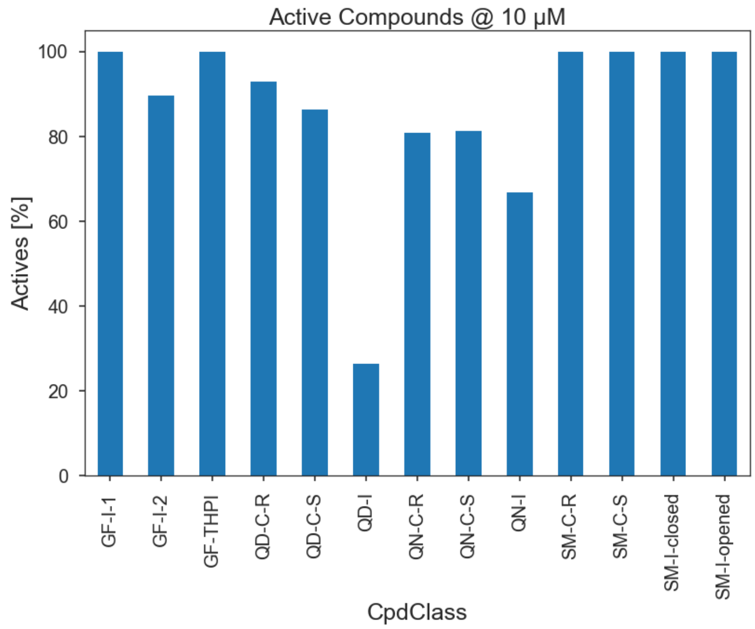

b)

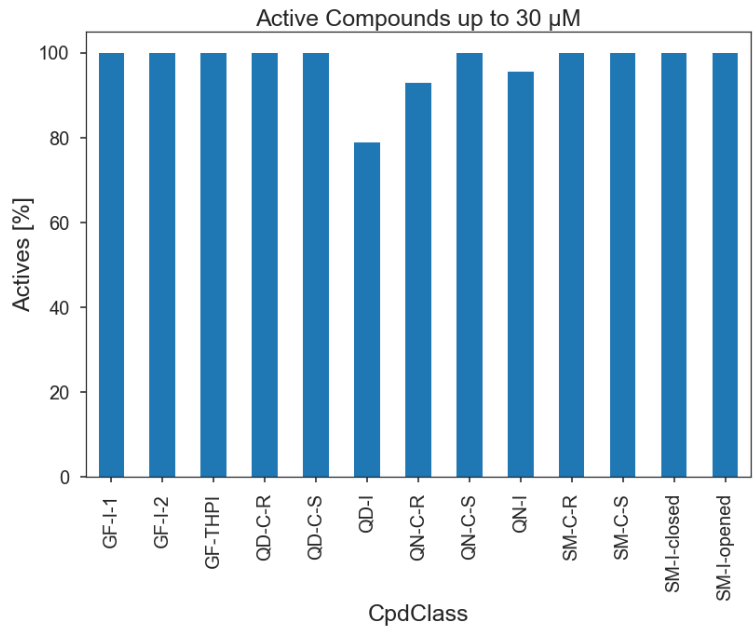

Supplementary Figure 11: **Activity of PNP collection in the CPA.** Bar graphs of the intra-subclass percent of active compounds (induction > 5%) in the cell painting assay at a) 10 µM and b) up to 30 µM. Source data are provided in the online repository.

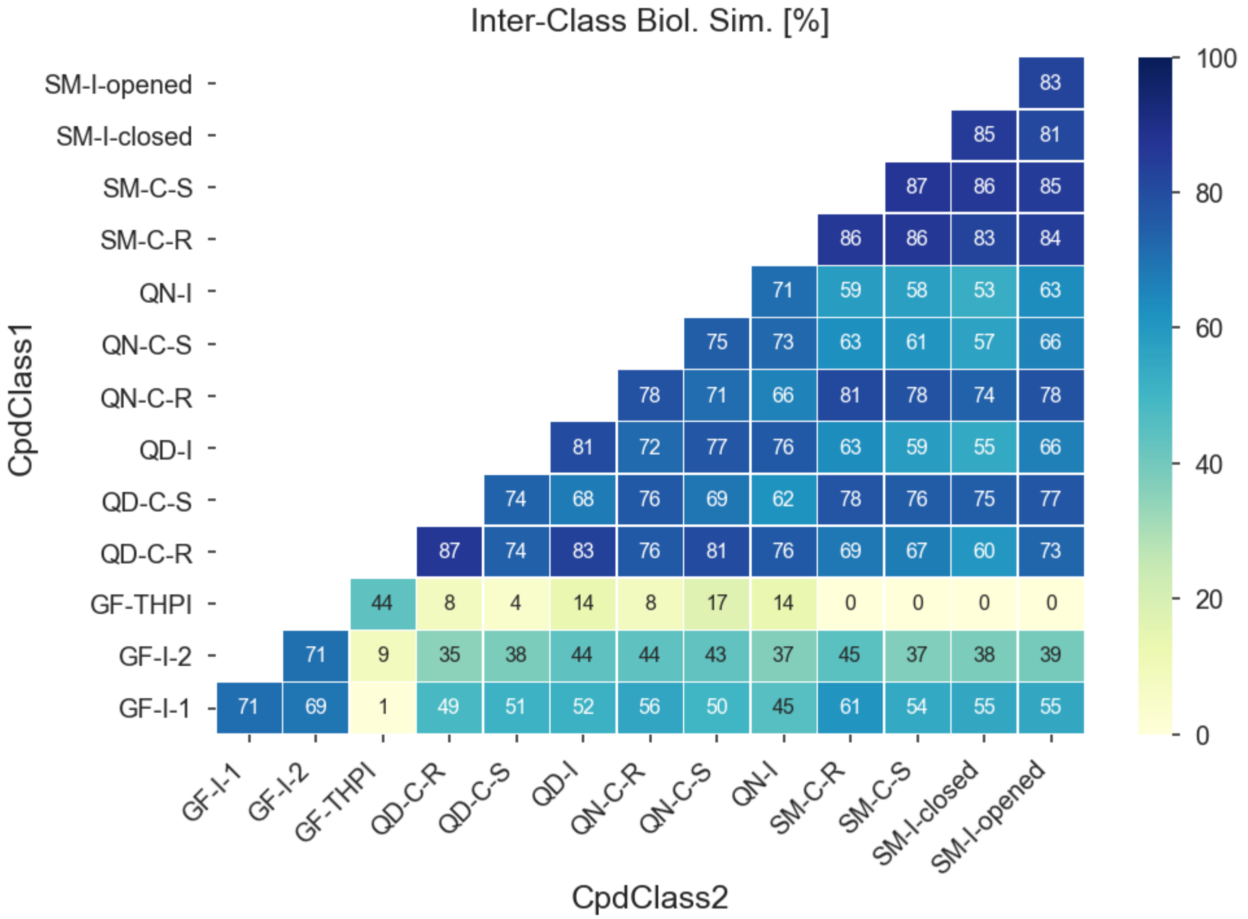

Supplementary Figure 12: **Cross-similarity analysis of median biosimilarity percentages of the PNP library.** Cross-similarities of inter- and intra-class biosimilarities of the PNP library. The color scale is on the right (blue = high biosimilarity, yellow = low biosimilarity). Source data are provided in the online repository.

a)

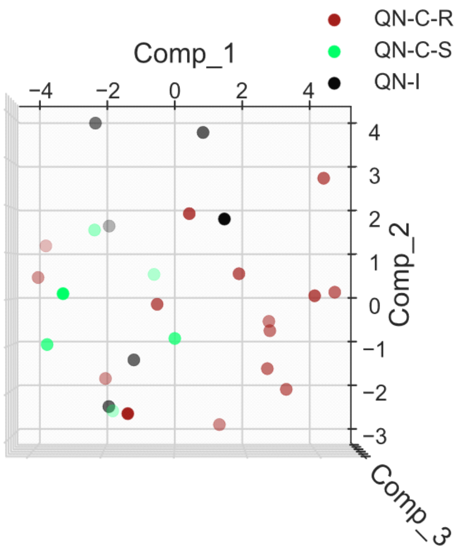

b)

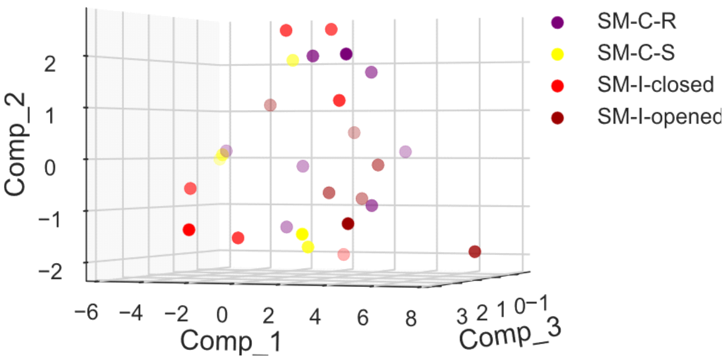

Supplementary Figure 13: **Principal component analysis of quinine- and sinomenine-derived compounds PNPs.** a) Principal component analysis of quinine-derived compounds. Expl. Var.: PC1 (35.6%), PC2 (18.0%), PC3 (10.0%). QN-I = quinine-indole (black), QN-C-S = quinine-chromanone (S)-isomer (light green), QN-C-R = quinine-chromanone (R)-isomer (dark red). b) Principal component analysis of sinomenine-derived compounds. Expl. Var.: PC1 (54.3%), PC2 (13.7%), PC3 (7.0%). SM-C-R = sinomenine-chromanone (R)-isomer (purple), SM-C-S = sinomenine-chromanone (S)-isomer (yellow), SM-I-closed = sinomenine-indole ring closed (red), SM-I-opened = sinomenine-indole ring closed (dark red). Source data are provided as a Source Data file.

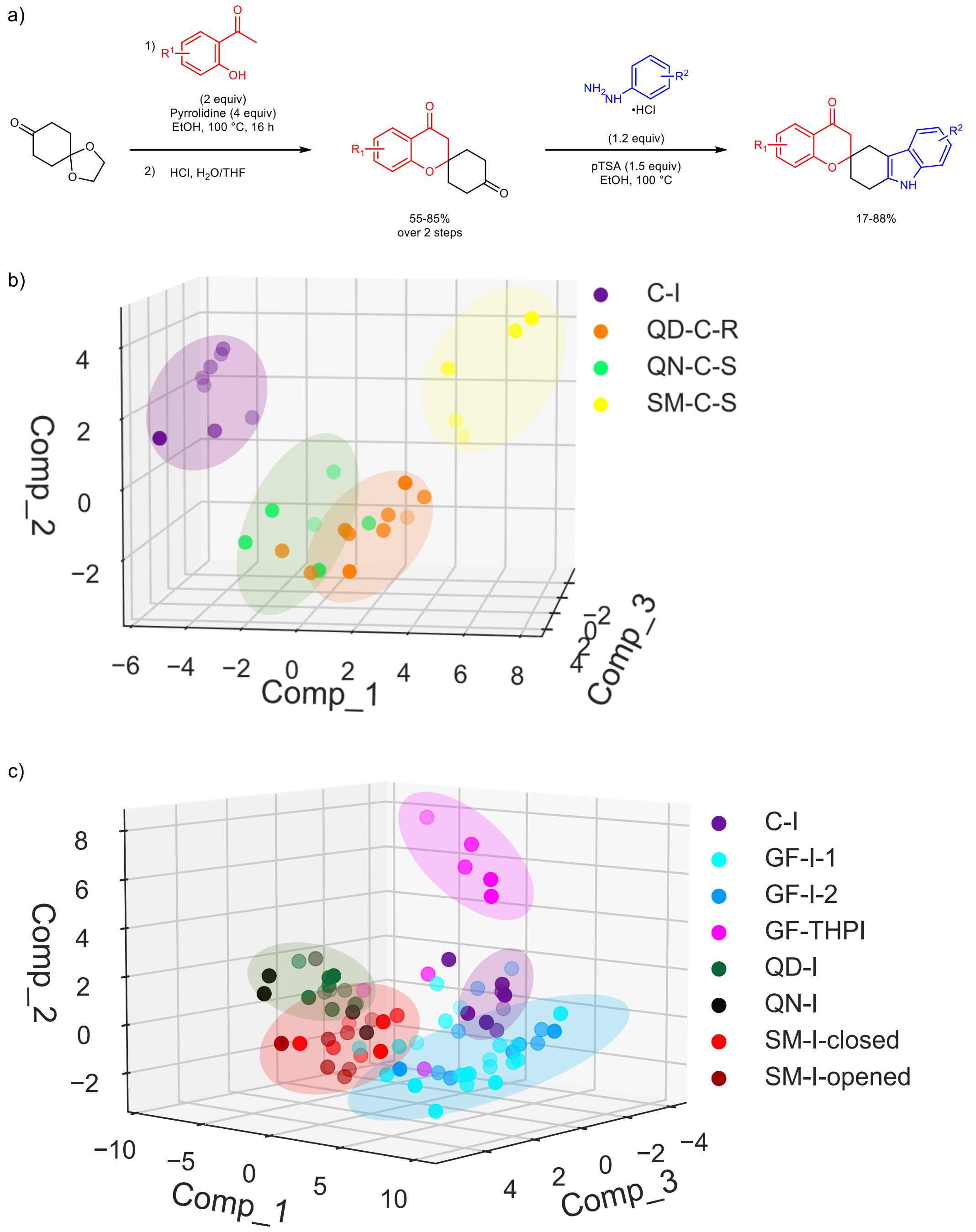

Supplementary Figure 14: **Synthesis and CPA analysis of the chromanone-indole class.** a) Synthesis of the chromanone-indole (**C-I**) compound class. b) Principal component analysis of **C-I** (purple) with chromanone-containing PNPs. Please see **Figure 2** in the manuscript for compound class abbreviations and structures. Expl. Var.: PC1 (49.7%), PC2 (19.6%), PC3 (6.6%). b) Principal component analysis of **C-I** (purple) with indole-containing PNPs. Expl. Var.: PC1 (52.3%), PC2 (12.4%), PC3 (9.1%). Please see **Figure 2** in the manuscript for compound class abbreviations and structures. Source data are provided as a Source Data file.

a)

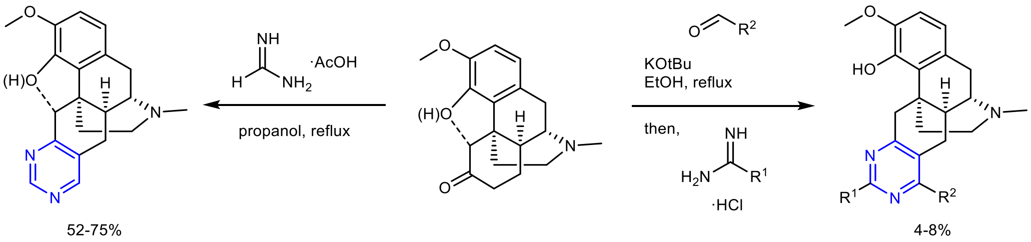

b)

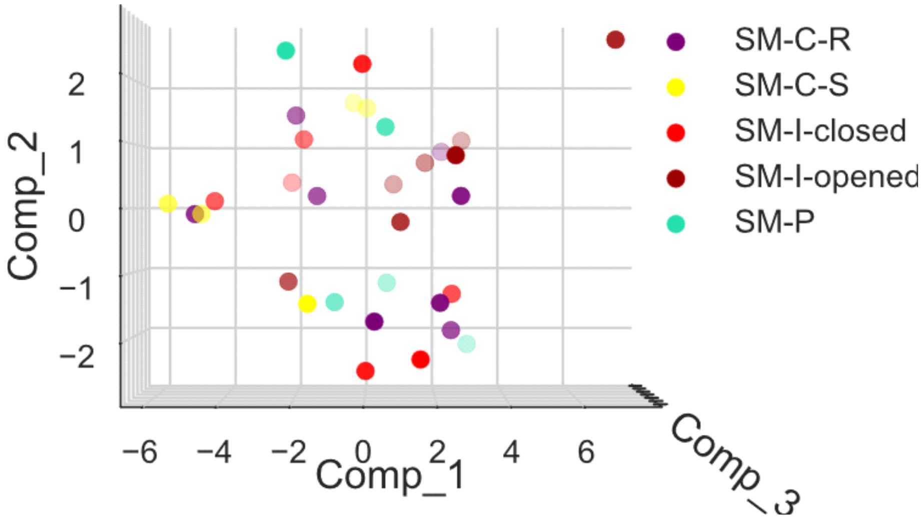

Supplementary Figure 15: **Synthesis and CPA analysis of the sinomenine-pyrimidine class.** a) Synthesis of the sinomenine-pyrimidine (**SM-P**) compound class. b) Principal component analysis of **SM-P** (turquoise) with other sinomenine-containing PNPs. Induction window: 20-53. Expl. Var.: PC1 (50.0%), PC2 (14.0%), PC3 (9.4%). Please see **Figure 2** in the manuscript for compound class abbreviations and structures. Source data are provided as a Source Data file.

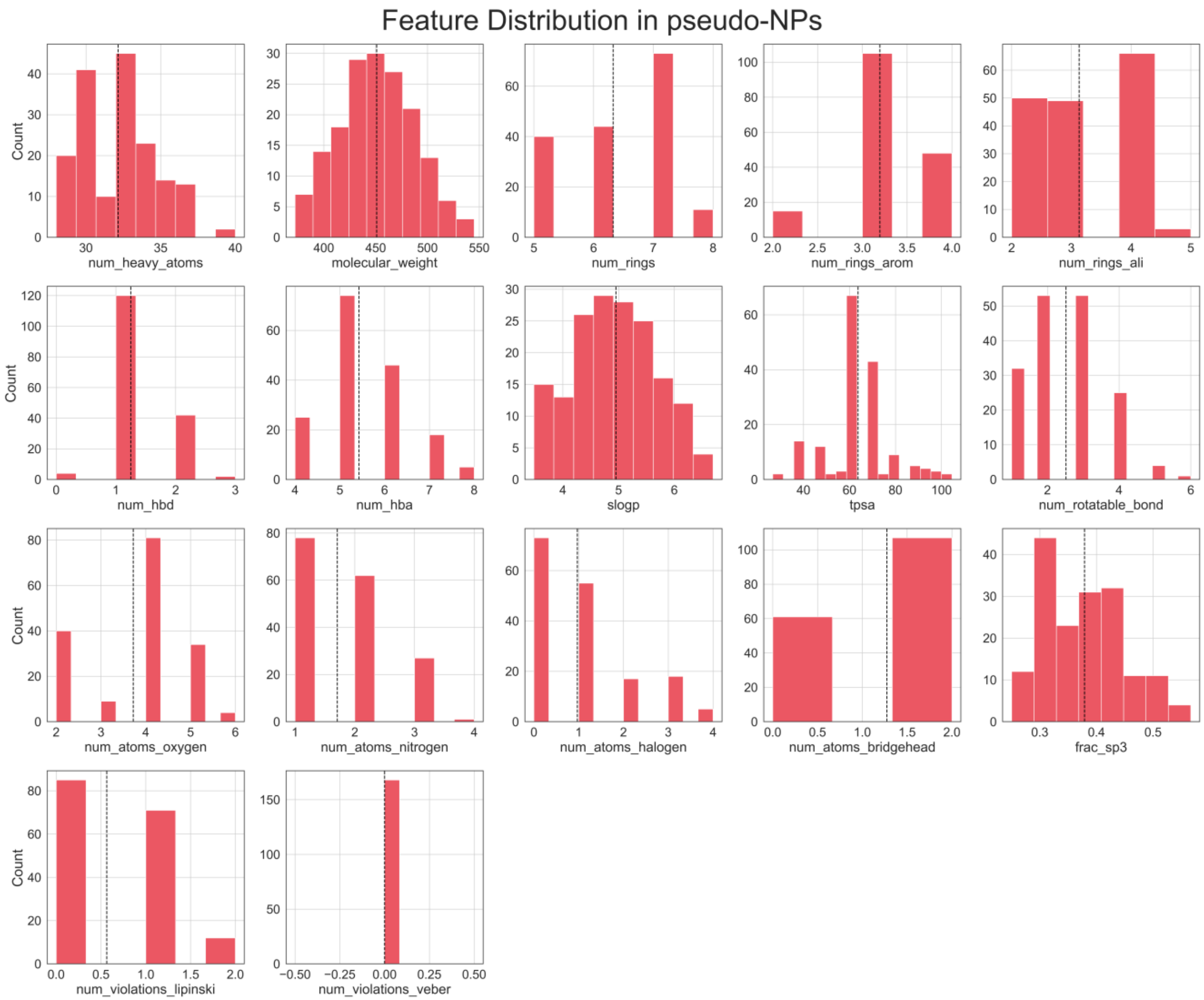

Supplementary Figure 16: **Feature distribution in the PNP collection in the PCA analysis.** Seventeen molecular features were used in the PCA analysis. Source data are provided in the online repository.

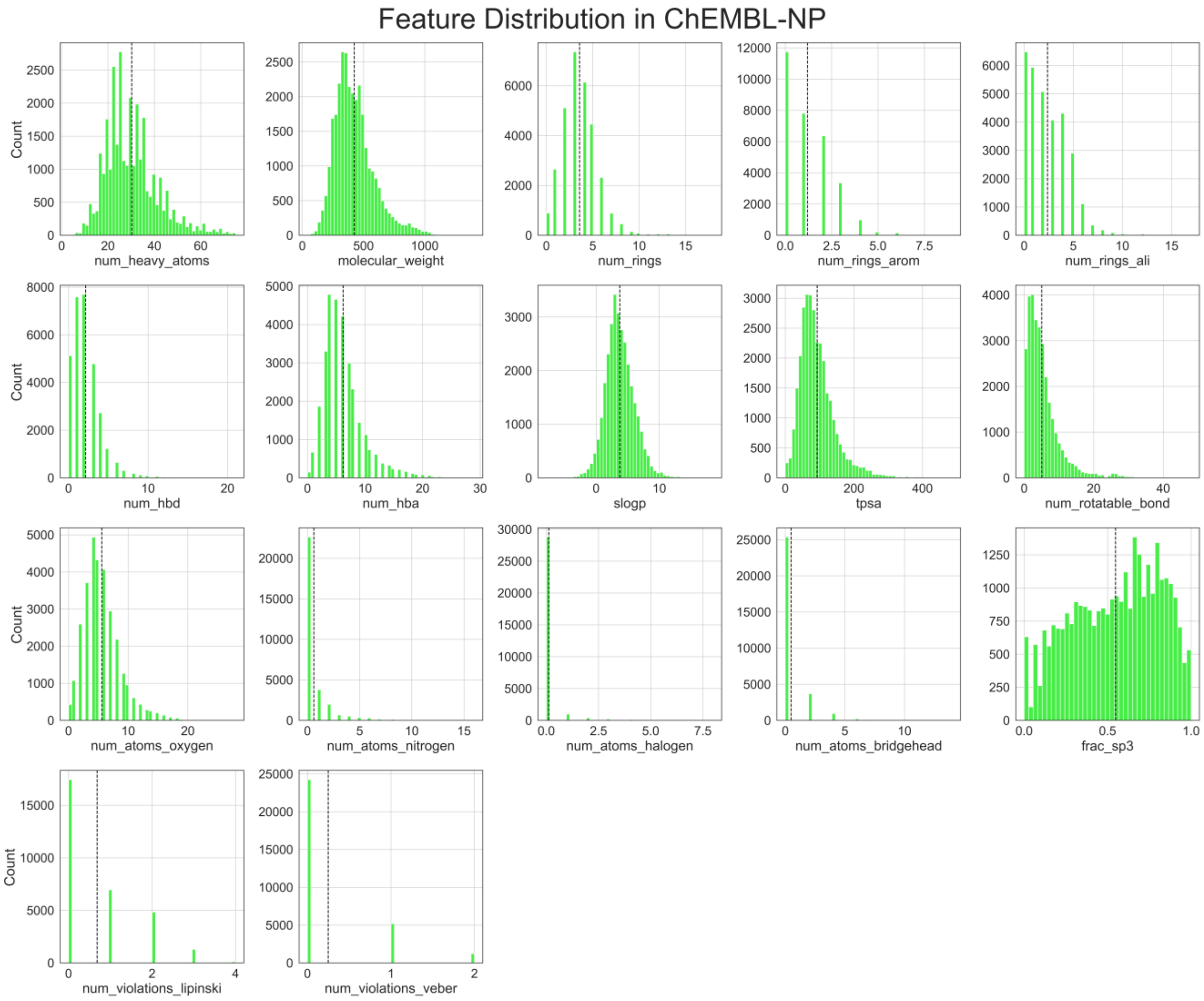

Supplementary Figure 17: **Feature distribution in the ChEMBL-NP dataset in the PCA analysis.** Seventeen molecular features were used in the PCA analysis. Source data are provided in the online repository.

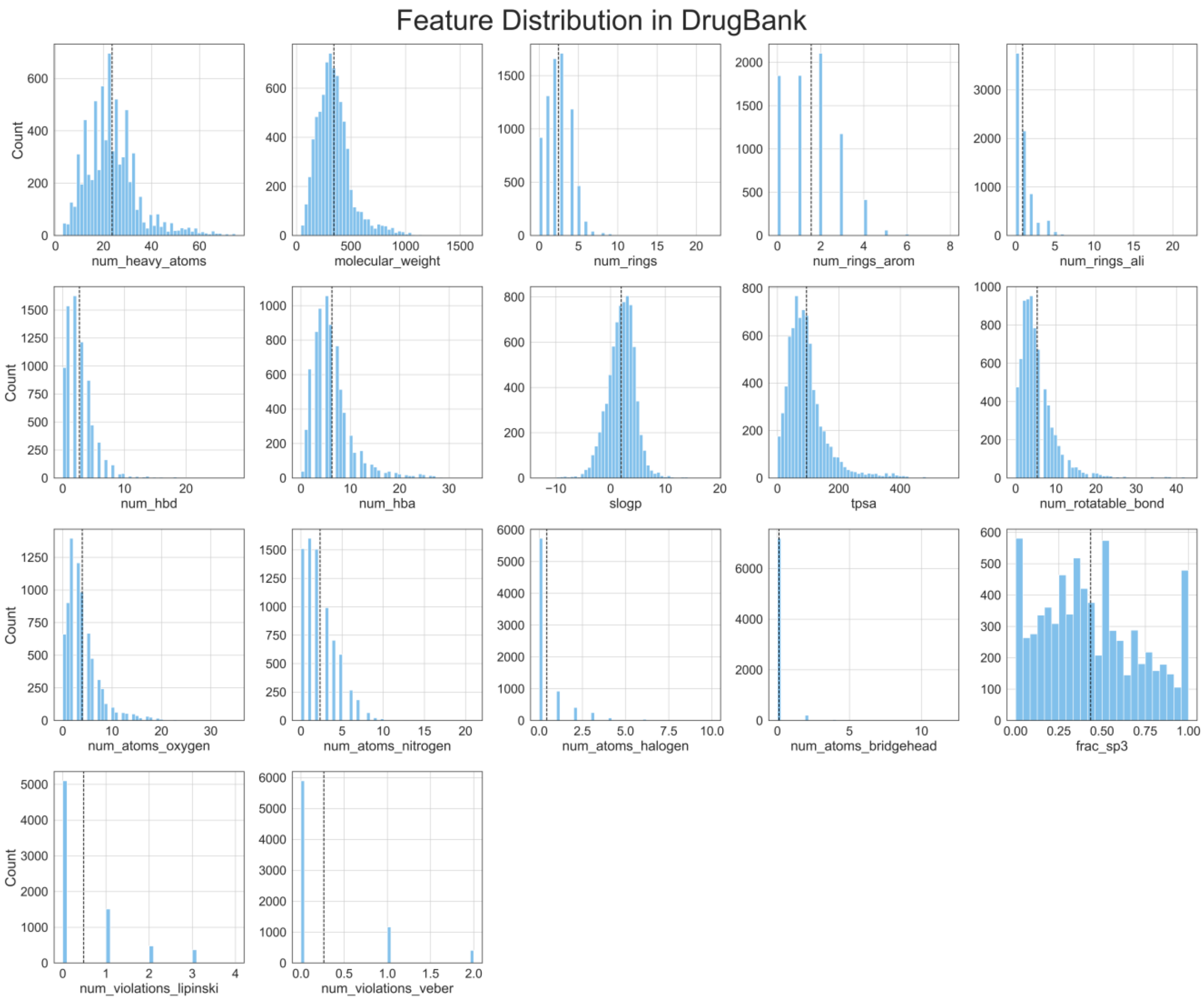

Supplementary Figure 18: Feature distribution in the DrugBank dataset in the PCA analysis. Seventeen molecular features were used in the PCA analysis. Source data are provided in the online repository.

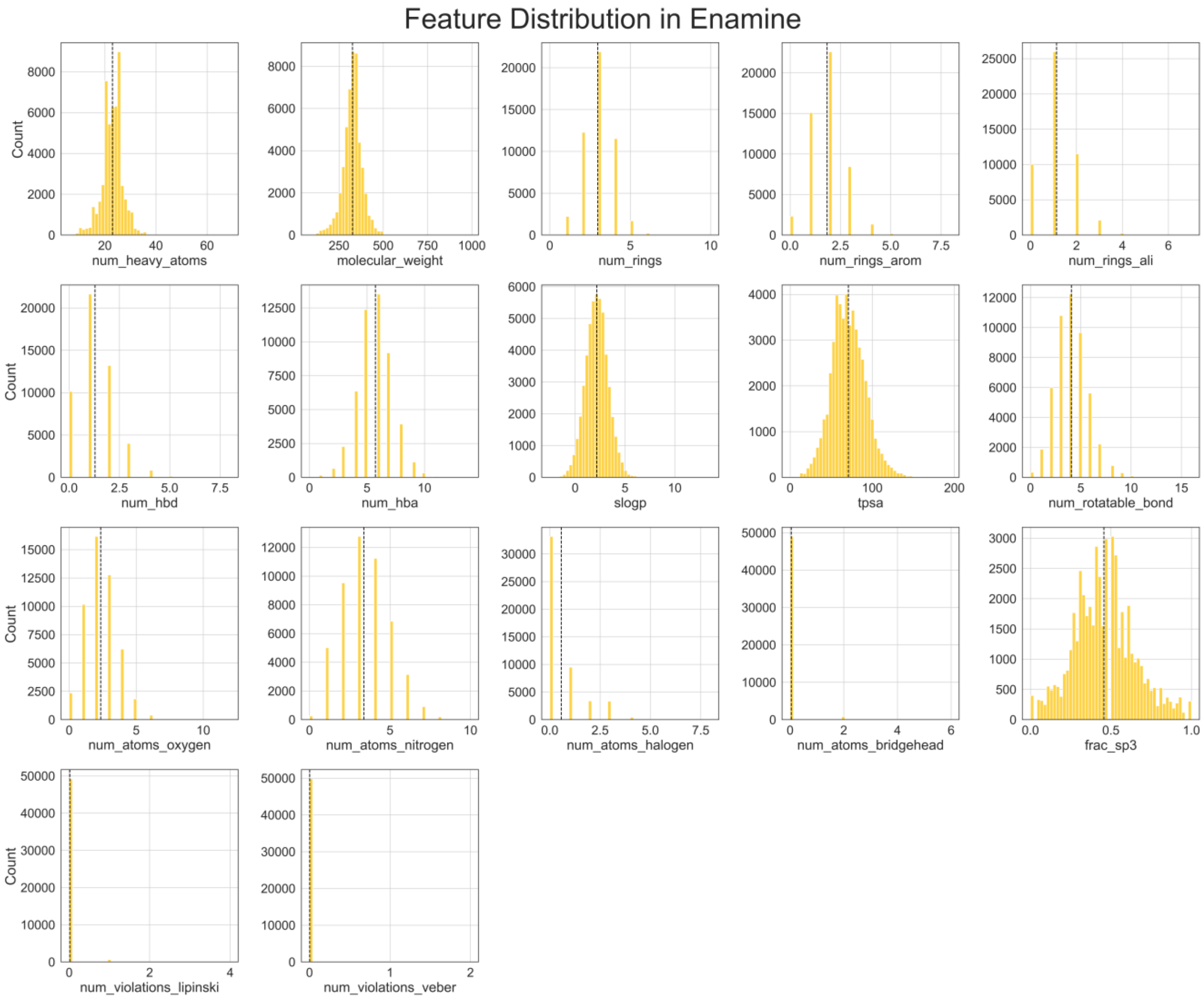

Supplementary Figure 19: Feature distribution in the Enamine dataset in the PCA analysis. Seventeen molecular features were used in the PCA analysis. Source data are provided in the online repository.

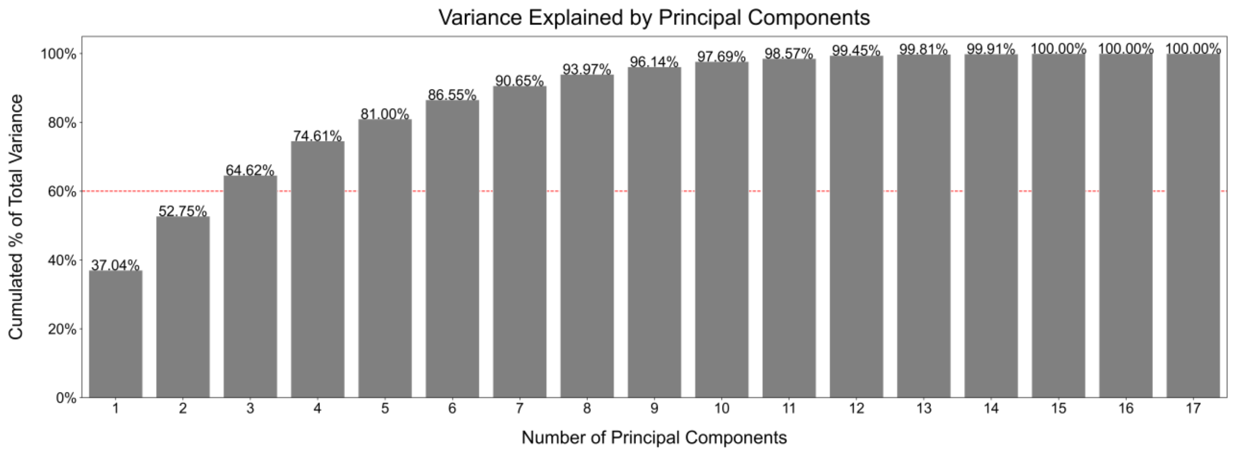

Supplementary Figure 20: Variance explained by principal components of the PCA of 17 molecular descriptors. Seventeen molecular features were used in the PCA analysis. Source data are provided in the online repository.

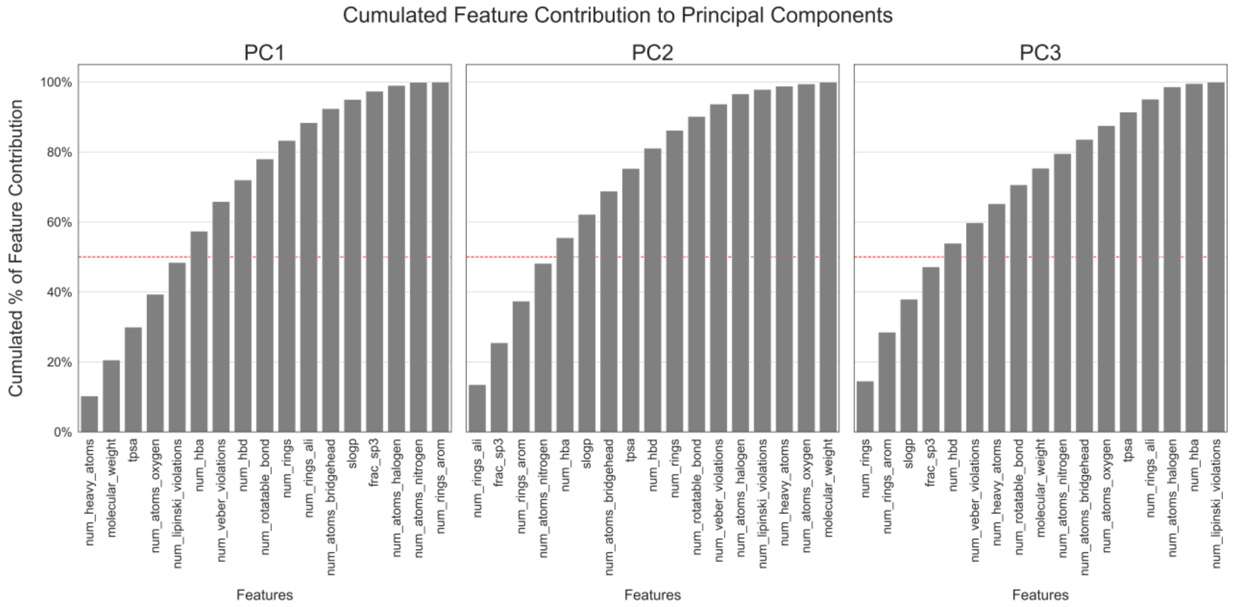

Supplementary Figure 21: Cumulated feature contribution to principal components of the PCA of 17 molecular descriptors. Seventeen molecular features were used in the PCA analysis. Source data are provided in the online repository.

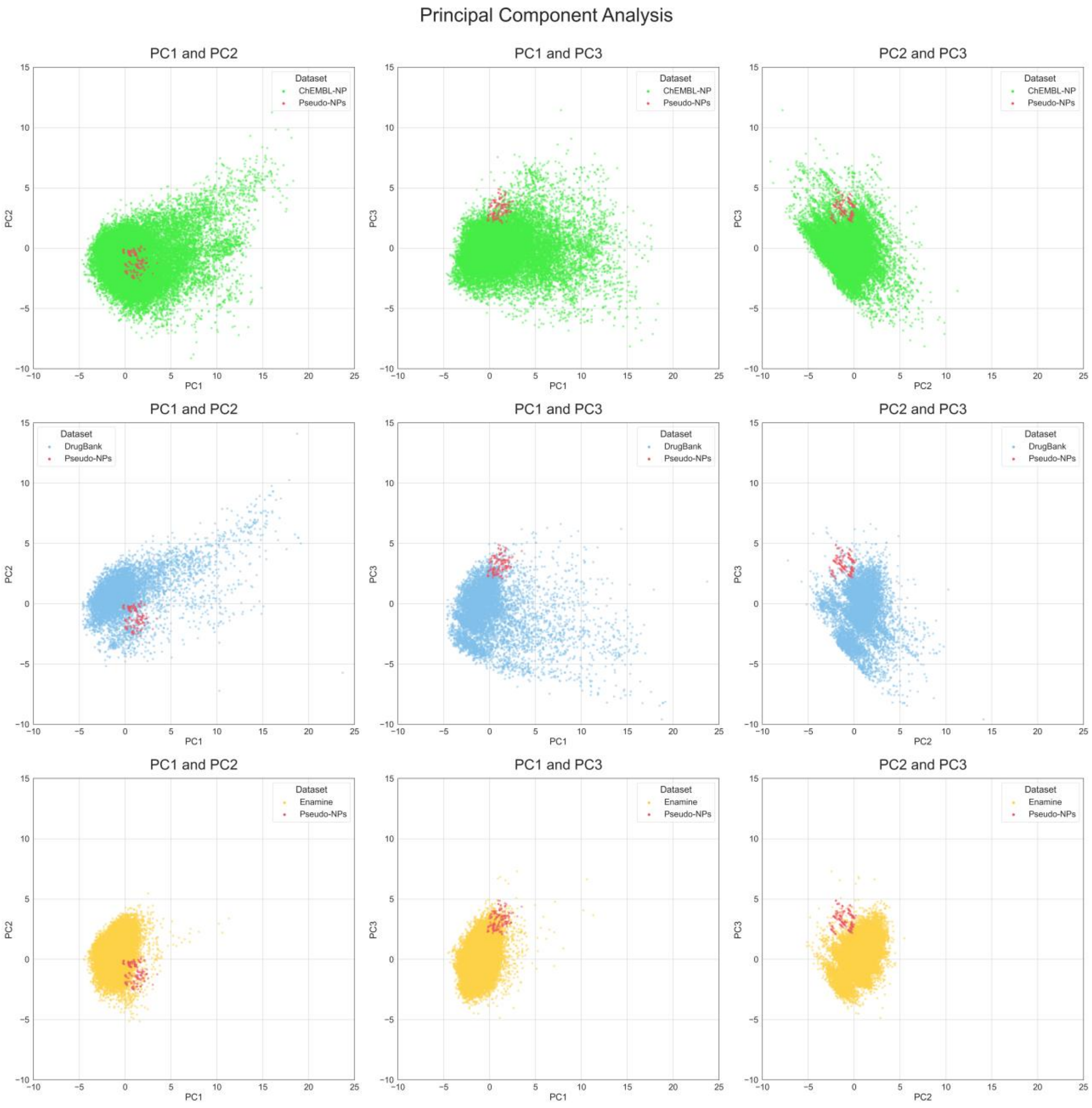

Supplementary Figure 22: Principal component analysis of 17 molecular descriptors showing the PNPs and a single reference set. ChEMBL-NP (green), Pseudo-NPs (red), DrugBank (blue), Enamine (yellow). Source data are provided in the online repository.

Supplementary Tables

In silico ADME-Tox predictions were carried out by using the free software SwissADME[3], ToxTree[4], and pkCSM-pharmacokinetics[5].

ADME									
	Predicted with SwissADME							predicted with pkCSM-pharmacokinetics (mean values)	
Class	BBB-permeability [%]	Pgp Substrate [%]	Inhibition of main Cyps [%]					Fraction unbound	Clearance (ml/min)
			CYP1A2 inhibitor	CYP2C19 inhibitor	CYP2C9 inhibitor	CYP2D6 inhibitor	CYP3A4 inhibitor		
QN-C-S	61.11	5.56	0.00	5.56	77.78	100.00	88.89	18.00	1.05
QN-C-R	64.29	7.14	0.00	64.29	64.29	64.29	64.29	17.15	1.02
QD-C-S	52.17	4.35	0.00	8.70	82.61	100.00	86.96	17.00	0.99
QD-C-R	42.86	0.00	0.00	7.14	78.57	100.00	85.71	17.14	0.98
QN-I	63.64	100.00	0.00	81.82	72.73	100.00	100.00	6.67	0.46
QD-I	63.16	100.00	5.26	89.47	78.95	100.00	100.00	4.58	0.36
SM-C-S	70.59	0.00	0.00	35.29	0.00	35.29	82.35	12.80	0.74
SM-C-R	90.91	0.00	0.00	36.36	9.09	27.27	90.91	10.65	0.71
SM-I-closed	100.00	0.00	0.00	5.56	100.00	100.00	77.78	17.65	1.13
SM-I-opened	100.00	15.38	0.00	0.00	100.00	100.00	7.69	8.70	1.09
GF-I-1	35.48	93.55	25.81	67.74	100.00	0.00	80.65	9.22	0.35
GF-I-2	10.53	94.74	31.58	57.89	100.00	0.00	78.95	6.62	0.41
GF-THPI	0.00	100.00	0.00	90.91	9.09	0.00	45.45	0.23	0.39

Supplementary Table 1: Mean values of ADME properties of the PNP subclasses predicted using Swiss ADME and pkCSM-pharmacokinetics. High mean percentages indicate high probability of the property calculated.

Toxicity										
Class	predicted with pkCSM-pharmacokinetics				ToxTree					
	hERG I [%]	hERG II [%]	hepatoxicity [%]	AMES toxicity [%]	revised Cramer Decision Tree (CDT)	alerts for S. typhimurium mutagenicity (AMES Test) [%]	Negative for genotoxic carcinogenicity [%]	Negative for nongenotoxic carcinogenicity [%]	not corrosive to skin [%]	not corrosive or irritating to skin [%]
QN-C-S	0.00	100.00	77.78	0.00	Class 3	5.56	94.44	100.00	94.44	5.56
QN-C-R	0.00	100.00	67.86	0.00	Class 3	3.57	96.43	100.00	82.14	17.86
QD-C-S	0.00	100.00	86.96	0.00	Class 3	4.35	95.65	100.00	86.96	13.04
QD-C-R	0.00	100.00	85.71	0.00	Class 3	0.00	100.00	100.00	85.71	14.29
QN-I	0.00	100.00	100.00	13.64	Class 3	4.55	95.45	100.00	50.00	50.00
QD-I	0.00	100.00	100.00	15.79	Class 3	5.26	94.74	100.00	57.89	42.11
SM-C-S	0.00	100.00	70.59	0.00	Class 3	5.88	94.12	100.00	82.35	17.65
SM-C-R	0.00	100.00	90.91	9.09	Class 3	18.18	81.82	100.00	90.91	18.18
SM-I-closed	0.00	100.00	100.00	38.89	Class 3	100.00	0.00	100.00	77.78	22.22
SM-I-opened	0.00	100.00	100.00	92.31	Class 3	100.00	0.00	100.00	84.62	15.38
GF-I-1	0.00	100.00	51.61	58.06	Class 3	100.00	0.00	100.00	0.00	100.00
GF-I-2	0.00	100.00	73.68	57.89	Class 3	100.00	0.00	100.00	0.00	100.00
GF-THPI	0.00	100.00	54.55	0.00	Class 3	100.00	0.00	100.00	0.00	100.00

Supplementary Table 2: Mean values of toxicity properties of the PNP subclasses predicted using pkCSM-pharmacokinetics and ToxTree. High mean percentages indicate high probability of the property calculated.

Compound Collection

Quinidine-chromanones (**QN-C**)

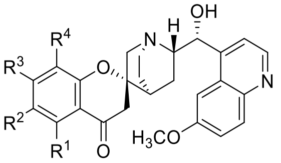

QN-C-S

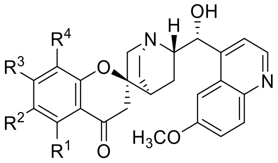

QN-C-R

Compound ID	Diastereomer	R1	R2	R3	R4	Yield
**QN-C-S-1**	S	H	H	H	H	29
**QN-C-R-1**	R	H	H	H	H	45
**QN-C-S-2**	S	H	Br	H	H	31
**QN-C-R-2**	R	H	Br	H	H	40
**QN-C-S-3**	S	H	Cl	H	H	5
**QN-C-S-4**	S	H	F	H	H	31
**QN-C-R-3**	R	H	F	H	H	43
**QN-C-R-4**	R	OMe	H	H	H	23
**QN-C-R-5**	R	H	OMe	H	H	34
**QN-C-S-5**	S	H	H	OMe	H	22
**QN-C-R-6**	R	H	H	OMe	H	41
**QN-C-S-6**	S	OMe	H	OMe	H	29
**QN-C-R-7**	R	OMe	H	OMe	H	51
**QN-C-S-7**	S	H	H	OMe	OMe	27
**QN-C-R-8**	R	H	H	OMe	OMe	42
**QN-C-S-8**	S	OH	H	H	H	16
**QN-C-R-9**	R	OH	H	H	H	43
**QN-C-R-10**	R	H	OH	H	H	34
**QN-C-S-9**	S	H	H	OH	H	22
**QN-C-R-11**	R	H	H	OH	H	48
**QN-C-R-12**	R	H	H	OH	OMe	37
**QN-C-S-10**	S	H	Me	H	H	27
**QN-C-R-13**	R	H	Me	H	H	35
**QN-C-R-14**	R	H	H	$N(C_4H_8)$	H	33
**QN-C-S-11**	S	$-C_4H_4-$		H	H	26
**QN-C-R-15**	R	$-C_4H_4-$		H	H	38
**QN-C-R-16**	R	H	H	$-C_4H_4-$		47
**QN-C-S-12**	S	H	N(CO)Me	H	H	33
**QN-C-R-17**	R	H	N(CO)Me	H	H	59
**QN-C-S-13**	S	H	F	H	F	28
**QN-C-R-18**	R	H	F	H	F	39
**QN-C-S-14**	S	H	Me	Me	H	26

**QN-C-R-19**	R	H	Me	Me	H	63
**QN-C-R-20**	R	H	H	Me	H	37
**QN-C-S-15**	S	H	Me	H	Me	20
**QN-C-R-21**	R	H	Me	H	Me	43
**QN-C-S-16**	S	H	Et	H	H	8
**QN-C-R-22**	R	H	Et	H	H	28
**QN-C-R-23**	R	H	H	H	Cl	44
**QN-C-R-24**	R	H	Cl	Me	H	12
**QN-C-S-17**	S	H	H	OBn	H	33
**QN-C-R-25**	R	$N(C_4H_8)$	H	H	H	37
**QN-C-R-26**	R	H	H	H	Br	34
**QN-C-S-18**	S	H	iPr	H	H	14
**QN-C-R-27**	R	H	Cl	H	Br	4

Quinidine-chromanones (**QD-C**):

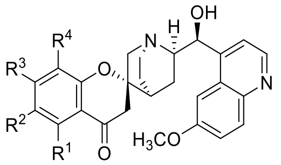

QD-C-S

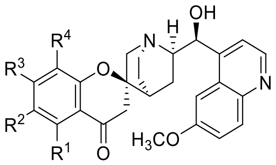

QD-C-R

Compound ID	Diastereomer	R1	R2	R3	R4	Yield
**QD-C-S-1**	S	H	H	H	H	64
**QD-C-S-2**	S	OH	H	H	H	45
**QD-C-R-1**	R	OH	H	H	H	8
**QD-C-S-3**	S	H	OH	H	H	32
**QD-C-S-4**	S	H	H	OH	H	48
**QD-C-R-2**	R	H	H	OH	H	8
**QD-C-S-5**	S	OMe	H	H	H	47
**QD-C-R-3**	R	OMe	H	H	H	19
**QD-C-S-6**	S	H	OMe	H	H	47
**QD-C-R-4**	R	H	OMe	H	H	27
**QD-C-S-7**	S	H	H	OMe	H	54
**QD-C-R-5**	R	H	H	OMe	H	24
**QD-C-S-8**	S	$-C_4H_4-$		H	H	31
**QD-C-R-6**	R	$-C_4H_4-$		H	H	21
**QD-C-S-9**	S	H	H	$-C_4H_4-$		43
**QD-C-S-10**	S	H	H	$N(C_4H_8)$	H	40
**QD-C-S-11**	S	H	H	OMe	OMe	9
**QD-C-R-7**	R	H	H	OMe	OMe	9
**QD-C-S-12**	S	H	Me	Me	H	42
**QD-C-R-8**	R	H	Me	Me	H	7
**QD-C-S-13**	S	H	Br	H	H	61
**QD-C-R-9**	R	H	Br	H	H	13
**QD-C-S-14**	S	H	Me	H	Me	52
**QD-C-R-10**	R	H	Me	H	Me	5
**QD-C-S-15**	S	H	OCF_3	H	H	30
**QD-C-R-11**	R	H	OCF_3	H	H	4
**QD-C-S-16**	S	H	H	OBn	H	42
**QD-C-R-12**	R	H	H	OBn	H	10
**QD-C-S-17**	S	H	iPr	H	H	54
**QD-C-R-13**	R	H	iPr	H	H	8
**QD-C-S-18**	S	H	Me	H	H	48
**QD-C-S-19**	S	H	F	H	H	57
**QD-C-S-20**	S	H	Cl	H	H	34

**QD-C-R-14**	R	H	Cl	H	H	9
**QD-C-S-21**	S	H	N(CO)Me	H	H	59
**QD-C-S-22**	S	H	CF_3	H	H	61
**QD-C-S-23**	S	H	Et	H	H	51

Quinine-indoles (**QN-I**):

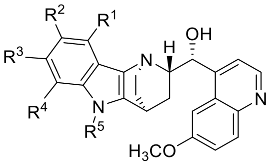

QN-I

Compound ID	R1	R2	R3	R4	R5	Yield
**QN-I-1**	H	H	H	H	H	17
**QN-I-2**	H	Me	H	H	H	4
**QN-I-3**	H	Cl	H	H	H	39
**QN-I-4**	H	H	Cl	H	H	21
**QN-I-5**	H	F	H	H	H	47
**QN-I-6**	CO_2Me	H	H	H	H	12
**QN-I-7**	H	CF_3	H	H	H	23
**QN-I-8**	H	NO_2	H	H	H	3
**QN-I-9**	H	H	Br	H	H	17
**QN-I-10**	H	OMe	H	H	H	6
**QN-I-11**	Cl	H	H	H	H	11
**QN-I-12**	Me	H	H	H	H	46
**QN-I-13**	H	H	CF_3	H	H	70
**QN-I-14**	H	H	Me	H	H	4
**QN-I-15**	H	H	CO_2H	H	H	14
**QN-I-16**	H	H	H	OMe	H	33
**QN-I-17**	H	CO_2H	H	H	H	9
**QN-I-18**	H	OCF_3	H	H	H	65
**QN-I-19**	H	H	H	F	H	18
**QN-I-20**	H	H	H	Cl	H	52
**QN-I-21**	H	CO_2Me	H	OMe	H	1
**QN-I-22**	H	Br	Me	H	H	19

Quinidine-indoles (**QD-I**):

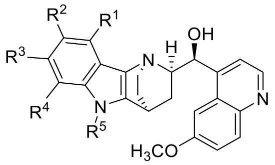

QD-I

Compound ID	R1	R2	R3	R4	R5	Yield
**QD-I-1**	H	H	H	H	H	11
**QD-I-2**	H	F	H	H	H	19
**QD-I-3**	H	H	Cl	H	H	26
**QD-I-4**	H	Me	H	H	H	45
**QD-I-5**	H	H	COOMe	H	H	8
**QD-I-6**	H	COOMe	H	H	H	3
**QD-I-7**	H	COOH	H	H	H	14
**QD-I-8**	H	H	COOH	H	H	11
**QD-I-9**	H	H	CF_3	H	H	24
**QD-I-10**	H	H	H	OMe	H	16
**QD-I-11**	H	H	Me	H	H	13
**QD-I-12**	H	Br	H	H	H	8
**QD-I-13**	H	CF_3	H	H	H	25
**QD-I-14**	H	NO_2	H	H	H	12
**QD-I-15**	H	H	Br	H	H	9
**QD-I-16**	H	Cl	H	H	H	48
**QD-I-17**	H	OMe	H	H	H	23
**QD-I-18**	Cl	H	H	H	H	76
**QD-I-19**	Me	H	H	H	H	49

Sinomenine-chromanones (**SM-C**):

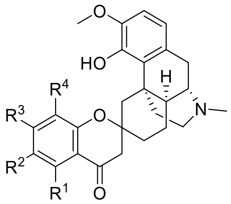

Compound ID	Diastereomer	R1	R2	R3	R4	Yield
**SM-C-S-1**	S	H	H	H	H	34
**SM-C-R-1**	R	H	H	H	H	39
**SM-C-S-2**	S	OH	H	H	H	31
**SM-C-S-3**	S	H	OH	H	H	37
**SM-C-S-4**	S	H	OMe	H	H	35
**SM-C-R-2**	R	H	OMe	H	H	44
**SM-C-S-5**	S	H	H	OMe	H	13
**SM-C-R-3**	R	H	H	OMe	H	42
**SM-C-S-6**	S	H	H	OMe	OMe	31
**SM-C-S-7**	S	H	Me	H	H	6
**SM-C-R-4**	R	H	Me	H	H	19
**SM-C-S-8**	S	H	H	Me	H	27
**SM-C-R-5**	R	H	H	Me	H	52
**SM-C-S-9**	S	H	Me	Me	H	31
**SM-C-R-6**	R	H	Me	Me	H	47
**SM-C-S-10**	S	H	F	H	H	20
**SM-C-R-7**	R	H	F	H	H	32
**SM-C-S-11**	S	H	H	$N(C_4H_8)$	H	15
**SM-C-R-8**	S	H	Cl	H	H	39
**SM-C-S-12**	S	H	Br	H	H	29
**SM-C-R-9**	R	H	Br	H	H	33
**SM-C-S-13**	S	$-C_4H_4-$		H	H	22
**SM-C-R-10**	R	$-C_4H_4-$		H	H	52
**SM-C-R-11**	R	H	H	$-C_4H_4-$		35
**SM-C-S-14**	S	H	Cl	Me	H	26
**SM-C-R-12**	R	H	Cl	Me	H	57
**SM-C-S-15**	S	H	Et	H	H	24
**SM-C-S-16**	S	H	H	OBn	H	12
**SM-C-R-13**	R	H	H	OBn	H	47

Sinomenine-indoles (**SM-I**):

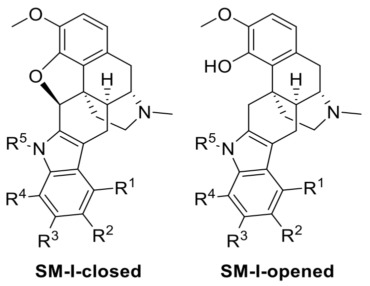

Compound ID	R1	R2	R3	R4	R5	Yield
**SM-I-closed-1**	H	H	H	H	H	95
**SM-I-closed-2**	H	Me	H	H	H	92
**SM-I-closed-3**	H	OMe	H	H	H	57
**SM-I-closed-4**	H	H	H	Me	H	93
**SM-I-closed-5**	H	iPr	H	H	H	87
**SM-I-closed-6**	H	Cl	H	H	H	52
**SM-I-closed-7**	H	H	H	H	Ph	95
**SM-I-closed-8**	H	H	Me	Me	H	20
**SM-I-closed-9**	H	H	H	H	Bn	53
**SM-I-closed-10**	H	Me	H	Me	H	9
**SM-I-closed-11**	Cl	H	H	Cl	H	34
**SM-I-closed-12**	H	OPh	H	H	H	71
**SM-I-closed-13**	$-C_4H_4-$		H	H	H	57
**SM-I-closed-14**	Cl	H	H	Me	H	34
**SM-I-closed-15**	Br	H	H	F	H	19
**SM-I-closed-16**	H	F	H	H	H	90
**SM-I-closed-17**	H	H	$-C_4H_4-$		H	20
**SM-I-closed-18**	H	OBn	H	H	H	74
**SM-I-opened-1**	H	H	H	H	H	68
**SM-I-opened-2**	H	Me	H	H	H	66
**SM-I-opened-3**	H	OMe	H	H	H	71
**SM-I-opened-4**	H	H	H	Me	H	42
**SM-I-opened-5**	H	iPr	H	H	H	62
**SM-I-opened-6**	H	Cl	H	H	H	48
**SM-I-opened-7**	Me	H	Me	H	H	64
**SM-I-opened-8**	H	H	H	H	Bn	66
**SM-I-opened-9**	Cl	H	H	Cl	H	35
**SM-I-opened-10**	H	OPh	H	H	H	50
**SM-I-opened-11**	$-C_4H_4-$		H	H	H	35
**SM-I-opened-12**	H	H	$-C_4H_4-$		H	35
**SM-I-opened-13**	H	OiPr	H	H	H	28

Griseofulvin-indoles (**GF-I**):

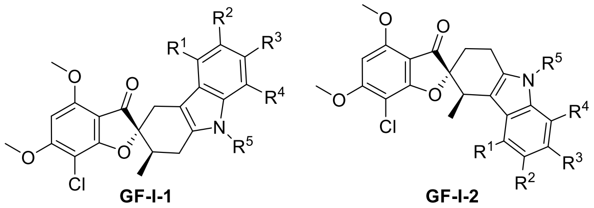

Compound ID	Regioisomer	R1	R2	R3	R4	R5	Yield
**GF-I-1-1**	1	H	H	H	H	H	63
**GF-I-2-1**	2	H	H	H	H	H	22
**GF-I-1-2**	1	H	Me	H	H	H	45
**GF-I-2-2**	2	H	Me	H	H	H	10
**GF-I-1-3**	1	H	iPr	H	H	H	66
**GF-I-2-3**	2	H	iPr	H	H	H	21
**GF-I-1-4**	1	H	OMe	H	H	H	55
**GF-I-1-5**	1	H	OPh	H	H	H	66
**GF-I-2-4**	2	H	OPh	H	H	H	12
**GF-I-1-6**	1	H	OiPr	H	H	H	27
**GF-I-2-5**	2	H	OiPr	H	H	H	13
**GF-I-1-7**	1	H	OBn	H	H	H	68
**GF-I-2-6**	2	H	OBn	H	H	H	7
**GF-I-1-8**	1	H	Ph	H	H	H	38
**GF-I-2-7**	2	H	Ph	H	H	H	13
**GF-I-1-9**	1	H	I	H	H	H	18
**GF-I-1-10**	1	H	Cl	H	H	H	68
**GF-I-2-8**	2	H	Cl	H	H	H	12
**GF-I-1-11**	1	H	F	H	H	H	75
**GF-I-2-9**	2	H	F	H	H	H	9
**GF-I-1-12**	1	H	OCF_3	H	H	H	59
**GF-I-2-10**	2	H	OCF_3	H	H	H	13
**GF-I-2-11**	2	H	CF_3	H	H	H	14
**GF-I-1-13**	1	H	H	H	Me	H	40
**GF-I-1-14**	1	H	Me	H	Me	H	51
**GF-I-1-15**	1	Me	H	H	Me	H	42
**GF-I-1-16**	1	H	H	Me	Me	H	48
**GF-I-2-12**	2	H	H	Me	Me	H	6
**GF-I-1-17**	1	Me	H	Me	H	H	36
**GF-I-1-18**	1	H	F	H	F	H	43
**GF-I-1-19**	1	F	H	H	Me	H	43
**GF-I-2-13**	2	F	H	H	Me	H	3

**GF-I-1-20**	1	F	H	F	H	H	23
**GF-I-2-14**	2	F	H	F	H	H	8
**GF-I-1-21**	1	F	H	H	F	H	16
**GF-I-1-22**	1	Cl	H	Cl	H	H	42
**GF-I-2-15**	2	Cl	H	Cl	H	H	10
**GF-I-1-23**	1	F	H	H	Cl	H	47
**GF-I-2-16**	2	F	H	H	Cl	H	19
**GF-I-1-24**	1	Br	H	H	F	H	32
**GF-I-1-25**	1	H	Cl	H	F	H	27
**GF-I-2-17**	2	H	Cl	H	F	H	32
**GF-I-1-26**	1	H	Br	H	F	H	24
**GF-I-2-18**	2	H	Br	H	F	H	9
**GF-I-1-27**	1	F	Br	H	F	H	32
**GF-I-2-19**	2	F	Br	H	F	H	13
**GF-I-1-28**	1	H	H	$-C_4H_4-$		H	77
**GF-I-2-20**	2	H	H	$-C_4H_4-$		H	17
**GF-I-1-29**	1	$-C_4H_4-$		H	H	H	43
**GF-I-1-30**	1	H	H	H	H	Ph	37
**GF-I-1-31**	1	H	H	H	H	Bn	42

Griseofulvin-4*H*-pyranoindoles (**GF-THPI**):

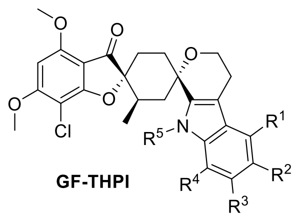

Compound ID	R1	R2	R3	R4	R5	Yield
**GF-THPI-1**	H	H	H	H	H	87
**GF-THPI-2**	Me	H	H	H	H	35
**GF-THPI-3**	Cl	H	H	H	H	17
**GF-THPI-4**	H	Me	H	H	H	76
**GF-THPI-5**	H	OMe	H	H	H	71
**GF-THPI-6**	H	Br	H	H	H	40
**GF-THPI-7**	H	Cl	H	H	H	96
**GF-THPI-8**	H	F	H	H	H	45
**GF-THPI-9**	H	H	iPr	H	H	74
**GF-THPI-10**	H	H	Cl	H	H	33
**GF-THPI-11**	H	H	H	Et	H	41

Predictive compounds:

Chromanone-indoles (**C-I**):

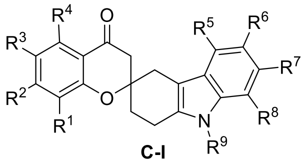

C-I

Compound ID	R1	R2	R3	R4	R5	R6	R7	R8	R9	Yield
**C-I-1**	H	OMe	H	OMe	F	H	H	Me	H	33
**C-I-2**	H	H	H	OH	F	H	H	Me	H	55
**C-I-3**	H	H	H	OMe	F	H	H	Me	H	21
**C-I-4**	H	OMe	H	H	F	H	H	Me	H	60
**C-I-5**	H	OMe	H	H	H	H	H	H	H	57
**C-I-6**	H	OMe	H	H	H	iPr	H	H	H	54
**C-I-7**	H	OMe	H	H	H	Cl	H	H	H	17
**C-I-8**	H	OMe	H	H	H	Me	H	Me	H	29
**C-I-9**	H	OMe	H	H	H	H	H	H	Ph	49
**C-I-10**	H	OMe	H	H	H	H	H	H	Bn	66
**C-I-11**	H	OMe	H	H	H	F	H	F	H	44
**C-I-12**	H	OMe	H	H	Cl	H	Cl	H	H	48
**C-I-13**	H	OMe	H	H	$-C_4H_4-$		H	H	H	33
**C-I-14**	H	OMe	H	H	H	H	$-C_4H_4-$		H	67
**C-I-15**	H	OMe	H	H	H	OBn	H	H	H	47
**C-I-16**	H	OMe	H	H	Cl	H	H	Me	H	23
**C-I-17**	H	OMe	H	H	Br	H	H	F	H	30
**C-I-18**	H	OMe	H	H	H	Br	H	F	H	42
**C-I-19**	H	OMe	H	H	H	Me	H	H	H	19
**C-I-20**	H	OMe	H	H	H	OCF_3	H	H	H	83
**C-I-21**	H	OMe	H	H	H	OPh	H	H	H	84
**C-I-22**	H	OMe	H	H	H	OiPr	H	H	H	77
**C-I-23**	H	OMe	H	H	H	CF_3	H	H	H	80
**C-I-24**	H	OMe	H	H	H	H	H	Me	H	70
**C-I-25**	H	OMe	H	H	H	F	H	H	H	69
**C-I-26**	H	OMe	H	H	Cl	H	H	Cl	H	50
**C-I-27**	H	OMe	H	H	F	H	H	Cl	H	47
**C-I-28**	H	OMe	H	H	H	C(O)OEt	H	H	H	80
**C-I-29**	H	OMe	H	H	H	SO_2Me	H	H	H	88
**C-I-30**	H	OMe	H	H	H	SO_2NH_2	H	H	H	49
**C-I-31**	H	OMe	H	H	H	H	H	C(O)OEt	H	50

Sinomenine-pyrimidines (**SM-P**):

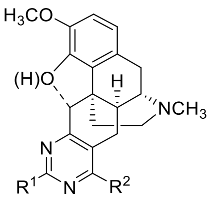

SM-P

Compound ID	Opened or Closed	R1	R2	Yield
**SM-P-1**	closed	H	H	52
**SM-P-2**	opened	H	H	75
**SM-P-3**	opened	NH_2	*p*(Br)C_6H_5	4
**SM-P-4**	opened	Ph	*p*(Br)C_6H_5	8

Supplementary Methods

Cheminformatics Analysis

The cheminformatics analysis was performed using Python 3.8, the cheminformatics toolkit RDKit [RDKit: Open-Source Cheminformatics Software, v 2020.03.6, http://www.rdkit.org/, last accessed 17-Dec-2020] and KNIME [v 4.2.3, https://www.knime.com, last accessed 17-Dec-2020]. The Python part of the analysis was performed in JupyterLab Notebooks in a conda environment.

The code and the KNIME workflow are available at http://dx.doi.org/10.5281/zenodo.4529728. The exact versions of the utilized programs and libraries are listed in the `environment.yaml` file of the repo.

Data Preparation

Three external data sets were prepared for the comparisons in this paper.

1. Natural products (NPs) from ChEMBL 26
2. Approved and experimental drugs from DrugBank
3. Enamine advanced screening collection

The retrieval and preparation of these data sets is described in detail below. The Python code for the preparation can be found in the "/1. Data Prep" sub-folder of the repository. To avoid licensing issues, the external data sets themselves are not included in the repository, all data of the internal compounds described in this paper is included.

The data sets were standardized using the MolVS functionality of the RDKit. In general, the molecules were standardized in this order (MolVS classes in parentheses):

- choose the largest fragment (LargestFragmentChooser)
- uncharge the molecule (Uncharger)
- standardize the molecule (Standardizer)
- remove stereo information (Standardizer)
- generate canonical tautomer (TautomerCanonicalizer)

The Natural product data set was additionally subjected to a deglycosylation step, in which all sugars contained in a molecule are removed. This step was performed in a KNIME workflow using the Sugar Remover component, which is part of the CDK [https://github.com/cdk/cdk, last accessed 17-Dec-2020] community module. The workflow is included in the repository ("/knime-workspace")

In addition to the standardization, the data sets were filtered for their suitable properties:

- Isotopes: remove molecules that contain isotopes
- MedChemAtoms: remove molecules with non-MedChem atoms
  (atoms other than: H, B, N, C, O, F, P, S, Cl, Br, I)

- MinHeavyAtoms: remove molecules with less than 3 heavy atoms
- MaxHeavyAtoms: remove molecules with more than 75 heavy atoms
- Duplicates: remove duplicated molecules
  (determined by comparison of InChIKeys)

The code for the standardization and the filters is included in the utils.py file and called in the Notebook located at "/1. Data Prep".
The detailed order of steps is described below for each data set.

Selection of the Natural Product Data Set
The chemical structures of sets of NPs are available from multiple sources.[6,7] For the purpose of this paper, a medium-sized representative data set was required and the following sources were evaluated:

- COCONUT, 412k cpds.[8]
- UNPD, 213k cpds.[9]
- ChEMBL NPs, 40.5k cpds.

The data processing steps are described in detail for the data set that was finally chosen for the analysis in this paper (ChEMBL NPs). The other evaluated data sets were processed accordingly.
The COCONUT database was downloaded as gzipped SD file.
The Universal Natural Products Dictionary (UNPD) is no longer available on the internet, an unofficial download link was used to obtain this data set.
After standardization and filtering, a distribution of the Natural Product Likeness Score was calculated for all evaluated data sets.

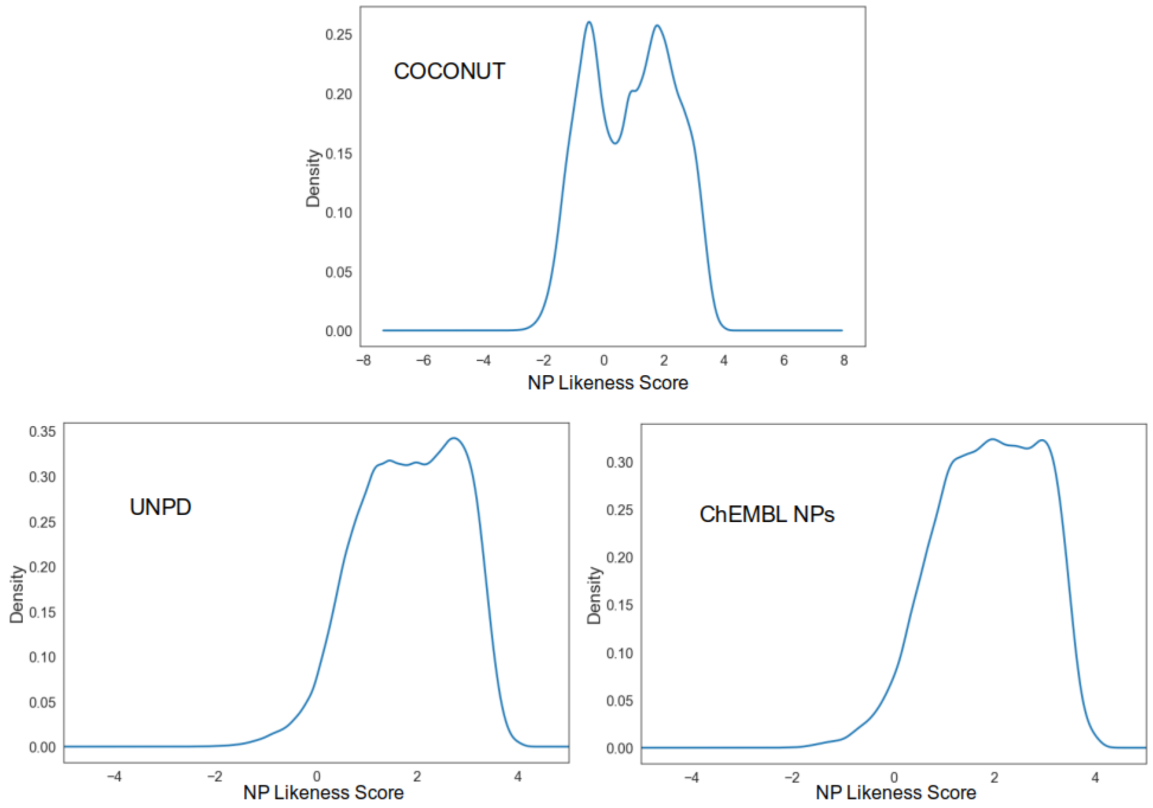

PCA Figure 1: NP likeness score distribution for different NP libraries.

In addition to NPs, the COCONUT data set contains NP analogs and derivatives,[10] which most likely explains the bimodal distribution of the NP Likeness Score distribution. Since the analogs and derivatives cannot be removed from the downloaded data set, the decision was made to not use it as a source for representative structures of NPs. The distributions of UNPD and ChEMBL look very similar. Although the ChEMBL data set is smaller than the UNPD, it seems to be representative of the NP chemical space and since it can be obtained from official sources, it was chosen for the analysis in this paper. The procedure for obtaining and processing this data set is described next.

Natural products from ChEMBL 26

Two versions of ChEMBL 26 were downloaded from the FTP server [http://ftp.ebi.ac.uk/pub/databases/chembl/ChEMBLdb/releases/chembl_26/] on 25-Mar-2020:

- chembl_26_postgresql.tar.gz
- chembl_26.sdf.gz

The PostgreSQL database was installed locally and all entries which had ("J. Nat. Prod" OR "J Nat Prod") as the journal source were extracted with the following SQL statement:

select distinct(md.chembl_id) as Chembl_Id

```
from docs d,
     molecule_dictionary md,
     compound_records cr
where md.molregno = cr.molregno
  and cr.doc_id = d.doc_id
  and d.journal in ('J. Nat. Prod.', 'J Nat Prod')
```

The obtained list of 40547 IDs was saved as chembl_26_natprot_ids_only.tsv in the "/Input Data" folder of this project. The downloaded SD file was then filtered for the IDs of the identified Natural products.

This set of natural product structures was the submitted to the following steps:

- choose the largest fragment
- uncharge the molecule
- standardize the molecule
- remove stereo information
- filter: Isotopes, MedChemAtoms, MinHeavyAtoms, Duplicates
- deglycosylate
- choose the largest fragment
- standardize
- filter: MinHeavyAtoms, MaxHeavyAtoms, Duplicates
- generate canonical tautomer
- filter: Duplicates

The final processed data set contains 30655 natural product structures ("chembl_np_std.tsv").

Approved and Experimental Drugs

As drugs reference set the approved and experimental data sets from DrugBank were used [v 5.1.5, https://www.drugbank.com, downloaded on 25-Mar-2020]. These were downloaded as two files, combined to give 8462 cpds. and submitted to the following standardization pipeline.

- choose the largest fragment
- uncharge the molecule
- standardize the molecule
- remove stereo information
- generate canonical tautomer
- filter: Isotopes, MedChemAtoms, MinHeavyAtoms, MaxHeavyAtoms, Duplicates

The resulting data set consists of 7472 unique structures (drugbank_std.tsv).

Enamine Advanced Screening Collection

The full Advanced Screening Collection was obtained from the Enamine site [https://enamine.net/hit-finding/compound-collections/screening-collection/advanced-collection), downloaded on 07-Dec-2020] as an SD file containing 527411 compounds. Because of the size of the full data set, a subset of 50k compounds was randomly chosen from the set and submitted to the following processing:

- choose the largest fragment
- uncharge the molecule
- standardize the molecule
- remove stereo information
- generate canonical tautomer
- filter: Isotopes, MedChemAtoms, MinHeavyAtoms, MaxHeavyAtoms, Duplicates

The processed data set consists of 49968 unqiue structures (enamine_subset_std.tsv). The IDs of the structures are included in the "/Input_Data" folder (enamine_subset_std_ids.tsv).

Pseudo-NPs (internal data set)
The set of 244 internal compounds discussed in this paper was submitted to the following standardization and filtering:

- choose the largest fragment
- uncharge the molecule
- standardize the molecule
- remove stereo information
- generate canonical tautomer
- filter: Isotopes, MedChemAtoms, MinHeavyAtoms, MaxHeavyAtoms

The result of this pipeline was stored in internal_cpds_mc.tsv. This version of the data set still contains duplicates based on different stereo chemistries and is used in per-compound-class comparisons.

In addition, the duplicates were removed (internal_cpds_std.tsv). This data set was used in the global PCA analyses and NP Likeness distributions.

NP Likeness and QED Scores
NP Likeness scores[11] (as implemented in the RDKit) were calculated using the implementation in the RDKit and compared to the NP class probability calculated by NP-Scout[12].

The NP-Scout scores were obtained by submitting the respective data sets to the web service [https://nerdd.zbh.uni-hamburg.de/npscout]. The code for the calculations and the comparisons can be found in "/2. NP Likeness Scores" of the repository.

In addition, the Quantitave Estimation of Drug-Likeness (QED) scores[13] were calculated (again, using the implementation in the RDKit).

Principal Component Analysis of 17 Molecular Descriptors

A Principal Component Analysis (PCA) was performed to investigate whether the pseudo-Natural Products (PNPs) developed internally explored different chemical space than the other datasets. The work for this step can be found in the “3. PCA” subdirectory.

Feature Computation

A set of 17 molecular descriptors were computed with the RDKit to use as features (**PCA Table S1**), using the standardized structures from previous step of (PNPs, NPs, drugs and commercial compounds). The Python code can be found in the “compute_features” Jupyter notebook, whereas output tsv files and plots are respectively located in the “results” and “plots” subfolders.

PCA Table S1: Description of features used in the PCA

Feature no	Feature Name	Description
1	frac_sp3	Fraction of sp^3 hybridized carbons
2	molecular_weight	Molecular weight (Da)
3	num_atoms_bridgehead	Number of bridgehead atoms
4	num_atoms_halogen	Number of halogen atoms
5	num_atoms_nitrogen	Number of nitrogen atoms
6	num_atoms_oxygen	Number of oxygen atoms
7	num_hba	Number of hydrogen-bond acceptors
8	num_hbd	Number of hydrogen-bond donors
9	num_heavy_atoms	Number of heavy atoms
10	num_rings	Number of rings
11	num_rings_ali	Number of aliphatic rings
12	num_rings_arom	Number of aromatic rings
13	nun_rotatable_bonds	Number of rotatable bonds
14	num_violations_lipinski	Number of violations to the Lipinski’s rule[14]
15	num_violations_veber	Number of violations to the Veber’s rule[15]
16	slogp	Log of the partition coefficient between octanol and water
17	tpsa	Topological Polar Surface Area ($Å^2$)

The distribution of each feature was plotted for each subset to gain insight about possible trends across in our datasets (**Supplementary Figures 16-19**). Mean values of the feature distributions (PCA Table S2) indicate that PNPs are typically larger than the synthetic and commercial subsets but comparable to NPs (higher molecular weight and number of heavy atoms). Moreover, PNPs tend to be more lipophilic (higher slogp and lower tpsa) and more planar and rigid as well

(higher number of saturated and aromatic rings, lower number of rotatable bonds and lower fraction of sp^3 hybridized carbons than observed in other datasets).

It has been previously observed that NPs typically contain more oxygen atoms and less nitrogen atoms than classical synthetic compounds.[16] This is the case in our study as well, with NPs having respectively the greatest and lowest number of oxygen and nitrogen atoms of all datasets. Interestingly, PNPs contain in average a much higher number of nitrogen atoms than NPs (x2.8) but a lower number of oxygen atoms comparable to the DrugBank dataset (respectively 3.75 vs 5.58 and 3.95). Additionally, PNPs tend to have significantly more halogen and bridgehead atoms than the other datasets.

Moreover, the PNPs of our study have in average less hydrogen-bond donors and acceptors than other NPs and synthetic compounds (respectively 1.25 vs 2.15 - 2.72 and 5.43 vs 6.19 - 6.23), but comparable values to commercial compounds (respectively 1.25 vs 1.29 and 5.43 vs 5.73).

The rules from Lipinski[14] and Veber[15] are commonly used filters to indicate if a molecule can be a good candidate for an oral drug. Most datasets are in very good compliance with both rules, with a higher number of violations in PNPs and NPs for the rule of 5, certainly due to a higher molecular weight (respectively 450.97 Da and 423.97 Da vs 326.15 - 343.32 Da).

PCA Table S2: Average values of features per dataset. Source data are provided in the online repository.

Feature	PNPs	Chembl-NP	Drugbank	Enamine
frac_sp3	0.38	0.55	0.43	0.46
molecular_weight (Da)	450.97	423.97	343.32	326.15
num_atoms_bridgehead	1.27	0.49	0.11	0.04
num_atoms_halogen	0.97	0.12	0.44	0.57
num_atoms_nitrogen	1.71	0.61	2.32	3.38
num_atoms_oxygen	3.72	5.58	3.95	2.36
num_hba	5.43	6.19	6.28	5.73
num_hbd	1.25	2.15	2.72	1.29
num_heavy_atoms	32.15	30.23	23.55	22.96
num_rings	6.33	3.58	2.46	2.98
num_rings_ali	3.13	2.38	0.89	1.14
num_rings_arom	3.20	1.20	1.56	1.84
nun_rotatable_bonds	2.52	5.07	5.35	4.07
num_violations_lipinski	0.57	0.68	0.49	0.02
num_violations_veber	0.00	0.25	0.27	0.00
slogp	4.95	3.76	1.95	2.17
tpsa (Å^2)	63.71	93.15	94.73	70.88

Principal Component Analysis

The Principal Component Analysis was performed on the assembled data from each dataset, using the scikit-learn Python package, as indicated in the "compute_pca.ipynb" notebook. The first three Principal Components represent non-less than 64.62% of the variance explained of the data (**Supplementary Figure 20**), which make them suitable for further analyzing the data.[17]

The most contributing features for each considered Principal Component were then investigated (**Supplementary Figure 21**).

The first Principal Component explains 37.04% if the data variance is composed in majority (>50%) of 6 features:

- number of heavy atoms
- molecular weight
- tpsa
- number of oxygen atoms
- number of violations to the Lipinski rule
- number of hydrogen bond donors

The second Principal Component explains 15.71% of the variance and is composed in majority of 5 features:

- number of aliphatic rings
- fraction of sp3 carbon atoms
- number of aromatic rings
- number of nitrogen atoms
- number of hydrogen bond donors

Finally, the third Principal Component explains 11.87% of the variance and is composed in majority of 5 features:

- number of rings
- number of aromatic rings
- slogp
- fraction of sp3 carbon atoms
- number of hydrogen bond donors

The position and spread of the datasets in the chemical space defined by Principal Components 1-3 were then compared (**Figure 3d** and **Supplementary Figure 10**). For better visualization, it was preferred to represent each combination of Principal Components (PC1 and PC2, PC1 and PC3 and PC2 and PC3) instead of a single 3D PCA plot. However, due to the large number of dots being overlaid, it was still not straightforward to accurately delimit each cluster. For this reason, the data was broken down into comparing the PNPs with each other datasets individually (**Supplementary Figure 22**).

Although the Enamine subset contained 10k more compounds than the ChEMBL-NP subset, the latter exhibited the broadest spread area of all datasets. This was in good agreement with the expected structural diversity of NPs.

On the contrary, the Enamine subset appeared to be very dense, in spite of having randomly selected 50k compounds of the 527k from the original file, indicating that most retained molecules shared a narrower range of physicochemical properties.

The DrugBank dataset distribution was found to be in between both previous cases, which could potentially be explained by the fact that drugs are the results from both the inspiration by the Nature and the synthesis efforts of medicinal chemists.

Finally, no large overlap between the PNPs and the other datasets in more than 1 Principal Component could be observed, indicating that the PNPs occupy a closely related but still

distinguishable chemical space than the Natural Products, drugs and commercially available compounds of this study.

Chemical Similarities
The chemical class similarities were calculated and compared using two different fingerprints, implemented in the RDKit.

- ECFC4, a count fingerprint with radius 2
- ECFP6, a bit fingerprint of length 1024 with radius 3

For a comparison of fingerprints in the RDKit, see e.g. Riniker et al.[18]

As a reference, a random set of 100 compounds from the processed Enamine data set is used. The IDs can be found in the file enamine_sample_100.tsv.
The inter- and intra-class similarities were determined by calculating all individual similarities between the compounds, then taking the median value.

The code for the calculation of chemical similarities can be found in "/4. Chem Class Similarity".

Biological Similarities
Class-based similarities of the Cell Painting Assay (CPA) profiles were calculated.
The profiles have a length of 579 features (all columns starting with "Median_") and are compared by Distance Correlation (`scipy.spatial.distance`).
The Similarity is then (1 - Distance Correlation). Negative values are set to zero.
The Similarity is expressed in percent.
In addition, the Cell Painting data contains an Induction parameter, which describes the percentage of significantly changed features in the profile (i.e. the percentage of features with abs. values ≥ 3.0).
This parameter is used to estimate the extent to which the morphological profile of a given test compound is changed compared to the controls.
The respective chapter in the Supporting Information describes the Cell Painting assay more in detail.

The list of 579 features and the code for the biological similarity calculation can be found in the cpa module of the repository. The Notebook performing the calculations is located in the folder "/5. Bio Class Similarity".

Principal Moment of Inertia Details

PMIs were calculated using RDKit (RDKit: Open-source cheminformatics; http://www.rdkit.org) and PMI plots were produced using matplotlib (J. D. Hunter, Computing in Science & Engineering, 2007, 9, 3, 90) running in Python 3.8. Raw data set of PMI analysis can be found at http://dx.doi.org/10.5281/zenodo.4529728.

Reference compounds:

Chromane-containing NPs

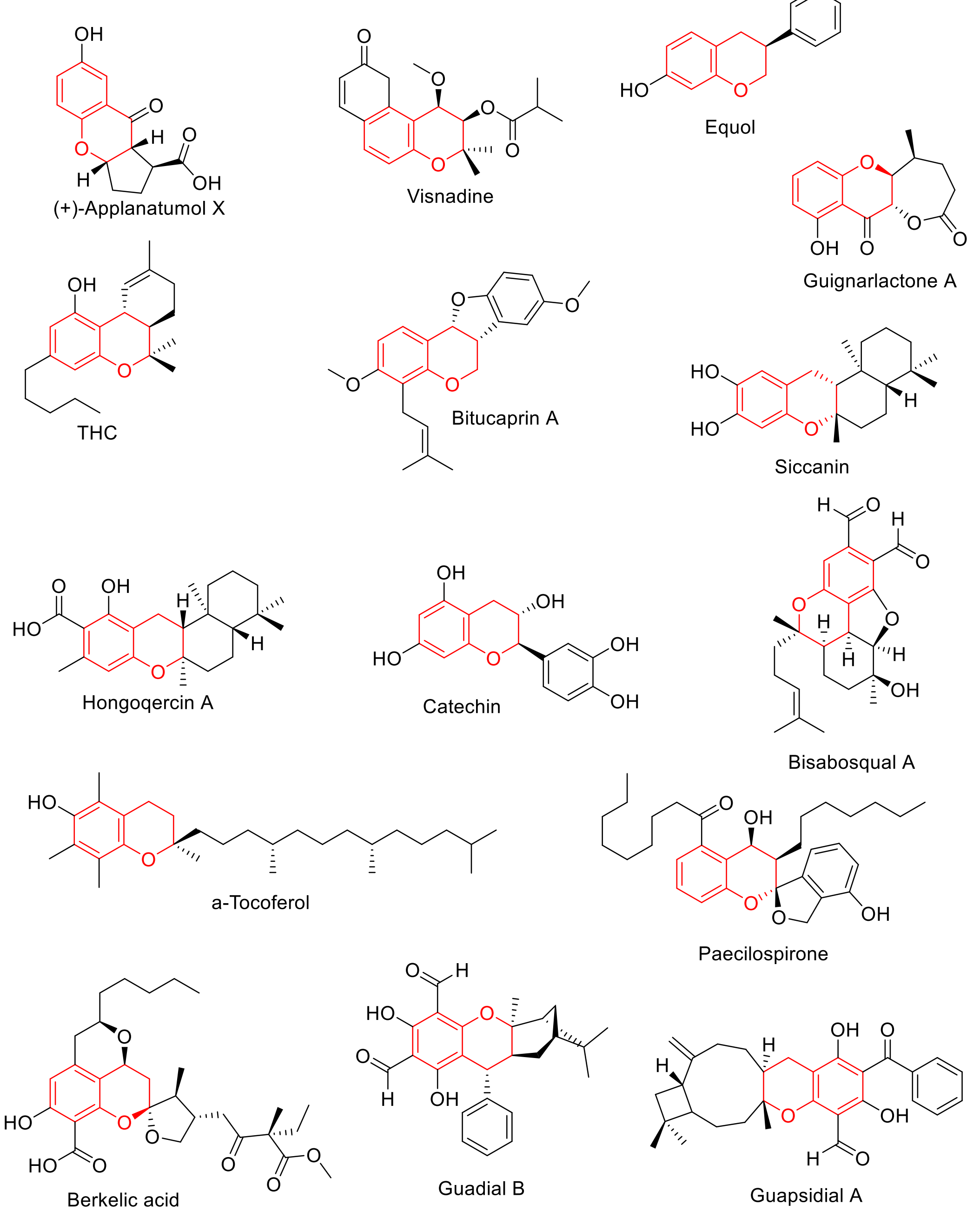

Indole-containing NPs

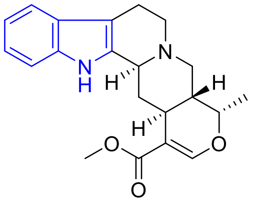

Ajmalicine

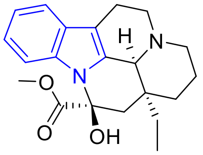

Vincamine

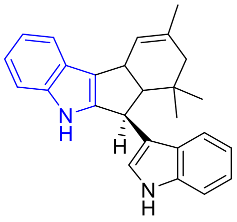

Yuehchukene

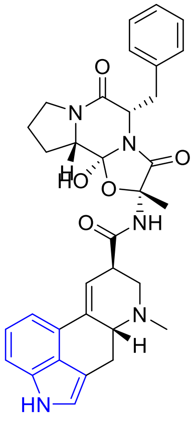

Ergotamine

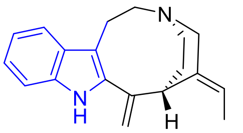

Pericine

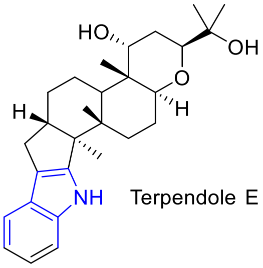

Terpendole E

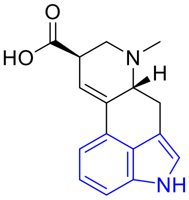

Lysergic Acid

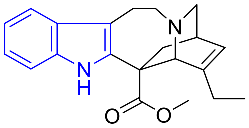

Catharanthine

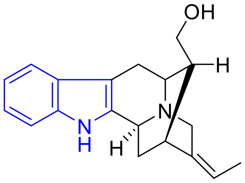

Koumidine

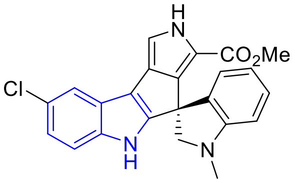

Spiroindimicin B

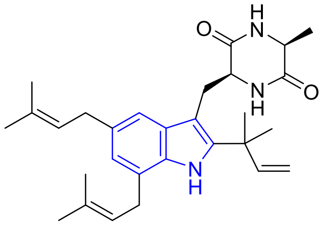

Echinulin

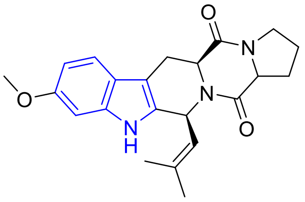

Fumitremorgin C

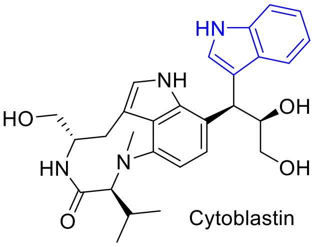

Cytoblastin

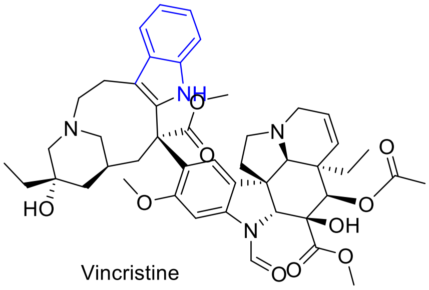

Vincristine

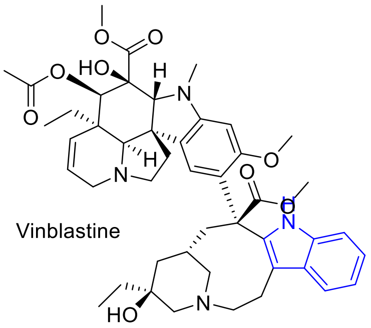

Vinblastine

Chromane/chromene-containing synthetic compounds

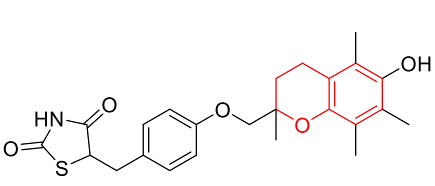

Troglitazone

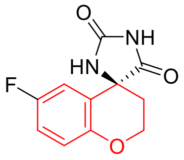

Sorbinil

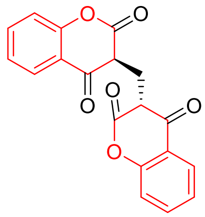

Bishydroxy[2h-1-Benzopyran
-2-one,1,2-Benzopyrone]

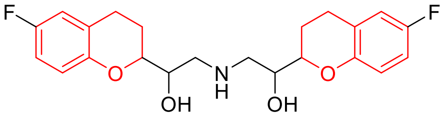

Nebivolol

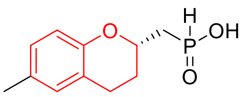

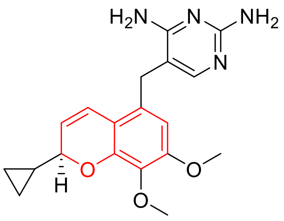

Iclaprim

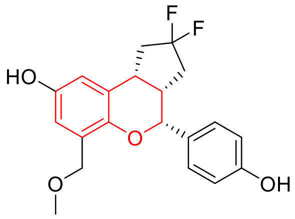

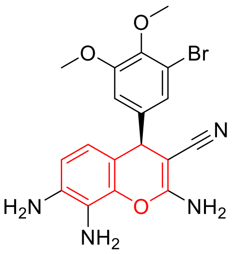

Crolibulin

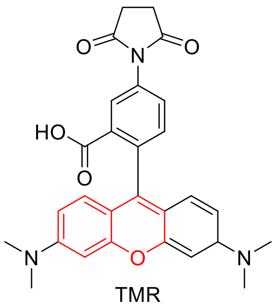

TMR

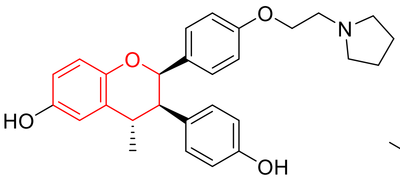

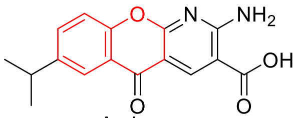

Amlexanox

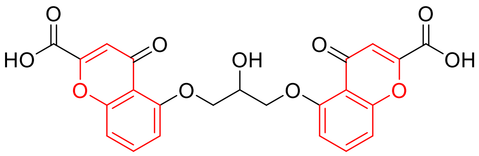

Cromoglicic acid

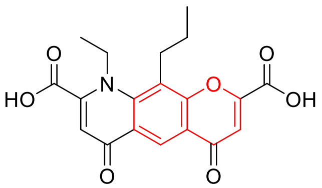

Nedocromil

Indole-containing synthetic compounds

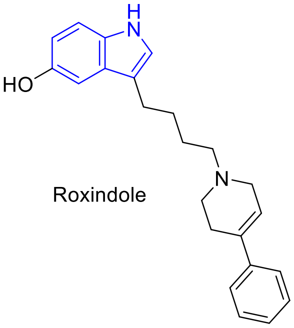

Roxindole

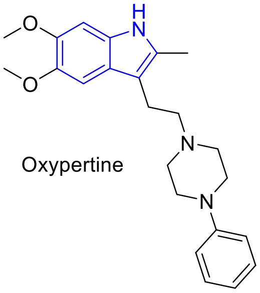

Oxypertine

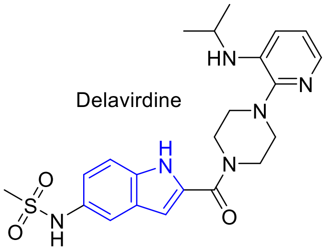

Delavirdine

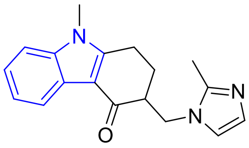

Ondansetron

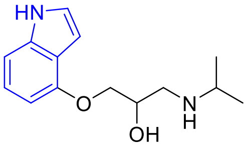

Pindolol

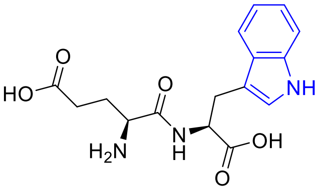

Oglufanide

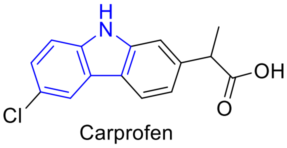

Carprofen

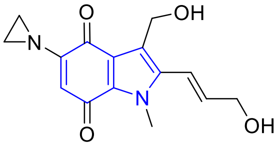

Apaziquone

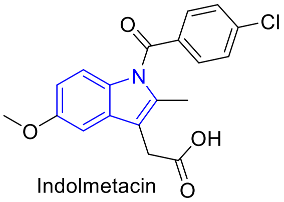

Indolmetacin

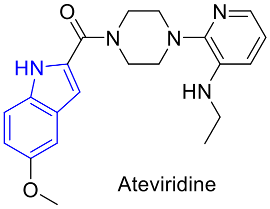

Ateviridine

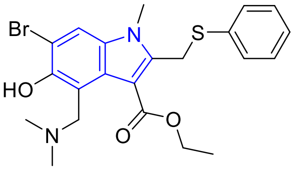

Arbidol

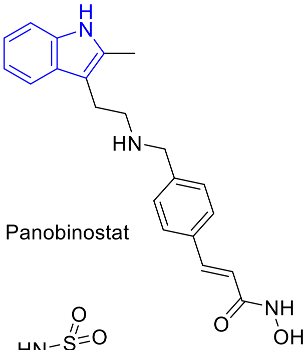

Panobinostat

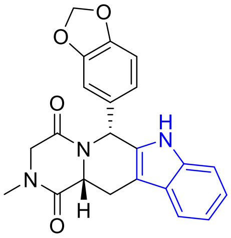

Tadalafil

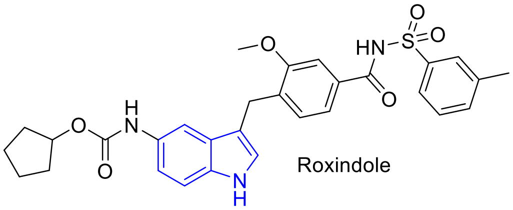

Roxindole

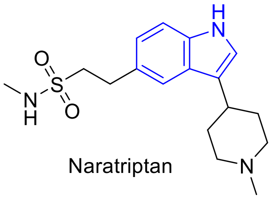

Naratriptan

Cell Painting Assay Experimental and Analyses

The described assay follows closely the method described by Bray et al.[19]

Initially, 5 µl U2OS medium were added to each well of a 384-well plate (PerkinElmer CellCarrier-384 Ultra). Subsequently, U2OS cell were seeded with a density of 1600 cells per well in 20 µl medium. The plate was incubated for 10 min at the ambient temperature, followed by an additional 4 h incubation (37 °C, 5% CO2). Compound treatment was performed with the Echo 520 acoustic dispenser (Labcyte) at final concentrations of 10 µM, 3 µM or 1 µM. Incubation with compound was performed for 20 h (37 °C, 5% CO2). Subsequently, mitochondria were stained with Mito Tracker Deep Red (Thermo Fisher Scientific, Cat. No. M22426). The Mito Tracker Deep Red stock solution (1 mM) was diluted to a final concentration of 100 nM in prewarmed medium. The medium was removed from the plate leaving 10 µl residual volume and 25 µl of the Mito Tracker solution were added to each well. The plate was incubated for 30 min in darkness (37 °C, 5% CO2). To fix the cells 7 µl of 18.5 % formaldehyde in PBS were added, resulting in a final formaldehyde concentration of 3.7 %. Subsequently, the plate was incubated for another 20 min in darkness (RT) and washed three times with 70 µl of PBS. (Biotek Washer Elx405). Cells were permeabilized by addition of 25 µl 0.1% Triton X-100 to each well, followed by 15 min incubation (RT) in darkness. The cells were washed three times with PBS leaving a final volume of 10 µl. To each well 25 µl of a staining solution were added, which contains 1% BSA, 5 µl/ml Phalloidin (Alexa594 conjugate, Thermo Fisher Scientific, A12381), 25 µg/ml Concanavalin A (Alexa488 conjugate, Thermo Fisher Scientific, Cat. No. C11252), 5 µg/ml Hoechst 33342 (Sigma, Cat. No. B2261-25mg), 1.5 µg/ml WGA-Alexa594 conjugate (Thermo Fisher Scientific, Cat. No. W11262) and 1.5 µM SYTO 14 solution (Thermo Fisher Scientific, Cat. No. S7576). The plate is incubated for 30 min (RT) in darkness and washed three times with 70 µl PBS. After the final washing step, the PBS was not aspirated. The plates were sealed and centrifuged for 1 min at 500 rpm.

The plates were prepared in triplicates with shifted layouts to reduce plate effects and imaged using a Micro XL High-Content Screening System (Molecular Devices) in 5 channels (DAPI: Ex350-400/ Em410-480; FITC: Ex470-500/ Em510-540; Spectrum Gold: Ex520-545/ Em560-585; TxRed: Ex535-585/ Em600-650; Cy5: Ex605-650/ Em670-715) with 9 sites per well and 20x magnification (binning 2).

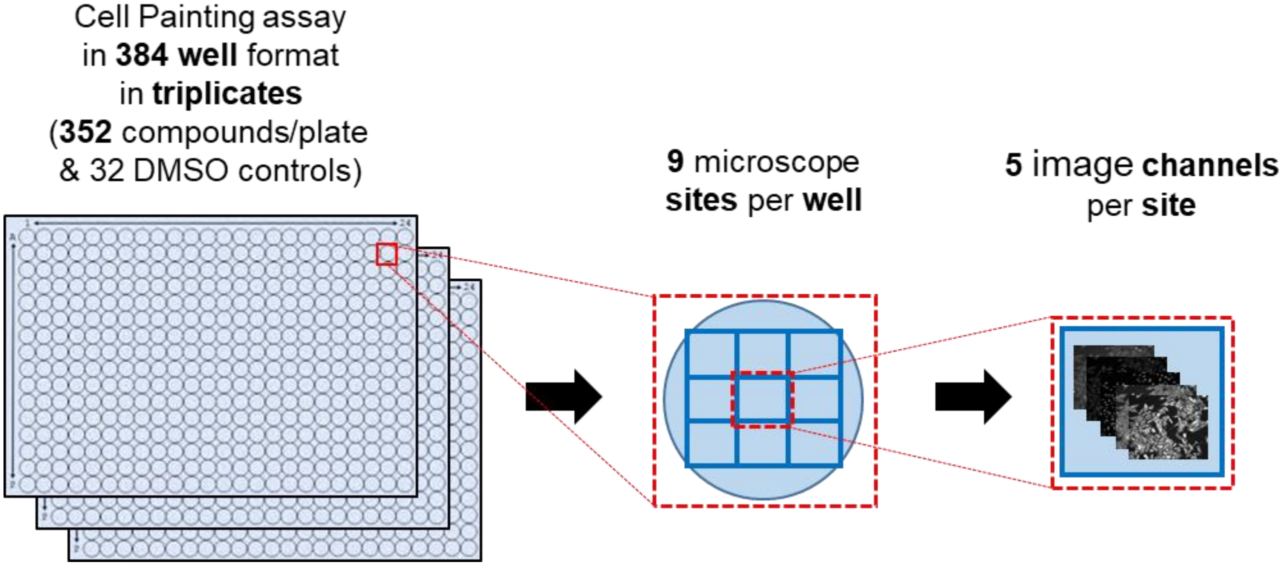

The generated images were processed with the *CellProfiler* package (https://cellprofiler.org/, version 3.0.0) on a computing cluster of the Max Planck Society to extract 1716 cell features (parameters) per microscope site. The data was then further aggregated as medians per well (9 sites -> 1 well), then over the three replicates.

Further analysis was performed with custom *Python* (https://www.python.org/) scripts using the *Pandas* (https://pandas.pydata.org/) and *Dask* (https://dask.org/) data processing libraries as well as the *Scientific Python* (https://scipy.org/) package (separate publication to follow).

From the total set of 1716 features, a subset of highly reproducible and robust features was determined using the procedure described by Woehrmann et al.[20] in the following way: Two biological repeats of one plate containing reference compounds were analysed. For every feature, its full profile over each whole plate was calculated. If the profiles from the two repeats showed a similarity >= 0.8 (see below), the feature was added to the set.

This procedure was only performed once and resulted in a set of 579 robust features out of the total of 1716 that was used for all further analyses.

Determination of reproducible Features

1716 — *Determined by CellProfiler*

Keep features that have a minimum correlation of 0.80 between repeats for all cpds.

579 — *Final set of relevant features. Used for all further analyses*

To determine the phenotypic profiles for each test compound Z-scores were then calculated for each features as how many times the Median Absolute Deviation (MAD) of the controls the measured feature value of a test compound deviates from the Median of the controls:

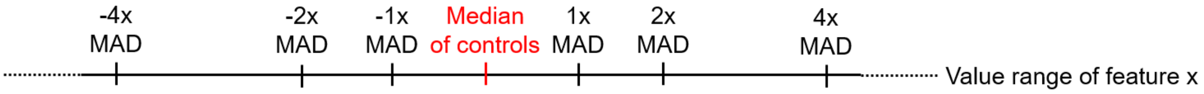

$$z - score = \frac{value_{meas.} - Median_{Controls}}{MAD_{Controls}}$$

The phenotypic compound profile is then determined as the list of z-scores of all features for one compound.

In addition to the phenotypic profile, an induction value was determined for each compound as the fraction of significantly changed features, in percent:

$$Induction\ [\%] = \frac{number\ of\ features\ with\ abs.\ \ values > 3}{total\ number\ of\ features}$$

Similarities of phenotypic profiles were calculated from the correlation distances between two profiles (https://docs.scipy.org/doc/scipy/reference/generated/scipy.spatial.distance.correlation.html; Similarity = 1 - Correlation Distance) and the compounds with the most similar profiles were determined from a set of 3000 reference compounds that was also measured in the assay.

An example for two compounds with highly similar profiles (96% similarity):

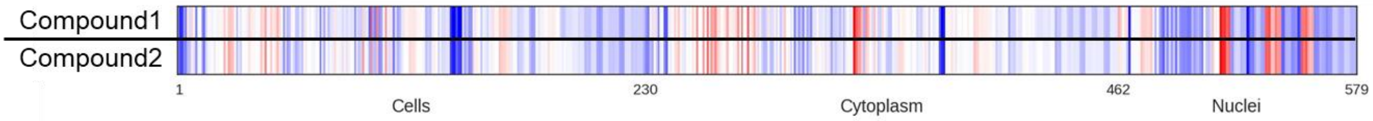

An example for two compounds with low similarity profiles (0% similarity):

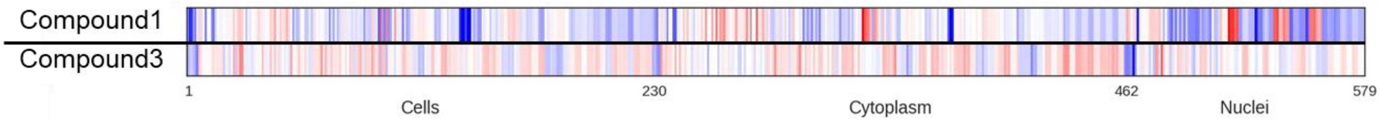

Each colored band represents one Z-score of a feature.

In order to analyze the data and to draw comparisons, the term ‘biosimilarity’ was introduced which describes the resemblance between morphological profiles. Compounds that show a biosimilarity above 75% are defined as ‘similar’, whereas comparisons with a value below 75% are defined as ‘dissimilar’.

Different methods of analysis were applied for the comparison of compounds’ profiles. The cross-similarity analysis equally compares the phenotypic profile of all entered compounds to each other and allows a statement consistency of a compound set via a table which shows all comparative biosimilarity percentages. From these one to one comparisons, median biosimilarities between compound sets can be calculated and is a quantitative representation of the overall comparison.

Additionally, a dimension reduction analysis (Principal Component Analysis, short PCA) was employed to evaluate the compound sets. Thereby, all morphological information from the profiles are reduced to the three-dimensional space. Each spot represents one finger print of a compound from the CPA. Profile similarity is depicted in the distance of the spots to each other. A close proximity translates to a high biosimilarity, whereas a greater distance between compound profiles is indicative of major differences in the morphological effect. For the PCA, we employed 'row' normalization in which each profile was normalized according to the parameter with the highest z-score.

In order to provide a meaningful analysis, it is important to exclude false positive effects. One example that was identified is a dominating profile. This feature is especially present for compounds with high induction values. In fact, as percent induction increases, the similarity to the dominating profile simultaneously increases. This effect is not completely understood yet and is still under investigation. In order to avoid misrepresentation of the data, the induction limit for analysis was set to 40%, where this effect is not as pronounced.

As a consequence, an induction-dependent biosimilarity between compounds was found. While comparing classes with broad induction ranges, compounds were found to cluster together due to similar induction rather than true biosimilarity. Component 1 is highly induction-dependent if a wide induction range is used (**SI Figure 9a**). Typically, induction ranges of 25% or less had sufficient mixing relative to the PCs and induction (**SI Figure 9b**). However, it was found that many datasets required smaller induction ranges while others tolerated large ranges (up to 50%) with adequate mixing. Therefore, it was necessary to compare compounds in a variety of induction windows for the best comparisons of each dataset.

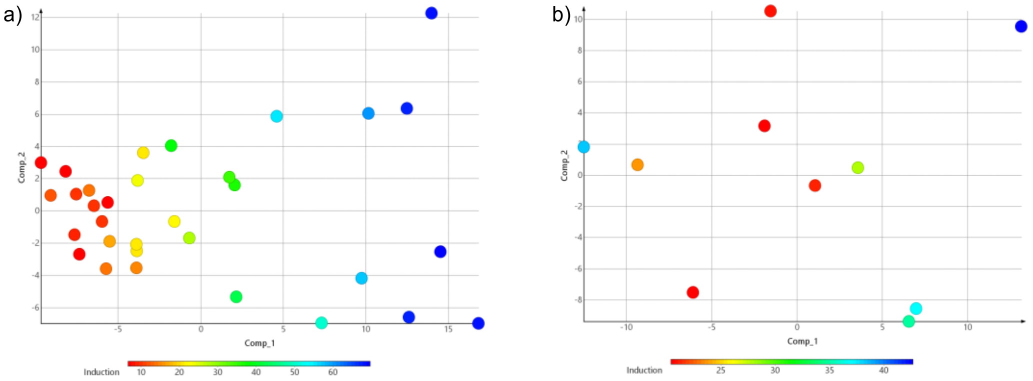

Supplementary Figure 23: PCA analyses of different induction ranges. Two-dimensional PCA plot of the quinine indoles (**QN-I**) with a broad induction range from 6.6% to 69.8% (**Supplementary Figure 23a**) and a defined induction window of 20-45% (**Supplementary Figure 23b**). Color represents the induction differences and shows that component 1 is mainly influenced through this value for a broad induction window. Good mixing is achieved when the induction is kept within a range of 25%. Explained variance for principal components (left): PC1 (48.9%), PC2 (13.8%), PC3 (6.6%), explained variance for principal components (right): PC1 (27.3%) PC2 (20.9%), PC3 (17.9%). Only the first two components are shown.

Synthetic Experimental Details

General Experimental Details

All reactions were conducted open to air without precautions to exclude water unless specifically stated. Dry solvents were purchased from Fisher Scientific and/or Acros and used without further treatment. Thin layer chromatography (TLC) was performed on silica coated aluminium plates (Merck 60 F254) and visualization was achieved under UV irradiation (254 nm) or by a potassium permanganate stain (1.5 g $KMnO_4$, 10 g K_2CO_3, 1.25 mL of 10% aqueous NaOH solution and 200 mL of water). Purification of crude products was achieved through flash column chromatography (FC, silica gel 60, 0.035-0.070 mm) or automated medium pressure liquid chromatography (MPLC, Grace Reveleris X2) using the indicated solvents. In certain cases, 'magic mix' (50 DCM: 8 EtOH: 1 NH_4OH (50% aq.)) or 'strong magic mix' (25 DCM: 8 EtOH: 1 NH_4OH (50% aq.)) was used as a cosolvent for silica chromatography. Challenging separations were carried out on an Agilent 1100 preparative HPLC system equipped with a mass detector (columns: Nuleodur C18 gravity VP 125/10 5 µm, Nucleodur C18 gravity VP 125/21 5 µm, Nucleodur C4 gravity VP 125/10 5 µm). Appropriate gradient systems were applied by mixing water (+ 0.1% TFA) and either acetonitrile or methanol. NMR spectra were recorded on Bruker AV 400 Avance III HD (NanoBay), Agilent Technologies DD2, Bruker AV 500 Avance III HD (Prodigy), Bruker AV 600 Avance III HD (CryoProbe) or Bruker AV 700 Avance III HD (CryoProbe) spectrometers. Data is reported in ppm with reference to the used deuterated solvent.[21] Isolated products as well as diastereomers could be assigned based on 2D NMR correlations ($^1H/^1H$ COSY, $^1H/^1H$ NOESY, $^1H/^{13}C$ HSQC, $^1H/^{13}C$ HMBC). High-resolution mass spectrometry (HRMS) was performed on an LTQ Orbitrap mass spectrometer coupled to an Accela HPLC-System (HPLC column: Hypersyl GOLD, 50 mm x 1 mm, particle size 1.9 µm, ionization method: electron spray ionization (ESI)). Microwave reactions were carried out in a CEM Discover SP Activent machine.

Synthesis of quinine- and quinidine-based ketones:

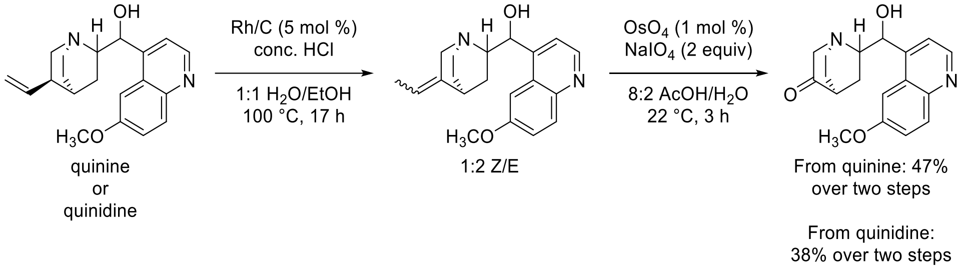

The quinine and quinidine-based ketones were synthesized according to Waldmann et. al.[1]

In two batches, quinidine or quinine (2 × 6.0 g, 2 × 18.4 mmol [18.4 mmol in each batch, 36.8 mmol over 2 batches], 1.0 eq) was dissolved in 1:1 H_2O/EtOH (2 × 120 mL, [120 mL in each batch, 240 mL overall]) and concentrated HCl (2 × 15.6 mL, 181 mmol [181 mmol in each batch, 362 mmol over 2 batches], 10 eq.) was added. Rh/C (5 wt%, 2 × 100 mg [100 mg in each batch, 200 mg overall], 1.7 w/w%) was added and the reaction mixtures were heated at reflux (101.5°C) under Ar for 17 h. The reaction mixtures were cooled to rt and the two batches were combined and filtered through celite, flushing through with MeOH. The filtrate was concentrated in vacuo. To the resulting oil was added H_2O (100 mL). Lyophilisation (72 h) gave the internal alkenes as the hydrochloride salts, which were carried forward to the next step without further purification.

The relevant cinchona-alkenyl hydrochloride salt (5.0 mmol) was dissolved in 8:2 AcOH/H2O (24 mL) and $K_2OsO_4{\bullet}2H_2O$ (18 mg, 0.05 mmol, 1.0 mol%) was added. The reaction mixture was stirred at rt for 10 min, then cooled to 0 °C. $NaIO_4$ (2.14 g, 10.0 mmol, 2.0 eq.) was added. The mixture was warmed to rt and stirred for 17 h. The mixture obtained was quenched with aqueous NaOH (10 M, ~50 mL) at 0°C until it was basic (tested with pH indicator paper). Sat. aq. Na_2SO_3 solution (10 mL) was added to destroy OsO_4. The resulting solution was extracted with 9:1 $CHCl_3$/MeOH (4 x 50 mL) and 8:2 $CHCl_3$/MeOH (2 x 50 mL). The combined organic fractions were dried over Na_2SO_4, filtered, and concentrated in vacuo. Flash column chromatography gave the desired cinchona ketones (quinine-derived = 47%; quinidine-derived = 38%).

General procedure for quinine- and quinidine-based indoles (**QN-I** and **QD-I**):

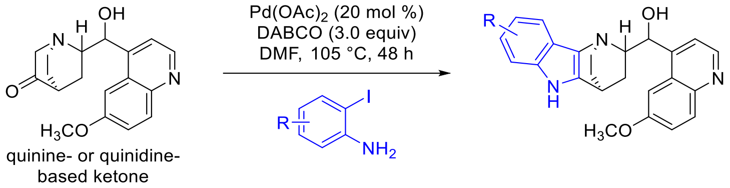

All experimental details for the **QN-I** and **QD-I** compound classes and were obtained according Waldmann et. al.[1]

The quinine- or quinidine-derived ketone (100 mg, 0.32 mmol, 1.0 eq.), 2-iodoaniline (0.96 mmol, 3.0 eq.), DABCO (108 mg, 0.96 mmol, 3.0 eq.) and $Pd(OAc)_2$ (7 mg, 10 mol%) were added to a 7.0 mL reaction vial equipped with a screw-cap lid bearing a PTFE/silicone septum. The reactants were dissolved in anhydrous DMF (1.0 mL, 0.3 M) and degassed under a stream of Argon for 10 min. The lid was additionally sealed with parafilm and transferred to a pre-heated heating block at 105 °C (the solvent level in the vial was submerged below level of the heating block). The reaction progress was monitored every 24 h by HPLC-MS. In case of incomplete conversion, the reaction mixture was cooled to rt, and additional $Pd(OAc)_2$ (7 mg, 10 mol%) was added. The resulting solution was degassed under a steam of argon for 10 min and returned to the heating block for additional 24 h. Following complete conversion of starting ketone (or after a maximum 72 h reaction time) the reaction mixture was cooled to rt, diluted in MeOH (5 mL) and passed through a syringe filter. After removal of the solvents in vacuo the cinchona-indole products were purified by column chromatography and/or by mass-directed preparative HPLC (MeCN + 0.1% TFA/H_2O +0.1%TFA).

General procedure for quinine-based chromanones (QN-C):

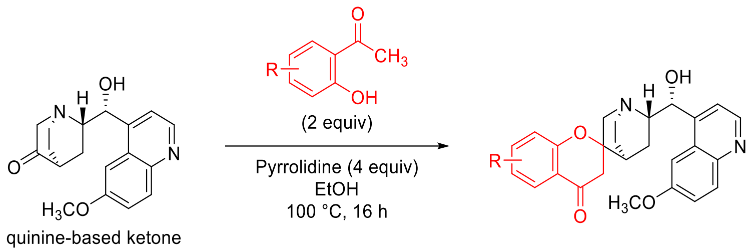

To an oven-dried pressure vessel equipped with a stir bar that was cooled under an atm of Ar was added the quinine-derived ketone (100 mg, 0.33 mmol, 1 equiv). Then, anhydrous EtOH (1 ml) was added followed by a 2-hydroxyacetophenone derivative (0.66 mmol, 2 equiv) and pyrrolidine (104 ul, 1.33 mmol, 4 equiv). The pressure vessel was flushed with Ar and sealed with the proper cap. The reaction was then heated to 100 °C for 16 h. After cooling to room temperature, the reaction was diluted with DCM, transferred to round-bottomed flask, and concentrated. Purification could be achieved by silica chromatography (0-12% MeOH in DCM) to afford pure isomers. All of the reactions produced two diastereomers; however, after purification, sometimes only one of the isomers could be obtained in pure form.

Compound data for quinine-based chromanones (**QN-C-S** and **QN-C-R**):

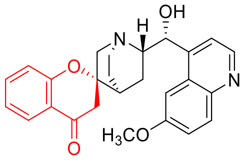

QN-C-S-1

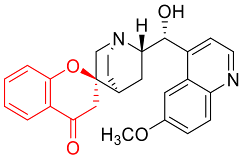

QN-C-R-1

Following the representative procedure for the quinine-based ketone, 40 mg (29%) of **QN-C-S-1** and 63 mg (45%) of **QN-C-R-1** were isolated as yellow solids.

Minor diastereomer **QN-C-S-1**:

^{1}H NMR (700 MHz, CD_2Cl_2) δ 8.57 (d, *J* = 4.5 Hz, 1H), 7.90 (d, *J* = 9.1 Hz, 1H), 7.75 (dd, *J* = 7.9, 1.8 Hz, 1H), 7.49 (ddd, *J* = 8.8, 7.2, 1.8 Hz, 1H), 7.46 (d, *J* = 4.5 Hz, 1H), 7.29 – 7.22 (m, 2H), 7.02 – 6.94 (m, 2H), 5.60 (s, 1H), 3.85 (s, 3H), 3.58 – 3.41 (m, 1H), 3.15 (d, *J* = 14.6 Hz, 1H), 3.09 – 3.04 (m, 1H), 2.88 – 2.80 (m, 2H), 2.78 (d, *J* = 16.4 Hz, 1H), 2.71 (d, *J* = 16.4 Hz, 1H), 2.26 (s, 1H), 2.03 – 1.94 (m, 2H), 1.59 – 1.46 (m, 2H).

^{13}C NMR (176 MHz, CD_2Cl_2) δ 191.89, 160.04, 158.21, 147.82, 147.61, 144.58, 136.72, 131.79, 126.94, 126.63, 121.80, 121.58, 121.25, 118.95, 118.76, 101.84, 80.99, 71.45, 64.27, 60.19, 56.17, 46.33, 43.01, 29.51, 23.29, 20.91.

HRMS (ESI) calcd for $C_{26}H_{26}N_2O_4$ $[M + H]^+$ 431.19653, found 431.19616.

$[\alpha]_D^{20}$ −126 (*c* 0.1, MeOH).

Major diastereomer **QN-C-R-1**:

^{1}H NMR (700 MHz, CD_2Cl_2) δ 8.59 (d, *J* = 4.5 Hz, 1H), 7.94 (d, *J* = 9.0 Hz, 1H), 7.76 (dd, *J* = 7.8, 1.8 Hz, 1H), 7.46 (d, *J* = 4.5 Hz, 1H), 7.42 (ddd, *J* = 8.6, 7.1, 1.8 Hz, 1H), 7.35 – 7.28 (m, 2H), 6.96 (t, *J* = 7.5 Hz, 1H), 6.80 (d, *J* = 8.3 Hz, 1H), 5.49 (d, *J* = 5.7 Hz, 1H), 3.89 (s, 3H), 3.53 – 3.46 (m, 1H), 3.42 – 3.34 (m, 1H), 3.16 – 3.06 (m, 1H), 2.88 – 2.79 (m, 2H), 2.71 (d, *J* = 14.7 Hz, 1H), 2.54 (td, *J* = 12.4, 10.5, 4.3 Hz, 1H), 2.29 – 2.23 (m, 1H), 1.92 – 1.82 (m, 1H), 1.71 – 1.64 (m, 2H), 1.57 – 1.47 (m, 1H).

^{13}C NMR (176 MHz, CD_2Cl_2) δ 192.20, 160.02, 158.06, 148.15, 147.91, 144.70, 136.66, 131.81, 127.27, 126.57, 121.75, 121.50, 121.36, 119.10, 118.69, 102.08, 81.52, 72.20, 63.81, 59.89, 56.02, 45.08, 42.94, 29.72, 23.25, 22.67.

HRMS (ESI) calcd for $C_{26}H_{26}N_2O_4$ $[M + H]^+$ 431.19653, found 431.19606.

$[\alpha]_D^{20}$ −233 (*c* 0.1, MeOH).

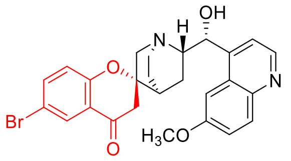

QN-C-S-2

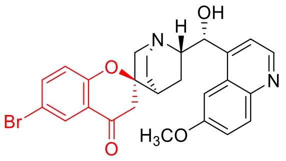

QN-C-R-2

Following the representative procedure for the quinine-based ketone, 50 mg (31%) of **QN-C-S-2** and 65 mg (40%) of **QN-C-R-2** were isolated as brown solids.

Minor diastereomer **QN-C-S-2**:

^{1}H NMR (700 MHz, $CDCl_3$) δ 8.57 (d, J = 4.4 Hz, 1H), 7.95 (d, J = 9.2 Hz, 1H), 7.89 (d, J = 2.5 Hz, 1H), 7.54 (dd, J = 8.8, 2.6 Hz, 1H), 7.42 (d, J = 4.5 Hz, 1H), 7.32 (dd, J = 9.2, 2.7 Hz, 1H), 7.17 (d, J = 2.7 Hz, 1H), 6.87 (d, J = 8.8 Hz, 1H), 5.49 (d, J = 4.2 Hz, 1H), 3.88 (s, 3H), 3.52 – 3.39 (m, 1H), 3.12 (d, J = 14.6 Hz, 1H), 3.03 – 2.93 (m, 1H), 2.84 – 2.76 (m, 2H), 2.74 (d, J = 16.5 Hz, 1H), 2.68 (d, J = 16.5 Hz, 1H), 2.25 – 2.17 (m, 1H), 1.98 (ddd, J = 14.0, 7.5, 2.7 Hz, 1H), 1.94 – 1.85 (m, 1H), 1.56 – 1.45 (m, 1H), 1.42 (ddt, J = 13.5, 9.9, 3.4 Hz, 1H).

^{13}C NMR (176 MHz, CD_3OD) δ 192.55, 160.06, 159.66, 150.43, 148.17, 144.75, 140.14, 131.34, 129.52, 129.51, 128.11, 123.32, 121.86, 120.04, 114.65, 102.57, 82.44, 71.73, 64.61, 60.48, 56.45, 43.68, 30.17, 30.07, 22.75, 21.40.

HRMS (ESI) calcd for $C_{26}H_{25}N_2O_4Br$ $[M + H]^+$ 509.10705 and 511.10500, found 509.10676 and 511.10527.

$[\alpha]_D^{20}$ −157 (c 0.1, MeOH).

Major diastereomer **QN-C-R-2**:

^{1}H NMR (700 MHz, $CDCl_3$) δ 8.72 (d, J = 4.5 Hz, 1H), 8.04 (d, J = 9.2 Hz, 1H), 7.91 (d, J = 2.5 Hz, 1H), 7.50 (d, J = 4.5 Hz, 1H), 7.48 (dd, J = 8.8, 2.5 Hz, 1H), 7.38 (dd, J = 9.2, 2.7 Hz, 1H), 7.26 (s, 1H), 6.68 (d, J = 8.8 Hz, 1H), 5.56 (s, 1H), 3.92 (s, 3H), 3.53 – 3.39 (m, 2H), 3.23 – 3.14 (m, 1H), 2.86 (s, 2H), 2.77 (d, J = 14.7 Hz, 1H), 2.65 – 2.55 (m, 1H), 2.30 – 2.22 (m, 1H), 1.75 – 1.65 (m, 3H), 1.55 – 1.46 (m, 1H).

^{13}C NMR (176 MHz, $CDCl_3$) δ 191.03, 158.58, 158.01, 147.88, 147.20, 144.56, 139.15, 131.97, 129.14, 126.75, 122.23, 121.77, 120.49, 118.42, 114.07, 101.38, 81.80, 72.31, 63.92, 59.18, 55.82, 44.59, 42.97, 29.30, 22.43, 21.97.

HRMS (ESI) calcd for $C_{26}H_{25}N_2O_4Br$ $[M + H]^+$ 509.10705, found 509.10690. The dideuterated compound was also observed from exchange with CD_3OD during NMR analysis: HRMS (ESI) calcd for $C_{26}H_{23}D_2N_2O_4Br$ $[M + H]^+$ 511.12015 and 513.11810, found 511.11783 and 513.11648.

$[\alpha]_D^{20}$ −249 (c 0.1, MeOH).

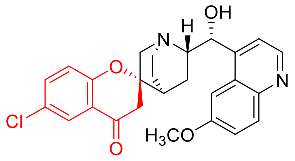

QN-C-S-3

Following the representative procedure for the quinine-based ketone, 7 mg (5%) of **QN-C-S-3** was isolated a brown solid. The other diastereomer was not isolated in pure form.

QN-C-S-3:

^{1}H NMR (700 MHz, CD_3OD) δ 8.90 (d, *J* = 5.2 Hz, 1H), 8.17 – 8.07 (m, 2H), 7.72 (d, *J* = 2.7 Hz, 1H), 7.69 (dd, *J* = 9.3, 2.5 Hz, 1H), 7.61 – 7.56 (m, 2H), 7.16 (d, *J* = 8.8 Hz, 1H), 6.21 (s, 1H), 4.24 (td, *J* = 11.7, 5.8 Hz, 1H), 4.05 (s, 3H), 3.85 – 3.75 (m, 2H), 3.59 (dd, *J* = 14.4, 2.6 Hz, 1H), 3.51 (td, *J* = 11.6, 6.4 Hz, 1H), 3.08 (d, *J* = 17.1 Hz, 1H), 2.92 (d, *J* = 17.1 Hz, 1H), 2.57 (t, *J* = 3.3 Hz, 1H), 2.43 (ddd, *J* = 14.1, 6.4, 3.4 Hz, 1H), 2.28 – 2.20 (m, 1H), 2.17 – 2.08 (m, 1H), 1.54 (ddt, *J* = 14.0, 10.9, 3.1 Hz, 1H).

^{13}C NMR (176 MHz, CD_3OD) δ 190.64, 162.95 (q, *J* = 35.5 Hz), 161.61, 158.47, 153.25, 144.59, 139.20, 137.69, 128.81, 128.42, 127.34, 126.91, 126.61, 122.87, 121.69, 120.97, 117.96 (q, *J* = 291.7 Hz), 102.75, 80.42, 67.83, 62.07, 60.78, 57.15, 45.12, 44.85, 29.61, 19.50, 19.32.

HRMS (ESI) calcd for $C_{26}H_{26}N_2O_4Cl$ $[M + H]^+$ 465.15756 and 467.15461, found 465.15867 and 467.16742.

$[\alpha]_D^{20}$ – 152 (*c* 0.1, MeOH).

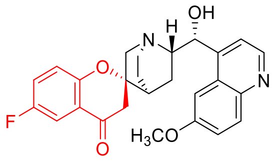

QN-C-S-4

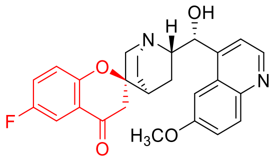

QN-C-R-3

Following the representative procedure for the quinine-based ketone, 44 mg (31%) of **QN-C-S-4** and 61 mg (43%) of **QN-C-R3** were isolated as brown solids.

Minor diastereomer **QN-C-S-4**:

^{1}H NMR (700 MHz, CD_3OD) δ 8.64 (d, J = 4.5 Hz, 1H), 7.91 (d, J = 9.2 Hz, 1H), 7.66 (d, J = 4.6 Hz, 1H), 7.41 (s, 1H), 7.38 – 7.35 (m, 1H), 7.34 (dd, J = 8.1, 2.8 Hz, 1H), 7.29 (td, J = 8.4, 2.8 Hz, 1H), 7.03 (dd, J = 9.0, 3.0 Hz, 1H), 5.58 (d, J = 3.6 Hz, 1H), 3.95 (s, 3H), 3.64 (t, J = 11.8 Hz, 1H), 3.14 (d, J = 14.7 Hz, 1H), 3.11 – 3.06 (m, 1H), 2.92 (d, J = 14.6 Hz, 1H), 2.87 – 2.80 (m, 1H), 2.70 (brs, 1H), 2.14 (brs, 1H), 2.08 – 1.99 (m, 1H), 1.97 – 1.88 (m, 1H), 1.67 – 1.58 (m, 1H), 1.37 – 1.29 (m, 1H).

^{13}C NMR (176 MHz, CD_3OD) δ 193.12, 159.65, 159.14, 157.58 (d, *J* = 68.6 Hz), 150.48, 148.17, 144.76 131.35, 128.13, 124.90 (d, *J* = 24.7 Hz), 123.31, 122.42 (d, *J* = 6.3 Hz), 121.46 (d, *J* = 7.3 Hz), 120.04, 111.96 (d, *J* = 23.7 Hz), 102.57, 82.21, 71.80, 64.66, 60.50, 56.43, 43.66, 30.14, 30.03, 22.80, 21.43.

^{19}F NMR (565 MHz, CD_3OD) δ -123.72 (tt, *J* = 8.0, 4.0 Hz).

HRMS (ESI) calcd for $C_{26}H_{25}N_2O_4F$ $[M + H]^+$ 449.18711, found 449.18685.

$[\alpha]_D^{20}$ −120 (*c* 0.1, MeOH).

Major diastereomer **QN-C-R3**:

^{1}H NMR (700 MHz, CD_3OD) δ 8.65 (d, *J* = 4.6 Hz, 1H), 7.94 (d, *J* = 9.3 Hz, 1H), 7.67 (d, *J* = 4.7 Hz, 1H), 7.45 (s, 1H), 7.41 (d, *J* = 9.4 Hz, 1H), 7.36 (dd, *J* = 8.2, 2.5 Hz, 1H), 7.15 (td, *J* = 7.7, 3.4 Hz, 1H), 6.69 (dd, *J* = 9.1, 4.2 Hz, 1H), 5.63 (d, *J* = 3.9 Hz, 1H), 3.95 (s, 3H), 3.77 – 3.68 (m, 1H), 3.48 – 3.41 (m, 1H), 3.18 (d, *J* = 14.7 Hz, 1H), 2.96 – 2.85 (m, 2H), 2.73 – 2.63 (m, 1H), 2.26 – 2.18 (m, 1H), 1.90 – 1.83 (m, 1H), 1.81 – 1.73 (m, 1H), 1.67 – 1.55 (m, 2H).

^{13}C NMR (176 MHz, CD_3OD) δ 192.96, 159.64, 158.47 (d, *J* = 240.7 Hz), 157.04, 150.37, 148.20, 144.77, 131.42, 128.15, 124.85 (d, *J* = 24.7 Hz), 123.35, 122.49 (d, *J* = 6.3 Hz), 121.29 (d, *J* = 7.4 Hz), 120.10, 111.95 (d, *J* = 23.5 Hz), 102.52, 82.38, 71.94, 63.99, 60.33, 56.45, 43.73, 30.32, 30.21, 22.58, 22.10.

^{19}F NMR (565 MHz, CD_3OD) δ -123.51 (tt, *J* = 8.1, 4.0 Hz).

HRMS (ESI) calcd for $C_{26}H_{25}N_2O_4F$ $[M + H]^+$ 449.18711, found 449.18682.

$[\alpha]_D^{20}$ −277 (*c* 0.1, MeOH).

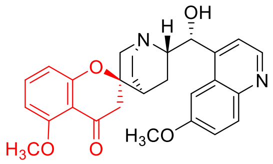

QN-C-R-4

Following the representative procedure for the quinine-based ketone, 34 mg (23%) of **QN-C-R-4** was isolated as an orange solid. The other diastereomer was not isolated in pure form.

QN-C-R-4:

^{1}H NMR (500 MHz, CD_3OD) δ 8.60 (d, *J* = 4.6 Hz, 1H), 7.92 (d, *J* = 9.2 Hz, 1H), 7.60 (d, *J* = 4.6 Hz, 1H), 7.34 (dd, *J* = 9.2, 2.7 Hz, 1H), 7.30 (d, *J* = 2.7 Hz, 1H), 7.26 (t, *J* = 8.4 Hz, 1H), 6.45 (d, *J* = 8.4 Hz, 1H), 6.23 (d, *J* = 8.3 Hz, 1H), 5.54 (d, *J* = 3.5 Hz, 1H), 4.25 – 4.00 (m, 2H), 3.88 (s, 3H), 3.80 (s, 3H), 3.71 – 3.58 (m, 1H), 3.39 – 3.28 (m, 1H), 3.20 – 3.05 (m, 1H), 2.82 – 2.74 (m, 2H), 2.66 – 2.51 (m, 1H), 2.30 – 2.21 (m, 1H), 1.88 – 1.68 (m, 2H), 1.62 – 1.48 (m, 2H).

^{13}C NMR (126 MHz, CD_3OD) δ 191.68, 161.59, 160.70, 158.42, 149.34, 147.48, 143.90, 136.94, 130.87, 127.11, 122.37, 119.10, 111.21, 110.65, 104.24, 101.70, 80.52, 71.15, 63.68, 59.21, 56.13, 56.03, 46.53, 43.11, 29.67, 22.22, 20.91.

HRMS (ESI) calcd for $C_{27}H_{28}N_2O_5$ $[M + H]^+$ 461.20710, found 461.20667.

$[\alpha]_D^{20}$ −253 (*c* 0.1, MeOH)

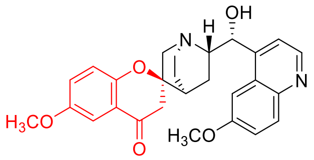

QN-C-R-5

Following the representative procedure for the quinine-based ketone, 50 mg (34%) of **QN-C-R-5** was isolated as a yellow solid. The other diastereomer was not isolated in pure form.

QN-C-R-5:

^{1}H NMR (500 MHz, $CO(CD_3)_2$) δ 8.67 (d, J = 4.5 Hz, 1H), 7.95 (d, J = 9.2 Hz, 1H), 7.58 (dd, J = 6.4, 3.6 Hz, 2H), 7.35 (dd, J = 9.2, 2.7 Hz, 1H), 7.17 (d, J = 3.2 Hz, 1H), 7.06 (dd, J = 9.0, 3.2 Hz, 1H), 6.75 (d, J = 9.0 Hz, 1H), 5.64 (s, 1H), 3.93 (s, 3H), 3.75 (s, 3H), 3.61 – 3.48 (m, 2H), 3.08 (d, J = 14.5 Hz, 1H), 2.87 (d, J = 14.4 Hz, 1H), 2.79 (d, J = 14.5 Hz, 1H), 2.68 – 2.54 (m, 1H), 2.28 – 2.18 (m, 1H), 2.09 – 2.06 (m, 2H), 1.90 – 1.54 (m, 4H).

^{13}C NMR (126 MHz, $CO(CD_3)_2$) δ 192.20, 158.38, 154.79, 154.71, 149.50, 148.31, 145.29, 132.26, 127.92, 125.19, 122.07, 121.76, 120.35, 119.71, 107.91, 102.78, 81.78, 71.82, 63.65, 60.60, 56.11, 55.91, 49.62, 44.95, 42.94, 23.49, 22.72.

HRMS (ESI) calcd for $C_{27}H_{28}N_2O_5$ $[M + H]^+$ 461.20710, found 461.20681.

$[\alpha]_D^{20}$ −322 (c 0.1, MeOH).

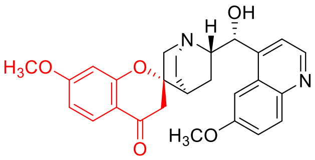

QN-C-S-5

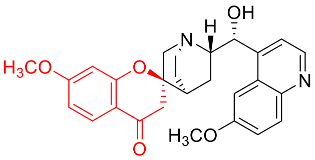

QN-C-R-6

Following the representative procedure for the quinine-based ketone, 33 mg (22%) of **QN-C-S-5** and 60 mg (41%) of **QN-C-R-6** were isolated as brown solids.

Minor diastereomer **QN-C-S-5**:

^{1}H NMR (500 MHz, CD_2Cl_2) δ 8.47 (s, 1H), 7.86 (d, *J* = 9.5 Hz, 1H), 7.66 (d, *J* = 8.8 Hz, 1H), 7.40 (d, *J* = 4.6 Hz, 1H), 7.26 (d, *J* = 7.5 Hz, 2H), 6.51 (dd, *J* = 8.8, 2.4 Hz, 1H), 6.41 (d, *J* = 2.4 Hz, 1H), 5.45 (d, *J* = 4.9 Hz, 1H), 3.86 (s, 3H), 3.81 (s, 3H), 3.44 – 3.29 (m, 1H), 3.07 – 2.94 (m, 2H), 2.84 – 2.57 (m, 4H), 2.20 (s, 1H), 2.00 – 1.86 (m, 2H), 1.60 – 1.39 (m, 2H).

^{13}C NMR (126 MHz, CD_2Cl_2) δ 190.10, 166.24, 161.68, 157.56, 147.70, 147.24, 143.96, 131.14, 127.78, 126.54, 121.17, 118.42, 114.57, 109.20, 101.42, 101.27, 81.06, 71.39, 63.93, 59.57, 55.56, 55.51, 45.57, 42.30, 29.25, 23.39, 20.65.

HRMS (ESI) calcd for $C_{27}H_{28}N_2O_5$ $[M + H]^+$ 461.20710, found 461.20658.

$[\alpha]_D^{20}$ −128 (*c* 0.1, MeOH).

Major diastereomer **QN-C-R-6**:

^{1}H NMR (500 MHz, CD_2Cl_2) δ 8.49 (d, *J* = 4.5 Hz, 1H), 7.87 (d, *J* = 9.0 Hz, 1H), 7.66 (d, *J* = 8.8 Hz, 1H), 7.46 (d, *J* = 4.5 Hz, 1H), 7.30 – 7.21 (m, 2H), 6.49 (dd, *J* = 8.7, 2.4 Hz, 1H), 6.18 (d, *J* = 2.4 Hz, 1H), 5.53 (d, *J* = 5.2 Hz, 1H), 3.83 (s, 3H), 3.70 (s, 3H), 3.54 – 3.39 (m, 2H), 3.08 (d, *J* = 14.6 Hz, 1H), 2.79 – 2.65 (m, 3H), 2.58 – 2.48 (m, 1H), 2.24 (s, 1H), 1.83 – 1.74 (m, 1H), 1.73 – 1.63 (m, 2H), 1.56 – 1.43 (m, 1H).

^{13}C NMR (126 MHz, CD_2Cl_2) δ 189.99, 166.24, 161.41, 157.47, 147.91, 147.27, 143.89, 131.05, 127.77, 126.63, 121.21, 118.58, 114.58, 109.38, 101.46, 101.11, 81.19, 71.08, 63.15, 59.29, 55.52, 44.32, 42.48, 29.49, 22.17, 22.12. A methoxy carbon could not be detected in the ^{13}C spectrum; however, HSQC showed that the ^{13}C peak at 55.52 ppm corresponded to a methoxy ^{1}H peak at 3.84 ppm and a second methoxy ^{1}H peak at 3.71 ppm.

HRMS (ESI) calcd for $C_{27}H_{28}N_2O_5$ $[M + H]^+$ 461.20710, found 461.20657.

$[\alpha]_D^{20}$ −367 (*c* 0.1, MeOH).

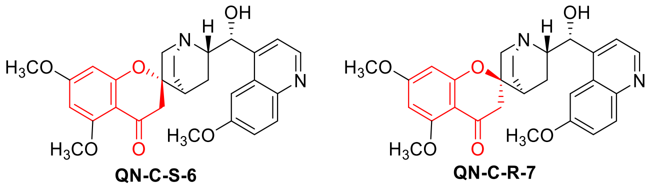

Following the representative procedure for the quinine-based ketone, 23 mg (29%) of **QN-C-S-6** and 40 mg (51%) of **QN-C-R-7** were isolated as light brown solids.

Minor diastereomer **QN-C-S-6**:

The NMR sample for this spectra was solvated in CD_3OD:CD_2Cl_2 (3:1) and referenced according to CD_3OD. **^{1}H NMR** (500 MHz, CD_3OD) δ 8.65 (d, *J* = 4.6 Hz, 1H), 7.96 (d, *J* = 9.2 Hz, 1H), 7.67 (d, *J* = 4.6 Hz, 1H), 7.49 – 7.27 (m, 2H), 6.15 (d, *J* = 2.3 Hz, 1H), 6.10 (d, *J* = 2.3 Hz, 1H), 5.63 (d, *J* = 3.1 Hz, 1H), 3.97 (s, 3H), 3.84 (s, 3H), 3.80 (s, 3H), 3.77 – 3.66 (m, 1H), 3.17 (d, *J* = 14.4 Hz, 1H), 3.10 – 3.02 (m, 1H), 2.96 – 2.84 (m, 2H), 2.71 (d, *J* = 16.3 Hz, 1H), 2.60 (d, *J* = 16.3 Hz, 1H), 2.32 – 2.24 (m, 1H), 2.15 – 2.07 (m, 1H), 2.07 – 1.98 (m, 1H), 1.73 – 1.61 (m, 1H), 1.42 – 1.32 (m, 1H).

^{13}C NMR (176 MHz, CD_3OD) δ 190.74, 167.87, 164.18, 162.91, 159.25, 149.61, 147.79, 144.39, 131.19, 127.58, 122.94, 119.55, 106.07, 102.15, 95.14, 93.37, 80.72, 71.10, 64.31, 60.12, 56.41, 56.23, 56.21, 47.47, 43.55, 30.20, 22.24, 20.96.

HRMS (ESI) calcd for $C_{28}H_{30}N_2O_6$ $[M + H]^+$ 491.21766, found 491.21709.

$[\alpha]^{20}_{D}$ −113 (*c* 0.1, MeOH).

Major diastereomer **QN-C-R-7**:

^{1}H NMR (500 MHz, CD_2Cl_2) δ 8.57 (d, *J* = 4.5 Hz, 1H), 7.88 (d, *J* = 9.9 Hz, 1H), 7.49 (d, *J* = 4.5 Hz, 1H), 7.24 (d, *J* = 8.0 Hz, 2H), 5.98 (d, *J* = 2.3 Hz, 1H), 5.79 (d, *J* = 2.3 Hz, 1H), 5.61 (s, 1H), 3.79 (s, 3H), 3.77 (s, 3H), 3.66 (s, 3H), 3.57 – 3.39 (m, 2H), 3.07 (d, *J* = 14.5 Hz, 1H), 2.74 – 2.62 (m, 3H), 2.61 – 2.51 (m, 1H), 2.24 (s, 1H), 1.82 – 1.65 (m, 3H), 1.57 – 1.46 (m, 1H).

^{13}C NMR (176 MHz, CD_2Cl_2) δ 187.98, 166.13, 162.72, 161.85, 157.66, 147.47, 147.34, 144.19, 131.37, 126.61, 121.37, 118.68, 105.60, 101.43, 93.88, 92.69, 80.15, 70.74, 62.80, 59.38, 55.86, 55.74, 55.56, 46.02, 42.69, 29.76, 21.94, 21.76.

HRMS (ESI) calcd for $C_{28}H_{30}N_2O_6$ $[M + H]^+$ 491.21766, found 491.21700.

$[\alpha]^{20}_{D}$ −132 (*c* 0.1, MeOH).

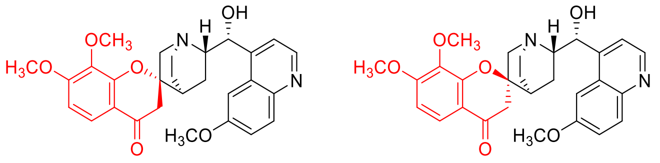

QN-C-S-7 QN-C-R-8

Following the representative procedure for the quinine-based ketone, 21 mg (27%) of **QN-C-S-7** and 33 mg (42%) of **QN-C-R-8** were isolated as brown solids.

Minor diastereomer **QN-C-S-7**:

The NMR sample for these spectra was solvated in CD_3OD:CD_2Cl_2 (3:1) and referenced according to CD_3OD. **^{1}H NMR** (700 MHz, CD_3OD) δ 8.65 (d, J = 4.6 Hz, 1H), 7.96 (d, J = 9.0 Hz, 1H), 7.67 (d, J = 4.3 Hz, 1H), 7.55 (d, J = 8.9 Hz, 1H), 7.40 (dd, J = 11.2, 2.2 Hz, 2H), 6.70 (d, J = 8.9 Hz, 1H), 5.61 (d, J = 3.3 Hz, 1H), 3.97 (s, 3H), 3.91 (s, 3H), 3.86 (s, 3H), 3.73 – 3.66 (m, 1H), 3.22 (d, J = 14.6 Hz, 1H), 3.10 – 3.03 (m, 1H), 2.96 – 2.88 (m, 2H), 2.78 (d, J = 16.6 Hz, 1H), 2.70 (d, J = 16.6 Hz, 1H), 2.30 – 2.25 (m, 1H), 2.16 – 2.06 (m, 2H), 1.71 – 1.63 (m, 1H), 1.43 – 1.36 (m, 1H).

^{13}C NMR (176 MHz, CD_3OD) δ 191.50, 159.48, 158.30, 153.56, 148.84, 146.84, 143.45, 137.35, 130.21, 126.69, 122.28, 121.99, 118.62, 115.75, 105.51, 101.23, 81.14, 70.28, 63.54, 60.38, 59.20, 55.69, 55.39, 45.32, 42.56, 29.66, 21.48, 20.18.

HRMS (ESI) calcd for $C_{28}H_{30}N_2O_6$ $[M + H]^+$ 491.21766, found 491.21716.

$[\alpha]_D^{20}$ −85 (c 0.1, MeOH).

Major diastereomer **QN-C-R-8**:

^{1}H NMR (700 MHz, CD_2Cl_2) δ 8.50 (d, J = 4.4 Hz, 1H), 7.83 (d, J = 9.0 Hz, 1H), 7.43 – 7.39 (m, 2H), 7.22 – 7.16 (m, 2H), 5.60 (s, 1H), 3.74 (s, 3H), 3.73 (s, 3H), 3.58 – 3.44 (m, 2H), 3.12 (d, J = 14.5 Hz, 1H), 3.05 (s, 3H), 2.71 – 2.65 (m, 2H), 2.63 (d, J = 14.7 Hz, 1H), 2.57 – 2.50 (m, 1H), 2.17 (s, 1H), 1.68 – 1.57 (m, 3H), 1.48 – 1.38 (m, 1H).

^{13}C NMR (176 MHz, CD_2Cl_2) δ 190.66, 159.53, 158.12, 153.60, 147.84, 147.62, 144.59, 137.62, 131.80, 126.90, 122.42, 121.71, 118.99, 116.37, 105.80, 101.94, 81.89, 71.67, 63.40, 60.33, 59.63, 56.43, 56.11, 44.45, 43.15, 30.17, 22.41, 22.34.

HRMS (ESI) calcd for $C_{28}H_{30}N_2O_6$ $[M + H]^+$ 491.21766, found 491.21710.

$[\alpha]_D^{20}$ −295 (c 0.1, MeOH).

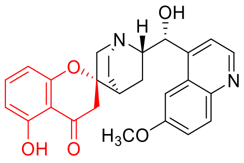

QN-C-S-8

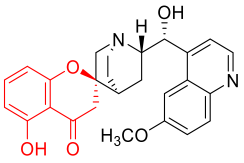

QN-C-R-9

Following the representative procedure for the quinine-based ketone, 23 mg (16%) of **QN-C-S-8** and 61 mg (43%) of **QN-C-R-9** were isolated as tan solids.

Minor diastereomer **QN-C-S-8**:

^{1}H NMR (700 MHz, CD_3OD) δ 8.66 (d, J = 3.2 Hz, 1H), 7.94 (d, J = 9.2 Hz, 1H), 7.68 (d, J = 4.4 Hz, 1H), 7.45 (s, 1H), 7.41 (d, J = 9.2 Hz, 1H), 7.38 (t, J = 8.3 Hz, 1H), 6.47 (d, J = 8.2 Hz, 1H), 6.43 (d, J = 8.3 Hz, 1H), 5.59 (d, J = 3.7 Hz, 1H), 3.97 (s, 3H), 3.69 – 3.60 (m, 1H), 3.17 – 3.08 (m, 2H), 2.93 (d, J = 14.8 Hz, 1H), 2.88 – 2.81 (m, 1H), 2.25 (s, 1H), 2.14 – 2.06 (m, 1H), 2.02 – 1.93 (m, 1H), 1.70 – 1.62 (m, 1H), 1.48 – 1.39 (m, 1H). Two aliphatic protons are missing from the spectra. This is most likely due to exchange of the α-carbonyl protons with deuterium.

^{13}C NMR (176 MHz, CD_3OD) δ 199.61, 162.84, 161.22, 159.72, 150.50, 148.19, 144.83, 139.58, 131.38, 128.21, 123.34, 120.11, 109.99, 109.04, 109.03, 102.62, 81.54, 71.83, 64.64, 60.60, 56.45, 43.65, 30.50, 23.04, 21.46. An aliphatic carbon is missing from the spectra. This is most likely due to exchange of the a-carbonyl protons with deuterium, resulting in a weak carbon signal.

HRMS (ESI) calcd for $C_{26}H_{26}N_2O_5$ $[M + H]^+$ 447.19145, found 447.19102

$[\alpha]_D^{20}$ –120 (c 0.1, MeOH).

Major diastereomer **QN-C-R-9**:

^{1}H NMR (500 MHz, CD_3OD) δ 8.64 (d, J = 4.6 Hz, 1H), 7.94 (d, J = 9.2 Hz, 1H), 7.66 (d, J = 4.6 Hz, 1H), 7.49 – 7.36 (m, 2H), 7.21 (t, J = 8.3 Hz, 1H), 6.36 (d, J = 8.3 Hz, 1H), 6.06 (d, J = 8.2 Hz, 1H), 5.60 (d, J = 3.9 Hz, 1H), 3.94 (s, 3H), 3.77 – 3.64 (m, 1H), 3.46 – 3.37 (m, 1H), 3.13 (d, J = 14.5 Hz, 1H), 2.93 – 2.81 (m, 1H), 2.72 – 2.58 (m, 1H), 2.23 (s, 1H), 1.89 – 1.68 (m, 2H), 1.68 – 1.52 (m, 2H). Two aliphatic protons are missing from the spectra. This is most likely due to exchange of the α-carbonyl protons with deuterium.

^{13}C NMR (126 MHz, CD_3OD) δ 198.13, 161.38, 159.42, 158.18, 149.09, 146.76, 143.31, 138.08, 129.96, 126.73, 121.97, 118.67, 108.65, 107.60, 107.39, 101.02, 80.34, 70.67, 62.53, 58.78, 54.99, 42.24, 29.35, 21.35, 20.73. An aliphatic carbon is missing from the spectra. This is most likely due to exchange of the a-carbonyl protons with deuterium, resulting in a weak carbon signal.

HRMS (ESI) calcd for $C_{26}H_{26}N_2O_5$ $[M + H]^+$ 447.19145, found 447.19095

$[\alpha]_D^{20}$ –351 (c 0.1, MeOH).

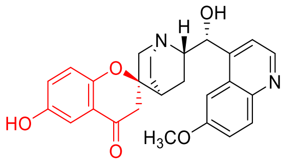

QN-C-R-10

Following the representative procedure for the quinine-based ketone, 48 mg (34%) of **QN-C-R-10** was isolated as a yellow solid. The other diastereomer was not isolated in pure form.

QN-C-R-10:

^{1}H NMR (700 MHz, CD_3OD) δ 8.65 (d, J = 4.4 Hz, 1H), 7.96 (d, J = 9.2 Hz, 1H), 7.66 (d, J = 4.5 Hz, 1H), 7.45 (s, 1H), 7.42 (d, J = 9.2 Hz, 1H), 7.09 (d, J = 2.0 Hz, 1H), 6.88 (dd, J = 8.8, 2.3 Hz, 1H), 6.51 (d, J = 8.9 Hz, 1H), 5.59 (d, J = 4.0 Hz, 1H), 3.95 (s, 3H), 3.74 – 3.60 (m, 1H), 3.42 (td, J = 8.8, 3.8 Hz, 1H), 3.12 (d, J = 14.6 Hz, 1H), 2.81 (d, J = 14.6 Hz, 1H), 2.69 – 2.55 (m, 1H), 2.21 (s, 1H), 1.86 – 1.79 (m, 1H), 1.74 (ddt, J = 14.4, 9.4, 4.3 Hz, 1H), 1.68 – 1.53 (m, 2H). Two aliphatic protons are missing from the spectra. This is most likely due to exchange of the α-carbonyl protons with deuterium.

^{13}C NMR (176 MHz, CD_3OD) δ 194.38, 159.61, 154.37, 152.85, 150.60, 148.20, 144.78, 131.39, 128.21, 126.03, 123.36, 122.23, 120.30, 120.09, 111.00, 102.54, 81.63, 72.28, 64.21, 60.23, 56.40, 43.71, 30.36, 22.78, 22.32. An aliphatic carbon is missing from the spectra. This is most likely due to exchange of the a-carbonyl protons with deuterium, resulting in a weak carbon signal.

HRMS (ESI) calcd for $C_{26}H_{26}N_2O_5$ $[M + H]^+$ 447.19145, found 447.19090.

$[\alpha]_D^{20}$ –415 (c 0.1, MeOH).

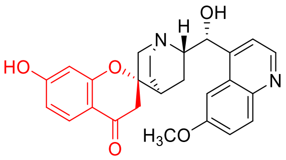

QN-C-S-9

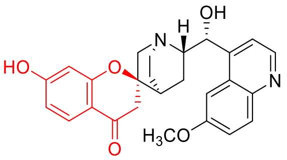

QN-C-R-11

Following the representative procedure for the quinine-based ketone, 31 mg (22%) of **QN-C-S-9** and 40 mg (48%) of **QN-C-R-11** were isolated as brown solids.

211-1, 211-2

Minor isomer **QN-C-S-9**:

^{1}H NMR (700 MHz, CD_3OD) δ 8.69 (d, J = 4.4 Hz, 1H), 7.96 (d, J = 9.2 Hz, 1H), 7.74 (d, J = 4.4 Hz, 1H), 7.61 (d, J = 8.6 Hz, 1H), 7.46 (s, 1H), 7.43 (d, J = 9.2 Hz, 1H), 6.48 (d, J = 8.6 Hz, 1H), 6.39 (s, 1H), 5.86 (s, 1H), 4.05 – 3.88 (m, 4H), 3.46 – 3.36 (m, 2H), 3.22 (d, J = 14.4 Hz, 1H), 3.18 – 3.07 (m, 1H), 2.36 (s, 1H), 2.28 – 2.19 (m, 1H), 2.16 – 2.07 (m, 1H), 1.92 – 1.82 (m, 1H), 1.50 – 1.40 (m, 1H). Two aliphatic protons are missing from the spectra. This is most likely due to exchange of the α-carbonyl protons with deuterium.

^{13}C NMR (176 MHz, CD_3OD) δ 191.84, 167.23, 162.89, 160.00, 148.82, 148.16, 144.79, 131.49, 129.48, 127.86, 123.53, 120.30, 114.79, 111.89, 104.43, 102.50, 80.90, 69.90, 63.56, 60.82, 56.83, 44.44, 30.21, 21.40, 20.58. An aliphatic carbon is missing from the spectra. This is most likely due to exchange of the a-carbonyl protons with deuterium, resulting in a weak carbon signal.

HRMS (ESI) calcd for $C_{26}H_{26}N_2O_5$ $[M + H]^+$ 447.19145, found 447.19102

$[\alpha]_D^{20}$ –59.2 (*c* 0.5, DMSO).

Major isomer **QN-C-R-11**:

^{1}H NMR (500 MHz, CD_3OD) δ 8.69 (d, J = 4.6 Hz, 1H), 7.98 (d, J = 9.2 Hz, 1H), 7.72 (d, J = 4.6 Hz, 1H), 7.61 (d, J = 8.7 Hz, 1H), 7.48 (d, J = 2.6 Hz, 1H), 7.45 (dd, J = 9.2, 2.6 Hz, 1H), 6.41 (dd, J = 8.7, 2.2 Hz, 1H), 5.91 (d, J = 2.2 Hz, 1H), 5.88 (d, J = 3.1 Hz, 1H), 4.05 – 3.92 (m, 4H), 3.68 – 3.57 (m, 1H), 3.40 (d, J = 15.2 Hz, 1H), 3.10 (d, J = 14.4 Hz, 1H), 2.99 – 2.87 (m, 1H), 2.35 (s, 1H), 2.06 – 1.95 (m, 1H), 1.95 – 1.84 (m, 1H), 1.80 – 1.66 (m, 1H), 1.66 – 1.54 (m, 1H). Two aliphatic protons are missing from the spectra. This is most likely due to exchange of the α-carbonyl protons with deuterium.

^{13}C NMR (126 MHz, CD_3OD) δ 190.41, 165.66, 161.21, 158.50, 147.71, 146.77, 143.29, 130.07, 128.04, 126.43, 122.21, 118.84, 113.39, 110.47, 102.62, 100.86, 80.02, 69.15, 61.72, 59.00, 55.33, 42.82, 29.13, 20.38, 19.75. An aliphatic carbon is missing from the spectra. This is most likely due to exchange of the a-carbonyl protons with deuterium, resulting in a weak carbon signal.

HRMS (ESI) calcd for $C_{26}H_{26}N_2O_5$ $[M + H]^+$ 447.19145, found 447.19098

$[\alpha]_D^{20}$ –231 (*c* 0.1, MeOH).

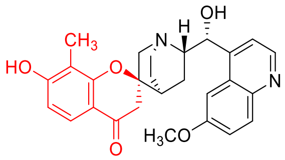

QN-C-R-12

Following the representative procedure for the quinine-based ketone, 27 mg (37%) of **QN-C-R-12** was isolated as a brown solid. The other diastereomer was not isolated in pure form.

Major diastereomer **QN-C-R-12**:

^{1}H NMR (700 MHz, CD_3OD) δ 8.68 (d, J = 4.6 Hz, 1H), 7.99 (d, J = 9.0 Hz, 1H), 7.70 (d, J = 4.5 Hz, 1H), 7.47 – 7.45 (m, 2H), 6.39 (d, J = 8.6 Hz, 1H), 5.74 (d, J = 3.3 Hz, 1H), 3.97 (s, 3H), 3.91 – 3.81 (m, 1H), 3.66 – 3.56 (m, 1H), 3.25 (d, J = 14.4 Hz, 1H), 2.91 (d, J = 14.5 Hz, 1H), 2.87 – 2.74 (m, 2H), 2.22 (s, 1H), 1.89 – 1.82 (m, 1H), 1.80 – 1.73 (m, 1H), 1.66 – 1.58 (m, 1H), 1.35 – 1.25 (m, 1H), 1.15 (s, 3H). An aliphatic proton is missing from the spectra. This is most likely due to exchange of an α-carbonyl proton with deuterium.

^{13}C NMR (176 MHz, CD_3OD) δ 193.24, 164.46, 160.40, 159.77, 150.01, 148.20, 144.82, 131.51, 127.87, 126.01, 123.38, 119.95, 114.86, 112.94, 110.36, 102.50, 82.14, 72.18, 63.79, 59.95, 56.46, 44.11, 30.81, 30.74, 22.54, 21.92. An aliphatic carbon is missing from the spectra. This is most likely due to exchange of the a-carbonyl protons with deuterium, resulting in a weak carbon signal.

HRMS (ESI) calcd for $C_{27}H_{28}N_2O_5$ $[M + H]^+$ 461.20710, found 461.20689;

$[\alpha]^{20}_{D}$ –94 (c 0.75, MeOH).

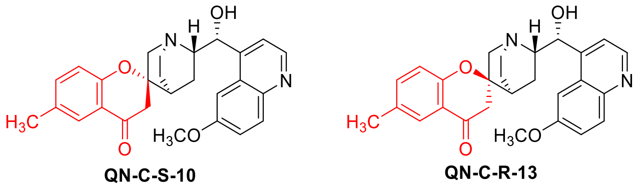

Following the representative procedure for the quinine-based ketone, 38 mg (27%) of **QN-C-S-10** and 50 mg (35%) of **QN-C-R-13** were isolated as brown solids.

Minor diastereomer **QN-C-S-10**:

^{1}H NMR (700 MHz, CD_2Cl_2) δ 8.48 (d, *J* = 4.4 Hz, 1H), 7.88 (d, *J* = 9.8 Hz, 1H), 7.55 (d, *J* = 2.3 Hz, 1H), 7.41 (d, *J* = 4.5 Hz, 1H), 7.30 (d, *J* = 8.5 Hz, 1H), 7.28 – 7.25 (m, 2H), 6.87 (d, *J* = 8.5 Hz, 1H), 5.48 (d, *J* = 4.9 Hz, 1H), 3.87 (s, 3H), 3.43 – 3.32 (m, 1H), 3.09 – 2.97 (m, 2H), 2.81 – 2.62 (m, 4H), 2.28 (s, 3H), 2.21 – 2.19 (m, 1H), 1.98 – 1.90 (m, 2H), 1.56 – 1.40 (m, 2H).

^{13}C NMR (176 MHz, CD_2Cl_2) δ 191.82, 157.61, 157.56, 147.71, 147.17, 143.91, 137.11, 131.06, 130.47, 126.49, 125.65, 121.13, 120.31, 118.36, 117.95, 101.44, 80.42, 71.34, 63.91, 59.60, 55.48, 45.86, 42.29, 28.95, 23.42, 23.11, 19.92.

HRMS (ESI) calcd for $C_{27}H_{28}N_2O_4$ $[M + H]^+$ 445.21218, found 445.21173.

$[\alpha]_D^{20}$ −131 (*c* 0.1, MeOH).

Major diastereomer **QN-C-R-13**:

^{1}H NMR (700 MHz, CD_2Cl_2) δ 8.49 (d, *J* = 4.5 Hz, 1H), 7.88 (d, *J* = 9.2 Hz, 1H), 7.54 (s, 1H), 7.43 (d, *J* = 4.5 Hz, 1H), 7.29 (d, *J* = 2.7 Hz, 1H), 7.26 (dd, *J* = 9.2, 2.6 Hz, 1H), 7.20 (dd, *J* = 8.5, 2.3 Hz, 1H), 6.65 (d, *J* = 8.4 Hz, 1H), 5.50 (d, *J* = 5.4 Hz, 1H), 3.84 (s, 3H), 3.49 – 3.37 (m, 2H), 3.08 (dd, *J* = 14.7, 2.3 Hz, 1H), 2.82 – 2.73 (m, 2H), 2.66 (d, *J* = 14.6 Hz, 1H), 2.55 – 2.46 (m, 1H), 2.27 – 2.18 (m, 4H), 1.81 – 1.73 (m, 1H), 1.71 – 1.60 (m, 2H), 1.53 – 1.45 (m, 1H).

^{13}C NMR (176 MHz, CD_2Cl_2) δ 192.33, 158.03, 157.93, 148.40, 147.75, 144.48, 137.66, 131.57, 131.13, 127.19, 126.19, 121.75, 120.92, 119.11, 118.43, 102.06, 81.17, 71.85, 63.70, 59.81, 56.02, 45.11, 42.99, 29.66, 22.88, 22.55, 20.45.

HRMS (ESI) calcd for $C_{27}H_{28}N_2O_4$ $[M + H]^+$ 445.21218, found 445.21168.

$[\alpha]_D^{20}$ −280 (*c* 0.1, MeOH).

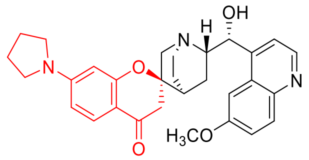

QN-C-R-14

Following the representative procedure for the quinine-based ketone employing 4-fluoro-2-hydroxyacetophenone, 33 mg (22%) of an S_NAr product **QN-C-R-14** was isolated. The other diastereomer was not isolated in pure form.

Major diastereomer **QN-C-R-14**:

^{1}H NMR (400 MHz,CD_2Cl_2) δ 8.56 (d, J = 4.5 Hz, 1H), 7.92 (d, J = 9.0 Hz, 1H), 7.57 (d, J = 8.8 Hz, 1H), 7.47 (d, J = 4.5 Hz, 1H), 7.36 – 7.24 (m, 2H), 6.16 (dd, J = 8.8, 2.3 Hz, 1H), 5.73 (d, J = 2.3 Hz, 1H), 5.46 (d, J = 5.5 Hz, 1H), 3.86 (s, 3H), 3.53 – 3.41 (m, 1H), 3.41 – 3.28 (m, 1H), 3.28 – 3.15 (m, 4H), 3.03 (d, J = 14.7 Hz, 1H), 2.73 – 2.57 (m, 3H), 2.54 – 2.43 (m, 1H), 2.21 (brs, 1H), 2.03 – 1.91 (m, 4H), 1.91 – 1.80 (m, 1H), 1.70 – 1.55 (m, 2H), 1.55 – 1.40 (m, 1H).

^{13}C NMR (101 MHz, CD_2Cl_2) δ 189.55, 161.76, 157.95, 154.06, 148.56, 147.88, 144.60, 131.69, 128.16, 127.30, 121.66, 119.09, 110.63, 106.88, 102.12, 98.14, 81.04, 72.20, 63.93, 59.78, 55.94, 48.06, 44.94, 42.95, 30.53, 25.69, 23.21, 23.06.

HRMS (ESI) calcd for $C_{30}H_{33}N_3O_4$ $[M + H]^+$ 500.25438, found 500.25362.

$[\alpha]_D^{20}$ –368 (c 0.1, MeOH).

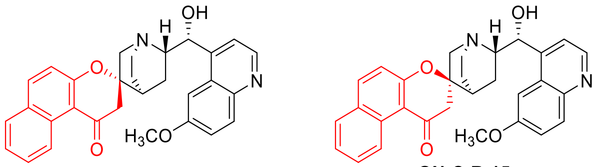

QN-C-S-11 **QN-C-R-15**

Following the representative procedure for the quinine-based ketone, 40 mg (26%) of **QN-C-S-11** and 59 mg (38%) of **QN-C-R-15** were isolated as brown solids.

Minor diastereomer **QN-C-S-11**:

^{1}H NMR (700 MHz, CD_2Cl_2) δ 9.31 (d, J = 8.7 Hz, 1H), 8.49 (d, J = 4.5 Hz, 1H), 7.92 (d, J = 9.0 Hz, 1H), 7.87 (d, J = 9.1 Hz, 1H), 7.74 (d, J = 8.1 Hz, 1H), 7.57 (t, J = 7.8 Hz, 1H), 7.42 (d, J = 4.5 Hz, 1H), 7.39 (t, J = 7.5 Hz, 1H), 7.29 – 7.24 (m, 2H), 7.10 (d, J = 9.0 Hz, 1H), 5.48 (d, J = 4.9 Hz, 1H), 3.86 (s, 3H), 3.45 – 3.36 (m, 1H), 3.10 – 3.02 (m, 2H), 2.88 – 2.75 (m, 3H), 2.77 – 2.71 (m, 1H), 2.33 (s, 1H), 2.02 – 1.93 (m, 2H), 1.64 – 1.54 (m, 1H), 1.54 – 1.47 (m, 1H).

^{13}C NMR (176 MHz, CD_2Cl_2) δ 193.29, 162.23, 158.13, 148.27, 147.74, 144.47, 137.87, 131.63, 131.52, 129.85, 129.39, 128.72, 127.07, 125.67, 125.00, 121.69, 119.63, 118.94, 112.48, 102.00, 81.39, 71.87, 64.22, 60.08, 56.03, 47.56, 42.85, 29.46, 23.62, 21.04.

HRMS (ESI) calcd for $C_{30}H_{28}N_2O_4$ $[M + H]^+$ 481.21218, found 481.21161.

$[\alpha]_D^{20}$ −172 (c 0.1, MeOH).

Major diastereomer **QN-C-R-15**:

^{1}H NMR (700 MHz, CD_2Cl_2) δ 9.33 (d, J = 8.7 Hz, 1H), 8.49 (d, J = 4.5 Hz, 1H), 7.87 (d, J = 9.2 Hz, 1H), 7.82 (d, J = 9.0 Hz, 1H), 7.70 (d, J = 8.1 Hz, 1H), 7.57 (t, J = 7.8 Hz, 1H), 7.46 (d, J = 4.5 Hz, 1H), 7.38 (t, J = 7.5 Hz, 1H), 7.30 (s, 1H), 7.25 (d, J = 9.3 Hz, 1H), 6.85 (d, J = 8.9 Hz, 1H), 5.58 (s, 1H), 3.82 (s, 3H), 3.57 – 3.44 (m, 2H), 3.20 – 3.11 (m, 1H), 2.98 – 2.84 (m, 2H, 2.78 (d, J = 14.8 Hz, 1H), 2.62 – 2.52 (m, 1H), 2.36 (brs, 1H), 1.84 – 1.67 (m, 3H), 1.60 – 1.52 (m, 1H).

^{13}C NMR (176 MHz, CD_2Cl_2) δ 192.75, 161.50, 157.70, 147.96, 147.33, 144.01, 137.57, 131.13, 131.08, 129.53, 129.04, 128.35, 126.74, 125.32, 124.69, 121.42, 119.06, 118.75, 112.19, 101.62, 81.08, 71.11, 63.08, 59.46, 55.71, 45.87, 42.63, 29.13, 22.07, 21.97.

HRMS (ESI) calcd for $C_{30}H_{28}N_2O_4$ $[M + H]^+$ 481.21218, found 481.481.21166.

$[\alpha]_D^{20}$ −366 (c 0.1, MeOH).

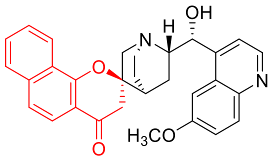

QN-C-R-16

Following the representative procedure for the quinine-based ketone, 72 mg (47%) of **QN-C-R-16** was isolated as an orange-red solid. The other diastereomer was not isolated in pure form.

Major diastereomer **QN-C-R-16**:

^{1}H NMR (700 MHz, CD_2Cl_2) δ 8.48 (d, J = 4.5 Hz, 1H), 7.99 (d, J = 9.7 Hz, 1H), 7.71 (t, J = 7.7 Hz, 2H), 7.50 – 7.42 (m, 3H), 7.32 (d, J = 8.0 Hz, 3H), 6.96 (t, J = 7.6 Hz, 1H), 5.65 (d, J = 4.7 Hz, 1H), 3.75 (s, 3H), 3.73 – 3.68 (m, 1H), 3.65 – 3.56 (m, 1H), 3.30 (dd, J = 14.8, 2.3 Hz, 1H), 2.96 – 2.85 (m, 2H), 2.79 (d, J = 14.7 Hz, 1H), 2.67 – 2.57 (m, 1H), 2.35 (s, 1H), 1.73 – 1.67 (m, 2H), 1.63 – 1.57 (m, 1H), 1.57 – 1.49 (m, 1H).

^{13}C NMR (176 MHz, CD_2Cl_2) δ 191.48, 158.09, 157.77, 148.06, 147.78, 144.53, 138.00, 131.76, 129.82, 128.23, 126.90, 126.65, 125.30, 122.70, 121.81, 121.43, 121.14, 119.09, 115.84, 102.02, 83.03, 72.23, 63.67, 59.55, 55.91, 44.16, 43.19, 30.30, 22.84, 22.59.

HRMS (ESI) calcd for $C_{30}H_{28}N_2O_4$ $[M + H]^+$ 481.21218, found 481.21158.

$[\alpha]_D^{20}$ −320 (c 0.1, MeOH).

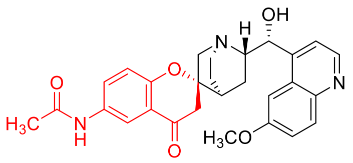

QN-C-S-12

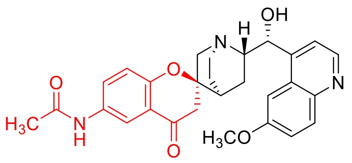

QN-C-R-17

Following the representative procedure for the quinine-based ketone on a 0.16 mmol scale, 26 mg (33%) of **QN-C-S-12** and 46 mg (59%) of **QN-C-R-17** were isolated as yellow solids.

Minor diastereomer **QN-C-S-12**:

^{1}H NMR (500 MHz, CD_3OD) δ 8.66 (d, J = 4.6 Hz, 1H), 7.94 (d, J = 9.1 Hz, 1H), 7.90 (d, J = 2.7 Hz, 1H), 7.71 – 7.68 (m, 2H), 7.44 – 7.39 (m, 2H), 7.00 (d, J = 8.9 Hz, 1H), 5.61 (d, J = 3.4 Hz, 1H), 3.97 (s, 3H), 3.74 – 3.62 (m, 1H), 3.23 – 3.09 (m, 2H), 3.00 – 2.82 (m, 3H), 2.75 (d, J = 16.8 Hz, 1H), 2.21 (s, 1H), 2.14 – 2.03 (m, 4H), 2.03 – 1.91 (m, 1H), 1.72 – 1.59 (m, 1H), 1.45 – 1.33 (m, 1H).

^{13}C NMR (126 MHz, CD^3OD) δ 193.68, 171.59, 159.74, 157.58, 150.33, 148.18, 144.76, 133.78, 131.36, 130.40, 128.13, 123.40, 121.71, 120.12, 119.83, 118.18, 102.51, 81.88, 71.59, 64.65, 60.56, 56.47, 46.24, 43.77, 30.13, 23.58, 22.65, 21.38.

HRMS (ESI) calcd for $C_{28}H_{29}N_3O_5$ $[M + H]^+$ 488.21800, found 488.21770.

$[\alpha]_D^{20}$ −141 (c 0.1, MeOH).

Major diastereomer **QN-C-R-17**:

^{1}H NMR (500 MHz, CD_3OD) δ 8.66 (d, J = 4.6 Hz, 1H), 7.96 (d, J = 9.2 Hz, 1H), 7.92 (d, J = 2.7 Hz, 1H), 7.67 (d, J = 4.6 Hz, 1H), 7.53 (dd, J = 8.9, 2.7 Hz, 1H), 7.47 – 7.40 (m, 2H), 6.63 (d, J = 8.9 Hz, 1H), 5.62 (d, J = 3.9 Hz, 1H), 3.96 (s, 3H), 3.77 – 3.66 (m, 1H), 3.48 – 3.38 (m, 1H), 3.16 (d, J = 14.4 Hz, 1H), 2.99 – 2.84 (m, 3H), 2.74 – 2.63 (m, 1H), 2.23 (s, 1H), 2.07 (s, 3H), 1.90 – 1.81 (m, 1H), 1.81 – 1.71 (m, 1H), 1.67 – 1.54 (m, 2H).

^{13}C NMR (126 MHz, CD_3OD) δ 192.25, 170.16, 158.28, 155.92, 149.11, 146.82, 143.37, 132.38, 130.02, 128.90, 126.79, 122.04, 120.37, 118.74, 118.27, 116.77, 101.08, 80.80, 70.64, 62.76, 58.90, 55.07, 43.90, 42.35, 29.00, 22.20, 21.27, 20.80.

HRMS (ESI) calcd for $C_{28}H_{29}N_3O_5$ $[M + H]^+$ 488.21800, found 488.21775.

$[\alpha]_D^{20}$ −239 (c 0.1, MeOH).

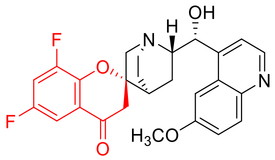

QN-C-S-13

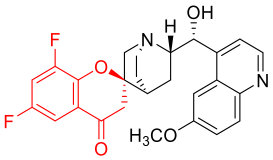

QN-C-R-18

Following the representative procedure for the quinine-based ketone, 21 mg (28%) of **QN-C-S-13** and 29 mg (39%) of **QN-C-R-18** were isolated as brown solids.

Minor diastereomer **QN-C-S-13**:

^{1}H NMR (600 MHz, CD_2Cl_2) δ 8.48 (d, *J* = 4.5 Hz, 1H), 7.83 (d, *J* = 9.2 Hz, 1H), 7.36 (d, *J* = 4.5 Hz, 1H), 7.21 (dd, *J* = 9.2, 2.7 Hz, 1H), 7.19 – 7.15 (m, 2H), 7.02 (ddd, *J* = 10.7, 8.1, 3.1 Hz, 1H), 5.43 (d, *J* = 4.8 Hz, 1H), 3.79 (s, 3H), 3.39 – 3.25 (m, 1H), 3.07 (d, *J* = 14.7 Hz, 1H), 3.00 – 2.90 (m, 1H), 2.80 – 2.66 (m, 4H), 2.16 (s, 1H), 1.97 – 1.80 (m, 2H), 1.50 – 1.37 (m, 2H).

One carbon could not be found in the ^{13}C NMR spectra: **^{13}C NMR** (176 MHz, CD_2Cl_2) δ 190.34, 158.21, 155.96 (dd, *J* = 243.6, 9.7 Hz), 152.67 (dd, *J* = 251.9, 11.2 Hz), 147.85, 145.11 (dd, *J* = 11.4, 3.0 Hz), 144.61, 131.83, 126.98, 123.02 (d, *J* = 7.5 Hz), 121.75, 118.84, 111.33 (dd, *J* = 28.0, 21.4 Hz), 107.12 (dd, *J* = 23.1, 4.0 Hz), 101.89, 83.00, 71.95, 64.16, 60.13, 56.04, 46.32, 42.77, 29.49, 23.46, 20.99.

^{19}F NMR (565 MHz, CD_2Cl_2) δ -119.57, -130.81 (d, *J* = 10.2 Hz).

HRMS (ESI) calcd for $C_{26}H_{24}N_2O_4F_2$ $[M + H]^+$ 467.17769, found 467.17736.

$[\alpha]_D^{20}$ −110 (*c* 0.1, MeOH).

Major diastereomer **QN-C-R-18**:

^{1}H NMR (600 MHz, CD_2Cl_2) δ 8.51 (d, *J* = 4.4 Hz, 1H), 7.89 (d, *J* = 9.2 Hz, 1H), 7.38 (d, *J* = 2.7 Hz, 2H), 7.36 (d, *J* = 4.5 Hz, 2H), 7.31 – 7.23 (m, 2H), 7.04 (ddd, *J* = 11.0, 8.1, 3.1 Hz, 1H), 5.36 (d, *J* = 6.5 Hz, 1H), 3.88 (s, 3H), 3.55 (q, *J* = 8.1 Hz, 1H), 3.35 – 3.27 (m, 1H), 3.10 (d, *J* = 14.8 Hz, 1H), 2.87 (s, 2H), 2.67 (d, *J* = 14.8 Hz, 1H), 2.56 – 2.47 (m, 1H), 2.25 (s, 1H), 1.87 (ddd, *J* = 12.8, 8.3, 3.9 Hz, 1H), 1.66 (dddd, *J* = 26.9, 13.3, 7.3, 3.1 Hz, 2H), 1.48 (tdd, *J* = 13.8, 6.0, 2.9 Hz, 1H).

^{13}C NMR (151 MHz, CD_2Cl_2) δ 190.05, 157.57, 155.56 (dd, *J* = 243.7, 9.6 Hz), 152.11 (dd, *J* = 252.0, 11.1 Hz), 147.60, 147.33, 144.56 (dd, *J* = 11.4, 2.9 Hz), 144.29, 131.26, 126.88, 122.69 (d, *J* = 7.3 Hz), 121.43, 119.02, 110.91 (dd, *J* = 28.0, 21.4 Hz), 106.71 (dd, *J* = 23.3, 4.1 Hz), 101.85, 82.94, 72.32, 62.94, 59.28, 55.50, 44.48, 42.19, 29.16, 23.45, 22.20.

^{19}F NMR (565 MHz, CD_2Cl_2) δ -119.45, -130.99 (d, *J* = 9.9 Hz).

HRMS (ESI) calcd for $C_{26}H_{24}N_2O_4F_2$ $[M + H]^+$ 467.17769, found 467.17714.

$[\alpha]_D^{20}$ −222 (*c* 0.1, MeOH.

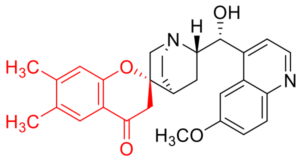

QN-C-S-14

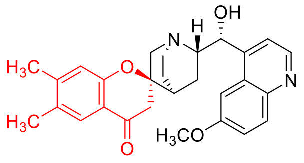

QN-C-R-19

Following the representative procedure for the quinine-based ketone, 19 mg (26%) of **QN-C-S-14** and 46 mg (63%) of **QN-C-R-19** were isolated as light brown solids.

Minor diastereomer **QN-C-S-14**:

^{1}H NMR (500 MHz, CD_2Cl_2) δ 8.56 (d, *J* = 4.4 Hz, 1H), 7.89 (d, *J* = 9.2 Hz, 1H), 7.47 (s, 1H), 7.44 (d, *J* = 4.5 Hz, 1H), 7.26 (dd, *J* = 9.2, 2.7 Hz, 1H), 7.23 (d, *J* = 2.7 Hz, 1H), 6.76 (s, 1H), 5.55 (s, 1H), 3.84 (s, 3H), 3.51 – 3.38 (m, 1H), 3.15 – 3.01 (m, 2H), 2.85 – 2.74 (m, 2H), 2.71 (d, *J* = 16.5 Hz, 1H), 2.63 (d, *J* = 16.5 Hz, 1H), 2.29 – 2.20 (m, 4H), 2.18 (s, 3H), 2.00 – 1.89 (m, 2H), 1.56 – 1.44 (m, 2H).

^{13}C NMR (176 MHz, CD_2Cl_2) δ 191.72, 158.33, 158.19, 147.86, 147.57, 147.35, 144.69, 131.88, 130.38, 126.96, 126.57, 121.77, 119.23, 119.05, 118.92, 101.83, 80.74, 71.65, 64.36, 60.17, 56.15, 46.39, 42.94, 30.12, 29.58, 20.98, 20.64, 18.87.

HRMS (ESI) calcd for $C_{28}H_{30}N_2O_4$ $[M + H]^+$ 459.22783, found 459.22783.

$[\alpha]_D^{20}$ −129 (*c* 0.1, MeOH).

Major diastereomer **QN-C-R-19**:

^{1}H NMR (500 MHz, CD_2Cl_2) δ 8.44 (d, *J* = 4.6 Hz, 1H), 7.80 (d, *J* = 9.8 Hz, 1H), 7.40 (s, 1H), 7.37 (d, *J* = 4.5 Hz, 1H), 7.25 – 7.13 (m, 2H), 6.45 (s, 1H), 5.46 (s, 1H), 3.74 (s, 3H), 3.44 – 3.31 (m, 2H), 3.01 (d, *J* = 14.5 Hz, 1H), 2.73 – 2.62 (m, 2H), 2.59 (d, *J* = 14.5 Hz, 1H), 2.51 – 2.40 (m, 1H), 2.15 (s, 1H), 2.07 (s, 6H), 1.74 – 1.64 (m, 1H), 1.65 – 1.53 (m, 2H), 1.48 – 1.36 (m, 1H).

^{13}C NMR (126 MHz, CD_2Cl_2) δ 191.47, 157.73, 157.61, 147.81, 147.41, 146.99, 144.10, 131.24, 130.02, 126.75, 126.12, 121.39, 118.74, 118.72, 118.68, 101.53, 80.65, 71.27, 63.18, 59.40, 55.64, 44.70, 42.65, 29.37, 22.39, 22.10, 20.16, 18.48.

HRMS (ESI) calcd for $C_{28}H_{30}N_2O_4$ $[M + H]^+$ 459.22783, found 459.22726.

$[\alpha]_D^{20}$ −273 (*c* 0.1, MeOH).

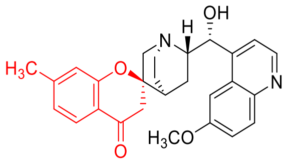

QN-C-R-20

Following the representative procedure for the quinine-based ketone, 52 mg (37%) of **QN-C-R-20** was isolated. The other diastereomer was not isolated in pure form.

QN-C-R-20:

^{1}H NMR (700 MHz, $CDCl_3$) δ 8.65 (d, *J* = 4.5 Hz, 1H), 7.99 (d, *J* = 9.2 Hz, 1H), 7.68 (d, *J* = 8.0 Hz, 1H), 7.50 (d, *J* = 4.5 Hz, 1H), 7.34 (dd, *J* = 9.2, 2.7 Hz, 1H), 7.22 (d, *J* = 2.7 Hz, 1H), 6.76 (d, *J* = 8.0 Hz, 1H), 6.51 (s, 1H), 5.60 (s, 1H), 3.87 (s, 3H), 3.54 (s, 1H), 3.46 (td, *J* = 8.8, 4.5 Hz, 1H), 3.17 (d, *J* = 14.1 Hz, 1H), 2.85 – 2.77 (m, 2H), 2.75 (d, *J* = 14.7 Hz, 1H), 2.63 – 2.56 (m, 1H), 2.29 – 2.24 (m, 4H), 1.75 – 1.65 (m, 3H), 1.56 – 1.47 (m, 1H).

^{13}C NMR (176 MHz, $CDCl_3$) δ 191.75, 159.63, 157.93, 148.24, 147.75, 147.41, 144.37, 131.71, 126.71, 126.50, 122.80, 121.77, 118.71, 118.54, 118.41, 101.36, 80.99, 71.84, 63.83, 59.13, 55.80, 44.89, 43.07, 29.50, 22.35, 21.98, 21.79.

HRMS (ESI) calcd for $C_{27}H_{28}N_2O_4$ $[M + H]^+$ 445.21218, found 445.21306.

$[\alpha]_D^{20}$ −126 (*c* 0.1, MeOH).

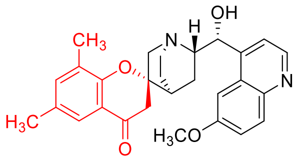

QN-C-S-15

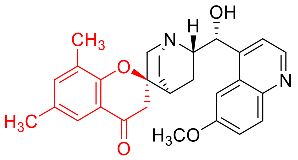

QN-C-R-21

Following the representative procedure for the quinine-based ketone, 29 mg (20%) of **QN-C-S-15** and 62 mg (43%) of **QN-C-R-21** were isolated.

Minor diastereomer **QN-C-S-15**:

^{1}H NMR (700 MHz, $CDCl_3$) δ 8.65 (d, *J* = 4.4 Hz, 1H), 7.98 (d, *J* = 9.2 Hz, 1H), 7.47 (d, *J* = 4.5 Hz, 1H), 7.43 (s, 1H), 7.33 (dd, *J* = 9.2, 2.6 Hz, 1H), 7.18 (d, *J* = 11.5 Hz, 2H), 5.60 (s, 1H), 3.88 (s, 3H), 3.53 (bs, 1H), 3.15 (d, *J* = 14.6 Hz, 1H), 3.08 – 2.98 (m, 1H), 2.89 – 2.79 (m, 2H), 2.73 (d, *J* = 16.3 Hz, 1H), 2.67 (d, *J* = 16.3 Hz, 1H), 2.27 – 2.23 (m, 4H), 2.21 (s, 3H), 2.04 – 1.91 (m, 2H), 1.61 – 1.52 (m, 1H), 1.44 (td, *J* = 10.3, 5.4 Hz, 1H).

^{13}C NMR (176 MHz, $CDCl_3$) δ 192.56, 158.09, 155.80, 147.68, 146.98, 144.42, 138.63, 131.88, 130.14, 127.36, 126.49, 123.85, 121.74, 120.23, 118.37, 101.24, 80.36, 71.55, 64.49, 59.63, 55.92, 46.13, 42.98, 29.49, 24.58, 21.11, 20.48, 15.70.

HRMS (ESI) calcd for $C_{28}H_{30}N_2O_4$ $[M + H]^+$ 459.22783, found 459.22709.

$[\alpha]_D^{20}$ −182 (*c* 0.1, MeOH)

Major diastereomer **QN-C-R-21**:

^{1}H NMR (700 MHz, $CDCl_3$) δ 8.66 (d, *J* = 4.4 Hz, 1H), 7.96 (d, *J* = 9.2 Hz, 1H), 7.51 (d, *J* = 4.5 Hz, 1H), 7.41 (s, 1H), 7.30 (dd, *J* = 9.2, 2.7 Hz, 1H), 7.21 (d, *J* = 2.7 Hz, 1H), 6.98 (d, *J* = 2.3 Hz, 1H), 5.75 (s, 1H), 3.83 (s, 3H), 3.77 – 3.65 (m, 1H), 3.65 – 3.57 (m, 1H), 3.26 (d, *J* = 14.6 Hz, 1H), 2.84 – 2.74 (m, 3H), 2.74 – 2.64 (m, 1H), 2.25 (s, 1H), 2.18 (s, 3H), 1.80 – 1.64 (m, 2H), 1.60 – 1.32 (m, 5H).

^{13}C NMR (176 MHz, $CDCl_3$) δ 192.30, 157.85, 155.28, 147.55, 146.70, 144.24, 138.50, 131.64, 130.19, 126.82, 126.20, 123.69, 121.68, 120.09, 118.38, 101.03, 80.60, 71.71, 63.38, 58.78, 55.76, 44.08, 43.15, 29.23, 21.90, 21.67, 20.31, 14.38.

HRMS (ESI) calcd for $C_{28}H_{30}N_2O_4$ $[M + H]^+$ 459.22783, found 459.22972

$[\alpha]_D^{20}$ −331 (*c* 0.1, MeOH)

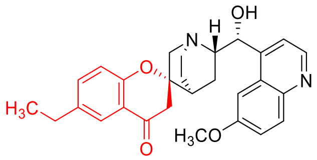

QN-C-S-16

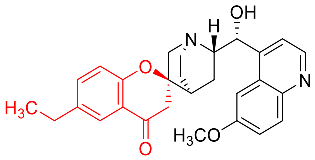

QN-C-R-22

Following the representative procedure for the quinine-based ketone, 11 mg (8%) of **QN-C-S-16** and 41 mg (28%) of **QN-C-R-22** were isolated.

Minor diastereomer **QN-C-S-16**:

^{1}H NMR (700 MHz, CD_3OD) δ 8.90 (d, *J* = 5.2 Hz, 1H), 8.15 – 8.05 (m, 2H), 7.69 (dd, *J* = 9.3, 2.5 Hz, 1H), 7.61 (d, *J* = 2.3 Hz, 1H), 7.58 (d, *J* = 2.6 Hz, 1H), 7.47 (dd, *J* = 8.5, 2.3 Hz, 1H), 7.05 (d, *J* = 8.5 Hz, 1H), 6.21 (s, 1H), 4.24 (tt, *J* = 11.6, 3.0 Hz, 1H), 4.05 (s, 3H), 3.83 – 3.74 (m, 2H), 3.58 (dd, *J* = 14.2, 2.6 Hz, 1H), 3.51 (td, *J* = 11.5, 7.2 Hz, 1H), 3.03 (d, *J* = 17.1 Hz, 1H), 2.87 (d, *J* = 17.0 Hz, 1H), 2.62 (q, *J* = 7.6 Hz, 2H), 2.58 – 2.52 (m, 1H), 2.42 (ddd, *J* = 14.1, 6.4, 3.4 Hz, 1H), 2.27 (ddd, *J* = 13.6, 10.7, 3.4 Hz, 1H), 2.15 – 2.05 (m, 1H), 1.58 – 1.49 (m, 1H), 1.20 (t, *J* = 7.6 Hz, 3H).

^{13}C NMR (176 MHz, CD_3OD) δ 192.11, 161.60, 158.20, 153.20, 144.62, 139.74, 139.25, 138.11, 128.42, 127.38, 126.87, 125.82, 121.64, 120.99, 119.57, 102.79, 79.73, 67.87, 62.26, 60.82, 57.17, 45.25, 45.19, 29.63, 28.82, 19.52, 19.37, 15.98.

HRMS (ESI) calcd for $C_{28}H_{30}N_2O_4$ $[M + H]^+$ 459.22783, found 459.22967.

$[\alpha]_D^{20}$ –116 (*c* 0.1, MeOH).

Major diastereomer **QN-C-R-22**:

^{1}H NMR (700 MHz, $CDCl_3$) δ 8.57 (d, *J* = 4.5 Hz, 1H), 7.94 (d, *J* = 9.2 Hz, 1H), 7.60 (d, *J* = 2.3 Hz, 1H), 7.47 (d, *J* = 4.5 Hz, 1H), 7.29 (dd, *J* = 9.2, 2.7 Hz, 1H), 7.23 – 7.16 (m, 2H), 6.59 (d, *J* = 8.4 Hz, 1H), 5.60 (s, 1H), 3.84 (s, 3H), 3.63 – 3.51 (m, 1H), 3.48 – 3.39 (m, 1H), 3.17 (d, *J* = 14.5 Hz, 1H), 2.85 – 2.77 (m, 2H), 2.74 (d, *J* = 14.7 Hz, 1H), 2.62 – 2.56 (m, 1H), 2.54 (q, *J* = 7.6 Hz, 2H), 2.28 – 2.22 (m, 1H), 1.76 – 1.67 (m, 2H), 1.68 – 1.61 (m, 1H), 1.54 – 1.47 (m, 1H), 1.17 (t, *J* = 7.6 Hz, 3H).

^{13}C NMR (176 MHz, $CDCl_3$) δ 192.32, 157.88, 157.70, 147.59, 147.56, 144.18, 137.34, 136.59, 131.52, 126.63, 125.01, 121.72, 120.57, 118.58, 118.20, 101.33, 80.76, 71.43, 63.66, 59.06, 55.79, 44.90, 43.00, 29.29, 27.93, 22.18, 21.58, 15.54.

HRMS (ESI) calcd for $C_{28}H_{30}N_2O_4$ $[M + H]^+$ 459.22783, found 459.22840

$[\alpha]_D^{20}$ –261 (*c* 0.1, MeOH)

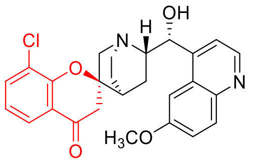

QN-C-R-23

Following the representative procedure for the quinine-based ketone, 66 mg (44%) of **QN-C-R-23** was isolated. The other diastereomer was not isolated in pure form.

QN-C-R-23:

^{1}H NMR (700 MHz, $CDCl_3$) δ 8.59 (d, *J* = 4.5 Hz, 1H), 7.97 (d, *J* = 9.2 Hz, 1H), 7.68 (dd, *J* = 7.8, 1.6 Hz, 1H), 7.45 (d, *J* = 4.5 Hz, 1H), 7.39 (dd, *J* = 7.8, 1.6 Hz, 1H), 7.32 (dd, *J* = 9.2, 2.7 Hz, 1H), 7.28 (d, *J* = 2.8 Hz, 1H), 6.86 (t, *J* = 7.8 Hz, 1H), 5.56 (d, *J* = 4.8 Hz, 1H), 3.88 (s, 3H), 3.68 (td, *J* = 9.0, 4.5 Hz, 1H), 3.61 – 3.48 (m, 1H), 3.26 (d, *J* = 14.5 Hz, 1H), 2.90 – 2.79 (m, 2H), 2.71 (d, *J* = 14.8 Hz, 1H), 2.65 – 2.52 (m, 1H), 2.23 – 2.18 (m, 1H), 1.72 – 1.59 (m, 2H), 1.60 – 1.53 (m, 1H), 1.50 – 1.37 (m, 1H).

^{13}C NMR (176 MHz, $CDCl_3$) δ 191.55, 157.79, 155.13, 147.56, 147.34, 144.32, 136.39, 131.55, 126.70, 125.05, 123.38, 122.23, 121.68, 121.49, 118.61, 101.70, 82.91, 72.57, 63.48, 58.56, 55.73, 44.00, 43.01, 29.45, 22.49, 22.23.

HRMS (ESI) calcd for $C_{26}H_{25}N_2O_4Cl$ $[M + H]^+$ 465.15756 and 467.15422, found 465.15689 and 467.15422

$[\alpha]^{20}_{D}$ −217 (*c* 0.1, MeOH)

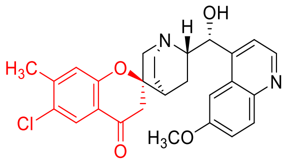

QN-C-R-24

Following the representative procedure for the quinine-based ketone, 19 mg (12%) of **QN-C-R-24** was isolated. The other diastereomer was not isolated in pure form.

QN-C-R-24:

^{1}H NMR (700 MHz, $CDCl_3$) δ 8.62 (d, *J* = 4.5 Hz, 1H), 7.98 (d, *J* = 9.2 Hz, 1H), 7.74 (s, 1H), 7.48 (d, *J* = 4.5 Hz, 1H), 7.34 (dd, *J* = 9.2, 2.7 Hz, 1H), 7.21 (d, *J* = 2.7 Hz, 1H), 6.62 (s, 1H), 5.57 (s, 1H), 3.87 (s, 3H), 3.53 (bs, 1H), 3.41 (td, *J* = 8.8, 4.5 Hz, 1H), 3.15 (d, *J* = 14.7 Hz, 1H), 2.80 (s, 2H), 2.74 (d, *J* = 14.7 Hz, 1H), 2.58 (ddt, *J* = 14.4, 10.8, 4.4 Hz, 1H), 2.27 (s, 3H), 2.26 – 2.21 (m, 1H), 1.77 – 1.60 (m, 3H), 1.54 – 1.45 (m, 1H).

^{13}C NMR (176 MHz, $CDCl_3$) δ 190.86, 157.94, 157.86, 147.73, 147.51, 145.69, 144.31, 131.67, 127.66, 126.71, 126.34, 121.74, 120.46, 119.91, 118.51, 101.39, 81.48, 71.83, 63.79, 59.16, 55.82, 44.63, 43.01, 29.38, 22.33, 21.72, 20.87.

HRMS (ESI) calcd for $C_{27}H_{26}N_2O_4Cl$ $[M + H]^+$ 479.17488 and 481.17211, found 479.17488 and 481.17211.

$[\alpha]_D^{20}$ −255 (*c* 0.1, MeOH).

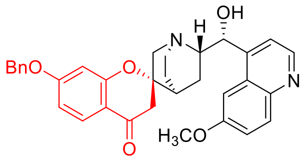

QN-C-S-17

Following the representative procedure for the quinine-based ketone, 57 mg (33%) of **QN-C-S-17** was isolated.

QN-C-S-17:

^{1}H NMR (700 MHz, $CDCl_3$) δ 8.54 (s, 1H), 7.89 (d, J = 9.2 Hz, 1H), 7.71 (d, J = 8.7 Hz, 1H), 7.45 (d, J = 4.5 Hz, 1H), 7.43 – 7.37 (m, 4H), 7.36 – 7.32 (m, 1H), 7.24 (d, J = 9.3 Hz, 1H), 7.14 (s, 1H), 6.60 (dd, J = 8.7, 2.4 Hz, 1H), 6.49 (d, J = 2.4 Hz, 1H), 5.66 (s, 1H), 5.08 (s, 2H), 3.82 (s, 3H), 3.63 (bs, 1H), 3.16 (d, J = 14.5 Hz, 1H), 3.02 (s, 1H), 2.85 (d, J = 14.3 Hz, 2H), 2.67 (d, J = 16.4 Hz, 1H), 2.60 (d, J = 16.5 Hz, 1H), 2.29 – 2.17 (m, 1H), 2.08 – 1.90 (m, 2H), 1.59 (bs, 1H), 1.43 – 1.33 (m, 1H).

^{13}C NMR (176 MHz, $CDCl_3$) δ 190.13, 165.69, 161.55, 158.02, 147.41, 146.99, 144.09, 135.89, 131.52, 128.82, 128.46, 127.64, 126.36, 121.73, 118.49, 114.86, 110.29, 102.54, 101.17, 80.84, 70.57, 70.44, 64.02, 59.56, 56.02, 45.71, 42.98, 29.35, 22.25, 20.49. An aromatic proton is missing.

HRMS (ESI) calcd for $C_{33}H_{32}N_2O_5$ $[M + H]^+$ 537.23840, found 537.23943.

[α]$^{20}_{D}$ −126 (c 0.1, MeOH).

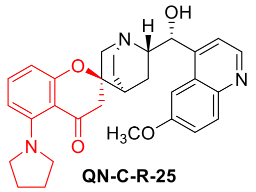

QN-C-R-25

Following the representative procedure for the quinine-based ketone employing 6-fluoro-2-hydroxyacetophenone, 54 mg (37%) of the S_NAr product **QN-C-R-25** was isolated. The other diastereomer was not isolated in pure form.

QN-C-R-25:

^{1}H NMR (600 MHz, $CDCl_3$) δ 8.57 (d, *J* = 4.5 Hz, 1H), 7.89 (d, *J* = 9.2 Hz, 1H), 7.48 (d, *J* = 4.5 Hz, 1H), 7.24 (dd, *J* = 9.2, 2.6 Hz, 1H), 7.16 (d, *J* = 2.7 Hz, 1H), 7.09 (t, *J* = 8.2 Hz, 1H), 6.30 (dd, *J* = 8.6, 1.0 Hz, 1H), 5.91 (d, *J* = 7.9 Hz, 1H), 5.72 (bs, 1H), 3.78 (s, 3H), 3.72 (bs, 1H), 3.46 (td, *J* = 8.9, 3.9 Hz, 1H), 3.23 (d, *J* = 14.5 Hz, 1H), 3.19 – 3.07 (m, 4H), 2.86 – 2.73 (m, 3H), 2.70 – 2.60 (m, 1H), 2.42 – 2.34 (m, 1H), 1.98 – 1.86 (m, 4H), 1.84 – 1.66 (m, 3H), 1.62 – 1.47 (m, 1H).

^{13}C NMR (151 MHz, $CDCl_3$) δ 187.68, 160.58, 157.88, 150.08, 147.43, 147.02, 144.08, 135.39, 131.42, 126.47, 121.81, 118.70, 109.97, 106.86, 104.25, 101.17, 78.10, 70.58, 63.19, 59.05, 55.97, 52.45, 46.57, 43.09, 30.32, 25.94, 22.03, 21.21.

HRMS (ESI) calcd for $C_{30}H_{33}N_3O_4$ $[M + H]^+$ 500.25438, found 500.25631.

$[\alpha]_D^{20}$ +181 (*c* 0.1, MeOH).

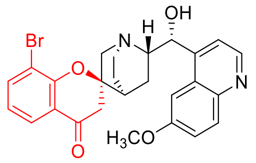

QN-C-R-26

Following the representative procedure for the quinine-based ketone, 56 mg (34%) of **QN-C-R-26** was isolated. The other diastereomer was not isolated in pure form.

QN-C-R-26:

^{1}H NMR (700 MHz, $CDCl_3$) δ 8.56 (d, *J* = 4.5 Hz, 1H), 7.91 (d, *J* = 9.2 Hz, 1H), 7.69 (dd, *J* = 7.7, 1.6 Hz, 1H), 7.49 (d, *J* = 4.5 Hz, 1H), 7.47 (dd, *J* = 7.9, 1.6 Hz, 1H), 7.26 (dd, *J* = 9.3, 2.5 Hz, 1H), 7.20 (d, *J* = 2.7 Hz, 1H), 6.78 (t, *J* = 7.8 Hz, 1H), 5.80 (s, 1H), 3.87 – 3.76 (m, 4H), 3.76 – 3.66 (m, 1H), 3.35 (d, *J* = 14.7 Hz, 1H), 2.86 (d, *J* = 16.9 Hz, 2H), 2.83 (d, *J* = 16.9 Hz, 2H), 2.78 (d, *J* = 14.7 Hz, 1H), 2.72 (t, *J* = 12.2 Hz, 1H), 2.19 (s, 1H), 1.76 – 1.68 (m, 2H), 1.53 – 1.43 (m, 1H), 1.41 – 1.32 (m, 1H).

^{13}C NMR (176 MHz, $CDCl_3$) δ 191.07, 157.88, 155.68, 147.47, 146.96, 144.03, 139.56, 131.37, 126.49, 125.80, 122.32, 121.97, 121.73, 118.52, 112.13, 101.58, 82.49, 71.30, 62.99, 58.52, 55.96, 43.54, 43.27, 29.21, 21.64, 21.44.

HRMS (ESI) calcd for $C_{26}H_{25}N_2O_4Br$ $[M + H]^+$ 509.10705 and 511.10500 found 509.10887 and 511.10691.

$[\alpha]_D^{20}$ −192 (*c* 0.1, MeOH).

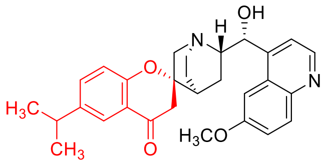

QN-C-S-18

Following the representative procedure for the quinine-based ketone, 21 mg (14%) of **QN-C-S-18** was isolated. The other diastereomer was not isolated in pure form.

QN-C-S-18:

^{1}H NMR (600 MHz, $CDCl_3$) δ 8.62 (d, *J* = 4.5 Hz, 1H), 7.98 (d, *J* = 9.2 Hz, 1H), 7.64 (d, *J* = 2.3 Hz, 1H), 7.47 (d, *J* = 4.5 Hz, 1H), 7.34 (dd, *J* = 9.2, 2.7 Hz, 1H), 7.28 – 7.26 (m, 1H), 7.23 (d, *J* = 2.7 Hz, 1H), 6.65 (d, *J* = 8.5 Hz, 1H), 5.51 (d, *J* = 4.6 Hz, 1H), 3.88 (s, 3H), 3.52 – 3.38 (m, 2H), 3.15 (dd, *J* = 14.7, 2.3 Hz, 1H), 2.82 (s, 2H), 2.73 (d, *J* = 14.7 Hz, 1H), 2.60 – 2.52 (m, 1H), 2.28 – 2.23 (m, 1H), 1.71 – 1.66 (m, 2H), 1.54 – 1.46 (m, 1H), 1.28 – 1.21 (m, 2H), 1.19 (d, *J* = 6.9 Hz, 6H).

^{13}C NMR (151 MHz, **$CDCl_3$**) δ 192.68, 157.94, 157.85, 147.73, 147.71, 144.35, 141.86, 135.28, 131.68, 126.79, 123.57, 121.68, 120.57, 118.55, 118.22, 101.46, 81.02, 72.16, 64.03, 59.10, 55.74, 44.97, 43.00, 33.36, 29.33, 24.00, 22.50, 22.08.

HRMS (ESI) calcd for $C_{29}H_{32}N_2O_4$ $[M + H]^+$ 473.24348, found 473.24348.

$[\alpha]_D^{20}$ −191 (*c* 0.1, MeOH).

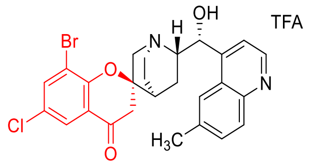

QN-C-R-27

Following the representative procedure for the quinine-based ketone, 9 mg (4%) of **QN-C-R-27** was isolated as the TFA salt following preparative HPLC (ACN/water). The other diastereomer was not isolated in pure form.

The sample was dissolved in a mixture of $CD_3OD/CDCl_3$. **^{1}H NMR** (700 MHz, CD_3OD) δ 8.81 (d, *J* = 5.0 Hz, 1H), 8.10 (d, *J* = 9.9 Hz, 1H), 7.99 (d, *J* = 5.0 Hz, 1H), 7.68 (d, *J* = 2.5 Hz, 1H), 7.60 (d, *J* = 8.2 Hz, 2H), 7.49 (d, *J* = 2.5 Hz, 1H), 6.38 (s, 1H), 4.53 (dddd, *J* = 12.9, 10.4, 7.8, 2.4 Hz, 1H), 4.16 (t, *J* = 9.4 Hz, 1H), 4.06 (s, 3H), 3.90 (dd, *J* = 14.4, 2.5 Hz, 1H), 3.48 (d, *J* = 14.5 Hz, 1H), 3.36 (ddt, *J* = 12.4, 10.9, 2.3 Hz, 1H), 3.12 (d, *J* = 17.5 Hz, 1H), 3.08 (d, *J* = 17.4 Hz, 1H), 2.47 – 2.41 (m, 1H), 2.17 – 2.05 (m, 2H), 1.96 – 1.82 (m, 1H), 1.21 – 1.10 (m, 1H).

The sample was dissolved in a mixture of $CD_3OD/CDCl_3$. **^{13}C NMR** (176 MHz, CD_3OD) δ 188.90, 160.78, 153.81, 151.02, 144.53, 139.57, 139.46, 128.95, 127.76, 127.59, 126.14, 122.67, 119.90, 112.78, 102.03, 81.68, 67.59, 60.75, 59.57, 57.07, 44.36, 42.37, 28.89, 19.57, 19.48.

HRMS (ESI) calcd for $C_{26}H_{24}N_2O_4BrCl$ $[M + H]^+$ 543.06807 and 545.06603, found 543.06868 and 545.06655, respectively.

$[\alpha]_D^{20}$ −292.9 (*c* 0.098, MeOH).

General procedure for quinidine-based chromanones (QD-C):

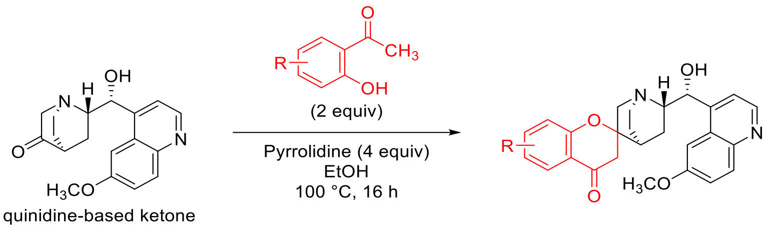

To an oven-dried pressure vessel equipped with a stir bar that was cooled under an atm of Ar was added the quinidine-derived ketone (50 mg, 0.16 mmol, 1 equiv). Then, anhydrous EtOH (0.5 ml) was added followed by a 2-hydroxyacetophenone derivative (0.33 mmol, 2 equiv) and pyrrolidine (58 ul, 0.66 mmol, 4 equiv). The pressure vessel was flushed with Ar and sealed with the proper cap. The reaction was then heated to 100 °C for 16 h. After cooling to room temperature, the reaction was diluted with DCM, transferred to round-bottomed flask, and concentrated. Purification could be achieved by silica chromatography (0-12% MeOH in DCM) to afford the pure isomers. All of the reactions produced two diastereomers; however, after purification, sometimes only one of the isomers could be obtained in pure form.

Compound data for quinine-based chromanones (**QD-C-S** and **QD-C-R**):

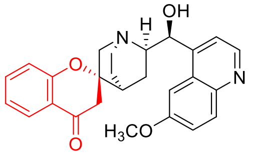

QD-C-S-1

Following the representative procedure for the quinidine-based ketone, 44 mg (64%) of **QD-C-S-1** was isolated. The other diastereomer was not isolated in pure form.

QD-C-S-1:

^{1}H NMR (500 MHz, CD_3OD) δ 8.64 (d, J = 4.6 Hz, 1H), 7.92 (d, J = 9.2 Hz, 1H), 7.79 (dd, J = 8.0, 1.8 Hz, 1H), 7.69 (d, J = 4.6 Hz, 1H), 7.51 (ddd, J = 8.8, 7.2, 1.7 Hz, 1H), 7.36 (dd, J = 9.2, 2.6 Hz, 1H), 7.27 (d, J = 2.7 Hz, 1H), 7.04 – 6.96 (m, 2H), 5.70 (s, 1H), 4.01 – 3.96 (m, 1H), 3.95 (s, 3H), 3.27 – 2.96 (m, 6H), 2.25 (brs, 1H), 2.15 – 2.03 (m, 1H), 1.90 (brs, 1H), 1.32 – 1.23 (m, 1H), 1.19 – 1.10 (m, 1H).

^{13}C NMR (126 MHz, CD_3OD) δ 193.39, 159.77, 158.33, 148.40, 146.82, 143.29, 136.72, 130.34, 126.31, 126.18, 122.24, 121.25, 120.83, 118.34, 118.32, 100.72, 80.69, 70.26, 58.17, 56.46, 55.51, 49.33, 43.72, 28.93, 20.22, 19.89.

HRMS (ESI) calcd for $C_{26}H_{26}N_2O_4$ $[M + H]^+$ 431.19653, found 431.19622.

$[\alpha]_D^{20}$ +263 (c 0.1, MeOH).

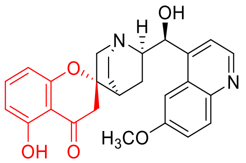

QD-C-S-2

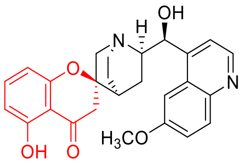

QD-C-R-1

Following the representative procedure for the quinidine-based ketone, 32 mg (45%) of **QD-C-S-2** and 6 mg (8%) of **QD-C-R-1** were isolated as yellow solids.

Major diastereomer **QD-C-S-2**:

This NMR sample was dissolved in a mixture of CD_3OD:CD_2Cl_2 (3:2) and was referenced to CD_3OD. **^{1}H NMR** (700 MHz, CD_3OD) δ 8.66 (d, *J* = 4.7 Hz, 1H), 7.95 (d, *J* = 9.2 Hz, 1H), 7.69 (d, *J* = 4.8 Hz, 1H), 7.43 – 7.33 (m, 2H), 7.29 (s, 1H), 6.52 – 6.40 (m, 2H), 5.68 (s, 1H), 4.01 – 3.88 (m, 4H), 3.22 (d, *J* = 17.2 Hz, 1H), 3.12 (d, *J* = 17.2 Hz, 1H), 3.09 – 2.95 (m, 4H), 2.27 (s, 1H), 2.09 – 2.03 (m, 1H), 1.94 – 1.87 (m, 1H), 1.30 – 1.24 (m, 1H), 1.21 – 1.12 (m, 1H).

^{13}C NMR (176 MHz, CD_3OD) δ 199.48, 162.23, 160.61, 159.08, 149.32, 147.73, 144.14, 139.22, 131.13, 127.16, 122.86, 119.14, 109.64, 108.82, 108.71, 101.71, 81.39, 71.23, 58.93, 57.14, 56.30, 50.09, 44.04, 30.19, 21.27, 20.69.

HRMS (ESI) calcd for $C_{26}H_{26}N_2O_5$ $[M + H]^+$ 447.19145, found 447.19108.

$[\alpha]_D^{20}$ +186 (*c* 0.26, DMSO).

Minor diastereomer **QD-C-R-1**:

^{1}H NMR (700 MHz, CD_2Cl_2) δ 11.54 (bs, 1H), 8.68 (d, *J* = 4.4 Hz, 1H), 7.98 (d, *J* = 9.0 Hz, 1H), 7.49 (d, *J* = 4.5 Hz, 1H), 7.41 (t, *J* = 8.3 Hz, 1H), 7.39 – 7.29 (m, 2H), 6.51 (dd, *J* = 23.5, 8.3 Hz, 2H), 5.56 (d, *J* = 6.6 Hz, 1H), 3.85 (s, 3H), 3.52 (d, *J* = 15.1 Hz, 1H), 3.24 (q, *J* = 7.5 Hz, 1H), 2.90 (s, 2H), 2.84 – 2.76 (m, 1H), 2.66 (d, *J* = 15.0 Hz, 1H), 2.60 (dt, *J* = 13.7, 8.5 Hz, 1H), 2.34 (dd, *J* = 13.6, 7.5 Hz, 1H), 2.30 – 2.24 (m, 1H), 1.61 – 1.57 (m, 2H), 1.49 (ddd, *J* = 13.7, 9.8, 3.8 Hz, 1H).

^{13}C NMR (176 MHz, CD_2Cl_2) δ 197.73, 161.69, 159.45, 157.51, 147.49, 147.13, 144.40, 138.30, 131.54, 126.79, 121.21, 118.62, 109.07, 107.96, 107.78, 101.42, 80.59, 72.02, 59.54, 56.54, 55.32, 49.11, 45.41, 29.65, 22.40, 21.86.

HRMS (ESI) calcd for $C_{26}H_{26}N_2O_5$ $[M + H]^+$ 447.19145, found 447.19096.

$[\alpha]_D^{20}$ +44 (*c* 0.7, MeOH).

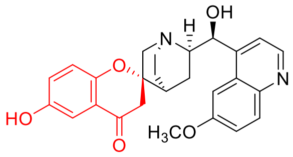

QD-C-S-3

Following the representative procedure for the quinidine-based ketone, 23 mg (32%) of **QD-C-S-3** was isolated a dark brown solid. The other diastereomer was not isolated in pure form.

QD-C-S-3:

^{1}H NMR (700 MHz, CD_3OD) δ 8.67 (d, J = 3.7 Hz, 1H), 7.94 (d, J = 9.3 Hz, 1H), 7.72 (d, J = 4.3 Hz, 1H), 7.40 (d, J = 9.3 Hz, 1H), 7.35 (s, 1H), 7.16 (s, 1H), 7.03 (d, J = 8.9 Hz, 1H), 6.90 (d, J = 8.9 Hz, 1H), 5.76 (s, 1H), 4.03 – 3.92 (m, 4H), 3.24 – 3.01 (m, 6H), 2.24 (s, 1H), 2.13 – 2.05 (m, 1H), 1.97 – 1.87 (m, 1H), 1.39 – 1.29 (m, 1H), 1.26 – 1.18 (m, 1H).

^{13}C NMR (176 MHz, CD_3OD) δ 194.48, 159.84, 154.41, 153.02, 149.60, 148.20, 144.67, 131.44, 127.74, 126.18, 123.47, 122.41, 120.53, 119.83, 111.09, 102.15, 81.29, 71.28, 59.78, 57.50, 56.54, 50.45, 44.86, 30.32, 21.49, 20.71.

HRMS (ESI) calcd for $C_{26}H_{26}N_2O_5$ $[M + H]^+$ 447.19145, found 447.19130.

$[\alpha]^{20}_{D}$ +250 (c 0.1, MeOH).

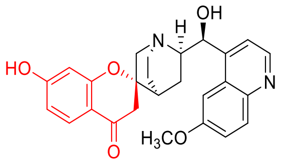

QD-C-S-4

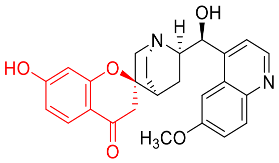

QD-C-R-2

Following the representative procedure for the quinidine-based ketone, 34 mg (48%) of **QD-C-S-4** and 6 mg (8%) of **QD-C-R-2** were isolated as brown solids.

Major diastereomer **QD-C-S-4**:

^{1}H NMR (700 MHz, CD_3OD) δ 8.67 (d, *J* = 4.7 Hz, 1H), 7.94 (d, *J* = 9.2 Hz, 1H), 7.72 (d, *J* = 4.6 Hz, 1H), 7.67 (d, *J* = 8.7 Hz, 1H), 7.40 (d, *J* = 9.2 Hz, 1H), 7.36 (s, 1H), 6.47 (d, *J* = 8.7 Hz, 1H), 6.36 (s, 1H), 5.78 (s, 1H), 4.06 – 3.94 (m, 4H), 3.25 – 3.19 (m, 1H), 3.18 – 3.00 (m, 5H), 2.26 (s, 1H), 2.16 – 2.07 (m, 1H), 1.98 – 1.90 (m, 1H), 1.37 (q, *J* = 10.1 Hz, 1H), 1.27 – 1.19 (m, 1H).

^{13}C NMR (176 MHz, CD_3OD) δ 190.76, 165.09, 161.14, 157.75, 147.37, 146.10, 142.57, 129.35, 127.33, 125.62, 121.37, 117.74, 112.97, 109.58, 102.29, 100.04, 79.64, 69.05, 57.66, 55.42, 54.46, 48.33, 42.34, 28.52, 19.48, 18.60.

HRMS (ESI) calcd for $C_{26}H_{26}N_2O_5$ $[M + H]^+$ 447.19145, found 447.19134.

$[\alpha]_D^{20}$ +234 (*c* 0.1, MeOH)

Minor diastereomer **QD-C-R-2**:

^{1}H NMR (700 MHz, CD_3OD) δ 8.67 (d, *J* = 4.6 Hz, 1H), 7.94 (d, *J* = 9.1 Hz, 1H), 7.71 – 7.63 (m, 2H), 7.48 (s, 1H), 7.40 (dd, *J* = 9.0, 2.4 Hz, 1H), 6.55 (s, 1H), 6.50 (d, *J* = 8.7 Hz, 1H), 5.62 (d, *J* = 6.2 Hz, 1H), 3.88 (s, 3H), 3.72 (d, *J* = 14.6 Hz, 1H), 3.23 (q, *J* = 7.7 Hz, 1H), 2.91 – 2.79 (m, 3H), 2.71 – 2.61 (m, 2H), 2.60 – 2.53 (m, 1H), 2.23 (s, 1H), 1.71 – 1.57 (m, 2H), 1.48 – 1.38 (m, 1H).

^{13}C NMR (176 MHz, MeOD) δ 192.84, 167.33, 163.62, 159.53, 150.45, 148.26, 144.98, 131.39, 129.49, 128.57, 123.38, 120.47, 115.14, 111.60, 104.74, 102.44, 81.91, 72.13, 60.60, 57.37, 56.19, 50.10, 46.92, 31.31, 23.18, 22.60.

HRMS (ESI) calcd for $C_{26}H_{26}N_2O_5$ $[M + H]^+$ 447.19145, found 447.19118.

$[\alpha]_D^{20}$ +40 (*c* 0.11, MeOH).

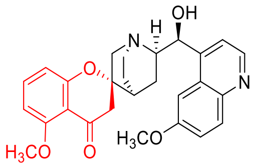

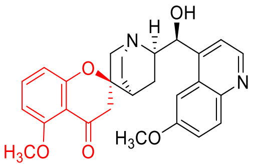

Following the representative procedure for the quinidine-based ketone, 35 mg (47%) of **QD-C-S-5** and 14 mg (19%) of **QD-C-R-3** were isolated as brown solids.

Major diastereomer **QD-C-S-5**:

^{1}H NMR (700 MHz, CD_2Cl_2) δ 8.59 (d, *J* = 4.5 Hz, 1H), 7.90 (d, *J* = 9.2 Hz, 1H), 7.54 (d, *J* = 4.5 Hz, 1H), 7.37 (t, *J* = 8.3 Hz, 1H), 7.25 (dd, *J* = 9.1, 2.7 Hz, 1H), 7.17 (d, *J* = 2.8 Hz, 1H), 6.56 (dd, *J* = 8.3, 1.0 Hz, 1H), 6.49 (d, *J* = 8.3 Hz, 1H), 5.76 (s, 1H), 3.81 (s, 3H), 3.80 (s, 3H), 3.79 – 3.76 (m, 1H), 3.15 – 3.07 (m, 1H), 3.07 – 2.87 (m, 5H), 2.24 (s, 1H), 2.04 – 1.96 (m, 1H), 1.91 – 1.82 (m, 1H), 1.28 – 1.23 (m, 2H).

^{13}C NMR (176 MHz, CD_2Cl_2) δ 190.93, 161.65, 160.75, 158.18, 147.87, 147.51, 144.47, 136.54, 131.79, 126.62, 121.78, 118.77, 111.62, 110.73, 104.28, 101.56, 80.47, 71.13, 59.18, 56.66, 56.29, 56.15, 49.73, 45.94, 29.80, 21.84, 20.38.

HRMS (ESI) calcd for $C_{27}H_{28}N_2O_5$ $[M + H]^+$ 461.20710, found 461.20658.

$[\alpha]_D^{20}$ +265 (*c* 0.1, MeOH).

Minor diastereomer **QD-C-R-3**:

^{1}H NMR (700 MHz, CD_2Cl_2) δ 8.67 (d, *J* = 4.5 Hz, 1H), 7.97 (d, *J* = 9.1 Hz, 1H), 7.49 (d, *J* = 4.4 Hz, 1H), 7.42 (t, *J* = 8.3 Hz, 1H), 7.36 – 7.32 (m, 2H), 6.65 (dd, *J* = 8.3, 1.0 Hz, 1H), 6.54 (dd, *J* = 8.4, 1.0 Hz, 1H), 5.56 (d, *J* = 6.5 Hz, 1H), 3.87 (s, 3H), 3.84 (s, 3H), 3.48 (dd, *J* = 15.1, 2.3 Hz, 1H), 3.24 – 3.20 (m, 1H), 2.83 – 2.74 (m, 3H), 2.64 (dd, *J* = 15.0, 1.4 Hz, 1H), 2.62 – 2.56 (m, 1H), 2.34 – 2.29 (m, 1H), 2.24 (t, *J* = 3.2 Hz, 1H), 1.62 – 1.58 (m, 1H), 1.56 – 1.52 (m, 1H), 1.47 (ddd, *J* = 13.7, 9.9, 3.9 Hz, 1H).

^{13}C NMR (176 MHz, CD_2Cl_2) δ 189.93, 161.07, 160.38, 157.62, 147.61, 147.38, 144.51, 136.06, 131.64, 126.95, 121.34, 118.74, 111.19, 110.29, 103.94, 101.56, 80.18, 72.10, 59.78, 56.62, 55.95, 55.45, 49.30, 47.68, 29.50, 22.53, 21.82.

HRMS (ESI) calcd for $C_{27}H_{28}N_2O_5$ $[M + H]^+$ 461.20710, found 461.20674.

$[\alpha]_D^{20}$ +45 (*c* 0.58, MeOH).

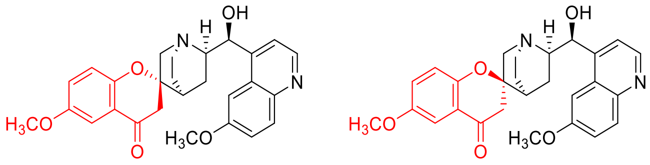

QD-C-S-6 **QD-C-R-4**

Following the representative procedure for the quinidine-based ketone, 35 mg (47%) of **QD-C-S-6** and 20 mg (27%) of **QD-C-R-4** were isolated as yellow solids.

Major diastereomer **QD-C-S-6**:

This NMR sample was dissolved in a mixture of CD_3OD:CD_2Cl_2 (3:1) and was referenced to CD_3OD. **^{1}H NMR** (700 MHz, CD_3OD) δ 8.66 (d, *J* = 4.6 Hz, 1H), 7.94 (d, *J* = 9.2 Hz, 1H), 7.71 (d, *J* = 4.6 Hz, 1H), 7.39 (dd, *J* = 9.2, 2.6 Hz, 1H), 7.32 (d, *J* = 2.7 Hz, 1H), 7.26 (d, *J* = 3.2 Hz, 1H), 7.14 (dd, *J* = 9.0, 3.2 Hz, 1H), 6.96 (d, *J* = 9.0 Hz, 1H), 5.72 (s, 1H), 3.98 – 3.94 (m, 4H), 3.78 (s, 3H), 3.20 – 3.14 (m, 1H), 3.14 – 2.98 (m, 5H), 2.24 (s, 1H), 2.12 – 2.04 (m, 1H), 1.94 – 1.85 (m, 1H), 1.32 – 1.26 (m, 1H), 1.22 – 1.13 (m, 1H).

^{13}C NMR (176 MHz, CD_3OD) δ 194.17, 159.41, 155.23, 149.48, 147.90, 144.37, 131.26, 127.42, 126.13, 123.13, 121.83, 120.56, 119.43, 108.13, 101.90, 81.54, 71.27, 59.36, 57.34, 57.27, 56.42, 56.19, 50.25, 44.65, 30.06, 21.35, 20.75.

HRMS (ESI) calcd for $C_{27}H_{28}N_2O_5$ $[M + H]^+$ 461.20710, found 461.20680.

$[\alpha]_D^{20}$ +252 (*c* 0.1, MeOH).

Minor diastereomer **QD-C-R-4**:

^{1}H NMR (700 MHz, CD_2Cl_2) δ 8.67 (d, *J* = 4.5 Hz, 1H), 7.97 (d, *J* = 9.1 Hz, 1H), 7.51 (d, *J* = 4.5 Hz, 1H), 7.35 (d, *J* = 2.7 Hz, 1H), 7.32 (dd, *J* = 9.1, 2.7 Hz, 1H), 7.27 (d, *J* = 3.2 Hz, 1H), 7.14 (dd, *J* = 8.9, 3.2 Hz, 1H), 7.00 (d, *J* = 8.9 Hz, 1H), 5.62 (s, 1H), 3.83 (s, 3H), 3.80 (s, 3H), 3.57 (d, *J* = 14.9 Hz, 1H), 3.25 – 3.21 (m, 1H), 2.89 – 2.77 (m, 3H), 2.67 (d, *J* = 14.9 Hz, 1H), 2.64 – 2.59 (m, 1H), 2.38 – 2.32 (m, 1H), 2.25 (s, 1H), 1.63 – 1.54 (m, 2H), 1.43 (ddd, *J* = 13.7, 10.0, 3.9 Hz, 1H), 1.33 – 1.21 (m, 1H).

^{13}C NMR (176 MHz, CD_2Cl_2) δ 191.99, 158.06, 154.61, 154.25, 148.04, 147.67, 144.91, 132.07, 127.29, 125.34, 121.75, 121.42, 120.01, 119.15, 107.82, 101.97, 81.12, 72.29, 60.17, 57.16, 56.21, 55.91, 49.71, 46.55, 29.80, 22.72, 22.19.

HRMS (ESI) calcd for $C_{27}H_{28}N_2O_5$ $[M + H]^+$ 461.20710, found 461.20675.

$[\alpha]_D^{20}$ +63 (*c* 0.11, MeOH).

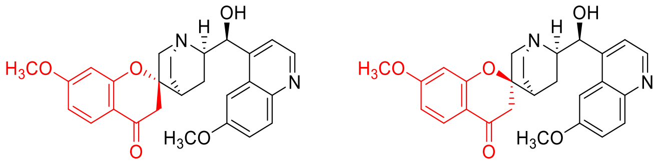

QD-C-S-7 **QD-C-R-5**

Following the representative procedure for the quinidine-based ketone, 40 mg (54%) of **QD-C-S-7** and 18 mg (24%) of **QD-C-R-5** were isolated as brown solids.

Major diastereomer **QD-C-S-7**:

This NMR sample was dissolved in a mixture of CD_3OD:CD_2Cl_2 (3:1) and was referenced to CD_3OD. **^{1}H NMR** (700 MHz, CD_3OD) δ 8.66 (d, J = 4.5 Hz, 1H), 7.94 (d, J = 9.3 Hz, 1H), 7.73 (d, J = 8.8 Hz, 1H), 7.71 (d, J = 4.6 Hz, 1H), 7.39 (dd, J = 9.2, 2.6 Hz, 1H), 7.32 (d, J = 2.7 Hz, 1H), 6.59 (dd, J = 8.8, 2.4 Hz, 1H), 6.51 (d, J = 2.4 Hz, 1H), 5.74 (s, 1H), 4.02 – 3.91 (m, 4H), 3.84 (s, 3H), 3.19 – 2.98 (m, 6H), 2.27 (s, 1H), 2.14 – 2.05 (m, 1H), 2.00 – 1.88 (m, 1H), 1.36 – 1.29 (m, 1H), 1.23 – 1.13 (m, 1H).

^{13}C NMR (176 MHz, CD_3OD) δ 192.75, 167.99, 162.90, 159.39, 149.29, 147.87, 144.34, 131.25, 128.85, 127.37, 123.12, 119.41, 115.61, 110.60, 102.35, 101.87, 81.95, 71.07, 59.32, 57.32, 56.44, 56.25, 50.22, 44.35, 30.36, 21.40, 20.71.

HRMS (ESI) calcd for $C_{27}H_{28}N_2O_5$ $[M + H]^+$ 461.20710, found 461.20667.

$[\alpha]_D^{20}$ +194 (c 0.29, CH_2Cl_2).

Minor diastereomer **QD-C-R-5**:

^{1}H NMR (700 MHz, CD_2Cl_2) δ 8.67 (d, J = 4.5 Hz, 1H), 7.97 (d, J = 9.1 Hz, 1H), 7.74 (d, J = 8.8 Hz, 1H), 7.51 (d, J = 4.5 Hz, 1H), 7.35 (d, J = 2.7 Hz, 1H), 7.33 (dd, J = 9.1, 2.7 Hz, 1H), 6.58 (dd, J = 8.8, 2.4 Hz, 1H), 6.47 (d, J = 2.4 Hz, 1H), 5.57 (d, J = 6.5 Hz, 1H), 3.87 (s, 3H), 3.84 (s, 3H), 3.51 (d, J = 14.6 Hz, 1H), 3.27 – 3.18 (m, 1H), 2.82 – 2.74 (m, 3H), 2.65 (d, J = 14.9 Hz, 1H), 2.59 (ddd, J = 13.7, 9.8, 7.2 Hz, 1H), 2.38 – 2.31 (m, 1H), 2.24 (s, 1H), 1.63 – 1.53 (m, 2H), 1.48 (ddd, J = 13.7, 9.9, 4.0 Hz, 1H).

^{13}C NMR (176 MHz, CD_2Cl_2) δ 190.46, 166.84, 161.91, 158.03, 148.03, 147.79, 144.90, 132.04, 128.52, 127.39, 121.72, 119.22, 115.36, 109.72, 102.06, 102.01, 81.70, 72.38, 60.12, 57.27, 56.19, 55.85, 49.68, 46.42, 30.19, 23.02, 22.42.

HRMS (ESI) calcd for $C_{27}H_{28}N_2O_5$ $[M + H]^+$ 461.20710, found 461.20710.

$[\alpha]_D^{20}$ +71 (c 0.1, MeOH).

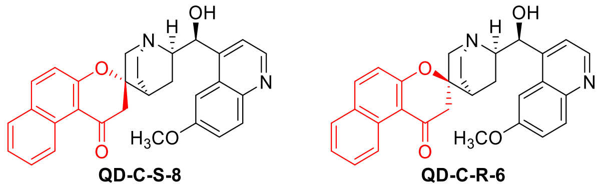

Following the representative procedure for the quinidine-based ketone, 24 mg (31%) of **QD-C-S-8** and 16 mg (21%) of **QD-C-R-6** were isolated as red-orange solids.

Major diastereomer **QD-C-S-8**:

This NMR sample was dissolved in a mixture of CD_3OD:CD_2Cl_2 (3:1) and was referenced to CD_3OD. **^{1}H NMR** (700 MHz, CD_3OD) δ 9.35 (dd, J = 8.7, 1.0 Hz, 1H), 8.67 (d, J = 4.6 Hz, 1H), 7.99 (d, J = 9.0 Hz, 1H), 7.95 (d, J = 9.2 Hz, 1H), 7.77 (d, J = 8.0 Hz, 1H), 7.72 (dd, J = 4.6, 0.9 Hz, 1H), 7.59 (ddd, J = 8.5, 6.9, 1.5 Hz, 1H), 7.43 – 7.36 (m, 2H), 7.31 (d, J = 2.7 Hz, 1H), 7.15 (d, J = 8.9 Hz, 1H), 5.68 (s, 1H), 4.00 (dd, J = 14.9, 2.3 Hz, 1H), 3.96 (s, 3H), 3.27 – 3.17 (m, 2H), 3.14 – 3.03 (m, 3H), 3.03 – 2.95 (m, 1H), 2.36 (s, 1H), 2.17 – 2.09 (m, 1H), 1.97 – 1.88 (m, 1H), 1.32 – 1.24 (m, 1H), 1.19 (ddd, J = 14.0, 9.6, 4.8 Hz, 1H).

^{13}C NMR (176 MHz, CD_3OD) δ 195.15, 162.87, 159.19, 149.56, 147.82, 144.24, 138.82, 131.98, 131.17, 130.29, 130.08, 129.22, 127.32, 126.08, 125.48, 122.96, 119.92, 119.29, 113.11, 101.82, 82.00, 71.41, 59.15, 57.22, 56.32, 50.20, 45.90, 29.91, 21.40, 20.81.

HRMS (ESI) calcd for $C_{30}H_{28}N_2O_4$ $[M + H]^+$ 481.21218, found 481.21165.

$[\alpha]^{20}_{D}$ +248 (c 0.12, CH_2Cl_2).

Minor diastereomer **QD-C-R-6**:

^{1}H NMR (700 MHz, CD_2Cl_2) δ 9.36 (d, J = 8.7 Hz, 1H), 8.63 (d, J = 4.5 Hz, 1H), 7.98 (d, J = 8.8 Hz, 1H), 7.94 (d, J = 9.1 Hz, 1H), 7.78 (d, J = 8.0 Hz, 1H), 7.61 (ddd, J = 8.5, 6.9, 1.5 Hz, 1H), 7.49 (d, J = 4.5 Hz, 1H), 7.43 (ddd, J = 8.1, 6.9, 1.2 Hz, 1H), 7.33 (d, J = 2.8 Hz, 1H), 7.29 (dd, J = 9.1, 2.7 Hz, 1H), 7.19 (d, J = 8.9 Hz, 1H), 5.61 (d, J = 6.4 Hz, 1H), 3.75 (s, 3H), 3.63 (d, J = 13.8 Hz, 1H), 3.28 – 3.19 (m, 1H), 2.95 (q, J = 15.9 Hz, 2H), 2.85 – 2.78 (m, 1H), 2.74 (d, J = 14.9 Hz, 1H), 2.63 (ddd, J = 13.5, 9.9, 7.2 Hz, 1H), 2.45 – 2.39 (m, 1H), 2.35 (s, 1H), 1.71 – 1.64 (m, 1H), 1.62 – 1.53 (m, 1H), 1.46 (ddd, J = 13.7, 9.8, 3.9 Hz, 1H).

^{13}C NMR (176 MHz, CD_2Cl_2) δ 193.19, 162.01, 158.03, 147.96, 147.82, 144.81, 137.98, 131.94, 131.59, 129.97, 129.50, 128.76, 127.30, 125.74, 125.14, 121.75, 119.63, 119.25, 112.74, 101.99, 81.41, 72.25, 60.08, 57.03, 55.83, 49.65, 47.88, 29.85, 22.76, 22.14.

HRMS (ESI) calcd for $C_{30}H_{28}N_2O_4$ $[M + H]^+$ 481.21171.

$[\alpha]^{20}_{D}$ +554 (c 0.14, MeOH).

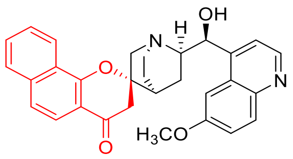

QD-C-S-9

Following the representative procedure for the quinidine-based ketone, 33 mg (43%) of **QD-C-S-9** was isolated as a dark brown solid. The other diastereomer was not isolated in pure form.

Major diastereomer **QD-C-S-9**:

This NMR sample was dissolved in a mixture of CD_3OD:CD_2Cl_2 (3:1) and was referenced to CD_3OD. **^{1}H NMR** (700 MHz, CD_3OD) δ 8.66 (d, *J* = 4.6 Hz, 1H), 8.30 (dd, *J* = 8.4, 1.1 Hz, 1H), 7.94 (d, *J* = 9.2 Hz, 1H), 7.81 (dd, *J* = 8.3, 1.1 Hz, 1H), 7.77 (d, *J* = 8.7 Hz, 1H), 7.72 (dd, *J* = 4.6, 0.9 Hz, 1H), 7.63 (ddd, *J* = 8.2, 6.9, 1.3 Hz, 1H), 7.56 (ddd, *J* = 8.3, 6.9, 1.3 Hz, 1H), 7.41 – 7.36 (m, 2H), 7.31 (d, *J* = 2.7 Hz, 1H), 5.70 (d, *J* = 2.2 Hz, 1H), 4.05 (dd, *J* = 15.0, 2.2 Hz, 1H), 3.96 (s, 3H), 3.28 – 3.15 (m, 4H), 3.12 – 3.07 (m, 1H), 3.07 – 3.01 (m, 1H), 2.37 (s, 1H), 2.15 – 2.09 (m, 1H), 1.97 – 1.90 (m, 1H), 1.31 – 1.26 (m, 1H), 1.19 (ddd, *J* = 14.1, 9.6, 4.8 Hz, 1H).

^{13}C NMR (176 MHz, CD_3OD) δ 193.66, 159.15, 158.77, 149.49, 147.79, 144.21, 138.83, 131.16, 130.54, 128.78, 127.31, 127.27, 126.05, 123.79, 122.91, 121.71, 121.61, 119.24, 116.23, 101.80, 83.19, 71.38, 59.00, 57.43, 56.31, 50.20, 44.22, 30.52, 21.46, 21.26.

HRMS (ESI) calcd for $C_{30}H_{28}N_2O_4$ $[M + H]^+$ 481.21218, found 481.21161.

$[\alpha]^{20}_{D}$ +125 (*c* 0.4, DMSO).

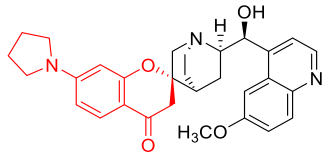

QD-C-S-10

Following the representative procedure for the quinidine-based ketone employing 4-fluoro-2-hydroxyacetophenone, 32 mg (40%) of the S_NAr product **QD-C-S-10** was isolated as a brown solid. The other diastereomer was not isolated in pure form.

Major diastereomer **QD-C-S-10**:

^{1}H NMR (700 MHz, CD_3OD) δ 8.66 (d, *J* = 4.6 Hz, 1H), 7.94 (d, *J* = 9.2 Hz, 1H), 7.70 (dd, *J* = 4.6, 0.9 Hz, 1H), 7.63 (d, *J* = 8.9 Hz, 1H), 7.39 (dd, *J* = 9.2, 2.7 Hz, 1H), 7.34 (d, *J* = 2.7 Hz, 1H), 6.26 (dd, *J* = 8.9, 2.3 Hz, 1H), 6.00 (d, *J* = 2.3 Hz, 1H), 5.81 (s, 1H), 4.06 – 3.99 (m, 1H), 3.97 (s, 3H), 3.40 – 3.32 (m, 4H), 3.24 – 3.05 (m, 4H), 3.05 – 2.94 (m, 2H), 2.30 (s, 1H), 2.16 – 2.08 (m, 1H), 2.06 – 1.97 (m, 5H), 1.38 – 1.30 (m, 1H), 1.21 (ddd, *J* = 14.1, 9.6, 4.9 Hz, 1H).

^{13}C NMR (176 MHz, CD_3OD) δ 191.48, 162.52, 159.34, 155.24, 148.89, 147.81, 144.28, 131.23, 128.82, 127.26, 123.08, 119.40, 110.75, 107.95, 101.81, 98.62, 80.71, 70.59, 59.40, 57.25, 56.52, 50.20, 48.54, 44.12, 30.54, 26.03, 21.33, 20.53.

HRMS (ESI) calcd for $C_{30}H_{33}N_3O_4$ $[M + H]^+$ 500.25438, found 500.25381.

$[\alpha]_D^{20}$ +364 (*c* 0.1, MeOH).

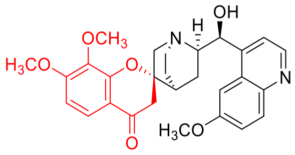

QD-C-S-11

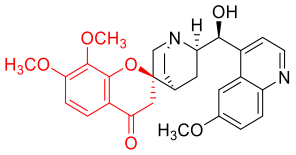

QD-C-R-7

Following the representative procedure for the quinidine-based ketone on a 0.24 mmol scale, 11 mg (9%) of **QD-C-S-11** and 11 mg (9%) of **QD-C-R-7** were isolated.

QD-C-S-11:

^{1}H **NMR** (700 MHz, $CDCl_3$) δ 8.69 (d, *J* = 4.5 Hz, 1H), 7.99 (d, *J* = 9.1 Hz, 1H), 7.58 (d, *J* = 8.9 Hz, 1H), 7.55 (d, *J* = 4.5 Hz, 1H), 7.33 (dd, *J* = 9.1, 2.7 Hz, 1H), 7.12 (d, *J* = 2.7 Hz, 1H), 6.58 (d, *J* = 9.0 Hz, 1H), 5.71 (bs, 1H), 3.90 (s, 3H), 3.88 (s, 3H), 3.87 (s, 3H), 3.81 (d, *J* = 14.8 Hz, 1H), 3.11 – 2.99 (m, 5H), 2.95 (t, *J* = 11.7 Hz, 1H), 2.26 – 2.20 (m, 1H), 2.05 – 1.92 (m, 2H), 1.26 – 1.16 (m, 2H).

^{13}C NMR (176 MHz, $CDCl_3$) δ 191.83, 159.22, 158.06, 153.79, 147.78, 147.14, 144.29, 137.58, 131.88, 126.20, 122.58, 121.76, 118.12, 116.42, 105.38, 100.97, 81.97, 72.05, 61.11, 58.55, 57.07, 56.31, 55.83, 49.97, 44.20, 30.04, 21.39, 20.60.

HRMS (ESI) calcd for $C_{28}H_{30}N_2O_6$ $[M + H]^+$ 491.21766, found 491.21719

$[\alpha]_D^{20}$ +200 (*c* 0.164, MeOH)

QD-C-R-7:

^{1}H NMR (700 MHz, $CDCl_3$) δ 8.75 (d, *J* = 4.5 Hz, 1H), 8.01 (d, *J* = 9.1 Hz, 1H), 7.65 (d, *J* = 8.9 Hz, 1H), 7.58 (d, *J* = 4.5 Hz, 1H), 7.34 (dd, *J* = 9.1, 2.7 Hz, 1H), 7.27 (d, *J* = 2.7 Hz, 1H), 6.66 (d, *J* = 8.9 Hz, 1H), 5.82 (d, *J* = 3.9 Hz, 1H), 4.09 – 4.03 (m, 4H), 3.97 (s, 3H), 3.89 (s, 3H), 3.27 (ddd, *J* = 10.8, 6.6, 3.9 Hz, 1H), 2.92 – 2.83 (m, 3H), 2.82 – 2.72 (m, 2H), 2.26 (p, *J* = 3.1 Hz, 1H), 2.24 – 2.19 (m, 1H), 1.62 – 1.57 (m, 1H), 1.54 – 1.49 (m, 1H), 1.14 (ddd, *J* = 13.9, 10.7, 3.3 Hz, 1H).

^{13}C NMR (176 MHz, $CDCl_3$) δ 190.81, 159.31, 157.95, 153.02, 147.94, 147.20, 144.50, 137.48, 131.95, 126.54, 122.94, 121.64, 118.54, 116.23, 105.82, 101.27, 81.98, 72.77, 61.57, 59.17, 57.48, 56.36, 55.80, 50.38, 45.40, 29.07, 21.84, 20.93.

HRMS (ESI) calcd for $C_{28}H_{30}N_2O_6$ $[M + H]^+$ 491.21766, found 491.21733

$[\alpha]_D^{20}$ +51.6 (*c* 0.124, MeOH)

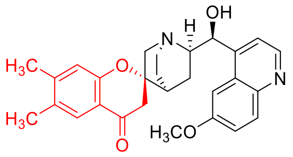

QD-C-S-12

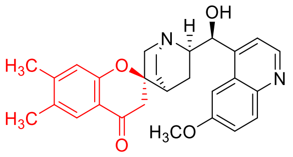

QD-C-R-8

Following the representative procedure for the quinidine-based ketone on a 0.24 mmol scale, 46 mg (42%) of **QD-C-S-12** and 8 mg (7%) of **QD-C-R-8** were isolated.

Major diastereomer **QD-C-S-12**:

The sample was dissolved in a mixture of $CD_3OD/CDCl_3$. **^{1}H NMR** (700 MHz, CD_3OD) δ 8.63 (d, *J* = 4.6 Hz, 1H), 7.91 (d, *J* = 9.1 Hz, 1H), 7.67 (dd, *J* = 4.5, 0.8 Hz, 1H), 7.51 (s, 1H), 7.35 (dd, *J* = 9.2, 2.7 Hz, 1H), 7.25 (d, *J* = 2.7 Hz, 1H), 6.78 (s, 1H), 5.67 (s, 1H), 3.96 – 3.89 (m, 4H), 3.16 – 3.09 (m, 1H), 3.09 – 2.95 (m, 5H), 2.27 – 2.21 (m, 4H), 2.18 (s, 3H), 2.06 (ddt, *J* = 13.4, 8.5, 2.1 Hz, 1H), 1.94 – 1.85 (m, 1H), 1.28 – 1.22 (m, 1H), 1.13 (ddd, *J* = 14.4, 9.5, 4.8 Hz, 1H).

The sample was dissolved in a mixture of $CD_3OD/CDCl_3$. **^{13}C NMR** (176 MHz, CD_3OD) δ 194.11, 159.04, 158.89, 149.17, 148.16, 147.53, 144.03, 131.05, 130.70, 127.04, 126.91, 122.89, 119.56, 119.35, 119.03, 101.50, 81.22, 71.05, 58.97, 57.22, 56.21, 50.09, 44.46, 29.76, 21.08, 20.70, 20.67, 18.93.

HRMS (ESI) calcd for $C_{28}H_{30}N_2O_4$ $[M + H]^+$ 459.22783, found 459.22764

$[\alpha]_D^{20}$ +307.5 (*c* 0.093, MeOH)

Minor diastereomer **QD-C-R-8**:

^{1}H NMR (700 MHz, $CDCl_3$) δ 8.74 (d, *J* = 4.5 Hz, 1H), 8.02 (d, *J* = 9.1 Hz, 1H), 7.60 – 7.55 (m, 2H), 7.35 (dd, *J* = 9.2, 2.7 Hz, 1H), 7.28 (d, *J* = 2.7 Hz, 1H), 6.83 (s, 1H), 5.71 (bs, 1H), 3.86 (s, 3H), 3.71 (d, *J* = 7.1 Hz, 1H), 3.28 – 3.19 (m, 1H), 2.85 (d, *J* = 16.4 Hz, 1H), 2.81 (d, *J* = 16.4 Hz, 1H), 2.74 (d, *J* = 14.9 Hz, 1H), 2.68 (ddd, *J* = 13.7, 10.0, 7.0 Hz, 1H), 2.43 – 2.34 (m, 1H), 2.31 (s, 3H), 2.28 – 2.25 (m, 1H), 2.22 (s, 3H), 1.67 – 1.59 (m, 1H), 1.57 – 1.51 (m, 1H), 1.32 – 1.27 (m, 2H).

^{13}C NMR (176 MHz, $CDCl_3$) δ 191.78, 157.93, 157.65, 147.85, 147.30, 144.60, 131.97, 130.42, 126.82, 121.71, 118.97, 118.89, 118.58, 101.41, 59.57, 57.28, 55.74, 49.70, 46.35, 29.46, 20.71, 18.92.

HRMS (ESI) calcd for $C_{28}H_{30}N_2O_4$ $[M + H]^+$ 459.22783, found 459.22728

$[\alpha]_D^{20}$ +57.5 (*c* 0.08, MeOH)

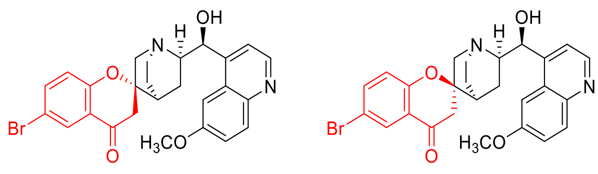

Following the representative procedure for the quinidine-based ketone on a 0.24 mmol scale, 74 mg (61%) of **QD-C-S-13** and 16 mg (13%) of **QD-C-R-9** were isolated.

Major diastereomer **QD-C-S-13**:

The sample was dissolved in a mixture of $CD_3OD/CDCl_3$. **^{1}H NMR** (700 MHz, CD_3OD) δ 8.61 (d, *J* = 4.6 Hz, 1H), 7.91 (d, *J* = 9.2 Hz, 1H), 7.88 (d, *J* = 2.6 Hz, 1H), 7.64 (dd, *J* = 4.6, 0.9 Hz, 1H), 7.55 (dd, *J* = 8.8, 2.6 Hz, 1H), 7.33 (dd, *J* = 9.2, 2.7 Hz, 1H), 7.18 (d, *J* = 2.7 Hz, 1H), 6.89 (d, *J* = 8.8 Hz, 1H), 5.62 (s, 1H), 3.95 – 3.89 (m, 4H), 3.24 – 3.16 (m, 1H), 3.06 – 2.92 (m, 5H), 2.20 (dt, *J* = 4.7, 2.5 Hz, 1H), 2.05 – 1.98 (m, 1H), 1.81 (ddt, *J* = 10.4, 7.4, 3.3 Hz, 1H), 1.26 – 1.18 (m, 1H), 1.12 – 1.06 (m, 1H).

The sample was dissolved in a mixture of $CD_3OD/CDCl_3$. **^{13}C NMR** (176 MHz, CD_3OD) δ 192.58, 159.09, 158.60, 148.75, 147.31, 143.72, 139.55, 130.91, 129.19, 126.62, 122.58, 122.50, 120.86, 118.64, 114.19, 101.17, 81.89, 70.90, 58.45, 56.83, 55.96, 49.83, 29.36, 23.80, 20.70, 20.47.

HRMS (ESI) calcd for $C_{26}H_{25}N_2O_4Br$ $[M + H]^+$ 511.10500, found 511.10491.

$[\alpha]_D^{20}$ +304.9 (*c* 0.144, MeOH)

Minor diastereomer **QD-C-R-9**:

^{1}H NMR (700 MHz, CD_3OD) δ 8.67 (d, *J* = 4.5 Hz, 1H), 7.99 (d, *J* = 9.2 Hz, 1H), 7.94 (d, *J* = 2.5 Hz, 1H), 7.58 (dd, *J* = 8.8, 2.6 Hz, 1H), 7.51 (d, *J* = 4.5 Hz, 1H), 7.33 (dd, *J* = 9.2, 2.7 Hz, 1H), 7.24 (d, *J* = 2.7 Hz, 1H), 6.94 (d, *J* = 8.7 Hz, 1H), 5.61 (d, *J* = 5.2 Hz, 1H), 3.86 (s, 3H), 3.68 (dd, *J* = 15.1, 2.3 Hz, 1H), 3.21 – 3.12 (m, 1H), 2.90 – 2.78 (m, 3H), 2.70 (d, *J* = 14.8 Hz, 1H), 2.62 (ddd, *J* = 13.8, 9.8, 7.3 Hz, 1H), 2.39 – 2.31 (m, 1H), 2.27 – 2.19 (m, 1H), 1.65 – 1.48 (m, 2H), 1.32 (ddd, *J* = 13.7, 9.9, 4.0 Hz, 1H).

^{13}C NMR (176 MHz, CD_3OD) δ 190.90, 158.52, 157.90, 147.71, 147.21, 144.43, 139.20, 131.80, 129.27, 126.83, 122.36, 121.65, 120.64, 118.64, 114.22, 101.49, 81.58, 72.24, 59.24, 57.06, 55.68, 49.60, 46.18, 29.53, 22.02, 21.55.

HRMS (ESI) calcd for $C_{26}H_{25}N_2O_4$ $[M + H]^+$ 509.10705, found 509.10675 .

$[\alpha]_D^{20}$ +60 (*c* 0.11, MeOH).

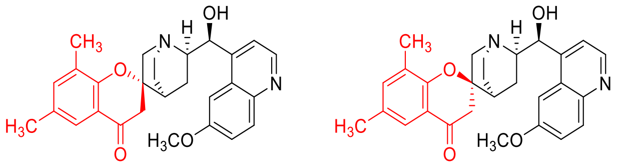

QD-C-S-14 **QD-C-R-10**

Following the representative procedure for the quinidine-based ketone on a 0.24 mmol scale, 57 mg (52%) of **QD-C-S-14** and 6 mg (5%) of **QD-C-R-10** were isolated.

Major diastereomer **QD-C-S-14:**

^{1}H NMR (500 MHz, $CDCl_3$) δ 8.55 (d, J = 4.5 Hz, 1H), 7.91 (d, J = 9.2 Hz, 1H), 7.52 (d, J = 4.5 Hz, 1H), 7.42 (d, J = 2.3 Hz, 1H), 7.28 – 7.26 (m, 1H), 7.14 (d, J = 2.3 Hz, 1H), 7.08 (d, J = 2.8 Hz, 1H), 5.69 (s, 1H), 3.94 – 3.74 (m, 4H), 3.11 – 2.85 (m, 6H), 2.24 – 2.20 (m, 4H), 2.17 (s, 3H), 2.05 – 1.95 (m, 1H), 1.82 – 1.74 (m, 1H), 1.26 – 1.18 (m, 1H), 1.14 (ddt, J = 14.0, 9.4, 4.7 Hz, 1H).

^{13}C NMR (126 MHz, $CDCl_3$) δ 193.50, 157.89, 155.77, 147.53, 143.93, 138.56, 131.49, 129.96, 127.16, 126.15, 125.29, 123.78, 121.56, 120.34, 118.26, 101.03, 80.69, 71.39, 58.48, 56.92, 55.76, 49.68, 44.12, 29.45, 21.09, 20.71, 20.44, 15.57.

HRMS (ESI) calcd for $C_{28}H_{30}N_2O_4$ $[M + H]^+$ 459.22783, found 459.22728.

$[\alpha]_D^{20}$ +200 (c 0.156, MeOH).

Minor diastereomer **QD-C-R-10**:

^{1}H NMR (500 MHz, $CDCl_3$) δ 8.72 (d, J = 4.5 Hz, 1H), 7.99 (d, J = 9.2 Hz, 1H), 7.55 (d, J = 4.6 Hz, 1H), 7.48 (d, J = 2.3 Hz, 1H), 7.32 (dd, J = 9.2, 2.7 Hz, 1H), 7.20 (bs, 2H), 5.82 – 5.69 (m, 1H), 3.90 – 3.72 (m, 4H), 3.20 (td, J = 8.8, 4.7 Hz, 1H), 2.95 – 2.87 (m, 1H), 2.87 – 2.79 (m, 2H), 2.79 – 2.61 (m, 2H), 2.46 – 2.40 (m, 1H), 2.33 (s, 3H), 2.30 – 2.22 (m, 4H), 1.69 – 1.61 (m, 1H), 1.59 – 1.51 (m, 1H), 1.26 (ddd, J = 13.7, 9.7, 4.1 Hz, 1H).

^{13}C NMR (126 MHz, $CDCl_3$) δ 192.64, 157.95, 155.76, 147.78, 146.75, 144.48, 138.73, 131.92, 130.27, 127.59, 126.69, 123.91, 121.83, 120.32, 118.67, 100.99, 80.26, 71.56, 59.07, 57.37, 55.72, 49.70, 46.51, 29.64, 21.74, 21.46, 20.51, 15.89.

HRMS (ESI) calcd for $C_{28}H_{30}N_2O_4$ $[M + H]^+$ 459.22783, found 459.22712.

$[\alpha]_D^{20}$ +112.5 (c 0.088, MeOH).

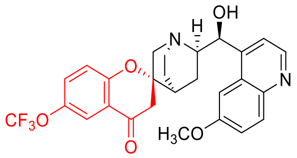

QD-C-S-15

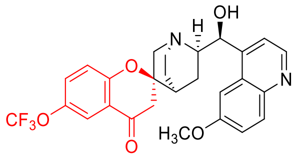

QD-C-R-11

Following the representative procedure for the quinidine-based ketone on a 0.24 mmol scale, 37 mg (30%) of **QD-C-S-15** and 5 mg (4%) of **QD-C-R-11** were isolated.

Major diastereomer **QD-C-S-15**:

^{1}H NMR (600 MHz, $CDCl_3$) δ 8.56 (d, *J* = 4.5 Hz, 1H), 7.93 (d, *J* = 9.2 Hz, 1H), 7.66 (dd, *J* = 3.0, 1.3 Hz, 1H), 7.51 (d, *J* = 4.5 Hz, 1H), 7.32 (dd, *J* = 9.0, 3.0 Hz, 1H), 7.29 (dd, *J* = 9.2, 2.7 Hz, 1H), 7.05 (d, *J* = 2.6 Hz, 1H), 6.99 (d, *J* = 9.0 Hz, 1H), 5.65 (bs, 1H), 4.52 (bs, 1H), 3.86 (s, 3H), 3.83 (d, *J* = 15.5 Hz, 1H), 3.20 (d, *J* = 16.8 Hz, 1H), 3.07 – 2.89 (m, 5H), 2.27 – 2.18 (m, 1H), 2.03 – 1.93 (m, 1H), 1.79 (tt, *J* = 10.1, 3.6 Hz, 1H), 1.24 – 1.17 (m, 1H), 1.14 (ddd, *J* = 14.0, 9.5, 4.8 Hz, 1H).

^{13}C NMR (151 MHz, $CDCl_3$) δ 191.98, 158.22, 158.01, 147.57, 147.41, 143.97, 143.05 (q, *J* = 2.1 Hz), 131.57, 129.65, 126.11, 121.68, 121.44, 120.60 (q, *J* = 257 Hz), 120.01, 118.76, 118.10, 100.96, 82.08, 71.97, 58.45, 57.04, 55.75, 49.82, 43.83, 29.21, 21.03, 20.53.

^{19}F NMR (565 MHz, $CDCl_3$) δ -58.42.

HRMS (ESI) calcd for $C_{27}H_{25}N_2O_5F_3$ $[M + H]^+$ 515.17883, found 515.17841.

$[\alpha]_D^{20}$ +243.8 (*c* 0.137, MeOH)

Minor diastereomer **QD-C-R-11**:

^{1}H NMR (600 MHz, $CDCl_3$) δ 8.72 (d, *J* = 4.5 Hz, 1H), 8.02 (d, *J* = 9.2 Hz, 1H), 7.69 (d, *J* = 2.4 Hz, 1H), 7.54 (d, *J* = 4.5 Hz, 1H), 7.37 (td, *J* = 9.4, 3.0 Hz, 2H), 7.27 (d, *J* = 2.8 Hz, 1H), 7.08 (d, *J* = 8.9 Hz, 1H), 5.65 (d, *J* = 5.1 Hz, 1H), 3.89 (s, 3H), 3.72 (dd, *J* = 15.0, 2.3 Hz, 1H), 3.20 (td, *J* = 8.7, 4.7 Hz, 1H), 2.92 – 2.84 (m, 3H), 2.74 (d, *J* = 15.5 Hz, 1H), 2.65 (ddd, *J* = 13.7, 9.7, 7.3 Hz, 1H), 2.35 (ddt, *J* = 13.1, 7.8, 2.4 Hz, 1H), 2.29 – 2.23 (m, 1H), 1.66 – 1.51 (m, 2H), 1.33 (ddd, *J* = 13.7, 9.9, 4.0 Hz, 1H).

^{13}C NMR (151 MHz, $CDCl_3$) δ 191.06, 157.95, 157.93, 147.77, 147.03, 144.52, 143.28 (q, *J* = 2.2 Hz), 131.92, 129.76, 126.81, 121.69, 121.40, 120.53 (q, *J* = 272 Hz), 120.20, 118.91, 118.60, 101.45, 81.77, 72.39, 59.21, 57.17, 55.70, 49.65, 46.14, 29.42, 22.03, 21.45.

^{19}F NMR (565 MHz, $CDCl_3$) δ -58.41.

HRMS (ESI) calcd for $C_{27}H_{25}N_2O_5F_3$ $[M + H]^+$ 515.17883, found 515.17863.

$[\alpha]_D^{20}$ +90 (*c* 0.09, MeOH).

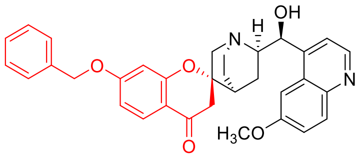

QD-C-S-16

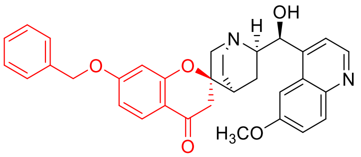

QD-C-R-12

Following the representative procedure for the quinidine-based ketone on a 0.24 mmol scale, 54 mg (42%) of **QD-C-S-16** and 13 mg (10%) of **QD-C-R-12** were isolated.

Major diastereomer **QD-C-S-16**:

^{1}H NMR (500 MHz, $CDCl_3$) δ 8.56 (d, *J* = 4.5 Hz, 1H), 7.92 (d, *J* = 9.2 Hz, 1H), 7.73 (d, *J* = 8.8 Hz, 1H), 7.54 (d, *J* = 4.6 Hz, 1H), 7.42 – 7.36 (m, 4H), 7.35 – 7.31 (m, 1H), 7.28 (dd, *J* = 9.2, 2.6 Hz, 1H), 7.09 (d, *J* = 2.7 Hz, 1H), 6.59 (dd, *J* = 8.8, 2.4 Hz, 1H), 6.48 (d, *J* = 2.4 Hz, 1H), 5.68 (s, 1H), 5.06 (s, 2H), 3.92 – 3.79 (m, 4H), 3.10 (d, *J* = 16.8 Hz, 1H), 3.06 – 2.85 (m, 5H), 2.27 – 2.17 (m, 1H), 2.05 – 1.95 (m, 1H), 1.87 – 1.75 (m, 1H), 1.27 – 1.07 (m, 2H).

^{13}C NMR (126 MHz, $CDCl_3$) δ 191.56, 165.62, 161.86, 157.88, 147.57, 143.95, 135.92, 131.51, 128.78, 128.40, 128.39, 127.64, 126.15, 121.57, 118.23, 115.17, 110.04, 102.43, 101.02, 81.67, 71.62, 70.37, 58.51, 57.06, 55.76, 49.78, 43.82, 29.43, 21.25, 20.53. An aromatic carbon is missing from the spectra.

HRMS (ESI) calcd for $C_{33}H_{32}N_2O_5$ $[M + H]^+$ 537.23840, found 537.23824.

$[\alpha]_D^{20}$ +237.2 (*c* 0.137, MeOH).

Minor diastereomer **QD-C-R-12**:

^{1}H NMR (700 MHz, $CDCl_3$) δ 8.71 (d, *J* = 4.5 Hz, 1H), 8.00 (d, *J* = 9.1 Hz, 1H), 7.79 (d, *J* = 8.7 Hz, 1H), 7.54 (d, *J* = 4.5 Hz, 1H), 7.46 – 7.43 (m, 2H), 7.41 (t, *J* = 7.6 Hz, 2H), 7.37 – 7.31 (m, 2H), 7.25 (s, 1H), 6.65 (dd, *J* = 8.8, 2.4 Hz, 1H), 6.56 (d, *J* = 2.3 Hz, 1H), 5.69 (s, 1H), 5.12 (s, 2H), 3.84 (s, 3H), 3.72 (d, *J* = 14.7 Hz, 1H), 3.20 (td, *J* = 8.7, 5.0 Hz, 1H), 2.90 – 2.84 (m, 1H), 2.82 (d, *J* = 16.4 Hz, 1H), 2.79 (d, *J* = 16.5 Hz, 1H), 2.73 (d, *J* = 14.9 Hz, 1H), 2.65 (ddd, *J* = 13.5, 9.9, 7.2 Hz, 1H), 2.44 – 2.35 (m, 1H), 2.26 (t, *J* = 3.4 Hz, 1H), 1.70 – 1.59 (m, 1H), 1.59 – 1.48 (m, 1H), 1.30 (ddd, *J* = 13.7, 9.9, 3.9 Hz, 1H).

^{13}C NMR (176 MHz, $CDCl_3$) δ 190.41, 165.74, 161.46, 157.93, 147.77, 147.01, 144.51, 136.01, 131.88, 128.88, 128.62, 128.51, 127.74, 126.80, 121.71, 118.66, 115.18, 110.28, 102.82, 101.44, 81.20, 71.84, 70.56, 59.41, 57.14, 55.78, 49.61, 46.23, 29.80, 21.96, 21.41.

HRMS (ESI) calcd for $C_{33}H_{32}N_2O_5$ $[M + H]^+$ 537.23840, found 537.23806

$[\alpha]_D^{20}$ +38.5 (*c* 0.166, MeOH)

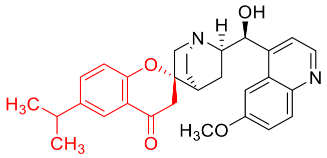

QD-C-S-17

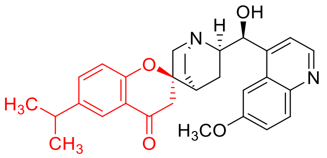

QD-C-R-13

Following the representative procedure for the quinidine-based ketone on a 0.24 mmol scale, 61 mg (54%) of **QD-C-S-17** and 9 mg (8%) of **QD-C-R-13** were isolated.

Major diastereomer **QD-C-S-17**:

^{1}H NMR (700 MHz, $CDCl_3$) δ 8.55 (d, *J* = 4.5 Hz, 1H), 7.90 (d, *J* = 9.2 Hz, 1H), 7.64 (d, *J* = 2.4 Hz, 1H), 7.52 (d, *J* = 4.6 Hz, 1H), 7.34 (dd, *J* = 8.6, 2.4 Hz, 1H), 7.27 (d, *J* = 2.6 Hz, 1H), 7.08 (d, *J* = 2.8 Hz, 1H), 6.87 (d, *J* = 8.5 Hz, 1H), 5.69 (s, 1H), 3.86 – 3.81 (m, 4H), 3.15 (d, *J* = 16.7 Hz, 1H), 3.05 – 2.93 (m, 4H), 2.92 – 2.88 (m, 1H), 2.84 (hept, *J* = 6.8 Hz, 1H), 2.25 – 2.19 (m, 1H), 2.03 – 1.96 (m, 1H), 1.86 – 1.78 (m, 1H), 1.21 – 1.18 (m, 7H), 1.12 (ddd, *J* = 14.0, 9.5, 4.8 Hz, 1H).

^{13}C NMR (176 MHz, $CDCl_3$) δ 193.32, 158.09, 157.93, 147.56, 147.53, 143.94, 141.76, 135.23, 131.48, 126.16, 123.57, 121.62, 120.74, 118.23, 118.20, 101.03, 81.00, 71.58, 58.57, 57.06, 55.76, 49.79, 44.17, 33.34, 29.14, 23.98, 21.09, 20.51.

HRMS (ESI) calcd for $C_{29}H_{32}N_2O_4$ $[M + H]^+$ 473.24348, found 473.24275.

$[\alpha]_D^{20}$ +184.8 (*c* 0.138, MeOH).

Minor diastereomer **QD-C-R-13**:

^{1}H NMR (500 MHz, $CDCl_3$) δ 8.71 (d, *J* = 4.6 Hz, 1H), 8.01 (d, *J* = 9.2 Hz, 1H), 7.69 (d, *J* = 2.4 Hz, 1H), 7.55 (d, *J* = 4.5 Hz, 1H), 7.41 (dd, *J* = 8.5, 2.4 Hz, 1H), 7.34 (dd, *J* = 9.2, 2.7 Hz, 1H), 7.27 (d, *J* = 2.9 Hz, 1H), 6.99 (d, *J* = 8.4 Hz, 1H), 5.68 (d, *J* = 4.8 Hz, 1H), 3.86 (s, 3H), 3.72 (d, *J* = 15.0 Hz, 1H), 3.26 – 3.15 (m, 1H), 2.92 – 2.82 (m, 4H), 2.73 (d, *J* = 15.0 Hz, 1H), 2.71 – 2.63 (m, 1H), 2.40 – 2.32 (m, 1H), 2.30 – 2.25 (m, 1H), 1.67 – 1.58 (m, 1H), 1.58 – 1.49 (m, 1H), 1.30 (ddd, *J* = 13.8, 10.0, 3.8 Hz, 1H), 1.24 (d, *J* = 6.9 Hz, 6H).

^{13}C NMR (126 MHz, $CDCl_3$) δ 192.37, 157.88, 157.62, 147.81, 147.12, 144.51, 142.25, 135.44, 131.90, 126.79, 123.81, 121.72, 120.69, 118.57, 118.30, 101.31, 80.81, 72.18, 59.44, 57.26, 55.72, 49.70, 46.39, 33.44, 29.33, 24.06, 24.04, 21.98, 21.51.

HRMS (ESI) calcd for $C_{29}H_{32}N_2O_4$ $[M + H]^+$ 473.24348, found 473.24278.

$[\alpha]_D^{20}$ +57.1 (*c* 0.091, MeOH).

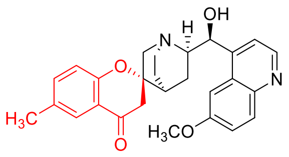

QD-C-S-18

Following the representative procedure for the quinidine-based ketone, 52 mg (48%) of **QD-C-S-18** was isolated. The other diastereomer was not isolated in pure form.

QD-C-S-18:

^{1}H NMR (500 MHz, $CDCl_3$) δ 8.62 (d, J = 4.6 Hz, 1H), 7.77 (d, J = 9.2 Hz, 1H), 7.70 (d, J = 8.0 Hz, 1H), 7.59 (d, J = 4.6 Hz, 1H), 7.07 (dd, J = 9.1, 2.5 Hz, 1H), 6.90 (d, J = 2.7 Hz, 1H), 6.81 (dd, J = 8.1, 1.5 Hz, 1H), 6.76 (s, 1H), 6.17 (bs, 1H), 4.39 – 4.22 (m, 1H), 3.59 (s, 3H), 3.28 (d, J = 14.4 Hz, 1H), 3.16 (tt, J = 11.3, 4.6 Hz, 4H), 3.02 (d, J = 16.9 Hz, 1H), 2.38 – 2.28 (m, 4H), 2.18 – 2.08 (m, 1H), 2.04 – 1.90 (m, 1H), 1.40 – 1.28 (m, 1H), 1.18 – 1.10 (m, 1H).

^{13}C NMR (126 MHz, $CDCl_3$) δ 191.00, 159.00, 158.07, 148.49, 147.30, 145.35, 143.80, 131.42, 126.65, 125.56, 123.32, 122.08, 118.71, 118.44, 118.31, 100.10, 79.63, 68.42, 58.92, 56.29, 56.21, 49.32, 43.65, 28.77, 22.06, 19.85, 19.28.

HRMS (ESI) calcd for $C_{27}H_{28}N_2O_4$ $[M + H]^+$ 445.21218, found 445.21193.

$[\alpha]_D^{20}$ +172 (c 0.1, DMSO).

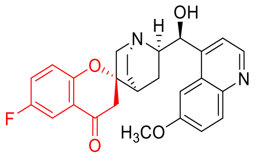

QD-C-S-19

Following the representative procedure for the quinidine-based ketone, 61 mg (57%) of **QD-C-S-19** was isolated. The other diastereomer was not isolated in pure form.

QD-C-S-19:

The NMR sample was dissolved in 3 $CDCl_3$: 1 CD_3OD. **^{1}H NMR** (600 MHz, CD_3OD) δ 8.61 (d, *J* = 4.6 Hz, 1H), 7.88 (d, *J* = 9.2 Hz, 1H), 7.64 (d, *J* = 4.6 Hz, 1H), 7.44 (dd, *J* = 8.1, 3.2 Hz, 1H), 7.29 (dd, *J* = 9.2, 2.7 Hz, 1H), 7.21 (ddd, *J* = 9.0, 7.7, 3.2 Hz, 1H), 7.18 (d, *J* = 2.7 Hz, 1H), 6.97 (dd, *J* = 9.1, 4.1 Hz, 1H), 5.83 (bs, 1H), 4.10 – 4.06 (m, 1H), 3.89 (s, 3H), 3.27 – 2.98 (m, 5H), 2.27 (s, 1H), 2.12 – 2.03 (m, 1H), 1.99 (d, *J* = 13.5 Hz, 1H), 1.95 – 1.84 (m, 1H), 1.35 – 1.26 (m, 1H), 1.14 (ddd, *J* = 12.3, 9.6, 5.5 Hz, 1H).

^{13}C NMR (151 MHz, CD_3OD) δ 192.17, 158.59, 158.33, 156.28 (d, *J* = 132.7 Hz), 147.65, 147.15, 143.62, 130.87, 126.33, 124.41 (d, *J* = 24.4 Hz), 122.56, 121.54 (d, *J* = 6.4 Hz), 120.29 (d, *J* = 7.3 Hz), 118.60, 111.78 (d, *J* = 23.4 Hz), 100.89, 80.82, 69.55, 58.59, 56.32, 56.12, 49.51, 29.04, 28.92, 20.14, 19.82.

^{19}F NMR (565 MHz, CD_3OD) δ -121.36.

HRMS (ESI) calcd for $C_{26}H_{25}N_2O_4F$ $[M + H]^+$ 449.18711, found 449.18697.

$[\alpha]^{20}_{D}$ +201 (*c* 0.1, DMSO).

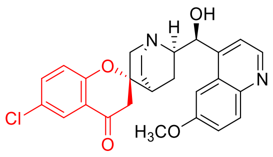

QD-C-S-20

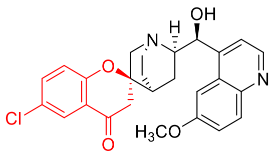

QD-C-R-14

Following the representative procedure for the quinidine-based ketone on a 0.24 mmol scale, 38 mg (34%) of **QD-C-S-20** and 10 mg (9%) of **QD-C-R-14** were isolated.

Major diastereomer **QD-C-S-20**:

The NMR sample was dissolved in 3 $CDCl_3$: 1 CD_3OD. **^{1}H NMR** (700 MHz, CD_3OD) δ 8.63 (d, *J* = 4.6 Hz, 1H), 7.92 (d, *J* = 9.2 Hz, 1H), 7.74 (d, *J* = 2.7 Hz, 1H), 7.66 (d, *J* = 4.5 Hz, 1H), 7.43 (dd, *J* = 8.8, 2.7 Hz, 1H), 7.35 (dd, *J* = 9.2, 2.7 Hz, 1H), 7.23 (d, *J* = 2.7 Hz, 1H), 6.97 (d, *J* = 8.8 Hz, 1H), 5.66 (bs, 1H), 4.00 – 3.88 (m, 4H), 3.25 – 3.18 (m, 1H), 3.11 – 2.93 (m, 4H), 2.22 (bs, 1H), 2.08 – 2.02 (m, 1H), 1.89 – 1.79 (m, 1H), 1.30 – 1.22 (m, 1H), 1.13 (ddd, *J* = 14.0, 9.4, 4.7 Hz, 1H).

^{13}C NMR (176 MHz, CD_3OD) δ 192.79, 158.83, 158.75, 148.88, 147.41, 143.87, 136.92, 130.99, 127.29, 126.81, 126.15, 122.71, 122.22, 120.67, 118.81, 101.33, 81.92, 70.91, 58.66, 56.91, 56.11, 49.92, 44.04, 29.51, 20.80, 20.51.

HRMS (ESI) calcd for $C_{26}H_{25}N_2O_4Cl$ $[M + H]^+$ 465.15756, found 465.15734.

$[\alpha]_D^{20}$ +165 (*c* 0.1, DMSO).

Minor diastereomer **QD-C-R-14**:

^{1}H NMR (700 MHz, $CDCl_3$) δ 8.69 (d, *J* = 4.5 Hz, 1H), 7.97 (d, *J* = 9.2 Hz, 1H), 7.79 (d, *J* = 2.7 Hz, 1H), 7.54 (d, *J* = 4.5 Hz, 1H), 7.46 (dd, *J* = 8.7, 2.7 Hz, 1H), 7.31 (dd, *J* = 9.2, 2.6 Hz, 1H), 7.22 (d, *J* = 2.7 Hz, 1H), 7.02 (d, *J* = 8.8 Hz, 1H), 5.75 (s, 1H), 3.83 (s, 4H), 3.26 – 3.16 (m, 1H), 2.94 – 2.82 (m, 3H), 2.76 (d, *J* = 14.8 Hz, 1H), 2.71 – 2.63 (m, 1H), 2.45 – 2.36 (m, 1H), 2.26 (bs, 1H), 1.68 – 1.60 (m, 1H), 1.60 – 1.53 (m, 1H), 1.32 – 1.25 (m, 1H).

^{13}C NMR (176 MHz, $CDCl_3$) δ 190.72, 157.98, 157.92, 147.70, 146.71, 144.43, 136.51, 131.84, 127.26, 126.64, 126.17, 121.82, 121.76, 120.35, 118.65, 101.28, 81.14, 71.52, 59.28, 56.90, 55.84, 49.50, 46.25, 29.47, 21.60, 21.05.

HRMS (ESI) calcd for $C_{26}H_{25}N_2O_4Cl$ $[M + H]^+$ 465.15756 and 467.15461, found 465.15731 and 467.15460.

$[\alpha]_D^{20}$ +86 (*c* 0.1, MeOH).

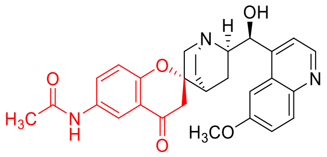

QD-C-S-21

Following the representative procedure for the quinidine-based ketone, 69 mg (59%) of **QD-C-S-21** was isolated. The other diastereomer was not isolated in pure form.

QD-C-S-21:

The NMR sample was dissolved in 3 $CDCl_3$: 1 CD_3OD. **^{1}H NMR** (500 MHz, CD_3OD) δ 8.65 (d, *J* = 4.6 Hz, 1H), 7.94 (d, *J* = 9.2 Hz, 1H), 7.83 (d, *J* = 2.7 Hz, 1H), 7.78 (dd, *J* = 8.9, 2.7 Hz, 1H), 7.68 (d, *J* = 4.6 Hz, 1H), 7.37 (dd, *J* = 9.2, 2.6 Hz, 1H), 7.28 (d, *J* = 2.7 Hz, 1H), 6.98 (d, *J* = 8.9 Hz, 1H), 5.74 (bs, 1H), 4.03 – 3.90 (m, 4H), 3.23 – 2.97 (m, 5H), 2.26 (bs, 1H), 2.15 – 2.01 (m, 4H), 1.96 – 1.84 (m, 1H), 1.32 – 1.25 (m, 1H), 1.20 – 1.10 (m, 1H).

^{13}C NMR (126 MHz, CD_3OD) δ 193.43, 170.61, 158.81, 156.65, 148.66, 147.55, 143.90, 133.05, 131.00, 129.99, 126.82, 122.68, 121.19, 119.19, 118.95, 117.52, 101.41, 81.21, 70.51, 58.77, 56.86, 56.79, 56.29, 29.62, 29.54, 23.64, 20.70, 20.31.

HRMS (ESI) calcd for $C_{28}H_{29}N3_2O_5$ $[M + H]^+$ 488.21800, found 488.21754.

$[\alpha]^{20}_{D}$ +165 (*c* 0.23, DMSO).

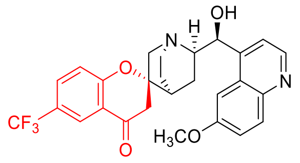

QD-C-S-22

Following the representative procedure for the quinidine-based ketone, 73 mg (61%) of **QD-C-S-22** was isolated. The other diastereomer was not isolated in pure form.

QD-C-S-22:

^{1}H NMR (600 MHz, $CDCl_3$) δ 8.53 (d, J = 4.5 Hz, 1H), 8.10 (d, J = 2.3 Hz, 1H), 7.90 (d, J = 9.2 Hz, 1H), 7.69 (dd, J = 8.8, 2.4 Hz, 1H), 7.51 (d, J = 4.5 Hz, 1H), 7.29 – 7.26 (m, 1H), 7.07 (d, J = 8.7 Hz, 1H), 7.05 (d, J = 2.7 Hz, 1H), 5.69 (bs, 1H), 3.91 (d, J = 14.8 Hz, 1H), 3.84 (s, 3H), 3.24 (d, J = 16.8 Hz, 1H), 3.11 – 2.89 (m, 5H), 2.22 (bs, 1H), 2.06 – 1.96 (m, 1H), 1.85 – 1.75 (m, 1H), 1.26 – 1.21 (m, 1H), 1.17 – 1.11 (m, 1H).

^{13}C NMR (151 MHz, $CDCl_3$) δ 191.70, 161.93, 158.01, 147.51, 147.34, 143.90, 132.84 (q, J = 3.3 Hz), 131.50, 126.07, 124.48 (q, J = 3.9 Hz), 123.83 (q, J = 33.5 Hz), 123.86 (q, J = 271.7 Hz), 121.67, 120.73, 119.28, 118.14, 100.95, 82.41, 71.62, 58.44, 56.94, 55.78, 49.75, 43.86, 29.30, 20.93, 20.41.

^{19}F NMR (565 MHz, $CDCl_3$) δ -62.12.

HRMS (ESI) calcd for $C_{27}H_{25}N_2O_4F_3$ $[M + H]^+$ 499.18392, found 499.18340

$[\alpha]_D^{20}$ +222 (c 0.1, MeOH)

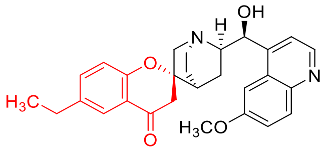

QD-C-S-23

Following the representative procedure for the quinidine-based ketone, 56 mg (51%) of **QD-C-S-23** was isolated. The other diastereomer was not isolated in pure form.

QD-C-S-23:

^{1}H NMR (700 MHz, $CDCl_3$) δ 8.56 (d, J = 4.5 Hz, 1H), 7.91 (d, J = 9.2 Hz, 1H), 7.60 (d, J = 2.3 Hz, 1H), 7.53 (d, J = 4.5 Hz, 1H), 7.30 (dd, J = 8.5, 2.3 Hz, 1H), 7.28 – 7.26 (m, 1H), 7.08 (d, J = 2.7 Hz, 1H), 6.87 (d, J = 8.4 Hz, 1H), 5.69 (bs, 1H), 3.90 – 3.78 (m, 4H), 3.14 (d, J = 16.7 Hz, 1H), 3.09 – 2.94 (m, 4H), 2.94 – 2.87 (m, 1H), 2.56 (q, J = 7.6 Hz, 2H), 2.22 (bs, 1H), 2.04 – 1.97 (m, 1H), 1.86 – 1.76 (m, 1H), 1.21 – 1.09 (m, 5H).

^{13}C NMR (176 MHz, $CDCl_3$) δ 193.24, 158.02, 157.95, 147.57, 147.45, 144.00, 137.14, 136.55, 131.55, 126.16, 125.01, 121.62, 120.80, 118.24, 118.23, 101.01, 80.97, 71.57, 58.61, 57.04, 55.78, 53.54, 49.80, 44.19, 29.20, 27.94, 21.11, 20.46, 15.54.

HRMS (ESI) calcd for $C_{28}H_{30}N_2O_4$ $[M + H]^+$ 459.22783, found 459.22761.

$[\alpha]^{20}_{D}$ +297 (c 0.1, MeOH).

Synthesis of sinomenine-based ketones:

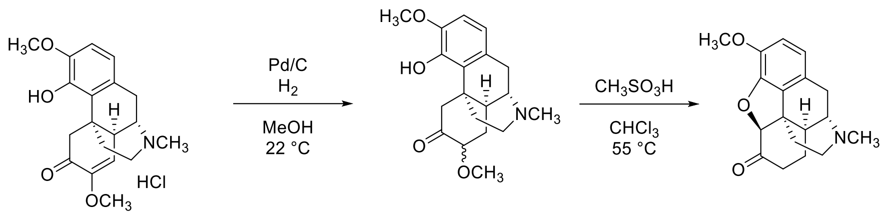

Sinomenine-HCl (12 g, 36.4 mmol) was dissolved in 100 ml methanol and then 8.5 ml of HCl (aq, 37%) was added dropwise. The flask was evacuated and refilled with argon (3x). Pd/C (300 mg, 10% Pd by weight) was then added. The reaction mixture was evacuated and refilled with H_2 (3x) and stirred for 2 h. After this time, LCMS showed the reaction to be complete. The reaction was evacuated and backfilled with argon (3x). The reaction was then filtered through Celite and evaporated to dryness. Diethyl ether was added to the crude material and concentrated. The dry solid was then dissolved in water, K_2CO_3 (aq) was added, and was extracted with DCM (3x). The combined organic layers were dried over Na_2SO_4 and concentrated to afford dihydrosinomenine in quantitative yield as a white powder. The crude material was used in the next step without further purification.

Dihydrosinomenine (4 g, 12 mmol, 1 equiv) was dissolved in $CHCl_3$ (60 ml) 250 ml round-bottomed flask. $MeSO_3H$ (15 ml) was added dropwise to the stirred solution. The reaction was heated to 55 ºC and stirred for 3 h. After cooling to room temperature, the mixture was quenched by slowly adding K_2CO_3 (aq) dropwise from an addition funnel until the pH was > 9. The phases were separated and the aqueous phase was extracted with $CHCl_3$ (3X). The organic phases were combined, dried, and concentrated. Purification was achieved by silica chromatography (50% magic mix (50:8:1 DCM:EtOH:NH_3) in 50% DCM) to afford 2.5 g (69%) of the desired product as a white powder. ^{1}H NMR (700 MHz, Chloroform-*d*) δ 6.67 (d, *J* = 8.1 Hz, 1H), 6.60 (d, *J* = 8.5 Hz, 1H), 4.63 (s, 1H), 3.86 (s, 3H), 3.15 (dd, *J* = 5.6, 2.9 Hz, 1H), 2.99 (d, *J* = 18.4 Hz, 1H), 2.59 – 2.51 (m, 2H), 2.40 (s, 3H), 2.38 – 2.34 (m, 2H), 2.30 (dd, *J* = 18.5, 5.6 Hz, 1H), 2.17 (td, *J* = 12.3, 3.6 Hz, 1H), 2.05 (td, *J* = 12.3, 4.8 Hz, 1H), 1.84 – 1.79 (m, 1H), 1.77 (dd, *J* = 12.5, 1.9 Hz, 1H), 1.21 (qd, *J* = 12.7, 4.8 Hz, 1H). ^{13}C NMR (176 MHz, Chloroform-*d*) δ 208.06, 145.45, 142.90, 127.21, 126.15, 119.87, 114.75, 91.37, 59.26, 56.85, 46.90, 46.83, 42.80, 42.35, 40.18, 35.31, 25.58, 20.05; HRMS (ESI) calcd for $C_{18}H_{21}NO_3$ $[M + H]^+$ 300.15942, found 300.15904; $[\alpha]^{20}_D$ +183.4 (*c* 1.0, $CHCl_3$).

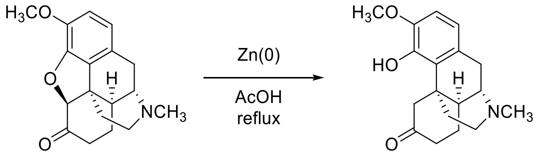

To a 100 ml RBF was added the ketone (2 g, 6.68 mmol) and 40 ml of AcOH. The solution was brought to reflux and freshly activated zinc (3.92 g, 60.1 mmol, 9 equiv) was added. The reaction was refluxed for 4 more hours followed by a second addition of activated zinc (3.92 g, 60.1 mmol, 9 equiv). The reaction was further refluxed for 16 h. The reaction was then cooled to 0 °C, made basic with NH_4OH (aq.), and extracted with DCM (3x). The organic layers were combined, dried and concentrated. The crude product was sufficiently pure and did not require further purification. 1.9 g (94%) of the desired product as a white powder. In some cases, purification was necessary and could be achieved by silica chromatography (50% magic mix (50:8:1 DCM:EtOH:NH_3) in 50% DCM). ^{1}H NMR (700 MHz, CD_2Cl_2) δ 6.70 (d, *J* = 8.2 Hz, 1H), 6.62 (d, *J* = 8.2 Hz, 1H), 6.14 (s, 1H), 4.12 (dd, *J* = 13.6, 2.3 Hz, 1H), 3.83 (s, 3H), 2.98 – 2.91 (m, 2H), 2.63 (dd, *J* = 18.3, 6.0 Hz, 1H), 2.45 – 2.37 (m, 2H), 2.35 (s, 3H), 2.20 (dd, *J* = 13.6, 1.1 Hz, 1H), 2.19 – 2.13 (m, 2H), 1.98 (td, *J* = 12.2, 3.3 Hz, 1H), 1.87 – 1.79 (m, 2H), 1.75 (td, *J* = 12.5, 4.6 Hz, 1H), 1.63 (qd, *J* = 13.3, 5.0 Hz, H); ^{13}C NMR (176 MHz, CD_2Cl_2) δ 205.94, 140.41, 140.18, 126.63, 118.85, 114.14, 104.36, 52.54, 51.63, 45.99, 42.09, 40.75, 38.05, 36.85, 36.70, 34.30, 22.83, 19.23; HRMS (ESI) calcd for $C_{18}H_{23}NO_3$ $[M + H]^+$ 302.17507, found 302.17559; $[\alpha]_D^{20}$ +51.2 (*c* 0.12, MeOH).

General procedure for sinomenine-based chromanones (**SM-C-S** and **SM-C-R**):

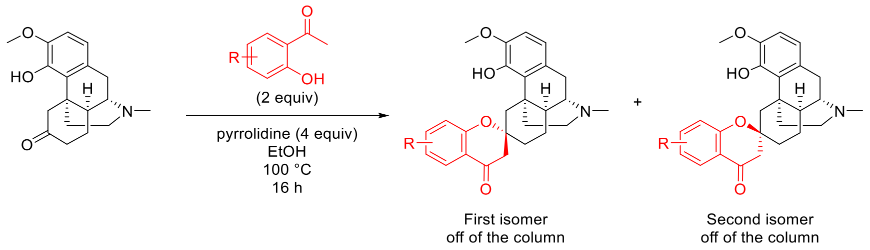

To an oven-dried pressure vessel equipped with a stir bar that was cooled under an atm of Ar was added the sinomenine-derived ketone (100 mg, 0.33 mmol, 1 equiv). Then, anhydrous EtOH (1 ml) was added followed by a 2-hydroxyacetophenone derivative (0.66 mmol, 2 equiv) and pyrrolidine (104 ul, 1.33 mmol, 4 equiv). The pressure vessel was flushed with Ar and sealed with the proper cap. The reaction was then heated to 10 °C for 16 h. After cooling to room temperature, the reaction was diluted with DCM, transferred to round-bottomed flask, and concentrated. Purification could be achieved by silica chromatography (50-100% magic mix (50 DCM: 8 EtOH: 1 NH_4OH) in DCM) to afford the pure isomers. All of the reactions produced two diastereomers; however, after purification, sometimes only one of the isomers could be obtained in pure form.

Compound data for sinomenine-based chromanones (**SM-C-S** and **SM-C-R**):

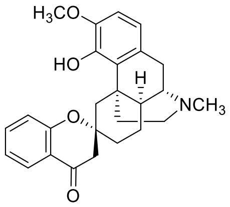

SM-C-S-1

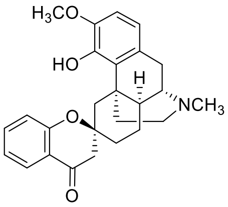

SM-C-R-1

Following the general procedure for sinomenine-based chromanones, **SM-C-S-1** (48 mg, 34%) and **SM-C-R-1** (54 mg, 39%) were obtained.

SM-C-S-1:

^{1}H NMR (700 MHz, $CDCl_3$) δ 7.80 (dd, *J* = 8.1, 1.7 Hz, 1H), 7.47 – 7.42 (m, 1H), 6.96 – 6.91 (m, 2H), 6.75 (d, *J* = 8.3 Hz, 1H), 6.64 (d, *J* = 8.3 Hz, 1H), 5.91 (s, 1H), 4.04 (dd, *J* = 13.7, 2.5 Hz, 1H), 3.91 (s, 3H), 3.00 (bs, 1H), 2.94 (d, *J* = 18.2 Hz, 1H), 2.76 (dd, *J* = 17.8, 5.2 Hz, 1H), 2.69 (d, *J* = 17.0 Hz, 1H), 2.59 – 2.51 (m, 1H), 2.43 (s, 3H), 2.39 (d, *J* = 17.0 Hz, 1H), 2.10 (dq, *J* = 12.7, 3.1 Hz, 1H), 2.05 (t, *J* = 11.7 Hz, 1H), 1.92 (d, *J* = 12.4 Hz, 1H), 1.87 (d, *J* = 12.7 Hz, 1H), 1.77 (td, *J* = 13.3, 4.1 Hz, 1H), 1.74 – 1.68 (m, 1H), 1.60 – 1.52 (m, 2H), 1.41 (qd, *J* = 13.7, 3.5 Hz, 1H).

^{13}C NMR (151 MHz, $CDCl_3$) δ 193.16, 160.04, 145.22, 144.39, 136.11, 130.23, 126.49, 124.61, 120.68, 120.63, 119.32, 118.49, 109.45, 81.97, 57.50, 56.56, 47.45, 46.32, 44.32, 43.76, 42.59, 38.87, 37.35, 36.69, 24.37, 24.09.

$[\alpha]_D^{20}$ –6.9 (*c* 0.12, MeOH).

HRMS (ESI) calcd for $C_{26}H_{29}NO_4$ $[M + H]^+$ 420.21693, found 420.21693.

SM-C-R-1:

^{1}H NMR (700 MHz, $CDCl_3$) δ 7.79 (dd, *J* = 7.8, 1.8 Hz, 1H), 7.21 (ddd, *J* = 8.6, 7.0, 1.8 Hz, 1H), 6.89 (t, *J* = 7.4 Hz, 1H), 6.67 (d, *J* = 8.2 Hz, 1H), 6.65 (d, *J* = 8.3 Hz, 1H), 5.94 (d, *J* = 8.3 Hz, 1H), 4.97 (s, 1H), 4.14 (dd, *J* = 14.9, 2.7 Hz, 1H), 3.79 (s, 3H), 3.06 (bs, 1H), 2.93 (d, *J* = 7.2 Hz, 2H), 2.73 (d, *J* = 16.2 Hz, 1H), 2.61 – 2.53 (m, 2H), 2.45 (s, 3H), 2.13 – 2.04 (m, 2H), 1.98 – 1.88 (m, 2H), 1.82 (d, *J* = 12.8 Hz, 1H), 1.69 – 1.56 (m, 2H), 1.49 – 1.40 (m, 1H), 1.17 (d, *J* = 14.9 Hz, 1H).

^{13}C NMR (176 MHz, $CDCl_3$) δ 192.81, 159.96, 144.75, 144.21, 135.37, 129.00, 126.03, 125.45, 120.42, 119.94, 118.83, 117.89, 107.96, 80.44, 58.32, 55.89, 49.31, 47.87, 45.14, 42.30, 40.20, 37.73, 36.92, 36.14, 24.32, 22.79.

$[\alpha]_D^{20}$ –3.0 (*c* 0.1, MeOH).

HRMS (ESI) calcd for $C_{26}H_{29}NO_4$ $[M + H]^+$ 420.21693, found 420.21632.

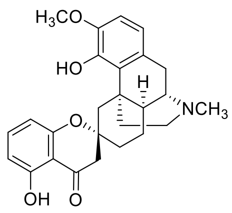

SM-C-S-2

Following the general procedure for sinomenine-based chromanones, **SM-C-S-2** (45 mg, 31%) was obtained. The other diastereomer could not be obtained in sufficient purity.

SM-C-S-2:

^{1}H NMR (600 MHz, $CDCl_3$) δ 11.79 (s, 1H), 7.32 (t, *J* = 8.3 Hz, 1H), 6.74 (d, *J* = 8.3 Hz, 1H), 6.64 (d, *J* = 8.3 Hz, 1H), 6.43 (dd, *J* = 8.3, 0.9 Hz, 1H), 6.39 (dd, *J* = 8.2, 0.9 Hz, 1H), 5.92 (s, 1H), 4.03 (dd, *J* = 13.7, 2.6 Hz, 1H), 3.90 (s, 3H), 2.93 (d, *J* = 18.3 Hz, 2H), 2.72 – 2.62 (m, 2H), 2.49 – 2.43 (m, 1H), 2.41 (d, *J* = 17.4 Hz, 4H), 2.38 (s, 3H), 2.08 (dq, *J* = 12.6, 3.1 Hz, 1H), 1.98 (td, *J* = 12.3, 3.3 Hz, 1H), 1.87 – 1.75 (m, 3H), 1.62 (td, *J* = 12.6, 4.6 Hz, 1H), 1.57 (dq, *J* = 13.8, 3.5 Hz, 1H), 1.51 (d, *J* = 13.7 Hz, 1H), 1.40 (qd, *J* = 13.8, 3.5 Hz, 1H).

^{13}C NMR (151 MHz, $CDCl_3$) δ 199.14, 161.87, 160.19, 145.17, 144.30, 138.25, 130.44, 124.58, 119.37, 109.49, 108.61, 108.02, 107.84, 81.75, 57.37, 56.61, 47.40, 46.41, 43.79, 43.21, 42.66, 38.89, 37.41, 37.26, 24.40, 24.03.

HRMS (ESI) calcd for $C_{26}H_{29}NO_5$ $[M + H]^+$ 436.21185, found 436.21185.

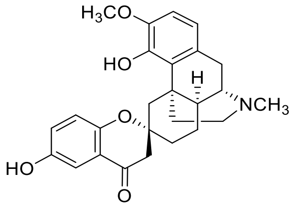

SM-C-S-3

Following the general procedure for sinomenine-based chromanones on a 0.17 mmol scale, **SM-C-S-3** (27 mg, 37%) was obtained. The other diastereomer could not be obtained in sufficient purity.

SM-C-S-3:

^{1}H NMR (700 MHz, $CDCl_3$) δ 7.20 (d, *J* = 3.1 Hz, 1H), 7.09 (dd, *J* = 8.9, 3.1 Hz, 1H), 6.82 (d, *J* = 8.9 Hz, 1H), 6.72 (d, *J* = 8.3 Hz, 1H), 6.61 (d, *J* = 8.3 Hz, 1H), 4.00 (dd, *J* = 13.8, 2.5 Hz, 1H), 3.82 (s, 3H), 3.08 (s, 1H), 2.91 (d, *J* = 18.4 Hz, 1H), 2.80 (dd, *J* = 18.5, 5.5 Hz, 1H), 2.66 – 2.58 (m, 2H), 2.45 (s, 3H), 2.30 (d, *J* = 17.1 Hz, 1H), 2.09 (t, *J* = 11.3 Hz, 1H), 1.98 (d, *J* = 12.4 Hz, 1H), 1.90 (d, *J* = 12.2 Hz, 1H), 1.85 (d, *J* = 12.9 Hz, 1H), 1.71 (td, *J* = 13.0, 4.5 Hz, 1H), 1.56 (td, *J* = 13.3, 4.0 Hz, 1H), 1.49 – 1.42 (m, 2H), 1.30 (qd, *J* = 13.5, 3.4 Hz, 1H).

^{13}C NMR (176 MHz, $CDCl_3$) δ 193.51, 153.85, 150.71, 145.47, 144.51, 129.03, 125.41, 124.09, 120.68, 119.62, 119.33, 111.04, 109.76, 81.27, 57.88, 56.54, 47.58, 45.23, 44.28, 43.27, 42.08, 37.99, 36.94, 36.39, 24.19, 24.16.

$[\alpha]_D^{20}$ −77 (*c* 0.1, MeOH).

HRMS (ESI) calcd for $C_{26}H_{29}NO_5$ $[M + H]^+$ 436.21185, found 436.21117.

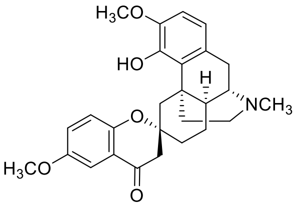

SM-C-S-4

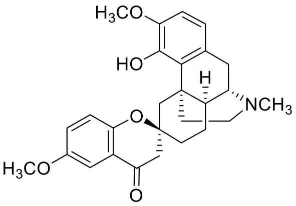

SM-C-R-2

Following the general procedure for sinomenine-based chromanones, **SM-C-S-4** (52 mg, 35%) and **SM-C-R-2** (65 mg, 44%) were obtained.

SM-C-S-4:

^{1}H NMR (700 MHz, $CDCl_3$) δ 7.24 (d, *J* = 3.2 Hz, 1H), 7.07 (dd, *J* = 9.0, 3.2 Hz, 1H), 6.87 (d, *J* = 9.0 Hz, 1H), 6.74 (d, *J* = 8.3 Hz, 1H), 6.63 (d, *J* = 8.3 Hz, 1H), 5.91 (s, 1H), 4.03 (dd, *J* = 13.6, 2.5 Hz, 1H), 3.91 (s, 3H), 3.79 (s, 3H), 2.98 – 2.90 (m, 2H), 2.72 (dd, *J* = 18.1, 5.2 Hz, 1H), 2.66 (d, *J* = 17.1 Hz, 1H), 2.50 (dd, *J* = 12.5, 4.1 Hz, 1H), 2.41 (s, 3H), 2.37 (d, *J* = 17.1 Hz, 1H), 2.09 (dq, *J* = 12.7, 3.2 Hz, 1H), 2.02 (td, *J* = 12.4, 3.2 Hz, 1H), 1.90 – 1.82 (m, 2H), 1.74 (td, *J* = 13.3, 4.1 Hz, 1H), 1.67 (td, *J* = 12.7, 4.6 Hz, 1H), 1.57 – 1.50 (m, 2H), 1.39 (qd, *J* = 13.7, 3.4 Hz, 1H).

^{13}C NMR (176 MHz, $CDCl_3$) δ 193.12, 154.61, 153.67, 145.26, 144.41, 130.10, 125.18, 124.58, 120.41, 119.79, 119.33, 109.51, 107.18, 81.69, 57.58, 56.60, 55.96, 47.47, 46.21, 44.27, 43.55, 42.53, 38.76, 37.31, 36.78, 24.38, 24.13.

$[\alpha]_D^{20}$ −32 (*c* 0.1, MeOH).

HRMS (ESI) calcd for $C_{27}H_{31}NO_5$ $[M + H]^+$ 450.22750, found 450.22680.

SM-C-R-2:

^{1}H NMR (700 MHz, $CDCl_3$) δ 7.23 (d, *J* = 3.2 Hz, 1H), 6.84 (dd, *J* = 9.0, 3.2 Hz, 1H), 6.65 (d, *J* = 8.2 Hz, 1H), 6.63 (d, *J* = 8.3 Hz, 1H), 5.88 (d, *J* = 9.0 Hz, 1H), 5.03 (s, 1H), 4.13 (dd, *J* = 14.9, 2.7 Hz, 1H), 3.79 (s, 3H), 3.78 (s, 3H), 2.98 – 2.94 (m, 1H), 2.92 (d, *J* = 18.0 Hz, 1H), 2.83 (dd, *J* = 18.0, 5.5 Hz, 1H), 2.71 (d, *J* = 16.3 Hz, 1H), 2.55 (d, *J* = 16.3 Hz, 1H), 2.50 – 2.45 (m, 1H), 2.39 (s, 3H), 2.07 (dq, *J* = 13.7, 3.1 Hz, 1H), 2.01 (td, *J* = 12.3, 3.3 Hz, 1H), 1.91 (qd, *J* = 13.0, 3.6 Hz, 1H), 1.84 – 1.77 (m, 2H), 1.60 – 1.52 (m, 2H), 1.42 (dq, *J* = 13.0, 3.2 Hz, 1H), 1.11 (d, *J* = 14.9 Hz, 1H).

^{13}C NMR (176 MHz, $CDCl_3$) δ 192.91, 154.80, 153.09, 144.55, 144.14, 129.73, 125.81, 124.75, 119.90, 119.27, 118.77, 107.77, 106.31, 80.39, 58.01, 55.90, 55.87, 49.30, 47.78, 45.74, 42.54, 40.21, 38.16, 37.06, 36.30, 24.24, 22.91.

$[\alpha]_D^{20}$ +49 (*c* 0.1, MeOH).

HRMS (ESI) calcd for $C_{27}H_{31}NO_5$ $[M + H]^+$ 450.22750, found 450.22664.

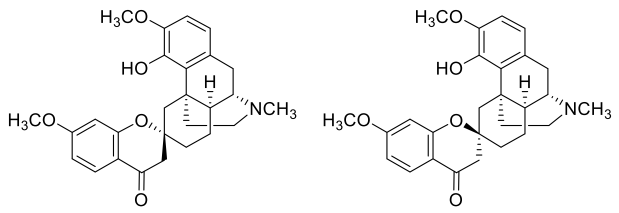

SM-C-S-5 **SM-C-R-3**

Following the general procedure for sinomenine-based chromanones, **SM-C-S-5** (20 mg, 13%) and **SM-C-R-3** (62 mg, 42%) were obtained.

SM-C-S-5:

^{1}H NMR (700 MHz, $CDCl_3$) δ 7.73 (d, *J* = 8.7 Hz, 1H), 6.77 (d, *J* = 8.3 Hz, 1H), 6.66 (d, *J* = 8.3 Hz, 1H), 6.51 (dd, *J* = 8.7, 2.4 Hz, 1H), 6.39 (d, *J* = 2.4 Hz, 1H), 5.96 (s, 1H), 4.05 (dd, *J* = 13.7, 2.5 Hz, 1H), 3.91 (s, 3H), 3.83 (s, 3H), 3.21 (bs, 1H), 2.95 (bs, 2H), 2.77 (bs, 1H), 2.62 (d, *J* = 17.0 Hz, 1H), 2.56 (s, 3H), 2.32 (d, *J* = 17.0 Hz, 1H), 2.28 – 2.05 (m, 3H), 1.98 – 1.86 (m, 2H), 1.80 (td, *J* = 13.3, 4.1 Hz, 1H), 1.65 – 1.54 (m, 2H), 1.40 (qd, *J* = 13.6, 3.5 Hz, 1H).

^{13}C NMR (176 MHz, $CDCl_3$) δ 191.41, 166.35, 161.96, 145.71, 144.56, 128.22, 123.62, 119.49, 114.48, 109.92, 109.53, 101.24, 81.99, 58.49, 56.59, 55.73, 47.78, 44.52, 43.97, 43.16, 41.85, 37.54, 36.83, 36.31, 24.29, 24.11. An aromatic proton is missing.

$[\alpha]_D^{20}$ –9 (*c* 0.1, MeOH).

HRMS (ESI) calcd for $C_{27}H_{31}NO_5$ $[M + H]^+$ 450.22750, found 450.22687.

SM-C-R-3:

^{1}H NMR (700 MHz, $CDCl_3$) δ 7.75 (d, *J* = 8.7 Hz, 1H), 6.68 (d, *J* = 8.4 Hz, 1H), 6.66 (d, *J* = 8.3 Hz, 1H), 6.47 (dd, *J* = 8.7, 2.4 Hz, 1H), 5.38 (d, *J* = 2.4 Hz, 1H), 5.07 (s, 1H), 4.18 (dd, *J* = 15.0, 2.7 Hz, 1H), 3.77 (s, 3H), 3.67 (s, 3H), 3.20 (bs, 1H), 3.11 – 3.02 (m, 1H), 2.99 – 2.92 (m, 1H), 2.74 – 2.67 (m, 2H), 2.53 (s, 3H), 2.50 (d, *J* = 16.3 Hz, 1H), 2.21 – 2.14 (m, 1H), 2.12 – 2.04 (m, 2H), 1.95 (qd, *J* = 13.0, 3.6 Hz, 1H), 1.88 – 1.83 (m, 1H), 1.77 (bs, 1H), 1.65 (td, *J* = 13.4, 4.1 Hz, 1H), 1.49 (dd, *J* = 13.0, 3.4 Hz, 1H), 1.19 (d, *J* = 15.0 Hz, 1H).

^{13}C NMR (176 MHz, $CDCl_3$) δ 191.13, 165.83, 161.72, 145.12, 144.38, 127.84, 125.06, 118.93, 114.46, 109.20, 108.15, 107.99, 101.07, 80.55, 58.84, 55.88, 55.33, 48.90, 48.07, 44.17, 41.90, 41.15, 39.79, 37.05, 35.89, 24.48, 22.62.

$[\alpha]_D^{20}$ -23 (*c* 0.1, MeOH).

HRMS (ESI) calcd for $C_{27}H_{31}NO_5$ $[M + H]^+$ 450.22750, found 450.22692.

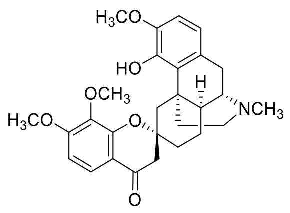

SM-C-S-6

Following the general procedure for sinomenine-based chromanones, **SM-C-S-6** (50 mg, 31%) was obtained. The other diastereomer could not be obtained in sufficient purity.

SM-C-S-6:

^{1}H NMR (600 MHz, MeOD) δ 7.56 (d, *J* = 8.8 Hz, 1H), 6.93 (d, *J* = 8.4 Hz, 1H), 6.77 – 6.71 (m, 2H), 4.23 (dd, *J* = 13.5, 2.5 Hz, 1H), 3.91 (s, 3H), 3.89 (s, 3H), 3.82 (s, 3H), 3.62 – 3.55 (m, 1H), 3.28 – 3.17 (m, 1H), 3.14 – 3.02 (m, 2H), 2.84 (s, 3H), 2.67 (d, *J* = 17.2 Hz, 1H), 2.55 (t, *J* = 11.6 Hz, 1H), 2.33 (d, *J* = 17.3 Hz, 1H), 2.15 (dd, *J* = 12.7, 3.0 Hz, 1H), 2.08 (d, *J* = 14.1 Hz, 1H), 2.03 – 1.98 (m, 1H), 1.82 (qd, *J* = 15.5, 15.0, 4.7 Hz, 2H), 1.72 – 1.61 (m, 2H), 1.49 – 1.39 (m, 1H).

^{13}C NMR (151 MHz, MeOD) δ 193.26, 160.66, 154.97, 148.02, 146.55, 138.43, 127.57, 123.76, 123.69, 120.38, 116.74, 111.99, 106.50, 83.01, 61.29, 61.06, 56.82, 56.74, 48.86, 44.69, 43.98, 43.91, 41.33, 37.50, 36.94, 36.80, 25.03, 24.69.

$[\alpha]_D^{20}$ +34.6 (*c* 0.18, MeOH).

HRMS (ESI) calcd for $C_{28}H_{34}O_6N$ $[M + H]^+$ 480.23806, found 480.23699.

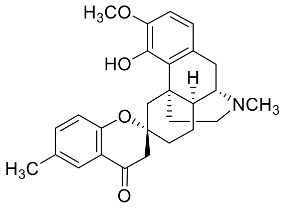

SM-C-S-7

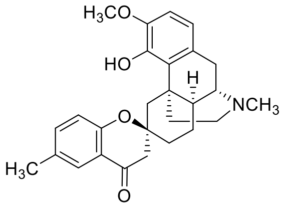

SM-C-R-4

Following the general procedure for sinomenine-based chromanones, **S-C-S-7** (9 mg, 6%) and **S-C-R-4** (27 mg, 19%) were obtained.

SM-C-S-7:

^{1}H NMR (700 MHz, $CDCl_3$) δ 7.60 (d, *J* = 1.3 Hz, 1H), 7.28 – 7.26 (m, 1H), 6.84 (d, *J* = 8.4 Hz, 1H), 6.76 (d, *J* = 8.3 Hz, 1H), 6.65 (d, *J* = 8.3 Hz, 1H), 5.92 (bs, 1H), 4.04 (dd, *J* = 13.7, 2.5 Hz, 1H), 3.91 (s, 3H), 3.08 (bs, 1H), 2.94 (d, *J* = 18.2 Hz, 1H), 2.88 – 2.79 (m, 1H), 2.64 (d, *J* = 17.0 Hz, 2H), 2.54 – 2.44 (m, 2H), 2.40 – 2.31 (m, 2H), 2.28 (s, 3H), 2.16 – 2.05 (m, 2H), 2.05 – 1.95 (m, 1H), 1.88 (d, *J* = 12.8 Hz, 1H), 1.83 – 1.72 (m, 2H), 1.57 (d, *J* = 13.6 Hz, 2H), 1.40 (qd, *J* = 13.6, 3.5 Hz, 1H).

^{13}C NMR (176 MHz, $CDCl_3$) δ 193.23, 157.98, 145.48, 144.47, 137.26, 130.09, 126.09, 124.09, 120.24, 119.41, 118.35, 109.71, 81.48, 70.72, 58.04, 56.59, 47.62, 44.39, 43.25, 42.17, 38.13, 37.05, 36.61, 29.84, 24.22, 20.50. An aromatic carbon could not be found in the spectra.

$[\alpha]_D^{20}$ –19 (*c* 0.1, MeOH).

HRMS (ESI) calcd for $C_{27}H_{31}O_4N$ $[M + H]^+$ 434.23258, found 434.23387.

SM-C-R-4:

^{1}H NMR (700 MHz, $CDCl_3$) δ 7.59 (d, *J* = 1.2 Hz, 1H), 7.02 (dd, *J* = 8.4, 2.3 Hz, 1H), 6.71 (d, *J* = 8.3 Hz, 1H), 6.67 (d, *J* = 8.3 Hz, 1H), 5.81 (d, *J* = 8.4 Hz, 1H), 5.08 (bs, 1H), 4.15 (dd, *J* = 14.9, 2.7 Hz, 1H), 3.81 (s, 3H), 3.37 (s, 1H), 3.19 (d, *J* = 18.8 Hz, 1H), 3.02 – 2.85 (m, 2H), 2.70 (d, *J* = 16.3 Hz, 1H), 2.63 (s, 3H), 2.56 (d, *J* = 16.3 Hz, 1H), 2.38 – 2.23 (m, 5H), 2.07 (dd, *J* = 13.6, 3.1 Hz, 1H), 1.97 – 1.84 (m, 3H), 1.63 (td, *J* = 13.5, 4.1 Hz, 1H), 1.48 (dd, *J* = 13.0, 3.4 Hz, 1H), 1.23 (d, *J* = 14.9 Hz, 1H).

^{13}C NMR (176 MHz, $CDCl_3$) δ 192.59, 157.70, 145.27, 144.36, 136.51, 129.42, 125.70, 124.33, 119.94, 119.02, 117.59, 108.51, 79.85, 70.71, 59.42, 55.95, 49.08, 48.27, 41.44, 39.54, 36.24, 35.52, 31.07, 24.51, 22.38, 20.57. An aromatic carbon could not be found in the spectra.

$[\alpha]_D^{20}$ +20 (*c* 0.1, MeOH).

HRMS (ESI) calcd for $C_{27}H_{31}O_4N$ $[M + H]^+$ 434.23258, found 434.23418.

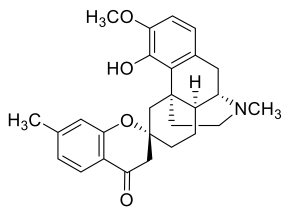

SM-C-S-8

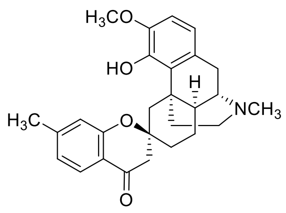

SM-C-R-5

Following the general procedure for sinomenine-based chromanones, **SM-C-S-8** (39 mg, 27%) and **SM-C-R-5** (74 mg, 52%) were obtained.

SM-C-S-8:

^{1}H NMR (600 MHz, $CDCl_3$) δ 7.67 (d, *J* = 7.8 Hz, 1H), 6.76 – 6.70 (m, 3H), 6.62 (d, *J* = 8.3 Hz, 1H), 6.02 (s, 1H), 4.02 (dd, *J* = 13.7, 2.5 Hz, 1H), 3.88 (s, 3H), 3.06 – 2.99 (m, 1H), 2.92 (d, *J* = 18.3 Hz, 1H), 2.77 (dd, *J* = 18.4, 5.5 Hz, 1H), 2.63 (d, *J* = 17.1 Hz, 1H), 2.57 (dd, *J* = 12.6, 4.1 Hz, 1H), 2.44 (s, 3H), 2.36 – 2.28 (m, 4H), 2.10 – 2.02 (m, 2H), 1.93 (d, *J* = 12.1 Hz, 1H), 1.89 – 1.82 (m, 1H), 1.78 – 1.68 (m, 2H), 1.57 – 1.49 (m, 2H), 1.37 (qd, *J* = 13.6, 3.4 Hz, 1H).

^{13}C NMR (151 MHz, CDCl3) δ 192.64, 159.95, 147.50, 145.34, 144.42, 129.34, 126.32, 124.21, 122.00, 119.28, 118.46, 118.33, 109.57, 81.66, 57.76, 56.50, 47.48, 45.56, 44.16, 43.51, 42.22, 38.28, 37.07, 36.49, 24.21, 24.11, 22.02.

$[\alpha]_D^{20}$ – 8 (*c* 0.1, MeOH).

HRMS (ESI) calcd for $C_{27}H_{31}O_4N$ $[M + H]^+$ 434.23258, found 434.23331.

SM-C-R-5:

^{1}H NMR (700 MHz, $CDCl_3$) δ 7.63 (d, *J* = 7.9 Hz, 1H), 6.70 – 6.65 (m, 2H), 6.64 (d, *J* = 8.3 Hz, 1H), 5.70 (s, 1H), 5.07 (bs, 1H), 4.11 (dd, *J* = 14.9, 2.7 Hz, 1H), 3.75 (s, 3H), 3.25 (s, 1H), 3.08 (dd, *J* = 18.5, 5.6 Hz, 1H), 2.93 (d, *J* = 18.5 Hz, 1H), 2.76 (d, *J* = 11.9 Hz, 1H), 2.66 (d, *J* = 16.3 Hz, 1H), 2.55 (s, 3H), 2.47 (d, *J* = 16.2 Hz, 1H), 2.20 (td, *J* = 12.5, 3.8 Hz, 1H), 2.14 (s, 3H), 2.10 (d, *J* = 12.5 Hz, 1H), 2.02 (dq, *J* = 13.7, 3.1 Hz, 1H), 1.89 (qd, *J* = 13.0, 3.7 Hz, 1H), 1.85 – 1.73 (m, 2H), 1.59 (td, *J* = 13.5, 4.1 Hz, 1H), 1.44 (dq, *J* = 13.1, 3.1 Hz, 1H), 1.15 (d, *J* = 14.9 Hz, 1H).

^{13}C NMR (176 MHz, $CDCl_3$) δ 192.06, 159.74, 146.89, 145.09, 144.23, 127.22, 125.82, 124.57, 121.32, 118.88, 117.90, 117.74, 108.16, 79.93, 58.94, 56.06, 48.96, 47.97, 43.57, 41.57, 39.65, 36.66, 36.52, 35.59, 24.43, 22.39, 21.88.

$[\alpha]_D^{20}$ – 11 (*c* 0.1, MeOH).

HRMS (ESI) calcd for $C_{27}H_{31}O_4N$ $[M + H]^+$ 434.23258, found 434.23382.

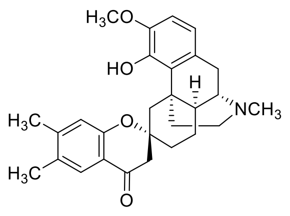

SM-C-S-9

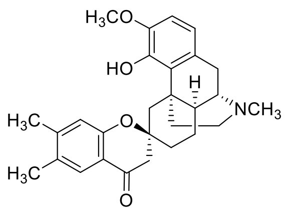

SM-C-R-6

Following the general procedure for sinomenine-based chromanones on a 0.17 mmol scale, **SM-C-S-9** (23 mg, 31%) and **SM-C-R-6** (35 mg, 47%) were obtained.

SM-C-S-9:

^{1}H NMR (600 MHz, $CDCl_3$) δ 7.55 (s, 1H), 6.75 – 6.70 (m, 2H), 6.62 (d, *J* = 8.2 Hz, 1H), 5.88 (s, 1H), 4.03 (dd, *J* = 13.6, 2.5 Hz, 1H), 3.90 (s, 3H), 2.96 – 2.86 (m, 2H), 2.71 – 2.60 (m, 2H), 2.47 – 2.41 (m, 1H), 2.38 (s, 3H), 2.34 (d, *J* = 17.1 Hz, 1H), 2.25 (s, 3H), 2.19 (s, 3H), 2.09 (dq, *J* = 12.6, 3.2 Hz, 1H), 1.98 (td, *J* = 12.3, 3.3 Hz, 1H), 1.86 – 1.78 (m, 2H), 1.73 (td, *J* = 13.2, 4.1 Hz, 1H), 1.62 (td, *J* = 12.6, 4.6 Hz, 1H), 1.56 – 1.48 (m, 2H), 1.45 – 1.37 (m, 1H).

^{13}C NMR (151 MHz, $CDCl_3$) δ 193.08, 158.31, 146.46, 145.17, 144.40, 130.47, 129.20, 126.52, 124.77, 119.25, 119.02, 118.50, 109.38, 81.75, 57.43, 56.55, 47.43, 46.59, 44.36, 43.77, 42.68, 39.04, 37.40, 36.93, 24.46, 24.07, 20.61, 18.84.

$[\alpha]_D^{20}$ -6.3 (*c* 1.3, MeOH).

HRMS (ESI) calcd for $C_{28}H_{33}NO_4$ $[M + H]^+$ 448.24824, found 448.24747.

SM-C-R-6:

^{1}H NMR (600 MHz, $CDCl_3$) δ 7.53 (d, *J* = 1.0 Hz, 1H), 6.68 – 6.62 (m, 2H), 5.77 (s, 1H), 4.92 (s, 1H), 4.14 (dd, *J* = 14.8, 2.7 Hz, 1H), 3.77 (s, 3H), 2.96 – 2.89 (m, 2H), 2.79 (dd, *J* = 17.9, 5.4 Hz, 1H), 2.69 (d, *J* = 16.2 Hz, 1H), 2.51 (d, *J* = 16.2 Hz, 1H), 2.44 – 2.38 (m, 1H), 2.37 (s, 3H), 2.19 (s, 3H), 2.10 (s, 3H), 2.06 (dq, *J* = 13.5, 3.1 Hz, 1H), 1.96 (td, *J* = 12.3, 3.2 Hz, 1H), 1.90 (td, *J* = 13.0, 3.6 Hz, 1H), 1.80 – 1.73 (m, 2H), 1.56 (td, *J* = 13.4, 4.2 Hz, 1H), 1.51 (td, *J* = 12.6, 4.6 Hz, 1H), 1.44 – 1.39 (m, 1H), 1.10 (d, *J* = 14.8 Hz, 1H).

^{13}C NMR (151 MHz, $CDCl_3$) δ 192.90, 158.47, 145.90, 144.60, 144.23, 130.25, 128.26, 126.21, 125.97, 118.66, 118.51, 118.10, 107.49, 80.35, 57.84, 56.07, 49.46, 47.71, 46.16, 42.72, 40.64, 38.48, 37.40, 36.43, 24.25, 23.04, 20.66, 18.90.

$[\alpha]_D^{20}$ +12 (*c* 0.1, MeOH).

HRMS (ESI) calcd for $C_{28}H_{33}NO_4$ $[M + H]^+$ 448.24824, found 448.24739.

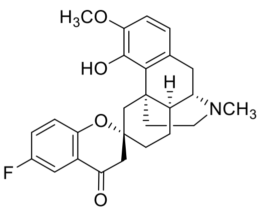

SM-C-S-10

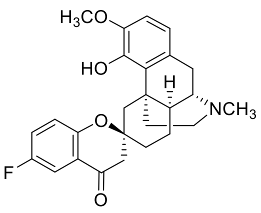

SM-C-R-7

Following the general procedure for sinomenine-based chromanones, **SM-C-S-10** (29 mg, 20%) and **SM-C-R-7** (47 mg, 32%) were obtained.

SM-C-S-10: **^{1}H NMR** (600 MHz, MeOD) δ 7.42 (dd, *J* = 8.3, 3.2 Hz, 1H), 7.29 (ddd, *J* = 9.1, 8.0, 3.2 Hz, 1H), 6.98 (dd, *J* = 9.1, 4.2 Hz, 1H), 6.94 (d, *J* = 8.4 Hz, 1H), 6.77 (d, *J* = 8.3 Hz, 1H), 4.21 (dd, *J* = 13.6, 2.5 Hz, 1H), 3.89 (s, 3H), 3.74 – 3.68 (m, 1H), 3.26 (dd, *J* = 19.6, 5.8 Hz, 1H), 3.22 – 3.05 (m, 2H), 2.87 (s, 3H), 2.64 (d, *J* = 17.2 Hz, 1H), 2.57 (td, *J* = 13.2, 3.5 Hz, 1H), 2.38 (d, *J* = 17.3 Hz, 1H), 2.16 – 2.06 (m, 2H), 1.98 (d, *J* = 12.4 Hz, 1H), 1.84 – 1.60 (m, 3H), 1.52 (d, *J* = 13.6 Hz, 1H), 1.44 (q, *J* = 13.9, 13.5 Hz, 1H).

^{13}C NMR (151 MHz, MeOD) δ 193.09, 158.34 (d, *J* = 239.8 Hz), 157.43 (d, *J* = 1.6 Hz), 148.15, 146.54, 127.09, 124.68 (d, *J* = 24.7 Hz), 123.36, 122.07 (d, *J* = 6.4 Hz), 121.42 (d, *J* = 7.2 Hz), 120.45, 112.17, 112.10 (d, *J* = 23.4 Hz) 82.42, 61.33, 56.88, 49.34, 45.39, 44.66, 43.14, 41.11, 37.62, 37.18, 37.12, 24.49, 24.17.

^{19}F NMR (470 MHz, MeOD) δ -122.73 – -126.49 (m).

$[\alpha]_D^{20}$ –29 (*c* 0.1, MeOH).

HRMS (ESI) calcd for $C_{26}H_{29}O_4NF$ $[M + H]^+$ 438.20751, found 438.20808.

SM-C-R-7: **^{1}H NMR** (700 MHz, $CDCl_3$) δ 7.42 (dd, *J* = 8.3, 3.2 Hz, 1H), 6.91 (ddd, *J* = 9.0, 7.8, 3.2 Hz, 1H), 6.66 (d, *J* = 8.3 Hz, 1H), 6.64 (d, *J* = 8.4 Hz, 1H), 5.86 (dd, *J* = 9.0, 4.2 Hz, 1H), 5.13 (bs, 1H), 4.10 (dd, *J* = 15.0, 2.7 Hz, 1H), 3.78 (s, 3H), 3.16 (bs, 1H), 3.00 (dd, *J* = 18.4, 5.5 Hz, 1H), 2.92 (d, *J* = 18.4 Hz, 1H), 2.73 – 2.63 (m, 2H), 2.54 (d, *J* = 16.4 Hz, 1H), 2.49 (s, 3H), 2.13 (td, *J* = 12.5, 3.4 Hz, 1H), 2.08 – 1.99 (m, 2H), 1.90 (qd, *J* = 13.0, 3.6 Hz, 1H), 1.83 (d, *J* = 12.7 Hz, 1H), 1.71 (td, *J* = 13.0, 4.5 Hz, 1H), 1.60 (td, *J* = 13.4, 4.1 Hz, 1H), 1.45 (dd, *J* = 13.2, 3.5 Hz, 1H), 1.15 (d, *J* = 15.0 Hz, 1H).

^{13}C NMR (176 MHz, $CDCl_3$) δ 191.83 (d, *J* = 1.9 Hz), 156.58 (d, *J* = 202.0 Hz), 155.90 (d, *J* = 36.7 Hz), 144.76, 144.06, 128.12, 124.85, 122.74 (d, *J* = 24.4 Hz), 120.46 (d, *J* = 6.3 Hz), 119.38 (d, *J* = 7.1 Hz), 118.97, 110.86 (d, *J* = 22.9 Hz), 108.12, 80.50, 58.58, 55.87, 48.87, 47.93, 44.23, 41.91, 39.51, 37.04, 36.71, 35.85, 24.32, 22.51.

^{19}F NMR (377 MHz, $CDCl_3$) δ -123.21 – -123.36 (m).

$[\alpha]_D^{20}$ – (*c* 0.1, MeOH)

HRMS (ESI) calcd for $C_{26}H_{29}O_4NF$ $[M + H]^+$ 438.20751, found 438.20956

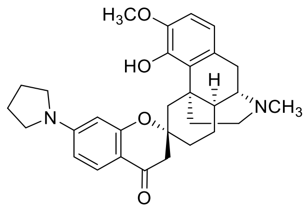

SM-C-S-11

Following the general procedure for sinomenine-based chromanones employing 4-fluoro-2-hydroxyacetophenone, the S_NAr product **SM-C-S-11** (24 mg, 15%) was obtained. The other diastereomer could not be obtained in sufficient purity.

SM-C-S-11:

^{1}H NMR (600 MHz, $CDCl_3$) δ 7.65 (d, *J* = 8.8 Hz, 1H), 6.78 (d, *J* = 8.3 Hz, 1H), 6.65 (d, *J* = 8.3 Hz, 1H), 6.17 (dd, *J* = 8.8, 2.3 Hz, 1H), 6.05 (bs, 1H), 5.92 (d, *J* = 2.3 Hz, 1H), 4.05 (dd, *J* = 13.9, 2.4 Hz, 1H), 3.89 (s, 3H), 3.52 (bs, 1H), 3.38 – 3.28 (m, 4H), 3.18 (dd, *J* = 19.1, 5.7 Hz, 1H), 3.11 – 3.03 (m, 1H), 3.00 – 2.90 (m, 1H), 2.74 (s, 3H), 2.52 (d, *J* = 16.9 Hz, 1H), 2.47 – 2.34 (m, 2H), 2.24 (d, *J* = 16.9 Hz, 1H), 2.14 – 2.06 (m, 2H), 2.04 – 1.94 (m, 5H), 1.79 (td, *J* = 13.3, 4.1 Hz, 1H), 1.66 – 1.55 (m, 2H), 1.37 (qd, *J* = 13.6, 3.5 Hz, 1H).

^{13}C NMR (151 MHz, $CDCl_3$) δ 190.15, 161.37, 153.64, 146.16, 144.71, 128.20, 125.61, 122.59, 119.56, 110.32, 109.96, 106.43, 97.93, 80.67, 59.60, 56.54, 48.17, 47.76, 43.77, 42.74, 42.63, 41.03, 36.16, 35.89, 25.49, 24.35, 23.79. An aliphatic carbon could not be found in the spectra.

$[\alpha]_D^{20}$ +7 (*c* 0.1, MeOH).

HRMS (ESI) calcd for $C_{30}H_{37}O_4N_2$ $[M + H]^+$ 489.27478, found 489.27532.

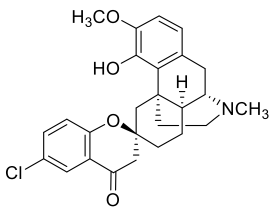

SM-C-R-8

Following the general procedure for sinomenine-based chromanones, **SM-C-R-8** (59 mg, 39%) was obtained. The other diastereomer could not be obtained in sufficient purity.

SM-C-R-8:

^{1}H NMR (700 MHz, $CDCl_3$) δ 7.75 (d, *J* = 2.7 Hz, 1H), 7.13 (dd, *J* = 8.8, 2.7 Hz, 1H), 6.67 – 6.64 (m, 2H), 5.88 (d, *J* = 8.8 Hz, 1H), 5.05 (bs, 1H), 4.11 (dd, *J* = 15.0, 2.8 Hz, 1H), 3.80 (s, 3H), 3.07 (bs, 1H), 2.97 – 2.86 (m, 2H), 2.72 (d, *J* = 16.3 Hz, 1H), 2.63 – 2.53 (m, 2H), 2.45 (s, 3H), 2.14 – 2.03 (m, 2H), 1.98 – 1.87 (m, 2H), 1.83 (d, *J* = 12.4 Hz, 1H), 1.69 – 1.59 (m, 2H), 1.50 – 1.42 (m, 1H), 1.16 (d, *J* = 15.0 Hz, 1H).

^{13}C NMR (176 MHz, $CDCl_3$) δ 191.62, 158.39, 144.65, 144.04, 135.12, 125.41, 125.32, 125.21, 121.13, 119.62, 118.96, 118.65, 107.97, 80.92, 58.33, 55.90, 48.97, 47.89, 44.94, 42.26, 39.84, 37.60, 36.99, 36.11, 24.29, 22.69.

$[\alpha]_D^{20}$ −10 (*c* 0.1, MeOH).

HRMS (ESI) calcd for $C_{26}H_{29}O_4NCl$ $[M + H]^+$ 454.17796 and 456.17501, found 454.17840 and 456.17586.

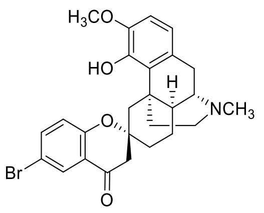

SM-C-S-12

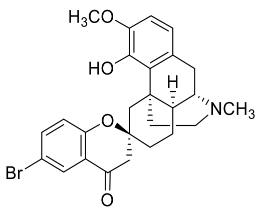

SM-C-R-9

Following the general procedure for sinomenine-based chromanones, **SM-C-S-12** (48 mg, 29%) and **SM-C-R-9** (38 mg, 33%) were obtained.

SM-C-S-12:

^{1}H NMR (700 MHz, $CDCl_3$) δ 7.88 (d, *J* = 2.6 Hz, 1H), 7.51 (dd, *J* = 8.8, 2.6 Hz, 1H), 6.83 (d, *J* = 8.8 Hz, 1H), 6.79 (d, *J* = 8.3 Hz, 1H), 6.67 (d, *J* = 8.3 Hz, 1H), 6.01 (bs, 1H), 4.01 (dd, *J* = 13.9, 2.5 Hz, 1H), 3.91 (s, 3H), 3.46 (s, 1H), 3.14 (dd, *J* = 19.0, 5.7 Hz, 1H), 3.03 – 2.93 (m, 2H), 2.69 (s, 3H), 2.58 (d, *J* = 17.1 Hz, 1H), 2.43 – 2.31 (m, 3H), 2.11 – 2.02 (m, 2H), 1.95 (d, *J* = 12.7 Hz, 1H), 1.84 (td, *J* = 13.4, 4.1 Hz, 1H), 1.62 (d, *J* = 14.0 Hz, 2H), 1.35 (qd, *J* = 13.7, 3.6 Hz, 1H).

^{13}C NMR (176 MHz, $CDCl_3$) δ 191.29, 158.71, 146.06, 144.49, 138.86, 128.87, 126.03, 122.42, 121.71, 120.74, 119.70, 113.50, 110.37, 81.56, 70.64, 59.20, 56.62, 47.94, 43.97, 42.72, 41.95, 41.13, 36.26, 36.12, 24.35, 23.62.

$[\alpha]^{20}_{D}$ −15 (*c* 0.1, MeOH).

HRMS (ESI) calcd for $C_{26}H_{29}O_4NBr$ $[M + H]^+$ 498.12745 and 500.12761, found 498.12700 and 500.12444.

SM-C-R-9:

^{1}H NMR (700 MHz, Chloroform-*d*) δ 7.89 (d, *J* = 2.5 Hz, 1H), 7.27 – 7.24 (m, 1H), 6.63 (s, 2H), 5.82 (d, *J* = 8.8 Hz, 1H), 5.07 (bs, 1H), 4.10 (dd, *J* = 15.0, 2.8 Hz, 1H), 3.79 (s, 3H), 2.95 – 2.89 (m, 2H), 2.80 (dd, *J* = 18.0, 5.5 Hz, 1H), 2.70 (d, *J* = 16.3 Hz, 1H), 2.55 (d, *J* = 16.3 Hz, 1H), 2.48 – 2.42 (m, 1H), 2.37 (s, 3H), 2.05 (dq, *J* = 14.0, 3.2 Hz, 1H), 1.98 (td, *J* = 12.3, 3.2 Hz, 1H), 1.90 (td, *J* = 13.1, 3.6 Hz, 1H), 1.82 – 1.76 (m, 2H), 1.59 (td, *J* = 13.4, 4.1 Hz, 1H), 1.52 (td, *J* = 12.6, 4.6 Hz, 1H), 1.46 – 1.41 (m, 1H), 1.11 (d, *J* = 14.9 Hz, 1H).

^{13}C NMR (176 MHz, $CDCl_3$) δ 191.63, 158.91, 144.42, 143.97, 137.87, 128.30, 125.63, 121.65, 120.05, 118.83, 118.57, 112.38, 107.70, 81.05, 70.70, 57.86, 55.82, 48.99, 47.72, 45.71, 42.58, 40.08, 37.18, 36.33, 24.17, 22.82.

$[\alpha]^{20}_{D}$ +73 (*c* 0.1, MeOH).

HRMS (ESI) calcd for $C_{26}H_{29}O_4NBr$ $[M + H]^+$ 498.12745 and 500.12761, found 498.12702 and 500.12462.

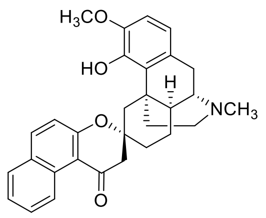

SM-C-S-13

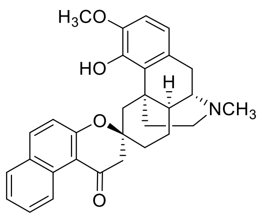

SM-C-R-10

Following the general procedure for sinomenine-based chromanones, **SM-C-S-13** (34 mg, 22%) and **SM-C-R-10** (80 mg, 52%) were obtained.

SM-C-S-13:

^{1}H NMR (700 MHz, $CDCl_3$) δ 9.34 (d, *J* = 8.6 Hz, 1H), 7.90 (d, *J* = 9.0 Hz, 1H), 7.72 (d, *J* = 7.9 Hz, 1H), 7.58 (ddd, *J* = 8.5, 6.8, 1.4 Hz, 1H), 7.38 (ddd, *J* = 8.0, 6.8, 1.2 Hz, 1H), 7.06 (d, *J* = 9.0 Hz, 1H), 6.82 (d, *J* = 8.3 Hz, 1H), 6.69 (d, *J* = 8.3 Hz, 1H), 6.08 (bs, 1H), 4.12 (dd, *J* = 13.8, 2.5 Hz, 1H), 3.94 (s, 3H), 3.50 (s, 1H), 3.18 (dd, *J* = 19.1, 5.7 Hz, 1H), 3.07 – 2.94 (m, 2H), 2.77 (d, *J* = 16.6 Hz, 1H), 2.72 (s, 3H), 2.48 – 2.38 (m, 3H), 2.21 (dd, *J* = 12.8, 3.0 Hz, 1H), 2.15 – 2.07 (m, 1H), 1.98 (d, *J* = 12.1 Hz, 1H), 1.84 (td, *J* = 13.3, 4.1 Hz, 1H), 1.71 (d, *J* = 13.8 Hz, 1H), 1.64 (dd, *J* = 13.7, 3.5 Hz, 1H), 1.42 (qd, *J* = 13.6, 3.4 Hz, 1H).

^{13}C NMR (176 MHz, $CDCl_3$) δ 193.51, 161.88, 146.11, 144.63, 137.58, 131.40, 129.57, 129.04, 128.50, 125.96, 125.55, 124.59, 122.64, 119.69, 119.64, 111.71, 110.39, 81.29, 70.67, 59.39, 56.66, 48.07, 45.47, 42.81, 42.29, 41.11, 36.29, 35.19, 24.38, 23.69.

$[\alpha]_D^{20}$ –25 (*c* 0.1, MeOH).

HRMS (ESI) calcd for $C_{30}H_{32}O_4N$ $[M + H]^+$ 470.23258, found 470.23390.

SM-C-R-10:

^{1}H NMR (700 MHz, $CDCl_3$) δ 9.40 (d, *J* = 8.6 Hz, 1H), 7.65 (d, *J* = 7.8 Hz, 1H), 7.60 (d, *J* = 9.0 Hz, 1H), 7.56 (ddd, *J* = 8.5, 6.8, 1.5 Hz, 1H), 7.34 (ddd, *J* = 8.0, 6.8, 1.2 Hz, 1H), 6.65 (d, *J* = 8.3 Hz, 1H), 6.63 (d, *J* = 8.2 Hz, 1H), 6.03 (d, *J* = 9.0 Hz, 1H), 5.11 (bs, 1H), 4.25 (dd, *J* = 14.9, 2.7 Hz, 1H), 3.73 (s, 3H), 3.00 (s, 1H), 2.94 – 2.85 (m, 2H), 2.83 (d, *J* = 15.7 Hz, 1H), 2.61 (d, *J* = 15.6 Hz, 1H), 2.54 – 2.47 (m, 1H), 2.38 (s, 3H), 2.09 (dd, *J* = 13.6, 2.9 Hz, 1H), 2.04 (td, *J* = 12.4, 3.3 Hz, 1H), 1.95 – 1.87 (m, 2H), 1.80 – 1.75 (m, 1H), 1.65 – 1.56 (m, 2H), 1.46 – 1.41 (m, 1H), 1.17 (d, *J* = 14.9 Hz, 1H).

^{13}C NMR (176 MHz, $CDCl_3$) δ 193.64, 161.99, 144.56, 144.12, 136.31, 131.16, 129.18, 129.11, 128.50, 128.08, 125.61, 125.46, 124.24, 119.12, 118.66, 111.38, 107.89, 80.57, 70.59, 58.06, 55.70, 50.36, 47.67, 45.08, 42.21, 39.90, 36.58, 36.10, 24.17, 22.71.

$[\alpha]_D^{20}$ +152 (*c* 0.1, MeOH).

HRMS (ESI) calcd for $C_{30}H_{32}O_4N$ $[M + H]^+$ 470.23258, found 470.23367.

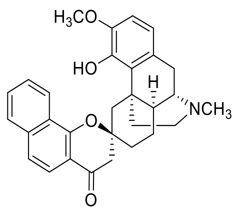

SM-C-R-11

Following the general procedure for sinomenine-based chromanones, **SM-C-R-11** (49 mg, 35%) was obtained. The other diastereomer could not be obtained in sufficient purity.

SM-C-R-11:

^{1}H NMR (700 MHz, $CDCl_3$) δ 7.78 (d, *J* = 8.6 Hz, 1H), 7.64 (d, *J* = 8.1 Hz, 1H), 7.43 (ddd, *J* = 8.1, 6.9, 1.3 Hz, 1H), 7.26 (d, *J* = 8.5 Hz, 1H), 7.02 (ddd, *J* = 8.2, 6.8, 1.2 Hz, 1H), 6.69 (d, *J* = 8.3 Hz, 1H), 6.62 (d, *J* = 8.3 Hz, 1H), 6.58 (d, *J* = 8.3 Hz, 1H), 5.48 (bs, 1H), 4.31 (dd, *J* = 14.8, 2.8 Hz, 1H), 3.47 (s, 3H), 3.01 – 2.91 (m, 2H), 2.88 – 2.76 (m, 2H), 2.70 (d, *J* = 16.3 Hz, 1H), 2.52 – 2.44 (m, 1H), 2.39 (s, 3H), 2.27 (dq, *J* = 13.6, 3.0 Hz, 1H), 2.02 (td, *J* = 12.3, 3.2 Hz, 1H), 1.95 – 1.81 (m, 3H), 1.61 (td, *J* = 12.6, 4.6 Hz, 1H), 1.52 (td, *J* = 13.4, 4.0 Hz, 1H), 1.41 (dq, *J* = 12.7, 2.9 Hz, 1H), 1.31 (d, *J* = 14.8 Hz, 1H).

^{13}C NMR (176 MHz, $CDCl_3$) δ 192.28, 158.35, 144.96, 144.53, 137.60, 130.09, 128.97, 127.26, 126.17, 125.42, 125.23, 124.19, 121.48, 119.79, 118.67, 114.67, 107.93, 81.98, 70.69, 57.81, 55.67, 48.79, 47.74, 46.20, 42.88, 42.65, 38.71, 36.61, 35.05, 24.28, 23.53.

$[\alpha]_D^{20}$ −110 (*c* 0.1, MeOH).

HRMS (ESI) calcd for $C_{30}H_{32}O_4N$ $[M + H]^+$ 470.23258, found 470.23209.

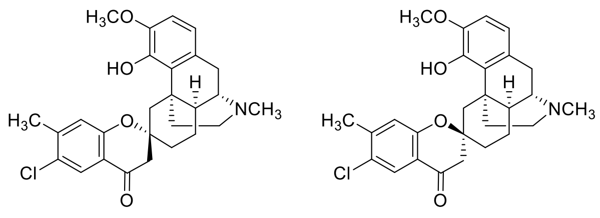

SM-C-S-14 **SM-C-R-12**

Following the general procedure for sinomenine-based chromanones, **SM-C-S-14** (41 mg, 26%) and **SM-C-R-12** (88 mg, 57%) were obtained.

SM-C-S-14:

^{1}H NMR (700 MHz, $CDCl_3$) δ 7.74 (s, 1H), 6.82 (s, 1H), 6.75 (d, *J* = 8.3 Hz, 1H), 6.64 (d, *J* = 8.3 Hz, 1H), 5.99 (bs, 1H), 4.01 (dd, *J* = 13.7, 2.6 Hz, 1H), 3.90 (s, 3H), 3.12 (s, 1H), 2.93 (d, *J* = 18.4 Hz, 1H), 2.84 (dd, *J* = 19.0, 5.3 Hz, 1H), 2.70 – 2.64 (m, 1H), 2.60 (d, *J* = 17.1 Hz, 1H), 2.50 (s, 3H), 2.36 – 2.30 (m, 4H), 2.17 – 2.10 (m, 1H), 2.08 – 1.98 (m, 2H), 1.90 – 1.85 (m, 1H), 1.81 – 1.73 (m, 2H), 1.59 – 1.50 (m, 2H), 1.37 (qd, *J* = 13.7, 3.5 Hz, 1H).

^{13}C NMR (176 MHz, $CDCl_3$) δ 191.58, 158.23, 145.53, 145.04, 144.41, 128.65, 126.91, 126.18, 123.73, 120.65, 119.62, 119.43, 109.81, 81.98, 58.10, 56.57, 47.62, 45.01, 44.04, 42.96, 42.04, 37.87, 36.93, 36.61, 24.16, 24.08, 20.89.

$[\alpha]_D^{20}$ −5.3 (*c* 0.17, MeOH).

HRMS (ESI) calcd for $C_{27}H_{31}O_4NCl$ $[M + H]^+$ 468.19361 and 470.19066, found 468.19390 and 470.19088.

SM-C-R-12:

^{1}H NMR (700 MHz, $CDCl_3$) δ 7.68 (s, 1H), 6.65 (d, *J* = 8.3 Hz, 1H), 6.63 (d, *J* = 8.3 Hz, 1H), 5.78 (s, 1H), 5.16 (bs, 1H), 4.08 (dd, *J* = 15.0, 2.7 Hz, 1H), 3.74 (s, 3H), 3.16 (s, 1H), 3.01 (dd, *J* = 18.5, 5.5 Hz, 1H), 2.91 (d, *J* = 18.3 Hz, 1H), 2.70 – 2.62 (m, 2H), 2.51 – 2.44 (m, 4H), 2.15 (s, 3H), 2.14 – 2.10 (m, 1H), 2.05 – 1.98 (m, 2H), 1.87 (qd, *J* = 13.0, 3.6 Hz, 1H), 1.81 – 1.77 (m, 1H), 1.70 (td, *J* = 13.0, 4.5 Hz, 1H), 1.59 (td, *J* = 13.4, 4.1 Hz, 1H), 1.44 (dt, *J* = 13.1, 3.3 Hz, 1H), 1.12 (d, *J* = 15.0 Hz, 1H).

^{13}C NMR (176 MHz, $CDCl_3$) δ 191.26, 158.17, 144.97, 144.34, 144.14, 127.93, 126.11, 125.65, 124.81, 119.96, 119.24, 119.02, 108.09, 80.57, 58.72, 56.13, 48.86, 47.96, 44.00, 41.84, 39.61, 37.02, 36.89, 35.82, 24.43, 22.49, 20.93.

$[\alpha]_D^{20}$ +47 (*c* 0.1, MeOH).

HRMS (ESI) calcd for $C_{27}H_{31}O_4NCl$ $[M + H]^+$ 468.19361 and 470.19066, found 468.19390 and 470.18994.

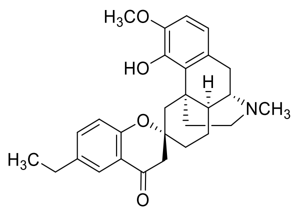

SM-C-S-15

Following the general procedure for sinomenine-based chromanones, **SM-C-S-15** (33 mg, 24%) was obtained. The other diastereomer could not be obtained in sufficient purity.

SM-C-S-15:

^{1}H NMR (700 MHz, $CDCl_3$) δ 7.60 (d, *J* = 2.4 Hz, 1H), 7.29 (dd, *J* = 8.5, 2.4 Hz, 1H), 6.85 (d, *J* = 8.4 Hz, 1H), 6.76 (d, *J* = 8.3 Hz, 1H), 6.64 (d, *J* = 8.3 Hz, 1H), 5.99 (bs, 1H), 4.04 (dd, *J* = 13.7, 2.5 Hz, 1H), 3.90 (s, 3H), 3.15 (s, 1H), 2.94 (d, *J* = 18.4 Hz, 1H), 2.91 – 2.84 (m, 1H), 2.70 (d, *J* = 10.6 Hz, 1H), 2.64 (d, *J* = 17.1 Hz, 1H), 2.58 (q, *J* = 7.6 Hz, 2H), 2.52 (s, 3H), 2.35 (d, *J* = 17.0 Hz, 1H), 2.20 – 2.13 (m, 1H), 2.11 – 2.02 (m, 2H), 1.89 (d, *J* = 12.5 Hz, 1H), 1.85 – 1.73 (m, 2H), 1.60 – 1.53 (m, 2H), 1.38 (qd, *J* = 13.8, 3.5 Hz, 1H), 1.20 (t, *J* = 7.6 Hz, 3H).

^{13}C NMR (176 MHz, $CDCl_3$) δ 193.16, 158.09, 145.57, 144.49, 136.53, 136.20, 128.49, 124.86, 123.82, 120.19, 119.41, 118.36, 109.80, 81.33, 58.25, 56.56, 47.67, 44.89, 44.33, 43.14, 41.99, 37.77, 36.89, 36.46, 27.97, 24.25, 24.10, 15.63.

$[\alpha]_D^{20}$ −16 (*c* 0.1, MeOH).

HRMS (ESI) calcd for $C_{28}H_{34}O_4N$ $[M + H]^+$ 448.24824, found 448.24932.

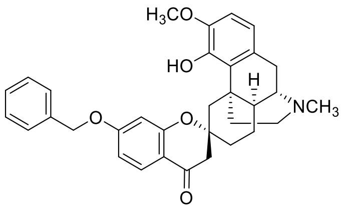

SM-C-S-16

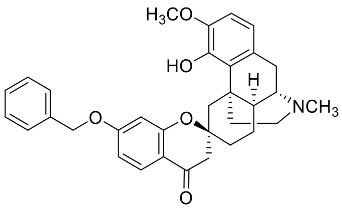

SM-C-R-13

Following the general procedure for sinomenine-based chromanones, **SM-C-S-16** (20 mg, 12%) and **SM-C-R-13** (82 mg, 47%) were obtained.

SM-C-S-16: **^{1}H NMR** (500 MHz, MeOD) δ 7.70 (d, *J* = 8.8 Hz, 1H), 7.46 – 7.42 (m, 2H), 7.41 – 7.36 (m, 2H), 7.35 – 7.31 (m, 1H), 6.94 (d, *J* = 8.3 Hz, 1H), 6.76 (d, *J* = 8.3 Hz, 1H), 6.64 (dd, *J* = 8.8, 2.4 Hz, 1H), 6.52 (d, *J* = 2.4 Hz, 1H), 5.13 (s, 2H), 4.22 (dd, *J* = 13.6, 2.4 Hz, 1H), 3.88 (s, 3H), 3.70 (s, 1H), 3.29 – 3.07 (m, 3H), 2.87 (s, 3H), 2.64 (d, *J* = 17.2 Hz, 1H), 2.57 (td, *J* = 13.2, 3.5 Hz, 1H), 2.30 (d, *J* = 17.2 Hz, 1H), 2.18 – 2.09 (m, 2H), 1.95 (d, *J* = 12.1 Hz, 1H), 1.82 – 1.66 (m, 3H), 1.55 (d, *J* = 13.6 Hz, 1H), 1.44 (qd, *J* = 14.0, 3.4 Hz, 1H).

^{13}C NMR (126 MHz, MeOD) δ 193.01, 167.20, 163.28, 148.10, 146.56, 137.80, 129.63, 129.19, 128.66, 127.05, 123.45, 120.43, 115.42, 112.02, 111.01, 103.44, 82.57, 71.35, 61.33, 56.78, 45.49, 44.48, 43.72, 41.10, 37.69, 37.12, 36.79, 24.58, 24.49. There is an aromatic and an aliphatic carbon that could not be found in the spectra.

$[\alpha]^{20}_{D}$ +2 (*c* 0.1, MeOH).

HRMS (ESI) calcd for $C_{33}H_{36}O_5N$ $[M + H]^+$ 526.25880, found 526.25934.

SM-C-R-13: **^{1}H NMR** (700 MHz, $CDCl_3$) δ 7.73 (d, *J* = 8.7 Hz, 1H), 7.39 – 7.30 (m, 5H), 6.68 – 6.64 (m, 2H), 6.54 (dd, *J* = 8.7, 2.4 Hz, 1H), 5.47 (d, *J* = 2.4 Hz, 1H), 4.88 (d, *J* = 11.5 Hz, 1H), 4.82 (d, *J* = 11.6 Hz, 1H), 4.17 (dd, *J* = 15.0, 2.7 Hz, 1H), 3.68 (s, 3H), 3.26 (s, 1H), 3.08 (dd, *J* = 18.5, 5.5 Hz, 1H), 2.95 (d, *J* = 18.4 Hz, 1H), 2.76 (d, *J* = 10.8 Hz, 1H), 2.66 (d, *J* = 16.3 Hz, 1H), 2.55 (s, 3H), 2.48 (d, *J* = 16.3 Hz, 1H), 2.21 (t, *J* = 10.8 Hz, 1H), 2.12 (d, *J* = 11.8 Hz, 1H), 2.04 (dq, *J* = 13.6, 3.1 Hz, 1H), 1.91 (qd, *J* = 13.0, 3.6 Hz, 1H), 1.86 (d, *J* = 12.2 Hz, 1H), 1.79 (td, *J* = 13.2, 4.5 Hz, 1H), 1.61 (td, *J* = 13.4, 4.1 Hz, 1H), 1.47 (dq, *J* = 13.1, 3.3 Hz, 1H), 1.18 (d, *J* = 15.0 Hz, 1H).

^{13}C NMR (176 MHz, $CDCl_3$) δ 190.94, 164.90, 161.59, 145.17, 144.40, 136.12, 128.73, 128.26, 127.75, 127.22, 124.76, 118.93, 114.48, 108.88, 108.26, 101.71, 80.40, 69.85, 58.94, 56.13, 48.75, 48.04, 43.68, 41.66, 39.74, 36.70, 35.69, 24.44, 22.44. There is an aromatic and an aliphatic carbon that could not be found in the spectra.

$[\alpha]^{20}_{D}$ –23 (*c* 0.1, MeOH).

HRMS (ESI) calcd for $C_{33}H_{36}O_5N$ $[M + H]^+$ 526.25880, found 526.26000.

General procedure for ring closed sinomenine-based indoles (SM-I-closed)

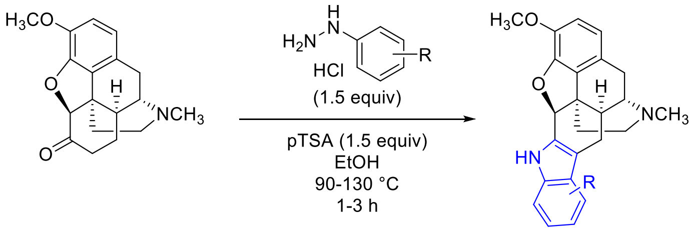

To an oven-dried microwave vial under an atmosphere of argon was added the sinomenine ketone (40 mg, 0.13 mmol, 1 equiv.), phenylhydrazine HCl salt (0.20 mmol, 1.5 equiv), and paratolylsulfonic acid (*p*TSA) (35 mg, 0.20 mmol, 1.5 equiv). Then anhydrous EtOH (1.5 ml) was added and the vial flushed with argon again. A microwave cap was placed on the vial and the reaction was heated to 90-130 °C for 1-3 h in the microwave. After the reaction had cooled to room temperature, it was diluted with dichloromethane and transferred to a sept funnel and saturated $NaHCO_3$ was added. The aqueous layer was extracted with dichloromethane (3 x 10 ml). The combined organic layers were dried and concentrated. A flashmaster column was manual equilibrated with 1% triethylamine in DCM and then purified by silica chromatography (0-12% MeOH in DCM).

Compound data for ring closed sinomenine-based indoles (SM-I-closed):

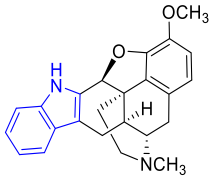

SM-I-closed-1

Following the general procedure for ring closed sinomenine-based indoles on a 1.5 mmol scale in EtOH (11 ml) with a reaction time of 2 h at 100 °C, 531 mg (95%) of **SM-I-closed-1** was isolated after silica chromatography.

^{1}H NMR (600 MHz, $CDCl_3$) δ 8.26 (s, 1H), 7.40 (d, *J* = 7.9 Hz, 1H), 7.30 (d, *J* = 8.2 Hz, 1H), 7.16 (t, *J* = 7.6 Hz, 1H), 7.04 (t, *J* = 7.5 Hz, 1H), 6.66 – 6.60 (m, 2H), 5.67 (s, 1H), 3.76 (s, 3H), 3.36 (dd, *J* = 6.2, 2.8 Hz, 1H), 3.11 (d, *J* = 18.6 Hz, 1H), 2.81 (dd, *J* = 15.1, 5.9 Hz, 1H), 2.75 – 2.67 (m, 1H), 2.66 – 2.56 (m, 2H), 2.50 (s, 3H), 2.38 (td, *J* = 12.1, 3.6 Hz, 1H), 2.20 – 2.06 (m, 2H), 2.03 – 1.97 (m, 1H).

^{13}C NMR (151 MHz, $CDCl_3$) δ 144.50, 143.20, 137.00, 130.29, 129.07, 127.13, 126.50, 123.05, 119.54, 119.07, 119.03, 113.71, 112.85, 111.41, 85.82, 60.04, 56.14, 46.93, 43.71, 43.19, 41.12, 35.90, 21.21, 20.54.

HRMS (ESI) calcd for $C_{24}H_{24}N_2O_2$ $[M + H]^+$ 373.19105, found 373.19055.

$[\alpha]_D^{20}$ +422 (*c* 0.1, MeOH).

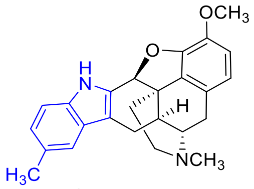

SM-I-closed-2

Following the general procedure for ring closed sinomenine-based indoles on a 0.08 mmol scale with a reaction time of 2 h at 100 °C, 30 mg (92%) of **SM-I-closed-2** was isolated after silica chromatography.

^{1}H NMR (700 MHz, $CDCl_3$) δ 8.09 (s, 1H), 7.23 – 7.15 (m, 2H), 6.99 (dd, *J* = 8.2, 1.7 Hz, 1H), 6.63 (d, *J* = 2.3 Hz, 2H), 5.65 (s, 1H), 3.76 (s, 3H), 3.40 – 3.31 (m, 1H), 3.10 (d, *J* = 18.5 Hz, 1H), 2.77 (dd, *J* = 15.1, 5.9 Hz, 1H), 2.73 – 2.55 (m, 3H), 2.50 (s, 3H), 2.39 (s, 4H), 2.16 – 2.05 (m, 2H), 2.02 – 1.93 (m, 1H).

^{13}C NMR (176 MHz, $CDCl_3$) δ 144.56, 143.23, 135.35, 130.41, 129.09, 128.84, 127.08, 126.75, 124.65, 119.02, 118.70, 113.27, 112.92, 111.06, 85.90, 60.09, 56.17, 46.96, 43.68, 43.17, 41.05, 35.85, 21.52, 21.21, 20.58.

HRMS (ESI) calcd for $C_{25}H_{26}N_2O_2$ $[M + H]^+$ 387.20670, found 387.20654.

$[\alpha]^{20}_{D}$ +214 (*c* 0.1, MeOH).

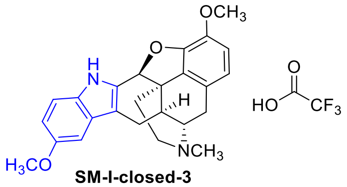

SM-I-closed-3

Following the general procedure for ring closed sinomenine-based indoles on a 0.08 mmol scale with a reaction time of 1 h at 100 °C, 19 mg (57%) of **SM-I-closed-3** was isolated after silica chromatography and preparative HPLC (acetonitrile/water).

^{1}H NMR (700 MHz, $CDCl_3$) δ 13.36 (s, 1H), 8.15 (s, 1H), 7.19 (d, *J* = 8.8 Hz, 1H), 6.84 (dd, *J* = 8.9, 2.4 Hz, 1H), 6.76 (d, *J* = 2.4 Hz, 1H), 6.74 – 6.69 (m, 2H), 5.68 (s, 1H), 4.10 – 4.04 (m, 1H), 3.79 (s, 7H), 3.78 (s, 7H), 3.43 (dd, *J* = 12.4, 4.6 Hz, 1H), 3.15 (dd, *J* = 11.7, 5.4 Hz, 3H), 2.92 (s, 3H), 2.86 (dd, *J* = 15.0, 5.9 Hz, 2H), 2.48 (td, *J* = 13.4, 4.8 Hz, 2H), 2.17 – 2.07 (m, 2H).

^{13}C NMR (176 MHz, $CDCl_3$) δ 154.30, 144.94, 144.32, 132.20, 129.70, 127.20, 126.31, 122.33, 119.56, 114.38, 113.91, 112.28, 111.94, 100.76, 85.05, 61.22, 56.34, 56.01, 47.48, 42.08, 41.32, 38.33, 33.35, 21.54, 20.74.

HRMS (ESI) calcd for $C_{25}H_{26}N_2O_3$ [M + H] 403.20162, found 403.20162.

$[\alpha]_D^{20}$ +298 (*c* 0.1, MeOH).

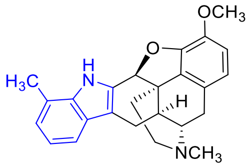

SM-I-closed-4

Following the general procedure for ring closed sinomenine-based indoles on a 0.08 mmol scale with a reaction time of 2 h at 100 °C, 30 mg (93%) of **SM-I-closed-4** was isolated after silica chromatography.

^{1}H NMR (400 MHz, CD_3OD) δ 7.21 – 7.17 (m, 1H), 6.93 – 6.85 (m, 2H), 6.80 (d, *J* = 8.3 Hz, 1H), 6.76 (d, *J* = 8.4 Hz, 1H), 5.80 (s, 1H), 4.18 – 4.07 (m, 1H), 3.76 (s, 3H), 3.38 – 3.32 (m, 1H), 3.20 (d, *J* = 6.6 Hz, 1H), 3.17 – 3.09 (m, 1H), 3.00 (s, 3H), 2.99 – 2.75 (m, 3H), 2.46 (s, 3H), 2.35 – 2.23 (m, 1H), 2.22 – 2.05 (m, 2H).

^{13}C NMR (176 MHz, $CDCl_3$) δ 146.38, 145.27, 138.19, 130.58, 128.42, 126.76, 124.73, 124.24, 122.03, 120.72, 120.33, 117.13, 116.75, 112.16, 86.15, 63.20, 57.23, 43.20, 41.63, 40.91, 34.83, 22.20, 21.65, 16.97. One aliphatic carbon is missing.

HRMS (ESI) calcd for $C_{25}H_{26}N_2O_2$ $[M + H]^+$ 387.20670, found 387.20685.

$[\alpha]_D^{20}$ +271 (*c* 0.1, MeOH).

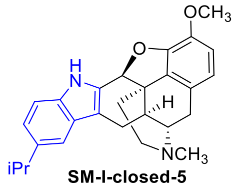

SM-I-closed-5

Following the general procedure for ring closed sinomenine-based indoles on a 0.08 mmol scale with a reaction time of 1 h at 100 °C, 30 mg (87%) of **SM-I-closed-5** was isolated after silica chromatography.

^{1}H NMR (700 MHz, $CDCl_3$) δ 8.28 (s, 1H), 7.25 (s, 2H), 7.23 (d, *J* = 8.4 Hz, 2H), 7.06 (dd, *J* = 8.4, 1.7 Hz, 1H), 6.63 (s, 2H), 5.68 (s, 1H), 3.75 (s, 3H), 3.37 (dd, *J* = 6.3, 2.8 Hz, 1H), 3.11 (d, *J* = 18.6 Hz, 1H), 2.96 (hept, *J* = 6.8 Hz, 1H), 2.82 (dd, *J* = 15.1, 5.9 Hz, 1H), 2.70 (ddd, *J* = 11.5, 5.9, 2.8 Hz, 1H), 2.67 – 2.58 (m, 2H), 2.50 (s, 3H), 2.38 (td, *J* = 12.2, 3.6 Hz, 1H), 2.15 (dd, *J* = 14.1, 11.6 Hz, 1H), 2.10 (td, *J* = 12.4, 4.9 Hz, 1H), 2.00 (ddd, *J* = 12.4, 3.6, 1.8 Hz, 1H), 1.26 (dd, *J* = 6.9, 2.3 Hz, 6H).

^{13}C NMR (176 MHz, $CDCl_3$) δ 144.55, 143.21, 140.29, 135.61, 130.52, 129.14, 127.15, 126.50, 122.32, 118.99, 115.70, 113.45, 112.79, 111.26, 85.97, 60.05, 56.09, 46.91, 43.67, 43.19, 41.12, 35.90, 34.31, 24.78, 24.76, 21.27, 20.55.

HRMS (ESI) calcd for $C_{27}H_{30}N_2O_2$ $[M + H]^+$ 415.23800, found 415.23670.

$[\alpha]_D^{20}$ +402 (*c* 0.1, MeOH).

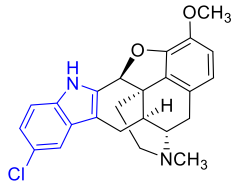

SM-I-closed-6

Following the general procedure for ring closed sinomenine-based indoles on a 0.13 mmol scale with a reaction time of 2 h at 100 °C, 28 mg (52%) of **SM-I-closed-6** was isolated after silica chromatography.

^{1}H NMR (700 MHz, $CDCl_3$) δ 8.52 (s, 1H), 7.30 (d, *J* = 2.0 Hz, 1H), 7.11 (d, *J* = 8.6 Hz, 1H), 7.06 (dd, *J* = 8.7, 2.0 Hz, 1H), 6.66 (s, 2H), 5.65 (s, 1H), 3.75 (s, 3H), 3.38 – 3.28 (m, 1H), 3.11 (d, *J* = 18.6 Hz, 1H), 2.70 (dd, *J* = 14.9, 6.0 Hz, 1H), 2.67 – 2.56 (m, 3H), 2.48 (s, 3H), 2.36 (td, *J* = 12.1, 3.6 Hz, 1H), 2.12 – 2.03 (m, 2H), 2.01 – 1.95 (m, 1H).

^{13}C NMR (176 MHz, $CDCl_3$) δ 144.41, 143.12, 135.29, 131.71, 128.98, 127.55, 127.34, 125.06, 123.09, 119.26, 118.55, 113.29, 112.94, 112.36, 85.62, 59.87, 56.16, 46.84, 43.74, 43.19, 41.11, 35.97, 21.07, 20.47.

HRMS (ESI) calcd for $C_{24}H_{23}N_2O_2Cl$ $[M + H]^+$ 407.15208 and 409.14913, found 407.15124 and 409.14789.

$[\alpha]_D^{20}$ +176 (*c* 0.1, MeOH).

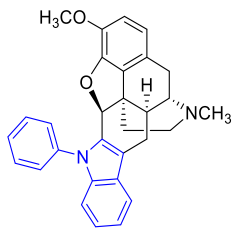

SM-I-closed-7

Following the general procedure for ring closed sinomenine-based indoles on a 0.13 mmol scale with a reaction time of 2 h at 100 °C, 57 mg (95%) of **SM-I-closed-7** was isolated after silica chromatography.

The proton and carbon NMRs are a mixture of rotamers. The HPLC trace for this product is pure.

^{1}H NMR (700 MHz, $CDCl_3$) δ 7.68 – 7.56 (m, 3H), 7.50 – 7.47 (m, 2H), 7.33 – 7.26 (m, 1H), 7.21 – 7.15 (m, 2H), 7.12 – 7.09 (m, 1H), 6.67 (d, J = 8.1 Hz, 1H), 6.63 (d, J = 8.2 Hz, 1H), 5.54 (s, 1H), 3.78 (s, 3H), 3.38 (dd, J = 6.4, 2.9 Hz, 1H), 3.12 (d, J = 18.5 Hz, 1H), 2.86 (dd, J = 15.2, 5.6 Hz, 1H), 2.73 – 2.66 (m, 1H), 2.65 – 2.57 (m, 2H), 2.50 (s, 3H), 2.38 (td, J = 12.3, 3.6 Hz, 1H), 2.25 (dd, J = 14.8, 12.0 Hz, 1H), 2.04 (td, J = 12.4, 5.0 Hz, 1H), 1.94 – 1.87 (m, 1H).

^{13}C NMR (176 MHz, $CDCl_3$) δ 149.31, 144.85, 143.17, 138.71, 137.89, 132.43, 129.46, 129.43, 129.33, 129.15, 128.45, 127.85, 127.24, 126.26, 123.20, 122.03, 121.06, 119.92, 119.56, 118.96, 118.89, 117.90, 114.63, 114.58, 110.86, 84.82, 59.89, 56.86, 46.79, 43.86, 43.12, 41.45, 36.04, 21.60, 20.65. All carbons that appear in the spectra are reported.

HRMS (ESI) calcd for $C_{30}H_{28}N_2O_2$ $[M + H]^+$ 449.22235, found 449.22114.

$[\alpha]_D^{20}$ +421 (*c* 0.1, MeOH).

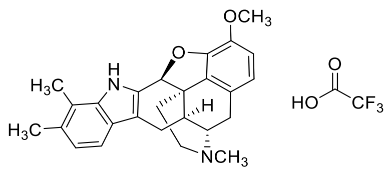

SM-I-closed-8

Following the general procedure for ring closed sinomenine-based indoles on a 0.13 mmol scale with a reaction time of 2 h at 100 °C, 14 mg (20%) of **SM-I-closed-8** was isolated after silica chromatography.

^{1}H NMR (700 MHz, CD_3OD) δ 7.88 (s, 1H), 7.07 (d, *J* = 8.0 Hz, 1H), 6.83 – 6.77 (m, 2H), 6.76 (d, *J* = 8.3 Hz, 1H), 5.79 (s, 1H), 4.10 (dd, *J* = 6.8, 2.8 Hz, 1H), 3.76 (s, 3H), 3.16 (dd, *J* = 20.0, 6.9 Hz, 1H), 3.13 – 3.07 (m, 1H), 2.99 (s, 3H), 2.95 (td, *J* = 13.1, 3.9 Hz, 1H), 2.83 (dd, *J* = 15.0, 5.8 Hz, 1H), 2.80 – 2.75 (m, 1H), 2.38 (s, 3H), 2.32 (s, 3H), 2.27 (td, *J* = 13.7, 4.8 Hz, 1H), 2.18 – 2.13 (m, 2H), 2.07 (dd, *J* = 15.0, 11.2 Hz, 1H).

^{13}C NMR (176 MHz, CD_3OD) δ 146.39, 145.26, 138.89, 131.13, 130.10, 128.47, 125.25, 124.73, 123.03, 120.67, 119.66, 116.70, 116.58, 112.16, 86.30, 79.46, 63.19, 57.22, 43.14, 41.61, 40.91, 34.84, 22.21, 21.62, 19.47, 13.23.

HRMS (ESI) calcd for $C_{26}H_{28}N_2O_2$ $[M + H]^+$ 401.22235, found 401.22196.

$[\alpha]_D^{20}$ +475 (*c* 0.15, MeOH).

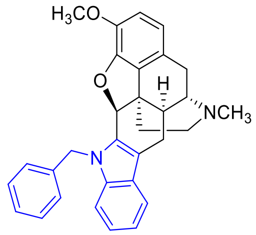

SM-I-closed-9

Following the general procedure for ring closed sinomenine-based indoles on a 0.13 mmol scale with a reaction time of 2 h at 100 °C, 33 mg (53%) of **SM-I-closed-9** was isolated after silica chromatography.

^{1}H NMR (700 MHz, $CDCl_3$) δ 7.32 (d, *J* = 8.0 Hz, 1H), 7.20 – 7.16 (m, 3H), 7.16 – 7.12 (m, 2H), 7.07 (d, *J* = 8.3 Hz, 1H), 7.05 – 7.00 (m, 1H), 6.99 (d, *J* = 7.0 Hz, 2H), 6.94 (ddd, *J* = 7.9, 6.8, 1.1 Hz, 1H), 6.56 (q, *J* = 8.2 Hz, 2H), 5.54 (s, 1H), 5.44 (q, *J* = 16.8 Hz, 2H), 3.64 (s, 3H), 3.48 – 3.39 (m, 1H), 3.03 (d, *J* = 18.8 Hz, 1H), 2.91 – 2.63 (m, 4H), 2.51 (s, 3H), 2.42 (d, *J* = 14.4 Hz, 1H), 2.11 (ddd, *J* = 15.0, 11.5, 1.4 Hz, 2H), 1.91 – 1.85 (m, 1H).

^{13}C NMR (176 MHz, $CDCl_3$) δ 144.65, 143.56, 138.00, 137.55, 137.02, 131.18, 128.80, 127.35, 126.46, 126.03, 122.99, 119.43, 119.22, 119.07, 114.09, 112.66, 111.45, 110.13, 84.81, 60.46, 56.42, 47.30, 47.01, 43.37, 40.02, 34.95, 21.26, 21.06.

HRMS (ESI) calcd for $C_{31}H_{30}N_2O_2$ $[M + H]^+$ 463.23800, found 463.23700.

$[\alpha]^{20}_{D}$ +367 (*c* 0.1, MeOH).

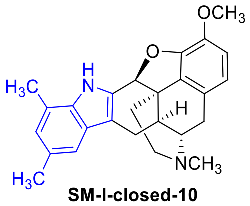

SM-I-closed-10

Following the general procedure for ring closed sinomenine-based indoles on a 0.13 mmol scale with a reaction time of 2 h at 100 °C, 6 mg (9%) of **SM-I-closed-10** was isolated after silica chromatography and preparative HPLC (acetonitrile/water).

^{1}H NMR (700 MHz, CD_3OD) δ 6.97 (s, 1H), 6.80 (d, *J* = 8.2 Hz, 1H), 6.78 – 6.74 (m, 2H), 5.78 (s, 1H), 4.14 – 4.07 (m, 1H), 3.76 (s, 3H), 3.36 – 3.32 (m, 2H), 3.18 (dd, *J* = 20.0, 6.9 Hz, 1H), 3.05 – 2.92 (m, 4H), 2.85 (dd, *J* = 15.0, 5.7 Hz, 1H), 2.81 – 2.75 (m, 1H), 2.43 (s, 3H), 2.32 (s, 3H), 2.28 (td, *J* = 14.0, 5.0 Hz, 1H), 2.21 – 2.14 (m, 1H), 2.08 (dd, *J* = 15.0, 11.3 Hz, 1H).

^{13}C NMR (176 MHz, CD_3OD) δ 144.17, 143.03, 134.35, 128.38, 127.22, 126.20, 124.75, 123.82, 122.49, 119.46, 118.45, 114.49, 114.43, 109.45, 84.01, 61.00, 54.99, 46.40, 40.95, 39.39, 38.73, 32.64, 19.97, 19.44, 19.29, 14.68. By 2D-NMR, an aliphatic carbon can be seen underneath the solvent peak.

HRMS (ESI) calcd for $C_{26}H_{28}N_2O_2$ $[M + H]^+$ 401.22235, found 401.22203.

$[\alpha]_D^{20}$ +306 (*c* 0.16, MeOH).

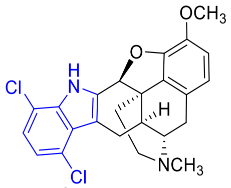

SM-I-closed-11

Following the general procedure for ring closed sinomenine-based indoles on a 0.13 mmol scale with a reaction time of 3 h at 100 °C, 20 mg (34%) of **SM-I-closed-11** was isolated after silica chromatography.

^{1}H NMR (700 MHz, $CDCl_3$) δ 8.53 (s, 1H), 6.99 (d, *J* = 8.1 Hz, 1H), 6.87 (d, *J* = 8.1 Hz, 1H), 6.67 (s, 2H), 5.62 (s, 1H), 3.78 (s, 3H), 3.36 – 3.30 (m, 2H), 3.10 (d, *J* = 18.6 Hz, 1H), 2.64 (ddd, *J* = 11.6, 6.0, 2.8 Hz, 1H), 2.63 – 2.57 (m, 2H), 2.47 (s, 3H), 2.35 (td, *J* = 12.1, 3.6 Hz, 1H), 2.28 (ddd, *J* = 15.9, 11.6, 1.4 Hz, 1H), 2.07 (td, *J* = 12.3, 4.9 Hz, 1H), 2.01 – 1.96 (m, 1H).

^{13}C NMR (176 MHz, $CDCl_3$) δ 144.28, 143.14, 135.07, 131.95, 128.81, 127.36, 125.43, 124.83, 122.45, 120.82, 119.38, 115.51, 114.95, 112.90, 85.02, 59.81, 56.16, 46.79, 43.31, 43.21, 41.07, 35.93, 22.68, 20.40.

HRMS (ESI) calcd for $C_{24}H_{22}N_2O_2Cl_2$ $[M + H]^+$ 441.11311 and 443.11016, found 441.11254 and 443.10866.

$[\alpha]^{20}_{D}$ +434(*c* 0.1, MeOH).

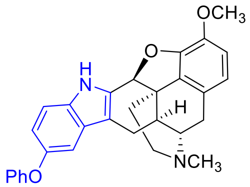

SM-I-closed-12

Following the general procedure for ring closed sinomenine-based indoles on a 0.13 mmol scale with a reaction time of 1 h at 100 °C, 44 mg (71%) of **SM-I-closed-12** was isolated after silica chromatography.

^{1}H NMR (700 MHz, $CDCl_3$) δ 8.55 (s, 1H), 7.29 – 7.23 (m, 3H), 7.04 (d, *J* = 2.3 Hz, 1H), 7.00 (tt, *J* = 7.3, 1.1 Hz, 1H), 6.94 – 6.89 (m, 3H), 6.67 (d, *J* = 8.2 Hz, 2H), 6.65 (d, *J* = 8.2 Hz, 2H), 5.69 (s, 1H), 3.77 (s, 3H), 3.35 (d, *J* = 3.7 Hz, 1H), 3.11 (d, *J* = 18.6 Hz, 1H), 2.73 – 2.66 (m, 2H), 2.65 (dd, *J* = 12.1, 4.5 Hz, 1H), 2.59 (dd, *J* = 18.6, 6.3 Hz, 1H), 2.50 (s, 3H), 2.39 (td, *J* = 12.2, 3.6 Hz, 1H), 2.15 – 2.06 (m, 2H), 2.03 – 1.95 (m, 1H).

^{13}C NMR (176 MHz, $CDCl_3$) δ 159.38, 149.81, 144.49, 143.16, 133.94, 131.60, 129.59, 129.00, 127.12, 122.04, 119.13, 117.44, 116.67, 113.62, 112.93, 112.33, 109.63, 85.76, 59.95, 56.15, 46.87, 43.67, 43.11, 41.04, 35.87, 21.17, 20.53. An aromatic carbon is missing.

HRMS (ESI) calcd for $C_{30}H_{28}N_2O_3$ $[M + H]^+$ 465.21727, found 465.21609.

$[\alpha]_D^{20}$ +275 (*c* 0.1, MeOH).

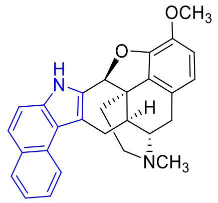

SM-I-closed-13

Following the general procedure for ring closed sinomenine-based indoles on a 0.13 mmol scale with a reaction time of 1 h at 100 °C, 32 mg (57%) of **SM-I-closed-13** was isolated after silica chromatography.

^{1}H NMR (700 MHz, $CDCl_3$) δ 8.92 (s, 1H), 8.21 (d, *J* = 7.8 Hz, 1H), 7.82 (dd, *J* = 8.1, 1.3 Hz, 1H), 7.49 (d, *J* = 8.8 Hz, 1H), 7.46 (ddd, *J* = 8.3, 6.9, 1.3 Hz, 1H), 7.37 (d, *J* = 8.8 Hz, 1H), 7.34 (ddd, *J* = 8.0, 6.9, 1.2 Hz, 1H), 6.67 (d, *J* = 8.2 Hz, 2H), 6.66 (d, *J* = 8.2 Hz, 2H), 5.79 (s, 1H), 3.74 (s, 3H), 3.50 (dd, *J* = 6.5, 2.9 Hz, 1H), 3.36 (dd, *J* = 15.0, 6.1 Hz, 1H), 3.16 (d, *J* = 18.7 Hz, 1H), 2.86 – 2.80 (m, 1H), 2.74 (dd, *J* = 18.8, 6.4 Hz, 1H), 2.69 (dd, *J* = 12.0, 4.7 Hz, 1H), 2.59 – 2.51 (m, 4H), 2.44 (td, *J* = 12.2, 3.5 Hz, 1H), 2.17 (td, *J* = 12.4, 4.9 Hz, 1H), 2.06 – 2.01 (m, 1H).

^{13}C NMR (176 MHz, $CDCl_3$) δ 144.41, 143.25, 133.73, 129.61, 129.36, 129.15, 128.87, 128.29, 126.95, 125.71, 123.97, 123.12, 122.93, 119.46, 119.15, 115.40, 113.19, 112.98, 85.90, 60.14, 56.10, 46.91, 43.10, 42.90, 40.95, 35.70, 24.44, 20.67.

HRMS (ESI) calcd for $C_{28}H_{26}N_2O_2$ $[M + H]^+$ 423.20670, found 423.20788.

$[\alpha]_D^{20}$ +217 (*c* 0.1, MeOH).

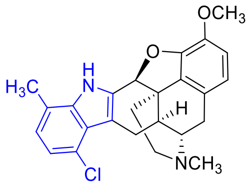

SM-I-closed-14

Following the general procedure for ring closed sinomenine-based indoles on a 0.13 mmol scale with a reaction time of 2 h at 100 °C, 19 mg (34%) of **SM-I-closed-14** was isolated after silica chromatography.

^{1}H NMR (700 MHz, $CDCl_3$) δ 8.23 (s, 1H), 6.88 (d, *J* = 7.6 Hz, 1H), 6.81 (d, *J* = 7.6 Hz, 1H), 6.66 (s, 2H), 5.65 (s, 1H), 3.77 (s, 3H), 3.37 (dd, *J* = 15.8, 6.0 Hz, 2H), 3.10 (d, *J* = 18.6 Hz, 1H), 2.66 (ddd, *J* = 11.3, 5.7, 2.7 Hz, 1H), 2.65 – 2.59 (m, 2H), 2.48 (s, 3H), 2.40 – 2.33 (m, 4H), 2.29 (ddd, *J* = 15.9, 11.6, 1.4 Hz, 1H), 2.08 (td, *J* = 12.3, 4.9 Hz, 1H), 1.98 (ddd, *J* = 12.3, 3.6, 1.8 Hz, 1H).

^{13}C NMR (176 MHz, $CDCl_3$) δ 144.30, 143.13, 137.60, 130.86, 128.99, 127.35, 124.34, 123.83, 123.14, 120.35, 119.33, 119.27, 114.32, 112.65, 85.53, 59.92, 56.05, 46.87, 43.26, 43.20, 41.12, 35.91, 22.81, 20.46, 16.29.

HRMS (ESI) calcd for $C_{25}H_{25}N_2O_2Cl$ [M + H] 421.16773 and 423.16478, found 421.16845 and 423.16548

$[\alpha]_D^{20}$ +394 (*c* 0.1, MeOH).

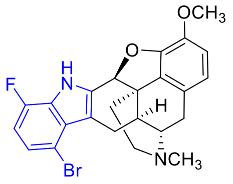

SM-I-closed-15

Following the general procedure for ring closed sinomenine-based indoles on a 0.13 mmol scale with a reaction time of 3 h at 100 °C, 12 mg (19%) of **SM-I-closed-15** was isolated after silica chromatography.

^{1}H NMR (600 MHz, $CDCl_3$) δ 8.53 (s, 1H), 7.04 (dd, *J* = 8.3, 4.1 Hz, 1H), 6.71 (dd, *J* = 10.2, 8.3 Hz, 1H), 6.67 (s, 2H), 5.60 (s, 1H), 3.78 (s, 3H), 3.44 (dd, *J* = 15.9, 6.0 Hz, 1H), 3.36 (dd, *J* = 6.3, 2.8 Hz, 1H), 3.10 (d, *J* = 18.6 Hz, 1H), 2.69 – 2.57 (m, 3H), 2.48 (s, 3H), 2.35 (td, *J* = 12.1, 3.6 Hz, 1H), 2.26 (ddd, *J* = 15.9, 11.6, 1.4 Hz, 1H), 2.07 (td, *J* = 12.3, 4.9 Hz, 1H), 1.98 (ddd, *J* = 10.6, 3.6, 1.8 Hz, 1H).

^{13}C NMR (151 MHz, $CDCl_3$) δ 149.15 (d, *J* = 244.4 Hz), 144.24, 143.15, 132.26, 128.83, 128.07 (d, *J* = 5.2 Hz), 127.33, 126.50 (d, *J* = 14.7 Hz), 123.39 (d, *J* = 6.3 Hz), 119.42, 115.15 (d, *J* = 1.8 Hz), 113.00, 108.81, 108.72 (d, *J* = 17.7 Hz), 85.06, 59.85, 56.21, 46.80, 43.25, 43.20, 40.86, 35.88, 22.55, 20.41.

^{19}F NMR (565 MHz, $CDCl_3$) δ -137.27 (dd, *J* = 10.2, 4.2 Hz).

HRMS (ESI) calcd for $C_{24}H_{22}BrFN_2O_2$ $[M + H]^+$ 469.09215 and 471.09010, found 469.09217 and 471.08971.

$[\alpha]_D^{20}$ +291 (*c* 0.156, MeOH).

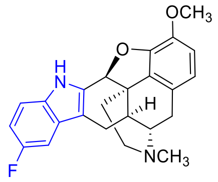

SM-I-closed-16

Following the general procedure for ring closed sinomenine-based indoles on a 0.11 mmol scale with a reaction time of 2 h at 100 °C, 40 mg (90%) of **SM-I-closed-16** was isolated after silica chromatography.

^{1}H NMR (600 MHz, $CDCl_3$) δ 8.52 (s, 1H), 7.13 (dd, *J* = 8.8, 4.3 Hz, 1H), 7.00 (dd, *J* = 9.4, 2.5 Hz, 1H), 6.87 (td, *J* = 9.1, 2.5 Hz, 1H), 6.67 (s, 2H), 5.68 (s, 1H), 3.75 (s, 3H), 3.36 (dd, *J* = 6.3, 2.6 Hz, 1H), 3.12 (d, *J* = 18.6 Hz, 1H), 2.75 – 2.65 (m, 2H), 2.65 – 2.55 (m, 2H), 2.49 (s, 3H), 2.37 (td, *J* = 12.2, 3.6 Hz, 1H), 2.15 – 2.05 (m, 2H), 1.99 (ddd, *J* = 12.4, 3.6, 1.7 Hz, 1H).

^{13}C NMR (151 MHz, $CDCl_3$) δ157.68 (d, *J* = 234.8 Hz), 144.43, 143.12, 133.46, 132.12, 129.00, 127.29, 126.74 (d, *J* = 9.8 Hz), 119.20, 113.64 (d, *J* = 4.8 Hz), 112.86, 112.01 (d, *J* = 9.6 Hz), 111.17 (d, *J* = 26.3 Hz), 103.89 (d, *J* = 23.4 Hz), 85.72, 59.89, 56.11, 46.84, 43.72, 43.18, 41.14, 35.97, 21.16, 20.48.

^{19}F NMR (565 MHz, $CDCl_3$) δ -124.69 (td, *J* = 9.2, 4.3 Hz).

HRMS (ESI) calcd for $C_{24}H_{23}FN_2O_2$ $[M + H]^+$ 391.18163, found 391.18037 .

$[\alpha]_D^{20}$ +459 (*c* 0.142, MeOH).

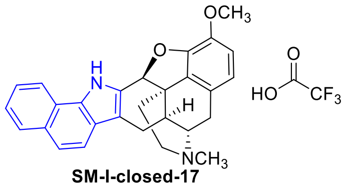

SM-I-closed-17

Following the general procedure for ring closed sinomenine-based indoles on a 0.13 mmol scale with a reaction time of 1 h at 40 °C, 14 mg (20%) of **SM-I-closed-17** was isolated after silica chromatography and preparative HPLC (acetonitrile/water).

^{1}H NMR (700 MHz, CD_3OD) δ 8.24 (d, *J* = 8.2 Hz, 1H), 7.84 (d, *J* = 8.1 Hz, 1H), 7.49 (t, *J* = 7.4 Hz, 1H), 7.44 (d, *J* = 8.6 Hz, 1H), 7.41 – 7.34 (m, 2H), 6.81 (d, *J* = 8.1 Hz, 1H), 6.78 (d, *J* = 8.3 Hz, 1H), 5.88 (s, 1H), 4.17 – 4.10 (m, 1H), 3.76 (s, 3H), 3.39 – 3.31 (m, 2H), 3.24 – 3.09 (m, 2H), 3.05 – 2.93 (m, 4H), 2.88 – 2.80 (m, 1H), 2.33 (td, *J* = 13.6, 4.7 Hz, 1H), 2.24 – 2.07 (m, 2H).

^{13}C NMR (176 MHz, CD_3OD) δ 146.42, 145.32, 133.64, 132.41, 129.60, 129.18, 128.43, 126.44, 125.14, 124.78, 123.59, 122.63, 121.65, 121.04, 120.74, 119.45, 116.85, 113.58, 86.18, 63.23, 57.28, 48.64, 43.27, 41.65, 41.04, 34.86, 22.22, 21.68. By 2D-NMR, an aliphatic carbon can be observed underneath the solvent peak.

HRMS (ESI) calcd for $C_{28}H_{26}N_2O_2$ $[M + H]^+$ 423.20670, found 423.20640.

$[\alpha]_D^{20}$ +584 (*c* 0.1, MeOH).

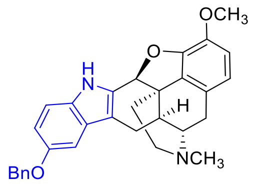

SM-I-closed-18

Following the general procedure for ring closed sinomenine-based indoles on a 0.13 mmol scale with a reaction time of 1 h at 100 °C, 47 mg (74%) of **SM-I-closed-18** was isolated after silica chromatography.

^{1}H NMR (600 MHz, $CDCl_3$) δ 8.41 (s, 1H), 7.44 (d, *J* = 7.0 Hz, 2H), 7.37 (t, *J* = 7.6 Hz, 2H), 7.31 (t, *J* = 7.3 Hz, 1H), 7.17 (d, *J* = 8.7 Hz, 1H), 6.94 (d, *J* = 2.4 Hz, 1H), 6.90 (dd, *J* = 8.8, 2.4 Hz, 1H), 6.69 – 6.61 (m, 2H), 5.68 (s, 1H), 5.09 – 5.01 (m, 2H), 3.76 (s, 3H), 3.36 (dd, *J* = 6.2, 2.8 Hz, 1H), 3.12 (d, *J* = 18.6 Hz, 1H), 2.75 (dd, *J* = 14.8, 5.9 Hz, 1H), 2.72 – 2.67 (m, 1H), 2.66 – 2.58 (m, 2H), 2.50 (s, 3H), 2.38 (td, *J* = 12.1, 3.6 Hz, 1H), 2.18 – 2.06 (m, 2H), 2.02 – 1.97 (m, 1H).

^{13}C NMR (151 MHz, $CDCl_3$) δ 153.18, 144.51, 143.13, 137.74, 132.34, 131.15, 129.09, 128.58, 127.83, 127.57, 127.19, 126.73, 119.00, 113.81, 113.32, 112.82, 112.16, 102.45, 85.92, 71.03, 59.96, 56.09, 46.85, 43.65, 43.16, 41.14, 35.91, 21.26, 20.51.

HRMS (ESI) calcd for $C_{31}H_{30}N_2O_3$ $[M + H]^+$ 479.23292, found 479.23338.

$[\alpha]^{20}_{D}$ +277 (*c* 0.1, MeOH).

General procedure for ring opened sinomenine-based indoles (SM-I-opened):

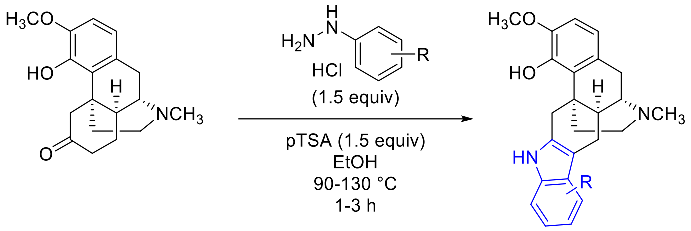

To an oven-dried microwave vial under an atmosphere of argon was added the sinomenine ketone (35 mg, 0.12 mmol, 1 equiv.), phenylhydrazine HCl salt (0.17 mmol, 1.5 equiv), and paratolylsulfonic acid (*p*TSA) (30 mg, 0.17 mmol, 1.5 equiv). Then anhydrous EtOH (2 ml) was added and the vial flushed with argon again. A microwave cap was placed on the vial and the reaction was heated to 90-130 °C for 1-3 h in the microwave. After the reaction had cooled to room temperature, it was diluted with dichloromethane and transferred to a sept funnel and saturated $NaHCO_3$ was added. The aqueous layer was extracted with dichloromethane (3 x 10 ml). The combined organic layers were dried and concentrated. A MPLC column was manual equilibrated with 1% triethylamine in DCM and then purified by silica chromatography (0-12% MeOH in DCM).

Compound data for ring opened sinomenine-based indoles (**S-I-opened**):

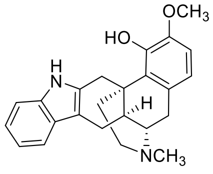

SM-I-opened-1

Following the general procedure for ring opened sinomenine-based indoles on a 0.33 mmol scale with a reaction time of 1 h at 100 °C, 85 mg (68%) of **SM-I-opened-1** were isolated after silica chromatography.

^{1}H NMR (700 MHz, $(CD_3)_2SO$) δ 10.57 (s, 1H), 8.21 (s, 1H), 7.17 (d, *J* = 8.1 Hz, 2H), 6.90 (t, *J* = 7.5 Hz, 1H), 6.82 (t, *J* = 7.4 Hz, 1H), 6.64 (d, *J* = 8.3 Hz, 1H), 6.54 (d, *J* = 8.3 Hz, 1H), 4.87 (d, *J* = 16.4 Hz, 1H), 3.65 (s, 3H), 3.05 – 3.00 (m, 1H), 2.92 – 2.87 (m, 2H), 2.60 (dd, *J* = 15.3, 5.8 Hz, 1H), 2.55 – 2.50 (m, 5H), 2.43 – 2.33 (m, 2H), 2.31 (s, 3H), 2.14 (ddd, *J* = 11.6, 5.8, 3.1 Hz, 1H), 2.02 (td, *J* = 12.1, 3.0 Hz, 1H), 1.88 (dt, *J* = 12.5, 2.6 Hz, 1H), 1.76 (td, *J* = 12.6, 4.6 Hz, 1H).

^{13}C NMR (176 MHz, $(CD_3)_2SO$) δ 145.00, 144.76, 135.52, 134.48, 131.31, 127.01, 125.02, 119.61, 117.99, 117.86, 116.76, 110.49, 109.17, 105.88, 56.56, 55.67, 46.59, 42.59, 42.32, 38.34, 36.71, 33.14, 23.67, 22.57.

HRMS (ESI) calcd for $C_{24}H_{26}N_2O_2$ $[M + H]^+$ 375.20670, found 375.20787.

$[\alpha]_D^{20}$ +74.4 (*c* 0.121, MeOH).

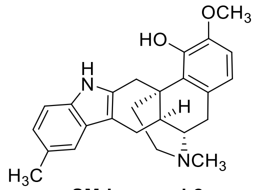

SM-I-opened-2

Following the general procedure for ring opened sinomenine-based indoles on a 0.13 mmol scale with a reaction time of 1 h at 100 °C, 34 mg (66%) of **SM-I-opened-2** were isolated after silica chromatography.

The NMR sample was prepared in a 3:1 $CDCl_3$:CD_3OD solution. **^{1}H NMR** (400 MHz, CD_3OD) δ 7.06 (d, *J* = 8.2 Hz, 1H), 7.04 – 7.00 (m, 1H), 6.77 (dd, *J* = 8.3, 1.7 Hz, 1H), 6.51 (s, 2H), 4.74 (d, *J* = 16.3 Hz, 1H), 3.67 (s, 3H), 3.13 – 3.06 (m, 1H), 3.00 – 2.89 (m, 2H), 2.66 (ddd, *J* = 15.4, 5.9, 1.5 Hz, 1H), 2.59 (dt, *J* = 16.4, 2.1 Hz, 1H), 2.51 – 2.40 (m, 2H), 2.38 (s, 3H), 2.34 – 2.23 (m, 4H), 2.18 (td, *J* = 12.5, 3.2 Hz, 1H), 1.97 (dt, *J* = 12.8, 2.5 Hz, 1H), 1.82 (td, *J* = 12.9, 4.7 Hz, 1H).

The NMR sample was prepared in a 3:1 $CDCl_3$:CD_3OD solution. **^{13}C NMR** (176 MHz, CD_3OD) δ 144.93, 144.69, 134.59, 134.22, 131.33, 127.94, 127.74, 124.55, 121.71, 118.62, 117.16, 110.33, 108.94, 106.68, 57.85, 56.06, 47.20, 42.71, 42.64, 38.45, 37.32, 33.99, 24.33, 23.23, 21.45.

HRMS (ESI) calcd for $C_{25}H_{28}N_2O_2$ $[M + H]^+$ 389.22235, found 389.22240.

$[\alpha]_D^{20}$ +52.1 (*c* 0.165, MeOH).

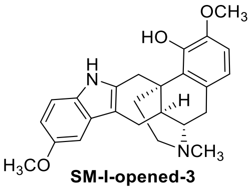

SM-I-opened-3

Following the general procedure for ring opened sinomenine-based indoles on a 0.13 mmol scale with a reaction time of 1 h at 100 °C, 38 mg (71%) of **SM-I-opened-3** were isolated after silica chromatography.

The NMR sample was prepared in a 3:1 $CDCl_3$:CD_3OD solution. **^{1}H NMR** (700 MHz, CD_3OD) δ 7.06 (d, *J* = 8.7 Hz, 1H), 6.73 (d, *J* = 2.5 Hz, 1H), 6.61 – 6.59 (m, 1H), 6.53 (d, *J* = 1.8 Hz, 2H), 4.76 (d, *J* = 16.3 Hz, 1H), 3.73 (s, 3H), 3.67 (s, 3H), 3.11 – 3.08 (m, 1H), 3.01 – 2.94 (m, 2H), 2.65 (ddd, *J* = 15.3, 5.9, 1.4 Hz, 1H), 2.59 (dt, *J* = 16.4, 1.9 Hz, 1H), 2.49 – 2.42 (m, 2H), 2.38 (s, 3H), 2.29 – 2.24 (m, 1H), 2.18 (td, *J* = 12.4, 3.1 Hz, 1H), 1.97 (dt, *J* = 12.7, 2.5 Hz, 1H), 1.82 (td, *J* = 12.9, 4.7 Hz, 1H).

The NMR sample was prepared in a 3:1 $CDCl_3$:CD_3OD solution. **^{13}C NMR** (176 MHz, CD_3OD) δ 153.47, 144.96, 144.71, 135.51, 131.31, 131.22, 127.99, 124.51, 118.64, 111.26, 109.82, 108.95, 107.06, 100.20, 57.84, 56.22, 56.05, 47.19, 42.71, 42.65, 38.50, 37.30, 34.03, 24.33, 23.26.

HRMS (ESI) calcd for $C_{25}H_{28}N_2O_3$ $[M + H]^+$ 405.21727, found 405.21738.

$[\alpha]_D^{20}$ +56.3 (*c* 0.158, MeOH).

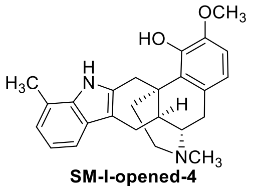

SM-I-opened-4

Following the general procedure for ring opened sinomenine-based indoles on a 0.12 mmol scale with a reaction time of 1 h at 90 °C, 19mg (42%) of **SM-I-opened-4** were isolated after silica chromatography.

^{1}H NMR (600 MHz,CDCl$_3$) δ 7.65 (s, 1H), 7.17 (d, *J* = 7.8 Hz, 1H), 6.93 (t, *J* = 7.5 Hz, 1H), 6.85 (dt, *J* = 7.1, 1.0 Hz, 1H), 6.61 – 6.55 (m, 2H), 5.98 (s, 1H), 4.78 (d, *J* = 16.2 Hz, 1H), 3.76 (s, 3H), 3.17 (dd, *J* = 6.3, 3.2 Hz, 1H), 3.04 (d, *J* = 18.5 Hz, 1H), 2.98 (dd, *J* = 18.6, 6.4 Hz, 1H), 2.75 (ddd, *J* = 15.4, 5.8, 1.5 Hz, 1H), 2.69 (dt, *J* = 16.4, 2.1 Hz, 1H), 2.57 – 2.50 (m, 2H), 2.47 (s, 3H), 2.44 (s, 3H), 2.35 (ddd, *J* = 11.4, 5.8, 3.2 Hz, 1H), 2.21 (td, *J* = 12.3, 3.2 Hz, 1H), 2.03 (dt, *J* = 12.7, 2.6 Hz, 1H), 1.93 (td, *J* = 12.7, 4.7 Hz, 1H).

^{13}C NMR (151 MHz, CDCl$_3$) δ 144.41, 144.37, 135.06, 133.82, 131.89, 127.29, 124.65, 121.37, 119.64, 119.23, 118.63, 115.26, 108.55, 108.51, 57.59, 56.09, 47.19, 43.13, 43.09, 39.06, 37.45, 34.14, 24.03, 23.15, 16.80.

HRMS (ESI) calcd for $C_{25}H_{28}N_2O_2$ $[M + H]^+$ 389.22235, found 389.22227.

$[\alpha]_D^{20}$ +164.8 (*c* 0.162, MeOH).

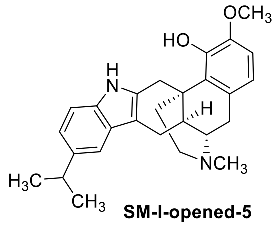

SM-I-opened-5

Following the general procedure for ring opened sinomenine-based indoles on a 0.12 mmol scale with a reaction time of 1 h at 100 °C, 30 mg (62%) of **SM-I-opened-5** were isolated after silica chromatography.

^{1}H NMR (600 MHz, $CDCl_3$) δ 7.77 (bs, 1H), 7.16 (d, *J* = 1.7 Hz, 1H), 7.06 – 6.99 (m, 1H), 6.91 (dd, *J* = 8.3, 1.7 Hz, 1H), 6.59 (d, *J* = 2.3 Hz, 2H), 6.24 (bs, 1H), 4.79 (d, *J* = 16.3 Hz, 1H), 3.74 (s, 3H), 3.18 (dd, *J* = 6.2, 3.1 Hz, 1H), 3.06 (d, *J* = 18.4 Hz, 1H), 2.99 (dd, *J* = 18.5, 6.2 Hz, 1H), 2.94 (sep, *J* = 6.9 Hz, 1H), 2.76 (ddd, *J* = 15.3, 5.8, 1.4 Hz, 1H), 2.69 (dt, *J* = 16.3, 2.1 Hz, 1H), 2.60 – 2.50 (m, 2H), 2.48 (s, 3H), 2.40 (ddd, *J* = 11.5, 5.7, 3.2 Hz, 1H), 2.23 (td, *J* = 12.3, 3.3 Hz, 1H), 2.05 (dt, *J* = 12.6, 2.7 Hz, 1H), 1.98 (td, *J* = 12.7, 4.7 Hz, 1H), 1.26 (dd, *J* = 6.9, 1.2 Hz, 6H).

^{13}C NMR (151 MHz, $CDCl_3$) δ 144.48, 139.74, 134.30, 134.22, 131.79, 127.75, 124.67, 119.68, 118.51, 114.42, 110.34, 108.54, 107.76, 57.66, 56.04, 47.22, 43.13, 42.89, 38.90, 37.41, 34.38, 34.12, 24.95, 24.86, 24.05, 23.12.

HRMS (ESI) calcd for $C_{27}H_{32}N_2O_2$ [M + H] 417.25365, found 417.25365.

$[\alpha]^{20}_{D}$ +6.0 (*c* 0.15, MeOH).

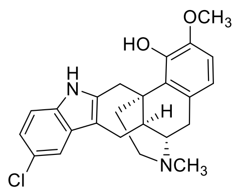

SM-I-opened-6

Following the general procedure for ring opened sinomenine-based indoles on a 0.12 mmol scale with a reaction time of 1 h at 100 °C, 23 mg (48%) of **SM-I-opened-6** were isolated after silica chromatography.

^{1}H NMR (600 MHz, $(CD_3)_2SO$) δ 10.84 (bs, 1H), 8.25 (bs, 1H), 7.18 (d, *J* = 2.1 Hz, 1H), 7.17 (d, *J* = 8.5 Hz, 1H), 6.89 (dd, *J* = 8.5, 2.1 Hz, 1H), 6.66 (d, *J* = 8.3 Hz, 1H), 6.55 (d, *J* = 8.3 Hz, 1H), 4.87 (d, *J* = 16.5 Hz, 1H), 3.65 (s, 3H), 3.03 (bs, 1H), 2.95 – 2.84 (m, 2H), 2.59 (dd, *J* = 15.6, 5.8 Hz, 1H), 2.53 (d, *J* = 16.8 Hz, 1H), 2.46 – 2.26 (m, 5H), 2.18 – 2.10 (m, 1H), 2.07 – 1.96 (m, 1H), 1.91 – 1.84 (m, 1H), 1.76 (td, *J* = 12.1, 11.6, 4.2 Hz, 1H).

^{13}C NMR (151 MHz, $(CD_3)_2SO$) δ 144.99, 144.69, 136.59, 133.93, 131.19, 128.10, 124.74, 122.51, 119.29, 118.00, 116.06, 111.80, 109.19, 105.94, 79.18, 56.49, 55.63, 46.51, 42.51, 38.24, 36.59, 33.06, 23.56, 22.24.

HRMS (ESI) calcd for $C_{24}H_{25}N_2O_2Cl$ $[M + H]^+$ 409.16773 and 411.16478, found 409.16742 and 411.16476, respectively.

$[\alpha]_D^{20}$ −14.3 (*c* 0.126, MeOH).

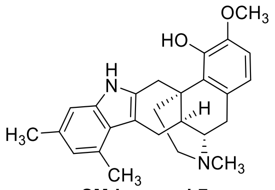

SM-I-opened-7

Following the general procedure for ring opened sinomenine-based indoles on a 0.12 mmol scale with a reaction time of 1 h at 100 °C, 30 mg (64%) of **SM-I-opened-7** were isolated after silica chromatography.

^{1}H NMR (600 MHz, $CDCl_3$) δ 7.63 (s, 1H), 6.74 (s, 1H), 6.59 (d, *J* = 1.4 Hz, 2H), 6.56 – 6.52 (m, 1H), 6.13 (s, 1H), 4.72 (d, *J* = 16.1 Hz, 1H), 3.74 (s, 3H), 3.15 (dd, *J* = 6.3, 3.2 Hz, 1H), 3.07 – 2.93 (m, 3H), 2.72 (ddd, *J* = 15.0, 11.4, 2.7 Hz, 1H), 2.67 (dt, *J* = 16.2, 2.1 Hz, 1H), 2.56 (ddd, *J* = 12.0, 4.8, 2.0 Hz, 1H), 2.50 (s, 3H), 2.47 (s, 3H), 2.39 – 2.33 (m, 1H), 2.32 (s, 3H), 2.21 (td, *J* = 12.3, 3.2 Hz, 1H), 2.02 (dt, *J* = 12.6, 2.7 Hz, 1H), 1.94 (td, *J* = 12.7, 4.7 Hz, 1H).

^{13}C NMR (151 MHz, $CDCl_3$) δ 144.47, 144.43, 136.08, 132.68, 131.67, 130.34, 129.33, 124.62, 124.56, 121.93, 118.52, 108.54, 108.35, 108.09, 57.67, 56.04, 47.22, 43.18, 43.09, 38.80, 36.79, 34.09, 25.72, 23.99, 21.51, 19.67.

HRMS (ESI) calcd for $C_{26}H_{30}N_2O_2$ $[M + H]^+$ 403.23800, found 403.23763.

$[\alpha]_D^{20}$ +64.8 (*c* 0.128, MeOH).

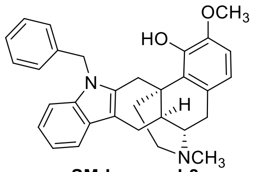

SM-I-opened-8

Following the general procedure for ring opened sinomenine-based indoles on a 0.12 mmol scale with a reaction time of 2 h at 100 °C, 36 mg (66%) of **SM-I-opened-8** were isolated after silica chromatography.

^{1}H NMR (700 MHz, CD_2Cl_2) δ 7.22 (d, *J* = 7.3 Hz, 1H), 7.17 – 7.11 (m, 3H), 7.02 (d, *J* = 8.1 Hz, 1H), 6.90 (ddd, *J* = 7.9, 5.9, 1.6 Hz, 3H), 6.86 (ddd, *J* = 8.0, 7.0, 1.1 Hz, 1H), 6.55 – 6.48 (m, 2H), 5.60 (s, 1H), 5.22 (s, 2H), 4.70 (d, *J* = 16.3 Hz, 1H), 3.64 (s, 3H), 3.02 (dd, *J* = 6.3, 3.2 Hz, 1H), 2.92 (d, *J* = 18.4 Hz, 1H), 2.87 (dd, *J* = 18.5, 6.4 Hz, 1H), 2.68 (ddd, *J* = 15.7, 6.2, 1.6 Hz, 1H), 2.44 (ddd, *J* = 15.7, 11.5, 2.7 Hz, 1H), 2.41 – 2.34 (m, 2H), 2.29 (s, 3H), 2.21 (ddd, *J* = 11.5, 6.2, 3.1 Hz, 1H), 2.02 (td, *J* = 12.2, 3.1 Hz, 1H), 1.83 (dt, *J* = 12.5, 2.6 Hz, 1H), 1.75 (td, *J* = 12.6, 4.7 Hz, 1H).

^{13}C NMR (176 MHz, CD_2Cl_2) δ 144.39, 144.01, 138.98, 136.63, 135.59, 132.13, 128.49, 127.23, 126.95, 126.37, 124.63, 120.27, 118.70, 118.60, 117.39, 108.99, 108.35, 107.48, 57.28, 55.87, 46.98, 46.20, 42.79, 42.79, 38.81, 37.39, 32.48, 23.84, 22.93.

HRMS (ESI) calcd for $C_{31}H_{33}O_2N_2$ $[M + H]^+$ 465.25365, found 465.25345.

$[\alpha]_D^{20}$ +81 (*c* 0.1, MeOH).

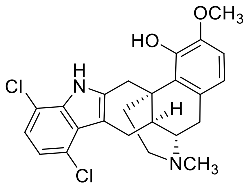

SM-I-opened-9

Following the general procedure for ring opened sinomenine-based indoles on a 0.12 mmol scale with a reaction time of 2 h at 100 °C, 18 mg (35%) of **SM-I-opened-9** were isolated after silica chromatography.

^{1}H NMR (700 MHz, CD_2Cl_2) δ 8.23 (bs, 1H), 6.91 (d, *J* = 8.1 Hz, 1H), 6.84 (d, *J* = 8.1 Hz, 1H), 6.64 (s, 2H), 6.06 (bs, 1H), 4.78 (d, *J* = 16.4 Hz, 1H), 3.76 (s, 3H), 3.17 – 3.08 (m, 2H), 3.02 (d, *J* = 18.6 Hz, 1H), 2.95 (dd, *J* = 18.7, 6.4 Hz, 1H), 2.72 – 2.63 (m, 2H), 2.49 (ddd, *J* = 11.6, 4.9, 2.0 Hz, 1H), 2.40 (s, 3H), 2.26 (ddd, *J* = 11.6, 6.1, 3.2 Hz, 1H), 2.13 (td, *J* = 12.2, 3.1 Hz, 1H), 1.96 (dt, *J* = 12.6, 2.6 Hz, 1H), 1.86 (td, *J* = 12.6, 4.8 Hz, 1H).

^{13}C NMR (176 MHz, CD_2Cl_2) δ 144.78, 144.53, 136.63, 133.81, 132.43, 126.42, 124.48, 124.13, 120.54, 120.31, 119.19, 114.88, 109.59, 108.89, 57.55, 56.38, 47.23, 43.16, 43.11, 39.19, 37.07, 34.24, 24.88, 24.10.

HRMS (ESI) calcd $C_{24}H_{25}O_2N_2{}^{37}Cl_2$ $[M + H]^+$ 447.12286, found 447.12267.

$[\alpha]_D^{20}$ +85 (*c* 0.1, MeOH).

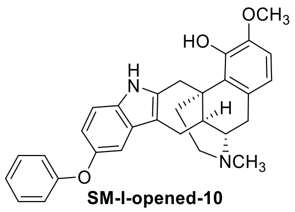

SM-I-opened-10

Following the general procedure for ring opened sinomenine-based indoles on a 0.12 mmol scale with a reaction time of 1 h at 100 °C, 27 mg (50%) of **SM-I-opened-10** were isolated after silica chromatography.

^{1}H NMR (700 MHz, CD_2Cl_2) δ 8.26 (bs, 1H), 7.23 (dd, *J* = 8.7, 7.3 Hz, 2H), 7.15 (d, *J* = 8.6 Hz, 1H), 6.99 – 6.94 (m, 2H), 6.88 (d, *J* = 7.6 Hz, 2H), 6.76 (dd, *J* = 8.6, 2.4 Hz, 1H), 6.66 – 6.62 (m, 2H), 6.29 (bs, 1H), 4.81 (d, *J* = 16.4 Hz, 1H), 3.74 (s, 3H), 3.13 (dd, *J* = 6.6, 3.1 Hz, 1H), 3.03 (d, *J* = 18.6 Hz, 1H), 2.95 (dd, *J* = 18.6, 6.5 Hz, 1H), 2.69 (dt, *J* = 16.4, 2.1 Hz, 1H), 2.65 (dd, *J* = 15.7, 5.7 Hz, 1H), 2.54 (ddd, *J* = 12.0, 4.8, 2.0 Hz, 1H), 2.42 (s, 4H), 2.35 (ddd, *J* = 11.4, 5.8, 3.1 Hz, 1H), 2.17 (td, *J* = 12.2, 3.2 Hz, 1H), 2.01 (dt, *J* = 12.7, 2.7 Hz, 1H), 1.94 (td, *J* = 12.7, 4.7 Hz, 1H).

^{13}C NMR (176 MHz, CD_2Cl_2) δ 160.12, 149.67, 144.87, 144.67, 136.21, 133.09, 132.29, 129.79, 128.61, 124.83, 122.01, 119.10, 117.45, 114.28, 111.56, 108.82, 108.75, 108.21, 57.71, 56.31, 47.34, 43.07, 42.96, 39.09, 37.68, 34.30, 24.25, 23.14.

HRMS (ESI) calcd for $C_{30}H_{31}O_3N_2$ [M + H] 467.23292, found 467.23264.

$[\alpha]_D^{20}$ –23 (*c* 0.1, MeOH).

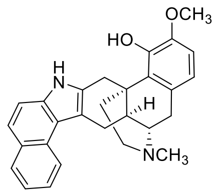

SM-I-opened-11

Following the general procedure for ring opened sinomenine-based indoles on a 0.12 mmol scale with a reaction time of 1 h at 100 °C, 17 mg (35%) of **SM-I-opened-11** were isolated after silica chromatography.

^{1}H NMR (700 MHz, CD_2Cl_2) δ 8.57 (bs, 1H), 8.16 (d, *J* = 8.3 Hz, 1H), 7.82 (d, *J* = 7.9 Hz, 1H), 7.42 (s, 2H), 7.41 – 7.39 (m, 1H), 7.31 (ddd, *J* = 8.0, 6.8, 1.2 Hz, 1H), 6.63 (d, *J* = 8.3 Hz, 1H), 6.60 (d, *J* = 8.3 Hz, 1H), 4.83 (d, *J* = 16.0 Hz, 1H), 3.70 (s, 3H), 3.27 (dd, *J* = 6.7, 3.2 Hz, 1H), 3.19 (dd, *J* = 15.2, 6.0 Hz, 1H), 3.13 (dd, *J* = 18.9, 6.5 Hz, 1H), 3.07 (d, *J* = 18.8 Hz, 1H), 2.88 (ddd, *J* = 14.8, 11.3, 2.4 Hz, 1H), 2.77 (d, *J* = 16.0 Hz, 1H), 2.61 (ddd, *J* = 12.0, 4.7, 2.1 Hz, 1H), 2.48 (s, 4H), 2.24 (td, *J* = 12.1, 3.5 Hz, 1H), 2.03 (dt, *J* = 12.7, 3.2 Hz, 1H), 1.98 (td, *J* = 12.5, 4.6 Hz, 1H).

^{13}C NMR (176 MHz, CD_2Cl_2) δ 144.51, 144.23, 132.25, 131.53, 131.46, 129.41, 128.74, 128.31, 124.86, 124.26, 123.49, 122.34, 121.04, 120.57, 118.83, 112.72, 109.74, 108.54, 57.67, 55.93, 47.02, 42.76, 42.56, 38.40, 36.70, 34.00, 26.12, 24.00.

HRMS (ESI) calcd for $C_{28}H_{29}O_2N_2$ [M + H] 425.22235, found 425.22212.

$[\alpha]_D^{20}$ −24 (*c* 0.1, MeOH).

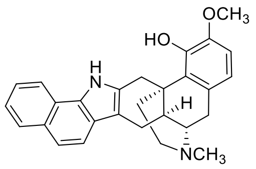

SM-I-opened-12

Following the general procedure for ring opened sinomenine-based indoles on a 0.12 mmol scale with a reaction time of 1 h at 100 °C, 17 mg (35%) of **SM-I-opened-12** were isolated after silica chromatography.

^{1}H NMR (700 MHz, CD_2Cl_2) δ 8.80 (bs, 1H), 7.90 (d, *J* = 8.2 Hz, 1H), 7.75 (d, *J* = 8.0 Hz, 1H), 7.36 (ddd, *J* = 8.2, 6.8, 1.3 Hz, 1H), 7.32 (d, *J* = 8.4 Hz, 1H), 7.28 (d, *J* = 8.5 Hz, 1H), 7.23 (ddd, *J* = 8.0, 6.8, 1.2 Hz, 1H), 6.55 – 6.49 (m, 2H), 4.78 (d, *J* = 16.0 Hz, 1H), 3.61 (s, 4H), 3.02 (dd, *J* = 6.6, 3.2 Hz, 1H), 2.93 (d, *J* = 18.6 Hz, 1H), 2.88 (dd, *J* = 18.7, 6.5 Hz, 1H), 2.67 – 2.58 (m, 2H), 2.47 – 2.39 (m, 2H), 2.33 (s, 3H), 2.23 (ddd, *J* = 11.7, 6.0, 3.2 Hz, 1H), 2.08 (td, *J* = 12.1, 3.2 Hz, 1H), 1.90 (dt, *J* = 12.5, 2.7 Hz, 1H), 1.84 (td, *J* = 12.6, 4.9 Hz, 1H).

^{13}C NMR (176 MHz, CD_2Cl_2) δ 144.84, 144.62, 132.77, 132.21, 130.15, 129.82, 129.07, 125.40, 124.91, 123.40, 123.21, 121.97, 119.77, 119.73, 119.09, 118.71, 109.80, 108.82, 57.84, 56.29, 47.37, 43.10, 43.02, 39.06, 37.73, 34.32, 24.31, 23.21.

HRMS (ESI) calcd for $C_{28}H_{28}N_2O_2$ $[M + H]^+$ 425.22235, found 425.22195.

$[\alpha]_D^{20}$ +350 (*c* 0.1, MeOH).

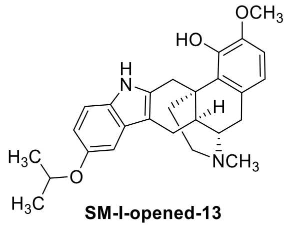

SM-I-opened-13

Following the general procedure for ring opened sinomenine-based indoles on a 0.12 mmol scale with a reaction time of 1 h at 100 °C, 14 mg (28%) of **SM-I-opened-13** were isolated after silica chromatography.

^{1}H NMR (700 MHz, CD_2Cl_2) δ 7.83 (s, 1H), 7.07 (d, *J* = 8.7 Hz, 1H), 6.77 (d, *J* = 2.4 Hz, 1H), 6.66 – 6.59 (m, 3H), 6.11 (s, 1H), 4.71 (d, *J* = 16.3 Hz, 1H), 4.42 (hept, *J* = 6.0 Hz, 1H), 3.74 (s, 3H), 3.11 (dd, *J* = 6.6, 3.2 Hz, 1H), 3.03 (d, *J* = 18.5 Hz, 1H), 2.96 (dd, *J* = 18.6, 6.5 Hz, 1H), 2.69 – 2.61 (m, 2H), 2.50 (ddd, *J* = 11.9, 4.7, 2.0 Hz, 1H), 2.47 – 2.39 (m, 4H), 2.31 (ddd, *J* = 11.6, 5.9, 3.2 Hz, 1H), 2.18 – 2.12 (m, 1H), 1.96 (dt, *J* = 12.7, 2.7 Hz, 1H), 1.89 (td, *J* = 12.6, 4.7 Hz, 1H), 1.26 (dd, *J* = 8.8, 6.0 Hz, 6H).

^{13}C NMR (176 MHz, CD_2Cl_2) δ 151.98, 144.79, 144.58, 135.50, 132.43, 131.39, 128.46, 124.98, 119.07, 112.75, 111.15, 108.73, 107.83, 104.40, 71.63, 57.74, 56.31, 47.34, 43.17, 43.12, 39.16, 37.74, 34.38, 24.27, 23.21, 22.47, 22.39.

HRMS (ESI) calcd for $C_{27}H_{32}N_2O_3$ $[M + H]^+$ 433.24857, found 433.24823.

$[\alpha]_D^{20}$ +6 (*c* 0.1, MeOH).

Synthesis of the griseofulvin-based ketone:

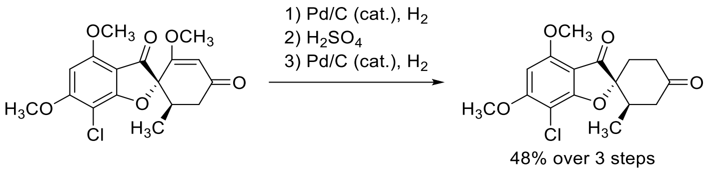

To an oven-dried three-neck flask was added griseofulvin (10.583 g, 30 mmol), 10% by weight Pd/C (500mg) and anhydrous ethyl acetate (100 ml). The reaction was evacuated and backfilled with H_2 (3x). A balloon of H_2 was attached and the reaction was stirred at 22 °C. After 54 h, the reaction was evacuated and backfilled with Ar (3x). The mixture was filtered through Celite and washed with dichloromethane. The eluent was concentrated and used in the next step without further purification.

To 500 ml flask containing the crude hydrogenated product was added AcOH (200 ml) and an aqueous solution of 2*M* H_2SO_4 (60 ml). The reaction was heated to 80 °C for 16 h. After this time, the reaction was cooled to room temperature and diluted with EtOAc (200 ml) and poured into ice water (200 ml). The organic layer was washed three times with water, dried over Na_2SO_4 and concentrated. The crude product was used in the next step without further purification.

To an oven-dried three-neck flask was added the above crude enone, 10% by weight Pd/C (600mg) and anhydrous ethyl acetate (80 ml) under an atmosphere of Ar. The reaction was evacuated and backfilled with H_2 (3x). A balloon of H_2 was attached and the reaction was stirred at 22 °C. After 6 h, the reaction was evacuated and backfilled with Ar (3x). The mixture was filtered through Celite, washed with dichloromethane, and concentrated. Purification by MPLC (silica gel, 10-36% EtOAc in CyHex) afforded 4.7g (48% over three steps) of the title compound as a white solid. ^{1}H NMR (400 MHz, Chloroform-*d*) δ 6.10 (s, 1H), 4.00 (s, 3H), 3.97 (s, 3H), 2.98 (dt, J = 15.2, 8.9 Hz, 1H), 2.87 (dd, J = 16.0, 12.5 Hz, 1H), 2.54 – 2.42 (m, 3H), 2.20 (dd, J = 8.8, 5.2 Hz, 2H), 0.92 (d, J = 6.5 Hz, 3H); ^{13}C NMR (101 MHz, $CDCl_3$) δ 209.12, 197.49, 168.07, 164.44, 157.84, 105.51, 97.43, 90.55, 89.24, 57.03, 56.42, 43.96, 38.44, 36.22, 31.18, 15.19; HRMS (ESI) calcd for $C_{16}H_{16}ClO_5$ [M + H] 325.08373 and 327.08078, found 325.08375 and 327.08073; $[\alpha]_D^{20}$ +30.1 (*c* 0.3 , $CHCl_3$).

General procedures for griseofulvin-derived indoles (**GF-I-1** and **GF-I-2**):

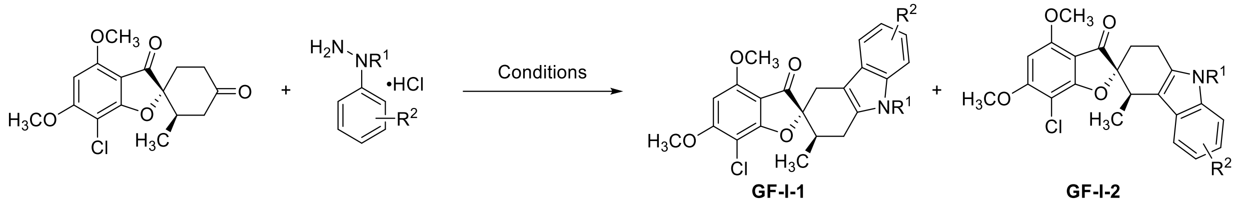

Procedure A: This procedure was adapted from a protocol reported by König et. al.[22]

To a microwave vial equipped with a stir bar was added tartaric acid (300 mg) and dimethylurea (700 mg). The microwave vial was stirred and heated to 90 °C. After all of the solid had melted (about 10 min), griseofulvin ketone (100 mg, 0.31 mmol, 1 equiv.) and a phenylhydrazine•HCl salt (0.37 mmol, 1.2 equiv) were added simultaneously to the heated solution. The reaction was stirred at 90 °C and monitored by LCMS. After all of the starting griseofulvin ketone and hydrazone intermediate were consumed (1-24 h), the reaction was quenched by adding water to the reaction while still heating. The reaction was then cooled to room temperature, and the aqueous layer was extracted with dichloromethane (3 x 10 ml). The combined organic layers were dried over Na_2SO_4, concentrated. Purification was achieved by MPLC (silica gel, hexane/EtOAc) to afford a major and minor isomer.

Procedure B:

To an oven-dried microwave vial under an atmosphere of Ar was added the griseofulvin-derived ketone (100 mg, 0.31 mmol, 1 equiv.), *p*-toluenesulfonic acid (80 mg, 0.46 mmol, 1.5 equiv.), and a phenylhydrazine•HCl salt (0.46 mmol, 1.5 equiv). Then, anhydrous EtOH (3 ml) was added and the reaction vessel purged with Ar for 5 min. The microwave vial was then sealed and reacted in the microwave at 90-130 ºC for 1-4 h. Once the starting material and hydrazone intermediate had been consumed (determined by LCMS), the reaction was diluted with water and DCM. The organic layer was washed with sat. $NaHCO_3$, dried over Na_2SO_4, and concentrated. Purification was achieved by MPLC (silica gel, 25-100% DCM in cyclohexane) to separately afford two isomers.

Compound data for griseofulvin-derived indoles (**GF-I**):

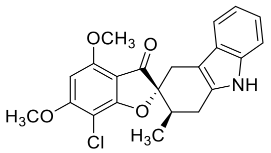

G-I-1-1

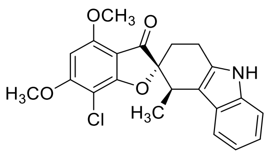

G-I-2-1

Following the general procedure A for griseofulvin-derived indoles, the starting materials were reacted for 1 h. After purification, 77 mg (63%) of the major isomer (**GF-I-1-1**) and 26 mg (22%) of the minor isomer (**GF-I-2-1**) were isolated as yellow solids.

GF-I-1-1:

^{1}H NMR (700 MHz, $CDCl_3$) δ 7.99 (s, 1H), 7.39 (d, *J* = 7.8 Hz, 1H), 7.28 (d, *J* = 8.1 Hz, 1H), 7.14 – 7.09 (m, 1H), 7.06 (t, *J* = 7.5 Hz, 1H), 6.09 (s, 1H), 3.99 (s, 3H), 3.94 (s, 3H), 3.26 (d, *J* = 16.2 Hz, 1H), 3.19 (dd, *J* = 15.1, 6.0 Hz, 1H), 3.03 (d, *J* = 16.2 Hz, 1H), 2.78 (dd, *J* = 16.1, 5.4 Hz, 1H), 2.48 – 2.40 (m, 1H), 1.15 (d, *J* = 7.0 Hz, 3H).

^{13}C NMR (176 MHz, $CDCl_3$) δ 197.60, 168.01, 164.07, 157.95, 136.40, 132.13, 127.57, 121.28, 119.35, 117.69, 110.73, 105.40, 105.07, 97.51, 92.48, 89.07, 56.96, 56.36, 34.68, 28.43, 27.53, 14.25.

HRMS (ESI) calcd for $C_{22}H_{20}ClNO_4$ $[M + H]^+$ 398.11536 and 400.11241, found 398.11506 and 400.11218.

$[\alpha]_D^{20}$ +49.0 (*c* 0.15, $CHCl_3$).

GF-I-2-1:

^{1}H NMR (700 MHz, $CDCl_3$) δ 7.86 (s, 1H), 7.48 (d, *J* = 7.8 Hz, 1H), 7.31 (d, *J* = 8.0 Hz, 1H), 7.13 (t, *J* = 7.6 Hz, 1H), 7.06 (t, *J* = 7.4 Hz, 1H), 6.10 (s, 1H), 3.99 (s, 3H), 3.98 (s, 3H), 3.36 (q, *J* = 7.1 Hz, 1H), 3.09 – 2.96 (m, 2H), 2.39 (m, 1H), 2.07 (dt, *J* = 13.9, 5.2 Hz, 1H), 1.45 (d, *J* = 7.2 Hz, 3H).

^{13}C NMR (176 MHz, $CDCl_3$) δ 197.73, 168.08, 164.05, 158.03, 136.46, 131.76, 127.15, 121.33, 119.33, 118.27, 112.52, 110.87, 105.60, 97.72, 92.44, 89.08, 56.99, 56.46, 35.35, 26.51, 19.82, 16.64.

HRMS (ESI) calcd for $C_{22}H_{20}ClNO_4$ $[M + H]^+$ 398.11536 and 400.11241, found 398.11498 and 400.11241.

$[\alpha]_D^{20}$ +21.3 (*c* 0.075, $CHCl_3$).

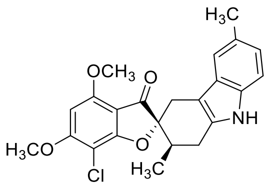

GF-I-1-2

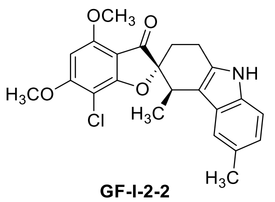

GF-I-2-2

Following the general procedure A for griseofulvin-derived indoles, the starting materials were reacted for 1 h. After purification, 57 mg (45%) of the major isomer (**GF-I-1-2**) and 12 mg (10%) of the minor isomer (**GF-I-2-2**) were isolated as yellow solids.

GF-I-1-2:

^{1}H NMR (500 MHz, $CDCl_3$) δ 7.90 (s, 1H), 7.22 – 7.13 (m, 2H), 6.93 (d, *J* = 8.3 Hz, 1H), 6.09 (s, 1H), 3.99 (s, 3H), 3.94 (s, 3H), 3.23 (d, *J* = 14.4 Hz, 1H), 3.17 (dd, *J* = 16.2, 5.7 Hz, 1H), 2.99 (d, *J* = 16.3 Hz, 1H), 2.74 (dd, *J* = 16.1, 5.2 Hz, 1H), 2.42 (m, 4H), 1.15 (d, *J* = 6.9 Hz, 3H).

^{13}C NMR (176 MHz, $CDCl_3$) δ 196.61, 167.00, 163.03, 156.94, 133.68, 131.21, 127.56, 126.83, 121.72, 116.52, 109.37, 104.40, 103.61, 96.52, 91.52, 88.05, 55.96, 55.38, 33.64, 27.49, 26.44, 20.56, 13.23.

HRMS (ESI) calcd for $C_{23}H_{22}ClNO_4$ $[M + H]^+$ 412.13101 and 414.12806, found 412.13081 and 414.12806.

$[\alpha]_D^{20}$ +60.8 (*c* 0.097, $CHCl_3$).

GF-I-2-2:

^{1}H NMR (500 MHz, $CDCl_3$) δ 7.77 (s, 1H), 7.26 (s, 1H), 7.20 (d, *J* = 8.2 Hz, 1H), 6.95 (d, *J* = 8.8 Hz, 1H), 6.09 (s, 1H), 3.99 (s, 3H), 3.98 (s, 3H), 3.31 (q, *J* = 7.1 Hz, 1H), 3.08 – 2.92 (m, 2H), 2.45 – 2.33 (m, 4H), 2.08 – 1.99 (m, 1H), 1.44 (d, *J* = 7.1 Hz, 3H).

^{13}C NMR (126 MHz, $CDCl_3$) δ 197.86, 168.02, 163.99, 157.98, 134.66, 131.83, 128.48, 127.32, 122.72, 118.03, 112.09, 110.50, 105.52, 97.62, 92.47, 88.95, 56.97, 56.43, 35.26, 26.31, 21.67, 19.83, 16.73.

HRMS (ESI) calcd for $C_{23}H_{22}ClNO_4$ $[M + H]^+$ 412.13101 and 414.12806, found 412.13079 and 414.12796.

$[\alpha]_D^{20}$ -7.8 (*c* 0.051, $CHCl_3$).

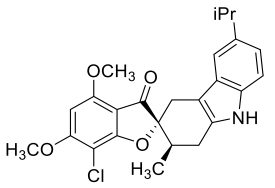

GF-I-1-3

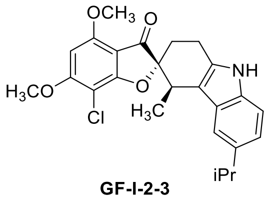

GF-I-2-3

Following the general procedure A for griseofulvin-derived indoles, the starting materials were reacted for 1 h. After purification, 90 mg (66%) of the major isomer (**G-I-1-3**) and 28 mg (21%) of the minor isomer (**G-I-2-3**) were isolated as yellow solids.

GF-I-1-3:

^{1}H NMR (700 MHz, $CDCl_3$) δ 7.78 (s, 1H), 7.24 – 7.21 (m, 2H), 7.01 (d, *J* = 8.4 Hz, 1H), 6.10 (s, 1H), 4.00 (s, 3H), 3.96 (s, 3H), 3.25 (d, *J* = 16.3 Hz, 1H), 3.20 (dd, *J* = 16.2, 5.4 Hz, 1H), 3.04 – 2.94 (m, 2H), 2.76 (dd, *J* = 16.0, 5.1 Hz, 1H), 2.43 (h, *J* = 6.5 Hz, 1H), 1.28 (d, *J* = 6.9 Hz, 6H), 1.15 (d, *J* = 7.6 Hz, 3H).

^{13}C NMR (176 MHz, $CDCl_3$) δ 197.61, 168.03, 164.03, 157.96, 140.21, 134.94, 132.22, 127.69, 120.44, 114.75, 110.47, 105.44, 104.98, 92.54, 89.05, 56.98, 56.41, 34.65, 34.37, 28.55, 27.49, 24.85, 14.22.

HRMS (ESI) calcd for $C_{25}H_{26}ClNO_4$ $[M + H]^+$ 440.16231 and 442.15936, found 440.16203 and 442.15924.

$[\alpha]_D^{20}$ +52.8 (*c* 0.12, $CHCl_3$).

GF-I-2-3:

^{1}H NMR (600 MHz, $CDCl_3$) δ 7.80 (s, 1H), 7.30 (s, 1H), 7.24 (d, *J* = 8.3 Hz, 1H), 7.03 (d, *J* = 8.4 Hz, 1H), 6.10 (s, 1H), 3.99 (s, 3H), 3.98 (s, 3H), 3.35 (q, *J* = 7.1 Hz, 1H), 3.06 – 2.93 (m, 3H), 2.39 (m, 1H), 2.07 – 1.98 (m, 1H), 1.46 (d, *J* = 7.1 Hz, 3H), 1.29 (d, *J* = 6.9 Hz, 6H).

^{13}C NMR (151 MHz, $CDCl_3$) δ 197.86, 168.05, 164.01, 158.00, 140.02, 134.95, 131.86, 127.16, 120.27, 115.24, 112.32, 110.63, 105.55, 92.49, 89.01, 56.96, 56.43, 35.28, 34.40, 26.30, 24.90, 24.83, 19.83, 16.79.

HRMS (ESI) calcd for $C_{25}H_{26}ClNO_4$ $[M + H]^+$ 440.16231 and 442.15936, found 440.16204 and 442.15936.

$[\alpha]_D^{20}$ -37.7 (*c* 0.077, $CHCl_3$).

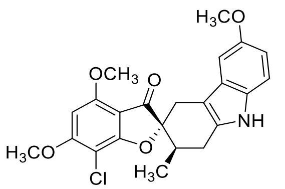

GF-I-1-4

Following the general procedure A for griseofulvin-derived indoles, the starting materials were reacted for 1 h. After purification, 73 mg (55%) of the major isomer (**GF-I-1-4**) was isolated as a yellow solid. The minor isomer was not obtained in an analytically pure form.

GF-I-1-4:

^{1}H NMR (500 MHz, $CDCl_3$) δ 7.81 (s, 1H), 7.18 (d, *J* = 8.7 Hz, 1H), 6.85 (d, *J* = 2.3 Hz, 1H), 6.77 (dd, *J* = 8.7, 2.4 Hz, 1H), 6.09 (s, 1H), 4.00 (s, 3H), 3.96 (s, 3H), 3.82 (s, 3H), 3.26 – 3.14 (m, 2H), 2.99 (d, *J* = 16.2 Hz, 1H), 2.77 (dd, *J* = 16.1, 5.3 Hz, 1H), 2.44 (h, *J* = 6.4 Hz, 1H), 1.14 (d, *J* = 6.9 Hz, 3H).

^{13}C NMR (126 MHz, $CDCl_3$) δ 197.59, 167.98, 164.04, 157.92, 154.02, 133.01, 131.42, 127.96, 111.36, 110.90, 105.36, 104.97, 100.19, 97.47, 92.46, 88.98, 56.98, 56.39, 56.04, 34.66, 28.51, 27.54, 14.25

HRMS (ESI) calcd for $C_{23}H_{22}ClNO_5$ $[M + H]^+$ 428.12593 and 430.12298, found 428.12570 and 430.12288.

$[\alpha]^{20}_{D}$ +55.5 (*c* 0.21, $CHCl_3$).

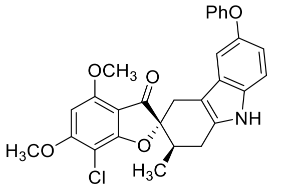

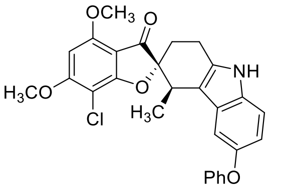

Following the general procedure A for griseofulvin-derived indoles, the starting materials were reacted for 1 h. After purification, 99 mg (66%) of the major isomer (**GF-I-1-5**) and 18 mg (12%) of the minor isomer (**GF-I-2-4**) were isolated as white solids.

GF-I-1-5:

^{1}H NMR (600 MHz, $CDCl_3$) δ 7.99 (s, 1H), 7.29 – 7.23 (m, 3H), 7.04 (d, *J* = 2.3 Hz, 1H), 6.99 (t, *J* = 7.3 Hz, 1H), 6.95 (d, *J* = 8.2 Hz, 2H), 6.86 (dd, *J* = 8.6, 2.3 Hz, 1H), 6.09 (s, 1H), 3.99 (s, 3H), 3.94 (s, 3H), 3.24 – 3.12 (m, 2H), 2.97 (d, *J* = 16.2 Hz, 1H), 2.82 (dd, *J* = 16.2, 5.9 Hz, 1H), 2.47 (h, *J* = 6.5 Hz, 1H), 1.14 (d, *J* = 6.9 Hz, 3H).

^{13}C NMR (151 MHz, $CDCl_3$) δ 197.50, 167.99, 164.11, 159.56, 157.94, 149.76, 133.68, 133.36, 129.57, 128.29, 121.92, 117.36, 114.79, 111.51, 108.74, 105.38, 105.34, 97.49, 92.31, 89.08, 56.98, 56.37, 34.74, 28.48, 27.61, 14.31.

HRMS (ESI) calcd for $C_{28}H_{24}ClNO_5$ [M + H] 490.14158 and 492.13863, found 490.14137 and 492.13862.

$[\alpha]_D^{20}$ +53.5 (*c* 0.56, $CHCl_3$).

GF-I-2-4:

^{1}H NMR (600 MHz, $CDCl_3$) δ 7.88 (s, 1H), 7.31 – 7.22 (m, 3H), 7.16 (d, *J* = 2.3 Hz, 1H), 6.99 (tt, *J* = 7.4, 1.1 Hz, 1H), 6.97 – 6.92 (m, 2H), 6.87 (dd, *J* = 8.6, 2.3 Hz, 1H), 6.09 (s, 1H), 3.99 (s, 3H), 3.97 (s, 3H), 3.31 (q, *J* = 7.1 Hz, 1H), 3.07 – 2.99 (m, 2H), 2.45 – 2.31 (m, 1H), 2.09 (dt, *J* = 13.8, 5.3 Hz, 1H), 1.38 (d, *J* = 7.1 Hz, 3H).

^{13}C NMR (151 MHz, $CDCl_3$) δ 197.47, 167.92, 163.95, 159.52, 157.90, 149.37, 133.33, 133.17, 129.47, 127.71, 121.76, 117.08, 114.74, 112.50, 111.48, 109.43, 105.45, 97.52, 92.17, 88.97, 56.87, 56.32, 35.20, 26.49, 19.76, 16.24.

HRMS (ESI) calcd for $C_{28}H_{24}ClNO_5$ $[M + H]^+$ 490.14158 and 492.13863, found 490.14132 and 492.13873.

$[\alpha]_D^{20}$ -35.4 (*c* 014, $CHCl_3$).

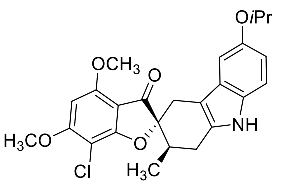

GF-I-1-6

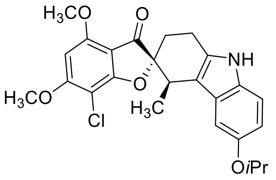

GF-I-2-5

Following the general procedure A for griseofulvin-derived indoles, the starting materials were reacted for 1 h. After purification, 38 mg (27%) of the major isomer (**GF-I-1-6**) and 18 mg (13%) of the minor isomer (**GF-I-2-5**) were isolated as white solids.

GF-I-1-6:

^{1}H NMR (700 MHz, $CDCl_3$) δ 7.82 (s, 1H), 7.16 (d, *J* = 8.7 Hz, 1H), 6.88 (d, *J* = 2.3 Hz, 1H), 6.76 (dd, *J* = 8.7, 2.4 Hz, 1H), 6.09 (s, 1H), 4.46 (hept, *J* = 6.1 Hz, 1H), 3.99 (s, 3H), 3.95 (s, 3H), 3.20 (d, *J* = 16.4 Hz, 1H), 3.17 (dd, *J* = 16.2, 5.7 Hz, 1H), 2.98 (d, *J* = 15.9 Hz, 1H), 2.76 (dd, *J* = 16.0, 5.4 Hz, 1H), 2.43 (h, *J* = 6.8 Hz, 1H), 1.32 (d, *J* = 6.1 Hz, 6H), 1.14 (d, *J* = 6.9 Hz, 3H).

^{13}C NMR (176 MHz, $CDCl_3$) δ 197.60, 168.01, 164.07, 157.94, 151.93, 133.01, 131.80, 128.11, 113.21, 111.21, 105.41, 104.89, 104.43, 97.53, 92.49, 89.06, 71.57, 56.97, 56.38, 34.72, 28.51, 27.57, 22.41, 22.38, 14.26.

HRMS (ESI) calcd for $C_{25}H_{26}ClNO_5$ $[M + H]^+$ 456.15723 and 458.15428, found 456.15705 and 458.15412.

$[\alpha]_D^{20}$ +63.8 (*c* 0.68, $CHCl_3$).

GF-I-2-5:

^{1}H NMR (700 MHz, $CDCl_3$) δ 7.79 (s, 1H), 7.18 (d, *J* = 8.7 Hz, 1H), 6.97 (d, *J* = 2.3 Hz, 1H), 6.77 (dd, *J* = 8.7, 2.3 Hz, 1H), 6.09 (s, 1H), 4.48 (hept, *J* = 6.1 Hz, 1H), 3.98 (s, 3H), 3.97 (s, 3H), 3.31 (q, *J* = 7.1 Hz, 1H), 3.05 – 2.93 (m, 2H), 2.37 (ddd, *J* = 13.8, 9.2, 6.5 Hz, 1H), 2.08 – 2.01 (m, 1H), 1.42 (d, *J* = 7.1 Hz, 3H), 1.32 (dd, *J* = 6.1, 1.9 Hz, 6H).

^{13}C NMR (176 MHz, $CDCl_3$) δ 197.75, 168.05, 164.03, 158.00, 151.74, 132.68, 131.88, 127.62, 112.94, 112.19, 111.28, 105.56, 105.22, 97.66, 92.45, 89.05, 71.61, 56.97, 56.42, 35.28, 26.45, 22.44, 22.38, 19.86, 16.48.

HRMS (ESI) calcd for $C_{25}H_{26}ClNO_5$ $[M + H]^+$ 456.15723 and 458.15428, found 456.15703 and 458.15417.

$[\alpha]_D^{20}$ -25.7 (*c* 0.45, $CHCl_3$).

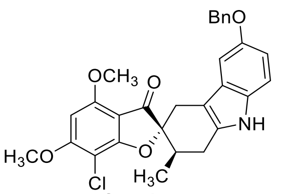

GF-I-1-7

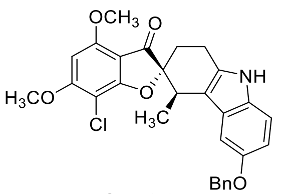

GF-I-2-6

Following the general procedure A for griseofulvin-derived indoles, the starting materials were reacted for 1 h. After purification, 105 mg (68%) of the major isomer (**GF-I-1-7**) and 11 mg (7%) of the minor isomer (**GF-I-2-6**) were isolated as solids.

GF-I-1-7:

^{1}H NMR (600 MHz, $CDCl_3$) δ 7.98 (s, 1H), 7.47 (d, *J* = 7.2 Hz, 2H), 7.38 (t, *J* = 7.5 Hz, 2H), 7.31 (t, *J* = 7.4 Hz, 1H), 7.15 (d, *J* = 8.7 Hz, 1H), 6.96 (d, *J* = 2.4 Hz, 1H), 6.84 (dd, *J* = 8.7, 2.4 Hz, 1H), 6.09 (s, 1H), 5.08 (s, 2H), 3.98 (s, 3H), 3.93 (s, 3H), 3.23 (d, *J* = 16.2 Hz, 1H), 3.14 (dd, *J* = 16.2, 5.6 Hz, 1H), 3.00 (d, *J* = 16.2 Hz, 1H), 2.75 (dd, *J* = 16.2, 5.5 Hz, 1H), 2.43 (h, *J* = 6.8 Hz, 1H), 1.14 (d, *J* = 6.9 Hz, 3H).

^{13}C NMR (151 MHz, $CDCl_3$) δ 197.55, 167.93, 164.03, 157.89, 153.16, 137.85, 133.13, 131.67, 128.54, 127.87, 127.76, 127.62, 111.51, 111.36, 105.31, 104.76, 101.80, 97.37, 92.45, 89.03, 71.08, 56.91, 56.30, 34.64, 28.40, 27.55, 14.23.

HRMS (ESI) calcd for $C_{29}H_{26}ClNO_5$ $[M + H]^+$ 504.15723 and 506.15428, found 504.15687 and 506.15423.

$[\alpha]_D^{20}$ +44.6 (*c* 0.28, $CHCl_3$).

GF-I-2-6:

^{1}H NMR (600 MHz, $CDCl_3$) δ 7.75 (s, 1H), 7.46 (d, *J* = 7.0 Hz, 2H), 7.37 (t, *J* = 7.5 Hz, 2H), 7.30 (t, *J* = 7.4 Hz, 1H), 7.20 (d, *J* = 8.7 Hz, 1H), 7.02 (d, *J* = 2.4 Hz, 1H), 6.86 (dd, *J* = 8.7, 2.4 Hz, 1H), 6.10 (s, 1H), 5.08 (s, 2H), 3.99 (s, 3H), 3.98 (s, 3H), 3.30 (q, *J* = 7.1 Hz, 1H), 3.08 – 2.92 (m, 2H), 2.37 (ddd, *J* = 13.8, 9.2, 6.6 Hz, 1H), 2.09 – 1.99 (m, 1H), 1.41 (d, *J* = 7.1 Hz, 3H).

^{13}C NMR (151 MHz, $CDCl_3$) δ 197.61, 167.93, 163.92, 157.89, 153.00, 137.77, 132.64, 131.64, 128.50, 127.75, 127.63, 127.41, 112.27, 111.35, 111.27, 105.44, 102.67, 97.57, 92.29, 88.93, 71.15, 56.87, 56.33, 35.15, 26.29, 19.77, 16.34.

HRMS (ESI) calcd for $C_{29}H_{26}ClNO_5$ $[M + H]^+$ 504.15723 and 506.15428, found 504.15681 and 506.15403.

$[\alpha]_D^{20}$ -35.0 (*c* 0.14, $CHCl_3$).

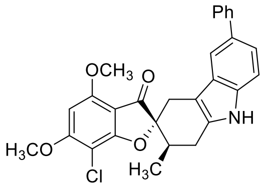

GF-I-1-8

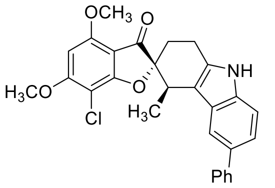

GF-I-2-7

Following the general procedure A for griseofulvin-derived indoles, starting materials were reacted for 1 h. After purification, 56 mg (38%) of the major isomer (**GF-I-1-8**) and 20 mg (13%) of the minor isomer (**GF-I-2-7**) were isolated as white solids.

GF-I-1-8:

^{1}H NMR (500 MHz, $CDCl_3$) δ 7.92 (s, 1H), 7.64 – 7.56 (m, 3H), 7.42 (t, *J* = 7.8 Hz, 2H), 7.39 – 7.33 (m, 2H), 7.31 – 7.26 (m, 1H), 6.10 (s, 1H), 4.00 (s, 3H), 3.97 (s, 3H), 3.29 (d, *J* = 16.6 Hz, 1H), 3.23 (dd, *J* = 16.3, 5.4 Hz, 1H), 3.06 (d, *J* = 16.3 Hz, 1H), 2.81 (dd, *J* = 16.2, 5.3 Hz, 1H), 2.47 (h, *J* = 6.8 Hz, 1H), 1.17 (d, *J* = 6.9 Hz, 3H).

^{13}C NMR (126 MHz, $CDCl_3$) δ 197.56, 168.01, 164.08, 157.96, 142.87, 135.90, 133.11, 132.91, 128.71, 128.10, 127.50, 126.30, 121.21, 116.39, 110.90, 105.57, 105.39, 97.54, 92.38, 89.04, 57.00, 56.41, 34.69, 28.53, 27.52, 14.27.

HRMS (ESI) calcd for $C_{28}H_{24}ClNO_4$ $[M + H]^+$ 474.14666 and 476.14371, found 474.14649 and 476.14365.

$[\alpha]_D^{20}$ +84.7 (*c* 0.77, $CHCl_3$).

GF-I-2-7:

^{1}H NMR (500 MHz, $CDCl_3$) δ 7.89 (s, 1H), 7.67 (s, 1H), 7.64 – 7.60 (m, 2H), 7.42 (t, *J* = 7.8 Hz, 3H), 7.38 – 7.36 (m, 2H), 7.31 – 7.27 (m, 1H), 6.10 (s, 1H), 4.00 (s, 4H), 3.99 (s, 3H), 3.40 (q, *J* = 7.1 Hz, 1H), 3.11 – 2.97 (m, 2H), 2.47 – 2.36 (m, 1H), 2.12 – 2.04 (m, 1H), 1.48 (d, *J* = 7.1 Hz, 3H).

^{13}C NMR (126 MHz, $CDCl_3$) δ 197.74, 168.04, 164.06, 158.02, 142.89, 135.94, 133.03, 132.52, 128.72, 127.61, 127.55, 126.35, 121.18, 116.90, 112.92, 111.04, 105.54, 97.68, 92.35, 89.03, 57.00, 56.46, 35.31, 26.39, 19.88, 16.75.

HRMS (ESI) calcd for $C_{28}H_{24}ClNO_4$ $[M + H]^+$ 474.14666 and 476.14371, found 474.14648 and 476.14369.

$[\alpha]_D^{20}$ -65.2 (*c* 0.38, $CHCl_3$).

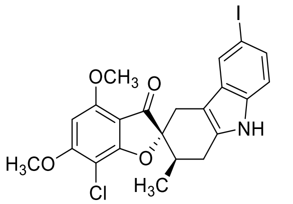

GF-I-1-9

Following the general procedure B for griseofulvin-derived indoles, the starting materials were reacted for 1 h. After purification, 29 mg (18%) of the major isomer (**GF-I-1-9**) was isolated as a solid. The minor isomer was not obtained in an analytically pure form.

GF-I-1-9:

^{1}H NMR (600 MHz, $CDCl_3$) δ 8.05 (s, 1H), 7.68 (s, 1H), 7.33 (d, *J* = 8.4 Hz, 1H), 7.02 (d, *J* = 8.4 Hz, 1H), 6.09 (s, 1H), 4.00 (s, 3H), 3.94 (s, 3H), 3.22 – 3.08 (m, 2H), 2.97 (d, *J* = 16.2 Hz, 1H), 2.78 (dd, *J* = 16.3, 5.7 Hz, 1H), 2.45 (h, *J* = 6.5 Hz, 1H), 1.12 (d, *J* = 6.9 Hz, 3H).

^{13}C NMR (151 MHz, $CDCl_3$) δ 197.39, 167.97, 164.16, 157.97, 135.50, 133.27, 130.11, 129.54, 126.63, 112.71, 105.34, 104.57, 97.51, 92.10, 89.13, 82.75, 57.01, 56.40, 34.67, 28.35, 27.47, 14.30.

HRMS (ESI) calcd for $C_{22}H_{19}ClINO_4$ $[M + H]^+$ 524.01201, and 526.00906, found 524.01184 and 526.00885.

$[\alpha]_D^{20}$ +65.0 (*c* 0.51, $CHCl_3$).

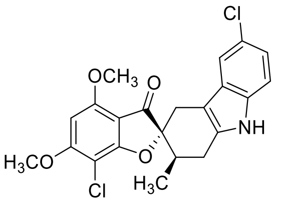

GF-I-1-10

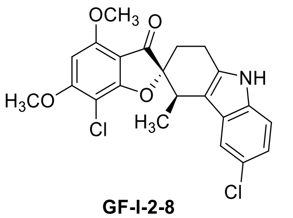

GF-I-2-8

Following the general procedure B for griseofulvin-derived indoles, starting materials were reacted for 1 h. After purification, 90 mg (68%) of the major isomer (**GF-I-1-10**) and 16 mg (12%) of the minor isomer (**GF-I-2-8**) were isolated as yellow solids.

GF-I-1-10:

^{1}H NMR (700 MHz, $CDCl_3$) δ 7.94 (s, 1H), 7.43 (d, *J* = 1.9 Hz, 1H), 7.20 (d, *J* = 8.5 Hz, 1H), 7.06 (dd, *J* = 8.6, 2.0 Hz, 1H), 6.10 (s, 1H), 3.99 (s, 3H), 3.97 (s, 3H), 3.32 (q, *J* = 7.1 Hz, 1H), 3.05 – 2.98 (m, 2H), 2.36 (dt, *J* = 13.8, 7.6 Hz, 1H), 2.08 (dt, *J* = 13.8, 5.3 Hz, 1H), 1.41 (d, *J* = 7.1 Hz, 3H).

^{13}C NMR (176 MHz, $CDCl_3$) δ 197.49, 168.02, 164.13, 158.05, 134.82, 133.43, 128.20, 125.01, 121.45, 117.87, 112.29, 111.75, 105.53, 97.68, 92.15, 89.16, 57.01, 56.45, 35.25, 26.51, 19.82, 16.45.

HRMS (ESI) calcd for $C_{22}H_{19}Cl_2NO_4$ $[M + H]^+$ 432.07639 and 434.07358, found 432.07630 and 434.07358.

$[\alpha]_D^{20}$ +200 (*c* 0.097, $CHCl_3$).

GF-I-2-8:

^{1}H NMR (700 MHz, $CDCl_3$) δ 7.97 (s, 1H), 7.32 (d, *J* = 2.0 Hz, 1H), 7.17 (d, *J* = 8.6 Hz, 1H), 7.05 (dd, *J* = 8.6, 2.0 Hz, 1H), 6.10 (s, 1H), 4.00 (s, 3H), 3.95 (s, 3H), 3.22 – 3.14 (m, 2H), 2.98 (d, *J* = 16.2 Hz, 1H), 2.80 (dd, *J* = 16.2, 5.7 Hz, 1H), 2.46 (h, *J* = 6.5 Hz, 1H), 1.13 (d, *J* = 6.9 Hz, 3H).

^{13}C NMR (176 MHz, $CDCl_3$) δ 197.39, 167.99, 164.17, 157.99, 134.77, 133.84, 128.68, 125.12, 121.47, 117.34, 111.64, 105.38, 105.05, 97.56, 92.12, 89.16, 57.01, 56.41, 34.70, 28.44, 27.49, 14.29.

HRMS (ESI) calcd for $C_{22}H_{19}Cl_2NO_4$ $[M + H]^+$ 432.07639 and 434.07358, found 432.07639 and 434.07344.

$[\alpha]_D^{20}$ -25.7 (*c* 0.11, $CHCl_3$).

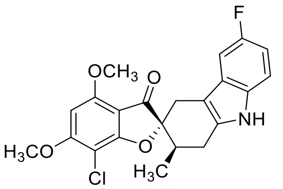

GF-I-1-11

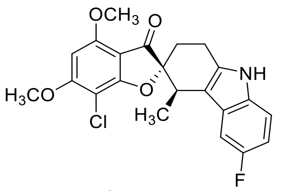

GF-I-2-9

Following the general procedure B for griseofulvin-derived indoles, the starting materials were reacted for 2 h. After purification, 96 mg (75%) of the major isomer (**GF-I-1-11**) and 12 mg (9%) of the minor isomer (**GF-I-2-9**) were isolated as solids.

GF-I-1-11:

^{1}H NMR (600 MHz, $CDCl_3$) δ 8.07 (s, 1H), 7.14 (dd, *J* = 8.8, 4.3 Hz, 1H), 7.00 (dd, *J* = 9.5, 2.5 Hz, 1H), 6.82 (td, *J* = 9.1, 2.5 Hz, 1H), 6.09 (s, 1H), 3.99 (s, 3H), 3.93 (s, 3H), 3.19 (d, *J* = 16.2 Hz, 1H), 3.14 (dd, *J* = 16.3, 5.7 Hz, 1H), 2.99 (d, *J* = 16.2 Hz, 1H), 2.79 (dd, *J* = 16.2, 5.8 Hz, 1H), 2.45 (q, *J* = 5.9 Hz, 1H), 1.13 (d, *J* = 6.9 Hz, 3H).

^{13}C NMR (151 MHz, $CDCl_3$) δ 197.48, 167.97, 164.15, 157.96, 157.83 (d, *J* = 233.8 Hz), 134.25, 132.86, 127.85 (d, *J* = 9.8 Hz), 111.18 (d, *J* = 9.5 Hz), 109.14 (d, *J* = 26.1 Hz), 105.34, 105.26 (d, *J* = 4.4 Hz), 102.81 (d, *J* = 23.5 Hz), 97.45, 92.24, 89.11, 56.98, 56.35, 34.70, 28.43, 27.57, 14.28.

^{19}F NMR (565 MHz, $CDCl_3$) δ -125.13 (td, *J* = 9.5, 4.4 Hz).

HRMS (ESI) calcd for $C_{22}H_{19}ClNO_4F$ $[M + H]^+$ 416.10594 and 418.10299, found 416.10554 and 418.10264.

$[\alpha]_D^{20}$ +51.3 (*c* 0.19, $CHCl_3$).

GF-I-2-9:

^{1}H NMR (600 MHz, $CDCl_3$) δ 7.89 (s, 1H), 7.20 (dd, *J* = 8.8, 4.4 Hz, 1H), 7.11 (dd, *J* = 9.7, 2.5 Hz, 1H), 6.85 (td, *J* = 9.0, 2.5 Hz, 1H), 6.10 (s, 1H), 3.99 (s, 3H), 3.97 (s, 3H), 3.32 (q, *J* = 7.1 Hz, 1H), 3.02 (dd, *J* = 7.3, 5.7 Hz, 2H), 2.36 (dt, *J* = 13.8, 7.6 Hz, 1H), 2.09 (dt, *J* = 13.7, 5.3 Hz, 1H), 1.41 (d, *J* = 7.1 Hz, 3H).

^{13}C NMR (151 MHz, $CDCl_3$) δ 197.55, 168.03, 164.10, 158.03, 157.73 (d, *J* = 233.7 Hz), 133.82, 132.91, 127.43 (d, *J* = 9.8 Hz), 112.63 (d, *J* = 4.4 Hz), 111.26 (d, *J* = 9.8 Hz), 109.24 (d, *J* = 25.9 Hz), 105.54, 103.53 (d, *J* = 23.7 Hz), 97.65, 92.23, 89.11, 57.00, 56.45, 35.29, 26.57, 19.86, 16.29.

^{19}F NMR (565 MHz, $CDCl_3$) δ -125.01 (td, *J* = 9.6, 4.5 Hz).

HRMS (ESI) calcd for $C_{22}H_{19}ClNO_4F$ $[M + H]^+$ 416.10594 and 418.10299, found 416.10559 and 418.10271.

$[\alpha]_D^{20}$ +14 (*c* 0.1, CH_2Cl_2).

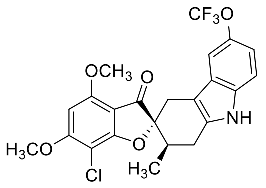

GF-I-1-12

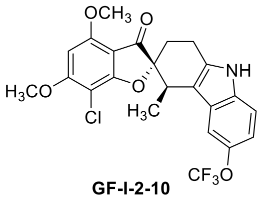

GF-I-2-10

Following the general procedure A for griseofulvin-derived indoles, the starting materials were reacted for 6 h. After purification, 88 mg (59%) of the major isomer (**GF-I-1-12**) and 20 mg (13%) of the minor isomer (**GF-I-2-10**) were isolated as yellow solids.

GF-I-1-12:

^{1}H NMR (600 MHz, $CDCl_3$) δ 8.19 (s, 1H), 7.20 (d, *J* = 8.7 Hz, 2H), 6.95 (d, *J* = 8.4 Hz, 1H), 6.10 (s, 1H), 4.00 (s, 3H), 3.94 (s, 3H), 3.22 (d, *J* = 16.2 Hz, 1H), 3.15 (dd, *J* = 16.3, 5.7 Hz, 1H), 3.02 (d, *J* = 16.2 Hz, 1H), 2.81 (dd, *J* = 16.3, 6.0 Hz, 1H), 2.47 (h, *J* = 6.5 Hz, 1H), 1.13 (d, *J* = 6.9 Hz, 3H).

^{13}C NMR (151 MHz, $CDCl_3$) δ 197.44, 167.99, 164.21, 157.98, 142.87, 134.70, 134.46, 127.71, 120.93 (q, *J* = 255.0 Hz), 115.02, 111.16, 110.28, 105.59, 105.35, 97.49, 92.12, 89.14, 57.00, 56.36, 34.72, 28.42, 27.58, 14.29.

^{19}F NMR (565 MHz, $CDCl_3$) δ -58.03.

HRMS (ESI) calcd for $C_{23}H_{19}ClNO_4F_3$ $[M + H]^+$ 482.09766 and 484.09445, found 482.09723 and 484.09445.

$[\alpha]_D^{20}$ +58.2 (*c* 0.24, $CHCl_3$).

GF-I-1-12:

^{1}H NMR (600 MHz, $CDCl_3$) δ 8.05 (s, 1H), 7.30 (s, 1H), 7.24 (d, *J* = 8.7 Hz, 1H), 6.98 (d, *J* = 8.7 Hz, 1H), 6.10 (s, 1H), 3.99 (s, 3H), 3.97 (s, 3H), 3.35 (q, *J* = 7.1 Hz, 1H), 3.06 – 2.99 (m, 2H), 2.36 (dt, *J* = 13.7, 7.5 Hz, 1H), 2.09 (dt, *J* = 13.8, 5.4 Hz, 1H), 1.42 (d, *J* = 7.1 Hz, 3H).

^{13}C NMR (151 MHz, $CDCl_3$) δ 197.39, 167.90, 164.04, 157.93, 142.66, 134.63, 133.90, 127.14, 120.83 (q, *J* = 255.2 Hz), 114.93, 112.71, 111.12, 110.83, 105.39, 97.53, 91.99, 89.03, 56.89, 56.32, 35.13, 26.42, 19.71, 16.24.

HRMS (ESI) calcd for $C_{23}H_{19}ClNO_4F_3$ $[M + H]^+$ 482.09766 and 484.09445, found 482.09725 and 484.09458.

$[\alpha]_D^{20}$ +10.6 (*c* 0.085, $CHCl_3$).

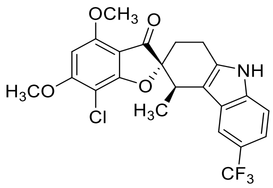

GF-I-2-11

Following the general procedure B for griseofulvin-derived indoles, the starting materials were reacted for 4 h. After purification, 20 mg (14%) of the minor isomer (**G-I-2-11**) was isolated. The other isomer was not obtained in pure form.

GF-I-2-11:

^{1}H NMR (600 MHz, $CDCl_3$) δ 8.15 (s, 1H), 7.74 (s, 1H), 7.38 – 7.28 (m, 2H), 6.11 (s, 1H), 4.00 (s, 3H), 3.97 (s, 3H), 3.39 (q, *J* = 7.1 Hz, 1H), 3.08 – 3.00 (m, 2H), 2.39 (dt, *J* = 13.8, 7.7 Hz, 1H), 2.11 (dt, *J* = 13.8, 5.3 Hz, 1H), 1.45 (d, *J* = 7.1 Hz, 3H).

^{13}C NMR (151 MHz, $CDCl_3$) δ 197.47, 168.00, 164.18, 158.07, 137.86, 133.72, 126.47, 125.55 (q, *J* = 271.4 Hz), 121.72 (q, *J* = 31.5 Hz), 118.17 (q, *J* = 3.6 Hz), 115.81 (q, *J* = 4.2 Hz); 113.30, 110.94, 105.49, 97.67, 92.03, 89.17, 57.02, 56.46, 35.22, 26.45, 19.82, 16.60.

^{19}F NMR (565 MHz, $CDCl_3$) δ -60.13.

HRMS (ESI) calcd for $C_{23}H_{19}ClF_3NO_4$ $[M + H]^+$ 466.10275 and 468.09980, found 466.10256, and 468.09988.

$[\alpha]_D^{20}$ -5.6 (*c* 0.25, $CHCl_3$).

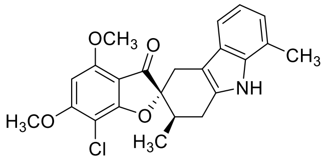

GF-I-1-13

Following the general procedure B for griseofulvin-derived indoles, the starting materials were reacted for 1 h. After purification, 51 mg (40%) of the major isomer (**GF-I-1-13**) was isolated as a yellow solid. The minor isomer was not obtained in an analytically pure form.

GF-I-1-13:

^{1}H NMR (700 MHz, $CDCl_3$) δ 7.86 (s, 1H), 7.25 (d, J = 7.9 Hz, 1H), 7.02 – 6.96 (m, 1H), 6.93 (d, J = 7.1 Hz, 1H), 6.09 (s, 1H), 3.99 (s, 3H), 3.95 (s, 3H), 3.25 (m, 2H), 3.02 (d, J = 16.3 Hz, 1H), 2.81 (dd, J = 16.0, 5.2 Hz, 1H), 2.47 (m, 4H), 1.17 (d, J = 7.0 Hz, 3H).

^{13}C NMR (176 MHz, $CDCl_3$) δ 197.62, 168.01, 164.06, 157.96, 135.87, 131.71, 127.11, 122.06, 119.86, 119.62, 115.48, 105.66, 105.41, 97.55, 92.50, 89.07, 56.97, 56.38, 34.69, 28.53, 27.57, 16.76, 14.25.

HRMS (ESI) calcd for $C_{23}H_{22}ClNO_4$ $[M + H]^+$ 412.13101 and 414.12806, found 412.13079 and 414.12794.

$[\alpha]_D^{20}$ +28.6 (c 0.14, $CHCl_3$).

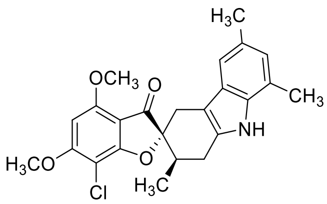

GF-I-1-14

Following the general procedure B for griseofulvin-derived indoles, the starting materials were reacted for 1 h. After purification, 67 mg (51%) of the major isomer (**GF-I-1-14**) was isolated as a brown solid. The minor isomer was not obtained in an analytically pure form.

GF-I-1-14:

^{1}H NMR (600 MHz, $CDCl_3$) δ 7.81 (s, 1H), 7.03 (s, 1H), 6.77 (s, 1H), 6.09 (s, 1H), 3.99 (s, 3H), 3.95 (s, 3H), 3.27 – 3.19 (m, 2H), 2.98 (d, J = 16.2 Hz, 1H), 2.77 (dd, J = 16.0, 5.0 Hz, 1H), 2.41 (m, 7H), 1.17 (d, J = 6.9 Hz, 3H).

^{13}C NMR (151 MHz, $CDCl_3$) δ 197.65, 167.96, 164.01, 157.92, 134.11, 131.78, 128.81, 127.34, 123.61, 119.49, 115.16, 105.35, 105.10, 97.48, 92.55, 89.01, 56.94, 56.34, 34.61, 28.54, 27.45, 21.49, 16.68, 14.20.

HRMS (ESI) calcd for $C_{24}H_{24}ClNO_4$ $[M + H]^+$ 426.14666 and 428.14371, found 426.14637 and 428.14357.

$[\alpha]_D^{20}$ +16.7 (*c* 0.12, $CHCl_3$).

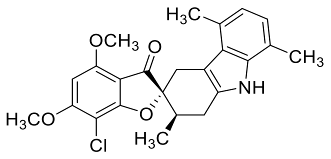

GF-I-1-15

Following the general procedure B for griseofulvin-derived indoles, the starting materials were reacted for 1 h. After purification, 55 mg (42%) of the major isomer (**GF-I-1-15**) was isolated as a yellow solid. The minor isomer was not obtained in an analytically pure form.

GF-I-1-15:

^{1}H NMR (600 MHz, $CDCl_3$) δ 7.87 (s, 1H), 6.79 (d, *J* = 7.2 Hz, 1H), 6.71 (d, *J* = 6.7 Hz, 1H), 6.10 (s, 1H), 4.00 (s, 3H), 3.96 (s, 3H), 3.53 (d, *J* = 16.0 Hz, 1H), 3.31 – 3.21 (m, 2H), 2.77 (dd, *J* = 16.0, 4.8 Hz, 1H), 2.56 (s, 3H), 2.44 – 2.37 (m, 4H), 1.18 (d, *J* = 7.0 Hz, 3H).

^{13}C NMR (151 MHz, $CDCl_3$) δ 197.75, 167.98, 164.08, 157.97, 135.69, 130.98, 127.42, 126.03, 121.86, 120.66, 117.38, 105.90, 105.33, 97.52, 92.66, 89.02, 56.95, 56.35, 34.02, 29.71, 28.43, 19.59, 16.43, 14.11.

HRMS (ESI) calcd for $C_{24}H_{24}ClNO_4$ $[M + H]^+$ 426.14666 and 428.14371, found 426.14641 and 428.14362.

$[\alpha]_D^{20}$ +12.1 (*c* 0.11, $CHCl_3$).

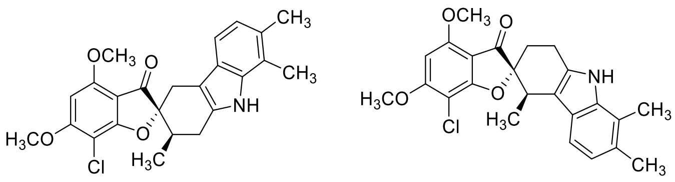

GF-I-1-16 **GF-I-2-12**

Following the general procedure B for griseofulvin-derived indoles, the starting materials were reacted for 1 h. After purification, 63 mg (48%) of the major isomer (**GF-I-1-16**) and 8 mg (6%) of the minor isomer (**GF-I-2-12**) were isolated as yellow solids.

GF-I-1-16:

^{1}H NMR (700 MHz, $CDCl_3$) δ 7.88 (s, 1H), 7.14 (d, *J* = 7.9 Hz, 1H), 6.89 (d, *J* = 7.9 Hz, 1H), 6.09 (s, 1H), 3.98 (s, 3H), 3.94 (s, 3H), 3.27 – 3.18 (m, 2H), 3.01 (d, *J* = 16.1 Hz, 1H), 2.79 (dd, *J* = 15.9, 5.1 Hz, 1H), 2.44 (h, *J* = 5.6 Hz, 1H), 2.37 (s, 3H), 2.34 (s, 3H), 1.17 (d, *J* = 7.0 Hz, 3H).

^{13}C NMR (176 MHz, $CDCl_3$) δ 197.67, 167.98, 164.02, 157.92, 136.52, 131.18, 128.69, 125.43, 121.96, 117.81, 114.68, 105.37, 105.35, 97.45, 92.60, 89.02, 56.93, 56.33, 34.65, 28.50, 27.51, 19.39, 14.23, 13.15.

HRMS (ESI) calcd for $C_{24}H_{24}ClNO_4$ $[M + H]^+$ 426.14666 and 428.14371, found 426.14642 and 428.14362.

$[\alpha]_D^{20}$ +30.6 (*c* 0.23, $CHCl_3$).

GF-I-2-12:

^{1}H NMR (700 MHz, $CDCl_3$) δ 7.66 (s, 1H), 7.22 (d, *J* = 7.9 Hz, 1H), 6.90 (d, *J* = 8.0 Hz, 1H), 6.09 (s, 1H), 3.99 (s, 3H), 3.98 (s, 3H), 3.32 (q, *J* = 7.1 Hz, 1H), 3.09 – 2.97 (m, 2H), 2.42 – 2.36 (m, 7H), 2.09 – 2.01 (m, 1H), 1.42 (d, *J* = 7.1 Hz, 3H).

^{13}C NMR (176 MHz, $CDCl_3$) δ 197.82, 168.09, 164.00, 158.00, 136.54, 130.86, 128.85, 124.98, 122.01, 117.97, 115.30, 113.00, 105.61, 92.53, 89.03, 56.97, 56.44, 35.36, 26.44, 19.87, 19.40, 16.70, 13.25.

HRMS (ESI) calcd for $C_{24}H_{24}ClNO_4$ $[M + H]^+$ 426.14666 and 428.14371, found 426.14639 and 428.14362.

$[\alpha]_D^{20}$ +44.3 (*c* 0.42, $CHCl_3$).

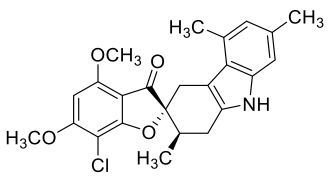

GF-I-1-17

Following the general procedure B for griseofulvin-derived indoles, the starting materials were reacted for 1 h. After purification, 47 mg (36%) of the major isomer (**GF-I-1-17**) was isolated as a yellow solid. The minor isomer was not obtained in an analytically pure form.

GF-I-1-17:

^{1}H NMR (700 MHz, $CDCl_3$) δ 7.79 (s, 1H), 6.90 (s, 1H), 6.62 (s, 1H), 6.10 (s, 1H), 3.99 (s, 3H), 3.96 (s, 3H), 3.49 (d, *J* = 16.4 Hz, 1H), 3.25 (d, *J* = 16.4 Hz, 1H), 3.17 (dd, *J* = 16.0, 5.7 Hz, 1H), 2.70 (dd, *J* = 16.0, 4.9 Hz, 1H), 2.53 (s, 3H), 2.40 – 2.34 (m, 4H), 1.15 (dd, *J* = 6.9, 1.2 Hz, 3H).

^{13}C NMR (176 MHz, $CDCl_3$) δ 197.76, 168.01, 164.07, 157.97, 136.80, 130.96, 130.60, 129.36, 124.44, 122.36, 108.52, 105.37, 105.07, 97.53, 92.73, 89.02, 56.95, 56.35, 34.06, 29.78, 28.36, 21.56, 19.71, 14.11.

HRMS (ESI) calcd for $C_{24}H_{24}ClNO_4$ $[M + H]^+$ 426.14666 and 428.14371, found 426.14641 and 428.14360.

$[\alpha]^{20}_{D}$ +28.5 (*c* 0.15, $CHCl_3$).

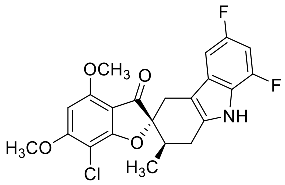

GF-I-1-18

Following the general procedure A for griseofulvin-derived indoles, the starting materials were reacted for 24 h. After purification, 57 mg (43%) of the major isomer (**GF-I-1-18**) was isolated as a yellow solid. The minor isomer was not obtained in an analytically pure form.

GF-I-1-18:

^{1}H NMR (600 MHz, $CDCl_3$) δ 8.35 (s, 1H), 6.79 (d, *J* = 9.0 Hz, 1H), 6.58 (t, *J* = 10.3 Hz, 1H), 6.09 (s, 1H), 3.99 (s, 3H), 3.92 (s, 3H), 3.20 – 3.12 (m, 2H), 2.97 (d, *J* = 16.2 Hz, 1H), 2.82 (dd, *J* = 16.5, 6.0 Hz, 1H), 2.45 (h, *J* = 6.6 Hz, 1H), 1.11 (d, *J* = 6.9 Hz, 3H).

^{13}C NMR (151 MHz, $CDCl_3$) δ 197.31, 167.85, 164.14, 157.90, 156.75 (dd, *J* = 236.2, 9.9 Hz), 148.04 (dd, *J* = 245.6, 14.3 Hz), 134.75, 129.83 (dd, *J* = 11.1, 6.7 Hz), 120.81 (d, *J* = 12.0 Hz)106.12 (dd, *J* = 4.9, 2.6 Hz), 105.20, 98.61 (dd, *J* = 23.3, 3.7 Hz), 97.34, 96.24 (dd, *J* = 30.0, 20.7 Hz), 91.90, 89.08, 56.90, 56.25, 34.61, 28.24, 27.53, 14.16.

^{19}F NMR (565 MHz, $CDCl_3$) δ -122.43 (td, *J* = 9.4, 2.2 Hz), -132.21 (dd, *J* = 10.9, 2.3 Hz).

HRMS (ESI) calcd for $C_{22}H_{18}ClNO_4F_2$ $[M + H]^+$ 434.09652 and 436.09357, found 434.09621 and 436.09339.

$[\alpha]_D^{20}$ +58.8 (*c* 0.25, $CHCl_3$).

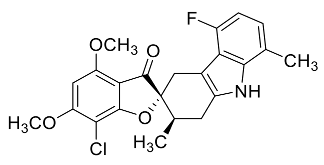

GF-I-1-19

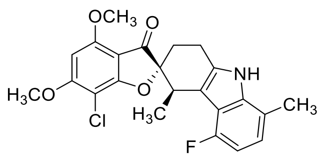

GF-I-2-13

Following the general procedure A for griseofulvin-derived indoles, the starting materials were reacted for 6 h. After purification, 57 mg (43%) of the major isomer (**GF-I-1-19**) and 4 mg (3%) of the minor isomer (**GF-I-2-13**) were isolated as yellow solids.

GF-I-1-19:

^{1}H NMR (700 MHz, $CDCl_3$) δ 7.89 (s, 1H), 6.67 (dd, *J* = 8.0, 4.7 Hz, 1H), 6.51 (dd, *J* = 10.5, 7.9 Hz, 1H), 6.01 (s, 1H), 3.92 (s, 3H), 3.86 (s, 3H), 3.34 (d, *J* = 16.7 Hz, 1H), 3.16 – 3.07 (m, 2H), 2.75 (dd, *J* = 16.0, 5.8 Hz, 1H), 2.39 (h, *J* = 6.5 Hz, 1H), 2.30 (s, 3H), 1.07 (d, *J* = 6.9 Hz, 3H).

^{13}C NMR (176 MHz, $CDCl_3$) δ 197.41, 167.93, 164.00, 157.86, 155.13 (d, *J* = 242.3 Hz), 137.82 (d, *J* = 11.8 Hz), 131.47, 121.72 (d, *J* = 7.3 Hz), 115.59, 115.54 (d, *J* = 24.2 Hz), 105.35, 104.27 (d, *J* = 18.7 Hz), 103.88 (d, *J* = 1.7 Hz), 97.41, 92.20, 89.00, 56.88, 56.26, 34.36, 28.78, 28.22, 16.06, 14.20.

^{19}F NMR (470 MHz, $CDCl_3$) δ -129.51 (dd, *J* = 10.4, 4.3 Hz).

HRMS (ESI) calcd for $C_{23}H_{21}ClNO_4F$ $[M + H]^+$ 430.12159 and 432.11864, found 430.12135 and 432.11852.

$[\alpha]_D^{20}$ +56.3 (*c* 0.15, $CHCl_3$).

GF-I-2-13:

^{1}H NMR (600 MHz, $CDCl_3$) δ 7.78 (s, 1H), 6.81 (dd, *J* = 7.7, 4.8 Hz, 1H), 6.64 (dd, *J* = 10.8, 7.9 Hz, 1H), 6.10 (s, 1H), 3.99 (s, 6H), 3.33 (q, *J* = 7.0 Hz, 1H), 3.15 – 3.06 (m, 1H), 2.95 (ddd, *J* = 16.4, 6.6, 2.0 Hz, 1H), 2.46 (ddd, *J* = 14.1, 11.2, 6.6 Hz, 1H), 2.42 (s, 3H), 1.97 (dd, *J* = 14.1, 5.7 Hz, 1H), 1.46 (d, *J* = 7.0 Hz, 3H).

^{13}C NMR (151 MHz, $CDCl_3$) δ 197.70, 167.75, 163.93, 157.94, 154.77 (d, *J* = 242.4 Hz), 137.67 (d, *J* = 11.9 Hz), 130.80, 121.96 (d, *J* = 7.3 Hz), 115.58 (d, *J* = 3.5 Hz), 115.06 (d, *J* = 21.2 Hz), 112.48 (d, *J* = 2.7 Hz), 105.31, 104.53 (d, *J* = 19.7 Hz), 97.65, 92.11, 88.93, 56.86, 56.33, 35.59, 24.90, 19.75, 18.20 (d, *J* = 3.7 Hz), 16.11.

^{19}F NMR (565 MHz, $CDCl_3$) δ -126.80 (dd, *J* = 11.6, 4.5 Hz).

HRMS (ESI) calcd for $C_{23}H_{21}ClNO_4F$ $[M + H]^+$ 430.12159 and 432.11864, found 430.12121 and 432.11859.

$[\alpha]_D^{20}$ +49.0 (*c* 0.16, $CHCl_3$).

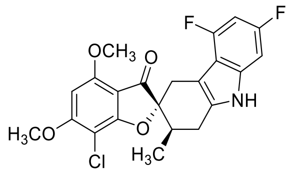

GF-I-1-20

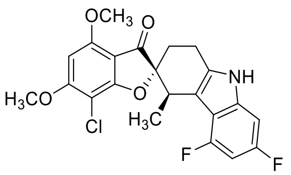

GF-I-2-14

Following the general procedure B for griseofulvin-derived indoles, the starting materials were reacted for 2.5 h. After purification, 30 mg (23%) of the major isomer (**GF-I-1-20**) 10 mg (8%) of the minor isomer (**GF-I-2-14**) were isolated as white solids.

GF-I-1-20:

^{1}H NMR (700 MHz, $CDCl_3$) δ 8.21 (s, 1H), 6.74 (dd, *J* = 9.2, 2.0 Hz, 1H), 6.50 (td, *J* = 10.3, 2.0 Hz, 1H), 6.12 (s, 1H), 4.03 (s, 3H), 3.97 (s, 3H), 3.39 (d, *J* = 16.7 Hz, 1H), 3.21 (d, *J* = 16.7 Hz, 1H), 3.12 (dd, *J* = 16.1, 5.7 Hz, 1H), 2.81 (dd, *J* = 16.2, 6.4 Hz, 1H), 2.49 (h, *J* = 6.6 Hz, 1H), 1.14 (d, *J* = 6.9 Hz, 3H).

^{13}C NMR (176 MHz, $CDCl_3$) δ 197.42, 167.95, 164.10, 158.73 (dd, *J* = 237.7, 11.8 Hz), 157.87, 155.58 (dd, *J* = 247.0, 15.0 Hz), 137.74 (t, *J* = 14.5 Hz), 112.80 (dd, *J* = 20.8, 1.5 Hz), 105.33, 103.24, 97.40, 94.78 (dd, *J* = 28.5, 23.3 Hz), 93.41 (dd, *J* = 26.2, 4.2 Hz), 92.02, 89.04, 56.90, 56.27, 34.43, 28.80, 28.06, 14.23;

^{19}F NMR (470 MHz, $CDCl_3$) δ -120.08 (td, *J* = 9.6, 3.5 Hz), -122.72 (d, *J* = 11.0 Hz).

HRMS (ESI) calcd for $C_{22}H_{18}ClNO_4F_2$ $[M + H]^+$ 434.09652 and 436.09357, found 434.09610 and 436.09345.

$[\alpha]_D^{20}$ +56.8 (*c* 0.85, $CHCl_3$).

GF-I-2-14:

^{1}H NMR (700 MHz, $CDCl_3$) δ 8.00 (s, 1H), 6.82 (d, *J* = 8.5 Hz, 1H), 6.56 (t, *J* = 10.3 Hz, 1H), 6.13 (s, 1H), 4.02 (s, 3H), 4.01 (s, 3H), 3.31 (q, *J* = 7.0 Hz, 1H), 3.10 – 3.02 (m, 1H), 2.92 (dd, *J* = 16.3, 5.6 Hz, 1H), 2.48 – 2.42 (m, 1H), 1.98 (dd, *J* = 14.0, 5.7 Hz, 1H), 1.46 (d, *J* = 7.2 Hz, 3H).

^{13}C NMR (176 MHz, $CDCl_3$) δ 197.65, 167.83, 164.14, 159.05 (dd, *J* = 238.0, 12.1 Hz), 158.12, 155.39 (dd, *J* = 247.2, 15.0 Hz), 137.84 (t, *J* = 14.6 Hz), 131.41, 112.50 (dd, *J* = 20.5, 1.8 Hz), 105.40, 97.76, 95.13 (dd, *J* = 28.2, 24.3 Hz), 93.56 (dd, *J* = 26.8, 3.4 Hz), 92.07, 89.15, 57.01, 56.46, 35.62, 25.06, 19.78, 18.20 (d, *J* = 3.4 Hz).

^{19}F NMR (470 MHz, $CDCl_3$) δ -119.78 – -119.96 (m).

HRMS (ESI) calcd for $C_{22}H_{18}ClNO_4F_2$ $[M + H]^+$ 434.09652 and 436.09357, found 434.09614 and 436.09326.

$[\alpha]_D^{20}$ +59.6 (*c* 0.60, $CHCl_3$).

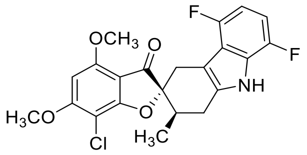

GF-I-1-21

Following the general procedure B for griseofulvin-derived indoles, the starting materials were reacted for 2.5 h. After purification, 22 mg (16%) of the major isomer (**GF-I-1-21**) was isolated as a solid. The minor isomer was not obtained in an analytically pure form.

Major isomer:

^{1}H NMR (600 MHz, $CDCl_3$) δ 8.11 (s, 1H), 6.68 (td, *J* = 10.8, 10.3, 3.3 Hz, 1H), 6.56 (td, *J* = 9.2, 3.0 Hz, 1H), 6.10 (s, 1H), 4.00 (s, 3H), 3.96 (s, 3H), 3.39 (d, *J* = 16.7 Hz, 1H), 3.23 – 3.16 (m, 2H), 2.84 (dd, *J* = 16.2, 6.0 Hz, 1H), 2.48 (h, *J* = 6.6 Hz, 1H), 1.14 (d, *J* = 6.9 Hz, 3H).

^{13}C NMR (151 MHz, $CDCl_3$) δ 197.31, 168.00, 164.18, 157.99, 152.43 (dd, *J* = 240.7, 2.2 Hz), 145.61 (dd, *J* = 238.0, 2.9 Hz), 132.80, 126.20 (dd, *J* = 15.8, 12.3 Hz), 119.10 (dd, *J* = 23.6, 5.5 Hz), 105.97 (dd, *J* = 19.3, 8.3 Hz), 105.40, 104.69, δ 103.99 (dd, *J* = 22.1, 7.1 Hz), 97.56, 91.91, 89.15, 57.02, 56.42, 34.40, 28.67, 28.25, 14.27.

^{19}F NMR (565 MHz, $CDCl_3$) δ -131.07 (ddd, *J* = 21.4, 9.9, 3.2 Hz), -141.24 (ddd, *J* = 21.5, 10.2, 3.3 Hz).

HRMS (ESI) calcd for $C_{22}H_{18}ClNO_4F_2$ $[M + H]^+$ 434.09652 and 436.09320, found 434.09617 and 434.09320.

$[\alpha]_D^{20}$ +203 (*c* 0.093, $CHCl_3$).

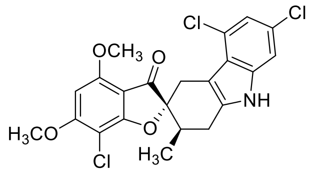

GF-I-1-22

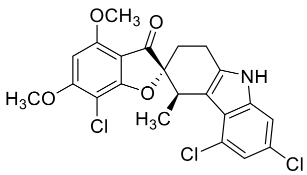

GF-I-2-15

Following the general procedure B for griseofulvin-derived indoles, the starting materials were reacted for 2 h. After purification, 61 mg (42%) of the major isomer (**GF-I-1-22**) and 14 mg (10%) of the minor isomer (**GF-I-2-15**) were isolated as white solids.

GF-I-1-22:

^{1}H NMR (600 MHz, $CDCl_3$) δ 8.54 (s, 1H), 6.94 (d, *J* = 1.7 Hz, 1H), 6.89 (d, *J* = 1.6 Hz, 1H), 6.09 (s, 1H), 4.00 (s, 3H), 3.93 (s, 3H), 3.57 (d, *J* = 16.7 Hz, 1H), 3.35 (d, *J* = 16.7 Hz, 1H), 3.03 (dd, *J* = 16.4, 5.7 Hz, 1H), 2.79 (dd, *J* = 16.4, 7.1 Hz, 1H), 2.44 (h, *J* = 6.8 Hz, 1H), 1.09 (d, *J* = 6.9 Hz, 3H).

^{13}C NMR (151 MHz, $CDCl_3$) δ 197.91, 168.13, 164.33, 157.98, 137.30, 133.87, 126.49, 125.33, 123.53, 119.96, 109.44, 105.46, 105.06, 97.41, 92.24, 89.13, 57.03, 56.35, 34.39, 29.60, 28.13, 14.40.

HRMS (ESI) calcd for $C_{22}H_{18}Cl_3NO_4$ $[M + H]^+$ 466.03742 and 468.03447, found 466.03714 and 468.03411.

$[\alpha]_D^{20}$ +50.2 (*c* 0.30, $CHCl_3$).

GF-I-2-15:

^{1}H NMR (600 MHz, Mixture of 10 CD_2Cl_2: 1 CD_3OD) δ 7.24 (d, *J* = 1.7 Hz, 1H), 6.99 (d, *J* = 1.8 Hz, 1H), 6.14 (s, 1H), 3.97 (s, 3H), 3.96 (s, 3H), 3.48 (q, *J* = 7.0 Hz, 1H), 3.10 – 2.99 (m, 1H), 2.93 (dd, *J* = 16.9, 6.1 Hz, 1H), 2.46 (ddd, *J* = 14.3, 11.7, 7.0 Hz, 1H), 1.90 (dd, *J* = 14.3, 6.8 Hz, 1H), 1.43 (d, *J* = 6.9 Hz, 3H).

^{13}C NMR (151 MHz, Mixture of 10 CD_2Cl_2: 1 CD_3OD) δ 197.73, 167.53, 164.31, 158.24, 137.62, 133.72, 126.26, 124.74, 122.91, 119.77, 112.77, 109.61, 105.01, 97.25, 92.08, 89.34, 57.00, 56.22, 34.91, 23.99, 19.63, 19.44.

HRMS (ESI) calcd for $C_{22}H_{18}Cl_3NO_4$ $[M + H]^+$ 466.03742 and 468.03447, found 466.03699 and 468.03396.

$[\alpha]_D^{20}$ +64.1 (*c* 0.13, $CHCl_3$).

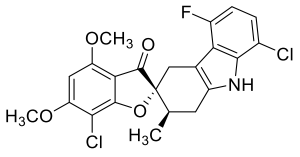

GF-I-1-23

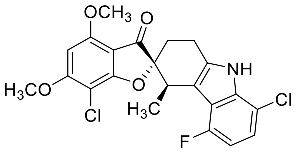

GF-I-2-16

Following the general procedure B for griseofulvin-derived indoles, the starting materials were reacted for 2.5 h. After purification, 65 mg (47%) of the major isomer (**GF-I-1-23**) and 27 mg (19%) of the minor isomer (**GF-I-2-16**) were isolated as white solids.

GF-I-1-23:

^{1}H NMR (600 MHz, $CDCl_3$) δ 8.14 (s, 1H), 6.97 (dd, *J* = 8.4, 4.0 Hz, 1H), 6.64 (dd, *J* = 10.0, 8.4 Hz, 1H), 6.10 (s, 1H), 4.00 (s, 3H), 3.96 (s, 3H), 3.39 (d, *J* = 17.0 Hz, 1H), 3.24 – 3.15 (m, 2H), 2.85 (dd, *J* = 16.3, 5.8 Hz, 1H), 2.48 (h, *J* = 6.7 Hz, 1H), 1.15 (d, *J* = 6.9 Hz, 3H).

^{13}C NMR (151 MHz, $CDCl_3$) δ 196.11, 166.83, 163.02, 156.83, 154.17 (d, *J* = 245.0 Hz), 134.34 (d, *J* = 12.6 Hz), 131.63, 119.56 (d, *J* = 7.9 Hz), 116.24 (d, *J* = 22.5 Hz), 110.15 (d, *J* = 3.4 Hz), 104.45 (d, *J* = 21.3 Hz), 104.22, 103.77 (d, *J* = 2.1 Hz), 96.41, 90.71, 88.00, 55.86, 55.27, 33.21, 27.54, 27.11, 13.10.

^{19}F NMR (565 MHz, $CDCl_3$) δ -127.39 (dd, *J* = 10.0, 2.2 Hz).

HRMS (ESI) calcd for $C_{22}H_{18}Cl_2FNO_4$ $[M + H]^+$ 450.06697 and 452.06402, found 450.06686 and 452.06379.

$[\alpha]_D^{20}$ +64.0 (*c* 0.63, $CHCl_3$).

GF-I-1-23:

^{1}H NMR (500 MHz, $CDCl_3$) δ 8.12 (s, 1H), 7.01 (dd, *J* = 8.4, 4.0 Hz, 1H), 6.67 (dd, *J* = 10.3, 8.4 Hz, 1H), 6.10 (s, 1H), 3.99 (s, 3H), 3.99 (s, 3H), 3.31 (q, *J* = 7.0 Hz, 1H), 3.17 – 3.05 (m, 1H), 2.97 (ddd, *J* = 16.6, 6.6, 2.1 Hz, 1H), 2.45 (ddd, *J* = 14.1, 11.1, 6.6 Hz, 1H), 1.98 (dd, *J* = 14.2, 5.7 Hz, 1H), 1.44 (d, *J* = 7.0 Hz, 3H).

^{13}C NMR (126 MHz, $CDCl_3$) δ 197.55, 167.78, 164.13, 158.09, 154.92 (d, *J* = 244.9 Hz), 135.41 (d, *J* = 12.7 Hz), 132.14, 120.87 (d, *J* = 8.1 Hz), 116.89 (d, *J* = 22.4 Hz), 113.30 (d, *J* = 3.1 Hz), 111.41 (d, *J* = 3.5 Hz), 105.75 (d, *J* = 21.9 Hz), 105.33, 97.74, 91.85, 89.09, 57.01, 56.46, 35.63, 24.88, 19.83, 18.26 (d, *J* = 3.6 Hz).

^{19}F NMR (470 MHz, $CDCl_3$) δ -124.90 (dd, *J* = 10.4, 4.0 Hz).

HRMS (ESI) calcd for $C_{22}H_{18}Cl_2FNO_4$ $[M + H]^+$ 450.06697 and 452.06402, found 450.06688 and 452.06381.

$[\alpha]_D^{20}$ +59.5 (*c* 0.48, $CHCl_3$).

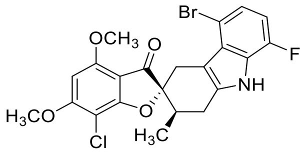

GF-I-1-24

Following the general procedure B for griseofulvin-derived indoles, the starting materials were reacted for 2.5 h. After purification, 48 mg (32%) of the major isomer (**GF-I-1-24**) was isolated as a solid. The minor isomer was not obtained in an analytically pure form.

GF-I-1-24:

^{1}H NMR (500 MHz, $CDCl_3$) δ 8.36 (s, 1H), 7.03 (dd, *J* = 8.4, 4.1 Hz, 1H), 6.65 (dd, *J* = 10.4, 8.4 Hz, 1H), 6.11 (s, 1H), 4.01 (s, 3H), 3.96 (s, 3H), 3.62 (d, *J* = 16.9 Hz, 1H), 3.40 (d, *J* = 17.1 Hz, 1H), 3.19 (dd, *J* = 16.3, 5.8 Hz, 1H), 2.83 (dd, *J* = 16.4, 5.8 Hz, 1H), 2.44 (h, *J* = 6.6 Hz, 1H), 1.15 (d, *J* = 7.0 Hz, 3H).

^{13}C NMR (126 MHz, $CDCl_3$) δ 197.46, 167.88, 164.12, 157.91, 148.64 (d, *J* = 242.8 Hz), 129.21 (d, *J* = 5.6 Hz), 129.19, 125.23 (d, *J* = 14.5 Hz), 122.82 (d, *J* = 6.4 Hz), 107.33 (d, *J* = 17.8 Hz), 107.31 (d, *J* = 2.8 Hz), 106.71 (d, *J* = 1.9 Hz), 105.24, 97.39, 91.80, 89.00, 56.93, 56.29, 33.92, 28.98, 28.23, 14.08.

^{19}F NMR (470 MHz, $CDCl_3$) δ -137.92 (dd, *J* = 10.5, 4.2 Hz).

HRMS (ESI) calcd for $C_{22}H_{18}ClBrFNO_4$ $[M + H]^+$ 494.01645, 496.01350 and 498.01146, found 494.01638, 496.01382, and 498.01123.

$[\alpha]_D^{20}$ +25.9 (*c* 0.54, $CHCl_3$).

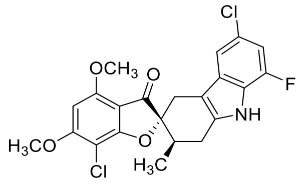

GF-I-1-25

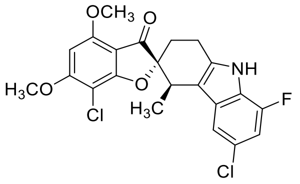

GF-I-2-17

Following the general procedure B for griseofulvin-derived indoles, the starting materials were reacted for 2.5 h. After purification, 37 mg (27%) of the major isomer (**GF-I-1-25**) and 44 mg (32%) of the minor isomer (**GF-I-2-17**) were isolated as white solids.

GF-I-1-25:

^{1}**H NMR** (500 MHz, $CDCl_3$) δ 8.24 (s, 1H), 7.10 (d, *J* = 1.7 Hz, 1H), 6.82 (dd, *J* = 10.6, 1.7 Hz, 1H), 6.10 (s, 1H), 4.00 (s, 3H), 3.95 (s, 3H), 3.22 – 3.13 (m, 2H), 2.97 (d, *J* = 16.4 Hz, 1H), 2.83 (dd, *J* = 16.3, 5.6 Hz, 1H), 2.47 (h, *J* = 6.9 Hz, 1H), 1.13 (d, *J* = 6.9 Hz, 3H).

^{13}**C NMR** (126 MHz, $CDCl_3$) δ 197.31, 167.93, 164.23, 157.99, 148.44 (d, *J* = 246.3 Hz), 134.49, 131.18 (d, *J* = 6.3 Hz), 124.41 (d, *J* = 8.7 Hz), 122.87 (d, *J* = 12.7 Hz), 113.37 (d, *J* = 3.4 Hz), 107.63 (d, *J* = 20.1 Hz), 105.91 (d, *J* = 2.6 Hz), 105.28, 97.48, 91.86, 89.14, 57.02, 56.41, 34.66, 28.33, 27.55, 14.26.

^{19}**F NMR** (470 MHz, $CDCl_3$) δ -133.27 (d, *J* = 10.6 Hz).

HRMS (ESI) calcd for $C_{22}H_{18}Cl_2FNO_4$ $[M + H]^+$ 450.06697 and 452.06402, found 450.06689 and 452.06392.

$[\alpha]_D^{20}$ +59.0 (*c* 0.59, $CHCl_3$).

GF-I-2-17:

^{1}**H NMR** (700 MHz, $CDCl_3$) δ 8.22 (s, 1H), 7.21 (d, *J* = 1.7 Hz, 1H), 6.83 (dd, *J* = 10.6, 1.7 Hz, 1H), 6.10 (s, 1H), 3.99 (s, 3H), 3.96 (s, 3H), 3.30 (q, *J* = 7.1 Hz, 1H), 3.08 – 2.99 (m, 2H), 2.36 (ddd, *J* = 13.8, 8.2, 7.0 Hz, 1H), 2.10 (dt, *J* = 13.8, 5.4 Hz, 1H), 1.40 (d, *J* = 7.1 Hz, 3H).

^{13}**C NMR** (176 MHz, $CDCl_3$) δ 197.44, 168.06, 164.29, 158.17, 148.71 (d, *J* = 246.6 Hz), 134.22, 130.85 (d, *J* = 6.2 Hz), 124.42 (d, *J* = 8.7 Hz), 123.13 (d, *J* = 12.8 Hz), 114.01 (d, *J* = 3.3 Hz), 113.18 (d, *J* = 2.2 Hz), 107.67 (d, *J* = 20.1 Hz), 105.57, 97.75, 92.02, 89.30, 57.12, 56.54, 35.40, 26.58, 19.89, 16.39.

^{19}**F NMR** (470 MHz, $CDCl_3$) δ -133.07 (d, *J* = 10.5 Hz).

HRMS (ESI) calcd for $C_{22}H_{18}Cl_2FNO_4$ $[M + H]^+$ 450.06697 and 452.06402, found 450.06696 and 452.06398.

$[\alpha]_D^{20}$ +33.3 (*c* 0.60, CH_2Cl_2).

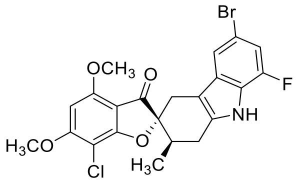

GF-I-1-26

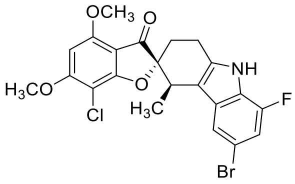

GF-I-2-18

Following the general procedure B for griseofulvin-derived indoles, the starting materials were reacted for 2.5 h. After purification, 37 mg (24%) of the major isomer (**GF-I-1-26**) and 13 mg (9%) of the minor isomer (**GF-I-2-18**) were isolated as white solids.

GF-I-1-26:

^{1}H NMR (500 MHz, $CDCl_3$) δ 8.21 (s, 1H), 7.26 (s, 2H), 6.95 (dd, *J* = 10.4, 1.6 Hz, 1H), 6.10 (s, 1H), 4.01 (s, 3H), 3.95 (s, 3H), 3.24 – 3.12 (m, 2H), 2.96 (d, *J* = 16.4 Hz, 1H), 2.83 (dd, *J* = 16.4, 5.8 Hz, 1H), 2.47 (h, *J* = 6.5 Hz, 1H), 1.13 (d, *J* = 6.9 Hz, 3H).

^{13}C NMR (126 MHz, $CDCl_3$) δ 197.28, 167.93, 164.23, 157.99, 148.61 (d, *J* = 247.4 Hz), 134.35, 131.92 (d, *J* = 6.2 Hz), 123.23 (d, *J* = 12.9 Hz), 116.47 (d, *J* = 3.3 Hz), 111.29 (d, *J* = 8.2 Hz), 110.15 (d, *J* = 20.0 Hz), 105.83 (d, *J* = 2.5 Hz), 105.28, 97.50, 91.82, 89.15, 57.03, 56.42, 34.65, 28.33, 27.54, 14.26.

^{19}F NMR (470 MHz, $CDCl_3$) δ -133.03 (d, *J* = 10.3 Hz); HRMS (ESI) calcd for $C_{22}H_{18}ClBrFNO_4$ $[M + H]^+$ 494.01645, 496.01350 and 498.01146, found 494.01638, 496.01395, and 498.01126.

$[\alpha]_D^{20}$ +56.8 (*c* 1.29, $CHCl_3$).

GF-I-2-18:

^{1}H NMR (500 MHz, $CDCl_3$) δ 8.12 (s, 1H), 7.36 (d, *J* = 1.5 Hz, 1H), 6.98 (dd, *J* = 10.3, 1.5 Hz, 1H), 6.10 (s, 1H), 4.00 (s, 3H), 3.98 (s, 3H), 3.29 (q, *J* = 7.1 Hz, 1H), 3.05 (dd, *J* = 7.6, 5.3 Hz, 2H), 2.37 (dt, *J* = 13.9, 7.7 Hz, 1H), 2.09 (dt, *J* = 13.8, 5.3 Hz, 1H), 1.40 (d, *J* = 7.1 Hz, 3H).

^{13}C NMR (126 MHz, $CDCl_3$) δ 197.33, 167.94, 164.18, 158.05, 148.78 (d, *J* = 247.5 Hz), 133.93, 131.51 (d, *J* = 5.9 Hz), 123.35 (d, *J* = 12.8 Hz), 116.96 (d, *J* = 3.3 Hz), 113.10 (d, *J* = 2.2 Hz), 111.25 (d, *J* = 8.1 Hz), 110.13 (d, *J* = 19.9 Hz), 105.43, 97.63, 91.84, 89.14, 57.03, 56.46, 35.26, 26.36, 19.78, 16.40.

^{19}F NMR (470 MHz, $CDCl_3$) δ -132.89 (d, *J* = 10.4 Hz).

HRMS (ESI) calcd for $C_{22}H_{18}ClBrFNO_4$ $[M + H]^+$ 494.01645, 496.01350 and 498.01146, found 494.01636, 496.01393, and 498.01125.

$[\alpha]_D^{20}$ +14.1 (*c* 0.62, $CHCl_3$).

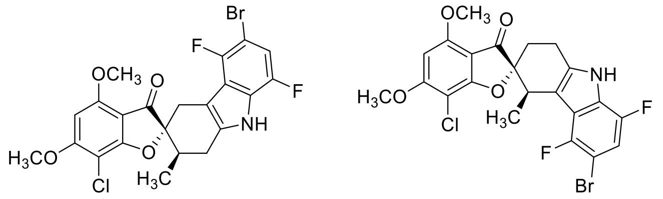

GF-I-1-27 GF-I-2-19

Following the general procedure B for griseofulvin-derived indoles, the starting materials were reacted for 2.5 h. After purification, 54 mg (32%) of the major isomer (**GF-I-1-27**) and 20 mg (13%) of the minor isomer (**GF-I-2-19**) were isolated as solids.

GF-I-1-27:

^{1}H NMR (600 MHz, $CDCl_3$) δ 8.77 (d, *J* = 5.4 Hz, 1H), 6.85 – 6.70 (m, 1H), 6.10 (s, 1H), 4.00 (s, 3H), 3.92 (s, 3H), 3.36 (d, *J* = 16.6 Hz, 1H), 3.19 (d, *J* = 16.5 Hz, 1H), 3.10 (dd, *J* = 16.4, 5.6 Hz, 1H), 2.86 (dd, *J* = 16.5, 7.0 Hz, 1H), 2.48 (h, *J* = 6.9 Hz, 1H), 1.10 (d, *J* = 6.9 Hz, 3H).

^{13}C NMR (151 MHz, $CDCl_3$) δ 197.65, 168.14, 164.46, 158.11, 148.84 (d, *J* = 241.8 Hz), 144.78 (d, *J* = 243.6 Hz), 134.11, 125.46 (dd, *J* = 15.4, 11.2 Hz), 119.17 (dd, *J* = 23.7, 5.9 Hz), 109.74 (d, *J* = 22.0 Hz), 105.47, 104.54, 97.53, 95.78 (dd, *J* = 22.1, 8.8 Hz), 91.89, 89.34, 57.13, 56.48, 34.64, 28.89, 28.13, 14.43.

^{19}F NMR (565 MHz, $CDCl_3$) δ -125.59 (d, *J* = 17.5 Hz), -137.76 – -138.66 (m).

HRMS (ESI) calcd for $C_{22}H_{17}ClBrF_2NO_4$ $[M + H]^+$ 512.00703, 514.00498, and 516.00203, found 512.00697, 514.00455, and 516.00178.

$[\alpha]_D^{20}$ +75.0 (*c* 0.088, $CHCl_3$).

GF-I-2-19:

^{1}H NMR (600 MHz, CD_2Cl_2) δ 8.38 (s, 1H), 6.99 (dd, *J* = 9.6, 4.7 Hz, 1H), 6.14 (s, 1H), 3.99 (s, 3H), 3.97 (s, 3H), 3.28 (q, *J* = 7.0 Hz, 1H), 3.13 – 3.03 (m, 1H), 3.02 – 2.93 (m, 1H), 2.41 (ddd, *J* = 14.2, 11.1, 6.7 Hz, 1H), 2.02 – 1.91 (m, 1H), 1.39 (d, *J* = 7.0 Hz, 3H).

^{13}C NMR (151 MHz, CD_2Cl_2) δ 196.92, 167.80, 164.45, 158.46, 148.82 (dd, *J* = 241.4, 2.9 Hz), 145.28 (dd, *J* = 243.1, 2.7 Hz), 134.02, 125.64 (dd, *J* = 15.4, 11.6 Hz), 119.11 (dd, *J* = 23.5, 5.8 Hz), 113.24 (d, *J* = 3.3 Hz), 110.29 (d, *J* = 22.0 Hz), 105.42, 97.58, 96.27 (dd, *J* = 22.7, 9.2 Hz), , 91.69, 89.74, 57.37, 56.71, 35.68, 24.87, 19.98, 18.28 (d, *J* = 3.3 Hz).

^{19}F NMR (565 MHz, CD_2Cl_2) δ -123.26 (dd, *J* = 18.9, 4.7 Hz), -138.94 (dd, *J* = 18.9, 9.6 Hz).

HRMS (ESI) calcd for $C_{22}H_{17}ClBrF_2NO_4$ $[M + H]^+$ 512.00703, 514.00498, and 516.00203, found 512.00701, 514.00451, and 516.00183;.

$[\alpha]_D^{20}$ -49.6 (*c* 0.40, $CHCl_3$).

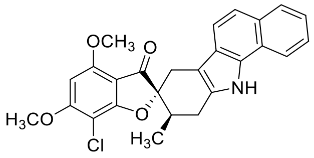

GF-I-1-28

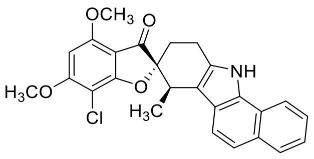

GF-I-2-20

Following the general procedure B for griseofulvin-derived indoles, starting materials were reacted for 1 h. After purification, 106 mg (77%) of the major isomer (**GF-I-1-28**) and 24 mg (17%) of the minor isomer (**GF-I-2-20**) were isolated as solids.

GF-I-1-28:

^{1}H NMR (600 MHz, $CDCl_3$) δ 9.02 (s, 1H), 7.90 (d, *J* = 8.1 Hz, 1H), 7.85 (d, *J* = 7.9 Hz, 1H), 7.45 – 7.35 (m, 3H), 7.33 (ddd, *J* = 8.0, 6.8, 1.2 Hz, 1H), 6.06 (s, 1H), 3.97 (s, 3H), 3.87 (s, 3H), 3.34 (d, *J* = 16.1 Hz, 1H), 3.22 (dd, *J* = 15.9, 5.6 Hz, 1H), 3.13 (d, *J* = 16.1 Hz, 1H), 2.90 (dd, *J* = 15.9, 6.0 Hz, 1H), 2.46 (h, *J* = 6.6 Hz, 1H), 1.15 (d, *J* = 6.9 Hz, 3H).

^{13}C NMR (151 MHz, $CDCl_3$) δ 197.87, 168.07, 164.14, 157.97, 130.62, 130.26, 130.06, 128.83, 125.12, 123.18, 122.91, 121.65, 119.86, 119.54, 118.12, 106.56, 105.43, 97.40, 92.66, 89.04, 56.95, 56.29, 34.88, 28.39, 27.86, 14.36.

HRMS (ESI) calcd for $C_{26}H_{22}ClNO_4$ $[M + H]^+$ 448.13101 and 450.12806, found 448.13055 and 450.12770.

$[\alpha]^{20}_{D}$ +13.2 (*c* 0.32, $CHCl_3$).

GF-I-1-28:

^{1}H NMR (600 MHz, $CDCl_3$) δ 8.78 (s, 1H), 7.95 (d, *J* = 8.2 Hz, 1H), 7.88 (d, *J* = 8.1 Hz, 1H), 7.59 (d, *J* = 8.6 Hz, 1H), 7.48 – 7.43 (m, 2H), 7.36 (ddd, *J* = 8.1, 6.9, 1.2 Hz, 1H), 6.09 (s, 1H), 3.98 (s, 3H), 3.94 (s, 3H), 3.45 (q, *J* = 7.1 Hz, 1H), 3.12 (dd, *J* = 7.8, 5.2 Hz, 2H), 2.43 (dt, *J* = 13.7, 8.0 Hz, 1H), 2.11 (dt, *J* = 13.7, 5.2 Hz, 1H), 1.50 (d, *J* = 7.1 Hz, 3H).

^{13}C NMR (151 MHz, $CDCl_3$) δ 197.84, 168.06, 164.08, 158.04, 130.68, 130.03, 129.80, 128.90, 125.35, 123.46, 122.58, 121.72, 119.97, 119.38, 118.66, 114.22, 105.57, 97.63, 92.56, 89.05, 56.97, 56.39, 35.43, 26.58, 19.85, 17.07.

HRMS (ESI) calcd for $C_{26}H_{22}ClNO_4$ $[M + H]^+$ 448.13101 and 450.12806, found 448.13050 and 450.12767.

$[\alpha]^{20}_{D}$ +98.9 (*c* 0.27, $CHCl_3$).

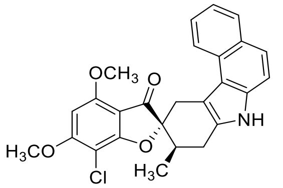

GF-I-1-29

Following the general procedure B for griseofulvin-derived indoles, the starting materials were reacted for 1 h. After purification, 59 mg (43%) of the major isomer (**GF-I-1-29**) was isolated as a solid. The minor isomer was not obtained in an analytically pure form.

GF-I-1-29:

^{1}H NMR (700 MHz, $CDCl_3$) δ 8.37 (s, 1H), 8.17 (d, *J* = 8.3 Hz, 1H), 7.87 (d, *J* = 8.0 Hz, 1H), 7.49 (d, *J* = 8.7 Hz, 1H), 7.46 – 7.42 (m, 2H), 7.35 (ddd, *J* = 8.0, 6.8, 1.2 Hz, 1H), 7.26 (s, 1H), 6.09 (s, 1H), 3.99 (s, 3H), 3.95 (s, 3H), 3.72 (d, *J* = 16.0 Hz, 1H), 3.48 (d, *J* = 16.0 Hz, 1H), 3.28 (dd, *J* = 15.8, 5.6 Hz, 1H), 2.86 (dd, *J* = 15.8, 5.3 Hz, 1H), 2.47 (h, *J* = 6.7 Hz, 1H), 1.19 (d, *J* = 7.0 Hz, 3H).

^{13}C NMR (176 MHz, $CDCl_3$) δ 197.73, 168.05, 164.17, 158.03, 132.54, 130.05, 129.62, 128.74, 128.71, 125.44, 123.37, 122.77, 122.10, 120.81, 112.75, 107.17, 105.40, 97.56, 92.67, 89.12, 56.99, 56.39, 34.19, 30.55, 28.38, 14.18.

HRMS (ESI) calcd for $C_{26}H_{22}ClNO_4$ $[M + H]^+$ 448.13101 and 450.12806, found 448.13082 and 450.12805.

$[\alpha]_D^{20}$ +59.6 (*c* 0.66, $CHCl_3$).

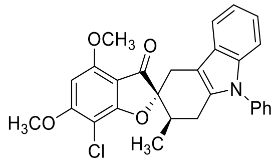

GF-I-1-30

Following the general procedure A for griseofulvin-derived indoles, the starting materials were reacted for 1 h. After purification, 54 mg (37%) of the major isomer (**GF-I-1-30**) was isolated as a yellow solid. The minor isomer was not isolated in analytically pure form.

GF-I-1-30:

^{1}H NMR (700 MHz, $CDCl_3$) δ 7.55 (t, *J* = 7.8 Hz, 2H), 7.49 – 7.40 (m, 4H), 7.27 (m, 1H), 7.13 (m, 2H), 6.12 (s, 1H), 4.02 (s, 3H), 3.99 (s, 3H), 3.35 (d, *J* = 16.3 Hz, 1H), 3.16 (d, *J* = 16.3 Hz, 1H), 3.07 (dd, *J* = 16.5, 5.5 Hz, 1H), 2.76 (dd, *J* = 16.5, 5.9 Hz, 1H), 2.48 (h, *J* = 6.4 Hz, 1H), 1.12 (d, *J* = 7.0 Hz, 3H).

^{13}C NMR (176 MHz, $CDCl_3$) δ 197.43, 168.03, 164.06, 157.90, 137.92, 137.90, 134.01, 129.52, 127.43, 127.39, 127.36, 121.60, 119.89, 117.75, 110.13, 105.99, 105.47, 97.53, 92.29, 89.09, 56.97, 56.38, 35.02, 28.16, 27.89, 14.21.

HRMS (ESI) calcd for $C_{28}H_{24}ClNO_4$ $[M + H]^+$ 474.14666 and 476.14371, found 474.14632 and 476.14376.

$[\alpha]_D^{20}$ +39.8 (*c* 0.22, $CHCl_3$).

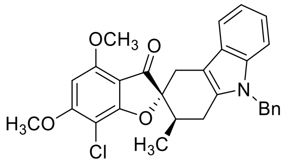

GF-I-1-31

Following the general procedure A for griseofulvin-derived indoles, the starting materials were reacted for 1 h. After purification, 63 mg (42%) of the major isomer (**GF-I-1-31**) was isolated as a yellow solid. The minor isomer was not obtained in an analytically pure form.

GF-I-1-31:

^{1}H NMR (700 MHz, $CDCl_3$) δ 7.45 (d, *J* = 7.6 Hz, 1H), 7.30 (t, *J* = 7.5 Hz, 2H), 7.25 – 7.20 (m, 2H), 7.11 (t, *J* = 7.5 Hz, 1H), 7.08 (t, *J* = 7.3 Hz, 1H), 7.04 (d, *J* = 7.7 Hz, 2H), 6.10 (s, 1H), 5.32 (s, 2H), 3.99 (s, 3H), 3.96 (s, 3H), 3.33 (d, *J* = 16.2 Hz, 1H), 3.15 – 3.10 (m, 2H), 2.76 (dd, *J* = 16.2, 5.6 Hz, 1H), 2.49 (h, *J* = 6.5 Hz, 1H), 1.14 (d, *J* = 7.0 Hz, 3H).

^{13}C NMR (176 MHz, $CDCl_3$) δ 197.36, 167.91, 163.96, 157.82, 137.96, 137.16, 133.76, 128.81, 127.26, 127.22, 126.03, 121.06, 119.18, 117.74, 109.31, 104.66, 92.25, 89.00, 56.88, 56.31, 46.48, 34.66, 27.73, 27.43, 14.25.

HRMS (ESI) calcd for $C_{29}H_{26}ClNO_4$ $[M + H]^+$ 488.16231 and 490.15936, found 488.16196 and 490.15938.

$[\alpha]_D^{20}$ +31.8 (*c* 0.19, $CHCl_3$).

General procedure for the synthesis of 3-ethanolic indoles and compound data:

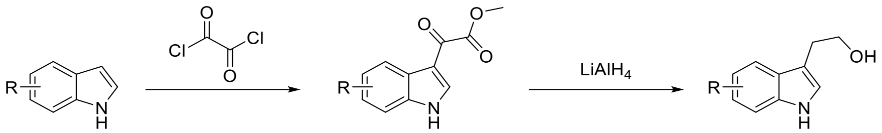

In a 50 ml oven-dried round-bottomed flask was added the indole (1.75 mmol) and dry Et_2O (10 ml) under an atmosphere of argon. The reaction was cooled to at 0 °C and oxalyl chloride (450 µl, 5.25 mmol, 3 equiv) was added dropwise. The ice bath was removed and the reaction was stirred at 22 °C for 16 h. The reaction was then cooled to 0 °C and MeOH (0.5 ml) was added dropwise and stirred for 30 min. The crude reaction mixture was filtered with a Buchner funnel, washed with cold Et_2O, and dried. The solid product was used in the next step without further purification.

The solid product was dissolved in dry THF (4 ml) and added dropwise to a suspension of $LiAlH_4$ (332 mg, 10 mmol, 5 equiv) in dry THF (8 mL) in a 50 ml RBF at 0 °C. The mixture was stirred at 80 °C for 2.5 h and then cooled to 0 °C. The reaction was quenched by slowly adding H_2O (1.5 mL), 10% aqueous NaOH (3.0 mL), H_2O (4.5 mL) at 0 °C and stirred for 30 min. The solution was then filtered and extracted with EtOAc (10 ml x 3). The combined organic layers were dried over Na2SO4 and the solvent was removed under reduced pressure to give the crude product. Silica chromatography was performed (30-90% EtOAc in CyHex) to afford the desired product.

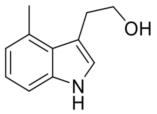

Following the general procedure, 115 mg (38%) of the desired product was obtained.

^{1}H NMR (400 MHz, $CDCl_3$) δ 8.06 (bs, 1H), 7.20 (ddd, *J* = 8.2, 1.1, 0.6 Hz, 1H), 7.08 (dd, *J* = 8.2, 7.1 Hz, 1H), 7.03 (s, 1H), 6.87 (dt, *J* = 7.1, 0.9 Hz, 1H), 3.91 (t, *J* = 6.4 Hz, 2H), 3.20 (t, *J* = 6.4 Hz, 2H), 2.71 (d, *J* = 0.8 Hz, 3H).

^{13}C NMR (101 MHz, $CDCl_3$) δ 137.18, 131.04, 126.03, 122.95, 122.44, 121.49, 113.10, 109.41, 63.71, 30.64, 20.61.

HRMS (ESI) calcd for $C_{11}H_{13}NO$ $[M + H]^+$ 176.10699, found 176.10688.

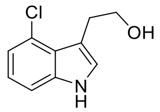

Following the general procedure, 40 mg (18%) of the desired product was obtained.

^{1}H NMR (400 MHz, $CDCl_3$) δ 8.39 (bs, 1H), 7.21 (dd, *J* = 6.5, 2.5 Hz, 1H), 7.08 – 7.04 (m, 2H), 7.01 (s, 1H), 3.95 (t, *J* = 6.4 Hz, 2H), 3.24 (td, *J* = 6.4, 0.8 Hz, 2H).

^{13}C NMR (101 MHz, $CDCl_3$) δ 138.23, 126.49, 124.49, 124.27, 122.77, 120.69, 112.63, 110.30, 63.77, 29.91.

HRMS (ESI) calcd for $C_{10}H_{10}NOCl$ $[M + H]^+$ 196.05237 and 198.04942, found 196.05230 and 198.04935.

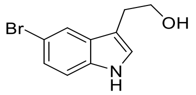

Following the general procedure, 216 mg (51%) of the desired product was obtained.

^{1}H NMR (400 MHz, $CDCl_3$) δ 8.57 (bs, 1H), 7.72 (d, *J* = 1.9 Hz, 1H), 7.26 – 7.23 (m, 1H), 7.13 (d, *J* = 8.6 Hz, 1H), 6.91 (s, 1H), 3.83 (t, *J* = 6.4 Hz, 2H), 2.91 (td, *J* = 6.4, 0.9 Hz, 2H).

^{13}C NMR (101 MHz, $CDCl_3$) δ 135.29, 129.49, 124.93, 124.21, 121.55, 113.10, 112.72, 112.06, 62.74, 28.69.

HRMS (ESI) calcd for $C_{10}H_{10}NOBr$ $[M + H]^+$ 240.00185 and 241.99981, found 240.00196 and 241.99983.

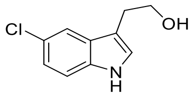

Following the general procedure, 51 mg (15%) of the desired product was obtained.

^{1}H NMR (400 MHz, $CDCl_3$) δ 8.32 (bs, 1H), 7.57 (dd, *J* = 2.0, 0.6 Hz, 1H), 7.23 (d, *J* = 8.6 Hz, 1H), 7.14 (dd, *J* = 8.6, 2.0 Hz, 1H), 7.02 (s, 1H), 3.87 (t, *J* = 6.4 Hz, 2H), 2.95 (td, *J* = 6.4, 0.9 Hz, 2H).

^{13}C NMR ^{13}C NMR (101 MHz, $CDCl_3$) δ 135.00, 128.80, 125.34, 124.21, 122.58, 118.54, 112.53, 112.30, 62.73, 28.72.

HRMS (ESI) calcd for $C_{10}H_{10}NOCl$ $[M + H]^+$ 196.05237 and 198.04942, found 196.05229 and 198.04932.

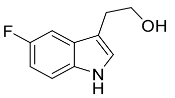

Following the general procedure, 233 mg (71%) of the desired product was obtained.

^{1}H NMR (500 MHz, $CDCl_3$) δ 8.12 (bs, 1H), 7.33 – 7.25 (m, 2H), 7.15 (s, 1H), 6.98 (td, J = 9.0, 2.5 Hz, 1H), 3.92 (t, J = 6.3 Hz, 2H), 3.01 (t, J = 6.3 Hz, 2H).

^{13}C NMR ^{13}C NMR (126 MHz, $CDCl_3$) δ 157.81 (d, J = 234.7 Hz), 132.90, 127.83 (d, J = 9.6 Hz), 124.28, 127.83 (d, J = 9.6 Hz), 112.57 (d, J = 4.9 Hz), 111.85 (d, J = 9.6 Hz), 110.62 (d, J = 26.4 Hz), 103.80 (d, J = 23.5 Hz), 62.51, 28.68.

^{19}F NMR (470 MHz, $CDCl_3$) δ -124.58 (td, J = 9.4, 4.3 Hz).

HRMS (ESI) calcd for $C_{10}H_{10}NOF$ $[M + H]^+$ 180.08192, found 180.08179.

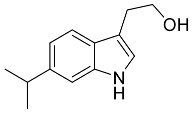

Following the general procedure, 49 mg (14%) of the desired product was obtained.

^{1}H NMR (400 MHz, $CDCl_3$) δ 8.22 (bs, 1H), 7.65 (d, J = 8.2 Hz, 1H), 7.25 (dt, J = 1.5, 0.7 Hz, 1H), 7.17 (dd, J = 8.3, 1.6 Hz, 1H), 6.95 (s, 1H), 3.97 (t, J = 6.5 Hz, 2H), 3.21 – 3.03 (m, 3H), 2.41 (s, 1H), 1.45 (d, J = 7.0 Hz, 6H).

^{13}C NMR (101 MHz, $CDCl_3$) δ 143.67, 137.19, 125.99, 122.63, 119.06, 118.88, 112.09, 108.92, 62.91, 34.71, 29.11, 24.93.

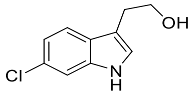

Following the general procedure, 19 mg (6%) of the desired product was obtained.

^{1}H NMR (400 MHz, $CDCl_3$) δ 7.48 (dd, J = 8.4, 0.6 Hz, 1H), 7.29 (dd, J = 1.9, 0.6 Hz, 1H), 7.06 (dd, J = 8.5, 1.8 Hz, 1H), 6.98 (s, 1H), 3.86 (t, J = 6.4 Hz, 2H), 2.96 (td, J = 6.4, 0.9 Hz, 2H).

^{13}C NMR (101 MHz, $CDCl_3$) δ 137.02, 128.12, 126.31, 123.43, 120.21, 119.85, 112.60, 111.40, 62.79, 28.76.

HRMS (ESI) calcd for $C_{10}H_{10}NOCl$ 196.05237 and 198.04942 $[M + H]^+$, found 196.05230 and 198.04932.

General procedure for griseofulvin-based 4H-pyranoindoles and compound data (GF-THPI):

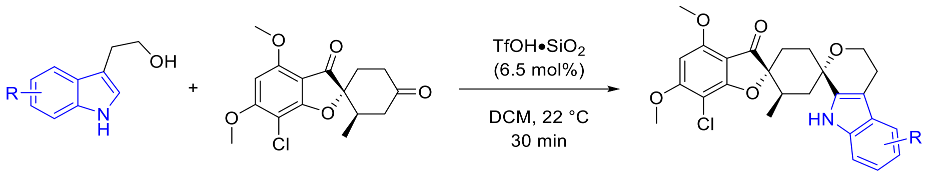

To a 5 ml vial was added the ethanol-derived indole (0.10 mmol, 1 equiv), DCM (1 ml), the griseofulvin-derived ketone (39 mg, 0.12 mmol, 1.2 equiv), and TfOH-SiO_2 (11 mg, 0.0065 mmol, 0.065 equiv), successively. The reaction was stirred for 30 min at 22 °C. The reaction was filtered, concentrated, and purified by silica chromatography (15-50% EtOAc in CyHex). NMRs of the crude reaction mixture indicated that only a single diastereoisomer was present for each reaction.

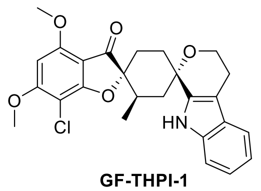

GF-THPI-1

Following the general procedure, 33 mg (87%) of **GF-THPI-1** was obtained.

^{1}H NMR (400 MHz, $CDCl_3$) δ 8.06 (s, 1H), 7.49 (ddt, J = 7.7, 1.3, 0.7 Hz, 1H), 7.33 (m, 1H), 7.17 (ddd, J = 8.1, 7.1, 1.3 Hz, 1H), 7.10 (ddd, J = 8.1, 7.1, 1.1 Hz, 1H), 6.10 (s, 1H), 4.02 (s, 3H), 3.99 (s, 3H), 2.82 (m, 2H), 2.72 (ddd, J = 13.0, 6.7, 4.2 Hz, 1H), 2.54 (m, 3H), 2.02 (m, 3H), 1.79 (ddd, J = 12.8, 4.0, 2.6 Hz, 1H), 1.43 (s, 1H), 0.82 (d, J = 6.7 Hz, 3H) ppm.

^{13}C NMR (176 MHz, $CDCl_3$) δ 199.4, 168.7, 164.3, 157.7, 138.1, 135.9, 127.0, 121.8, 119.5, 118.3, 111.1, 107.3, 106.1, 92.6, 88.9, 72.4, 60.2, 57.0, 56.3, 38.8, 33.9, 30.1, 28.7, 27.0, 22.6, 14.8 ppm.

HRMS (ESI) calcd for $C_{26}H_{27}NO_5Cl$ $[M + H]^+$ 468.1572, found 468.1568.

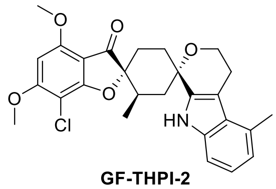

GF-THPI-2

Following the general procedure, 16 mg (35%) of **GF-THPI-2** was obtained.

^{1}H NMR (700 MHz, $CDCl_3$) δ 7.99 (s, 1H), 7.16 (d, J = 8.0 Hz, 1H), 7.03 (t, J = 7.6 Hz, 1H), 6.82 (d, J = 7.2 Hz, 1H), 6.10 (s, 1H), 4.06 – 3.96 (m, 6H), 3.07 (qt, J = 10.0, 5.3 Hz, 2H), 2.74 – 2.66 (m, 1H), 2.67 – 2.57 (m, 3H), 2.53 (t, J = 13.7 Hz, 1H), 2.51 – 2.41 (m, 1H), 2.06 (d, J = 14.6 Hz, 1H), 1.98 (d, J = 13.7 Hz, 1H), 1.78 (d, J = 12.9 Hz, 1H), 1.62 (s, 1H), 0.82 (d, J = 6.8 Hz, 3H).

^{13}C NMR (176 MHz, $CDCl_3$) δ 199.32, 168.55, 164.22, 157.58, 137.40, 135.63, 130.39, 125.84, 121.76, 120.59, 108.71, 107.48, 106.00, 97.32, 92.51, 88.78, 72.20, 60.25, 56.91, 56.25, 38.82, 33.82, 30.02, 28.69, 24.94, 19.58, 14.73.

HRMS (ESI) calcd for $C_{27}H_{28}NO_5Cl$ $[M + H]^+$ 482.17288 and 484.16993, found 482.17237 and 484.16977, respectively.

$[\alpha]_D^{20}$ +29.2 (c 0.30, DCM).

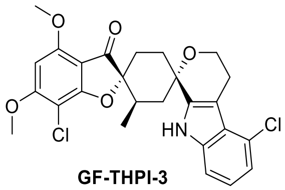

GF-THPI-3

Following the general procedure, 9 mg (17%) of **GF-THPI-3** was obtained.

^{1}H NMR (700 MHz, $CDCl_3$) δ 8.15 (s, 1H), 7.21 (t, *J* = 4.4 Hz, 1H), 7.03 (d, *J* = 4.5 Hz, 2H), 6.10 (s, 1H), 4.05 – 3.96 (m, 8H), 3.14 (t, *J* = 5.4 Hz, 2H), 2.74 – 2.67 (m, 1H), 2.60 (td, *J* = 14.2, 4.2 Hz, 1H), 2.54 – 2.44 (m, 2H), 2.07 (d, *J* = 12.9 Hz, 1H), 1.99 (d, *J* = 14.4 Hz, 1H), 1.79 (d, *J* = 12.9 Hz, 1H), 0.82 (d, *J* = 6.8 Hz, 3H).

^{13}C NMR (176 MHz, $CDCl_3$) δ 199.51, 168.71, 164.43, 157.72, 138.98, 136.96, 126.16, 124.52, 122.30, 120.13, 109.73, 107.57, 106.10, 97.50, 92.53, 88.94, 72.20, 60.37, 57.06, 56.40, 38.89, 33.92, 30.13, 28.77, 24.37, 14.84.

HRMS (ESI) calcd for $C_{26}H_{25}NO_5Cl_2$ $[M + H]^+$ 502.11825, 504.11530, 506.11235, found 502.11787, 504.11502, 506.11196, respectively.

$[\alpha]_D^{20}$ +35.9 (*c* 0.11, DCM).

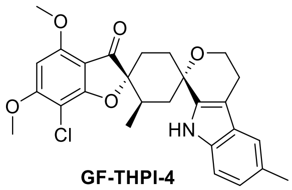

GF-THPI-4

Following the general procedure, 28 mg (76%) of **G-THPI-4** was obtained.

^{1}H NMR (400 MHz, $CDCl_3$) δ 7.84 (s, 1H), 7.20 (dd, *J* = 1.6, 0.8 Hz, 1H), 7.14 (dd, *J* = 8.1, 0.6 Hz, 1H), 6.91 (ddd, *J* = 8.2, 1.6, 0.6 Hz, 1H), 6.02 (s, 1H), 3.94 – 3.92 (m, 8H), 2.74 – 2.68 (m, 2H), 2.63 (dtd, *J* = 13.4, 7.0, 4.5 Hz, 1H), 2.54 – 2.47 (m, 1H), 2.45 – 2.38 (m, 1H), 2.37 (s, 3H), 1.97 (dd, *J* = 13.1, 2.7 Hz, 1H), 1.90 (ddd, *J* = 14.2, 4.2, 2.4 Hz, 1H), 1.71 (ddd, *J* = 12.8, 4.0, 2.6 Hz, 1H), 0.74 (d, *J* = 6.8 Hz, 3H).

^{13}C NMR (101 MHz, $CDCl_3$) *δ* 199.5, 168.8, 164.4, 157.8, 138.3, 134.2, 128.9, 127.3, 123.4, 118.1, 110.8, 106.9, 92.7, 89.0, 72.5, 60.4, 57.1, 56.5, 38.9, 34.0, 30.2, 28.9, 22.7, 21.7, 14.9.

HRMS (ESI) calcd for $C_{27}H_{28}NO_5Cl_2$ $[M + H]^+$ 482.1656 and 484.1627, found 482.1652 and 484.1626, respectively.

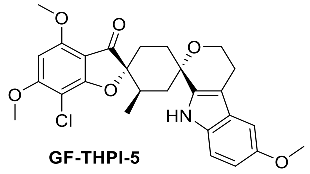

Following the general procedure, 35 mg (71%) of **GF-THPI-5** was obtained.

^{1}H NMR (700 MHz, $CDCl_3$) δ 8.00 (bs, 1H), 7.21 (d, *J* = 8.7 Hz, 1H), 6.95 (d, *J* = 2.4 Hz, 1H), 6.82 (dd, *J* = 8.7, 2.5 Hz, 1H), 6.09 (s, 1H), 4.05 – 3.96 (m, 8H), 3.85 (s, 3H), 2.84 – 2.67 (m, 3H), 2.61 (td, *J* = 14.3, 4.2 Hz, 1H), 2.55 – 2.44 (m, 2H), 2.05 (d, *J* = 13.0 Hz, 1H), 1.98 (d, *J* = 14.2 Hz, 1H), 1.79 (d, *J* = 12.9 Hz, 1H), 0.81 (d, *J* = 6.8 Hz, 3H).

^{13}C NMR (176 MHz, $CDCl_3$) δ 199.40, 168.65, 164.33, 157.71, 154.25, 139.01, 130.96, 127.36, 111.76, 111.68, 107.12, 106.09, 100.65, 97.39, 92.60, 88.90, 72.43, 60.23, 57.01, 56.33, 56.16, 38.87, 33.89, 30.11, 28.76, 22.65, 14.83.

HRMS (ESI) calcd for $C_{27}H_{28}NO_6Cl$ $[M + H]^+$ 498.16779 and 500.16484, found 498.16739 and 500.16483, respectively.

$[\alpha]_D^{20}$ +31.1 (*c* 0.36, DCM).

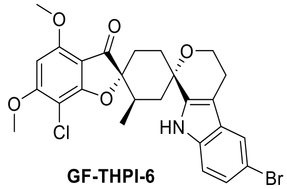

Following the general procedure, 22 mg (40%) of **GF-THPI-6** was obtained.

^{1}H NMR (700 MHz, $CDCl_3$) δ 8.14 (s, 1H), 7.60 (s, 1H), 7.23 (d, *J* = 8.5 Hz, 1H), 7.18 (d, *J* = 8.5 Hz, 1H), 6.09 (s, 1H), 4.03 – 3.96 (m, 8H), 2.81 – 2.67 (m, 3H), 2.61 (td, *J* = 14.2, 4.2 Hz, 1H), 2.55 – 2.44 (m, 2H), 2.05 (d, *J* = 14.5 Hz, 1H), 1.98 (d, *J* = 14.2 Hz, 1H), 1.79 (d, *J* = 12.8 Hz, 1H), 0.82 (d, *J* = 6.7 Hz, 3H).

^{13}C NMR (176 MHz, $CDCl_3$) δ 199.45, 168.68, 164.42, 157.72, 139.51, 134.53, 128.82, 124.58, 121.01, 112.77, 112.48, 107.10, 106.06, 97.45, 92.48, 88.93, 72.30, 60.12, 57.04, 56.35, 38.76, 33.87, 30.05, 28.73, 22.44, 14.82.

HRMS (ESI) calcd for $C_{26}H_{25}NO_5ClBr$ $[M + H]^+$ 546.06774, 548.06479, and 550.06274, found 546.06755, 548.06507, and 550.06244, respectively.

$[\alpha]_D^{20}$ +27.7 (*c* 0.70, DCM).

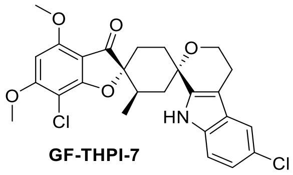

GF-THPI-7

Following the general procedure, 48 mg (96%) of **GF-THPI-7** was obtained. Crystals for x-ray analysis were grown using a mixture of ethanol/water.

^{1}H NMR (700 MHz, $CDCl_3$ δ 8.19 (s, 1H), 7.44 (s, 1H), 7.20 (d, *J* = 8.5 Hz, 1H), 7.09 (d, *J* = 8.5 Hz, 1H), 6.08 (s, 1H), 4.05 – 3.96 (m, 6H), 3.94 (s, 3H), 2.81 – 2.68 (m, 3H), 2.62 (td, *J* = 14.3, 4.3 Hz, 1H), 2.53 (t, *J* = 13.6 Hz, 1H), 2.48 (td, *J* = 13.5, 4.2 Hz, 1H), 2.05 (d, *J* = 14.2 Hz, 1H), 1.99 (d, *J* = 14.1 Hz, 1H), 1.80 (d, *J* = 12.9 Hz, 1H), 0.82 (d, *J* = 6.8 Hz, 3H).

^{13}C NMR (176 MHz, $CDCl_3$) δ 199.43, 168.66, 164.40, 157.71, 139.69, 134.25, 128.15, 125.23, 121.97, 117.88, 112.01, 107.13, 106.04, 97.41, 92.48, 88.91, 72.32, 60.10, 57.03, 56.29, 38.73, 33.85, 30.05, 28.71, 22.44, 14.82.

HRMS (ESI) calcd for $C_{26}H_{25}NO_5Cl_2$ $[M + H]^+$ 502.11825, 504.11530, and 506.11235, found 502.11789, 504.11500, and 506.11197, respectively.

$[\alpha]_D^{20}$ +26.6 (*c* 0.45, DCM).

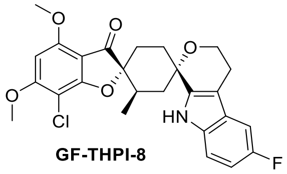

GF-THPI-8

Following the general procedure, 22 mg (45%) of **GF-THPI-8** was obtained.

^{1}H NMR (700 MHz, $CDCl_3$) δ 8.10 (s, 1H), 7.22 (dd, *J* = 8.7, 4.2 Hz, 1H), 7.12 (d, *J* = 9.2 Hz, 1H), 6.89 (t, *J* = 8.9 Hz, 1H), 6.09 (s, 1H), 4.05 – 3.96 (m, 8H), 2.82 – 2.68 (m, 3H), 2.61 (td, *J* = 14.2, 4.1 Hz, 1H), 2.53 (t, *J* = 13.7 Hz, 1H), 2.48 (td, *J* = 13.5, 4.0 Hz, 1H), 2.05 (d, *J* = 14.1 Hz, 1H), 1.99 (d, *J* = 14.2 Hz, 1H), 1.79 (d, *J* = 13.0 Hz, 1H), 0.82 (d, *J* = 6.8 Hz, 3H).

^{13}C NMR (176 MHz, $CDCl_3$) δ 199.45, 168.68, 164.40, 158.01 (d, *J* = 234.5 Hz), 157.72, 140.08, 132.34, 127.38 (d, *J* = 9.7 Hz), 111.58 (d, *J* = 9.7 Hz), 109.90 (d, *J* = 26.2 Hz), 107.57 (d, *J* = 4.4 Hz), 106.07, 103.41 (d, *J* = 23.4 Hz), 97.44, 92.52, 88.92, 72.37, 60.16, 57.03, 56.34, 38.81, 33.88, 30.08, 28.74, 22.53, 14.83.

^{19}F NMR (565 MHz, $CDCl_3$) δ -124.84 (td, *J* = 9.4, 4.3 Hz).

HRMS (ESI) calcd for $C_{26}H_{25}NO_5ClF$ $[M + H]^+$ 486.14781 and 488.14486, found 486.14732 and 488.14475, respectively.

$[\alpha]_D^{20}$ +29.7 (*c* 0.16, DCM).

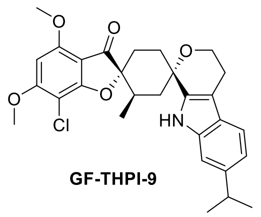

GF-THPI-9

Following the general procedure, 38 mg (74%) of **GF-THPI-9** was obtained.

^{1}H NMR (700 MHz, $CDCl_3$) δ 8.03 (s, 1H), 7.41 (d, *J* = 8.0 Hz, 1H), 7.15 (s, 1H), 7.01 (d, *J* = 8.1 Hz, 1H), 6.09 (s, 1H), 4.05 – 3.97 (m, 5H), 3.95 (s, 3H), 3.00 (hept, *J* = 6.9 Hz, 1H), 2.80 (qt, *J* = 15.0, 5.3 Hz, 2H), 2.76 – 2.68 (m, 1H), 2.63 (td, *J* = 14.2, 4.2 Hz, 1H), 2.54 (t, *J* = 13.7 Hz, 1H), 2.49 (td, *J* = 13.5, 4.2 Hz, 1H), 2.04 (d, *J* = 14.5 Hz, 1H), 1.97 (d, *J* = 14.1 Hz, 1H), 1.80 (d, *J* = 12.9 Hz, 1H), 1.29 (d, *J* = 6.4 Hz, 6H), 0.83 (d, *J* = 6.8 Hz, 3H).

^{13}C NMR (176 MHz, $CDCl_3$) δ 199.44, 168.65, 164.31, 157.70, 143.23, 137.58, 136.16, 125.21, 118.79, 118.04, 108.40, 107.08, 106.11, 97.39, 92.65, 88.88, 72.39, 60.24, 57.00, 56.29, 38.81, 34.55, 33.87, 30.09, 28.75, 24.70, 24.67, 22.66, 14.85.

HRMS (ESI) calcd for $C_{29}H_{32}NO_5Cl$ $[M + H]^+$ 510.20418 and 512.20123, found 510.20352 and 512.20099, respectively.

$[\alpha]_D^{20}$ +29.4 (*c* 0.43, DCM).

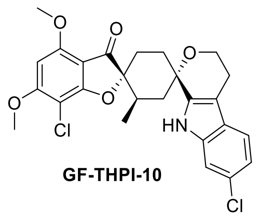

GF-THPI-10

Following the general procedure, 17 mg (33%) of **GF-THPI-10** was obtained.

^{1}H NMR (700 MHz, $CDCl_3$) δ 8.10 (s, 1H), 7.38 (d, *J* = 8.3 Hz, 1H), 7.30 (s, 1H), 7.06 (d, *J* = 8.4 Hz, 1H), 6.10 (s, 1H), 4.06 – 3.96 (m, 8H), 2.83 – 2.67 (m, 3H), 2.60 (td, *J* = 14.2, 4.2 Hz, 1H), 2.56 – 2.44 (m, 2H), 2.04 (d, *J* = 14.1 Hz, 1H), 1.97 (d, *J* = 14.2 Hz, 1H), 1.79 (d, *J* = 13.0 Hz, 1H), 0.82 (d, *J* = 6.8 Hz, 3H).

^{13}C NMR (176 MHz, $CDCl_3$) δ 199.45, 168.68, 164.42, 157.73, 138.81, 136.24, 127.58, 125.67, 120.24, 119.09, 111.09, 107.48, 106.07, 97.44, 92.50, 88.94, 72.30, 60.12, 57.04, 56.37, 38.75, 33.89, 30.03, 28.74, 22.47, 14.83.

HRMS (ESI) calcd for $C_{26}H_{25}NO_5Cl_2$ $[M + H]^+$ 502.11825, 504.11530, and 506.11235, found 502.11788, 504.11501, and 506.11195, respectively.

$[\alpha]_D^{20}$ +30.9 (*c* 0.20, DCM).

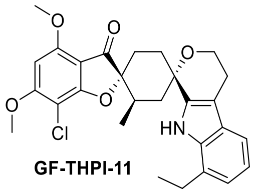

GF-THPI-11

Following the general procedure, 20 mg (41%) of **GF-THPI-11** was obtained.

^{1}H NMR (700 MHz, $CDCl_3$) δ 7.81 (s, 1H), 7.35 (d, *J* = 7.7 Hz, 1H), 7.07 (t, *J* = 7.5 Hz, 1H), 7.01 (d, *J* = 7.2 Hz, 1H), 6.11 (s, 1H), 4.06 – 3.96 (m, 8H), 2.90 – 2.70 (m, 5H), 2.64 (td, *J* = 14.2, 4.1 Hz, 1H), 2.56 (t, *J* = 13.6 Hz, 1H), 2.50 (td, *J* = 13.5, 4.1 Hz, 1H), 2.06 (d, *J* = 16.5 Hz, 1H), 1.98 (d, *J* = 14.0 Hz, 1H), 1.79 (d, *J* = 12.9 Hz, 1H), 1.37 (t, *J* = 7.6 Hz, 3H), 0.83 (d, *J* = 6.8 Hz, 3H).

^{13}C NMR (176 MHz, $CDCl_3$) δ 199.61, 168.71, 164.37, 157.68, 137.64, 134.57, 126.81, 126.66, 120.66, 120.01, 116.04, 107.99, 106.07, 97.50, 92.62, 88.90, 72.44, 60.20, 57.05, 56.42, 38.80, 33.81, 30.05, 28.69, 24.19, 22.71, 14.85, 14.16.

HRMS (ESI) calcd for $C_{28}H_{30}NO_5Cl$ $[M + H]^+$ 496.18853 and 498.18558, found 496.18800 and 498.18536, respectively.

$[\alpha]^{20}_{D}$ +27.2 (*c* 0.42, DCM).

General procedure for chromanone-based ketones:

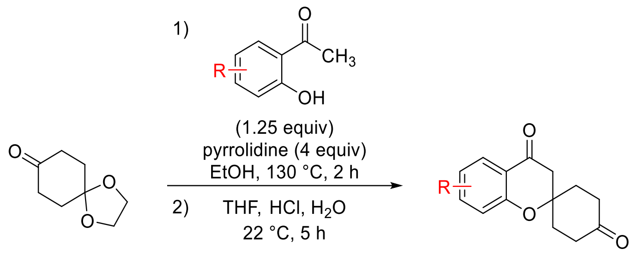

To a dry, large microwave vial equipped with a stirring bar was added the monoprotected diketone (468 mg, 3 mmol), a 2-hydroxyacetophenone (3.75 mmol, 1.25 equiv) followed by 7 ml of anhydrous EtOH and pyrrolidine (1.2 ml, 12 mmol, 4 equiv). The vial was capped with a septum and purged with Ar for 15 min. After this time, the septum was replaced with a microwave cap, and the reaction was heated in the microwave at 130 C for 2 hours. The reaction was diluted with DCM and concentrated. The crude mixture was purified by silica chromatography (10-50% EA in CyHex) to afford the chromanone acetal-protected ketone.

The purified product was dissolved in 10 ml of THF in a 250 ml flask. 15 ml of 3M HCl (aq) was added and the reaction was stirred. After the ketone had been completely deprotected (monitored by LCMS – typically 3-6 hours), the reaction was cooled to 0C and 200 ml of $NaHCO_3$ (aq) was added very slowly via an addition funnel. The formed precipitate was the filtered using a Buchner funnel, washed with cold water, and dried for several hours to afford the pure, desired product. If the product did not precipitate, the aqueous phase was extracted with DCM (3x), dried over Na_2SO_4, and concentrated to afford the desired product.

Compound data for chromanone-based ketones:

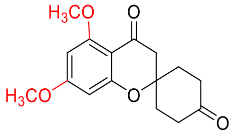

Following the general procedure on a 2 mmol scale, 300 mg (52%) was obtained as a white solid.

^{1}H NMR (700 MHz, $CDCl_3$) δ 6.12 (d, *J* = 1.4 Hz, 1H), 6.09 (d, *J* = 1.4 Hz, 1H), 3.88 (s, 3H), 3.84 (s, 3H), 2.75 – 2.67 (m, 4H), 2.46 – 2.39 (m, 2H), 2.34 – 2.27 (m, 2H), 1.88 (td, *J* = 13.6, 5.0 Hz, 2H).

^{13}C NMR (176 MHz, $CDCl_3$) δ 209.83, 188.56, 166.39, 162.49, 162.27, 105.64, 94.05, 93.10, 77.80, 56.29, 55.77, 48.58, 36.45, 34.17.

HRMS (ESI) calcd for $C_{16}H_{18}O_5$ $[M + H]^+$ 291.12270, found 291.12297.

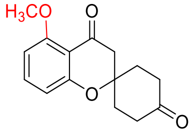

Following the general procedure, 434 mg (75%) was obtained as a white solid.

^{1}H NMR (500 MHz, $CDCl_3$) δ 7.41 (t, *J* = 8.3 Hz, 1H), 6.62 (d, *J* = 8.4 Hz, 1H), 6.55 (d, *J* = 8.4 Hz, 1H), 3.92 (s, 3H), 2.79 – 2.67 (m, 4H), 2.47 – 2.38 (m, 2H), 2.34 – 2.26 (m, 2H), 1.88 (td, *J* = 13.7, 5.0 Hz, 2H).

^{13}C NMR (126 MHz, $CDCl_3$) δ 209.85, 190.18, 160.65, 160.64, 136.48, 111.05, 110.42, 104.21, 77.55, 56.35, 48.88, 36.40, 34.09.

HRMS (ESI) calcd for $C_{15}H_{16}O_4$ $[M + H]^+$ 261.11214, found 261.11239.

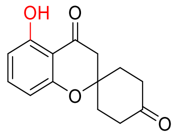

Following the general procedure on a 2 mmol scale, 420 mg (85%) was obtained as a tan solid.

^{1}H NMR (500 MHz, $CDCl_3$) δ 11.60 (s, 1H), 7.40 (t, J = 8.3 Hz, 1H), 6.55 (dd, J = 8.4, 0.9 Hz, 1H), 6.50 (dd, J = 8.2, 1.0 Hz, 1H), 2.82 (s, 2H), 2.80 – 2.69 (m, 2H), 2.52 – 2.43 (m, 2H), 2.32 (ddt, J = 15.2, 4.6, 2.1 Hz, 2H), 1.90 (td, J = 13.8, 5.1 Hz, 2H).

^{13}C NMR (126 MHz, $CDCl_3$) δ 209.49, 197.34, 162.15, 158.90, 138.84, 109.96, 107.93, 107.89, 77.85, 46.94, 36.32, 34.32.

HRMS (ESI) calcd for $C_{14}H_{14}O_4$ $[M + H]^+$ 247.09649, found 247.09670.

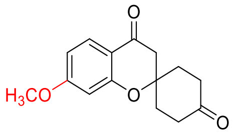

Following the general procedure on a 5 mmol scale, 1 g (77%) was obtained as a yellow solid.

^{1}H NMR (700 MHz, $CDCl_3$) δ 7.84 (d, J = 8.7 Hz, 1H), 6.61 (dd, J = 8.8, 2.4 Hz, 1H), 6.48 (d, J = 2.4 Hz, 1H), 3.86 (s, 3H), 2.79 – 2.69 (m, 4H), 2.46 (ddt, J = 14.9, 6.3, 3.3 Hz, 2H), 2.32 (ddt, J = 15.2, 4.8, 2.1 Hz, 2H), 1.89 (td, J = 13.8, 5.0 Hz, 2H).

^{13}C NMR (176 MHz, $CDCl_3$) δ 209.76, 189.96, 166.68, 161.01, 128.76, 114.72, 110.00, 101.52, 78.60, 55.85, 47.30, 36.45, 34.37.

HRMS (ESI) calcd for $C_{15}H_{16}O_4$ $[M + H]^+$ 261.11214, found 261.11233.

General procedure for chromanone-based indoles (C-I):

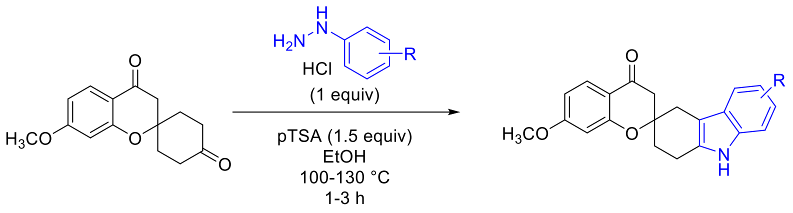

To an oven-dried microwave vial under an atmosphere of argon was added the phenylhydrazine HCl salt (0.23 mmol, 1 equiv), ketone (0.23 mmol, 1 equiv.), and paratolylsulfonic acid (PTSA) (0.35 mmol, 1.5 equiv). Then anhydrous EtOH (1.5 ml) was added and the vial flushed with argon again. A microwave cap was placed on the vial and the reaction was heated to 80-130 °C for 1-4 h in a microwave. After the reaction had cooled to room temperature, and LCMS was done to determine if all of the starting ketone and hydrazone intermediate (mass of desired product + 17) were consumed. Once complete, the reaction was diluted with dichloromethane and transferred to a sept funnel and saturated $NaHCO_3$ was added. The aqueous layer was extracted with dichloromethane (3 x 10 ml). The combined organic layers were dried and concentrated. The compound was purified by silica chromatography (10-50% EtOAc in CyHex).

Compound data for chromanone-based indoles (C-I):

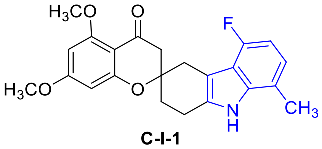

C-I-1

Following the general procedure on a 0.26 mmol scale with a reaction time of 2 h at 100 °C, 34 mg (33%) of **C-I-1** was obtained as a white solid.

^{1}H NMR (700 MHz, $CDCl_3$) δ 7.82, 6.80, 6.79, 6.79, 6.78, 6.63, 6.62, 6.62, 6.61, 6.05, 6.05, 6.02, 6.02, 3.88, 3.78, 3.37, 3.35, 3.15, 3.13, 2.93, 2.90, 2.76, 2.73, 2.40, 2.39, 2.39, 2.38, 2.37, 2.04, 2.04, 2.03, 2.02, 2.02, 2.01, 2.00.

^{13}C NMR (176 MHz, $CDCl_3$) δ 189.49, 166.21, 163.31, 162.07, 155.32 (d, *J* = 242.9 Hz), 137.94 (d, *J* = 11.7 Hz), 131.58, 122.13 (d, *J* = 7.4 Hz), 115.79 (d, *J* = 21.1 Hz), 115.64 (d, *J* = 3.6 Hz), 105.65, 105.64, 104.61 (d, *J* = 18.8 Hz), 94.06, 92.78, 79.44, 56.24, 55.69, 48.09, 33.76, 30.73, 19.99, 16.21.

^{19}F NMR (470 MHz, $CDCl_3$) δ -128.81 (dd, *J* = 11.0, 4.7 Hz).

HRMS (ESI) calcd for $C_{23}H_{22}NO_4F$ $[M + H]^+$ 396.16056, found 396.16012.

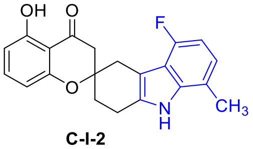

C-I-2

Following the general procedure on a 0.2 mmol scale with a reaction time of 1 h at 90 °C, 39 mg (55%) of **C-I-2** was obtained as a tan solid.

^{1}H NMR (500 MHz, $CDCl_3$) δ 11.71 (s, 1H), 7.84 (bzs, 1H), 7.34 (t, *J* = 8.3 Hz, 1H), 6.81 (dd, *J* = 7.9, 4.8 Hz, 1H), 6.64 (dd, *J* = 10.6, 7.9 Hz, 1H), 6.50 (dd, *J* = 8.3, 0.9 Hz, 1H), 6.37 (dd, *J* = 8.3, 0.9 Hz, 1H), 3.39 (d, *J* = 16.4 Hz, 1H), 3.15 (d, *J* = 16.5 Hz, 1H), 3.01 (d, *J* = 17.1 Hz, 1H), 2.98 – 2.87 (m, 1H), 2.82 (d, *J* = 17.1 Hz, 1H), 2.73 (dt, *J* = 16.4, 5.6 Hz, 1H), 2.47 – 2.37 (m, 4H), 2.01 (ddd, *J* = 13.5, 8.9, 5.9 Hz, 1H).

^{13}C NMR (126 MHz, $CDCl_3$) δ 198.09, 161.90, 159.75, 155.23 (d, *J* = 242.6 Hz), 138.61, 137.89 (d, *J* = 11.7 Hz), 131.41, 122.19 (d, *J* = 7.4 Hz), 115.75, 115.72, 115.55, 108.64 (d, *J* = 129.0 Hz), 107.85, 105.19, 104.62 (d, *J* = 18.7 Hz), 79.40, 46.24, 33.75, 30.83, 19.82, 16.19.

^{19}F NMR (377 MHz, $CDCl_3$) δ -128.94 (dd, *J* = 11.2, 4.8 Hz).

HRMS (ESI) calcd for $C_{21}H_{18}NO_3F$ $[M + H]^+$ 352.13435, found 352.13448.

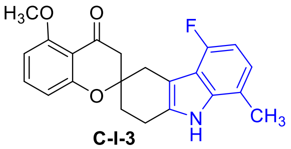

C-I-3

Following the general procedure on a 0.2 mmol scale with a reaction time of 1 h at 90 °C, 15 mg (21%) of **C-I-3** was obtained as a white solid.

^{1}H NMR (700 MHz, $(CD_3)_2SO$) δ 10.99 (s, 1H), 7.41 (t, *J* = 8.3 Hz, 1H), 6.72 (dd, *J* = 7.8, 5.0 Hz, 1H), 6.63 (d, *J* = 8.1 Hz, 1H), 6.55 (dd, *J* = 10.9, 7.8 Hz, 1H), 6.49 (dd, *J* = 8.4, 0.9 Hz, 1H), 3.80 (s, 3H), 3.13 (d, *J* = 16.0 Hz, 1H), 3.03 (d, *J* = 16.0 Hz, 1H), 2.90 (d, *J* = 15.9 Hz, 1H), 2.85 – 2.78 (m, 2H), 2.74 (dt, *J* = 16.6, 5.5 Hz, 1H), 2.37 (s, 3H), 2.28 – 2.22 (m, 1H), 1.99 (ddd, *J* = 13.5, 9.2, 6.0 Hz, 1H).

^{13}C NMR (176 MHz, $(CD_3)_2SO$) δ 189.64, 160.53, 159.76, 154.20 (d, *J* = 239.2 Hz), 137.83 (d, *J* = 12.0 Hz), 136.13, 132.87, 120.73 (d, *J* = 7.4 Hz), 116.29 (d, *J* = 3.2 Hz), 114.78 (d, *J* = 20.6 Hz), 110.33, 109.93, 103.99, 103.14, 103.03, 78.87, 55.80, 47.83, 32.32, 30.17, 19.12, 16.16.

^{19}F NMR (470 MHz, $(CD_3)_2SO$) δ -130.34.

HRMS (ESI) calcd for $C_{22}H_{21}NO_3F$ $[M + H]^+$ 366.15000, found 366.15005.

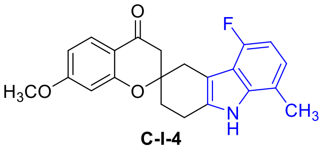

C-I-4

Following the general procedure with a reaction time of 2 h at 100 °C, 42 mg (60%) of **C-I-4** was obtained as a tan solid.

^{1}H NMR (700 MHz, $CDCl_3$) δ 7.89 (s, 1H), 7.84 (d, *J* = 8.8 Hz, 1H), 6.79 (dd, *J* = 7.7, 4.8 Hz, 1H), 6.63 (dd, *J* = 10.6, 7.9 Hz, 1H), 6.57 (dd, *J* = 8.8, 2.4 Hz, 1H), 6.36 (d, *J* = 2.4 Hz, 1H), 3.79 (s, 3H), 3.40 (d, *J* = 16.4 Hz, 1H), 3.15 (d, *J* = 16.4 Hz, 1H), 2.97 – 2.90 (m, 2H), 2.79 – 2.71 (m, 2H), 2.43 – 2.37 (m, 4H), 2.01 (ddd, *J* = 13.5, 9.0, 5.9 Hz, 1H).

^{13}C NMR (176 MHz, $CDCl_3$) δ 190.84, 166.50, 161.83, 155.31 (d, *J* = 242.6 Hz), 137.94 (d, *J* = 11.7 Hz), 131.56, 128.45, 122.15 (d, *J* = 7.3 Hz), 115.76 (d, *J* = 19.4 Hz), 115.69 (d, *J* = 2.2 Hz), 114.61, 109.74, 105.49 (d, *J* = 1.9 Hz), 104.60 (d, *J* = 18.8 Hz), 101.43, 80.16, 55.72, 46.76, 33.92, 30.81, 19.94, 16.21.

^{19}F NMR (470 MHz, $CDCl_3$) δ -125.31 (q, *J* = 11.1, 10.0 Hz).

HRMS (ESI) calcd for $C_{22}H_{20}NO_3F$ $[M + H]^+$ 366.15000, found 366.15003.

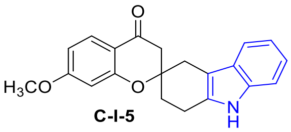

C-I-5

Following the general procedure on a 0.2 mmol scale with a reaction time of 1 h at 100 °C, 36 mg (57%) of **C-I-5** was obtained as a white solid.

^{1}H NMR (600 MHz, $CDCl_3$) δ 7.94 (s, 1H), 7.85 (d, *J* = 8.8 Hz, 1H), 7.40 (d, *J* = 7.8 Hz, 1H), 7.29 (d, *J* = 8.0 Hz, 1H), 7.14 (t, *J* = 7.4 Hz, 1H), 7.08 (t, *J* = 7.7 Hz, 1H), 6.58 (dd, *J* = 8.8, 2.4 Hz, 1H), 6.36 (d, *J* = 2.4 Hz, 1H), 3.79 (s, 3H), 3.21 (d, *J* = 15.9 Hz, 1H), 3.03 – 2.89 (m, 3H), 2.80 (d, *J* = 16.6 Hz, 1H), 2.73 (dt, *J* = 16.4, 5.6 Hz, 1H), 2.40 (dt, *J* = 11.8, 5.5 Hz, 1H), 2.05 (ddd, *J* = 13.9, 8.5, 5.9 Hz, 1H).

^{13}C NMR (151 MHz, $CDCl_3$) δ 190.94, 166.48, 161.87, 136.47, 132.06, 128.37, 127.57, 121.63, 119.52, 117.79, 114.60, 110.74, 109.76, 106.55, 101.41, 80.38, 55.69, 46.66, 32.41, 31.45, 20.03.

HRMS (ESI) calcd for $C_{21}H_{19}NO_3$ $[M + H]^+$ 334.14377, found 334.14394.

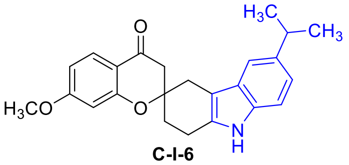

C-I-6

Following the general procedure on a 0.23 mmol scale with a reaction time of 1 h at 100 °C, 46 mg (54%) of **C-I-6** was obtained as a tan solid.

^{1}H NMR (700 MHz, $CDCl_3$) δ 7.87 (d, *J* = 8.8 Hz, 1H), 7.82 (s, 1H), 7.27 (d, *J* = 10.2 Hz, 1H), 7.24 (d, *J* = 8.3 Hz, 1H), 7.06 (dd, *J* = 8.4, 1.7 Hz, 1H), 6.59 (dd, *J* = 8.8, 2.4 Hz, 1H), 6.38 (d, *J* = 2.4 Hz, 1H), 3.81 (s, 3H), 3.22 (d, *J* = 15.8 Hz, 1H), 3.05 – 2.98 (m, 2H), 2.98 – 2.90 (m, 2H), 2.82 (d, *J* = 16.5 Hz, 1H), 2.74 (dt, *J* = 16.4, 5.8 Hz, 1H), 2.41 (dt, *J* = 12.2, 5.7 Hz, 1H), 2.07 (ddd, *J* = 13.8, 8.3, 5.9 Hz, 1H), 1.31 (d, *J* = 7.0 Hz, 6H).

^{13}C NMR (176 MHz, $CDCl_3$) δ 190.92, 166.46, 161.90, 140.34, 135.03, 132.18, 128.36, 127.65, 120.79, 114.79, 114.65, 110.49, 109.73, 106.39, 101.43, 80.46, 55.68, 46.65, 34.35, 32.41, 31.66, 24.84, 24.82, 20.09.

HRMS (ESI) calcd for $C_{24}H_{25}NO_3$ $[M + H]^+$ 376.19072, found 376.19076.

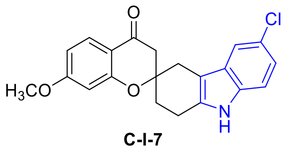

C-I-7

Following the general procedure on a 0.23 mmol scale with a reaction time of 1 h at 110 °C, 15 mg (17%) of **C-I-7** was obtained as a brown solid.

^{1}H NMR (700 MHz, $(CD_3)_2SO$) δ 11.03 (s, 1H), 7.70 (d, *J* = 8.8 Hz, 1H), 7.36 (s, 1H), 7.27 (d, *J* = 8.5 Hz, 1H), 6.99 (dd, *J* = 8.5, 2.1 Hz, 1H), 6.61 (dd, *J* = 8.8, 2.4 Hz, 1H), 6.47 (d, *J* = 2.4 Hz, 1H), 3.76 (s, 3H), 3.05 (d, *J* = 15.9 Hz, 1H), 2.95 – 2.88 (m, 2H), 2.88 – 2.80 (m, 2H), 2.74 (dt, *J* = 16.6, 5.4 Hz, 1H), 2.28 (dt, *J* = 13.5, 5.2 Hz, 1H), 2.02 (ddd, *J* = 13.6, 9.3, 6.0 Hz, 1H).

^{13}C NMR (176 MHz, $(CD_3)_2SO$) δ 190.00, 165.87, 161.19, 134.82, 134.66, 128.21, 127.58, 122.92, 120.15, 116.51, 114.03, 112.08, 109.46, 104.78, 101.25, 80.07, 55.75, 46.02, 31.25, 30.55, 19.30.

HRMS (ESI) calcd for $C_{21}H_{18}NO_3Cl$ $[M + H]^+$ 368.10480 and 370.10185, found 368.10493 and 370.10196.

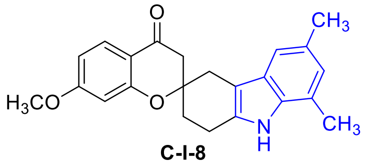

C-I-8

Following the general procedure on a 0.23 mmol scale with a reaction time of 1 h at 100 °C, 25 mg (29%) of **C-I-8** was obtained as a white solid.

^{1}H NMR (700 MHz, $CDCl_3$) δ 7.85 (s, 1H), 7.72 (s, 1H), 7.05 (s, 1H), 6.80 (s, 1H), 6.57 (dd, *J* = 8.8, 2.4 Hz, 1H), 6.36 (d, *J* = 2.3 Hz, 1H), 3.80 (s, 3H), 3.18 (d, *J* = 15.7 Hz, 1H), 3.00 – 2.93 (m, 2H), 2.91 (d, *J* = 16.5 Hz, 1H), 2.84 – 2.73 (m, 2H), 2.46 – 2.36 (m, 7H), 2.07 (ddd, *J* = 14.0, 8.5, 5.8 Hz, 1H).

^{13}C NMR (176 MHz, $CDCl_3$) δ 190.90, 166.44, 161.90, 134.26, 131.80, 129.04, 128.36, 127.40, 123.98, 119.51, 115.30, 114.65, 109.71, 106.71, 101.41, 80.46, 55.69, 46.65, 32.47, 31.65, 21.50, 20.16, 16.70.

HRMS (ESI) calcd for $C_{23}H_{23}NO_3$ $[M + H]^+$ 362.17507, found 362.17507.

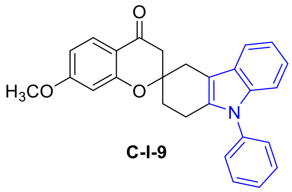

C-I-9

Following the general procedure on a 0.23 mmol scale with a reaction time of 1 h at 100 °C, 46 mg (49%) of **C-I-9** was obtained as a white solid.

^{1}H NMR (700 MHz, $CDCl_3$) δ 7.86 (d, *J* = 8.8 Hz, 1H), 7.53 (t, *J* = 7.7 Hz, 2H), 7.47 (dd, *J* = 6.5, 2.3 Hz, 1H), 7.45 – 7.38 (m, 3H), 7.26 (t, *J* = 4.4 Hz, 1H), 7.14 (tt, *J* = 7.2, 5.5 Hz, 2H), 6.59 (dd, *J* = 8.8, 2.4 Hz, 1H), 6.42 (d, *J* = 2.4 Hz, 1H), 3.82 (s, 3H), 3.31 (d, *J* = 16.0 Hz, 1H), 3.09 (d, *J* = 16.0 Hz, 1H), 2.98 (d, *J* = 16.5 Hz, 1H), 2.90 – 2.81 (m, 2H), 2.64 (dt, *J* = 16.8, 5.7 Hz, 1H), 2.43 – 2.37 (m, 1H), 2.04 (ddd, *J* = 13.8, 8.5, 5.8 Hz, 1H).

^{13}C NMR (176 MHz, $CDCl_3$) δ 190.77, 166.46, 161.82, 137.91, 137.78, 133.82, 129.60, 128.42, 127.45, 127.40, 127.21, 121.93, 120.04, 117.84, 114.65, 110.24, 109.65, 107.34, 101.54, 80.27, 55.72, 46.71, 32.56, 31.70, 19.91.

HRMS (ESI) calcd for $C_{27}H_{23}NO_3$ $[M + H]^+$ 410.17507, found 410.17467.

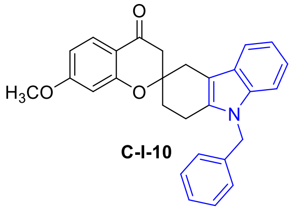

C-I-10

Following the general procedure on a 0.23 mmol scale with a reaction time of 1 h at 100 °C, 65 mg (66%) of **C-I-10** was obtained as a brown solid.

^{1}H NMR (700 MHz, $CDCl_3$) δ 7.86 (d, *J* = 8.9 Hz, 1H), 7.47 (d, *J* = 7.8 Hz, 1H), 7.30 (t, *J* = 7.5 Hz, 2H), 7.27 – 7.22 (m, 2H), 7.15 (t, *J* = 7.5 Hz, 1H), 7.11 (t, *J* = 7.4 Hz, 1H), 7.01 (d, *J* = 7.7 Hz, 2H), 6.58 (dd, *J* = 8.8, 2.4 Hz, 1H), 6.33 (d, *J* = 2.4 Hz, 1H), 5.29 (s, 2H), 3.79 (s, 3H), 3.27 (d, *J* = 15.9 Hz, 1H), 3.07 (d, *J* = 15.9 Hz, 1H), 2.94 (d, *J* = 16.6 Hz, 1H), 2.86 – 2.77 (m, 2H), 2.68 (dt, *J* = 16.5, 5.7 Hz, 1H), 2.41 (dt, *J* = 13.8, 5.6 Hz, 1H), 2.05 (ddd, *J* = 13.9, 8.4, 6.0 Hz, 1H).

^{13}C NMR (176 MHz, $CDCl_3$) δ 190.74, 166.40, 161.80, 138.00, 137.37, 133.65, 128.88, 128.38, 127.45, 127.23, 126.16, 121.43, 119.36, 117.90, 114.64, 109.69, 109.33, 106.17, 101.37, 80.21, 55.64, 46.58, 46.56, 32.56, 31.38, 19.12.

HRMS (ESI) calcd for $C_{28}H_{25}NO_3$ $[M + H]^+$ 424.19072, found 424.19032.

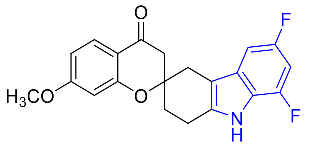

C-I-11

Following the general procedure on a 0.38 mmol scale with a reaction time of 2 h at 120 °C, 63 mg (44%) of **C-I-11** was obtained as a white solid.

^{1}H NMR (400 MHz, $CDCl_3$) δ 8.32 (s, 1H), 7.85 (d, J = 8.8 Hz, 1H), 6.83 (dd, J = 9.0, 2.2 Hz, 1H), 6.65 (ddd, J = 11.0, 9.5, 2.2 Hz, 1H), 6.57 (dd, J = 8.8, 2.4 Hz, 1H), 6.35 (d, J = 2.4 Hz, 1H), 3.78 (s, 3H), 3.14 (d, J = 15.2 Hz, 1H), 3.02 – 2.86 (m, 3H), 2.84 – 2.70 (m, 2H), 2.47 – 2.38 (m, 1H), 2.02 (ddd, J = 14.1, 9.2, 6.1 Hz, 1H).

^{13}C NMR (176 MHz, $CDCl_3$) δ 190.55, 166.57, 161.65, 157.04 (d, J = 237.1 Hz), 148.24 (dd, J = 244.5, 14.5 Hz), 134.72, 130.15, 128.48, 120.98 (d, J = 12.9 Hz), 114.60, 109.87, 107.83, 101.45, 98.97 (d, J = 23.7 Hz), 96.82 (dd, J = 29.8, 20.7 Hz), 79.94, 55.75, 46.85, 32.44, 31.17, 20.04.

^{19}F NMR (470 MHz, $CDCl_3$) δ -121.88, -132.22.

HRMS (ESI) calcd for $C_{21}H_{17}NO_3F_2$ $[M + H]^+$ 370.12493, found 370.12505.

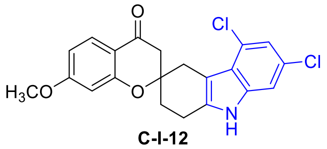

C-I-12

Following the general procedure on a 0.23 mmol scale with a reaction time of 2 h at 120 °C, 44 mg (48%) of **C-I-12** was obtained as a brown solid.

^{1}H NMR (700 MHz, $CDCl_3$) δ 7.74 (d, J = 8.8 Hz, 1H), 7.10 (d, J = 1.8 Hz, 1H), 6.90 (d, J = 1.6 Hz, 1H), 6.50 (d, J = 8.8 Hz, 1H), 6.30 (s, 1H), 3.73 (s, 3H), 3.49 (d, J = 16.5 Hz, 1H), 3.43 – 3.32 (m, 2H), 3.17 (d, J = 16.5 Hz, 1H), 2.85 – 2.76 (m, 1H), 2.66 – 2.59 (m, 1H), 2.34 – 2.26 (m, 1H), 1.93 – 1.86 (m, 1H).

^{13}C NMR (176 MHz, $CDCl_3$) δ 191.59, 166.64, 161.86, 137.55, 134.12, 128.27, 126.35, 125.45, 123.66, 119.70, 114.27, 109.73, 109.49, 105.89, 101.33, 79.90, 55.60, 46.65, 34.31, 30.00, 19.62.

HRMS (ESI) calcd for $C_{21}H_{19}NO_3Cl_2$ $[M + H]^+$ 402.06583, 404.06288, and 406.05993, found 402.06566, 404.06259, and 406.05936.

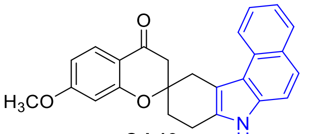

C-I-13

Following the general procedure on a 0.23 mmol scale with a reaction time of 1 h at 100 °C, 29 mg (33%) of **C-I-13** was obtained as a white solid.

^{1}H NMR (700 MHz, $CDCl_3$) δ 8.23 – 8.17 (m, 2H), 7.89 (d, *J* = 8.1 Hz, 1H), 7.87 (d, *J* = 9.2 Hz, 1H), 7.55 (d, *J* = 8.7 Hz, 1H), 7.50 (t, *J* = 7.6 Hz, 1H), 7.47 (d, *J* = 8.7 Hz, 1H), 7.38 (t, *J* = 7.4 Hz, 1H), 6.58 (dd, *J* = 8.8, 2.2 Hz, 1H), 6.38 (d, *J* = 2.3 Hz, 1H), 3.79 (s, 3H), 3.71 (d, *J* = 15.7 Hz, 1H), 3.42 (d, *J* = 15.6 Hz, 1H), 3.09 – 3.01 (m, 2H), 2.89 – 2.79 (m, 2H), 2.50 – 2.42 (m, 1H), 2.09 (ddd, *J* = 14.2, 9.2, 5.8 Hz, 1H).

^{13}C NMR (176 MHz, $CDCl_3$) δ 190.86, 166.56, 161.83, 132.64, 130.15, 129.72, 128.83, 128.48, 125.66, 123.44, 122.99, 122.58, 120.90, 114.62, 112.64, 109.82, 108.77, 101.47, 80.49, 55.74, 47.21, 35.82, 30.80, 20.04. One aromatic carbon is missing in the spectra.

HRMS (ESI) calcd for $C_{25}H_{21}NO_3$ $[M + H]^+$ 384.15942, found 384.15960.

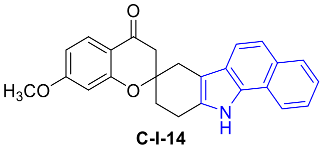

C-I-14

Following the general procedure on a 0.23 mmol scale with a reaction time of 1 h at 100 °C, 59 mg (67%) of **C-I-14** was obtained as a white solid.

^{1}H NMR (700 MHz, $(CD_3)_2SO$) δ 11.72 (s, 1H), 8.27 (d, *J* = 8.2 Hz, 1H), 7.87 (d, *J* = 8.1 Hz, 1H), 7.71 (dd, *J* = 8.8, 1.0 Hz, 1H), 7.52 – 7.46 (m, 2H), 7.39 (d, *J* = 8.5 Hz, 1H), 7.34 (t, *J* = 7.5 Hz, 1H), 6.62 (dd, *J* = 8.9, 2.1 Hz, 1H), 6.49 (d, *J* = 2.4 Hz, 1H), 3.76 (s, 3H), 3.15 (d, *J* = 15.7 Hz, 1H), 3.03 (d, *J* = 15.7 Hz, 1H), 3.00 – 2.93 (m, 2H), 2.91 – 2.83 (m, 2H), 2.34 (dt, *J* = 13.2, 5.3 Hz, 1H), 2.13 – 2.05 (m, 1H).

^{13}C NMR (176 MHz, $(CD_3)_2SO$) δ 190.13, 165.88, 161.29, 130.90, 130.30, 129.33, 128.35, 127.58, 125.05, 122.91, 122.44, 121.72, 120.25, 118.85, 118.23, 114.05, 109.45, 106.68, 101.26, 80.32, 55.75, 46.04, 31.56, 30.80, 19.41.

HRMS (ESI) calcd for $C_{25}H_{21}NO_3$ $[M + H]^+$ 384.15942, found 384.15968.

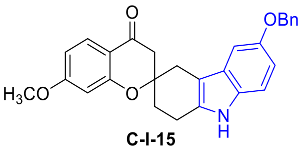

C-I-15

Following the general procedure on a 0.23 mmol scale with a reaction time of 1 h at 100 °C, 48 mg (47%) of **C-I-15** was obtained as a white solid.

^{1}H NMR (400 MHz, $CDCl_3$) δ 7.90 – 7.80 (m, 2H), 7.51 – 7.44 (m, 2H), 7.43 – 7.34 (m, 2H), 7.36 – 7.28 (m, 1H), 7.17 (d, J = 8.7 Hz, 1H), 6.97 (d, J = 2.4 Hz, 1H), 6.88 (dd, J = 8.7, 2.4 Hz, 1H), 6.59 (dd, J = 8.8, 2.4 Hz, 1H), 6.38 (d, J = 2.4 Hz, 1H), 5.09 (s, 2H), 3.79 (s, 3H), 3.17 (d, J = 15.5 Hz, 1H), 3.00 – 2.85 (m, 3H), 2.80 (d, J = 16.6 Hz, 1H), 2.70 (dt, J = 16.5, 5.7 Hz, 1H), 2.44 – 2.33 (m, 1H), 2.03 (ddd, J = 13.8, 8.4, 5.9 Hz, 1H).

^{13}C NMR (176 MHz, $CDCl_3$) δ 190.89, 166.47, 161.85, 153.30, 137.79, 133.01, 131.72, 128.58, 128.34, 127.96, 127.84, 127.65, 114.59, 112.03, 111.39, 109.74, 106.38, 101.79, 101.41, 80.36, 71.04, 55.67, 46.62, 32.38, 31.48, 20.06.

HRMS (ESI) calcd for $C_{28}H_{25}NO_4$ $[M + H]^+$ 440.18563, found 440.18534.

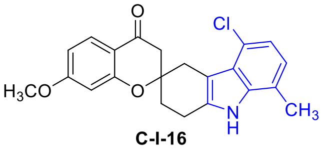

C-I-16

Following the general procedure on a 0.23 mmol scale with a reaction time of 2 h at 120 °C, 19 mg (23%) of **C-I-16** was obtained as a white solid.

^{1}H NMR (500 MHz, $CDCl_3$) δ 7.96 (s, 1H), 7.84 (d, J = 8.8 Hz, 1H), 6.93 (d, J = 7.8 Hz, 1H), 6.81 (d, J = 7.7 Hz, 1H), 6.57 (dd, J = 8.8, 2.4 Hz, 1H), 6.37 (d, J = 2.4 Hz, 1H), 3.79 (s, 3H), 3.62 (dd, J = 16.9, 1.6 Hz, 1H), 3.28 (d, J = 16.9 Hz, 1H), 2.98 (d, J = 16.7 Hz, 1H), 2.95 – 2.87 (m, 1H), 2.80 – 2.68 (m, 2H), 2.45 – 2.36 (m, 4H), 1.97 (ddd, J = 13.5, 9.4, 5.9 Hz, 1H).

^{13}C NMR (126 MHz, $CDCl_3$) δ 190.94, 166.48, 161.79, 136.81, 132.68, 128.43, 124.35, 123.19, 122.73, 120.10, 118.54, 114.53, 109.72, 107.18, 101.38, 80.10, 55.72, 46.94, 34.67, 30.17, 20.01, 16.37.

HRMS (ESI) calcd for $C_{22}H_{20}NO_3Cl$ $[M + H]^+$ 382.12045 and 384.11750, found 382.12076 and 384.11780.

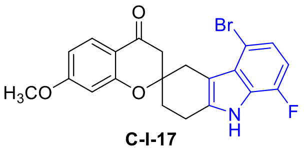

C-I-17

Following the general procedure on a 0.23 mmol scale with a reaction time of 2 h at 120 °C, 29 mg (30%) of **C-I-17** was obtained as a white solid.

^{1}H NMR (700 MHz, $CDCl_3$) δ 8.32 (s, 1H), 7.85 (d, *J* = 8.9 Hz, 1H), 7.07 (dd, *J* = 8.4, 4.0 Hz, 1H), 6.69 (dd, *J* = 10.3, 8.4 Hz, 1H), 6.57 (dd, *J* = 8.9, 2.2 Hz, 1H), 6.36 (d, *J* = 2.4 Hz, 1H), 3.79 (s, 3H), 3.68 (d, *J* = 16.5 Hz, 1H), 3.27 (d, *J* = 16.5 Hz, 1H), 3.00 (d, *J* = 16.6 Hz, 1H), 2.94 – 2.86 (m, 1H), 2.76 (d, *J* = 16.9 Hz, 1H), 2.72 (dt, *J* = 16.6, 5.2 Hz, 1H), 2.45 – 2.38 (m, 1H), 1.94 (ddd, *J* = 13.8, 9.7, 5.8 Hz, 1H).

^{13}C NMR (176 MHz, $CDCl_3$) δ 190.80, 166.56, 161.67, 148.78 (d, *J* = 242.9 Hz), 134.28, 129.43 (d, *J* = 5.3 Hz), 128.48, 125.41 (d, *J* = 14.4 Hz), 123.11 (d, *J* = 6.3 Hz), 114.51, 109.80, 108.20 (d, *J* = 1.9 Hz), 107.65 (d, *J* = 17.8 Hz), 107.57 (d, *J* = 2.8 Hz), 101.41, 79.72, 55.73, 47.06, 34.51, 29.89, 19.98.

^{19}F NMR (470 MHz, $CDCl_3$) δ -137.74 (dd, *J* = 10.3, 4.2 Hz).

HRMS (ESI) calcd for $C_{21}H_{17}NO_3BrF$ $[M + H]^+$ 430.04486 and 432.04281, found 430.04477 and 432.04246.

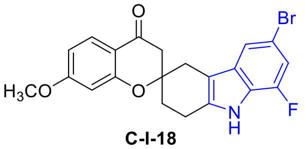

C-I-18

Following the general procedure on a 0.23 mmol scale with a reaction time of 1 h at 90 °C, 42 mg (42%) of **C-I-18** was obtained as a white solid.

^{1}H NMR (700 MHz, $CDCl_3$) δ 8.05 (s, 1H), 7.84 (dd, *J* = 8.7, 1.1 Hz, 1H), 7.30 (s, 1H), 7.01 (d, *J* = 10.3 Hz, 1H), 6.58 (dd, *J* = 8.8, 2.2 Hz, 1H), 6.34 (d, *J* = 2.1 Hz, 1H), 3.80 (s, 3H), 3.16 (d, *J* = 15.9 Hz, 1H), 3.00 – 2.93 (m, 1H), 2.93 – 2.88 (m, 2H), 2.82 – 2.74 (m, 2H), 2.44 (dt, *J* = 13.6, 5.3 Hz, 1H), 2.04 (ddd, *J* = 14.3, 9.2, 5.9 Hz, 1H).

^{13}C NMR (176 MHz, $CDCl_3$) δ 190.46, 166.57, 161.61, 148.68 (d, *J* = 247.6 Hz), 134.32, 132.12 (d, *J* = 6.0 Hz), 128.50, 123.32 (d, *J* = 12.8 Hz), 116.65 (d, *J* = 3.4 Hz), 114.60, 111.52 (d, *J* = 8.1 Hz), 110.48 (d, *J* = 19.9 Hz), 109.88, 107.35 (d, *J* = 2.3 Hz), 101.46, 79.85, 55.76, 46.88, 32.29, 31.21, 19.97.

^{19}F NMR (470 MHz, $CDCl_3$) δ -132.94 (d, *J* = 10.4 Hz).

HRMS (ESI) calcd for $C_{21}H_{17}NO_3BrF[M + H]^+$ 430.04486 and 432.04281, found 430.04477 and 432.04281.

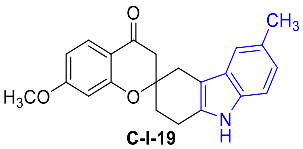

C-I-19

Following the general procedure on a 0.23 mmol scale with a reaction time of 1 h at 80 °C, 15 mg (19%) of **C-I-19** was obtained as a white solid.

^{1}H NMR (500 MHz, $CDCl_3$) δ 7.84 (d, *J* = 8.8 Hz, 1H), 7.73 (s, 1H), 7.20 – 7.17 (m, 2H), 6.96 (dd, *J* = 8.4, 1.5 Hz, 1H), 6.57 (dd, *J* = 8.8, 2.4 Hz, 1H), 6.35 (d, *J* = 2.4 Hz, 1H), 3.79 (s, 3H), 3.18 (dd, *J* = 15.9, 1.5 Hz, 1H), 3.00 – 2.88 (m, 3H), 2.80 (d, *J* = 16.6 Hz, 1H), 2.74 (dt, *J* = 16.3, 5.5 Hz, 1H), 2.45 – 2.35 (m, 4H), 2.12 – 2.01 (m, 1H).

^{13}C NMR (126 MHz, $CDCl_3$) δ 190.90, 166.44, 161.88, 134.74, 132.13, 128.82, 128.38, 127.83, 123.11, 117.66, 114.63, 110.36, 109.75, 106.21, 101.39, 80.42, 55.72, 46.68, 32.36, 31.58, 21.59, 20.11.

HRMS (ESI) calcd for $C_{22}H_{21}NO_3$ $[M + H]^+$ 348.15942, found 348.15952.

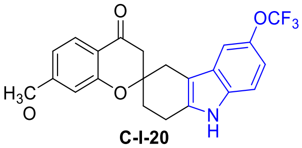

C-I-20

Following the general procedure on a 0.23 mmol scale with a reaction time of 1 h at 100 °C, 80 mg (83%) of **C-I-20** was obtained as a white solid.

^{1}H NMR (500 MHz, $CDCl_3$) δ 8.27 (s, 1H), 7.87 (d, *J* = 8.8 Hz, 1H), 7.28 – 7.24 (m, 2H), 7.03 – 6.97 (m, 1H), 6.60 (dd, *J* = 8.8, 2.4 Hz, 1H), 6.37 (d, *J* = 2.4 Hz, 1H), 3.79 (s, 3H), 3.19 (d, *J* = 14.6 Hz, 1H), 3.00 – 2.89 (m, 3H), 2.80 (d, *J* = 16.6 Hz, 1H), 2.73 (dt, *J* = 16.5, 5.5 Hz, 1H), 2.42 (dt, *J* = 12.8, 5.4 Hz, 1H), 2.03 (ddd, *J* = 14.0, 8.7, 5.8 Hz, 1H).

^{13}C NMR (126 MHz, $CDCl_3$) δ 190.87, 166.57, 161.76, 142.90 (q, *J* = 2.1 Hz), 134.72, 134.34, 128.38, 127.76, 120.90 (q, *J* = 255.2 Hz), 115.34, 114.49, 111.20, 110.39, 109.84, 106.94, 101.42, 80.09, 55.69, 46.62, 32.31, 31.14, 19.98.

^{19}F NMR (470 MHz, $CDCl_3$) δ -57.98.

HRMS (ESI) calcd for $C_{22}H_{18}NO_4F_3$ $[M + H]^+$ 418.12607, found 418.12567.

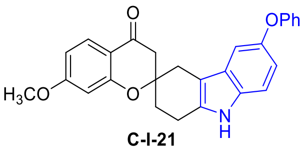

C-I-21

Following the general procedure on a 0.23 mmol scale with a reaction time of 1 h at 80 °C, 83 mg (84%) of **C-I-21** was obtained as a white solid.

^{1}H NMR (700 MHz, $CDCl_3$) δ 8.07 (s, 1H), 7.85 (d, *J* = 8.8 Hz, 1H), 7.28 (t, *J* = 7.8 Hz, 4H), 7.25 (d, *J* = 8.6 Hz, 3H), 7.07 (s, 1H), 7.02 (t, *J* = 7.3 Hz, 1H), 6.96 (d, *J* = 8.1 Hz, 2H), 6.90 (d, *J* = 8.6 Hz, 1H), 6.58 (d, *J* = 8.8 Hz, 1H), 6.37 (s, 1H), 3.78 (s, 3H), 3.13 (d, *J* = 15.8 Hz, 1H), 2.97 – 2.88 (m, 3H), 2.78 (d, *J* = 16.6 Hz, 1H), 2.72 (dt, *J* = 16.4, 5.8 Hz, 1H), 2.44 – 2.37 (m, 1H), 2.06 – 1.98 (m, 1H).

^{13}C NMR (176 MHz, $CDCl_3$) δ 190.85, 166.48, 161.78, 159.46, 149.88, 133.58, 133.38, 129.58, 128.35, 128.28, 122.01, 117.40, 115.07, 114.53, 111.55, 109.75, 108.70, 106.67, 101.42, 80.24, 55.66, 46.58, 32.46, 31.23, 20.06.

HRMS (ESI) calcd for $C_{27}H_{23}NO_4$ $[M + H]^+$ 426.16998, found 426.16965.

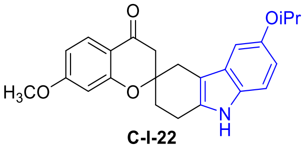

C-I-22

Following the general procedure on a 0.23 mmol scale with a reaction time of 1 h at 80 °C, 69 mg (77%) of **C-I-22** was obtained as a brown solid.

^{1}H NMR (700 MHz, $CDCl_3$) δ 7.92 (s, 1H), 7.85 (d, *J* = 8.8 Hz, 1H), 7.15 (d, *J* = 8.7 Hz, 1H), 6.90 (s, 1H), 6.78 (d, *J* = 8.7 Hz, 1H), 6.57 (dd, *J* = 8.8, 2.0 Hz, 1H), 6.37 (s, 1H), 4.49 (hept, *J* = 6.0 Hz, 1H), 3.78 (s, 3H), 3.15 (d, *J* = 15.8 Hz, 1H), 2.98 – 2.87 (m, 3H), 2.79 (d, *J* = 16.5 Hz, 1H), 2.69 (dt, *J* = 16.3, 5.7 Hz, 1H), 2.38 (dt, *J* = 13.0, 5.6 Hz, 1H), 2.07 – 1.97 (m, 1H), 1.34 (d, *J* = 6.2 Hz, 6H).

^{13}C NMR (176 MHz, $CDCl_3$) δ 190.92, 166.46, 161.85, 151.97, 132.95, 131.84, 128.32, 128.05, 114.57, 113.56, 111.27, 109.73, 106.21, 104.25, 101.40, 80.38, 71.53, 55.66, 46.63, 32.40, 31.48, 22.38, 22.37, 20.05.

HRMS (ESI) calcd for $C_{24}H_{25}NO_4$ $[M + H]^+$ 392.18563, found 392.18526.

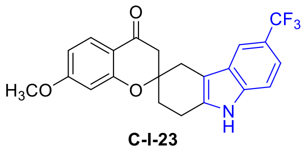

C-I-23

Following the general procedure on a 0.23 mmol scale with a reaction time of 2 h at 130 °C, 74 mg (80%) of **C-I-23** was obtained as a tan solid.

^{1}**H NMR** (500 MHz, $CDCl_3$) δ 8.19 (s, 1H), 7.85 (d, *J* = 8.8 Hz, 1H), 7.68 (s, 1H), 7.39 – 7.33 (m, 2H), 6.58 (dd, *J* = 8.8, 2.4 Hz, 1H), 6.35 (d, *J* = 2.3 Hz, 1H), 3.79 (s, 3H), 3.23 (d, *J* = 15.7 Hz, 1H), 3.04 – 2.90 (m, 3H), 2.83 – 2.72 (m, 2H), 2.47 – 2.39 (m, 1H), 2.05 (ddd, *J* = 13.4, 8.8, 5.9 Hz, 1H).

^{13}**C NMR** (126 MHz, $CDCl_3$) δ 190.71, 166.56, 161.71, 137.83, 134.06, 128.46, 127.01, 125.49 (q, *J* = 271.4 Hz), 121.94 (q, *J* = 31.8 Hz), 118.43 (q, *J* = 3.6 Hz), 115.50 (q, *J* = 4.1 Hz), 114.55, 110.83, 109.85, 107.50, 101.45, 80.02, 55.74, 46.70, 32.22, 31.22, 20.01.

^{19}**F NMR** (470 MHz, $CDCl_3$) δ -60.17.

HRMS (ESI) calcd for $C_{22}H_{18}NO_3F_3$ $[M + H]^+$ 402.13115, found 402.13073.

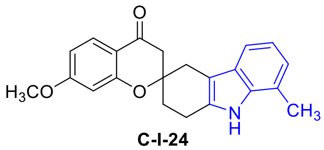

C-I-24

Following the general procedure on a 0.23 mmol scale with a reaction time of 1 h at 90 °C, 56 mg (70%) of **C-I-24** was obtained as a tan solid.

^{1}**H NMR** (500 MHz, $CDCl_3$) δ 7.96 (s, 1H), 7.86 (d, *J* = 8.8 Hz, 1H), 7.27 (d, *J* = 7.1 Hz, 1H), 7.02 (t, *J* = 7.5 Hz, 1H), 6.96 (d, *J* = 7.1 Hz, 1H), 6.58 (dd, *J* = 8.8, 2.4 Hz, 1H), 6.37 (d, *J* = 2.4 Hz, 1H), 3.80 (s, 3H), 3.21 (d, *J* = 16.2 Hz, 1H), 3.03 – 2.89 (m, 3H), 2.83 – 2.72 (m, 2H), 2.48 (s, 3H), 2.45 – 2.37 (m, 1H), 2.06 (ddd, *J* = 13.9, 8.4, 5.9 Hz, 1H).

^{13}**C NMR** (126 MHz, $CDCl_3$) δ 190.95, 166.43, 161.86, 135.92, 131.70, 128.33, 127.05, 122.29, 119.88, 119.70, 115.50, 114.55, 109.72, 107.00, 101.36, 80.37, 55.67, 46.61, 32.47, 31.46, 20.05, 16.76.

HRMS (ESI) calcd for $C_{22}H_{21}NO_3$ $[M + H]^+$ 348.15942, found 348.15951.

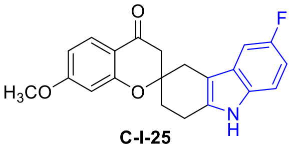

C-I-25

Following the general procedure on a 0.23 mmol scale with a reaction time of 1 h at 90 °C, 56 mg (69%) of **C-I-25** was obtained as a white solid.

^{1}H NMR (500 MHz, $CDCl_3$) δ 7.90 (s, 1H), 7.84 (d, *J* = 8.8 Hz, 1H), 7.20 (dd, *J* = 8.7, 4.3 Hz, 1H), 7.03 (dd, *J* = 9.5, 2.5 Hz, 1H), 6.87 (td, *J* = 9.1, 2.5 Hz, 1H), 6.57 (dd, *J* = 8.8, 2.4 Hz, 1H), 6.36 (d, *J* = 2.4 Hz, 1H), 3.79 (s, 3H), 3.16 (d, *J* = 14.5 Hz, 1H), 2.98 – 2.89 (m, 3H), 2.83 – 2.69 (m, 2H), 2.45 – 2.37 (m, 1H), 2.04 (ddd, *J* = 13.5, 8.9, 5.9 Hz, 1H).

^{13}C NMR (126 MHz, $CDCl_3$) δ 190.78, 166.51, 161.76, 157.93 (d, *J* = 234.4 Hz), 134.10, 132.87, 128.43, 127.98 (d, *J* = 9.7 Hz), 114.57, 111.19 (d, *J* = 9.7 Hz), 109.81, 109.62 (d, *J* = 26.1 Hz), 106.85 (d, *J* = 4.5 Hz), 103.05 (d, *J* = 23.6 Hz), 101.41, 80.18, 55.74, 46.72, 32.41, 31.24, 20.10.

^{19}F NMR (470 MHz, $CDCl_3$) δ -124.69 (td, *J* = 9.4, 4.3 Hz).

HRMS (ESI) calcd for $C_{21}H_{18}NO_3F$ $[M + H]^+$ 352.13435, found 352.13435.

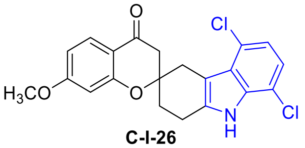

C-I-26

Following the general procedure on a 0.23 mmol scale with a reaction time of 2 h at 100 °C, 46 mg (50%) of **C-I-26** was obtained as a white solid.

^{1}H NMR (400 MHz, $CDCl_3$) δ 8.36 (s, 1H), 7.85 (d, *J* = 8.8 Hz, 1H), 6.99 (d, *J* = 8.2 Hz, 1H), 6.92 (d, *J* = 8.2 Hz, 1H), 6.57 (dd, *J* = 8.8, 2.4 Hz, 1H), 6.36 (d, *J* = 2.4 Hz, 1H), 3.78 (s, 3H), 3.60 (d, *J* = 16.6 Hz, 1H), 3.22 (d, *J* = 16.7 Hz, 1H), 3.03 – 2.86 (m, 2H), 2.80 – 2.68 (m, 2H), 2.47 – 2.36 (m, 1H), 1.94 (ddd, *J* = 13.5, 9.6, 5.9 Hz, 1H).

^{13}C NMR (126 MHz, $CDCl_3$) δ 190.70, 166.53, 161.64, 134.29, 133.99, 128.50, 126.11, 124.24, 121.29, 120.72, 114.80, 114.51, 109.84, 107.98, 101.36, 79.77, 55.74, 47.06, 34.43, 30.01, 19.93.

HRMS (ESI) calcd for $C_{21}H_{17}NO_3Cl_2$ $[M + H]^+$ 402.06583, 404.06288, and 406.05993, found 402.06565, 404.06255, and 406.05931.

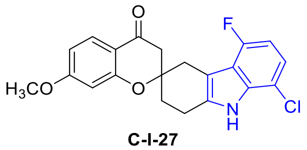

C-I-27

Following the general procedure on a 0.23 mmol scale with a reaction time of 2 h at 100 °C, 41 mg (47%) of **C-I-27** was obtained as a white solid.

^{1}H NMR (400 MHz, $CDCl_3$) δ 8.26 (s, 1H), 7.85 (d, *J* = 8.8 Hz, 1H), 6.98 (dd, *J* = 8.4, 4.1 Hz, 1H), 6.65 (dd, *J* = 10.1, 8.4 Hz, 1H), 6.57 (dd, *J* = 8.8, 2.4 Hz, 1H), 6.36 (d, *J* = 2.4 Hz, 1H), 3.79 (s, 3H), 3.38 (d, *J* = 16.6 Hz, 1H), 3.10 (d, *J* = 16.6 Hz, 1H), 3.00 – 2.87 (m, 2H), 2.83 – 2.69 (m, 2H), 2.47 – 2.38 (m, 1H), 1.99 (ddd, *J* = 13.6, 9.3, 5.9 Hz, 1H).

^{13}C NMR (126 MHz, $CDCl_3$) δ 190.66, 166.53, 161.66, 155.32 (d, *J* = 245.1 Hz), 135.47 (d, *J* = 12.5 Hz), 132.83, 128.49, 120.91 (d, *J* = 8.0 Hz), 117.43 (d, *J* = 22.3 Hz), 114.53, 111.30 (d, *J* = 3.5 Hz), 109.86, 106.18, 105.69 (d, *J* = 21.2 Hz), 101.36, 79.82, 55.74, 46.86, 33.67, 30.59, 19.82.

^{19}F NMR (470 MHz, $CDCl_3$) δ -126.93 (d, *J* = 10.1 Hz).

HRMS (ESI) calcd for $C_{21}H_{17}NO_3ClF$ $[M + H]^+$ 386.09538 and 388.09243, found 386.09589 and 388.09288.

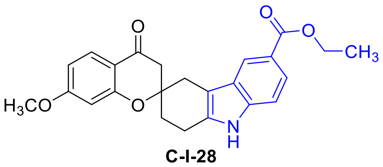

C-I-28

Following the general procedure on a 0.23 mmol scale with a reaction time of 2 h at 130 °C employing 4-hydrazinobenzoic acid HCl, an esterification occurred with ethanol to afford 75 mg (80%) of the ethyl ester **C-I-28** as a white solid.

^{1}H NMR (700 MHz, $CDCl_3$) δ 8.35 (s, 1H), 8.15 (s, 1H), 7.85 (t, J = 7.9 Hz, 2H), 7.29 (d, J = 8.5 Hz, 1H), 6.57 (dd, J = 8.8, 2.4 Hz, 1H), 6.35 (d, J = 2.4 Hz, 1H), 4.38 (q, J = 7.1 Hz, 2H), 3.78 (s, 3H), 3.23 (d, J = 16.0 Hz, 1H), 3.00 (d, J = 16.0 Hz, 1H), 2.98 – 2.90 (m, 2H), 2.79 (d, J = 16.5 Hz, 1H), 2.74 (dt, J = 16.5, 5.6 Hz, 1H), 2.41 (dt, J = 13.4, 5.5 Hz, 1H), 2.05 (ddd, J = 14.0, 8.6, 5.9 Hz, 1H), 1.40 (t, J = 7.1 Hz, 3H).

^{13}C NMR (176 MHz, $CDCl_3$) δ 190.84, 168.04, 166.64, 161.86, 139.28, 133.72, 128.51, 127.34, 123.29, 121.92, 120.66, 114.69, 110.45, 109.90, 107.97, 101.55, 80.22, 60.81, 55.81, 46.78, 32.33, 31.46, 20.11, 14.69.

HRMS (ESI) calcd for $C_{24}H_{23}NO_5$ $[M + H]^+$ 406.16490, found 406.16468.

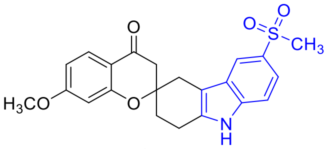

C-I-29

Following the general procedure on a 0.23 mmol scale with a reaction time of 4 h at 130 °C, 83 mg (88%) of **C-I-29** was obtained as a white solid.

^{1}H NMR (700 MHz, $CDCl_3$) δ 8.45 (s, 1H), 8.01 (s, 1H), 7.85 (d, J = 8.8 Hz, 1H), 7.66 (d, J = 8.5 Hz, 1H), 7.40 (d, J = 8.5 Hz, 1H), 6.58 (dd, J = 8.8, 2.3 Hz, 1H), 6.35 (d, J = 2.4 Hz, 1H), 3.79 (d, J = 0.9 Hz, 3H), 3.22 (d, J = 16.0 Hz, 1H), 3.06 (s, 3H), 3.02 – 2.94 (m, 2H), 2.92 (d, J = 16.9 Hz, 1H), 2.78 (dd, J = 16.6, 6.0 Hz, 2H), 2.44 (dt, J = 13.5, 5.5 Hz, 1H), 2.09 – 2.03 (m, 1H).

^{13}C NMR (176 MHz, $CDCl_3$) δ 190.48, 166.59, 161.60, 138.92, 135.24, 131.43, 128.52, 127.39, 120.19, 118.55, 114.61, 111.31, 109.82, 108.21, 101.55, 79.89, 55.76, 46.65, 45.40, 32.20, 31.15, 20.06.

HRMS (ESI) calcd for $C_{22}H_{21}NO_5S$ $[M + H]^+$ 412.12132, found 412.12113.

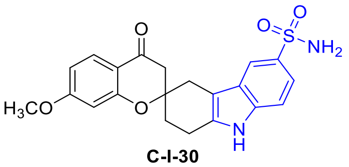

C-I-30

Following the general procedure on a 0.23 mmol with a reaction time of 2 h at 130 °C, 47 mg (49%) of **C-I-30** was obtained as a brown solid.

A mixture of 3 $CDCl_3$:1 CD_3OD was used as NMR solvent. **^{1}H NMR** (700 MHz, CD_3OD) δ 7.91 (s, 1H), 7.75 (d, J = 8.9 Hz, 1H), 7.55 (dd, J = 8.5, 1.8 Hz, 1H), 7.31 (d, J = 8.5 Hz, 1H), 6.52 (dd, J = 8.9, 2.4 Hz, 1H), 6.30 (d, J = 2.3 Hz, 1H), 3.74 (s, 3H), 3.14 (d, J = 15.3 Hz, 1H), 2.95 – 2.87 (m, 2H), 2.84 (d, J = 16.6 Hz, 1H), 2.76 – 2.68 (m, 2H), 2.35 (dt, J = 13.8, 5.7 Hz, 1H), 2.03 – 1.99 (m, 1H).

^{13}C NMR (176 MHz, CD_3OD) δ 191.36, 166.62, 161.76, 138.38, 135.00, 132.43, 128.22, 126.75, 118.72, 116.74, 114.25, 110.96, 109.72, 107.00, 101.32, 79.96, 55.54, 46.30, 31.93, 31.04, 19.67.

HRMS (ESI) calcd for $C_{21}H_{20}N_2O_5S$ $[M + H]^+$ 413.11657, found 413.11646.

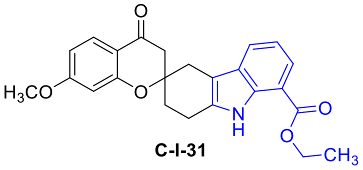

C-I-31

Following the general procedure on a 0.23 mmol scale with a reaction time of 2 h at 130 °C employing 2-hydrazinobenzoic acid HCl, an esterification occurred with ethanol to afford 47 mg (50%) of the ethyl ester **C-I-31** as a white solid.

^{1}H NMR (500 MHz, $CDCl_3$) δ 9.58 (s, 1H), 7.87 – 7.78 (m, 2H), 7.59 (d, J = 7.7 Hz, 1H), 7.10 (t, J = 7.7 Hz, 1H), 6.57 (dd, J = 8.8, 2.4 Hz, 1H), 6.35 (d, J = 2.4 Hz, 1H), 4.44 (q, J = 7.1 Hz, 2H), 3.78 (s, 3H), 3.22 (d, J = 15.9 Hz, 1H), 3.04 – 2.96 (m, 2H), 2.93 (d, J = 16.6 Hz, 1H), 2.85 – 2.75 (m, 2H), 2.49 – 2.39 (m, 1H), 2.05 (ddd, J = 14.1, 8.9, 6.0 Hz, 1H), 1.45 (t, J = 7.1 Hz, 3H).

^{13}C NMR (126 MHz, $CDCl_3$) δ 190.69, 167.72, 166.44, 161.73, 136.54, 133.40, 128.70, 128.36, 123.54, 123.19, 118.67, 114.56, 112.25, 109.82, 106.39, 101.32, 80.14, 60.83, 55.69, 46.79, 32.18, 31.37, 19.95, 14.55.

HRMS (ESI) calcd for $C_{24}H_{23}NO_5$ $[M + H]^+$ 406.16490, found 406.16459.

Synthesis and compound data of sinomenine-based pyrimadines (**SM-P**):

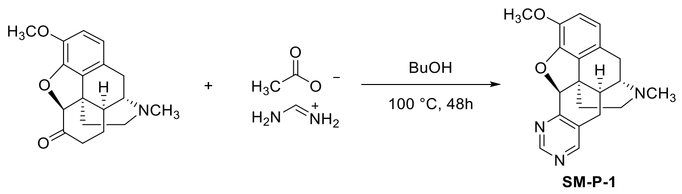

The synthetic procedure was adapted from Baran et. al.[23]

The sinomenine-derived ketone (50 mg, 0.17 mmol, 1 equiv) and formamidine acetate (347 mg, 3.34 mmol, 20 equiv) were suspended in BuOH (17 ml) and heated to 100 °C for 48 hours. The reaction was cooled and concentrated. The crude residue was suspended in EtOAc (15 ml) and triethylamine (1.42 ml, 60 equiv) was added dropwise with stirring. The solution was then concentrated and purified by silica chromatography (45% *strong* magic mix (25 DCM: 8 EtOH: 1 NH_3) in DCM) to obtain 29 mg (52%) of **SM-P-1** as a white solid.

^{1}H NMR (700 MHz, $CDCl_3$) δ 9.13 (s, 1H), 8.41 (s, 1H), 6.68 (d, *J* = 8.2 Hz, 1H), 6.65 (d, *J* = 8.1 Hz, 1H), 5.38 (s, 1H), 3.80 (s, 3H), 3.28 (dd, *J* = 6.2, 2.9 Hz, 1H), 3.12 (d, *J* = 18.7 Hz, 1H), 2.63 – 2.58 (m, 2H), 2.54 (dd, *J* = 15.9, 5.1 Hz, 1H), 2.48 – 2.44 (m, 4H), 2.37 (td, *J* = 12.3, 3.7 Hz, 1H), 2.21 (dd, *J* = 15.8, 12.5 Hz, 1H), 2.07 (td, *J* = 12.4, 5.0 Hz, 1H), 2.00 – 1.96 (m, 1H).

^{13}C NMR (176 MHz, $CDCl_3$) δ 161.02, 157.92, 157.90, 145.29, 143.08, 130.16, 128.17, 126.86, 119.61, 114.62, 89.42, 59.11, 56.82, 46.49, 43.18, 42.59, 39.64, 35.91, 26.14, 20.21.

HRMS (ESI) calcd for $C_{20}H_{21}N_3O_2$ $[M + H]^+$ 336.17065, found 336.17084.

$[\alpha]_D^{20}$ +316 (*c* 0.1, MeOH).

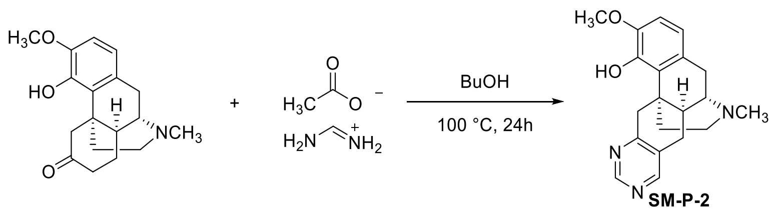

The synthetic procedure was adapted from Baran et. al.[23]

The sinomenine-derived ketone (50 mg, 0.17 mmol, 1 equiv) and formamidine acetate (347 mg, 3.34 mmol, 20 equiv) were suspended in BuOH (17 ml) and heated to 100 °C for 24 hours. The reaction was cooled and concentrated. The crude residue was suspended in EtOAc (15 ml) and triethylamine (1.42 ml, 60 equiv) was added dropwise with stirring. The solution was then concentrated and purified by silica chromatography (60% *strong* magic mix (25 DCM: 8 EtOH: 1 NH_3) in DCM) to obtain 42 mg (75%) of **SM-P-2** as a white solid.

^{1}H NMR (700 MHz, $CDCl_3$) δ 8.85 (s, 1H), 8.21 (s, 1H), 6.86 (s, 1H), 6.62 (d, J = 8.3 Hz, 1H), 6.59 (d, J = 8.1 Hz, 1H), 4.93 (d, J = 17.9 Hz, 1H), 3.75 (s, 3H), 3.10 (dd, J = 6.4, 3.2 Hz, 1H), 3.05 (d, J = 18.7 Hz, 1H), 2.85 (dd, J = 18.7, 6.5 Hz, 1H), 2.73 (td, J = 17.6, 3.8 Hz, 2H), 2.62 (dd, J = 17.0, 12.2 Hz, 1H), 2.56 – 2.51 (m, 1H), 2.43 (s, 3H), 2.34 – 2.28 (m, 1H), 2.17 (td, J = 12.4, 3.1 Hz, 1H), 2.08 – 2.03 (m, 1H), 1.84 (td, J = 12.8, 4.7 Hz, 1H).

^{13}C NMR (176 MHz, $CDCl_3$) δ 165.72, 156.16, 155.95, 144.93, 144.84, 130.89, 129.01, 123.48, 118.48, 109.01, 56.62, 56.07, 46.96, 43.01, 42.79, 41.92, 38.49, 35.71, 26.46, 23.42.

HRMS (ESI) calcd for $C_{20}H_{23}N3_2O_2$ $[M + H]^+$ 338.18630, found 338.18643.

$[\alpha]_D^{20}$ +106 (*c* 0.2, MeOH).

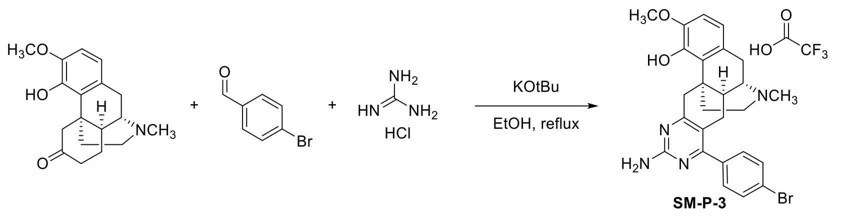

The synthetic procedure was adapted from Saikia et. al.[24]

In a flame-dried RBF under an atmosphere of argon was added the sinomenine-derived ketone (90 mg, 0.30 mmol, 1 equiv), 4-bromobenzaldehyde (55 mg, 0.30 mmol, 1 equiv), KO*t*Bu (67 mg, 0.60 mmol, 2 equiv), and EtOH (3 ml). The mixture was refluxed of 30 min, followed by the addition of amidine HCl (29 mg, 0.30 mmol, 1 equiv) and refluxed for another 16 h. After this time, the reaction was cooled and passed through a silica plug with 9 DCM: 1 MeOH (20 ml) and concentrated. Purification by silica chromatography (80% *strong* magic mix (25 DCM: 8 EtOH: 1 NH_3) in DCM) afforded a mixture of compounds. The reaction was further purified by preparative HPLC (MeOH/water) to afford 7 mg (4%) of **SM-P-3** as a TFA salt.

^{1}H NMR (700 MHz, $CDCl_3$) δ 13.24 (bs, 1H), 7.50 (d, *J* = 8.5 Hz, 2H), 7.18 – 7.15 (m, 2H), 6.68 (d, *J* = 8.4 Hz, 1H), 6.60 (d, *J* = 8.3 Hz, 1H), 4.99 (d, *J* = 18.7 Hz, 1H), 3.76 (s, 3H), 3.69 – 3.62 (m, 1H), 3.23 – 3.15 (m, 2H), 2.93 (d, *J* = 19.7 Hz, 1H), 2.80 – 2.68 (m, 5H), 2.64 (dd, *J* = 16.5, 5.7 Hz, 1H), 2.57 – 2.49 (m, 1H), 2.21 (dd, *J* = 16.2, 11.7 Hz, 1H), 2.18 – 2.08 (m, 2H).

^{13}C NMR (176 MHz, $CDCl_3$) δ 155.96, 146.15, 144.90, 133.73, 132.21, 130.13, 126.02, 125.47, 120.06, 119.36, 117.39, 115.74, 114.21, 110.43, 58.04, 56.25, 47.44, 41.09, 38.86, 38.08, 35.67, 33.77, 25.44, 23.44.

HRMS (ESI) calcd for $C_{26}H_{27}N_4O_2Br$ $[M + H]^+$ 507.13902 and 509.13697, found 507.13882 and 509.13680.

$[\alpha]_D^{20}$ +21 (*c* 0.1, MeOH).

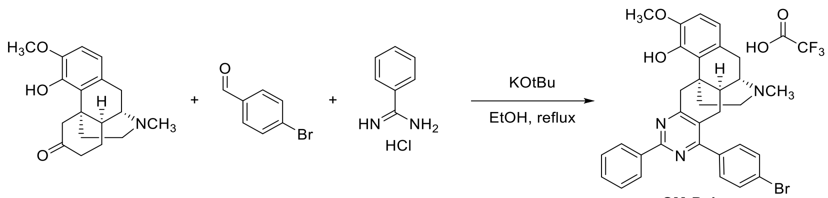

SM-P-4

The synthetic procedure was adapted from Saikia et. al.[24]

In a flame-dried RBF under an atmosphere of argon was added the sinomenine-derived ketone (100 mg, 0.33 mmol, 1 equiv), 4-bromobenzaldehyde (61 mg, 0.33 mmol, 1 equiv), KO*t*Bu (112 mg, 1.0 mmol, 3 equiv), and EtOH (3 ml). The mixture was refluxed for 1 h, followed by the addition of benzamidine HCl (78 mg, 0.50 mmol, 1.5 equiv) and refluxed for another 16 h. After this time, the reaction was cooled, diluted with DCM and water, and extracted. The water phase was extracted 2 more times. The organic phases were combined, dried, and concentrated. Purification by silica chromatography (30% *strong* magic mix (25 DCM: 8 EtOH: 1 NH_3) in DCM) afforded a mixture of compounds. The reaction was further purified by preparative HPLC (ACN/water) to afford 18 mg (8%) of **S-PM-4** as a TFA salt.

The NMR sample was dissolved in 3 $CDCl_3$: 1 CD_3OD. **^{1}H NMR** (700 MHz, CD_3OD) δ 7.67 – 7.63 (m, 3H), 7.53 – 7.49 (m, 2H), 7.27 (d, *J* = 8.4 Hz, 2H), 6.87 (d, *J* = 8.3 Hz, 1H), 6.75 (d, *J* = 8.5 Hz, 2H), 6.72 (d, *J* = 8.3 Hz, 1H), 5.30 (s, 1H), 5.21 (s, 1H), 3.90 – 3.85 (m, 4H), 3.41 – 3.31 (m, 1H), 3.10 – 2.99 (m, 2H), 2.90 – 2.78 (m, 4H), 2.48 – 2.34 (m, 1H), 2.09 (d, *J* = 14.2 Hz, 1H), 1.90 (d, *J* = 17.7 Hz, 1H), 1.23 – 1.19 (m, 1H).

^{13}C NMR (176 MHz, CD_3OD) δ 157.19, 144.50, 144.42, 137.44, 134.70, 132.68, 129.54, 128.91, 128.28, 127.01, 126.24, 126.07, 123.89, 122.78, 121.12, 116.06, 115.72, 84.42, 60.10, 57.06, 47.33, 41.19, 40.55, 35.15, 32.29, 25.16, 20.87.

HRMS (ESI) calcd for $C_{232}H_{30}N_3O_2Br$ $[M + H]^+$ 568.15942 and 568.15942, found 568.15942 and 570.15734.

$[\alpha]_D^{20}$ +174 (*c* 0.2, DMSO).

Representative NOESY correlations for the determination of diastereomers:

QN-C-S-5

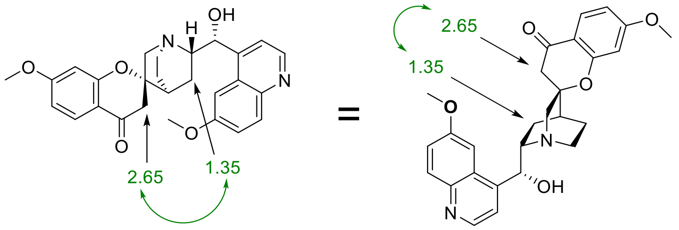

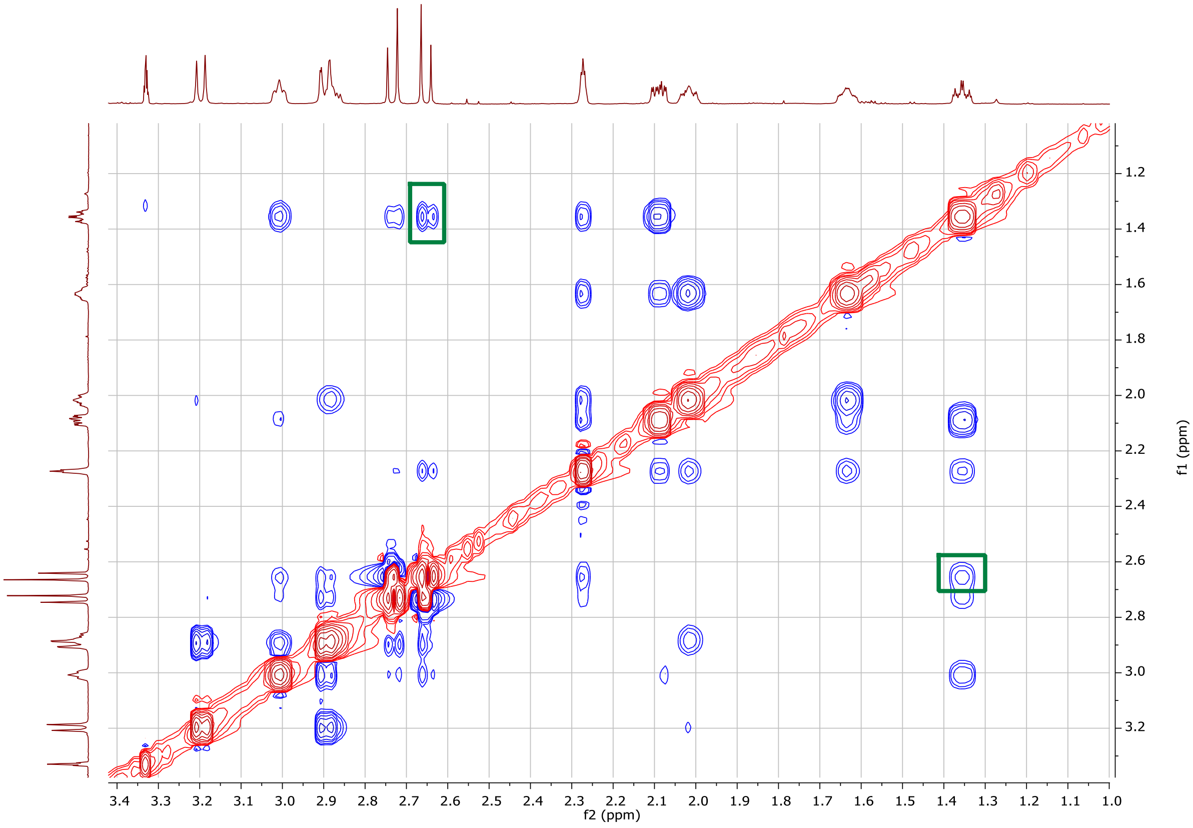

QN-C-R-6

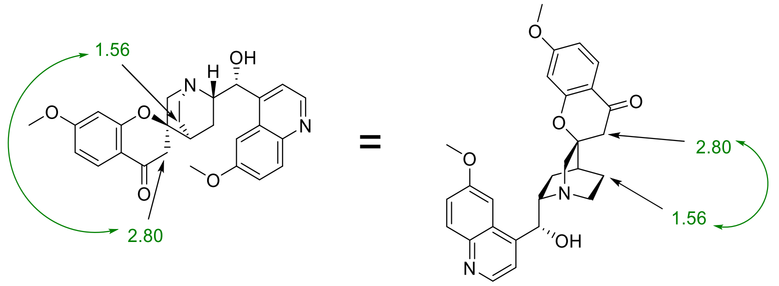

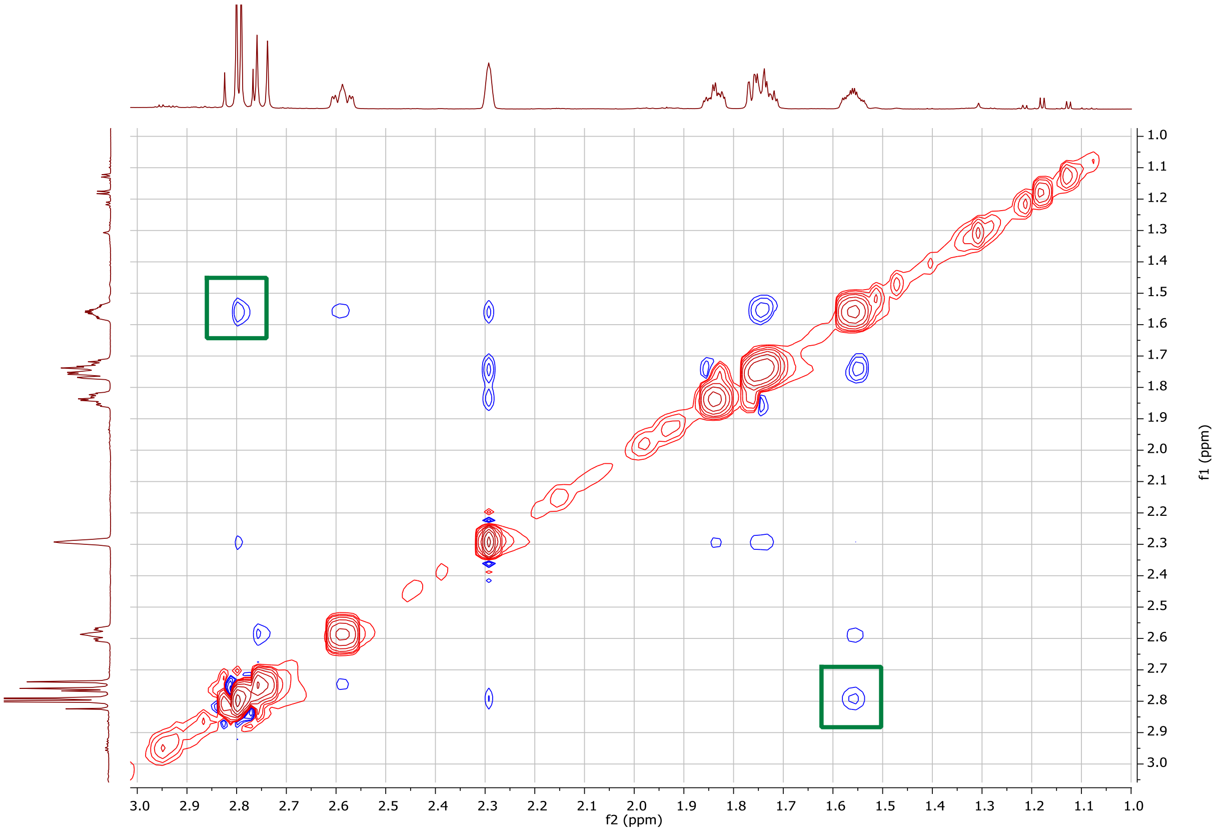

QD-C-S-8

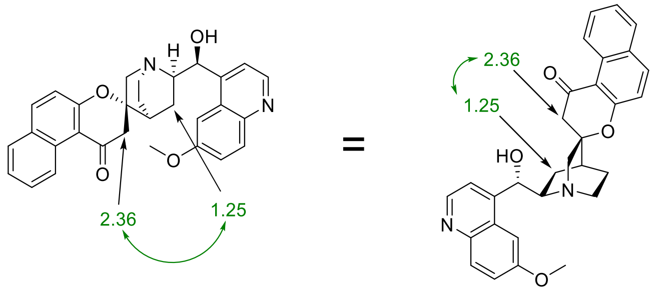

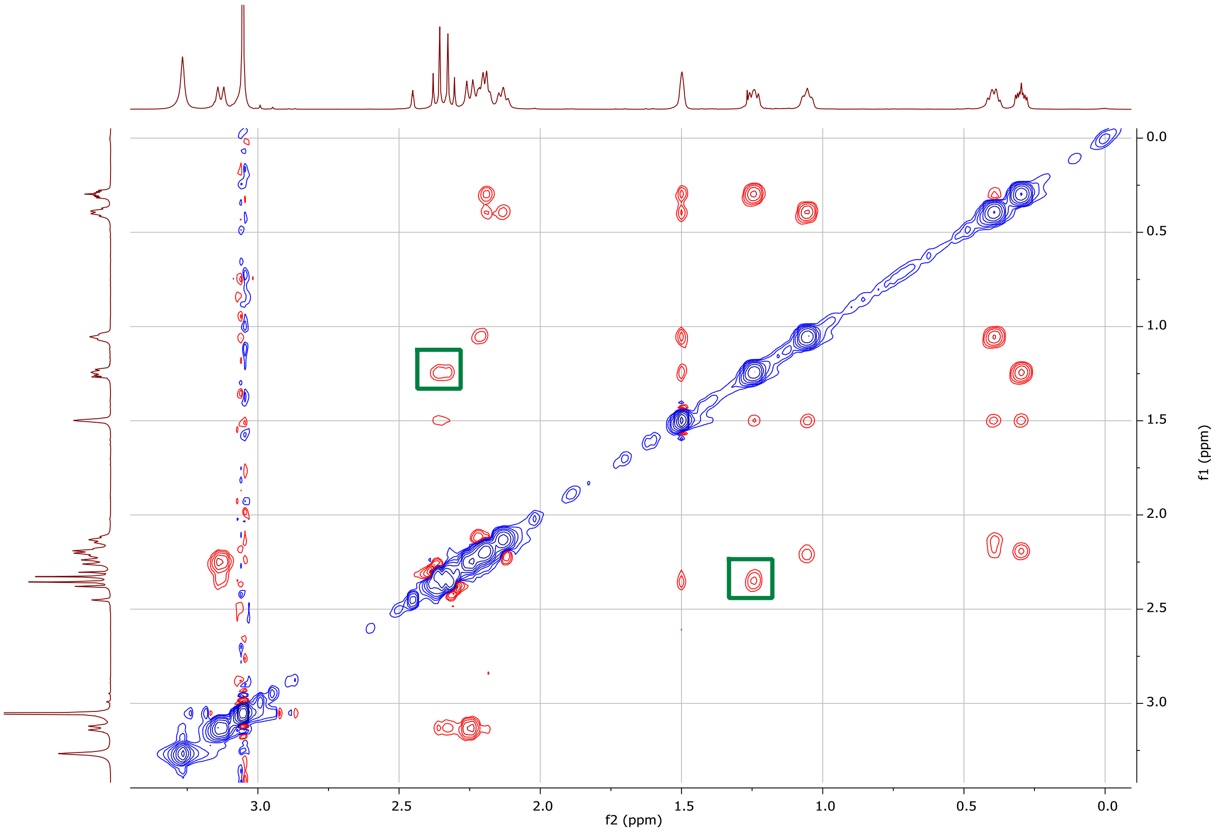

QD-C-R-6

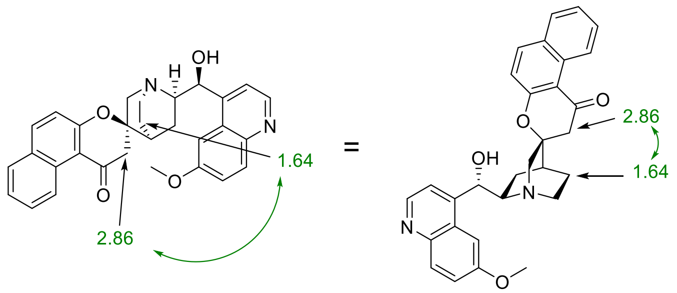

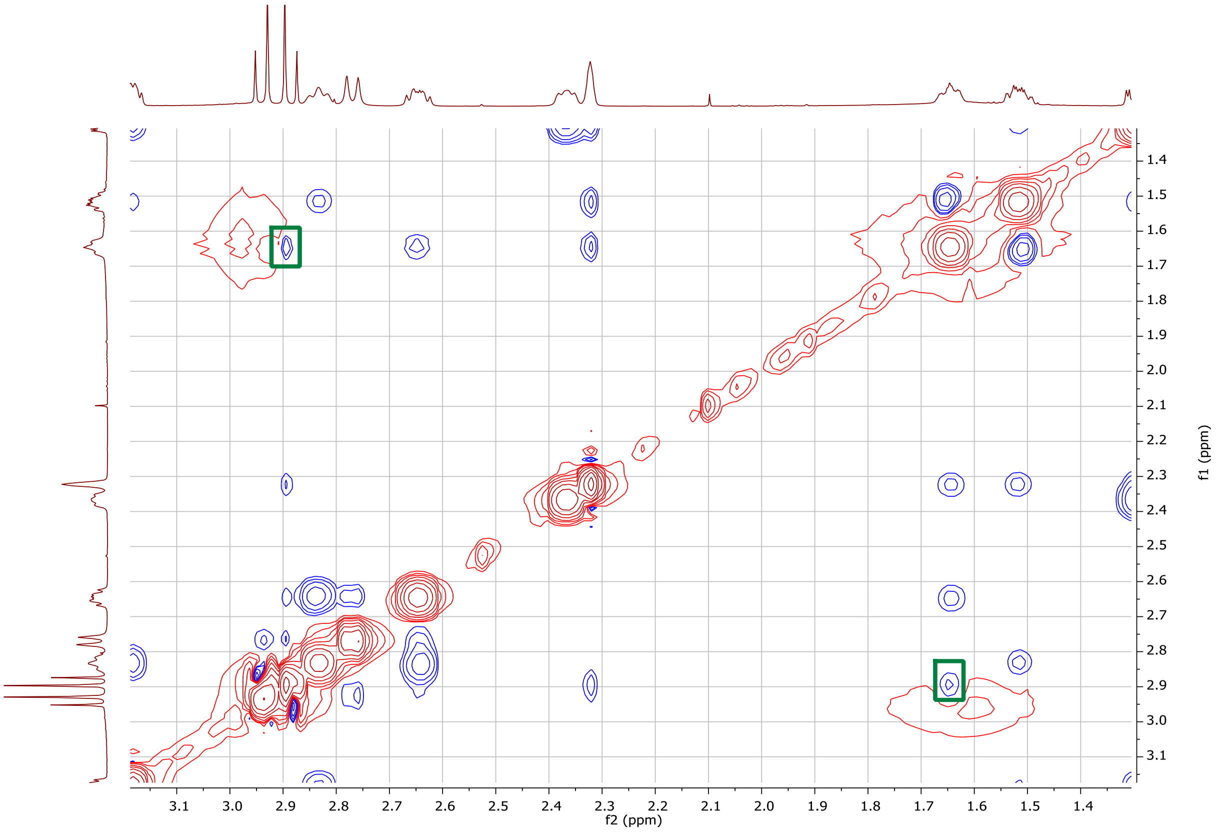

SM-C-S-9

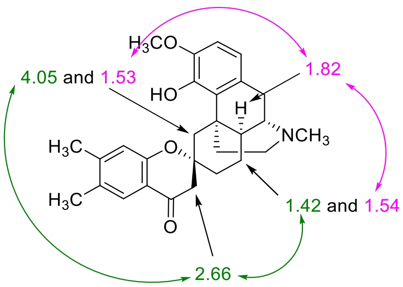
H3CO
HO
H3C
H3C
O
NCH3
H
4.05 and 1.53
1.82
1.42 and 1.54
2.66

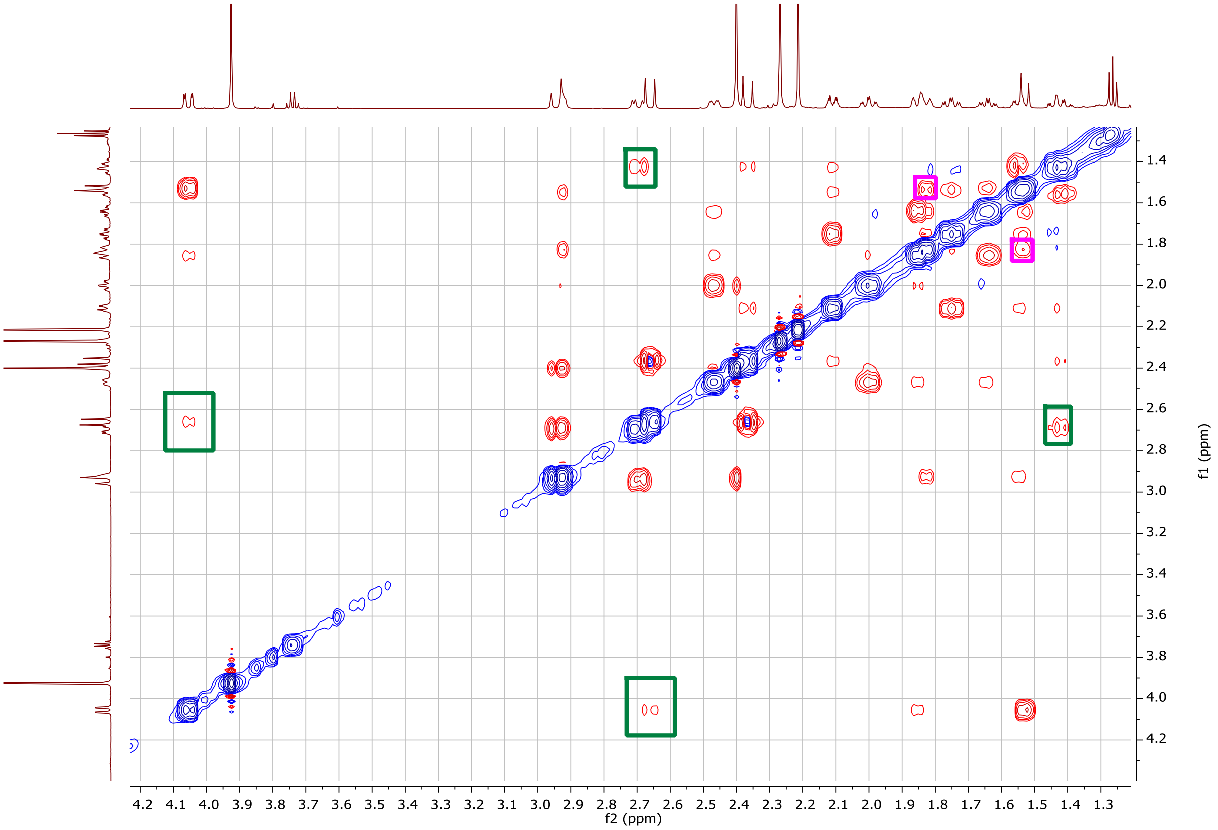
f2 (ppm)
f1 (ppm)

SM-C-R-6

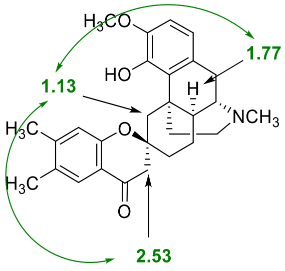

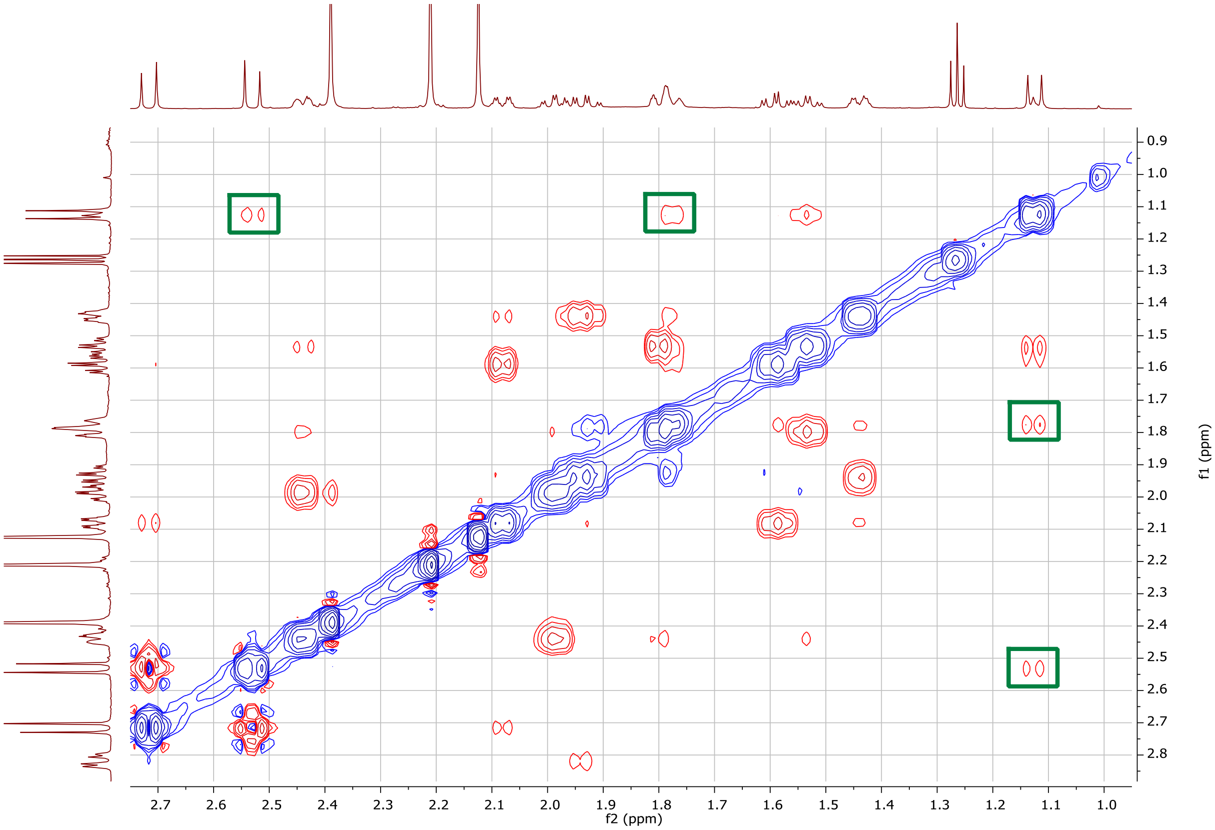

X-ray analysis of compound GF-THPI-7:

The crystal structure of compound **GF-THPI-7** was determined using the *Bruker D8 Venture* four-circle diffractometer equipped with a *PHOTON II* CPAD detector by *Bruker AXS GmbH*. The X-ray radiation was generated by the *IµS/IµS* 3.0 microfocus source Mo (λ = 0.71073 Å) from *Incoatec GmbH* equipped with HELIOS mirror optics and a single-hole collimator by *Bruker AXS GmbH*. The selected single crystal of **GF-THPI-7** was covered with an inert oil (perfluoropolyalkyl ether) and mounted on the *MicroMount* from *MiTeGen*. The APEX 3 Suite (v.2018.7-2) software integrated with SAINT (integration) and SADABS (adsorption correction) programs by *Bruker AXS GmbH* were used for data collection. The processing and finalization of the crystal structure were performed using the Olex2 program.[25] The crystal structures were solved by the ShelXT[26] structure solution program using the Intrinsic Phasing option, which were further refined by the ShelXL[27] refinement package using Least Squares minimization. The non-hydrogen atoms were anisotropically refined. The C-bound H atoms were placed in geometrically calculated positions, and a fixed isotropic displacement parameter was assigned to each atom according to the riding-model: C–H = 0.95–0.99 Å with *U*iso(H) = 1.5*U*eq(CH3) and 1.2*U*eq(CH_2, CH) for other hydrogen atoms. The O-bound hydrogen atoms on O11 and O12 as well as the N-bound hydrogen atoms on N1 and N2 were located on the Difference-Fourier-Map and refined independently. The crystallographic data for the structure of **GF-THPI-7** has been published as supplementary publication number 2047701 in the Cambridge Crystallographic Data Centre. A copy of these data can be obtained for free by applying to CCDC, 12 Union Road, Cambridge CB2 IEZ, UK, fax: 144-(0)1223-336033 or e-mail: deposit@ccdc.cam.ac.uk.

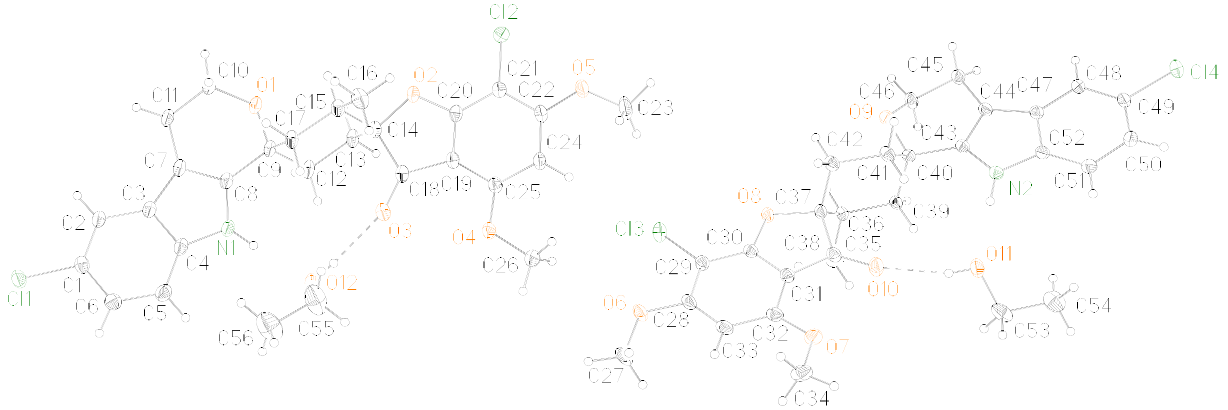

GF-THPI-7

X-ray Figure S1: Ortep plot of the molecular structure in the crystal of compound **GF-THPI-7**.[28] The displacement ellipsoids are drawn at the 50% probability level. Numbering scheme of hydrogen atoms are omitted for clarity.

X-ray Table S1: Crystallographic data of compound GF-THPI-7.

Compound	**GF-THPI-7**
Empirical formula	$C_{28}H_{31}Cl_2NO_6$
Formula weight	548.44
Temperature/K	100.0
Crystal system	triclinic
Space group	*P*1
a/Å	10.2617(18)
b/Å	10.7903(16)
c/Å	12.735(2)
α/°	85.787(9)
β/°	71.947(8)
γ/°	83.796(8)
Volume/Å^3	1331.6(4)
Z	2
ρ_{calc}g/cm^3	1.368
μ/mm^{-1}	0.287
F(000)	576.0
Crystal size/mm^3	0.708 × 0.397 × 0.22
Radiation	MoKα (λ = 0.71073)
2Θ range for data collection/°	3.8 to 61.124
Index ranges	$-14 \le h \le 14$,<br>$-15 \le k \le 15$,<br>$-18 \le l \le 18$
Reflections collected	149561
Independent reflections	16263 [R_{int} = 0.0467, R_{sigma} = 0.0257]
Data/restraints/parameters	16263/3/691
Goodness-of-fit on F^2	1.007
Final R indexes [I>=2σ (I)]	R_1 = 0.0321,<br>wR_2 = 0.0836
Final R indexes [all data]	R_1 = 0.0353,<br>wR_2 = 0.0858
Largest diff. peak/hole / e Å^{-3}	0.51/–0.25
Flack parameter	0.002(9)

HPLC Spectra

QN-I-1

Shimadzu Open Solution

Project: Test
Experiment: 382_180131_Zimmerm_Foley_Christo_0201_Li
Experiment Description:
Sample: 408276:01:01
Sample Description: C24H23N3O2
Data File Name: C:\LabSolutions\Data\382_180131_Zimmermmann_180131_Foley_180131_Christoforow_180201_Li\1046410347_11180201382.lcd
Sample Location: Plate Number: 2 - Position: 2
Run By: admin
Run Started: 12 February 2018 19:19:51
Run Finished: 13 February 2018 04:57:29
Method: Purity_Check+Pause

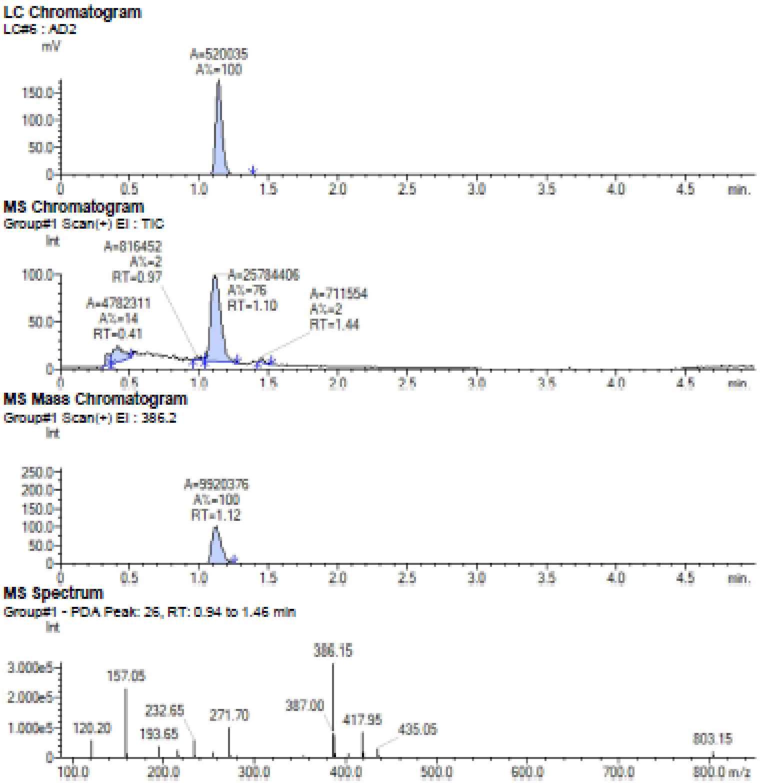

QN-I-2

Shimadzu Open Solution

Project: Test
Experiment: 382_180131_Zimmerm_Foley_Christo_0201_LI
Experiment Description:
Sample: 408277:01:01
Sample Description: C25H25N3O2
Data File Name: C:\LabSolutions\Data\382_180131_Zimmermmann_180131_Foley_180131_Christoforow_180201_LI\1046410419_11180201382.lcd
Sample Location: Plate Number: 2 - Position: 50
Run By: admin
Run Started: 12 February 2018 19:19:51
Run Finished: 13 February 2018 04:57:29
Method: Purity_Check+Pause

LC Chromatogram
LC#6 : AD2

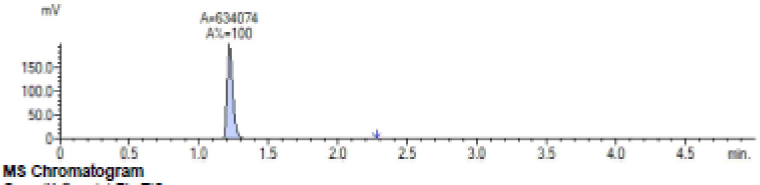

MS Chromatogram
Group#1 Scan(+) EI : TIC

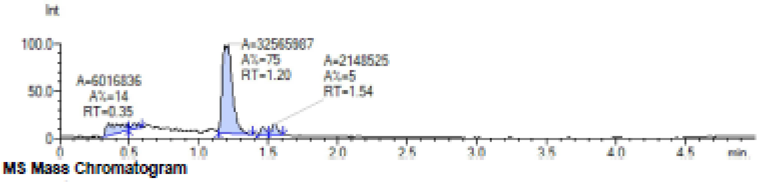

MS Mass Chromatogram
Group#1 Scan(+) EI : 400.2

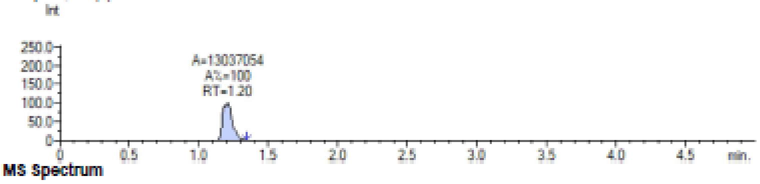

MS Spectrum
Group#1 - PDA Peak: 20, RT: 1.08 to 1.30 min

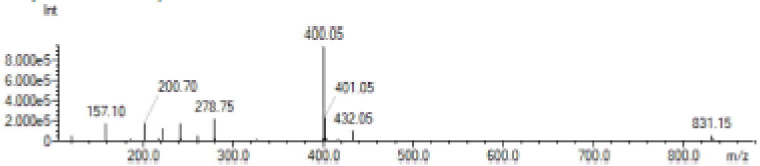

QN-I-3

Shimadzu Open Solution

Project: Test
Experiment: 382_180131_Zimmerm_Foley_Christo_0201_LI
Experiment Description:
Sample: 408278:01:01
Sample Description: C24H22ClN3O2
Data File Name: C:\LabSolutions\Data\382_180131_Zimmermmann_180131_Foley_180131_Christoforow_180201_LI\1046410737_11180201382.lcd
Sample Location: Plate Number: 2 - Position: 98
Run By: admin
Run Started: 12 February 2018 19:19:51
Run Finished: 13 February 2018 04:57:29
Method: Purity_Check+Pause

LC Chromatogram
LC#6 : AD2

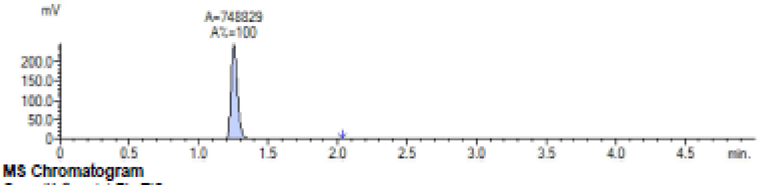

MS Chromatogram
Group#1 Scan(+) EI : TIC

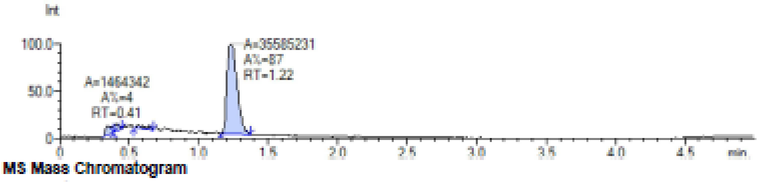

MS Mass Chromatogram
Group#1 Scan(+) EI : 420.1

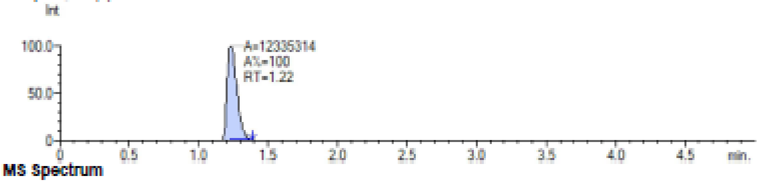

MS Spectrum
Group#1 - PDA Peak: 15, RT: 1.11 to 1.35 min

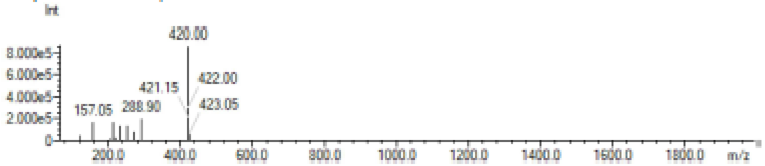

QN-I-4

Shimadzu Open Solution

Project: Test
Experiment: 382_180131_Zimmerm_Foley_Christo_0201_LI
Experiment Description:
Sample: 408279:01:01
Sample Description: C24H22ClN3O2
Data File Name: C:\LabSolutions\Data\382_180131_Zimmermmann_180131_Foley_180131_Christoforow_180201_LM046410807_11180201382.lcd
Sample Location: Plate Number: 2 - Position: 146
Run By: admin
Run Started: 12 February 2018 19:19:51
Run Finished: 13 February 2018 04:57:29
Method: Purity_Check+Pause

LC Chromatogram
LC#6 : AD2

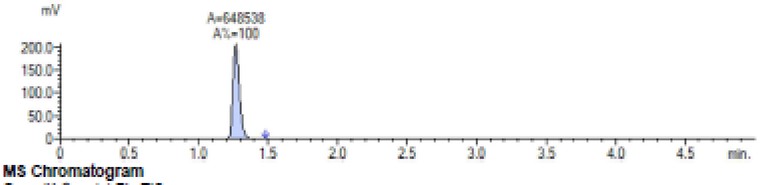

MS Chromatogram
Group#1 Scan(+) EI : TIC

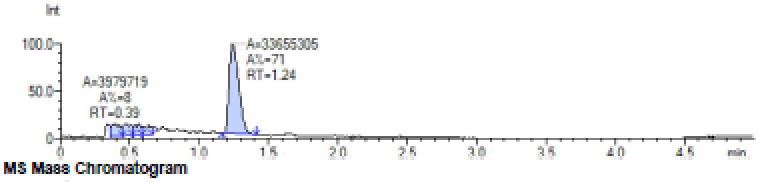

MS Mass Chromatogram
Group#1 Scan(+) EI : 420.1

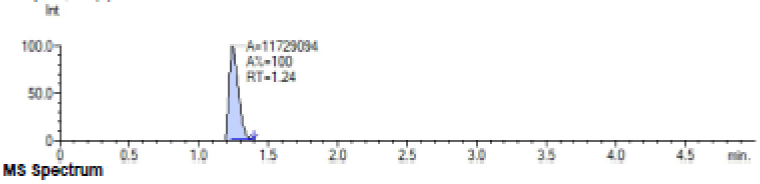

MS Spectrum
Group#1 - PDA Peak: 17, RT: 1.09 to 1.33 min

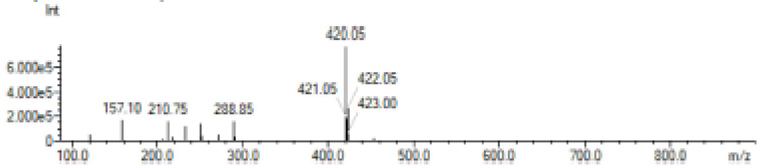

QN-I-5

Shimadzu Open Solution

Project: Test
Experiment: 382_180131_Zimmerm_Foley_Christo_0201_LI
Experiment Description:
Sample: 408280:01:02
Sample Description: C24H22FN3O2
Data File Name: C:\LabSolutions\Data\382_180131_Zimmermmann_180131_Foley_180131_Christoforow_180201_LI\1046409966_11180201382.lcd
Sample Location: Plate Number: 2 - Position: 194
Run By: admin
Run Started: 12 February 2018 19:19:51
Run Finished: 13 February 2018 04:57:29
Method: Purity_Check+Pause

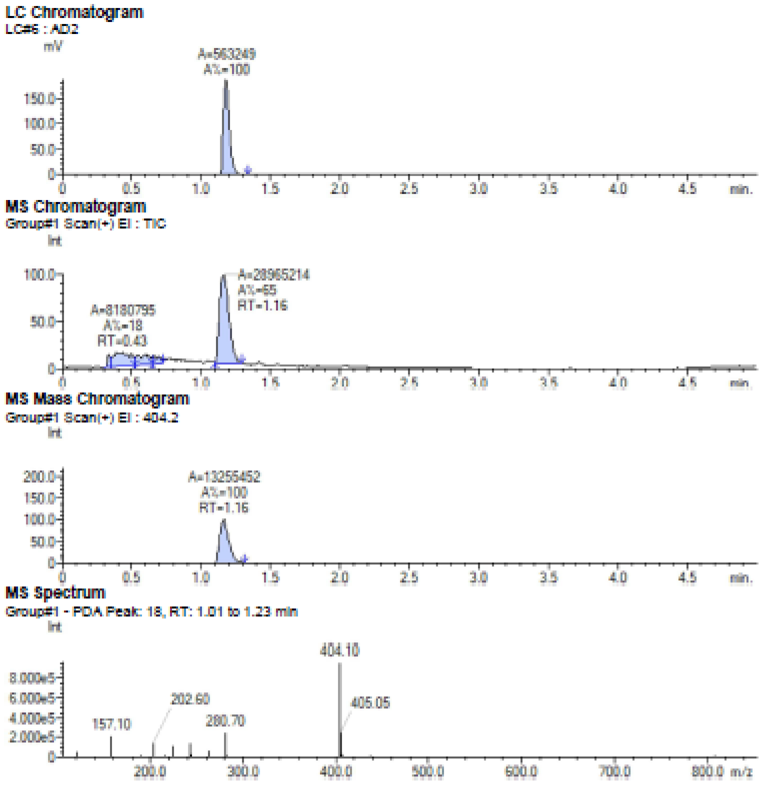

QN-I-6

Shimadzu Open Solution

Project:	Test
Experiment:	382_180131_Zimmerm_Foley_Christo_0201_LI
Experiment Description:	
Sample:	408281:01:01
Sample Description:	C26H25N3O4
Data File Name:	C:\LabSolutions\Data\382_180131_Zimmermmann_180131_Foley_180131_Christoforow_180201_LI\1046410709_11180201382.lcd
Sample Location:	Plate Number: 2 - Position: 242
Run By:	admin
Run Started:	12 February 2018 19:19:51
Run Finished:	13 February 2018 04:57:29
Method:	Purity_Check+Pause

LC Chromatogram

LC#6 : AD2

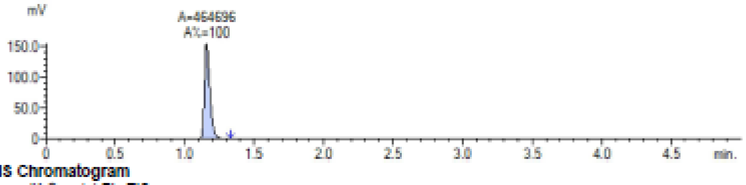

MS Chromatogram

Group#1 Scan(+) EI : TIC

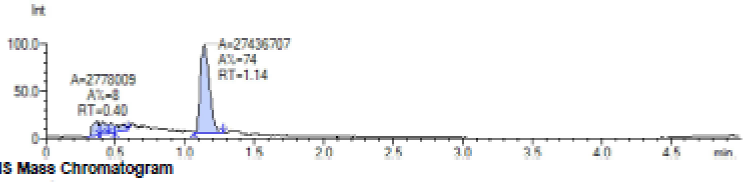

MS Mass Chromatogram

Group#1 Scan(+) EI : 444.2

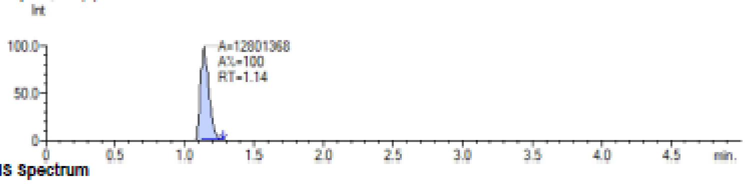

MS Spectrum

Group#1 - PDA Peak: 18, RT: 1.01 to 1.16 min

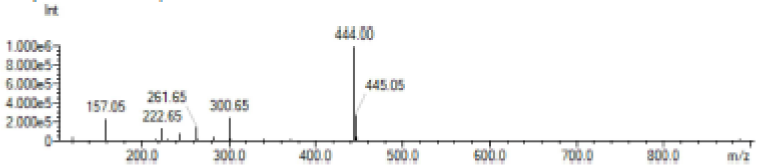

QN-I-7

Shimadzu Open Solution

Project: Test
Experiment: 382_180131_Zimmerm_Foley_Christo_0201_LI
Experiment Description:
Sample: 408285:01:01
Sample Description: C25H22F3N3O2
Data File Name: C:\LabSolutions\Data\382_180131_Zimmermmann_180131_Foley_180131_Christoforow_180201_LI\1046410657_11180201382.lcd
Sample Location: Plate Number: 2 - Position: 52
Run By: admin
Run Started: 12 February 2018 19:19:51
Run Finished: 13 February 2018 04:57:29
Method: Purity_Check+Pause

LC Chromatogram
LC#6 : AD2

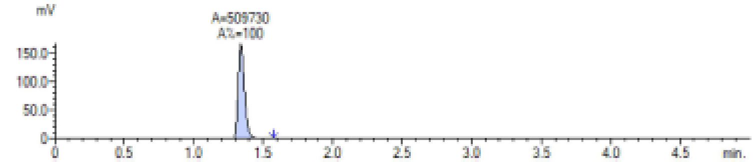

MS Chromatogram
Group#1 Scan(+) EI : TIC

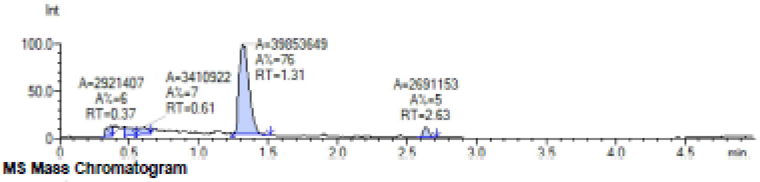

MS Mass Chromatogram
Group#1 Scan(+) EI : 454.2

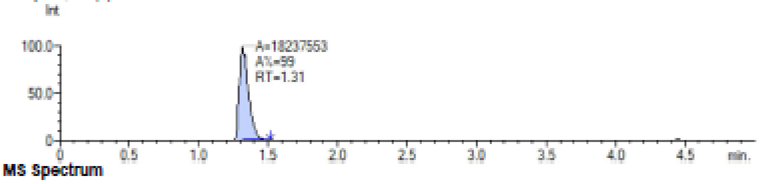

MS Spectrum
Group#1 - PDA Peak: 19, RT: 1.17 to 1.39 min

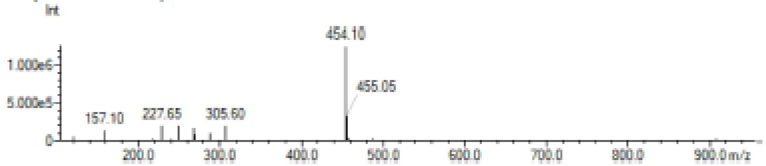

QN-I-8

Shimadzu Open Solution

Project: Test
Experiment: 382_180131_Zimmerm_Foley_Christo_0201_Li
Experiment Description:
Sample: 408286:01:01
Sample Description: C24H22N4O4
Data File Name: C:\LabSolutions\Data\382_180131_Zimmermmann_180131_Foley_180131_Christoforow_180201_Li\1046410344_11180201382.lcd
Sample Location: Plate Number: 2 - Position: 100
Run By: admin
Run Started: 12 February 2018 19:19:51
Run Finished: 13 February 2018 04:57:29
Method: Purity_Check+Pause

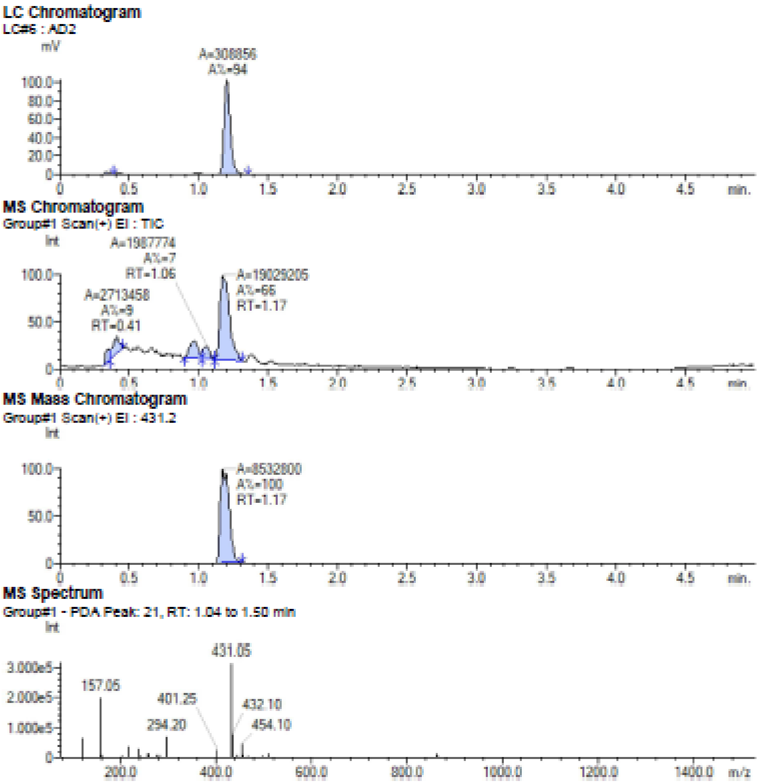

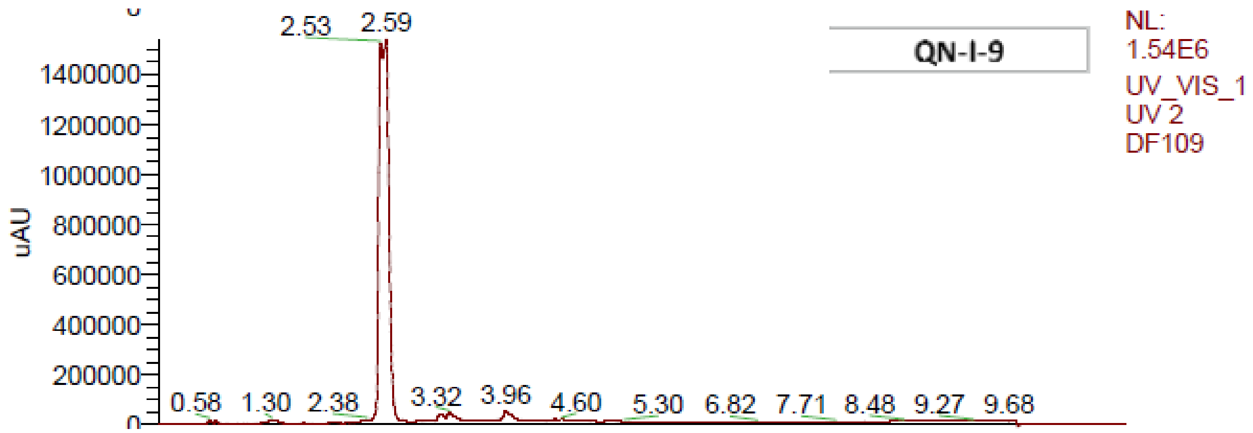

PEAK LIST
DF109.raw
RT: 0.00 - 11.00
Number of detected peaks: 1

Apex RT	Start RT	End RT	Area	%Area	Height	%Height
2.55	2.44	2.75	4051178.515	100.00	717497.954	100.00

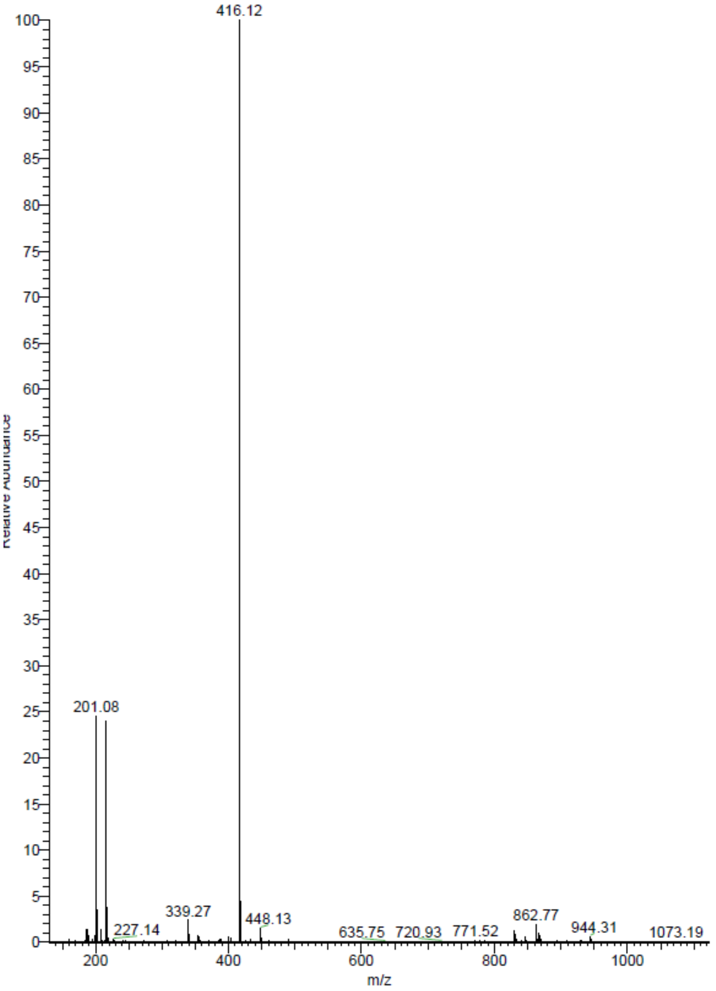

QN-I-10

Shimadzu Open Solution

**Project:**	Test
**Experiment:**	382_180131_Zimmerm_Foley_Christo_0201_LI
**Experiment Description:**	
**Sample:**	408291:01:01
**Sample Description:**	C25H25N3O3
**Data File Name:**	C:\LabSolutions\Data\382_180131_Zimmermmann_180131_Foley_180131_Christoforow_180201_LI\1046410279_11180201382.lcd
**Sample Location:**	Plate Number: 2 - Position: 340
**Run By:**	admin
**Run Started:**	12 February 2018 19:19:51
**Run Finished:**	13 February 2018 04:57:29
**Method:**	Purity_Check+Pause

LC Chromatogram

LC#6 : AD2

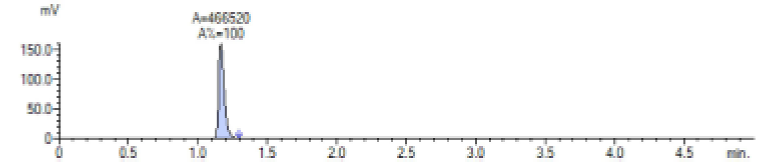

MS Chromatogram

Group#1 Scan(+) EI : TIC

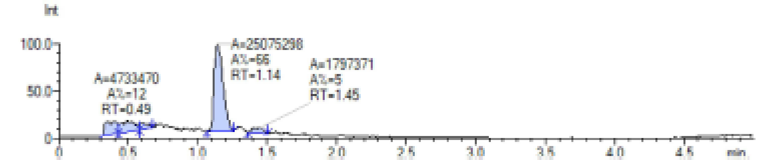

MS Mass Chromatogram

Group#1 Scan(+) EI : 416.2

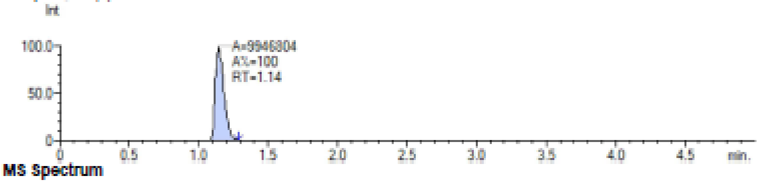

MS Spectrum

Group#1 - PDA Peak: 20, RT: 0.98 to 1.83 min

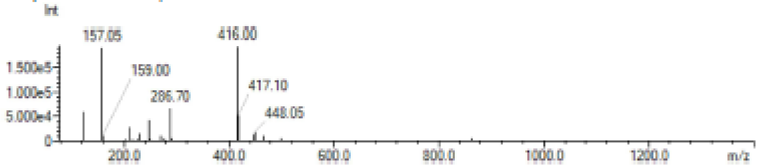

QN-I-11

Shimadzu Open Solution

Project:	Test
Experiment:	382_180131_Zimmerm_Foley_Christo_0201_LI
Experiment Description:	
Sample:	408298:01:01
Sample Description:	C24H22ClN3O2
Data File Name:	C:\LabSolutions\Data\382_180131_Zimmermmann_180131_Foley_180131_Christoforow_180201_LI\1046410170_11180201382.lcd
Sample Location:	Plate Number: 2 - Position: 294
Run By:	admin
Run Started:	12 February 2018 19:19:51
Run Finished:	13 February 2018 04:57:29
Method:	Purity_Check+Pause

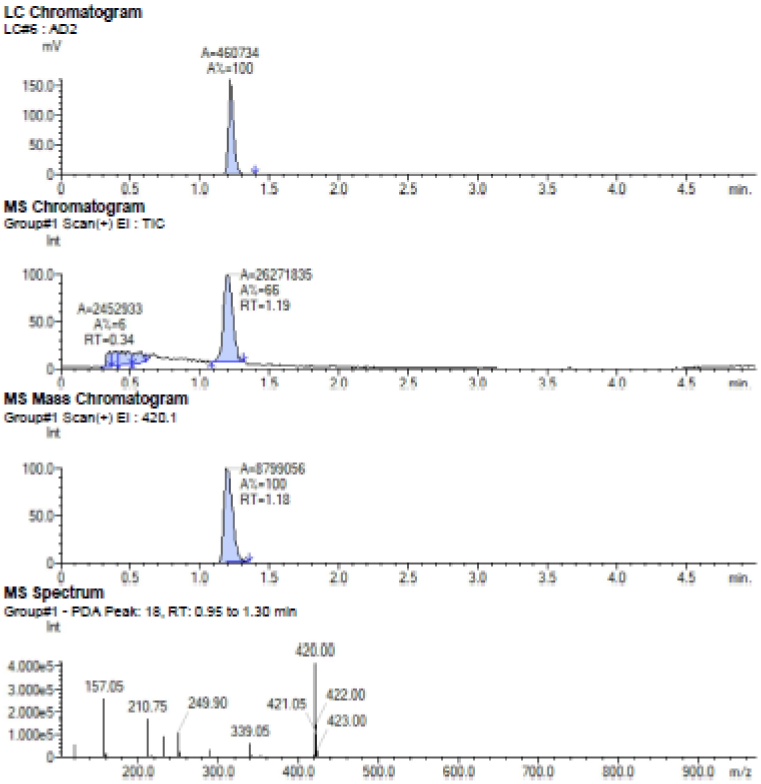

QN-I-12

Shimadzu Open Solution

Project: Test
Experiment: 382_180131_Zimmerm_Foley_Christo_0201_LI
Experiment Description:
Sample: 408299:01:01
Sample Description: C25H25N3O2
Data File Name: C:\LabSolutions\Data\382_180131_Zimmermmann_180131_Foley_180131_Christoforow_180201_LI\1046410082_11180201382.lcd
Sample Location: Plate Number: 2 - Position: 342
Run By: admin
Run Started: 12 February 2018 19:19:51
Run Finished: 13 February 2018 04:57:29
Method: Purity_Check+Pause

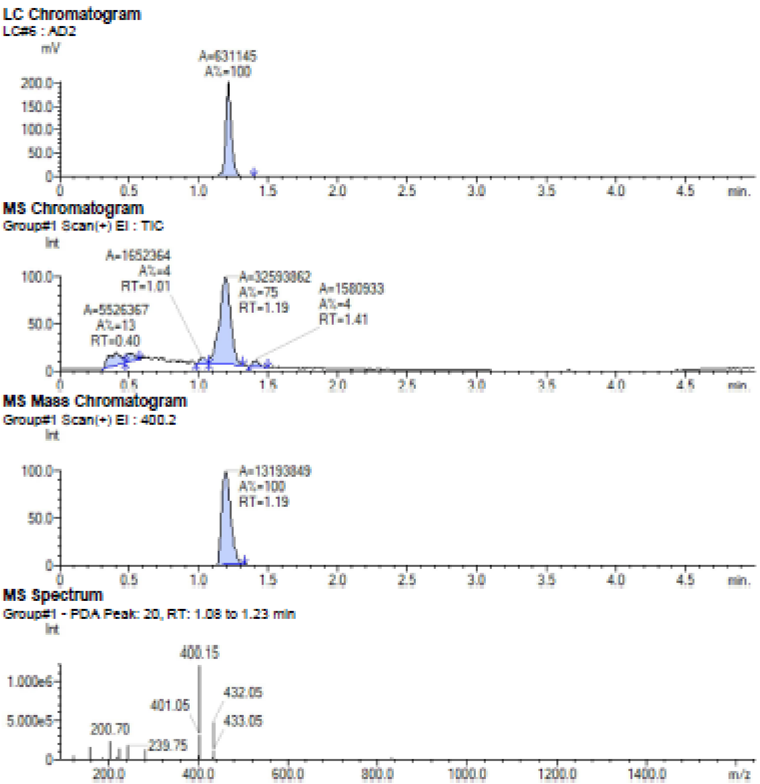

QN-I-13

Shimadzu Open Solution

Project: Test
Experiment: 382_180131_Zimmerm_Foley_Christo_0201_LI
Experiment Description:
Sample: 408301:01:01
Sample Description: C25H22F3N3O2
Data File Name: C:\LabSolutions\Data\382_180131_Zimmermmann_180131_Foley_180131_Christoforow_180201_LI\1046410367_11180201382.lcd
Sample Location: Plate Number: 2 - Position: 56
Run By: admin
Run Started: 12 February 2018 19:19:51
Run Finished: 13 February 2018 04:57:29
Method: Purity_Check+Pause

LC Chromatogram
LC#6 : AD2

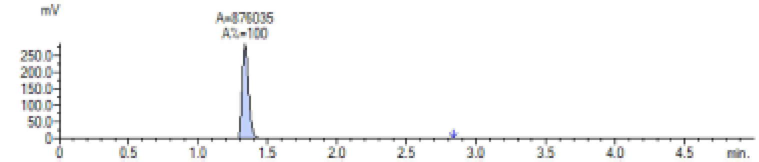

MS Chromatogram
Group#1 Scan(+) EI : TIC

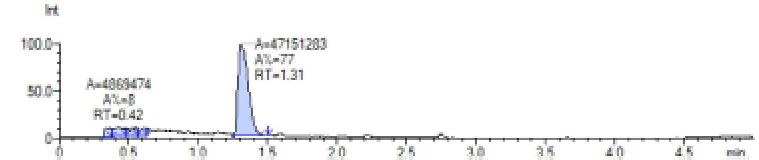

MS Mass Chromatogram
Group#1 Scan(+) EI : 454.2

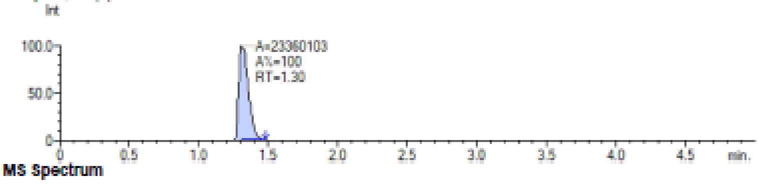

MS Spectrum
Group#1 - PDA Peak: 19, RT: 1.19 to 1.61 min

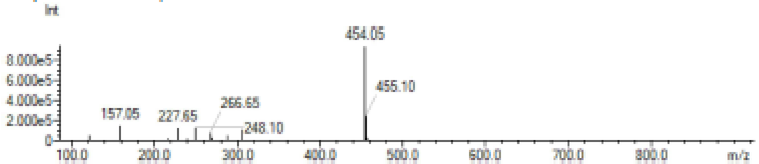

QN-I-14

Shimadzu Open Solution

Project:	Test
Experiment:	382_180131_Zimmerm_Foley_Christo_0201_LI
Experiment Description:	
Sample:	408302:01:01
Sample Description:	C25H25N3O2
Data File Name:	C:\LabSolutions\Data\382_180131_Zimmermmann_180131_Foley_180131_Christoforow_180201_LI\1046409963_11180201382.lcd
Sample Location:	Plate Number: 2 - Position: 104
Run By:	admin
Run Started:	12 February 2018 19:19:51
Run Finished:	13 February 2018 04:57:29
Method:	Purity_Check+Pause

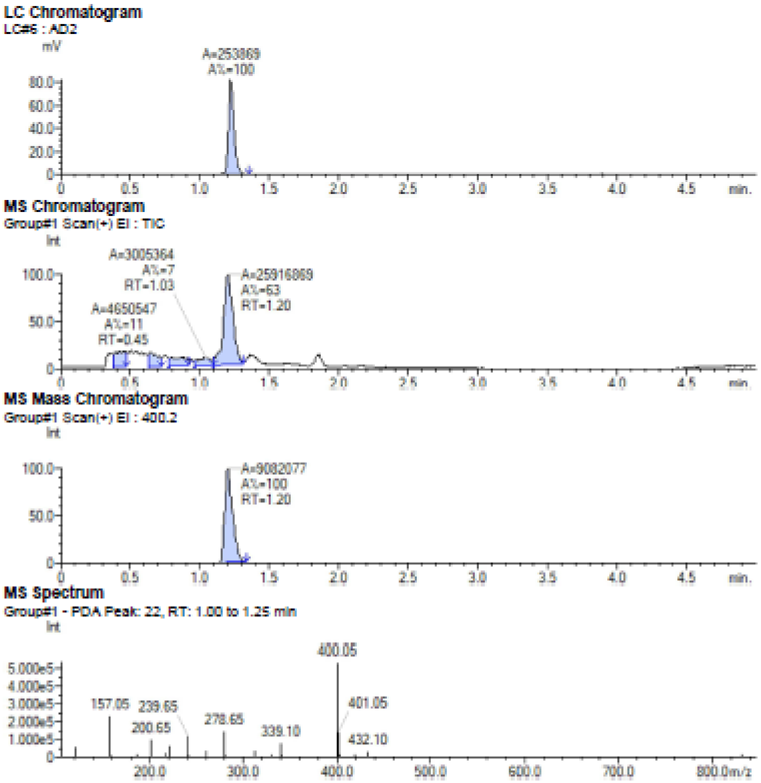

QN-I-15

Shimadzu Open Solution

Project:	Test
Experiment:	382_180131_Zimmerm_Foley_Christo_0201_LI
Experiment Description:	
Sample:	408306:01:01
Sample Description:	C25H23N3O4
Data File Name:	C:\LabSolutions\Data\382_180131_Zimmermmann_180131_Foley_180131_Christoforow_180201_LI\1046409354_11180201382.lcd
Sample Location:	Plate Number: 2 - Position: 296
Run By:	admin
Run Started:	12 February 2018 19:19:51
Run Finished:	13 February 2018 04:57:29
Method:	Purity_Check+Pause

LC Chromatogram

LC#6 : AD2

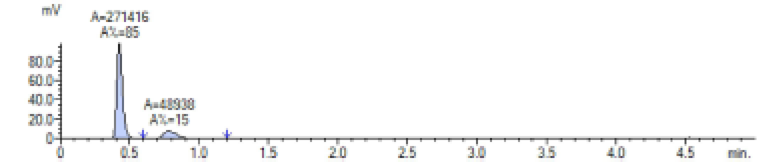

MS Chromatogram

Group#1 Scan(+) EI : TIC

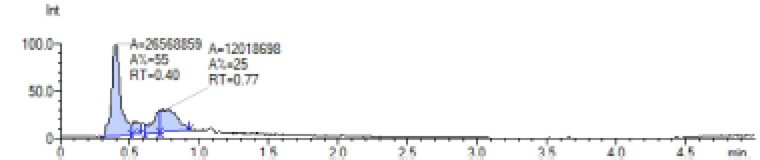

MS Mass Chromatogram

Group#1 Scan(+) EI : 430.2

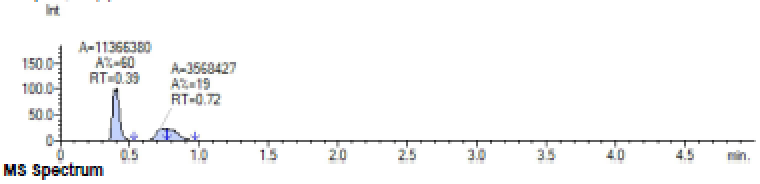

MS Spectrum

Group#1 - PDA Peak: 26, RT: 0.61 to 0.84 min

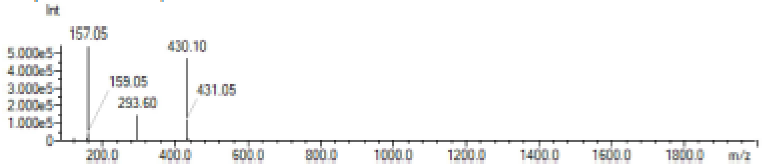

QN-I-16

Shimadzu Open Solution

Project:	Test
Experiment:	383_180131_NatA_TEAD_180201_Foley_Shan
Experiment Description:	
Sample:	408311:01:01
Sample Description:	C25H25N3O3
Data File Name:	C:\LabSolutions\Data\383_180131_NatA_180131_TEAD_180201_Foley_180201_Shan\1046410359_11180201383.lcd
Sample Location:	Plate Number: 1 - Position: 73
Run By:	admin
Run Started:	12 February 2018 10:27:31
Run Finished:	12 February 2018 12:52:26
Method:	Purity_Check_2018

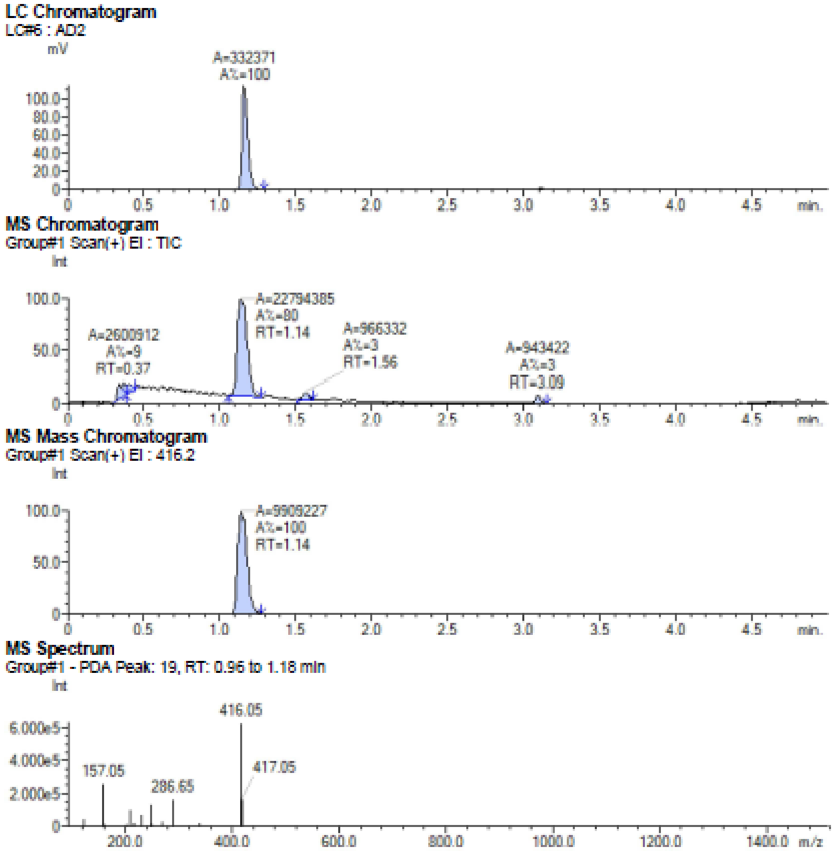

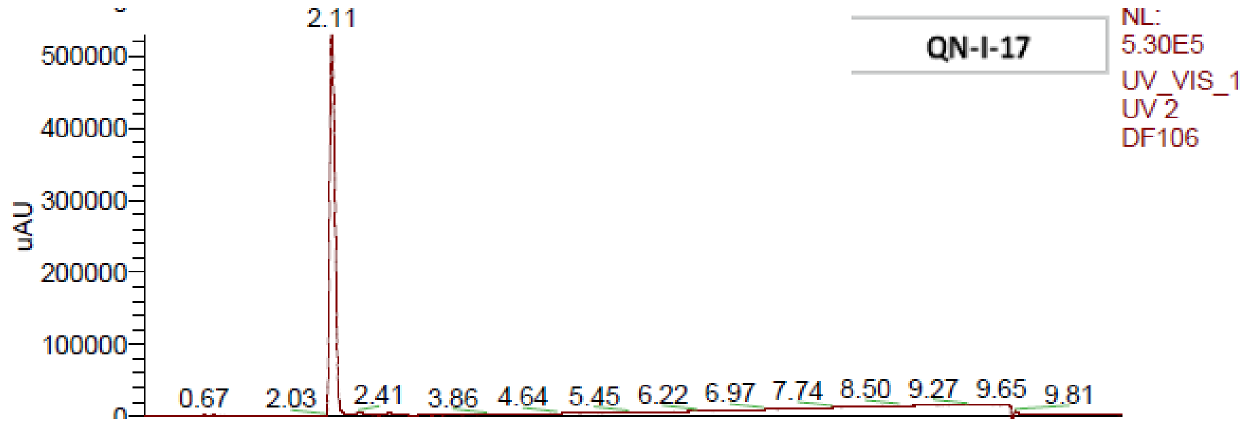

PEAK LIST
DF106.raw
RT: 0.00 - 11.00
Number of detected peaks: 1

Apex RT	Start RT	End RT	Area	%Area	Height	%Height
2.10	2.08	2.16	251947.851	100.00	71537.031	100.00

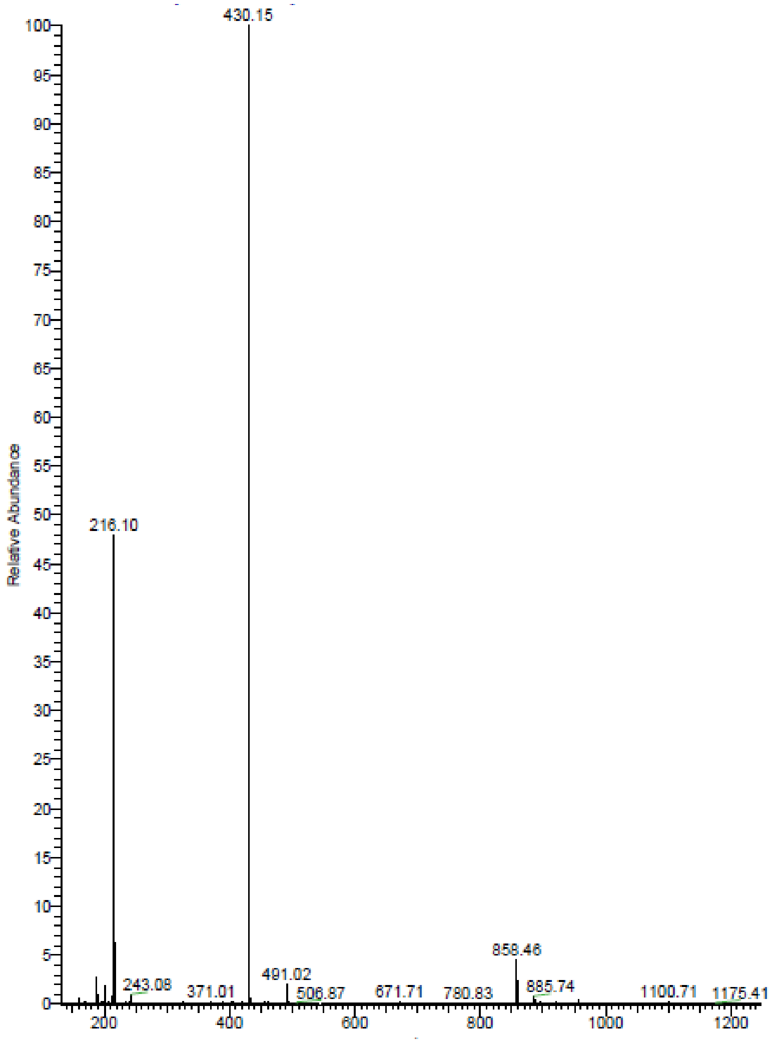

QN-I-18

Shimadzu Open Solution

Project: Test
Experiment: 423_0507_Foley_Pahl_08_Gerdes_09_Schade
Experiment Description:
Sample: 408559:01:01
Sample Description: C25H22F3N3O3
Data File Name: C:\LabSolutions\Data\423_180507_Foley_180507_Pahl_180508_Gerdes_180509_Schade\1046413857_11180507423.lcd
Sample Location: Plate Number: 1 - Position: 3
Run By: admin
Run Started: 11 September 2018 12:10:30
Run Finished: 11 September 2018 14:18:29
Method: Purity_Check_2018

LC Chromatogram
LC#6 : AD2

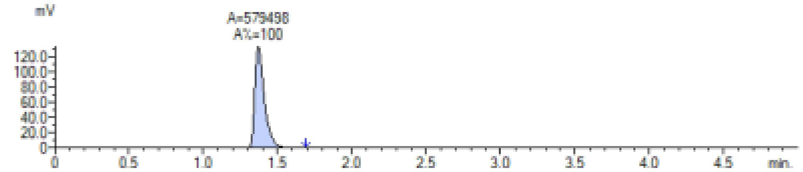

MS Chromatogram
Group#1 Scan(+) EI : TIC

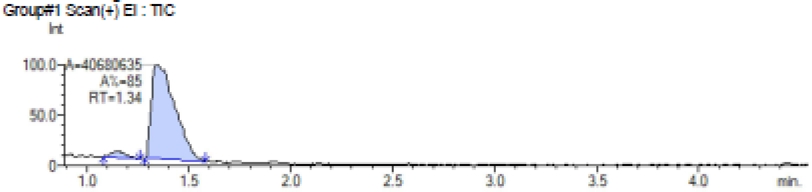

MS Mass Chromatogram
Group#1 Scan(+) EI : 470.2

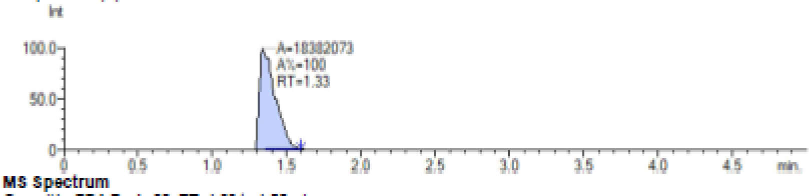

MS Spectrum
Group#1 - PDA Peak: 23, RT: 1.22 to 1.55 min

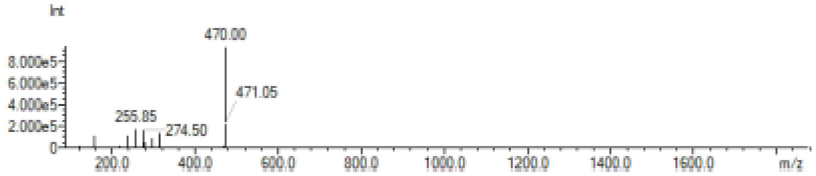

QN-I-19

Shimadzu Open Solution

Project: Test
Experiment: 431_0606_Zimmer_Heitk_Kaiser_Zinken
Experiment Description:
Sample: 406802:01:01
Sample Description: C24H22FN3O2
Data File Name: C:\LabSolutions\Data\431_180606_Zimmermann_180606_Heitkamp_180606_Kaiser_180606_Zinken\1045324771_11180606431.lcd
Sample Location: Plate Number: 2 - Position: 74
Run By: admin
Run Started: 13 September 2018 10:41:38
Run Finished: 13 September 2018 13:00:54
Method: Purity_Check_2018

LC Chromatogram
LC#6 : AD2

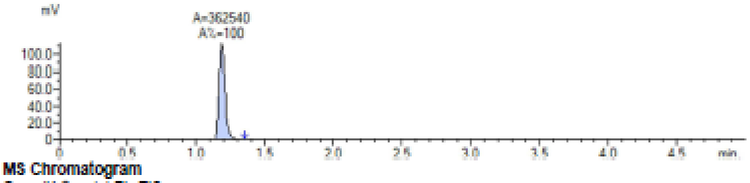

MS Chromatogram
Group#1 Scan(+) EI : TIC

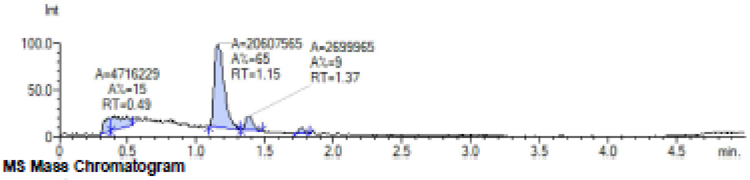

MS Mass Chromatogram
Group#1 Scan(+) EI : 404.2

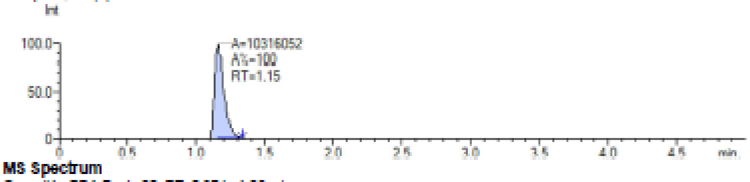

MS Spectrum
Group#1 - PDA Peak: 20, RT: 0.97 to 1.26 min

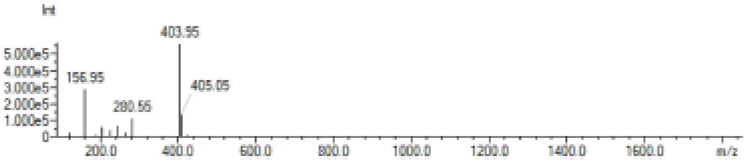

QN-I-20

Shimadzu Open Solution

**Project:**	Test
**Experiment:**	431_0606_Zimmer_Heitk_Kaiser_Zinken
**Experiment Description:**	
**Sample:**	408803:01:01
**Sample Description:**	C24H22ClN3O2
**Data File Name:**	C:\LabSolutions\Data\431_180606_Zimmermann_180606_Heitkamp_180606_Kaiser_180606_Zinken\1046325079_11180606431.lcd
**Sample Location:**	Plate Number: 2 - Position: 122
**Run By:**	admin
**Run Started:**	13 September 2018 10:41:38
**Run Finished:**	13 September 2018 13:00:54
**Method:**	Purity_Check_2018

LC Chromatogram

LC#6 : AD2

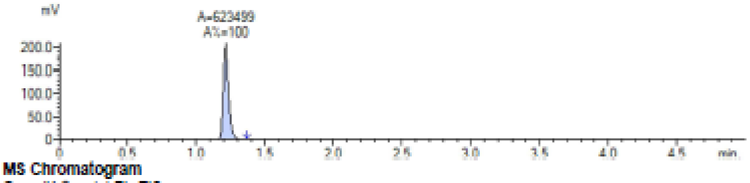

MS Chromatogram

Group#1 Scan(+) EI : TIC

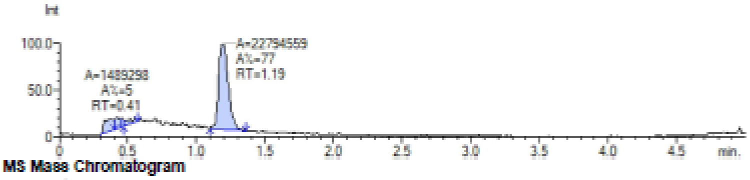

MS Mass Chromatogram

Group#1 Scan(+) EI : 420.1

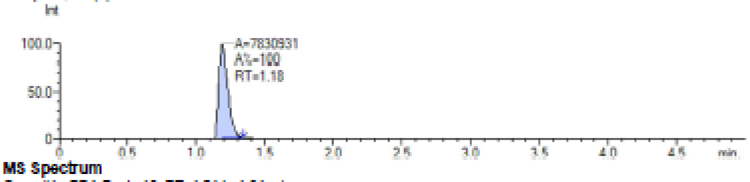

MS Spectrum

Group#1 - PDA Peak: 18, RT: 1.01 to 1.31 min

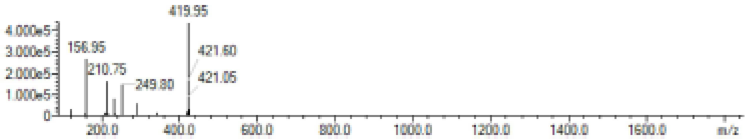

QN-I-21

Shimadzu Open Solution

**Project:**	Test
**Experiment:**	519_LCMS
**Experiment Description:**	
**Sample:**	408910:01:04
**Sample Description:**	C27H28N3O5
**Data File Name:**	C:\LabSolutions\Data\519_LCMS\1046360007_11190129519.lcd
**Sample Location:**	Plate Number: 2 - Position: 218
**Run By:**	admin
**Run Started:**	02 May 2019 19:41:55
**Run Finished:**	03 May 2019 13:04:26
**Method:**	190321_Purity-Check

LC Chromatogram

LC#6 : AD2

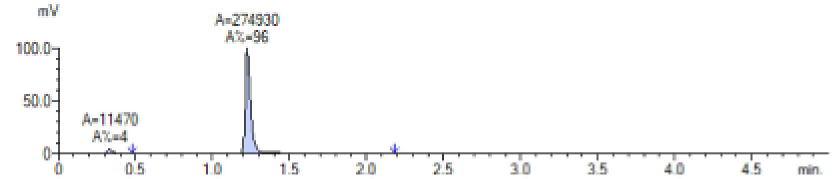

MS Chromatogram

Group#1 Scan(+) EI : TIC

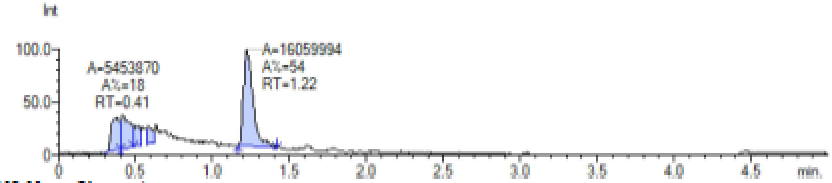

MS Mass Chromatogram

Group#1 Scan(+) EI : 474.2

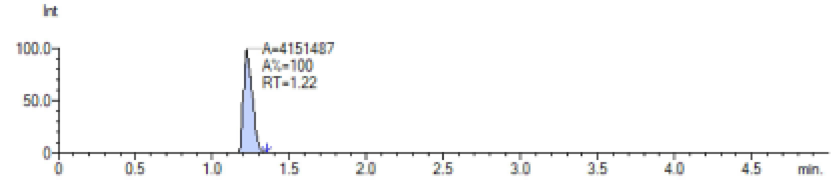

MS Spectrum

Group#1 - PDA Peak: 22, RT: 1.04 to 1.46 min

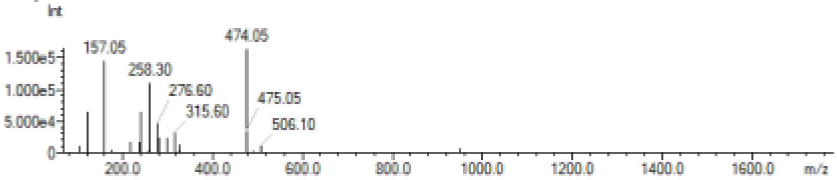

QN-I-22

Shimadzu Open Solution

**Project:**	Test
**Experiment:**	524_0205_Wess_06_Zink_07_Conc-10_08_Grig
**Experiment Description:**	
**Sample:**	409739:01:01
**Sample Description:**	C25H24BrN3O2
**Data File Name:**	C:\LabSolutions\Data\524_190205_Wesserler_190602_Zinken_190207_Conc-10_190208_Grigalunas\1046361064_11190207524.lcd
**Sample Location:**	Plate Number: 1 - Position: 50
**Run By:**	admin
**Run Started:**	23 April 2019 15:21:47
**Run Finished:**	23 April 2019 18:20:12
**Method:**	190321_Purity-Check

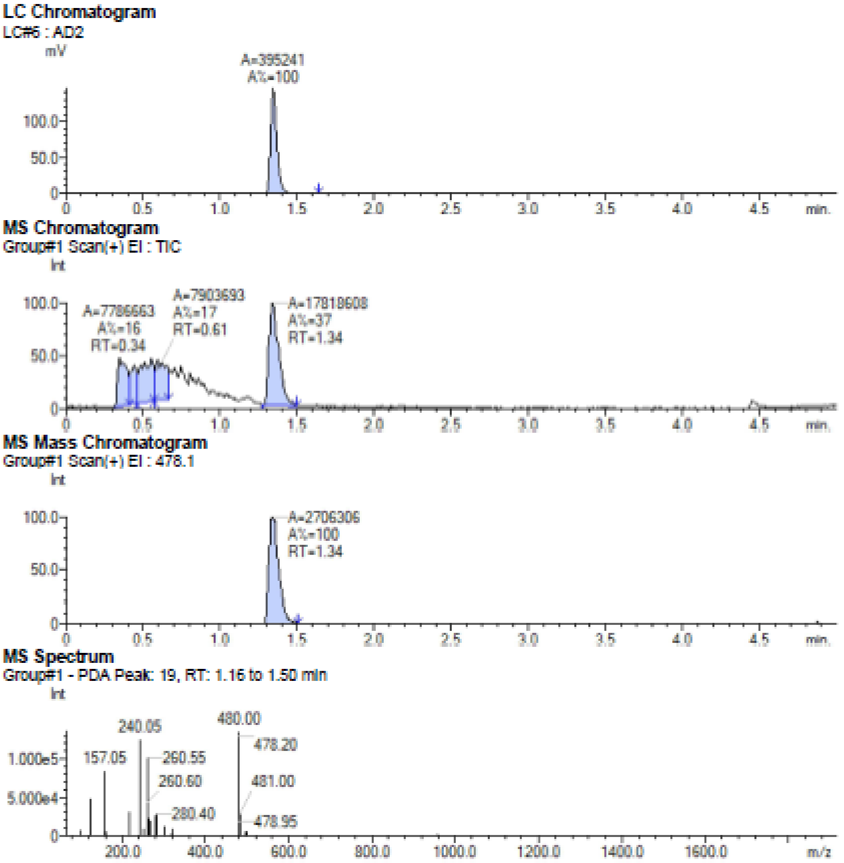

QD-I-1

Shimadzu Open Solution

Project:	Test
Experiment:	691_200707_CP20_03_0710_Christiane
Experiment Description:	
Sample:	407156:01:06
Sample Description:	C24H23N3O2
Data File Name:	C:\LabSolutions\Data\691_200707_CP20_03_0710_Christiane\1046409587_11200713691.lcd
Sample Location:	Plate Number: 1 - Position: 260
Run By:	admin
Run Started:	10 August 2020 13:38:24
Run Finished:	11 August 2020 11:22:27
Method:	190321_Purity-Check

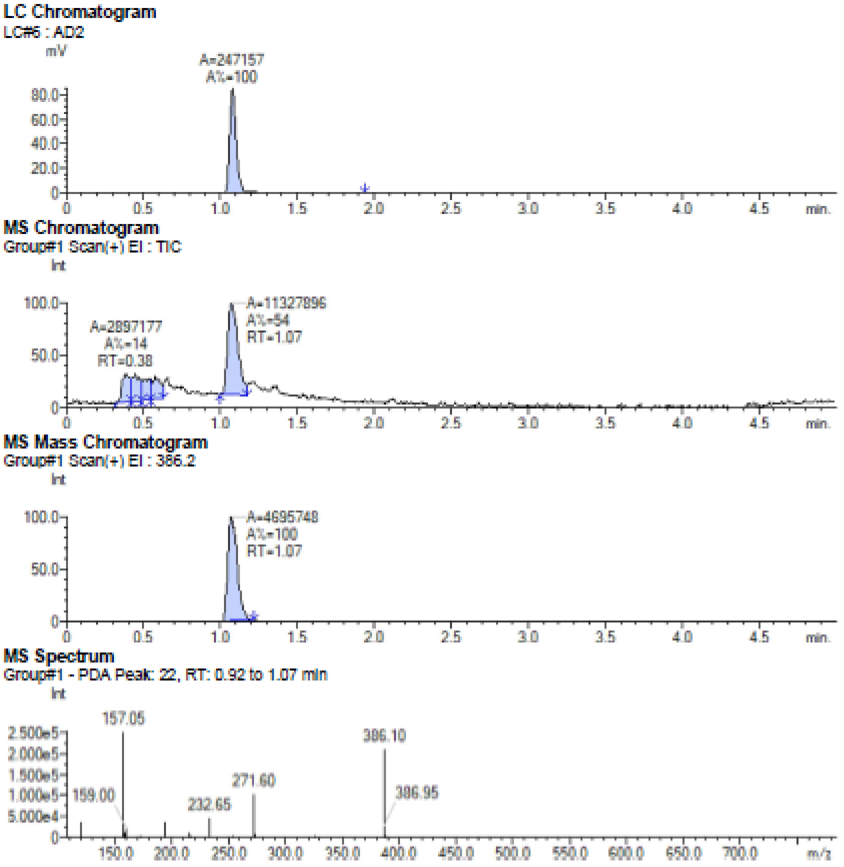

QD-I-2

Shimadzu Open Solution

Project: Test
Experiment: 691_200707_CP20_03_0710_Christiane
Experiment Description:
Sample: 407631:01:06
Sample Description: C24H22FN3O2
Data File Name: C:\LabSolutions\Data\691_200707_CP20_03_0710_Christiane\1046409426_11200713691.lcd
Sample Location: Plate Number: 1 - Position: 168
Run By: admin
Run Started: 10 August 2020 13:38:24
Run Finished: 11 August 2020 11:22:27
Method: 190321_Purity-Check

LC Chromatogram
LC#6 : AD2

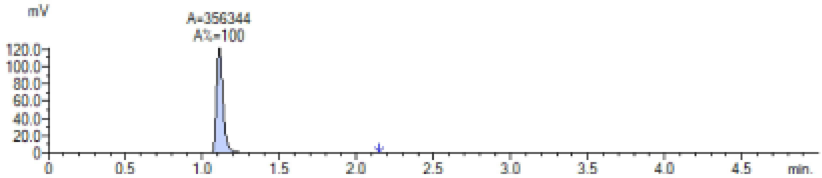

MS Chromatogram
Group#1 Scan(+) EI : TIC

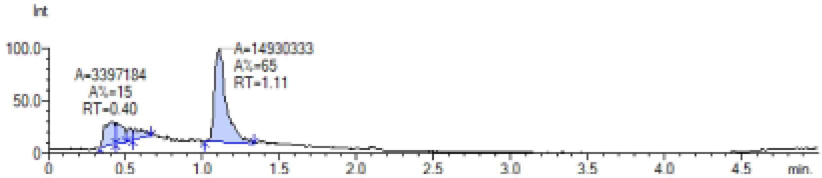

MS Mass Chromatogram
Group#1 Scan(+) EI : 404.2

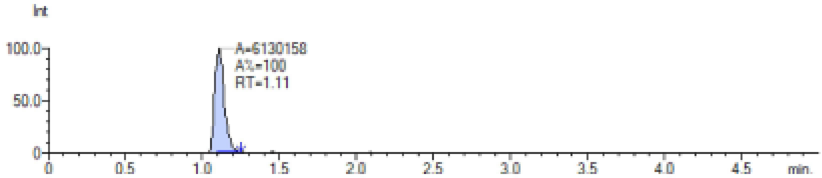

MS Spectrum
Group#1 - PDA Peak: 20, RT: 0.87 to 1.07 min

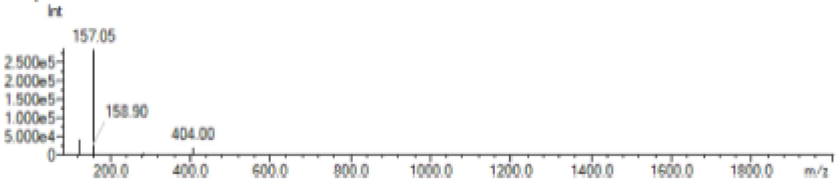

QD-I-3

Shimadzu Open Solution

**Project:**	Test
**Experiment:**	691_200707_CP20_03_0710_Christiane
**Experiment Description:**	
**Sample:**	407637:01:05
**Sample Description:**	C24H22ClN3O2
**Data File Name:**	C:\LabSolutions\Data\691_200707_CP20_03_0710_Christiane\1046409693_11200713691.lcd
**Sample Location:**	Plate Number: 1 - Position: 211
**Run By:**	admin
**Run Started:**	10 August 2020 13:38:24
**Run Finished:**	11 August 2020 11:22:27
**Method:**	190321_Purity-Check

LC Chromatogram

LC#6 : AD2

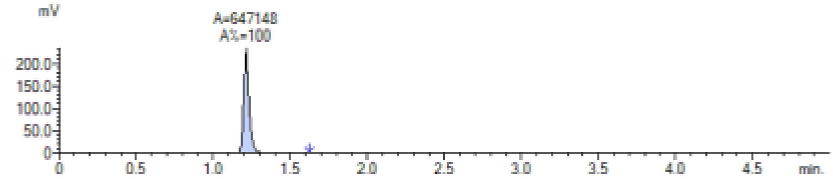

MS Chromatogram

Group#1 Scan(+) EI : TIC

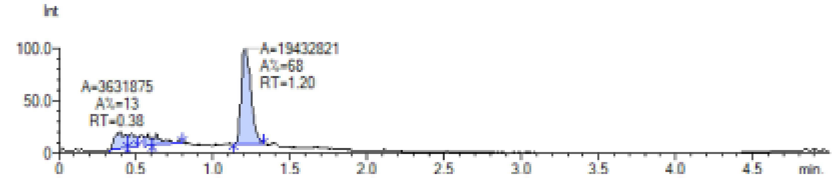

MS Mass Chromatogram

Group#1 Scan(+) EI : 420.1

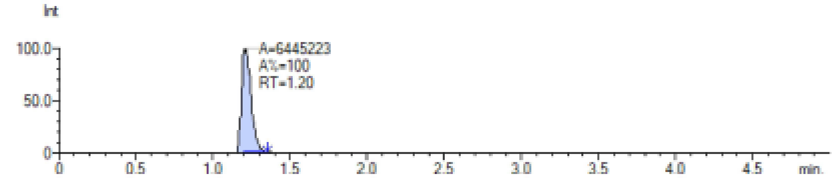

MS Spectrum

Group#1 - PDA Peak: 20, RT: 1.05 to 1.56 min

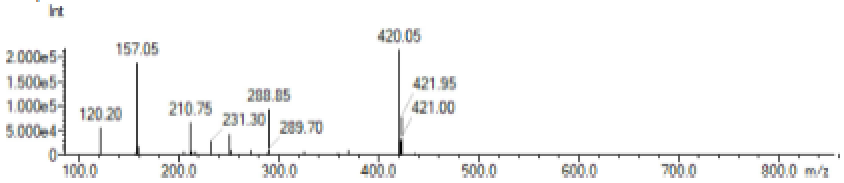

QD-I-4

Shimadzu Open Solution

**Project:**	Test
**Experiment:**	623_191008_CP-H_191016_Doetsch_1017_3077
**Experiment Description:**	
**Sample:**	407638:01:05
**Sample Description:**	C25H25N3O2
**Data File Name:**	C:\LabSolutions\Data\623_191008_CP-H_191016_Doetsch_191017_3077 \1046409414_11191017623.lcd
**Sample Location:**	Plate Number: 1 - Position: 205
**Run By:**	admin
**Run Started:**	28 October 2019 15:14:10
**Run Finished:**	29 October 2019 01:02:51
**Method:**	190321_Purity-Check

LC Chromatogram

LC#6 : AD2

MS Chromatogram

Group#1 Scan(+) EI : TIC

MS Mass Chromatogram

Group#1 Scan(+) EI : 400.2

MS Spectrum

Group#1 - PDA Peak: 19, RT: 0.90 to 1.35 min

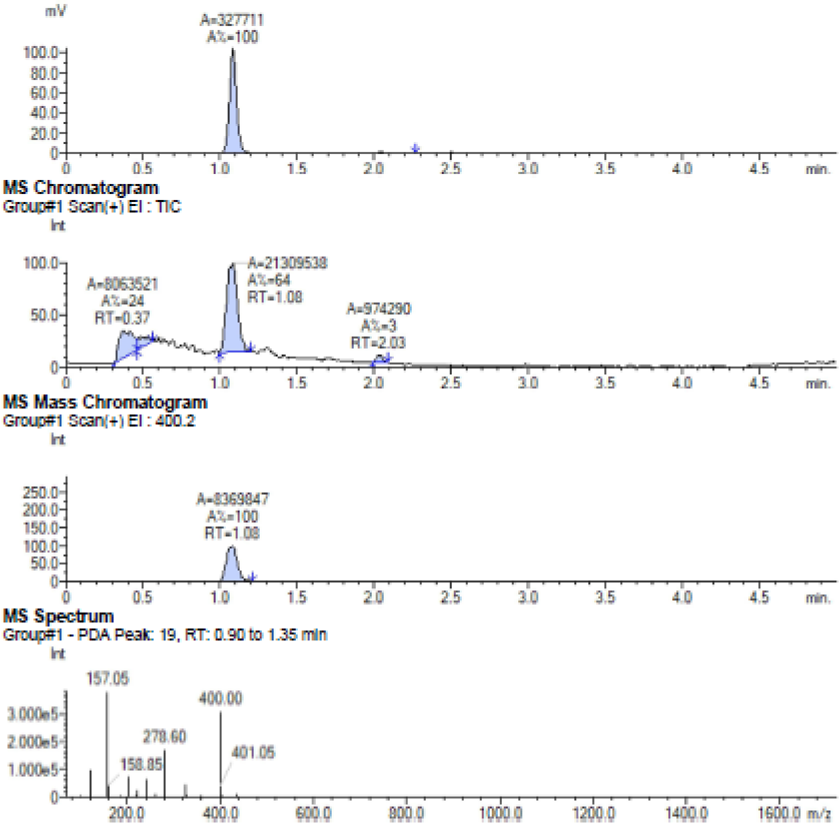

QD-I-5

Shimadzu Open Solution

Project:	Test
Experiment:	382_180131_Zimmerm_Foley_Christo_0201_LI
Experiment Description:	
Sample:	406267:01:01
Sample Description:	C26H25N3O4
Data File Name:	C:\LabSolutions\Data\382_180131_Zimmermmann_180131_Foley_180131_Christoforow_180201_LI\1046410326_11180201382.lcd
Sample Location:	Plate Number: 2 - Position: 148
Run By:	admin
Run Started:	12 February 2018 19:19:51
Run Finished:	13 February 2018 04:57:29
Method:	Purity_Check+Pause

LC Chromatogram

LC#6 : AD2

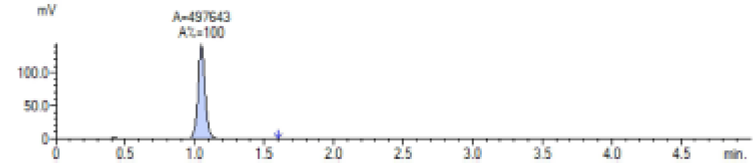

MS Chromatogram

Group#1 Scan(+) EI : TIC

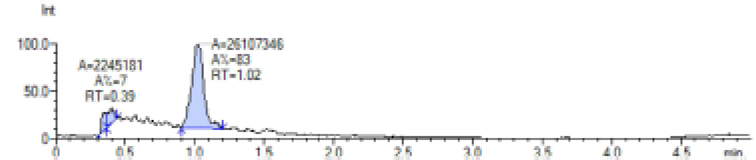

MS Mass Chromatogram

Group#1 Scan(+) EI : 444.2

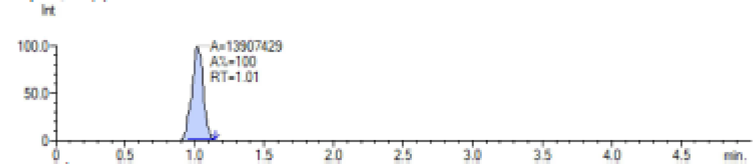

MS Spectrum

Group#1 - PDA Peak: 17, RT: 0.78 to 1.03 min

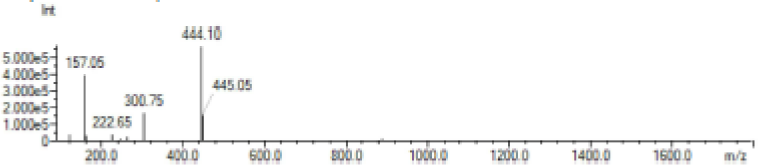

QD-I-6

Shimadzu Open Solution

**Project:**	Test
**Experiment:**	382_180131_Zimmerm_Foley_Christo_0201_LI
**Experiment Description:**	
**Sample:**	408288:01:01
**Sample Description:**	C26H25N3O4
**Data File Name:**	C:\LabSolutions\Data\382_180131_Zimmermmann_180131_Foley_180131_Christoforow_180201_LI\1046409941_11180201382.lcd
**Sample Location:**	Plate Number: 2 - Position: 196
**Run By:**	admin
**Run Started:**	12 February 2018 19:19:51
**Run Finished:**	13 February 2018 04:57:29
**Method:**	Purity_Check+Pause

LC Chromatogram
LC#6 : AD2

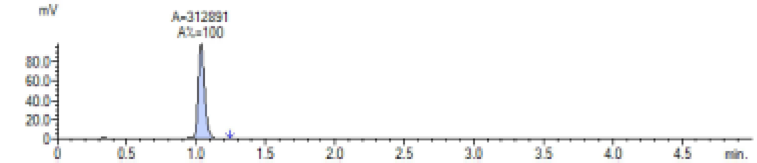

MS Chromatogram
Group#1 Scan(+) EI : TIC

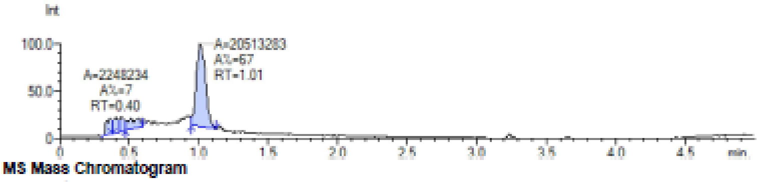

MS Mass Chromatogram
Group#1 Scan(+) EI : 444.2

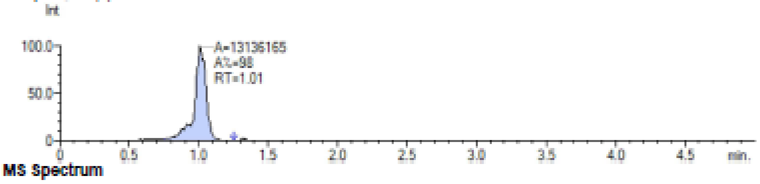

MS Spectrum
Group#1 - PDA Peak: 22, RT: 0.88 to 1.03 min

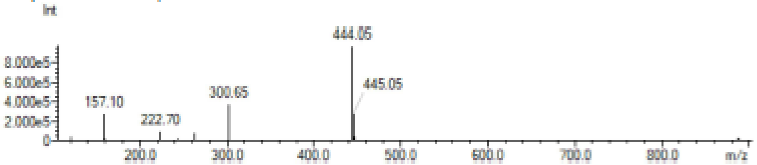

QD-I-7

Shimadzu Open Solution

Project: Test
Experiment: 382_180131_Zimmerm_Foley_Christo_0201_LI
Experiment Description:
Sample: 408289:01:01
Sample Description: C25H23N3O4
Data File Name: C:\LabSolutions\Data\382_180131_Zimmermmann_180131_Foley_180131_Christoforow_180201_LI\1046410734_11180201382.lcd
Sample Location: Plate Number: 2 - Position: 244
Run By: admin
Run Started: 12 February 2018 19:19:51
Run Finished: 13 February 2018 04:57:29
Method: Purity_Check+Pause

LC Chromatogram
LC#6 : AD2

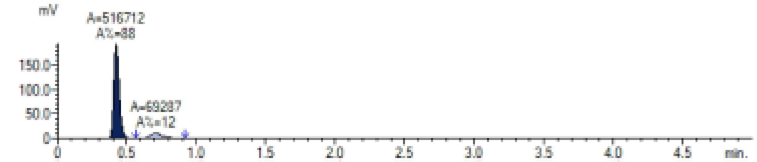

MS Chromatogram
Group#1 Scan(+) EI : TIC

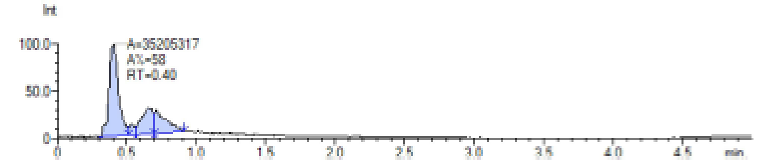

MS Mass Chromatogram
Group#1 Scan(+) EI : 430.2

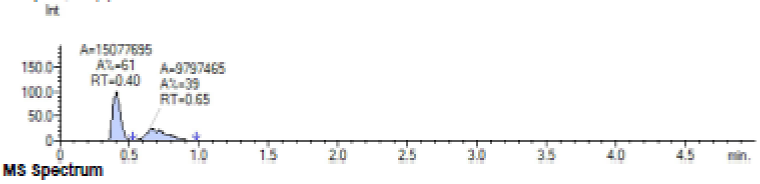

MS Spectrum
Group#1 - LC Peak: 1, RT: 0.36 to 0.56 min

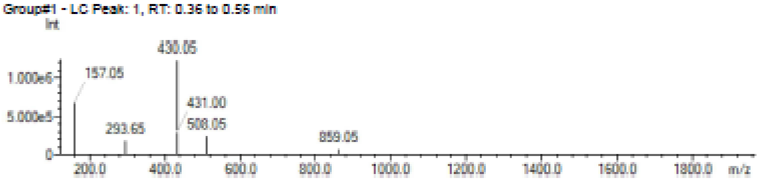

QD-I-8

Shimadzu Open Solution

Project: Test
Experiment: 382_180131_Zimmerm_Foley_Christo_0201_LI
Experiment Description:
Sample: 408293:01:01
Sample Description: C25H23N3O4
Data File Name: C:\LabSolutions\Data\382_180131_Zimmermmann_180131_Foley_180131_Christoforow_180201_LI\1046410689_11180201382.lcd
Sample Location: Plate Number: 2 - Position: 54
Run By: admin
Run Started: 12 February 2018 19:19:51
Run Finished: 13 February 2018 04:57:29
Method: Purity_Check+Pause

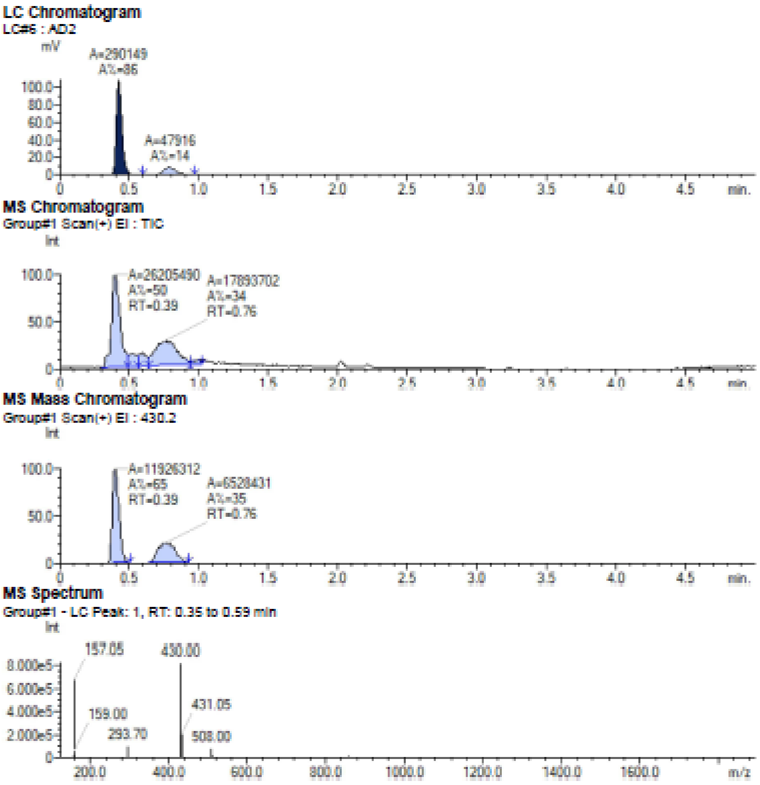

QD-I-9

Shimadzu Open Solution

Project: Test
Experiment: 382_180131_Zimmerm_Foley_Christo_0201_LI
Experiment Description:
Sample: 408294:01:01
Sample Description: C25H22F3N3O2
Data File Name: C:\LabSolutions\Data\382_180131_Zimmermmann_180131_Foley_180131_Christoforow_180201_LI\1046409949_11180201382.lcd
Sample Location: Plate Number: 2 - Position: 102
Run By: admin
Run Started: 12 February 2018 19:19:51
Run Finished: 13 February 2018 04:57:29
Method: Purity_Check+Pause

LC Chromatogram
LC#6 : AD2

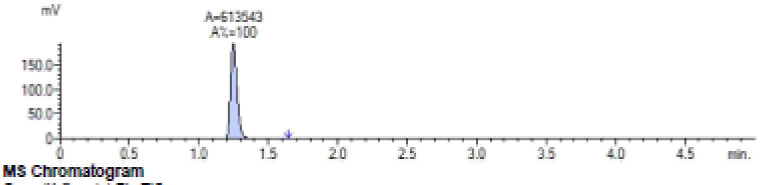

MS Chromatogram
Group#1 Scan(+) EI : TIC

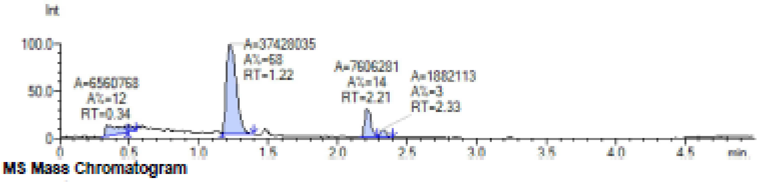

MS Mass Chromatogram
Group#1 Scan(+) EI : 454.2

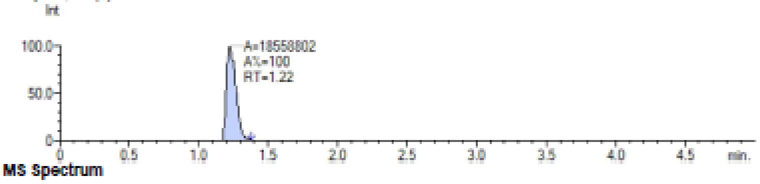

MS Spectrum
Group#1 - PDA Peak: 21, RT: 1.11 to 1.37 min

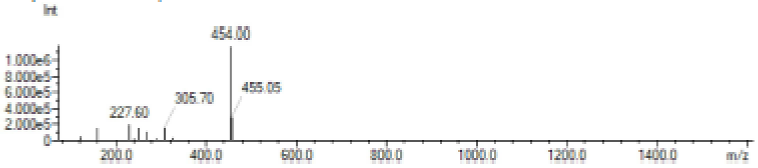

QD-I-10

Shimadzu Open Solution

**Project:**	Test
**Experiment:**	382_180131_Zimmerm_Foley_Christo_0201_Li
**Experiment Description:**	
**Sample:**	406295:01:01
**Sample Description:**	C25H25N3O3
**Data File Name:**	C:\LabSolutions\Data\382_180131_Zimmermmann_180131_Foley_180131_Christoforow_180201_Li\1046410146_11180201382.lcd
**Sample Location:**	Plate Number: 2 - Position: 150
**Run By:**	admin
**Run Started:**	12 February 2018 19:19:51
**Run Finished:**	13 February 2018 04:57:29
**Method:**	Purity_Check+Pause

LC Chromatogram
LC#6 : AD2

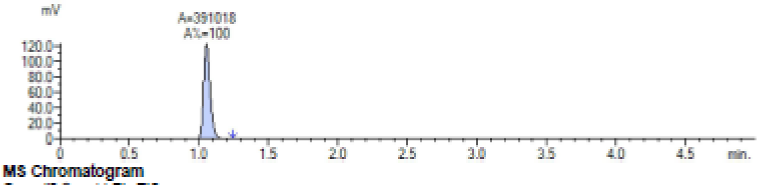

MS Chromatogram
Group#2 Scan(-) EI : TIC

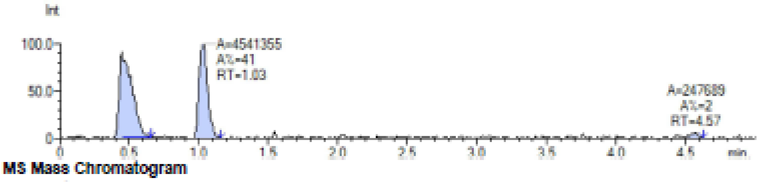

MS Mass Chromatogram
Group#2 Scan(-) EI : 414.2

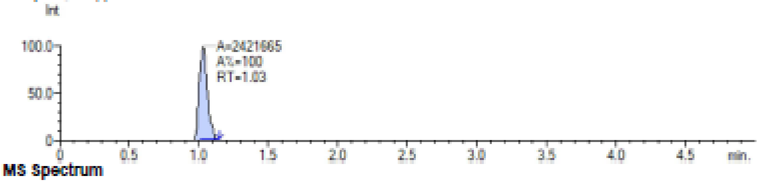

MS Spectrum
Group#2 - PDA Peak: 18, RT: 0.85 to 1.17 min

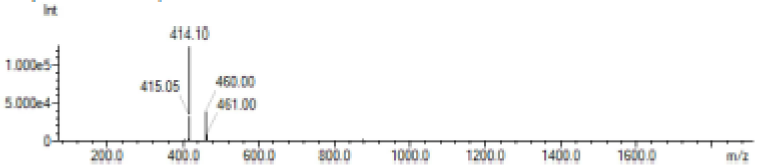

QD-I-11

Shimadzu Open Solution

Project: Test
Experiment: 382_180131_Zimmerm_Foley_Christo_0201_Li
Experiment Description:
Sample: 406297:01:01
Sample Description: C25H25N3O2
Data File Name: C:\LabSolutions\Data\382_180131_Zimmermmann_180131_Foley_180131_Christoforow_180201_Li\1046410636_11180201382.lcd
Sample Location: Plate Number: 2 - Position: 246
Run By: admin
Run Started: 12 February 2018 19:19:51
Run Finished: 13 February 2018 04:57:29
Method: Purity_Check+Pause

LC Chromatogram
LC#6 : AD2

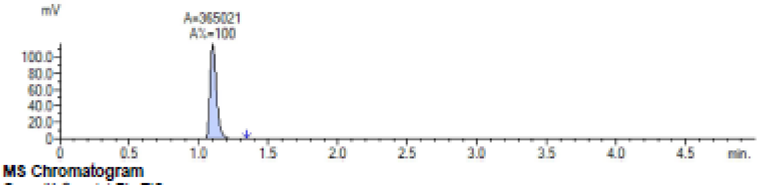

MS Chromatogram
Group#1 Scan(+) EI : TIC

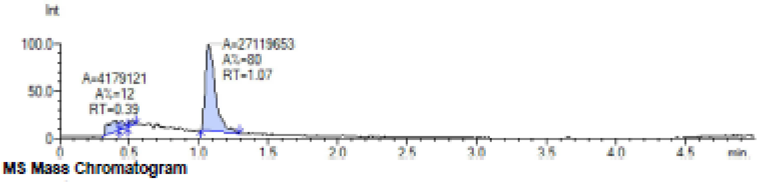

MS Mass Chromatogram
Group#1 Scan(+) EI : 400.2

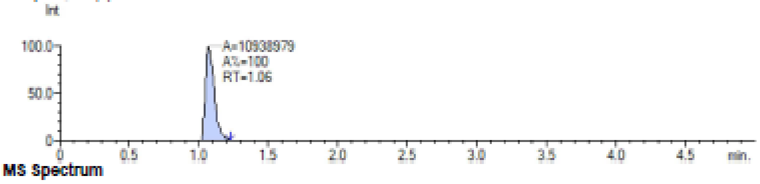

MS Spectrum
Group#1 - PDA Peak: 19, RT: 0.92 to 1.22 min

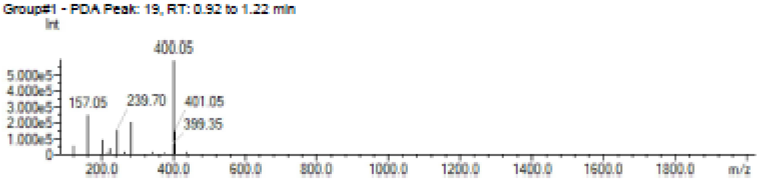

QD-I-12

Shimadzu Open Solution

**Project:**	Test
**Experiment:**	382_180131_Zimmerm_Foley_Christo_0201_LI
**Experiment Description:**	
**Sample:**	408304:01:01
**Sample Description:**	C24H22BrN3O2
**Data File Name:**	C:\LabSolutions\Data\382_180131_Zimmermmann_180131_Foley_180131_Christoforow_180201_LI\1046410911_11180201382.lcd
**Sample Location:**	Plate Number: 2 - Position: 200
**Run By:**	admin
**Run Started:**	12 February 2018 19:19:51
**Run Finished:**	13 February 2018 04:57:29
**Method:**	Purity_Check+Pause

LC Chromatogram

LC#6 : AD2

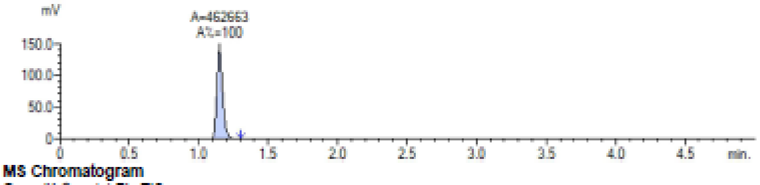

MS Chromatogram

Group#1 Scan(+) EI : TIC

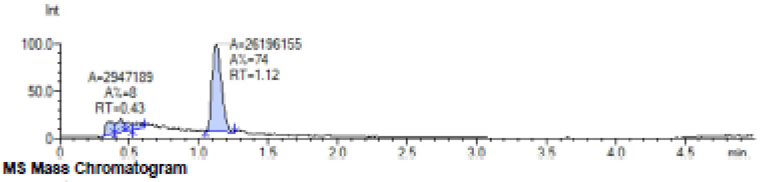

MS Mass Chromatogram

Group#1 Scan(+) EI : 464.1

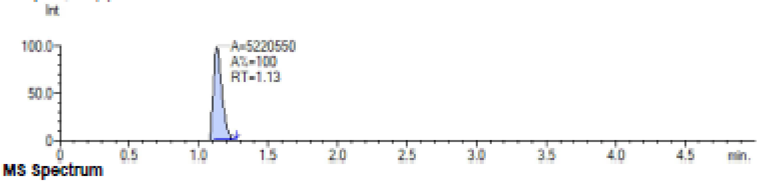

MS Spectrum

Group#1 - PDA Peak: 17, RT: 0.98 to 1.17 min

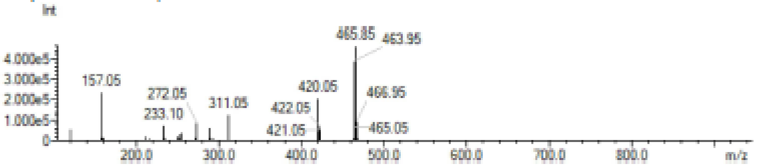

QD-I-13

Shimadzu Open Solution

Project: Test
Experiment: 382_180131_Zimmerm_Foley_Christo_0201_LI
Experiment Description:
Sample: 408305:01:01
Sample Description: C25H22F3N3O2
Data File Name: C:\LabSolutions\Data\382_180131_Zimmermmann_180131_Foley_180131_Christoforow_180201_LI\1046410417_11180201382.lcd
Sample Location: Plate Number: 2 - Position: 248
Run By: admin
Run Started: 12 February 2018 19:19:51
Run Finished: 13 February 2018 04:57:29
Method: Purity_Check+Pause

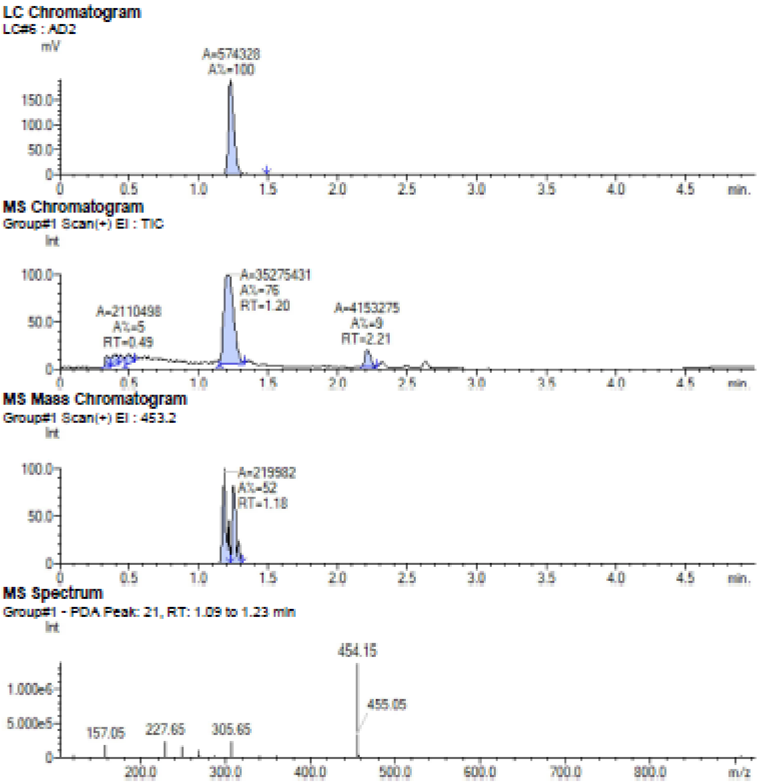

QD-I-14

Shimadzu Open Solution

Project: Test
Experiment: 382_180131_Zimmerm_Foley_Christo_0201_LI
Experiment Description:
Sample: 406307:01:01
Sample Description: C24H22N4O4
Data File Name: C:\LabSolutions\Data\382_180131_Zimmermmann_180131_Foley_180131_Christoforow_180201_LI\1046410234_11180201382.lcd
Sample Location: Plate Number: 2 - Position: 344
Run By: admin
Run Started: 12 February 2018 19:19:51
Run Finished: 13 February 2018 04:57:29
Method: Purity_Check+Pause

LC Chromatogram
LC#6 : AD2

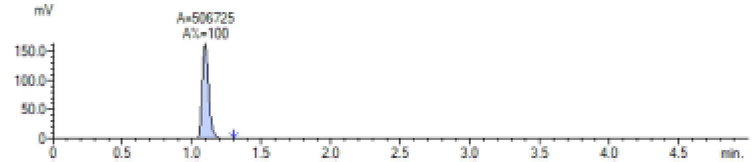

MS Chromatogram
Group#1 Scan(+) EI : TIC

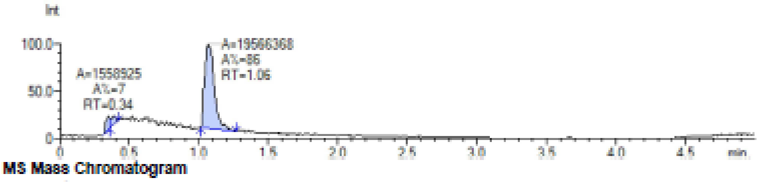

MS Mass Chromatogram
Group#1 Scan(+) EI : 431.2

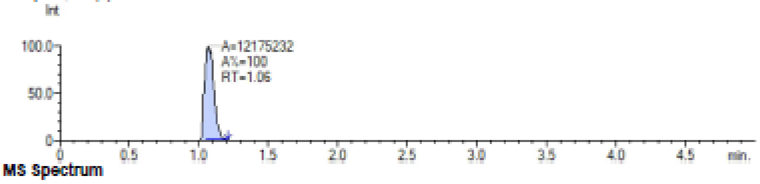

MS Spectrum
Group#1 - PDA Peak: 14, RT: 0.93 to 1.18 min

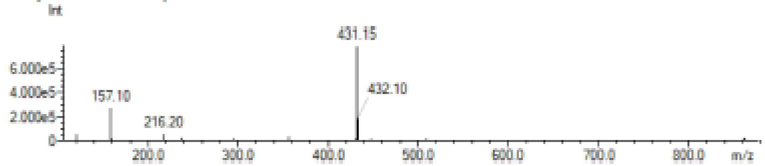

QD-I-15

Shimadzu Open Solution

Project: Test
Experiment: 382_180131_Zimmerm_Foley_Christo_0201_LI
Experiment Description:
Sample: 408308:01:01
Sample Description: C24H22BrN3O2
Data File Name: C:\LabSolutions\Data\382_180131_Zimmermmann_180131_Foley_180131_Christoforow_180201_LI\1046410910_11180201382.lcd
Sample Location: Plate Number: 2 - Position: 10
Run By: admin
Run Started: 12 February 2018 19:19:51
Run Finished: 13 February 2018 04:57:29
Method: Purity_Check+Pause

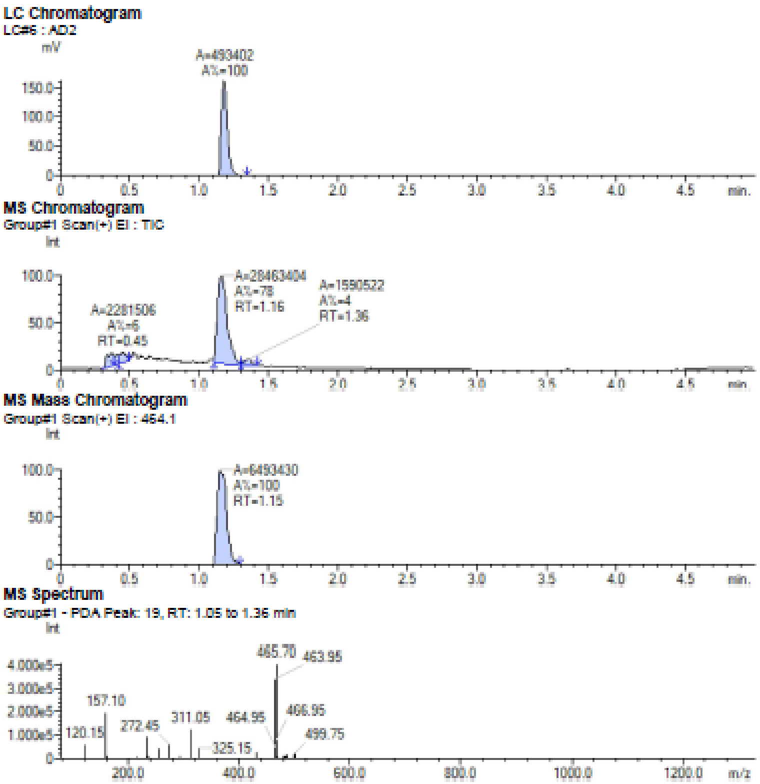

QD-I-16

Shimadzu Open Solution

Project: Test
Experiment: 383_180131_NatA_TEAD_180201_Foley_Shan
Experiment Description:
Sample: 408310:01:01
Sample Description: C24H22ClN3O2
Data File Name: C:\LabSolutions\Data\383_180131_NatA_180131_TEAD_180201_Foley_180201_Shan\1046410971_11180201383.lcd
Sample Location: Plate Number: 1 - Position: 25
Run By: admin
Run Started: 12 February 2018 10:27:31
Run Finished: 12 February 2018 12:52:26
Method: Purity_Check_2018

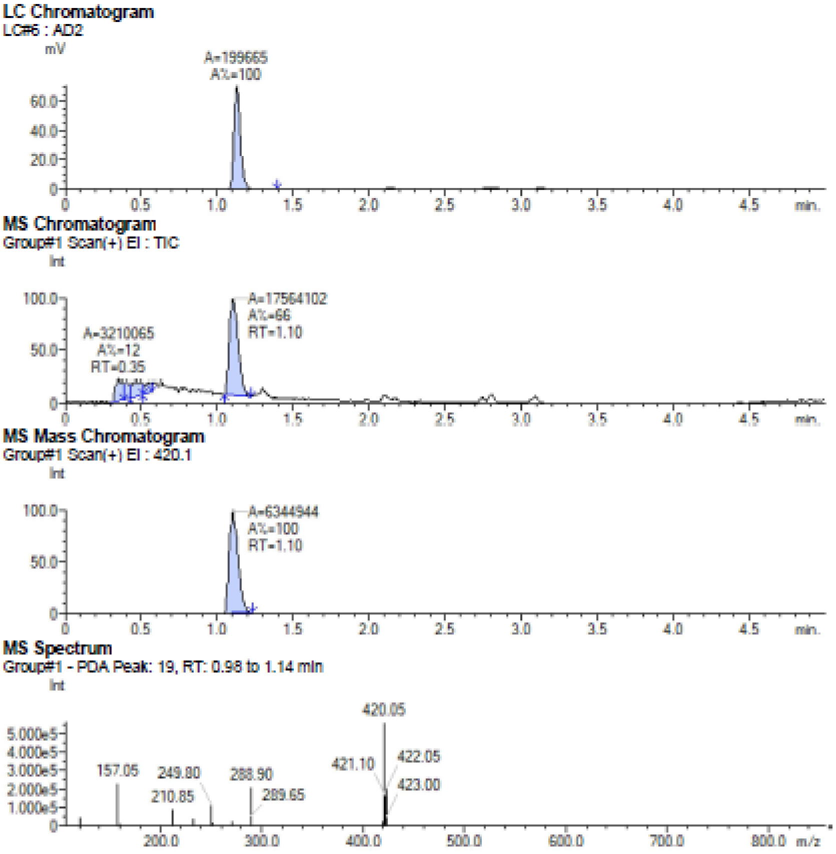

QD-I-17

Shimadzu Open Solution

Project:	Test
Experiment:	383_180131_NatA_TEAD_180201_Foley_Shan
Experiment Description:	
Sample:	408312:01:01
Sample Description:	C25H25N3O3
Data File Name:	C:\LabSolutions\Data\383_180131_NatA_180131_TEAD_180201_Foley_180201_Shan\1046410445_11180201383.lcd
Sample Location:	Plate Number: 1 - Position: 121
Run By:	admin
Run Started:	12 February 2018 10:27:31
Run Finished:	12 February 2018 12:52:26
Method:	Purity_Check_2018

LC Chromatogram

LC#6 : AD2

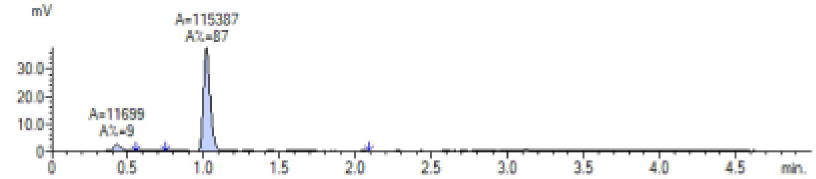

MS Chromatogram

Group#1 Scan(+) EI : TIC

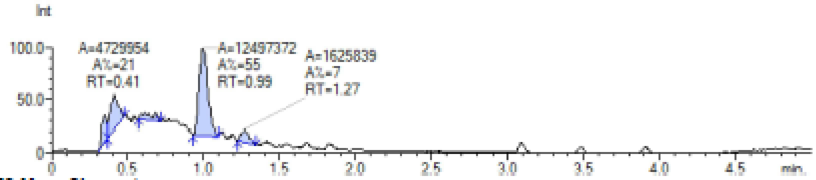

MS Mass Chromatogram

Group#1 Scan(+) EI : 416.2

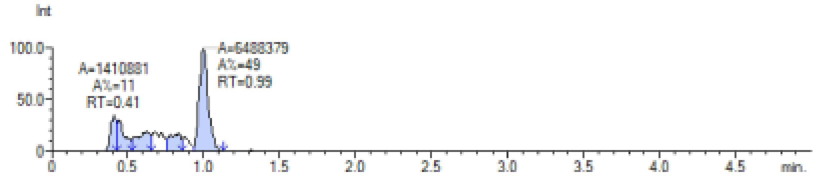

MS Spectrum

Group#1 - PDA Peak: 42, RT: 0.87 to 0.99 min

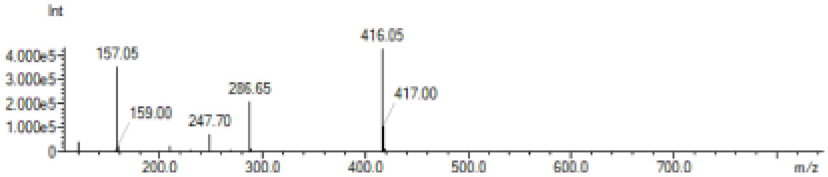

QD-I-18

Shimadzu Open Solution

Project:	Test
Experiment:	383_180131_NatA_TEAD_180201_Foley_Shan
Experiment Description:	
Sample:	408313:01:01
Sample Description:	C24H22ClN3O2
Data File Name:	C:\LabSolutions\Data\383_180131_NatA_180131_TEAD_180201_Foley_180201_Shan\1046410161_11180201383.lcd
Sample Location:	Plate Number: 1 - Position: 169
Run By:	admin
Run Started:	12 February 2018 10:27:31
Run Finished:	12 February 2018 12:52:26
Method:	Purity_Check_2018

LC Chromatogram

LC#6 : AD2

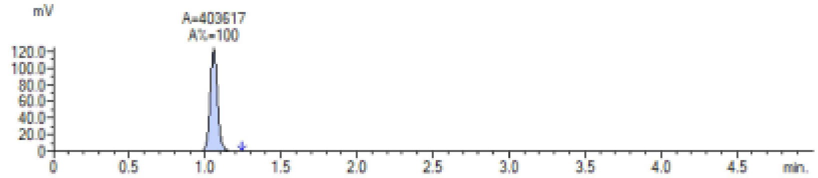

MS Chromatogram

Group#1 Scan(+) EI : TIC

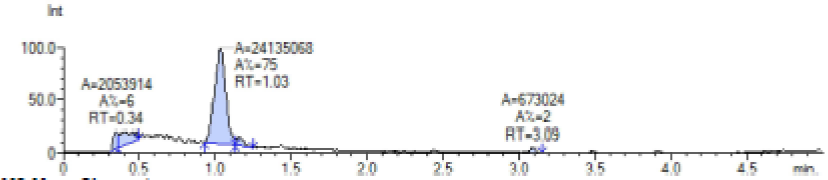

MS Mass Chromatogram

Group#1 Scan(+) EI : 420.1

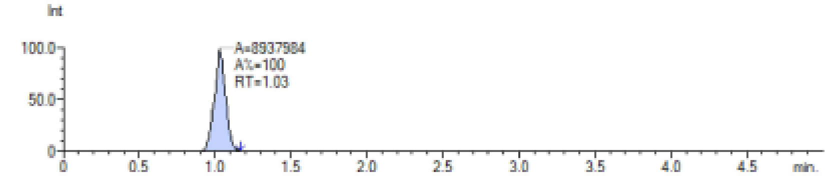

MS Spectrum

Group#1 - PDA Peak: 17, RT: 0.82 to 1.04 min

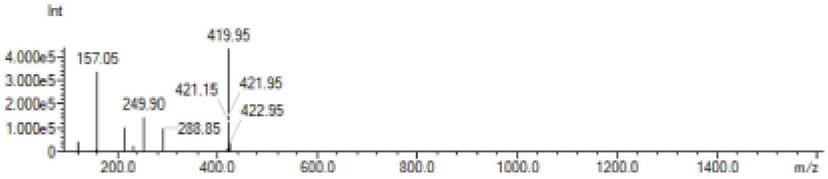

QD-I-19

Shimadzu Open Solution

Project:	Test
Experiment:	383_180131_NatA_TEAD_180201_Foley_Shan
Experiment Description:	
Sample:	408314:01:01
Sample Description:	C25H25N3O2
Data File Name:	C:\LabSolutions\Data\383_180131_NatA_180131_TEAD_180201_Foley_180201_Shan\1046410792_11180201383.lcd
Sample Location:	Plate Number: 1 - Position: 217
Run By:	admin
Run Started:	12 February 2018 10:27:31
Run Finished:	12 February 2018 12:52:26
Method:	Purity_Check_2018

LC Chromatogram

LC#6 : AD2

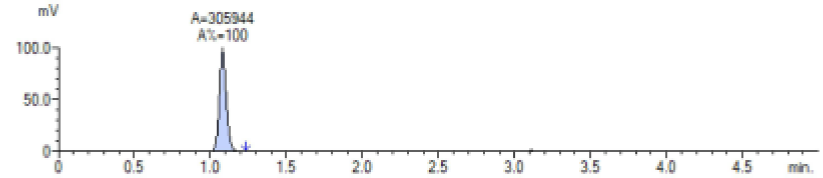

MS Chromatogram

Group#1 Scan(+) EI : TIC

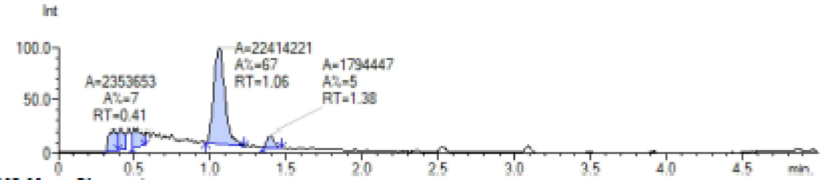

MS Mass Chromatogram

Group#1 Scan(+) EI : 400.2

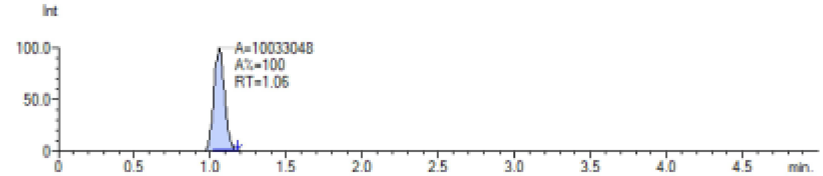

MS Spectrum

Group#1 - PDA Peak: 19, RT: 0.87 to 1.38 min

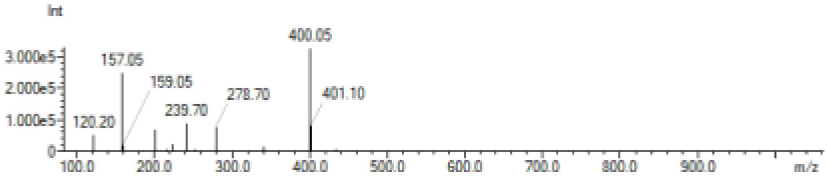

Shimadzu Open Solution

QN-C-S-1

**Project:**	Test
**Experiment:**	430_0530_Grigalu_0605_3101_TEAD_Wu
**Experiment Description:**	
**Sample:**	408580:01:01
**Sample Description:**	C26H26N2O4
**Data File Name:**	C:\LabSolutions\Data\430_180530_Grigalunas_180605_3101_180605_Tead_180605_Wu\1046325506_11180530431.lcd
**Sample Location:**	Plate Number: 2 - Position: 1
**Run By:**	admin
**Run Started:**	24 September 2018 14:46:57
**Run Finished:**	25 September 2018 00:19:16
**Method:**	Purity_Check_2018

LC Chromatogram

LC#6 : AD2

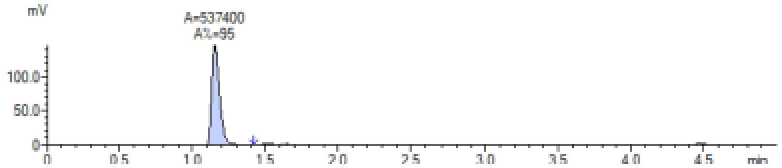

MS Chromatogram

Group#1 Scan(+) EI : TIC

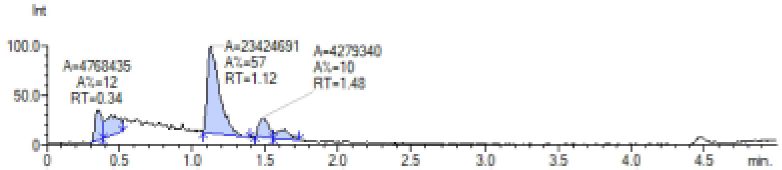

MS Mass Chromatogram

Group#1 Scan(+) EI : 431.2

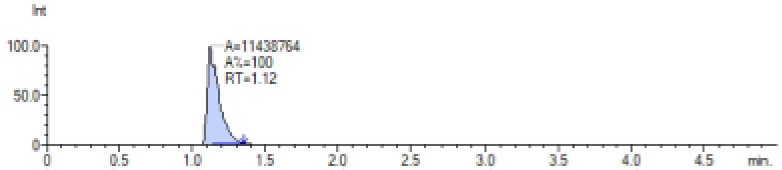

MS Spectrum

Group#1 - PDA Peak: 18, RT: 0.99 to 1.27 min

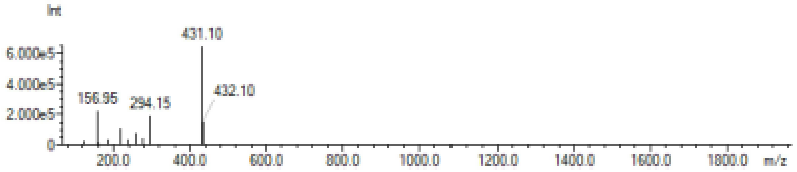

QN-C-R-1

Shimadzu Open Solution

**Project:**	Test
**Experiment:**	430_0530_Grigalu_0605_3101_TEAD_Wu
**Experiment Description:**	
**Sample:**	408581:01:02
**Sample Description:**	C26H26N2O4
**Data File Name:**	C:\LabSolutions\Data\430_180530_Grigalunas_180605_3101_180605_Tead_180605_Wu\1046325586_11180530431.lcd
**Sample Location:**	Plate Number: 2 - Position: 49
**Run By:**	admin
**Run Started:**	24 September 2018 14:46:57
**Run Finished:**	25 September 2018 00:19:16
**Method:**	Purity_Check_2018

LC Chromatogram

LC#6 : AD2

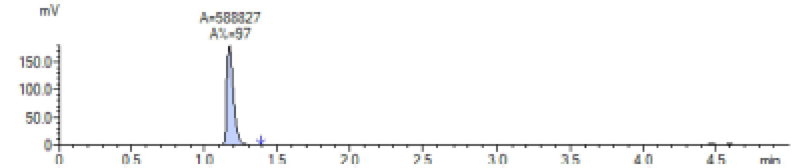

MS Chromatogram

Group#1 Scan(+) EI : TIC

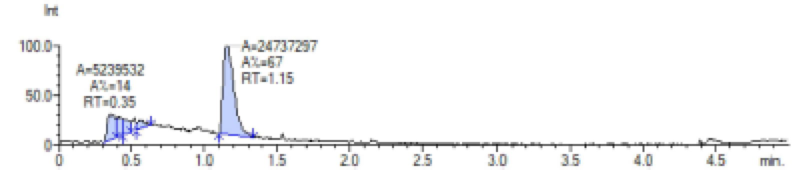

MS Mass Chromatogram

Group#1 Scan(+) EI : 431.2

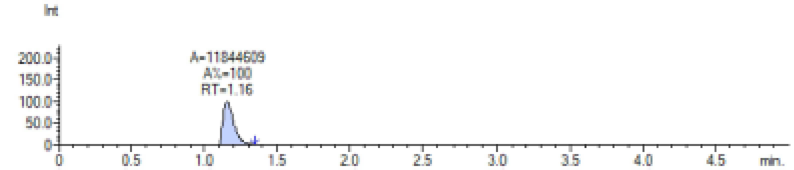

MS Spectrum

Group#1 - PDA Peak: 22, RT: 1.00 to 1.29 min

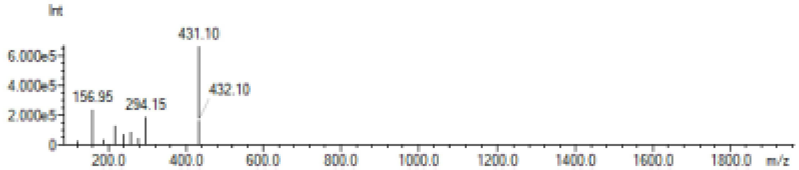

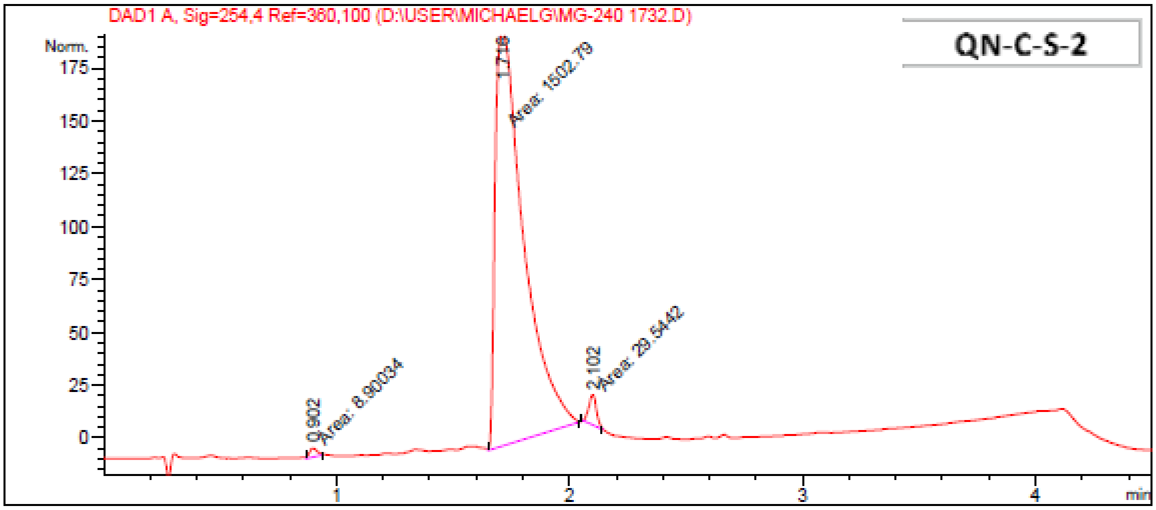

#	Time	Area	Height	Width	Area%	Symmetry
1	0.902	8.9	3.9	0.0383	0.577	0.849
2	1.716	1502.8	175.9	0.1424	97.506	0.33
3	2.102	29.5	12.9	0.038	1.917	1.019

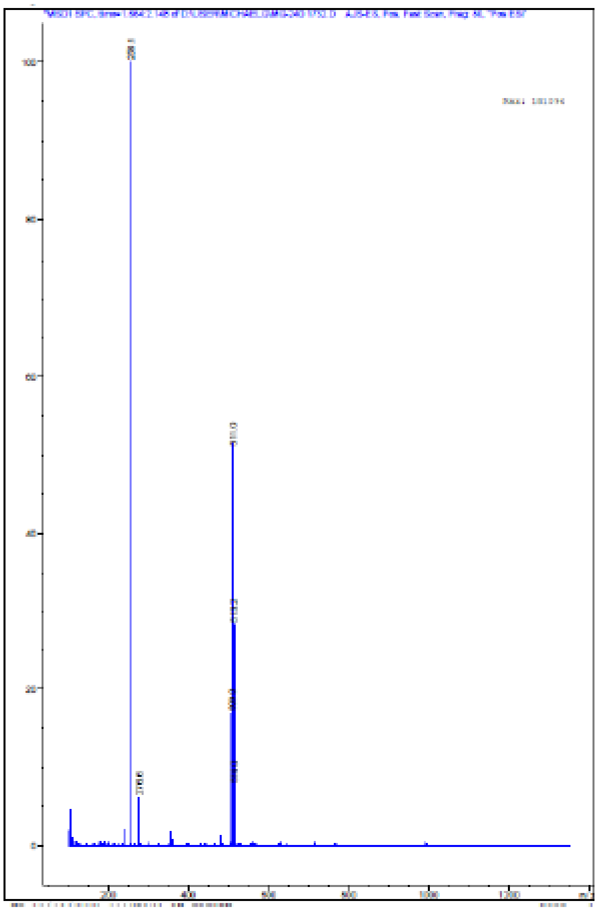

Shimadzu Open Solution

QN-C-R-2

Project:	Test
Experiment:	430_0530_Grigalu_0605_3101_TEAD_Wu
Experiment Description:	
Sample:	408615:01:01
Sample Description:	C26H25BrN2O4
Data File Name:	C:\LabSolutions\Data\430_180530_Grigalunas_180605_3101_180605_Tead_180605_Wu\1046325127_11180530431.lcd
Sample Location:	Plate Number: 2 - Position: 53
Run By:	admin
Run Started:	24 September 2018 14:46:57
Run Finished:	25 September 2018 00:19:16
Method:	Purity_Check_2018

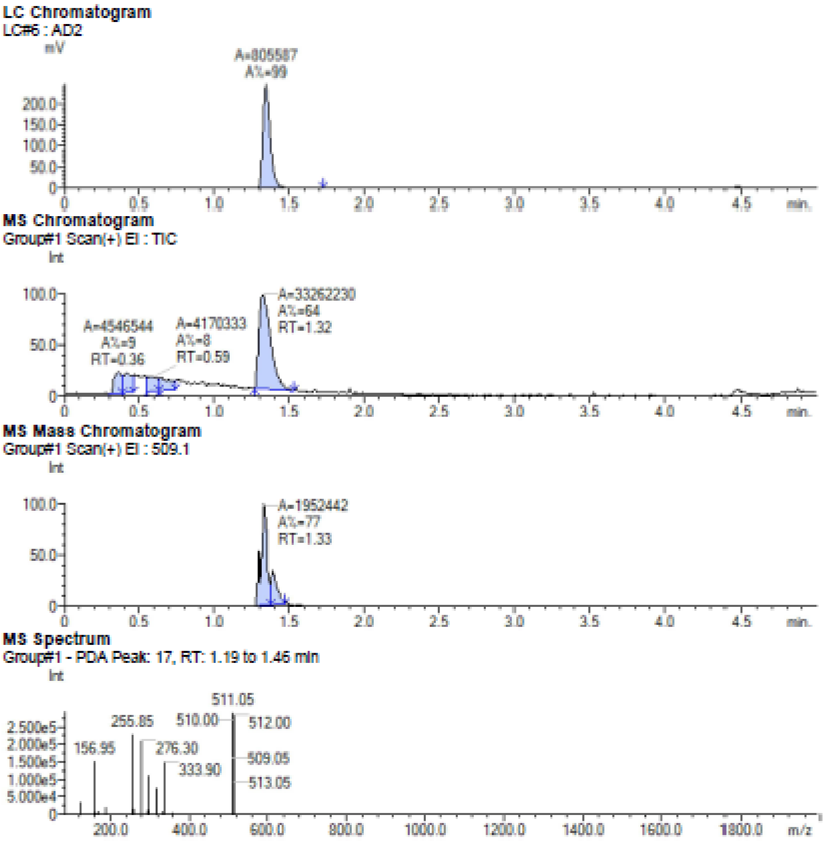

QN-C-S-3

Shimadzu Open Solution

Project: Test
Experiment: 525_0206_Christo_Grigalu_Burhop_Wilke
Experiment Description:
Sample: 409679:01:01
Sample Description: C26H25ClN2O4
Data File Name: C:\LabSolutions\Data\525_190206_Christoforow_190206_Grigalunas_190206_Burhop_190206_Wilke\1046360323_11190207525.lcd
Sample Location: Plate Number: 2 - Position: 150
Run By: admin
Run Started: 23 April 2019 18:20:13
Run Finished: 24 April 2019 04:25:41
Method: 190321_Purity-Check+shut down

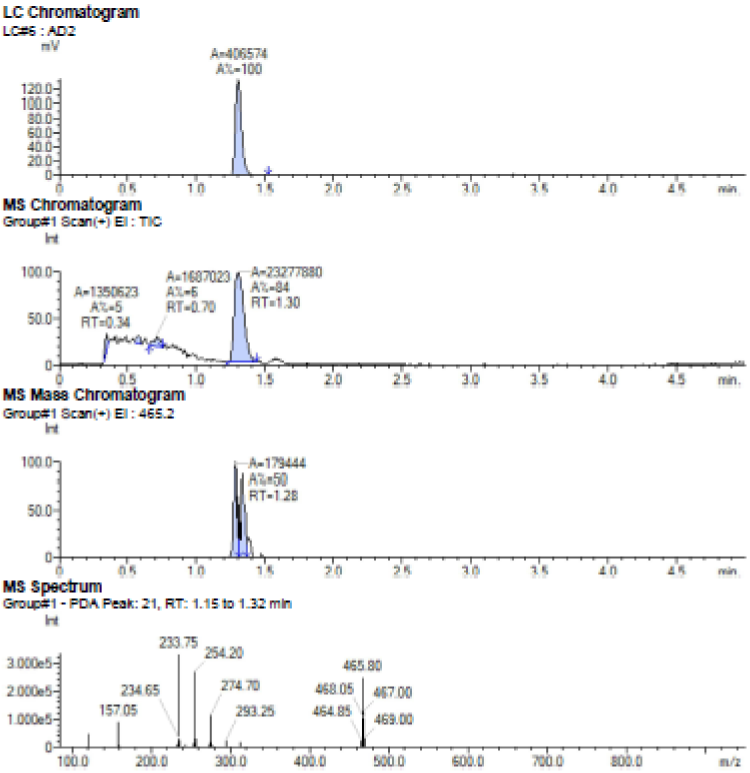

QN-C-S-4

Shimadzu Open Solution

Project: Test
Experiment: 430_0530_Grigalu_0605_3101_TEAD_Wu
Experiment Description:
Sample: 408605:01:01
Sample Description: C26H25FN2O4
Data File Name: C:\LabSolutions\Data\430_180530_Grigalunas_180605_3101_180605_Tead_180605_Wu\1046325545_11180530431.lcd
Sample Location: Plate Number: 2 - Position: 337
Run By: admin
Run Started: 24 September 2018 14:46:57
Run Finished: 25 September 2018 00:19:16
Method: Purity_Check_2018

LC Chromatogram
LC#6 : AD2

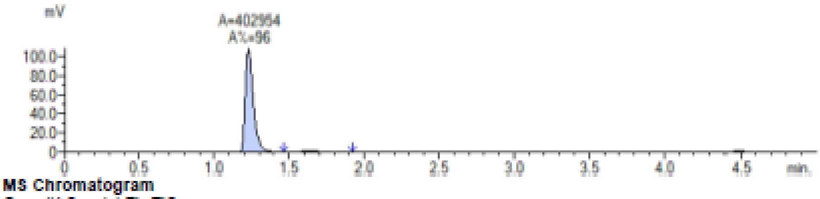

MS Chromatogram
Group#1 Scan(+) EI : TIC

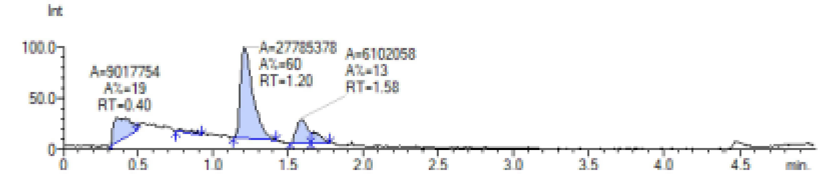

MS Mass Chromatogram
Group#1 Scan(+) EI : 449.2

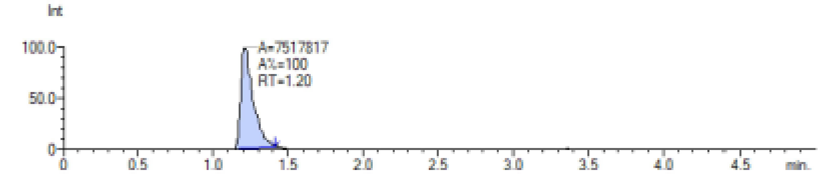

MS Spectrum
Group#1 - PDA Peak: 19, RT: 1.03 to 1.42 min

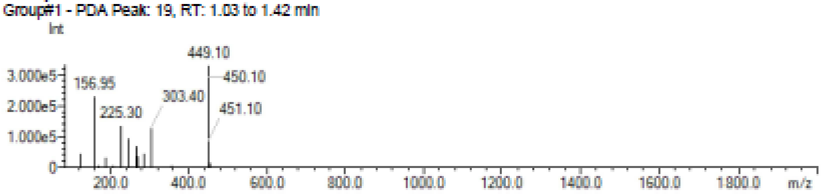

Shimadzu Open Solution

QN-C-R-3

Project: Test
Experiment: 430_0530_Grigalu_0605_3101_TEAD_Wu
Experiment Description:
Sample: 408606:01:01
Sample Description: C26H25FN2O4
Data File Name: C:\LabSolutions\Data\430_180530_Grigalunas_180605_3101_180605_Tead_180605_Wu\1046325524_11180530431.lcd
Sample Location: Plate Number: 2 - Position: 3
Run By: admin
Run Started: 24 September 2018 14:46:57
Run Finished: 25 September 2018 00:19:16
Method: Purity_Check_2018

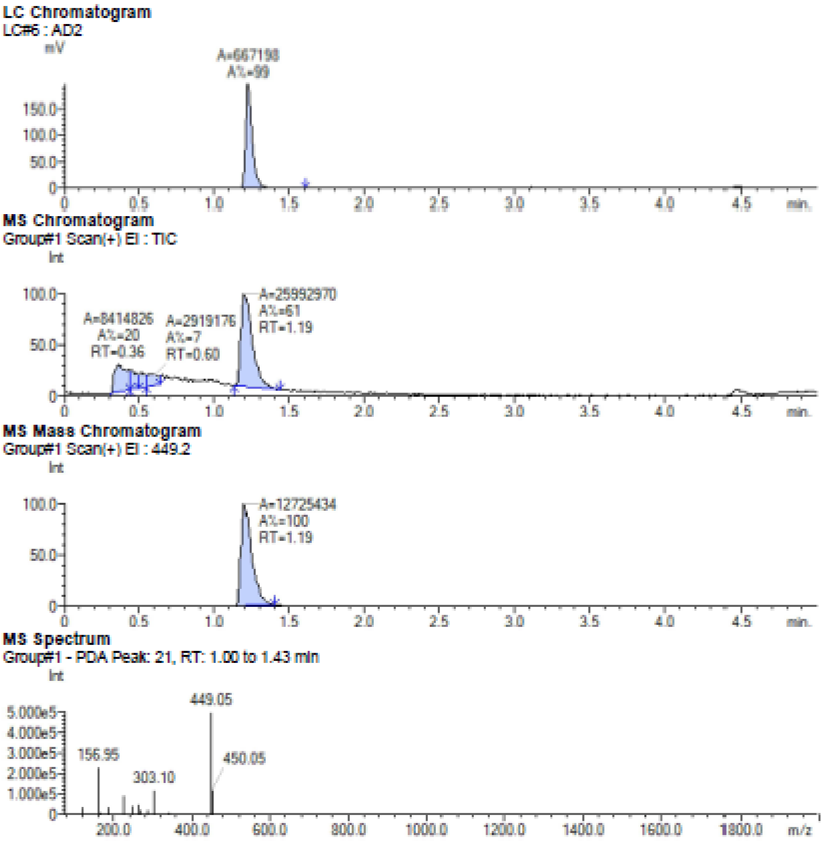

QN-C-R-4

Shimadzu Open Solution

Project: Test
Experiment: 430_0530_Grigalu_0605_3101_TEAD_Wu
Experiment Description:
Sample: 408608:01:01
Sample Description: C27H28N2O5
Data File Name: C:\LabSolutions\Data\430_180530_Grigalunas_180605_3101_180605_Tead_180605_Wu\1046325534_11180530431.lcd
Sample Location: Plate Number: 2 - Position: 99
Run By: admin
Run Started: 24 September 2018 14:46:57
Run Finished: 25 September 2018 00:19:16
Method: Purity_Check_2018

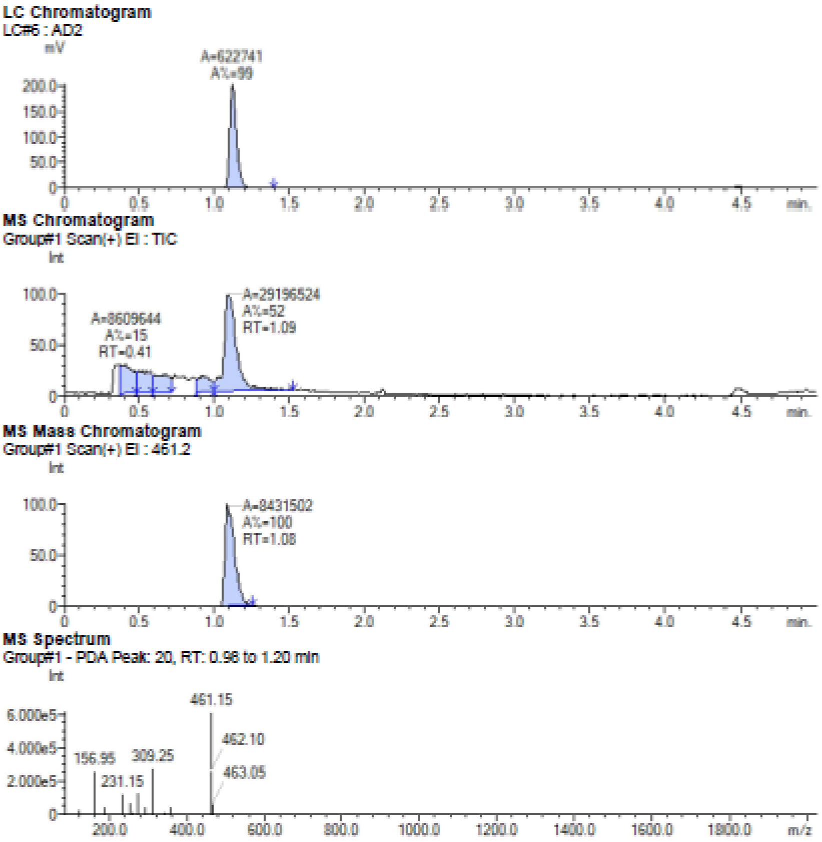

QN-C-R-5

Shimadzu Open Solution

**Project:**	Test
**Experiment:**	430_0530_Grigalu_0605_3101_TEAD_Wu
**Experiment Description:**	
**Sample:**	408607:01:02
**Sample Description:**	C27H28N2O5
**Data File Name:**	C:\LabSolutions\Data\430_180530_Grigalunas_180605_3101_180605_Tead_180605_Wu\1046325429_11180530431.lcd
**Sample Location:**	Plate Number: 2 - Position: 51
**Run By:**	admin
**Run Started:**	24 September 2018 14:46:57
**Run Finished:**	25 September 2018 00:19:16
**Method:**	Purity_Check_2018

LC Chromatogram

LC#6 : AD2

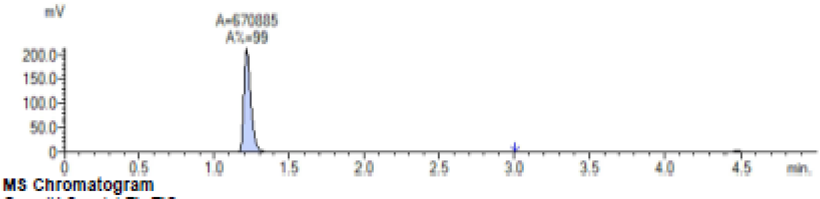

MS Chromatogram

Group#1 Scan(+) EI : TIC

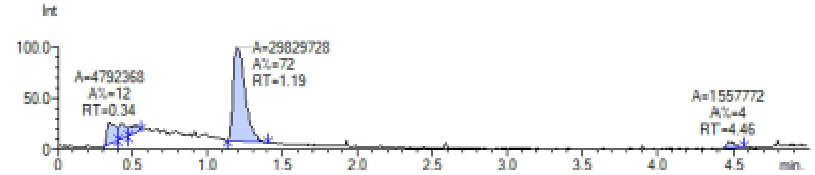

MS Mass Chromatogram

Group#1 Scan(+) EI : 461.2

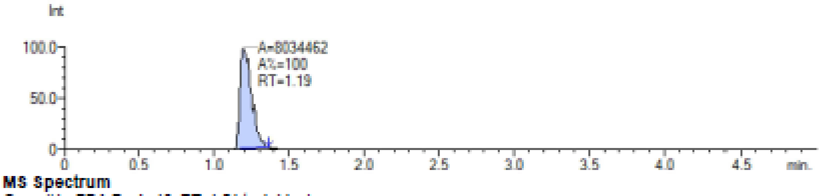

MS Spectrum

Group#1 - PDA Peak: 19, RT: 1.01 to 1.44 min

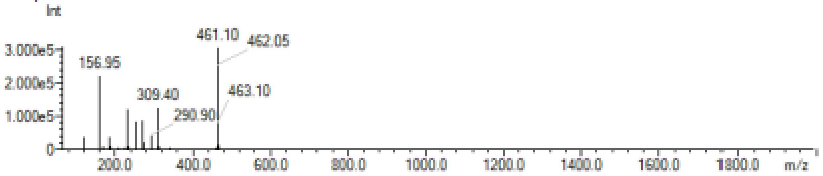

QN-C-S-5

Shimadzu Open Solution

Project: Test
Experiment: 430_0530_Grigalu_0605_3101_TEAD_Wu
Experiment Description:
Sample: 408610:01:01
Sample Description: C27H28N2O5
Data File Name: C:\LabSolutions\Data\430_180530_Grigalunas_180605_3101_180605_Tead_180605_Wu\1046325415_11180530431.lcd
Sample Location: Plate Number: 2 - Position: 195
Run By: admin
Run Started: 24 September 2018 14:46:57
Run Finished: 25 September 2018 00:19:16
Method: Purity_Check_2018

LC Chromatogram
LC#6 : AD2

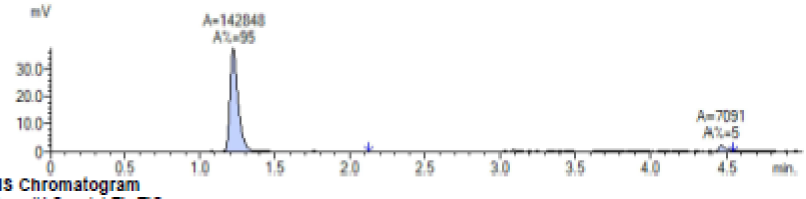

MS Chromatogram
Group#1 Scan(+) EI : TIC

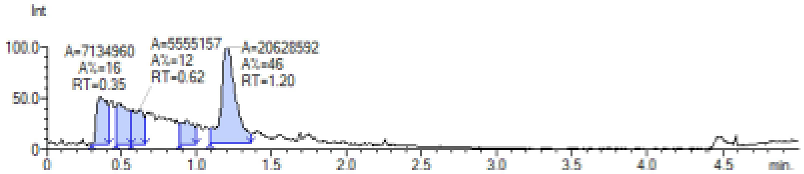

MS Mass Chromatogram
Group#1 Scan(+) EI : 461.2

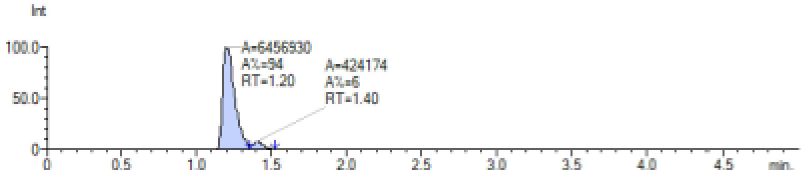

MS Spectrum
Group#1 - PDA Peak: 20, RT: 1.05 to 1.39 min

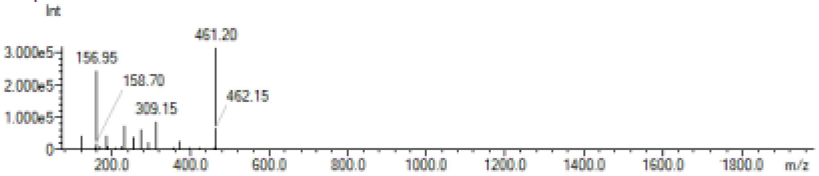

QN-C-R-6

Shimadzu Open Solution

Project: Test
Experiment: 430_0530_Grigalu_0605_3101_TEAD_Wu
Experiment Description:
Sample: 408611:01:01
Sample Description: C27H28N2O5
Data File Name: C:\LabSolutions\Data\430_180530_Grigalunas_180605_3101_180605_Tead_180605_Wu\1046325634_11180530431.lcd
Sample Location: Plate Number: 2 - Position: 243
Run By: admin
Run Started: 24 September 2018 14:46:57
Run Finished: 25 September 2018 00:19:16
Method: Purity_Check_2018

LC Chromatogram
LC#6 : AD2

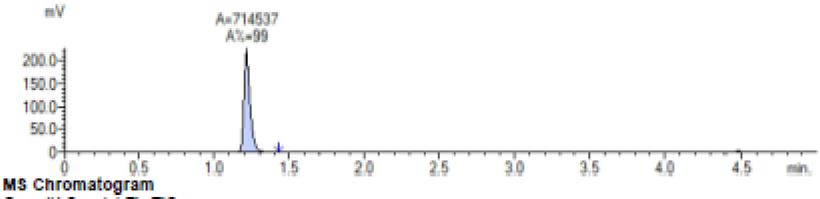

MS Chromatogram
Group#1 Scan(+) EI : TIC

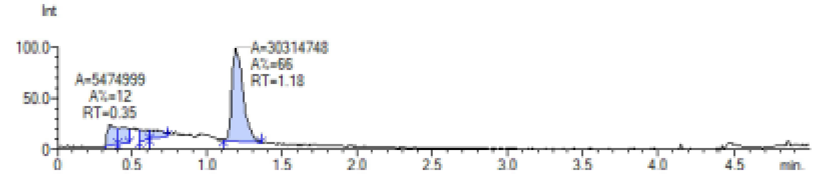

MS Mass Chromatogram
Group#1 Scan(+) EI : 461.2

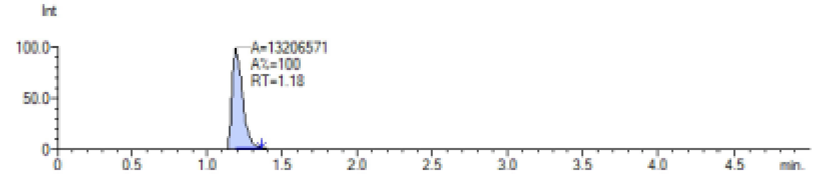

MS Spectrum
Group#1 - PDA Peak: 20, RT: 1.00 to 1.38 min

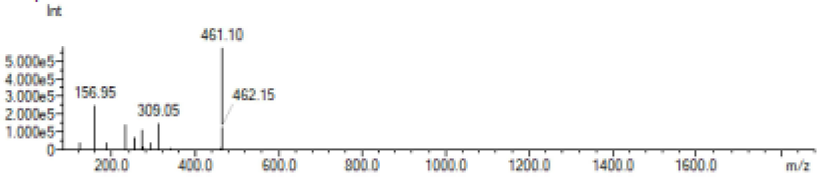

QN-C-S-6

Shimadzu Open Solution

Project: Test
Experiment: 430_0530_Grigalu_0605_3101_TEAD_Wu
Experiment Description:
Sample: 408643:01:02
Sample Description: C28H30N2O6
Data File Name: C:\LabSolutions\Data\430_180530_Grigalunas_180605_3101_180605_Tead_180605_Wu\1046325248_11180530431.lcd
Sample Location: Plate Number: 2 - Position: 247
Run By: admin
Run Started: 24 September 2018 14:46:57
Run Finished: 25 September 2018 00:19:16
Method: Purity_Check_2018

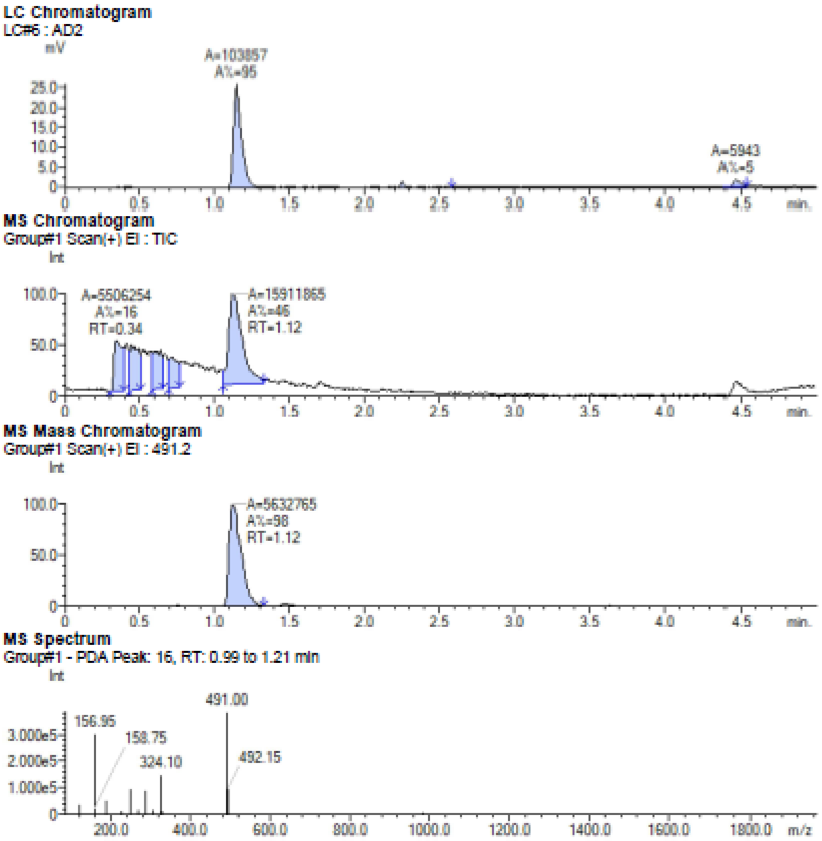

QN-C-R-7

Shimadzu Open Solution

**Project:**	Test
**Experiment:**	430_0530_Grigalu_0605_3101_TEAD_Wu
**Experiment Description:**	
**Sample:**	408644:01:01
**Sample Description:**	C28H30N2O6
**Data File Name:**	C:\LabSolutions\Data\430_180530_Grigalunas_180605_3101_180605_Tead_180605_Wu\1046325495_11180530431.lcd
**Sample Location:**	Plate Number: 2 - Position: 295
**Run By:**	admin
**Run Started:**	24 September 2018 14:46:57
**Run Finished:**	25 September 2018 00:19:16
**Method:**	Purity_Check_2018

LC Chromatogram
LC#6 : AD2

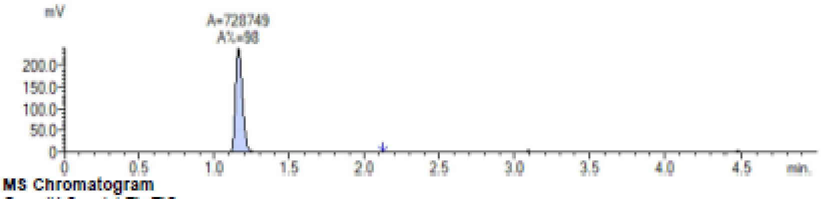

MS Chromatogram
Group#1 Scan(+) EI : TIC

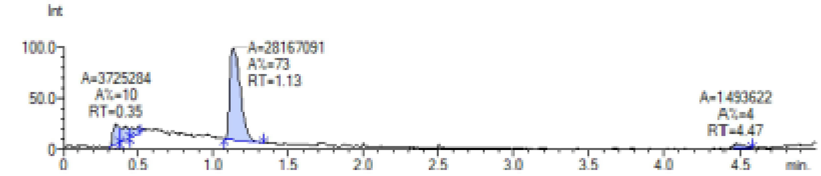

MS Mass Chromatogram
Group#1 Scan(+) EI : 491.2

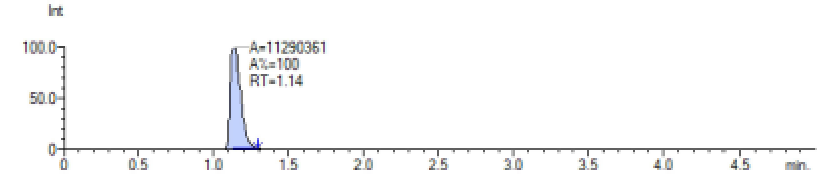

MS Spectrum
Group#1 - PDA Peak: 19, RT: 0.99 to 1.32 min

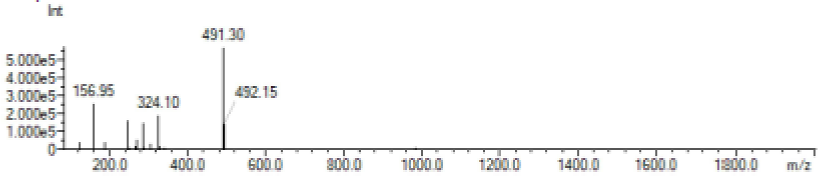

QN-C-S-7

Shimadzu Open Solution

Project: Test
Experiment: 430_0530_Grigalu_0605_3101_TEAD_Wu
Experiment Description:
Sample: 408636:01:01
Sample Description: C28H30N2O6
Data File Name: C:\LabSolutions\Data\430_180530_Grigalunas_180605_3101_180605_Tead_180605_Wu\1046325060_11180530431.lcd
Sample Location: Plate Number: 2 - Position: 341
Run By: admin
Run Started: 24 September 2018 14:46:57
Run Finished: 25 September 2018 00:19:16
Method: Purity_Check_2018

LC Chromatogram
LC#6 : AD2

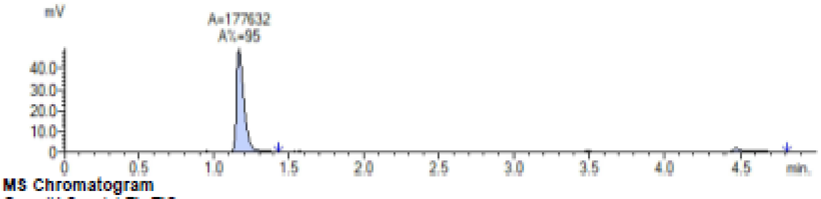

MS Chromatogram
Group#1 Scan(+) EI : TIC

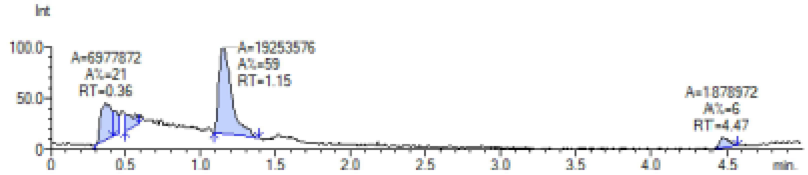

MS Mass Chromatogram
Group#1 Scan(+) EI : 491.2

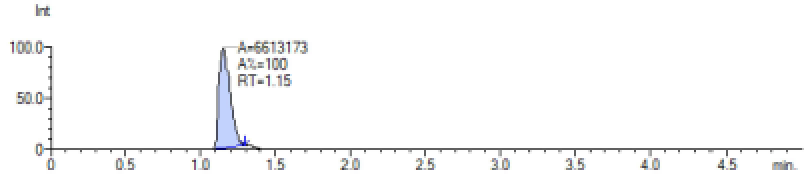

MS Spectrum
Group#1 - PDA Peak: 18, RT: 1.00 to 1.35 min

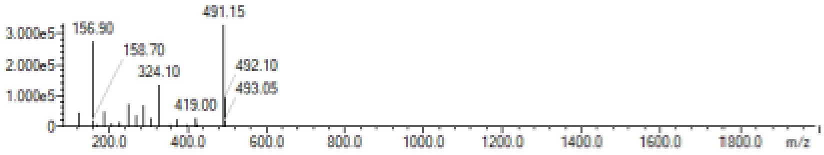

QN-C-R-8

Shimadzu Open Solution

Project: Test
Experiment: 430_0530_Grigalu_0605_3101_TEAD_Wu
Experiment Description:
Sample: 408637:01:01
Sample Description: C28H30N2O6
Data File Name: C:\LabSolutions\Data\430_180530_Grigalunas_180605_3101_180605_Tead_180605_Wu\1046324967_11180530431.lcd
Sample Location: Plate Number: 2 - Position: 7
Run By: admin
Run Started: 24 September 2018 14:46:57
Run Finished: 25 September 2018 00:19:16
Method: Purity_Check_2018

LC Chromatogram
LC#6 : AD2

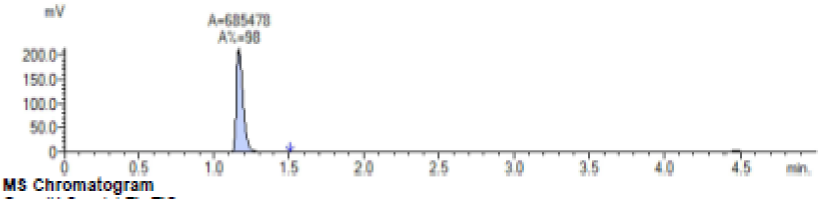

MS Chromatogram
Group#1 Scan(+) EI : TIC

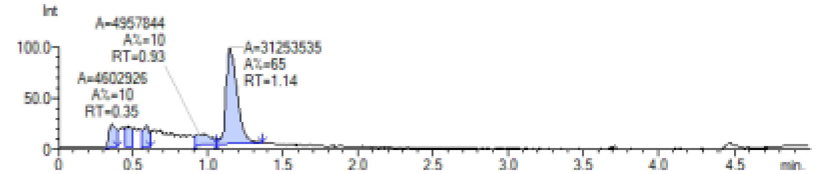

MS Mass Chromatogram
Group#1 Scan(+) EI : 491.2

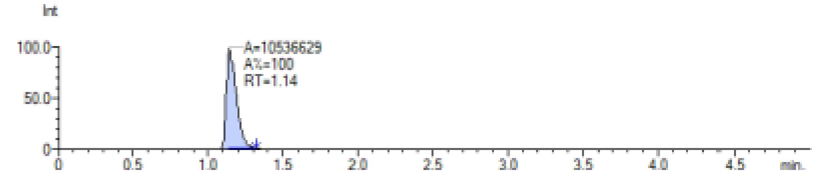

MS Spectrum
Group#1 - PDA Peak: 19, RT: 1.01 to 1.27 min

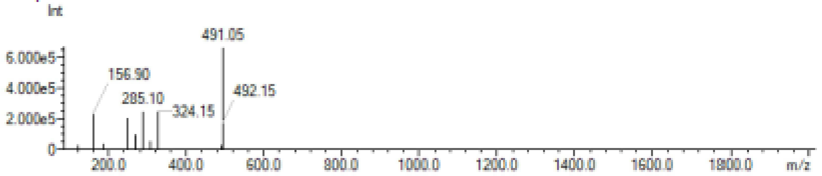

QN-C-S-8

Shimadzu Open Solution

Project:	Test
Experiment:	430_0530_Grigalu_0605_3101_TEAD_Wu
Experiment Description:	
Sample:	408584:01:01
Sample Description:	C26H26N2O5
Data File Name:	C:\LabSolutions\Data\430_180530_Grigalunas_180605_3101_180605_Tead_180605_Wu\1046325559_11180530431.lcd
Sample Location:	Plate Number: 2 - Position: 97
Run By:	admin
Run Started:	24 September 2018 14:46:57
Run Finished:	25 September 2018 00:19:16
Method:	Purity_Check_2018

LC Chromatogram
LC#6 : AD2

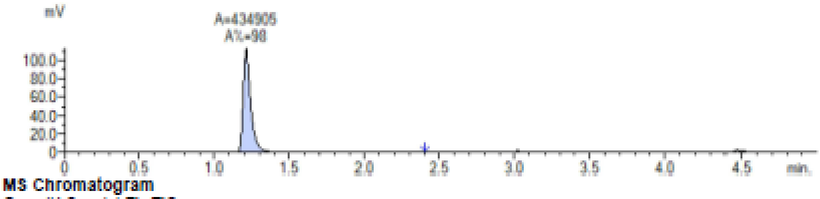

MS Chromatogram
Group#1 Scan(+) EI : TIC

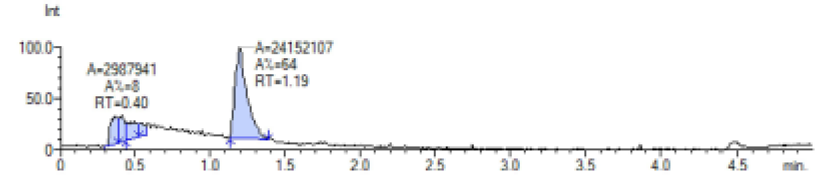

MS Mass Chromatogram
Group#1 Scan(+) EI : 447.2

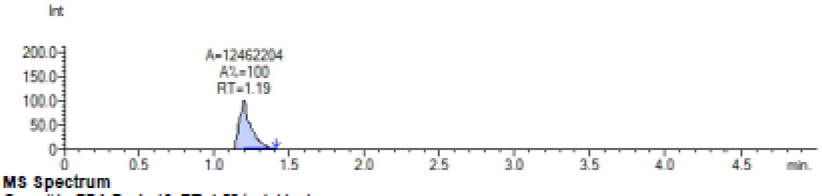

MS Spectrum
Group#1 - PDA Peak: 19, RT: 1.03 to 1.41 min

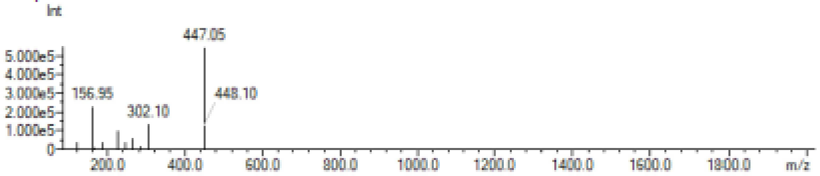

Shimadzu Open Solution

QN-C-R-9

Project: Test
Experiment: 430_0530_Grigalu_0605_3101_TEAD_Wu
Experiment Description:
Sample: 408585:01:02
Sample Description: C26H26N2O5
Data File Name: C:\LabSolutions\Data\430_180530_Grigalunas_180605_3101_180605_Tead_180605_Wu\1046325594_11180530431.lcd
Sample Location: Plate Number: 2 - Position: 145
Run By: admin
Run Started: 24 September 2018 14:46:57
Run Finished: 25 September 2018 00:19:16
Method: Purity_Check_2018

LC Chromatogram
LC#6 : AD2

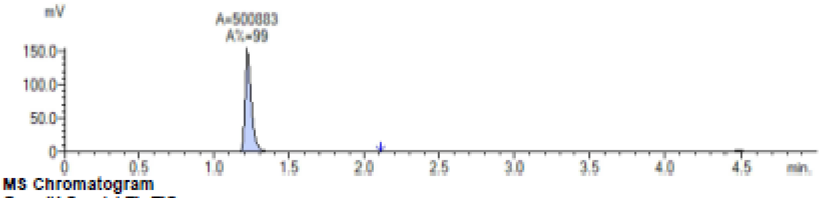

MS Chromatogram
Group#1 Scan(+) EI : TIC

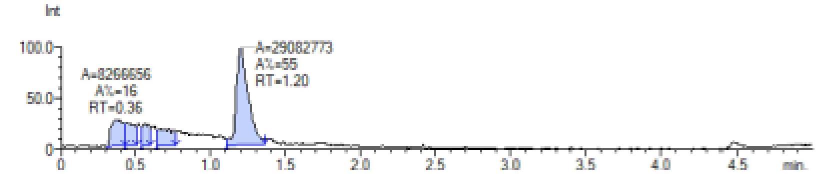

MS Mass Chromatogram
Group#1 Scan(+) EI : 447.2

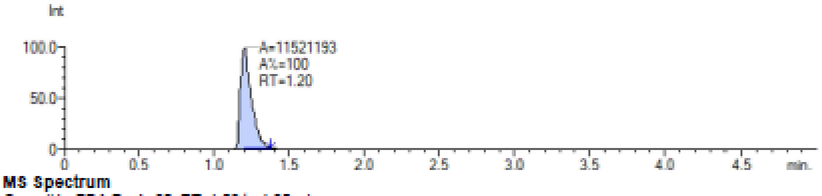

MS Spectrum
Group#1 - PDA Peak: 20, RT: 1.06 to 1.25 min

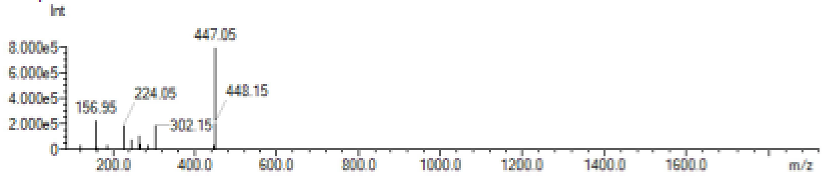

Shimadzu Open Solution

QN-C-R-10

**Project:**	Test
**Experiment:**	430_0530_Grigalu_0605_3101_TEAD_Wu
**Experiment Description:**	
**Sample:**	408588:01:02
**Sample Description:**	C26H26N2O5
**Data File Name:**	C:\LabSolutions\Data\430_180530_Grigalunas_180605_3101_180605_Tead_180605_Wu\1046324936_11180530431.lcd
**Sample Location:**	Plate Number: 2 - Position: 19
**Run By:**	admin
**Run Started:**	24 September 2018 14:46:57
**Run Finished:**	25 September 2018 00:19:16
**Method:**	Purity_Check_2018

LC Chromatogram
LC#6 : AD2

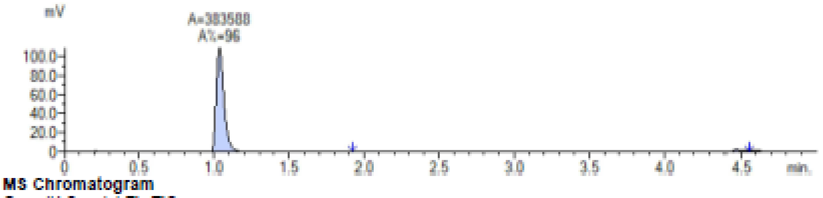

MS Chromatogram
Group#1 Scan(+) EI : TIC

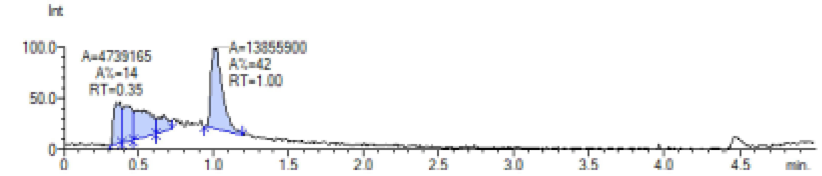

MS Mass Chromatogram
Group#1 Scan(+) EI : 447.2

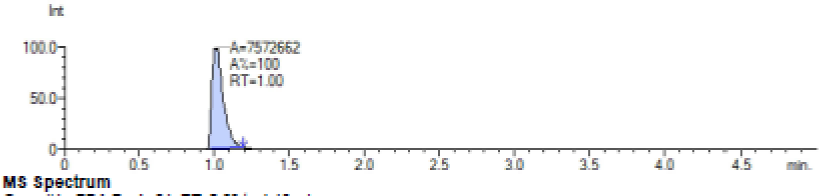

MS Spectrum
Group#1 - PDA Peak: 21, RT: 0.89 to 1.19 min

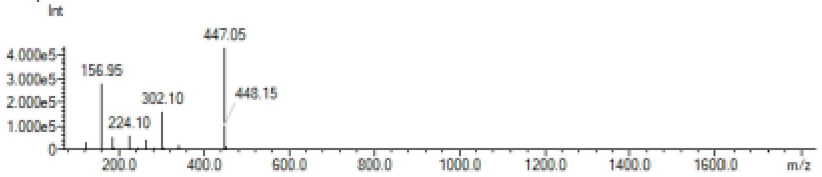

QN-C-S-9

Shimadzu Open Solution

Project: Test
Experiment: 430_0530_Grigalu_0605_3101_TEAD_Wu
Experiment Description:
Sample: 408586:01:01
Sample Description: C26H26N2O5
Data File Name: C:\LabSolutions\Data\430_180530_Grigalunas_180605_3101_180605_Tead_180605_Wu\1046325543_11180530431.lcd
Sample Location: Plate Number: 2 - Position: 193
Run By: admin
Run Started: 24 September 2018 14:46:57
Run Finished: 25 September 2018 00:19:16
Method: Purity_Check_2018

LC Chromatogram
LC#6 : AD2

MS Chromatogram
Group#1 Scan(+) EI : TIC

MS Mass Chromatogram
Group#1 Scan(+) EI : 447.2

MS Spectrum
Group#1 - PDA Peak: 18, RT: 0.88 to 1.37 min

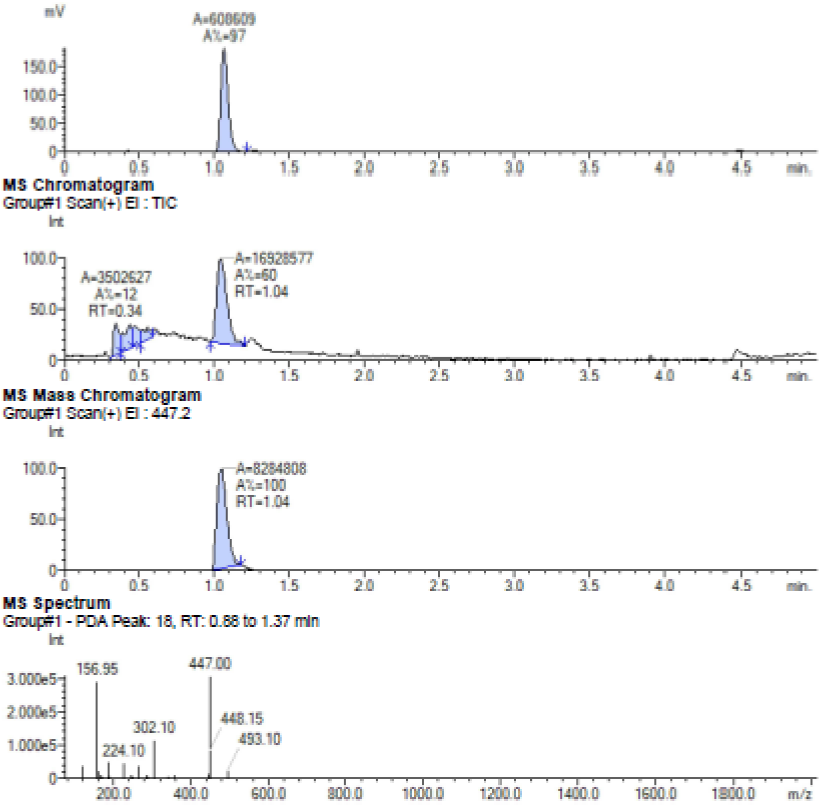

Shimadzu Open Solution

QN-C-R-11

Project: Test
Experiment: 430_0530_Grigalu_0605_3101_TEAD_Wu
Experiment Description:
Sample: 408587:01:02
Sample Description: C26H26N2O5
Data File Name: C:\LabSolutions\Data\430_180530_Grigalunas_180605_3101_180605_Tead_180605_Wu\1046325607_11180530431.lcd
Sample Location: Plate Number: 2 - Position: 241
Run By: admin
Run Started: 24 September 2018 14:46:57
Run Finished: 25 September 2018 00:19:16
Method: Purity_Check_2018

LC Chromatogram
LC#6 : AD2

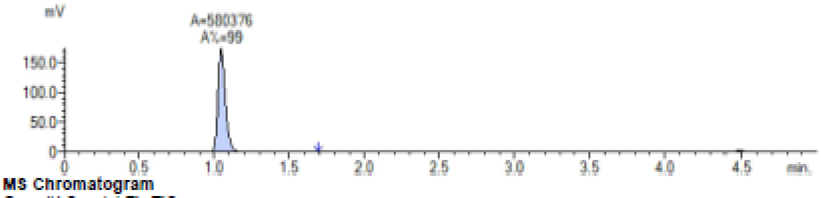

MS Chromatogram
Group#1 Scan(+) EI : TIC

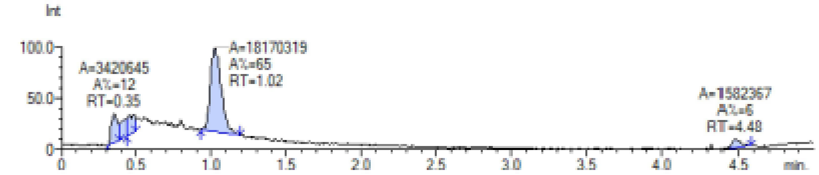

MS Mass Chromatogram
Group#1 Scan(+) EI : 447.2

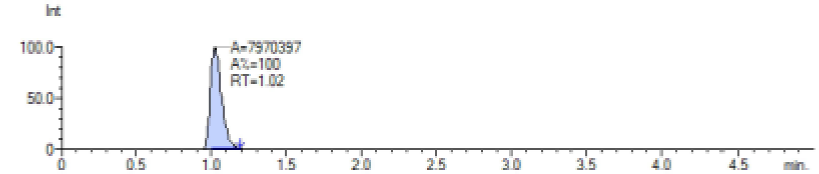

MS Spectrum
Group#1 - PDA Peak: 19, RT: 0.84 to 1.14 min

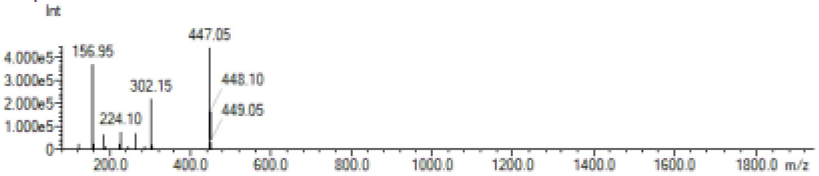

QN-C-R-12

Shimadzu Open Solution

Project: Test
Experiment: 430_0530_Grigalu_0605_3101_TEAD_Wu
Experiment Description:
Sample: 408638:01:01
Sample Description: C27H28N2O5
Data File Name: C:\LabSolutions\Data\430_180530_Grigalunas_180605_3101_180605_Tead_180605_Wu\1046325595_11180530431.lcd
Sample Location: Plate Number: 2 - Position: 55
Run By: admin
Run Started: 24 September 2018 14:46:57
Run Finished: 25 September 2018 00:19:16
Method: Purity_Check_2018

LC Chromatogram
LC#6 : AD2

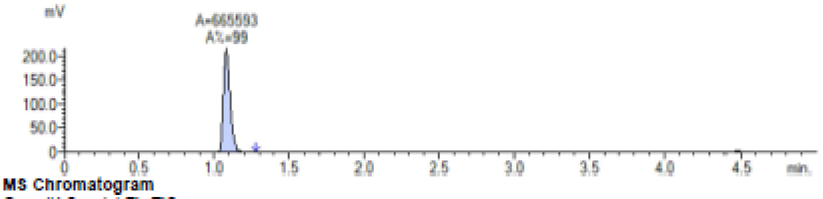

MS Chromatogram
Group#1 Scan(+) EI : TIC

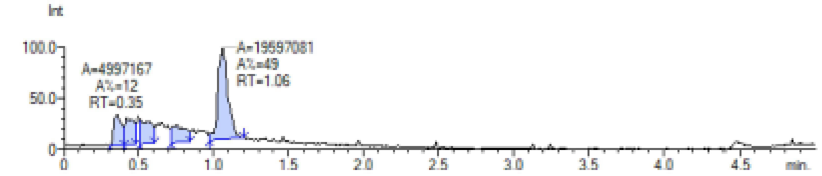

MS Mass Chromatogram
Group#1 Scan(+) EI : 461.2

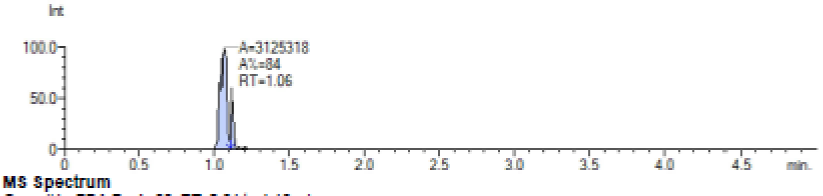

MS Spectrum
Group#1 - PDA Peak: 22, RT: 0.84 to 1.16 min

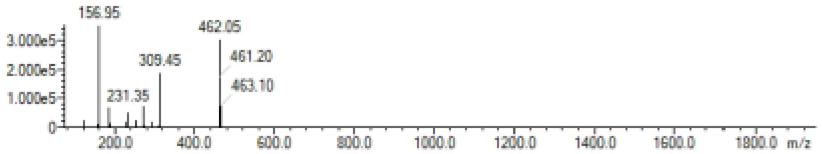

QN-C-S-10

Shimadzu Open Solution

Project: Test
Experiment: 430_0530_Grigalu_0605_3101_TEAD_Wu
Experiment Description:
Sample: 408612:01:02
Sample Description: C27H28N2O4
Data File Name: C:\LabSolutions\Data\430_180530_Grigalunas_180605_3101_180605_Tead_180605_Wu\1046325645_11180530431.lcd
Sample Location: Plate Number: 2 - Position: 291
Run By: admin
Run Started: 24 September 2018 14:46:57
Run Finished: 25 September 2018 00:19:16
Method: Purity_Check_2018

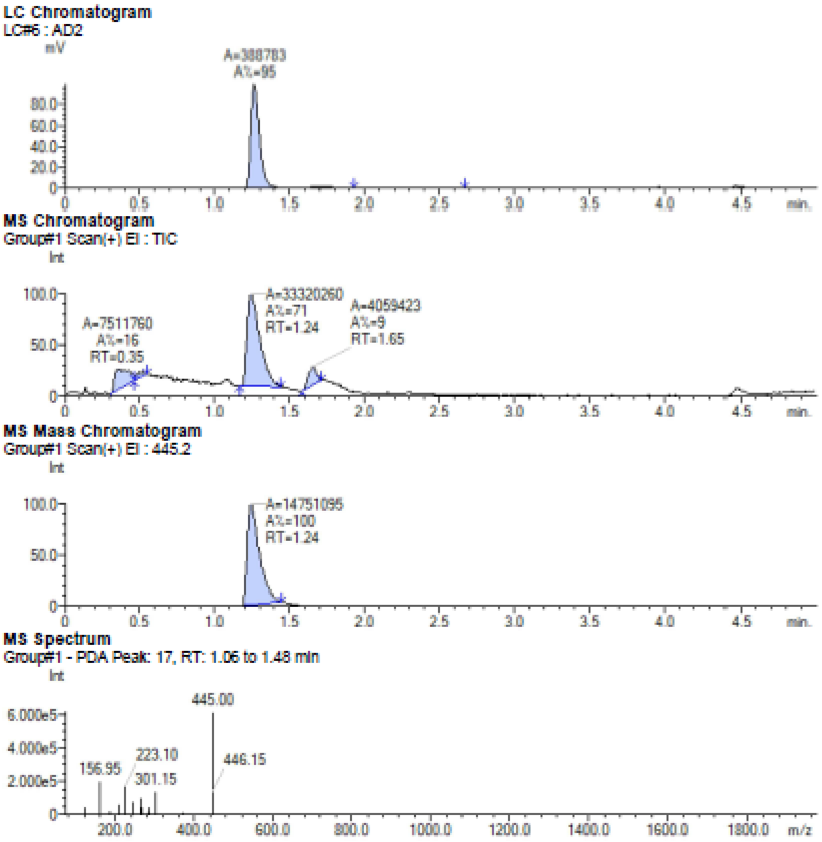

QN-C-R-13

Shimadzu Open Solution

**Project:**	Test
**Experiment:**	430_0530_Grigalu_0605_3101_TEAD_Wu
**Experiment Description:**	
**Sample:**	408613:01:01
**Sample Description:**	C27H28N2O4
**Data File Name:**	C:\LabSolutions\Data\430_180530_Grigalunas_180605_3101_180605_Tead_180605_Wu\1046325617_11180530431.lcd
**Sample Location:**	Plate Number: 2 - Position: 339
**Run By:**	admin
**Run Started:**	24 September 2018 14:46:57
**Run Finished:**	25 September 2018 00:19:16
**Method:**	Purity_Check_2018

LC Chromatogram
LC#6 : AD2

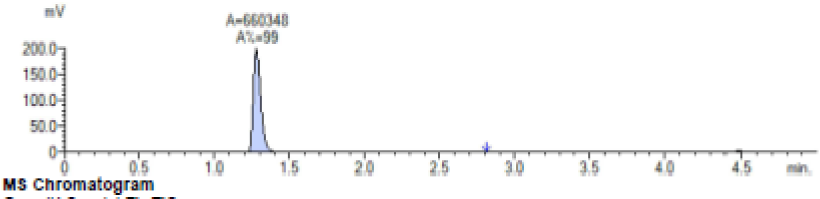

MS Chromatogram
Group#1 Scan(+) EI : TIC

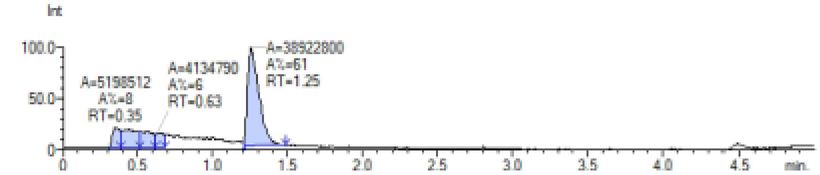

MS Mass Chromatogram
Group#1 Scan(+) EI : 445.2

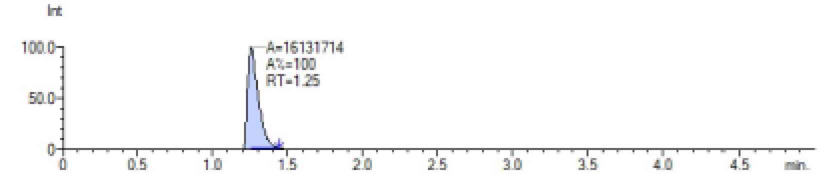

MS Spectrum
Group#1 - PDA Peak: 19, RT: 1.11 to 1.40 min

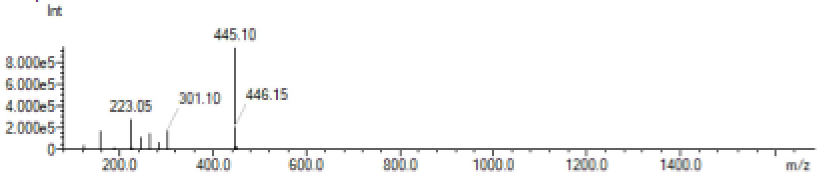

QN-C-R-14

Shimadzu Open Solution

Project: Test
Experiment: 430_0530_Grigalu_0605_3101_TEAD_Wu
Experiment Description:
Sample: 408609:01:01
Sample Description: C30H33N3O4
Data File Name: C:\LabSolutions\Data\430_180530_Grigalunas_180605_3101_180605_Tead_180605_Wu\1046324899_11180530431.lcd
Sample Location: Plate Number: 2 - Position: 147
Run By: admin
Run Started: 24 September 2018 14:46:57
Run Finished: 25 September 2018 00:19:16
Method: Purity_Check_2018

LC Chromatogram
LC#6 : AD2

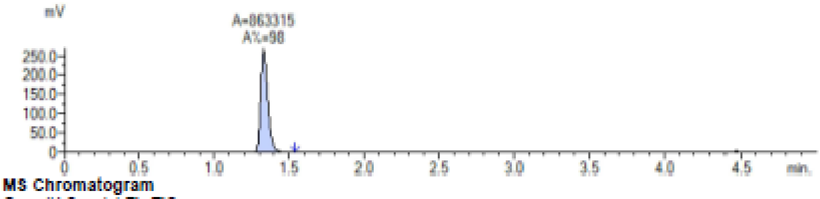

MS Chromatogram
Group#1 Scan(+) EI : TIC

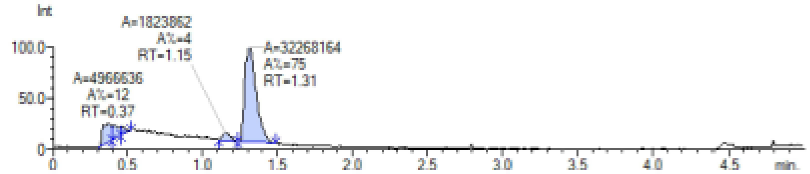

MS Mass Chromatogram
Group#1 Scan(+) EI : 500.2

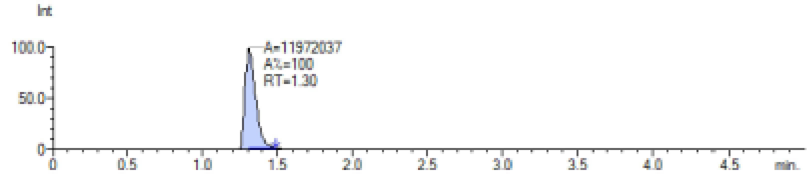

MS Spectrum
Group#1 - PDA Peak: 22, RT: 1.16 to 1.51 min

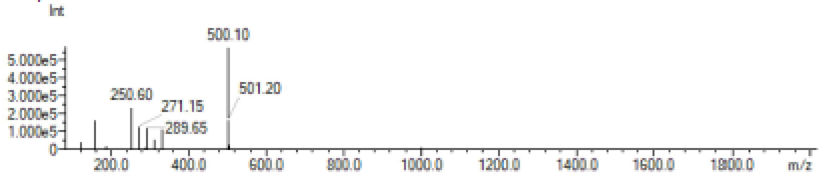

QN-C-S-11

Shimadzu Open Solution

**Project:**	Test
**Experiment:**	430_0530_Grigalu_0605_3101_TEAD_Wu
**Experiment Description:**	
**Sample:**	408616:01:01
**Sample Description:**	C30H28N2O4
**Data File Name:**	C:\LabSolutions\Data\430_180530_Grigalunas_180605_3101_180605_Tead_180605_Wu\1046324790_11180530431.lcd
**Sample Location:**	Plate Number: 2 - Position: 101
**Run By:**	admin
**Run Started:**	24 September 2018 14:46:57
**Run Finished:**	25 September 2018 00:19:16
**Method:**	Purity_Check_2018

LC Chromatogram

LC#6 : AD2

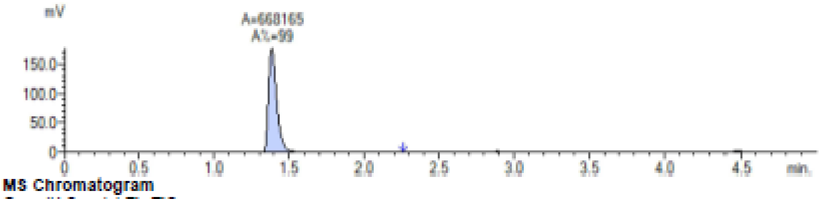

MS Chromatogram

Group#1 Scan(+) EI : TIC

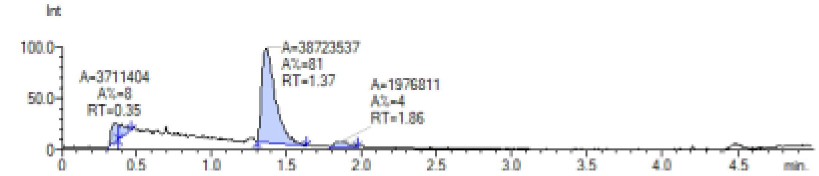

MS Mass Chromatogram

Group#1 Scan(+) EI : 481.2

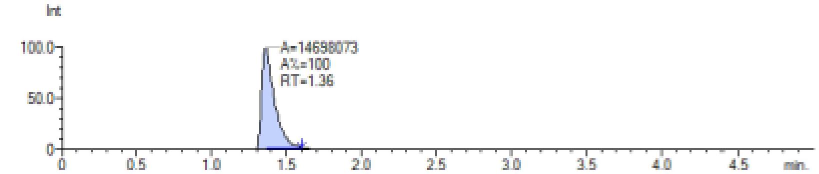

MS Spectrum

Group#1 - PDA Peak: 17, RT: 1.24 to 1.58 min

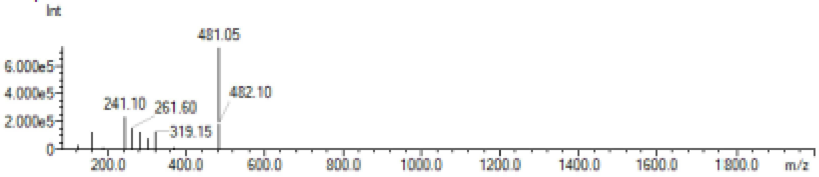

QN-C-R-15

Shimadzu Open Solution

Project: Test
Experiment: 430_0530_Grigalu_0605_3101_TEAD_Wu
Experiment Description:
Sample: 408617:01:01
Sample Description: C30H28N2O4
Data File Name: C:\LabSolutions\Data\430_180530_Grigalunas_180605_3101_180605_Tead_180605_Wu\1046325584_11180530431.lcd
Sample Location: Plate Number: 2 - Position: 149
Run By: admin
Run Started: 24 September 2018 14:46:57
Run Finished: 25 September 2018 00:19:16
Method: Purity_Check_2018

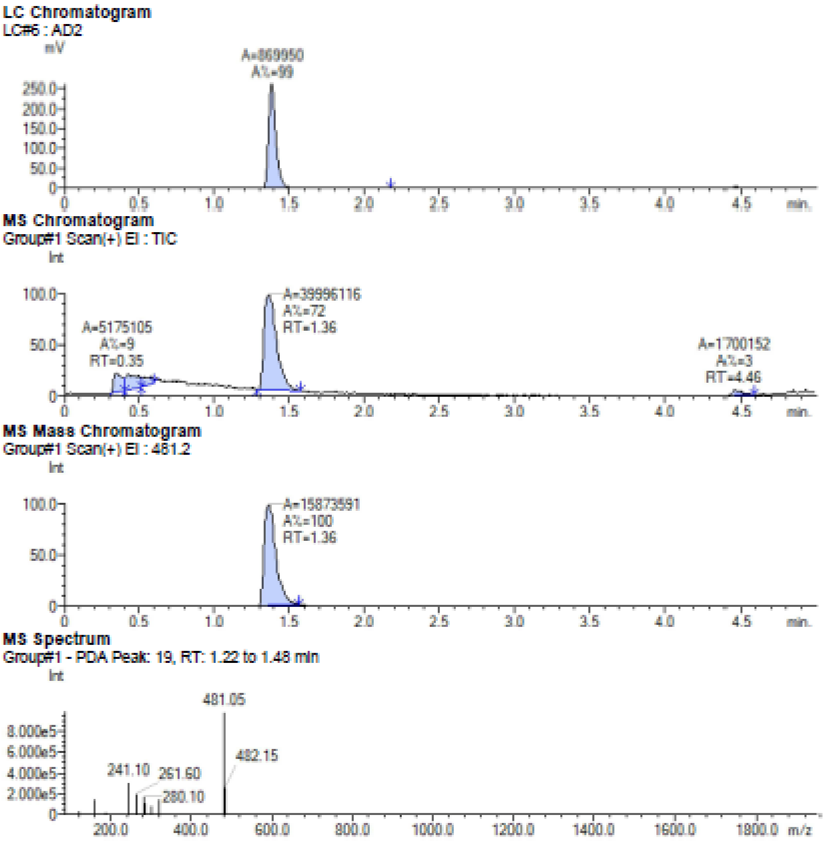

QN-C-R-16

Shimadzu Open Solution

Project: Test
Experiment: 430_0530_Grigalu_0605_3101_TEAD_Wu
Experiment Description:
Sample: 408618:01:01
Sample Description: C30H28N2O4
Data File Name: C:\LabSolutions\Data\430_180530_Grigalunas_180605_3101_180605_Tead_180605_Wu\1046325394_11180530431.lcd
Sample Location: Plate Number: 2 - Position: 197
Run By: admin
Run Started: 24 September 2018 14:46:57
Run Finished: 25 September 2018 00:19:16
Method: Purity_Check_2018

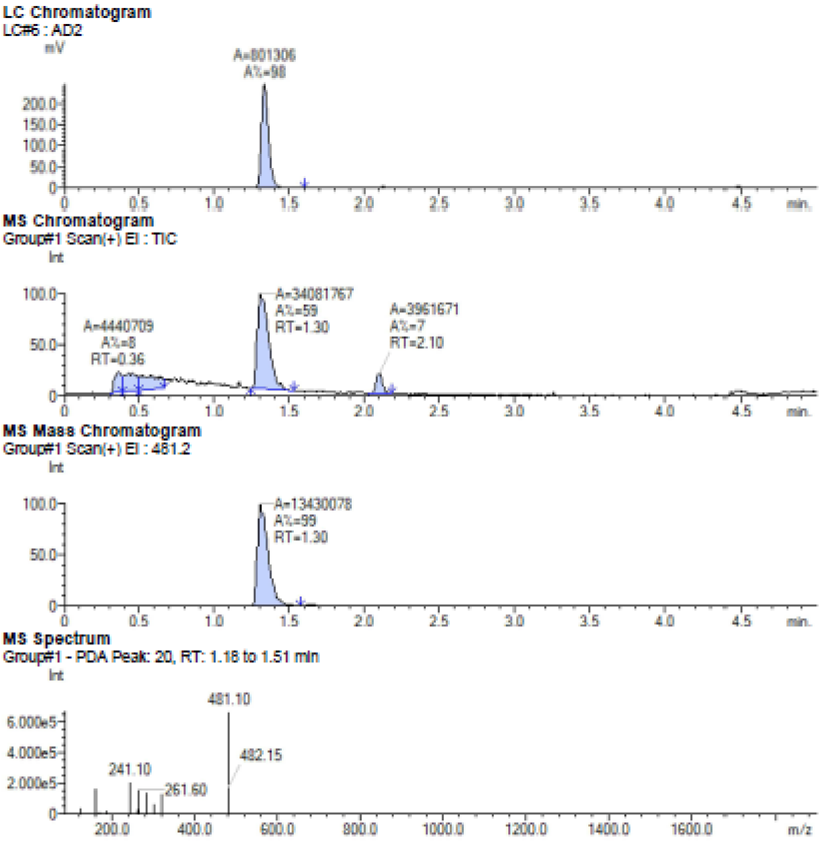

QN-C-S-12

Shimadzu Open Solution

Project: Test
Experiment: 430_0530_Grigalu_0605_3101_TEAD_Wu
Experiment Description:
Sample: 408634:01:02
Sample Description: C28H29N3O5
Data File Name: C:\LabSolutions\Data\430_180530_Grigalunas_180605_3101_180605_Tead_180605_Wu\1046325604_11180530431.lcd
Sample Location: Plate Number: 2 - Position: 245
Run By: admin
Run Started: 24 September 2018 14:46:57
Run Finished: 25 September 2018 00:19:16
Method: Purity_Check_2018

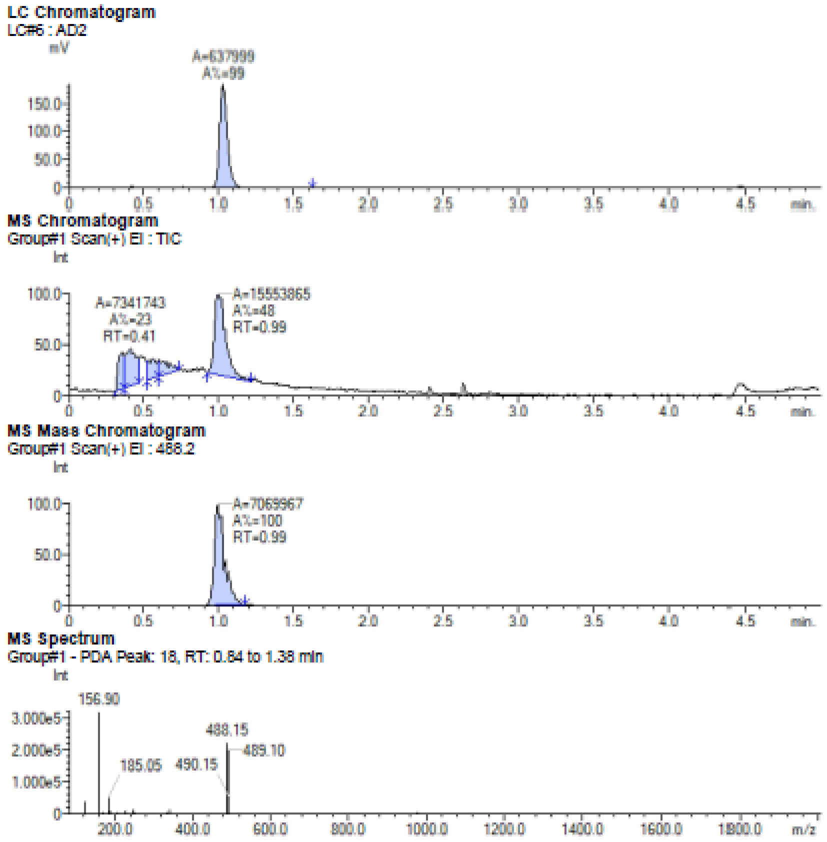

QN-C-R-17

Shimadzu Open Solution

Project: Test
Experiment: 430_0530_Grigalu_0605_3101_TEAD_Wu
Experiment Description:
Sample: 408635:01:02
Sample Description: C28H29N3O5
Data File Name: C:\LabSolutions\Data\430_180530_Grigalunas_180605_3101_180605_Tead_180605_Wu\1046325606_11180530431.lcd
Sample Location: Plate Number: 2 - Position: 293
Run By: admin
Run Started: 24 September 2018 14:46:57
Run Finished: 25 September 2018 00:19:16
Method: Purity_Check_2018

LC Chromatogram
LC#5 : AD2

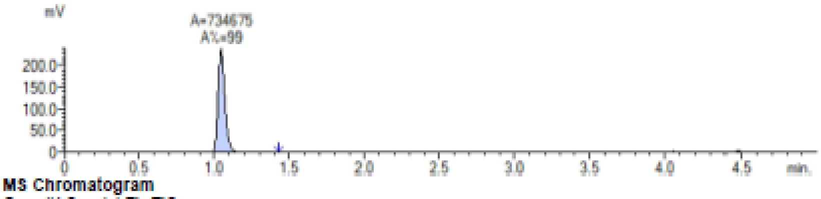

MS Chromatogram
Group#1 Scan(+) EI : TIC

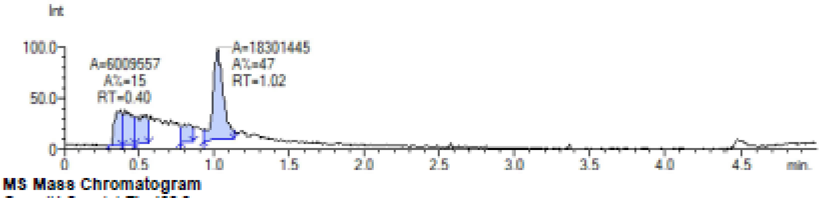

MS Mass Chromatogram
Group#1 Scan(+) EI : 488.2

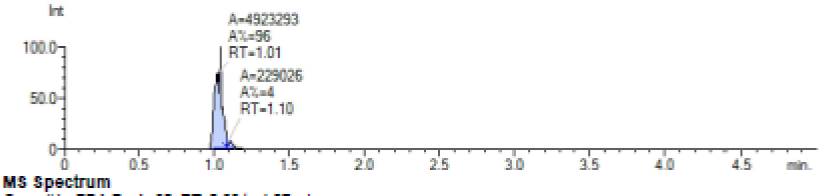

MS Spectrum
Group#1 - PDA Peak: 20, RT: 0.86 to 1.37 min

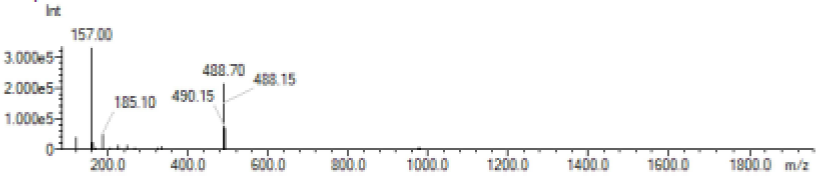

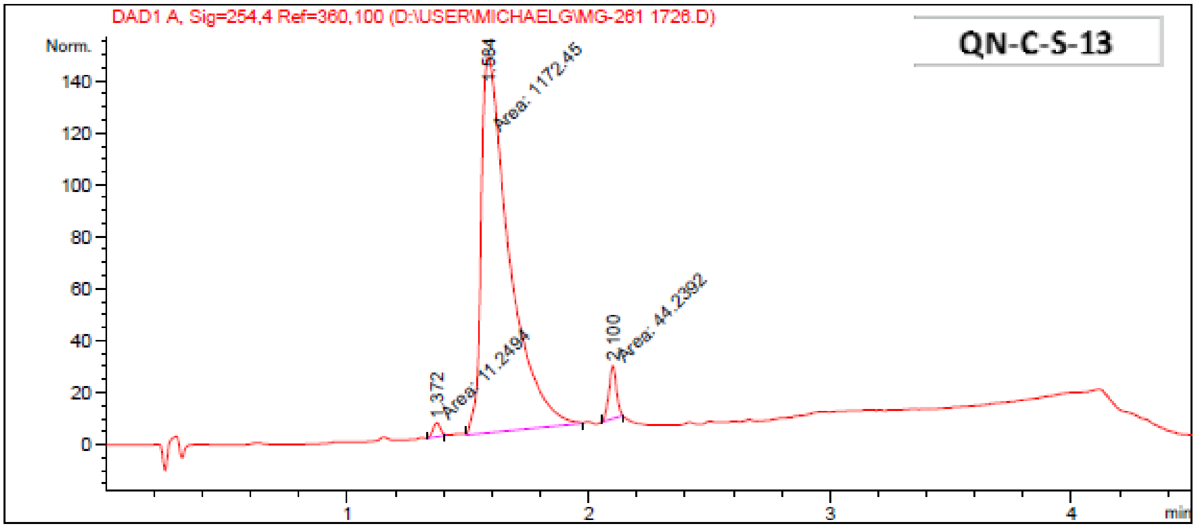

#	Time	Area	Height	Width	Area%	Symmetry
1	1.372	11.2	5.3	0.0351	0.916	1.072
2	1.584	1172.5	144.6	0.1351	95.481	0.326
3	2.1	44.2	20.3	0.0362	3.603	1.103

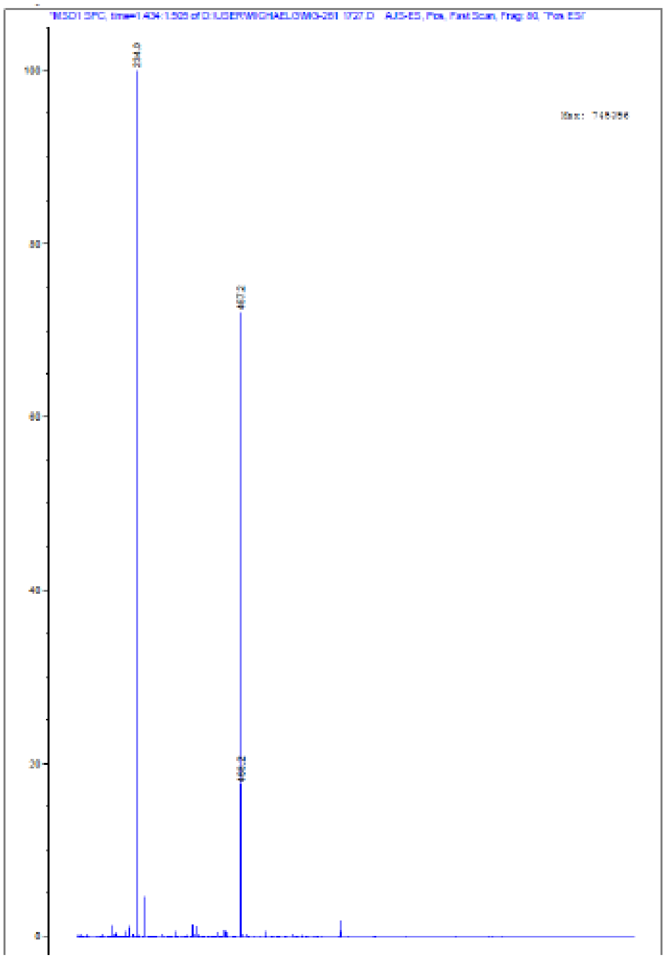

QN-C-R-18

Shimadzu Open Solution

**Project:**	Test
**Experiment:**	430_0530_Grigalu_0605_3101_TEAD_Wu
**Experiment Description:**	
**Sample:**	408640:01:02
**Sample Description:**	C26H24F2N2O4
**Data File Name:**	C:\LabSolutions\Data\430_180530_Grigalunas_180605_3101_180605_Tead_180605_Wu\1046325521_11180530431.lcd
**Sample Location:**	Plate Number: 2 - Position: 151
**Run By:**	admin
**Run Started:**	24 September 2018 14:46:57
**Run Finished:**	25 September 2018 00:19:16
**Method:**	Purity_Check_2018

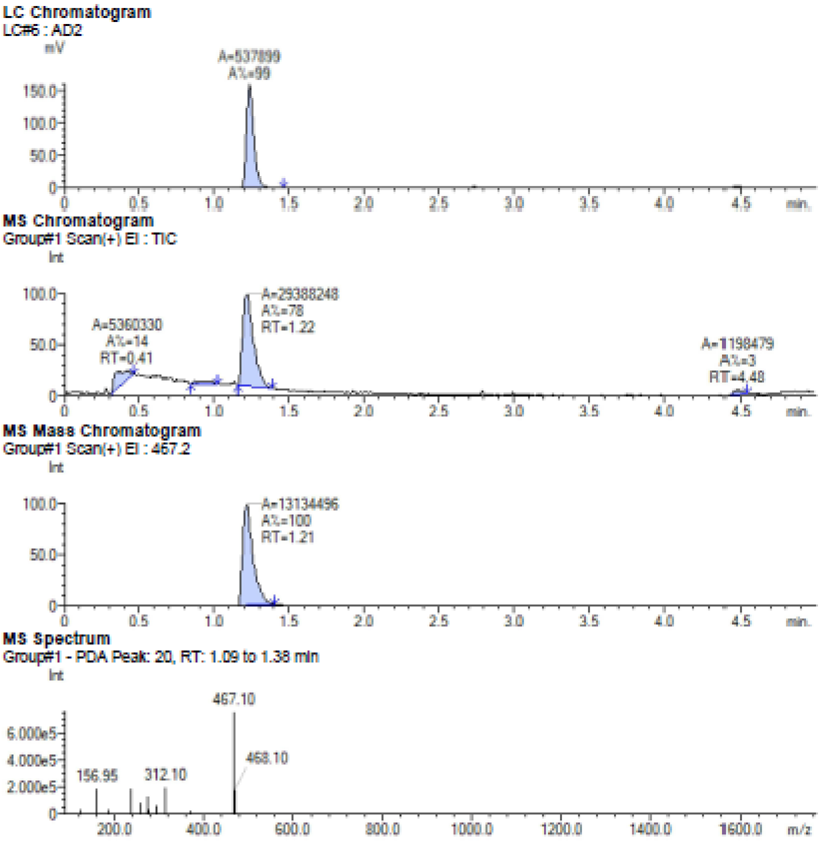

QN-C-S-14

Shimadzu Open Solution

Project: Test
Experiment: 430_0530_Grigalu_0605_3101_TEAD_Wu
Experiment Description:
Sample: 408641:01:02
Sample Description: C28H30N2O4
Data File Name: C:\LabSolutions\Data\430_180530_Grigalunas_180605_3101_180605_Tead_180605_Wu\1046325020_11180530431.lcd
Sample Location: Plate Number: 2 - Position: 15
Run By: admin
Run Started: 24 September 2018 14:46:57
Run Finished: 25 September 2018 00:19:16
Method: Purity_Check_2018

LC Chromatogram
LC#6 : AD2

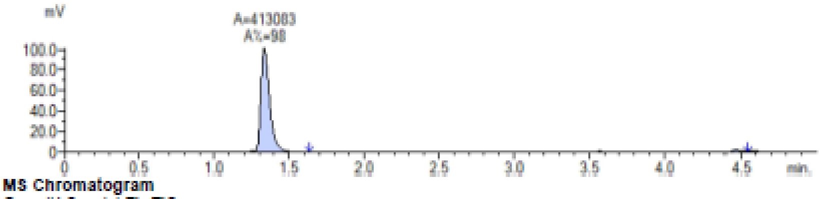

MS Chromatogram
Group#1 Scan(+) EI : TIC

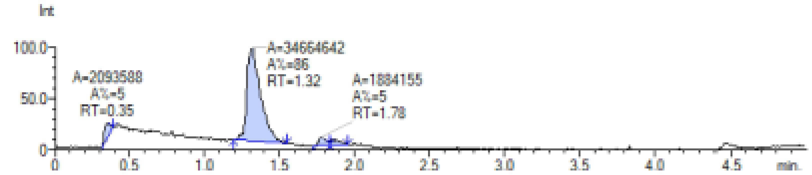

MS Mass Chromatogram
Group#1 Scan(+) EI : 459.2

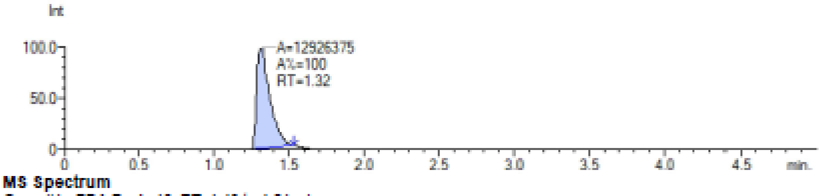

MS Spectrum
Group#1 - PDA Peak: 19, RT: 1.19 to 1.61 min

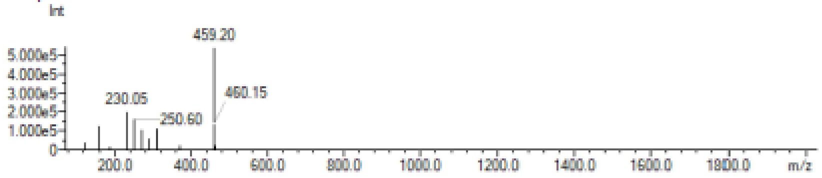

QN-C-R-19

Shimadzu Open Solution

Project:	Test
Experiment:	430_0530_Grigalu_0605_3101_TEAD_Wu
Experiment Description:	
Sample:	408642:01:02
Sample Description:	C28H30N2O4
Data File Name:	C:\LabSolutions\Data\430_180530_Grigalunas_180605_3101_180605_Tead_180605_Wu\1046325374_11180530431.lcd
Sample Location:	Plate Number: 2 - Position: 199
Run By:	admin
Run Started:	24 September 2018 14:46:57
Run Finished:	25 September 2018 00:19:16
Method:	Purity_Check_2018

LC Chromatogram
LC#6 : AD2

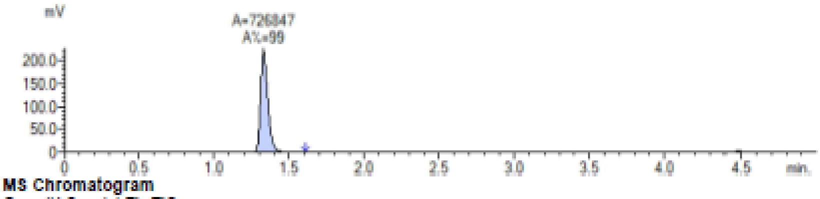

MS Chromatogram
Group#1 Scan(+) EI : TIC

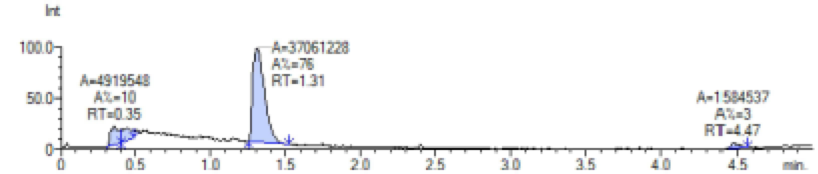

MS Mass Chromatogram
Group#1 Scan(+) EI : 459.2

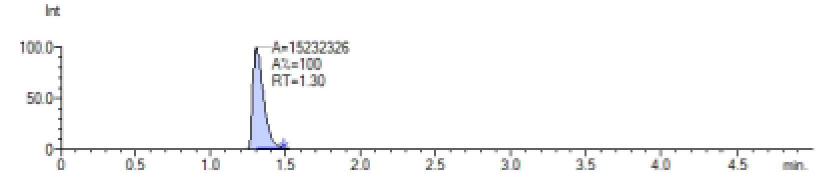

MS Spectrum
Group#1 - PDA Peak: 18, RT: 1.18 to 1.51 min

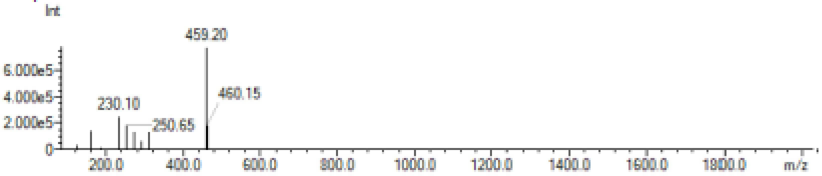

Shimadzu Open Solution

QN-C-R-20

Project: Test
Experiment: 525_0206_Christo_Grigalu_Burhop_Wilke
Experiment Description:
Sample: 409698:01:01
Sample Description: C27H28N2O4
Data File Name: C:\LabSolutions\Data\525_190206_Christoforow_190206_Grigalunas_190206_Burhop_190206_Wilke\1046360752_11190207525.lcd
Sample Location: Plate Number: 2 - Position: 198
Run By: admin
Run Started: 23 April 2019 18:20:13
Run Finished: 24 April 2019 04:25:41
Method: 190321_Purity-Check+shut down

LC Chromatogram
LC#6 : AD2

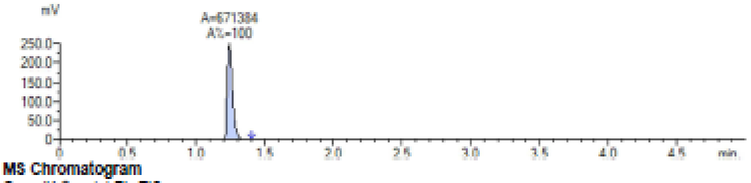

MS Chromatogram
Group#1 Scan(+) EI : TIC

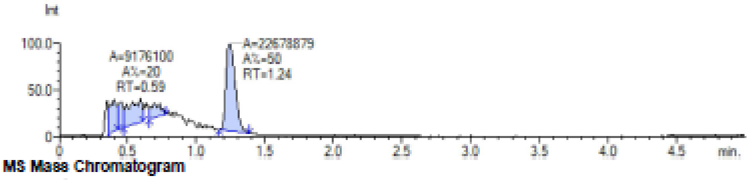

MS Mass Chromatogram
Group#1 Scan(+) EI : 445.2

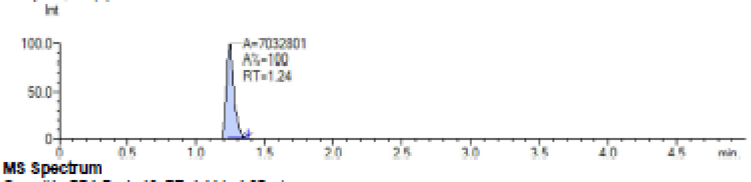

MS Spectrum
Group#1 - PDA Peak: 19, RT: 1.11 to 1.37 min

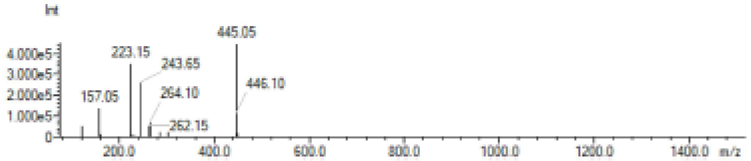

QN-C-S-15

Shimadzu Open Solution

**Project:**	Test
**Experiment:**	525_0206_Christo_Grigalu_Burhop_Wilke
**Experiment Description:**	
**Sample:**	409699:01:02
**Sample Description:**	C28H30N2O4
**Data File Name:**	C:\LabSolutions\Data\525_190206_Christoforow_190206_Grigalunas_190206_Burhop_190206_Wilke\1046360620_11190207525.lcd
**Sample Location:**	Plate Number: 2 - Position: 346
**Run By:**	admin
**Run Started:**	23 April 2019 18:20:13
**Run Finished:**	24 April 2019 04:25:41
**Method:**	190321_Purity-Check+shut down

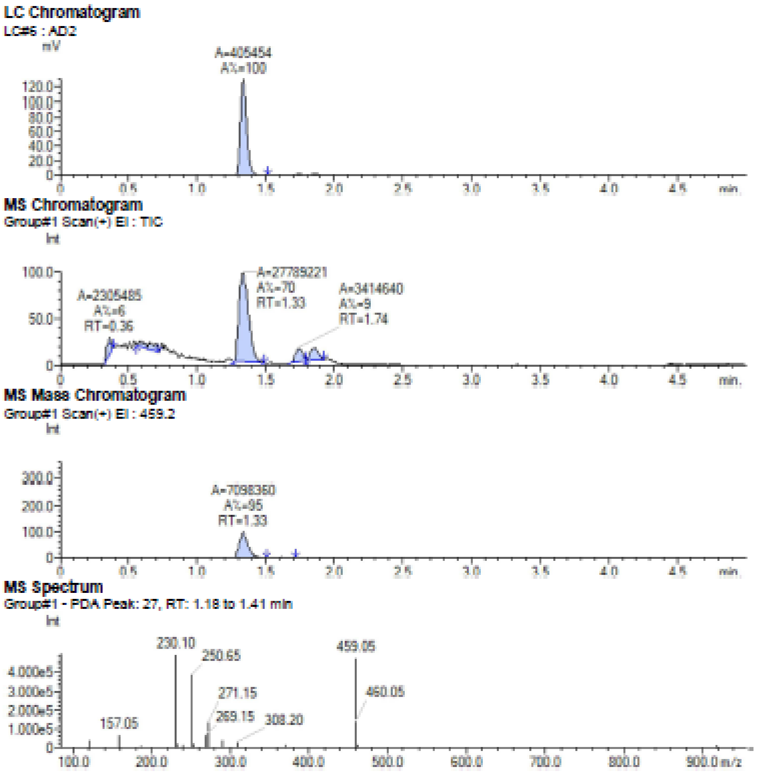

Shimadzu Open Solution

QN-C-R-21

Project:	Test
Experiment:	525_0206_Christo_Grigalu_Burhop_Wilke
Experiment Description:	
Sample:	409700:01:02
Sample Description:	C28H30N2O4
Data File Name:	C:\LabSolutions\Data\525_190206_Christoforow_190206_Grigalunas_190206_Burhop_190206_Wilke\1046361301_11190207525.lcd
Sample Location:	Plate Number: 2 - Position: 246
Run By:	admin
Run Started:	23 April 2019 18:20:13
Run Finished:	24 April 2019 04:25:41
Method:	190321_Purity-Check+shut down

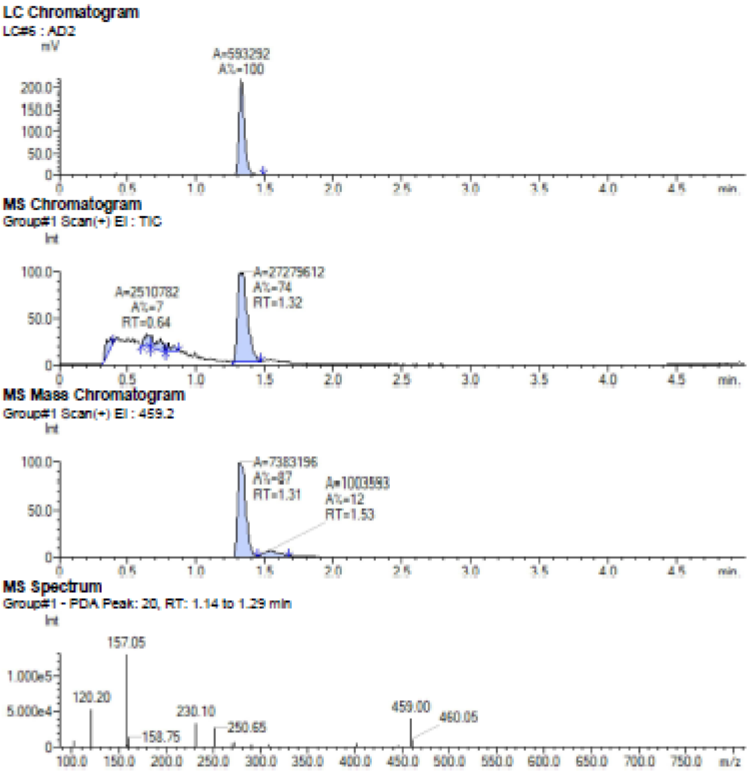

QN-C-S-16

Shimadzu Open Solution

**Project:**	Test
**Experiment:**	525_0206_Christo_Grigalu_Burhop_Wilke
**Experiment Description:**	
**Sample:**	409701:01:01
**Sample Description:**	C28H30N2O4
**Data File Name:**	C:\LabSolutions\Data\525_190206_Christoforow_190206_Grigalunas_190206_Burhop_190206_Wilke\1046361264_11190207525.lcd
**Sample Location:**	Plate Number: 2 - Position: 294
**Run By:**	admin
**Run Started:**	23 April 2019 18:20:13
**Run Finished:**	24 April 2019 04:25:41
**Method:**	190321_Purity-Check+shut down

LC Chromatogram

LC#6 : AD2

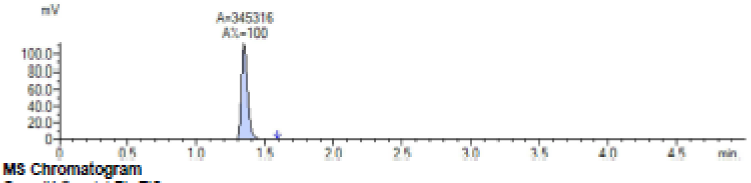

MS Chromatogram

Group#1 Scan(+) EI : TIC

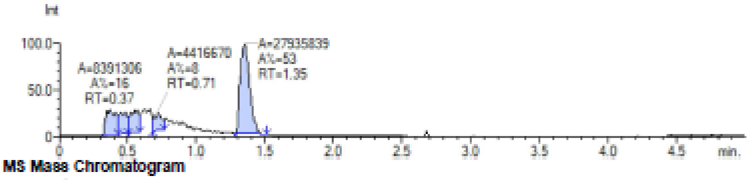

MS Mass Chromatogram

Group#1 Scan(+) EI : 459.2

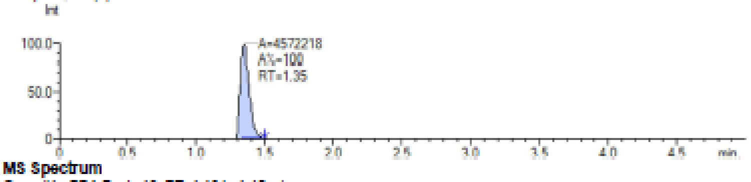

MS Spectrum

Group#1 - PDA Peak: 19, RT: 1.18 to 1.45 min

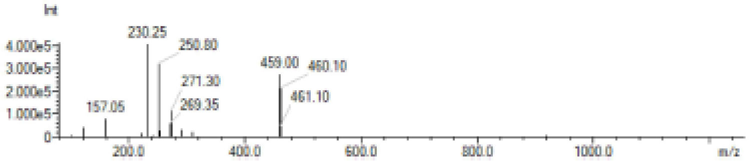

QN-C-R-22

Shimadzu Open Solution

Project: Test
Experiment: 525_0206_Christo_Grigalu_Burhop_Wilke
Experiment Description:
Sample: 409702:01:01
Sample Description: C28H30N2O4
Data File Name: C:\LabSolutions\Data\525_190206_Christoforow_190206_Grigalunas_190206_Burhop_190206_Wilke\1046360612_11190207525.lcd
Sample Location: Plate Number: 2 - Position: 342
Run By: admin
Run Started: 23 April 2019 18:20:13
Run Finished: 24 April 2019 04:25:41
Method: 190321_Purity-Check+shut down

LC Chromatogram
LC#5 : AD2

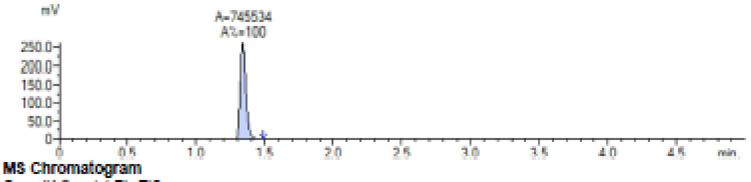

MS Chromatogram
Group#1 Scan(+) EI : TIC

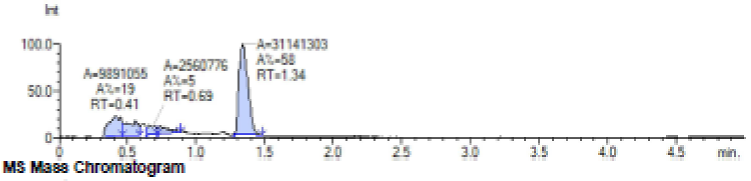

MS Mass Chromatogram
Group#1 Scan(+) EI : 459.2

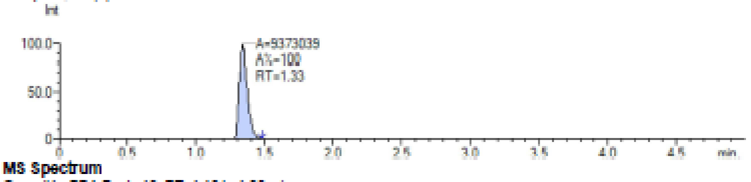

MS Spectrum
Group#1 - PDA Peak: 19, RT: 1.16 to 1.38 min

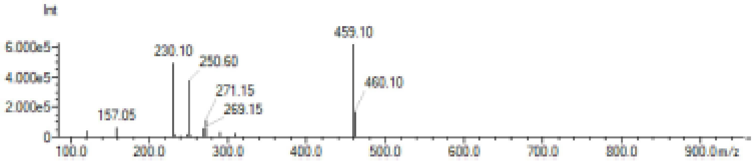

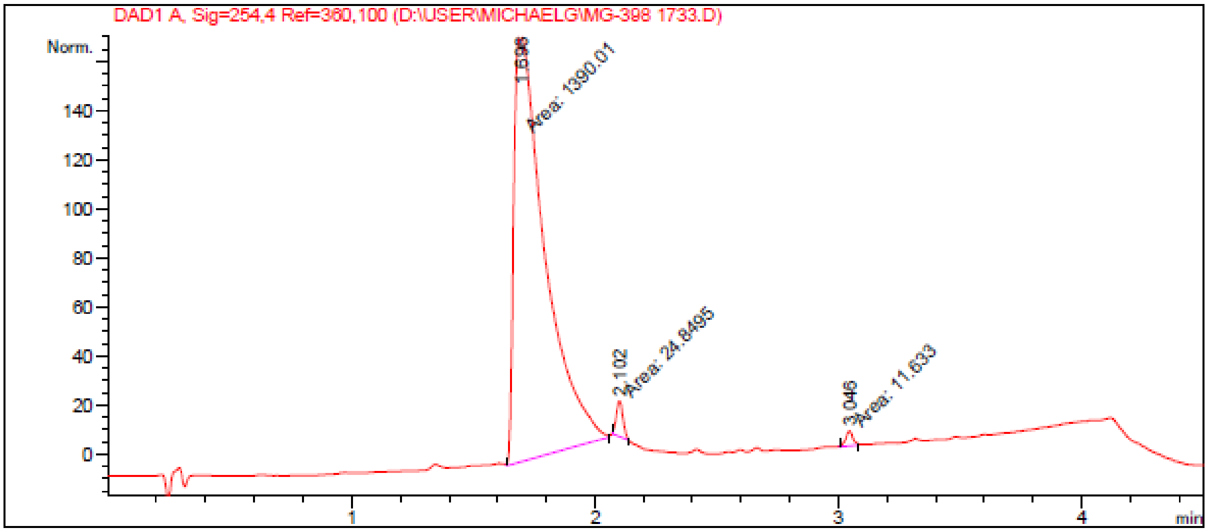

#	Time	Area	Height	Width	Area%	Symmetry
1	1.696	1390	156.9	0.1477	97.443	0.282
2	2.102	24.8	13	0.0319	1.742	0.955
3	3.046	11.6	5.5	0.0351	0.815	1.104

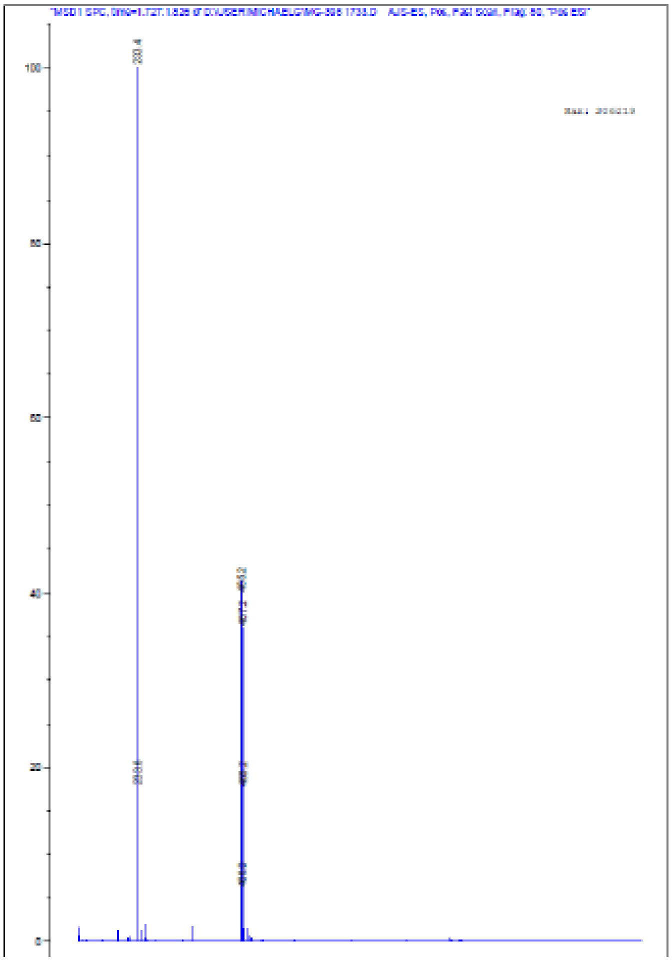

QN-C-R-24

Shimadzu Open Solution

Project: Test
Experiment: 525_0206_Christo_Grigalu_Burhop_Wilke
Experiment Description:
Sample: 409704:01:01
Sample Description: C27H27ClN2O4
Data File Name: C:\LabSolutions\Data\525_190206_Christoforow_190206_Grigalunas_190206_Burhop_190206_Wilke\1046361193_11190207525.lcd
Sample Location: Plate Number: 2 - Position: 56
Run By: admin
Run Started: 23 April 2019 18:20:13
Run Finished: 24 April 2019 04:25:41
Method: 190321_Purity-Check+shut down

LC Chromatogram

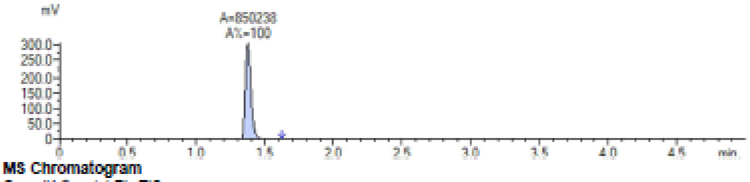

MS Chromatogram

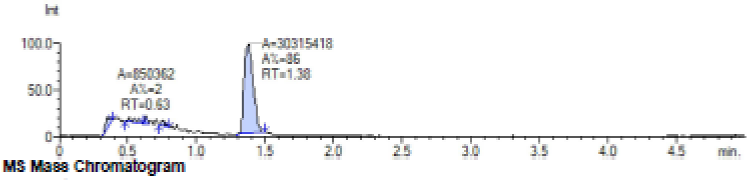

MS Mass Chromatogram

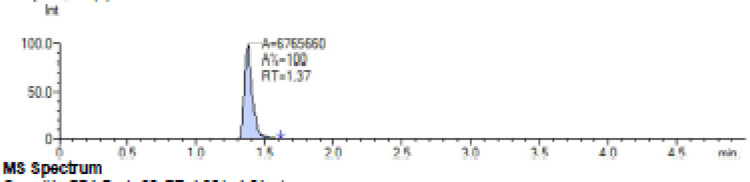

MS Spectrum

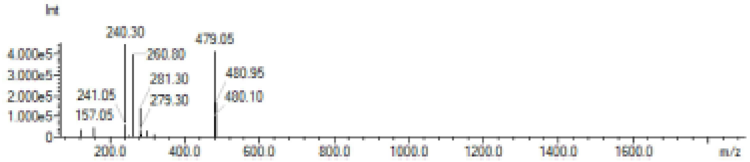

QN-C-S-17

Shimadzu Open Solution

**Project:**	Test
**Experiment:**	525_0206_Christo_Grigalu_Burhop_Wilke
**Experiment Description:**	
**Sample:**	409705:01:01
**Sample Description:**	C33H32N2O5
**Data File Name:**	C:\LabSolutions\Data\525_190206_Christoforow_190206_Grigalunas_190206_Burhop_190206_Wilke\1046361091_11190207525.lcd
**Sample Location:**	Plate Number: 2 - Position: 104
**Run By:**	admin
**Run Started:**	23 April 2019 18:20:13
**Run Finished:**	24 April 2019 04:25:41
**Method:**	190321_Purity-Check+shut down

LC Chromatogram

LC#6 : AD2

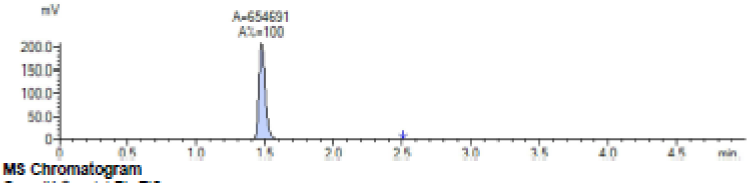

MS Chromatogram

Group#1 Scan(+) EI : TIC

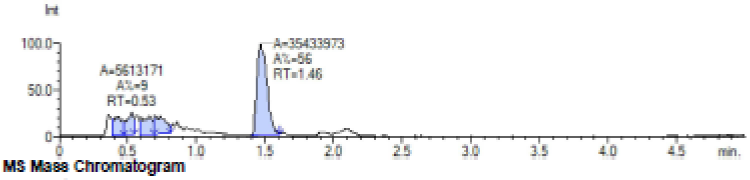

MS Mass Chromatogram

Group#1 Scan(+) EI : 537.2

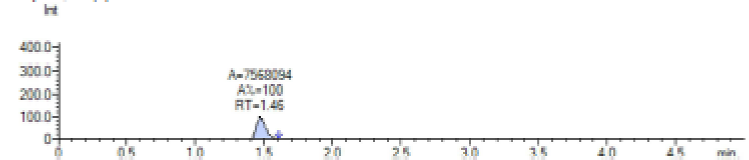

MS Spectrum

Group#1 - PDA Peak: 22, RT: 1.31 to 1.51 min

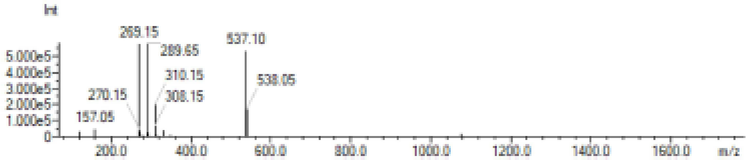

Shimadzu Open Solution

QN-C-R-25

Project: Test
Experiment: 525_0206_Christo_Grigalu_Burhop_Wilke
Experiment Description:
Sample: 409706:01:01
Sample Description: C30H33N3O4
Data File Name: C:\LabSolutions\Data\525_190206_Christoforow_190206_Grigalunas_190206_Burhop_190206_Wilke\1046361144_11190207525.lcd
Sample Location: Plate Number: 2 - Position: 152
Run By: admin
Run Started: 23 April 2019 18:20:13
Run Finished: 24 April 2019 04:25:41
Method: 190321_Purity-Check+shut down

LC Chromatogram
LC#6 : AD2

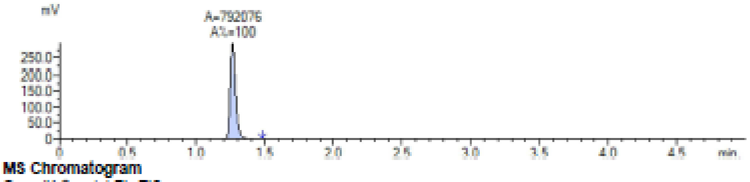

MS Chromatogram
Group#1 Scan(+) EI : TIC

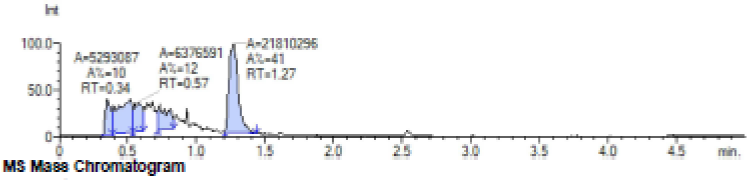

MS Mass Chromatogram
Group#1 Scan(+) EI : 500.2

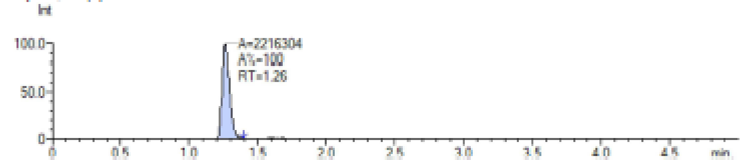

MS Spectrum
Group#1 - PDA Peak: 17, RT: 1.05 to 1.24 min

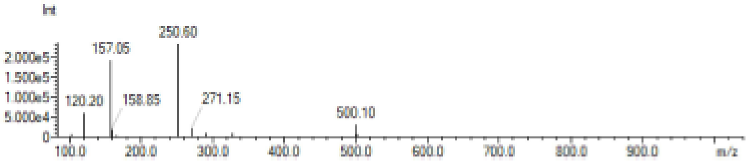

QN-C-R-26

Shimadzu Open Solution

Project: Test
Experiment: 525_0206_Christo_Grigalu_Burhop_Wilke
Experiment Description:
Sample: 409707:01:01
Sample Description: C26H25BrN2O4
Data File Name: C:\LabSolutions\Data\525_190206_Christoforow_190206_Grigalunas_190206_Burhop_190206_Wilke\1046360739_11190207525.lcd
Sample Location: Plate Number: 2 - Position: 200
Run By: admin
Run Started: 23 April 2019 18:20:13
Run Finished: 24 April 2019 04:25:41
Method: 190321_Purity-Check+shut down

LC Chromatogram
LC#6 : AD2

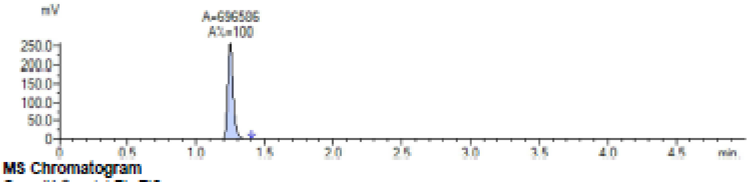

MS Chromatogram
Group#1 Scan(+) EI : TIC

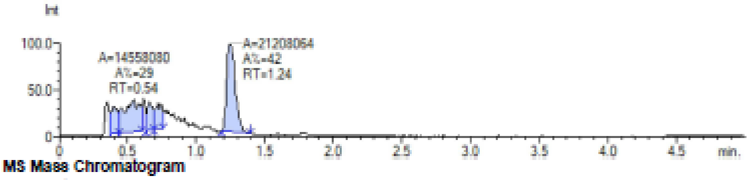

MS Mass Chromatogram
Group#1 Scan(+) EI : 508.1

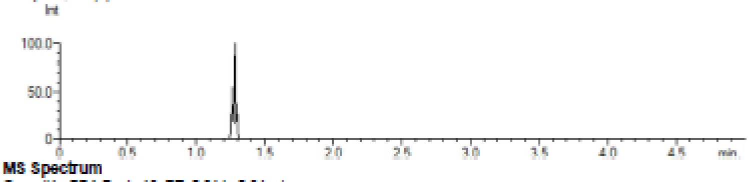

MS Spectrum
Group#1 - PDA Peak: 18, RT: 0.61 to 0.84 min

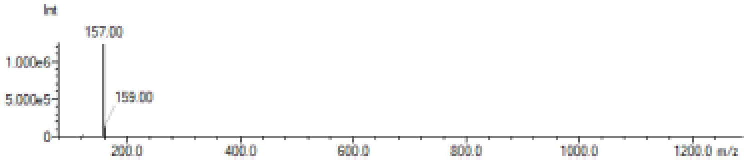

QN-C-S-18

Shimadzu Open Solution

**Project:**	Test
**Experiment:**	524_0205_Wess_06_Zink_07_Conc-10_08_Grig
**Experiment Description:**	
**Sample:**	409674:01:01
**Sample Description:**	C29H32N2O4
**Data File Name:**	C:\LabSolutions\Data\524_190205_Wesserler_190602_Zinken_190207_Conc-10_190208_Grigalunas\1046360714_11190208524.lcd
**Sample Location:**	Plate Number: 1 - Position: 74
**Run By:**	admin
**Run Started:**	23 April 2019 15:21:47
**Run Finished:**	23 April 2019 18:20:12
**Method:**	190321_Purity-Check

LC Chromatogram

LC#6 : AD2

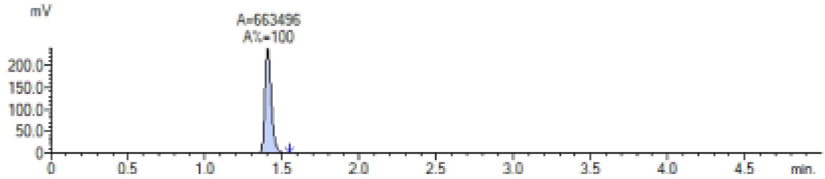

MS Chromatogram

Group#1 Scan(+) EI : TIC

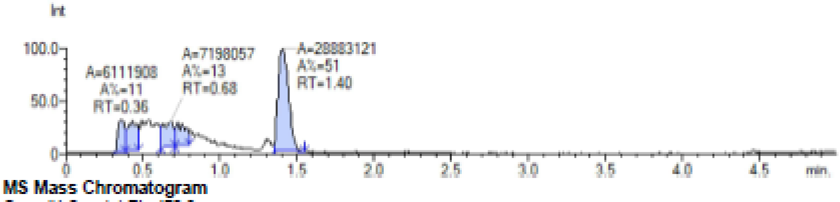

MS Mass Chromatogram

Group#1 Scan(+) EI : 473.2

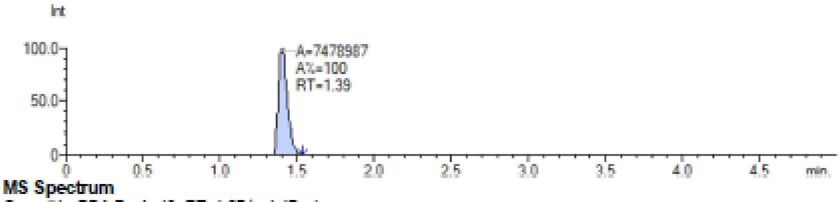

MS Spectrum

Group#1 - PDA Peak: 19, RT: 1.27 to 1.47 min

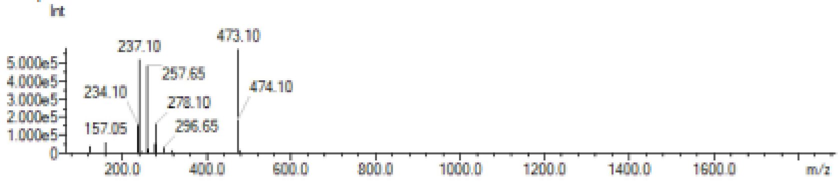

QN-C-R-27

Shimadzu Open Solution

**Project:**	Test
**Experiment:**	572_190612_Zinken_0607_Bischof_0612Griga
**Experiment Description:**	
**Sample:**	410427:01:01
**Sample Description:**	C26H24BrClN2O4
**Data File Name:**	C:\LabSolutions\Data\572_190612_Zinken_190607_Bischoff_190612_Grigalunas\1046364062_11190613572.lcd
**Sample Location:**	Plate Number: 1 - Position: 90
**Run By:**	admin
**Run Started:**	29 July 2019 19:27:00
**Run Finished:**	30 July 2019 04:41:49
**Method:**	190321_Purity-Check

LC Chromatogram
LC#6 : AD2

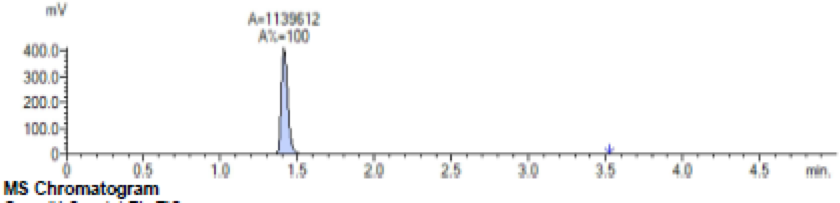

MS Chromatogram
Group#1 Scan(+) EI : TIC

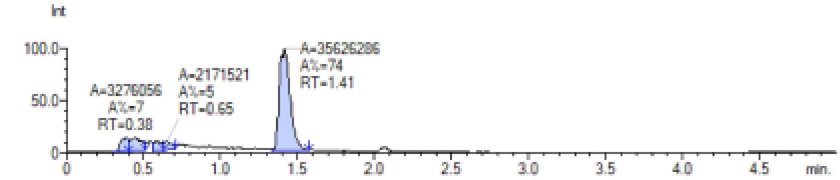

MS Mass Chromatogram
Group#1 Scan(+) EI : 543.1

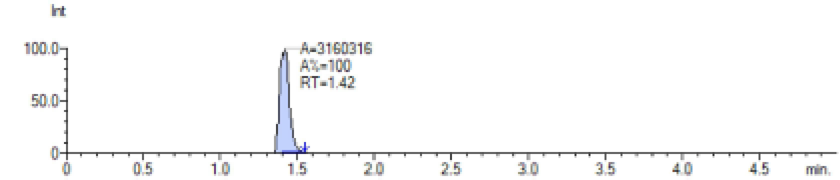

MS Spectrum
Group#1 - PDA Peak: 17, RT: 1.27 to 1.47 min

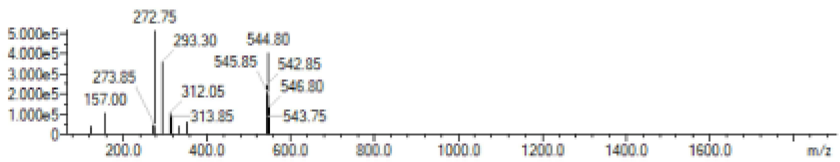

Shimadzu Open Solution

QN-C-R-28

Project: Test
Experiment: 572_190612_Zinken_0607_Bischof_0612Griga
Experiment Description:
Sample: 410428:01:01
Sample Description: C27H27BrN2O4
Data File Name: C:\LabSolutions\Data\572_190612_Zinken_190607_Bischoff_190612_Grigalunas\1046364478_11190613572.lcd
Sample Location: Plate Number: 1 - Position: 138
Run By: admin
Run Started: 29 July 2019 19:27:00
Run Finished: 30 July 2019 04:41:49
Method: 190321_Purity-Check

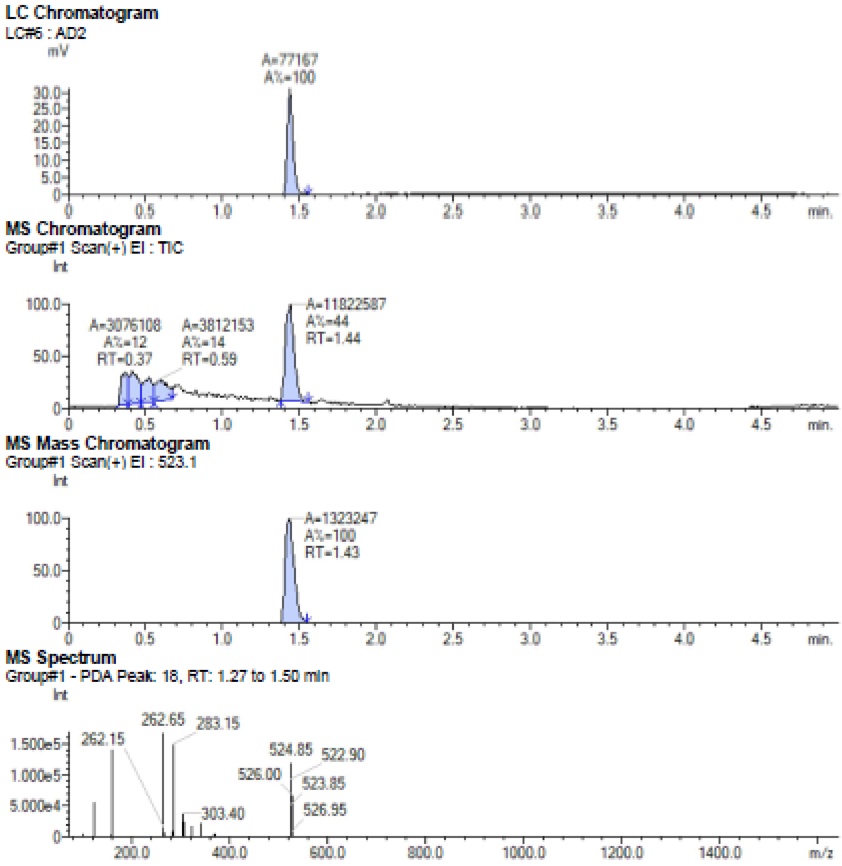

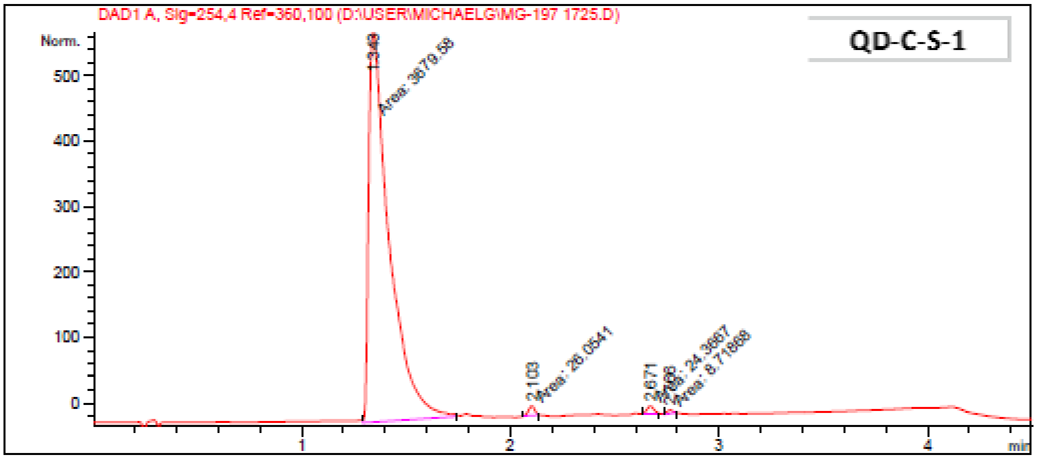

#	Time	Area	Height	Width	Area%	Symmetry
1	1.343	3679.6	539.6	0.1137	98.418	0.273
2	2.103	26.1	12.9	0.0338	0.697	1.094
3	2.671	24.4	10.5	0.0386	0.652	0.892
4	2.766	8.7	4.4	0.0329	0.233	1.339

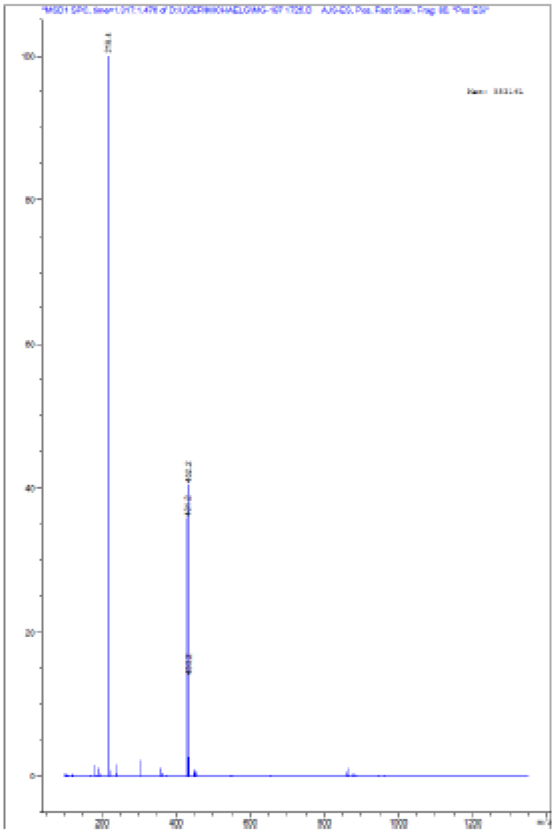

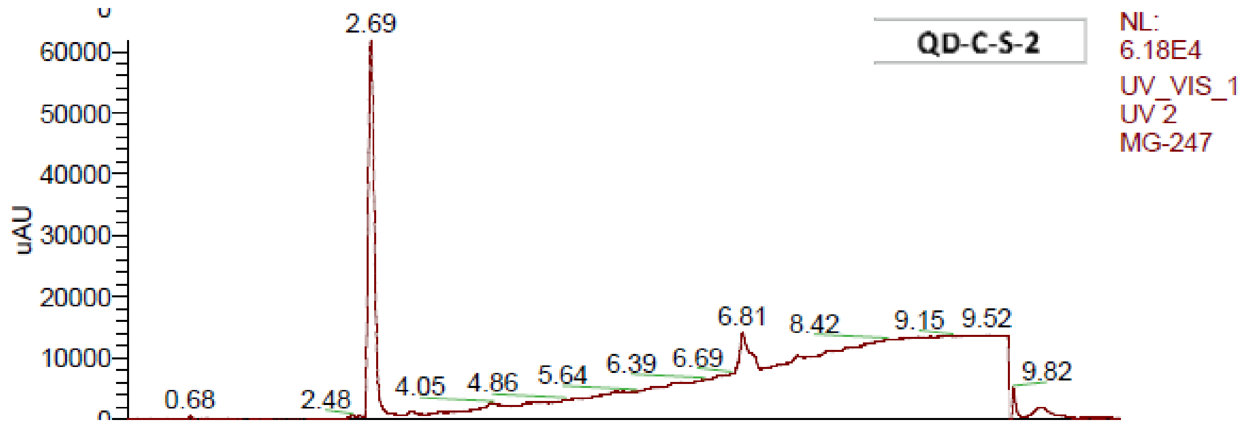

PEAK LIST
MG-247.raw
RT: 2.62 - 2.85
Number of detected peaks: 1

Apex RT	Start RT	End RT	Area	%Area	Height	%Height
2.69	2.63	2.79	114273.829	100.00	25938.152	100.00

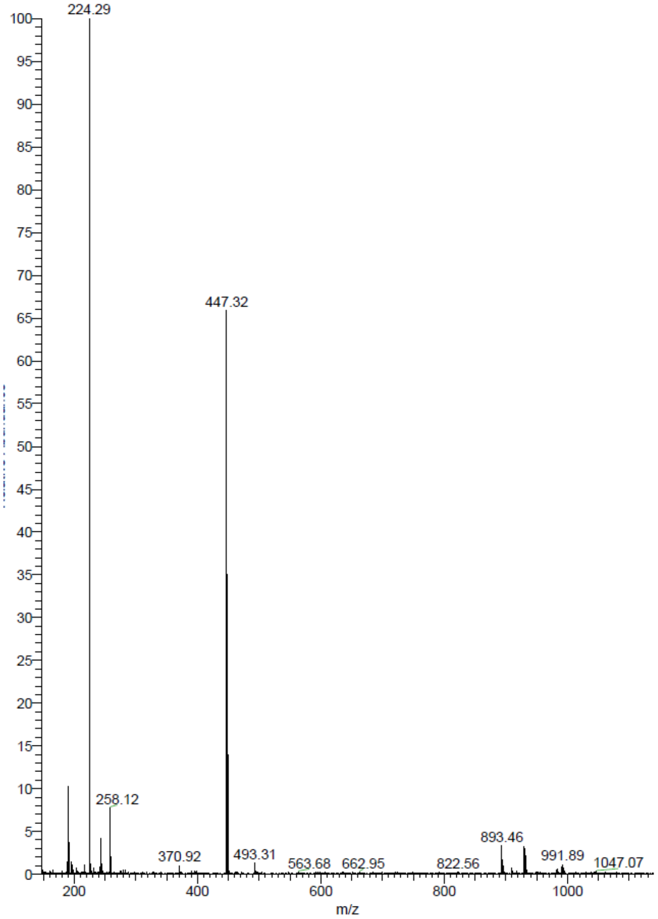

QD-C-R-1

Shimadzu Open Solution

Project: Test
Experiment: 430_0530_Grigalu_0605_3101_TEAD_Wu
Experiment Description:
Sample: 408620:01:01
Sample Description: C26H26N2O5
Data File Name: C:\LabSolutions\Data\430_180530_Grigalunas_180605_3101_180605_Tead_180605_Wu\1046325007_11180530431.lcd
Sample Location: Plate Number: 2 - Position: 57
Run By: admin
Run Started: 24 September 2018 14:46:57
Run Finished: 25 September 2018 00:19:16
Method: Purity_Check_2018

LC Chromatogram
LC#6 : AD2

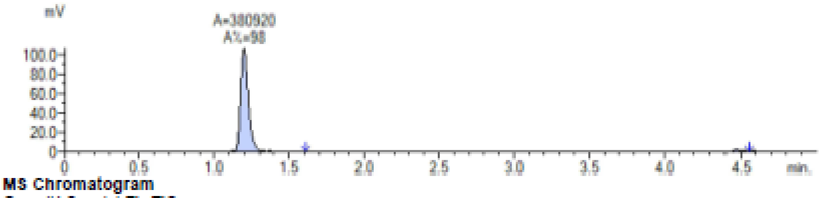

MS Chromatogram
Group#1 Scan(+) EI : TIC

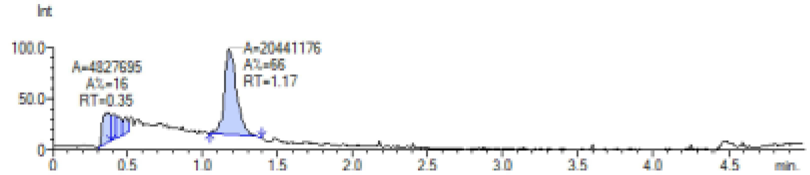

MS Mass Chromatogram
Group#1 Scan(+) EI : 447.2

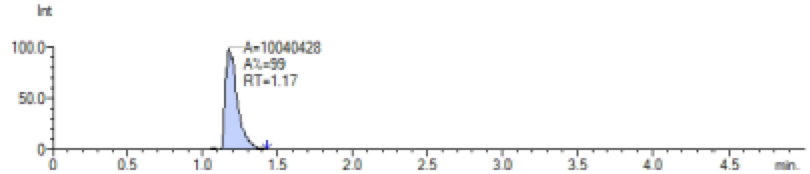

MS Spectrum
Group#1 - PDA Peak: 19, RT: 0.97 to 1.37 min

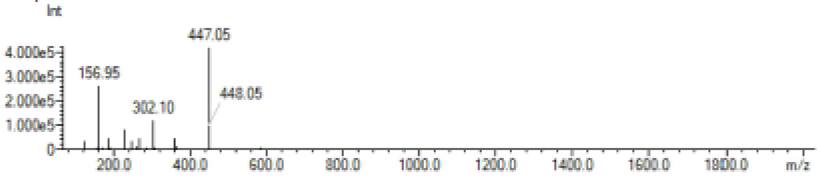

QD-C-S-3

Shimadzu Open Solution

Project: Test
Experiment: 430_0530_Grigalu_0605_3101_TEAD_Wu
Experiment Description:
Sample: 408621:01:02
Sample Description: C26H26N2O5
Data File Name: C:\LabSolutions\Data\430_180530_Grigalunas_180605_3101_180605_Tead_180605_Wu\1046325008_11180530431.lcd
Sample Location: Plate Number: 2 - Position: 105
Run By: admin
Run Started: 24 September 2018 14:46:57
Run Finished: 25 September 2018 00:19:16
Method: Purity_Check_2018

LC Chromatogram
LC#6 : AD2

MS Chromatogram
Group#1 Scan(+) EI : TIC

MS Mass Chromatogram
Group#1 Scan(+) EI : 447.2

MS Spectrum
Group#1 - PDA Peak: 19, RT: 0.89 to 1.15 min

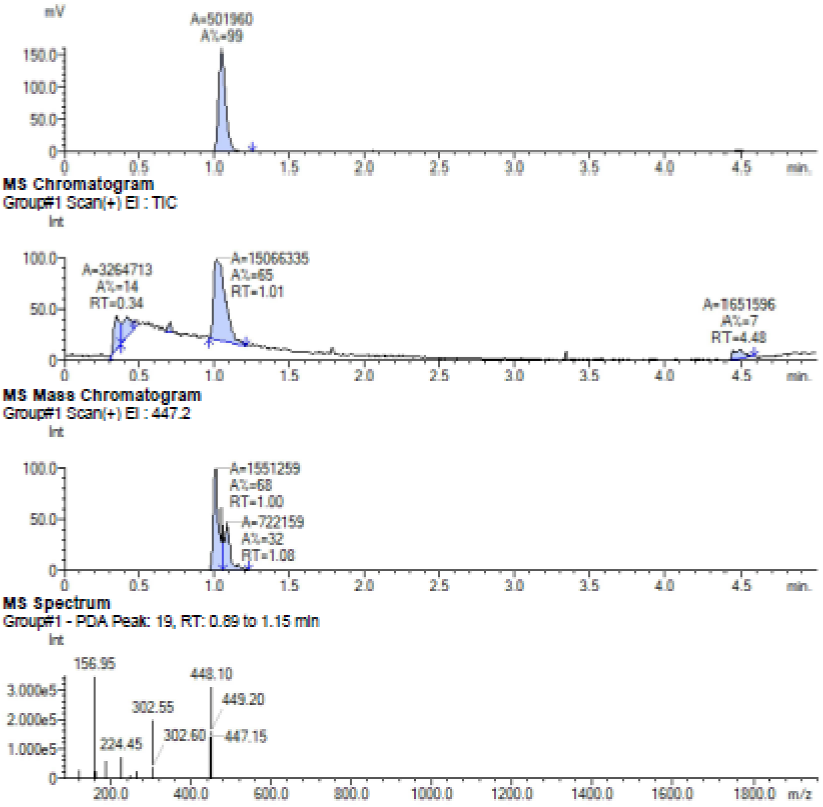

QD-C-S-4

Shimadzu Open Solution

Project: Test
Experiment: 430_0530_Grigalu_0605_3101_TEAD_Wu
Experiment Description:
Sample: 408622:01:01
Sample Description: C26H26N2O5
Data File Name: C:\LabSolutions\Data\430_180530_Grigalunas_180605_3101_180605_Tead_180605_Wu\1046325630_11180530431.lcd
Sample Location: Plate Number: 2 - Position: 153
Run By: admin
Run Started: 24 September 2018 14:46:57
Run Finished: 25 September 2018 00:19:16
Method: Purity_Check_2018

LC Chromatogram
LC#6 : AD2

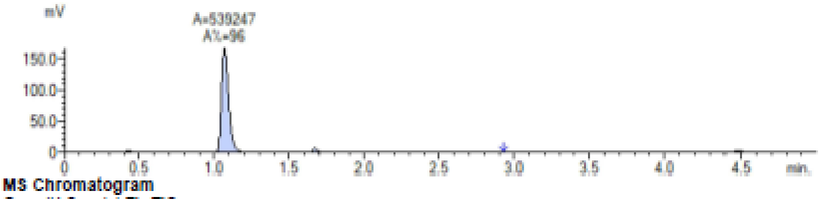

MS Chromatogram
Group#1 Scan(+) EI : TIC

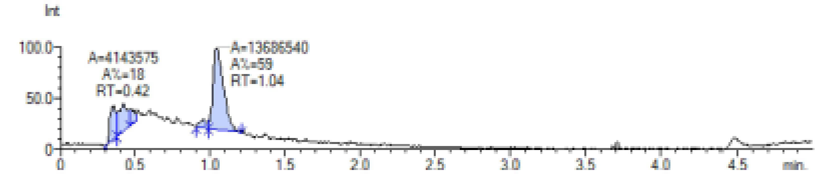

MS Mass Chromatogram
Group#1 Scan(+) EI : 447.2

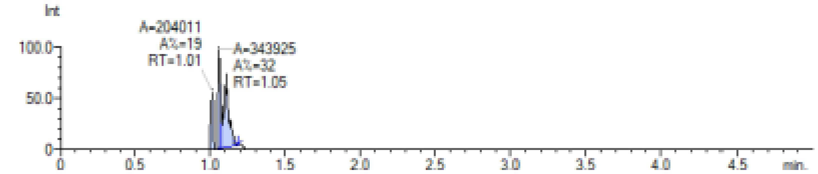

MS Spectrum
Group#1 - PDA Peak: 24, RT: 0.93 to 1.11 min

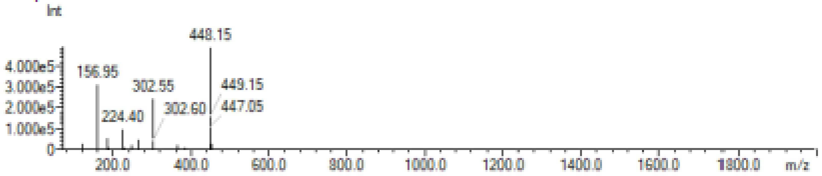

QD-C-R-2

Shimadzu Open Solution

Project: Test
Experiment: 430_0530_Grigalu_0605_3101_TEAD_Wu
Experiment Description:
Sample: 408623:01:01
Sample Description: C26H26N2O5
Data File Name: C:\LabSolutions\Data\430_180530_Grigalunas_180605_3101_180605_Tead_180605_Wu\1046325406_11180530431.lcd
Sample Location: Plate Number: 2 - Position: 201
Run By: admin
Run Started: 24 September 2018 14:46:57
Run Finished: 25 September 2018 00:19:16
Method: Purity_Check_2018

LC Chromatogram
LC#6 : AD2

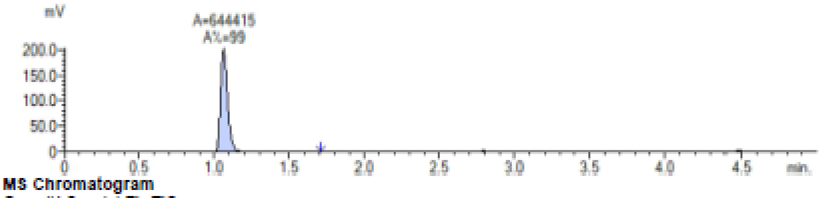

MS Chromatogram
Group#1 Scan(+) EI : TIC

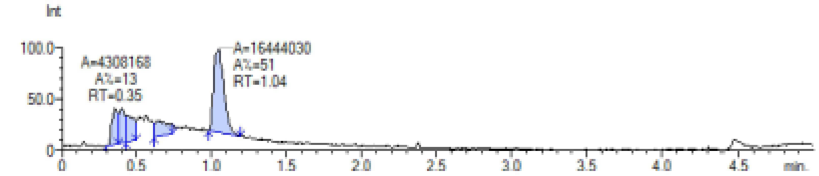

MS Mass Chromatogram
Group#1 Scan(+) EI : 447.2

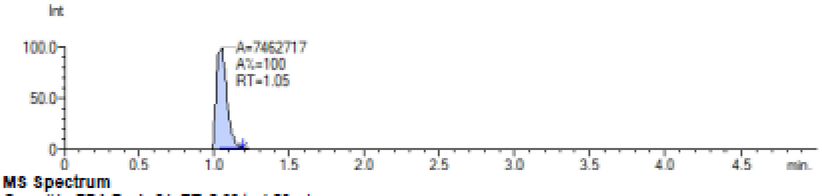

MS Spectrum
Group#1 - PDA Peak: 21, RT: 0.92 to 1.09 min

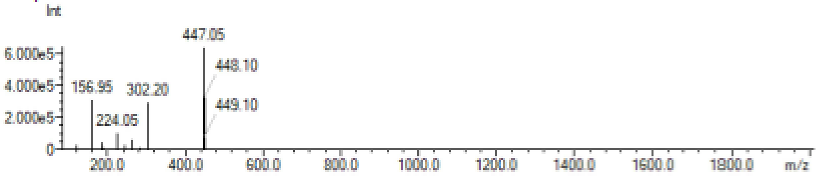

Shimadzu Open Solution

QD-C-S-5

**Project:**	Test
**Experiment:**	430_0530_Grigalu_0605_3101_TEAD_Wu
**Experiment Description:**	
**Sample:**	408624:01:01
**Sample Description:**	C27H28N2O5
**Data File Name:**	C:\LabSolutions\Data\430_180530_Grigalunas_180605_3101_180605_Tead_180605_Wu\1046325403_11180530431.lcd
**Sample Location:**	Plate Number: 2 - Position: 249
**Run By:**	admin
**Run Started:**	24 September 2018 14:46:57
**Run Finished:**	25 September 2018 00:19:16
**Method:**	Purity_Check_2018

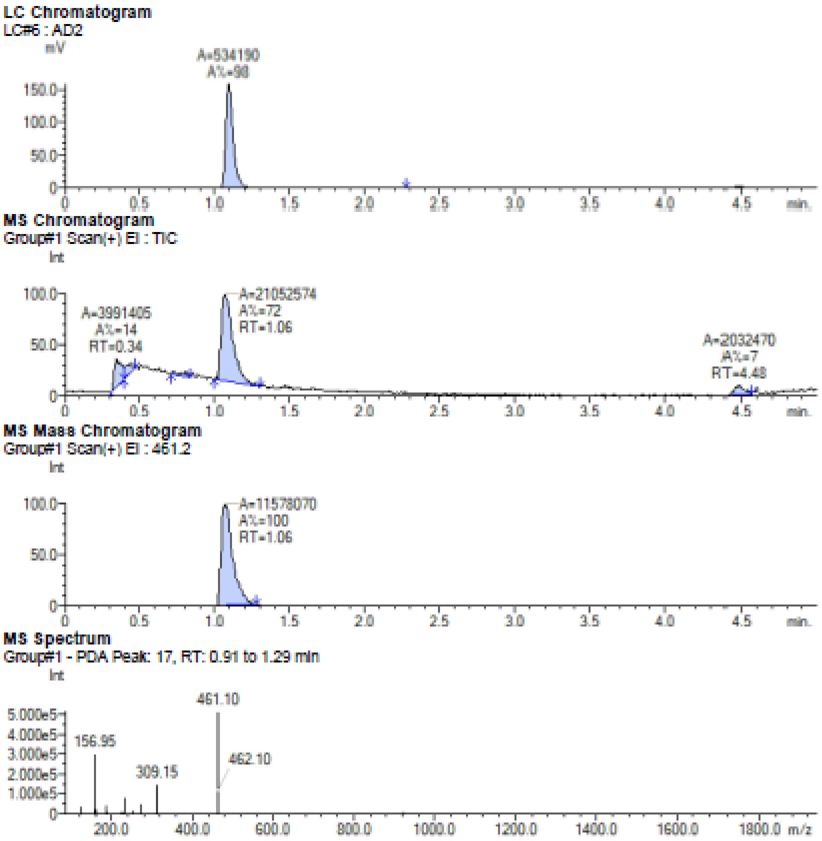

QD-C-R-3

Shimadzu Open Solution

**Project:**	Test
**Experiment:**	430_0530_Grigalu_0605_3101_TEAD_Wu
**Experiment Description:**	
**Sample:**	408625:01:01
**Sample Description:**	C27H28N2O5
**Data File Name:**	C:\LabSolutions\Data\430_180530_Grigalunas_180605_3101_180605_Tead_180605_Wu\1046324852_11180530431.lcd
**Sample Location:**	Plate Number: 2 - Position: 297
**Run By:**	admin
**Run Started:**	24 September 2018 14:46:57
**Run Finished:**	25 September 2018 00:19:16
**Method:**	Purity_Check_2018

LC Chromatogram
LC#6 : AD2

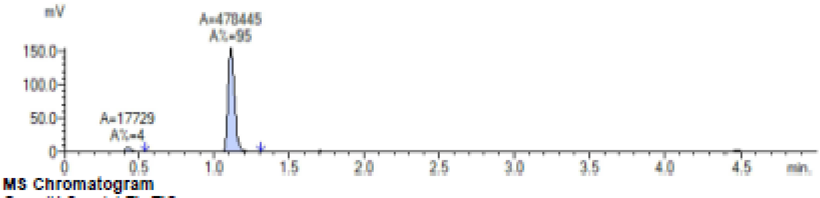

MS Chromatogram
Group#1 Scan(+) EI : TIC

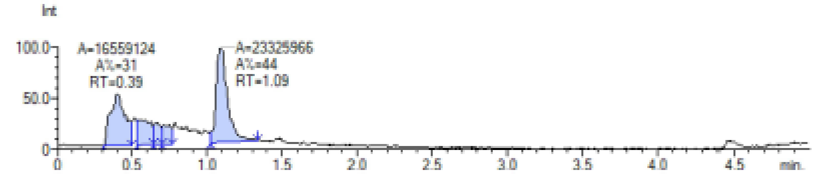

MS Mass Chromatogram
Group#1 Scan(+) EI : 461.2

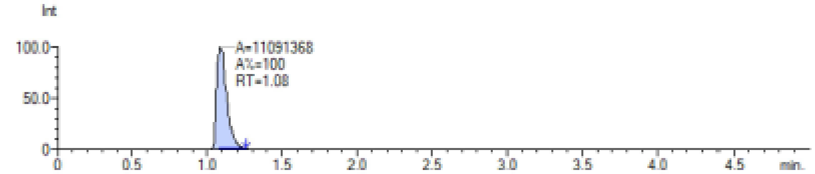

MS Spectrum
Group#1 - PDA Peak: 18, RT: 0.93 to 1.17 min

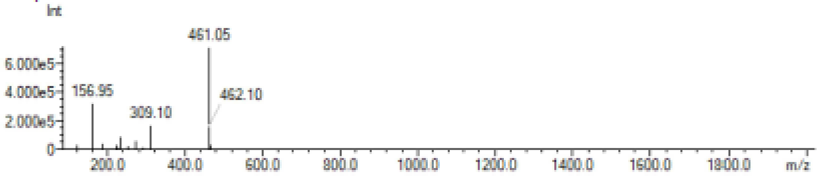

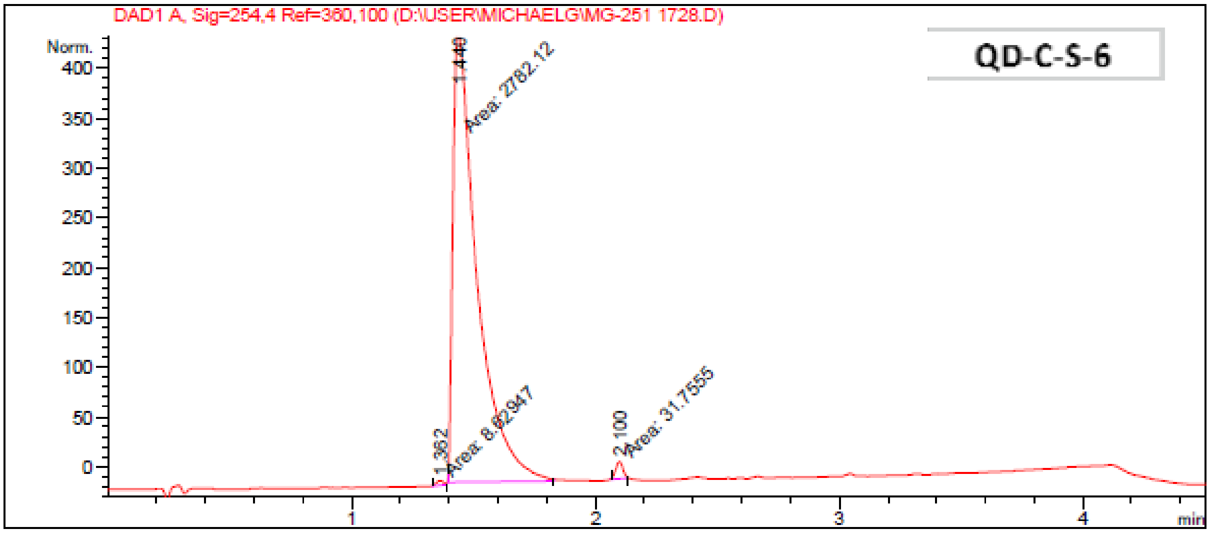

#	Time	Area	Height	Width	Area%	Symmetry
1	1.362	8.6	4.2	0.0343	0.306	0.996
2	1.44	2782.1	405.2	0.1144	98.569	0.288
3	2.1	31.8	15.4	0.0343	1.125	1.089

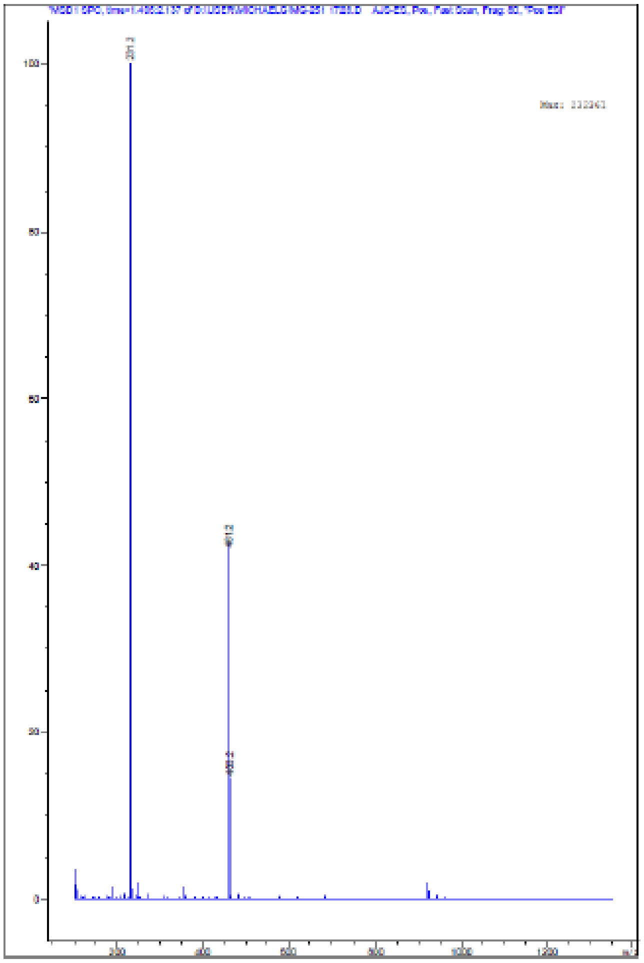

Shimadzu Open Solution

QD-C-R-4

Project: Test
Experiment: 430_0530_Grigalu_0605_3101_TEAD_Wu
Experiment Description:
Sample: 408627:01:01
Sample Description: C27H28N2O5
Data File Name: C:\LabSolutions\Data\430_180530_Grigalunas_180605_3101_180605_Tead_180605_Wu\1046324893_11180530431.lcd
Sample Location: Plate Number: 2 - Position: 11
Run By: admin
Run Started: 24 September 2018 14:46:57
Run Finished: 25 September 2018 00:19:16
Method: Purity_Check_2018

LC Chromatogram
LC#6 : AD2

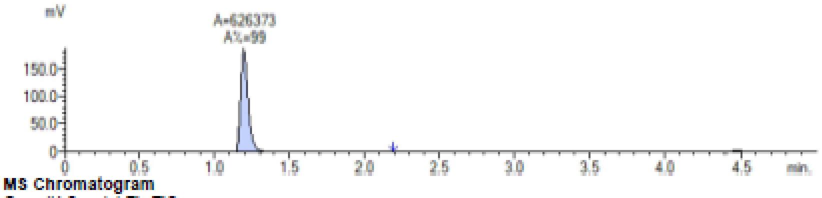

MS Chromatogram
Group#1 Scan(+) EI : TIC

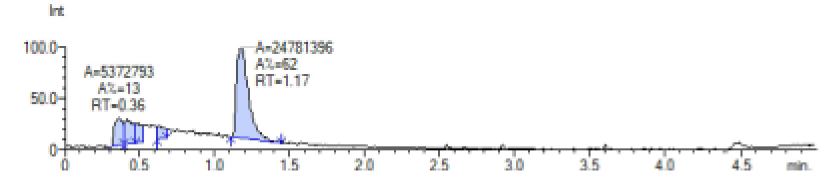

MS Mass Chromatogram
Group#1 Scan(+) EI : 461.2

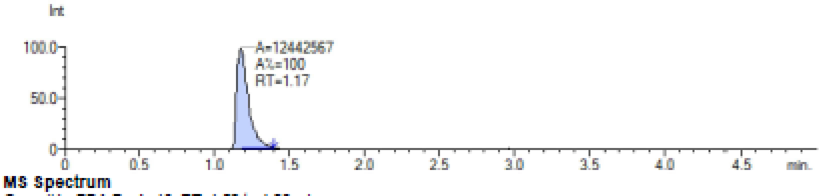

MS Spectrum
Group#1 - PDA Peak: 19, RT: 1.03 to 1.32 min

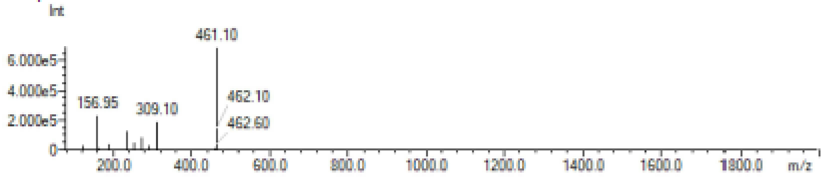

Shimadzu Open Solution

QD-C-S-7

Project: Test
Experiment: 430_0530_Grigalu_0605_3101_TEAD_Wu
Experiment Description:
Sample: 408628:01:02
Sample Description: C27H28N2O5
Data File Name: C:\LabSolutions\Data\430_180530_Grigalunas_180605_3101_180605_Tead_180605_Wu\1046325633_11180530431.lcd
Sample Location: Plate Number: 2 - Position: 59
Run By: admin
Run Started: 24 September 2018 14:46:57
Run Finished: 25 September 2018 00:19:16
Method: Purity_Check_2018

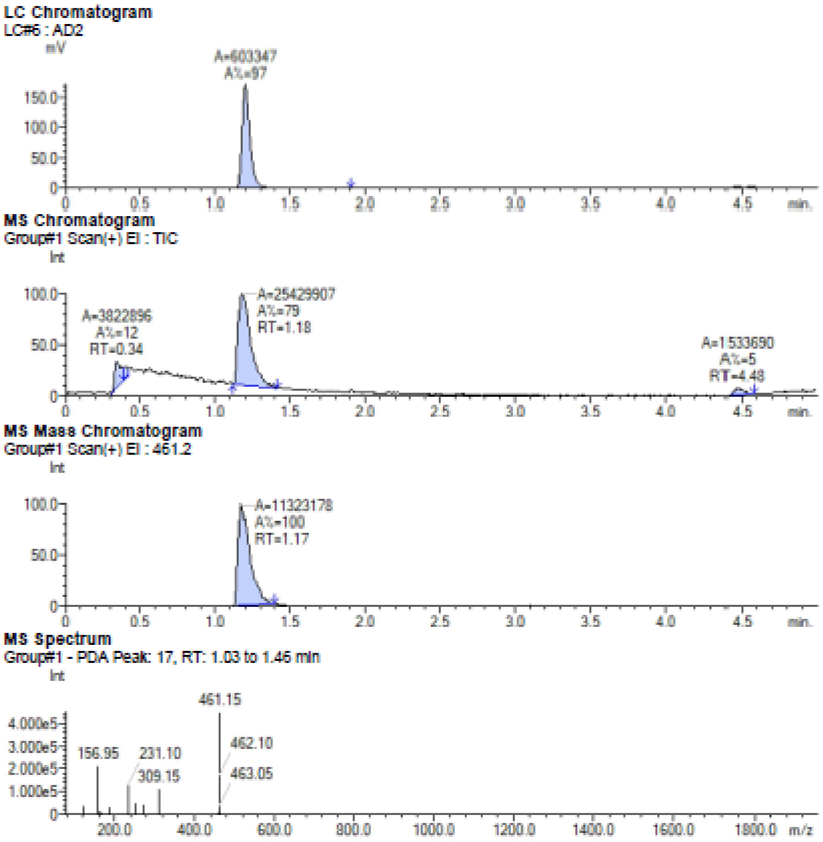

Shimadzu Open Solution

QD-C-R-5

Project: Test
Experiment: 430_0530_Grigalu_0605_3101_TEAD_Wu
Experiment Description:
Sample: 408629:01:01
Sample Description: C27H28N2O5
Data File Name: C:\LabSolutions\Data\430_180530_Grigalunas_180605_3101_180605_Tead_180605_Wu\1046325115_11180530431.lcd
Sample Location: Plate Number: 2 - Position: 107
Run By: admin
Run Started: 24 September 2018 14:46:57
Run Finished: 25 September 2018 00:19:16
Method: Purity_Check_2018

LC Chromatogram
LC#6 : AD2

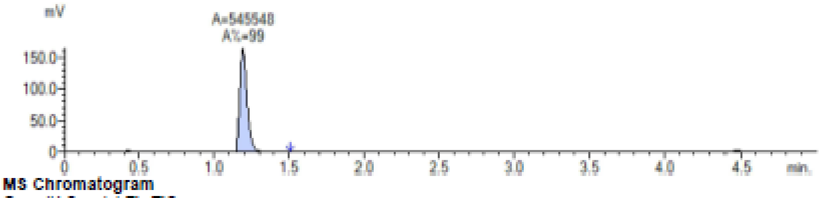

MS Chromatogram
Group#1 Scan(+) EI : TIC

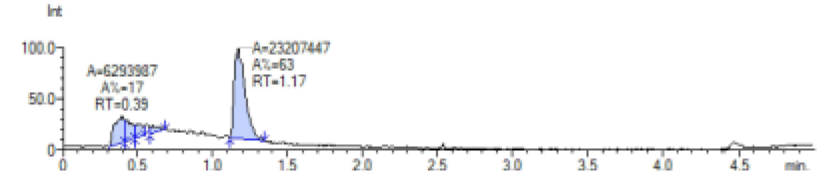

MS Mass Chromatogram
Group#1 Scan(+) EI : 461.2

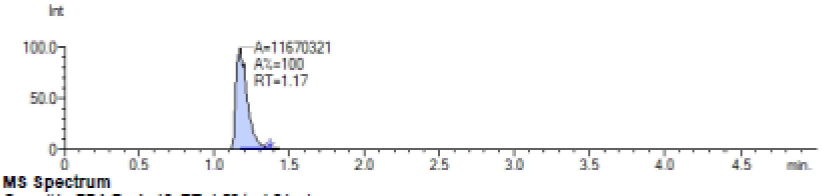

MS Spectrum
Group#1 - PDA Peak: 18, RT: 1.03 to 1.31 min

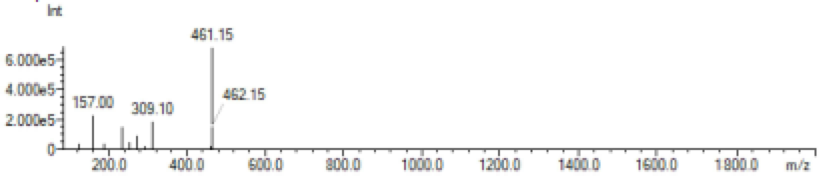

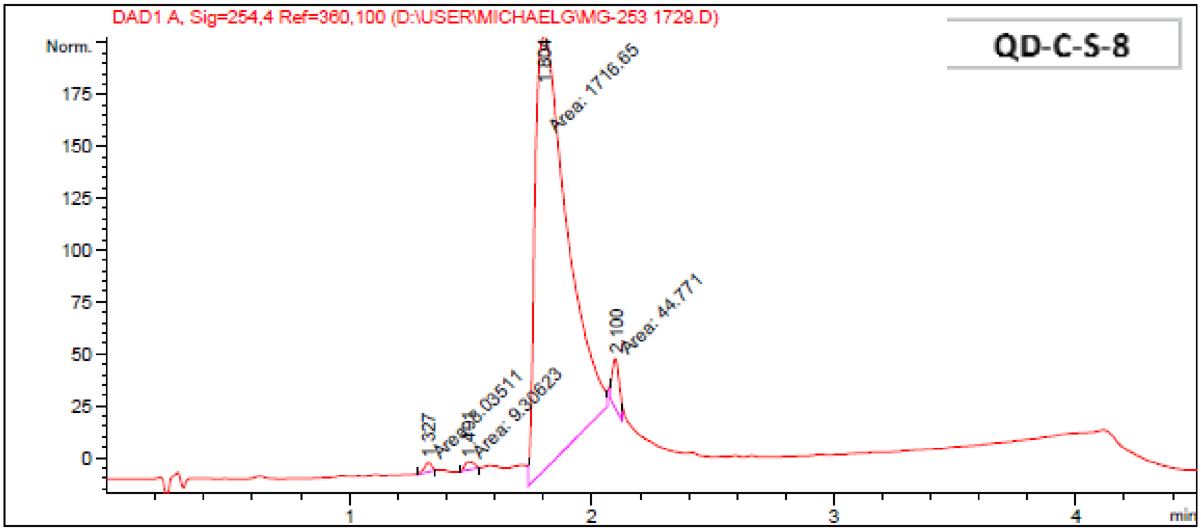

#	Time	Area	Height	Width	Area%	Symmetry
1	1.327	8	3.9	0.034	0.452	1.507
2	1.491	9.3	3.7	0.0416	0.523	0.569
3	1.804	1716.7	189.2	0.1512	96.508	0.377
4	2.1	44.8	21.9	0.0341	2.517	0.767

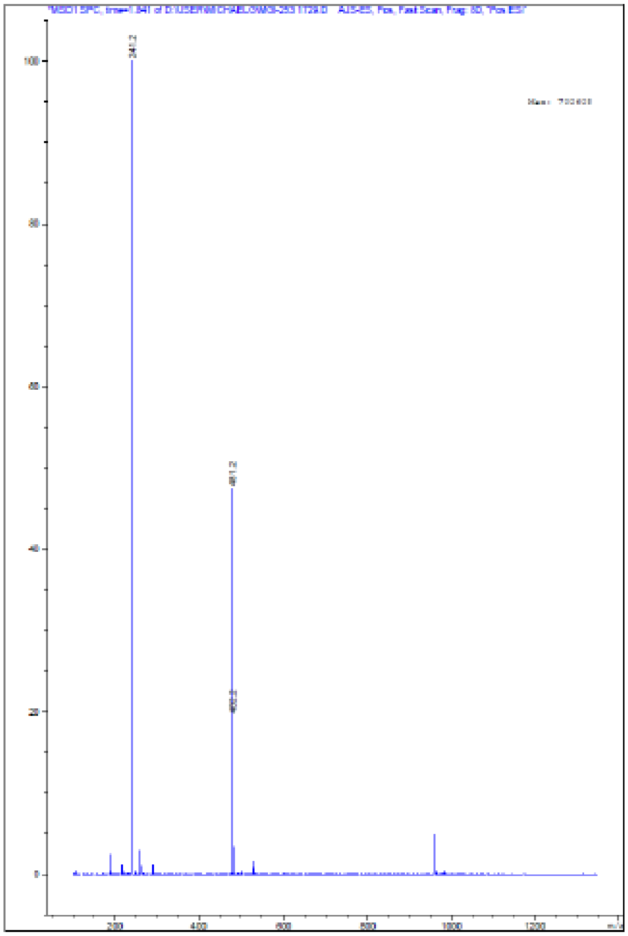

QD-C-R-6

Shimadzu Open Solution

**Project:**	Test
**Experiment:**	430_0530_Grigalu_0605_3101_TEAD_Wu
**Experiment Description:**	
**Sample:**	408631:01:01
**Sample Description:**	C30H28N2O4
**Data File Name:**	C:\LabSolutions\Data\430_180530_Grigalunas_180605_3101_180605_Tead_180605_Wu\1046325439_11180530431.lcd
**Sample Location:**	Plate Number: 2 - Position: 203
**Run By:**	admin
**Run Started:**	24 September 2018 14:46:57
**Run Finished:**	25 September 2018 00:19:16
**Method:**	Purity_Check_2018

LC Chromatogram
LC#6 : AD2

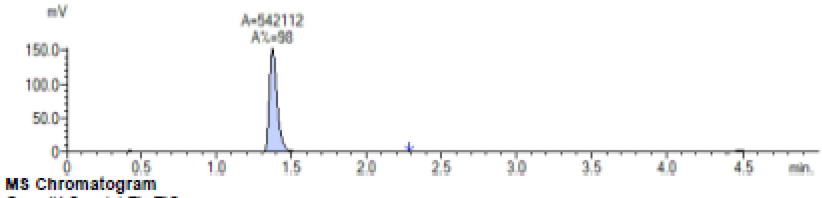

MS Chromatogram
Group#1 Scan(+) EI : TIC

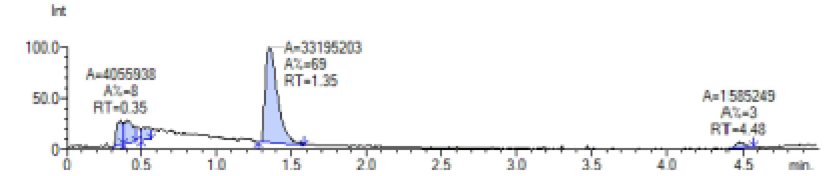

MS Mass Chromatogram
Group#1 Scan(+) EI : 481.2

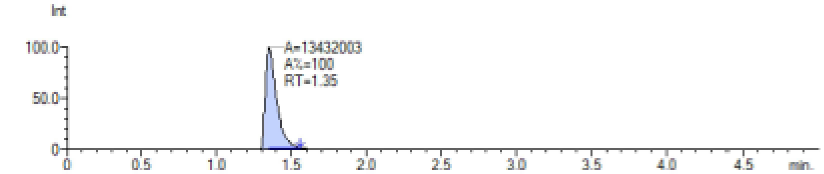

MS Spectrum
Group#1 - PDA Peak: 20, RT: 1.18 to 1.49 min

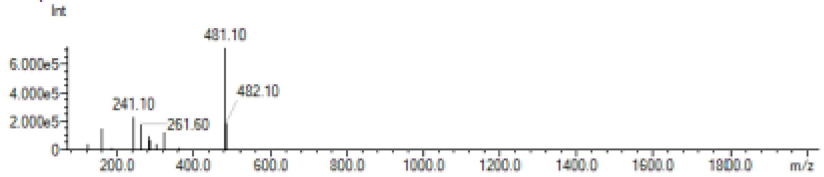

QD-C-S-9

Shimadzu Open Solution

Project: Test
Experiment: 430_0530_Grigalu_0605_3101_TEAD_Wu
Experiment Description:
Sample: 408632:01:01
Sample Description: C30H28N2O4
Data File Name: C:\LabSolutions\Data\430_180530_Grigalunas_180605_3101_180605_Tead_180605_Wu\1046324985_11180530431.lcd
Sample Location: Plate Number: 2 - Position: 251
Run By: admin
Run Started: 24 September 2018 14:46:57
Run Finished: 25 September 2018 00:19:16
Method: Purity_Check_2018

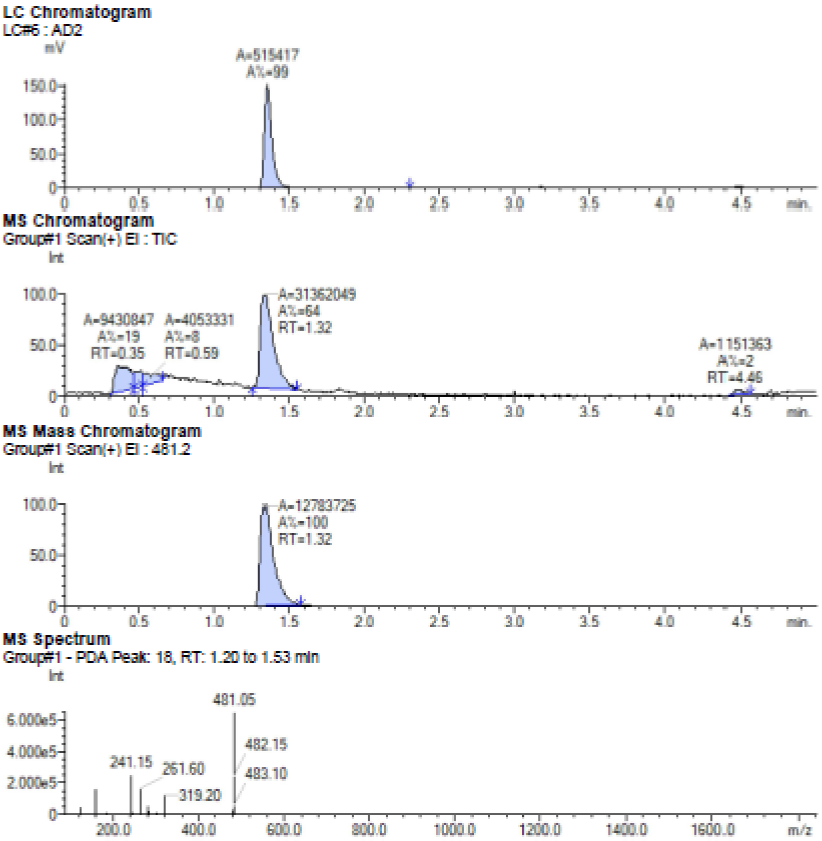

QD-C-S-10

Shimadzu Open Solution

Project: Test
Experiment: 430_0530_Grigalu_0605_3101_TEAD_Wu
Experiment Description:
Sample: 408633:01:01
Sample Description: C30H33N3O4
Data File Name: C:\LabSolutions\Data\430_180530_Grigalunas_180605_3101_180605_Tead_180605_Wu\1046324667_11180530431.lcd
Sample Location: Plate Number: 2 - Position: 299
Run By: admin
Run Started: 24 September 2018 14:46:57
Run Finished: 25 September 2018 00:19:16
Method: Purity_Check_2018

LC Chromatogram
LC#6 : AD2

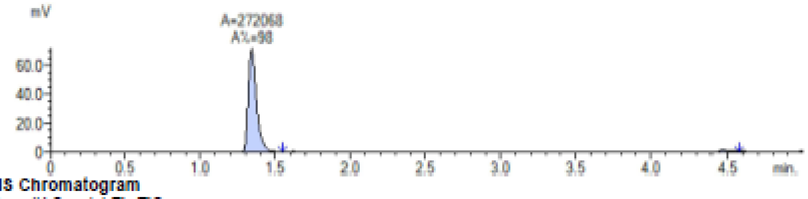

MS Chromatogram
Group#1 Scan(+) EI : TIC

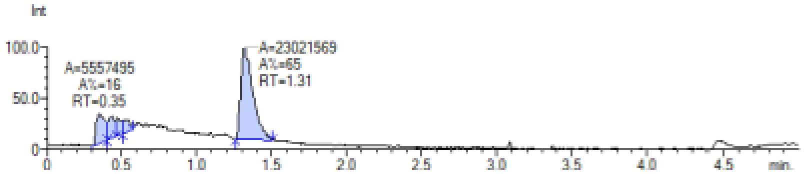

MS Mass Chromatogram
Group#1 Scan(+) EI : 500.2

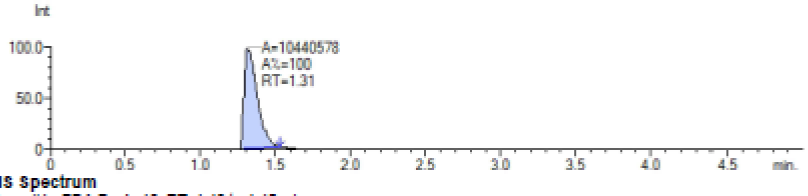

MS Spectrum
Group#1 - PDA Peak: 18, RT: 1.18 to 1.45 min

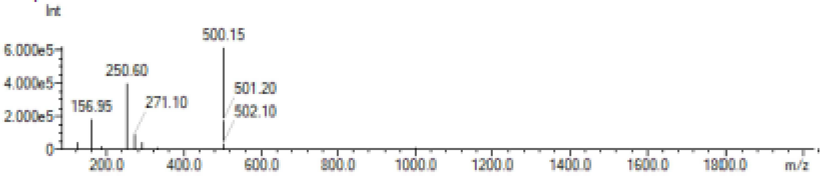

QD-C-S-11

Shimadzu Open Solution

Project: Test
Experiment: 572_190612_Zinken_0607_Bischof_0612Griga
Experiment Description:
Sample: 410477:01:01
Sample Description: C28H30N2O6
Data File Name: C:\LabSolutions\Data\572_190612_Zinken_190607_Bischoff_190612_Grigalunas\1046364494_11190613572.lcd
Sample Location: Plate Number: 1 - Position: 130
Run By: admin
Run Started: 29 July 2019 19:27:00
Run Finished: 30 July 2019 04:41:49
Method: 190321_Purity-Check

LC Chromatogram
LC#6 : AD2

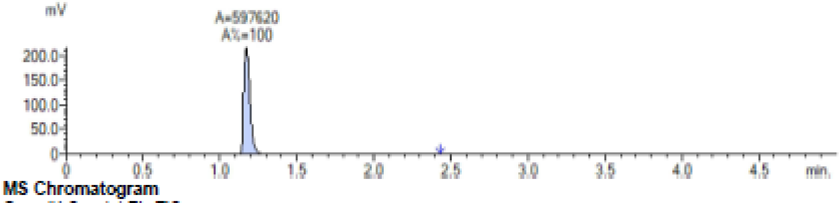

MS Chromatogram
Group#1 Scan(+) EI : TIC

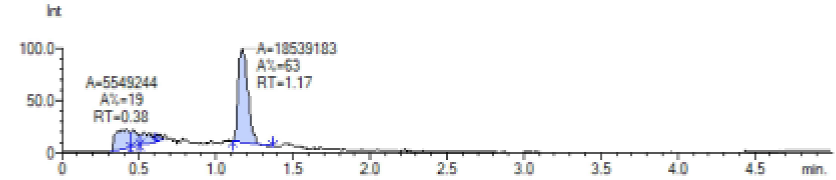

MS Mass Chromatogram
Group#1 Scan(+) EI : 491.2

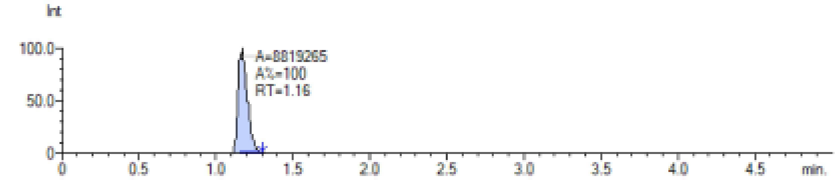

MS Spectrum
Group#1 - PDA Peak: 21, RT: 1.04 to 1.22 min

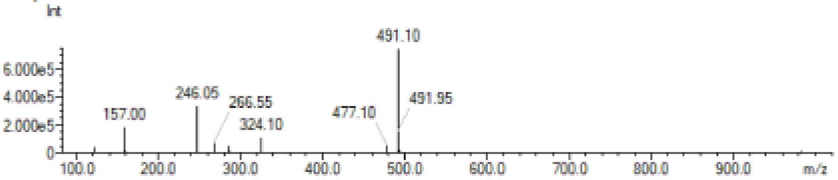

QD-C-R-7

Shimadzu Open Solution

**Project:**	Test
**Experiment:**	572_190612_Zinken_0607_Bischof_0612Griga
**Experiment Description:**	
**Sample:**	410478:01:01
**Sample Description:**	C28H30N2O6
**Data File Name:**	C:\LabSolutions\Data\572_190612_Zinken_190607_Bischoff_190612_Grigalunas\1046364477_11190613572.lcd
**Sample Location:**	Plate Number: 1 - Position: 178
**Run By:**	admin
**Run Started:**	29 July 2019 19:27:00
**Run Finished:**	30 July 2019 04:41:49
**Method:**	190321_Purity-Check

LC Chromatogram

LC#6 : AD2

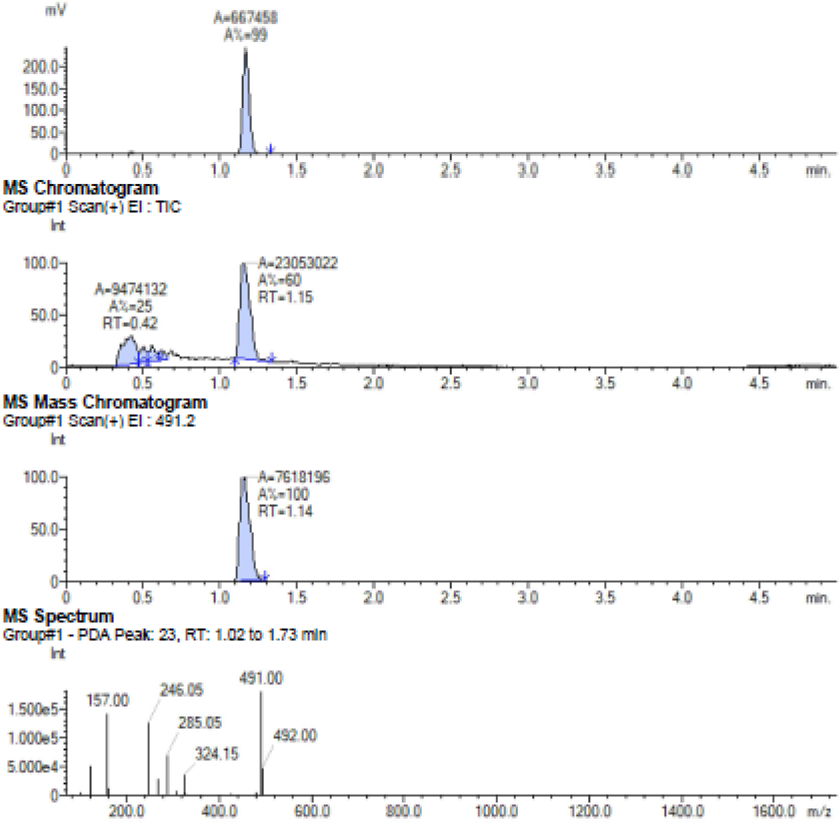

QD-C-S-12

Shimadzu Open Solution

**Project:**	Test
**Experiment:**	572_190612_Zinken_0607_Bischof_0612Griga
**Experiment Description:**	
**Sample:**	410479:01:01
**Sample Description:**	C28H30N2O4
**Data File Name:**	C:\LabSolutions\Data\572_190612_Zinken_190607_Bischoff_190612_Grigalunas\1046363972_11190613572.lcd
**Sample Location:**	Plate Number: 1 - Position: 226
**Run By:**	admin
**Run Started:**	29 July 2019 19:27:00
**Run Finished:**	30 July 2019 04:41:49
**Method:**	190321_Purity-Check

LC Chromatogram

LC#6 : AD2

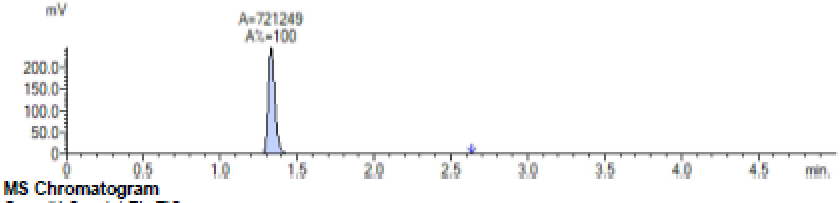

MS Chromatogram

Group#1 Scan(+) EI : TIC

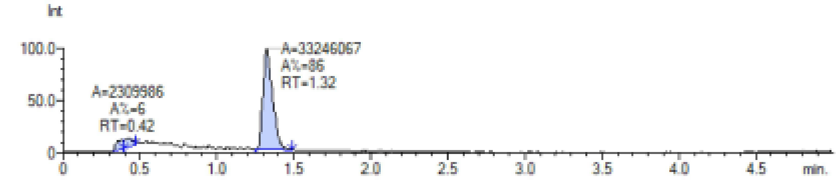

MS Mass Chromatogram

Group#1 Scan(+) EI : 459.2

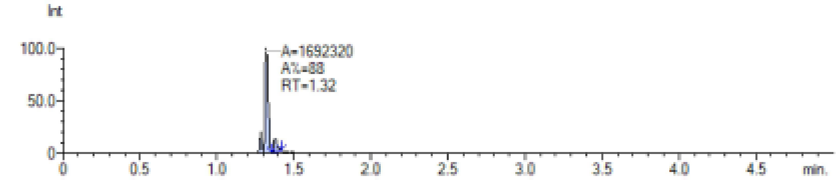

MS Spectrum

Group#1 - PDA Peak: 18, RT: 1.20 to 1.40 min

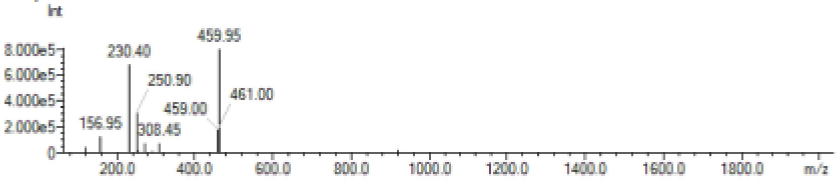

Shimadzu Open Solution

QD-C-R-8

Project: Test
Experiment: 572_190612_Zinken_0607_Bischof_0612Griga
Experiment Description:
Sample: 410480:01:01
Sample Description: C28H30N2O4
Data File Name: C:\LabSolutions\Data\572_190612_Zinken_190607_Bischoff_190612_Grigalunas\1046363953_11190613572.lcd
Sample Location: Plate Number: 1 - Position: 274
Run By: admin
Run Started: 29 July 2019 19:27:00
Run Finished: 30 July 2019 04:41:49
Method: 190321_Purity-Check

LC Chromatogram
LC#5 : AD2

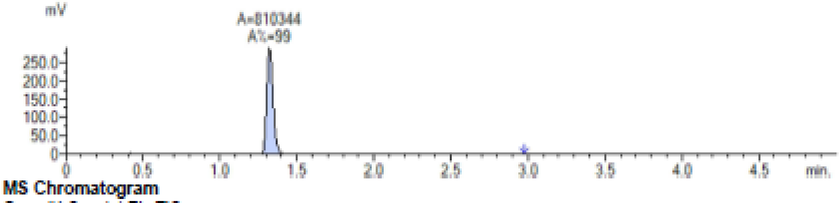

MS Chromatogram
Group#1 Scan(+) EI : TIC

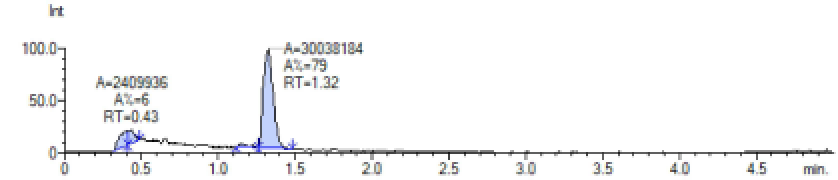

MS Mass Chromatogram
Group#1 Scan(+) EI : 459.2

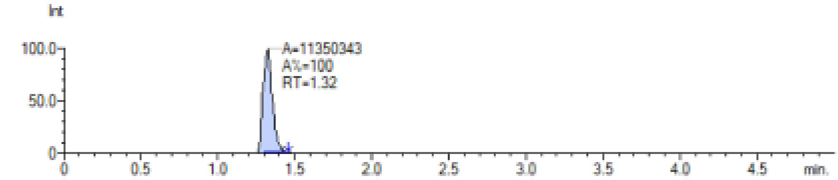

MS Spectrum
Group#1 - PDA Peak: 21, RT: 1.19 to 1.41 min

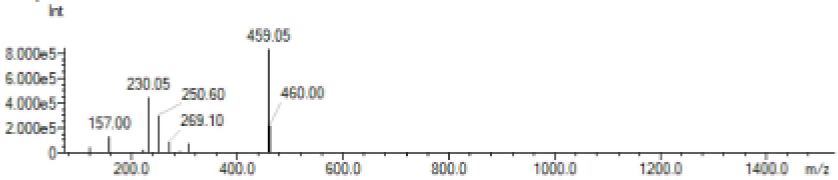

QD-C-S-13

Shimadzu Open Solution

Project: Test
Experiment: 572_190612_Zinken_0607_Bischof_0612Griga
Experiment Description:
Sample: 410481:01:01
Sample Description: C26H25BrN2O4
Data File Name: C:\LabSolutions\Data\572_190612_Zinken_190607_Bischoff_190612_Grigalunas\1046364796_11190613572.lcd
Sample Location: Plate Number: 1 - Position: 322
Run By: admin
Run Started: 29 July 2019 19:27:00
Run Finished: 30 July 2019 04:41:49
Method: 190321_Purity-Check

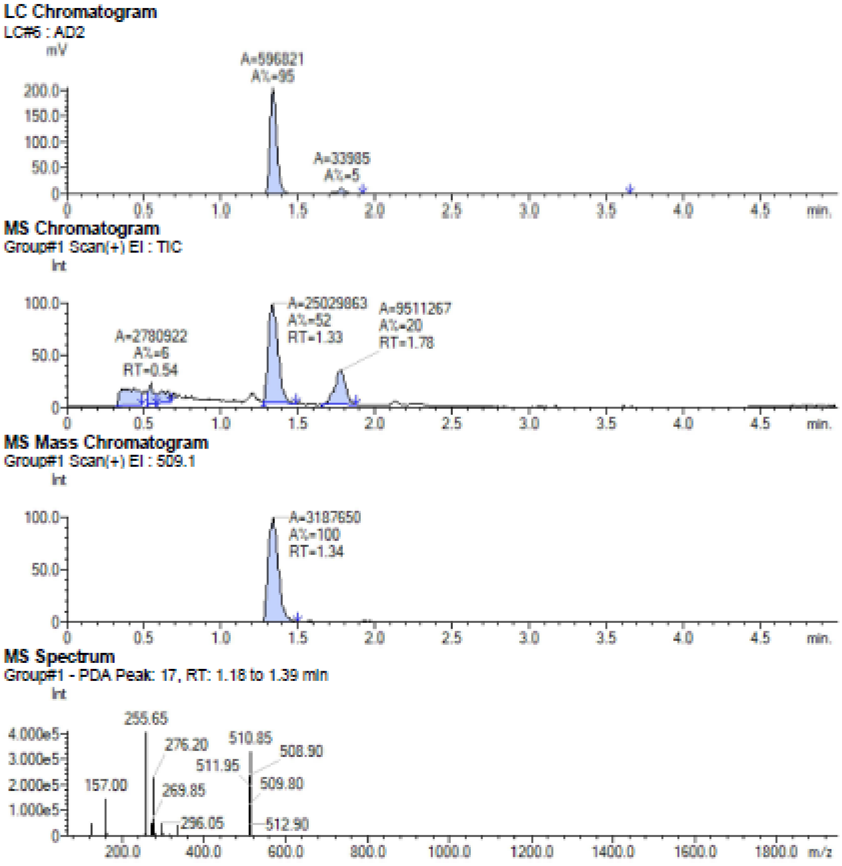

QD-C-R-9

Shimadzu Open Solution

Project: Test
Experiment: 572_190612_Zinken_0607_Bischof_0612Griga
Experiment Description:
Sample: 410482:01:01
Sample Description: C26H25BrN2O4
Data File Name: C:\LabSolutions\Data\572_190612_Zinken_190607_Bischoff_190612_Grigalunas\1046364036_11190613572.lcd
Sample Location: Plate Number: 1 - Position: 370
Run By: admin
Run Started: 29 July 2019 19:27:00
Run Finished: 30 July 2019 04:41:49
Method: 190321_Purity-Check

LC Chromatogram
LC#6 : AD2

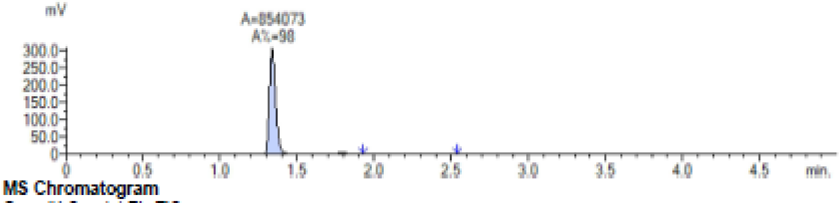

MS Chromatogram
Group#1 Scan(+) EI : TIC

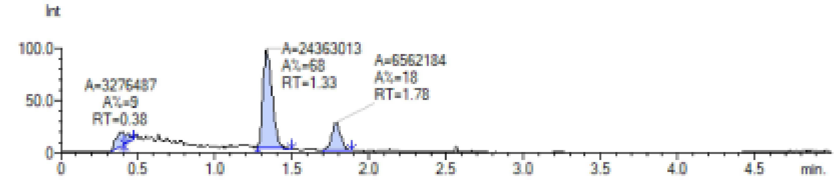

MS Mass Chromatogram
Group#1 Scan(+) EI : 509.1

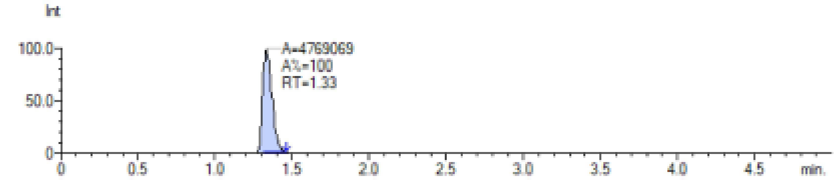

MS Spectrum
Group#1 - PDA Peak: 19, RT: 1.13 to 1.32 min

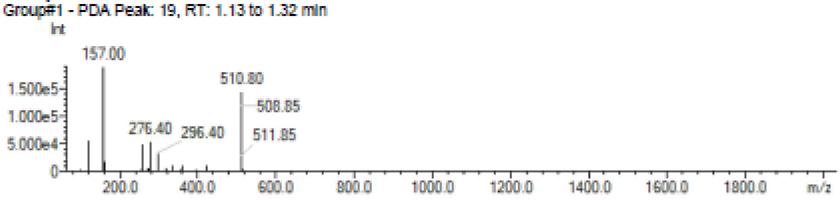

QD-C-S-14

Shimadzu Open Solution

Project: Test
Experiment: 572_190612_Zinken_0607_Bischof_0612Griga
Experiment Description:
Sample: 410483:01:01
Sample Description: C28H30N2O4
Data File Name: C:\LabSolutions\Data\572_190612_Zinken_190607_Bischoff_190612_Grigalunas\1046364637_11190613572.lcd
Sample Location: Plate Number: 1 - Position: 36
Run By: admin
Run Started: 29 July 2019 19:27:00
Run Finished: 30 July 2019 04:41:49
Method: 190321_Purity-Check

LC Chromatogram

LC#6 : AD2

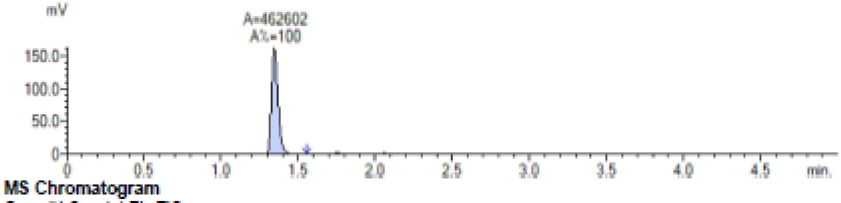

MS Chromatogram

Group#1 Scan(+) EI : TIC

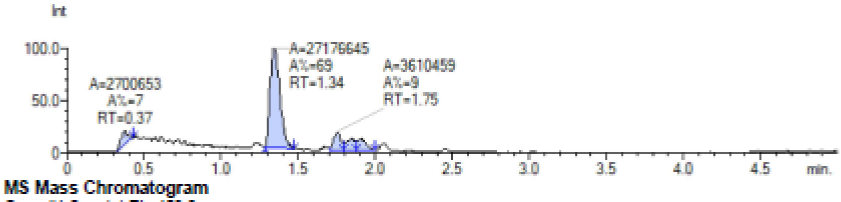

MS Mass Chromatogram

Group#1 Scan(+) EI : 459.2

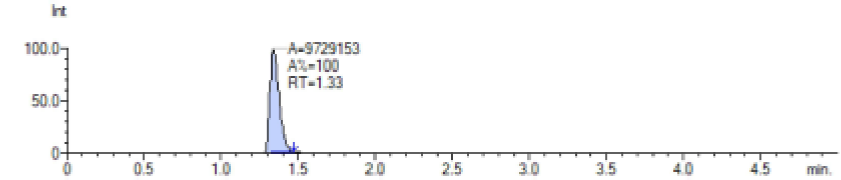

MS Spectrum

Group#1 - PDA Peak: 19, RT: 1.18 to 1.38 min

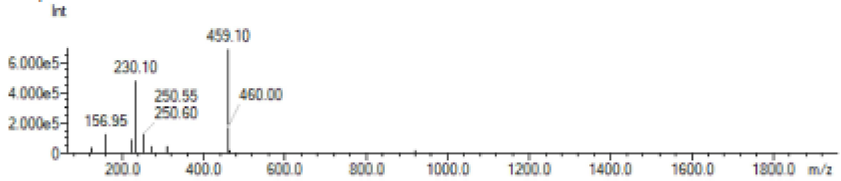

QD-C-R-10

Shimadzu Open Solution

Project: Test
Experiment: 572_190612_Zinken_0607_Bischof_0612Griga
Experiment Description:
Sample: 410484:01:01
Sample Description: C28H30N2O4
Data File Name: C:\LabSolutions\Data\572_190612_Zinken_190607_Bischoff_190612_Grigalunas\1046364749_11190613572.lcd
Sample Location: Plate Number: 1 - Position: 84
Run By: admin
Run Started: 29 July 2019 19:27:00
Run Finished: 30 July 2019 04:41:49
Method: 190321_Purity-Check

LC Chromatogram
LC#6 : AD2

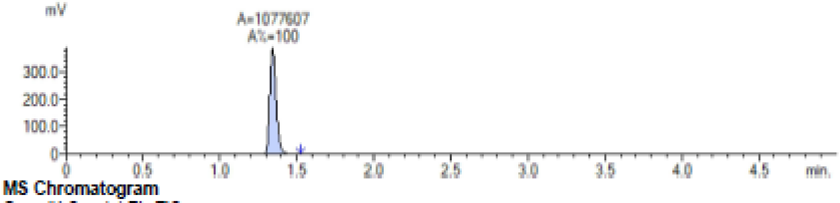

MS Chromatogram
Group#1 Scan(+) EI : TIC

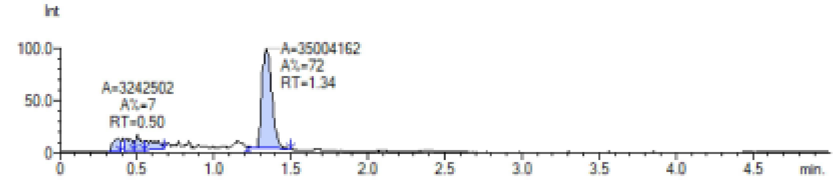

MS Mass Chromatogram
Group#1 Scan(+) EI : 459.2

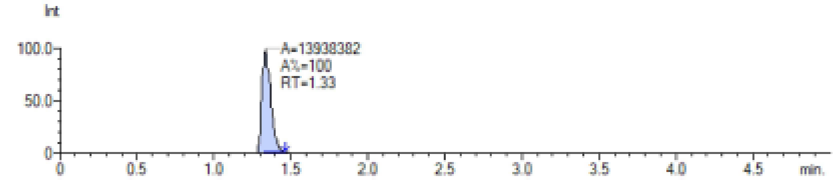

MS Spectrum
Group#1 - PDA Peak: 20, RT: 1.21 to 1.43 min

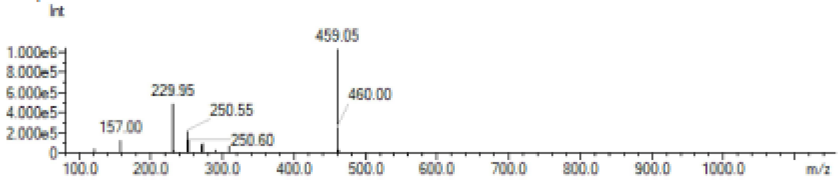

QD-C-S-15

Shimadzu Open Solution

Project: Test
Experiment: 572_190612_Zinken_0607_Bischof_0612Griga
Experiment Description:
Sample: 410485:01:01
Sample Description: C27H25F3N2O5
Data File Name: C:\LabSolutions\Data\572_190612_Zinken_190607_Bischoff_190612_Grigalunas\1046364103_11190613572.lcd
Sample Location: Plate Number: 1 - Position: 132
Run By: admin
Run Started: 29 July 2019 19:27:00
Run Finished: 30 July 2019 04:41:49
Method: 190321_Purity-Check

LC Chromatogram
LC#6 : AD2

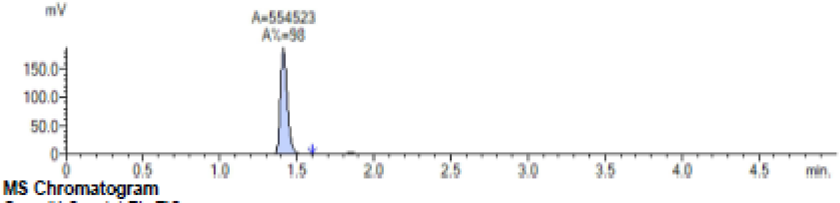

MS Chromatogram
Group#1 Scan(+) EI : TIC

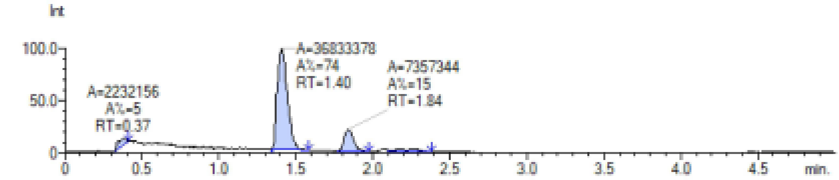

MS Mass Chromatogram
Group#1 Scan(+) EI : 515.2

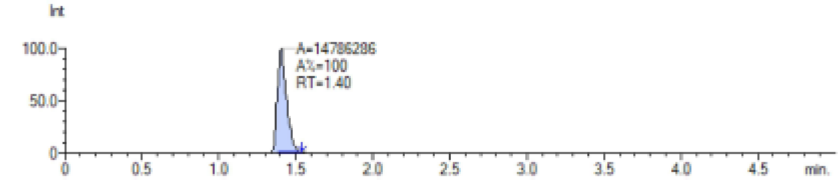

MS Spectrum
Group#1 - PDA Peak: 20, RT: 1.27 to 1.53 min

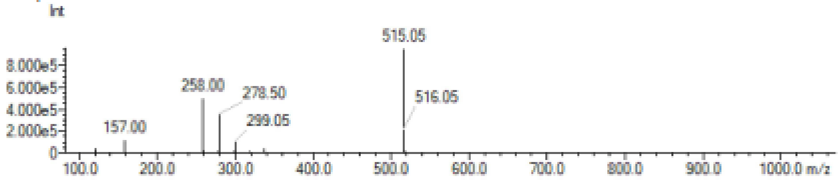

QD-C-R-11

Shimadzu Open Solution

**Project:**	Test
**Experiment:**	572_190612_Zinken_0607_Bischof_0612Griga
**Experiment Description:**	
**Sample:**	410486:01:01
**Sample Description:**	C27H25F3N2O5
**Data File Name:**	C:\LabSolutions\Data\572_190612_Zinken_190607_Bischoff_190612_Grigalunas\1046364662_11190613572.lcd
**Sample Location:**	Plate Number: 1 - Position: 180
**Run By:**	admin
**Run Started:**	29 July 2019 19:27:00
**Run Finished:**	30 July 2019 04:41:49
**Method:**	190321_Purity-Check

LC Chromatogram
LC#6 : AD2

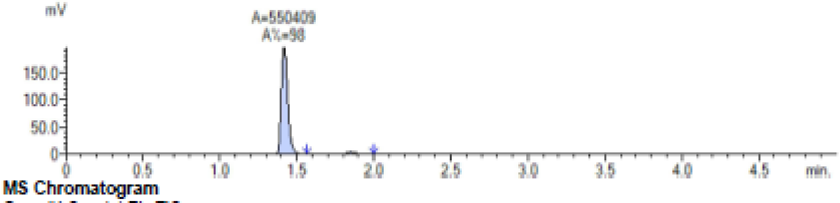

MS Chromatogram
Group#1 Scan(+) EI : TIC

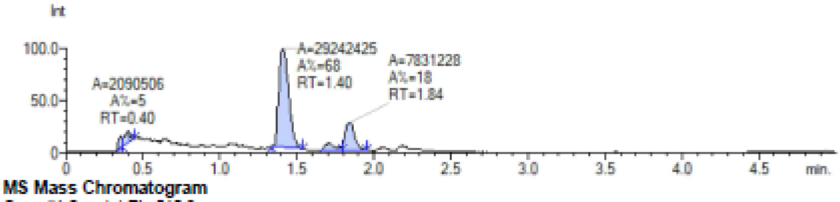

MS Mass Chromatogram
Group#1 Scan(+) EI : 515.2

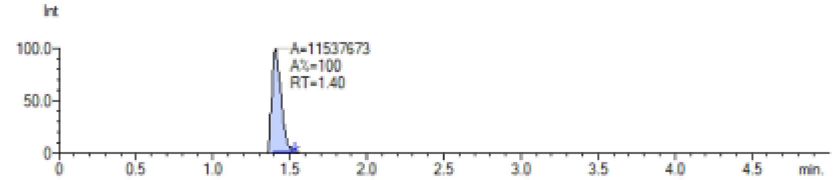

MS Spectrum
Group#1 - PDA Peak: 25, RT: 1.20 to 1.52 min

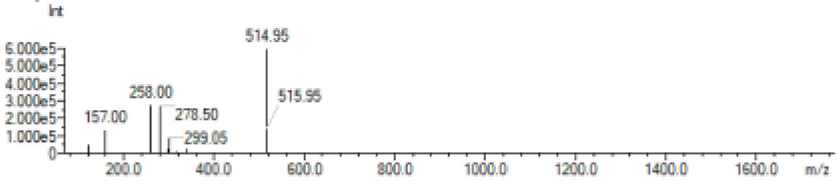

QD-C-S-16

Shimadzu Open Solution

**Project:**	Test
**Experiment:**	572_190612_Zinken_0607_Bischof_0612Griga
**Experiment Description:**	
**Sample:**	410487:01:01
**Sample Description:**	C33H32N2O5
**Data File Name:**	C:\LabSolutions\Data\572_190612_Zinken_190607_Bischoff_190612_Grigalunas\1046364715_11190613572.lcd
**Sample Location:**	Plate Number: 1 - Position: 228
**Run By:**	admin
**Run Started:**	29 July 2019 19:27:00
**Run Finished:**	30 July 2019 04:41:49
**Method:**	190321_Purity-Check

LC Chromatogram

LC#6 : AD2

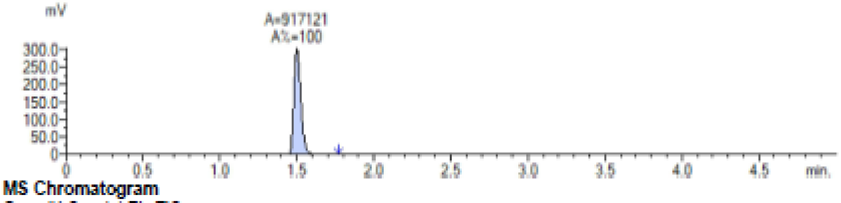

MS Chromatogram

Group#1 Scan(+) EI : TIC

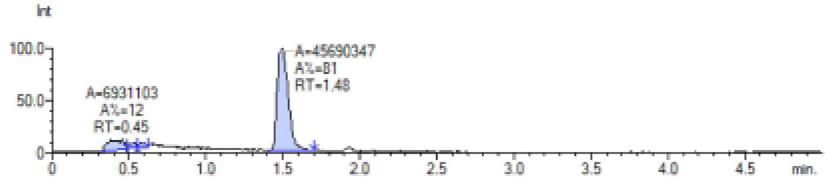

MS Mass Chromatogram

Group#1 Scan(+) EI : 537.2

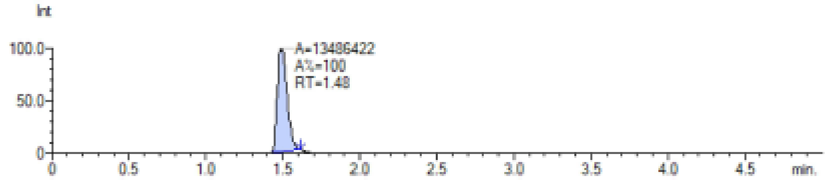

MS Spectrum

Group#1 - PDA Peak: 21, RT: 1.34 to 1.61 min

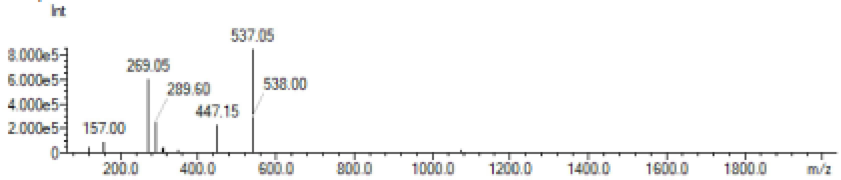

QD-C-R-12

Shimadzu Open Solution

Project:	Test
Experiment:	572_190612_Zinken_0607_Bischof_0612Griga
Experiment Description:	
Sample:	410488:01:01
Sample Description:	C33H32N2O5
Data File Name:	C:\LabSolutions\Data\572_190612_Zinken_190607_Bischoff_190612_Grigalunas\1046364800_11190613572.lcd
Sample Location:	Plate Number: 1 - Position: 276
Run By:	admin
Run Started:	29 July 2019 19:27:00
Run Finished:	30 July 2019 04:41:49
Method:	190321_Purity-Check

LC Chromatogram
LC#6 : AD2

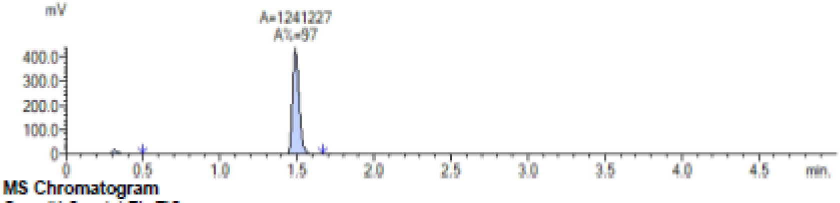

MS Chromatogram
Group#1 Scan(+) EI : TIC

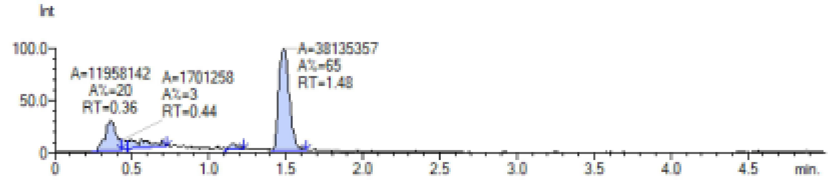

MS Mass Chromatogram
Group#1 Scan(+) EI : 537.2

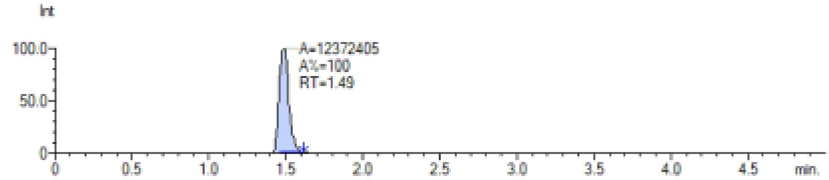

MS Spectrum
Group#1 - PDA Peak: 20, RT: 1.29 to 1.61 min

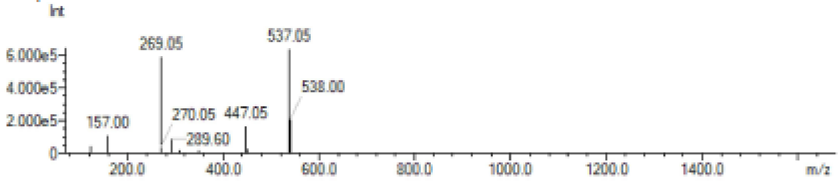

QD-C-S-17

Shimadzu Open Solution

**Project:**	Test
**Experiment:**	572_190612_Zinken_0607_Bischof_0612Griga
**Experiment Description:**	
**Sample:**	410489:01:01
**Sample Description:**	C29H32N2O4
**Data File Name:**	C:\LabSolutions\Data\572_190612_Zinken_190607_Bischoff_190612_Grigalunas\1046364507_11190613572.lcd
**Sample Location:**	Plate Number: 1 - Position: 324
**Run By:**	admin
**Run Started:**	29 July 2019 19:27:00
**Run Finished:**	30 July 2019 04:41:49
**Method:**	190321_Purity-Check

LC Chromatogram

LC#6 : AD2

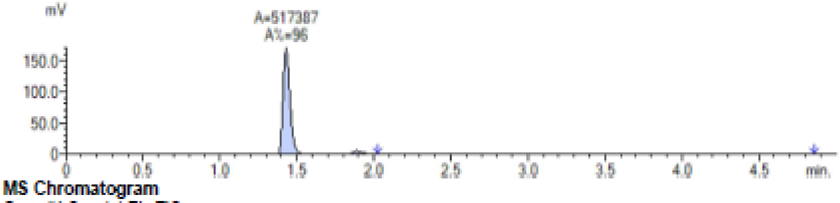

MS Chromatogram

Group#1 Scan(+) EI : TIC

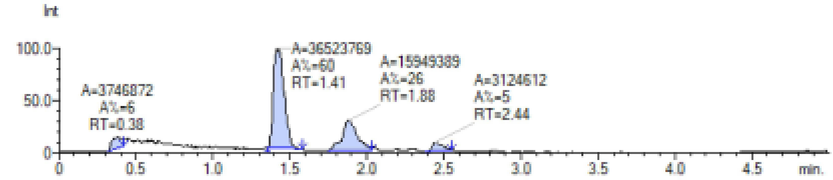

MS Mass Chromatogram

Group#1 Scan(+) EI : 473.2

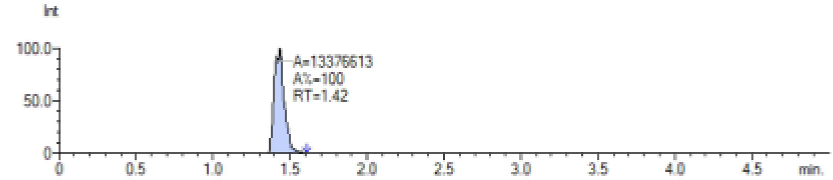

MS Spectrum

Group#1 - PDA Peak: 22, RT: 1.30 to 1.48 min

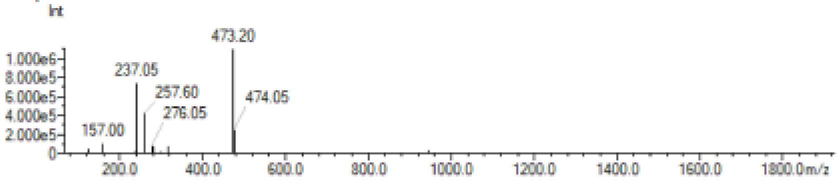

QD-C-R-13

Shimadzu Open Solution

Project:	Test
Experiment:	572_190612_Zinken_0607_Bischof_0612Griga
Experiment Description:	
Sample:	410490:01:01
Sample Description:	C29H32N2O4
Data File Name:	C:\LabSolutions\Data\572_190612_Zinken_190607_Bischoff_190612_Grigalunas\1046363914_11190613572.lcd
Sample Location:	Plate Number: 1 - Position: 372
Run By:	admin
Run Started:	29 July 2019 19:27:00
Run Finished:	30 July 2019 04:41:49
Method:	190321_Purity-Check

LC Chromatogram

LC#6 : AD2

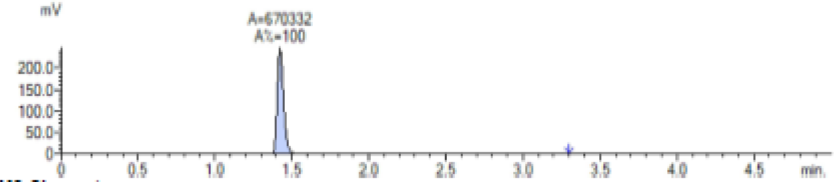

MS Chromatogram

Group#1 Scan(+) EI : TIC

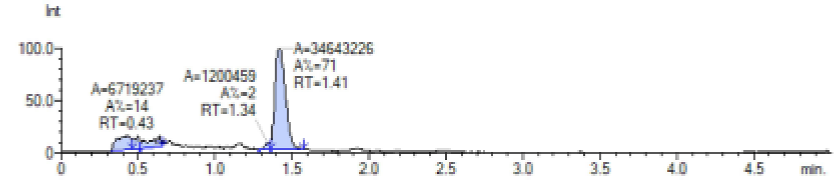

MS Mass Chromatogram

Group#1 Scan(+) EI : 473.2

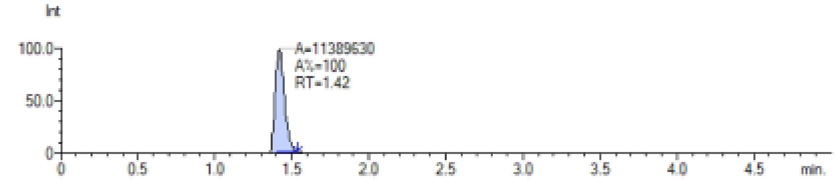

MS Spectrum

Group#1 - PDA Peak: 17, RT: 1.30 to 1.49 min

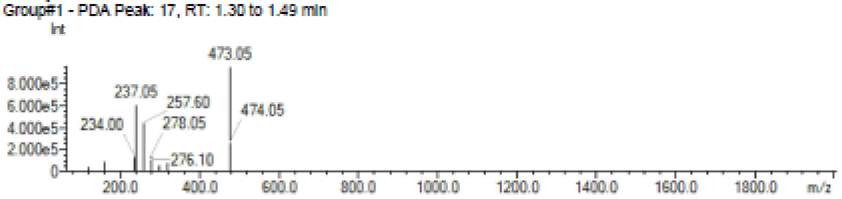

QD-C-S-18

Shimadzu Open Solution

Project: Test
Experiment: 669_0303_Xie_Wilke_0304_Grigalunas_Nigge
Experiment Description:
Sample: 412002:01:01
Sample Description: C27H28N2O4
Data File Name: C:\LabSolutions\Data\669_0303_Xie_Wilke_0304_Grigalunas_Niggemeyer\1046324898_11200304669.lcd
Sample Location: Plate Number: 1 - Position: 179
Run By: admin
Run Started: 14 May 2020 16:58:32
Run Finished: 15 May 2020 10:54:08
Method: Purity-Check+sleep

LC Chromatogram
LC#6 : AD2

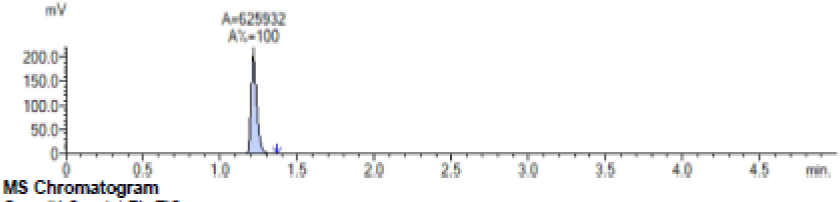

MS Chromatogram
Group#1 Scan(+) EI : TIC

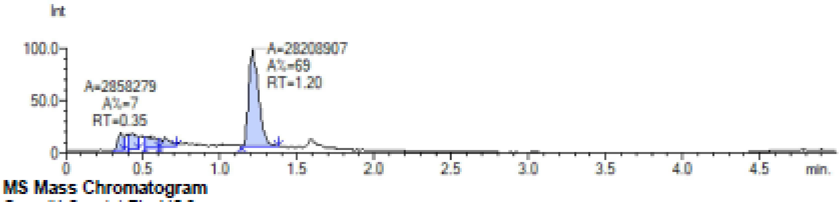

MS Mass Chromatogram
Group#1 Scan(+) EI : 445.2

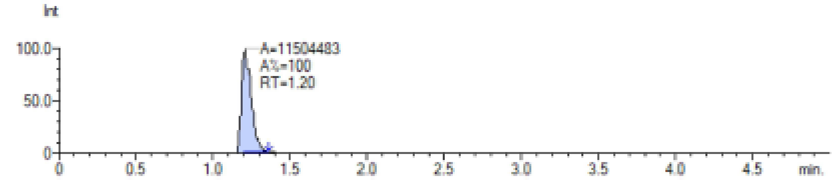

MS Spectrum
Group#1 - PDA Peak: 17, RT: 1.08 to 1.27 min

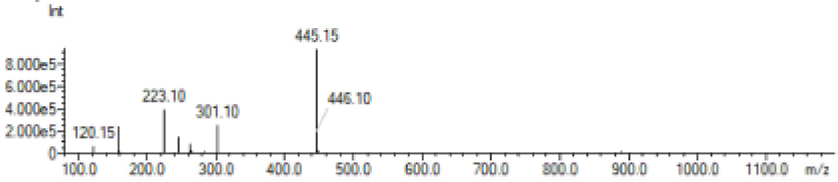

Shimadzu Open Solution

QD-C-S-19

Project: Test
Experiment: 669_0303_Xie_Wilke_0304_Grigalunas_Nigge
Experiment Description:
Sample: 412003:01:01
Sample Description: C26H25FN2O4
Data File Name: C:\LabSolutions\Data\669_0303_Xie_Wilke_0304_Grigalunas_Niggemeyer\1046324903_11200304669.lcd
Sample Location: Plate Number: 1 - Position: 227
Run By: admin
Run Started: 14 May 2020 16:58:32
Run Finished: 15 May 2020 10:54:08
Method: Purity-Check+sleep

LC Chromatogram
LC#6 : AD2

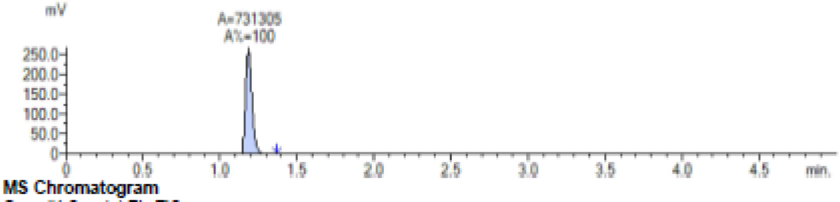

MS Chromatogram
Group#1 Scan(+) EI : TIC

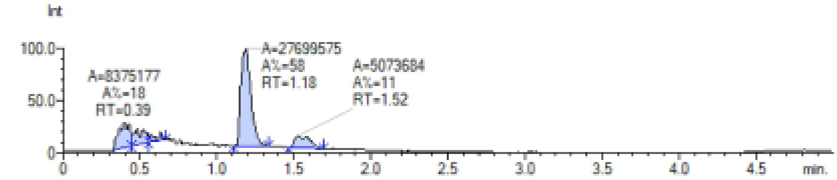

MS Mass Chromatogram
Group#1 Scan(+) EI : 449.2

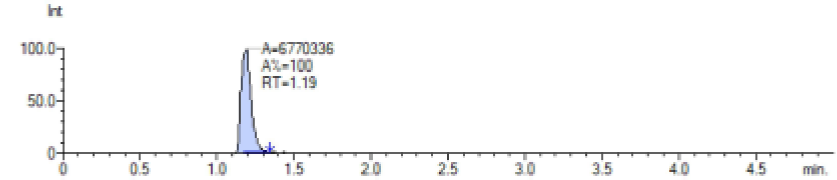

MS Spectrum
Group#1 - PDA Peak: 20, RT: 1.06 to 1.25 min

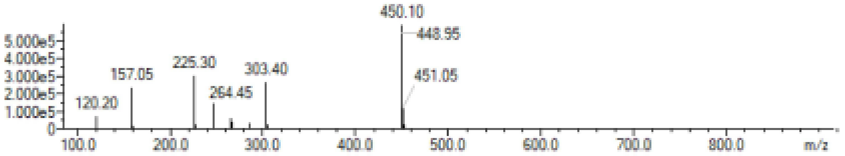

Shimadzu Open Solution

QD-C-S-20

Project: Test
Experiment: 669_0303_Xie_Wilke_0304_Grigalunas_Nigge
Experiment Description:
Sample: 412004:01:01
Sample Description: C26H25ClN2O4
Data File Name: C:\LabSolutions\Data\669_0303_Xie_Wilke_0304_Grigalunas_Niggemeyer\1046324804_11200304669.lcd
Sample Location: Plate Number: 1 - Position: 275
Run By: admin
Run Started: 14 May 2020 16:58:32
Run Finished: 15 May 2020 10:54:08
Method: Purity-Check+sleep

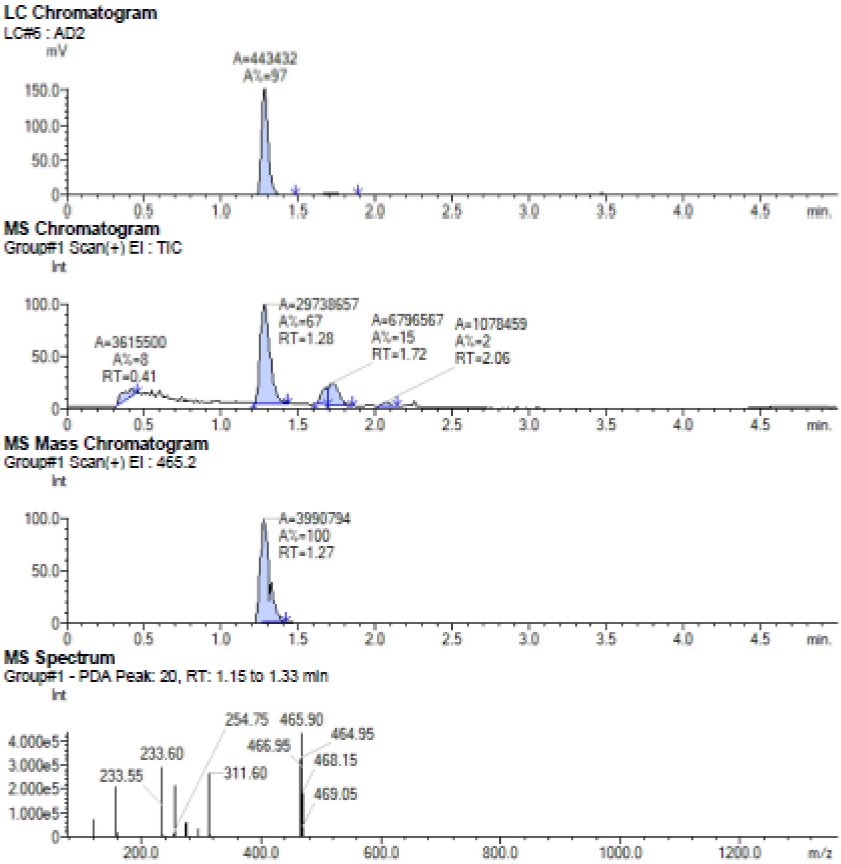

Shimadzu Open Solution

QD-C-R-14

Project: Test
Experiment: 669_0303_Xie_Wilke_0304_Grigalunas_Nigge
Experiment Description:
Sample: 412005:01:02
Sample Description: C26H25ClN2O4
Data File Name: C:\LabSolutions\Data\669_0303_Xie_Wilke_0304_Grigalunas_Niggemeyer\1046325349_11200304669.lcd
Sample Location: Plate Number: 1 - Position: 323
Run By: admin
Run Started: 14 May 2020 16:58:32
Run Finished: 15 May 2020 10:54:08
Method: Purity-Check+sleep

LC Chromatogram
LC#6 : AD2

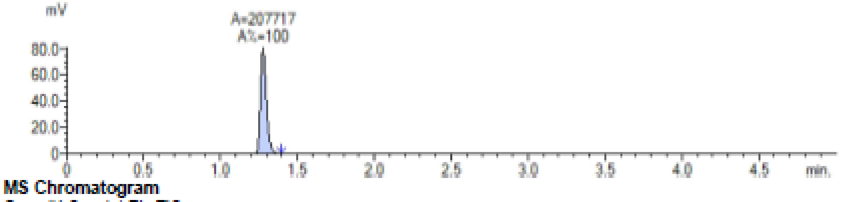

MS Chromatogram
Group#1 Scan(+) EI : TIC

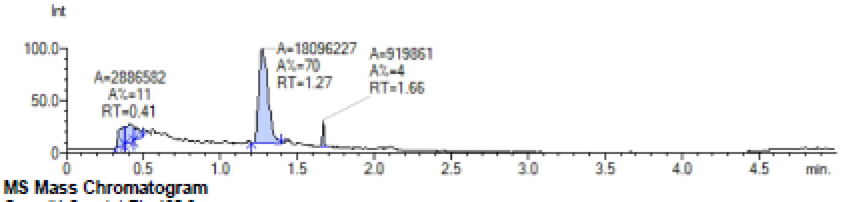

MS Mass Chromatogram
Group#1 Scan(+) EI : 465.2

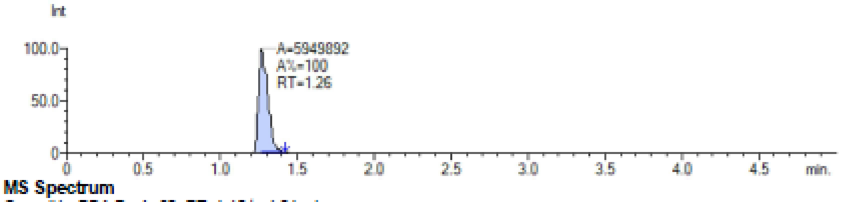

MS Spectrum
Group#1 - PDA Peak: 20, RT: 1.15 to 1.31 min

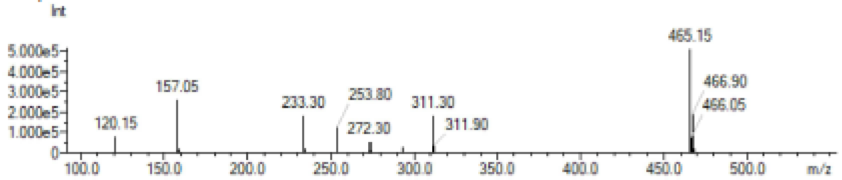

QD-C-S-21

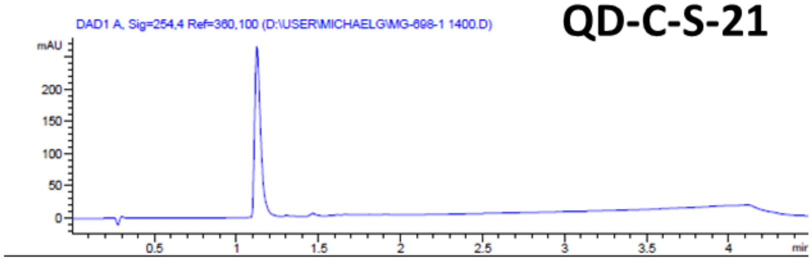
DAD1 A, Sig=254,4 Ref=360,100 (D:\USER\MICHAELG\MG-698-1 1400.D)
mAU
200
150
100
50
0
0.5
1
1.5
2
2.5
3
3.5
4
min

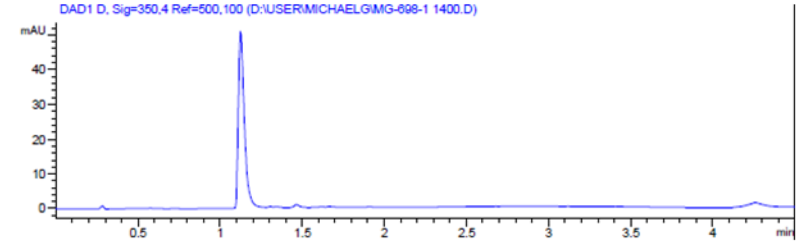
DAD1 D, Sig=350,4 Ref=500,100 (D:\USER\MICHAELG\MG-698-1 1400.D)
mAU
40
30
20
10
0
0.5
1
1.5
2
2.5
3
3.5
4
min

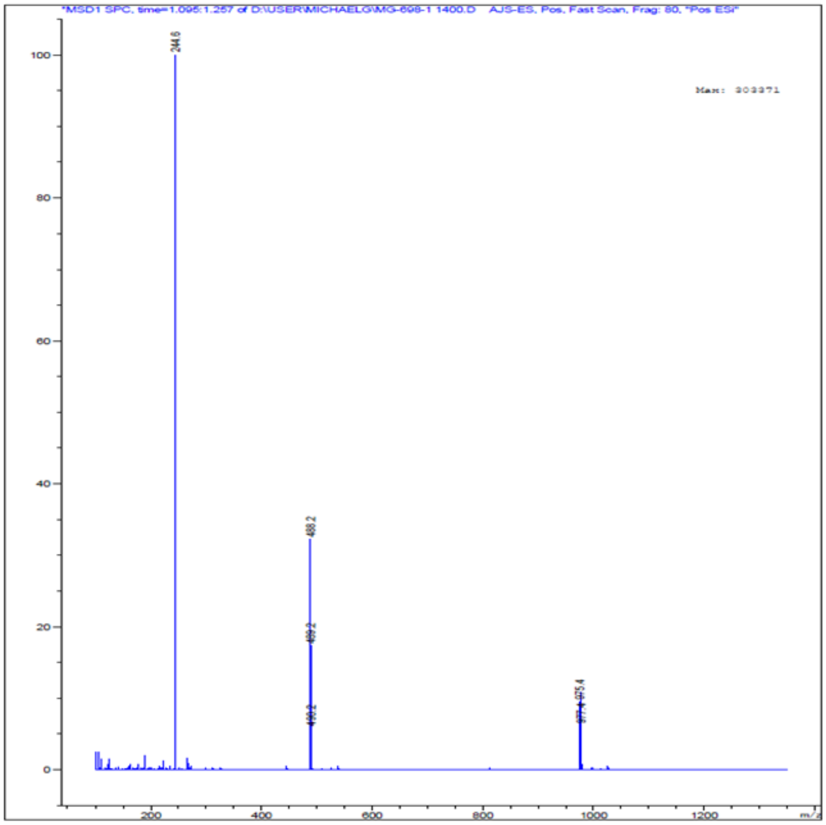
*MSD1 SPC, time=1.095:1.257 of D:\USER\MICHAELG\MG-698-1 1400.D AJS-ES, Pos, Fast Scan, Frag: 80, "Pos ESI"
244.6
488.2
975.4
100
80
60
40
20
0
200
400
600
800
1000
1200

Shimadzu Open Solution

QD-C-S-22

Project:	Test
Experiment:	669_0303_Xie_Wilke_0304_Grigalunas_Nigge
Experiment Description:	
Sample:	412007:01:02
Sample Description:	C27H25F3N2O4
Data File Name:	C:\LabSolutions\Data\669_0303_Xie_Wilke_0304_Grigalunas_Niggemeyer\1046325352_11200304669.lcd
Sample Location:	Plate Number: 1 - Position: 37
Run By:	admin
Run Started:	14 May 2020 16:58:32
Run Finished:	15 May 2020 10:54:08
Method:	Purity-Check+sleep

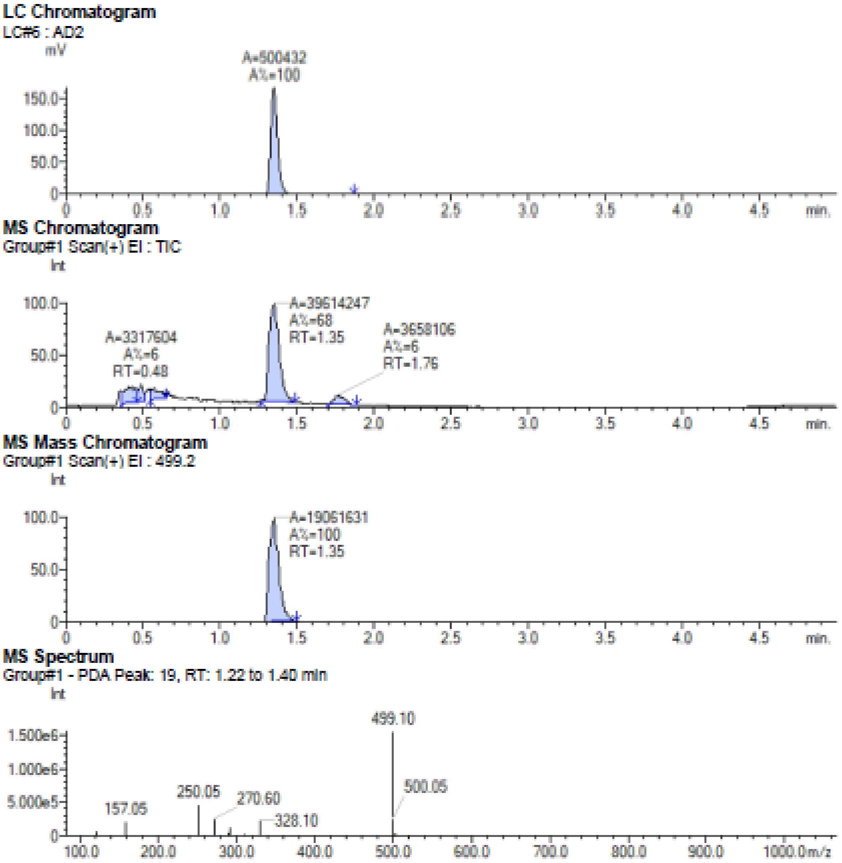

Shimadzu Open Solution

QD-C-S-23

**Project:**	Test
**Experiment:**	669_0303_Xie_Wilke_0304_Grigalunas_Nigge
**Experiment Description:**	
**Sample:**	412008:01:02
**Sample Description:**	C28H30N2O4
**Data File Name:**	C:\LabSolutions\Data\669_0303_Xie_Wilke_0304_Grigalunas_Niggemeyer\1046324743_11200304669.lcd
**Sample Location:**	Plate Number: 1 - Position: 85
**Run By:**	admin
**Run Started:**	14 May 2020 16:58:32
**Run Finished:**	15 May 2020 10:54:08
**Method:**	Purity-Check+sleep

LC Chromatogram

LC#6 : AD2

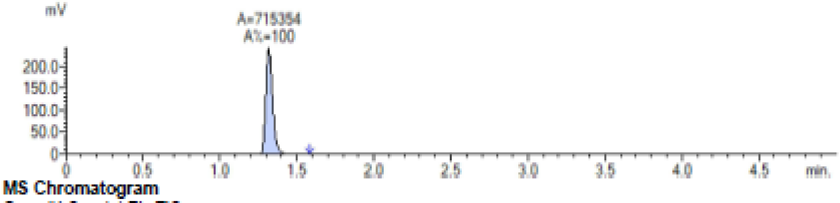

MS Chromatogram

Group#1 Scan(+) EI : TIC

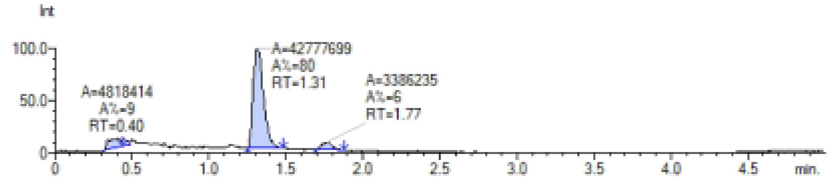

MS Mass Chromatogram

Group#1 Scan(+) EI : 459.2

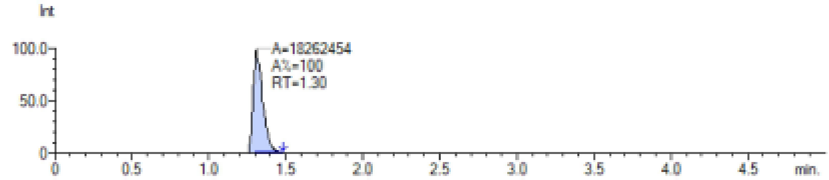

MS Spectrum

Group#1 - PDA Peak: 18, RT: 1.18 to 1.44 min

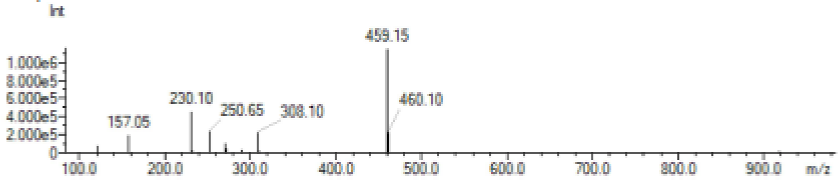

SM-C-S-1

Shimadzu Open Solution

**Project:**	Test
**Experiment:**	476_Grigalu_1011_Shaaban_Liu_1009_3076
**Experiment Description:**	
**Sample:**	409050:01:01
**Sample Description:**	C26H29NO4
**Data File Name:**	C:\LabSolutions\Data\476_Grigalunas_181011_Shaaban_181011_Liu_181009_3076 \1046359401_11181011476.lcd
**Sample Location:**	Plate Number: 2 - Position: 1
**Run By:**	admin
**Run Started:**	24 April 2019 18:14:10
**Run Finished:**	25 April 2019 04:58:04
**Method:**	190321_Purity-Check+shut down

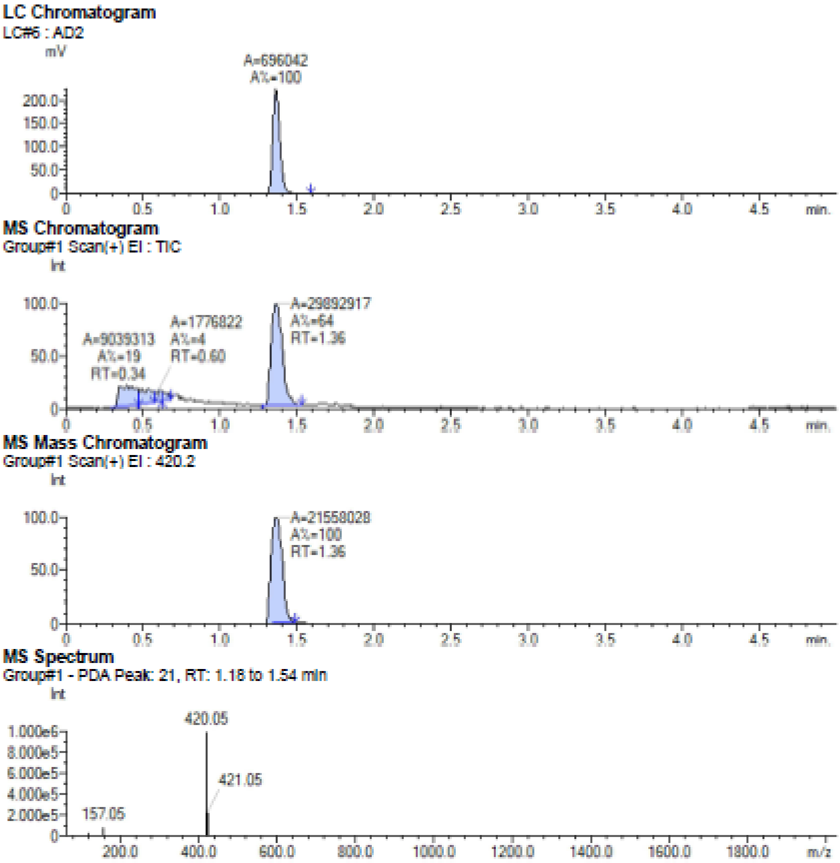

SM-C-R-1

Shimadzu Open Solution

Project: Test
Experiment: 476_Grigalu_1011_Shaaban_Liu_1009_3076
Experiment Description:
Sample: 409051:01:01
Sample Description: C26H29NO4
Data File Name: C:\LabSolutions\Data\476_Grigalunas_181011_Shaaban_181011_Liu_181009_3076 \1046413365_11181011476.lcd
Sample Location: Plate Number: 2 - Position: 49
Run By: admin
Run Started: 24 April 2019 18:14:10
Run Finished: 25 April 2019 04:58:04
Method: 190321_Purity-Check+shut down

LC Chromatogram
LC#6 : AD2

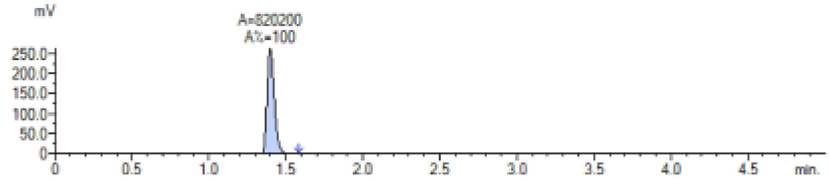

MS Chromatogram
Group#1 Scan(+) EI : TIC

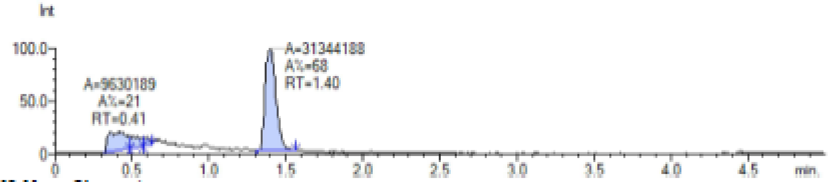

MS Mass Chromatogram
Group#1 Scan(+) EI : 420.2

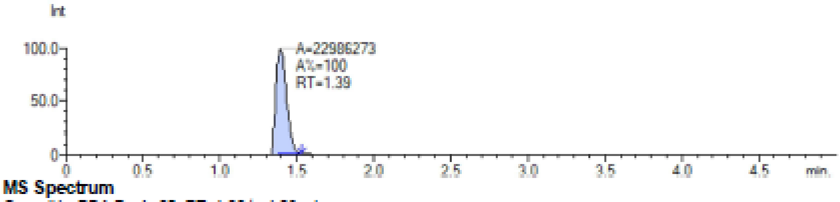

MS Spectrum
Group#1 - PDA Peak: 25, RT: 1.26 to 1.39 min

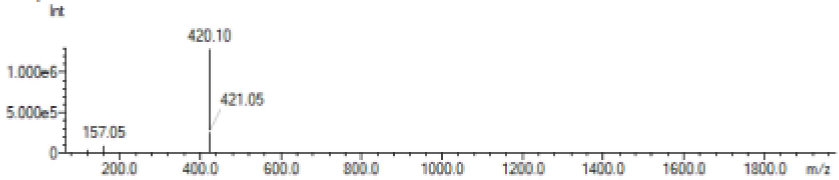

SM-C-S-2

Shimadzu Open Solution

**Project:**	Test
**Experiment:**	476_Grigalu_1011_Shaaban_Liu_1009_3076
**Experiment Description:**	
**Sample:**	409052:01:01
**Sample Description:**	C26H29NO5
**Data File Name:**	C:\LabSolutions\Data\476_Grigalunas_181011_Shaaban_181011_Liu_181009_3076 \1046360049_11181011476.lcd
**Sample Location:**	Plate Number: 2 - Position: 97
**Run By:**	admin
**Run Started:**	24 April 2019 18:14:10
**Run Finished:**	25 April 2019 04:58:04
**Method:**	190321_Purity-Check+shut down

LC Chromatogram

LC#6 : AD2

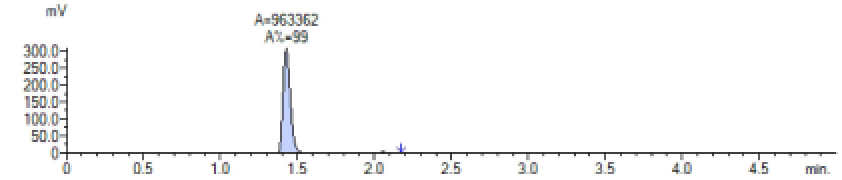

MS Chromatogram

Group#1 Scan(+) EI : TIC

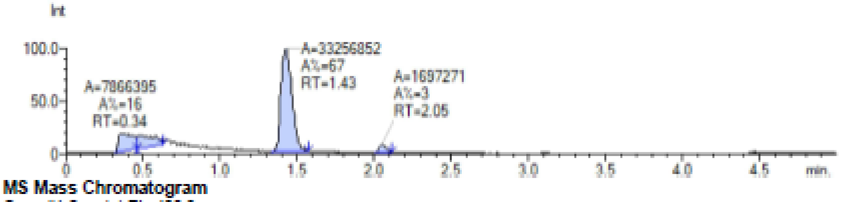

MS Mass Chromatogram

Group#1 Scan(+) EI : 436.2

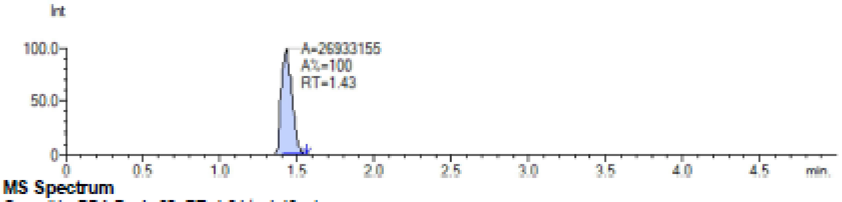

MS Spectrum

Group#1 - PDA Peak: 20, RT: 1.24 to 1.48 min

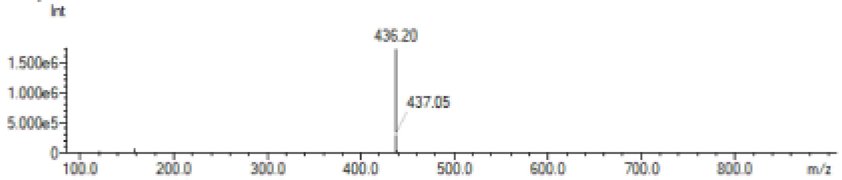

SM-C-S-3

Shimadzu Open Solution

Project:	Test
Experiment:	476_Grigalu_1011_Shaaban_Liu_1009_3076
Experiment Description:	
Sample:	409053:01:01
Sample Description:	C26H29NO5
Data File Name:	C:\LabSolutions\Data\476_Grigalunas_181011_Shaaban_181011_Liu_181009_3076 \1046359834_11181011476.lcd
Sample Location:	Plate Number: 2 - Position: 145
Run By:	admin
Run Started:	24 April 2019 18:14:10
Run Finished:	25 April 2019 04:58:04
Method:	190321_Purity-Check+shut down

LC Chromatogram

LC#6 : AD2

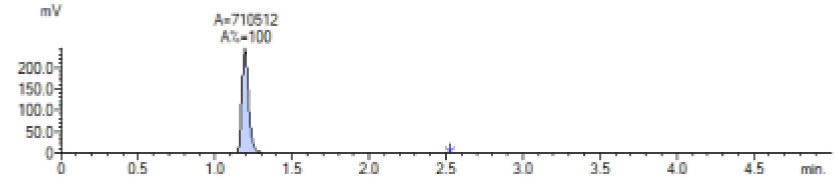

MS Chromatogram

Group#1 Scan(+) EI : TIC

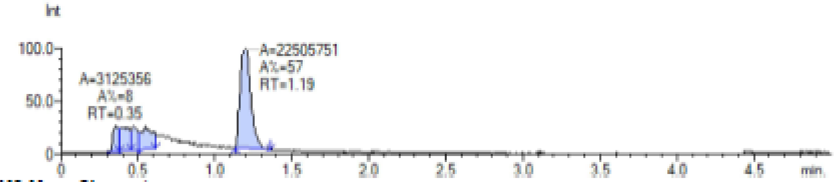

MS Mass Chromatogram

Group#1 Scan(+) EI : 436.2

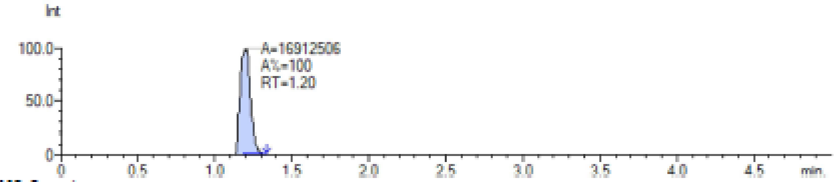

MS Spectrum

Group#1 - PDA Peak: 21, RT: 1.06 to 1.27 min

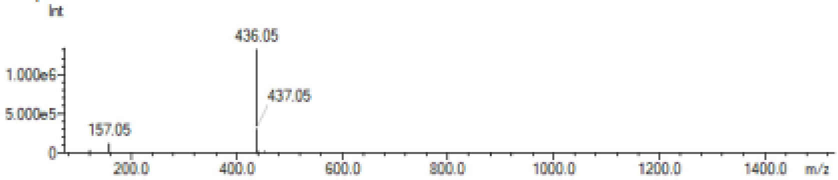

SM-C-S-4

Shimadzu Open Solution

Project: Test
Experiment: 476_Grigalu_1011_Shaaban_Liu_1009_3076
Experiment Description:
Sample: 409054:01:01
Sample Description: C27H31NO5
Data File Name: C:\LabSolutions\Data\476_Grigalunas_181011_Shaaban_181011_Liu_181009_3076 \1046359620_11181011476.lcd
Sample Location: Plate Number: 2 - Position: 193
Run By: admin
Run Started: 24 April 2019 18:14:10
Run Finished: 25 April 2019 04:58:04
Method: 190321_Purity-Check+shut down

LC Chromatogram
LC#6 : AD2

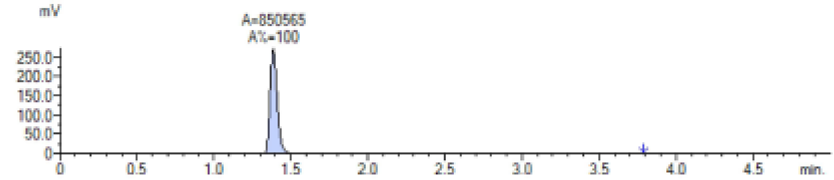

MS Chromatogram
Group#1 Scan(+) EI : TIC

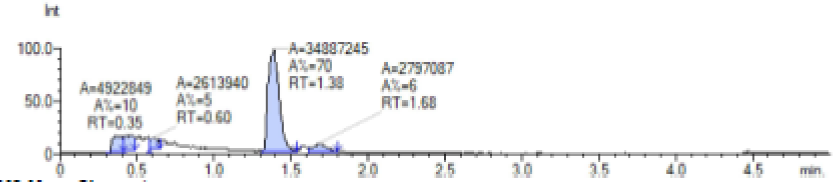

MS Mass Chromatogram
Group#1 Scan(+) EI : 450.2

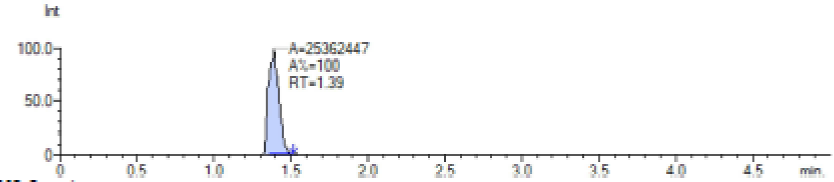

MS Spectrum
Group#1 - PDA Peak: 22, RT: 1.23 to 1.47 min

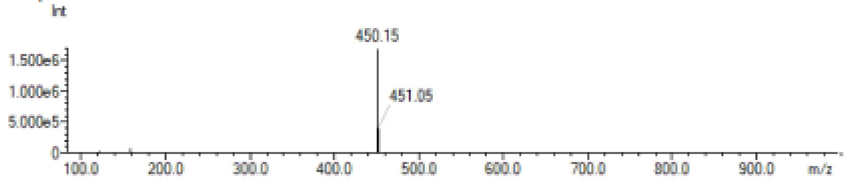

SM-C-R-2

Shimadzu Open Solution

**Project:**	Test
**Experiment:**	476_Grigalu_1011_Shaaban_Liu_1009_3076
**Experiment Description:**	
**Sample:**	409055:01:01
**Sample Description:**	C27H31NO5
**Data File Name:**	C:\LabSolutions\Data\476_Grigalunas_181011_Shaaban_181011_Liu_181009_3076 \1046359490_11181011476.lcd
**Sample Location:**	Plate Number: 2 - Position: 241
**Run By:**	admin
**Run Started:**	24 April 2019 18:14:10
**Run Finished:**	25 April 2019 04:58:04
**Method:**	190321_Purity-Check+shut down

LC Chromatogram

LC#6 : AD2

MS Chromatogram

Group#1 Scan(+) EI : TIC

MS Mass Chromatogram

Group#1 Scan(+) EI : 450.2

MS Spectrum

Group#1 - PDA Peak: 22, RT: 1.27 to 1.48 min

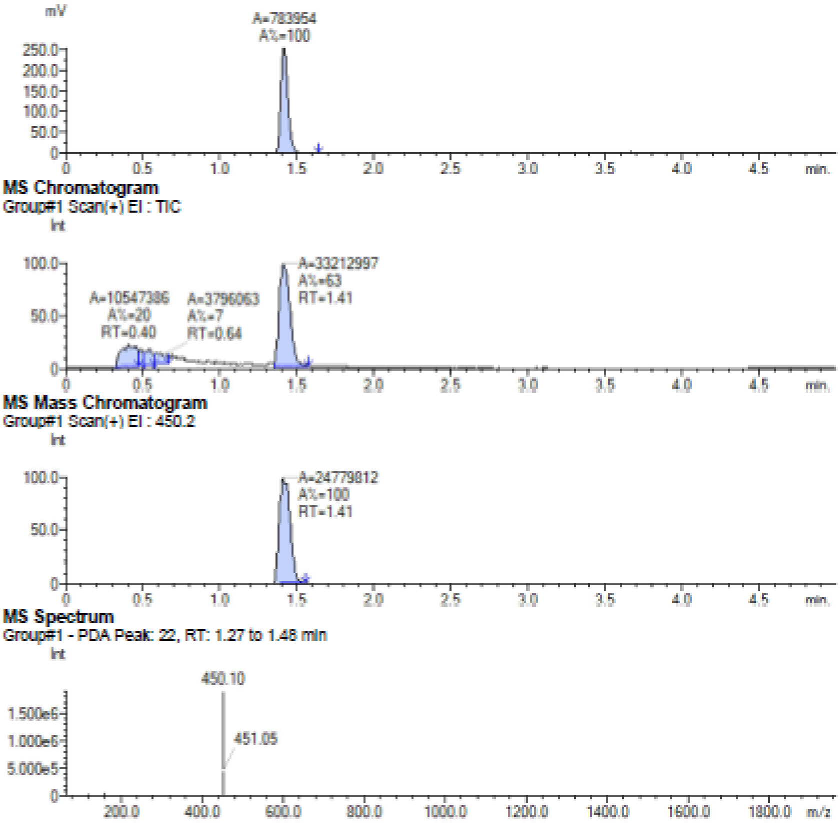

Shimadzu Open Solution

SM-C-S-5

**Project:**	Test
**Experiment:**	476_Grigalu_1011_Shaaban_Liu_1009_3076
**Experiment Description:**	
**Sample:**	409056:01:01
**Sample Description:**	C27H31NO5
**Data File Name:**	C:\LabSolutions\Data\476_Grigalunas_181011_Shaaban_181011_Liu_181009_3076 \1046359550_11181011476.lcd
**Sample Location:**	Plate Number: 2 - Position: 289
**Run By:**	admin
**Run Started:**	24 April 2019 18:14:10
**Run Finished:**	25 April 2019 04:58:04
**Method:**	190321_Purity-Check+shut down

LC Chromatogram

LC#6 : AD2

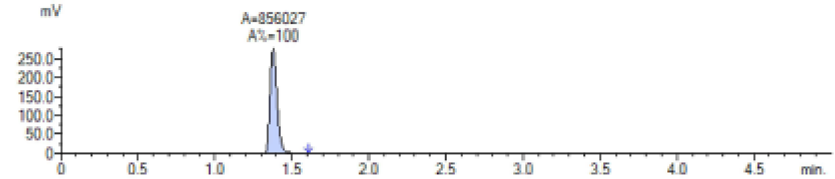

MS Chromatogram

Group#1 Scan(+) EI : TIC

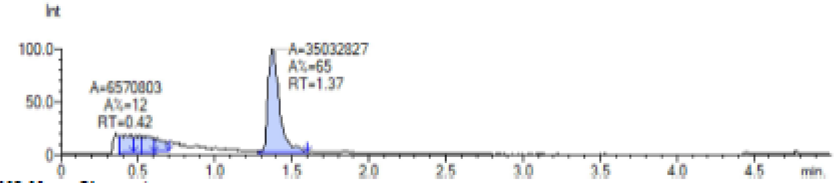

MS Mass Chromatogram

Group#1 Scan(+) EI : 450.2

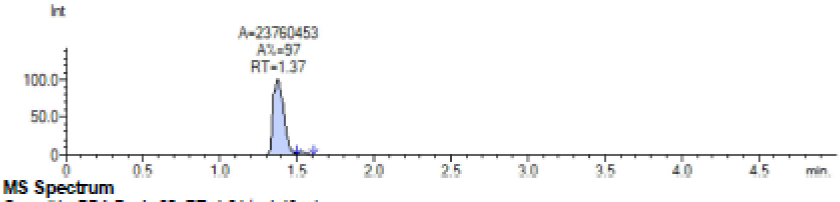

MS Spectrum

Group#1 - PDA Peak: 25, RT: 1.21 to 1.49 min

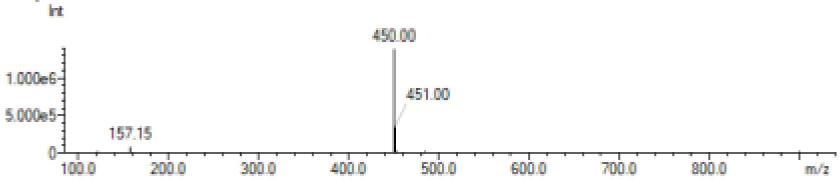

Shimadzu Open Solution

SM-C-R-3

**Project:**	Test
**Experiment:**	476_Grigalu_1011_Shaaban_Liu_1009_3076
**Experiment Description:**	
**Sample:**	409057:01:01
**Sample Description:**	C27H31NO5
**Data File Name:**	C:\LabSolutions\Data\476_Grigalunas_181011_Shaaban_181011_Liu_181009_3076 \1046360274_11181011476.lcd
**Sample Location:**	Plate Number: 2 - Position: 337
**Run By:**	admin
**Run Started:**	24 April 2019 18:14:10
**Run Finished:**	25 April 2019 04:58:04
**Method:**	190321_Purity-Check+shut down

LC Chromatogram
LC#6 : AD2

MS Chromatogram
Group#1 Scan(+) EI : TIC

MS Mass Chromatogram
Group#1 Scan(+) EI : 450.2

MS Spectrum
Group#1 - PDA Peak: 27, RT: 1.22 to 1.53 min

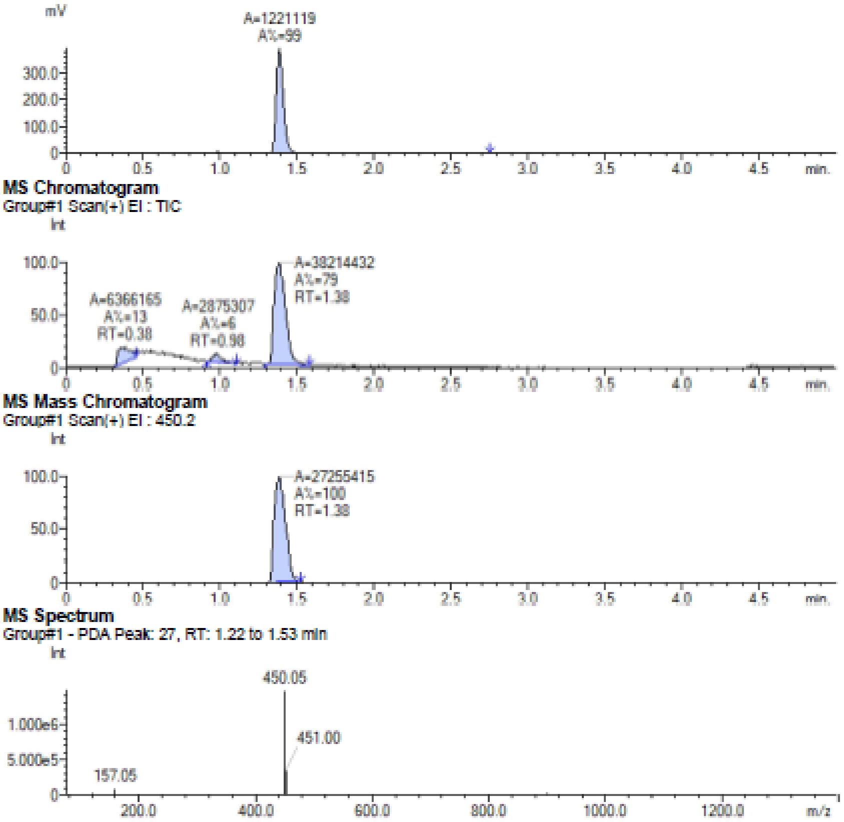

SM-C-S-6

Shimadzu Open Solution

Project: Test
Experiment: 525_0206_Christo_Grigalu_Burhop_Wilke
Experiment Description:
Sample: 409733:01:01
Sample Description: C28H33NO6
Data File Name: C:\LabSolutions\Data\525_190206_Christoforow_190206_Grigalunas_190206_Burhop_190206_Wilke\1046361127_11190207525.lcd
Sample Location: Plate Number: 2 - Position: 304
Run By: admin
Run Started: 23 April 2019 18:20:13
Run Finished: 24 April 2019 04:25:41
Method: 190321_Purity-Check+shut down

LC Chromatogram
LC#6 : AD2

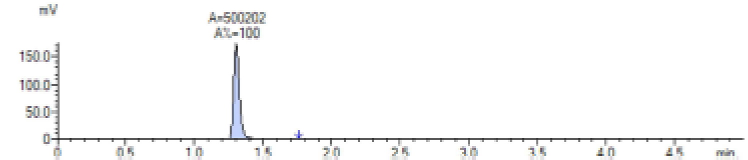

MS Chromatogram
Group#1 Scan(+) EI : TIC

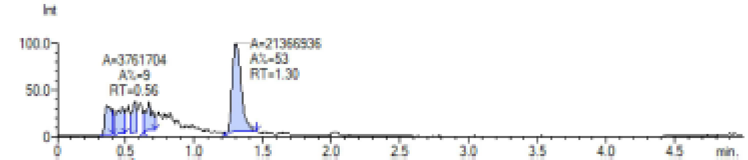

MS Mass Chromatogram
Group#1 Scan(+) EI : 480.2

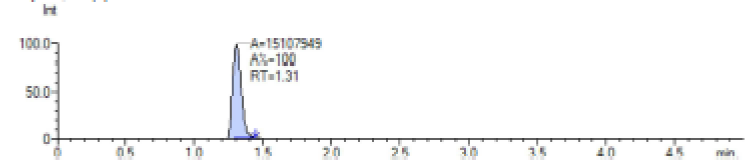

MS Spectrum
Group#1 - FDA Peak: 20, RT: 1.16 to 1.28 min

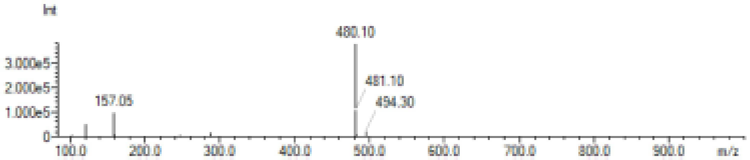

SM-C-S-7

Shimadzu Open Solution

**Project:**	Test
**Experiment:**	525_0206_Christo_Grigalu_Burhop_Wilke
**Experiment Description:**	
**Sample:**	409720:01:01
**Sample Description:**	C27H31NO4
**Data File Name:**	C:\LabSolutions\Data\525_190206_Christoforow_190206_Grigalunas_190206_Burhop_190206_Wilke\1046360560_11190207525.lcd
**Sample Location:**	Plate Number: 2 - Position: 62
**Run By:**	admin
**Run Started:**	23 April 2019 18:20:13
**Run Finished:**	24 April 2019 04:25:41
**Method:**	190321_Purity-Check+shut down

LC Chromatogram

LC#5 : AD2

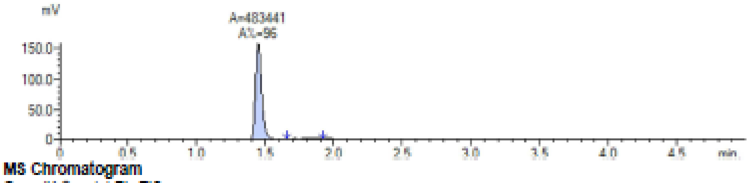

MS Chromatogram

Group#1 Scan(+) EI : TIC

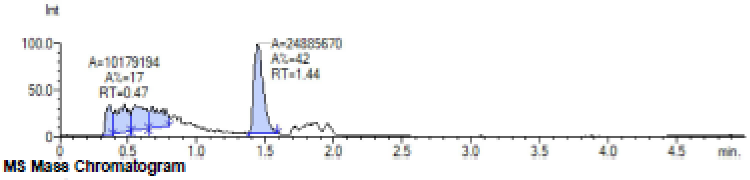

MS Mass Chromatogram

Group#1 Scan(+) EI : 434.2

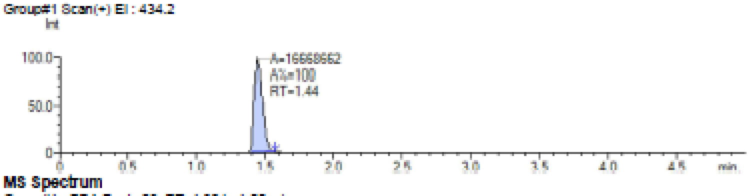

MS Spectrum

Group#1 - PDA Peak: 23, RT: 1.32 to 1.55 min

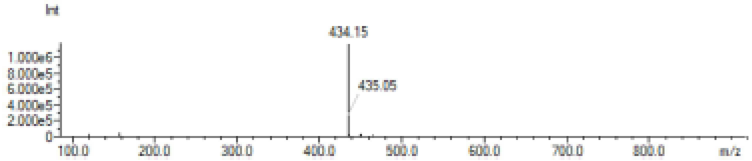

SM-C-R-4

Shimadzu Open Solution

Project: Test
Experiment: 525_0206_Christo_Grigalu_Burhop_Wilke
Experiment Description:
Sample: 409721:01:01
Sample Description: C27H31NO4
Data File Name: C:\LabSolutions\Data\525_190206_Christoforow_190206_Grigalunas_190206_Burhop_190206_Wilke\1046360893_11190207525.lcd
Sample Location: Plate Number: 2 - Position: 110
Run By: admin
Run Started: 23 April 2019 18:20:13
Run Finished: 24 April 2019 04:25:41
Method: 190321_Purity-Check+shut down

LC Chromatogram

LC#5 : AD2

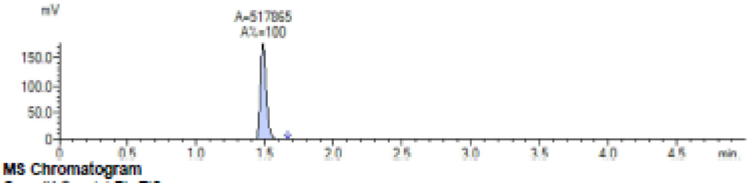

MS Chromatogram

Group#1 Scan(+) EI : TIC

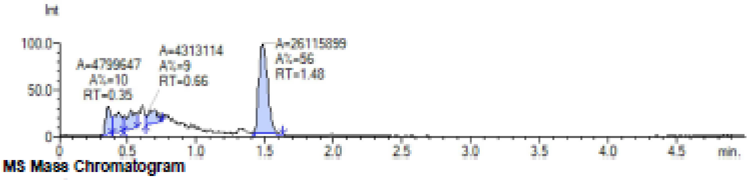

MS Mass Chromatogram

Group#1 Scan(+) EI : 434.2

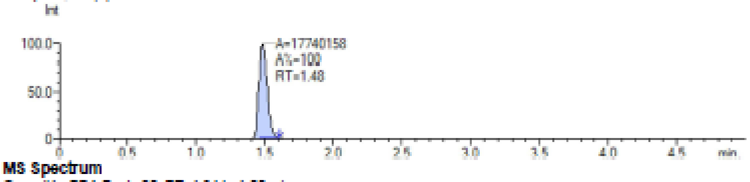

MS Spectrum

Group#1 - PDA Peak: 25, RT: 1.34 to 1.55 min

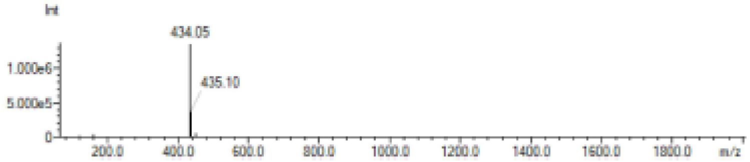

Shimadzu Open Solution

SM-C-S-8

**Project:**	Test
**Experiment:**	572_190612_Zinken_0607_Bischof_0612Griga
**Experiment Description:**	
**Sample:**	410414:01:01
**Sample Description:**	C27H31NO4
**Data File Name:**	C:\LabSolutions\Data\572_190612_Zinken_190607_Bischoff_190612_Grigalunas\1046363928_11190613572.lcd
**Sample Location:**	Plate Number: 1 - Position: 25
**Run By:**	admin
**Run Started:**	29 July 2019 19:27:00
**Run Finished:**	30 July 2019 04:41:49
**Method:**	190321_Purity-Check

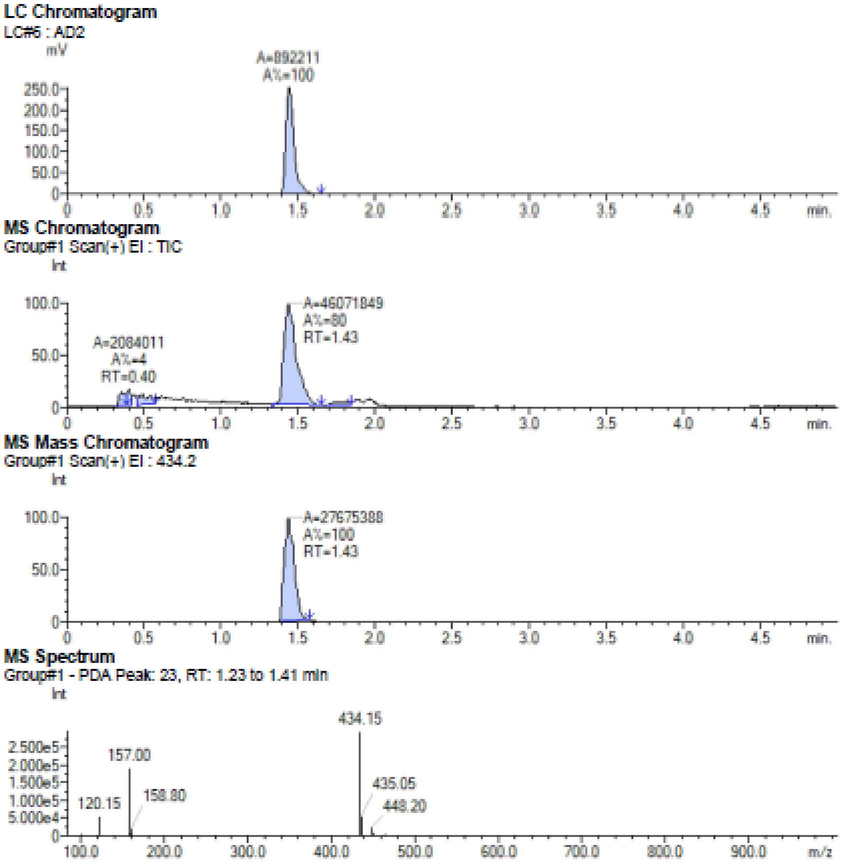

SM-C-R-5

Shimadzu Open Solution

**Project:**	Test
**Experiment:**	525_0206_Christo_Grigalu_Burhop_Wilke
**Experiment Description:**	
**Sample:**	409722:01:01
**Sample Description:**	C27H31NO4
**Data File Name:**	C:\LabSolutions\Data\525_190206_Christoforow_190206_Grigalunas_190206_Burhop_190206_Wilke\1046361207_11190207525.lcd
**Sample Location:**	Plate Number: 2 - Position: 158
**Run By:**	admin
**Run Started:**	23 April 2019 18:20:13
**Run Finished:**	24 April 2019 04:25:41
**Method:**	190321_Purity-Check+shut down

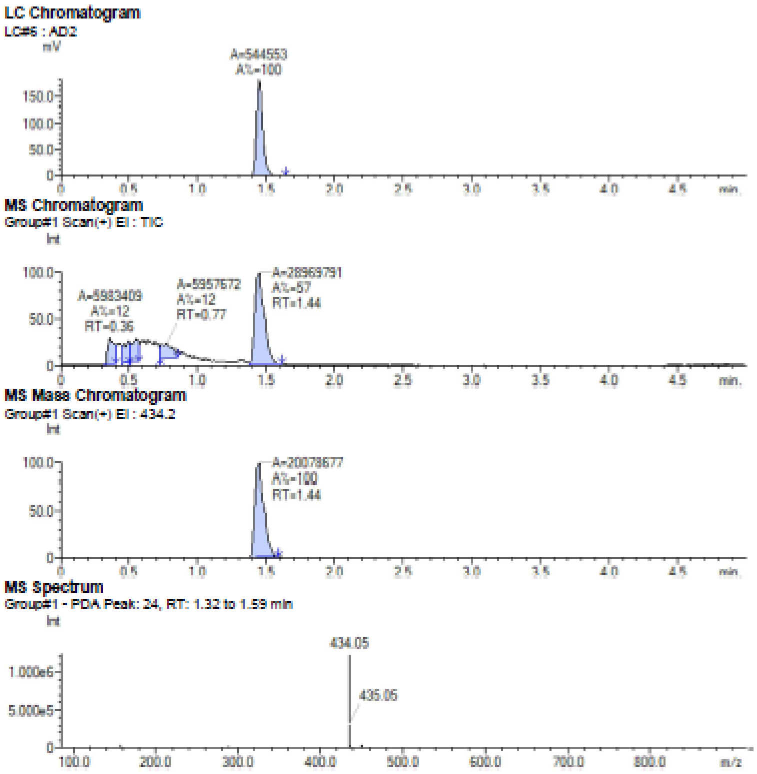

SM-C-S-9

Shimadzu Open Solution

**Project:**	Test
**Experiment:**	476_Grigalu_1011_Shaaban_Liu_1009_3076
**Experiment Description:**	
**Sample:**	409048:01:01
**Sample Description:**	C28H33NO4
**Data File Name:**	C:\LabSolutions\Data\476_Grigalunas_181011_Shaaban_181011_Liu_181009_3076 \1046357897_11181011476.lcd
**Sample Location:**	Plate Number: 2 - Position: 3
**Run By:**	admin
**Run Started:**	24 April 2019 18:14:10
**Run Finished:**	25 April 2019 04:58:04
**Method:**	190321_Purity-Check+shut down

LC Chromatogram

LC#6 : AD2

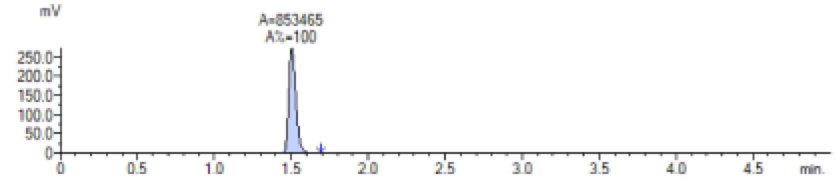

MS Chromatogram

Group#1 Scan(+) EI : TIC

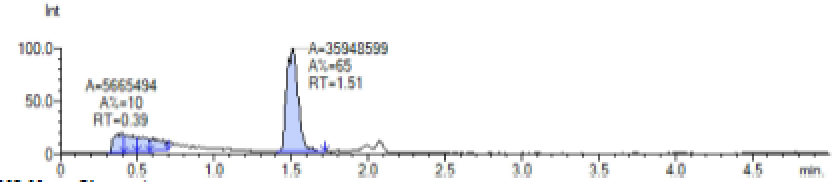

MS Mass Chromatogram

Group#1 Scan(+) EI : 448.2

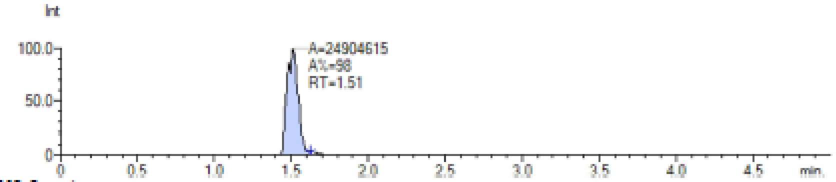

MS Spectrum

Group#1 - PDA Peak: 22, RT: 1.34 to 1.64 min

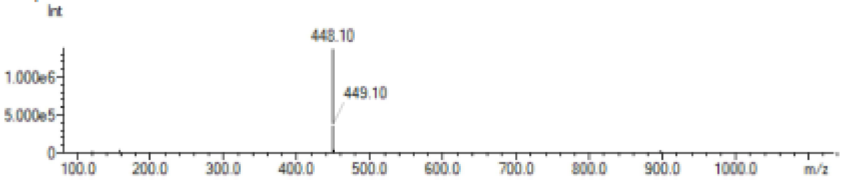

SM-C-R-6

Shimadzu Open Solution

**Project:**	Test
**Experiment:**	476_Grigalu_1011_Shaaban_Liu_1009_3076
**Experiment Description:**	
**Sample:**	409049:01:01
**Sample Description:**	C28H33NO4
**Data File Name:**	C:\LabSolutions\Data\476_Grigalunas_181011_Shaaban_181011_Liu_181009_3076 \1046359414_11181011476.lcd
**Sample Location:**	Plate Number: 2 - Position: 51
**Run By:**	admin
**Run Started:**	24 April 2019 18:14:10
**Run Finished:**	25 April 2019 04:58:04
**Method:**	190321_Purity-Check+shut down

LC Chromatogram
LC#6 : AD2

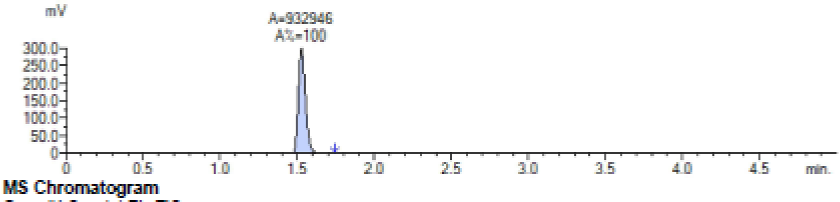

MS Chromatogram
Group#1 Scan(+) EI : TIC

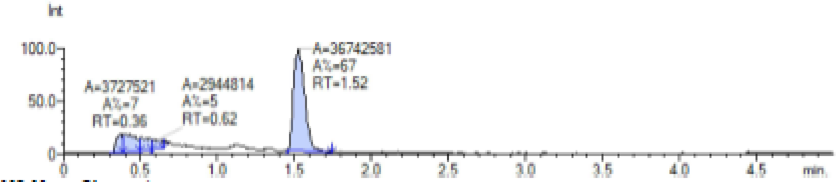

MS Mass Chromatogram
Group#1 Scan(+) EI : 448.2

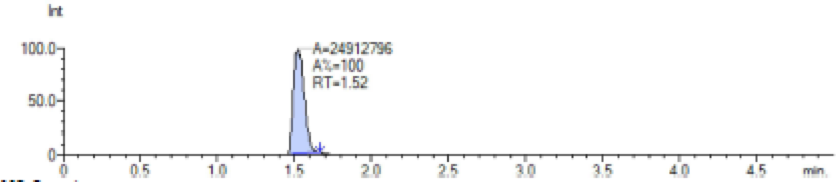

MS Spectrum
Group#1 - PDA Peak: 27, RT: 1.39 to 1.66 min

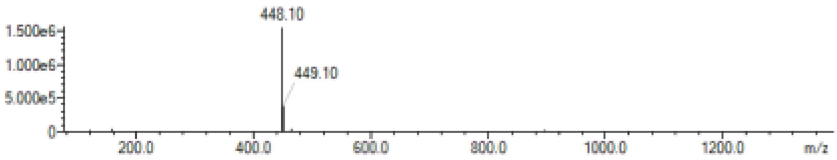

SM-C-S-10

Shimadzu Open Solution

**Project:**	Test
**Experiment:**	572_190612_Zinken_0607_Bischof_0612Griga
**Experiment Description:**	
**Sample:**	410415:01:01
**Sample Description:**	C26H28FNO4
**Data File Name:**	C:\LabSolutions\Data\572_190612_Zinken_190607_Bischoff_190612_Grigalunas\1046363747_11190613572.lcd
**Sample Location:**	Plate Number: 1 - Position: 73
**Run By:**	admin
**Run Started:**	29 July 2019 19:27:00
**Run Finished:**	30 July 2019 04:41:49
**Method:**	190321_Purity-Check

LC Chromatogram

LC#6 : AD2

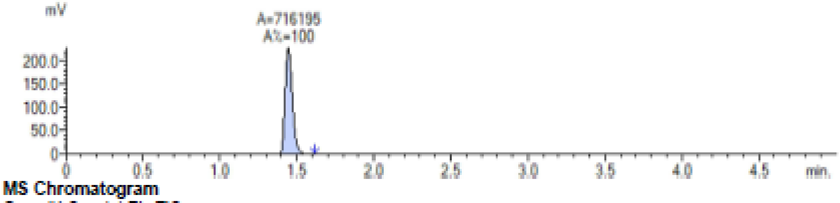

MS Chromatogram

Group#1 Scan(+) EI : TIC

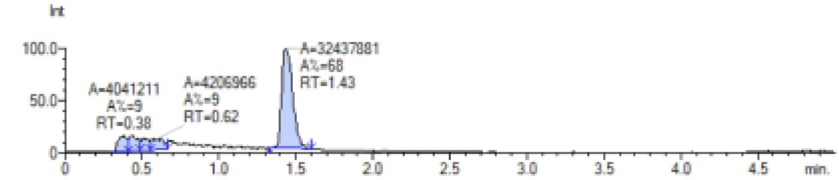

MS Mass Chromatogram

Group#1 Scan(+) EI : 438.2

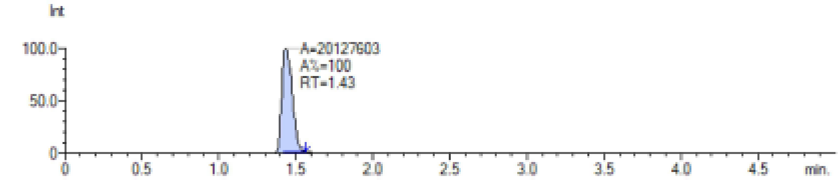

MS Spectrum

Group#1 - PDA Peak: 22, RT: 1.31 to 1.53 min

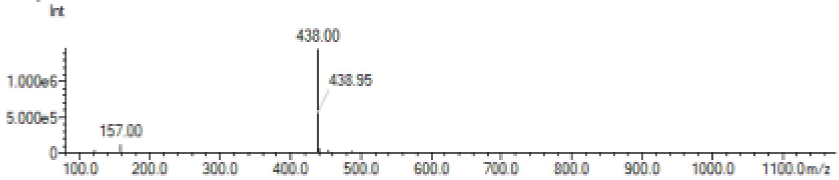

SM-C-R-7

Shimadzu Open Solution

Project:	Test
Experiment:	525_0206_Christo_Grigalu_Burhop_Wilke
Experiment Description:	
Sample:	409723:01:01
Sample Description:	C26H28FNO4
Data File Name:	C:\LabSolutions\Data\525_190206_Christoforow_190206_Grigalunas_190206_Burhop_190206_Wilke\1046361116_11190207525.lcd
Sample Location:	Plate Number: 2 - Position: 206
Run By:	admin
Run Started:	23 April 2019 18:20:13
Run Finished:	24 April 2019 04:25:41
Method:	190321_Purity-Check+shut down

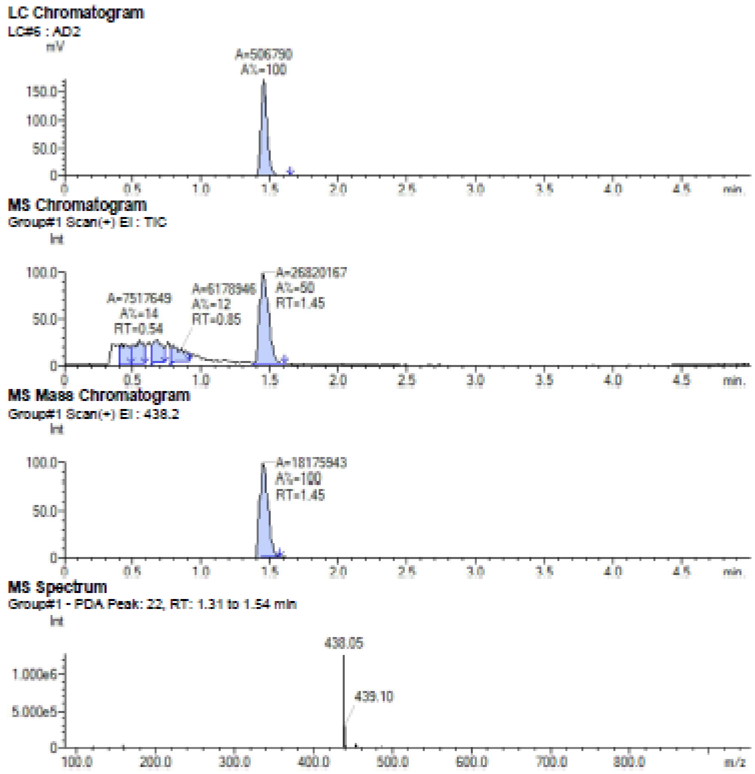

SM-C-S-11

Shimadzu Open Solution

Project: Test
Experiment: 572_190612_Zinken_0607_Bischof_0612Griga
Experiment Description:
Sample: 410416:01:01
Sample Description: C30H36N2O4
Data File Name: C:\LabSolutions\Data\572_190612_Zinken_190607_Bischoff_190612_Grigalunas\1046364803_11190613572.lcd
Sample Location: Plate Number: 1 - Position: 121
Run By: admin
Run Started: 29 July 2019 19:27:00
Run Finished: 30 July 2019 04:41:49
Method: 190321_Purity-Check

LC Chromatogram
LC#6 : AD2

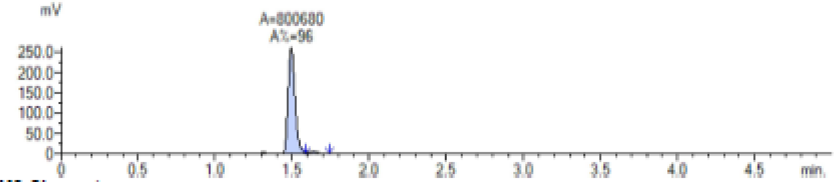

MS Chromatogram
Group#1 Scan(+) EI : TIC

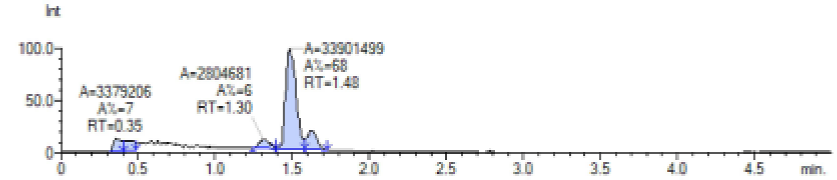

MS Mass Chromatogram
Group#1 Scan(+) EI : 489.3

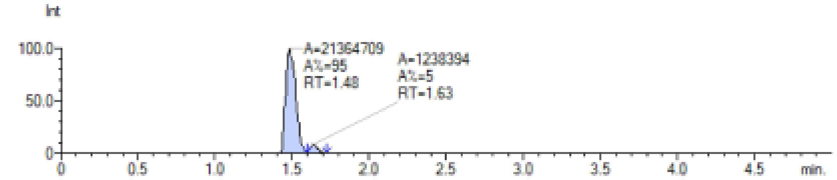

MS Spectrum
Group#1 - PDA Peak: 32, RT: 1.30 to 1.49 min

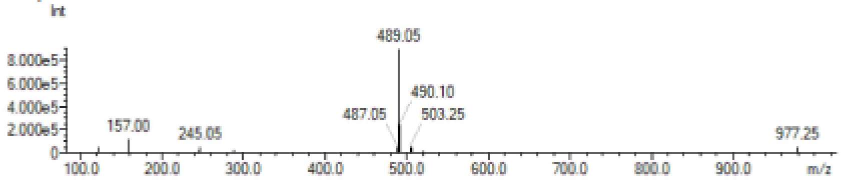

SM-C-R-8

Shimadzu Open Solution

**Project:**	Test
**Experiment:**	572_190612_Zinken_0607_Bischof_0612Griga
**Experiment Description:**	
**Sample:**	410417:01:01
**Sample Description:**	C26H28ClNO4
**Data File Name:**	C:\LabSolutions\Data\572_190612_Zinken_190607_Bischoff_190612_Grigalunas\1046364390_11190613572.lcd
**Sample Location:**	Plate Number: 1 - Position: 169
**Run By:**	admin
**Run Started:**	29 July 2019 19:27:00
**Run Finished:**	30 July 2019 04:41:49
**Method:**	190321_Purity-Check

LC Chromatogram

LC#6 : AD2

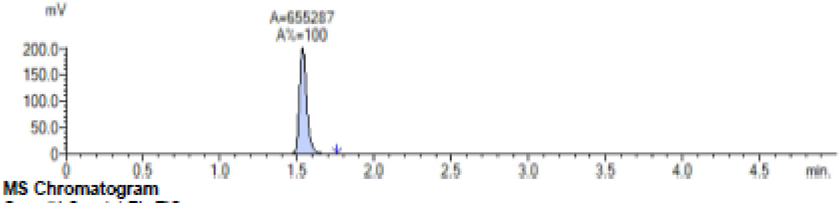

MS Chromatogram

Group#1 Scan(+) EI : TIC

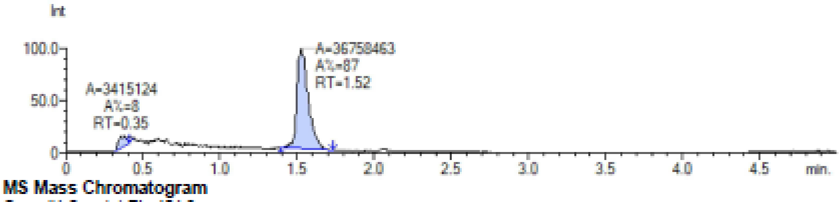

MS Mass Chromatogram

Group#1 Scan(+) EI : 454.2

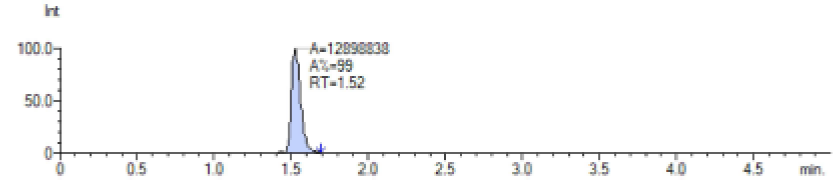

MS Spectrum

Group#1 - PDA Peak: 21, RT: 1.21 to 1.75 min

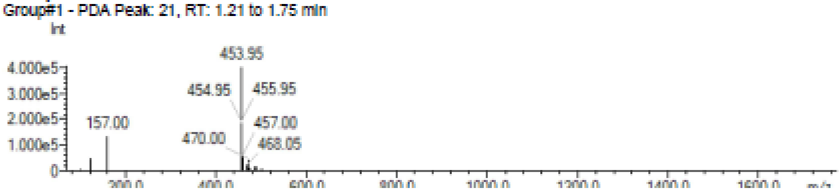

SM-C-S-12

Shimadzu Open Solution

**Project:**	Test
**Experiment:**	525_0206_Christo_Grigalu_Burhop_Wilke
**Experiment Description:**	
**Sample:**	409724:01:01
**Sample Description:**	C26H28BrNO4
**Data File Name:**	C:\LabSolutions\Data\525_190206_Christoforow_190206_Grigalunas_190206_Burhop_190206_Wilke\1046360891_11190207525.lcd
**Sample Location:**	Plate Number: 2 - Position: 254
**Run By:**	admin
**Run Started:**	23 April 2019 18:20:13
**Run Finished:**	24 April 2019 04:25:41
**Method:**	190321_Purity-Check+shut down

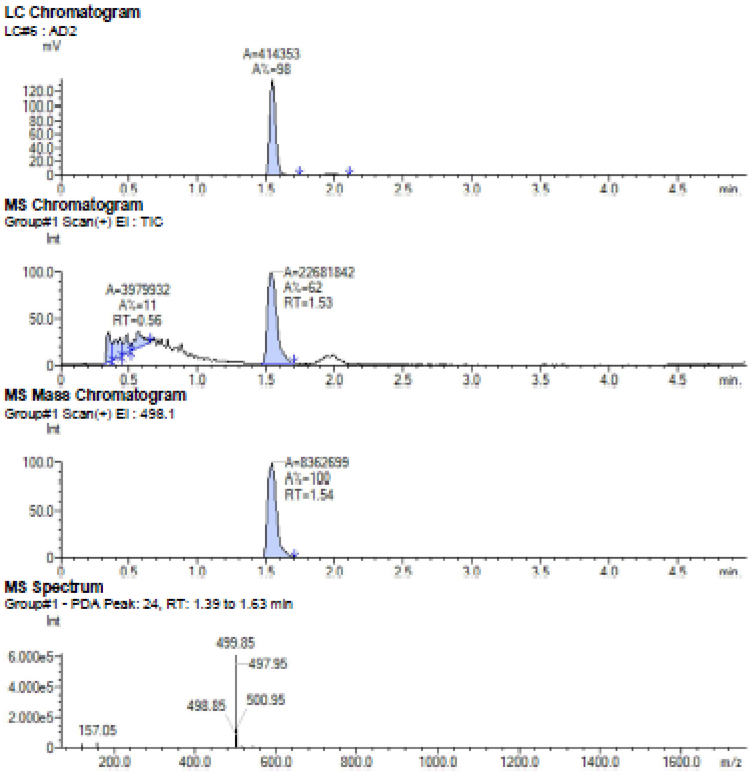

SM-C-R-9

Shimadzu Open Solution

Project:	Test
Experiment:	525_0206_Christo_Grigalu_Burhop_Wilke
Experiment Description:	
Sample:	409725:01:01
Sample Description:	C26H28BrNO4
Data File Name:	C:\LabSolutions\Data\525_190206_Christoforow_190206_Grigalunas_190206_Burhop_190206_Wilke\1046360536_11190207525.lcd
Sample Location:	Plate Number: 2 - Position: 302
Run By:	admin
Run Started:	23 April 2019 18:20:13
Run Finished:	24 April 2019 04:25:41
Method:	190321_Purity-Check+shut down

LC Chromatogram

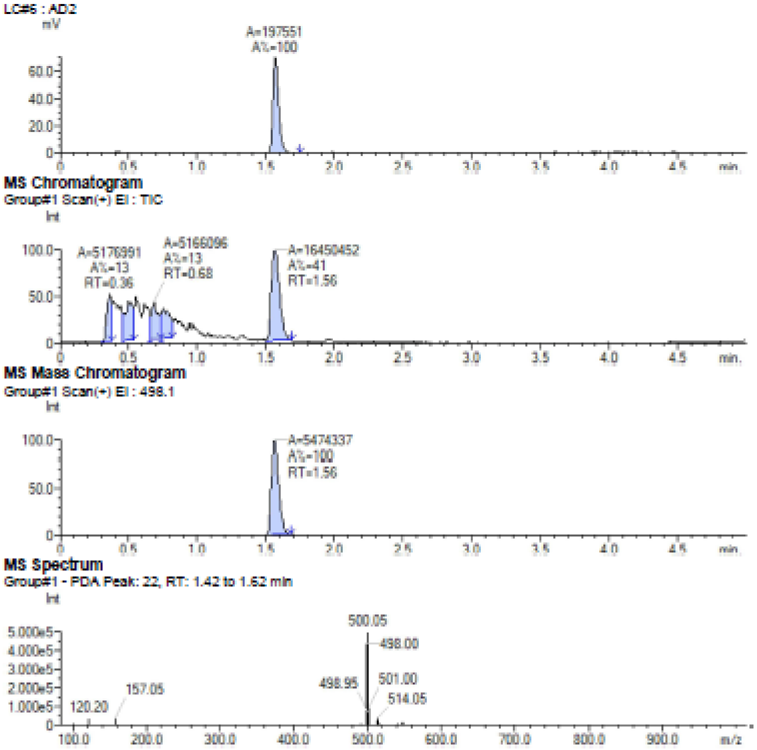

SM-C-S-13

Shimadzu Open Solution

Project: Test
Experiment: 525_0206_Christo_Grigalu_Burhop_Wilke
Experiment Description:
Sample: 409726:01:01
Sample Description: C30H31NO4
Data File Name: C:\LabSolutions\Data\525_190206_Christoforow_190206_Grigalunas_190206_Burhop_190206_Wilke\1046360904_11190207525.lcd
Sample Location: Plate Number: 2 - Position: 350
Run By: admin
Run Started: 23 April 2019 18:20:13
Run Finished: 24 April 2019 04:25:41
Method: 190321_Purity-Check+shut down

LC Chromatogram

LC#6 : AD2

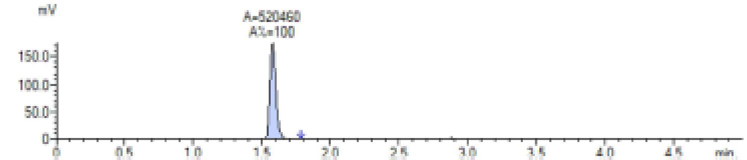

MS Chromatogram

Group#1 Scan(+) EI : TIC

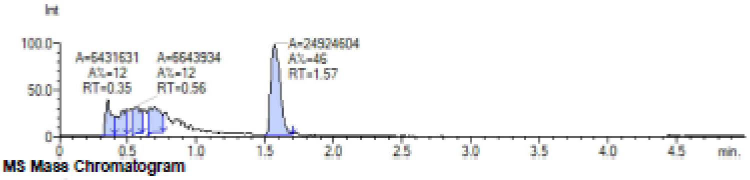

MS Mass Chromatogram

Group#1 Scan(+) EI : 470.2

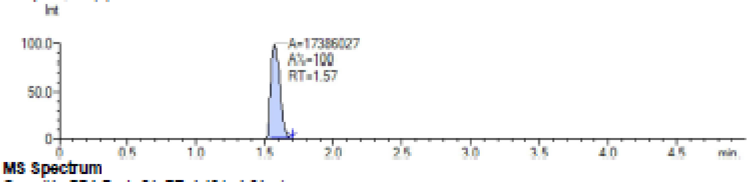

MS Spectrum

Group#1 - PDA Peak: 21, RT: 1.42 to 1.61 min

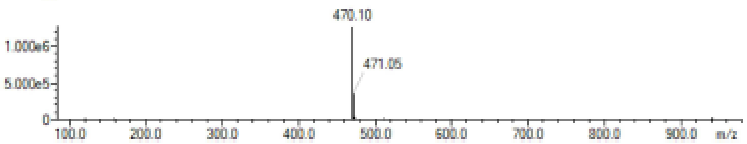

Shimadzu Open Solution

SM-C-R-10

**Project:**	Test
**Experiment:**	525_0206_Christo_Grigalu_Burhop_Wilke
**Experiment Description:**	
**Sample:**	409727:01:01
**Sample Description:**	C30H31NO4
**Data File Name:**	C:\LabSolutions\Data\525_190206_Christoforow_190206_Grigalunas_190206_Burhop_190206_Wilke\1046360654_11190207525.lcd
**Sample Location:**	Plate Number: 2 - Position: 16
**Run By:**	admin
**Run Started:**	23 April 2019 18:20:13
**Run Finished:**	24 April 2019 04:25:41
**Method:**	190321_Purity-Check+shut down

LC Chromatogram

LC#5 : AD2

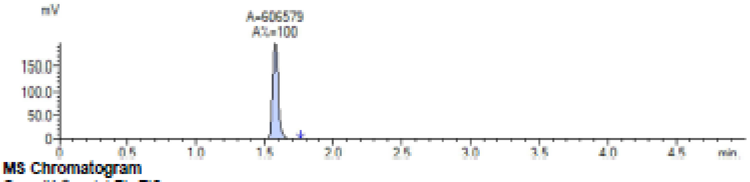

MS Chromatogram

Group#1 Scan(+) EI : TIC

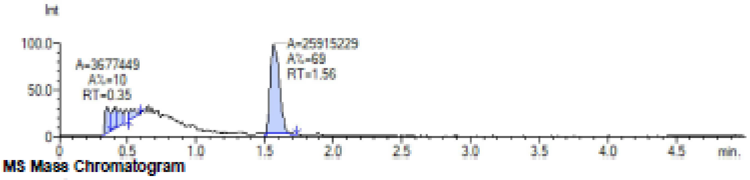

MS Mass Chromatogram

Group#1 Scan(+) EI : 470.2

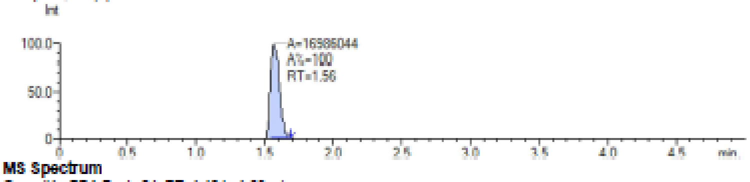

MS Spectrum

Group#1 - PDA Peak: 24, RT: 1.42 to 1.69 min

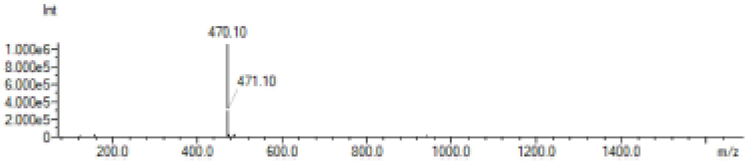

SM-C-R-11

Shimadzu Open Solution

Project: Test
Experiment: 525_0206_Christo_Grigalu_Burhop_Wilke
Experiment Description:
Sample: 409726:01:01
Sample Description: C30H31NO4
Data File Name: C:\LabSolutions\Data\525_190206_Christoforow_190206_Grigalunas_190206_Burhop_190206_Wilke\1046360853_11190207525.lcd
Sample Location: Plate Number: 2 - Position: 64
Run By: admin
Run Started: 23 April 2019 18:20:13
Run Finished: 24 April 2019 04:25:41
Method: 190321_Purity-Check+shut down

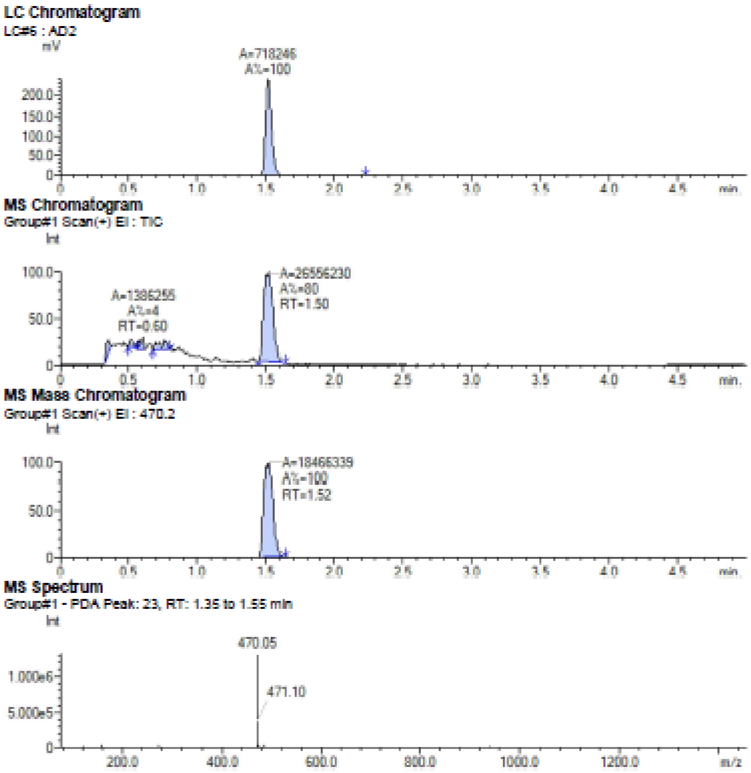

SM-C-S-14

Shimadzu Open Solution

**Project:**	Test
**Experiment:**	525_0206_Christo_Grigalu_Burhop_Wilke
**Experiment Description:**	
**Sample:**	409729:01:01
**Sample Description:**	C27H30ClNO4
**Data File Name:**	C:\LabSolutions\Data\525_190206_Christoforow_190206_Grigalunas_190206_Burhop_190206_Wilke\1046361039_11190207525.lcd
**Sample Location:**	Plate Number: 2 - Position: 112
**Run By:**	admin
**Run Started:**	23 April 2019 18:20:13
**Run Finished:**	24 April 2019 04:25:41
**Method:**	190321_Purity-Check+shut down

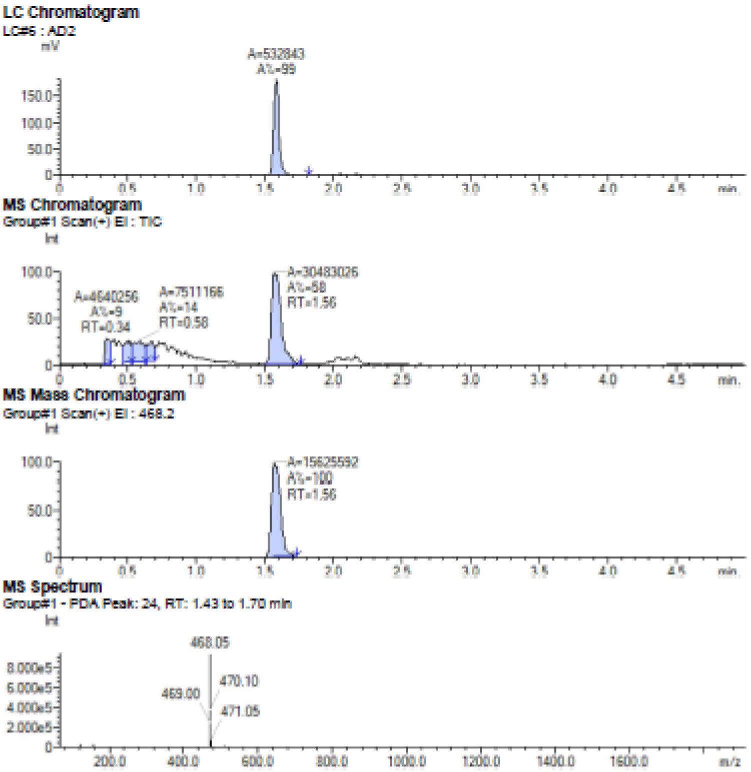

SM-C-R-12

Shimadzu Open Solution

Project: Test
Experiment: 525_0206_Christo_Grigalu_Burhop_Wilke
Experiment Description:
Sample: 409730:01:01
Sample Description: C27H30ClNO4
Data File Name: C:\LabSolutions\Data\525_190206_Christoforow_190206_Grigalunas_190206_Burhop_190206_Wilke\1046361295_11190207525.lcd
Sample Location: Plate Number: 2 - Position: 160
Run By: admin
Run Started: 23 April 2019 18:20:13
Run Finished: 24 April 2019 04:25:41
Method: 190321_Purity-Check+shut down

LC Chromatogram
LC#6 : AD2

MS Chromatogram
Group#1 Scan(+) EI : TIC

MS Mass Chromatogram
Group#1 Scan(+) EI : 468.2

MS Spectrum
Group#1 - PDA Peak: 23, RT: 1.46 to 1.67 min

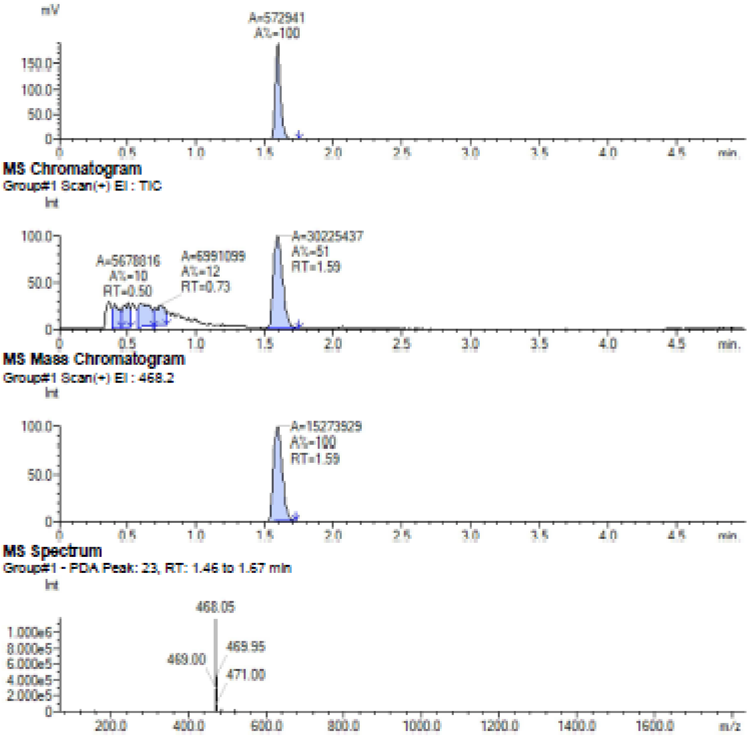

SM-C-S-15

Shimadzu Open Solution

Project: Test
Experiment: 525_0206_Christo_Grigalu_Burhop_Wilke
Experiment Description:
Sample: 409731:01:01
Sample Description: C28H33NO4
Data File Name: C:\LabSolutions\Data\525_190206_Christoforow_190206_Grigalunas_190206_Burhop_190206_Wilke\1046360661_11190207525.lcd
Sample Location: Plate Number: 2 - Position: 206
Run By: admin
Run Started: 23 April 2019 18:20:13
Run Finished: 24 April 2019 04:25:41
Method: 190321_Purity-Check+shut down

LC Chromatogram
LC#6 : AD2

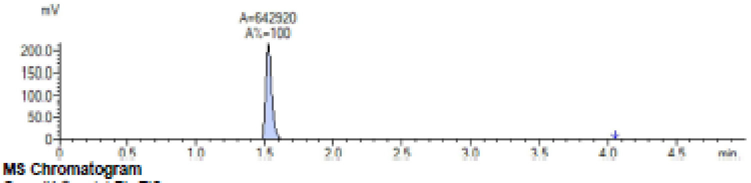

MS Chromatogram
Group#1 Scan(+) EI : TIC

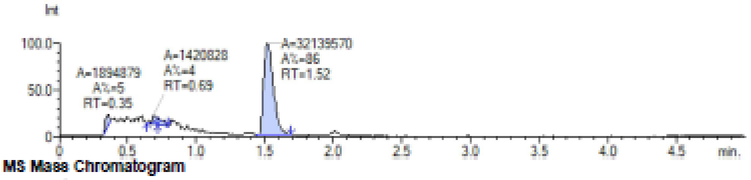

MS Mass Chromatogram
Group#1 Scan(+) EI : 448.2

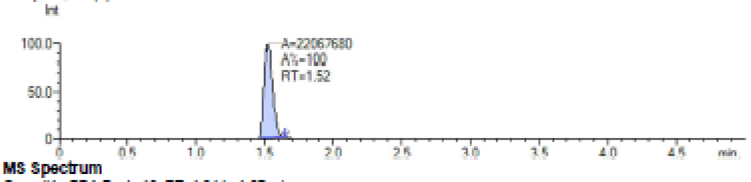

MS Spectrum
Group#1 - PDA Peak: 19, RT: 1.34 to 1.67 min

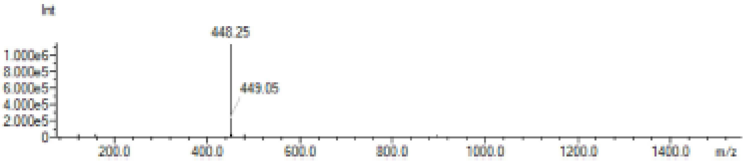

SM-C-S-16

Shimadzu Open Solution

**Project:**	Test
**Experiment:**	572_190612_Zinken_0607_Bischof_0612Griga
**Experiment Description:**	
**Sample:**	410418:01:01
**Sample Description:**	C33H35NO5
**Data File Name:**	C:\LabSolutions\Data\572_190612_Zinken_190607_Bischoff_190612_Grigalunas\1046363551_11190613572.lcd
**Sample Location:**	Plate Number: 1 - Position: 217
**Run By:**	admin
**Run Started:**	29 July 2019 19:27:00
**Run Finished:**	30 July 2019 04:41:49
**Method:**	190321_Purity-Check

LC Chromatogram

LC#6 : AD2

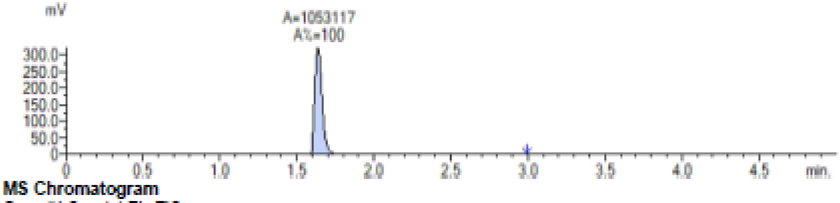

MS Chromatogram

Group#1 Scan(+) EI : TIC

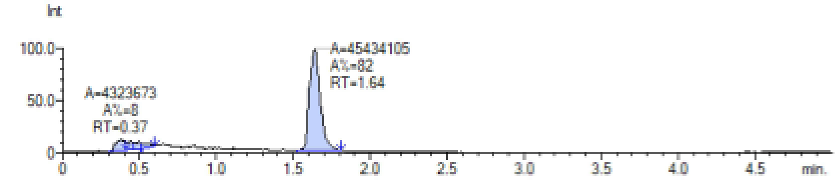

MS Mass Chromatogram

Group#1 Scan(+) EI : 526.3

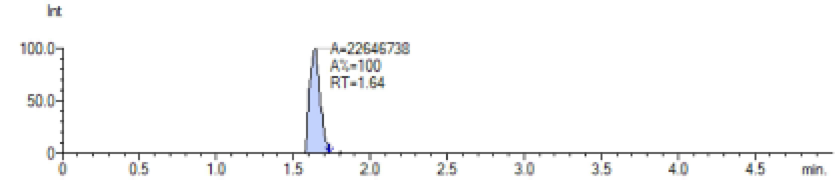

MS Spectrum

Group#1 - PDA Peak: 20, RT: 1.47 to 1.79 min

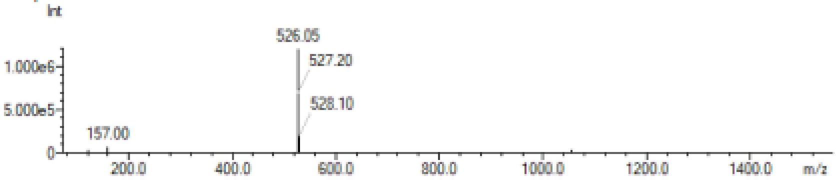

SM-C-R-13

Shimadzu Open Solution

**Project:**	Test
**Experiment:**	525_0206_Christo_Grigalu_Burhop_Wilke
**Experiment Description:**	
**Sample:**	409732:01:01
**Sample Description:**	C33H35NO5
**Data File Name:**	C:\LabSolutions\Data\525_190206_Christoforow_190206_Grigalunas_190206_Burhop_190206_Wilke\1046361366_11190207525.lcd
**Sample Location:**	Plate Number: 2 - Position: 256
**Run By:**	admin
**Run Started:**	23 April 2019 18:20:13
**Run Finished:**	24 April 2019 04:25:41
**Method:**	190321_Purity-Check+shut down

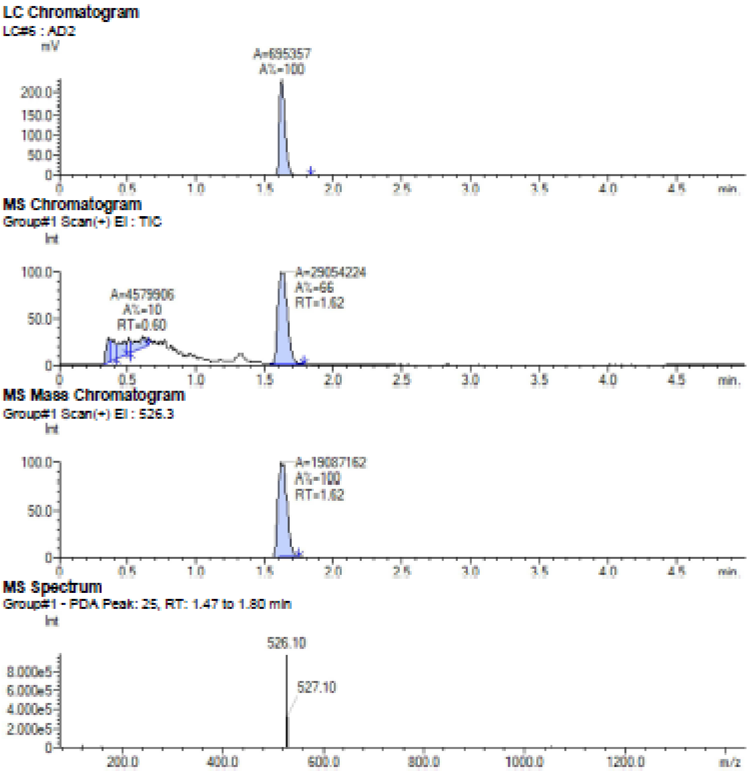

SM-I-closed-1

Shimadzu Open Solution

Project: Test
Experiment: 525_0206_Christo_Grigalu_Burhop_Wilke
Experiment Description:
Sample: 409709:01:01
Sample Description: C24H24N2O2
Data File Name: C:\LabSolutions\Data\525_190206_Christoforow_190206_Grigalunas_190206_Burhop_190206_Wilke\1046360950_11190207525.lcd
Sample Location: Plate Number: 2 - Position: 12
Run By: admin
Run Started: 23 April 2019 18:20:13
Run Finished: 24 April 2019 04:25:41
Method: 190321_Purity-Check+shut down

LC Chromatogram

LC#6 : AD2

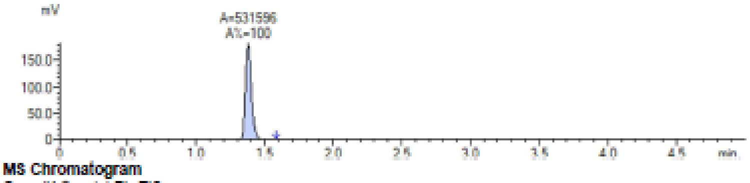

MS Chromatogram

Group#1 Scan(+) EI : TIC

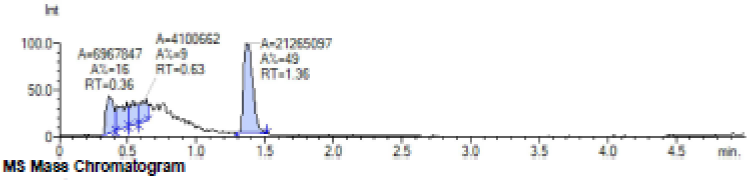

MS Mass Chromatogram

Group#1 Scan(+) EI : 373.2

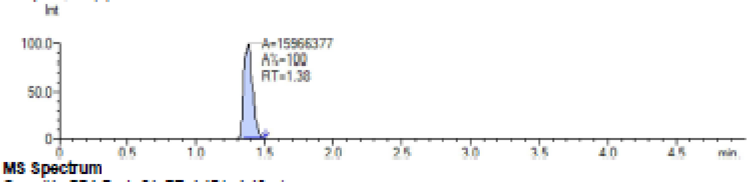

MS Spectrum

Group#1 - PDA Peak: 21, RT: 1.17 to 1.48 min

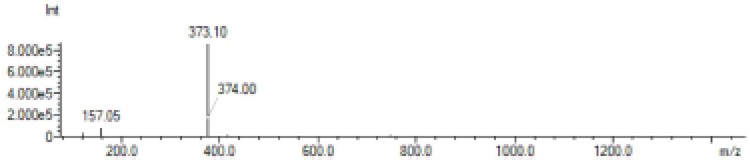

SM-I-closed-2

Shimadzu Open Solution

Project: Test
Experiment: 525_0206_Christo_Grigalu_Burhop_Wilke
Experiment Description:
Sample: 409712:01:01
Sample Description: C25H26N2O2
Data File Name: C:\LabSolutions\Data\525_190206_Christoforow_190206_Grigalunas_190206_Burhop_190206_Wilke\1046360623_11190207525.lcd
Sample Location: Plate Number: 2 - Position: 60
Run By: admin
Run Started: 23 April 2019 18:20:13
Run Finished: 24 April 2019 04:25:41
Method: 190321_Purity-Check+shut down

LC Chromatogram
LC#6 : AD2

MS Chromatogram
Group#1 Scan(+) EI : TIC

MS Mass Chromatogram
Group#1 Scan(+) EI : 387.2

MS Spectrum
Group#1 - PDA Peak: 22, RT: 1.27 to 1.54 min

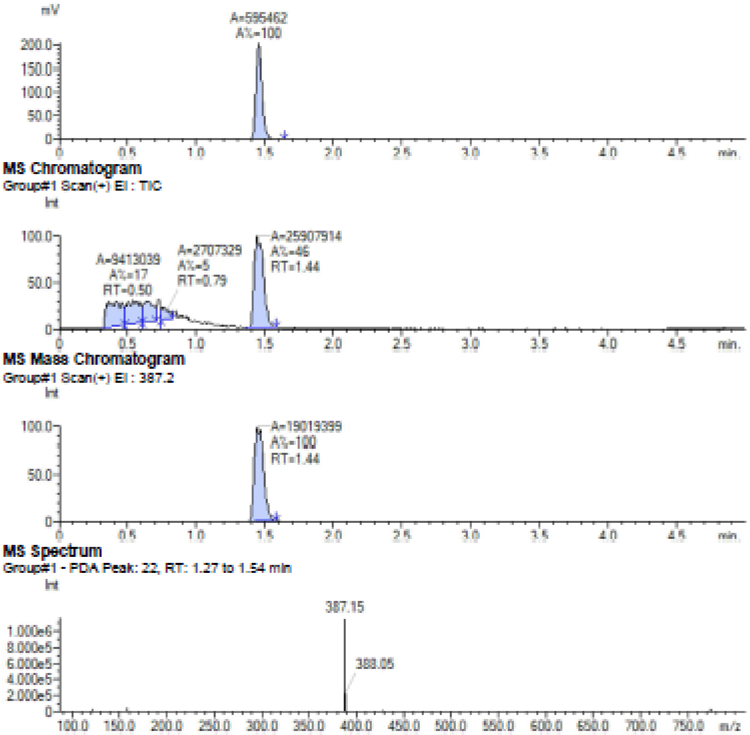

SM-I-closed-3

Shimadzu Open Solution

**Project:**	Test
**Experiment:**	525_0206_Christo_Grigalu_Burhop_Wilke
**Experiment Description:**	
**Sample:**	409713:01:01
**Sample Description:**	C25H26N2O3
**Data File Name:**	C:\LabSolutions\Data\525_190206_Christoforow_190206_Grigalunas_190206_Burhop_190206_Wilke\1046361253_11190207525.lcd
**Sample Location:**	Plate Number: 2 - Position: 108
**Run By:**	admin
**Run Started:**	23 April 2019 18:20:13
**Run Finished:**	24 April 2019 04:25:41
**Method:**	190321_Purity-Check+shut down

LC Chromatogram

LC#6 : AD2

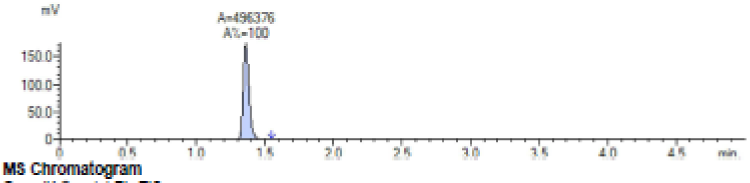

MS Chromatogram

Group#1 Scan(+) EI : TIC

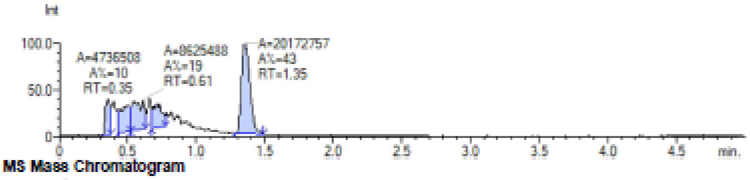

MS Mass Chromatogram

Group#1 Scan(+) EI : 403.2

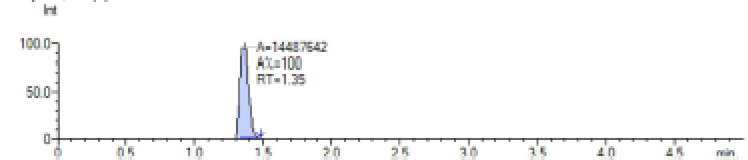

MS Spectrum

Group#1 - PDA Peak: 19, RT: 1.17 to 1.39 min

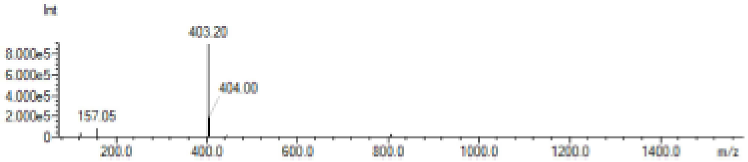

SM-I-closed-4

Shimadzu Open Solution

Project: Test
Experiment: 525_0206_Christo_Grigalu_Burhop_Wilke
Experiment Description:
Sample: 409714:01:01
Sample Description: C25H26N2O2
Data File Name: C:\LabSolutions\Data\525_190206_Christoforow_190206_Grigalunas_190206_Burhop_190206_Wilke\1046360557_11190207525.lcd
Sample Location: Plate Number: 2 - Position: 156
Run By: admin
Run Started: 23 April 2019 18:20:13
Run Finished: 24 April 2019 04:25:41
Method: 190321_Purity-Check+shut down

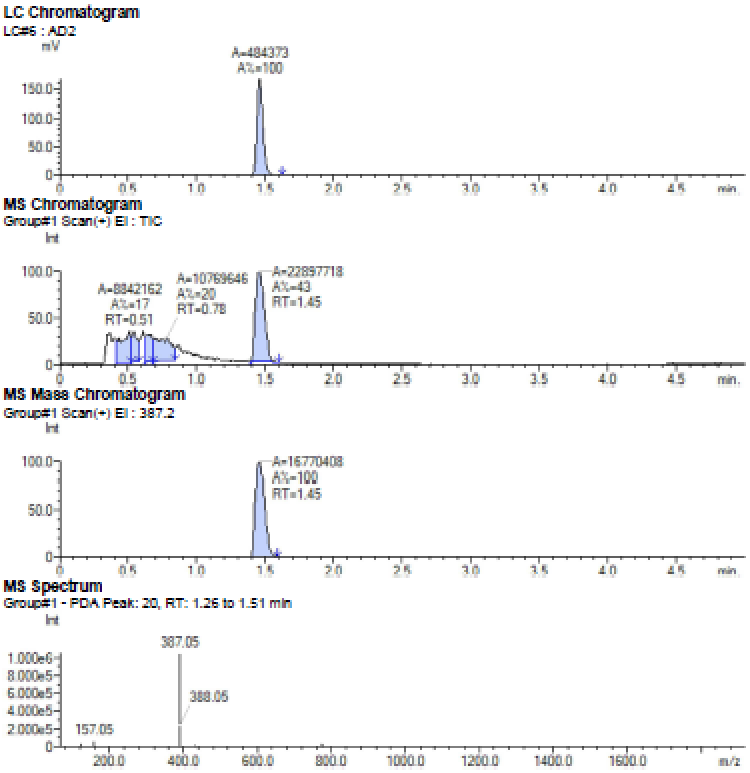

SM-I-closed-5

Shimadzu Open Solution

Project:	Test
Experiment:	525_0206_Christo_Grigalu_Burhop_Wilke
Experiment Description:	
Sample:	409715:01:01
Sample Description:	C27H30N2O2
Data File Name:	C:\LabSolutions\Data\525_190206_Christoforow_190206_Grigalunas_190206_Burhop_190206_Wilke\1046360602_11190207525.lcd
Sample Location:	Plate Number: 2 - Position: 204
Run By:	admin
Run Started:	23 April 2019 18:20:13
Run Finished:	24 April 2019 04:25:41
Method:	190321_Purity-Check+shut down

LC Chromatogram

LC#6 : AD2

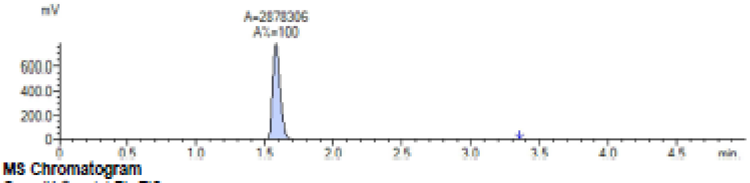

MS Chromatogram

Group#1 Scan(+) EI : TIC

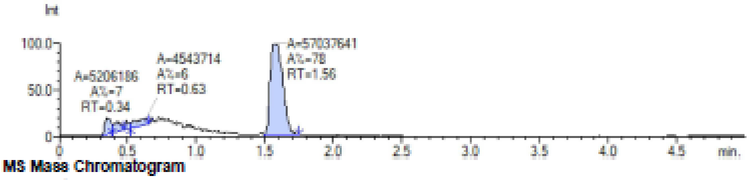

MS Mass Chromatogram

Group#1 Scan(+) EI : 415.2

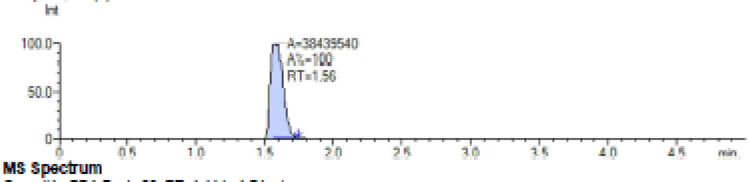

MS Spectrum

Group#1 - PDA Peak: 23, RT: 1.41 to 1.74 min

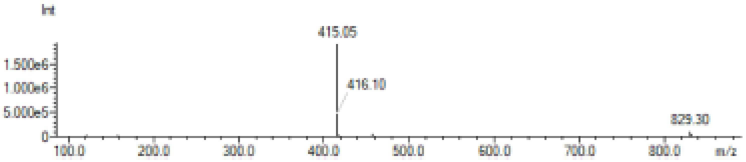

SM-I-closed-6

Shimadzu Open Solution

**Project:**	Test
**Experiment:**	525_0206_Christo_Grigalu_Burhop_Wilke
**Experiment Description:**	
**Sample:**	409716:01:01
**Sample Description:**	C24H23ClN2O2
**Data File Name:**	C:\LabSolutions\Data\525_190206_Christoforow_190206_Grigalunas_190206_Burhop_190206_Wilke\1046360600_11190207525.lcd
**Sample Location:**	Plate Number: 2 - Position: 252
**Run By:**	admin
**Run Started:**	23 April 2019 18:20:13
**Run Finished:**	24 April 2019 04:25:41
**Method:**	190321_Purity-Check+shut down

LC Chromatogram

LC#5 : AD2

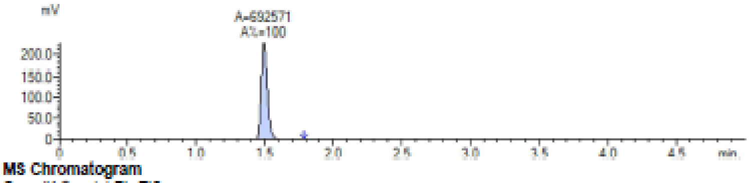

MS Chromatogram

Group#1 Scan(+) EI : TIC

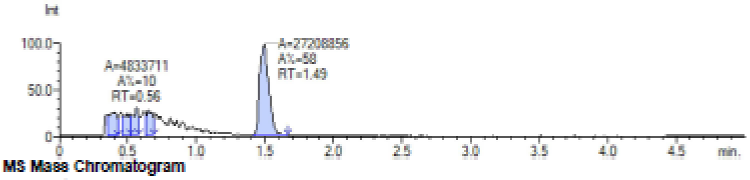

MS Mass Chromatogram

Group#1 Scan(+) EI : 407.1

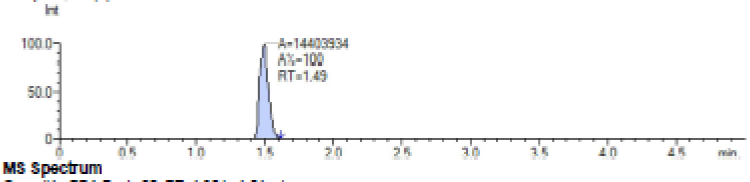

MS Spectrum

Group#1 - PDA Peak: 20, RT: 1.35 to 1.51 min

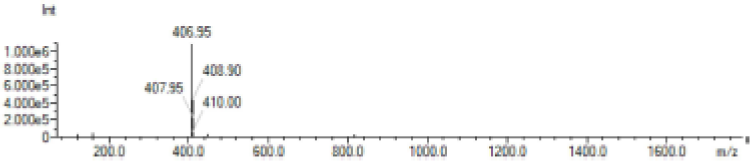

SM-I-closed-7

Shimadzu Open Solution

Project: Test
Experiment: 525_0206_Christo_Grigalu_Burhop_Wilke
Experiment Description:
Sample: 409717:01:02
Sample Description: C30H28N2O2
Data File Name: C:\LabSolutions\Data\525_190206_Christoforow_190206_Grigalunas_190206_Burhop_190206_Wilke\1046361149_11190207525.lcd
Sample Location: Plate Number: 2 - Position: 300
Run By: admin
Run Started: 23 April 2019 18:20:13
Run Finished: 24 April 2019 04:25:41
Method: 190321_Purity-Check+shut down

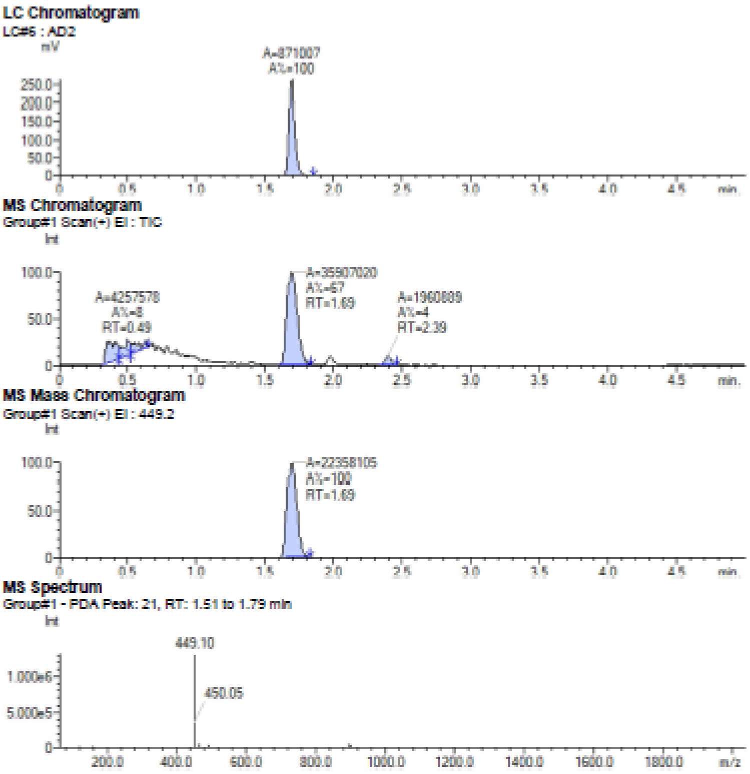

SM-I-closed-8

Shimadzu Open Solution

**Project:**	Test
**Experiment:**	572_190612_Zinken_0607_Bischof_0612Griga
**Experiment Description:**	
**Sample:**	410431:01:01
**Sample Description:**	C26H28N2O2
**Data File Name:**	C:\LabSolutions\Data\572_190612_Zinken_190607_Bischoff_190612_Grigalunas\1046364034_11190613572.lcd
**Sample Location:**	Plate Number: 1 - Position: 278
**Run By:**	admin
**Run Started:**	29 July 2019 19:27:00
**Run Finished:**	30 July 2019 04:41:49
**Method:**	190321_Purity-Check

LC Chromatogram

LC#5 : AD2

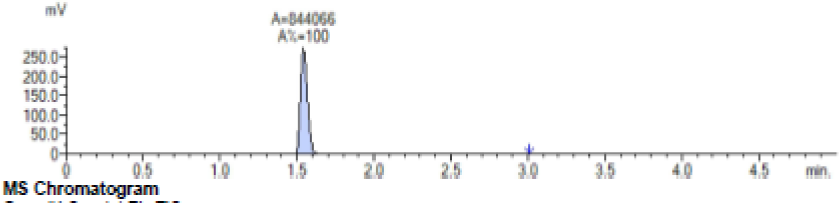

MS Chromatogram

Group#1 Scan(+) EI : TIC

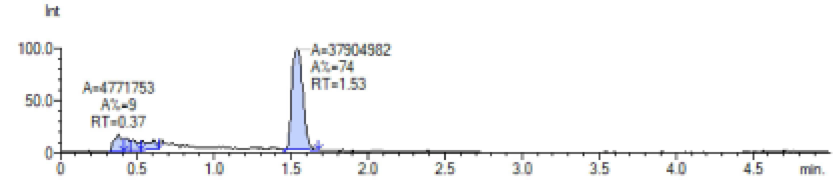

MS Mass Chromatogram

Group#1 Scan(+) EI : 401.2

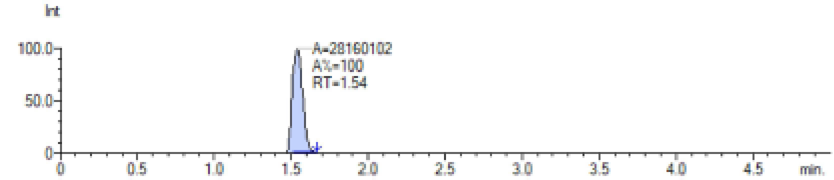

MS Spectrum

Group#1 - PDA Peak: 21, RT: 1.35 to 1.66 min

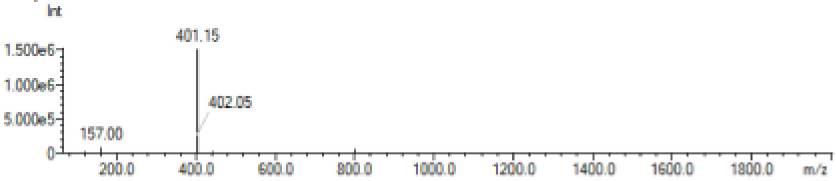

Shimadzu Open Solution

SM-I-closed-9

Project:	Test
Experiment:	525_0206_Christo_Grigalu_Burhop_Wilke
Experiment Description:	
Sample:	409718:01:01
Sample Description:	C31H30N2O2
Data File Name:	C:\LabSolutions\Data\525_190206_Christoforow_190206_Grigalunas_190206_Burhop_190206_Wilke\1046360164_11190207525.lcd
Sample Location:	Plate Number: 2 - Position: 348
Run By:	admin
Run Started:	23 April 2019 18:20:13
Run Finished:	24 April 2019 04:25:41
Method:	190321_Purity-Check+shut down

LC Chromatogram

LC#5 : AD2

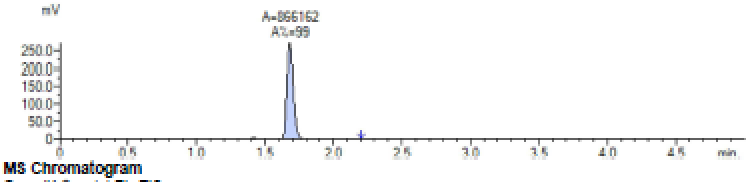

MS Chromatogram

Group#1 Scan(+) EI : TIC

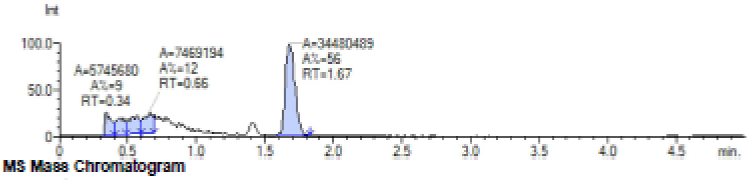

MS Mass Chromatogram

Group#1 Scan(+) EI : 463.2

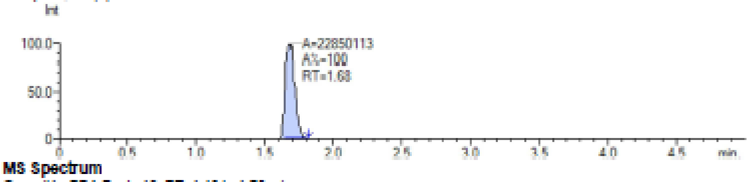

MS Spectrum

Group#1 - PDA Peak: 19, RT: 1.48 to 1.78 min

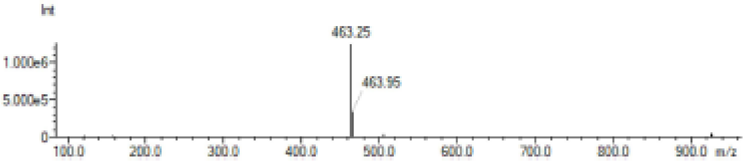

SM-I-closed-10

Shimadzu Open Solution

Project: Test
Experiment: 572_190612_Zinken_0607_Bischof_0612Griga
Experiment Description:
Sample: 410429:01:01
Sample Description: C26H28N2O2
Data File Name: C:\LabSolutions\Data\572_190612_Zinken_190607_Bischoff_190612_Grigalunas\1046363963_11190613572.lcd
Sample Location: Plate Number: 1 - Position: 182
Run By: admin
Run Started: 29 July 2019 19:27:00
Run Finished: 30 July 2019 04:41:49
Method: 190321_Purity-Check

LC Chromatogram
LC#6 : AD2

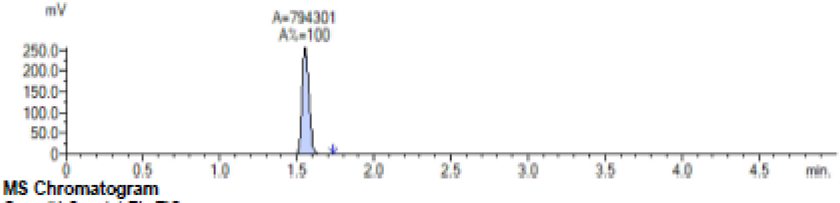

MS Chromatogram
Group#1 Scan(+) EI : TIC

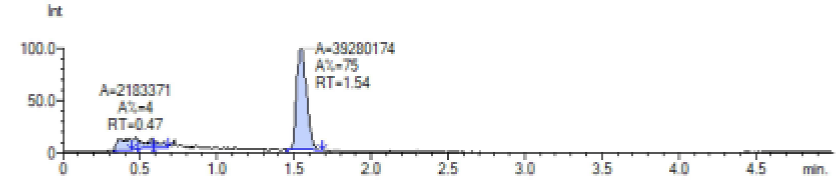

MS Mass Chromatogram
Group#1 Scan(+) EI : 400.2

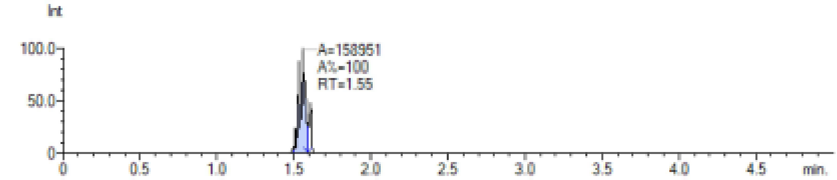

MS Spectrum
Group#1 - PDA Peak: 20, RT: 1.40 to 1.64 min

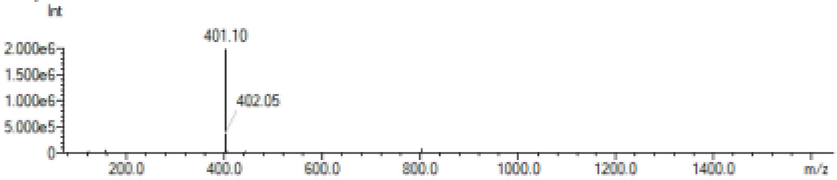

Shimadzu Open Solution

SM-I-closed-11

**Project:**	Test
**Experiment:**	524_0205_Wess_06_Zink_07_Conc-10_08_Grig
**Experiment Description:**	
**Sample:**	409675:01:01
**Sample Description:**	C24H22Cl2N2O2
**Data File Name:**	C:\LabSolutions\Data\524_190205_Wesserler_190602_Zinken_190207_Conc-10_190208_Grigalunas\1046361073_11190208524.lcd
**Sample Location:**	Plate Number: 1 - Position: 122
**Run By:**	admin
**Run Started:**	23 April 2019 15:21:47
**Run Finished:**	23 April 2019 18:20:12
**Method:**	190321_Purity-Check

LC Chromatogram

LC#5 : AD2

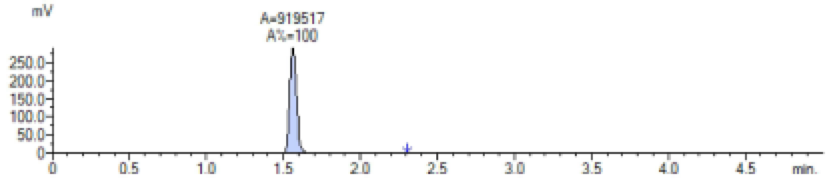

MS Chromatogram

Group#1 Scan(+) EI : TIC

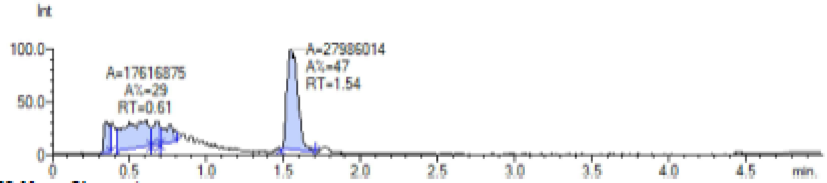

MS Mass Chromatogram

Group#1 Scan(+) EI : 441.1

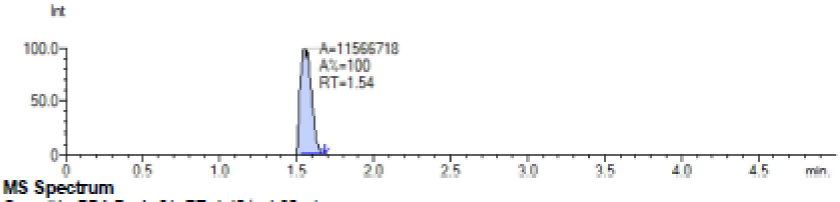

MS Spectrum

Group#1 - PDA Peak: 21, RT: 1.40 to 1.65 min

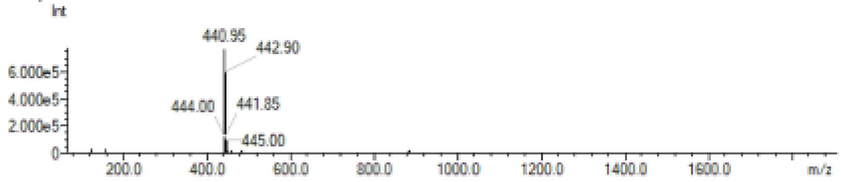

Shimadzu Open Solution

SM-I-closed-12

**Project:**	Test
**Experiment:**	524_0205_Wess_06_Zink_07_Conc-10_08_Grig
**Experiment Description:**	
**Sample:**	409676:01:01
**Sample Description:**	C30H28N2O3
**Data File Name:**	C:\LabSolutions\Data\524_190205_Wesserler_190602_Zinken_190207_Conc-10_190208_Grigalunas\1046361032_11190208524.lcd
**Sample Location:**	Plate Number: 1 - Position: 170
**Run By:**	admin
**Run Started:**	23 April 2019 15:21:47
**Run Finished:**	23 April 2019 18:20:12
**Method:**	190321_Purity-Check

LC Chromatogram

LC#6 : AD2

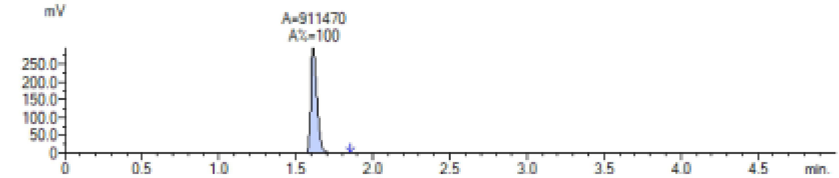

MS Chromatogram

Group#1 Scan(+) EI : TIC

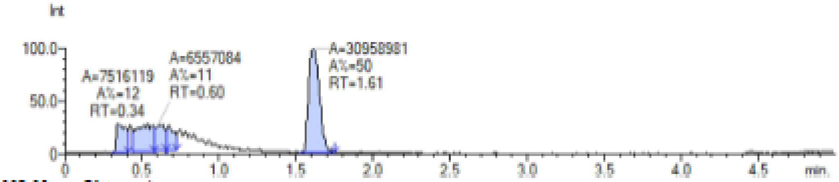

MS Mass Chromatogram

Group#1 Scan(+) EI : 465.2

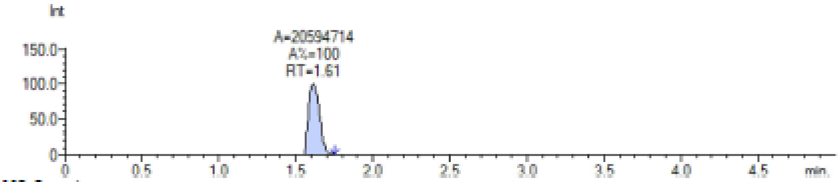

MS Spectrum

Group#1 - PDA Peak: 20, RT: 1.45 to 1.66 min

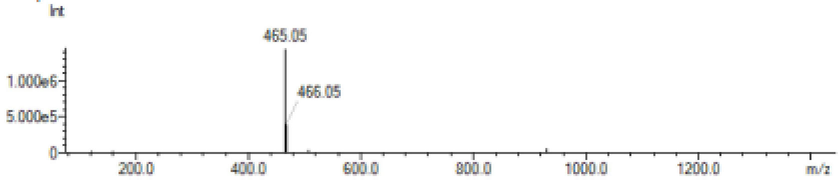

SM-I-closed-13

Shimadzu Open Solution

Project: Test
Experiment: 525_0206_Christo_Grigalu_Burhop_Wilke
Experiment Description:
Sample: 409719:01:01
Sample Description: C28H26N2O2
Data File Name: C:\LabSolutions\Data\525_190206_Christoforow_190206_Grigalunas_190206_Burhop_190206_Wilke\1046360750_11190207525.lcd
Sample Location: Plate Number: 2 - Position: 14
Run By: admin
Run Started: 23 April 2019 18:20:13
Run Finished: 24 April 2019 04:25:41
Method: 190321_Purity-Check+shut down

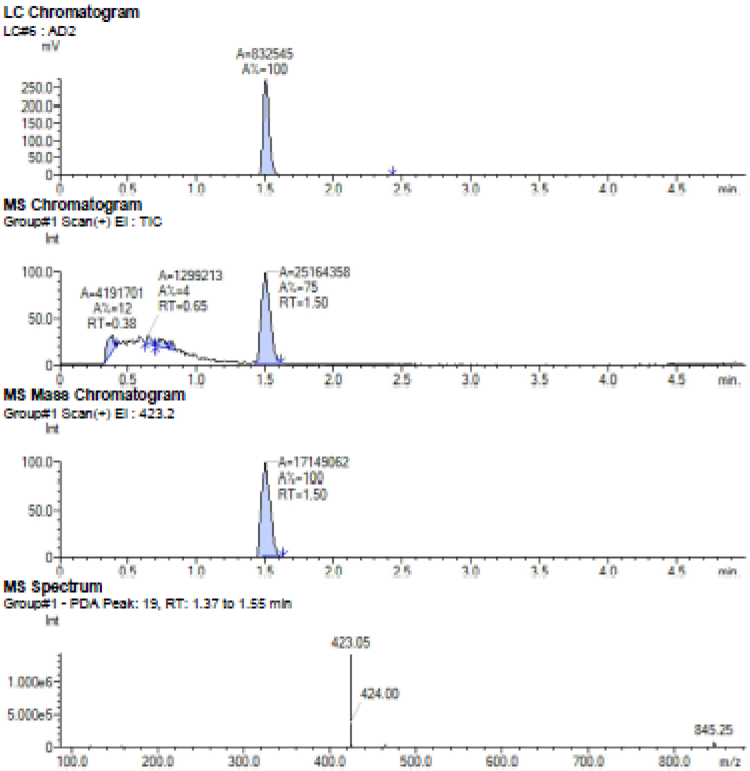

Shimadzu Open Solution

SM-I-closed-14

**Project:**	Test
**Experiment:**	524_0205_Wess_06_Zink_07_Conc-10_08_Grig
**Experiment Description:**	
**Sample:**	409677:01:01
**Sample Description:**	C25H25ClN2O2
**Data File Name:**	C:\LabSolutions\Data\524_190205_Wesserler_190602_Zinken_190207_Conc-10_190208_Grigalunas\1046361200_11190208524.lcd
**Sample Location:**	Plate Number: 1 - Position: 218
**Run By:**	admin
**Run Started:**	23 April 2019 15:21:47
**Run Finished:**	23 April 2019 18:20:12
**Method:**	190321_Purity-Check

LC Chromatogram

LC#6 : AD2

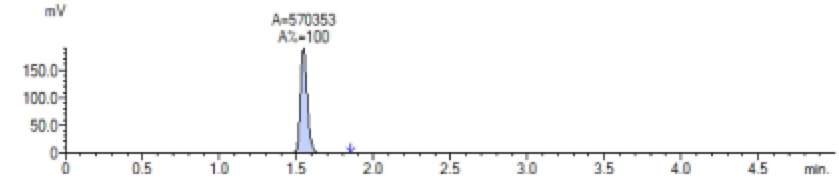

MS Chromatogram

Group#1 Scan(+) EI : TIC

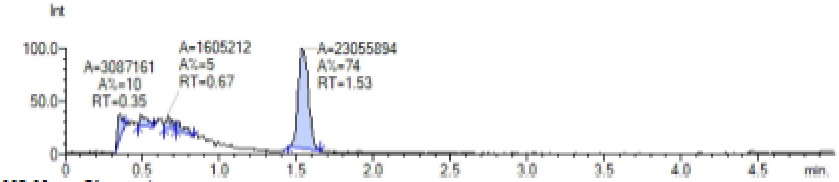

MS Mass Chromatogram

Group#1 Scan(+) EI : 421.2

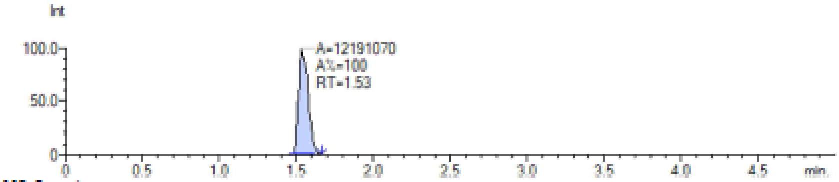

MS Spectrum

Group#1 - PDA Peak: 21, RT: 1.39 to 1.62 min

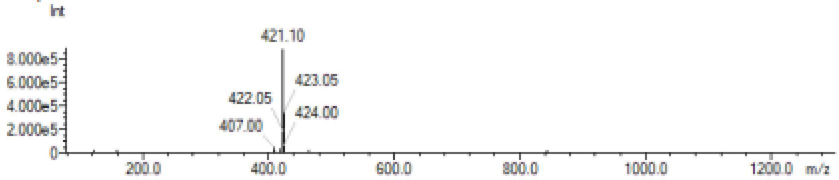

SM-I-closed-15

Shimadzu Open Solution

Project:	Test
Experiment:	572_190612_Zinken_0607_Bischof_0612Griga
Experiment Description:	
Sample:	410432:01:01
Sample Description:	C24H22BrFN2O2
Data File Name:	C:\LabSolutions\Data\572_190612_Zinken_190607_Bischoff_190612_Grigalunas\1046363814_11190613572.lcd
Sample Location:	Plate Number: 1 - Position: 374
Run By:	admin
Run Started:	29 July 2019 19:27:00
Run Finished:	30 July 2019 04:41:49
Method:	190321_Purity-Check

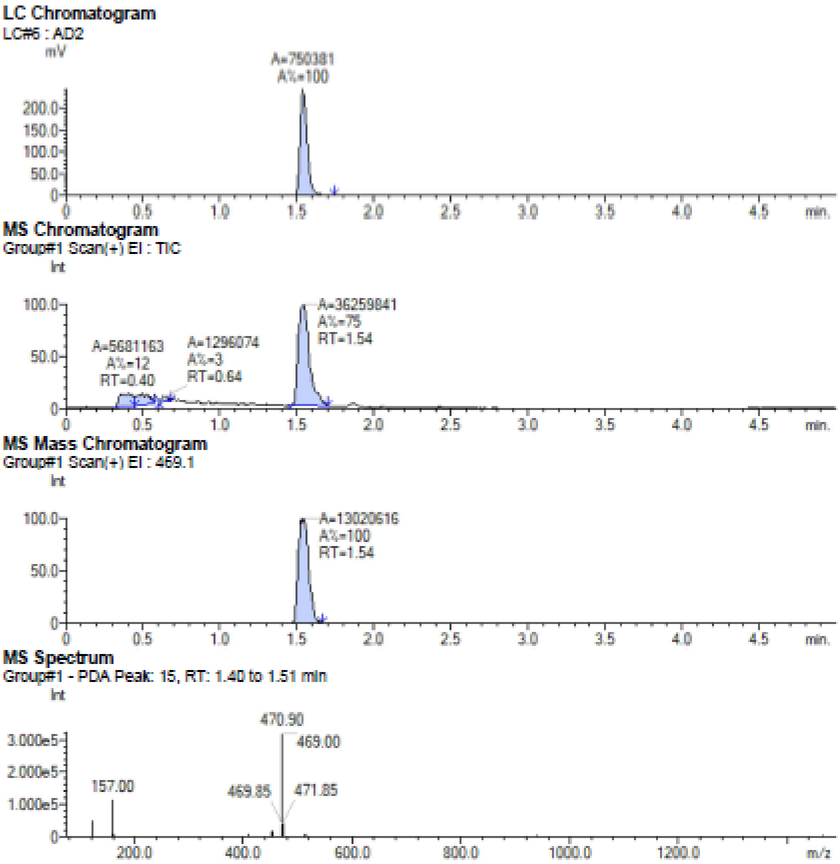

SM-I-closed-16

Shimadzu Open Solution

Project: Test
Experiment: 572_190612_Zinken_0607_Bischof_0612Griga
Experiment Description:
Sample: 410433:01:01
Sample Description: C24H23FN2O2
Data File Name: C:\LabSolutions\Data\572_190612_Zinken_190607_Bischoff_190612_Grigalunas\1046364647_11190613572.lcd
Sample Location: Plate Number: 1 - Position: 40
Run By: admin
Run Started: 29 July 2019 19:27:00
Run Finished: 30 July 2019 04:41:49
Method: 190321_Purity-Check

LC Chromatogram
LC#5 : AD2

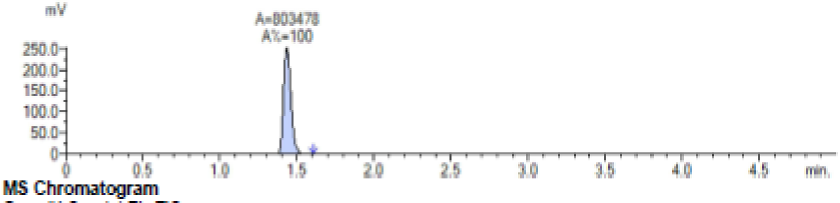

MS Chromatogram
Group#1 Scan(+) EI : TIC

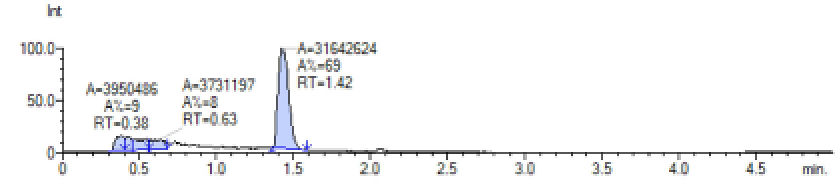

MS Mass Chromatogram
Group#1 Scan(+) EI : 391.2

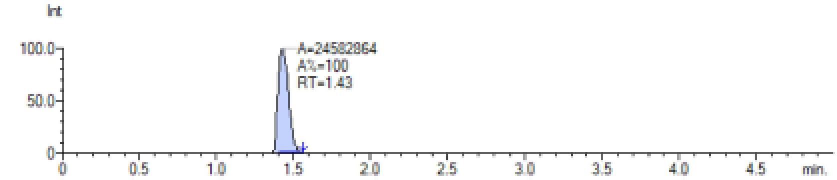

MS Spectrum
Group#1 - PDA Peak: 22, RT: 1.28 to 1.55 min

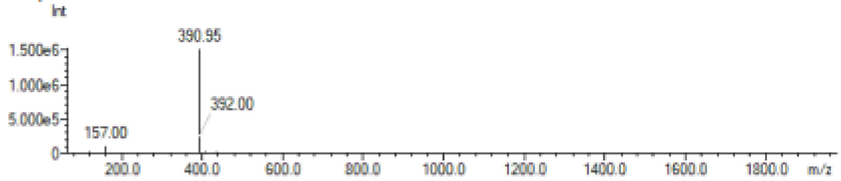

SM-I-closed-17

Shimadzu Open Solution

Project: Test
Experiment: 572_190612_Zinken_0607_Bischof_0612Griga
Experiment Description:
Sample: 410434:01:01
Sample Description: C28H26N2O2
Data File Name: C:\LabSolutions\Data\572_190612_Zinken_190607_Bischoff_190612_Grigalunas\1046364603_11190613572.lcd
Sample Location: Plate Number: 1 - Position: 88
Run By: admin
Run Started: 29 July 2019 19:27:00
Run Finished: 30 July 2019 04:41:49
Method: 190321_Purity-Check

LC Chromatogram
LC#6 : AD2

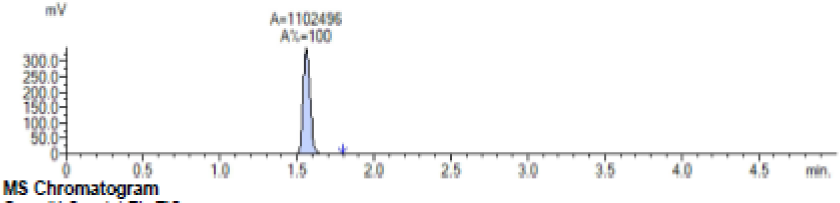

MS Chromatogram
Group#1 Scan(+) EI : TIC

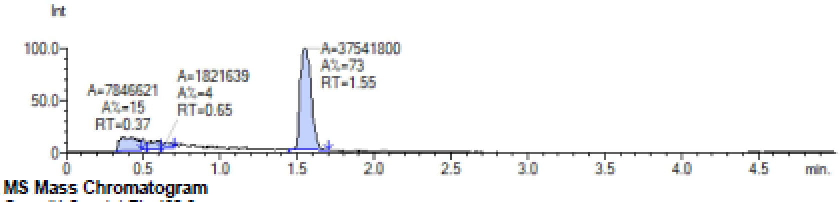

MS Mass Chromatogram
Group#1 Scan(+) EI : 423.2

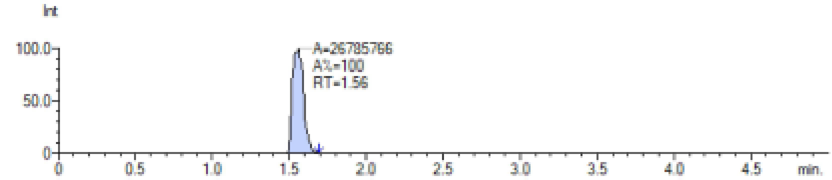

MS Spectrum
Group#1 - PDA Peak: 17, RT: 1.39 to 1.69 min

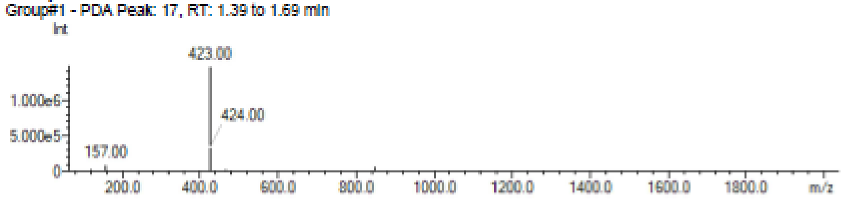

Shimadzu Open Solution

SM-I-closed-18

**Project:**	Test
**Experiment:**	524_0205_Wess_06_Zink_07_Conc-10_08_Grig
**Experiment Description:**	
**Sample:**	409678:01:01
**Sample Description:**	C31H30N2O3
**Data File Name:**	C:\LabSolutions\Data\524_190205_Wesserler_190602_Zinken_190207_Conc-10_190208_Grigalunas\1046361077_11190208524.lcd
**Sample Location:**	Plate Number: 1 - Position: 266
**Run By:**	admin
**Run Started:**	23 April 2019 15:21:47
**Run Finished:**	23 April 2019 18:20:12
**Method:**	190321_Purity-Check

LC Chromatogram

LC#6 : AD2

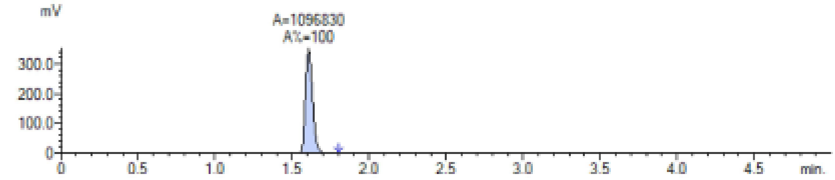

MS Chromatogram

Group#1 Scan(+) EI : TIC

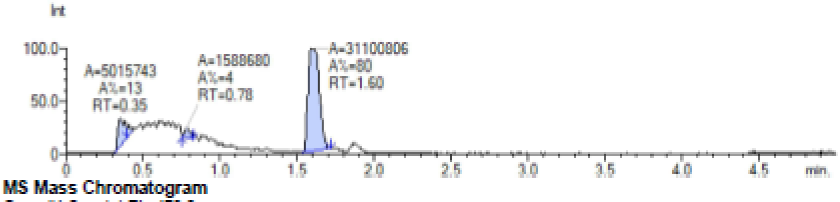

MS Mass Chromatogram

Group#1 Scan(+) EI : 479.2

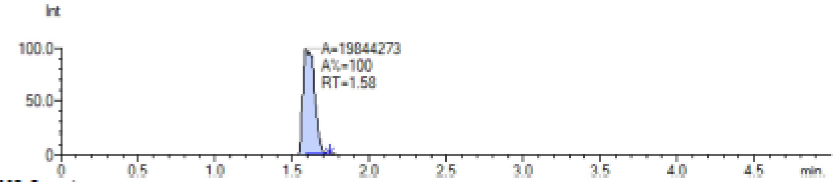

MS Spectrum

Group#1 - PDA Peak: 19, RT: 1.42 to 1.61 min

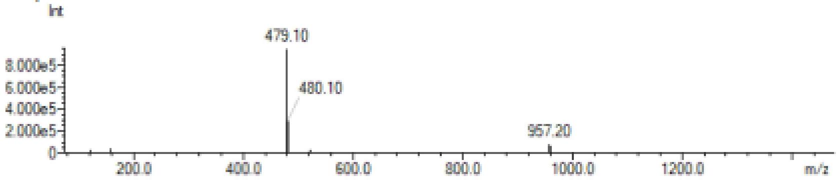

SM-I-opened-1

Shimadzu Open Solution

Project: Test
Experiment: 572_190612_Zinken_0607_Bischof_0612Griga
Experiment Description:
Sample: 410435:01:01
Sample Description: C24H26N2O2
Data File Name: C:\LabSolutions\Data\572_190612_Zinken_190607_Bischoff_190612_Grigalunas\1046364516_11190613572.lcd
Sample Location: Plate Number: 1 - Position: 136
Run By: admin
Run Started: 29 July 2019 19:27:00
Run Finished: 30 July 2019 04:41:49
Method: 190321_Purity-Check

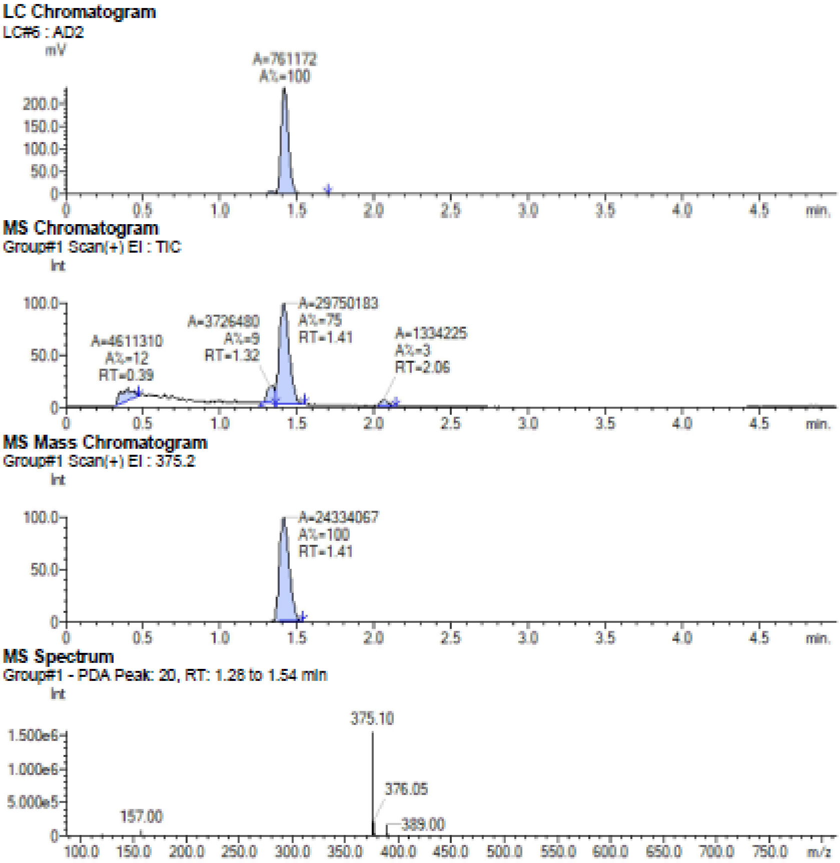

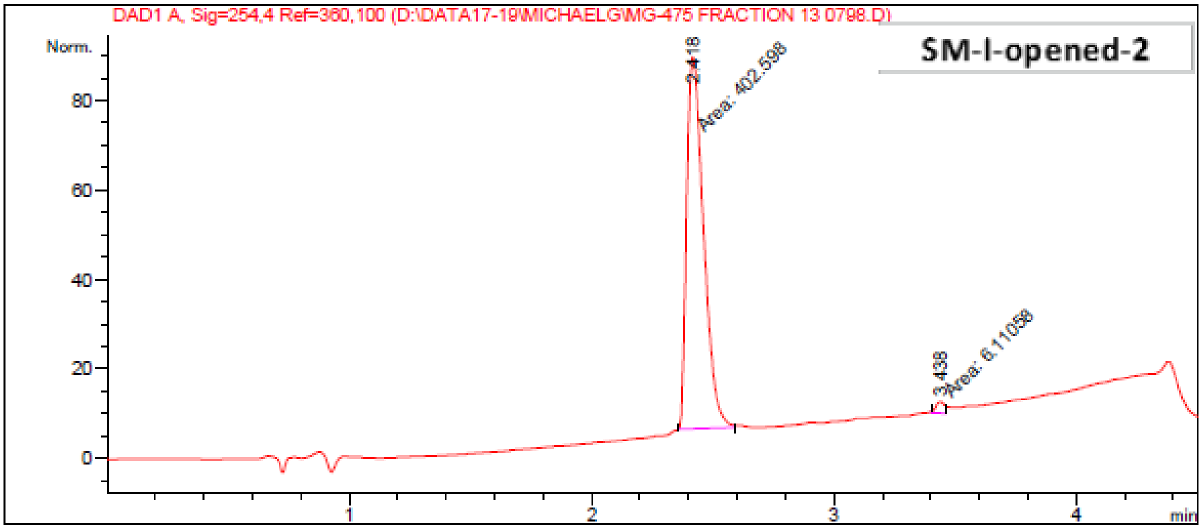

#	Time	Area	Height	Width	Area%	Symmetry
1	2.418	402.6	83.2	0.0806	98.505	0.532
2	3.438	6.1	2.5	0.0401	1.495	0.912

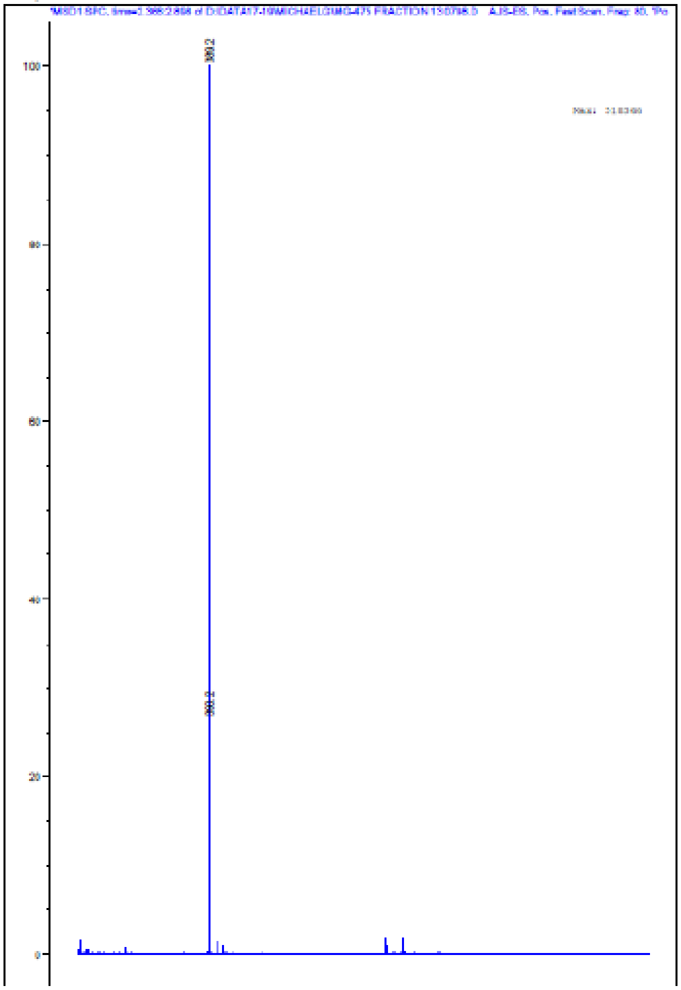

SM-I-opened-3

Shimadzu Open Solution

Project: Test
Experiment: 572_190612_Zinken_0607_Bischof_0612Griga
Experiment Description:
Sample: 410437:01:01
Sample Description: C25H28N2O3
Data File Name: C:\LabSolutions\Data\572_190612_Zinken_190607_Bischoff_190612_Grigalunas\1046363705_11190613572.lcd
Sample Location: Plate Number: 1 - Position: 232
Run By: admin
Run Started: 29 July 2019 19:27:00
Run Finished: 30 July 2019 04:41:49
Method: 190321_Purity-Check

LC Chromatogram
LC#6 : AD2

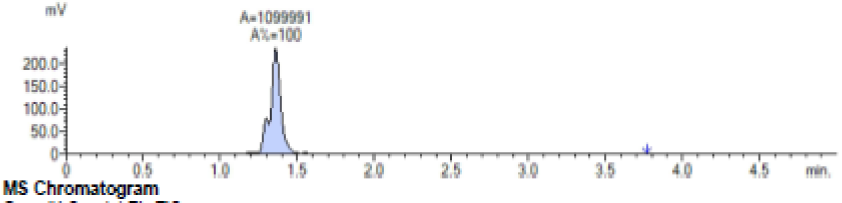

MS Chromatogram
Group#1 Scan(+) EI : TIC

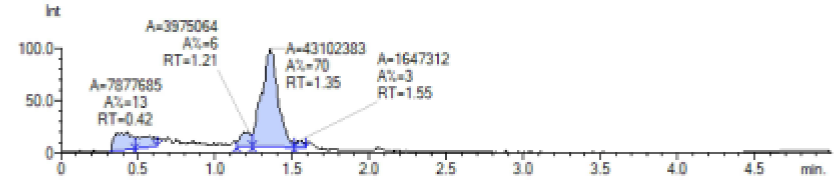

MS Mass Chromatogram
Group#1 Scan(+) EI : 405.2

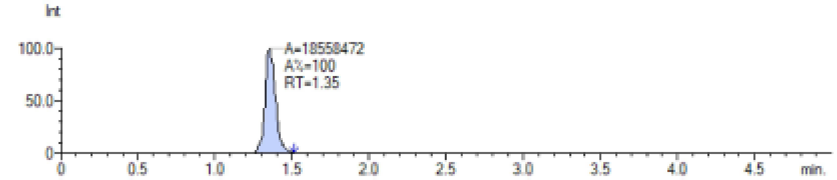

MS Spectrum
Group#1 - PDA Peak: 26, RT: 1.23 to 1.63 min

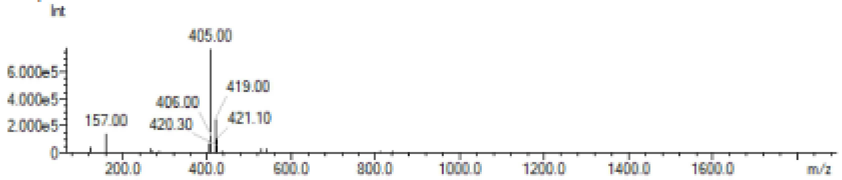

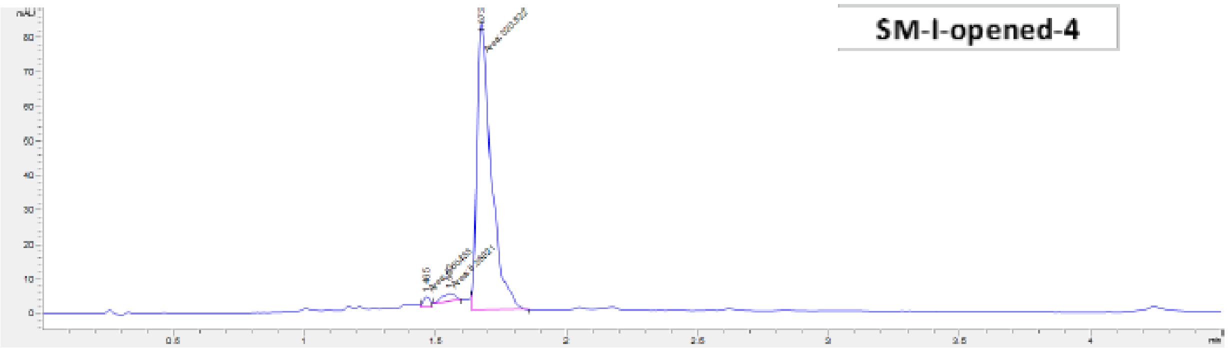

#	Time	Area	Height	Width	Area%	Symmetry
1	1.465	5.6	3.2	0.0296	1.674	0.889
2	1.552	8.4	2.4	0.0589	2.497	1.236
3	1.673	320.8	83.4	0.0641	95.829	0.433

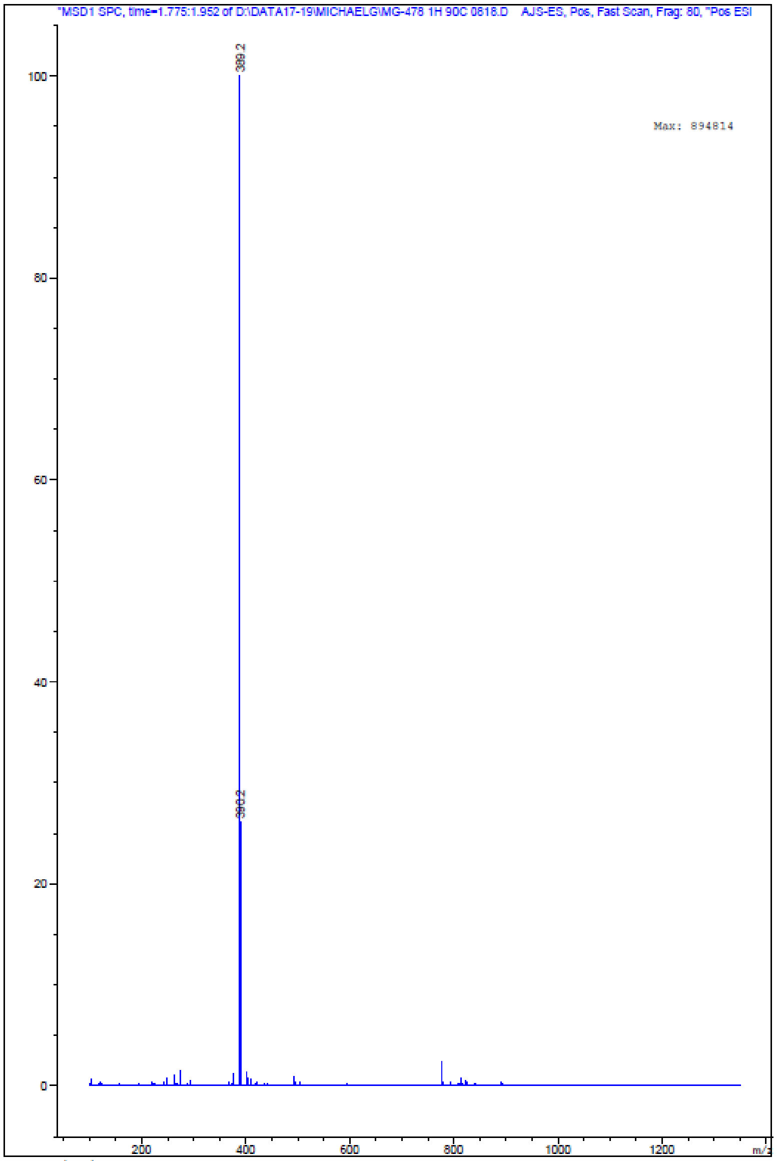

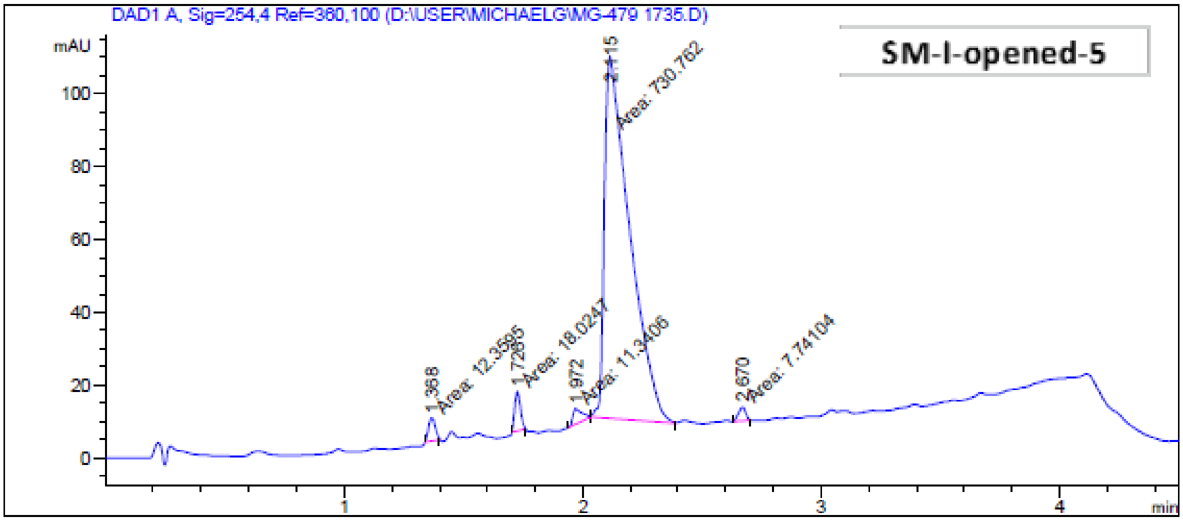

#	Time	Area	Height	Width	Area%	Symmetry
1	1.368	12.4	6.4	0.0323	1.584	0.911
2	1.726	18	10.8	0.0277	2.310	0.769
3	1.972	11.3	4.3	0.0444	1.453	0.518
4	2.115	730.8	99.4	0.1225	93.660	0.276
5	2.67	7.7	3.7	0.0345	0.992	1.186

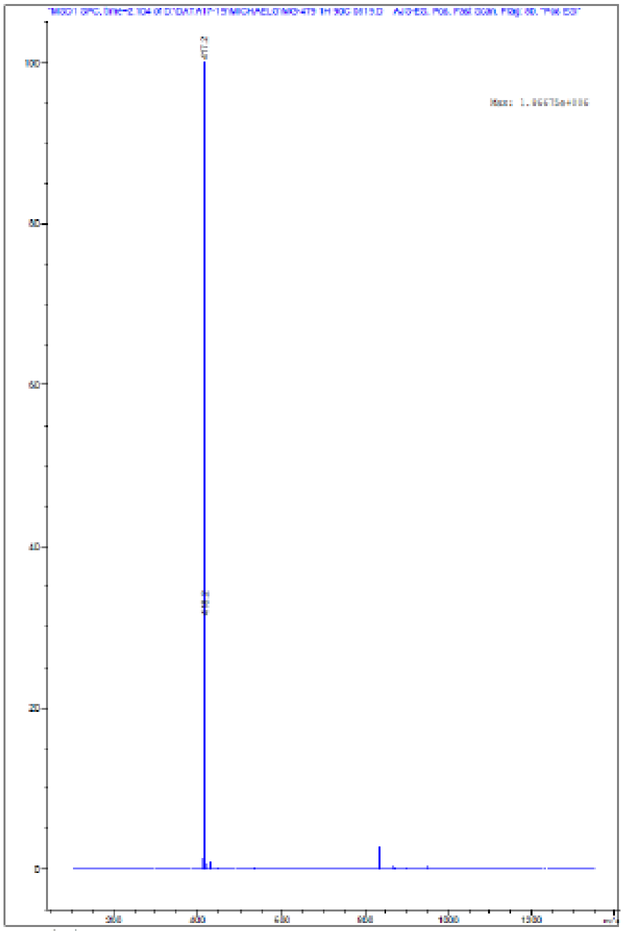

SM-I-opened-6

Shimadzu Open Solution

Project: Test
Experiment: 572_190612_Zinken_0607_Bischof_0612Griga
Experiment Description:
Sample: 410440:01:01
Sample Description: C24H25ClN2O2
Data File Name: C:\LabSolutions\Data\572_190612_Zinken_190607_Bischoff_190612_Grigalunas\1046364493_11190613572.lcd
Sample Location: Plate Number: 1 - Position: 376
Run By: admin
Run Started: 29 July 2019 19:27:00
Run Finished: 30 July 2019 04:41:49
Method: 190321_Purity-Check

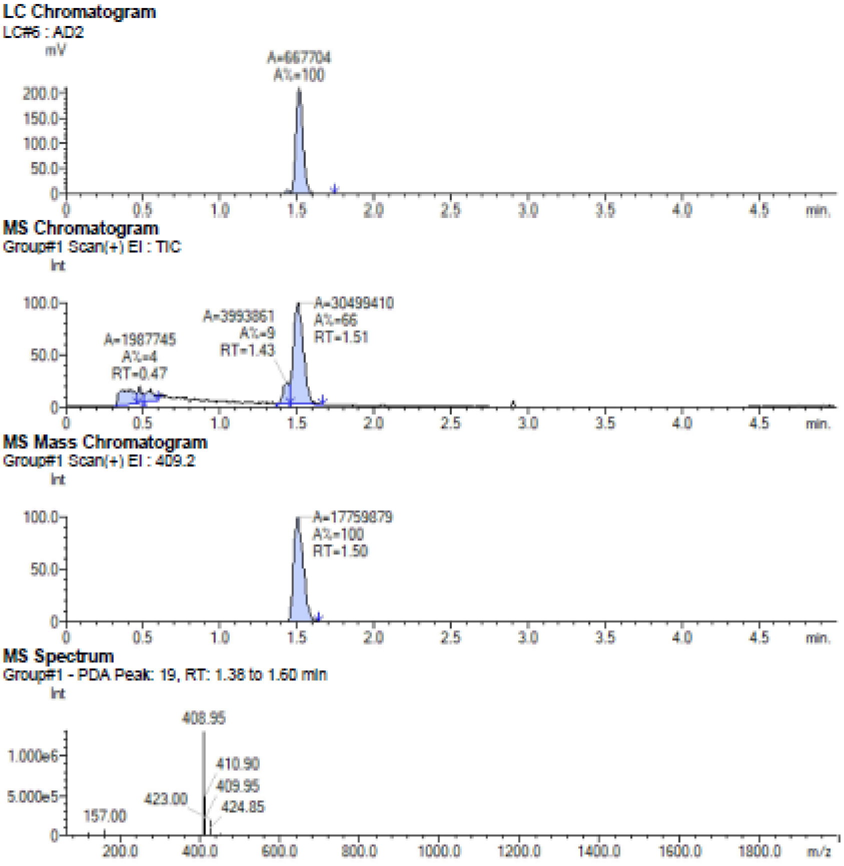

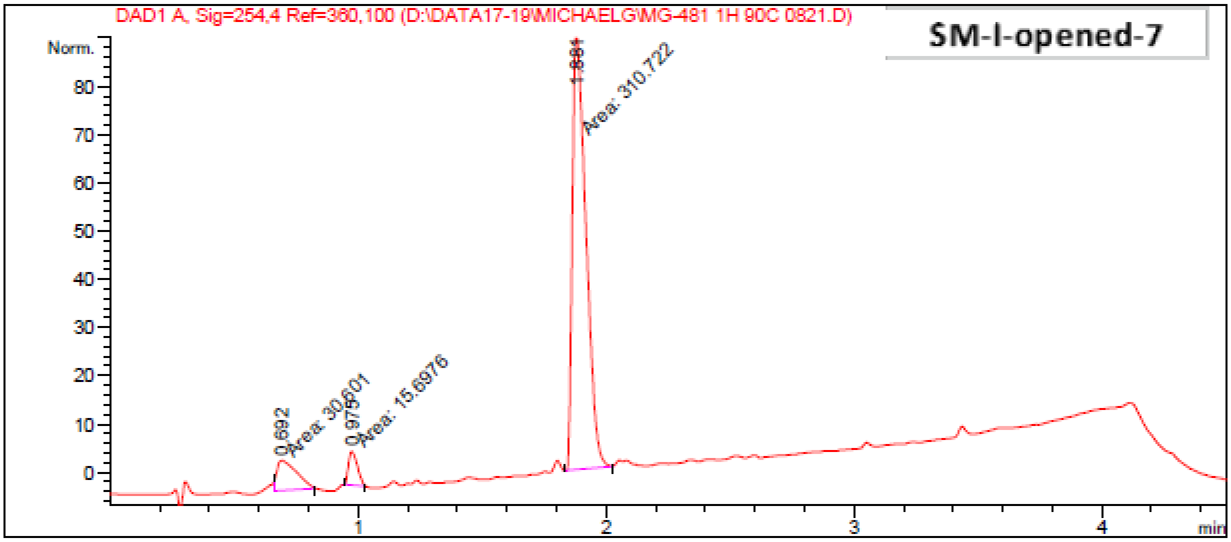

#	Time	Area	Height	Width	Area%	Symmetry
1	0.692	30.6	5.6	0.0911	8.571	0.315
2	0.975	15.7	6.4	0.0412	4.397	0.574
3	1.881	310.7	81.3	0.0637	87.032	0.428

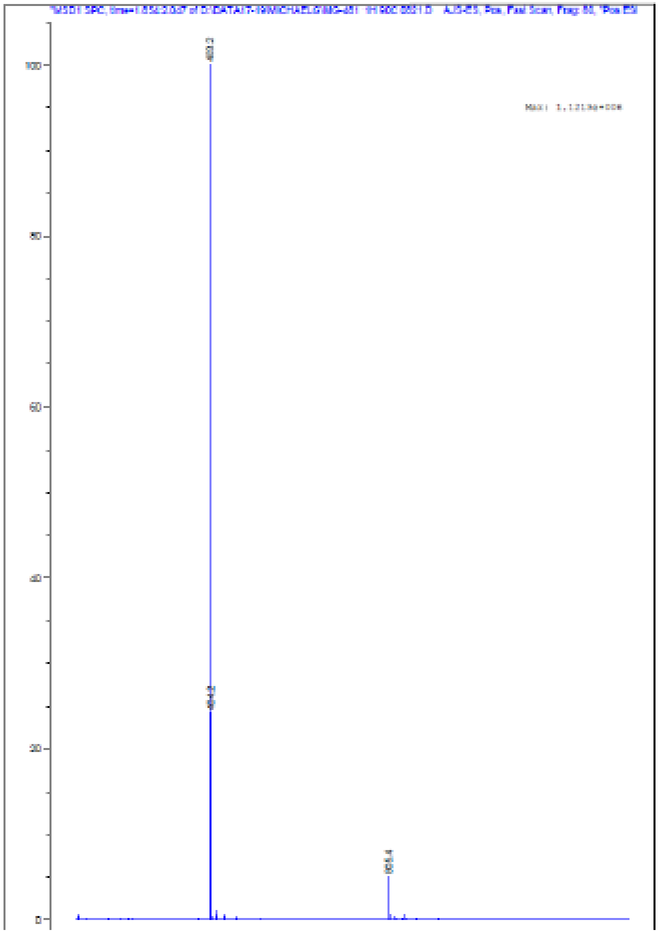

SM-I-opened-8

Shimadzu Open Solution

Project:	Test
Experiment:	572_190612_Zinken_0607_Bischof_0612Griga
Experiment Description:	
Sample:	410420:01:01
Sample Description:	C31H32N2O2
Data File Name:	C:\LabSolutions\Data\572_190612_Zinken_190607_Bischoff_190612_Grigalunas\1046364461_11190613572.lcd
Sample Location:	Plate Number: 1 - Position: 313
Run By:	admin
Run Started:	29 July 2019 19:27:00
Run Finished:	30 July 2019 04:41:49
Method:	190321_Purity-Check

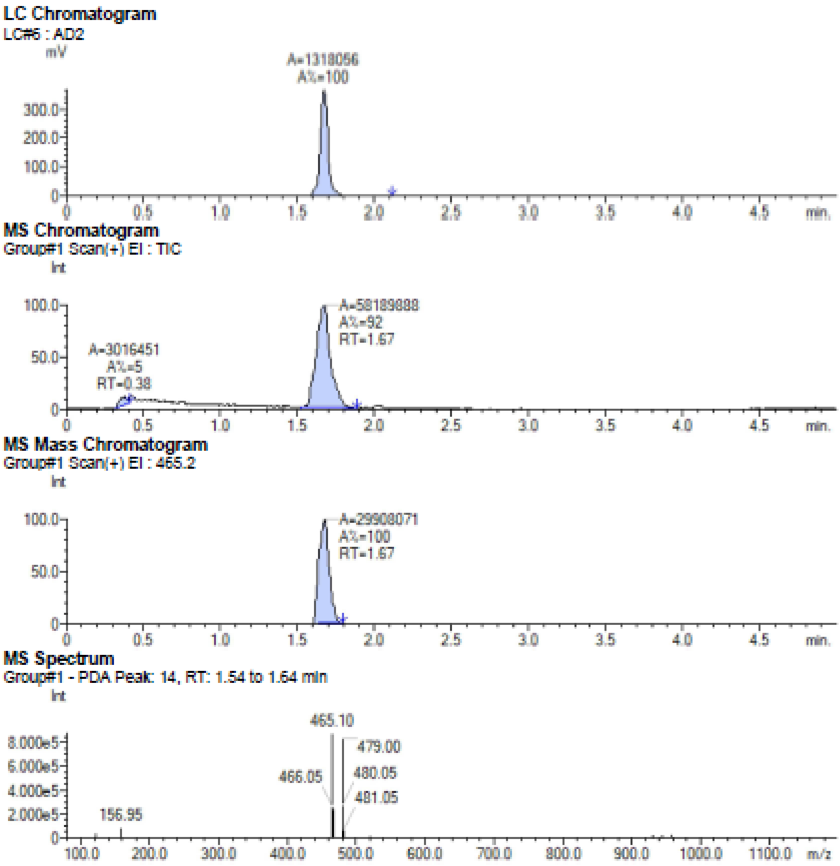

SM-I-opened-9

Shimadzu Open Solution

Project: Test
Experiment: 572_190612_Zinken_0607_Bischof_0612Griga
Experiment Description:
Sample: 410421:01:01
Sample Description: C24H24Cl2N2O2
Data File Name: C:\LabSolutions\Data\572_190612_Zinken_190607_Bischoff_190612_Grigalunas\1046364799_11190613572.lcd
Sample Location: Plate Number: 1 - Position: 361
Run By: admin
Run Started: 29 July 2019 19:27:00
Run Finished: 30 July 2019 04:41:49
Method: 190321_Purity-Check

LC Chromatogram
LC#6 : AD2

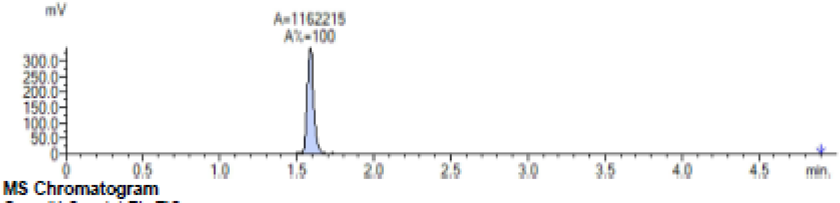

MS Chromatogram
Group#1 Scan(+) EI : TIC

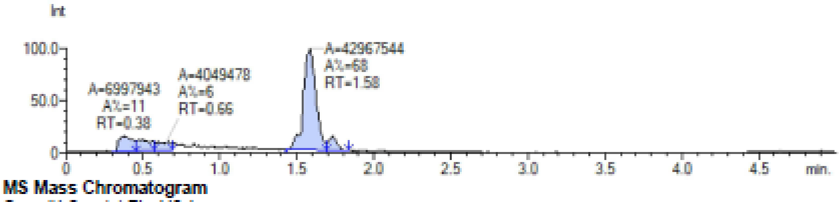

MS Mass Chromatogram
Group#1 Scan(+) EI : 443.1

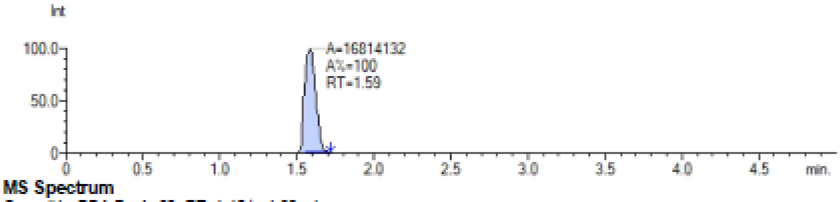

MS Spectrum
Group#1 - PDA Peak: 23, RT: 1.45 to 1.63 min

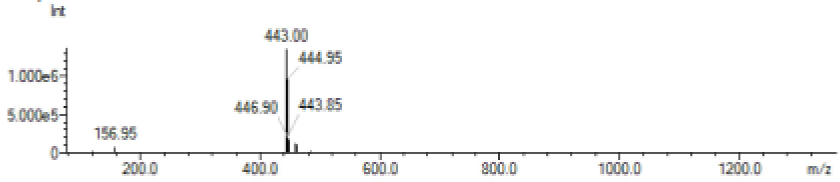

SM-I-opened-10

Shimadzu Open Solution

Project: Test
Experiment: 572_190612_Zinken_0607_Bischof_0612Griga
Experiment Description:
Sample: 410422:01:01
Sample Description: C30H30N2O3
Data File Name: C:\LabSolutions\Data\572_190612_Zinken_190607_Bischoff_190612_Grigalunas\1046363907_11190613572.lcd
Sample Location: Plate Number: 1 - Position: 27
Run By: admin
Run Started: 29 July 2019 19:27:00
Run Finished: 30 July 2019 04:41:49
Method: 190321_Purity-Check

LC Chromatogram
LC#6 : AD2

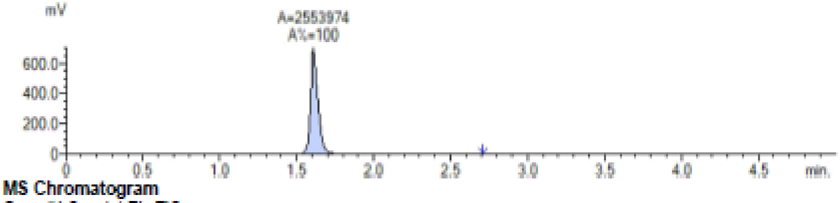

MS Chromatogram
Group#1 Scan(+) EI : TIC

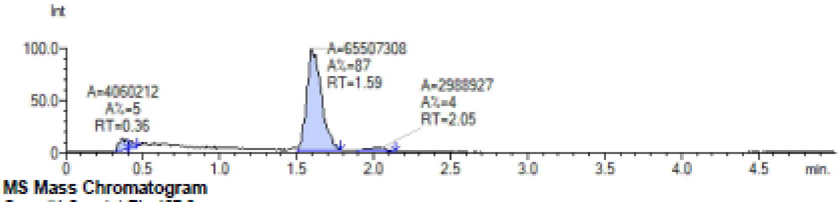

MS Mass Chromatogram
Group#1 Scan(+) EI : 467.2

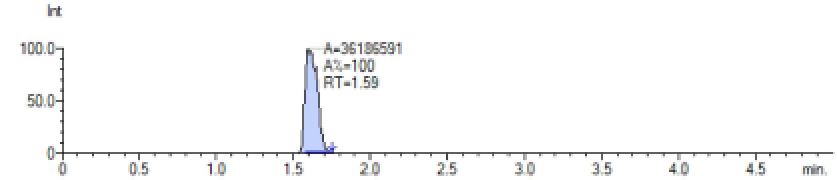

MS Spectrum
Group#1 - PDA Peak: 18, RT: 1.41 to 1.59 min

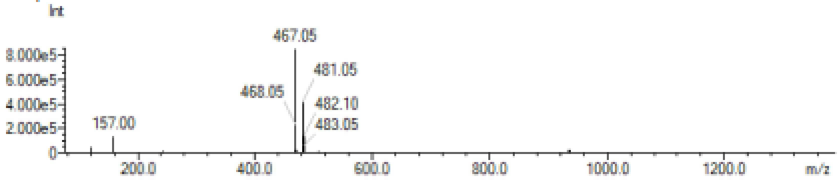

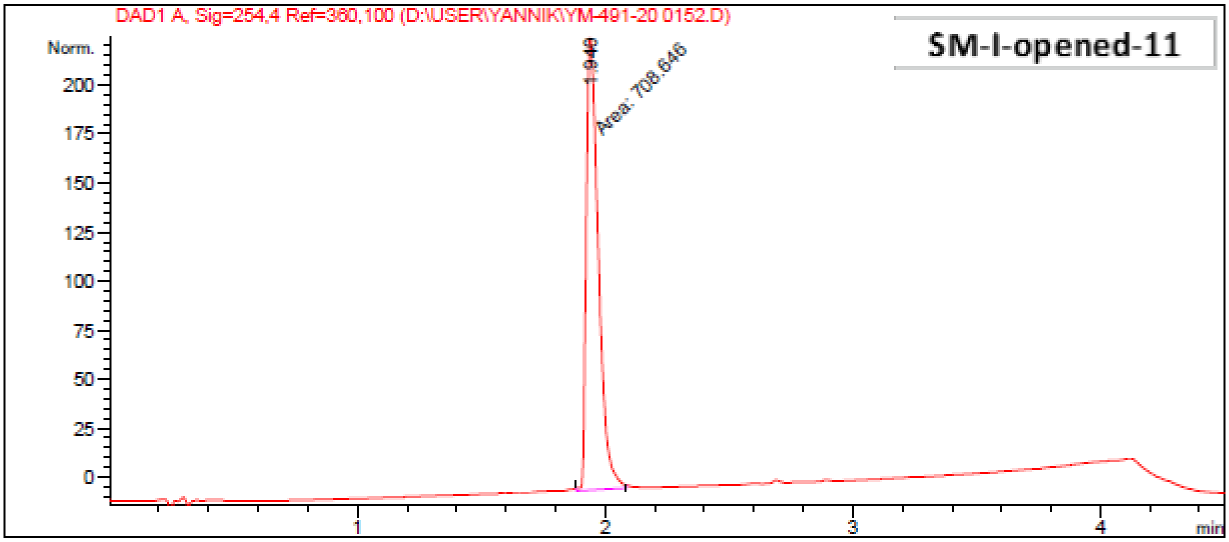

#	Time	Area	Height	Width	Area%	Symmetry
1	1.94	708.6	209.8	0.0563	100.000	0.448

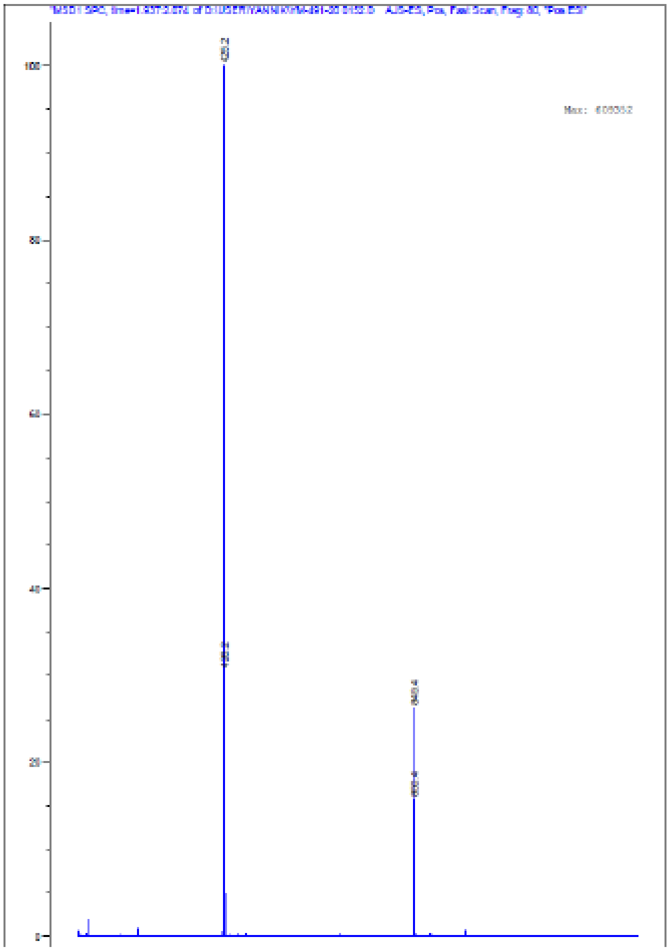

SM-I-opened-12

Shimadzu Open Solution

Project: Test
Experiment: 572_190612_Zinken_0607_Bischof_0612Griga
Experiment Description:
Sample: 410424:01:01
Sample Description: C28H28N2O2
Data File Name: C:\LabSolutions\Data\572_190612_Zinken_190607_Bischoff_190612_Grigalunas\1046364089_11190613572.lcd
Sample Location: Plate Number: 1 - Position: 123
Run By: admin
Run Started: 29 July 2019 19:27:00
Run Finished: 30 July 2019 04:41:49
Method: 190321_Purity-Check

LC Chromatogram
LC#6 : AD2

MS Chromatogram
Group#1 Scan(+) EI : TIC

MS Mass Chromatogram
Group#1 Scan(+) EI : 425.2

MS Spectrum
Group#1 - PDA Peak: 21, RT: 1.41 to 1.71 min

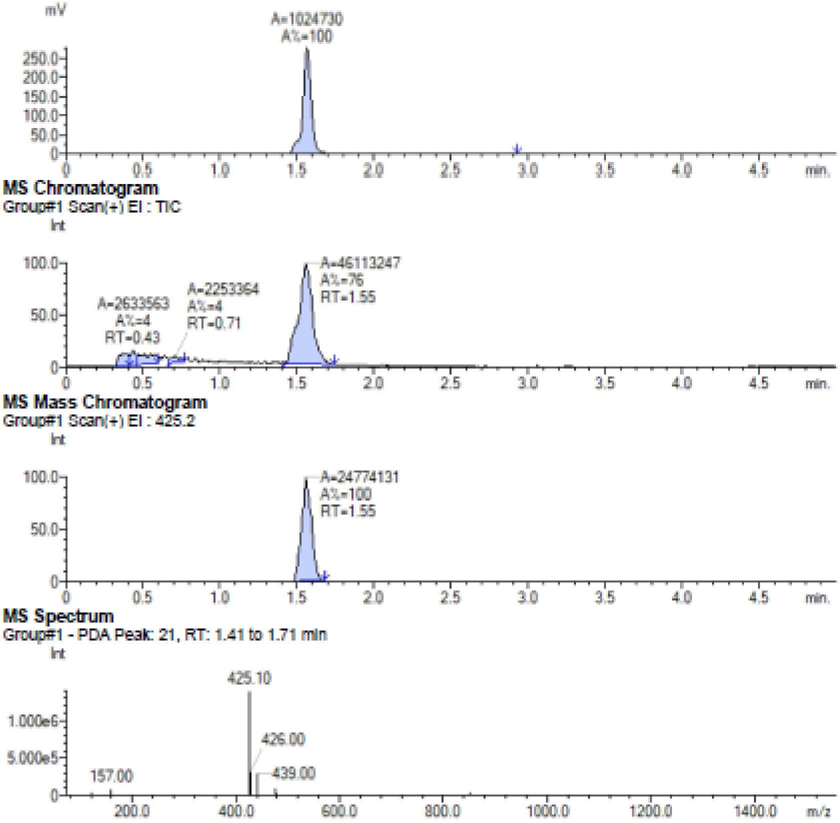

SM-I-opened-13

Shimadzu Open Solution

Project: Test
Experiment: 572_190612_Zinken_0607_Bischof_0612Griga
Experiment Description:
Sample: 410425:01:01
Sample Description: C27H32N2O3
Data File Name: C:\LabSolutions\Data\572_190612_Zinken_190607_Bischoff_190612_Grigalunas\1046364528_11190613572.lcd
Sample Location: Plate Number: 1 - Position: 171
Run By: admin
Run Started: 29 July 2019 19:27:00
Run Finished: 30 July 2019 04:41:49
Method: 190321_Purity-Check

LC Chromatogram
LC#5 : AD2

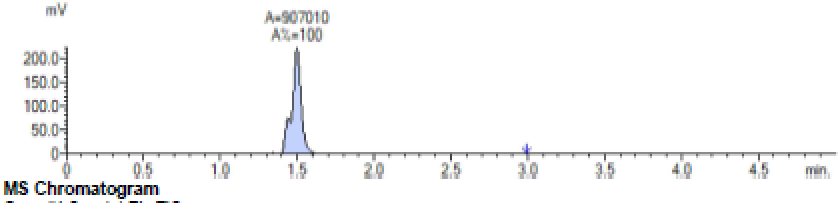

MS Chromatogram
Group#1 Scan(+) EI : TIC

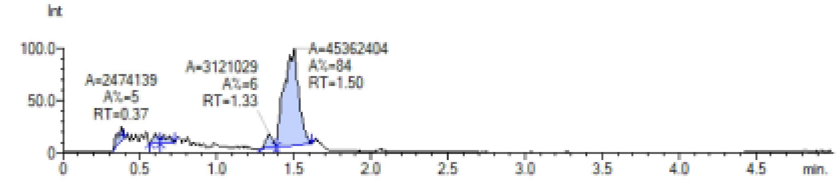

MS Mass Chromatogram
Group#1 Scan(+) EI : 433.2

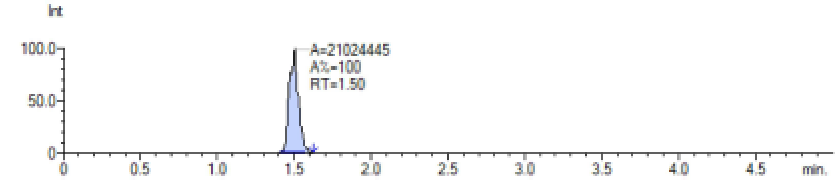

MS Spectrum
Group#1 - PDA Peak: 25, RT: 1.37 to 1.66 min

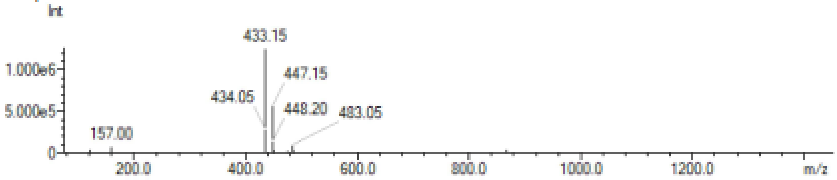

GF-I-1-1

Shimadzu Open Solution

Project: Test
Experiment: 430_0530_Grigalu_0605_3101_TEAD_Wu
Experiment Description:
Sample: 406582:01:02
Sample Description: C22H20ClNO4
Data File Name: C:\LabSolutions\Data\430_180530_Grigalunas_180605_3101_180605_Tead_180605_Wu\1046325015_11180530431.lcd
Sample Location: Plate Number: 2 - Position: 347
Run By: admin
Run Started: 24 September 2018 14:46:57
Run Finished: 25 September 2018 00:19:16
Method: Purity_Check_2018

LC Chromatogram
LC#6 : AD2

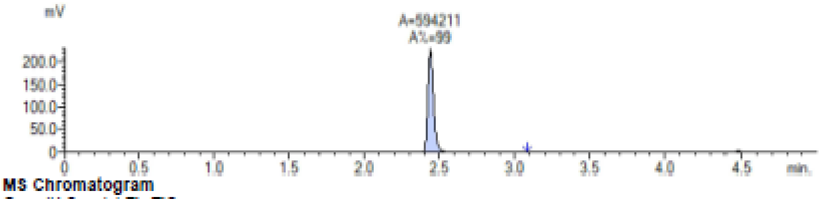

MS Chromatogram
Group#1 Scan(+) EI : TIC

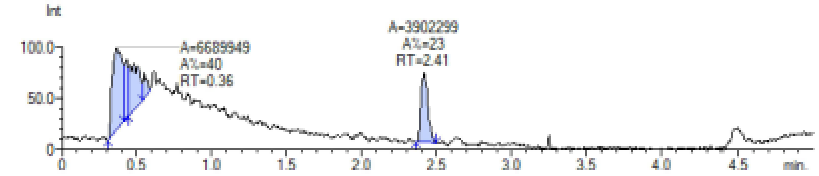

MS Mass Chromatogram
Group#1 Scan(+) EI : 398.1

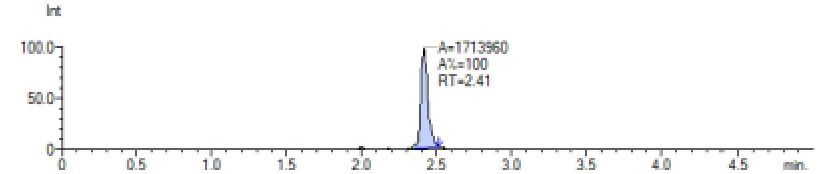

MS Spectrum
Group#1 - PDA Peak: 19, RT: 2.30 to 2.51 min

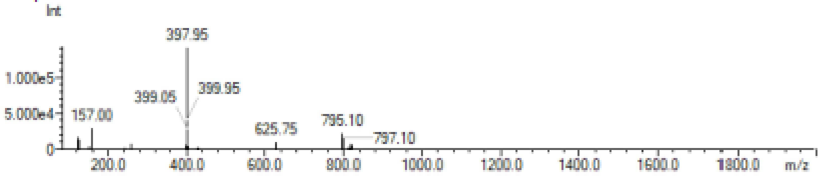

GF-I-2-1

Shimadzu Open Solution

**Project:**	Test
**Experiment:**	430_0530_Grigalu_0605_3101_TEAD_Wu
**Experiment Description:**	
**Sample:**	408583:01:02
**Sample Description:**	C22H20ClNO4
**Data File Name:**	C:\LabSolutions\Data\430_180530_Grigalunas_180605_3101_180605_Tead_180605_Wu\1046325130_11180530431.lcd
**Sample Location:**	Plate Number: 2 - Position: 13
**Run By:**	admin
**Run Started:**	24 September 2018 14:46:57
**Run Finished:**	25 September 2018 00:19:16
**Method:**	Purity_Check_2018

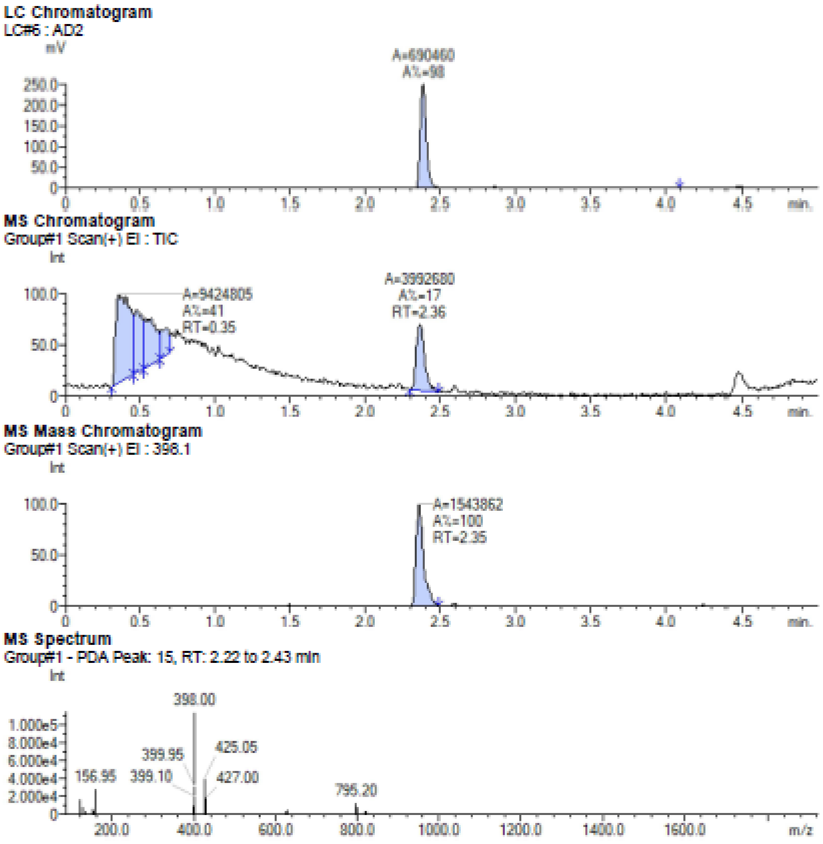

GF-I-1-2

Shimadzu Open Solution

Project: Test
Experiment: 430_0530_Grigalu_0605_3101_TEAD_Wu
Experiment Description:
Sample: 408590:01:01
Sample Description: C23H22ClNO4
Data File Name: C:\LabSolutions\Data\430_180530_Grigalunas_180605_3101_180605_Tead_180605_Wu\1046325018_11180530431.lcd
Sample Location: Plate Number: 2 - Position: 61
Run By: admin
Run Started: 24 September 2018 14:46:57
Run Finished: 25 September 2018 00:19:16
Method: Purity_Check_2018

LC Chromatogram
LC#6 : AD2

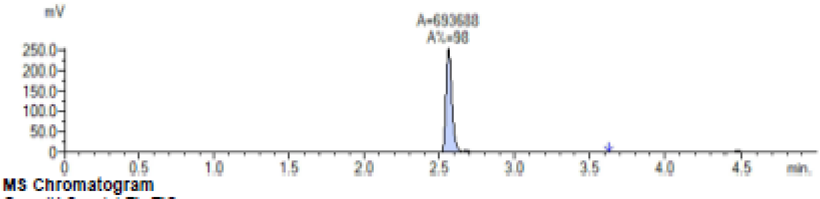

MS Chromatogram
Group#1 Scan(+) EI : TIC

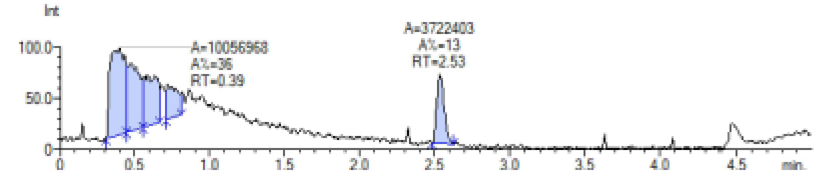

MS Mass Chromatogram
Group#1 Scan(+) EI : 412.1

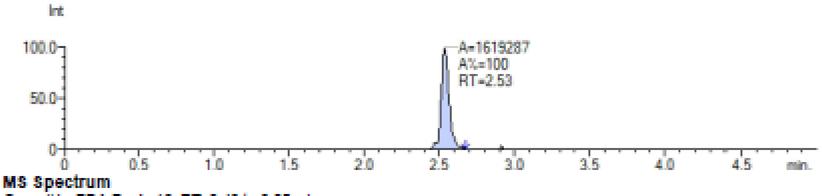

MS Spectrum
Group#1 - PDA Peak: 16, RT: 2.42 to 2.65 min

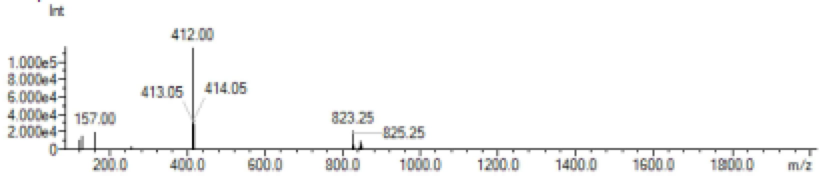

GF-I-2-2

Shimadzu Open Solution

Project: Test
Experiment: 430_0530_Grigalu_0605_3101_TEAD_Wu
Experiment Description:
Sample: 408591:01:01
Sample Description: C23H22ClNO4
Data File Name: C:\LabSolutions\Data\430_180530_Grigalunas_180605_3101_180605_Tead_180605_Wu\1046325412_11180530431.lcd
Sample Location: Plate Number: 2 - Position: 109
Run By: admin
Run Started: 24 September 2018 14:46:57
Run Finished: 25 September 2018 00:19:16
Method: Purity_Check_2018

LC Chromatogram
LC#6 : AD2

MS Chromatogram
Group#1 Scan(+) EI : TIC

MS Mass Chromatogram
Group#1 Scan(+) EI : 411.1

MS Spectrum
Group#1 - PDA Peak: 15, RT: 2.34 to 2.44 min

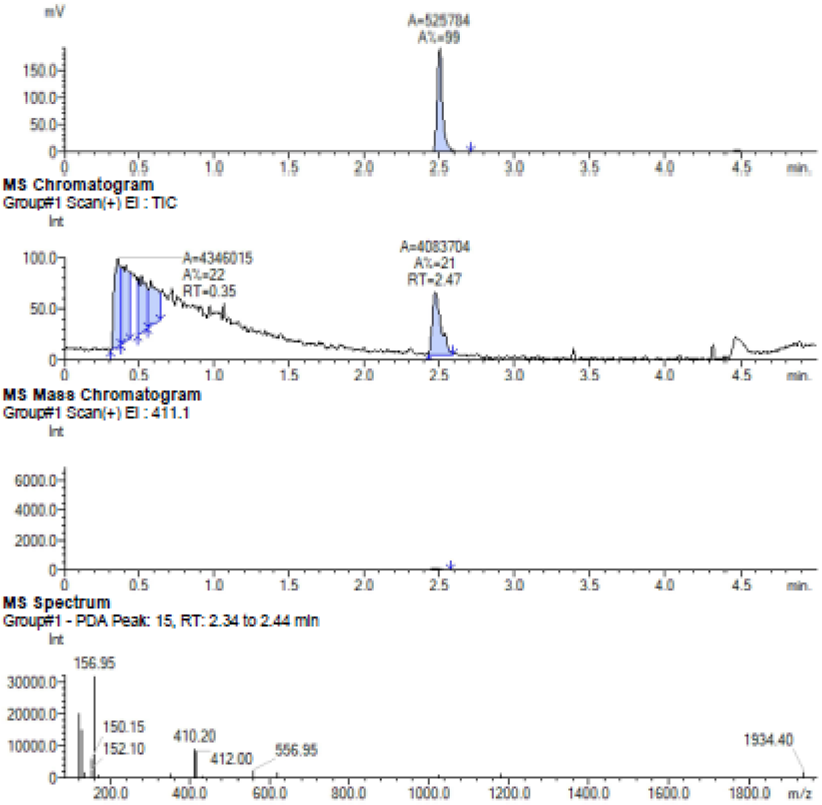

GF-I-1-3

Shimadzu Open Solution

Project: Test
Experiment: 430_0530_Grigalu_0605_3101_TEAD_Wu
Experiment Description:
Sample: 408595:01:01
Sample Description: C25H26ClNO4
Data File Name: C:\LabSolutions\Data\430_180530_Grigalunas_180605_3101_180605_Tead_180605_Wu\1046325542_11180530431.lcd
Sample Location: Plate Number: 2 - Position: 253
Run By: admin
Run Started: 24 September 2018 14:46:57
Run Finished: 25 September 2018 00:19:16
Method: Purity_Check_2018

LC Chromatogram
LC#6 : AD2

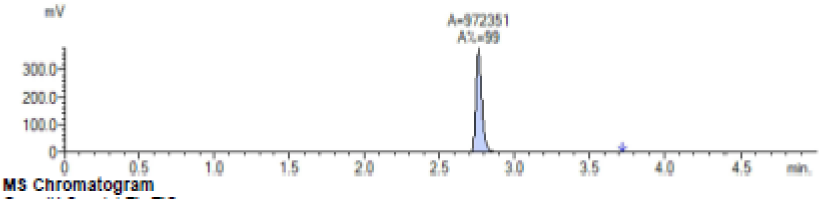

MS Chromatogram
Group#1 Scan(+) EI : TIC

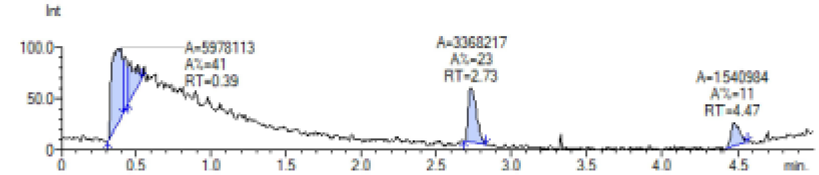

MS Mass Chromatogram
Group#1 Scan(+) EI : 440.2

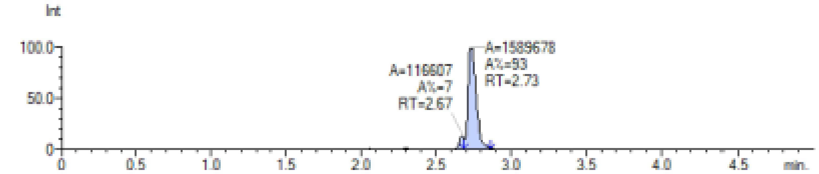

MS Spectrum
Group#1 - PDA Peak: 17, RT: 2.63 to 2.81 min

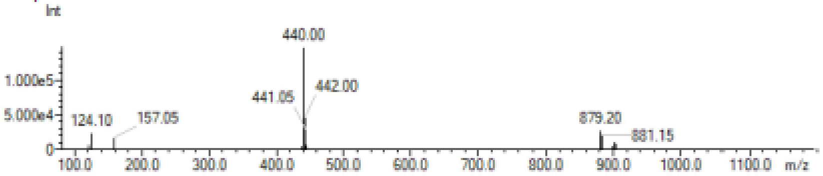

Shimadzu Open Solution

GF-I-2-3

Project: Test
Experiment: 430_0530_Grigalu_0605_3101_TEAD_Wu
Experiment Description:
Sample: 408596:01:01
Sample Description: C25H26ClNO4
Data File Name: C:\LabSolutions\Data\430_180530_Grigalunas_180605_3101_180605_Tead_180605_Wu\1046325438_11180530431.lcd
Sample Location: Plate Number: 2 - Position: 301
Run By: admin
Run Started: 24 September 2018 14:46:57
Run Finished: 25 September 2018 00:19:16
Method: Purity_Check_2018

LC Chromatogram
LC#6 : AD2

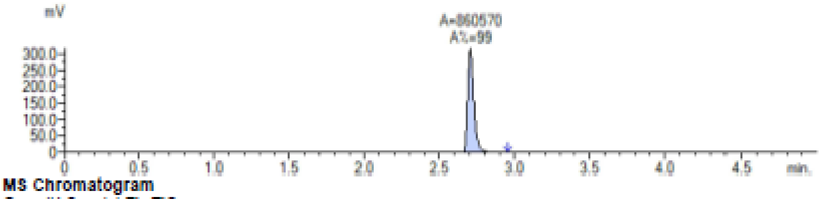

MS Chromatogram
Group#1 Scan(+) EI : TIC

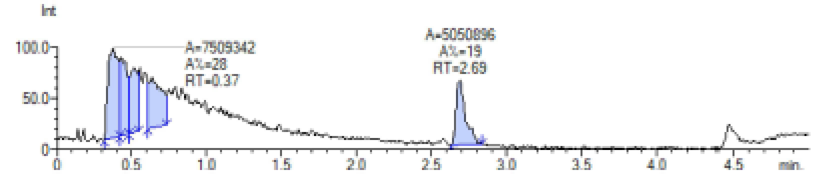

MS Mass Chromatogram
Group#1 Scan(+) EI : 440.2

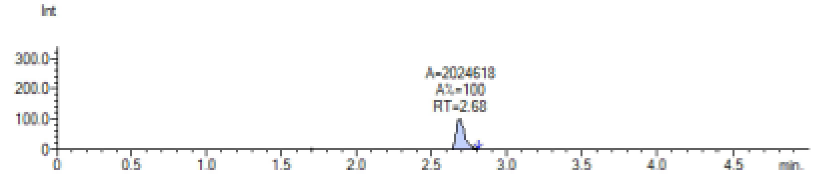

MS Spectrum
Group#1 - PDA Peak: 16, RT: 2.55 to 2.79 min

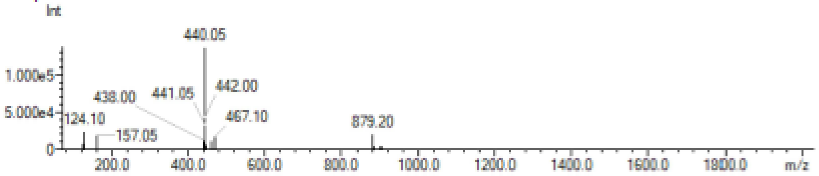

Shimadzu Open Solution

GF-I-1-4

Project: Test
Experiment: 430_0530_Grigalu_0605_3101_TEAD_Wu
Experiment Description:
Sample: 408592:01:01
Sample Description: C23H22ClNO5
Data File Name: C:\LabSolutions\Data\430_180530_Grigalunas_180605_3101_180605_Tead_180605_Wu\1046325585_11180530431.lcd
Sample Location: Plate Number: 2 - Position: 157
Run By: admin
Run Started: 24 September 2018 14:46:57
Run Finished: 25 September 2018 00:19:16
Method: Purity_Check_2018

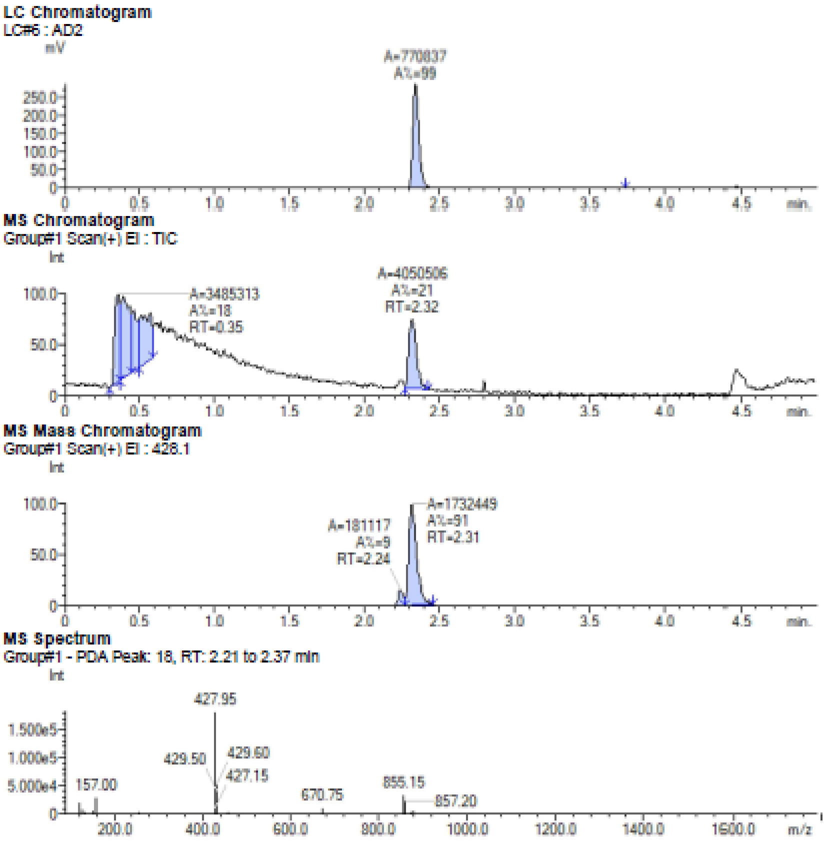

Shimadzu Open Solution

GF-I-1-5

**Project:**	Test
**Experiment:**	525_0206_Christo_Grigalu_Burhop_Wilke
**Experiment Description:**	
**Sample:**	409680:01:01
**Sample Description:**	C28H24ClNO5
**Data File Name:**	C:\LabSolutions\Data\525_190206_Christoforow_190206_Grigalunas_190206_Burhop_190206_Wilke\1046360506_11190207525.lcd
**Sample Location:**	Plate Number: 2 - Position: 2
**Run By:**	admin
**Run Started:**	23 April 2019 18:20:13
**Run Finished:**	24 April 2019 04:25:41
**Method:**	190321_Purity-Check+shut down

LC Chromatogram

LC#5 : AD2

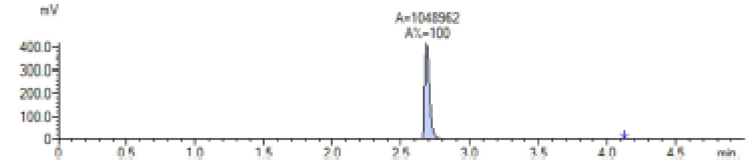

MS Chromatogram

Group#1 Scan(+) EI : TIC

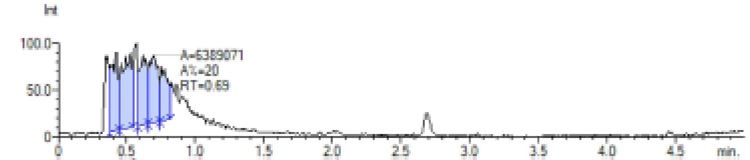

MS Mass Chromatogram

Group#1 Scan(+) EI : 490.1

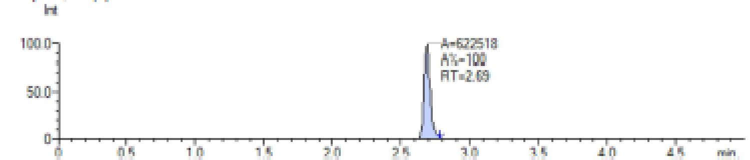

MS Spectrum

Group#1 - PDA Peak: 18, RT: 2.54 to 2.76 min

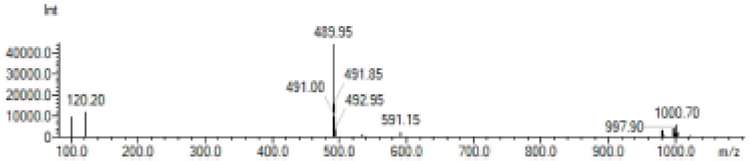

Shimadzu Open Solution

GF-I-2-4

Project: Test
Experiment: 525_0206_Christo_Grigalu_Burhop_Wilke
Experiment Description:
Sample: 409681:01:01
Sample Description: C28H24ClNO5
Data File Name: C:\LabSolutions\Data\525_190206_Christoforow_190206_Grigalunas_190206_Burhop_190206_Wilke\1046360695_11190207525.lcd
Sample Location: Plate Number: 2 - Position: 50
Run By: admin
Run Started: 23 April 2019 18:20:13
Run Finished: 24 April 2019 04:25:41
Method: 190321_Purity-Check+shut down

LC Chromatogram
LC#6 : AD2

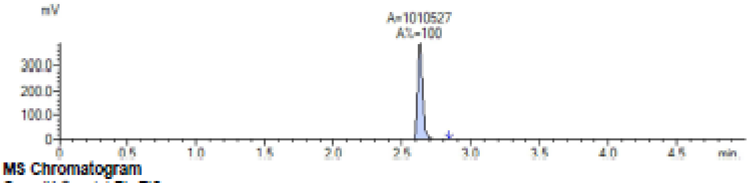

MS Chromatogram
Group#1 Scan(+) EI : TIC

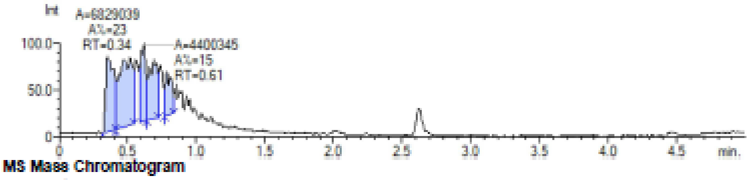

MS Mass Chromatogram
Group#1 Scan(+) EI : 490.1

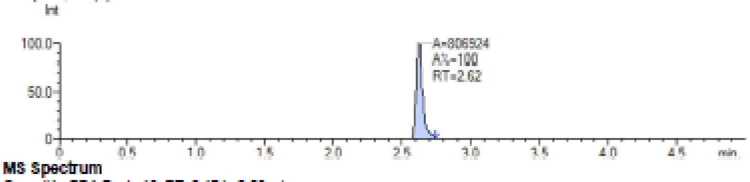

MS Spectrum
Group#1 - PDA Peak: 18, RT: 2.47 to 2.58 min

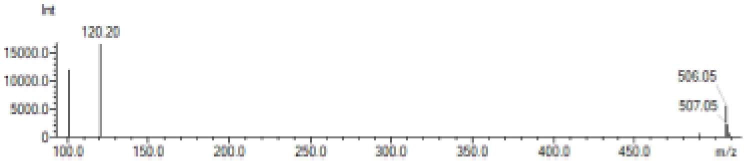

Shimadzu Open Solution

GF-I-1-6

**Project:**	Test
**Experiment:**	525_0206_Christo_Grigalu_Burhop_Wilke
**Experiment Description:**	
**Sample:**	409685:01:01
**Sample Description:**	C25H26ClNO5
**Data File Name:**	C:\LabSolutions\Data\525_190206_Christoforow_190206_Grigalunas_190206_Burhop_190206_Wilke\1046361005_11190207525.lcd
**Sample Location:**	Plate Number: 2 - Position: 242
**Run By:**	admin
**Run Started:**	23 April 2019 18:20:13
**Run Finished:**	24 April 2019 04:25:41
**Method:**	190321_Purity-Check+shut down

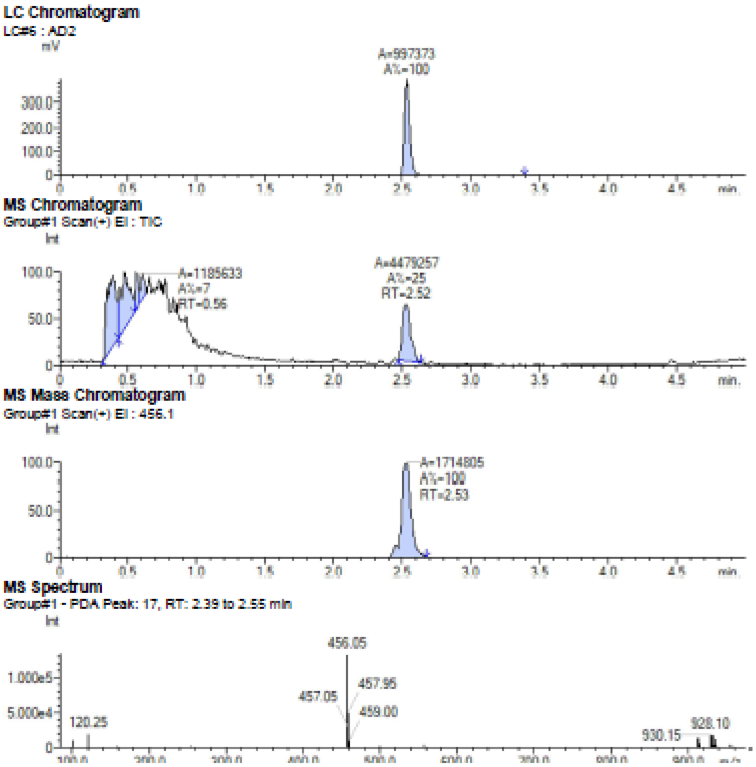

Shimadzu Open Solution

GF-I-2-5

**Project:**	Test
**Experiment:**	524_0205_Wess_06_Zink_07_Conc-10_08_Grig
**Experiment Description:**	
**Sample:**	409673:01:01
**Sample Description:**	C25H26ClNO5
**Data File Name:**	C:\LabSolutions\Data\524_190205_Wesserler_190602_Zinken_190207_Conc-10_190208_Grigalunas\1046360568_11190208524.lcd
**Sample Location:**	Plate Number: 1 - Position: 26
**Run By:**	admin
**Run Started:**	23 April 2019 15:21:47
**Run Finished:**	23 April 2019 18:20:12
**Method:**	190321_Purity-Check

LC Chromatogram

LC#6 : AD2

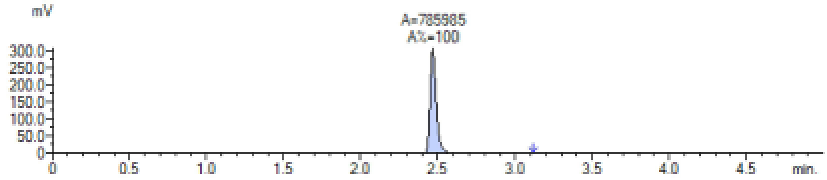

MS Chromatogram

Group#1 Scan(+) EI : TIC

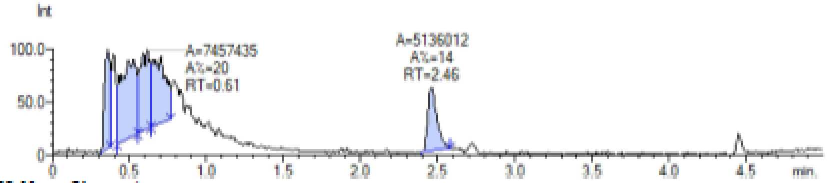

MS Mass Chromatogram

Group#1 Scan(+) EI : 456.1

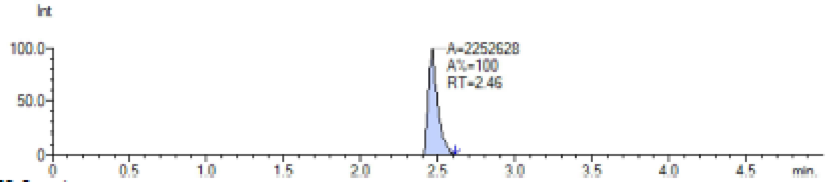

MS Spectrum

Group#1 - PDA Peak: 19, RT: 2.28 to 2.43 min

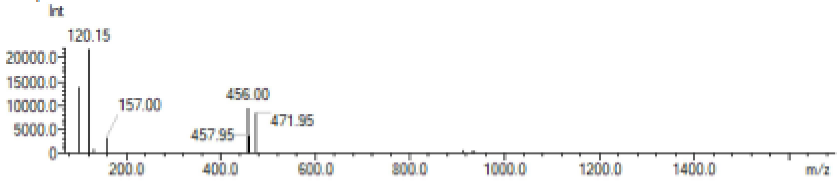

Shimadzu Open Solution

GF-I-1-7

**Project:**	Test
**Experiment:**	476_Grigalu_1011_Shaaban_Liu_1009_3076
**Experiment Description:**	
**Sample:**	409076:01:01
**Sample Description:**	C29H26ClNO5
**Data File Name:**	C:\LabSolutions\Data\476_Grigalunas_181011_Shaaban_181011_Liu_181009_3076 \1046359652_11181011476.lcd
**Sample Location:**	Plate Number: 2 - Position: 199
**Run By:**	admin
**Run Started:**	24 April 2019 18:14:10
**Run Finished:**	25 April 2019 04:58:04
**Method:**	190321_Purity-Check+shut down

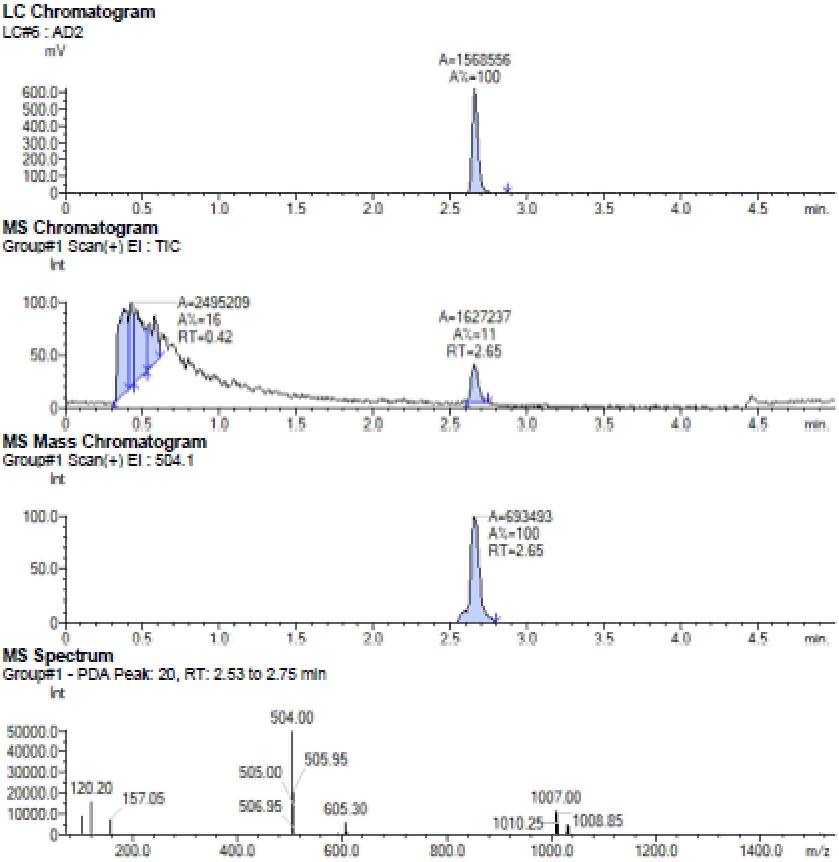

Shimadzu Open Solution

GF-I-2-6

**Project:**	Test
**Experiment:**	476_Grigalu_1011_Shaaban_Liu_1009_3076
**Experiment Description:**	
**Sample:**	409077:01:01
**Sample Description:**	C29H26ClNO5
**Data File Name:**	C:\LabSolutions\Data\476_Grigalunas_181011_Shaaban_181011_Liu_181009_3076 \1046359138_11181011476.lcd
**Sample Location:**	Plate Number: 2 - Position: 247
**Run By:**	admin
**Run Started:**	24 April 2019 18:14:10
**Run Finished:**	25 April 2019 04:58:04
**Method:**	190321_Purity-Check+shut down

LC Chromatogram

LC#6 : AD2

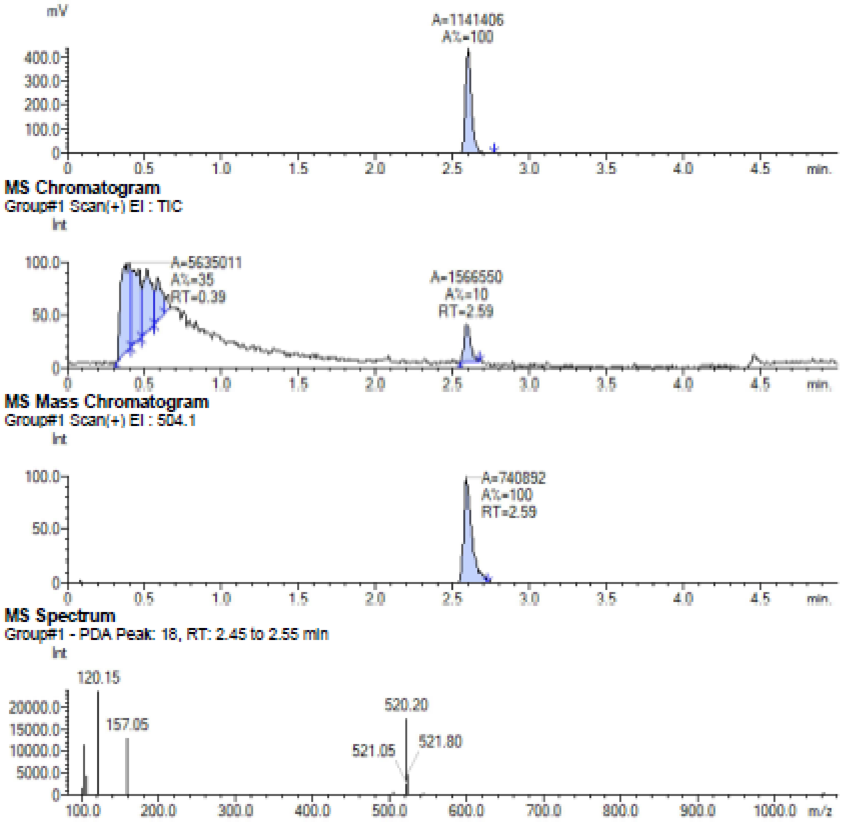

Shimadzu Open Solution

GF-I-1-8

Project:	Test
Experiment:	525_0206_Christo_Grigalu_Burhop_Wilke
Experiment Description:	
Sample:	409683:01:01
Sample Description:	C28H24ClNO4
Data File Name:	C:\LabSolutions\Data\525_190206_Christoforow_190206_Grigalunas_190206_Burhop_190206_Wilke\1046360601_11190207525.lcd
Sample Location:	Plate Number: 2 - Position: 146
Run By:	admin
Run Started:	23 April 2019 18:20:13
Run Finished:	24 April 2019 04:25:41
Method:	190321_Purity-Check+shut down

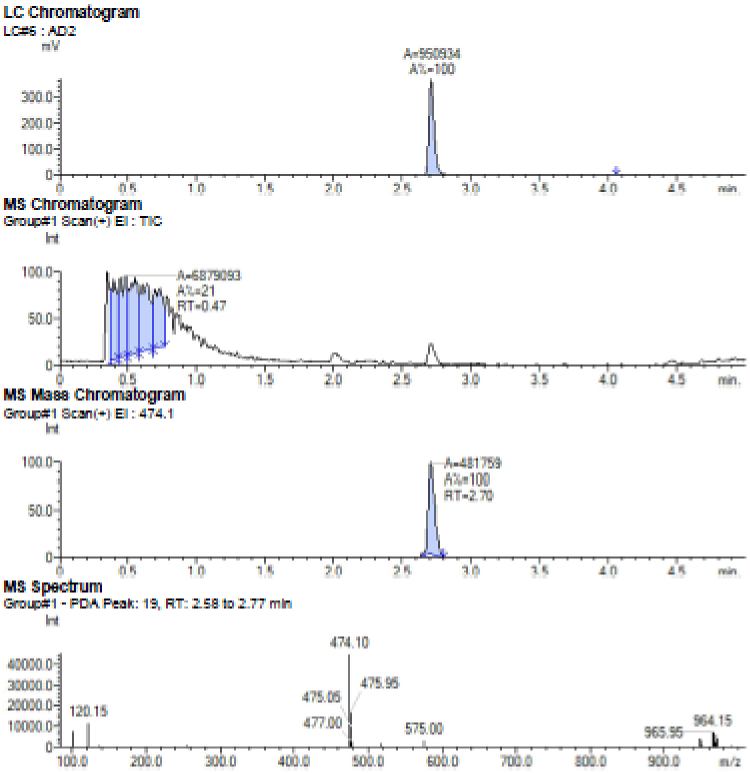

Shimadzu Open Solution

GF-I-2-7

Project:	Test
Experiment:	525_0206_Christo_Grigalu_Burhop_Wilke
Experiment Description:	
Sample:	409684:01:01
Sample Description:	C26H24ClNO4
Data File Name:	C:\LabSolutions\Data\525_190206_Christoforow_190206_Grigalunas_190206_Burhop_190206_Wilke\1046360756_11190207525.lcd
Sample Location:	Plate Number: 2 - Position: 194
Run By:	admin
Run Started:	23 April 2019 18:20:13
Run Finished:	24 April 2019 04:25:41
Method:	190321_Purity-Check+shut down

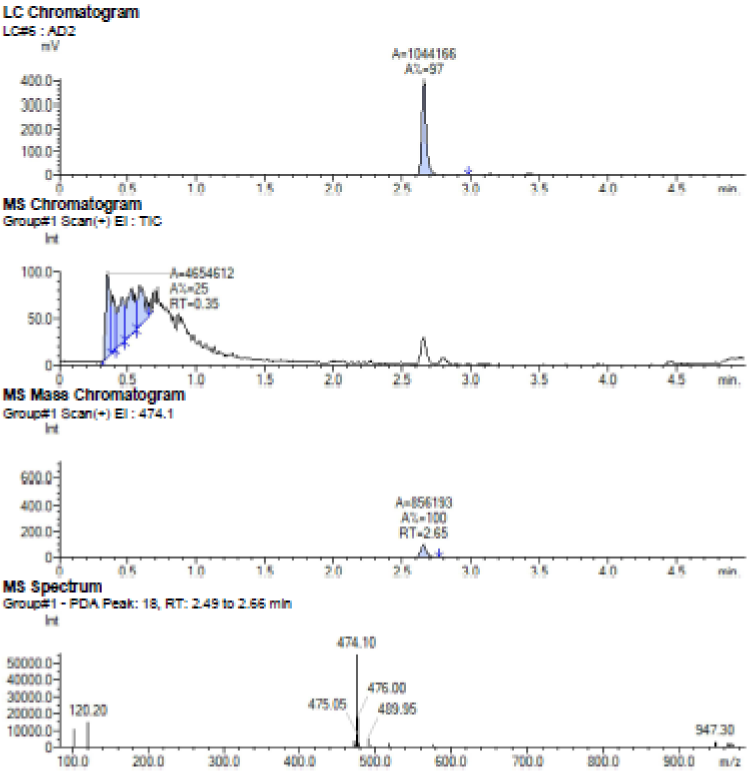

GF-I-1-9

Shimadzu Open Solution

**Project:**	Test
**Experiment:**	525_0206_Christo_Grigalu_Burhop_Wilke
**Experiment Description:**	
**Sample:**	409697:01:01
**Sample Description:**	C22H19ClINO4
**Data File Name:**	C:\LabSolutions\Data\525_190206_Christoforow_190206_Grigalunas_190206_Burhop_190206_Wilke\1046360618_11190207525.lcd
**Sample Location:**	Plate Number: 2 - Position: 102
**Run By:**	admin
**Run Started:**	23 April 2019 18:20:13
**Run Finished:**	24 April 2019 04:25:41
**Method:**	190321_Purity-Check+shut down

LC Chromatogram

LC#6 : AD2

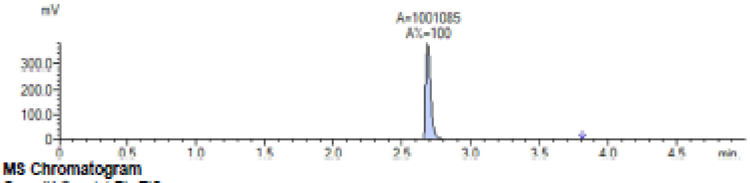

MS Chromatogram

Group#1 Scan(+) EI : TIC

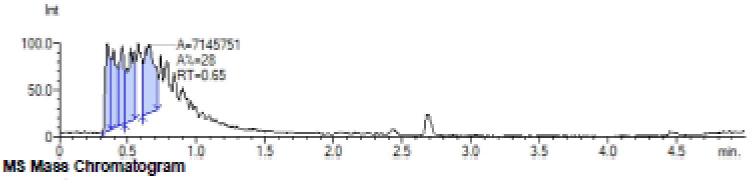

MS Mass Chromatogram

Group#1 Scan(+) EI : 524

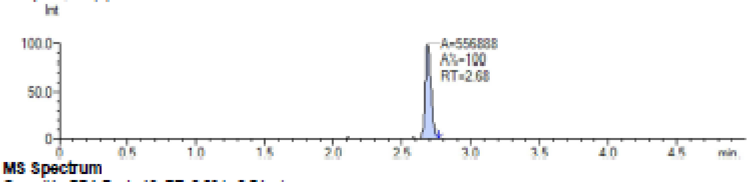

MS Spectrum

Group#1 - PDA Peak: 19, RT: 2.53 to 2.74 min

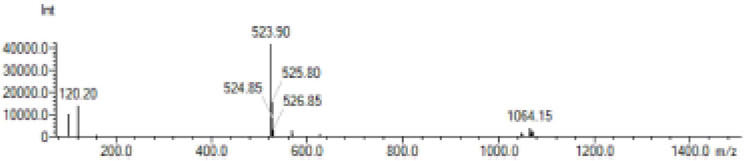

Shimadzu Open Solution

GF-I-1-10

**Project:**	Test
**Experiment:**	430_0530_Grigalu_0605_3101_TEAD_Wu
**Experiment Description:**	
**Sample:**	408579:01:02
**Sample Description:**	C22H19Cl2NO4
**Data File Name:**	C:\LabSolutions\Data\430_180530_Grigalunas_180605_3101_180605_Tead_180605_Wu\1046325618_11180530431.lcd
**Sample Location:**	Plate Number: 2 - Position: 67
**Run By:**	admin
**Run Started:**	24 September 2018 14:46:57
**Run Finished:**	25 September 2018 00:19:16
**Method:**	Purity_Check_2018

LC Chromatogram
LC#6 : AD2

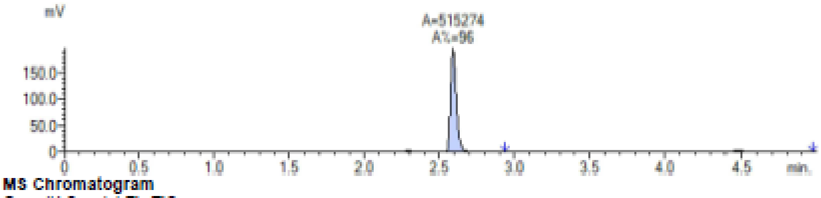

MS Chromatogram
Group#1 Scan(+) EI : TIC

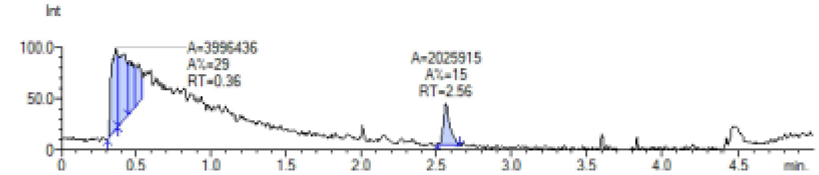

MS Mass Chromatogram
Group#1 Scan(+) EI : 432.1

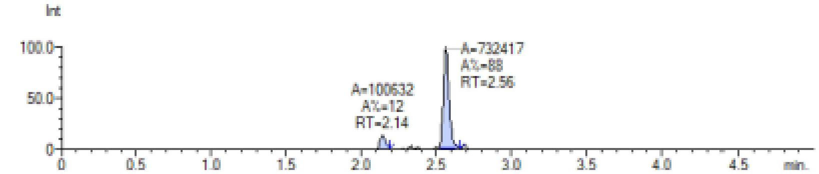

MS Spectrum
Group#1 - PDA Peak: 24, RT: 2.45 to 2.70 min

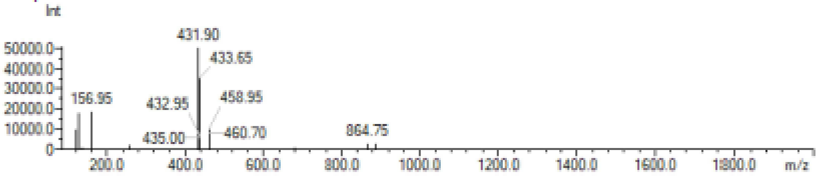

Shimadzu Open Solution

G-I-2-8

**Project:**	Test
**Experiment:**	476_Grigalu_1011_Shaaban_Liu_1009_3076
**Experiment Description:**	
**Sample:**	409045:01:01
**Sample Description:**	C22H19Cl2NO4
**Data File Name:**	C:\LabSolutions\Data\476_Grigalunas_181011_Shaaban_181011_Liu_181009_3076 \1046359875_11181011476.lcd
**Sample Location:**	Plate Number: 2 - Position: 153
**Run By:**	admin
**Run Started:**	24 April 2019 18:14:10
**Run Finished:**	25 April 2019 04:58:04
**Method:**	190321_Purity-Check+shut down

LC Chromatogram

LC#5 : AD2

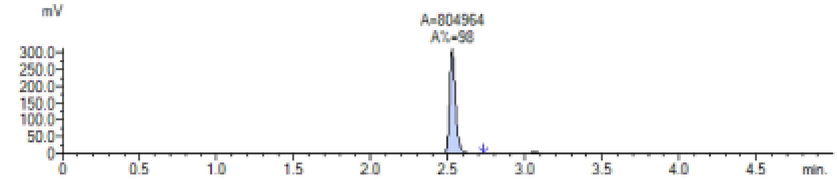

MS Chromatogram

Group#1 Scan(+) EI : TIC

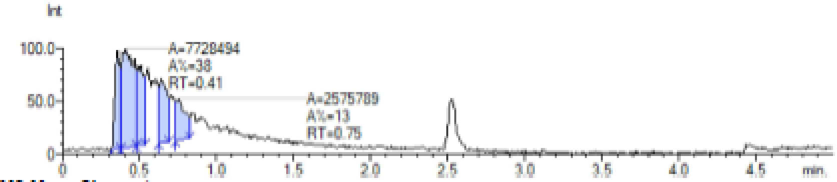

MS Mass Chromatogram

Group#1 Scan(+) EI : 432.1

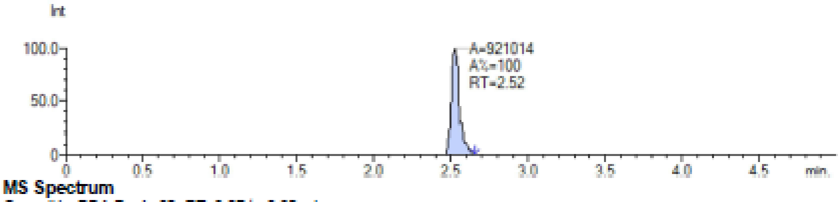

MS Spectrum

Group#1 - PDA Peak: 20, RT: 2.37 to 2.60 min

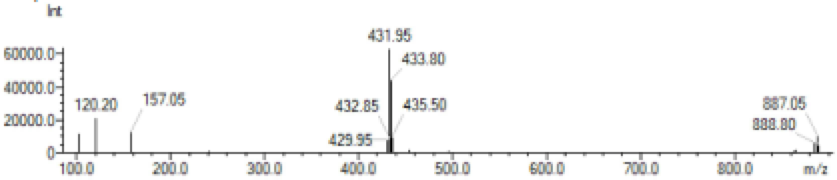

Shimadzu Open Solution

GF-I-1-11

**Project:**	Test
**Experiment:**	476_Grigalu_1011_Shaaban_Liu_1009_3076
**Experiment Description:**	
**Sample:**	409080:01:01
**Sample Description:**	C22H19ClFNO4
**Data File Name:**	C:\LabSolutions\Data\476_Grigalunas_181011_Shaaban_181011_Liu_181009_3076 \1046359357_11181011476.lcd
**Sample Location:**	Plate Number: 2 - Position: 9
**Run By:**	admin
**Run Started:**	24 April 2019 18:14:10
**Run Finished:**	25 April 2019 04:58:04
**Method:**	190321_Purity-Check+shut down

LC Chromatogram

LC#6 : AD2

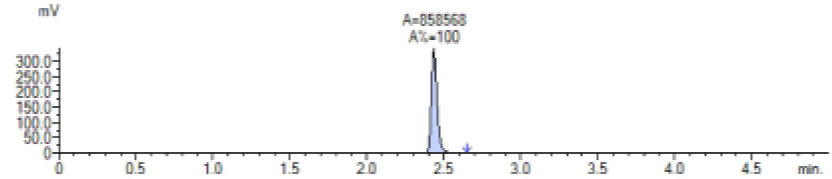

MS Chromatogram

Group#1 Scan(+) EI : TIC

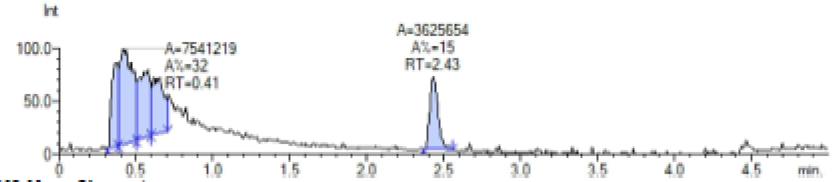

MS Mass Chromatogram

Group#1 Scan(+) EI : 416.1

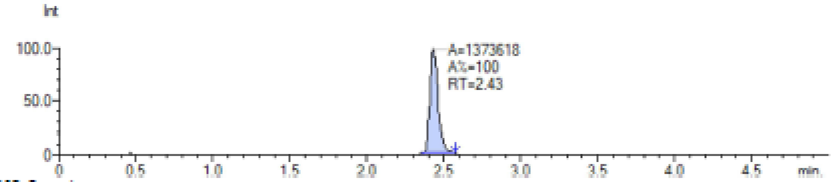

MS Spectrum

Group#1 - PDA Peak: 18, RT: 2.30 to 2.49 min

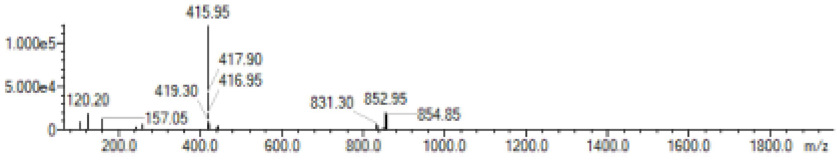

GF-I-2-9

Shimadzu Open Solution

**Project:**	Test
**Experiment:**	476_Grigalu_1011_Shaaban_Liu_1009_3076
**Experiment Description:**	
**Sample:**	409081:01:01
**Sample Description:**	C22H19ClFNO4
**Data File Name:**	C:\LabSolutions\Data\476_Grigalunas_181011_Shaaban_181011_Liu_181009_3076 \1046359674_11181011476.lcd
**Sample Location:**	Plate Number: 2 - Position: 57
**Run By:**	admin
**Run Started:**	24 April 2019 18:14:10
**Run Finished:**	25 April 2019 04:58:04
**Method:**	190321_Purity-Check+shut down

LC Chromatogram
LC#6 : AD2

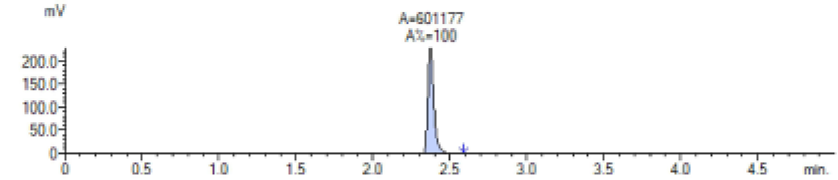

MS Chromatogram
Group#1 Scan(+) EI : TIC

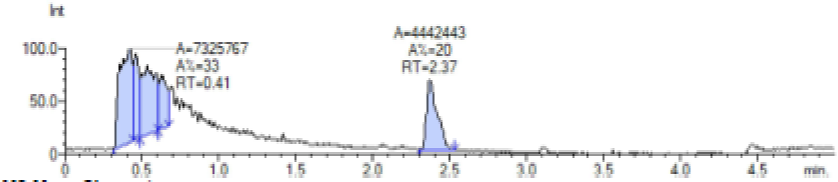

MS Mass Chromatogram
Group#1 Scan(+) EI : 416.1

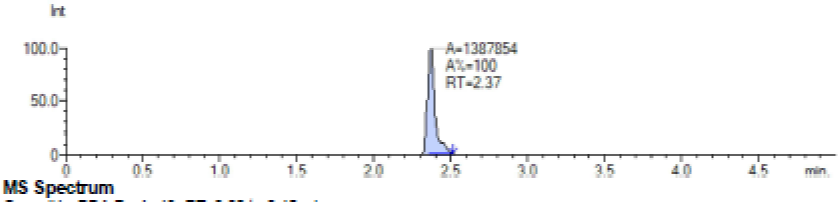

MS Spectrum
Group#1 - PDA Peak: 19, RT: 2.23 to 2.45 min

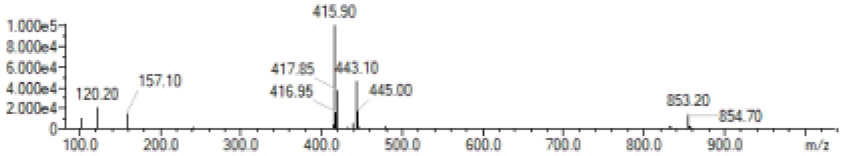

GF-I-1-12

Shimadzu Open Solution

**Project:**	Test
**Experiment:**	430_0530_Grigalu_0605_3101_TEAD_Wu
**Experiment Description:**	
**Sample:**	408647:01:02
**Sample Description:**	C23H19ClF3NO5
**Data File Name:**	C:\LabSolutions\Data\430_180530_Grigalunas_180605_3101_180605_Tead_180605_Wu\1046325566_11180530431.lcd
**Sample Location:**	Plate Number: 2 - Position: 161
**Run By:**	admin
**Run Started:**	24 September 2018 14:46:57
**Run Finished:**	25 September 2018 00:19:16
**Method:**	Purity_Check_2018

LC Chromatogram

LC#6 : AD2

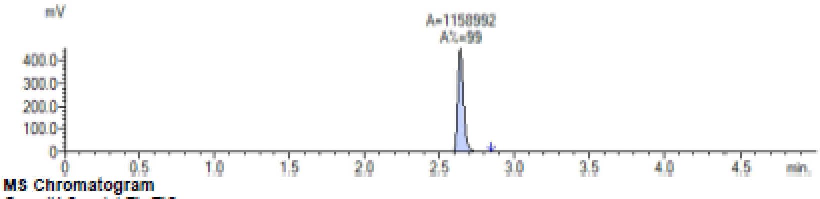

MS Chromatogram

Group#1 Scan(+) EI : TIC

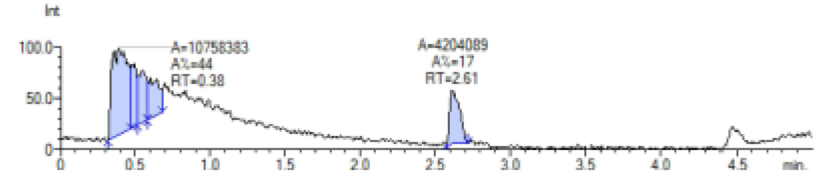

MS Mass Chromatogram

Group#1 Scan(+) EI : 482.1

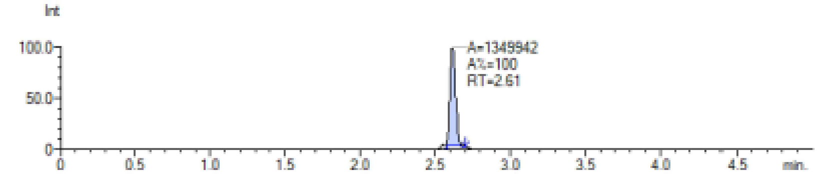

MS Spectrum

Group#1 - PDA Peak: 18, RT: 2.51 to 2.71 min

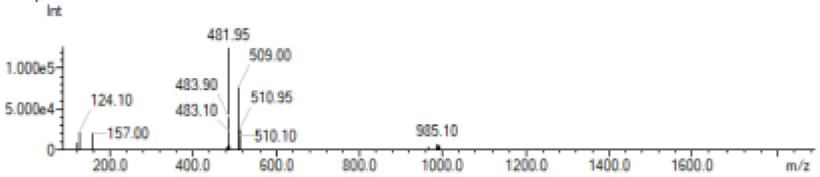

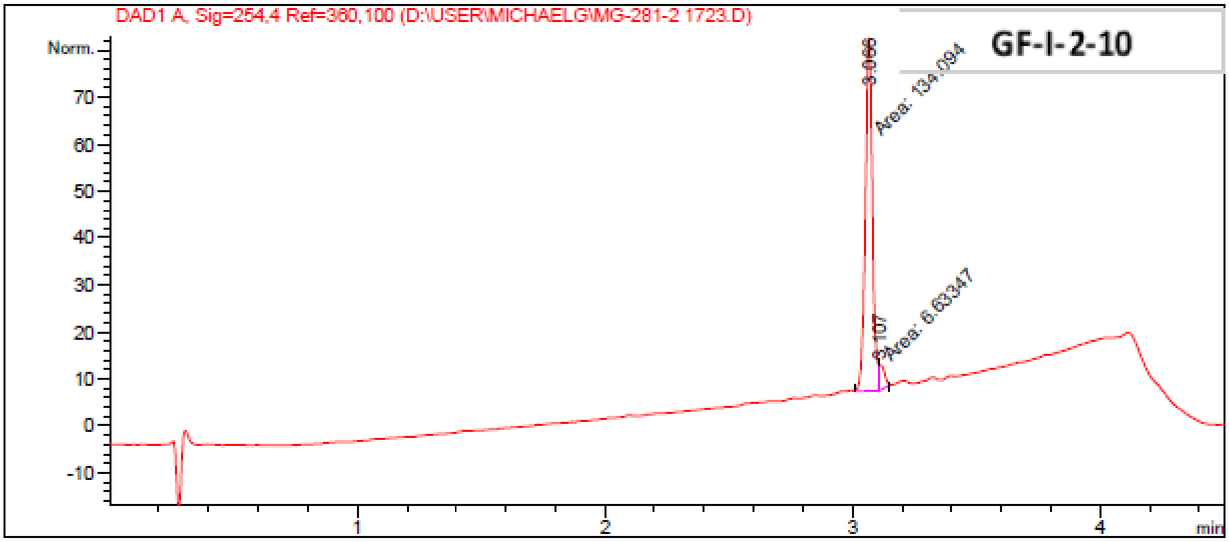

#	Time	Area	Height	Width	Area%	Symmetry
1	3.066	134.1	68.5	0.0326	95.286	1.003
2	3.107	6.6	5	0.0219	4.714	0

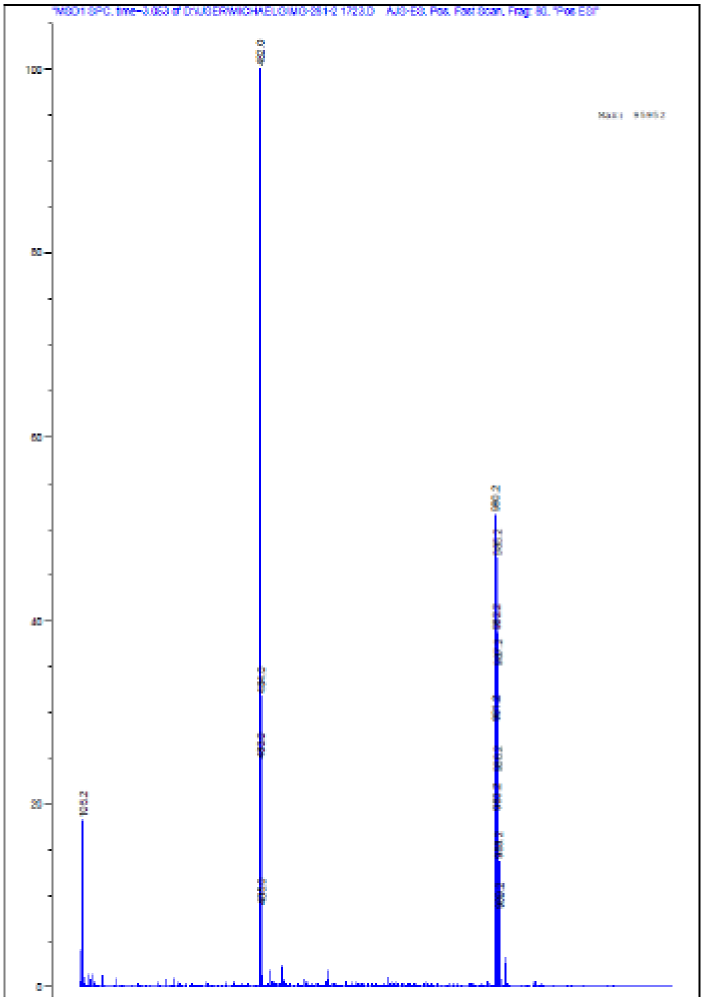

Shimadzu Open Solution

GF-I-2-11

Project:	Test
Experiment:	525_0206_Christo_Grigalu_Burhop_Wilke
Experiment Description:	
Sample:	409694:01:02
Sample Description:	C23H19ClF3NO4
Data File Name:	C:\LabSolutions\Data\525_190206_Christoforow_190206_Grigalunas_190206_Burhop_190206_Wilke\1046361333_11190207525.lcd
Sample Location:	Plate Number: 2 - Position: 340
Run By:	admin
Run Started:	23 April 2019 18:20:13
Run Finished:	24 April 2019 04:25:41
Method:	190321_Purity-Check+shut down

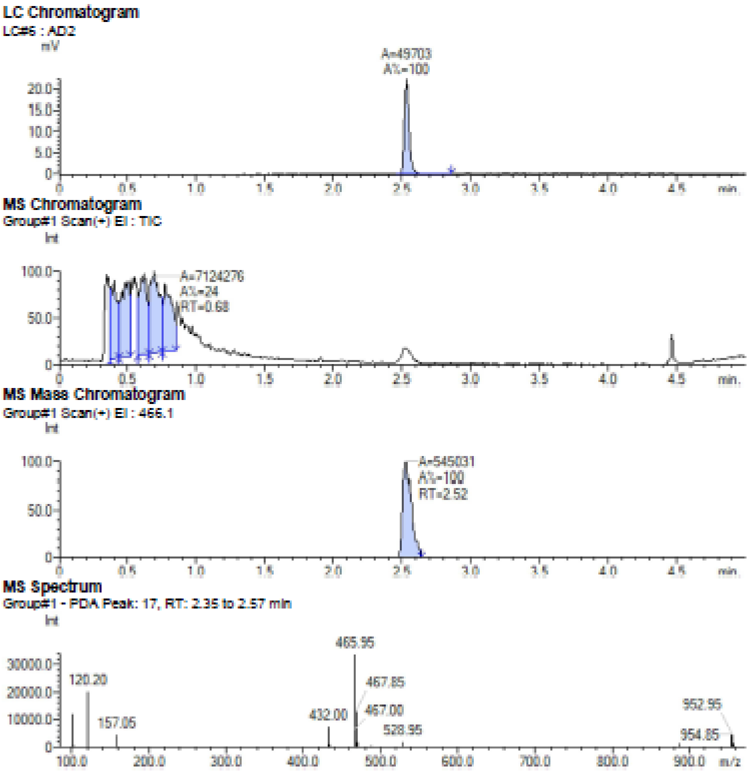

GF-I-1-13

Shimadzu Open Solution

Project: Test
Experiment: 430_0530_Grigalu_0605_3101_TEAD_Wu
Experiment Description:
Sample: 406594:01:01
Sample Description: C23H22ClNO4
Data File Name: C:\LabSolutions\Data\430_180530_Grigalunas_180605_3101_180605_Tead_180605_Wu\1046324697_11180530431.lcd
Sample Location: Plate Number: 2 - Position: 205
Run By: admin
Run Started: 24 September 2018 14:46:57
Run Finished: 25 September 2018 00:19:16
Method: Purity_Check_2018

LC Chromatogram
LC#6 : AD2

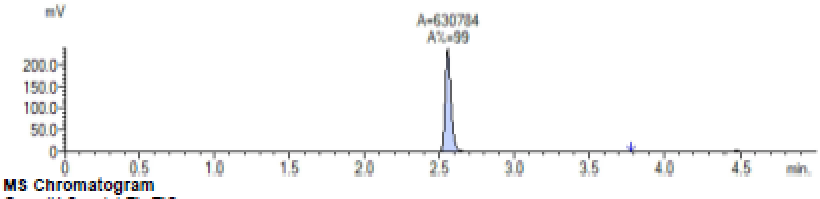

MS Chromatogram
Group#1 Scan(+) EI : TIC

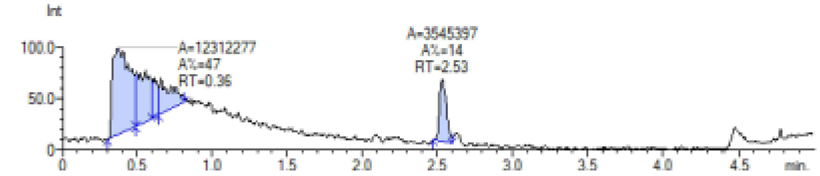

MS Mass Chromatogram
Group#1 Scan(+) EI : 412.1

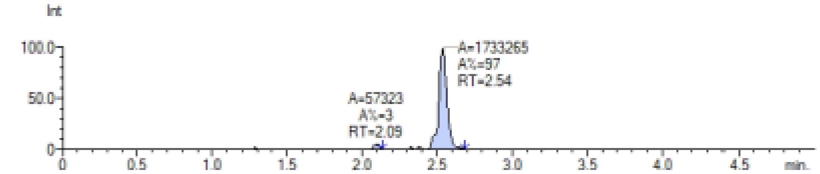

MS Spectrum
Group#1 - PDA Peak: 18, RT: 2.43 to 2.66 min

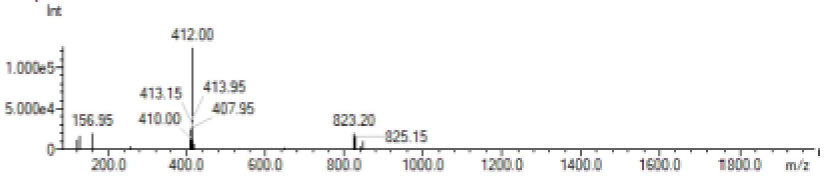

GF-I-1-14

Shimadzu Open Solution

Project: Test
Experiment: 430_0530_Grigalu_0605_3101_TEAD_Wu
Experiment Description:
Sample: 408597:01:01
Sample Description: C24H24ClNO4
Data File Name: C:\LabSolutions\Data\430_180530_Grigalunas_180605_3101_180605_Tead_180605_Wu\1046325022_11180530431.lcd
Sample Location: Plate Number: 2 - Position: 349
Run By: admin
Run Started: 24 September 2018 14:46:57
Run Finished: 25 September 2018 00:19:16
Method: Purity_Check_2018

LC Chromatogram
LC#6 : AD2

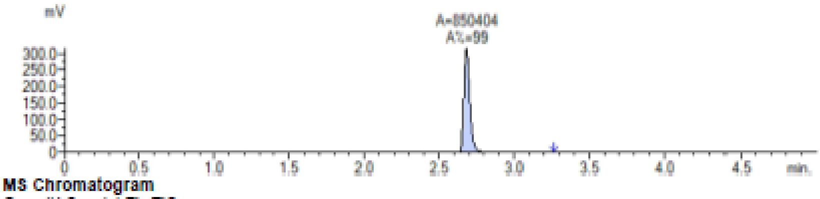

MS Chromatogram
Group#1 Scan(+) EI : TIC

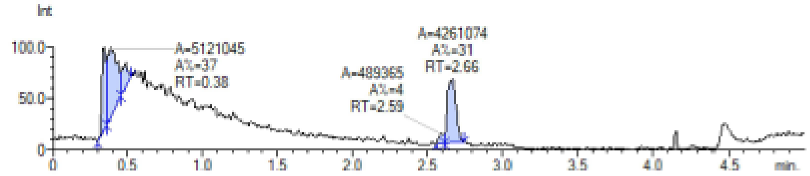

MS Mass Chromatogram
Group#1 Scan(+) EI : 426.1

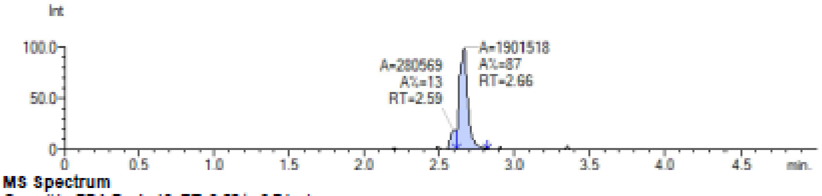

MS Spectrum
Group#1 - PDA Peak: 18, RT: 2.55 to 2.74 min

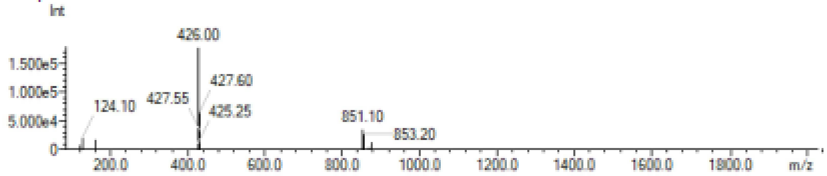

GF-I-1-15

Shimadzu Open Solution

Project: Test
Experiment: 430_0530_Grigalu_0605_3101_TEAD_Wu
Experiment Description:
Sample: 408596:01:01
Sample Description: C24H24ClNO4
Data File Name: C:\LabSolutions\Data\430_180530_Grigalunas_180605_3101_180605_Tead_180605_Wu\1046325132_11180530431.lcd
Sample Location: Plate Number: 2 - Position: 63
Run By: admin
Run Started: 24 September 2018 14:46:57
Run Finished: 25 September 2018 00:19:16
Method: Purity_Check_2018

LC Chromatogram
LC#6 : AD2

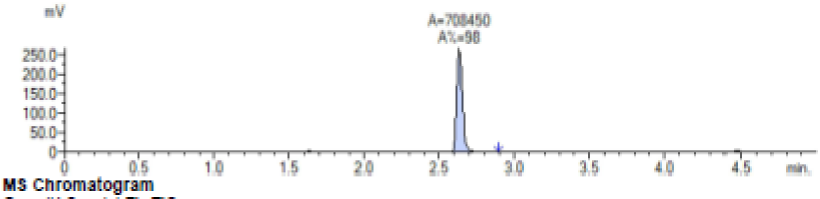

MS Chromatogram
Group#1 Scan(+) EI : TIC

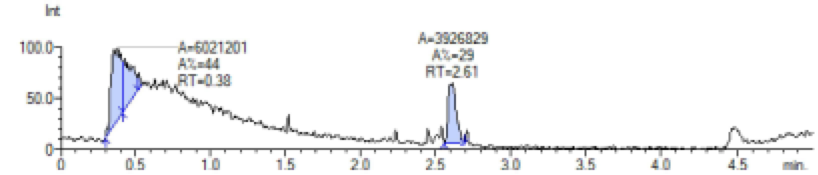

MS Mass Chromatogram
Group#1 Scan(+) EI : 426.1

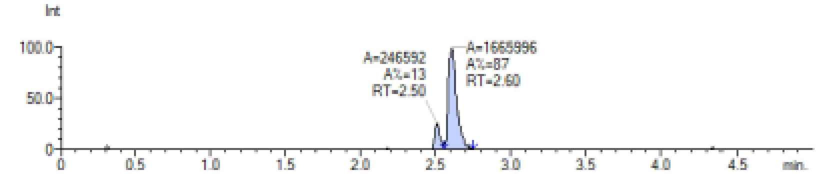

MS Spectrum
Group#1 - PDA Peak: 17, RT: 2.49 to 2.62 min

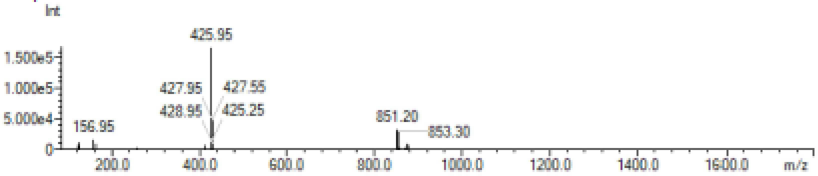

Shimadzu Open Solution

GF-I-1-16

Project: Test
Experiment: 430_0530_Grigalu_0605_3101_TEAD_Wu
Experiment Description:
Sample: 408601:01:02
Sample Description: C24H24ClNO4
Data File Name: C:\LabSolutions\Data\430_180530_Grigalunas_180605_3101_180605_Tead_180605_Wu\1046325598_11180530431.lcd
Sample Location: Plate Number: 2 - Position: 303
Run By: admin
Run Started: 24 September 2018 14:46:57
Run Finished: 25 September 2018 00:19:16
Method: Purity_Check_2018

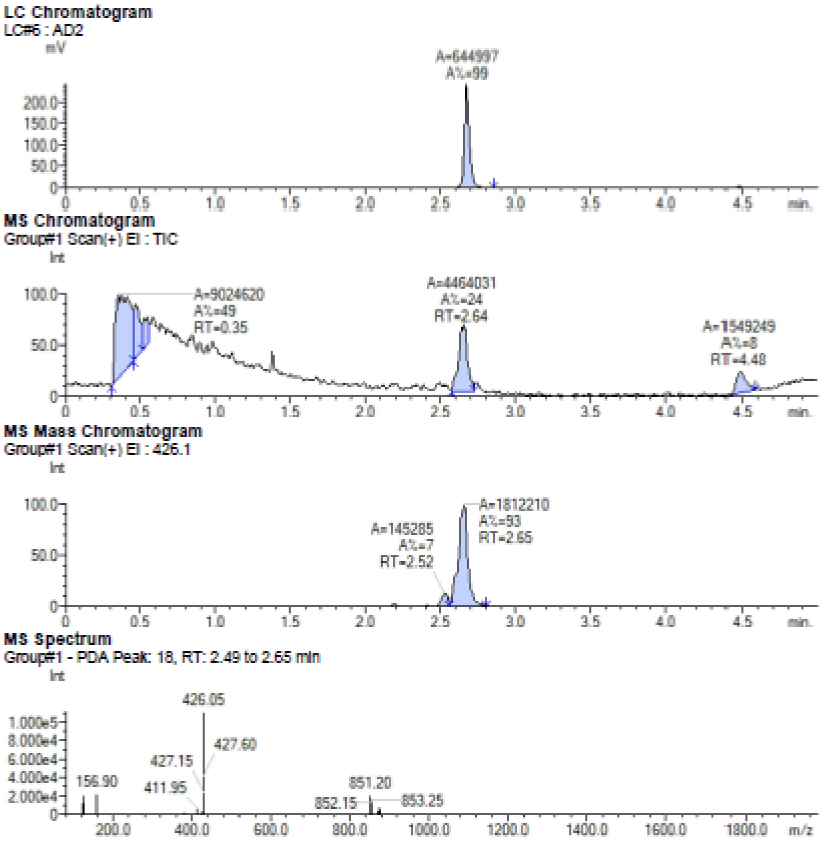

GF-I-2-12

Shimadzu Open Solution

Project:	Test
Experiment:	430_0530_Grigalu_0605_3101_TEAD_Wu
Experiment Description:	
Sample:	408602:01:01
Sample Description:	C24H24ClNO4
Data File Name:	C:\LabSolutions\Data\430_180530_Grigalunas_180605_3101_180605_Tead_180605_Wu\1046325188_11180530431.lcd
Sample Location:	Plate Number: 2 - Position: 351
Run By:	admin
Run Started:	24 September 2018 14:46:57
Run Finished:	25 September 2018 00:19:16
Method:	Purity_Check_2018

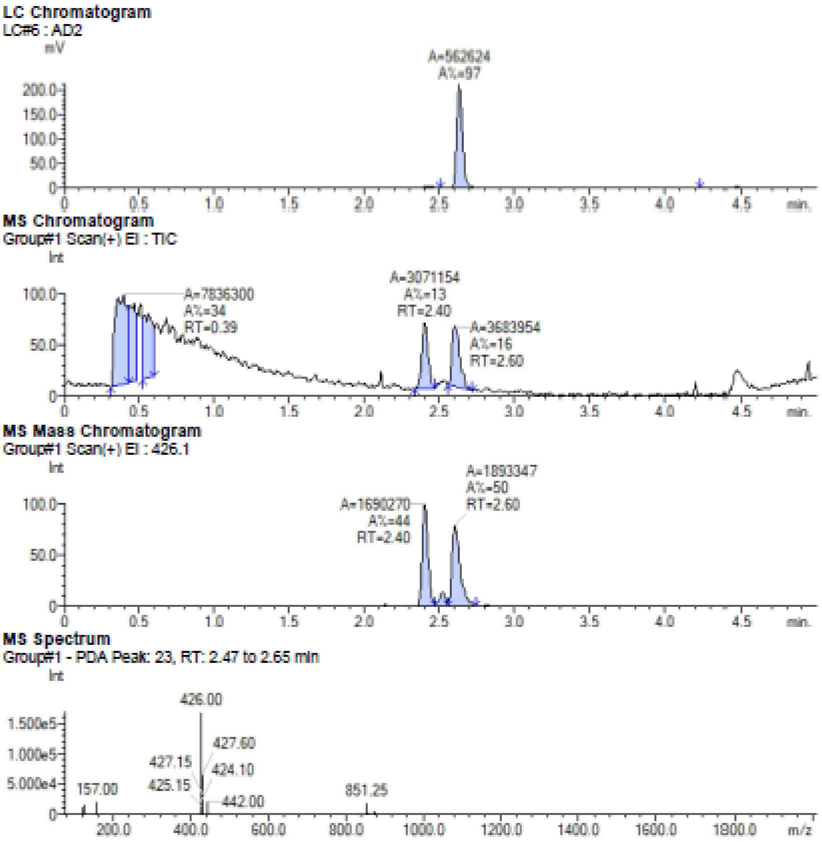

GF-I-1-18

Shimadzu Open Solution

**Project:**	Test
**Experiment:**	430_0530_Grigalu_0605_3101_TEAD_Wu
**Experiment Description:**	
**Sample:**	408600:01:01
**Sample Description:**	C22H18ClF2NO4
**Data File Name:**	C:\LabSolutions\Data\430_180530_Grigalunas_180605_3101_180605_Tead_180605_Wu\1046325508_11180530431.lcd
**Sample Location:**	Plate Number: 2 - Position: 207
**Run By:**	admin
**Run Started:**	24 September 2018 14:46:57
**Run Finished:**	25 September 2018 00:19:16
**Method:**	Purity_Check_2018

LC Chromatogram
LC#6 : AD2

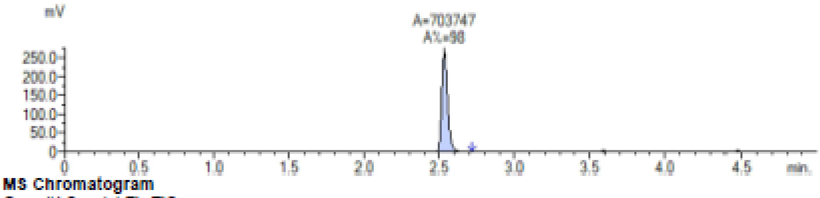

MS Chromatogram
Group#1 Scan(+) EI : TIC

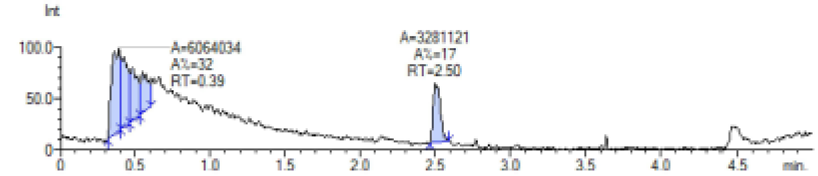

MS Mass Chromatogram
Group#1 Scan(+) EI : 434.1

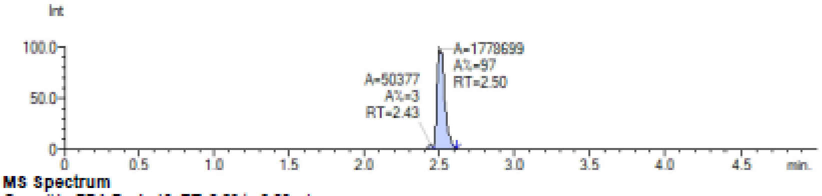

MS Spectrum
Group#1 - PDA Peak: 19, RT: 2.39 to 2.62 min

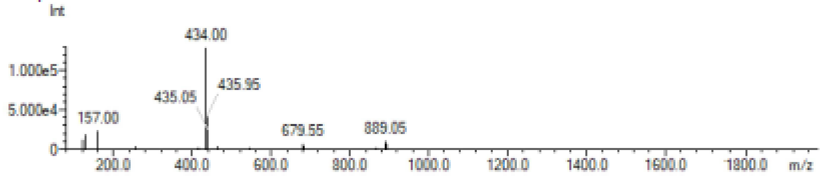

GF-I-1-19

Shimadzu Open Solution

**Project:**	Test
**Experiment:**	430_0530_Grigalu_0605_3101_TEAD_Wu
**Experiment Description:**	
**Sample:**	408604:01:01
**Sample Description:**	C23H21ClFNO4
**Data File Name:**	C:\LabSolutions\Data\430_180530_Grigalunas_180605_3101_180605_Tead_180605_Wu\1046325511_11180530431.lcd
**Sample Location:**	Plate Number: 2 - Position: 65
**Run By:**	admin
**Run Started:**	24 September 2018 14:46:57
**Run Finished:**	25 September 2018 00:19:16
**Method:**	Purity_Check_2018

LC Chromatogram
LC#6 : AD2

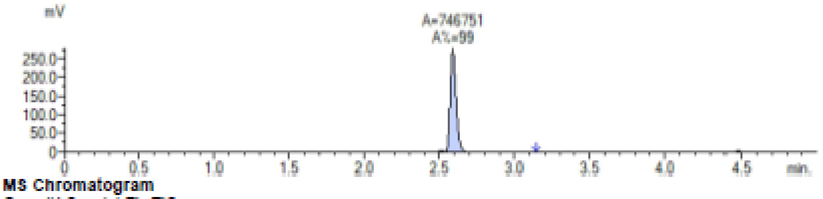

MS Chromatogram
Group#1 Scan(+) EI : TIC

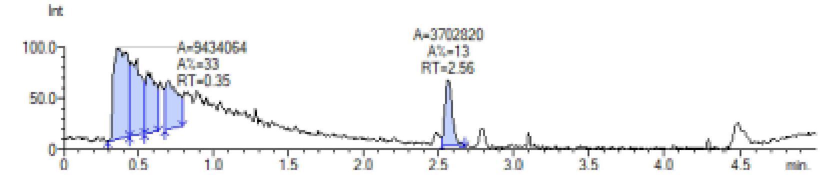

MS Mass Chromatogram
Group#1 Scan(+) EI : 430.1

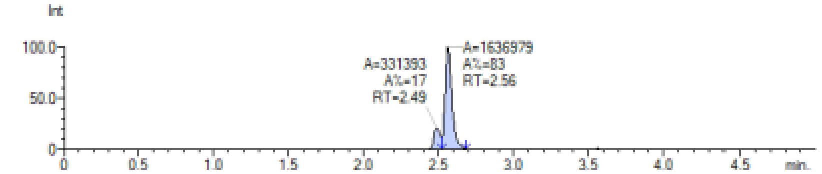

MS Spectrum
Group#1 - PDA Peak: 23, RT: 2.45 to 2.66 min

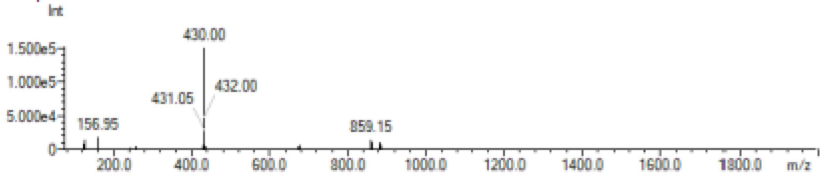

GF-I-2-13

Shimadzu Open Solution

**Project:**	Test
**Experiment:**	476_Grigalu_1011_Shaaban_Liu_1009_3076
**Experiment Description:**	
**Sample:**	409058:01:01
**Sample Description:**	C23H21ClFNO4
**Data File Name:**	C:\LabSolutions\Data\476_Grigalunas_181011_Shaaban_181011_Liu_181009_3076 \1046359086_11181011476.lcd
**Sample Location:**	Plate Number: 2 - Position: 291
**Run By:**	admin
**Run Started:**	24 April 2019 18:14:10
**Run Finished:**	25 April 2019 04:58:04
**Method:**	190321_Purity-Check+shut down

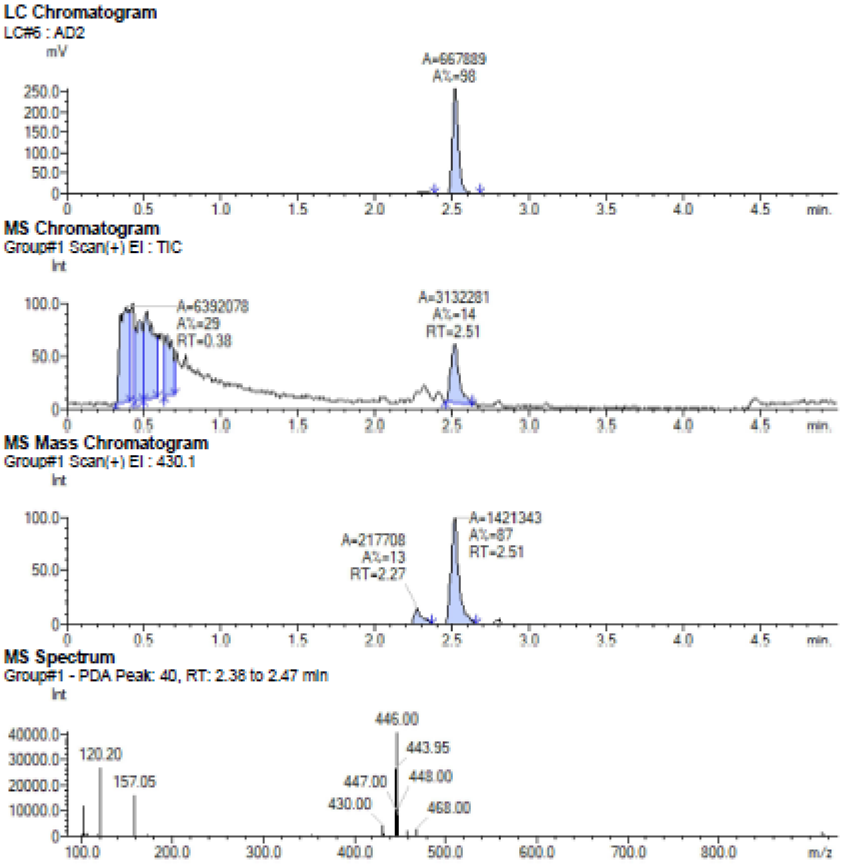

Shimadzu Open Solution

GF-I-1-20

**Project:**	Test
**Experiment:**	476_Grigalu_1011_Shaaban_Liu_1009_3076
**Experiment Description:**	
**Sample:**	409059:01:01
**Sample Description:**	C22H18ClF2NO4
**Data File Name:**	C:\LabSolutions\Data\476_Grigalunas_181011_Shaaban_181011_Liu_181009_3076 \1046359116_11181011476.lcd
**Sample Location:**	Plate Number: 2 - Position: 197
**Run By:**	admin
**Run Started:**	24 April 2019 18:14:10
**Run Finished:**	25 April 2019 04:58:04
**Method:**	190321_Purity-Check+shut down

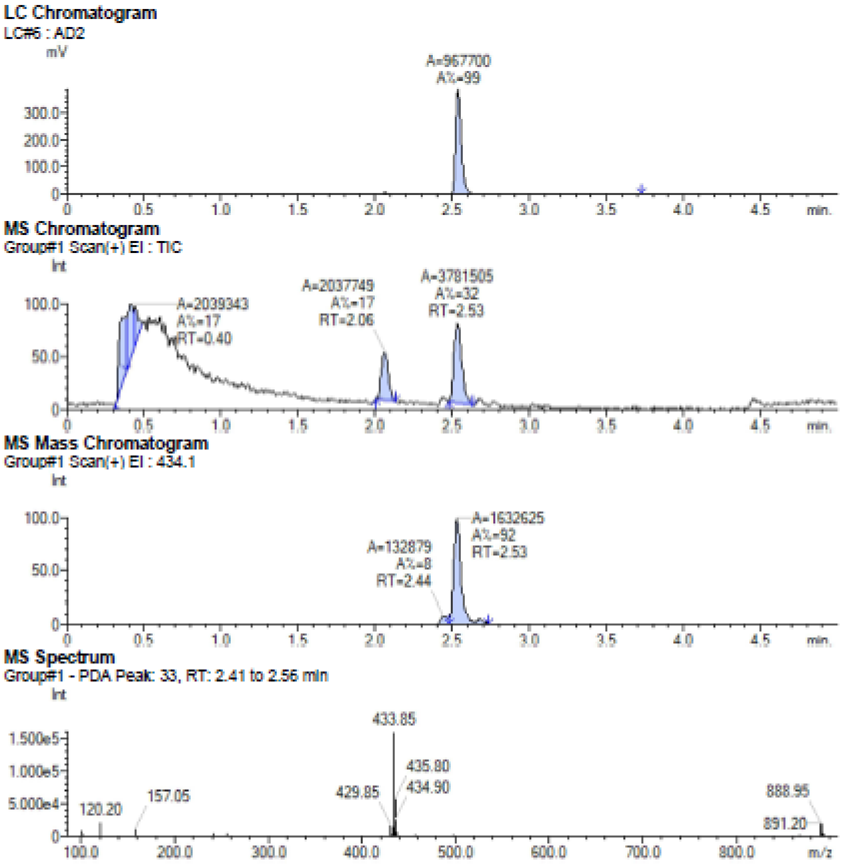

Shimadzu Open Solution

GF-I-2-14

**Project:**	Test
**Experiment:**	476_Grigalu_1011_Shaaban_Liu_1009_3076
**Experiment Description:**	
**Sample:**	409060:01:01
**Sample Description:**	C22H18ClF2NO4
**Data File Name:**	C:\LabSolutions\Data\476_Grigalunas_181011_Shaaban_181011_Liu_181009_3076 \1046359123_11181011476.lcd
**Sample Location:**	Plate Number: 2 - Position: 245
**Run By:**	admin
**Run Started:**	24 April 2019 18:14:10
**Run Finished:**	25 April 2019 04:58:04
**Method:**	190321_Purity-Check+shut down

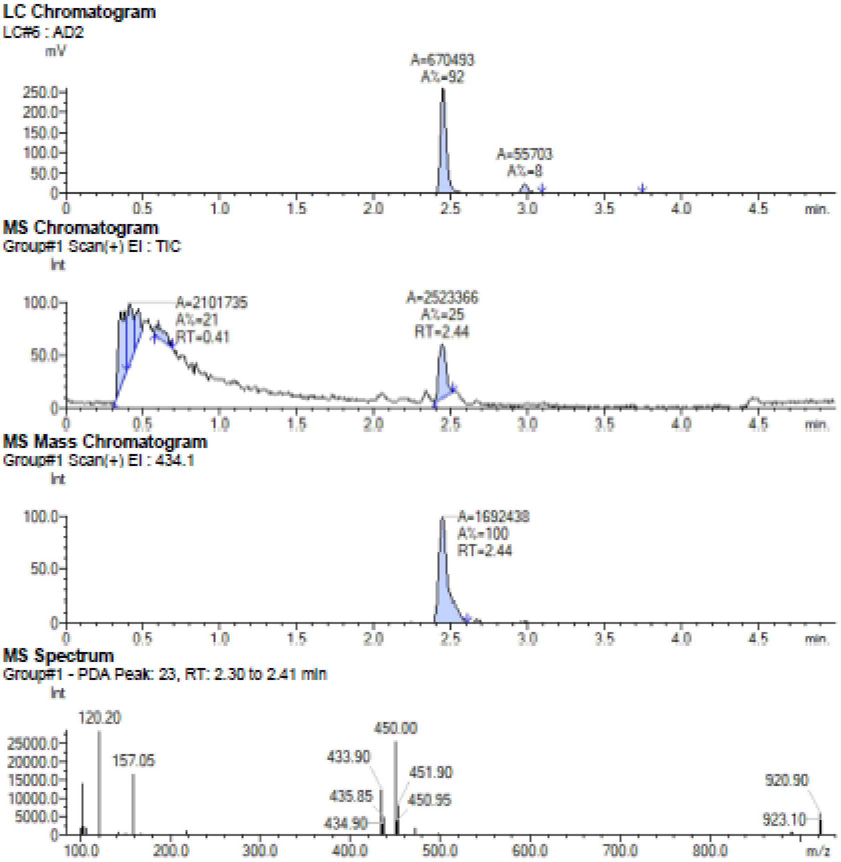

Shimadzu Open Solution

GF-I-1-21

**Project:**	Test
**Experiment:**	476_Grigalu_1011_Shaaban_Liu_1009_3076
**Experiment Description:**	
**Sample:**	409071:01:01
**Sample Description:**	C22H18ClF2NO4
**Data File Name:**	C:\LabSolutions\Data\476_Grigalunas_181011_Shaaban_181011_Liu_181009_3076 \1046359458_11181011476.lcd
**Sample Location:**	Plate Number: 2 - Position: 293
**Run By:**	admin
**Run Started:**	24 April 2019 18:14:10
**Run Finished:**	25 April 2019 04:58:04
**Method:**	190321_Purity-Check+shut down

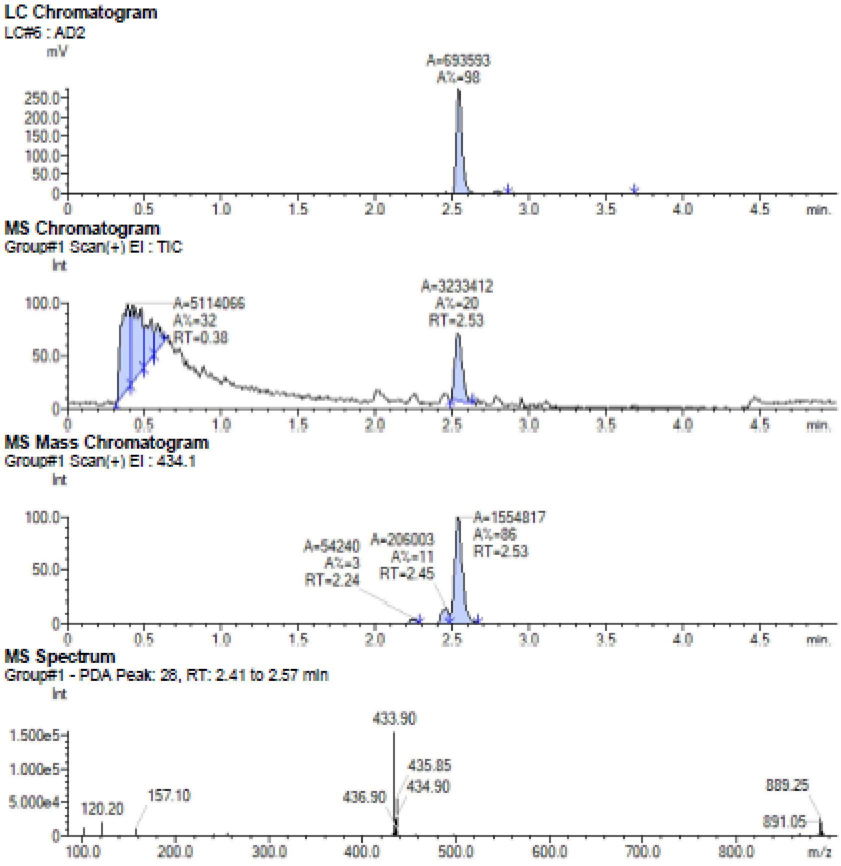

Shimadzu Open Solution

GF-I-1-22

**Project:**	Test
**Experiment:**	476_Grigalu_1011_Shaaban_Liu_1009_3076
**Experiment Description:**	
**Sample:**	409072:01:01
**Sample Description:**	C22H18Cl3NO4
**Data File Name:**	C:\LabSolutions\Data\476_Grigalunas_181011_Shaaban_181011_Liu_181009_3076 \1046360104_11181011476.lcd
**Sample Location:**	Plate Number: 2 - Position: 341
**Run By:**	admin
**Run Started:**	24 April 2019 18:14:10
**Run Finished:**	25 April 2019 04:58:04
**Method:**	190321_Purity-Check+shut down

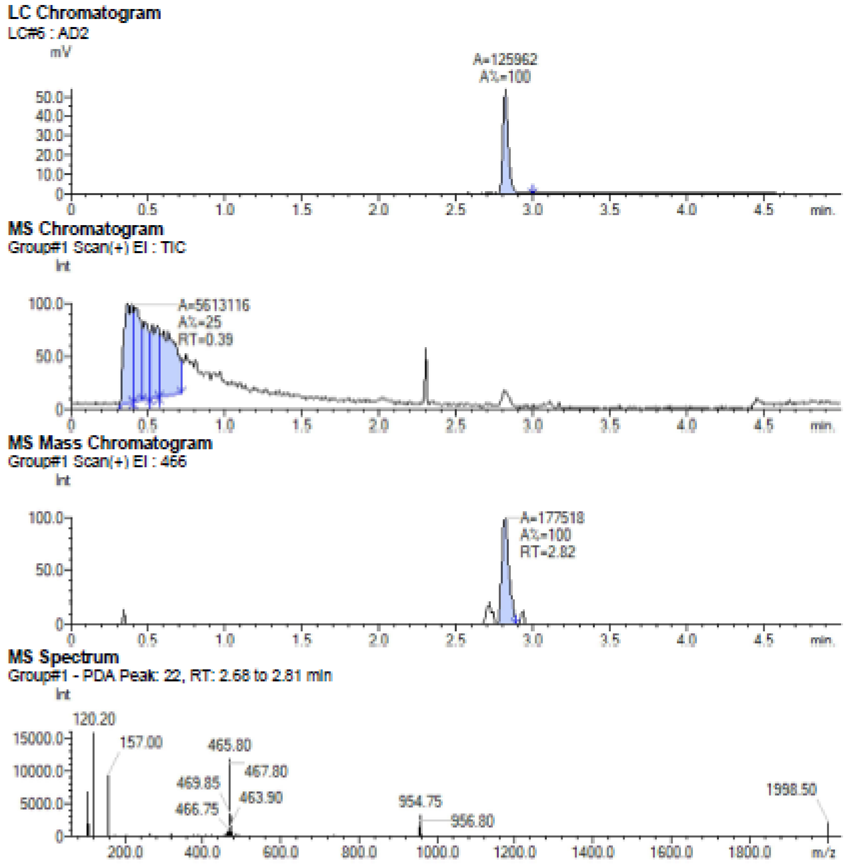

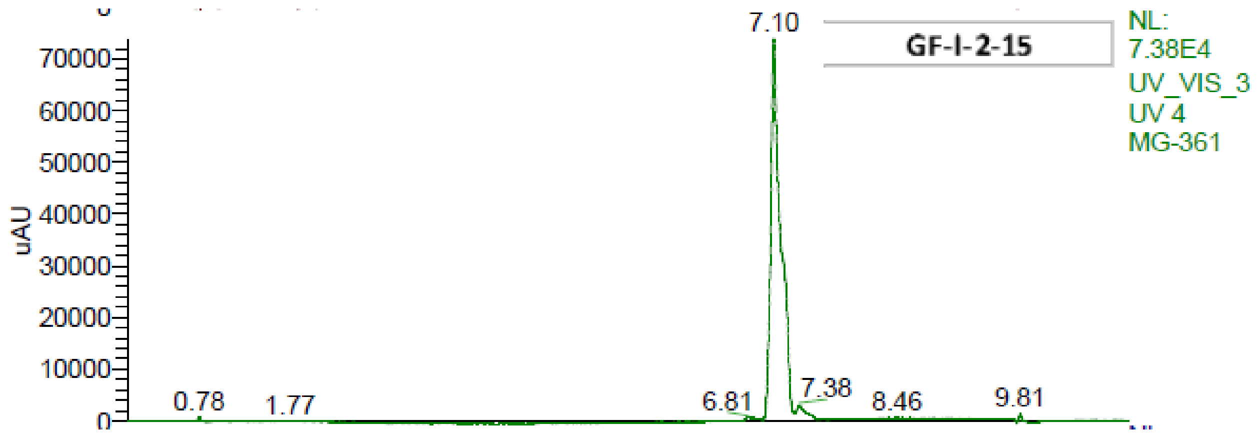

PEAK LIST
MG-361.raw
RT: 0.00 - 11.00
Number of detected peaks: 1

Apex RT	Start RT	End RT	Area	%Area	Height	%Height
7.10	7.04	7.25	15351.494	100.00	2799.519	100.00

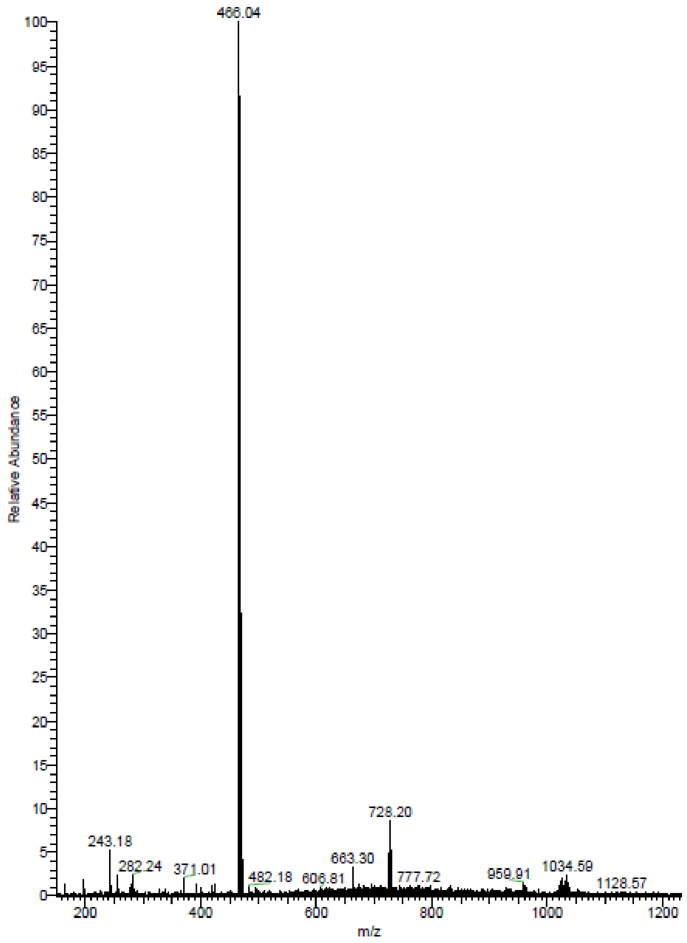

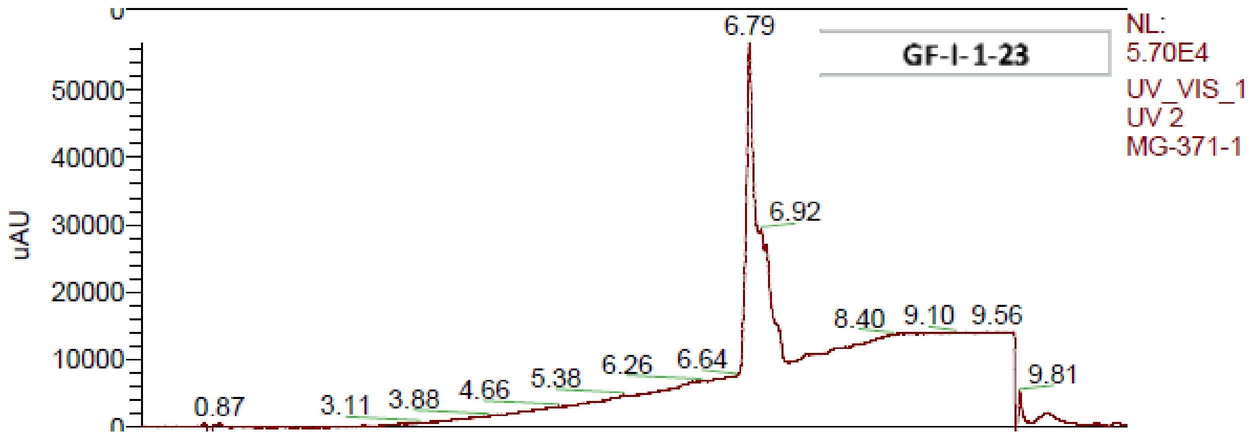

PEAK LIST
MG-371-1.raw
RT: 0.00 - 11.00
Number of detected peaks: 1

Apex RT	Start RT	End RT	Area	%Area	Height	%Height
6.79	6.65	7.21	110255.929	100.00	10734.076	100.00

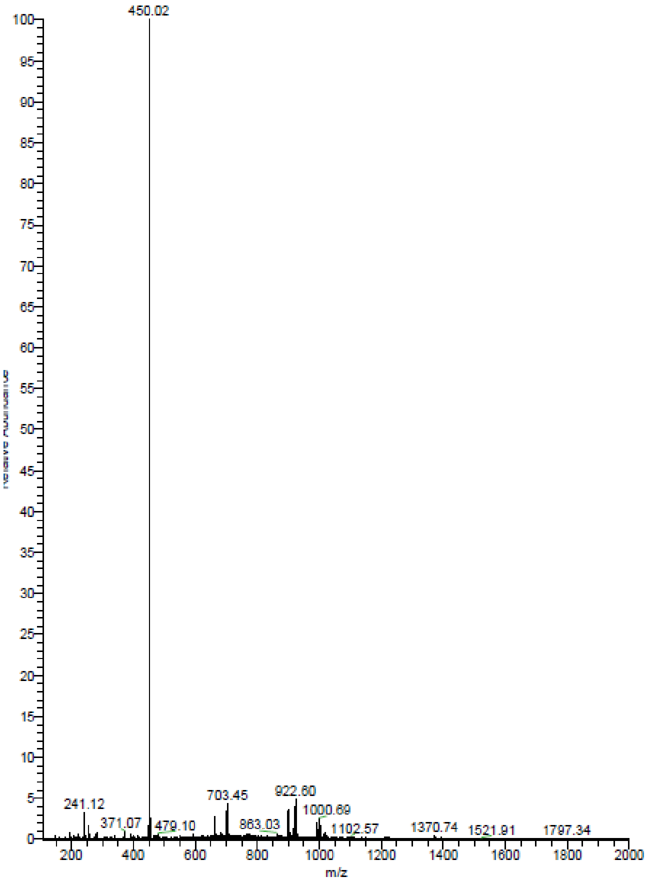

Shimadzu Open Solution

GF-I-1-24

Project: Test
Experiment: 525_0206_Christo_Grigalu_Burhop_Wilke
Experiment Description:
Sample: 409686:01:01
Sample Description: C22H18BrClFNO4
Data File Name: C:\LabSolutions\Data\525_190206_Christoforow_190206_Grigalunas_190206_Burhop_190206_Wilke\1046361179_11190207525.lcd
Sample Location: Plate Number: 2 - Position: 52
Run By: admin
Run Started: 23 April 2019 18:20:13
Run Finished: 24 April 2019 04:25:41
Method: 190321_Purity-Check+shut down

LC Chromatogram
LC#6 : AD2

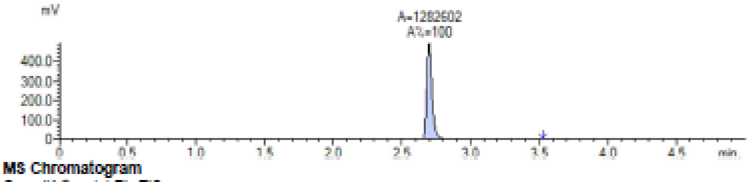

MS Chromatogram
Group#1 Scan(+) EI : TIC

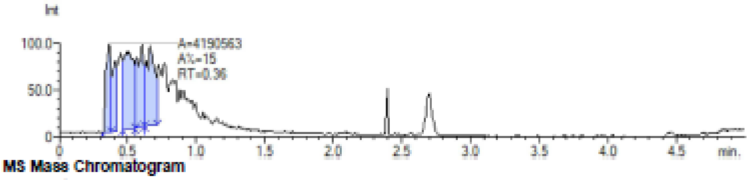

MS Mass Chromatogram
Group#1 Scan(+) EI : 494

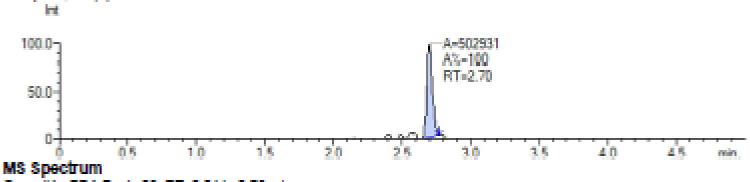

MS Spectrum
Group#1 - PDA Peak: 23, RT: 2.54 to 2.78 min

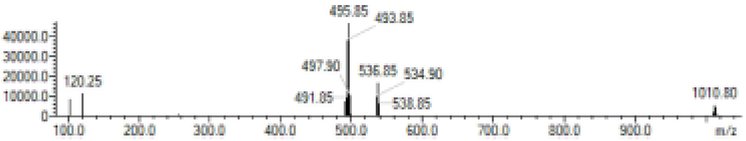

Shimadzu Open Solution

GF-I-1-25

Project: Test
Experiment: 525_0206_Christo_Grigalu_Burhop_Wilke
Experiment Description:
Sample: 409689:01:01
Sample Description: C22H18Cl2FNO4
Data File Name: C:\LabSolutions\Data\525_190206_Christoforow_190206_Grigalunas_190206_Burhop_190206_Wilke\1046360951_11190207525.lcd
Sample Location: Plate Number: 2 - Position: 100
Run By: admin
Run Started: 23 April 2019 18:20:13
Run Finished: 24 April 2019 04:25:41
Method: 190321_Purity-Check+shut down

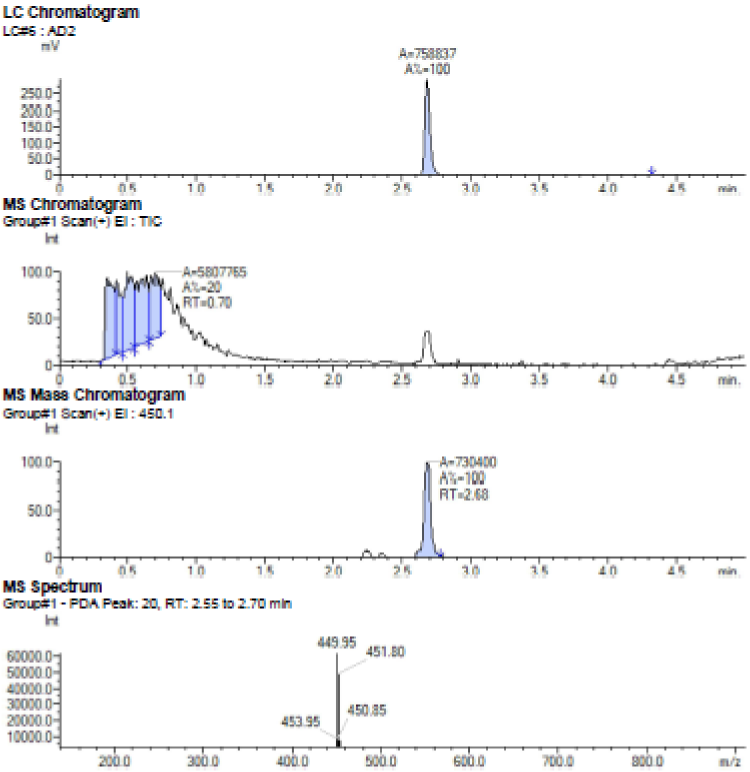

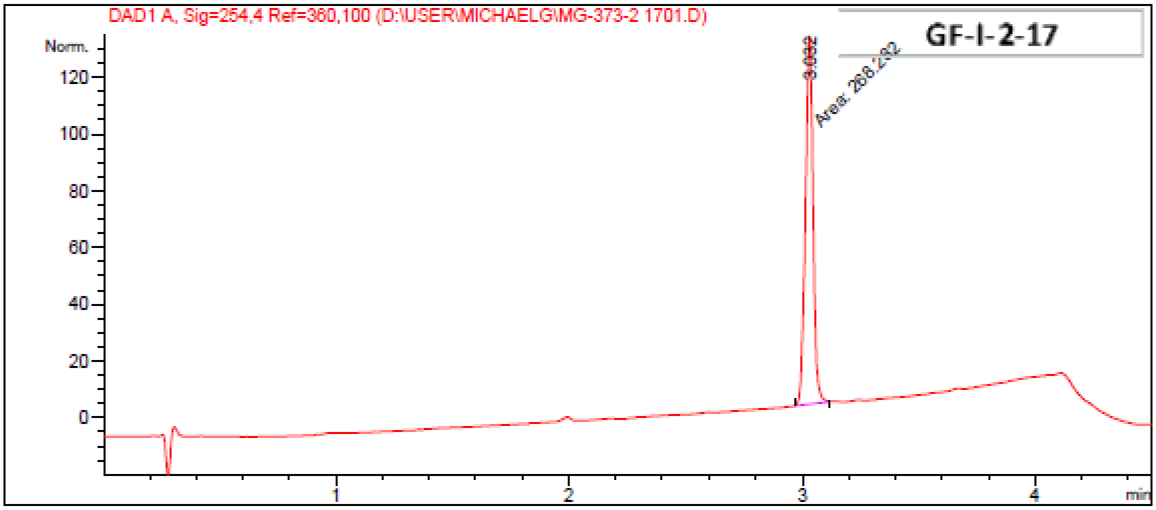

#	Time	Area	Height	Width	Area%	Symmetry
1	3.032	268.3	118.2	0.0378	100.000	1.145

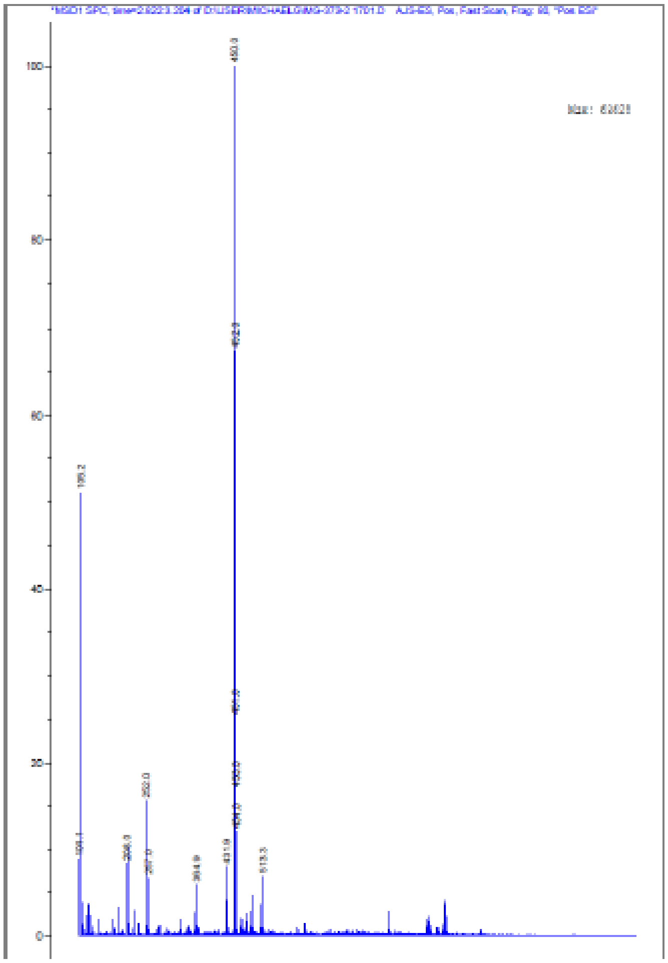

Shimadzu Open Solution

GF-I-1-26

Project:	Test
Experiment:	525_0206_Christo_Grigalu_Burhop_Wilke
Experiment Description:	
Sample:	409691:01:01
Sample Description:	C22H18BrClFNO4
Data File Name:	C:\LabSolutions\Data\525_190206_Christoforow_190206_Grigalunas_190206_Burhop_190206_Wilke\1046360758_11190207525.lcd
Sample Location:	Plate Number: 2 - Position: 196
Run By:	admin
Run Started:	23 April 2019 18:20:13
Run Finished:	24 April 2019 04:25:41
Method:	190321_Purity-Check+shut down

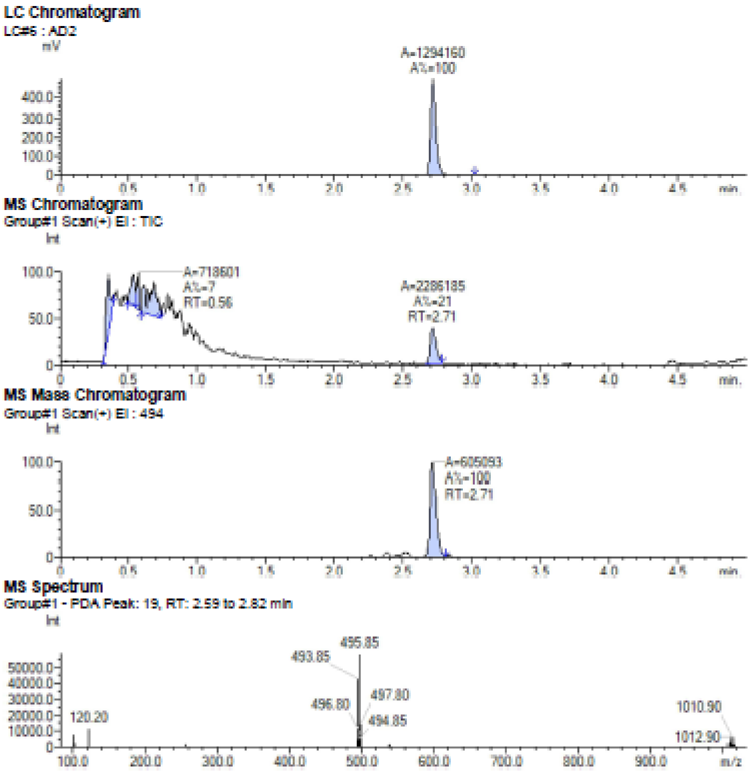

Shimadzu Open Solution

GF-I-2-18

Project:	Test
Experiment:	525_0206_Christo_Grigalu_Burhop_Wilke
Experiment Description:	
Sample:	409692:01:01
Sample Description:	C22H18BrClFNO4
Data File Name:	C:\LabSolutions\Data\525_190206_Christoforow_190206_Grigalunas_190206_Burhop_190206_Wilke\1046360615_11190207525.lcd
Sample Location:	Plate Number: 2 - Position: 244
Run By:	admin
Run Started:	23 April 2019 18:20:13
Run Finished:	24 April 2019 04:25:41
Method:	190321_Purity-Check+shut down

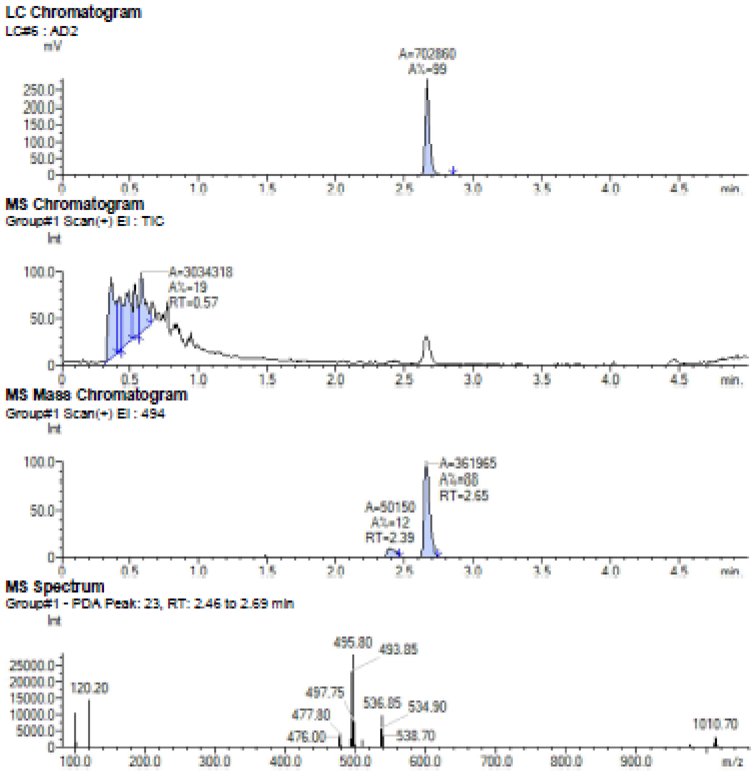

Shimadzu Open Solution

GF-I-1-27

**Project:**	Test
**Experiment:**	525_0206_Christo_Grigalu_Burhop_Wilke
**Experiment Description:**	
**Sample:**	409695:01:01
**Sample Description:**	C22H17BrClF2NO4
**Data File Name:**	C:\LabSolutions\Data\525_190206_Christoforow_190206_Grigalunas_190206_Burhop_190206_Wilke\1046361103_11190207525.lcd
**Sample Location:**	Plate Number: 2 - Position: 6
**Run By:**	admin
**Run Started:**	23 April 2019 18:20:13
**Run Finished:**	24 April 2019 04:25:41
**Method:**	190321_Purity-Check+shut down

LC Chromatogram

LC#6 : AD2

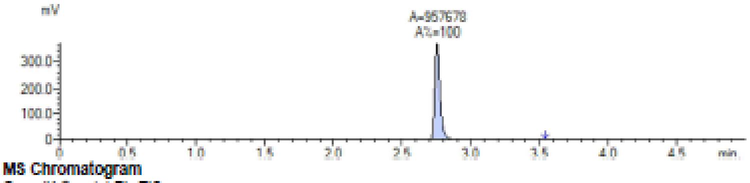

MS Chromatogram

Group#1 Scan(+) EI : TIC

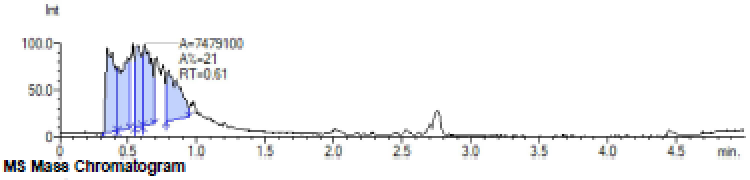

MS Mass Chromatogram

Group#1 Scan(+) EI : 512

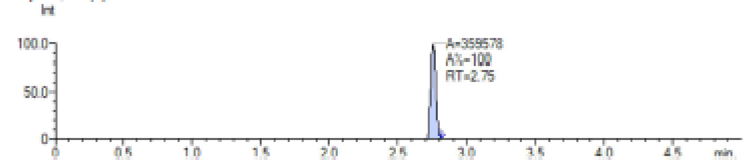

MS Spectrum

Group#1 - PDA Peak: 20, RT: 2.63 to 2.86 min

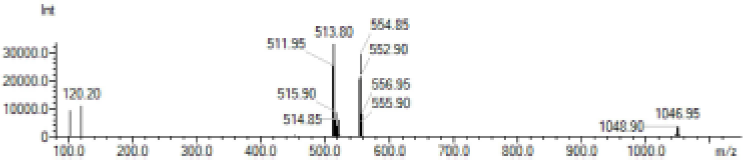

Shimadzu Open Solution

Project: Test
Experiment: 525_0206_Christo_Grigalu_Burhop_Wilke
Experiment Description:
Sample: 409696:01:01
Sample Description: C22H17BrClF2NO4
Data File Name: C:\LabSolutions\Data\525_190206_Christoforow_190206_Grigalunas_190206_Burhop_190206_Wilke\1046361107_11190207525.lcd
Sample Location: Plate Number: 2 - Position: 54
Run By: admin
Run Started: 23 April 2019 18:20:13
Run Finished: 24 April 2019 04:25:41
Method: 190321_Purity-Check+shut down

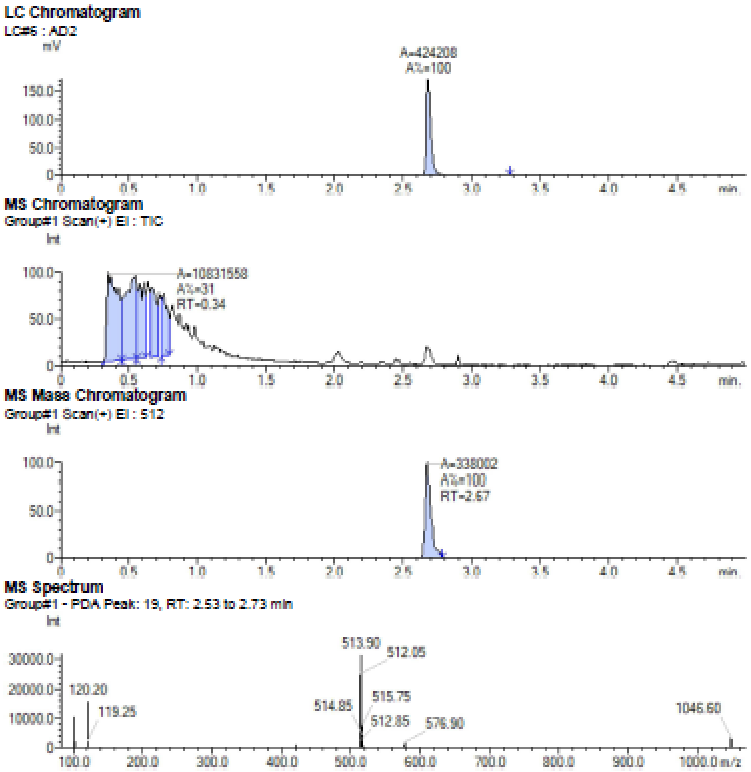

Shimadzu Open Solution

GF-I-1-28

**Project:**	Test
**Experiment:**	476_Grigalu_1011_Shaaban_Liu_1009_3076
**Experiment Description:**	
**Sample:**	409078:01:01
**Sample Description:**	C26H22ClNO4
**Data File Name:**	C:\LabSolutions\Data\476_Grigalunas_181011_Shaaban_181011_Liu_181009_3076 \1046359807_11181011476.lcd
**Sample Location:**	Plate Number: 2 - Position: 295
**Run By:**	admin
**Run Started:**	24 April 2019 18:14:10
**Run Finished:**	25 April 2019 04:58:04
**Method:**	190321_Purity-Check+shut down

LC Chromatogram

LC#6 : AD2

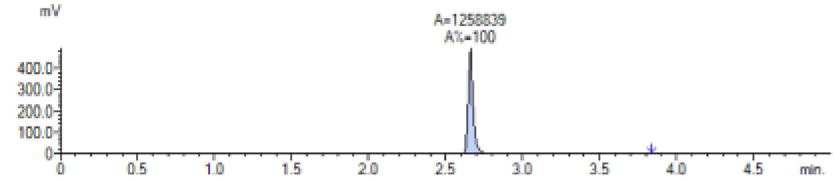

MS Chromatogram

Group#1 Scan(+) EI : TIC

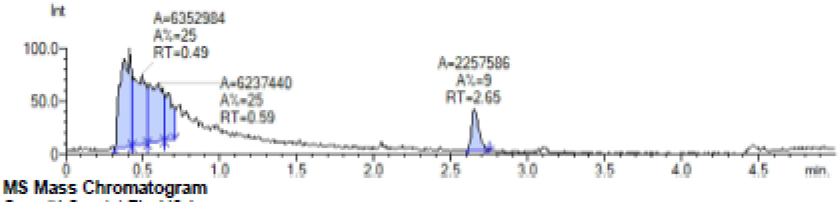

MS Mass Chromatogram

Group#1 Scan(+) EI : 448.1

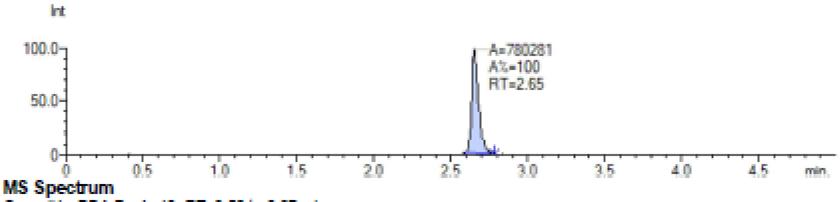

MS Spectrum

Group#1 - PDA Peak: 18, RT: 2.53 to 2.57 min

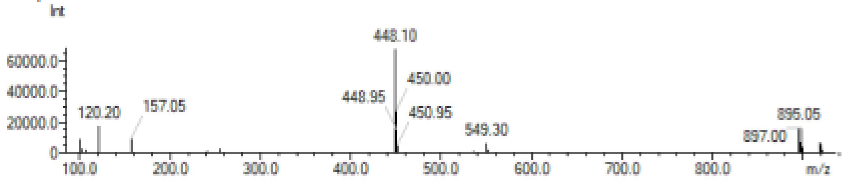

Shimadzu Open Solution

GF-I-2-20

**Project:**	Test
**Experiment:**	476_Grigalu_1011_Shaaban_Liu_1009_3076
**Experiment Description:**	
**Sample:**	409079:01:01
**Sample Description:**	C26H22ClNO4
**Data File Name:**	C:\LabSolutions\Data\476_Grigalunas_181011_Shaaban_181011_Liu_181009_3076 \1046359479_11181011476.lcd
**Sample Location:**	Plate Number: 2 - Position: 343
**Run By:**	admin
**Run Started:**	24 April 2019 18:14:10
**Run Finished:**	25 April 2019 04:58:04
**Method:**	190321_Purity-Check+shut down

LC Chromatogram

LC#5 : AD2

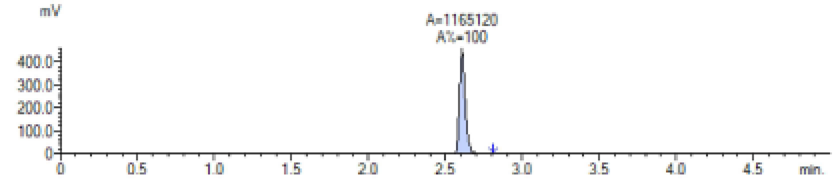

MS Chromatogram

Group#1 Scan(+) EI : TIC

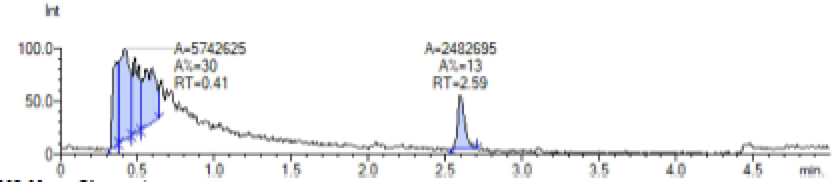

MS Mass Chromatogram

Group#1 Scan(+) EI : 448.1

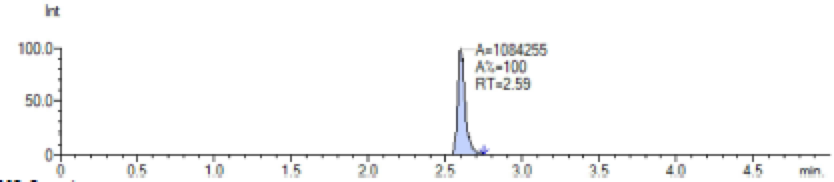

MS Spectrum

Group#1 - PDA Peak: 18, RT: 2.45 to 2.67 min

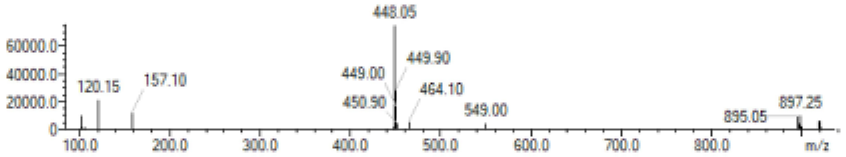

Shimadzu Open Solution

GF-I-1-29

Project:	Test
Experiment:	525_0206_Christo_Grigalu_Burhop_Wilke
Experiment Description:	
Sample:	409682:01:01
Sample Description:	C26H22ClNO4
Data File Name:	C:\LabSolutions\Data\525_190206_Christoforow_190206_Grigalunas_190206_Burhop_190206_Wilke\1046360570_11190207525.lcd
Sample Location:	Plate Number: 2 - Position: 98
Run By:	admin
Run Started:	23 April 2019 18:20:13
Run Finished:	24 April 2019 04:25:41
Method:	190321_Purity-Check+shut down

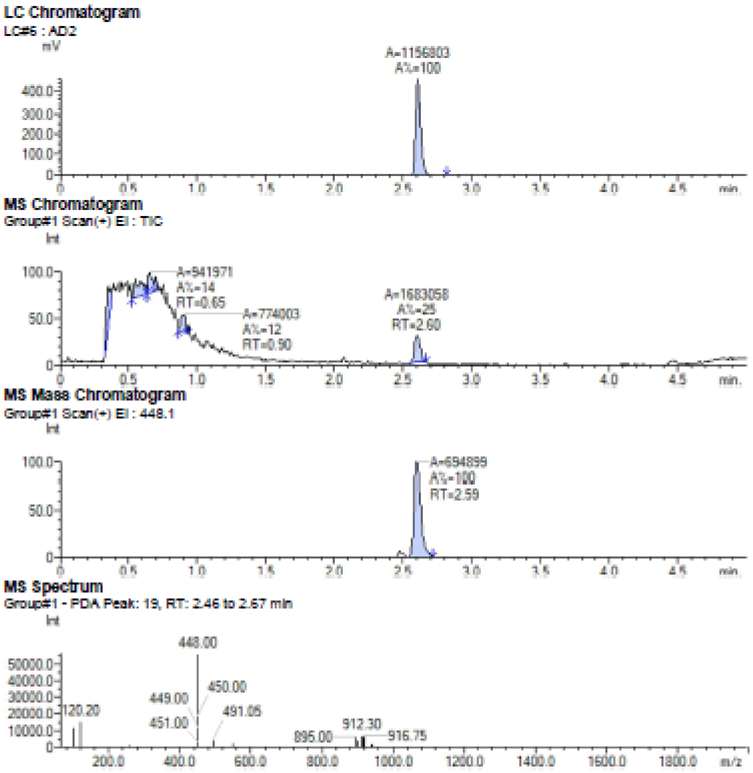

Shimadzu Open Solution

GF-I-1-30

Project: Test
Experiment: 430_0530_Grigalu_0605_3101_TEAD_Wu
Experiment Description:
Sample: 408599:01:01
Sample Description: C28H24ClNO4
Data File Name: C:\LabSolutions\Data\430_180530_Grigalunas_180605_3101_180605_Tead_180605_Wu\1046325173_11180530431.lcd
Sample Location: Plate Number: 2 - Position: 111
Run By: admin
Run Started: 24 September 2018 14:46:57
Run Finished: 25 September 2018 00:19:16
Method: Purity_Check_2018

LC Chromatogram
LC#6 : AD2

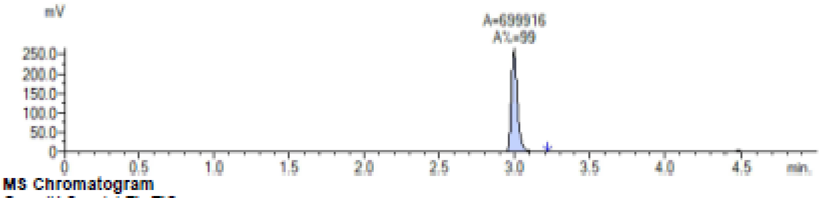

MS Chromatogram
Group#1 Scan(+) EI : TIC

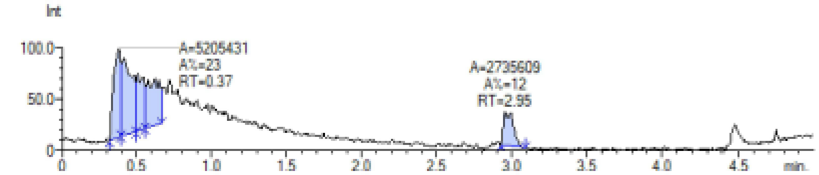

MS Mass Chromatogram
Group#1 Scan(+) EI : 474.1

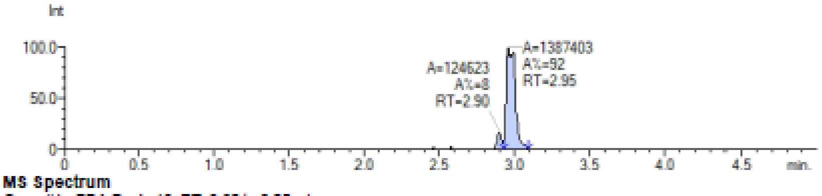

MS Spectrum
Group#1 - PDA Peak: 18, RT: 2.85 to 3.05 min

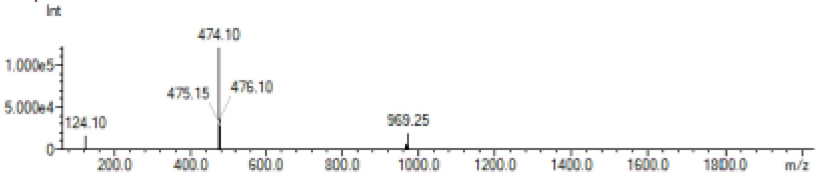

GF-I-1-31

Shimadzu Open Solution

Project: Test
Experiment: 430_0530_Grigalu_0605_3101_TEAD_Wu
Experiment Description:
Sample: 408603:01:02
Sample Description: C29H26ClNO4
Data File Name: C:\LabSolutions\Data\430_180530_Grigalunas_180605_3101_180605_Tead_180605_Wu\1046325019_11180530431.lcd
Sample Location: Plate Number: 2 - Position: 17
Run By: admin
Run Started: 24 September 2018 14:46:57
Run Finished: 25 September 2018 00:19:16
Method: Purity_Check_2018

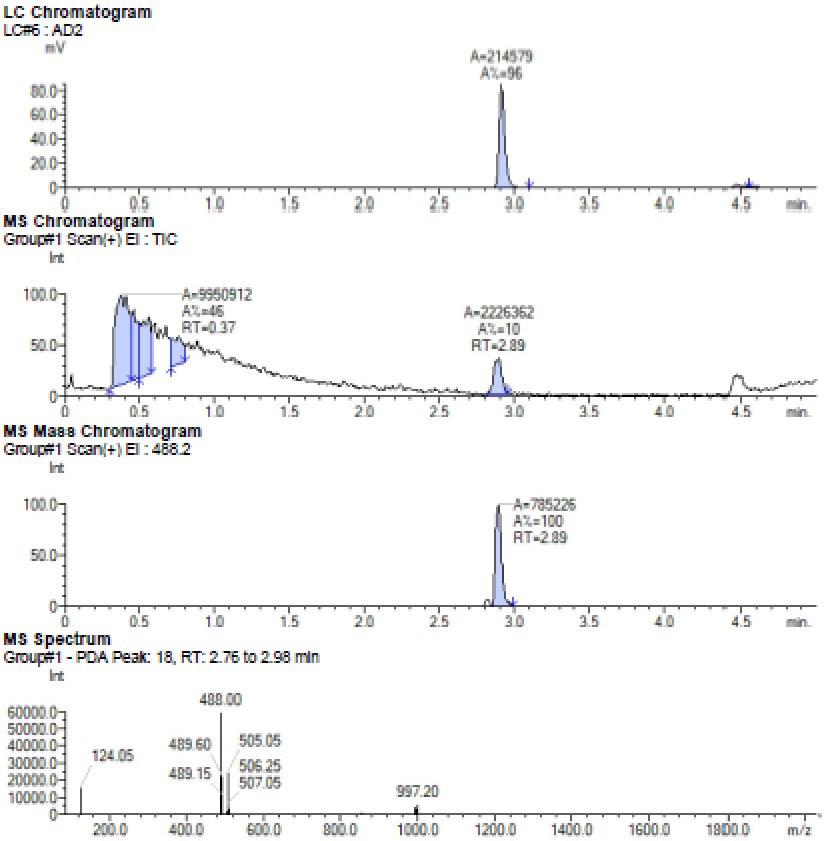

GF-THPI-1

Shimadzu Open Solution

Project: Test
Experiment: 433_0606_Burh_07_3069_08_Schoel_Liszewsk
Experiment Description:
Sample: 408746:01:02
Sample Description: C26H26ClNO5
Data File Name: C:\LabSolutions\Data\433_180606_Burhop_180607_3069_180608_Schoelermann_180608_Liszewski\1046324847_11180607433.lcd
Sample Location: Plate Number: 1 - Position: 5
Run By: admin
Run Started: 13 September 2018 13:00:55
Run Finished: 13 September 2018 16:04:31
Method: Purity_Check_2018

LC Chromatogram
LC#6 : AD2

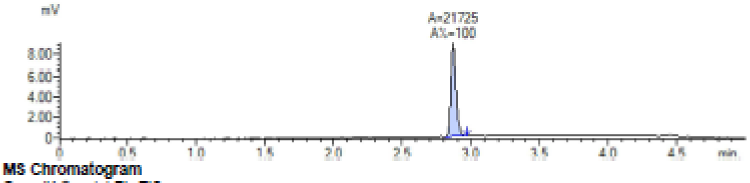

MS Chromatogram
Group#1 Scan(+) EI : TIC

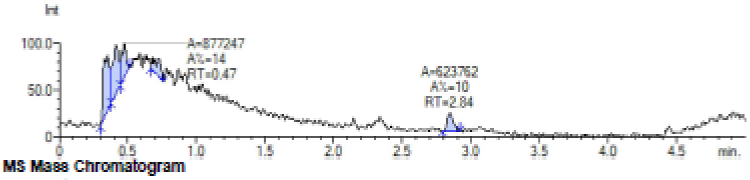

MS Mass Chromatogram
Group#1 Scan(+) EI : 468.1

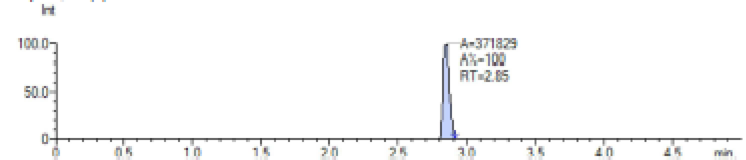

MS Spectrum
Group#1 - PDA Peak: 18, RT: 2.73 to 2.92 min

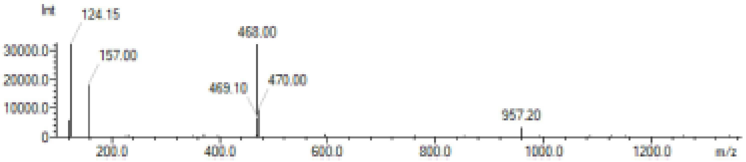

Shimadzu Open Solution

GF-THPI-2

Project: Test
Experiment: 676_200316_LDC_0518_Griga_REG_0515_Flege
Experiment Description:
Sample: 412366:01:01
Sample Description: C27H28ClNO5
Data File Name: C:\LabSolutions\Data\676_200316_LDC_0518_Grigalunas_REG_0515_Flegel\1046326890_11200518676.lcd
Sample Location: Plate Number: 1 - Position: 25
Run By: admin
Run Started: 20 July 2020 12:06:48
Run Finished: 20 July 2020 13:31:06
Method: 190321_Purity-Check

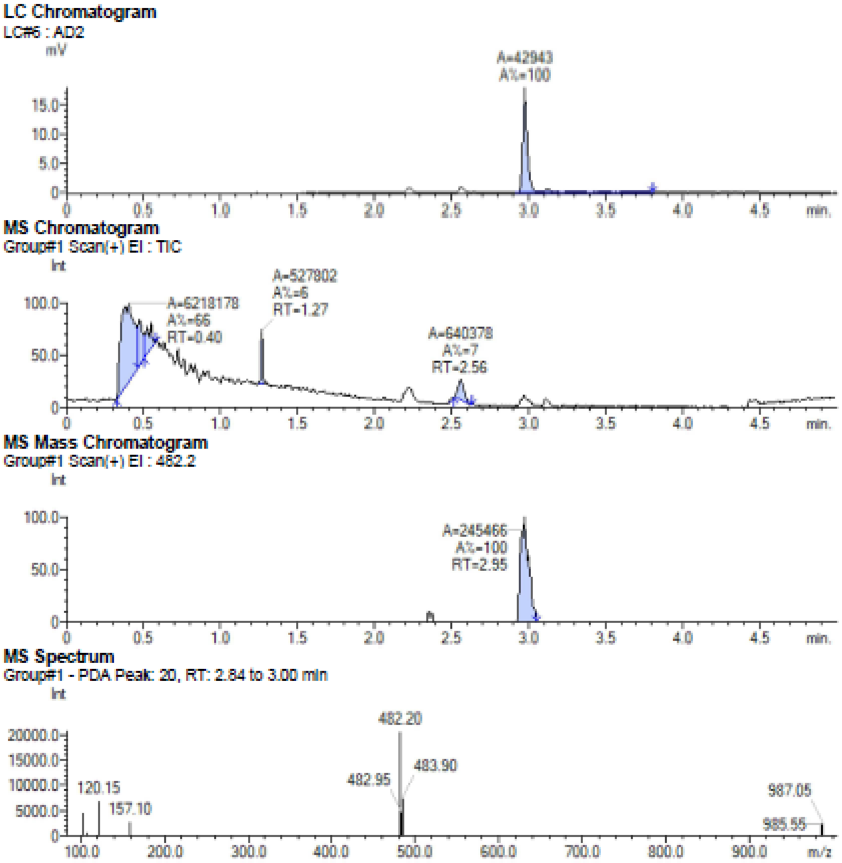

Shimadzu Open Solution

GF-THPI-3

**Project:**	Test
**Experiment:**	676_200316_LDC_0518_Griga_REG_0515_Flege
**Experiment Description:**	
**Sample:**	412367:01:01
**Sample Description:**	C26H25Cl2NO5
**Data File Name:**	C:\LabSolutions\Data\676_200316_LDC_0518_Grigalunas_REG_0515_Flegel\1046326485_11200518676.lcd
**Sample Location:**	Plate Number: 1 - Position: 73
**Run By:**	admin
**Run Started:**	20 July 2020 12:06:48
**Run Finished:**	20 July 2020 13:31:06
**Method:**	190321_Purity-Check

LC Chromatogram

LC#5 : AD2

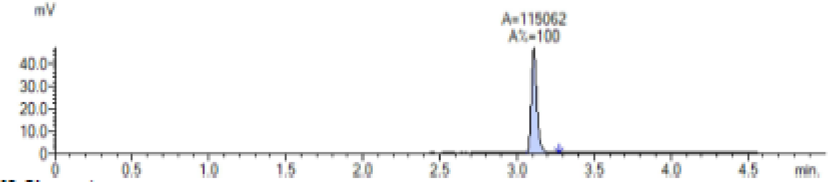

MS Chromatogram

Group#1 Scan(+) EI : TIC

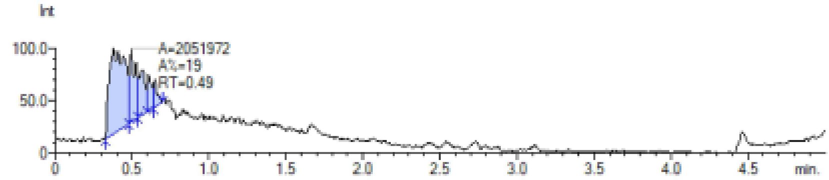

MS Mass Chromatogram

Group#1 Scan(+) EI : 502.1

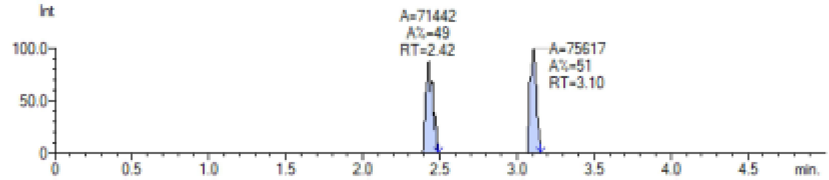

MS Spectrum

Group#1 - PDA Peak: 22, RT: 2.98 to 3.11 min

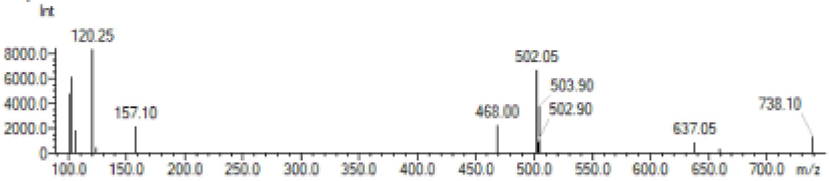

GF-THPI-4

Shimadzu Open Solution

Project: Test
Experiment: 560_0511_3103_15_Burhop_10_Wesseler
Experiment Description:
Sample: 409966:01:01
Sample Description: C27H28ClNO5
Data File Name: C:\LabSolutions\Data\560_190511_3103_190515_Burhop_190510_Wesseler\1046360651_11190516560.lcd
Sample Location: Plate Number: 1 - Position: 169
Run By: admin
Run Started: 03 June 2019 14:35:38
Run Finished: 03 June 2019 21:16:00
Method: 190321_Purity-Check

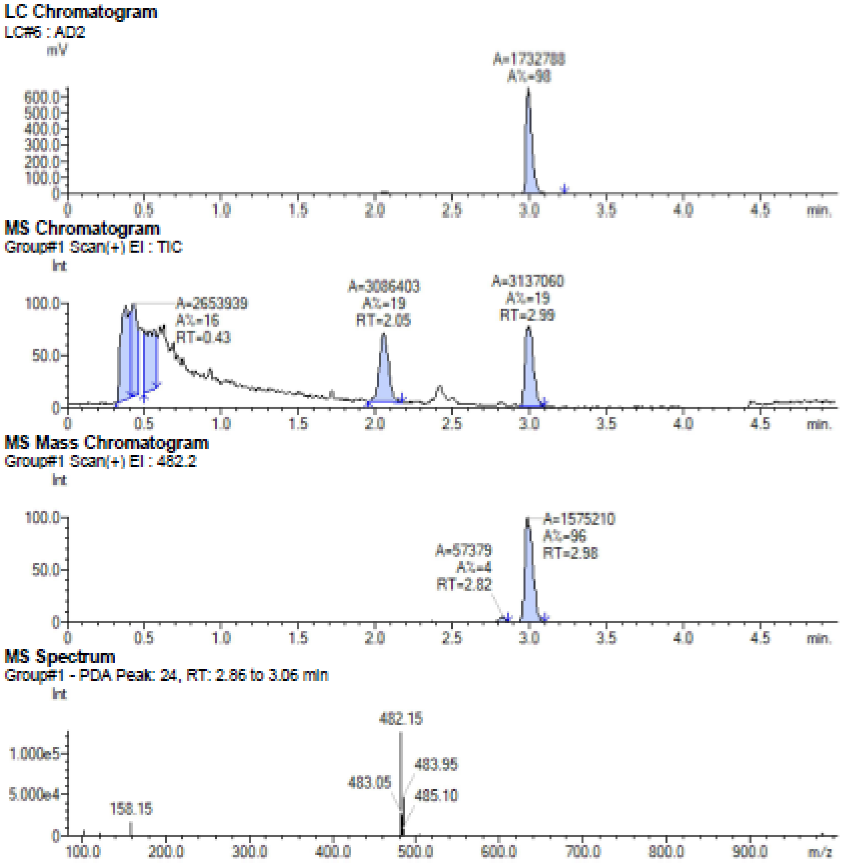

Shimadzu Open Solution

GF-THPI-5

**Project:**	Test
**Experiment:**	676_200316_LDC_0518_Griga_REG_0515_Flege
**Experiment Description:**	
**Sample:**	412368:01:01
**Sample Description:**	C27H28ClNO6
**Data File Name:**	C:\LabSolutions\Data\676_200316_LDC_0518_Grigalunas_REG_0515_Flege\1046326941_11200518676.lcd
**Sample Location:**	Plate Number: 1 - Position: 121
**Run By:**	admin
**Run Started:**	20 July 2020 12:06:48
**Run Finished:**	20 July 2020 13:31:06
**Method:**	190321_Purity-Check

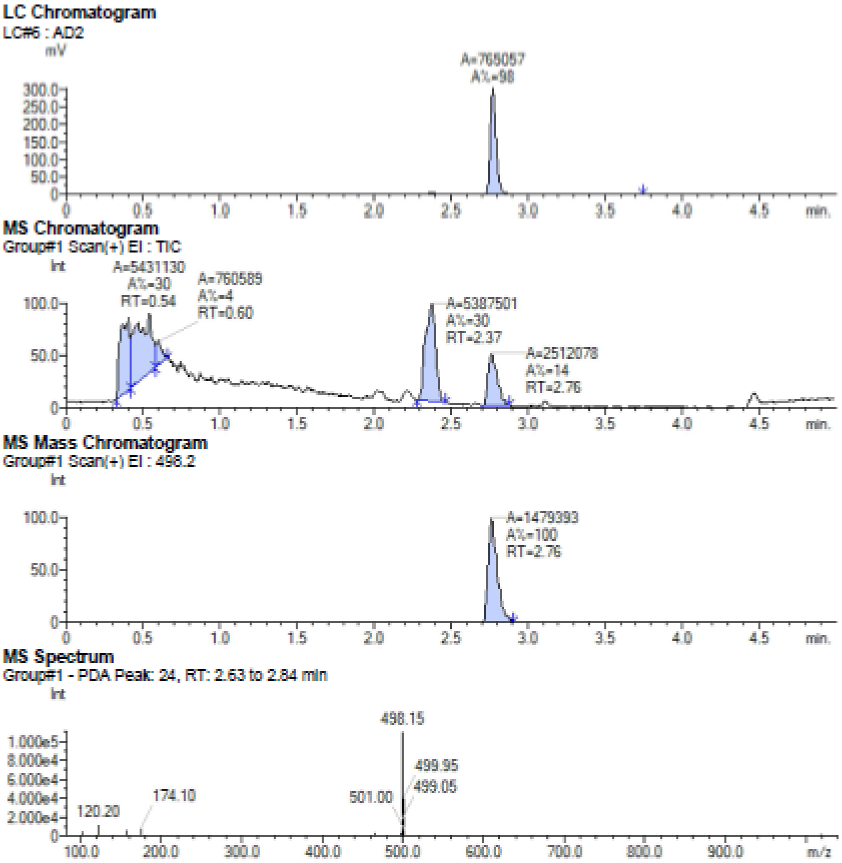

Shimadzu Open Solution

**Project:**	Test
**Experiment:**	676_200316_LDC_0518_Griga_REG_0515_Flege
**Experiment Description:**	
**Sample:**	412369:01:01
**Sample Description:**	C26H25BrClNO5
**Data File Name:**	C:\LabSolutions\Data\676_200316_LDC_0518_Grigalunas_REG_0515_Flege\1046326837_11200518676.lcd
**Sample Location:**	Plate Number: 1 - Position: 169
**Run By:**	admin
**Run Started:**	20 July 2020 12:06:48
**Run Finished:**	20 July 2020 13:31:06
**Method:**	190321_Purity-Check

GF-THPI-6

LC Chromatogram
LC#6 : AD2

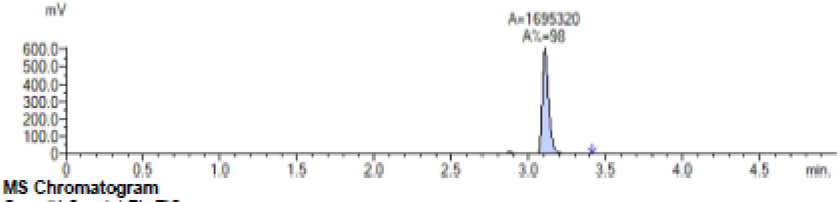

MS Chromatogram
Group#1 Scan(+) EI : TIC

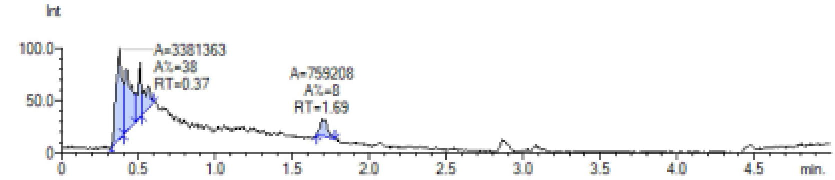

MS Mass Chromatogram
Group#1 Scan(+) EI : 546.1

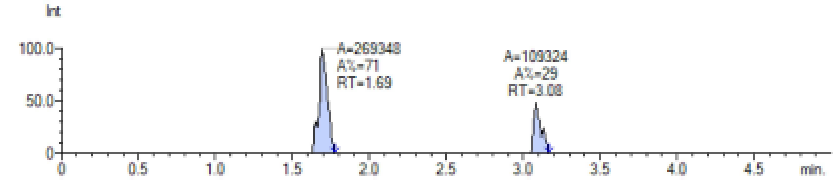

MS Spectrum
Group#1 - PDA Peak: 25, RT: 2.97 to 3.14 min

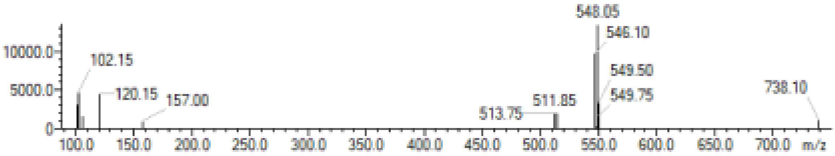

Shimadzu Open Solution

GF-THPI-7

Project:	Test
Experiment:	676_200316_LDC_0518_Griga_REG_0515_Flege
Experiment Description:	
Sample:	412370:01:01
Sample Description:	C26H25Cl2NO5
Data File Name:	C:\LabSolutions\Data\676_200316_LDC_0518_Grigalunas_REG_0515_Flegel\1046327064_11200518676.lcd
Sample Location:	Plate Number: 1 - Position: 217
Run By:	admin
Run Started:	20 July 2020 12:06:48
Run Finished:	20 July 2020 13:31:06
Method:	190321_Purity-Check

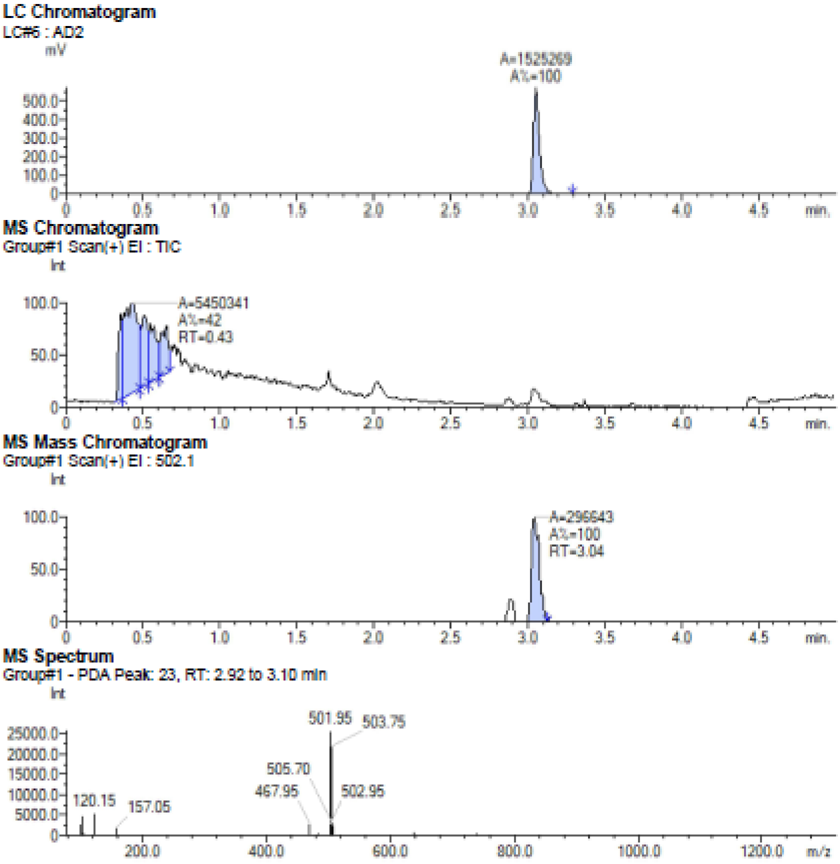

Shimadzu Open Solution

GF-THPI-8

**Project:**	Test
**Experiment:**	676_200316_LDC_0518_Griga_REG_0515_Flege
**Experiment Description:**	
**Sample:**	412371:01:01
**Sample Description:**	C26H25ClFNO5
**Data File Name:**	C:\LabSolutions\Data\676_200316_LDC_0518_Grigalunas_REG_0515_Flege\1046325721_11200518676.lcd
**Sample Location:**	Plate Number: 1 - Position: 265
**Run By:**	admin
**Run Started:**	20 July 2020 12:06:48
**Run Finished:**	20 July 2020 13:31:06
**Method:**	190321_Purity-Check

LC Chromatogram
LC#6 : AD2

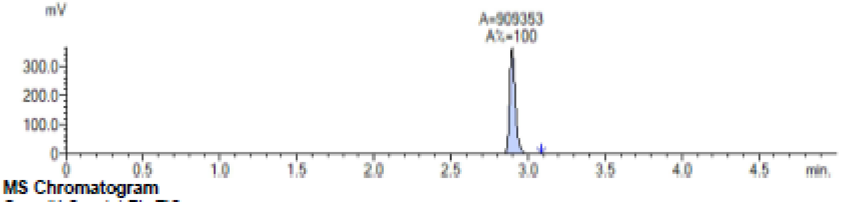

MS Chromatogram
Group#1 Scan(+) EI : TIC

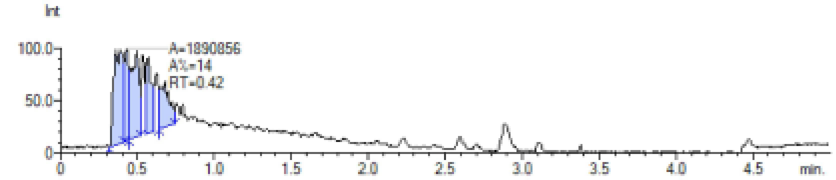

MS Mass Chromatogram
Group#1 Scan(+) EI : 486.1

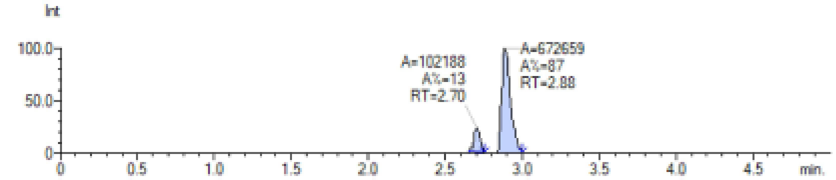

MS Spectrum
Group#1 - PDA Peak: 22, RT: 2.75 to 2.95 min

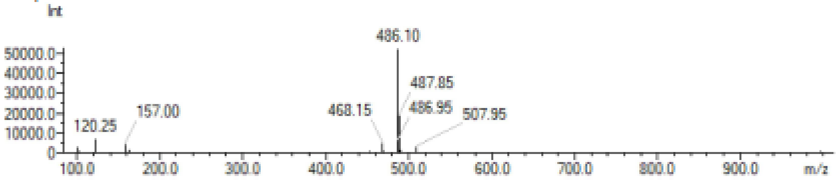

Shimadzu Open Solution

GF-THPI-9

Project: Test
Experiment: 676_200316_LDC_0518_Griga_REG_0515_Flege
Experiment Description:
Sample: 412372:01:01
Sample Description: C29H32ClNO5
Data File Name: C:\LabSolutions\Data\676_200316_LDC_0518_Grigalunas_REG_0515_Flege\1046326769_11200518676.lcd
Sample Location: Plate Number: 1 - Position: 313
Run By: admin
Run Started: 20 July 2020 12:06:48
Run Finished: 20 July 2020 13:31:06
Method: 190321_Purity-Check

LC Chromatogram
LC#5 : AD2

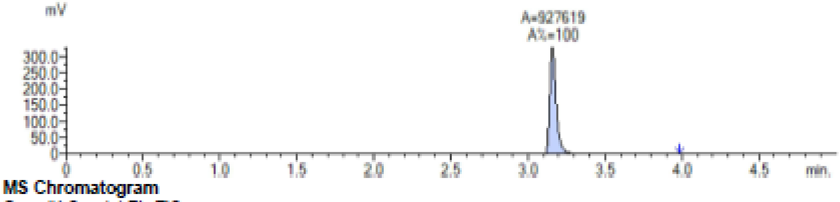

MS Chromatogram
Group#1 Scan(+) EI : TIC

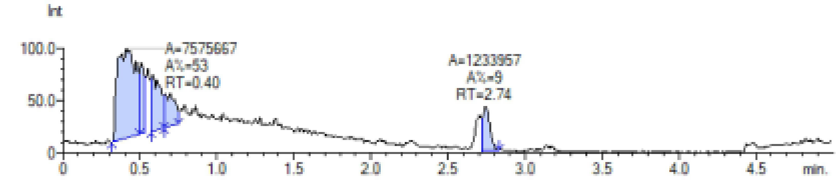

MS Mass Chromatogram
Group#1 Scan(+) EI : 510.2

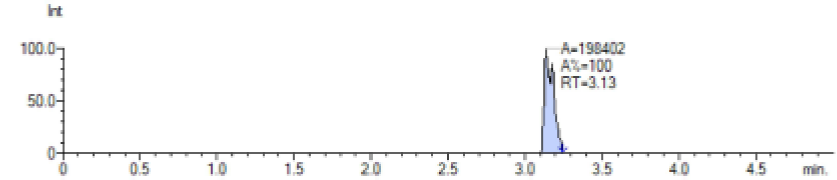

MS Spectrum
Group#1 - PDA Peak: 21, RT: 3.02 to 3.18 min

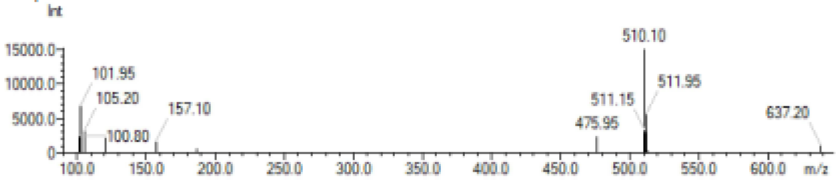

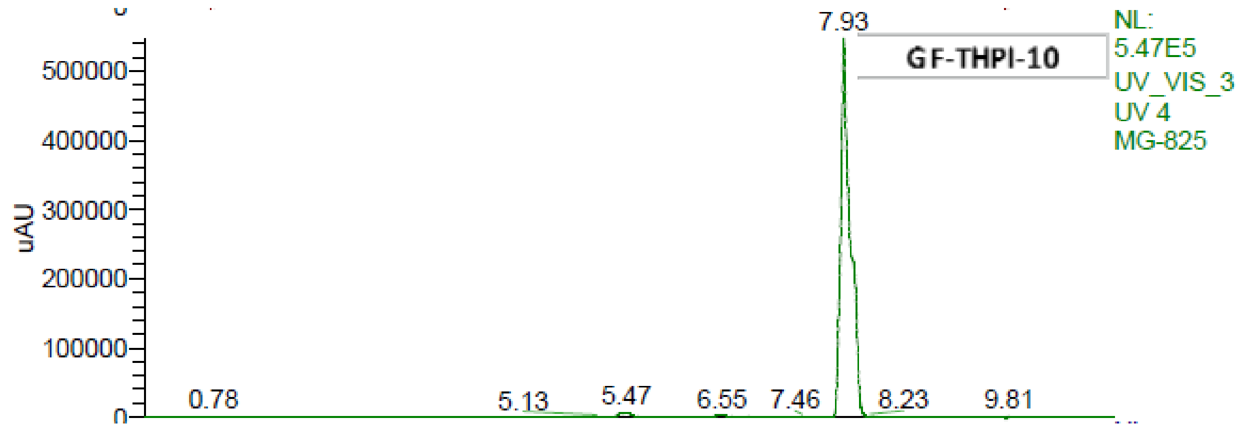

PEAK LIST
MG-825.raw
RT: 7.84 - 8.16
Number of detected peaks: 1

Apex RT	Start RT	End RT	Area	%Area	Height	%Height
7.93	7.86	8.13	153982.757	100.00	21949.126	100.00

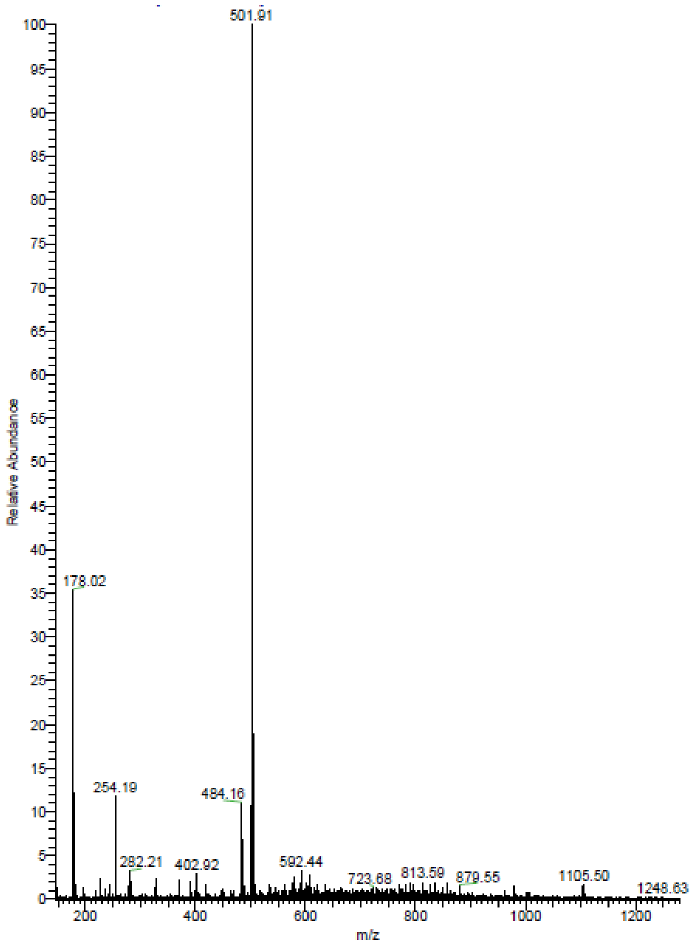

Shimadzu Open Solution

GF-THPI-11

**Project:**	Test
**Experiment:**	676_200316_LDC_0518_Griga_REG_0515_Flege
**Experiment Description:**	
**Sample:**	412374:01:01
**Sample Description:**	C28H30ClNO5
**Data File Name:**	C:\LabSolutions\Data\676_200316_LDC_0518_Grigalunas_REG_0515_Flege\1046327113_11200518676.lcd
**Sample Location:**	Plate Number: 1 - Position: 27
**Run By:**	admin
**Run Started:**	20 July 2020 12:06:48
**Run Finished:**	20 July 2020 13:31:06
**Method:**	190321_Purity-Check

LC Chromatogram

LC#6 : AD2

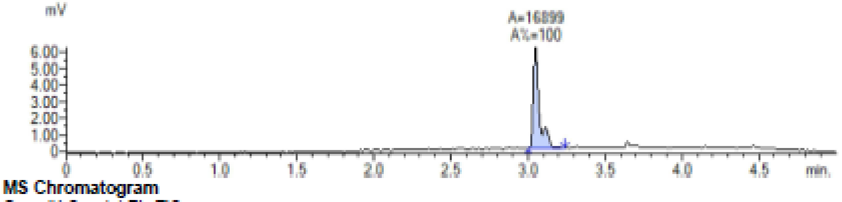

MS Chromatogram

Group#1 Scan(+) EI : TIC

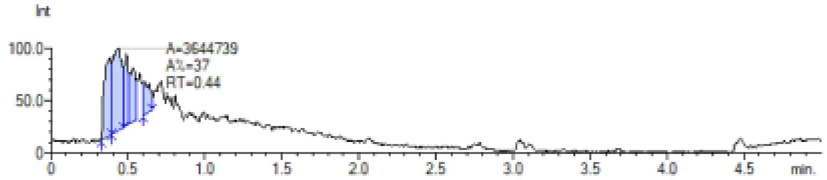

MS Mass Chromatogram

Group#1 Scan(+) EI : 496.2

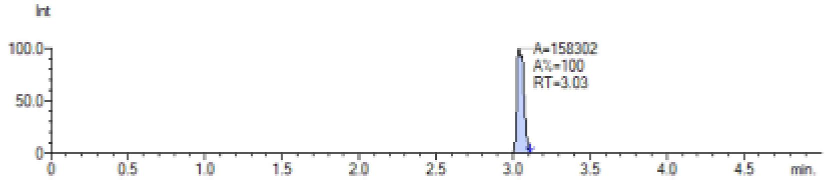

MS Spectrum

Group#1 - PDA Peak: 18, RT: 2.91 to 3.00 min

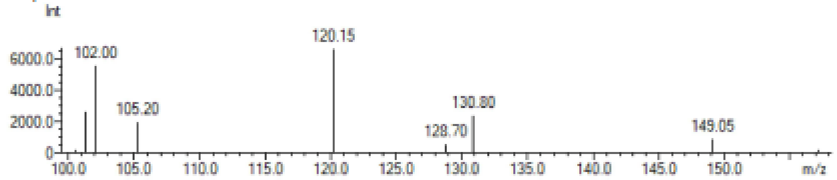

Shimadzu Open Solution

**Project:**	Test
**Experiment:**	476_Grigalu_1011_Shaaban_Liu_1009_3076
**Experiment Description:**	
**Sample:**	409062:01:01
**Sample Description:**	C23H22FNO4
**Data File Name:**	C:\LabSolutions\Data\476_Grigalunas_181011_Shaaban_181011_Liu_181009_3076 \1046359525_11181011476.lcd
**Sample Location:**	Plate Number: 2 - Position: 149
**Run By:**	admin
**Run Started:**	24 April 2019 18:14:10
**Run Finished:**	25 April 2019 04:58:04
**Method:**	190321_Purity-Check+shut down

C-I-1

LC Chromatogram

LC#6 : AD2

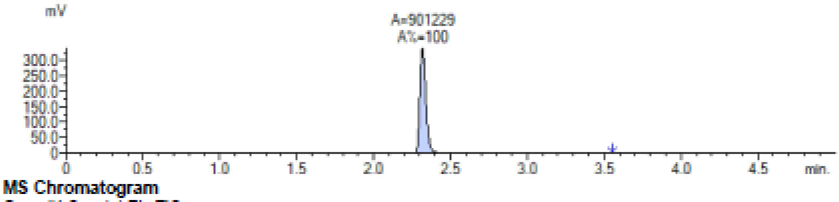

MS Chromatogram

Group#1 Scan(+) EI : TIC

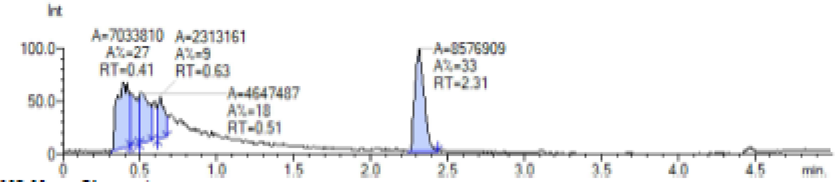

MS Mass Chromatogram

Group#1 Scan(+) EI : 396.2

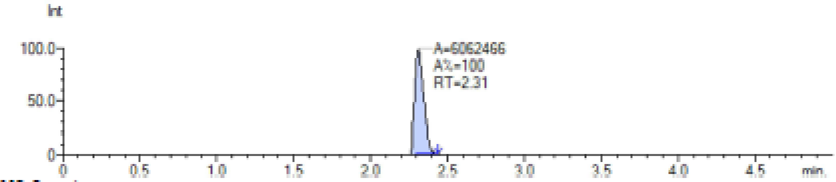

MS Spectrum

Group#1 - PDA Peak: 20, RT: 2.17 to 2.39 min

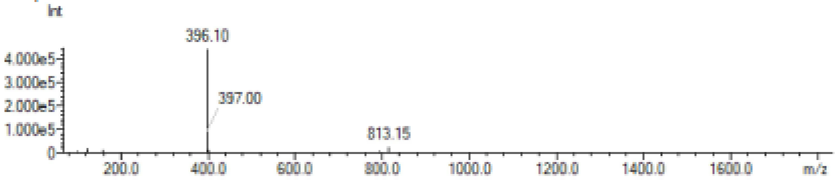

C-I-2

Shimadzu Open Solution

**Project:**	Test
**Experiment:**	572_190612_Zinken_0607_Bischof_0612Griga
**Experiment Description:**	
**Sample:**	410474:01:01
**Sample Description:**	C21H18FNO3
**Data File Name:**	C:\LabSolutions\Data\572_190612_Zinken_190607_Bischoff_190612_Grigalunas\1046364501_11190613572.lcd
**Sample Location:**	Plate Number: 1 - Position: 38
**Run By:**	admin
**Run Started:**	29 July 2019 19:27:00
**Run Finished:**	30 July 2019 04:41:49
**Method:**	190321_Purity-Check

LC Chromatogram

LC#5 : AD2

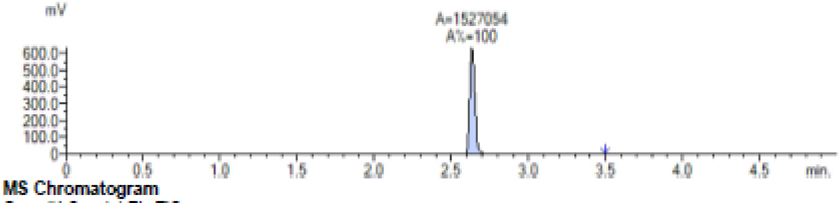

MS Chromatogram

Group#1 Scan(+) EI : TIC

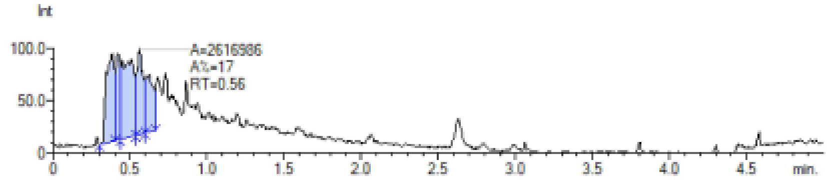

MS Mass Chromatogram

Group#1 Scan(+) EI : 352.1

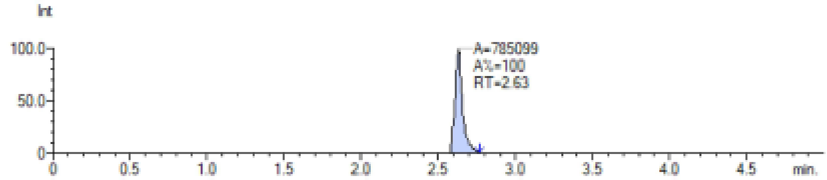

MS Spectrum

Group#1 - PDA Peak: 16, RT: 2.48 to 2.67 min

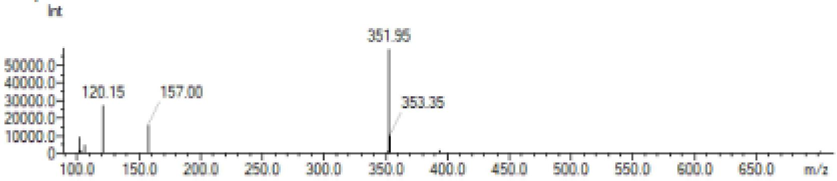

C-I-3

Shimadzu Open Solution

**Project:**	Test
**Experiment:**	572_190612_Zinken_0607_Bischof_0612Griga
**Experiment Description:**	
**Sample:**	410475:01:01
**Sample Description:**	C22H20FNO3
**Data File Name:**	C:\LabSolutions\Data\572_190612_Zinken_190607_Bischoff_190612_Grigalunas\1046364611_11190613572.lcd
**Sample Location:**	Plate Number: 1 - Position: 86
**Run By:**	admin
**Run Started:**	29 July 2019 19:27:00
**Run Finished:**	30 July 2019 04:41:49
**Method:**	190321_Purity-Check

LC Chromatogram
LC#6 : AD2

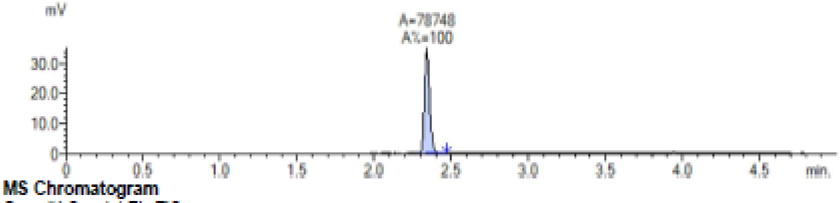

MS Chromatogram
Group#1 Scan(+) EI : TIC

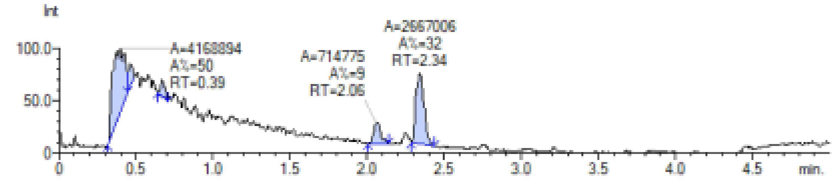

MS Mass Chromatogram
Group#1 Scan(+) EI : 366.1

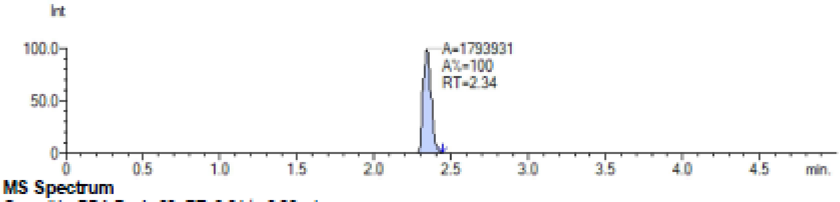

MS Spectrum
Group#1 - PDA Peak: 22, RT: 2.21 to 2.36 min

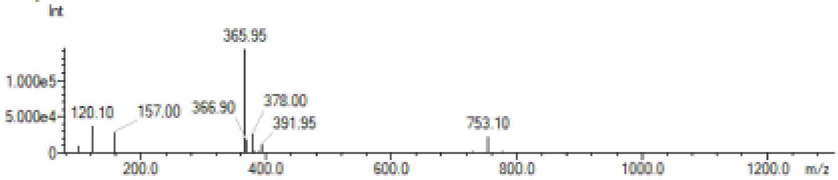

C-I-4

Shimadzu Open Solution

Project: Test
Experiment: 572_190612_Zinken_0607_Bischof_0612Griga
Experiment Description:
Sample: 410476:01:01
Sample Description: C22H20FNO3
Data File Name: C:\LabSolutions\Data\572_190612_Zinken_190607_Bischoff_190612_Grigalunas\1046364763_11190613572.lcd
Sample Location: Plate Number: 1 - Position: 134
Run By: admin
Run Started: 29 July 2019 19:27:00
Run Finished: 30 July 2019 04:41:49
Method: 190321_Purity-Check

LC Chromatogram
LC#6 : AD2

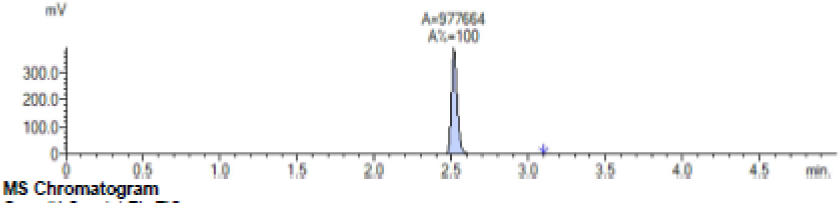

MS Chromatogram
Group#1 Scan(+) EI : TIC

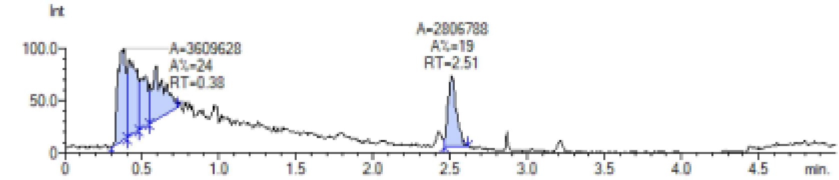

MS Mass Chromatogram
Group#1 Scan(+) EI : 366.1

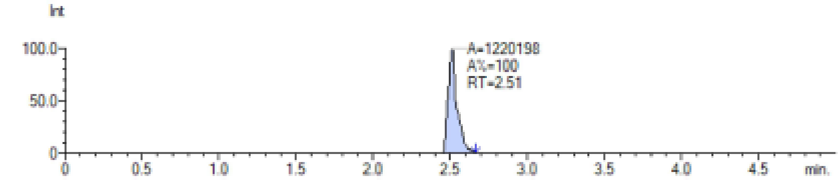

MS Spectrum
Group#1 - PDA Peak: 20, RT: 2.38 to 2.57 min

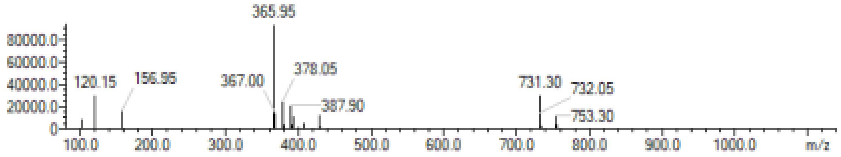

C-I-5

Shimadzu Open Solution

**Project:**	Test
**Experiment:**	669_0303_Xie_Wilke_0304_Grigalunas_Nigge
**Experiment Description:**	
**Sample:**	411973:01:01
**Sample Description:**	C21H19NO3
**Data File Name:**	C:\LabSolutions\Data\669_0303_Xie_Wilke_0304_Grigalunas_Niggemeyer\1046324744_11200304669.lcd
**Sample Location:**	Plate Number: 1 - Position: 25
**Run By:**	admin
**Run Started:**	14 May 2020 16:58:32
**Run Finished:**	15 May 2020 10:54:08
**Method:**	Purity-Check+sleep

LC Chromatogram

LC#6 : AD2

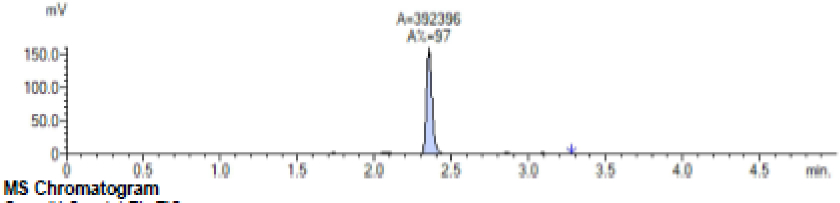

MS Chromatogram

Group#1 Scan(+) EI : TIC

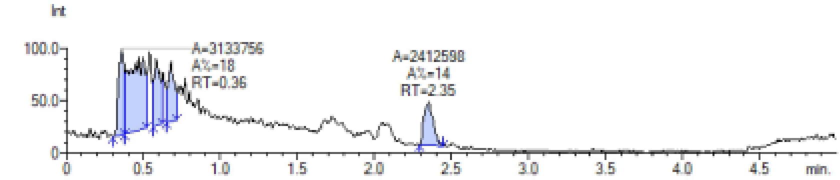

MS Mass Chromatogram

Group#1 Scan(+) EI : 334.1

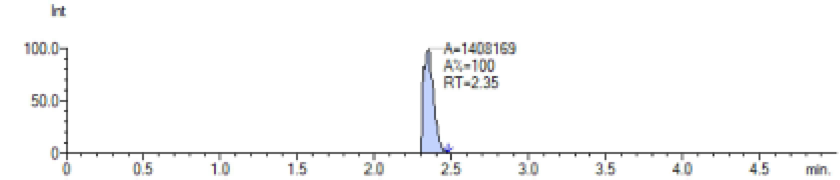

MS Spectrum

Group#1 - PDA Peak: 26, RT: 2.22 to 2.35 min

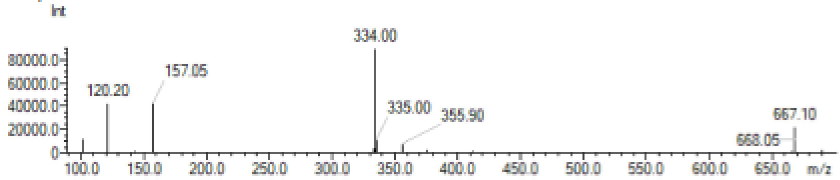

C-I-6

Shimadzu Open Solution

Project: Test
Experiment: 669_0303_Xie_Wilke_0304_Grigalunas_Nigge
Experiment Description:
Sample: 411974:01:02
Sample Description: C24H25NO3
Data File Name: C:\LabSolutions\Data\669_0303_Xie_Wilke_0304_Grigalunas_Niggemeyer\1046324880_11200304669.lcd
Sample Location: Plate Number: 1 - Position: 73
Run By: admin
Run Started: 14 May 2020 16:58:32
Run Finished: 15 May 2020 10:54:08
Method: Purity-Check+sleep

LC Chromatogram
LC#6 : AD2

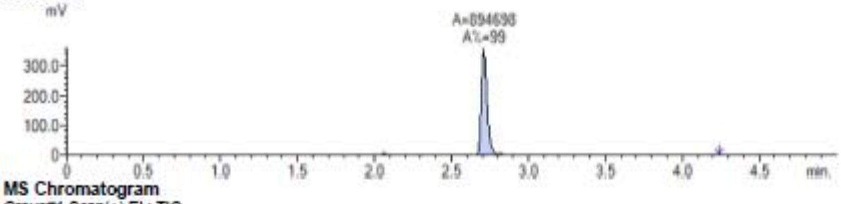

MS Chromatogram
Group#1 Scan(+) EI : TIC

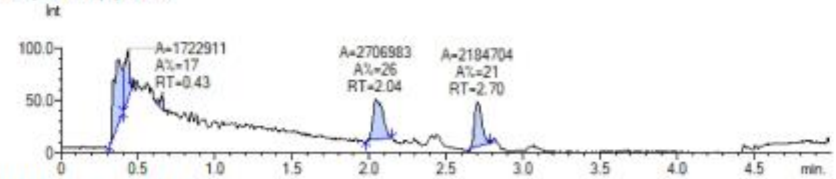

MS Mass Chromatogram
Group#1 Scan(+) EI : 376.2

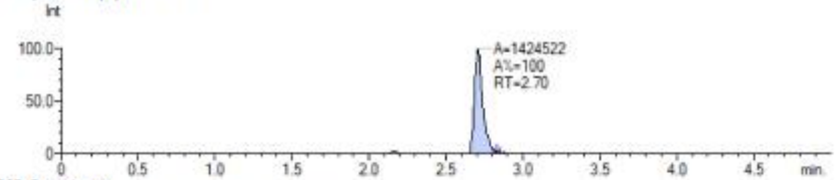

MS Spectrum
Group#1 - PDA Peak: 25, RT: 2.58 to 2.70 min

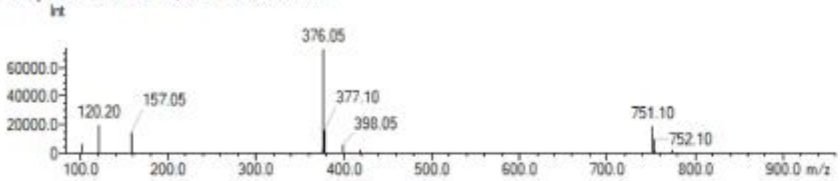

C-I-7

Shimadzu Open Solution

**Project:**	Test
**Experiment:**	669_0303_Xie_Wilke_0304_Grigalunas_Nigge
**Experiment Description:**	
**Sample:**	411975:01:01
**Sample Description:**	C21H18ClNO3
**Data File Name:**	C:\LabSolutions\Data\669_0303_Xie_Wilke_0304_Grigalunas_Niggemeyer\1046324802_11200304669.lcd
**Sample Location:**	Plate Number: 1 - Position: 121
**Run By:**	admin
**Run Started:**	14 May 2020 16:58:32
**Run Finished:**	15 May 2020 10:54:08
**Method:**	Purity-Check+sleep

LC Chromatogram

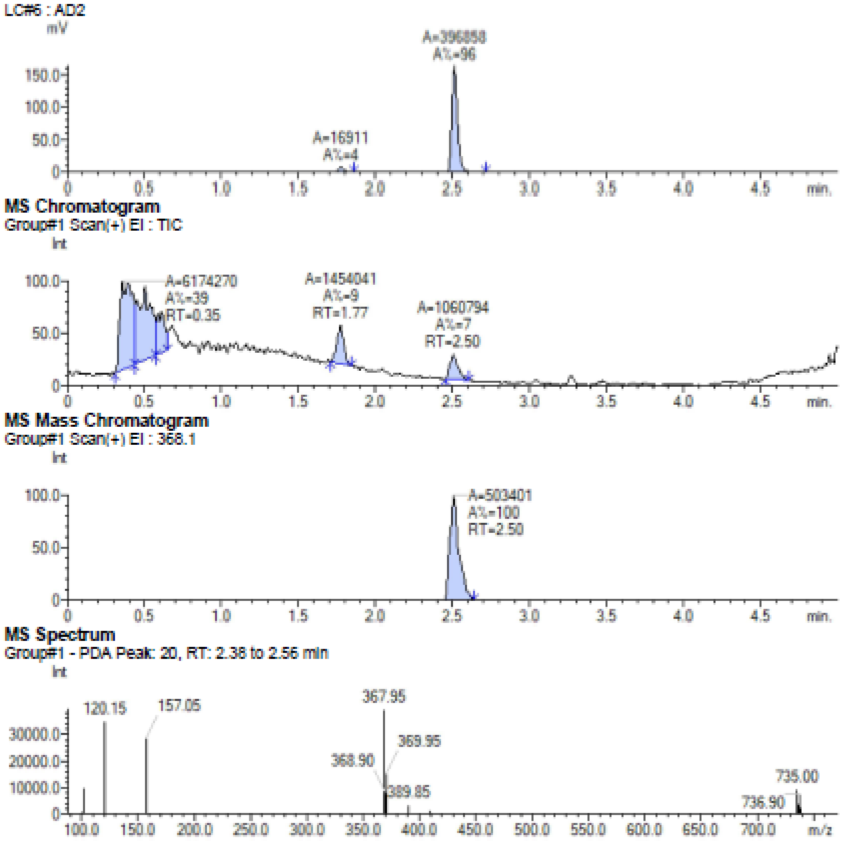

C-I-8

Shimadzu Open Solution

**Project:**	Test
**Experiment:**	669_0303_Xie_Wilke_0304_Grigalunas_Nigge
**Experiment Description:**	
**Sample:**	411976:01:02
**Sample Description:**	C23H23NO3
**Data File Name:**	C:\LabSolutions\Data\669_0303_Xie_Wilke_0304_Grigalunas_Niggemeyer\1046324826_11200304669.lcd
**Sample Location:**	Plate Number: 1 - Position: 169
**Run By:**	admin
**Run Started:**	14 May 2020 16:58:32
**Run Finished:**	15 May 2020 10:54:08
**Method:**	Purity-Check+sleep

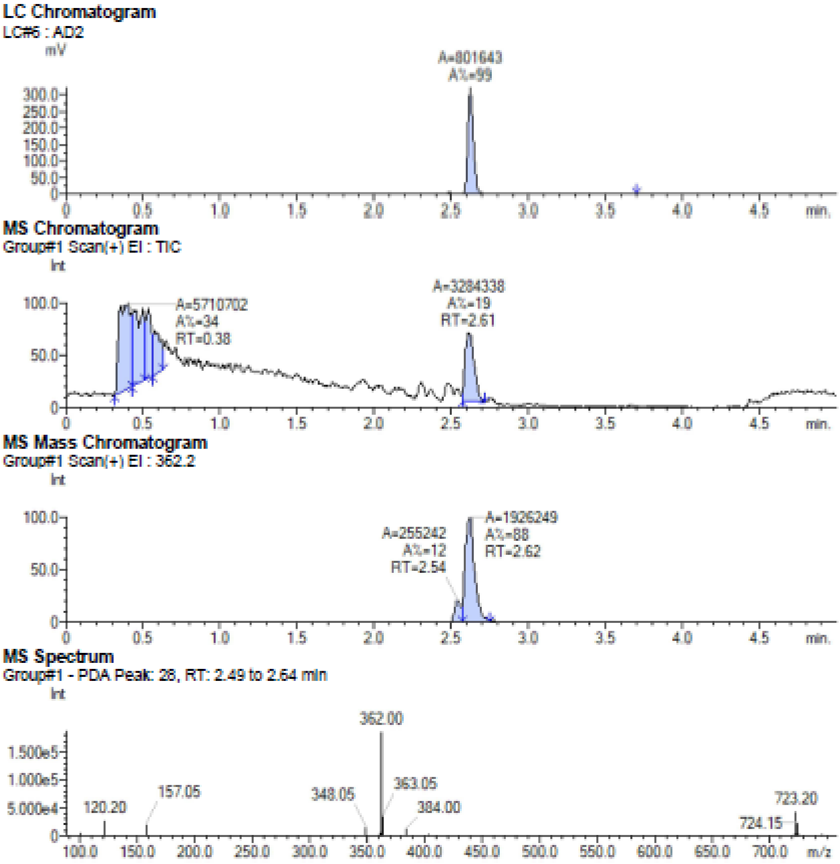

C-I-9

Shimadzu Open Solution

**Project:**	Test
**Experiment:**	669_0303_Xie_Wilke_0304_Grigalunas_Nigge
**Experiment Description:**	
**Sample:**	411977:01:01
**Sample Description:**	C27H23NO3
**Data File Name:**	C:\LabSolutions\Data\669_0303_Xie_Wilke_0304_Grigalunas_Niggemeyer\1046324874_11200304669.lcd
**Sample Location:**	Plate Number: 1 - Position: 217
**Run By:**	admin
**Run Started:**	14 May 2020 16:58:32
**Run Finished:**	15 May 2020 10:54:08
**Method:**	Purity-Check+sleep

LC Chromatogram

LC#5 : AD2

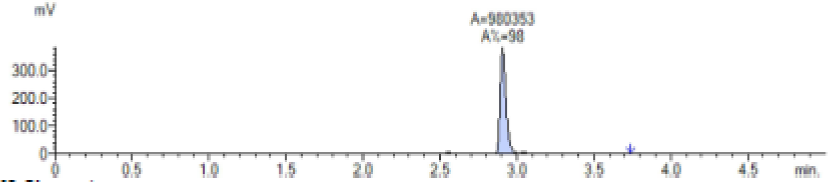

MS Chromatogram

Group#1 Scan(+) EI : TIC

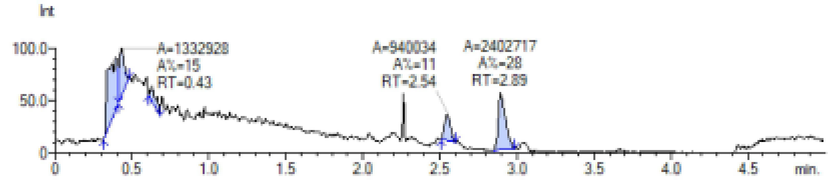

MS Mass Chromatogram

Group#1 Scan(+) EI : 410.2

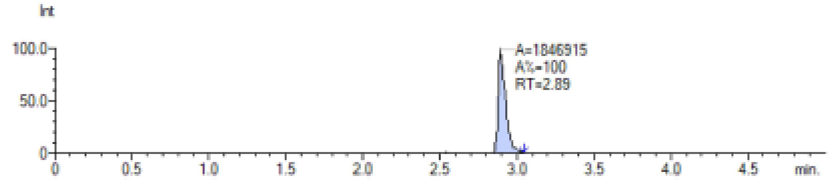

MS Spectrum

Group#1 - PDA Peak: 20, RT: 2.75 to 2.92 min

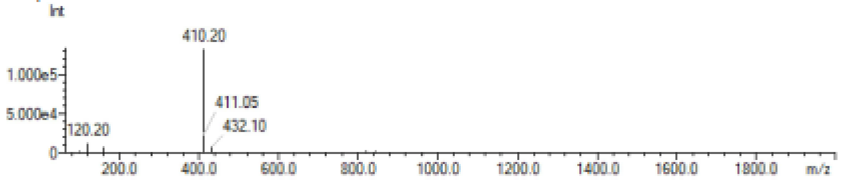

C-I-10

Shimadzu Open Solution

Project: Test
Experiment: 669_0303_Xie_Wilke_0304_Grigalunas_Nigge
Experiment Description:
Sample: 411978:01:01
Sample Description: C28H25NO3
Data File Name: C:\LabSolutions\Data\669_0303_Xie_Wilke_0304_Grigalunas_Niggemeyer\1046324631_11200304669.lcd
Sample Location: Plate Number: 1 - Position: 265
Run By: admin
Run Started: 14 May 2020 16:58:32
Run Finished: 15 May 2020 10:54:08
Method: Purity-Check+sleep

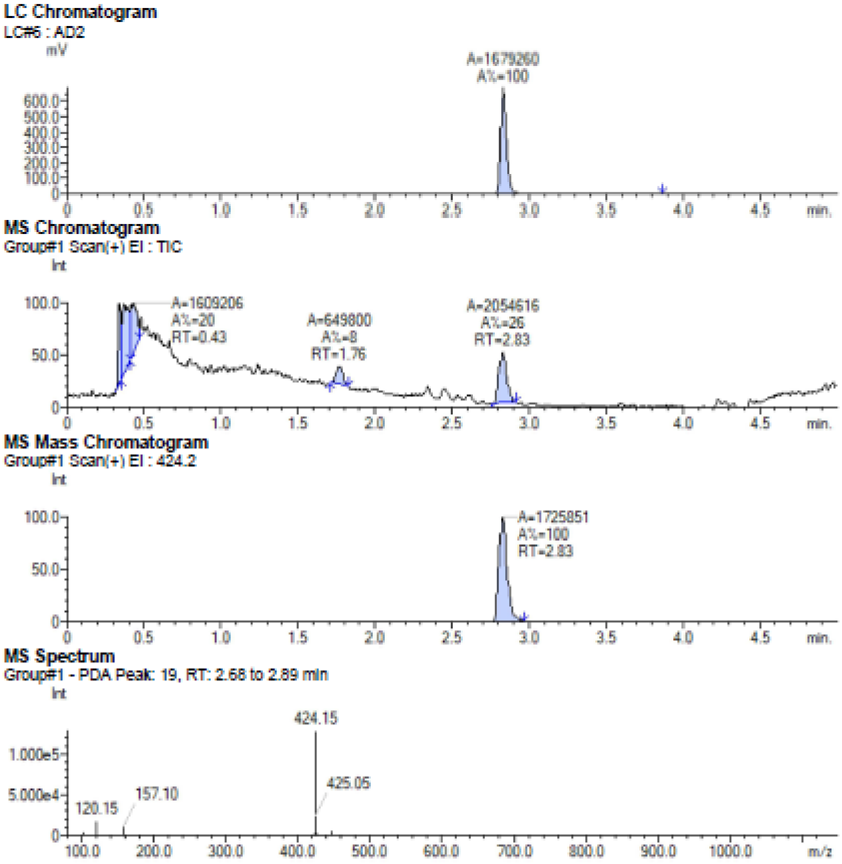

C-I-11

Shimadzu Open Solution

Project: Test
Experiment: 669_0303_Xie_Wilke_0304_Grigalunas_Nigge
Experiment Description:
Sample: 411979:01:01
Sample Description: C21H17F2NO3
Data File Name: C:\LabSolutions\Data\669_0303_Xie_Wilke_0304_Grigalunas_Niggemeyer\1046324658_11200304669.lcd
Sample Location: Plate Number: 1 - Position: 313
Run By: admin
Run Started: 14 May 2020 16:58:32
Run Finished: 15 May 2020 10:54:08
Method: Purity-Check+sleep

LC Chromatogram

LC#6 : AD2

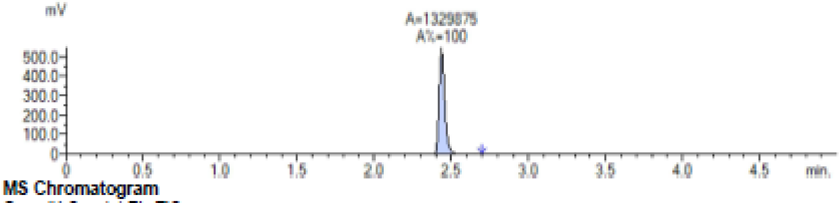

MS Chromatogram

Group#1 Scan(+) EI : TIC

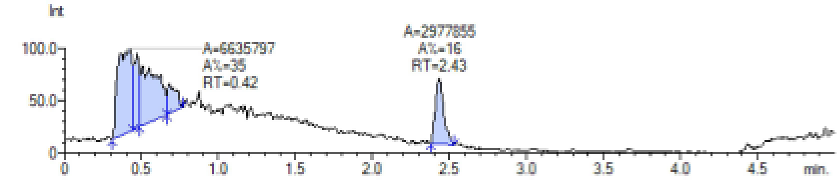

MS Mass Chromatogram

Group#1 Scan(+) EI : 370.1

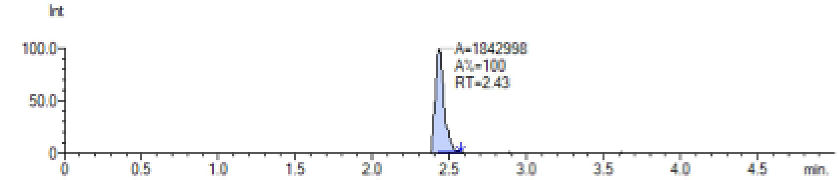

MS Spectrum

Group#1 - PDA Peak: 22, RT: 2.30 to 2.59 min

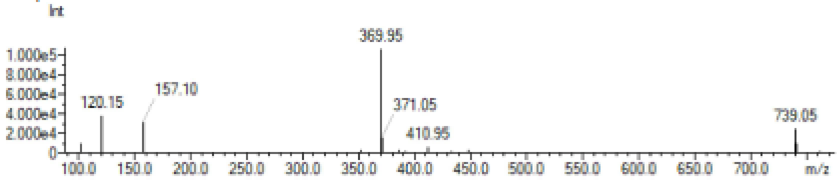

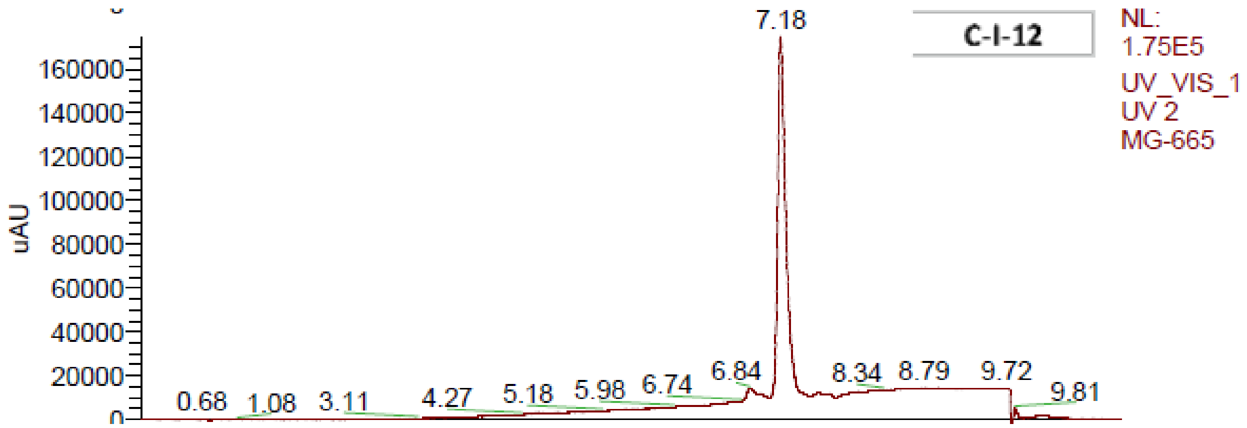

PEAK LIST
MG-665.raw
RT: 0.00 - 11.00
Number of detected peaks: 1

Apex RT	Start RT	End RT	Area	%Area	Height	%Height
7.18	7.07	7.44	4150835.062	100.00	623069.637	100.00

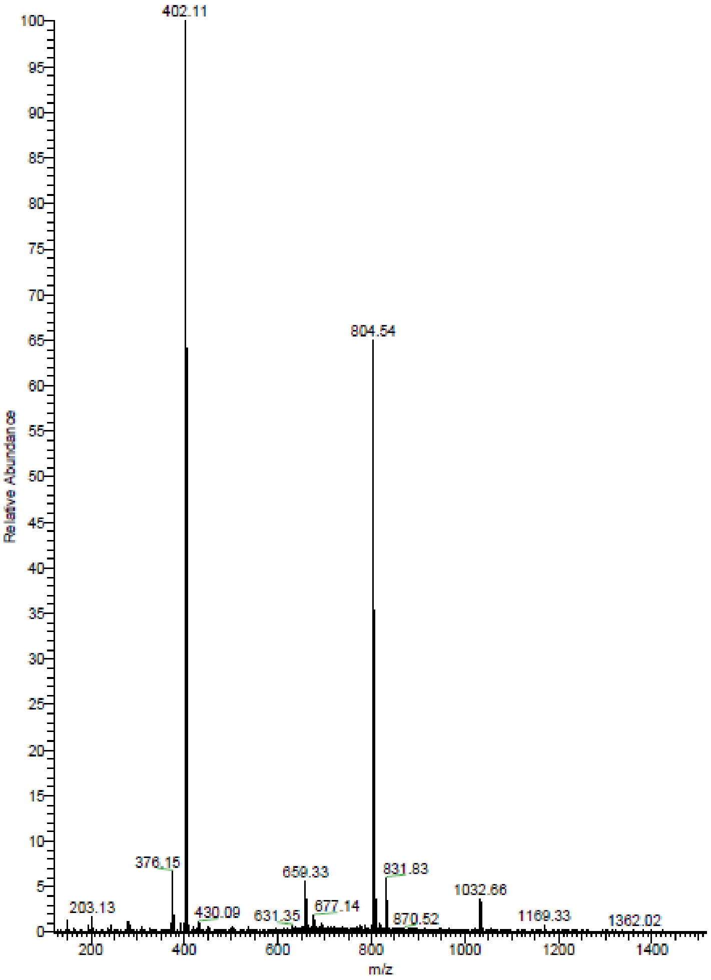

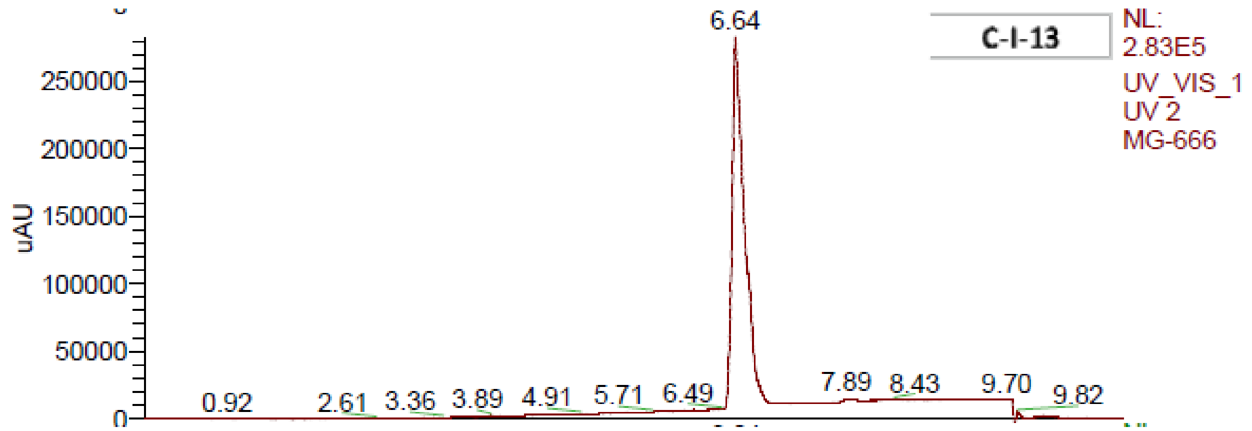

PEAK LIST
MG-666.raw
RT: 6.56 - 6.95
Number of detected peaks: 1

Apex RT	Start RT	End RT	Area	%Area	Height	%Height
6.64	6.56	6.86	122498.190	100.00	14648.324	100.00

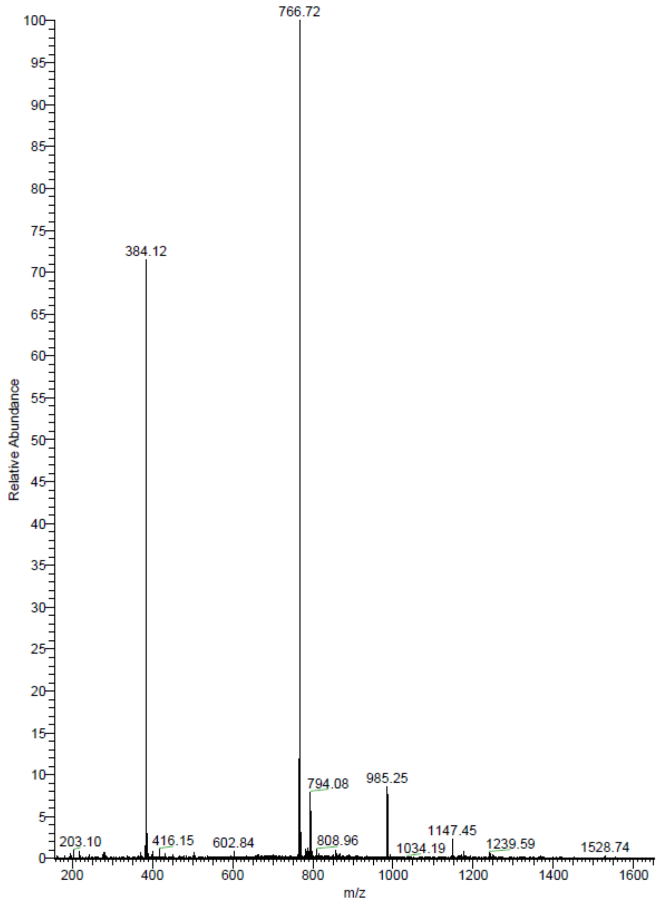

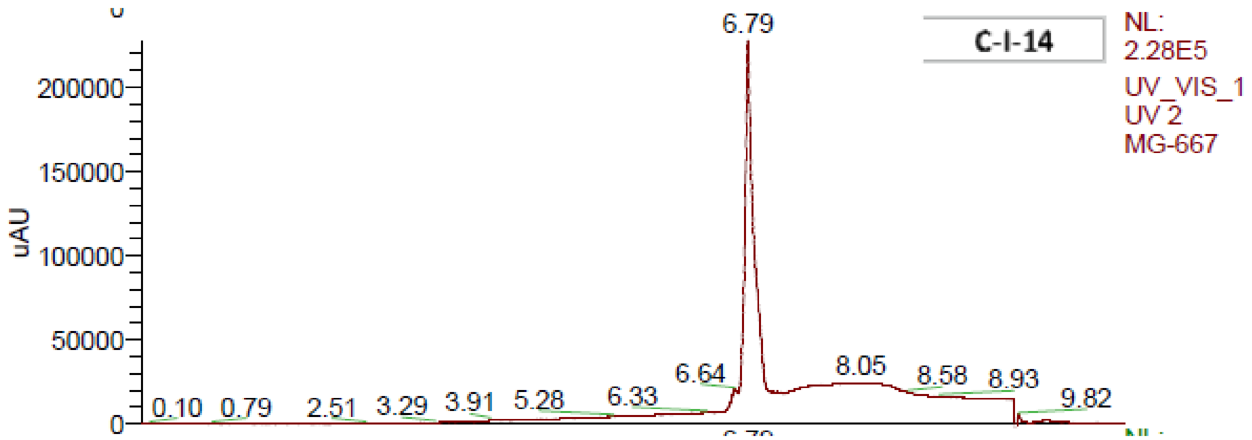

PEAK LIST
MG-667.raw
RT: 6.69 - 6.96
Number of detected peaks: 1

Apex RT	Start RT	End RT	Area	%Area	Height	%Height
6.79	6.71	6.96	39604.482	100.00	7044.159	100.00

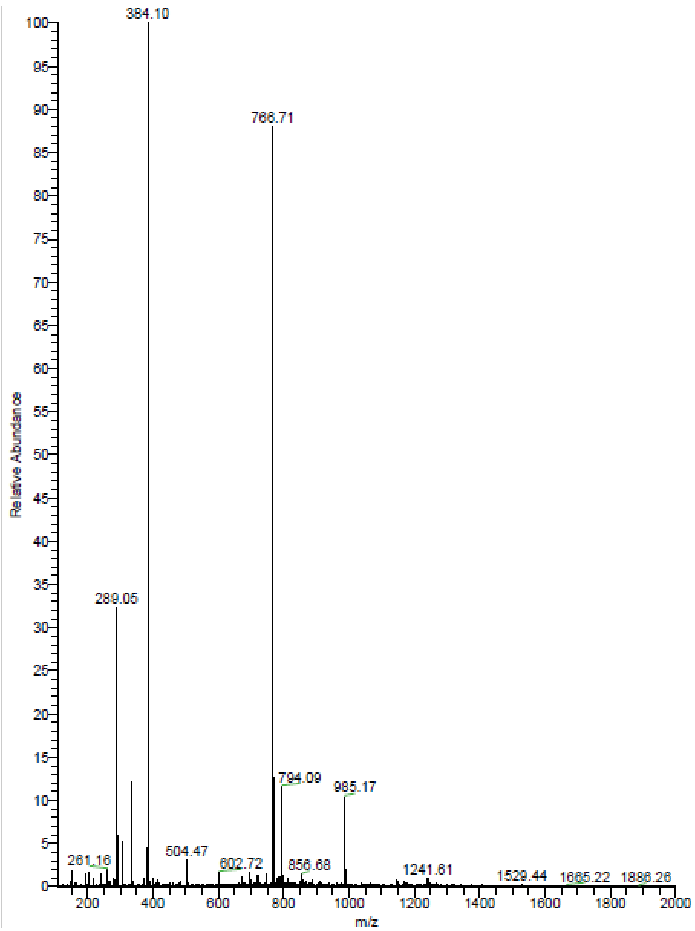

C-I-15

Shimadzu Open Solution

Project: Test
Experiment: 669_0303_Xie_Wilke_0304_Grigalunas_Nigge
Experiment Description:
Sample: 411983:01:01
Sample Description: C28H25NO4
Data File Name: C:\LabSolutions\Data\669_0303_Xie_Wilke_0304_Grigalunas_Niggemeyer\1046324969_11200304669.lcd
Sample Location: Plate Number: 1 - Position: 123
Run By: admin
Run Started: 14 May 2020 16:58:32
Run Finished: 15 May 2020 10:54:08
Method: Purity-Check+sleep

LC Chromatogram
LC#6 : AD2

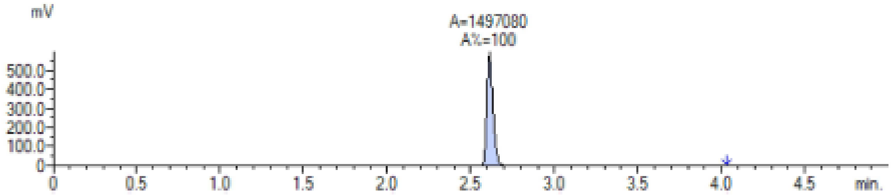

MS Chromatogram
Group#1 Scan(+) EI : TIC

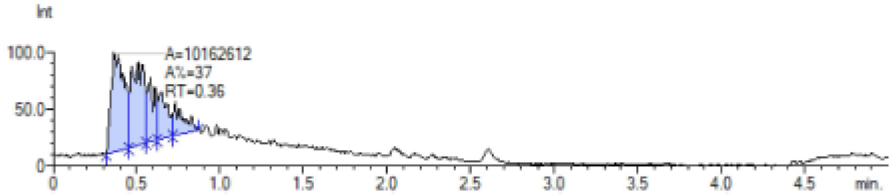

MS Mass Chromatogram
Group#1 Scan(+) EI : 440.2

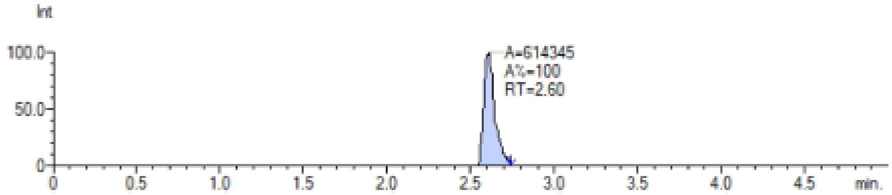

MS Spectrum
Group#1 - PDA Peak: 21, RT: 2.46 to 2.63 min

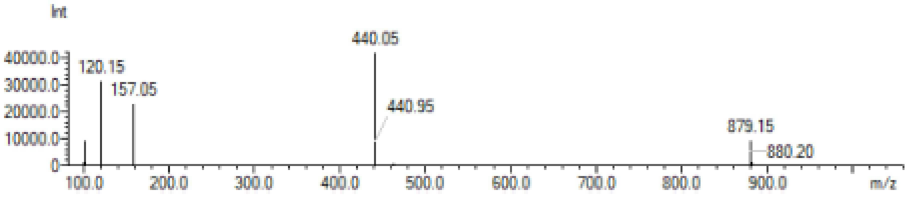

C-I-16

Shimadzu Open Solution

Project: Test
Experiment: 669_0303_Xie_Wilke_0304_Grigalunas_Nigge
Experiment Description:
Sample: 411984:01:01
Sample Description: C22H20ClNO3
Data File Name: C:\LabSolutions\Data\669_0303_Xie_Wilke_0304_Grigalunas_Niggemeyer\1046324588_11200304669.lcd
Sample Location: Plate Number: 1 - Position: 171
Run By: admin
Run Started: 14 May 2020 16:58:32
Run Finished: 15 May 2020 10:54:08
Method: Purity-Check+sleep

LC Chromatogram
LC#6 : AD2

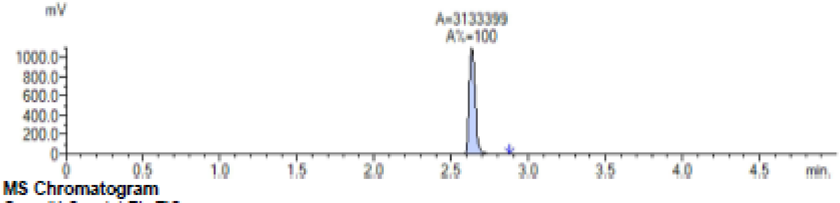

MS Chromatogram
Group#1 Scan(+) EI : TIC

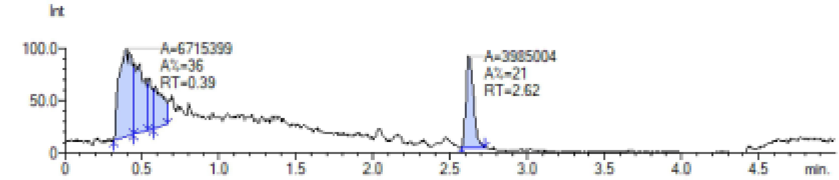

MS Mass Chromatogram
Group#1 Scan(+) EI : 382.1

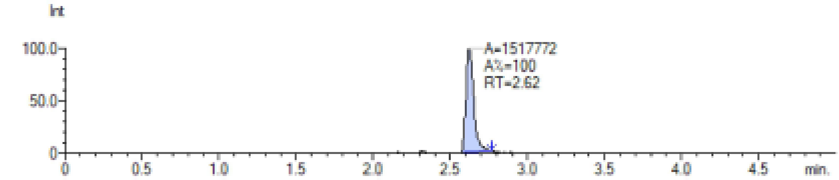

MS Spectrum
Group#1 - PDA Peak: 22, RT: 2.49 to 2.76 min

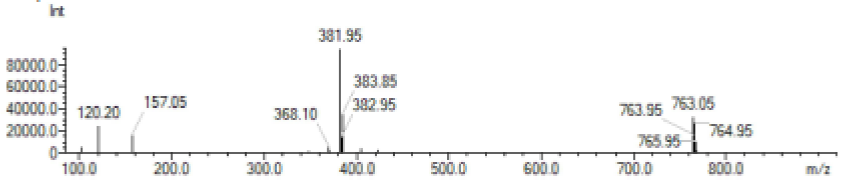

C-I-17

Shimadzu Open Solution

Project: Test
Experiment: 669_0303_Xie_Wilke_0304_Grigalunas_Nigge
Experiment Description:
Sample: 411985:01:01
Sample Description: C21H17BrFNO3
Data File Name: C:\LabSolutions\Data\669_0303_Xie_Wilke_0304_Grigalunas_Niggemeyer\1046324757_11200304669.lcd
Sample Location: Plate Number: 1 - Position: 219
Run By: admin
Run Started: 14 May 2020 16:58:32
Run Finished: 15 May 2020 10:54:08
Method: Purity-Check+sleep

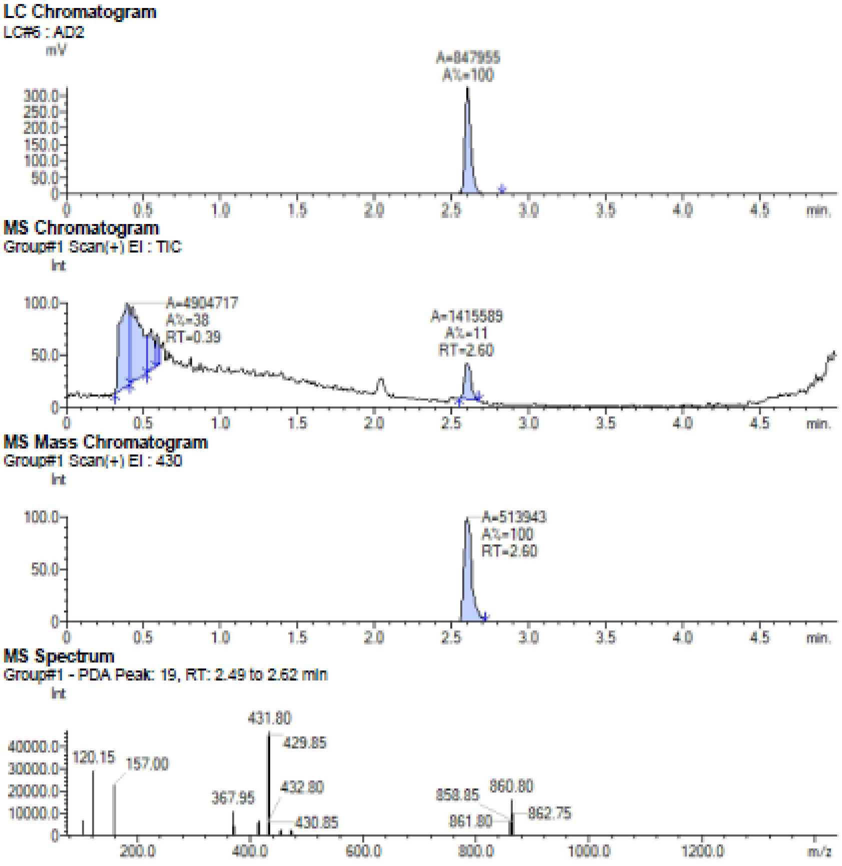

C-I-18

Shimadzu Open Solution

**Project:**	Test
**Experiment:**	669_0303_Xie_Wilke_0304_Grigalunas_Nigge
**Experiment Description:**	
**Sample:**	411986:01:01
**Sample Description:**	C21H16BrNO3
**Data File Name:**	C:\LabSolutions\Data\669_0303_Xie_Wilke_0304_Grigalunas_Niggemeyer\1046324978_11200304669.lcd
**Sample Location:**	Plate Number: 1 - Position: 267
**Run By:**	admin
**Run Started:**	14 May 2020 16:58:32
**Run Finished:**	15 May 2020 10:54:08
**Method:**	Purity-Check+sleep

LC Chromatogram

LC#5 : AD2

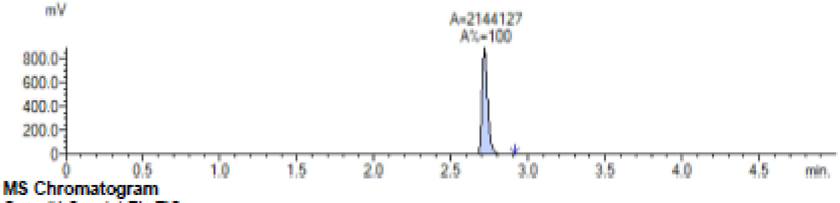

MS Chromatogram

Group#1 Scan(+) EI : TIC

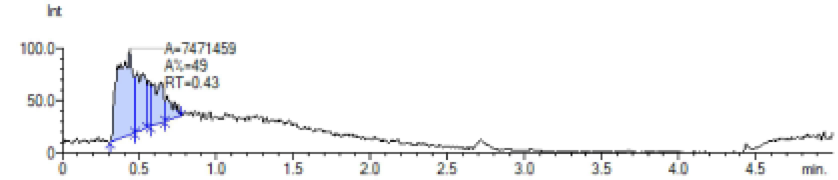

MS Mass Chromatogram

Group#1 Scan(+) EI : 410

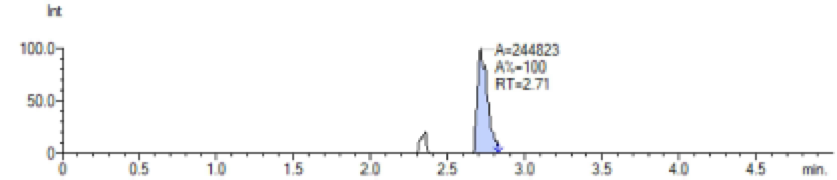

MS Spectrum

Group#1 - PDA Peak: 20, RT: 2.59 to 2.80 min

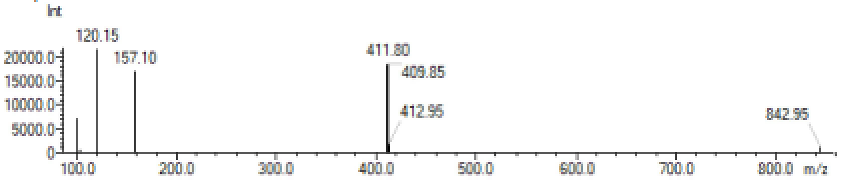

C-I-19

Shimadzu Open Solution

Project: Test
Experiment: 669_0303_Xie_Wilke_0304_Grigalunas_Nigge
Experiment Description:
Sample: 411989:01:01
Sample Description: C22H21NO3
Data File Name: C:\LabSolutions\Data\669_0303_Xie_Wilke_0304_Grigalunas_Niggemeyer\1046325339_11200304669.lcd
Sample Location: Plate Number: 1 - Position: 363
Run By: admin
Run Started: 14 May 2020 16:58:32
Run Finished: 15 May 2020 10:54:08
Method: Purity-Check+sleep

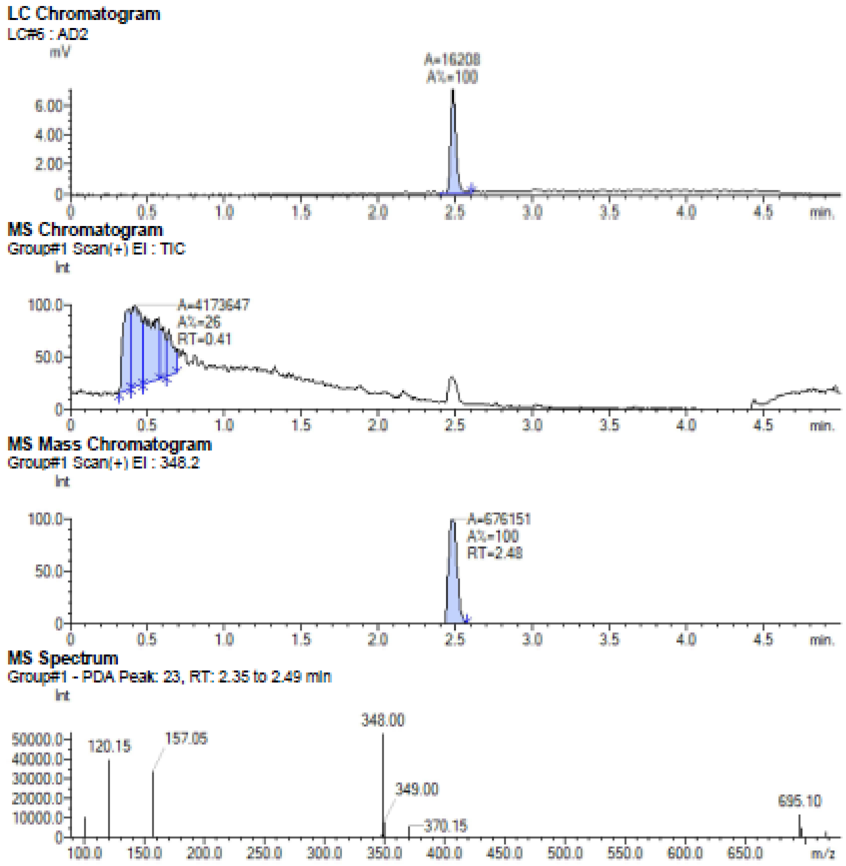

C-I-20

Shimadzu Open Solution

Project: Test
Experiment: 669_0303_Xie_Wilke_0304_Grigalunas_Nigge
Experiment Description:
Sample: 411990:01:01
Sample Description: C22H18F3NO4
Data File Name: C:\LabSolutions\Data\669_0303_Xie_Wilke_0304_Grigalunas_Niggemeyer\1046325344_11200304669.lcd
Sample Location: Plate Number: 1 - Position: 29
Run By: admin
Run Started: 14 May 2020 16:58:32
Run Finished: 15 May 2020 10:54:08
Method: Purity-Check+sleep

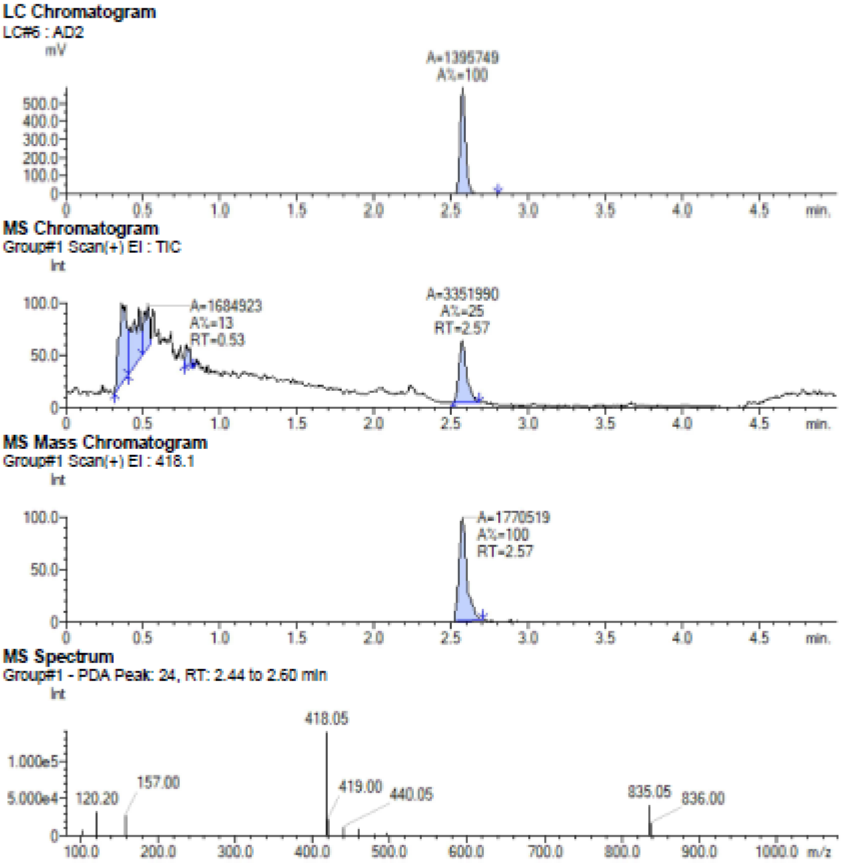

C-I-21

Shimadzu Open Solution

**Project:**	Test
**Experiment:**	669_0303_Xie_Wilke_0304_Grigalunas_Nigge
**Experiment Description:**	
**Sample:**	411991:01:02
**Sample Description:**	C27H23NO4
**Data File Name:**	C:\LabSolutions\Data\669_0303_Xie_Wilke_0304_Grigalunas_Niggemeyer\1046324657_11200304669.lcd
**Sample Location:**	Plate Number: 1 - Position: 77
**Run By:**	admin
**Run Started:**	14 May 2020 16:58:32
**Run Finished:**	15 May 2020 10:54:08
**Method:**	Purity-Check+sleep

LC Chromatogram

LC#6 : AD2

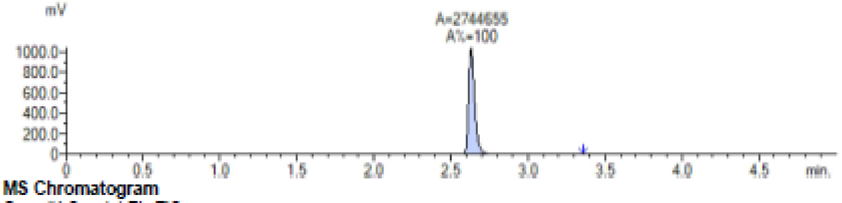

MS Chromatogram

Group#1 Scan(+) EI : TIC

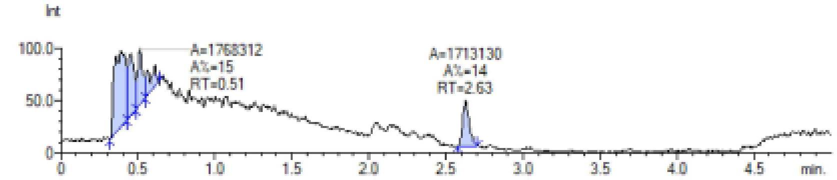

MS Mass Chromatogram

Group#1 Scan(+) EI : 426.2

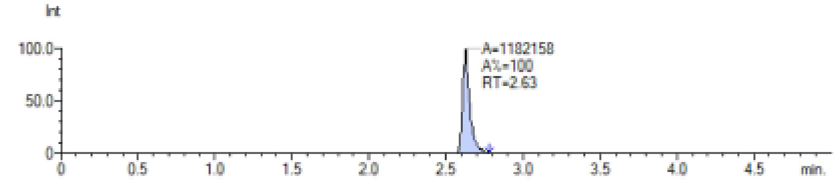

MS Spectrum

Group#1 - PDA Peak: 22, RT: 2.50 to 2.75 min

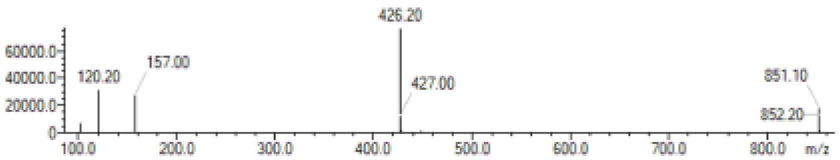

C-I-22

Shimadzu Open Solution

Project: Test
Experiment: 669_0303_Xie_Wilke_0304_Grigalunas_Nigge
Experiment Description:
Sample: 411992:01:02
Sample Description: C24H25NO4
Data File Name: C:\LabSolutions\Data\669_0303_Xie_Wilke_0304_Grigalunas_Niggemeyer\1046324883_11200304669.lcd
Sample Location: Plate Number: 1 - Position: 125
Run By: admin
Run Started: 14 May 2020 16:58:32
Run Finished: 15 May 2020 10:54:08
Method: Purity-Check+sleep

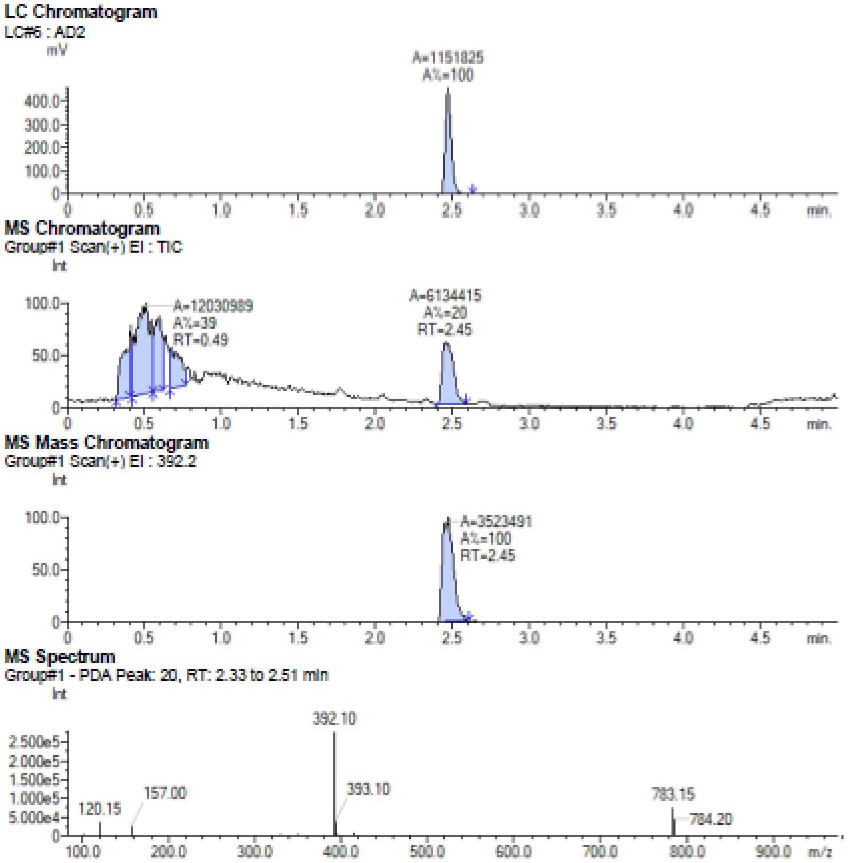

C-I-23

Shimadzu Open Solution

**Project:**	Test
**Experiment:**	669_0303_Xie_Wilke_0304_Grigalunas_Nigge
**Experiment Description:**	
**Sample:**	411993:01:02
**Sample Description:**	C22H18F3NO3
**Data File Name:**	C:\LabSolutions\Data\669_0303_Xie_Wilke_0304_Grigalunas_Niggemeyer\1046324642_11200304669.lcd
**Sample Location:**	Plate Number: 1 - Position: 173
**Run By:**	admin
**Run Started:**	14 May 2020 16:58:32
**Run Finished:**	15 May 2020 10:54:08
**Method:**	Purity-Check+sleep

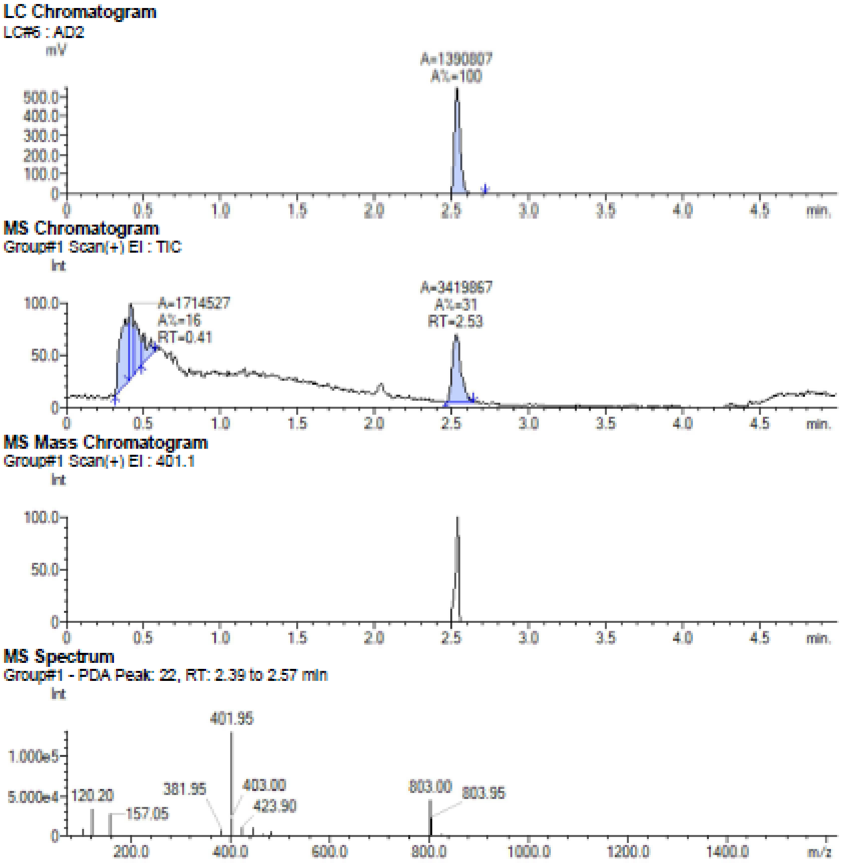

Shimadzu Open Solution

C-I-24

**Project:**	Test
**Experiment:**	669_0303_Xie_Wilke_0304_Grigalunas_Nigge
**Experiment Description:**	
**Sample:**	411994:01:02
**Sample Description:**	C22H21NO3
**Data File Name:**	C:\LabSolutions\Data\669_0303_Xie_Wilke_0304_Grigalunas_Niggemeyer\1046325155_11200304669.lcd
**Sample Location:**	Plate Number: 1 - Position: 221
**Run By:**	admin
**Run Started:**	14 May 2020 16:58:32
**Run Finished:**	15 May 2020 10:54:08
**Method:**	Purity-Check+sleep

LC Chromatogram

LC#6 : AD2

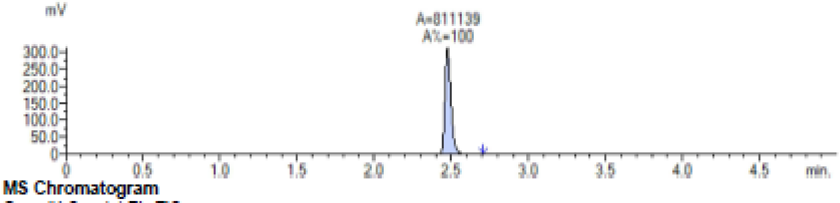

MS Chromatogram

Group#1 Scan(+) EI : TIC

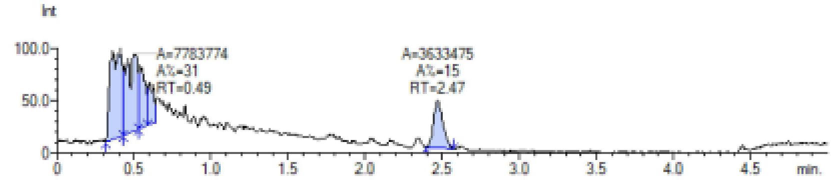

MS Mass Chromatogram

Group#1 Scan(+) EI : 348.2

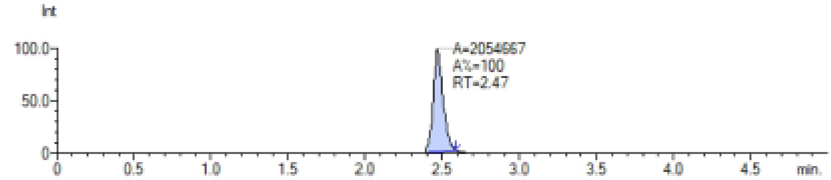

MS Spectrum

Group#1 - PDA Peak: 22, RT: 2.31 to 2.50 min

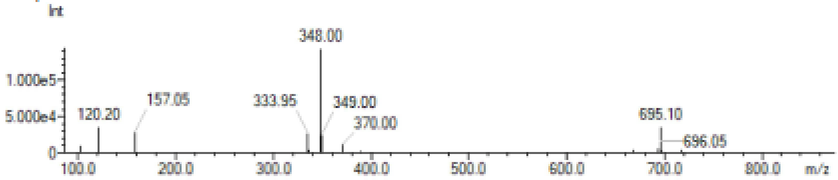

Shimadzu Open Solution

C-I-25

Project: Test
Experiment: 669_0303_Xie_Wilke_0304_Grigalunas_Nigge
Experiment Description:
Sample: 411995:01:01
Sample Description: C21H18FNO3
Data File Name: C:\LabSolutions\Data\669_0303_Xie_Wilke_0304_Grigalunas_Niggemeyer\1046324799_11200304669.lcd
Sample Location: Plate Number: 1 - Position: 269
Run By: admin
Run Started: 14 May 2020 16:58:32
Run Finished: 15 May 2020 10:54:08
Method: Purity-Check+sleep

LC Chromatogram
LC#6 : AD2

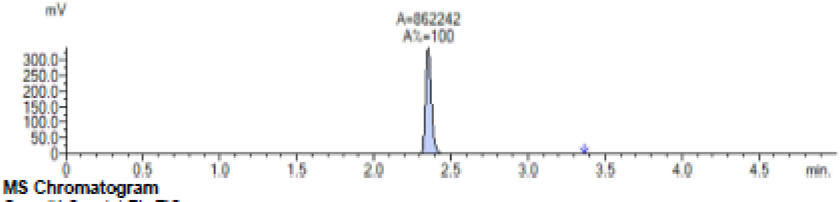

MS Chromatogram
Group#1 Scan(+) EI : TIC

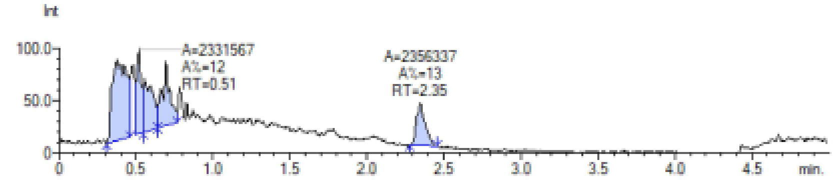

MS Mass Chromatogram
Group#1 Scan(+) EI : 352.1

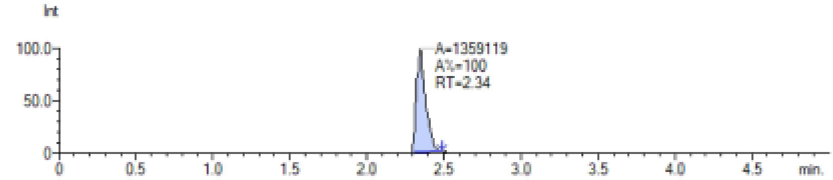

MS Spectrum
Group#1 - PDA Peak: 21, RT: 2.21 to 2.41 min

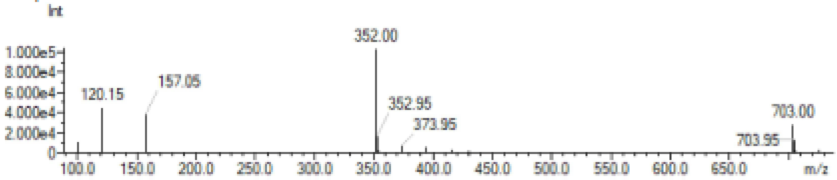

C-I-26

Shimadzu Open Solution

Project: Test
Experiment: 669_0303_Xie_Wilke_0304_Grigalunas_Nigge
Experiment Description:
Sample: 411996:01:01
Sample Description: C21H17Cl2NO3
Data File Name: C:\LabSolutions\Data\669_0303_Xie_Wilke_0304_Grigalunas_Niggemeyer\1046325070_11200304669.lcd
Sample Location: Plate Number: 1 - Position: 317
Run By: admin
Run Started: 14 May 2020 16:58:32
Run Finished: 15 May 2020 10:54:08
Method: Purity-Check+sleep

LC Chromatogram
LC#5 : AD2

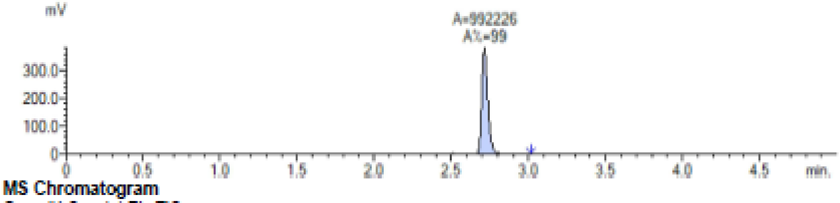

MS Chromatogram
Group#1 Scan(+) EI : TIC

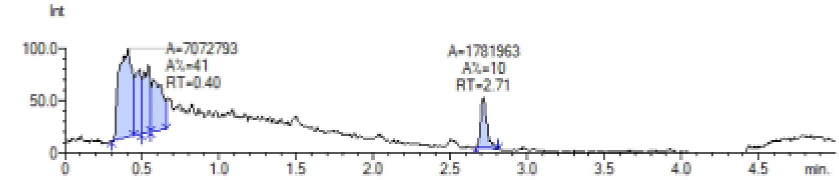

MS Mass Chromatogram
Group#1 Scan(+) EI : 402.1

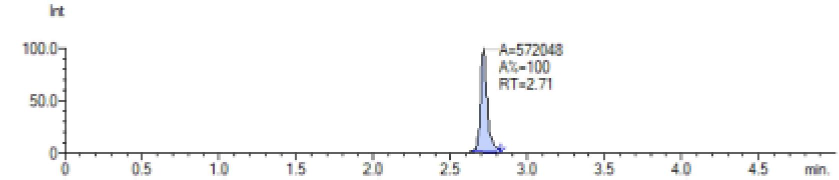

MS Spectrum
Group#1 - PDA Peak: 20, RT: 2.57 to 2.74 min

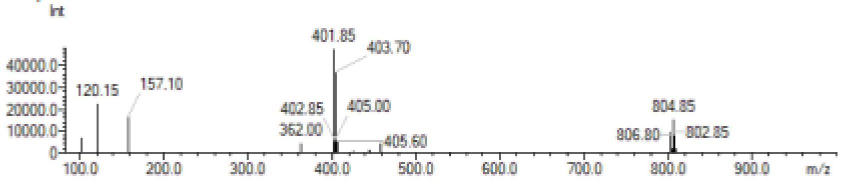

C-I-27

Shimadzu Open Solution

Project: Test
Experiment: 669_0303_Xie_Wilke_0304_Grigalunas_Nigge
Experiment Description:
Sample: 411997:01:01
Sample Description: C21H17ClFNO3
Data File Name: C:\LabSolutions\Data\669_0303_Xie_Wilke_0304_Grigalunas_Niggemeyer\1046324565_11200304669.lcd
Sample Location: Plate Number: 1 - Position: 365
Run By: admin
Run Started: 14 May 2020 16:58:32
Run Finished: 15 May 2020 10:54:08
Method: Purity-Check+sleep

LC Chromatogram
LC#5 : AD2

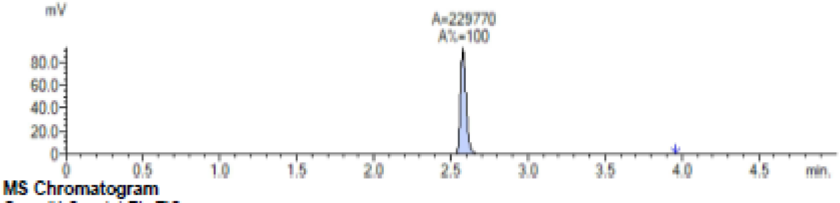

MS Chromatogram
Group#1 Scan(+) EI : TIC

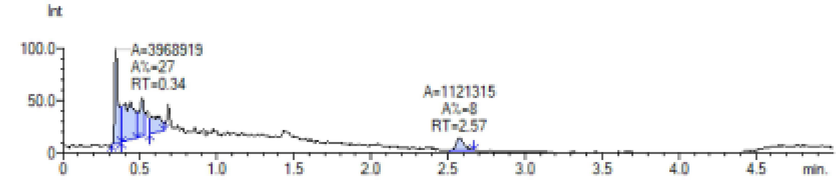

MS Mass Chromatogram
Group#1 Scan(+) EI : 386.1

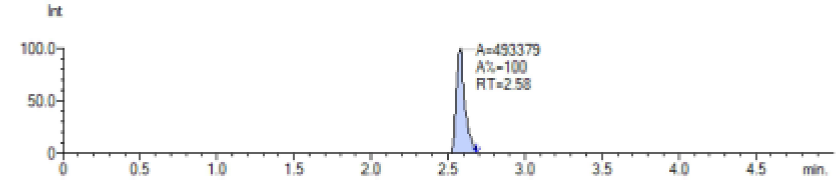

MS Spectrum
Group#1 - PDA Peak: 17, RT: 2.42 to 2.62 min

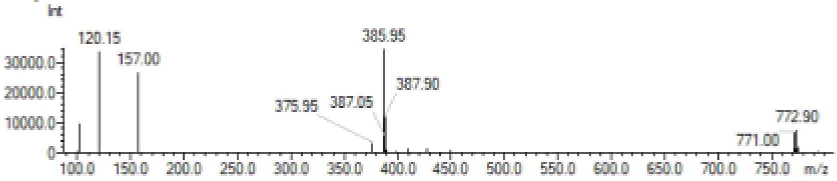

Shimadzu Open Solution

C-I-28

Project: Test
Experiment: 669_0303_Xie_Wilke_0304_Grigalunas_Nigge
Experiment Description:
Sample: 411998:01:01
Sample Description: C24H23NO5
Data File Name: C:\LabSolutions\Data\669_0303_Xie_Wilke_0304_Grigalunas_Niggemeyer\1046324885_11200304669.lcd
Sample Location: Plate Number: 1 - Position: 31
Run By: admin
Run Started: 14 May 2020 16:58:32
Run Finished: 15 May 2020 10:54:08
Method: Purity-Check+sleep

LC Chromatogram
LC#5 : AD2

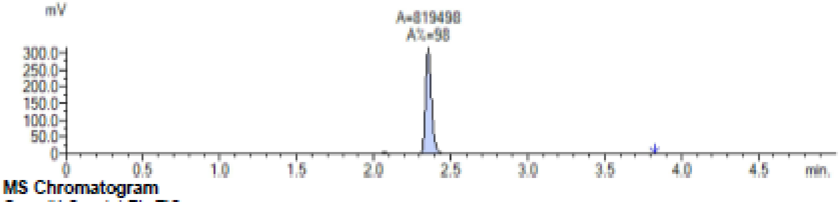

MS Chromatogram
Group#1 Scan(+) EI : TIC

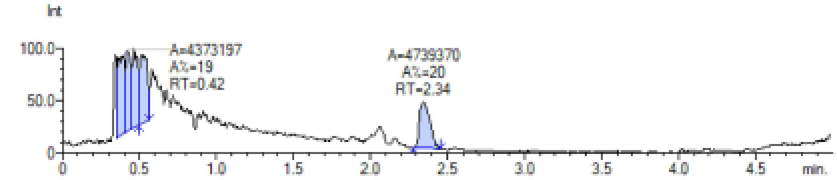

MS Mass Chromatogram
Group#1 Scan(+) EI : 406.2

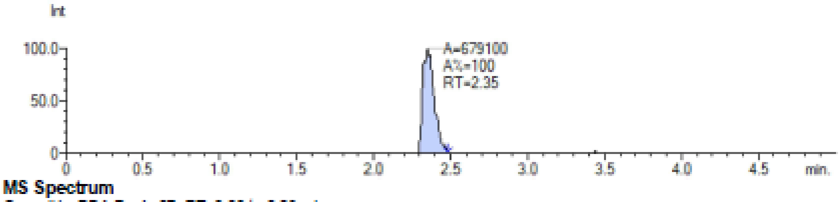

MS Spectrum
Group#1 - PDA Peak: 27, RT: 2.22 to 2.39 min

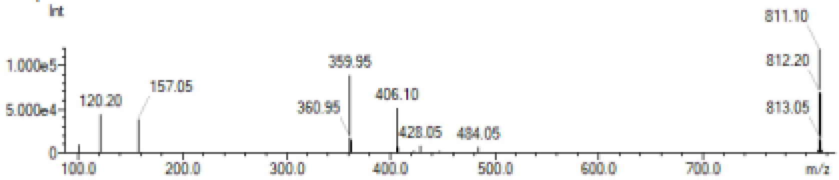

Shimadzu Open Solution

C-I-29

Project: Test
Experiment: 669_0303_Xie_Wilke_0304_Grigalunas_Nigge
Experiment Description:
Sample: 411999:01:01
Sample Description: C22H21NO5S
Data File Name: C:\LabSolutions\Data\669_0303_Xie_Wilke_0304_Grigalunas_Niggemeyer\1046324850_11200304669.lcd
Sample Location: Plate Number: 1 - Position: 79
Run By: admin
Run Started: 14 May 2020 16:58:32
Run Finished: 15 May 2020 10:54:08
Method: Purity-Check+sleep

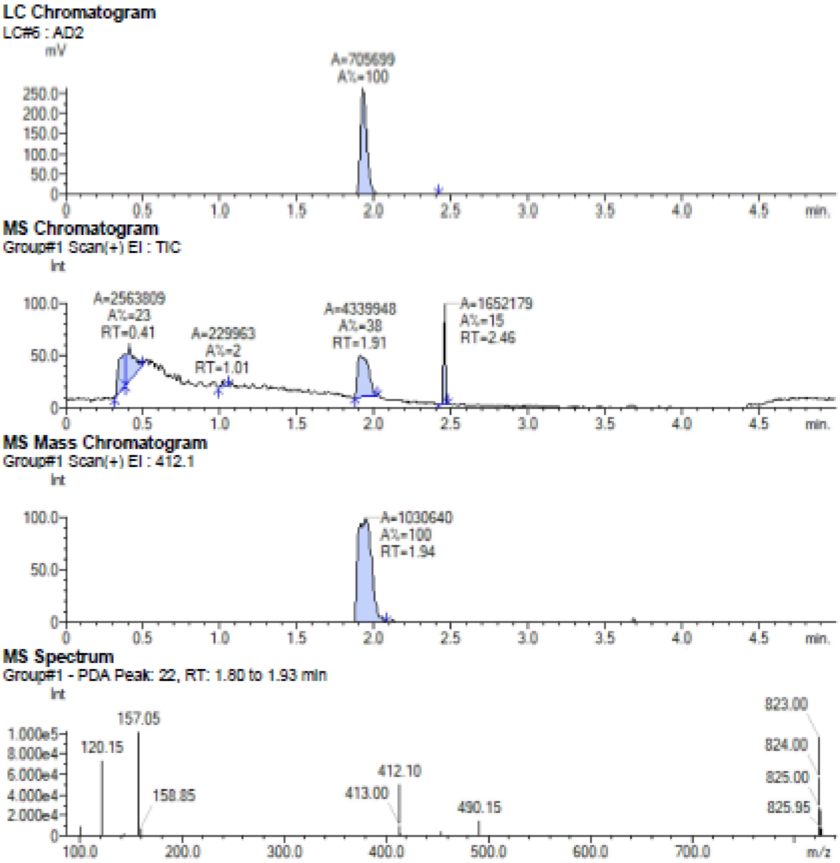

C-I-30

himadzu Open Solution

roject:	Test
kperiment:	669_0303_Xie_Wilke_0304_Grigalunas_Nigge
kperiment escription:	
ample:	412000:01:01
ample escription:	C21H20N2O5S
ata File ame:	C:\LabSolutions\Data\669_0303_Xie_Wilke_0304_Grigalunas_Niggemeyer\1046324996_11200304669.lcd
ample ocation:	Plate Number: 1 - Position: 127
un By:	admin
un tarted:	14 May 2020 16:58:32
un nished:	15 May 2020 10:54:08
ethod:	Purity-Check+sleep

C Chromatogram

:#6 : AD2

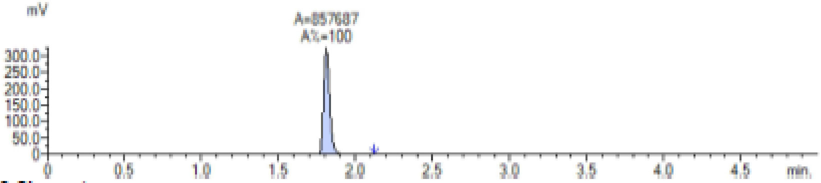

S Chromatogram

roup#1 Scan(+) EI : TIC

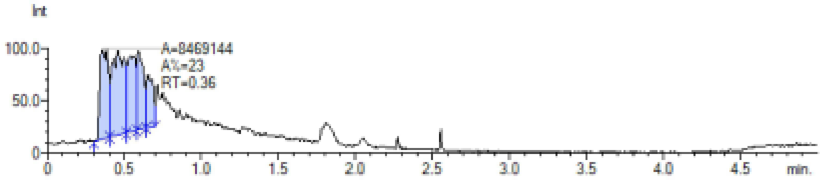

S Mass Chromatogram

roup#1 Scan(+) EI : 413.1

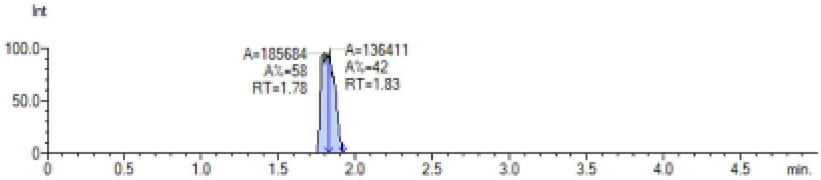

S Spectrum

roup#1 - PDA Peak: 22, RT: 1.64 to 1.81 min

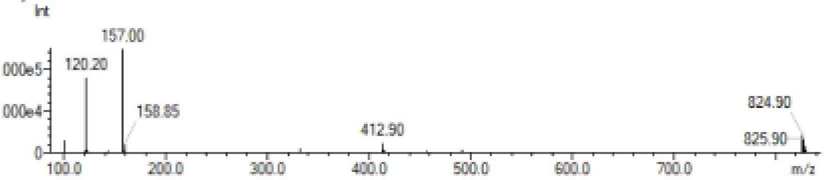

C-I-31

Shimadzu Open Solution

Project:	Test
Experiment:	669_0303_Xie_Wilke_0304_Grigalunas_Nigge
Experiment Description:	
Sample:	412001:01:01
Sample Description:	C24H23NO5
Data File Name:	C:\LabSolutions\Data\669_0303_Xie_Wilke_0304_Grigalunas_Niggemeyer\1046325330_11200304669.lcd
Sample Location:	Plate Number: 1 - Position: 175
Run By:	admin
Run Started:	14 May 2020 16:58:32
Run Finished:	15 May 2020 10:54:08
Method:	Purity-Check+sleep

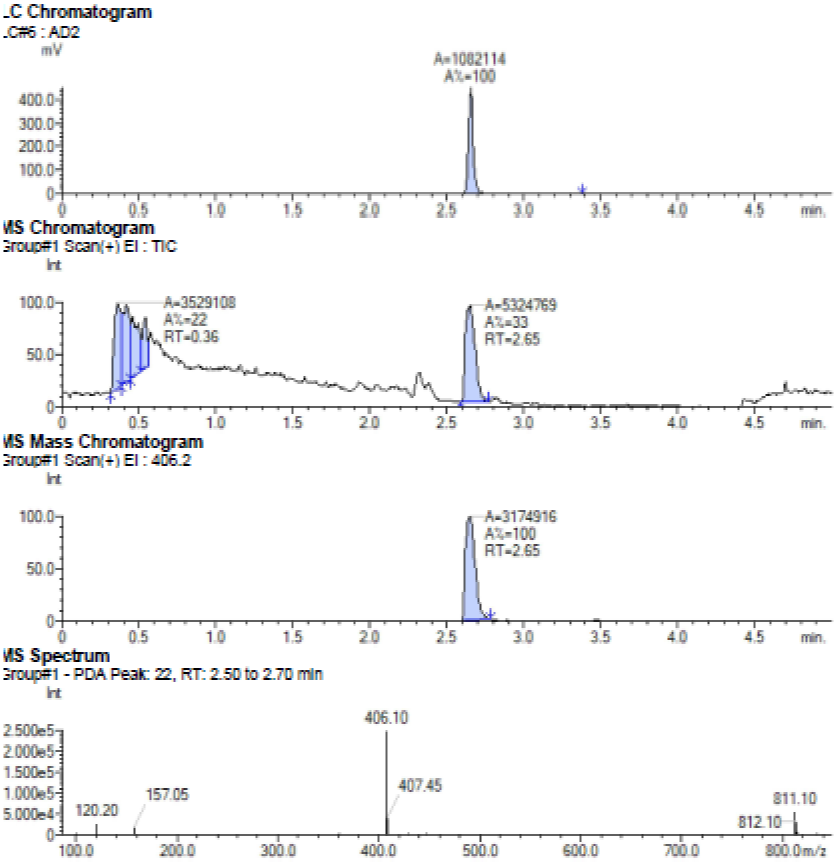

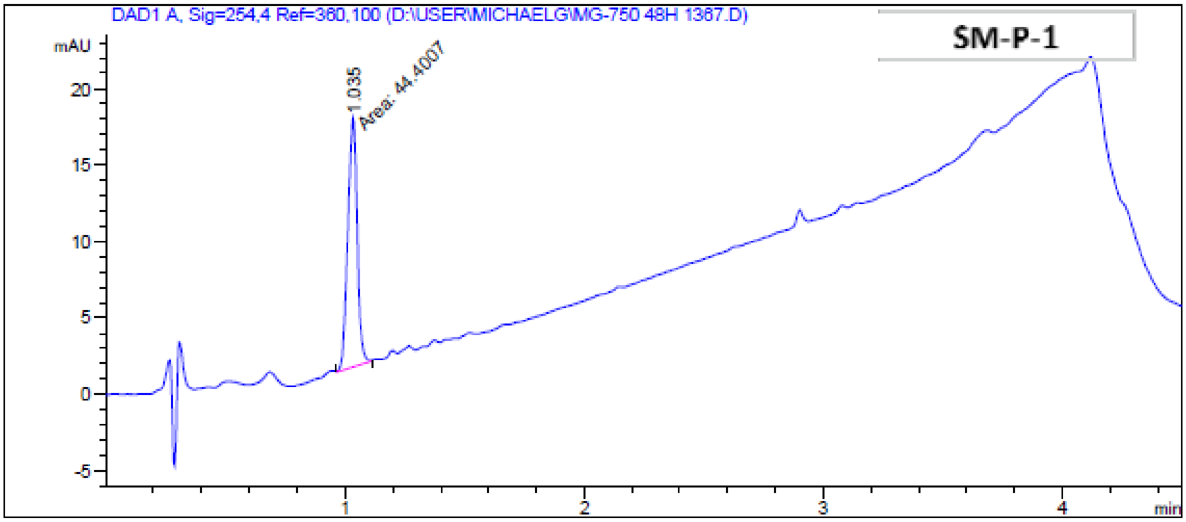

#	Time	Area	Height	Width	Area%	Symmetry
1	1.035	44.4	16.3	0.0454	100.000	1.249

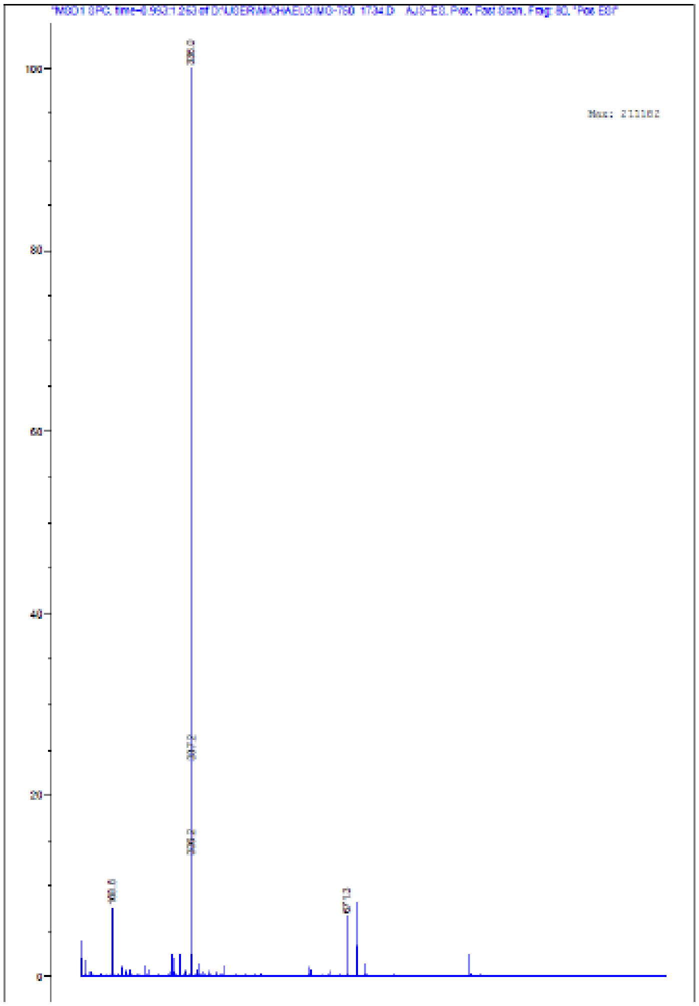

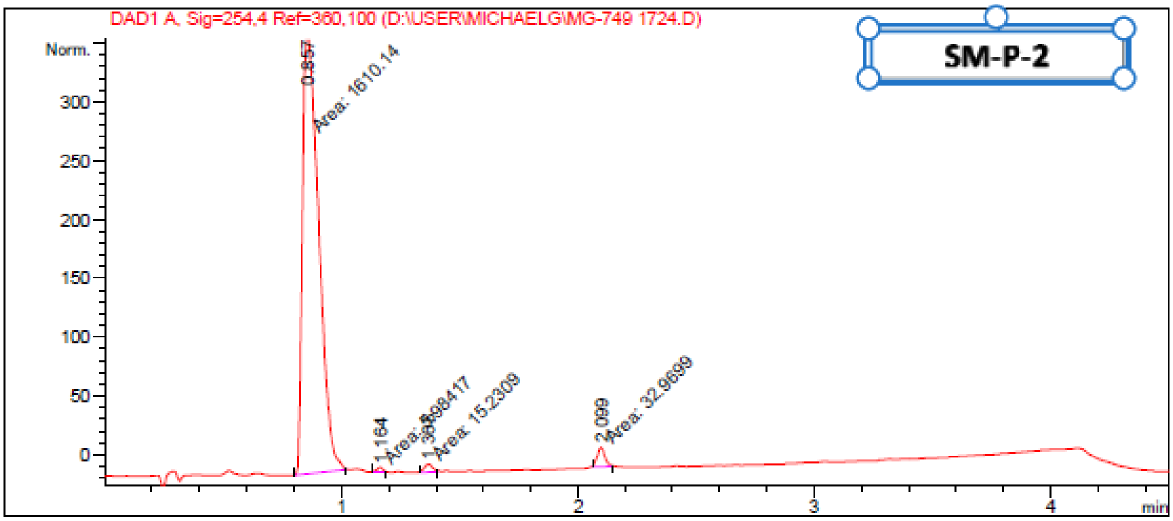

#	Time	Area	Height	Width	Area%	Symmetry
1	0.857	1610.1	334.5	0.0802	96.744	0.435
2	1.164	6	3.5	0.0288	0.360	1.087
3	1.368	15.2	6.2	0.0406	0.915	0.967
4	2.099	33	14.6	0.0376	1.981	0.995

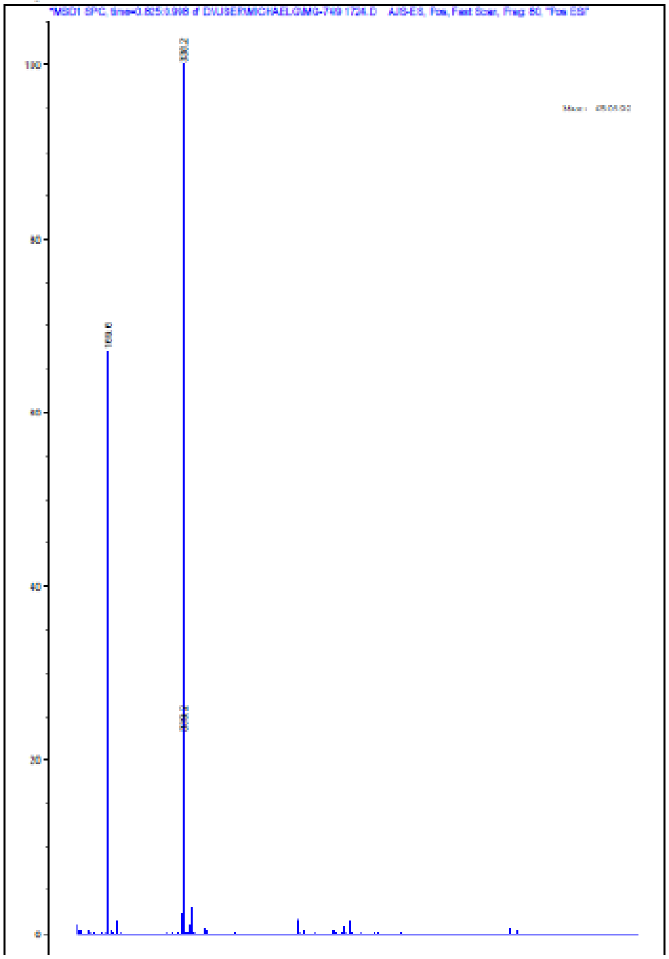

Shimadzu Open Solution

SM-P-3

Project: Test
Experiment: 669_0303_Xie_Wilke_0304_Grigalunas_Nigge
Experiment Description:
Sample: 412027:01:01
Sample Description: C26H27BrN4O2
Data File Name: C:\LabSolutions\Data\669_0303_Xie_Wilke_0304_Grigalunas_Niggemeyer\1046324644_11200304669.lcd
Sample Location: Plate Number: 1 - Position: 89
Run By: admin
Run Started: 14 May 2020 16:58:32
Run Finished: 15 May 2020 10:54:08
Method: Purity-Check+sleep

LC Chromatogram
LC#6 : AD2

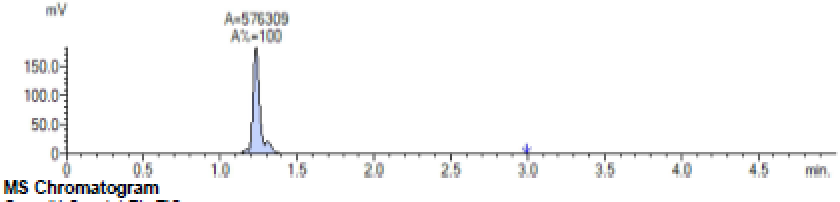

MS Chromatogram
Group#1 Scan(+) EI : TIC

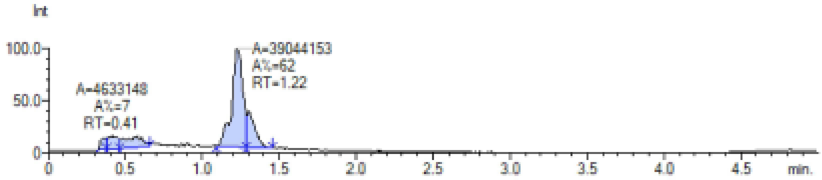

MS Mass Chromatogram
Group#1 Scan(+) EI : 507.1

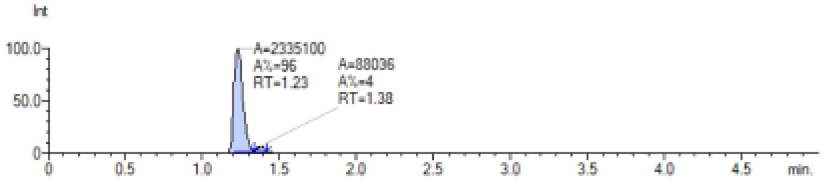

MS Spectrum
Group#1 - PDA Peak: 21, RT: 1.11 to 1.19 min

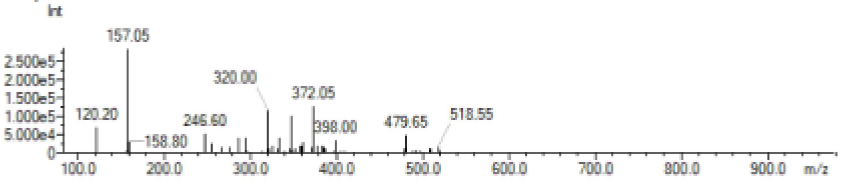

Shimadzu Open Solution

SM-P-4

Project: Test
Experiment: 669_0303_Xie_Wilke_0304_Grigalunas_Nigge
Experiment Description:
Sample: 412039:01:01
Sample Description: C32H30BrN3O2
Data File Name: C:\LabSolutions\Data\669_0303_Xie_Wilke_0304_Grigalunas_Niggemeyer\1046324949_11200304669.lcd
Sample Location: Plate Number: 1 - Position: 281
Run By: admin
Run Started: 14 May 2020 16:58:32
Run Finished: 15 May 2020 10:54:08
Method: Purity-Check+sleep

LC Chromatogram
LC#6 : AD2

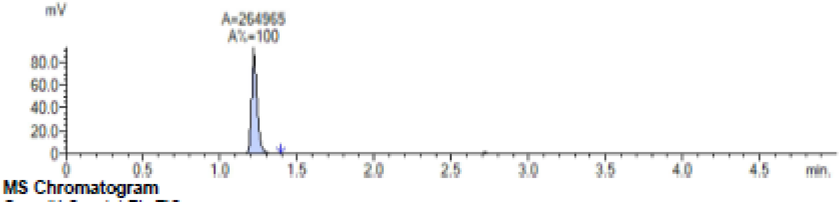

MS Chromatogram
Group#1 Scan(+) EI : TIC

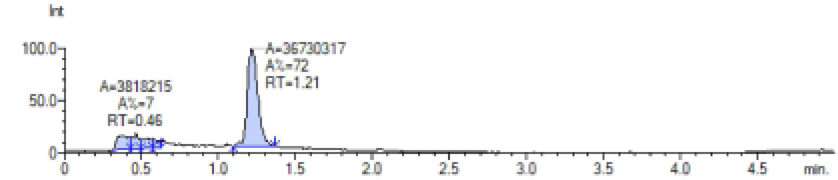

MS Mass Chromatogram
Group#1 Scan(+) EI : 568.2

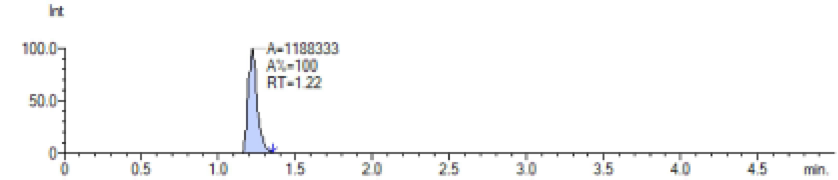

MS Spectrum
Group#1 - PDA Peak: 19, RT: 1.09 to 1.32 min

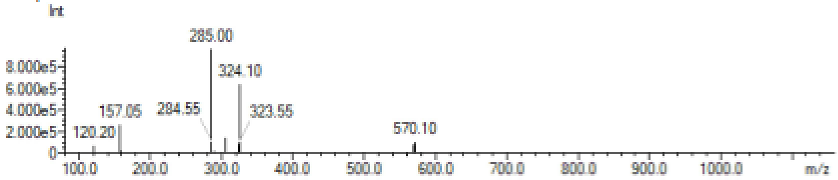

NMR Spectra

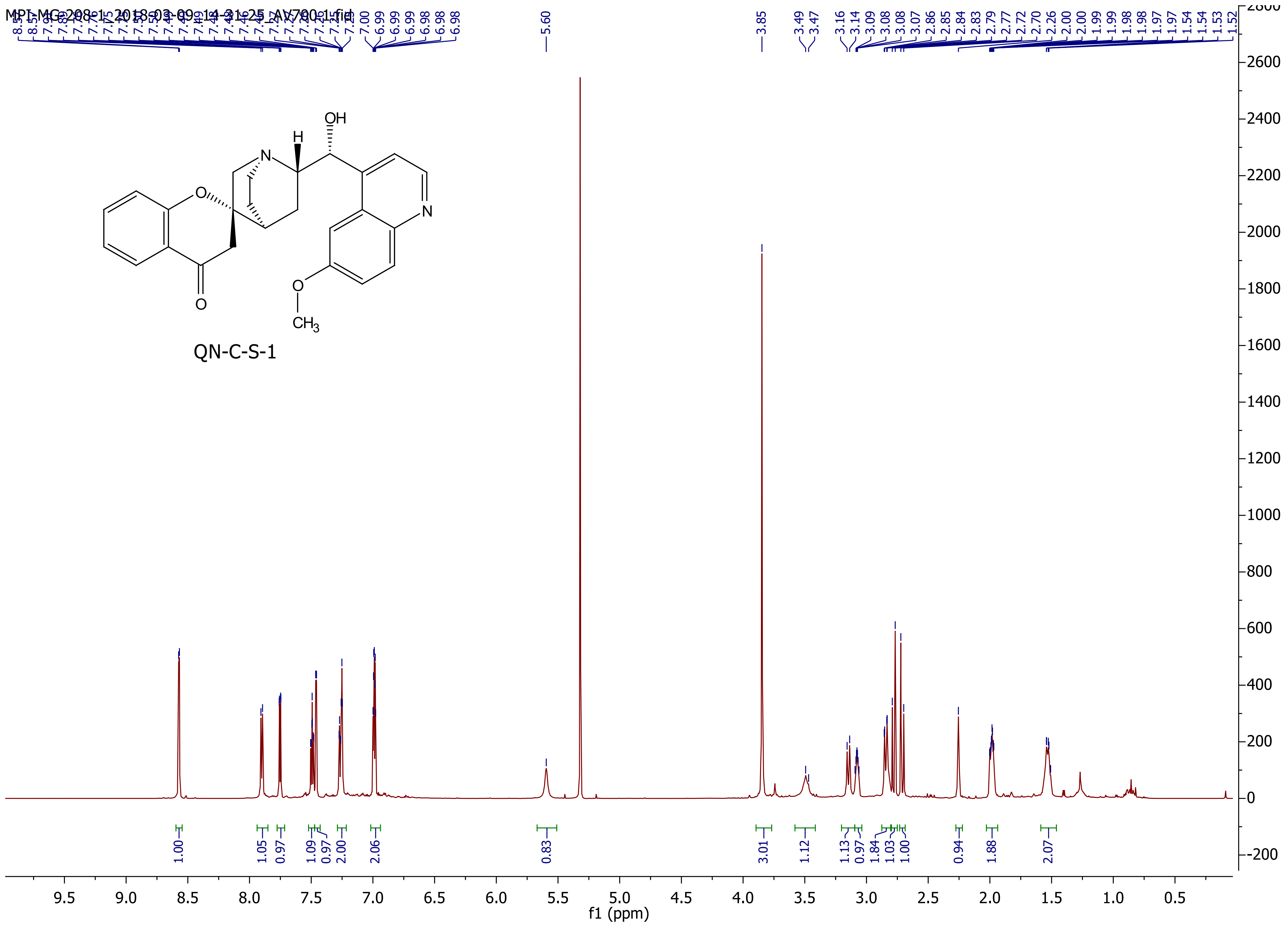

MPI-MG-208-1_2018-03-09_14-31-25_AV700-1.fid
7.00
6.99
6.99
6.99
6.98
6.98
6.98
5.60
3.85
3.49
3.47
3.16
3.14
3.09
3.08
3.08
3.07
2.86
2.85
2.84
2.83
2.79
2.77
2.72
2.70
2.26
2.00
2.00
1.99
1.99
1.98
1.98
1.97
1.97
1.54
1.54
1.53
1.52
OH
H
N
O
N
O
O
CH3
QN-C-S-1
1.00
1.05
0.97
1.09
0.97
2.00
2.06
0.83
3.01
1.12
1.13
0.97
1.84
1.03
1.00
0.94
1.88
2.07
f1 (ppm)

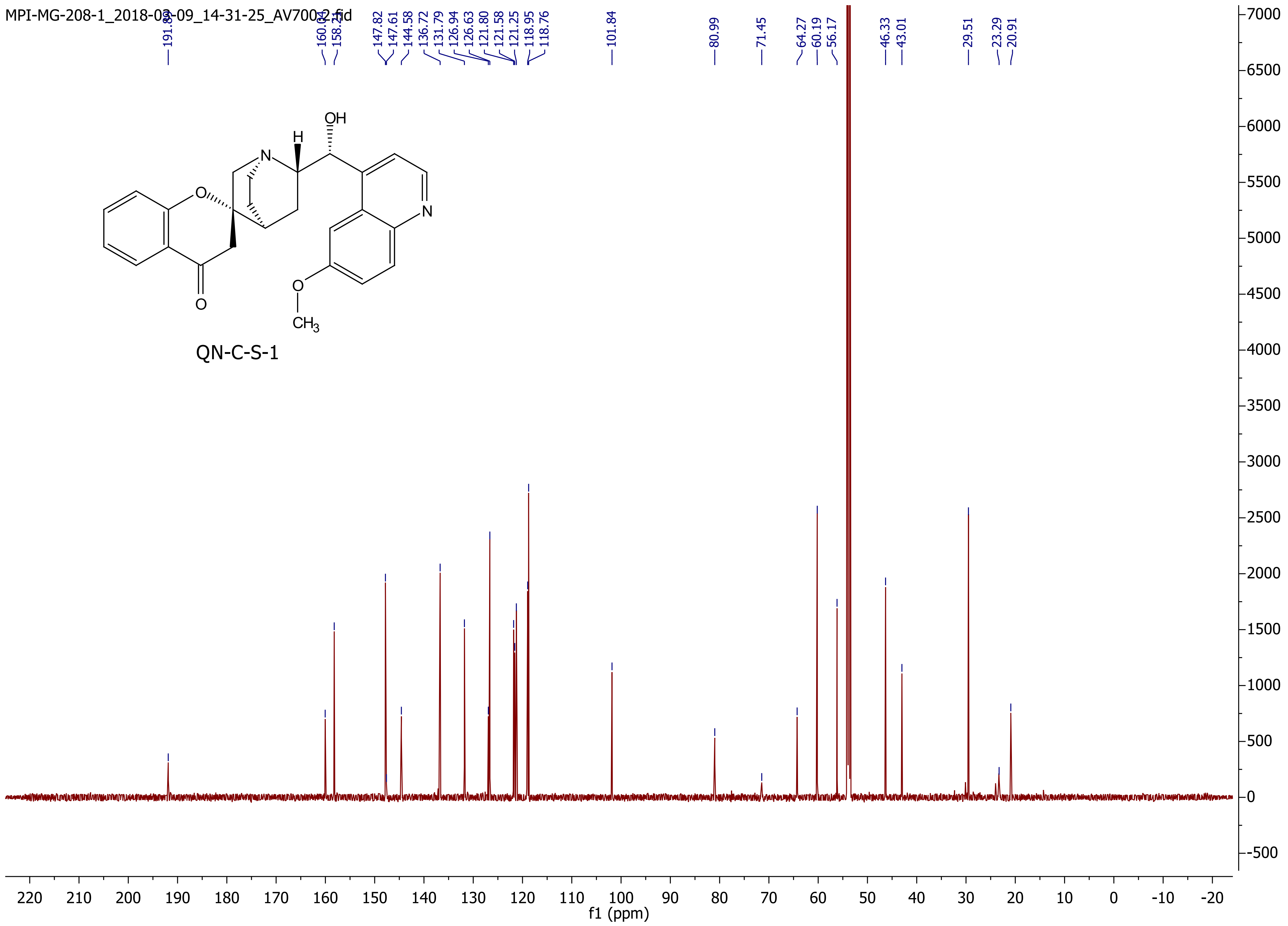
MPI-MG-208-1_2018-03-09_14-31-25_AV700 2.fid
160.04
158.21
147.82
147.61
144.58
136.72
131.79
126.94
126.63
121.80
121.58
121.25
118.95
118.76
101.84
80.99
71.45
64.27
60.19
56.17
46.33
43.01
29.51
23.29
20.91
OH
H
N
O
N
O
O
CH3
QN-C-S-1
f1 (ppm)

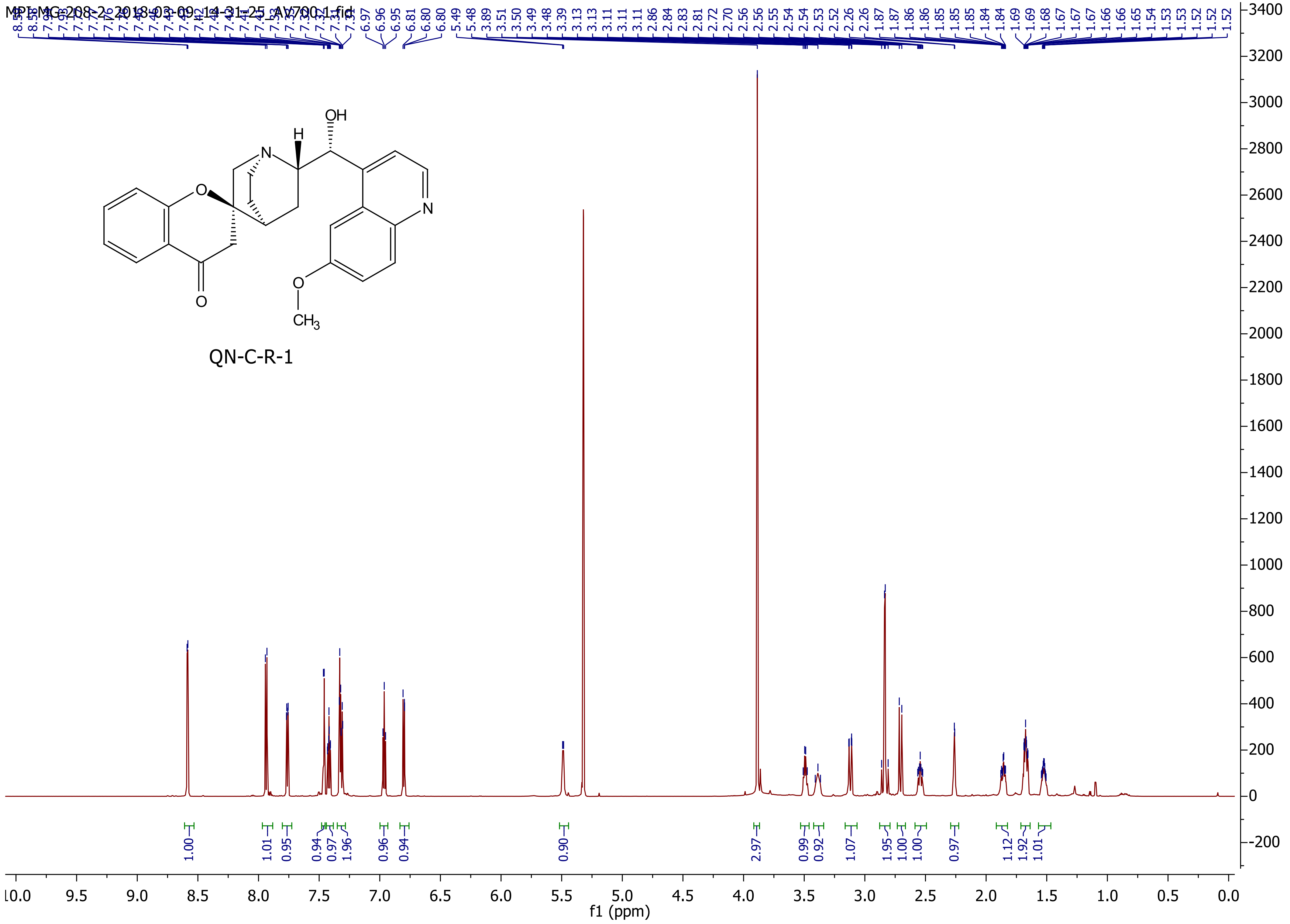

MPI-MG-208-2_2018-03-09_14-31-25_AV700 1-fid
6.97 6.96 6.95 6.81 6.80 6.80 5.49 5.48 3.89 3.51 3.50 3.49 3.48 3.39 3.13 3.13 3.11 3.11 3.11 2.86 2.84 2.83 2.81 2.72 2.70 2.56 2.56 2.55 2.54 2.54 2.53 2.52 2.26 2.26 1.87 1.87 1.86 1.86 1.85 1.85 1.85 1.84 1.84 1.69 1.69 1.68 1.67 1.67 1.67 1.66 1.66 1.65 1.54 1.53 1.53 1.52 1.52 1.52
OH
H
N
O
N
O
O
CH3
QN-C-R-1
1.00 1.01 0.95 0.94 0.97 1.96 0.96 0.94 0.90 2.97 0.99 0.92 1.07 1.95 1.00 1.00 0.97 1.12 1.92 1.01
f1 (ppm)

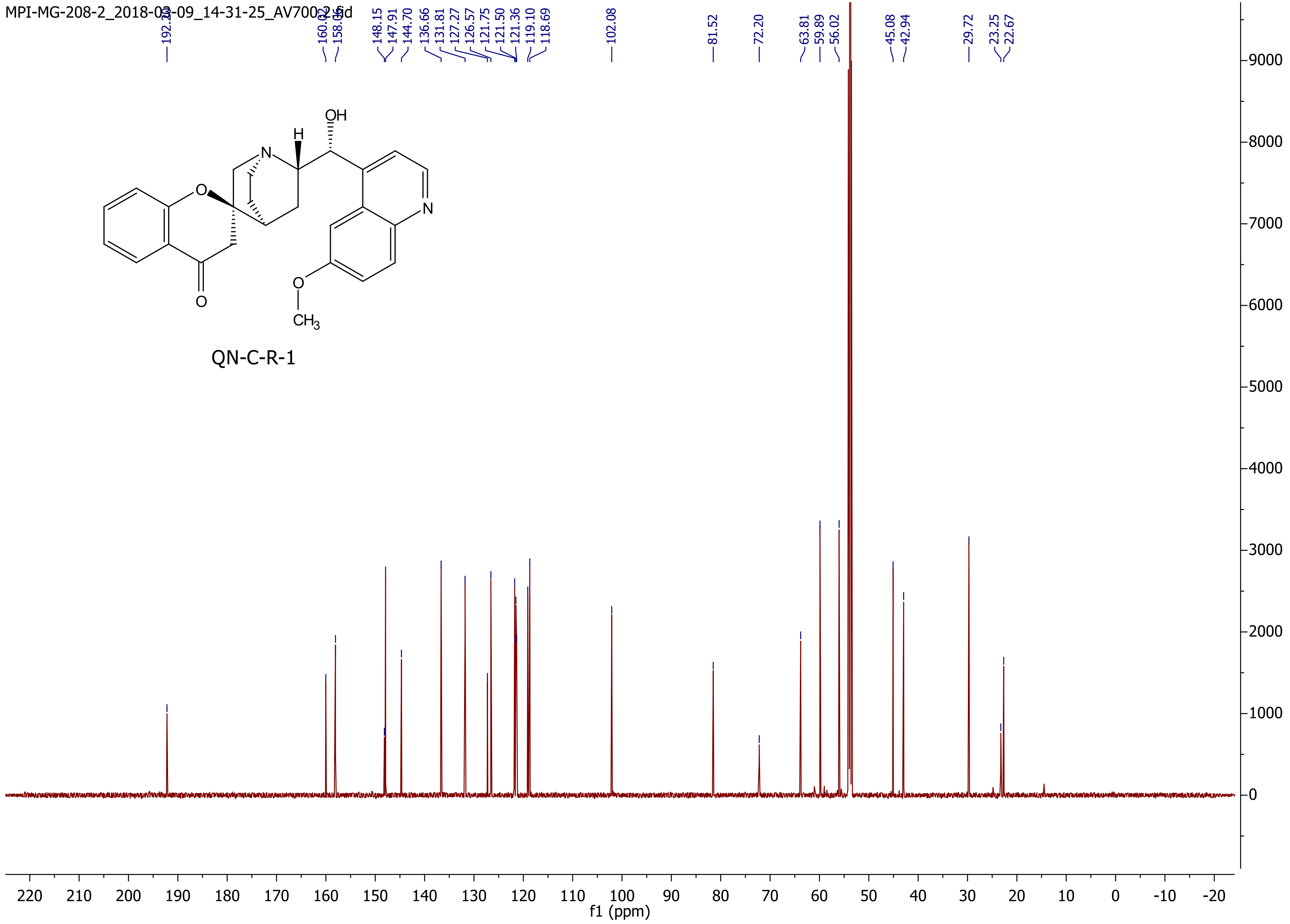
MPI-MG-208-2_2018-03-09_14-31-25_AV700.2.fid
192.20
160.02
158.06
148.15
147.91
144.70
136.66
131.81
127.27
126.57
121.75
121.50
121.36
119.10
118.69
102.08
81.52
72.20
63.81
59.89
56.02
45.08
42.94
29.72
23.25
22.67
OH
H
N
O
N
O
O
CH3
QN-C-R-1
f1 (ppm)

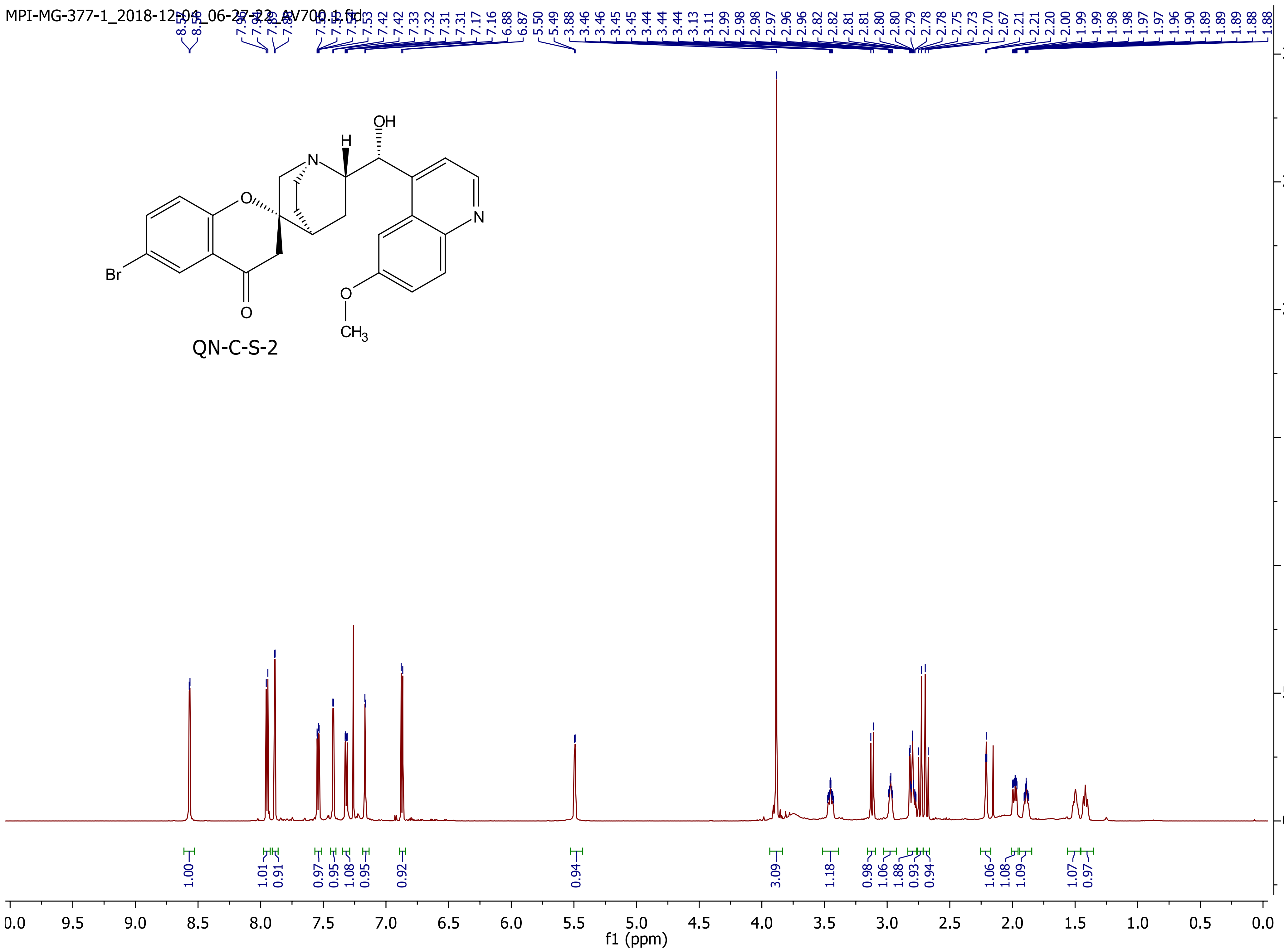
MPI-MG-377-1_2018-12-04_06-27-22_AV700.1.fid
QN-C-S-2
f1 (ppm)

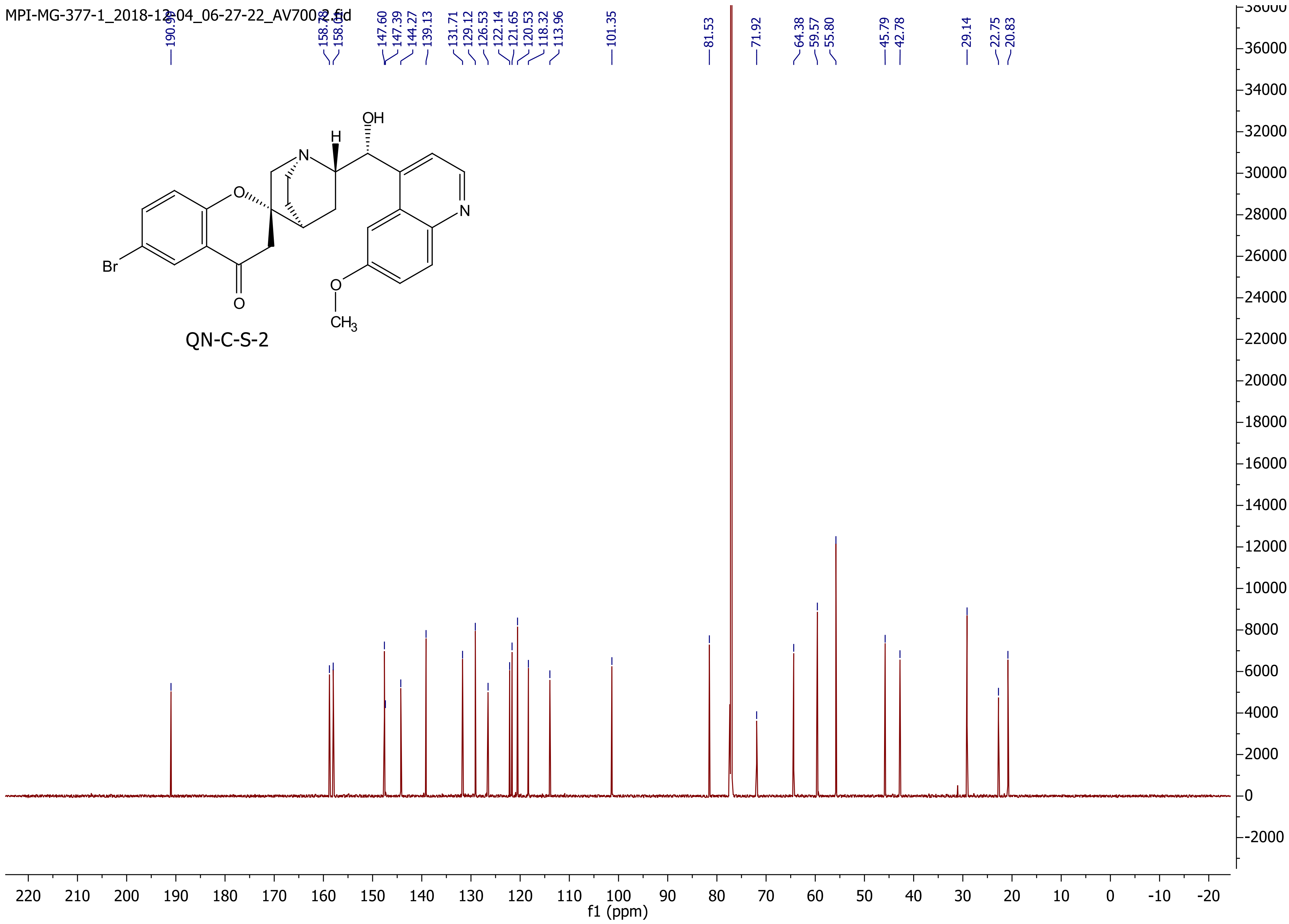
MPI-MG-377-1_2018-12-04_06-27-22_AV700.2.fid
147.60
147.39
144.27
139.13
131.71
129.12
126.53
122.14
121.65
120.53
118.32
113.96
101.35
81.53
71.92
64.38
59.57
55.80
45.79
42.78
29.14
22.75
20.83
OH
H
N
O
N
Br
O
O
CH3
QN-C-S-2
f1 (ppm)

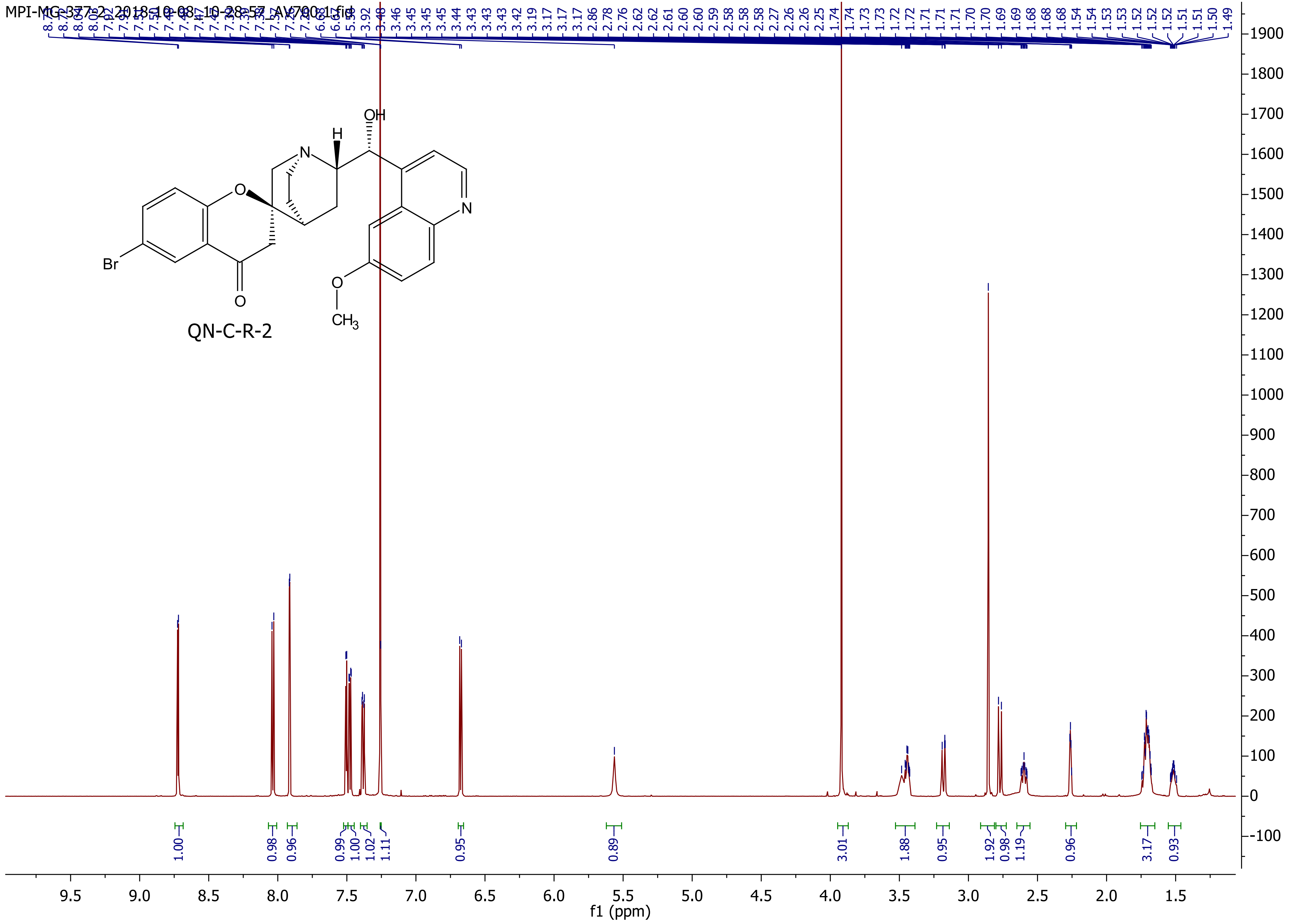
MPI-MG-377-2 2018-10-08_10-28-57 AV700 1.fid
OH
H
N
O
N
Br
O
O
CH3
QN-C-R-2
3.46 3.45 3.45 3.45 3.44 3.43 3.43 3.43 3.42 3.19 3.17 3.17 2.86 2.78 2.76 2.62 2.62 2.61 2.60 2.60 2.59 2.58 2.58 2.58 2.27 2.26 2.26 2.25 1.74 1.74 1.73 1.73 1.72 1.72 1.71 1.71 1.71 1.70 1.70 1.69 1.69 1.68 1.68 1.68 1.54 1.54 1.53 1.53 1.52 1.52 1.52 1.51 1.51 1.50 1.49
1.00 0.98 0.96 0.99 1.00 1.02 1.11 0.95 0.89 3.01 1.88 0.95 1.92 0.98 1.19 0.96 3.17 0.93
f1 (ppm)

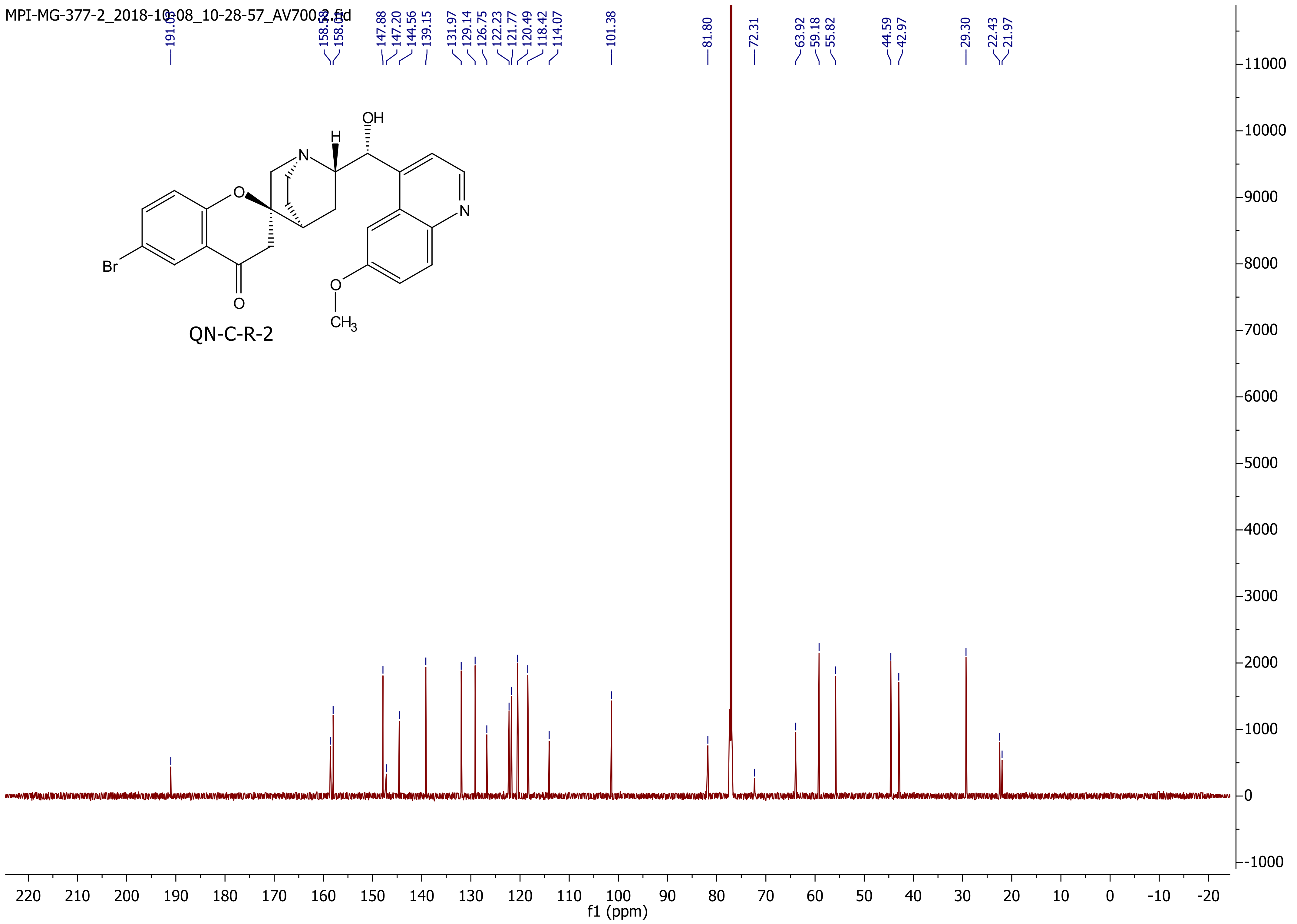

MPI-MG-377-2_2018-10-08_10-28-57_AV700.2.fid
191.0
158.5
158.0
147.88
147.20
144.56
139.15
131.97
129.14
126.75
122.23
121.77
120.49
118.42
114.07
101.38
81.80
72.31
63.92
59.18
55.82
44.59
42.97
29.30
22.43
21.97
OH
H
N
O
N
Br
O
O
CH3
QN-C-R-2
f1 (ppm)

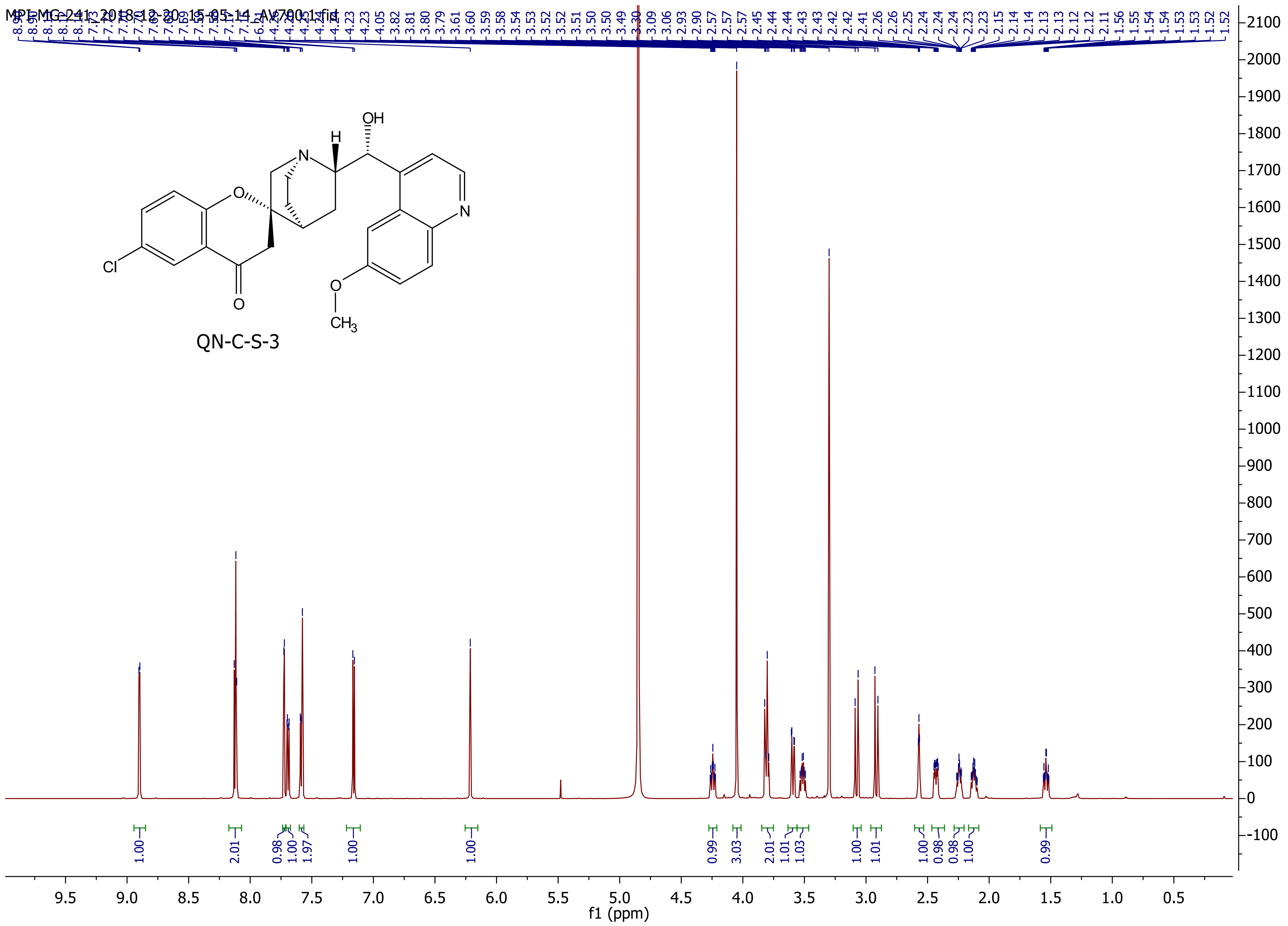
MPI-MG-241_2018-12-20_15-05-14_AV700-1.fid
QN-C-S-3
f1 (ppm)

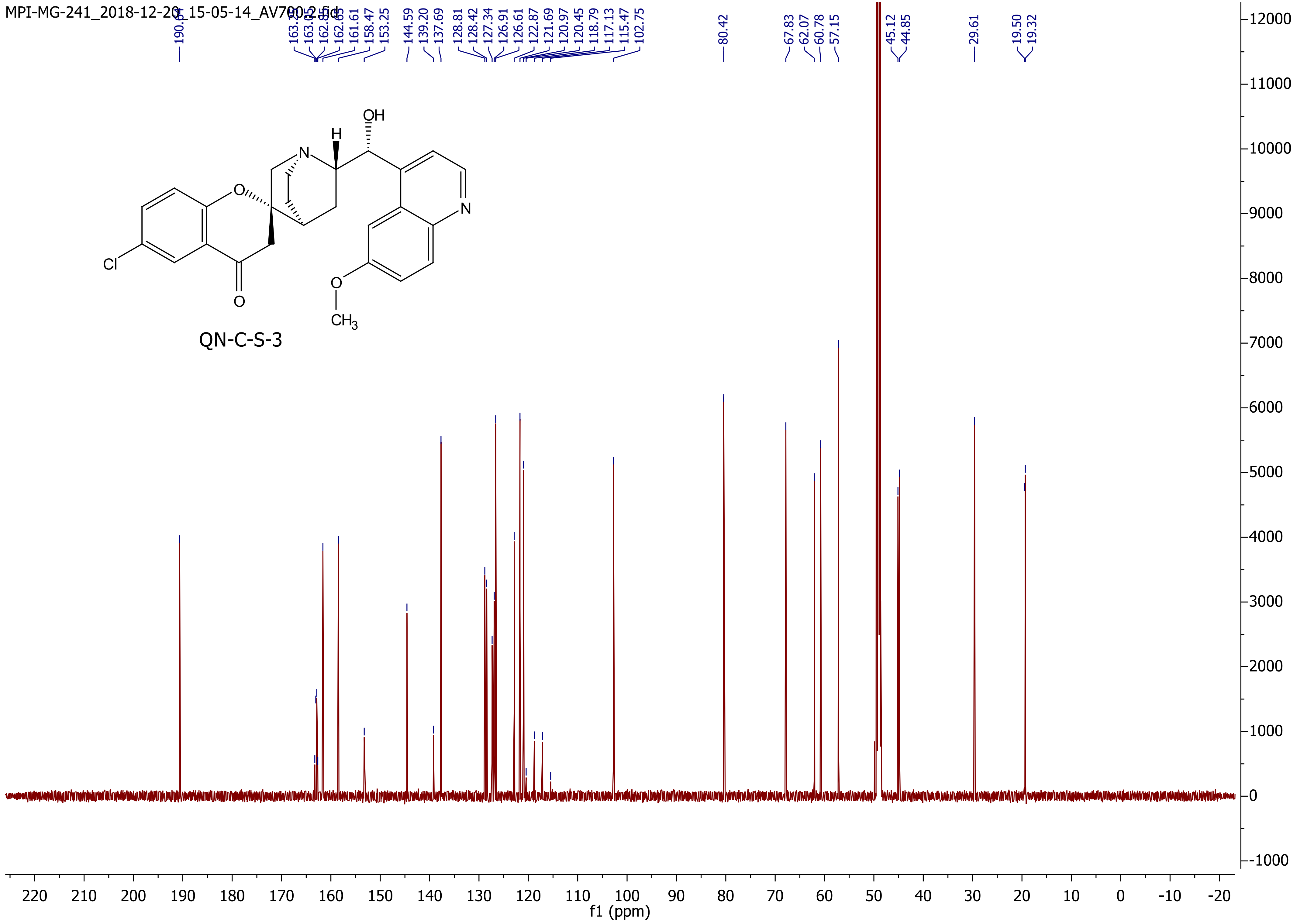
MPI-MG-241_2018-12-20_15-05-14_AV700-2.fid
162.65
161.61
158.47
153.25
144.59
139.20
137.69
128.81
128.42
127.34
126.91
126.61
122.87
121.69
120.97
120.45
118.79
117.13
115.47
102.75
80.42
67.83
62.07
60.78
57.15
45.12
44.85
29.61
19.50
19.32
OH
H
N
O
N
Cl
O
O
CH3
QN-C-S-3
f1 (ppm)

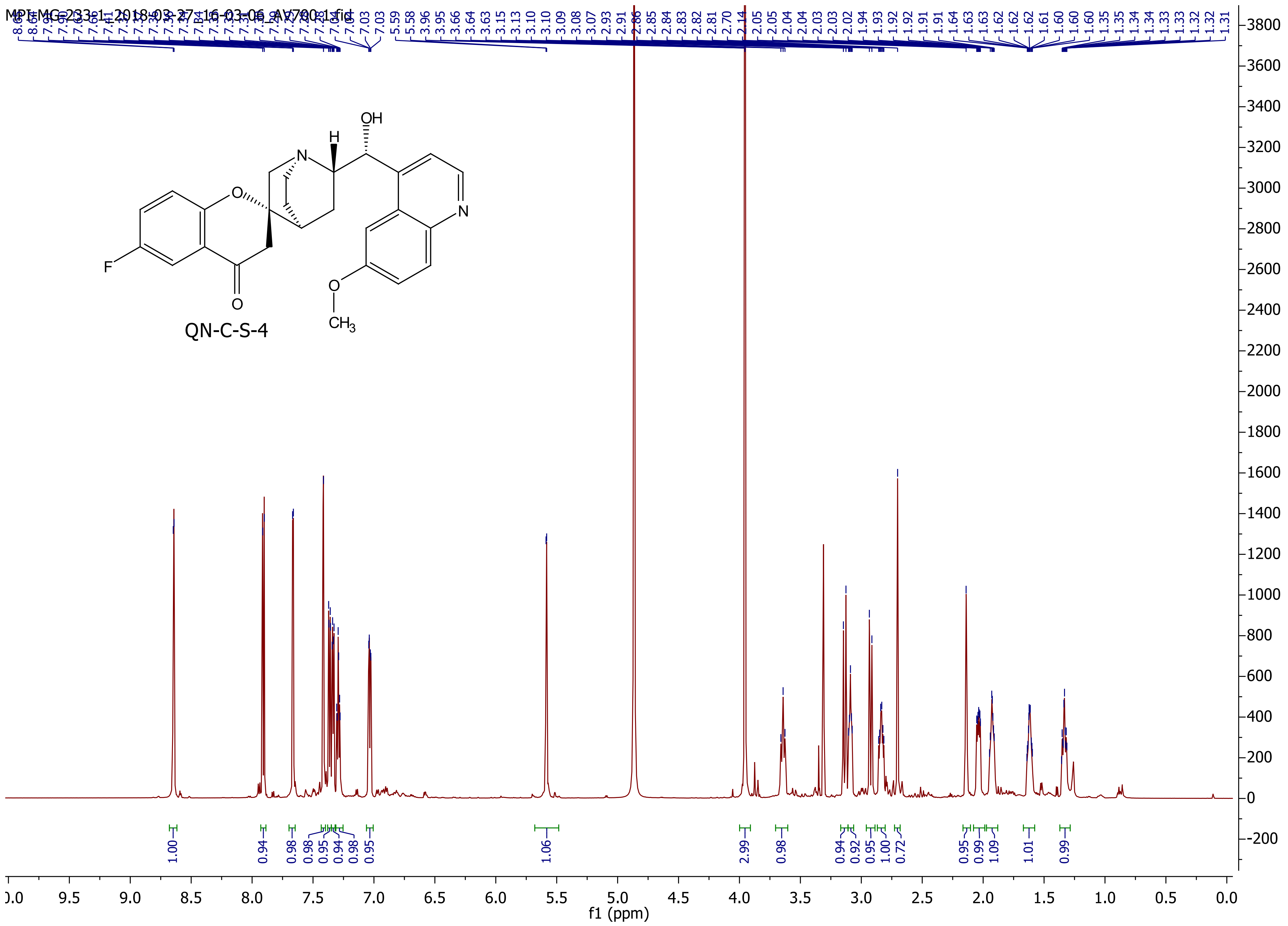
MPI-MG-233-1_2018-03-27-16-03-06_AV700-1.fid
OH
H
N
O
F
O
O
N
CH3
QN-C-S-4
f1 (ppm)

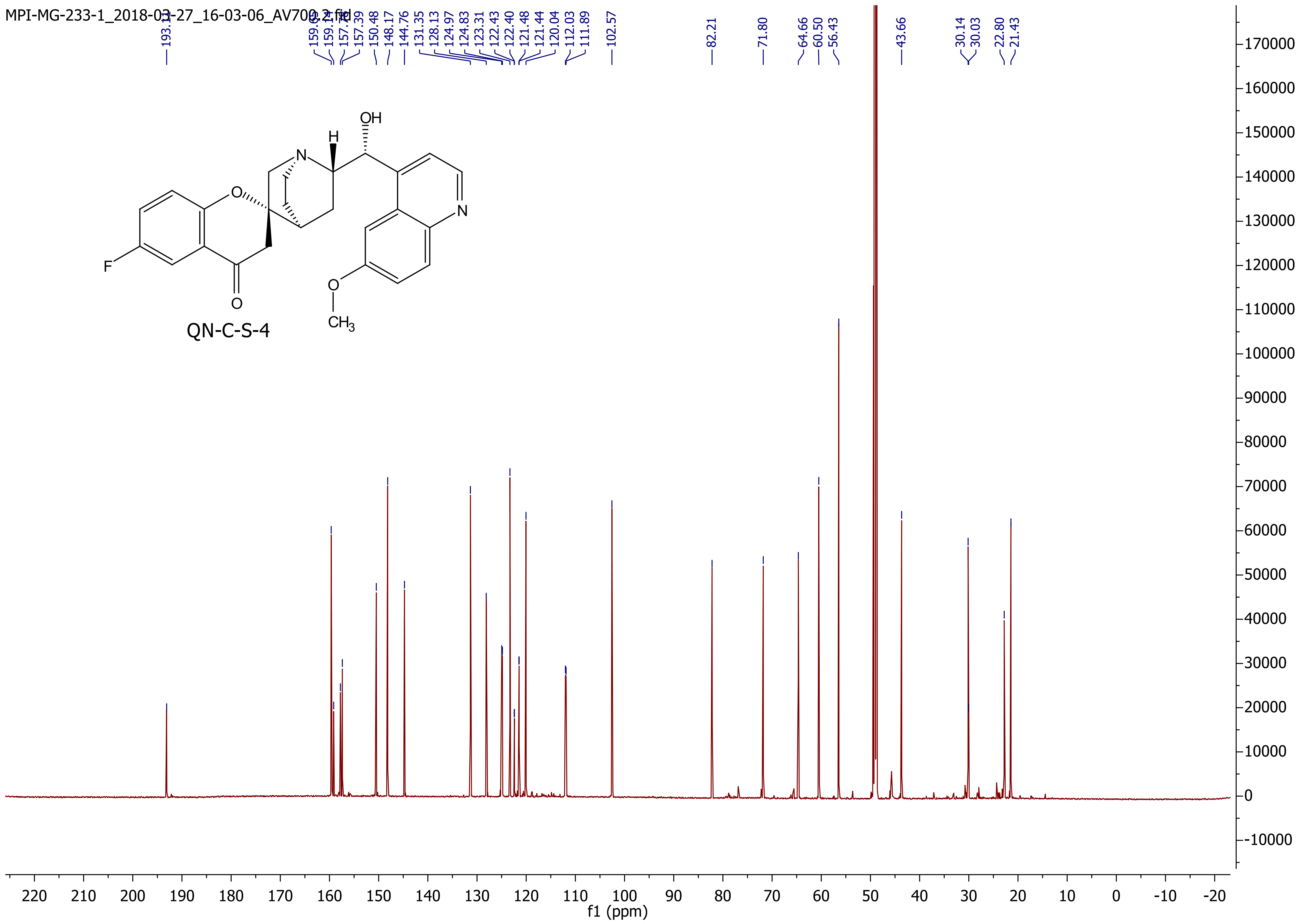

MPI-MG-233-1_2018-03-27_16-03-06_AV700.2.fid
193.1
159.1
157.39
150.48
148.17
144.76
131.35
128.13
124.97
124.83
123.31
122.43
122.40
121.48
121.44
120.04
112.03
111.89
102.57
82.21
71.80
64.66
60.50
56.43
43.66
30.14
30.03
22.80
21.43
OH
H
N
O
N
F
O
O
CH3
QN-C-S-4
f1 (ppm)

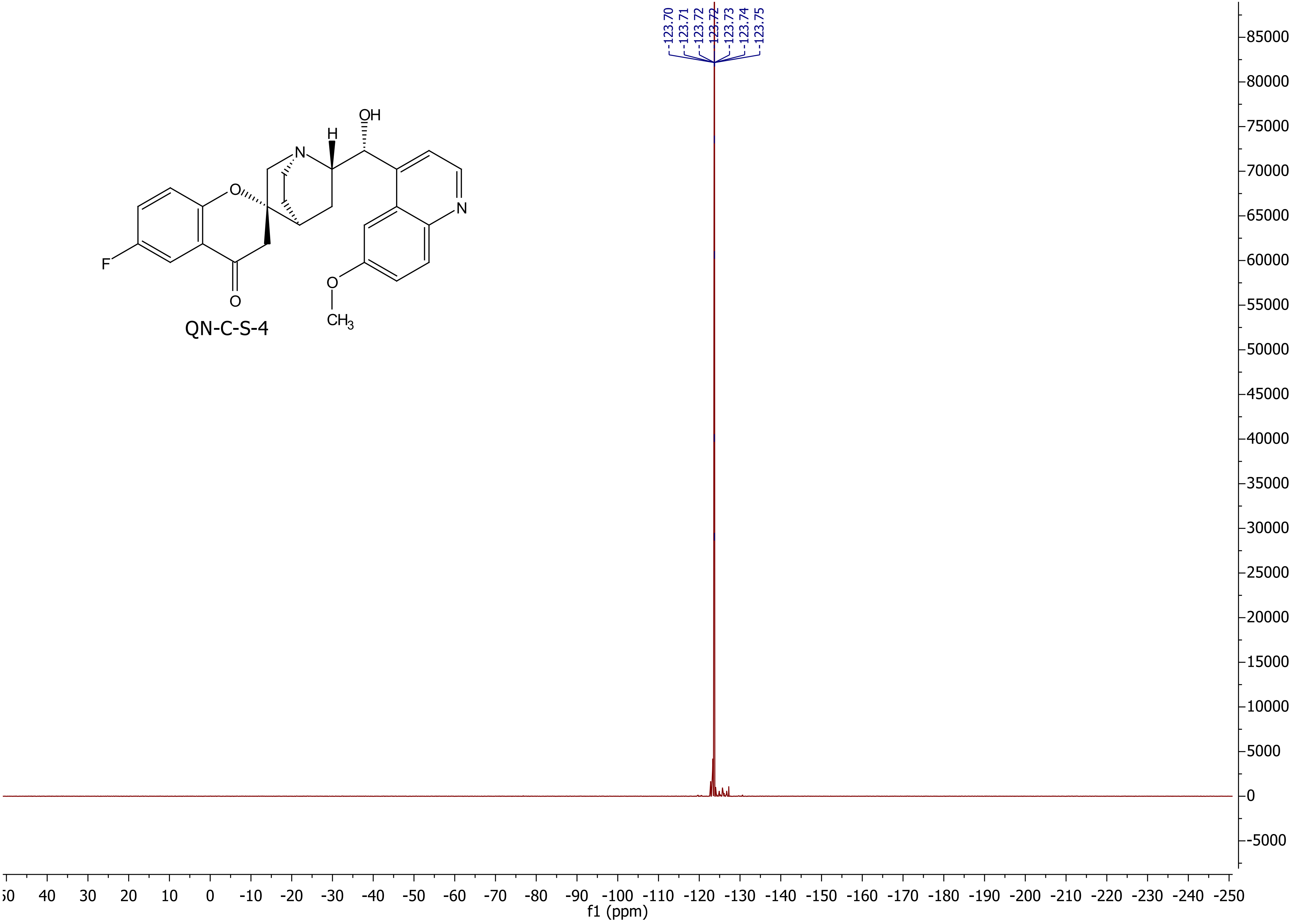

-123.70
-123.71
-123.72
-123.72
-123.73
-123.74
-123.75
OH
H
N
O
F
O
O
N
CH3
QN-C-S-4
85000
80000
75000
70000
65000
60000
55000
50000
45000
40000
35000
30000
25000
20000
15000
10000
5000
0
-5000
40 30 20 10 0 -10 -20 -30 -40 -50 -60 -70 -80 -90 -100 -110 -120 -130 -140 -150 -160 -170 -180 -190 -200 -210 -220 -230 -240 -250
f1 (ppm)

MPI-MG-233-2_2018-03-27_16-03-06_AV700.1.fid

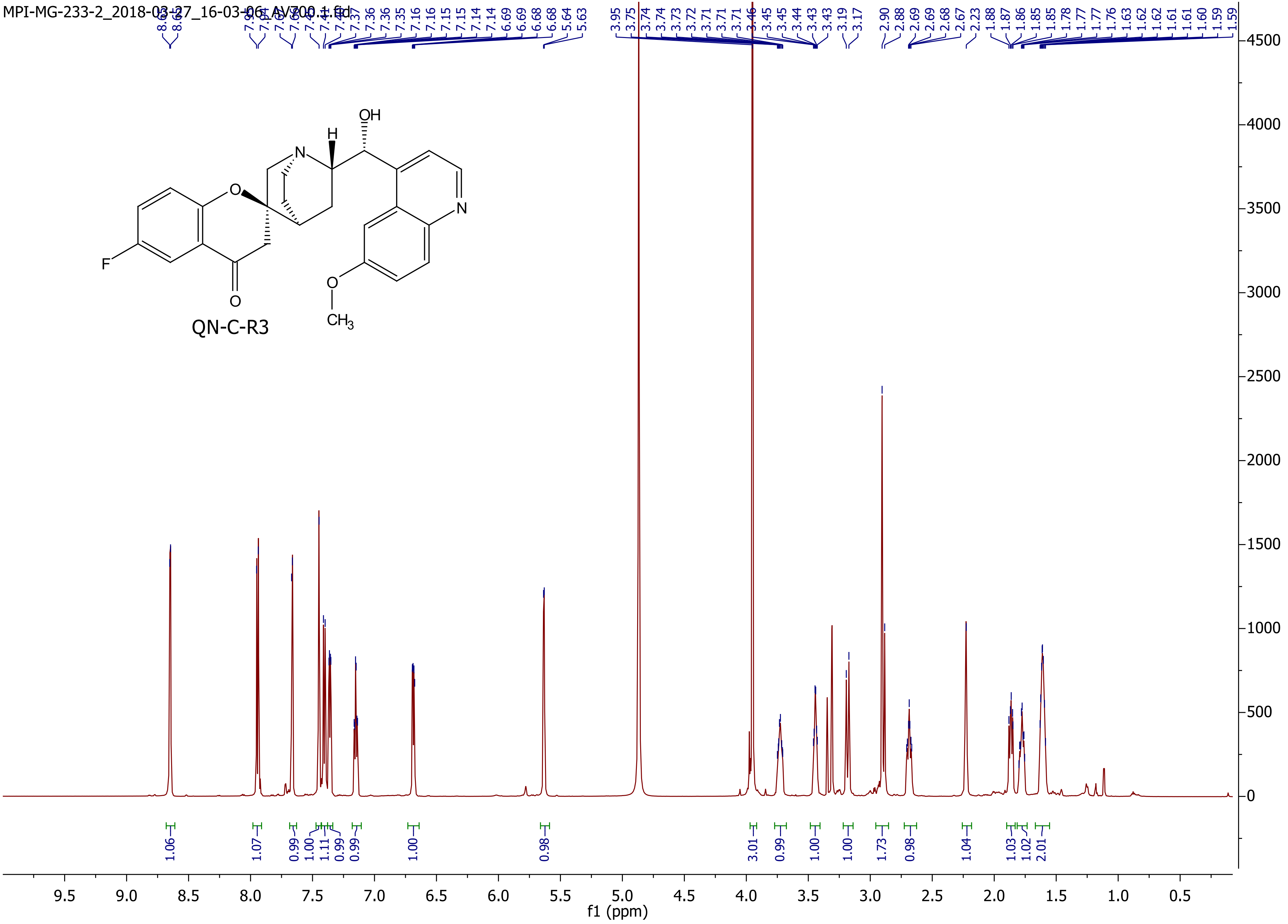

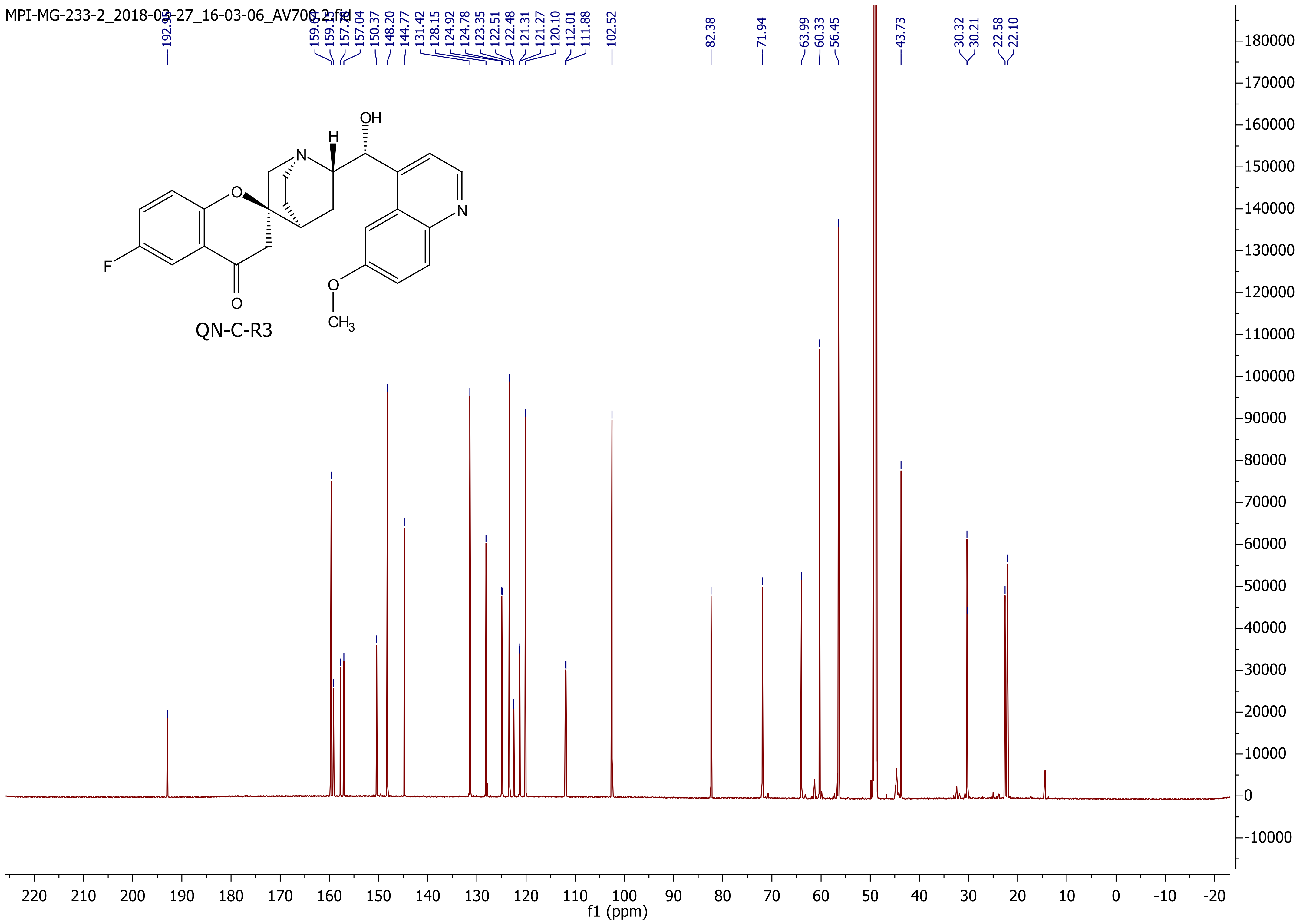

MPI-MG-233-2_2018-03-27_16-03-06_AV700.2.fid
159.64
159.15
157.78
157.04
150.37
148.20
144.77
131.42
128.15
124.92
124.78
123.35
122.51
122.48
121.31
121.27
120.10
112.01
111.88
102.52
82.38
71.94
63.99
60.33
56.45
43.73
30.32
30.21
22.58
22.10
OH
H
N
O
N
F
O
O
CH3
QN-C-R3
f1 (ppm)

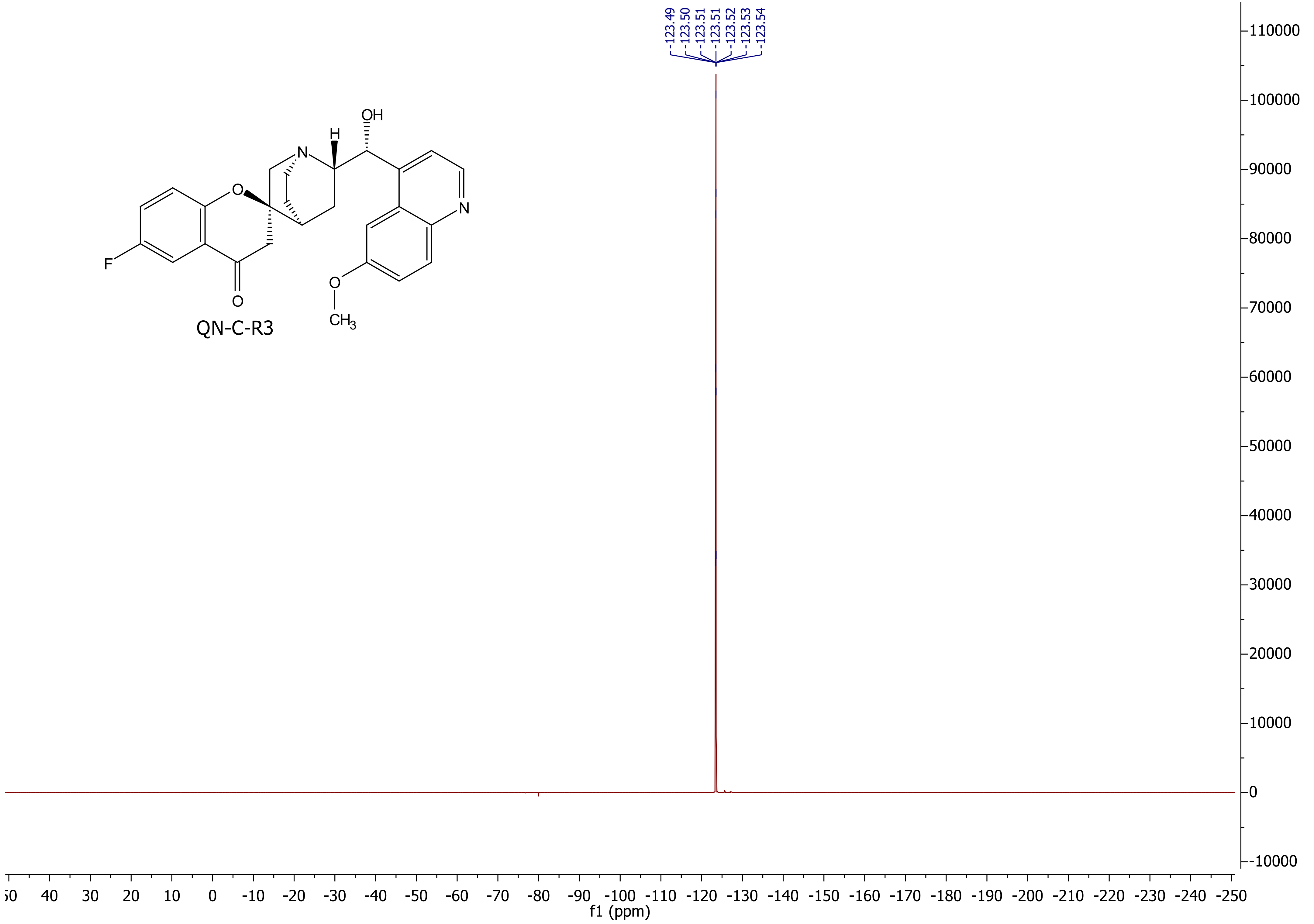

-123.49
-123.50
-123.51
-123.51
-123.52
-123.53
-123.54
OH
H
N
O
N
F
O
O
CH3
QN-C-R3
110000
100000
90000
80000
70000
60000
50000
40000
30000
20000
10000
0
-10000
50 40 30 20 10 0 -10 -20 -30 -40 -50 -60 -70 -80 -90 -100 -110 -120 -130 -140 -150 -160 -170 -180 -190 -200 -210 -220 -230 -240 -250
f1 (ppm)

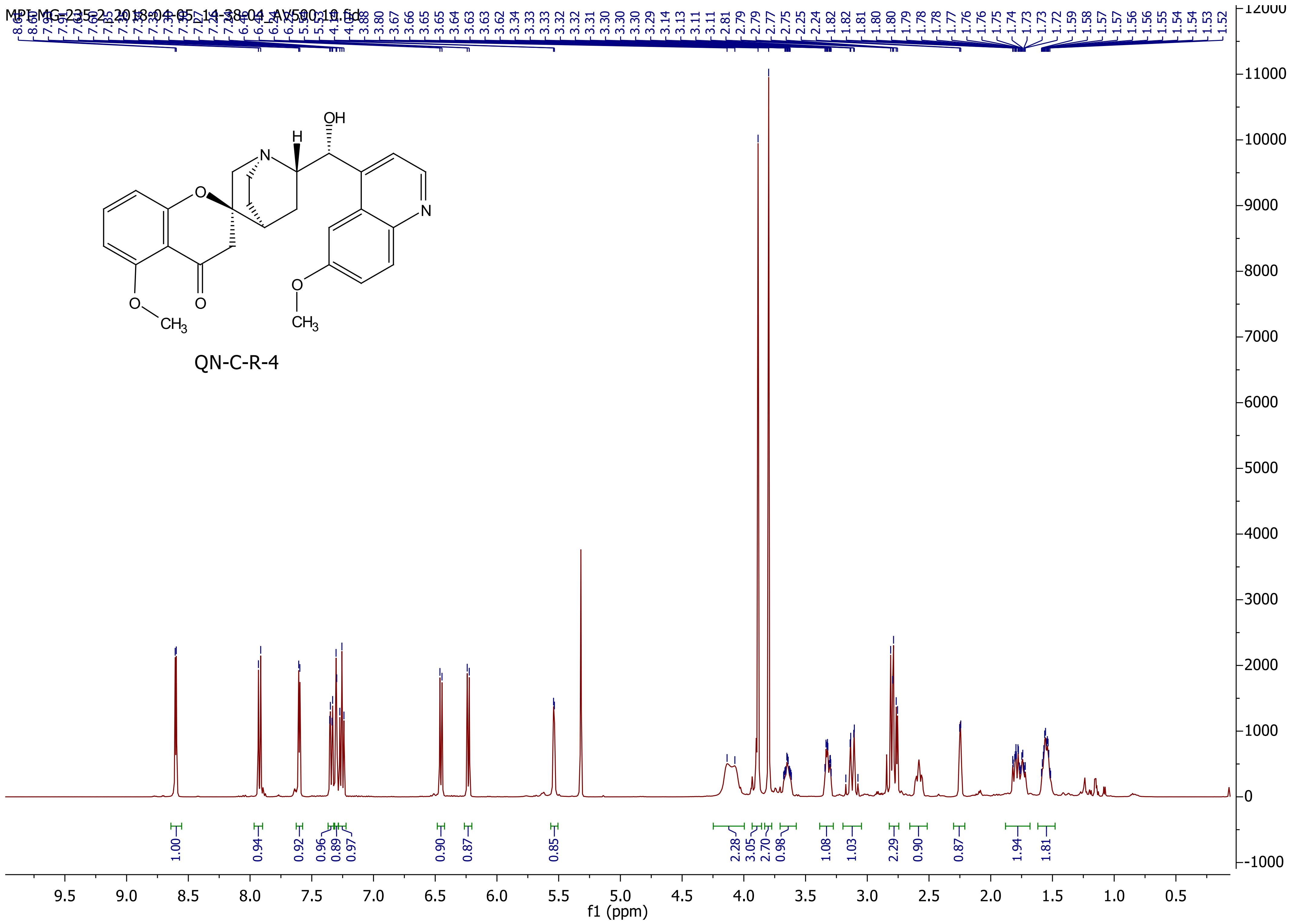
MPI-MG-235-2_2018-04-05_14-38-04_AV500.10.fid
QN-C-R-4
OH
H
N
O
O
O
CH3
O
CH3
N
1.00
0.94
0.92
0.96
0.89
0.97
0.90
0.87
0.85
2.28
3.05
2.70
0.98
1.08
1.03
2.29
0.90
0.87
1.94
1.81
f1 (ppm)

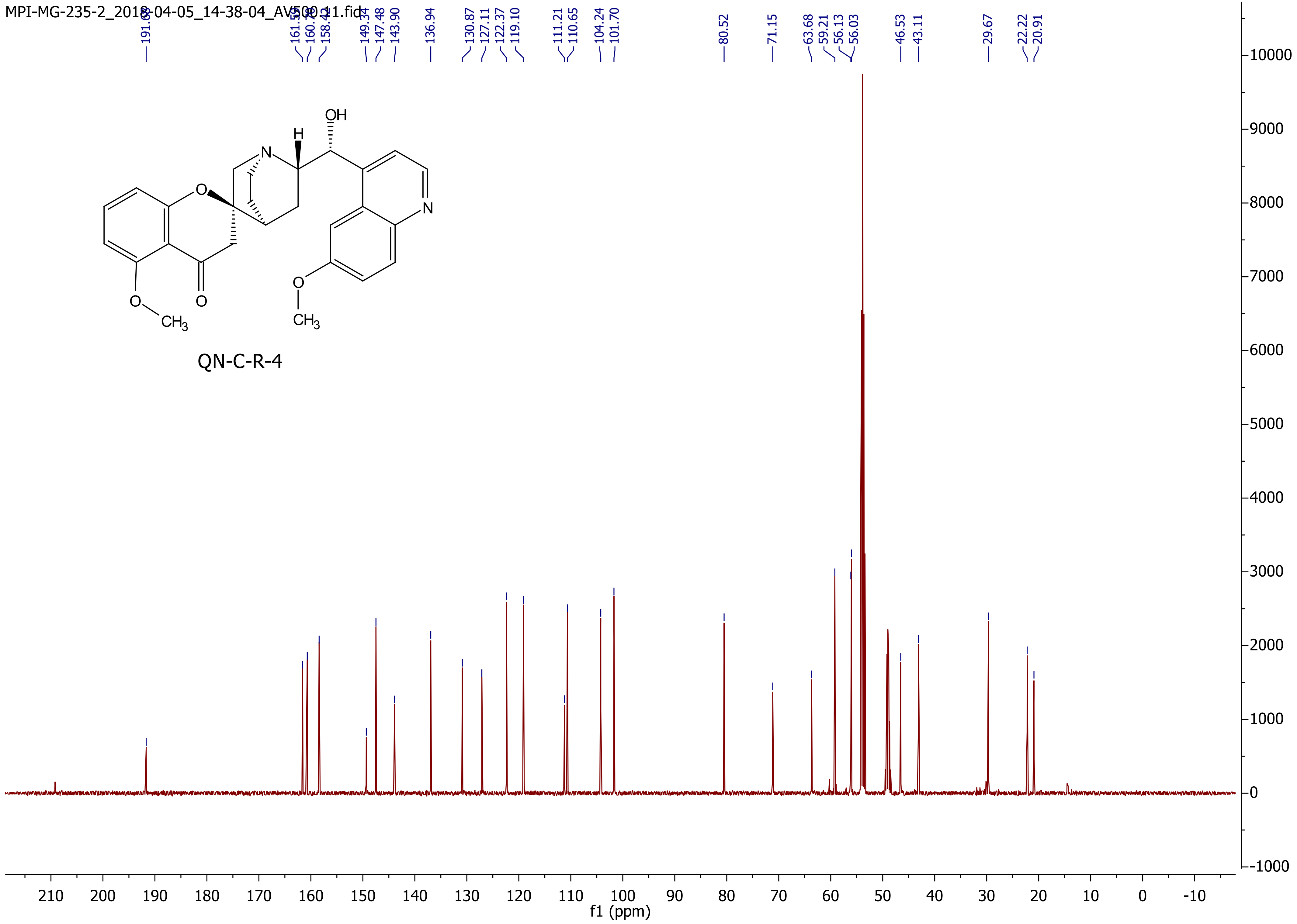

MPI-MG-235-2_2018-04-05_14-38-04_AV500 11.fid
191.68
161.59
160.78
158.42
149.34
147.48
143.90
136.94
130.87
127.11
122.37
119.10
111.21
110.65
104.24
101.70
80.52
71.15
63.68
59.21
56.13
56.03
46.53
43.11
29.67
22.22
20.91
OH
H
N
O
O
O
CH3
N
O
CH3
QN-C-R-4
10000
9000
8000
7000
6000
5000
4000
3000
2000
1000
0
-1000
210 200 190 180 170 160 150 140 130 120 110 100 90 80 70 60 50 40 30 20 10 0 -10
f1 (ppm)

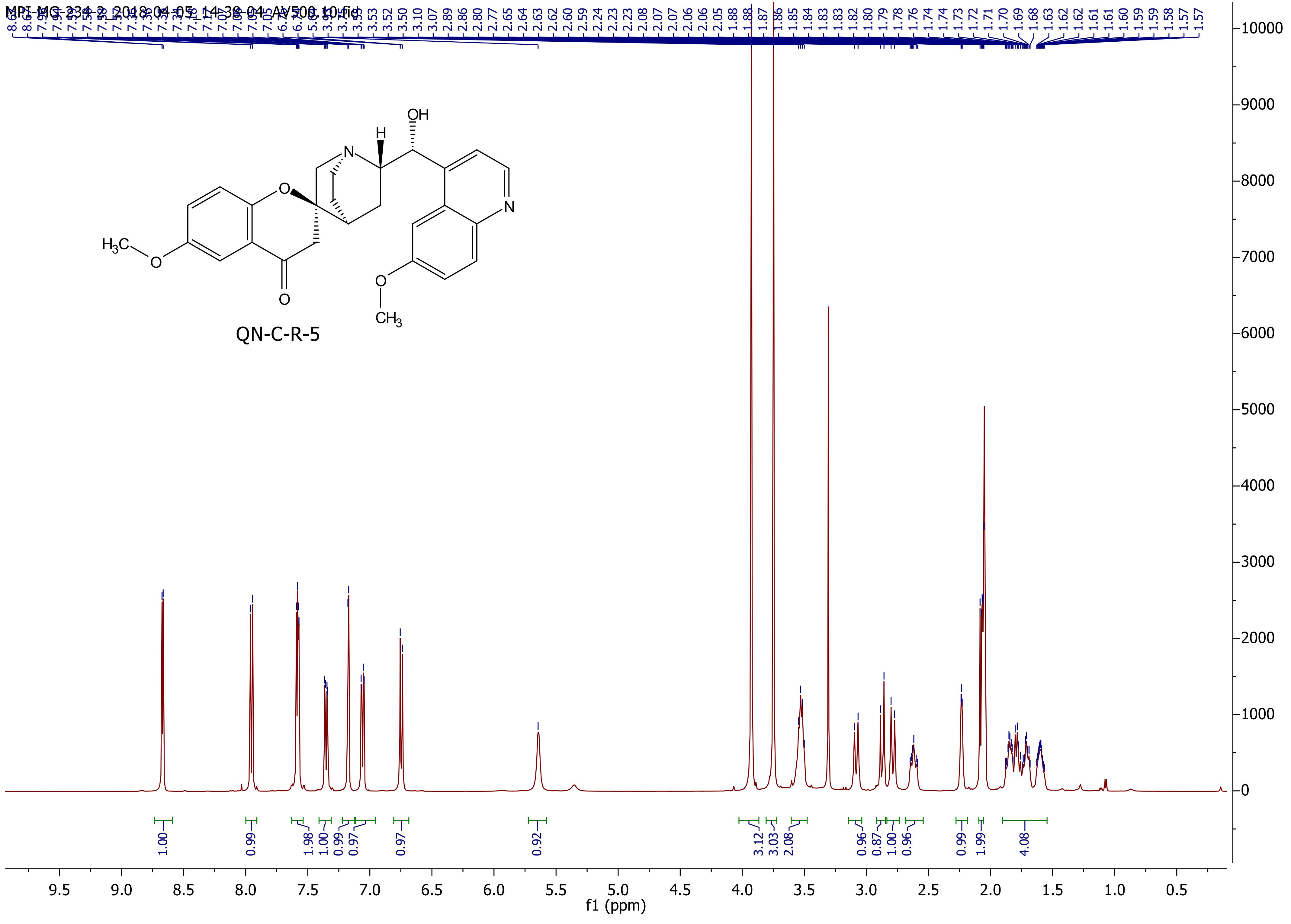
MPI-MG-234-2
3.53 3.52 3.50 3.10 3.07 2.89 2.86 2.80 2.77 2.65 2.64 2.63 2.62 2.60 2.59 2.24 2.23 2.23 2.08 2.07 2.07 2.06 2.06 2.05 1.88 1.88 1.87 1.86 1.85 1.84 1.83 1.83 1.82 1.80 1.79 1.78 1.76 1.74 1.74 1.73 1.72 1.71 1.70 1.69 1.68 1.63 1.62 1.62 1.61 1.61 1.60 1.59 1.59 1.58 1.57 1.57
OH
H
N
O
N
H_3C
O
O
O
CH_3
QN-C-R-5
1.00 0.99 1.98 1.00 0.99 0.97 0.97 0.92 3.12 3.03 2.08 0.96 0.87 1.00 0.96 0.99 1.99 4.08
9.5 9.0 8.5 8.0 7.5 7.0 6.5 6.0 5.5 5.0 4.5 4.0 3.5 3.0 2.5 2.0 1.5 1.0 0.5
f1 (ppm)
10000 9000 8000 7000 6000 5000 4000 3000 2000 1000 0

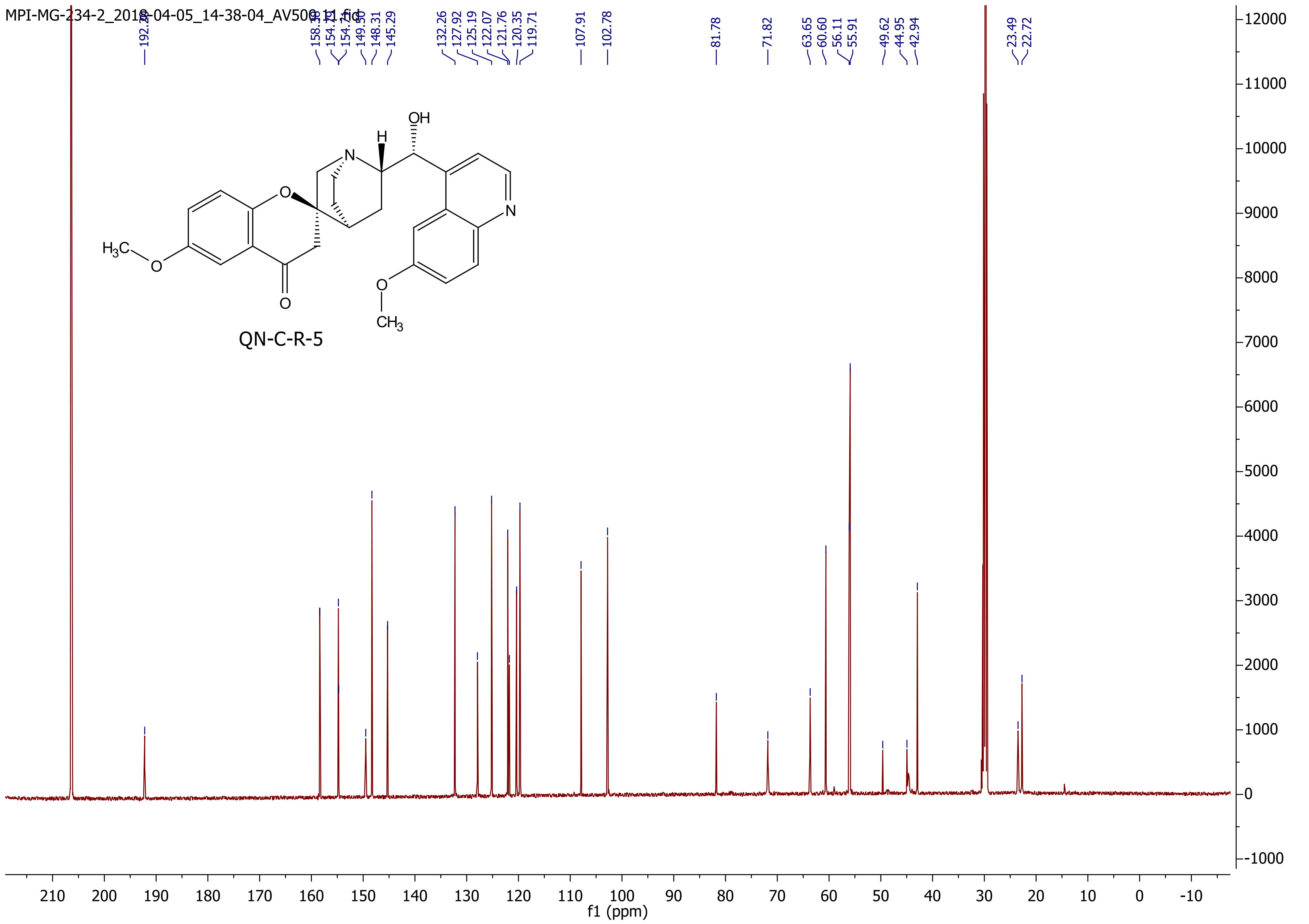

MPI-MG-234-2_2018-04-05_14-38-04_AV500.11.fid
149.50
148.31
145.29
132.26
127.92
125.19
122.07
121.76
120.35
119.71
107.91
102.78
81.78
71.82
63.65
60.60
56.11
55.91
49.62
44.95
42.94
23.49
22.72
OH
H
N
O
N
H_3C
O
O
O
CH_3
QN-C-R-5
f1 (ppm)

MPI-MG-237-1_2018-04-06_15-02-18-AV500.10.fid

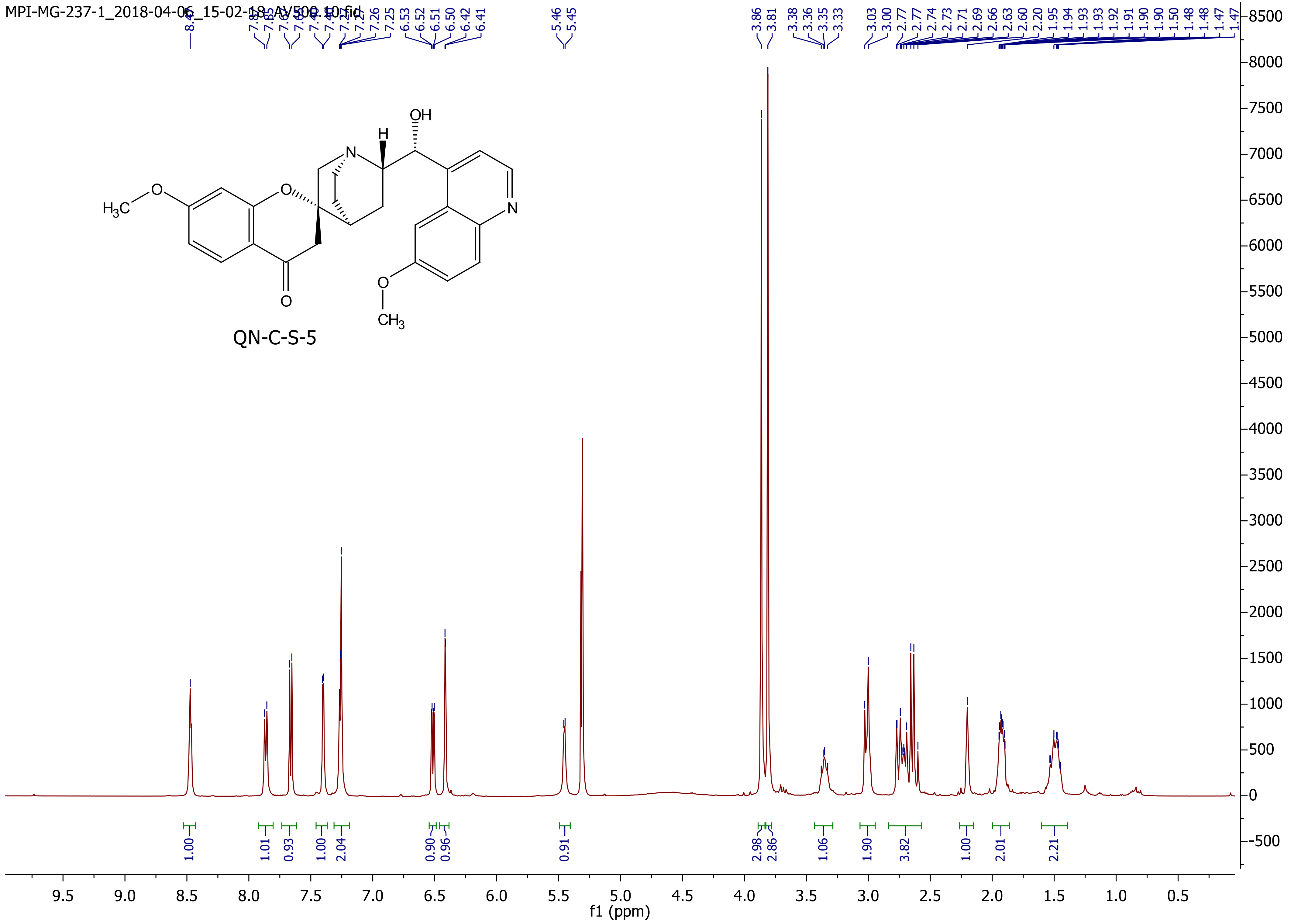

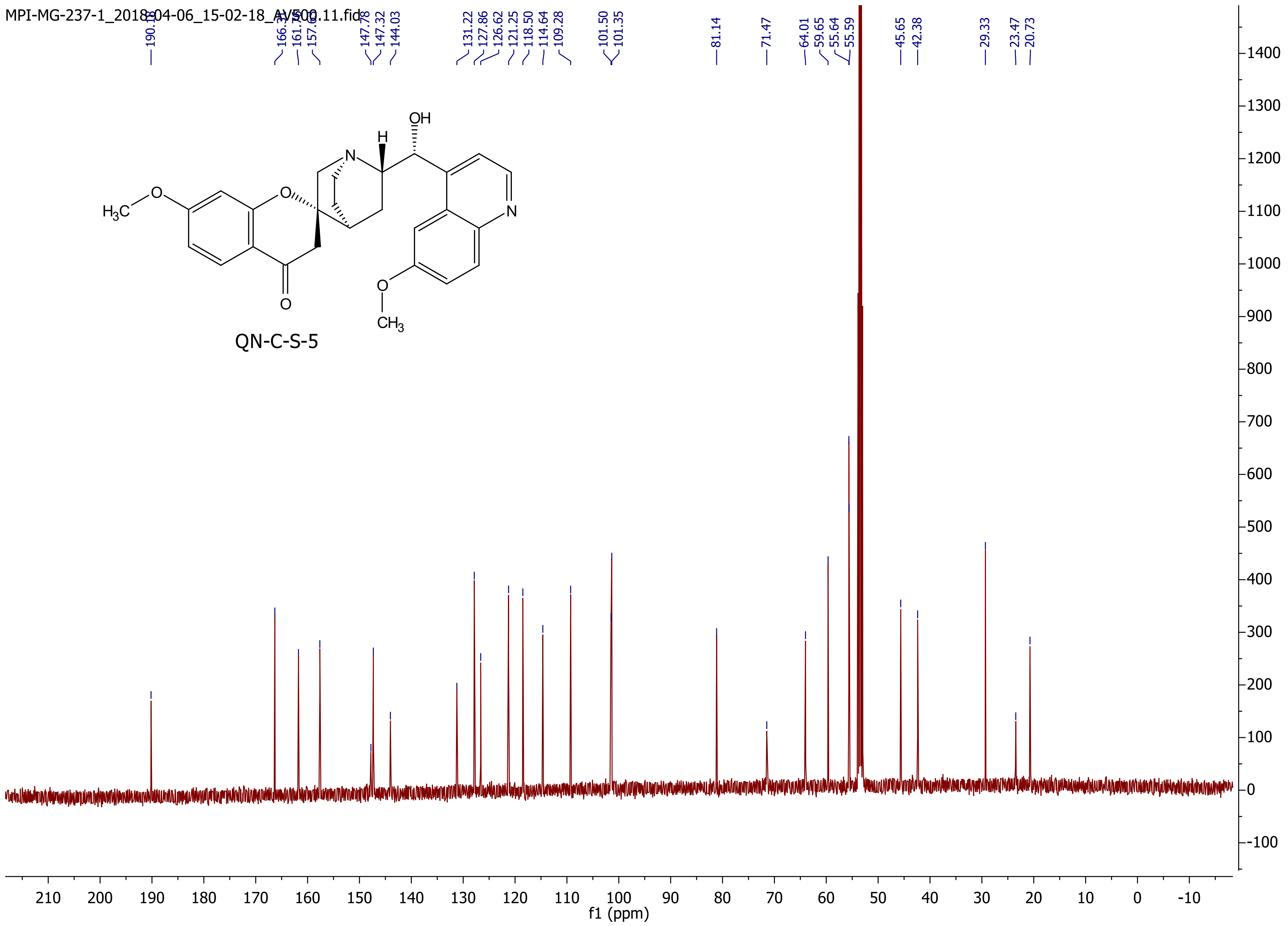

MPI-MG-237-1_2018-04-06_15-02-18_AV500.11.fid
190.18
147.78
147.32
144.03
131.22
127.86
126.62
121.25
118.50
114.64
109.28
101.50
101.35
81.14
71.47
64.01
59.65
55.64
55.59
45.65
42.38
29.33
23.47
20.73
OH
H
N
O
H3C
O
O
N
O
CH3
QN-C-S-5
f1 (ppm)

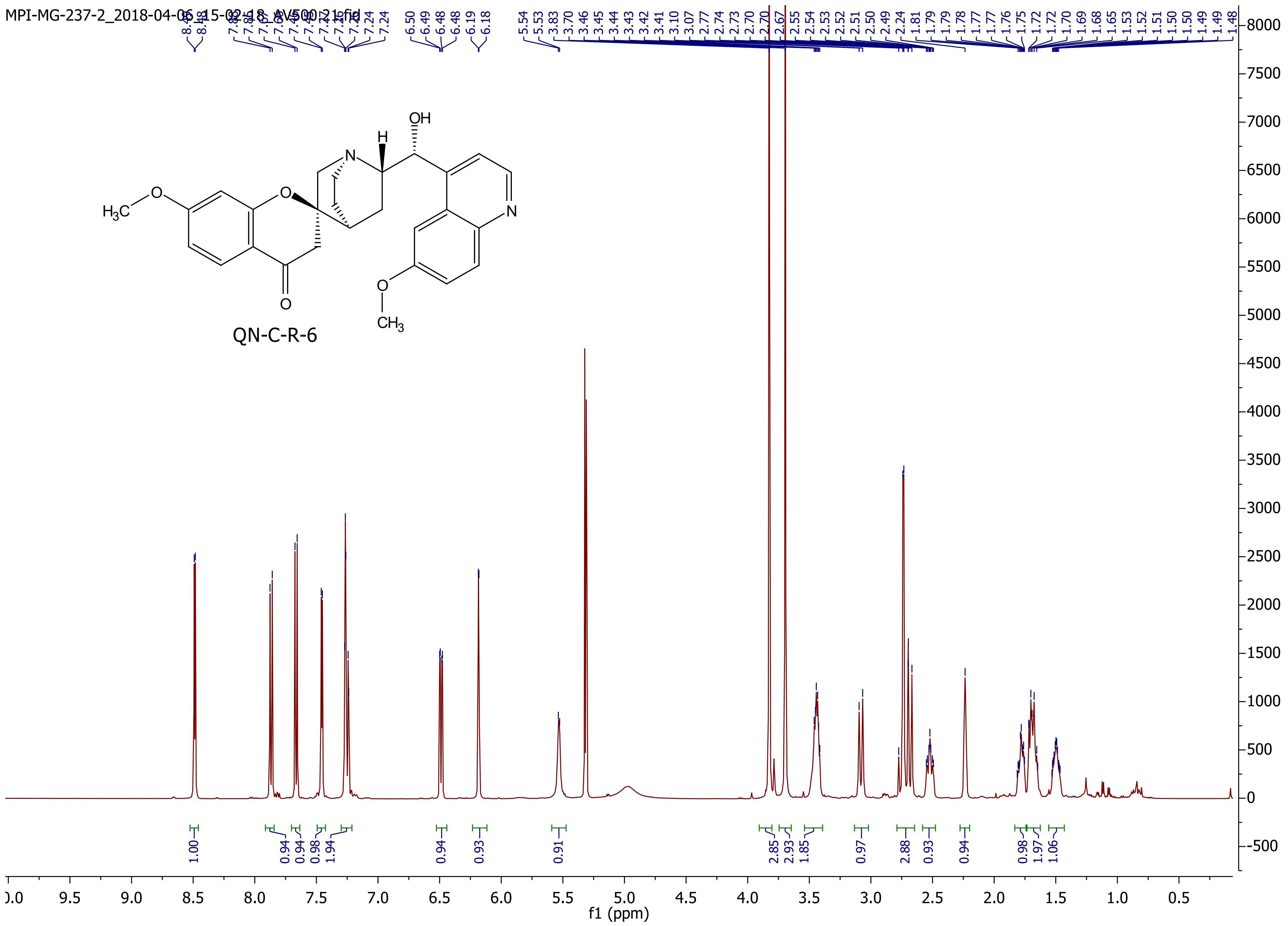
MPI-MG-237-2_2018-04-06_15-02-18_AV500.21.fid
OH
H
N
O
H_3C
O
O
N
O
CH_3
QN-C-R-6
1.00
0.94
0.94
0.98
1.94
0.94
0.93
0.91
2.85
2.93
1.85
0.97
2.88
0.93
0.94
0.98
1.97
1.06
f1 (ppm)

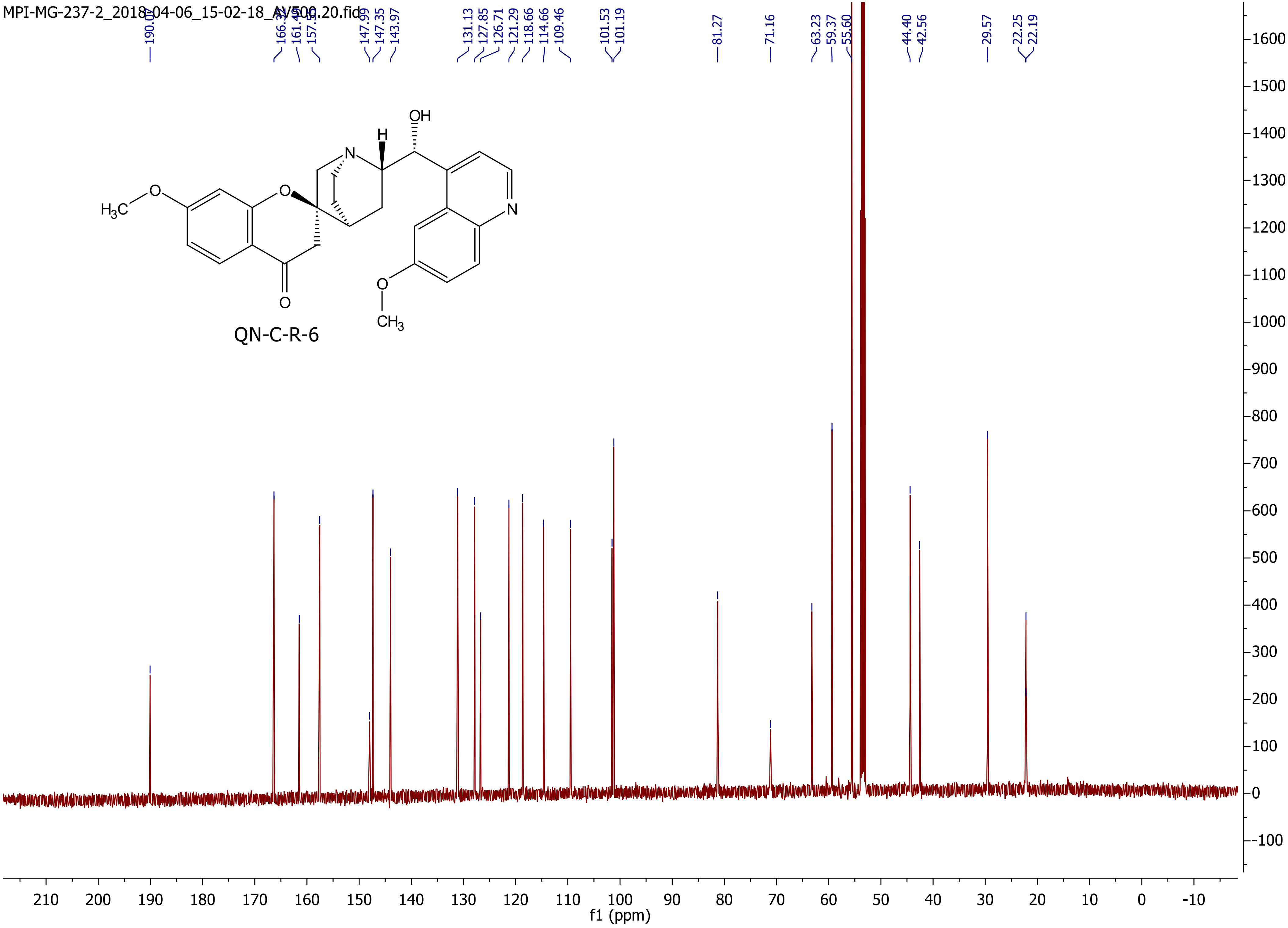
MPI-MG-237-2_2018-04-06_15-02-18_AV500.20.fid
147.99
147.35
143.97
131.13
127.85
126.71
121.29
118.66
114.66
109.46
101.53
101.19
81.27
71.16
63.23
59.37
55.60
44.40
42.56
29.57
22.25
22.19
OH
H
N
O
H_3C
O
O
N
O
CH_3
QN-C-R-6
f1 (ppm)

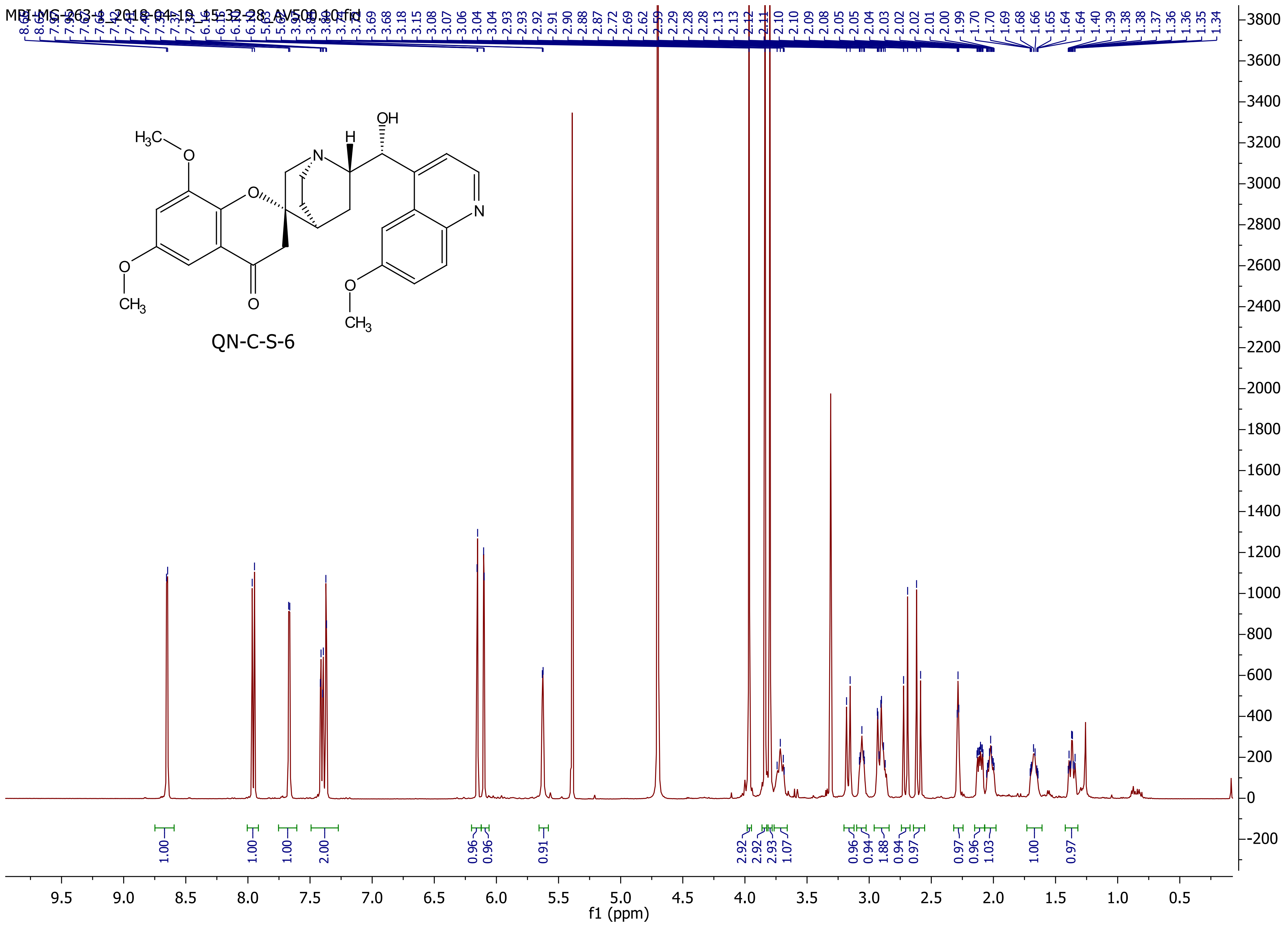

MPI-MG-263-1_2018-04-19_15-32-28_AV500.10.fid
QN-C-S-6
OH
H
N
O
H3C
CH3
1.00
1.00
1.00
2.00
0.96
0.96
0.91
2.92
2.92
2.93
1.07
0.96
0.94
1.88
0.94
0.97
0.97
0.96
1.03
1.00
0.97
f1 (ppm)

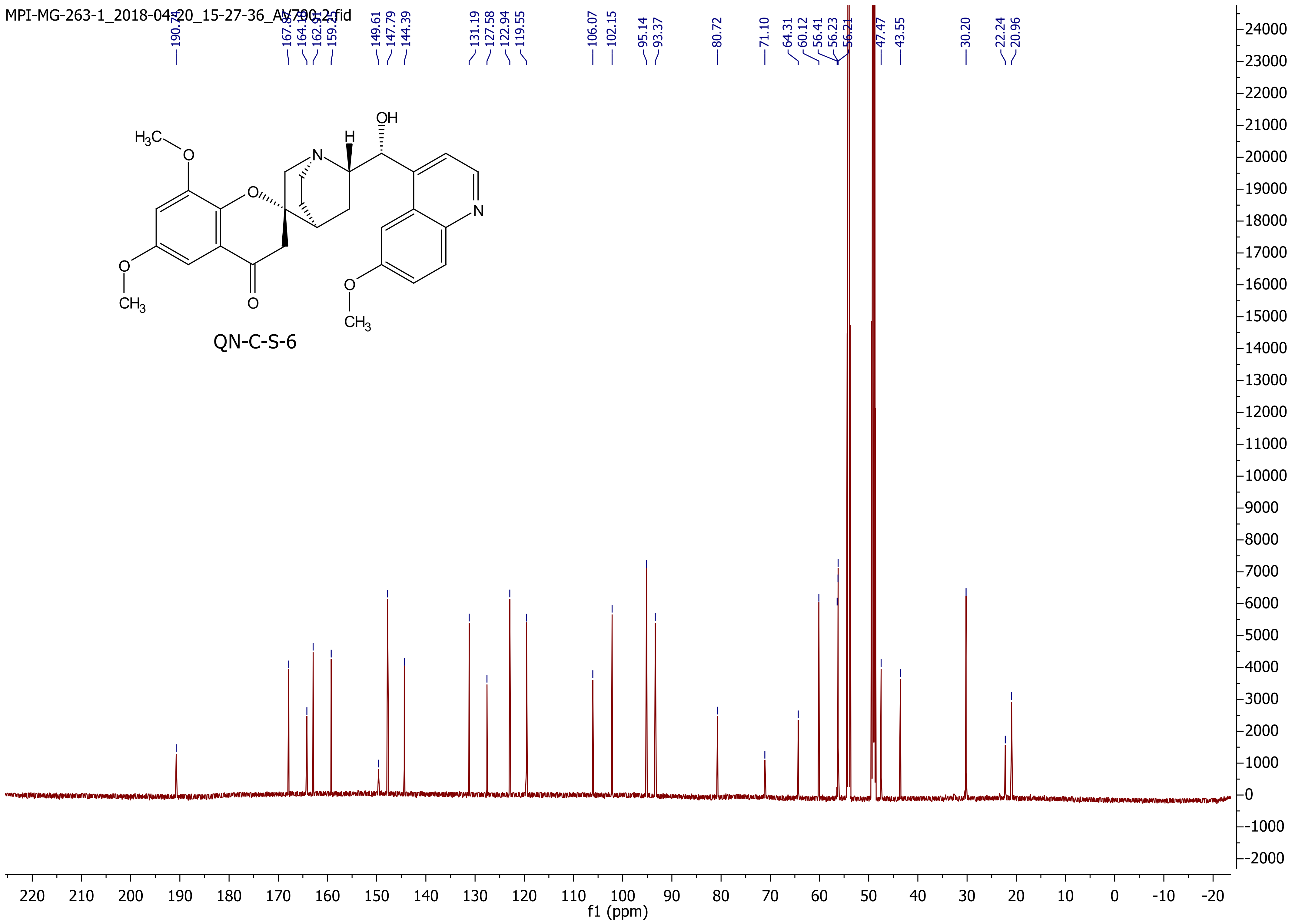

MPI-MG-263-1_2018-04-20_15-27-36_AV700-2.fid
149.61
147.79
144.39
131.19
127.58
122.94
119.55
106.07
102.15
95.14
93.37
80.72
71.10
64.31
60.12
56.41
56.23
56.21
47.47
43.55
30.20
22.24
20.96
QN-C-S-6
f1 (ppm)

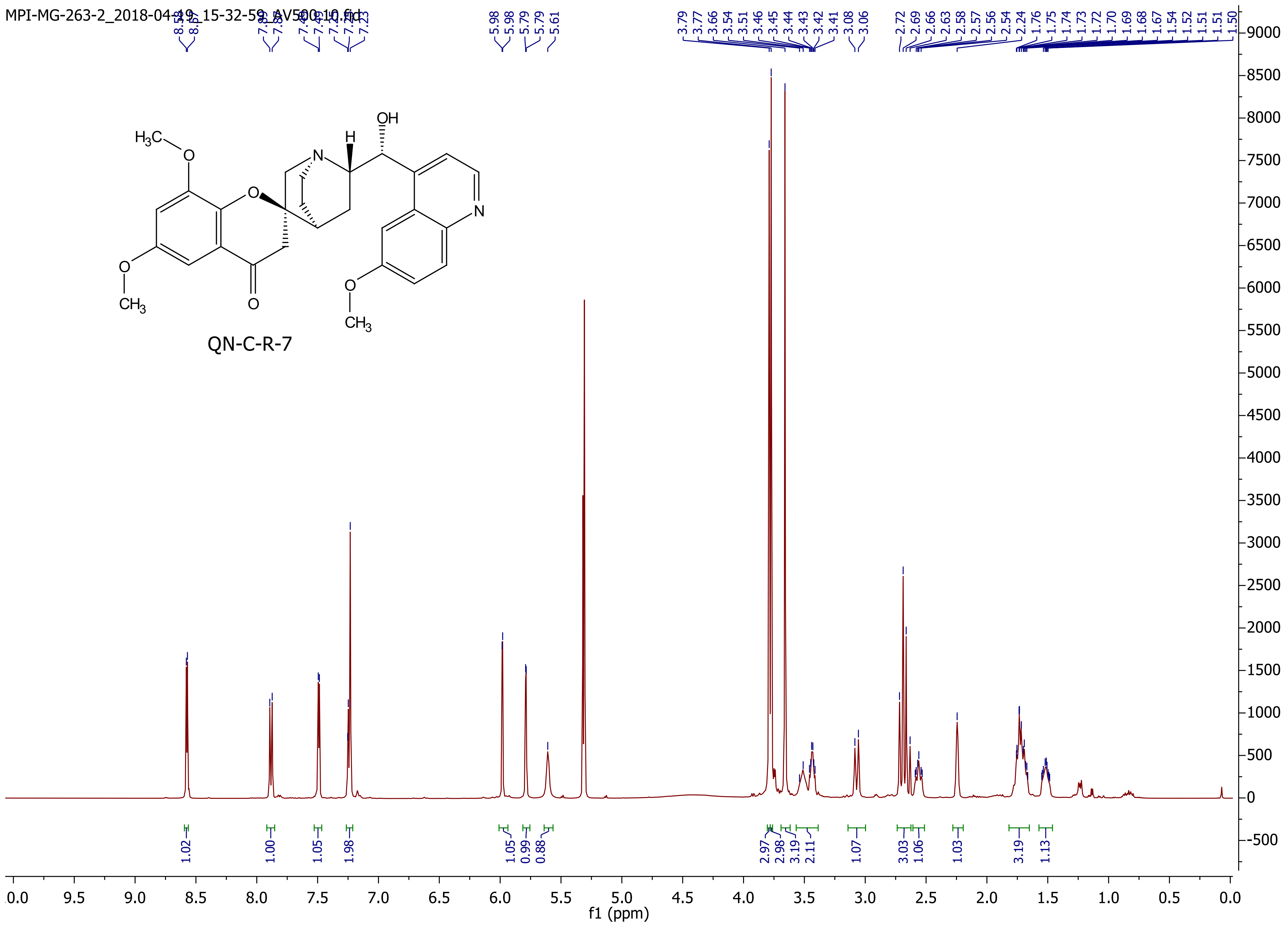

MPI-MG-263-2_2018-04-19_15-32-59_AV500 10.fid
QN-C-R-7
OH
H
N
N
O
O
O
O
H_3C
CH_3
CH_3
8.58
8.57
7.89
7.87
7.49
7.48
7.24
7.23
7.23
5.98
5.98
5.79
5.79
5.61
3.79
3.77
3.66
3.54
3.51
3.46
3.45
3.44
3.43
3.42
3.41
3.08
3.06
2.72
2.69
2.66
2.63
2.58
2.57
2.56
2.54
2.24
1.76
1.75
1.74
1.73
1.72
1.70
1.69
1.68
1.67
1.54
1.52
1.51
1.51
1.50
1.02
1.00
1.05
1.98
1.05
0.99
0.88
2.97
2.98
3.19
2.11
1.07
3.03
1.06
1.03
3.19
1.13
f1 (ppm)

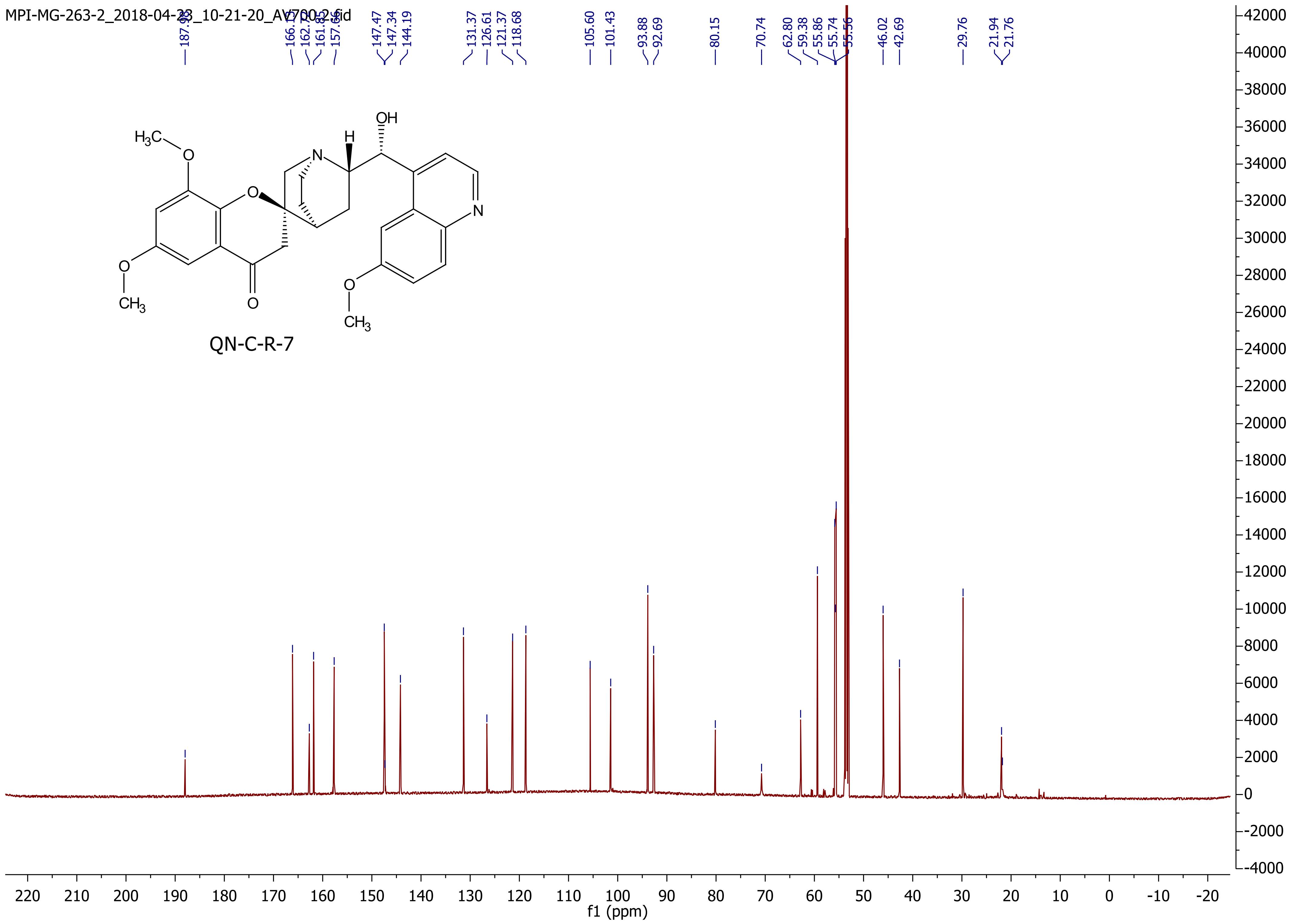
MPI-MG-263-2_2018-04-23_10-21-20_AV700.2.fid
187.9
147.47
147.34
144.19
131.37
126.61
121.37
118.68
105.60
101.43
93.88
92.69
80.15
70.74
62.80
59.38
55.86
55.74
55.56
46.02
42.69
29.76
21.94
21.76
OH
H
N
N
O
O
O
O
H3C
CH3
CH3
QN-C-R-7
f1 (ppm)

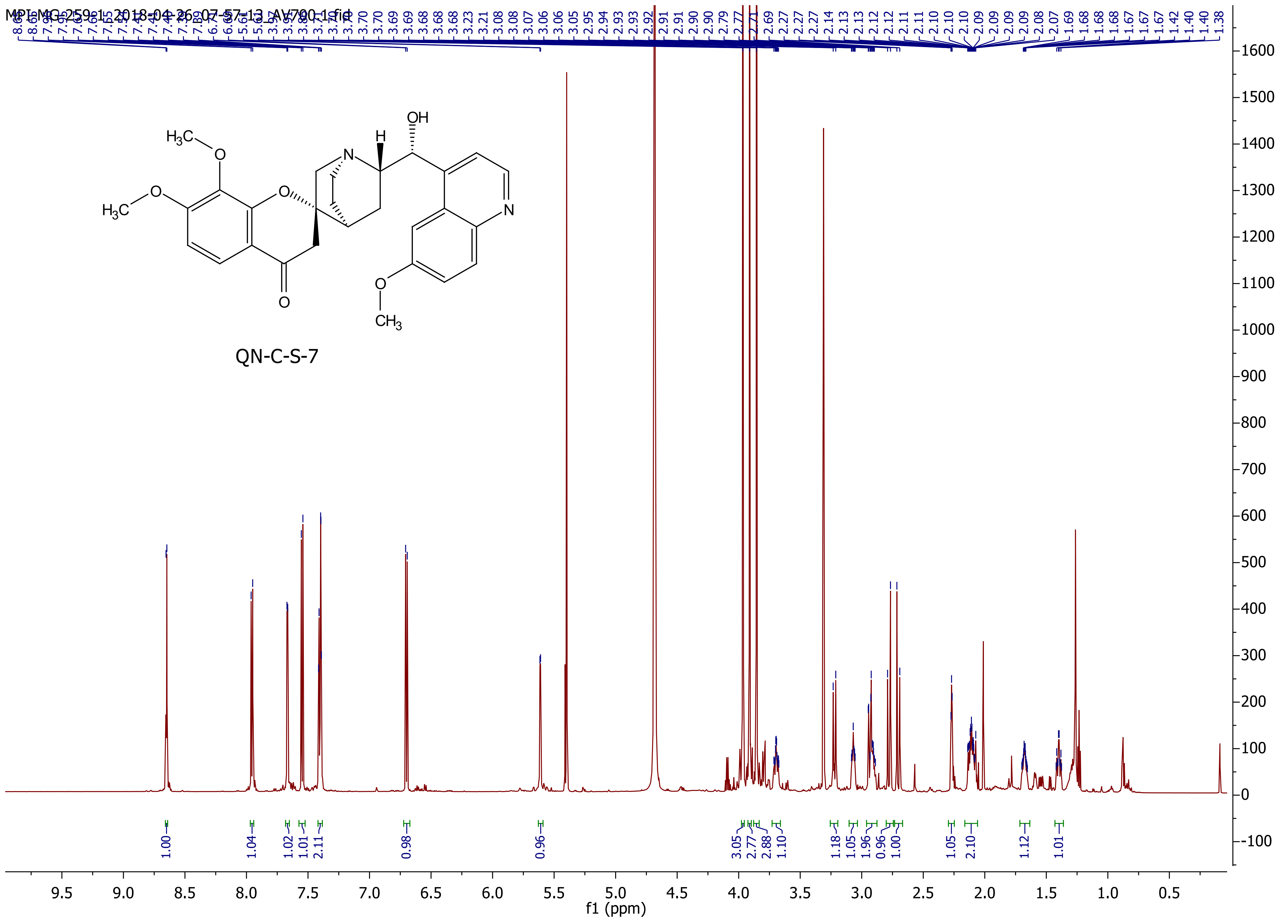
MPI-MG-259-1 2018-04-26 07-57-13 AV700 1.fid
QN-C-S-7
f1 (ppm)

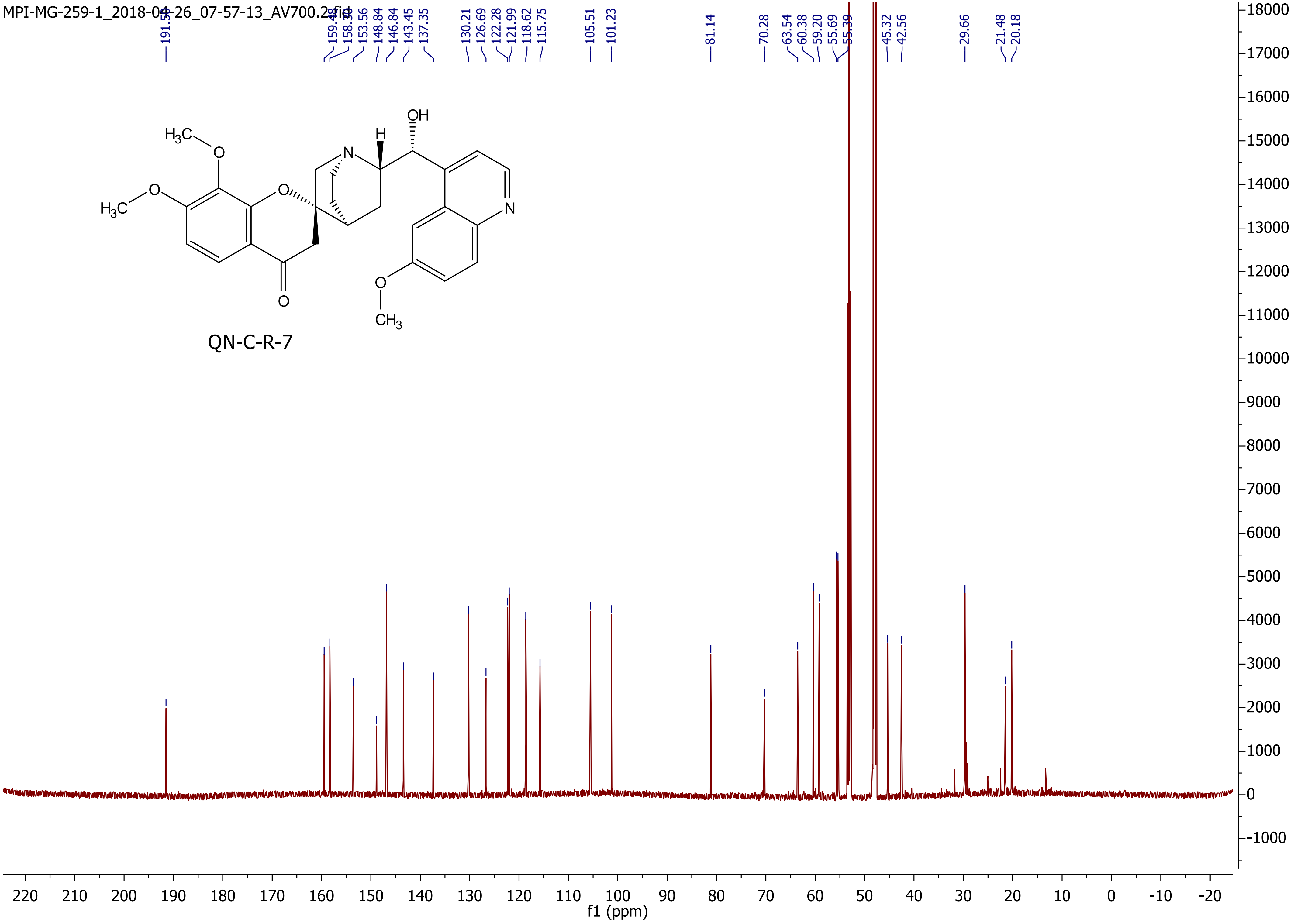

MPI-MG-259-1_2018-04-26_07-57-13_AV700.2.fid
159.48
153.56
148.84
146.84
143.45
137.35
130.21
126.69
122.28
121.99
118.62
115.75
105.51
101.23
81.14
70.28
63.54
60.38
59.20
55.69
55.39
45.32
42.56
29.66
21.48
20.18
OH
H
N
N
O
O
O
O
O
H_3C
H_3C
CH_3
QN-C-R-7
f1 (ppm)

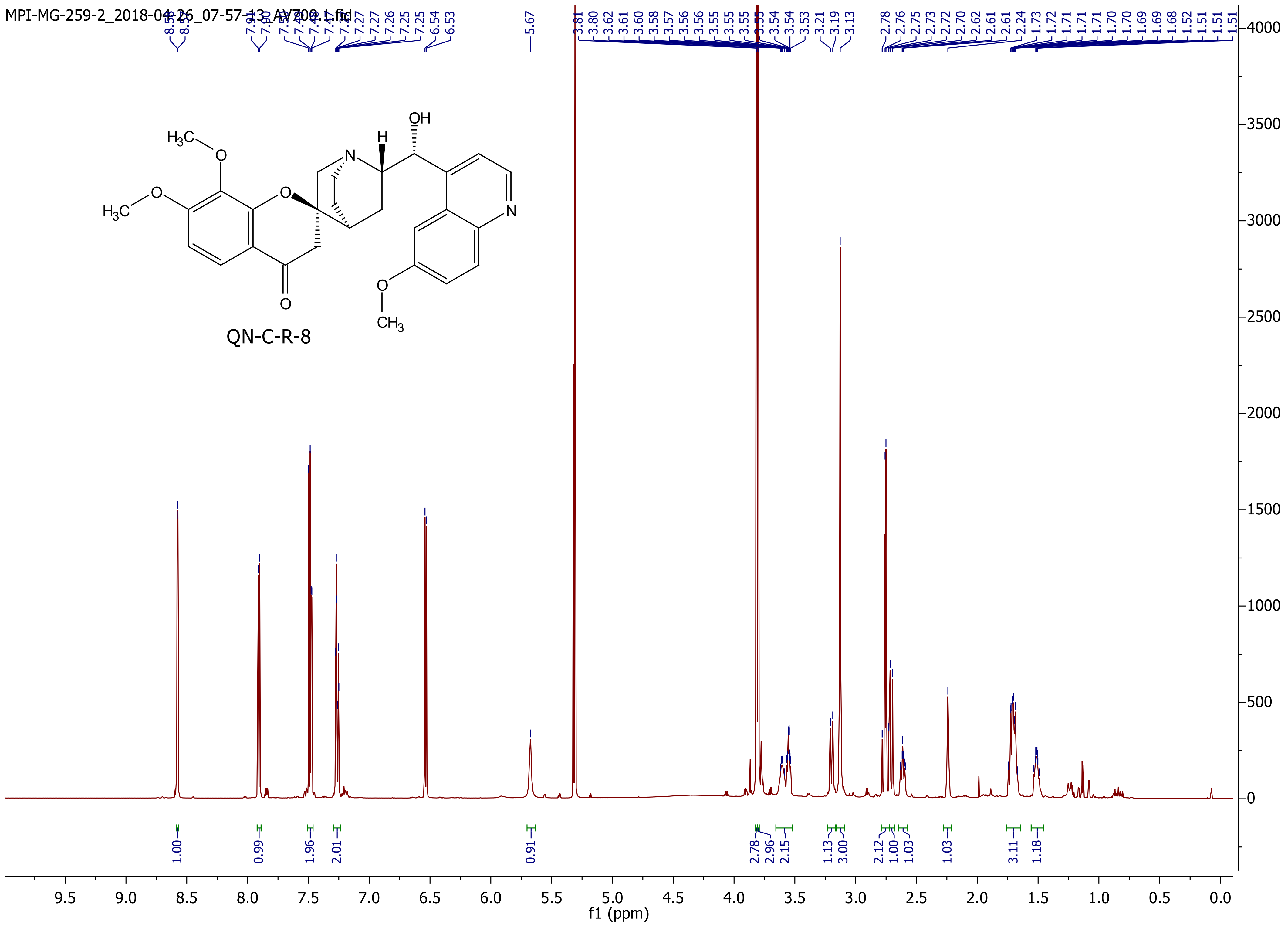
MPI-MG-259-2_2018-04-26_07-57-13_AV700.1.fid
QN-C-R-8
f1 (ppm)

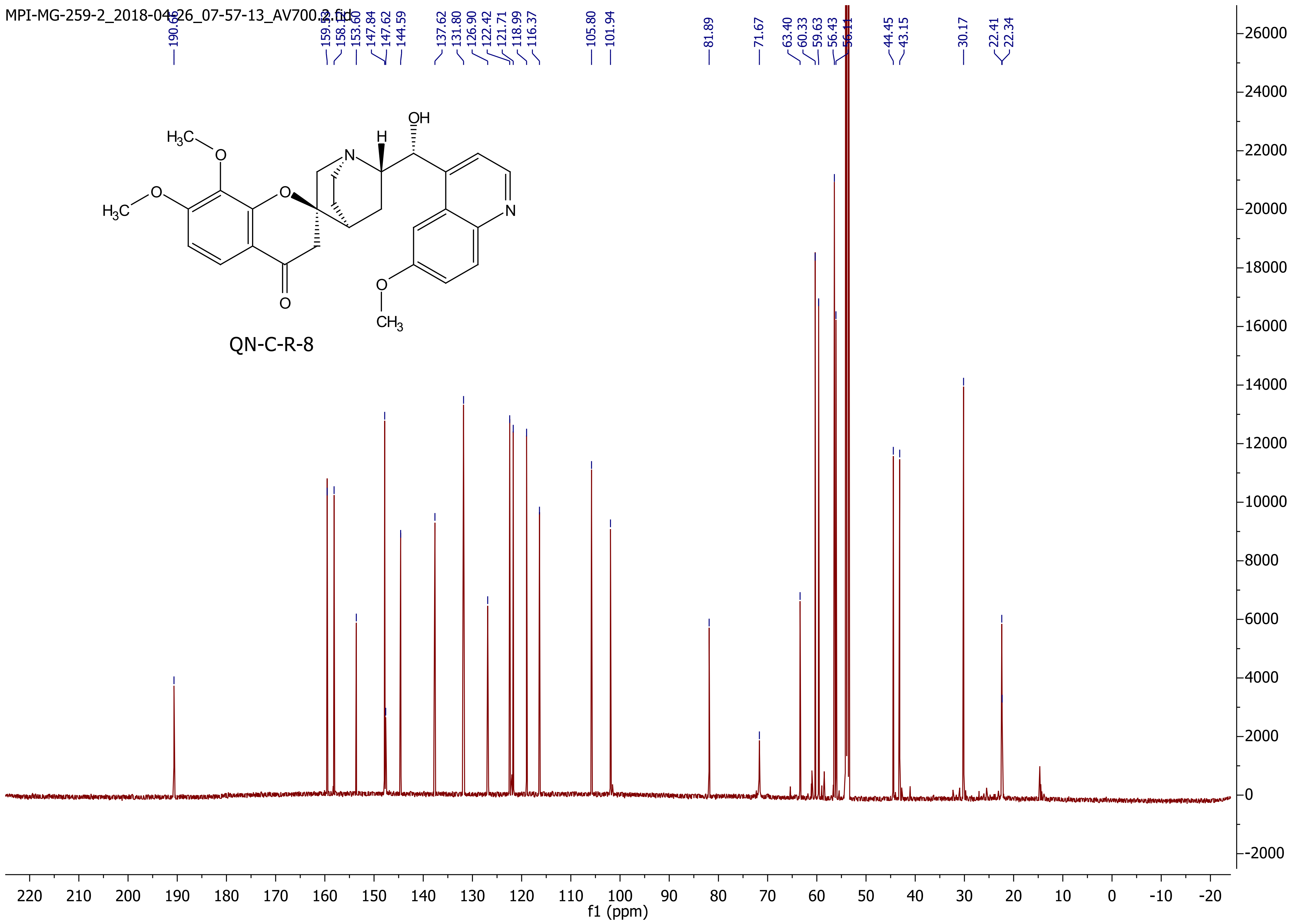
MPI-MG-259-2_2018-04-26_07-57-13_AV700.2.fid
153.60
147.84
147.62
144.59
137.62
131.80
126.90
122.42
121.71
118.99
116.37
105.80
101.94
81.89
71.67
63.40
60.33
59.63
56.43
56.11
44.45
43.15
30.17
22.41
22.34
OH
H
N
O
N
O
O
O
O
QN-C-R-8
f1 (ppm)

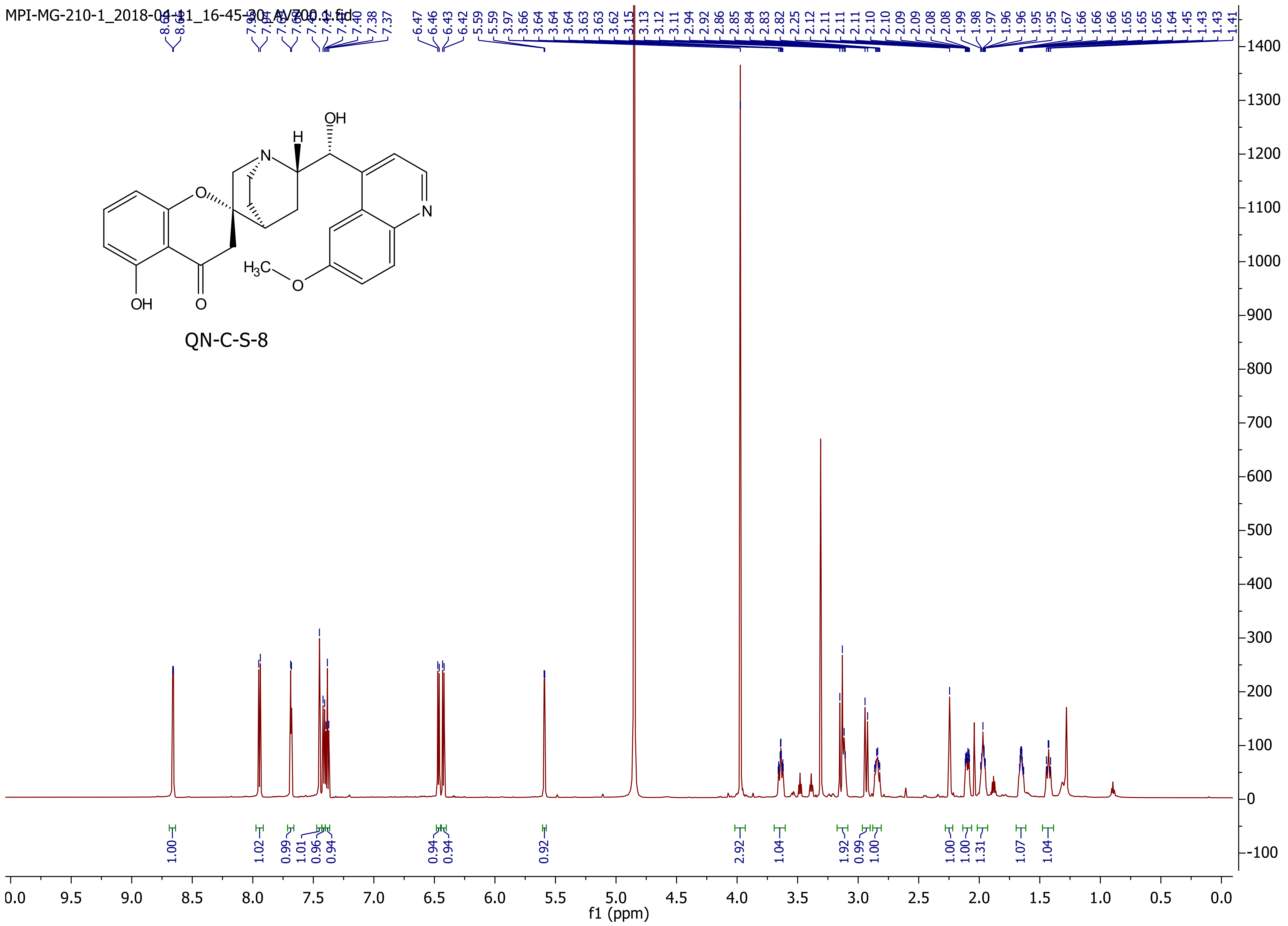
MPI-MG-210-1_2018-04-11_16-45-30: AV700.1.fid
QN-C-S-8
f1 (ppm)

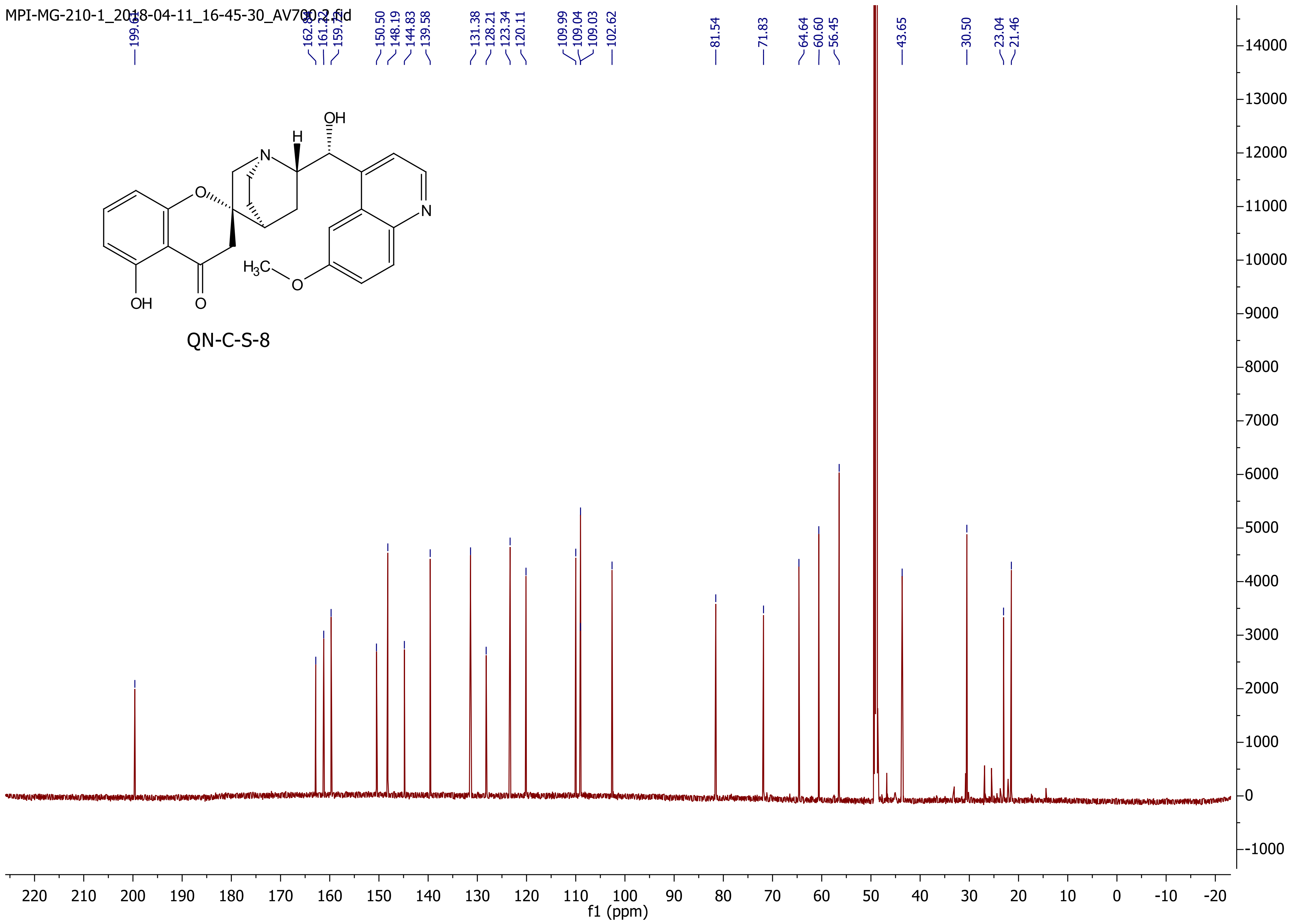

MPI-MG-210-1_2018-04-11_16-45-30_AV700.2.fid
199.6
162.8
161.2
159.7
150.50
148.19
144.83
139.58
131.38
128.21
123.34
120.11
109.99
109.04
109.03
102.62
81.54
71.83
64.64
60.60
56.45
43.65
30.50
23.04
21.46
OH
H
N
O
N
H3C
O
OH
O
QN-C-S-8
f1 (ppm)

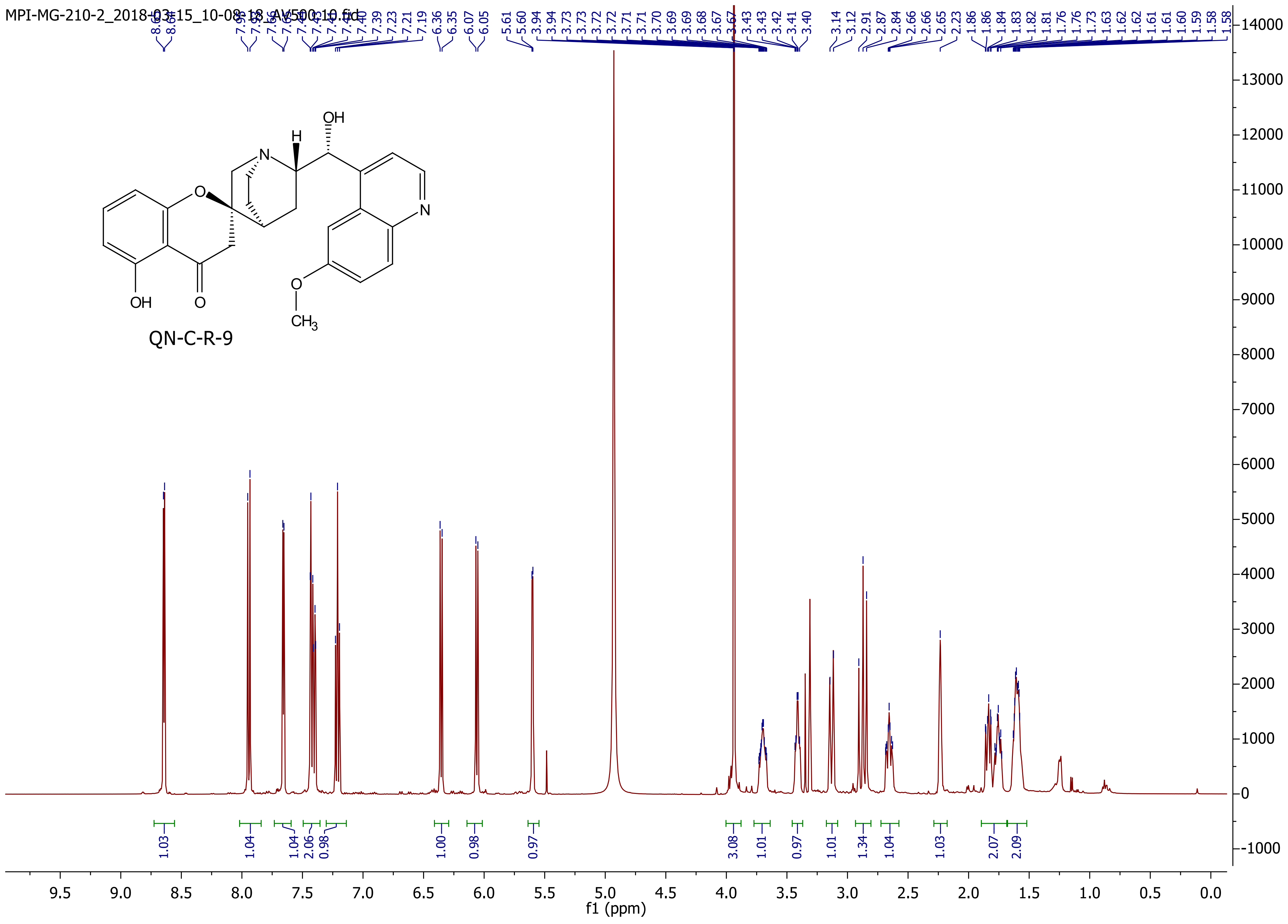
MPI-MG-210-2_2018-03-15_10-08-18_AV500.10.fid
OH
H
N
O
N
OH
O
O
CH3
QN-C-R-9
6.36
6.35
6.07
6.05
5.61
5.60
3.94
3.73
3.72
3.71
3.70
3.69
3.68
3.67
3.43
3.42
3.41
3.40
3.14
3.12
2.91
2.87
2.84
2.66
2.65
2.23
1.86
1.84
1.83
1.82
1.81
1.76
1.73
1.63
1.62
1.61
1.60
1.59
1.58
1.03
1.04
1.04
2.06
0.98
1.00
0.98
0.97
3.08
1.01
0.97
1.01
1.34
1.04
1.03
2.07
2.09
f1 (ppm)

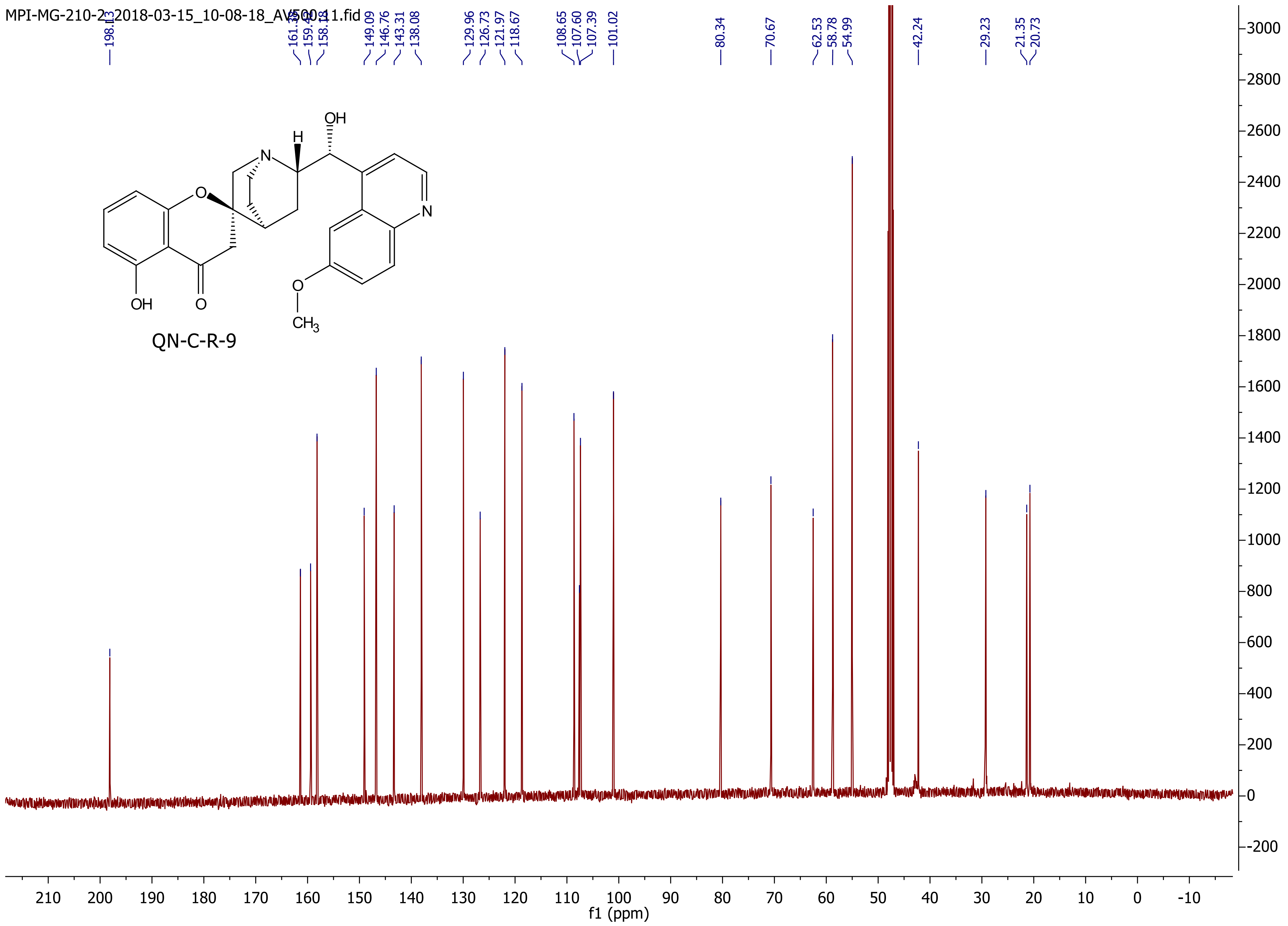
MPI-MG-210-2_2018-03-15_10-08-18_AV500_11.fid
198.13
161.3
159.4
158.1
149.09
146.76
143.31
138.08
129.96
126.73
121.97
118.67
108.65
107.60
107.39
101.02
80.34
70.67
62.53
58.78
54.99
42.24
29.23
21.35
20.73
QN-C-R-9
f1 (ppm)

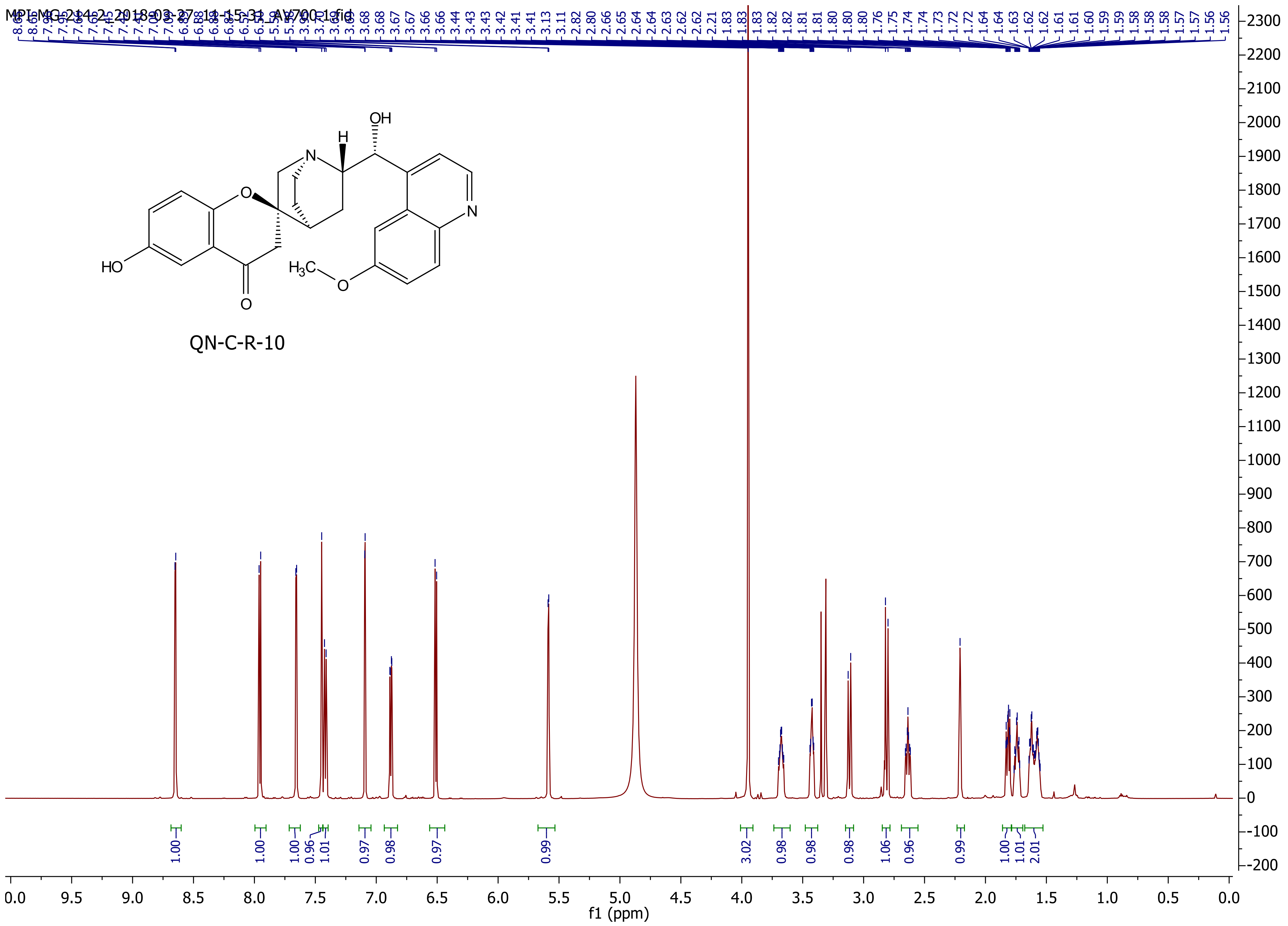
MPI-MG-214-2-2018-03-27 11-15-31 AV700 1.fid
OH
H
N
O
HO
O
H_3C
O
N
QN-C-R-10
f1 (ppm)

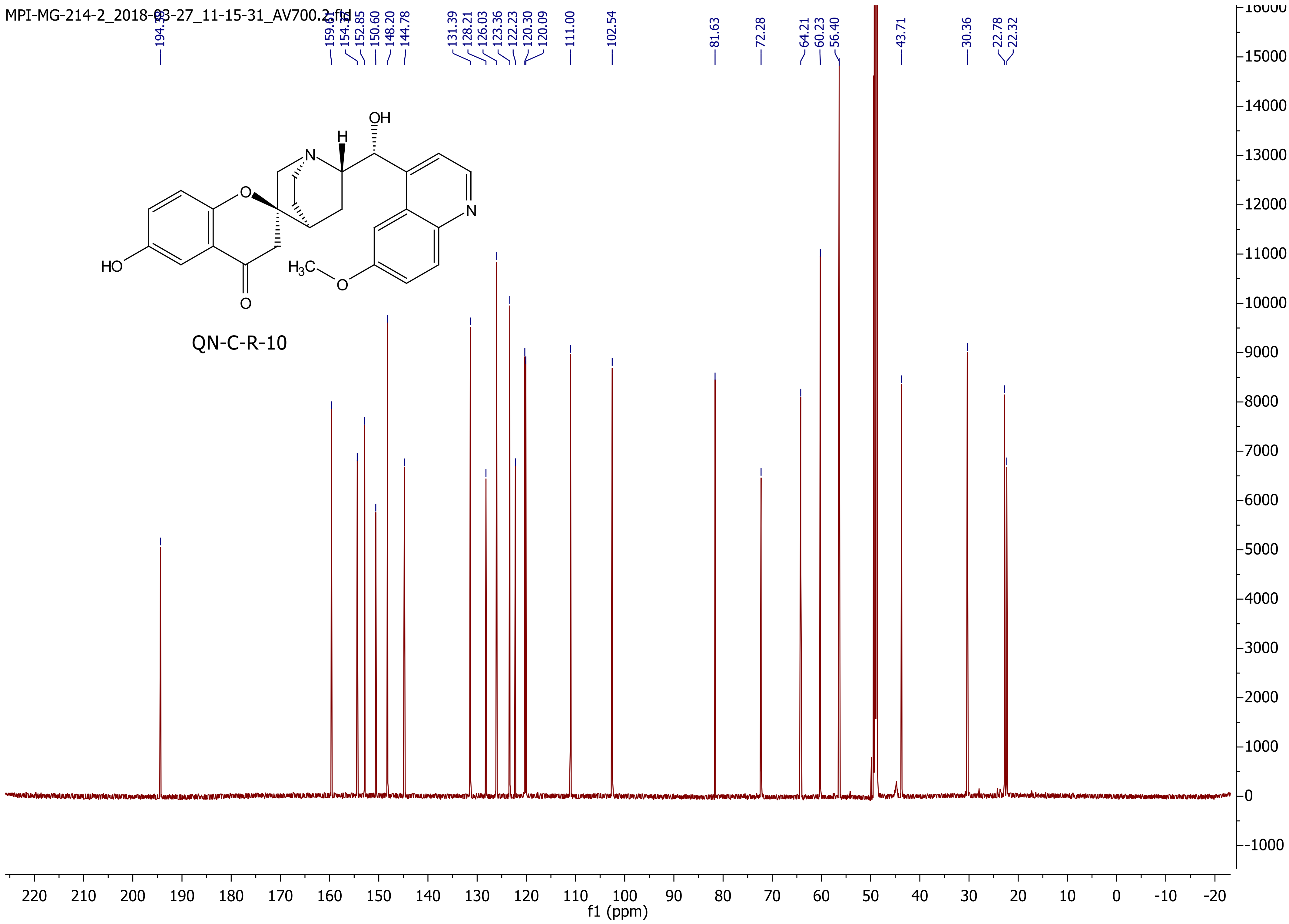
MPI-MG-214-2_2018-03-27_11-15-31_AV700.2.fid
194.38
159.61
154.37
152.85
150.60
148.20
144.78
131.39
128.21
126.03
123.36
122.23
120.30
120.09
111.00
102.54
81.63
72.28
64.21
60.23
56.40
43.71
30.36
22.78
22.32
OH
H
N
O
N
HO
O
H_3C
O
QN-C-R-10
f1 (ppm)

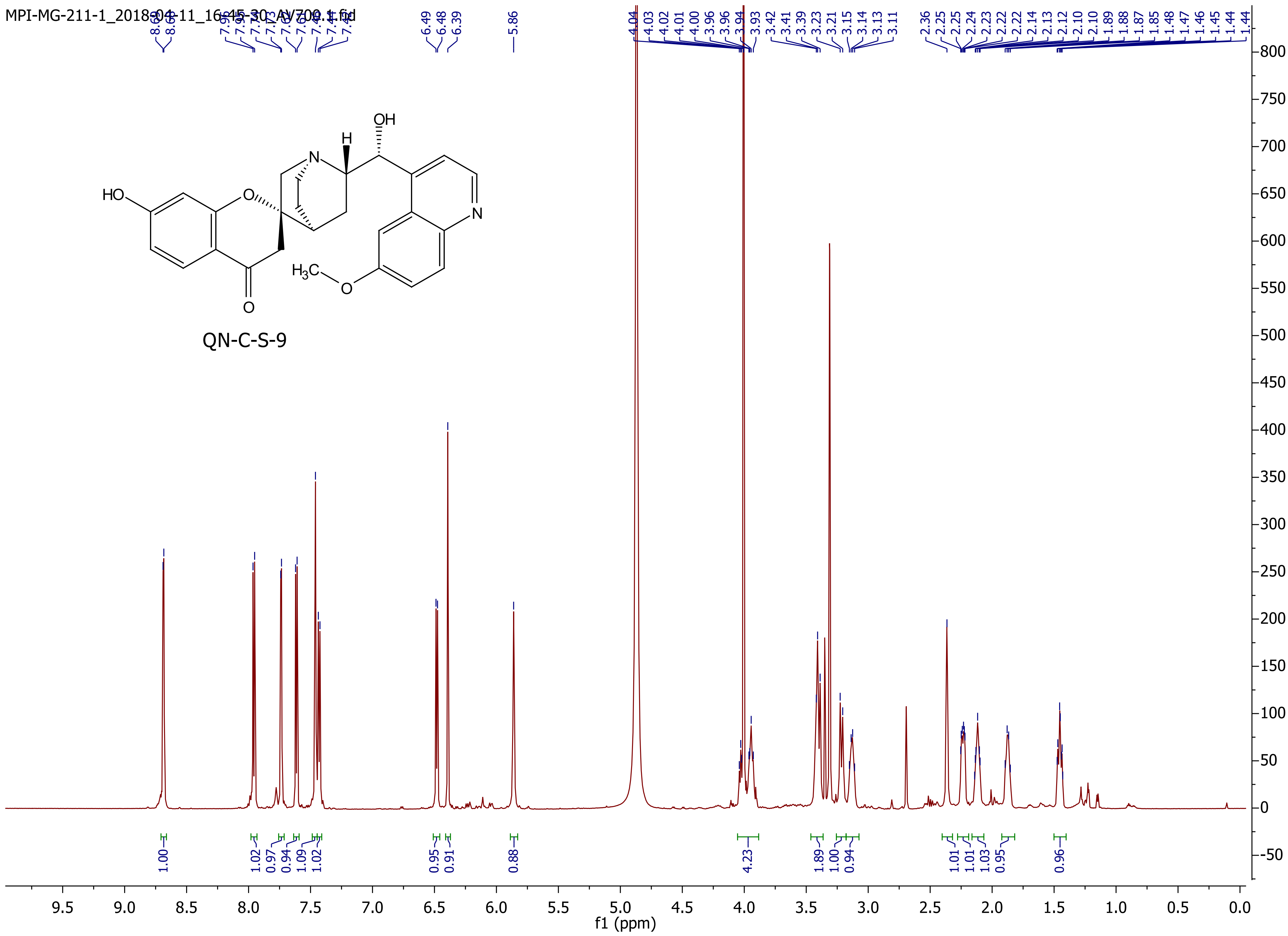

MPI-MG-211-1_2018-04-11_16-45-30_AV700.1.fid
6.49
6.48
6.39
5.86
4.04
4.03
4.02
4.01
4.00
3.96
3.96
3.94
3.93
3.42
3.41
3.39
3.23
3.21
3.15
3.14
3.13
3.11
2.36
2.25
2.25
2.24
2.23
2.22
2.22
2.14
2.13
2.12
2.10
2.10
1.89
1.88
1.87
1.85
1.48
1.47
1.46
1.45
1.44
1.44
OH
H
N
HO
O
N
H_3C
O
O
QN-C-S-9
1.00
1.02
0.97
0.94
1.09
1.02
0.95
0.91
0.88
4.23
1.89
1.00
0.94
1.01
1.01
1.03
0.95
0.96
f1 (ppm)

MPI-MG-211-1_2018-04-11_16-45-30_AV700.2.fid

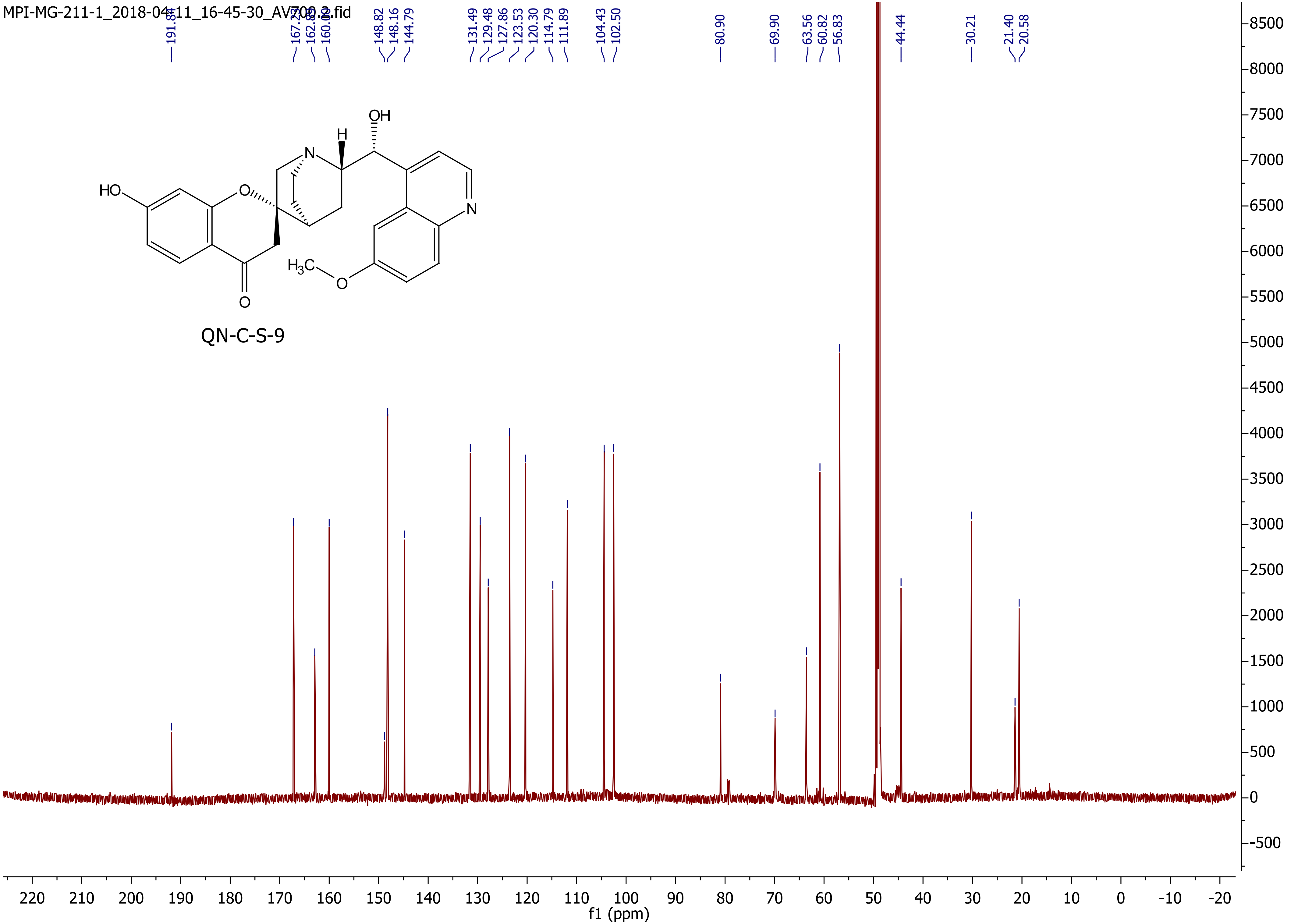

QN-C-S-9

MPI-MG-211-2_2018-03-15_10-08-18_AV500.10.fid

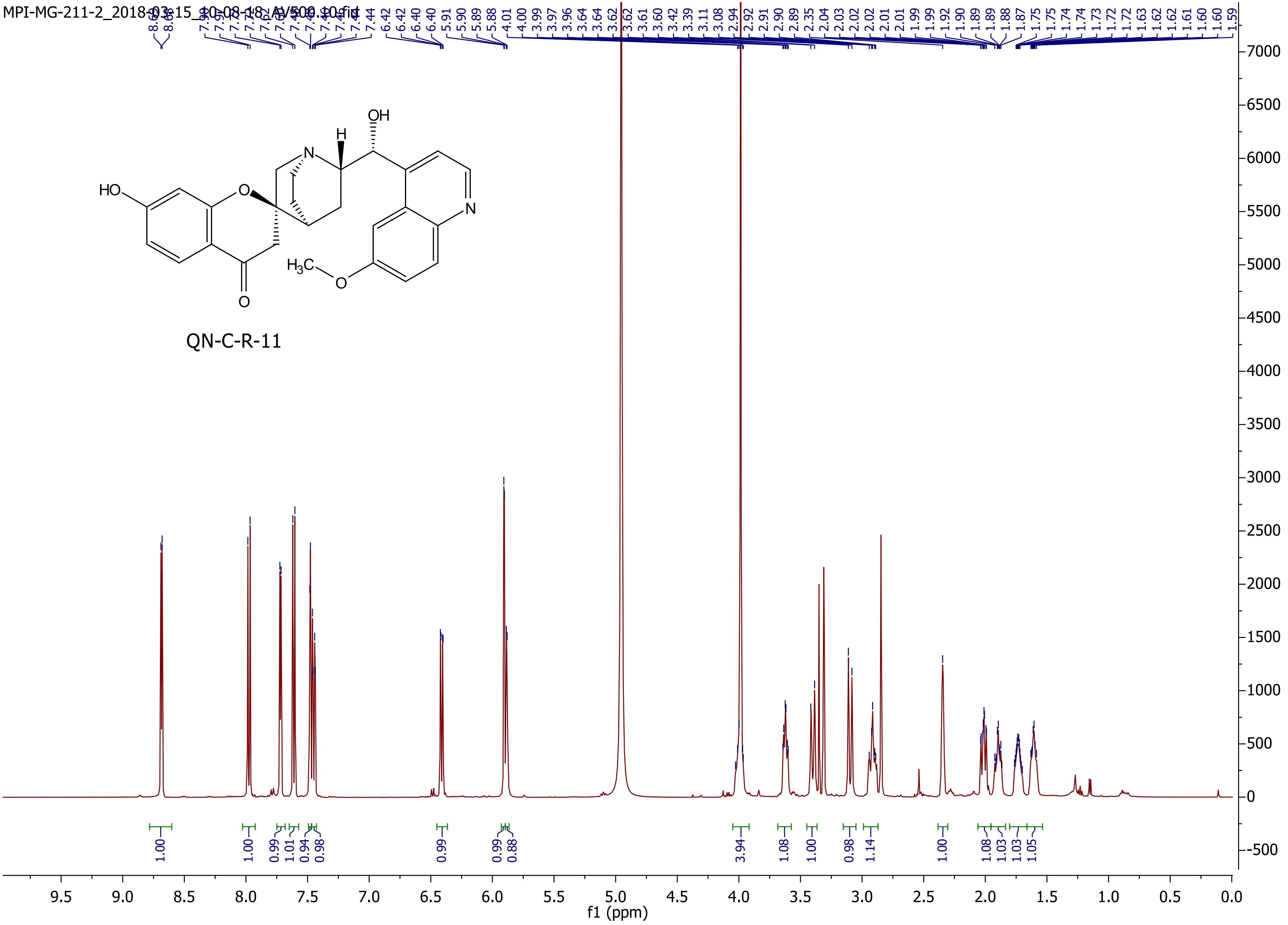

QN-C-R-11

MPI-MG-211-2_2018-03-15_10-08-18_AV500.11.fid

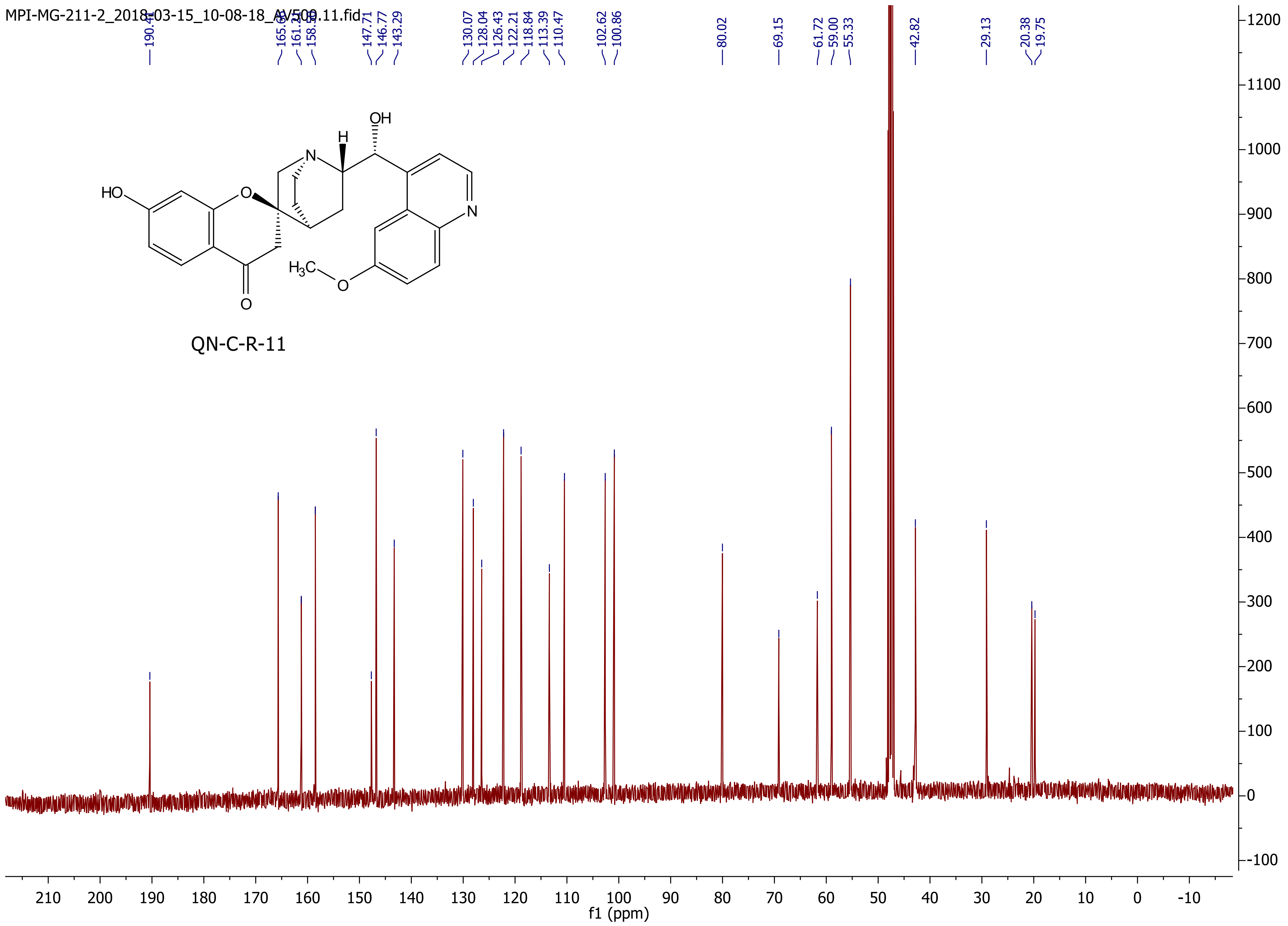

QN-C-R-11

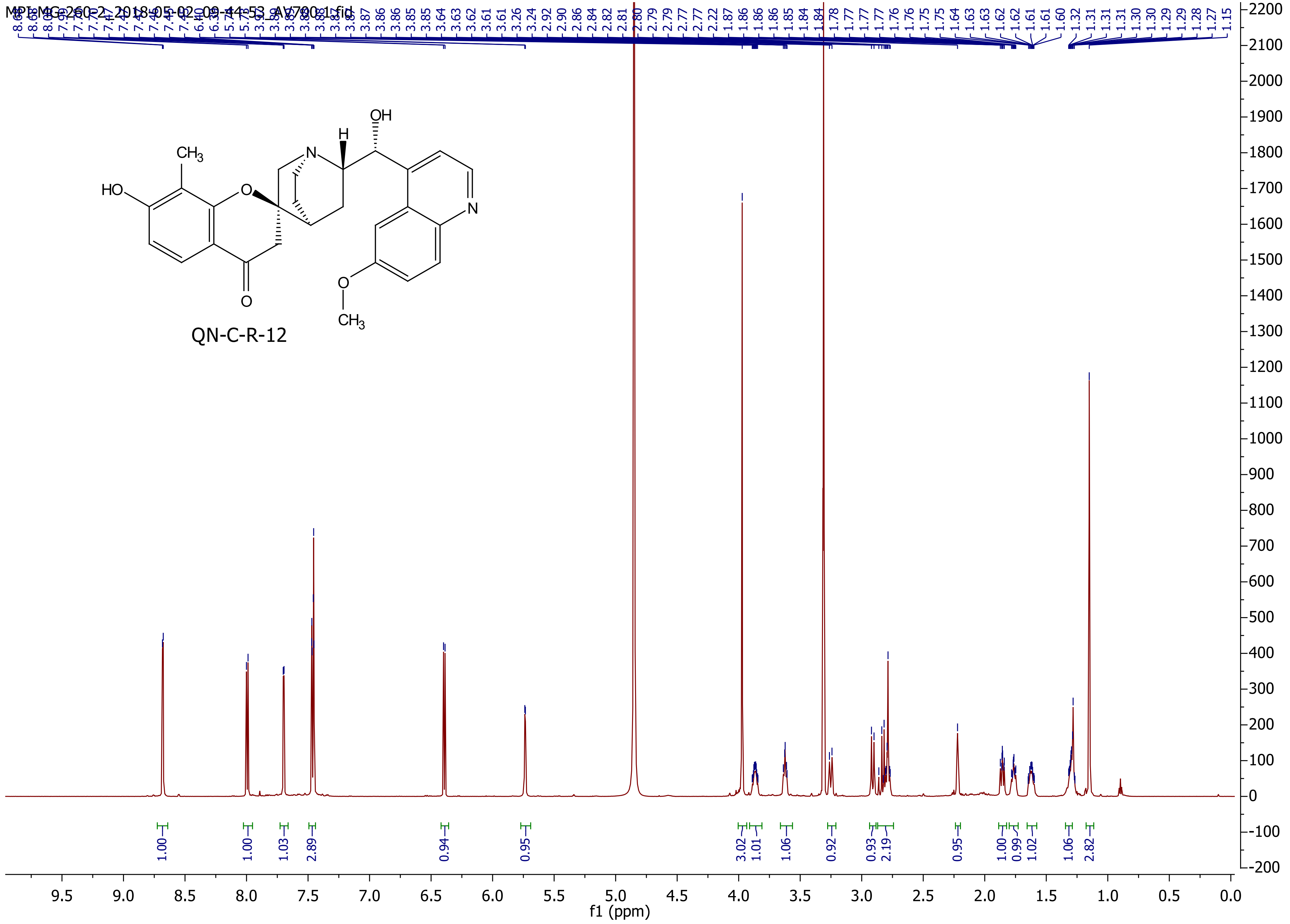

QN-C-R-12

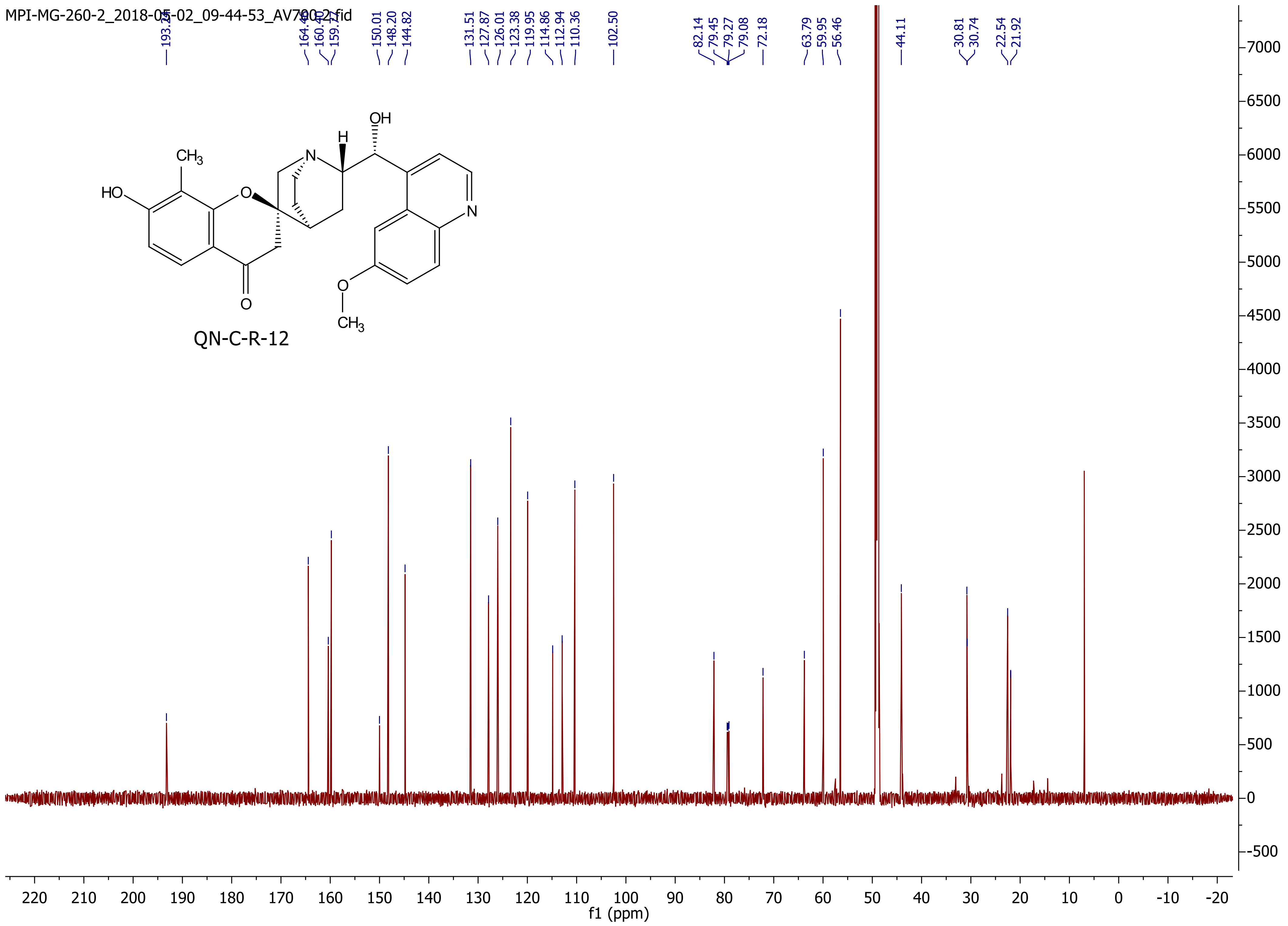
MPI-MG-260-2_2018-05-02_09-44-53_AV700-2.fid
150.01
148.20
144.82
131.51
127.87
126.01
123.38
119.95
114.86
112.94
110.36
102.50
82.14
79.45
79.27
79.08
72.18
63.79
59.95
56.46
44.11
30.81
30.74
22.54
21.92
HO
CH3
OH
QN-C-R-12
f1 (ppm)

MPI-MG-239-1_2018-04-11_07-58-29_AV700.1.fid

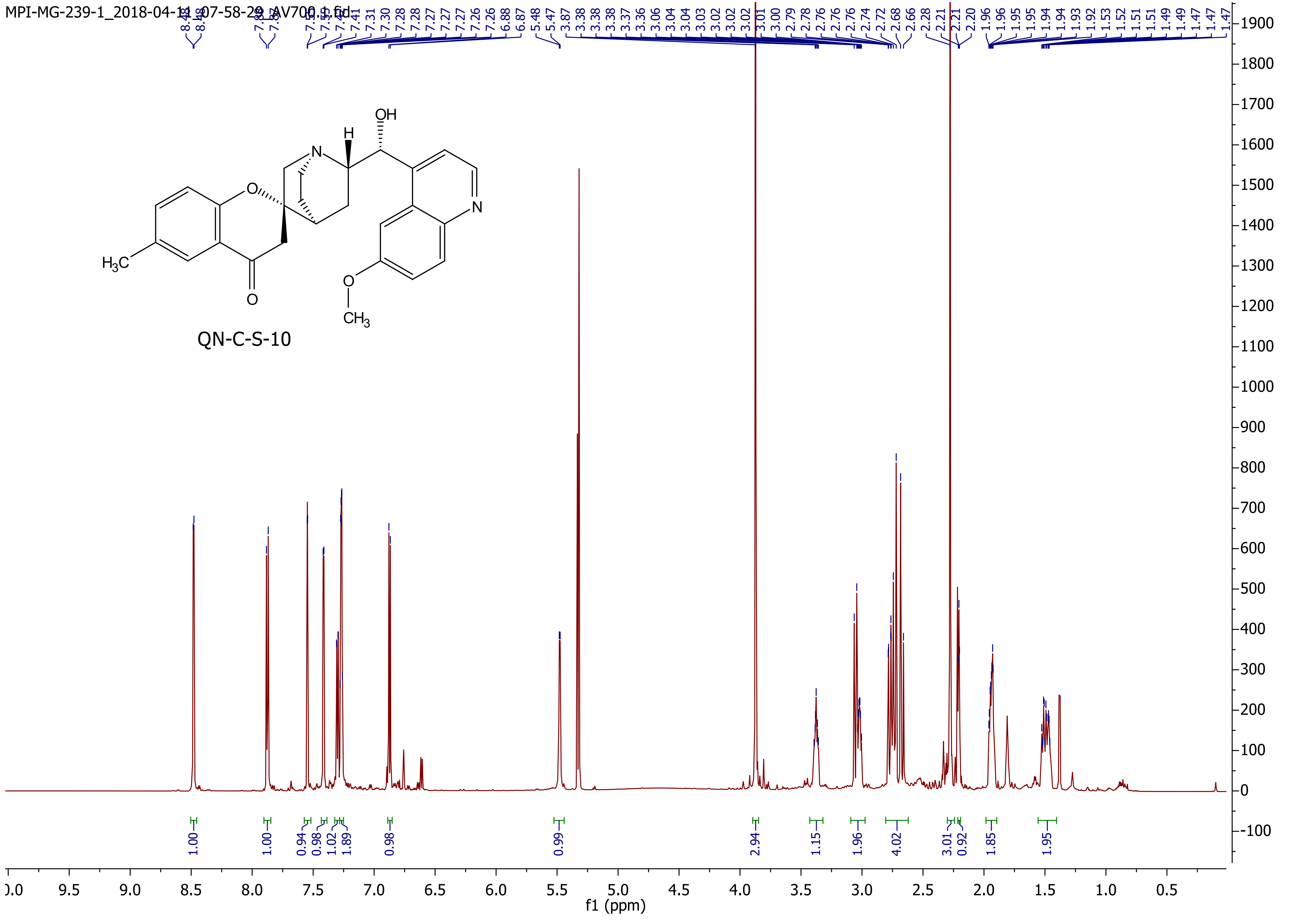

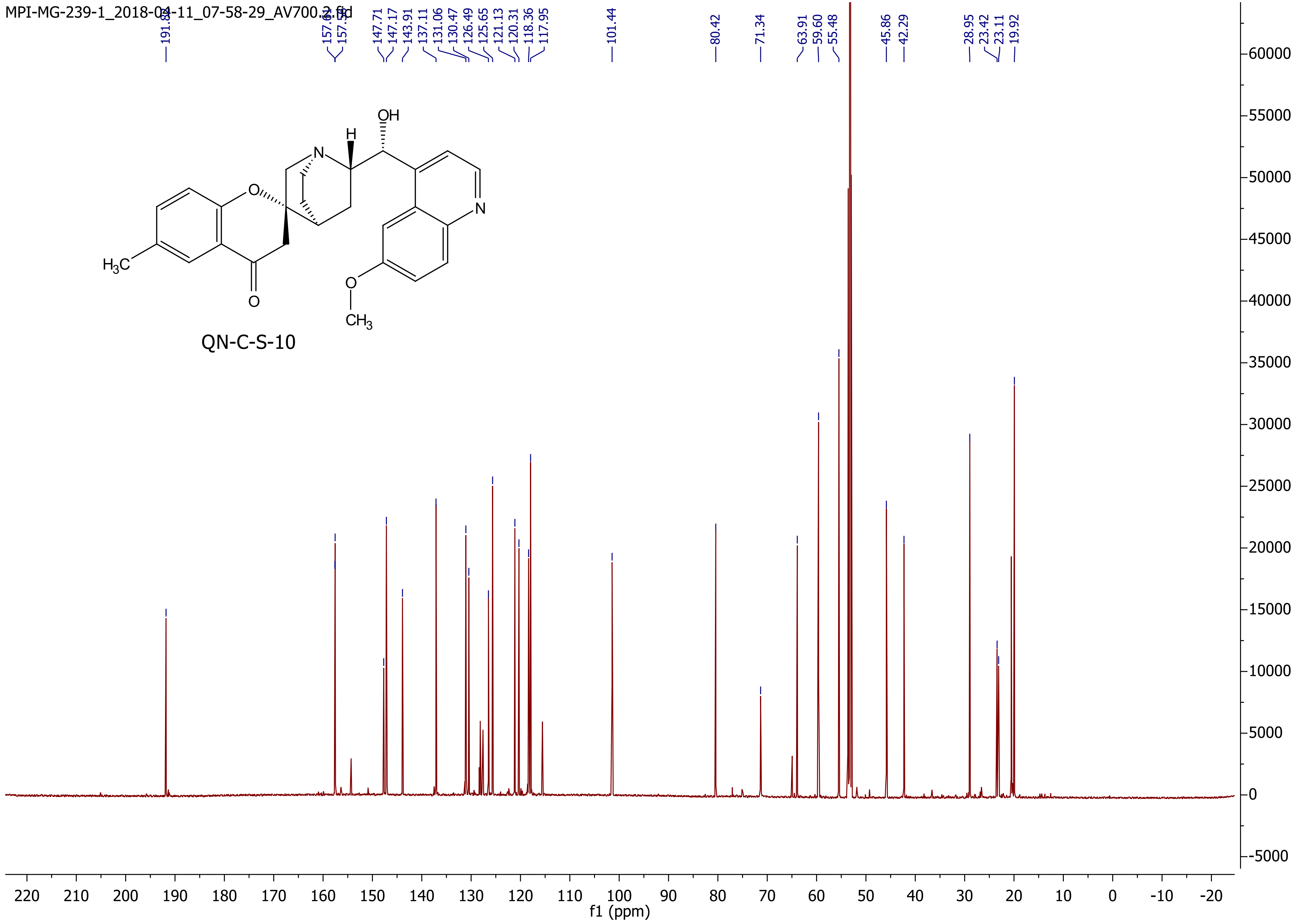

MPI-MG-239-1_2018-04-11_07-58-29_AV700.2.fid
147.71
147.17
143.91
137.11
131.06
130.47
126.49
125.65
121.13
120.31
118.36
117.95
101.44
80.42
71.34
63.91
59.60
55.48
45.86
42.29
28.95
23.42
23.11
19.92
OH
H
N
O
N
H_3C
O
O
CH_3
QN-C-S-10
f1 (ppm)

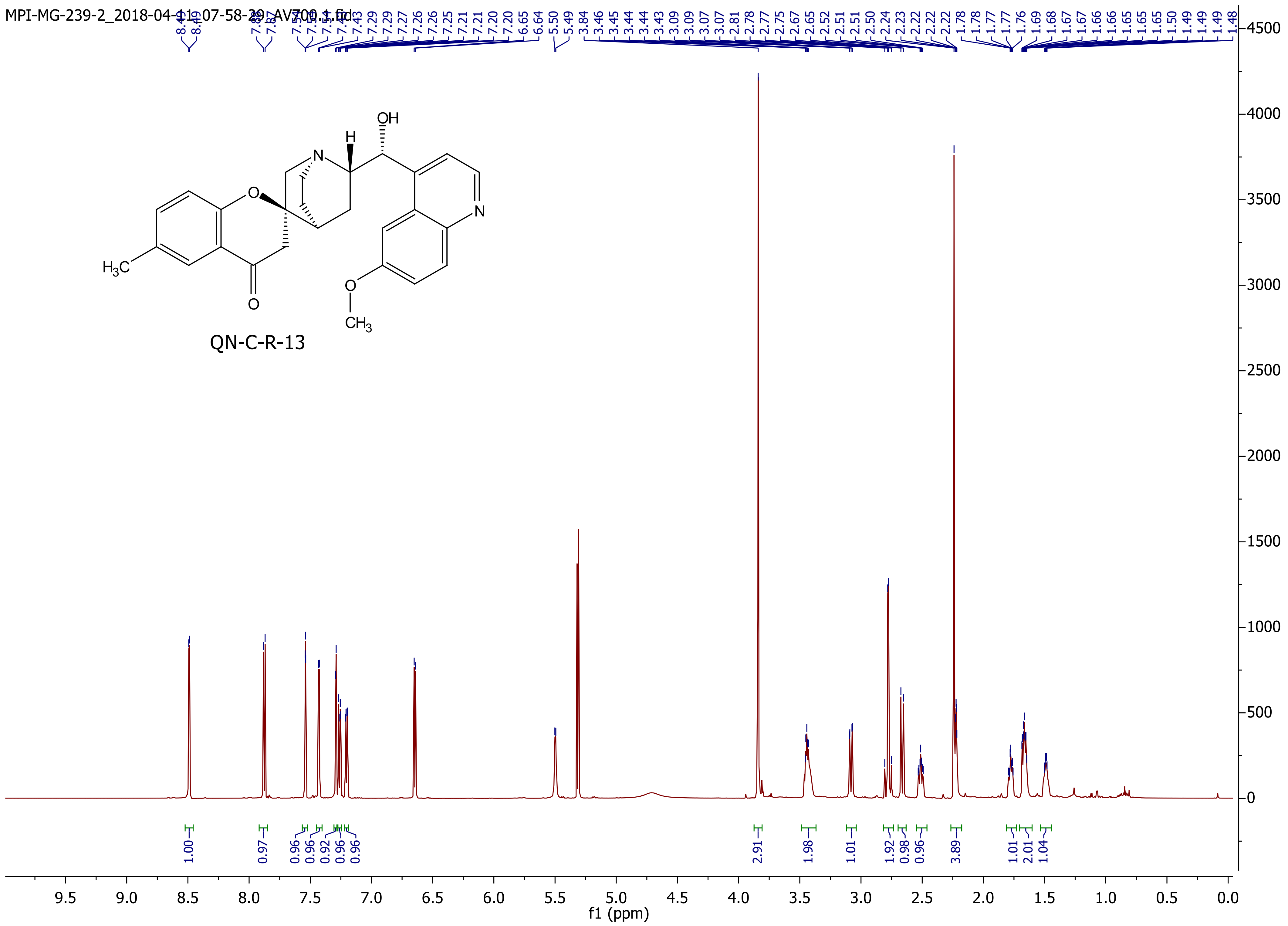
MPI-MG-239-2_2018-04-11_07-58-29_AV700.1.fid
QN-C-R-13
f1 (ppm)

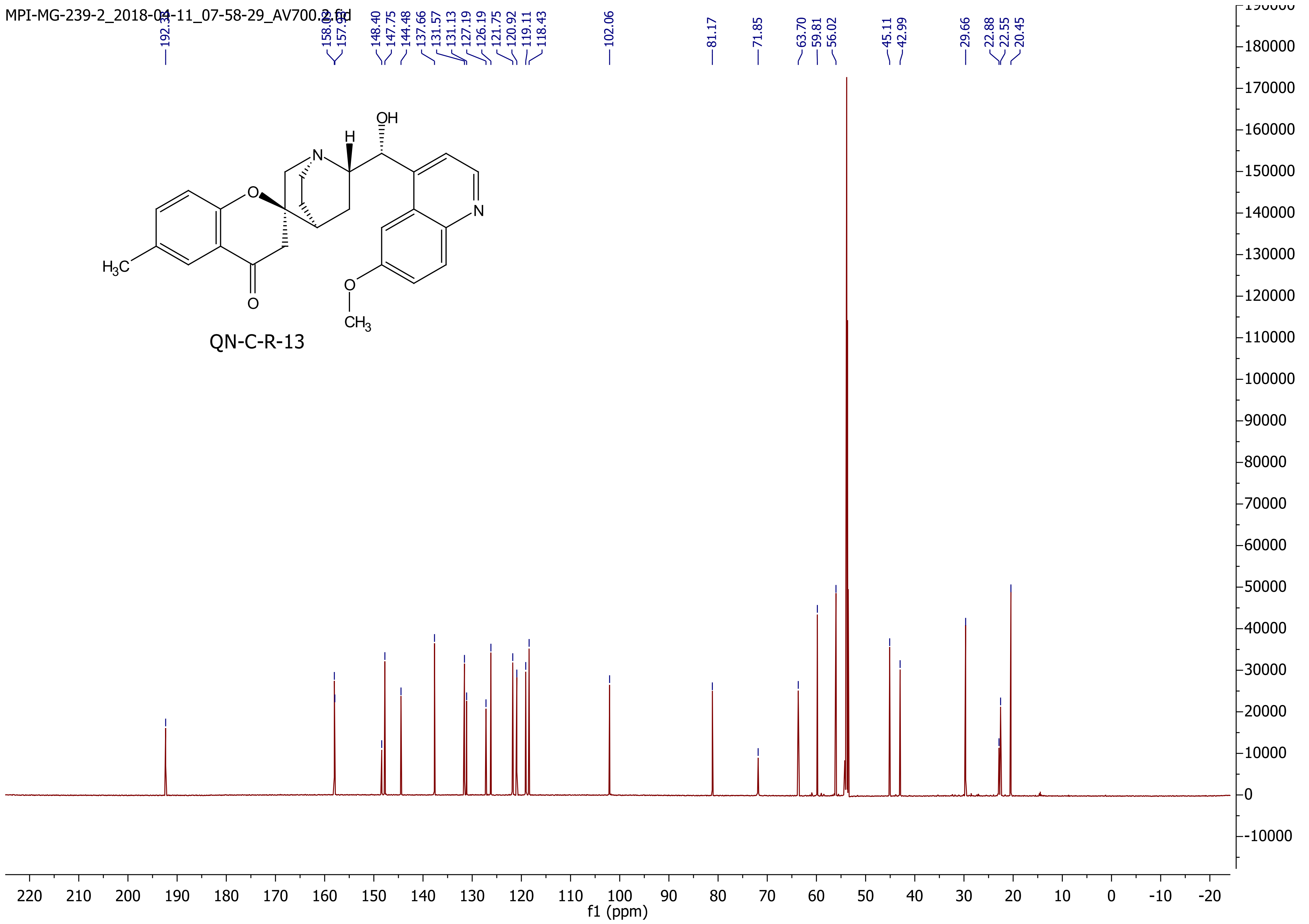
MPI-MG-239-2_2018-04-11_07-58-29_AV700.2.fid
192.31
158.02
157.91
148.40
147.75
144.48
137.66
131.57
131.13
127.19
126.19
121.75
120.92
119.11
118.43
102.06
81.17
71.85
63.70
59.81
56.02
45.11
42.99
29.66
22.88
22.55
20.45
OH
H
N
O
N
H3C
O
O
CH3
QN-C-R-13
f1 (ppm)

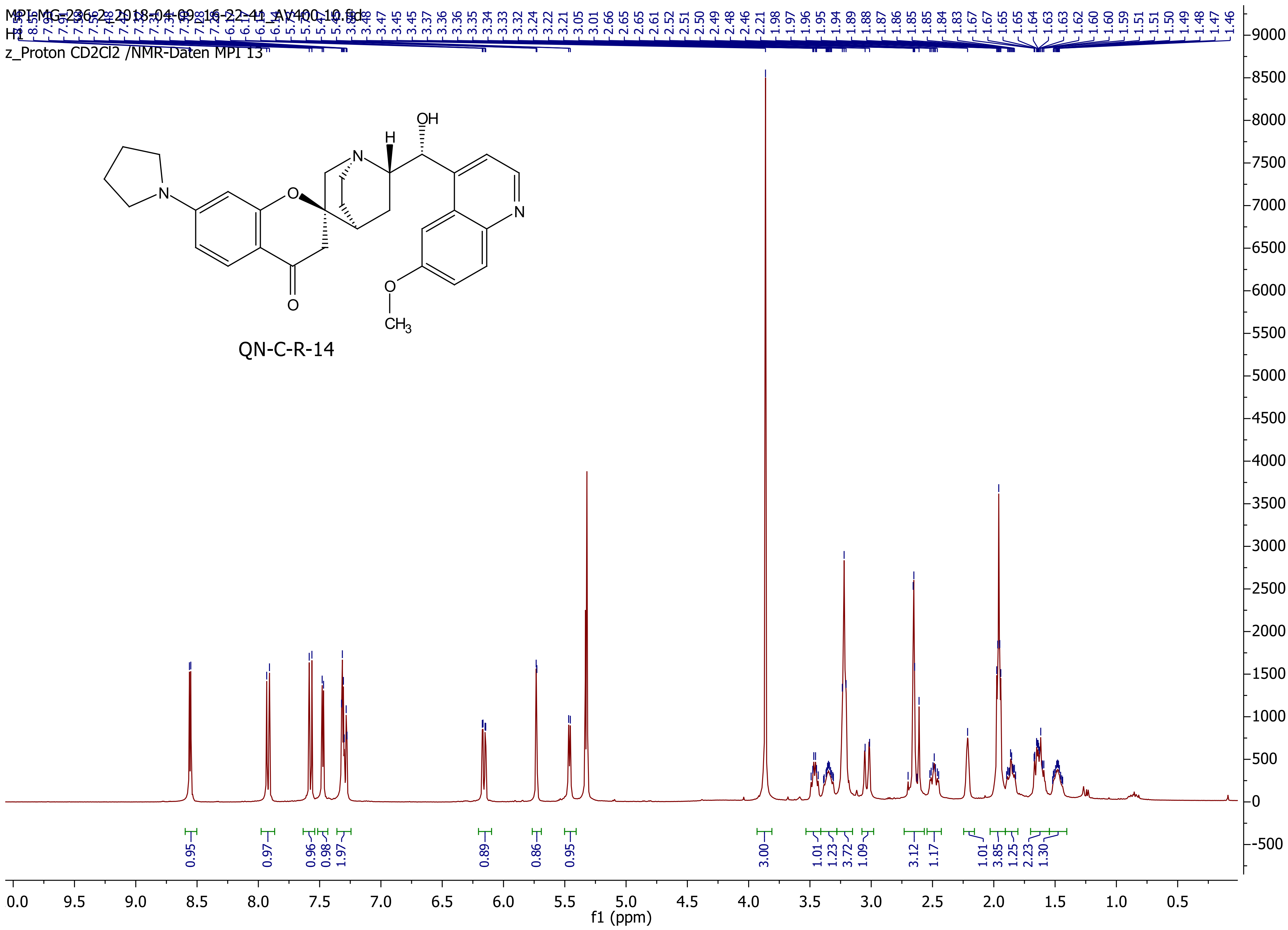
MPI-MG-236-2_2018-04-09_16-22-41_AV400_10.fid
H1
z_Proton CD2Cl2 /NMR-Daten MPI 13
QN-C-R-14
0.95
0.97
0.96
0.98
1.97
0.89
0.86
0.95
3.00
1.01
1.23
3.72
1.09
3.12
1.17
1.01
3.85
1.25
2.23
1.30
f1 (ppm)

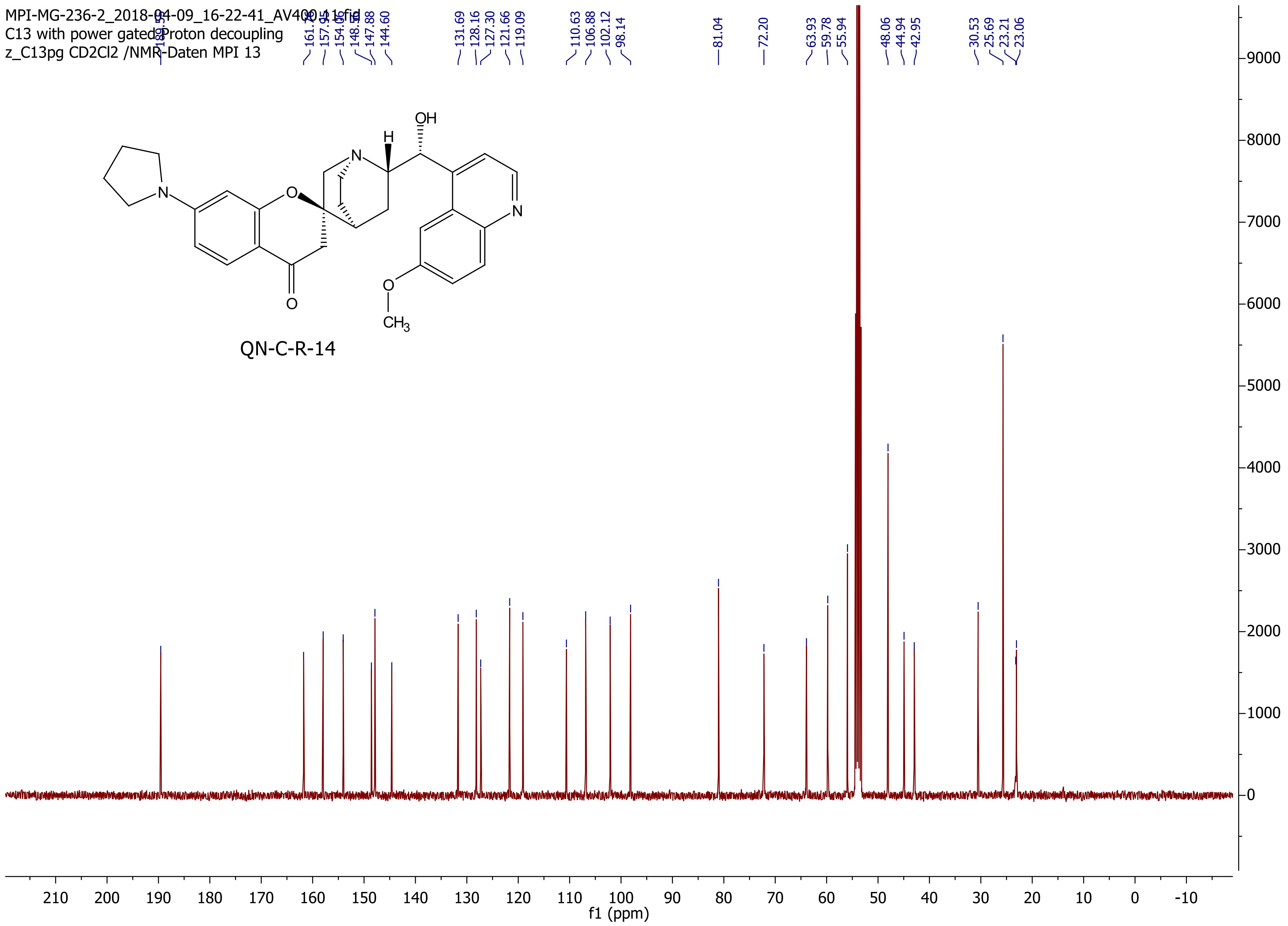
MPI-MG-236-2_2018-04-09_16-22-41_AV400.11.fid
C13 with power gated Proton decoupling
z_C13pg CD2Cl2 /NMR-Daten MPI 13
189.55
161.76
157.95
154.06
148.56
147.88
144.60
131.69
128.16
127.30
121.66
119.09
110.63
106.88
102.12
98.14
81.04
72.20
63.93
59.78
55.94
48.06
44.94
42.95
30.53
25.69
23.21
23.06
OH
H
N
O
N
N
O
O
CH3
QN-C-R-14
9000
8000
7000
6000
5000
4000
3000
2000
1000
0
210
200
190
180
170
160
150
140
130
120
110
100
90
80
70
60
50
40
30
20
10
0
-10
f1 (ppm)

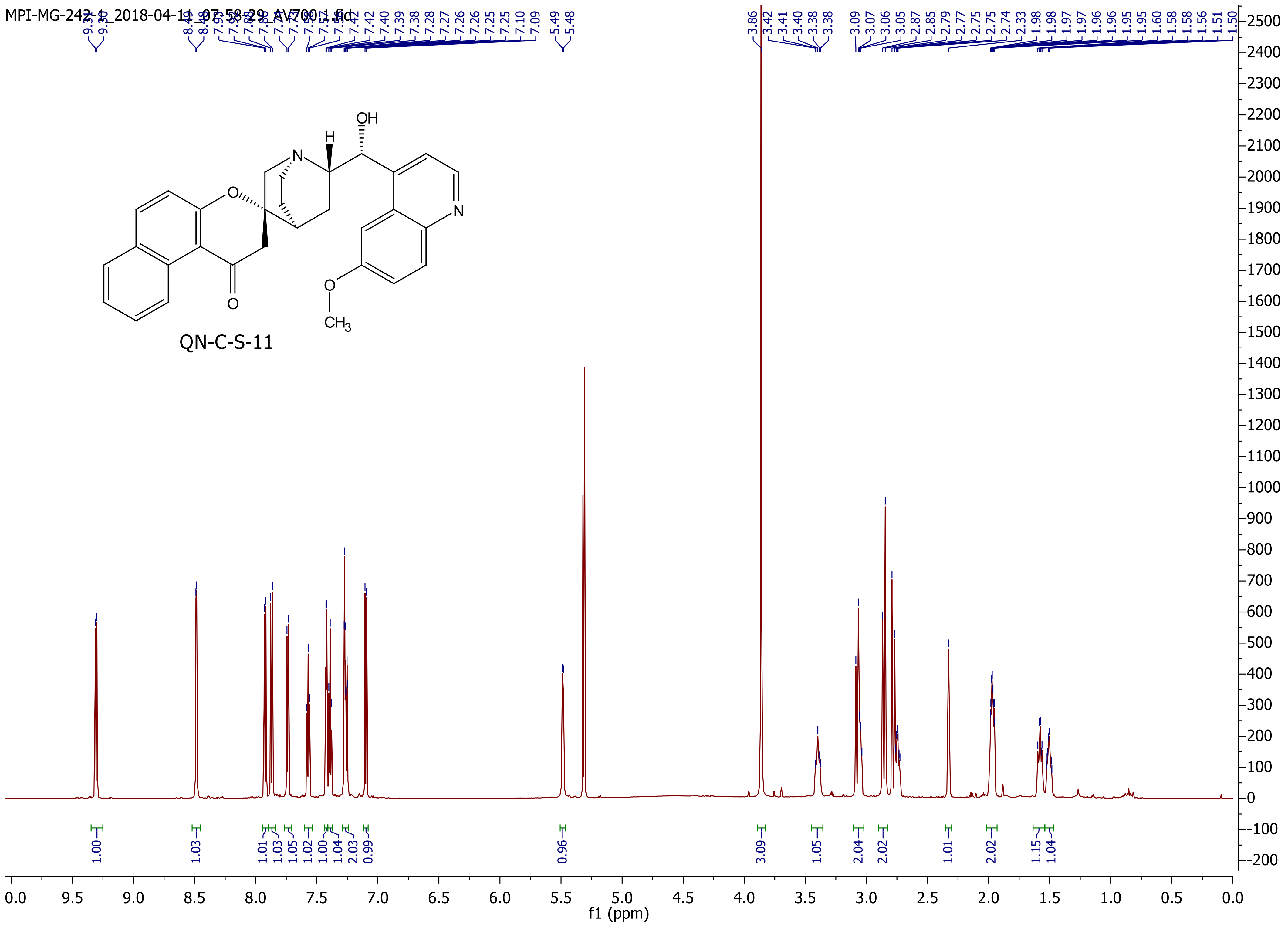

MPI-MG-242-1_2018-04-11_07-58-29_AV700 1.fid
QN-C-S-11
OH
H
N
O
N
O
O
CH3
3.86
3.42
3.41
3.40
3.38
3.38
3.09
3.07
3.06
3.05
2.87
2.85
2.79
2.77
2.75
2.75
2.74
2.33
1.98
1.98
1.97
1.97
1.96
1.96
1.95
1.95
1.60
1.58
1.58
1.56
1.51
1.50
7.42
7.40
7.39
7.38
7.28
7.27
7.26
7.26
7.25
7.25
7.10
7.09
5.49
5.48
1.00
1.03
1.01
1.03
1.05
1.02
1.00
1.04
2.03
0.99
0.96
3.09
1.05
2.04
2.02
1.01
2.02
1.15
1.04
f1 (ppm)

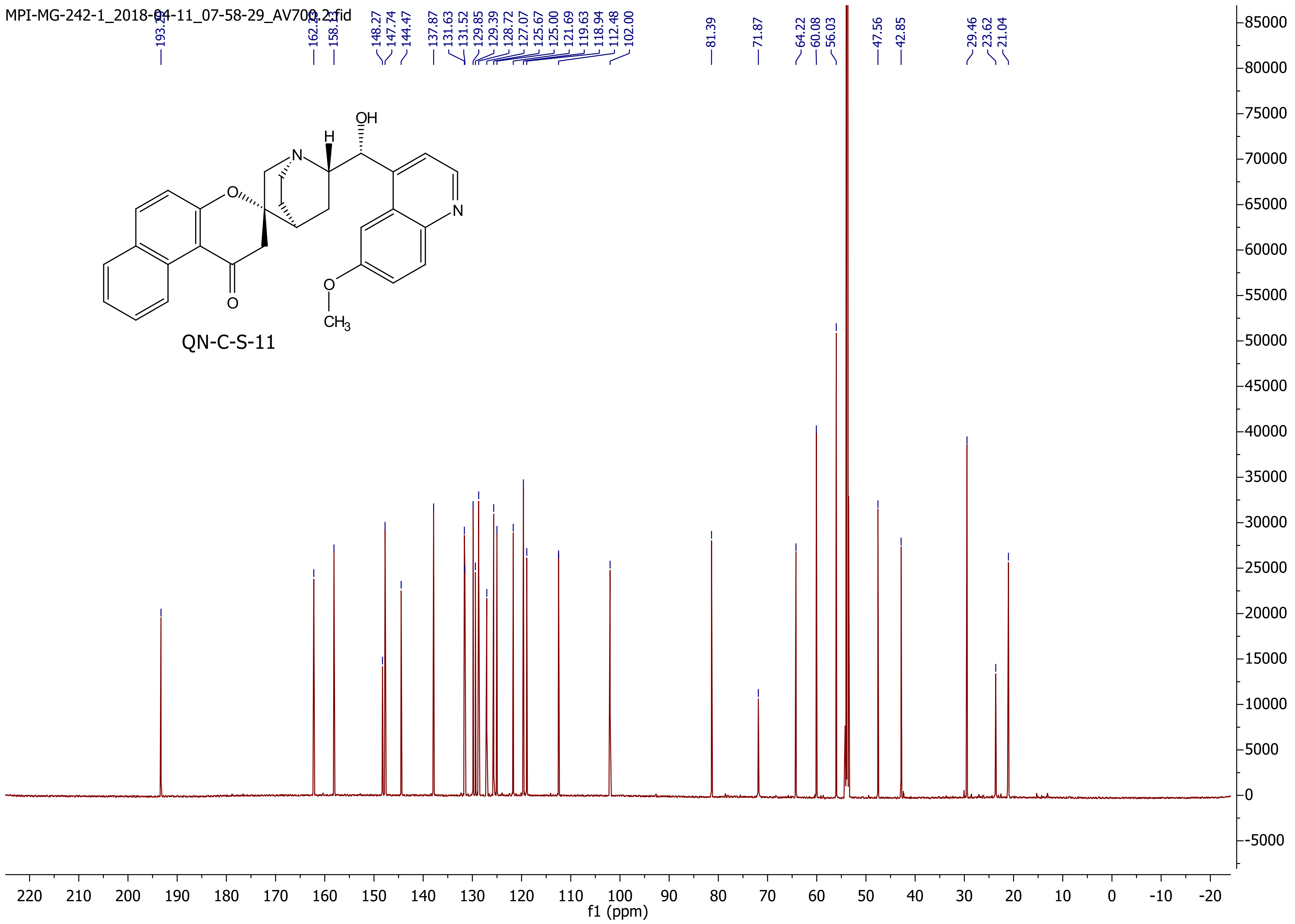

MPI-MG-242-1_2018-04-11_07-58-29_AV700.2.fid
148.27
147.74
144.47
137.87
131.63
131.52
129.85
129.39
128.72
127.07
125.67
125.00
121.69
119.63
118.94
112.48
102.00
81.39
71.87
64.22
60.08
56.03
47.56
42.85
29.46
23.62
21.04
QN-C-S-11
f1 (ppm)

MPI-MG-242-2_2018-04-11.07-58-29_AV700.1.fid

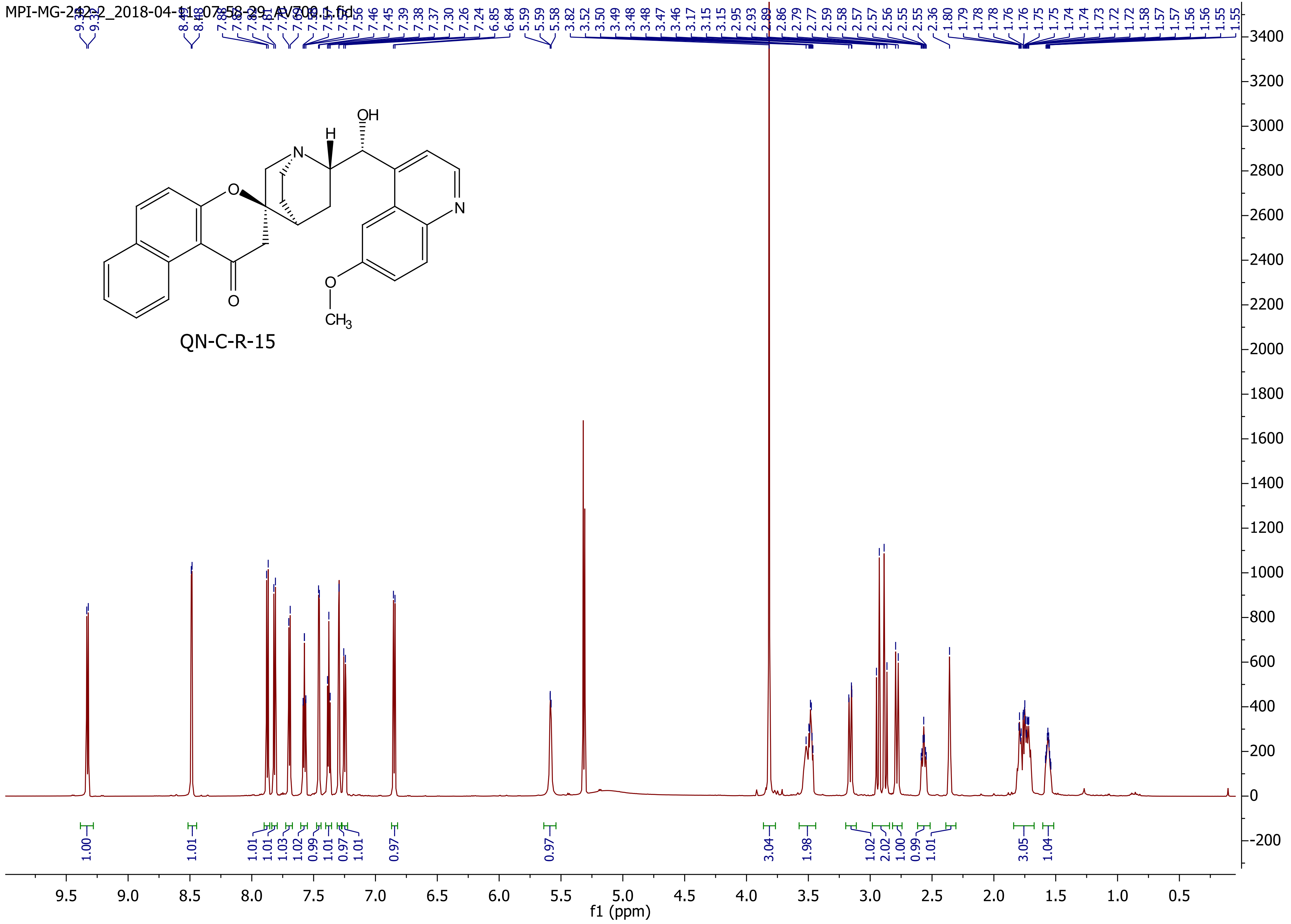

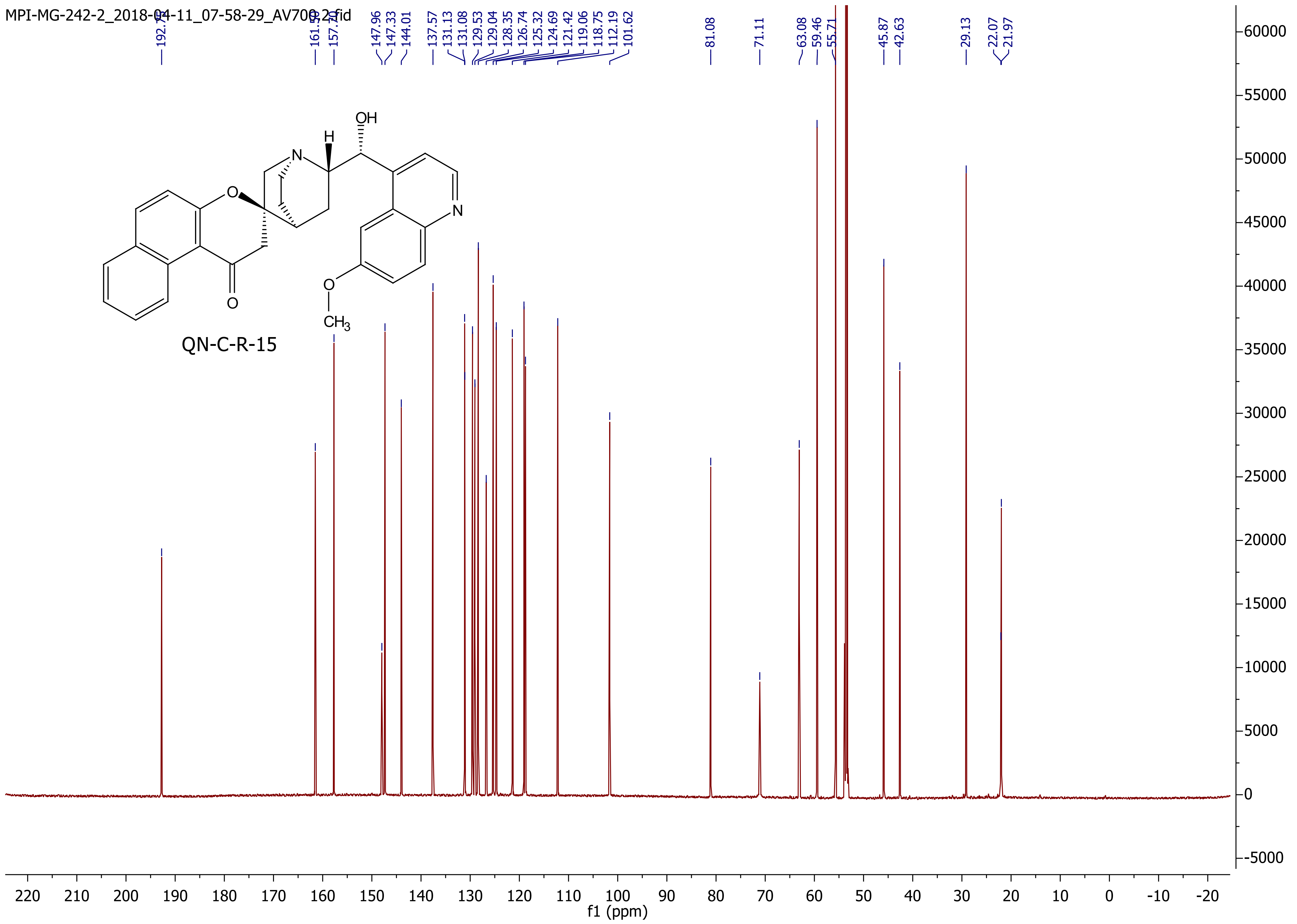

MPI-MG-242-2_2018-04-11_07-58-29_AV700.2.fid
192.7
161.5
157.7
147.96
147.33
144.01
137.57
131.13
131.08
129.53
129.04
128.35
126.74
125.32
124.69
121.42
119.06
118.75
112.19
101.62
81.08
71.11
63.08
59.46
55.71
45.87
42.63
29.13
22.07
21.97
OH
H
N
O
O
O
CH3
N
QN-C-R-15
f1 (ppm)

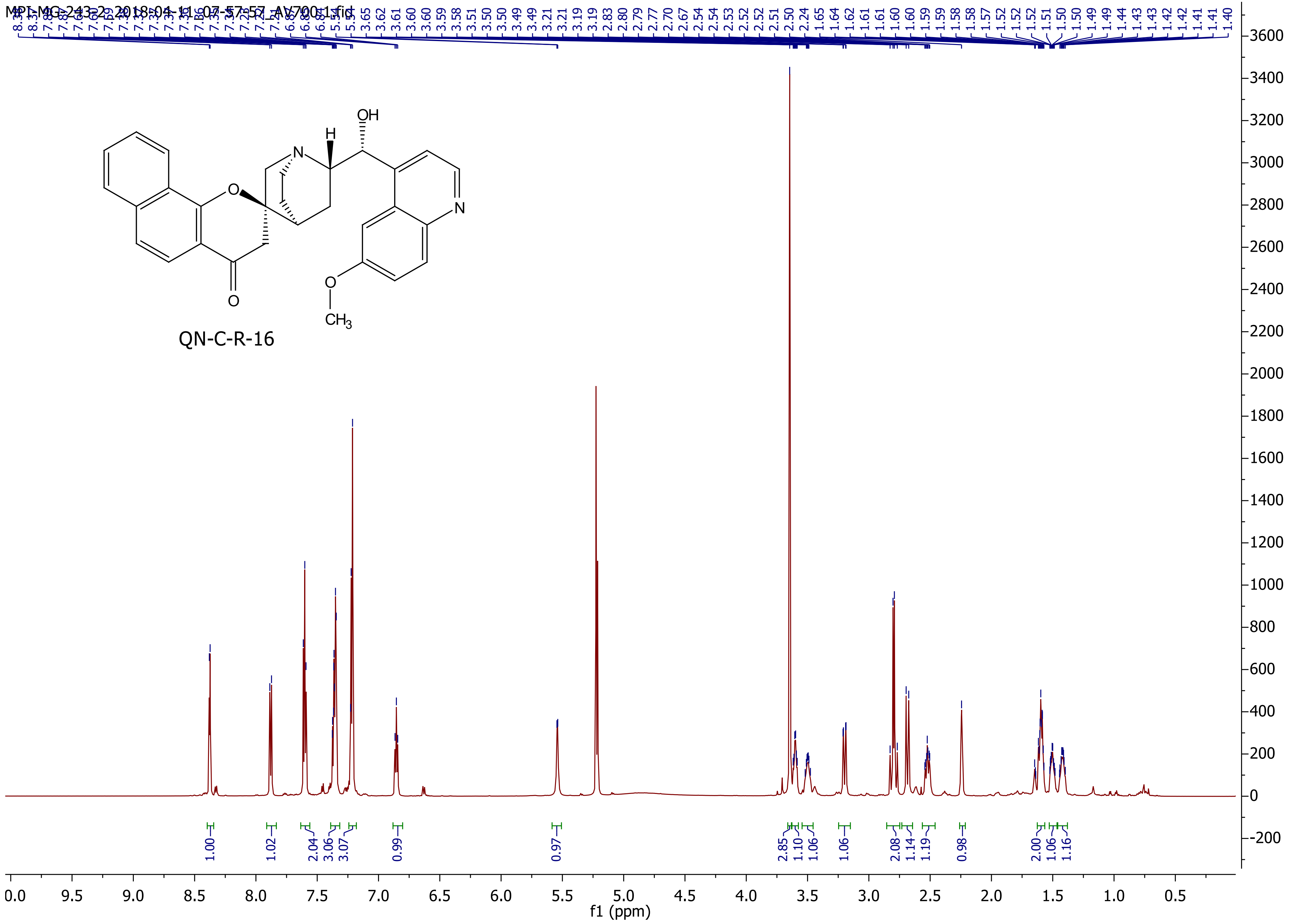

QN-C-R-16
OH
H
N
O
CH3
f1 (ppm)

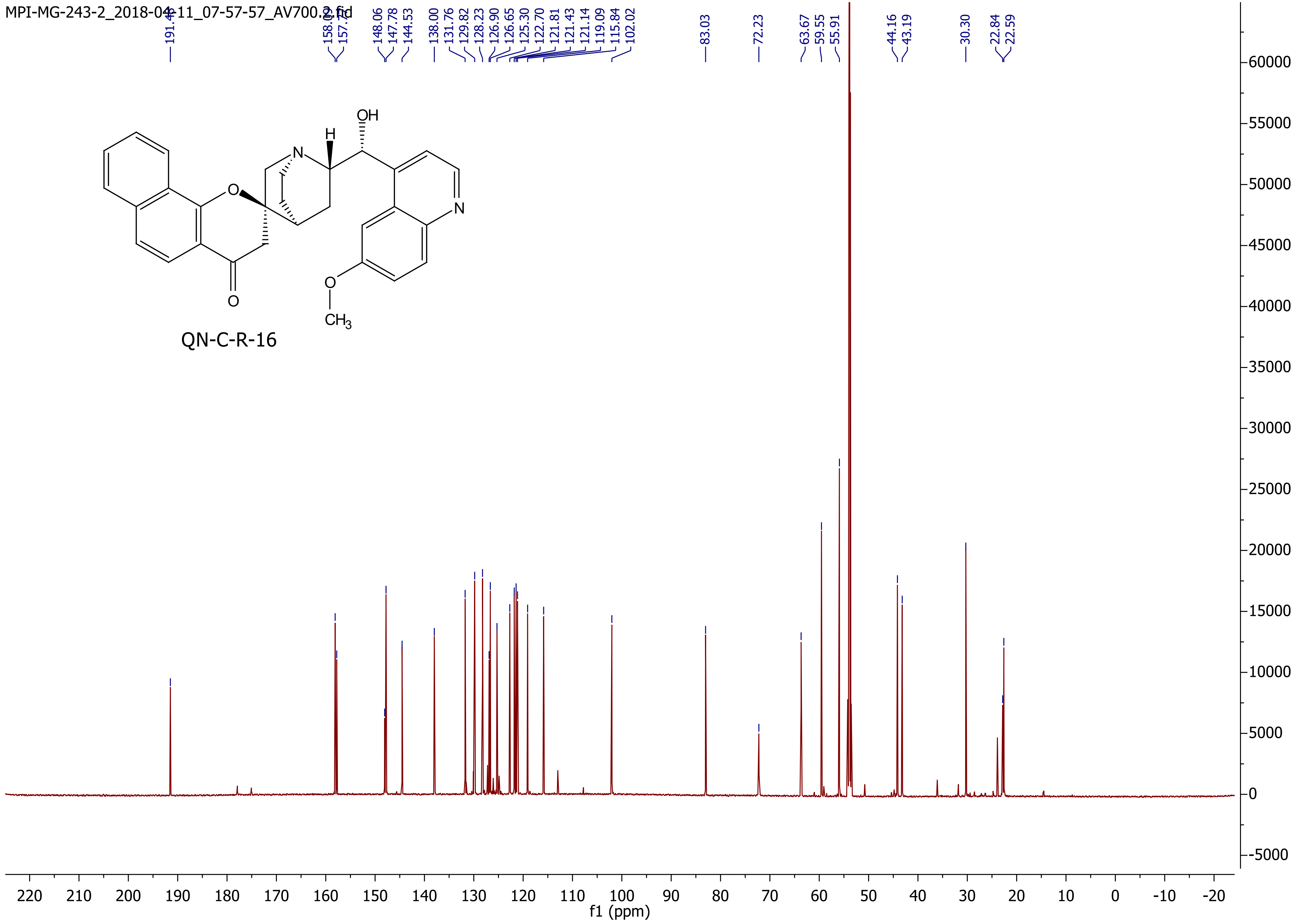

MPI-MG-243-2_2018-04-11_07-57-57_AV700.2.fid
148.06
147.78
144.53
138.00
131.76
129.82
128.23
126.90
126.65
125.30
122.70
121.81
121.43
121.14
119.09
115.84
102.02
83.03
72.23
63.67
59.55
55.91
44.16
43.19
30.30
22.84
22.59
OH
H
N
O
O
N
O
CH3
QN-C-R-16
f1 (ppm)

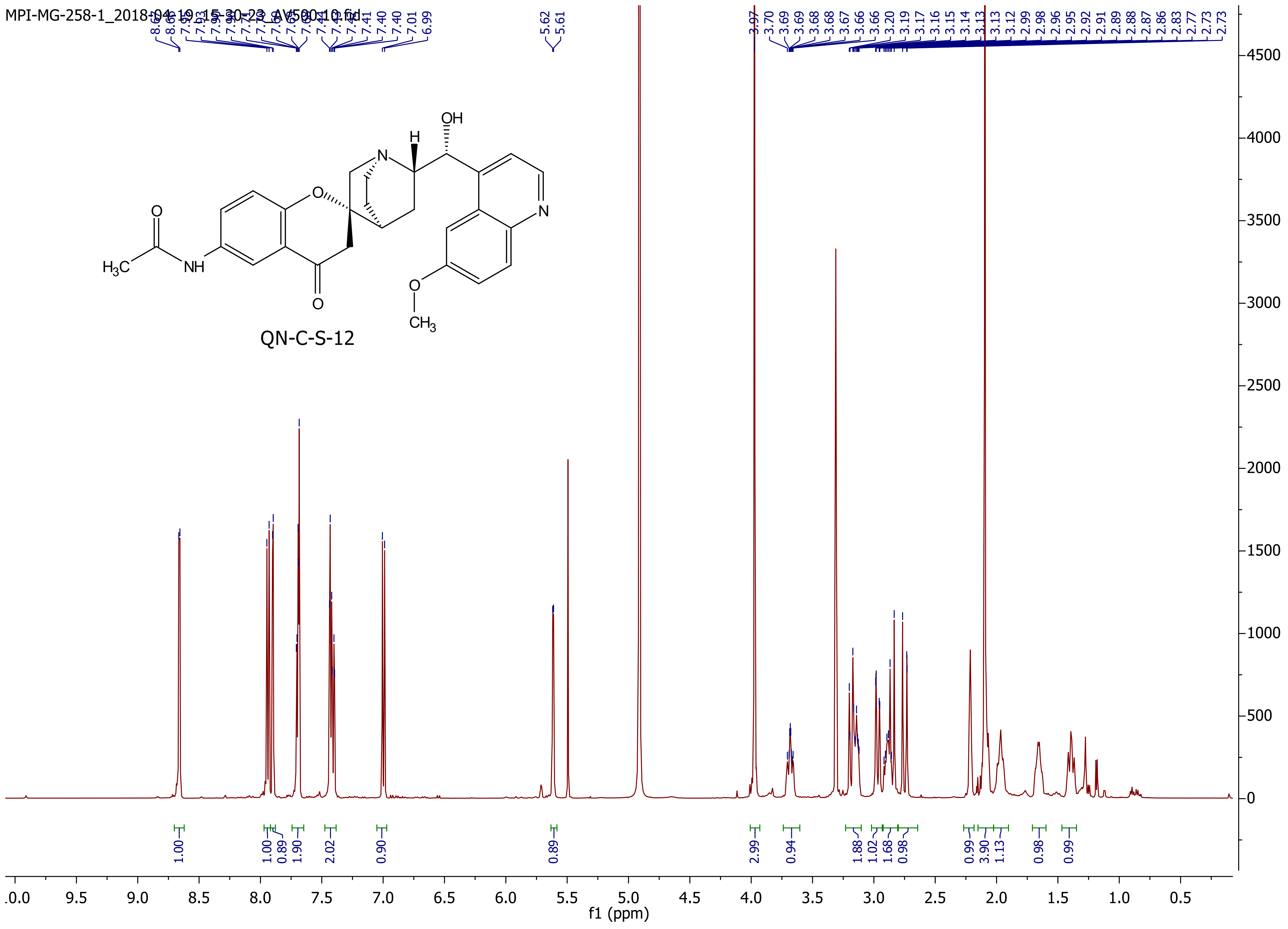

MPI-MG-258-1_2018-04-19_15-30-23_AV500.10.fid
QN-C-S-12
OH
H
N
O
NH
H3C
CH3
5.62
5.61
1.00
1.00
0.89
1.90
2.02
0.90
0.89
2.99
0.94
1.88
1.02
1.68
0.98
0.99
3.90
1.13
0.98
0.99
f1 (ppm)

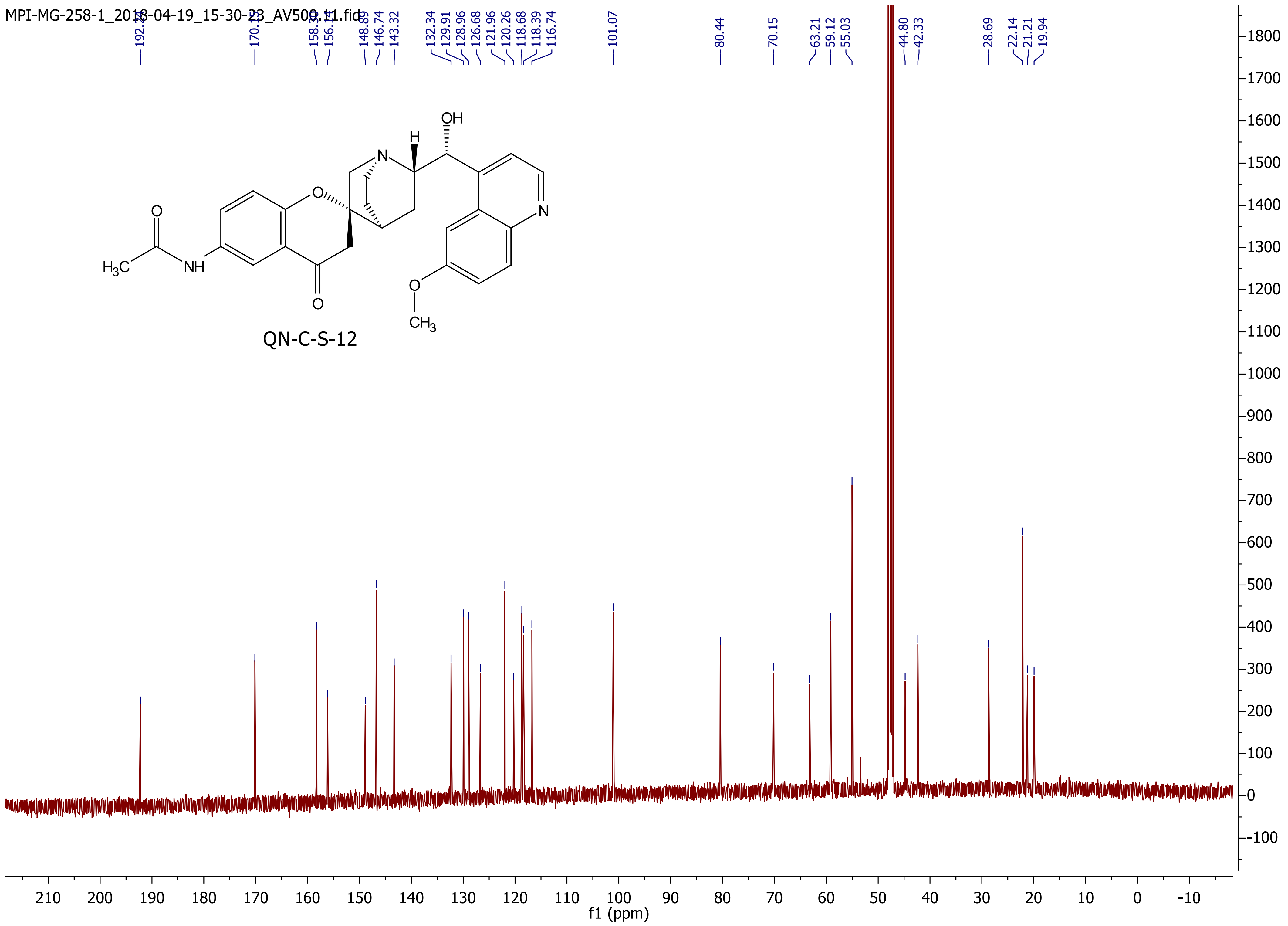

MPI-MG-258-1_2018-04-19_15-30-23_AV500.11.fid
148.89
146.74
143.32
132.34
129.91
128.96
126.68
121.96
120.26
118.68
118.39
116.74
101.07
80.44
70.15
63.21
59.12
55.03
44.80
42.33
28.69
22.14
21.21
19.94
OH
H
N
O
O
H3C
NH
O
N
O
CH3
QN-C-S-12
f1 (ppm)

MPI-MG-258-2_2018-04-19_15-30-23_AV500.21.fid

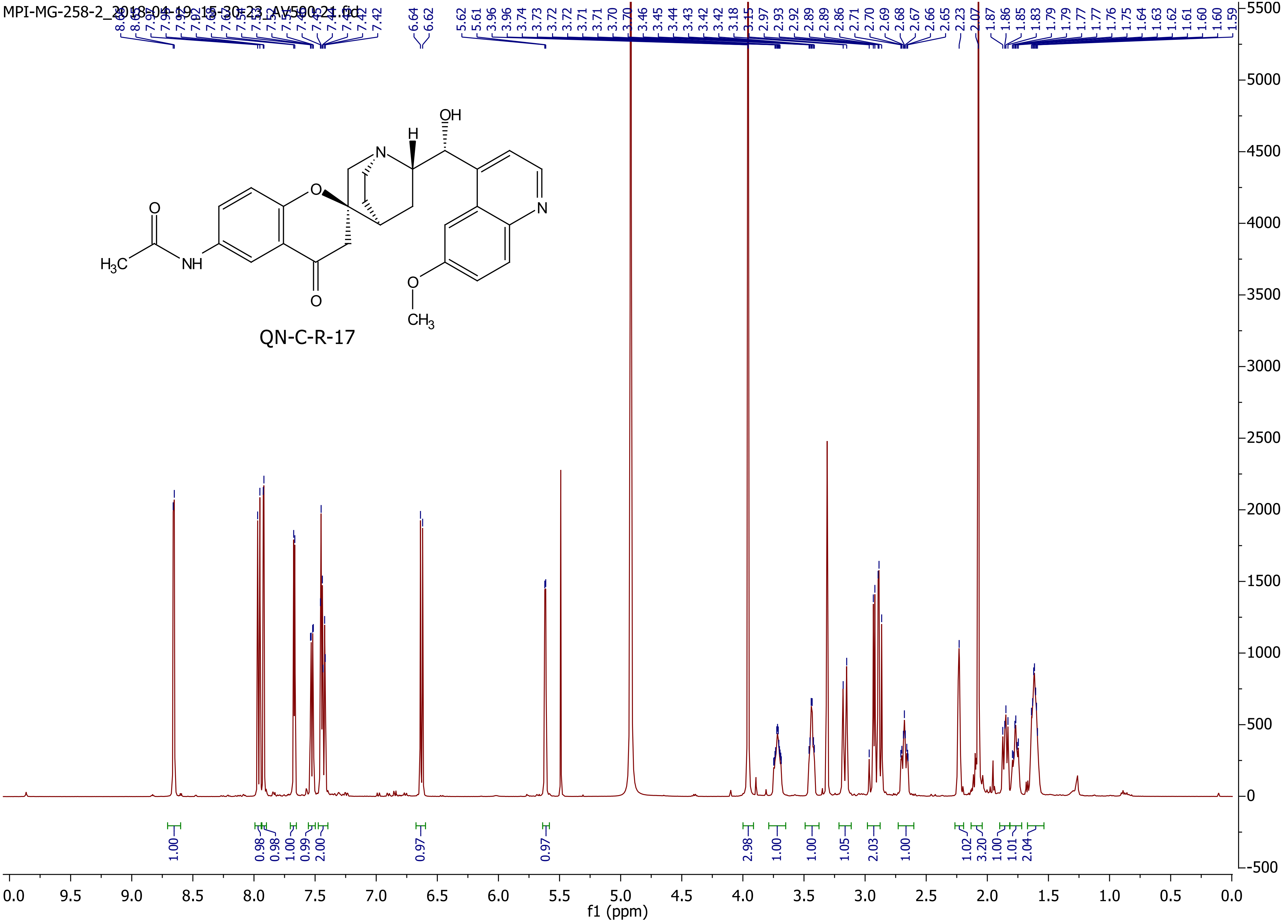

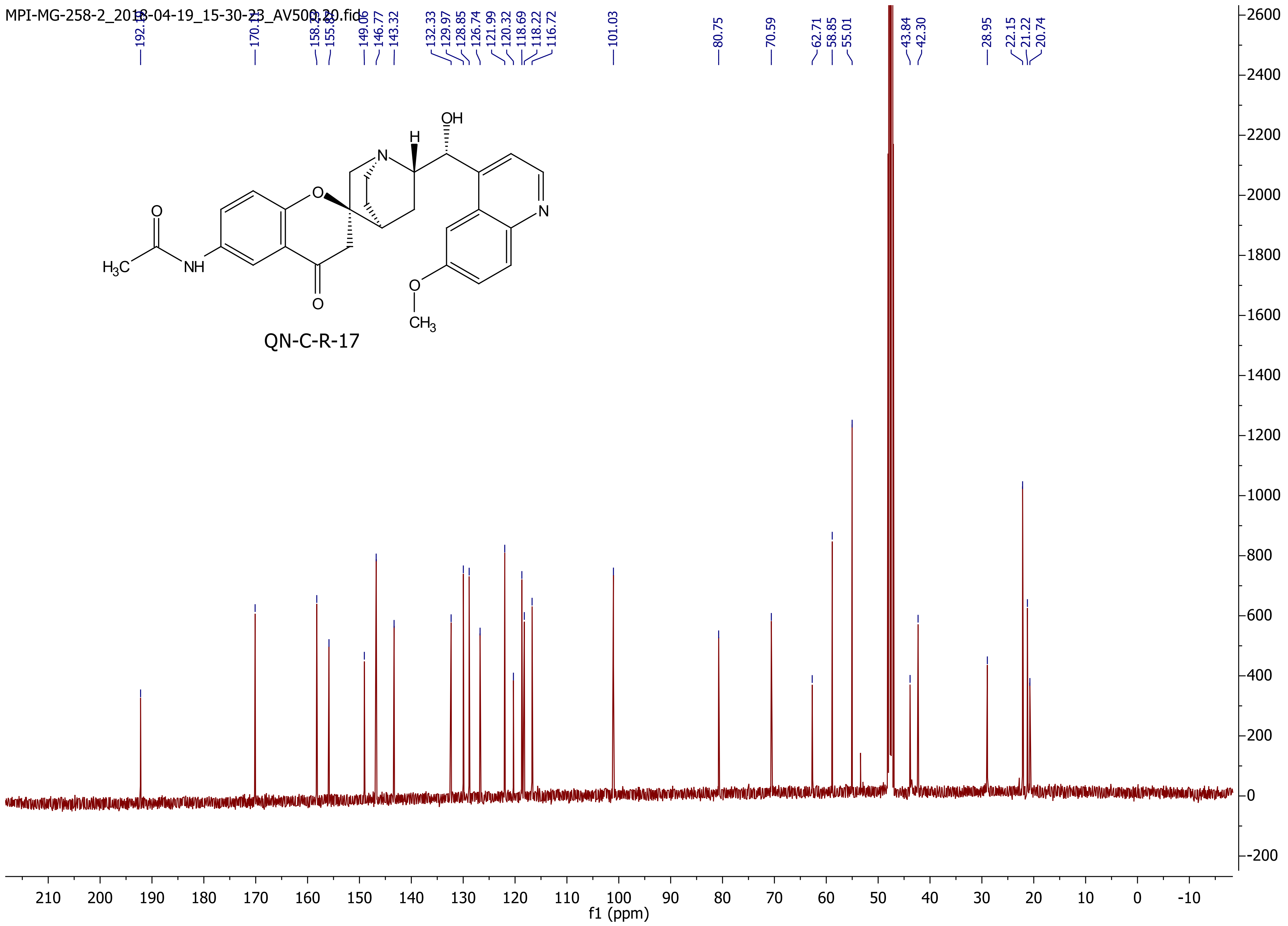

MPI-MG-258-2_2018-04-19_15-30-23_AV500.20.fid
149.06
146.77
143.32
132.33
129.97
128.85
126.74
121.99
120.32
118.69
118.22
116.72
101.03
80.75
70.59
62.71
58.85
55.01
43.84
42.30
28.95
22.15
21.22
20.74
QN-C-R-17
f1 (ppm)

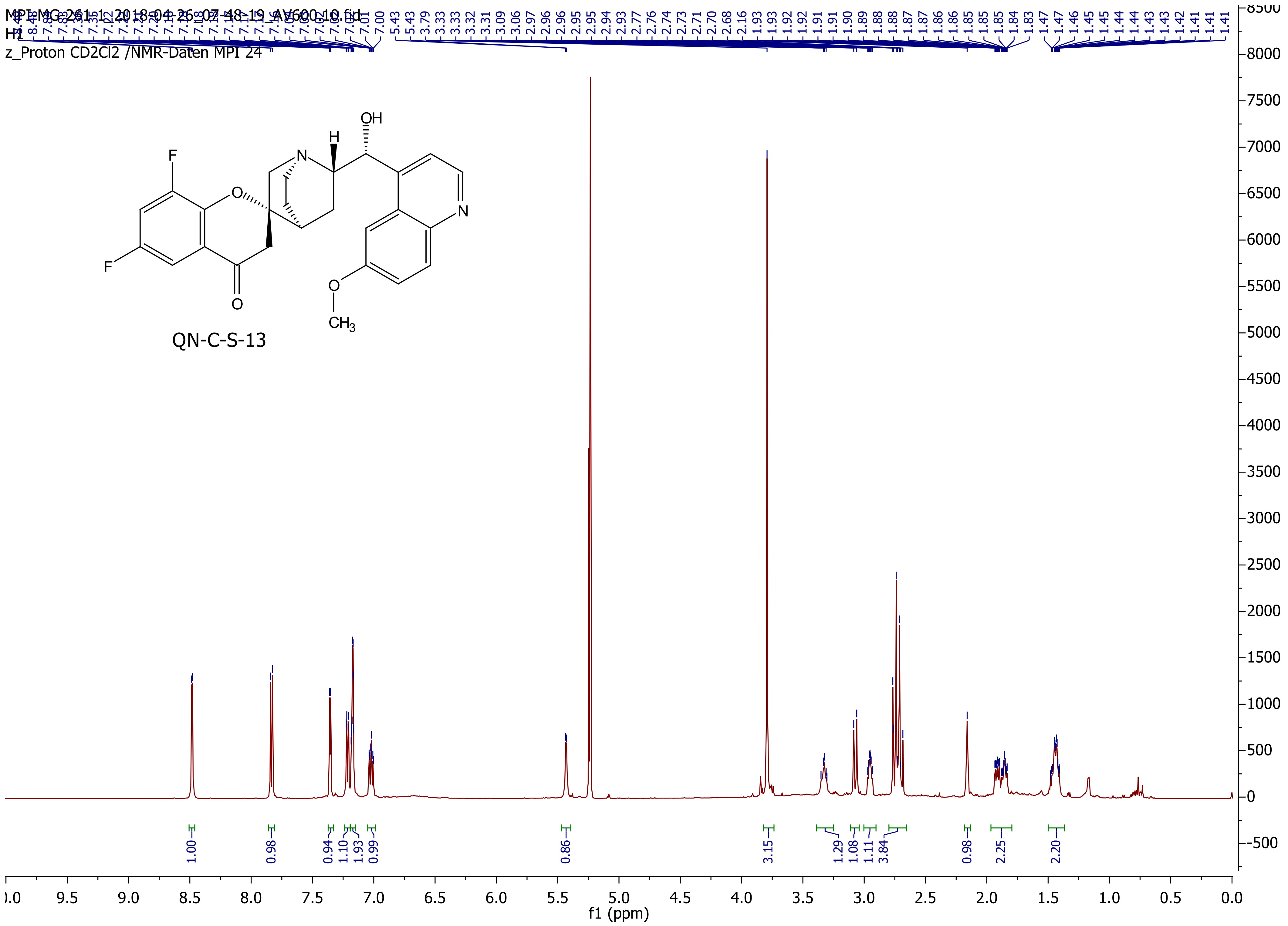

H1
z_Proton CD2Cl2 /NMR-Daten MPI 24
7.01
7.00
5.43
5.43
3.79
3.33
3.33
3.32
3.31
3.09
3.06
2.97
2.96
2.96
2.95
2.95
2.94
2.93
2.77
2.76
2.74
2.73
2.71
2.70
2.68
2.16
1.93
1.93
1.92
1.92
1.91
1.91
1.90
1.89
1.88
1.88
1.87
1.87
1.86
1.86
1.85
1.85
1.85
1.84
1.83
1.47
1.47
1.46
1.45
1.45
1.44
1.44
1.43
1.43
1.42
1.41
1.41
1.41
OH
H
N
F
O
F
O
N
O
CH3
QN-C-S-13
1.00
0.98
0.94
1.10
1.93
0.99
0.86
3.15
1.29
1.08
1.11
3.84
0.98
2.25
2.20
f1 (ppm)

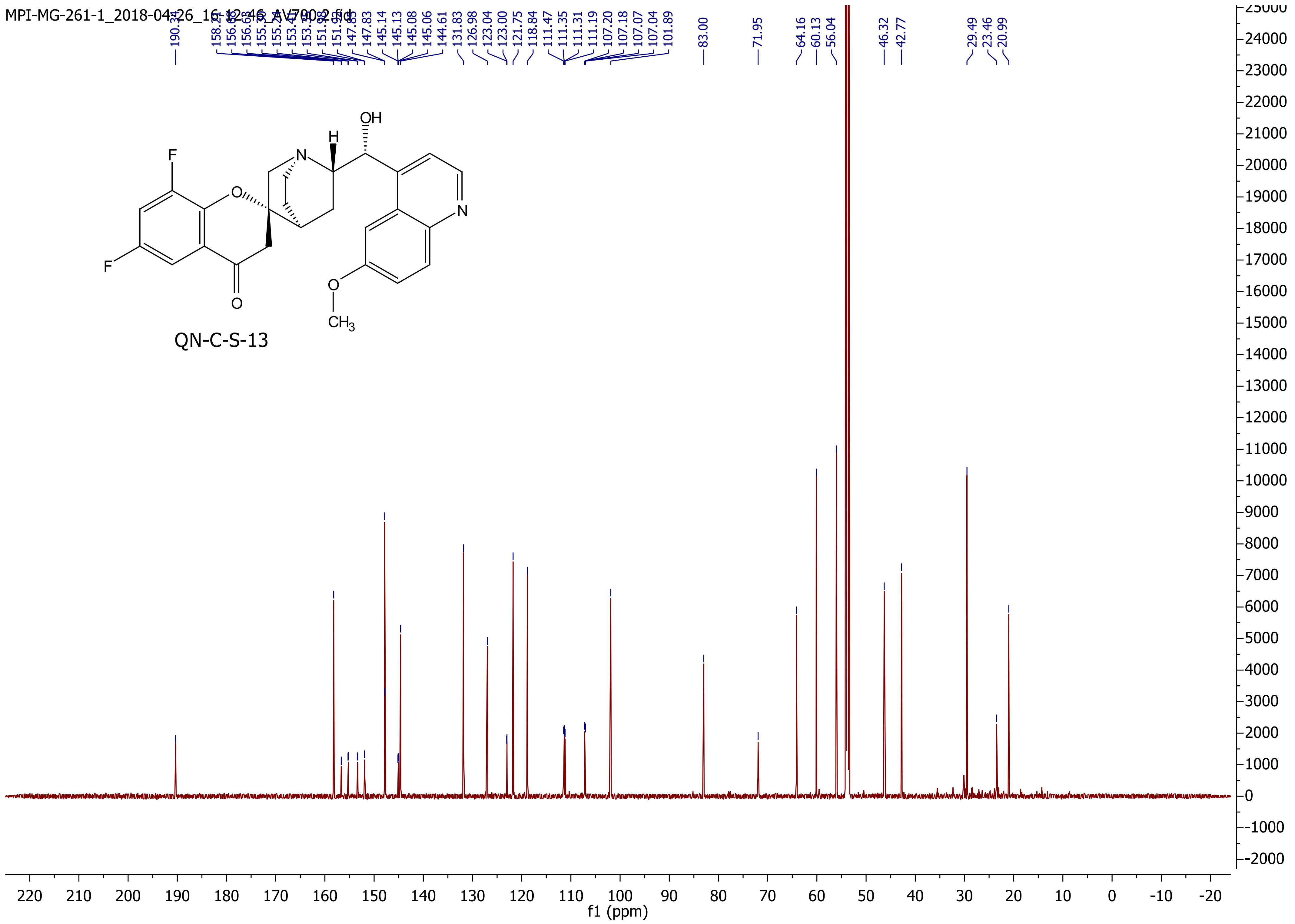

MPI-MG-261-1_2018-04-26_16-12-46_AV700-2.fid
190.34
147.83
145.14
145.13
145.08
145.06
144.61
131.83
126.98
123.04
123.00
121.75
118.84
111.47
111.35
111.31
111.19
107.20
107.18
107.07
107.04
101.89
83.00
71.95
64.16
60.13
56.04
46.32
42.77
29.49
23.46
20.99
OH
H
N
F
O
F
O
N
O
CH3
QN-C-S-13
f1 (ppm)

MPI-MG-261-1_2018-04-26_07-48-19_AV600.12.fid
F19

z_F19 CD2Cl2 /NMR-Daten MPI 24

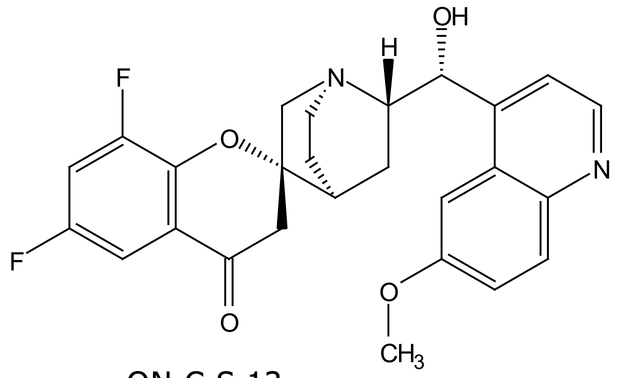

QN-C-S-13

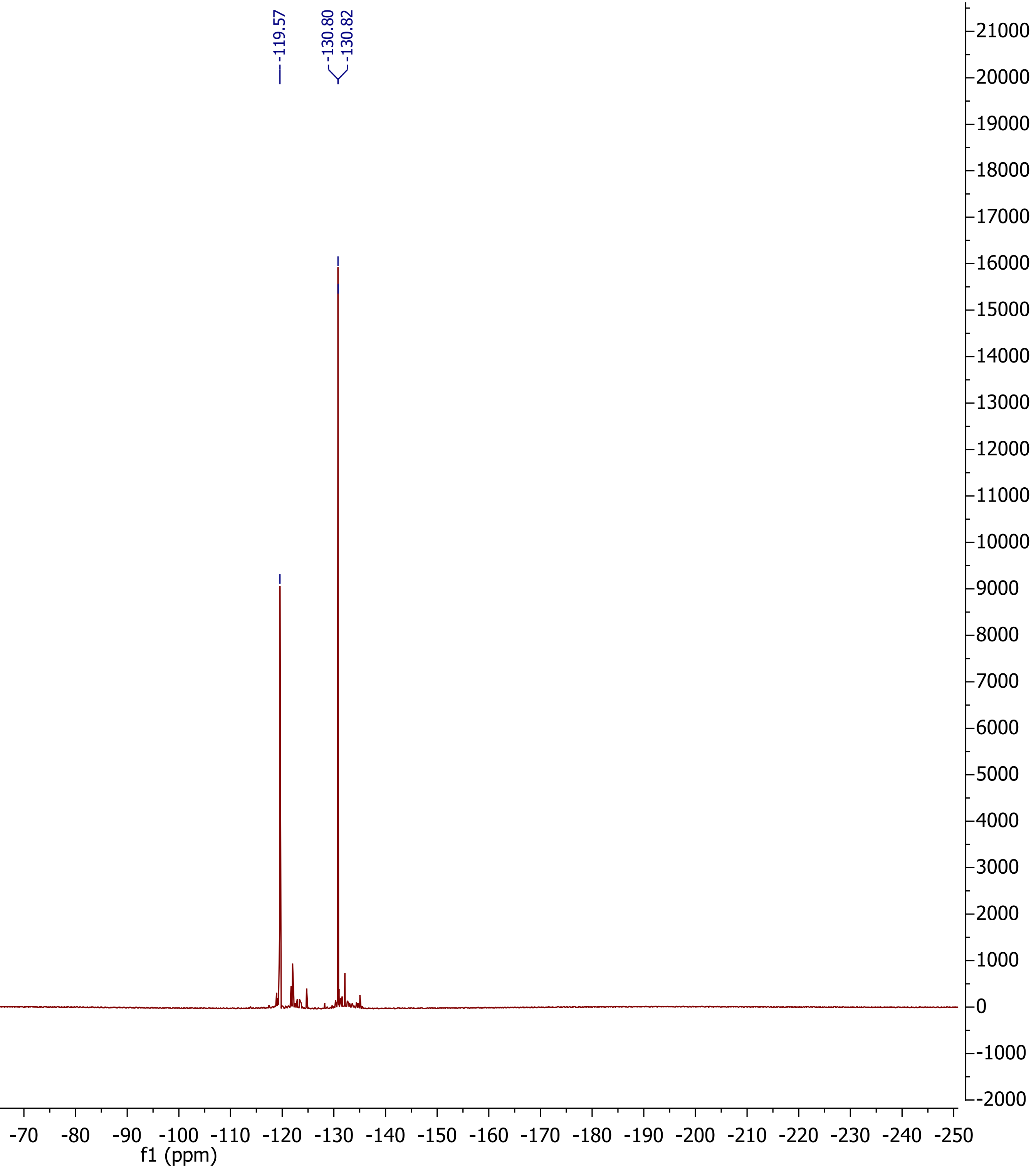

MPI-MG-261-2_2018-04-26_07-48-19_AV600-10.fid
H1
z_Proton CD2Cl2 /NMR-Daten MPI 25

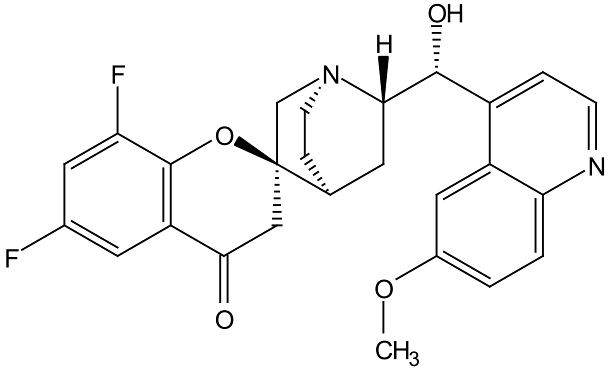

QN-C-R-18

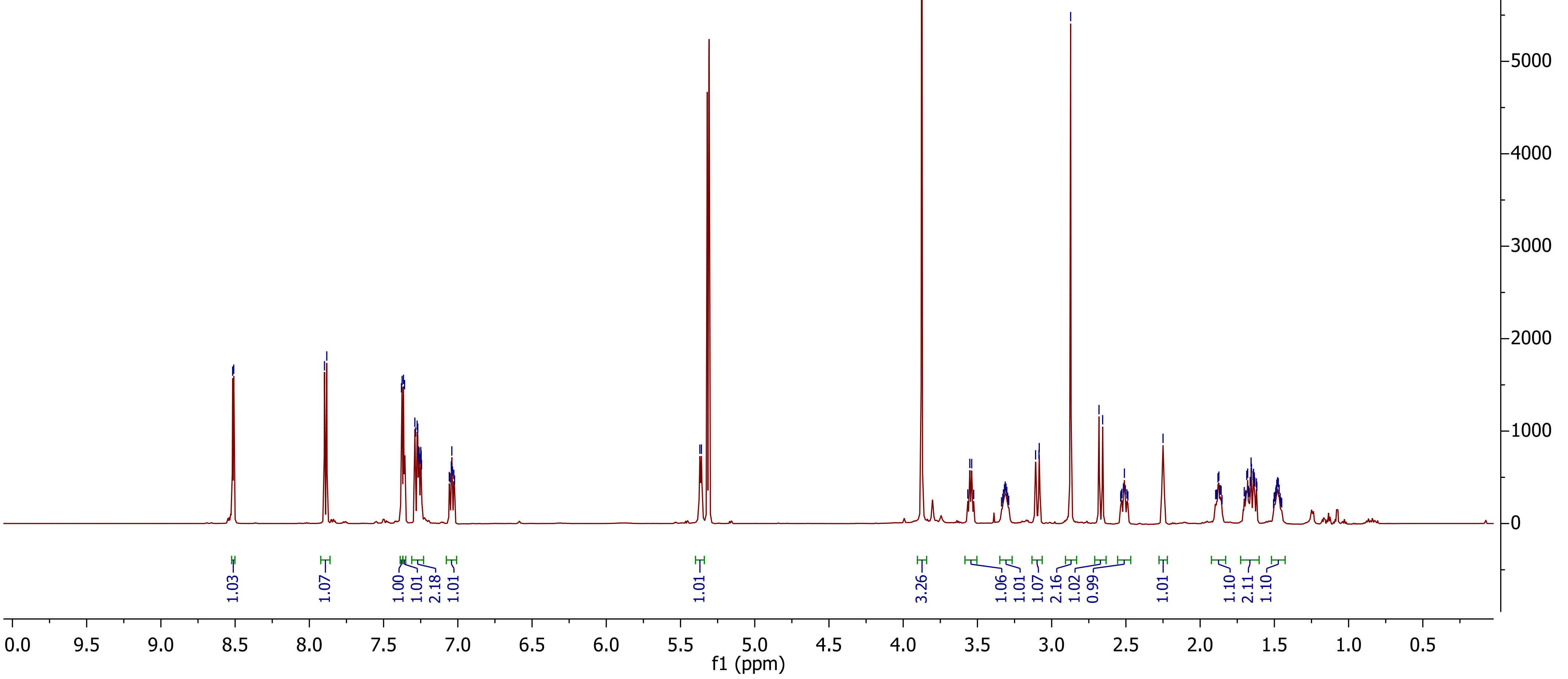

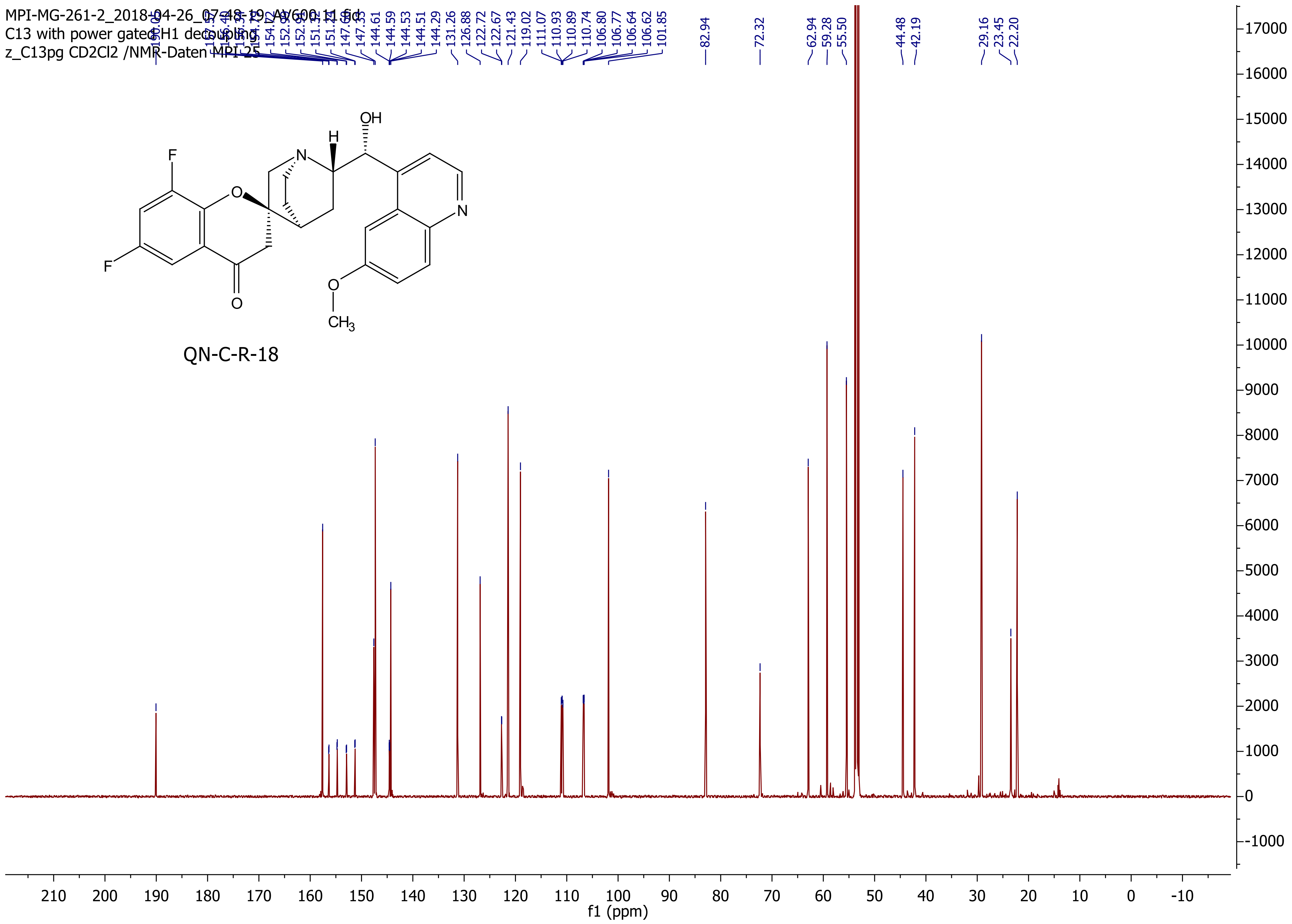

MPI-MG-261-2_2018-04-26_07-48-19_AV600.11.fid
C13 with power gated H1 decoupling
z_C13pg CD2Cl2 /NMR-Daten MPI-25
154.12
152.5
151.1
147.35
144.61
144.59
144.53
144.51
144.29
131.26
126.88
122.72
122.67
121.43
119.02
111.07
110.93
110.89
110.74
106.80
106.77
106.64
106.62
101.85
82.94
72.32
62.94
59.28
55.50
44.48
42.19
29.16
23.45
22.20
OH
H
N
F
O
N
F
O
O
CH_3
QN-C-R-18
f1 (ppm)

MPI-MG-261-2_2018-04-26_07-48-19_AV600.12.fid
F19

z_F19 CD2Cl2 /NMR-Daten MPI 25

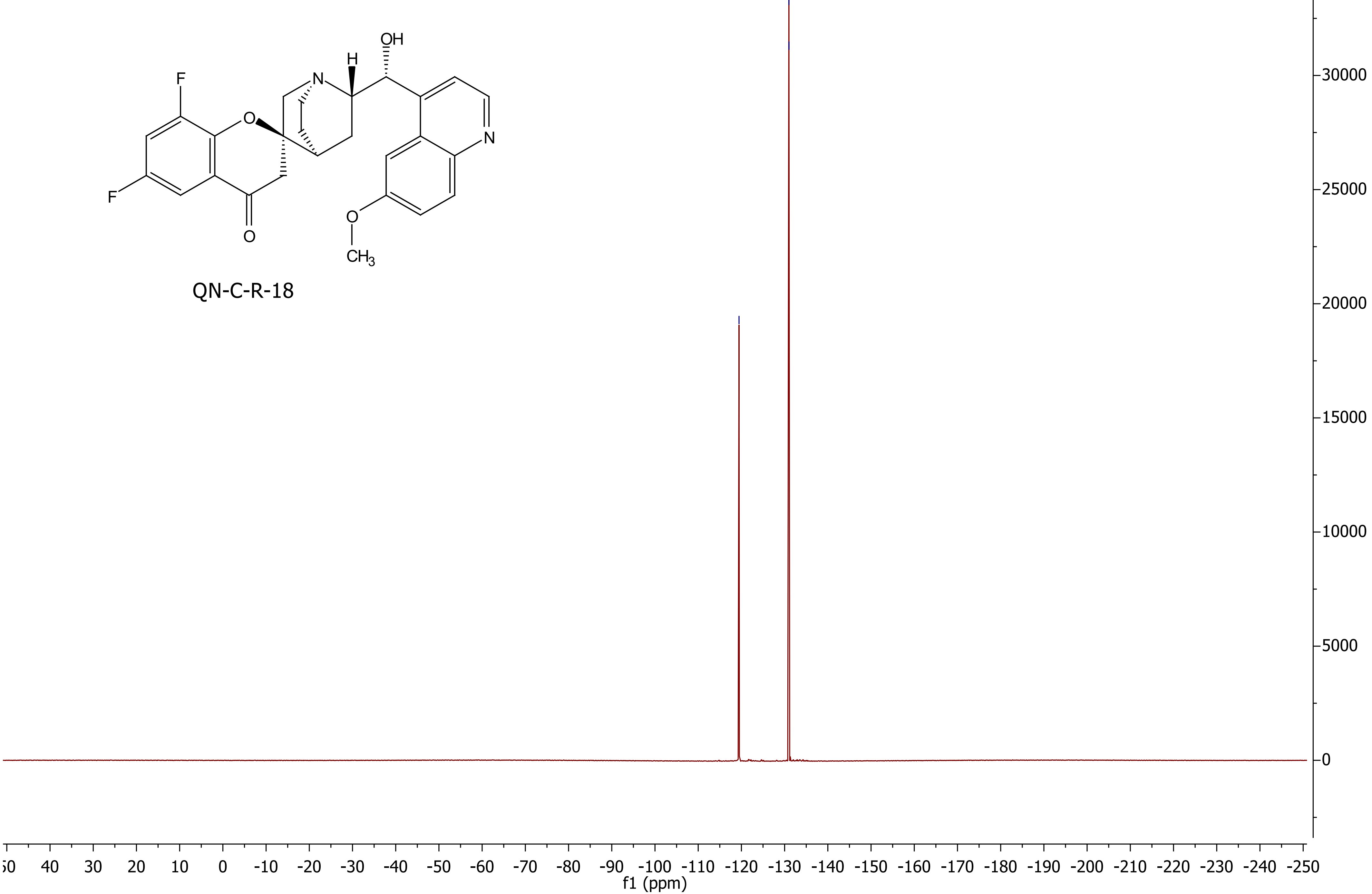

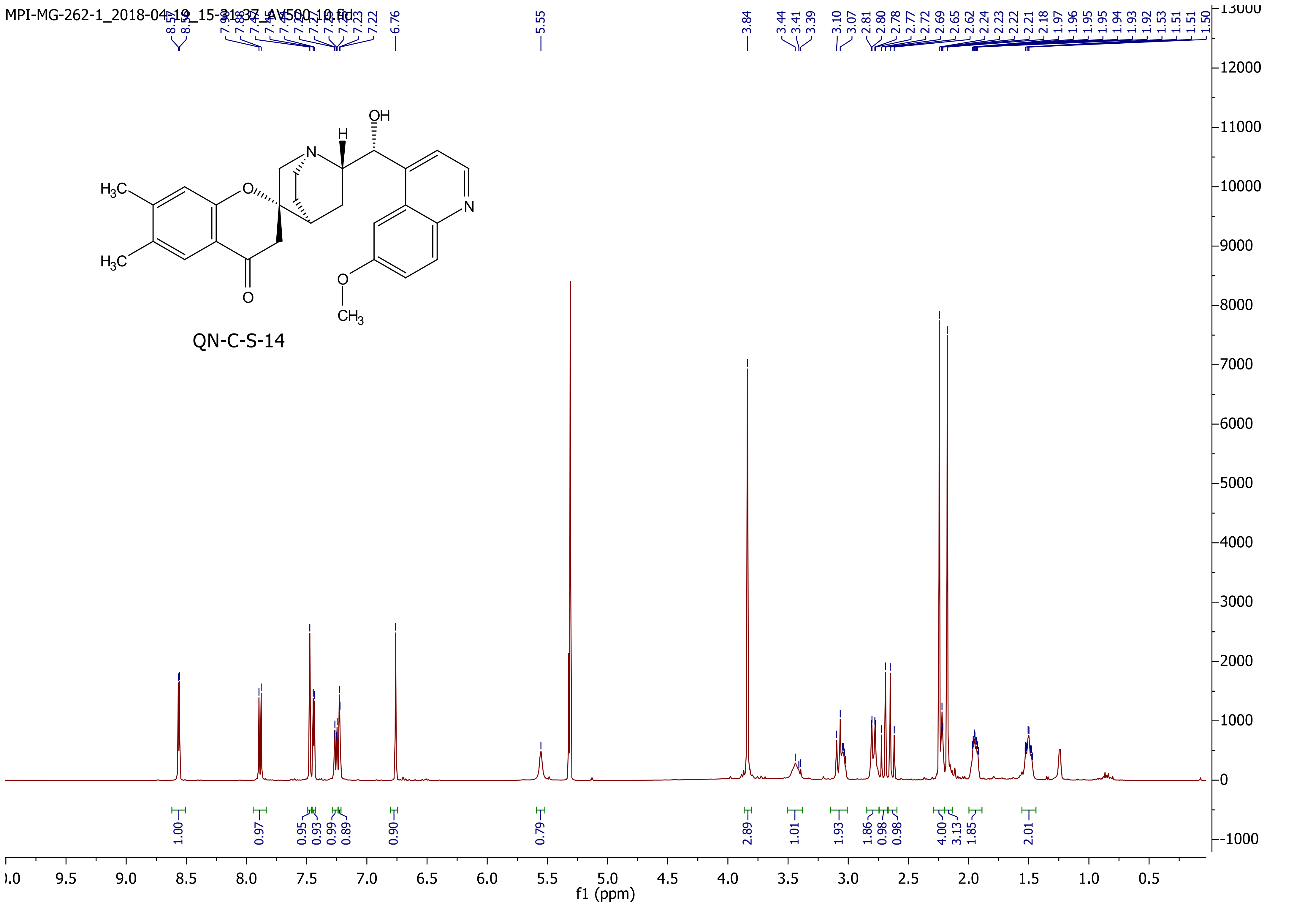
MPI-MG-262-1_2018-04-19_15-31-37_AV500.10.fid
QN-C-S-14
f1 (ppm)

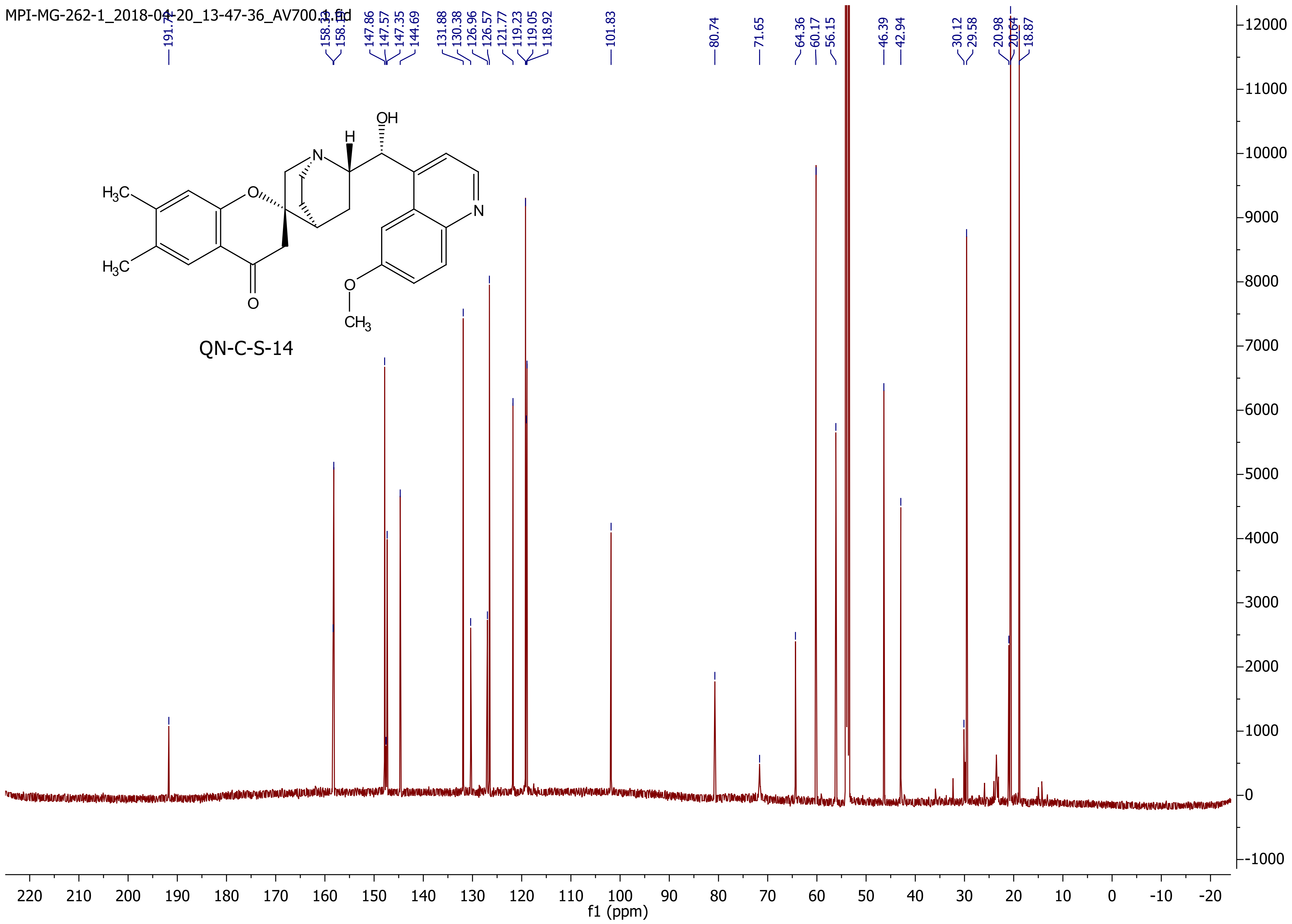

MPI-MG-262-1_2018-04-20_13-47-36_AV700.1.fid
147.86
147.57
147.35
144.69
131.88
130.38
126.96
126.57
121.77
119.23
119.05
118.92
101.83
80.74
71.65
64.36
60.17
56.15
46.39
42.94
30.12
29.58
20.98
18.87
OH
H
N
O
N
O
O
CH3
H3C
H3C
QN-C-S-14
f1 (ppm)

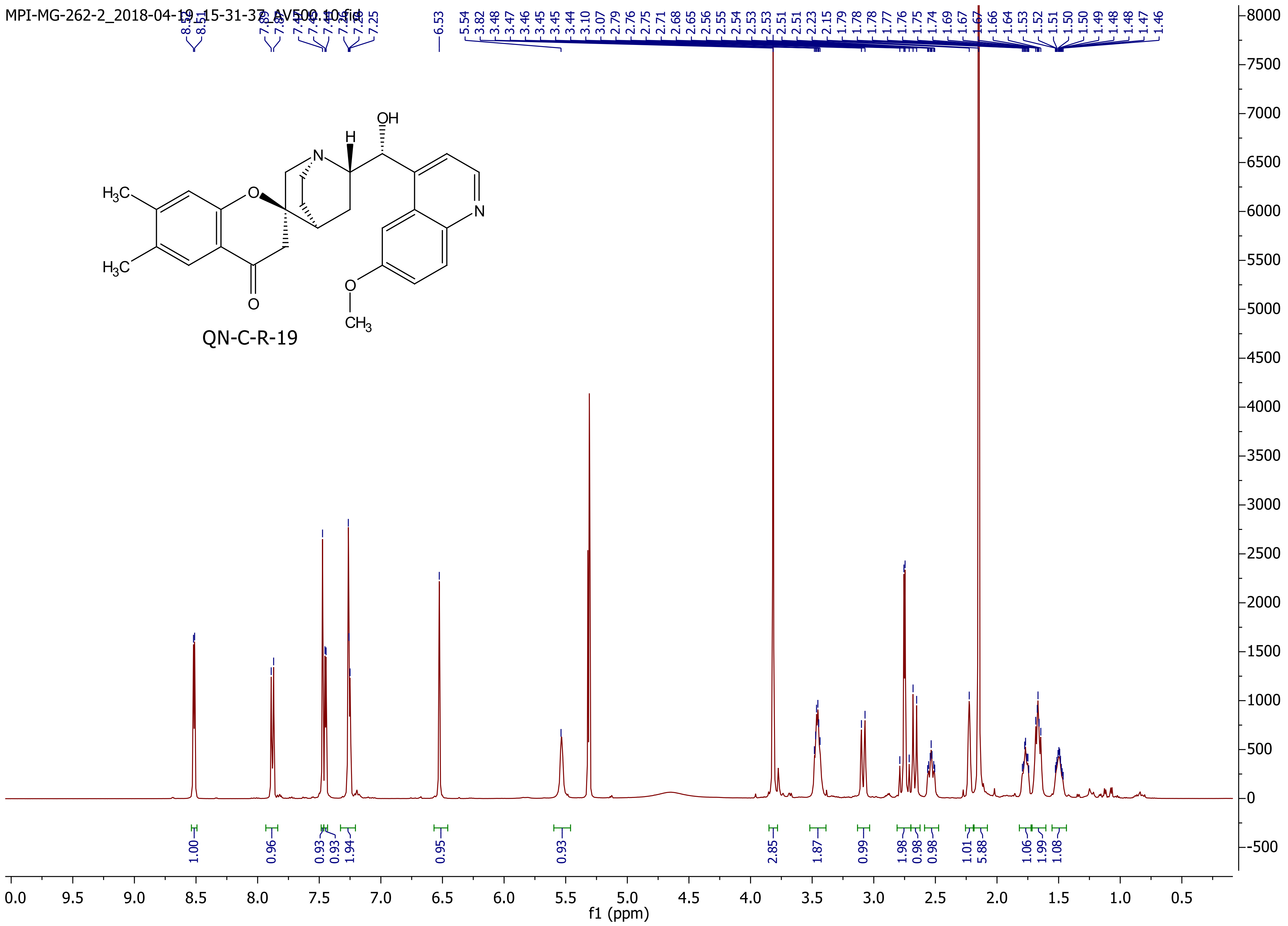

MPI-MG-262-2_2018-04-19_15-31-37_AV500.10.fid
6.53
5.54
3.82
3.48
3.47
3.46
3.45
3.45
3.44
3.10
3.07
2.79
2.76
2.75
2.71
2.68
2.65
2.56
2.55
2.54
2.53
2.51
2.51
2.23
2.15
1.79
1.78
1.78
1.77
1.76
1.75
1.74
1.69
1.67
1.67
1.66
1.64
1.53
1.52
1.51
1.50
1.50
1.49
1.48
1.47
1.46
OH
H
N
O
N
H3C
H3C
O
O
CH3
QN-C-R-19
1.00
0.96
0.93
0.93
1.94
0.95
0.93
2.85
1.87
0.99
1.98
0.98
0.98
1.01
5.88
1.06
1.99
1.08
f1 (ppm)

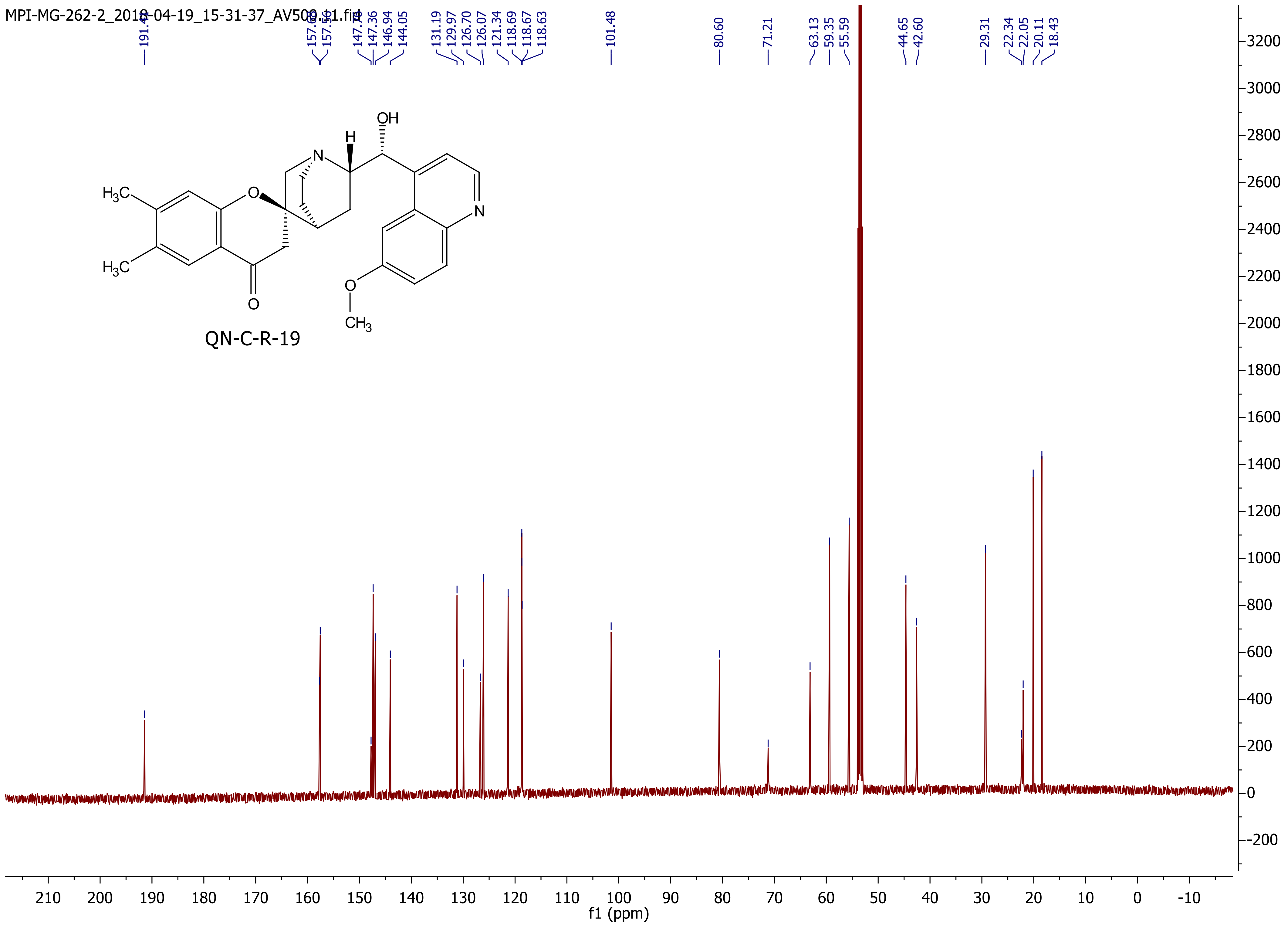
MPI-MG-262-2_2018-04-19_15-31-37_AV500.s1.fid
147.36
146.94
144.05
131.19
129.97
126.70
126.07
121.34
118.69
118.67
118.63
101.48
80.60
71.21
63.13
59.35
55.59
44.65
42.60
29.31
22.34
22.05
20.11
18.43
OH
H
N
O
N
H_3C
H_3C
O
O
CH_3
QN-C-R-19
f1 (ppm)

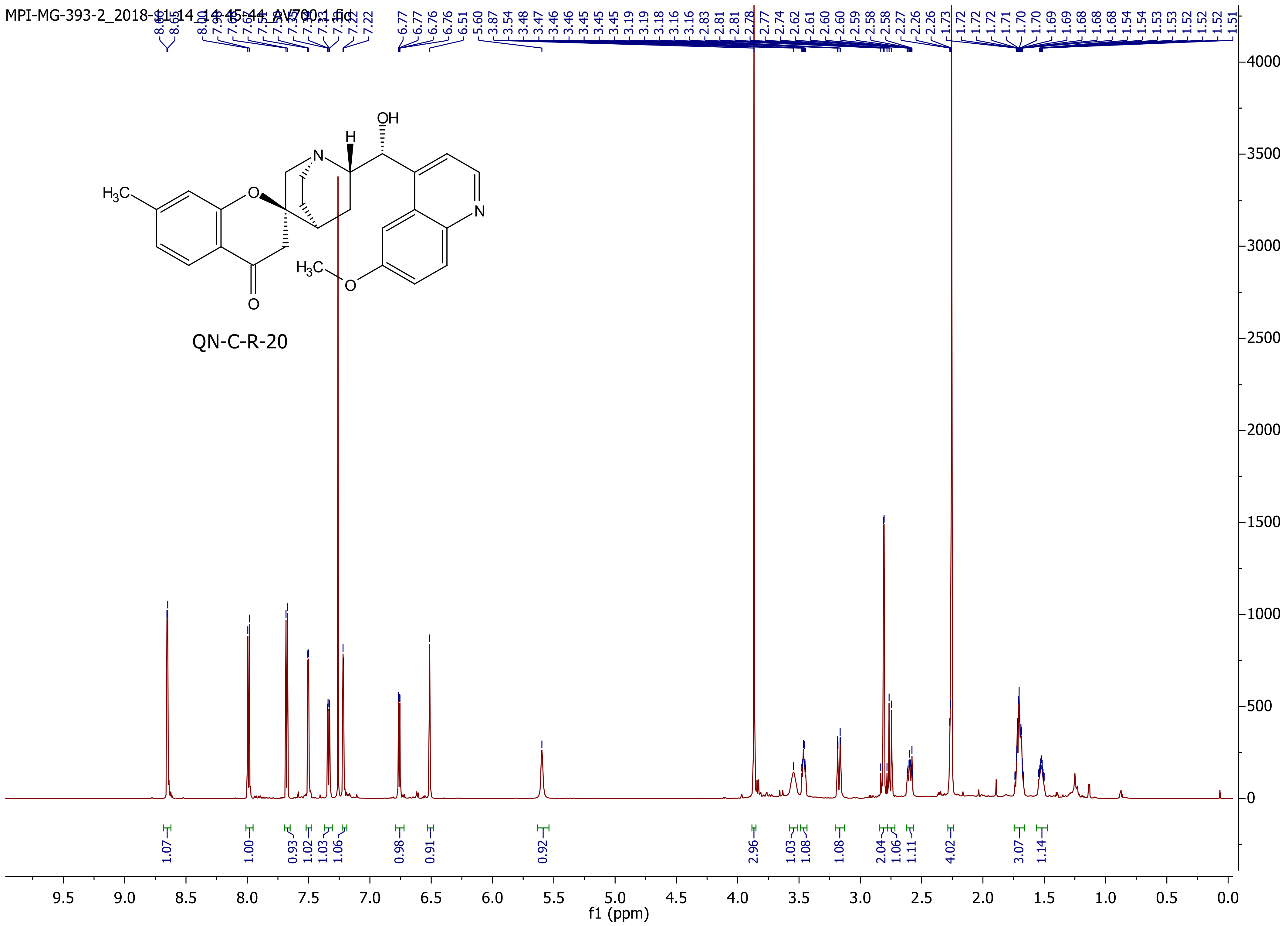
MPI-MG-393-2_2018-11-14_14-45-44_AV700.1.fid
QN-C-R-20
f1 (ppm)

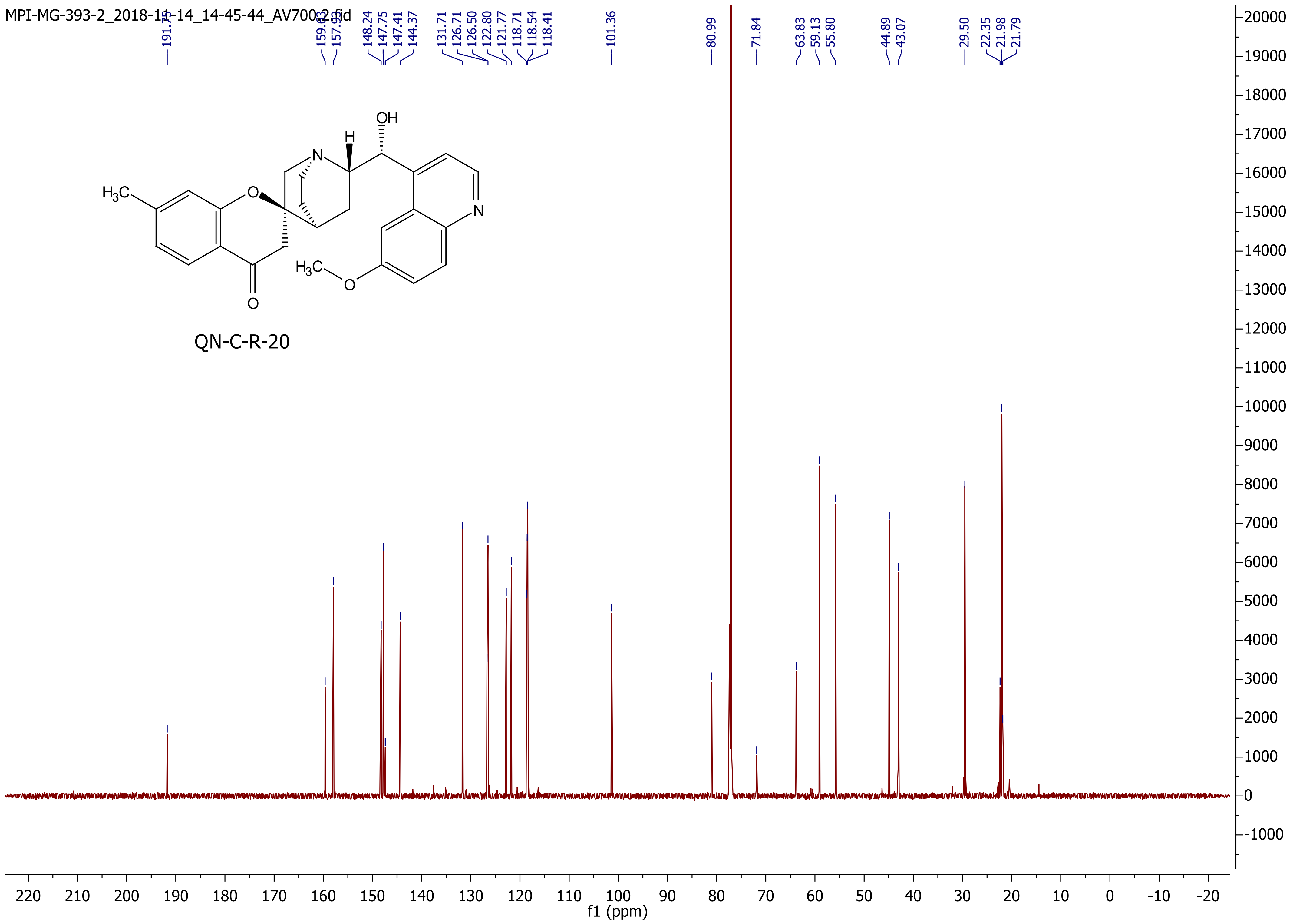

148.24
147.75
147.41
144.37
131.71
126.71
126.50
122.80
121.77
118.71
118.54
118.41
101.36
80.99
71.84
63.83
59.13
55.80
44.89
43.07
29.50
22.35
21.98
21.79
QN-C-R-20
f1 (ppm)

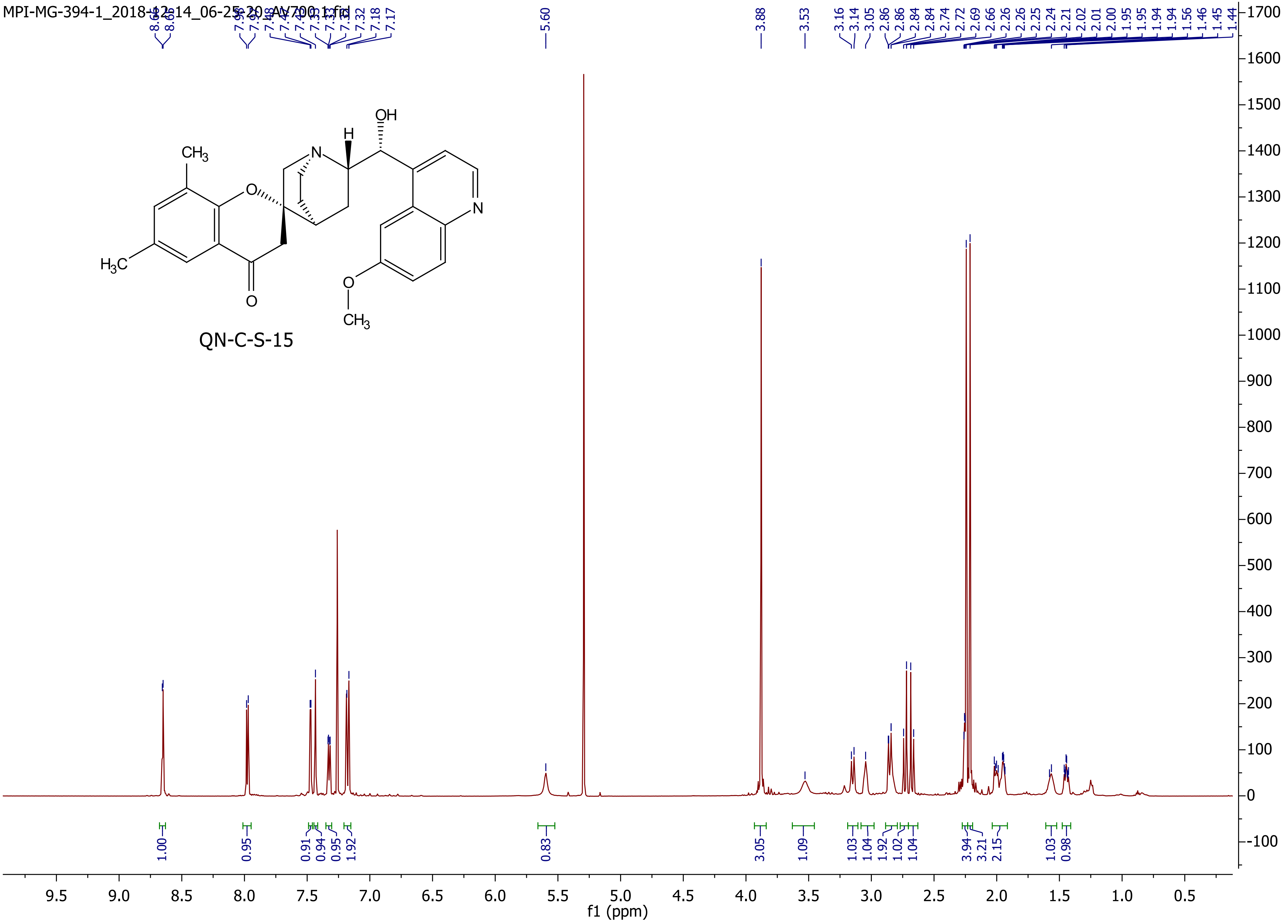
MPI-MG-394-1_2018-12-14_06-25-20_AV700 1.fid
5.60
3.88
3.53
3.16
3.14
3.05
2.86
2.86
2.84
2.84
2.74
2.72
2.69
2.66
2.26
2.26
2.25
2.24
2.21
2.02
2.01
2.00
1.95
1.95
1.94
1.94
1.56
1.46
1.45
1.44
7.32
7.18
7.17
OH
H
N
CH3
O
H3C
O
O
CH3
N
QN-C-S-15
1.00
0.95
0.91
0.94
0.95
1.92
0.83
3.05
1.09
1.03
1.04
1.92
1.02
1.04
3.94
3.21
2.15
1.03
0.98
f1 (ppm)

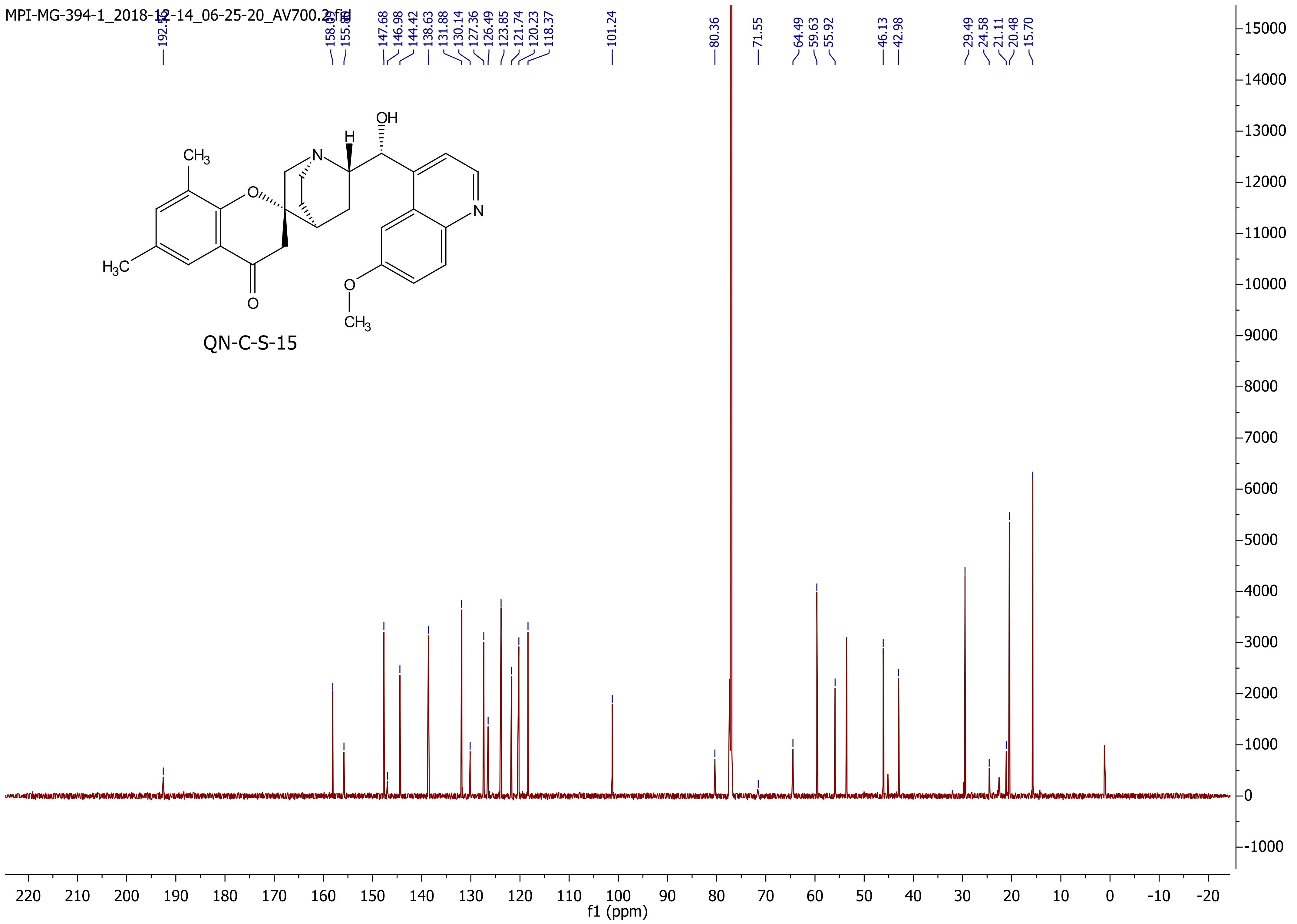

MPI-MG-394-1_2018-12-14_06-25-20_AV700.2.fid
147.68
146.98
144.42
138.63
131.88
130.14
127.36
126.49
123.85
121.74
120.23
118.37
101.24
80.36
71.55
64.49
59.63
55.92
46.13
42.98
29.49
24.58
21.11
20.48
15.70
OH
H
N
CH3
O
H3C
O
N
O
CH3
QN-C-S-15
f1 (ppm)

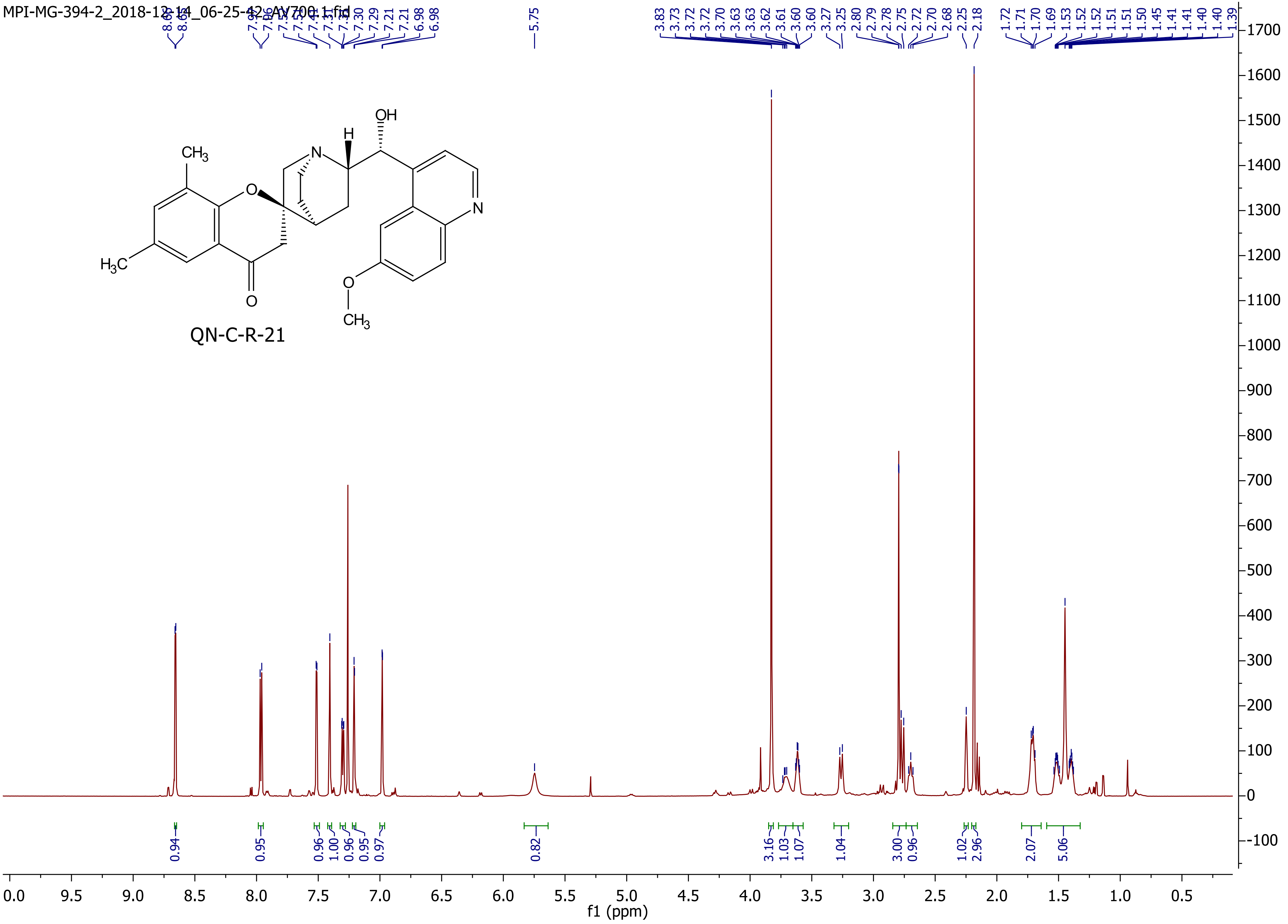
MPI-MG-394-2_2018-12-14_06-25-42_AV700 1 fid
QN-C-R-21
5.75
3.83 3.73 3.72 3.72 3.70 3.63 3.63 3.62 3.61 3.60 3.60 3.27 3.25 2.80 2.79 2.78 2.75 2.72 2.70 2.68 2.25 2.18
1.72 1.71 1.70 1.69 1.53 1.52 1.52 1.51 1.51 1.50 1.45 1.41 1.41 1.40 1.40 1.39
7.30 7.29 7.21 7.21 6.98 6.98
0.94 0.95 0.96 1.00 0.96 0.95 0.97 0.82 3.16 1.03 1.07 1.04 3.00 0.96 1.02 2.96 2.07 5.06
f1 (ppm)

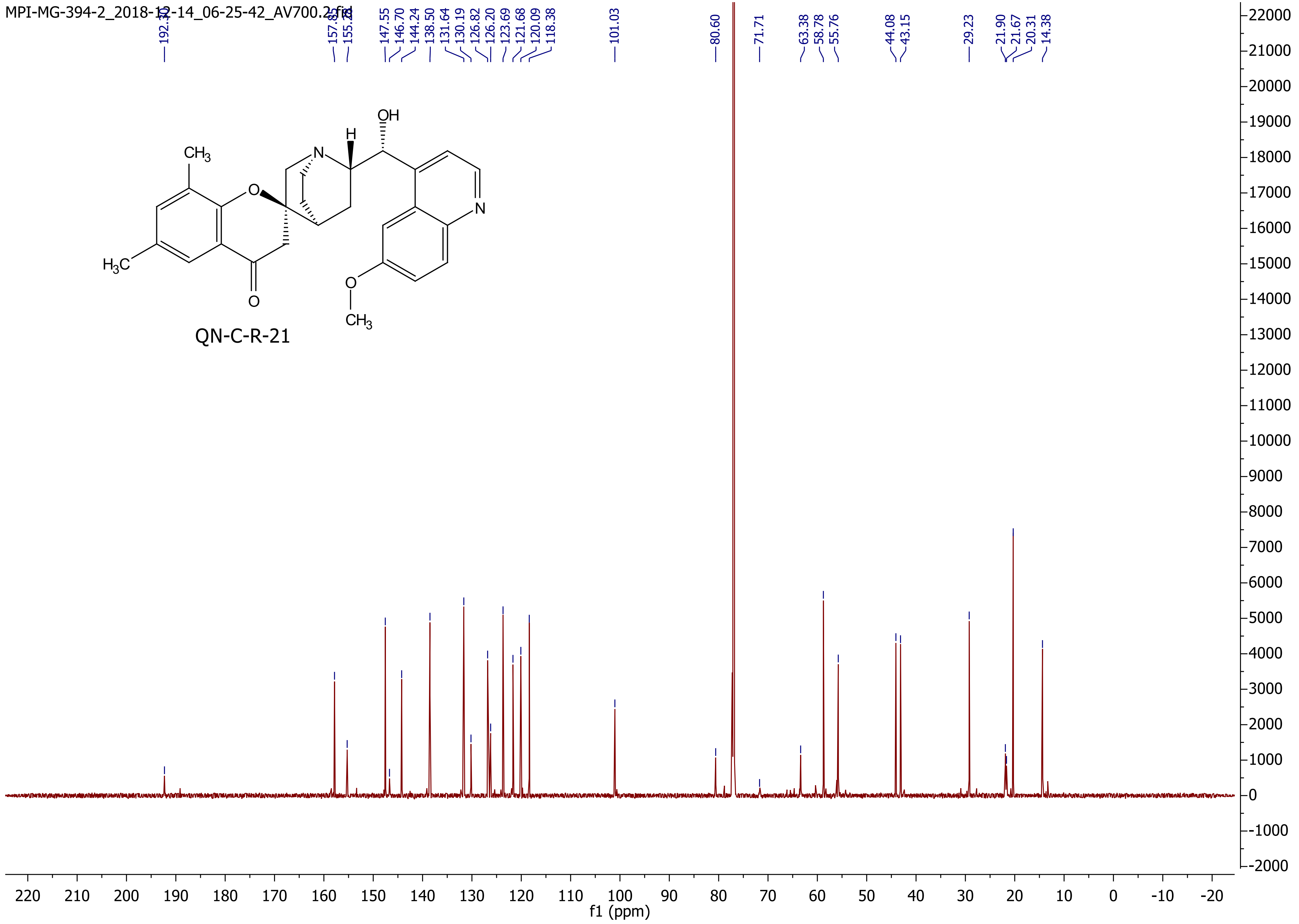

MPI-MG-394-2_2018-12-14_06-25-42_AV700.2.fid
147.55
146.70
144.24
138.50
131.64
130.19
126.82
126.20
123.69
121.68
120.09
118.38
101.03
80.60
71.71
63.38
58.78
55.76
44.08
43.15
29.23
21.90
21.67
20.31
14.38
OH
H
N
CH3
O
H3C
O
N
O
CH3
QN-C-R-21
f1 (ppm)

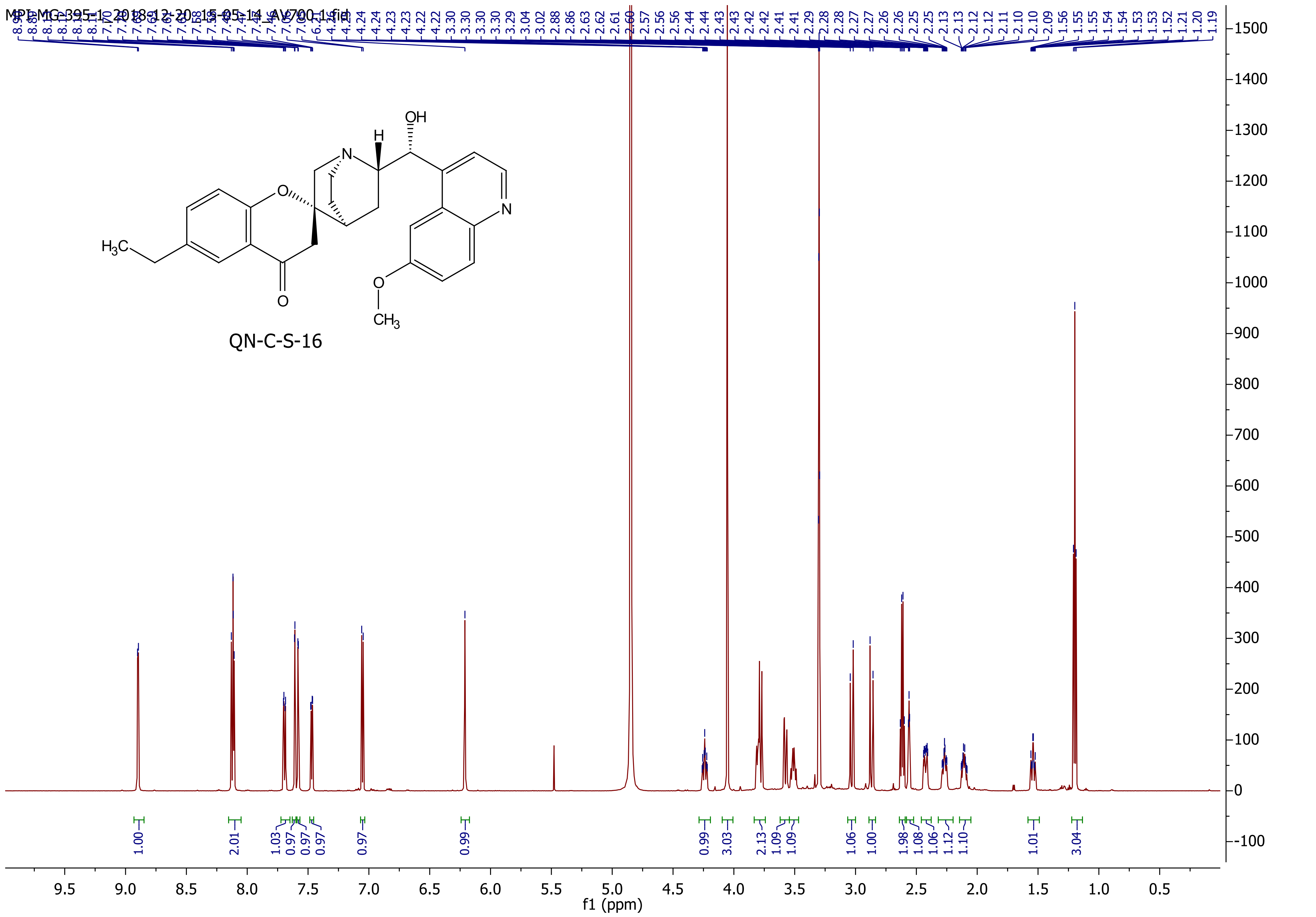

MPI-MG-395-1_2018-12-20_15-05-14_AV700-1.fid
QN-C-S-16
OH
H
N
O
H3C
O
O
CH3
N
1.00
2.01
1.03
0.97
0.97
0.97
0.97
0.99
0.99
3.03
2.13
1.09
1.09
1.06
1.00
1.98
1.08
1.06
1.12
1.10
1.01
3.04
f1 (ppm)

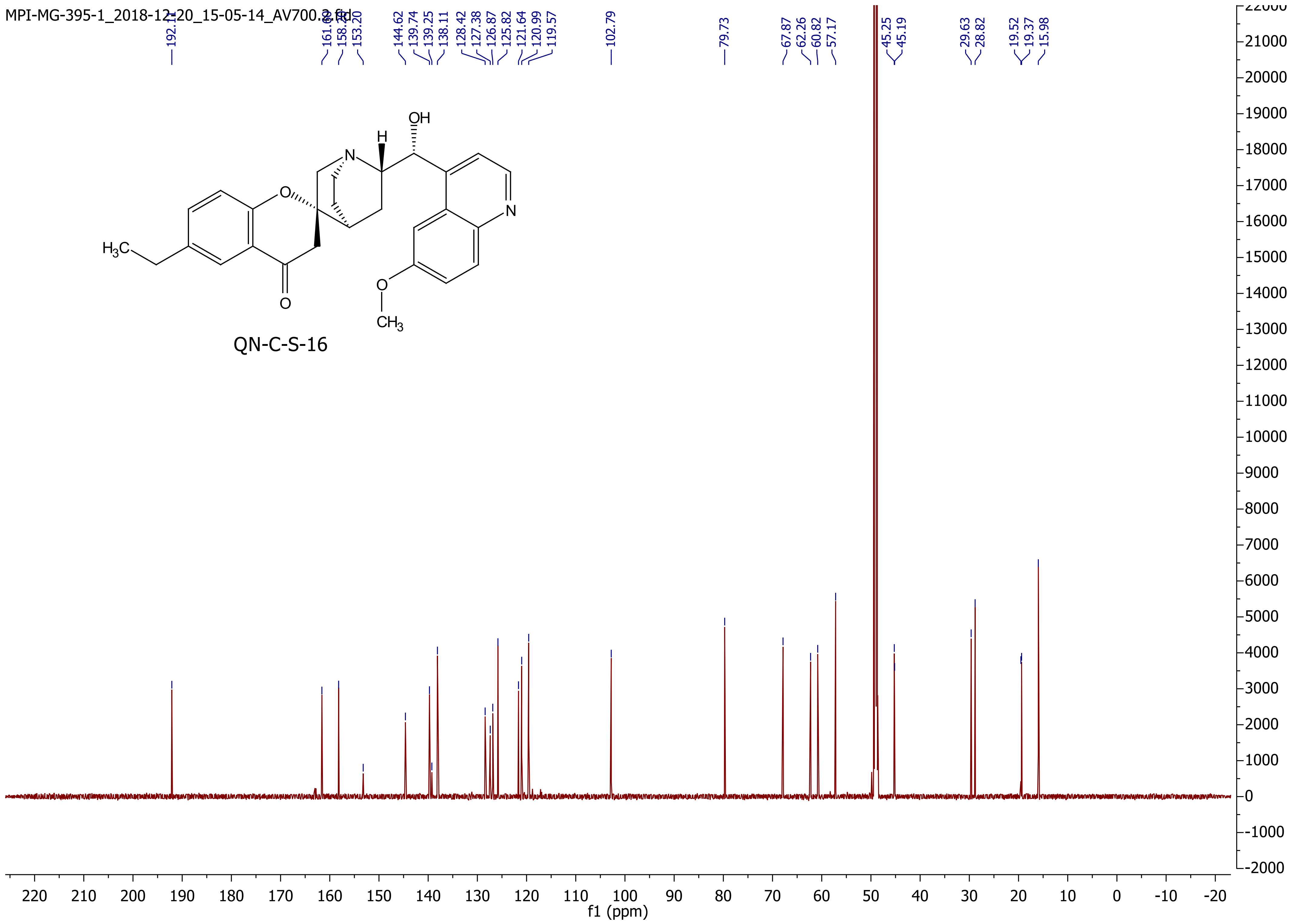
MPI-MG-395-1_2018-12-20_15-05-14_AV700.2.fid
153.20
144.62
139.74
139.25
138.11
128.42
127.38
126.87
125.82
121.64
120.99
119.57
102.79
79.73
67.87
62.26
60.82
57.17
45.25
45.19
29.63
28.82
19.52
19.37
15.98
QN-C-S-16
f1 (ppm)

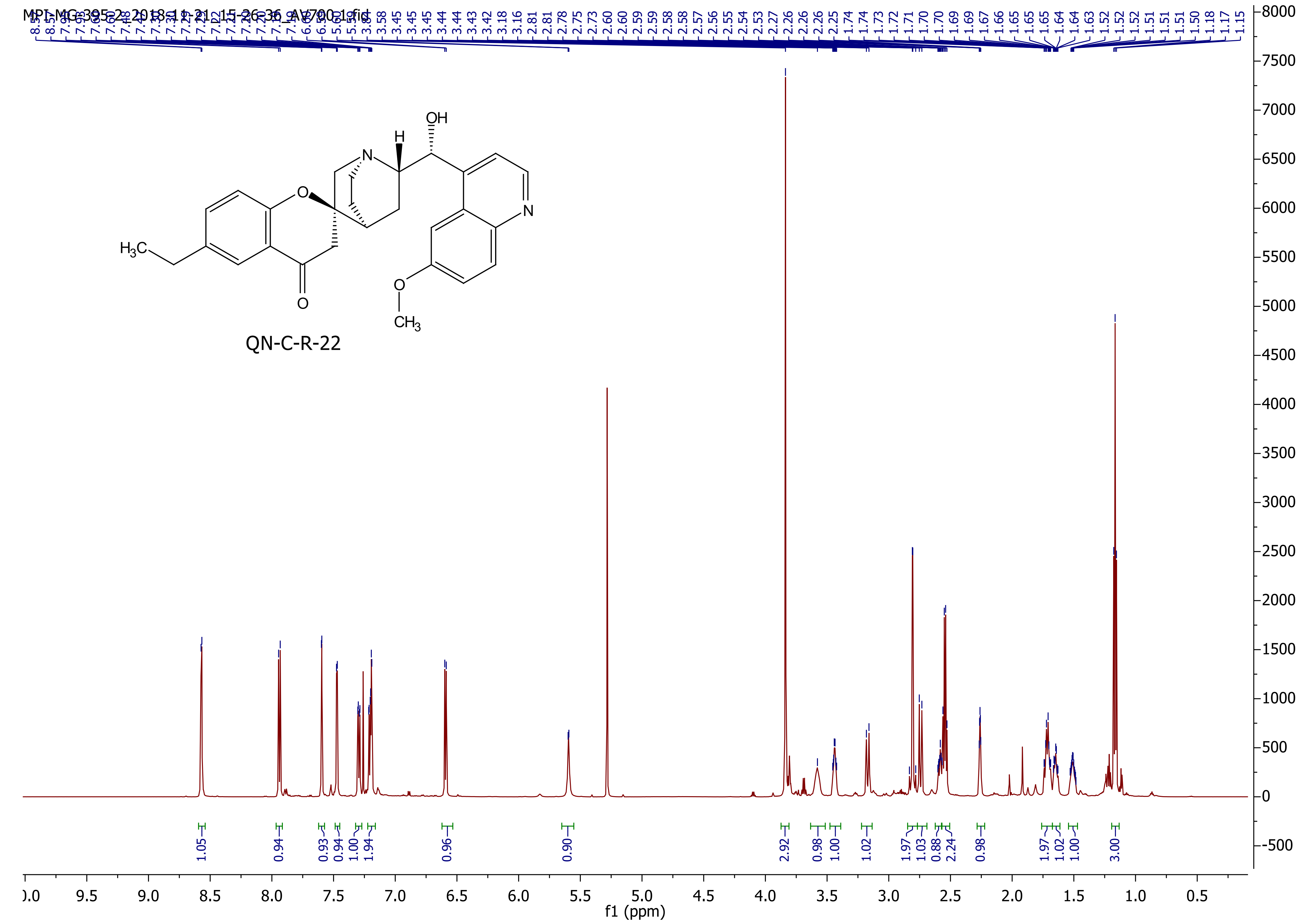
MPI-MG-395-2_2018-11-21_15-26-36_AV700_1.fid
QN-C-R-22
f1 (ppm)

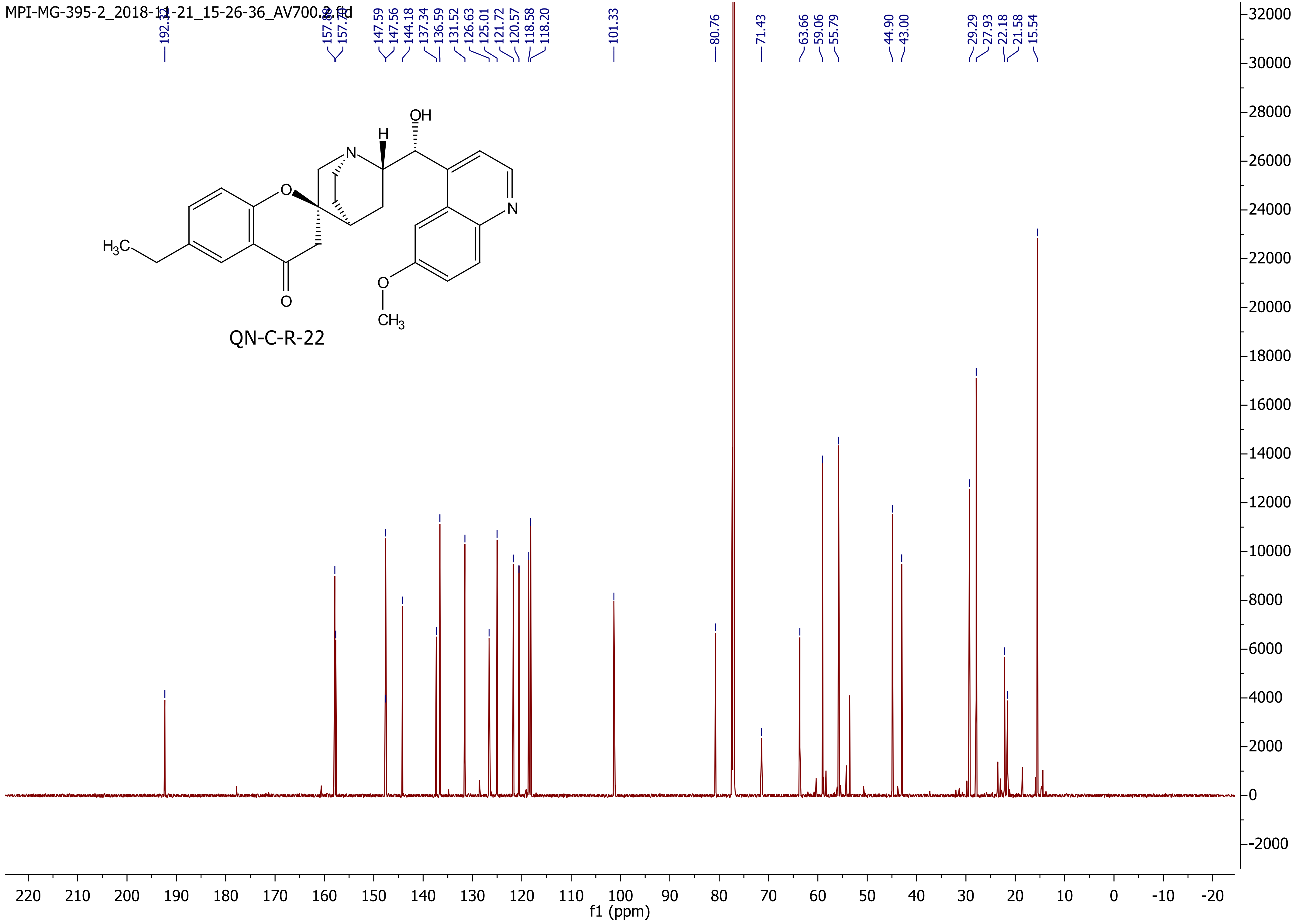

MPI-MG-395-2_2018-11-21_15-26-36_AV700.2.fid
192.32
157.88
157.70
147.59
147.56
144.18
137.34
136.59
131.52
126.63
125.01
121.72
120.57
118.58
118.20
101.33
80.76
71.43
63.66
59.06
55.79
44.90
43.00
29.29
27.93
22.18
21.58
15.54
OH
H
N
O
N
H_3C
O
O
CH_3
QN-C-R-22
32000
30000
28000
26000
24000
22000
20000
18000
16000
14000
12000
10000
8000
6000
4000
2000
0
-2000
220 210 200 190 180 170 160 150 140 130 120 110 100 90 80 70 60 50 40 30 20 10 0 -10 -20
f1 (ppm)

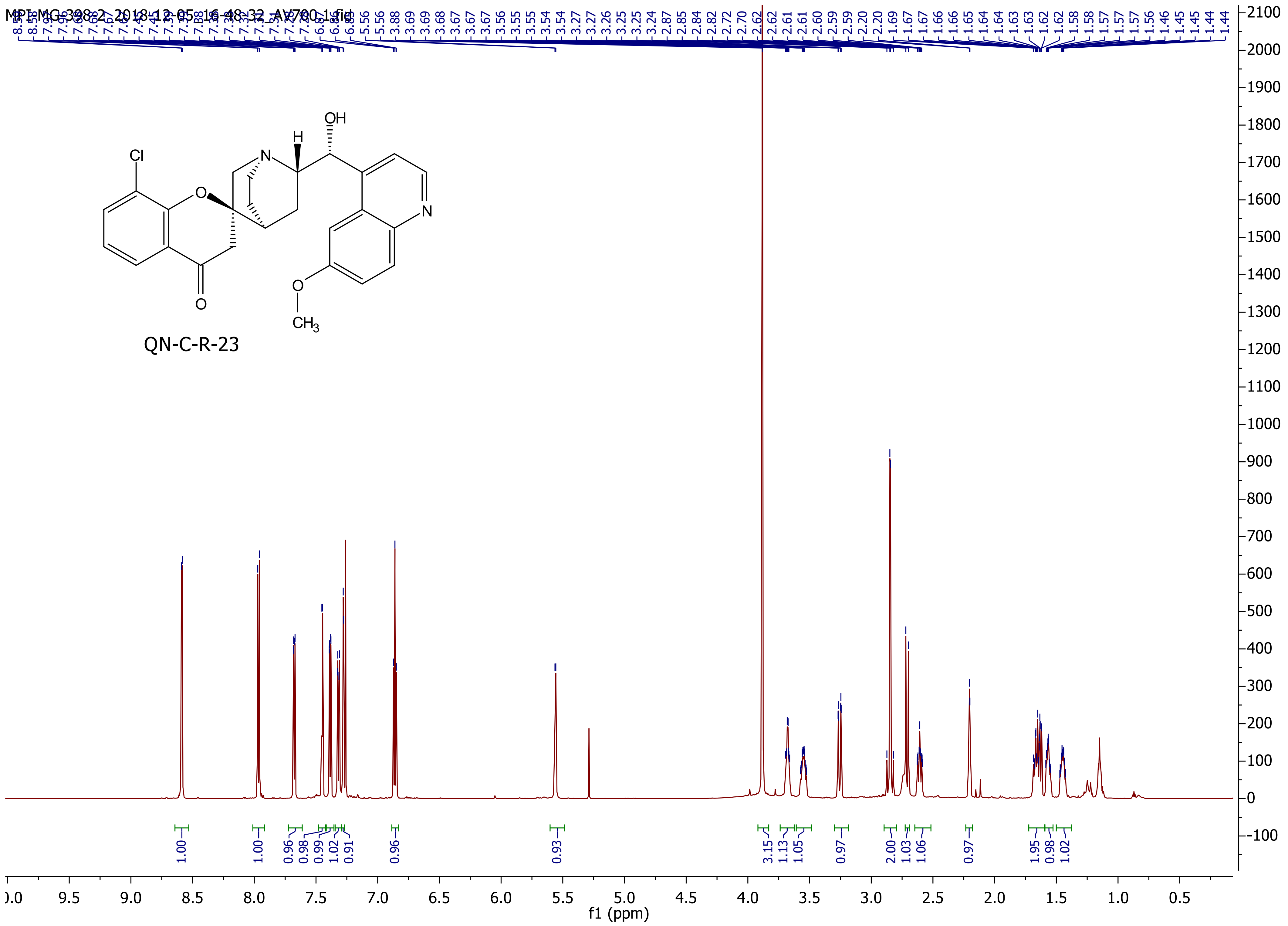

MPI-MG-398-2_2018-12-05_16-48-32_AV700.1.fid
QN-C-R-23
f1 (ppm)

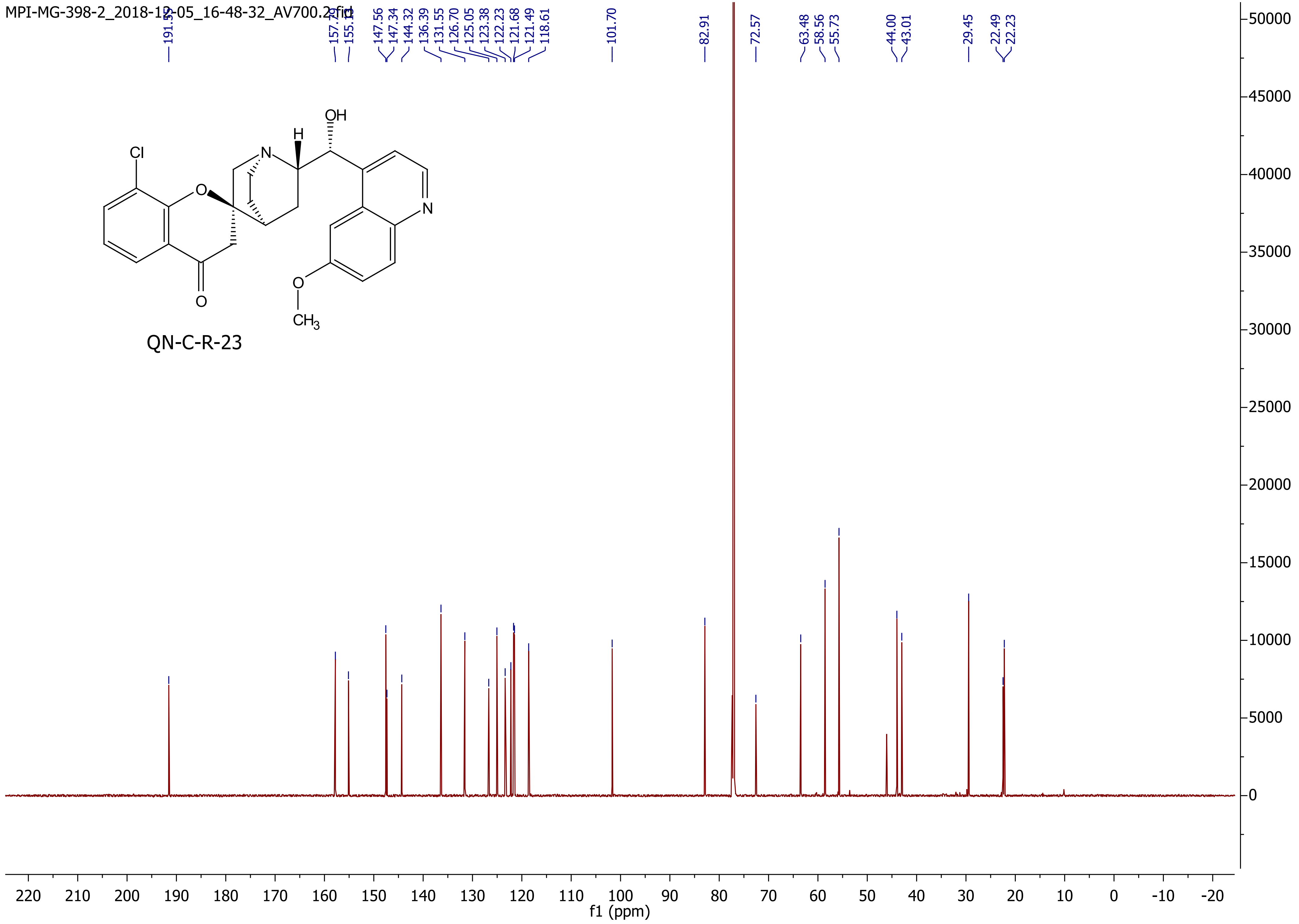

MPI-MG-398-2_2018-12-05_16-48-32_AV700.2.fid
191.55
157.79
155.13
147.56
147.34
144.32
136.39
131.55
126.70
125.05
123.38
122.23
121.68
121.49
118.61
101.70
82.91
72.57
63.48
58.56
55.73
44.00
43.01
29.45
22.49
22.23
Cl
O
N
H
OH
N
O
O
CH3
QN-C-R-23
50000
45000
40000
35000
30000
25000
20000
15000
10000
5000
0
220 210 200 190 180 170 160 150 140 130 120 110 100 90 80 70 60 50 40 30 20 10 0 -10 -20
f1 (ppm)

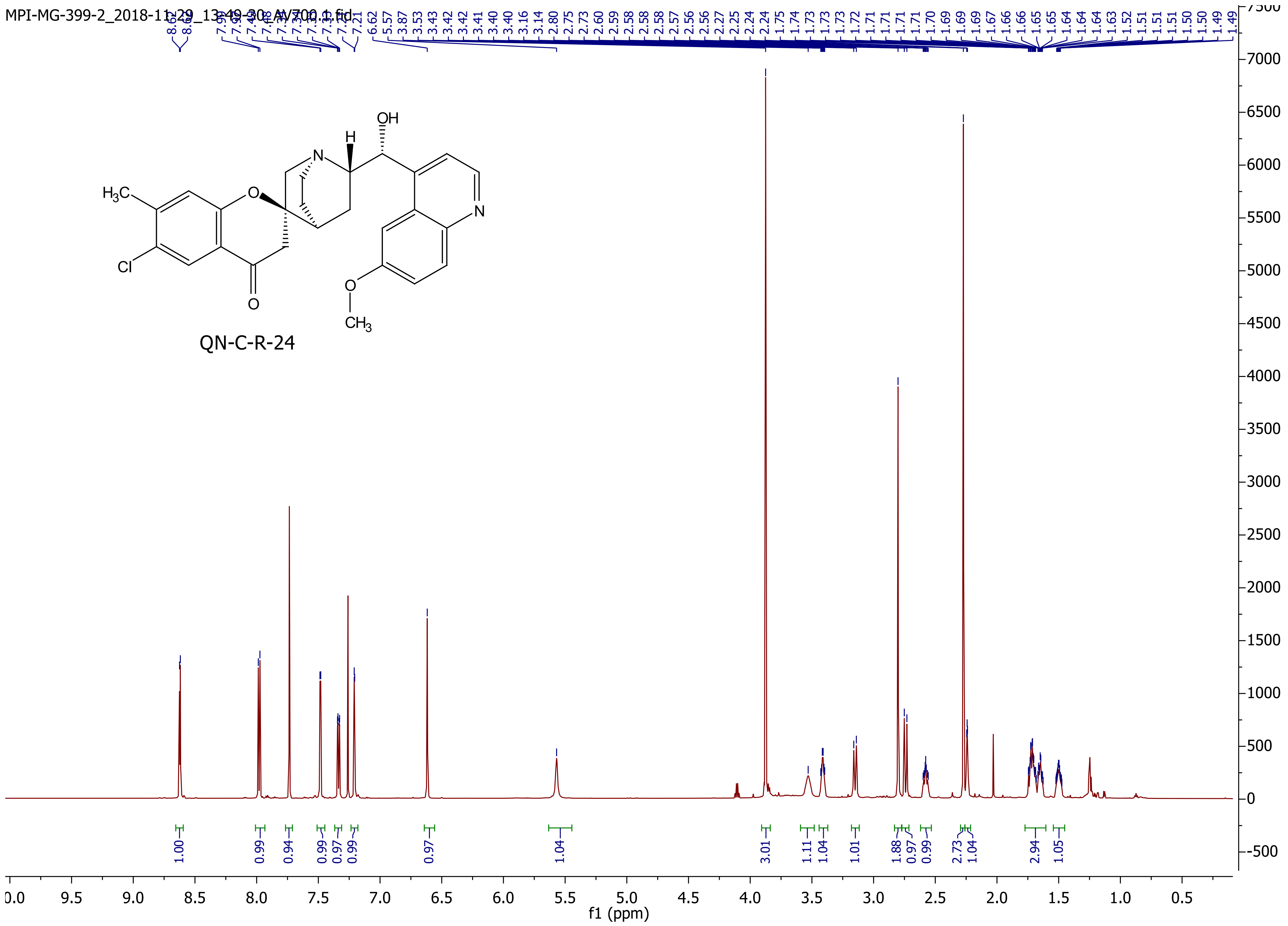

MPI-MG-399-2_2018-11-29_13-49-30_AV700.1.fid
QN-C-R-24
OH
H
N
O
H3C
Cl
O
N
O
CH3
1.00
0.99
0.94
0.99
0.97
0.99
0.97
1.04
3.01
1.11
1.04
1.01
1.88
0.97
0.99
2.73
1.04
2.94
1.05
f1 (ppm)

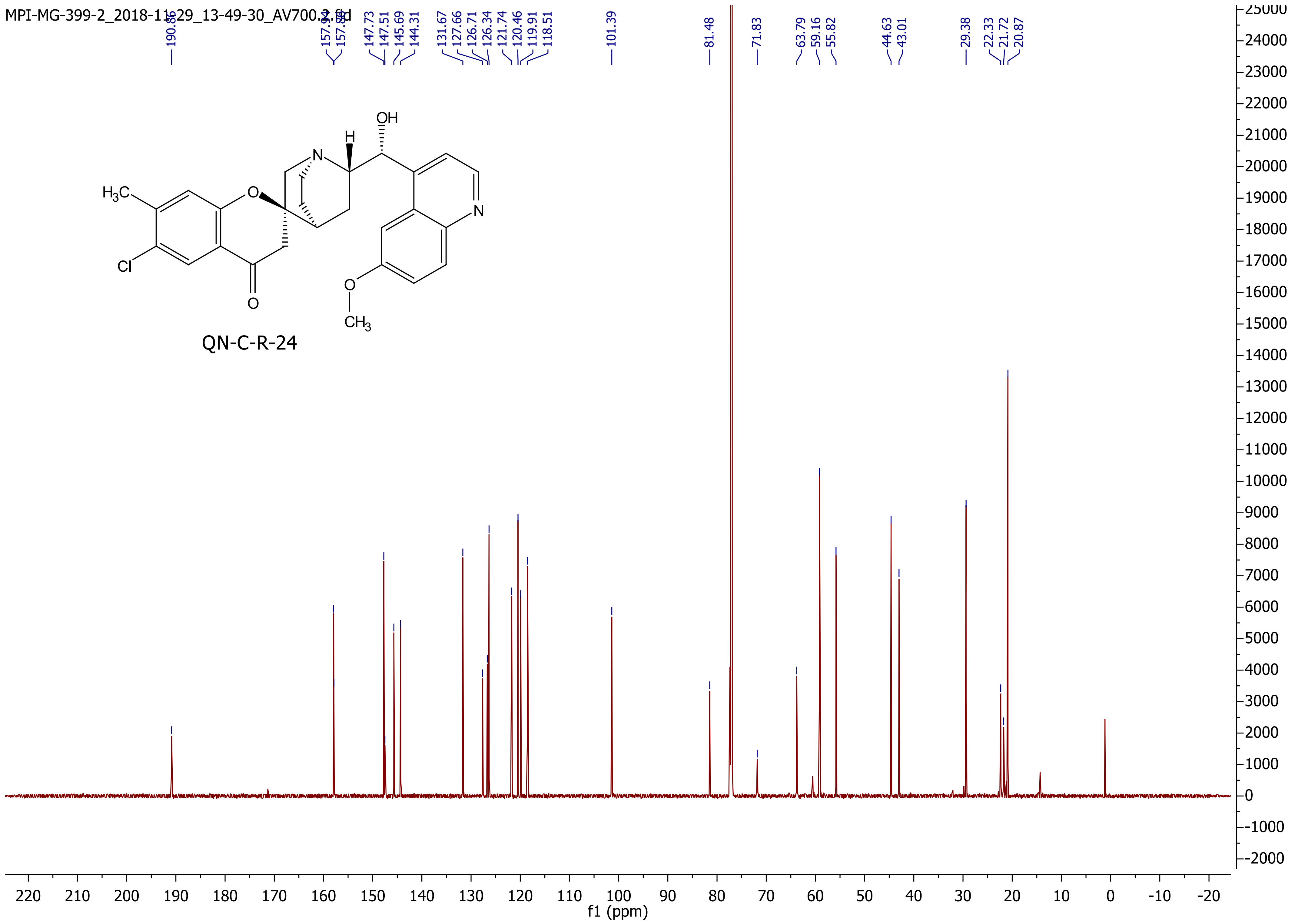
MPI-MG-399-2_2018-11-29_13-49-30_AV700
147.73
147.51
145.69
144.31
131.67
127.66
126.71
126.34
121.74
120.46
119.91
118.51
101.39
81.48
71.83
63.79
59.16
55.82
44.63
43.01
29.38
22.33
21.72
20.87
OH
H
N
O
H_3C
Cl
O
N
O
CH_3
QN-C-R-24
f1 (ppm)

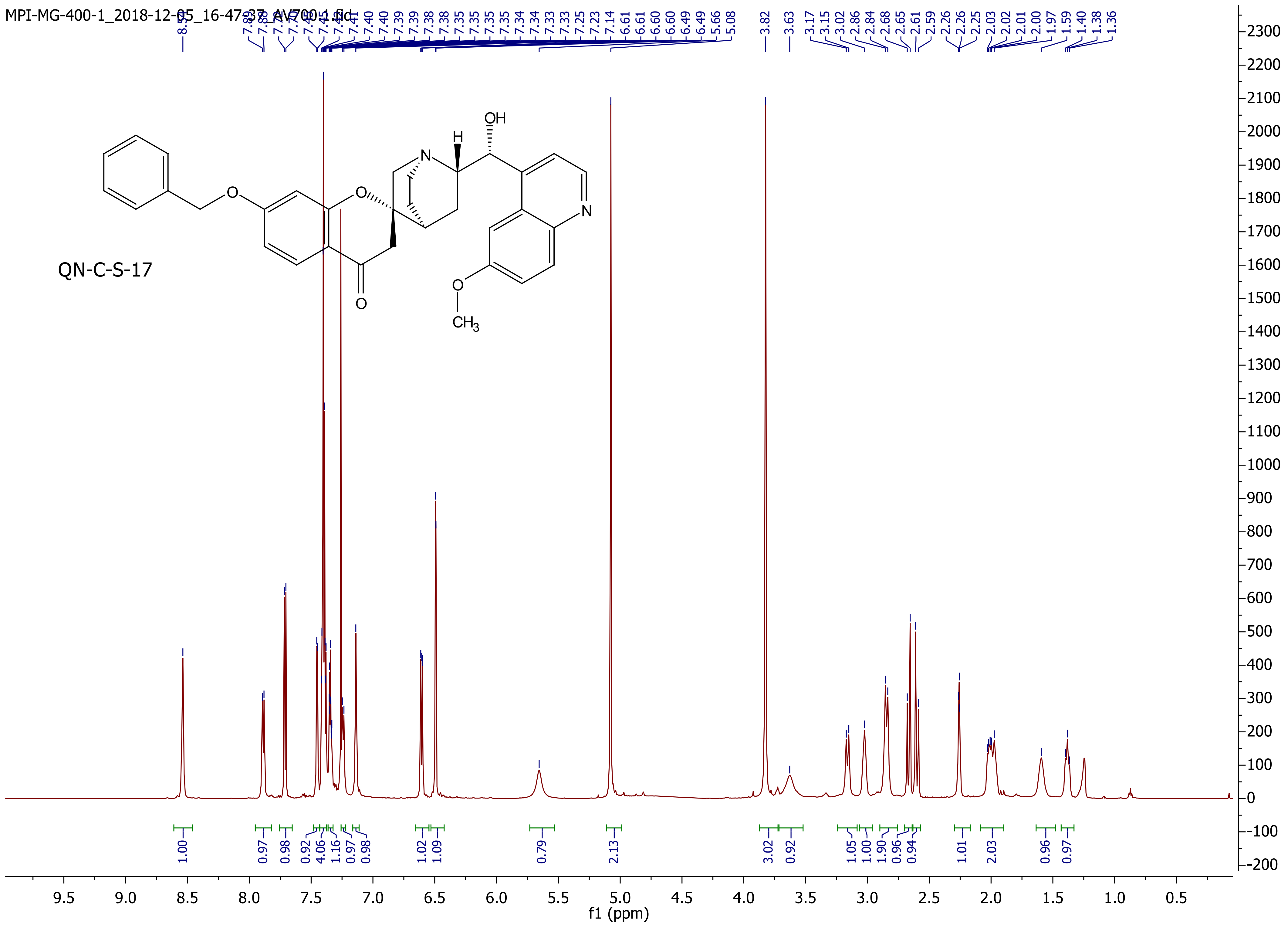

MPI-MG-400-1_2018-12-05_16-47-37_AV700-1.fid
QN-C-S-17
OH
H
N
O
O
N
O
CH3
7.41
7.40
7.40
7.39
7.39
7.38
7.38
7.35
7.35
7.35
7.35
7.34
7.34
7.33
7.33
7.25
7.23
7.14
6.61
6.61
6.60
6.60
6.49
6.49
5.66
5.08
3.82
3.63
3.17
3.15
3.02
2.86
2.84
2.68
2.65
2.61
2.59
2.26
2.26
2.25
2.03
2.02
2.01
2.00
1.97
1.59
1.40
1.38
1.36
1.00
0.97
0.98
0.92
4.06
1.16
0.97
0.98
1.02
1.09
0.79
2.13
3.02
0.92
1.05
1.00
1.90
0.96
0.94
1.01
2.03
0.96
0.97
f1 (ppm)

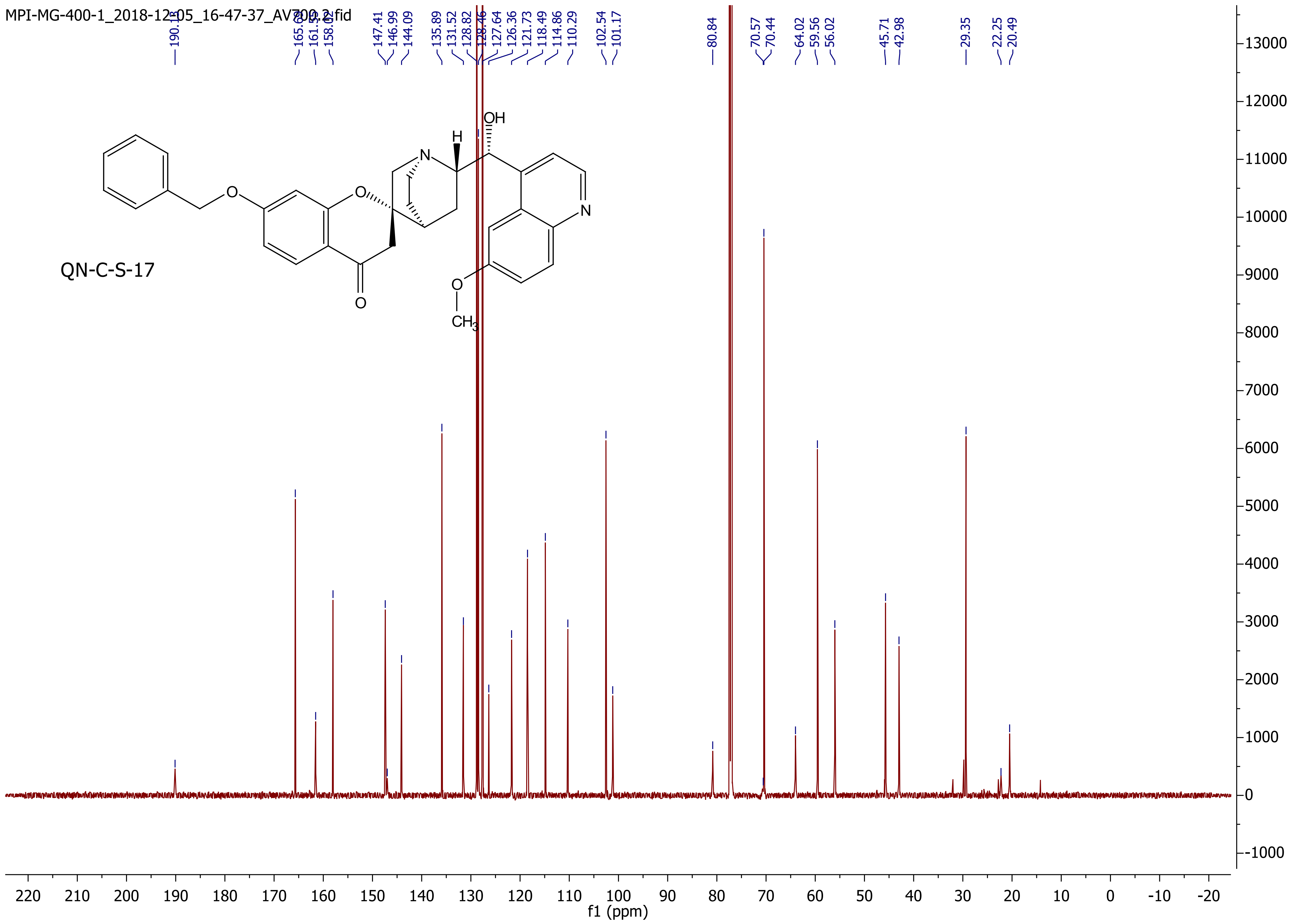

MPI-MG-400-1_2018-12-05_16-47-37_AV700.2.fid
158.02
147.41
146.99
144.09
135.89
131.52
128.82
128.46
127.64
126.36
121.73
118.49
114.86
110.29
102.54
101.17
80.84
70.57
70.44
64.02
59.56
56.02
45.71
42.98
29.35
22.25
20.49
QN-C-S-17
OH
H
N
O
O
O
N
O
CH3
f1 (ppm)

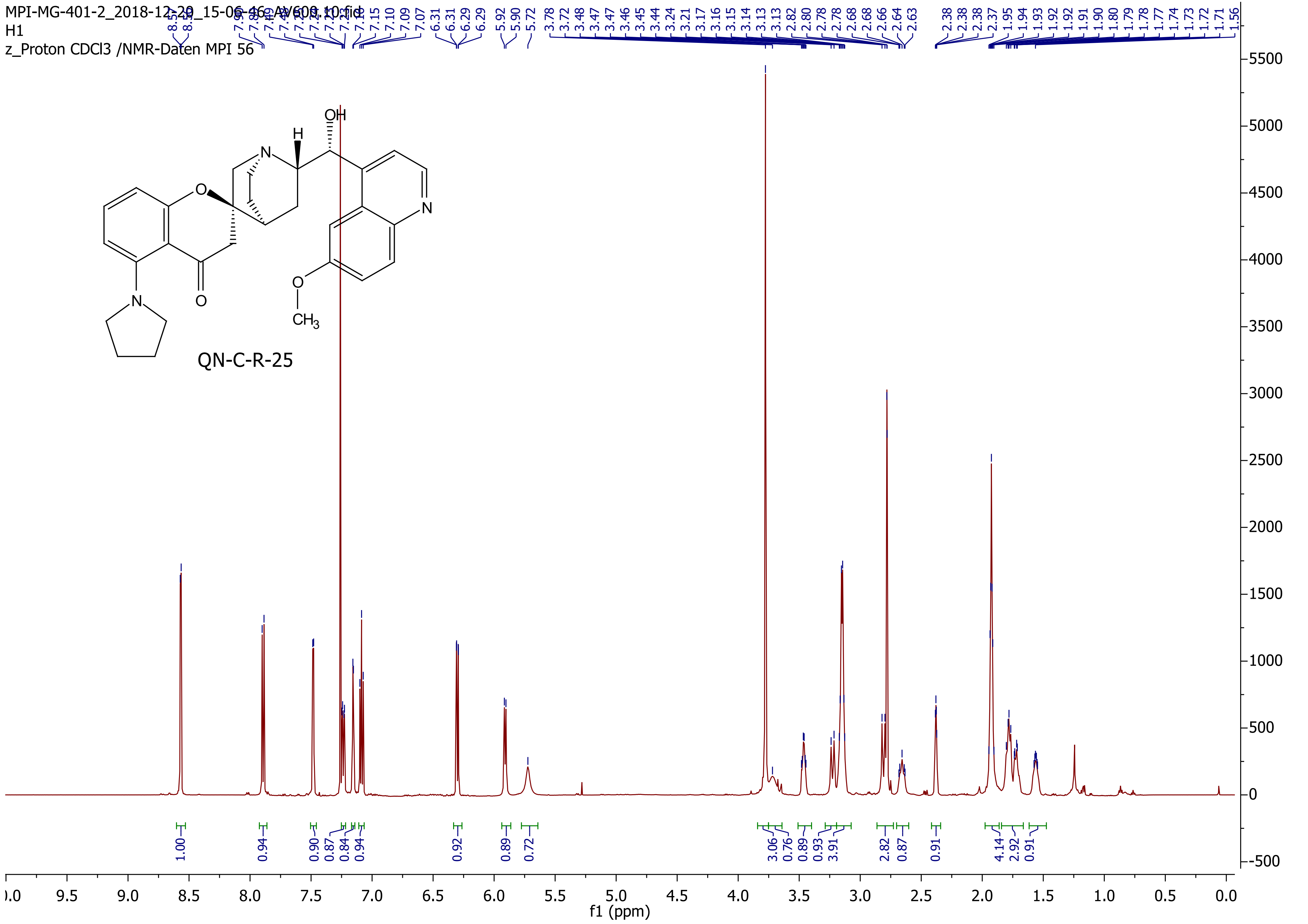
MPI-MG-401-2_2018-12-29_15-06-46_AV600.10.fid
H1
z_Proton CDCl3 /NMR-Daten MPI 56
QN-C-R-25
f1 (ppm)

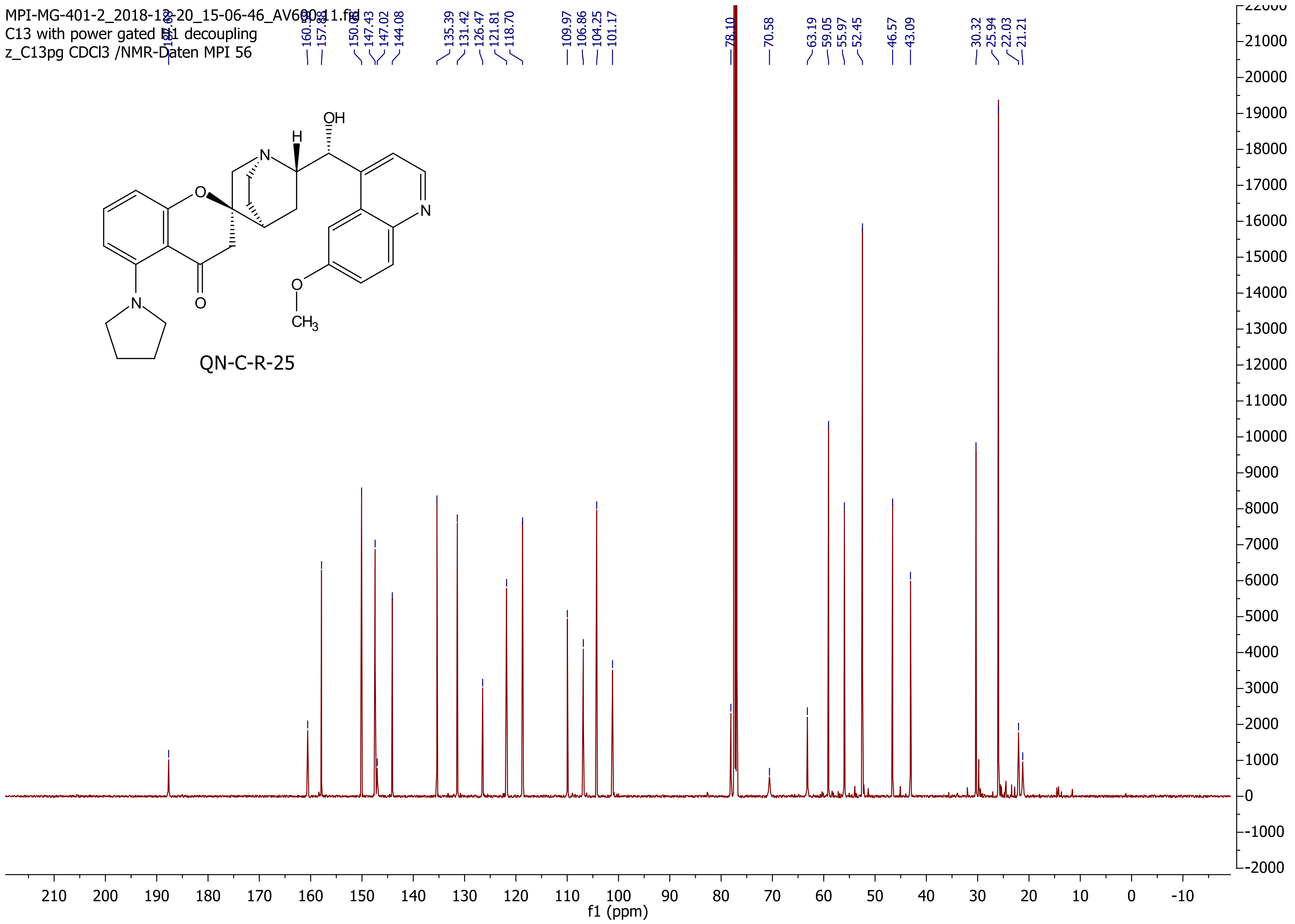

MPI-MG-401-2_2018-12-20_15-06-46_AV600-11.fid
C13 with power gated H1 decoupling
z_C13pg CDCl3 /NMR-Daten MPI 56
187.68
160.58
157.88
150.08
147.43
147.02
144.08
135.39
131.42
126.47
121.81
118.70
109.97
106.86
104.25
101.17
78.10
70.58
63.19
59.05
55.97
52.45
46.57
43.09
30.32
25.94
22.03
21.21
OH
H
N
O
N
O
N
O
CH3
QN-C-R-25
f1 (ppm)

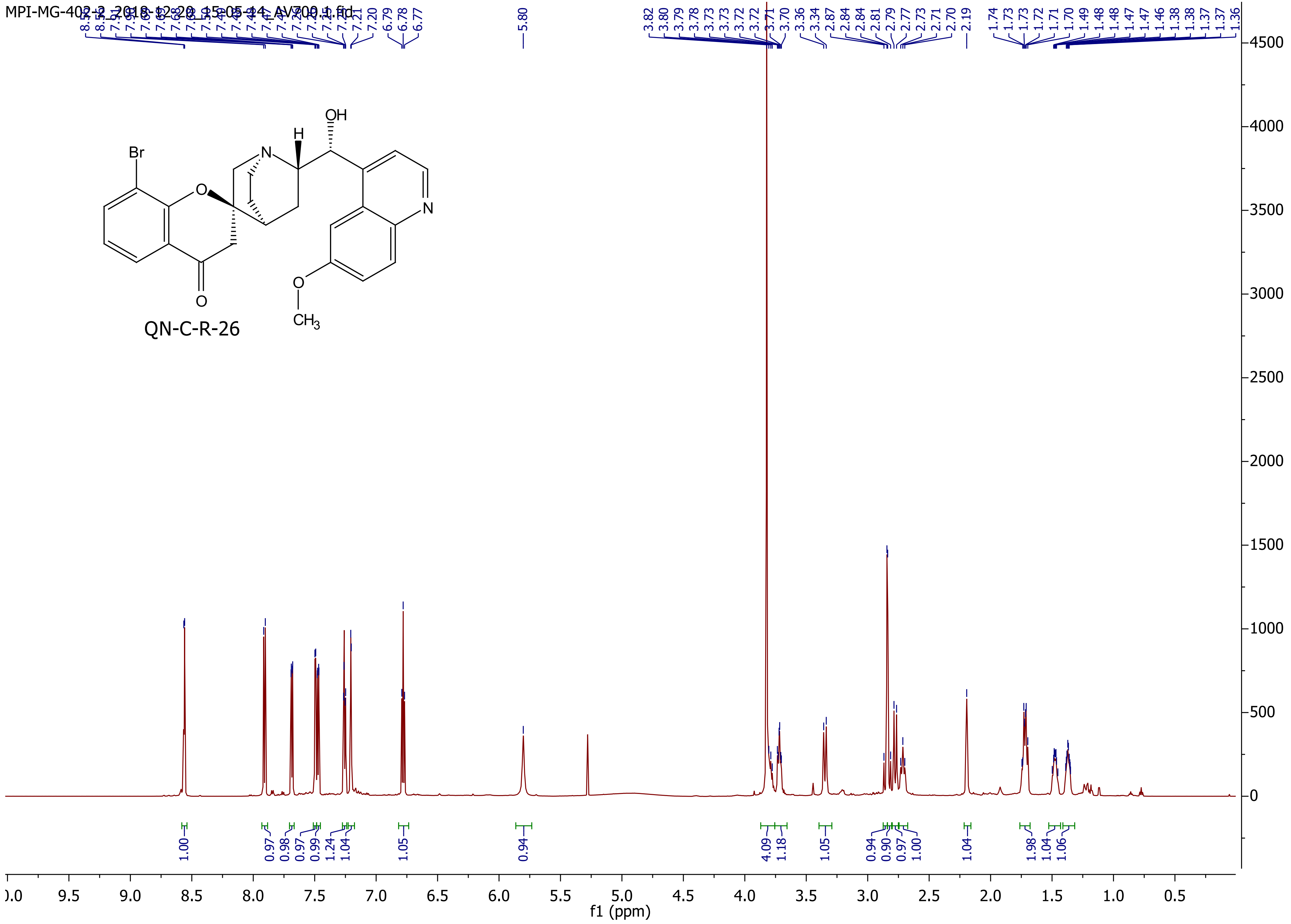

MPI-MG-402-2_2018-12-20_15-05-44_AV700.1.fid
QN-C-R-26
Br
O
N
H
OH
N
O
CH3
f1 (ppm)

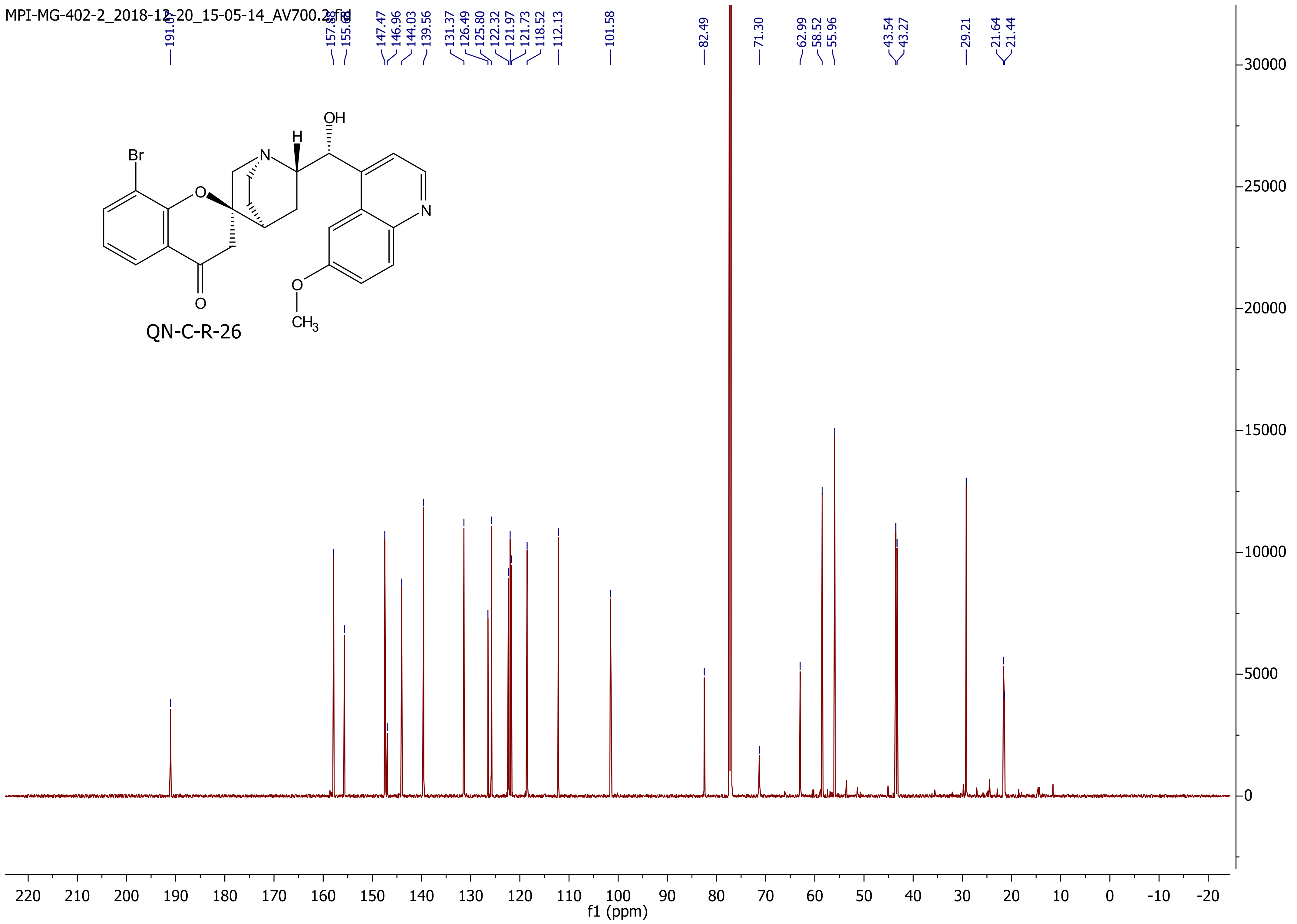

MPI-MG-402-2_2018-12-20_15-05-14_AV700.2.fid
147.47
146.96
144.03
139.56
131.37
126.49
125.80
122.32
121.97
121.73
118.52
112.13
101.58
82.49
71.30
62.99
58.52
55.96
43.54
43.27
29.21
21.64
21.44
Br
O
OH
H
N
N
O
O
CH3
QN-C-R-26
30000
25000
20000
15000
10000
5000
0
220 210 200 190 180 170 160 150 140 130 120 110 100 90 80 70 60 50 40 30 20 10 0 -10 -20
f1 (ppm)

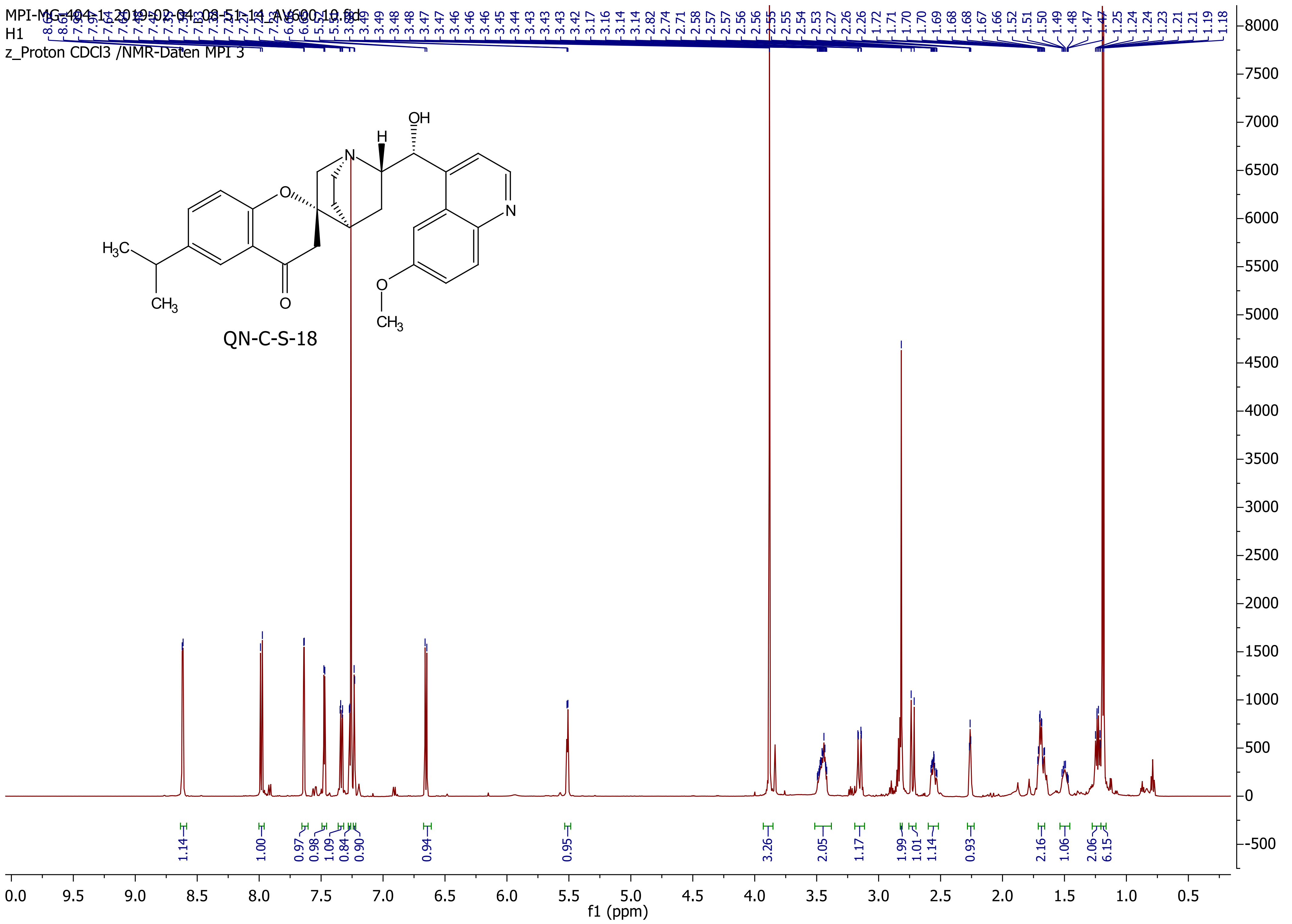
MPI-MG-404-1 2019-02-04 08-51-14 AV600 10.fid
H1
z_Proton CDCl3 /NMR-Daten MPI 3
OH
H
N
O
N
H3C
CH3
O
O
CH3
QN-C-S-18
1.14
1.00
0.97
0.98
1.09
0.84
0.90
0.94
0.95
3.26
2.05
1.17
1.99
1.01
1.14
0.93
2.16
1.06
2.06
6.15
f1 (ppm)

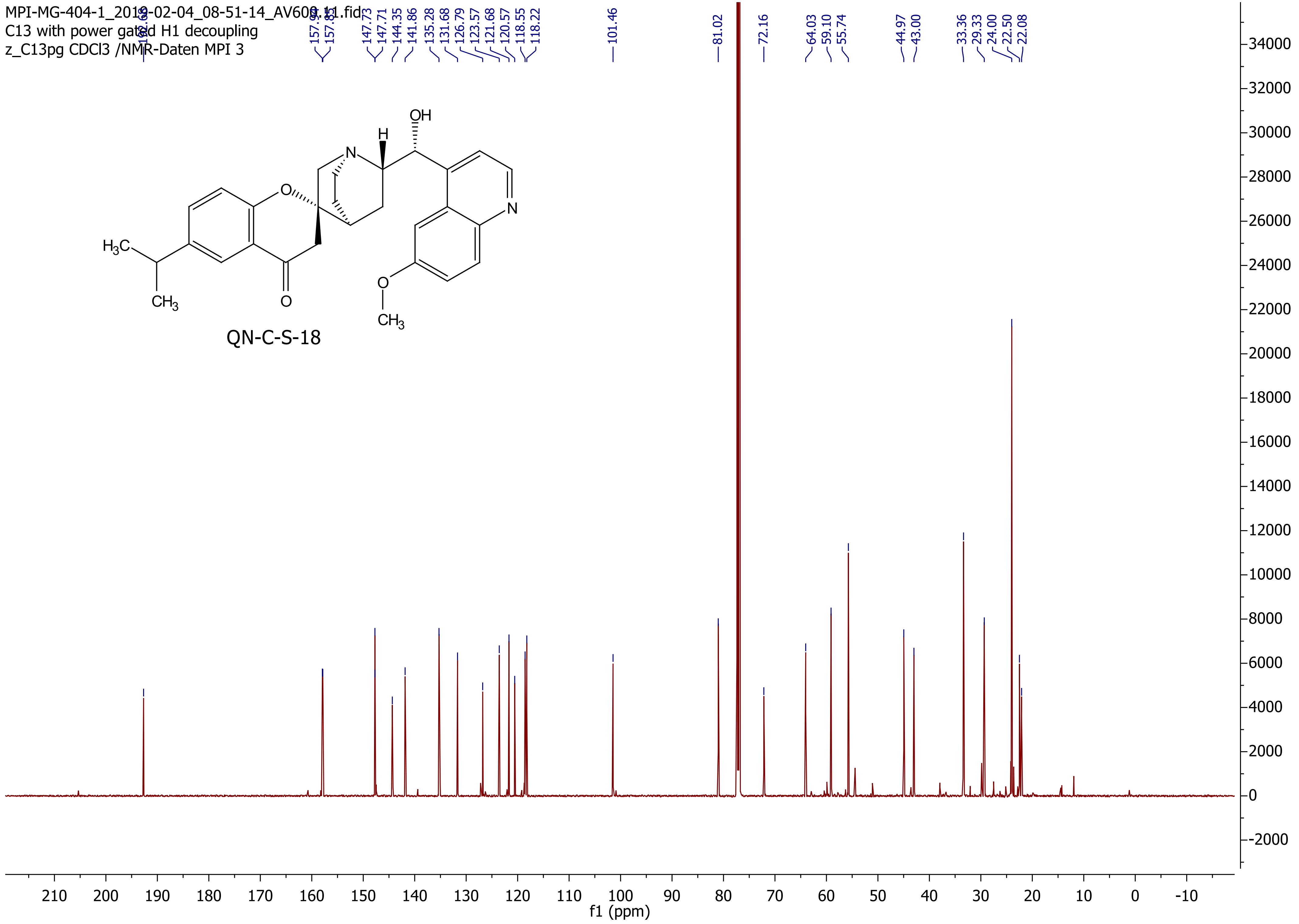
MPI-MG-404-1_2019-02-04_08-51-14_AV600.11.fid
C13 with power gated H1 decoupling
z_C13pg CDCl3 /NMR-Daten MPI 3
157.85
147.73
147.71
144.35
141.86
135.28
131.68
126.79
123.57
121.68
120.57
118.55
118.22
101.46
81.02
72.16
64.03
59.10
55.74
44.97
43.00
33.36
29.33
24.00
22.50
22.08
OH
H
N
O
N
H3C
CH3
O
O
CH3
QN-C-S-18
f1 (ppm)

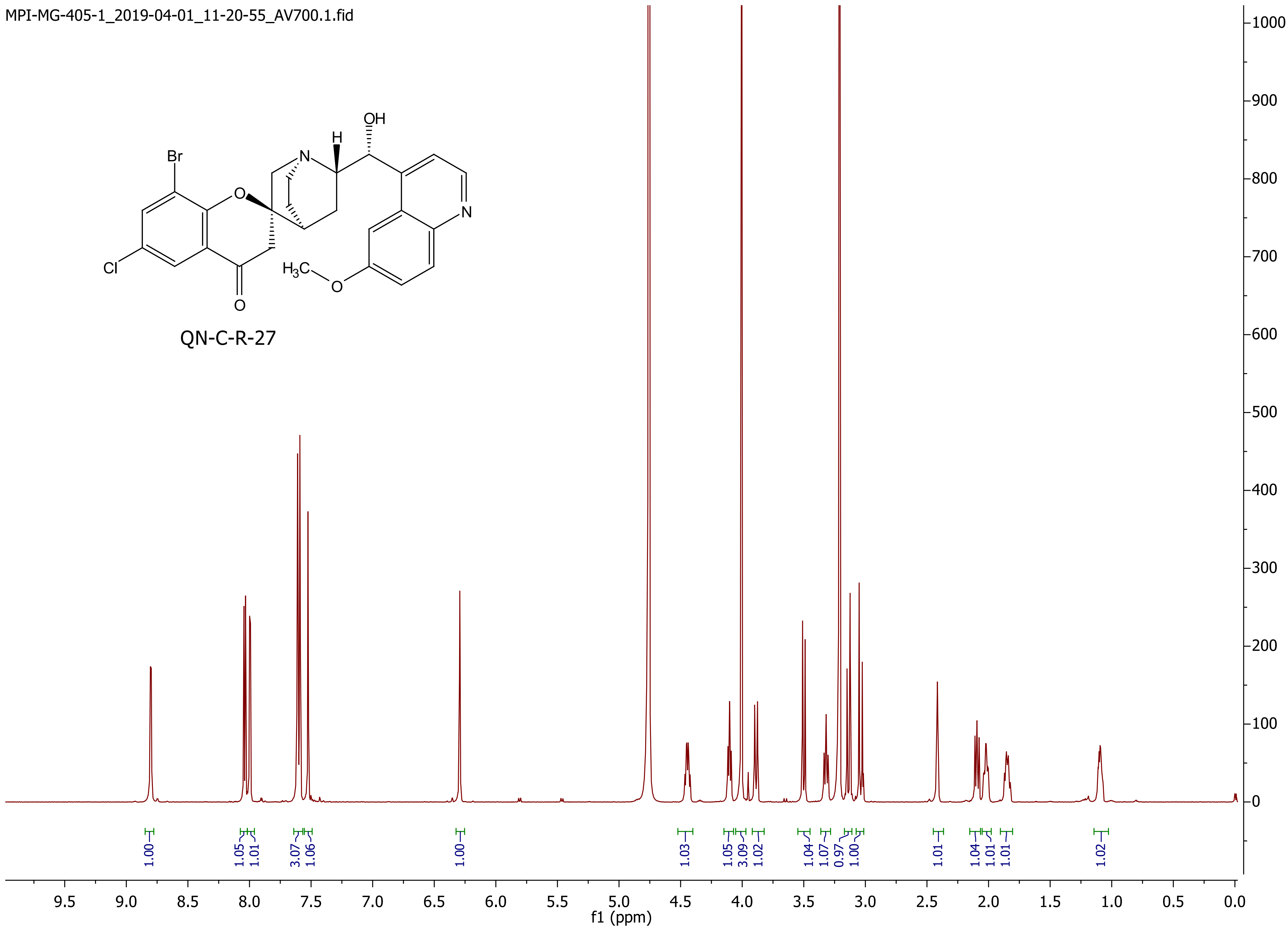
MPI-MG-405-1_2019-04-01_11-20-55_AV700.1.fid
QN-C-R-27
f1 (ppm)
1.00
1.05
1.01
3.07
1.06
1.00
1.03
1.05
3.09
1.02
1.04
1.07
0.97
1.00
1.01
1.04
1.01
1.01
1.02

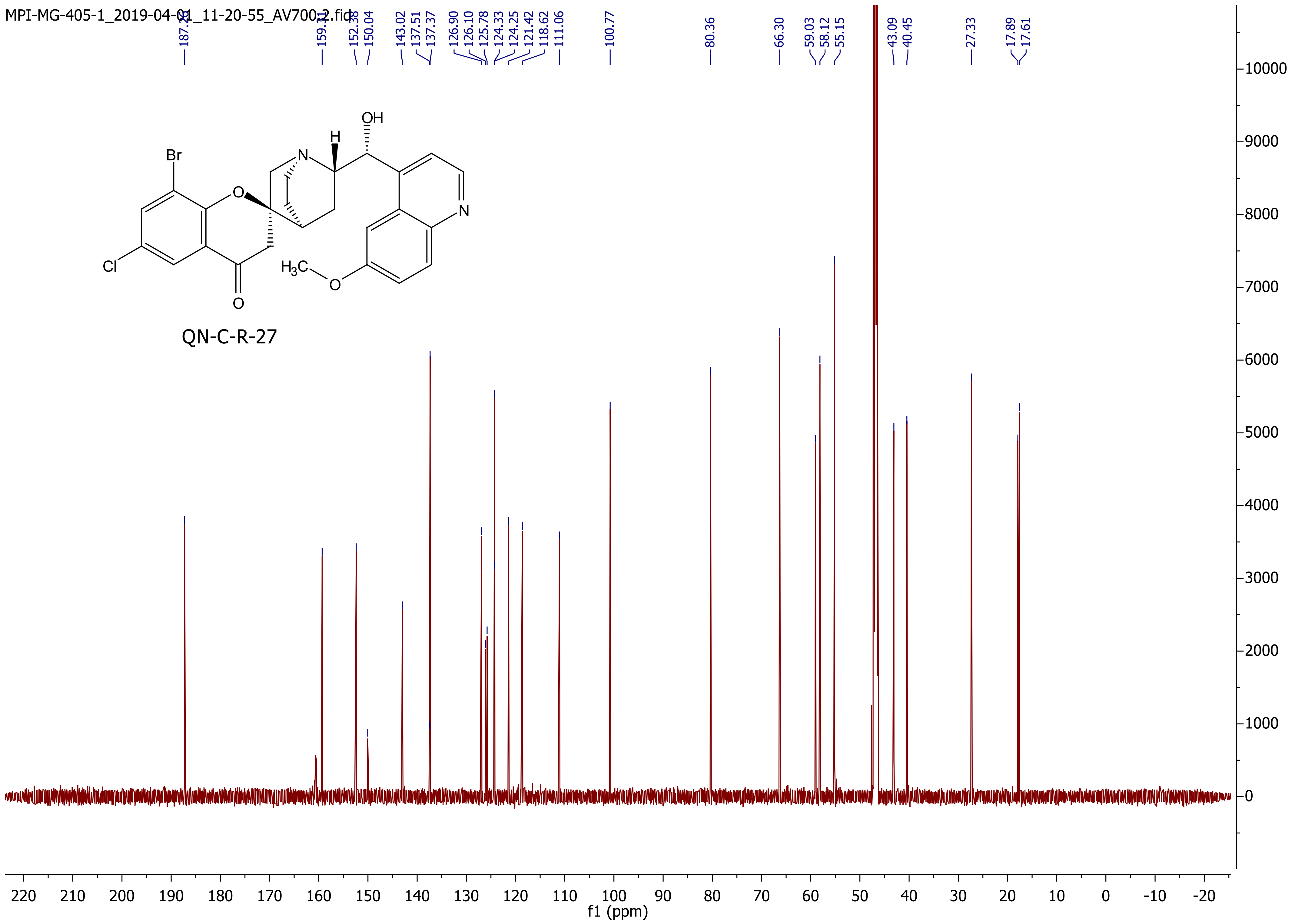

MPI-MG-405-1_2019-04-01_11-20-55_AV700_2.fid
152.38
150.04
143.02
137.51
137.37
126.90
126.10
125.78
124.33
124.25
121.42
118.62
111.06
100.77
80.36
66.30
59.03
58.12
55.15
43.09
40.45
27.33
17.89
17.61
Br
O
N
H
OH
N
Cl
O
H3C
O
QN-C-R-27
f1 (ppm)

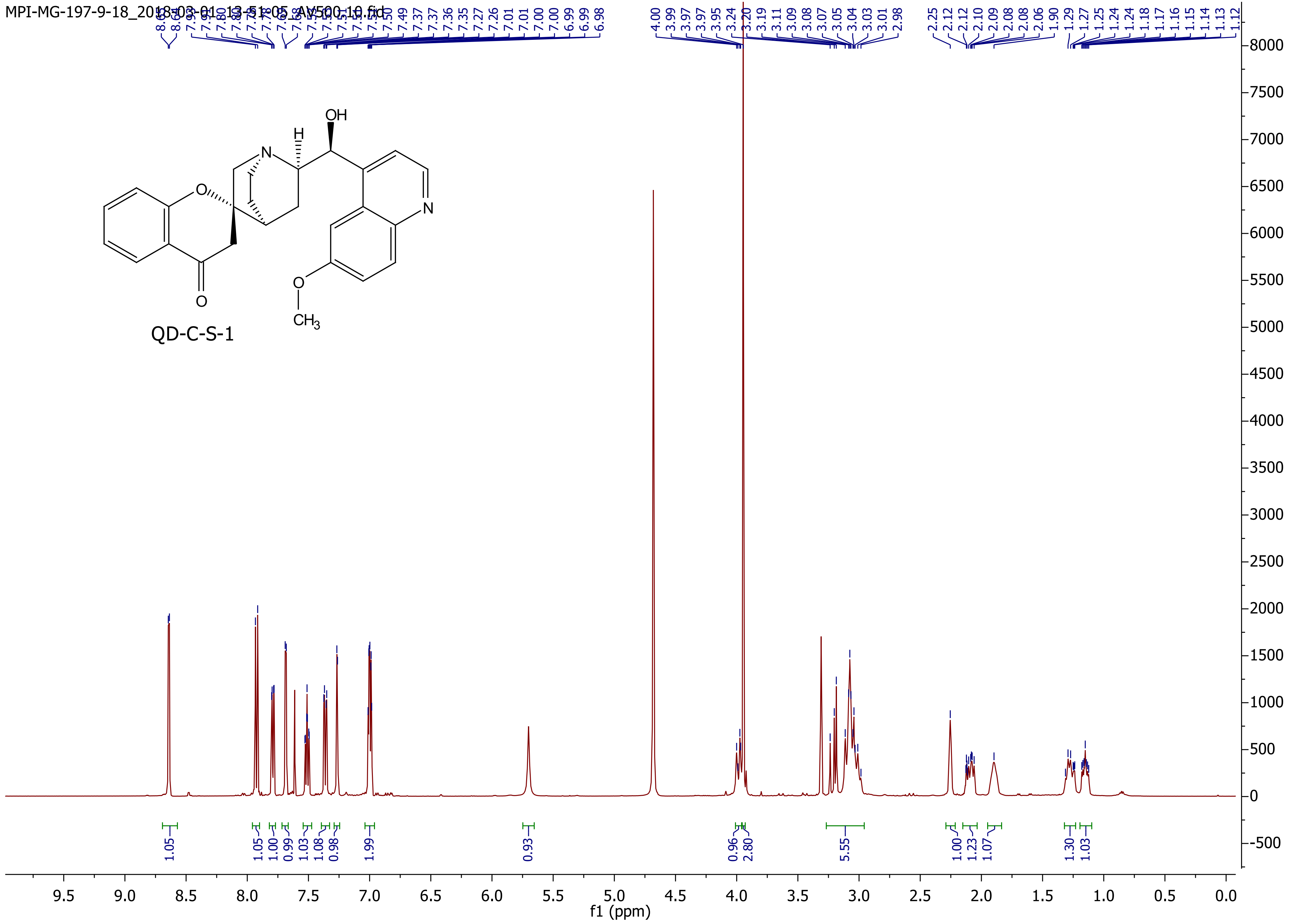
MPI-MG-197-9-18_20
AV500-10.fid
7.50
7.49
7.37
7.37
7.36
7.35
7.27
7.26
7.01
7.01
7.00
7.00
6.99
6.99
6.98
4.00
3.99
3.97
3.97
3.95
3.24
3.20
3.19
3.11
3.09
3.08
3.07
3.05
3.04
3.03
3.01
2.98
2.25
2.12
2.12
2.10
2.09
2.08
2.08
2.06
1.90
1.29
1.27
1.25
1.24
1.24
1.18
1.17
1.16
1.15
1.14
1.13
1.12
OH
H
N
O
N
O
O
CH3
QD-C-S-1
1.05
1.05
1.00
0.99
1.03
1.08
0.98
1.99
0.93
0.96
2.80
5.55
1.00
1.23
1.07
1.30
1.03
f1 (ppm)

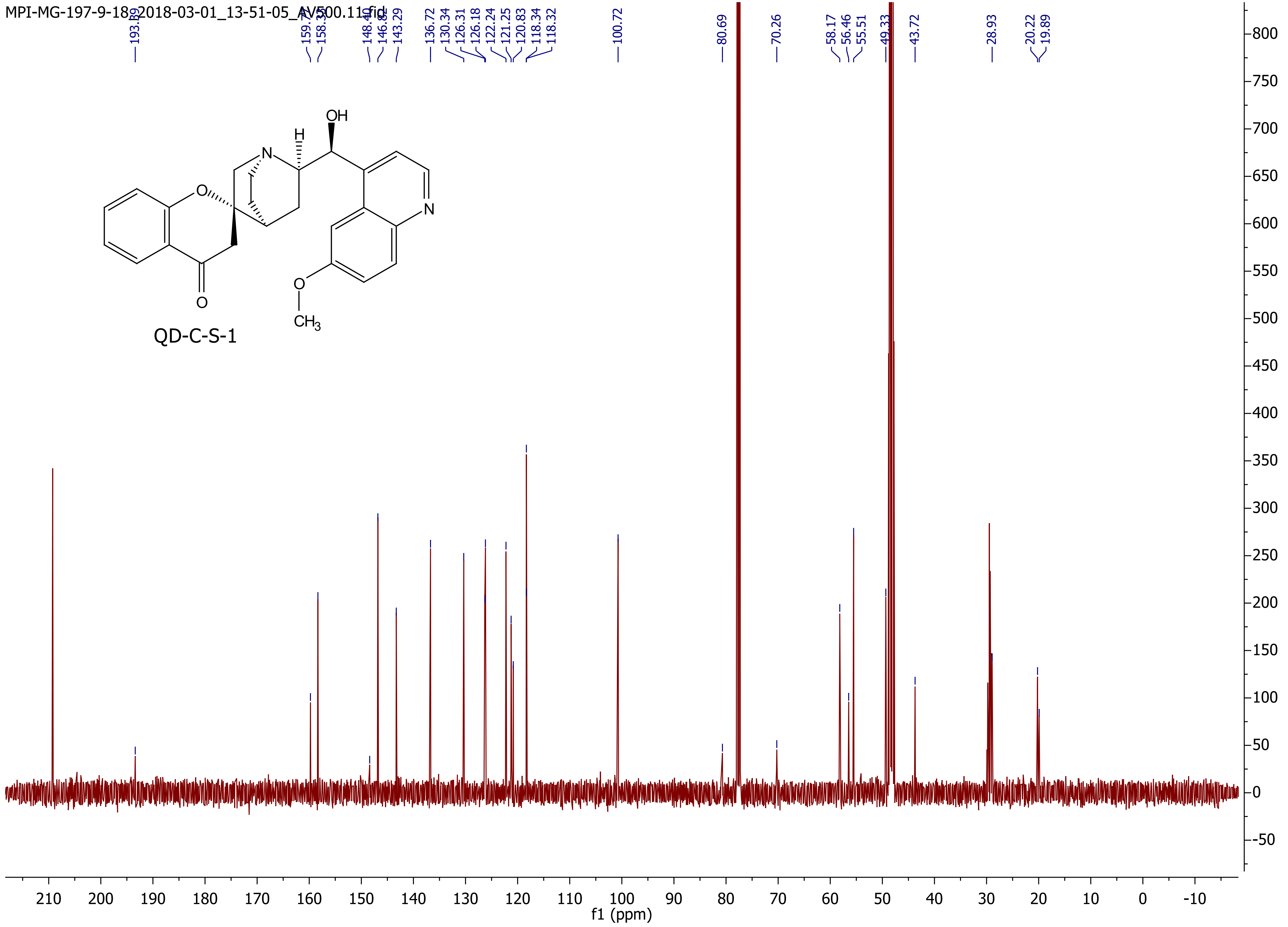

MPI-MG-197-9-18_2018-03-01_13-51-05_AV500.11.fid
143.29
136.72
130.34
126.31
126.18
122.24
121.25
120.83
118.34
118.32
100.72
80.69
70.26
58.17
56.46
55.51
49.33
43.72
28.93
20.22
19.89
OH
H
N
O
N
O
O
CH3
QD-C-S-1
f1 (ppm)

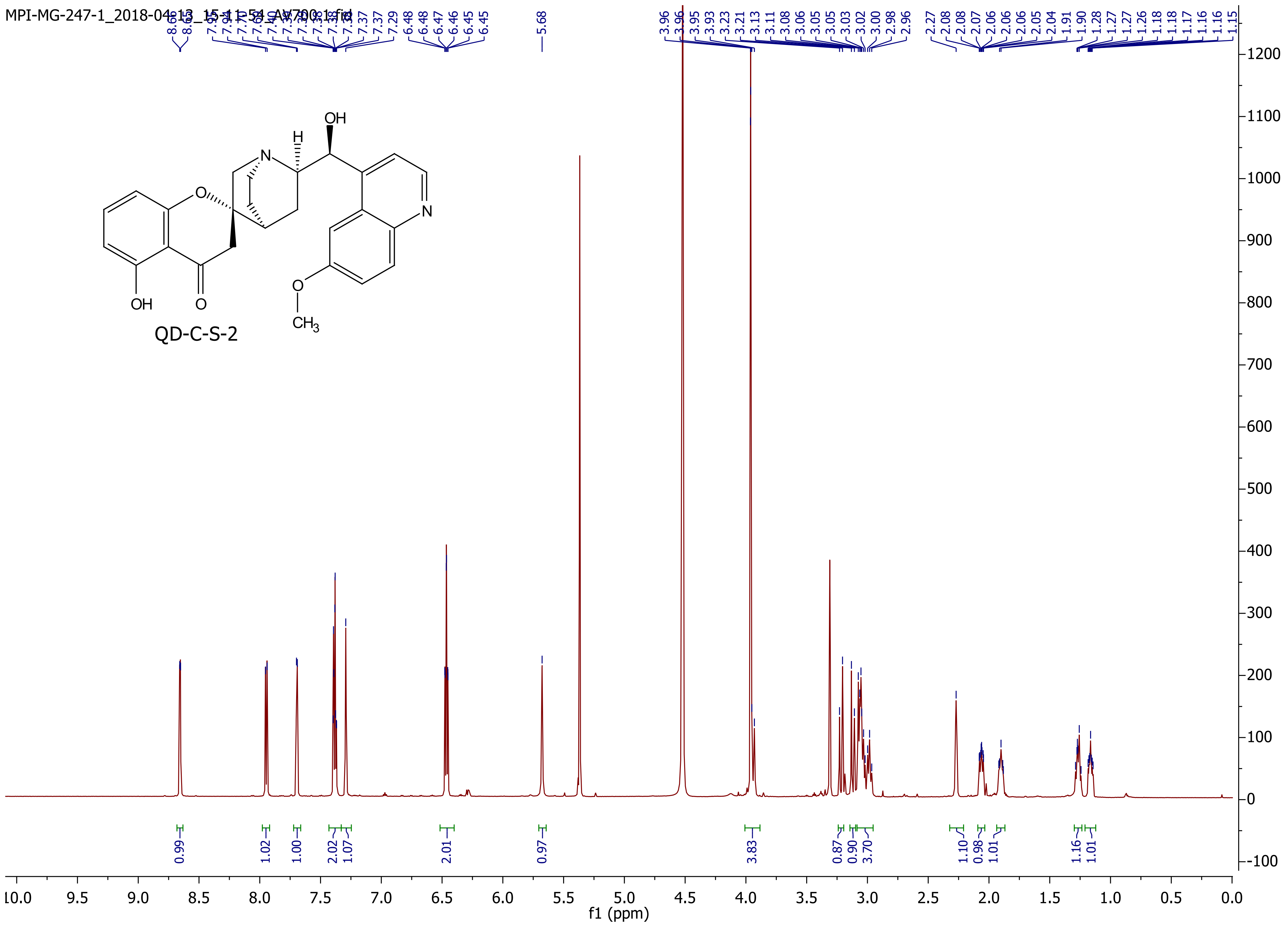

MPI-MG-247-1_2018-04-13_15-11-54_AV700 1.fid
QD-C-S-2
OH
H
N
O
N
OH
O
O
CH3
f1 (ppm)
0.99
1.02
1.00
2.02
1.07
2.01
0.97
3.83
0.87
0.90
3.70
1.10
0.98
1.01
1.16
1.01

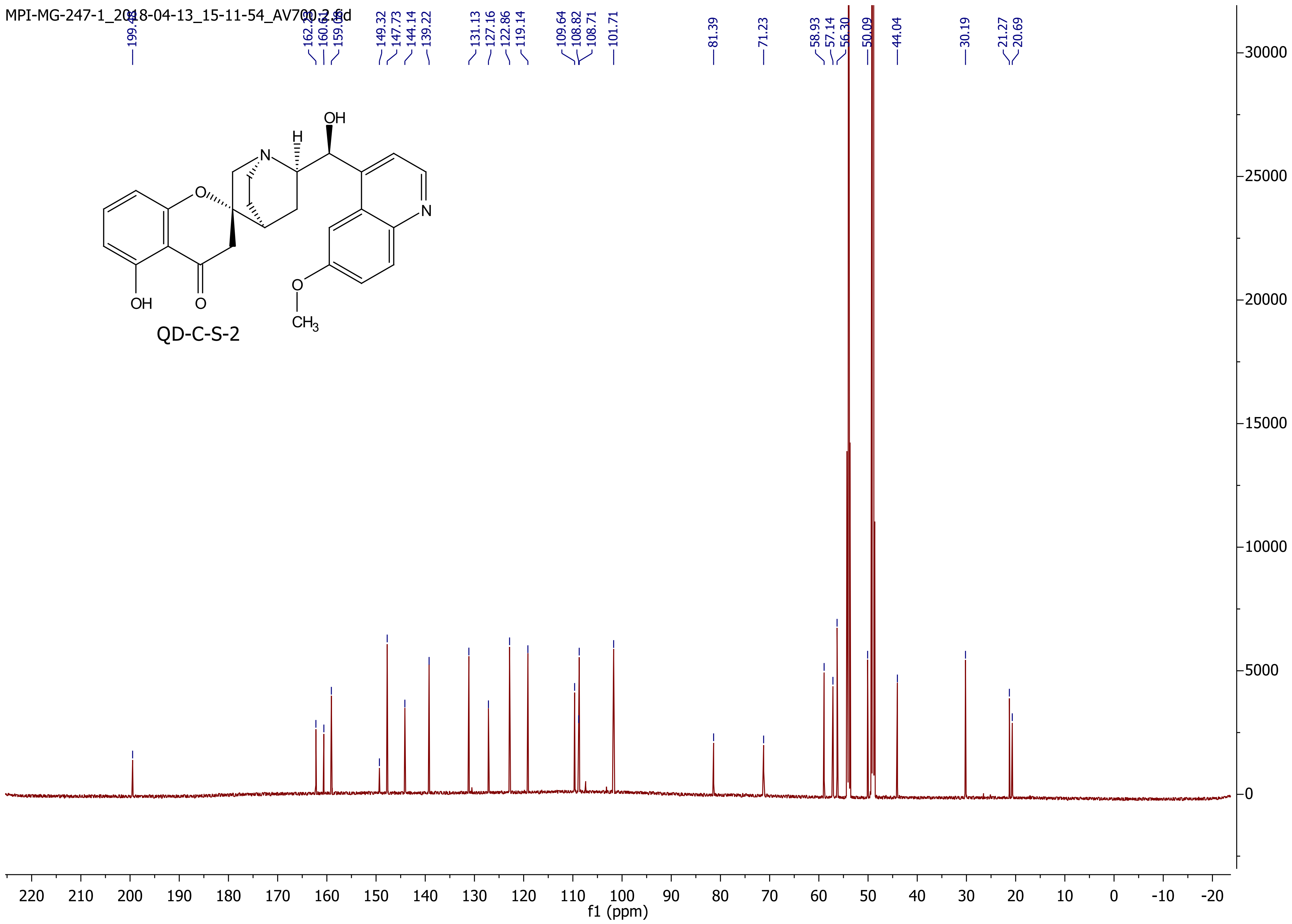

MPI-MG-247-1_2018-04-13_15-11-54_AV700.2.fid
149.32
147.73
144.14
139.22
131.13
127.16
122.86
119.14
109.64
108.82
108.71
101.71
81.39
71.23
58.93
57.14
56.30
50.09
44.04
30.19
21.27
20.69
OH
H
N
O
N
OH
O
O
CH3
QD-C-S-2
30000
25000
20000
15000
10000
5000
0
220 210 200 190 180 170 160 150 140 130 120 110 100 90 80 70 60 50 40 30 20 10 0 -10 -20
f1 (ppm)

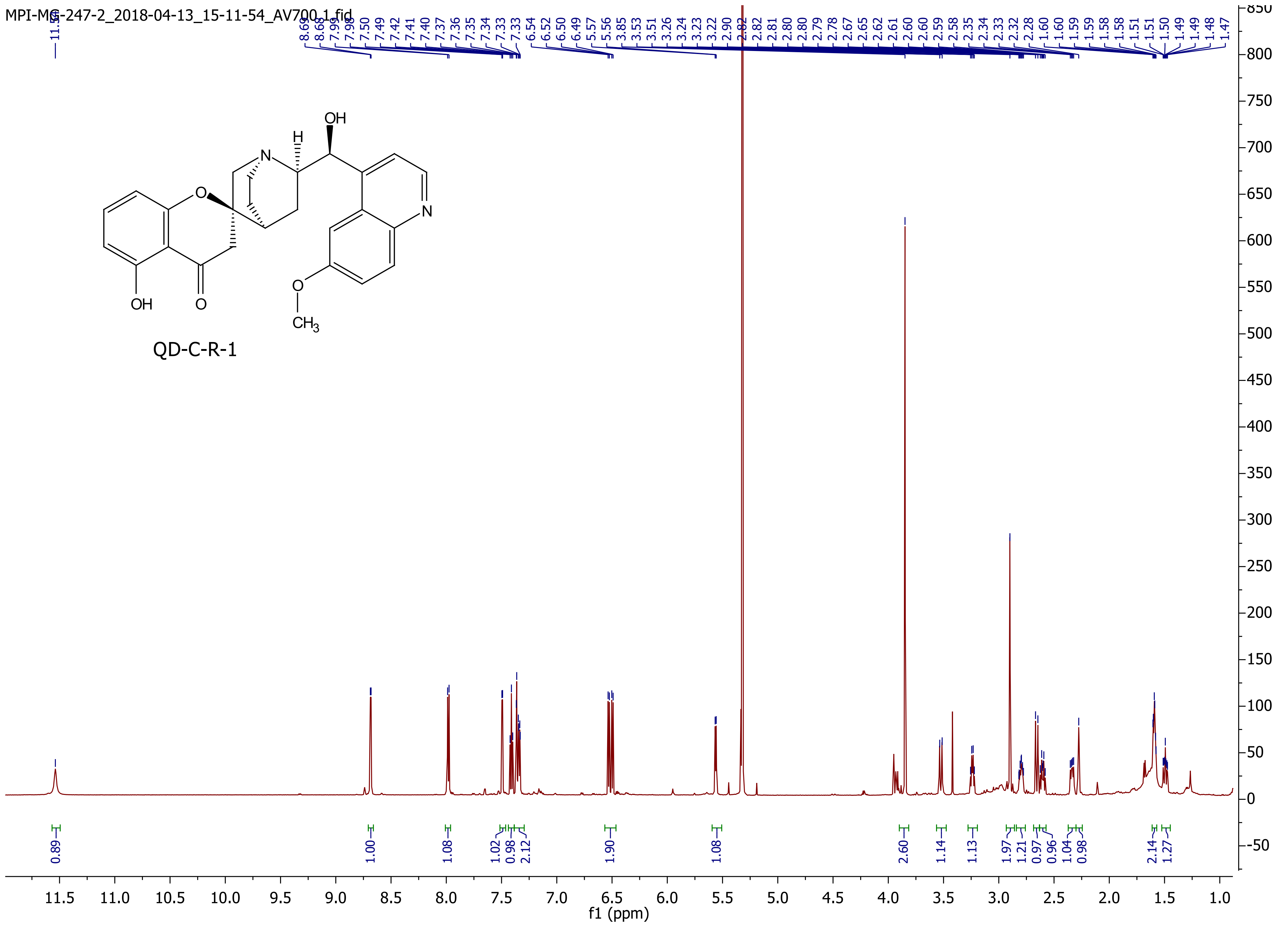
MPI-MG-247-2_2018-04-13_15-11-54_AV700_1.fid
QD-C-R-1
f1 (ppm)

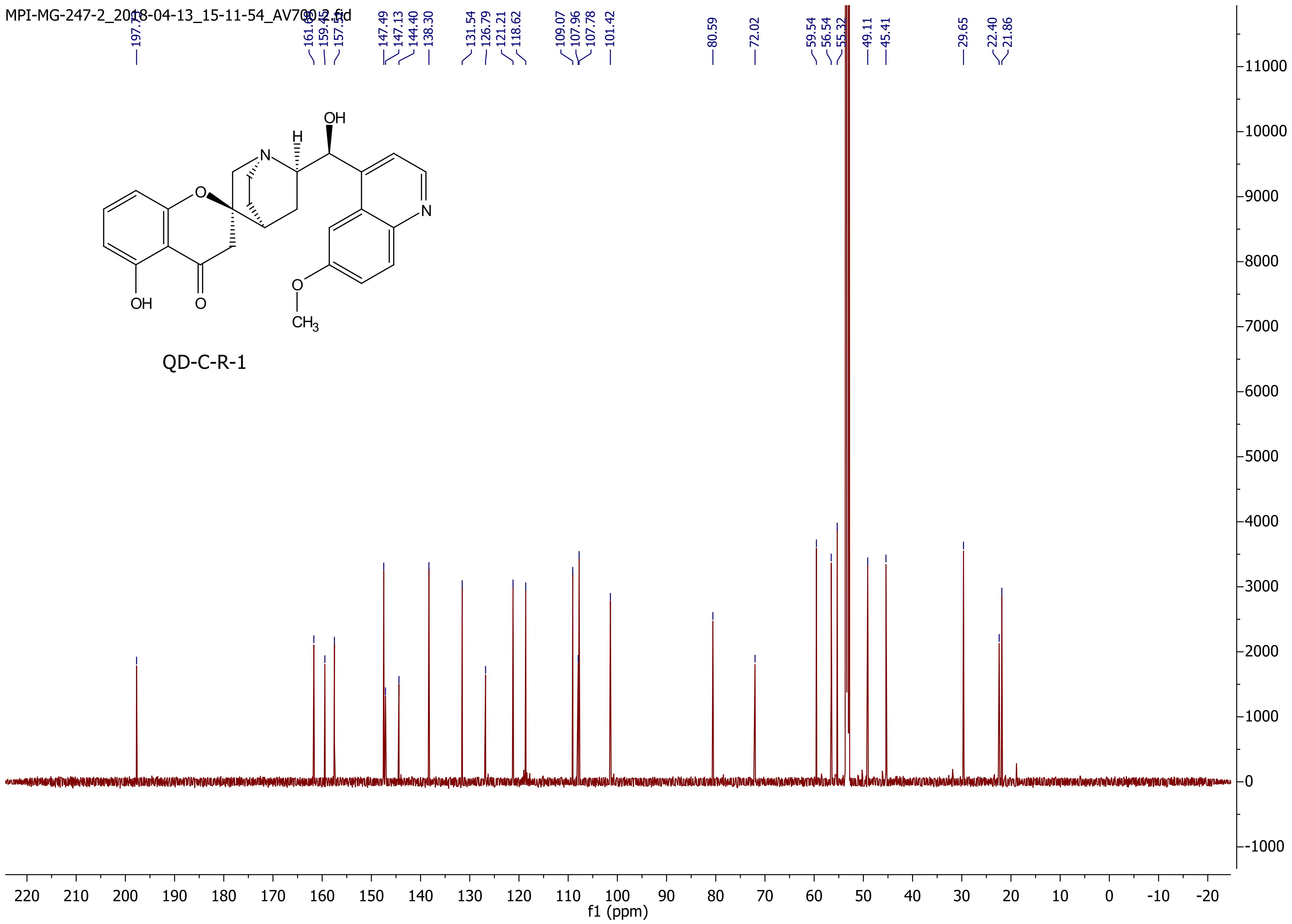

MPI-MG-247-2_2018-04-13_15-11-54_AV700 2.fid
147.49
147.13
144.40
138.30
131.54
126.79
121.21
118.62
109.07
107.96
107.78
101.42
80.59
72.02
59.54
56.54
55.32
49.11
45.41
29.65
22.40
21.86
OH
H
N
O
N
OH
O
O
CH3
QD-C-R-1
11000
10000
9000
8000
7000
6000
5000
4000
3000
2000
1000
0
-1000
220 210 200 190 180 170 160 150 140 130 120 110 100 90 80 70 60 50 40 30 20 10 0 -10 -20
f1 (ppm)

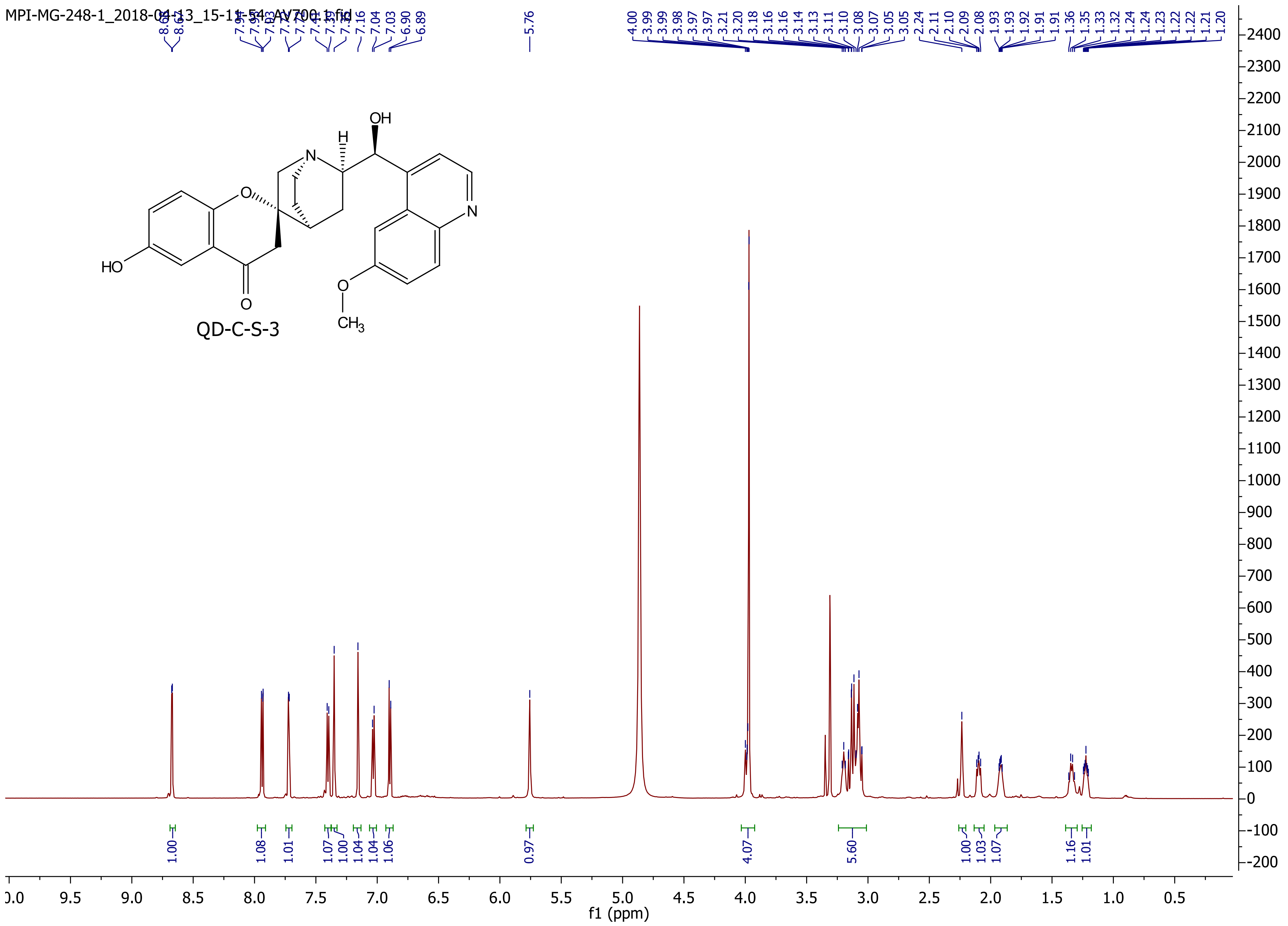
MPI-MG-248-1_2018-04-13_15-11-54_AV700 1.fid
QD-C-S-3
f1 (ppm)

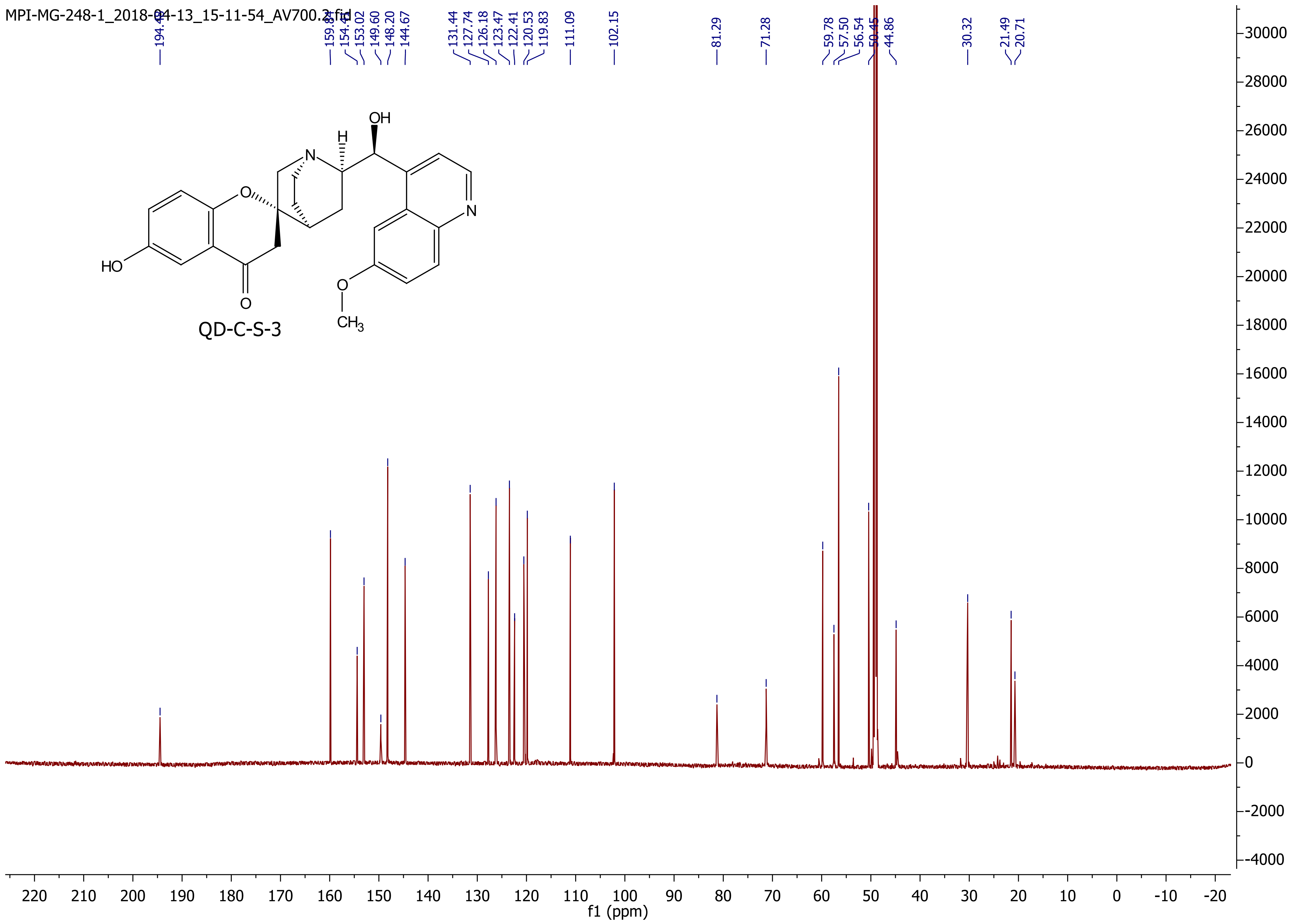

MPI-MG-248-1_2018-04-13_15-11-54_AV700.2.fid
153.02
149.60
148.20
144.67
131.44
127.74
126.18
123.47
122.41
120.53
119.83
111.09
102.15
81.29
71.28
59.78
57.50
56.54
44.86
30.32
21.49
20.71
OH
H
N
O
HO
O
N
O
CH3
QD-C-S-3
f1 (ppm)

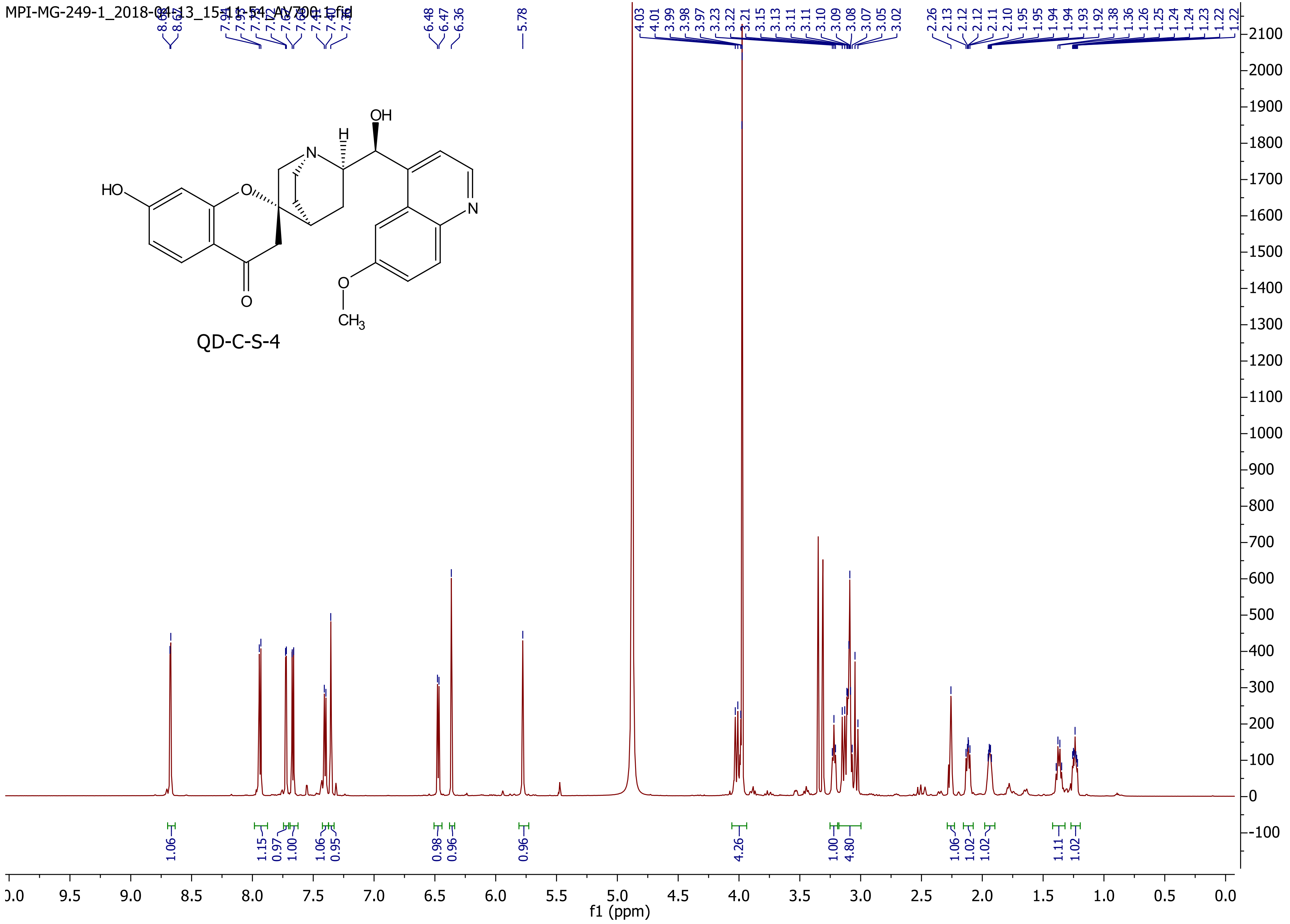

MPI-MG-249-1_2018-04-13_15-11-54_AV700 1.fid
HO
O
N
H
OH
N
O
CH3
QD-C-S-4
f1 (ppm)

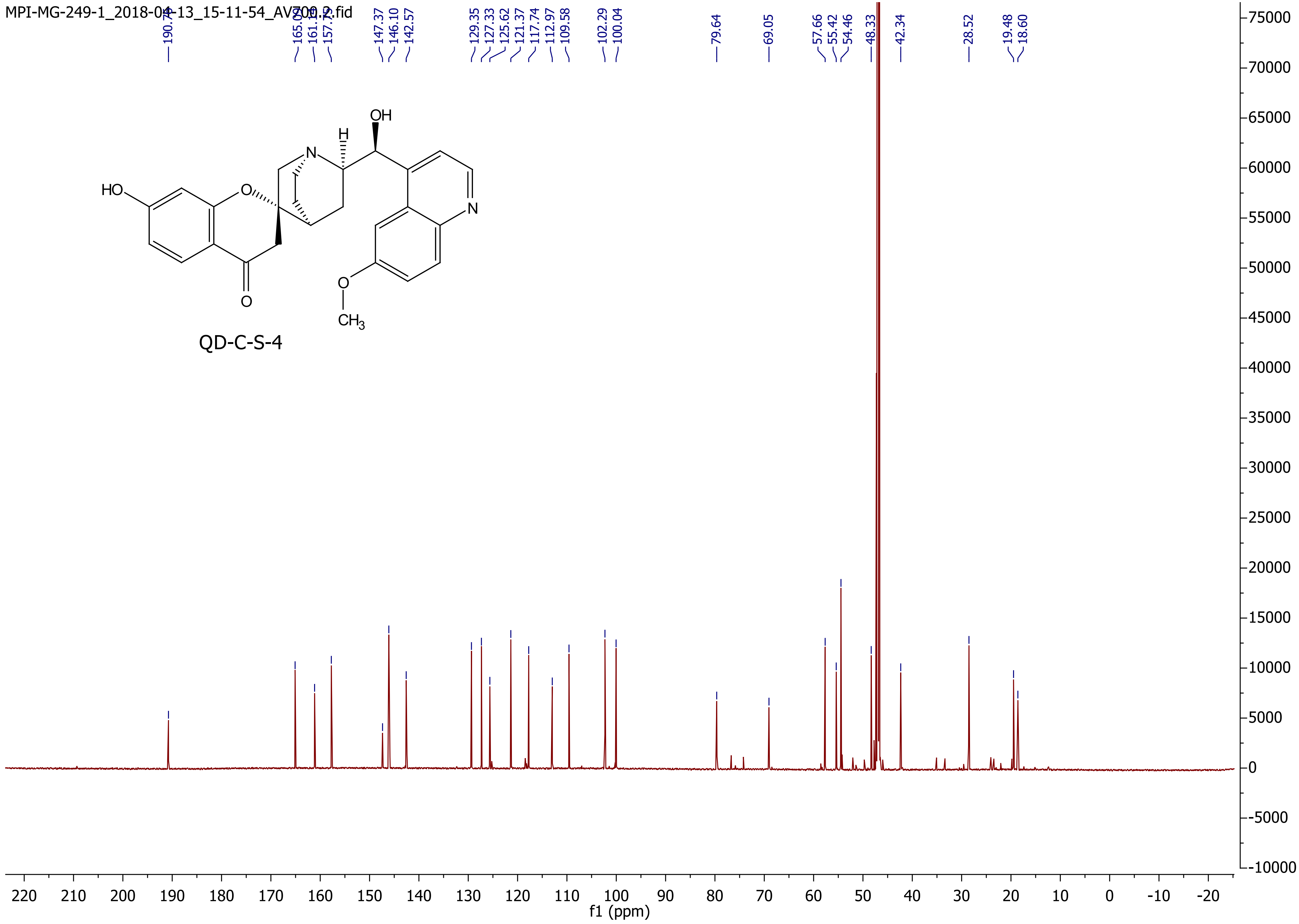

MPI-MG-249-1_2018-04-13_15-11-54_AV700.2.fid
190.76
165.08
161.14
157.75
147.37
146.10
142.57
129.35
127.33
125.62
121.37
117.74
112.97
109.58
102.29
100.04
79.64
69.05
57.66
55.42
54.46
48.33
42.34
28.52
19.48
18.60
HO
O
OH
H
N
N
O
O
CH3
QD-C-S-4
75000
70000
65000
60000
55000
50000
45000
40000
35000
30000
25000
20000
15000
10000
5000
0
-5000
-10000
220
210
200
190
180
170
160
150
140
130
120
110
100
90
80
70
60
50
40
30
20
10
0
-10
-20
f1 (ppm)

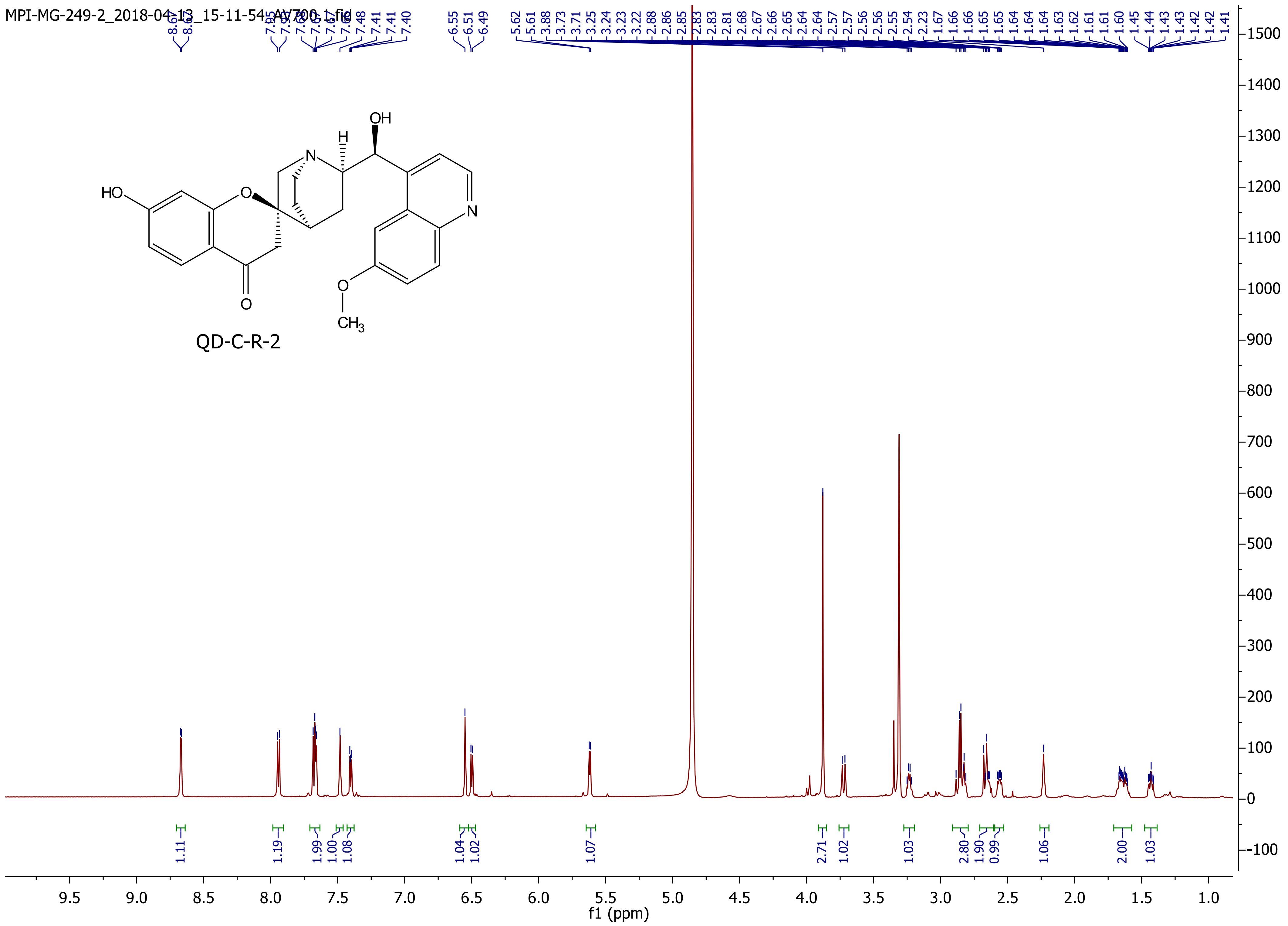
MPI-MG-249-2_2018-04-13_15-11-54_AV700 1.fid
7.48 7.41 7.41 7.40
6.55 6.51 6.49
5.62 5.61 3.88 3.73 3.71 3.25 3.24 3.23 3.22 2.88 2.86 2.85 2.83 2.83 2.81 2.68 2.67 2.66 2.65 2.64 2.64 2.57 2.57 2.56 2.56 2.55 2.54 2.23 1.67 1.66 1.66 1.65 1.65 1.64 1.64 1.64 1.63 1.62 1.61 1.61 1.60 1.45 1.44 1.43 1.43 1.42 1.42 1.41
OH
H
N
HO
O
N
O
O
CH3
QD-C-R-2
1.11 1.19 1.99 1.00 1.08 1.04 1.02 1.07 2.71 1.02 1.03 2.80 1.90 0.99 1.06 2.00 1.03
f1 (ppm)

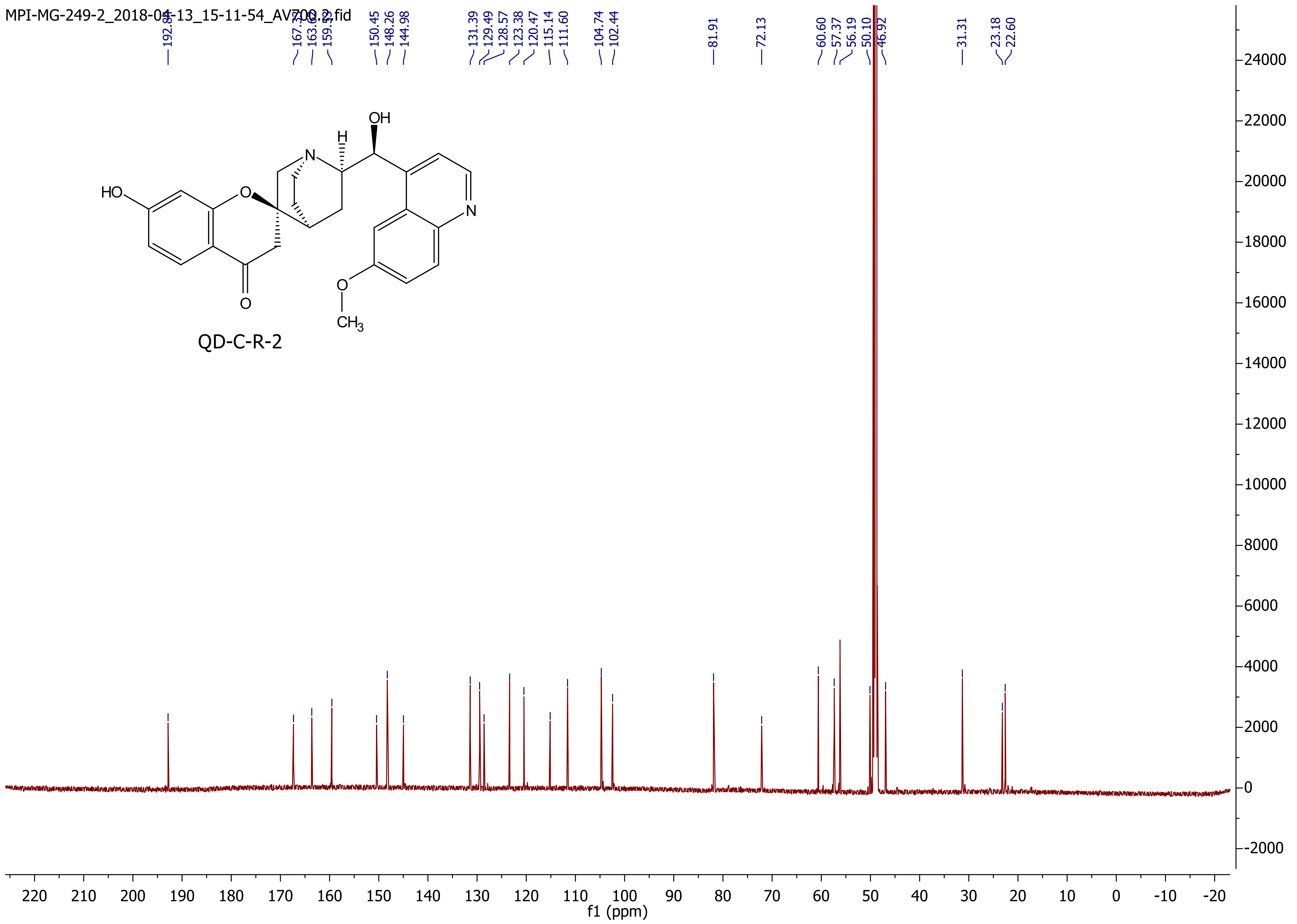
MPI-MG-249-2_2018-04-13_15-11-54_AV700.2.fid
150.45
148.26
144.98
131.39
129.49
128.57
123.38
120.47
115.14
111.60
104.74
102.44
81.91
72.13
60.60
57.37
56.19
50.10
46.92
31.31
23.18
22.60
HO
O
OH
H
N
N
O
CH3
QD-C-R-2
f1 (ppm)

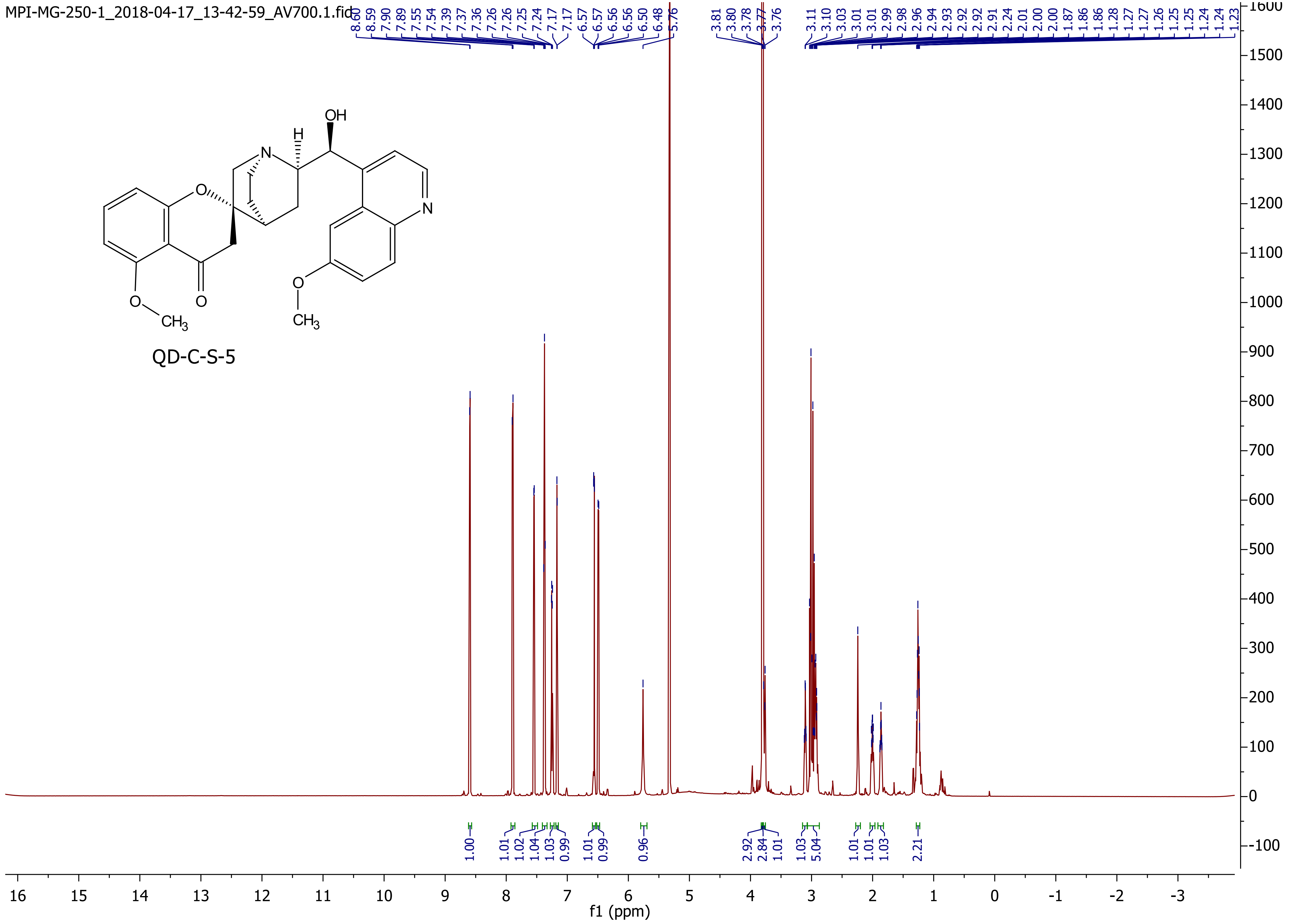

QD-C-S-5

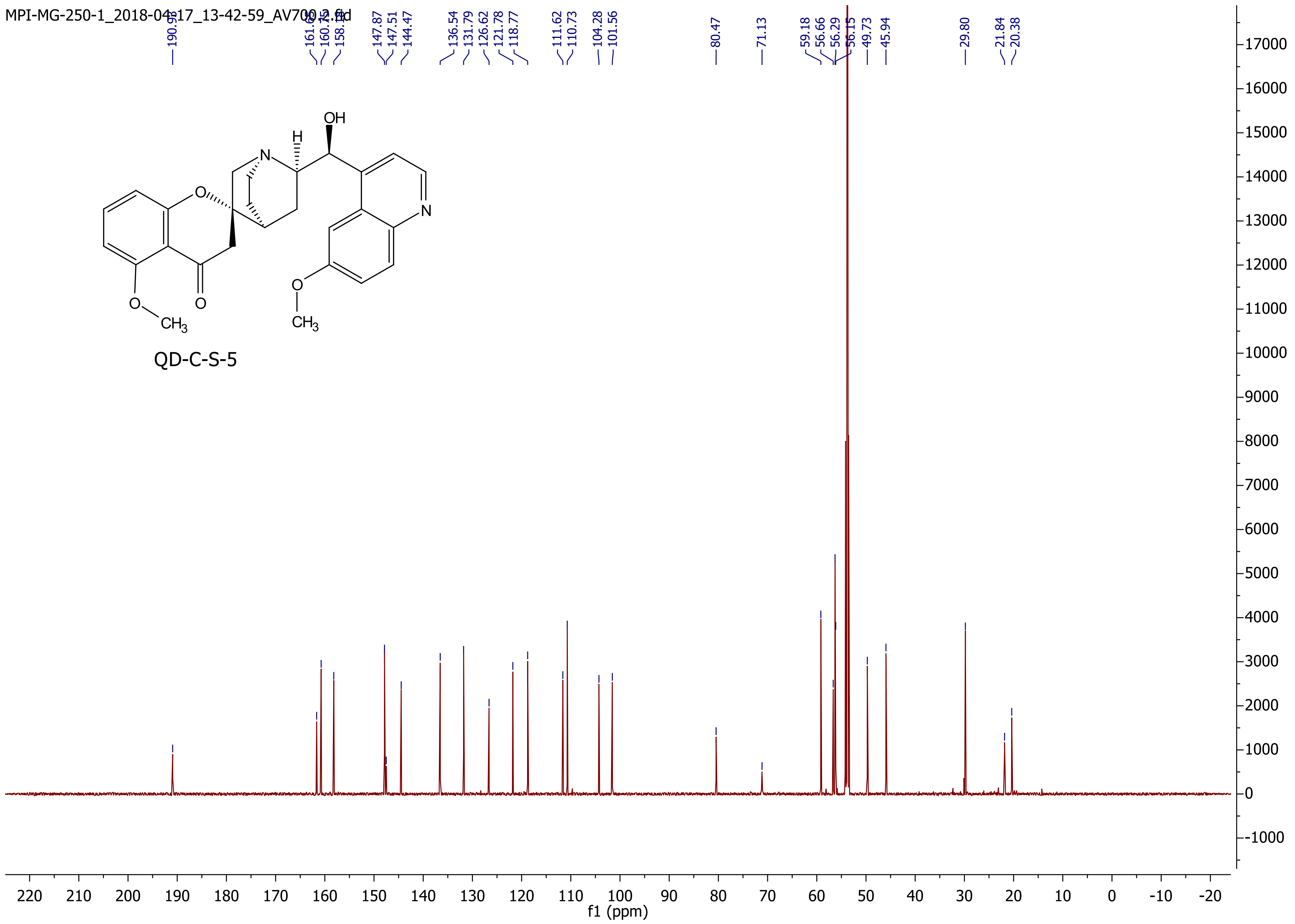

MPI-MG-250-1_2018-04-17_13-42-59_AV700
190.9
147.87
147.51
144.47
136.54
131.79
126.62
121.78
118.77
111.62
110.73
104.28
101.56
80.47
71.13
59.18
56.66
56.29
56.15
49.73
45.94
29.80
21.84
20.38
OH
H
N
O
N
O
CH3
O
O
CH3
QD-C-S-5
f1 (ppm)

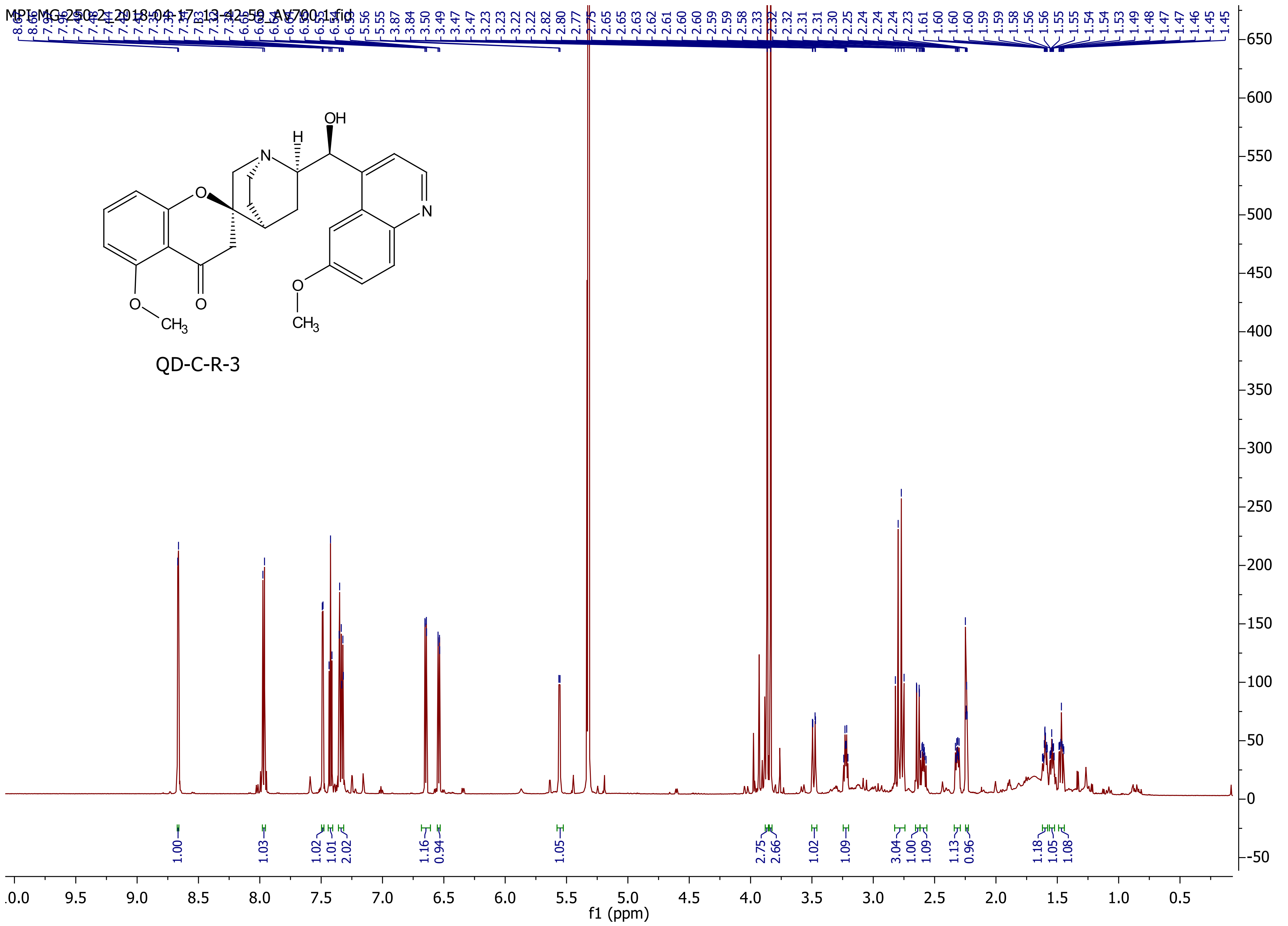

OH
H
N
O
N
O
O
CH3
O
CH3
QD-C-R-3
1.00
1.03
1.02
1.01
2.02
1.16
0.94
1.05
2.75
2.66
1.02
1.09
3.04
1.00
1.09
1.13
0.96
1.18
1.05
1.08
f1 (ppm)

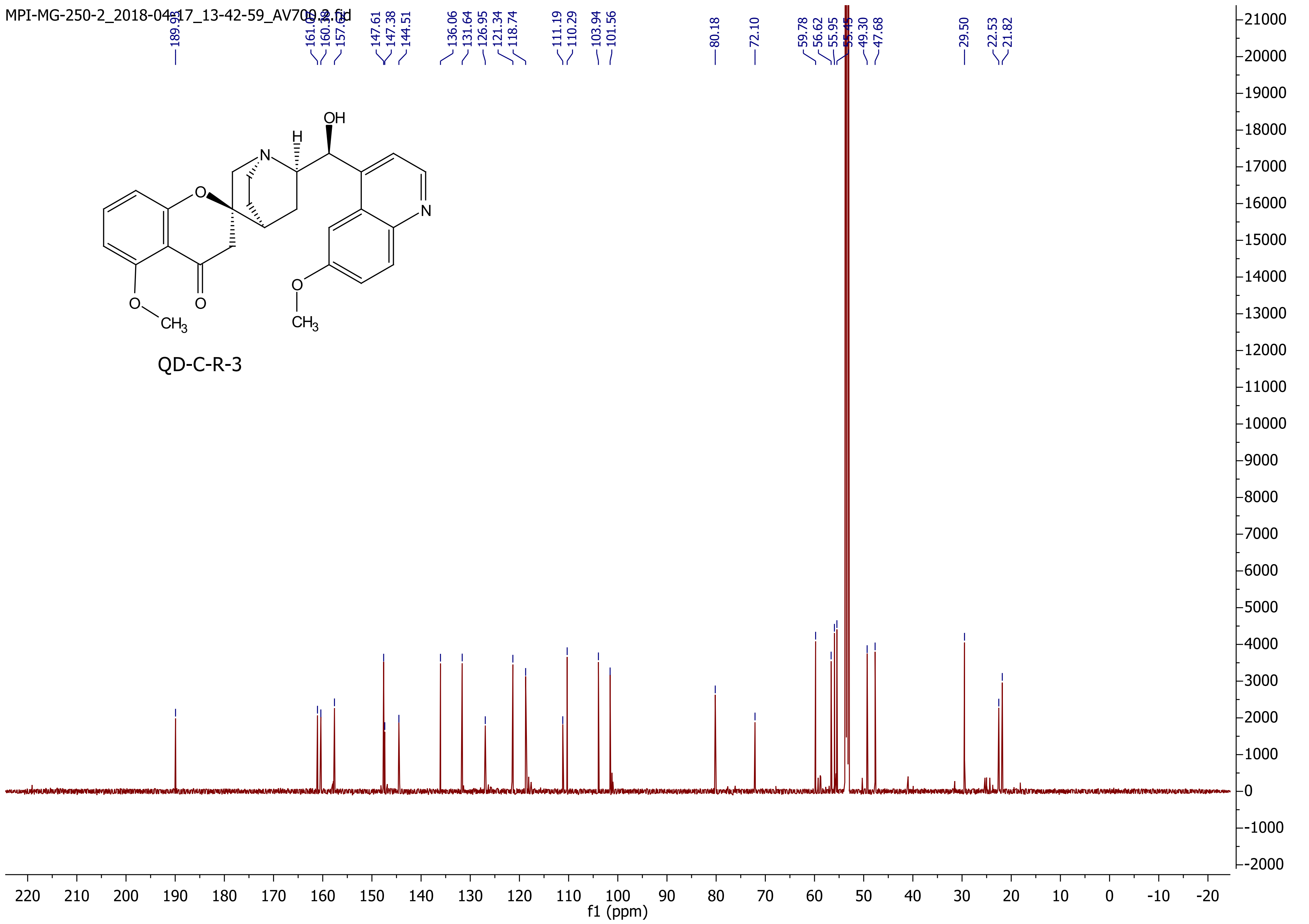
MPI-MG-250-2_2018-04-17_13-42-59_AV700.2.fid
189.93
147.61
147.38
144.51
136.06
131.64
126.95
121.34
118.74
111.19
110.29
103.94
101.56
80.18
72.10
59.78
56.62
55.95
49.30
47.68
29.50
22.53
21.82
OH
H
N
O
O
CH3
QD-C-R-3
f1 (ppm)

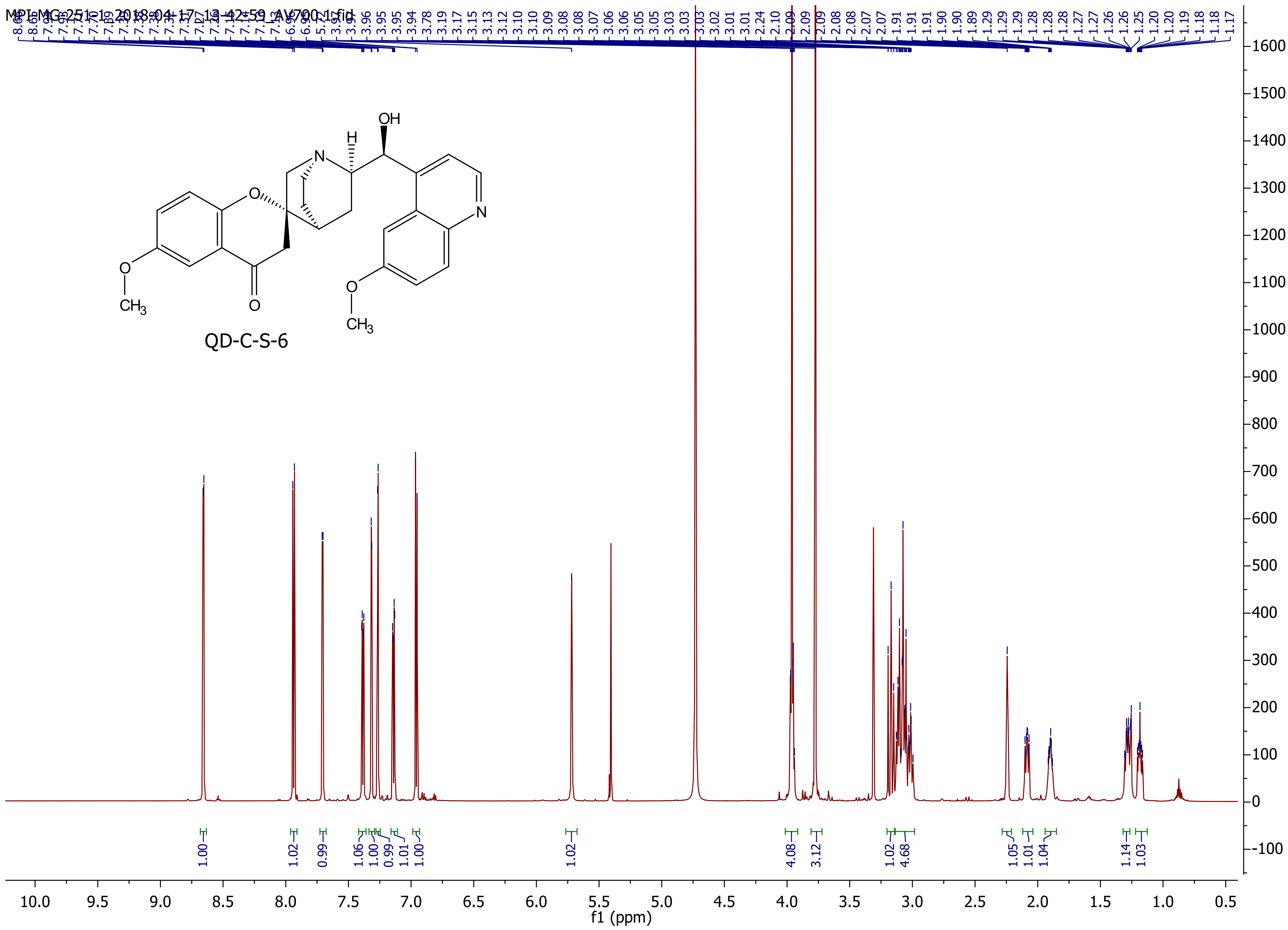
MPI-MG-251-1_2018-04-17_13-42-59_AV700 1.fid
OH
H
N
O
N
O
O
CH3
O
CH3
QD-C-S-6
1.00
1.02
0.99
1.06
1.00
0.99
1.01
1.00
1.02
4.08
3.12
1.02
4.68
1.05
1.01
1.04
1.14
1.03
f1 (ppm)

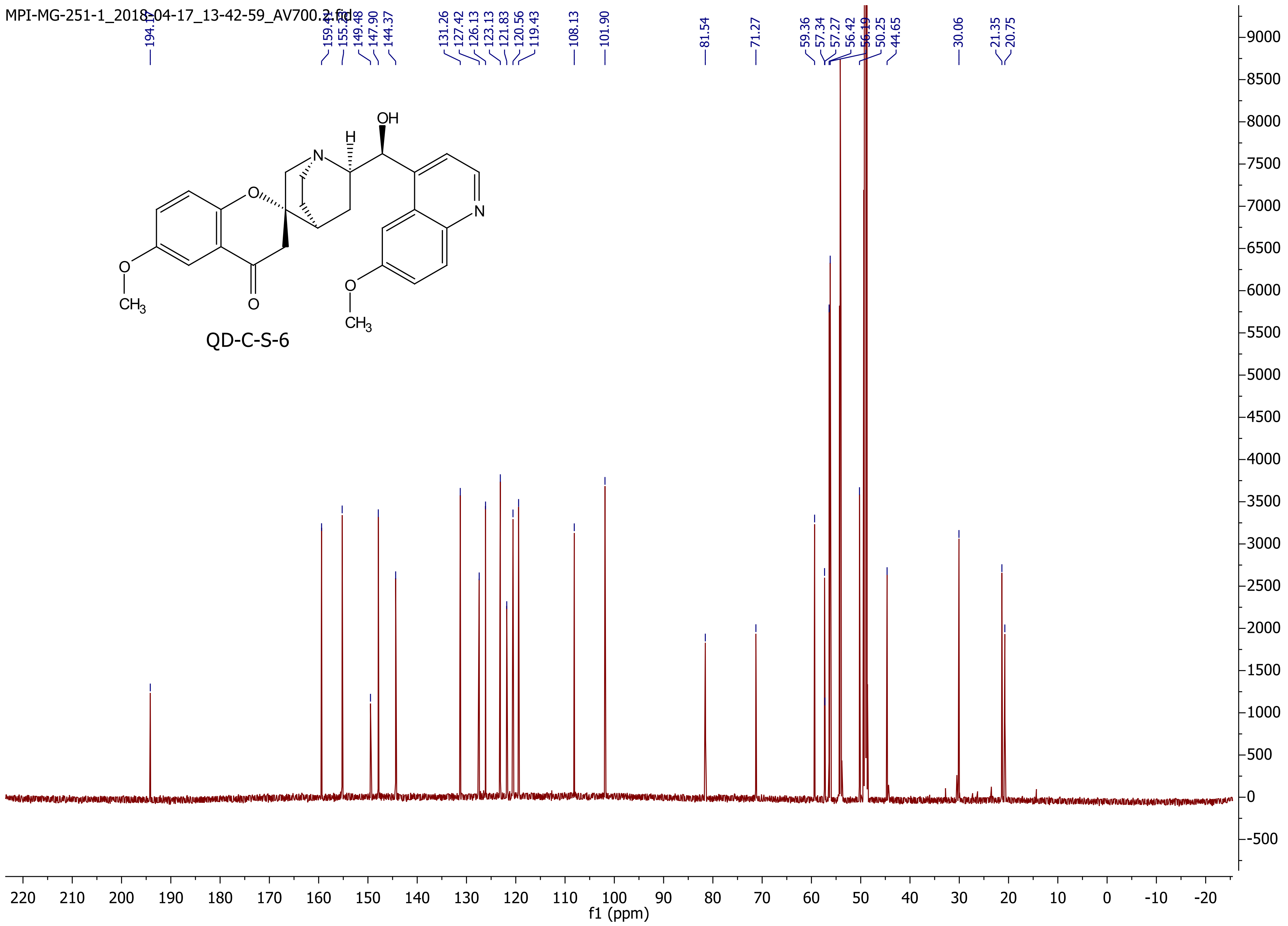

MPI-MG-251-1_2018-04-17_13-42-59_AV700.2.fid
149.48
147.90
144.37
131.26
127.42
126.13
123.13
121.83
120.56
119.43
108.13
101.90
81.54
71.27
59.36
57.34
57.27
56.42
56.19
50.25
44.65
30.06
21.35
20.75
OH
H
N
O
N
O
CH3
O
O
CH3
QD-C-S-6
f1 (ppm)

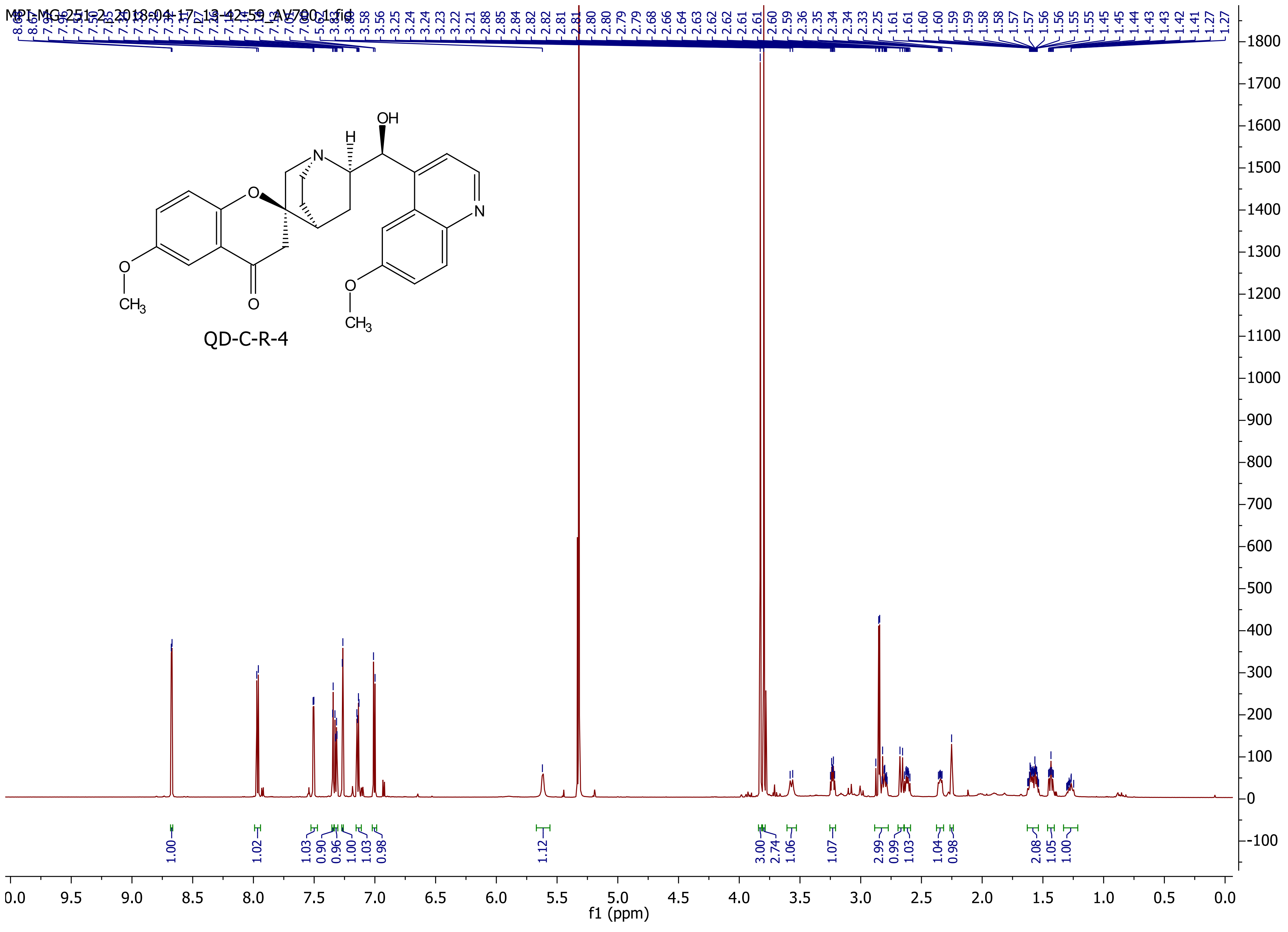
MPI-MG-251-2_2018-04-17_13-42-59_AV700 1 fid
OH
H
N
O
N
O
CH3
O
O
CH3
QD-C-R-4
1.00
1.02
1.03
0.90
0.96
1.00
1.03
0.98
1.12
3.00
2.74
1.06
1.07
2.99
0.99
1.03
1.04
0.98
2.08
1.05
1.00
f1 (ppm)

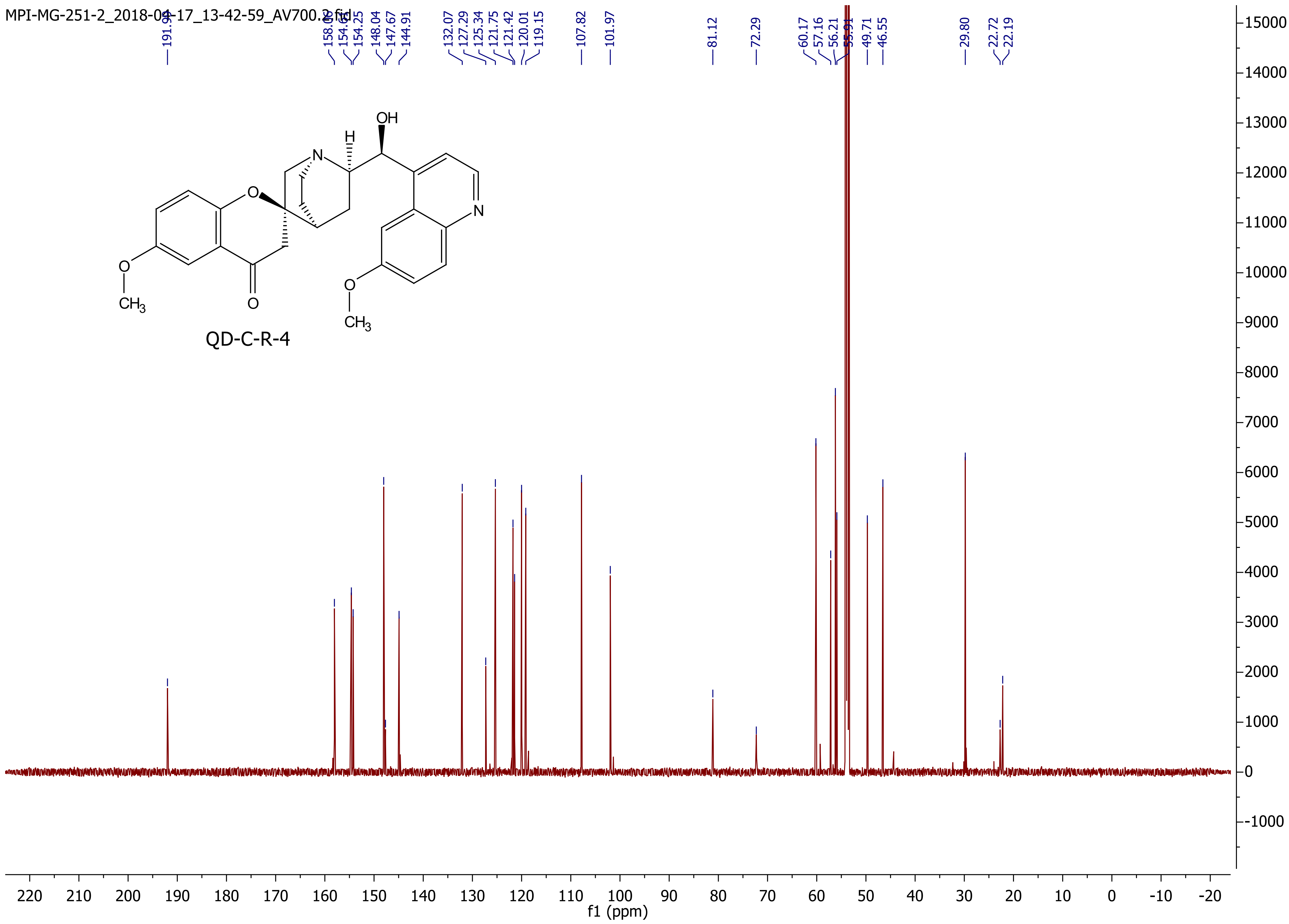

MPI-MG-251-2_2018-04-17_13-42-59_AV700.2.fid
191.94
158.06
154.61
154.25
148.04
147.67
144.91
132.07
127.29
125.34
121.75
121.42
120.01
119.15
107.82
101.97
81.12
72.29
60.17
57.16
56.21
55.91
49.71
46.55
29.80
22.72
22.19
QD-C-R-4
f1 (ppm)

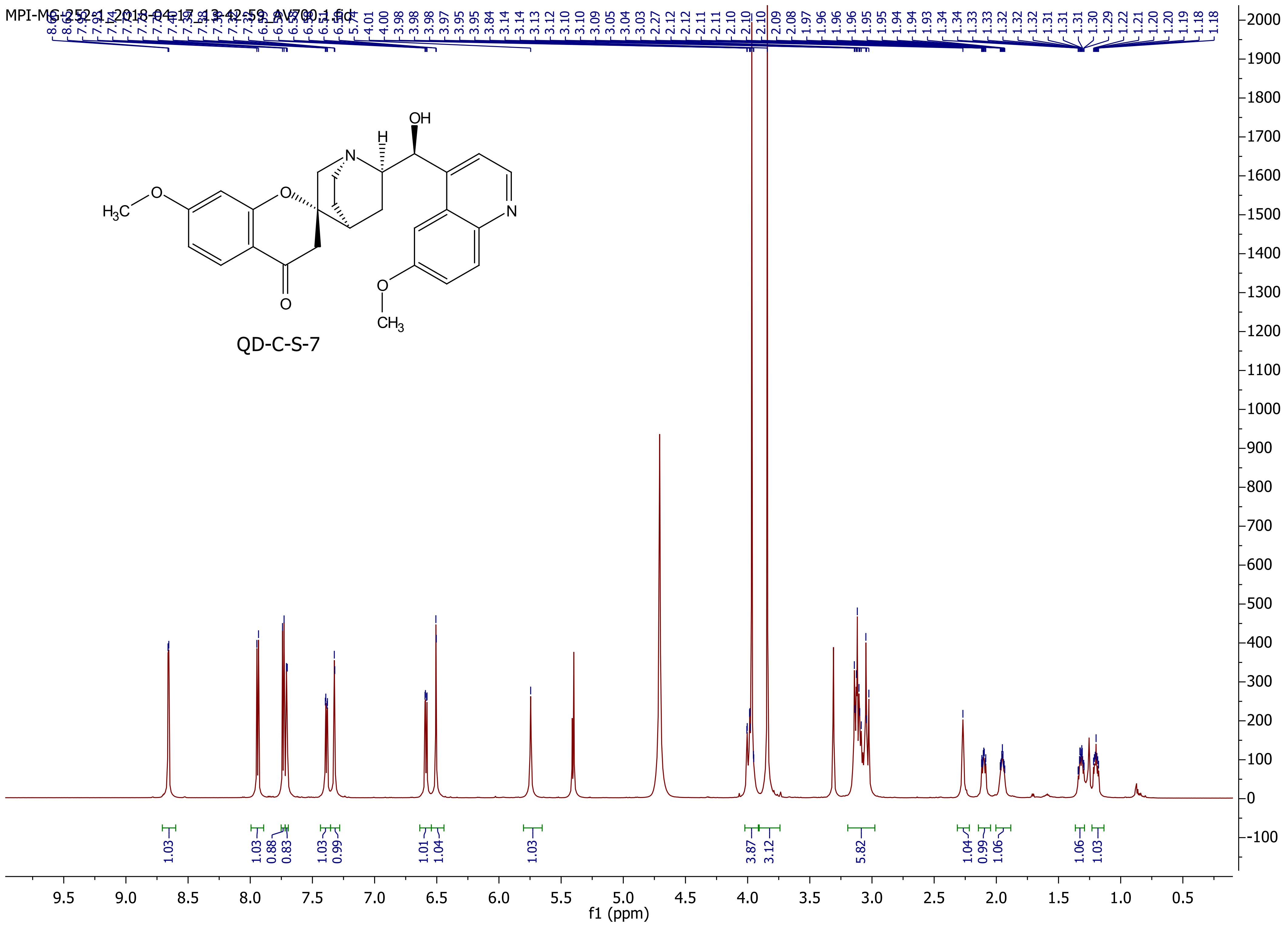

MPI-MC-252-1_2018-04-17_13-42-59_AV700-1.fid
4.01 4.00 3.98 3.98 3.97 3.95 3.95 3.84 3.14 3.14 3.13 3.12 3.10 3.10 3.09 3.05 3.04 3.03 2.27 2.12 2.12 2.11 2.11 2.10 2.10 2.10 2.09 2.08 1.97 1.96 1.96 1.96 1.95 1.95 1.94 1.94 1.93 1.34 1.34 1.33 1.33 1.32 1.32 1.32 1.31 1.31 1.31 1.30 1.29 1.22 1.21 1.20 1.20 1.19 1.18 1.18
OH
H
N
O
H3C
O
O
N
O
CH3
QD-C-S-7
1.03 1.03 0.88 0.83 1.03 0.99 1.01 1.04 1.03 3.87 3.12 5.82 1.04 0.99 1.06 1.06 1.03
9.5 9.0 8.5 8.0 7.5 7.0 6.5 6.0 5.5 5.0 4.5 4.0 3.5 3.0 2.5 2.0 1.5 1.0 0.5
f1 (ppm)
2000 1900 1800 1700 1600 1500 1400 1300 1200 1100 1000 900 800 700 600 500 400 300 200 100 0 -100

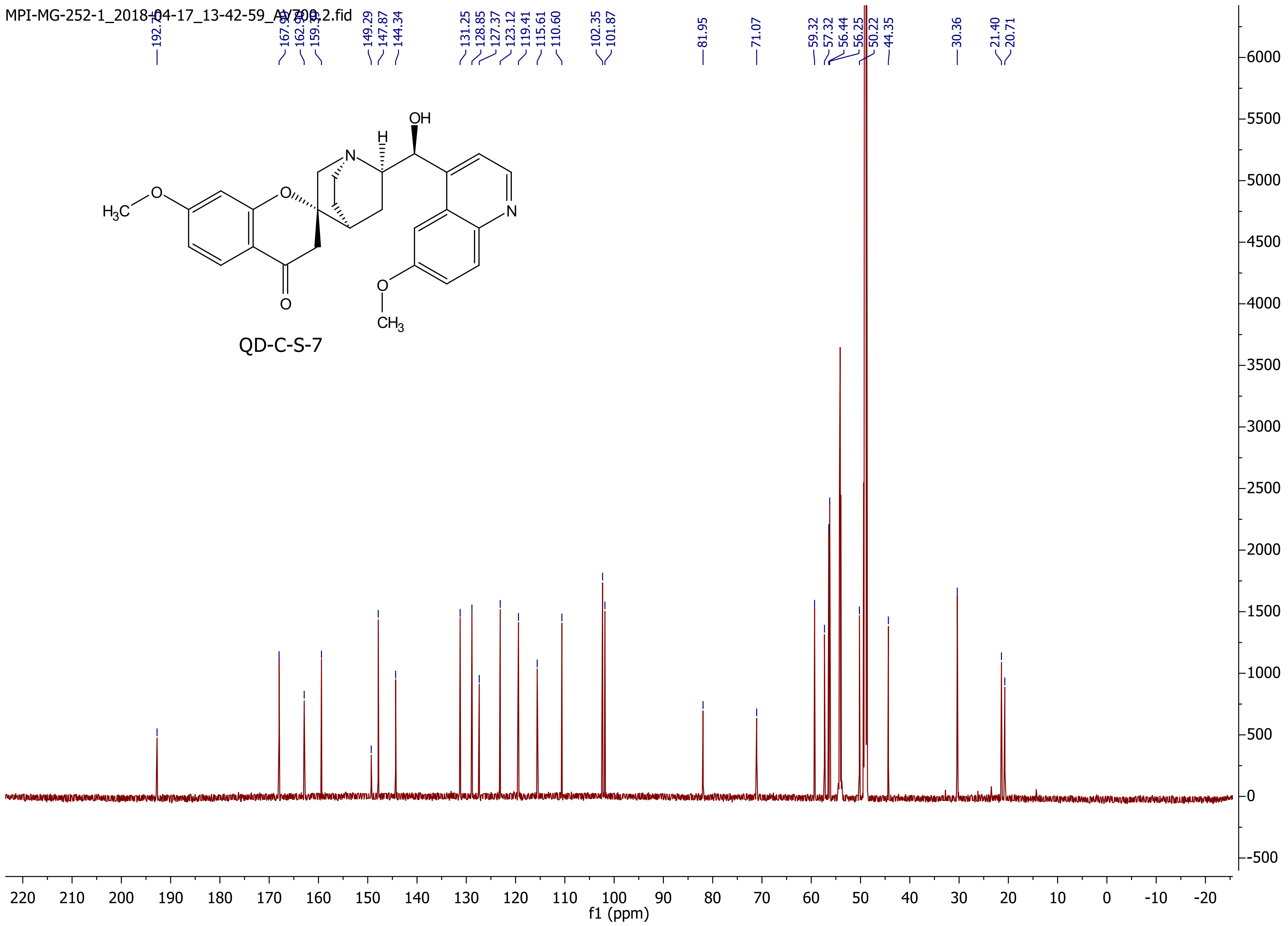

MPI-MG-252-1_2018-04-17_13-42-59_AV700.2.fid
149.29
147.87
144.34
131.25
128.85
127.37
123.12
119.41
115.61
110.60
102.35
101.87
81.95
71.07
59.32
57.32
56.44
56.25
50.22
44.35
30.36
21.40
20.71
OH
H
N
O
H3C
O
O
N
O
CH3
QD-C-S-7
6000
5500
5000
4500
4000
3500
3000
2500
2000
1500
1000
500
0
-500
220 210 200 190 180 170 160 150 140 130 120 110 100 90 80 70 60 50 40 30 20 10 0 -10 -20
f1 (ppm)

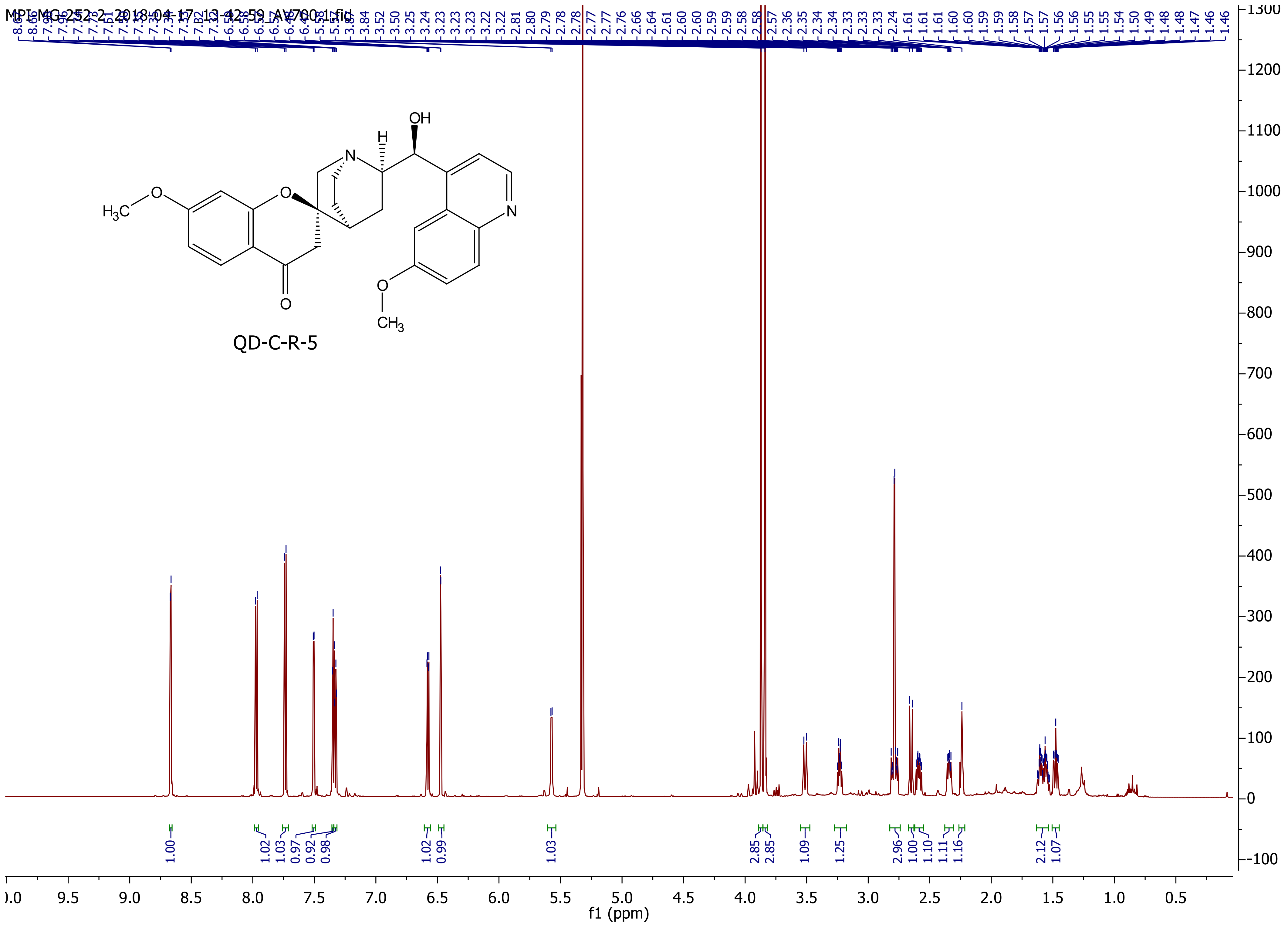
QD-C-R-5
OH
H
N
O
H3C
O
N
CH3
f1 (ppm)
1.00
1.02
1.03
0.97
0.92
0.98
1.02
0.99
1.03
2.85
2.85
1.09
1.25
2.96
1.00
1.10
1.11
1.16
2.12
1.07

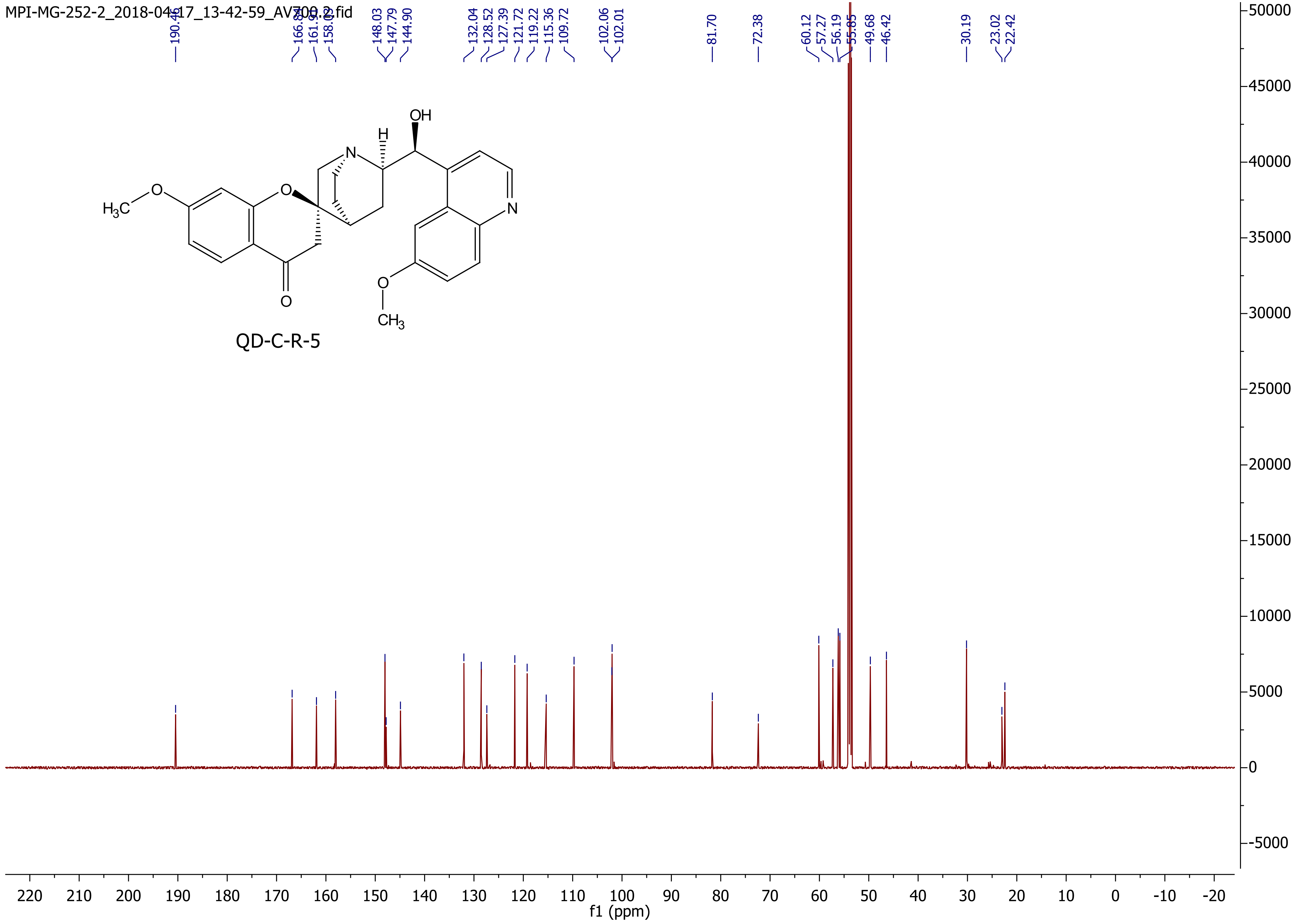

MPI-MG-252-2_2018-04-17_13-42-59_AV700.2.fid
190.46
148.03
147.79
144.90
132.04
128.52
127.39
121.72
119.22
115.36
109.72
102.06
102.01
81.70
72.38
60.12
57.27
56.19
55.85
49.68
46.42
30.19
23.02
22.42
OH
H
N
O
H_3C
O
O
N
O
CH_3
QD-C-R-5
f1 (ppm)

MPI-MG-543-1_2019-05-15_07-00-08_AV700.1.fid

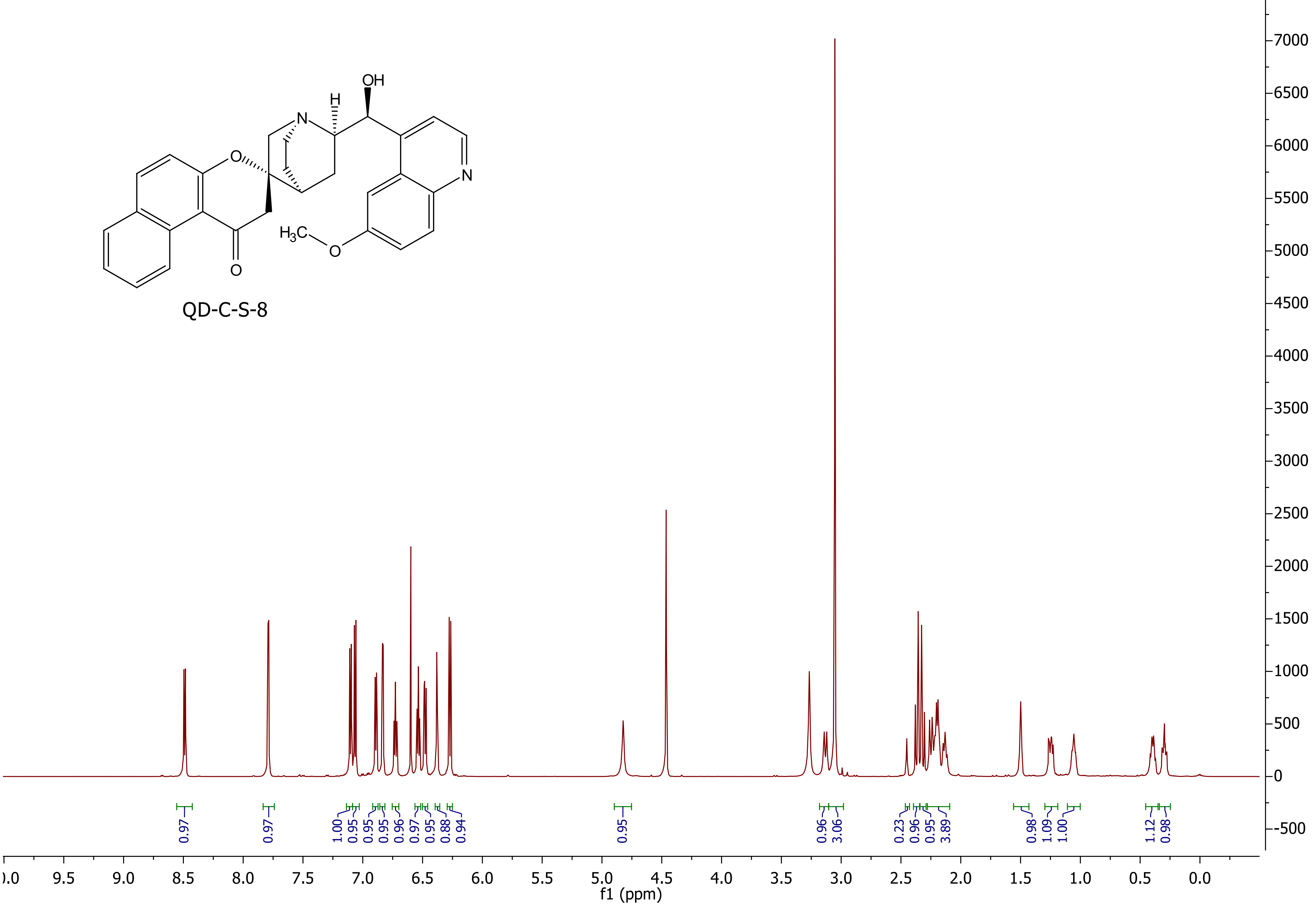

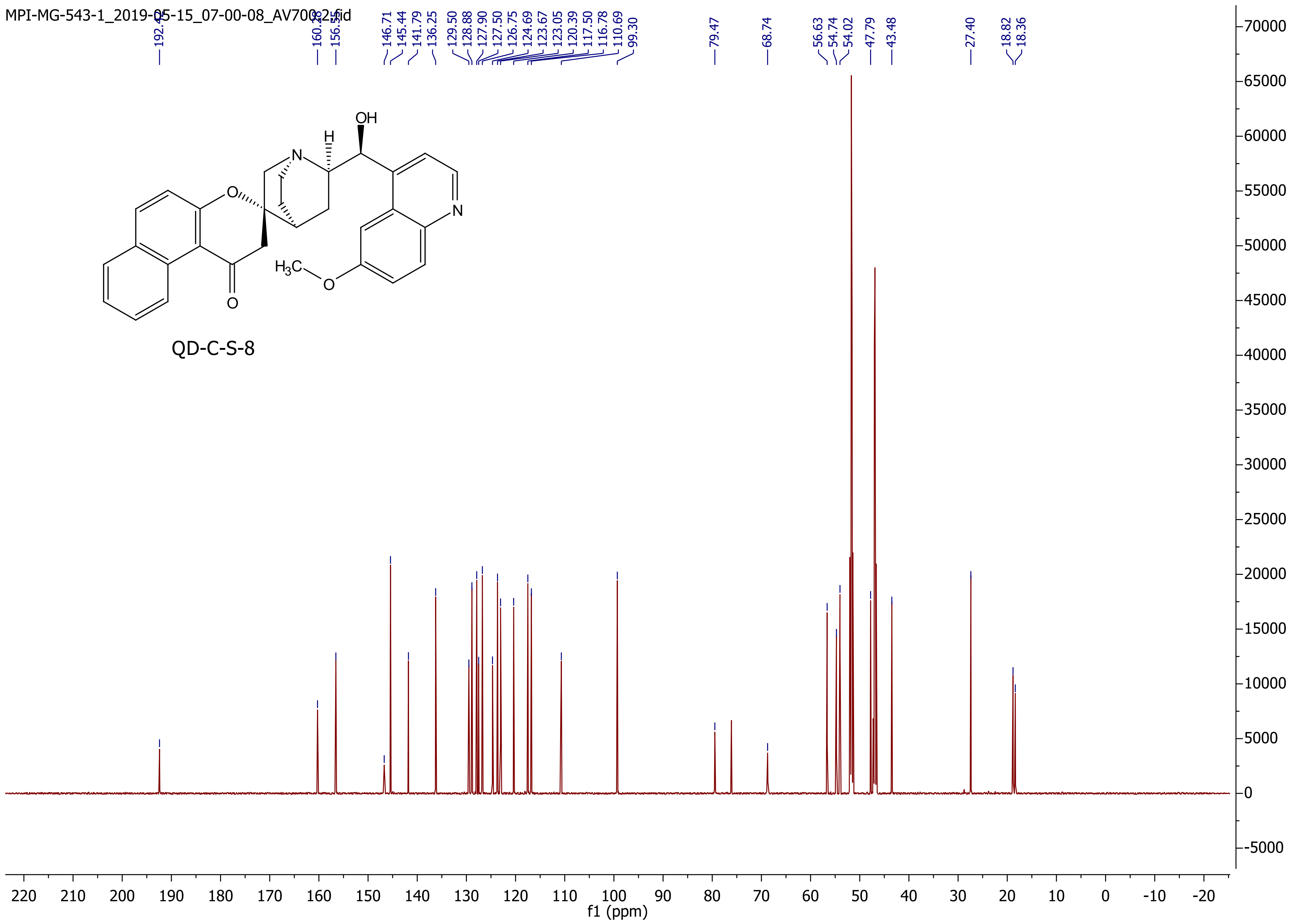

MPI-MG-543-1_2019-05-15_07-00-08_AV700.2.fid
146.71
145.44
141.79
136.25
129.50
128.88
127.90
127.50
126.75
124.69
123.67
123.05
120.39
117.50
116.78
110.69
99.30
79.47
68.74
56.63
54.74
54.02
47.79
43.48
27.40
18.82
18.36
OH
H
N
O
N
O
H3C
O
QD-C-S-8
f1 (ppm)

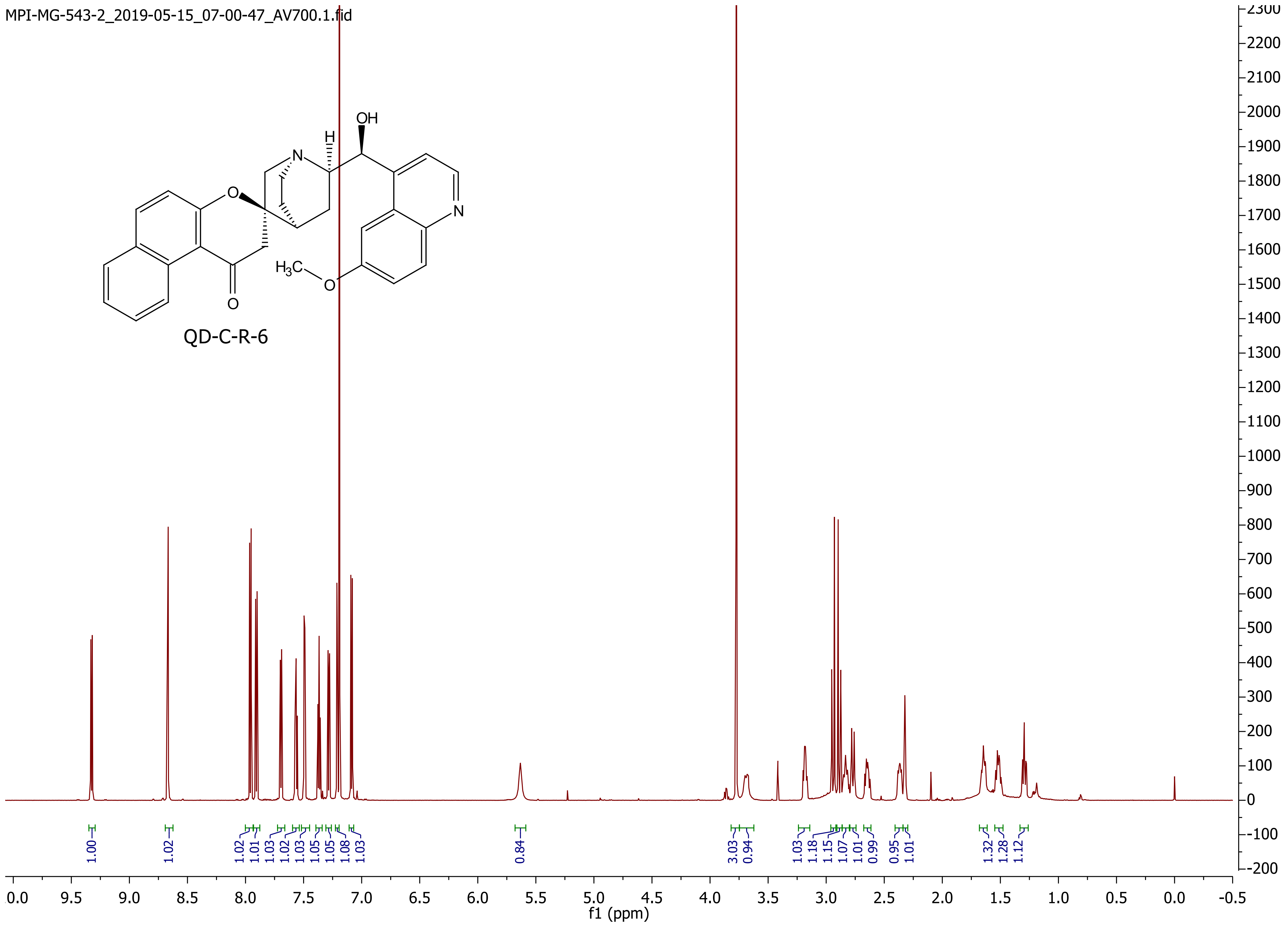
MPI-MG-543-2_2019-05-15_07-00-47_AV700.1.fid
QD-C-R-6
f1 (ppm)

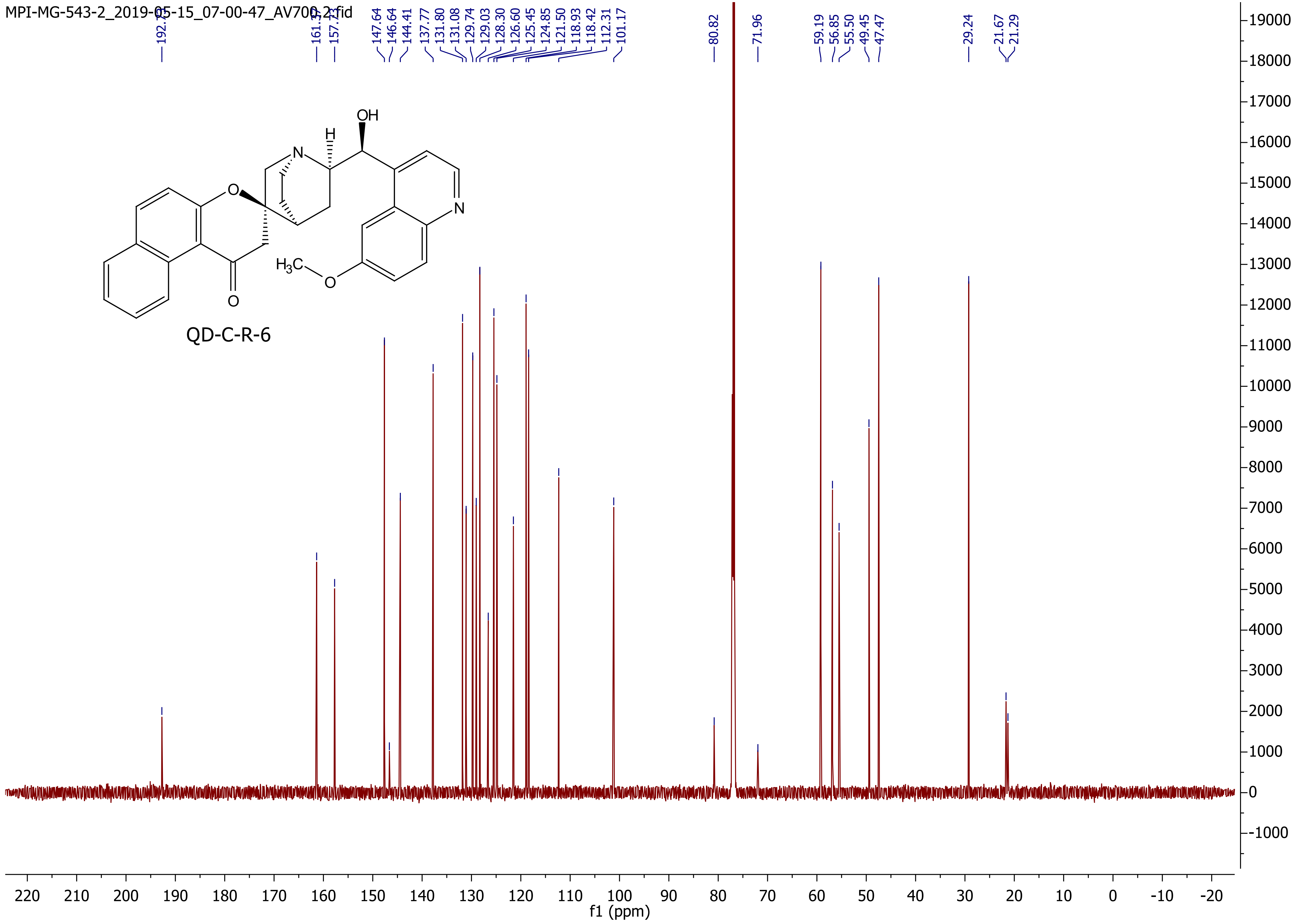
MPI-MG-543-2_2019-05-15_07-00-47_AV700.2.fid
192.73
161.37
157.73
147.64
146.64
144.41
137.77
131.80
131.08
129.74
129.03
128.30
126.60
125.45
124.85
121.50
118.93
118.42
112.31
101.17
80.82
71.96
59.19
56.85
55.50
49.45
47.47
29.24
21.67
21.29
OH
H
N
O
N
H_3C
O
O
QD-C-R-6
f1 (ppm)

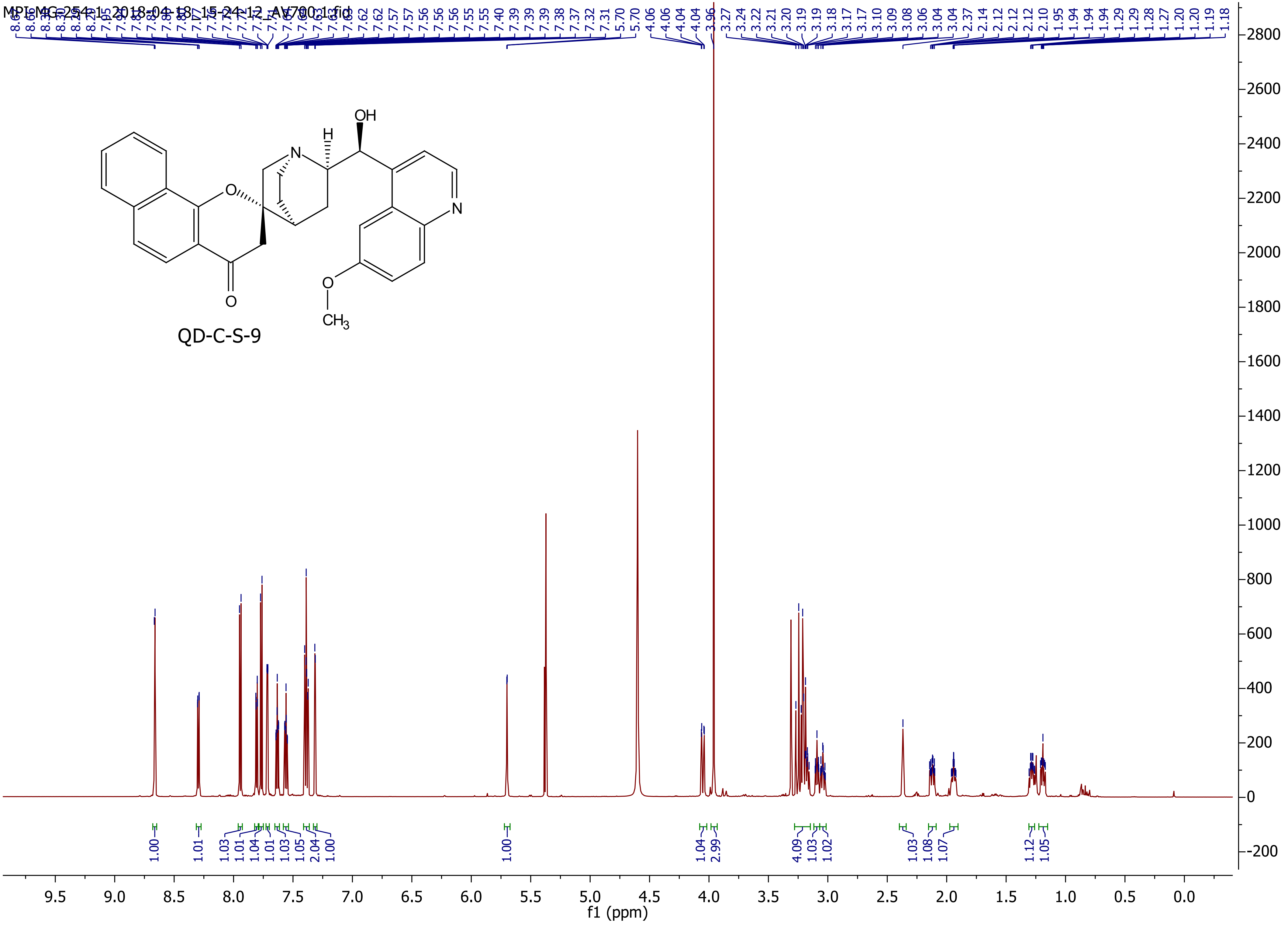

MPI-MG-254-1 2018-04-18 15-24-12 AV700 1.fid
OH
H
N
O
O
N
O
CH3
QD-C-S-9
f1 (ppm)

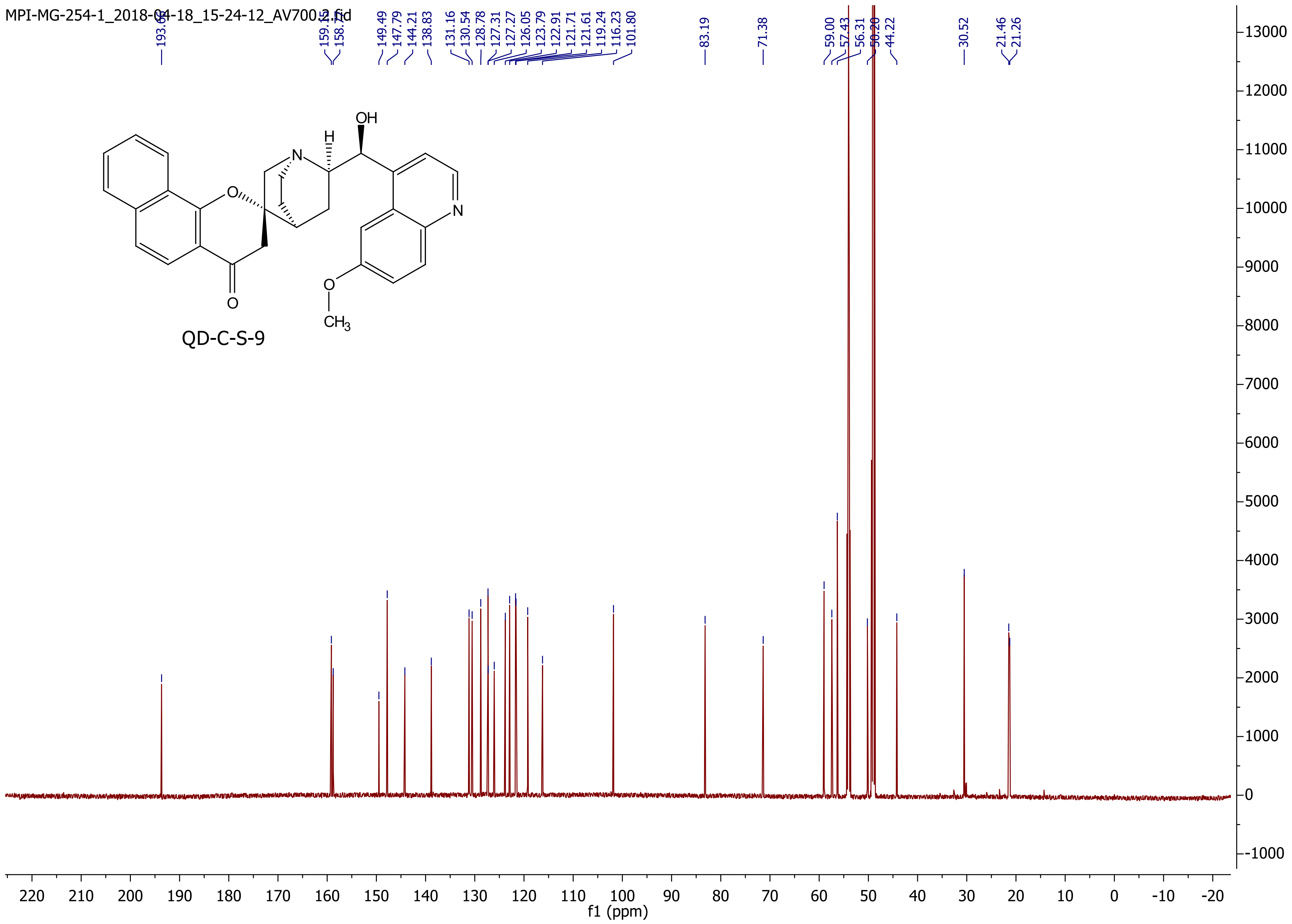

MPI-MG-254-1_2018-04-18_15-24-12_AV700.2.fid
149.49
147.79
144.21
138.83
131.16
130.54
128.78
127.31
127.27
126.05
123.79
122.91
121.71
121.61
119.24
116.23
101.80
83.19
71.38
59.00
57.43
56.31
50.20
44.22
30.52
21.46
21.26
OH
H
N
O
N
O
O
CH3
QD-C-S-9
f1 (ppm)

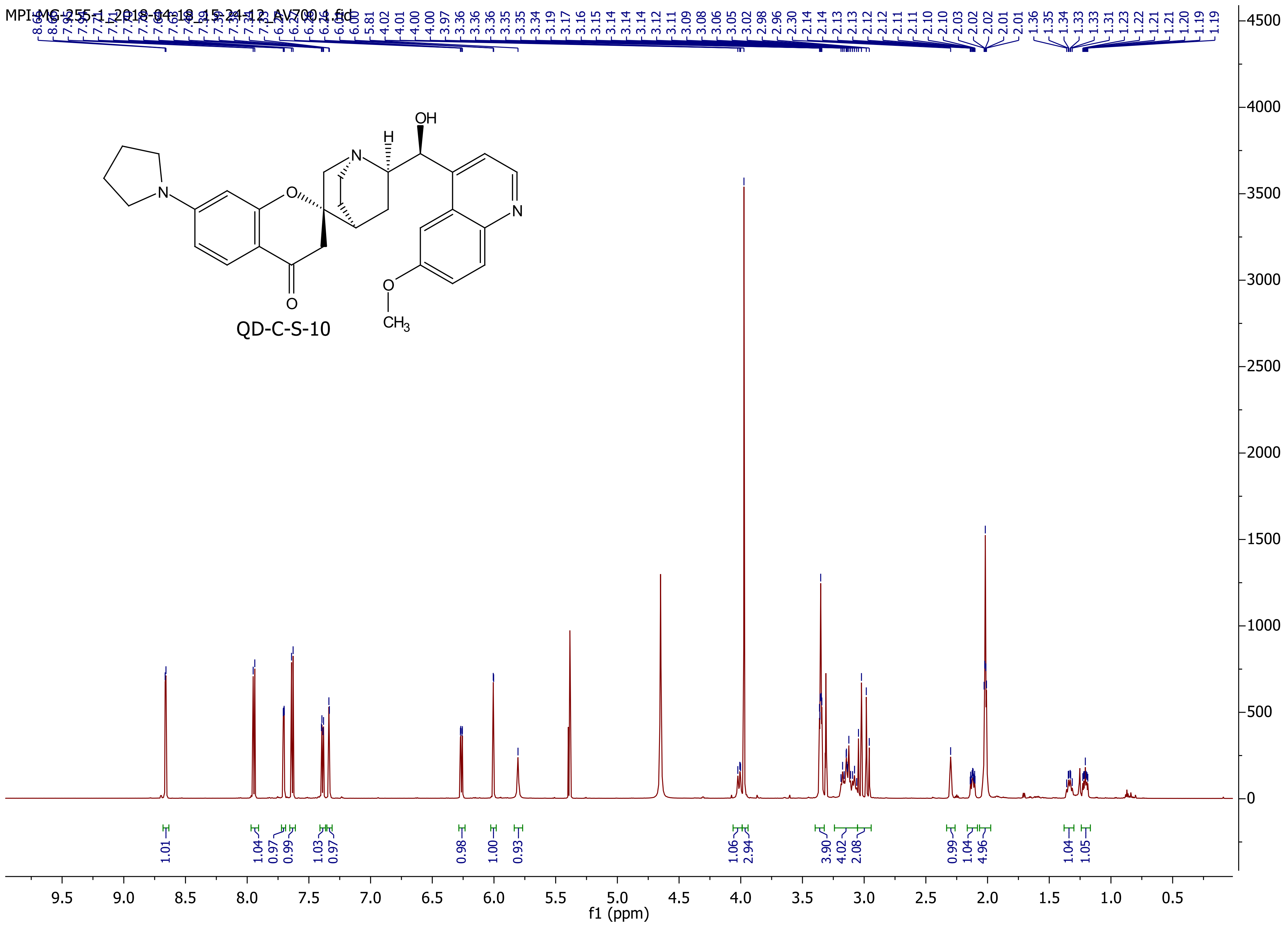

MPI-MG-255-1_2018-04-18_15-24-12_AV700 1.fid
QD-C-S-10
OH
H
N
O
CH3
f1 (ppm)

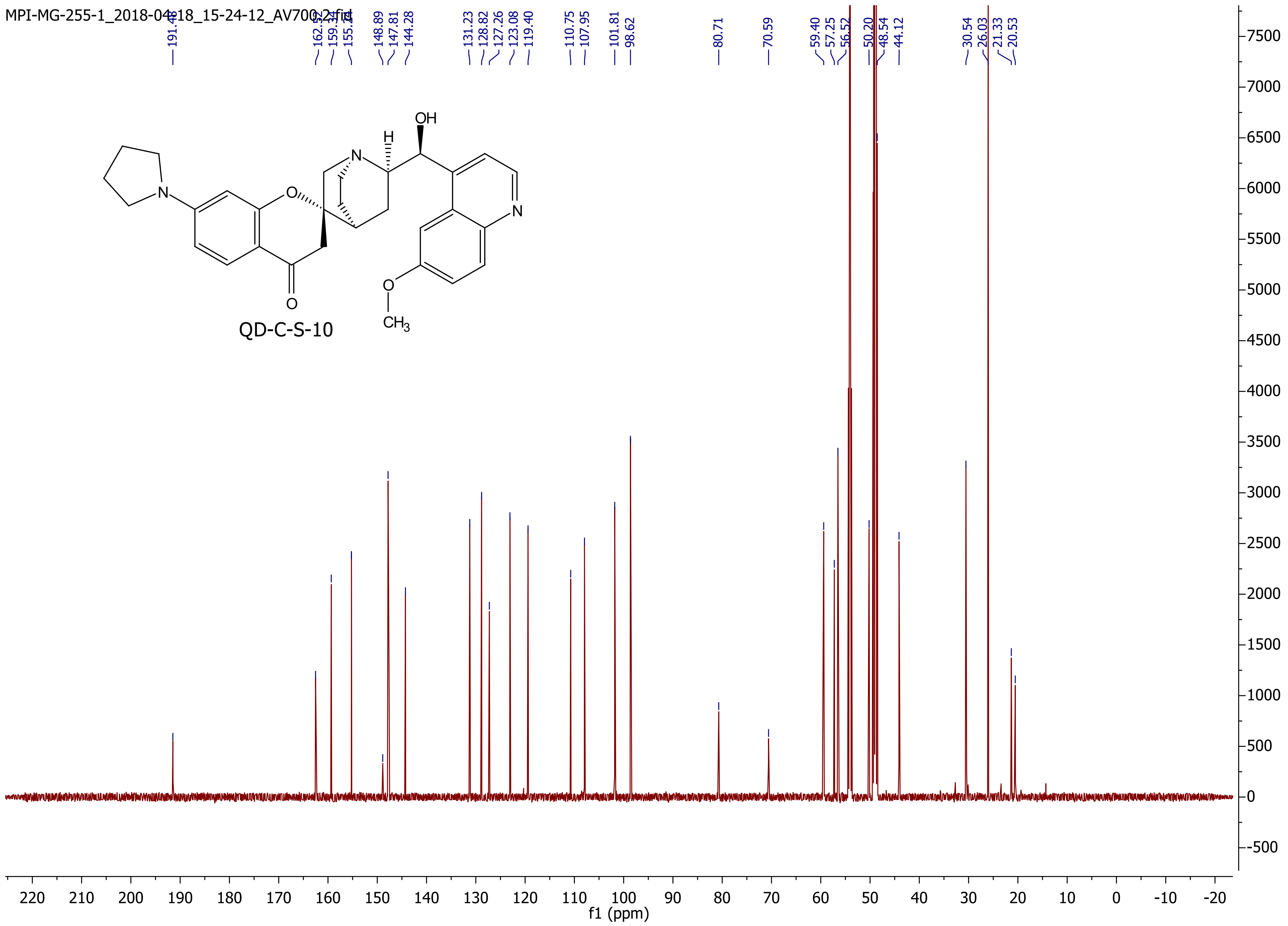
MPI-MG-255-1_2018-04-18_15-24-12_AV700.2.fid
191.4
148.89
147.81
144.28
131.23
128.82
127.26
123.08
119.40
110.75
107.95
101.81
98.62
80.71
70.59
59.40
57.25
56.52
50.20
48.54
44.12
30.54
26.03
21.33
20.53
OH
H
N
O
N
O
O
CH3
QD-C-S-10
f1 (ppm)

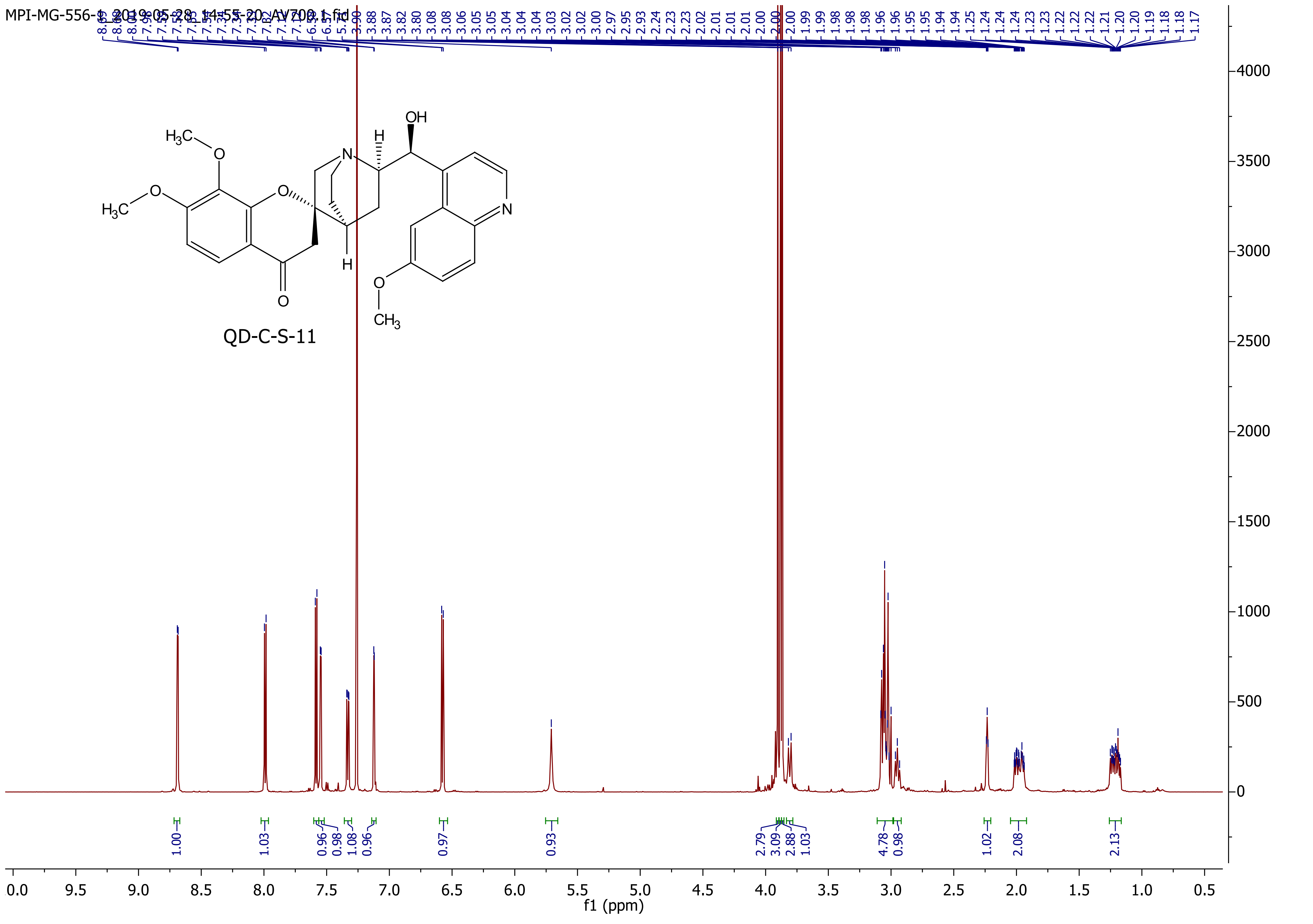

QD-C-S-11
OH
H
N
O
H
O
O
O
N
H_3C
H_3C
CH_3
1.00
1.03
0.96
0.98
1.08
0.96
0.97
0.93
2.79
3.09
2.88
1.03
4.78
0.98
1.02
2.08
2.13
f1 (ppm)

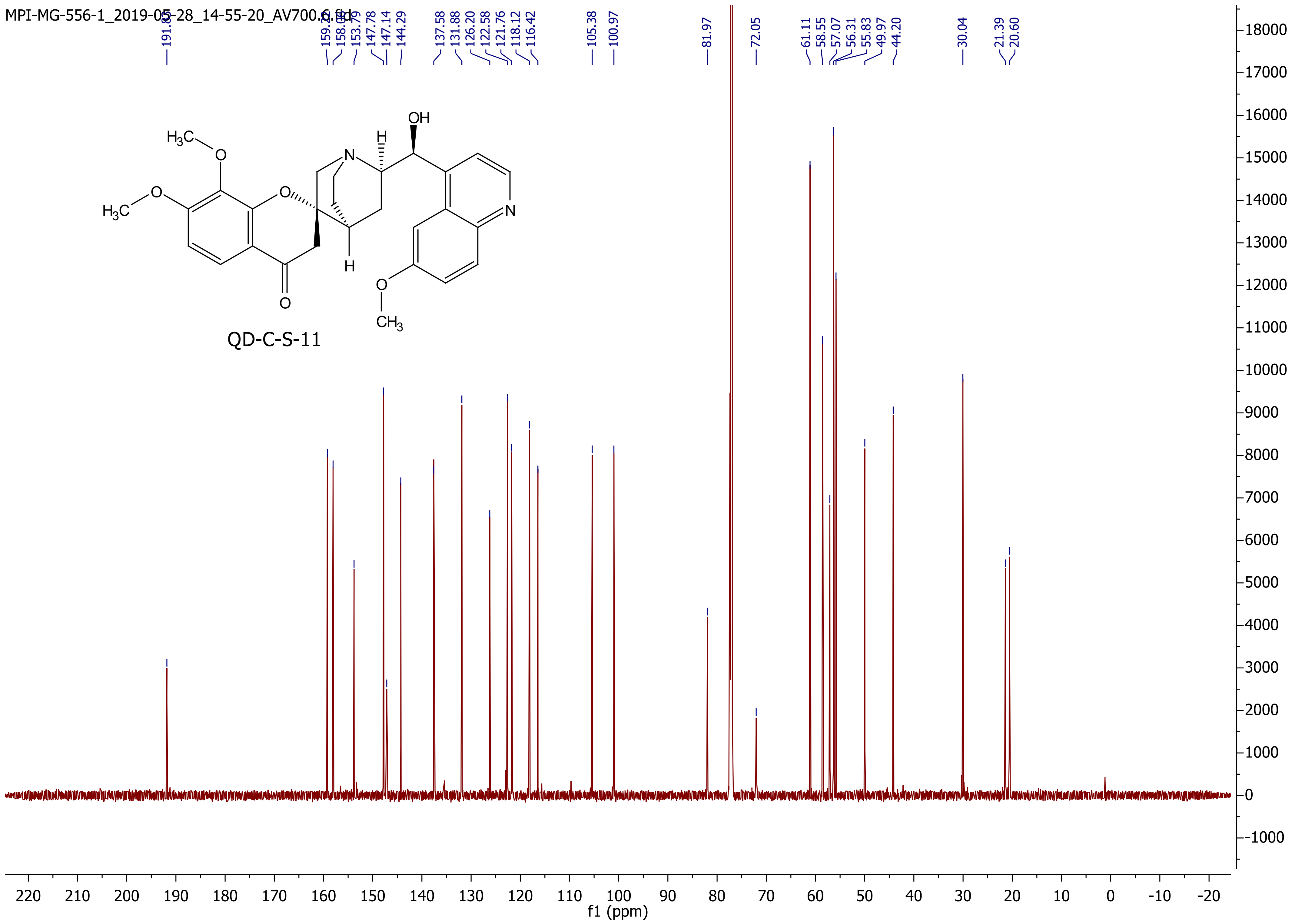

MPI-MG-556-1_2019-05-28_14-55-20_AV700.6.fid
153.79
147.78
147.14
144.29
137.58
131.88
126.20
122.58
121.76
118.12
116.42
105.38
100.97
81.97
72.05
61.11
58.55
57.07
56.31
55.83
49.97
44.20
30.04
21.39
20.60
OH
H
N
H3C
O
O
H3C
O
N
H
O
O
CH3
QD-C-S-11
f1 (ppm)

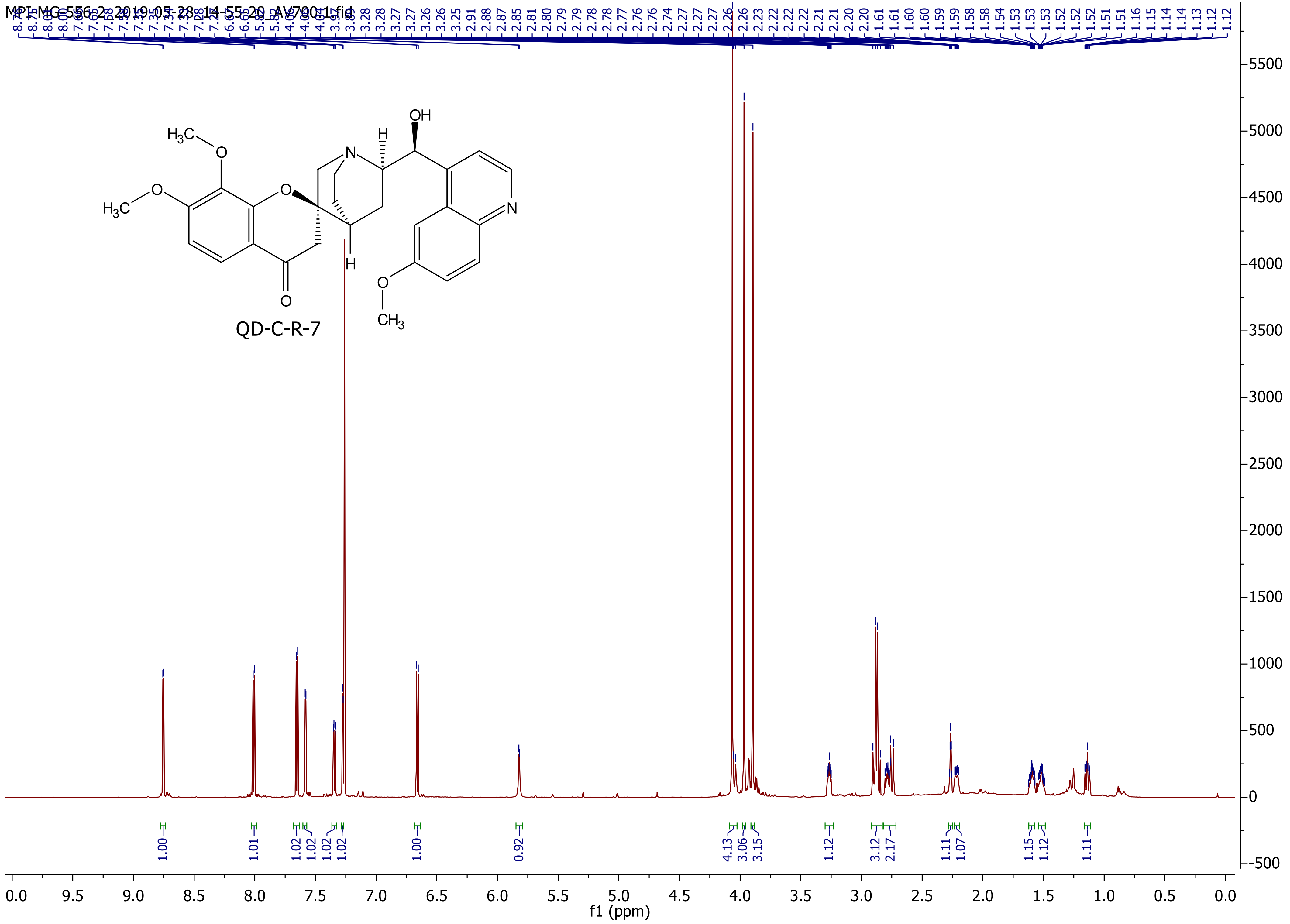
MPI-MG-556-2 2019-05-28 14-55-20 AV700 1.fid
3.28 3.28 3.27 3.26 3.26 3.25 2.91 2.88 2.87 2.85 2.81 2.80 2.79 2.79 2.78 2.78 2.77 2.76 2.76 2.74 2.27 2.27 2.27 2.26 2.26 2.23 2.22 2.22 2.22 2.21 2.21 2.20 2.20 1.61 1.61 1.60 1.60 1.59 1.59 1.58 1.58 1.54 1.53 1.53 1.53 1.52 1.52 1.52 1.51 1.51 1.16 1.15 1.14 1.14 1.13 1.12 1.12
OH
H
N
H_3C
O
O
O
H_3C
N
H
O
O
CH_3
QD-C-R-7
1.00 1.01 1.02 1.02 1.02 1.02 1.00 0.92 4.13 3.06 3.15 1.12 3.12 2.17 1.11 1.07 1.15 1.12 1.11
f1 (ppm)

MPI-MG-556-2_2019-05-28_14-55-20_AV700.6.fid

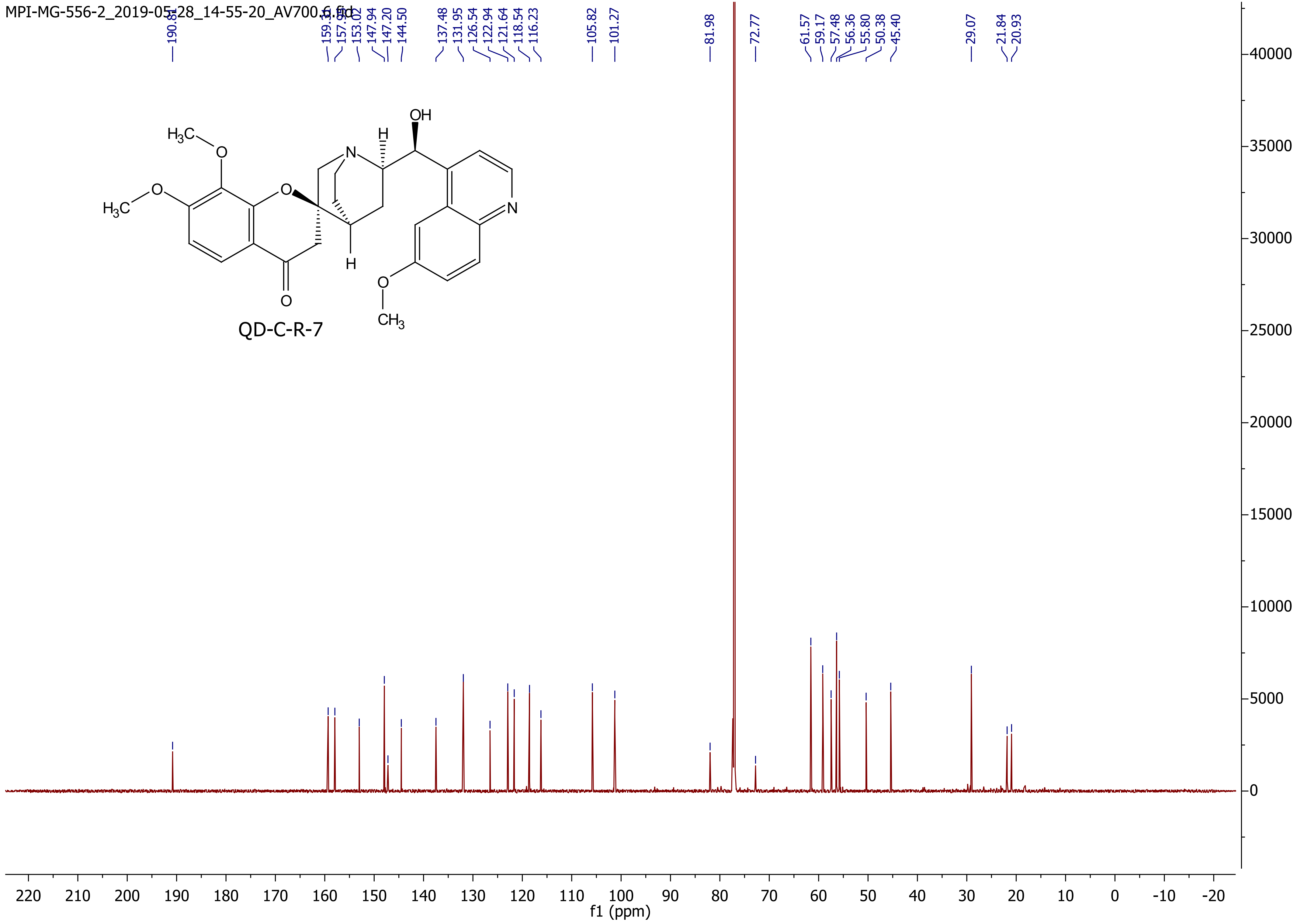

QD-C-R-7

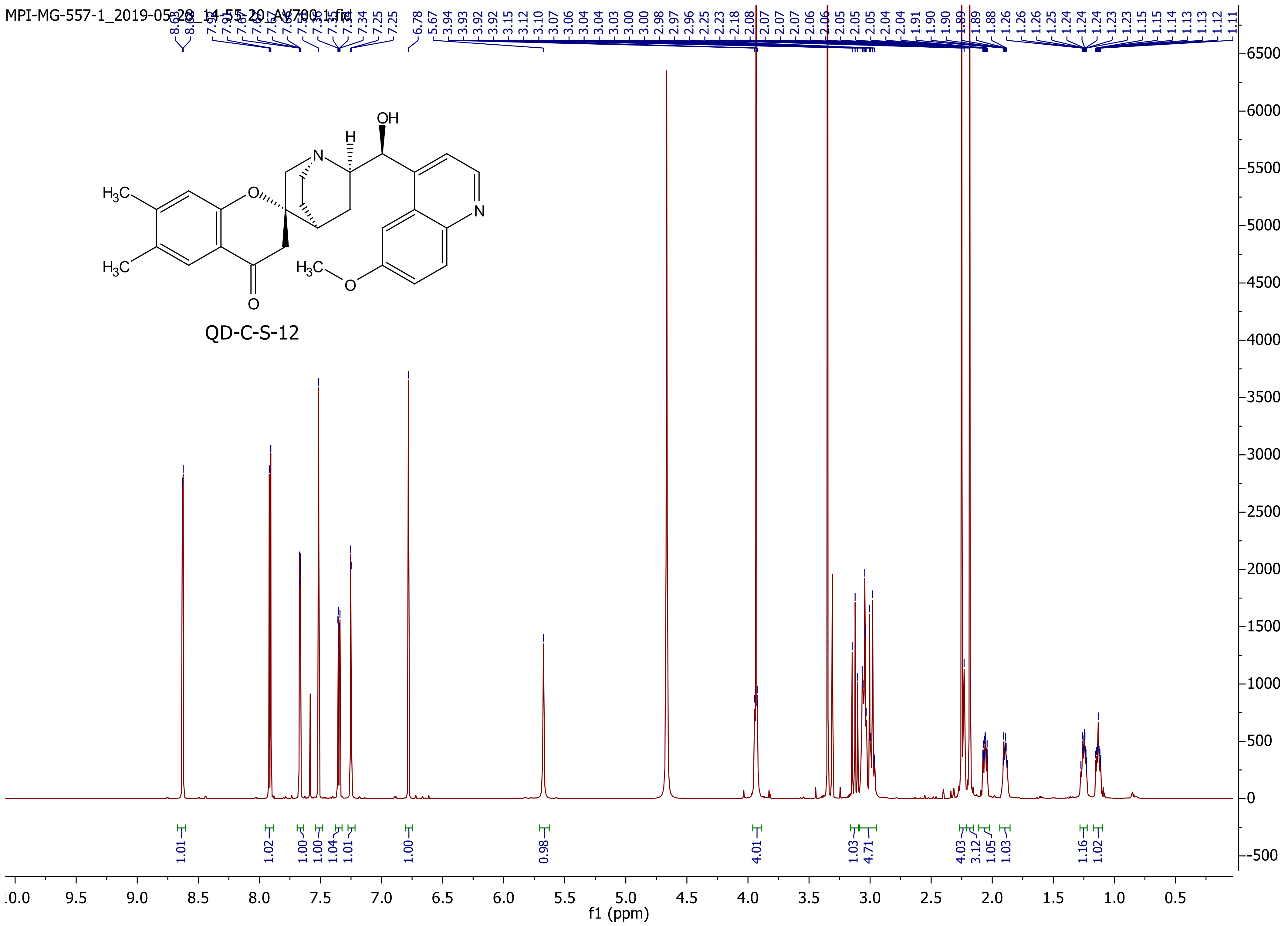
MPI-MG-557-1_2019-05-28_14-55-20_AV700 1.fid
OH
H
N
O
N
H_3C
H_3C
H_3C
O
O
QD-C-S-12
f1 (ppm)

MPI-MG-557-1_2019-05-28_14-55-20_AV700.6.fid

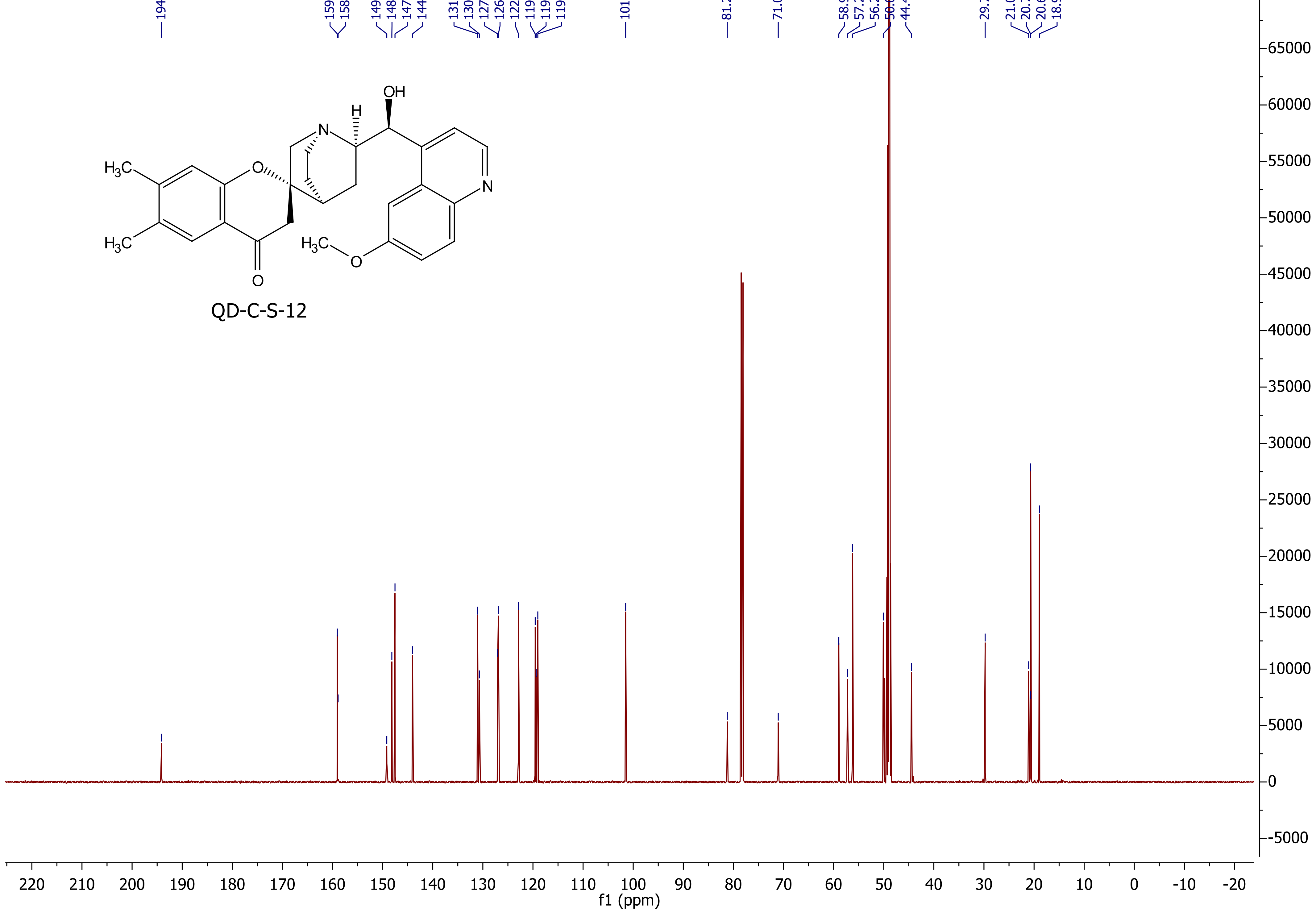

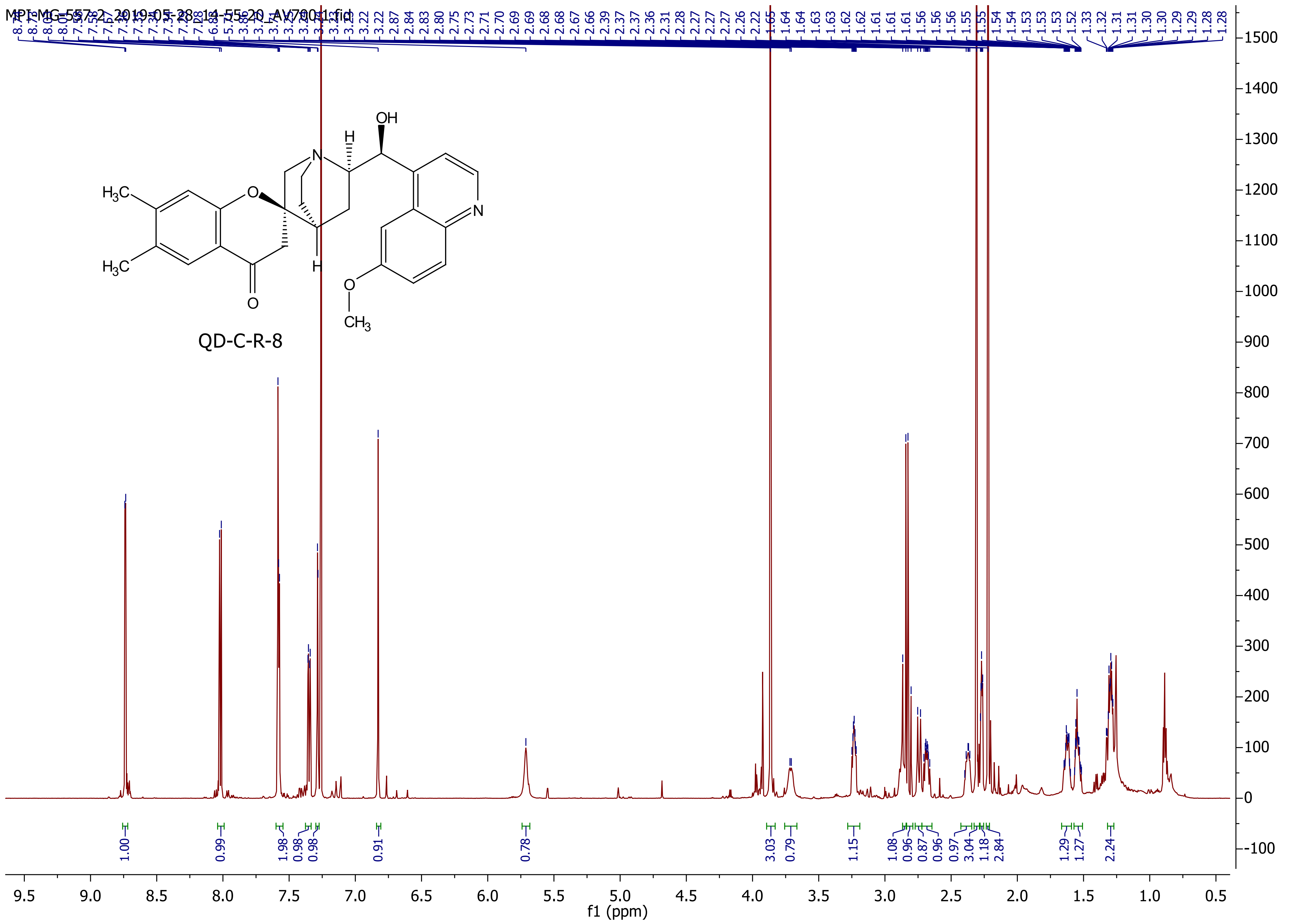

OH
H
N
O
H3C
H3C
N
H
O
O
CH3
QD-C-R-8
f1 (ppm)

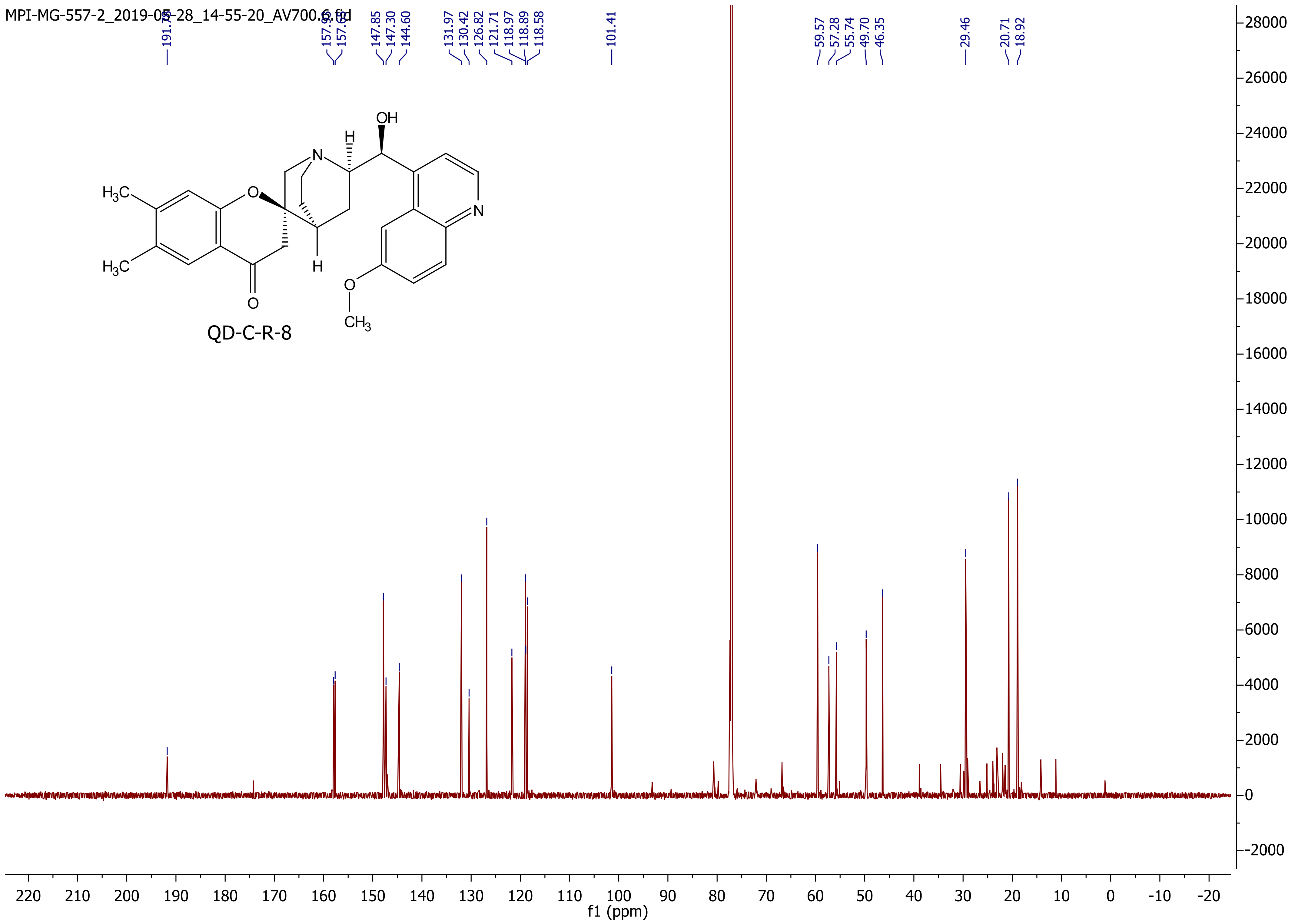

MPI-MG-557-2_2019-05-28_14-55-20_AV700.6.fid
147.85
147.30
144.60
131.97
130.42
126.82
121.71
118.97
118.89
118.58
101.41
59.57
57.28
55.74
49.70
46.35
29.46
20.71
18.92
OH
H
N
N
O
H3C
H3C
H
O
O
CH3
QD-C-R-8
f1 (ppm)

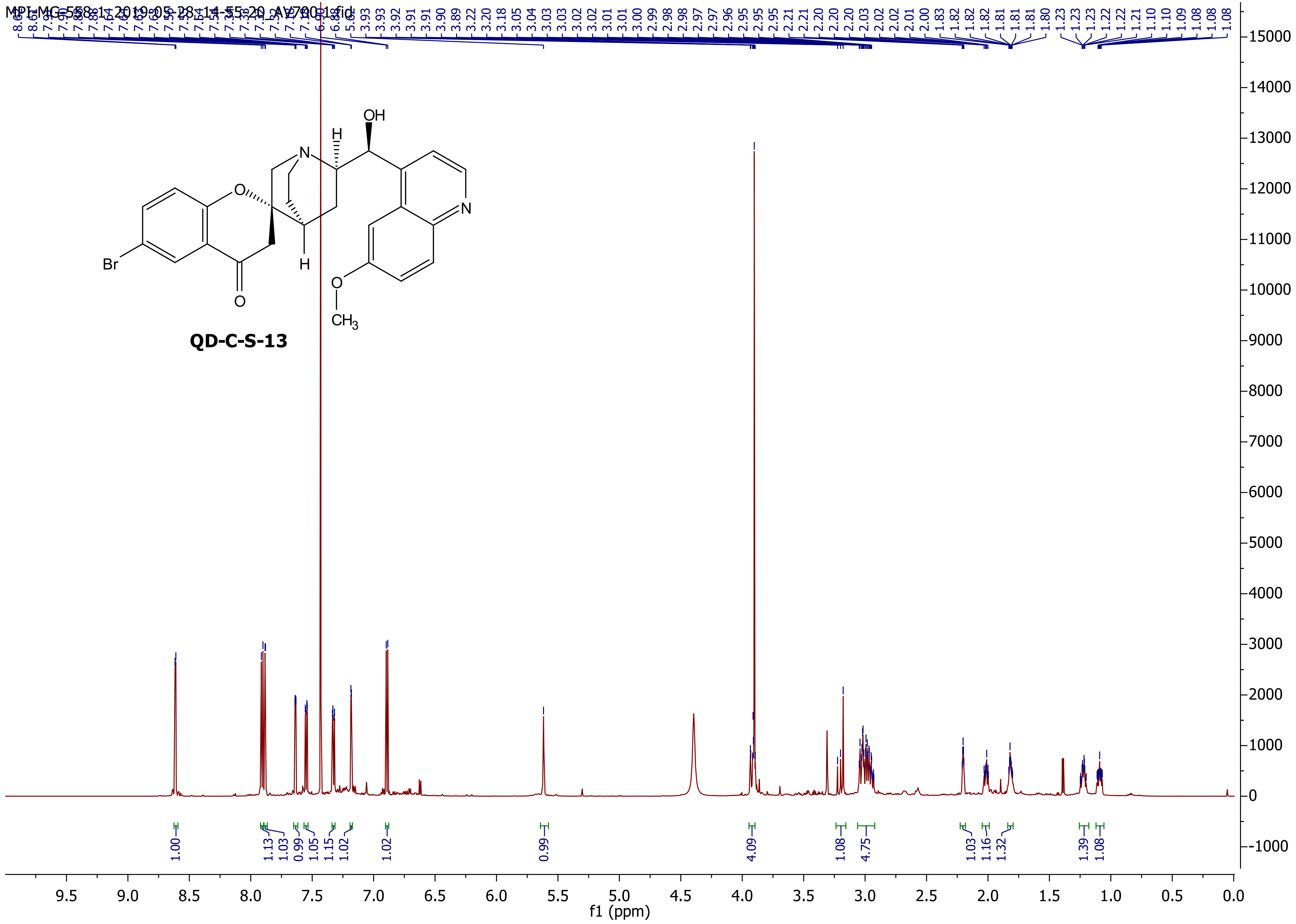
MPI-MG-558-1 2019-05-28 14-55-20 AV700 1 fid
3.93 3.93 3.92 3.91 3.91 3.90 3.89 3.22 3.20 3.18 3.05 3.04 3.03 3.03 3.02 3.02 3.01 3.01 3.00 2.99 2.98 2.98 2.97 2.97 2.96 2.95 2.95 2.95 2.21 2.21 2.20 2.20 2.20 2.03 2.02 2.02 2.01 2.00 1.83 1.82 1.82 1.82 1.81 1.81 1.81 1.80 1.23 1.23 1.23 1.22 1.22 1.21 1.10 1.10 1.09 1.08 1.08 1.08
OH
H
N
O
N
Br
O
H
O
CH3
QD-C-S-13
1.00 1.13 1.03 0.99 1.05 1.15 1.02 1.02 0.99 4.09 1.08 4.75 1.03 1.16 1.32 1.39 1.08
f1 (ppm)

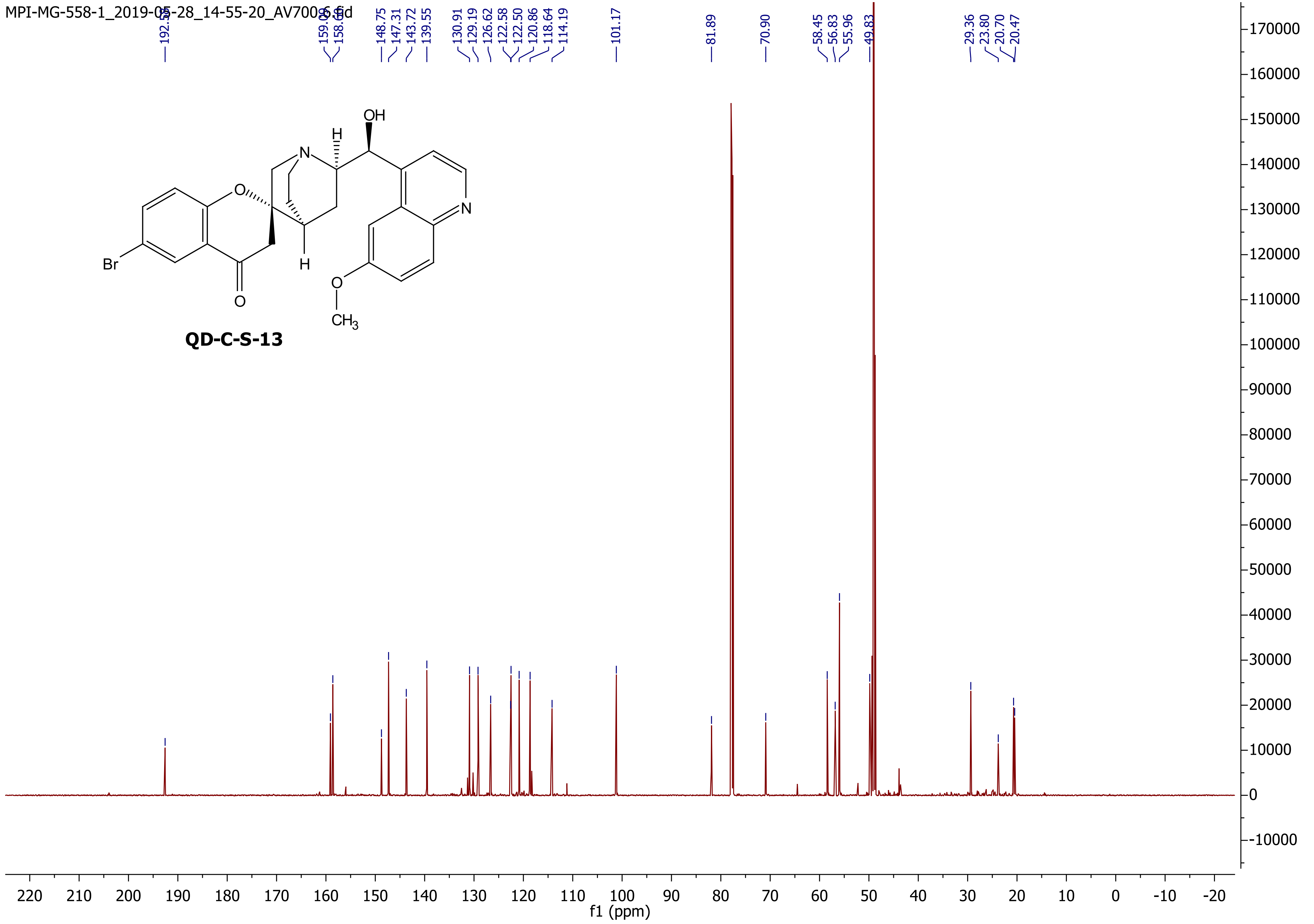

MPI-MG-558-1_2019-05-28_14-55-20_AV7006.fid
192.58
148.75
147.31
143.72
139.55
130.91
129.19
126.62
122.58
122.50
120.86
118.64
114.19
101.17
81.89
70.90
58.45
56.83
55.96
49.83
29.36
23.80
20.70
20.47
OH
H
N
O
N
Br
O
H
O
CH3
f1 (ppm)

QD-C-S-13

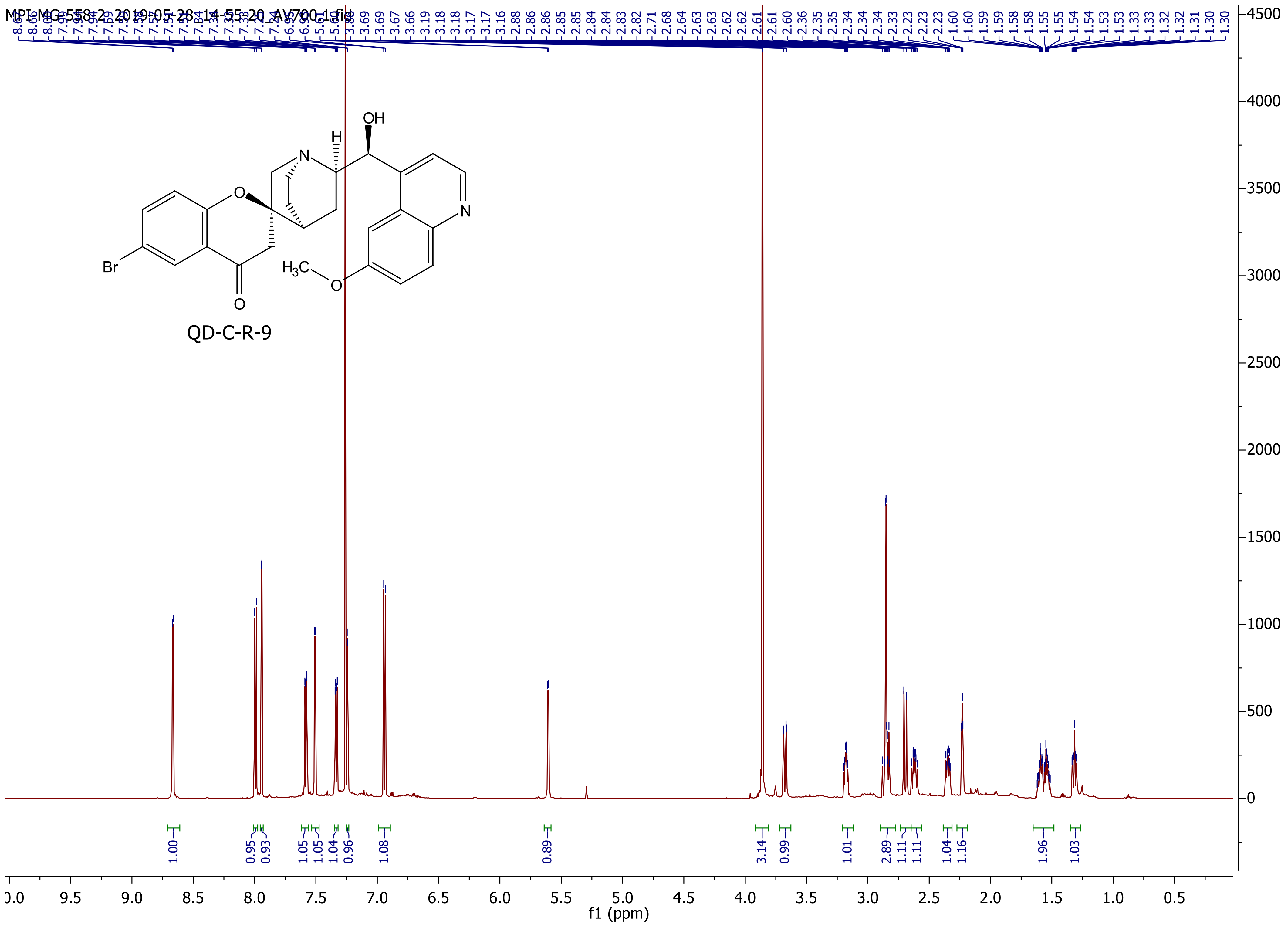
MPI-MG-558-2_2019-05-28_14-55-20_AV700_1.fid
OH
H
N
O
N
Br
O
H3C
O
QD-C-R-9
1.00
0.95
0.93
1.05
1.05
1.04
0.96
1.08
0.89
3.14
0.99
1.01
2.89
1.11
1.11
1.04
1.16
1.96
1.03
f1 (ppm)

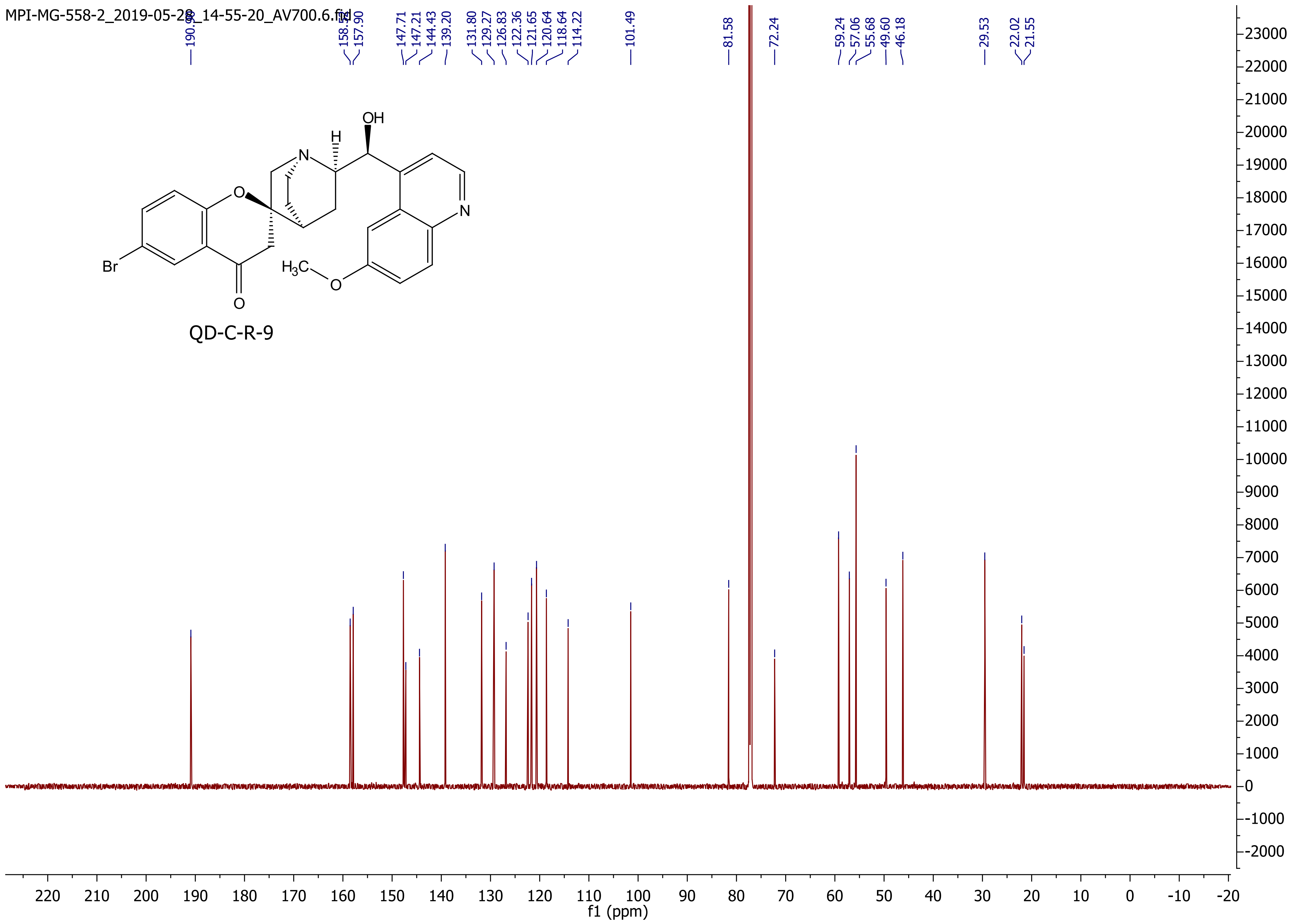

MPI-MG-558-2_2019-05-28_14-55-20_AV700.6.fid
147.71
147.21
144.43
139.20
131.80
129.27
126.83
122.36
121.65
120.64
118.64
114.22
101.49
81.58
72.24
59.24
57.06
55.68
49.60
46.18
29.53
22.02
21.55
157.90
OH
H
N
O
N
Br
O
H_3C
O
QD-C-R-9
f1 (ppm)

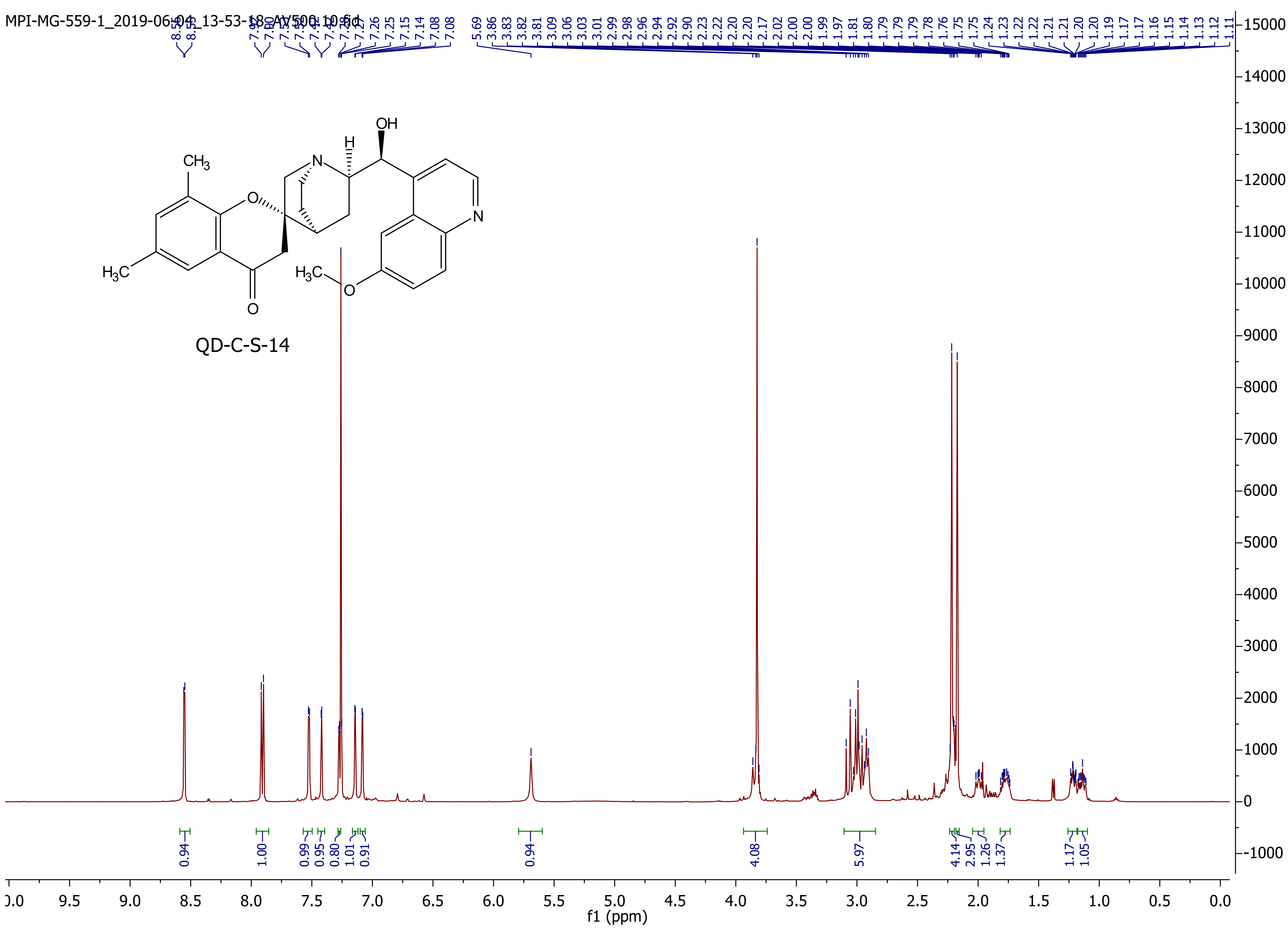

MPI-MG-559-1_2019-06-04_13-53-18_AV500 10.fid
QD-C-S-14
OH
H
N
CH3
O
H3C
O
H3C
O
N
f1 (ppm)

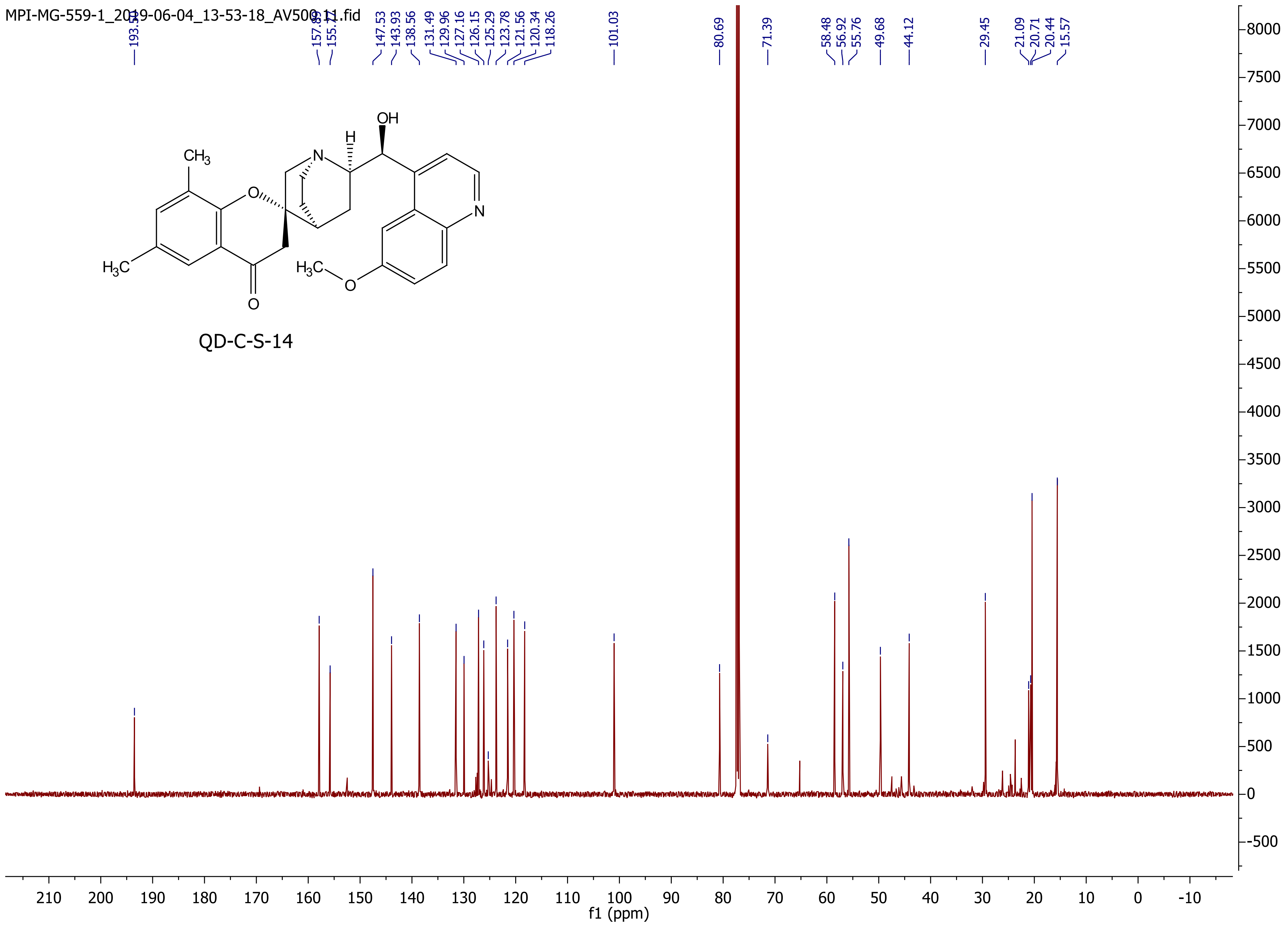

147.53
143.93
138.56
131.49
129.96
127.16
126.15
125.29
123.78
121.56
120.34
118.26
101.03
80.69
71.39
58.48
56.92
55.76
49.68
44.12
29.45
21.09
20.71
20.44
15.57
OH
H
N
CH3
O
N
H3C
H3C
O
O
QD-C-S-14
f1 (ppm)

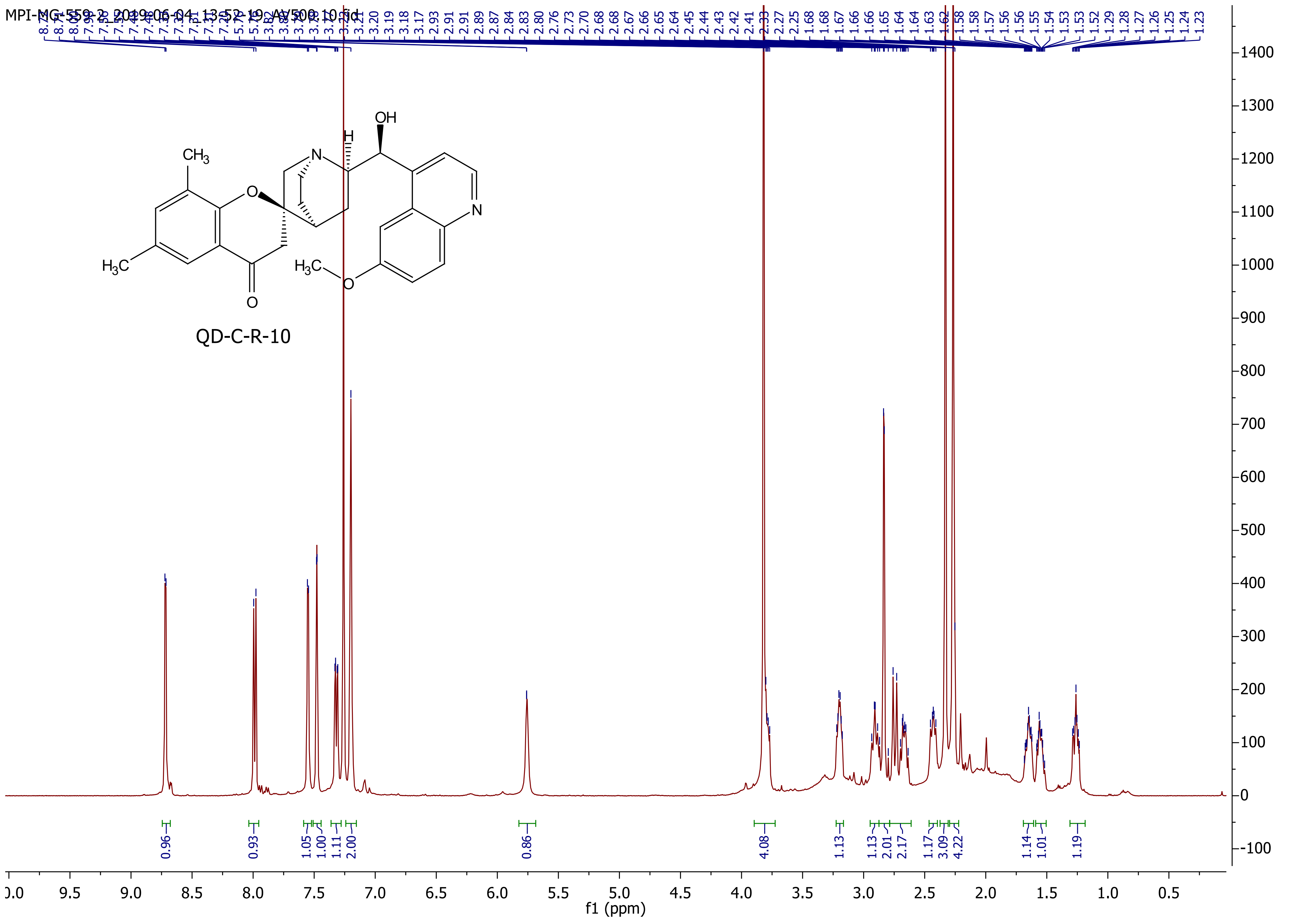
MPI-MG-559-2_2019-06-04_13-52-19_AV500 10.jid
QD-C-R-10
OH
H
N
O
CH3
H3C
H3C
O
f1 (ppm)
0.96
0.93
1.05
1.00
1.11
2.00
0.86
4.08
1.13
1.13
2.01
2.17
1.17
3.09
4.22
1.14
1.01
1.19

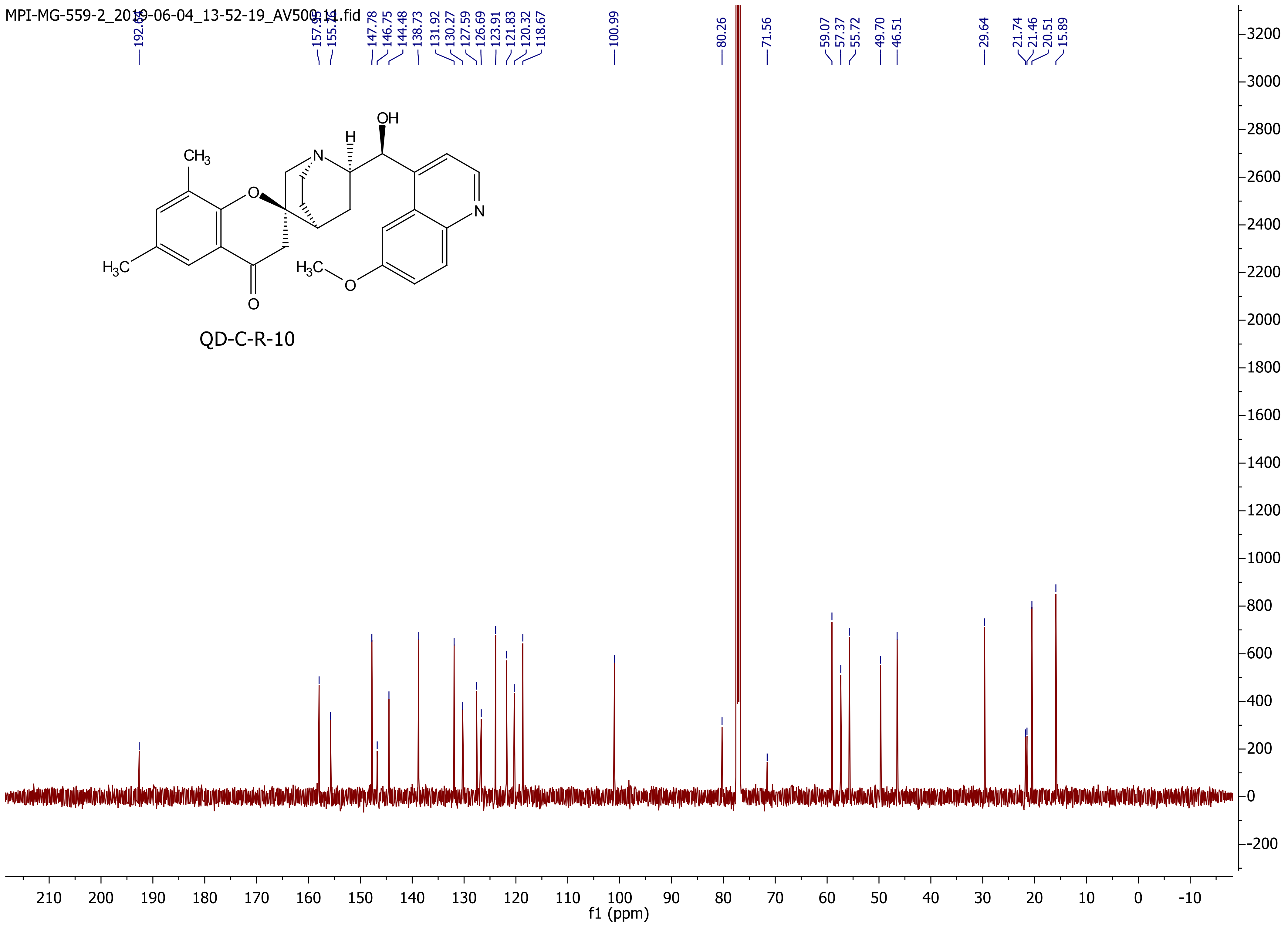

MPI-MG-559-2_2019-06-04_13-52-19_AV500
147.78
146.75
144.48
138.73
131.92
130.27
127.59
126.69
123.91
121.83
120.32
118.67
100.99
80.26
71.56
59.07
57.37
55.72
49.70
46.51
29.64
21.74
21.46
20.51
15.89
OH
H
N
CH3
O
H3C
H3C
O
O
N
QD-C-R-10
f1 (ppm)

MPI-MG-560-1_2019-06-05_06-26-03_AV600-10.fid
H1
z_Proton CDCl3 /NMR-Daten MPI 40

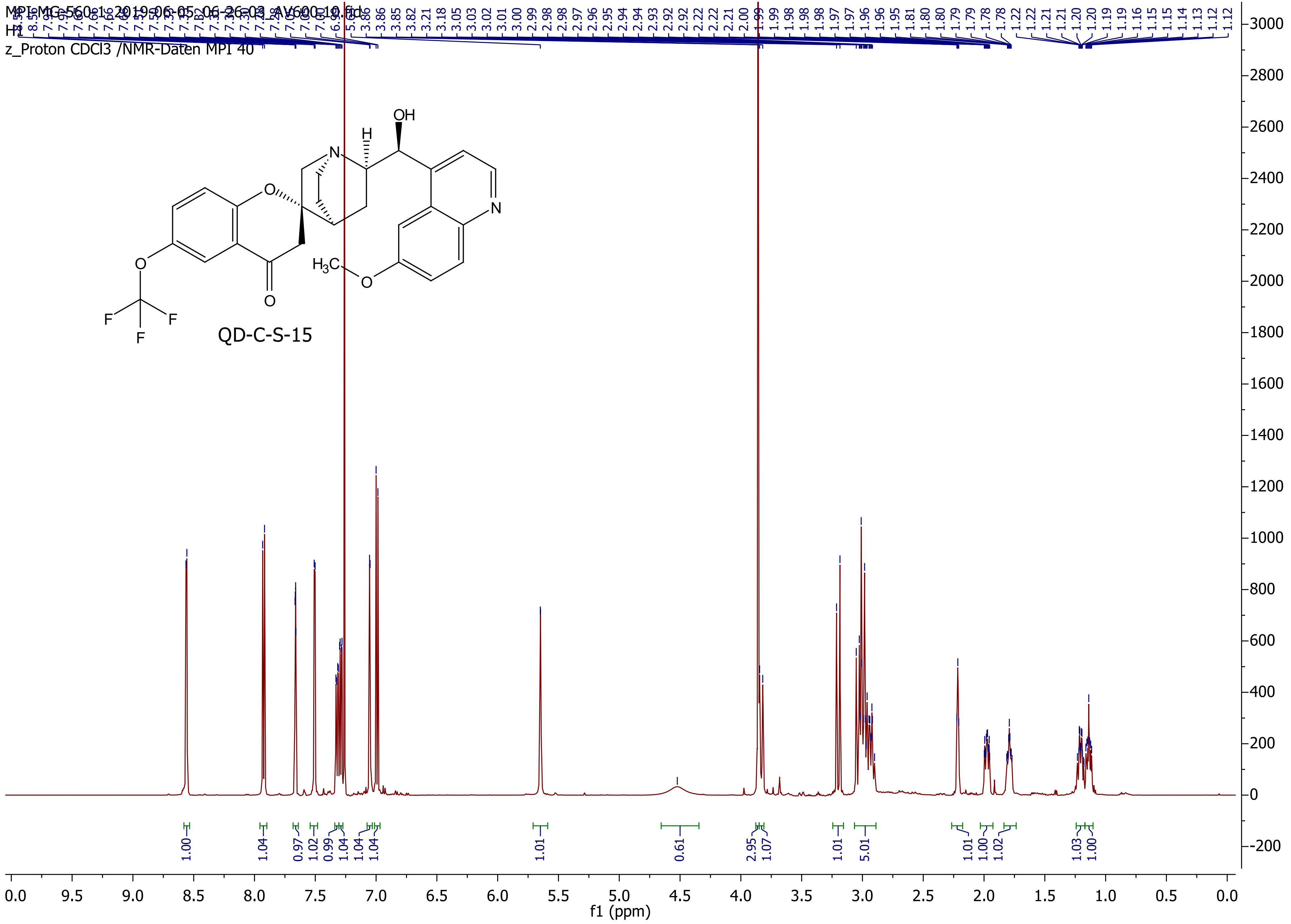

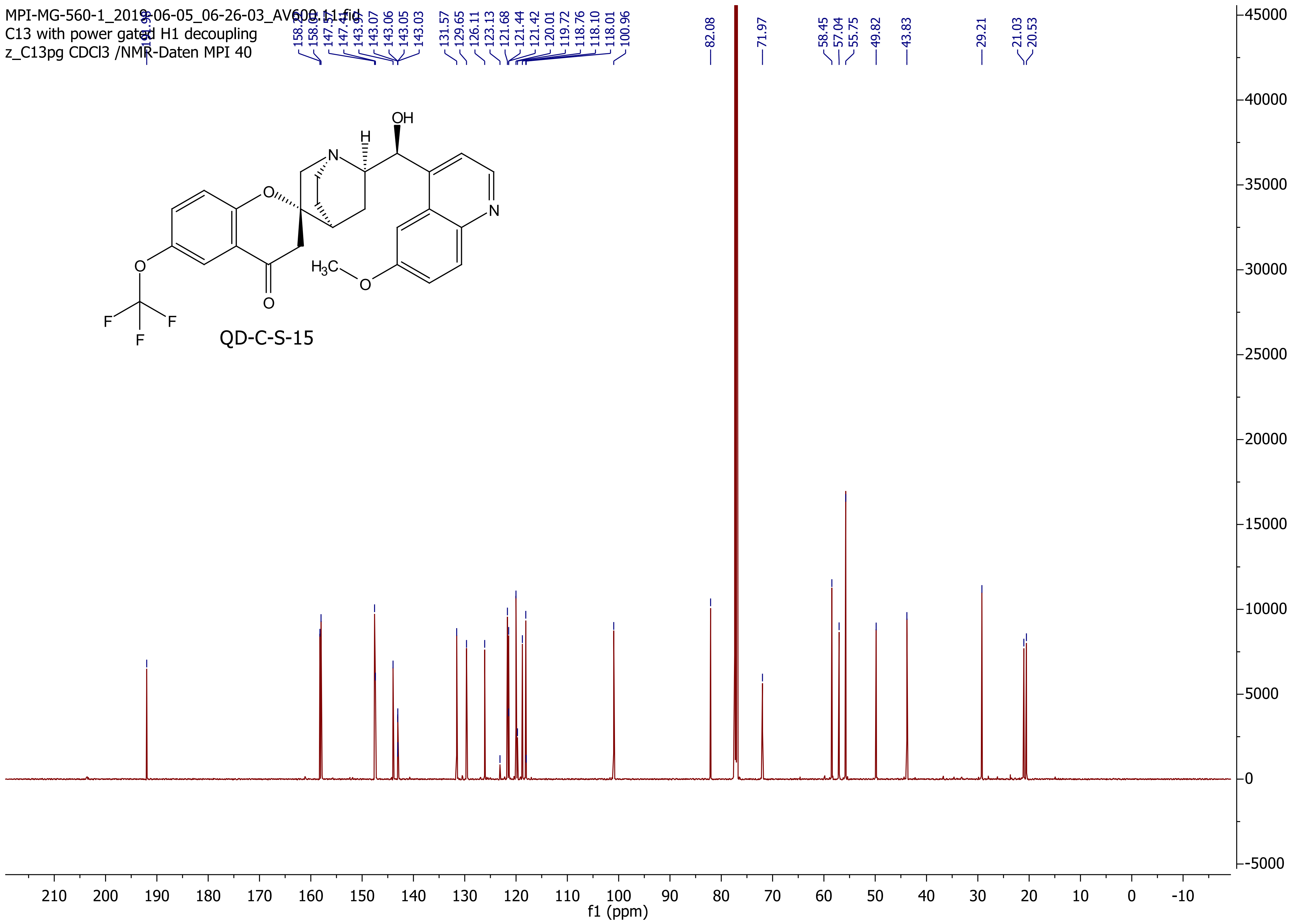

MPI-MG-560-1_2019-06-05_06-26-03_AV600.11.fid
C13 with power gated H1 decoupling
z_C13pg CDCl3 /NMR-Daten MPI 40
143.07
143.06
143.05
143.03
131.57
129.65
126.11
123.13
121.68
121.44
121.42
120.01
119.72
118.76
118.10
118.01
100.96
82.08
71.97
58.45
57.04
55.75
49.82
43.83
29.21
21.03
20.53
OH
H
N
O
N
H3C
O
O
O
F
F
F
QD-C-S-15
f1 (ppm)

MPI-MG-560-1_2019-06-05_06-26-03_AV600.12.fid
F19

z_F19 CDCl3 /NMR-Daten MPI 40

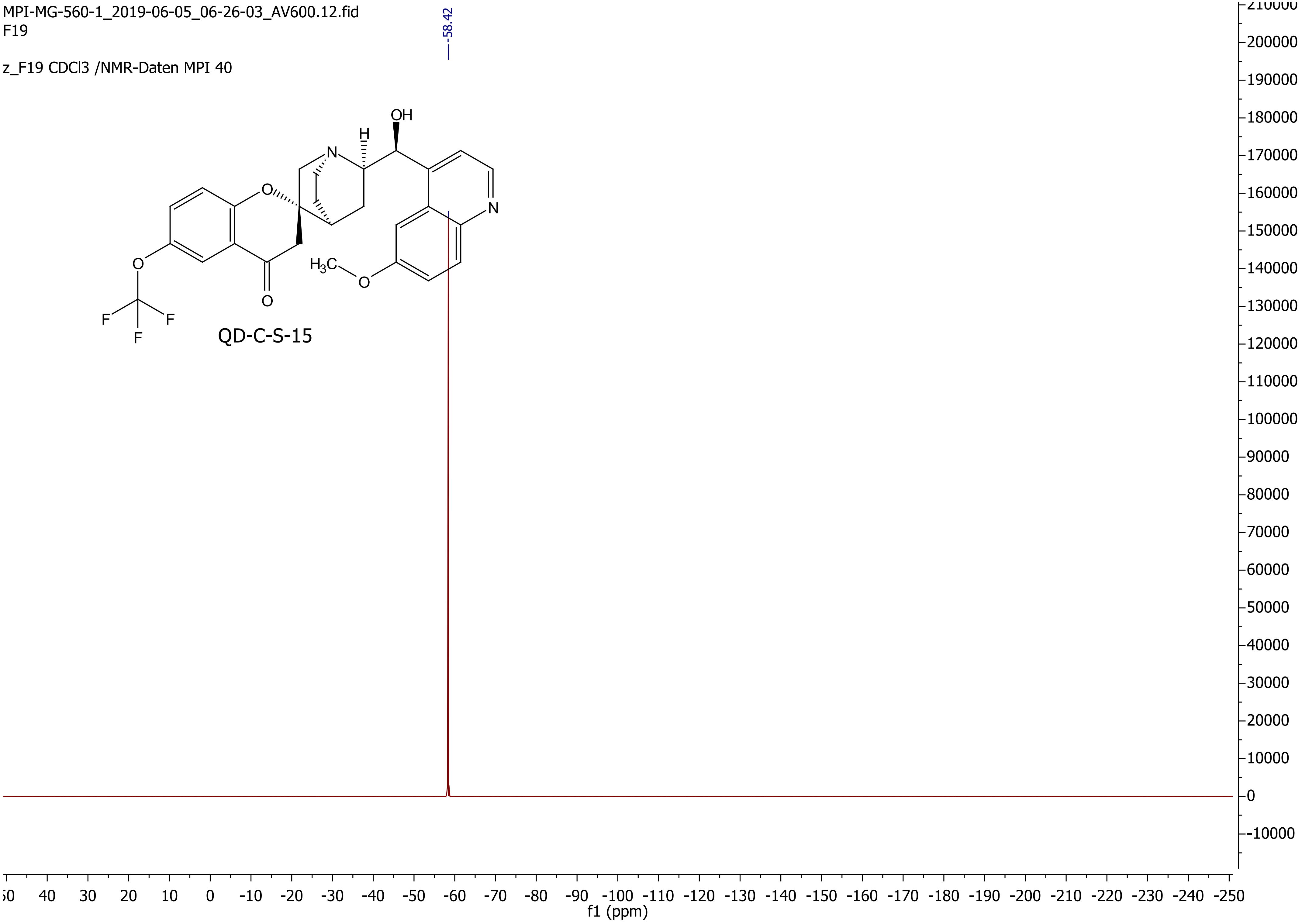

QD-C-S-15

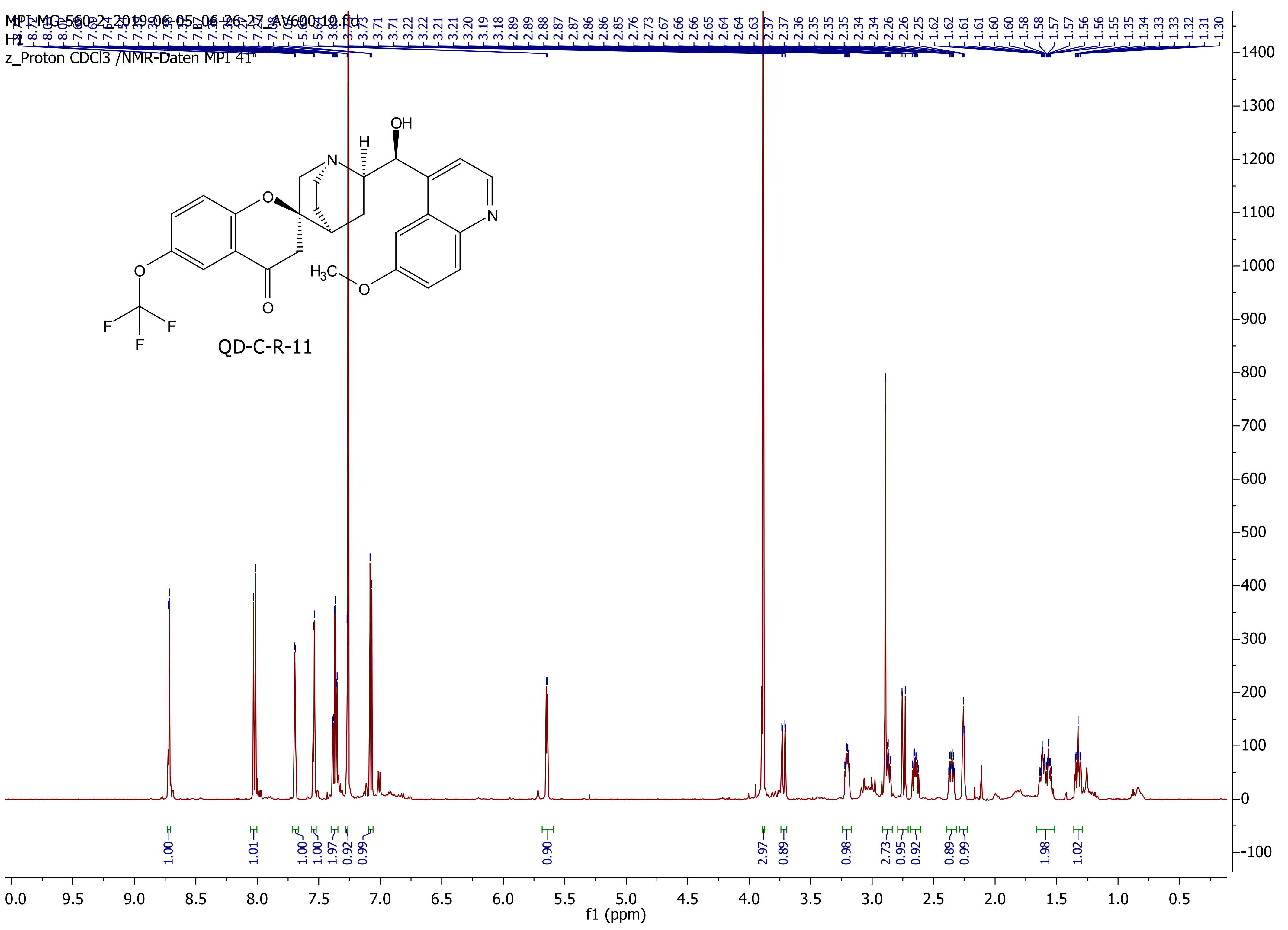
MPI-MG-560-2 2019-06-05 06-26-27 AV600 10.fid
H1
z_Proton CDCl3 /NMR-Daten MPI 41
QD-C-R-11
f1 (ppm)

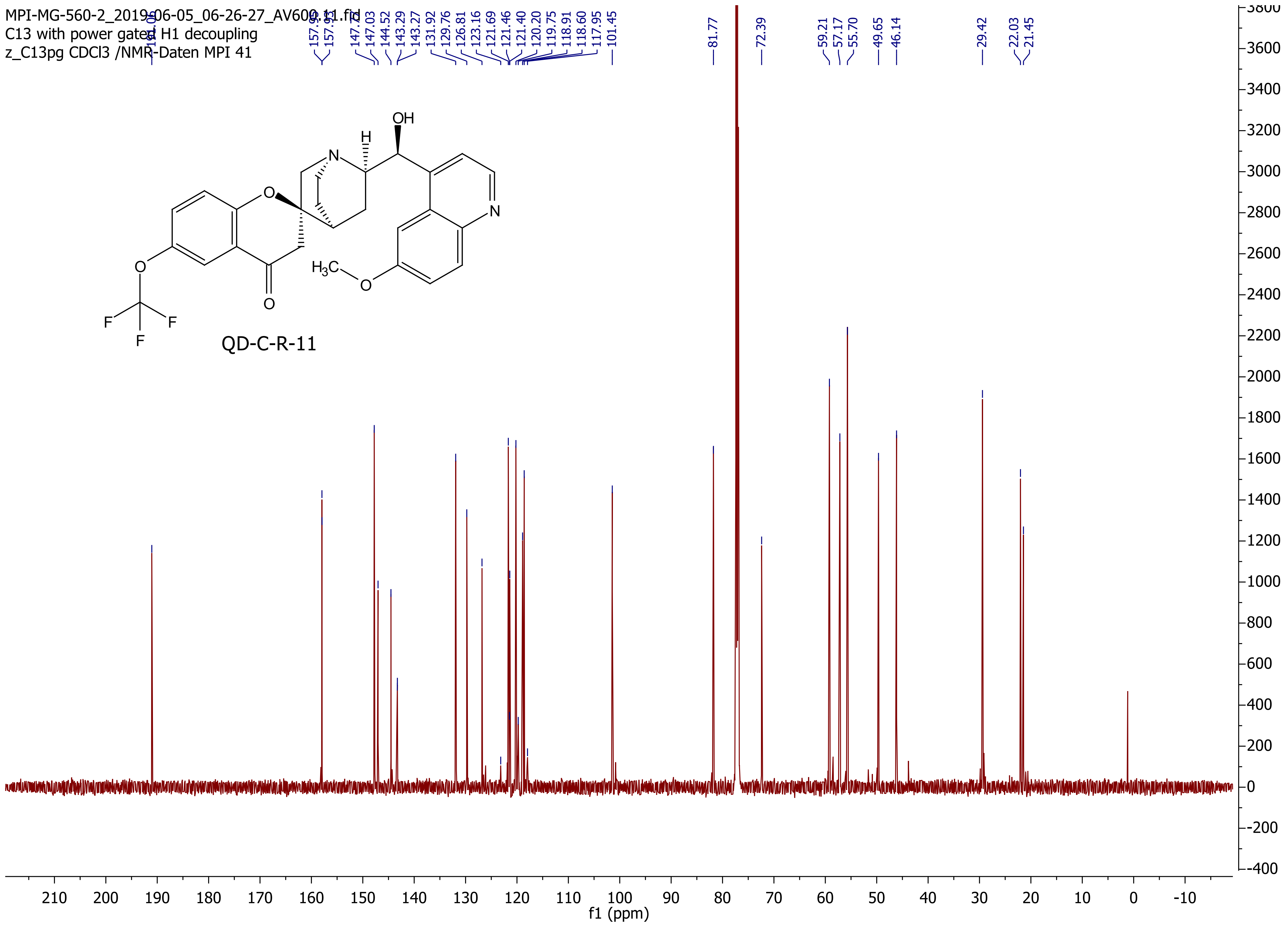
MPI-MG-560-2_2019-06-05_06-26-27_AV600.11.fid
C13 with power gated H1 decoupling
z_C13pg CDCl3 /NMR-Daten MPI 41
147.03
144.52
143.29
143.27
131.92
129.76
126.81
123.16
121.69
121.46
121.40
120.20
119.75
118.91
118.60
117.95
101.45
81.77
72.39
59.21
57.17
55.70
49.65
46.14
29.42
22.03
21.45
OH
H
N
O
N
O
H3C
O
O
F
F
F
QD-C-R-11
f1 (ppm)

MPI-MG-560-2_2019-06-05_06-26-27_AV600.12.fid
F19

z_F19 CDCl3 /NMR-Daten MPI 41

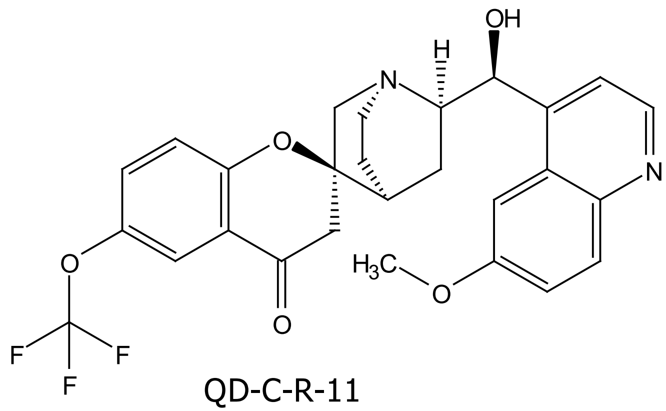

QD-C-R-11

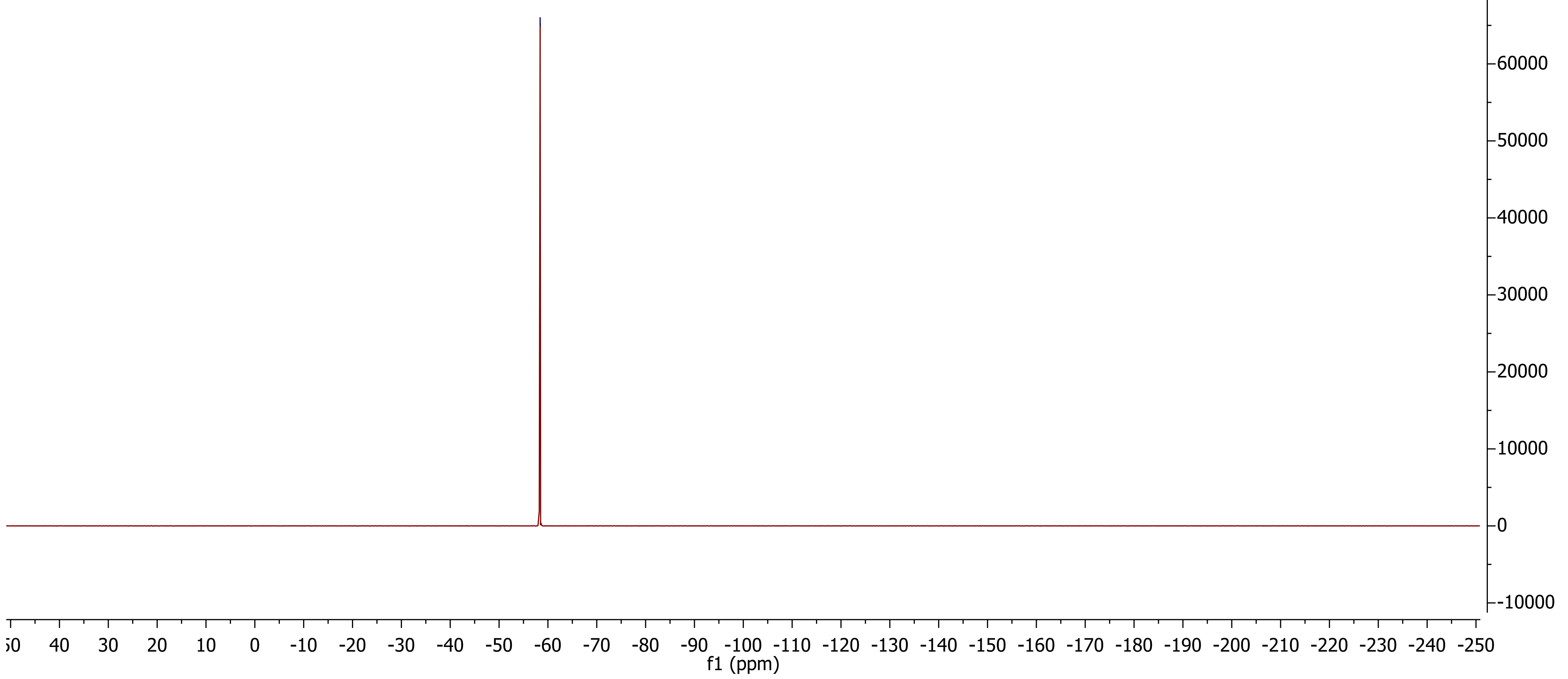

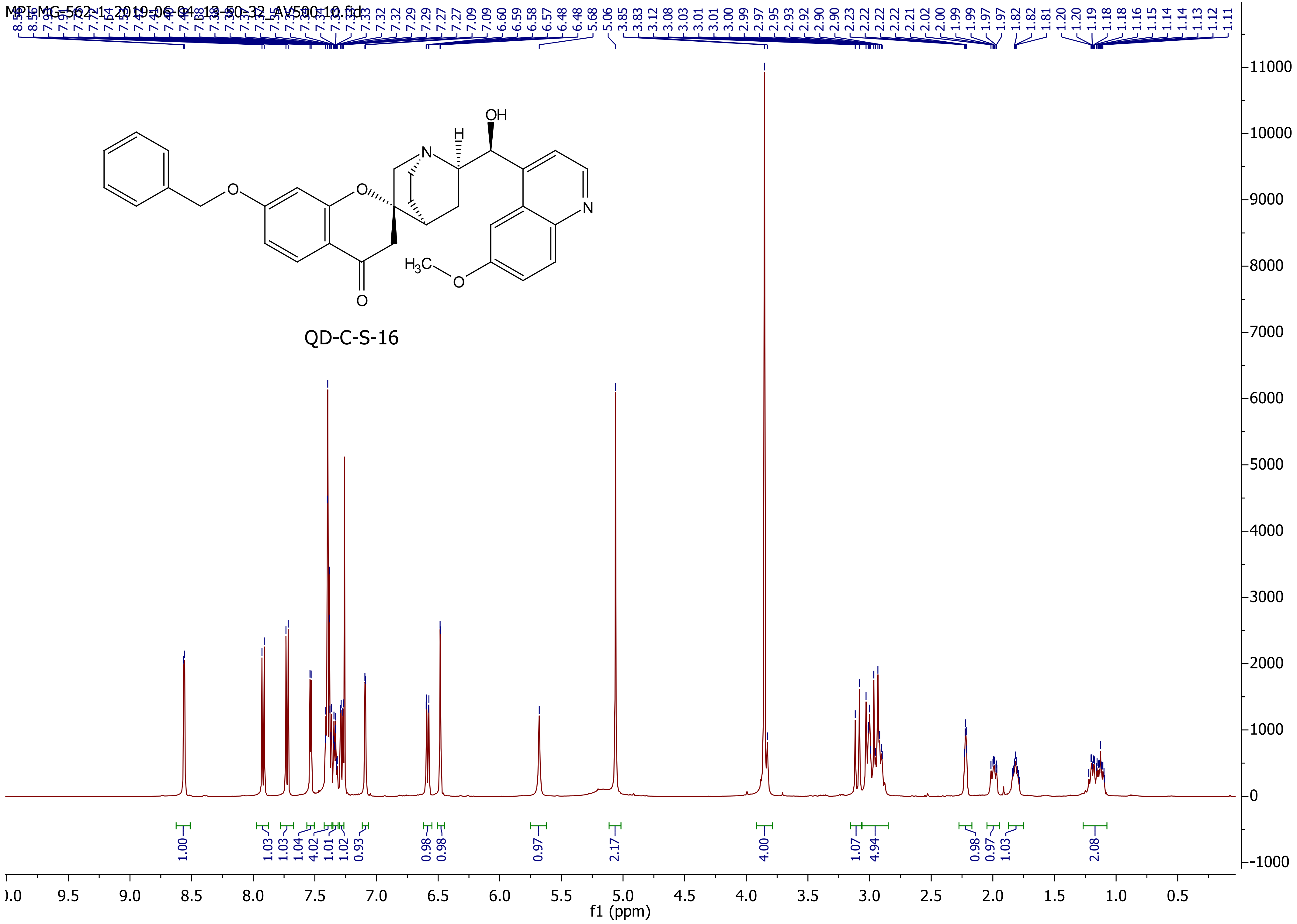

MPI-MG-562-1 2019-06-04 13-50-32 AV500 10.fid
OH
H
N
O
N
H3C
O
O
QD-C-S-16
f1 (ppm)

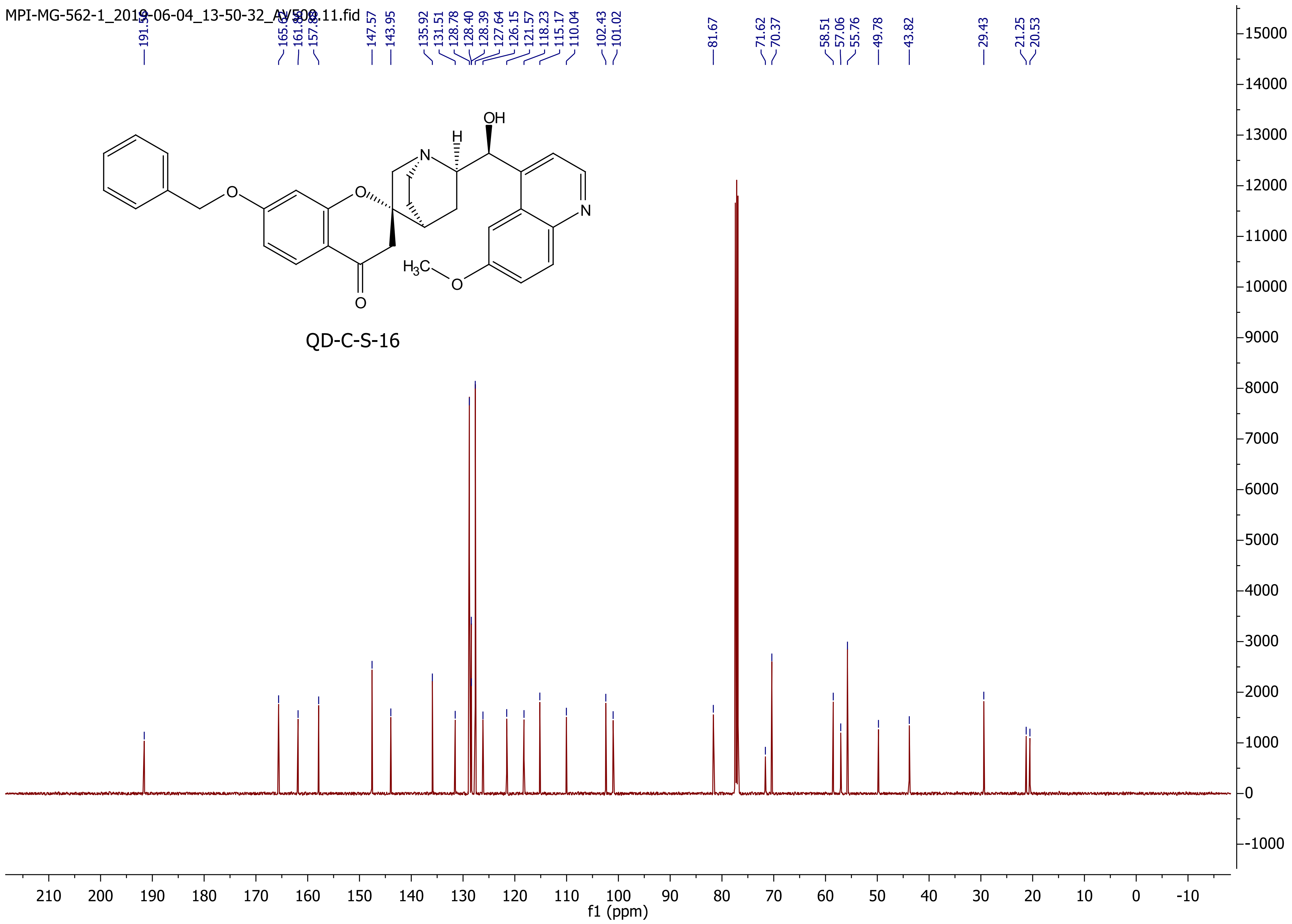

MPI-MG-562-1_2019-06-04_13-50-32_AV500.11.fid
147.57
143.95
135.92
131.51
128.78
128.40
128.39
127.64
126.15
121.57
118.23
115.17
110.04
102.43
101.02
81.67
71.62
70.37
58.51
57.06
55.76
49.78
43.82
29.43
21.25
20.53
OH
H
N
O
O
N
O
H3C
O
f1 (ppm)

QD-C-S-16

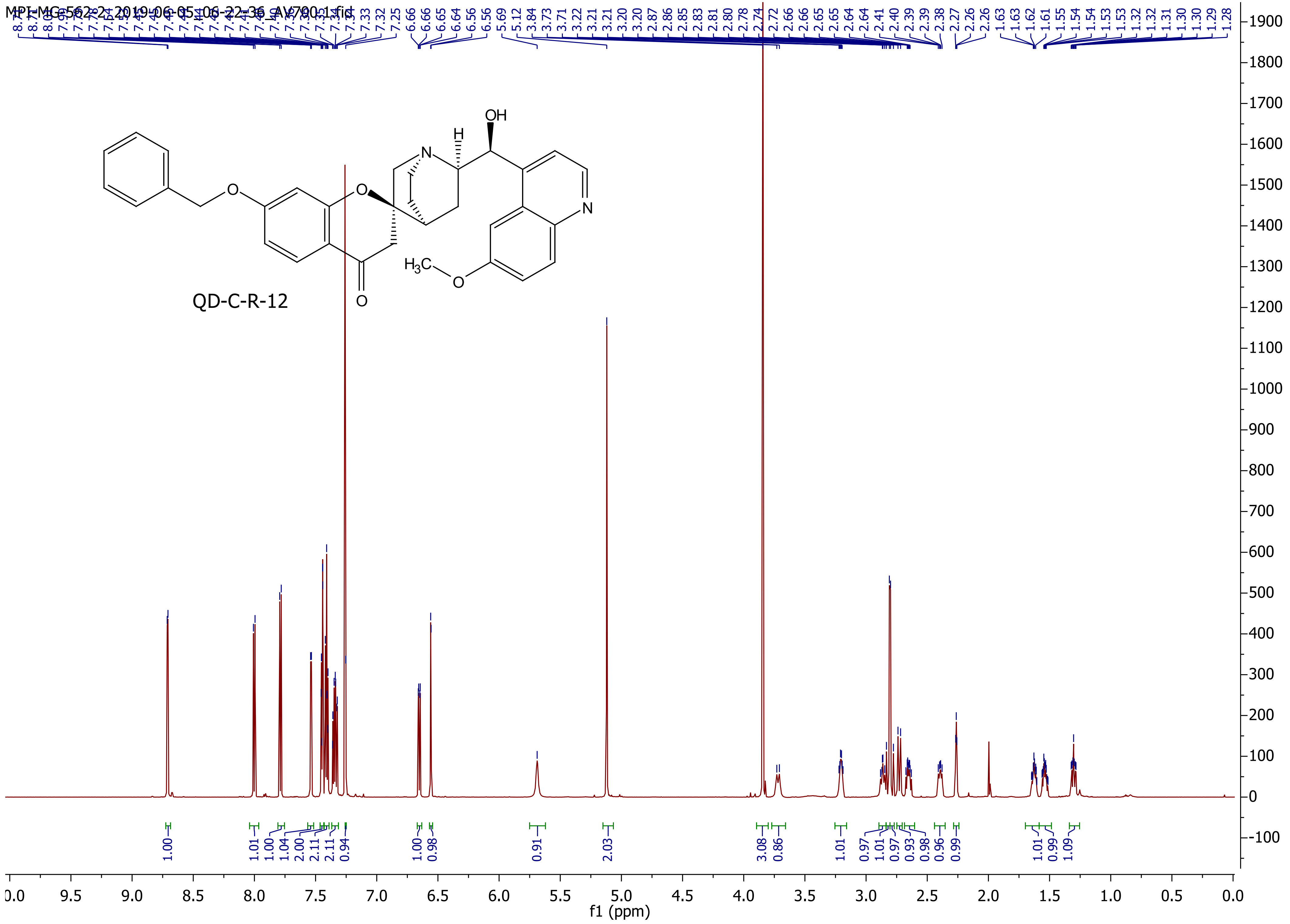
MPI-MG-562-2-2019-06-05_06-22-36_AV700-1.fid
QD-C-R-12
OH
H
N
O
H3C
f1 (ppm)

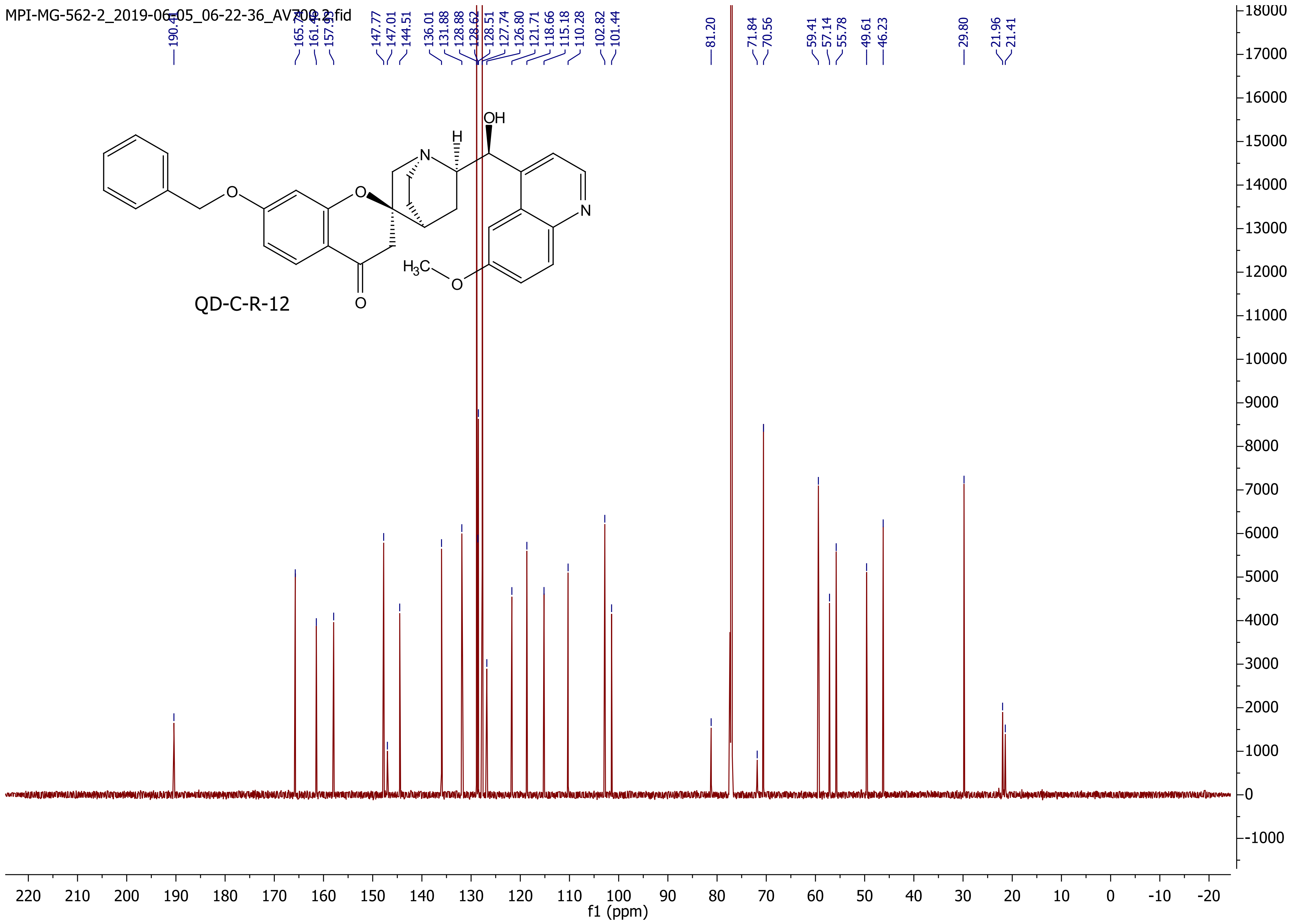

MPI-MG-562-2_2019-06-05_06-22-36_AV700.2.fid
147.77
147.01
144.51
136.01
131.88
128.88
128.62
128.51
127.74
126.80
121.71
118.66
115.18
110.28
102.82
101.44
81.20
71.84
70.56
59.41
57.14
55.78
49.61
46.23
29.80
21.96
21.41
OH
H
N
O
N
H3C
O
O
QD-C-R-12
f1 (ppm)

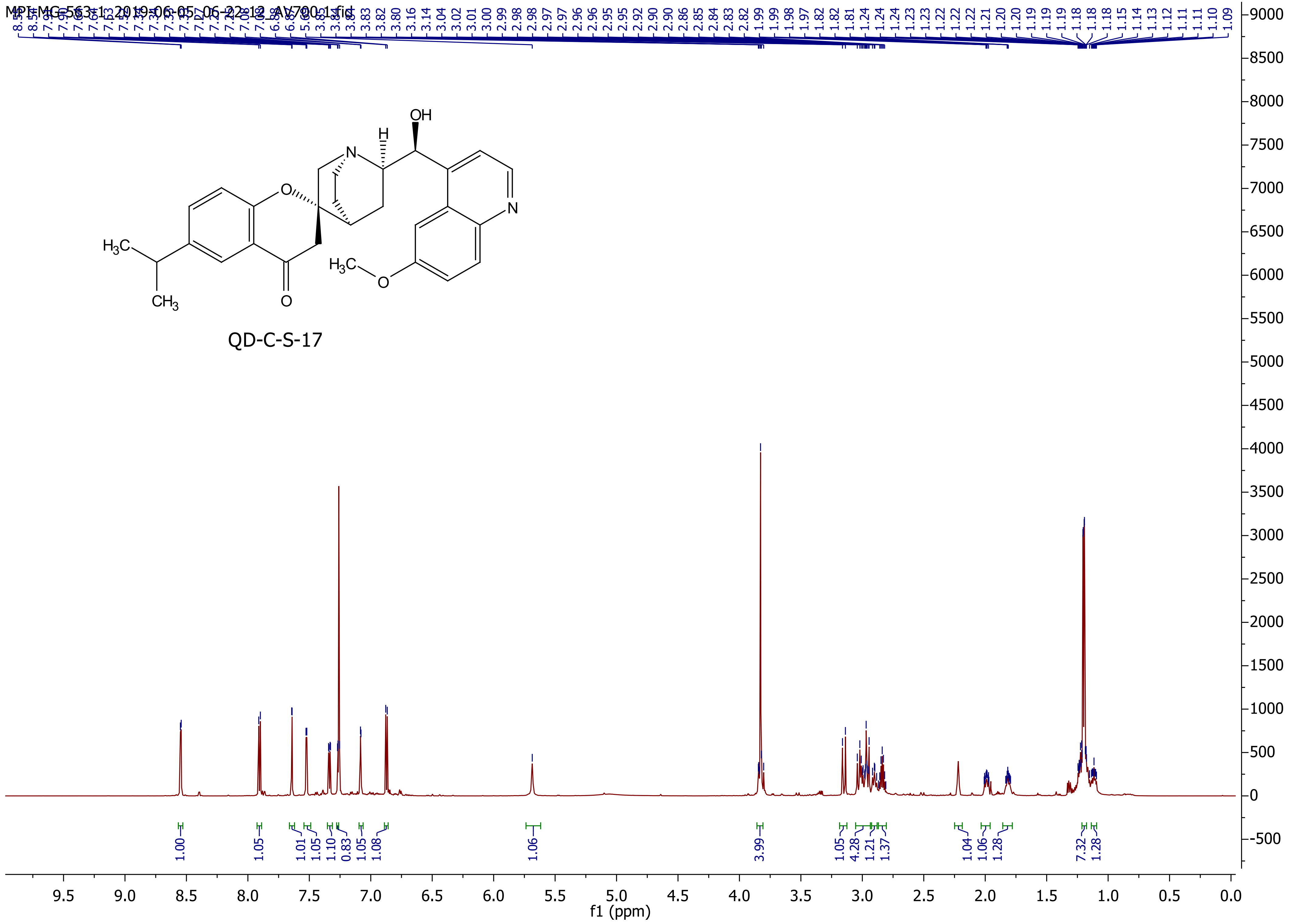

MPI-MG-563-1 2019-06-05 06-22-12 AV700 1.fid
QD-C-S-17
f1 (ppm)

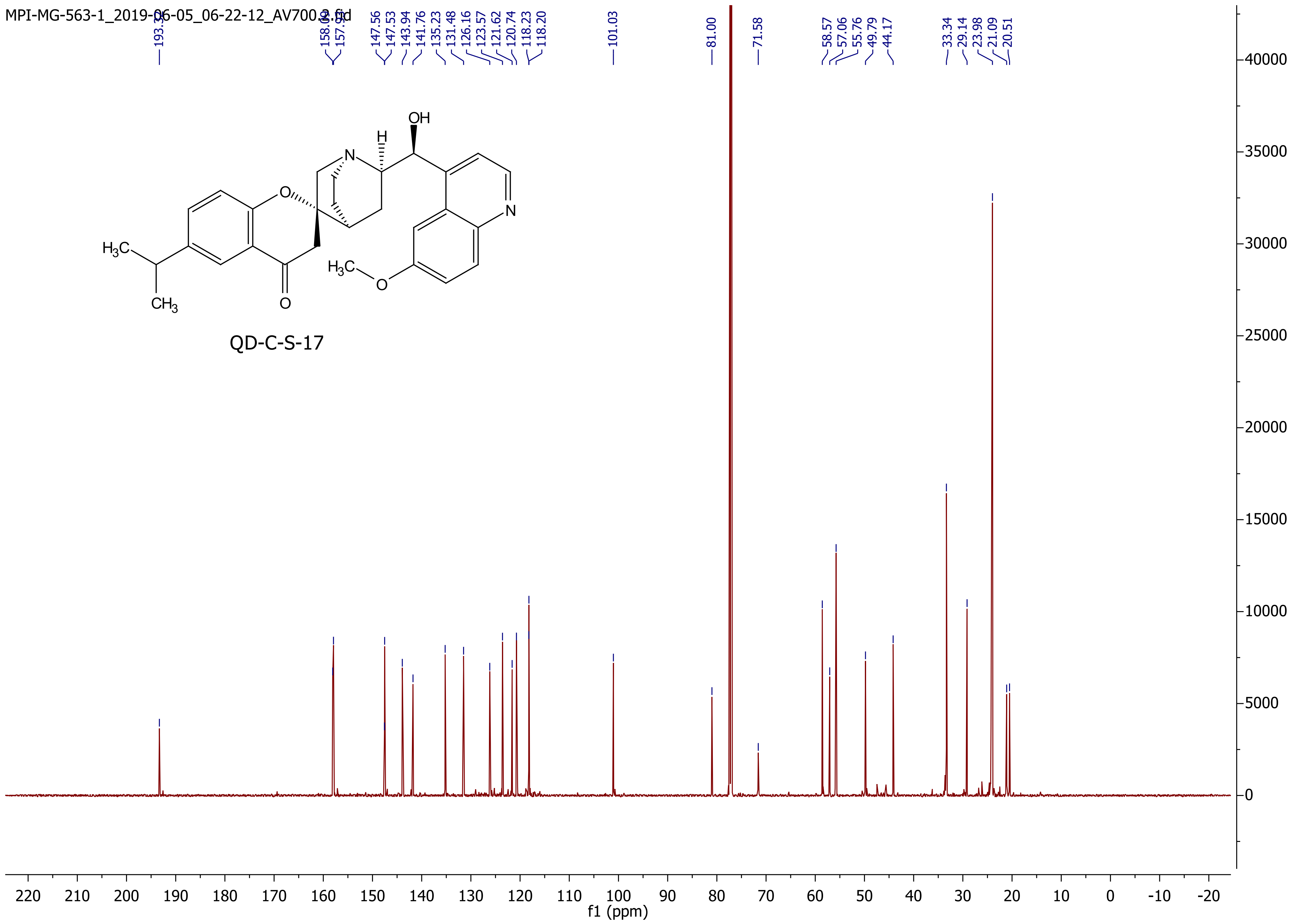
MPI-MG-563-1_2019-06-05_06-22-12_AV700.2.fid
147.56
147.53
143.94
141.76
135.23
131.48
126.16
123.57
121.62
120.74
118.23
118.20
101.03
81.00
71.58
58.57
57.06
55.76
49.79
44.17
33.34
29.14
23.98
21.09
20.51
OH
H
N
O
N
H_3C
CH_3
O
H_3C
O
QD-C-S-17
f1 (ppm)

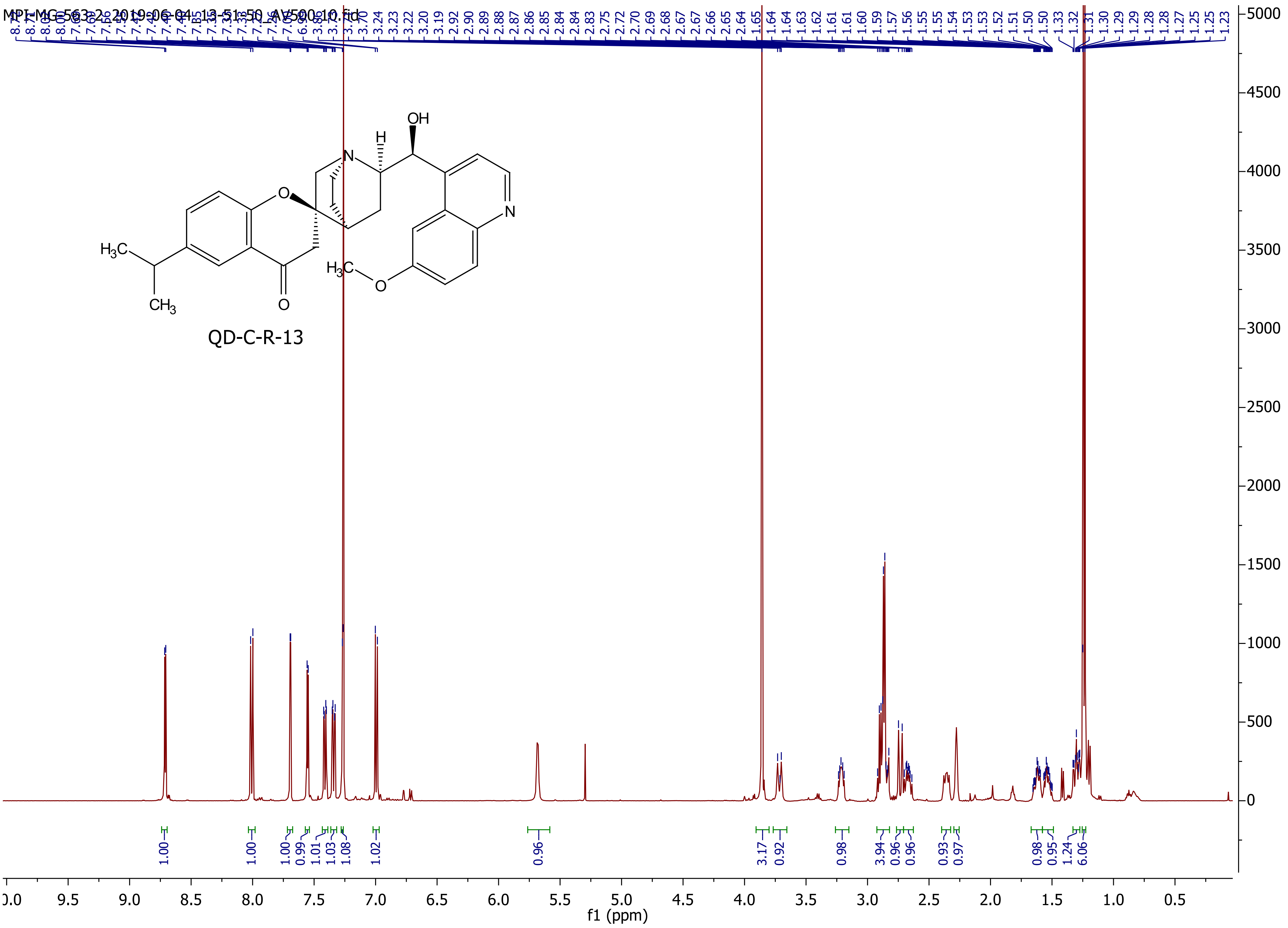

MPI-MG-563-2 2019-06-04 13-51-50 AV500 10.fid
QD-C-R-13
OH
H
N
O
H3C
CH3
O
H3C
O
N
1.00
1.00
1.00
0.99
1.01
1.03
1.08
1.02
0.96
3.17
0.92
0.98
3.94
0.96
0.96
0.93
0.97
0.98
0.95
1.24
6.06
f1 (ppm)

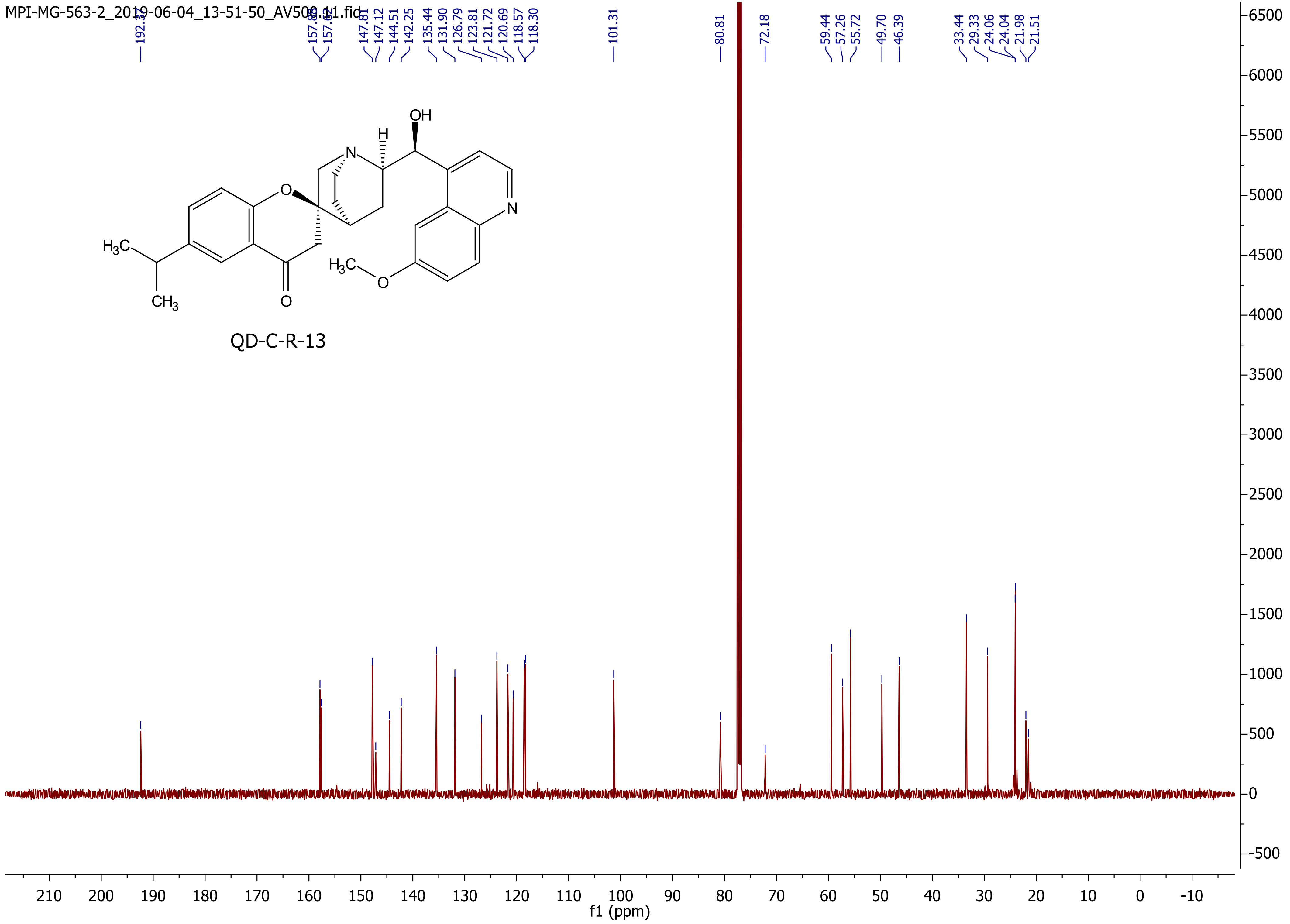

MPI-MG-563-2_2019-06-04_13-51-50_AV500.11.fid
192.37
157.88
157.62
147.81
147.12
144.51
142.25
135.44
131.90
126.79
123.81
121.72
120.69
118.57
118.30
101.31
80.81
72.18
59.44
57.26
55.72
49.70
46.39
33.44
29.33
24.06
24.04
21.98
21.51
OH
H
N
O
N
H_3C
CH_3
O
H_3C
O
QD-C-R-13
6500
6000
5500
5000
4500
4000
3500
3000
2500
2000
1500
1000
500
0
-500
210
200
190
180
170
160
150
140
130
120
110
100
90
80
70
60
50
40
30
20
10
0
-10
f1 (ppm)

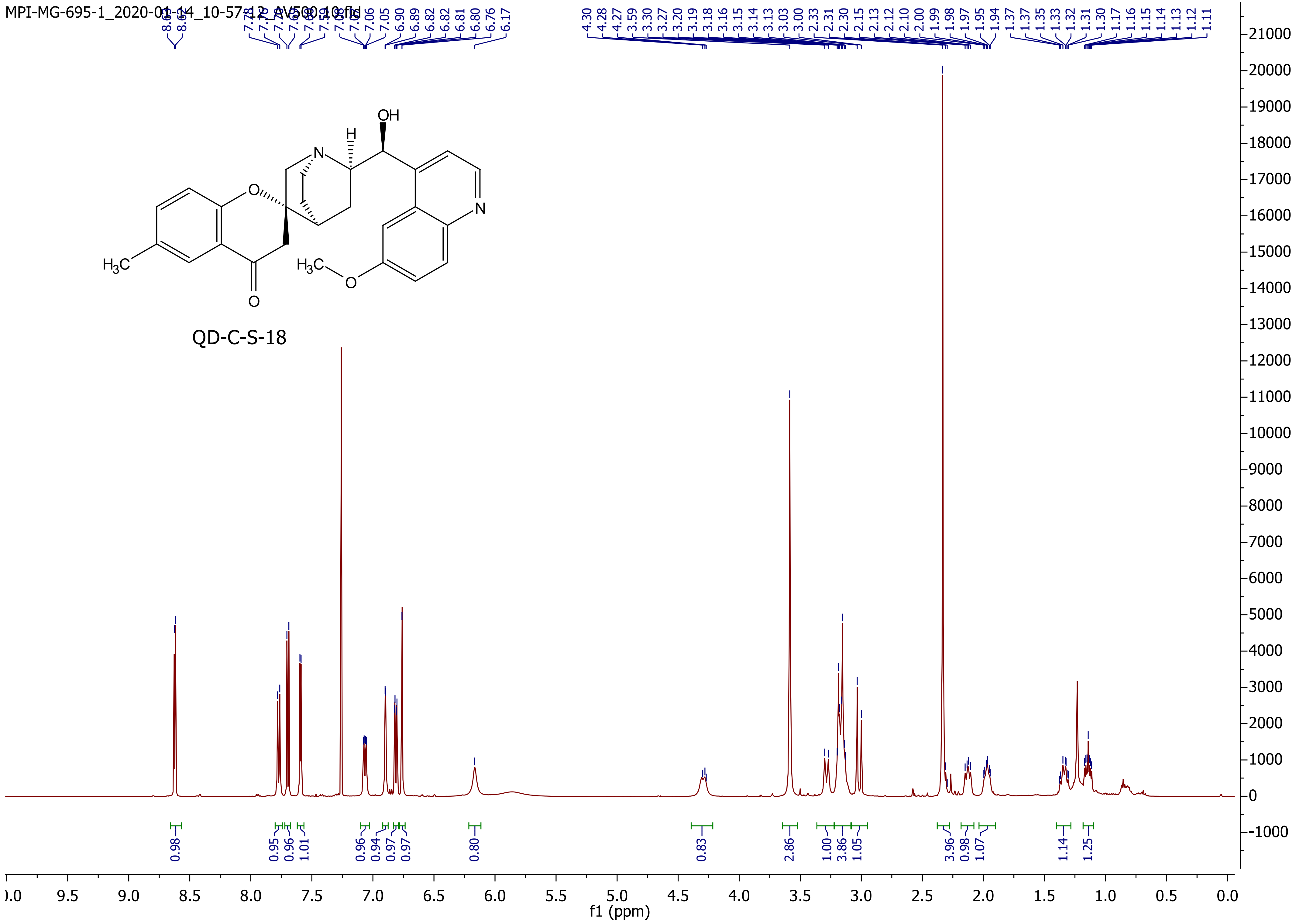
MPI-MG-695-1_2020-01-14_10-57-12_AV500 10 fid
QD-C-S-18
OH
H
N
O
N
H_3C
H_3C
O
O
7.06
7.05
6.90
6.89
6.82
6.82
6.81
6.80
6.76
6.17
4.30
4.28
4.27
3.59
3.30
3.27
3.20
3.19
3.18
3.16
3.15
3.14
3.13
3.03
3.00
2.33
2.31
2.30
2.15
2.13
2.12
2.10
2.00
1.99
1.98
1.97
1.95
1.94
1.37
1.37
1.35
1.33
1.32
1.31
1.30
1.17
1.16
1.15
1.14
1.13
1.12
1.11
0.98
0.95
0.96
1.01
0.96
0.94
0.97
0.97
0.80
0.83
2.86
1.00
3.86
1.05
3.96
0.98
1.07
1.14
1.25
f1 (ppm)

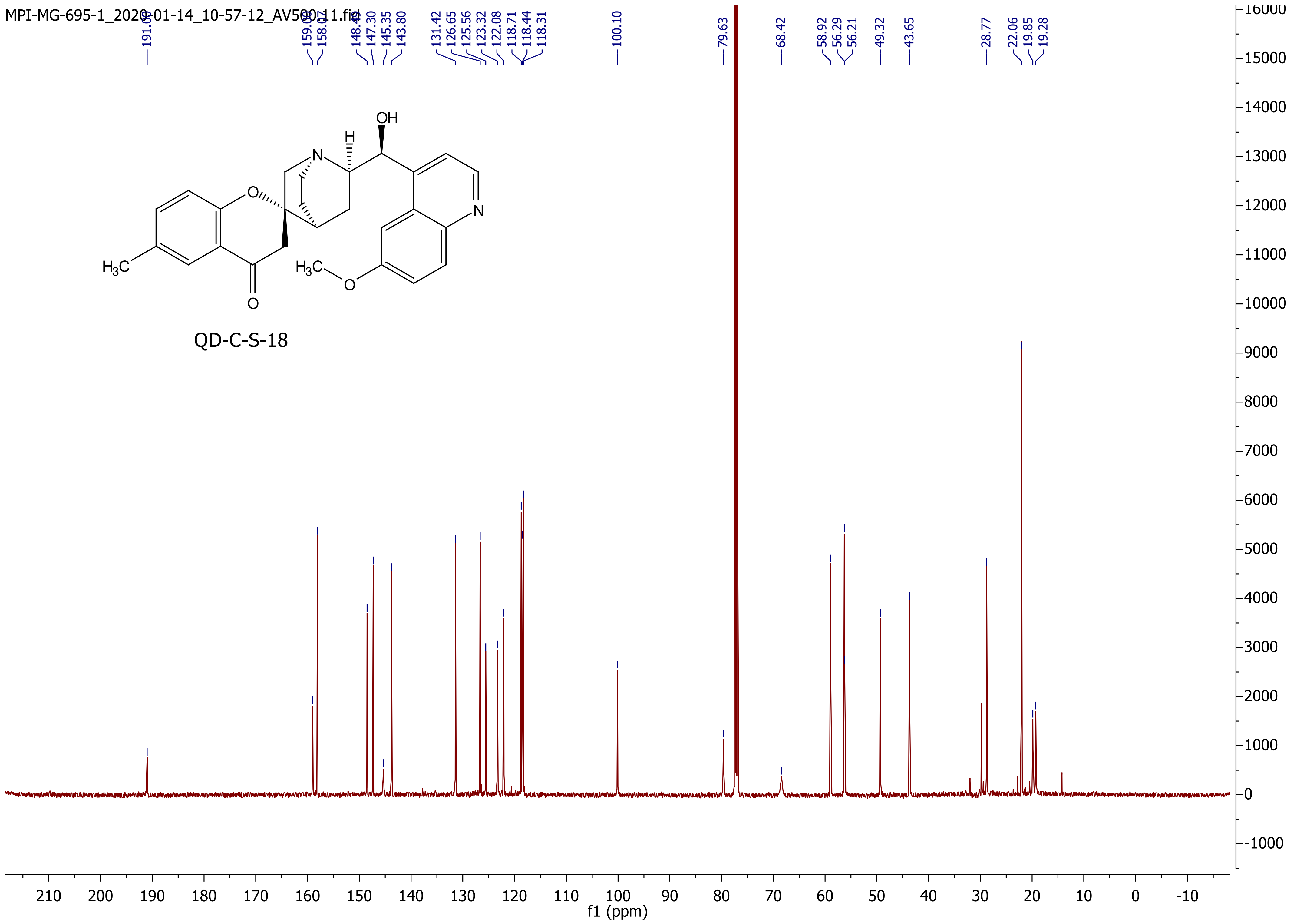

MPI-MG-695-1_2020-01-14_10-57-12_AV500_11.fid
191.0
159.0
158.0
148.4
147.30
145.35
143.80
131.42
126.65
125.56
123.32
122.08
118.71
118.44
118.31
100.10
79.63
68.42
58.92
56.29
56.21
49.32
43.65
28.77
22.06
19.85
19.28
OH
H
N
O
N
H3C
O
H3C
O
QD-C-S-18
f1 (ppm)

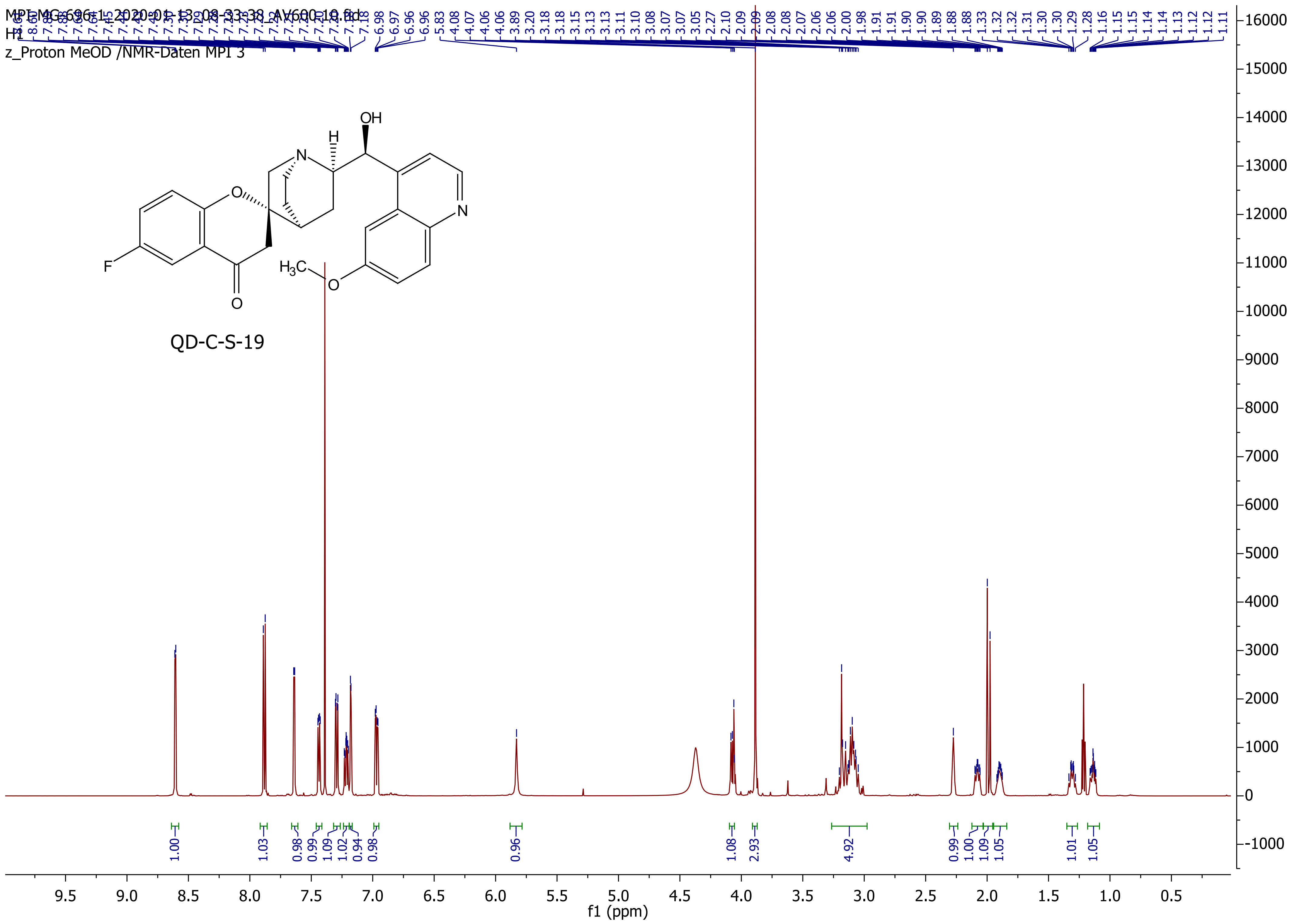
MPI-MG-696-1_2020-01-13_08-33-38_AV600-19.fid
H1
z_Proton MeOD /NMR-Daten MPI 3
QD-C-S-19
OH
H
N
O
F
O
H3C
O
N
f1 (ppm)

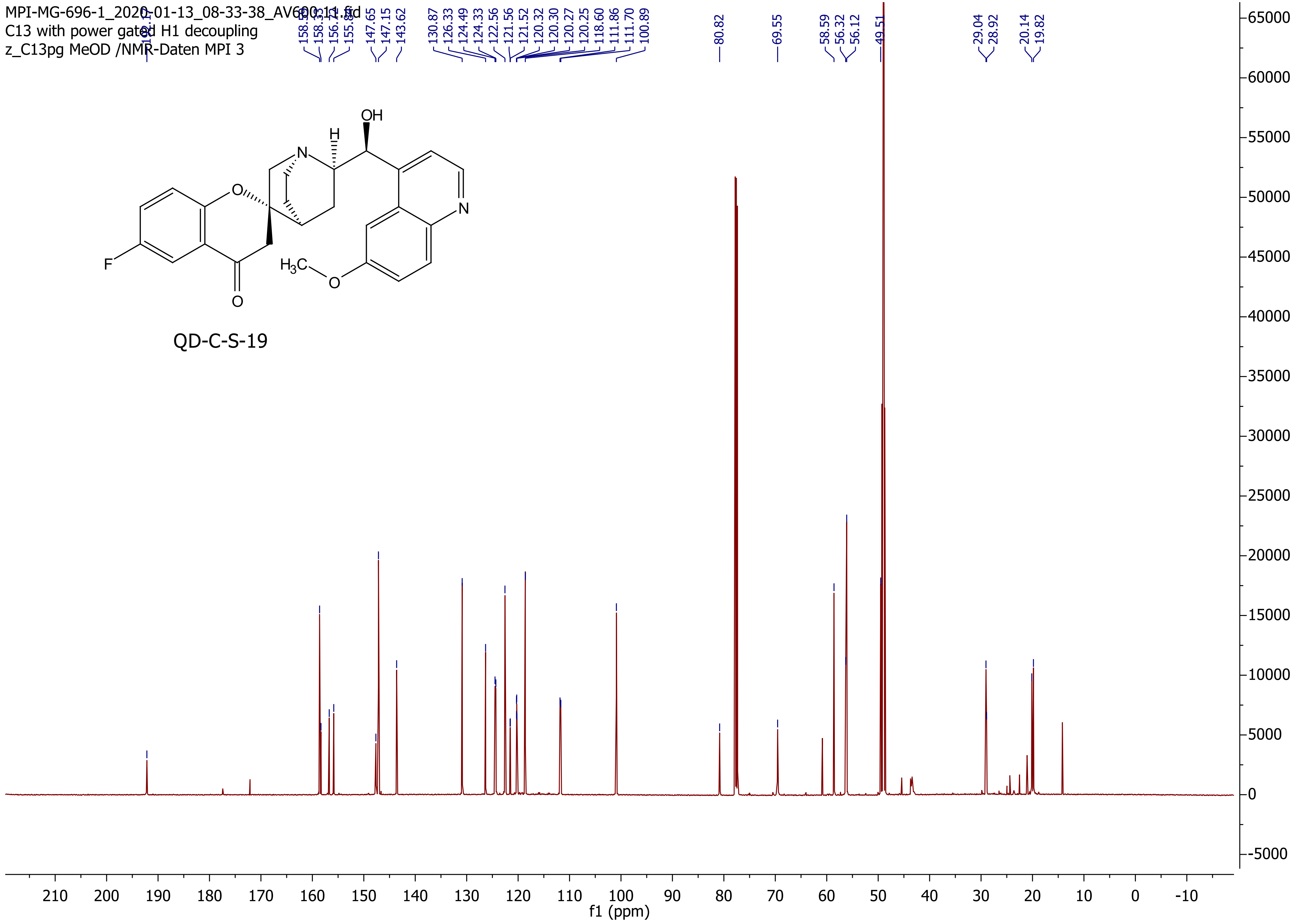
MPI-MG-696-1_2020-01-13_08-33-38_AV600.11.fid
C13 with power gated H1 decoupling
z_C13pg MeOD /NMR-Daten MPI 3
147.65
147.15
143.62
130.87
126.33
124.49
124.33
122.56
121.56
121.52
120.32
120.30
120.27
120.25
118.60
111.86
111.70
100.89
80.82
69.55
58.59
56.32
56.12
49.51
29.04
28.92
20.14
19.82
OH
H
N
O
N
F
O
H3C
O
QD-C-S-19
f1 (ppm)

MPI-MG-696-1_2020-01-13_08-33-38_AV600.12.fid
F19

z_F19 MeOD /NMR-Daten MPI 3

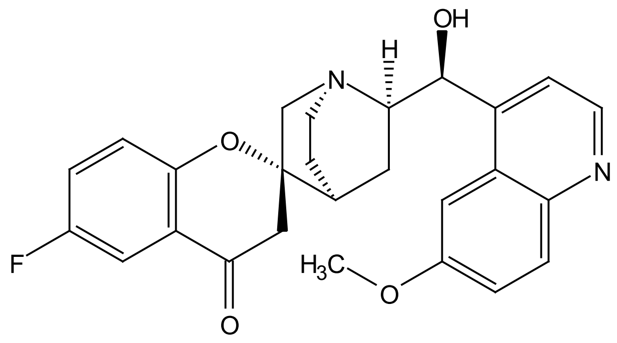

QD-C-S-19

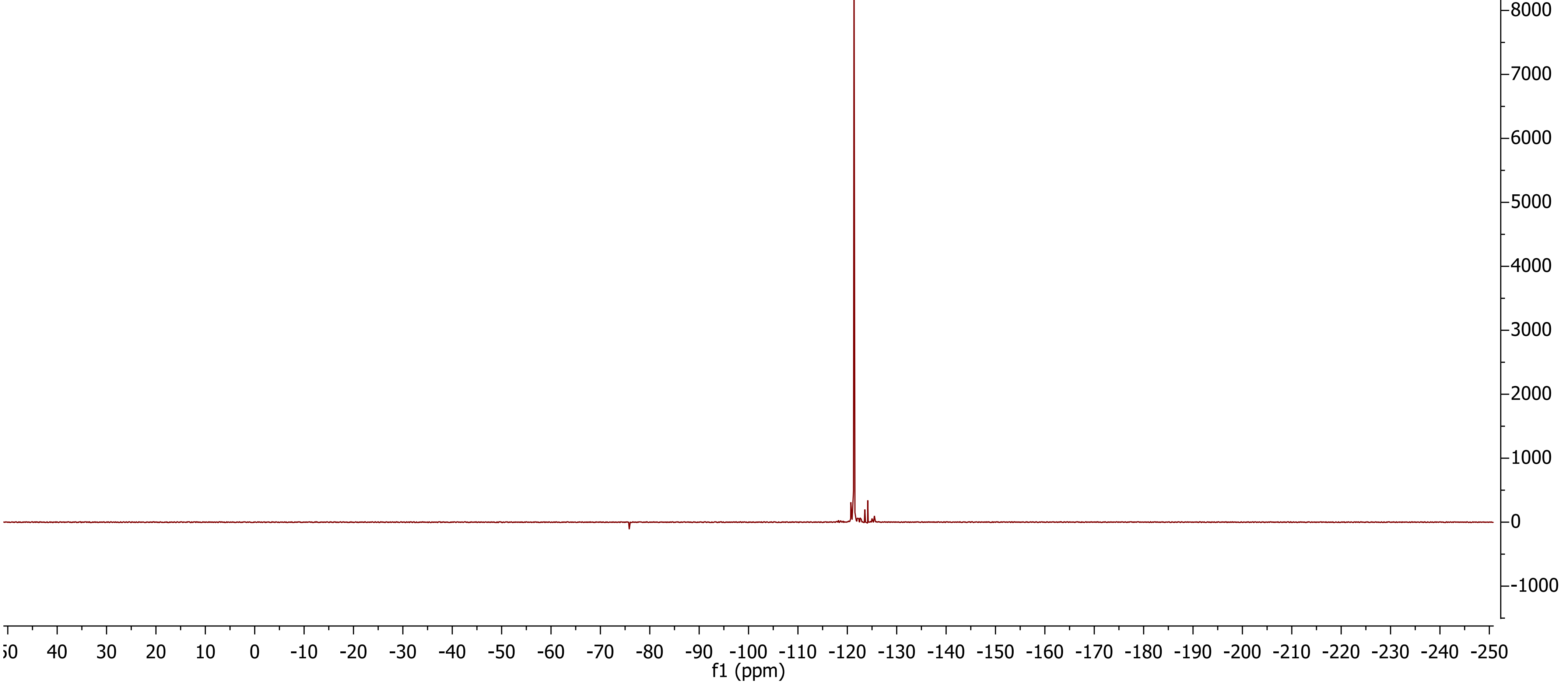

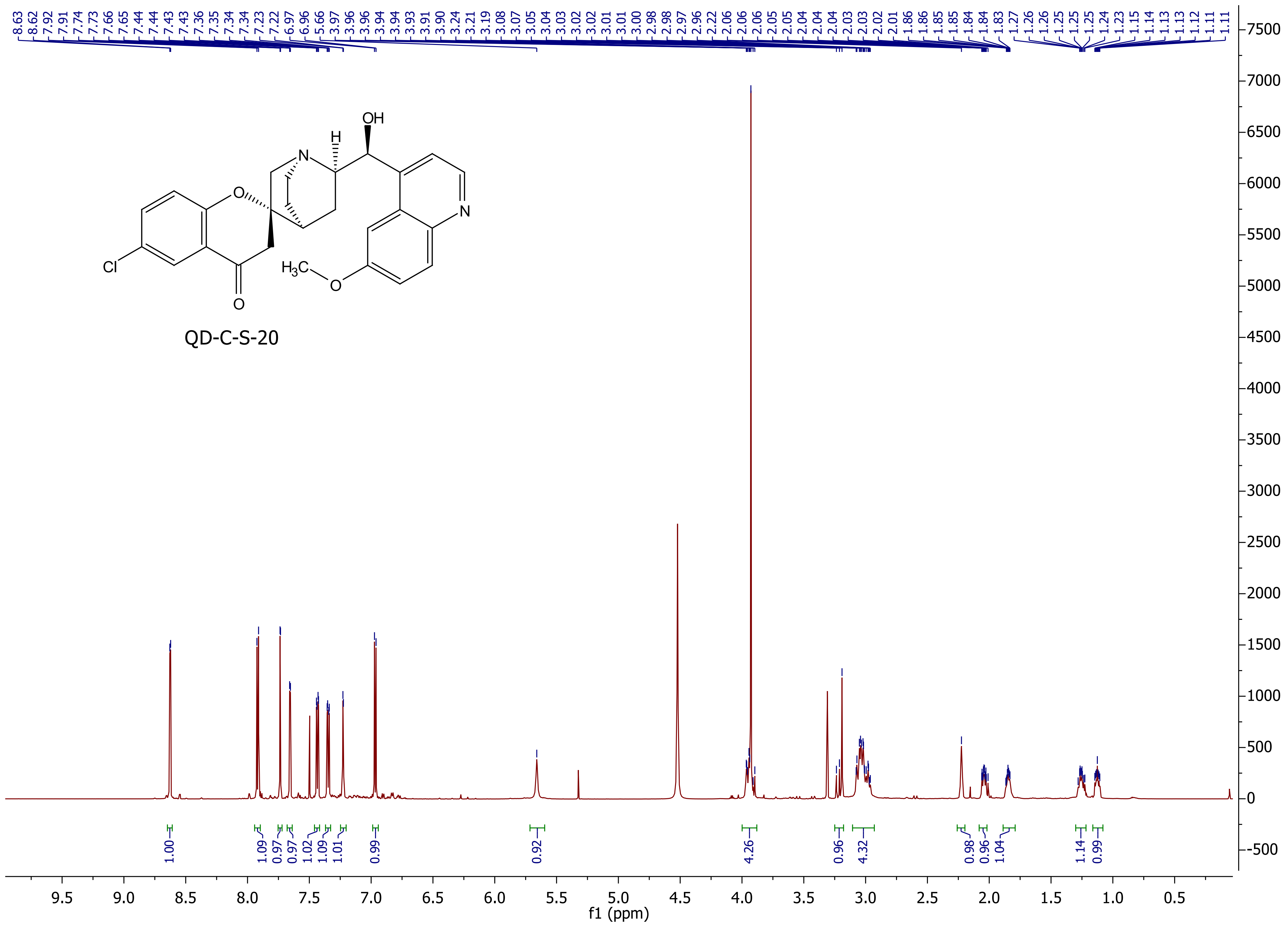
8.63
8.62
7.92
7.91
7.74
7.73
7.66
7.65
7.44
7.44
7.43
7.43
7.36
7.35
7.34
7.34
7.23
7.22
6.97
6.96
5.66
3.97
3.96
3.96
3.94
3.94
3.93
3.91
3.90
3.24
3.21
3.19
3.08
3.07
3.05
3.04
3.03
3.02
3.02
3.01
3.01
3.00
2.98
2.98
2.97
2.96
2.22
2.06
2.06
2.06
2.05
2.05
2.04
2.04
2.04
2.03
2.03
2.02
2.01
1.86
1.86
1.85
1.85
1.84
1.84
1.83
1.27
1.26
1.26
1.25
1.25
1.25
1.24
1.23
1.15
1.14
1.13
1.13
1.12
1.11
1.11
OH
H
N
O
N
Cl
O
H3C
O
QD-C-S-20
1.00
1.09
0.97
0.97
1.02
1.09
1.01
0.99
0.92
4.26
0.96
4.32
0.98
0.96
1.04
1.14
0.99
9.5
9.0
8.5
8.0
7.5
7.0
6.5
6.0
5.5
5.0
4.5
4.0
3.5
3.0
2.5
2.0
1.5
1.0
0.5
f1 (ppm)
7500
7000
6500
6000
5500
5000
4500
4000
3500
3000
2500
2000
1500
1000
500
0
-500

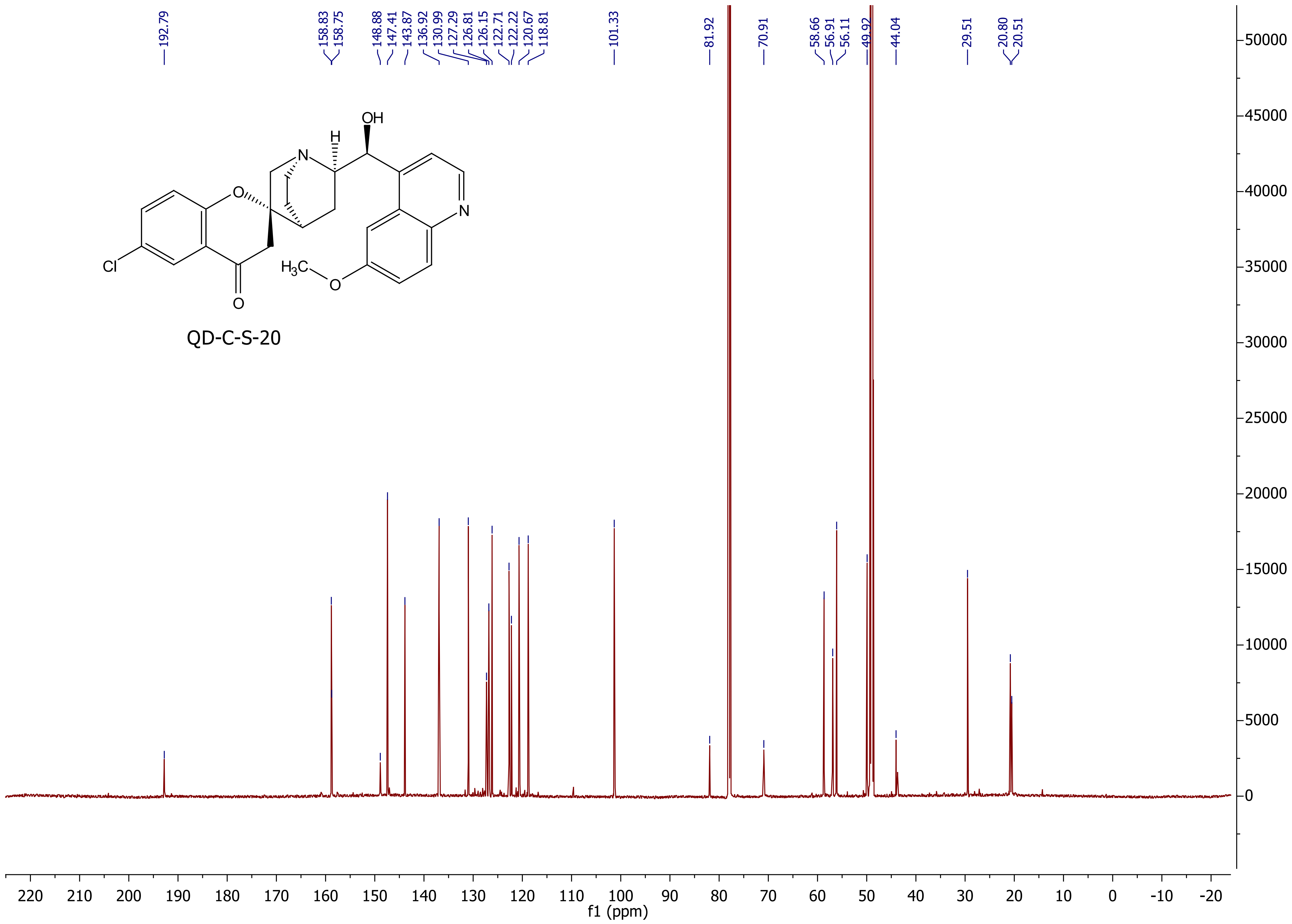

192.79
158.83
158.75
148.88
147.41
143.87
136.92
130.99
127.29
126.81
126.15
122.71
122.22
120.67
118.81
101.33
81.92
70.91
58.66
56.91
56.11
49.92
44.04
29.51
20.80
20.51
OH
H
N
O
N
Cl
O
H_3C
O
QD-C-S-20
f1 (ppm)

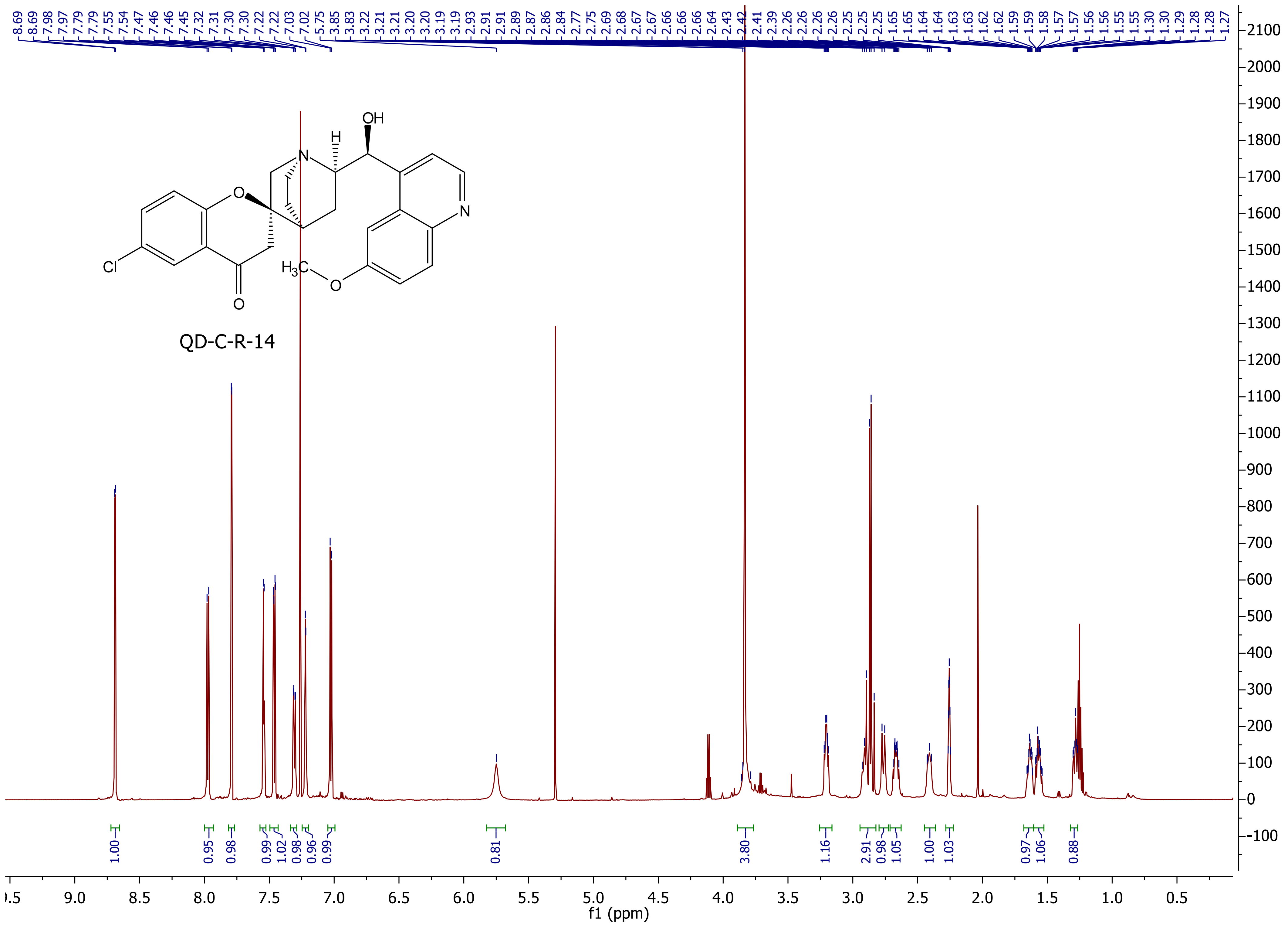
QD-C-R-14
f1 (ppm)

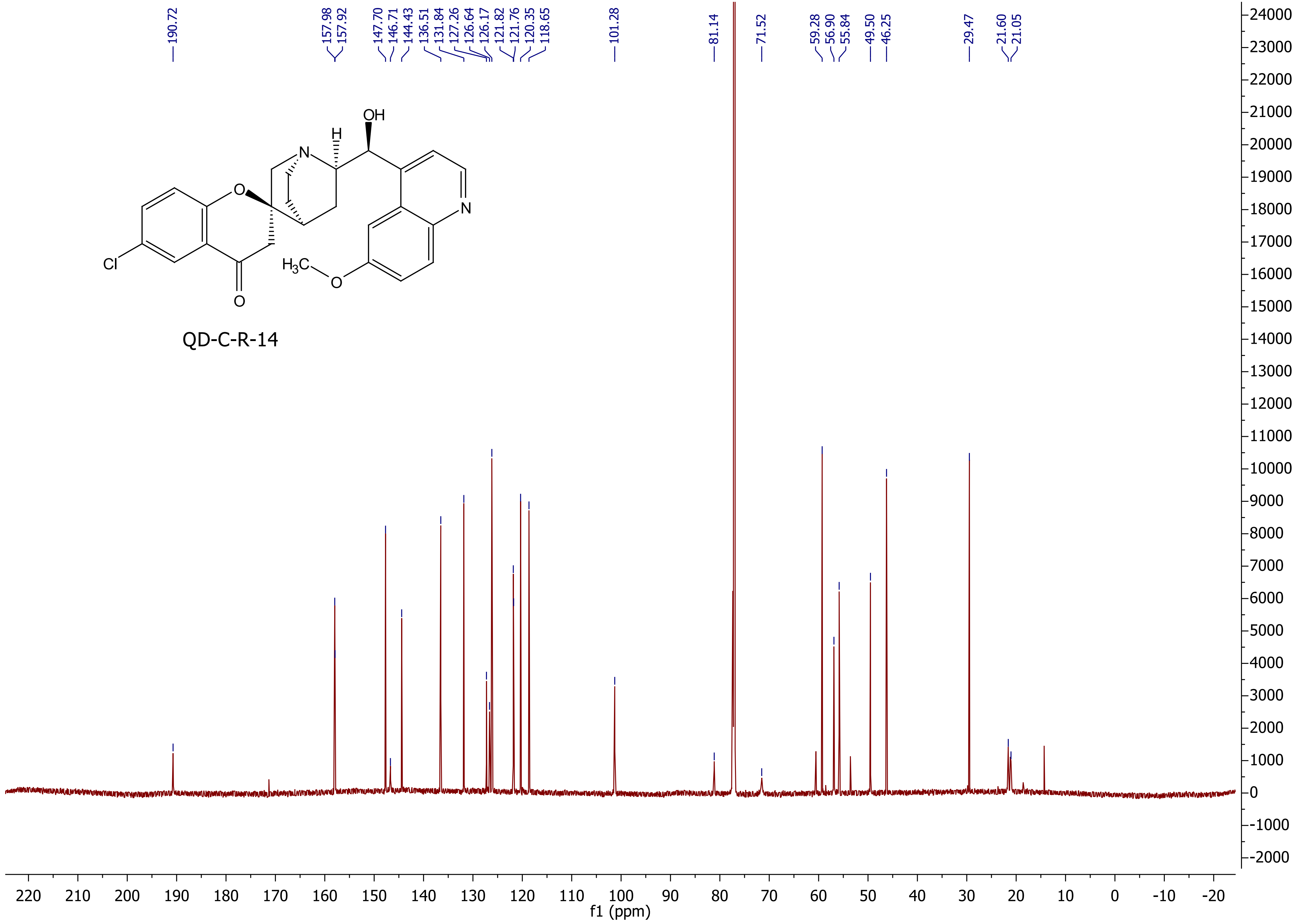

190.72
157.98
157.92
147.70
146.71
144.43
136.51
131.84
127.26
126.64
126.17
121.82
121.76
120.35
118.65
101.28
81.14
71.52
59.28
56.90
55.84
49.50
46.25
29.47
21.60
21.05
OH
H
N
O
N
Cl
O
H3C
O
f1 (ppm)

QD-C-R-14

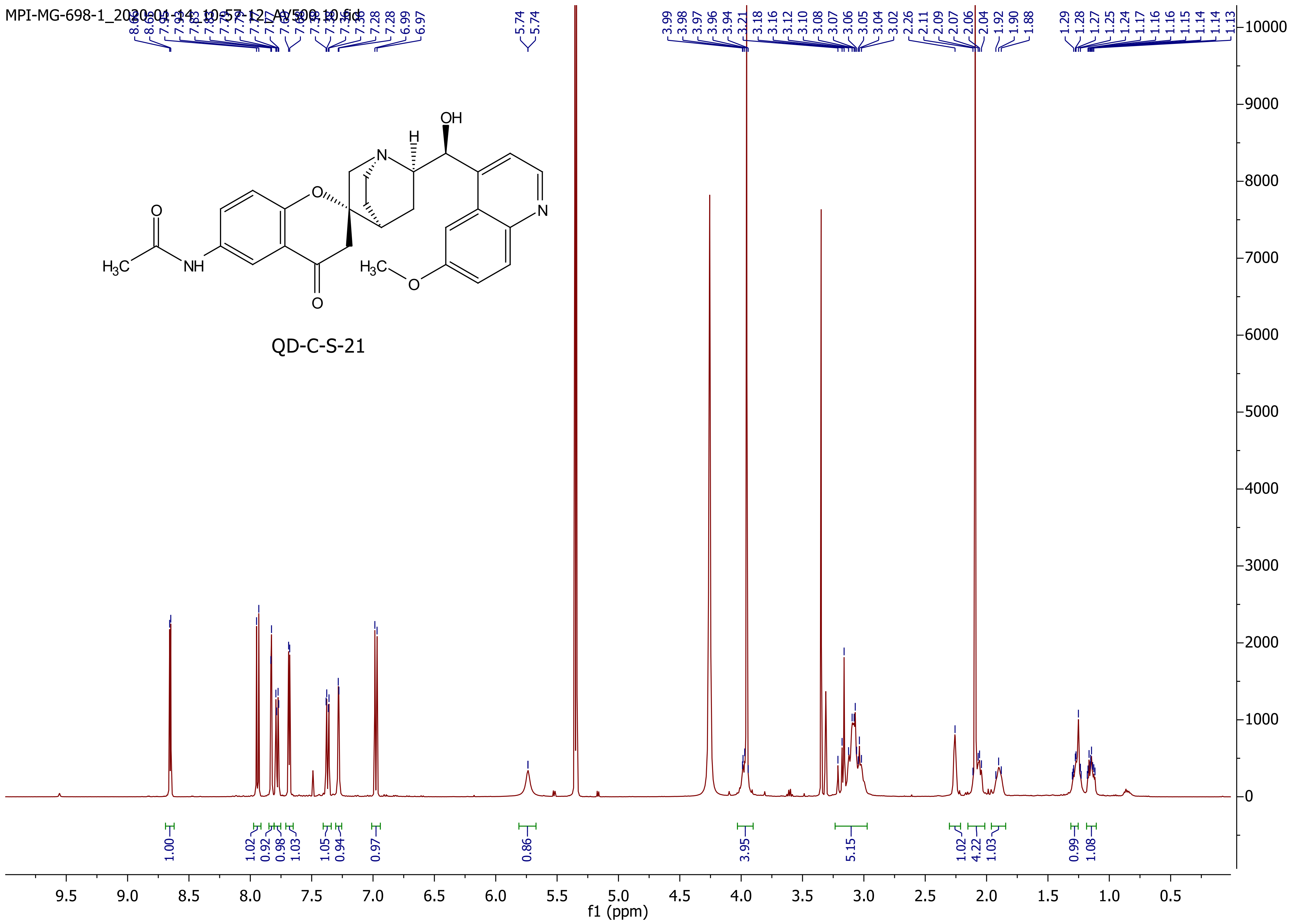

MPI-MG-698-1_2020-01-14_10-57-12_AV500.10.fid
5.74
5.74
3.99
3.98
3.97
3.96
3.94
3.21
3.18
3.16
3.12
3.10
3.08
3.07
3.06
3.05
3.04
3.02
2.26
2.11
2.09
2.07
2.06
2.04
1.92
1.90
1.88
1.29
1.28
1.27
1.25
1.24
1.17
1.16
1.16
1.15
1.14
1.14
1.13
7.28
7.28
6.99
6.97
OH
H
N
O
O
N
H_3C
NH
O
H_3C
O
O
QD-C-S-21
1.00
1.02
0.92
0.98
1.03
1.05
0.94
0.97
0.86
3.95
5.15
1.02
4.22
1.03
0.99
1.08
f1 (ppm)

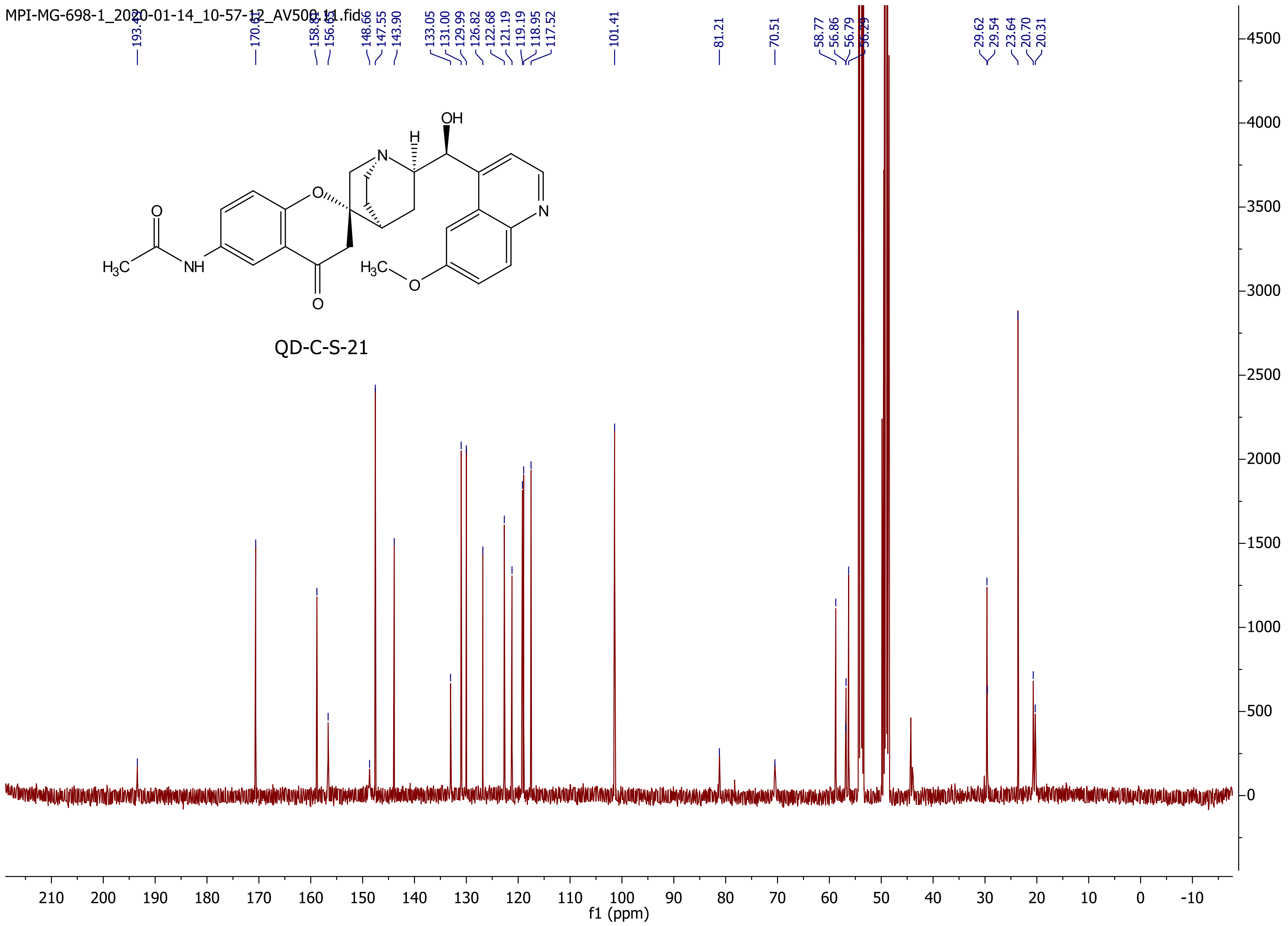

MPI-MG-698-1_2020-01-14_10-57-12_AV500.11.fid
148.66
147.55
143.90
133.05
131.00
129.99
126.82
122.68
121.19
119.19
118.95
117.52
101.41
81.21
70.51
58.77
56.86
56.79
29.62
29.54
23.64
20.70
20.31
QD-C-S-21
f1 (ppm)

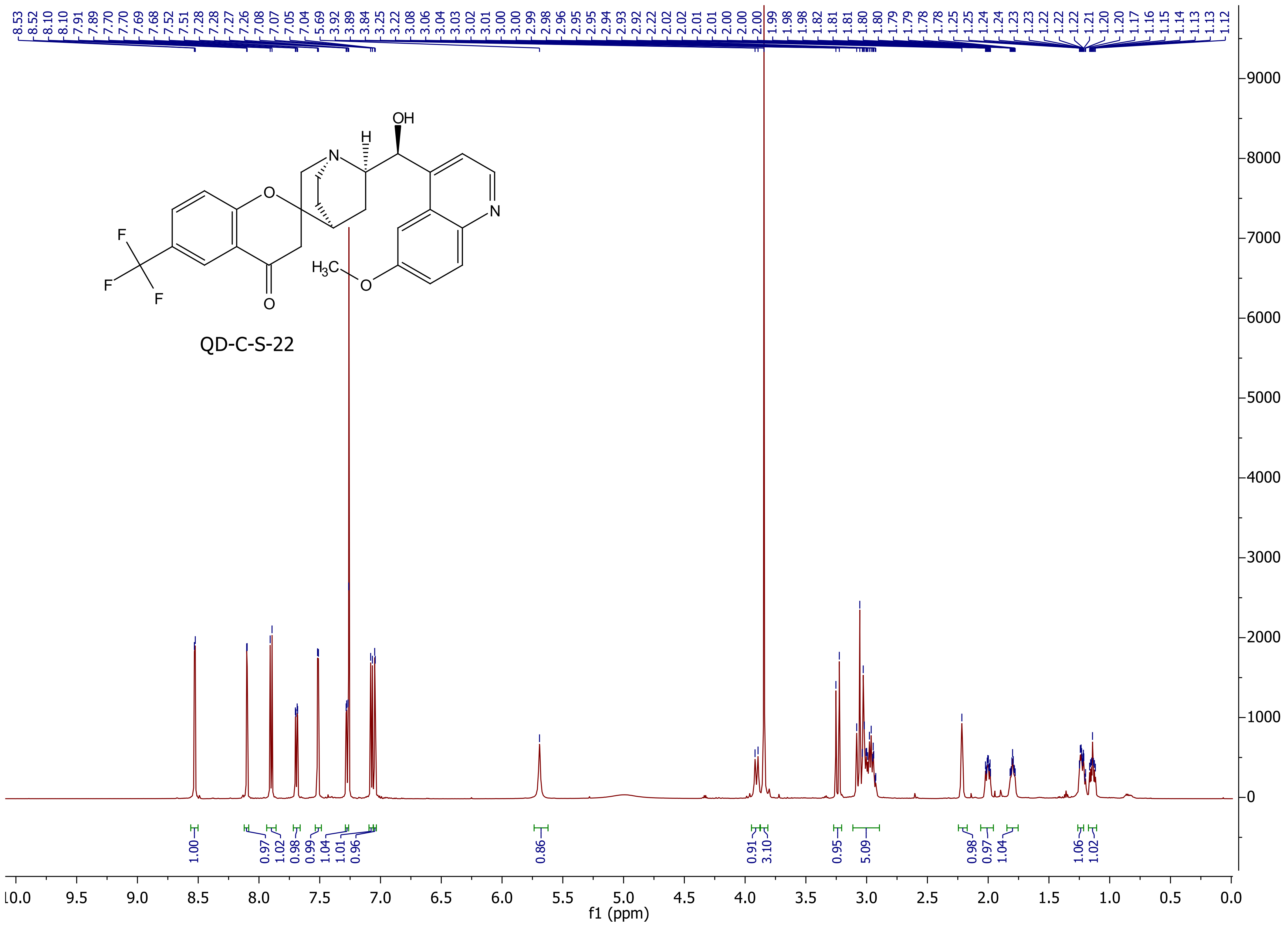
8.53 8.52 8.10 8.10 7.91 7.89 7.70 7.70 7.69 7.68 7.52 7.51 7.28 7.28 7.27 7.26 7.08 7.07 7.05 7.04 5.69 3.92 3.89 3.84 3.25 3.22 3.08 3.06 3.04 3.03 3.02 3.01 3.00 3.00 2.99 2.98 2.96 2.95 2.95 2.94 2.93 2.92 2.22 2.02 2.02 2.01 2.01 2.00 2.00 2.00 1.99 1.98 1.98 1.82 1.81 1.81 1.80 1.80 1.79 1.79 1.78 1.78 1.25 1.25 1.24 1.24 1.23 1.23 1.22 1.22 1.22 1.21 1.20 1.20 1.17 1.16 1.15 1.14 1.13 1.13 1.12
OH
H
N
O
N
F
F
F
O
H3C
O
QD-C-S-22
1.00 0.97 1.02 0.98 0.99 1.04 1.01 0.96 0.86 0.91 3.10 0.95 5.09 0.98 0.97 1.04 1.06 1.02
f1 (ppm)

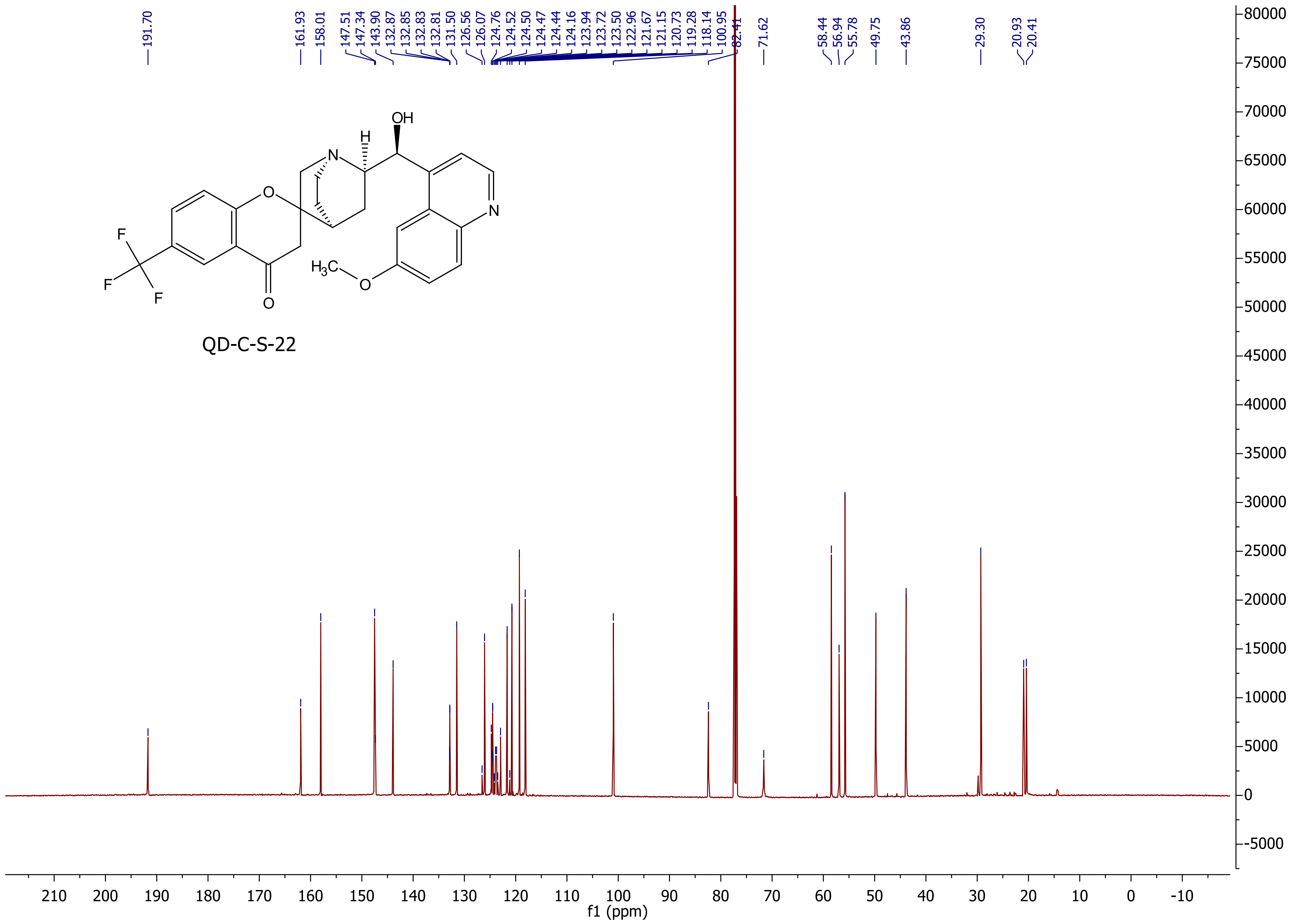
191.70
161.93
158.01
147.51
147.34
143.90
132.87
132.85
132.83
132.81
131.50
126.56
126.07
124.76
124.52
124.50
124.47
124.44
124.16
123.94
123.72
123.50
122.96
121.67
121.15
120.73
119.28
118.14
100.95
82.41
71.62
58.44
56.94
55.78
49.75
43.86
29.30
20.93
20.41
OH
H
N
O
N
F
F
F
O
H_3C
O
QD-C-S-22
f1 (ppm)

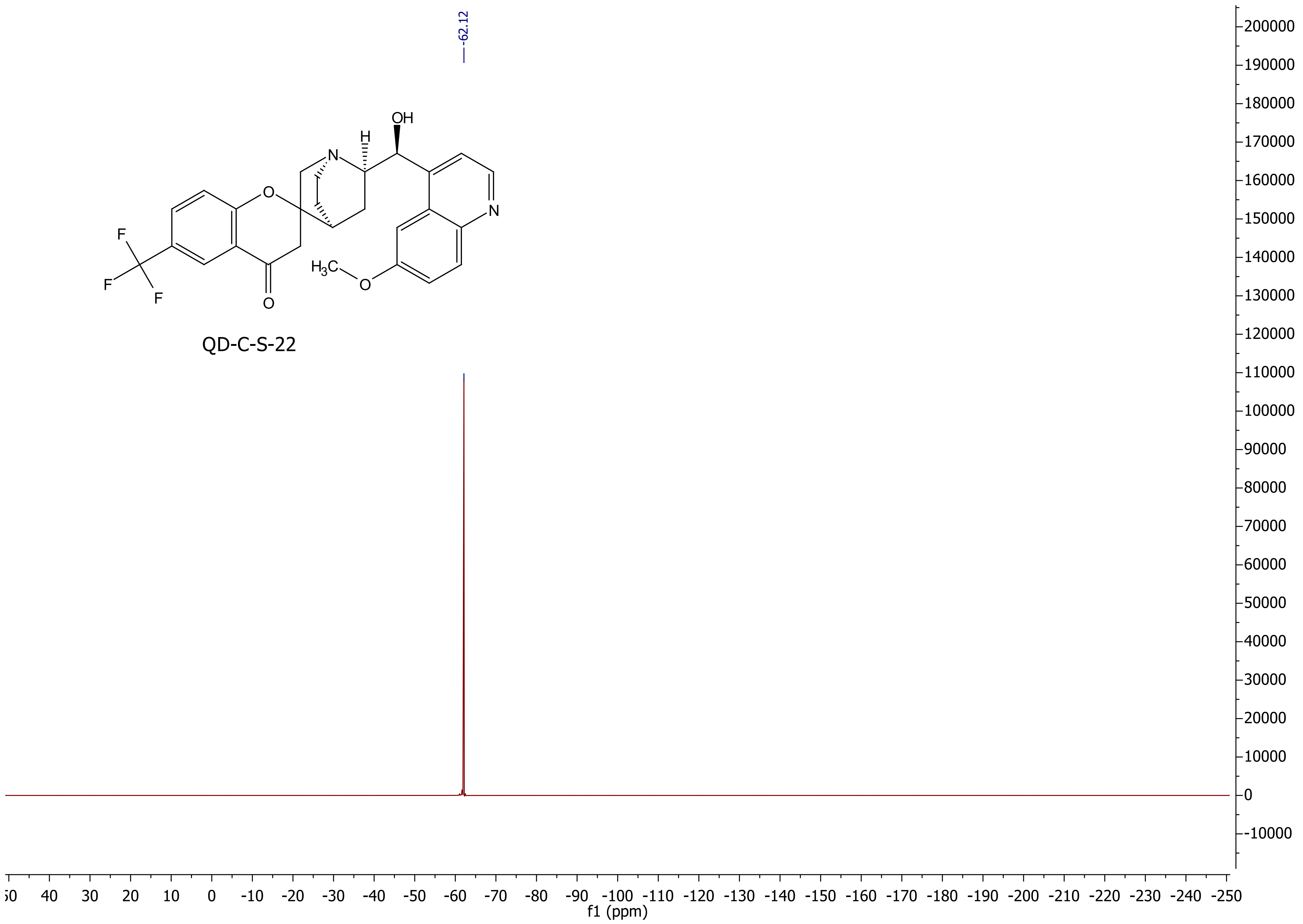

-62.12
OH
H
N
O
N
F
F
F
O
H3C
O
QD-C-S-22
f1 (ppm)

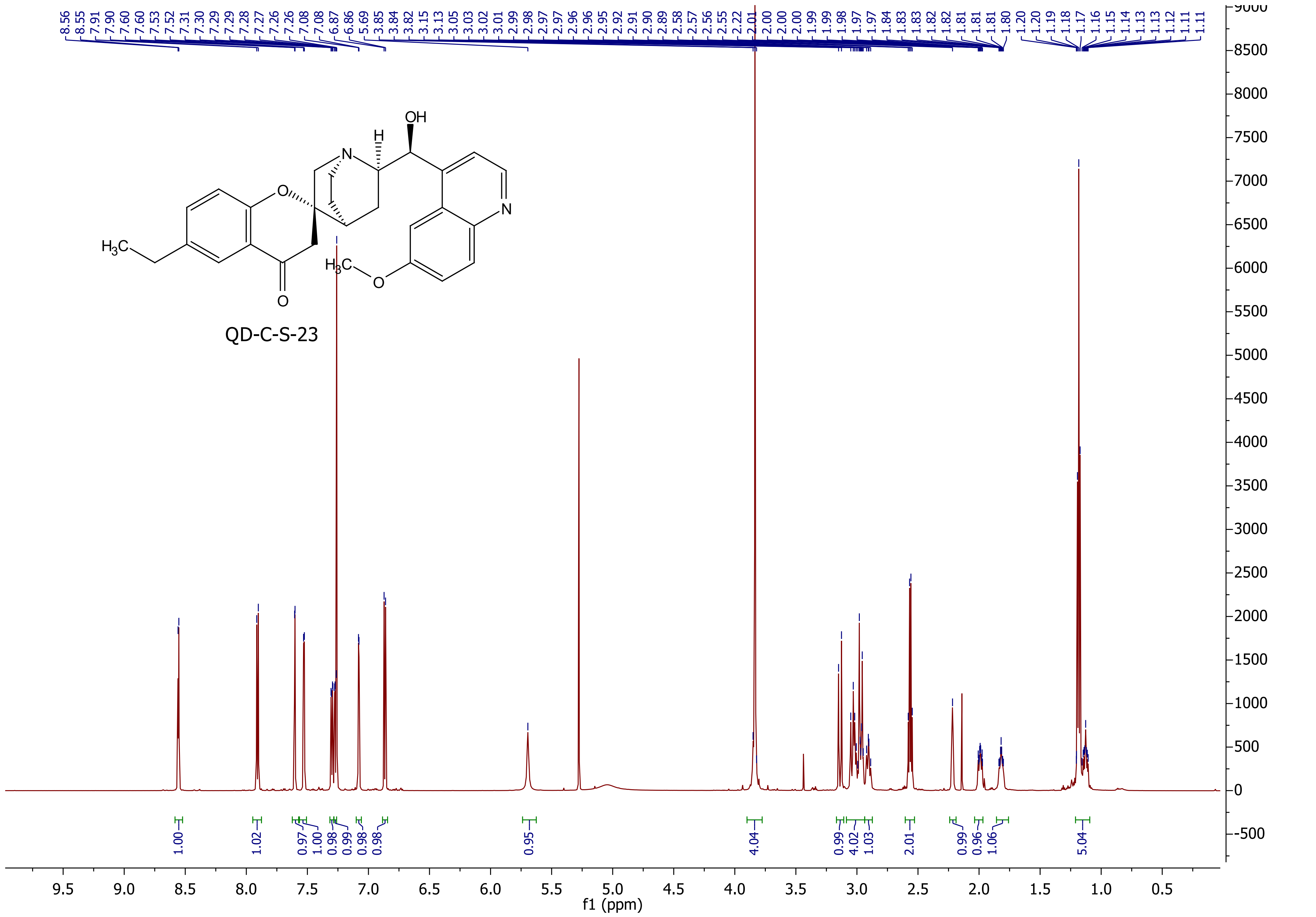

OH
H
N
O
N
H_3C
H_3C
O
O
QD-C-S-23
f1 (ppm)

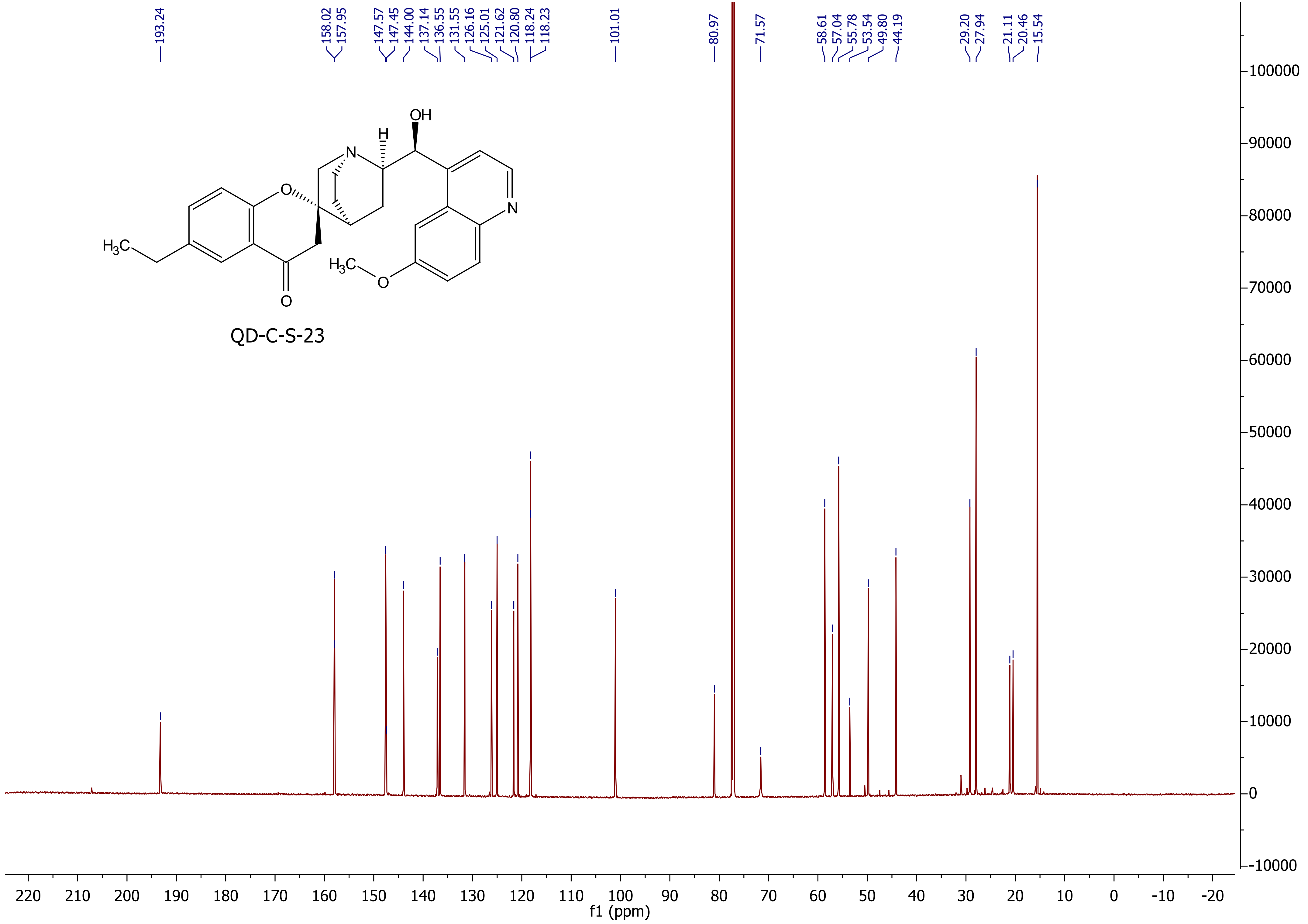

193.24
158.02
157.95
147.57
147.45
144.00
137.14
136.55
131.55
126.16
125.01
121.62
120.80
118.24
118.23
101.01
80.97
71.57
58.61
57.04
55.78
53.54
49.80
44.19
29.20
27.94
21.11
20.46
15.54
QD-C-S-23
f1 (ppm)

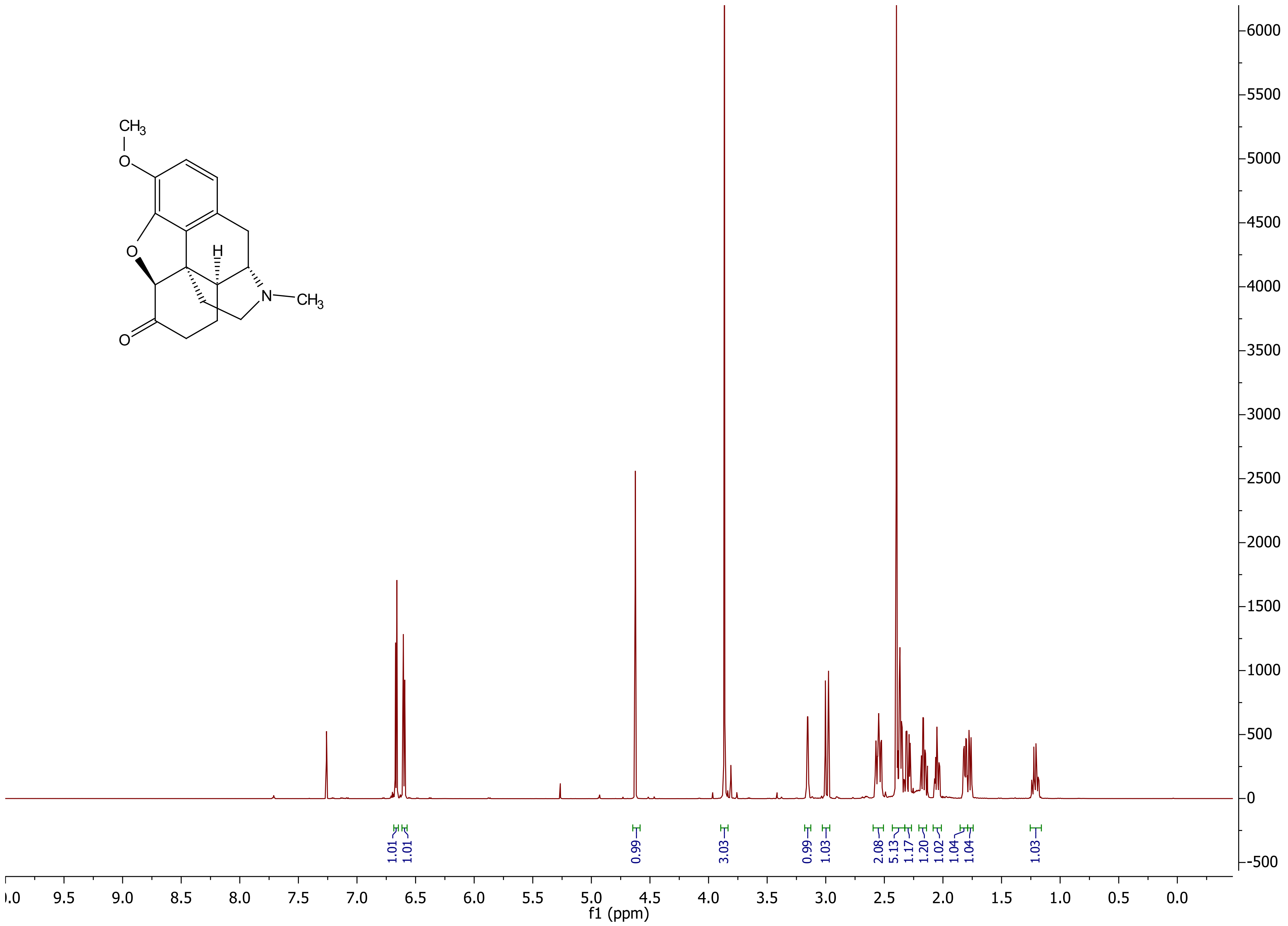

CH3
O
O
H
N
CH3
O
1.01
1.01
0.99
3.03
0.99
1.03
2.08
5.13
1.17
1.20
1.02
1.04
1.04
1.03
f1 (ppm)

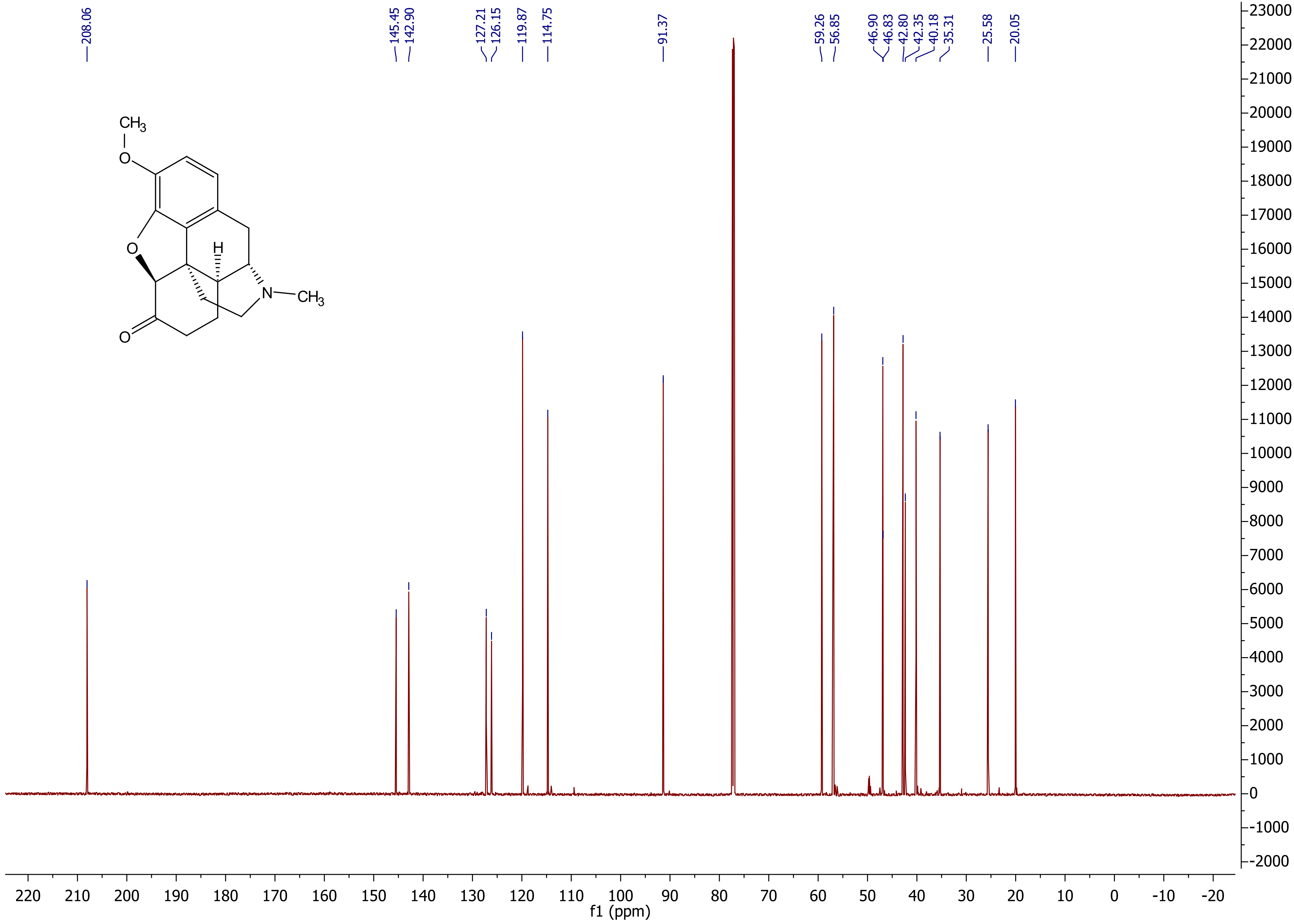

208.06
145.45
142.90
127.21
126.15
119.87
114.75
91.37
59.26
56.85
46.90
46.83
42.80
42.35
40.18
35.31
25.58
20.05
CH3
O
H
N
CH3
O
f1 (ppm)

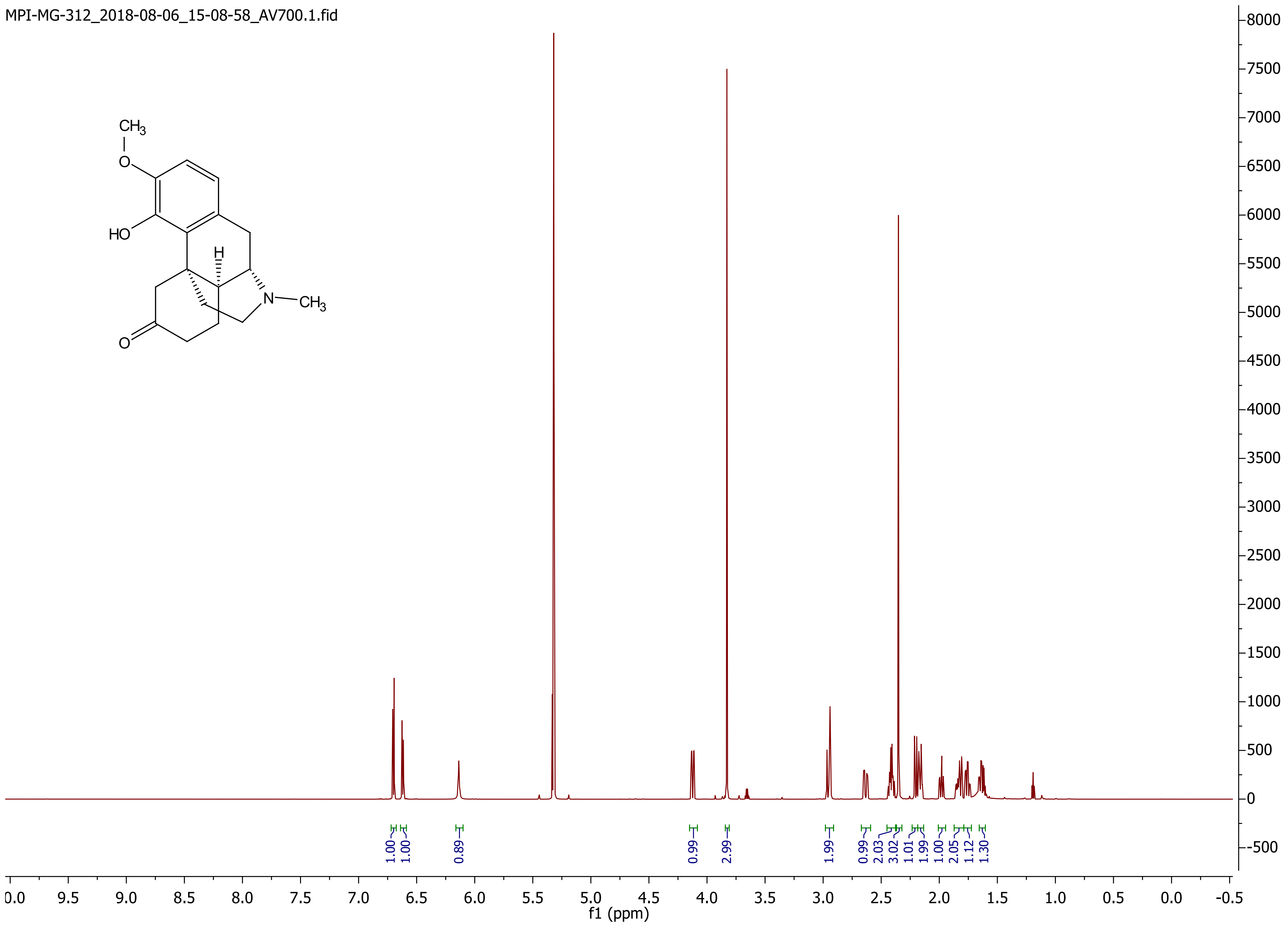
MPI-MG-312_2018-08-06_15-08-58_AV700.1.fid
CH3
O
HO
H
N
CH3
O
1.00
1.00
0.89
0.99
2.99
1.99
0.99
2.03
3.02
1.01
1.99
1.00
2.05
1.12
1.30
f1 (ppm)
8000
7500
7000
6500
6000
5500
5000
4500
4000
3500
3000
2500
2000
1500
1000
500
0
-500
9.5
9.0
8.5
8.0
7.5
7.0
6.5
6.0
5.5
5.0
4.5
4.0
3.5
3.0
2.5
2.0
1.5
1.0
0.5
0.0
-0.5

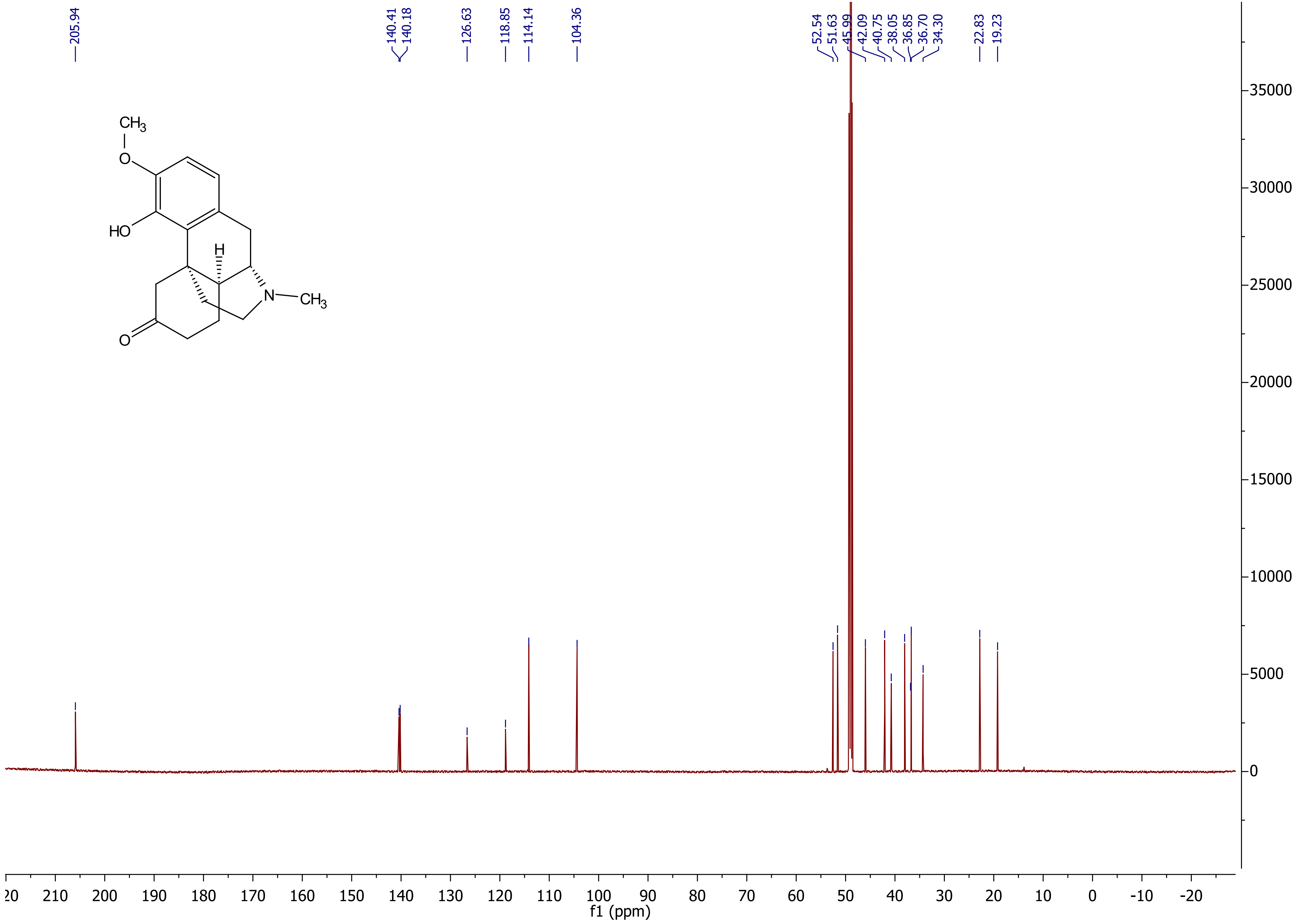

205.94
140.41
140.18
126.63
118.85
114.14
104.36
52.54
51.63
45.99
42.09
40.75
38.05
36.85
36.70
34.30
22.83
19.23
CH3
O
HO
H
N
CH3
O
35000
30000
25000
20000
15000
10000
5000
0
210
200
190
180
170
160
150
140
130
120
110
100
90
80
70
60
50
40
30
20
10
0
-10
-20
f1 (ppm)

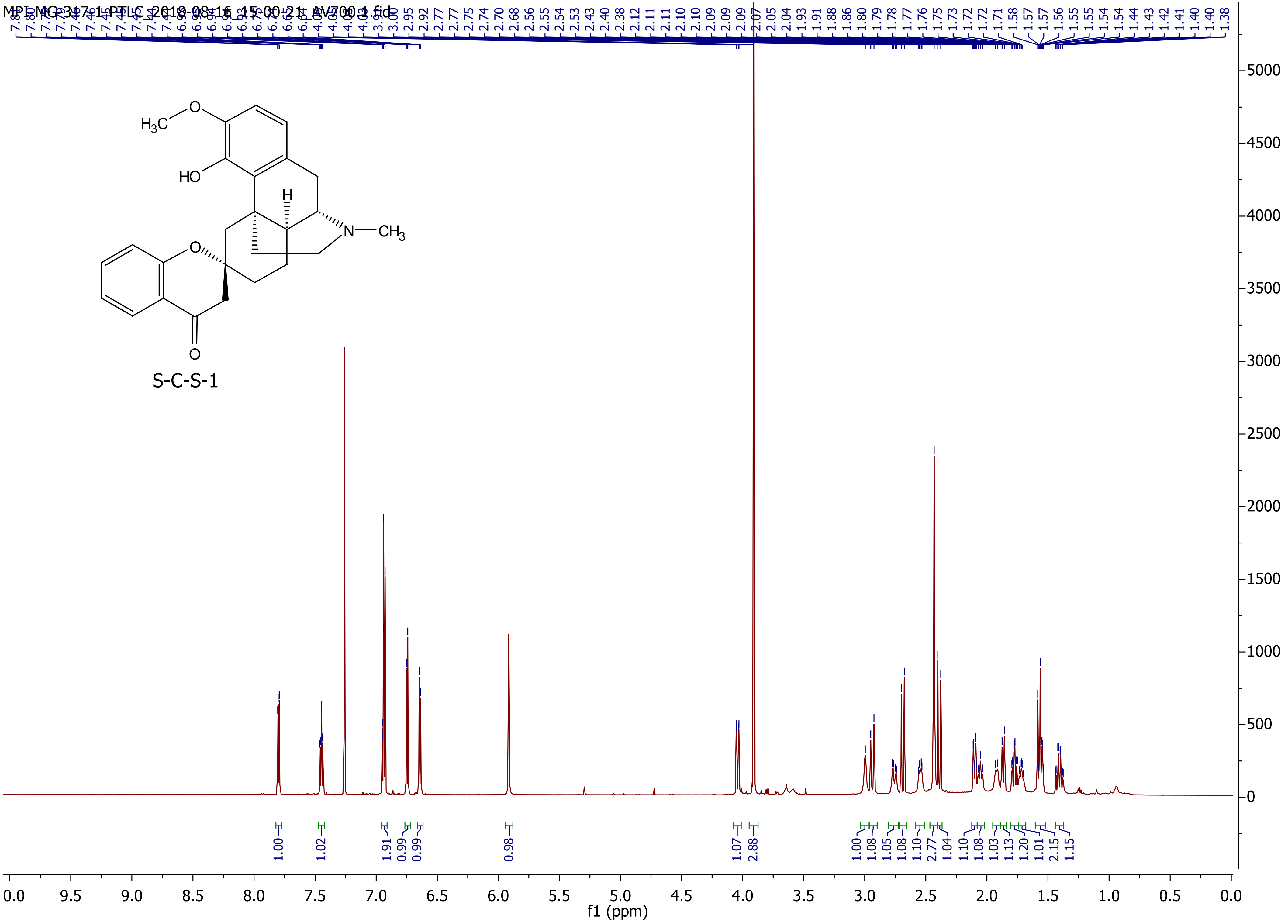

MPI-MG-317-1-PTLC_2018-08-16_15-00-21_AV700 1.fid
2.95 2.92 2.77 2.77 2.75 2.74 2.70 2.68 2.56 2.55 2.54 2.53 2.43 2.40 2.38 2.12 2.11 2.11 2.10 2.10 2.09 2.09 2.09 2.07 2.05 2.04 1.93 1.91 1.88 1.86 1.80 1.79 1.78 1.77 1.76 1.75 1.73 1.72 1.72 1.71 1.58 1.57 1.57 1.56 1.55 1.55 1.54 1.54 1.44 1.43 1.42 1.41 1.40 1.40 1.38
H3C
O
HO
H
N
CH3
O
O
S-C-S-1
1.00 1.02 1.91 0.99 0.99 0.98 1.07 2.88 1.00 1.08 1.05 1.08 1.10 2.77 1.04 1.10 1.08 1.03 1.13 1.20 1.01 2.15 1.15
f1 (ppm)

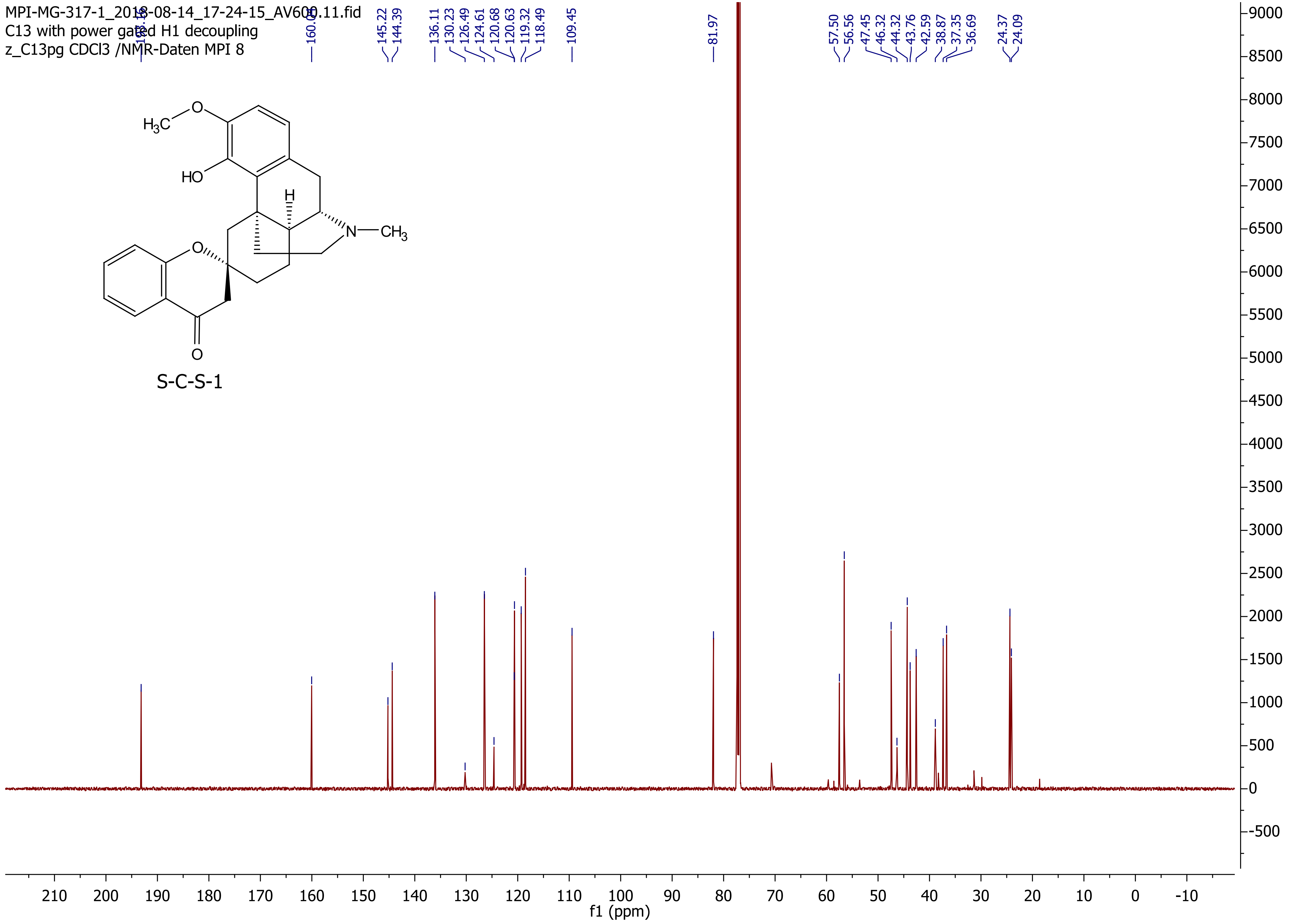

MPI-MG-317-1_2018-08-14_17-24-15_AV600.11.fid
C13 with power gated H1 decoupling
z_C13pg CDCl3 /NMR-Daten MPI 8
160.04
145.22
144.39
136.11
130.23
126.49
124.61
120.68
120.63
119.32
118.49
109.45
81.97
57.50
56.56
47.45
46.32
44.32
43.76
42.59
38.87
37.35
36.69
24.37
24.09
H₃C
O
HO
H
N
CH₃
O
O
S-C-S-1
f1 (ppm)

MPI-MG-317-2-PTLC_2018-08-16_15-00-21_AV700 1.fid

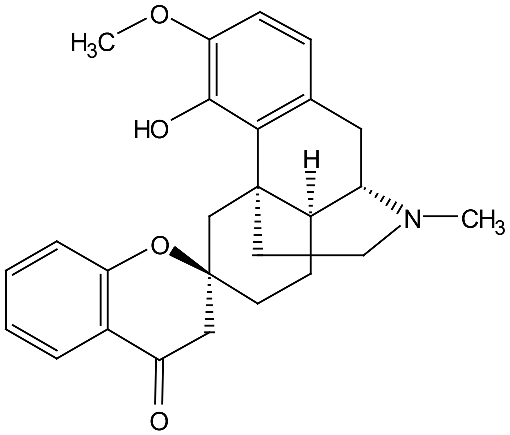

S-C-R-1

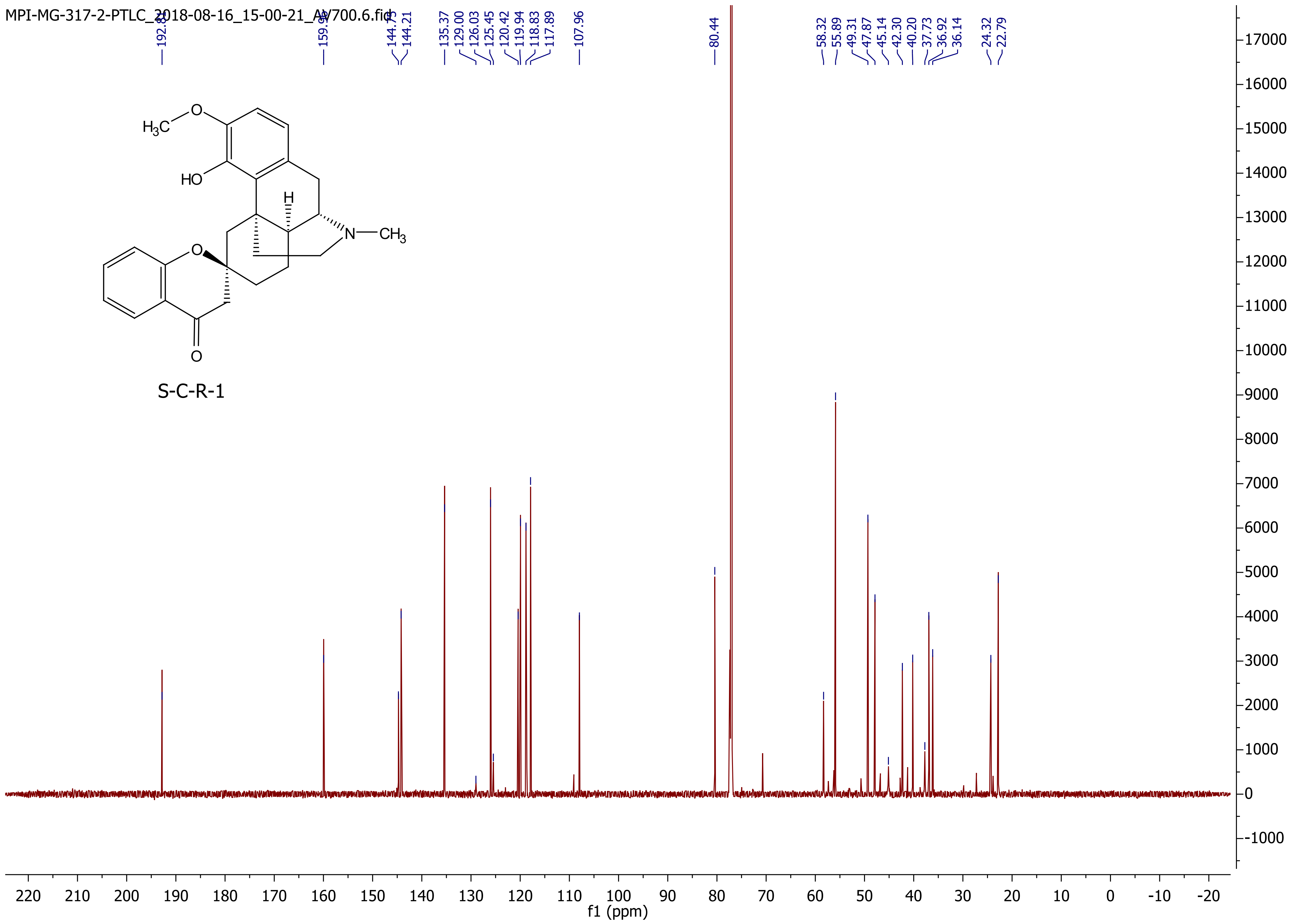

MPI-MG-317-2-PTLC_2018-08-16_15-00-21_AV700.6.fid
192.8
159.9
144.75
144.21
135.37
129.00
126.03
125.45
120.42
119.94
118.83
117.89
107.96
80.44
58.32
55.89
49.31
47.87
45.14
42.30
40.20
37.73
36.92
36.14
24.32
22.79
S-C-R-1
f1 (ppm)

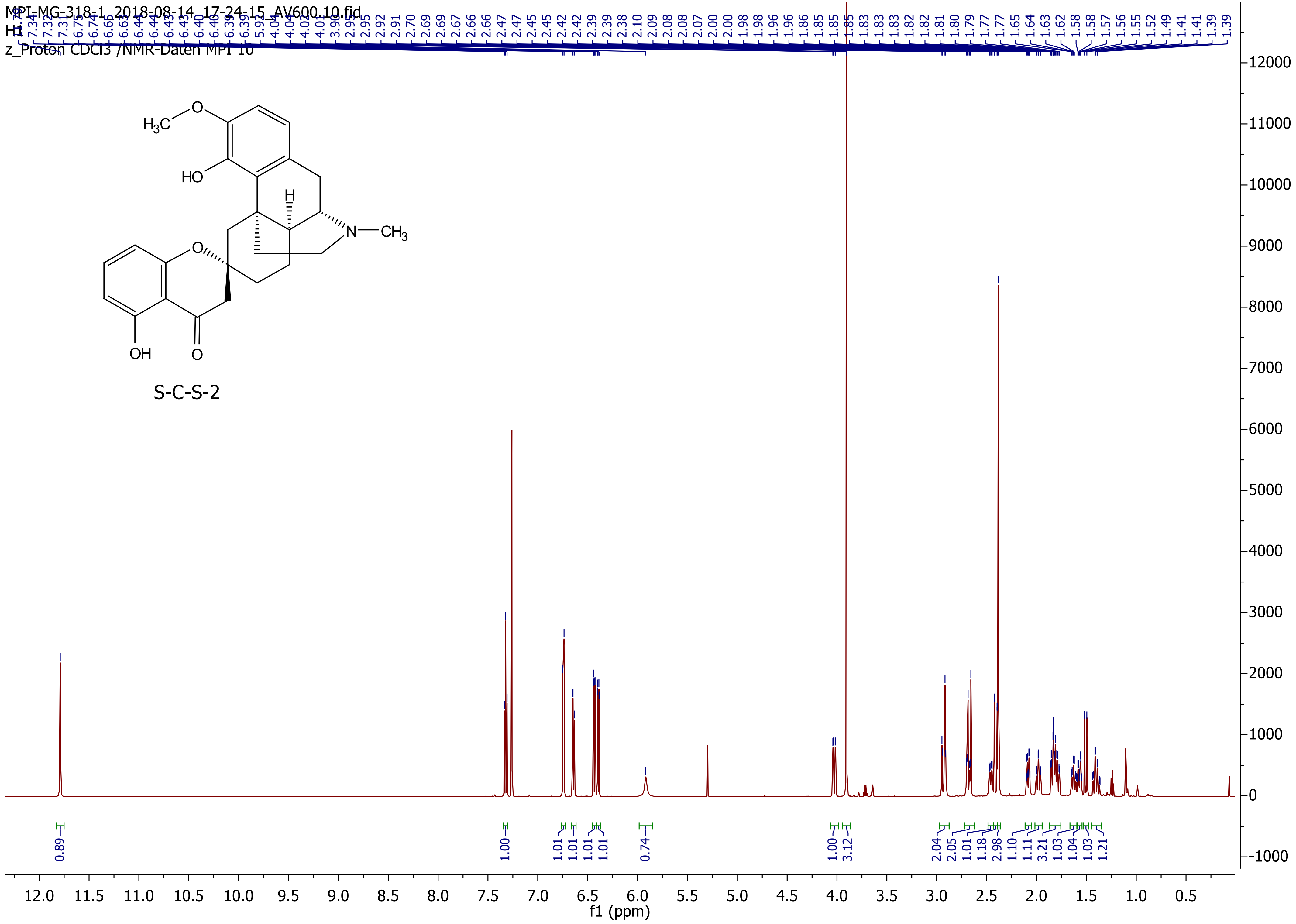

MPI-MG-318-1_2018-08-14_17-24-15_AV600.10.fid
H1
z_Proton CDCl3 /NMR-Daten MPI 10
H3C
O
HO
H
N
CH3
O
OH
O
S-C-S-2
f1 (ppm)

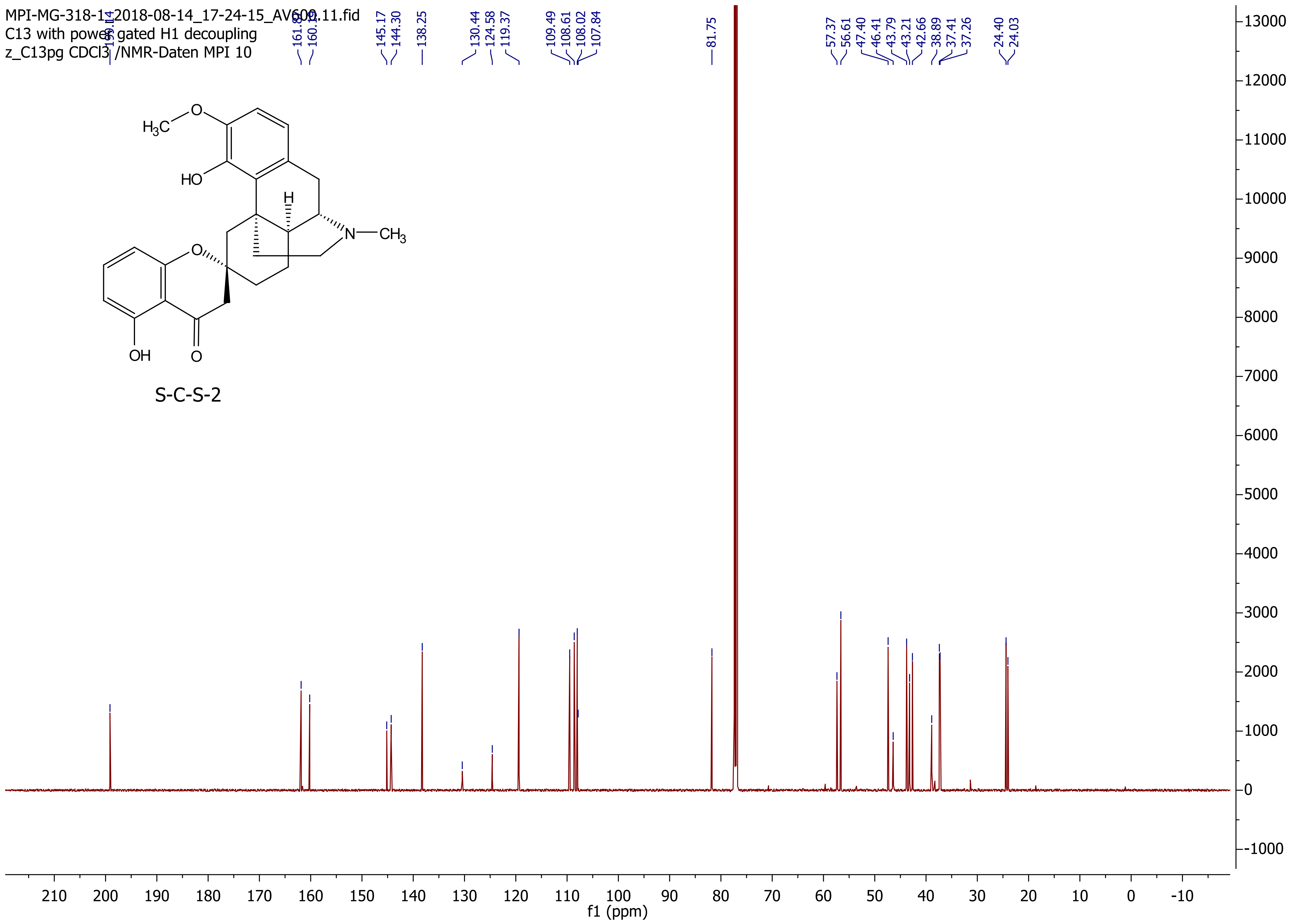
MPI-MG-318-1_2018-08-14_17-24-15_AV600.11.fid
C13 with power gated H1 decoupling
z_C13pg CDCl3 /NMR-Daten MPI 10
145.17
144.30
138.25
130.44
124.58
119.37
109.49
108.61
108.02
107.84
81.75
57.37
56.61
47.40
46.41
43.79
43.21
42.66
38.89
37.41
37.26
24.40
24.03
H_3C
O
HO
H
N
CH_3
O
OH
O
S-C-S-2
f1 (ppm)

MPI-MG-320-2_2018-08-20_06-23-16_AV700_1.fid

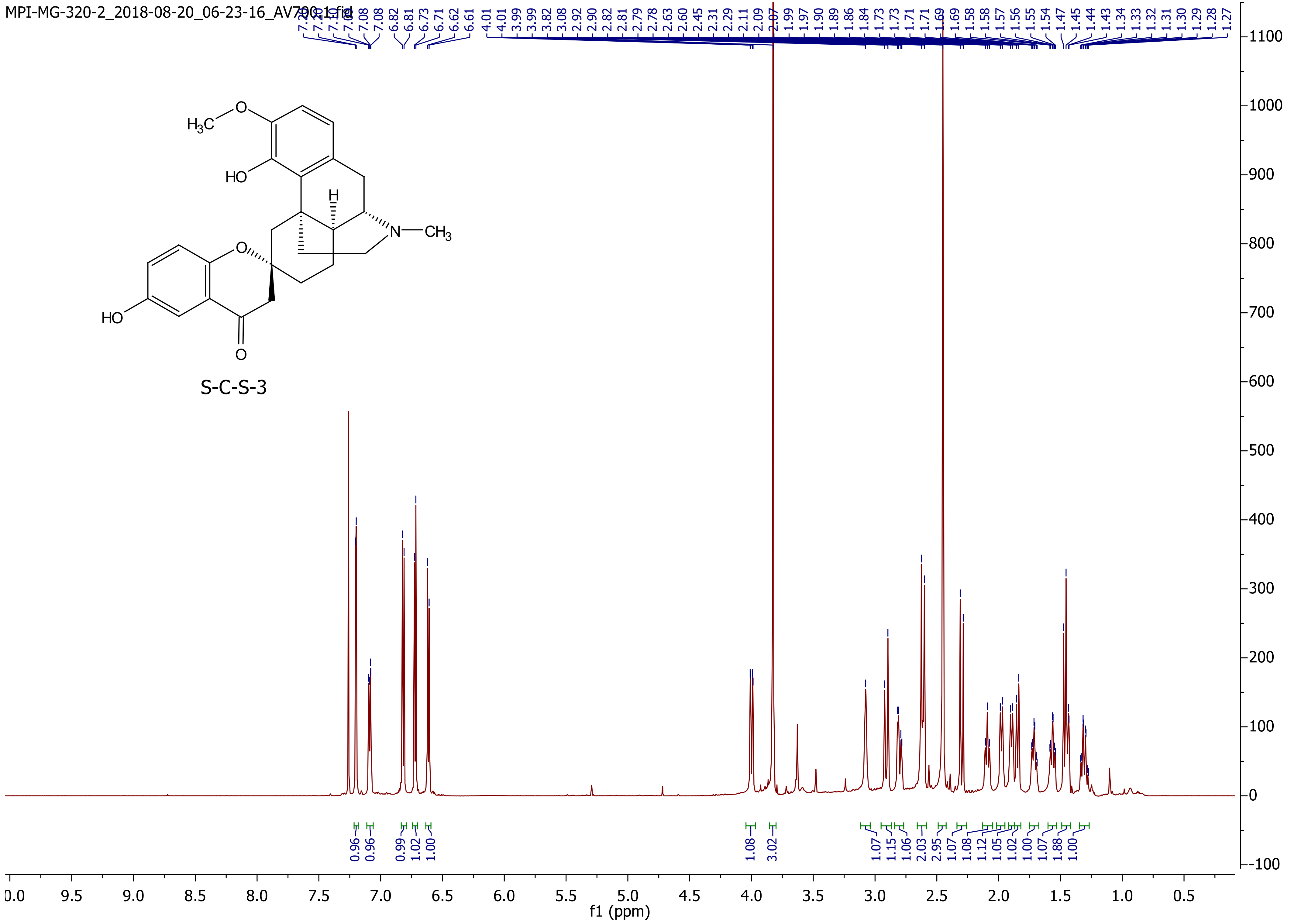

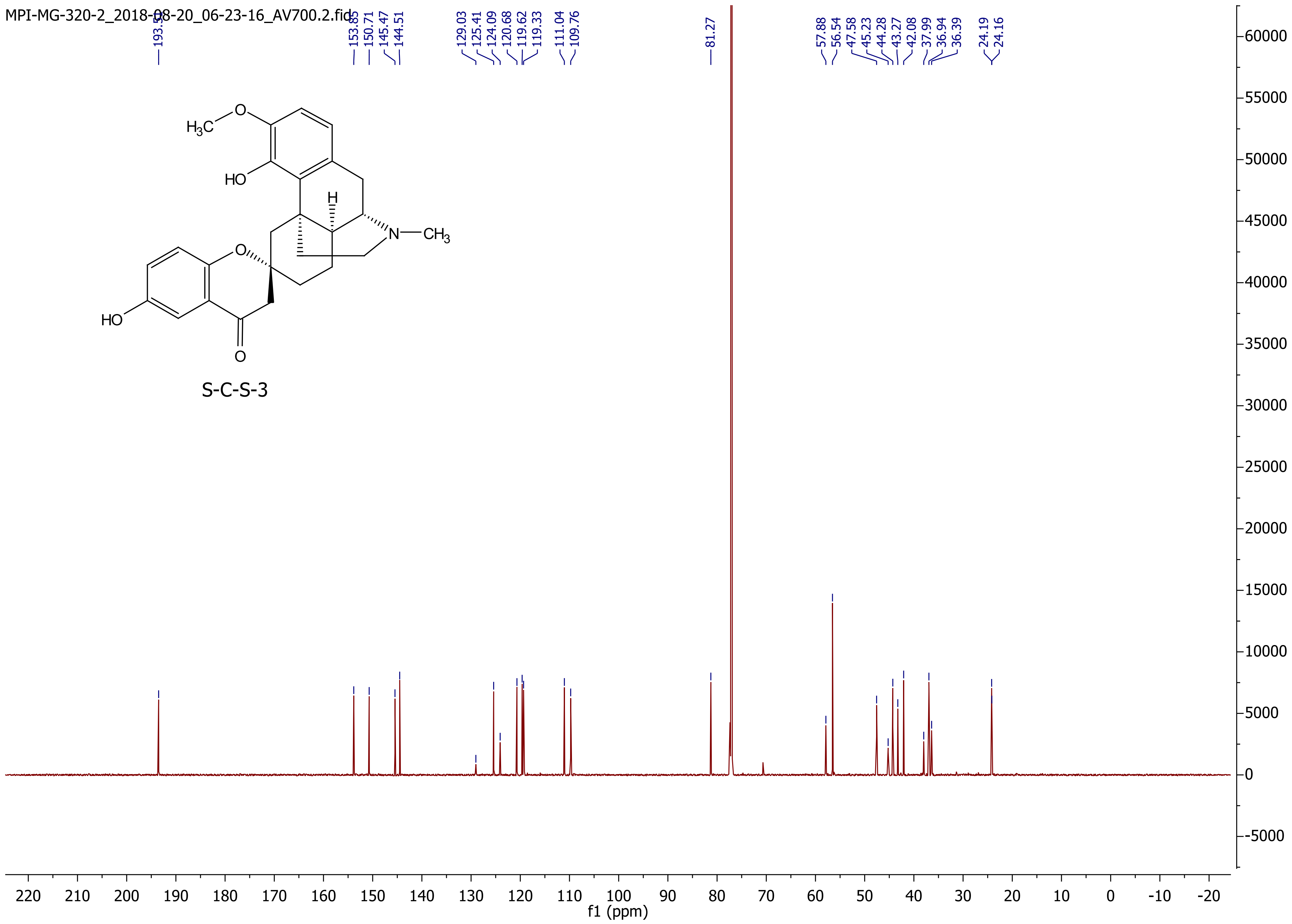

MPI-MG-320-2_2018-08-20_06-23-16_AV700.2.fid
153.85
150.71
145.47
144.51
129.03
125.41
124.09
120.68
119.62
119.33
111.04
109.76
81.27
57.88
56.54
47.58
45.23
44.28
43.27
42.08
37.99
36.94
36.39
24.19
24.16
H₃C
O
HO
H
N
CH₃
O
HO
O
S-C-S-3
f1 (ppm)

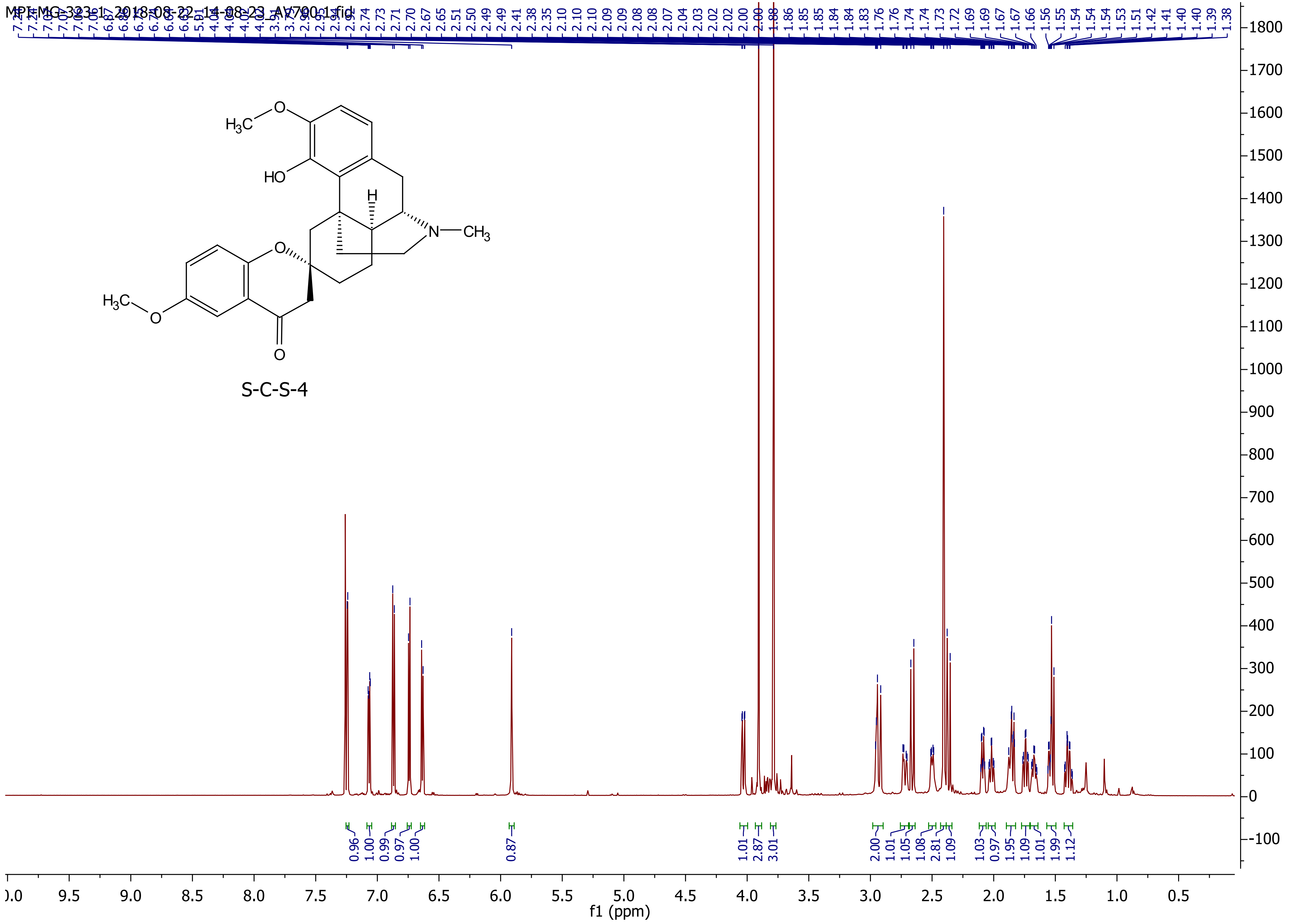
MPI-MG-323-1_2018-08-22_14-08-23_AV700.1.fid
S-C-S-4
f1 (ppm)

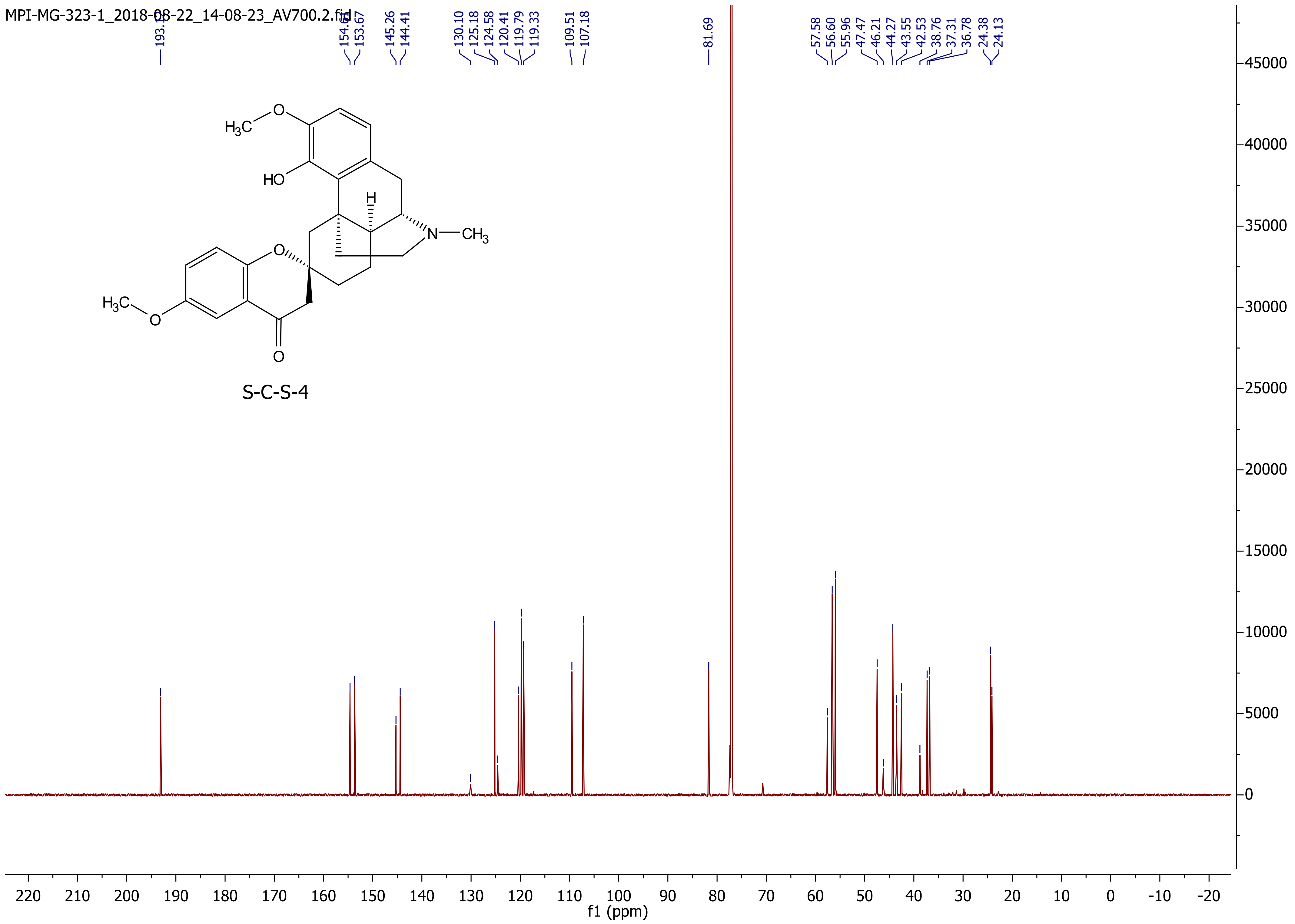

MPI-MG-323-1_2018-08-22_14-08-23_AV700.2.fid
153.67
145.26
144.41
130.10
125.18
124.58
120.41
119.79
119.33
109.51
107.18
81.69
57.58
56.60
55.96
47.47
46.21
44.27
43.55
42.53
38.76
37.31
36.78
24.38
24.13
H3C
O
HO
H
N
CH3
S-C-S-4
f1 (ppm)

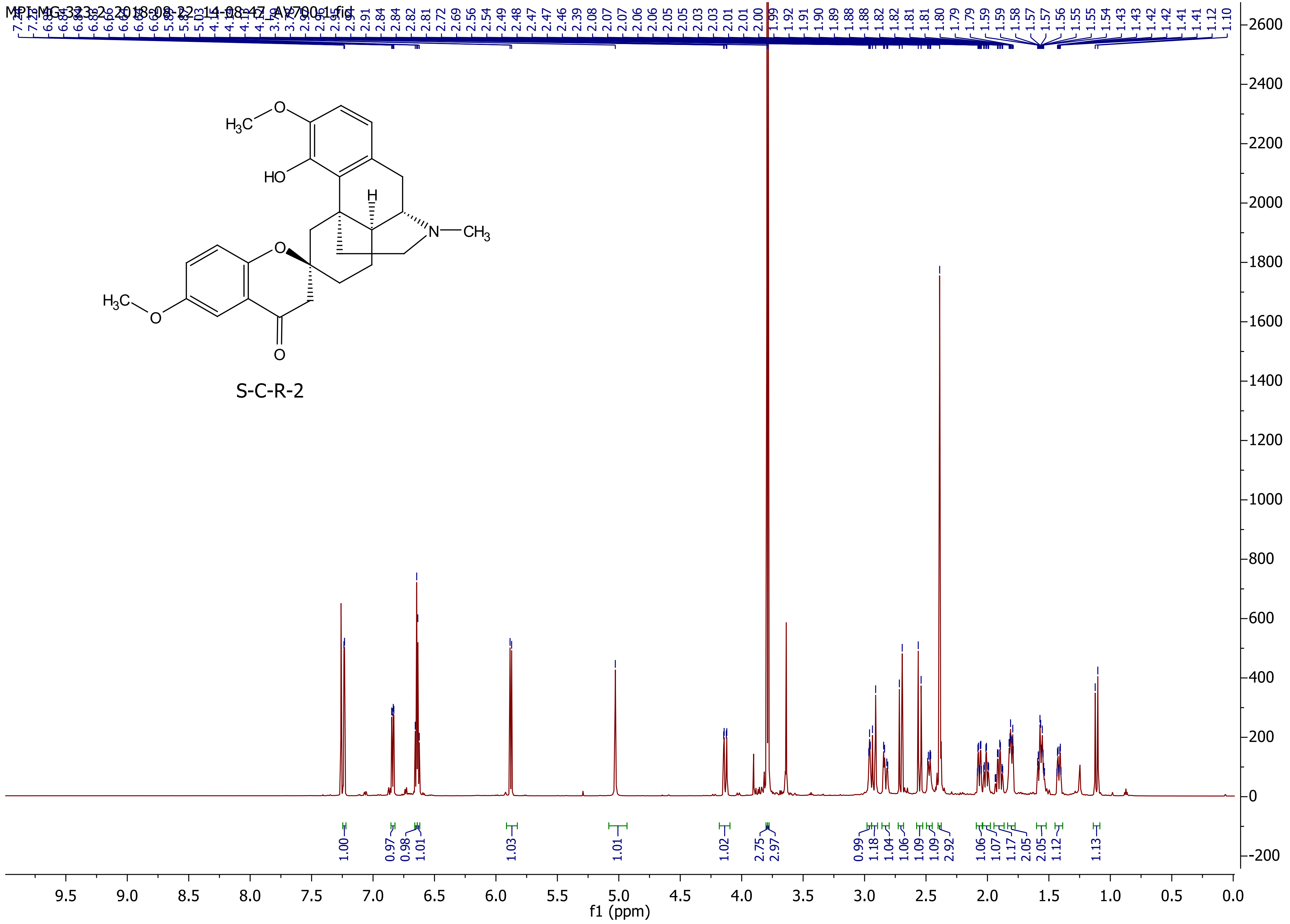

S-C-R-2
f1 (ppm)

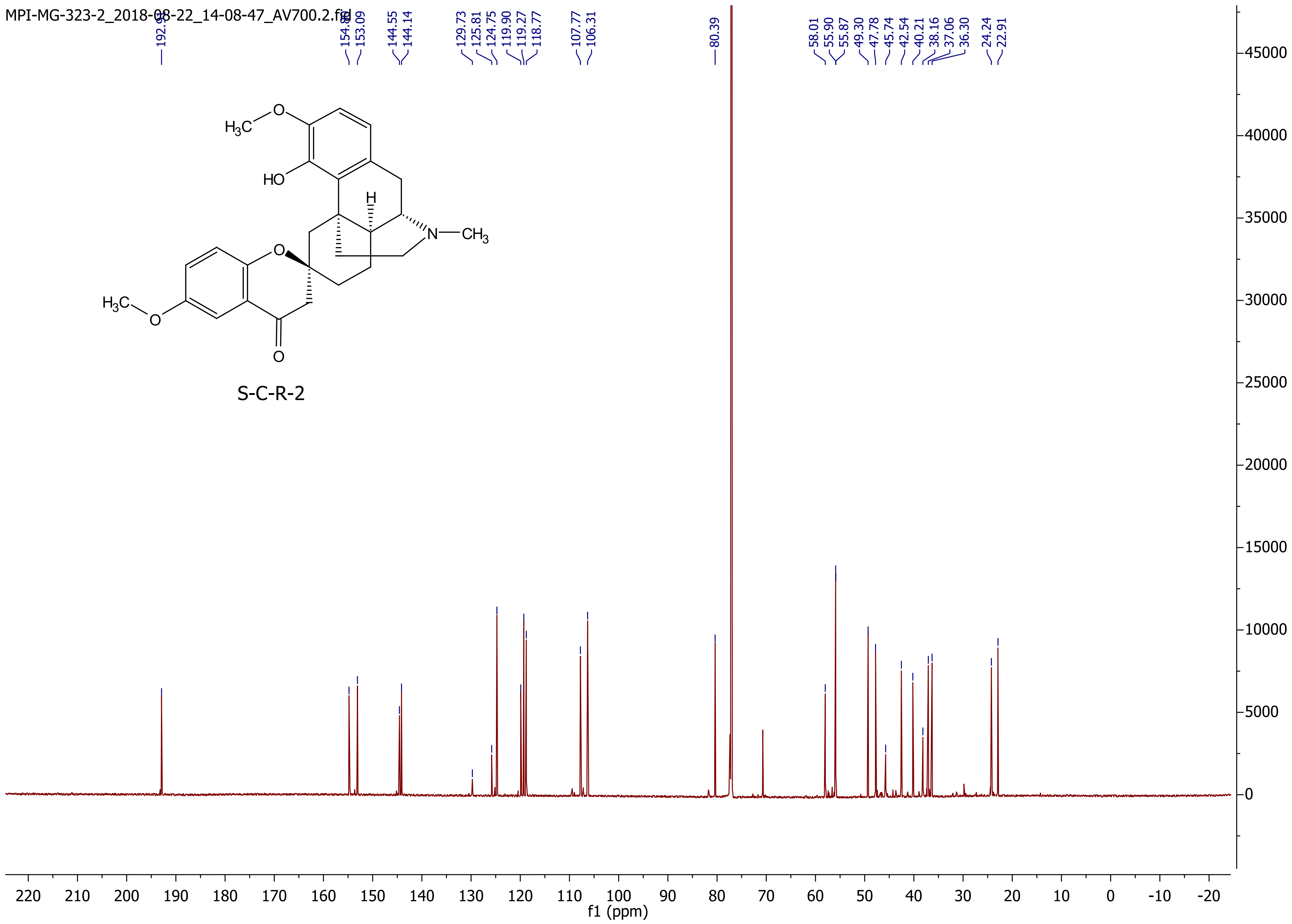

MPI-MG-323-2_2018-08-22_14-08-47_AV700.2.fid
153.09
144.55
144.14
129.73
125.81
124.75
119.90
119.27
118.77
107.77
106.31
80.39
58.01
55.90
55.87
49.30
47.78
45.74
42.54
40.21
38.16
37.06
36.30
24.24
22.91
S-C-R-2
f1 (ppm)

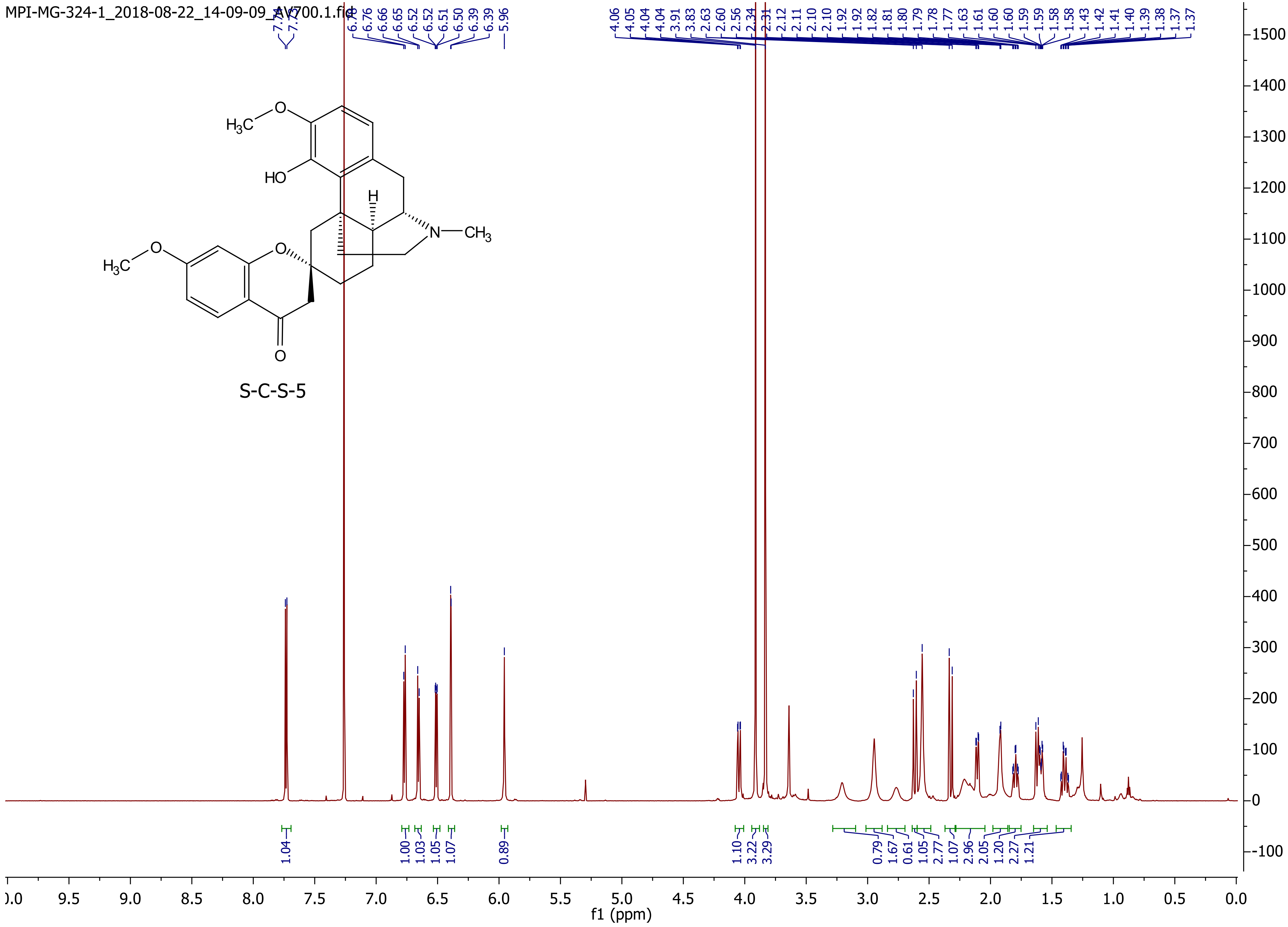

MPI-MG-324-1_2018-08-22_14-09-09_AV700.1.fid
6.78 6.76 6.66 6.65 6.52 6.52 6.51 6.50 6.39 6.39 5.96
4.06 4.05 4.04 4.04 3.91 3.83 2.63 2.60 2.56 2.34 2.31 2.12 2.11 2.10 2.10 1.92 1.92 1.82 1.81 1.80 1.79 1.78 1.77 1.63 1.61 1.60 1.60 1.59 1.59 1.58 1.58 1.43 1.42 1.41 1.40 1.39 1.38 1.37 1.37
H_3C
O
HO
H
N
CH_3
O
O
O
S-C-S-5
1.04
1.00 1.03 1.05 1.07
0.89
1.10 3.22 3.29
0.79 1.67 0.61 1.05 2.77 1.07 2.96 2.05 1.20 2.27 1.21
f1 (ppm)

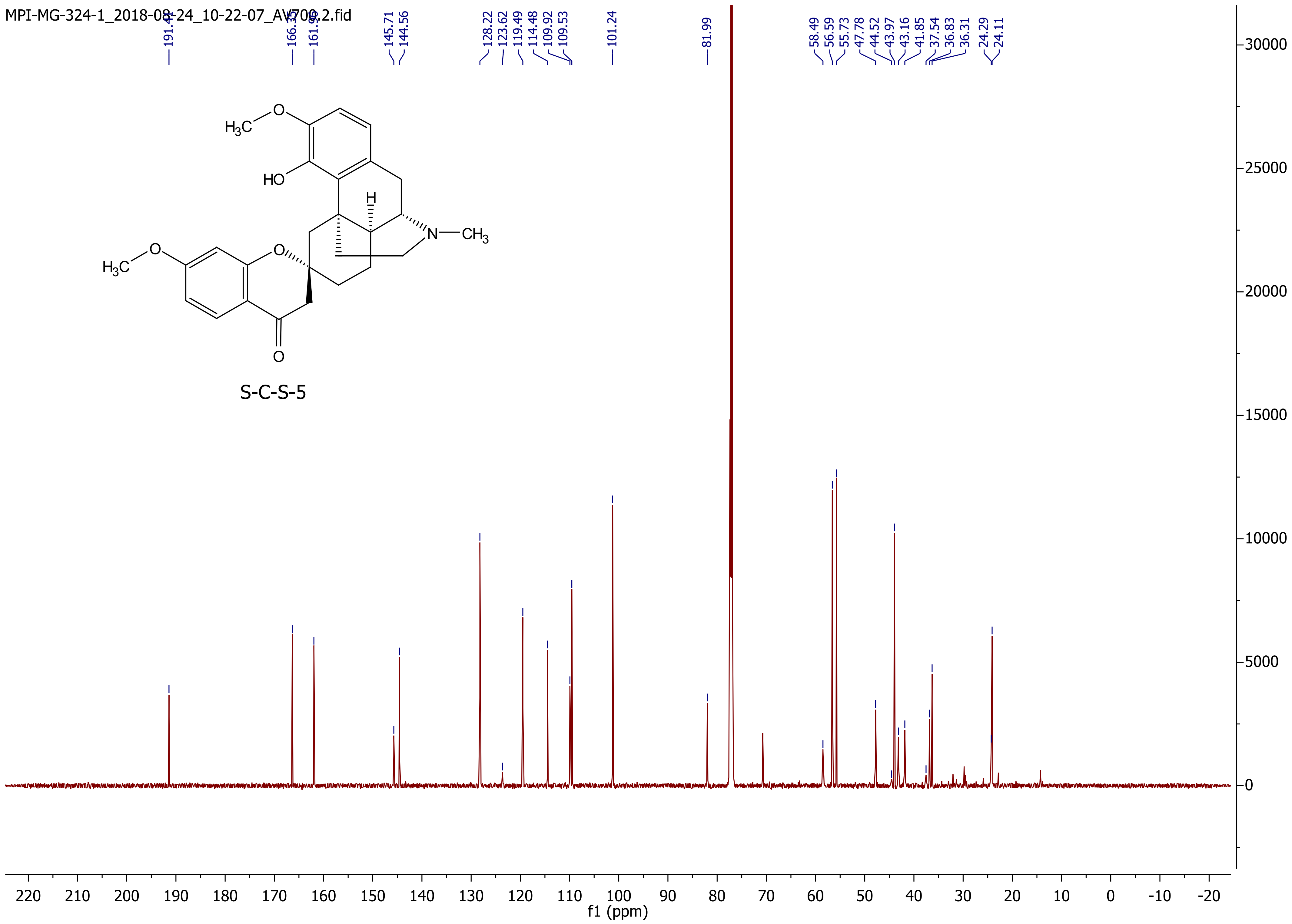
MPI-MG-324-1_2018-08-24_10-22-07_AV700.2.fid
145.71
144.56
128.22
123.62
119.49
114.48
109.92
109.53
101.24
81.99
58.49
56.59
55.73
47.78
44.52
43.97
43.16
41.85
37.54
36.83
36.31
24.29
24.11
H₃C
O
HO
H
N
CH₃
O
S-C-S-5
f1 (ppm)

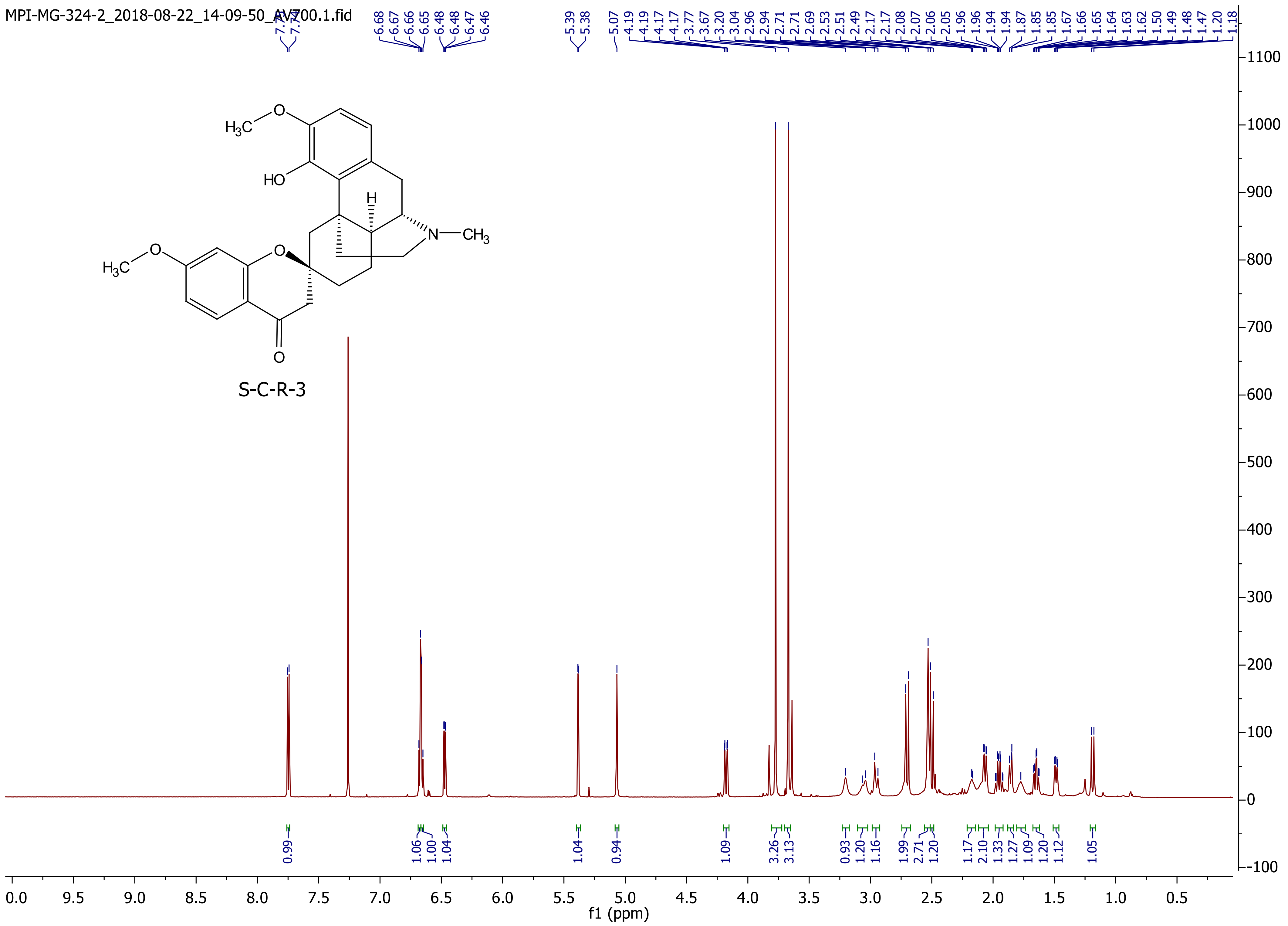
MPI-MG-324-2_2018-08-22_14-09-50_AV700.1.fid
H3C
O
HO
H
N
CH3
O
S-C-R-3
f1 (ppm)

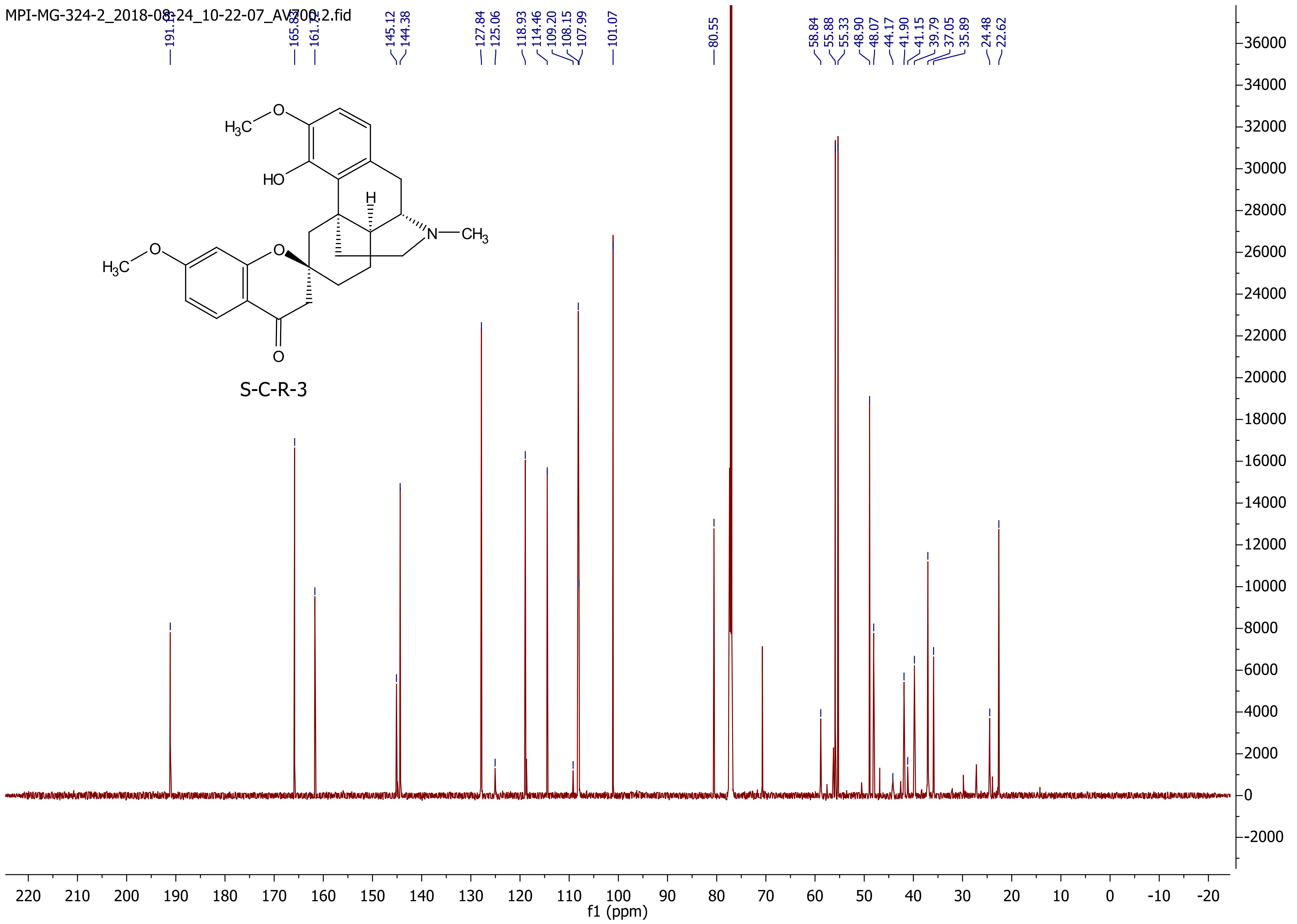

MPI-MG-324-2_2018-08-24_10-22-07_AV700.2.fid
145.12
144.38
127.84
125.06
118.93
114.46
109.20
108.15
107.99
101.07
80.55
58.84
55.88
55.33
48.90
48.07
44.17
41.90
41.15
39.79
37.05
35.89
24.48
22.62
H3C
O
HO
H
N
CH3
O
O
S-C-R-3
f1 (ppm)

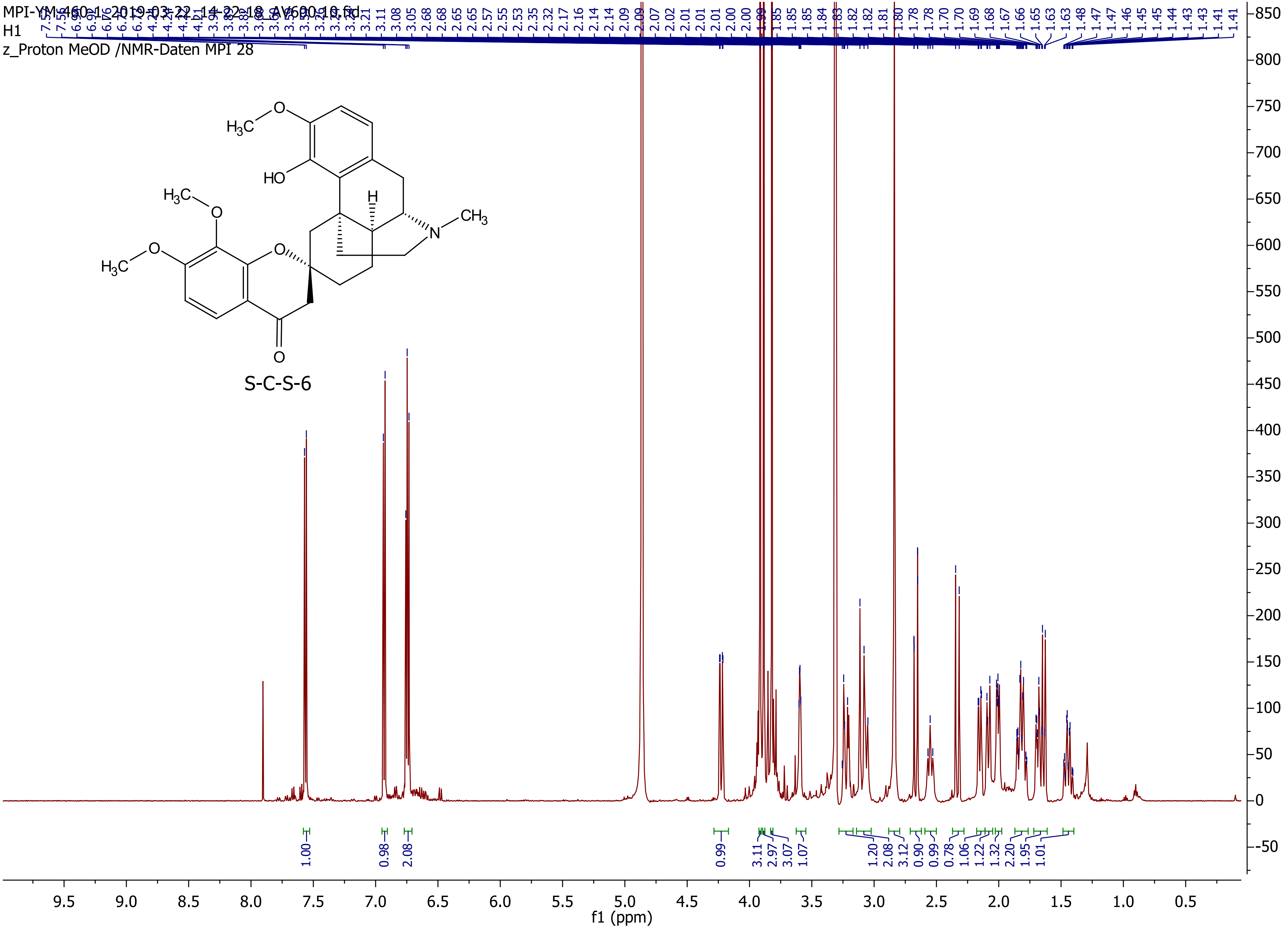
MPI-YM-460-1 2019-03-22 14-22-18 AV600 10.fid
H1
z_Proton MeOD /NMR-Daten MPI 28
H3C
O
HO
H
CH3
N
O
O
H3C
O
H3C
O
S-C-S-6
1.00
0.98
2.08
0.99
3.11
2.97
3.07
1.07
1.20
2.08
3.12
0.90
0.99
0.78
1.06
1.22
1.32
2.20
1.95
1.01
f1 (ppm)

MPI-YM-460-1_2019-03-22_14-22-18_AV600 11.fid
C13 with power gated H1 decoupling
z_C13pg MeOD /NMR-Daten MPI 28

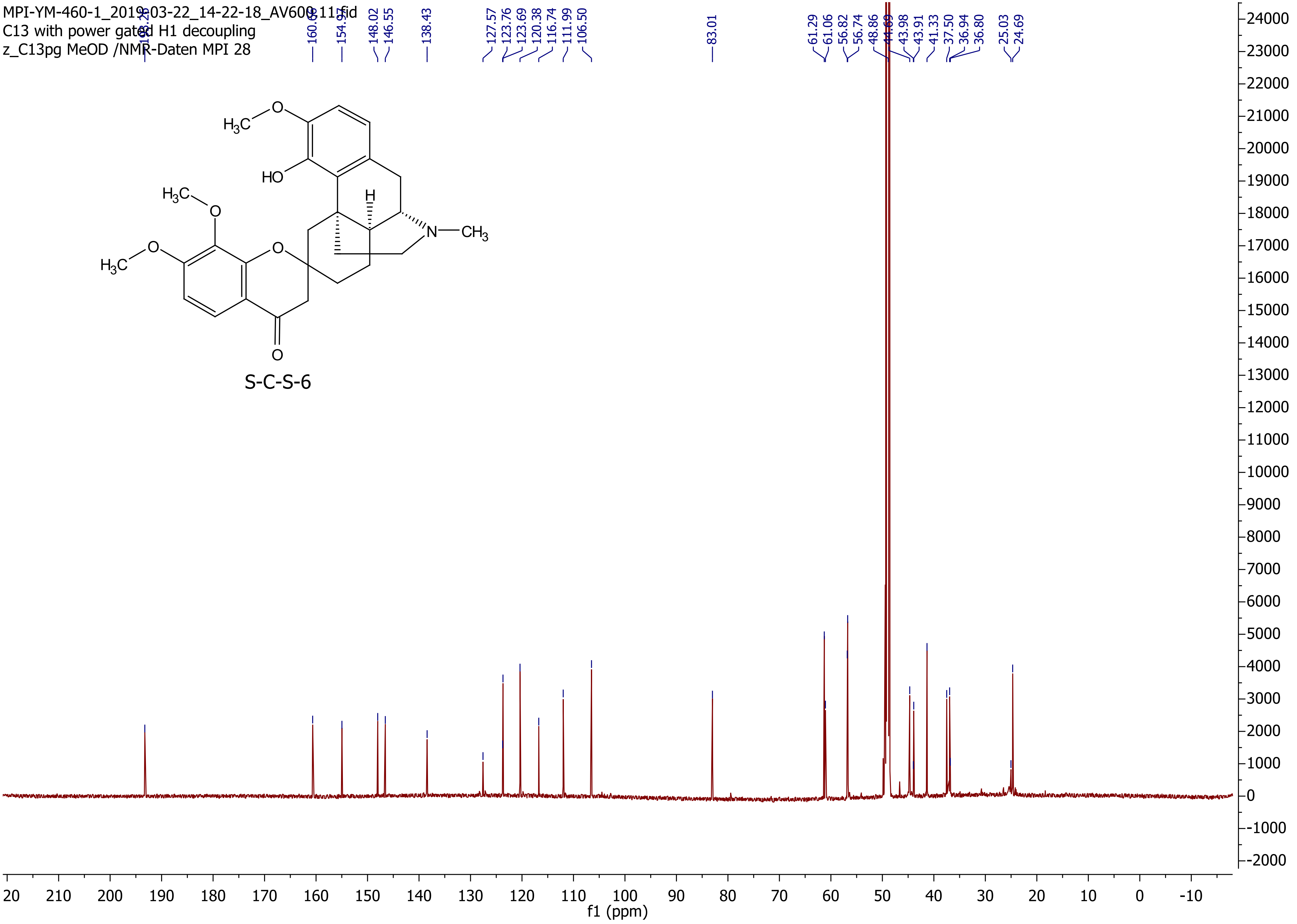

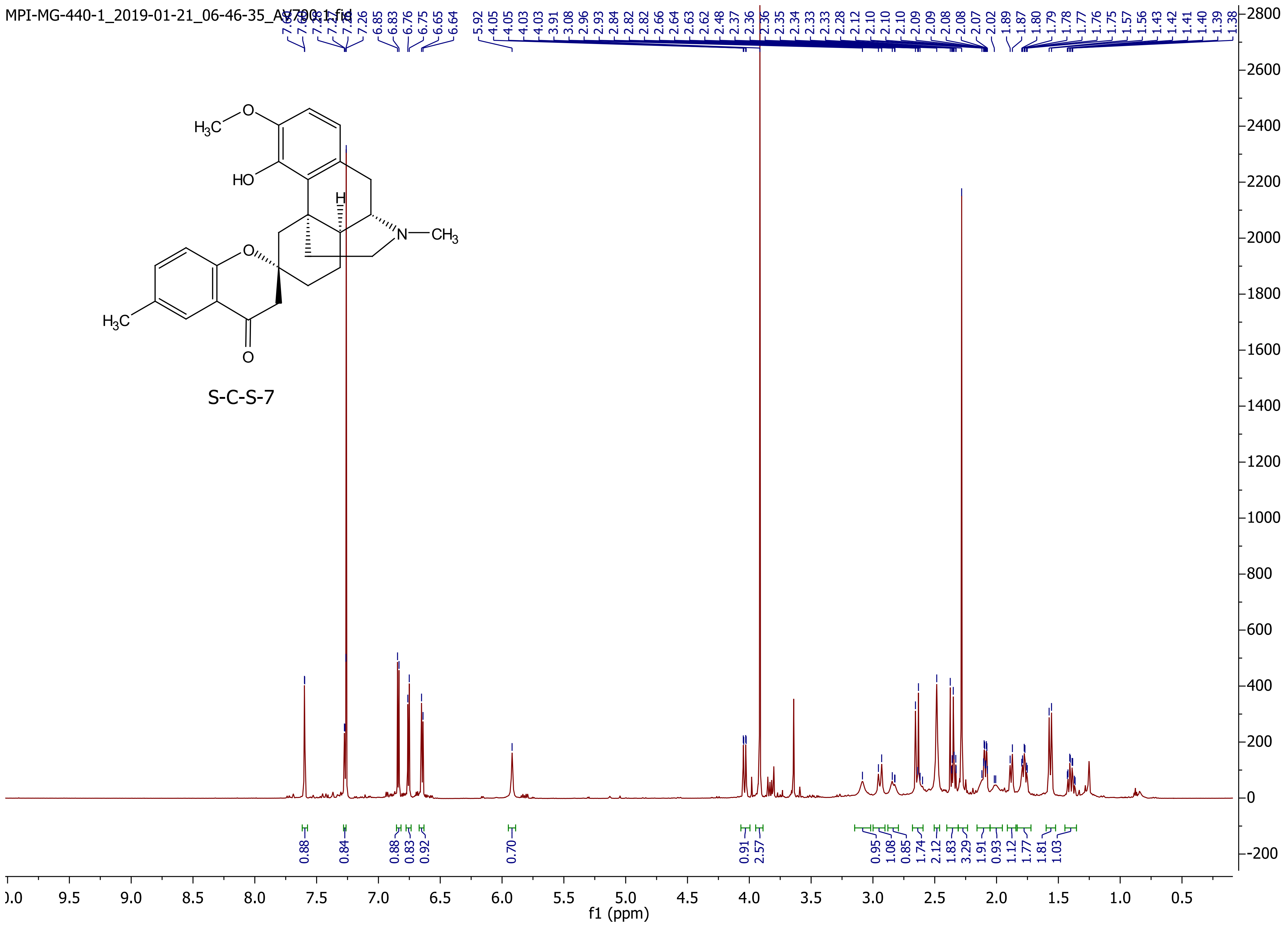

MPI-MG-440-1_2019-01-21_06-46-35_AV700 1.fid
7.26
6.85
6.83
6.76
6.75
6.65
6.64
5.92
4.05
4.05
4.03
4.03
3.91
3.08
2.96
2.93
2.84
2.82
2.82
2.66
2.64
2.63
2.62
2.48
2.37
2.36
2.36
2.35
2.34
2.33
2.33
2.28
2.12
2.10
2.10
2.10
2.09
2.09
2.08
2.08
2.07
2.02
1.89
1.87
1.80
1.79
1.78
1.77
1.76
1.75
1.57
1.56
1.43
1.42
1.41
1.40
1.39
1.38
H_3C
O
HO
H
N
CH_3
O
H_3C
O
S-C-S-7
0.88
0.84
0.88
0.83
0.92
0.70
0.91
2.57
0.95
1.08
0.85
1.74
2.12
1.83
3.29
1.91
0.93
1.12
1.77
1.81
1.03
f1 (ppm)

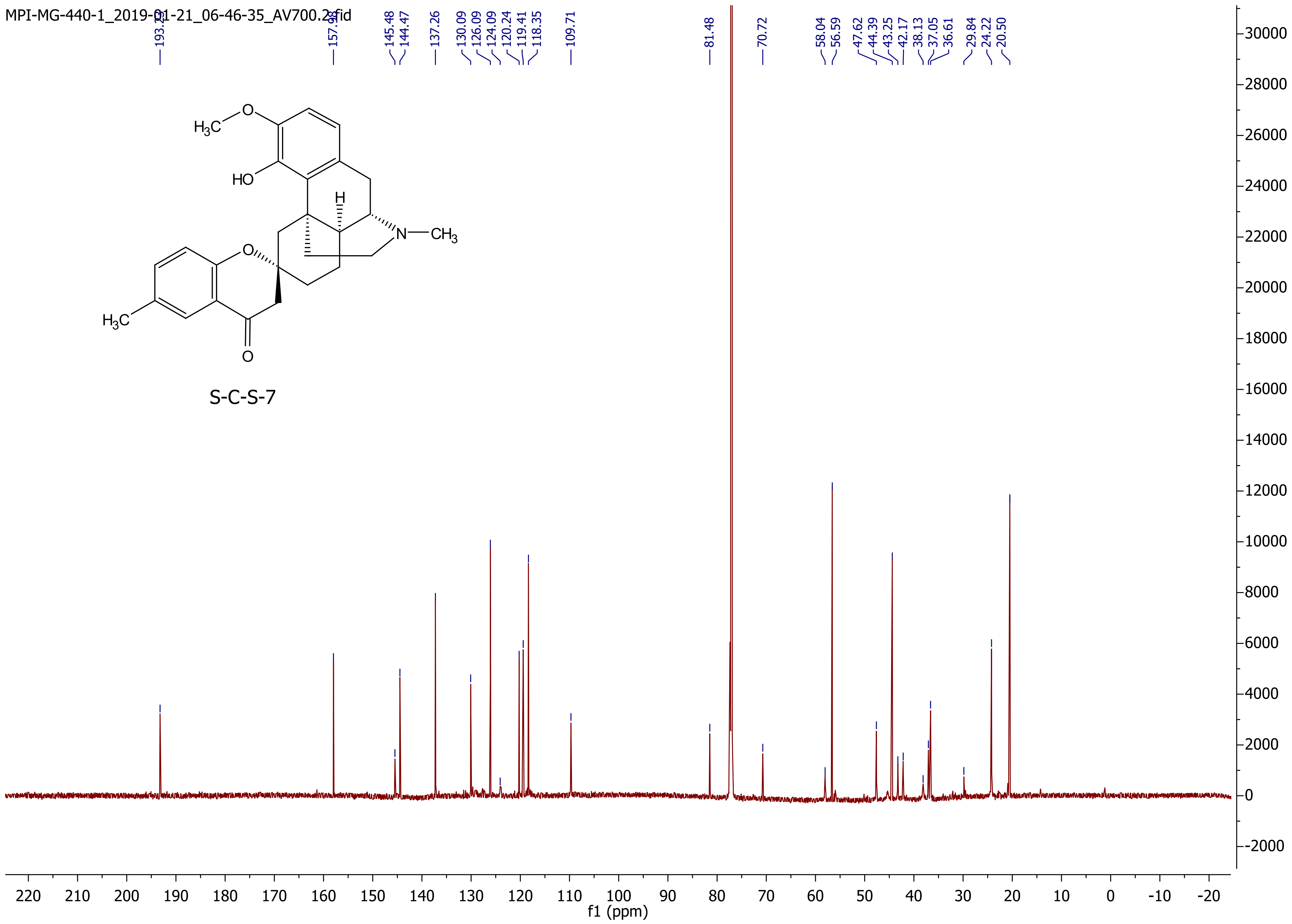
MPI-MG-440-1_2019-01-21_06-46-35_AV700.2.fid
193.22
157.98
145.48
144.47
137.26
130.09
126.09
124.09
120.24
119.41
118.35
109.71
81.48
70.72
58.04
56.59
47.62
44.39
43.25
42.17
38.13
37.05
36.61
29.84
24.22
20.50
H3C
O
HO
H
N
CH3
O
H3C
O
S-C-S-7
f1 (ppm)

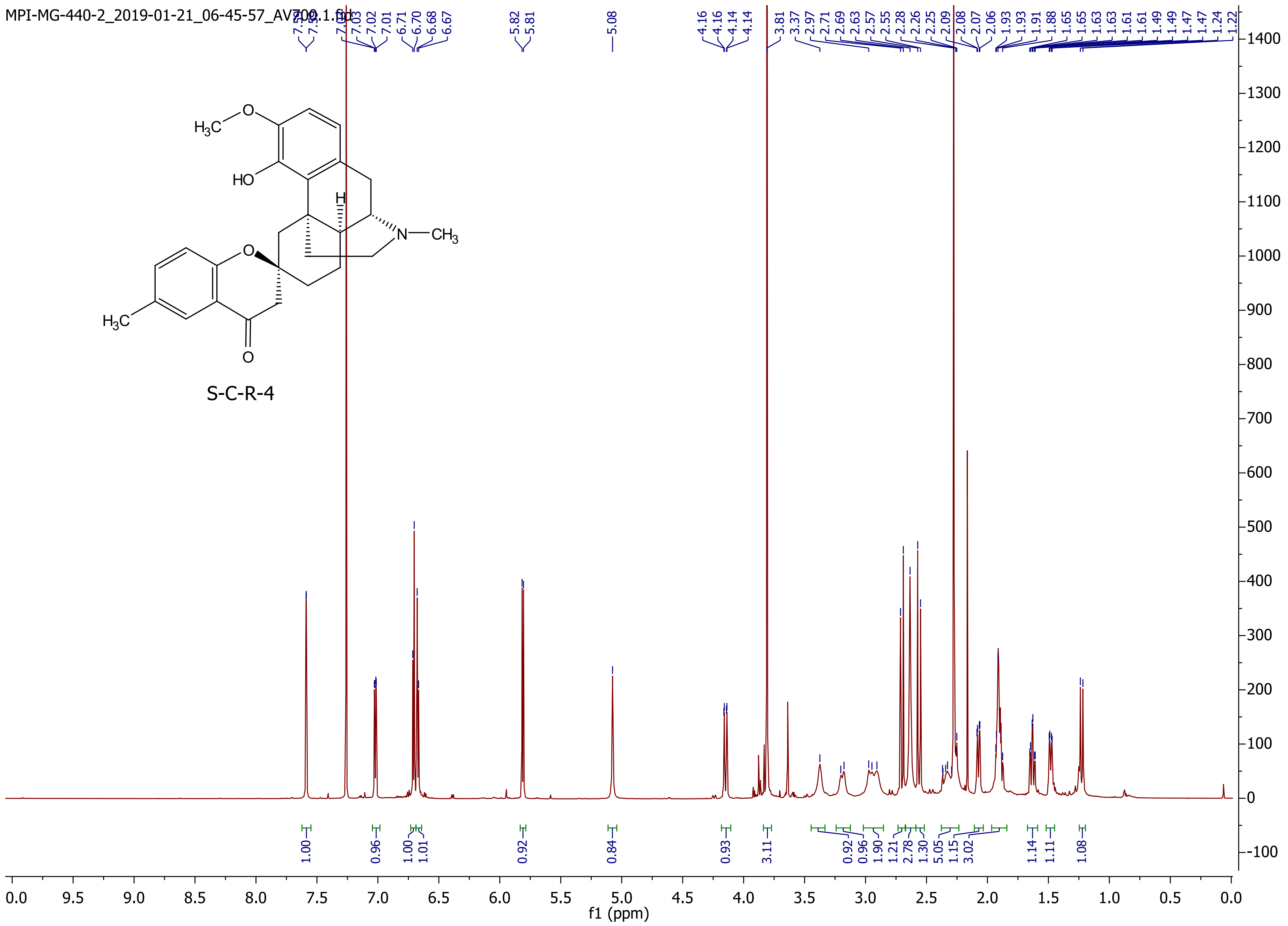

MPI-MG-440-2_2019-01-21_06-45-57_AV700.1.fid
S-C-R-4
f1 (ppm)

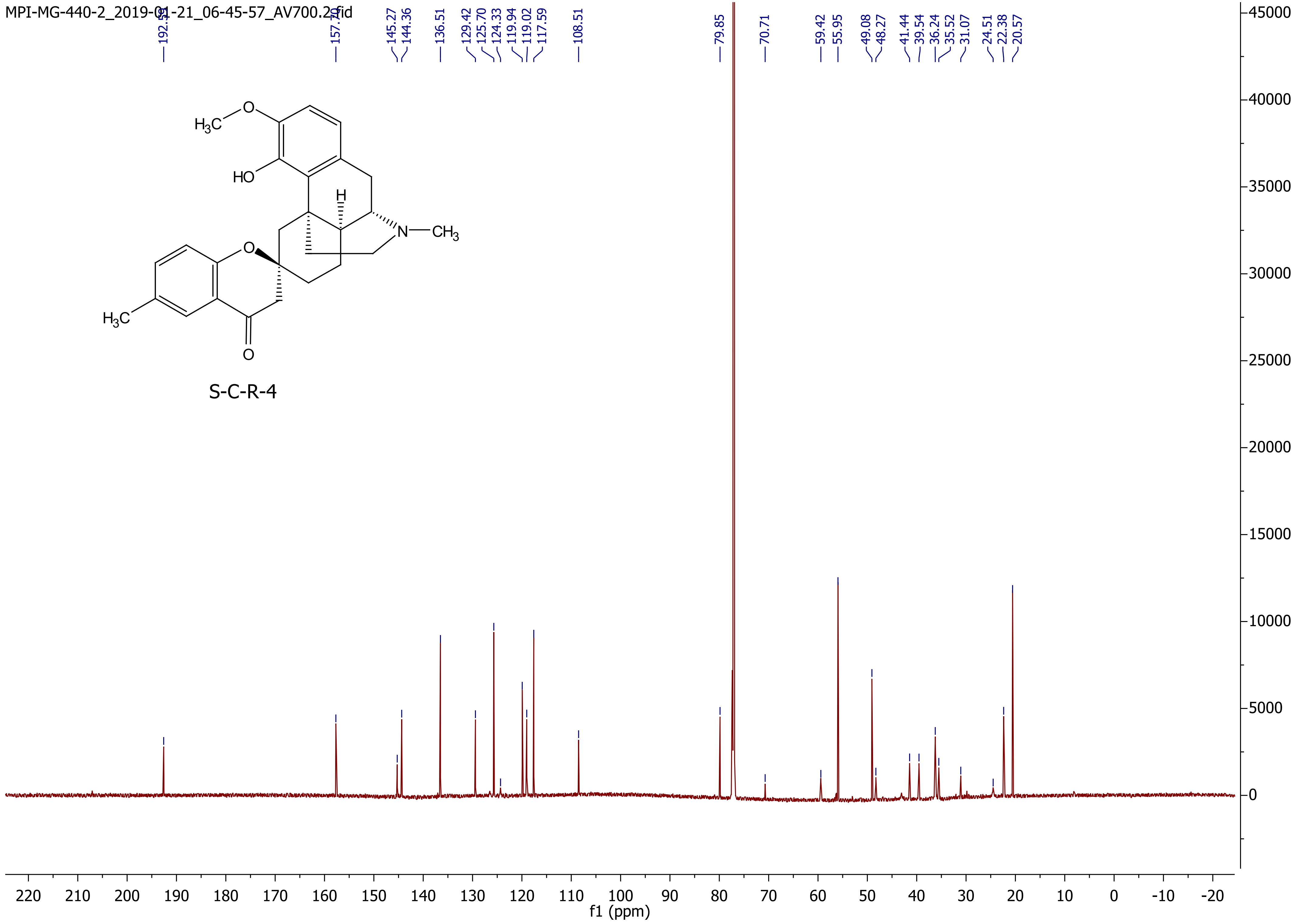
MPI-MG-440-2_2019-01-21_06-45-57_AV700.2.fid
192.59
157.70
145.27
144.36
136.51
129.42
125.70
124.33
119.94
119.02
117.59
108.51
79.85
70.71
59.42
55.95
49.08
48.27
41.44
39.54
36.24
35.52
31.07
24.51
22.38
20.57
H3C
O
HO
H
N
CH3
O
H3C
O
S-C-R-4
45000
40000
35000
30000
25000
20000
15000
10000
5000
0
220
210
200
190
180
170
160
150
140
130
120
110
100
90
80
70
60
50
40
30
20
10
0
-10
-20
f1 (ppm)

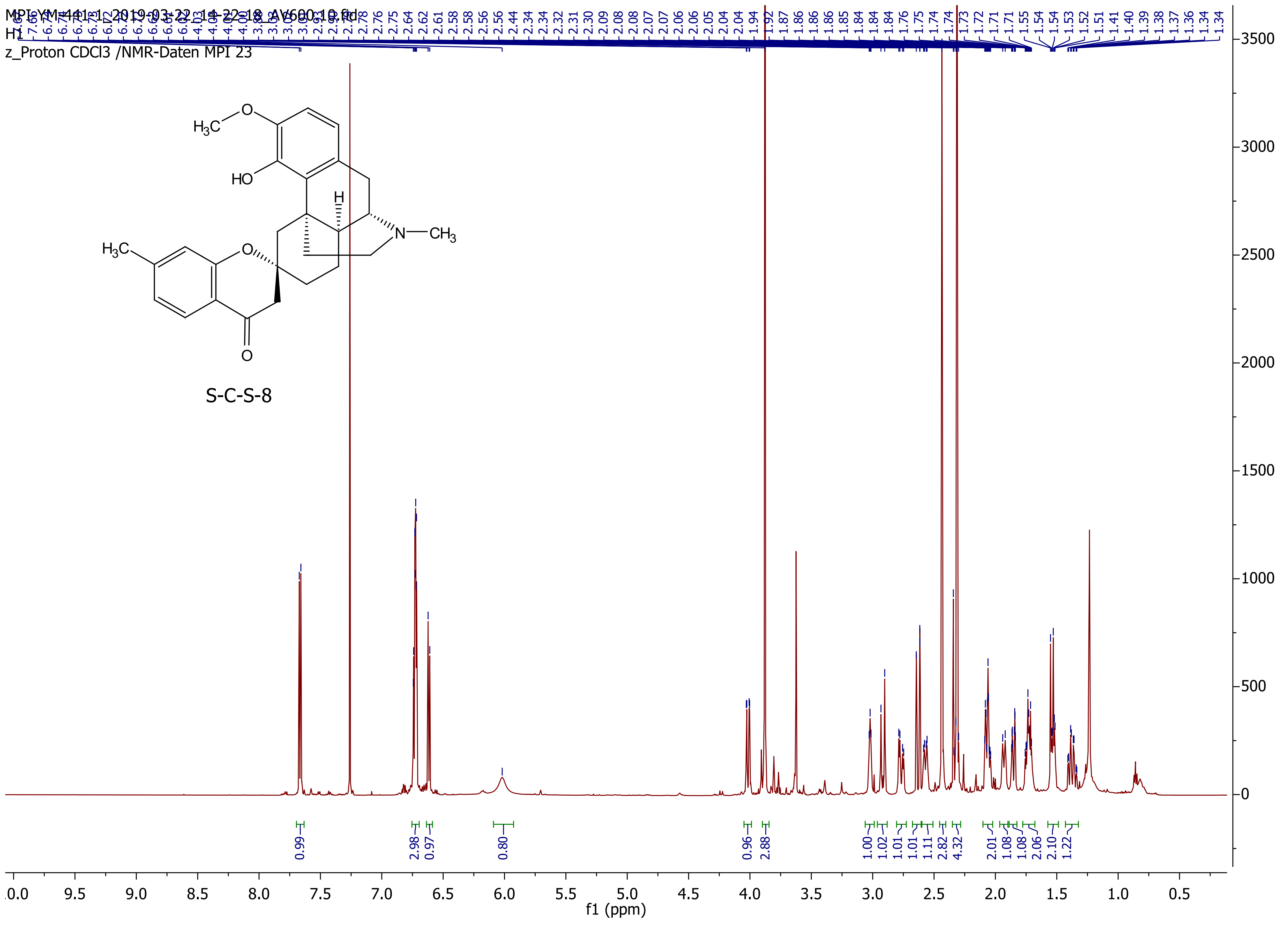

MPI-YM-441-1_2019-03-22_14-22-18_AV600.10.fid
H1
z_Proton CDCl3 /NMR-Daten MPI 23
2.78 2.76 2.75 2.64 2.62 2.61 2.58 2.58 2.56 2.56 2.44 2.34 2.34 2.32 2.31 2.30 2.09 2.08 2.08 2.07 2.07 2.06 2.06 2.05 2.04 2.04 1.94 1.92 1.87 1.86 1.86 1.86 1.85 1.84 1.84 1.84 1.76 1.75 1.74 1.74 1.73 1.72 1.71 1.71 1.55 1.54 1.54 1.53 1.52 1.51 1.41 1.40 1.39 1.38 1.37 1.36 1.34 1.34
S-C-S-8
0.99 2.98 0.97 0.80 0.96 2.88 1.00 1.02 1.01 1.01 1.11 2.82 4.32 2.01 1.08 1.08 2.06 2.10 1.22
f1 (ppm)

MPI-YM-441-1_2019-03-22_14-22-18_AV600.11.fid
C13 with power gated H1 decoupling
z_C13pg CDCl3 /NMR-Daten MPI 23

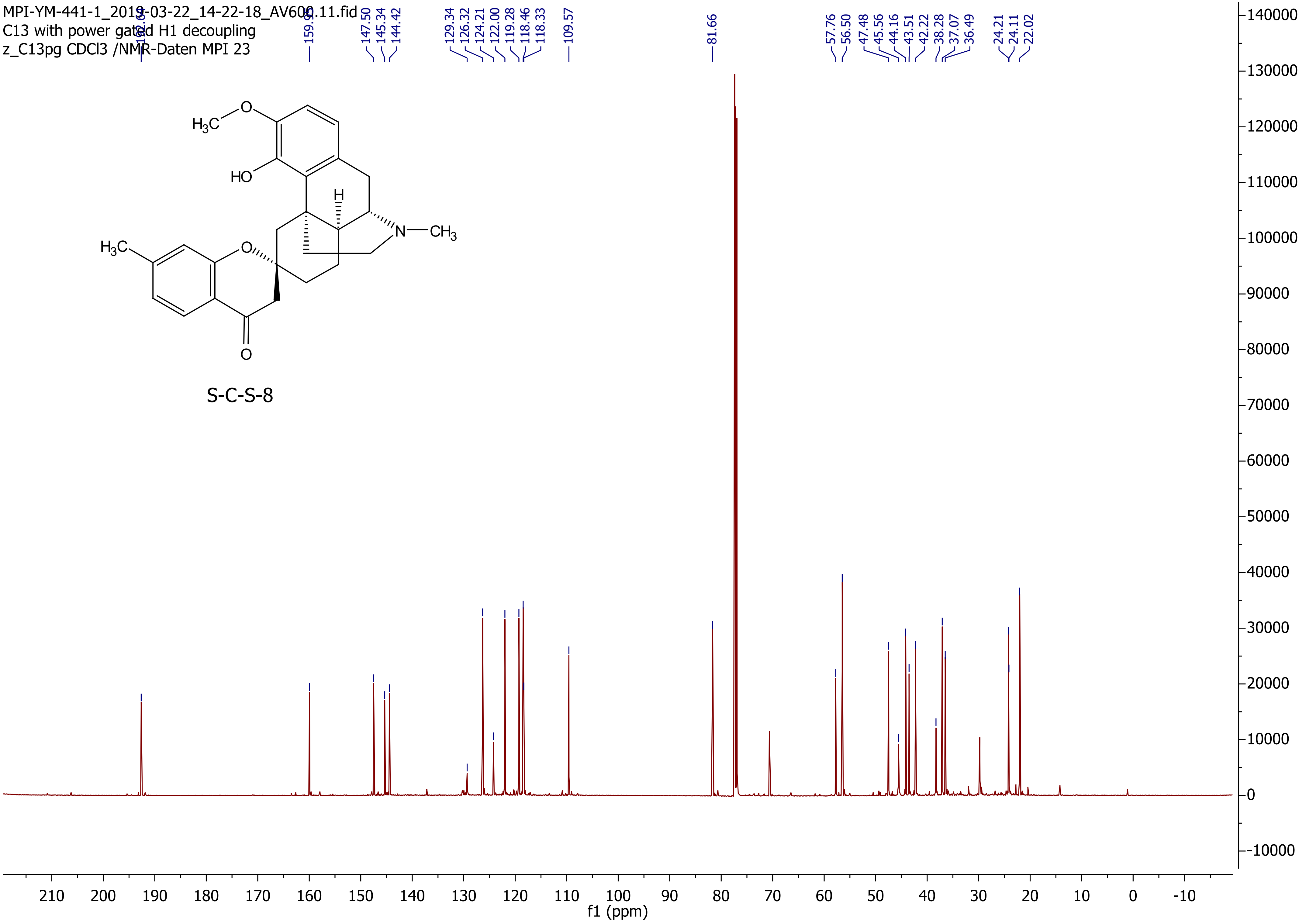

S-C-S-8

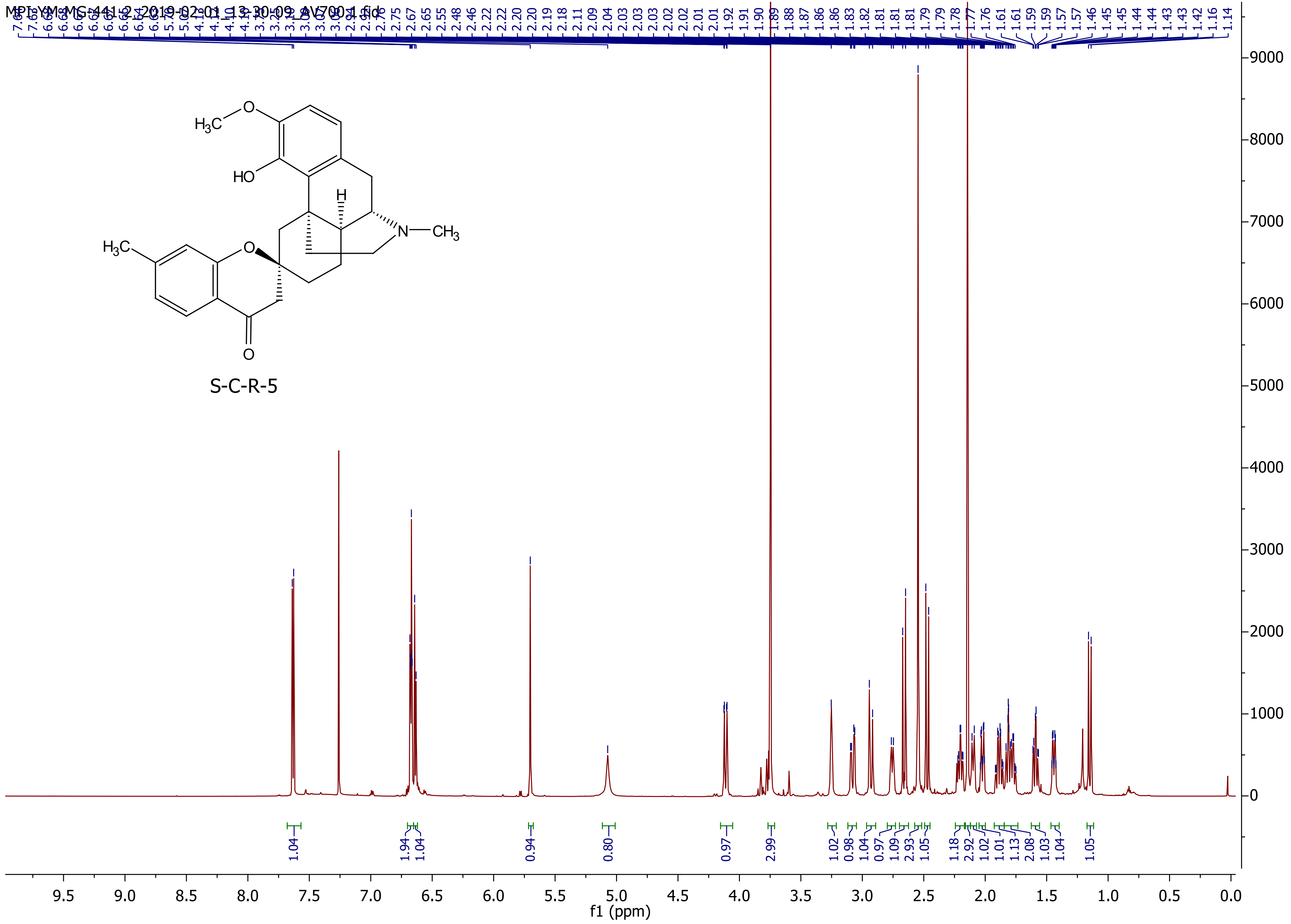
MPI-YM-MG-441-2_2019-02-01_13-30-09_AV700-1.fid
H3C
O
HO
H
N
CH3
H3C
O
O
S-C-R-5
1.04
1.94
1.04
0.94
0.80
0.97
2.99
1.02
0.98
1.04
0.97
1.09
2.93
1.05
1.18
2.92
1.02
1.01
1.13
2.08
1.03
1.04
1.05
f1 (ppm)

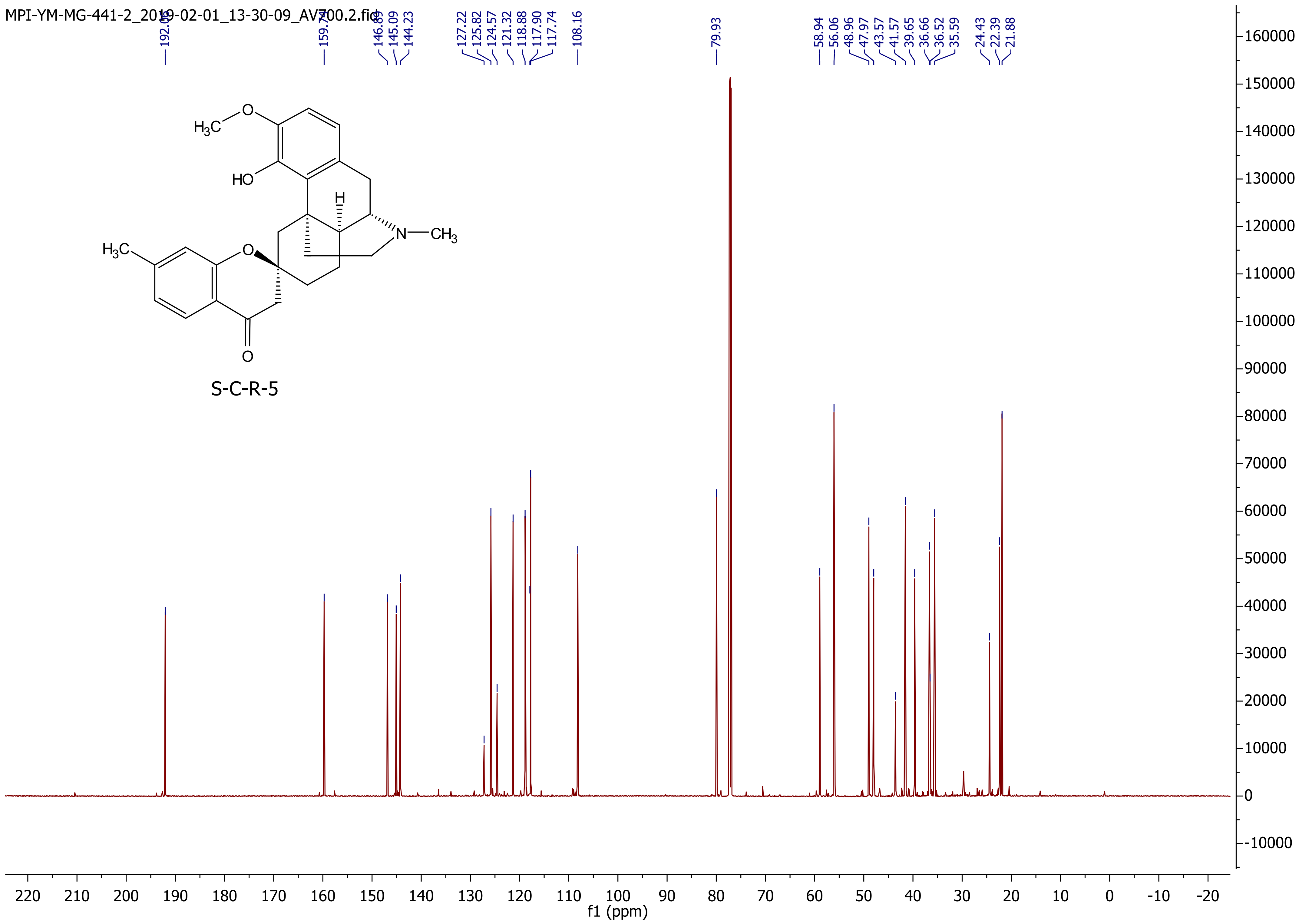

MPI-YM-MG-441-2_2019-02-01_13-30-09_AV700.2.fid
192.06
159.74
146.89
145.09
144.23
127.22
125.82
124.57
121.32
118.88
117.90
117.74
108.16
79.93
58.94
56.06
48.96
47.97
43.57
41.57
39.65
36.66
36.52
35.59
24.43
22.39
21.88
S-C-R-5
f1 (ppm)

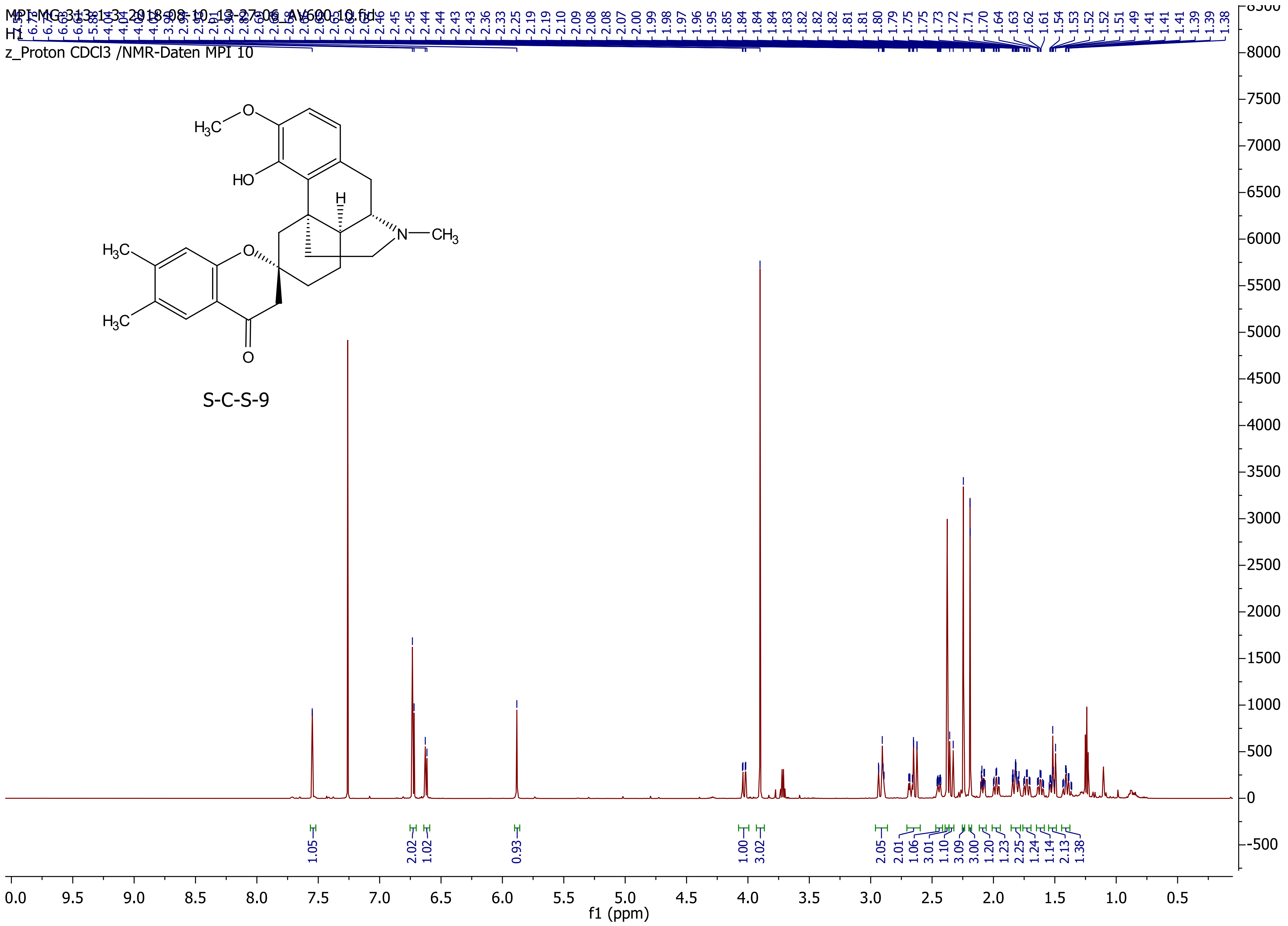
MPI-MG-313-1-3_2018-08-10_13-27-06_AV600-10.fid
H1
z_Proton CDCl3 /NMR-Daten MPI 10
S-C-S-9
1.05
2.02
1.02
0.93
1.00
3.02
2.05
2.01
1.06
3.01
1.10
3.09
3.00
1.20
1.23
2.25
1.24
1.14
2.13
1.38
f1 (ppm)

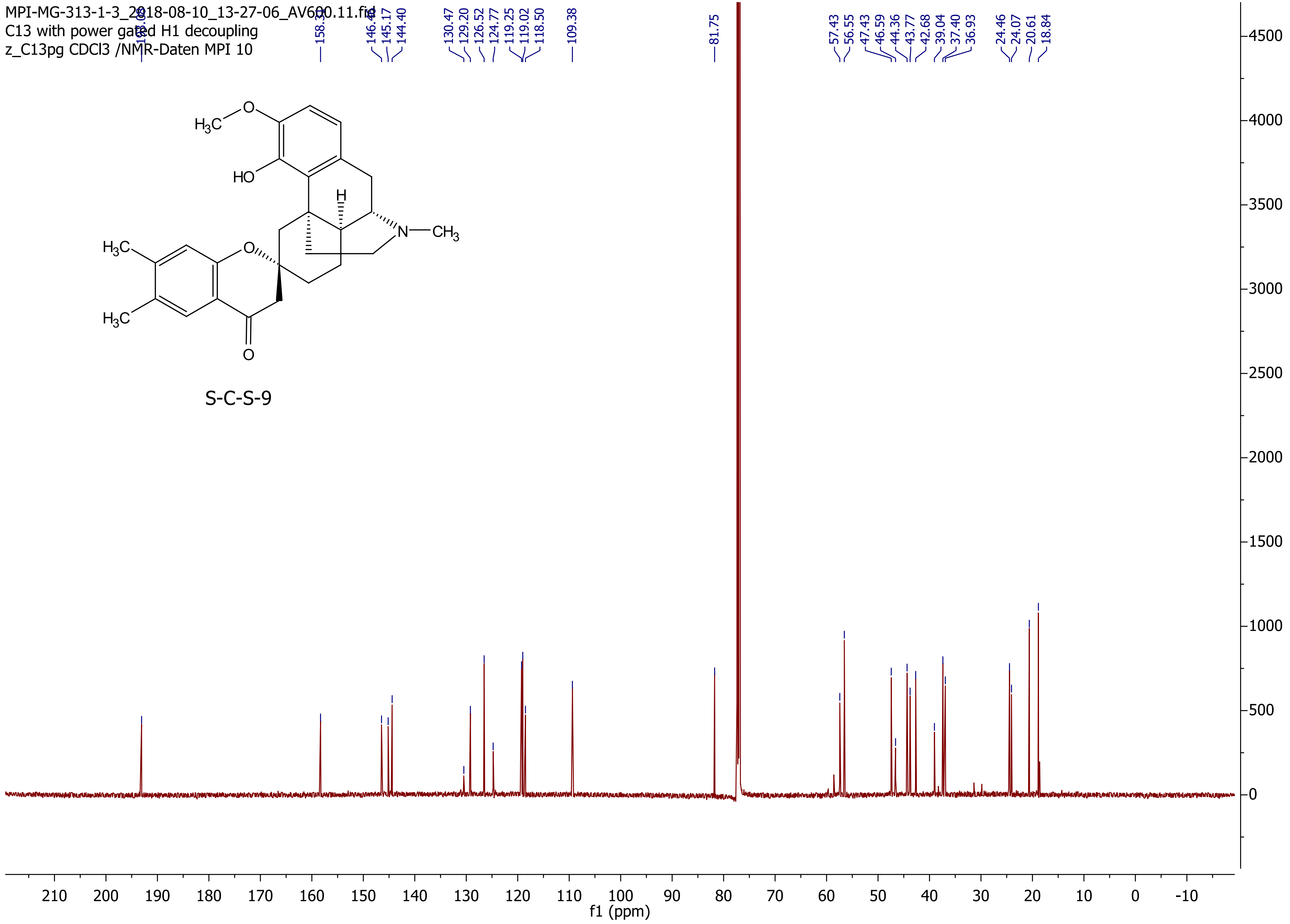
MPI-MG-313-1-3_2018-08-10_13-27-06_AV600.11.fid
C13 with power gated H1 decoupling
z_C13pg CDCl3 /NMR-Daten MPI 10
145.17
144.40
130.47
129.20
126.52
124.77
119.25
119.02
118.50
109.38
81.75
57.43
56.55
47.43
46.59
44.36
43.77
42.68
39.04
37.40
36.93
24.46
24.07
20.61
18.84
H_3C
O
HO
H
N
CH_3
H_3C
O
H_3C
O
S-C-S-9
f1 (ppm)

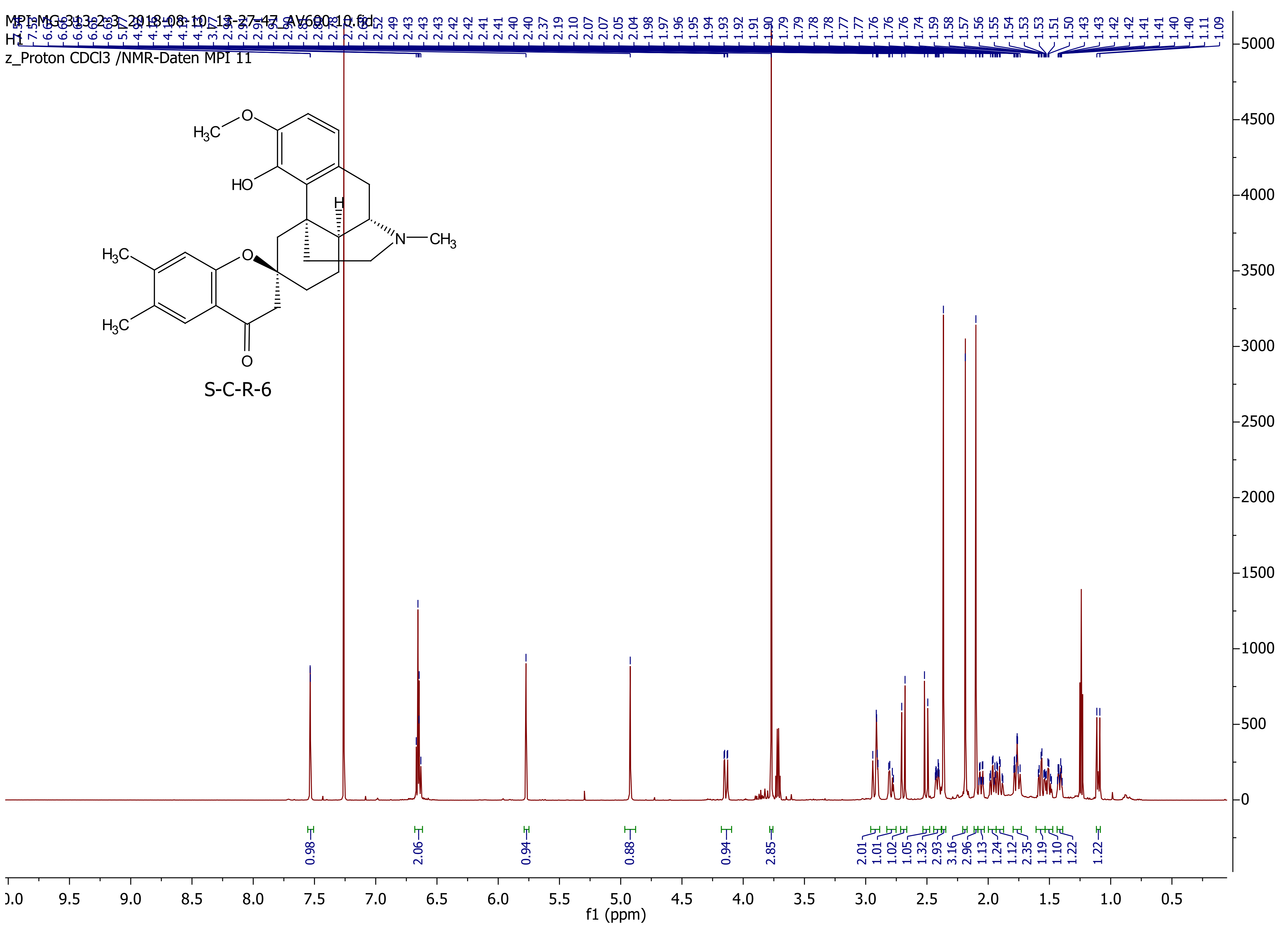

MPI-MG-313-2-3_2018-08-10_13-27-47_AV600-10.fid
H1
z_Proton CDCl3 /NMR-Daten MPI 11
2.52 2.49 2.43 2.43 2.43 2.42 2.42 2.41 2.41 2.40 2.40 2.37 2.19 2.10 2.07 2.07 2.05 2.04 1.98 1.97 1.96 1.95 1.94 1.93 1.92 1.91 1.90 1.79 1.79 1.78 1.78 1.77 1.77 1.76 1.76 1.76 1.74 1.59 1.58 1.57 1.56 1.55 1.54 1.53 1.53 1.51 1.50 1.43 1.43 1.42 1.42 1.41 1.41 1.40 1.40 1.11 1.09
H3C
O
HO
H
N
CH3
H3C
O
H3C
O
S-C-R-6
0.98
2.06
0.94
0.88
0.94
2.85
2.01
1.01
1.02
1.05
1.32
2.93
3.16
2.96
1.13
1.24
1.12
2.35
1.19
1.10
1.22
1.22
9.5 9.0 8.5 8.0 7.5 7.0 6.5 6.0 5.5 5.0 4.5 4.0 3.5 3.0 2.5 2.0 1.5 1.0 0.5
f1 (ppm)
5000 4500 4000 3500 3000 2500 2000 1500 1000 500 0

MPI-MG-313-2-3_2018-08-10_13-27-47_AV600.11.fid
C13 with power gated H1 decoupling
z_C13pg CDCl3 /NMR-Daten MPI 11

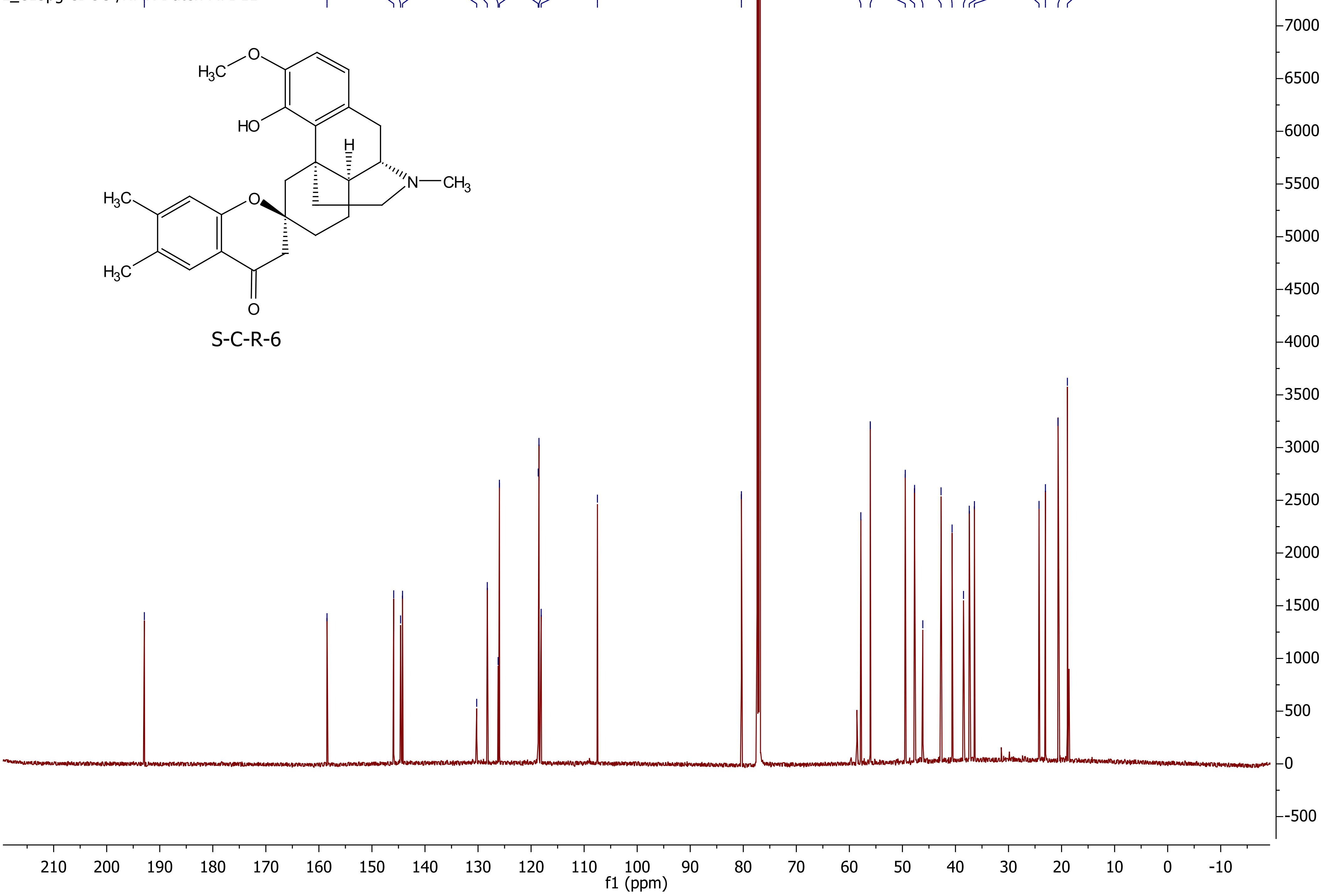

S-C-R-6

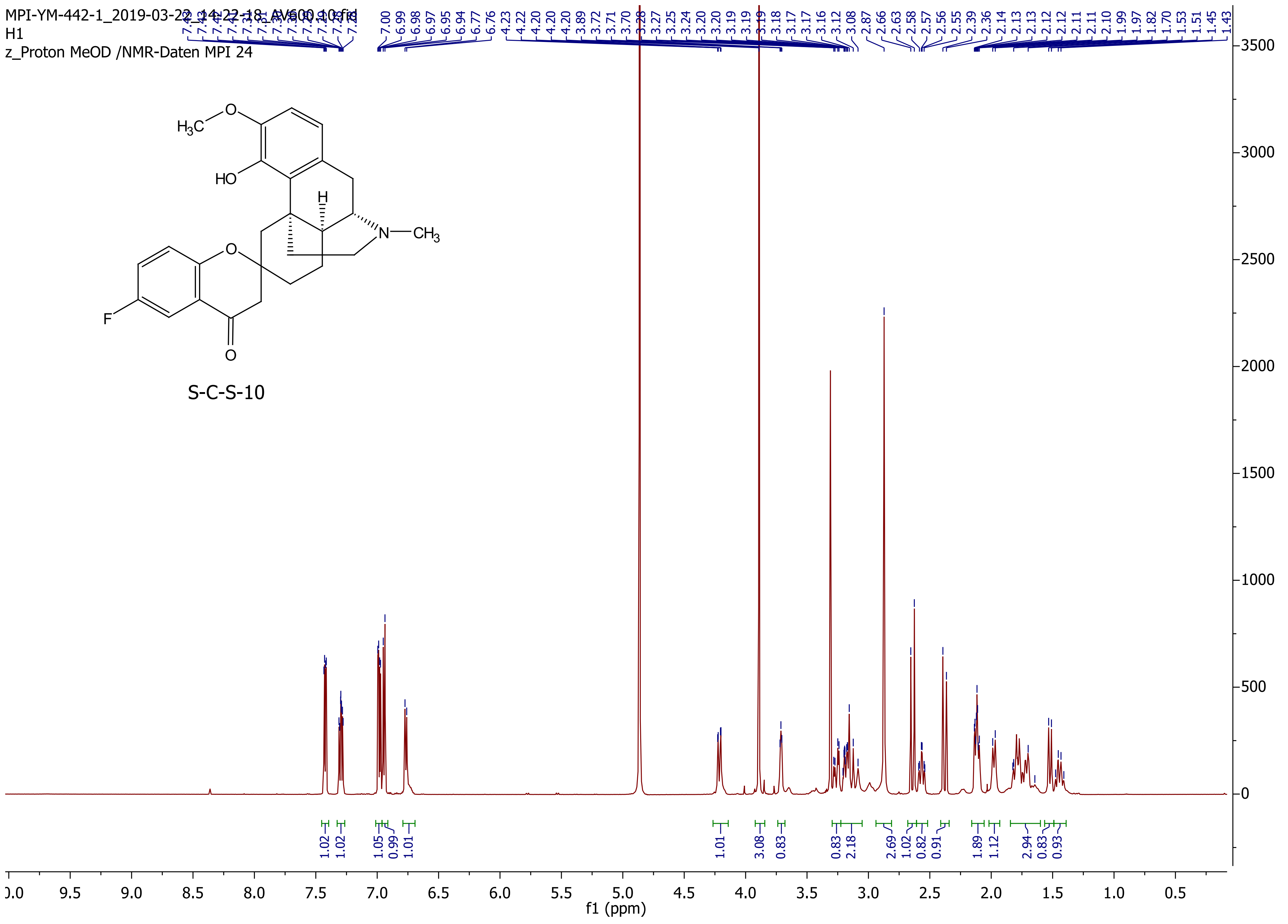
MPI-YM-442-1_2019-03-22_14-22-18_AV600.10.fid
H1
z_Proton MeOD /NMR-Daten MPI 24
S-C-S-10
f1 (ppm)

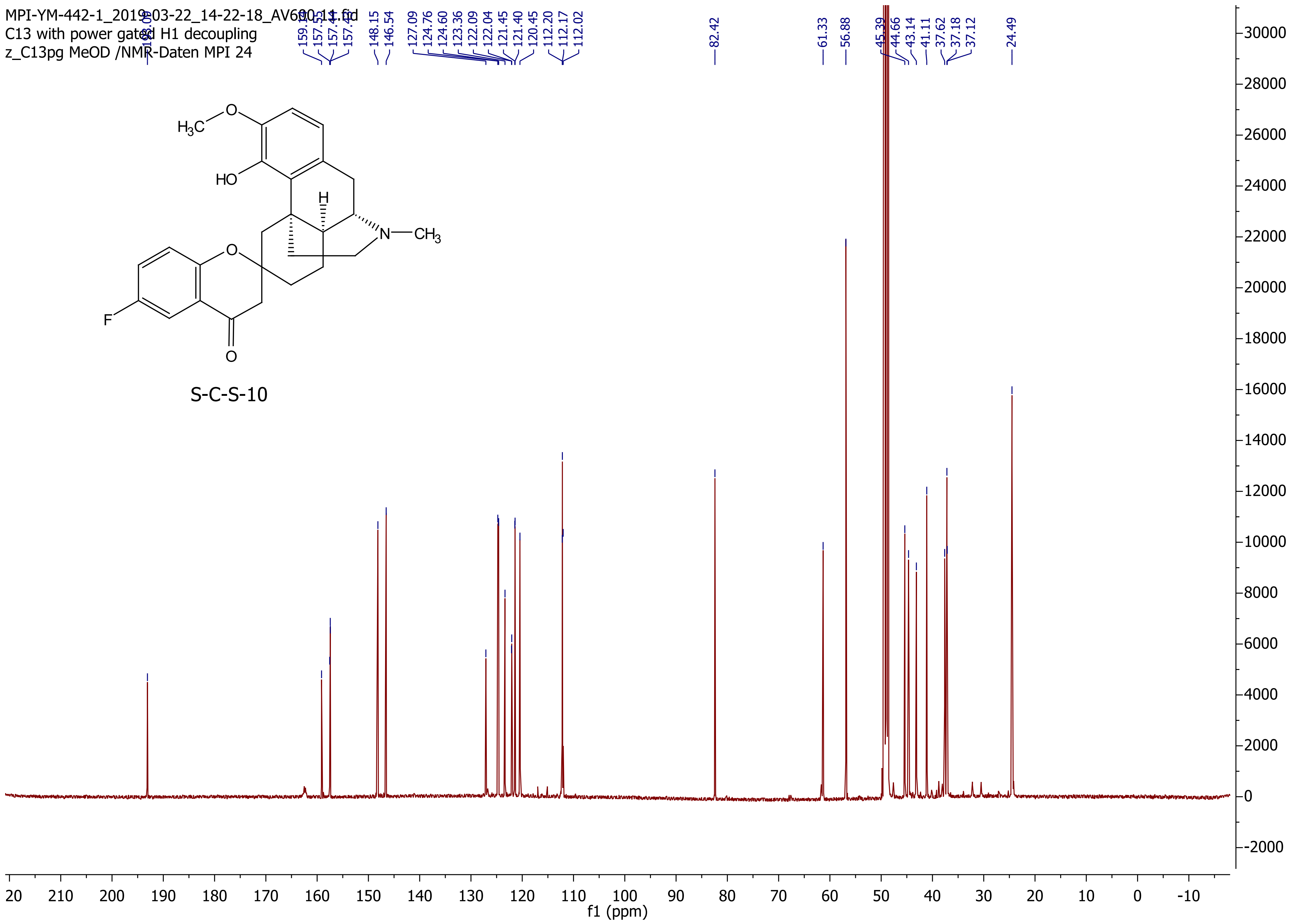

MPI-YM-442-1_2019-03-22_14-22-18_AV600.11.fid
C13 with power gated H1 decoupling
z_C13pg MeOD /NMR-Daten MPI 24
148.15
146.54
127.09
124.76
124.60
123.36
122.09
122.04
121.45
121.40
120.45
112.20
112.17
112.02
82.42
61.33
56.88
45.39
44.66
43.14
41.11
37.62
37.18
37.12
24.49
H3C
O
HO
H
N
CH3
O
F
O
S-C-S-10
f1 (ppm)

MPI-YM-442-1_2019-03-26_15-47-37_AV500.11.fid

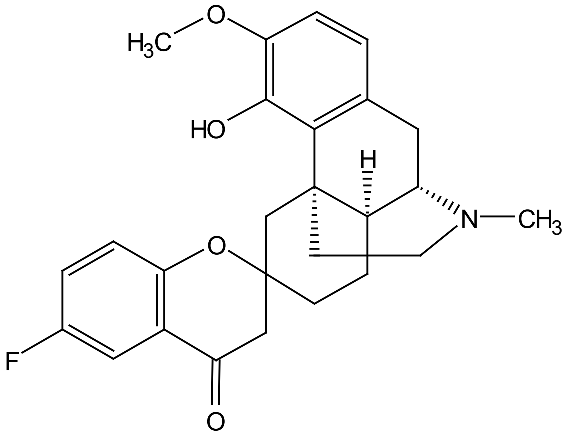

S-C-S-10

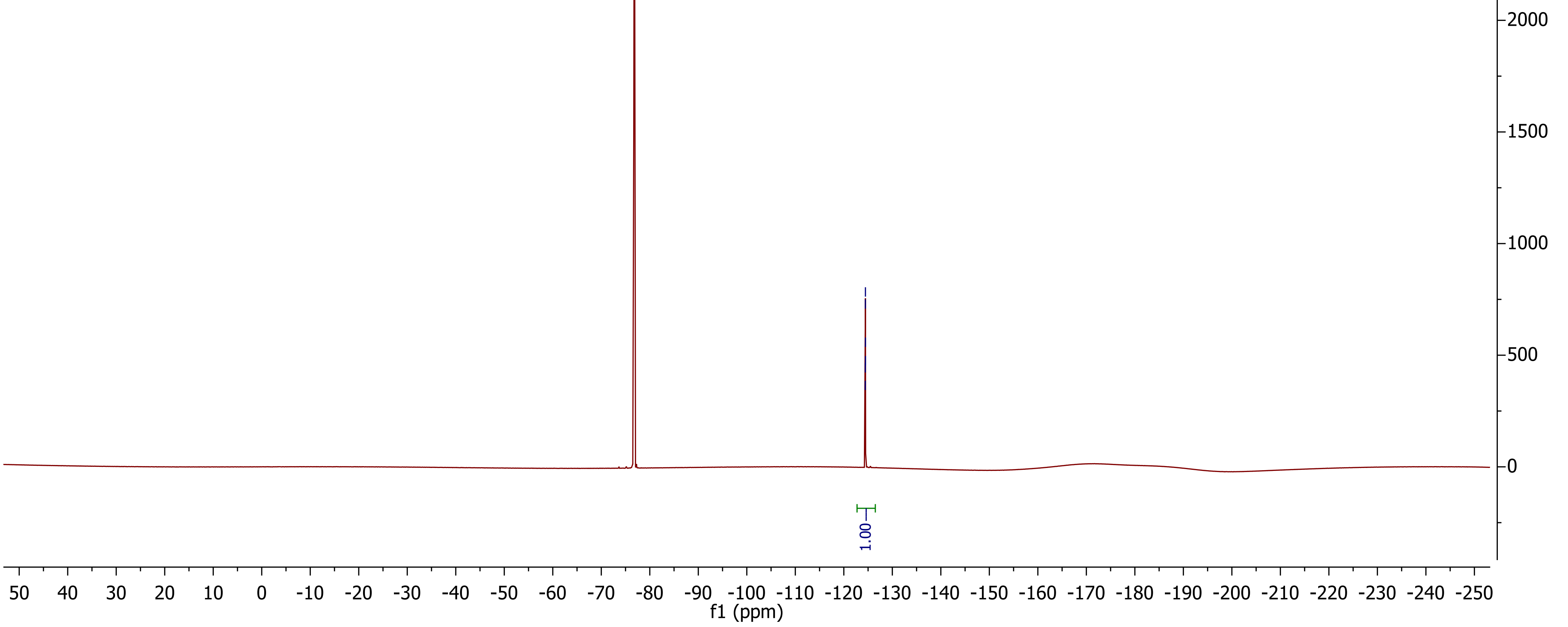

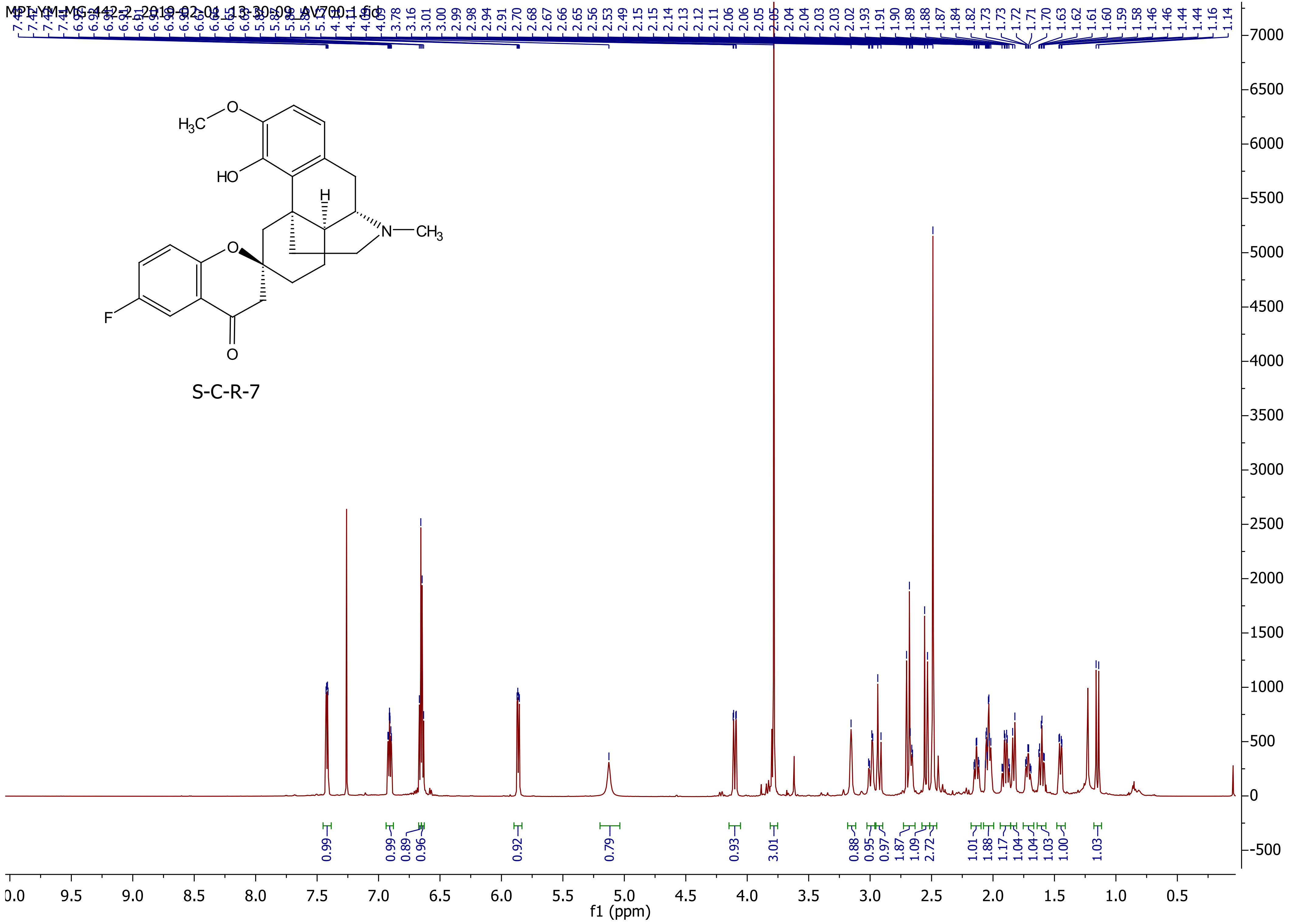
MPI-YM-MG-442-2_2019-02-01_13-30-09_AV700.1.fid
S-C-R-7
f1 (ppm)

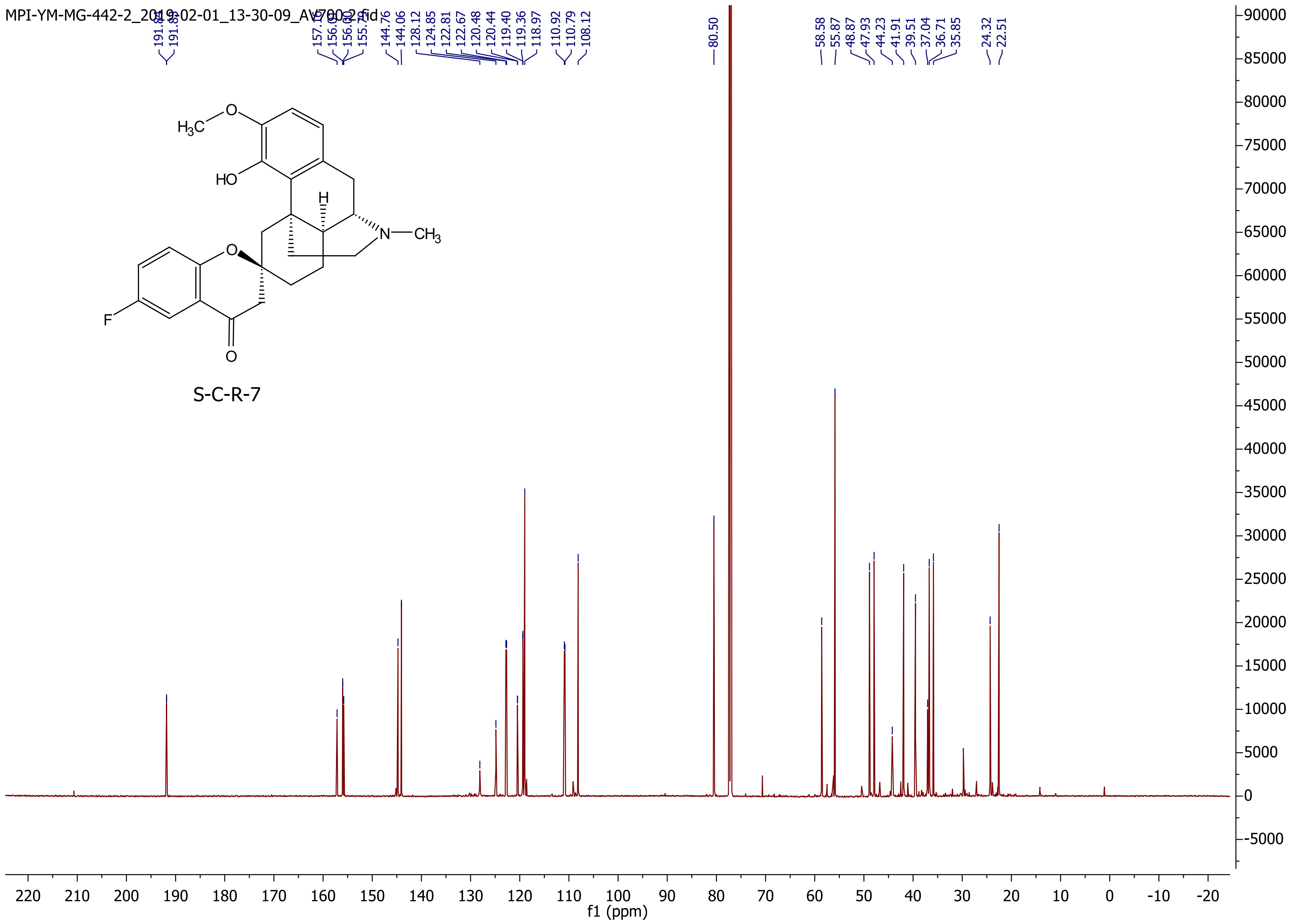

144.76
144.06
128.12
124.85
122.81
122.67
120.48
120.44
119.40
119.36
118.97
110.92
110.79
108.12
80.50
58.58
55.87
48.87
47.93
44.23
41.91
39.51
37.04
36.71
35.85
24.32
22.51
S-C-R-7
f1 (ppm)

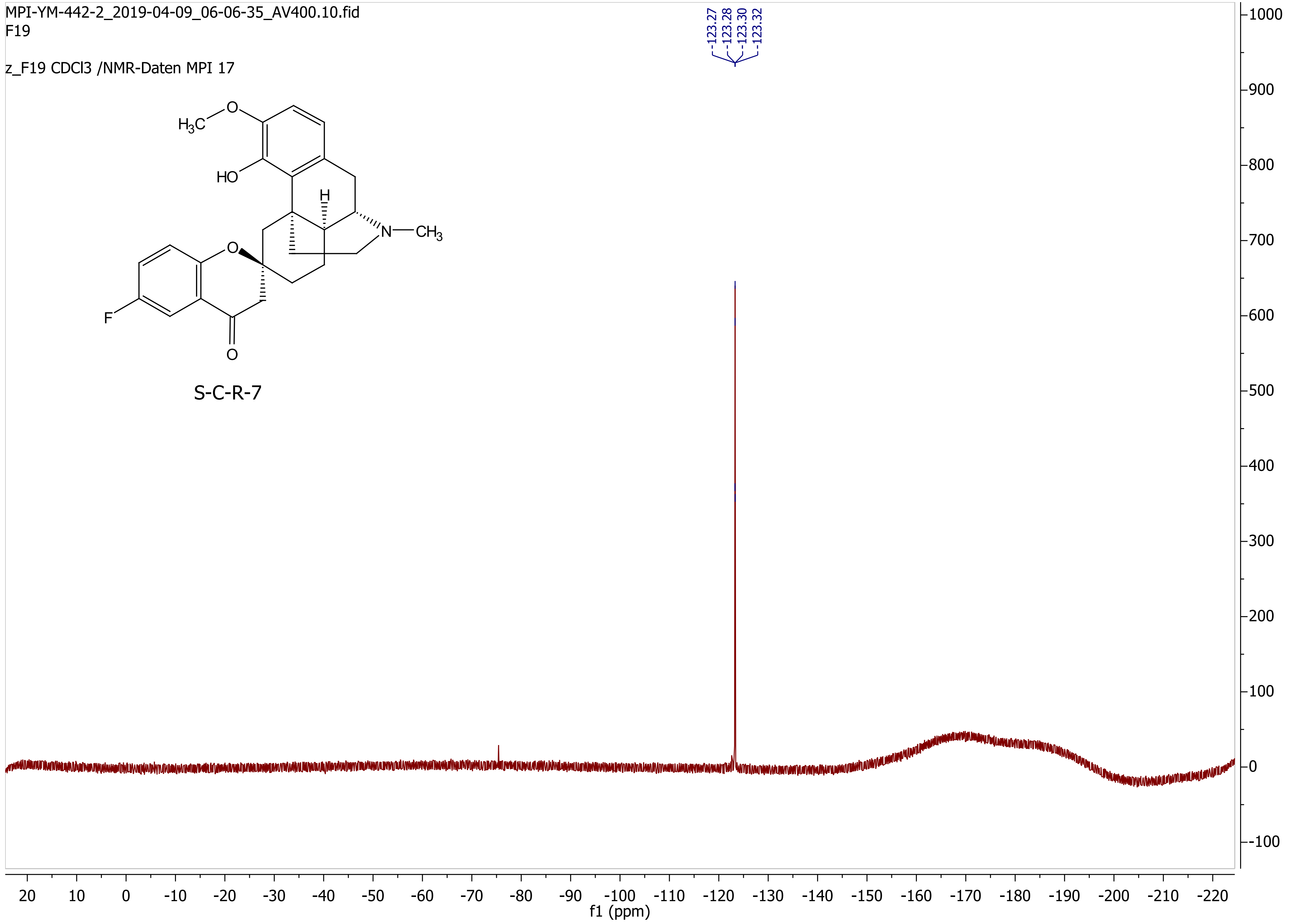
MPI-YM-442-2_2019-04-09_06-06-35_AV400.10.fid
F19
z_F19 CDCl3 /NMR-Daten MPI 17
-123.27
-123.28
-123.30
-123.32
H3C
O
HO
H
N
CH3
O
F
O
S-C-R-7
f1 (ppm)

MPI-YM-443-1_2019-03-22_14-22-18_AV600 10.fid
H1
z_Proton CDCl3 /NMR-Daten MPI 25

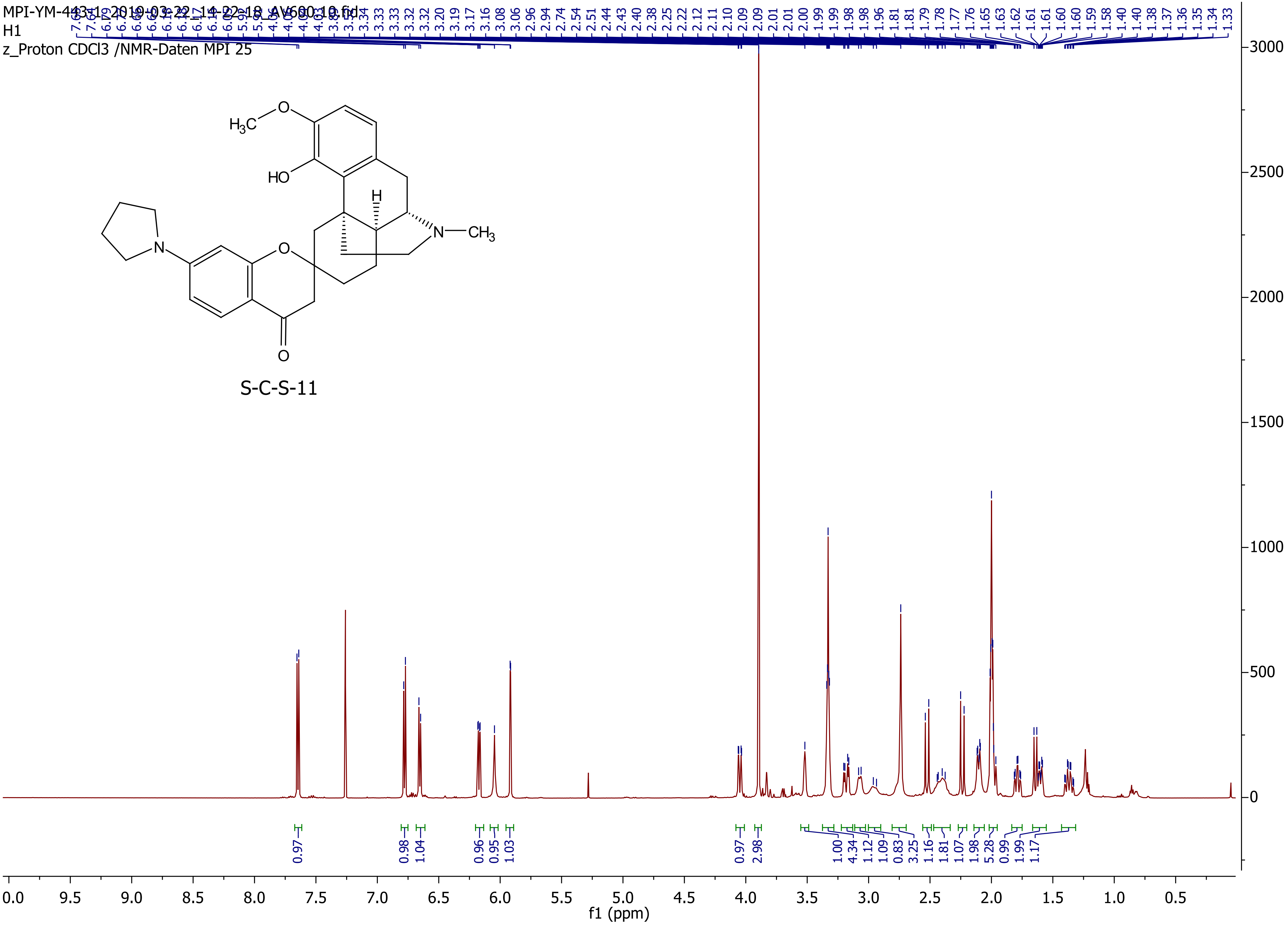

MPI-YM-443-1_2019-03-22_14-22-18_AV600.11.fid
C13 with power gated H1 decoupling
z_C13pg CDCl3 /NMR-Daten MPI 25

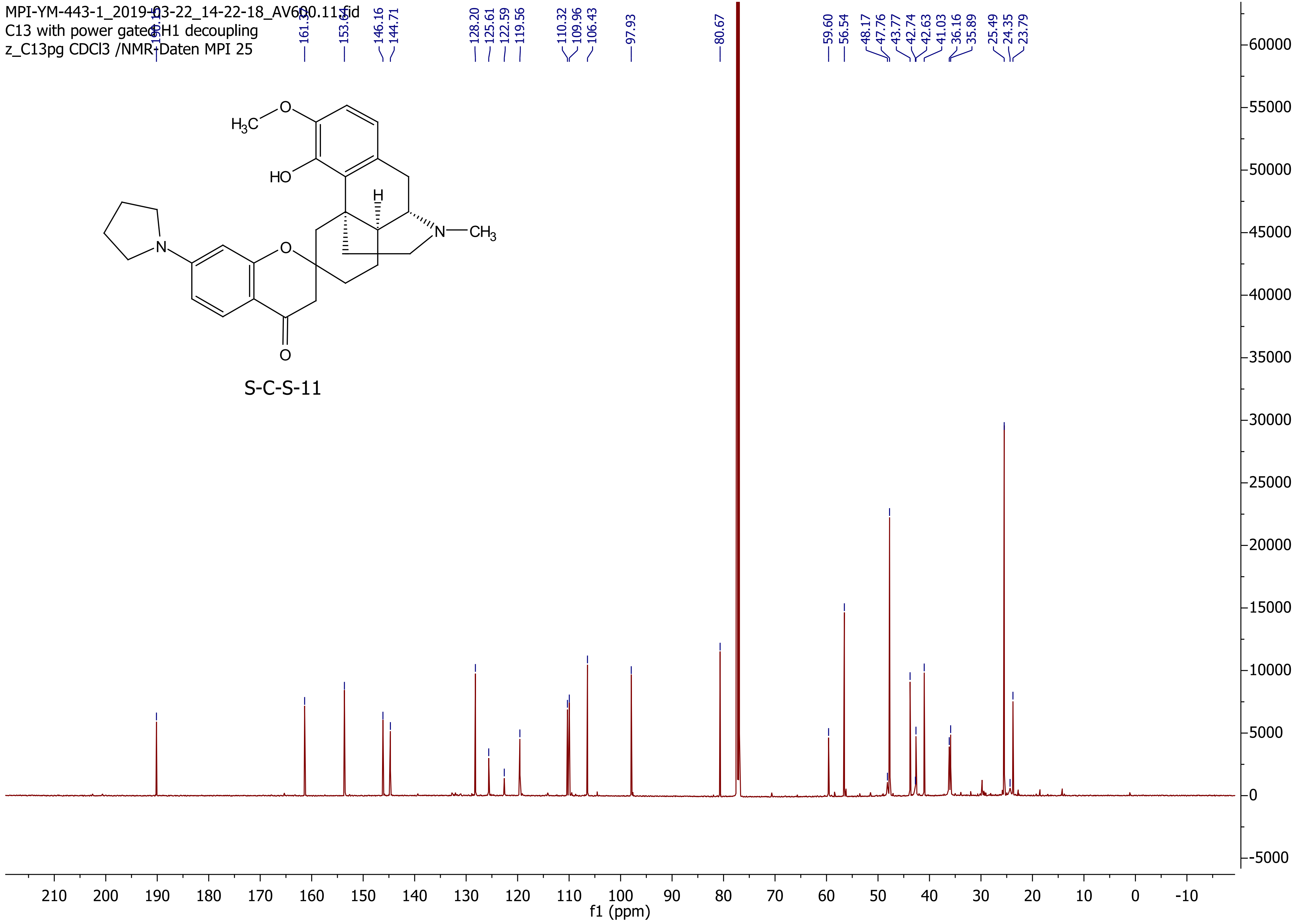

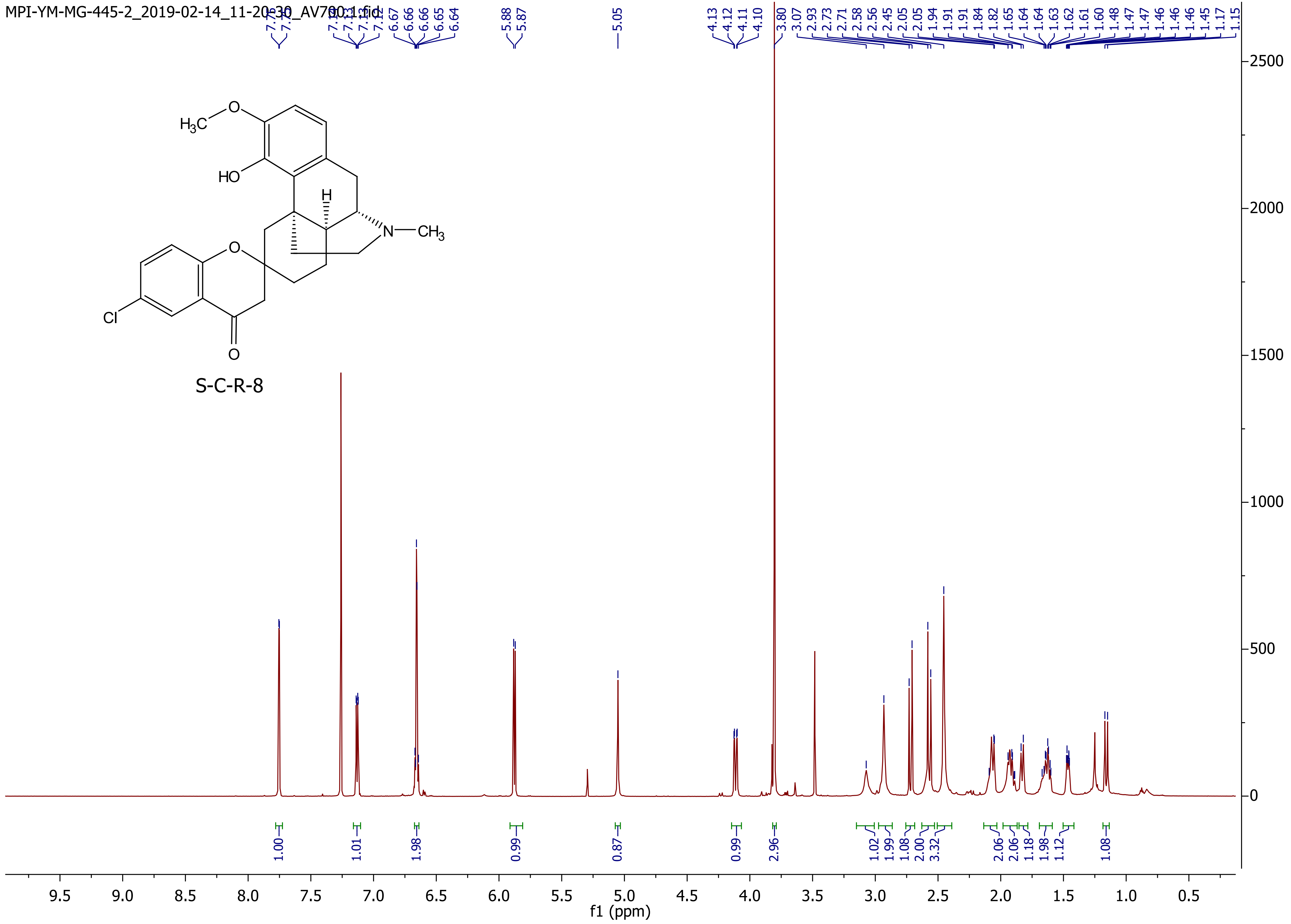
MPI-YM-MG-445-2_2019-02-14_11-20-30_AV700.1.fid
H3C
O
HO
H
N
CH3
O
Cl
O
S-C-R-8
5.88
5.87
5.05
4.13
4.12
4.11
4.10
3.80
1.00
1.01
1.98
0.99
0.87
0.99
2.96
1.02
1.99
1.08
2.00
3.32
2.06
2.06
1.18
1.98
1.12
1.08
f1 (ppm)

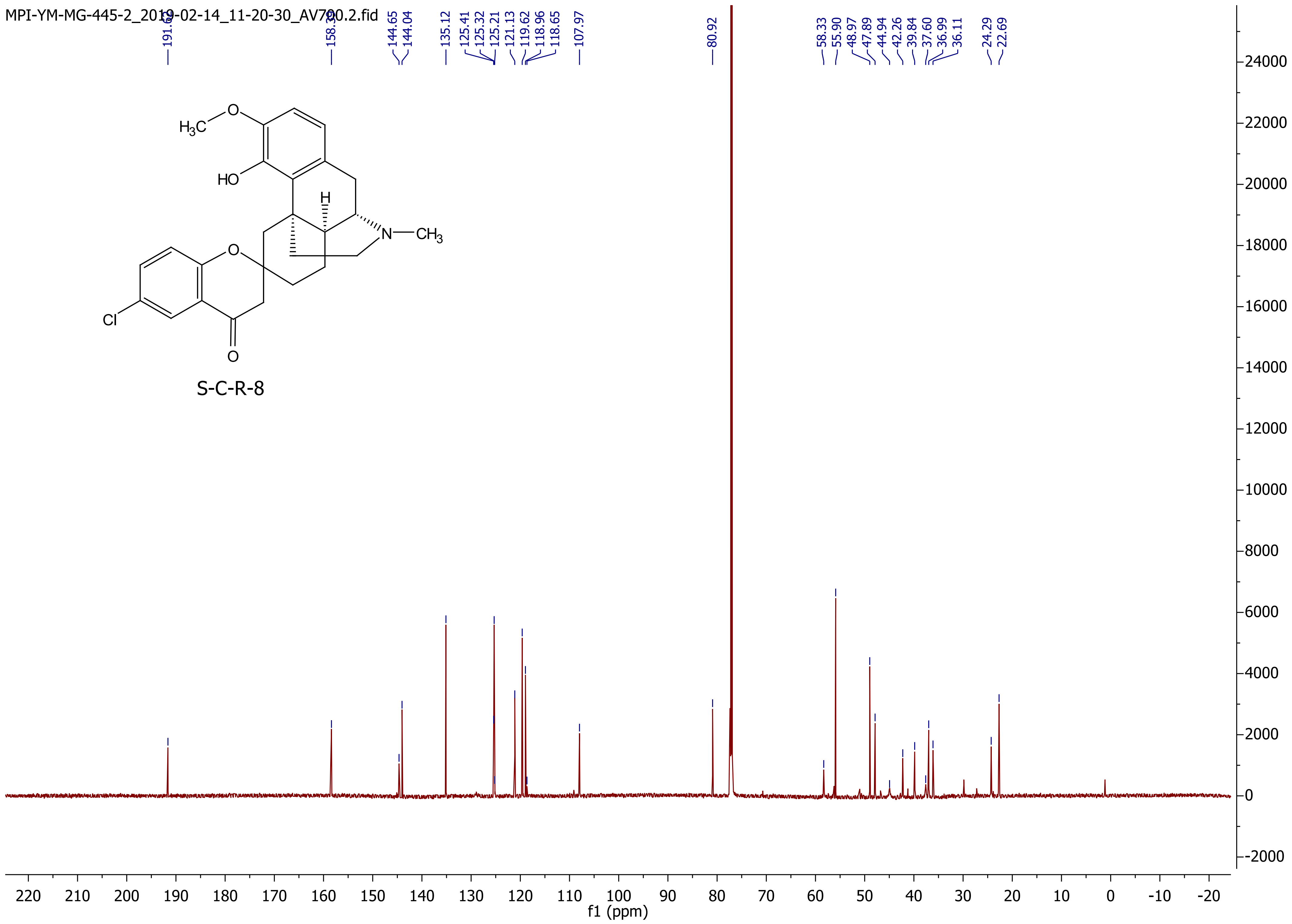

MPI-YM-MG-445-2_2019-02-14_11-20-30_AV700.2.fid
191.6
158.3
144.65
144.04
135.12
125.41
125.32
125.21
121.13
119.62
118.96
118.65
107.97
80.92
58.33
55.90
48.97
47.89
44.94
42.26
39.84
37.60
36.99
36.11
24.29
22.69
H3C
O
HO
H
N
CH3
O
Cl
O
S-C-R-8
f1 (ppm)

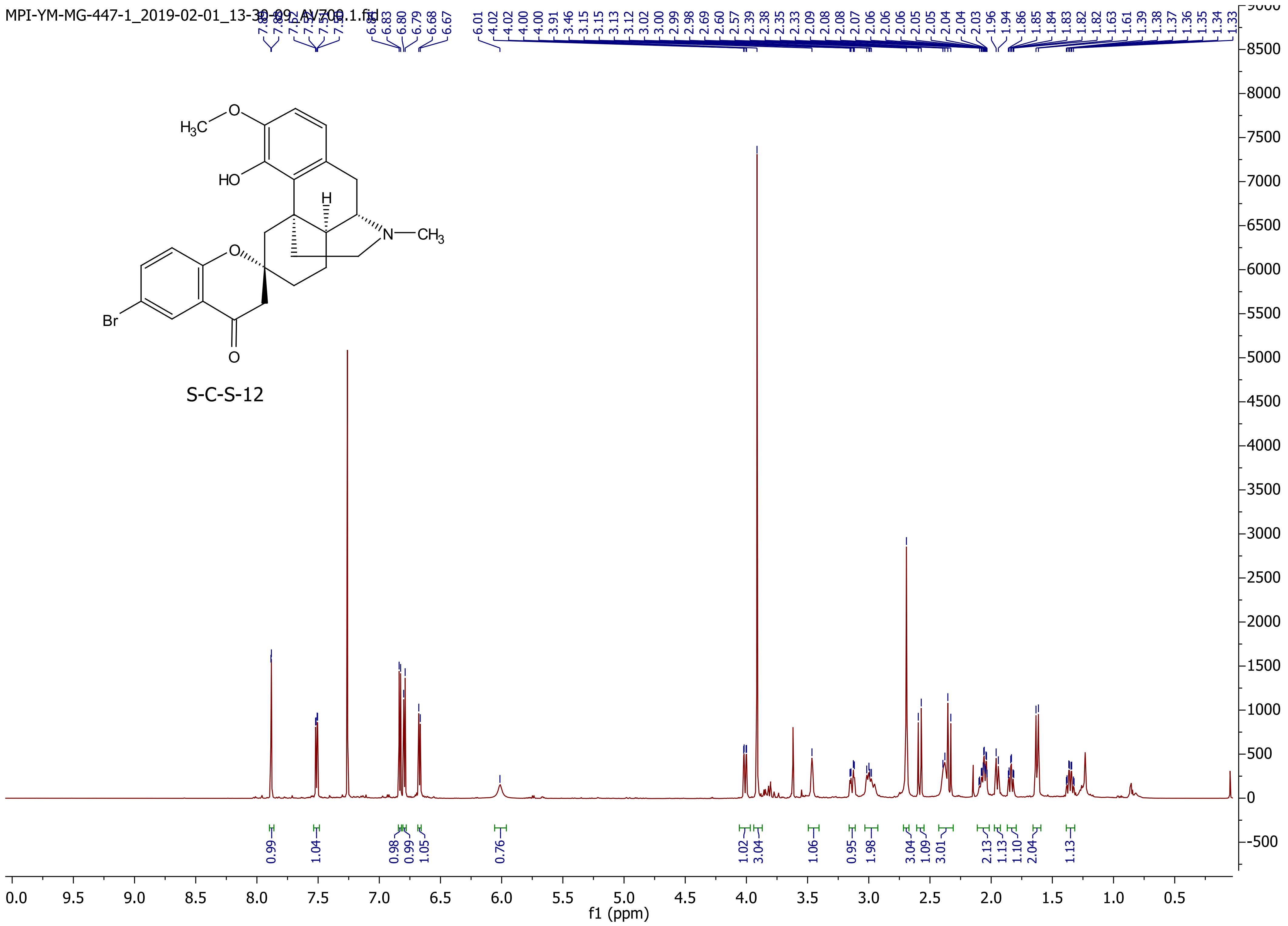
MPI-YM-MG-447-1_2019-02-01_13-30-09_AV700.1.fid
S-C-S-12
Br
HO
H_3C
O
N
CH_3
H
f1 (ppm)

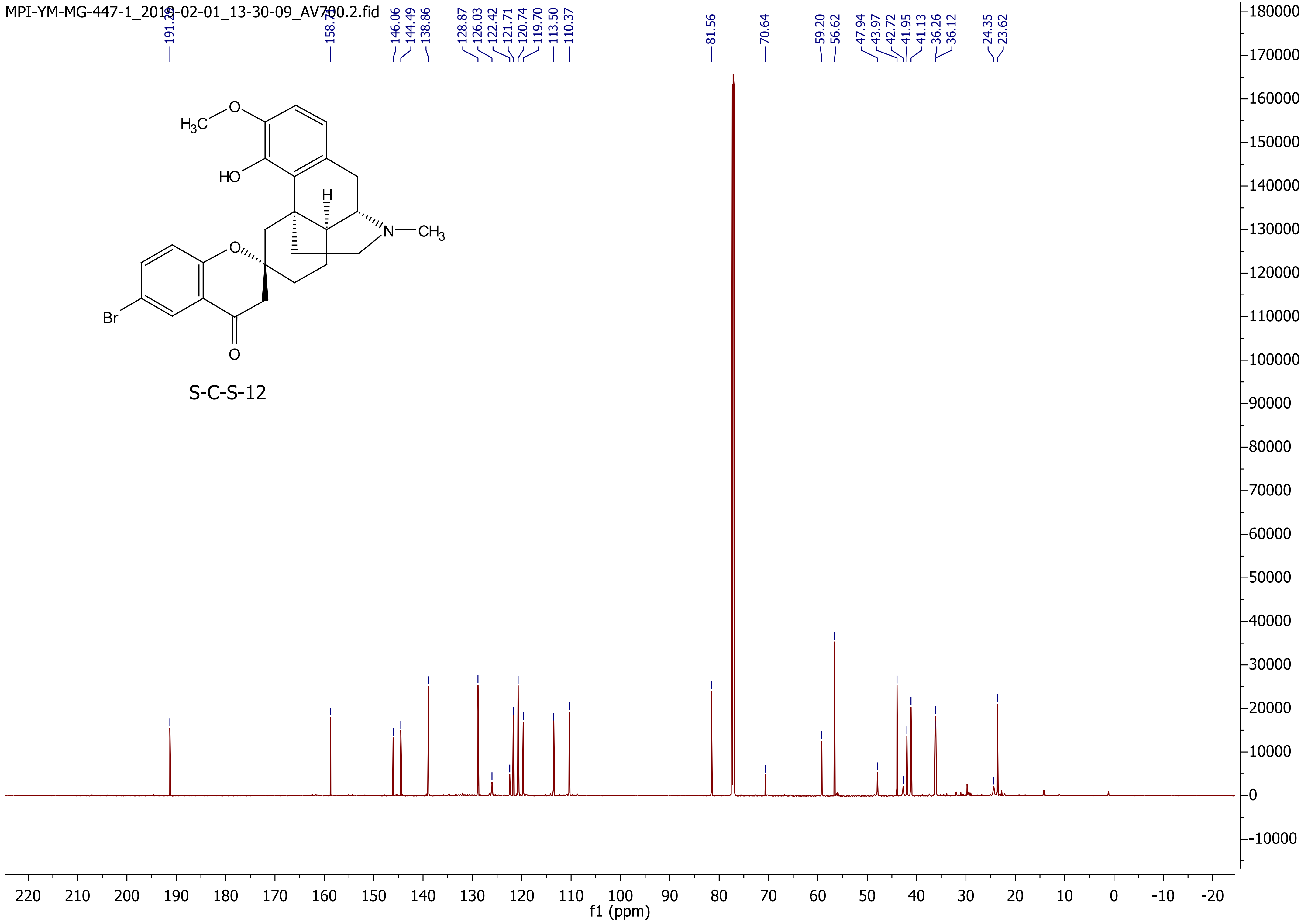
MPI-YM-MG-447-1_2019-02-01_13-30-09_AV700.2.fid
191.2
158.7
146.06
144.49
138.86
128.87
126.03
122.42
121.71
120.74
119.70
113.50
110.37
81.56
70.64
59.20
56.62
47.94
43.97
42.72
41.95
41.13
36.26
36.12
24.35
23.62
H3C
O
HO
H
N
CH3
O
Br
O
S-C-S-12
180000
170000
160000
150000
140000
130000
120000
110000
100000
90000
80000
70000
60000
50000
40000
30000
20000
10000
0
-10000
220 210 200 190 180 170 160 150 140 130 120 110 100 90 80 70 60 50 40 30 20 10 0 -10 -20
f1 (ppm)

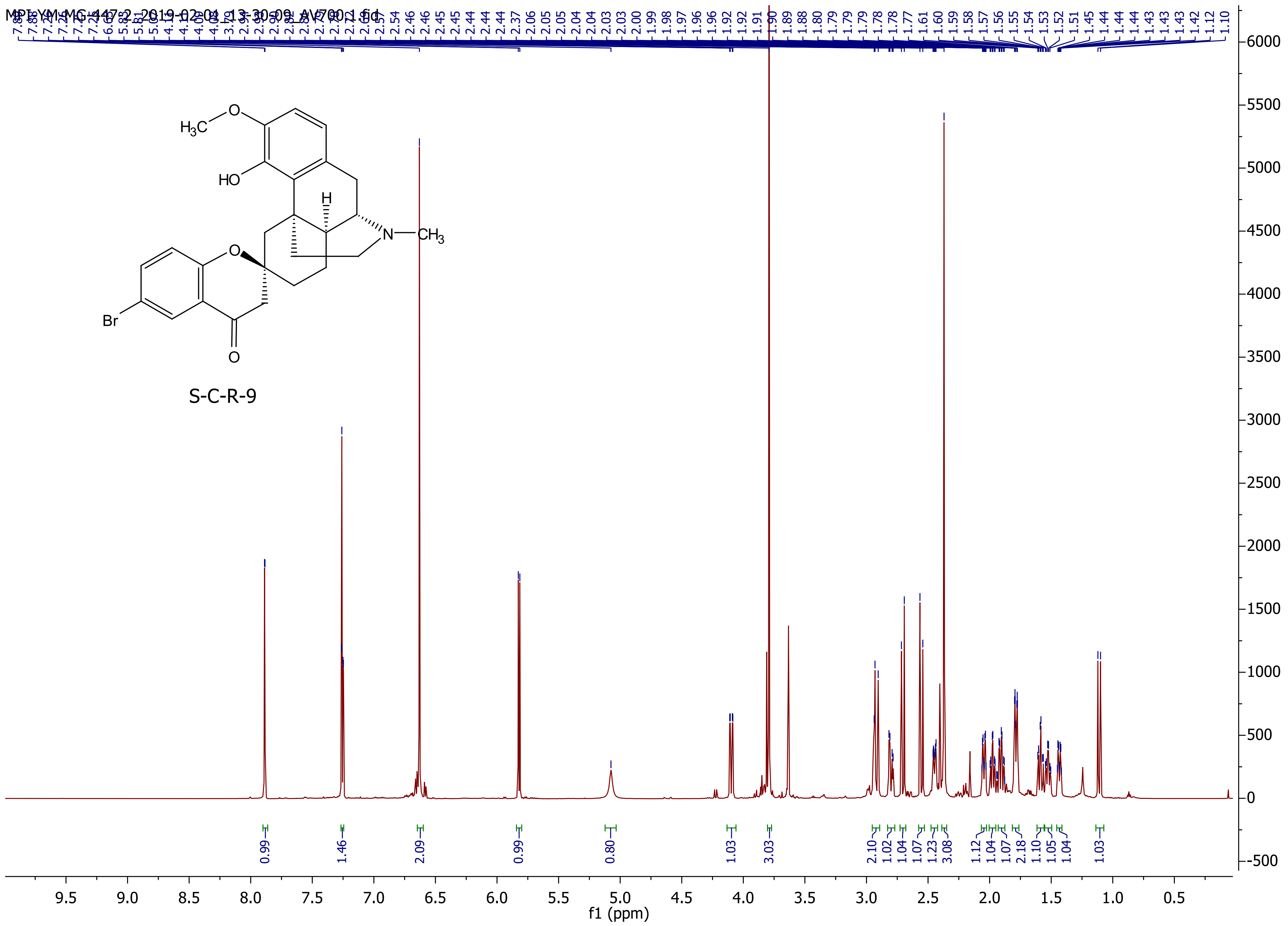
MPI-YM-MG-447-2_2019-02-04_13-30-09_AV700.1.fid
H3C
O
HO
H
N
CH3
O
Br
O
S-C-R-9
0.99
1.46
2.09
0.99
0.80
1.03
3.03
2.10
1.02
1.04
1.07
1.23
3.08
1.12
1.04
1.07
2.18
1.10
1.05
1.04
1.03
f1 (ppm)

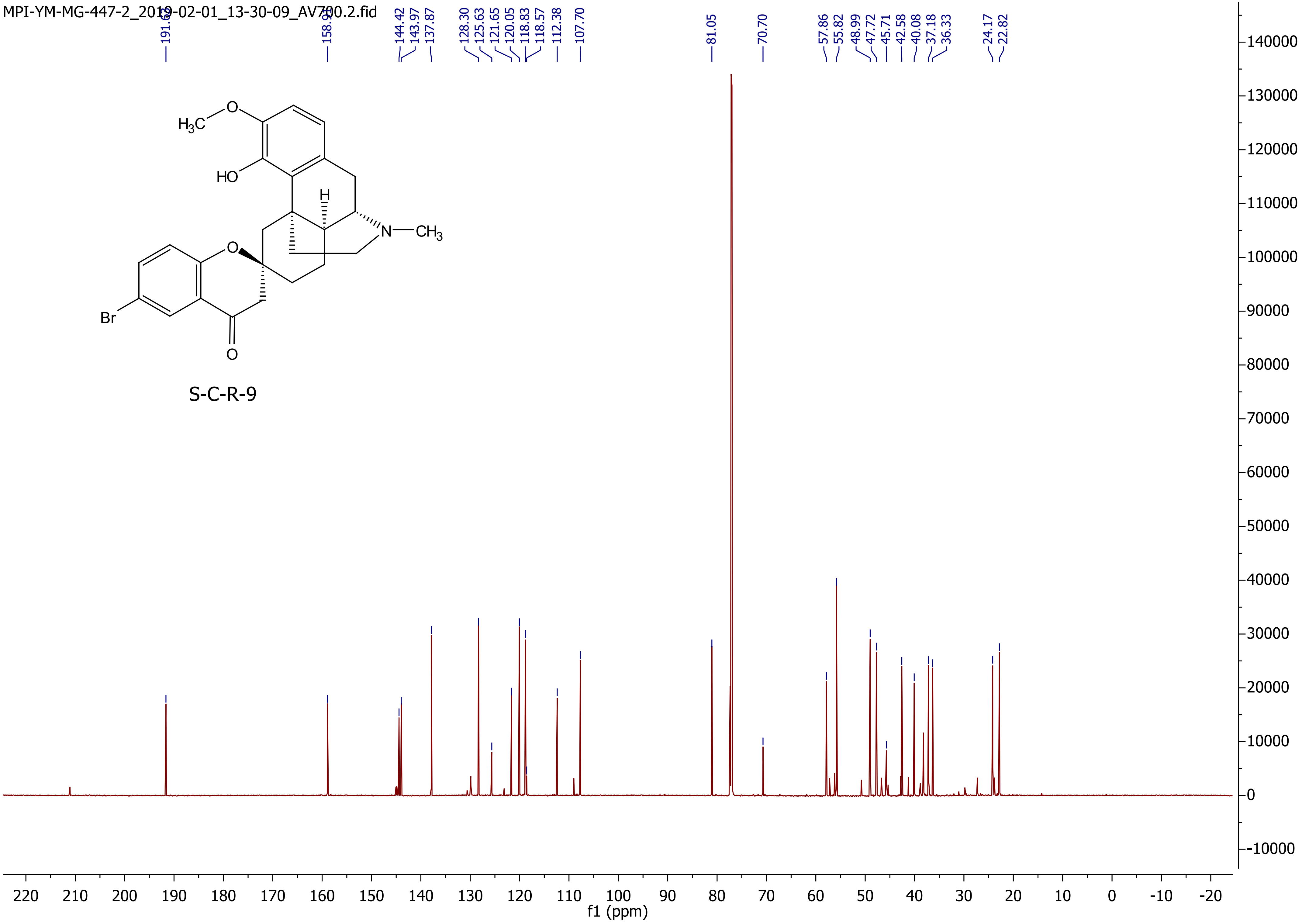
MPI-YM-MG-447-2_2019-02-01_13-30-09_AV700.2.fid
144.42
143.97
137.87
128.30
125.63
121.65
120.05
118.83
118.57
112.38
107.70
81.05
70.70
57.86
55.82
48.99
47.72
45.71
42.58
40.08
37.18
36.33
24.17
22.82
H3C
O
HO
H
N
CH3
O
Br
O
f1 (ppm)

S-C-R-9

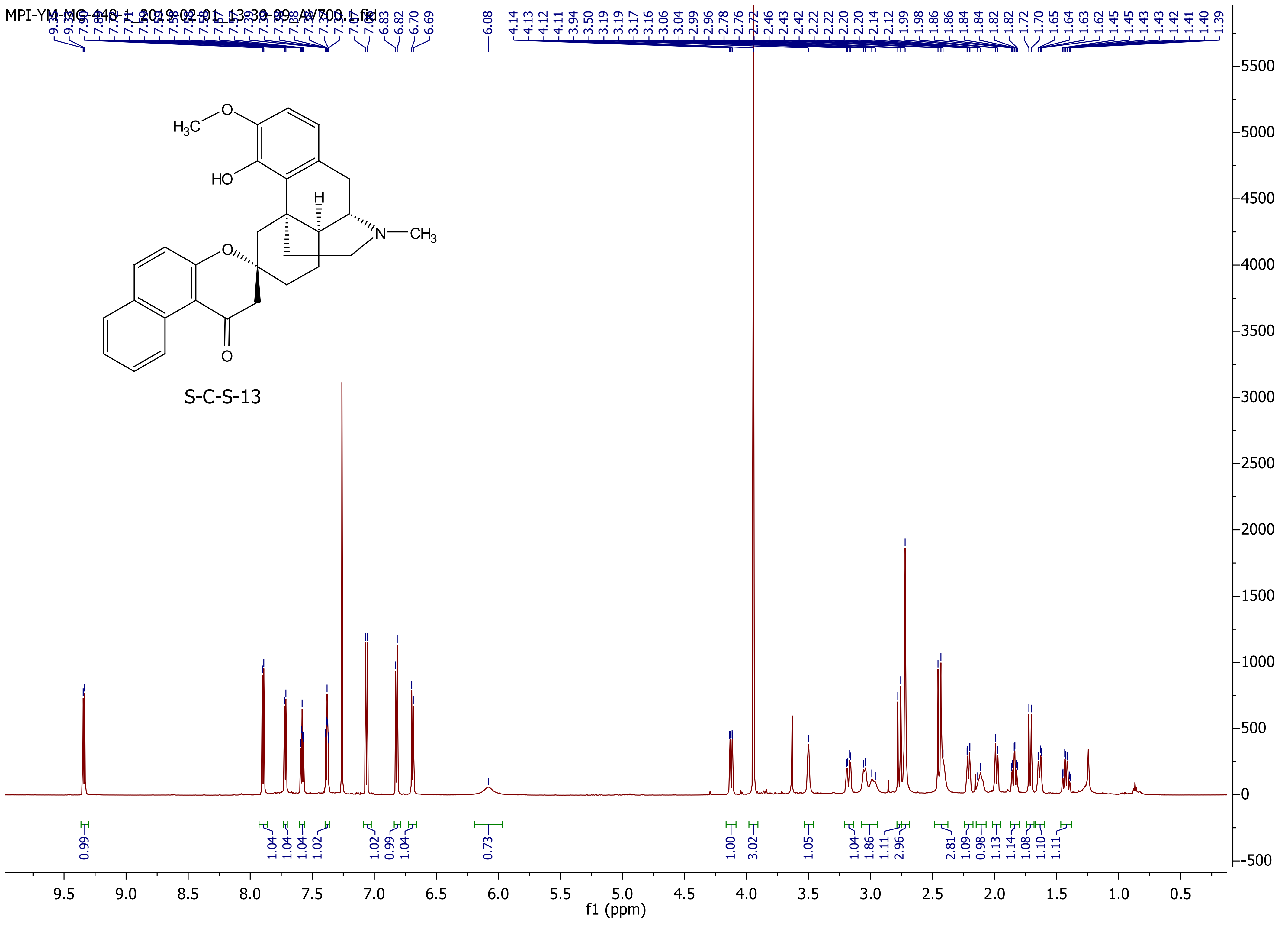

MPI-YM-MG-448-1_2019-02-01_13-30-09_AV700.1.fid
6.83
6.82
6.70
6.69
6.08
4.14
4.13
4.12
4.11
3.94
3.50
3.19
3.19
3.17
3.16
3.06
3.04
2.99
2.96
2.78
2.76
2.72
2.46
2.43
2.42
2.22
2.22
2.20
2.20
2.14
2.12
1.99
1.98
1.86
1.86
1.84
1.84
1.82
1.82
1.72
1.70
1.65
1.64
1.63
1.62
1.45
1.45
1.43
1.43
1.42
1.41
1.40
1.39
H3C
O
HO
H
N
CH3
O
O
S-C-S-13
0.99
1.04
1.04
1.04
1.02
1.02
0.99
1.04
0.73
1.00
3.02
1.05
1.04
1.86
1.11
2.96
2.81
1.09
0.98
1.13
1.14
1.08
1.10
1.11
f1 (ppm)

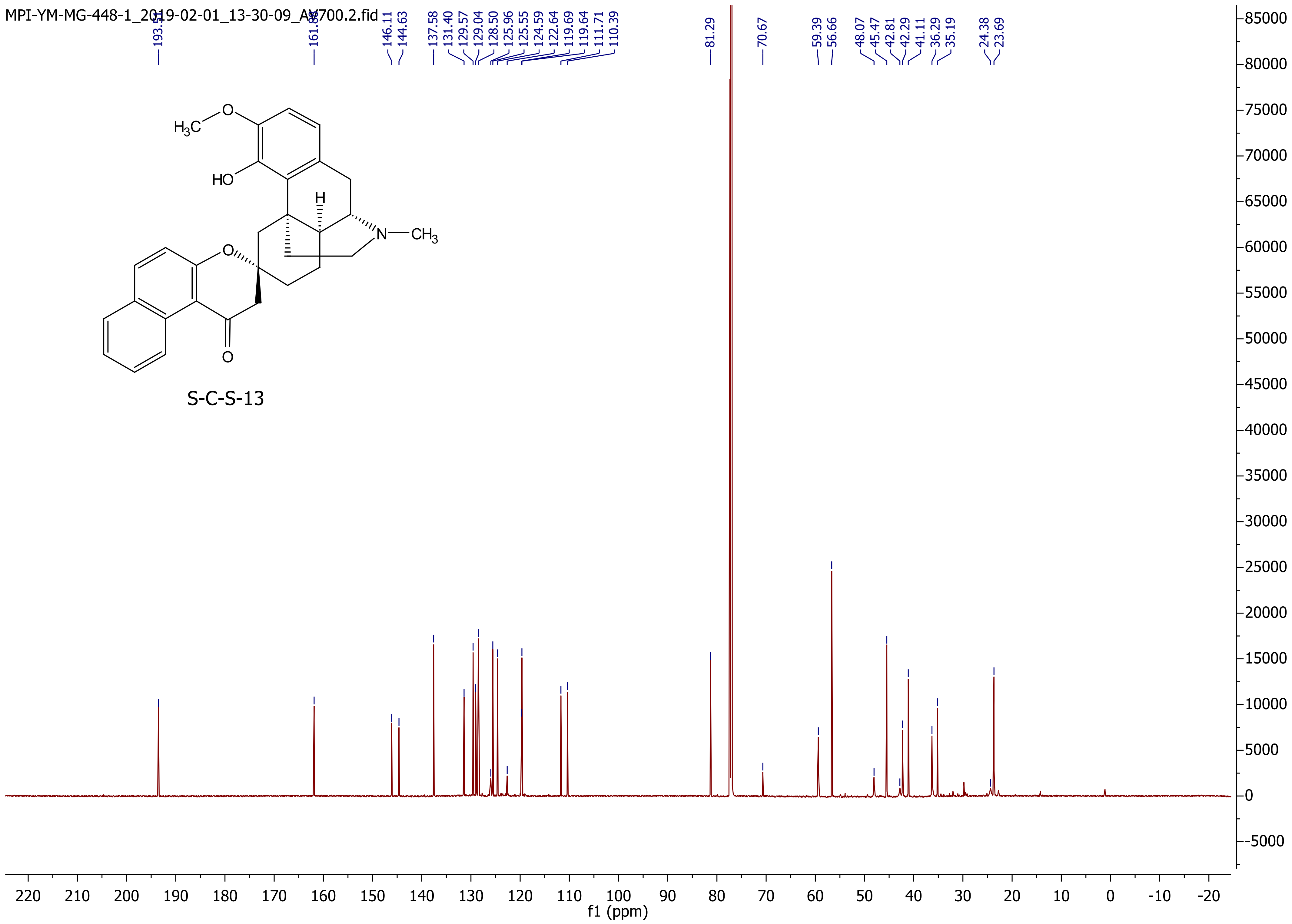

MPI-YM-MG-448-1_2019-02-01_13-30-09_AV700.2.fid
146.11
144.63
137.58
131.40
129.57
129.04
128.50
125.96
125.55
124.59
122.64
119.69
119.64
111.71
110.39
81.29
70.67
59.39
56.66
48.07
45.47
42.81
42.29
41.11
36.29
35.19
24.38
23.69
H3C
O
HO
H
N
CH3
O
O
S-C-S-13
f1 (ppm)

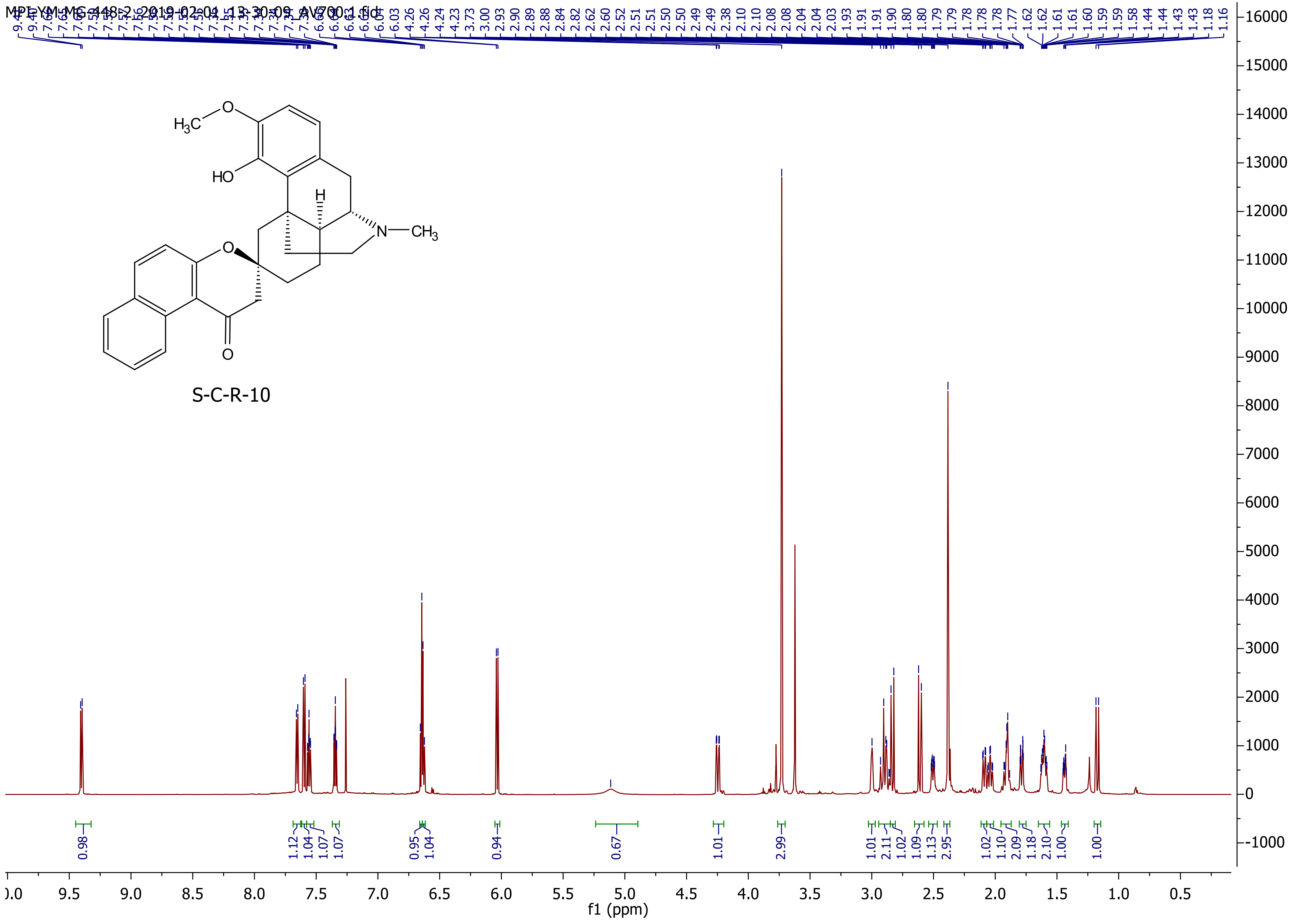

S-C-R-10
f1 (ppm)

MPI-YM-MG-448-2_2019-02-01_13-30-09_AV700.2.fid

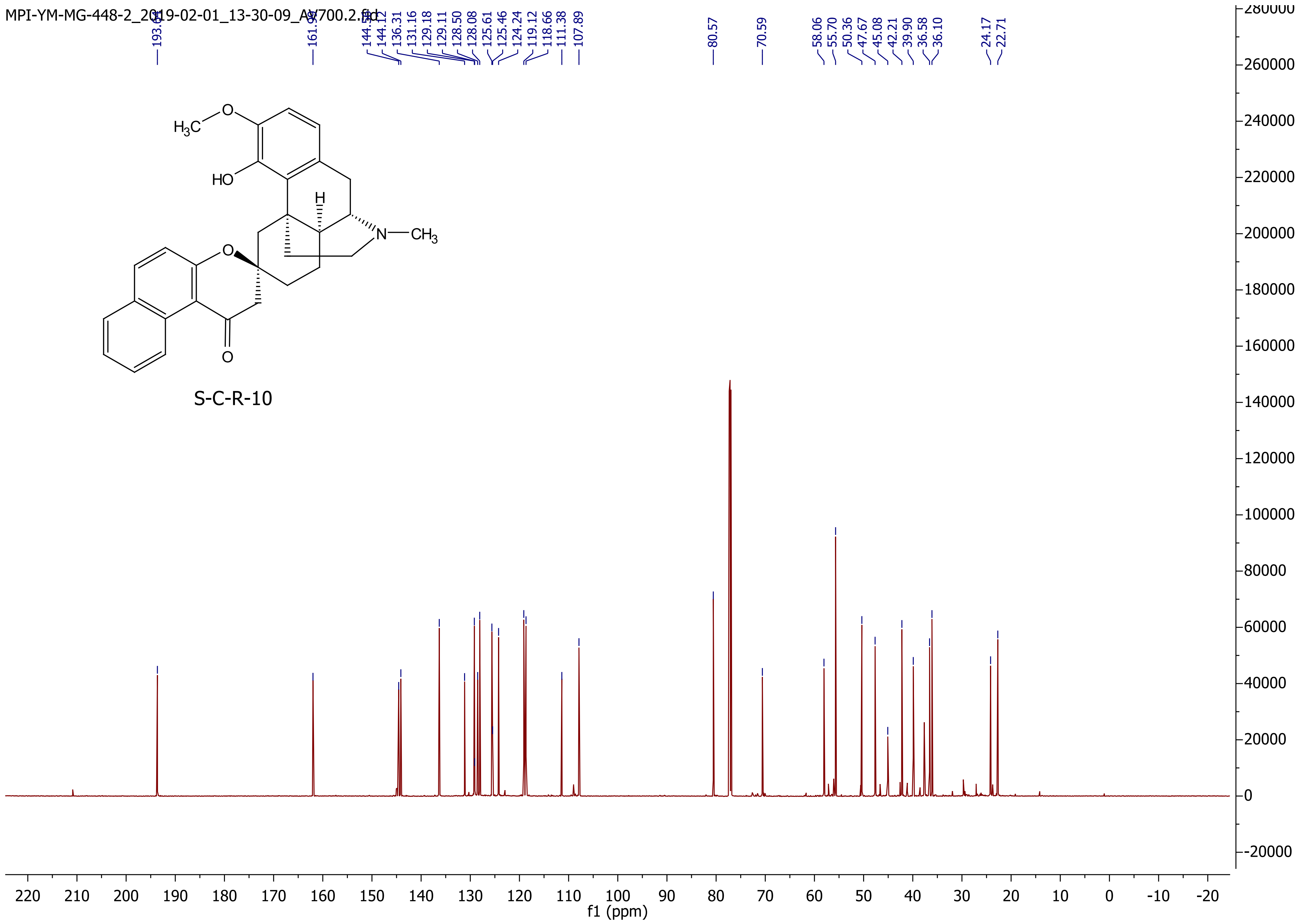

S-C-R-10

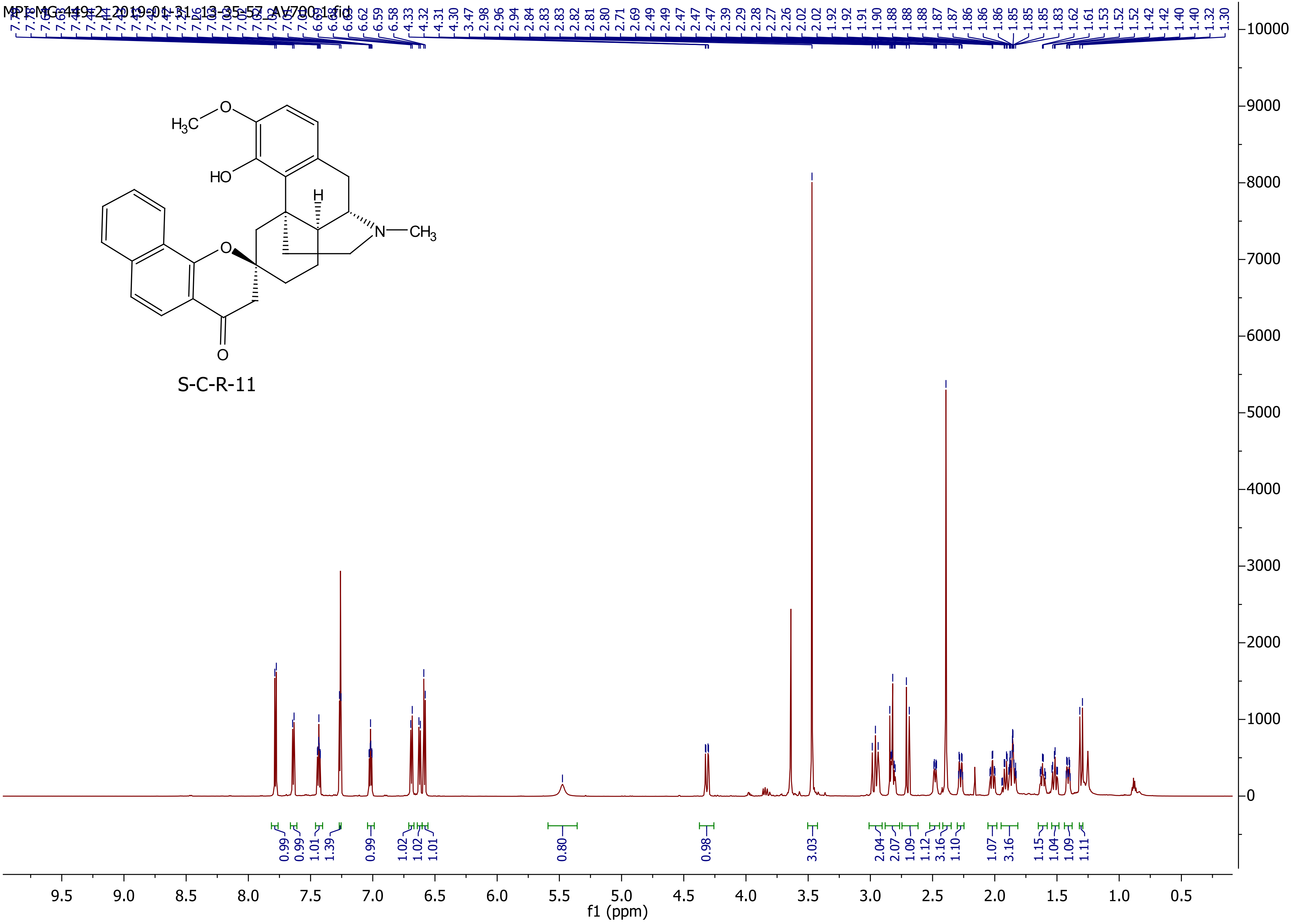

S-C-R-11

MPI-MG-449-2_2019-0[illegible]-31_13-35-57_AV700.2.fid

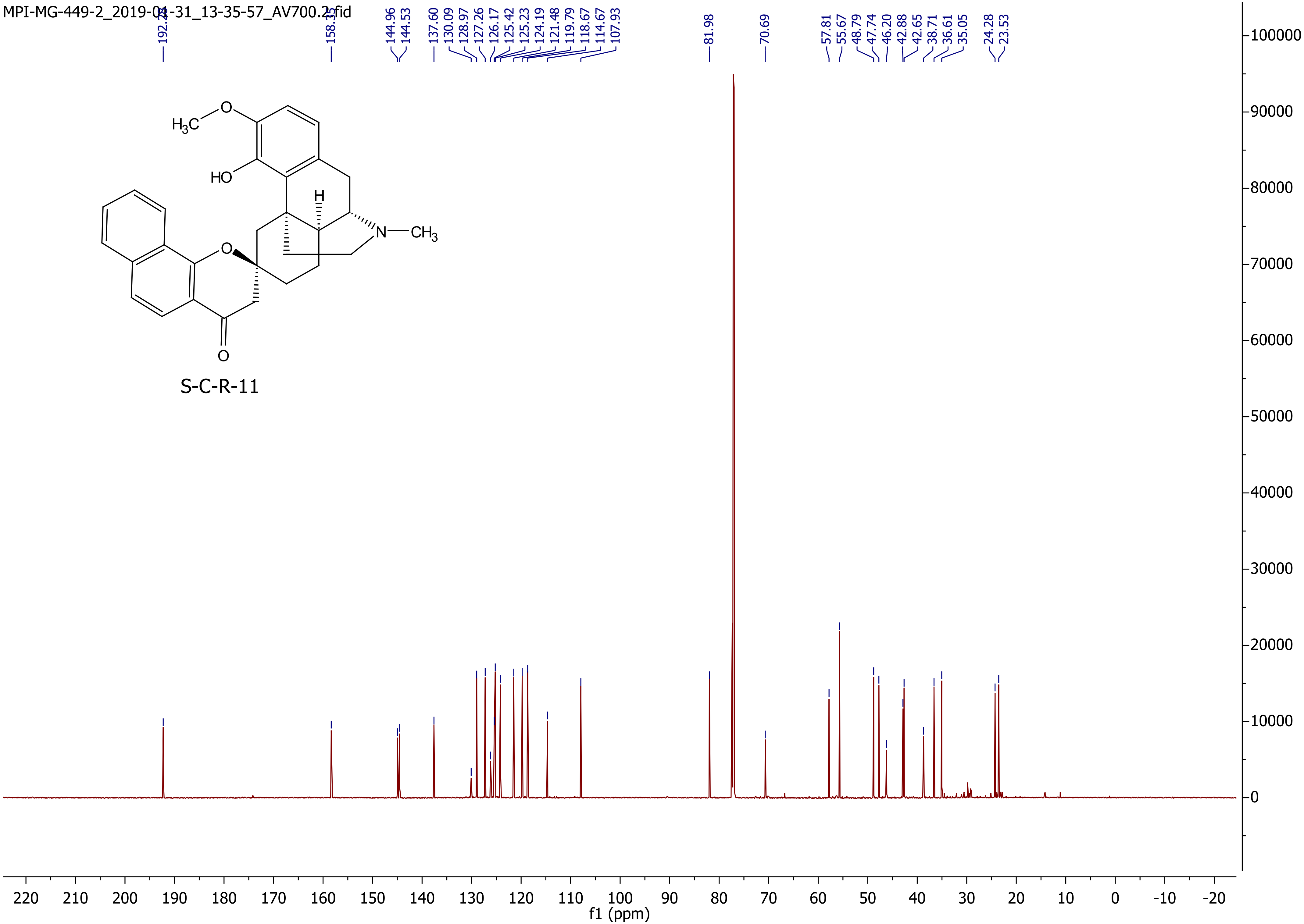

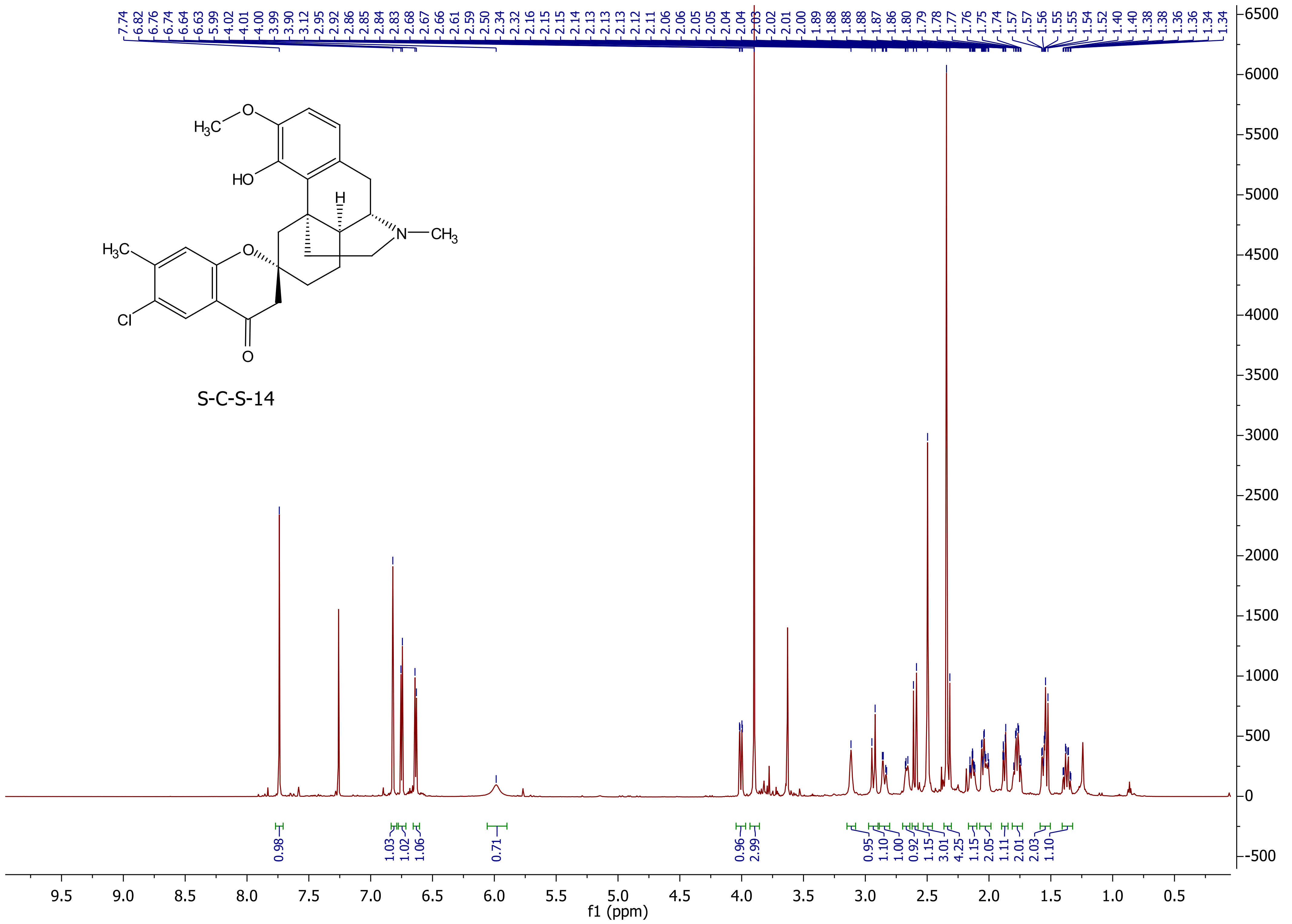
7.74 6.82 6.76 6.74 6.64 6.63 5.99 4.02 4.01 4.00 3.99 3.90 3.12 2.95 2.92 2.86 2.85 2.84 2.83 2.68 2.67 2.66 2.61 2.59 2.50 2.34 2.32 2.16 2.15 2.15 2.14 2.13 2.13 2.13 2.12 2.11 2.06 2.06 2.05 2.05 2.04 2.04 2.03 2.02 2.01 2.00 1.89 1.88 1.88 1.88 1.87 1.86 1.80 1.79 1.78 1.77 1.76 1.75 1.74 1.57 1.57 1.56 1.55 1.55 1.54 1.52 1.40 1.40 1.38 1.38 1.36 1.36 1.34 1.34
H3C
O
HO
H
N
CH3
H3C
O
Cl
O
S-C-S-14
0.98
1.03
1.02
1.06
0.71
0.96
2.99
0.95
1.10
1.00
0.92
1.15
3.01
4.25
1.15
2.05
1.11
2.01
2.03
1.10
f1 (ppm)

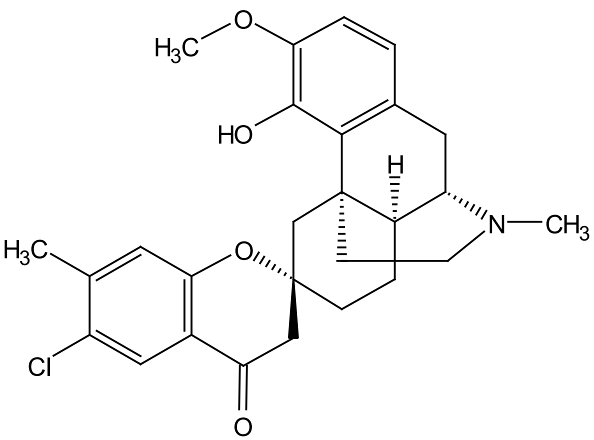

H3C
O
HO
H
N
CH3
H3C
O
Cl
O

S-C-S-14

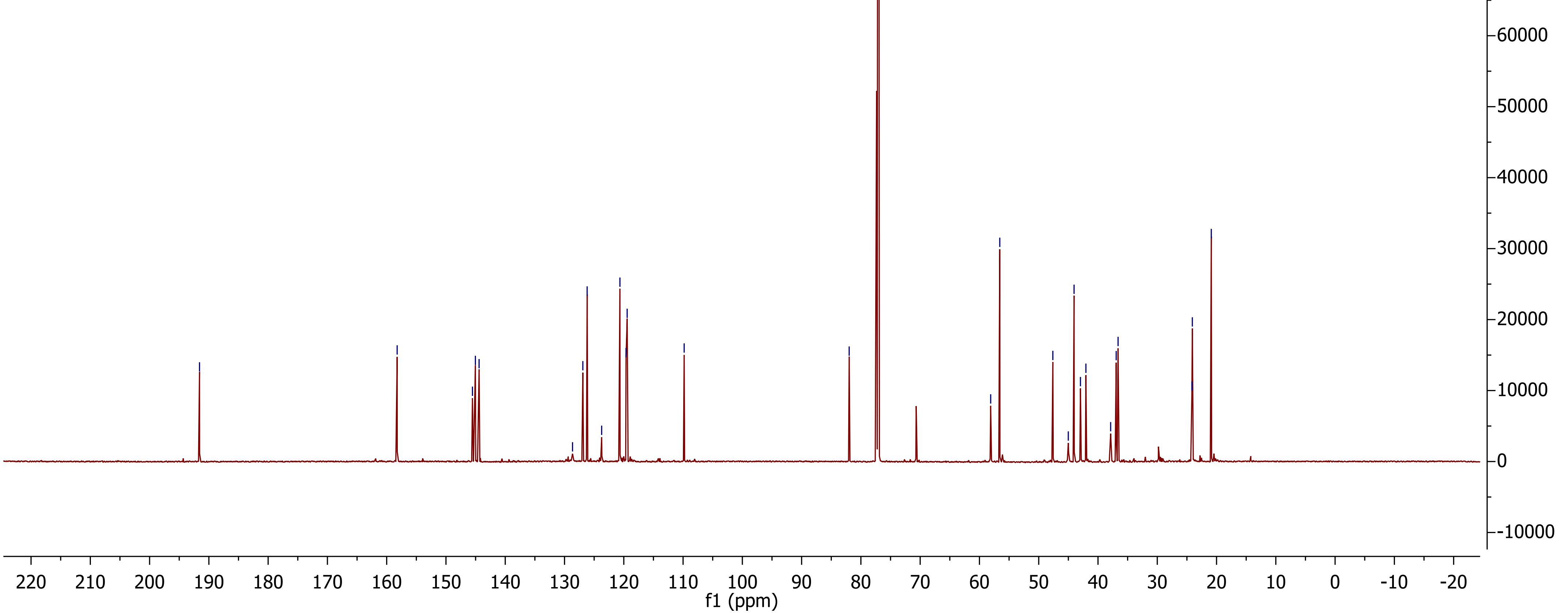

191.58
158.23
145.53
145.04
144.41
128.65
126.91
126.18
123.73
120.65
119.62
119.43
109.81
81.98
58.10
56.57
47.62
45.01
44.04
42.96
42.04
37.87
36.93
36.61
24.16
24.08
20.89
f1 (ppm)

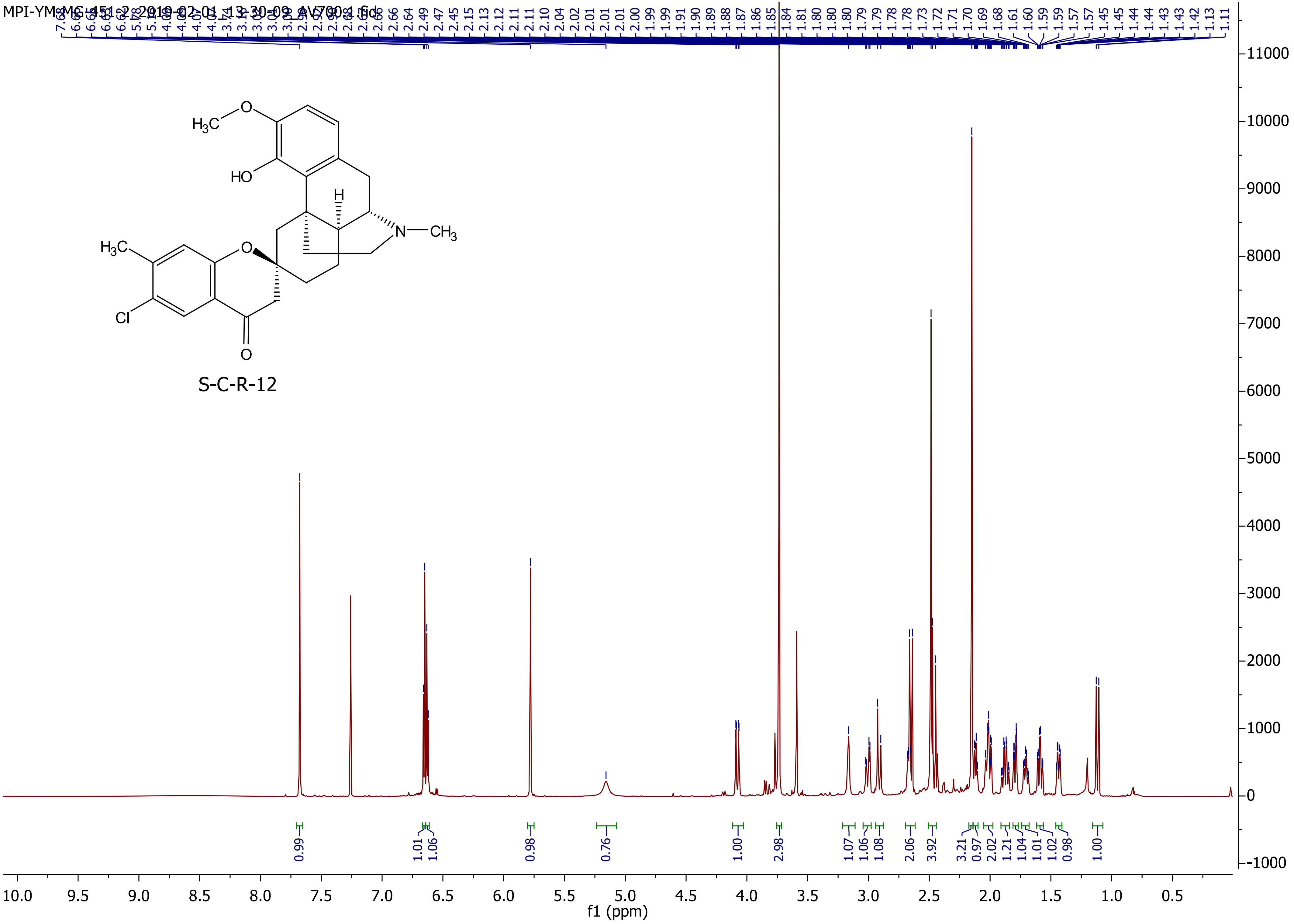
MPI-YM-MC-451-2 2019-02-01 13-30-09 AV700 1 fid
S-C-R-12
f1 (ppm)

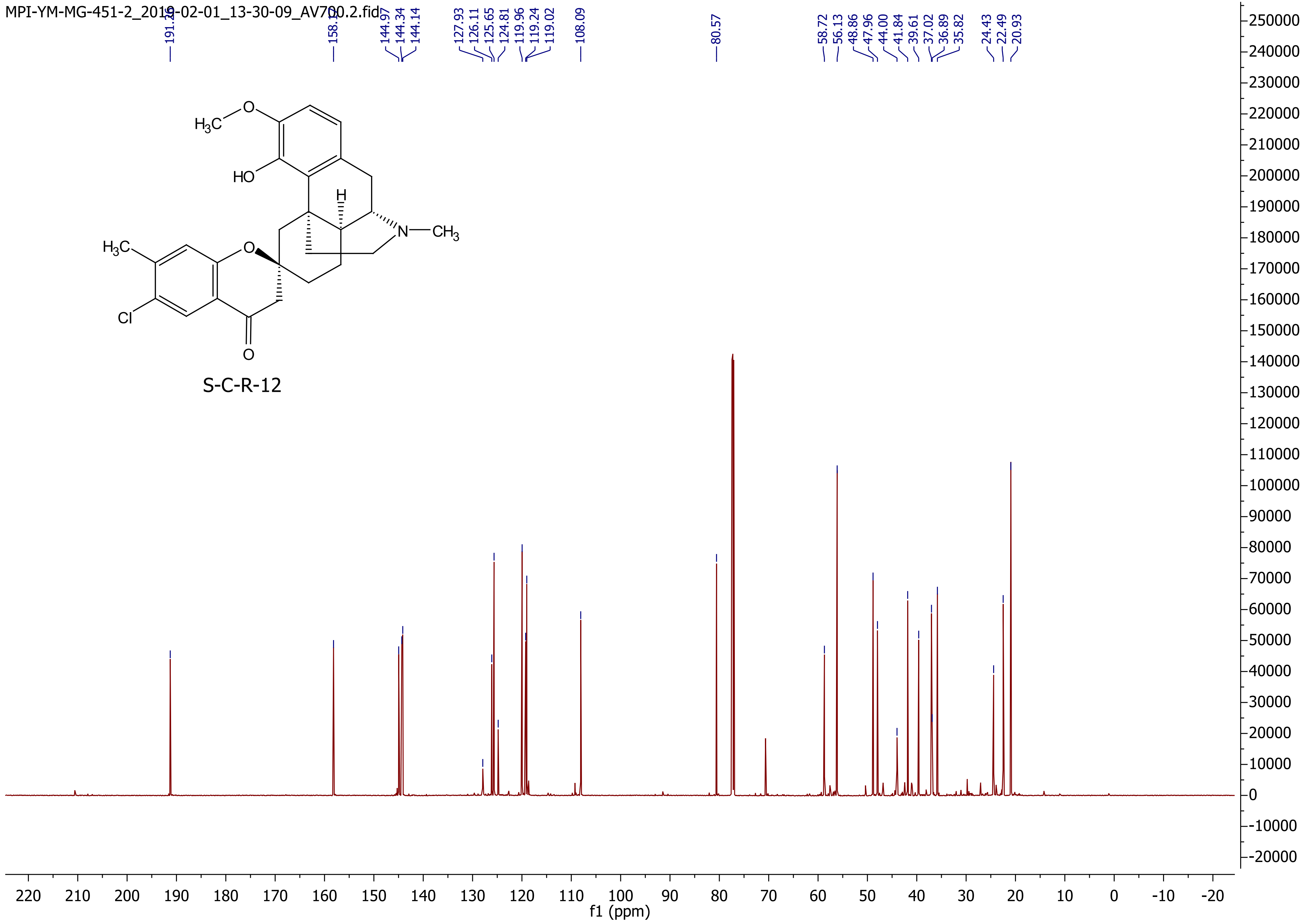

MPI-YM-MG-451-2_2019-02-01_13-30-09_AV700.2.fid
144.97
144.34
144.14
127.93
126.11
125.65
124.81
119.96
119.24
119.02
108.09
80.57
58.72
56.13
48.86
47.96
44.00
41.84
39.61
37.02
36.89
35.82
24.43
22.49
20.93
H3C
O
HO
H
N
CH3
H3C
O
Cl
O
S-C-R-12
f1 (ppm)

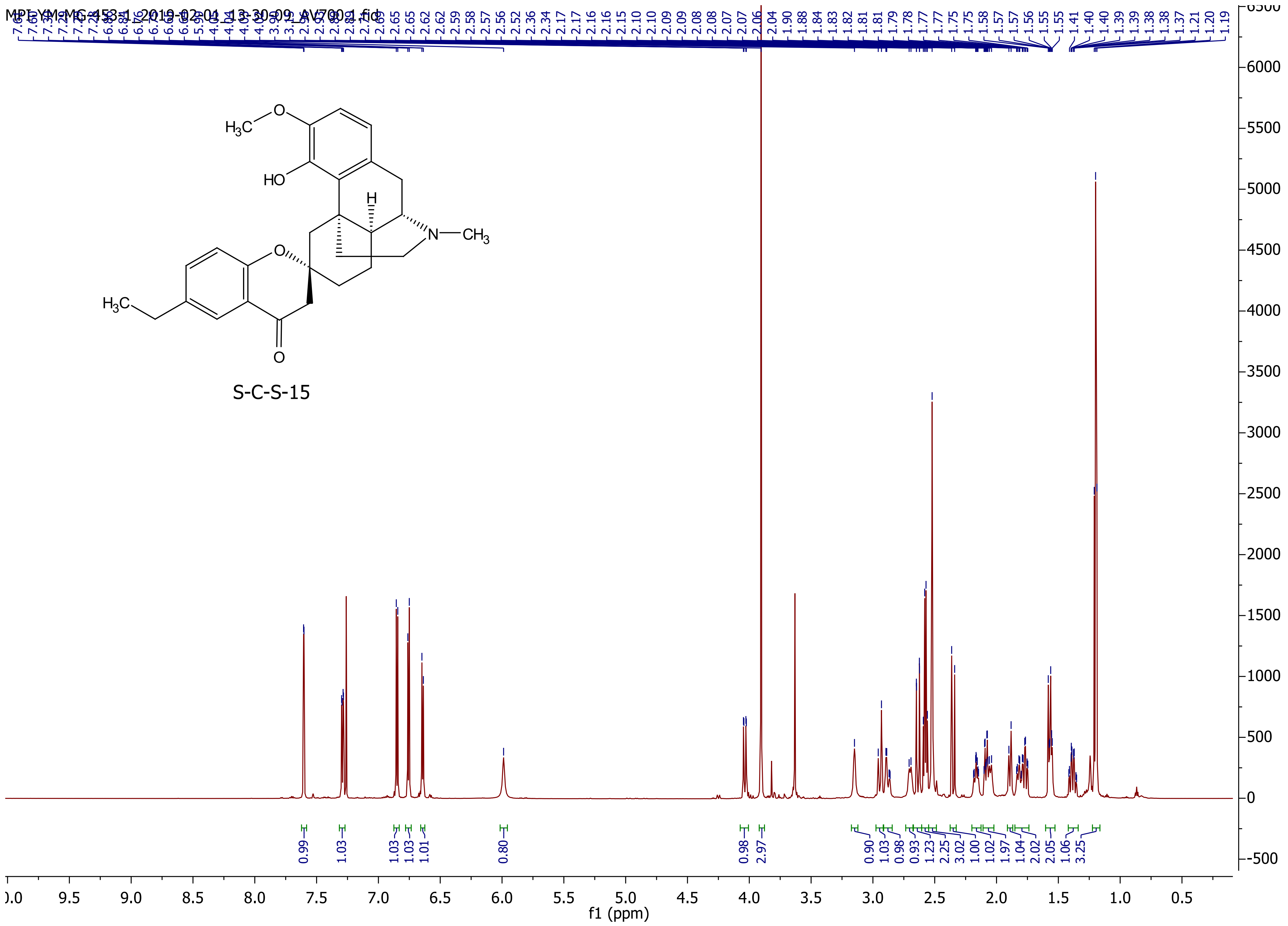

H3C
O
HO
H
N
CH3
O
H3C
O
S-C-S-15
f1 (ppm)
0.99
1.03
1.03
1.03
1.01
0.80
0.98
2.97
0.90
1.03
0.98
0.93
1.23
2.25
3.02
1.00
1.02
1.97
1.04
2.02
2.05
1.06
3.25

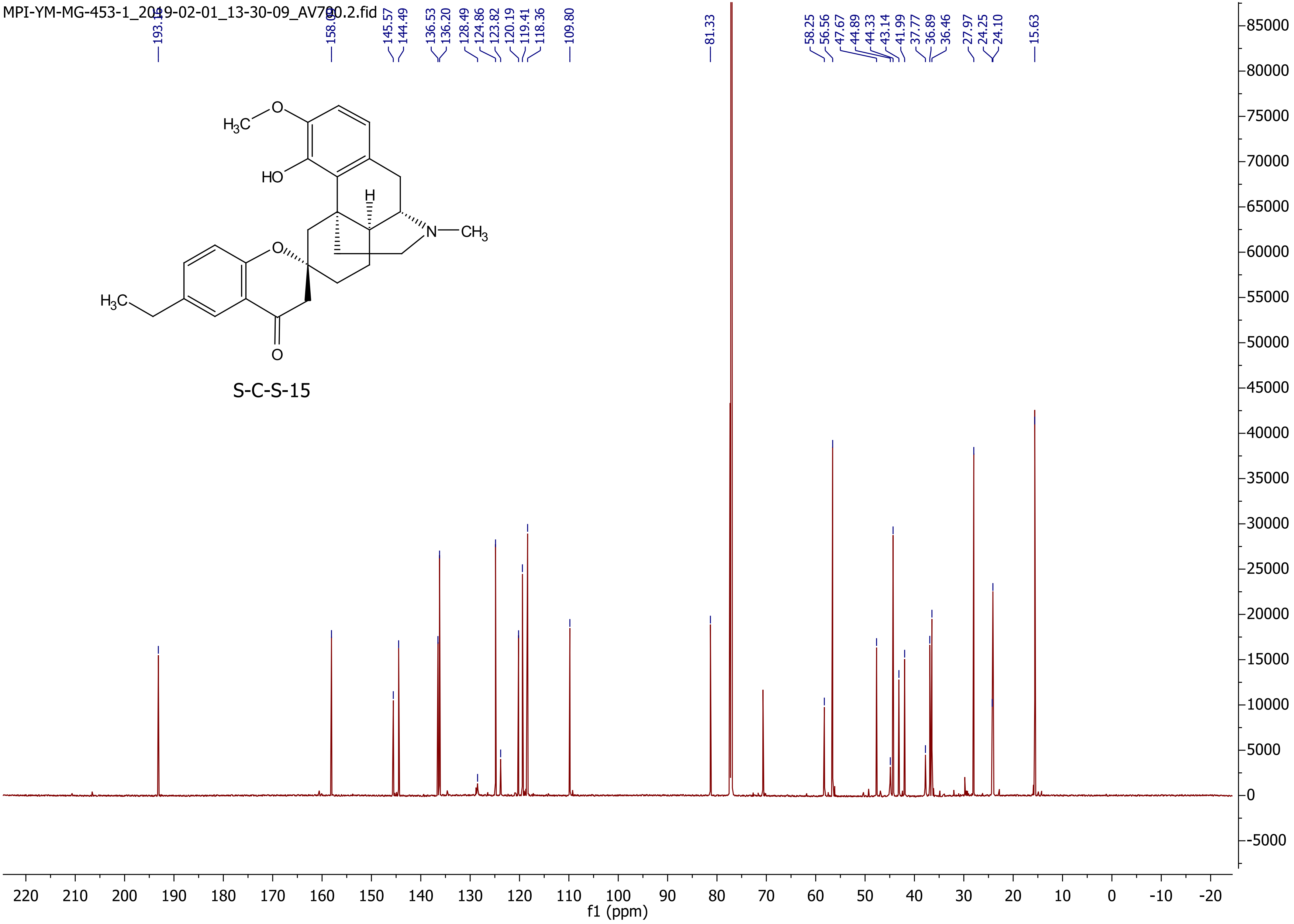
MPI-YM-MG-453-1_2019-02-01_13-30-09_AV700.2.fid
145.57
144.49
136.53
136.20
128.49
124.86
123.82
120.19
119.41
118.36
109.80
81.33
58.25
56.56
47.67
44.89
44.33
43.14
41.99
37.77
36.89
36.46
27.97
24.25
24.10
15.63
H3C
O
HO
H
N
CH3
O
H3C
O
S-C-S-15
f1 (ppm)

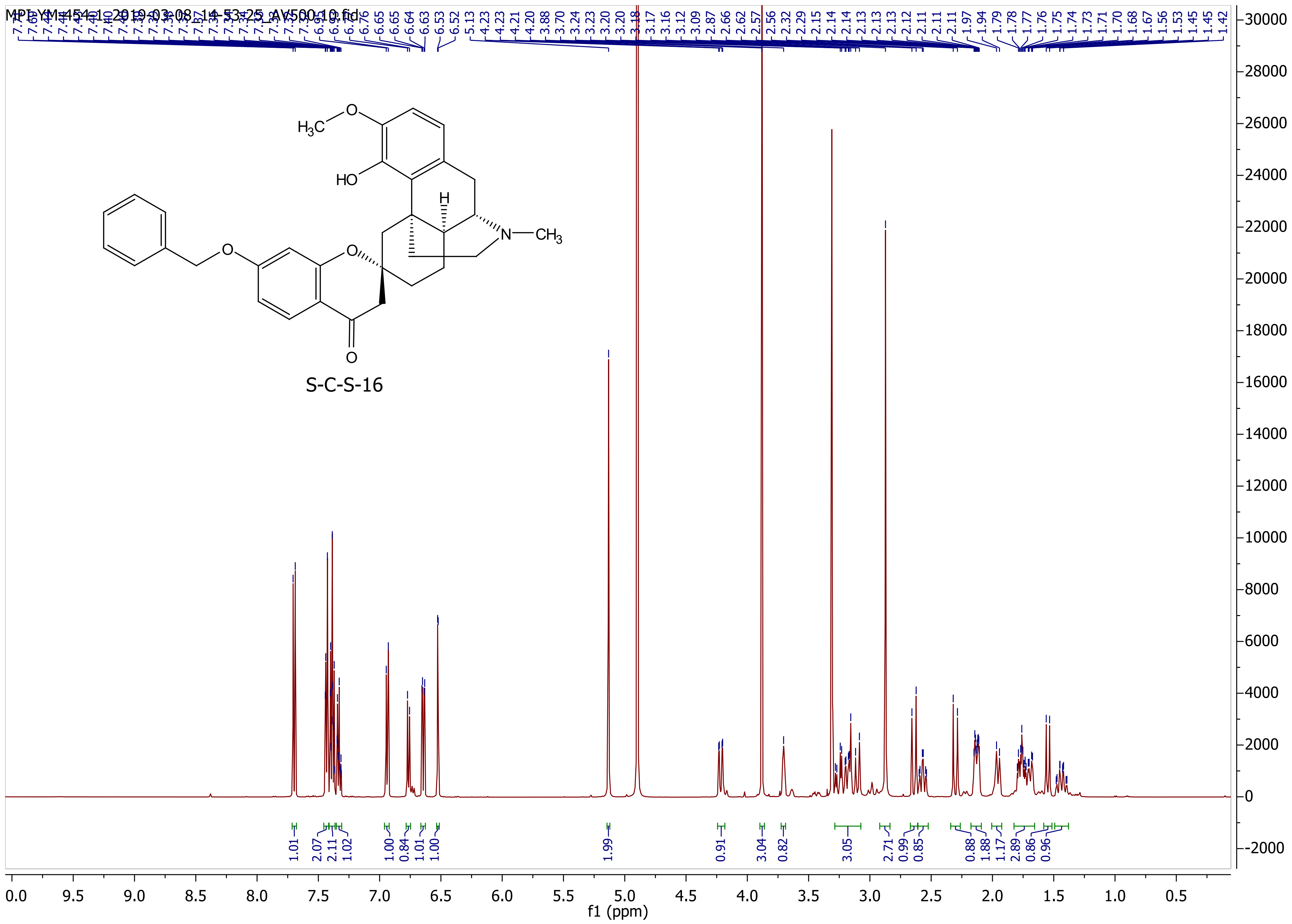

MPI-YM-454-1_2019-03-08_14-53-25_AV500.10.fid
S-C-S-16
f1 (ppm)

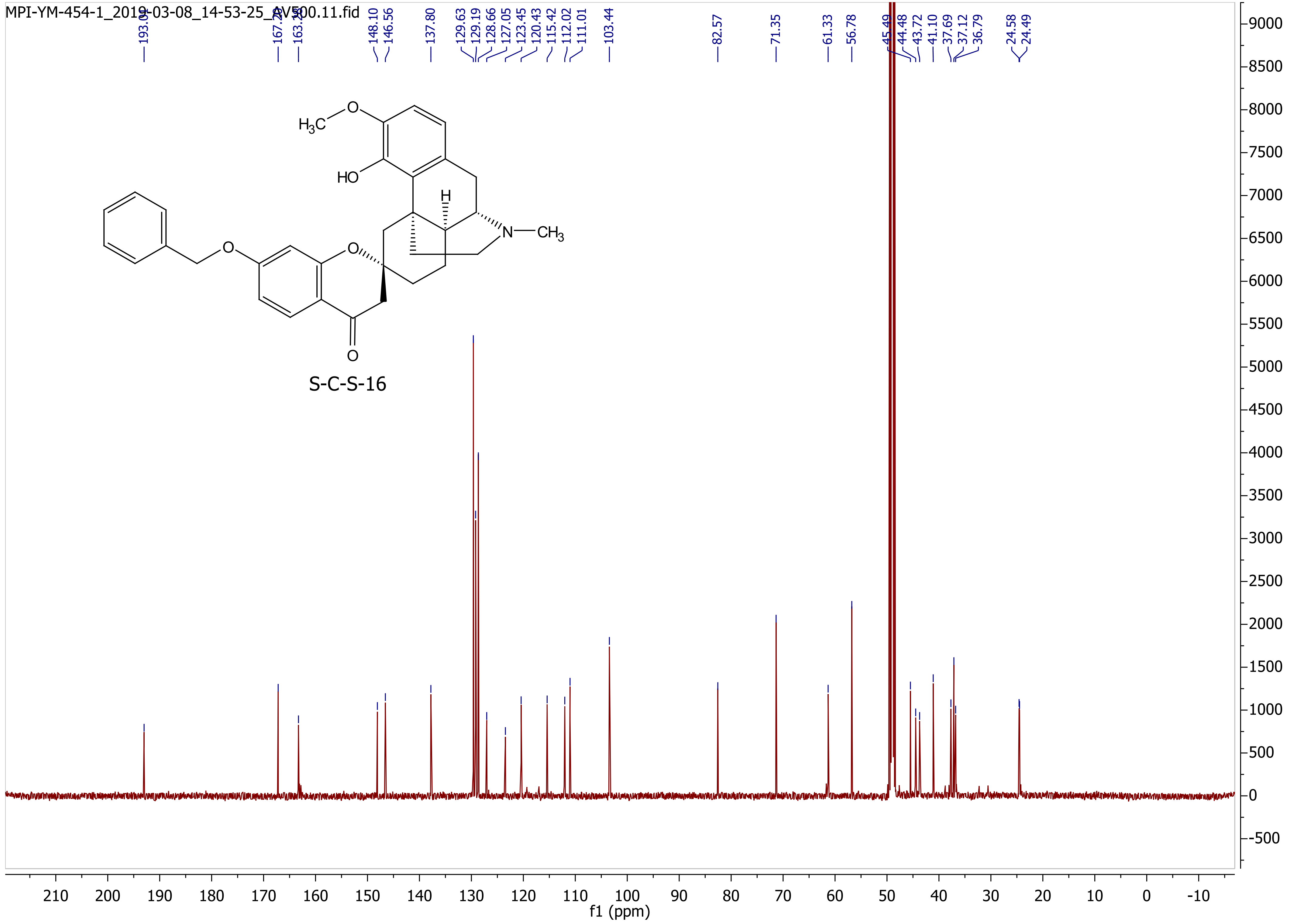
MPI-YM-454-1_2019-03-08_14-53-25_AV500.11.fid
148.10
146.56
137.80
129.63
129.19
128.66
127.05
123.45
120.43
115.42
112.02
111.01
103.44
82.57
71.35
61.33
56.78
45.49
44.48
43.72
41.10
37.69
37.12
36.79
24.58
24.49
H₃C
O
HO
H
N
CH₃
O
O
O
S-C-S-16
f1 (ppm)

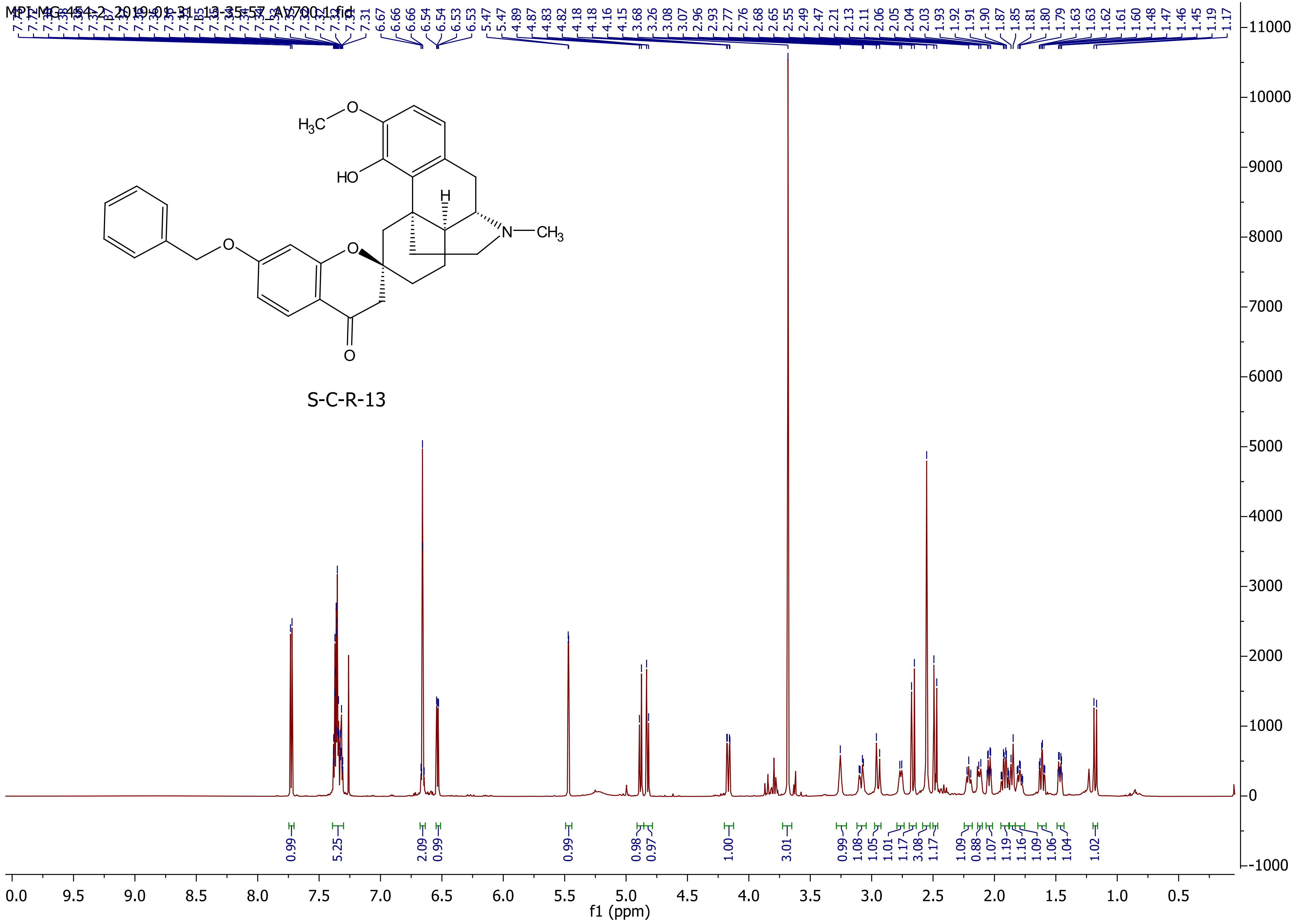

S-C-R-13

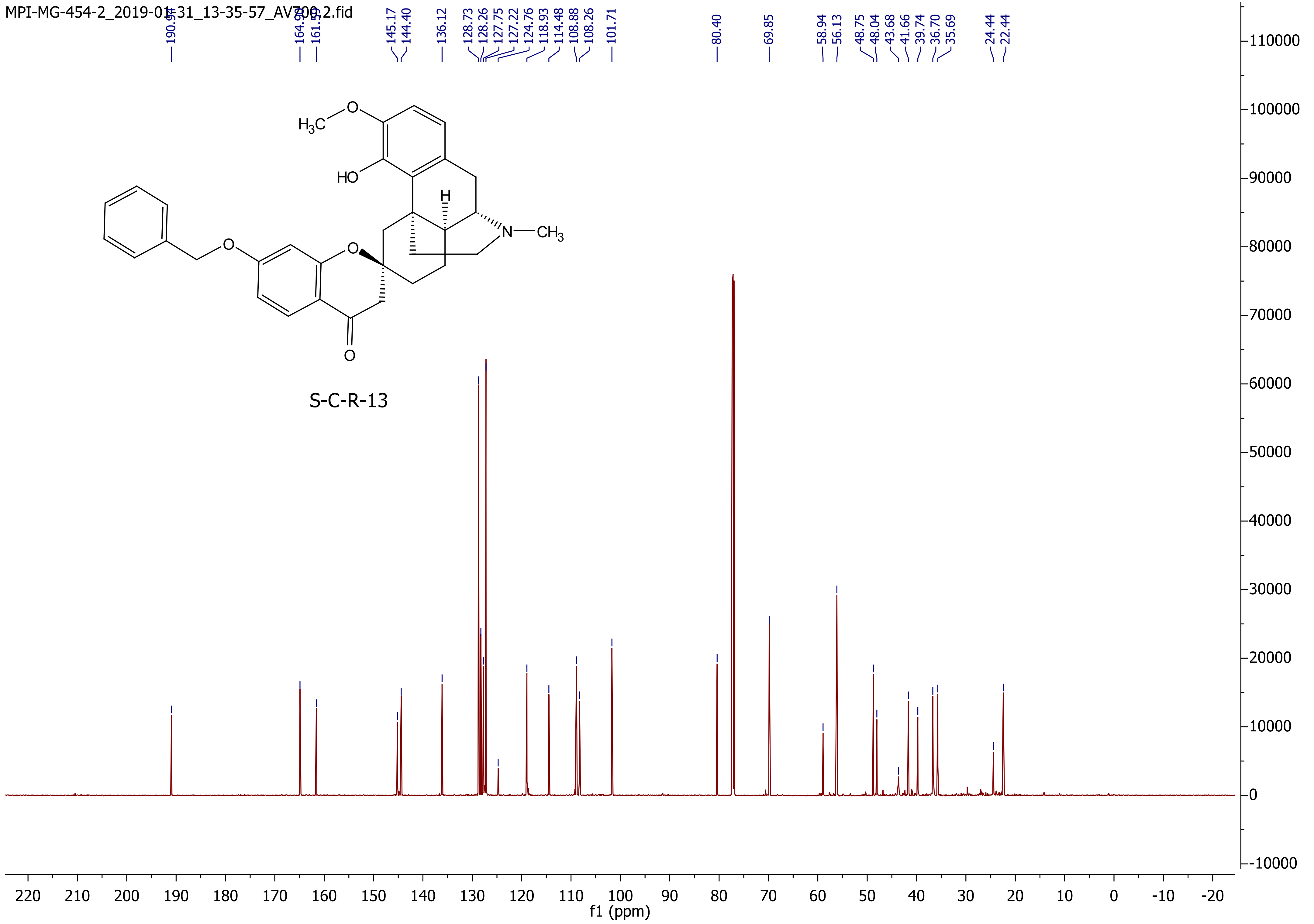
MPI-MG-454-2_2019-01-31_13-35-57_AV700_2.fid
190.94
164.90
161.59
145.17
144.40
136.12
128.73
128.26
127.75
127.22
124.76
118.93
114.48
108.88
108.26
101.71
80.40
69.85
58.94
56.13
48.75
48.04
43.68
41.66
39.74
36.70
35.69
24.44
22.44
H_3C
O
HO
H
N
CH_3
O
O
O
O
S-C-R-13
110000
100000
90000
80000
70000
60000
50000
40000
30000
20000
10000
0
-10000
220 210 200 190 180 170 160 150 140 130 120 110 100 90 80 70 60 50 40 30 20 10 0 -10 -20
f1 (ppm)

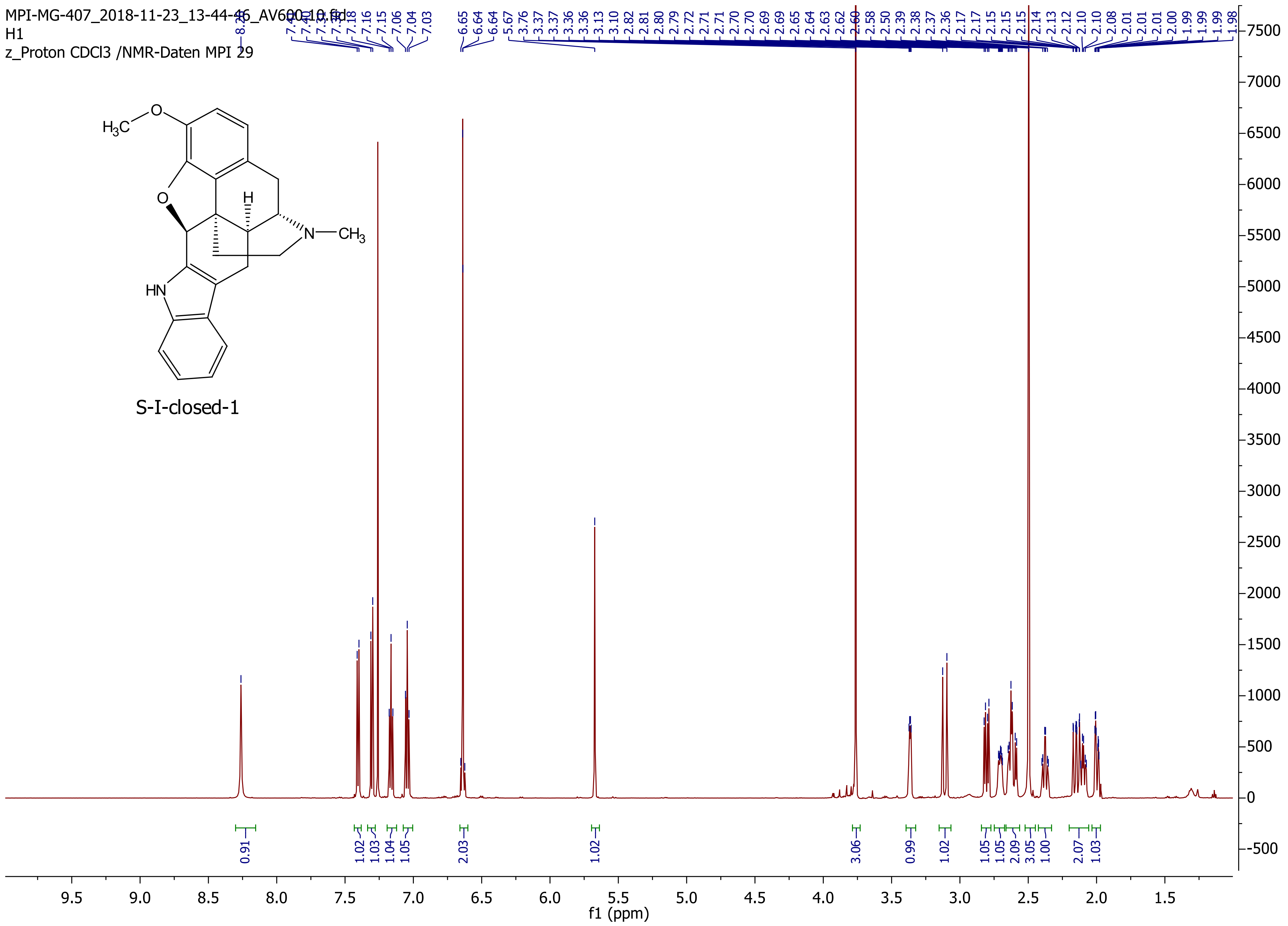
MPI-MG-407_2018-11-23_13-44-46_AV600_10.fid
H1
z_Proton CDCl3 /NMR-Daten MPI 29
H3C
O
O
H
N
CH3
HN
S-I-closed-1
f1 (ppm)
0.91
1.02
1.03
1.04
1.05
2.03
1.02
3.06
0.99
1.02
1.05
1.05
2.09
3.05
1.00
2.07
1.03

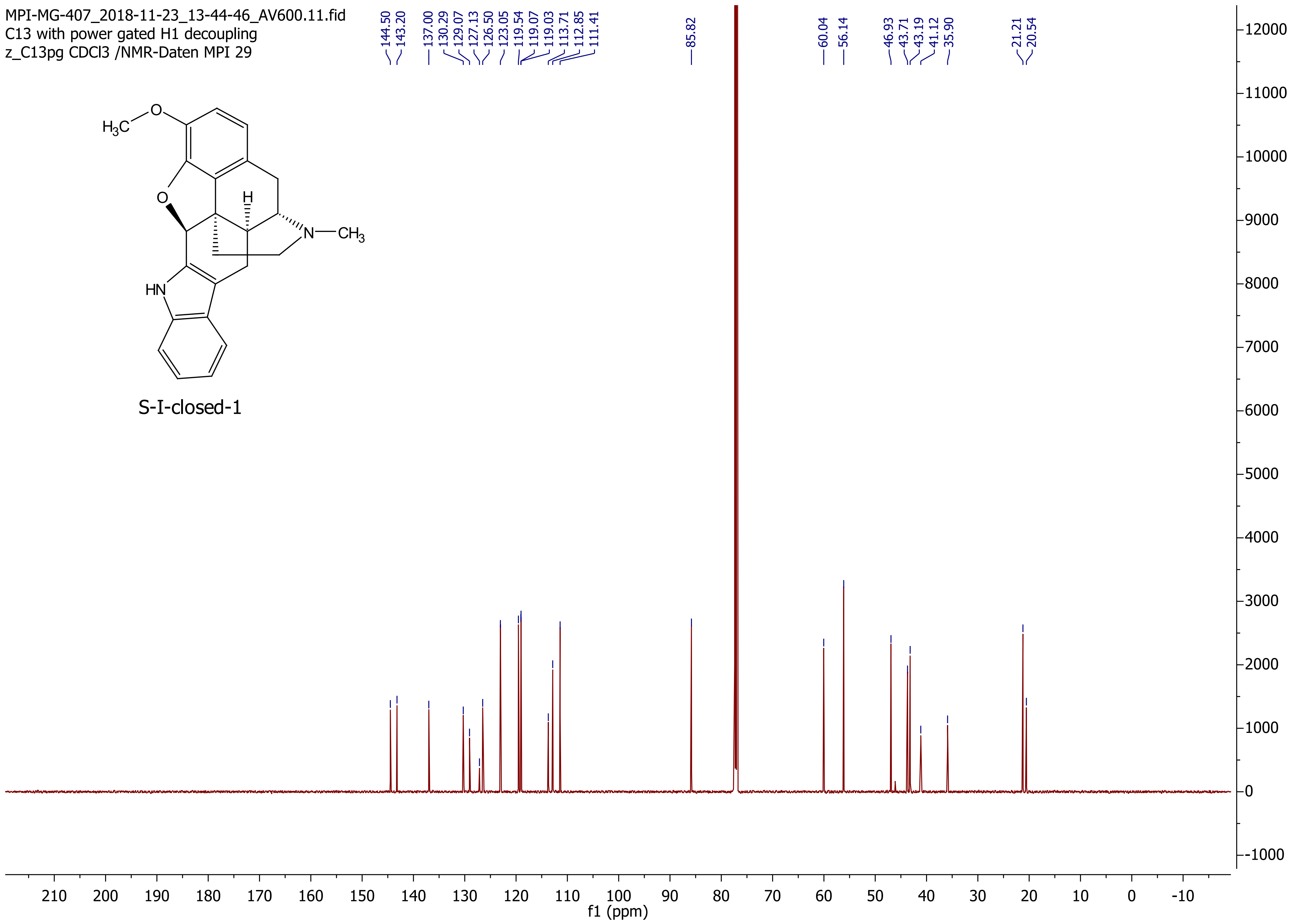
MPI-MG-407_2018-11-23_13-44-46_AV600.11.fid
C13 with power gated H1 decoupling
z_C13pg CDCl3 /NMR-Daten MPI 29
144.50
143.20
137.00
130.29
129.07
127.13
126.50
123.05
119.54
119.07
119.03
113.71
112.85
111.41
85.82
60.04
56.14
46.93
43.71
43.19
41.12
35.90
21.21
20.54
H_3C
O
O
H
N
CH_3
HN
f1 (ppm)
210 200 190 180 170 160 150 140 130 120 110 100 90 80 70 60 50 40 30 20 10 0 -10
12000 11000 10000 9000 8000 7000 6000 5000 4000 3000 2000 1000 0 -1000

S-I-closed-1

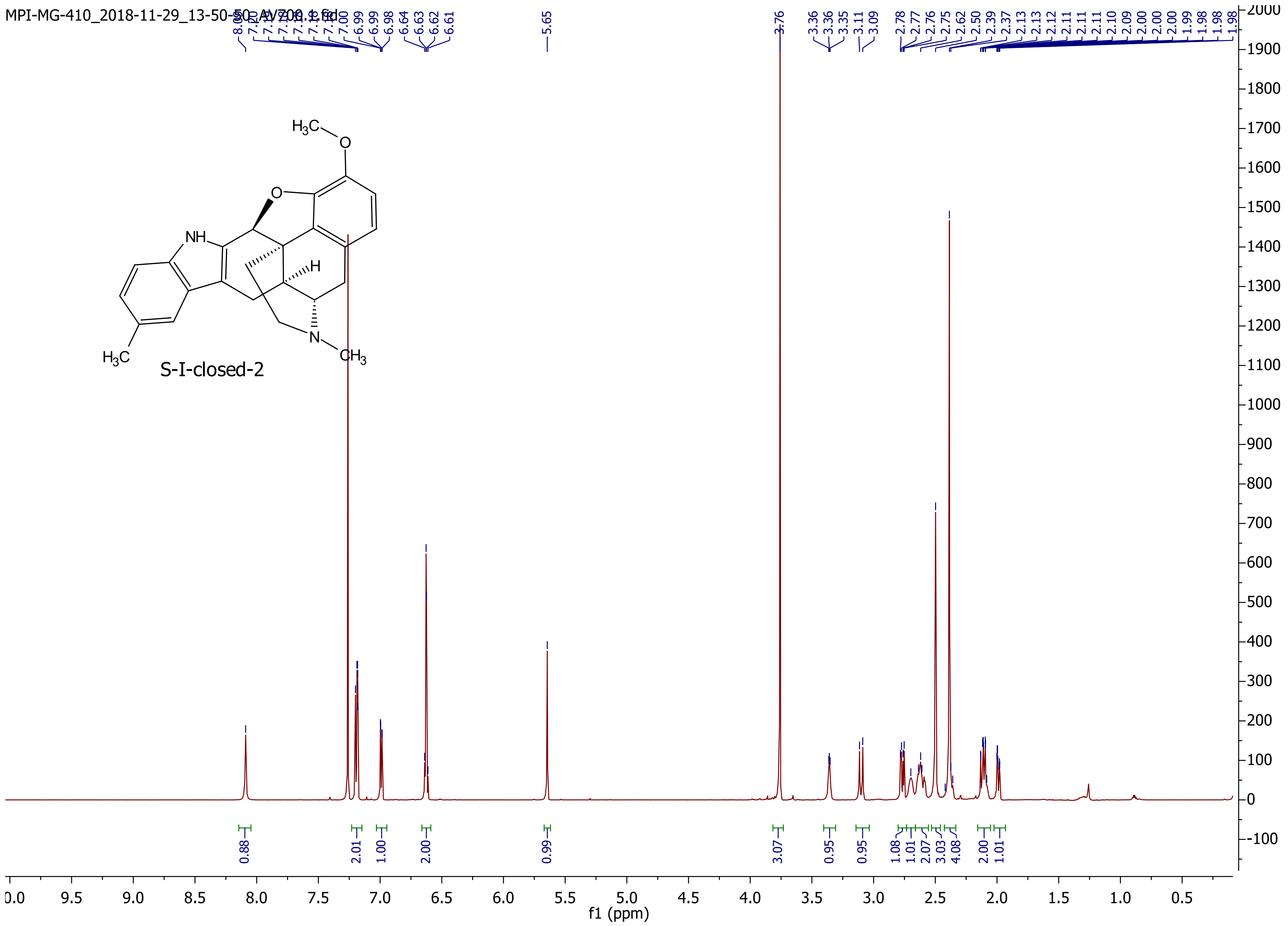

S-I-closed-2
f1 (ppm)

MPI-MG-410_2018-11-29_13-50-50_AV700.2.fid

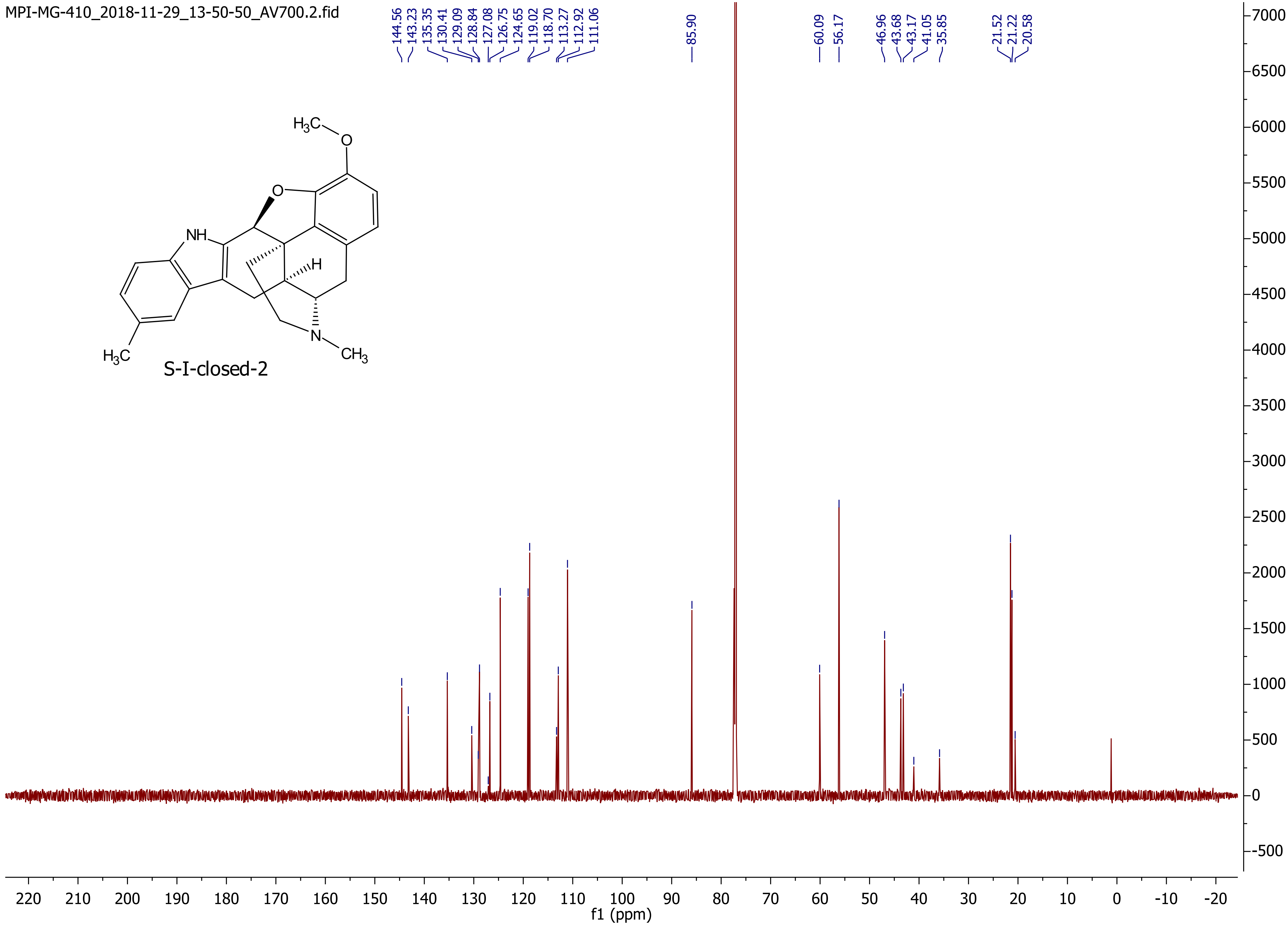

MPI-MG-411_2018-12-17_15-36-59_AV700.1.fid

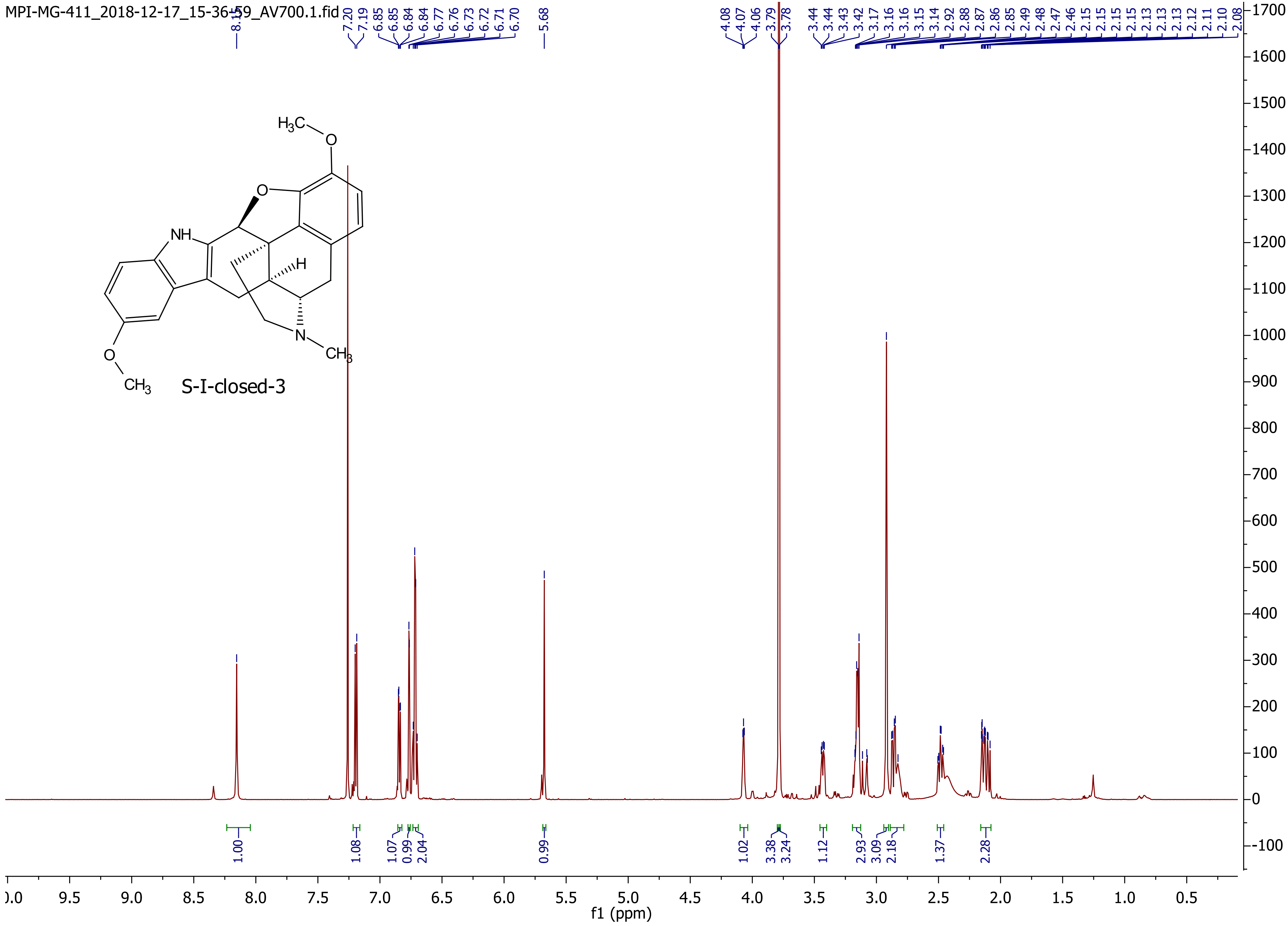

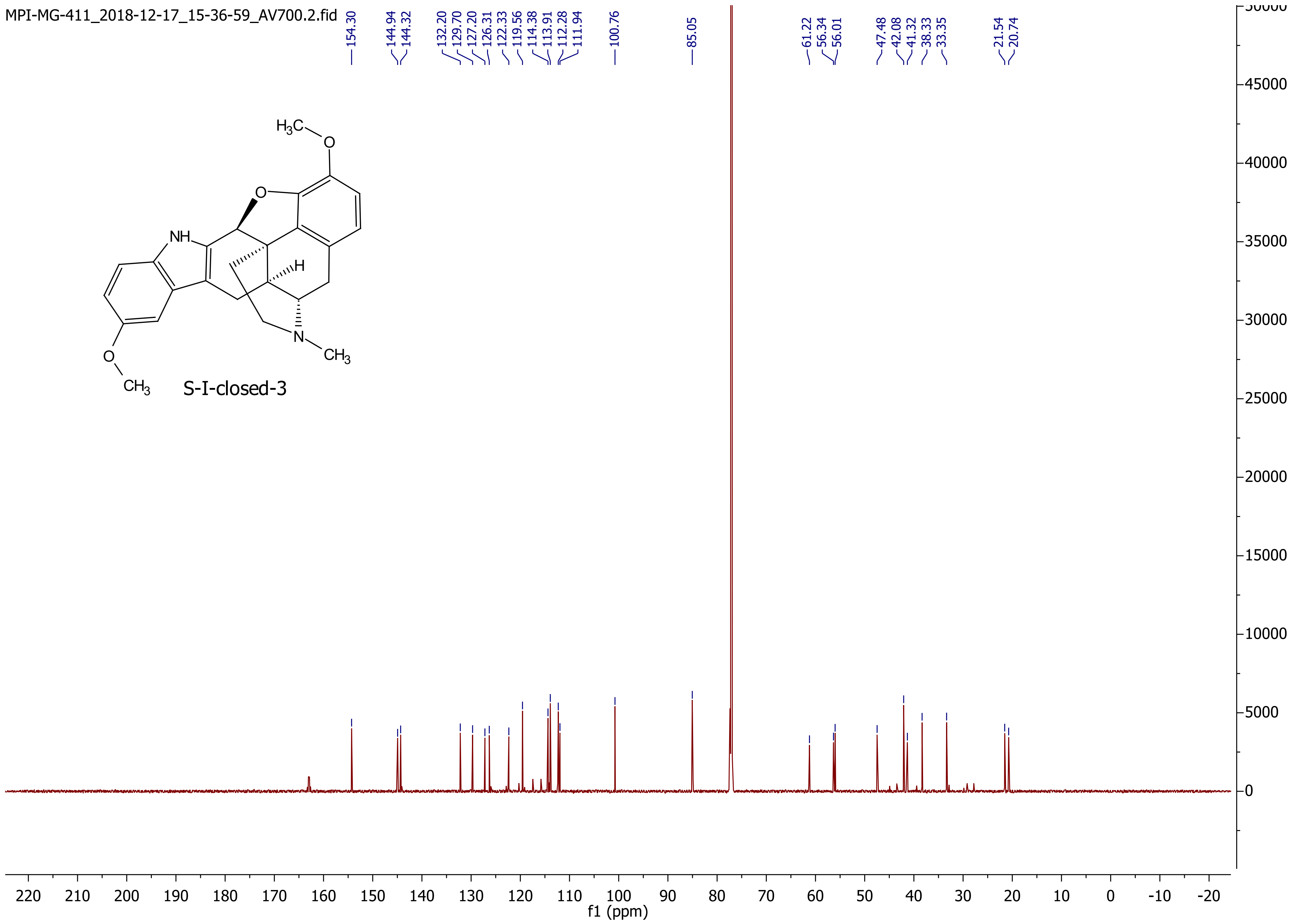
MPI-MG-411_2018-12-17_15-36-59_AV700.2.fid
154.30
144.94
144.32
132.20
129.70
127.20
126.31
122.33
119.56
114.38
113.91
112.28
111.94
100.76
85.05
61.22
56.34
56.01
47.48
42.08
41.32
38.33
33.35
21.54
20.74
S-I-closed-3
f1 (ppm)

MG-41201
MG-412 in MeOD
STANDARD 1H OBSERVE

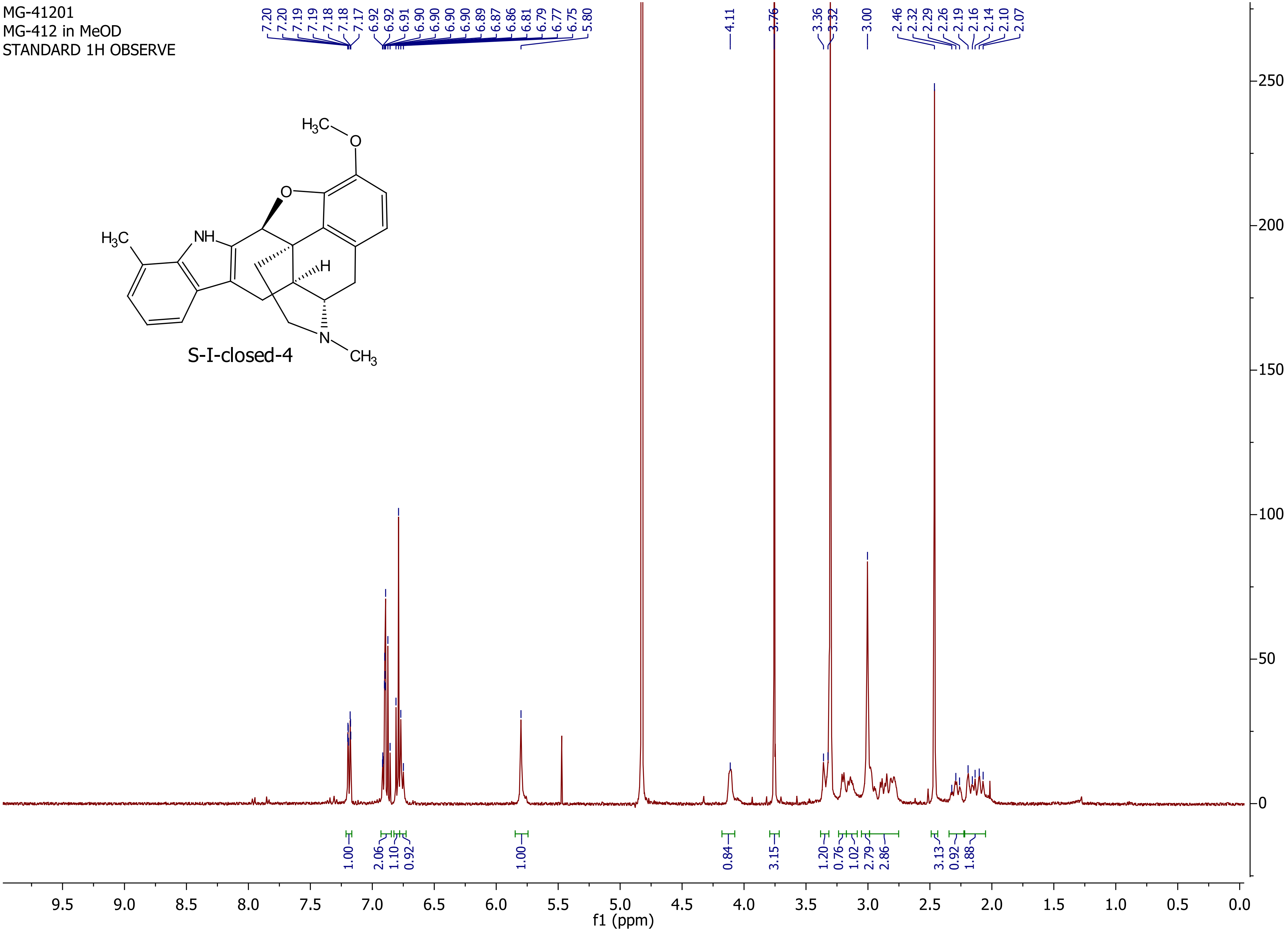

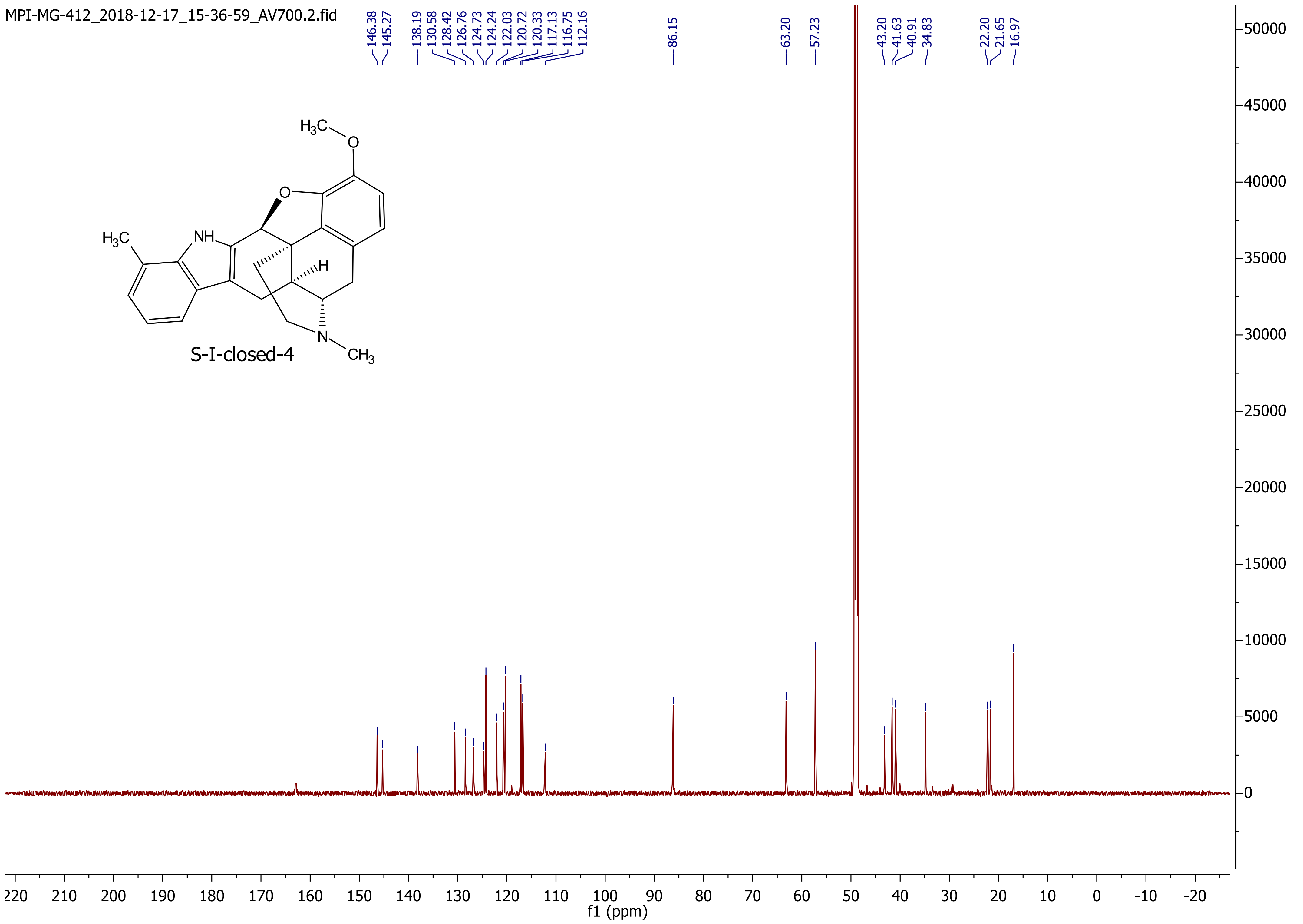

MPI-MG-412_2018-12-17_15-36-59_AV700.2.fid
146.38
145.27
138.19
130.58
128.42
126.76
124.73
124.24
122.03
120.72
120.33
117.13
116.75
112.16
86.15
63.20
57.23
43.20
41.63
40.91
34.83
22.20
21.65
16.97
S-I-closed-4
f1 (ppm)

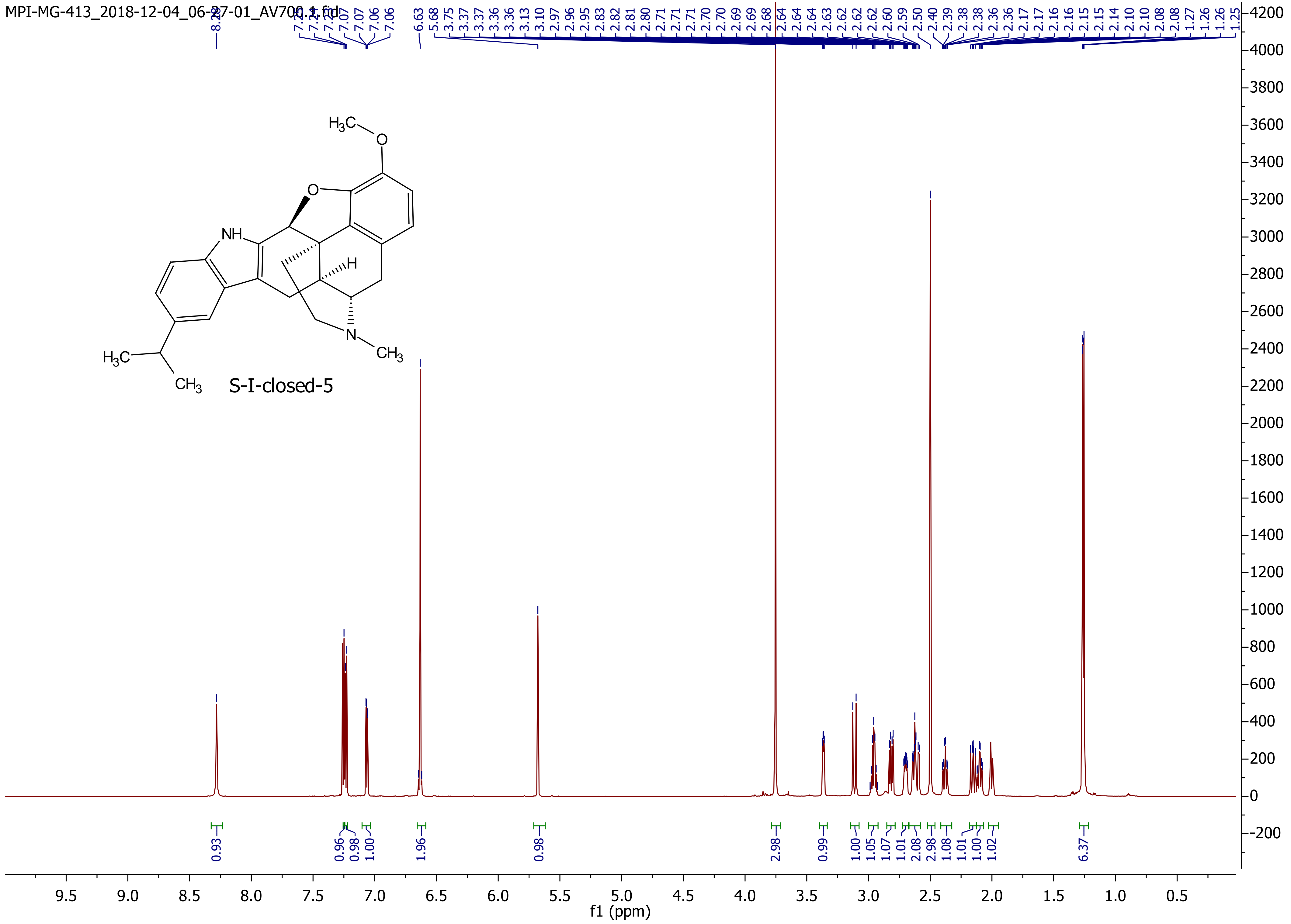
MPI-MG-413_2018-12-04_06-27-01_AV700.1.fid
7.07
7.07
7.06
7.06
6.63
5.68
3.75
3.37
3.37
3.36
3.36
3.13
3.10
2.97
2.96
2.95
2.83
2.82
2.81
2.80
2.71
2.71
2.71
2.70
2.70
2.69
2.69
2.68
2.64
2.64
2.64
2.63
2.62
2.62
2.62
2.60
2.59
2.50
2.40
2.39
2.38
2.38
2.36
2.36
2.17
2.17
2.16
2.16
2.15
2.15
2.14
2.10
2.10
2.08
2.08
1.27
1.26
1.26
1.25
H3C
O
O
NH
H
N
CH3
H3C
CH3
S-I-closed-5
0.93
0.96
0.98
1.00
1.96
0.98
2.98
0.99
1.00
1.05
1.07
1.01
2.08
2.98
1.08
1.01
1.00
1.02
6.37
f1 (ppm)

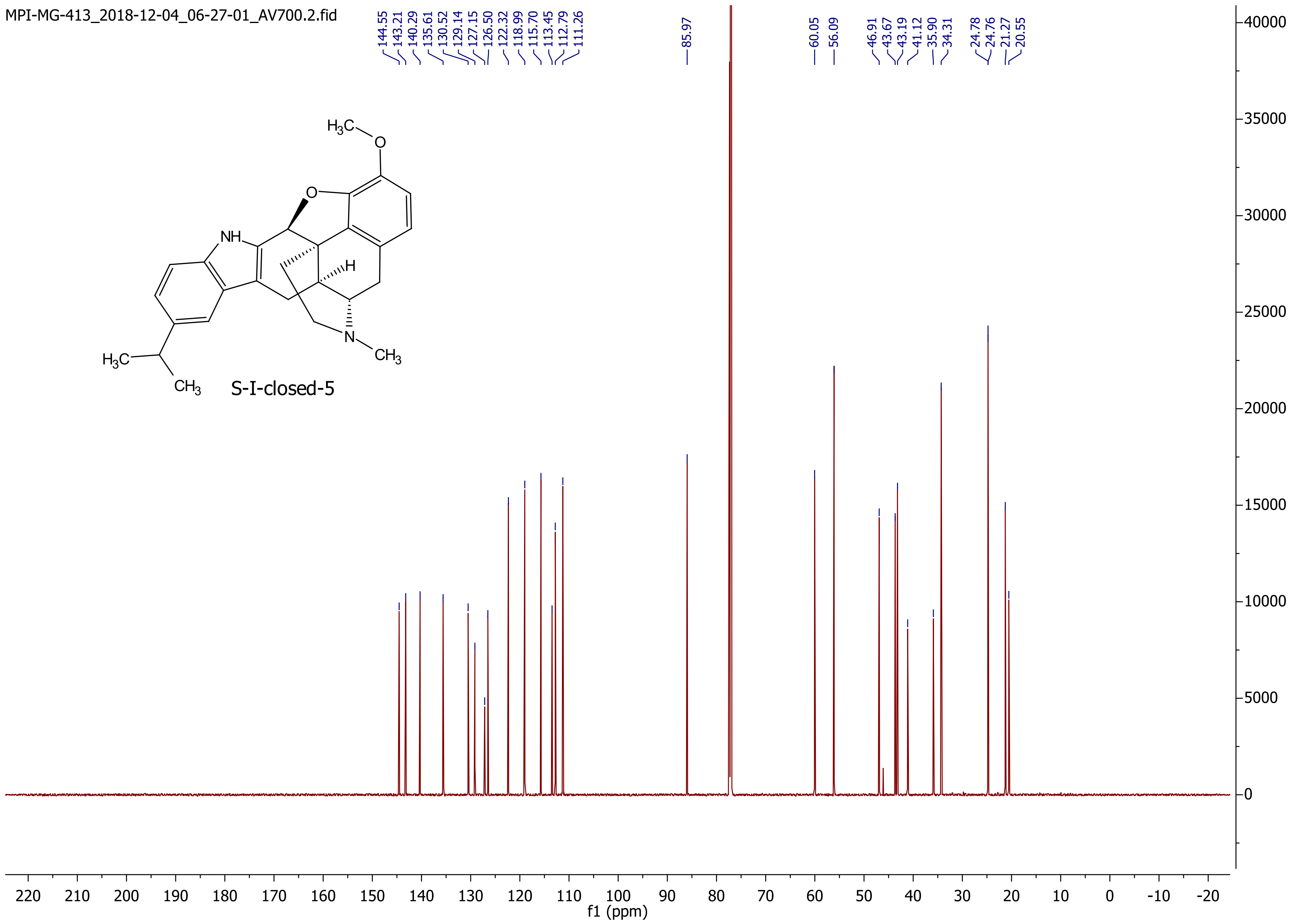
MPI-MG-413_2018-12-04_06-27-01_AV700.2.fid
144.55
143.21
140.29
135.61
130.52
129.14
127.15
126.50
122.32
118.99
115.70
113.45
112.79
111.26
85.97
60.05
56.09
46.91
43.67
43.19
41.12
35.90
34.31
24.78
24.76
21.27
20.55
S-I-closed-5
f1 (ppm)

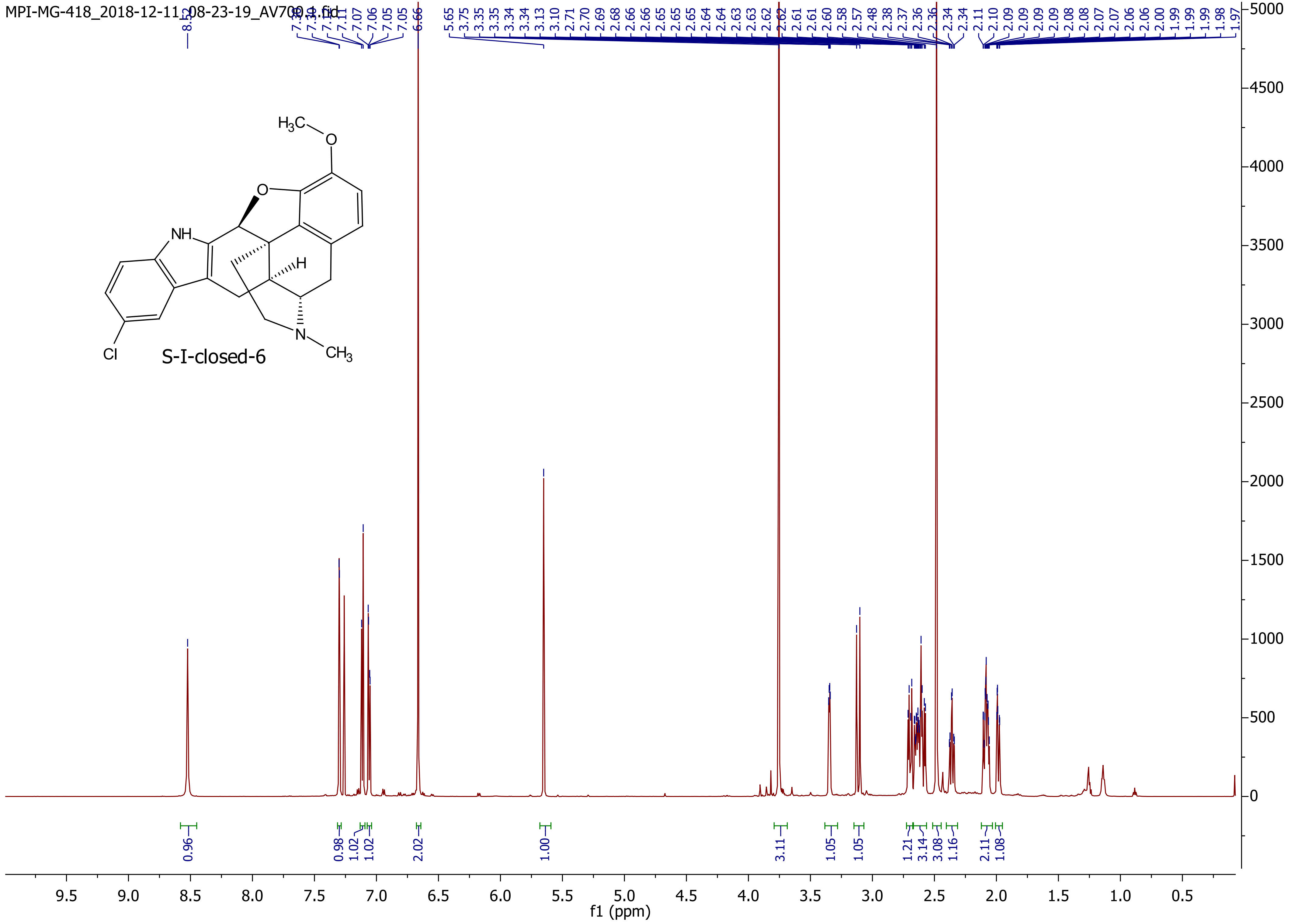
MPI-MG-418_2018-12-11_08-23-19_AV700.1.fid
S-I-closed-6
f1 (ppm)
0.96
0.98
1.02
1.02
2.02
1.00
3.11
1.05
1.05
1.21
3.14
3.08
1.16
2.11
1.08

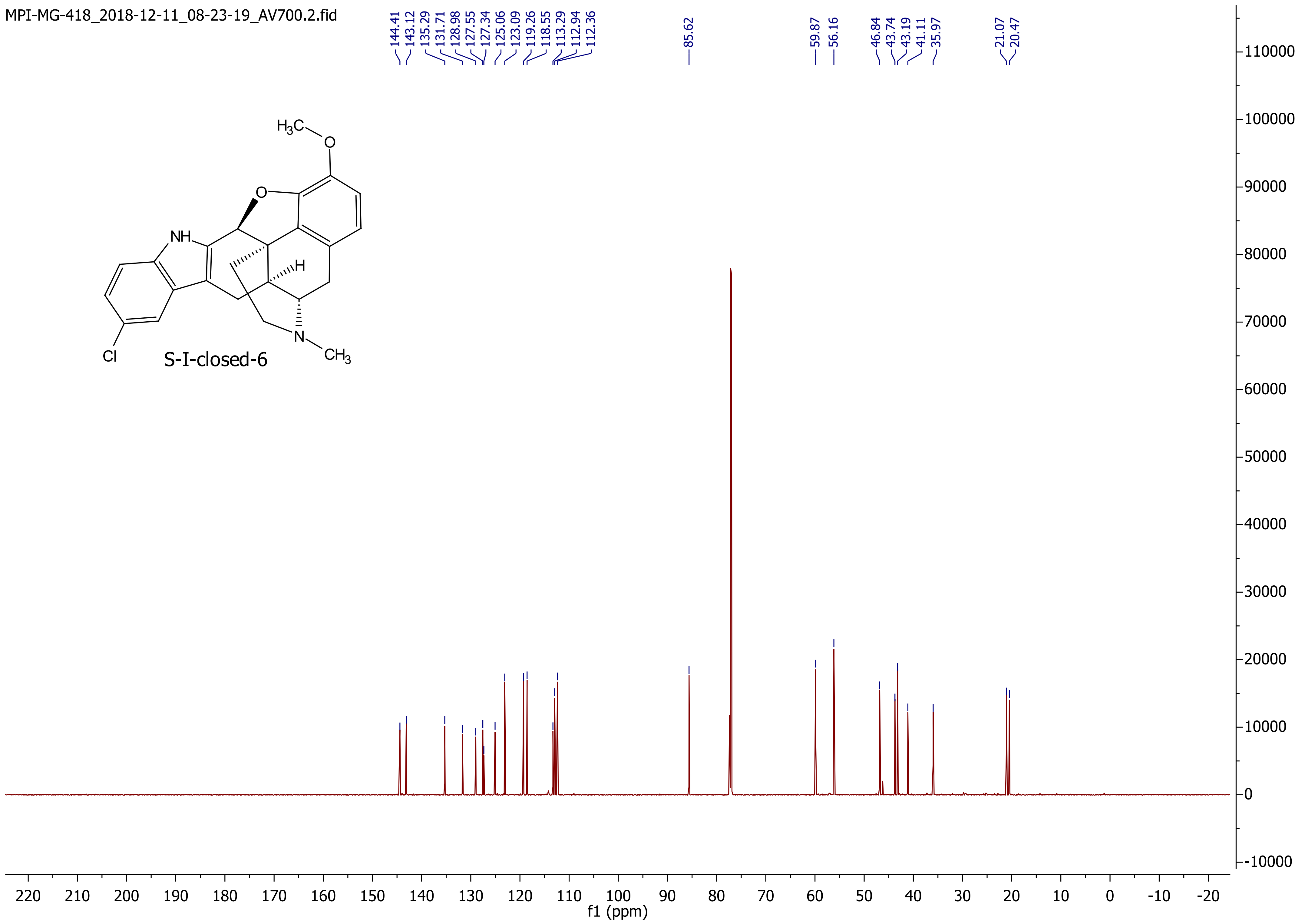
MPI-MG-418_2018-12-11_08-23-19_AV700.2.fid
144.41
143.12
135.29
131.71
128.98
127.55
127.34
125.06
123.09
119.26
118.55
113.29
112.94
112.36
85.62
59.87
56.16
46.84
43.74
43.19
41.11
35.97
21.07
20.47
S-I-closed-6
f1 (ppm)

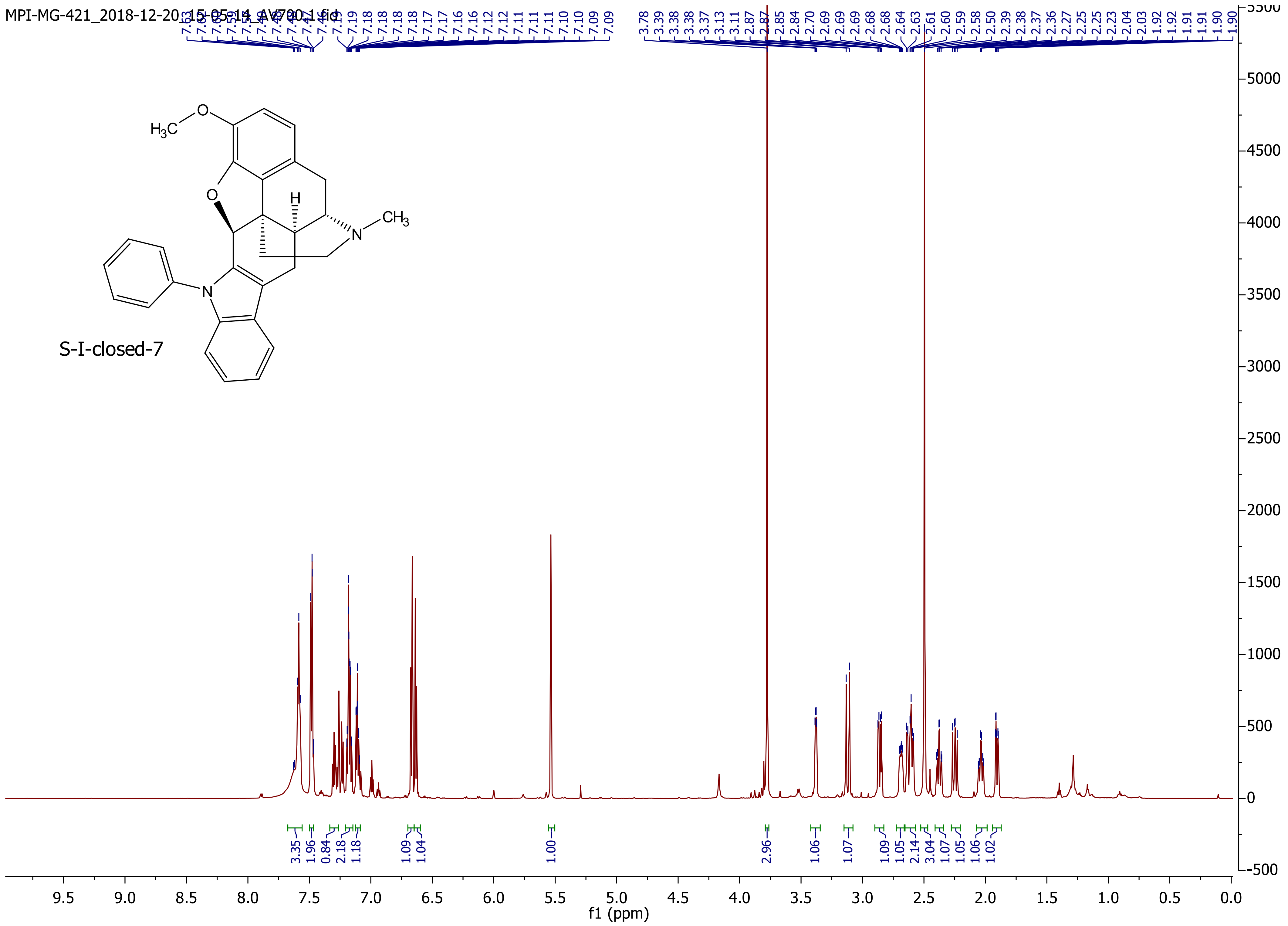

S-I-closed-7

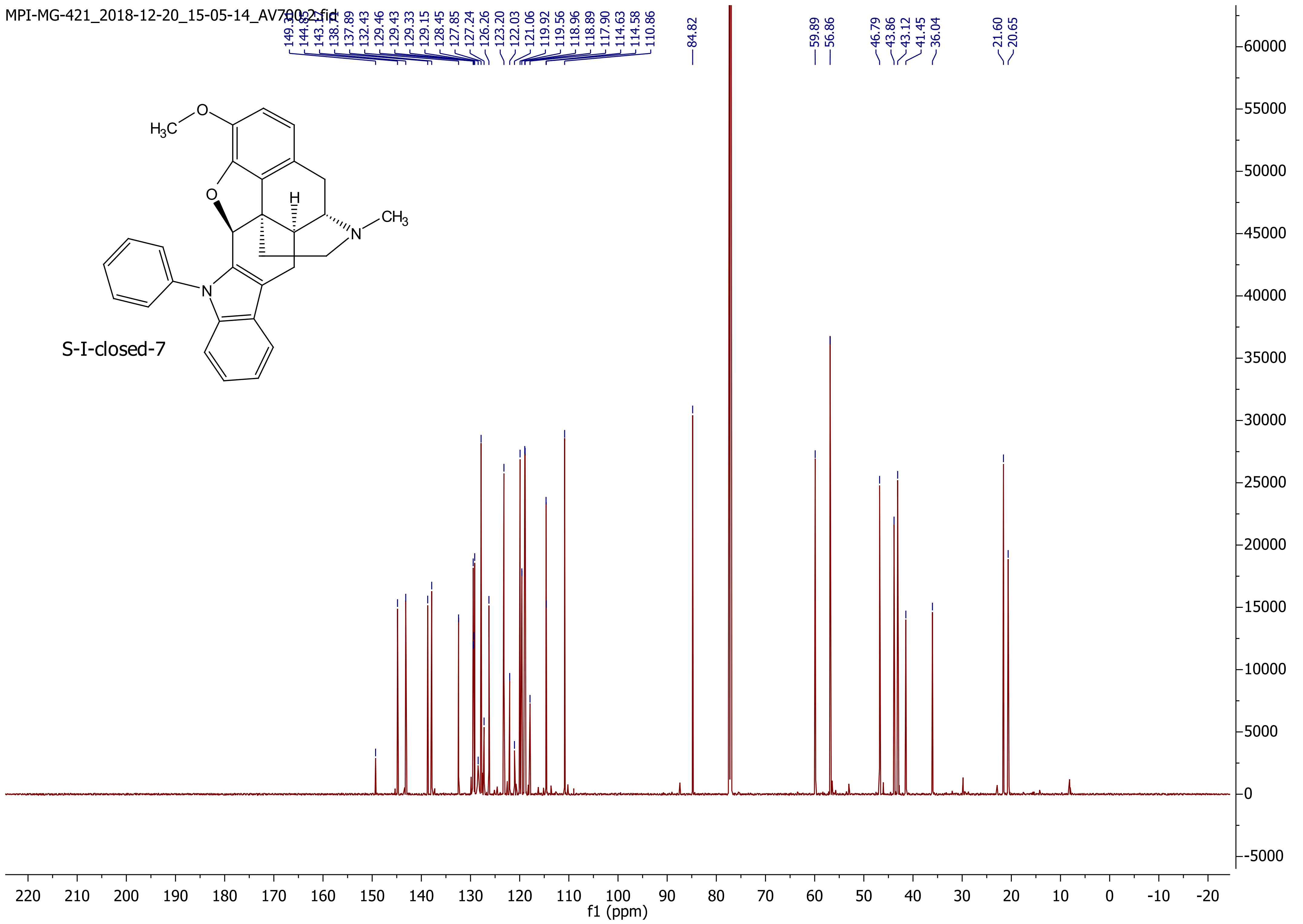

137.89
132.43
129.46
129.43
129.33
129.15
128.45
127.85
127.24
126.26
123.20
122.03
121.06
119.92
119.56
118.96
118.89
117.90
114.63
114.58
110.86
84.82
59.89
56.86
46.79
43.86
43.12
41.45
36.04
21.60
20.65
H3C
O
O
H
N
CH3
N
S-I-closed-7
f1 (ppm)

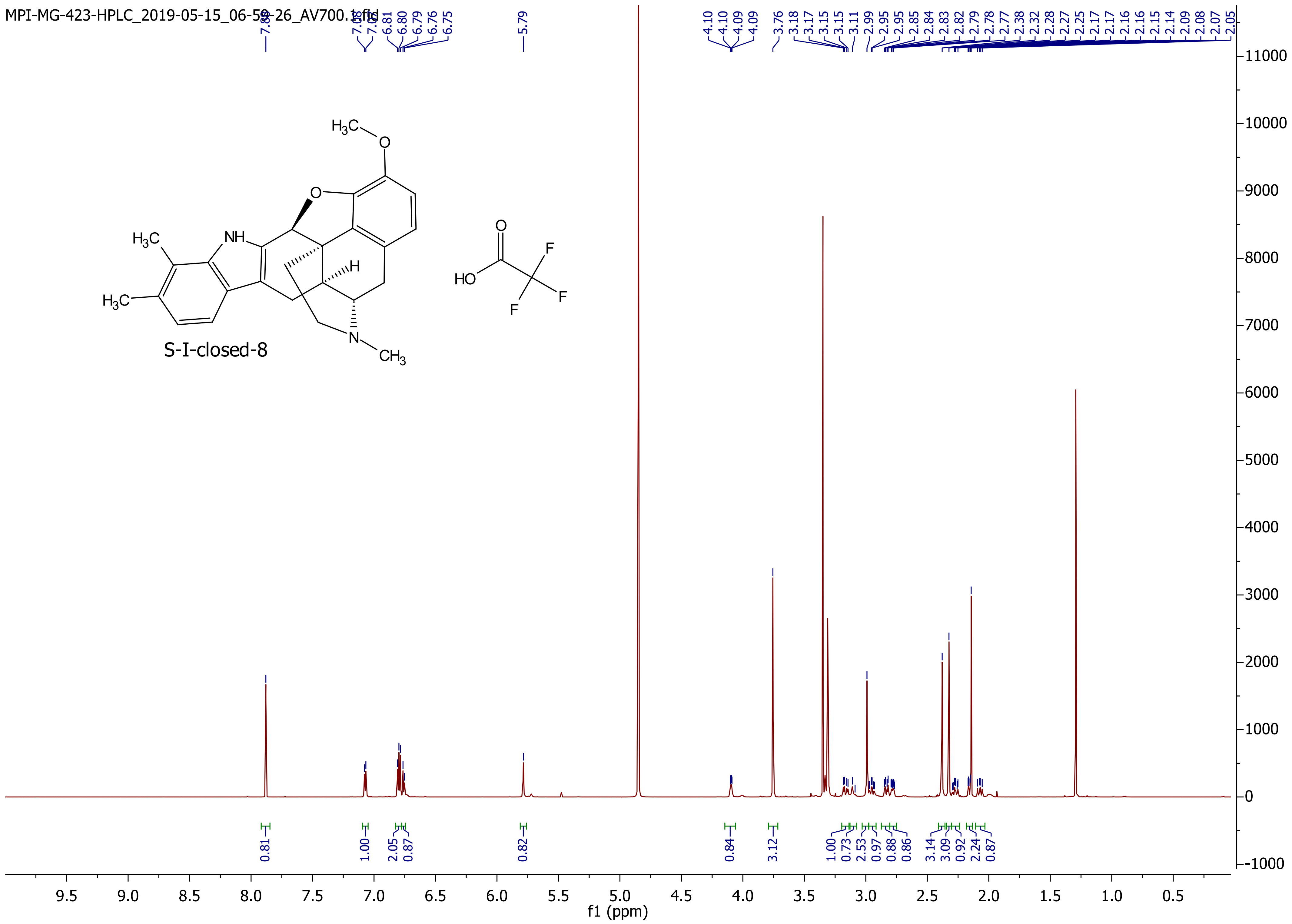
MPI-MG-423-HPLC_2019-05-15_06-59-26_AV700.1.fid
S-I-closed-8
H_3C
O
NH
H
N
CH_3
HO
F
f1 (ppm)

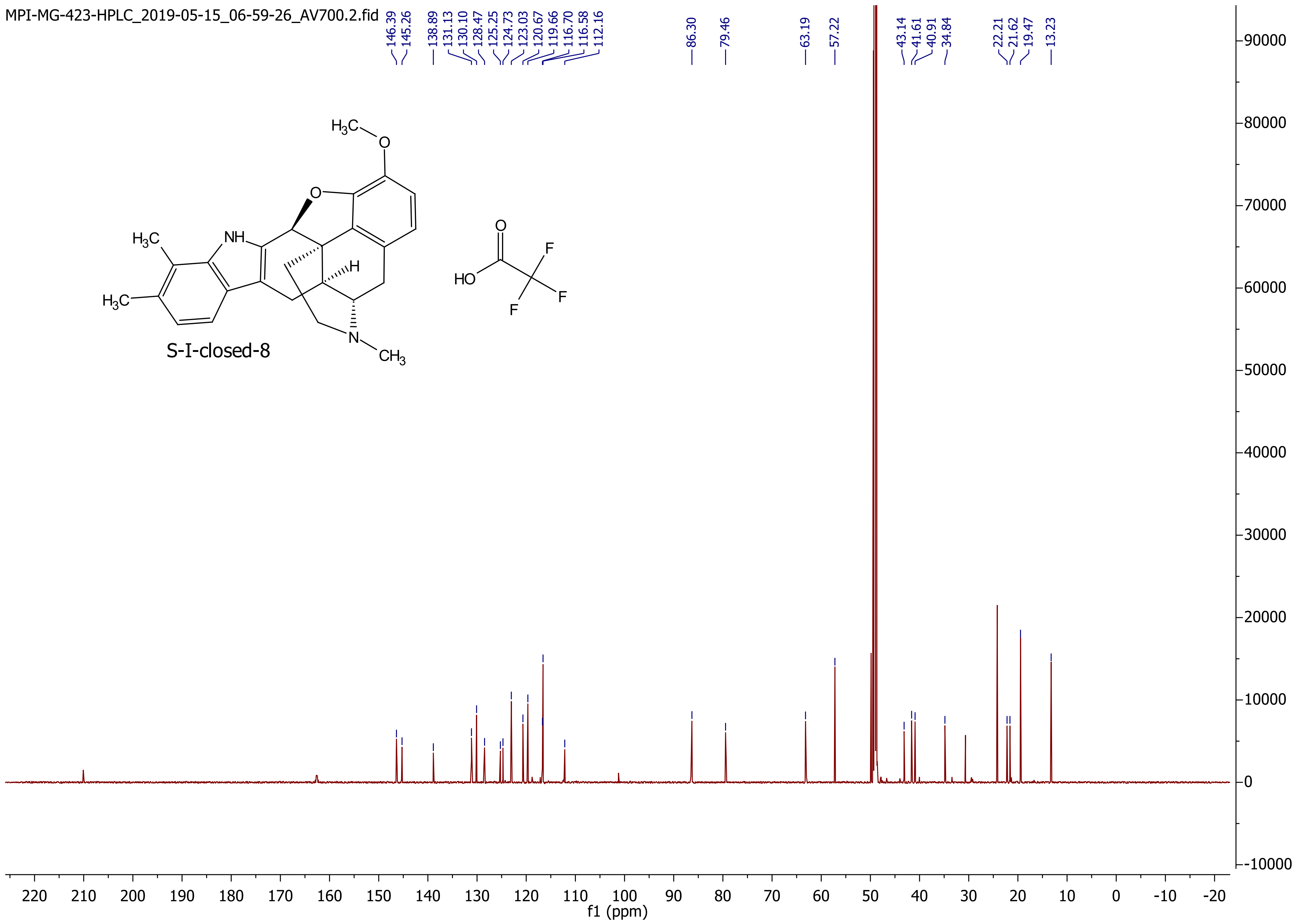

MPI-MG-423-HPLC_2019-05-15_06-59-26_AV700.2.fid
146.39
145.26
138.89
131.13
130.10
128.47
125.25
124.73
123.03
120.67
119.66
116.70
116.58
112.16
86.30
79.46
63.19
57.22
43.14
41.61
40.91
34.84
22.21
21.62
19.47
13.23
H3C
O
NH
H3C
H3C
H
N
CH3
O
HO
F
F
F
S-I-closed-8
90000
80000
70000
60000
50000
40000
30000
20000
10000
0
-10000
220 210 200 190 180 170 160 150 140 130 120 110 100 90 80 70 60 50 40 30 20 10 0 -10 -20
f1 (ppm)

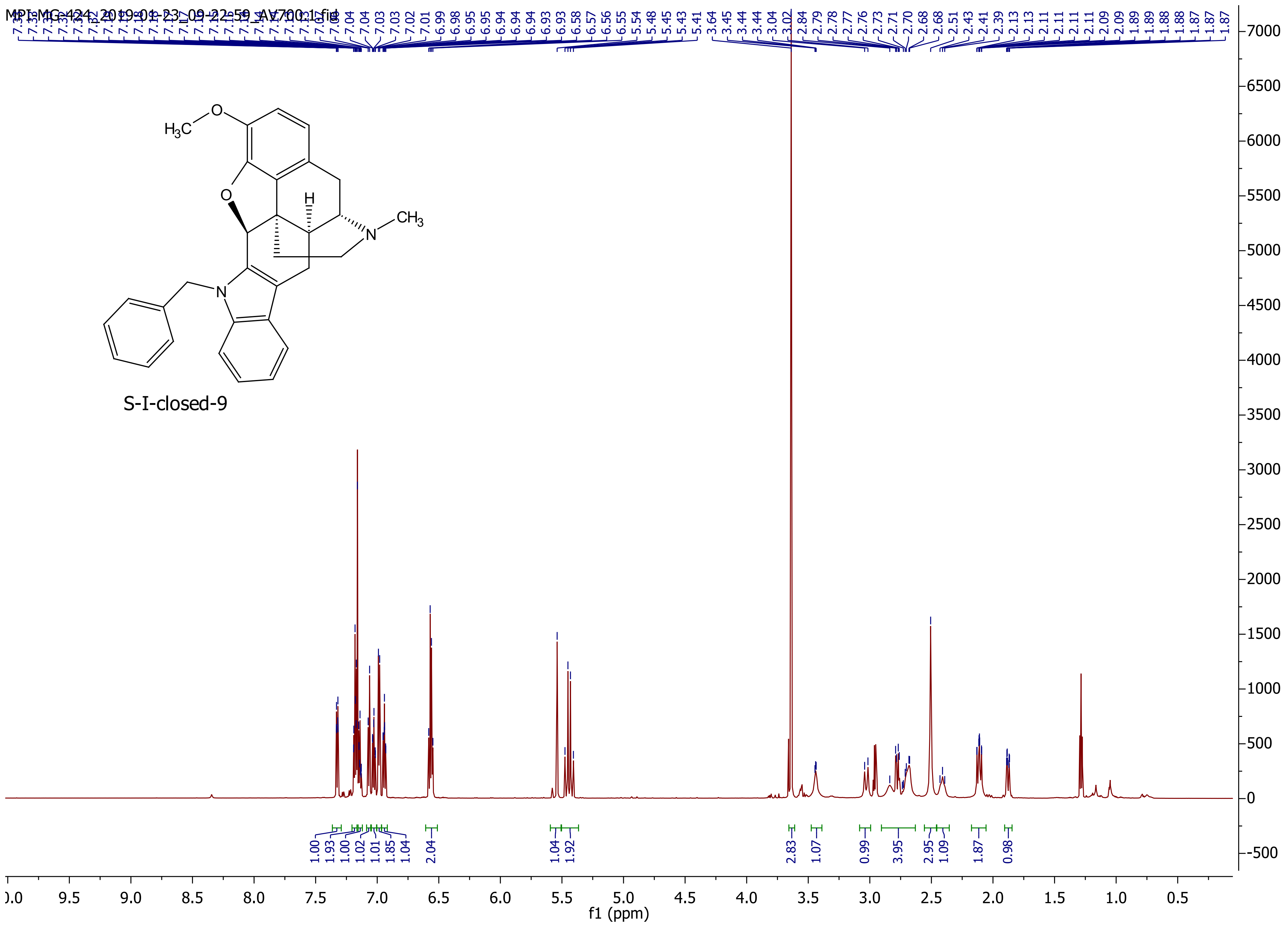
MPI-MG-424_2019-01-23_09-22-59_AV700.1.fid
H_3C
O
O
H
N
CH_3
N
S-I-closed-9
1.00
1.93
1.00
1.02
1.01
1.85
1.04
2.04
1.04
1.92
2.83
1.07
0.99
3.95
2.95
1.09
1.87
0.98
f1 (ppm)

MPI-MG-424_2019-01-23_09-22-59_AV700.2.fid

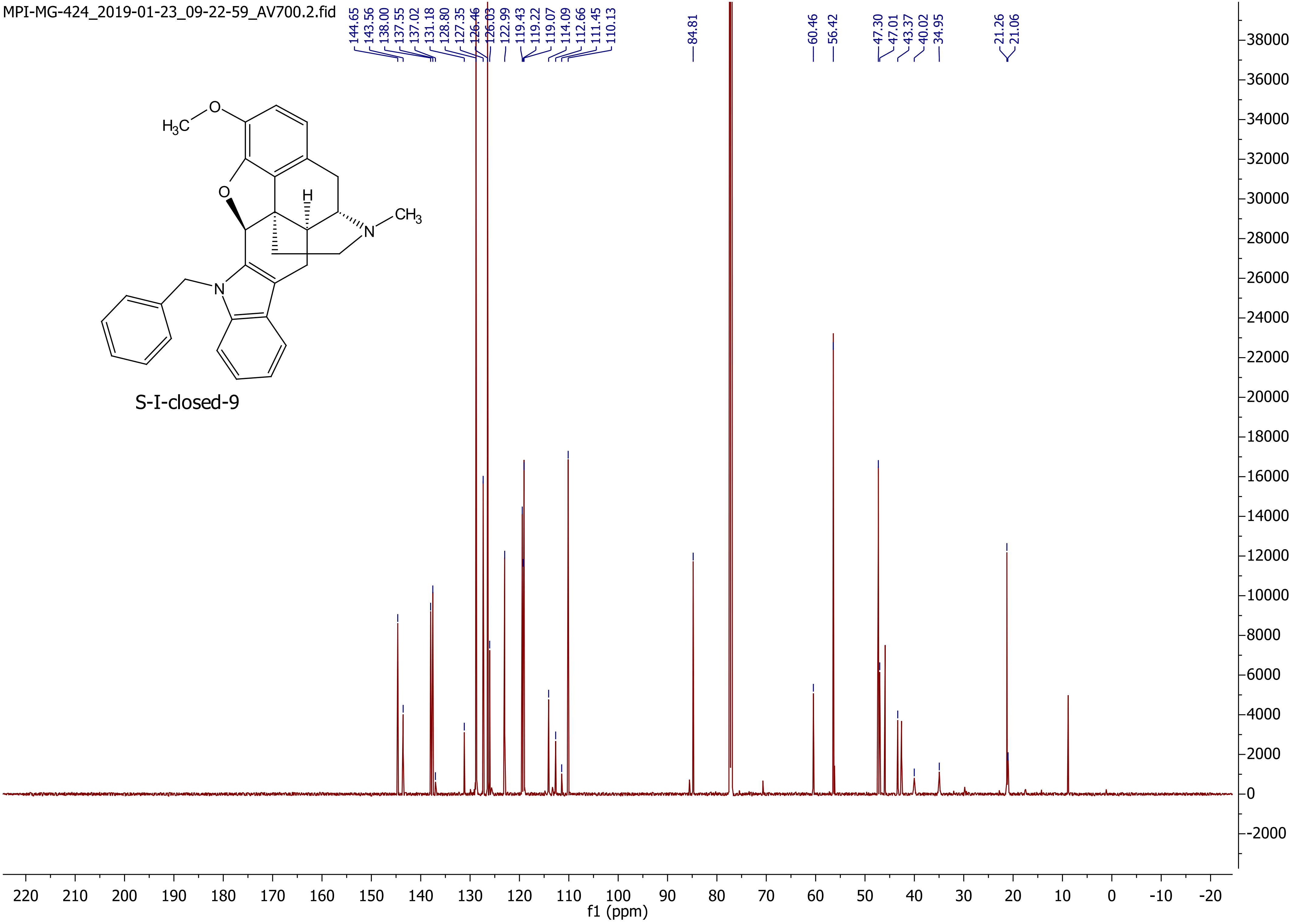

S-I-closed-9

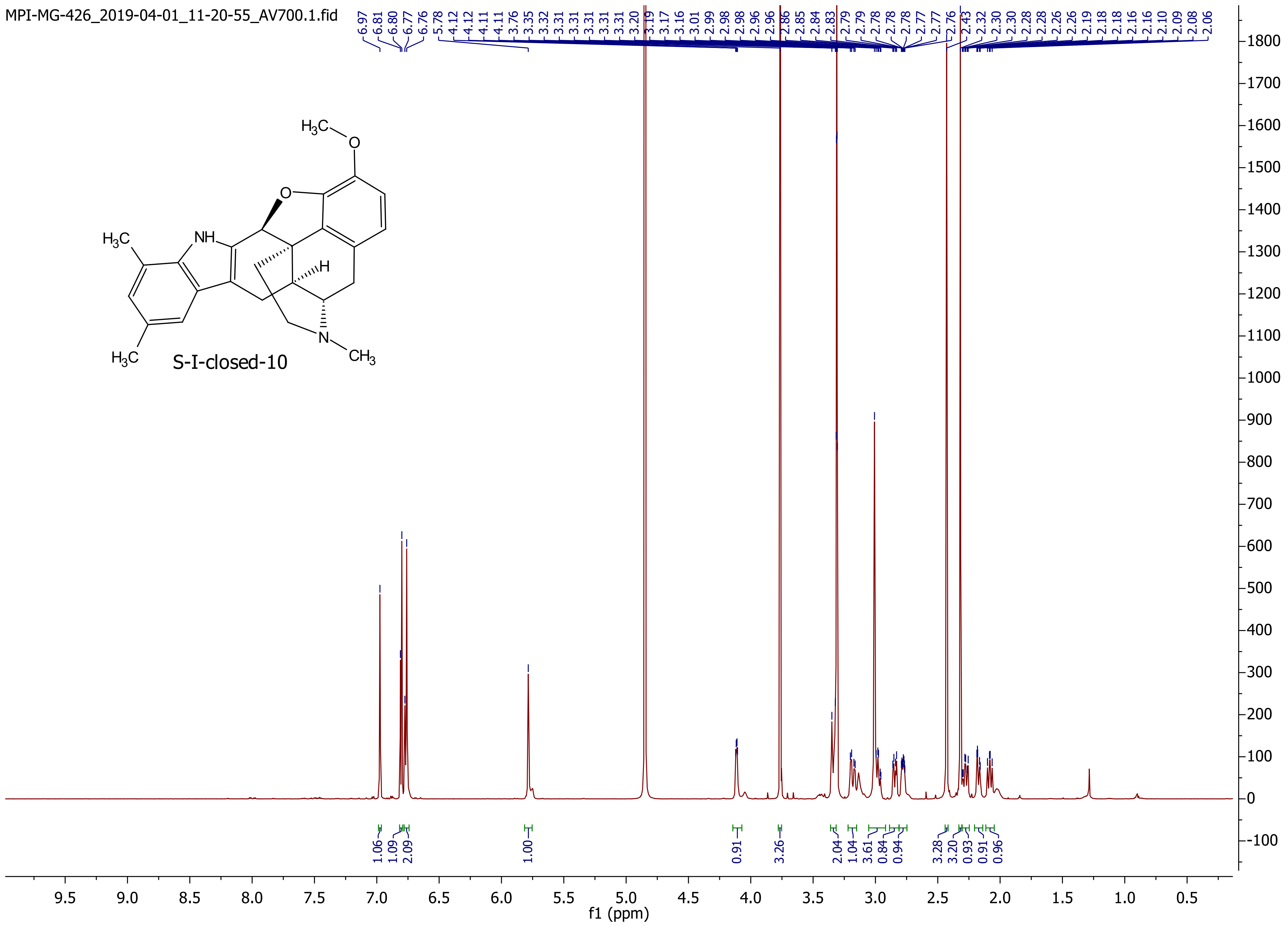
MPI-MG-426_2019-04-01_11-20-55_AV700.1.fid
6.97 6.81 6.80 6.77 6.76 5.78 4.12 4.12 4.11 4.11 3.76 3.35 3.32 3.31 3.31 3.31 3.31 3.31 3.20 3.19 3.17 3.16 3.01 2.99 2.98 2.98 2.96 2.96 2.86 2.85 2.84 2.83 2.79 2.79 2.78 2.78 2.78 2.77 2.77 2.76 2.43 2.32 2.30 2.30 2.28 2.28 2.26 2.26 2.19 2.18 2.18 2.16 2.16 2.10 2.09 2.08 2.06
S-I-closed-10
1.06 1.09 2.09 1.00 0.91 3.26 2.04 1.04 3.61 0.84 0.94 3.28 3.20 0.93 0.91 0.96
f1 (ppm)

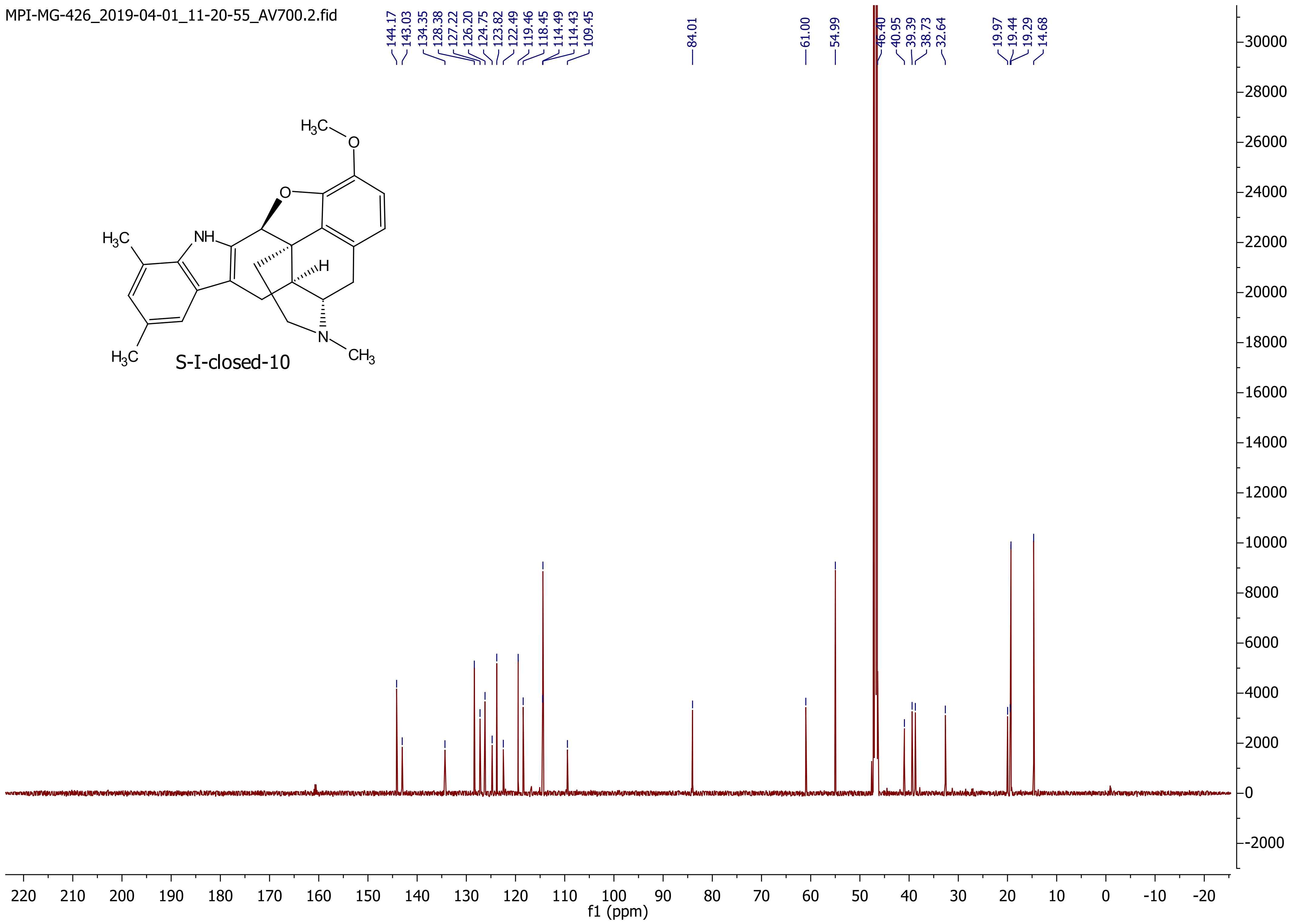

MPI-MG-426_2019-04-01_11-20-55_AV700.2.fid
144.17
143.03
134.35
128.38
127.22
126.20
124.75
123.82
122.49
119.46
118.45
114.49
114.43
109.45
84.01
61.00
54.99
46.40
40.95
39.39
38.73
32.64
19.97
19.44
19.29
14.68
S-I-closed-10
f1 (ppm)

MPI-MG-429_2019-02-08_06-36-59_AV700.1.fid

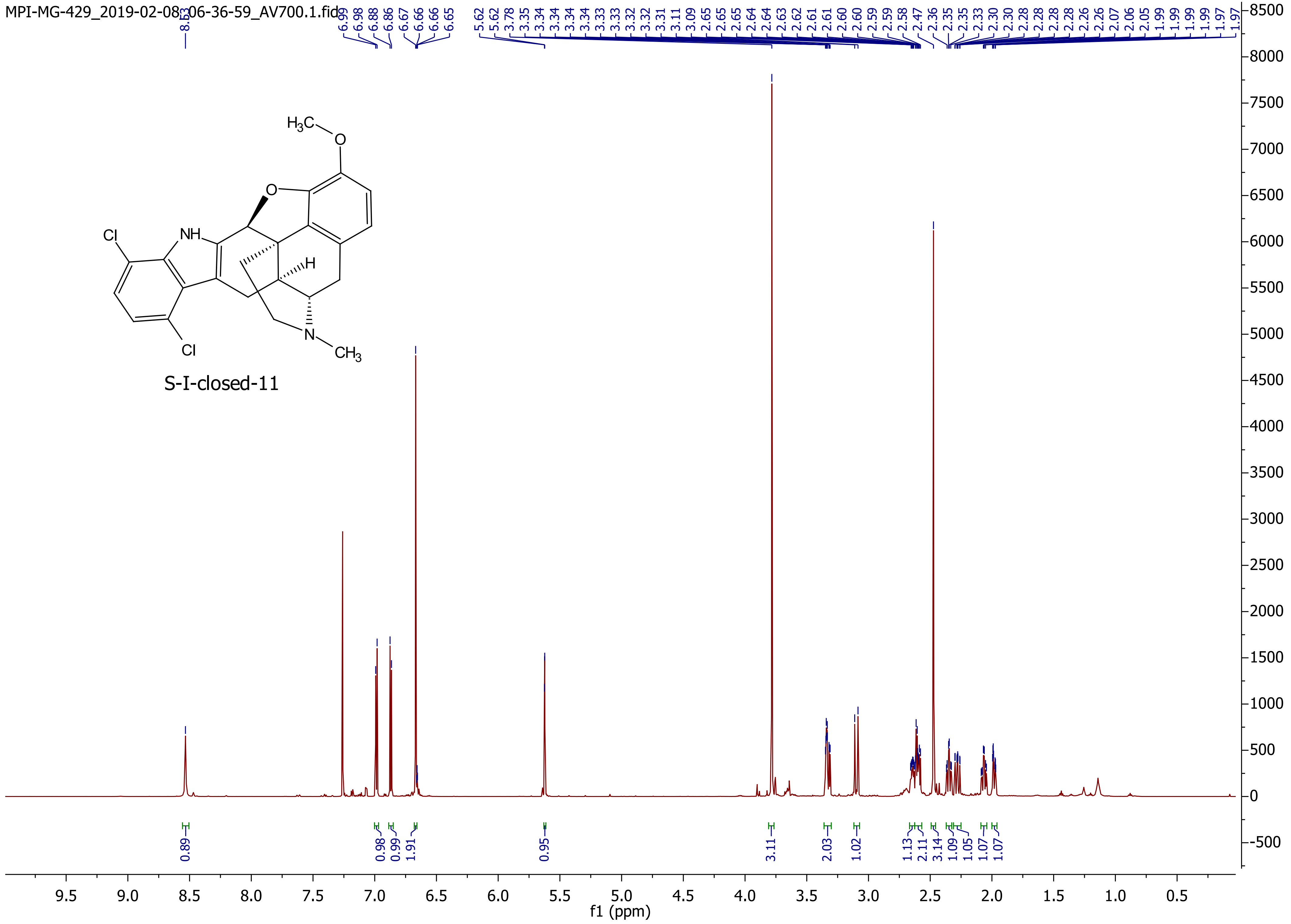

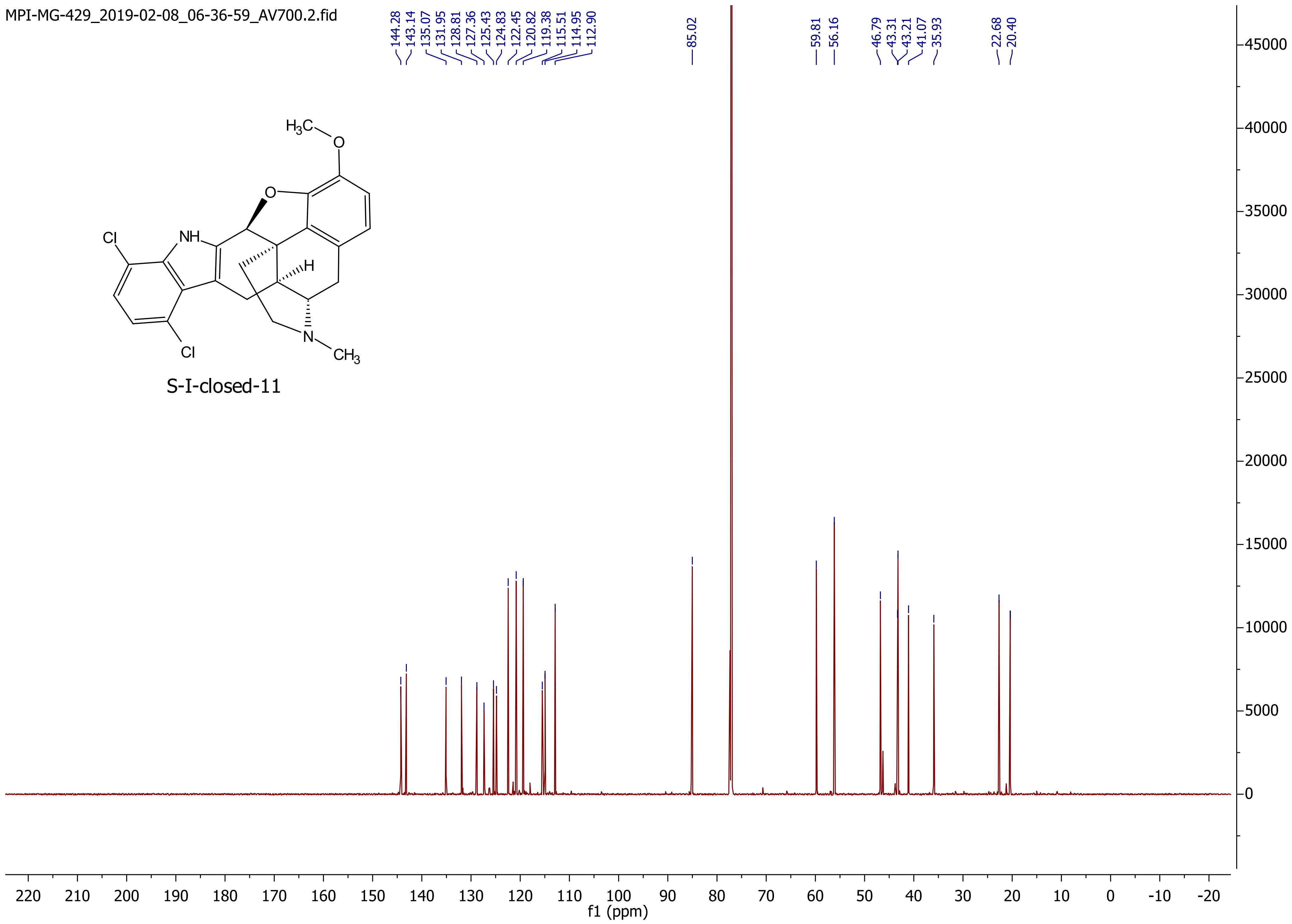

MPI-MG-429_2019-02-08_06-36-59_AV700.2.fid
144.28
143.14
135.07
131.95
128.81
127.36
125.43
124.83
122.45
120.82
119.38
115.51
114.95
112.90
85.02
59.81
56.16
46.79
43.31
43.21
41.07
35.93
22.68
20.40
S-I-closed-11
f1 (ppm)

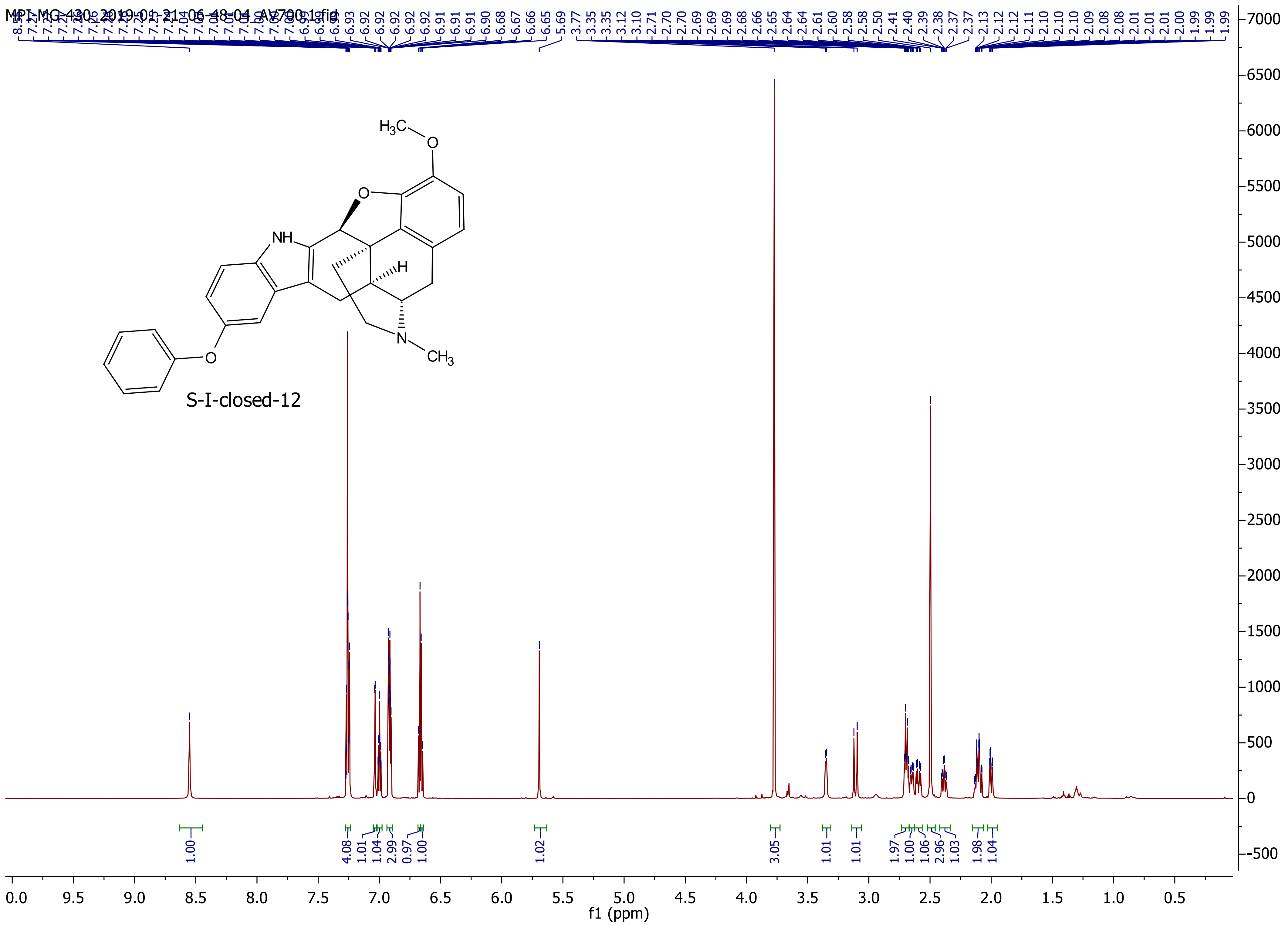
MPI-MG-430_2019-01-21_06-48-04_AV700 1.fid
S-I-closed-12
f1 (ppm)

MPI-MG-430_2019-01-21_06-48-04_AV700.2.fid

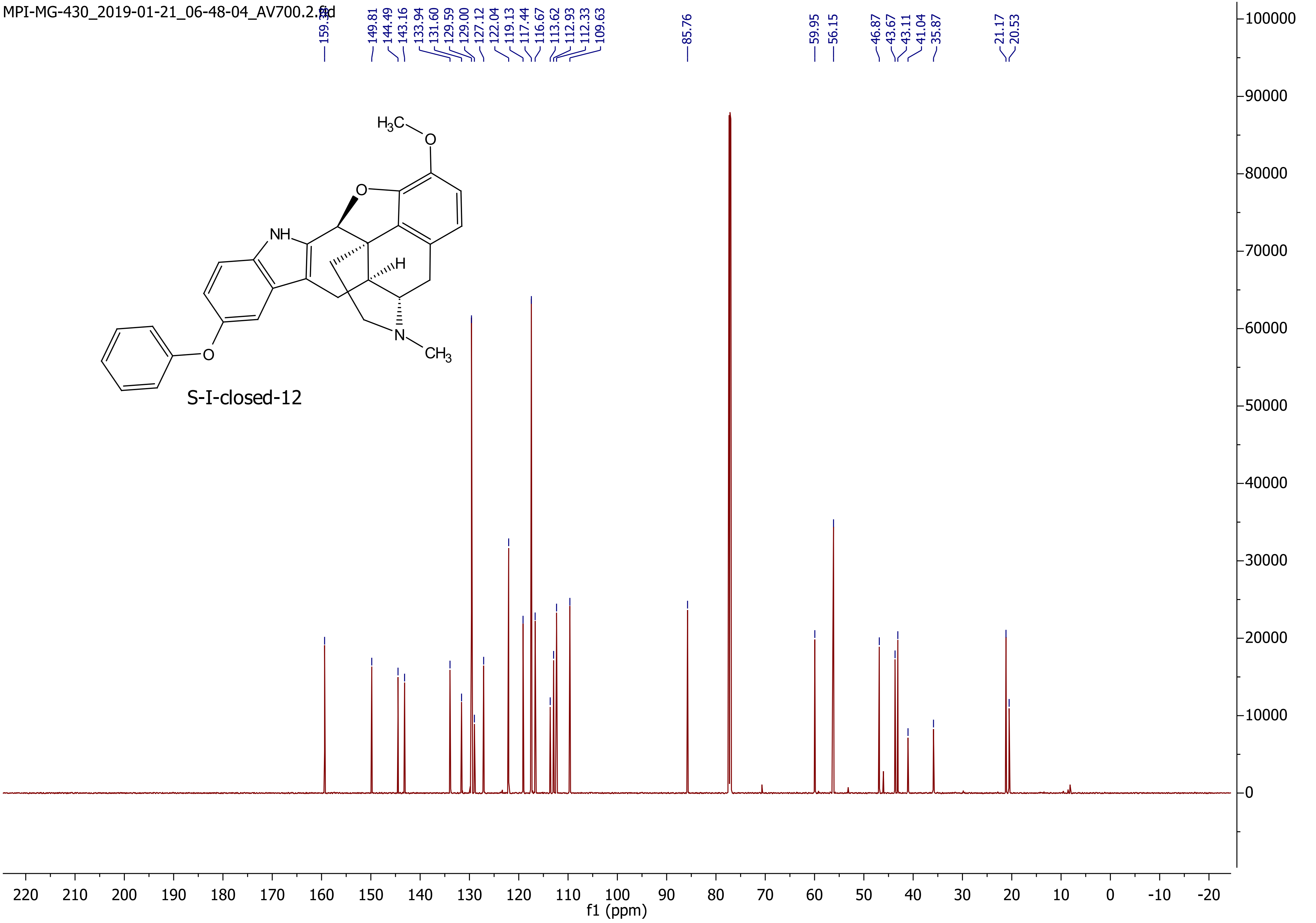

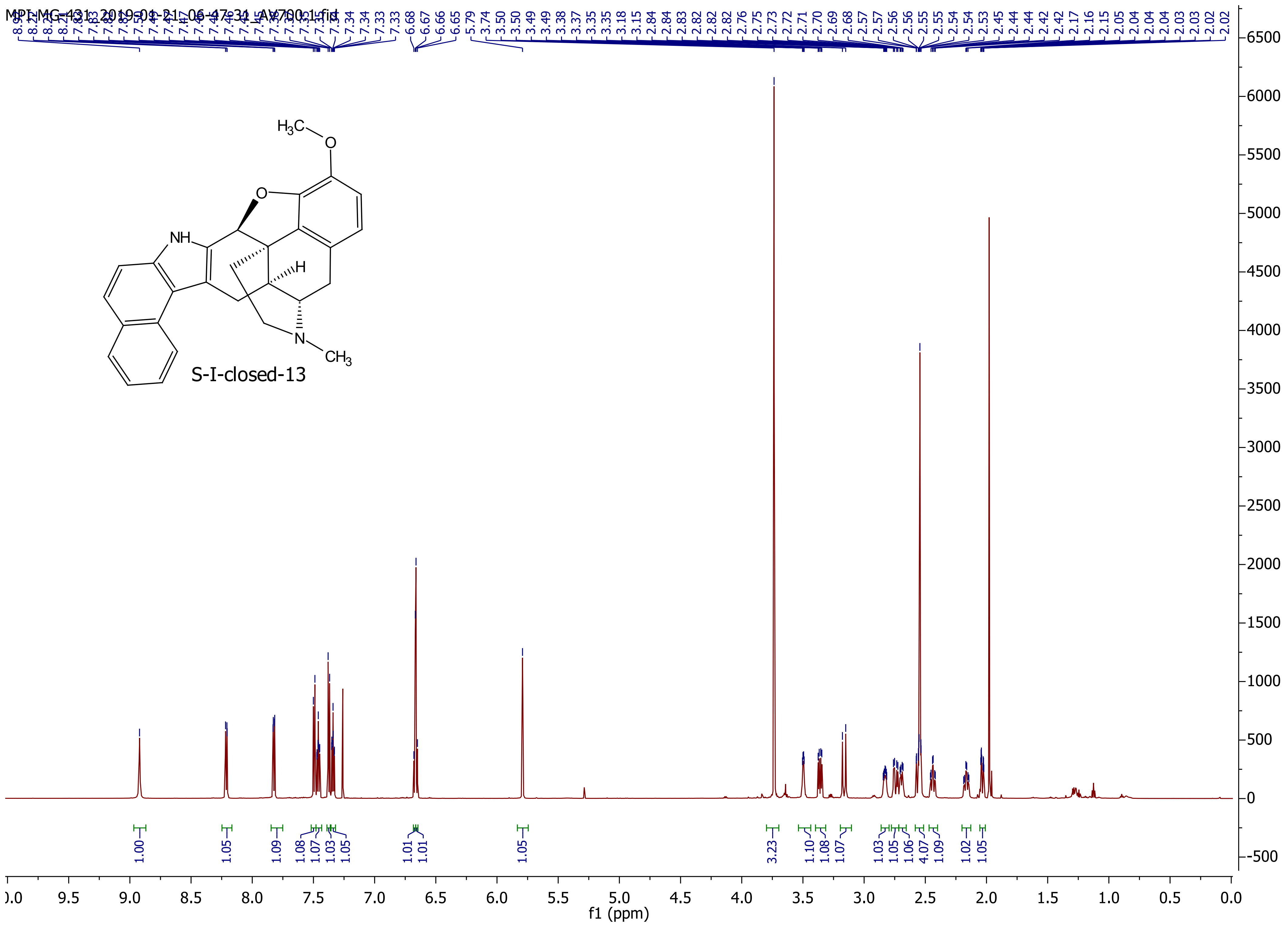

MPI-MG-431_2019-01-21_06-47-31_AV700-1.fid
7.34 7.34 7.33 7.33 6.68 6.67 6.66 6.65 5.79 3.74 3.50 3.50 3.49 3.38 3.37 3.35 3.35 3.18 3.15 2.84 2.84 2.83 2.82 2.82 2.82 2.76 2.75 2.73 2.72 2.71 2.70 2.69 2.68 2.57 2.57 2.56 2.56 2.55 2.55 2.54 2.54 2.53 2.45 2.44 2.44 2.42 2.42 2.17 2.16 2.15 2.05 2.04 2.04 2.04 2.03 2.03 2.02 2.02
H3C
O
NH
H
N
CH3
S-I-closed-13
1.00 1.05 1.09 1.08 1.07 1.03 1.05 1.01 1.01 1.05 3.23 1.10 1.08 1.07 1.03 1.05 1.06 4.07 1.09 1.02 1.05
f1 (ppm)

MPI-MG-431_2019-01-21_06-47-31_AV700.2.fid

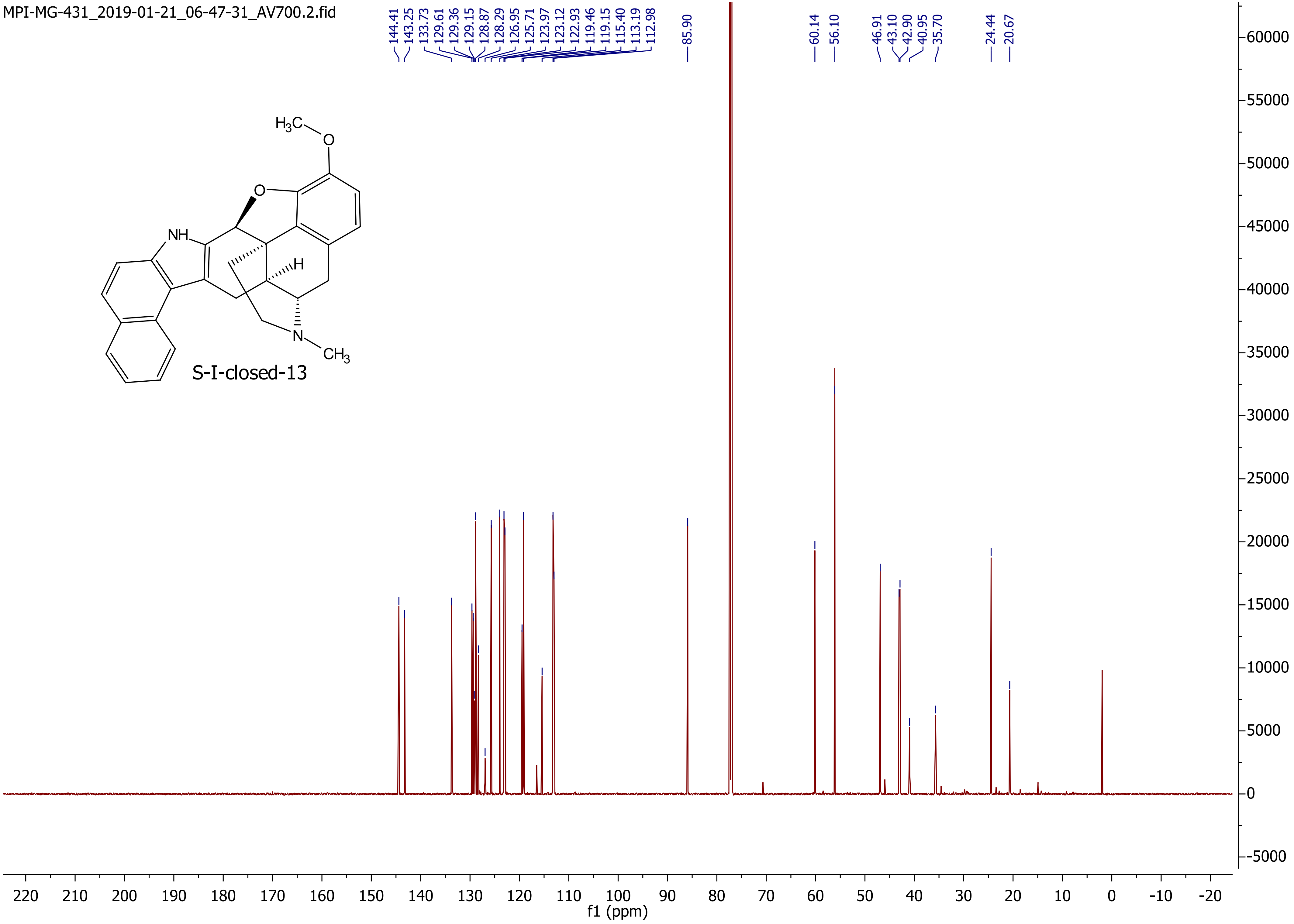

S-I-closed-13

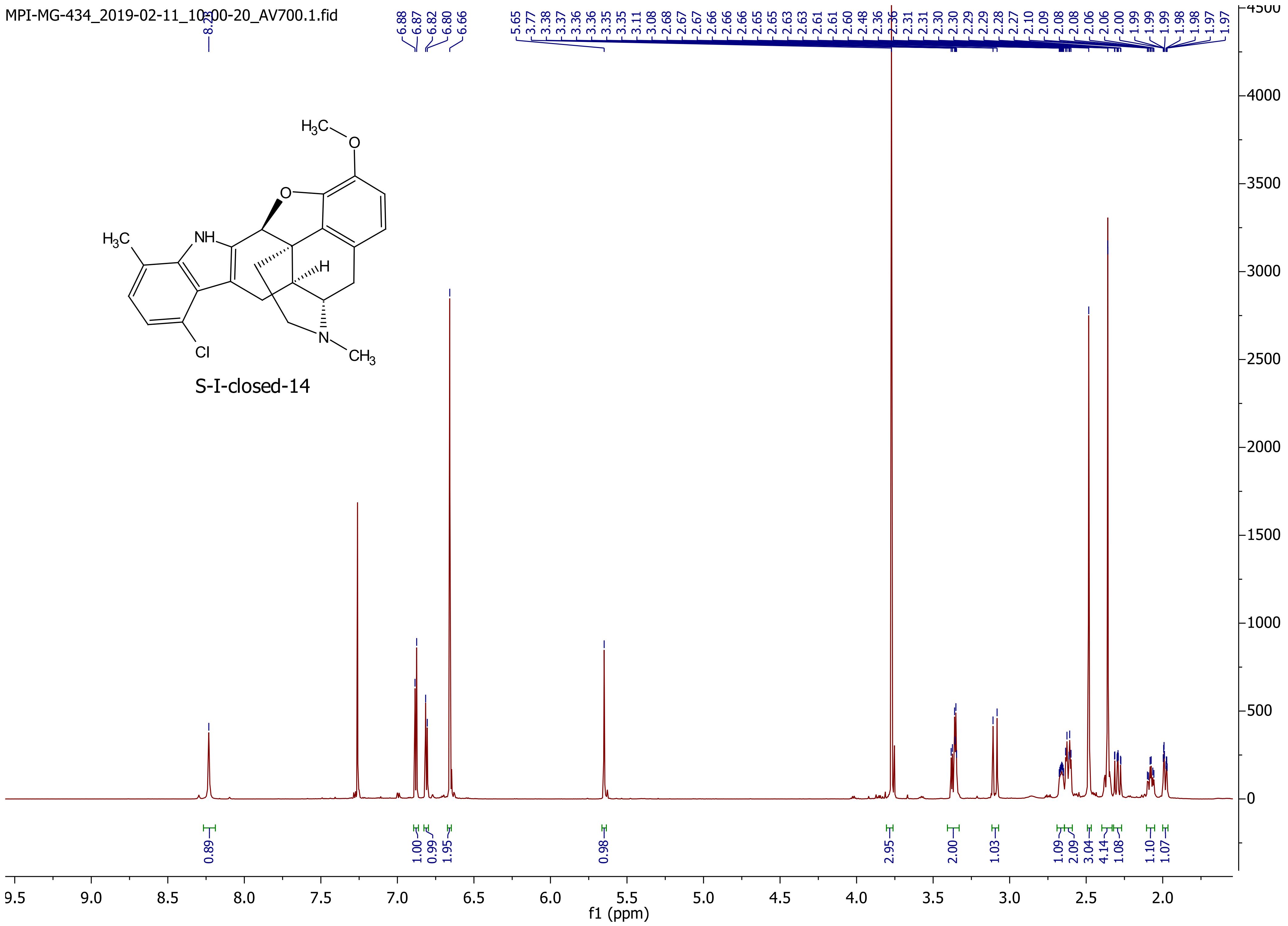
MPI-MG-434_2019-02-11_10-00-20_AV700.1.fid
S-I-closed-14
f1 (ppm)

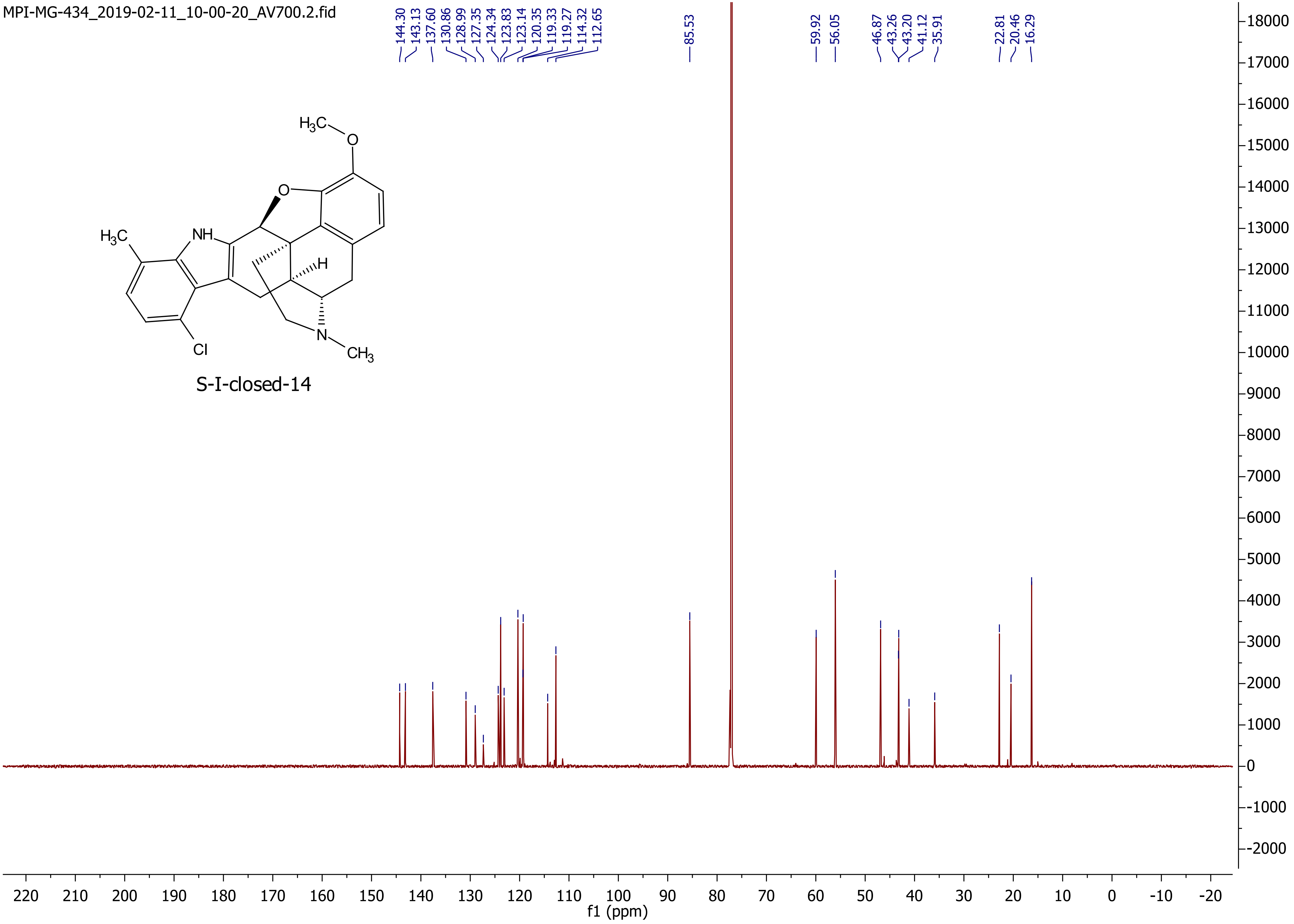
MPI-MG-434_2019-02-11_10-00-20_AV700.2.fid
144.30
143.13
137.60
130.86
128.99
127.35
124.34
123.83
123.14
120.35
119.33
119.27
114.32
112.65
85.53
59.92
56.05
46.87
43.26
43.20
41.12
35.91
22.81
20.46
16.29
H3C
O
NH
H
N
CH3
Cl
f1 (ppm)

S-I-closed-14

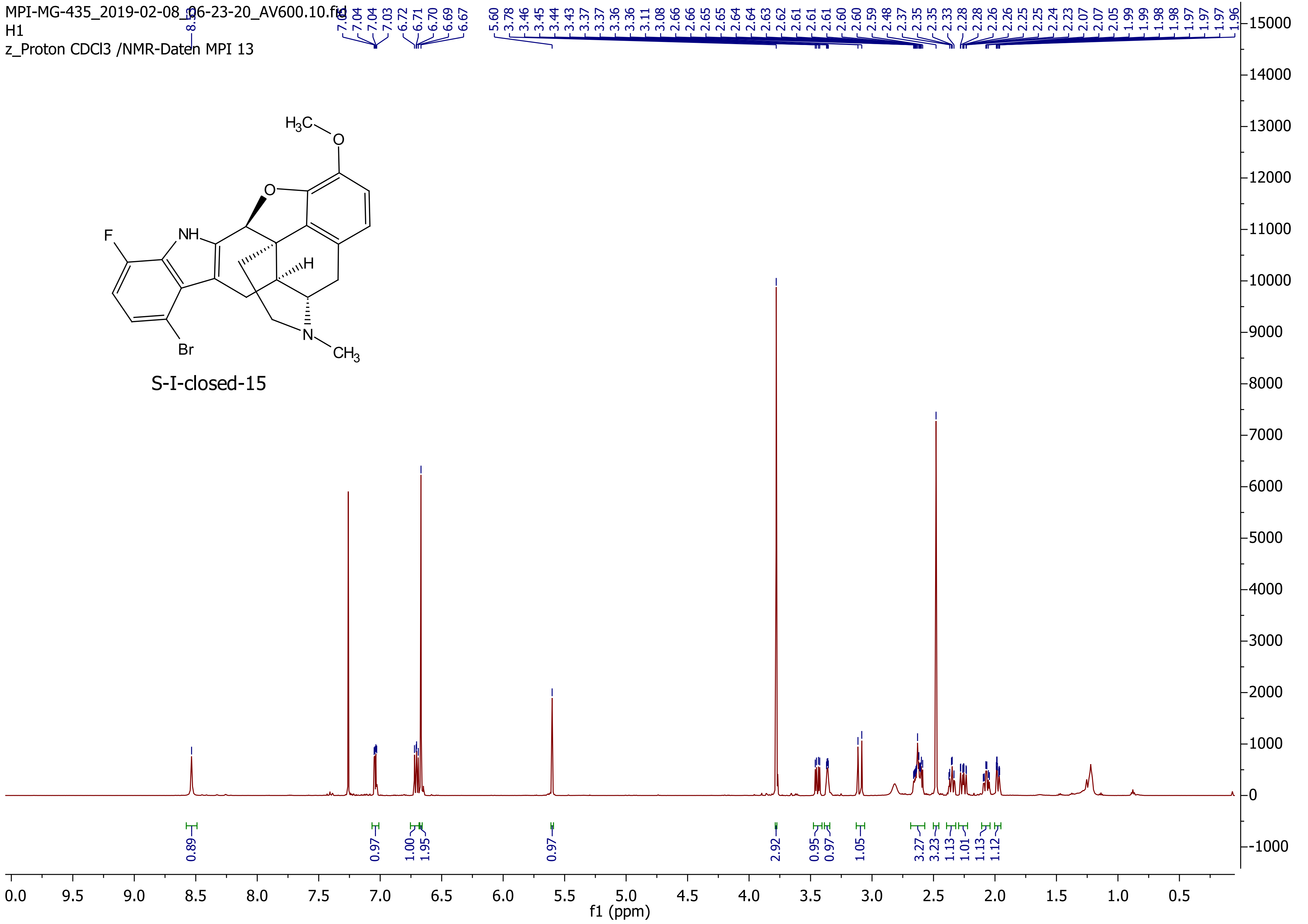
MPI-MG-435_2019-02-08_06-23-20_AV600.10.fid
H1
z_Proton CDCl3 /NMR-Daten MPI 13
7.04
7.04
7.03
6.72
6.71
6.70
6.69
6.67
5.60
3.78
3.46
3.45
3.44
3.43
3.37
3.37
3.36
3.36
3.11
3.08
2.66
2.66
2.65
2.65
2.64
2.64
2.63
2.62
2.61
2.61
2.61
2.60
2.60
2.59
2.48
2.37
2.35
2.35
2.33
2.28
2.28
2.26
2.26
2.25
2.25
2.24
2.23
2.07
2.07
2.05
1.99
1.99
1.98
1.98
1.97
1.97
1.97
1.96
H3C
O
NH
F
H
N
Br
CH3
S-I-closed-15
0.89
0.97
1.00
1.95
0.97
2.92
0.95
0.97
1.05
3.27
3.23
1.13
1.01
1.13
1.12
f1 (ppm)

MPI-MG-435_2019-02-08_06-23-20_AV600.11.fid
C13 with power gated H1 decoupling
z_C13pg CDCl3 /NMR-Daten MPI 13

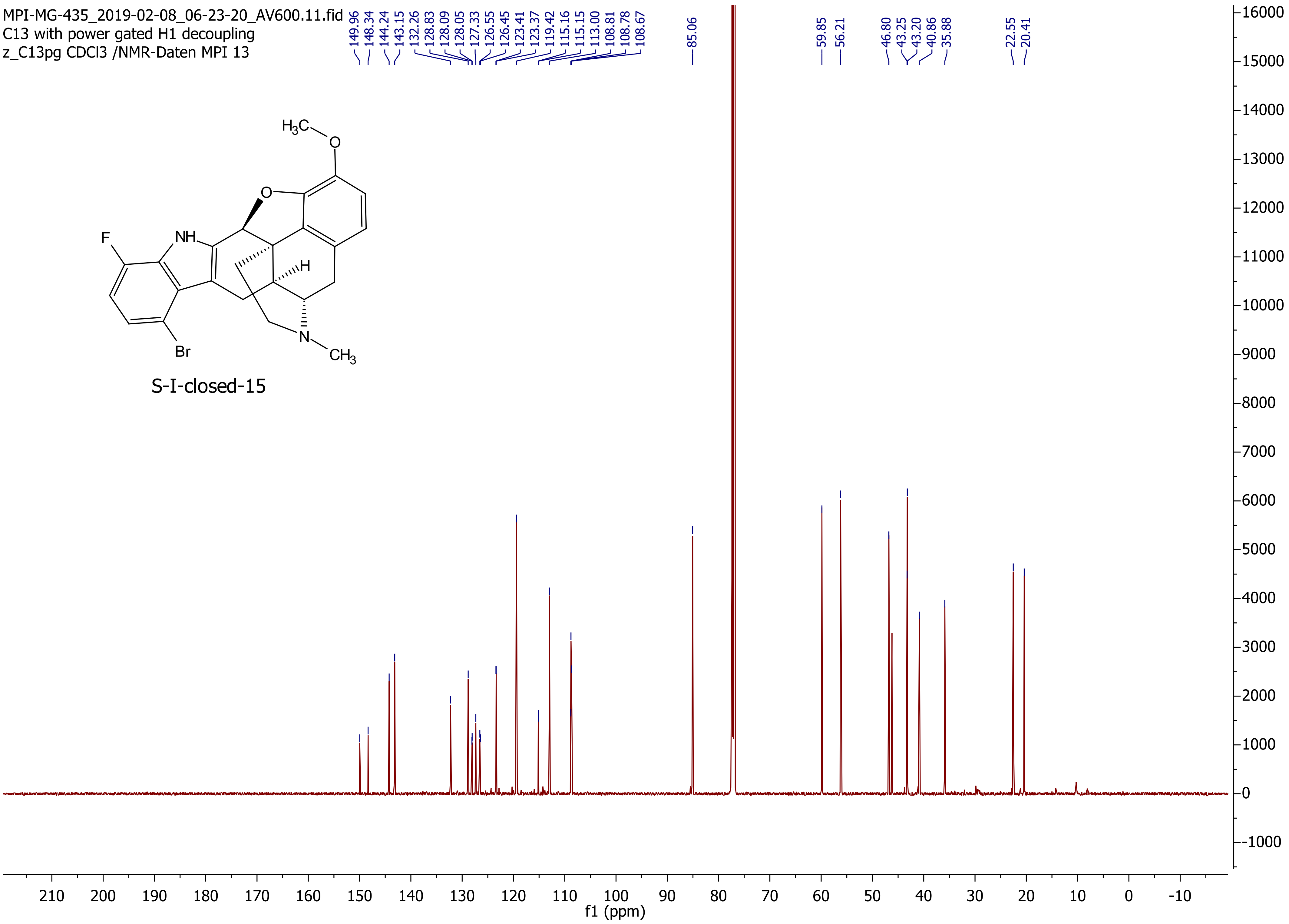

S-I-closed-15

MPI-MG-435_2019-02-08_06-23-20_AV600.12.fid
F19

z_F19 CDCl3 /NMR-Daten MPI 13

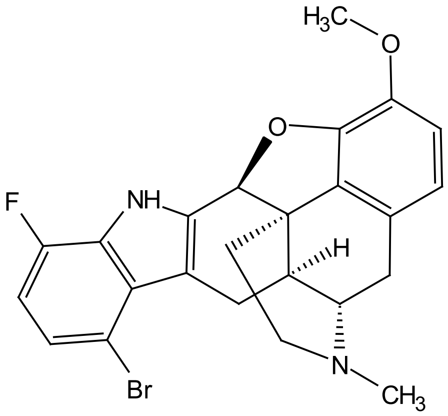

S-I-closed-15

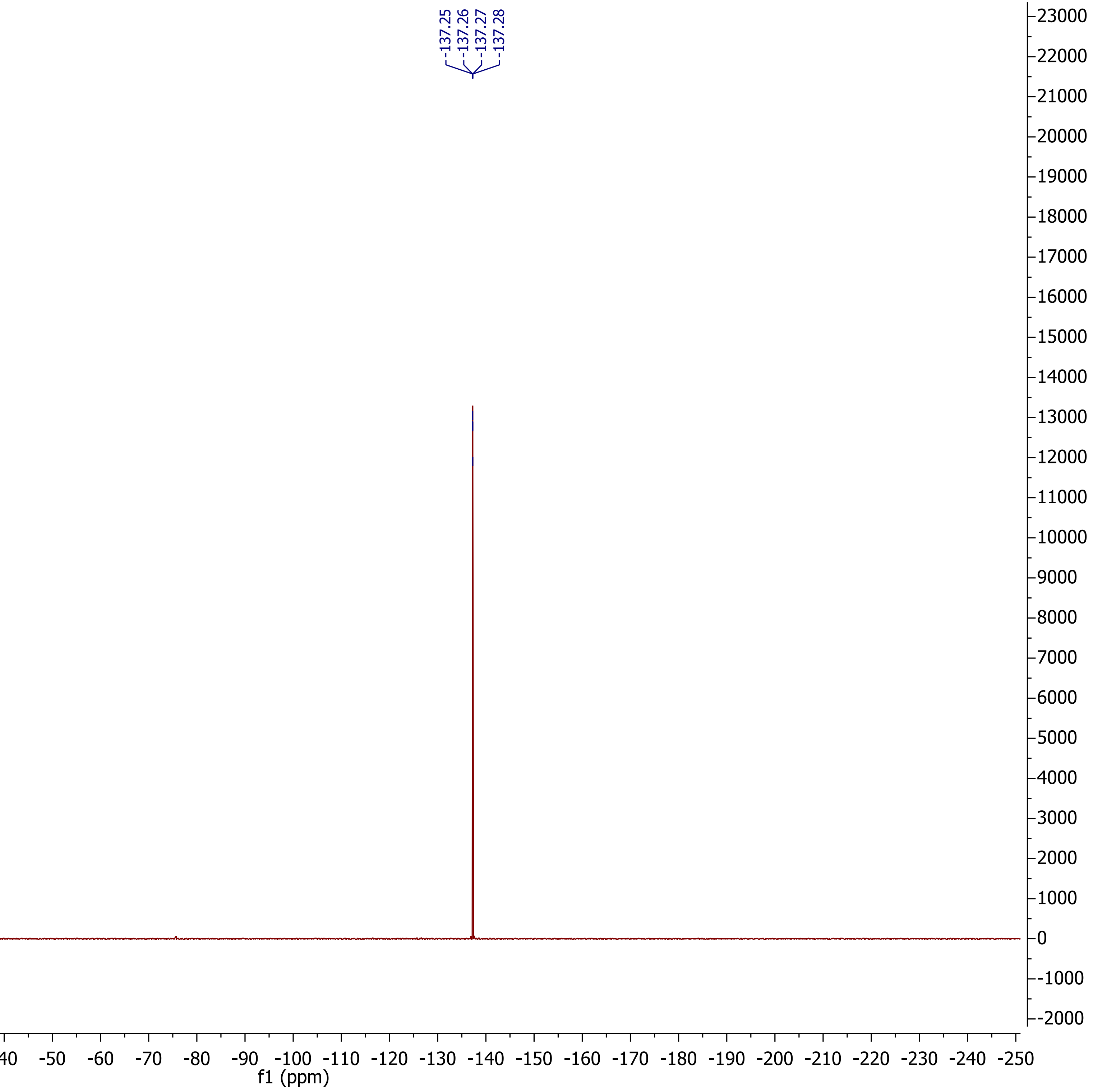

MPI-MG-436_2019-02-27_13-57-45_AV600.10.fid
H1
z_Proton CDCl3 /NMR-Daten MPI 40

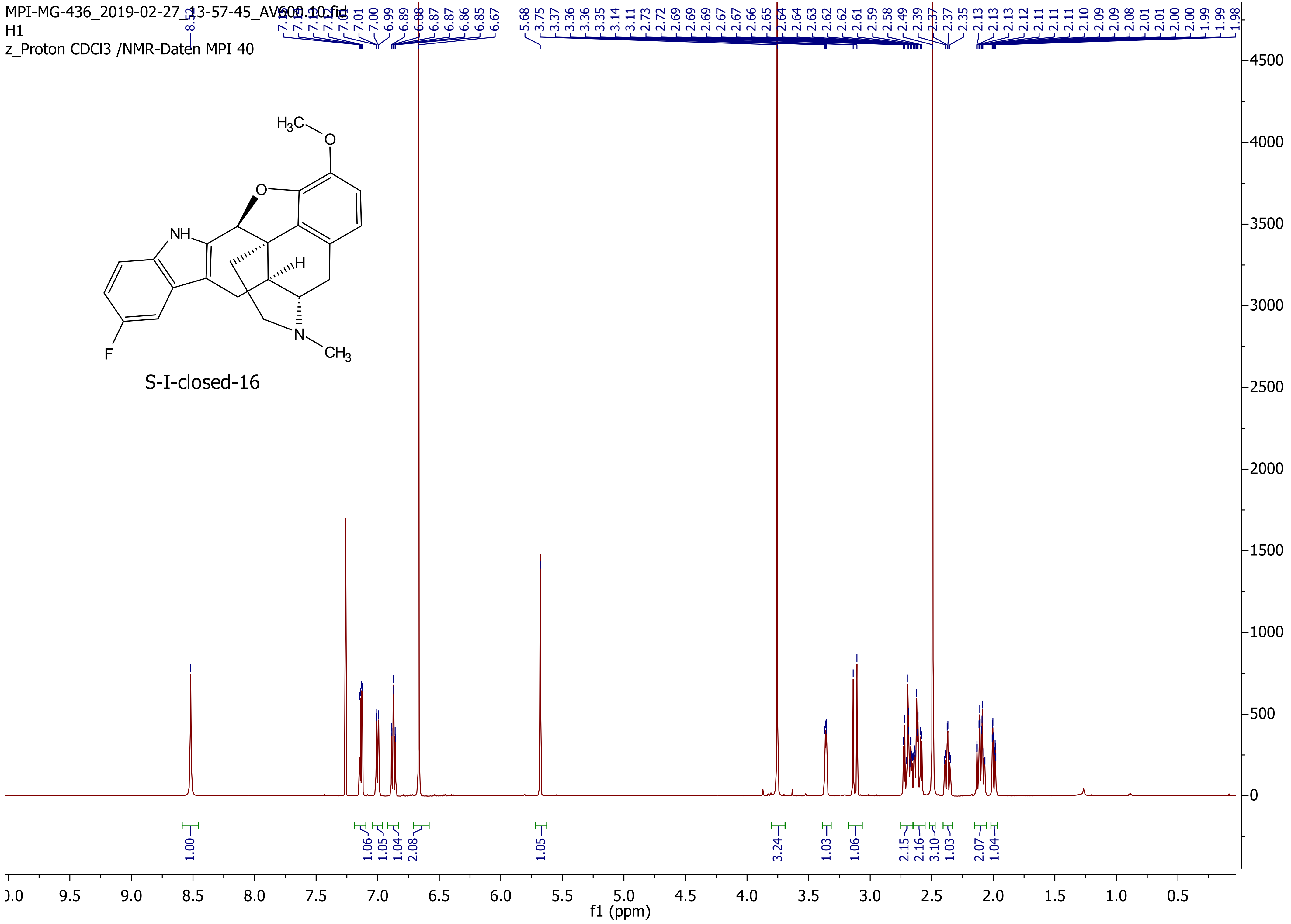

S-I-closed-16

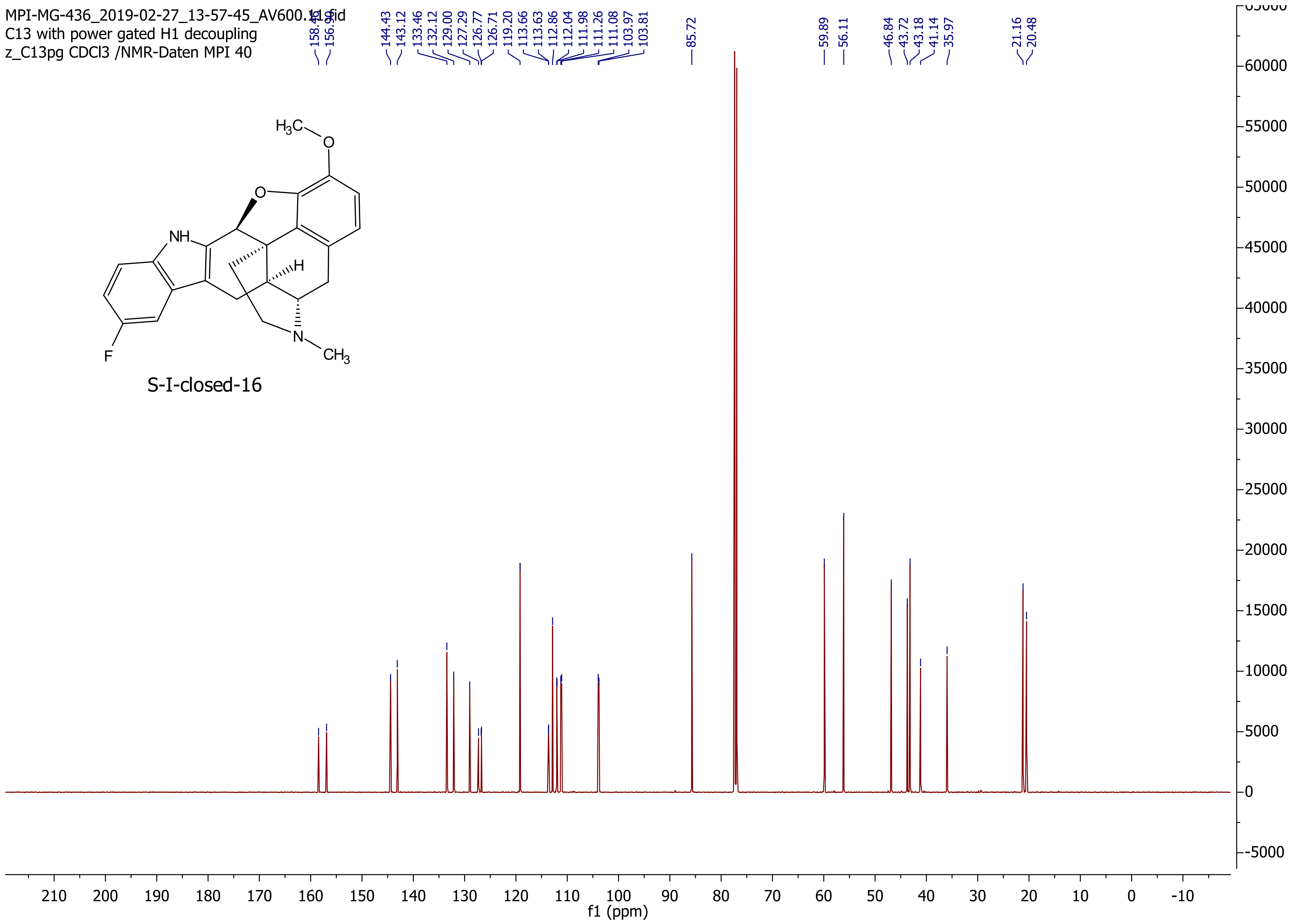

MPI-MG-436_2019-02-27_13-57-45_AV600.11.fid
C13 with power gated H1 decoupling
z_C13pg CDCl3 /NMR-Daten MPI 40
158.41
156.91
144.43
143.12
133.46
132.12
129.00
127.29
126.77
126.71
119.20
113.66
113.63
112.86
112.04
111.98
111.26
111.08
103.97
103.81
85.72
59.89
56.11
46.84
43.72
43.18
41.14
35.97
21.16
20.48
H3C
O
O
NH
H
N
CH3
F
S-I-closed-16
f1 (ppm)

MPI-MG-436_2019-02-27_13-57-45_AV600.12.fid
F19

z_F19 CDCl3 /NMR-Daten MPI 40

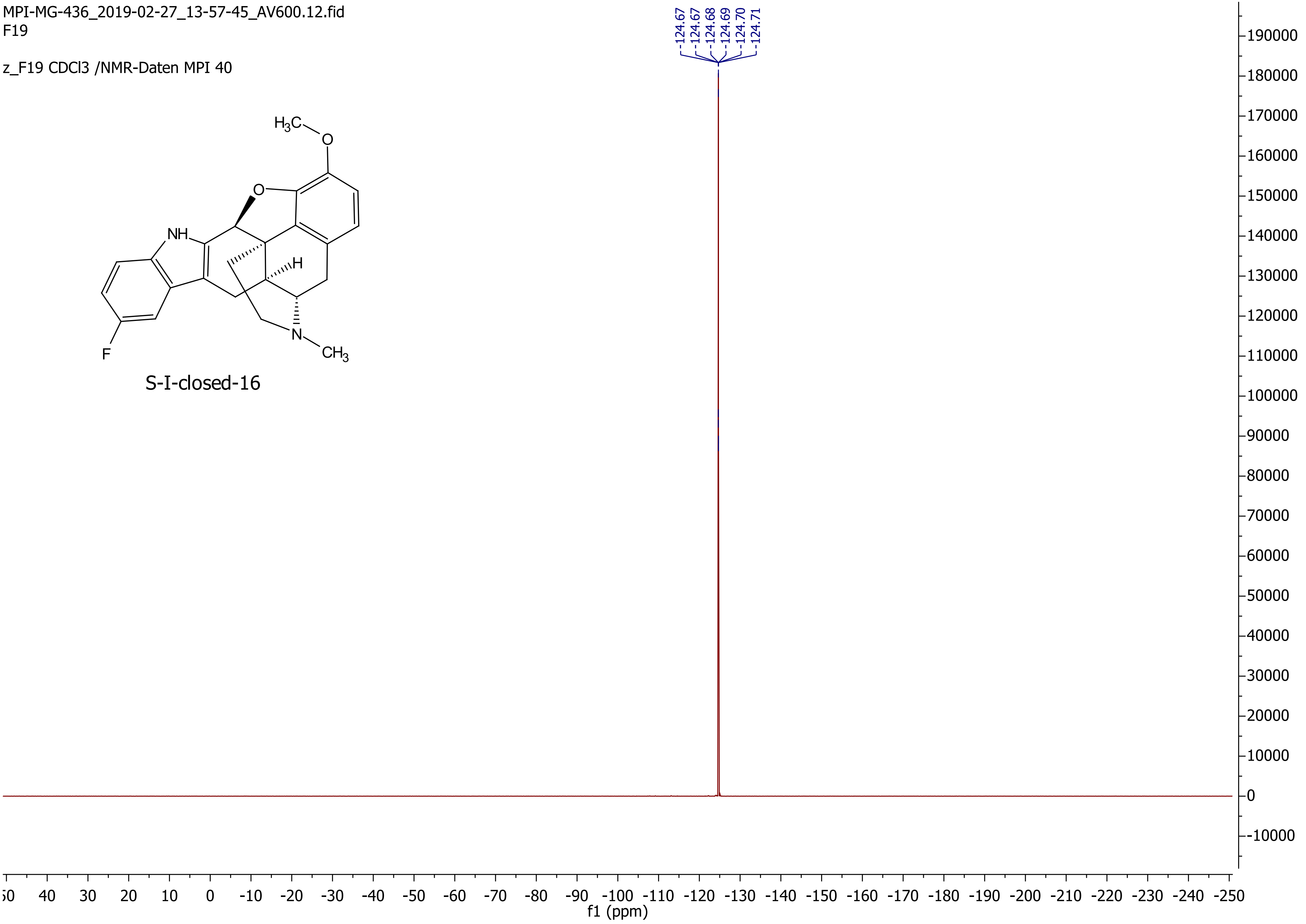

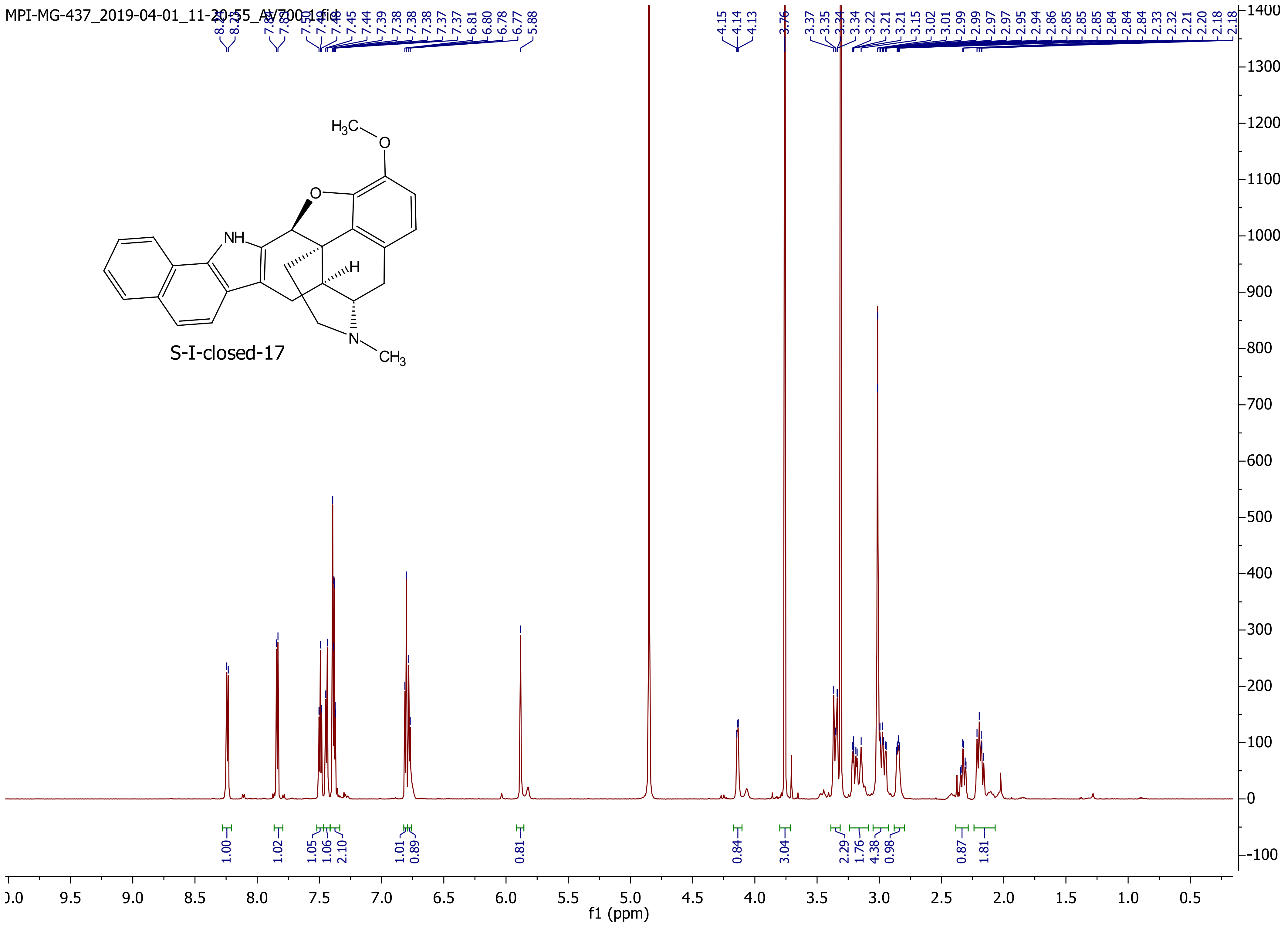
MPI-MG-437_2019-04-01_11-20-55_AV700_1.fid
S-I-closed-17
f1 (ppm)

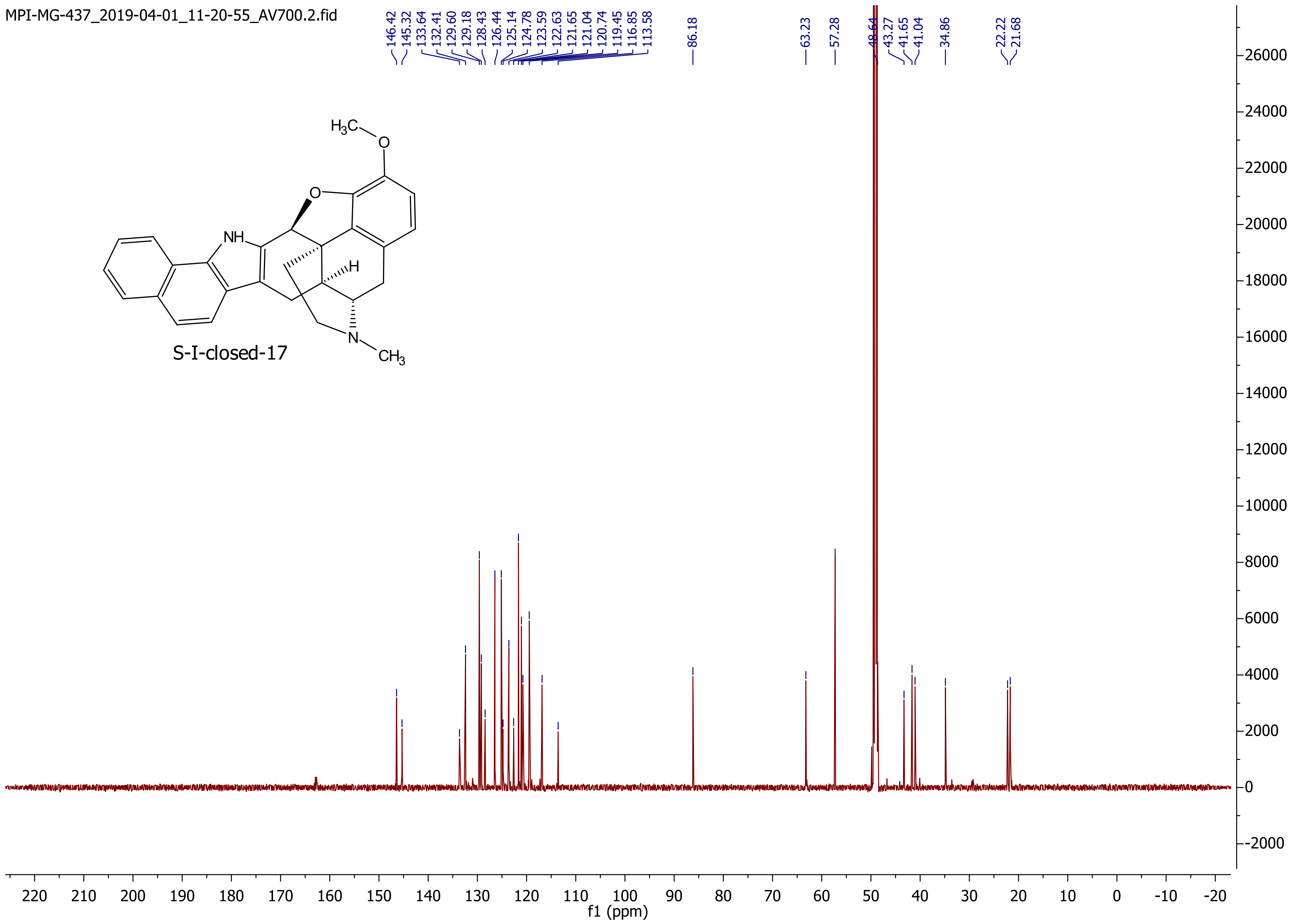

MPI-MG-437_2019-04-01_11-20-55_AV700.2.fid
146.42
145.32
133.64
132.41
129.60
129.18
128.43
126.44
125.14
124.78
123.59
122.63
121.65
121.04
120.74
119.45
116.85
113.58
86.18
63.23
57.28
48.64
43.27
41.65
41.04
34.86
22.22
21.68
S-I-closed-17
f1 (ppm)

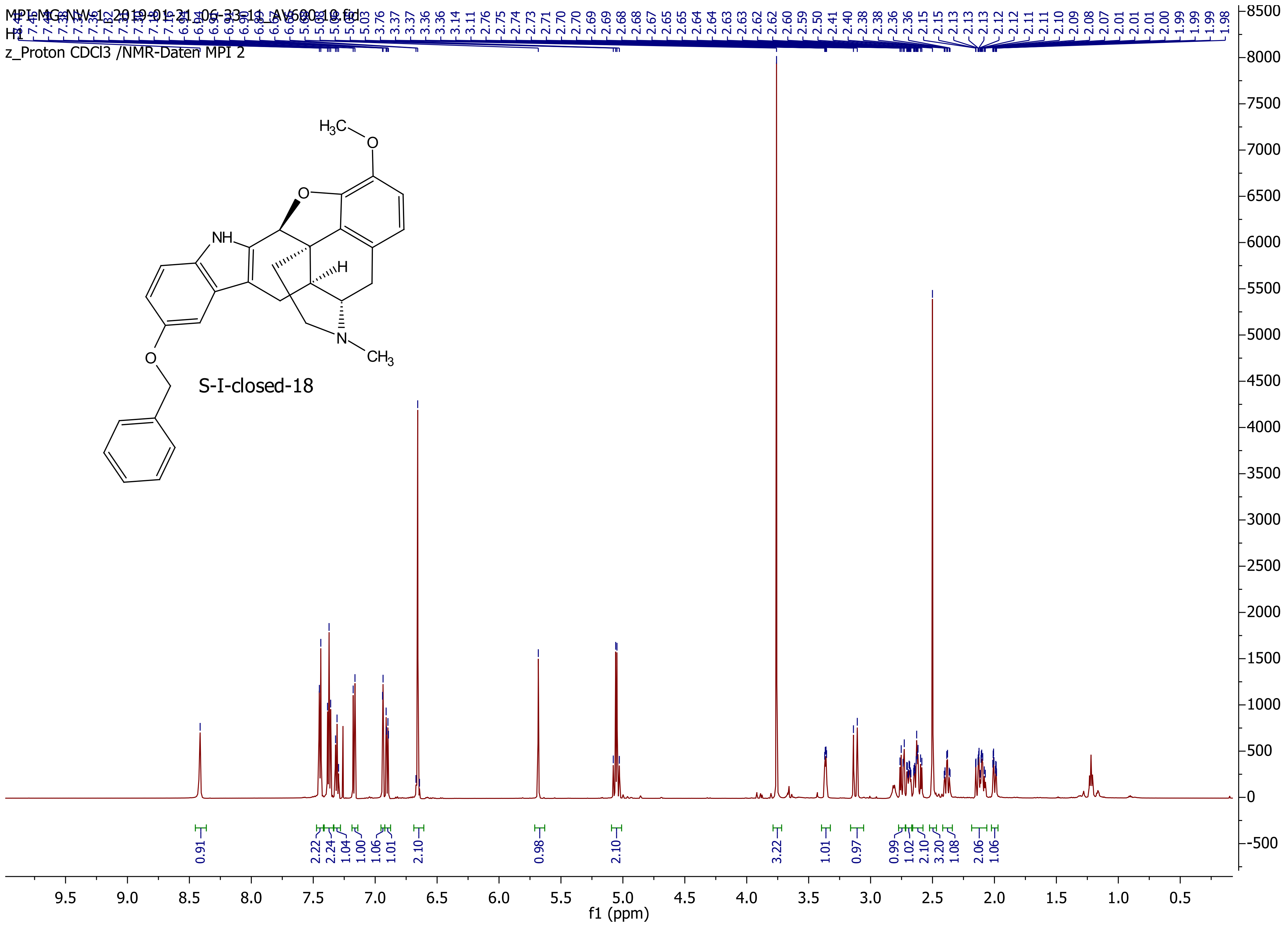
MPI-MG-NW-1_2019-01-21_06-33-11_AV600.19.fid
H1
z_Proton CDCl3 /NMR-Daten MPI 2
S-I-closed-18
f1 (ppm)

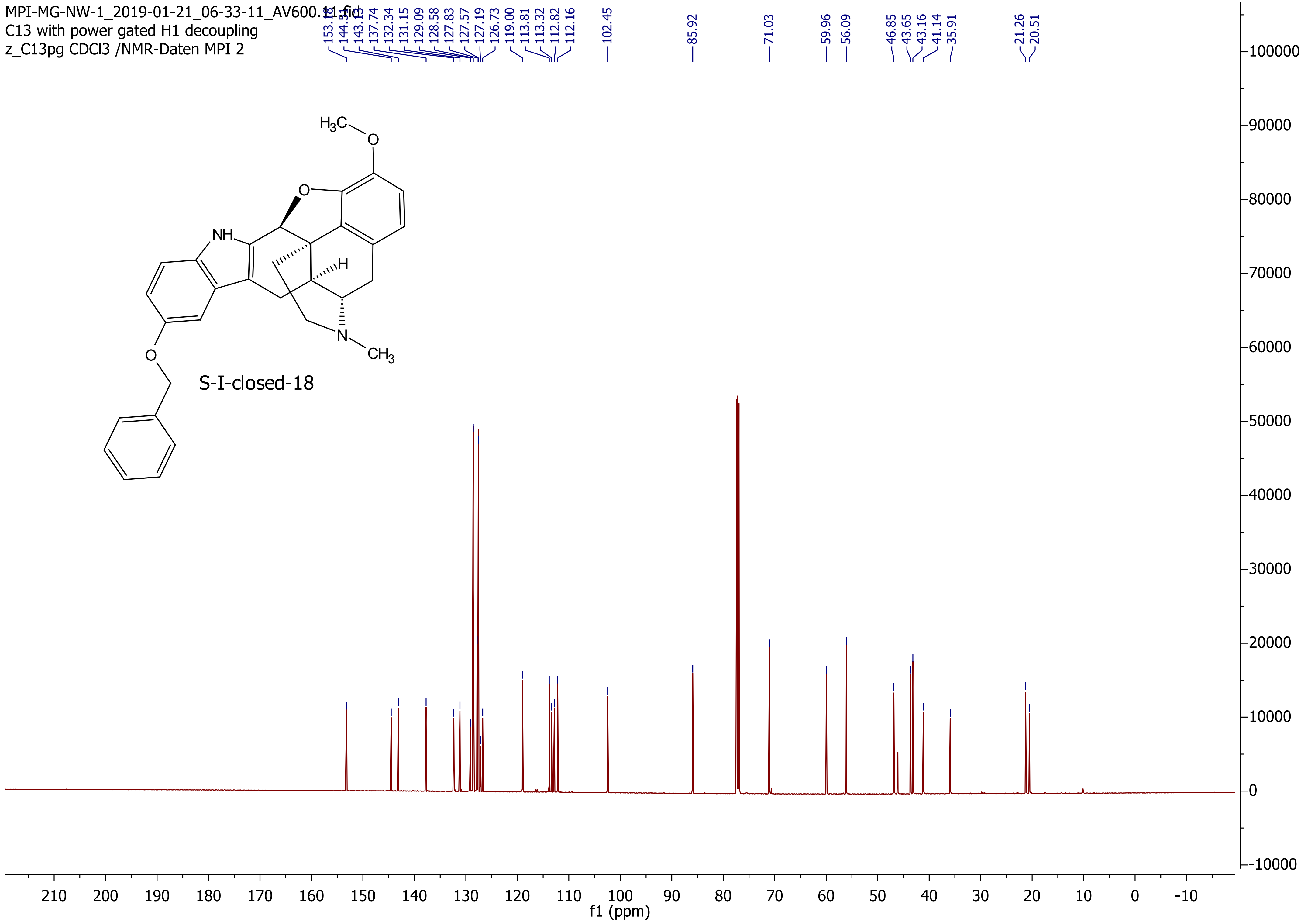

MPI-MG-NW-1_2019-01-21_06-33-11_AV600.11.fid
C13 with power gated H1 decoupling
z_C13pg CDCl3 /NMR-Daten MPI 2
153.18
144.51
143.15
137.74
132.34
131.15
129.09
128.58
127.83
127.57
127.19
126.73
119.00
113.81
113.32
112.82
112.16
102.45
85.92
71.03
59.96
56.09
46.85
43.65
43.16
41.14
35.91
21.26
20.51
H3C
O
O
NH
H
N
CH3
O
S-I-closed-18
f1 (ppm)

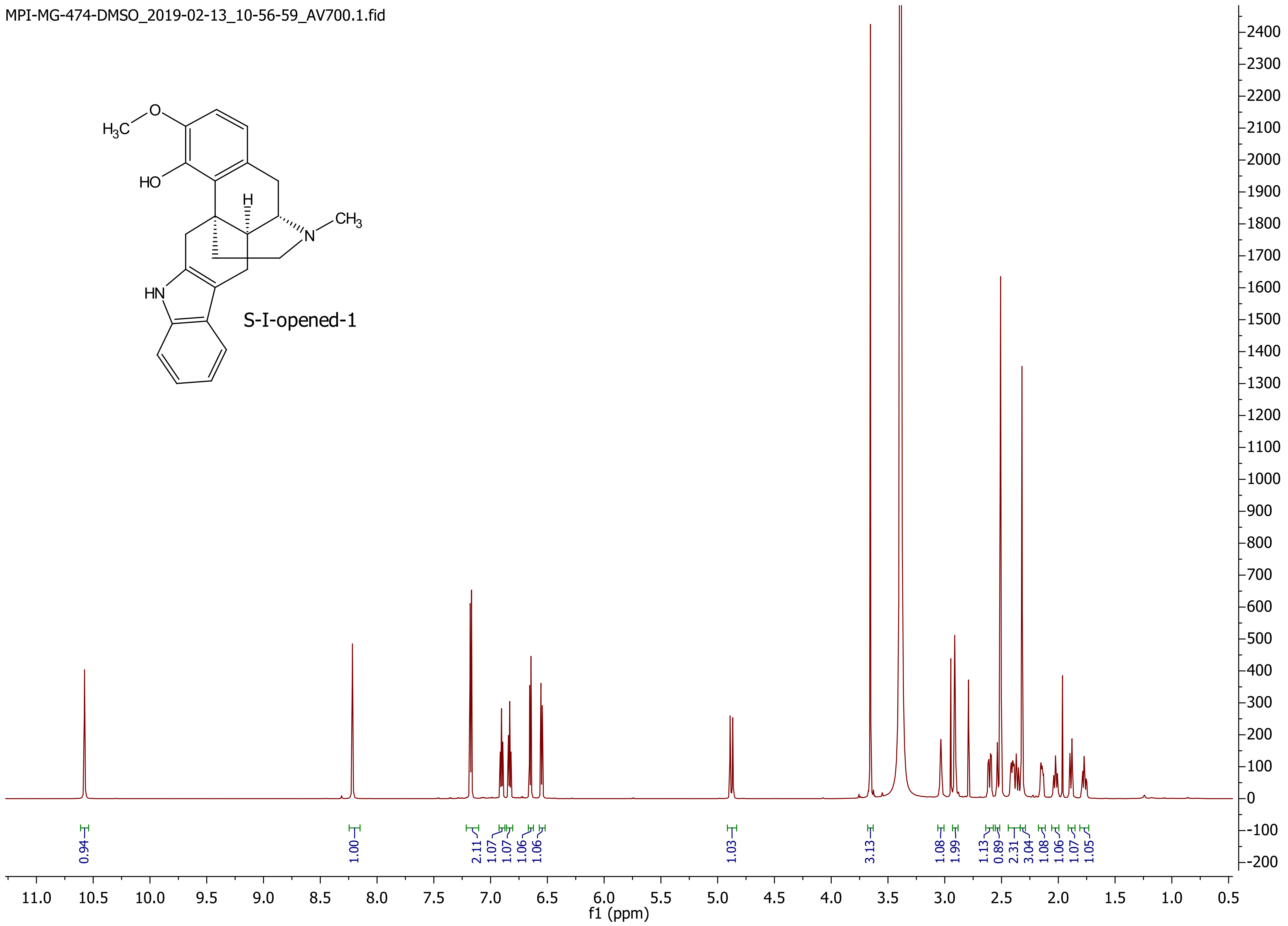
MPI-MG-474-DMSO_2019-02-13_10-56-59_AV700.1.fid
H3C
O
HO
H
CH3
N
HN
S-I-opened-1
0.94
1.00
2.11
1.07
1.07
1.06
1.06
1.03
3.13
1.08
1.99
1.13
0.89
2.31
3.04
1.08
1.06
1.07
1.05
f1 (ppm)

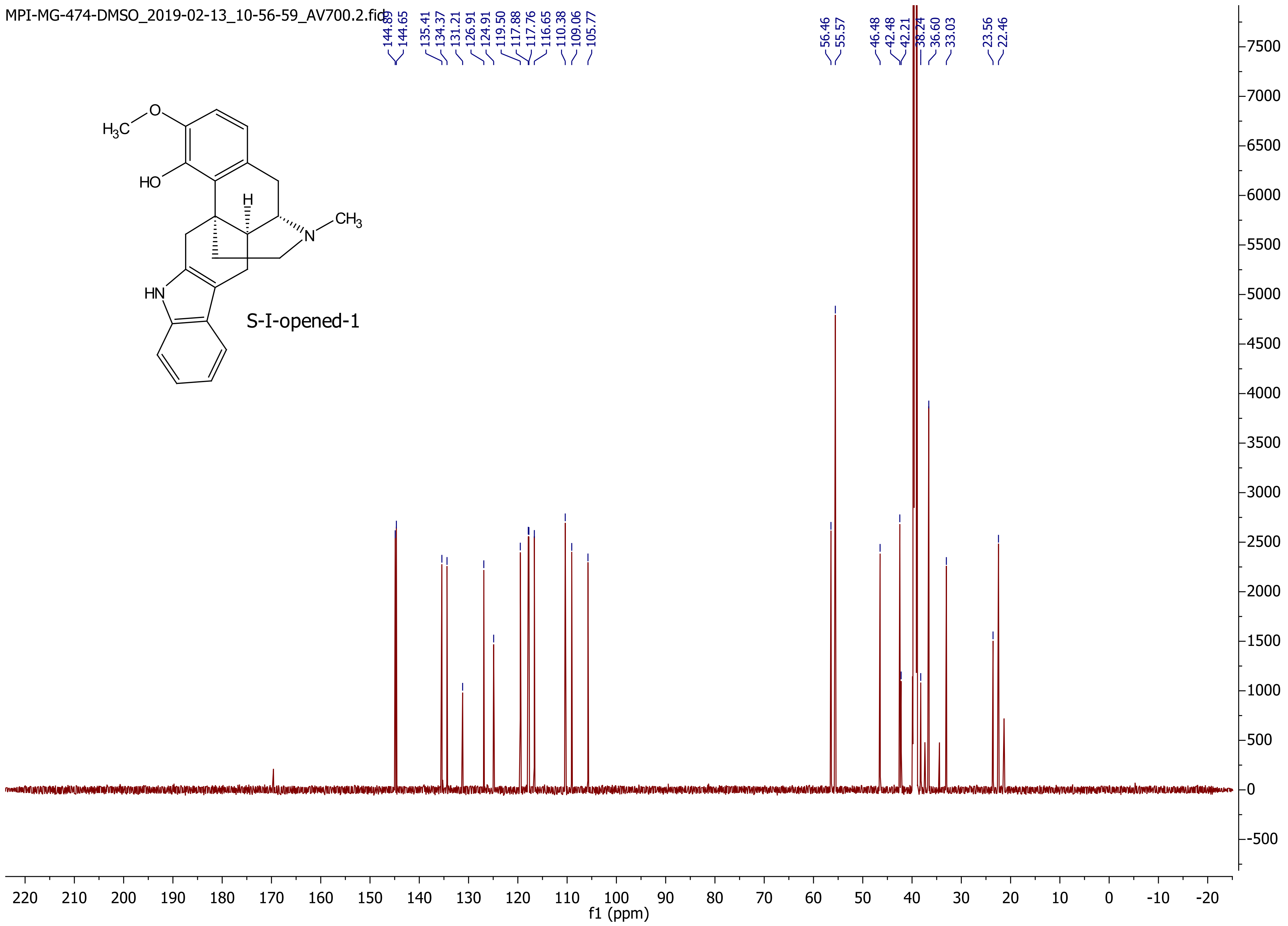

MPI-MG-474-DMSO_2019-02-13_10-56-59_AV700.2.fid
144.89
144.65
135.41
134.37
131.21
126.91
124.91
119.50
117.88
117.76
116.65
110.38
109.06
105.77
56.46
55.57
46.48
42.48
42.21
38.24
36.60
33.03
23.56
22.46
H3C
O
HO
H
N
CH3
HN
S-I-opened-1
f1 (ppm)

MG-47501
MG-475
STANDARD 1H OBSERVE

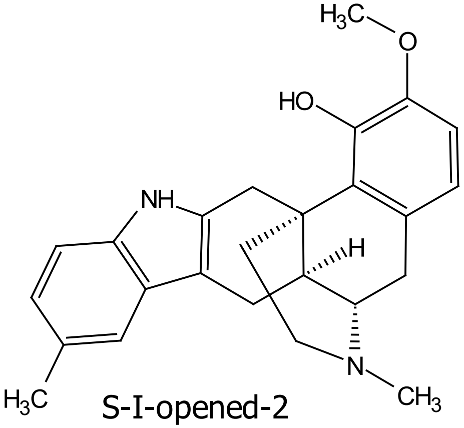

S-I-opened-2

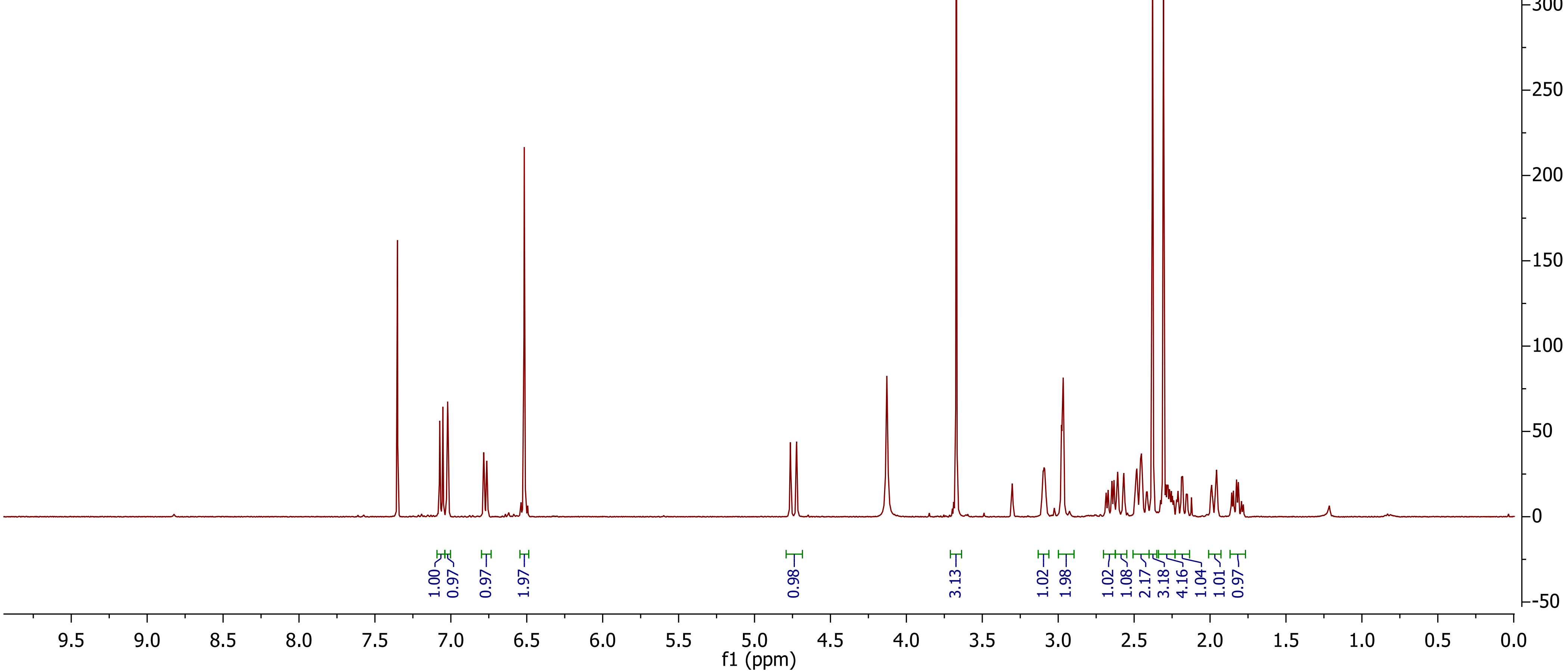

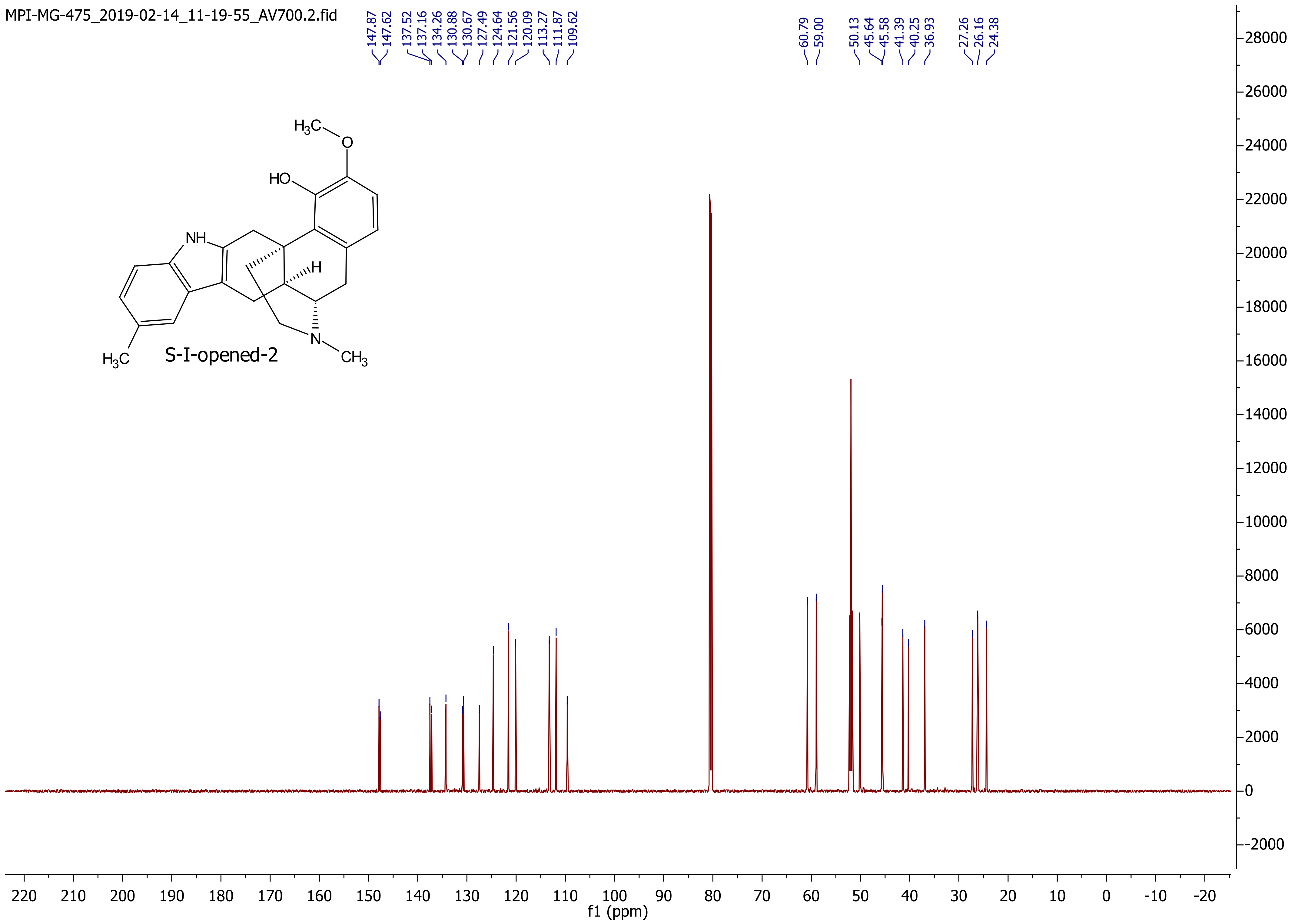
MPI-MG-475_2019-02-14_11-19-55_AV700.2.fid
147.87
147.62
137.52
137.16
134.26
130.88
130.67
127.49
124.64
121.56
120.09
113.27
111.87
109.62
60.79
59.00
50.13
45.64
45.58
41.39
40.25
36.93
27.26
26.16
24.38
S-I-opened-2
f1 (ppm)

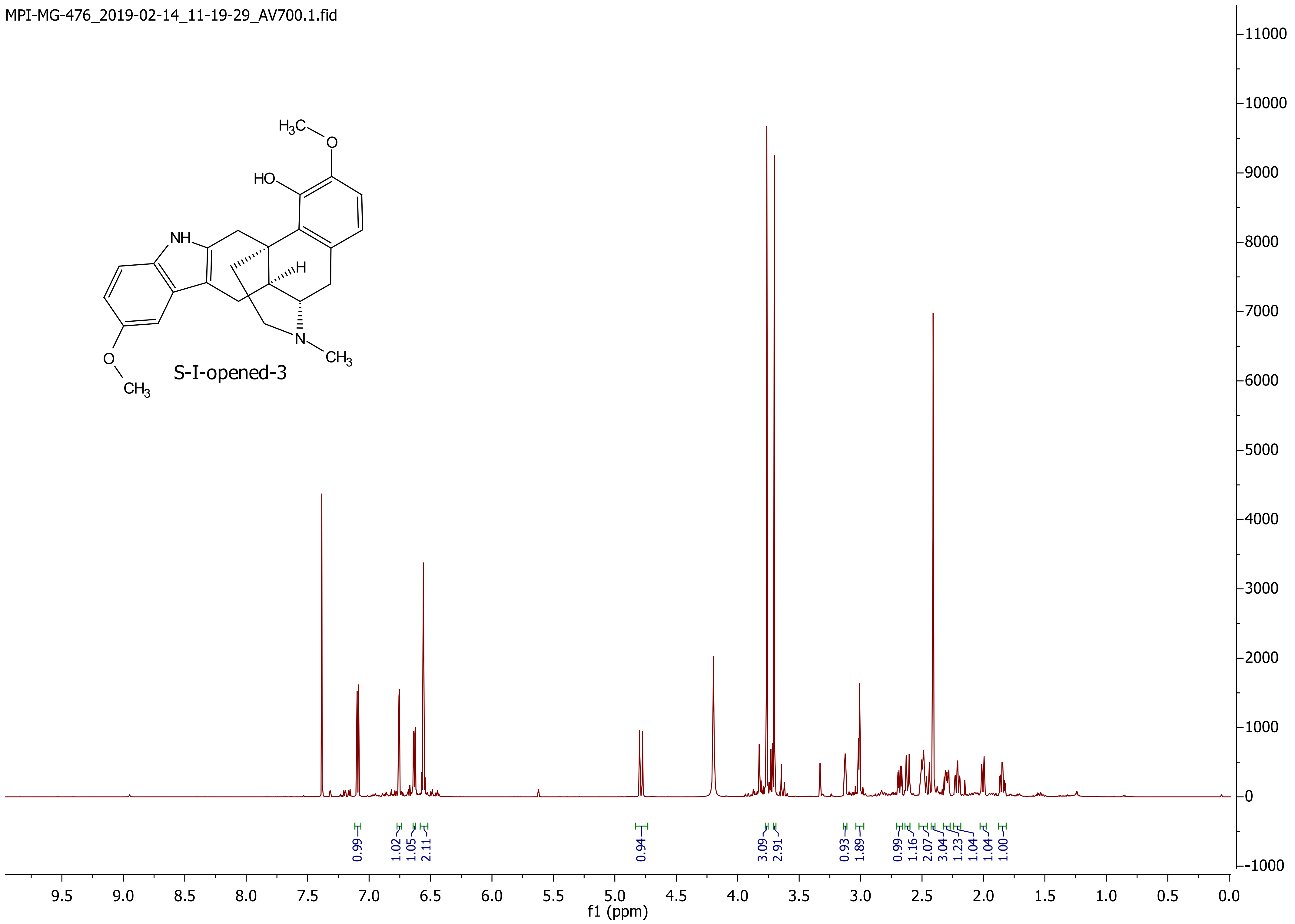
MPI-MG-476_2019-02-14_11-19-29_AV700.1.fid
S-I-opened-3
f1 (ppm)
0.99
1.02
1.05
2.11
0.94
3.09
2.91
0.93
1.89
0.99
1.16
2.07
3.04
1.23
1.04
1.04
1.00

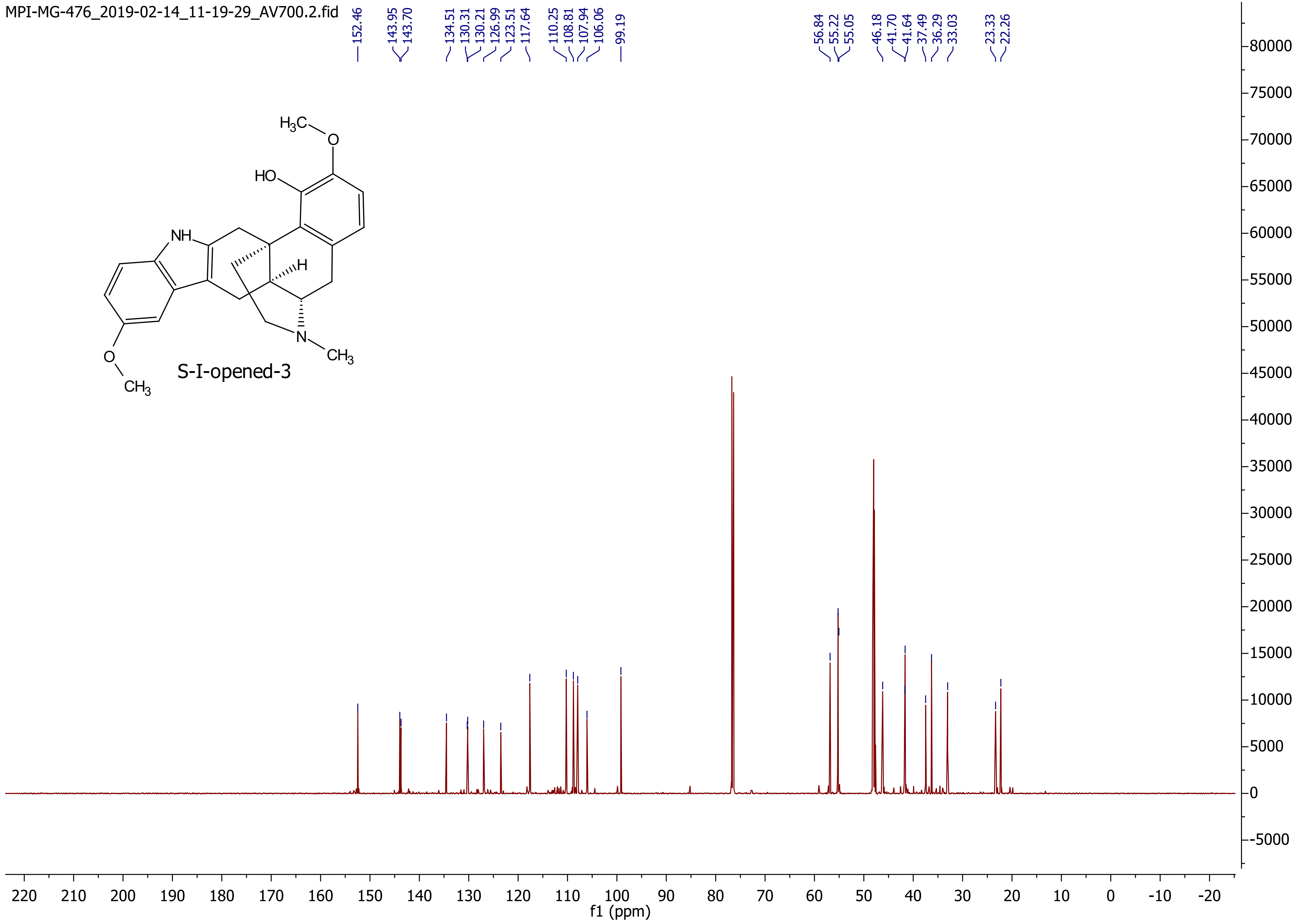

MPI-MG-476_2019-02-14_11-19-29_AV700.2.fid
152.46
143.95
143.70
134.51
130.31
130.21
126.99
123.51
117.64
110.25
108.81
107.94
106.06
99.19
56.84
55.22
55.05
46.18
41.70
41.64
37.49
36.29
33.03
23.33
22.26
H3C
O
HO
NH
H
N
CH3
O
CH3
S-I-opened-3
f1 (ppm)

MPI-MG-478_2019-02-27_13-58-16_AV600.10.fid
H1
z_Proton CDCl3 /NMR-Daten MPI 41

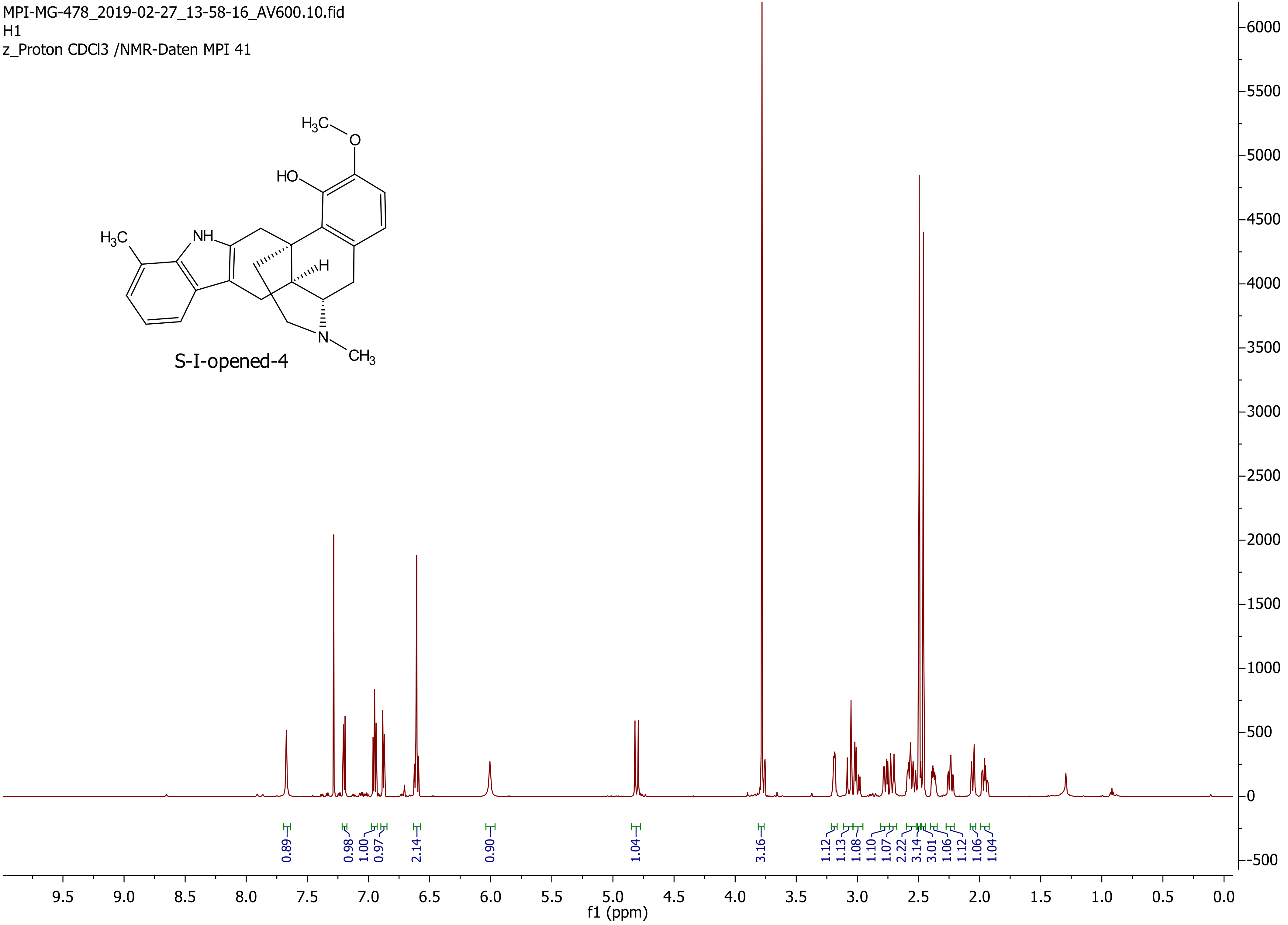

S-I-opened-4

MPI-MG-478_2019-02-27_13-58-16_AV600.11.fid
C13 with power gated H1 decoupling
z_C13pg CDCl3 /NMR-Daten MPI 41

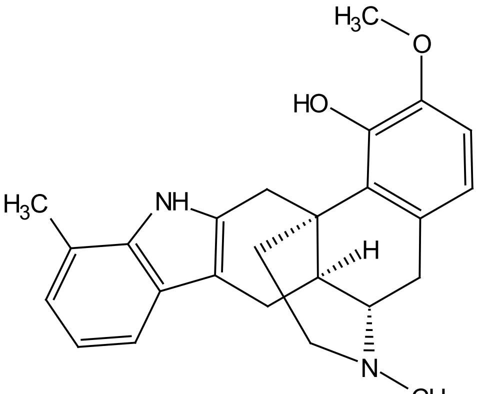

S-I-opened-4

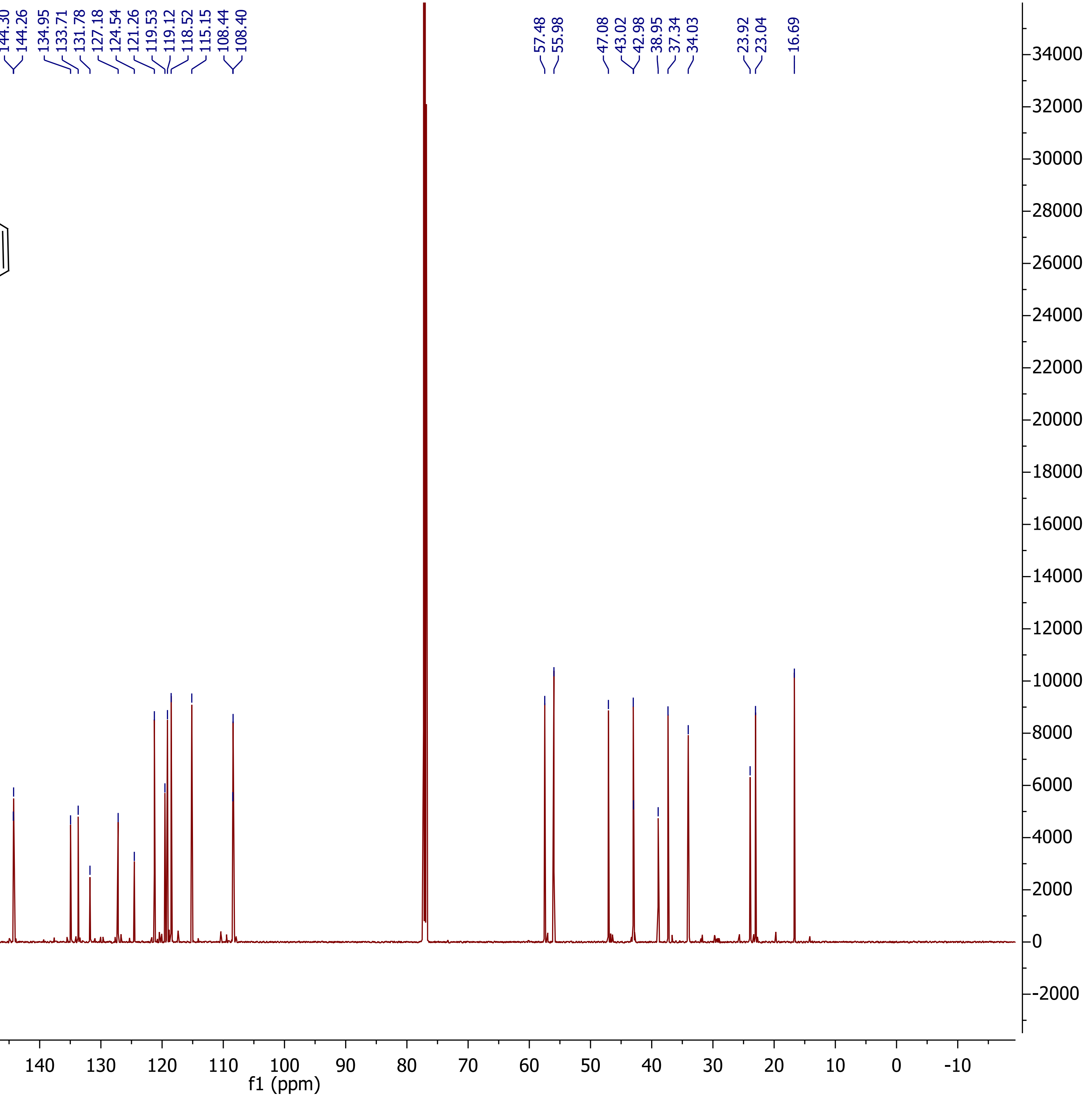

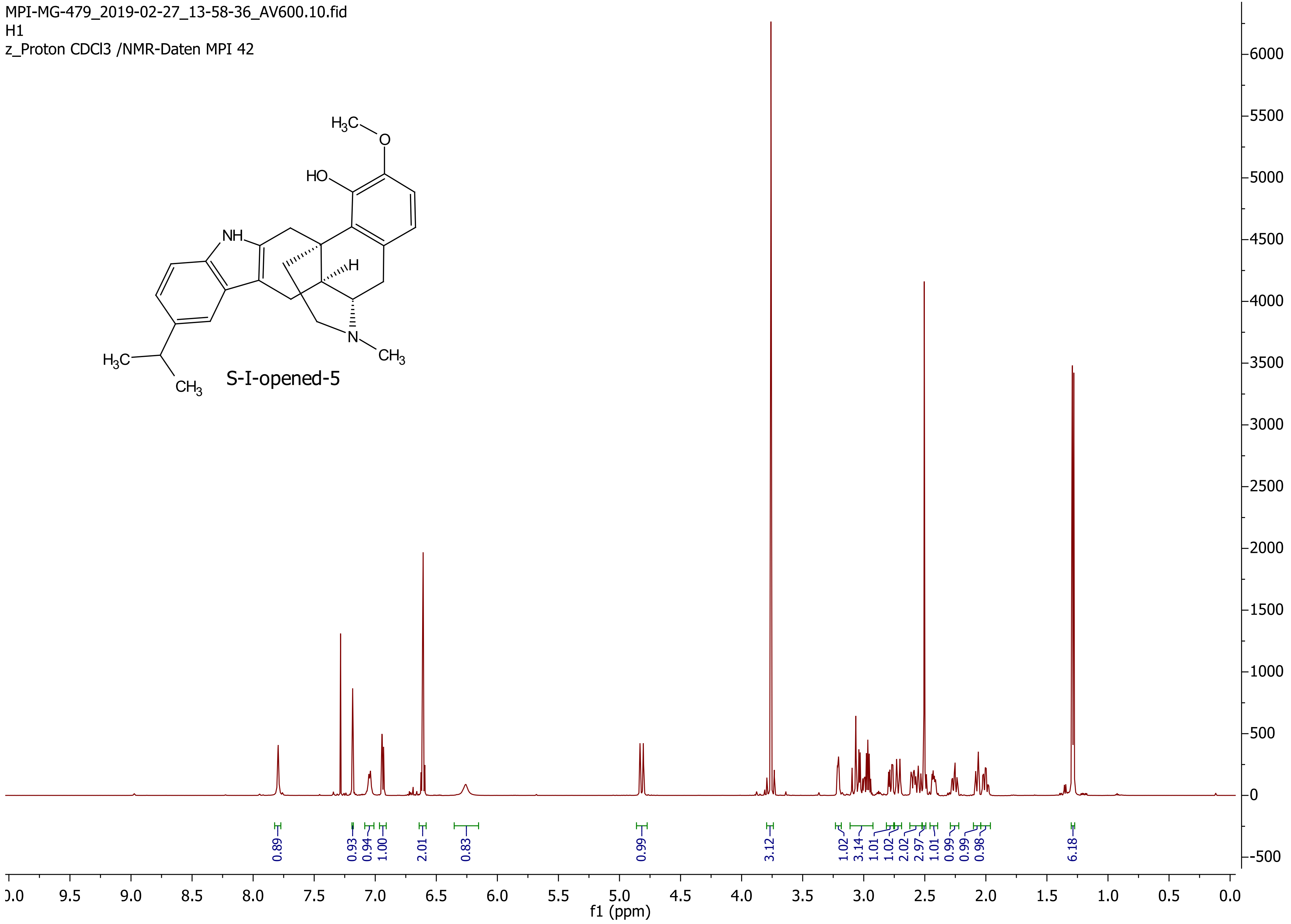
MPI-MG-479_2019-02-27_13-58-36_AV600.10.fid
H1
z_Proton CDCl3 /NMR-Daten MPI 42
S-I-opened-5
0.89
0.93
0.94
1.00
2.01
0.83
0.99
3.12
1.02
3.14
1.01
1.02
2.02
2.97
1.01
0.99
0.99
0.98
6.18
f1 (ppm)

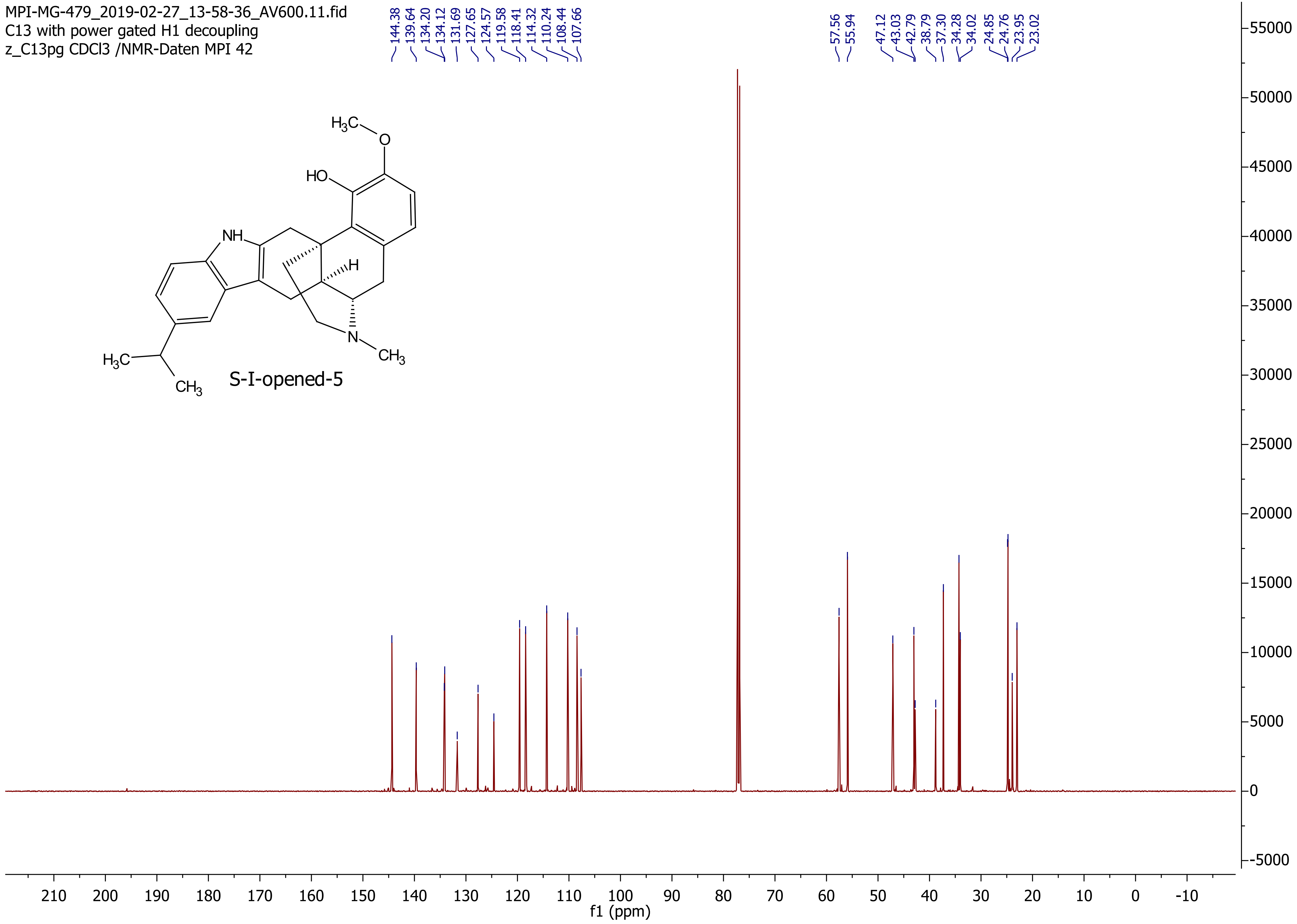
MPI-MG-479_2019-02-27_13-58-36_AV600.11.fid
C13 with power gated H1 decoupling
z_C13pg CDCl3 /NMR-Daten MPI 42
144.38
139.64
134.20
134.12
131.69
127.65
124.57
119.58
118.41
114.32
110.24
108.44
107.66
57.56
55.94
47.12
43.03
42.79
38.79
37.30
34.28
34.02
24.85
24.76
23.95
23.02
H3C
O
HO
NH
H
N
CH3
H3C
CH3
S-I-opened-5
f1 (ppm)

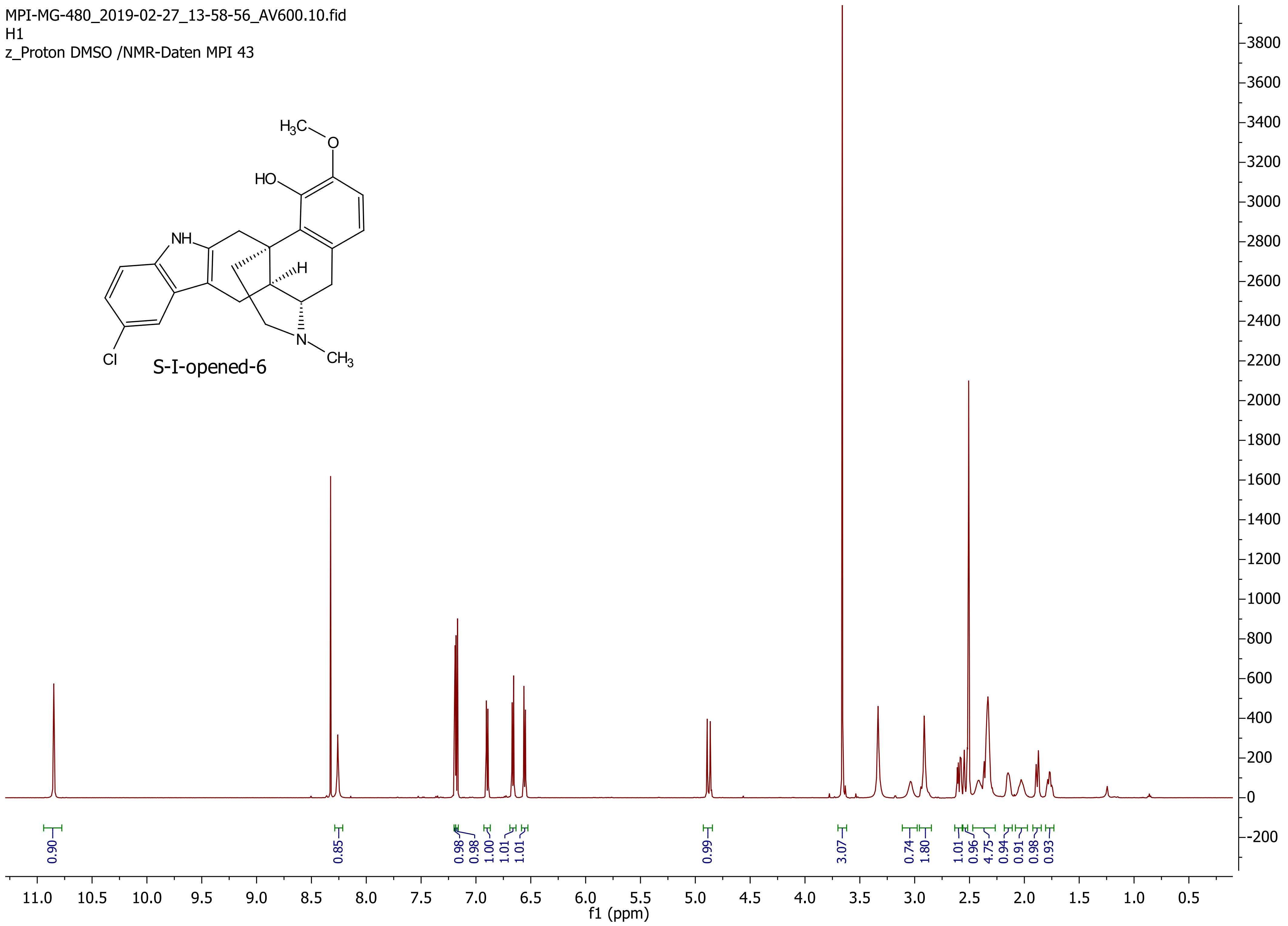
MPI-MG-480_2019-02-27_13-58-56_AV600.10.fid
H1
z_Proton DMSO /NMR-Daten MPI 43
H3C
O
HO
NH
H
N
CH3
Cl
S-I-opened-6
0.90
0.85
0.98
0.98
1.00
1.01
1.01
0.99
3.07
0.74
1.80
1.01
0.96
4.75
0.94
0.91
0.98
0.93
f1 (ppm)

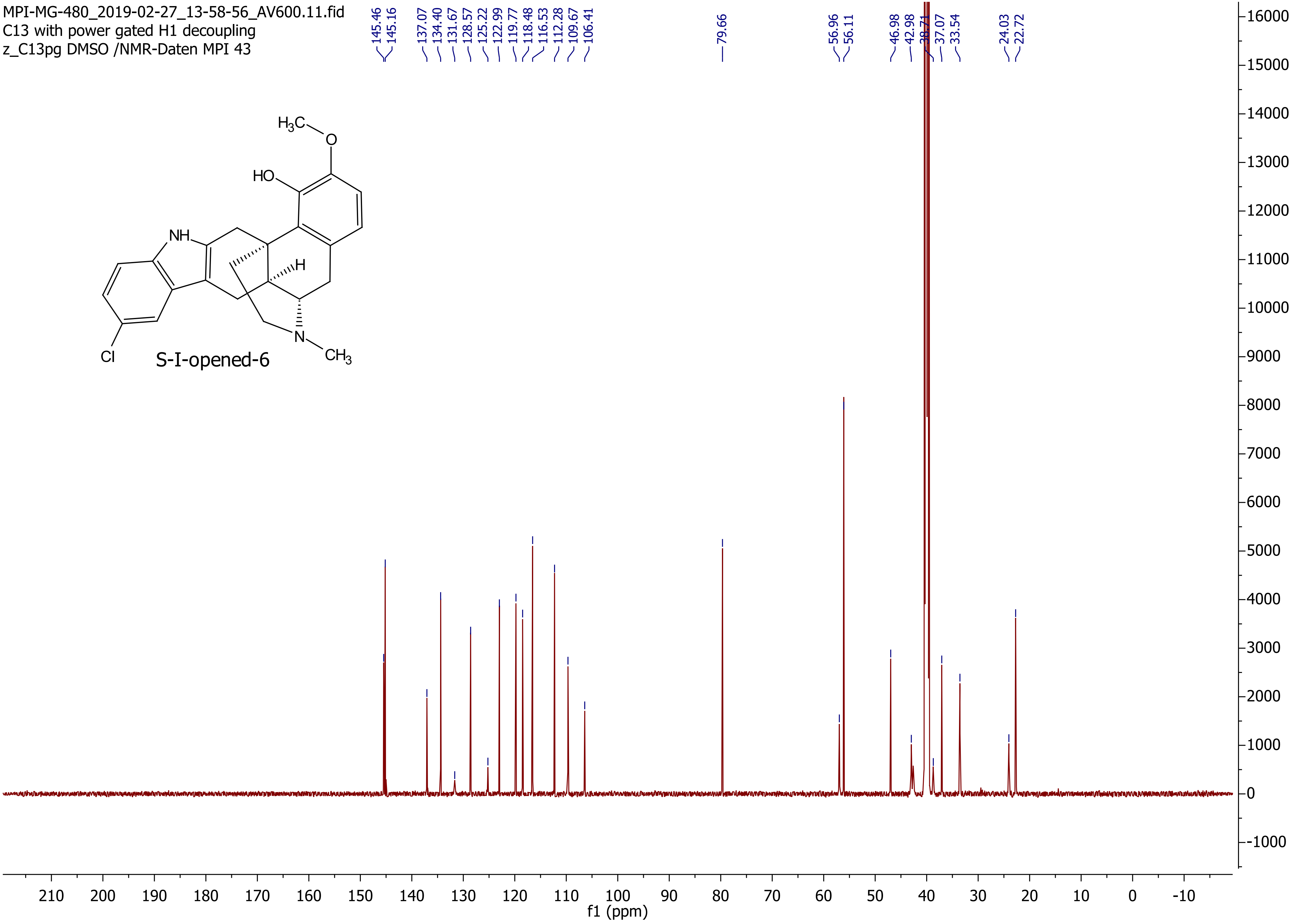
MPI-MG-480_2019-02-27_13-58-56_AV600.11.fid
C13 with power gated H1 decoupling
z_C13pg DMSO /NMR-Daten MPI 43
145.46
145.16
137.07
134.40
131.67
128.57
125.22
122.99
119.77
118.48
116.53
112.28
109.67
106.41
79.66
56.96
56.11
46.98
42.98
38.71
37.07
33.54
24.03
22.72
H3C
O
HO
NH
H
N
CH3
Cl
S-I-opened-6
f1 (ppm)

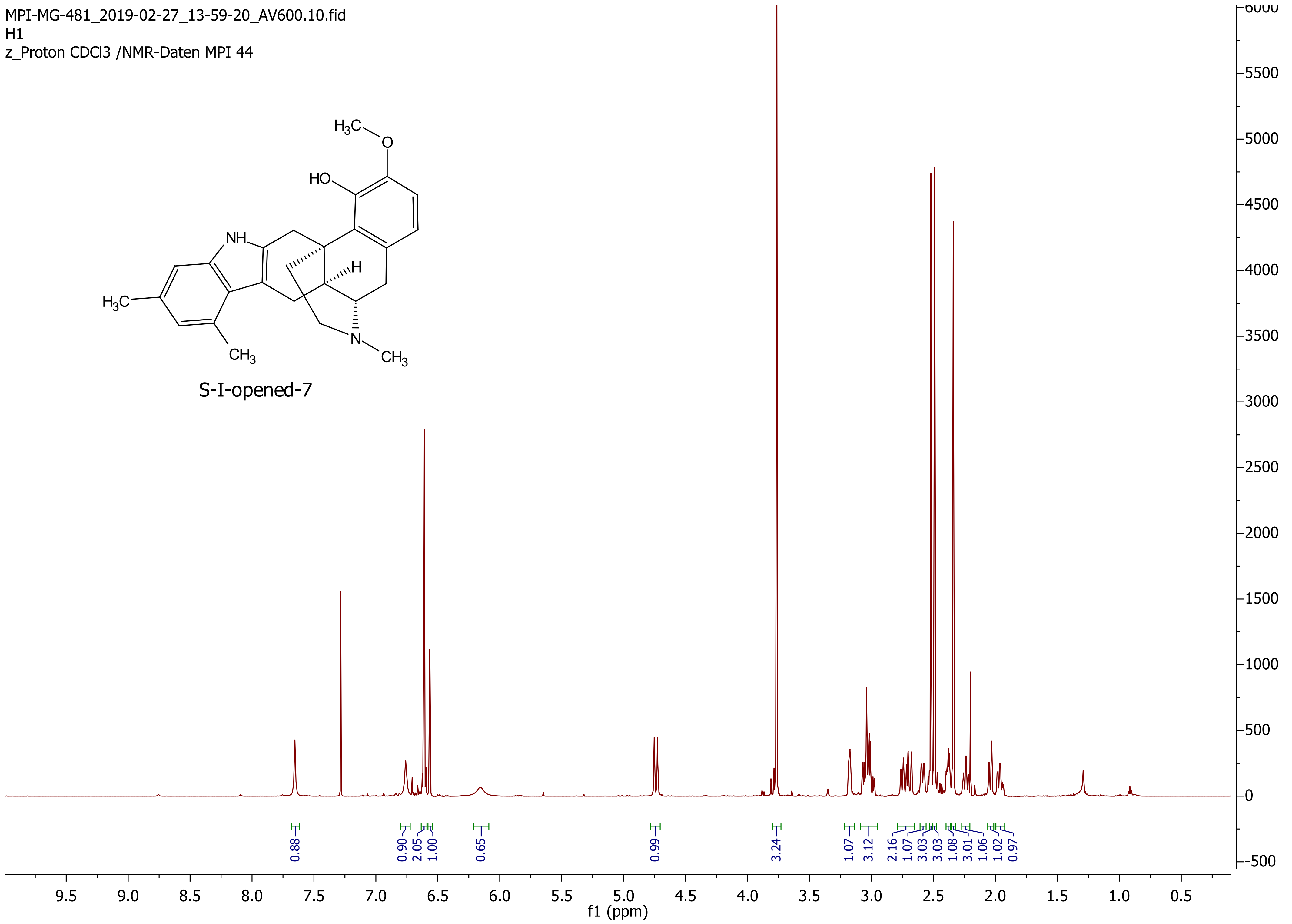
MPI-MG-481_2019-02-27_13-59-20_AV600.10.fid
H1
z_Proton CDCl3 /NMR-Daten MPI 44
S-I-opened-7
0.88
0.90
2.05
1.00
0.65
0.99
3.24
1.07
3.12
2.16
1.07
3.03
3.03
1.08
3.01
1.06
1.02
0.97
f1 (ppm)

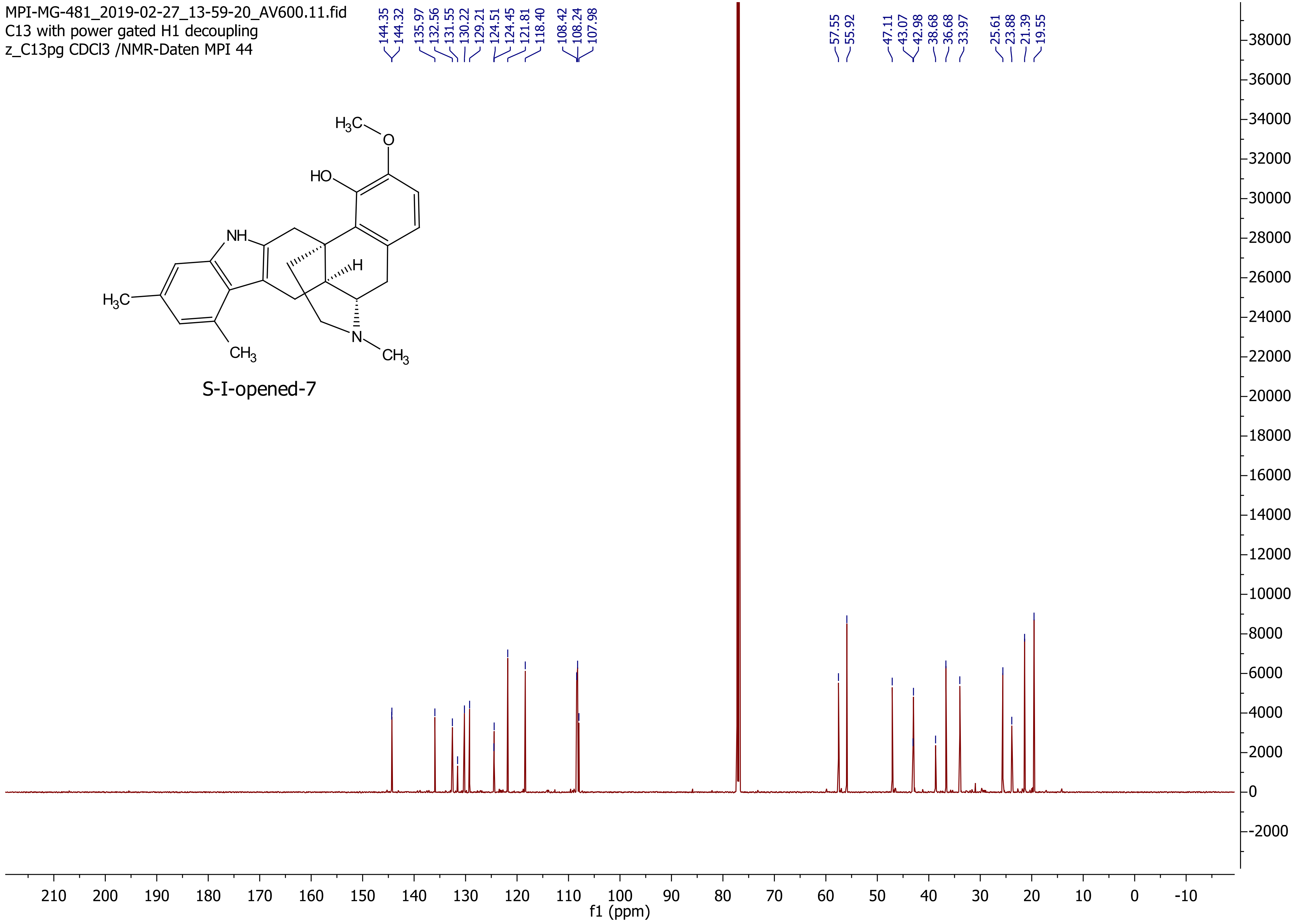
MPI-MG-481_2019-02-27_13-59-20_AV600.11.fid
C13 with power gated H1 decoupling
z_C13pg CDCl3 /NMR-Daten MPI 44
144.35
144.32
135.97
132.56
131.55
130.22
129.21
124.51
124.45
121.81
118.40
108.42
108.24
107.98
57.55
55.92
47.11
43.07
42.98
38.68
36.68
33.97
25.61
23.88
21.39
19.55
S-I-opened-7
f1 (ppm)

MPI-YM-485-pure_2019-06-05_06-23-08_AV700.1.fid

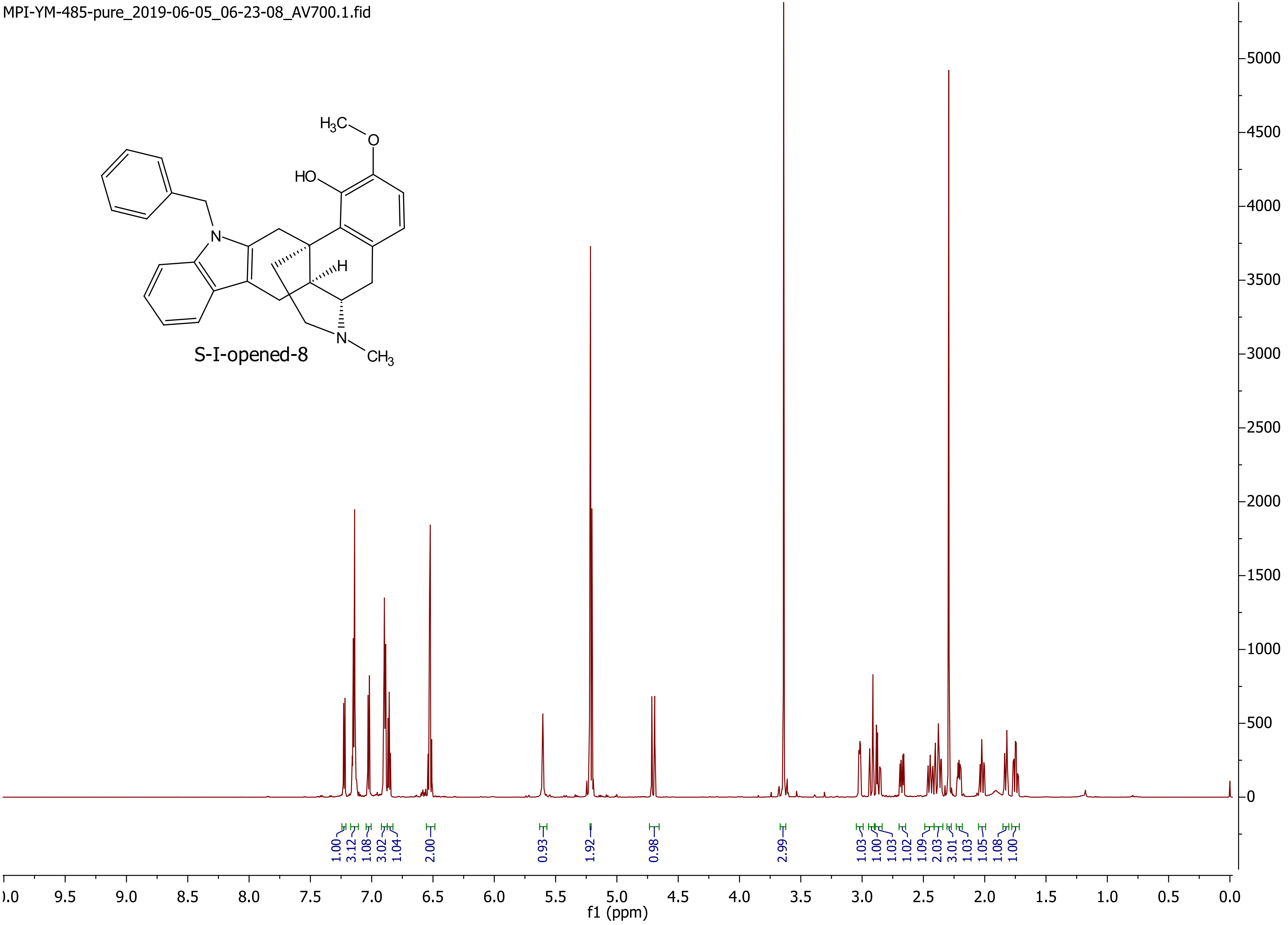

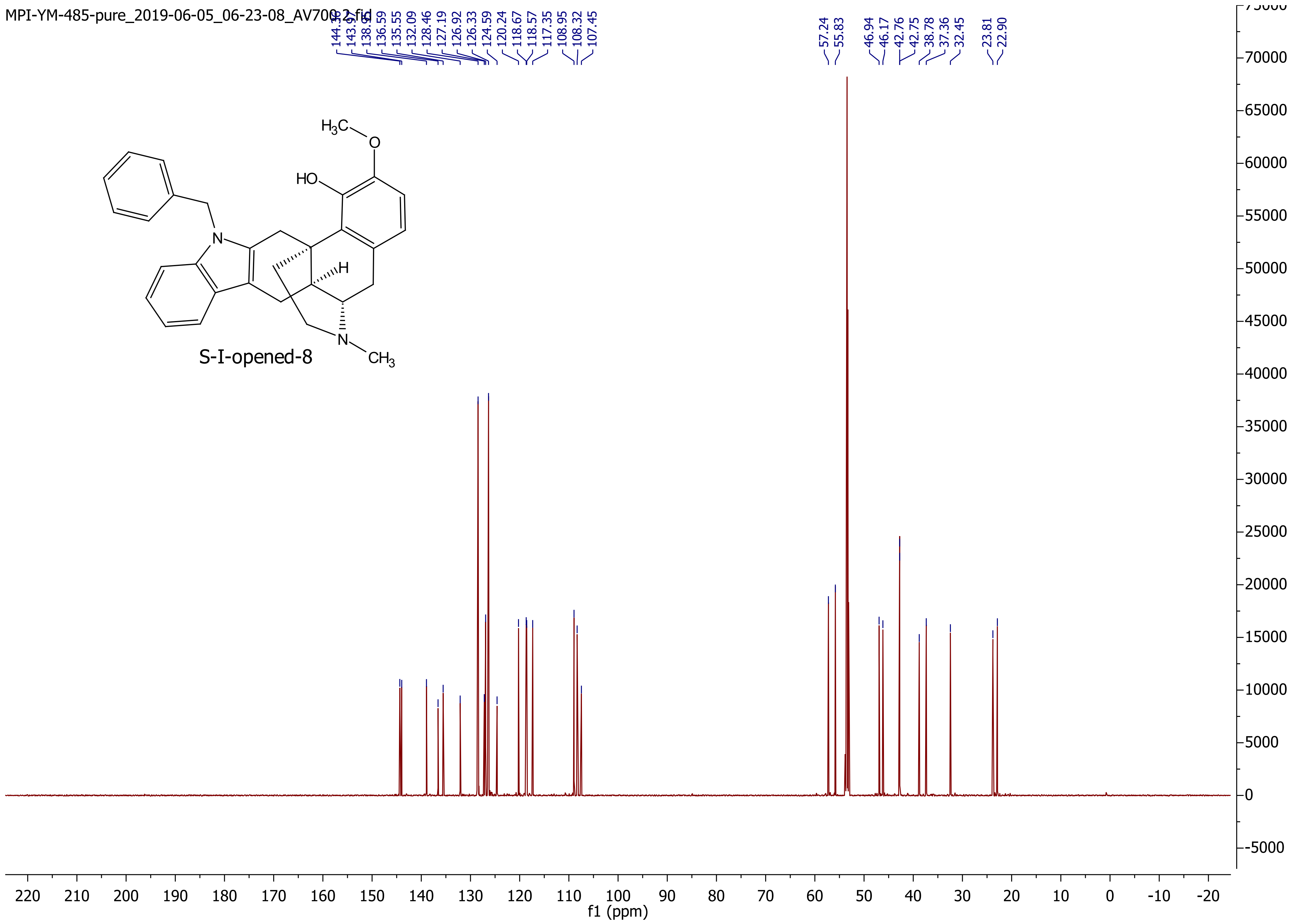

MPI-YM-485-pure_2019-06-05_06-23-08_AV700
136.59
135.55
132.09
128.46
127.19
126.92
126.33
124.59
120.24
118.67
118.57
117.35
108.95
108.32
107.45
57.24
55.83
46.94
46.17
42.76
42.75
38.78
37.36
32.45
23.81
22.90
H3C
O
HO
N
H
N
CH3
S-I-opened-8
f1 (ppm)

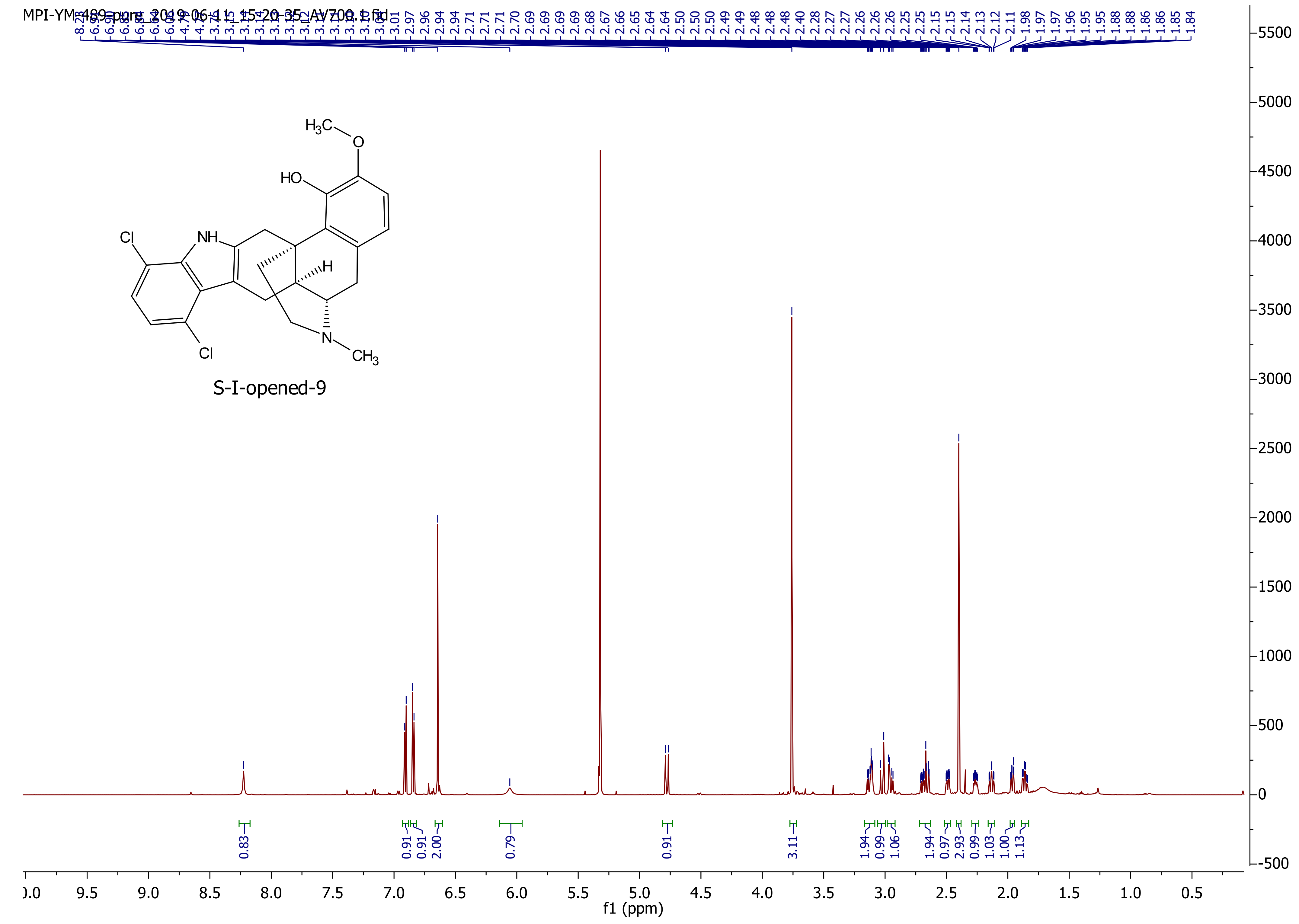
MPI-YM-489_pure_2019-06-11_15-20-35_AV700.1.fid
S-I-opened-9
f1 (ppm)

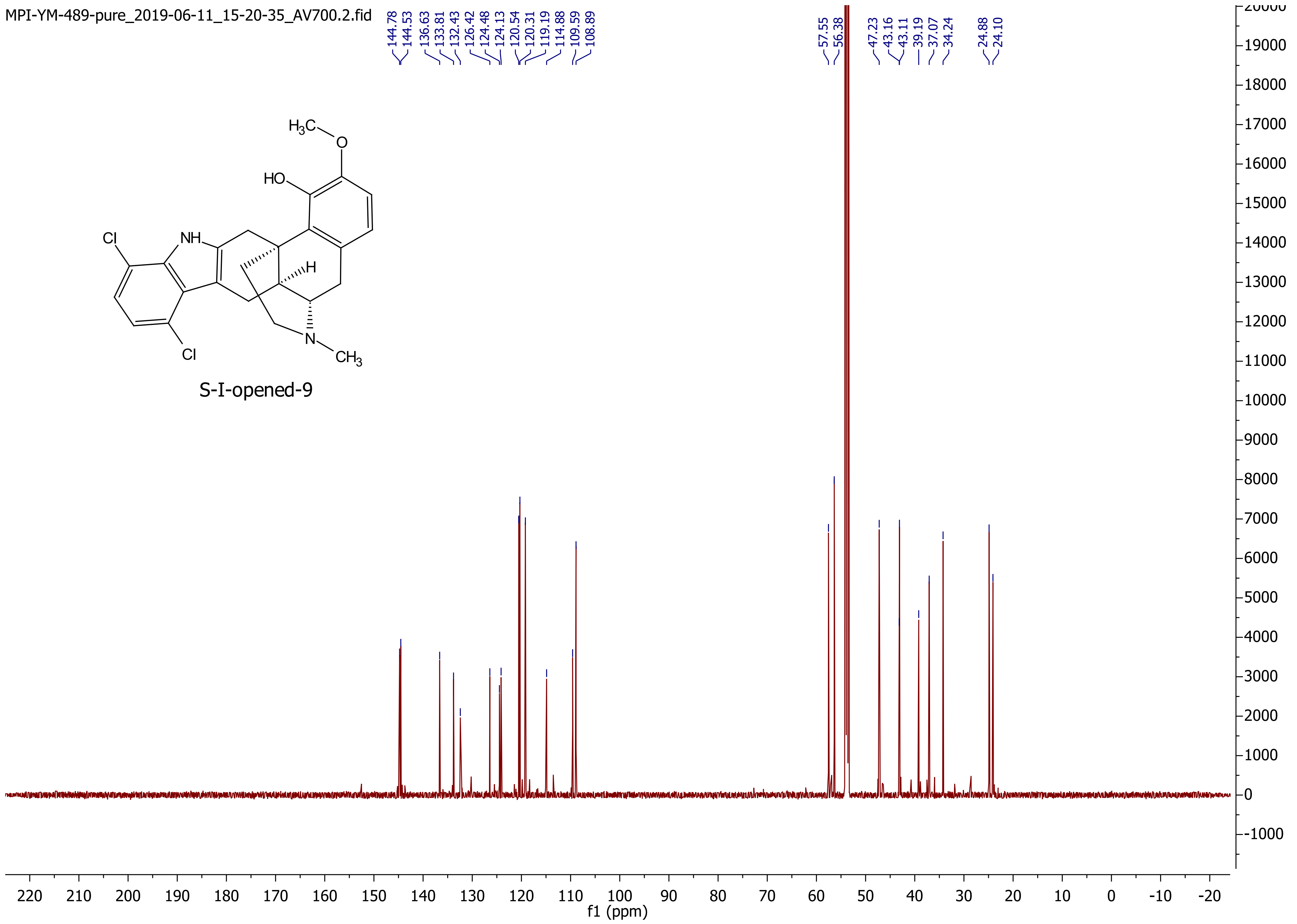
MPI-YM-489-pure_2019-06-11_15-20-35_AV700.2.fid
144.78
144.53
136.63
133.81
132.43
126.42
124.48
124.13
120.54
120.31
119.19
114.88
109.59
108.89
57.55
56.38
47.23
43.16
43.11
39.19
37.07
34.24
24.88
24.10
H3C
O
HO
NH
Cl
Cl
H
N
CH3
S-I-opened-9
f1 (ppm)

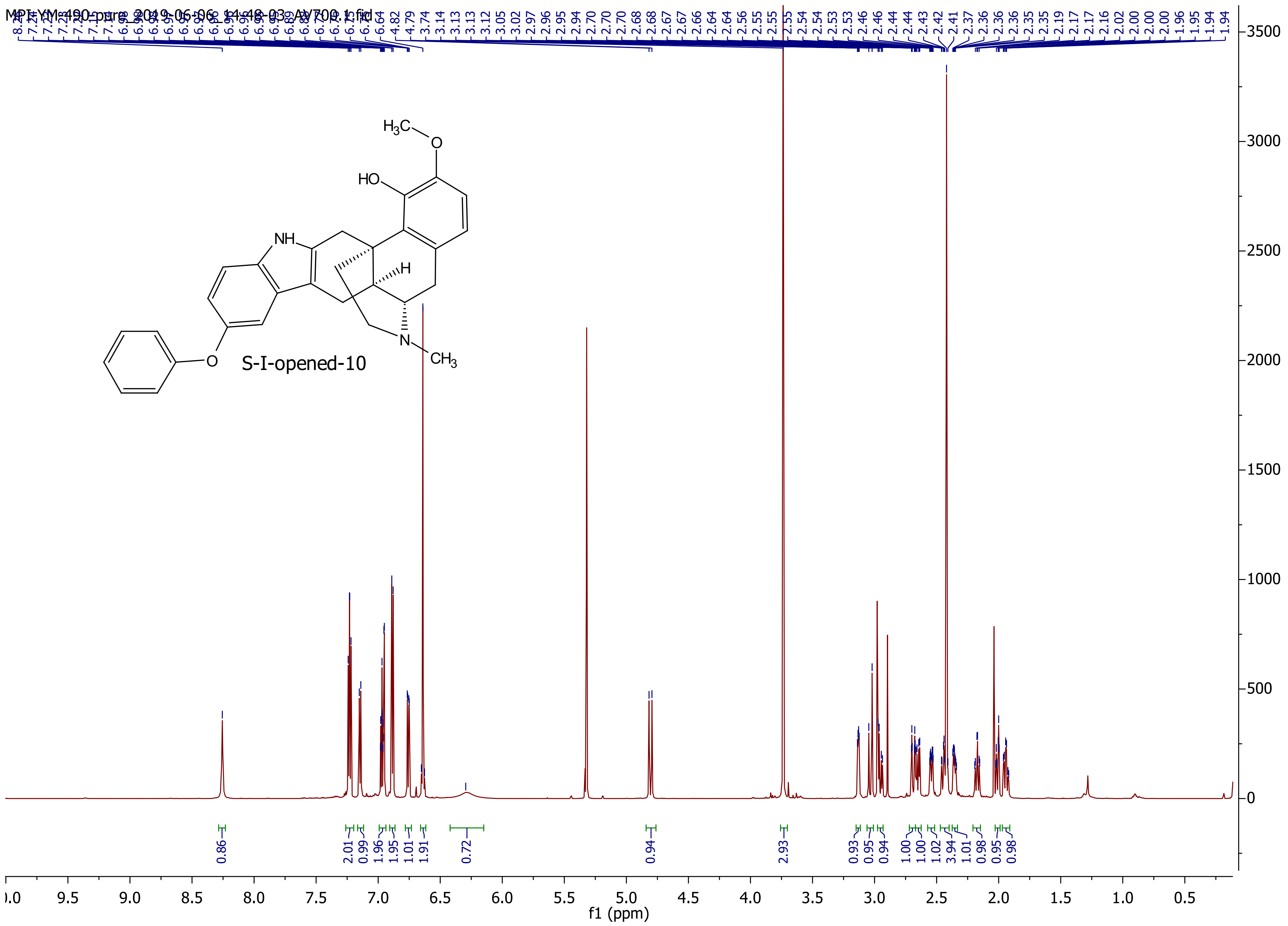

H3C
O
HO
NH
H
N
CH3
O
S-I-opened-10
0.86
2.01
0.99
1.96
1.95
1.01
1.91
0.72
0.94
2.93
0.93
0.95
0.94
1.00
1.00
1.02
3.94
1.01
0.98
0.95
0.98
f1 (ppm)

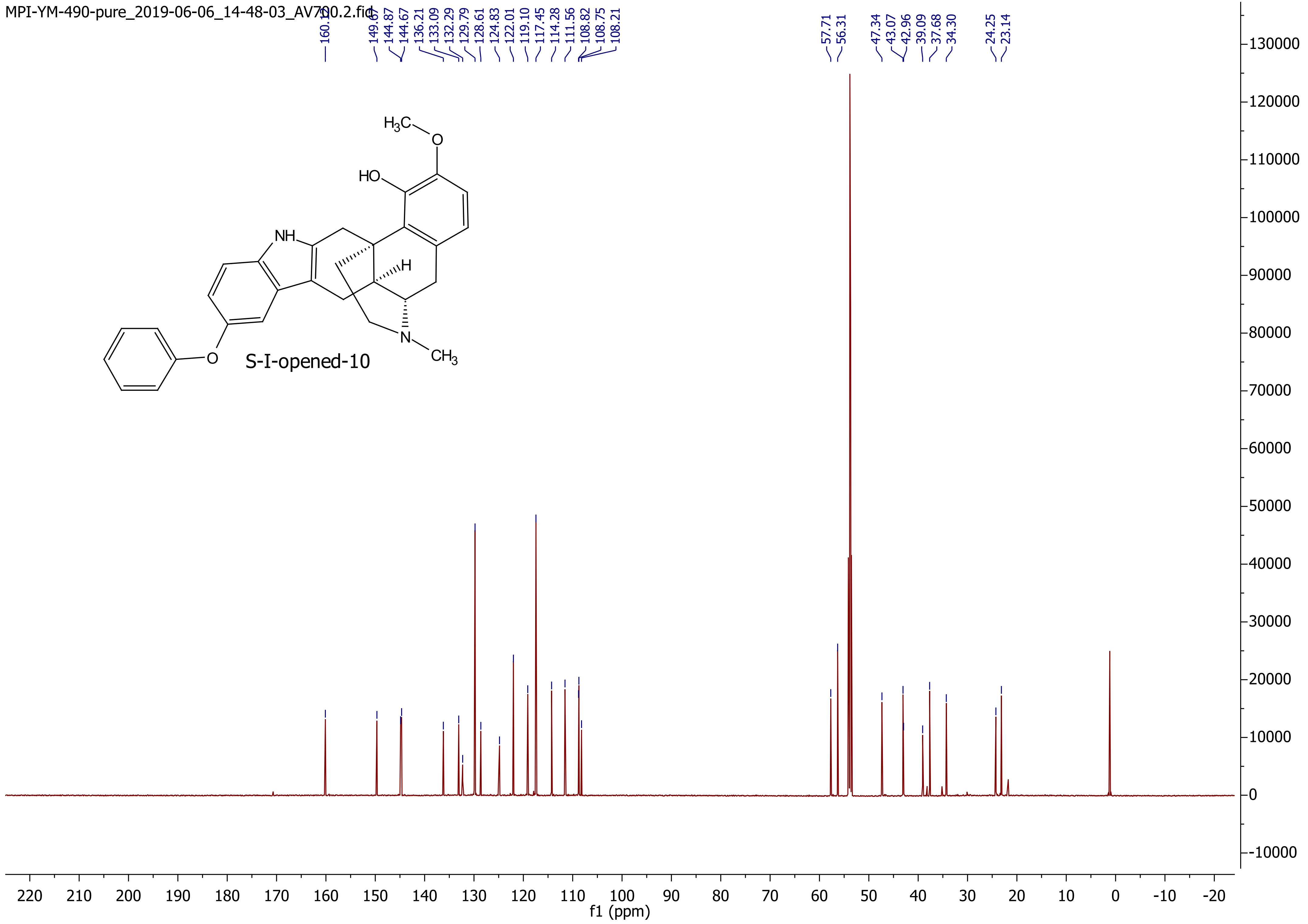

MPI-YM-490-pure_2019-06-06_14-48-03_AV700.2.fid
149.67
144.87
144.67
136.21
133.09
132.29
129.79
128.61
124.83
122.01
119.10
117.45
114.28
111.56
108.82
108.75
108.21
57.71
56.31
47.34
43.07
42.96
39.09
37.68
34.30
24.25
23.14
S-I-opened-10
f1 (ppm)

MPI-YM-491-pure_2019-06-05_06-23-40_AV700_1.fid

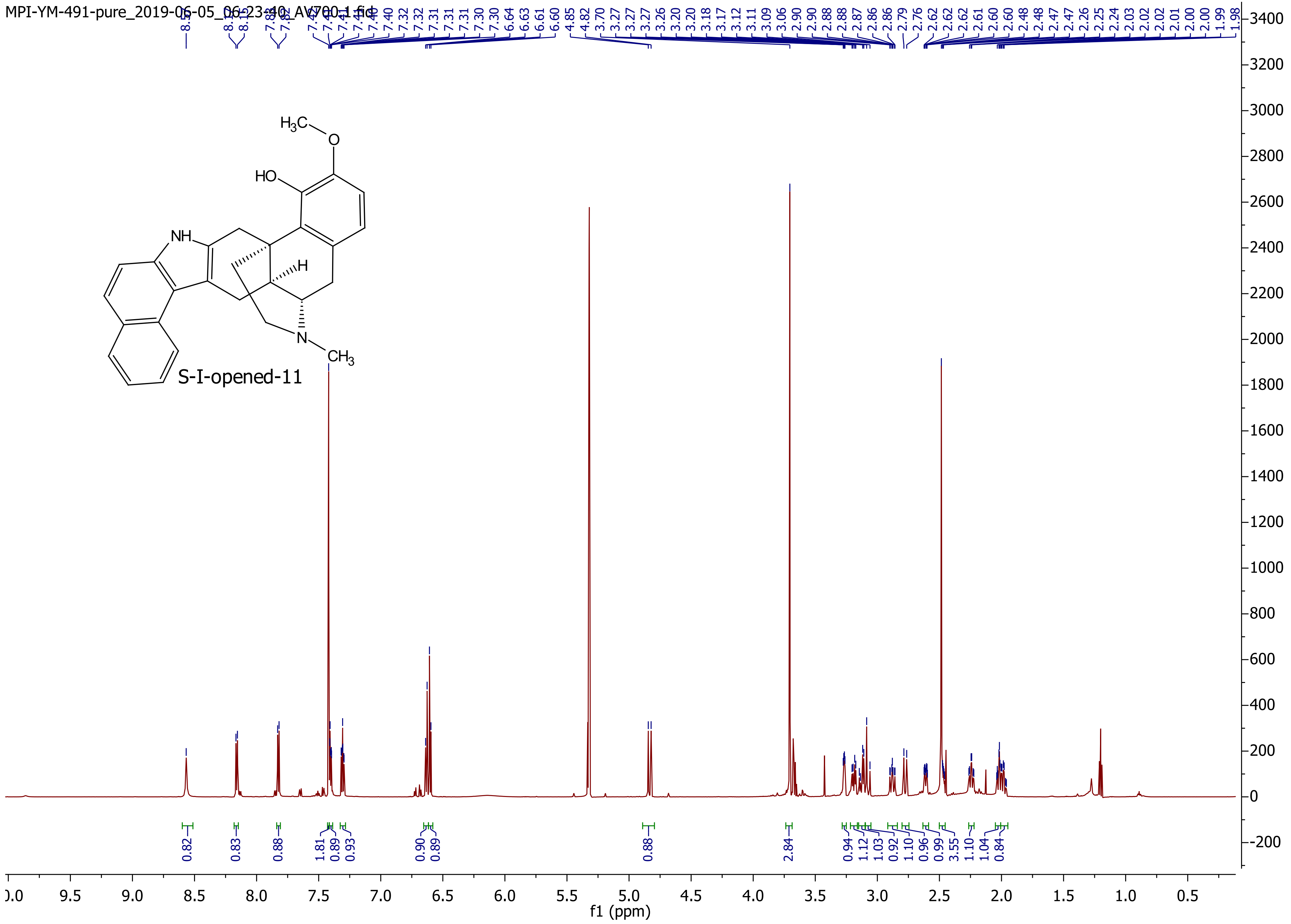

MPI-YM-491-pure_2019-06-05_06-23-40_AV700.2.fid

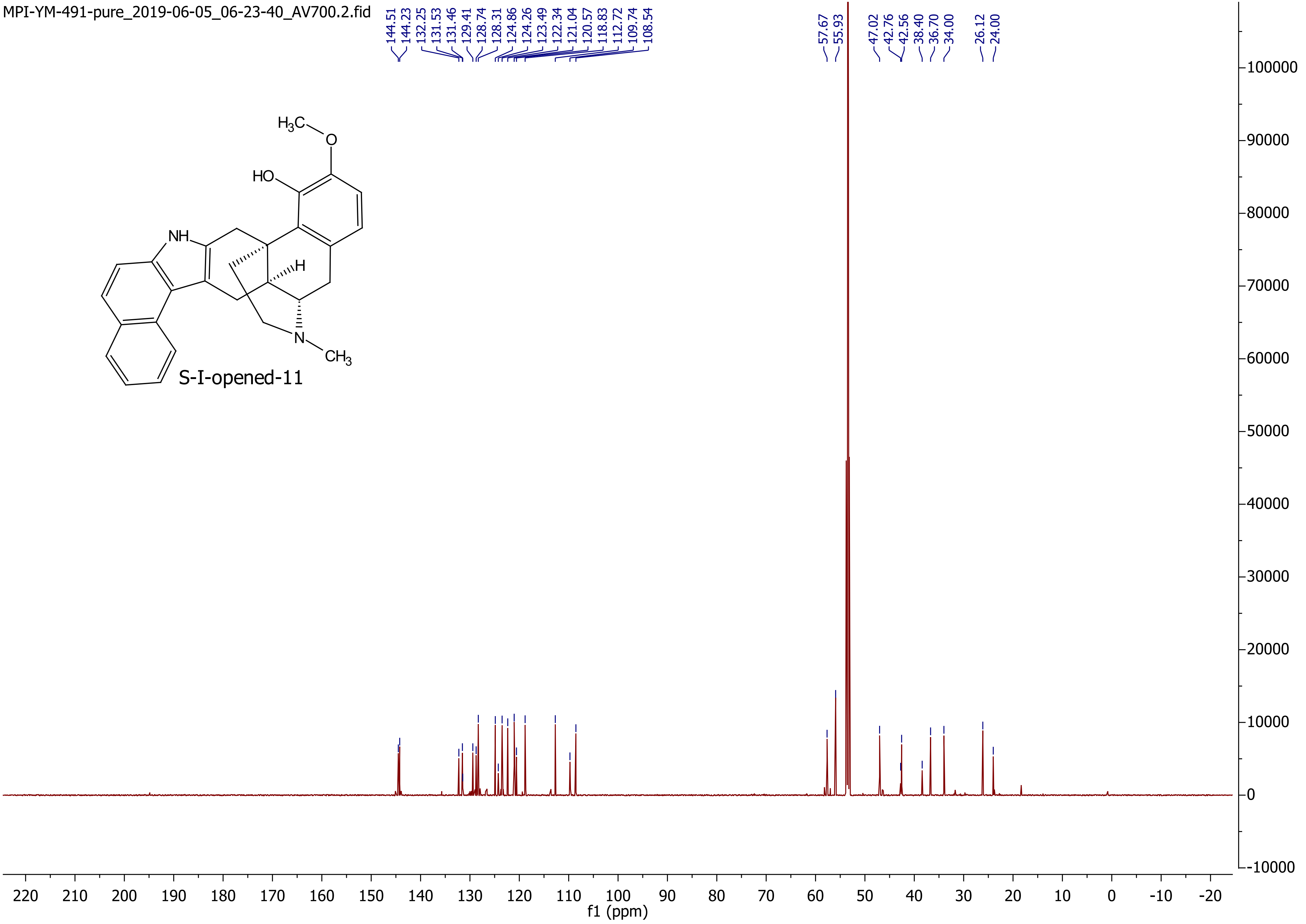

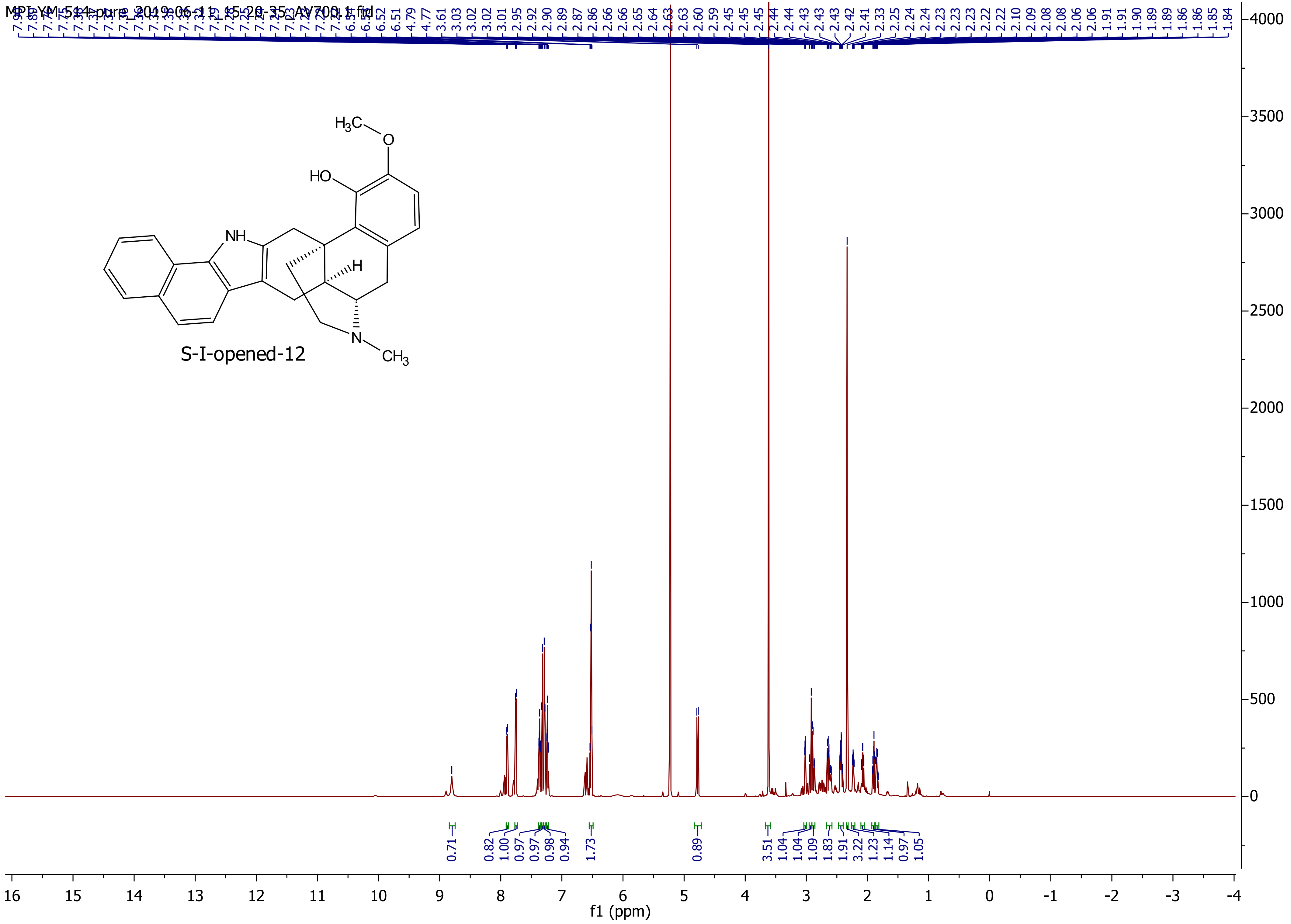
S-I-opened-12
H3C
O
HO
NH
H
N
CH3
0.71
0.82
1.00
0.97
0.97
0.98
0.94
1.73
0.89
3.51
1.04
1.09
1.83
1.91
3.22
1.23
1.14
0.97
1.05
f1 (ppm)

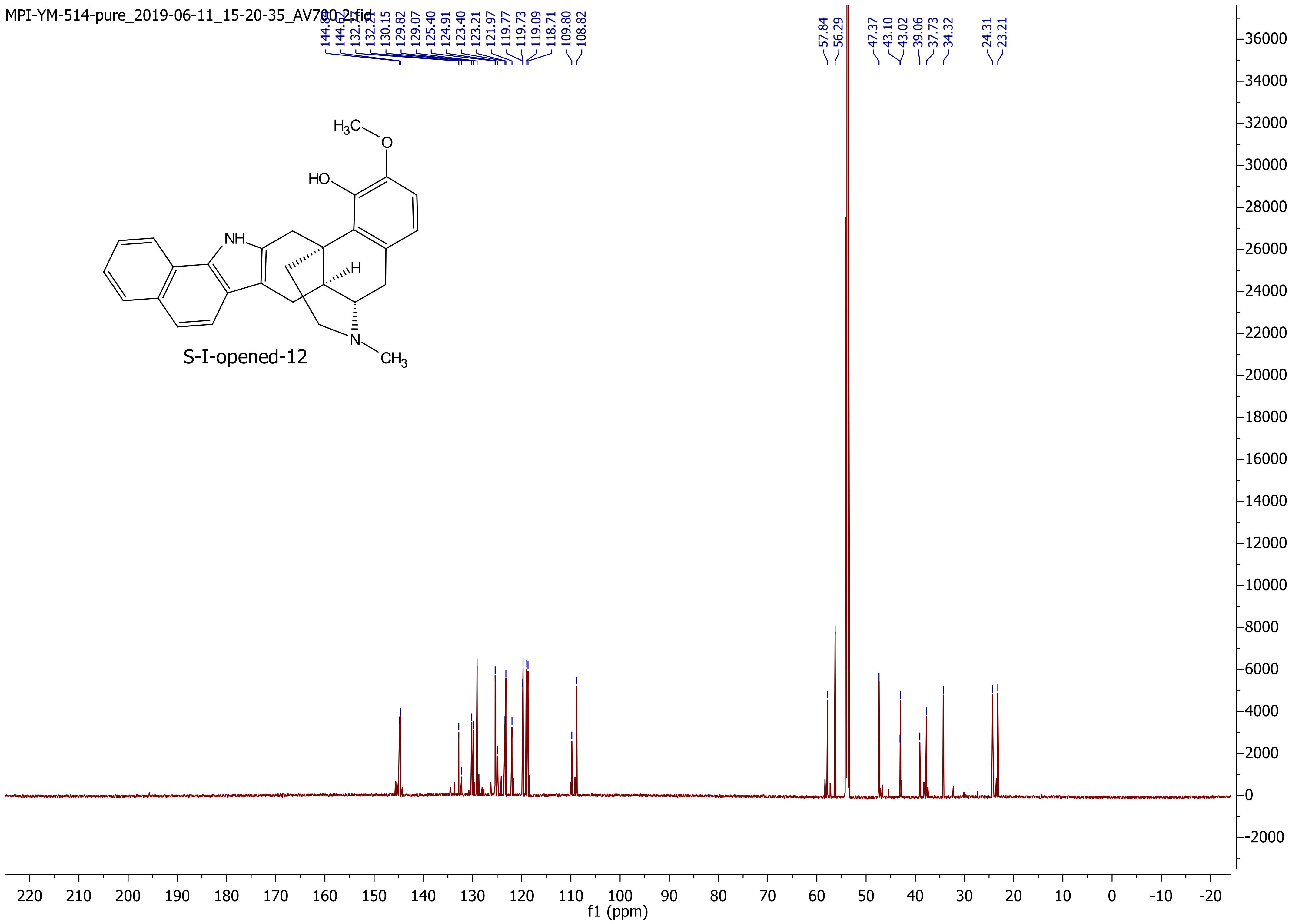
MPI-YM-514-pure_2019-06-11_15-20-35_AV700_2.fid
144.84
144.62
132.77
132.21
130.15
129.82
129.07
125.40
124.91
123.40
123.21
121.97
119.77
119.73
119.09
118.71
109.80
108.82
57.84
56.29
47.37
43.10
43.02
39.06
37.73
34.32
24.31
23.21
H3C
O
HO
NH
H
N
CH3
S-I-opened-12
36000
34000
32000
30000
28000
26000
24000
22000
20000
18000
16000
14000
12000
10000
8000
6000
4000
2000
0
-2000
220 210 200 190 180 170 160 150 140 130 120 110 100 90 80 70 60 50 40 30 20 10 0 -10 -20
f1 (ppm)

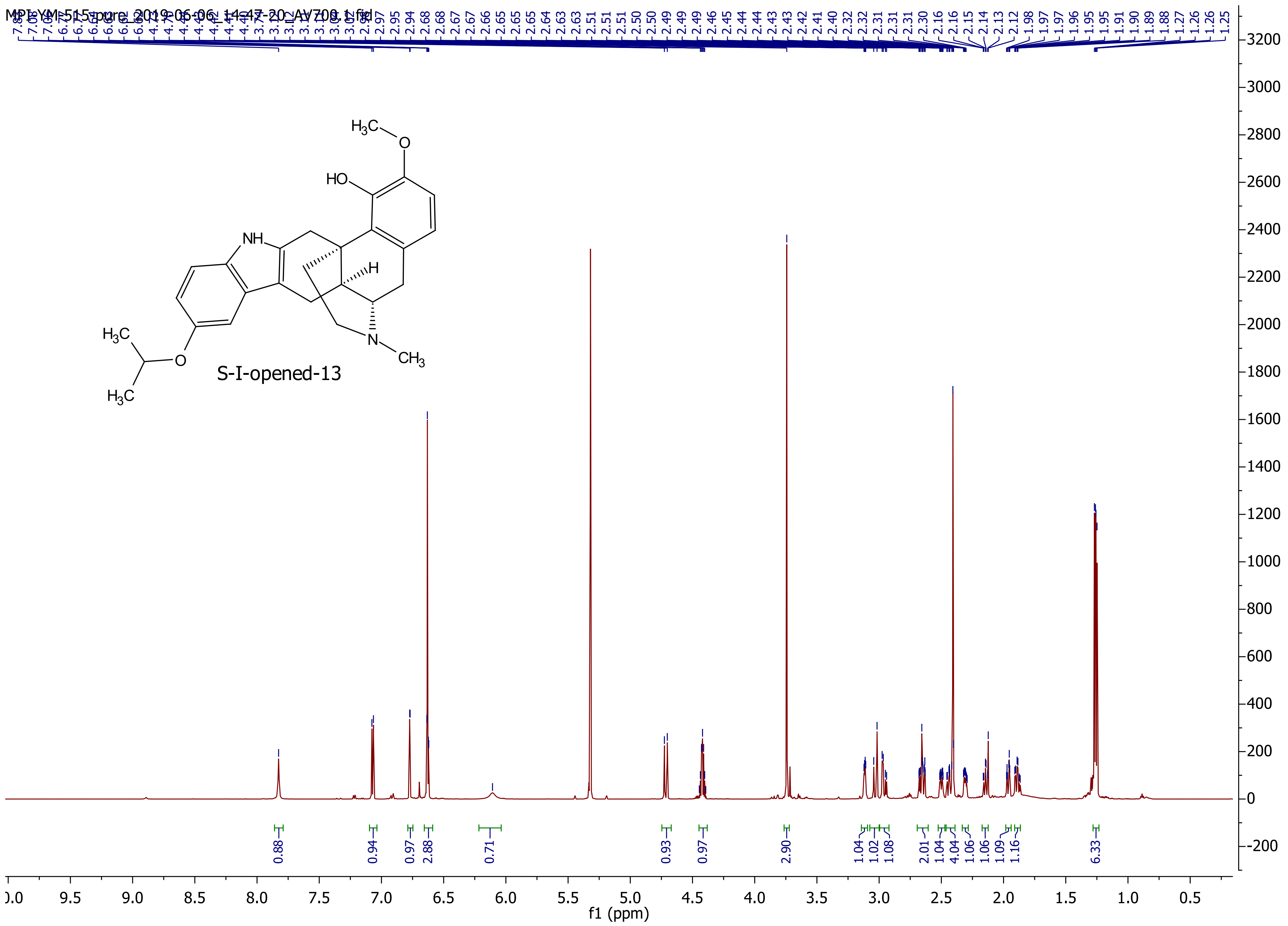

S-I-opened-13
HO
NH
H
N
H_3C
CH_3
f1 (ppm)
0.88
0.94
0.97
2.88
0.71
0.93
0.97
2.90
1.04
1.02
1.08
2.01
1.04
4.04
1.06
1.06
1.09
1.16
6.33

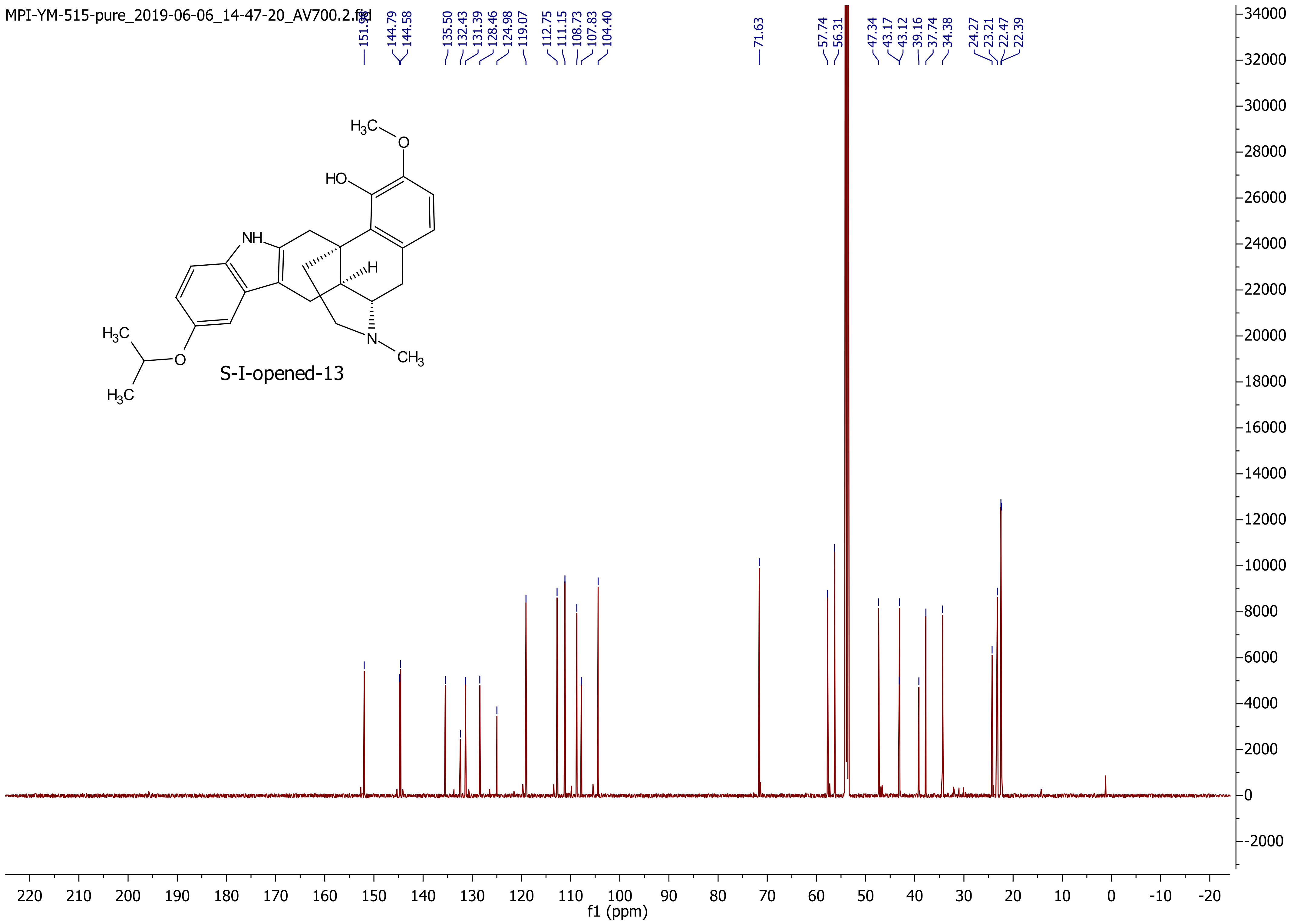
MPI-YM-515-pure_2019-06-06_14-47-20_AV700.2.fid
151.98
144.79
144.58
135.50
132.43
131.39
128.46
124.98
119.07
112.75
111.15
108.73
107.83
104.40
71.63
57.74
56.31
47.34
43.17
43.12
39.16
37.74
34.38
24.27
23.21
22.47
22.39
H_3C
O
HO
NH
H
H_3C
O
N
CH_3
H_3C
S-I-opened-13
34000
32000
30000
28000
26000
24000
22000
20000
18000
16000
14000
12000
10000
8000
6000
4000
2000
0
--2000
220 210 200 190 180 170 160 150 140 130 120 110 100 90 80 70 60 50 40 30 20 10 0 -10 -20
f1 (ppm)

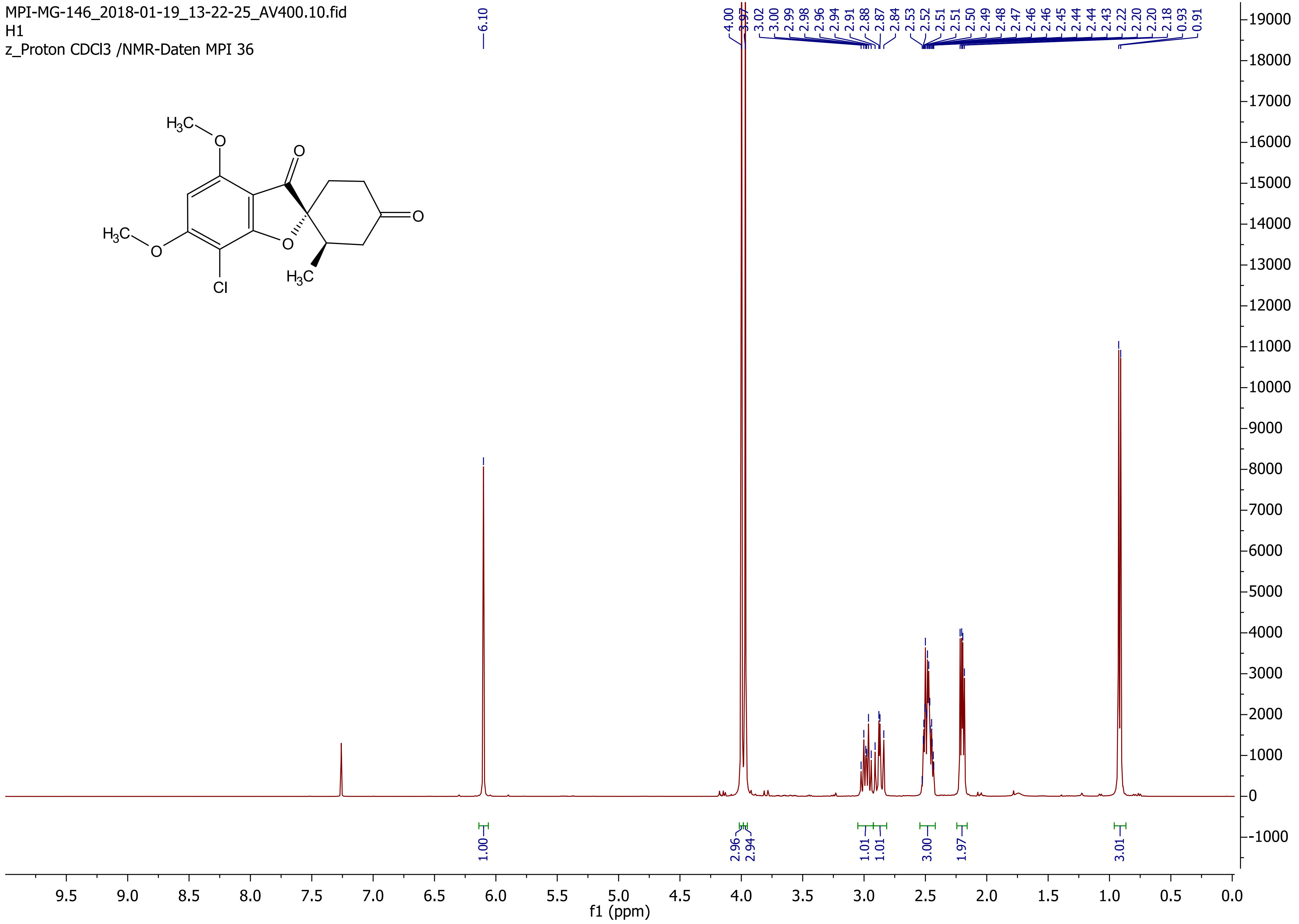
MPI-MG-146_2018-01-19_13-22-25_AV400.10.fid
H1
z_Proton CDCl3 /NMR-Daten MPI 36
H3C
O
O
O
H3C
O
O
Cl
H3C
6.10
4.00
3.97
3.02
3.00
2.99
2.98
2.96
2.94
2.91
2.88
2.87
2.84
2.53
2.52
2.51
2.51
2.50
2.49
2.48
2.47
2.46
2.46
2.45
2.44
2.44
2.43
2.22
2.20
2.20
2.18
0.93
0.91
1.00
2.96
2.94
1.01
1.01
3.00
1.97
3.01
f1 (ppm)

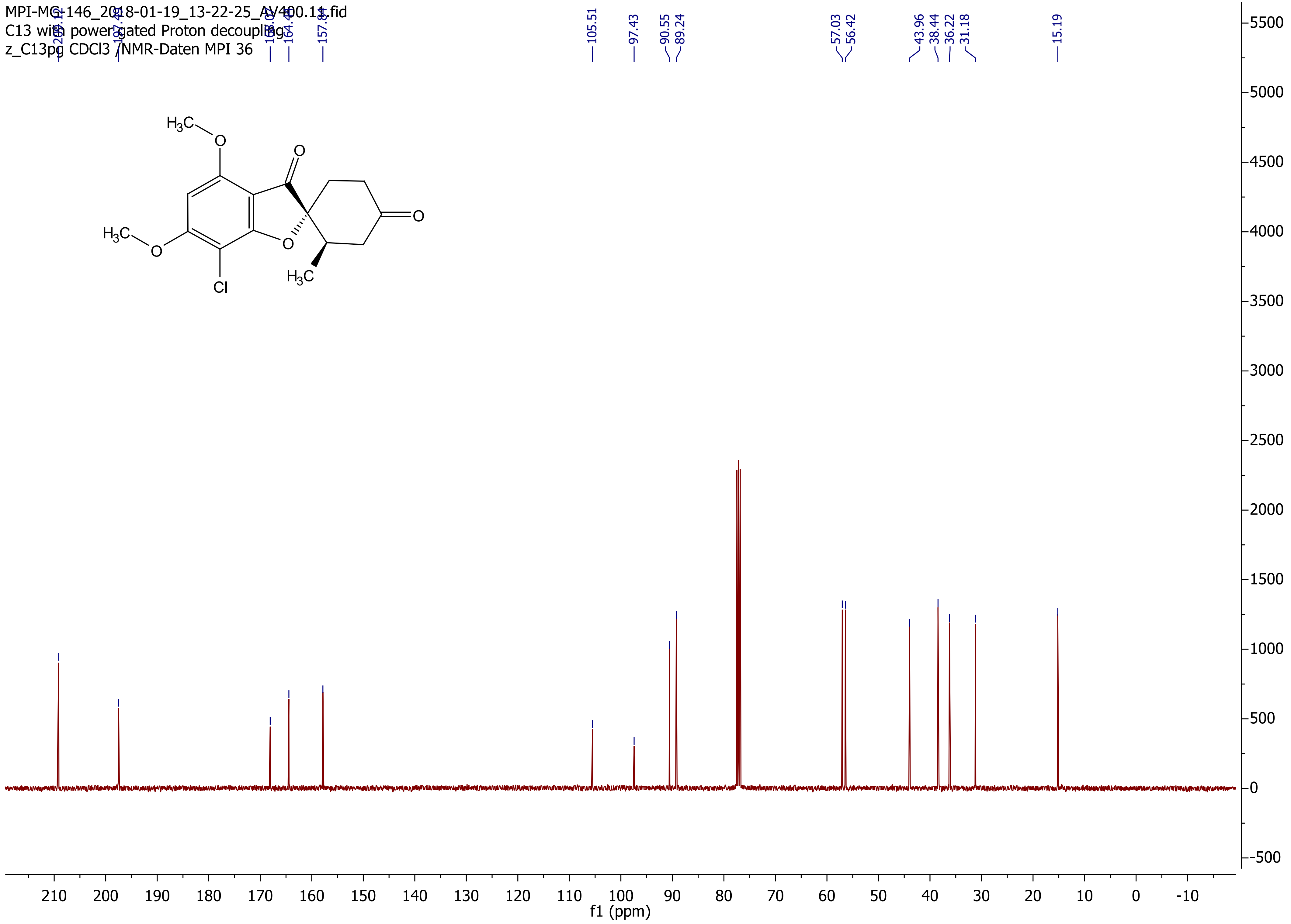
MPI-MG-146_2018-01-19_13-22-25_AV400.11.fid
C13 with power gated Proton decoupling
z_C13pg CDCl3 /NMR-Daten MPI 36
105.51
97.43
90.55
89.24
57.03
56.42
43.96
38.44
36.22
31.18
15.19
H3C
O
O
O
H3C
O
O
Cl
H3C
f1 (ppm)

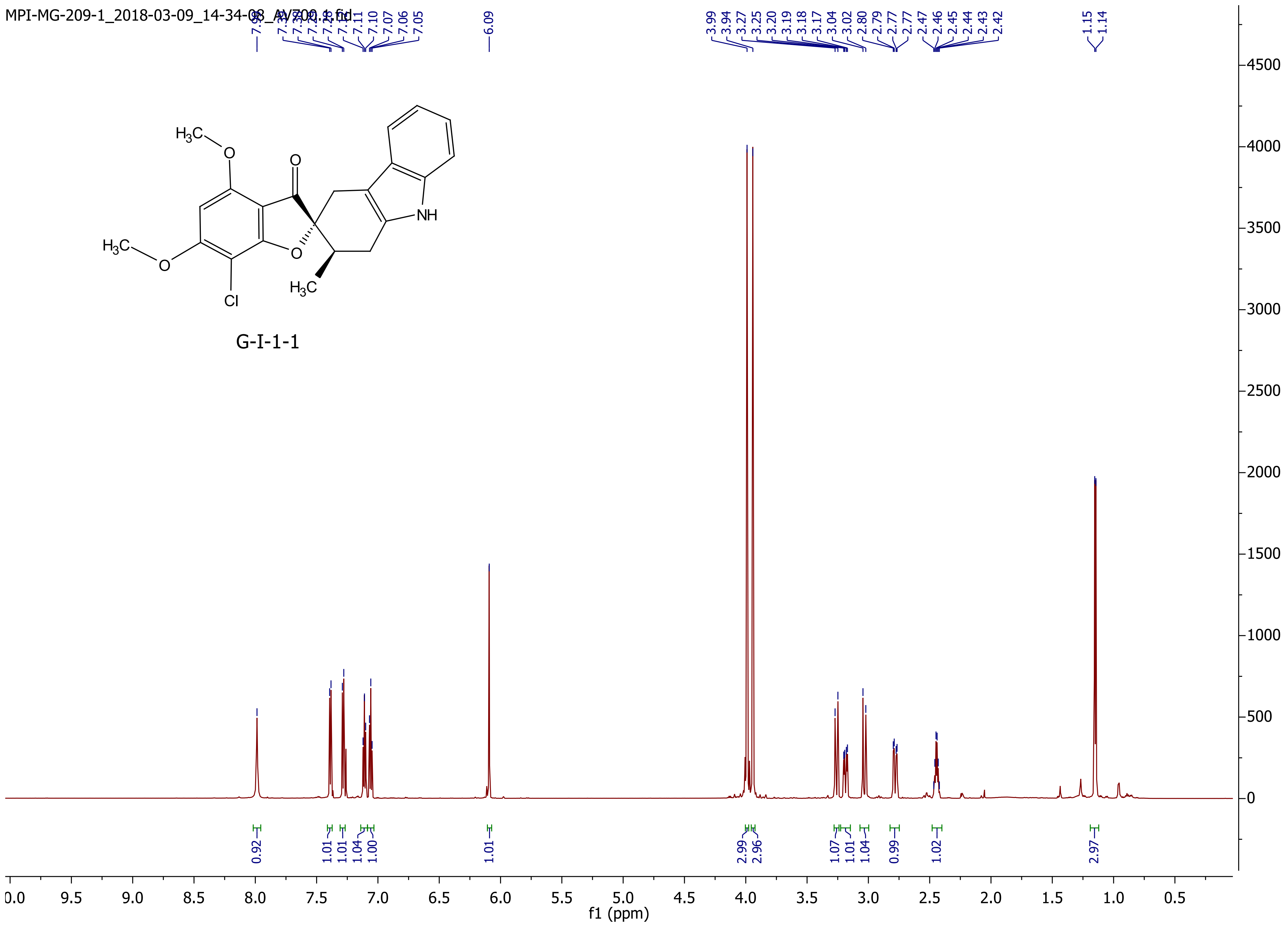

MPI-MG-209-1_2018-03-09_14-34-08_AV300.1.fid
7.11
7.10
7.07
7.06
7.05
6.09
3.99
3.94
3.27
3.25
3.20
3.19
3.18
3.17
3.04
3.02
2.80
2.79
2.77
2.77
2.47
2.46
2.45
2.44
2.43
2.42
1.15
1.14
H_3C
O
O
H_3C
O
O
NH
Cl
H_3C
G-I-1-1
0.92
1.01
1.01
1.04
1.00
1.01
2.99
2.96
1.07
1.01
1.04
0.99
1.02
2.97
f1 (ppm)

MPI-MG-209-1_2018-03-09_14-34-08_AV700-2.fid

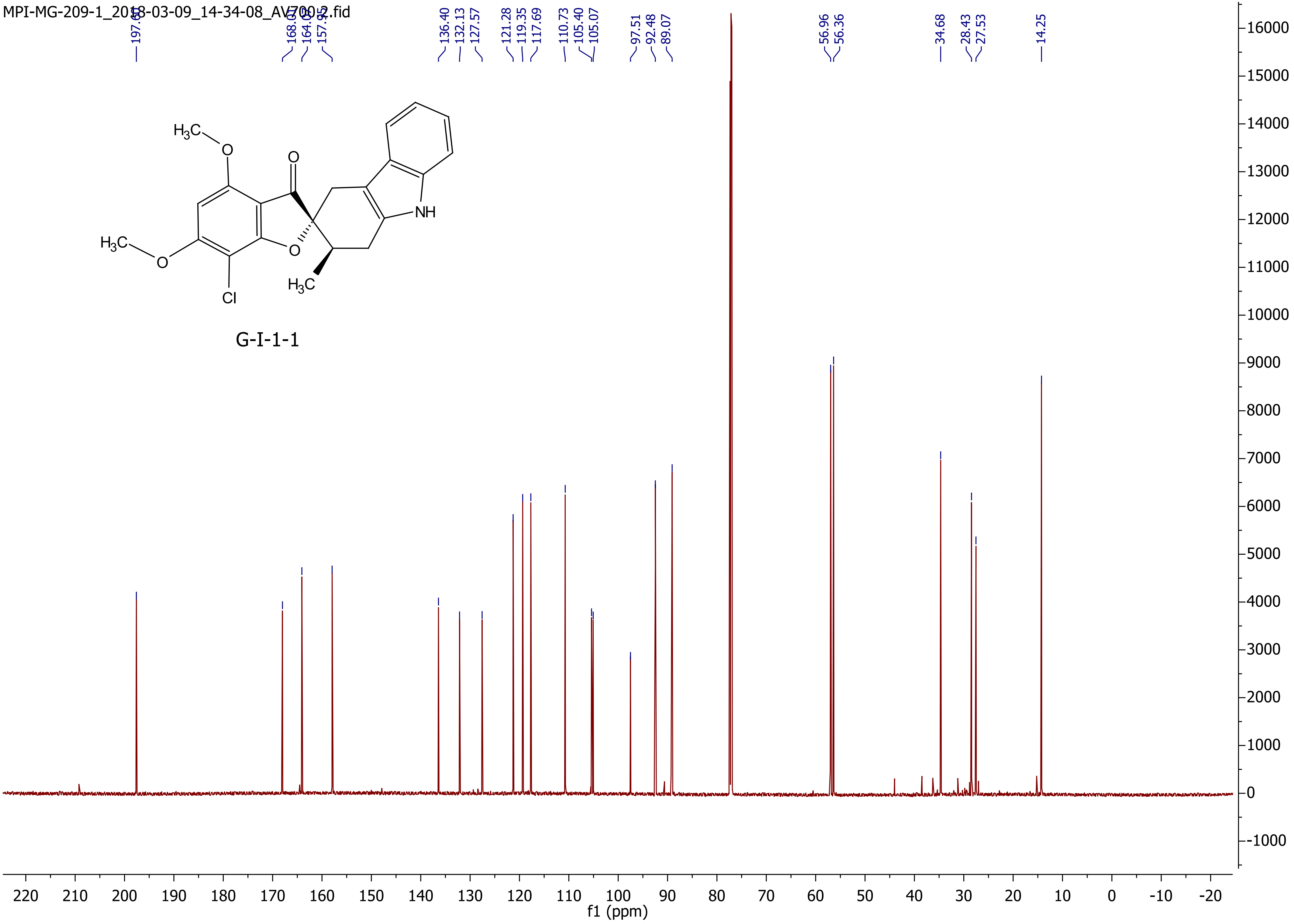

G-I-1-1

MPI-MG-209-2_2018-03-09_14-34-08_AV700.1.fid

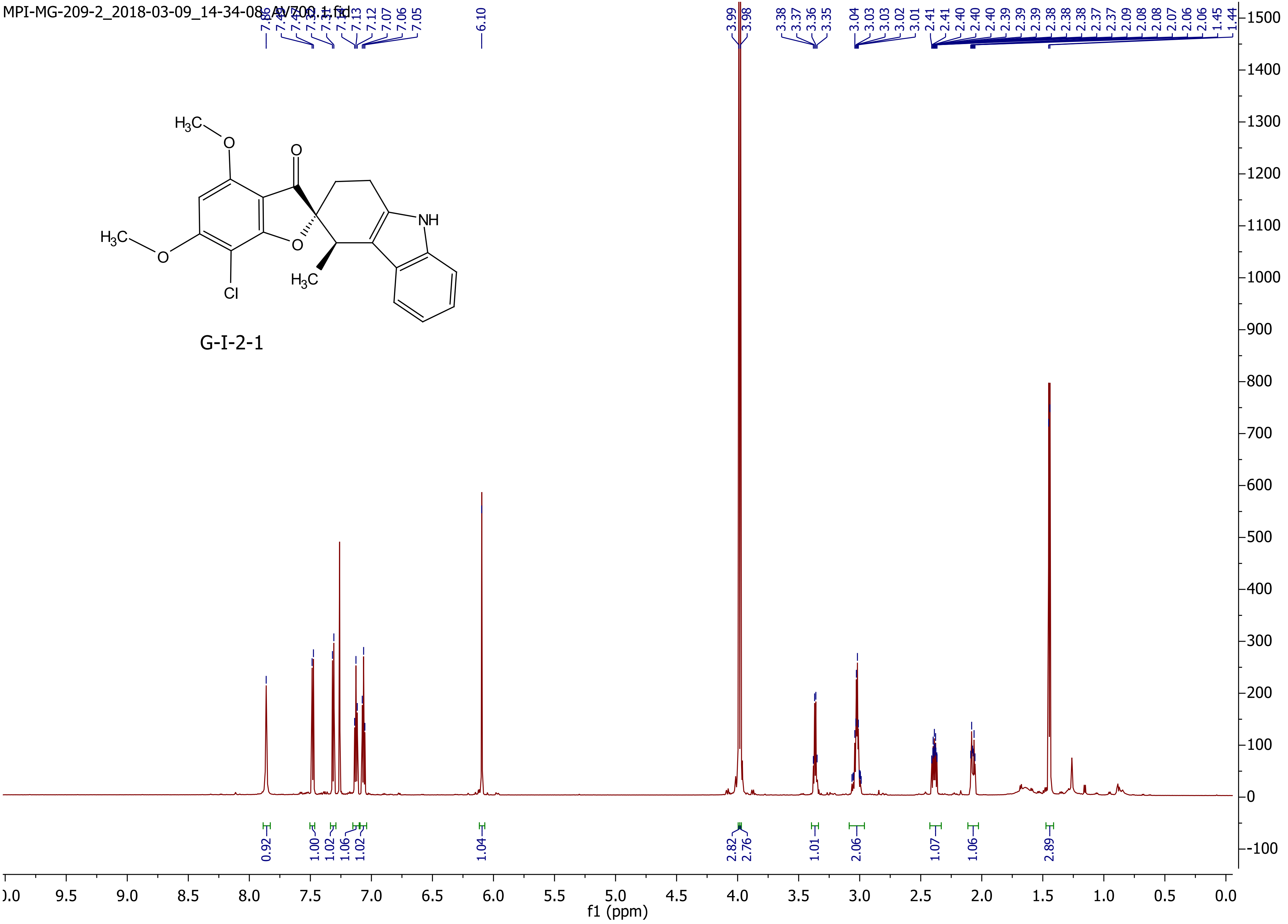

G-I-2-1

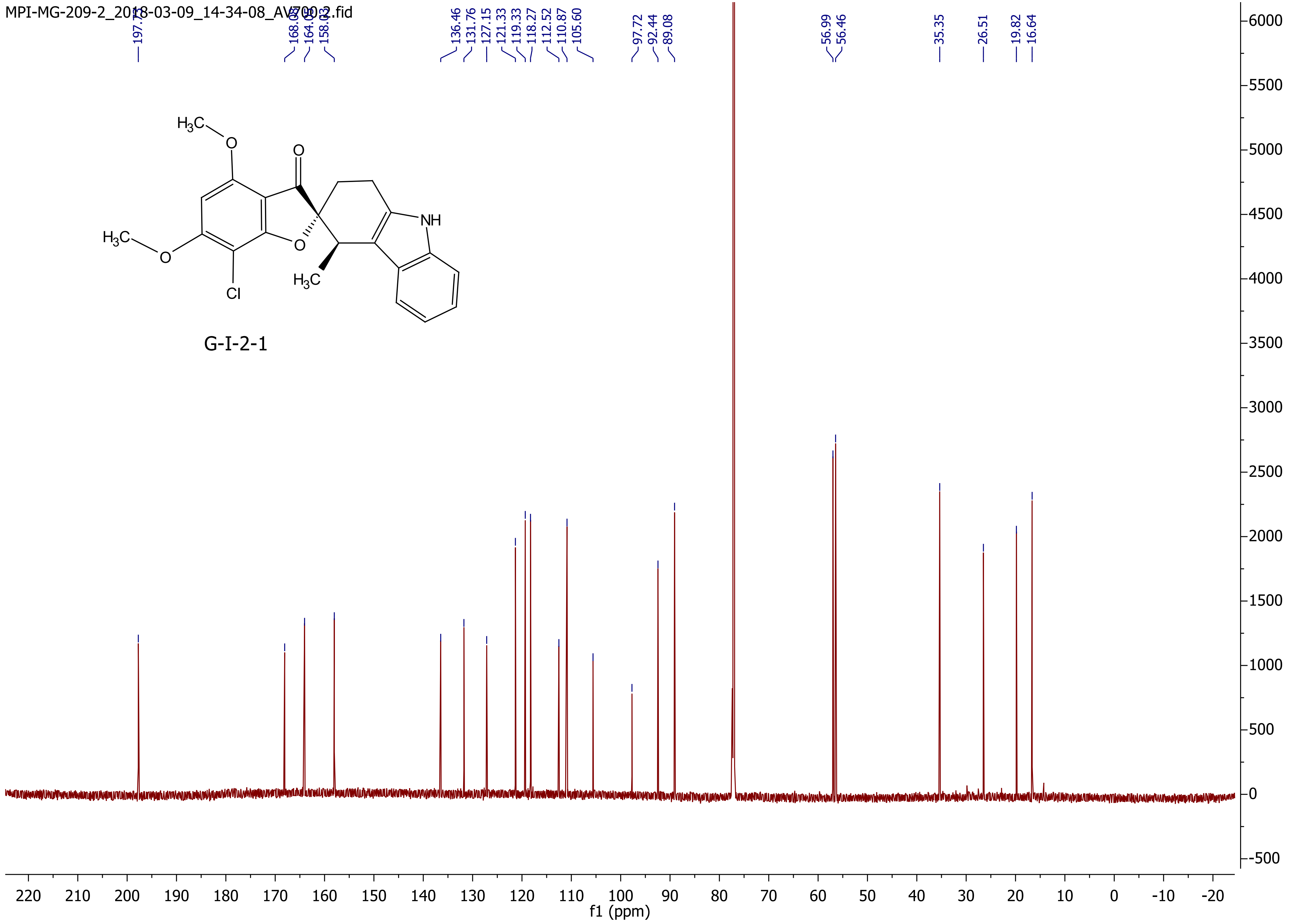
MPI-MG-209-2_2018-03-09_14-34-08_AV700 2.fid
197.7
168.0
164.0
158.0
136.46
131.76
127.15
121.33
119.33
118.27
112.52
110.87
105.60
97.72
92.44
89.08
56.99
56.46
35.35
26.51
19.82
16.64
G-I-2-1
f1 (ppm)

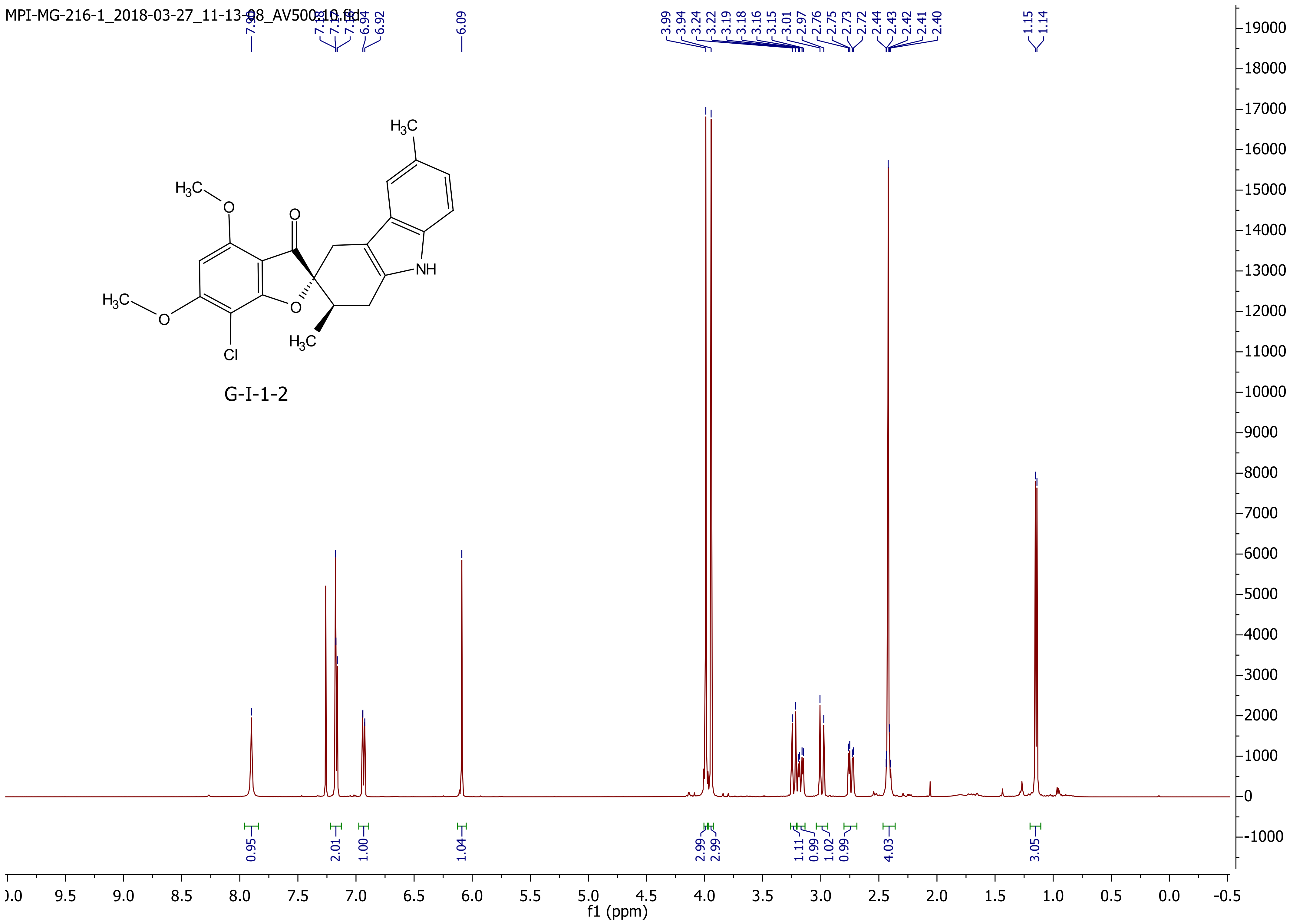

MPI-MG-216-1_2018-03-27_11-13-08_AV500.10.fid
6.94
6.92
6.09
3.99
3.94
3.24
3.22
3.19
3.18
3.16
3.15
3.01
2.97
2.76
2.75
2.73
2.72
2.44
2.43
2.42
2.41
2.40
1.15
1.14
H3C
O
NH
Cl
G-I-1-2
0.95
2.01
1.00
1.04
2.99
2.99
1.11
0.99
1.02
0.99
4.03
3.05
f1 (ppm)

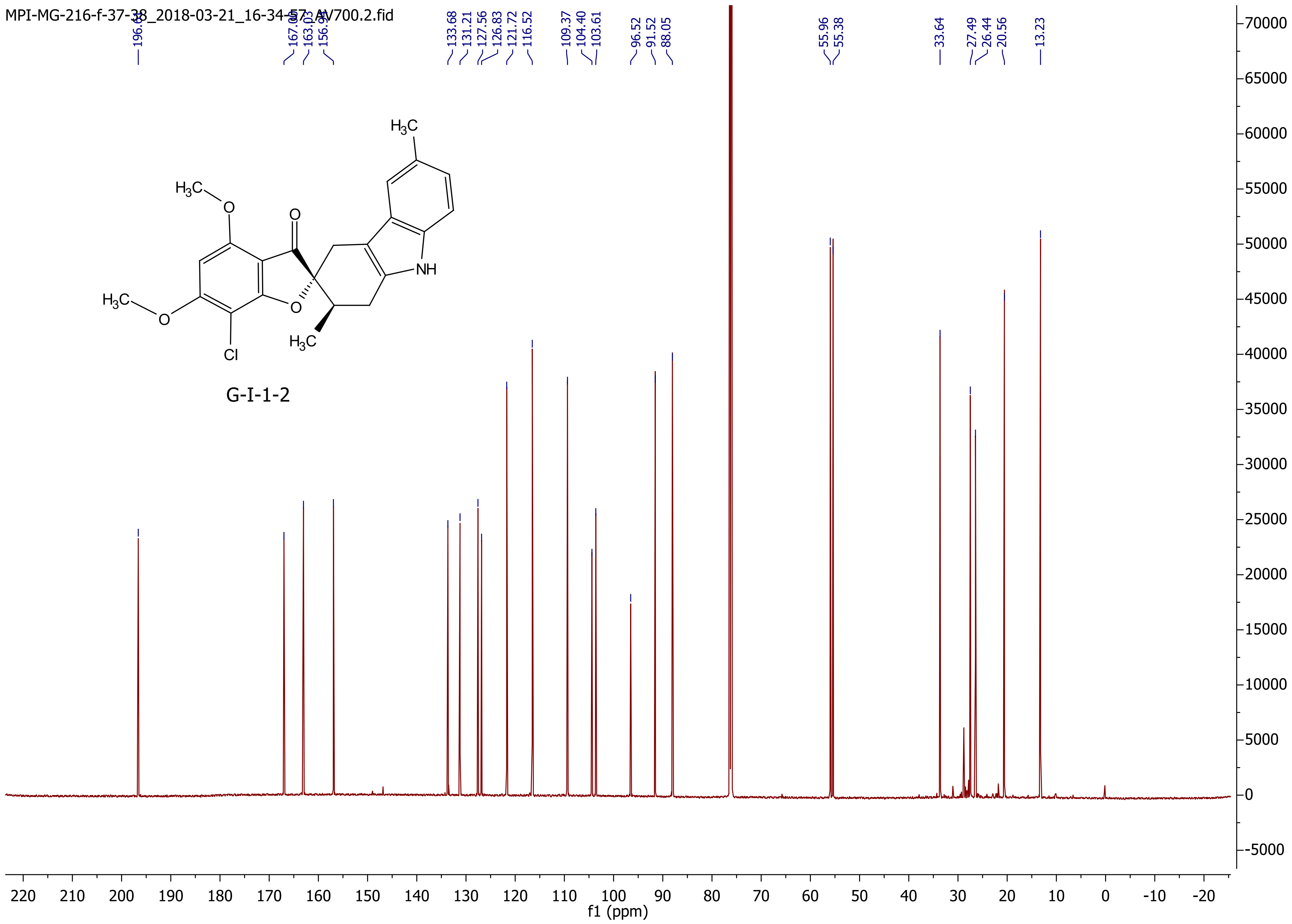
MPI-MG-216-f-37-38_2018-03-21_16-34-57_AV700.2.fid
163.03
133.68
131.21
127.56
126.83
121.72
116.52
109.37
104.40
103.61
96.52
91.52
88.05
55.96
55.38
33.64
27.49
26.44
20.56
13.23
H3C
H3C
O
O
H3C
O
O
NH
H3C
Cl
G-I-1-2
f1 (ppm)

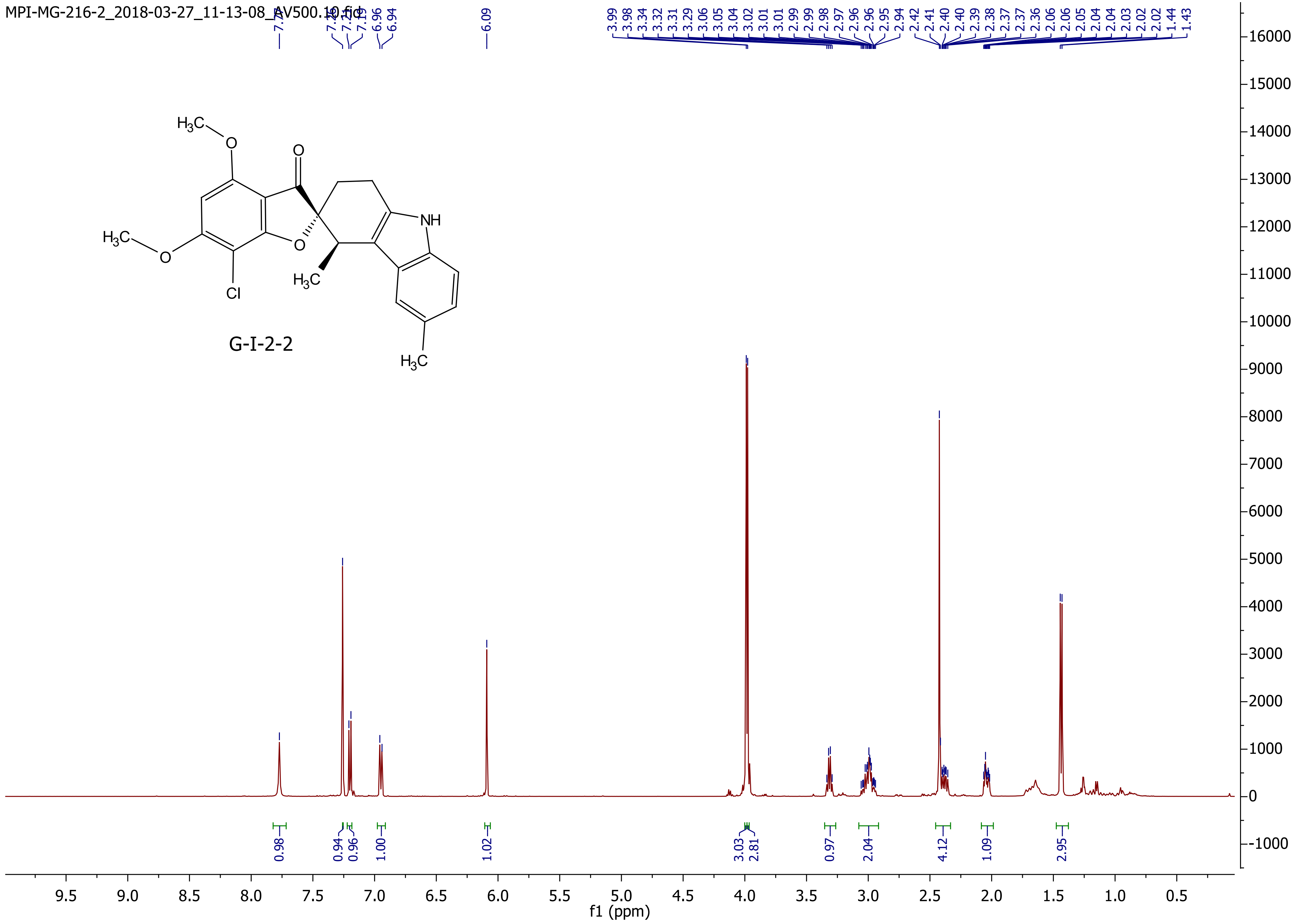

MPI-MG-216-2_2018-03-27_11-13-08_AV500.10.fid
6.96
6.94
6.09
3.99
3.98
3.34
3.32
3.31
3.29
3.06
3.05
3.04
3.02
3.01
3.01
2.99
2.99
2.98
2.97
2.96
2.96
2.95
2.94
2.42
2.41
2.40
2.40
2.39
2.38
2.37
2.37
2.36
2.06
2.06
2.05
2.04
2.04
2.03
2.02
2.02
1.44
1.43
H3C
O
O
H3C
O
NH
O
Cl
H3C
H3C
G-I-2-2
0.98
0.94
0.96
1.00
1.02
3.03
2.81
0.97
2.04
4.12
1.09
2.95
f1 (ppm)

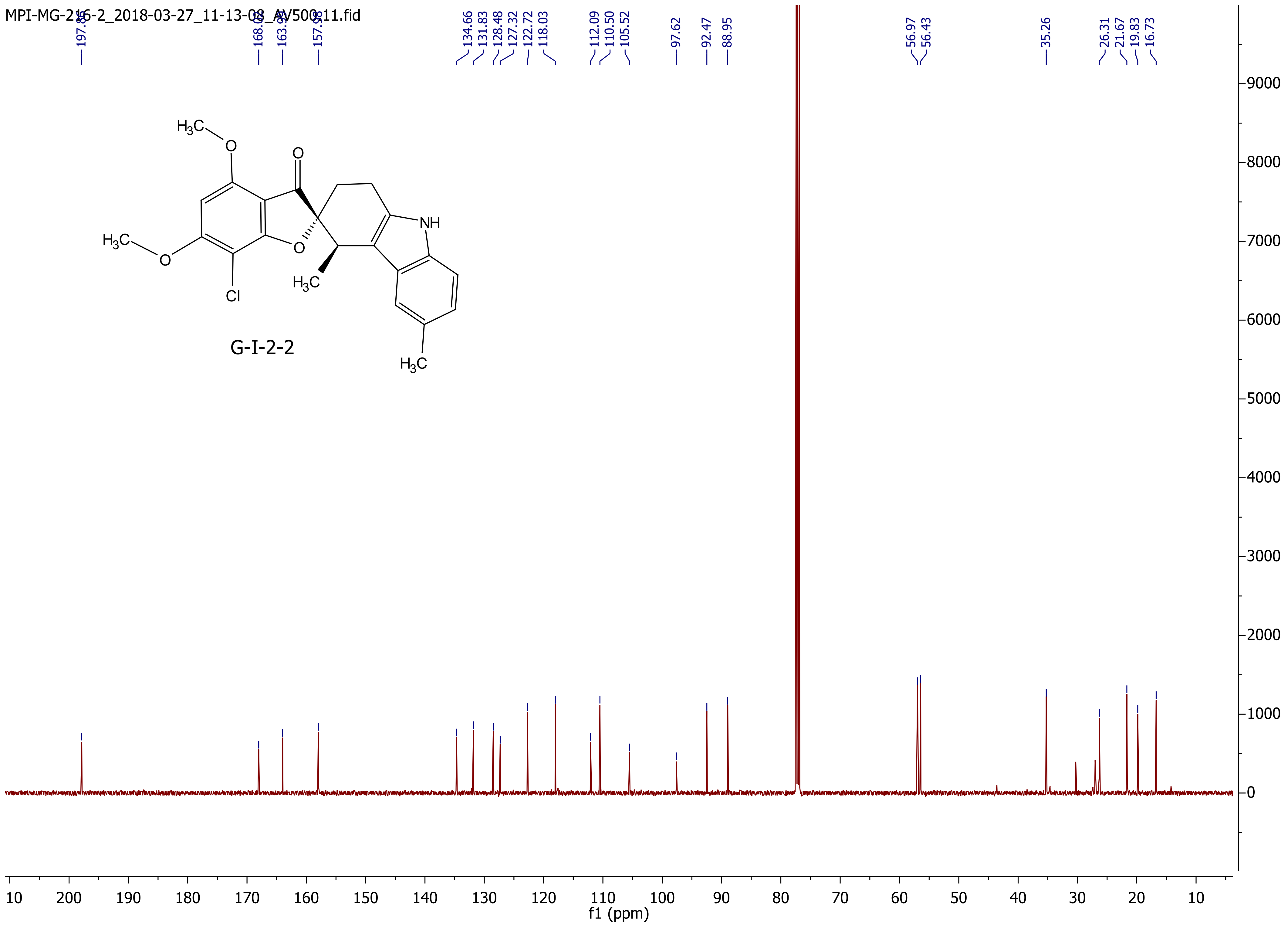

MPI-MG-216-2_2018-03-27_11-13-08_AV50011.fid
197.8
168.0
163.9
157.9
134.66
131.83
128.48
127.32
122.72
118.03
112.09
110.50
105.52
97.62
92.47
88.95
56.97
56.43
35.26
26.31
21.67
19.83
16.73
G-I-2-2
f1 (ppm)

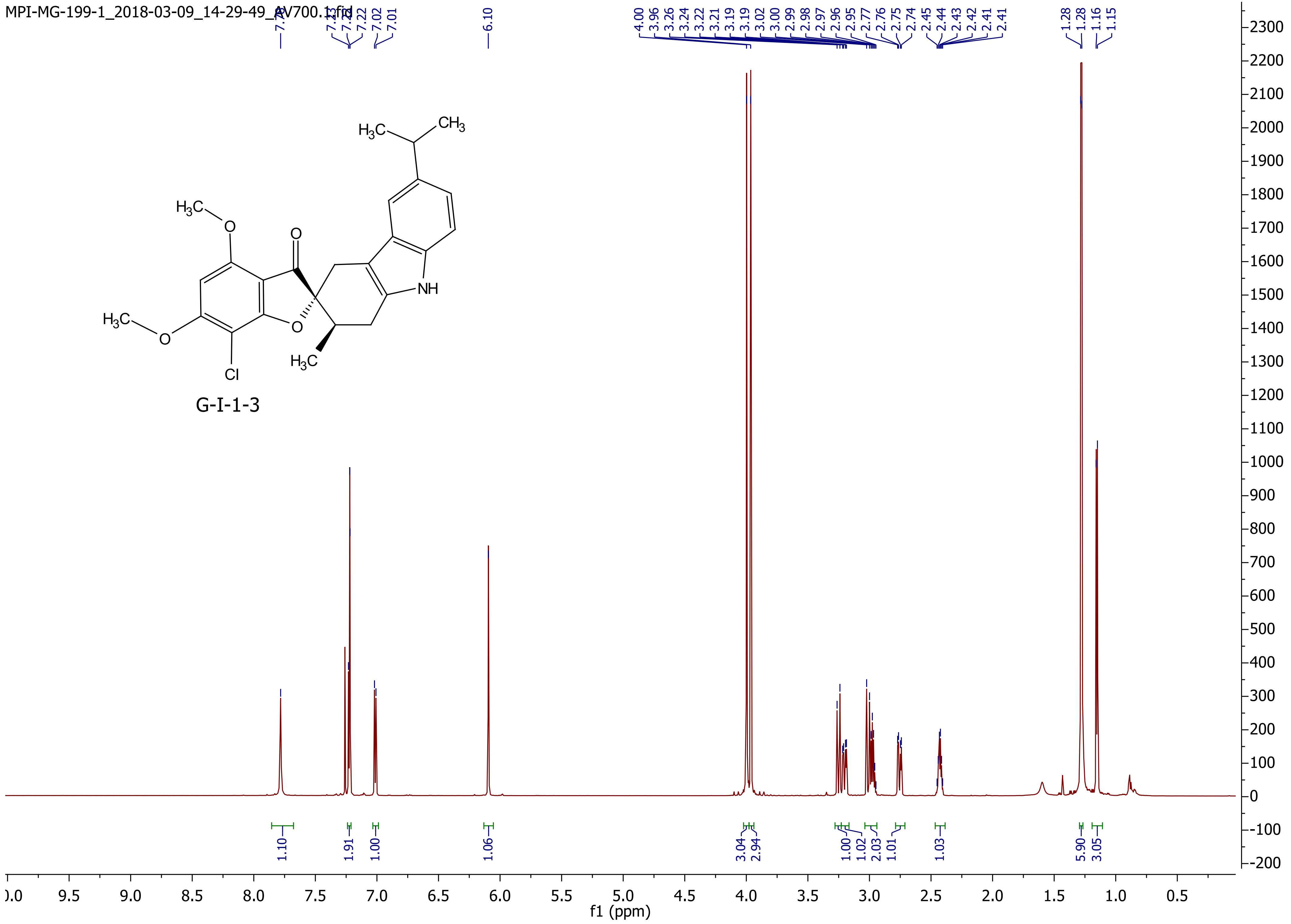

MPI-MG-199-1_2018-03-09_14-29-49_AV700.1.fid
7.78
7.22
7.22
7.02
7.01
6.10
4.00
3.96
3.26
3.24
3.22
3.21
3.19
3.19
3.02
3.00
2.99
2.98
2.97
2.96
2.95
2.77
2.76
2.75
2.74
2.45
2.44
2.43
2.42
2.41
2.41
1.28
1.28
1.16
1.15
H3C
CH3
H3C
O
O
NH
H3C
O
O
H3C
Cl
G-I-1-3
1.10
1.91
1.00
1.06
3.04
2.94
1.00
1.02
2.03
1.01
1.03
5.90
3.05
f1 (ppm)

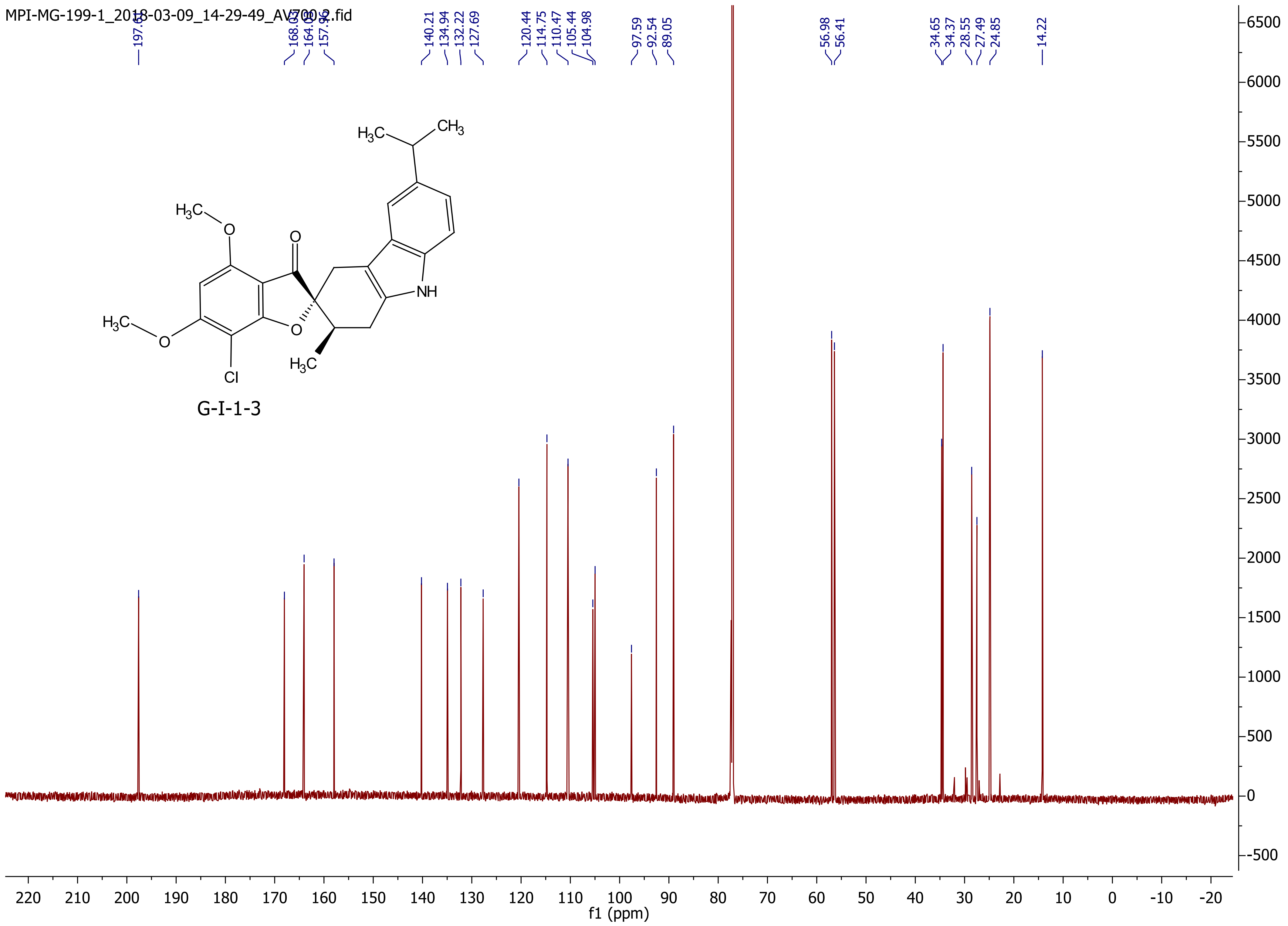

MPI-MG-199-1_2018-03-09_14-29-49_AV700 2.fid
140.21
134.94
132.22
127.69
120.44
114.75
110.47
105.44
104.98
97.59
92.54
89.05
56.98
56.41
34.65
34.37
28.55
27.49
24.85
14.22
H3C
CH3
O
NH
Cl
G-I-1-3
f1 (ppm)

MPI-MG-220-2_2018-05-09_07-49-32_AV600.10.fid
H1
z_Proton CDCl3 /NMR-Daten MPI 39

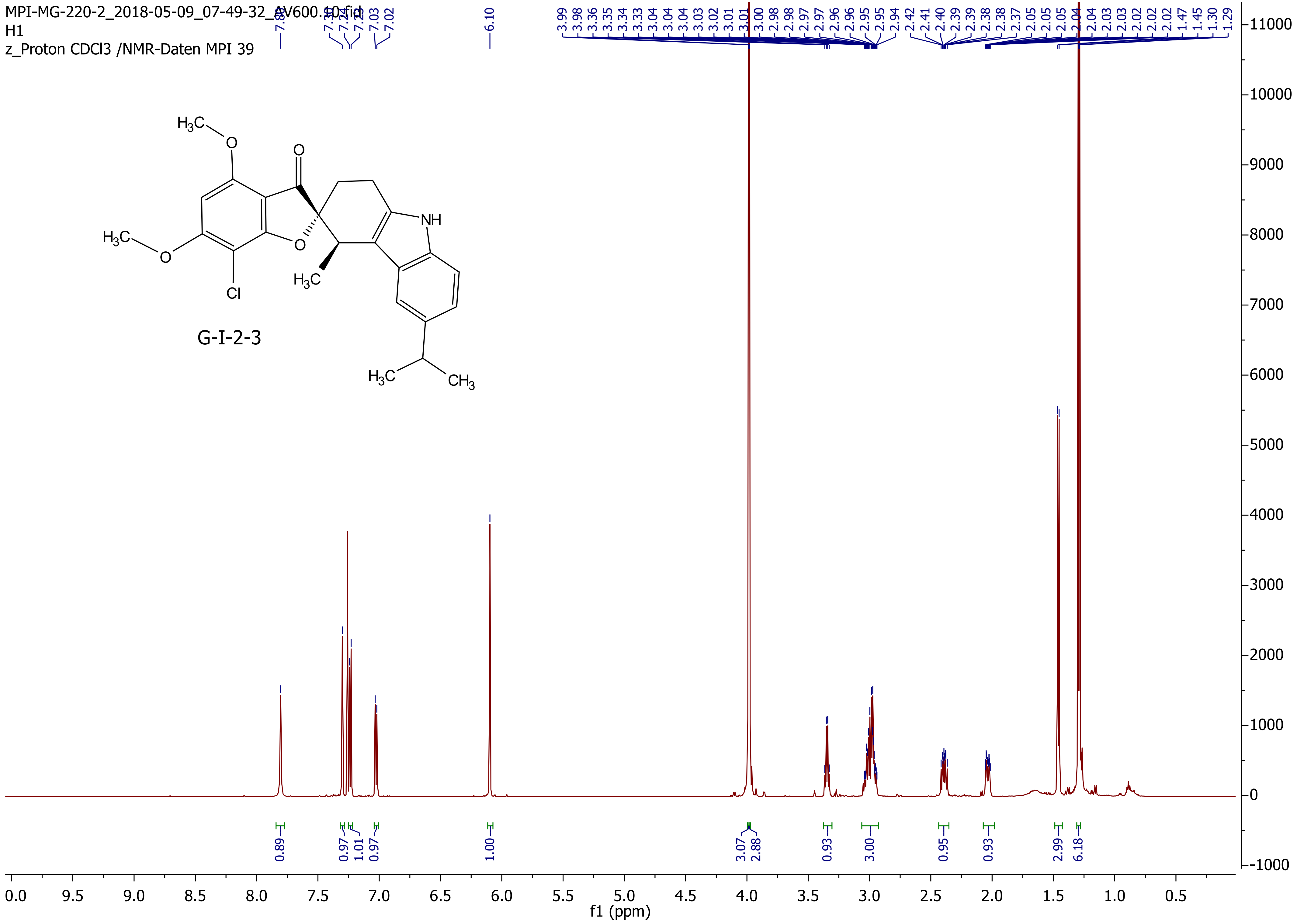

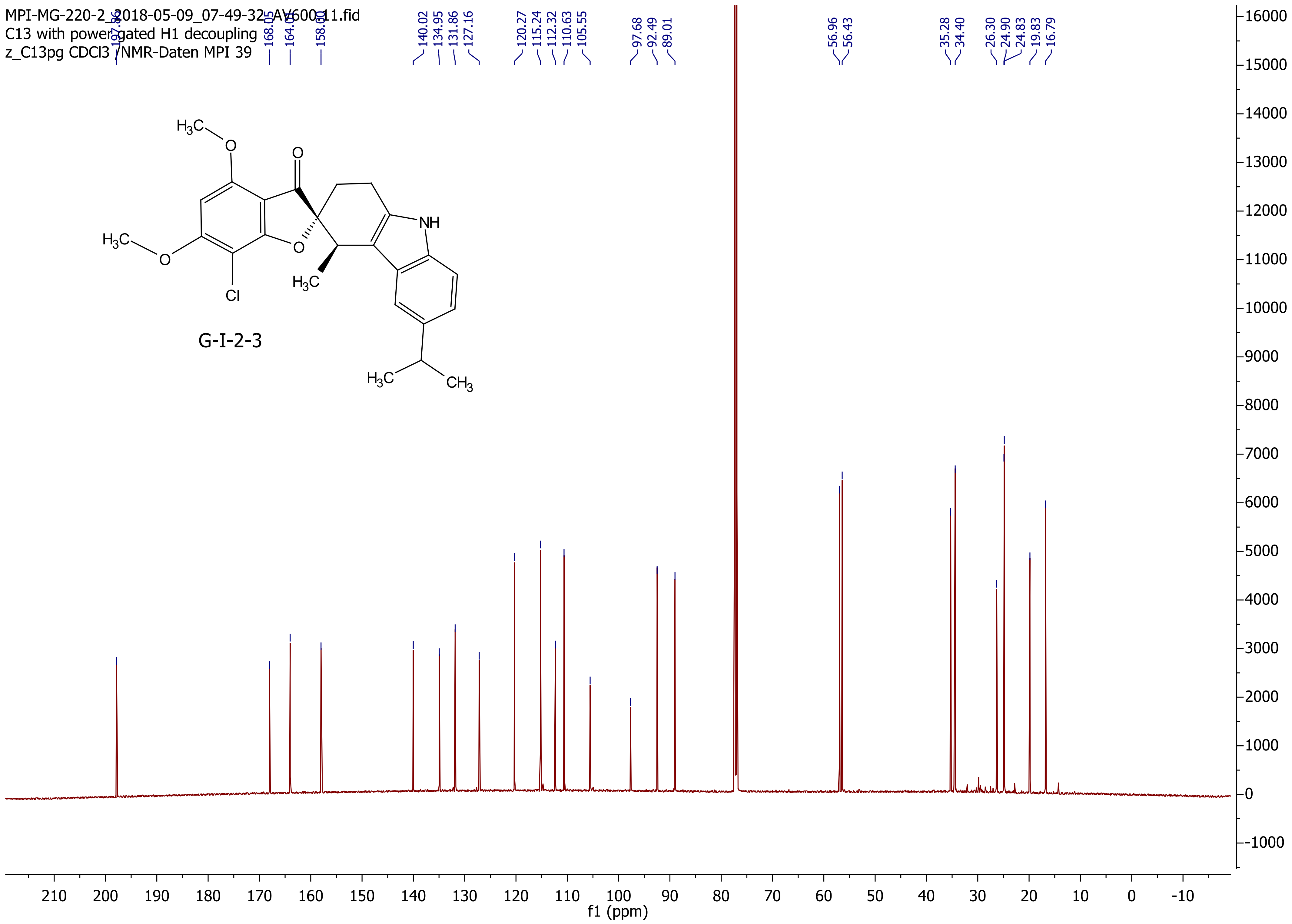
MPI-MG-220-2_2018-05-09_07-49-32_AV600_11.fid
C13 with power gated H1 decoupling
z_C13pg CDCl3 /NMR-Daten MPI 39
168.05
140.02
134.95
131.86
127.16
120.27
115.24
112.32
110.63
105.55
97.68
92.49
89.01
56.96
56.43
35.28
34.40
26.30
24.90
24.83
19.83
16.79
G-I-2-3
f1 (ppm)

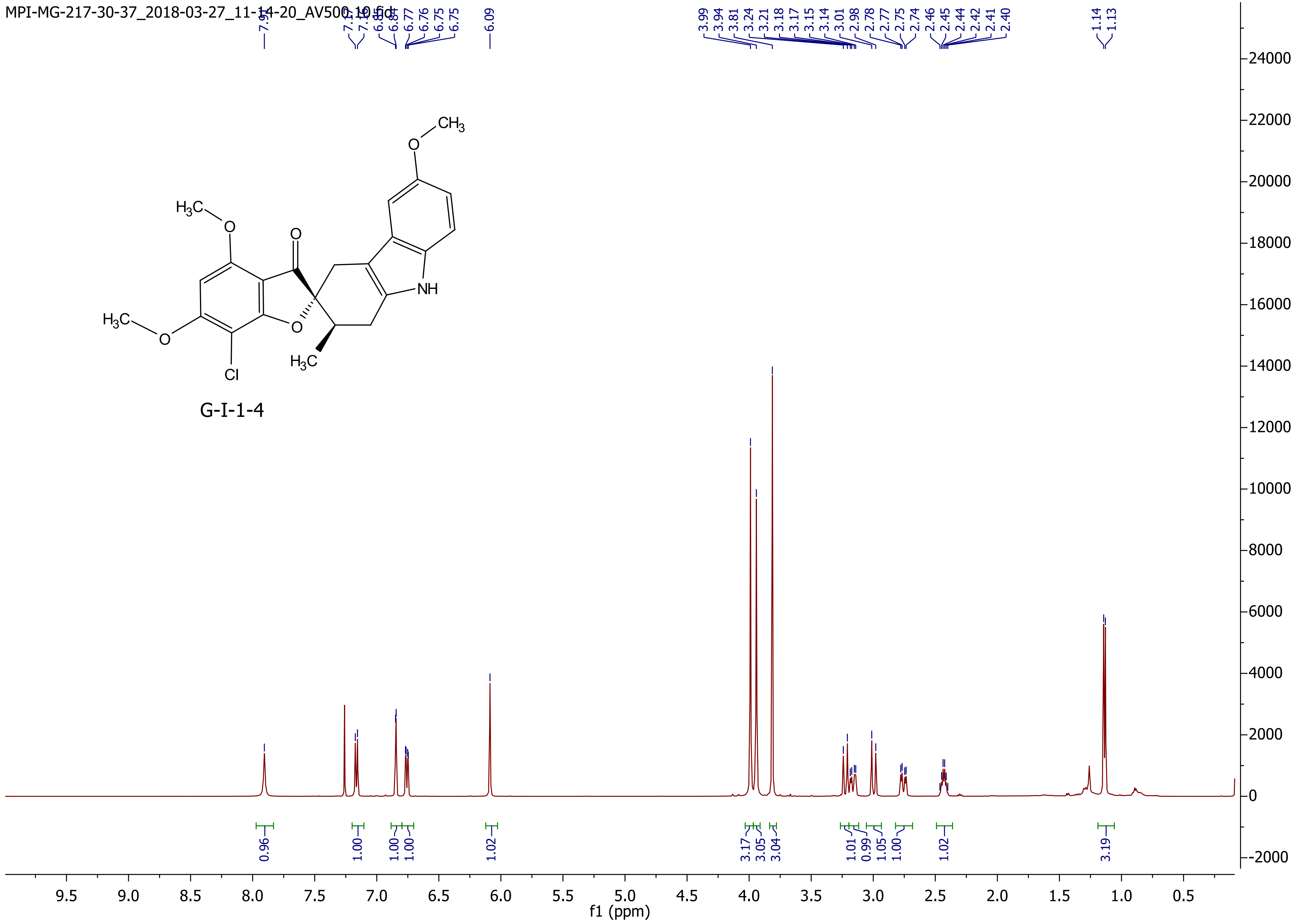

G-I-1-4

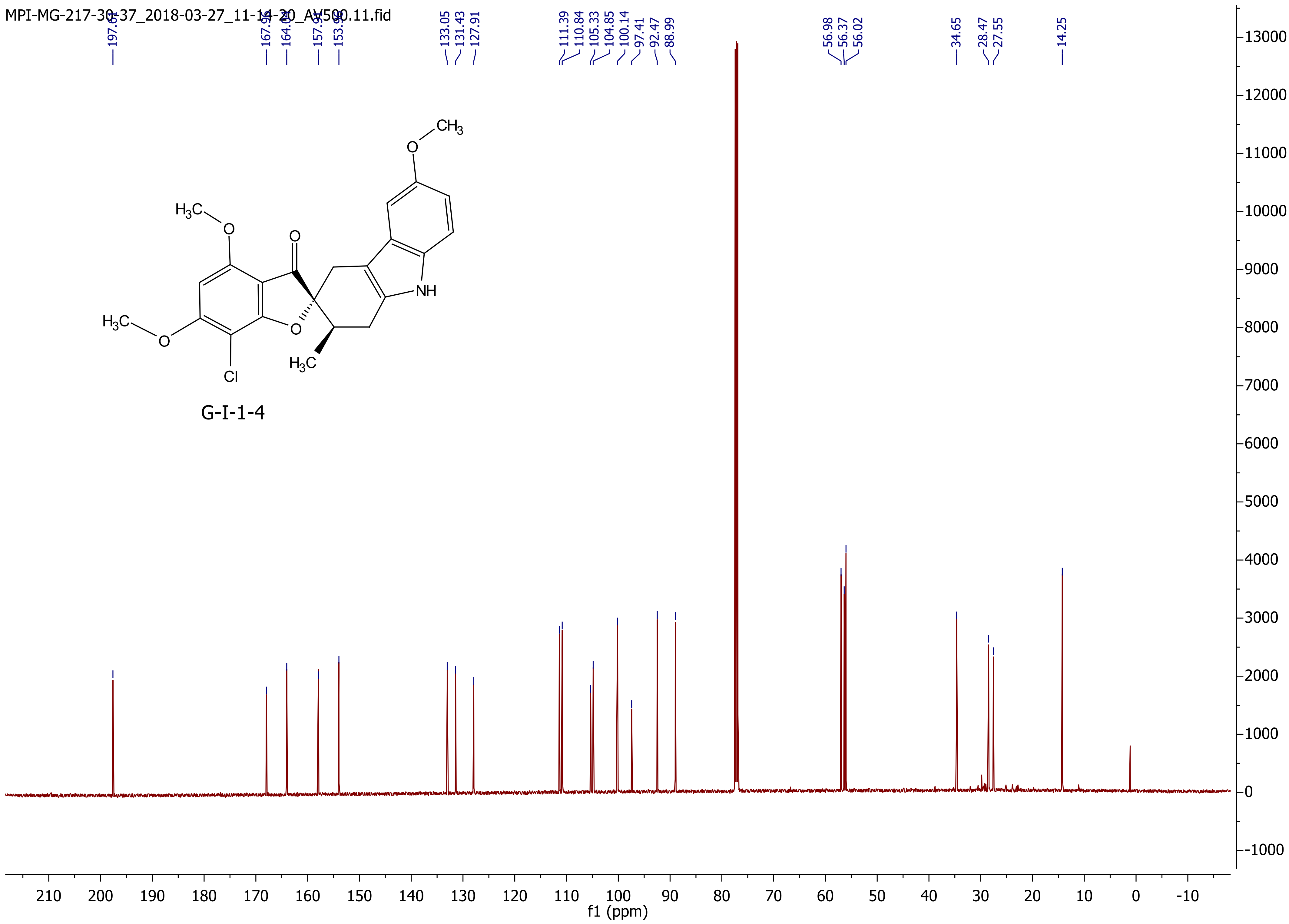
MPI-MG-217-30-37_2018-03-27_11-14-20_AV500.11.fid
133.05
131.43
127.91
111.39
110.84
105.33
104.85
100.14
97.41
92.47
88.99
56.98
56.37
56.02
34.65
28.47
27.55
14.25
CH3
O
H3C
O
O
O
NH
H3C
O
H3C
Cl
G-I-1-4
13000
12000
11000
10000
9000
8000
7000
6000
5000
4000
3000
2000
1000
0
-1000
210 200 190 180 170 160 150 140 130 120 110 100 90 80 70 60 50 40 30 20 10 0 -10
f1 (ppm)

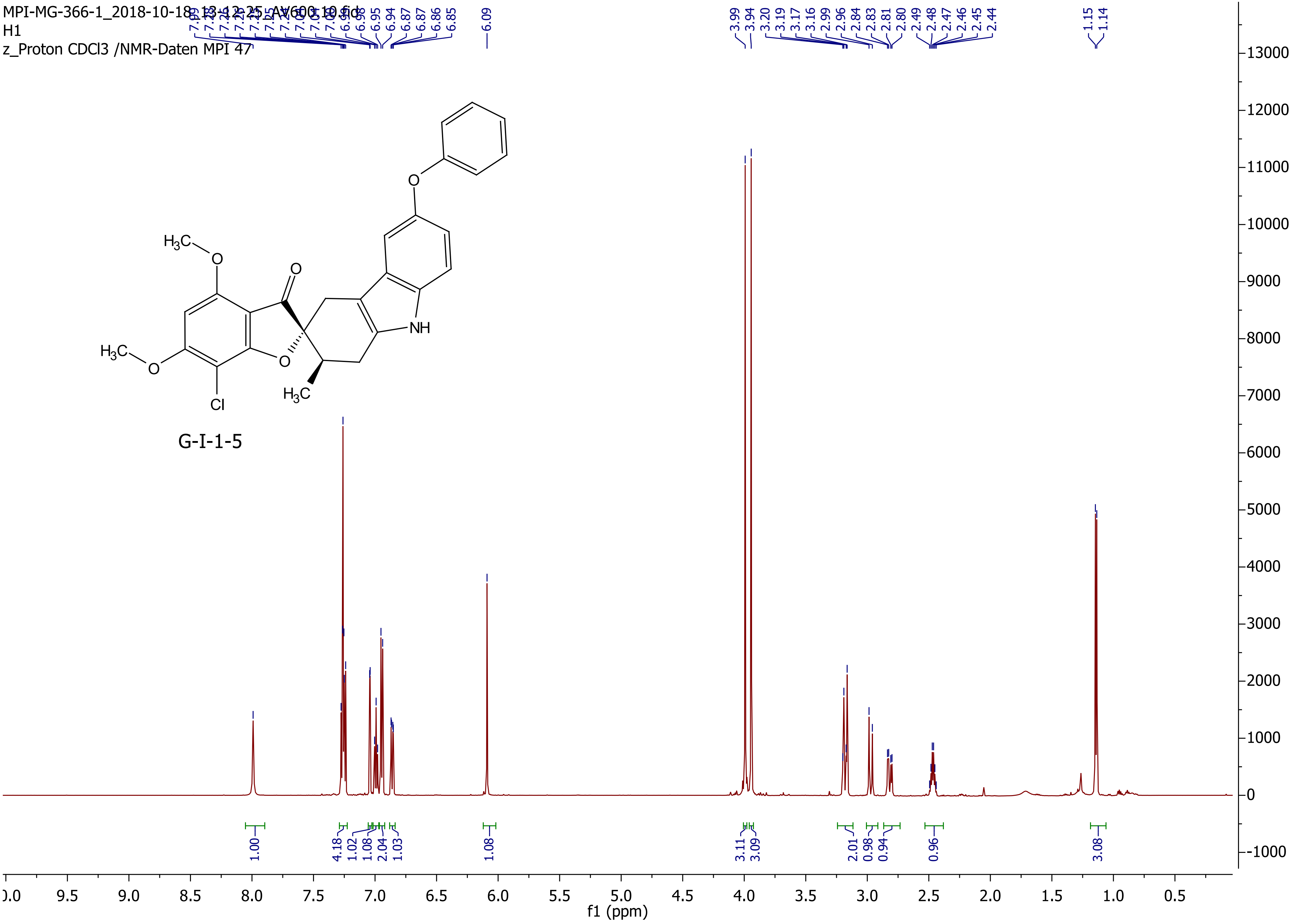

MPI-MG-366-1_2018-10-18_13-12-25_AV600 10.fid
H1
z_Proton CDCl3 /NMR-Daten MPI 47
6.95
6.94
6.87
6.87
6.86
6.85
6.09
3.99
3.94
3.20
3.19
3.17
3.16
2.99
2.96
2.84
2.83
2.81
2.80
2.49
2.48
2.47
2.46
2.45
2.44
1.15
1.14
H_3C
O
O
H_3C
O
Cl
O
H_3C
O
NH
1.00
4.18
1.02
1.08
2.04
1.03
1.08
3.11
3.09
2.01
0.98
0.94
0.96
3.08
f1 (ppm)

G-I-1-5

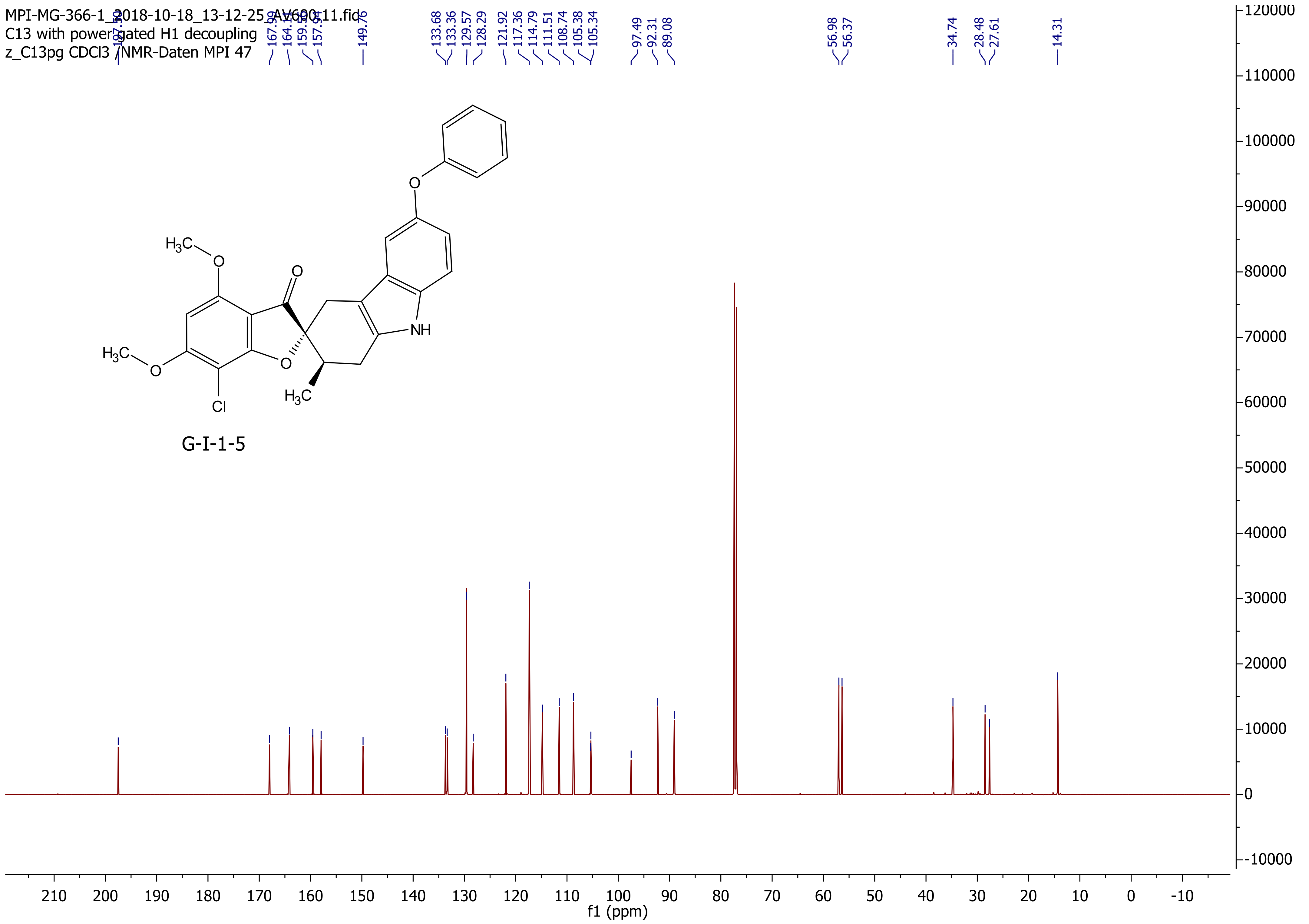

MPI-MG-366-1_2018-10-18_13-12-25_AV600.11.fid
C13 with power gated H1 decoupling
z_C13pg CDCl3 /NMR-Daten MPI 47
197.50
167.99
164.11
159.56
157.94
149.76
133.68
133.36
129.57
128.29
121.92
117.36
114.79
111.51
108.74
105.38
105.34
97.49
92.31
89.08
56.98
56.37
34.74
28.48
27.61
14.31
H_3C
O
O
H_3C
O
O
Cl
H_3C
NH
O
G-I-1-5
f1 (ppm)

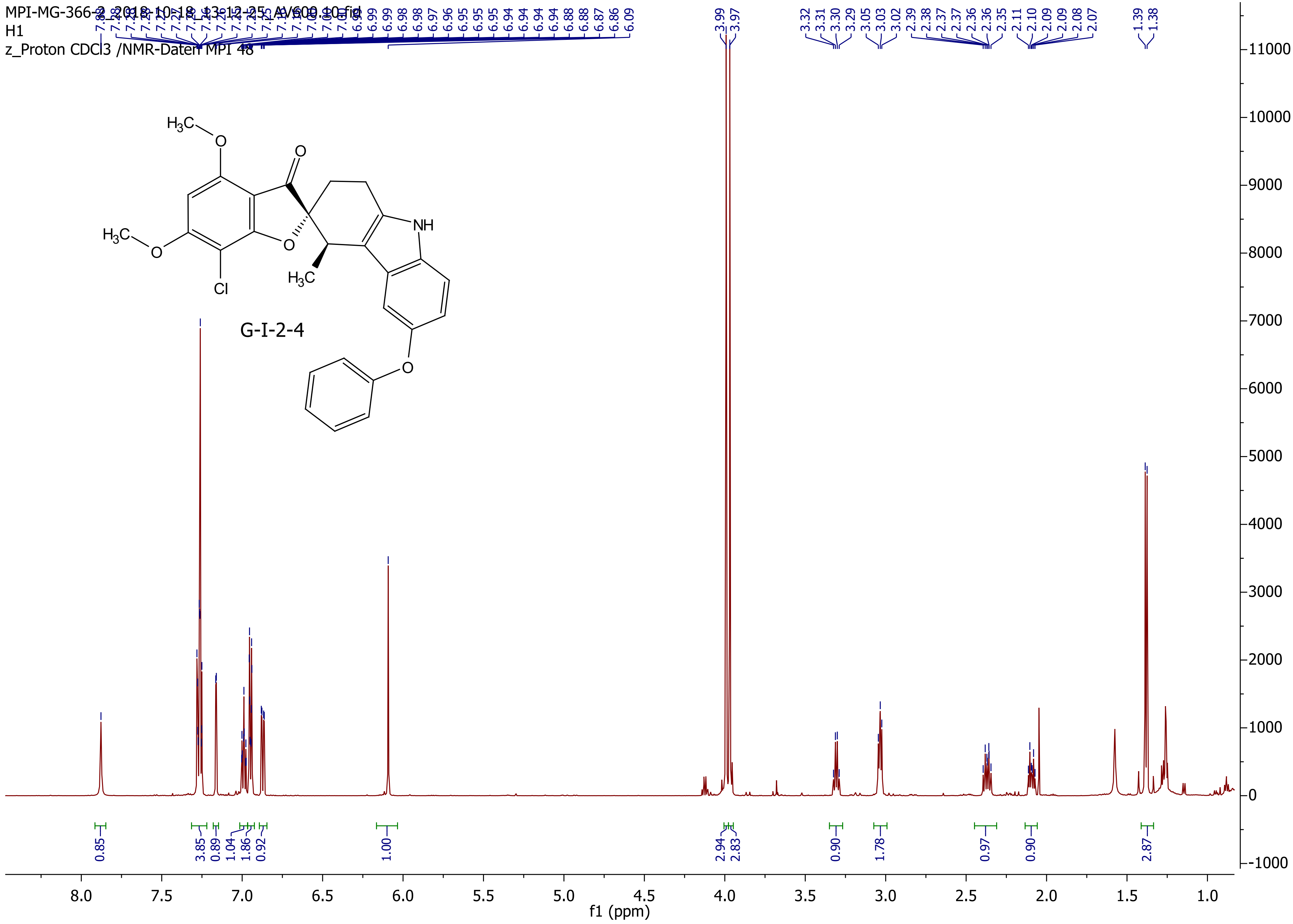

MPI-MG-366-2 2018-10-18 13-12-25 AV600.10.fid
H1
z_Proton CDCl3 /NMR-Daten MPI 48
6.99 6.98 6.98 6.97 6.96 6.95 6.95 6.95 6.94 6.94 6.94 6.94 6.88 6.88 6.87 6.86 6.09
3.99 3.97
3.32 3.31 3.30 3.29 3.05 3.03 3.02 2.39 2.38 2.37 2.37 2.36 2.36 2.35 2.11 2.10 2.09 2.09 2.08 2.07
1.39 1.38
G-I-2-4
0.85 3.85 0.89 1.04 1.86 0.92 1.00 2.94 2.83 0.90 1.78 0.97 0.90 2.87
f1 (ppm)

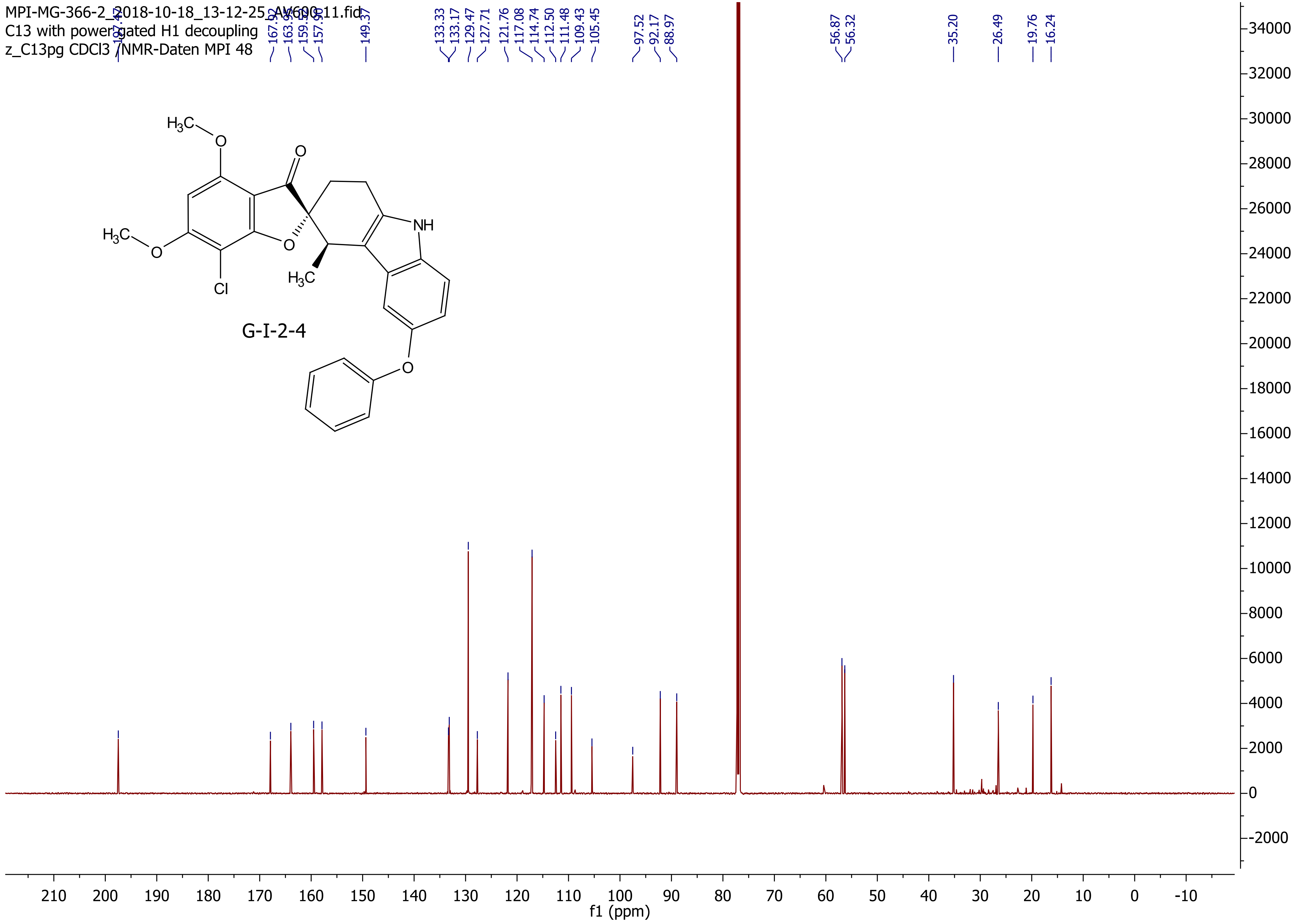
MPI-MG-366-2_2018-10-18_13-12-25_AV600 11.fid
C13 with power gated H1 decoupling
z_C13pg CDCl3 /NMR-Daten MPI 48
167.92
149.37
133.33
133.17
129.47
127.71
121.76
117.08
114.74
112.50
111.48
109.43
105.45
97.52
92.17
88.97
56.87
56.32
35.20
26.49
19.76
16.24
H3C
O
O
H3C
O
Cl
O
H3C
NH
O
G-I-2-4
f1 (ppm)

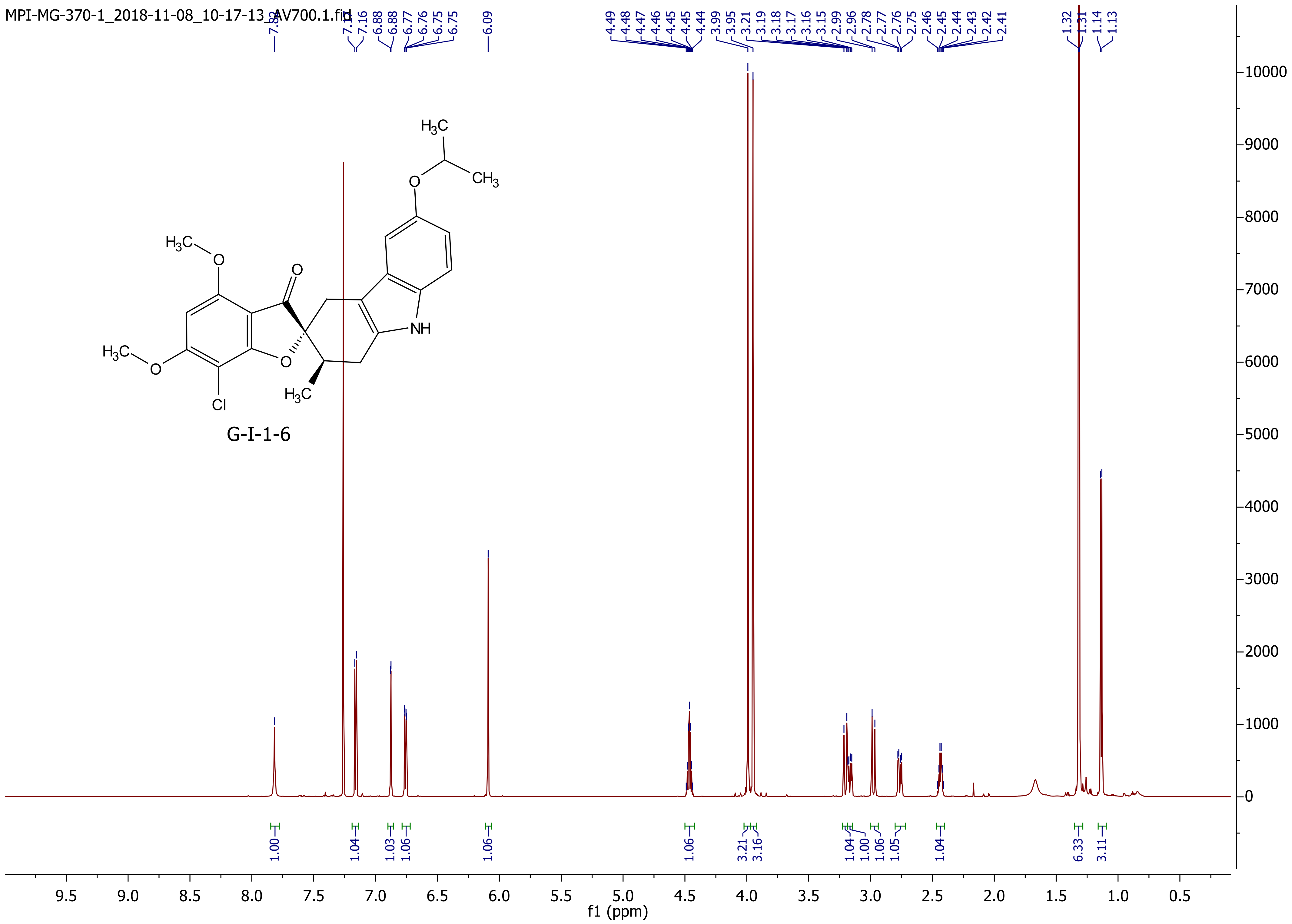
MPI-MG-370-1_2018-11-08_10-17-13_AV700.1.fid
G-I-1-6
f1 (ppm)

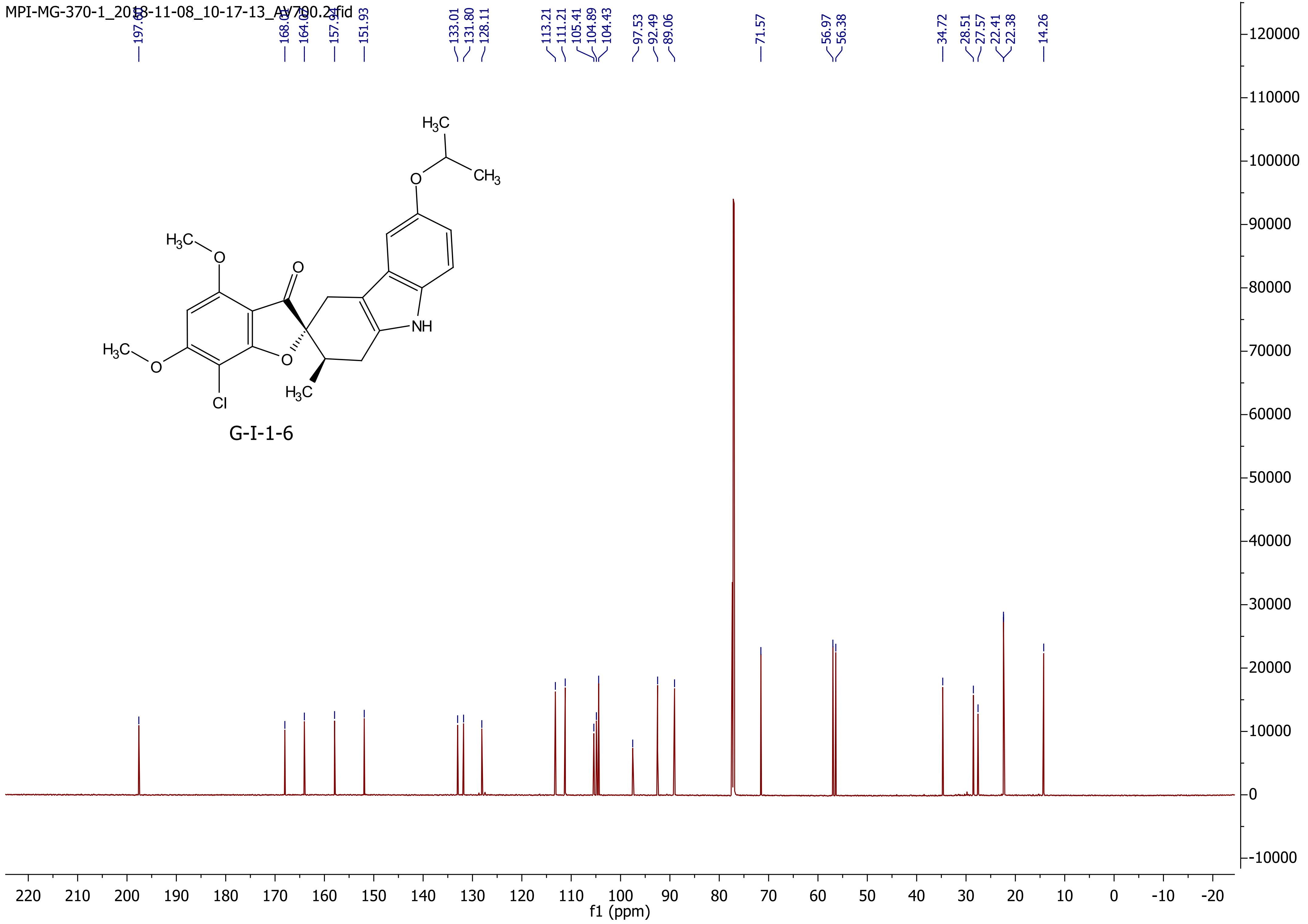

MPI-MG-370-1_2018-11-08_10-17-13_AV700.2.fid
151.93
133.01
131.80
128.11
113.21
111.21
105.41
104.89
104.43
97.53
92.49
89.06
71.57
56.97
56.38
34.72
28.51
27.57
22.41
22.38
14.26
H3C
CH3
O
H3C
O
O
NH
H3C
O
O
Cl
H3C
G-I-1-6
f1 (ppm)
220 210 200 190 180 170 160 150 140 130 120 110 100 90 80 70 60 50 40 30 20 10 0 -10 -20
120000
110000
100000
90000
80000
70000
60000
50000
40000
30000
20000
10000
0
-10000

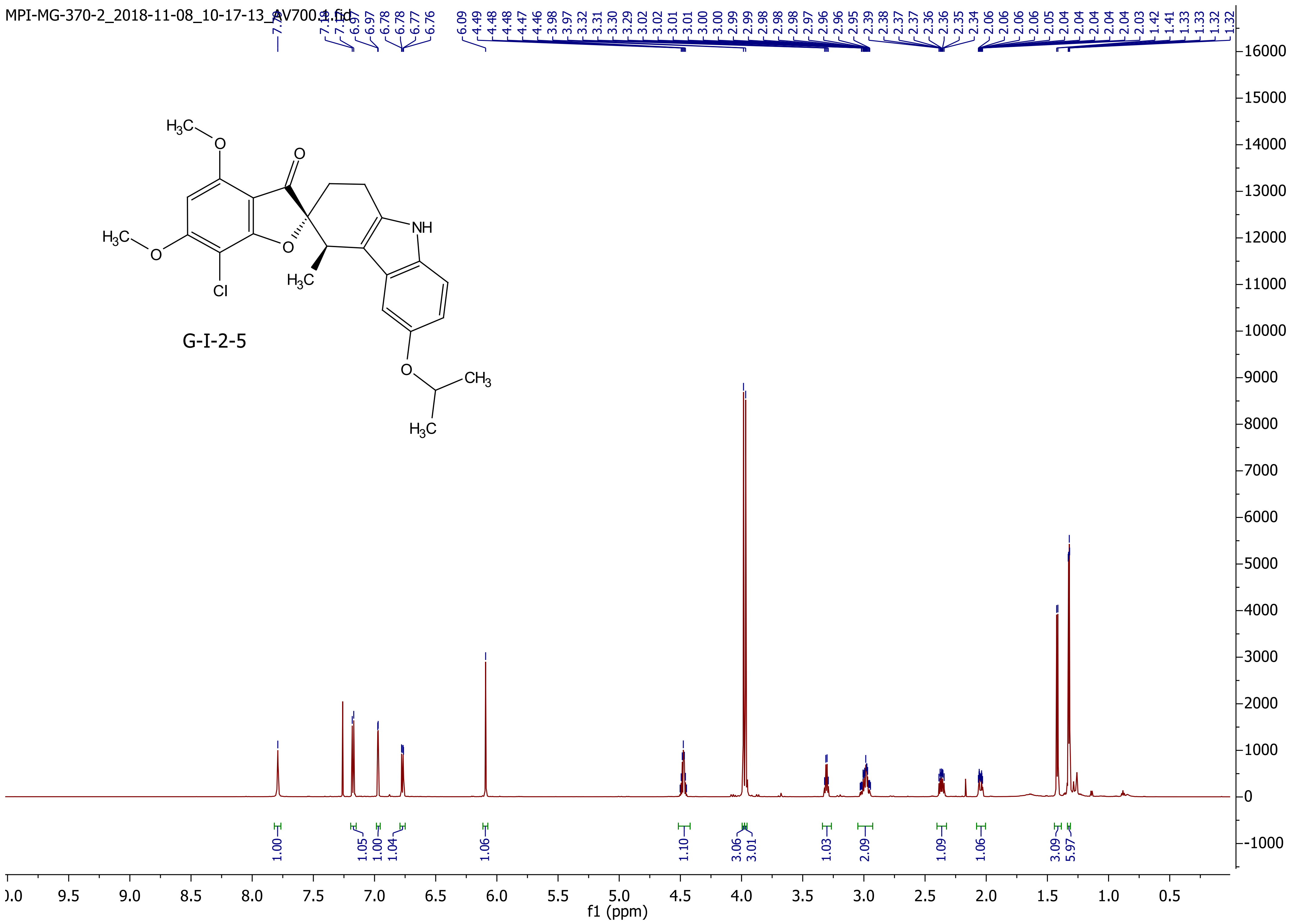

MPI-MG-370-2_2018-11-08_10-17-13_AV700.1.fid
6.97
6.97
6.78
6.78
6.77
6.76
6.09
4.49
4.48
4.48
4.47
4.46
3.98
3.97
3.32
3.31
3.30
3.29
3.02
3.02
3.01
3.01
3.00
3.00
2.99
2.99
2.98
2.98
2.98
2.97
2.96
2.96
2.95
2.39
2.38
2.37
2.37
2.36
2.36
2.35
2.34
2.06
2.06
2.06
2.06
2.05
2.04
2.04
2.04
2.04
2.04
2.03
1.42
1.41
1.33
1.33
1.32
1.32
H3C
O
O
H3C
O
O
NH
Cl
H3C
O
CH3
H3C
G-I-2-5
1.00
1.05
1.00
1.04
1.06
1.10
3.06
3.01
1.03
2.09
1.09
1.06
3.09
5.97
f1 (ppm)

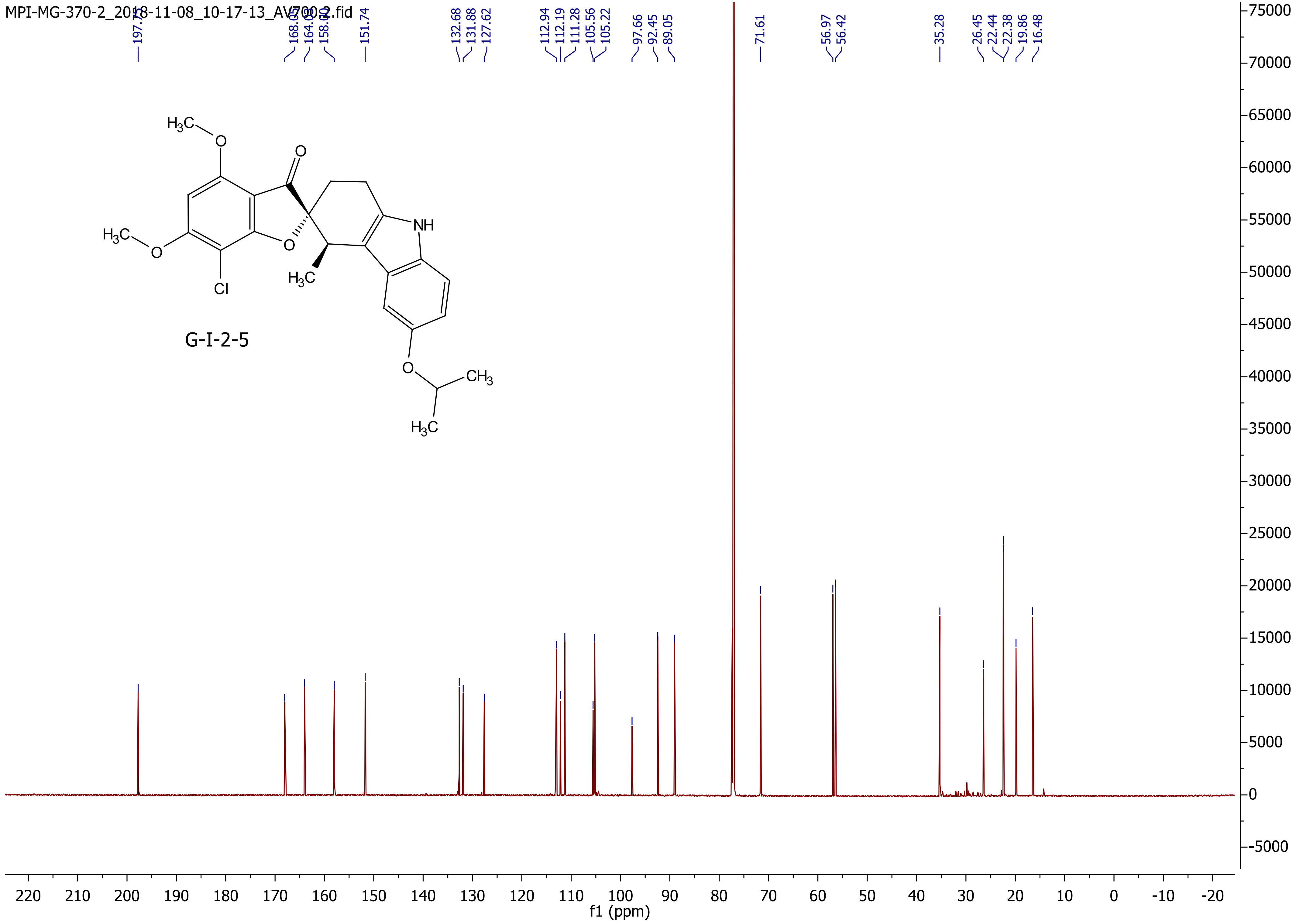
MPI-MG-370-2_2018-11-08_10-17-13_AV700 2.fid
151.74
132.68
131.88
127.62
112.94
112.19
111.28
105.56
105.22
97.66
92.45
89.05
71.61
56.97
56.42
35.28
26.45
22.44
22.38
19.86
16.48
H_3C
O
O
H_3C
O
O
NH
Cl
H_3C
O
CH_3
H_3C
G-I-2-5
f1 (ppm)

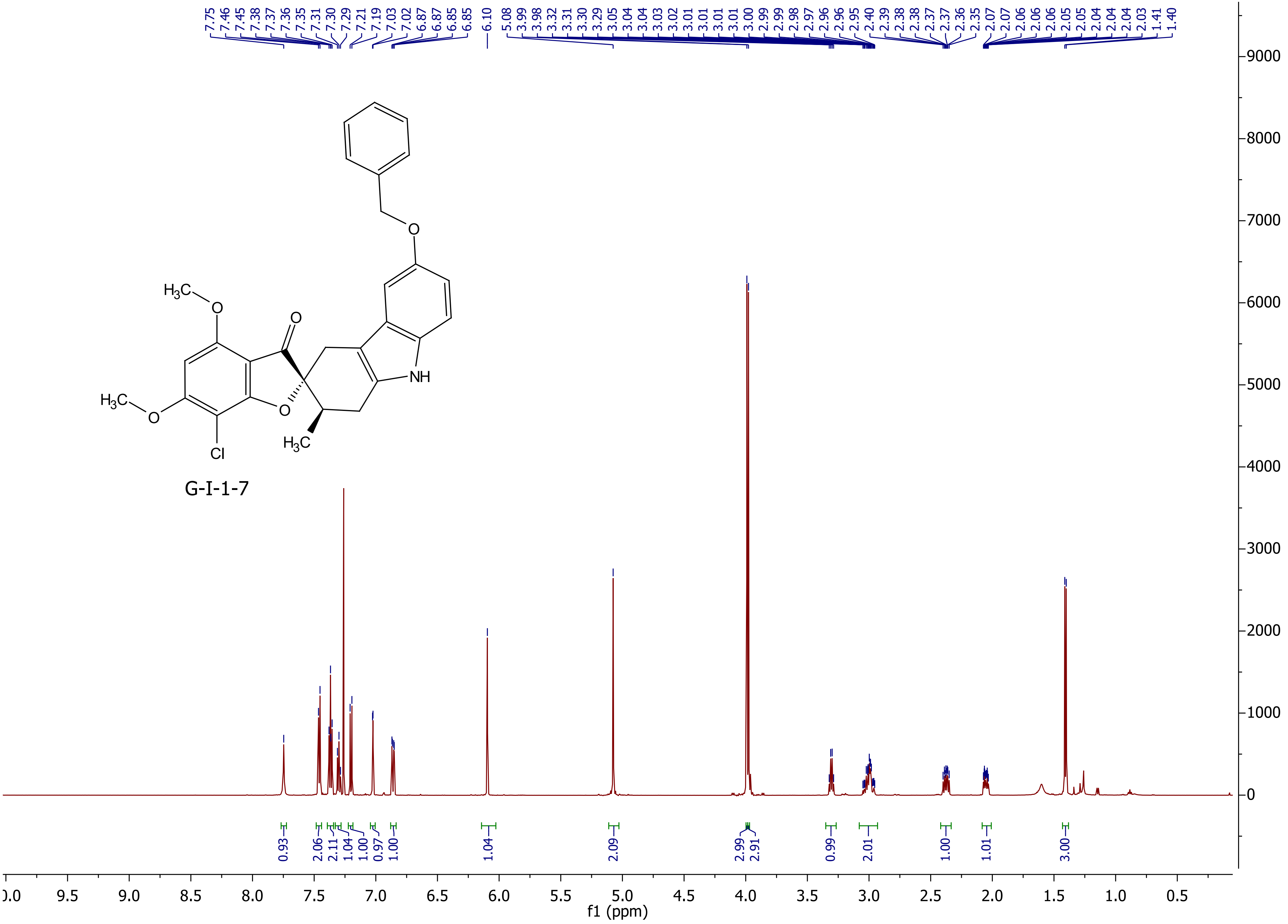

G-I-1-7

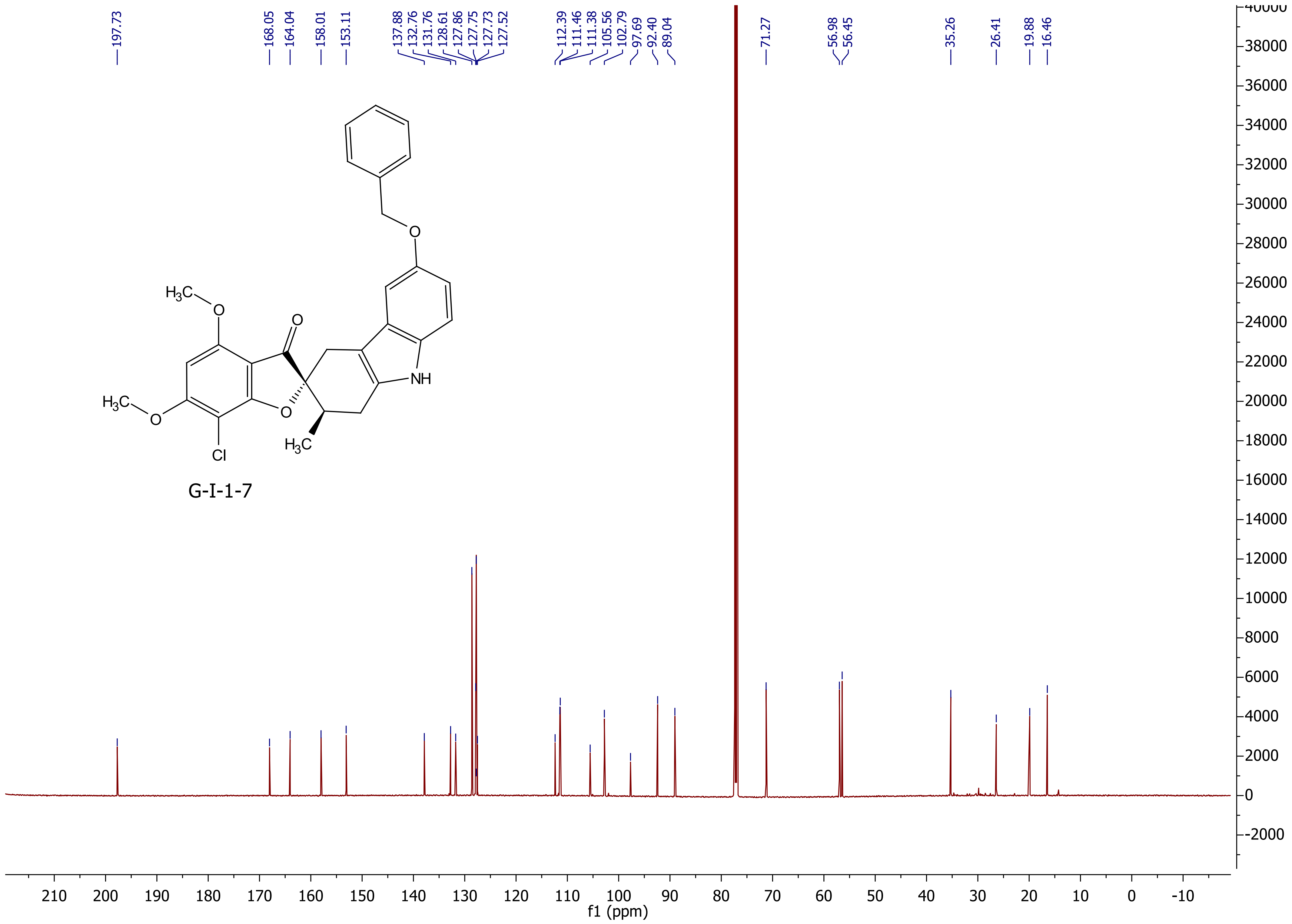

197.73
168.05
164.04
158.01
153.11
137.88
132.76
131.76
128.61
127.86
127.75
127.73
127.52
112.39
111.46
111.38
105.56
102.79
97.69
92.40
89.04
71.27
56.98
56.45
35.26
26.41
19.88
16.46
O
NH
H_3C
O
O
O
H_3C
O
Cl
H_3C
G-I-1-7
f1 (ppm)

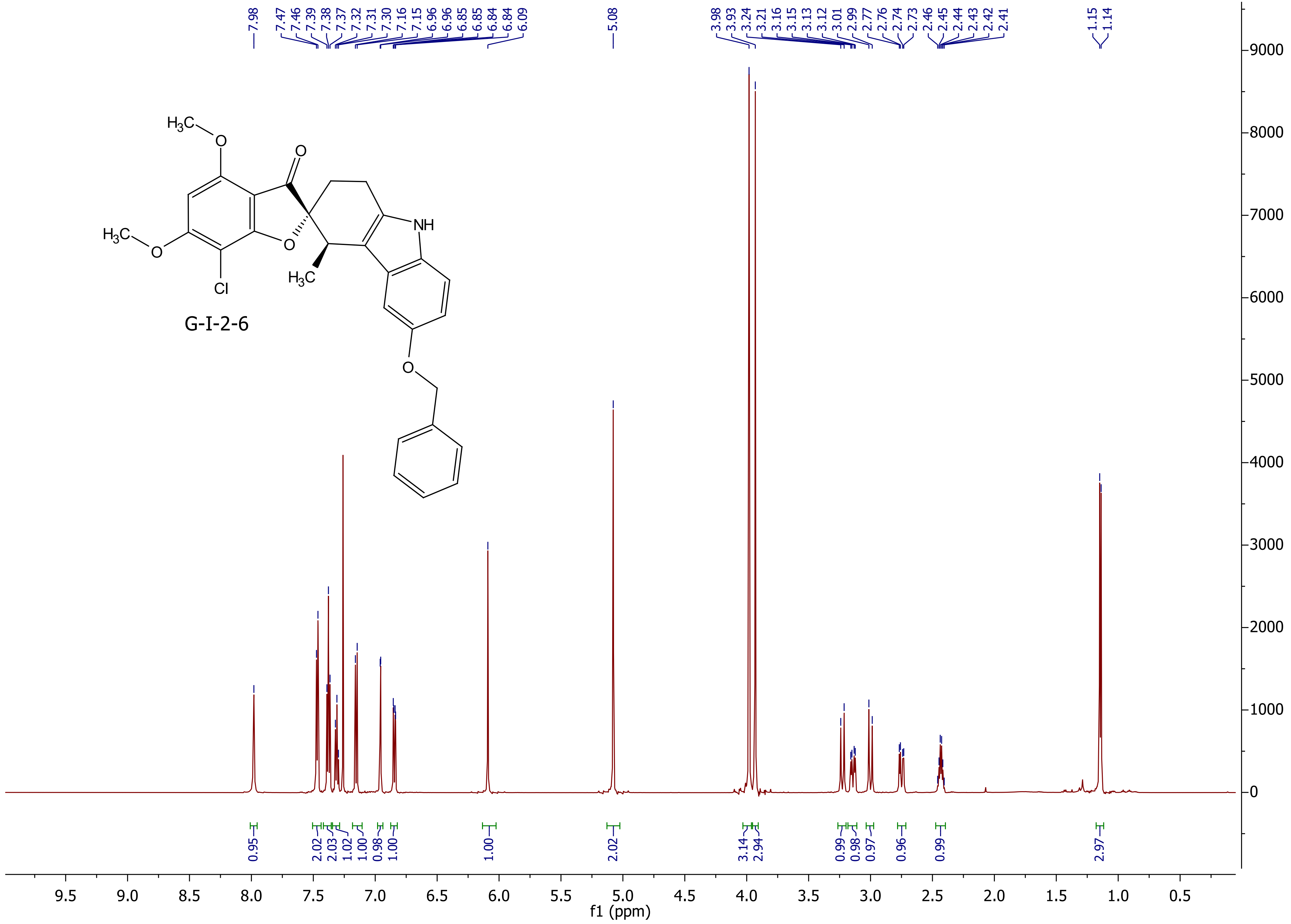
G-I-2-6
7.98
7.47 7.46 7.39 7.38 7.37 7.32 7.31 7.30 7.16 7.15 6.96 6.96 6.85 6.84 6.84 6.09
5.08
3.98 3.93 3.24 3.21 3.16 3.15 3.13 3.12 3.01 2.99 2.77 2.76 2.74 2.73 2.46 2.45 2.44 2.43 2.42 2.41
1.15 1.14
0.95 2.02 2.03 1.02 1.00 0.98 1.00 1.00 2.02 3.14 2.94 0.99 0.98 0.97 0.96 0.99 2.97
f1 (ppm)

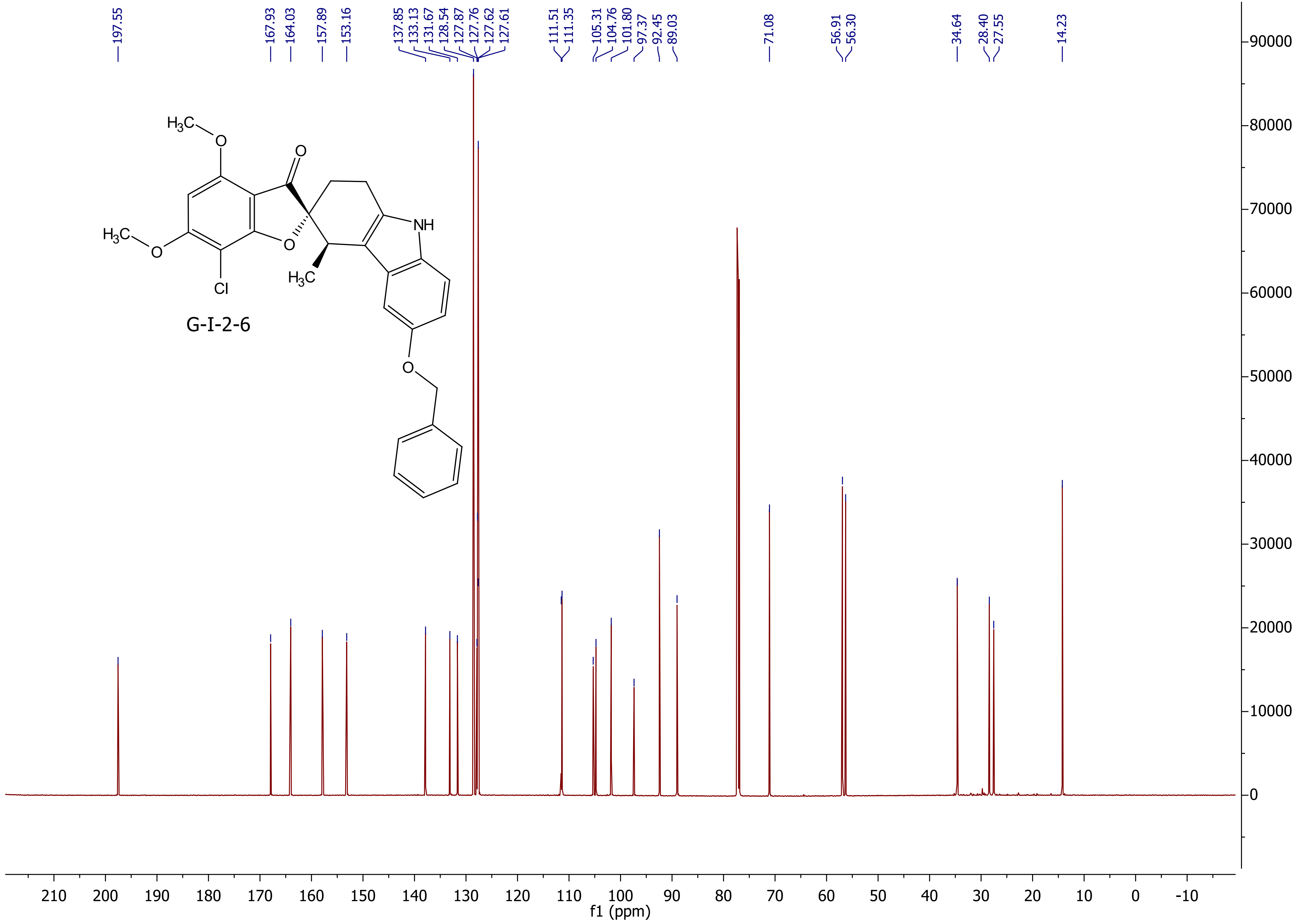

197.55
167.93
164.03
157.89
153.16
137.85
133.13
131.67
128.54
127.87
127.76
127.62
127.61
111.51
111.35
105.31
104.76
101.80
97.37
92.45
89.03
71.08
56.91
56.30
34.64
28.40
27.55
14.23
H_3C
O
O
H_3C
O
O
NH
Cl
H_3C
O
G-I-2-6
90000
80000
70000
60000
50000
40000
30000
20000
10000
0
210
200
190
180
170
160
150
140
130
120
110
100
90
80
70
60
50
40
30
20
10
0
-10
f1 (ppm)

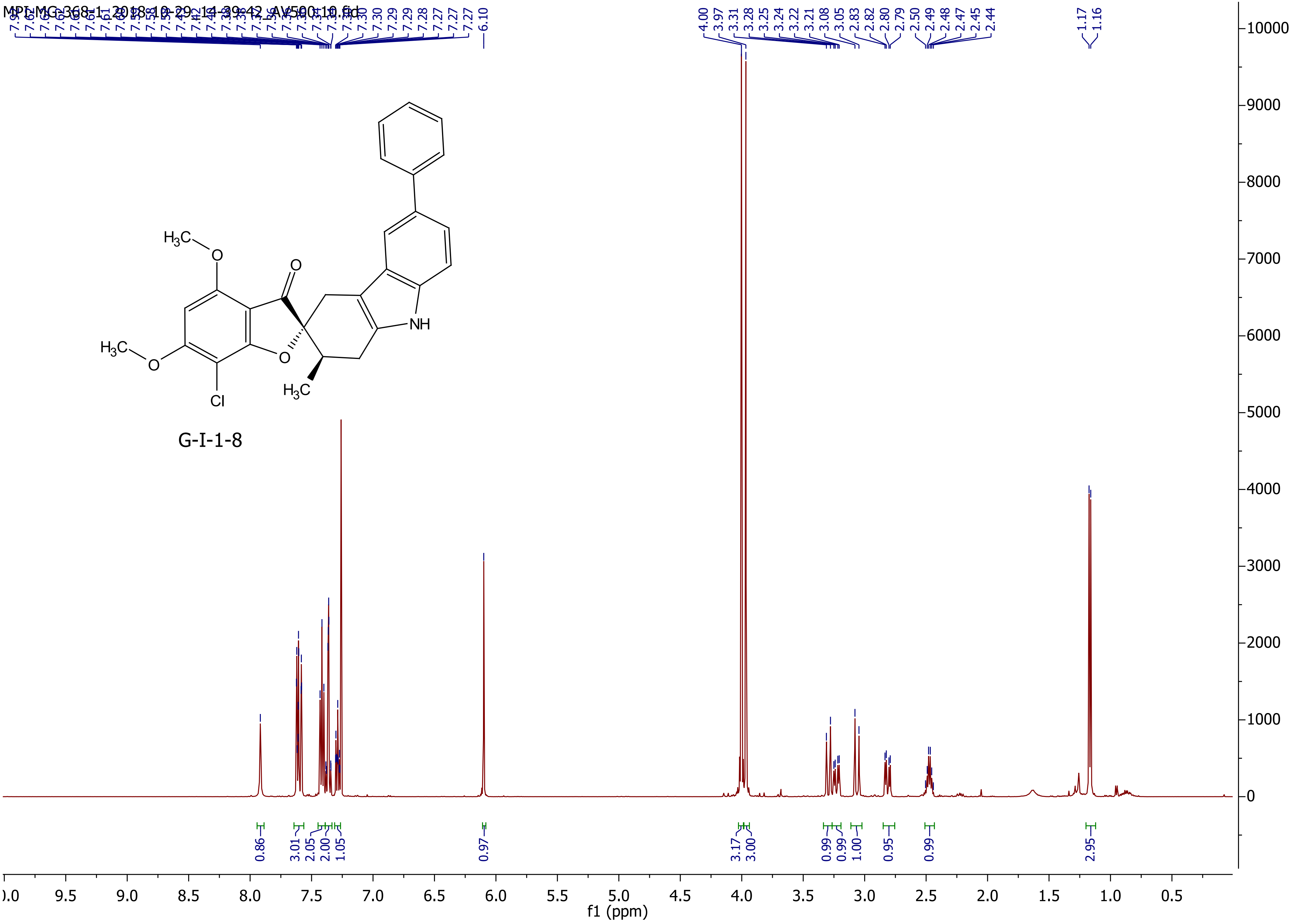
6.10
4.00 3.97 3.31 3.28 3.25 3.24 3.22 3.21 3.08 3.05 2.83 2.82 2.80 2.79 2.50 2.49 2.48 2.47 2.45 2.44
1.17 1.16
H_3C
O
O
H_3C
O
O
Cl
H_3C
NH
0.86 3.01 2.05 2.00 1.05 0.97 3.17 3.00 0.99 0.99 1.00 0.95 0.99 2.95
f1 (ppm)

G-I-1-8

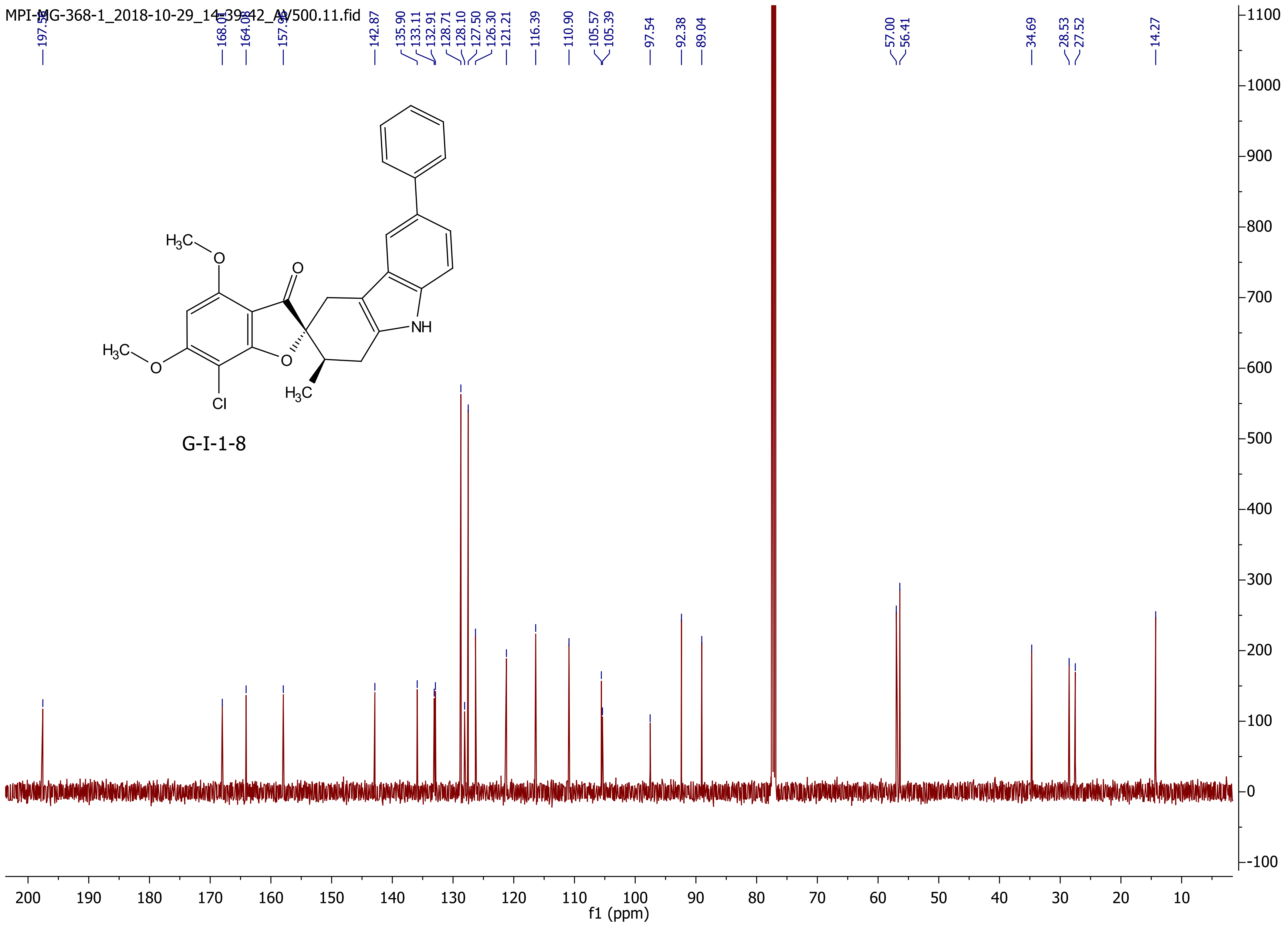
MPI-MG-368-1_2018-10-29_14-39-42_A/500.11.fid
142.87
135.90
133.11
132.91
128.71
128.10
127.50
126.30
121.21
116.39
110.90
105.57
105.39
97.54
92.38
89.04
57.00
56.41
34.69
28.53
27.52
14.27
H3C
O
O
H3C
O
Cl
O
NH
H3C
G-I-1-8
f1 (ppm)

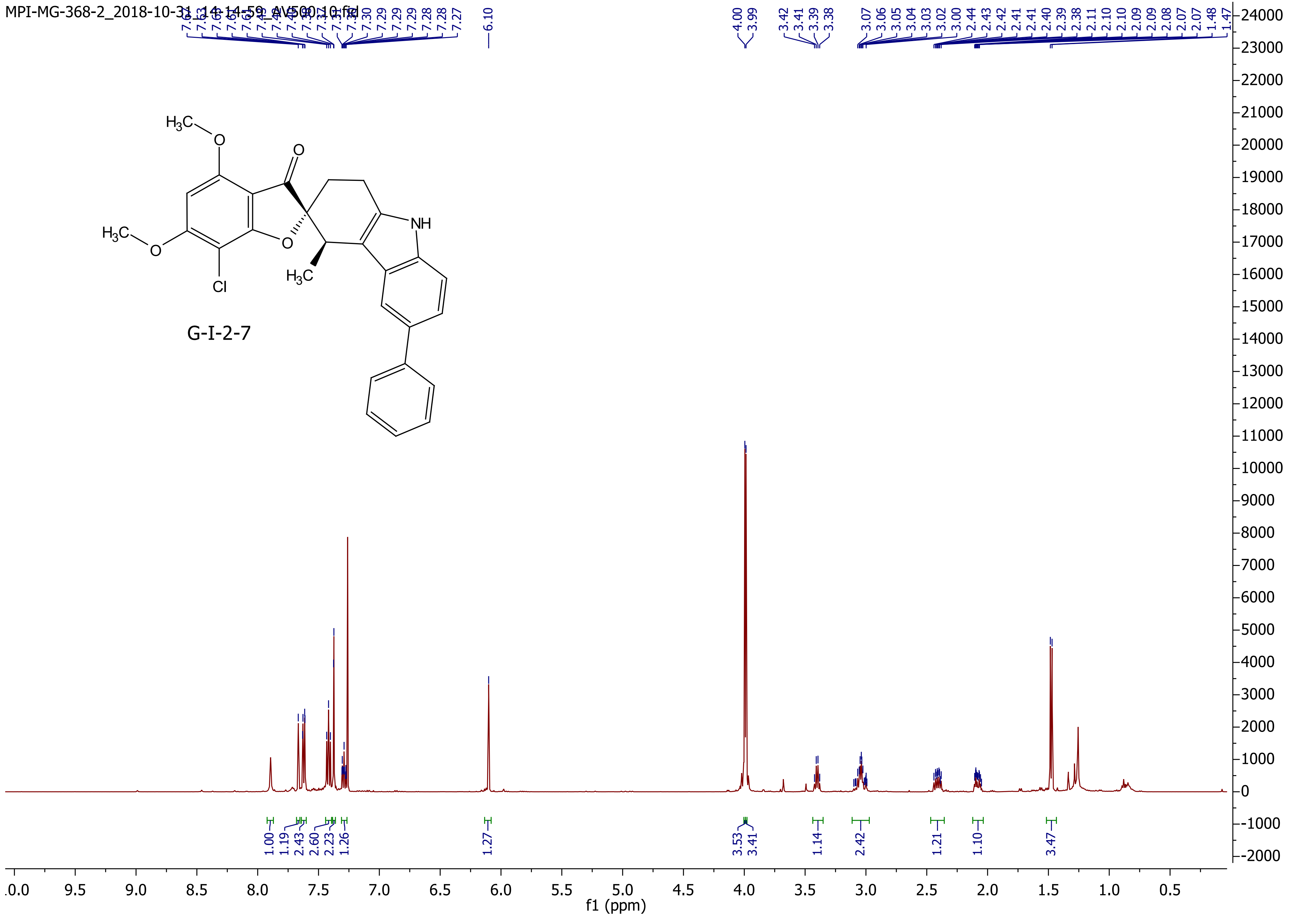
MPI-MG-368-2_2018-10-31_14-14-59_AV500 10.fid
H3C
O
O
NH
H3C
O
O
Cl
H3C
G-I-2-7
f1 (ppm)

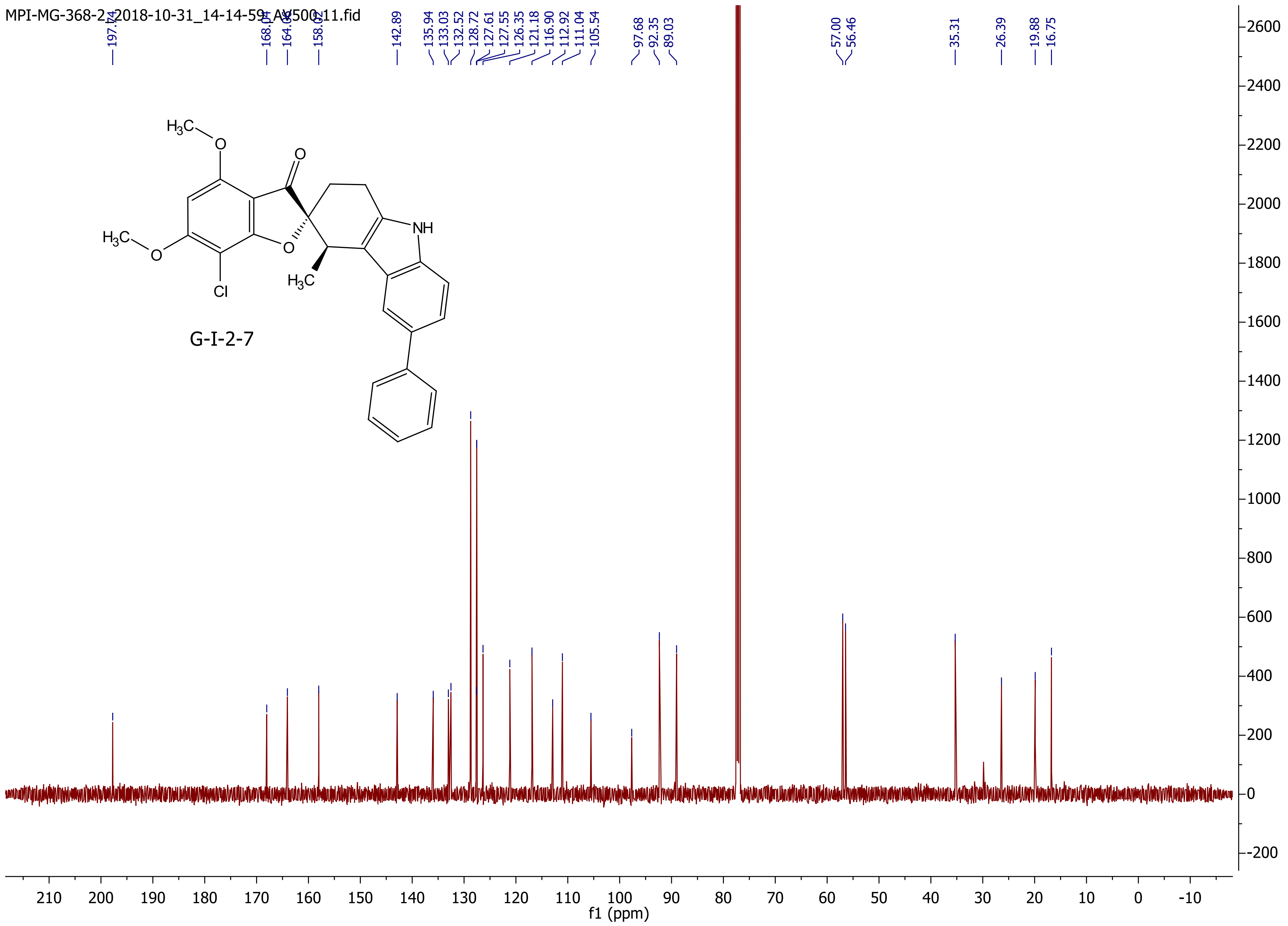

MPI-MG-368-2_2018-10-31_14-14-59_AV500_11.fid
197.74
168.04
164.04
158.02
142.89
135.94
133.03
132.52
128.72
127.61
127.55
126.35
121.18
116.90
112.92
111.04
105.54
97.68
92.35
89.03
57.00
56.46
35.31
26.39
19.88
16.75
H3C
O
O
NH
O
Cl
H3C
f1 (ppm)

G-I-2-7

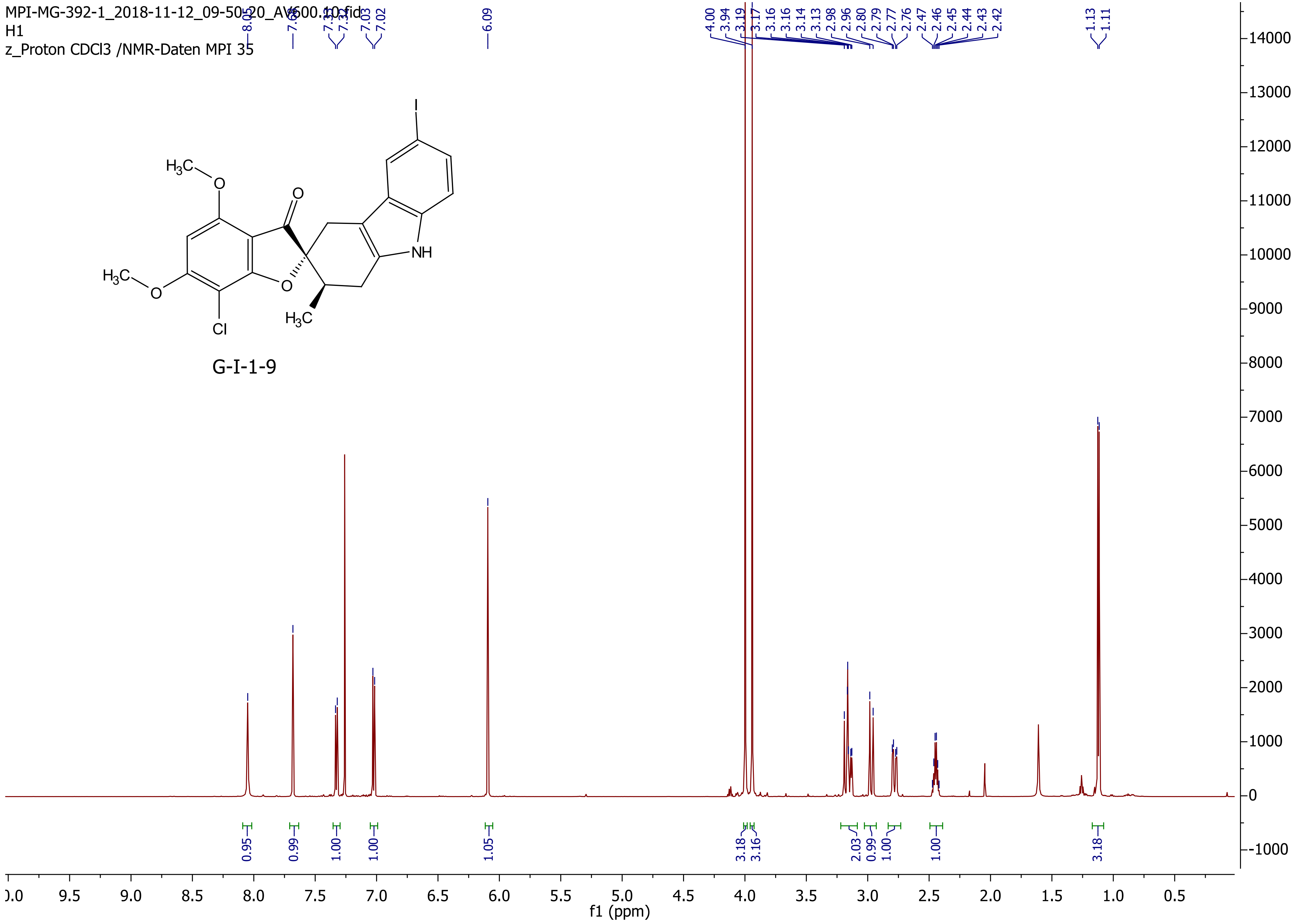
MPI-MG-392-1_2018-11-12_09-50-20_AV600.10.fid
H1
z_Proton CDCl3 /NMR-Daten MPI 35
8.05
6.09
4.00 3.94 3.19 3.17 3.16 3.16 3.14 3.13 2.98 2.96 2.80 2.79 2.77 2.76 2.47 2.46 2.45 2.44 2.43 2.42
1.13 1.11
G-I-1-9
0.95 0.99 1.00 1.00 1.05 3.18 3.16 2.03 0.99 1.00 1.00 3.18
f1 (ppm)

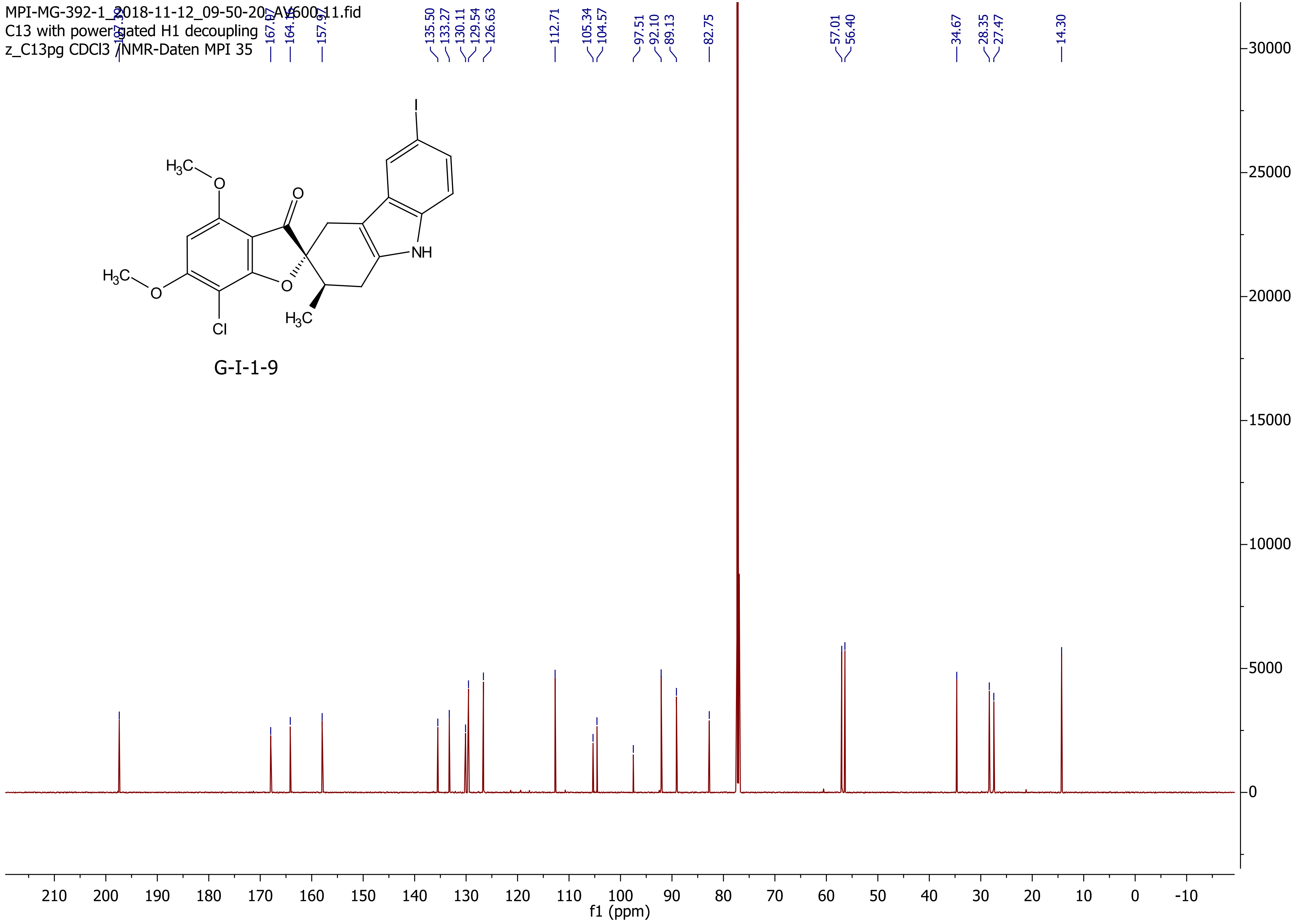
MPI-MG-392-1_2018-11-12_09-50-20_AV600_11.fid
C13 with power gated H1 decoupling
z_C13pg CDCl3 /NMR-Daten MPI 35
197.39
167.97
164.16
157.97
135.50
133.27
130.11
129.54
126.63
112.71
105.34
104.57
97.51
92.10
89.13
82.75
57.01
56.40
34.67
28.35
27.47
14.30
I
H_3C
O
O
H_3C
O
NH
O
H_3C
Cl
G-I-1-9
30000
25000
20000
15000
10000
5000
0
210
200
190
180
170
160
150
140
130
120
110
100
90
80
70
60
50
40
30
20
10
0
-10
f1 (ppm)

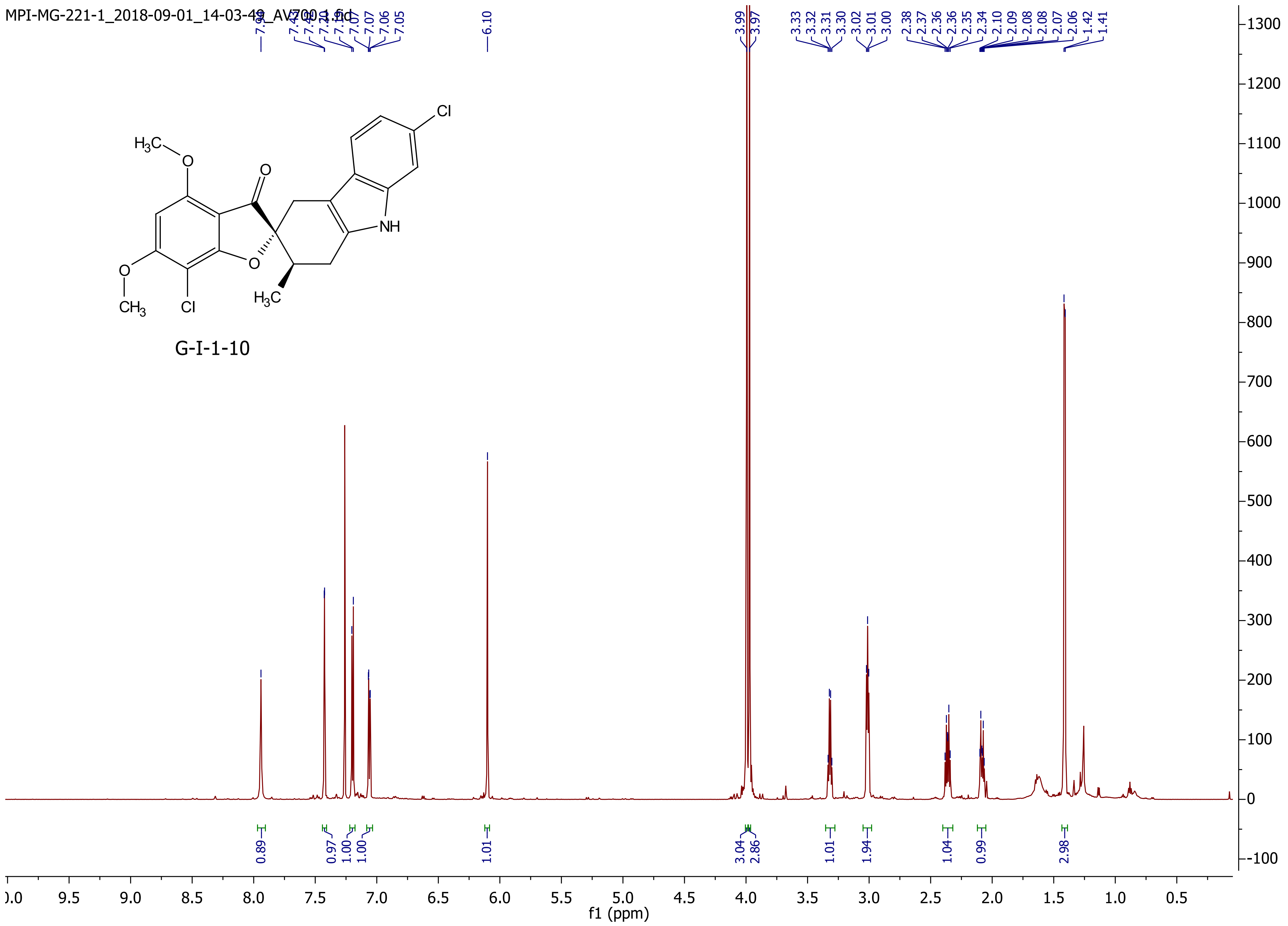

MPI-MG-221-1_2018-09-01_14-03-49_AV700 1.fid
6.10
3.99
3.97
3.33
3.32
3.31
3.30
3.02
3.01
3.00
2.38
2.37
2.36
2.36
2.35
2.34
2.10
2.09
2.08
2.08
2.07
2.06
1.42
1.41
7.07
7.06
7.05
Cl
H_3C
O
O
NH
O
O
CH_3
Cl
H_3C
G-I-1-10
0.89
0.97
1.00
1.00
1.01
3.04
2.86
1.01
1.94
1.04
0.99
2.98
f1 (ppm)

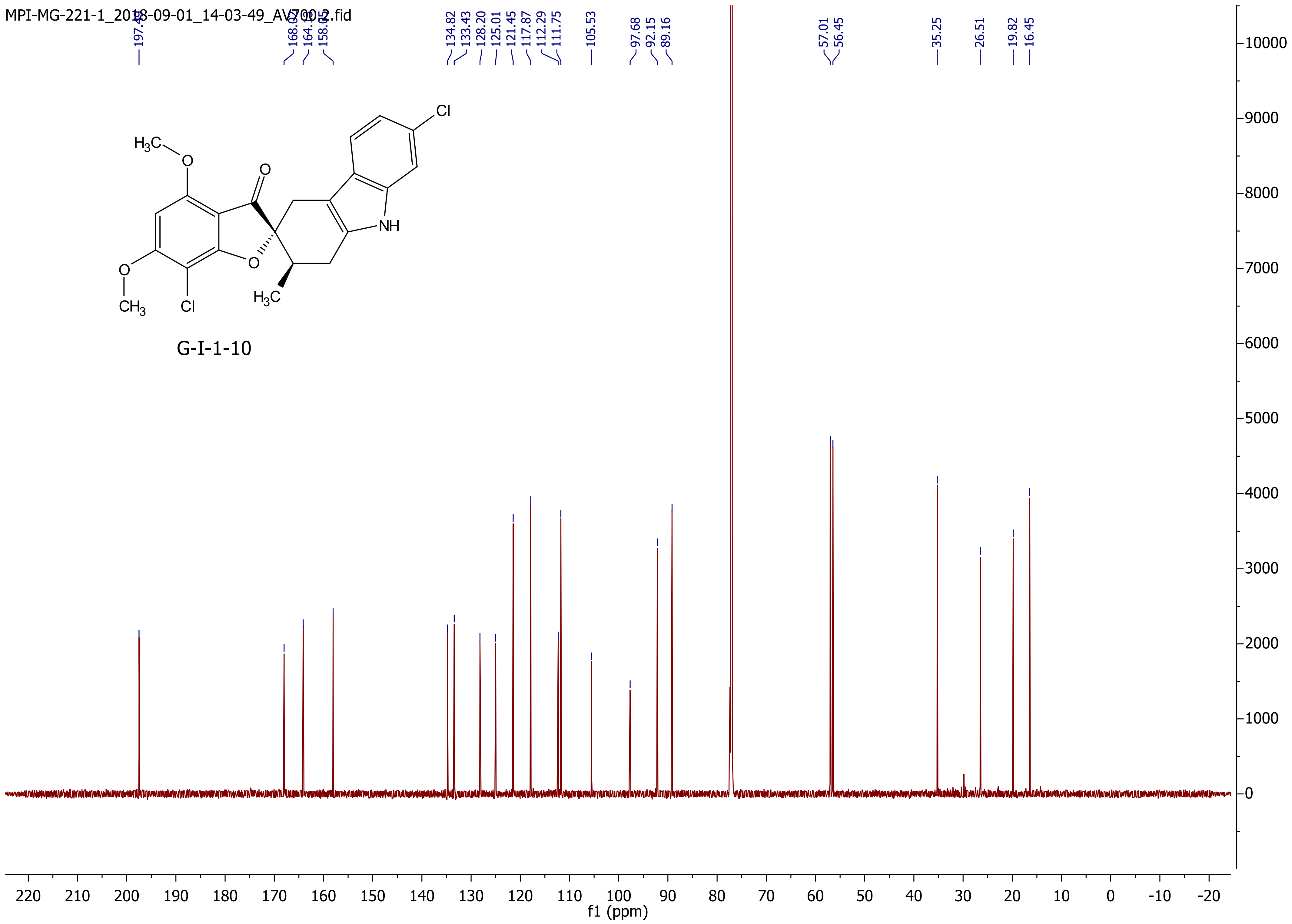
MPI-MG-221-1_2018-09-01_14-03-49_AV700-2.fid
197.4
168.0
164.1
158.0
134.82
133.43
128.20
125.01
121.45
117.87
112.29
111.75
105.53
97.68
92.15
89.16
57.01
56.45
35.25
26.51
19.82
16.45
G-I-1-10
f1 (ppm)

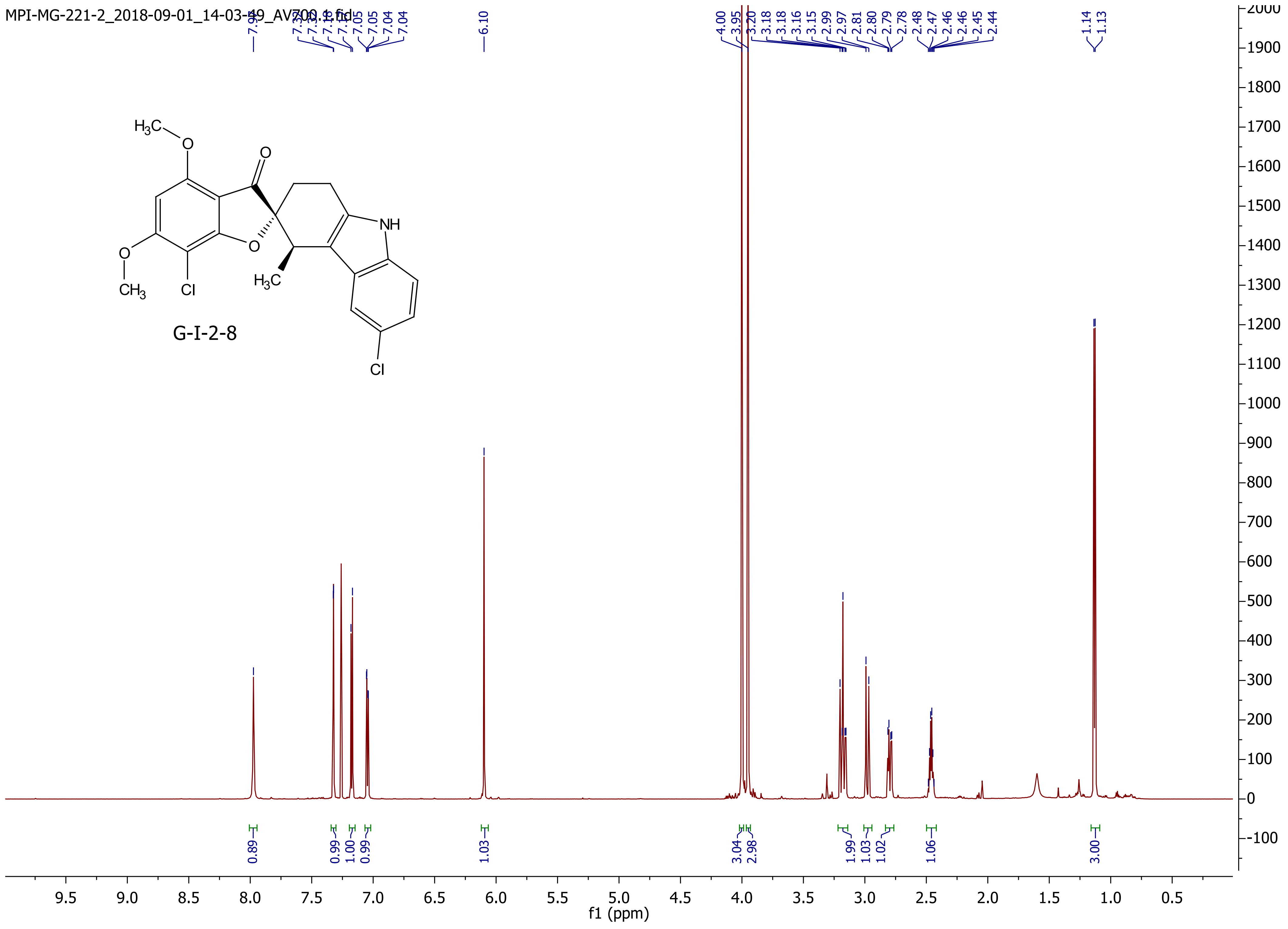

MPI-MG-221-2_2018-09-01_14-03-49_AV700.1.fid
7.05
7.05
7.04
7.04
6.10
4.00
3.95
3.20
3.18
3.18
3.16
3.15
2.99
2.97
2.81
2.80
2.79
2.78
2.48
2.47
2.46
2.46
2.45
2.44
1.14
1.13
H3C
O
O
NH
O
O
CH3
Cl
H3C
Cl
G-I-2-8
0.89
0.99
1.00
0.99
1.03
3.04
2.98
1.99
1.03
1.02
1.06
3.00
f1 (ppm)

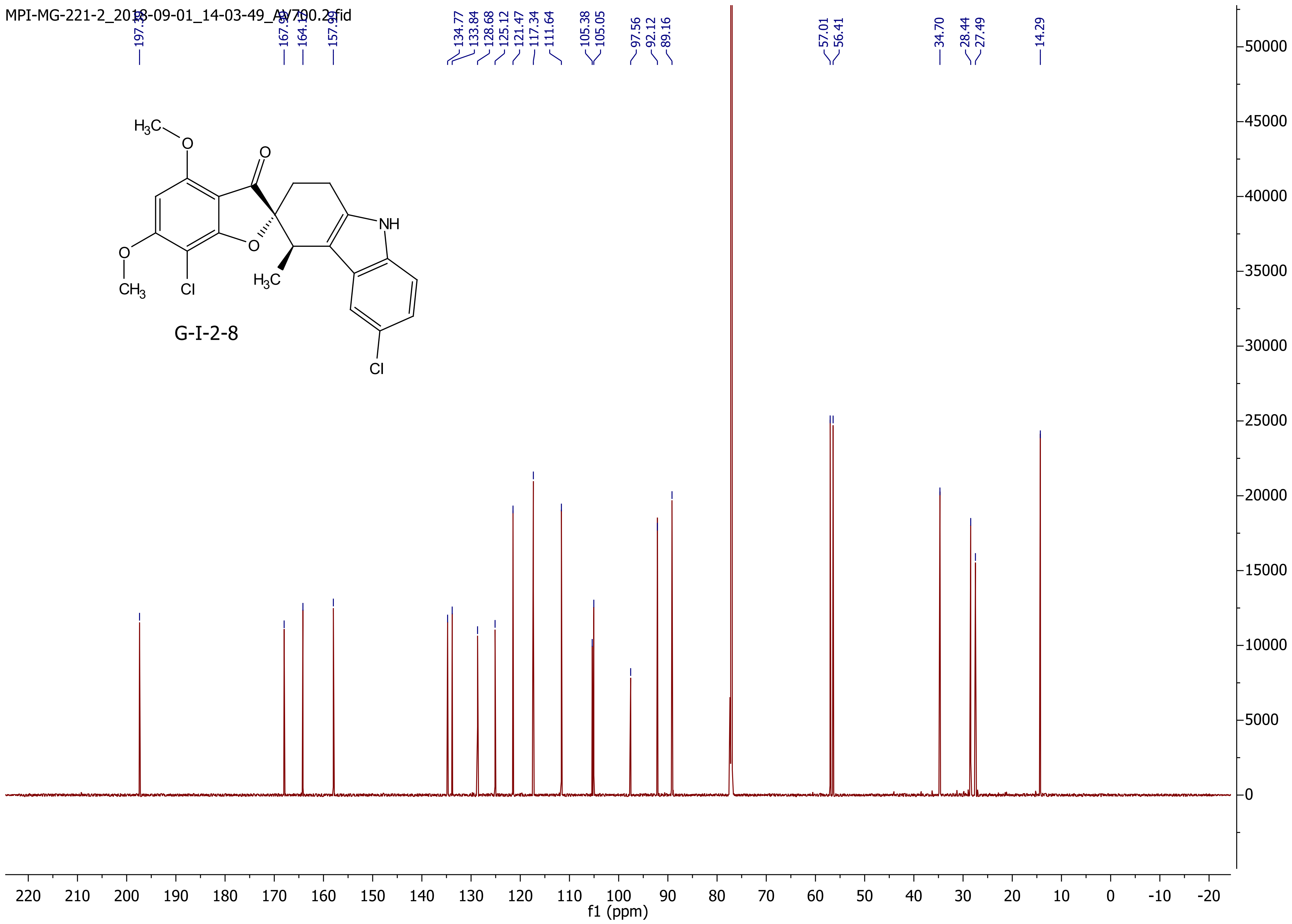
MPI-MG-221-2_2018-09-01_14-03-49_AV700.2.fid
134.77
133.84
128.68
125.12
121.47
117.34
111.64
105.38
105.05
97.56
92.12
89.16
57.01
56.41
34.70
28.44
27.49
14.29
H_3C
O
O
O
NH
O
CH_3
Cl
H_3C
Cl
G-I-2-8
f1 (ppm)

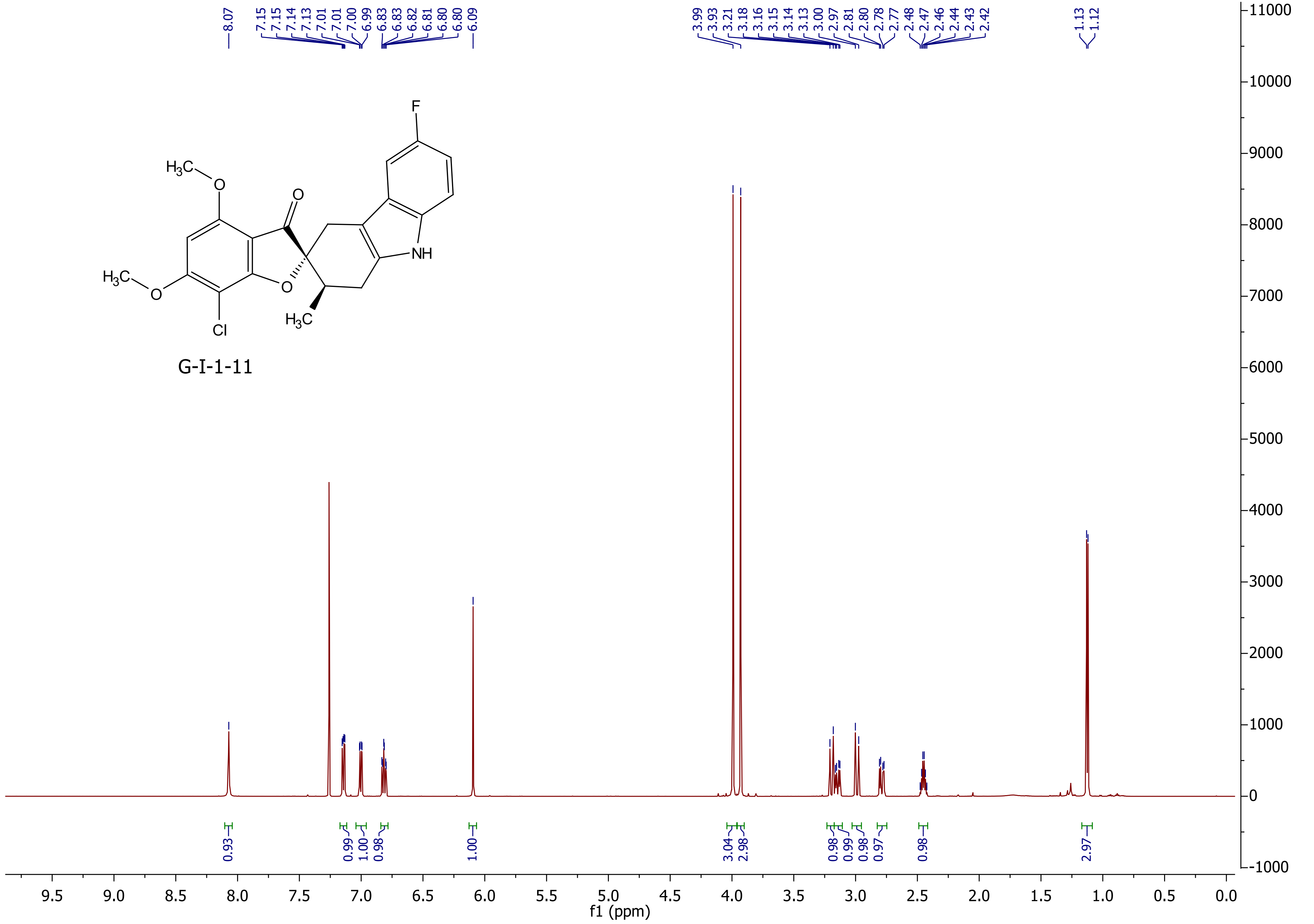

8.07
7.15
7.15
7.14
7.13
7.01
7.01
7.00
6.99
6.83
6.83
6.82
6.81
6.80
6.80
6.09
3.99
3.93
3.21
3.18
3.16
3.15
3.14
3.13
3.00
2.97
2.81
2.80
2.78
2.77
2.48
2.47
2.46
2.44
2.43
2.42
1.13
1.12
F
H3C
O
O
NH
H3C
O
O
Cl
H3C
G-I-1-11
0.93
0.99
1.00
0.98
1.00
3.04
2.98
0.98
0.99
0.98
0.97
0.98
2.97
f1 (ppm)

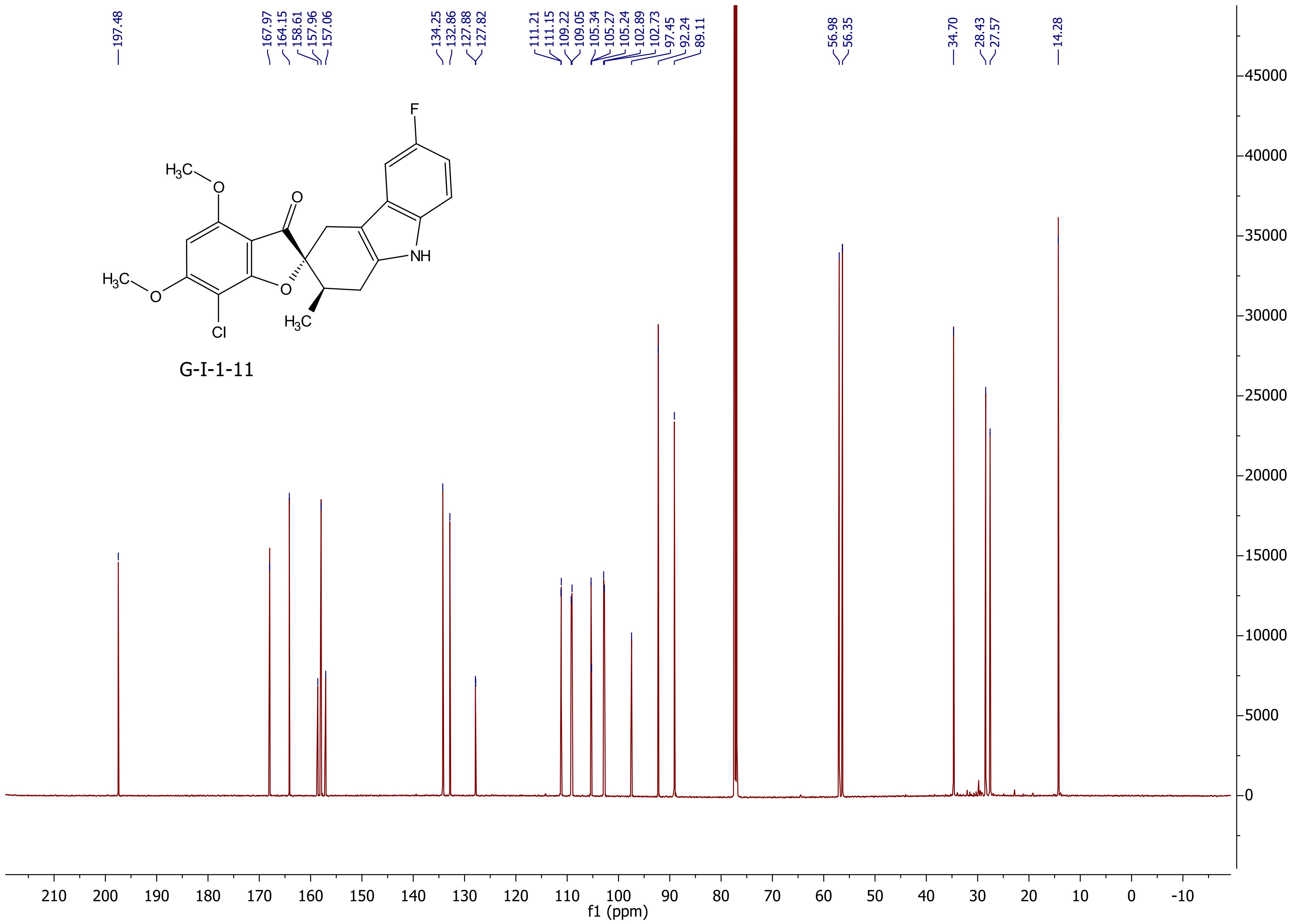
197.48
167.97
164.15
158.61
157.96
157.06
134.25
132.86
127.88
127.82
111.21
111.15
109.22
109.05
105.34
105.27
105.24
102.89
102.73
97.45
92.24
89.11
56.98
56.35
34.70
28.43
27.57
14.28
F
H_3C
O
O
NH
H_3C
O
O
H_3C
Cl
G-I-1-11
45000
40000
35000
30000
25000
20000
15000
10000
5000
0
210
200
190
180
170
160
150
140
130
120
110
100
90
80
70
60
50
40
30
20
10
0
-10
f1 (ppm)

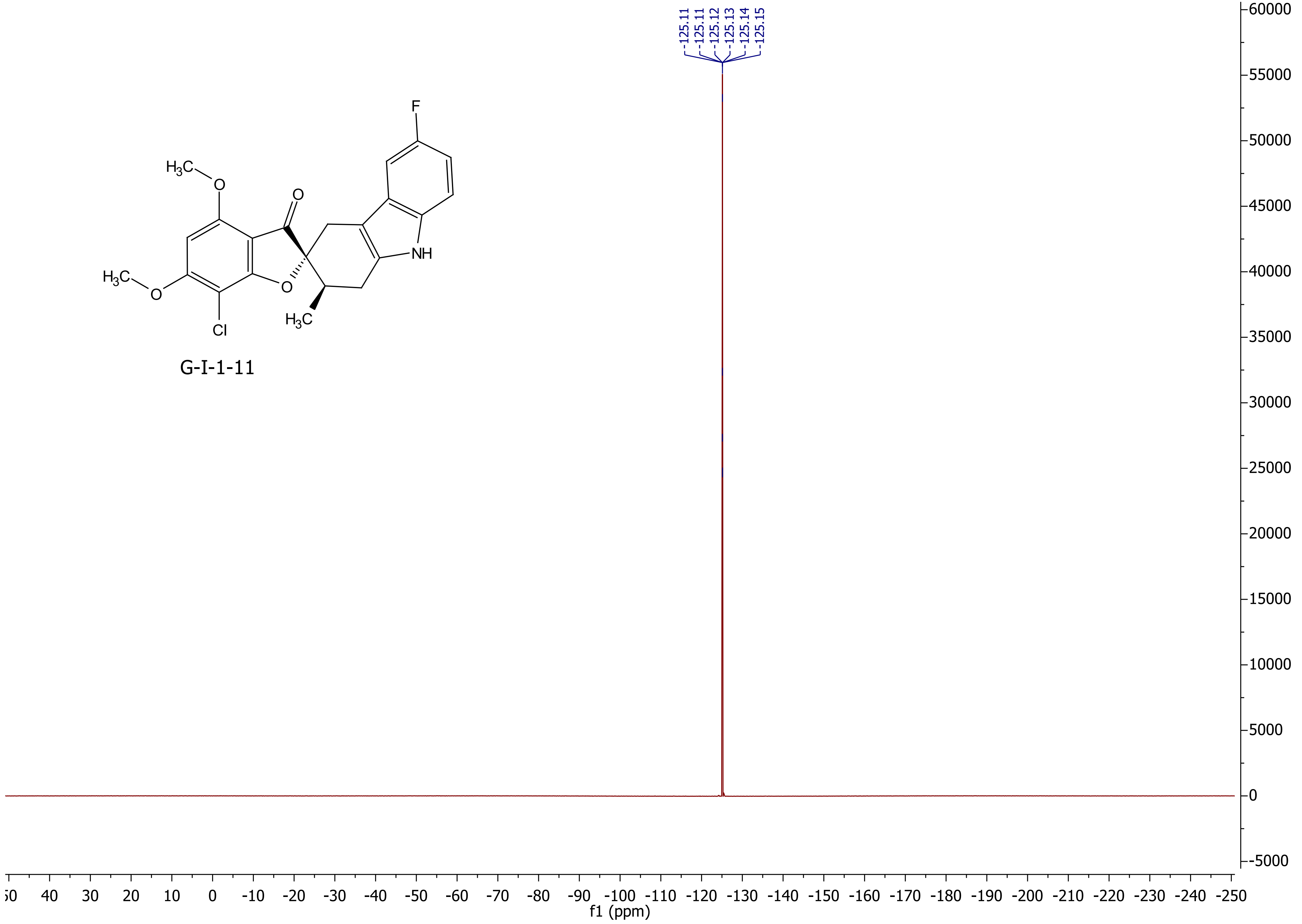

-125.11
-125.11
-125.12
-125.13
-125.14
-125.15
F
H_3C
O
O
NH
H_3C
O
O
Cl
H_3C
G-I-1-11
60000
55000
50000
45000
40000
35000
30000
25000
20000
15000
10000
5000
0
-5000
50 40 30 20 10 0 -10 -20 -30 -40 -50 -60 -70 -80 -90 -100 -110 -120 -130 -140 -150 -160 -170 -180 -190 -200 -210 -220 -230 -240 -250
f1 (ppm)

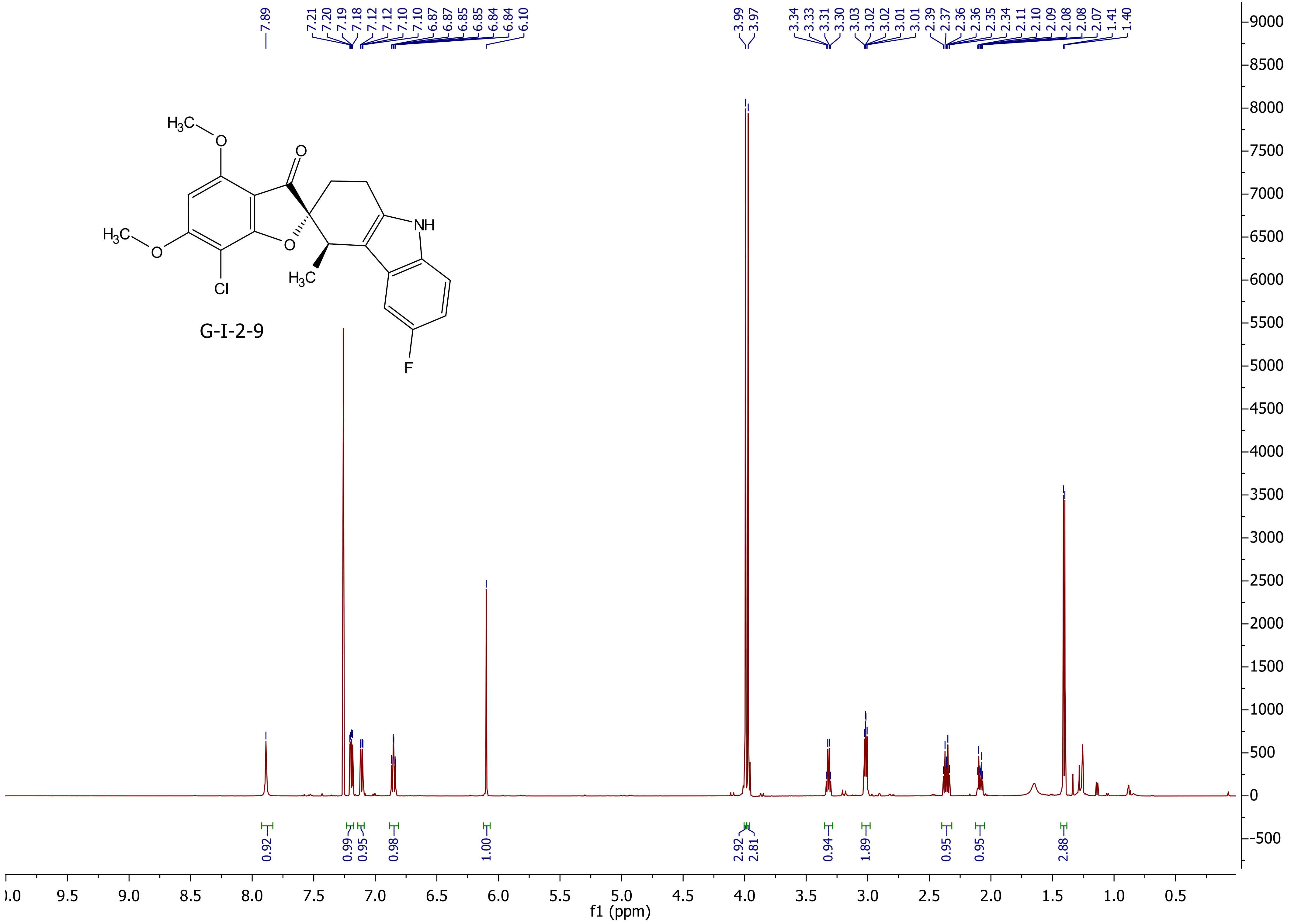
7.89
7.21
7.20
7.19
7.18
7.12
7.12
7.10
7.10
6.87
6.87
6.85
6.85
6.84
6.84
6.10
3.99
3.97
3.34
3.33
3.31
3.30
3.03
3.02
3.02
3.01
3.01
2.39
2.37
2.36
2.36
2.35
2.34
2.11
2.10
2.09
2.08
2.08
2.07
1.41
1.40
H_3C
O
O
H_3C
O
O
NH
Cl
H_3C
F
G-I-2-9
0.92
0.99
0.95
0.98
1.00
2.92
2.81
0.94
1.89
0.95
0.95
2.88
f1 (ppm)

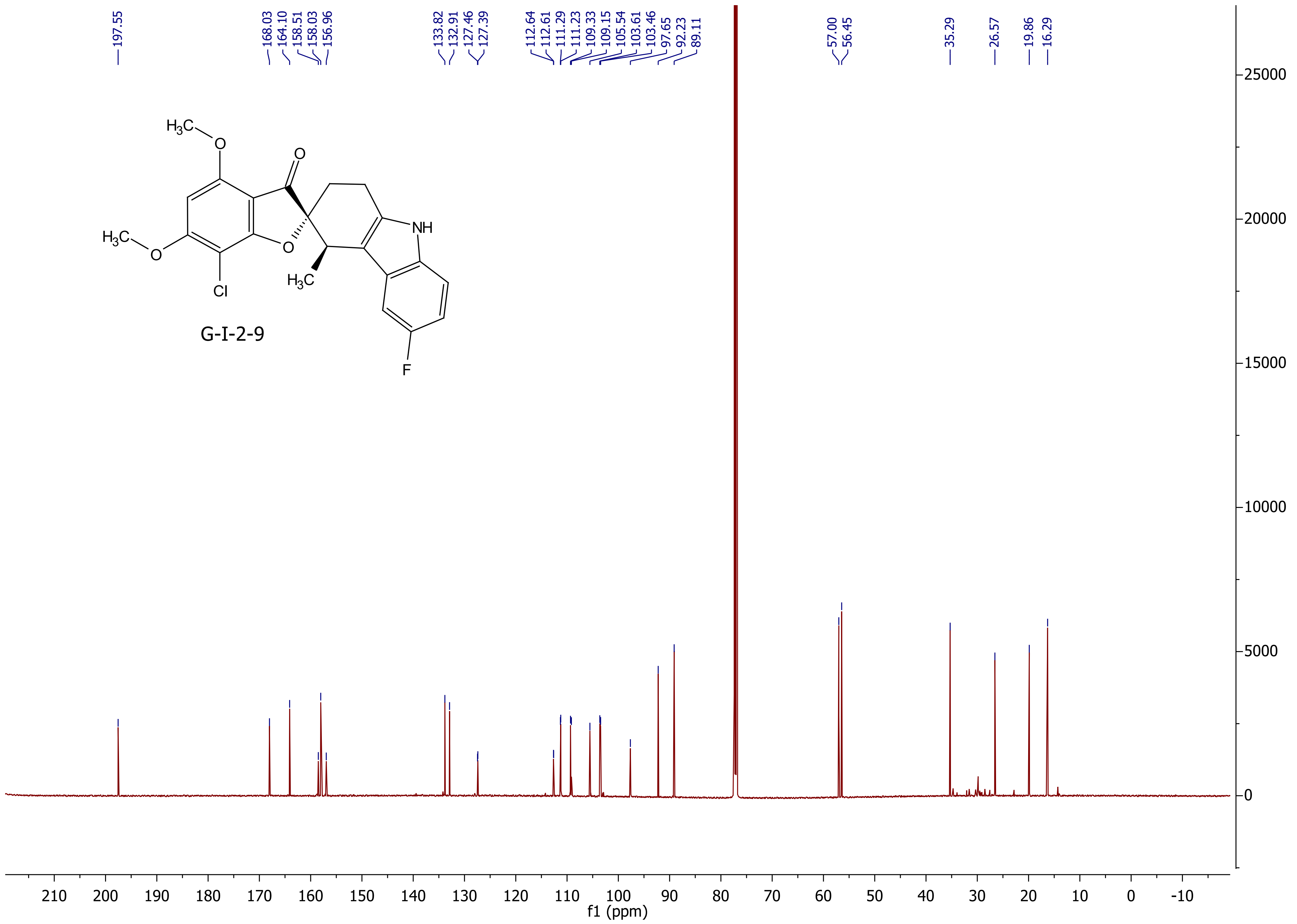

197.55
168.03
164.10
158.51
158.03
156.96
133.82
132.91
127.46
127.39
112.64
112.61
111.29
111.23
109.33
109.15
105.54
103.61
103.46
97.65
92.23
89.11
57.00
56.45
35.29
26.57
19.86
16.29
G-I-2-9
f1 (ppm)

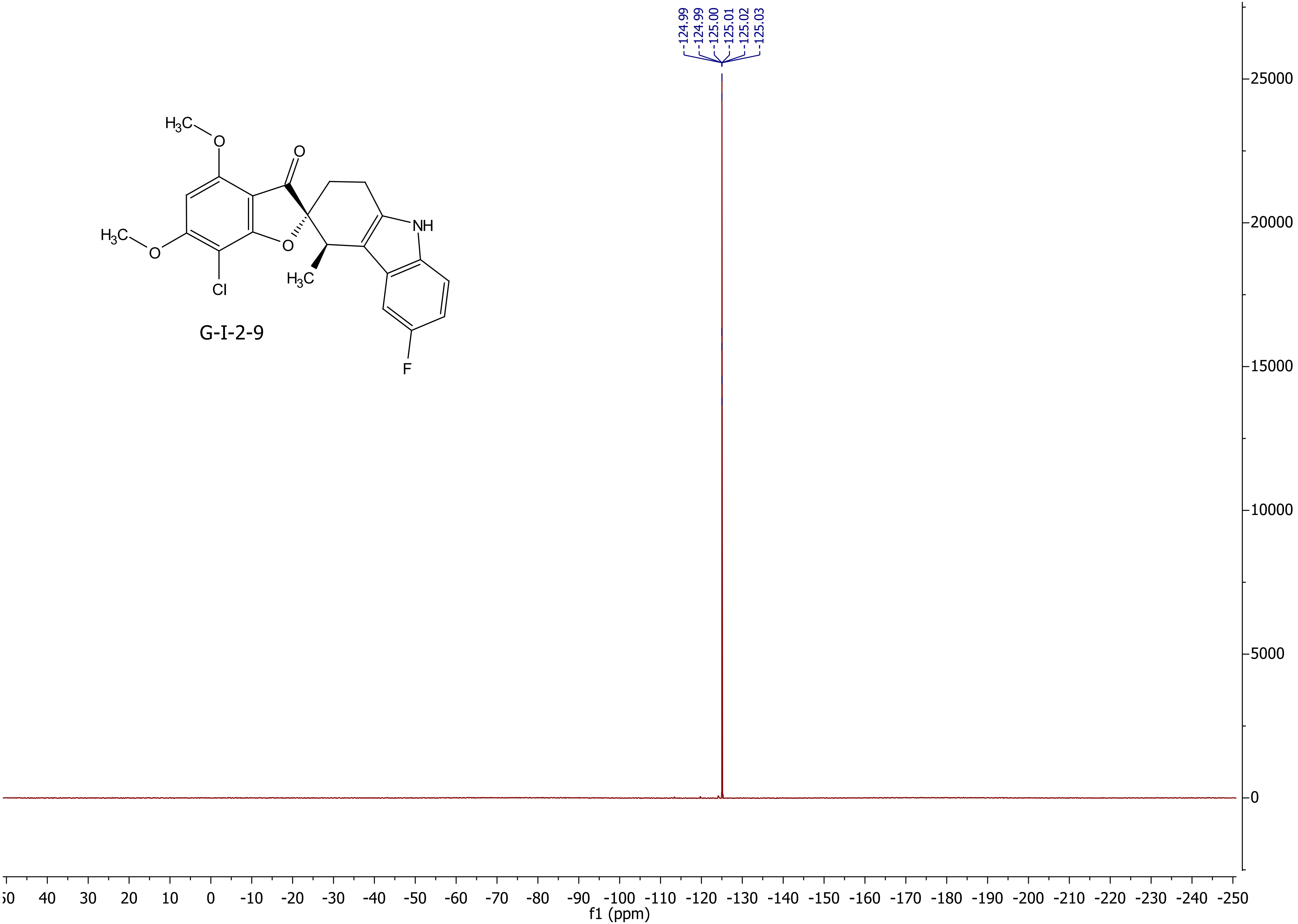

-124.99
-124.99
-125.00
-125.01
-125.02
-125.03
H3C
O
O
H3C
O
O
NH
Cl
H3C
F
25000
20000
15000
10000
5000
0
50
40
30
20
10
0
-10
-20
-30
-40
-50
-60
-70
-80
-90
-100
-110
-120
-130
-140
-150
-160
-170
-180
-190
-200
-210
-220
-230
-240
-250
f1 (ppm)

G-I-2-9

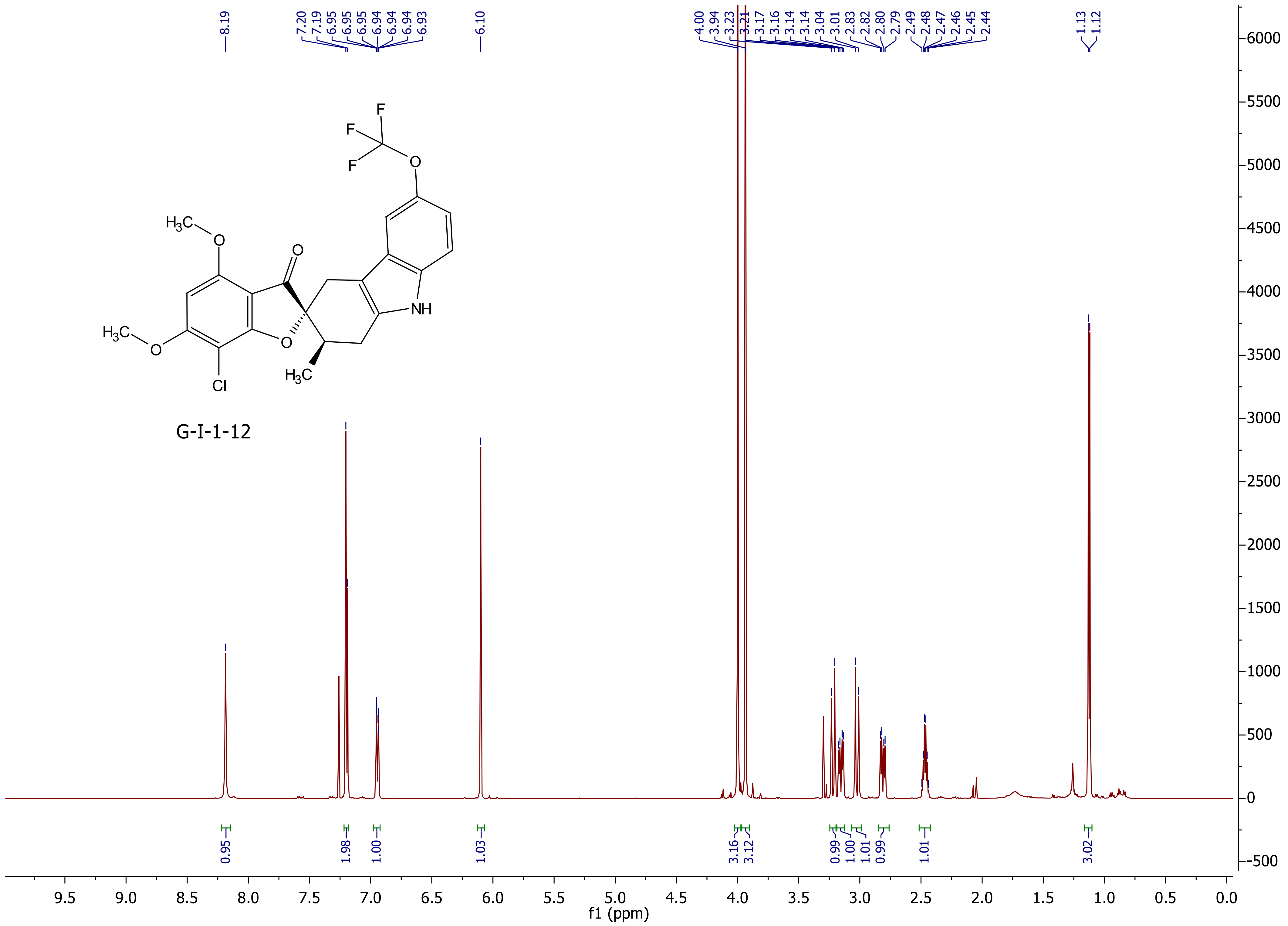

8.19
7.20
7.19
6.95
6.95
6.95
6.94
6.94
6.94
6.93
6.10
4.00
3.94
3.23
3.21
3.17
3.16
3.14
3.14
3.04
3.01
2.83
2.82
2.80
2.79
2.49
2.48
2.47
2.46
2.45
2.44
1.13
1.12
F
F
F
O
H_3C
O
O
NH
H_3C
O
O
Cl
H_3C
G-I-1-12
0.95
1.98
1.00
1.03
3.16
3.12
0.99
1.00
1.01
0.99
1.01
3.02
f1 (ppm)

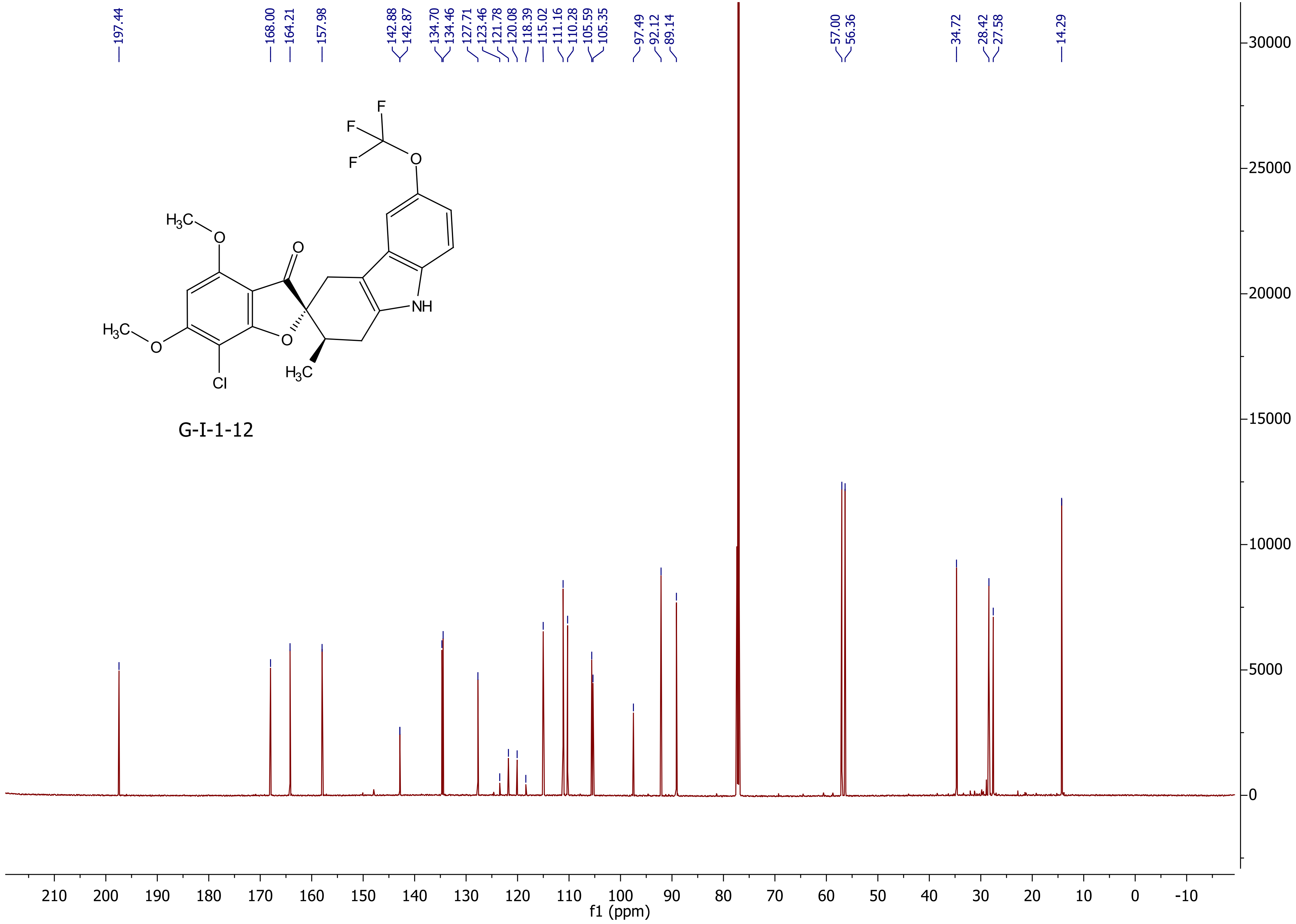
197.44
168.00
164.21
157.98
142.88
142.87
134.70
134.46
127.71
123.46
121.78
120.08
118.39
115.02
111.16
110.28
105.59
105.35
97.49
92.12
89.14
57.00
56.36
34.72
28.42
27.58
14.29
f1 (ppm)

G-I-1-12

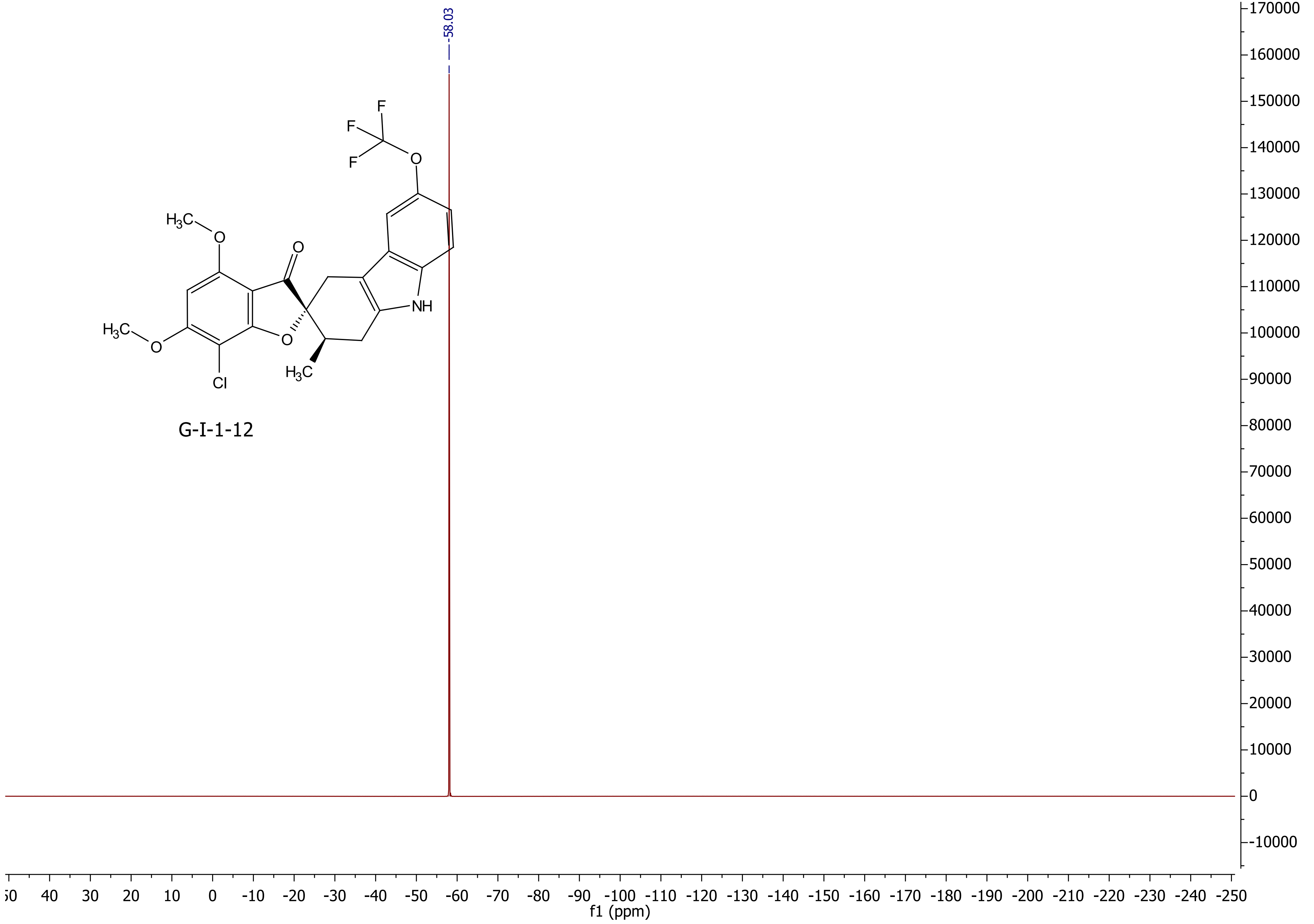

-58.03
G-I-1-12
f1 (ppm)

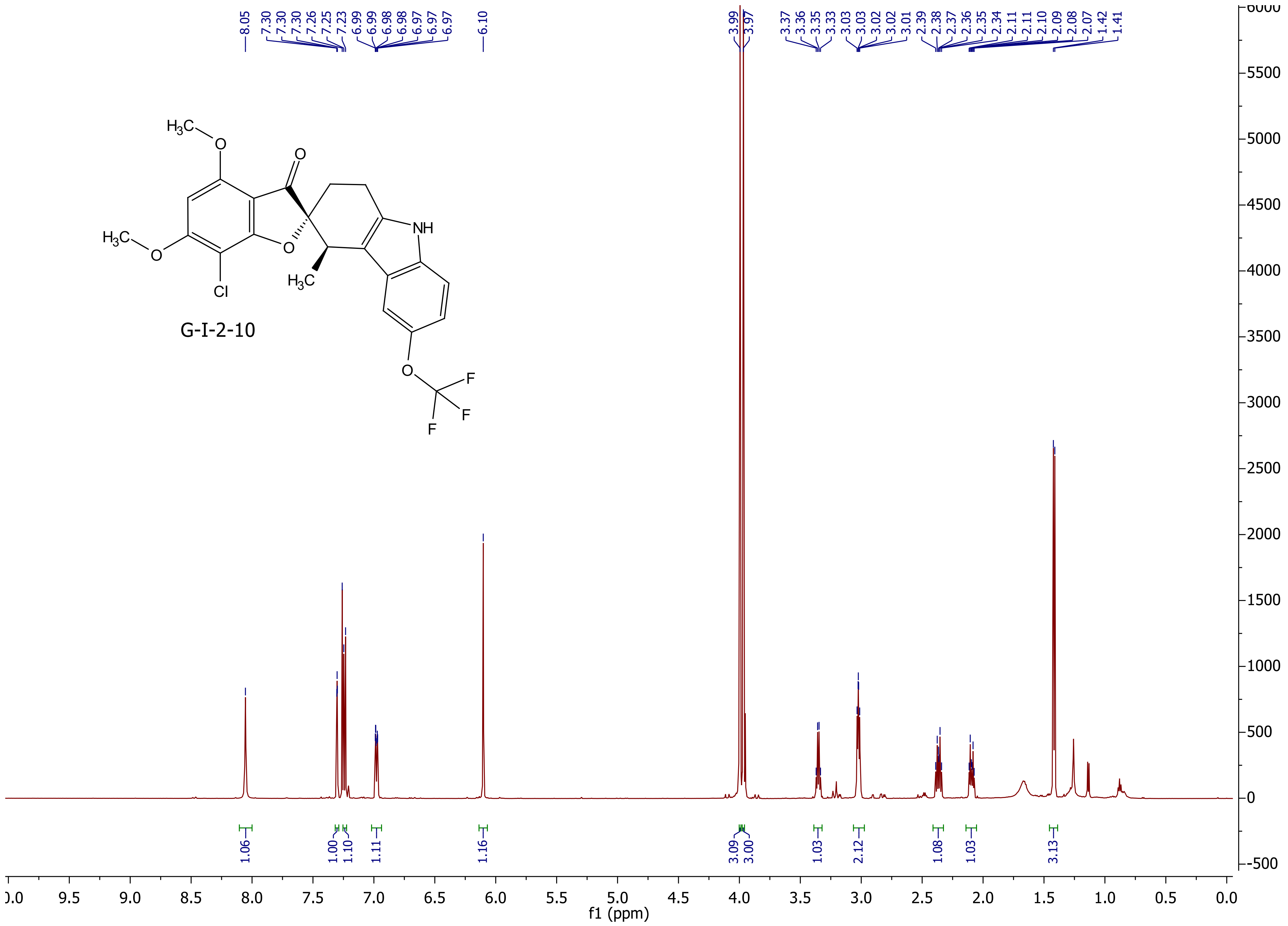
G-I-2-10
8.05
7.30
7.30
7.30
7.26
7.25
7.23
6.99
6.99
6.98
6.98
6.97
6.97
6.97
6.10
3.99
3.97
3.37
3.36
3.35
3.33
3.03
3.03
3.02
3.02
3.01
2.39
2.38
2.37
2.36
2.35
2.34
2.11
2.11
2.10
2.09
2.08
2.07
1.42
1.41
1.06
1.00
1.10
1.11
1.16
3.09
3.00
1.03
2.12
1.08
1.03
3.13
f1 (ppm)

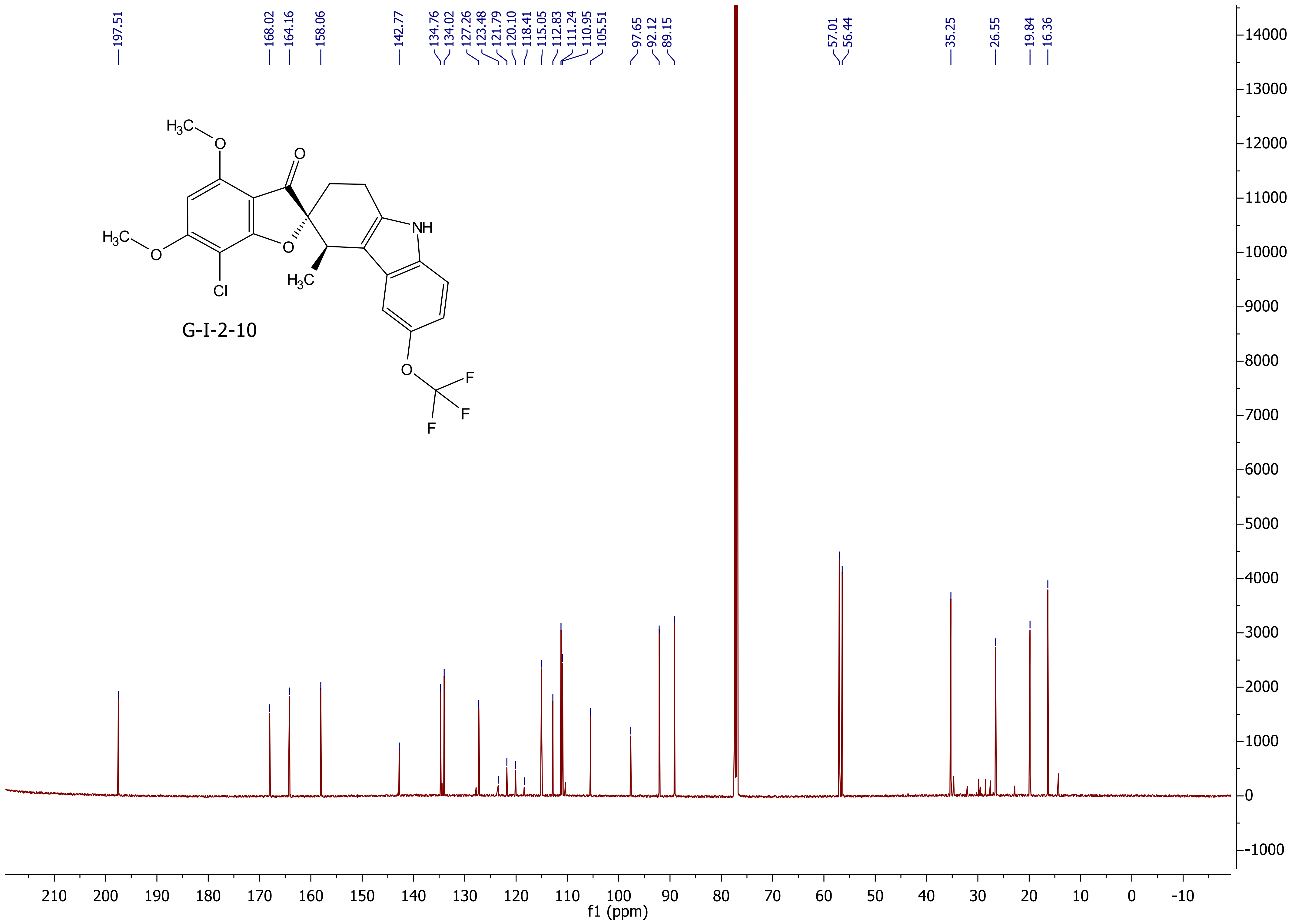

197.51
168.02
164.16
158.06
142.77
134.76
134.02
127.26
123.48
121.79
120.10
118.41
115.05
112.83
111.24
110.95
105.51
97.65
92.12
89.15
57.01
56.44
35.25
26.55
19.84
16.36
G-I-2-10
f1 (ppm)

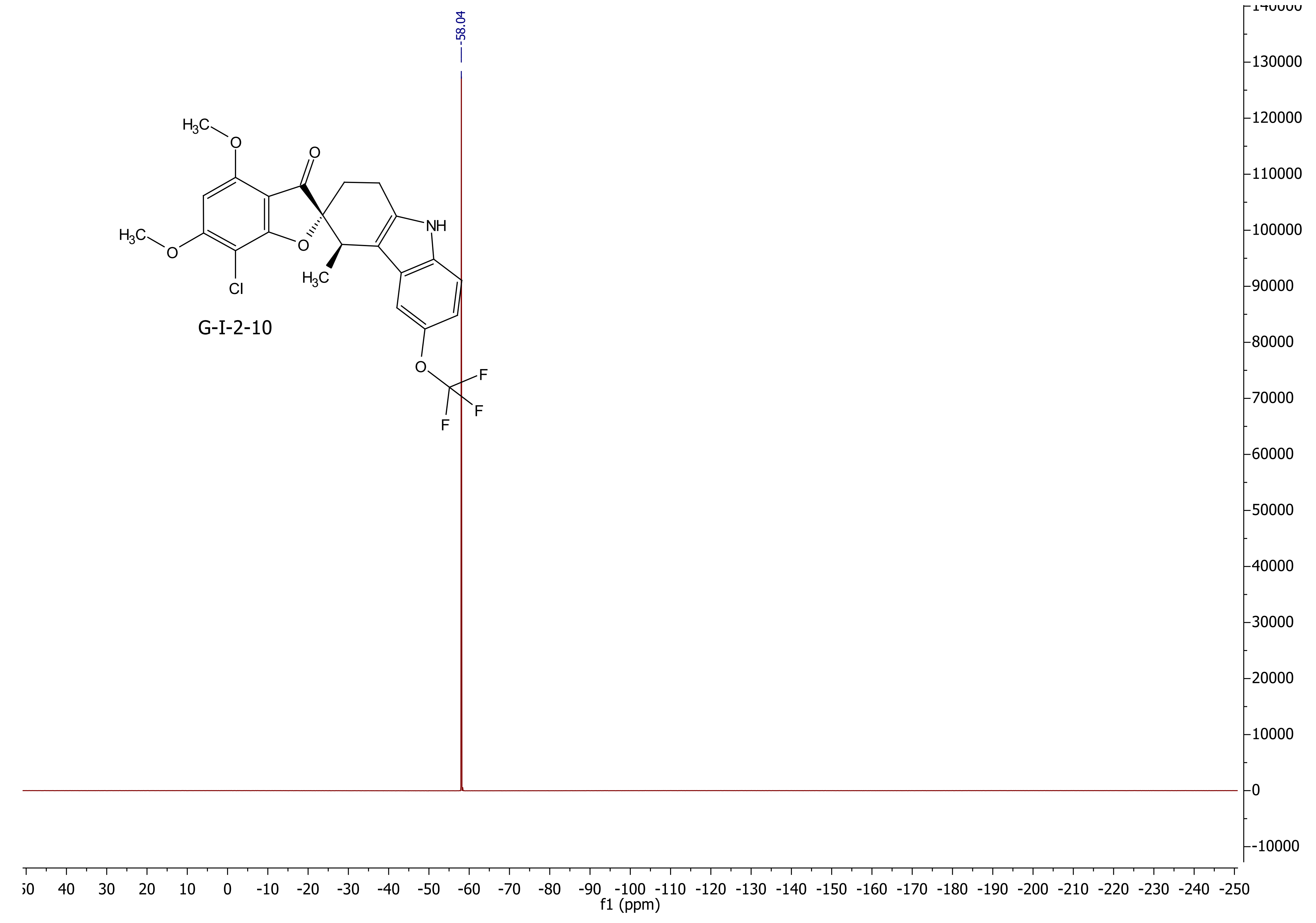
-58.04
H3C
O
O
H3C
O
NH
O
H3C
Cl
G-I-2-10
O
F
F
F
140000
130000
120000
110000
100000
90000
80000
70000
60000
50000
40000
30000
20000
10000
0
-10000
40 30 20 10 0 -10 -20 -30 -40 -50 -60 -70 -80 -90 -100 -110 -120 -130 -140 -150 -160 -170 -180 -190 -200 -210 -220 -230 -240 -250
f1 (ppm)

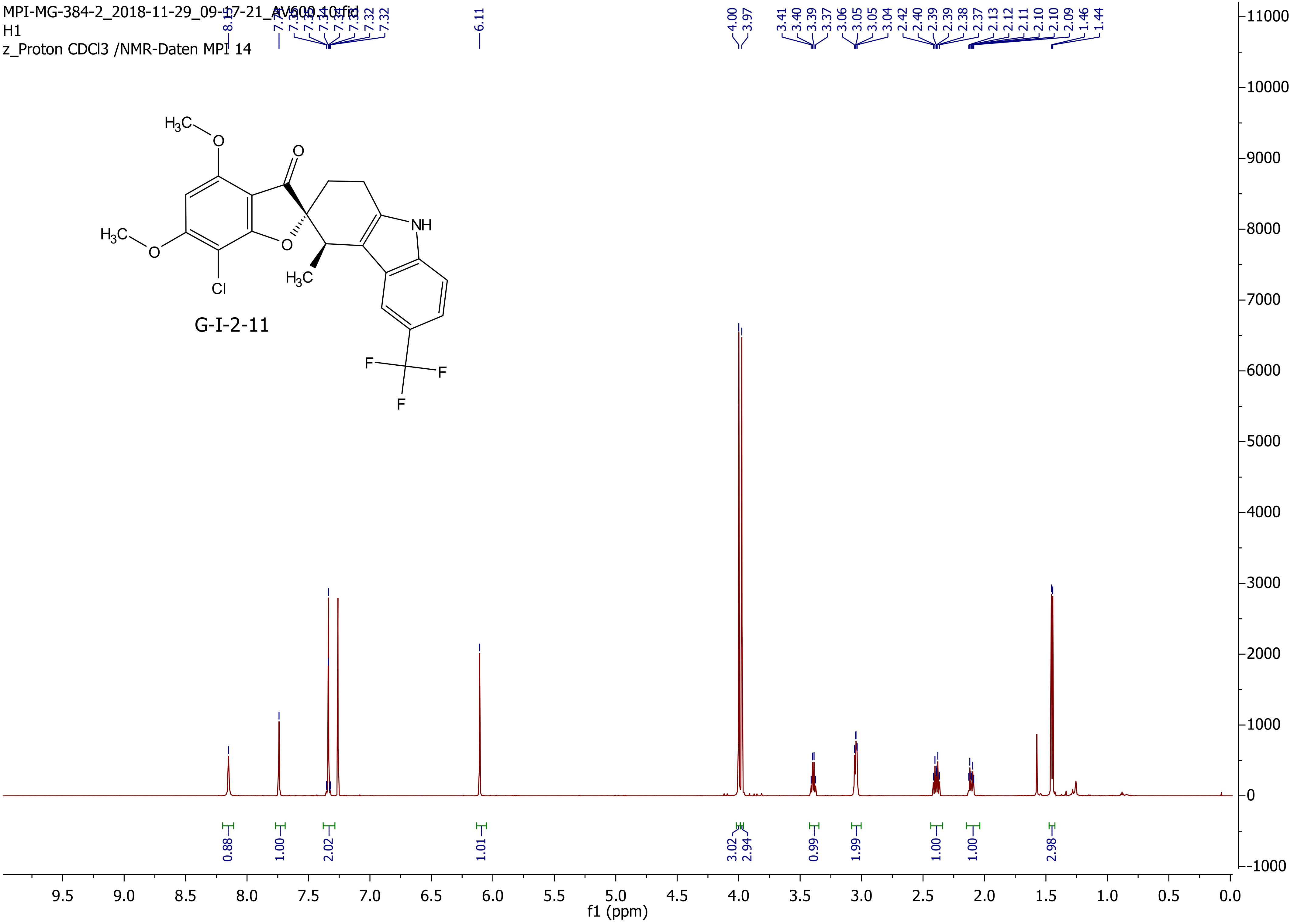
MPI-MG-384-2_2018-11-29_09-17-21_AV600.10.fid
H1
z_Proton CDCl3 /NMR-Daten MPI 14
G-I-2-11
4.00
3.97
3.41
3.40
3.39
3.37
3.06
3.05
3.05
3.04
2.42
2.40
2.39
2.39
2.38
2.37
2.13
2.12
2.11
2.10
2.10
2.09
1.46
1.44
6.11
7.32
7.32
0.88
1.00
2.02
1.01
3.02
2.94
0.99
1.99
1.00
1.00
2.98
f1 (ppm)

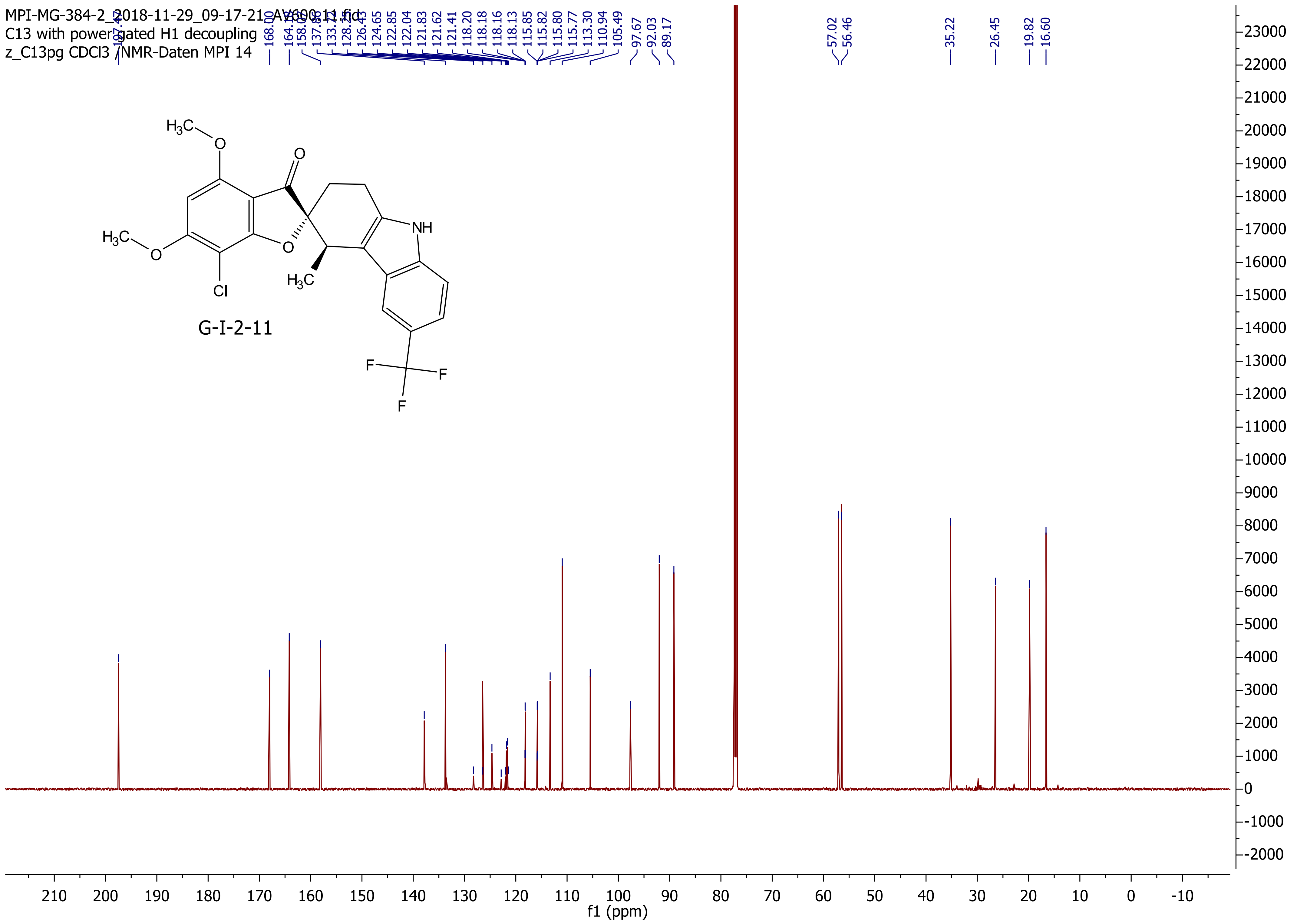

MPI-MG-384-2_2018-11-29_09-17-21_AV600 11.fid
C13 with power gated H1 decoupling
z_C13pg CDCl3 /NMR-Daten MPI 14
168.00
126.43
124.65
122.85
122.04
121.83
121.62
121.41
118.20
118.18
118.16
118.13
115.85
115.82
115.80
115.77
113.30
110.94
105.49
97.67
92.03
89.17
57.02
56.46
35.22
26.45
19.82
16.60
G-I-2-11
f1 (ppm)

MPI-MG-384-2_2018-11-29_09-17-21_AV600.12.fid
F19

z_F19 CDCl3 /NMR-Daten MPI 14

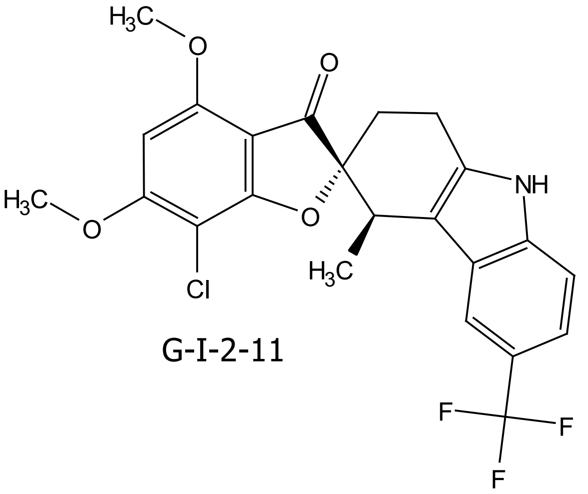

G-I-2-11

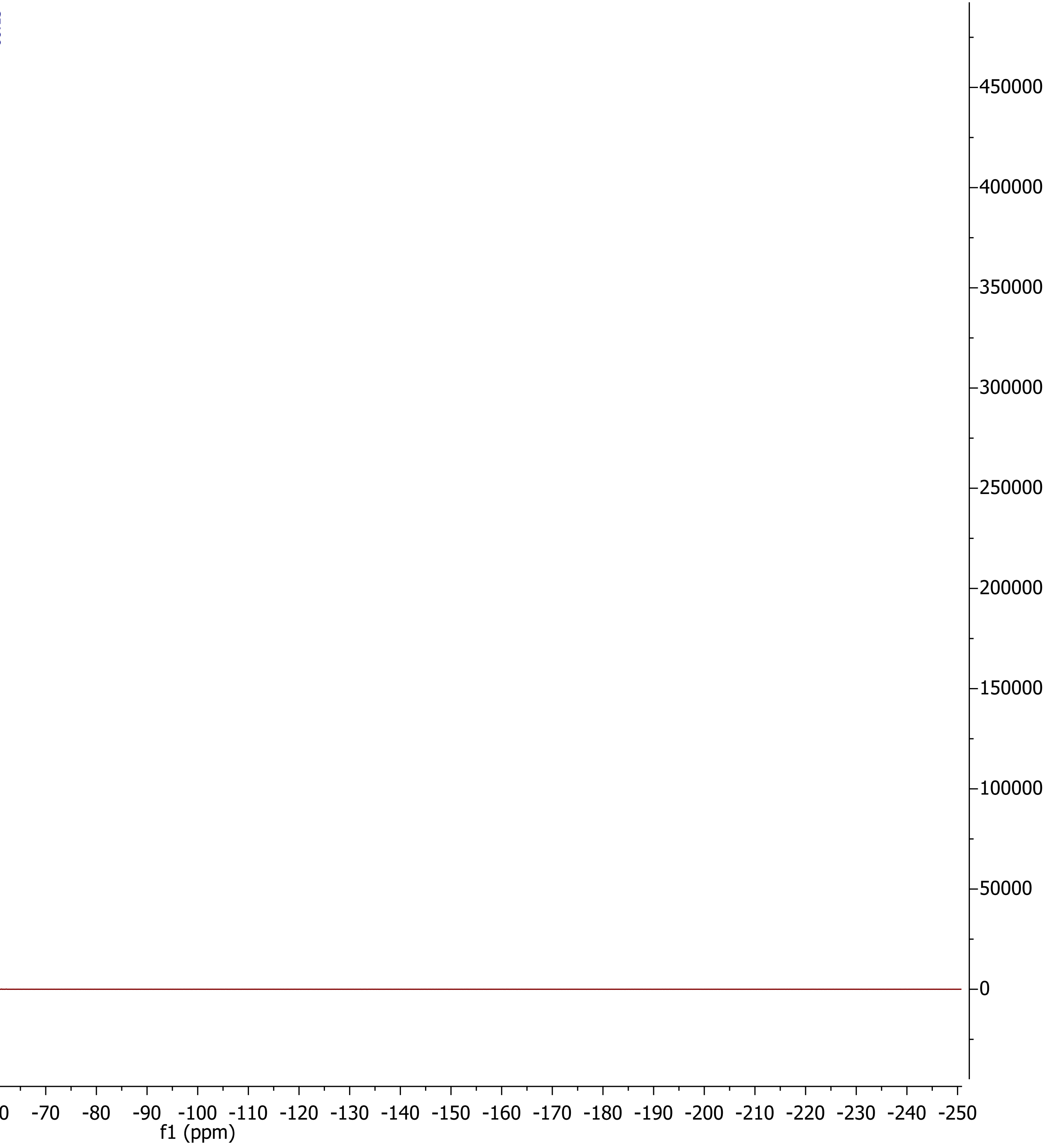

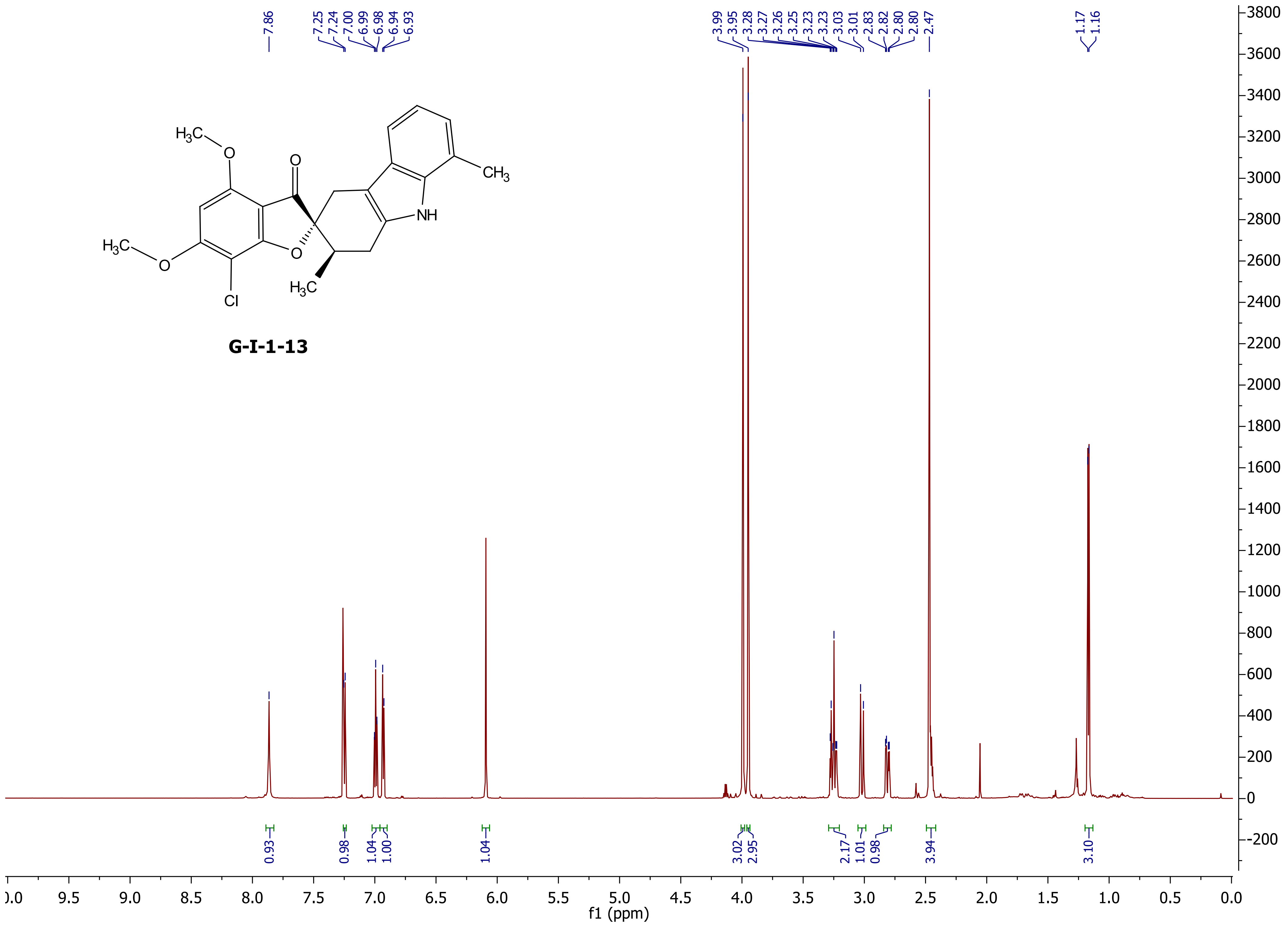
7.86
7.25
7.24
7.00
6.99
6.98
6.94
6.93
3.99
3.95
3.28
3.27
3.26
3.25
3.23
3.23
3.03
3.01
2.83
2.82
2.80
2.80
2.47
1.17
1.16
H3C
O
O
CH3
NH
H3C
O
O
H3C
Cl
G-I-1-13
0.93
0.98
1.04
1.00
1.04
3.02
2.95
2.17
1.01
0.98
3.94
3.10
f1 (ppm)

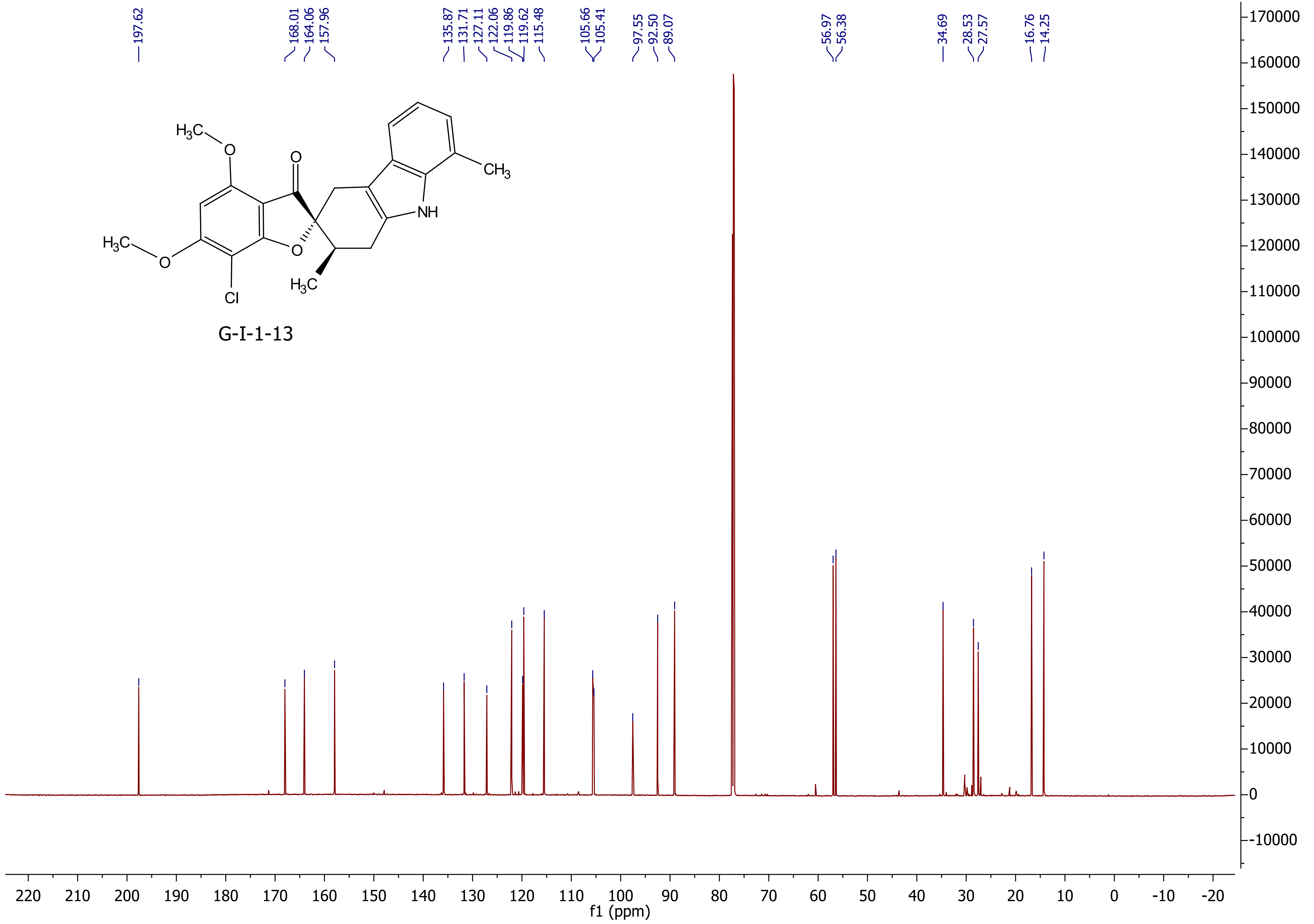
197.62
168.01
164.06
157.96
135.87
131.71
127.11
122.06
119.86
119.62
115.48
105.66
105.41
97.55
92.50
89.07
56.97
56.38
34.69
28.53
27.57
16.76
14.25
H3C
O
O
H3C
O
O
Cl
H3C
CH3
NH
G-I-1-13
f1 (ppm)

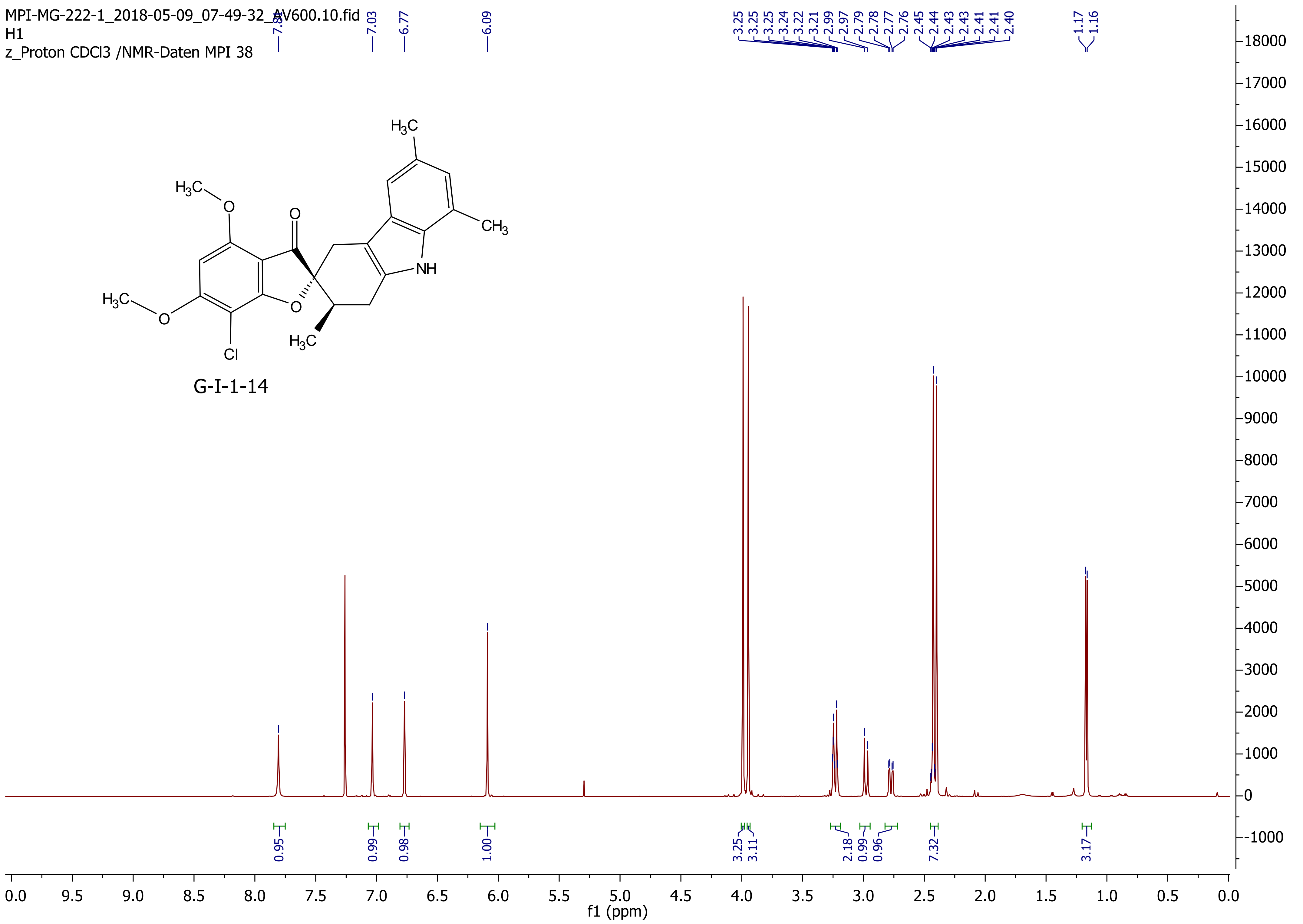
MPI-MG-222-1_2018-05-09_07-49-32_AV600.10.fid
H1
z_Proton CDCl3 /NMR-Daten MPI 38
7.03
6.77
6.09
3.25 3.25 3.25 3.24 3.22 3.21 2.99 2.97 2.79 2.78 2.77 2.76 2.45 2.44 2.43 2.43 2.41 2.41 2.40
1.17 1.16
0.95 0.99 0.98 1.00 3.25 3.11 2.18 0.99 0.96 7.32 3.17
G-I-1-14
f1 (ppm)

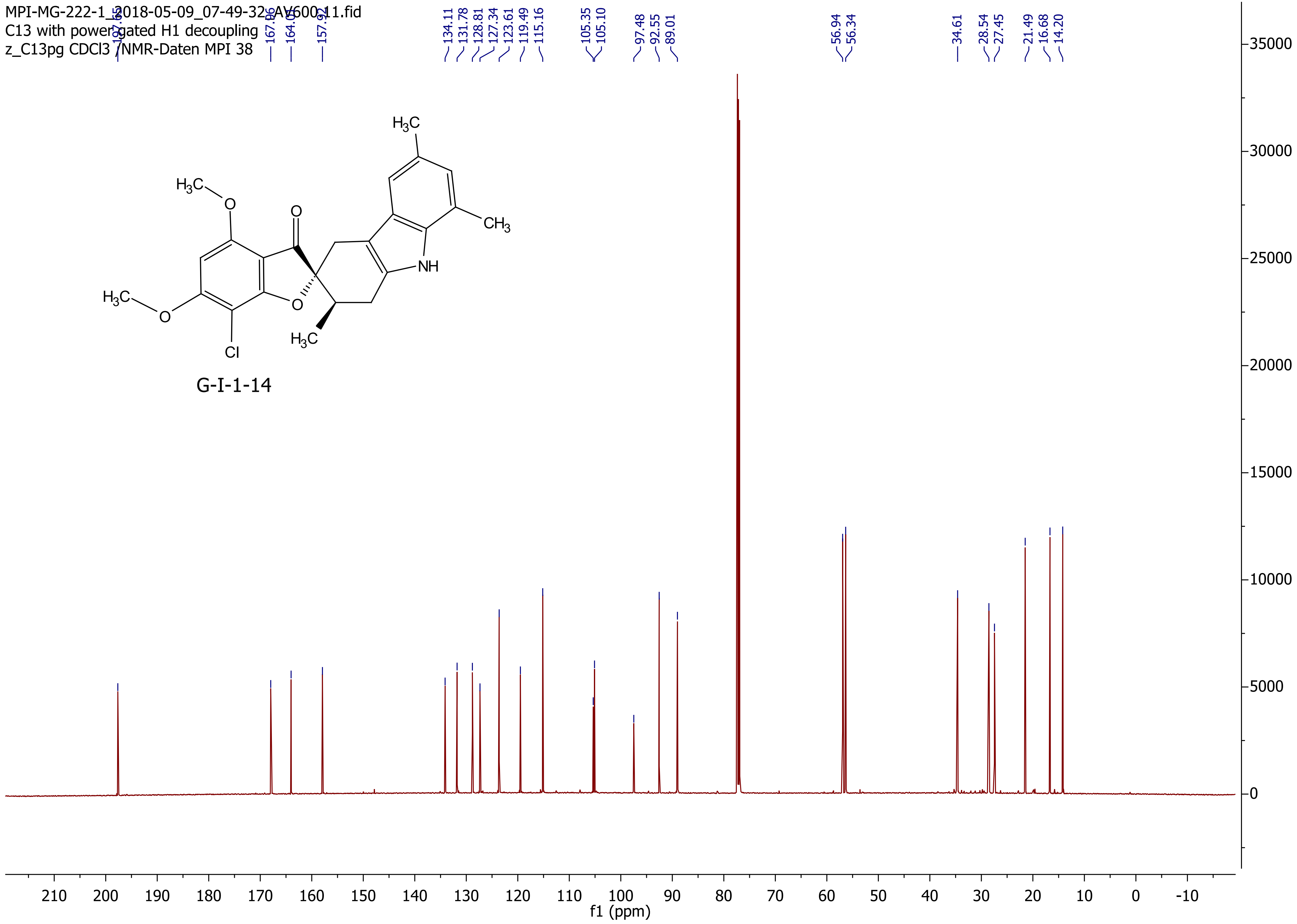
MPI-MG-222-1_2018-05-09_07-49-32_AV600_11.fid
C13 with power gated H1 decoupling
z_C13pg CDCl3 /NMR-Daten MPI 38
167.96
134.11
131.78
128.81
127.34
123.61
119.49
115.16
105.35
105.10
97.48
92.55
89.01
56.94
56.34
34.61
28.54
27.45
21.49
16.68
14.20
H_3C
O
O
CH_3
NH
H_3C
O
O
Cl
H_3C
H_3C
G-I-1-14
f1 (ppm)

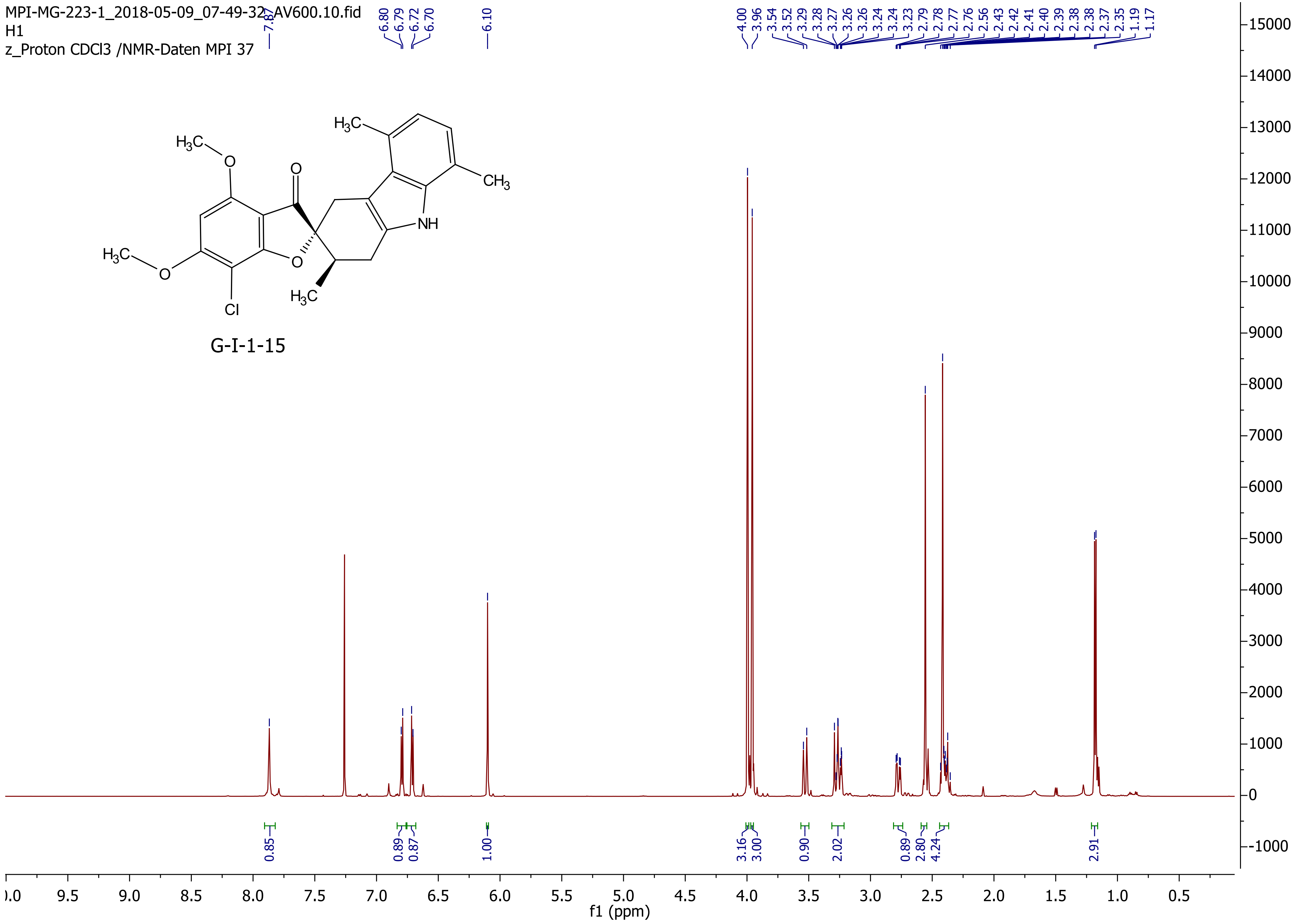
MPI-MG-223-1_2018-05-09_07-49-32_AV600.10.fid
H1
z_Proton CDCl3 /NMR-Daten MPI 37
7.87
6.80
6.79
6.72
6.70
6.10
4.00
3.96
3.54
3.52
3.29
3.28
3.27
3.26
3.26
3.24
3.24
3.23
2.79
2.78
2.77
2.76
2.56
2.43
2.42
2.41
2.40
2.39
2.38
2.38
2.37
2.35
1.19
1.17
H3C
O
O
CH3
NH
H3C
O
O
H3C
Cl
H3C
G-I-1-15
0.85
0.89
0.87
1.00
3.16
3.00
0.90
2.02
0.89
2.80
4.24
2.91
f1 (ppm)

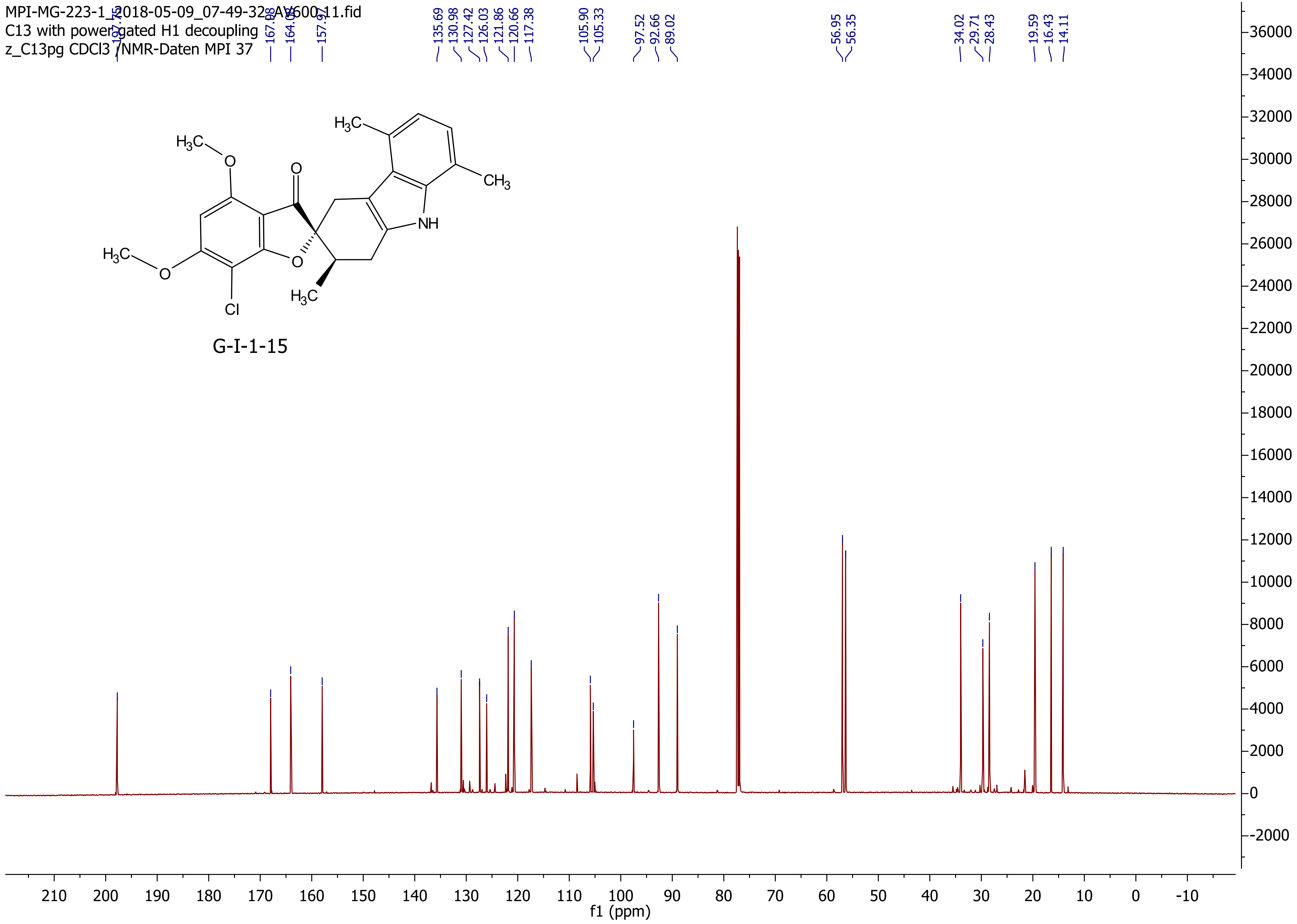
MPI-MG-223-1_2018-05-09_07-49-32_AV600_11.fid
C13 with power gated H1 decoupling
z_C13pg CDCl3 /NMR-Daten MPI 37
197.75
167.98
164.08
157.97
135.69
130.98
127.42
126.03
121.86
120.66
117.38
105.90
105.33
97.52
92.66
89.02
56.95
56.35
34.02
29.71
28.43
19.59
16.43
14.11
H3C
O
O
H3C
O
H3C
O
Cl
H3C
NH
CH3
H3C
f1 (ppm)

G-I-1-15

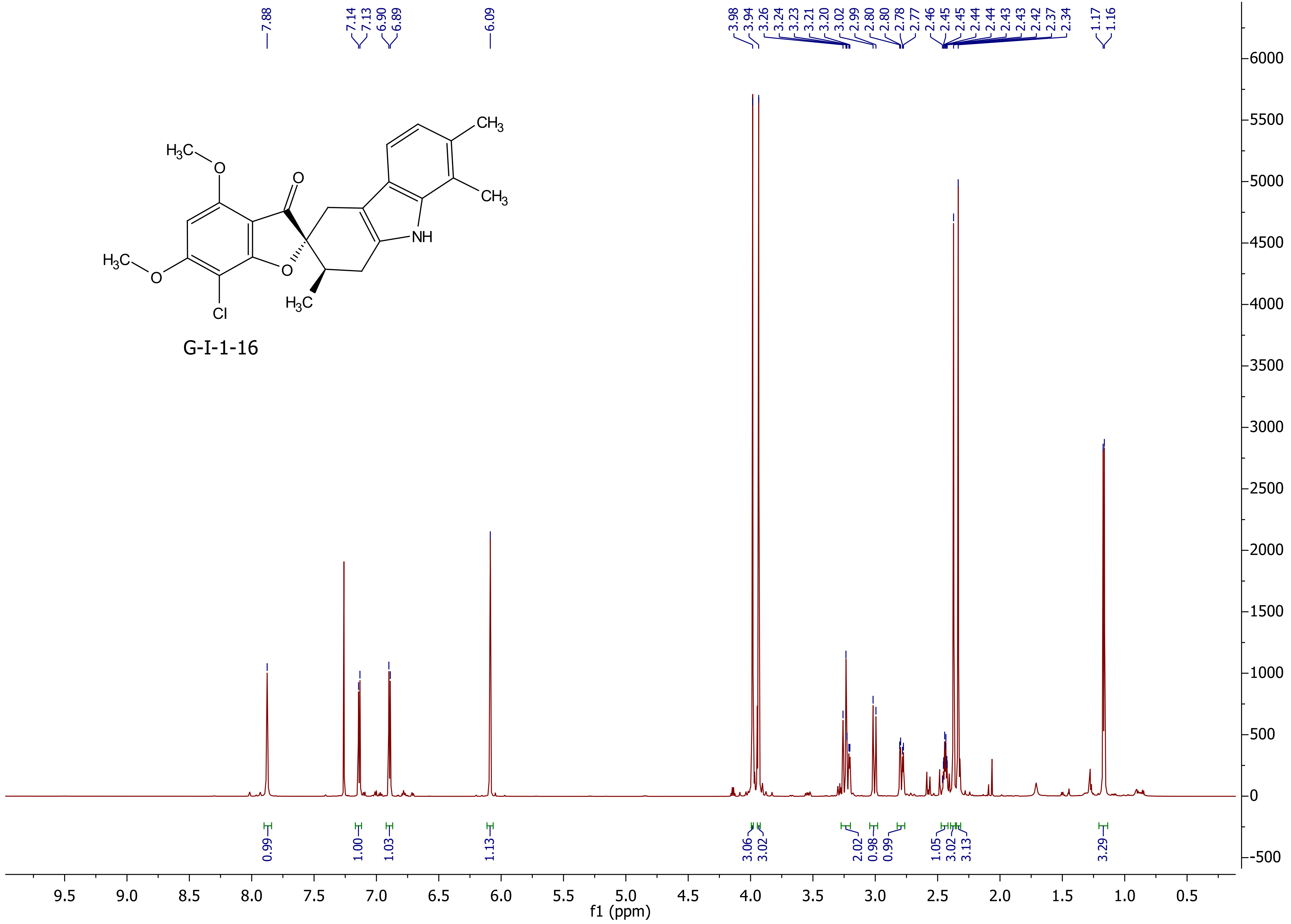

7.88
7.14
7.13
6.90
6.89
6.09
3.98
3.94
3.26
3.24
3.23
3.21
3.20
3.02
2.99
2.80
2.80
2.78
2.77
2.46
2.45
2.45
2.44
2.44
2.43
2.43
2.42
2.37
2.34
1.17
1.16
H_3C
O
O
CH_3
CH_3
NH
H_3C
O
O
H_3C
Cl
G-I-1-16
0.99
1.00
1.03
1.13
3.06
3.02
2.02
0.98
0.99
1.05
3.02
3.13
3.29
f1 (ppm)

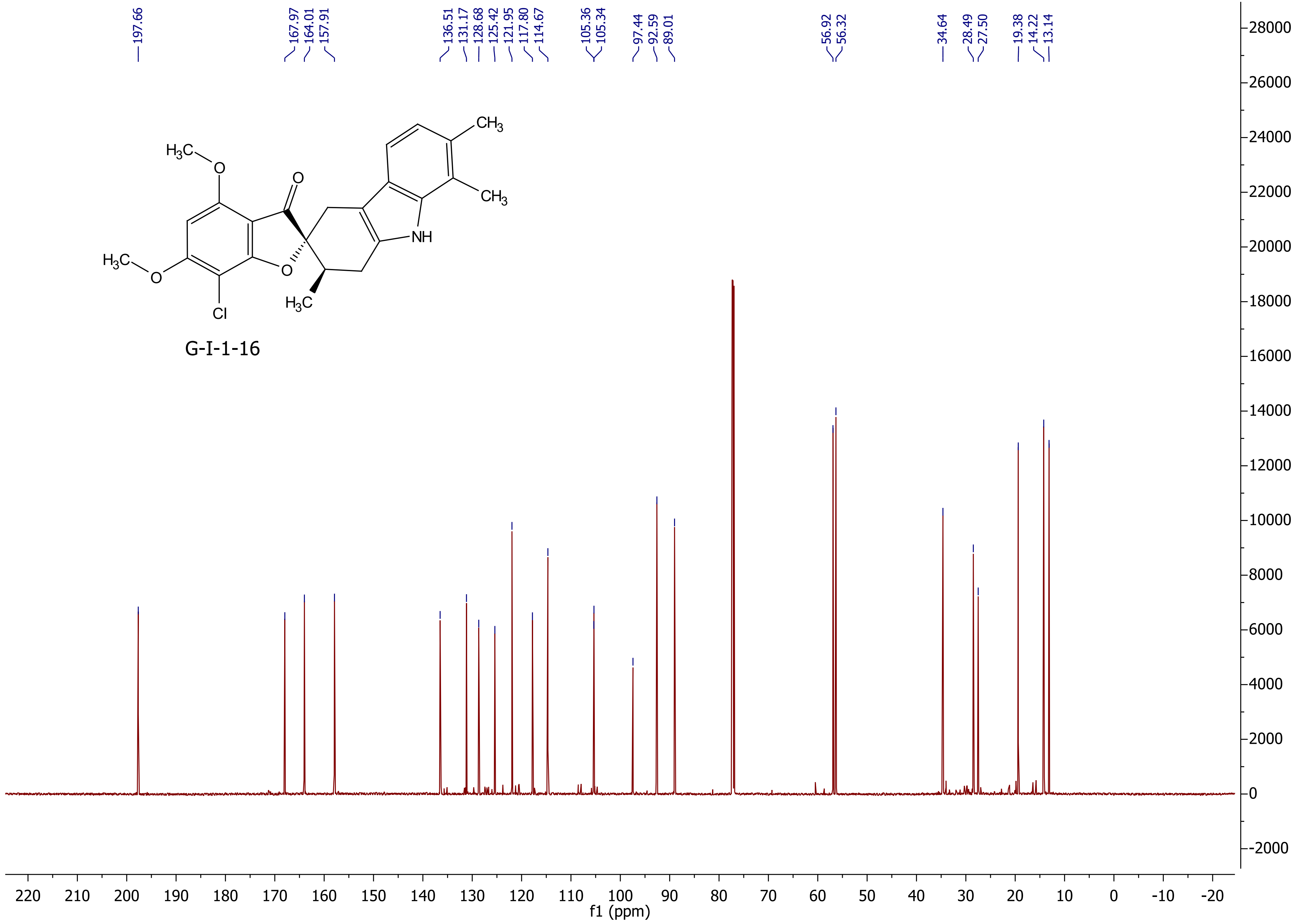

197.66
167.97
164.01
157.91
136.51
131.17
128.68
125.42
121.95
117.80
114.67
105.36
105.34
97.44
92.59
89.01
56.92
56.32
34.64
28.49
27.50
19.38
14.22
13.14
f1 (ppm)

G-I-1-16

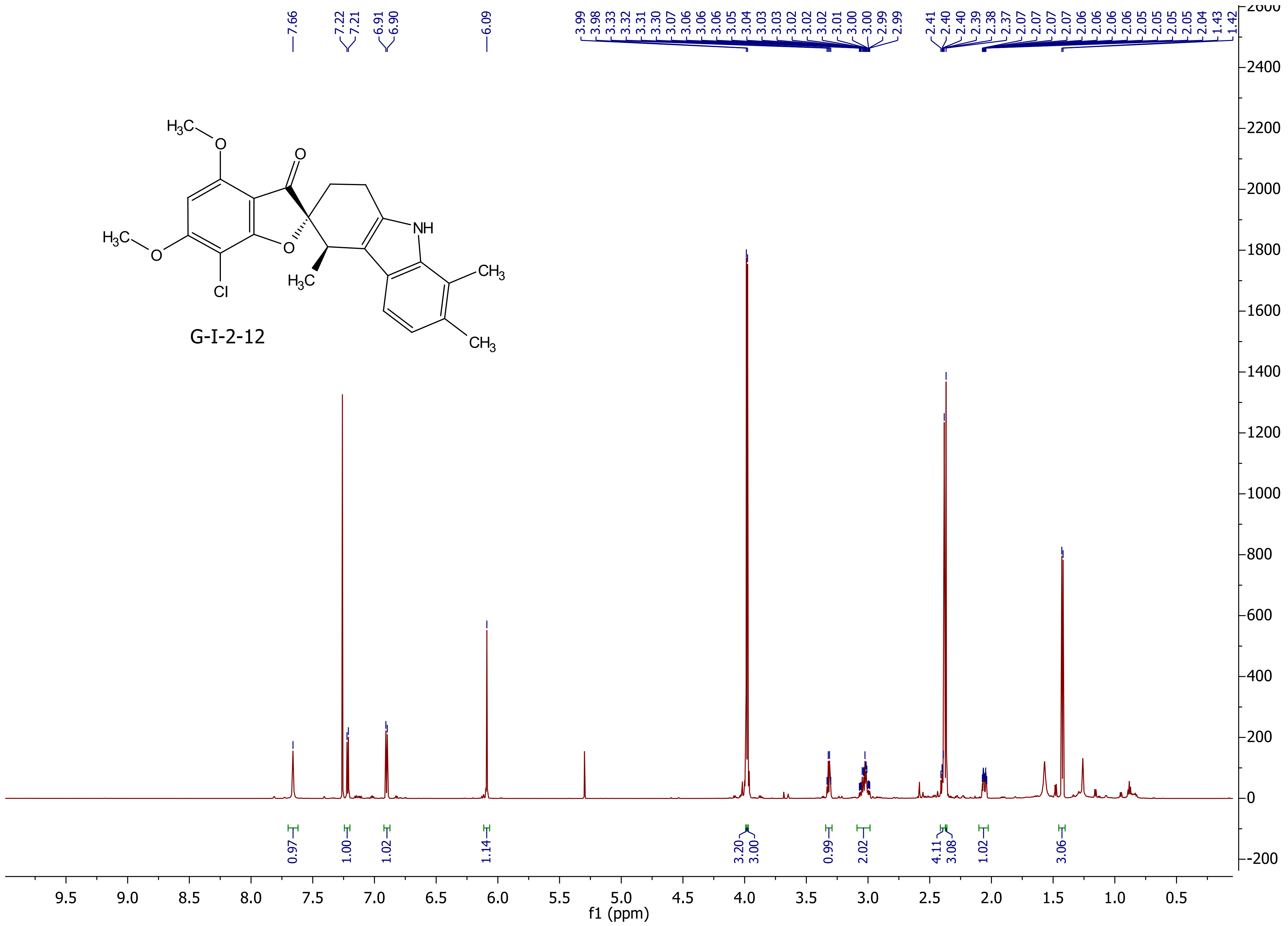

G-I-2-12
f1 (ppm)

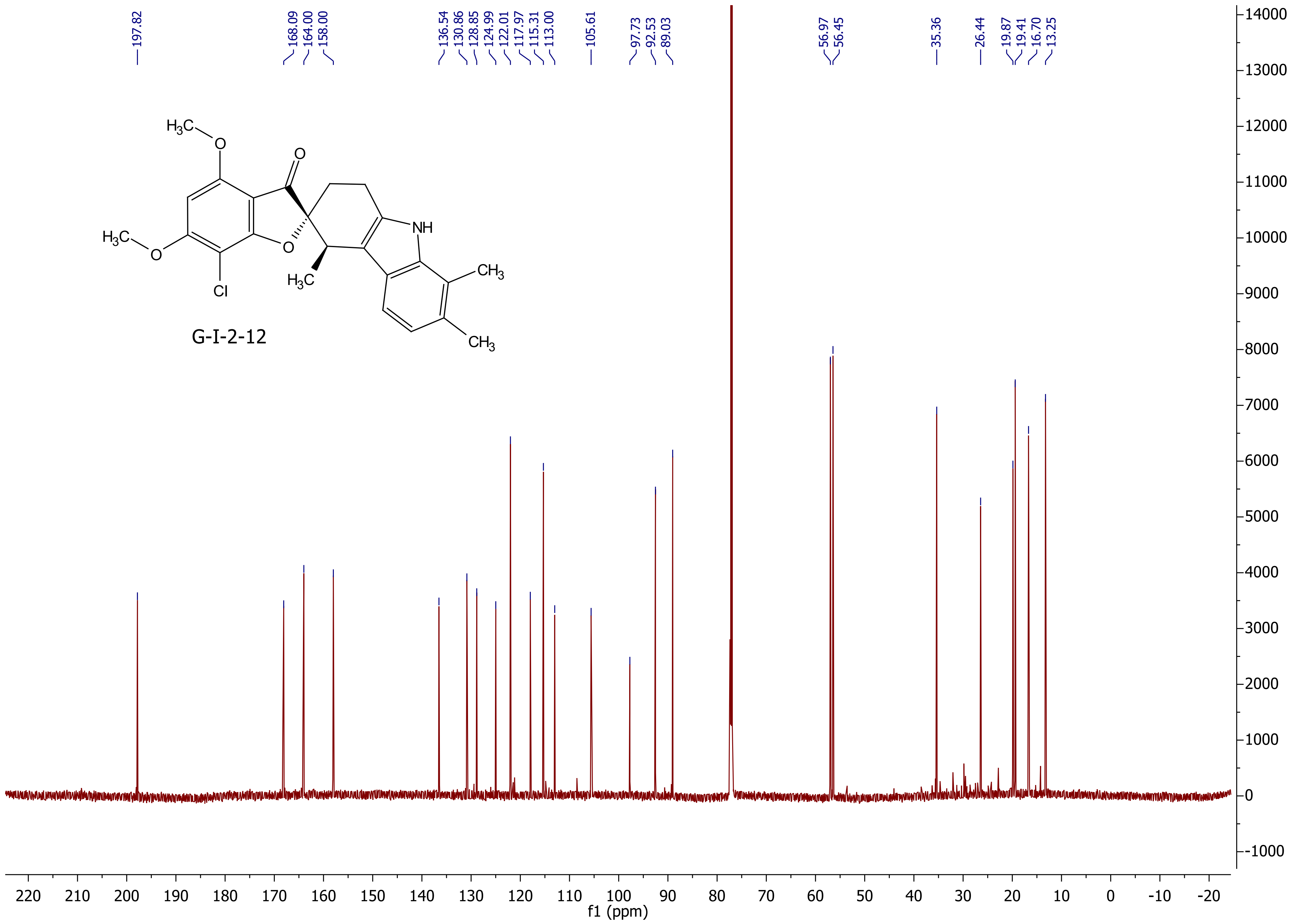

197.82
168.09
164.00
158.00
136.54
130.86
128.85
124.99
122.01
117.97
115.31
113.00
105.61
97.73
92.53
89.03
56.97
56.45
35.36
26.44
19.87
19.41
16.70
13.25
G-I-2-12
f1 (ppm)

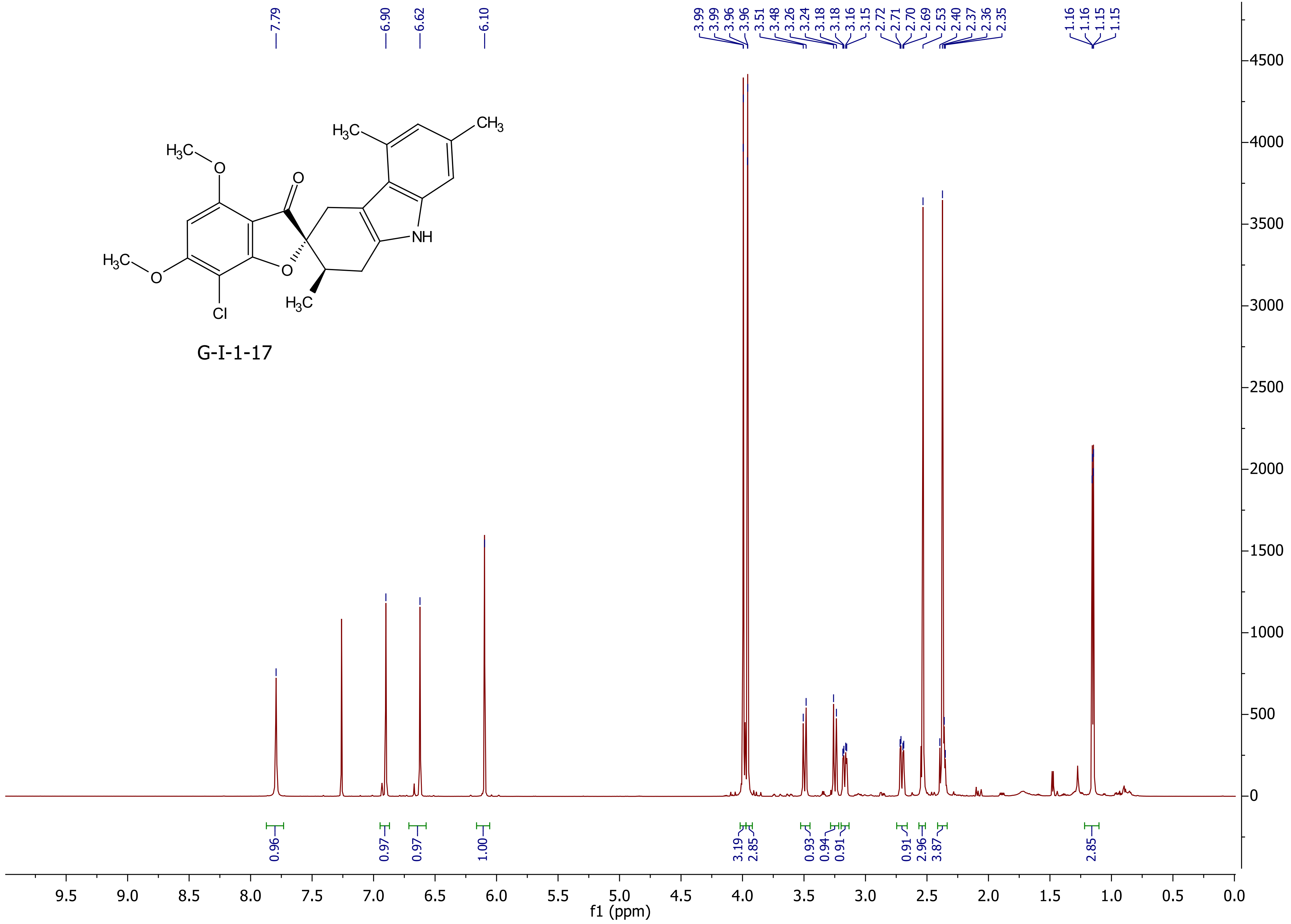

7.79
6.90
6.62
6.10
3.99
3.99
3.96
3.96
3.51
3.48
3.26
3.24
3.18
3.18
3.16
3.15
2.72
2.71
2.70
2.69
2.53
2.40
2.37
2.36
2.35
1.16
1.16
1.15
1.15
H_3C
CH_3
H_3C
O
O
NH
H_3C
O
O
H_3C
Cl
G-I-1-17
0.96
0.97
0.97
1.00
3.19
2.85
0.93
0.94
0.91
0.91
2.96
3.87
2.85
9.5
9.0
8.5
8.0
7.5
7.0
6.5
6.0
5.5
5.0
4.5
4.0
3.5
3.0
2.5
2.0
1.5
1.0
0.5
0.0
f1 (ppm)
4500
4000
3500
3000
2500
2000
1500
1000
500
0

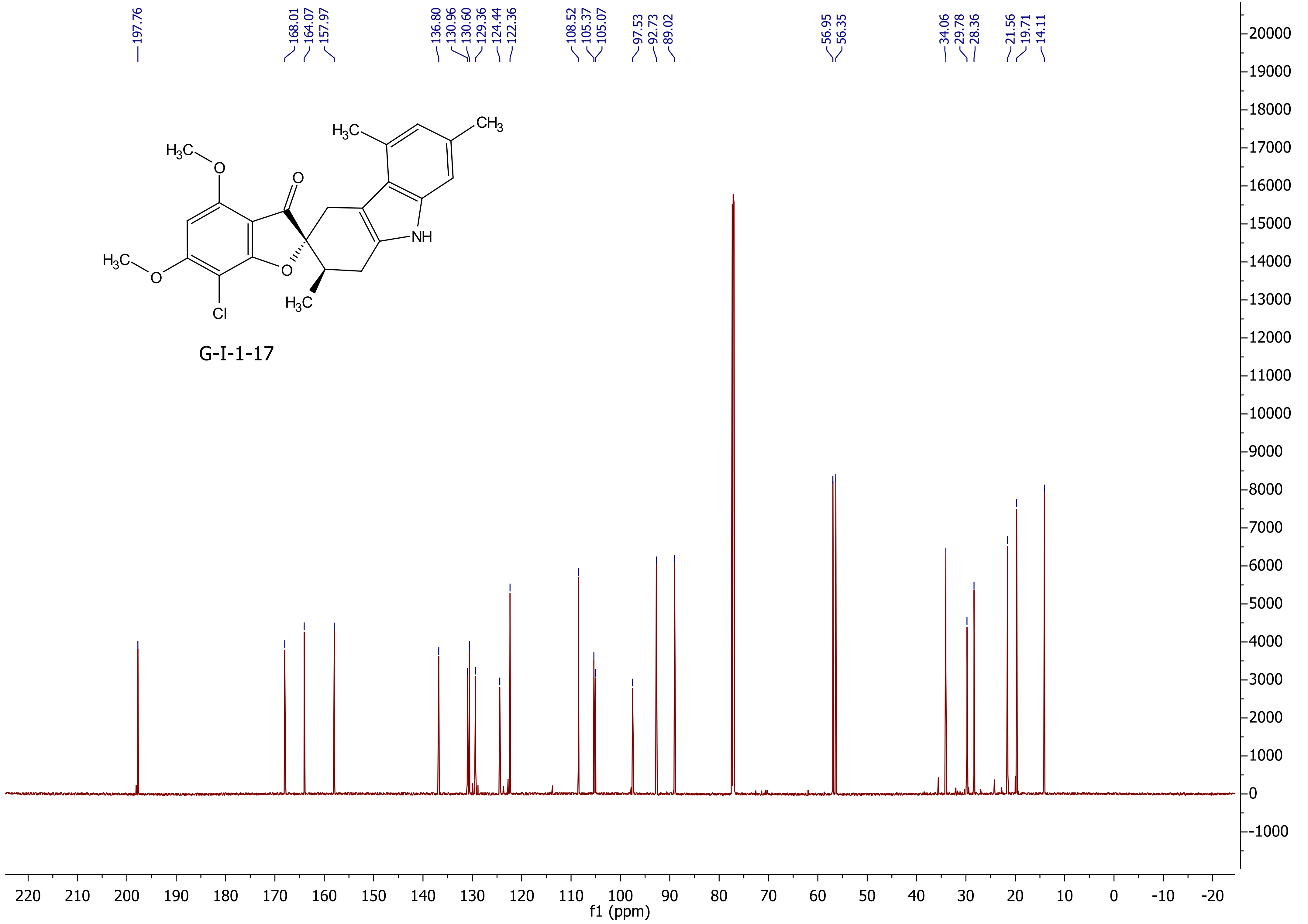

197.76
168.01
164.07
157.97
136.80
130.96
130.60
129.36
124.44
122.36
108.52
105.37
105.07
97.53
92.73
89.02
56.95
56.35
34.06
29.78
28.36
21.56
19.71
14.11
G-I-1-17
f1 (ppm)

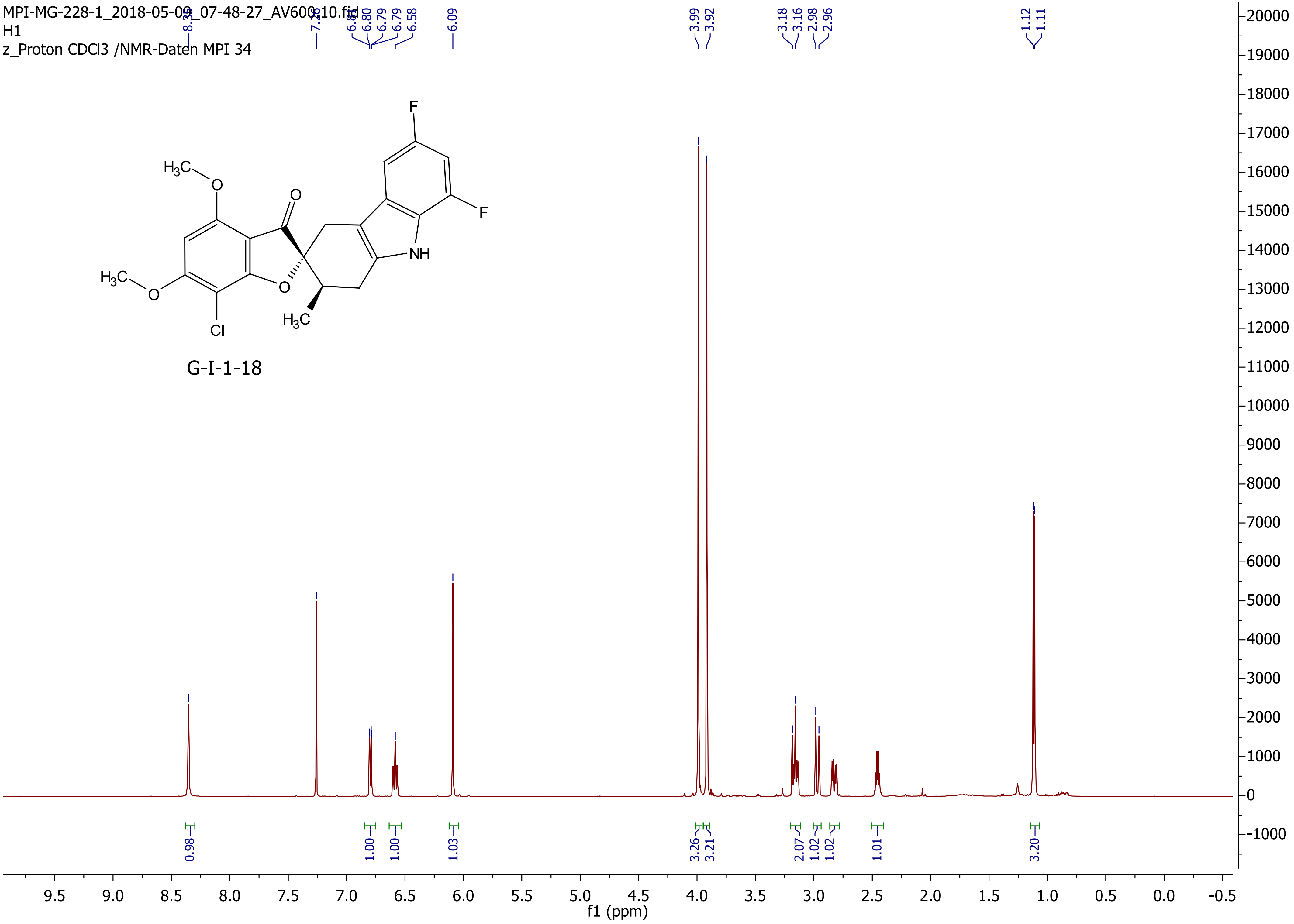
MPI-MG-228-1_2018-05-09_07-48-27_AV600_10.fid
H1
z_Proton CDCl3 /NMR-Daten MPI 34
G-I-1-18
3.99
3.92
3.18
3.16
2.98
2.96
1.12
1.11
6.80
6.79
6.79
6.58
6.09
0.98
1.00
1.00
1.03
3.26
3.21
2.07
1.02
1.02
1.01
3.20
f1 (ppm)

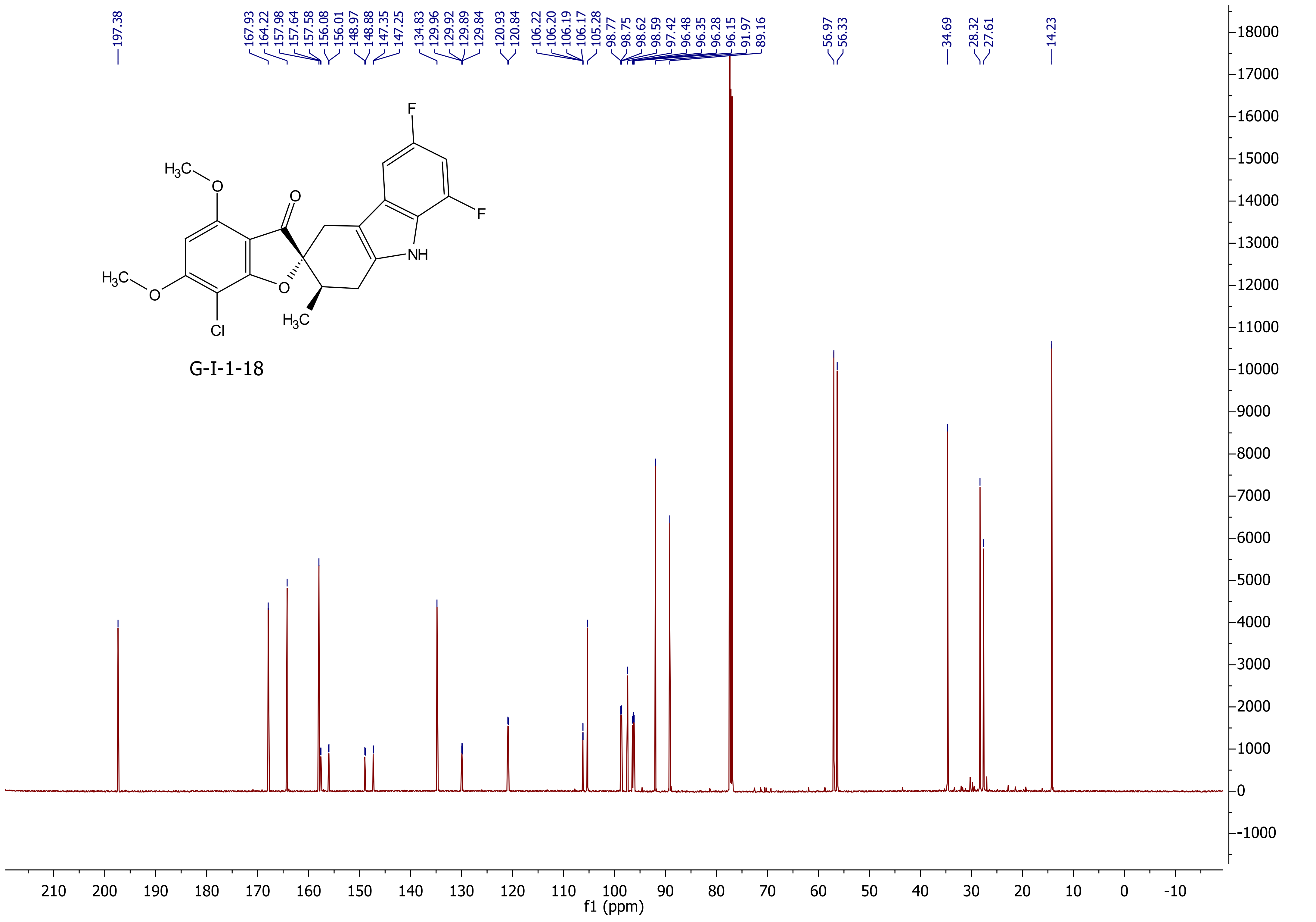
197.38
167.93
164.22
157.98
157.64
157.58
156.08
156.01
148.97
148.88
147.35
147.25
134.83
129.96
129.92
129.89
129.84
120.93
120.84
106.22
106.20
106.19
106.17
105.28
98.77
98.75
98.62
98.59
97.42
96.48
96.35
96.28
96.15
91.97
89.16
56.97
56.33
34.69
28.32
27.61
14.23
F
F
NH
O
O
O
O
H3C
H3C
H3C
Cl
G-I-1-18
18000
17000
16000
15000
14000
13000
12000
11000
10000
9000
8000
7000
6000
5000
4000
3000
2000
1000
0
-1000
210 200 190 180 170 160 150 140 130 120 110 100 90 80 70 60 50 40 30 20 10 0 -10
f1 (ppm)

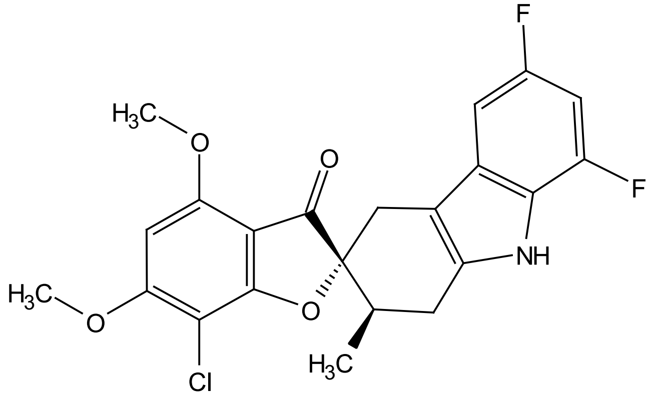
F
F
H3C
O
O
NH
H3C
O
O
Cl
H3C

G-I-1-18

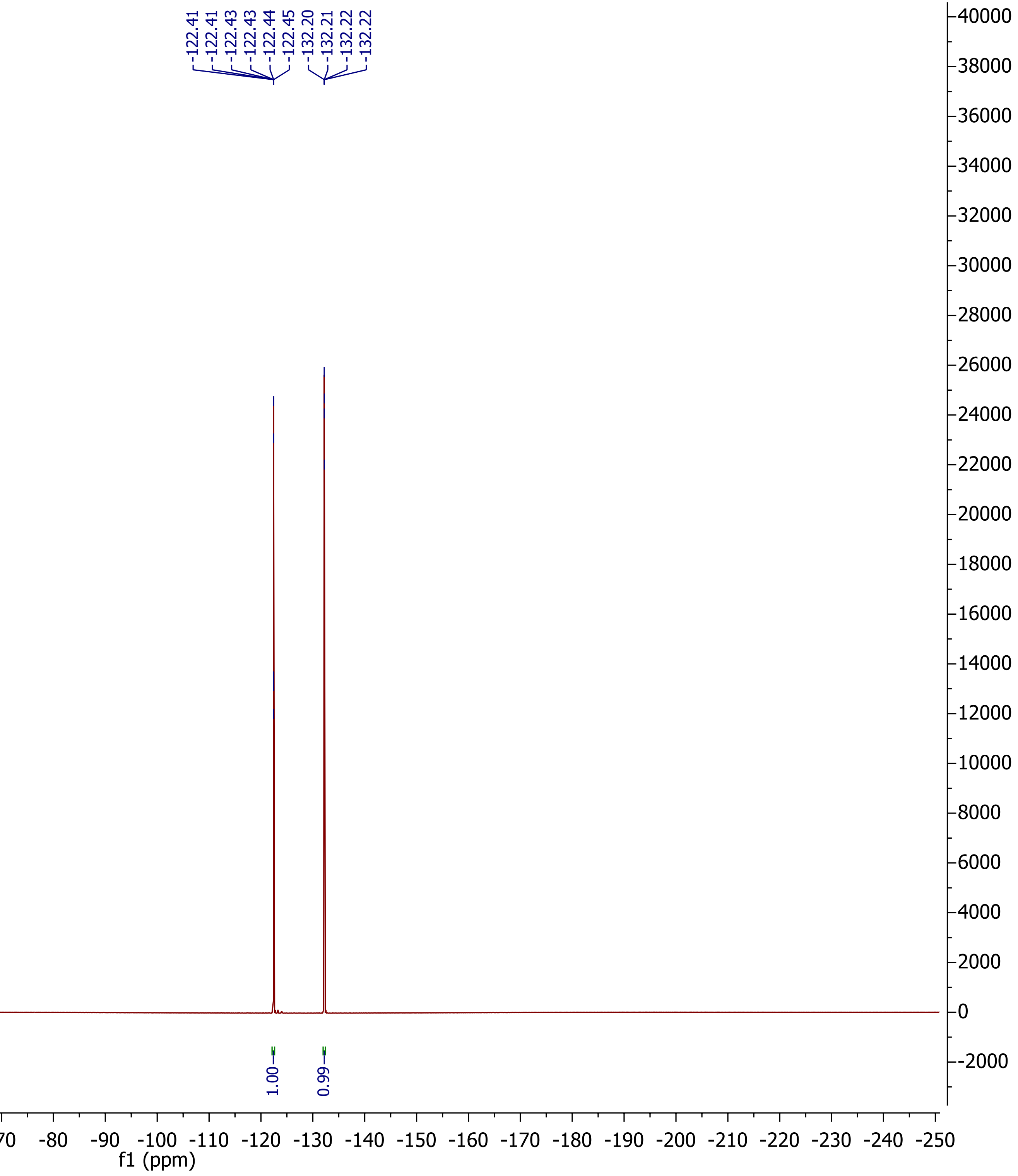
-122.41
-122.41
-122.43
-122.43
-122.44
-122.45
-132.20
-132.21
-132.22
-132.22
1.00
0.99
40000 38000 36000 34000 32000 30000 28000 26000 24000 22000 20000 18000 16000 14000 12000 10000 8000 6000 4000 2000 0 -2000
40 30 20 10 0 -10 -20 -30 -40 -50 -60 -70 -80 -90 -100 -110 -120 -130 -140 -150 -160 -170 -180 -190 -200 -210 -220 -230 -240 -250
f1 (ppm)

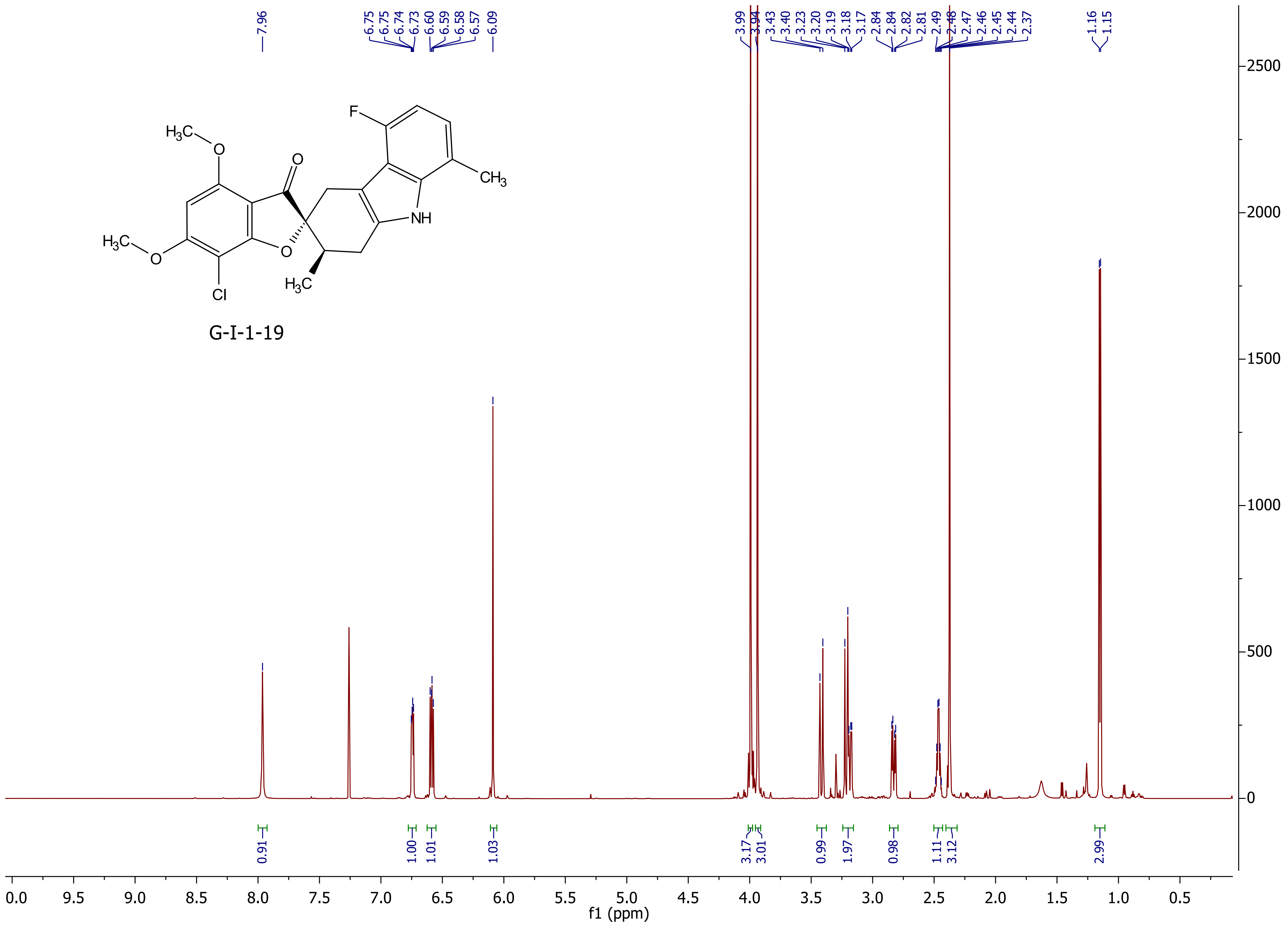

G-I-1-19
F
H_3C
O
O
CH_3
NH
H_3C
O
O
Cl
H_3C
7.96
6.75
6.75
6.74
6.73
6.60
6.59
6.58
6.57
6.09
3.99
3.94
3.43
3.40
3.23
3.20
3.19
3.18
3.17
2.84
2.84
2.82
2.81
2.49
2.48
2.47
2.46
2.45
2.44
2.37
1.16
1.15
0.91
1.00
1.01
1.03
3.17
3.01
0.99
1.97
0.98
1.11
3.12
2.99
2500
2000
1500
1000
500
0
0.0
9.5
9.0
8.5
8.0
7.5
7.0
6.5
6.0
5.5
5.0
4.5
4.0
3.5
3.0
2.5
2.0
1.5
1.0
0.5
f1 (ppm)

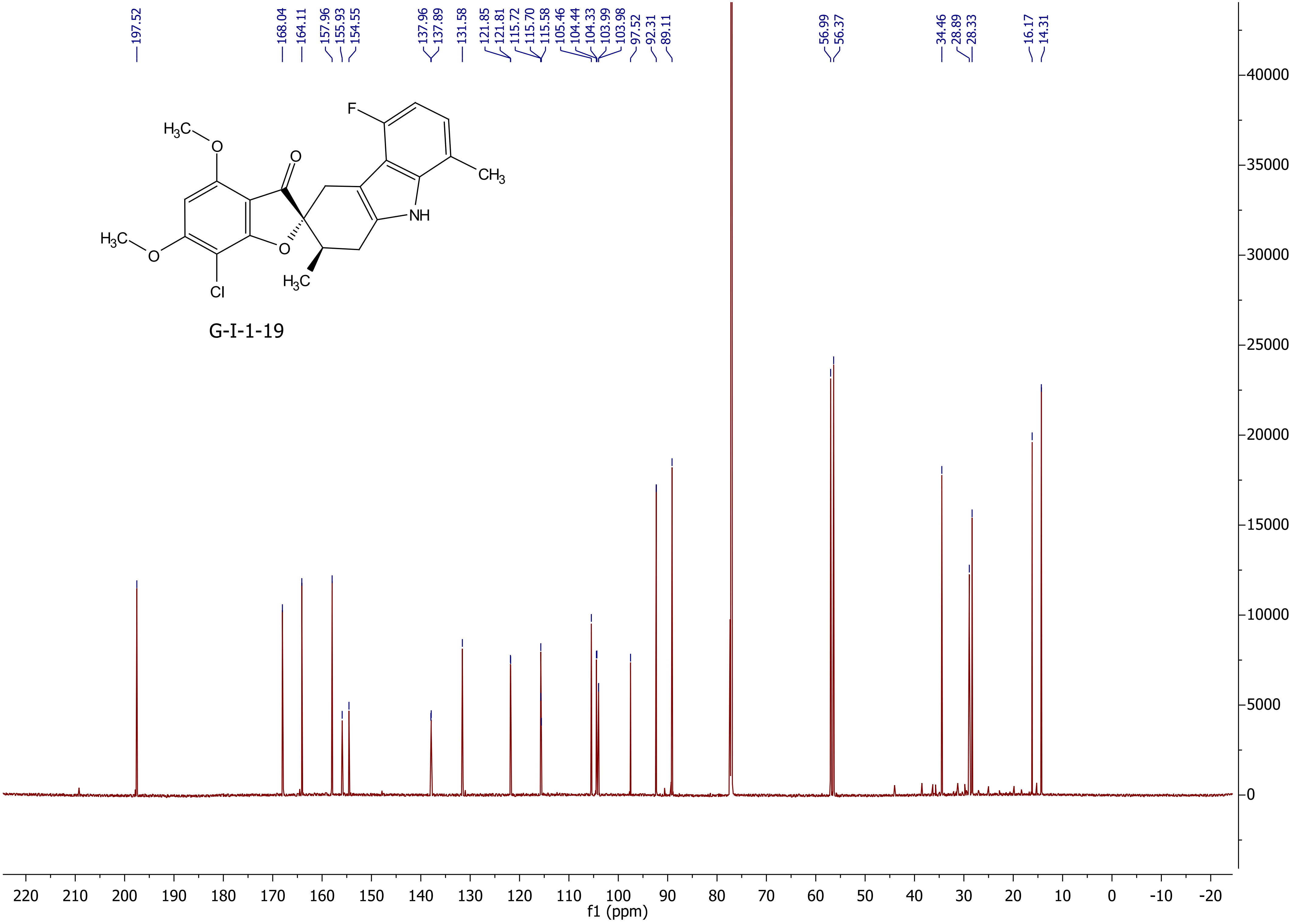

197.52
168.04
164.11
157.96
155.93
154.55
137.96
137.89
131.58
121.85
121.81
115.72
115.70
115.58
105.46
104.44
104.33
103.99
103.98
97.52
92.31
89.11
56.99
56.37
34.46
28.89
28.33
16.17
14.31
F
H_3C
O
O
CH_3
NH
H_3C
O
O
Cl
H_3C
f1 (ppm)

G-I-1-19

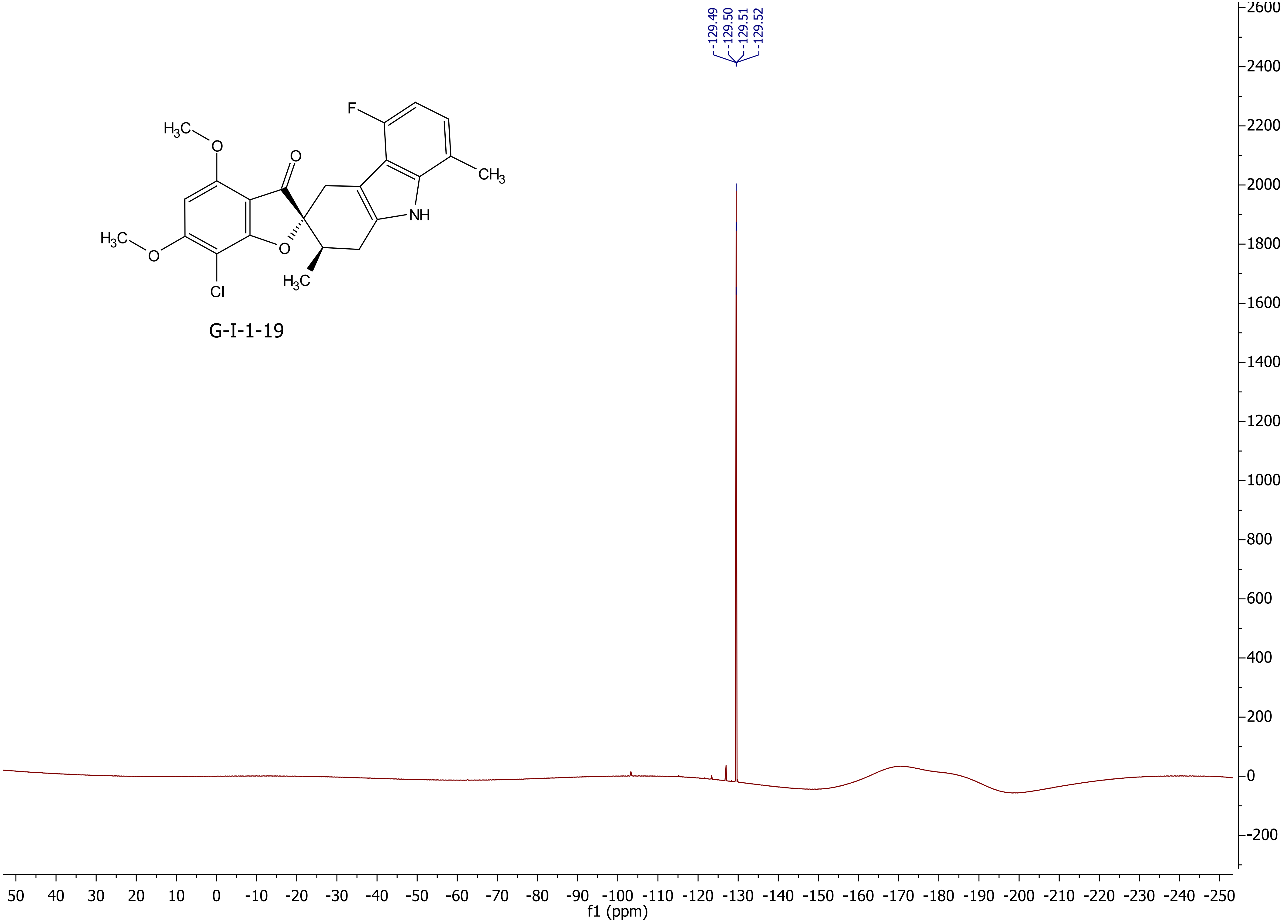

-129.49
-129.50
-129.51
-129.52
2600
2400
2200
2000
1800
1600
1400
1200
1000
800
600
400
200
0
-200
50 40 30 20 10 0 -10 -20 -30 -40 -50 -60 -70 -80 -90 -100 -110 -120 -130 -140 -150 -160 -170 -180 -190 -200 -210 -220 -230 -240 -250
f1 (ppm)

G-I-1-19

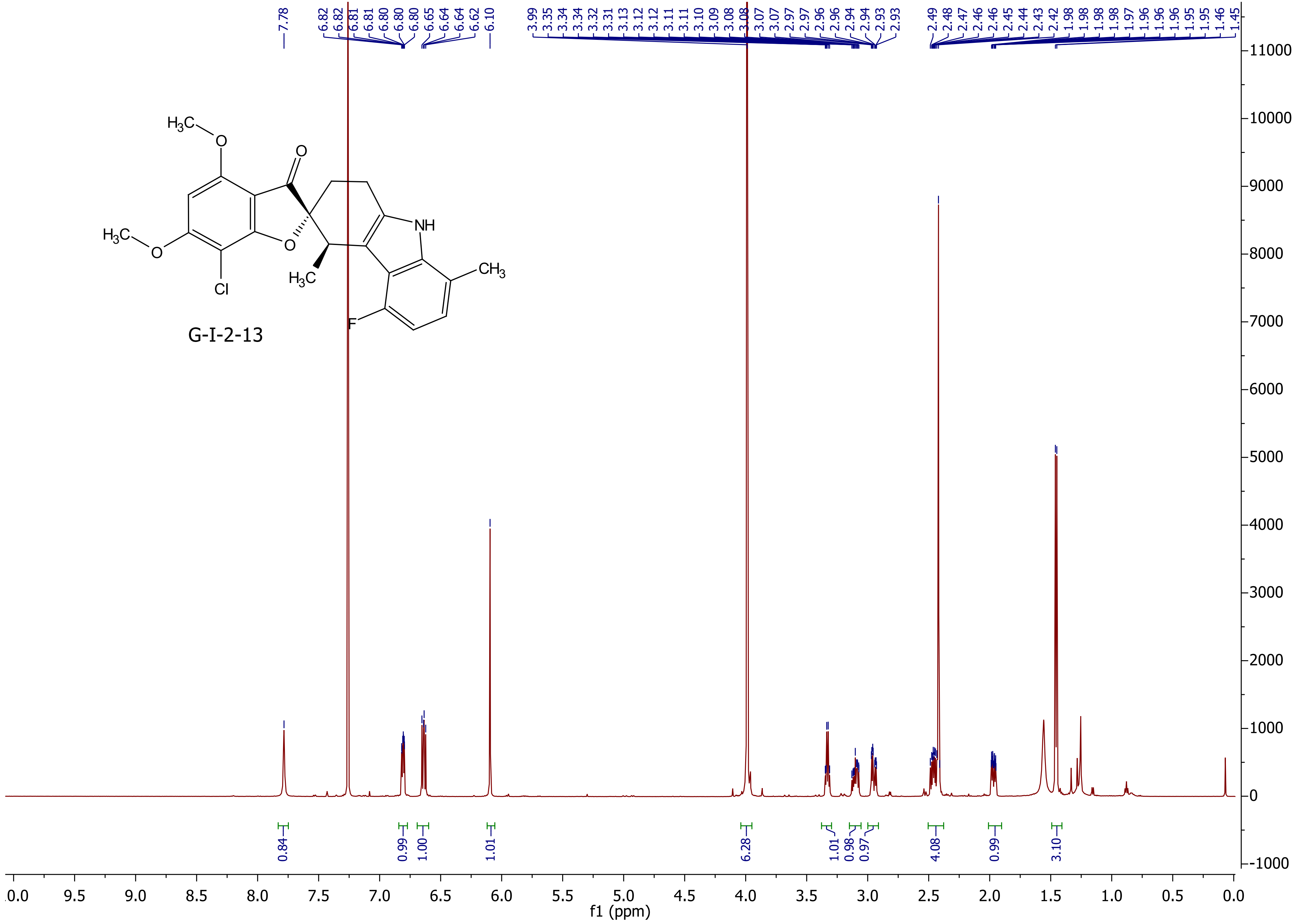

7.78
6.82 6.82 6.81 6.81 6.80 6.80 6.80 6.65 6.64 6.64 6.62 6.10
3.99 3.35 3.34 3.34 3.32 3.31 3.13 3.12 3.12 3.11 3.11 3.10 3.09 3.08 3.08 3.07 3.07 2.97 2.97 2.96 2.96 2.94 2.94 2.93 2.93
2.49 2.48 2.47 2.46 2.46 2.45 2.44 2.43 2.42 1.98 1.98 1.98 1.98 1.97 1.96 1.96 1.96 1.95 1.95 1.46 1.45
G-I-2-13
0.84 0.99 1.00 1.01 6.28 1.01 0.98 0.97 4.08 0.99 3.10
f1 (ppm)

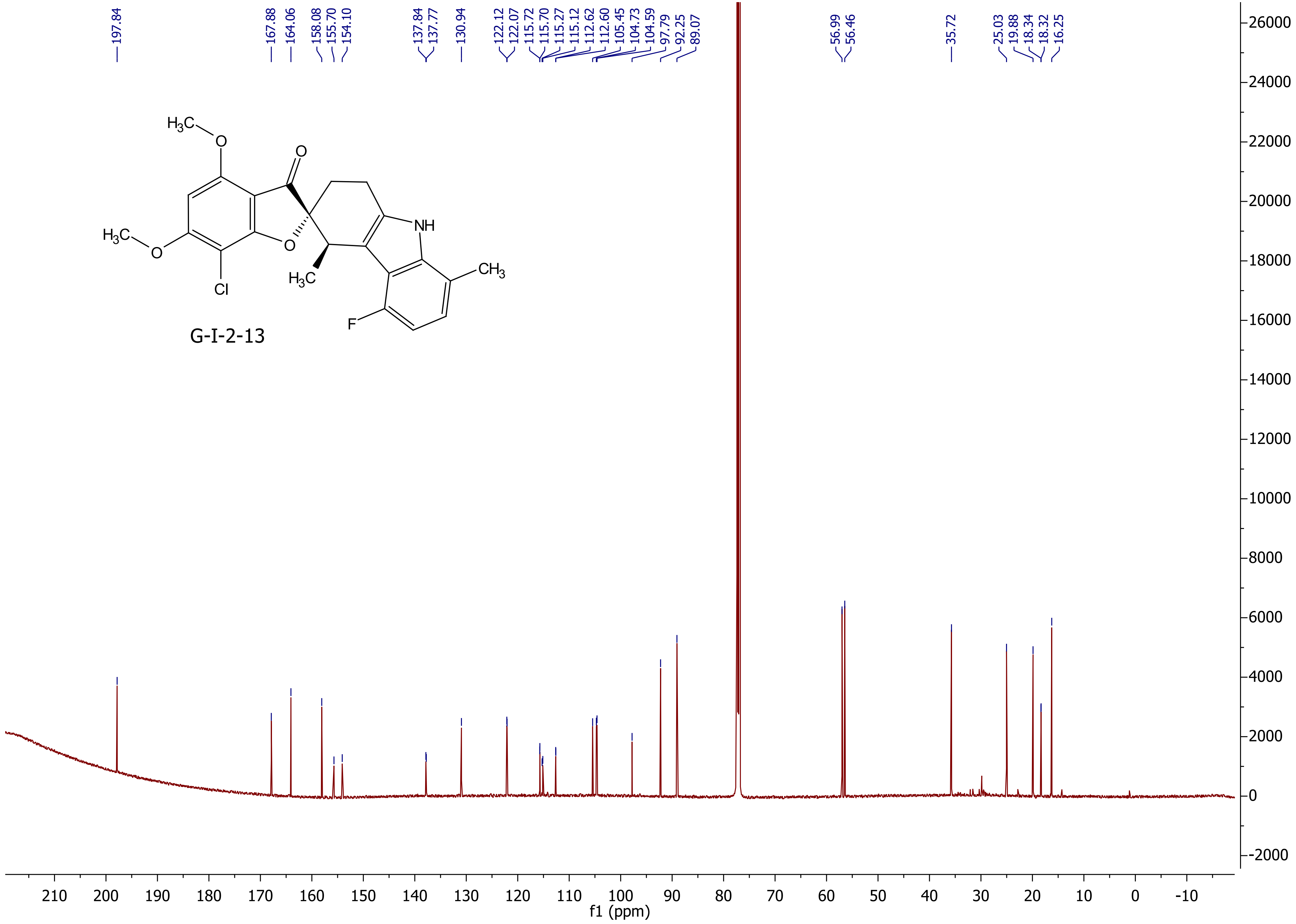
197.84
167.88
164.06
158.08
155.70
154.10
137.84
137.77
130.94
122.12
122.07
115.72
115.70
115.27
115.12
112.62
112.60
105.45
104.73
104.59
97.79
92.25
89.07
56.99
56.46
35.72
25.03
19.88
18.34
18.32
16.25
H_3C
O
O
H_3C
O
Cl
O
NH
H_3C
CH_3
F
G-I-2-13
f1 (ppm)

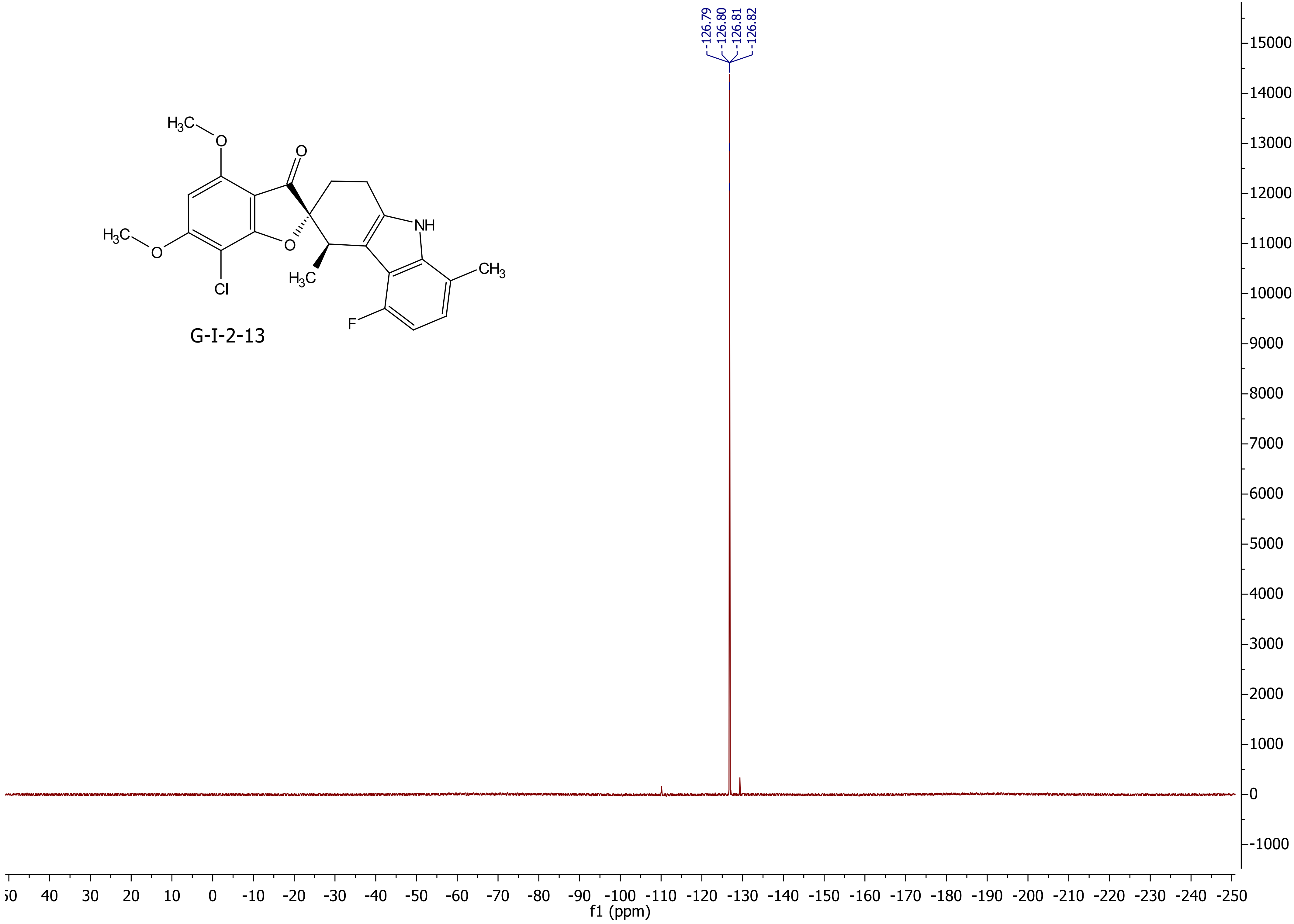

-126.79
-126.80
-126.81
-126.82
G-I-2-13
f1 (ppm)

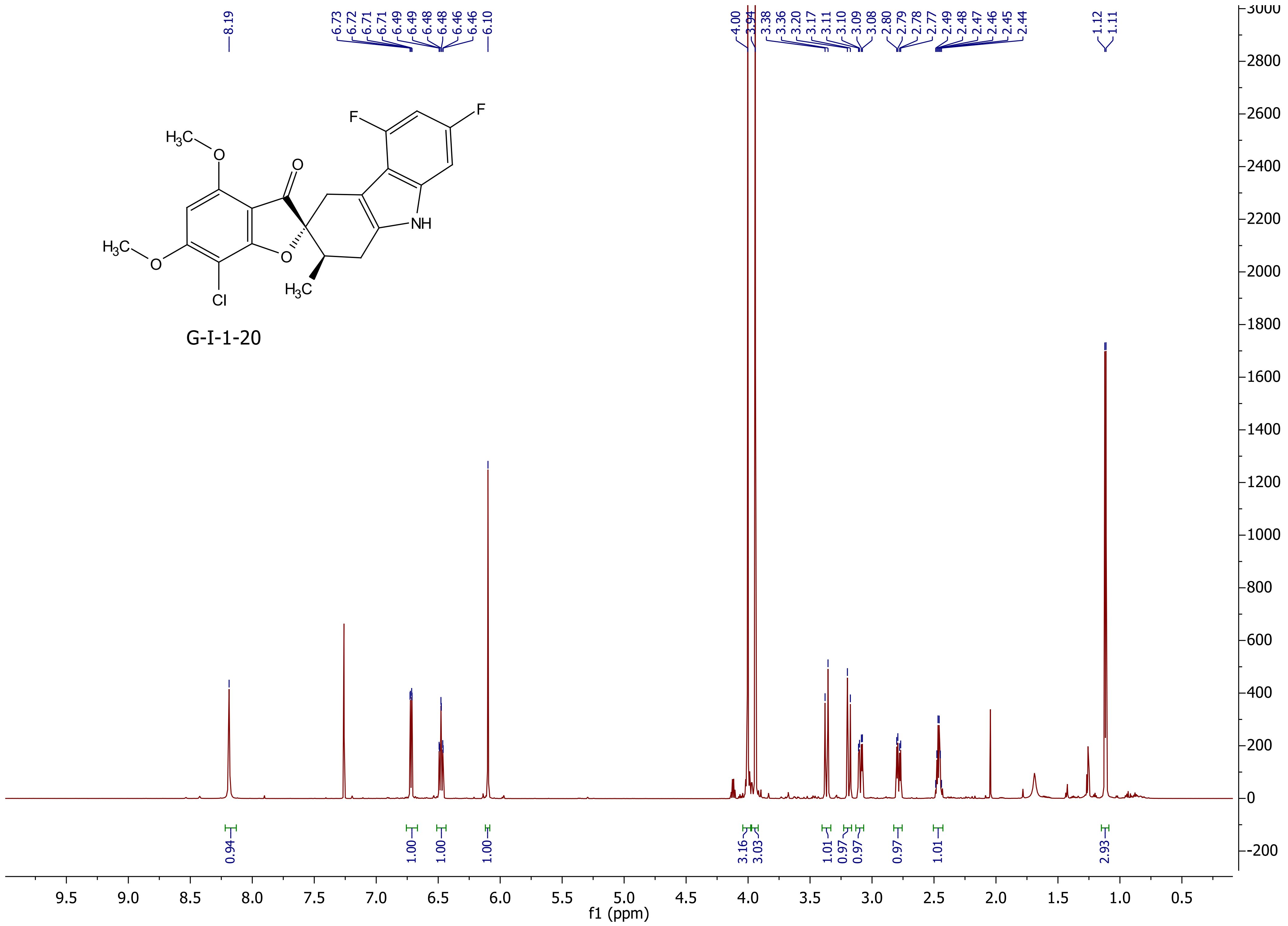

G-I-1-20
f1 (ppm)

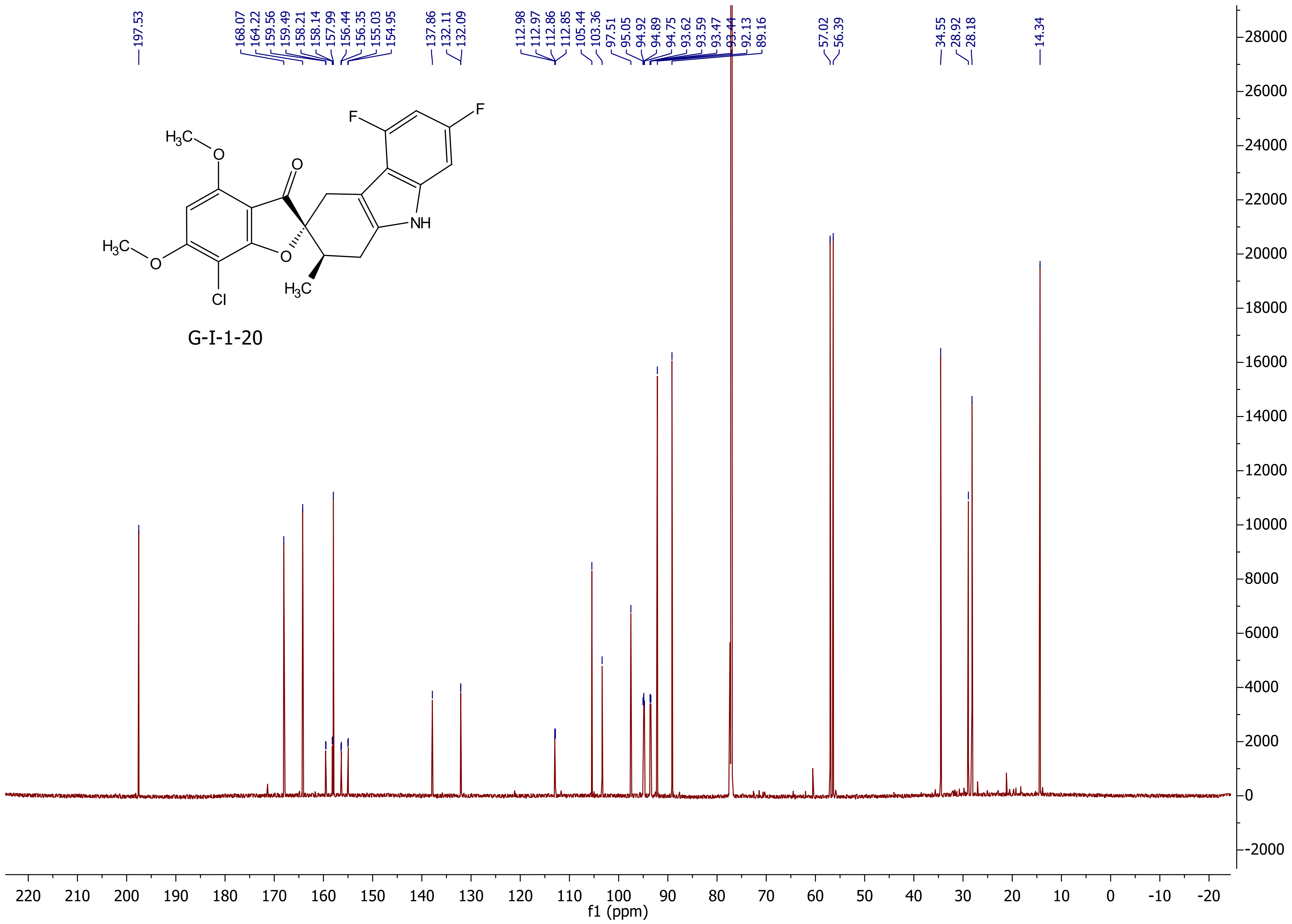

197.53
168.07
164.22
159.56
159.49
158.21
158.14
157.99
156.44
156.35
155.03
154.95
137.86
132.11
132.09
112.98
112.97
112.86
112.85
105.44
103.36
97.51
95.05
94.92
94.89
94.75
93.62
93.59
93.47
93.44
92.13
89.16
57.02
56.39
34.55
28.92
28.18
14.34
G-I-1-20
f1 (ppm)

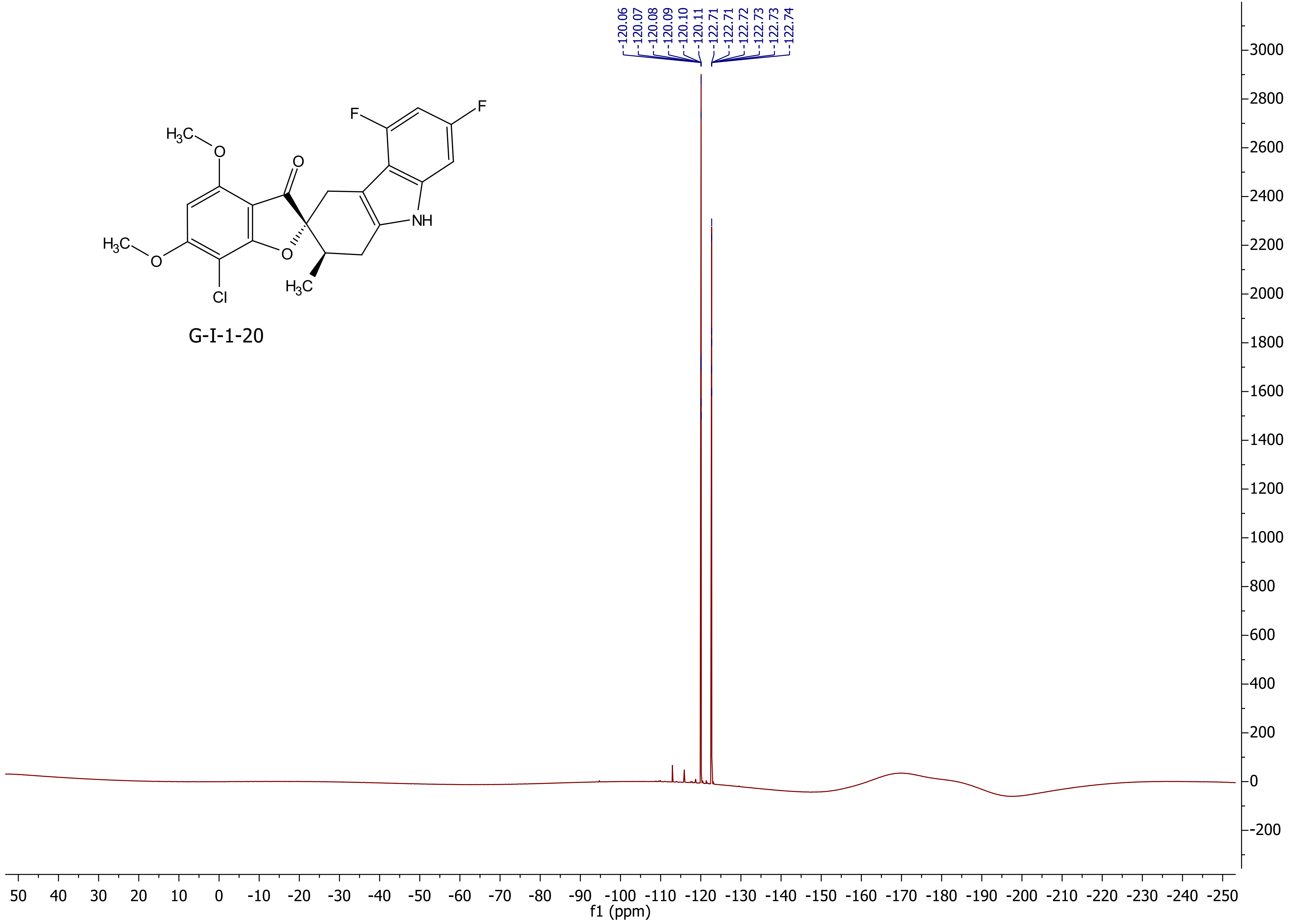

120.06
120.07
120.08
120.09
120.10
120.11
122.71
122.71
122.72
122.73
122.73
122.74
G-I-1-20
f1 (ppm)

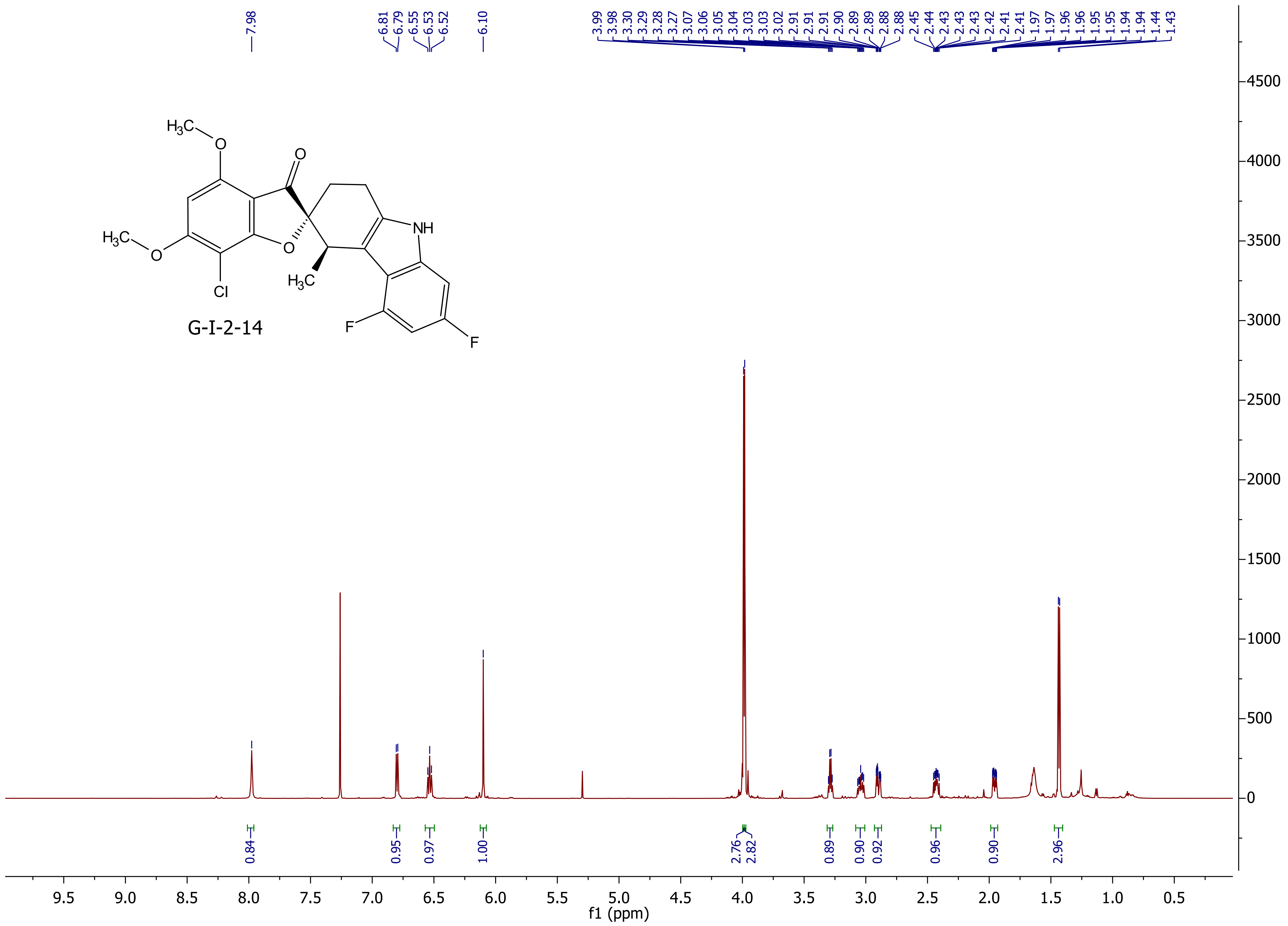

7.98
6.81
6.79
6.55
6.53
6.52
6.10
3.99
3.98
3.30
3.29
3.28
3.27
3.07
3.06
3.05
3.04
3.03
3.03
3.02
2.91
2.91
2.91
2.90
2.89
2.89
2.88
2.88
2.45
2.44
2.43
2.43
2.43
2.42
2.41
2.41
1.97
1.97
1.96
1.96
1.95
1.95
1.94
1.94
1.44
1.43
G-I-2-14
0.84
0.95
0.97
1.00
2.76
2.82
0.89
0.90
0.92
0.96
0.90
2.96
f1 (ppm)

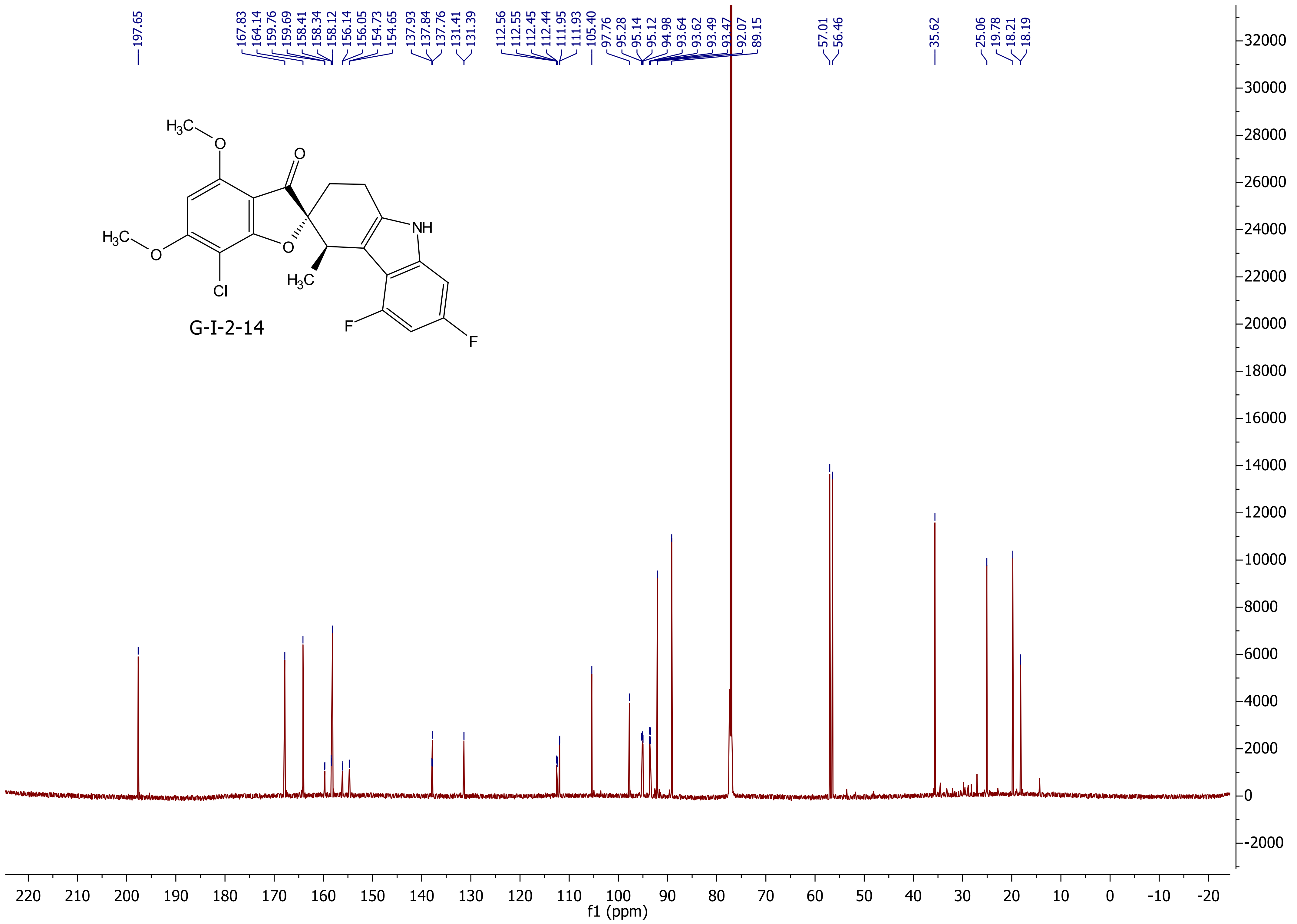

197.65
167.83
164.14
159.76
159.69
158.41
158.34
158.12
156.14
156.05
154.73
154.65
137.93
137.84
137.76
131.41
131.39
112.56
112.55
112.45
112.44
111.95
111.93
105.40
97.76
95.28
95.14
95.12
94.98
93.64
93.62
93.49
93.47
92.07
89.15
57.01
56.46
35.62
25.06
19.78
18.21
18.19
G-I-2-14
f1 (ppm)

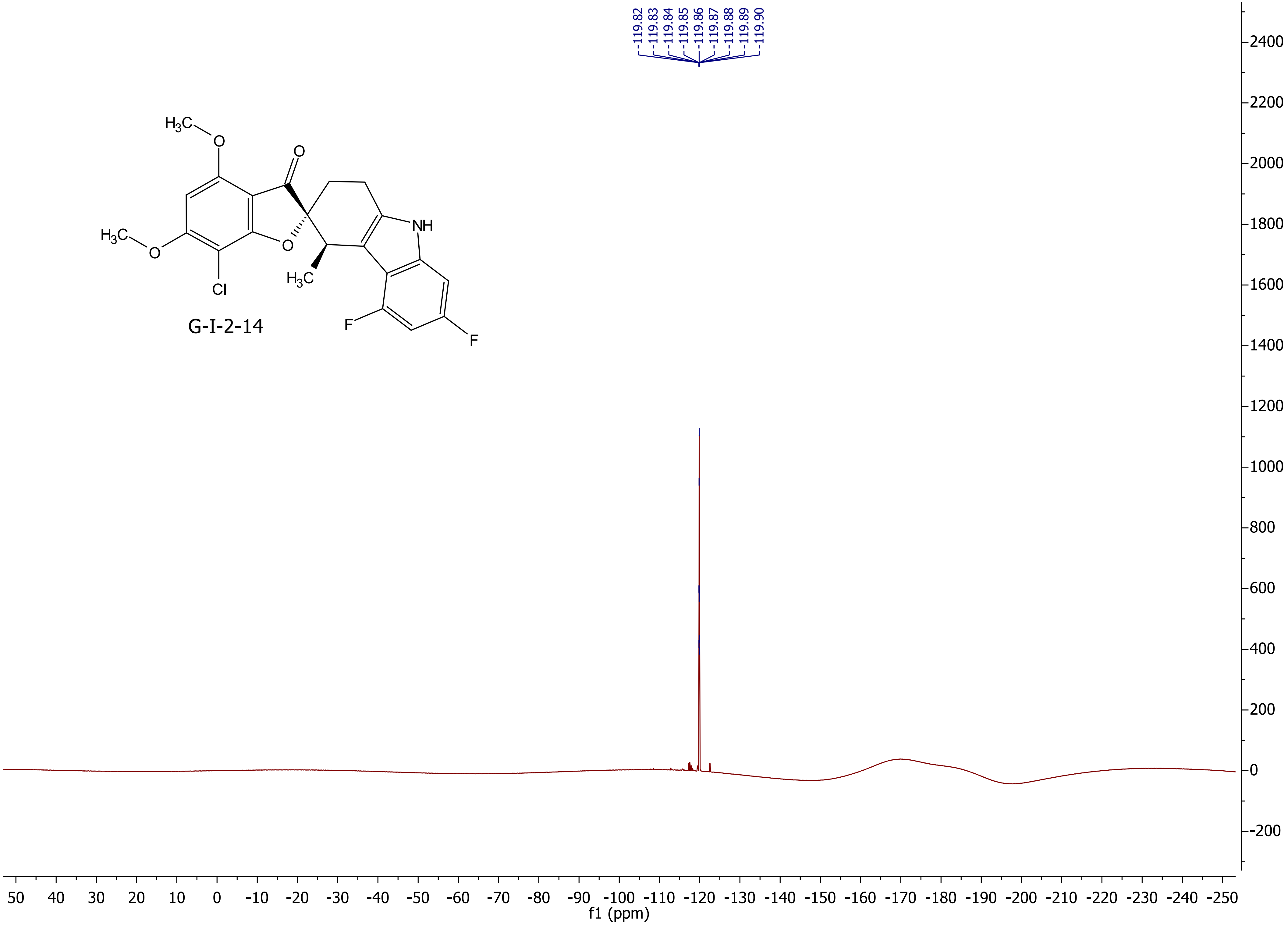
H3C
O
O
H3C
O
O
NH
Cl
H3C
F
F
G-I-2-14
-119.82
-119.83
-119.84
-119.85
-119.86
-119.87
-119.88
-119.89
-119.90
2400
2200
2000
1800
1600
1400
1200
1000
800
600
400
200
0
-200
50 40 30 20 10 0 -10 -20 -30 -40 -50 -60 -70 -80 -90 -100 -110 -120 -130 -140 -150 -160 -170 -180 -190 -200 -210 -220 -230 -240 -250
f1 (ppm)

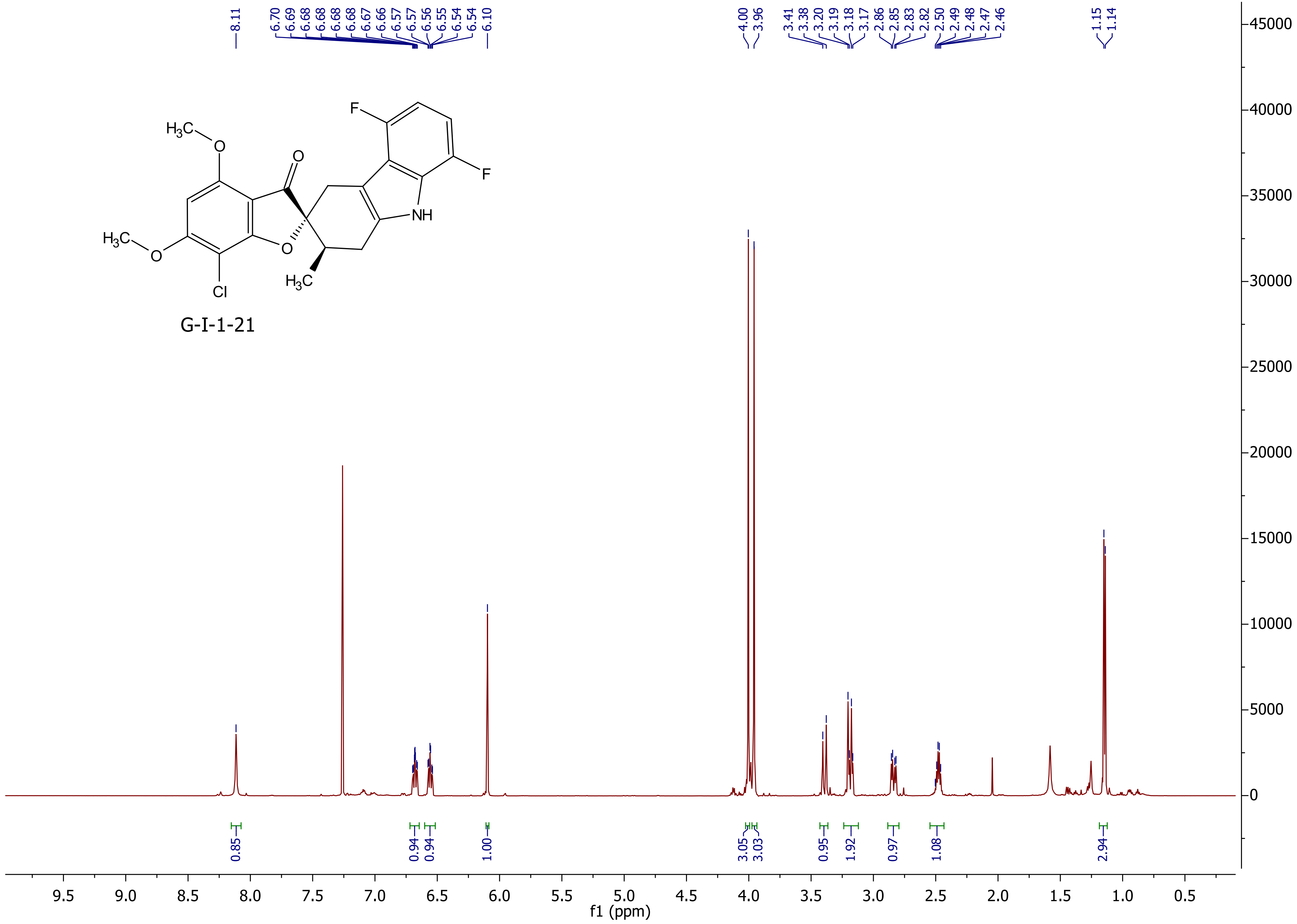

8.11
6.70
6.69
6.68
6.68
6.68
6.67
6.66
6.57
6.57
6.56
6.55
6.54
6.54
6.10
4.00
3.96
3.41
3.38
3.20
3.19
3.18
3.17
2.86
2.85
2.83
2.82
2.50
2.49
2.48
2.47
2.46
1.15
1.14
F
F
H_3C
O
O
NH
H_3C
O
O
Cl
H_3C
G-I-1-21
0.85
0.94
0.94
1.00
3.05
3.03
0.95
1.92
0.97
1.08
2.94
45000
40000
35000
30000
25000
20000
15000
10000
5000
0
9.5
9.0
8.5
8.0
7.5
7.0
6.5
6.0
5.5
5.0
4.5
4.0
3.5
3.0
2.5
2.0
1.5
1.0
0.5
f1 (ppm)

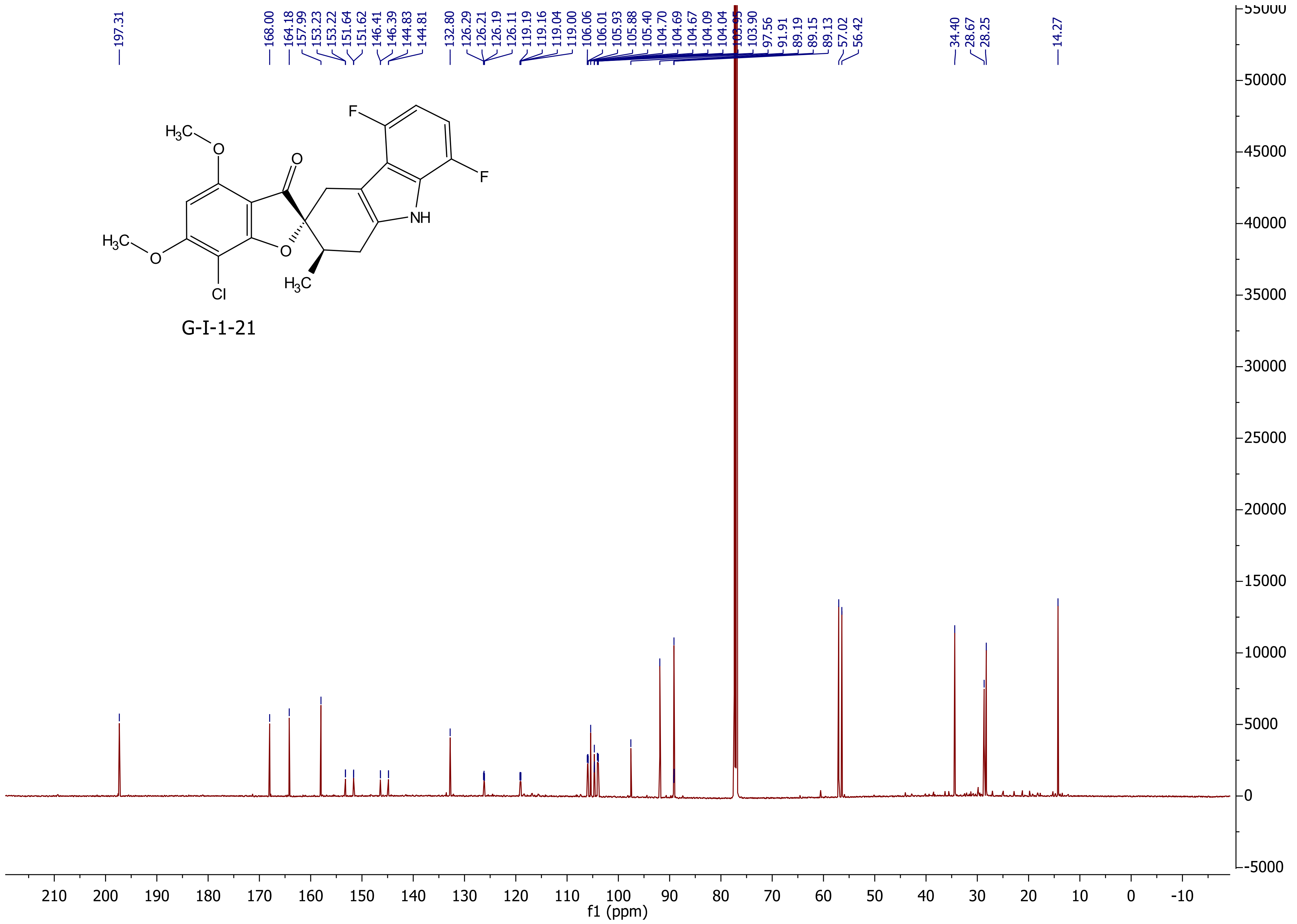
197.31
168.00
164.18
157.99
153.23
153.22
151.64
151.62
146.41
146.39
144.83
144.81
132.80
126.29
126.21
126.19
126.11
119.19
119.16
119.04
119.00
106.06
106.01
105.93
105.88
105.40
104.70
104.69
104.67
104.09
104.04
103.95
103.90
97.56
91.91
89.19
89.15
89.13
57.02
56.42
34.40
28.67
28.25
14.27
H3C
O
O
F
F
NH
H3C
O
O
Cl
H3C
G-I-1-21
f1 (ppm)

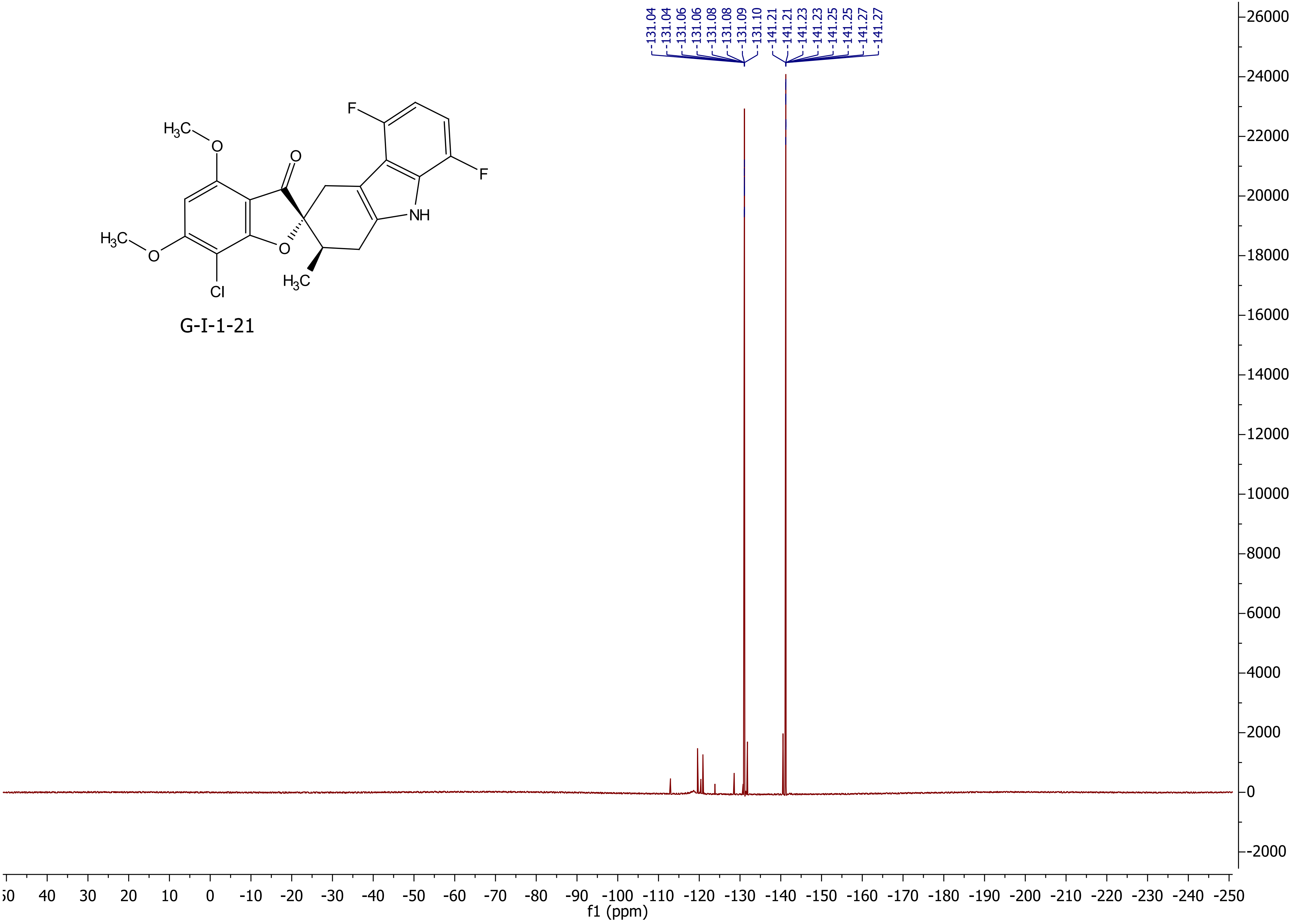
-131.04
-131.04
-131.06
-131.06
-131.08
-131.08
-131.09
-131.10
-141.21
-141.21
-141.23
-141.23
-141.25
-141.25
-141.27
-141.27
f1 (ppm)

G-I-1-21

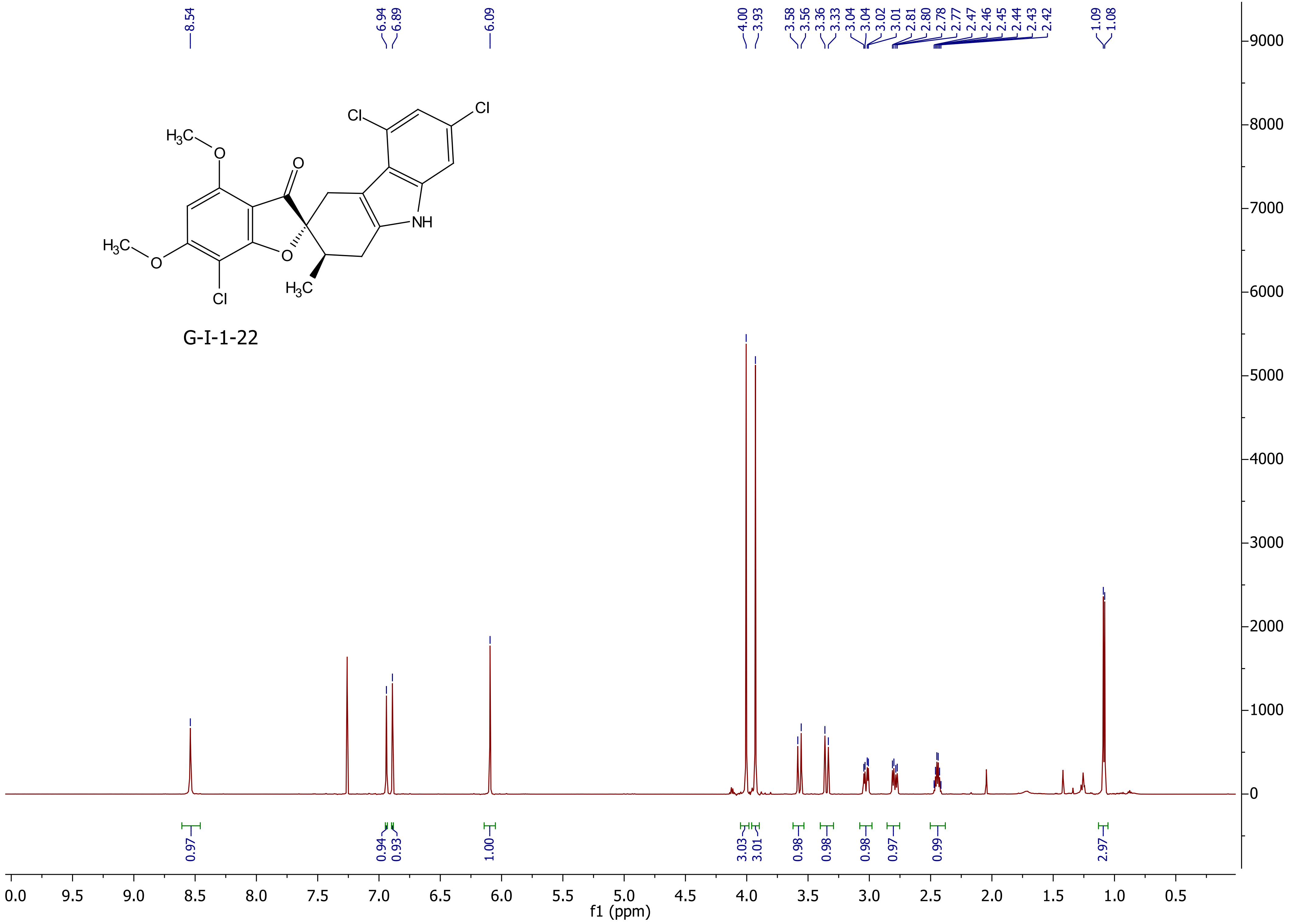

8.54
6.94
6.89
6.09
4.00
3.93
3.58
3.56
3.36
3.33
3.04
3.04
3.02
3.01
2.81
2.80
2.78
2.77
2.47
2.46
2.45
2.44
2.43
2.42
1.09
1.08
G-I-1-22
0.97
0.94
0.93
1.00
3.03
3.01
0.98
0.98
0.98
0.97
0.99
2.97
f1 (ppm)

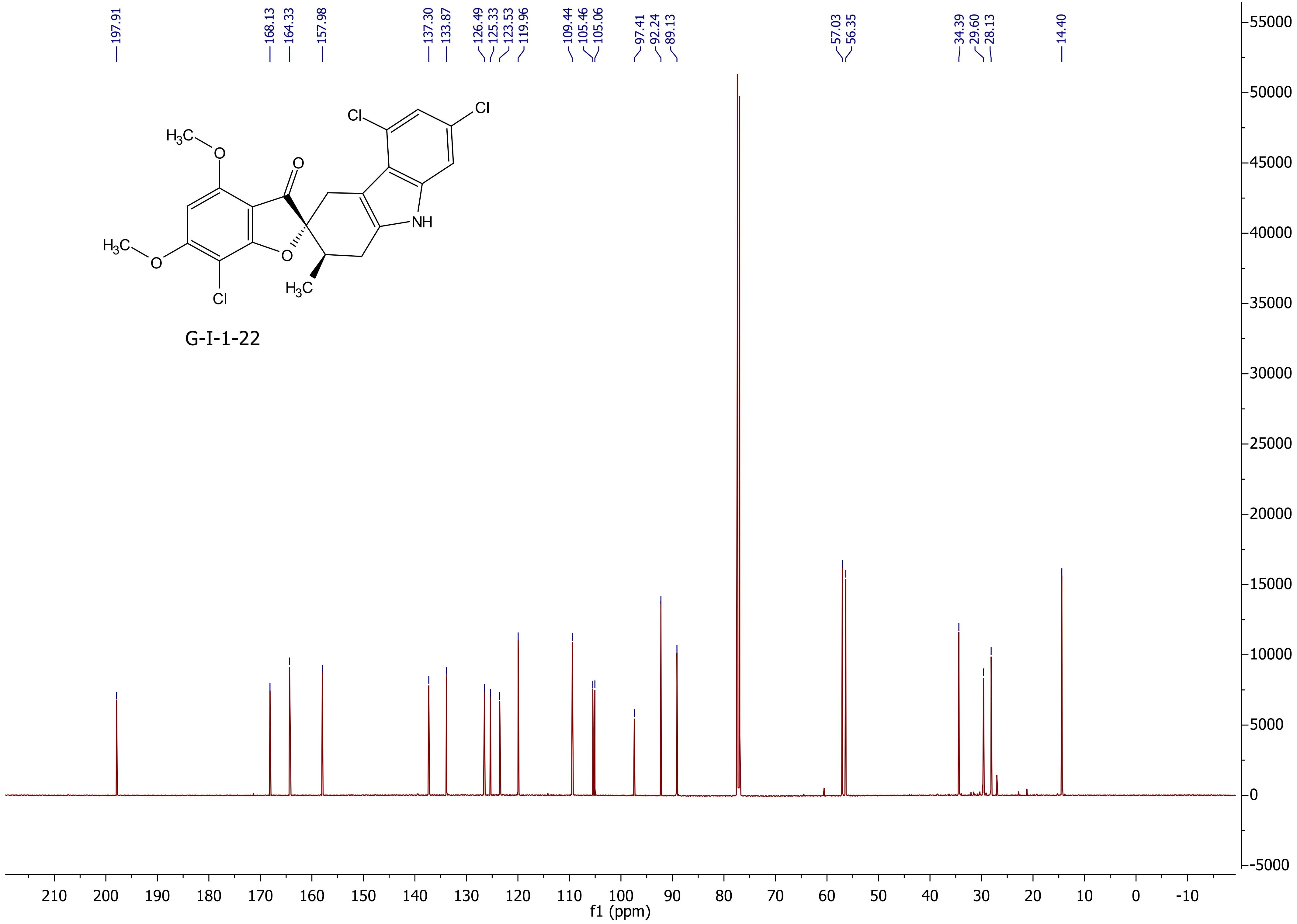
197.91
168.13
164.33
157.98
137.30
133.87
126.49
125.33
123.53
119.96
109.44
105.46
105.06
97.41
92.24
89.13
57.03
56.35
34.39
29.60
28.13
14.40
G-I-1-22
f1 (ppm)

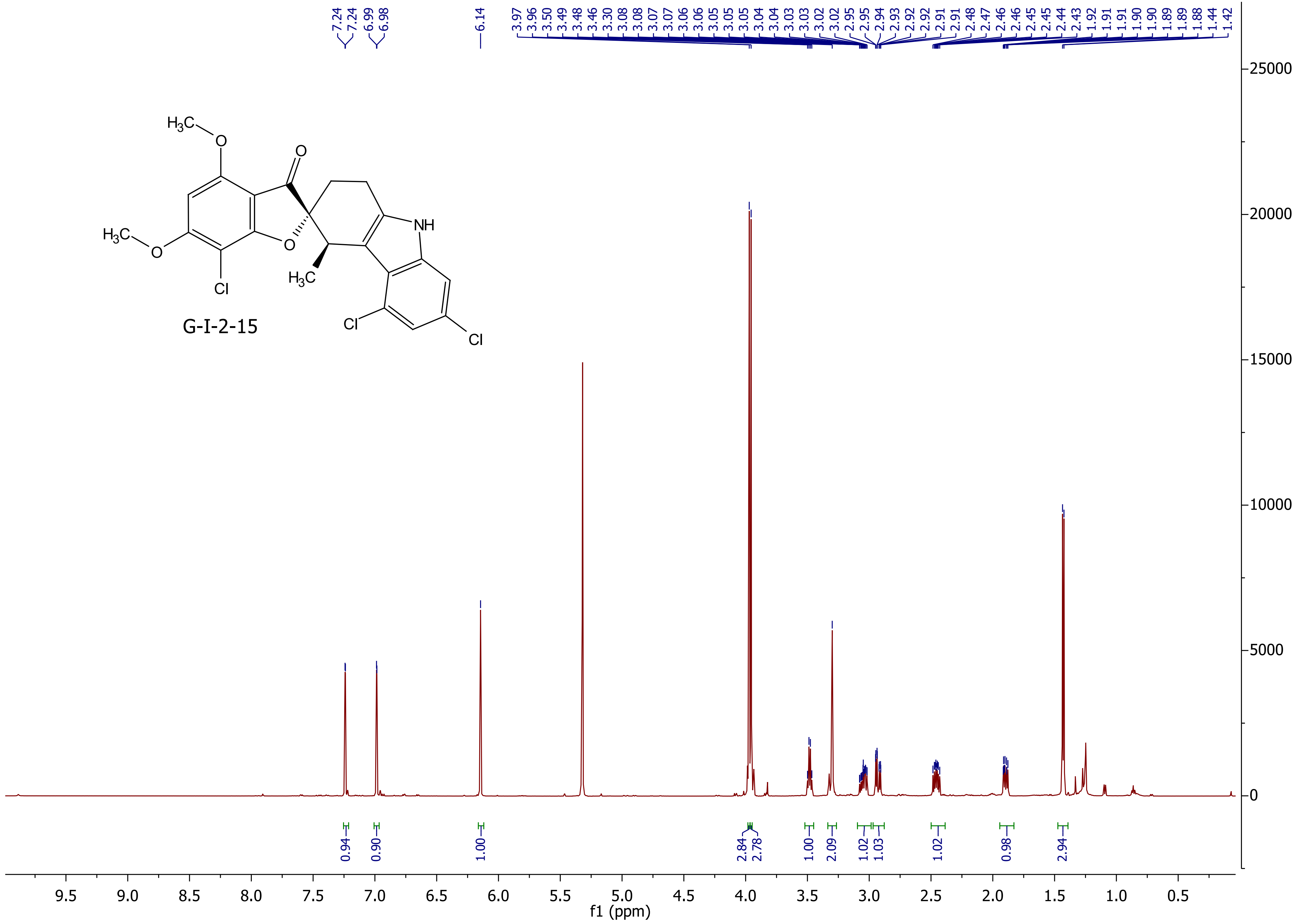

7.24 7.24 6.99 6.98
6.14
3.97 3.96 3.50 3.49 3.48 3.46 3.30 3.08 3.08 3.07 3.07 3.06 3.06 3.05 3.05 3.05 3.04 3.04 3.03 3.03 3.02 3.02 2.95 2.95 2.94 2.93 2.92 2.92 2.91 2.91 2.48 2.47 2.46 2.46 2.45 2.45 2.44 2.43 1.92 1.91 1.91 1.90 1.90 1.89 1.89 1.88 1.44 1.42
H3C
O
O
H3C
O
O
NH
Cl
H3C
Cl
Cl
G-I-2-15
0.94 0.90 1.00 2.84 2.78 1.00 2.09 1.02 1.03 1.02 0.98 2.94
25000
20000
15000
10000
5000
0
9.5 9.0 8.5 8.0 7.5 7.0 6.5 6.0 5.5 5.0 4.5 4.0 3.5 3.0 2.5 2.0 1.5 1.0 0.5
f1 (ppm)

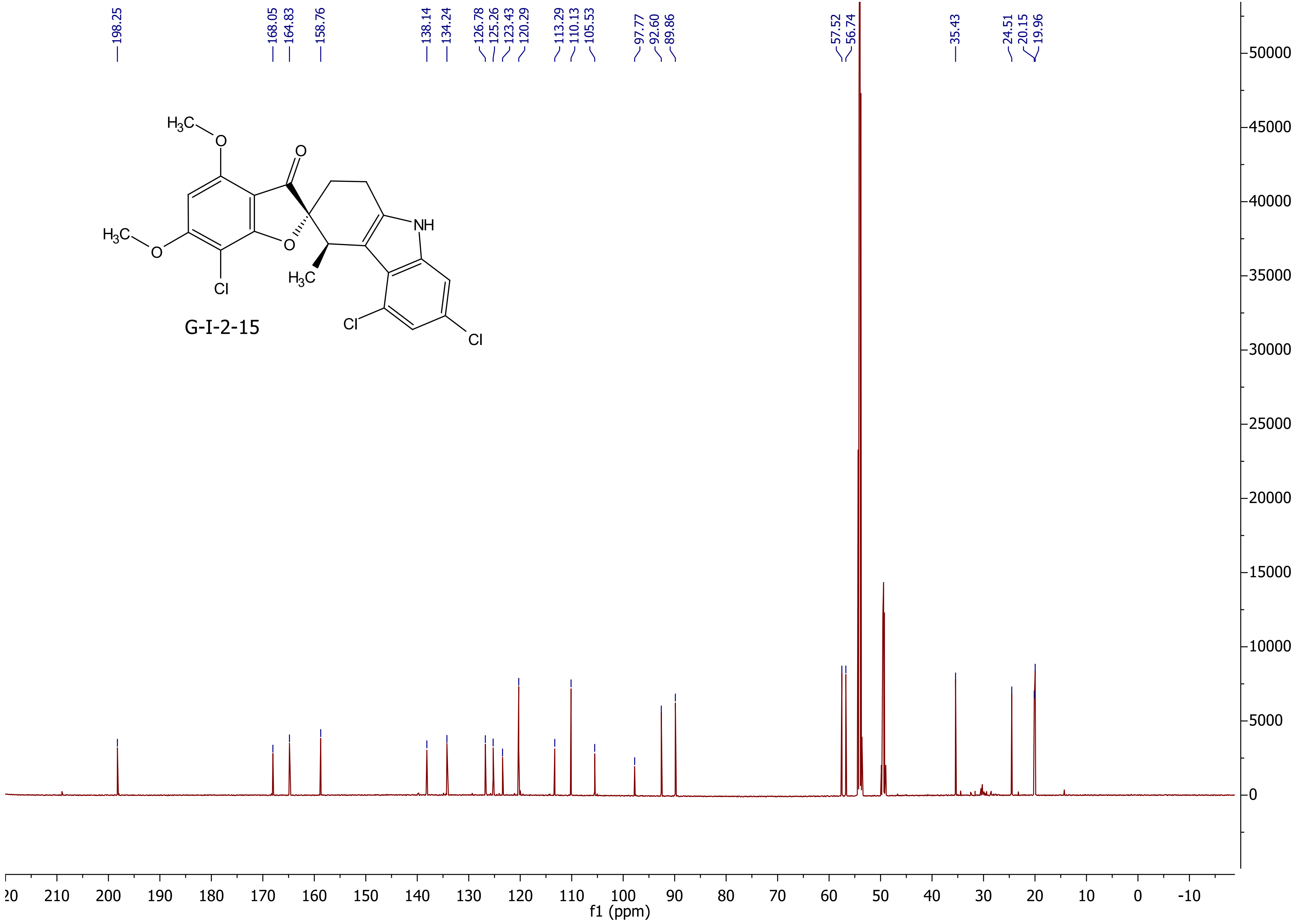

198.25
168.05
164.83
158.76
138.14
134.24
126.78
125.26
123.43
120.29
113.29
110.13
105.53
97.77
92.60
89.86
57.52
56.74
35.43
24.51
20.15
19.96
G-I-2-15
f1 (ppm)

MPI-MG-371-1_2018-11-08_10-22-07_AV600.10.fid
H1
z_Proton CDCl3 /NMR-Daten MPI 22

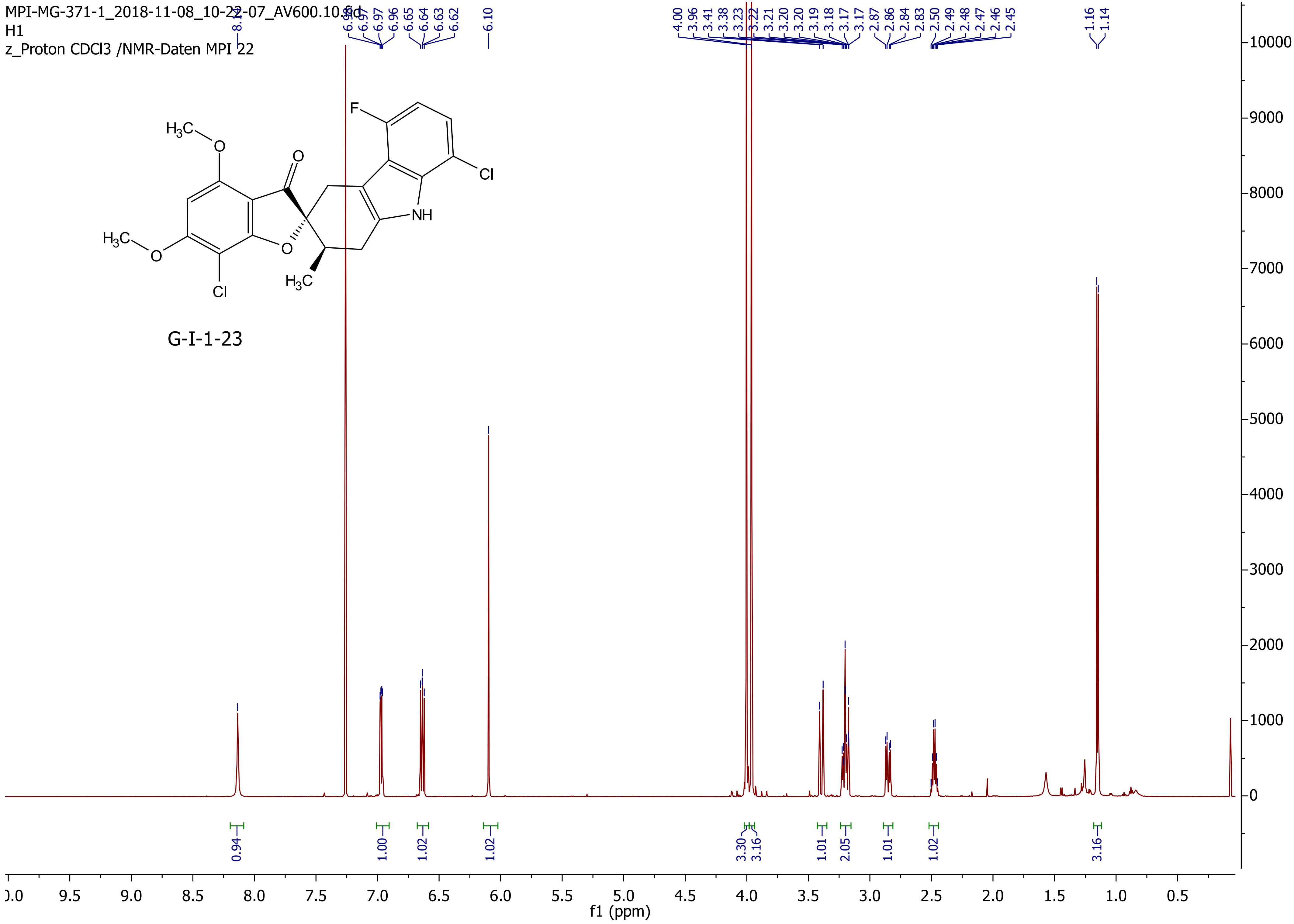

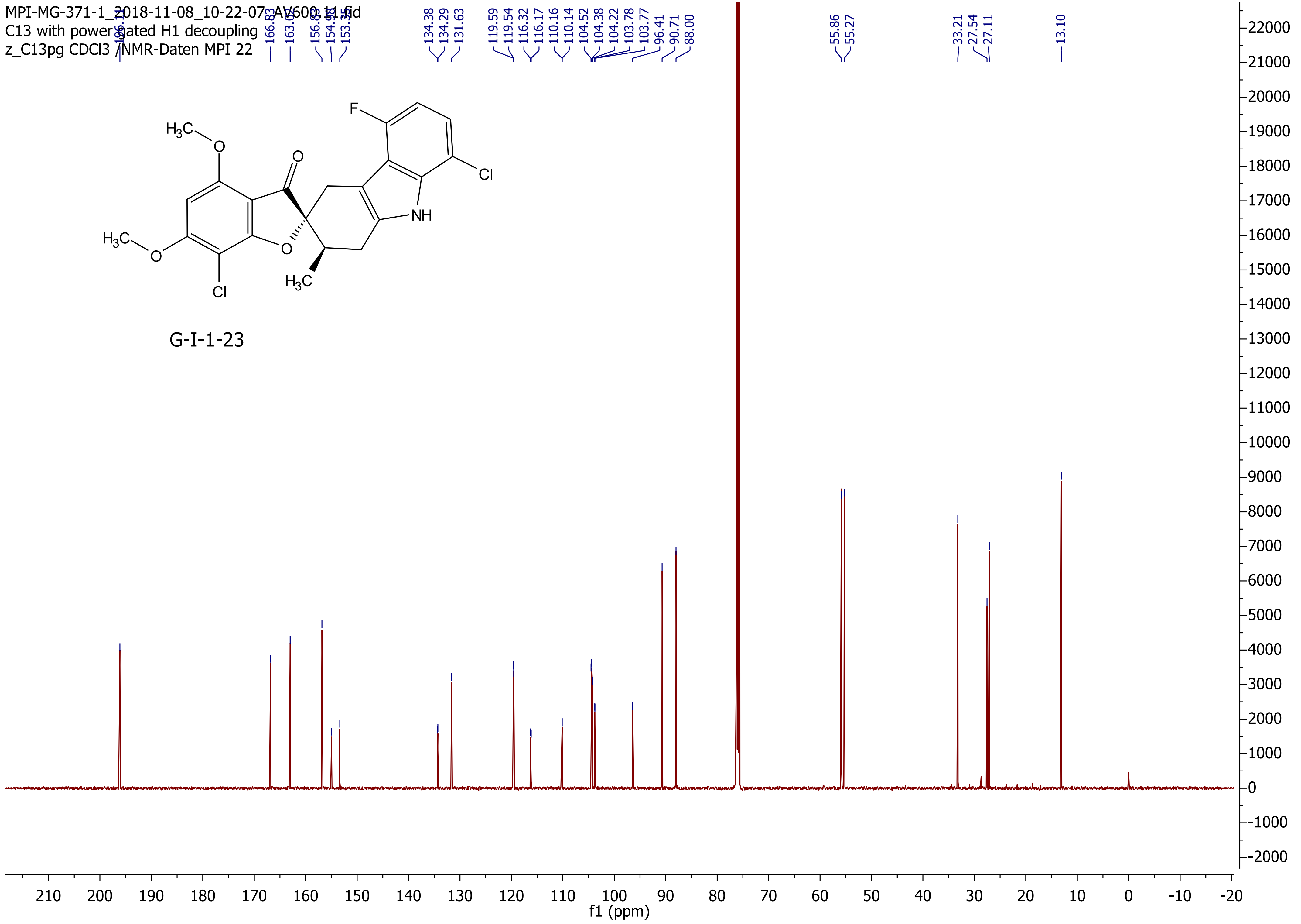

MPI-MG-371-1_2018-11-08_10-22-07_AV600 11.fid
C13 with power gated H1 decoupling
z_C13pg CDCl3 /NMR-Daten MPI 22
166.83
134.38
134.29
131.63
119.59
119.54
116.32
116.17
110.16
110.14
104.52
104.38
104.22
103.78
103.77
96.41
90.71
88.00
55.86
55.27
33.21
27.54
27.11
13.10
F
Cl
NH
O
H_3C
Cl
G-I-1-23
f1 (ppm)

MPI-MG-371-1_2018-11-08_10-22-07_AV600.12.fid
F19

z_F19 CDCl3 /NMR-Daten MPI 22

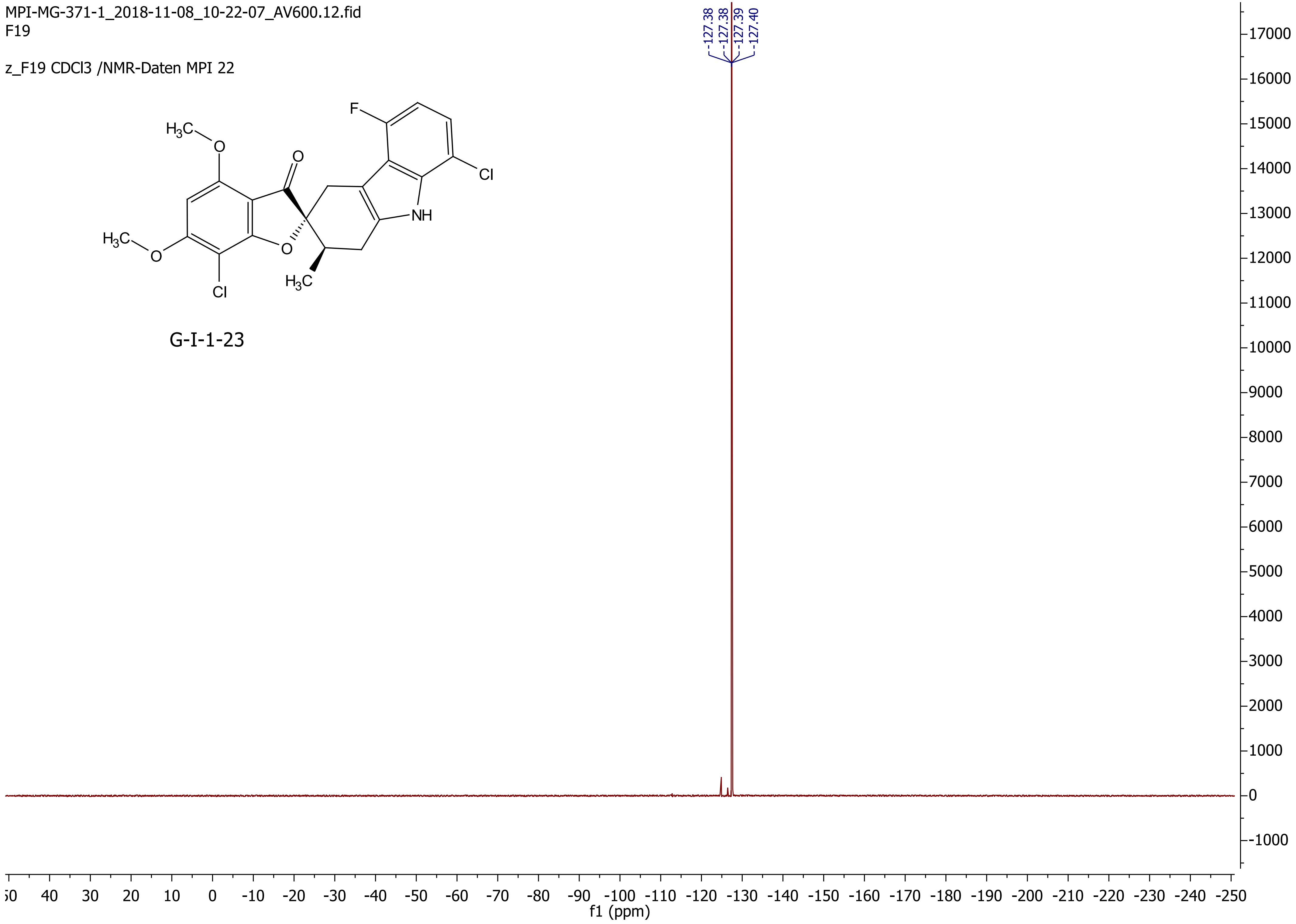

G-I-1-23

MPI-MG-371-2_2018-10-31_14-14-59_AV500.10.fid

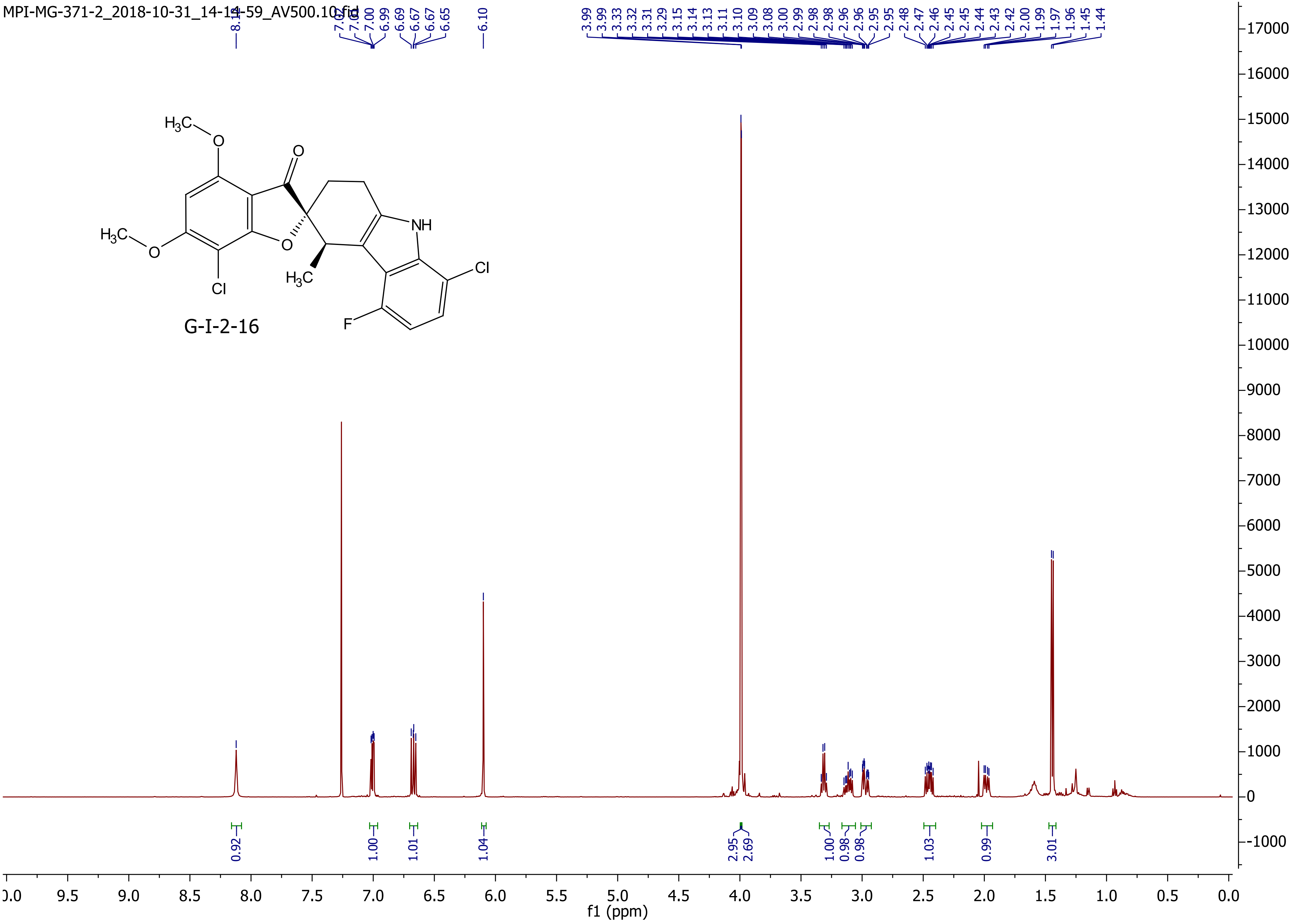

G-I-2-16

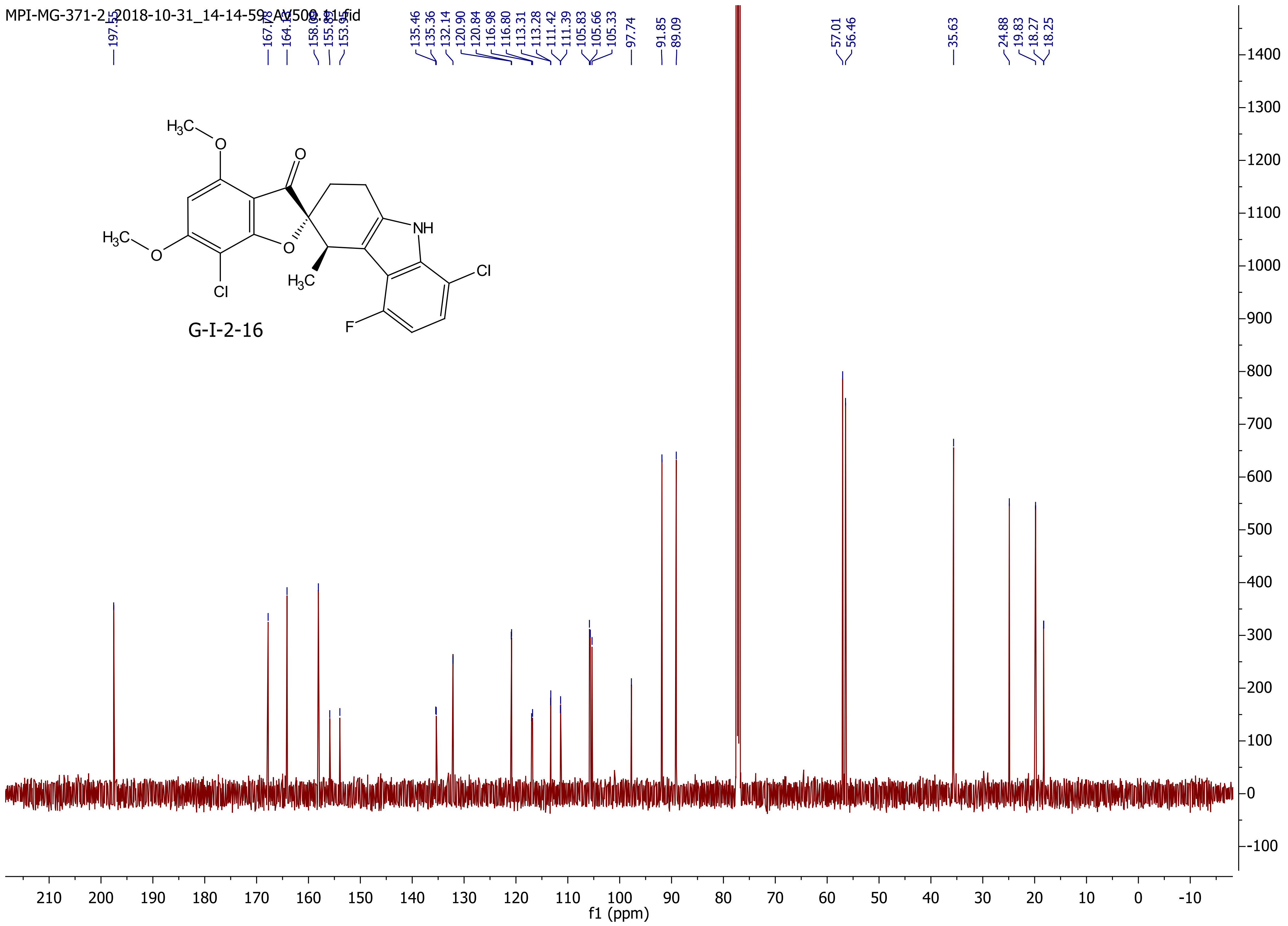

MPI-MG-371-2_2018-10-31_14-14-59_AV500.11.fid
197.55
167.78
164.1
158.0
155.8
153.9
135.46
135.36
132.14
120.90
120.84
116.98
116.80
113.31
113.28
111.42
111.39
105.83
105.66
105.33
97.74
91.85
89.09
57.01
56.46
35.63
24.88
19.83
18.27
18.25
G-I-2-16
f1 (ppm)

MPI-MG-371-2-A_2018-11-09_15-31-13_AV500.10.fid

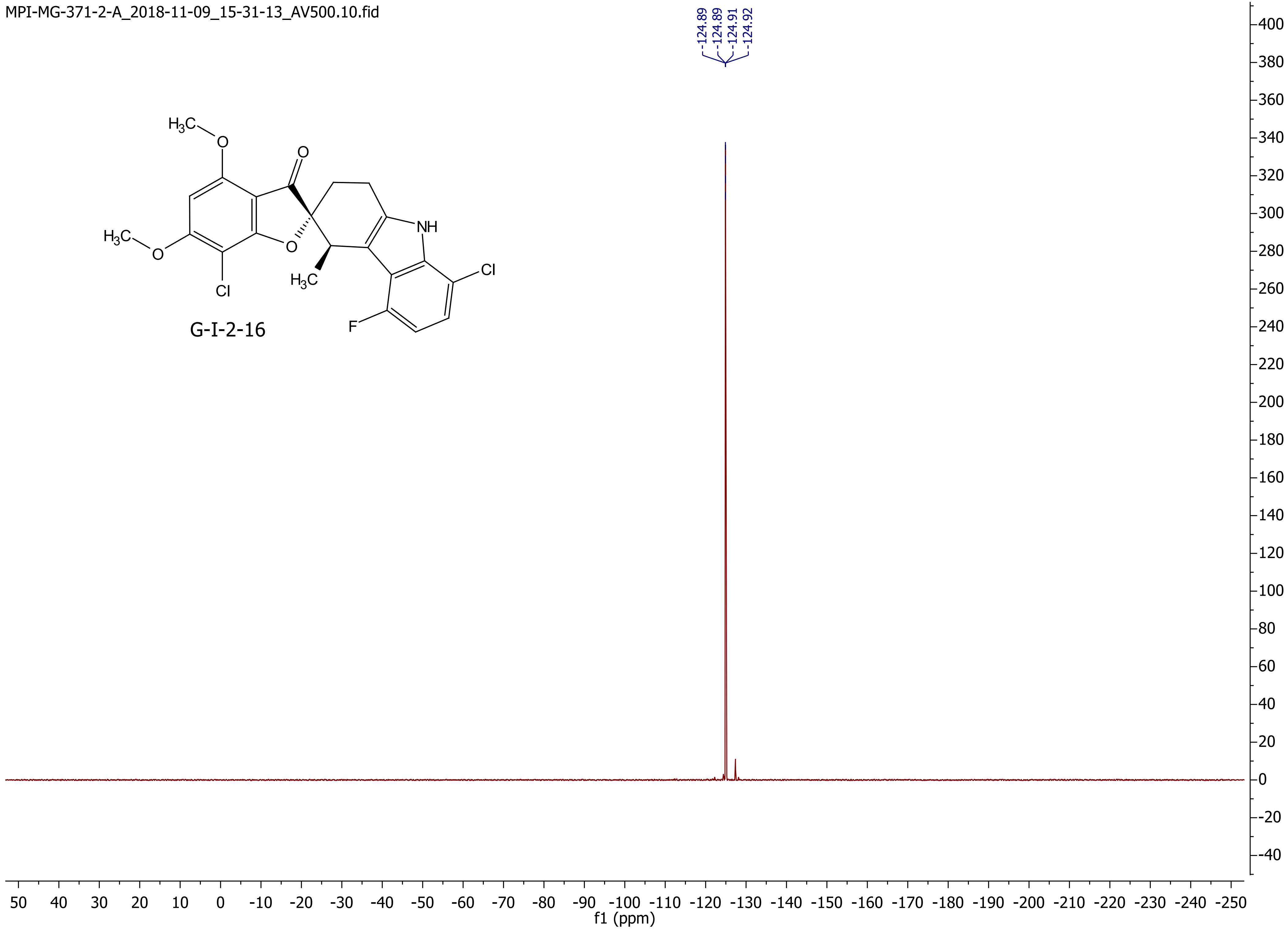

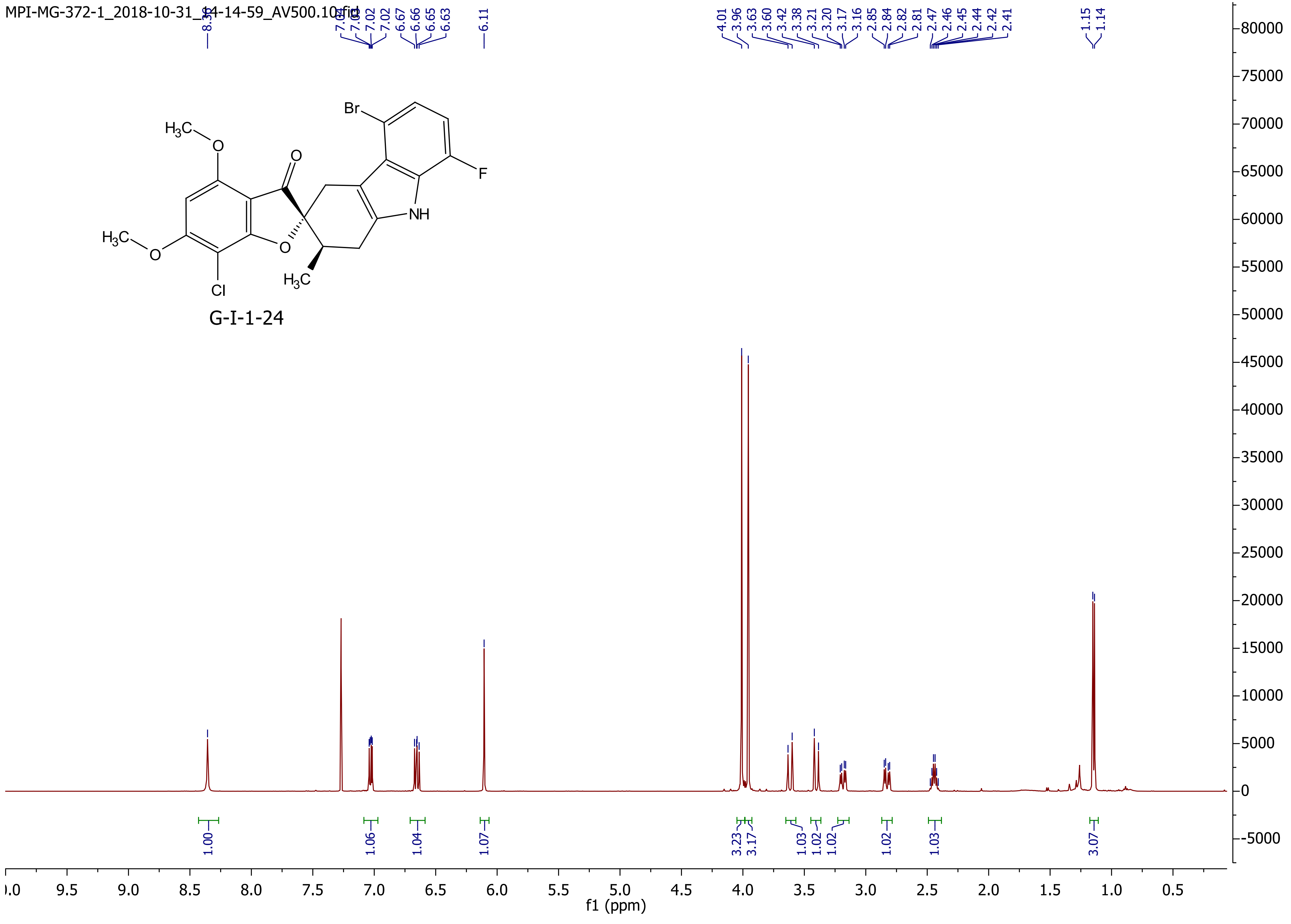

MPI-MG-372-1_2018-10-31_14-14-59_AV500.10.fid
Br
F
NH
O
H3C
Cl
G-I-1-24
7.02
7.02
6.67
6.66
6.65
6.63
6.11
4.01
3.96
3.63
3.60
3.42
3.38
3.21
3.20
3.17
3.16
2.85
2.84
2.82
2.81
2.47
2.46
2.45
2.44
2.42
2.41
1.15
1.14
1.00
1.06
1.04
1.07
3.23
3.17
1.03
1.02
1.02
1.02
1.03
3.07
f1 (ppm)

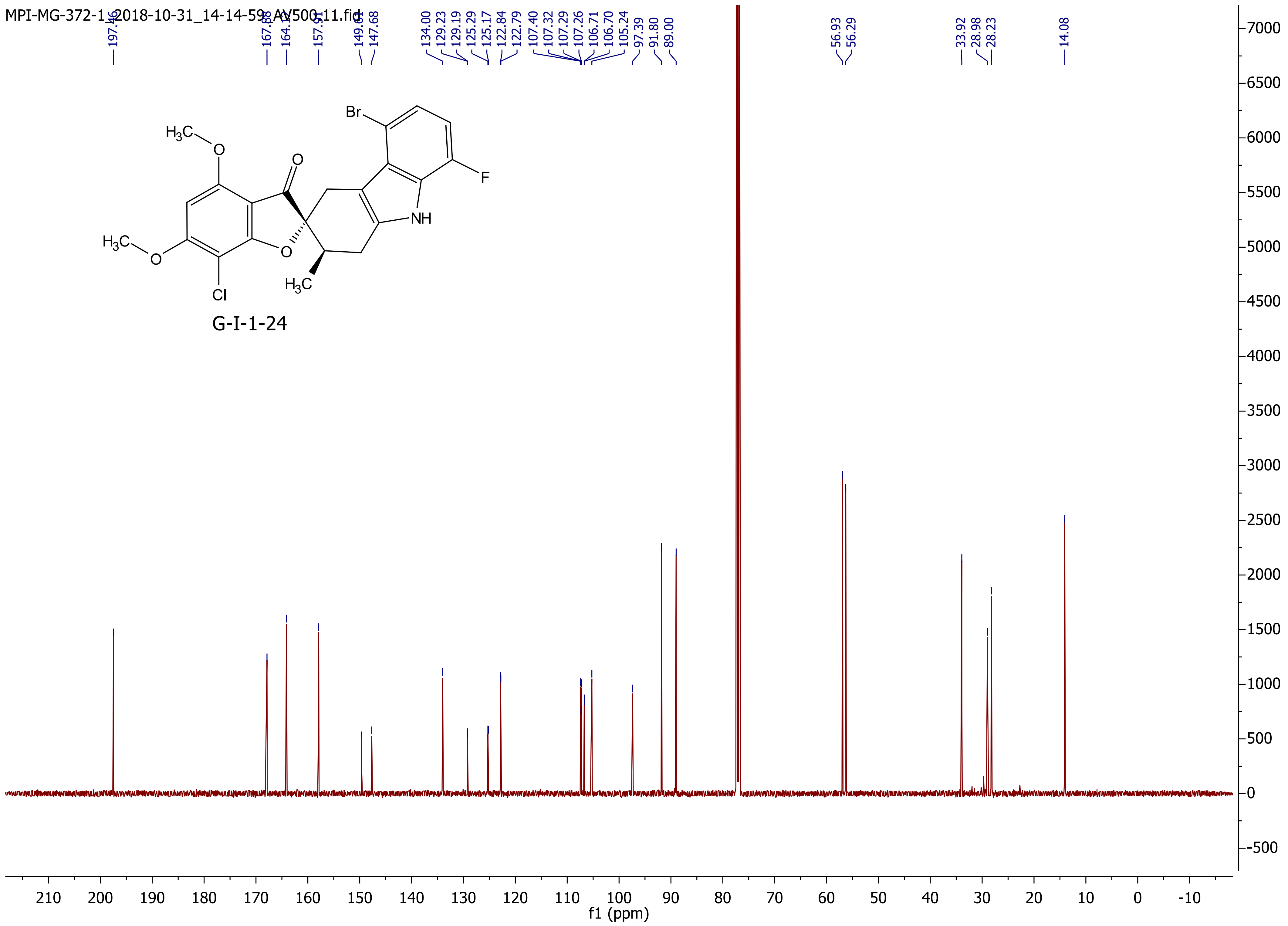
MPI-MG-372-1_2018-10-31_14-14-59_AV500_11.fid
167.88
147.68
134.00
129.23
129.19
125.29
125.17
122.84
122.79
107.40
107.32
107.29
107.26
106.71
106.70
105.24
97.39
91.80
89.00
56.93
56.29
33.92
28.98
28.23
14.08
Br
F
NH
O
Cl
G-I-1-24
f1 (ppm)

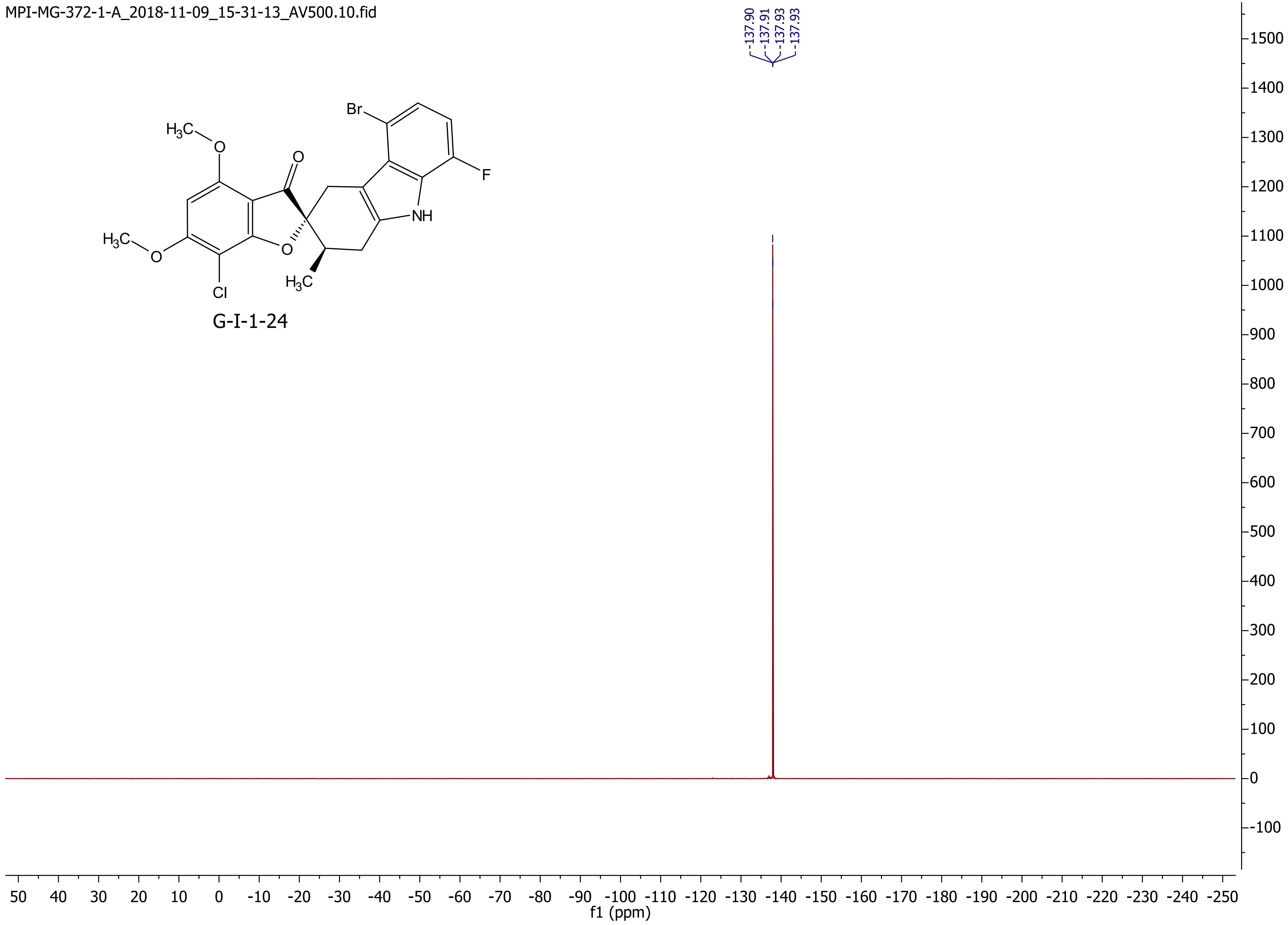
MPI-MG-372-1-A_2018-11-09_15-31-13_AV500.10.fid
-137.90
-137.91
-137.93
-137.93
Br
F
NH
O
H3C
O
O
H3C
O
Cl
H3C
G-I-1-24
1500
1400
1300
1200
1100
1000
900
800
700
600
500
400
300
200
100
0
-100
50 40 30 20 10 0 -10 -20 -30 -40 -50 -60 -70 -80 -90 -100 -110 -120 -130 -140 -150 -160 -170 -180 -190 -200 -210 -220 -230 -240 -250
f1 (ppm)

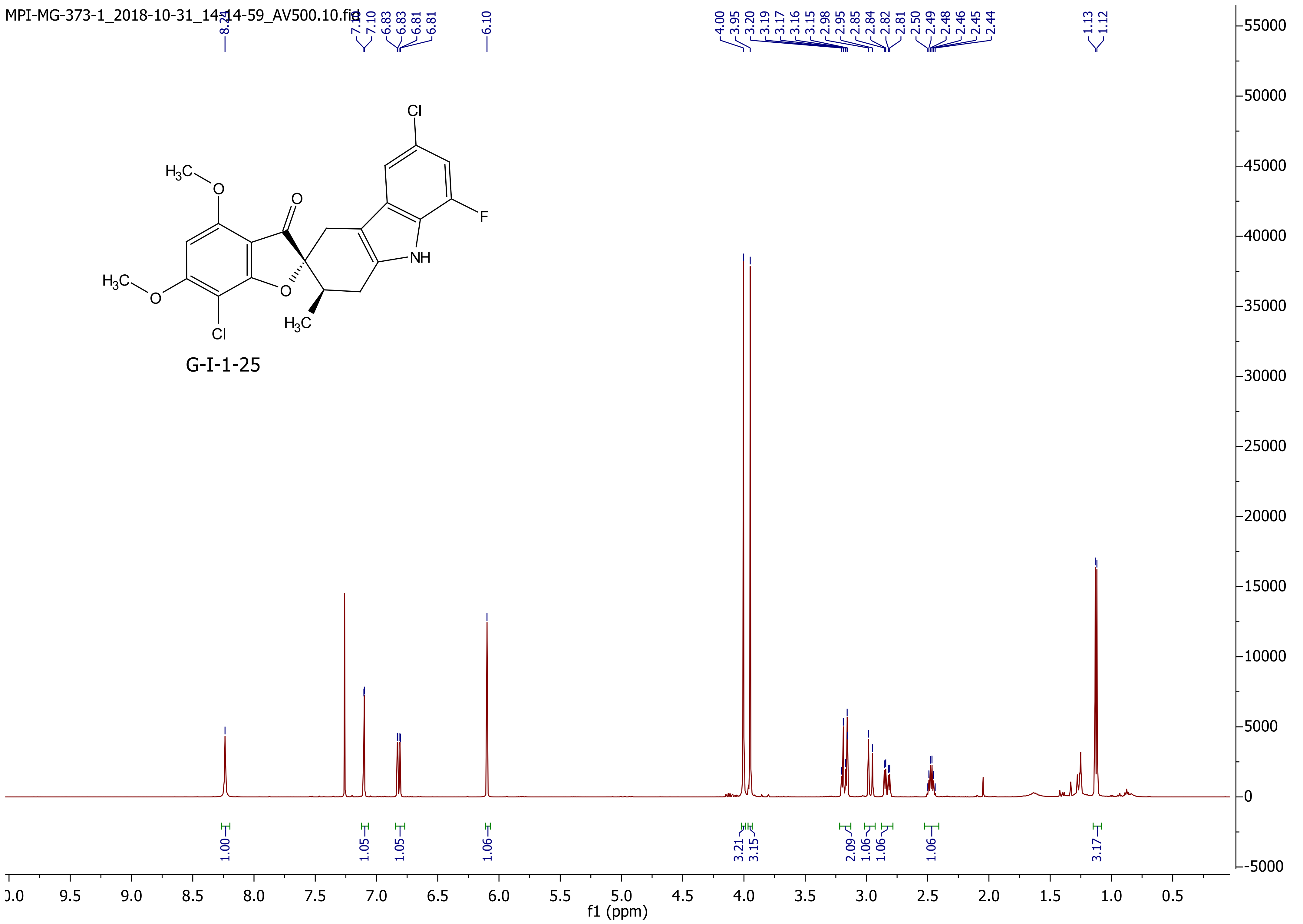
MPI-MG-373-1_2018-10-31_14-14-59_AV500.10.fid
8.24
7.10
7.10
6.83
6.83
6.81
6.81
6.10
4.00
3.95
3.20
3.19
3.17
3.16
3.15
2.98
2.95
2.85
2.84
2.82
2.81
2.50
2.49
2.48
2.46
2.45
2.44
1.13
1.12
Cl
H_3C
O
O
F
NH
O
H_3C
O
H_3C
Cl
G-I-1-25
1.00
1.05
1.05
1.06
3.21
3.15
2.09
1.06
1.06
1.06
3.17
f1 (ppm)

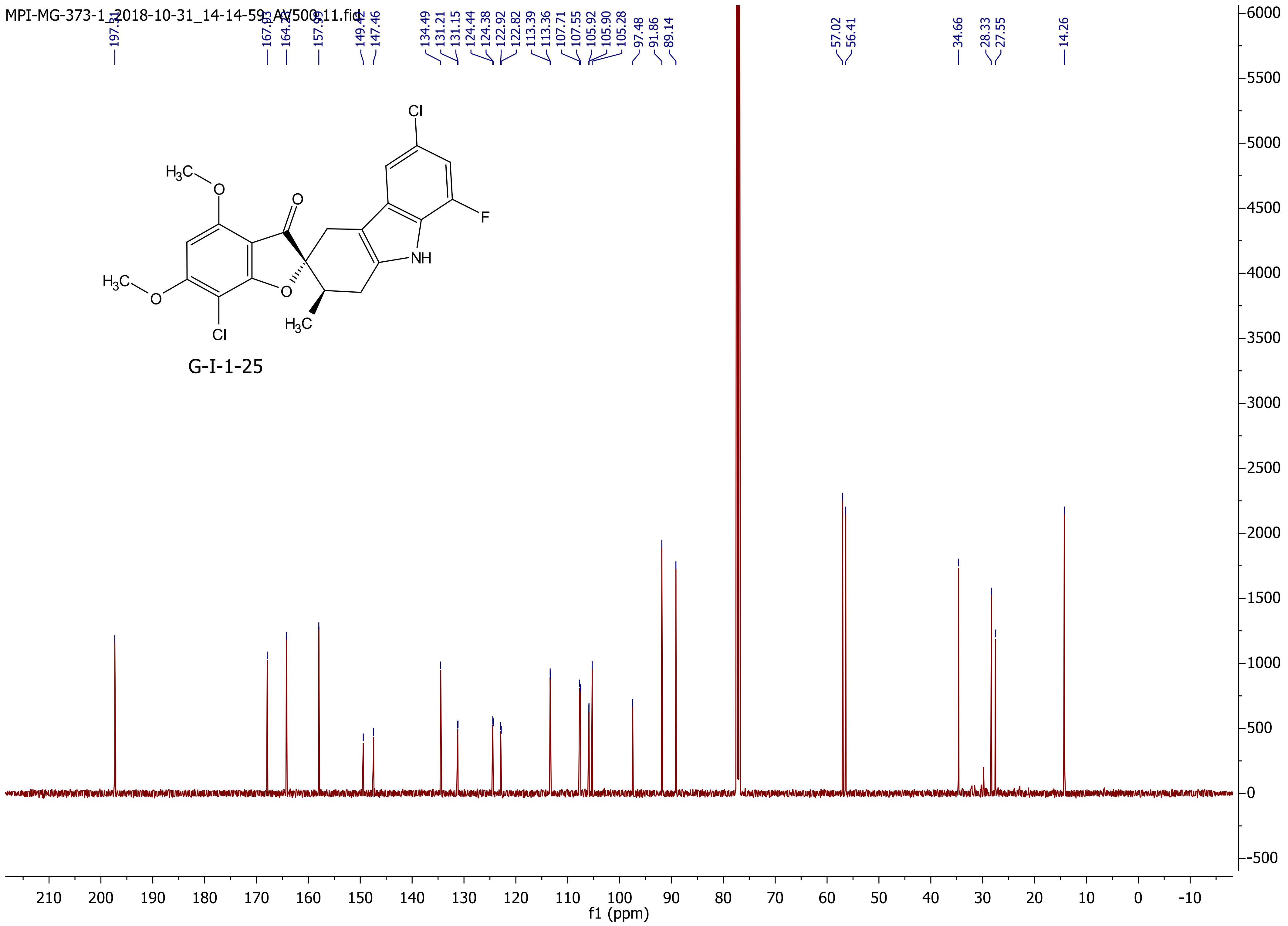
MPI-MG-373-1_2018-10-31_14-14-59_AV500_11.fid
197.31
167.93
164.21
157.93
149.42
147.46
134.49
131.21
131.15
124.44
124.38
122.92
122.82
113.39
113.36
107.71
107.55
105.92
105.90
105.28
97.48
91.86
89.14
57.02
56.41
34.66
28.33
27.55
14.26
G-I-1-25
f1 (ppm)

MPI-MG-373-1-A_2018-11-09_15-31-13_AV500.10.fid

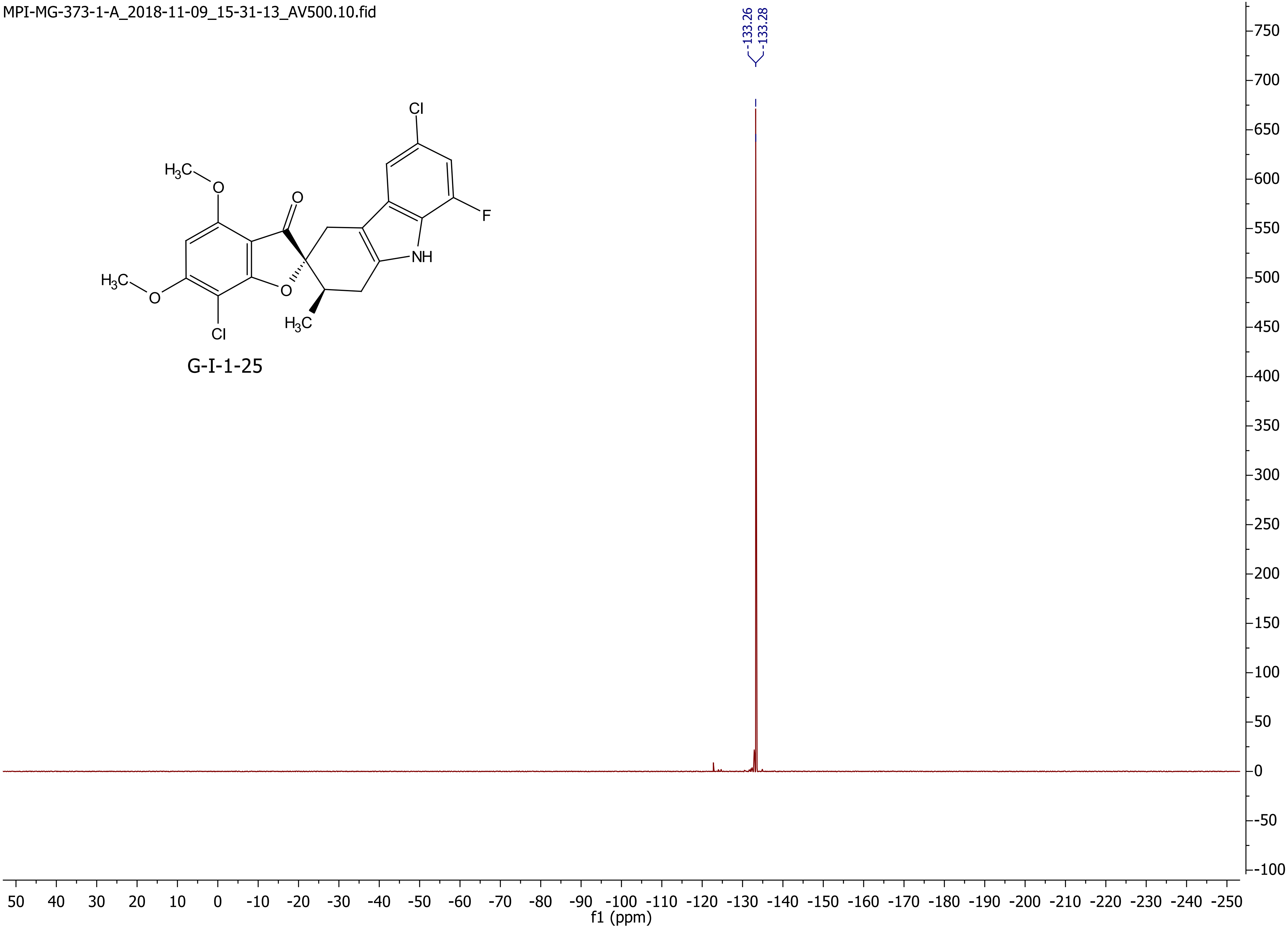

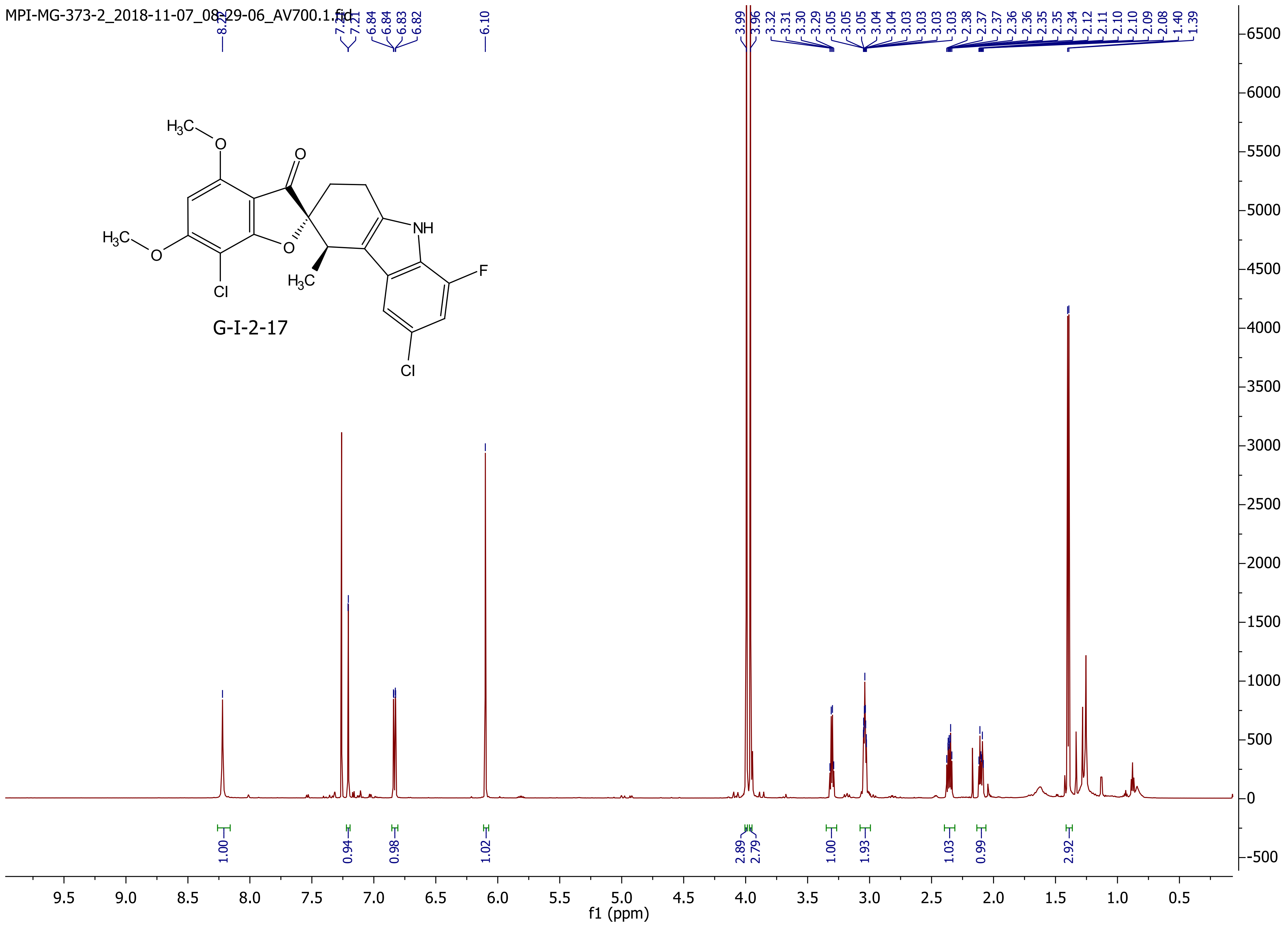
MPI-MG-373-2_2018-11-07_08-29-06_AV700.1.fid
G-I-2-17
NH
F
Cl
O
H_3C
f1 (ppm)
1.00
0.94
0.98
1.02
2.89
2.79
1.00
1.93
1.03
0.99
2.92

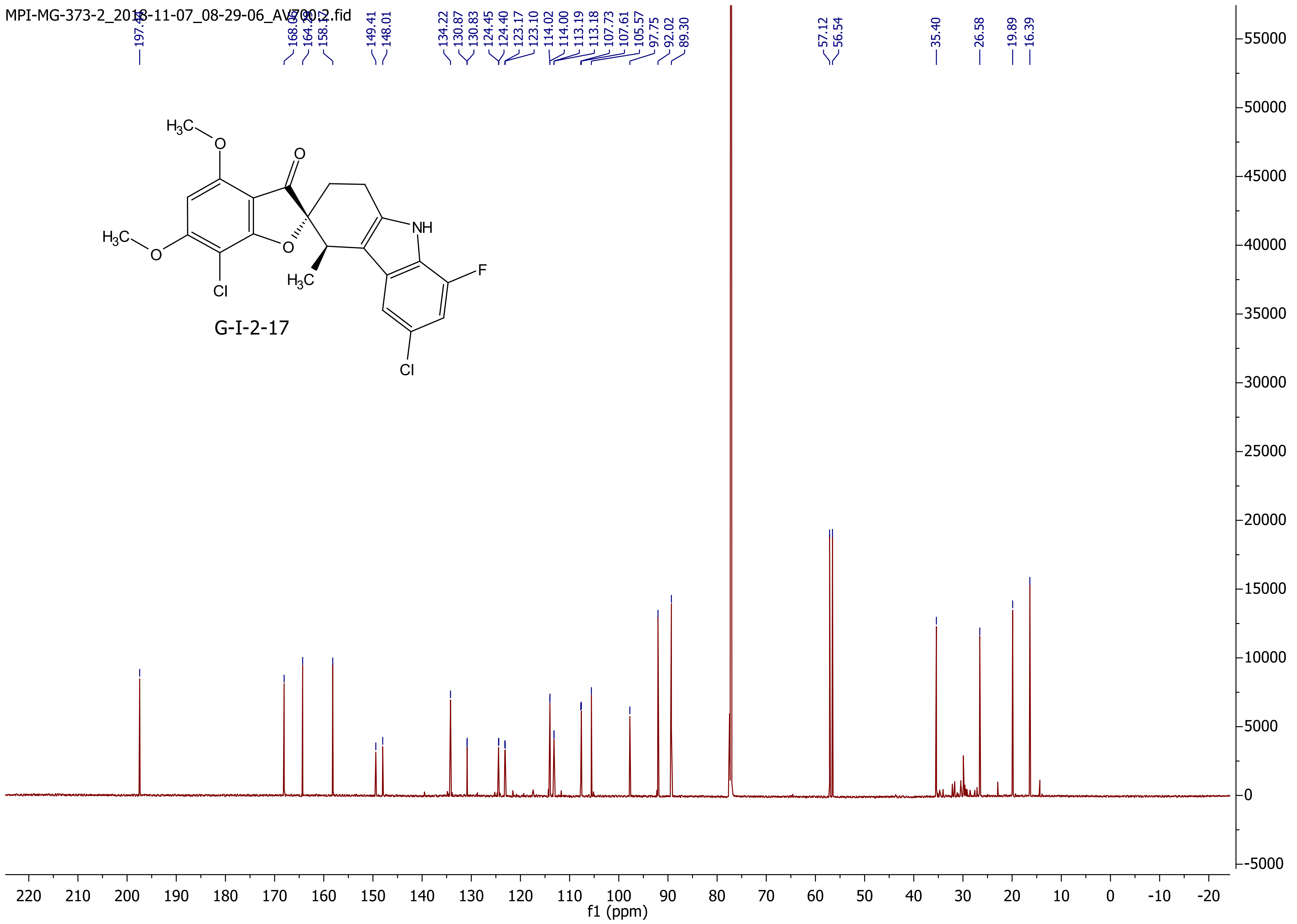
MPI-MG-373-2_2018-11-07_08-29-06_AV700.2.fid
197.4
168.0
164.2
158.1
149.41
148.01
134.22
130.87
130.83
124.45
124.40
123.17
123.10
114.02
114.00
113.19
113.18
107.73
107.61
105.57
97.75
92.02
89.30
57.12
56.54
35.40
26.58
19.89
16.39
G-I-2-17
f1 (ppm)

MPI-MG-373-2-A_2018-11-09_15-31-13_AV500.10.fid

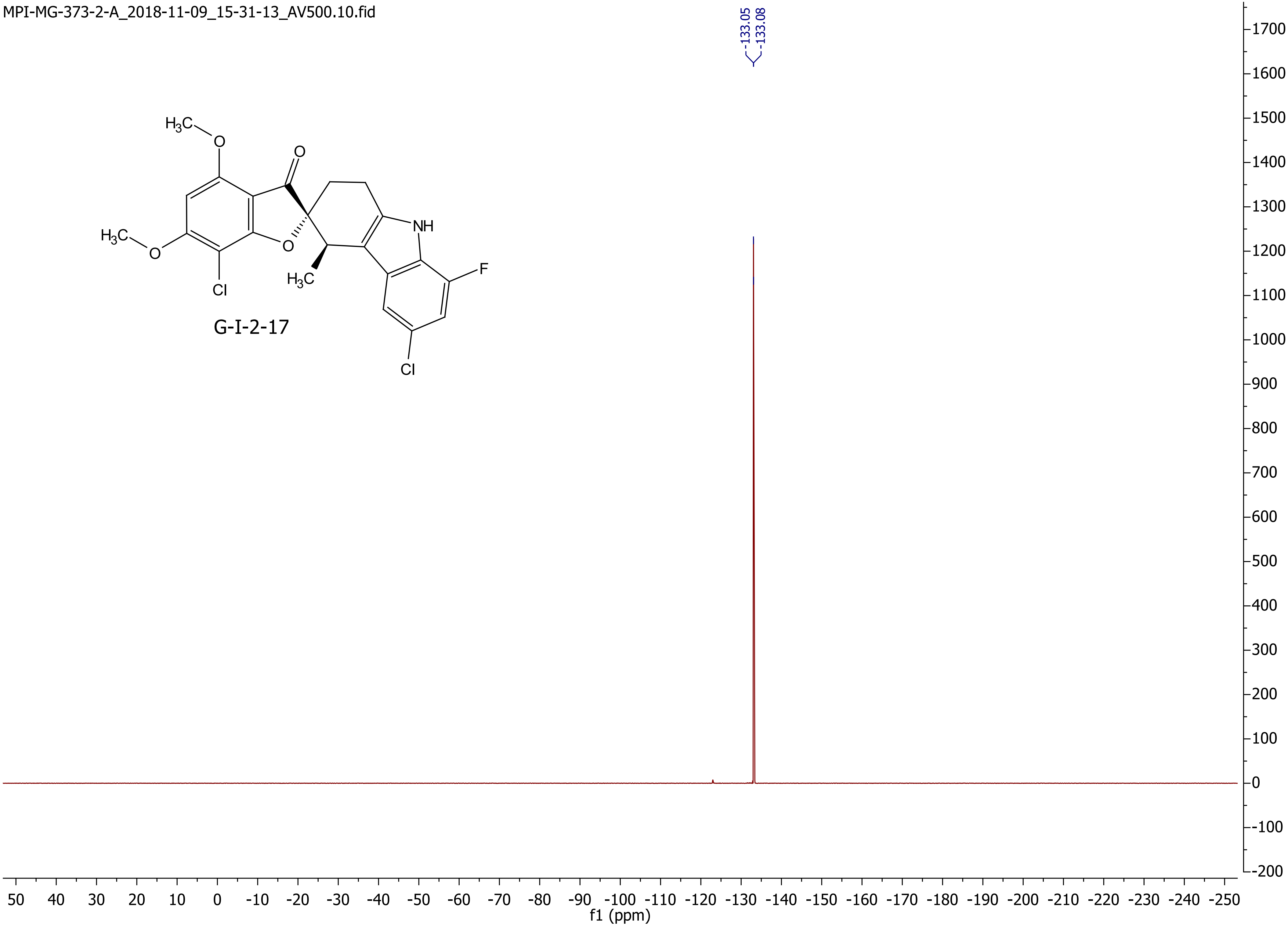

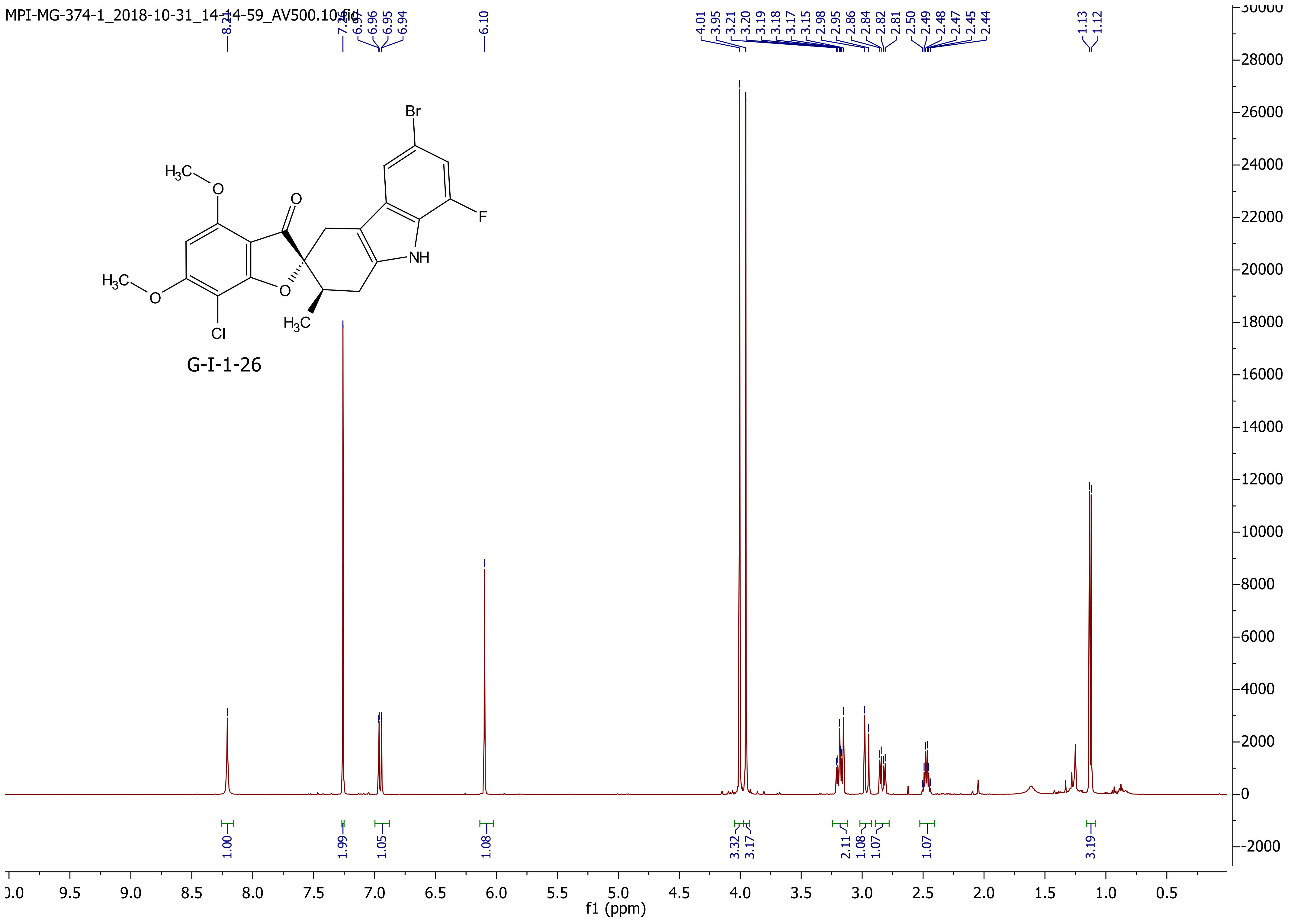
MPI-MG-374-1_2018-10-31_14-14-59_AV500.10.fid
8.21
7.26
6.97
6.96
6.95
6.94
6.10
4.01
3.95
3.21
3.20
3.19
3.18
3.17
3.15
2.98
2.95
2.86
2.84
2.82
2.81
2.50
2.49
2.48
2.47
2.45
2.44
1.13
1.12
Br
F
NH
O
H3C
O
O
H3C
O
Cl
H3C
G-I-1-26
1.00
1.99
1.05
1.08
3.32
3.17
2.11
1.08
1.07
1.07
3.19
f1 (ppm)

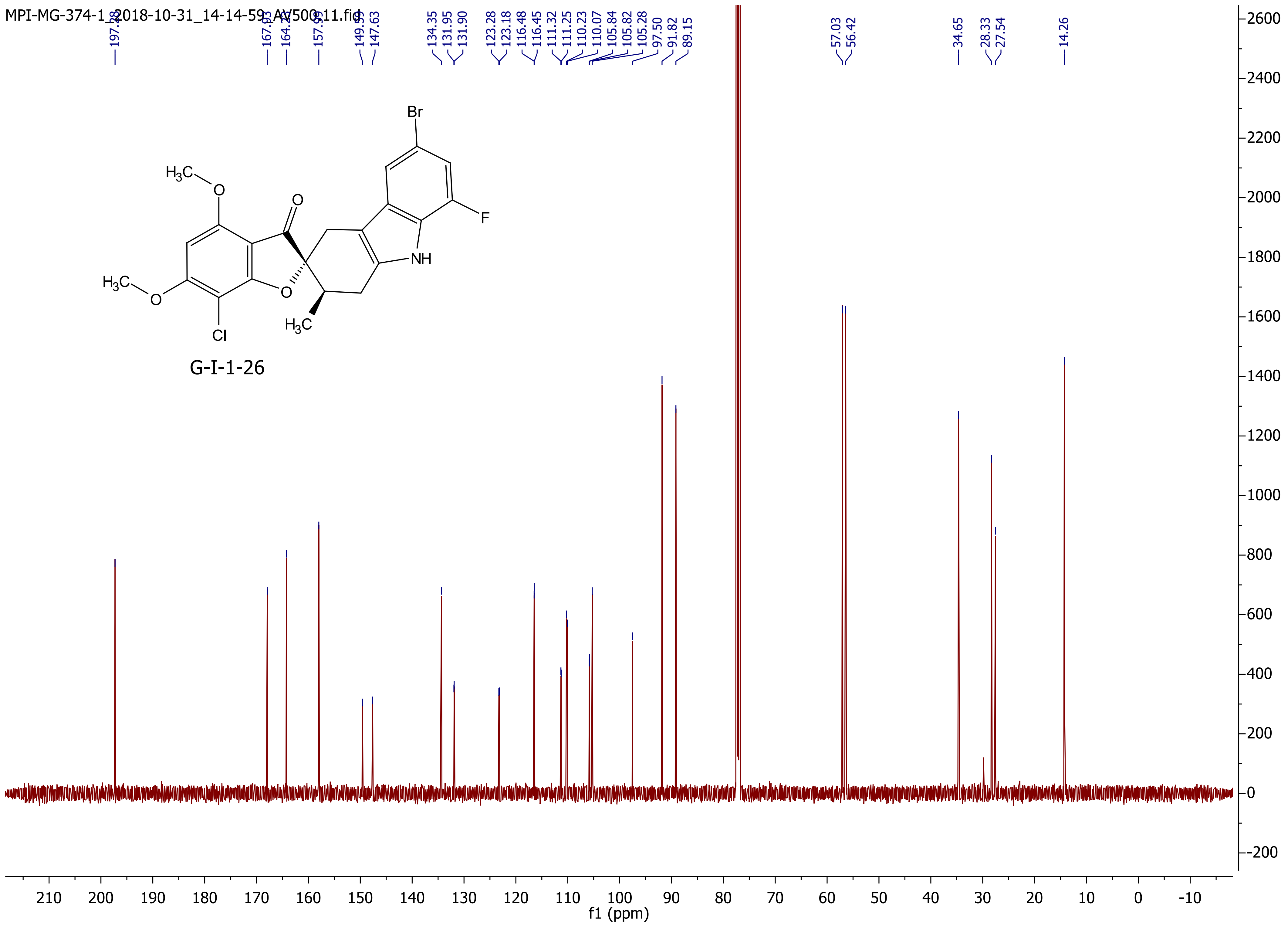

MPI-MG-374-1_2018-10-31_14-14-59_AV500_11.fid
197.28
167.93
164.23
157.99
149.59
147.63
134.35
131.95
131.90
123.28
123.18
116.48
116.45
111.32
111.25
110.23
110.07
105.84
105.82
105.28
97.50
91.82
89.15
57.03
56.42
34.65
28.33
27.54
14.26
Br
F
NH
H_3C
O
Cl
G-I-1-26
f1 (ppm)

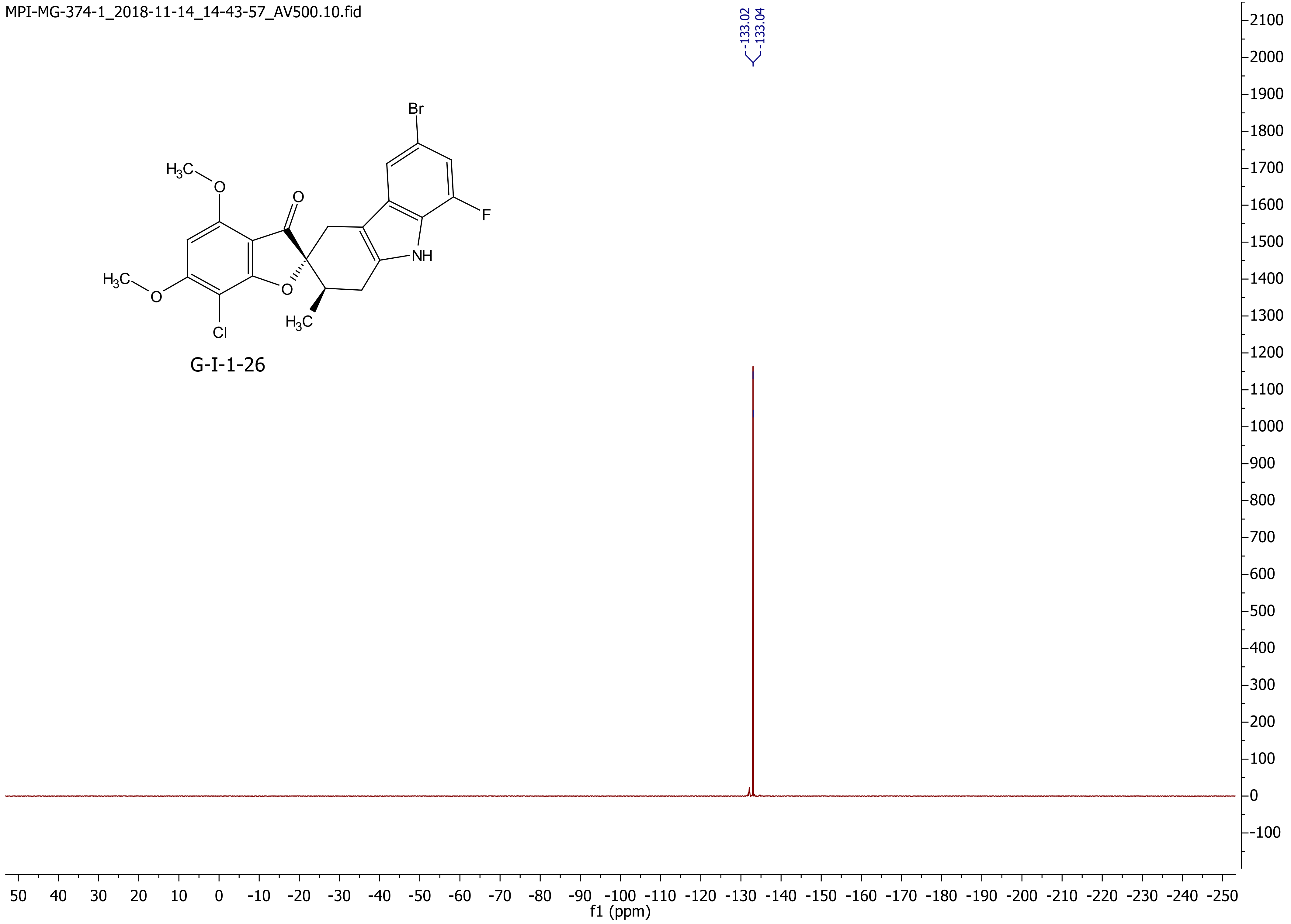

MPI-MG-374-1_2018-11-14_14-43-57_AV500.10.fid
-133.02
-133.04
Br
F
NH
O
O
O
O
Cl
f1 (ppm)

G-I-1-26

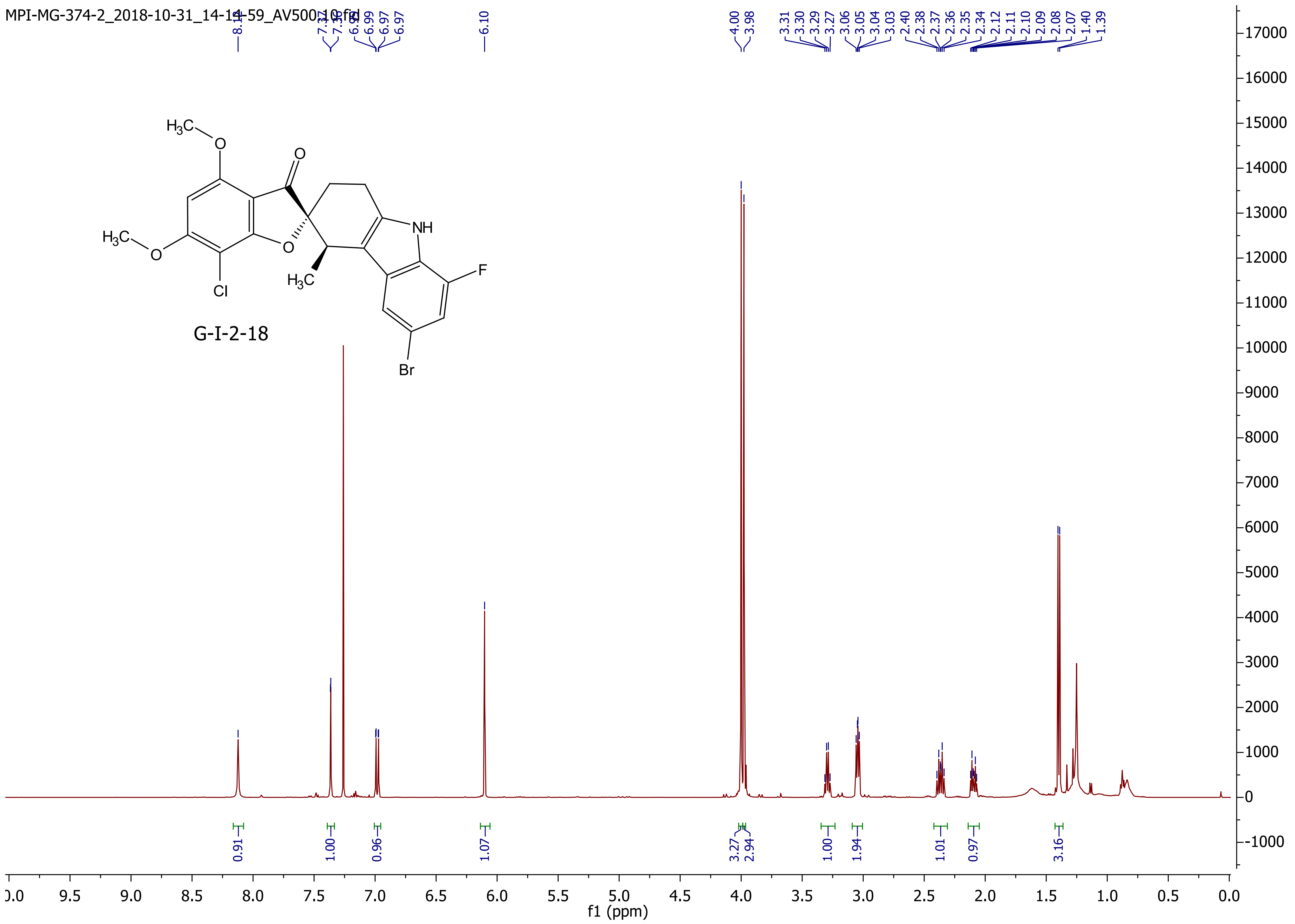

MPI-MG-374-2_2018-10-31_14-14-59_AV500_10.fid
G-I-2-18
H3C
O
O
H3C
O
O
NH
F
Cl
H3C
Br
6.99
6.97
6.97
6.10
4.00
3.98
3.31
3.30
3.29
3.27
3.06
3.05
3.04
3.03
2.40
2.38
2.37
2.36
2.35
2.34
2.12
2.11
2.10
2.09
2.08
2.07
1.40
1.39
0.91
1.00
0.96
1.07
3.27
2.94
1.00
1.94
1.01
0.97
3.16
f1 (ppm)

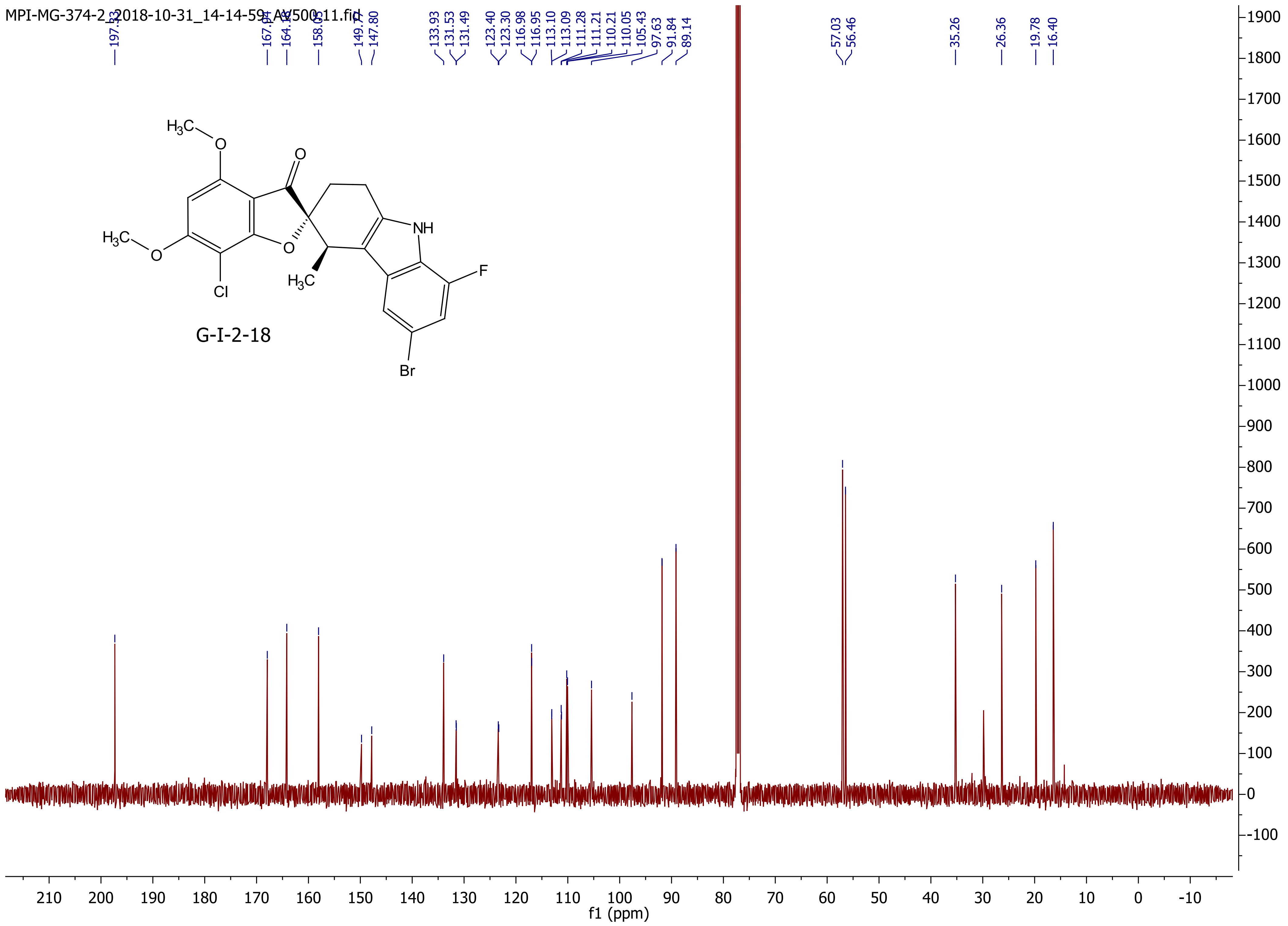

MPI-MG-374-2_2018-10-31_14-14-59_AV500_11.fid
147.80
133.93
131.53
131.49
123.40
123.30
116.98
116.95
113.10
113.09
111.28
111.21
110.21
110.05
105.43
97.63
91.84
89.14
57.03
56.46
35.26
26.36
19.78
16.40
H3C
O
O
NH
F
H3C
O
O
Cl
H3C
Br
G-I-2-18
f1 (ppm)
210 200 190 180 170 160 150 140 130 120 110 100 90 80 70 60 50 40 30 20 10 0 -10
1900 1800 1700 1600 1500 1400 1300 1200 1100 1000 900 800 700 600 500 400 300 200 100 0 -100

MPI-MG-374-2_2018-11-14_14-43-57_AV500.10.fid

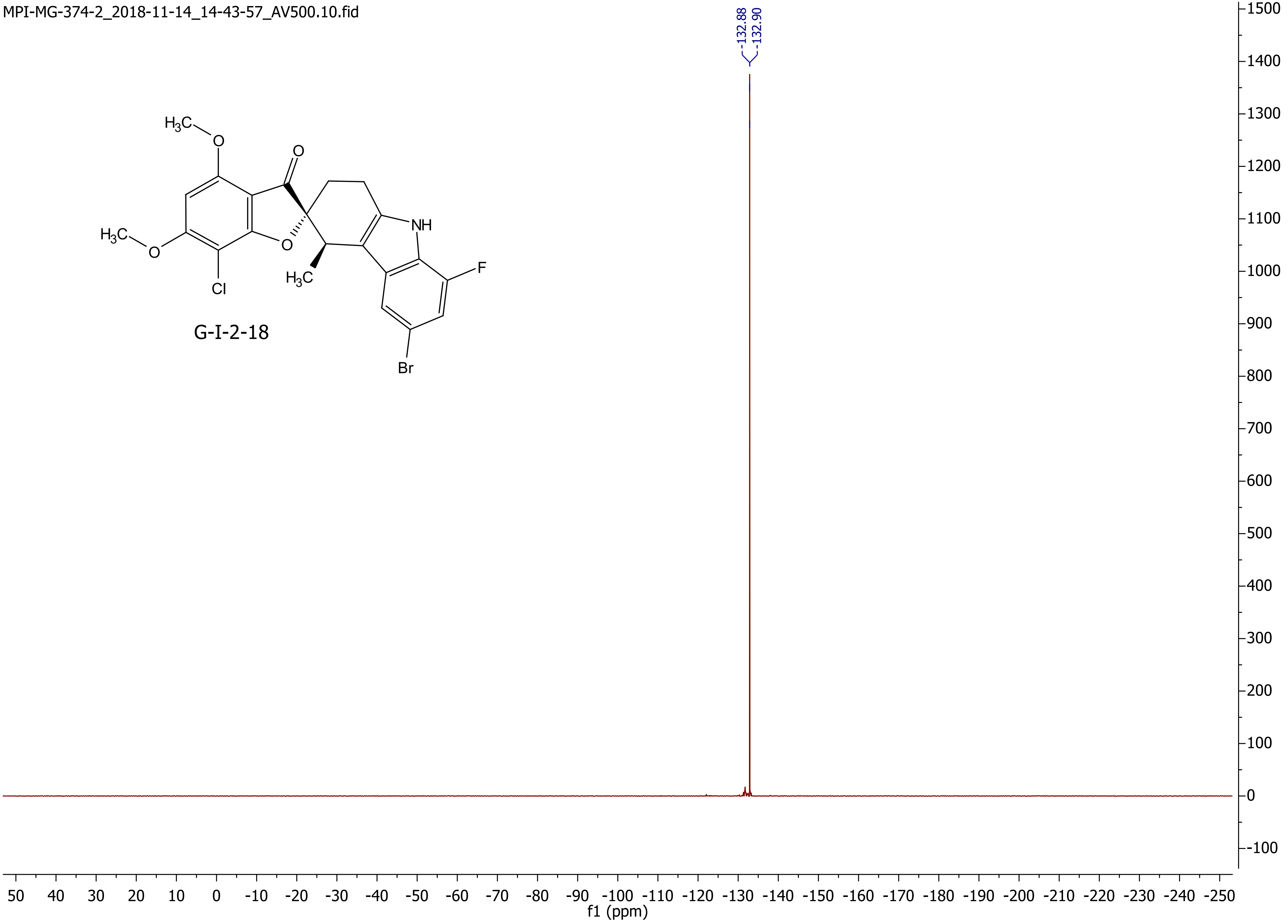

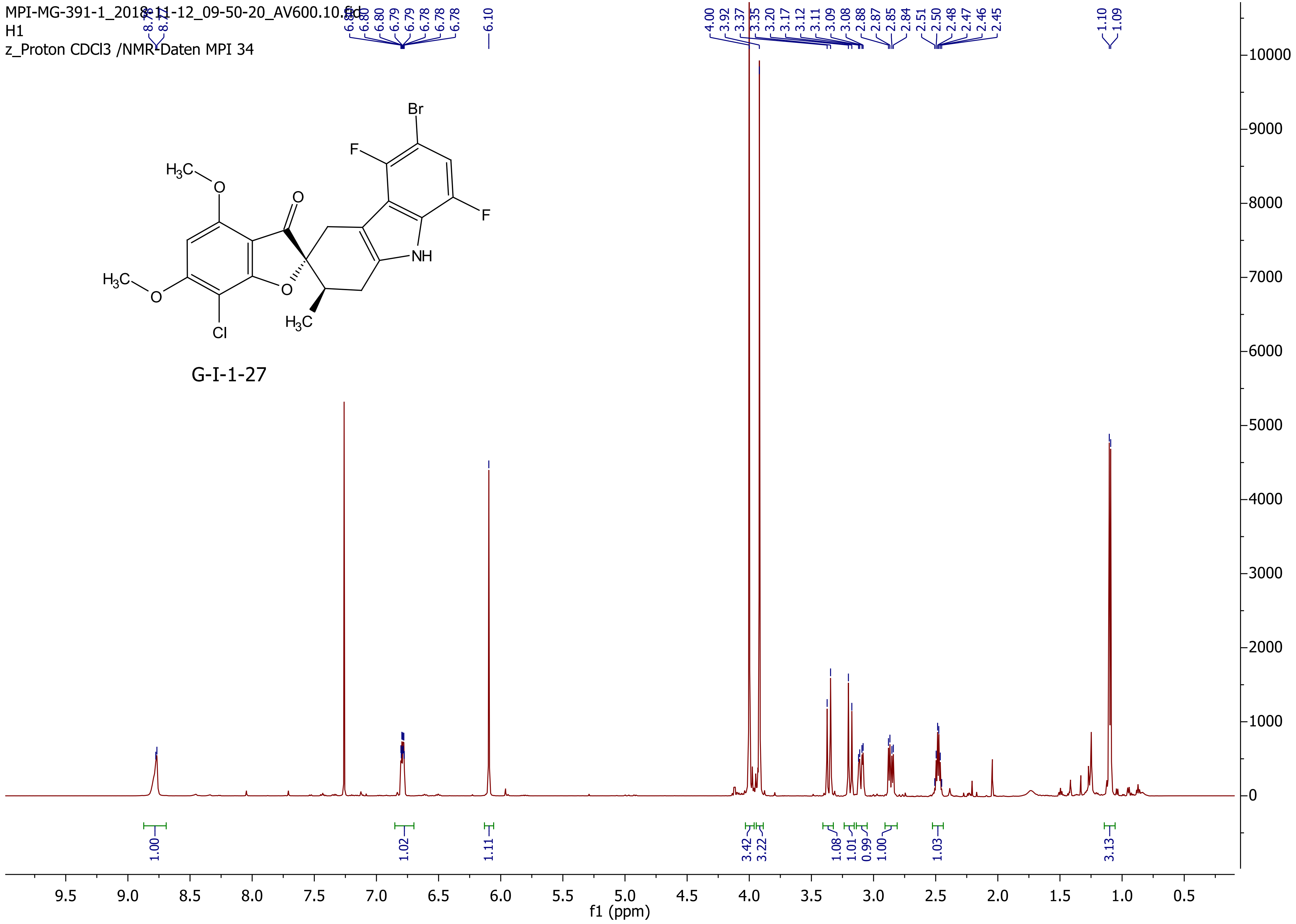
MPI-MG-391-1_2018-11-12_09-50-20_AV600.10.fid
H1
z_Proton CDCl3 /NMR-Daten MPI 34
8.77 8.77
6.80 6.80 6.80 6.79 6.79 6.78 6.78 6.78
6.10
4.00 3.92 3.37 3.35 3.20 3.17 3.12 3.11 3.09 3.08 2.88 2.87 2.85 2.84 2.51 2.50 2.48 2.47 2.46 2.45
1.10 1.09
Br
F
F
NH
O
O
O
O
H3C
H3C
H3C
Cl
G-I-1-27
1.00 1.02 1.11 3.42 3.22 1.08 1.01 0.99 1.00 1.03 3.13
f1 (ppm)
9.5 9.0 8.5 8.0 7.5 7.0 6.5 6.0 5.5 5.0 4.5 4.0 3.5 3.0 2.5 2.0 1.5 1.0 0.5
10000 9000 8000 7000 6000 5000 4000 3000 2000 1000 0

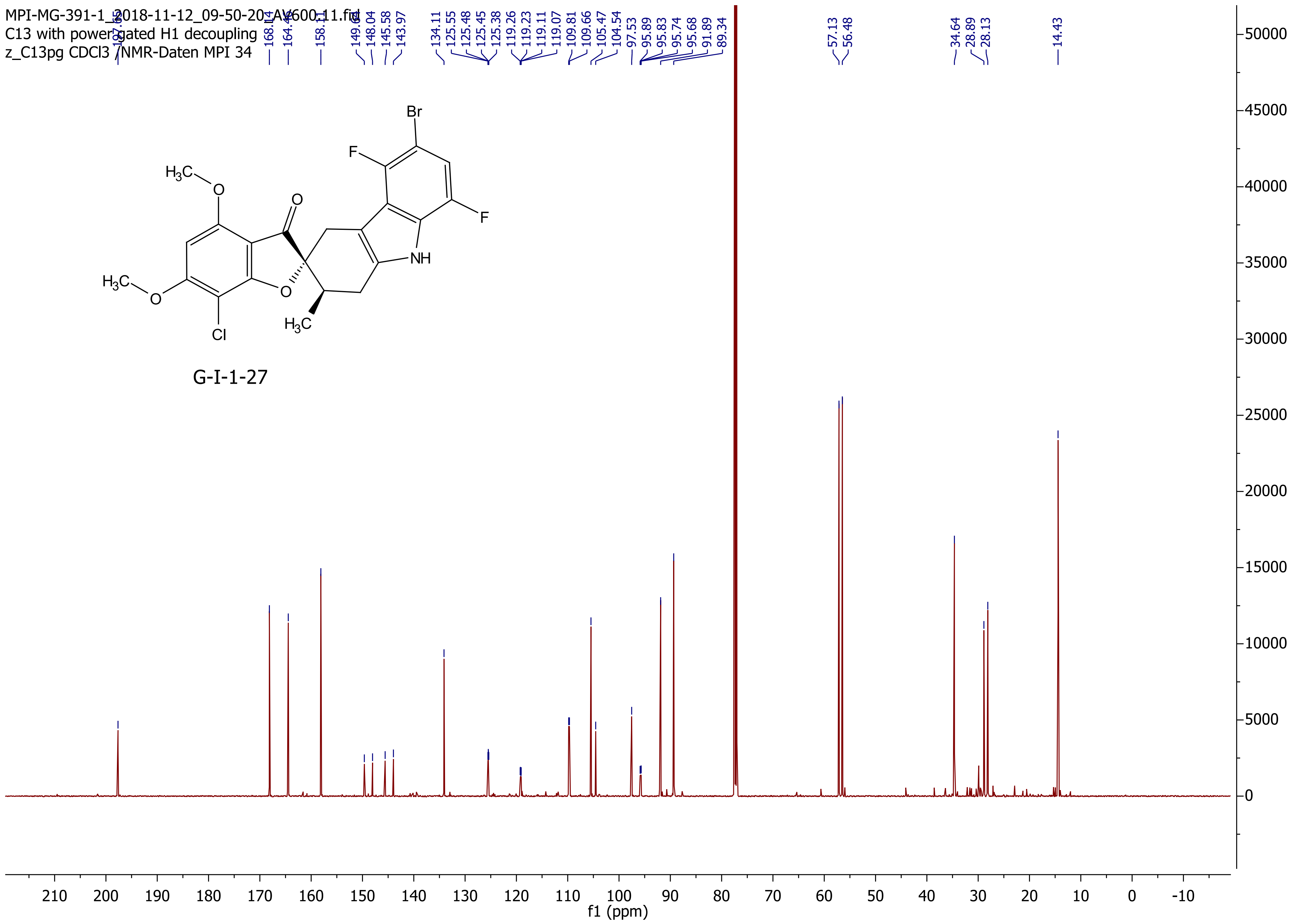

MPI-MG-391-1_2018-11-12_09-50-20_AV600_11.fid
C13 with power gated H1 decoupling
z_C13pg CDCl3 /NMR-Daten MPI 34
168.14
148.04
145.58
143.97
134.11
125.55
125.48
125.45
125.38
119.26
119.23
119.11
119.07
109.81
109.66
105.47
104.54
97.53
95.89
95.83
95.74
95.68
91.89
89.34
57.13
56.48
34.64
28.89
28.13
14.43
Br
F
F
H3C
O
O
NH
H3C
O
O
Cl
H3C
G-I-1-27
f1 (ppm)

MPI-MG-391-1_2018-11-12_09-50-20_AV600.12.fid
F19

z_F19 CDCl3 /NMR-Daten MPI 34

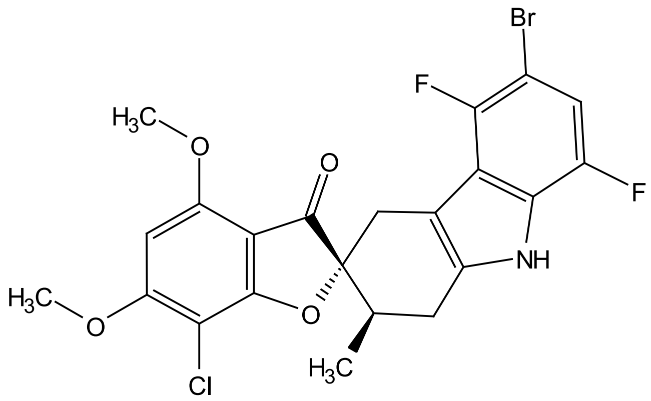

G-I-1-27

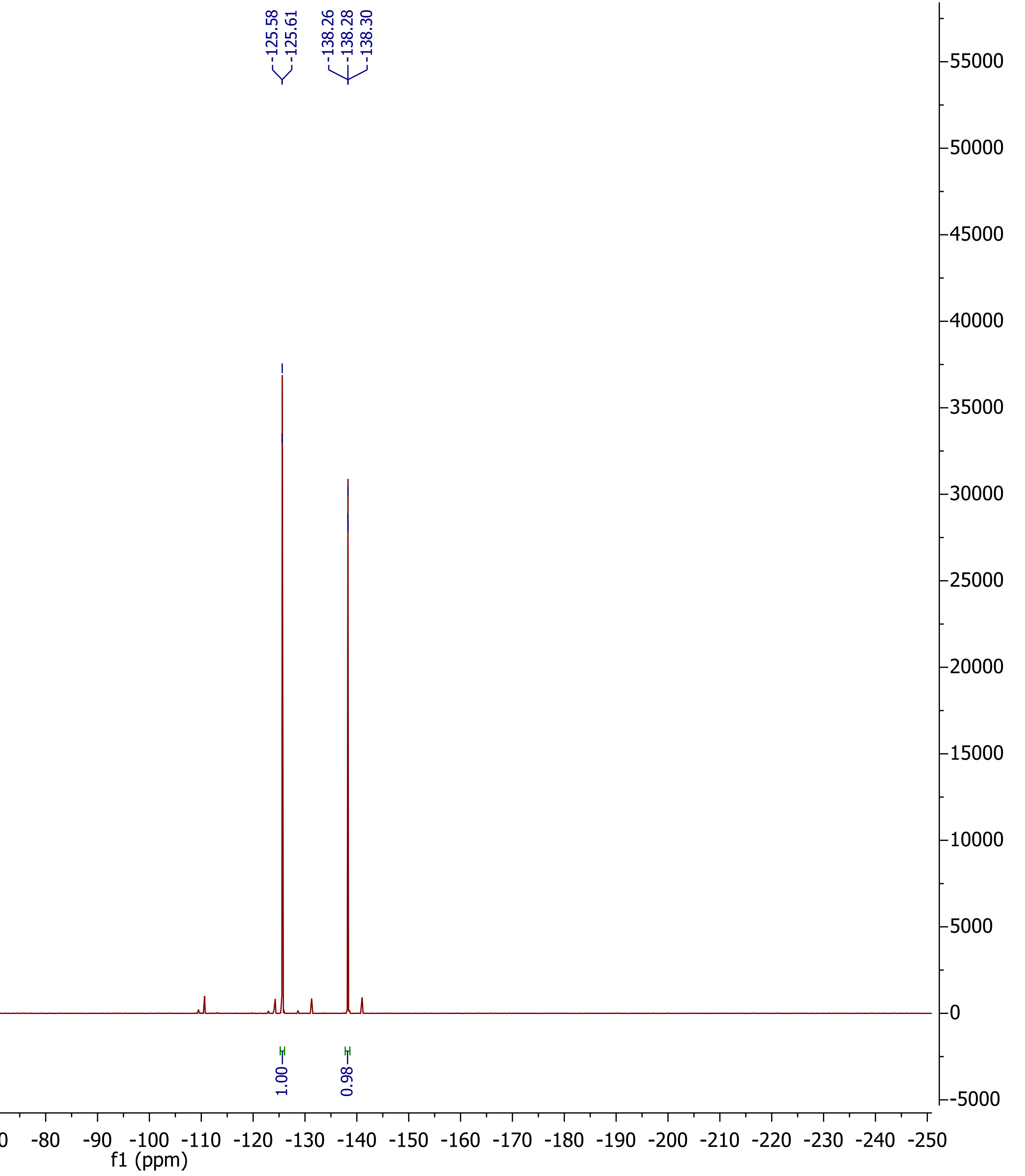

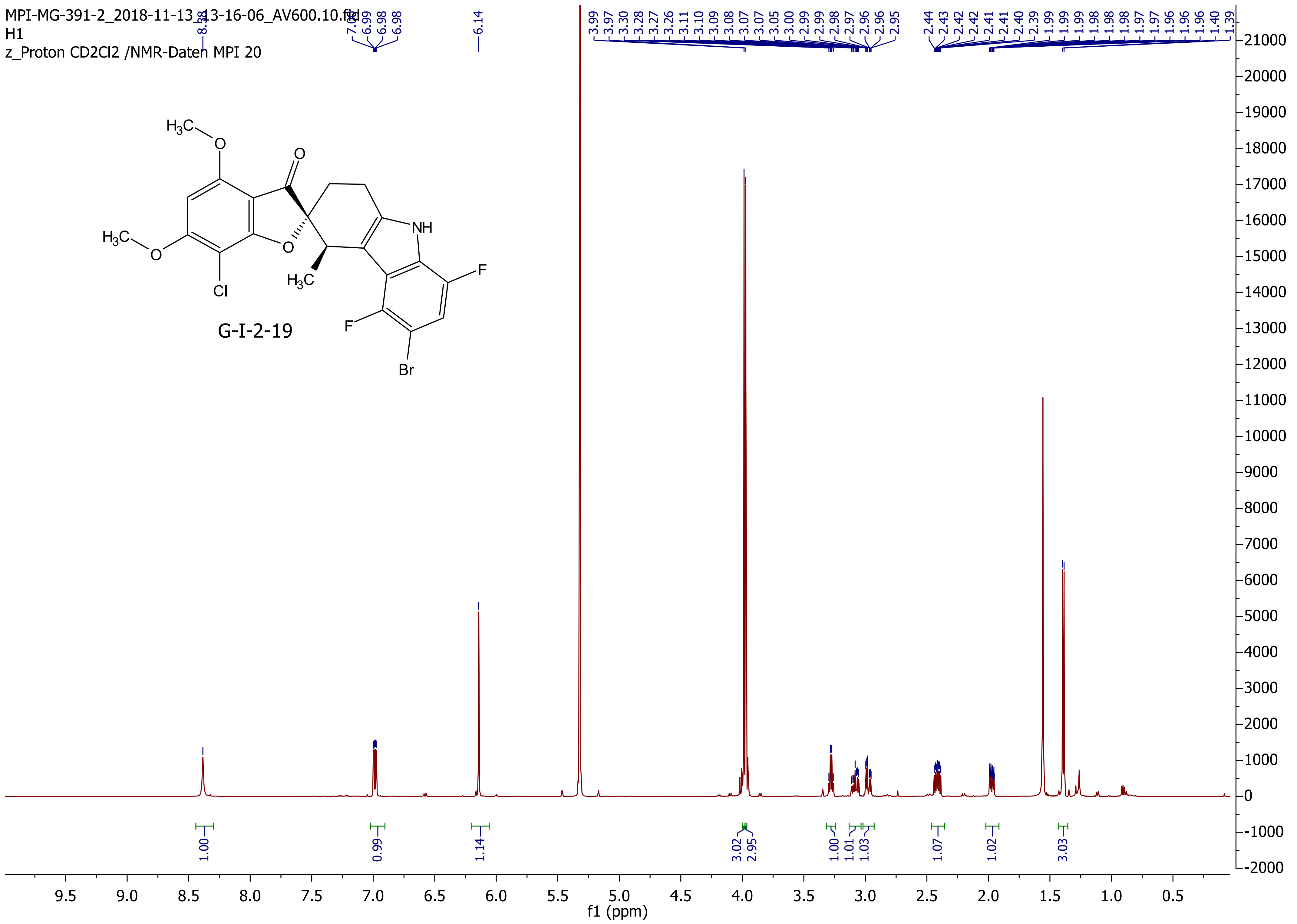
MPI-MG-391-2_2018-11-13_13-16-06_AV600.10.fid
H1
z_Proton CD2Cl2 /NMR-Daten MPI 20
8.38
7.00
6.99
6.98
6.98
6.14
3.99
3.97
3.30
3.28
3.27
3.26
3.11
3.10
3.09
3.08
3.07
3.07
3.05
3.00
2.99
2.99
2.98
2.97
2.96
2.96
2.95
2.44
2.43
2.42
2.42
2.41
2.41
2.40
2.39
1.99
1.99
1.99
1.98
1.98
1.98
1.97
1.97
1.96
1.96
1.96
1.40
1.39
H3C
O
O
H3C
O
O
NH
F
Cl
H3C
F
Br
G-I-2-19
1.00
0.99
1.14
3.02
2.95
1.00
1.01
1.03
1.07
1.02
3.03
f1 (ppm)

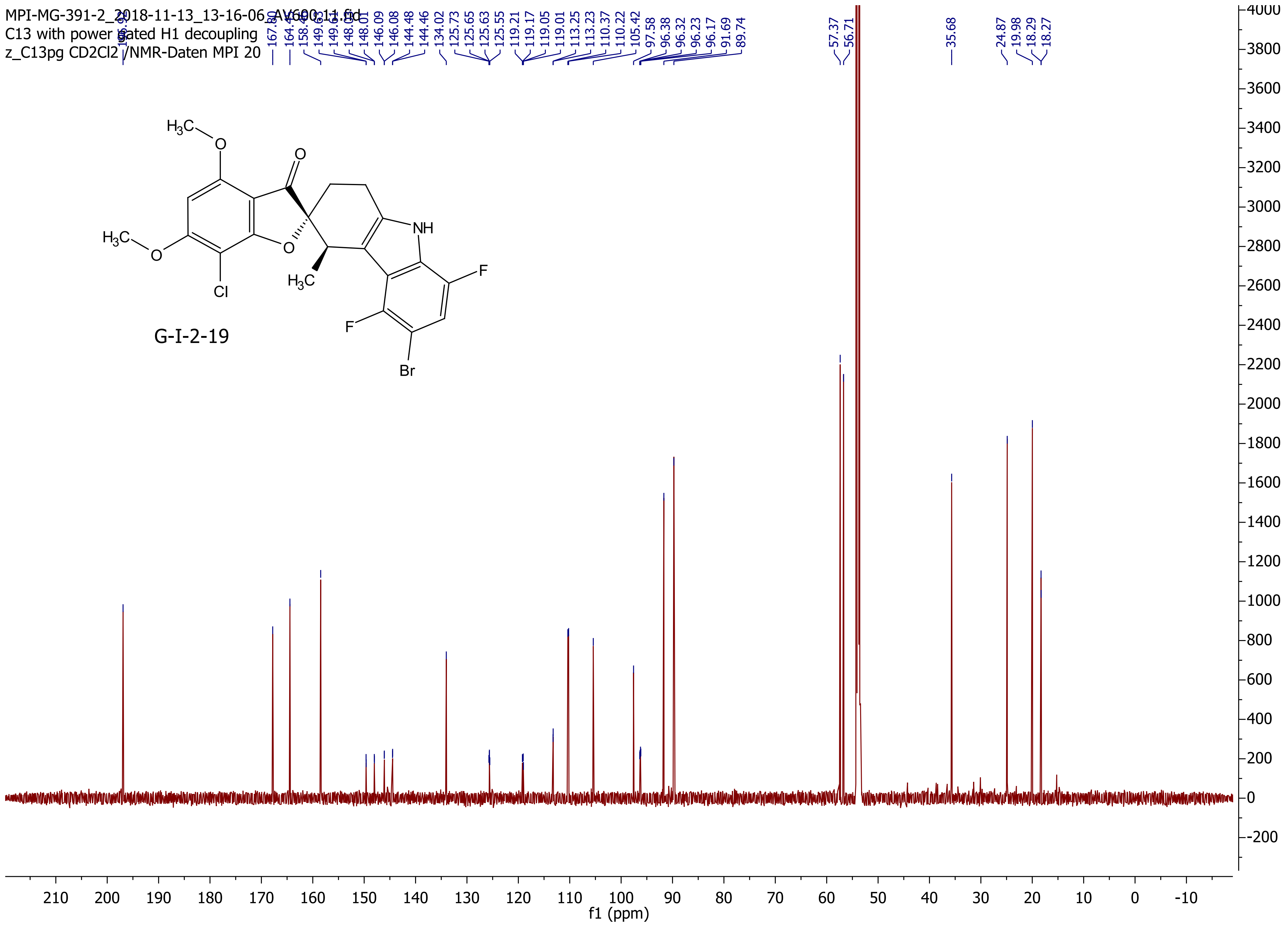

MPI-MG-391-2_2018-11-13_13-16-06_AV600.11.fid
C13 with power gated H1 decoupling
z_C13pg CD2Cl2 /NMR-Daten MPI 20
167.80
148.01
146.09
146.08
144.48
144.46
134.02
125.73
125.65
125.63
125.55
119.21
119.17
119.05
119.01
113.25
113.23
110.37
110.22
105.42
97.58
96.38
96.32
96.23
96.17
91.69
89.74
57.37
56.71
35.68
24.87
19.98
18.29
18.27
H_3C
O
O
H_3C
O
O
Cl
H_3C
NH
F
F
Br
G-I-2-19
f1 (ppm)

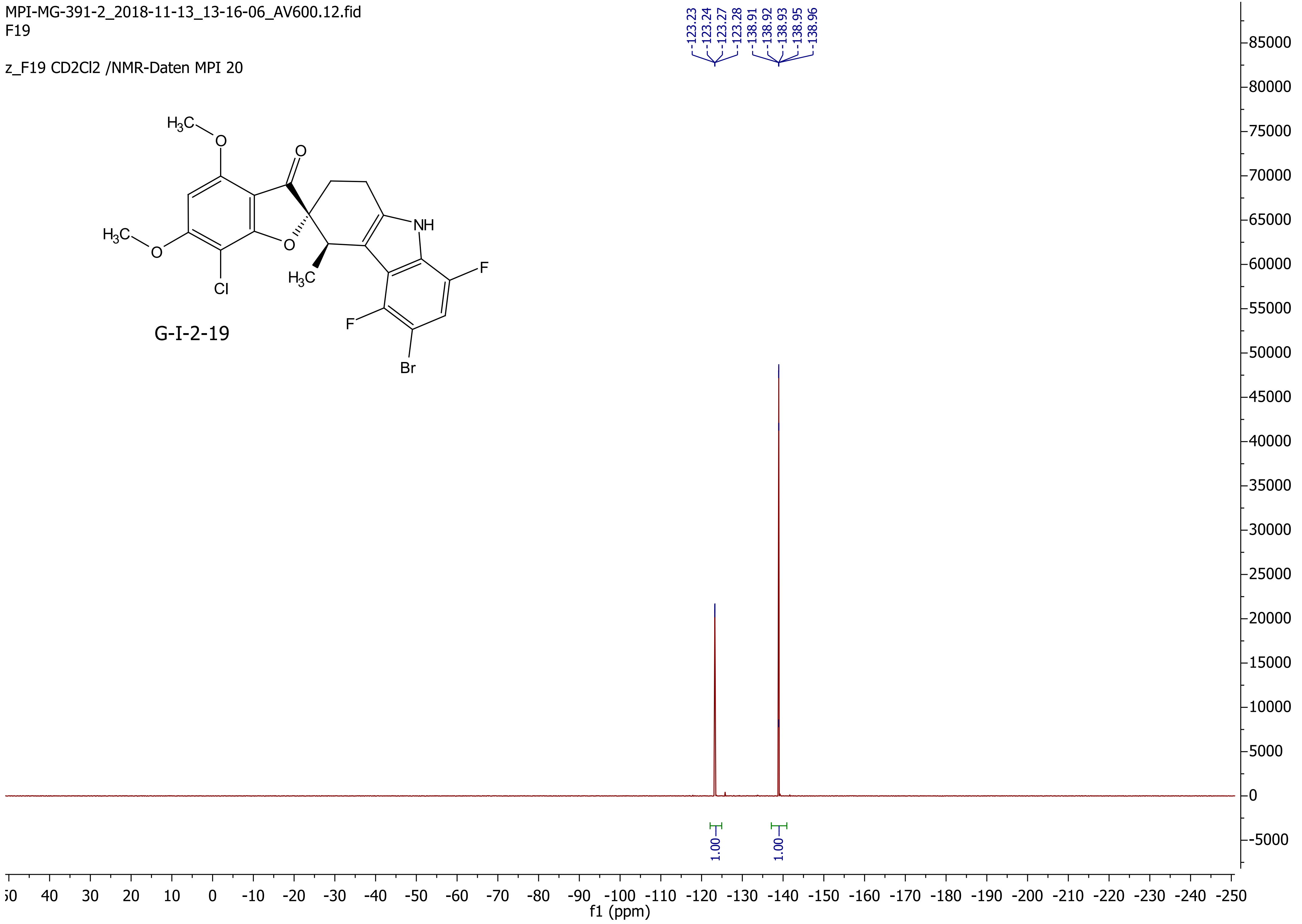
MPI-MG-391-2_2018-11-13_13-16-06_AV600.12.fid
F19
z_F19 CD2Cl2 /NMR-Daten MPI 20
G-I-2-19
-123.23
-123.24
-123.27
-123.28
-138.91
-138.92
-138.93
-138.95
-138.96
1.00
1.00
f1 (ppm)

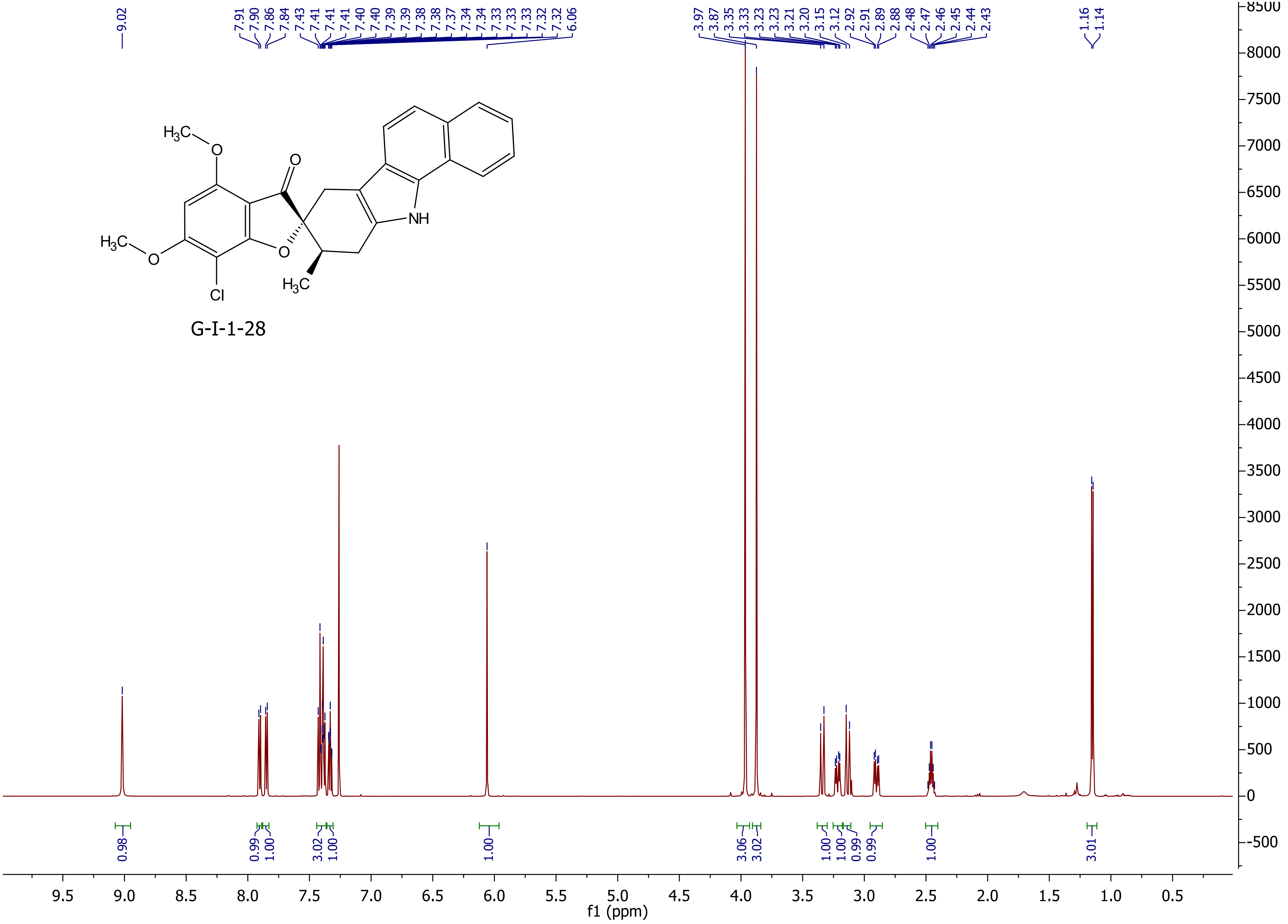
9.02
7.91 7.90 7.86 7.84 7.43 7.41 7.41 7.41 7.40 7.40 7.39 7.39 7.38 7.38 7.37 7.34 7.34 7.33 7.33 7.33 7.32 7.32 6.06
3.97 3.87 3.35 3.33 3.23 3.23 3.21 3.20 3.15 3.12 2.92 2.91 2.89 2.88 2.48 2.47 2.46 2.45 2.44 2.43
1.16 1.14
0.98 0.99 1.00 3.02 1.00 1.00 3.06 3.02 1.00 1.00 0.99 0.99 1.00 3.01
f1 (ppm)
H3C
O
O
H3C
O
Cl
O
NH
H3C

G-I-1-28

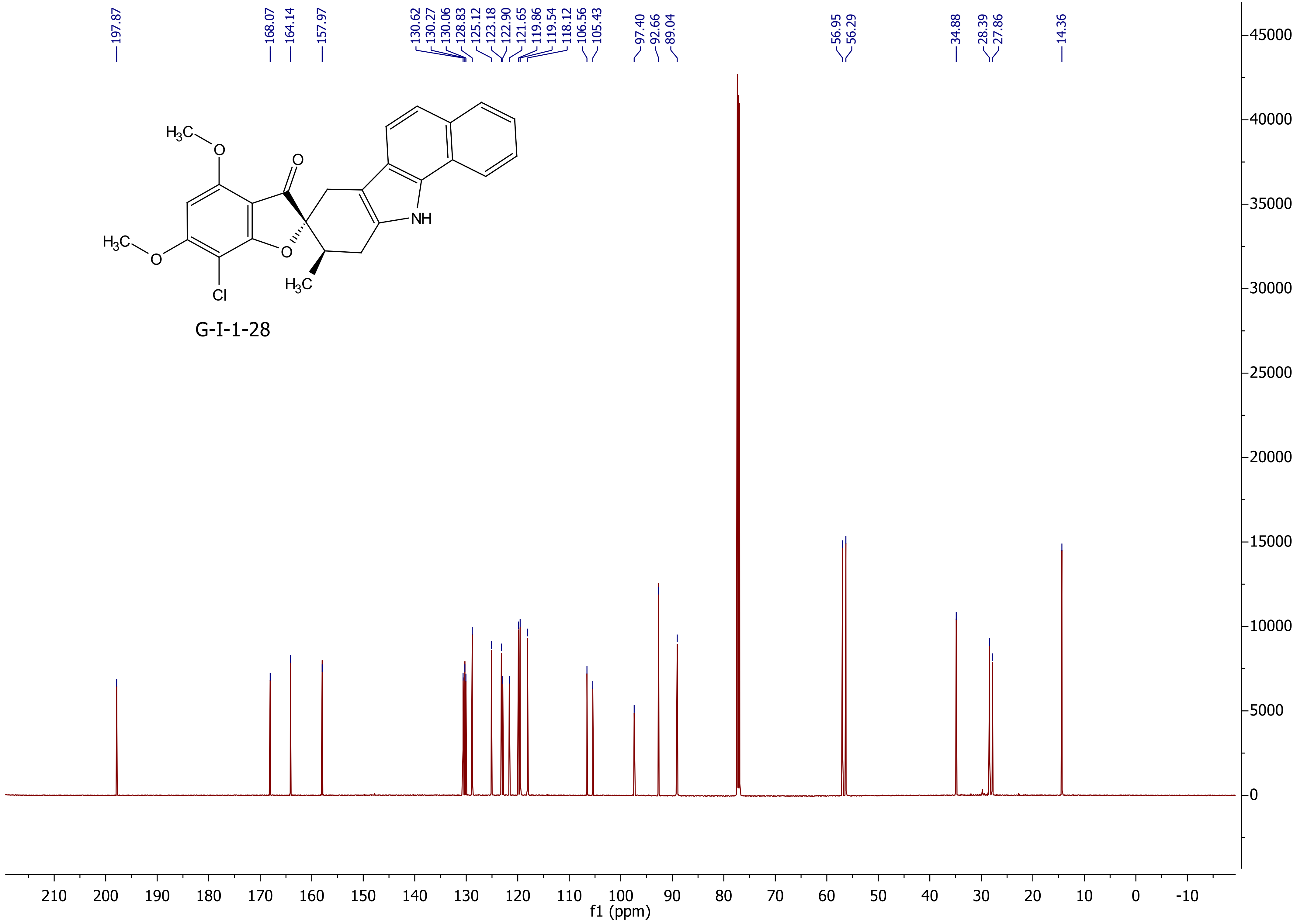
197.87
168.07
164.14
157.97
130.62
130.27
130.06
128.83
125.12
123.18
122.90
121.65
119.86
119.54
118.12
106.56
105.43
97.40
92.66
89.04
56.95
56.29
34.88
28.39
27.86
14.36
G-I-1-28
f1 (ppm)

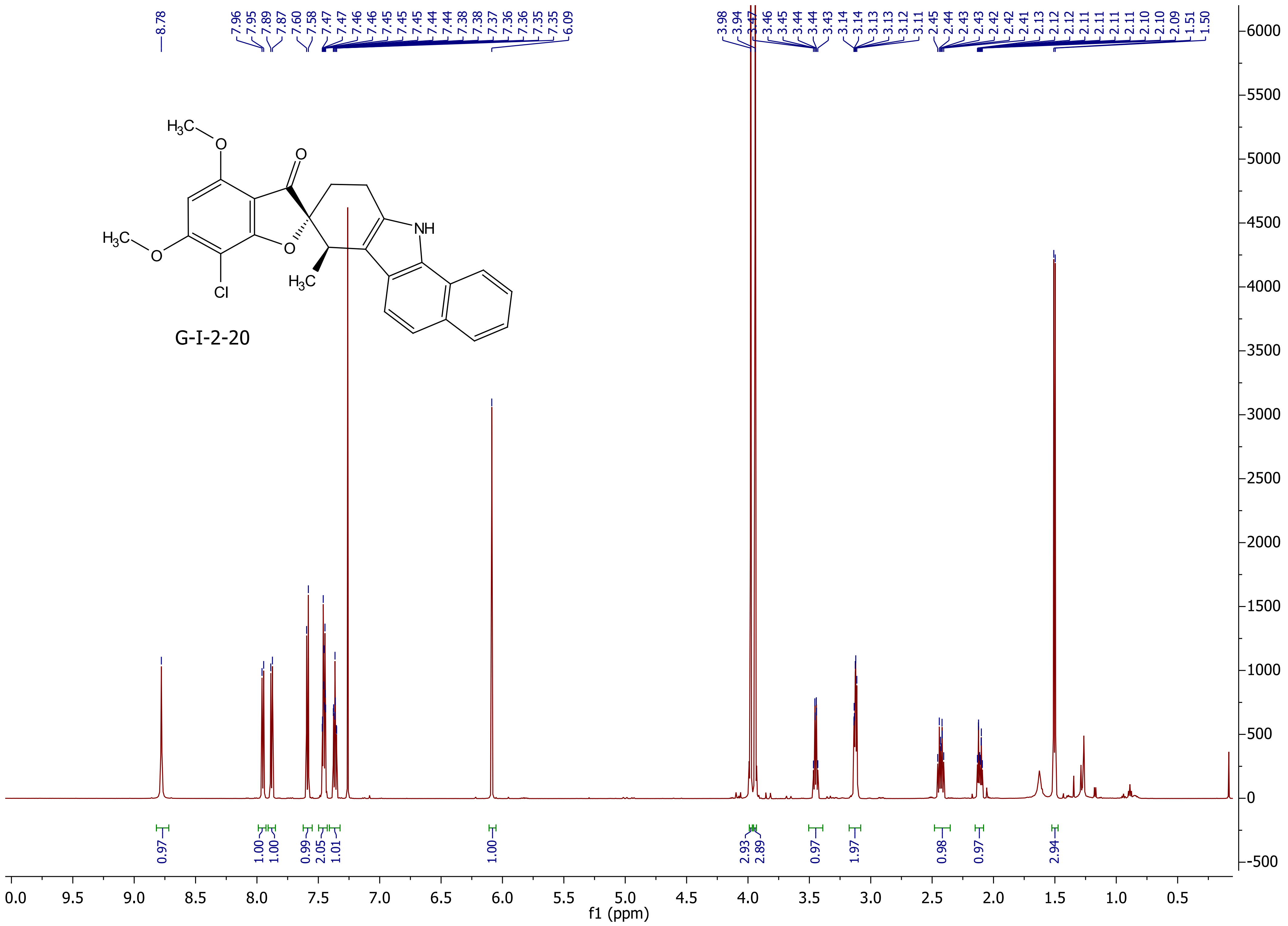
G-I-2-20
f1 (ppm)

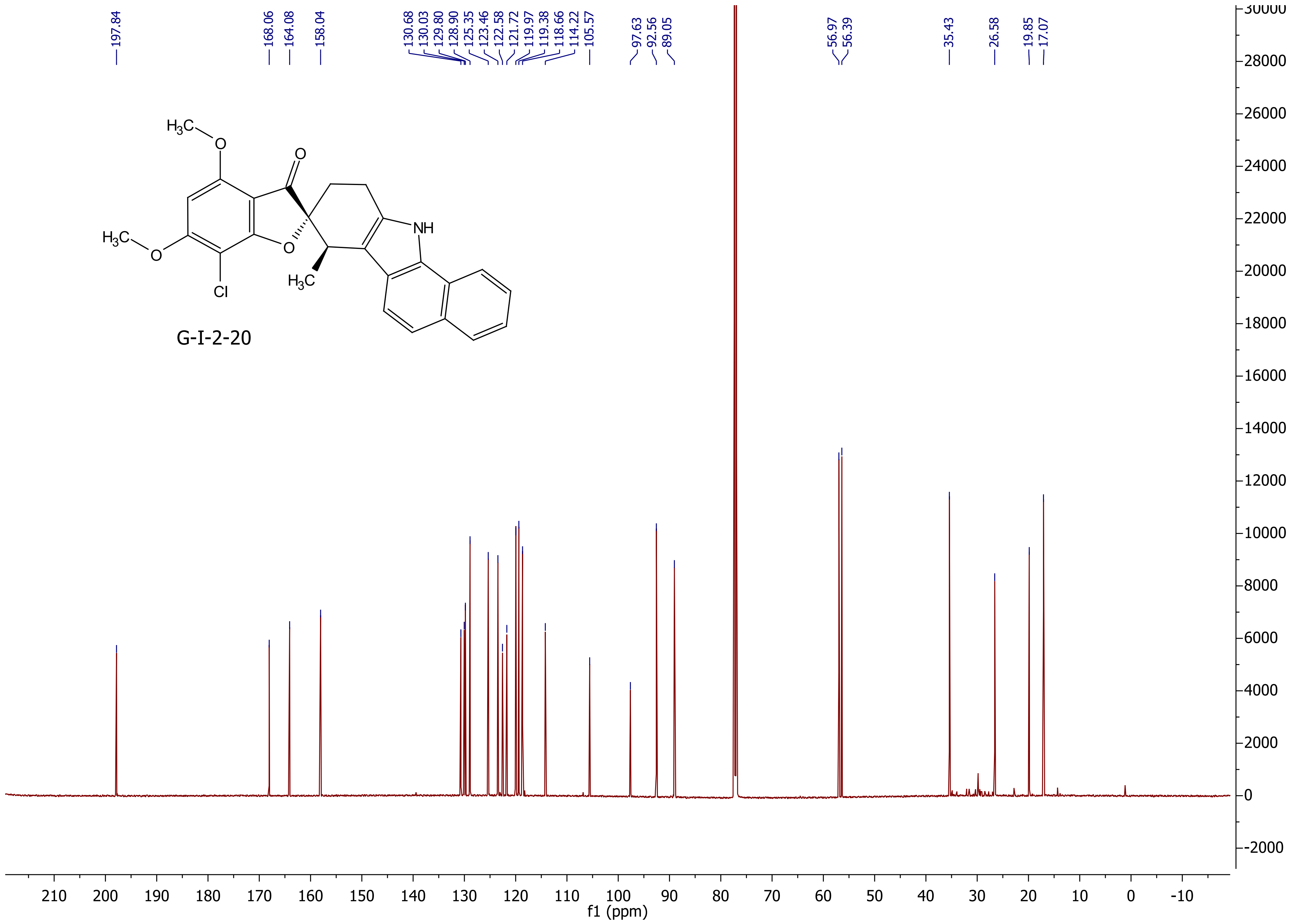

197.84
168.06
164.08
158.04
130.68
130.03
129.80
128.90
125.35
123.46
122.58
121.72
119.97
119.38
118.66
114.22
105.57
97.63
92.56
89.05
56.97
56.39
35.43
26.58
19.85
17.07
G-I-2-20
f1 (ppm)

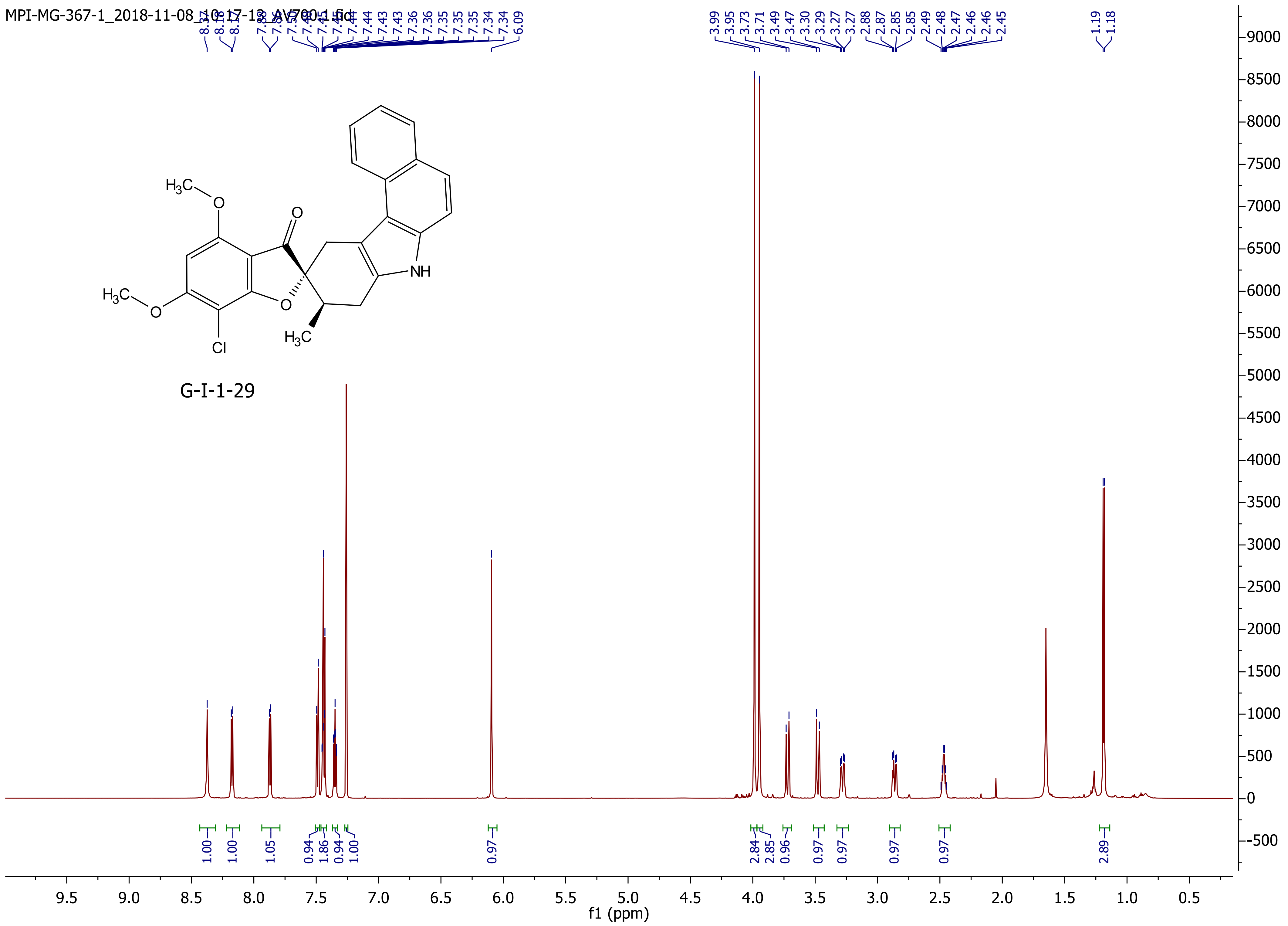
MPI-MG-367-1_2018-11-08_10-17-13_AV700.1.fid
7.44
7.43
7.43
7.36
7.36
7.35
7.35
7.35
7.34
7.34
6.09
3.99
3.95
3.73
3.71
3.49
3.47
3.30
3.29
3.27
3.27
2.88
2.87
2.85
2.85
2.49
2.48
2.47
2.46
2.46
2.45
1.19
1.18
H3C
O
O
H3C
O
O
Cl
H3C
NH
G-I-1-29
1.00
1.00
1.05
0.94
1.86
0.94
1.00
0.97
2.84
2.85
0.96
0.97
0.97
0.97
0.97
2.89
f1 (ppm)

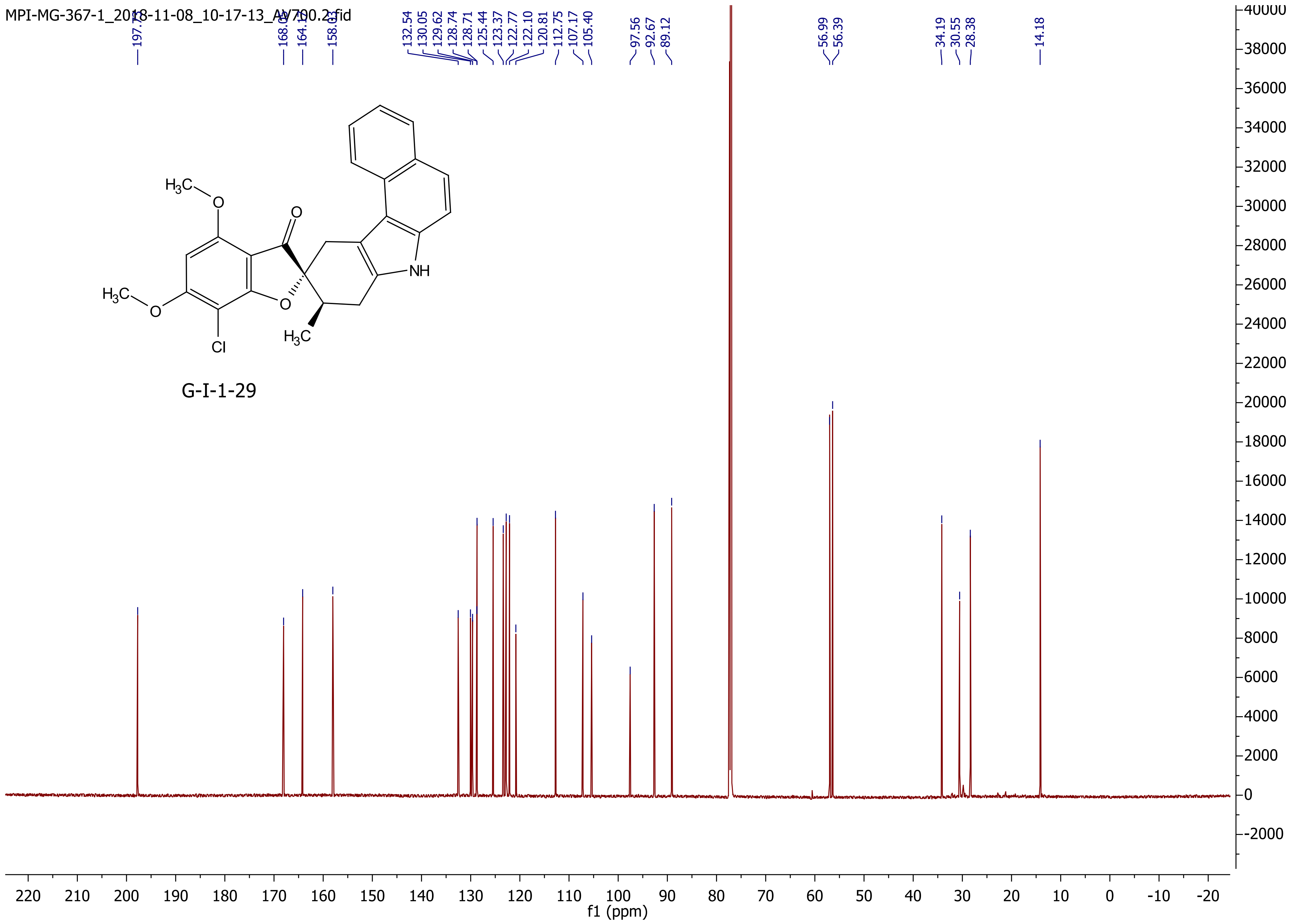

MPI-MG-367-1_2018-11-08_10-17-13_AV700.2.fid
132.54
130.05
129.62
128.74
128.71
125.44
123.37
122.77
122.10
120.81
112.75
107.17
105.40
97.56
92.67
89.12
56.99
56.39
34.19
30.55
28.38
14.18
H_3C
O
O
H_3C
O
O
NH
H_3C
Cl
G-I-1-29
f1 (ppm)

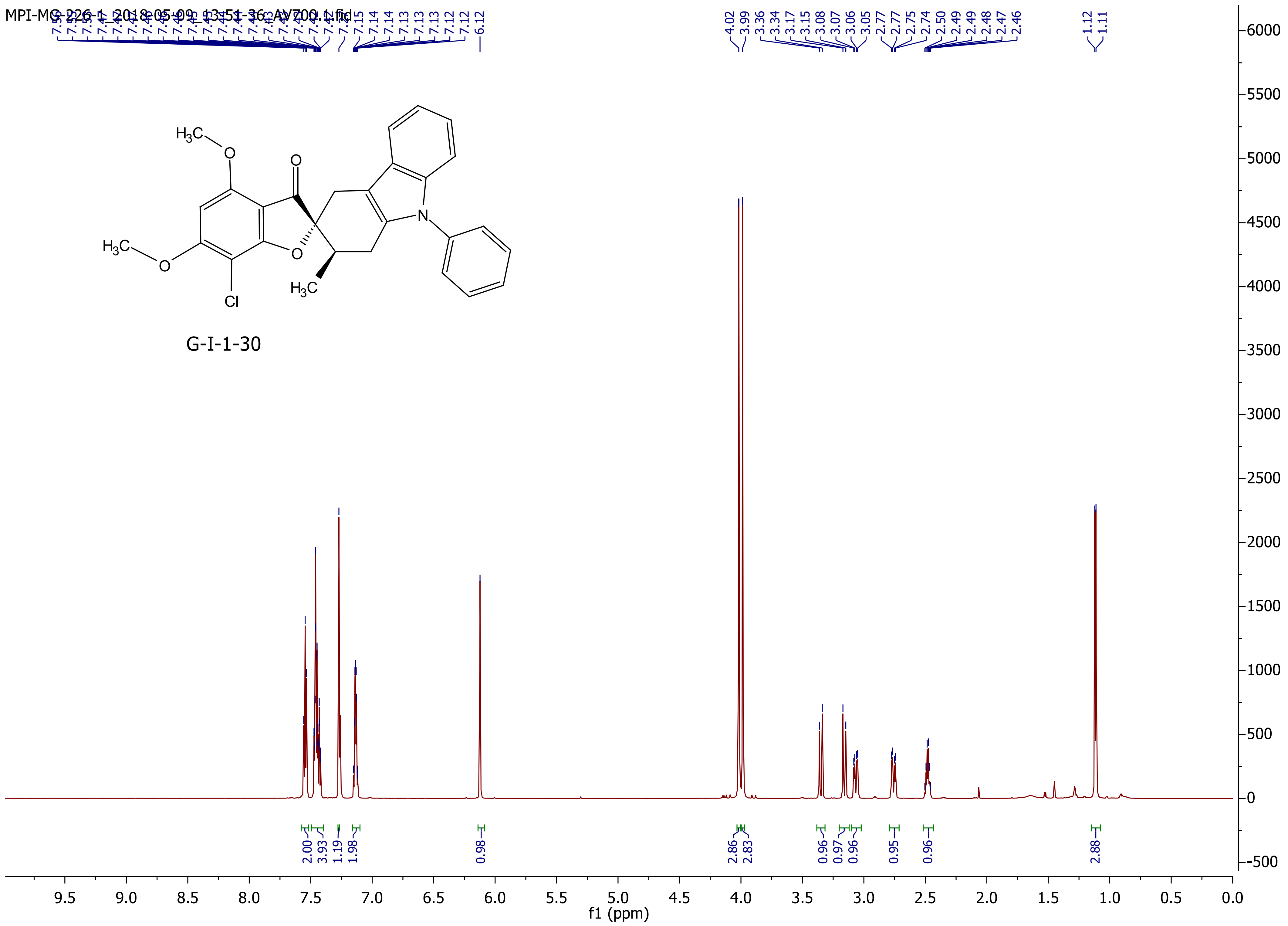
MPI-MG-226-1_2018-05-09_13-51-36_AV700.1.fid
7.15 7.14 7.14 7.13 7.13 7.13 7.12 7.12 6.12
4.02 3.99 3.36 3.34 3.17 3.15 3.08 3.07 3.06 3.05 2.77 2.77 2.75 2.74 2.50 2.49 2.49 2.48 2.47 2.46
1.12 1.11
H3C O O H3C O O Cl N H3C
G-I-1-30
2.00 3.93 1.19 1.98 0.98 2.86 2.83 0.96 0.97 0.96 0.95 0.96 2.88
f1 (ppm)

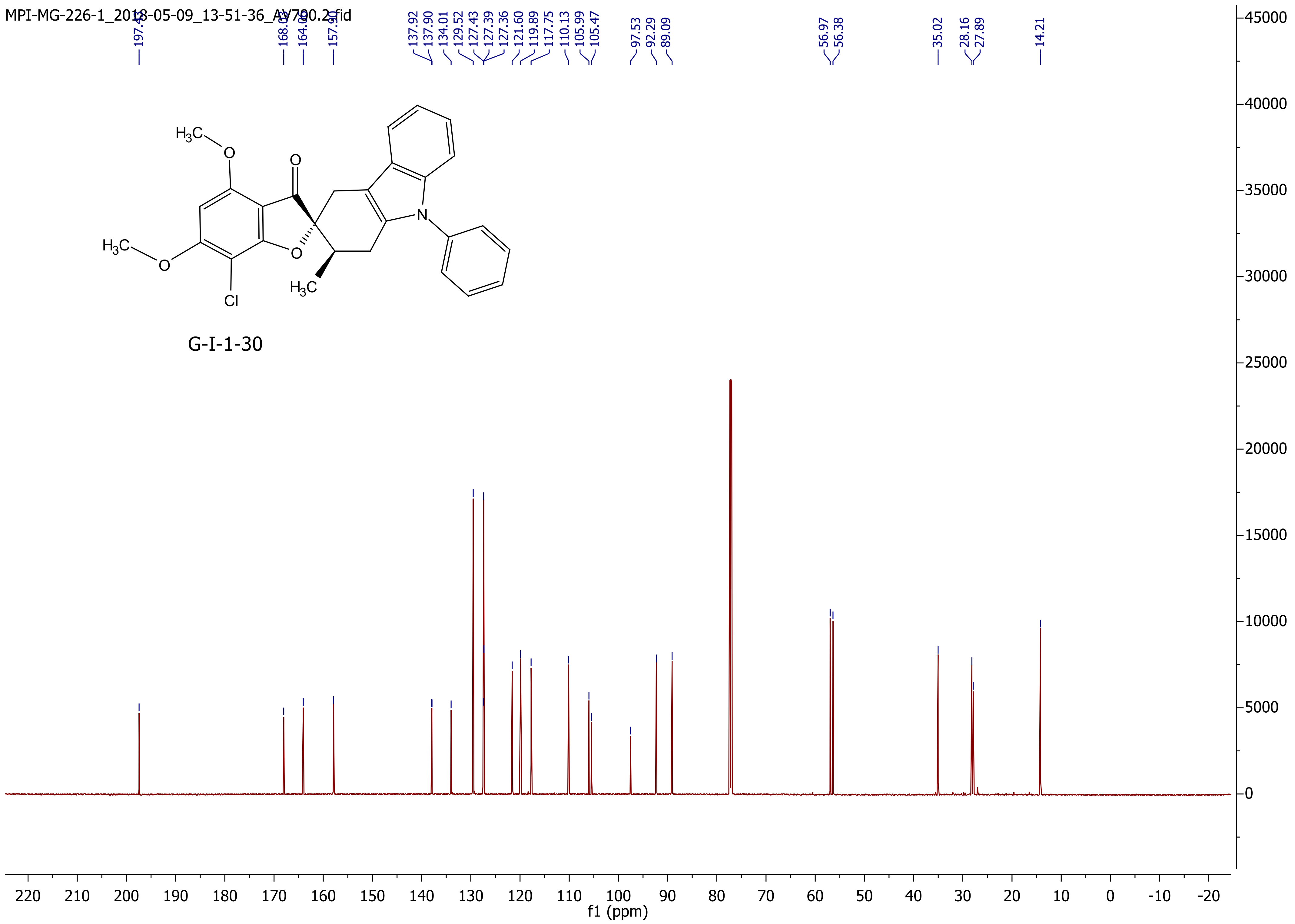

MPI-MG-226-1_2018-05-09_13-51-36_AV700.2.fid
197.4
168.0
164.0
157.9
137.92
137.90
134.01
129.52
127.43
127.39
127.36
121.60
119.89
117.75
110.13
105.99
105.47
97.53
92.29
89.09
56.97
56.38
35.02
28.16
27.89
14.21
G-I-1-30
f1 (ppm)

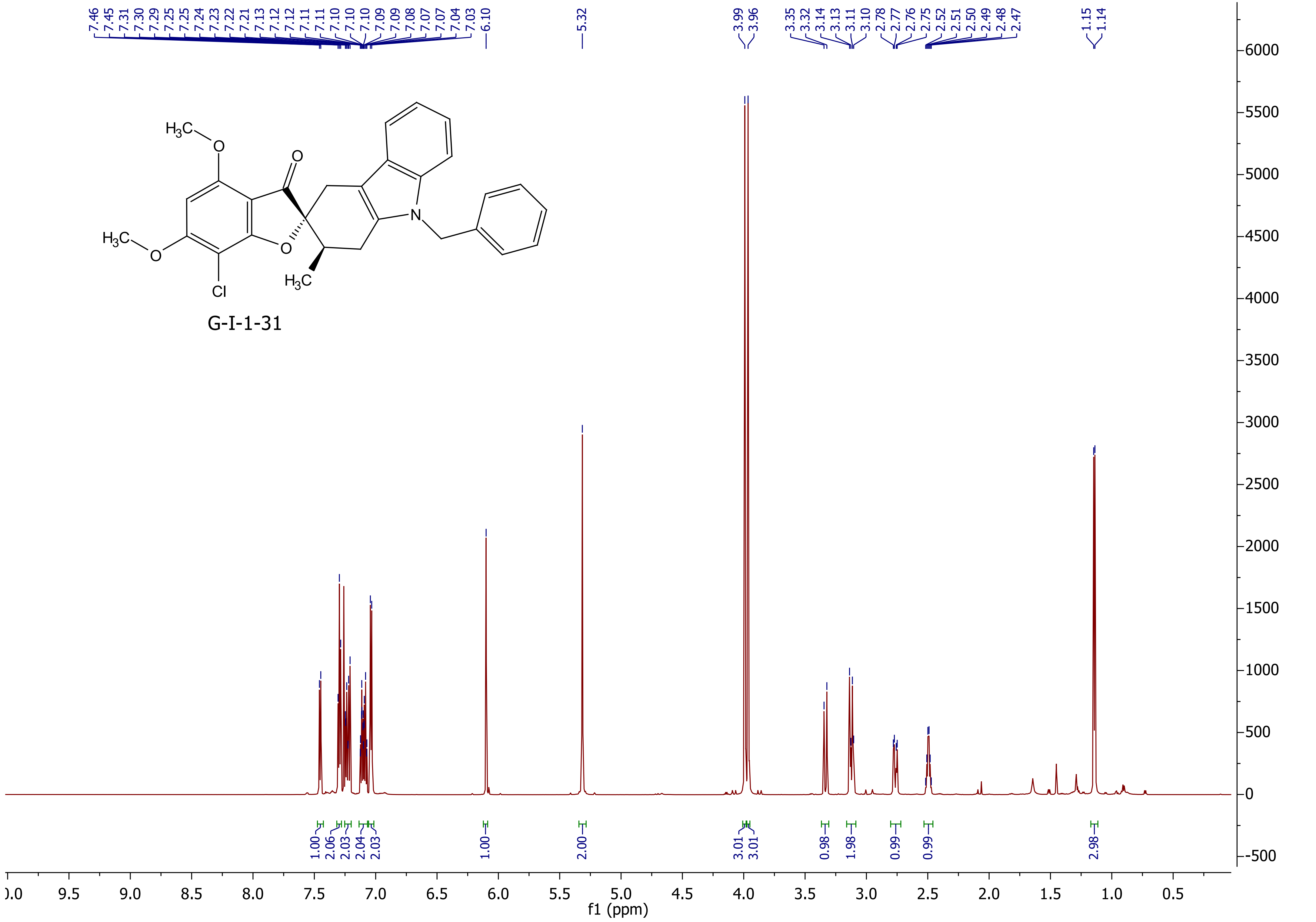
7.46 7.45 7.31 7.30 7.29 7.25 7.25 7.24 7.23 7.22 7.21 7.13 7.12 7.12 7.11 7.11 7.10 7.10 7.10 7.09 7.09 7.08 7.07 7.07 7.04 7.03 6.10
5.32
3.99 3.96
3.35 3.32 3.14 3.13 3.11 3.10 2.78 2.77 2.76 2.75 2.52 2.51 2.50 2.49 2.48 2.47
1.15 1.14
H3C
O
O
H3C
O
O
N
Cl
H3C
G-I-1-31
1.00 2.06 2.03 2.04 2.03
1.00
2.00
3.01 3.01
0.98 1.98 0.99 0.99
2.98
f1 (ppm)

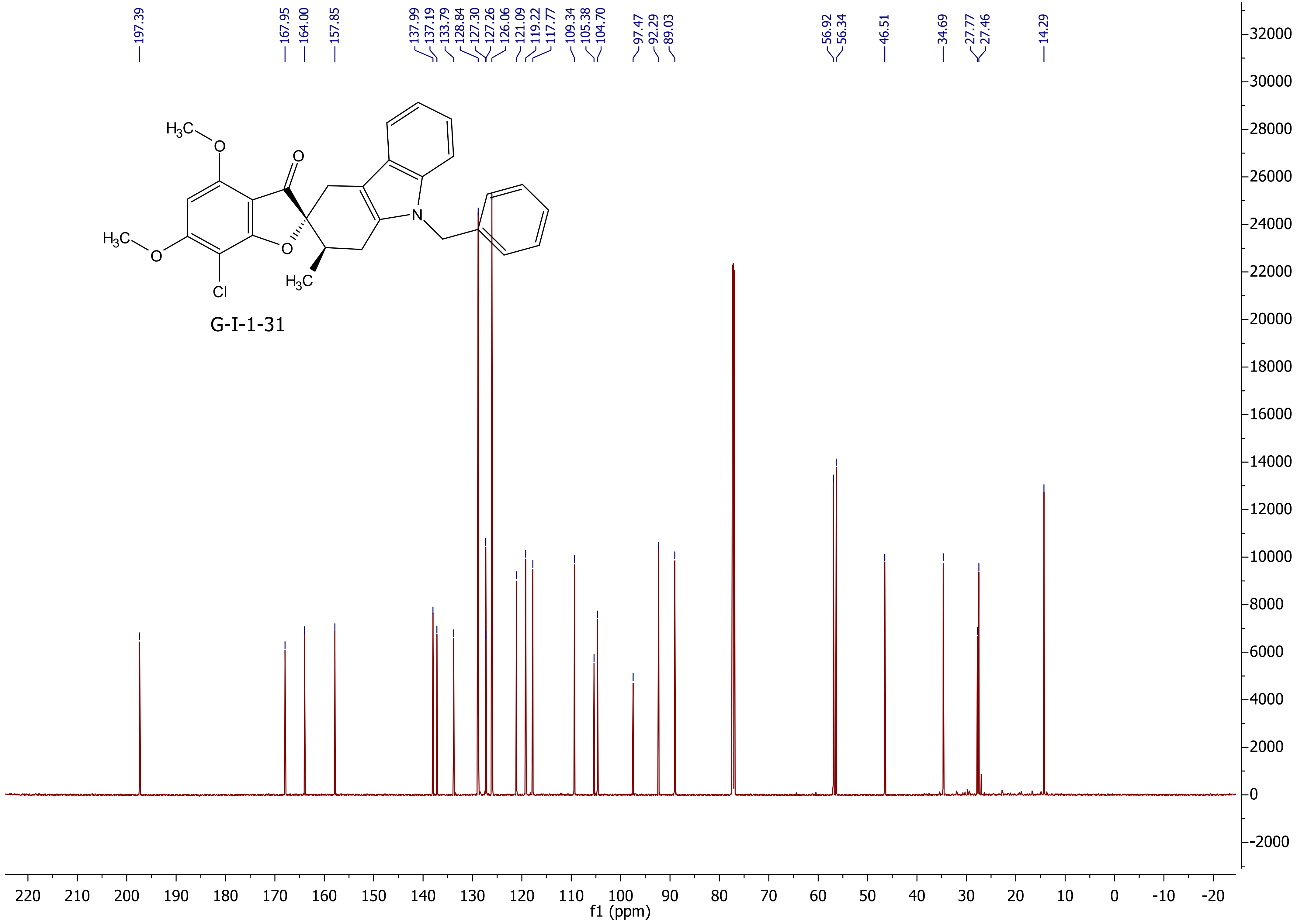
197.39
167.95
164.00
157.85
137.99
137.19
133.79
128.84
127.30
127.26
126.06
121.09
119.22
117.77
109.34
105.38
104.70
97.47
92.29
89.03
56.92
56.34
46.51
34.69
27.77
27.46
14.29
H3C
O
O
H3C
O
O
Cl
H3C
N
G-I-1-31
f1 (ppm)
220 210 200 190 180 170 160 150 140 130 120 110 100 90 80 70 60 50 40 30 20 10 0 -10 -20
32000 30000 28000 26000 24000 22000 20000 18000 16000 14000 12000 10000 8000 6000 4000 2000 0 -2000

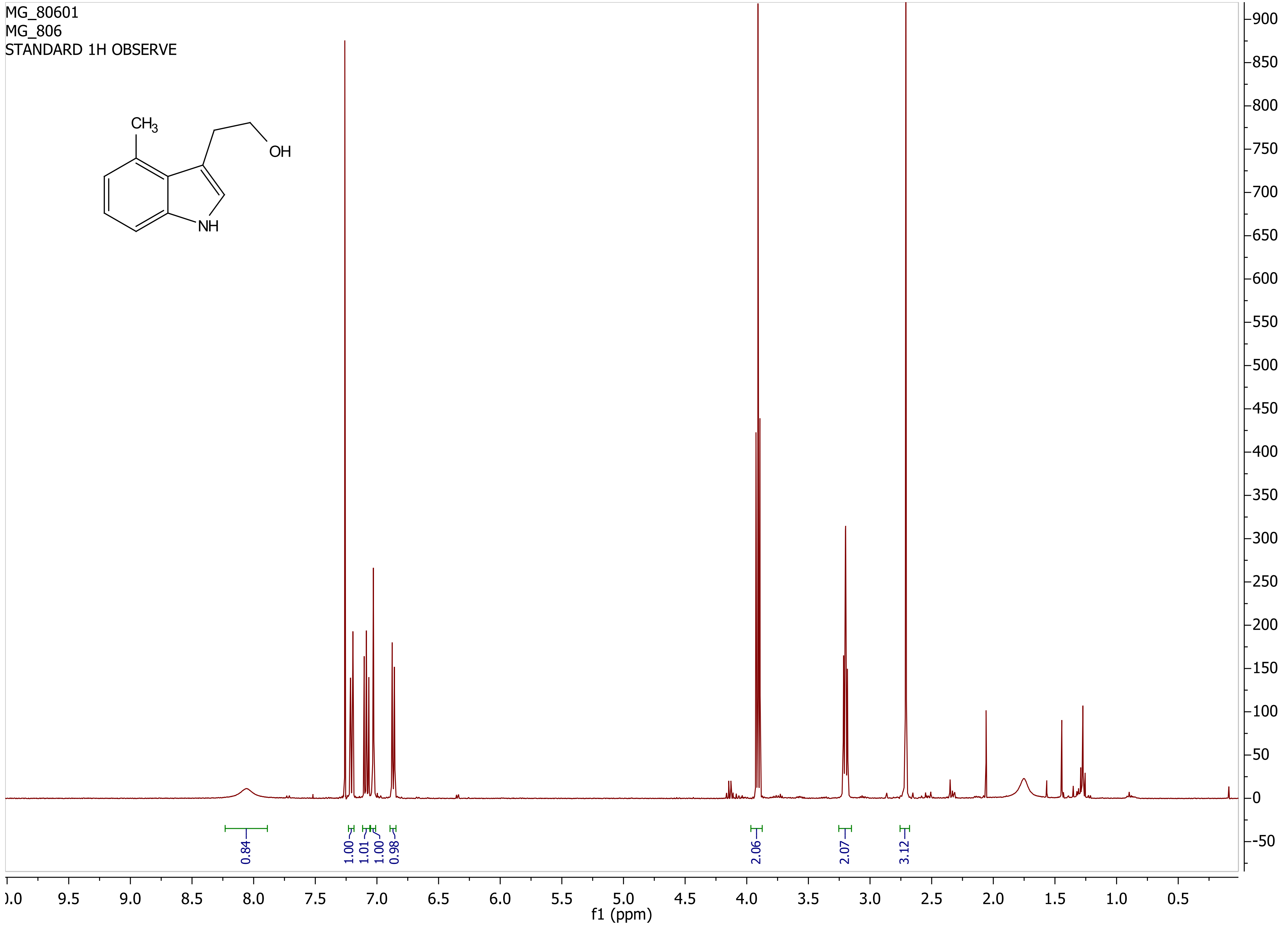
MG_80601
MG_806
STANDARD 1H OBSERVE
CH3
OH
NH
0.84
1.00
1.01
1.00
0.98
2.06
2.07
3.12
f1 (ppm)

MG_80602
MG_806
STANDARD 1H OBSERVE

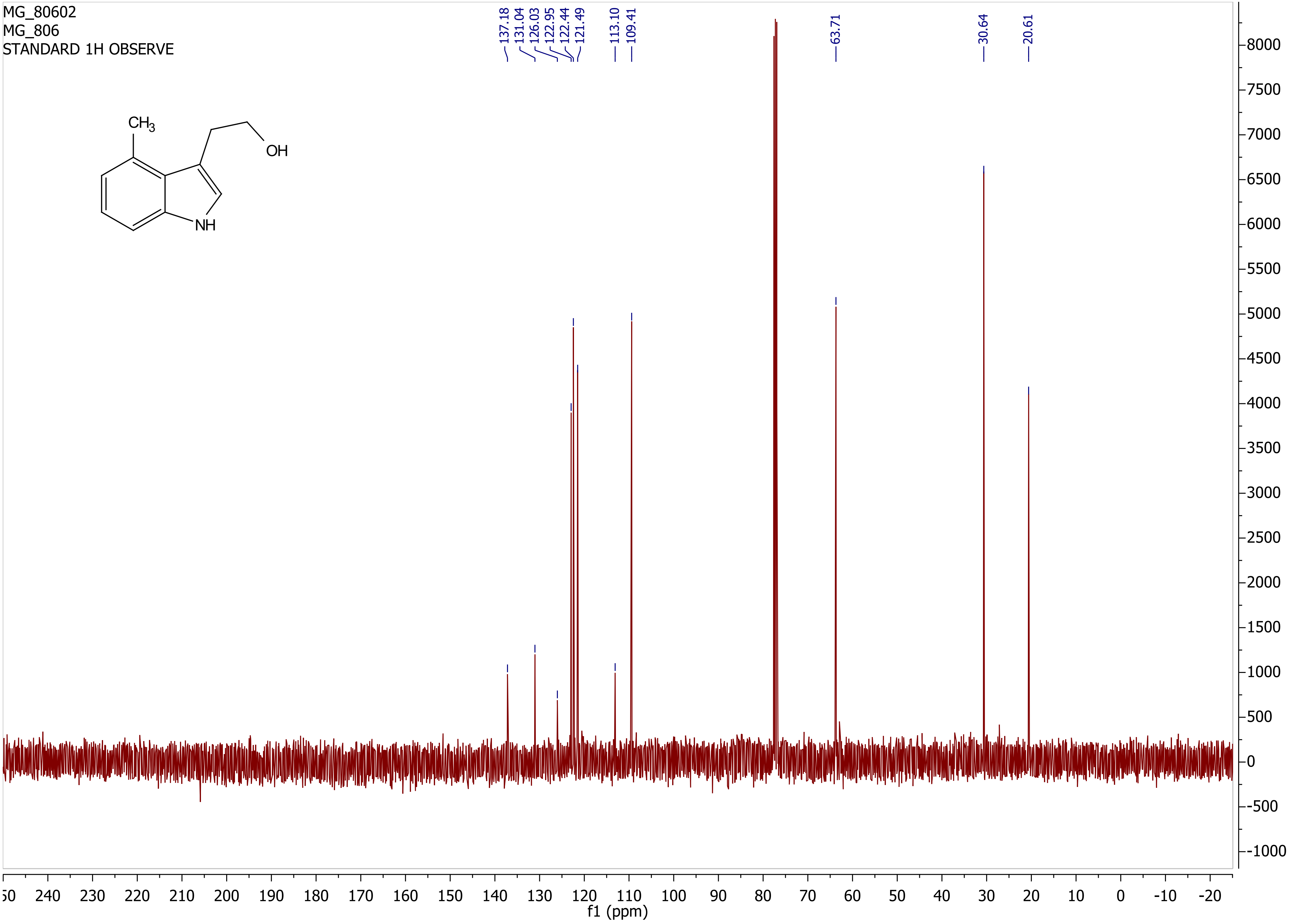

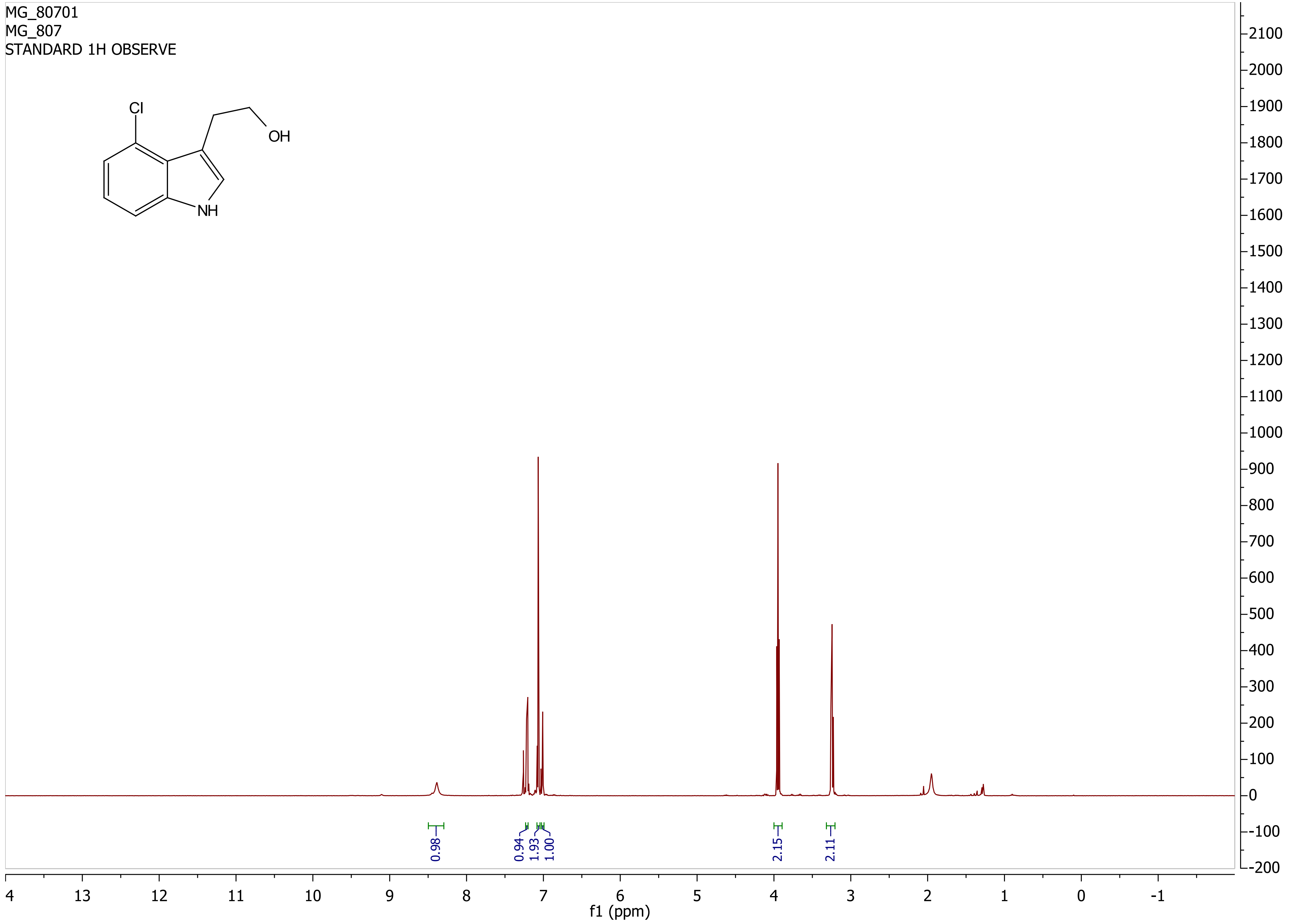
MG_80701
MG_807
STANDARD 1H OBSERVE
Cl
OH
NH
0.98
0.94
1.93
1.00
2.15
2.11
f1 (ppm)

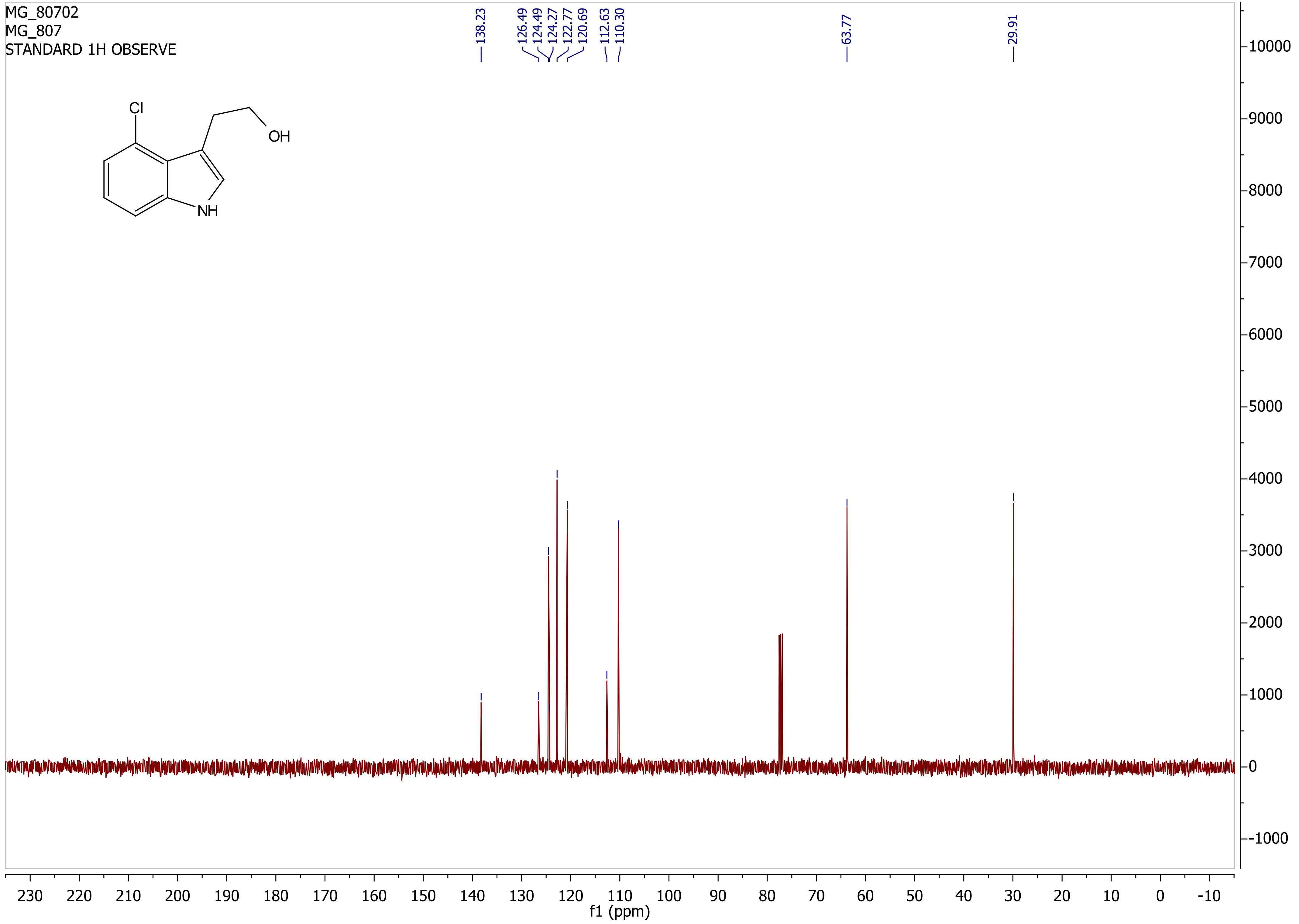
MG_80702
MG_807
STANDARD 1H OBSERVE
138.23
126.49
124.49
124.27
122.77
120.69
112.63
110.30
63.77
29.91
Cl
OH
NH
10000
9000
8000
7000
6000
5000
4000
3000
2000
1000
0
-1000
230 220 210 200 190 180 170 160 150 140 130 120 110 100 90 80 70 60 50 40 30 20 10 0 -10
f1 (ppm)

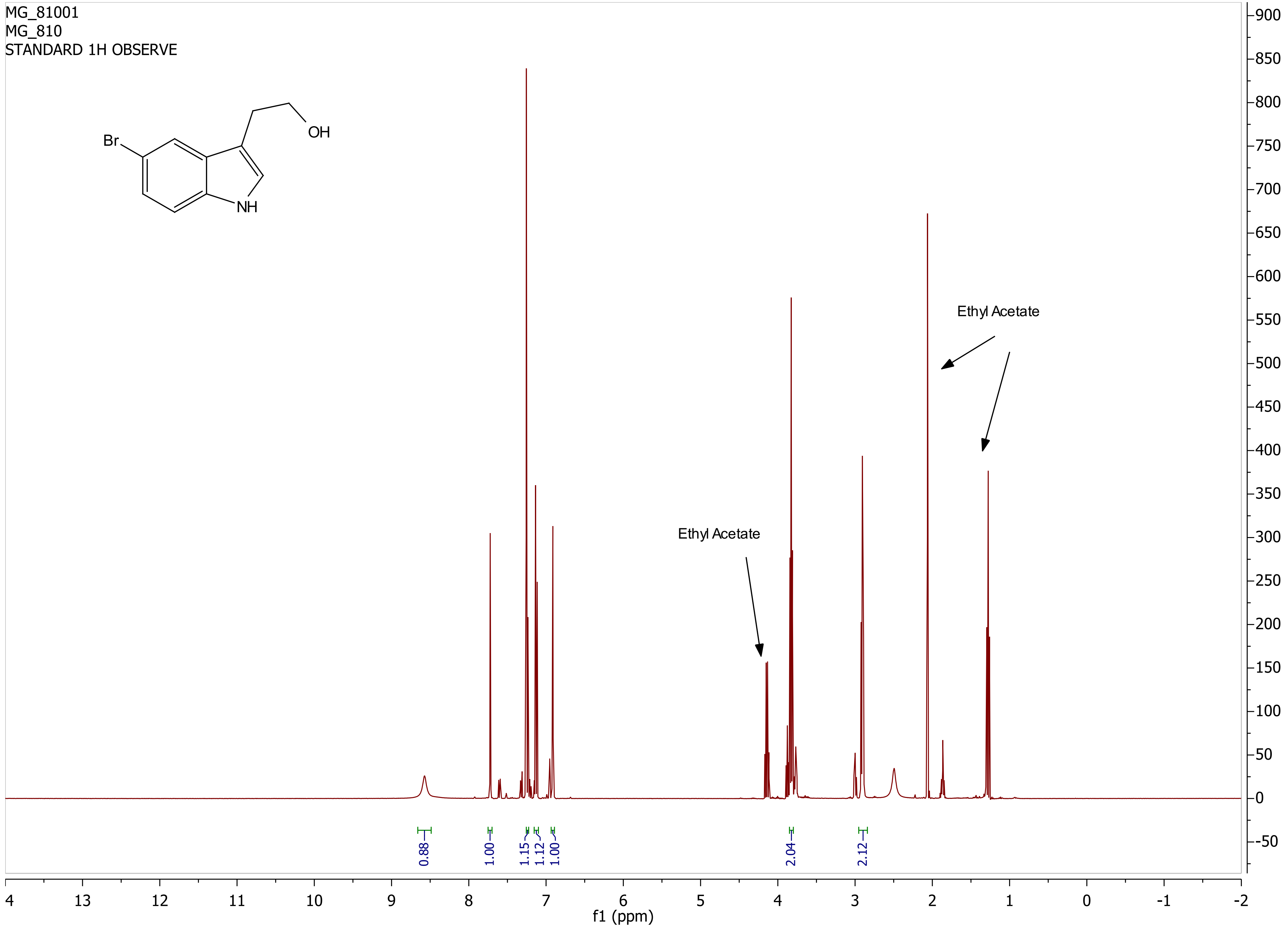

MG_81001
MG_810
STANDARD 1H OBSERVE
Br
OH
NH
Ethyl Acetate
Ethyl Acetate
0.88
1.00
1.15
1.12
1.00
2.04
2.12
f1 (ppm)

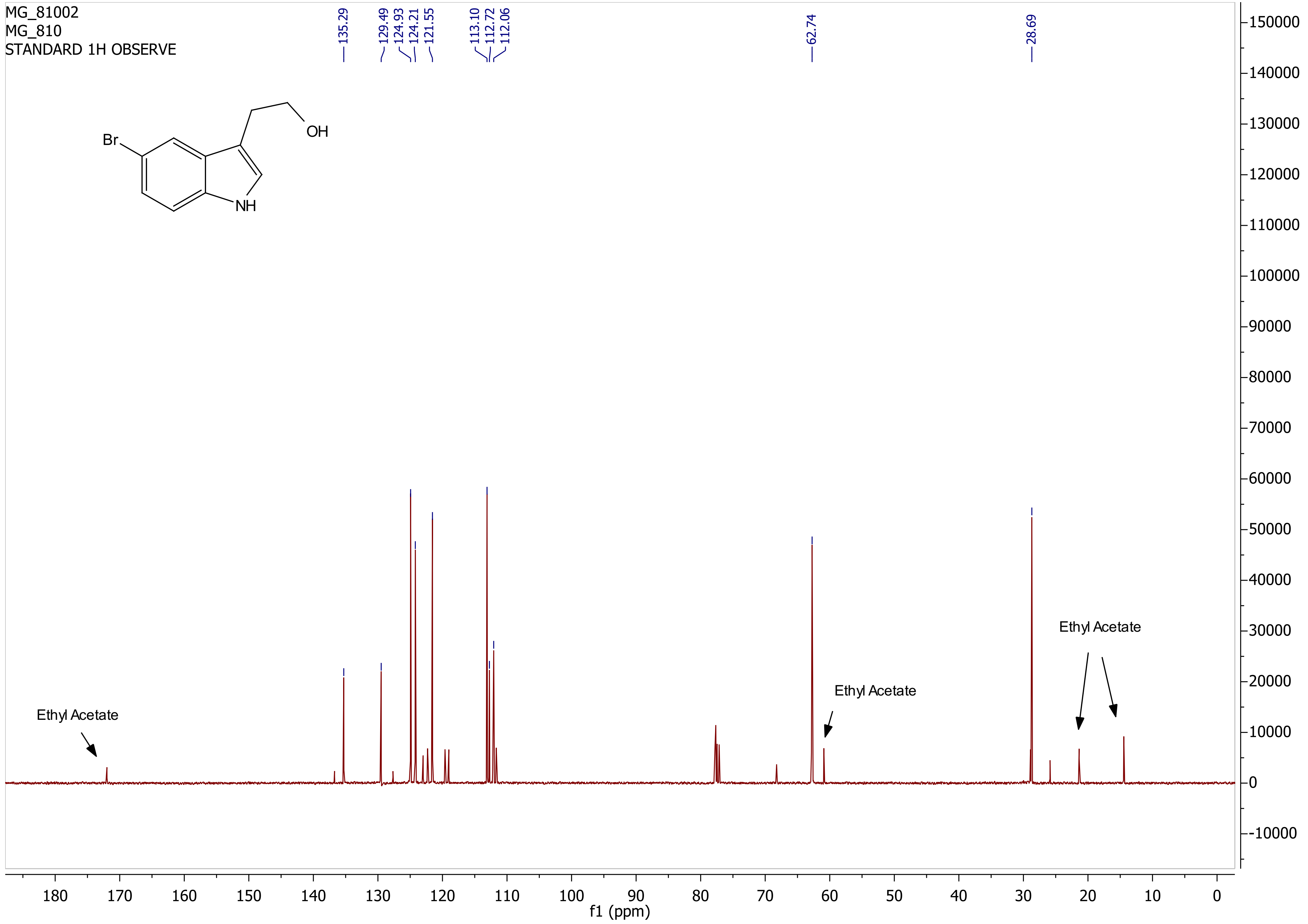

MG_81002
MG_810
STANDARD 1H OBSERVE
135.29
129.49
124.93
124.21
121.55
113.10
112.72
112.06
62.74
28.69
Br
OH
NH
Ethyl Acetate
Ethyl Acetate
Ethyl Acetate
f1 (ppm)

MG-81101
MG-811
STANDARD 1H OBSERVE

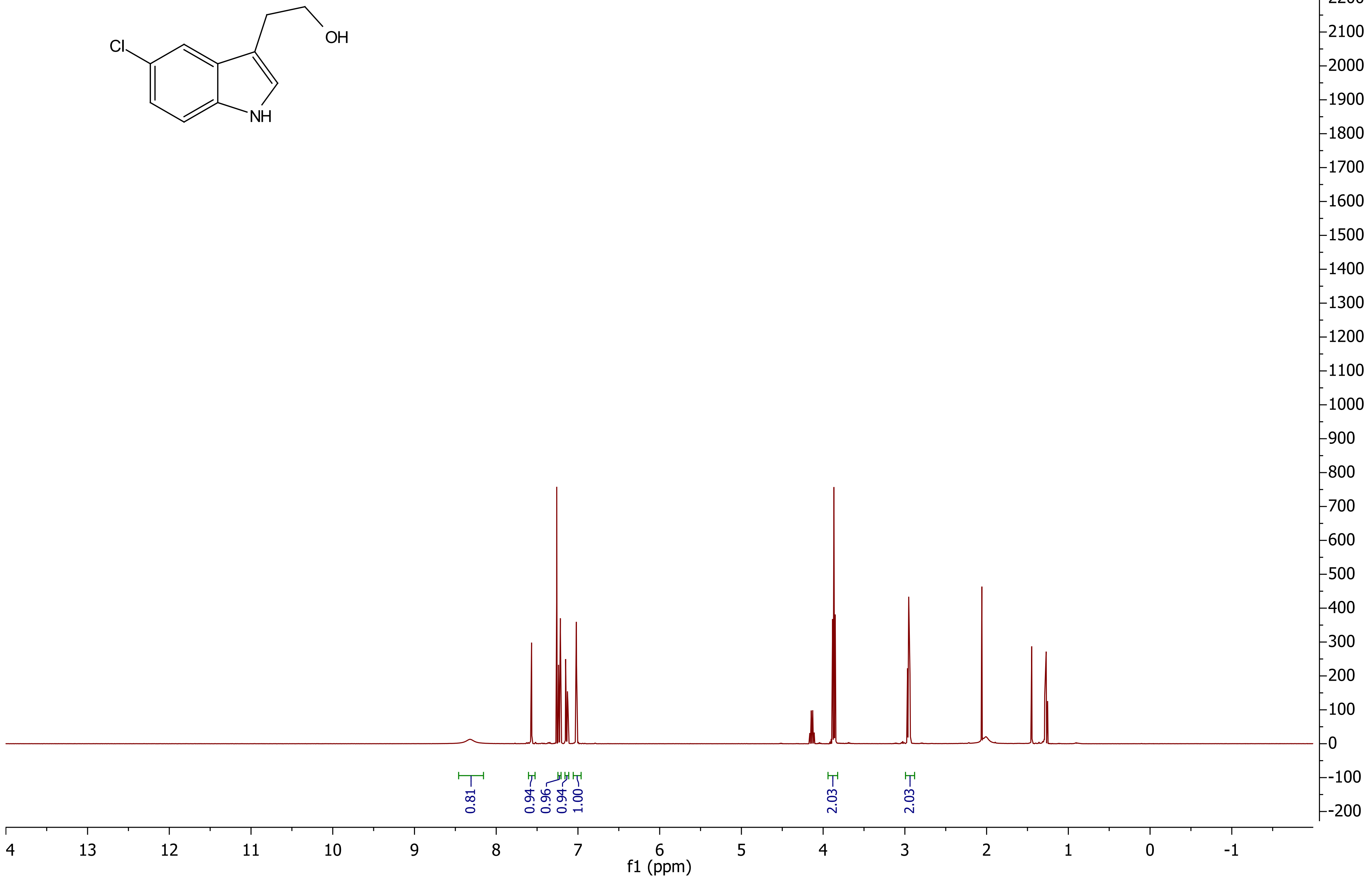

MG-81102
MG-811
STANDARD 1H OBSERVE

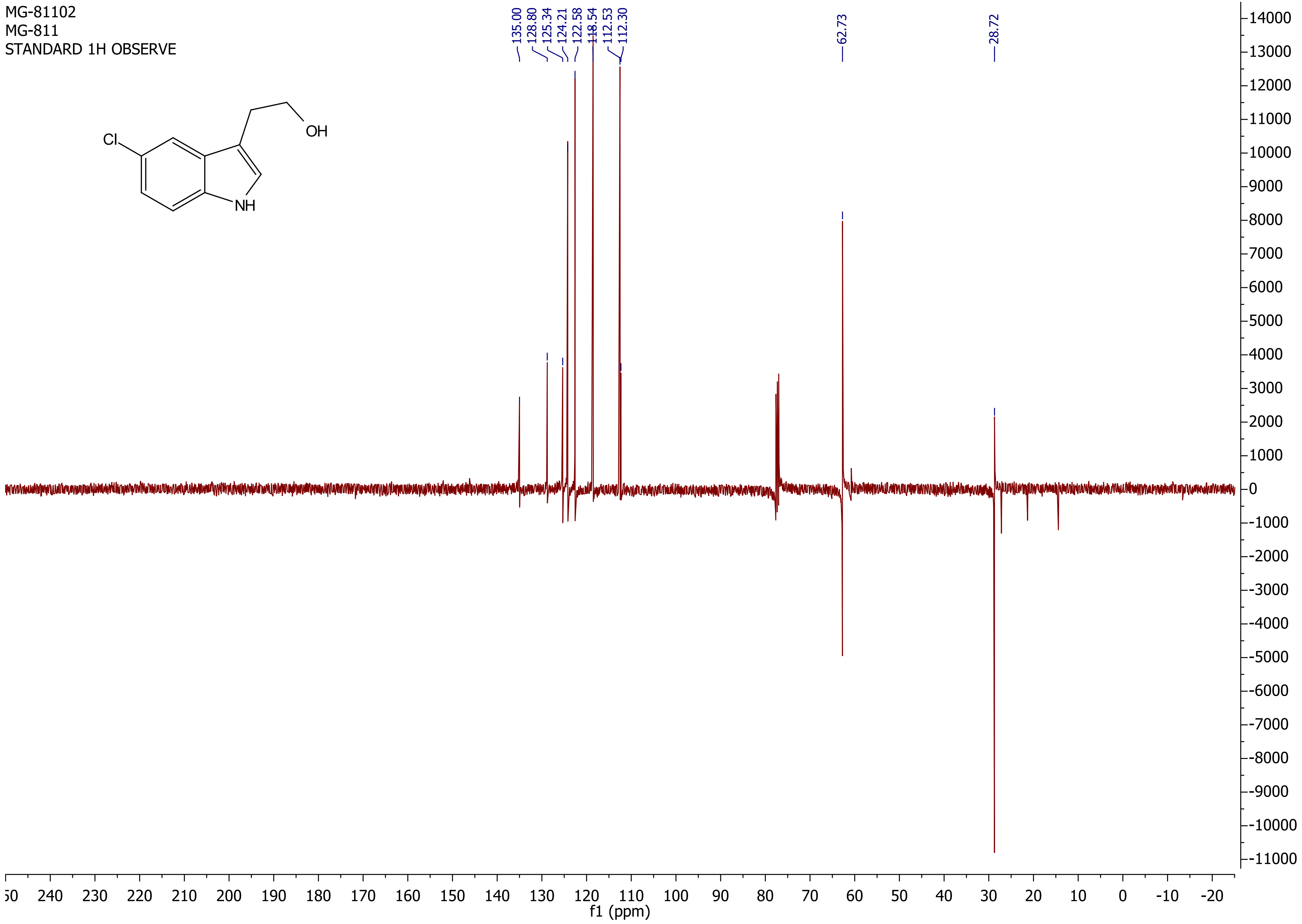

MPI-MG-812_2020-05-18_08-22-08_AV500.10.fid

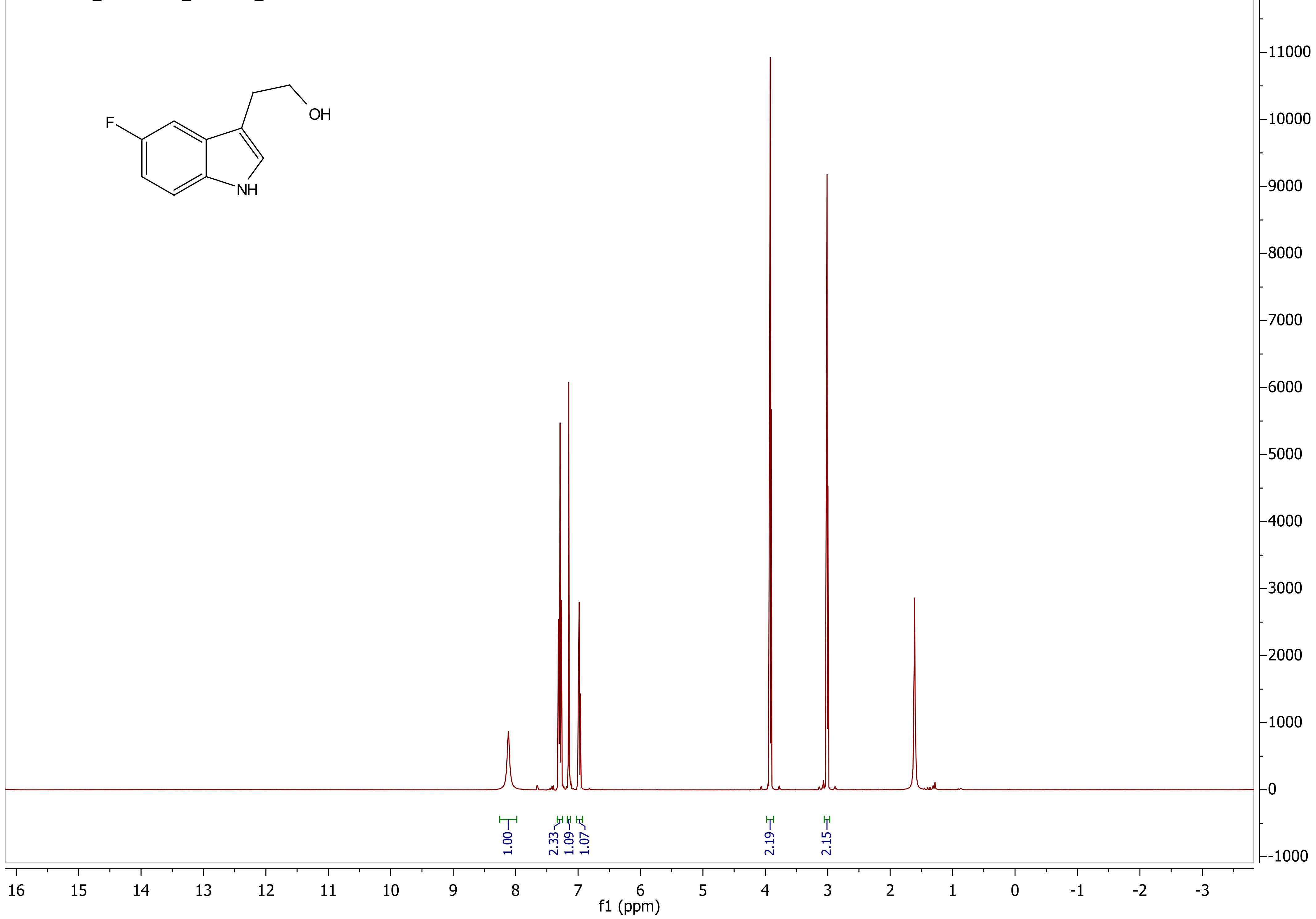

MPI-MG-812_2020-05-18_08-22-08_AV500.11.fid

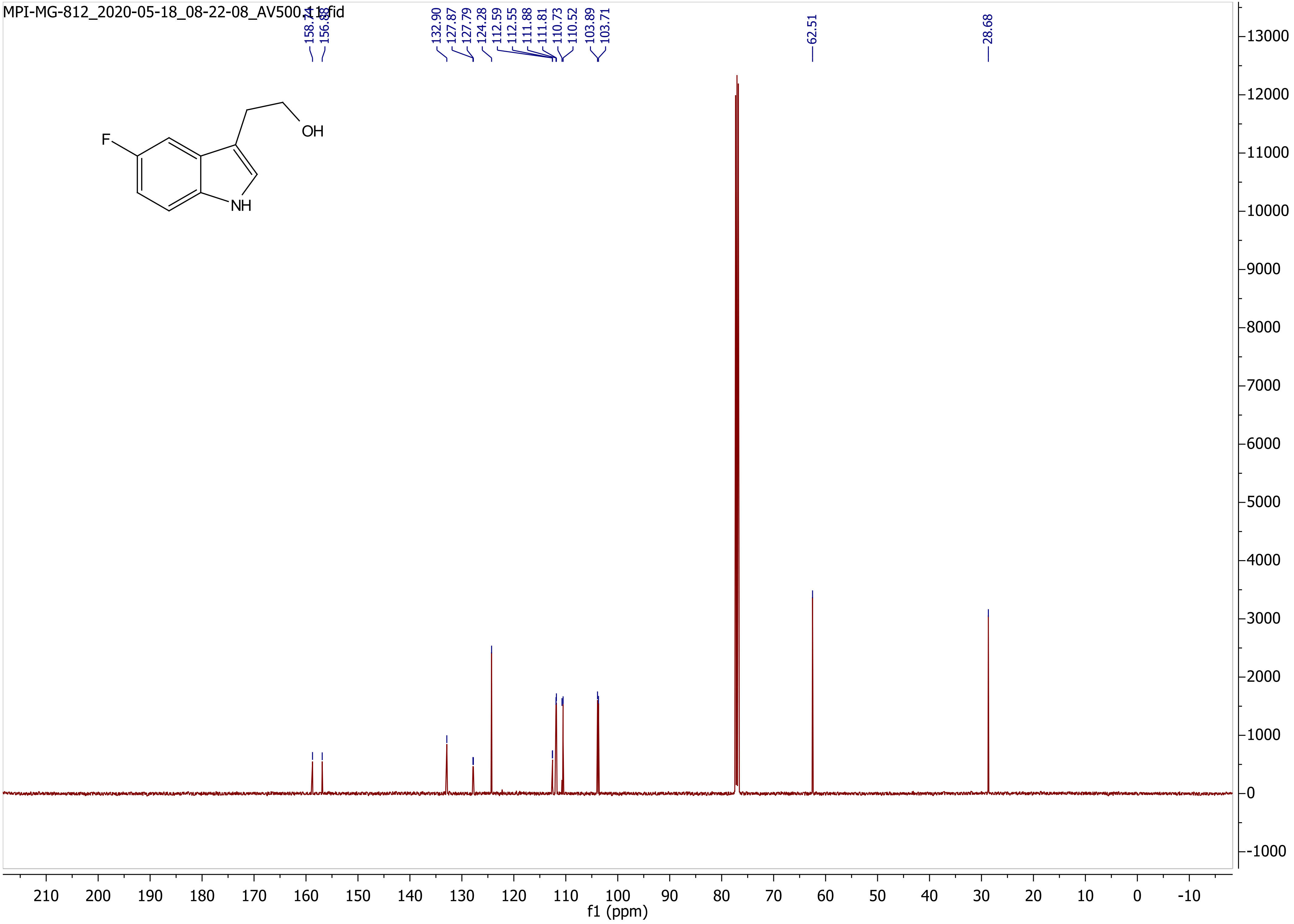

MPI-MG-812_2020-05-18_08-22-08_AV500.12.fid

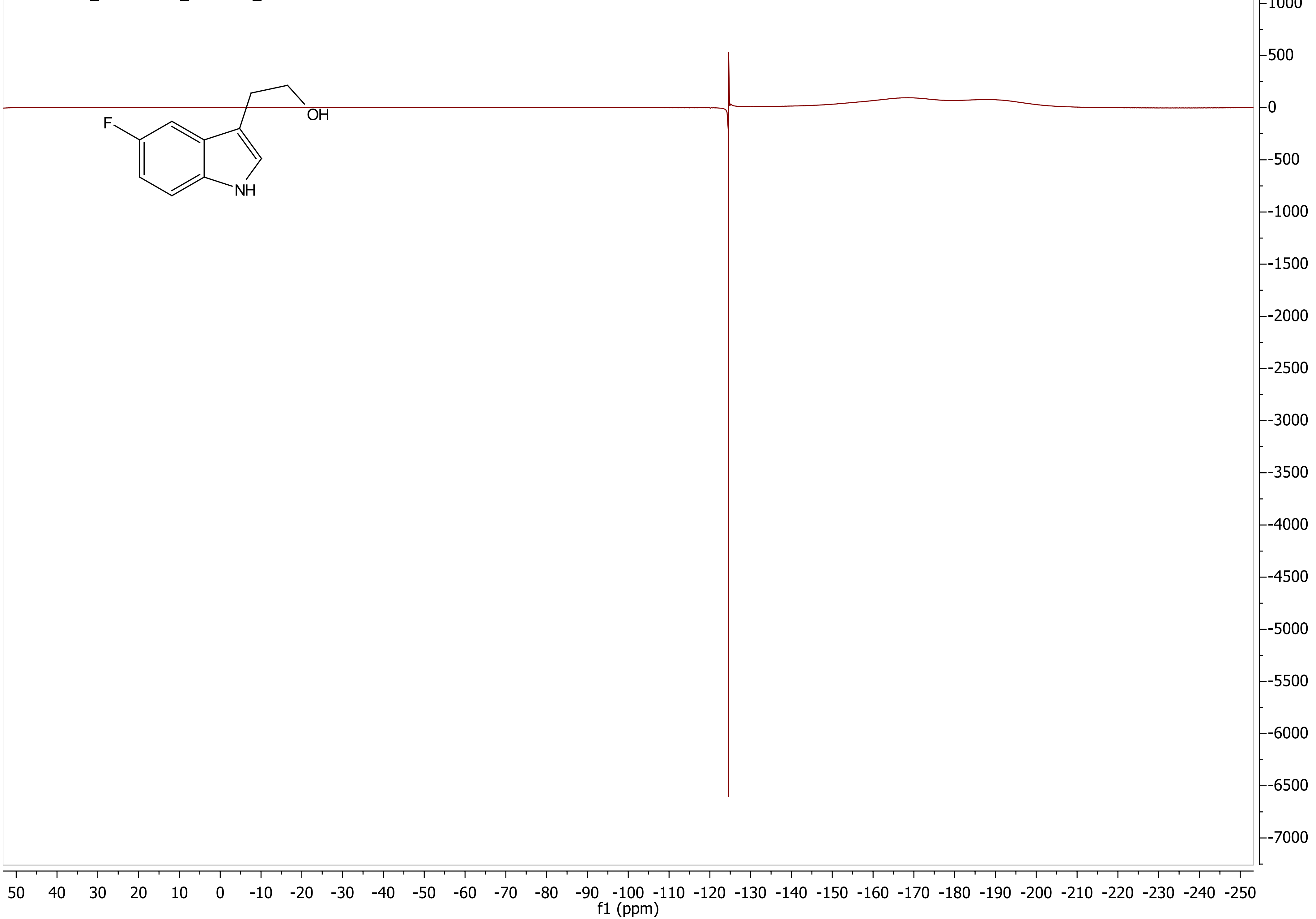

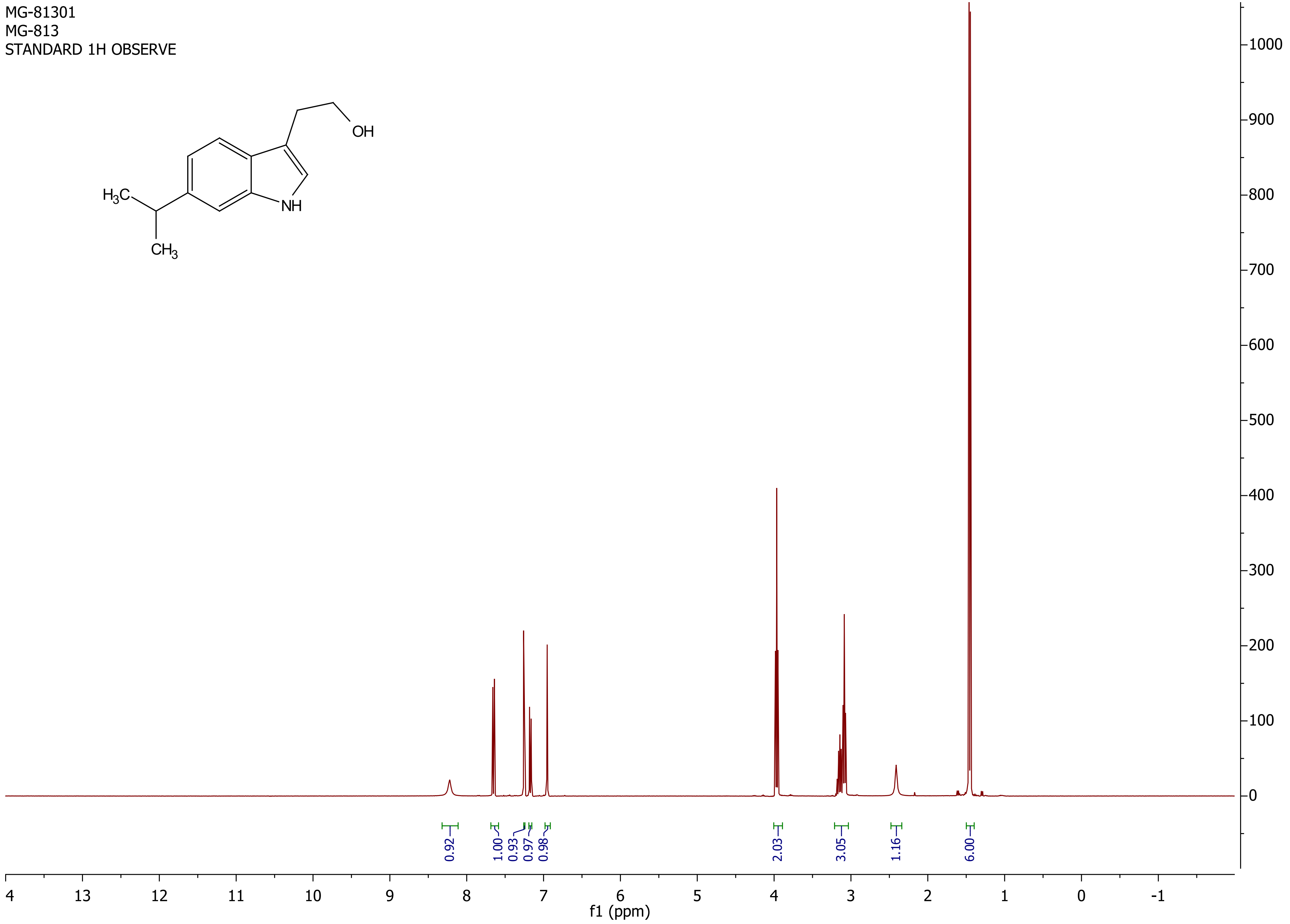
MG-81301
MG-813
STANDARD 1H OBSERVE
OH
NH
H3C
CH3
0.92
1.00
0.93
0.97
0.98
2.03
3.05
1.16
6.00
f1 (ppm)

MG-81302
MG-813
STANDARD 1H OBSERVE

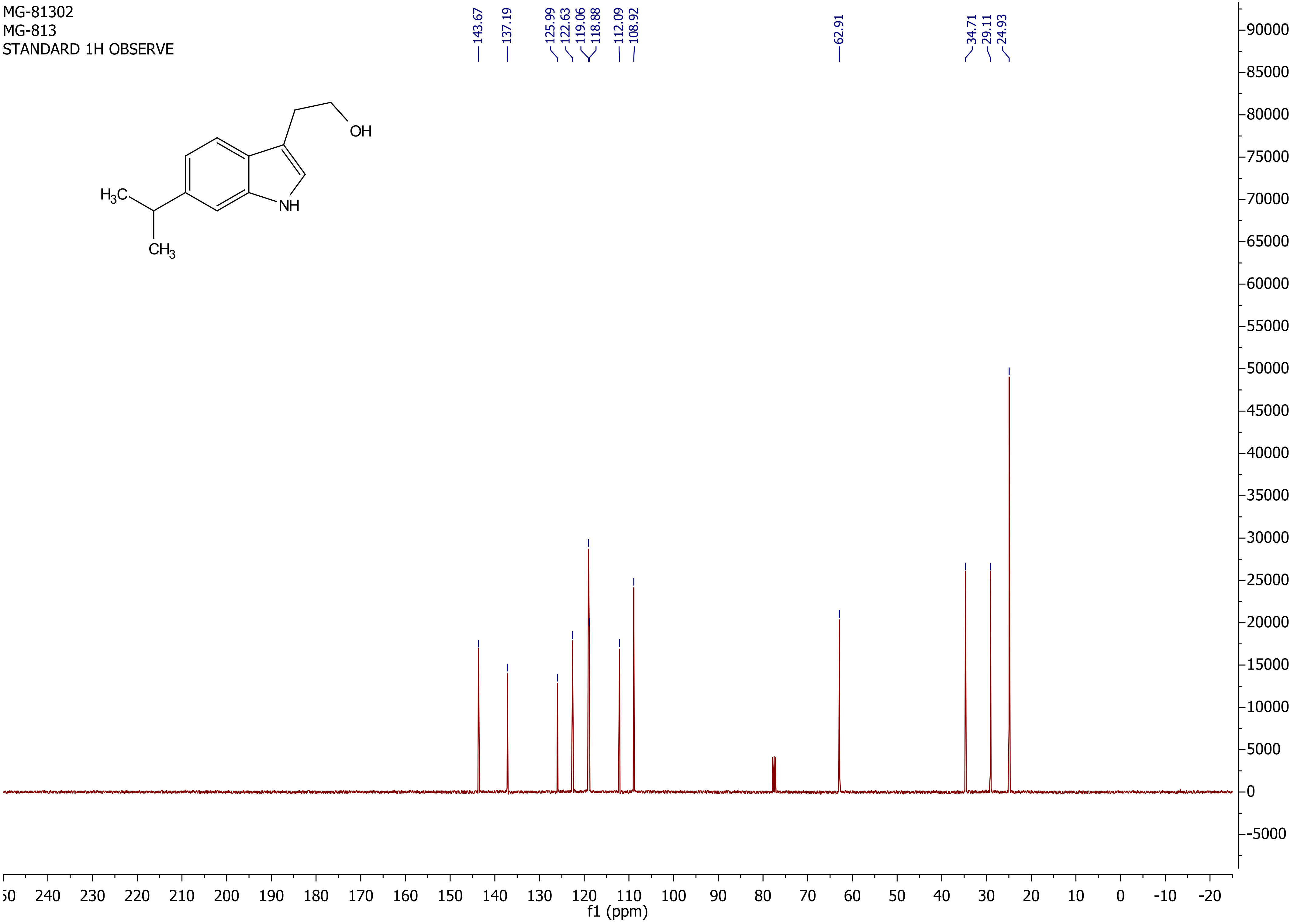

MG-81501
MG-815
STANDARD 1H OBSERVE

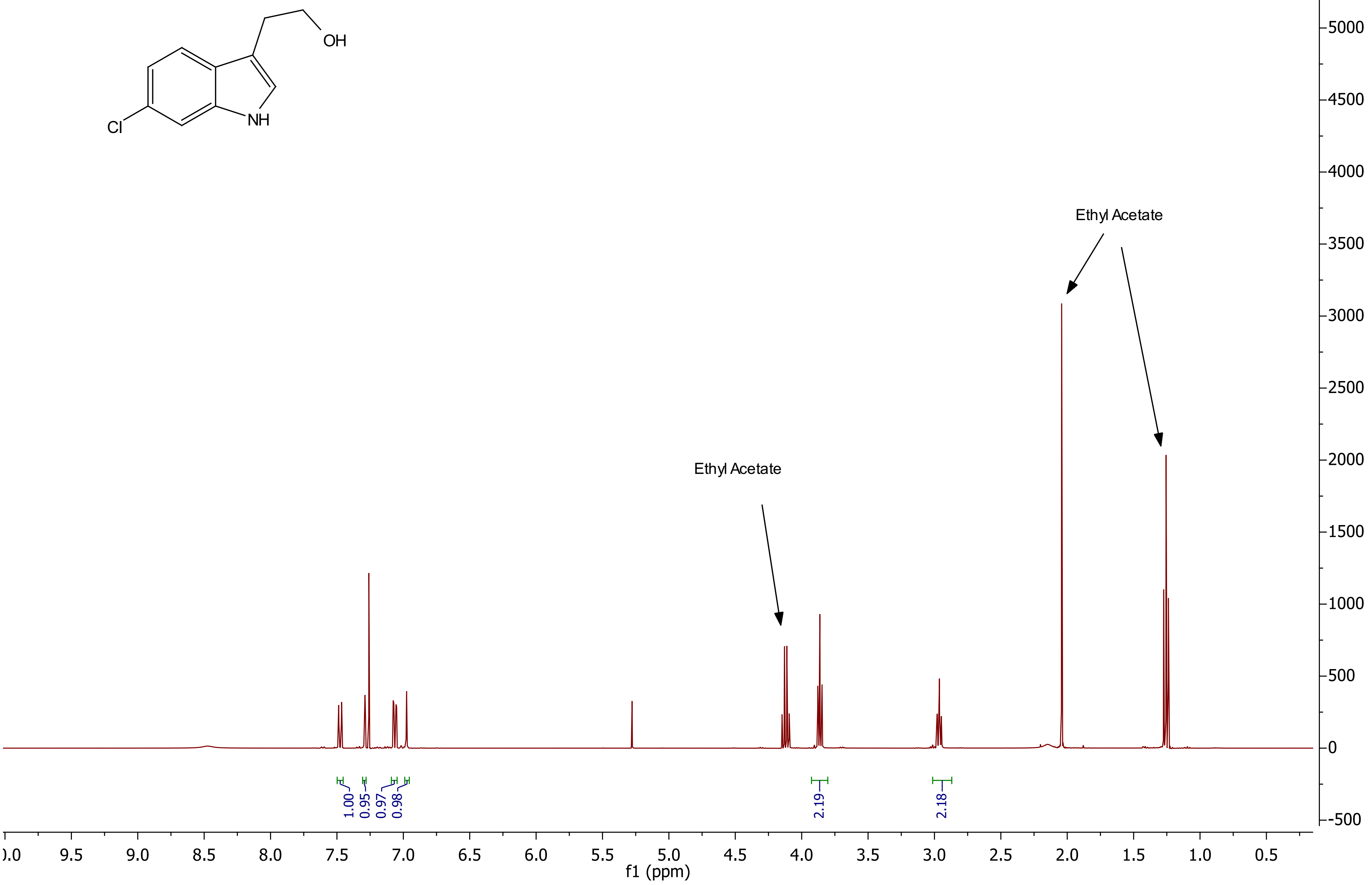

MG-81502
MG-815
STANDARD 1H OBSERVE

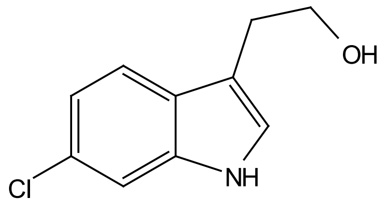

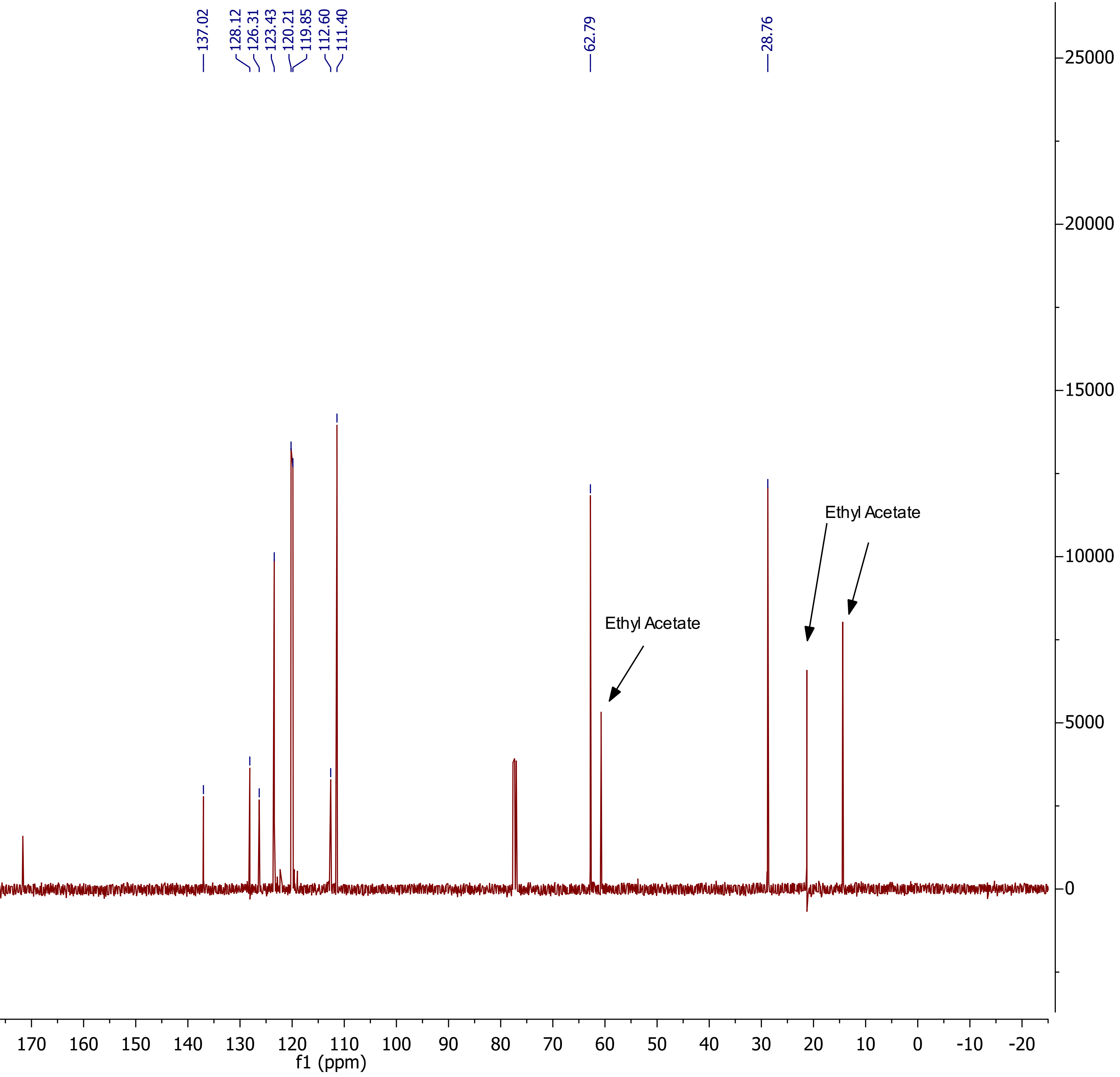

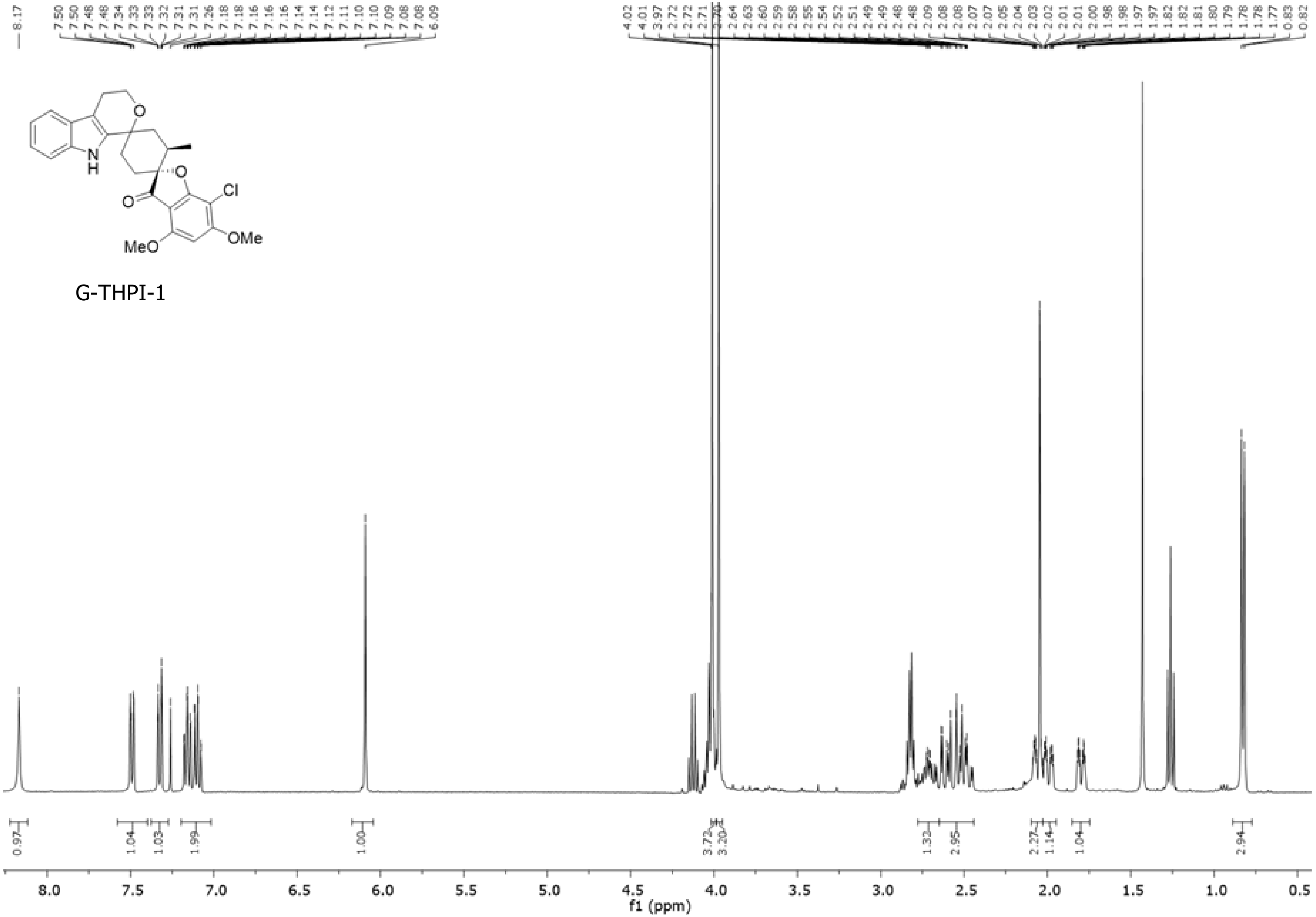
G-THPI-1
MeO
OMe
Cl
N
H
O
f1 (ppm)

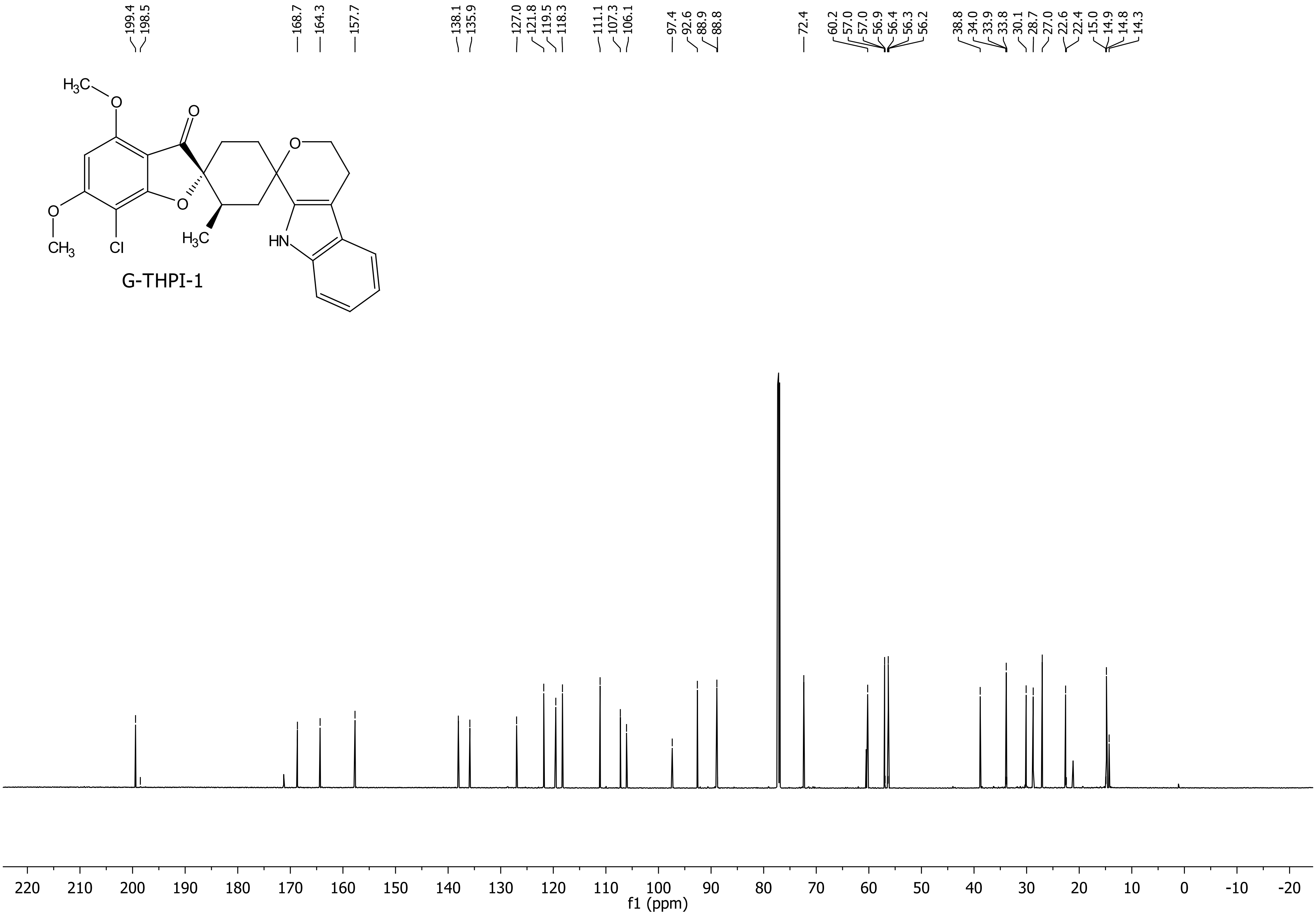
199.4
198.5
168.7
164.3
157.7
138.1
135.9
127.0
121.8
119.5
118.3
111.1
107.3
106.1
97.4
92.6
88.9
88.8
72.4
60.2
57.0
57.0
56.9
56.4
56.3
56.2
38.8
34.0
33.9
33.8
30.1
28.7
27.0
22.6
22.4
15.0
14.9
14.8
14.3
G-THPI-1
220 210 200 190 180 170 160 150 140 130 120 110 100 90 80 70 60 50 40 30 20 10 0 -10 -20
f1 (ppm)

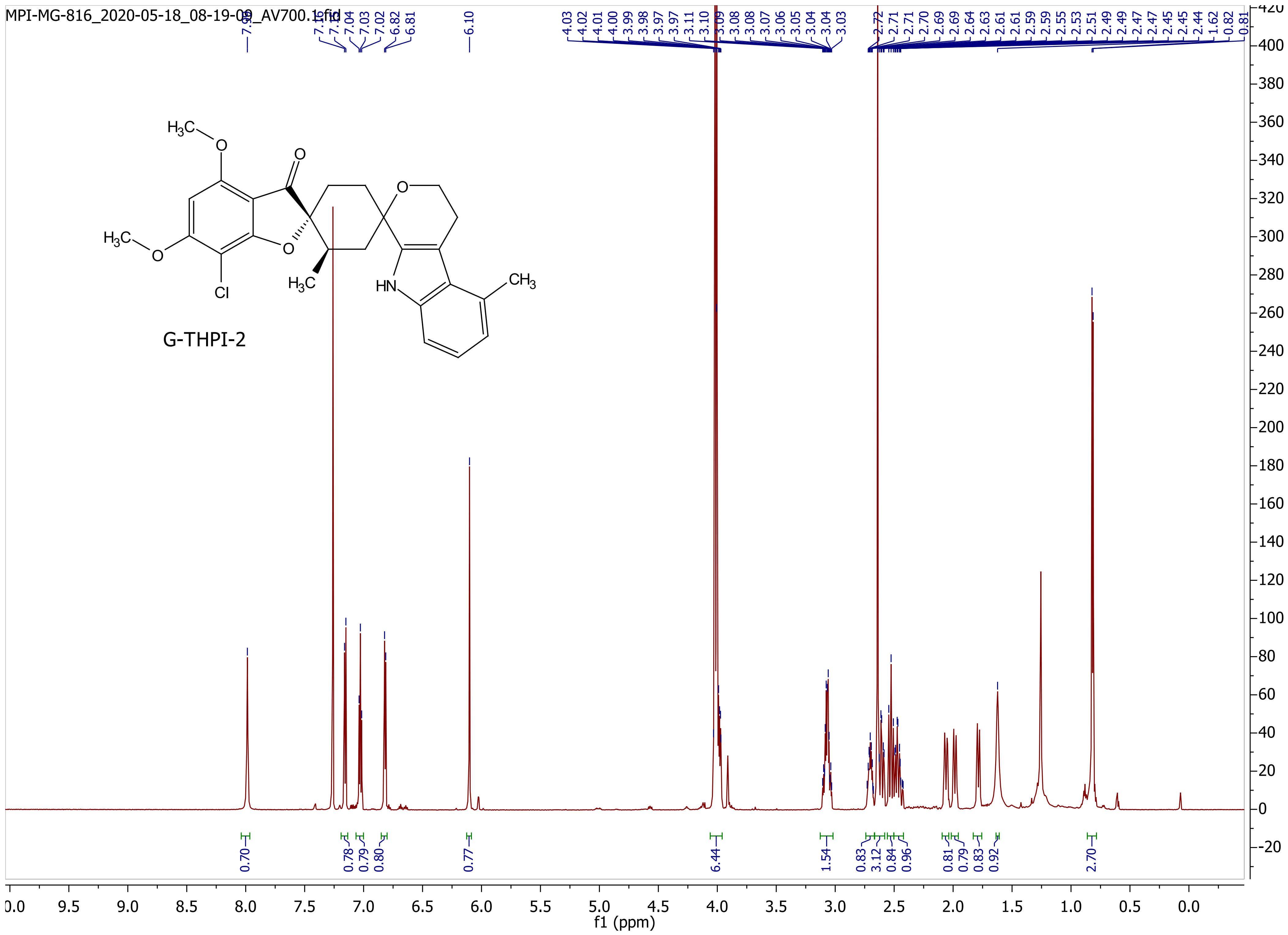

MPI-MG-816_2020-05-18_08-19-09_AV700.1.fid
G-THPI-2
H3C
O
O
H3C
O
O
O
Cl
H3C
O
HN
CH3
f1 (ppm)

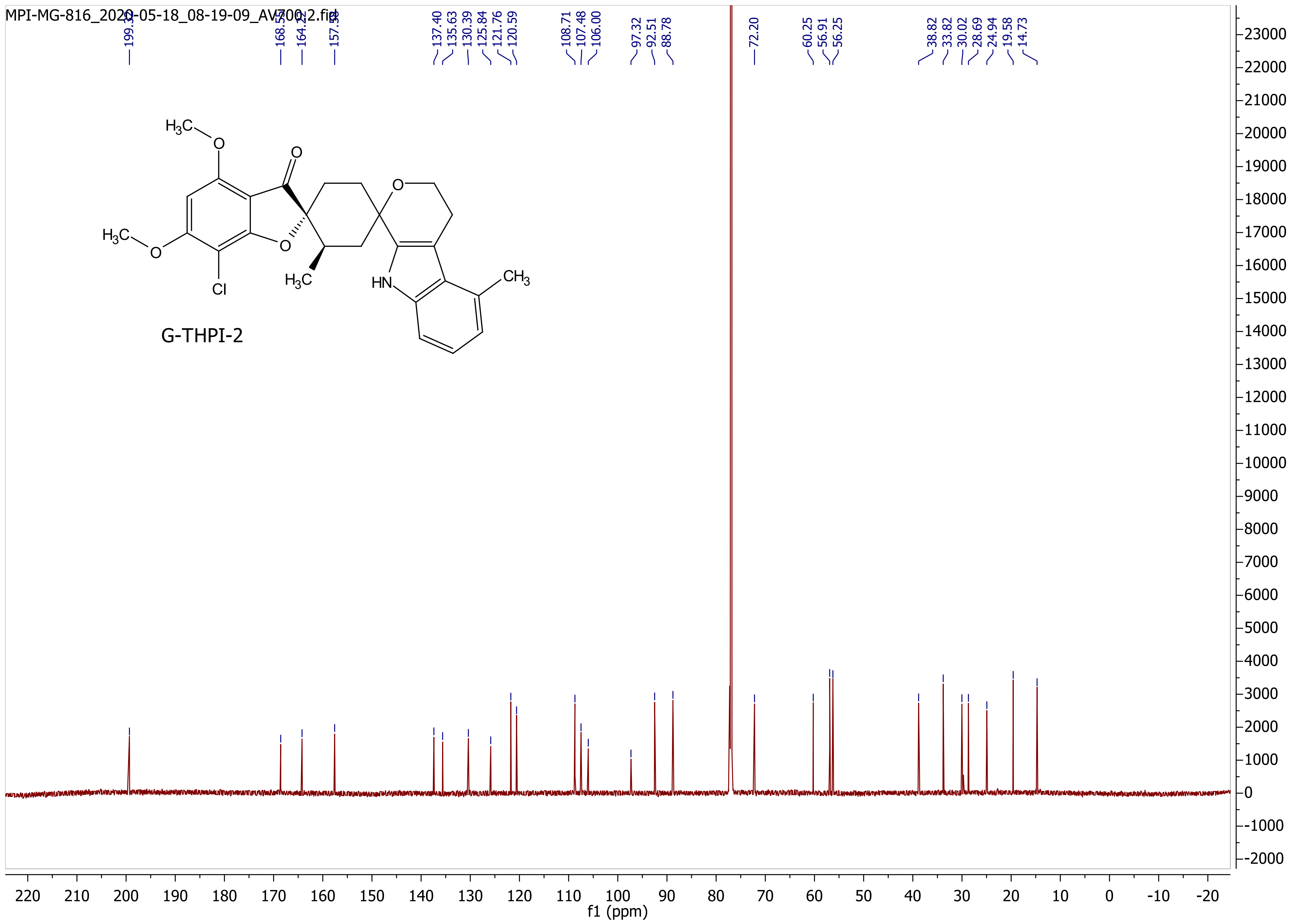
MPI-MG-816_2020-05-18_08-19-09_AV700-2.fid
137.40
135.63
130.39
125.84
121.76
120.59
108.71
107.48
106.00
97.32
92.51
88.78
72.20
60.25
56.91
56.25
38.82
33.82
30.02
28.69
24.94
19.58
14.73
G-THPI-2
f1 (ppm)

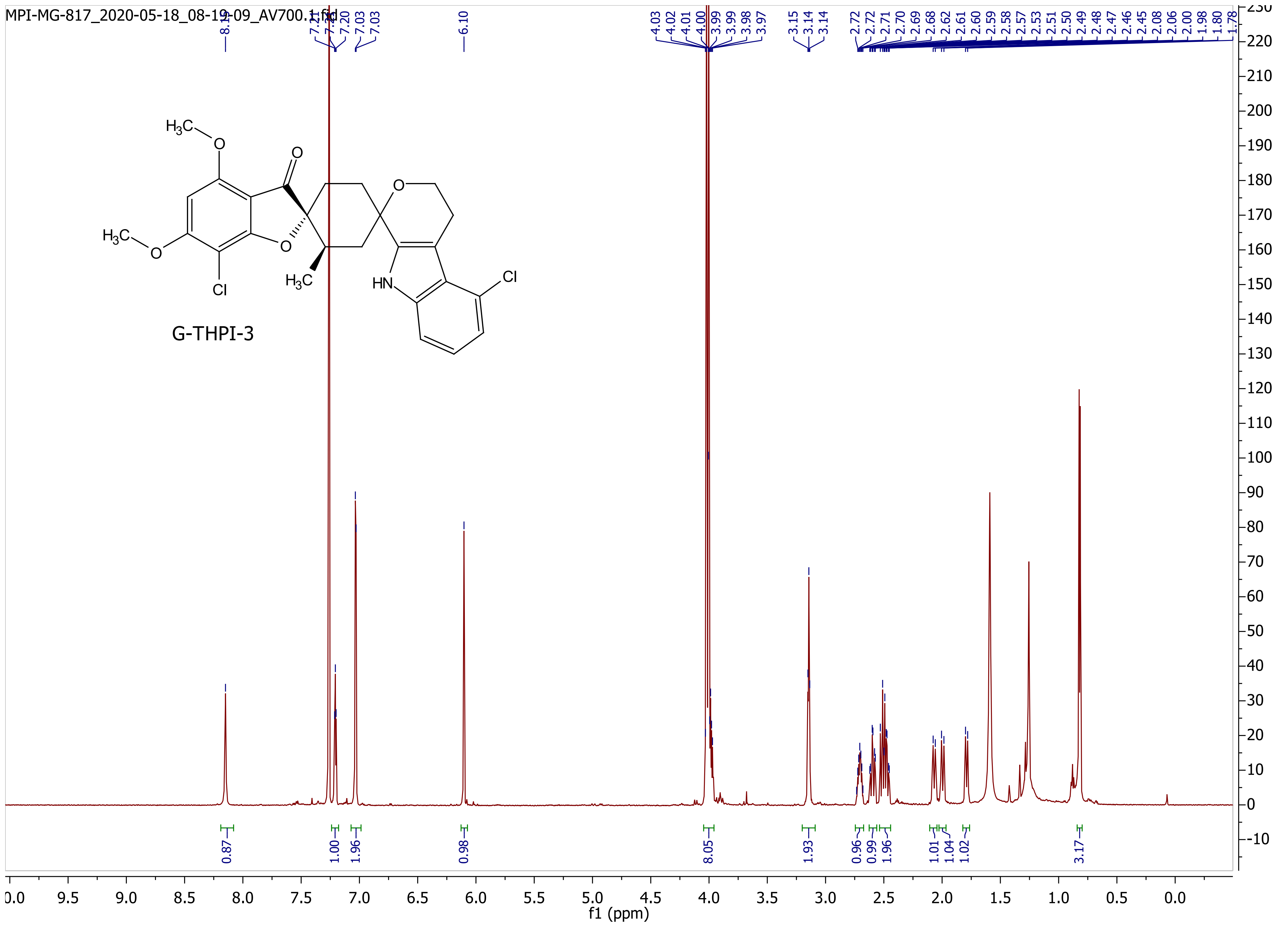
MPI-MG-817_2020-05-18_08-19-09_AV700.1.fid
G-THPI-3
H3C
O
O
H3C
O
O
Cl
H3C
O
HN
Cl
8.14
7.20
7.03
7.03
6.10
4.03
4.02
4.01
4.00
3.99
3.99
3.98
3.97
3.15
3.14
3.14
2.72
2.72
2.71
2.70
2.69
2.68
2.62
2.61
2.60
2.59
2.58
2.57
2.53
2.51
2.50
2.49
2.48
2.47
2.46
2.45
2.08
2.06
2.00
1.98
1.80
1.78
0.87
1.00
1.96
0.98
8.05
1.93
0.96
0.99
1.96
1.01
1.04
1.02
3.17
f1 (ppm)

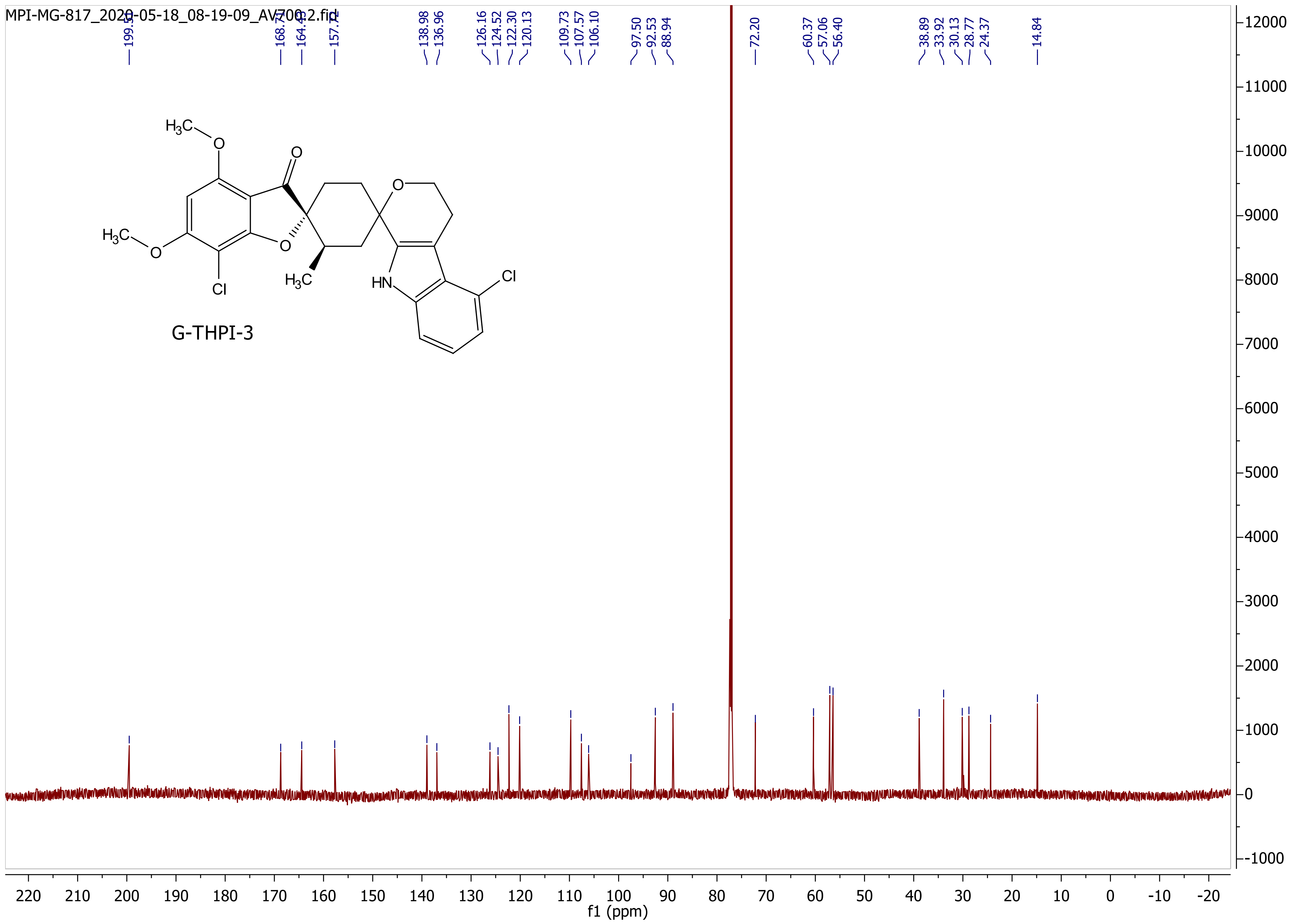

MPI-MG-817_2020-05-18_08-19-09_AV700c2.fid
199.5
168.7
164.4
157.7
138.98
136.96
126.16
124.52
122.30
120.13
109.73
107.57
106.10
97.50
92.53
88.94
72.20
60.37
57.06
56.40
38.89
33.92
30.13
28.77
24.37
14.84
G-THPI-3
f1 (ppm)

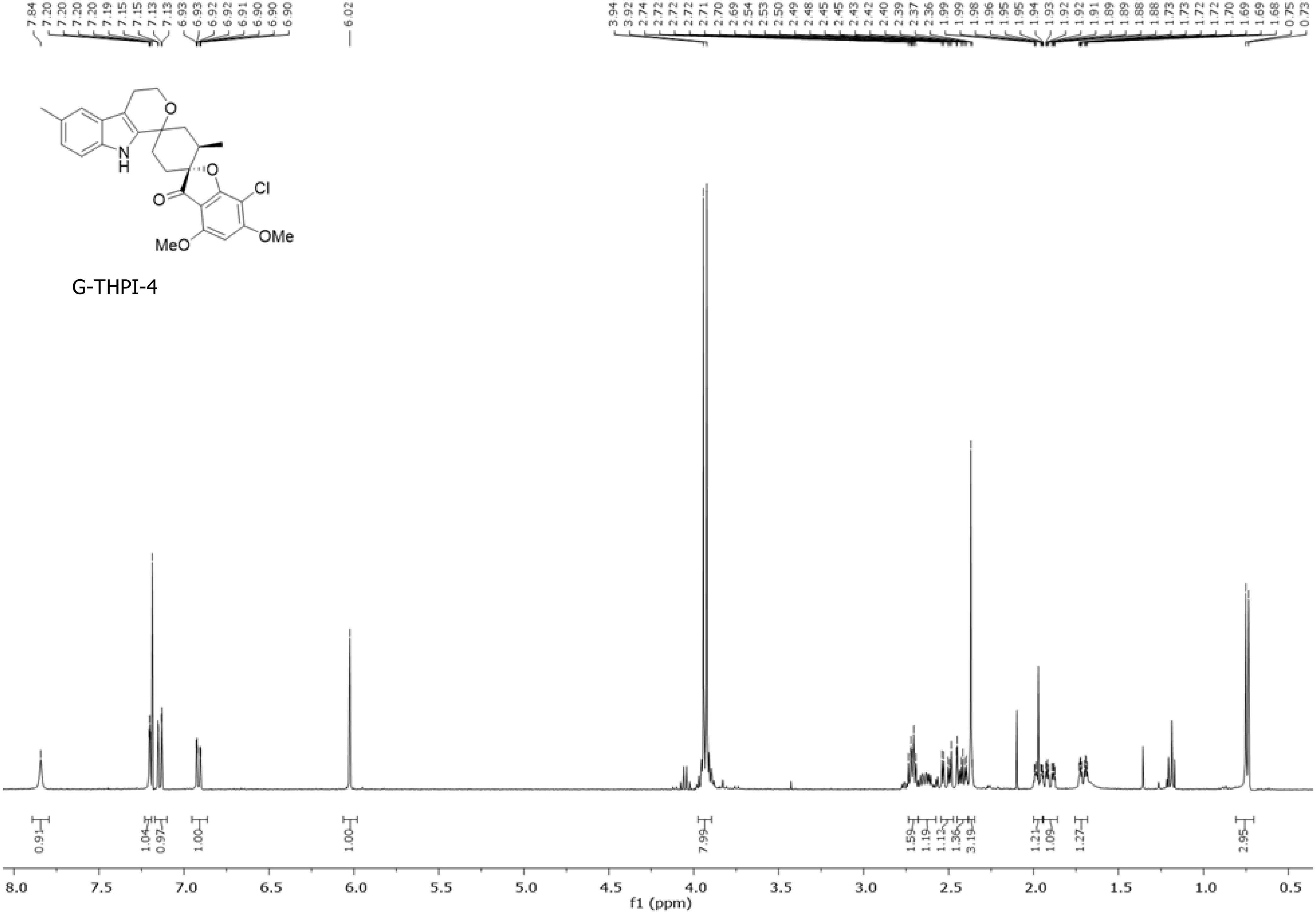

7.84 7.20 7.20 7.20 7.20 7.19 7.15 7.15 7.13 7.13 6.93 6.93 6.92 6.92 6.91 6.90 6.90 6.90
6.02
3.94 3.92 2.74 2.72 2.72 2.72 2.71 2.70 2.69 2.54 2.53 2.50 2.49 2.48 2.45 2.45 2.43 2.42 2.40 2.39 2.37 2.36 1.99 1.99 1.98 1.96 1.95 1.95 1.94 1.93 1.92 1.92 1.91 1.89 1.89 1.88 1.88 1.73 1.73 1.72 1.72 1.70 1.69 1.69 1.68 0.75 0.73
O
NH
O
Cl
O
MeO
OMe
0.91 1.04 0.97 1.00 1.00 7.99 1.59 1.19 1.12 1.36 3.19 1.21 1.09 1.27 2.95
8.0 7.5 7.0 6.5 6.0 5.5 5.0 4.5 4.0 3.5 3.0 2.5 2.0 1.5 1.0 0.5
f1 (ppm)

G-THPI-4

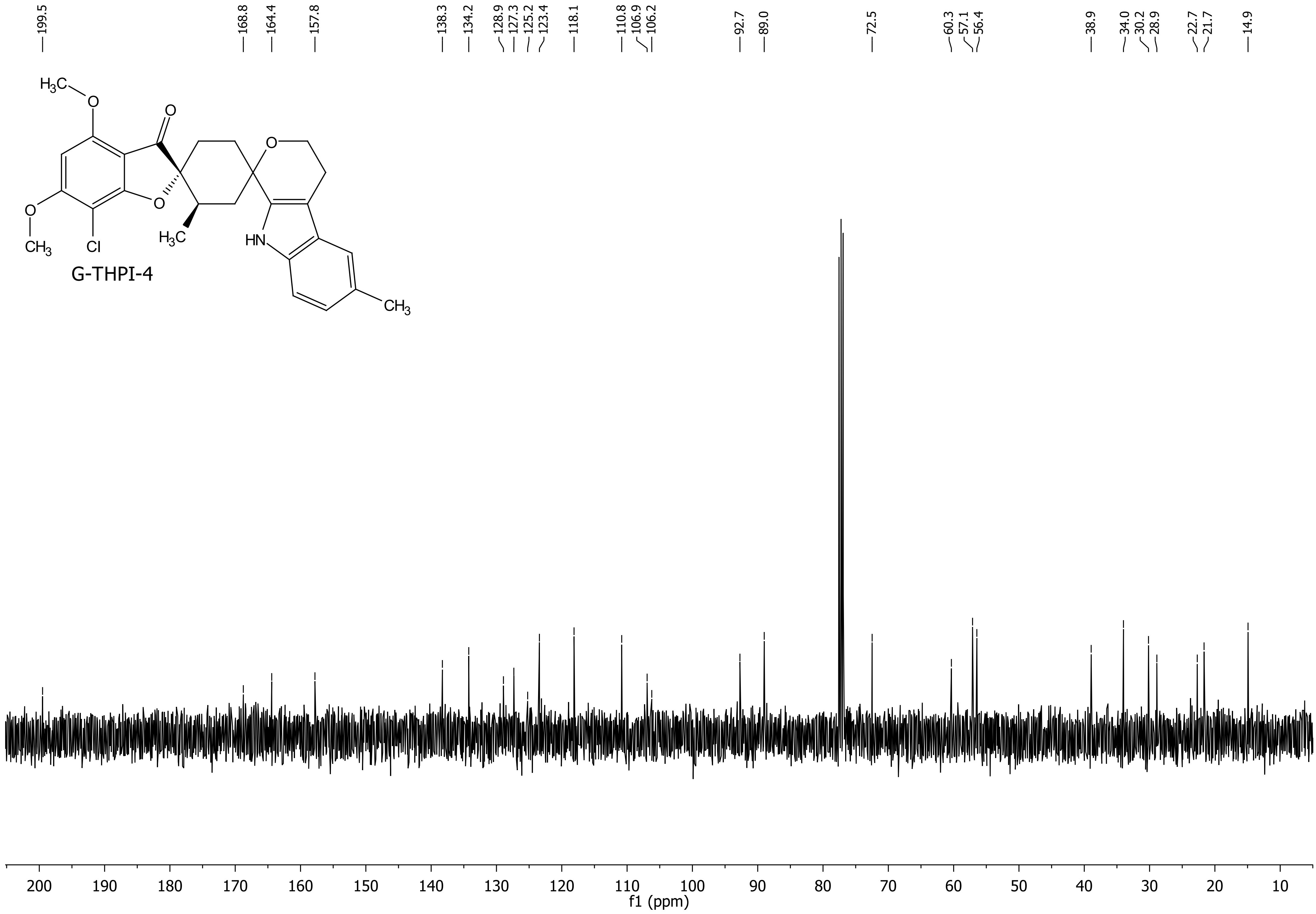

199.5
168.8
164.4
157.8
138.3
134.2
128.9
127.3
125.2
123.4
118.1
110.8
106.9
106.2
92.7
89.0
72.5
60.3
57.1
56.4
38.9
34.0
30.2
28.9
22.7
21.7
14.9
G-THPI-4
f1 (ppm)

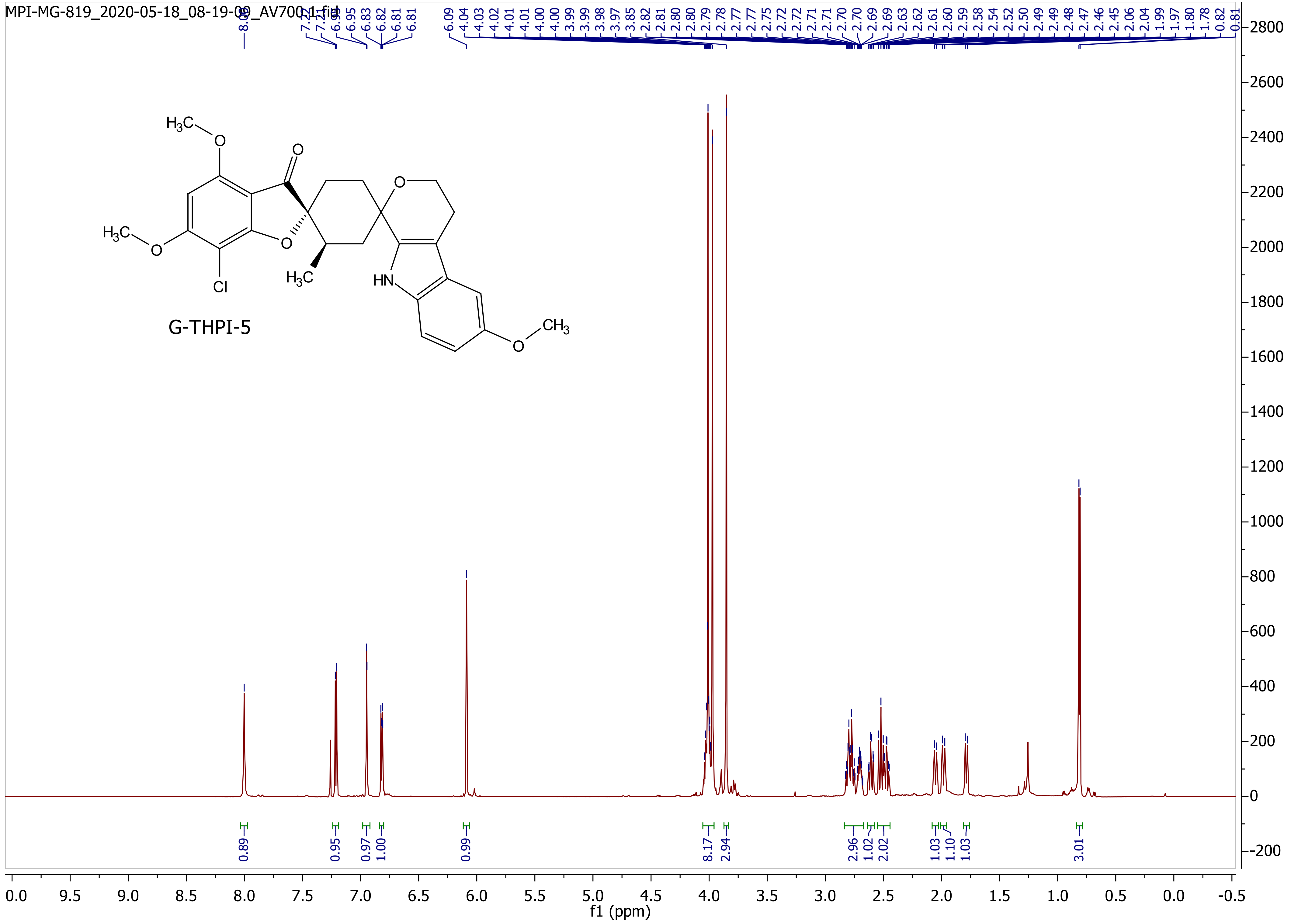

MPI-MG-819_2020-05-18_08-19-09_AV700 1.fid
G-THPI-5
f1 (ppm)

MPI-MG-819_2020-05-18_08-19-09_AV700 2.fid

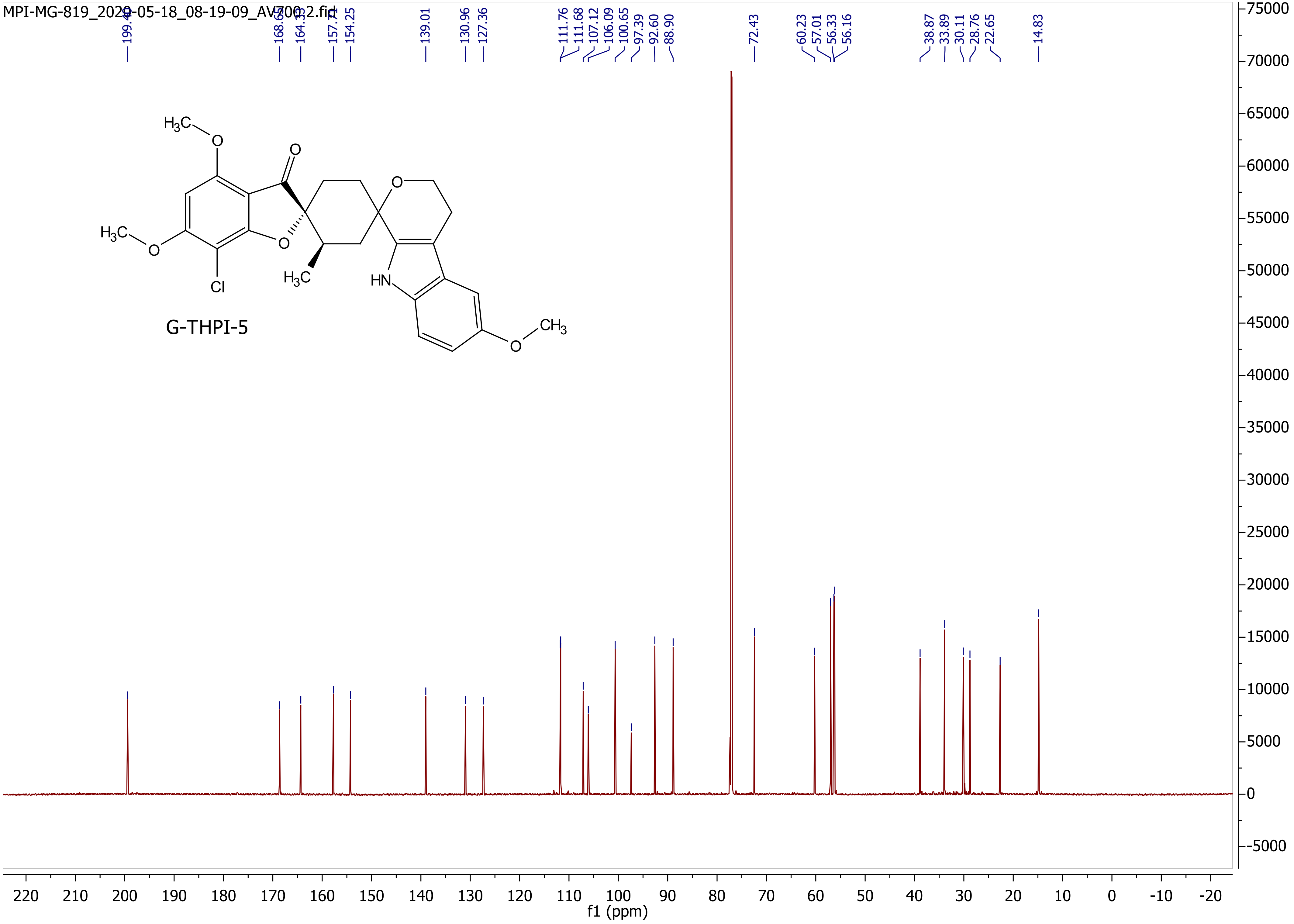

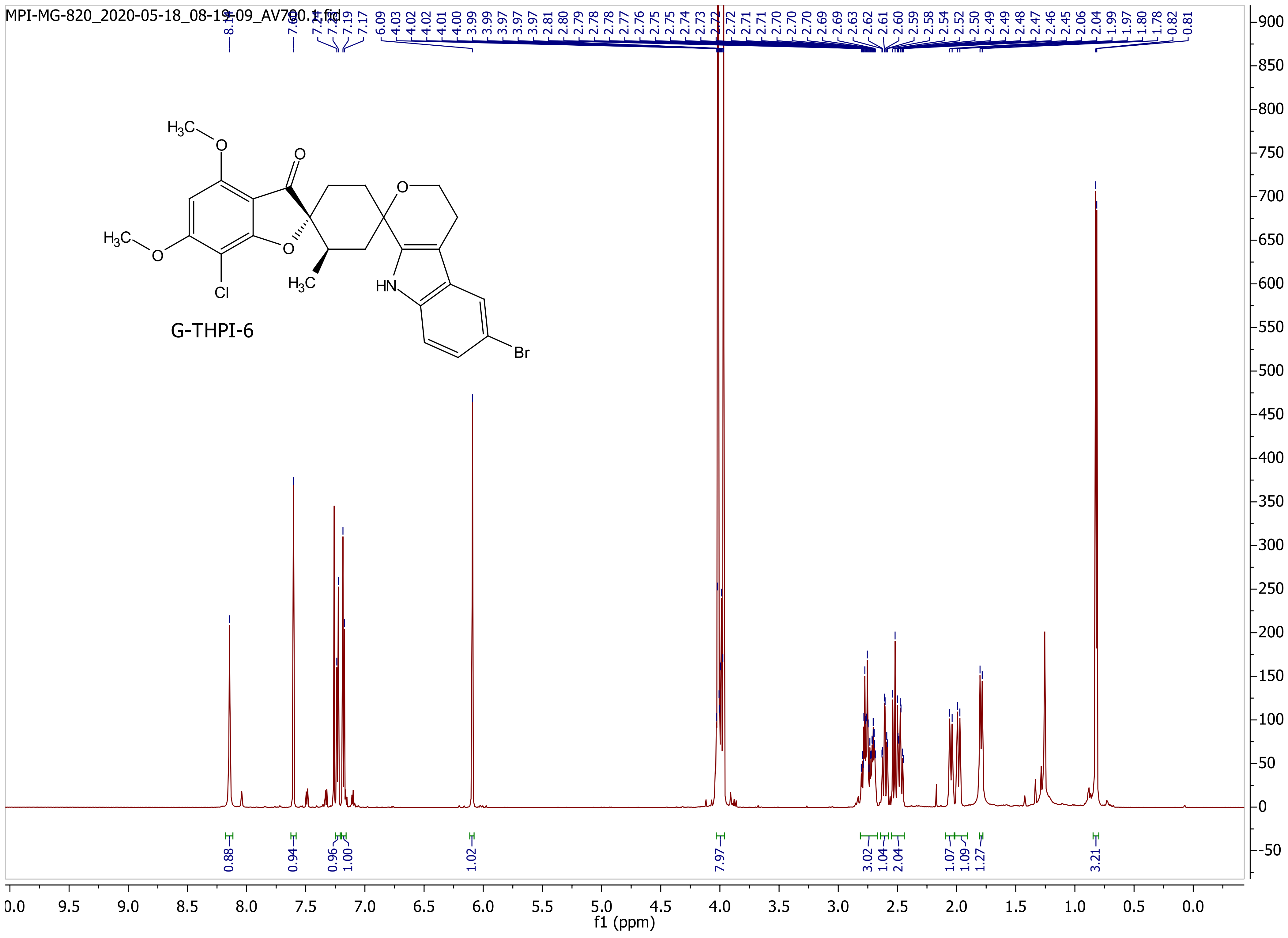

MPI-MG-820_2020-05-18_08-19-09_AV700.1.fid
G-THPI-6
f1 (ppm)

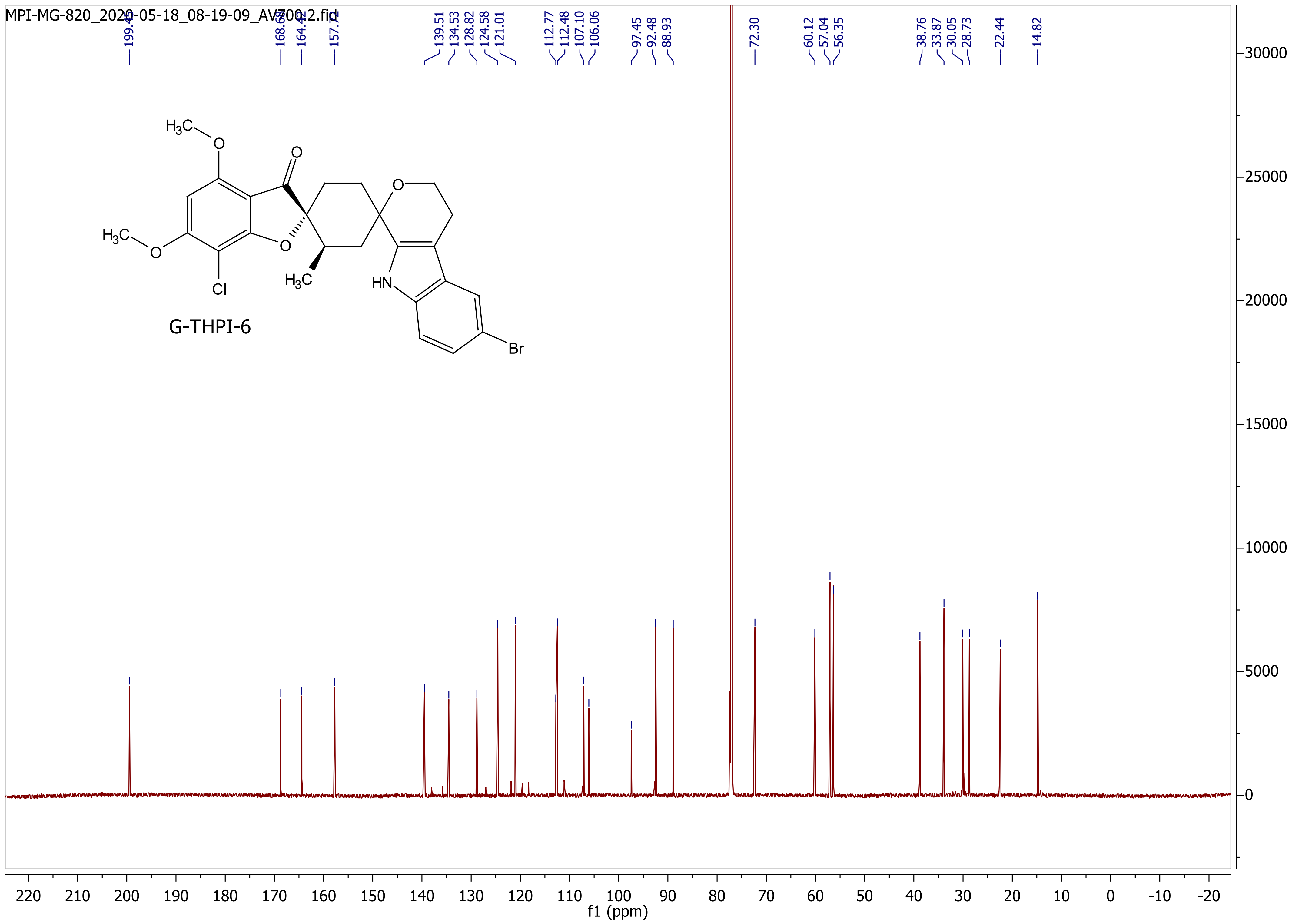

MPI-MG-820_2020-05-18_08-19-09_AV700-2.fid
199.4
168.6
164.4
157.7
139.51
134.53
128.82
124.58
121.01
112.77
112.48
107.10
106.06
97.45
92.48
88.93
72.30
60.12
57.04
56.35
38.76
33.87
30.05
28.73
22.44
14.82
G-THPI-6
f1 (ppm)

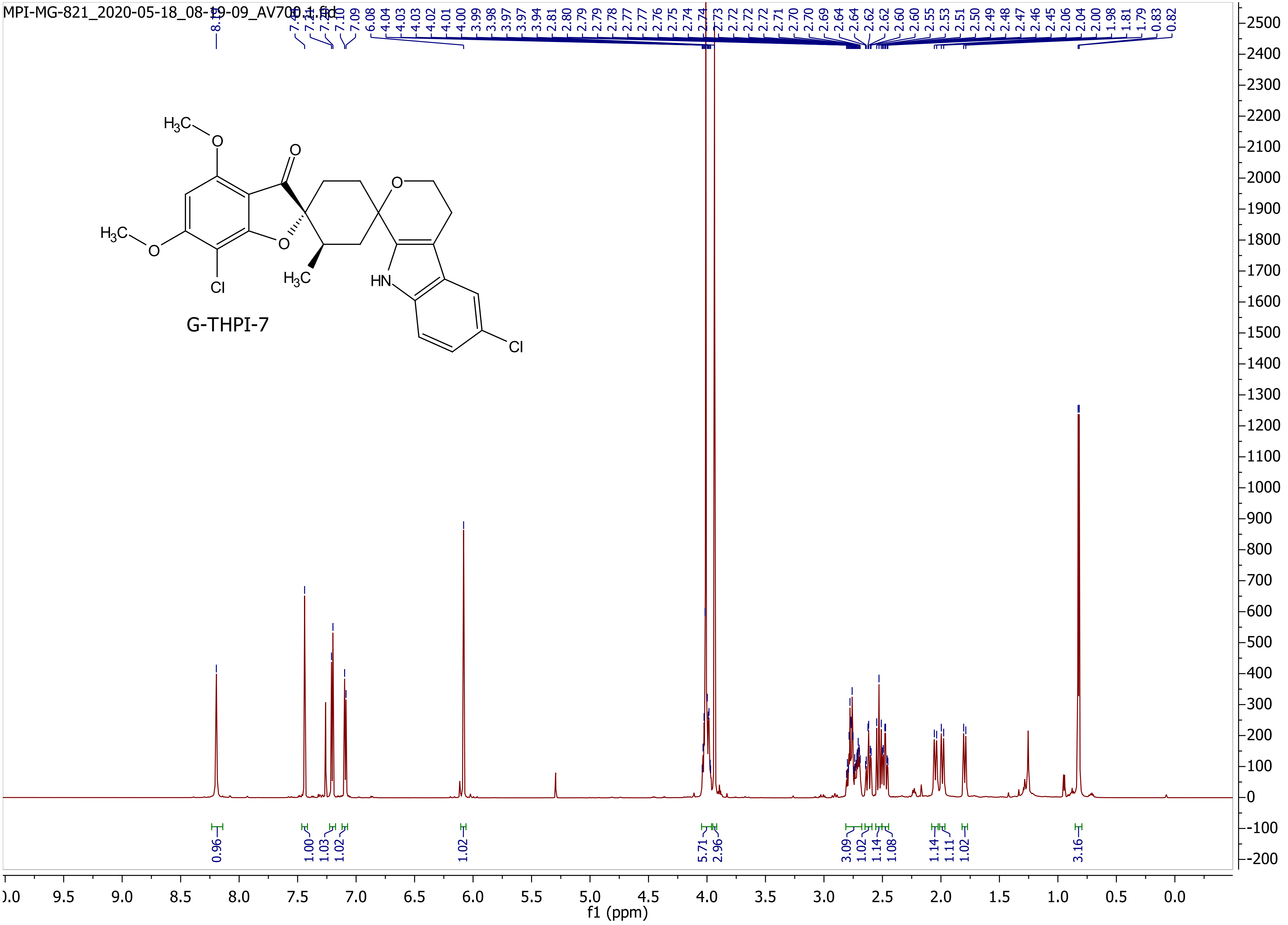

MPI-MG-821_2020-05-18_08-19-09_AV700.1.fid
G-THPI-7
H_3C
O
HN
Cl
f1 (ppm)
0.96
1.00
1.03
1.02
1.02
5.71
2.96
3.09
1.02
1.14
1.08
1.14
1.11
1.02
3.16

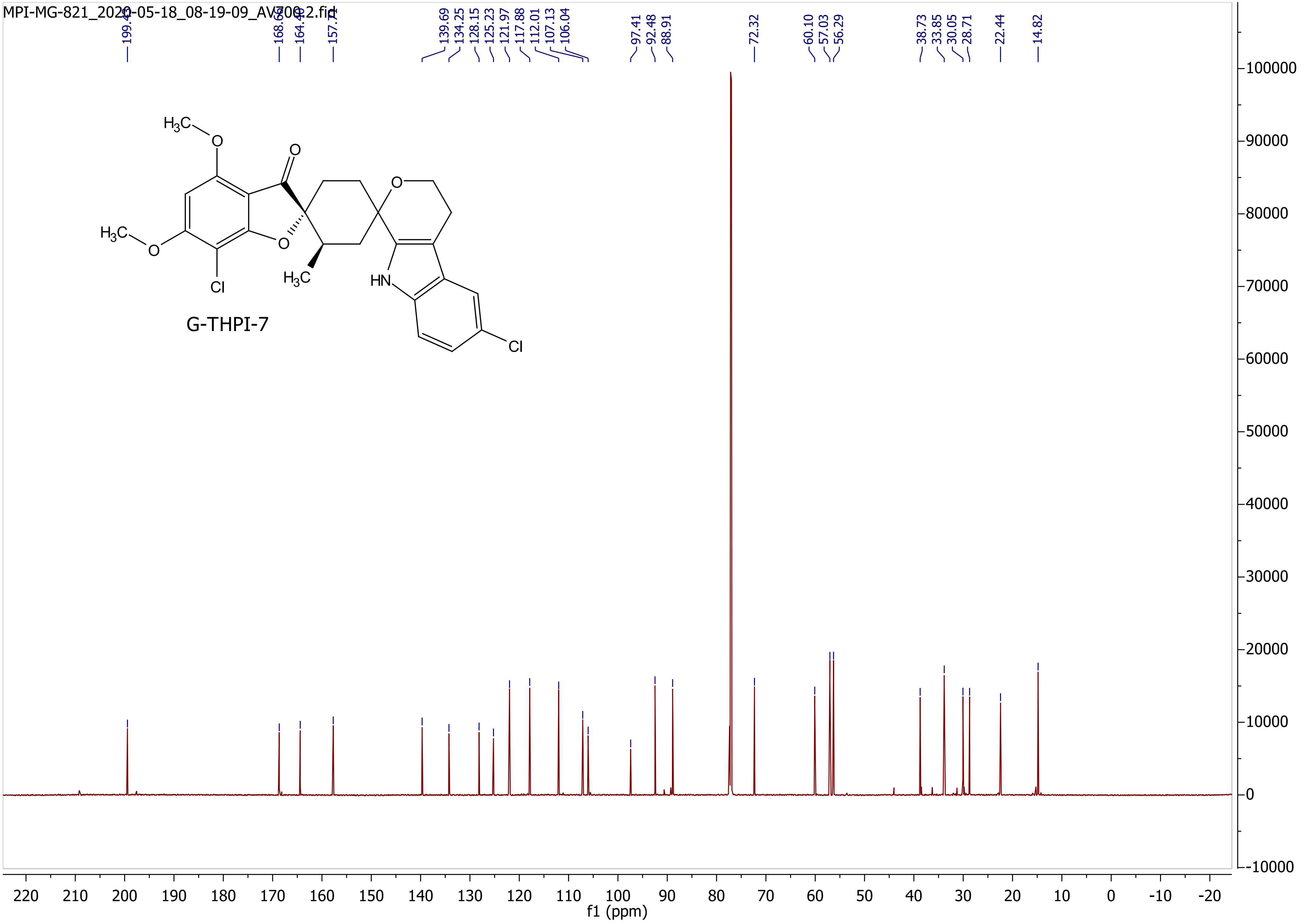

MPI-MG-821_2020-05-18_08-19-09_AV700e2.fid
139.69
134.25
128.15
125.23
121.97
117.88
112.01
107.13
106.04
97.41
92.48
88.91
72.32
60.10
57.03
56.29
38.73
33.85
30.05
28.71
22.44
14.82
G-THPI-7
f1 (ppm)

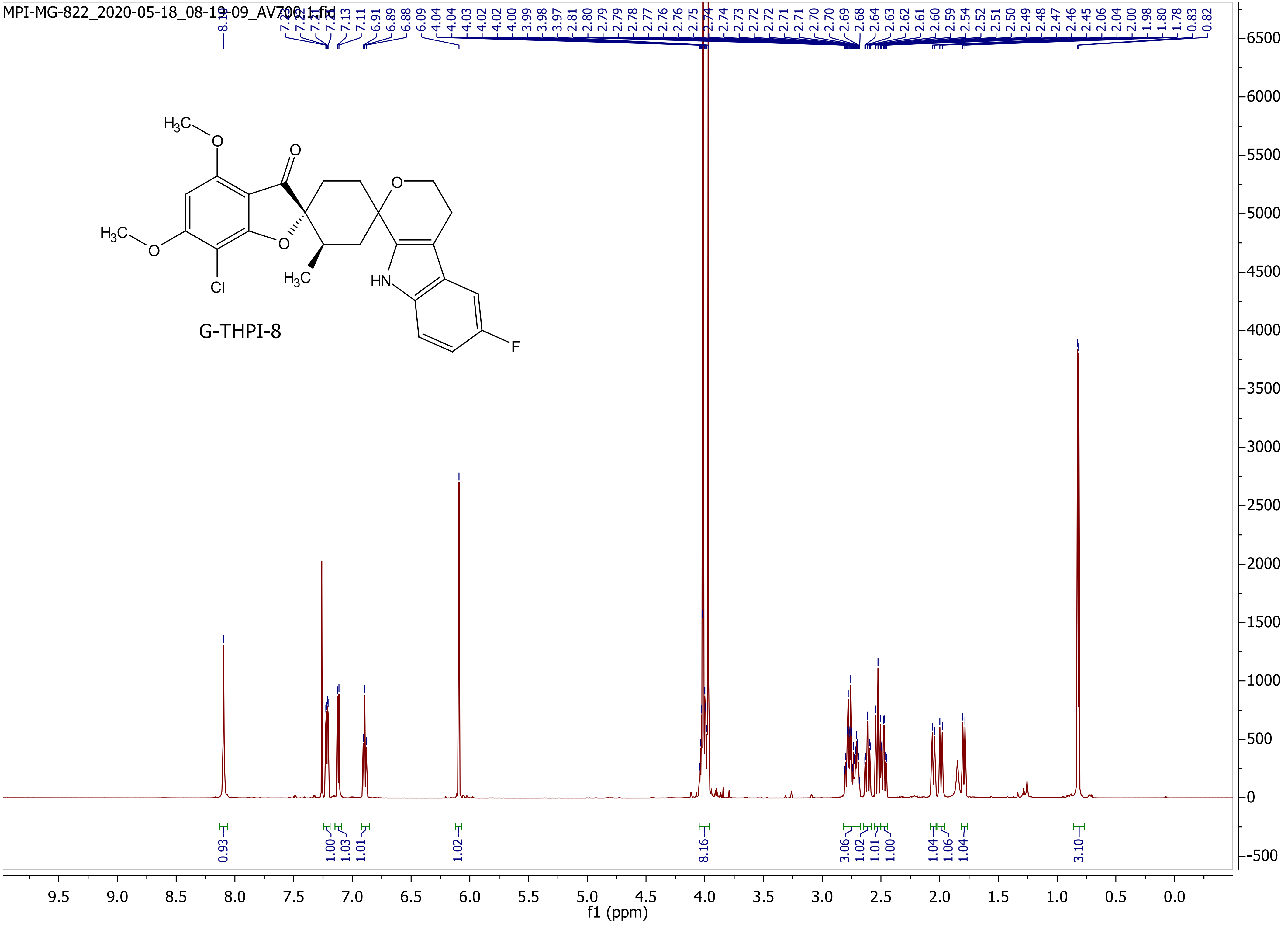

MPI-MG-822_2020-05-18_08-19-09_AV700 1.fid
G-THPI-8
f1 (ppm)

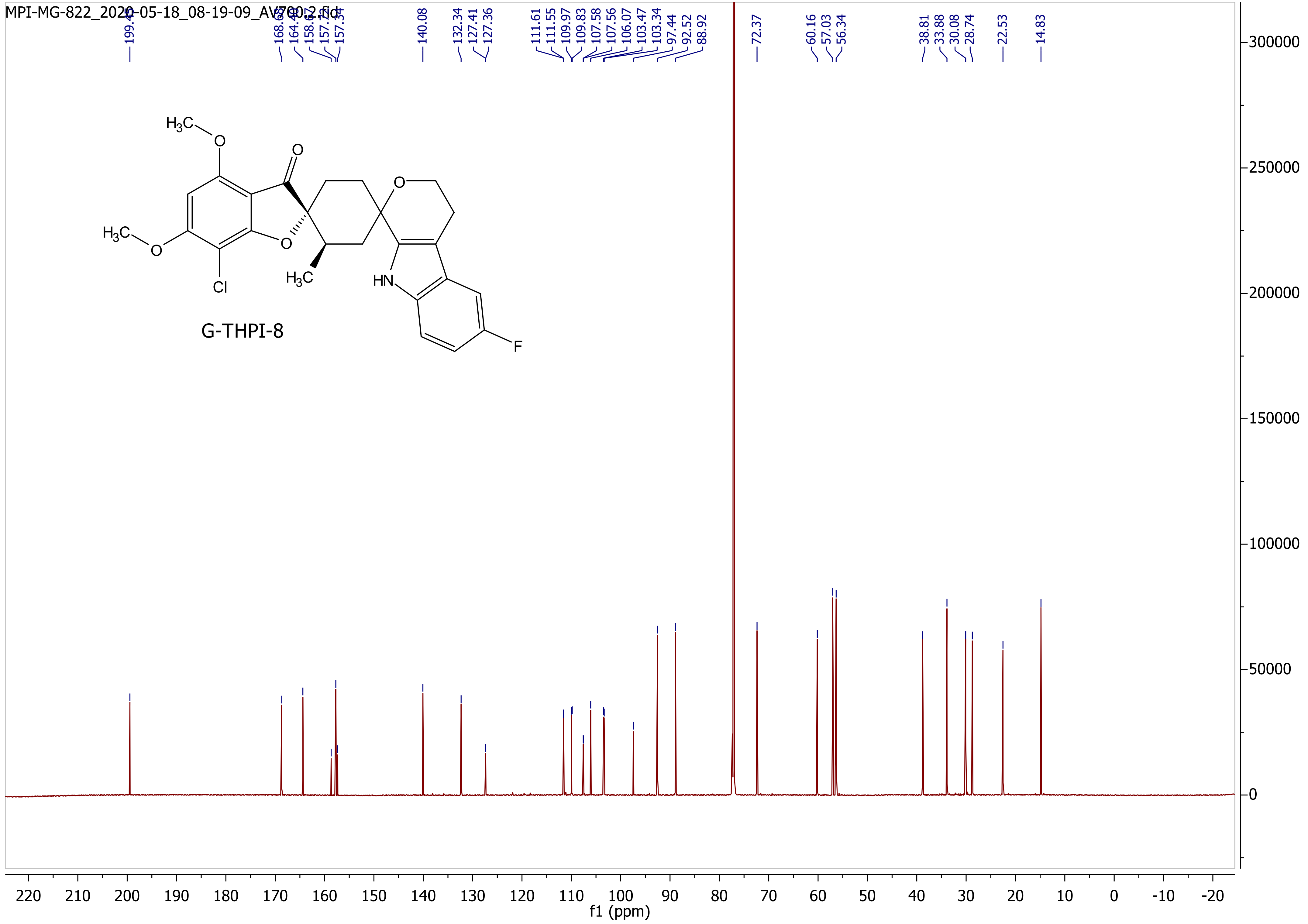

MPI-MG-822_2020-05-18_08-19-09_AV700.2.fid
140.08
132.34
127.41
127.36
111.61
111.55
109.97
109.83
107.58
107.56
106.07
103.47
103.34
97.44
92.52
88.92
72.37
60.16
57.03
56.34
38.81
33.88
30.08
28.74
22.53
14.83
G-THPI-8
f1 (ppm)

MPI-MG-822_2020-05-19_13-46-37_AV600.11.fid
F19

z_F19 CDCl3 /NMR-Daten MPI 18

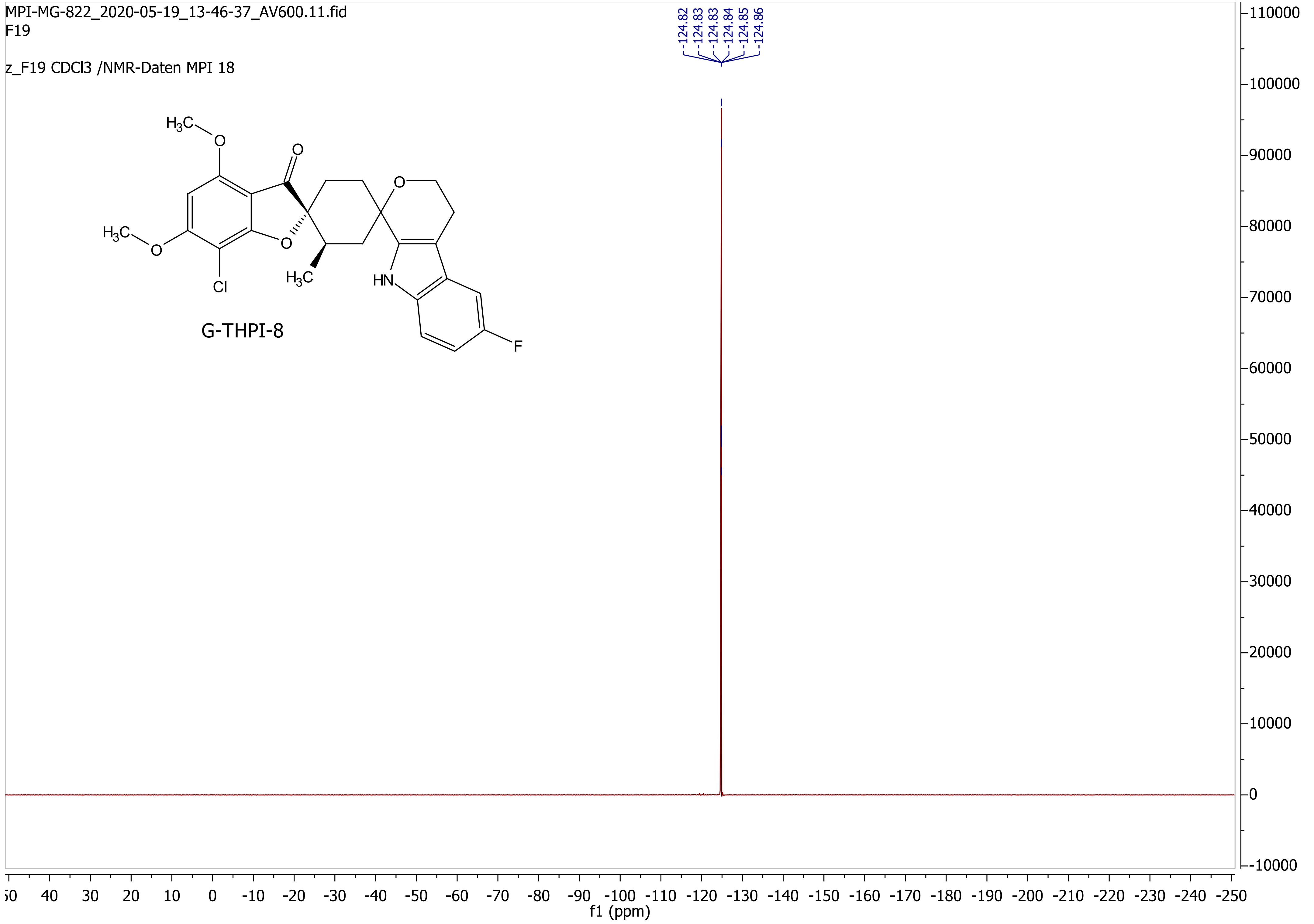

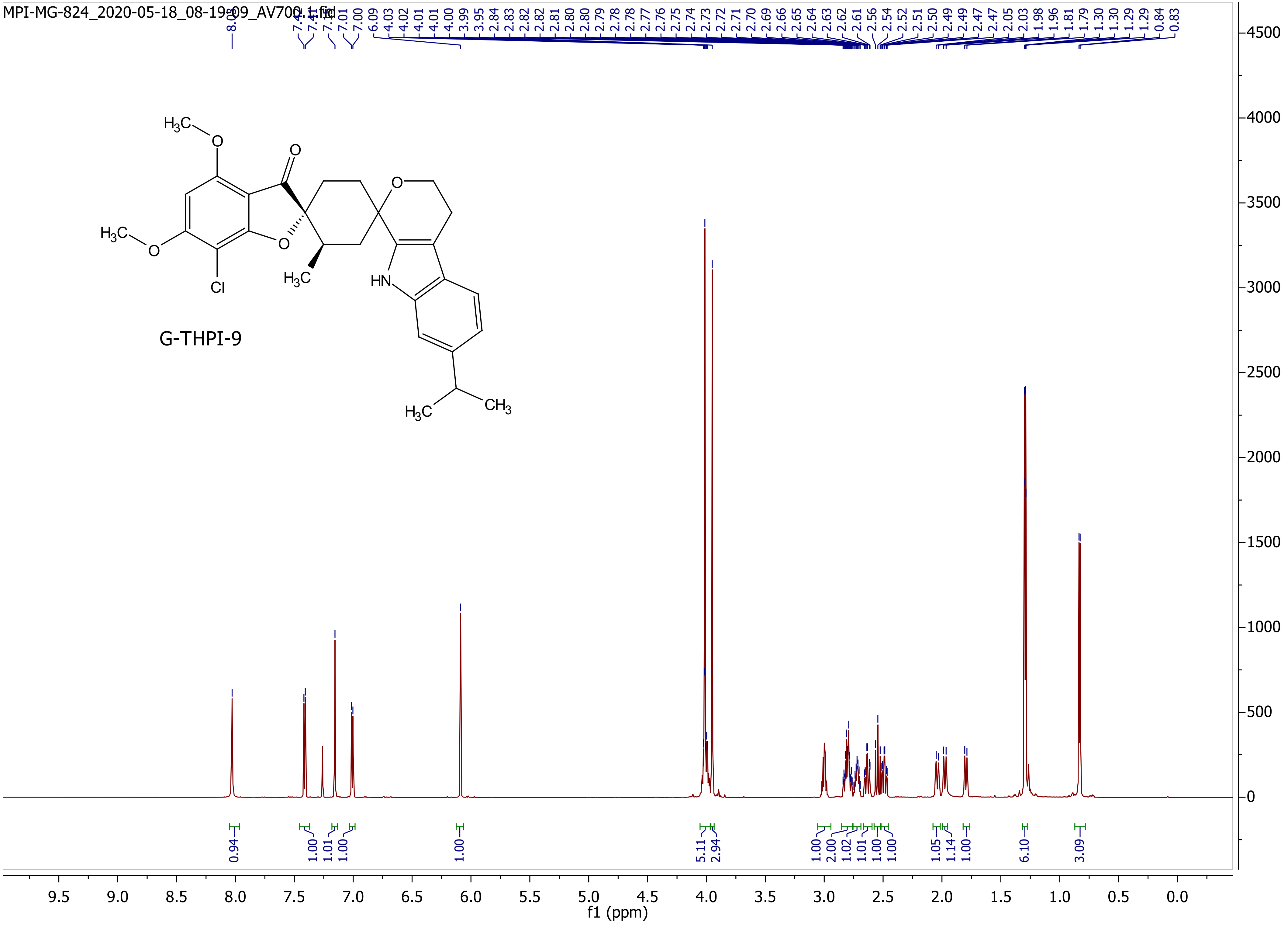
MPI-MG-824_2020-05-18_08-19-09_AV700.1.fid
G-THPI-9
f1 (ppm)

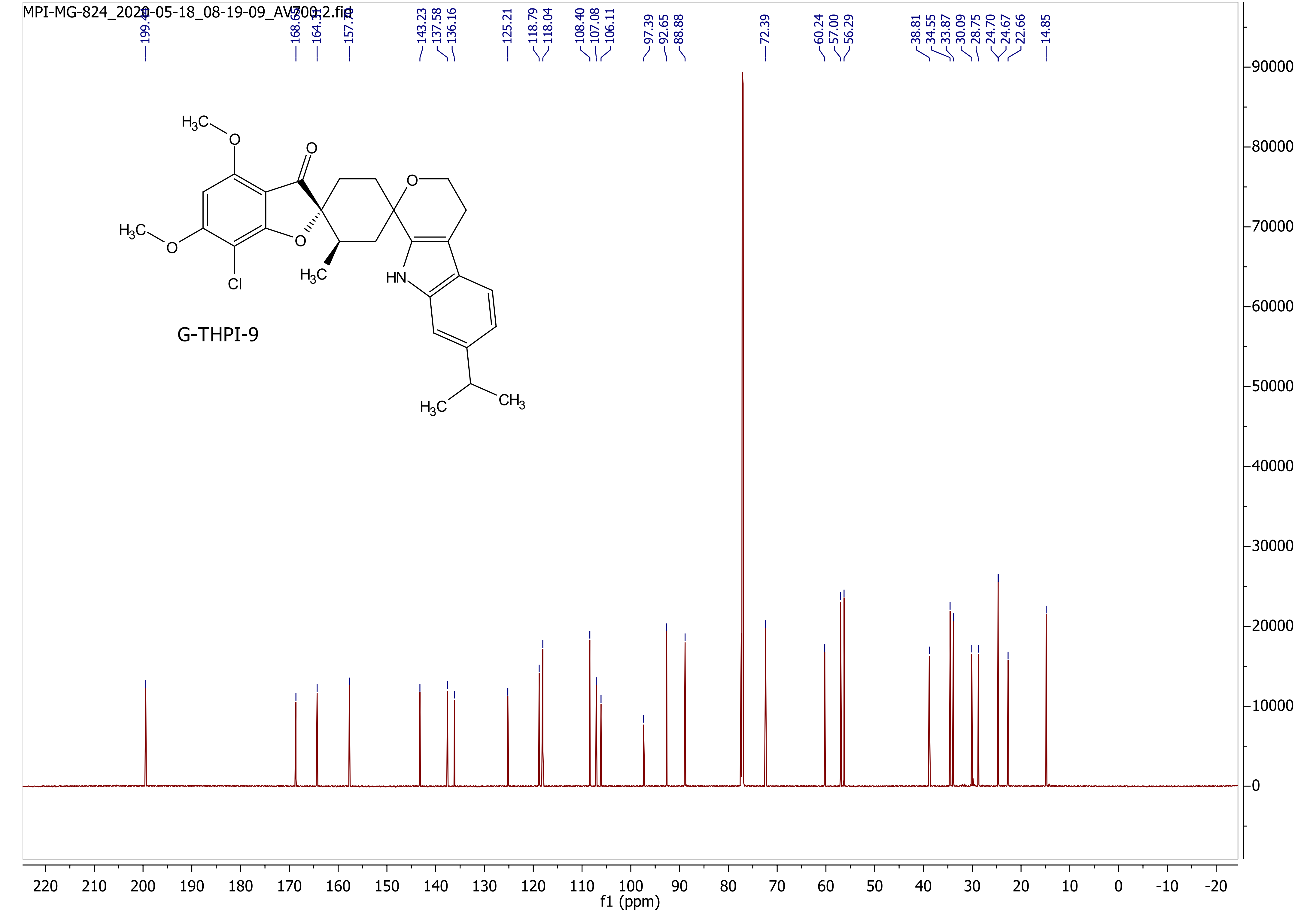

MPI-MG-824_2020-05-18_08-19-09_AV700_2.fid
143.23
137.58
136.16
125.21
118.79
118.04
108.40
107.08
106.11
97.39
92.65
88.88
72.39
60.24
57.00
56.29
38.81
34.55
33.87
30.09
28.75
24.70
24.67
22.66
14.85
G-THPI-9
f1 (ppm)

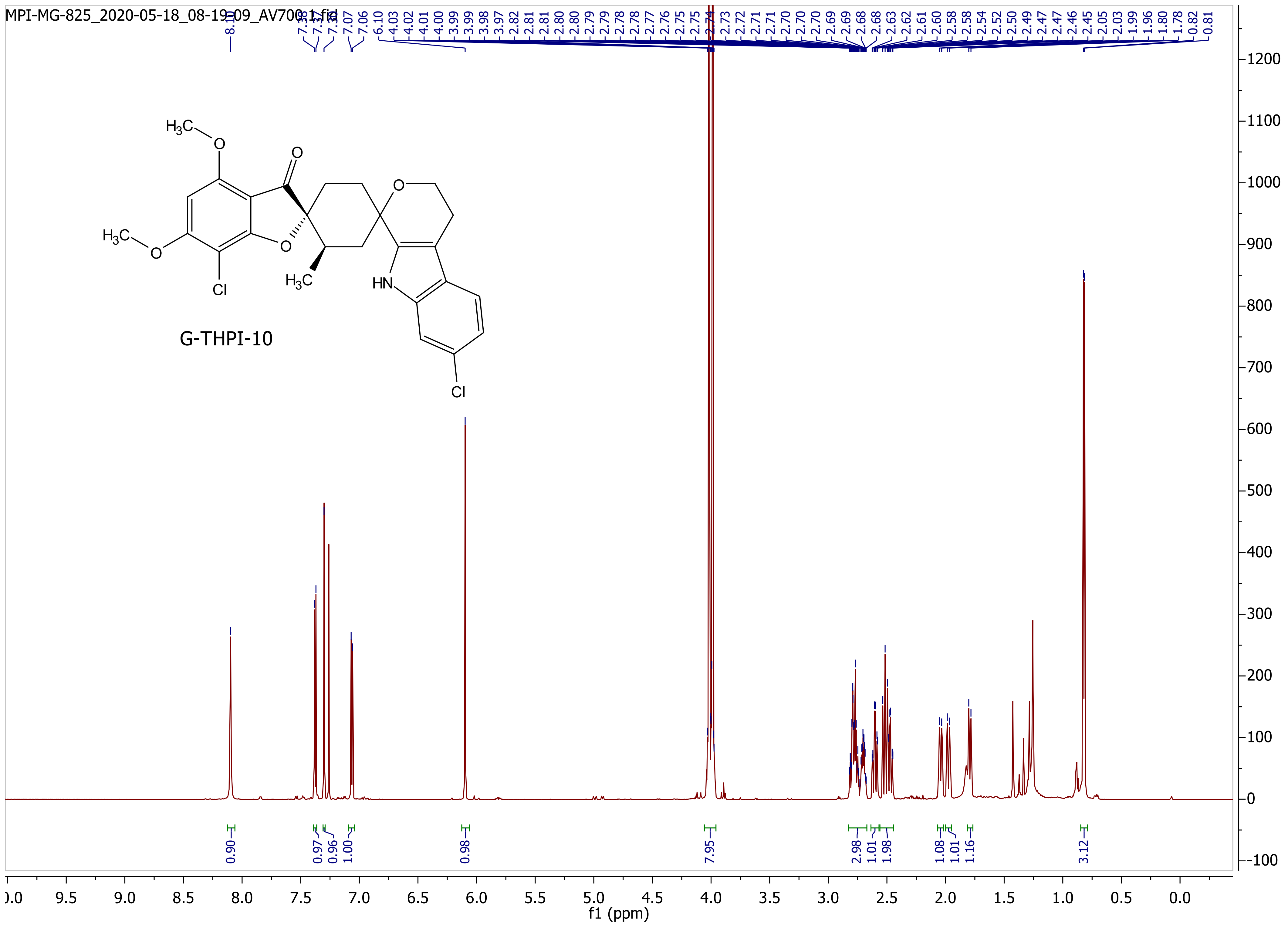

MPI-MG-825_2020-05-18_08-19-09_AV700-1.fid
8.1
7.37
7.3
7.07
7.06
6.10
4.03
4.02
4.01
4.00
3.99
3.99
3.98
3.97
2.82
2.81
2.81
2.80
2.80
2.79
2.79
2.78
2.78
2.77
2.76
2.75
2.75
2.74
2.73
2.72
2.71
2.71
2.70
2.70
2.70
2.69
2.69
2.68
2.68
2.63
2.62
2.61
2.60
2.58
2.58
2.54
2.52
2.50
2.49
2.47
2.47
2.46
2.45
2.05
2.03
1.99
1.96
1.80
1.78
0.82
0.81
H3C
O
O
O
H3C
O
O
Cl
H3C
HN
Cl
G-THPI-10
0.90
0.97
0.96
1.00
0.98
7.95
2.98
1.01
1.98
1.08
1.01
1.16
3.12
f1 (ppm)

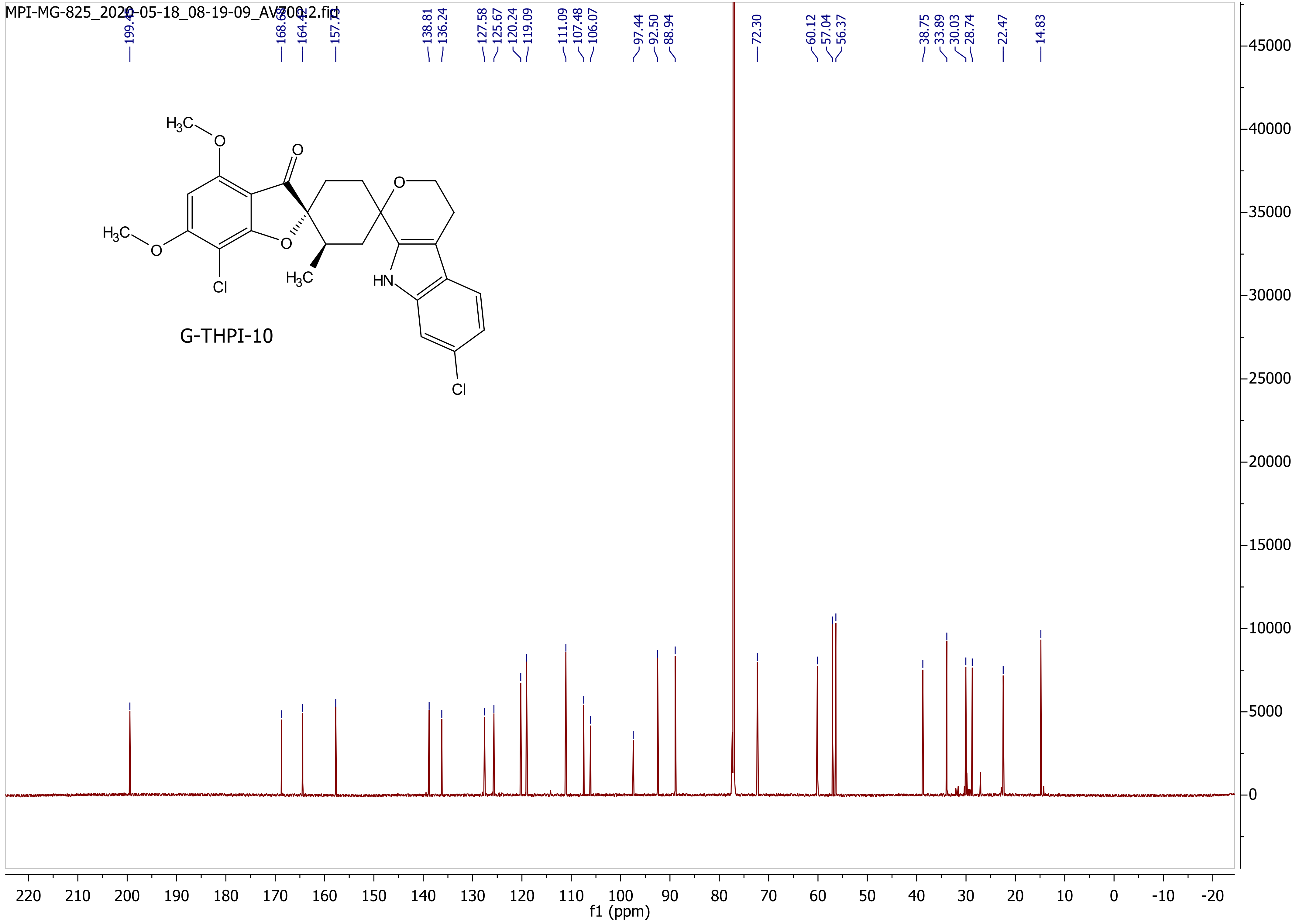

MPI-MG-825_202[illegible]-05-18_08-19-09_AV[illegible]_2.fid
138.81
136.24
127.58
125.67
120.24
119.09
111.09
107.48
106.07
97.44
92.50
88.94
72.30
60.12
57.04
56.37
38.75
33.89
30.03
28.74
22.47
14.83
G-THPI-10
f1 (ppm)

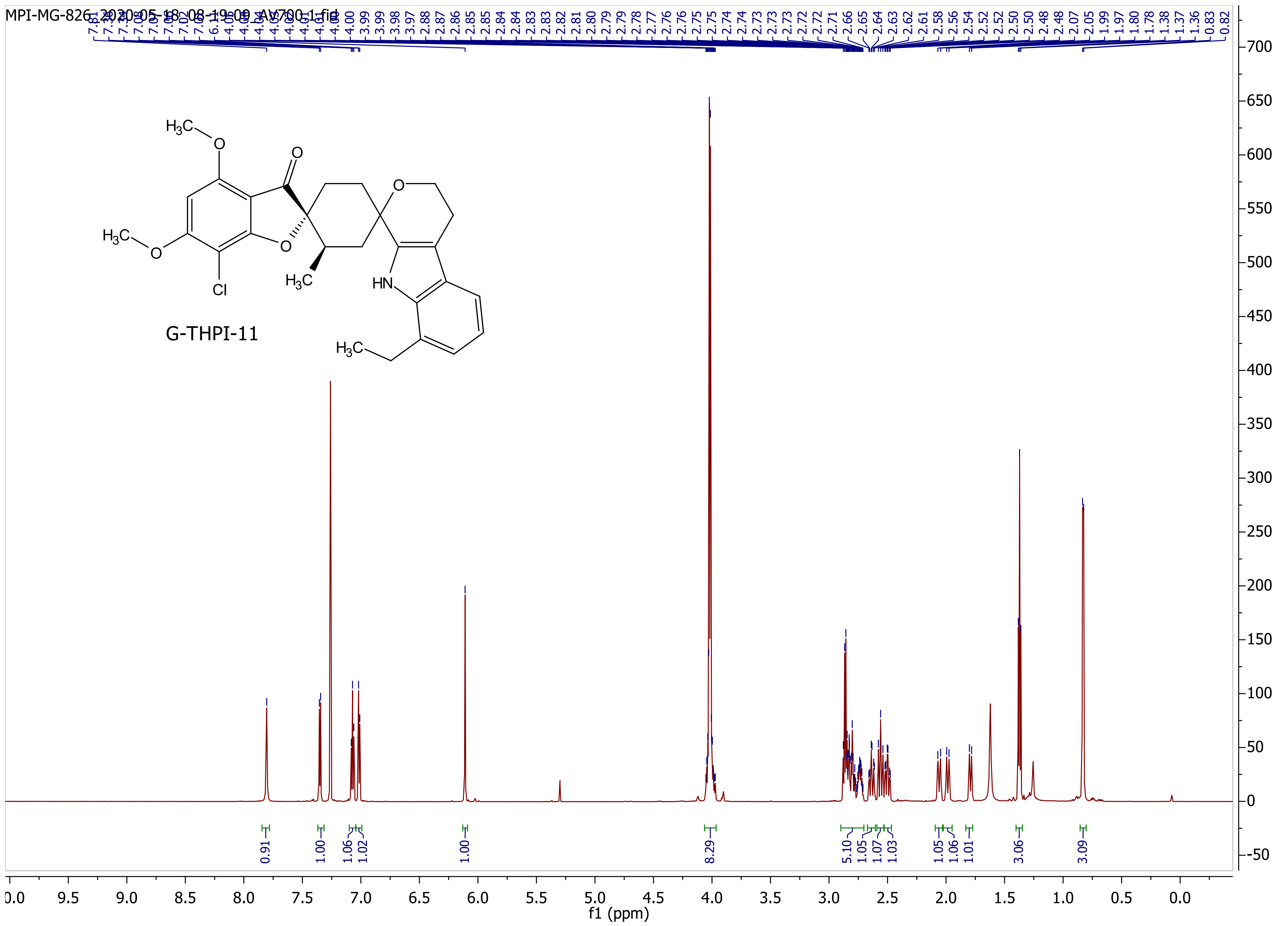

MPI-MG-826_2020-05-18_08-19-09_AV700-1.fid
G-THPI-11
H3C
O
Cl
HN
0.91
1.00
1.06
1.02
1.00
8.29
5.10
1.05
1.07
1.03
1.05
1.06
1.01
3.06
3.09
f1 (ppm)

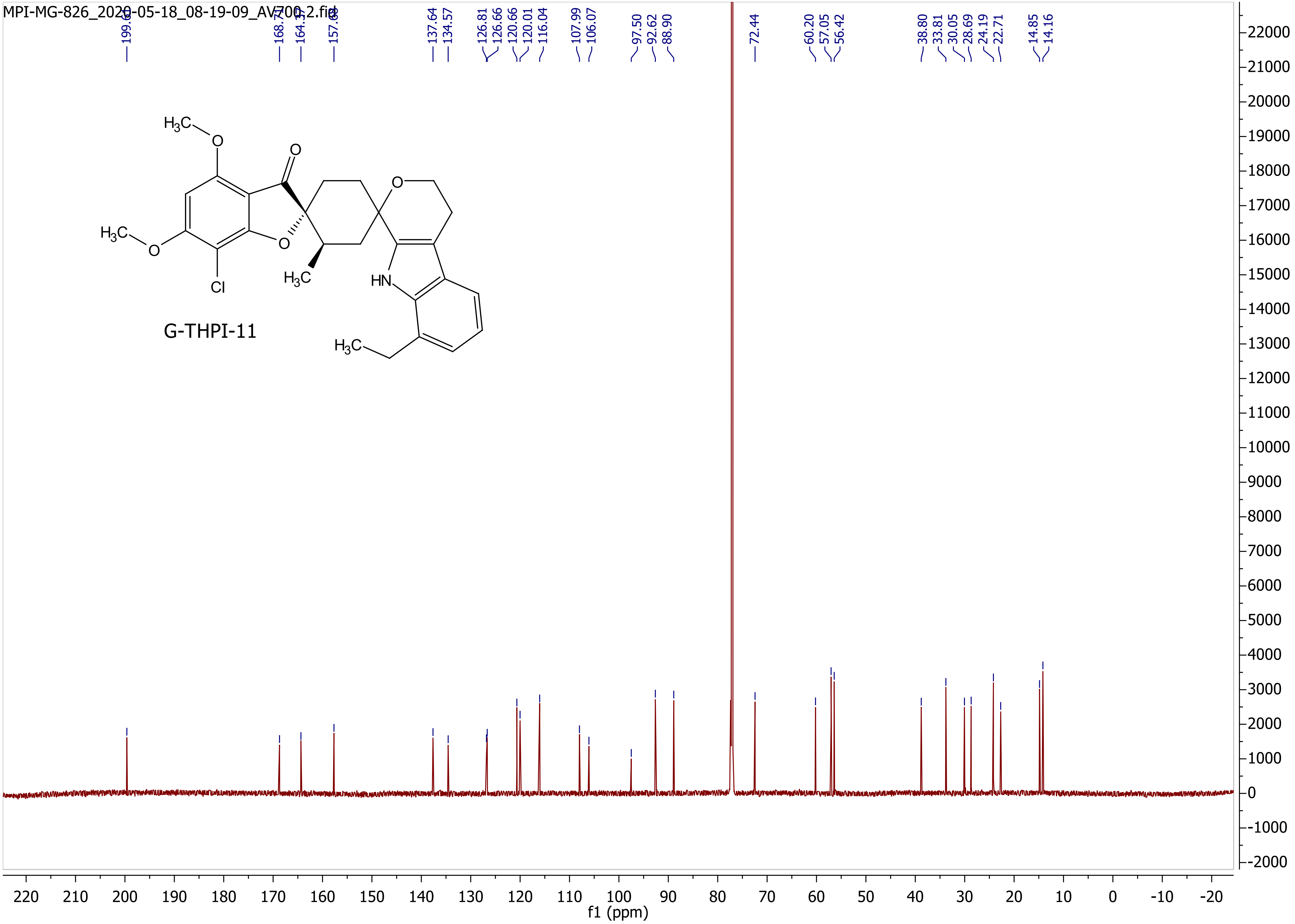
MPI-MG-826_2020-05-18_08-19-09_AV700-2.fid
137.64
134.57
126.81
126.66
120.66
120.01
116.04
107.99
106.07
97.50
92.62
88.90
72.44
60.20
57.05
56.42
38.80
33.81
30.05
28.69
24.19
22.71
14.85
14.16
H3C
O
O
H3C
O
O
Cl
H3C
O
HN
H3C
G-THPI-11
f1 (ppm)

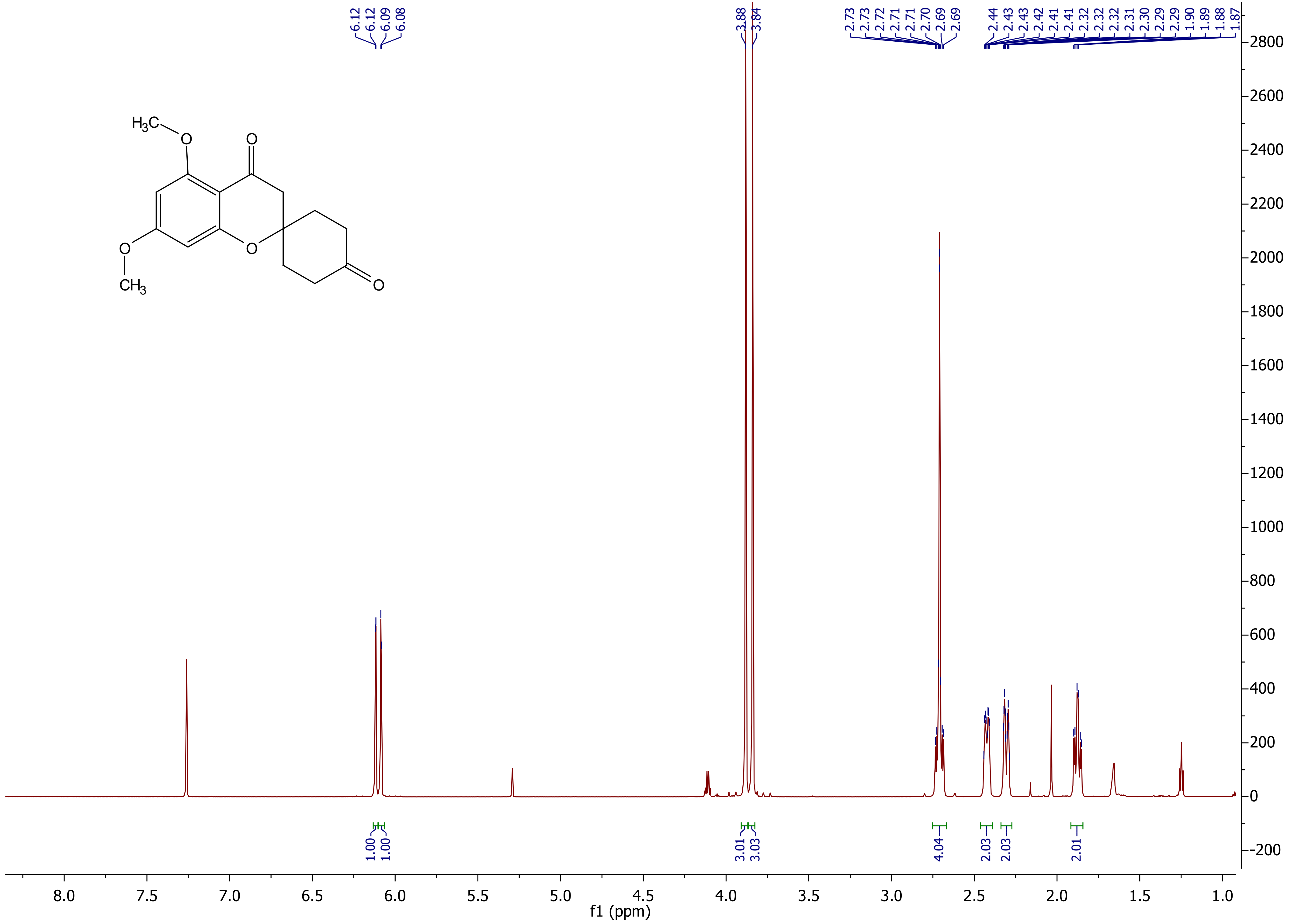

6.12
6.12
6.09
6.08
3.88
3.84
2.73
2.73
2.72
2.71
2.71
2.70
2.69
2.69
2.44
2.43
2.43
2.42
2.41
2.41
2.32
2.32
2.32
2.31
2.30
2.29
2.29
1.90
1.89
1.88
1.87
1.00
1.00
3.01
3.03
4.04
2.03
2.03
2.01
f1 (ppm)

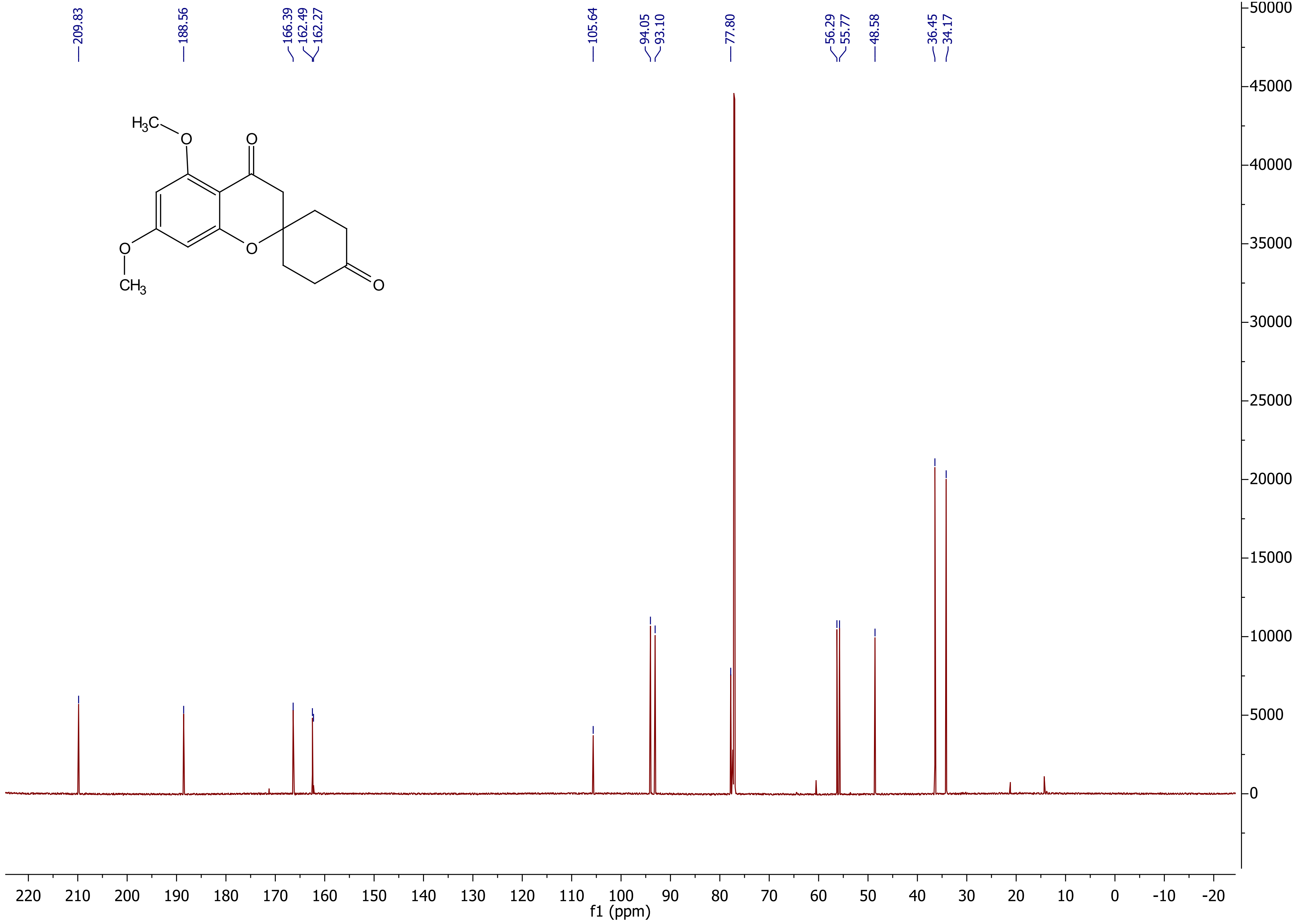
209.83
188.56
166.39
162.49
162.27
105.64
94.05
93.10
77.80
56.29
55.77
48.58
36.45
34.17
H3C
O
O
O
O
O
CH3
50000
45000
40000
35000
30000
25000
20000
15000
10000
5000
0
220 210 200 190 180 170 160 150 140 130 120 110 100 90 80 70 60 50 40 30 20 10 0 -10 -20
f1 (ppm)

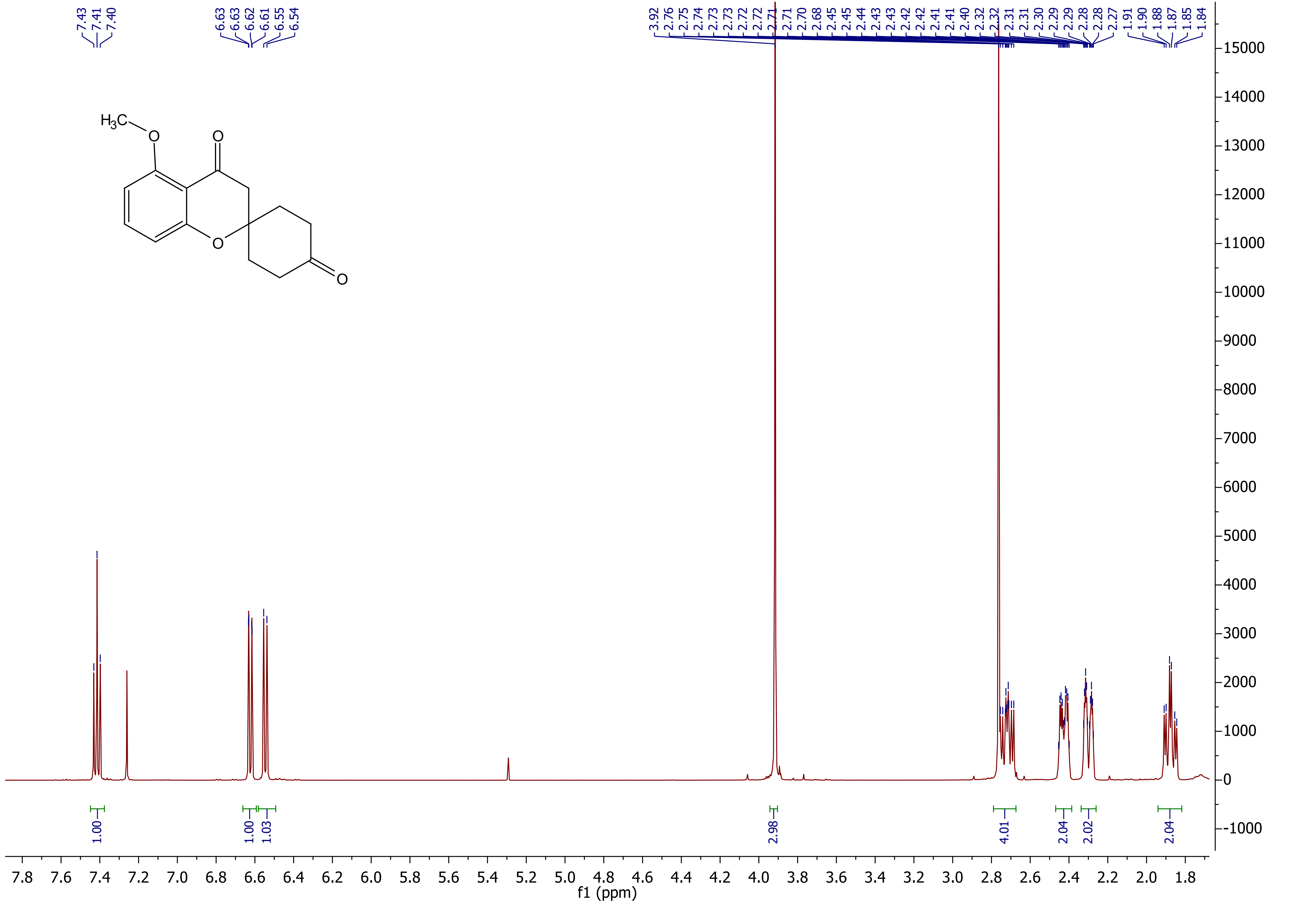

7.43
7.41
7.40
6.63
6.63
6.62
6.61
6.55
6.54
3.92
2.76
2.75
2.74
2.73
2.73
2.72
2.72
2.71
2.71
2.70
2.68
2.45
2.45
2.44
2.43
2.43
2.42
2.42
2.41
2.41
2.40
2.32
2.32
2.31
2.31
2.30
2.29
2.29
2.28
2.28
2.27
1.91
1.90
1.88
1.87
1.85
1.84
H_3C
O
O
O
O
1.00
1.00
1.03
2.98
4.01
2.04
2.02
2.04
f1 (ppm)

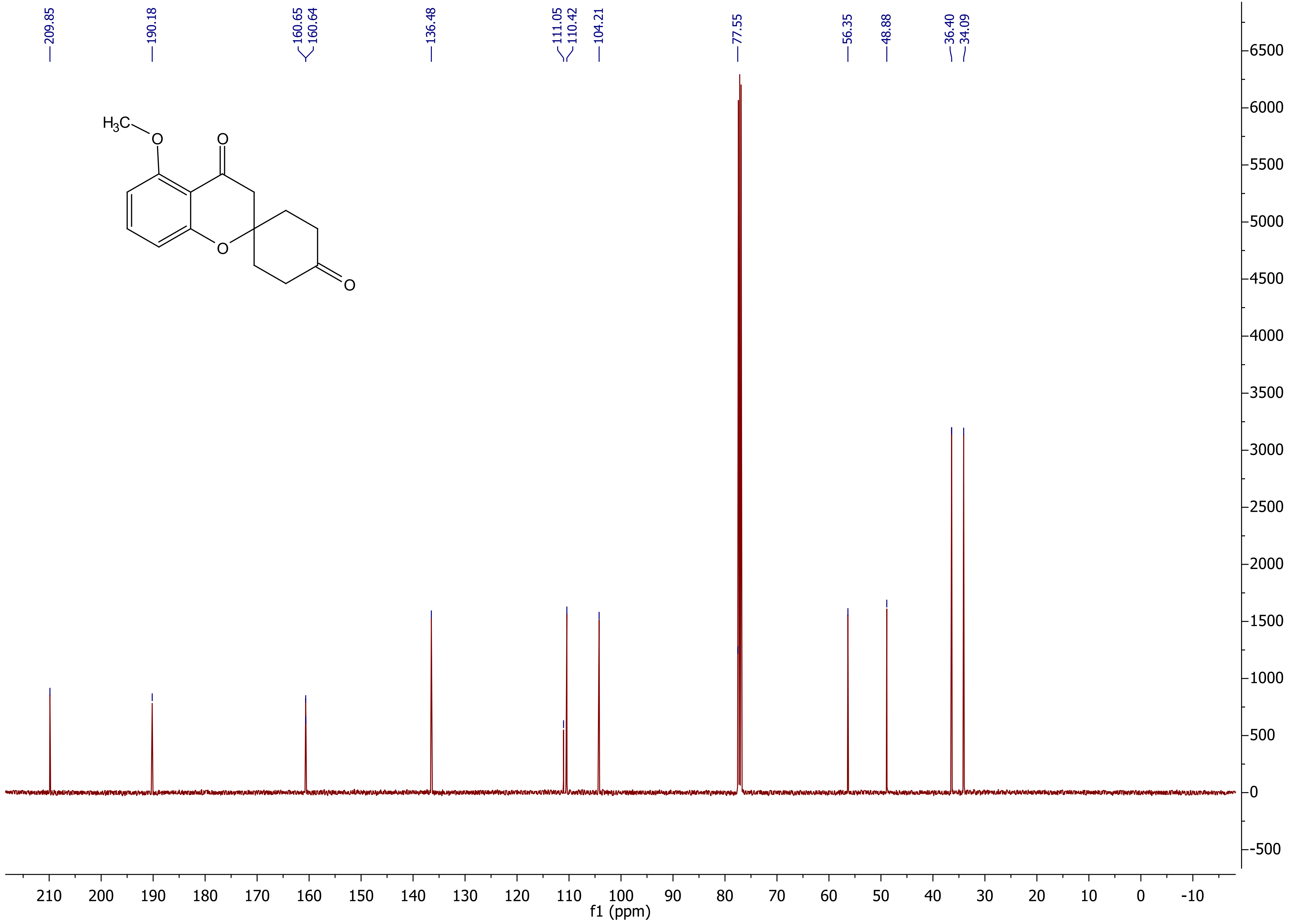
209.85
190.18
160.65
160.64
136.48
111.05
110.42
104.21
77.55
56.35
48.88
36.40
34.09
H3C
O
O
O
O
f1 (ppm)

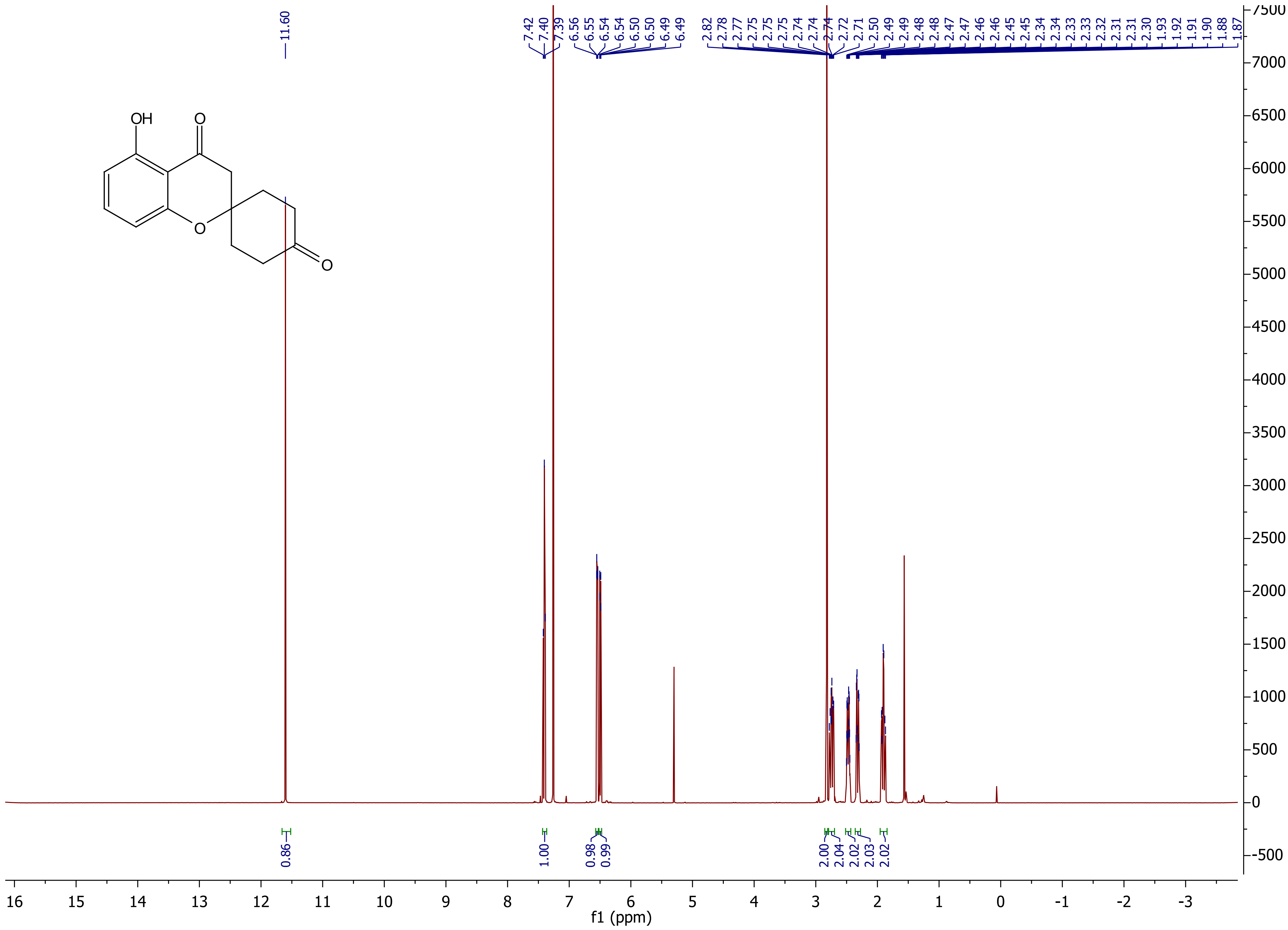

OH
O
O
O
11.60
7.42
7.40
7.39
6.56
6.55
6.54
6.54
6.50
6.50
6.49
6.49
2.82
2.78
2.77
2.75
2.75
2.75
2.74
2.74
2.74
2.72
2.71
2.50
2.49
2.49
2.48
2.48
2.47
2.47
2.46
2.46
2.45
2.45
2.34
2.34
2.33
2.33
2.32
2.31
2.31
2.30
1.93
1.92
1.91
1.90
1.88
1.87
0.86
1.00
0.98
0.99
2.00
2.04
2.02
2.03
2.02
f1 (ppm)

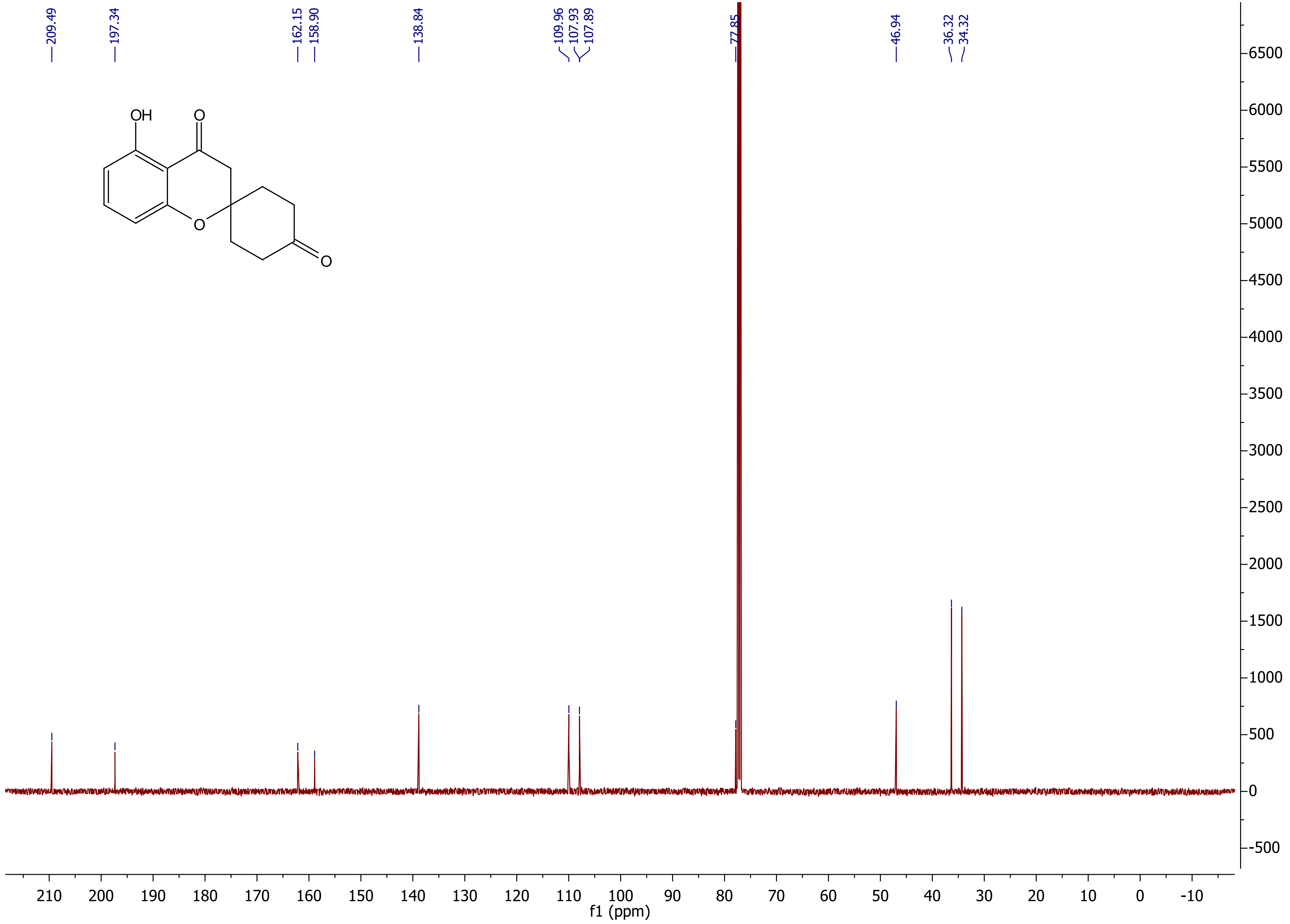

209.49
197.34
162.15
158.90
138.84
109.96
107.93
107.89
77.85
46.94
36.32
34.32
OH
O
O
O
f1 (ppm)

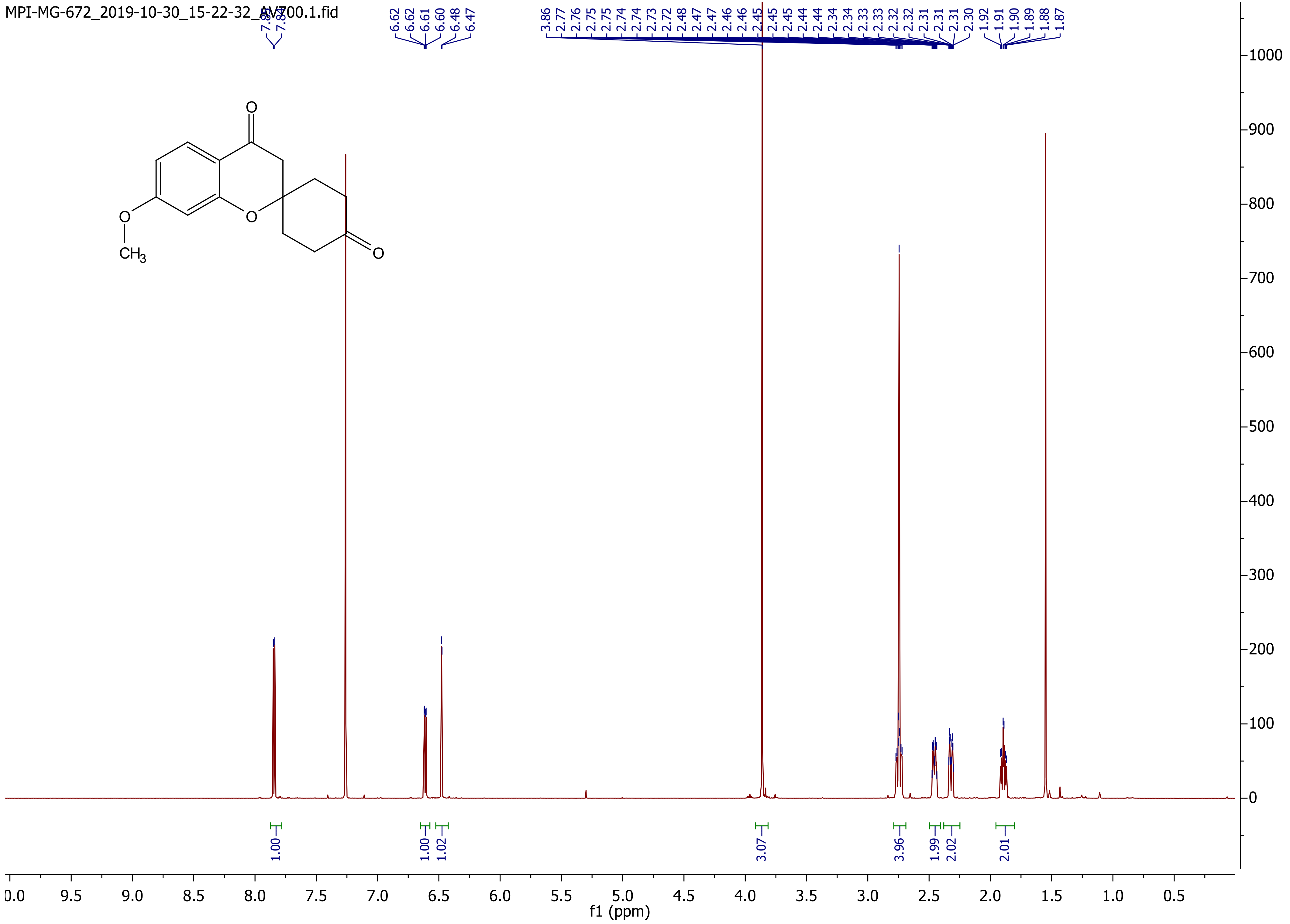
MPI-MG-672_2019-10-30_15-22-32_AV700.1.fid
7.85
7.84
6.62
6.62
6.61
6.60
6.48
6.47
3.86
2.77
2.76
2.75
2.75
2.74
2.74
2.73
2.72
2.48
2.47
2.47
2.46
2.46
2.45
2.45
2.45
2.44
2.44
2.34
2.34
2.33
2.33
2.32
2.32
2.31
2.31
2.31
2.30
1.92
1.91
1.90
1.89
1.88
1.87
O
O
O
O
CH3
1.00
1.00
1.02
3.07
3.96
1.99
2.02
2.01
1000
900
800
700
600
500
400
300
200
100
0
10.0
9.5
9.0
8.5
8.0
7.5
7.0
6.5
6.0
5.5
5.0
4.5
4.0
3.5
3.0
2.5
2.0
1.5
1.0
0.5
f1 (ppm)

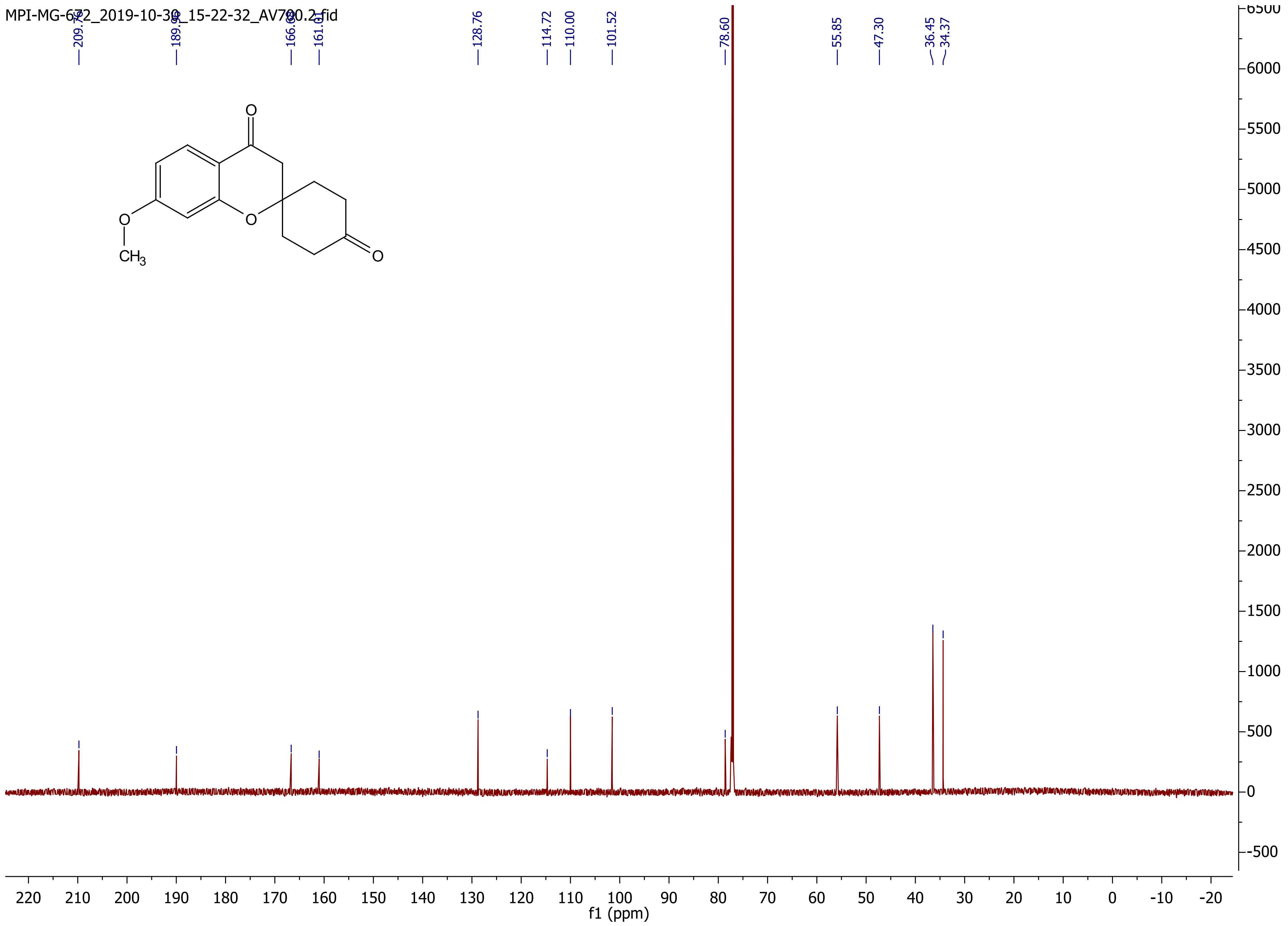
MPI-MG-6?2_2019-10-3?_15-22-32_AV7?0.2.fid
128.76
114.72
110.00
101.52
78.60
55.85
47.30
36.45
34.37
O
O
O
O
CH3
f1 (ppm)

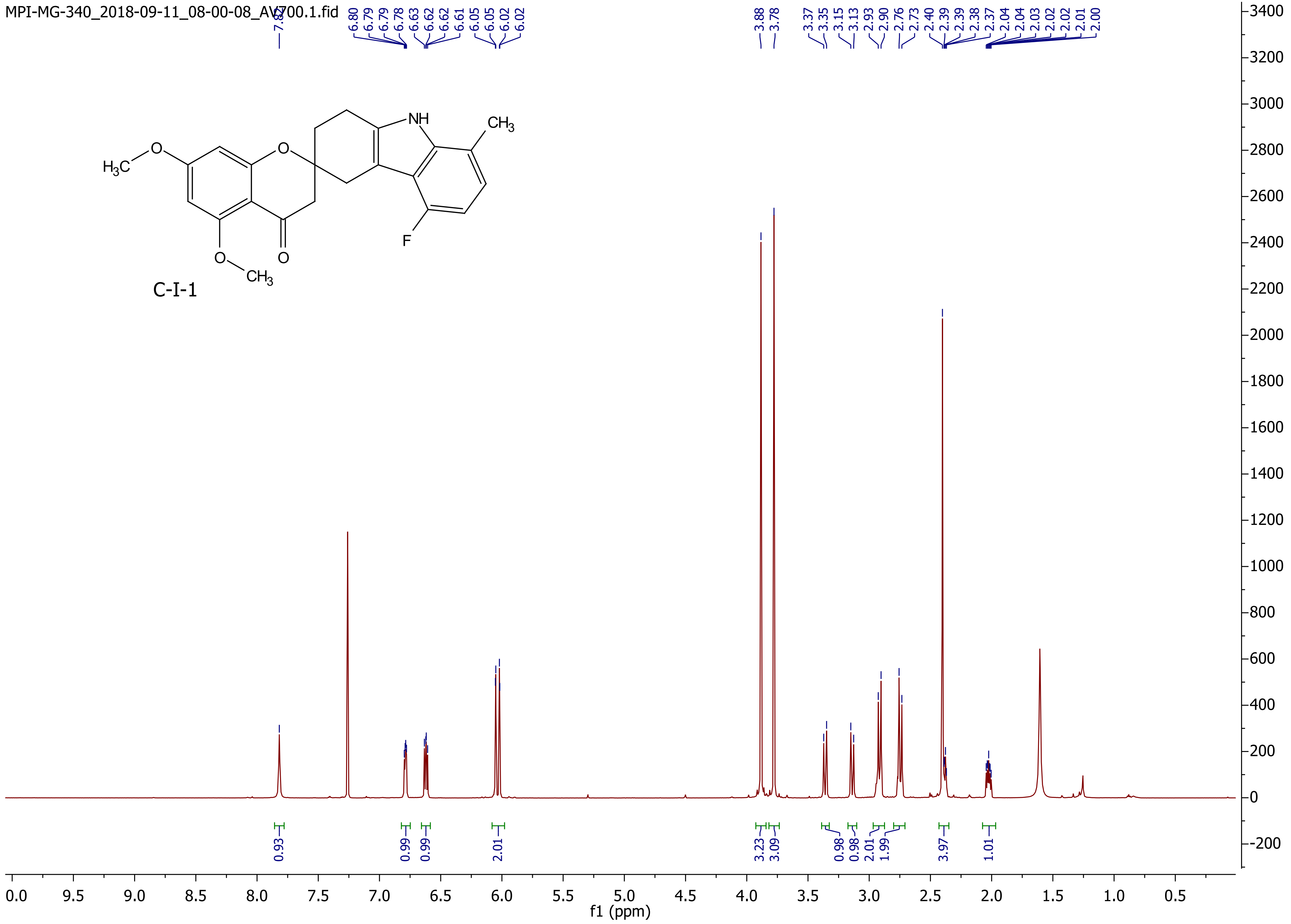
MPI-MG-340_2018-09-11_08-00-08_AV700.1.fid
7.82
6.80 6.79 6.79 6.78 6.63 6.62 6.62 6.61 6.05 6.05 6.02 6.02
3.88 3.78
3.37 3.35 3.15 3.13 2.93 2.90 2.76 2.73 2.40 2.39 2.39 2.38 2.37 2.04 2.04 2.03 2.02 2.02 2.01 2.00
NH
CH3
O
H3C
O
F
O
O
CH3
C-I-1
0.93 0.99 0.99 2.01 3.23 3.09 0.98 0.98 2.01 1.99 3.97 1.01
f1 (ppm)

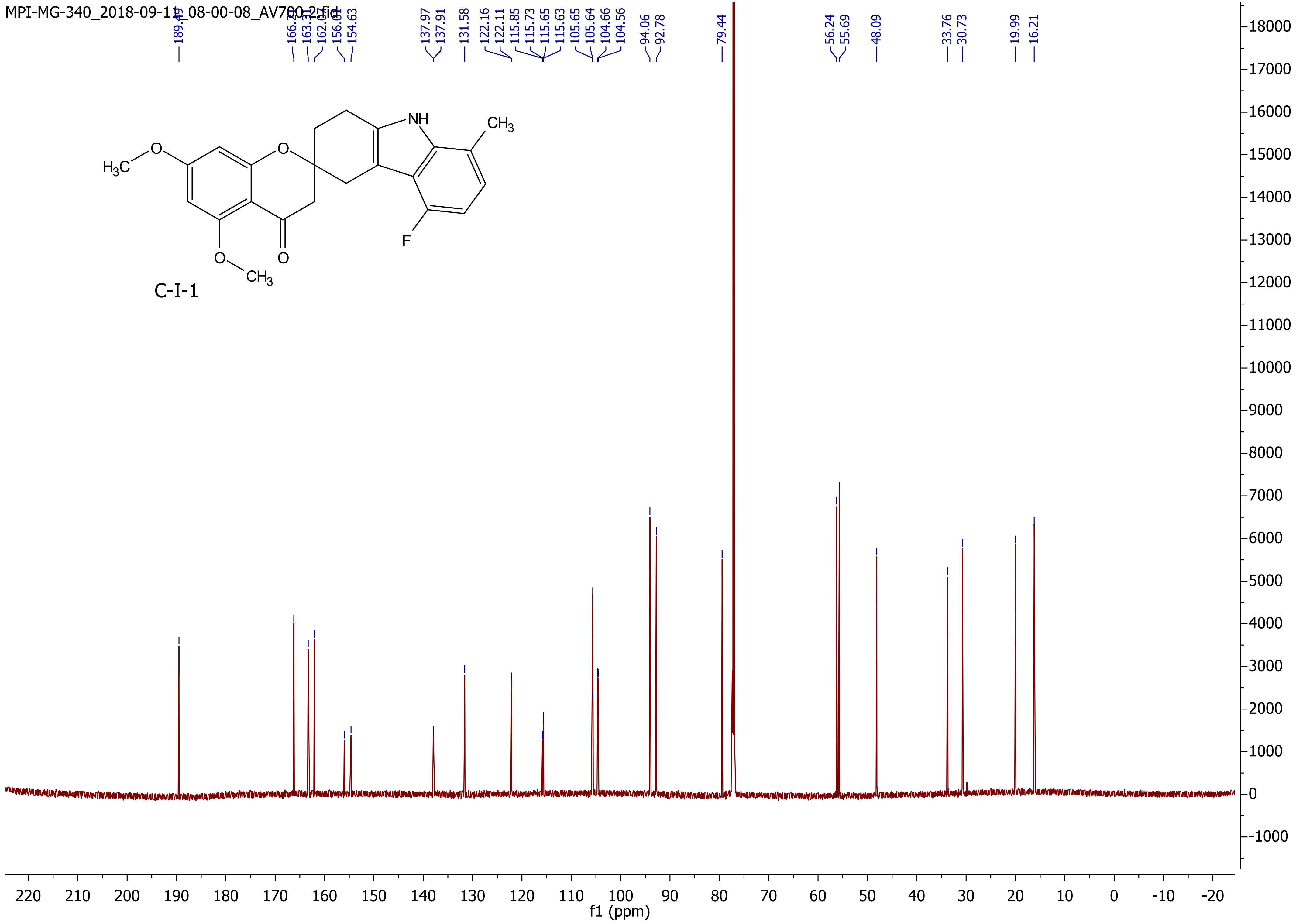

154.63
137.97
137.91
131.58
122.16
122.11
115.85
115.73
115.65
115.63
105.65
105.64
104.66
104.56
94.06
92.78
79.44
56.24
55.69
48.09
33.76
30.73
19.99
16.21
NH
CH3
O
H3C
O
O
O
CH3
F
C-I-1
f1 (ppm)

MPI-MG-340_2018-09-06_09-42-06_AV500.12.fid

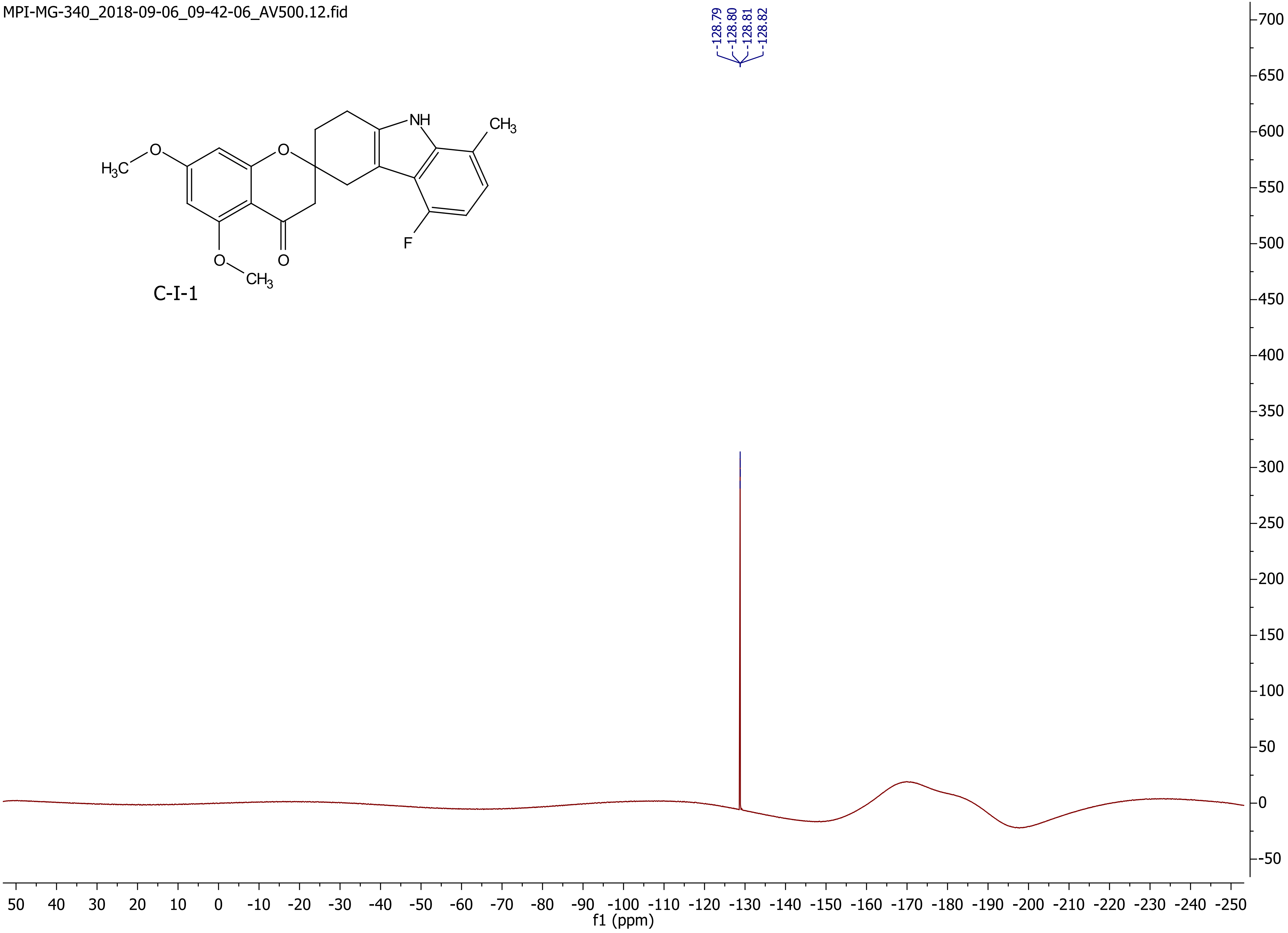

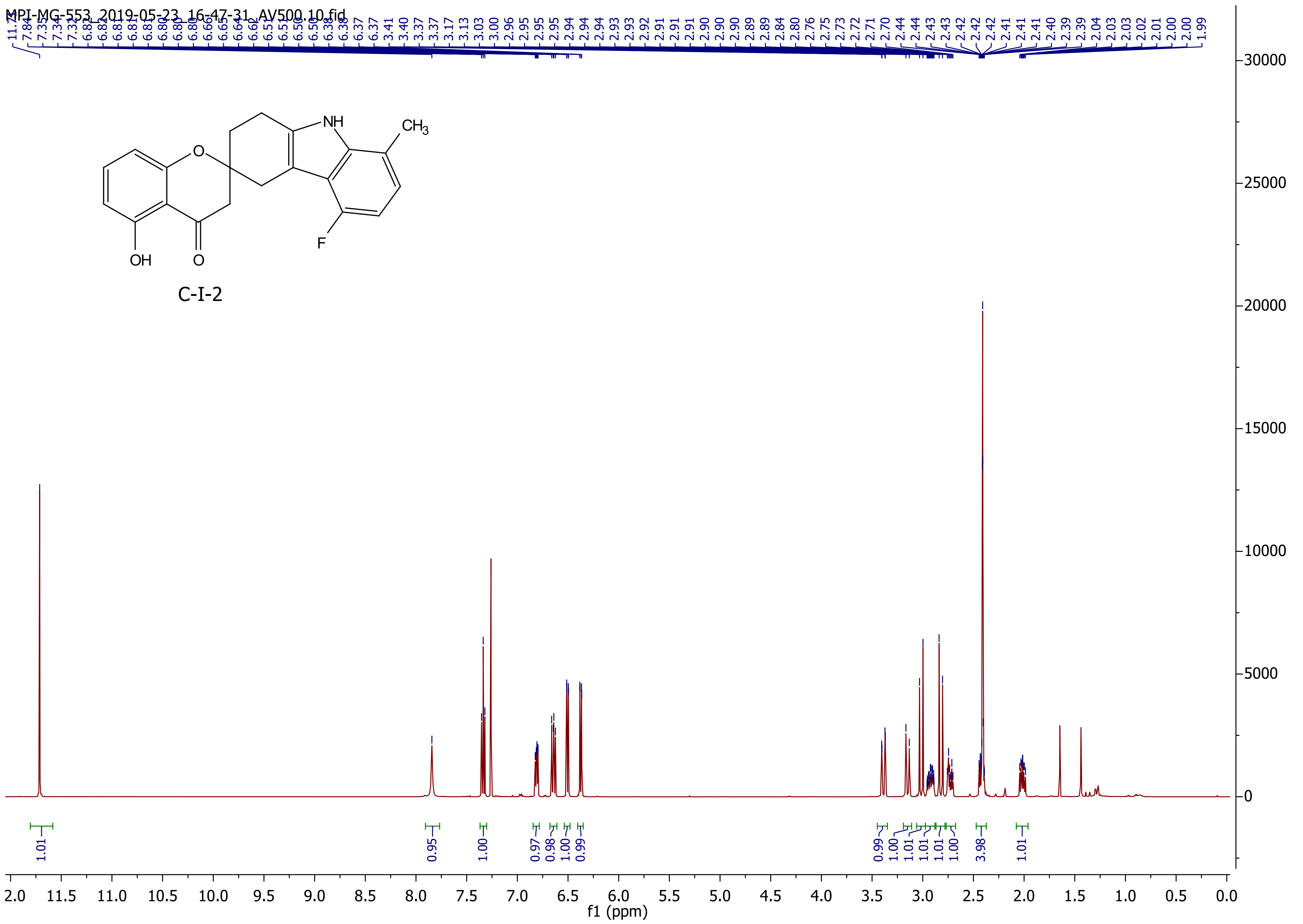

MPI-MG-553_2019-05-23_16-47-31_AV500.10.fid
NH
CH3
O
OH
O
F
C-I-2
f1 (ppm)

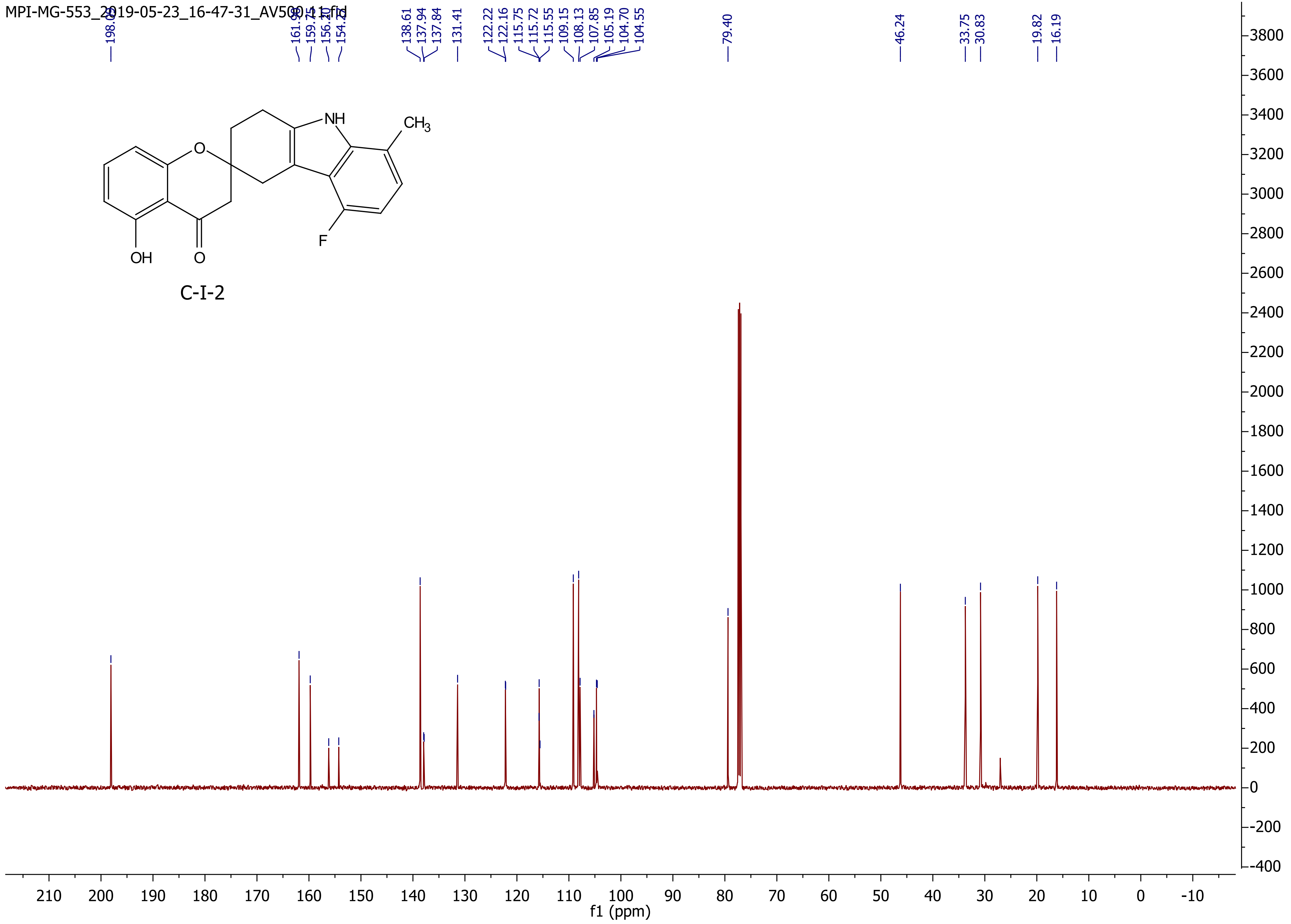
MPI-MG-553_2019-05-23_16-47-31_AV500.11.fid
198.09
161.90
159.75
156.20
154.27
138.61
137.94
137.84
131.41
122.22
122.16
115.75
115.72
115.55
109.15
108.13
107.85
105.19
104.70
104.55
79.40
46.24
33.75
30.83
19.82
16.19
NH
CH3
O
OH
O
F
3800
3600
3400
3200
3000
2800
2600
2400
2200
2000
1800
1600
1400
1200
1000
800
600
400
200
0
-200
-400
210
200
190
180
170
160
150
140
130
120
110
100
90
80
70
60
50
40
30
20
10
0
-10
f1 (ppm)

C-I-2

MPI-MG-553_2019-05-28_06-06-53_AV400.10.fid
F19

z_F19 CDCl3 /NMR-Daten MPI 11

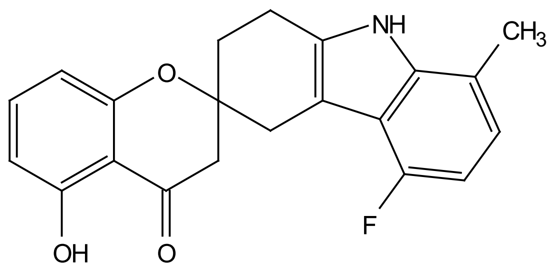

C-I-2

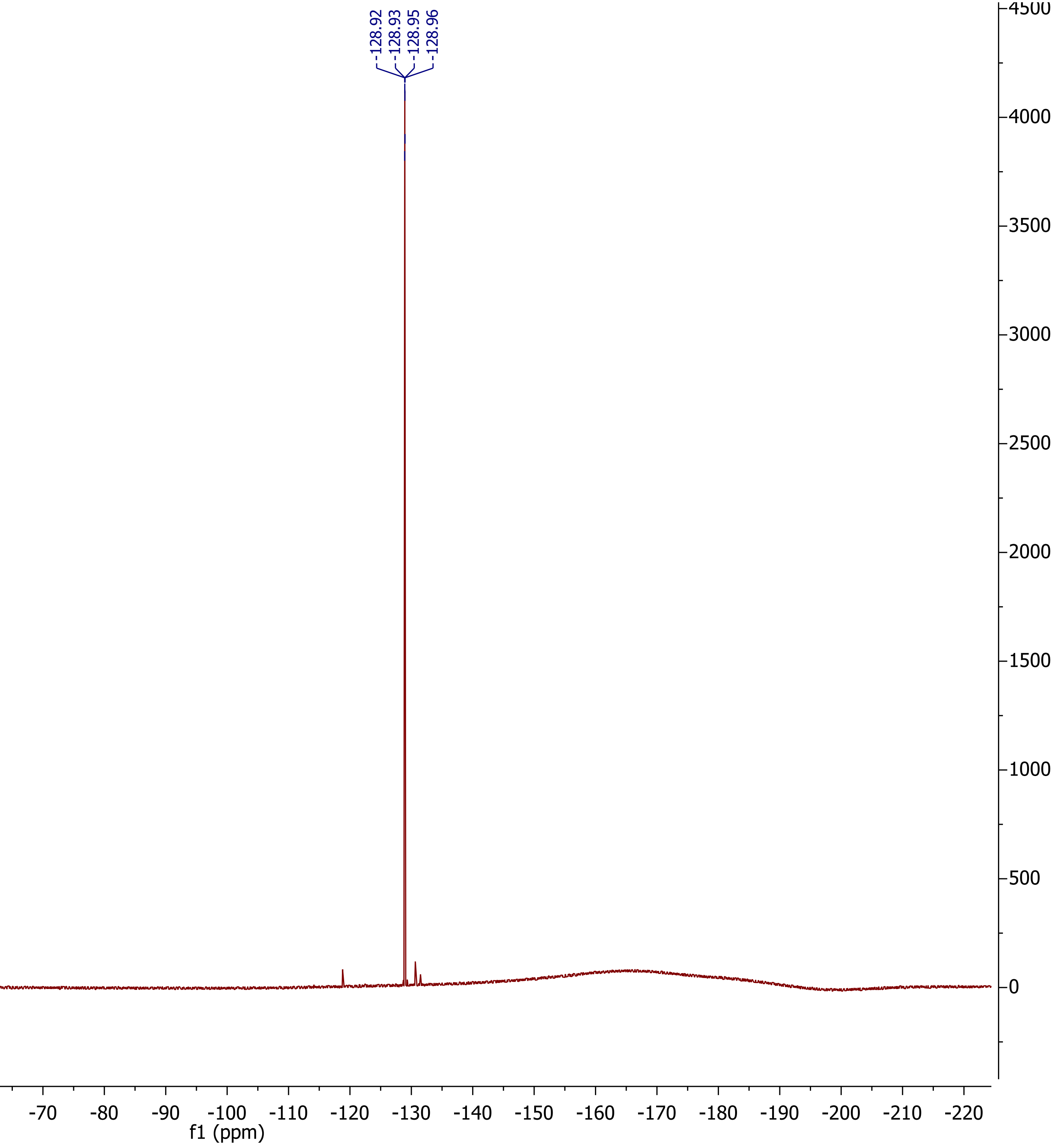

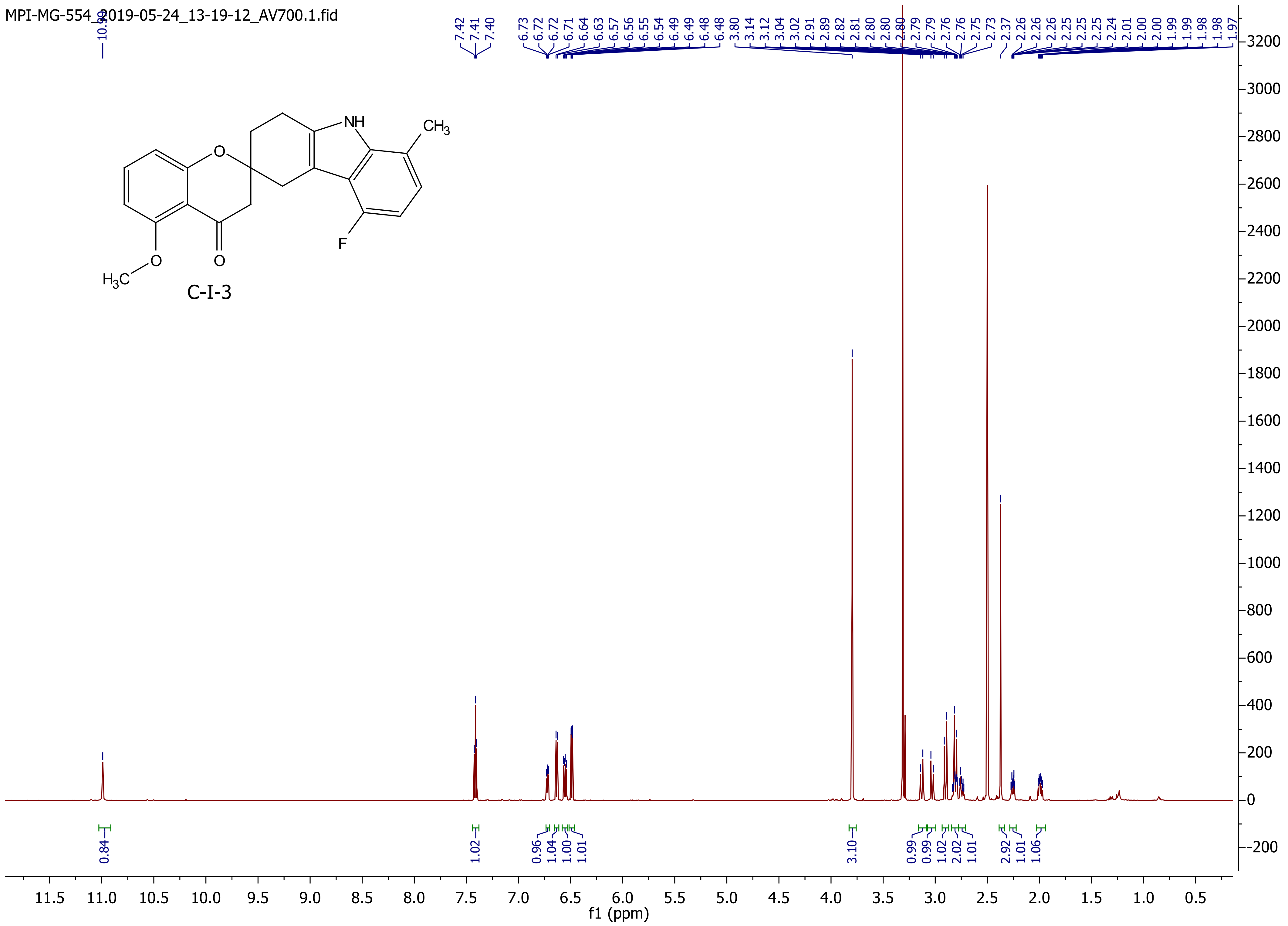

MPI-MG-554_2019-05-24_13-19-12_AV700.1.fid
7.42 7.41 7.40 6.73 6.72 6.72 6.71 6.64 6.63 6.57 6.56 6.55 6.54 6.49 6.49 6.48 6.48 3.80 3.14 3.12 3.04 3.02 2.91 2.89 2.82 2.81 2.80 2.80 2.80 2.79 2.79 2.76 2.76 2.75 2.73 2.37 2.26 2.26 2.26 2.25 2.25 2.25 2.24 2.01 2.00 2.00 1.99 1.99 1.98 1.98 1.97
NH
CH3
O
O
O
F
H3C
C-I-3
0.84
1.02
0.96
1.04
1.00
1.01
3.10
0.99
0.99
1.02
2.02
1.01
2.92
1.01
1.06
11.5 11.0 10.5 10.0 9.5 9.0 8.5 8.0 7.5 7.0 6.5 6.0 5.5 5.0 4.5 4.0 3.5 3.0 2.5 2.0 1.5 1.0 0.5
f1 (ppm)
3200 3000 2800 2600 2400 2200 2000 1800 1600 1400 1200 1000 800 600 400 200 0 -200

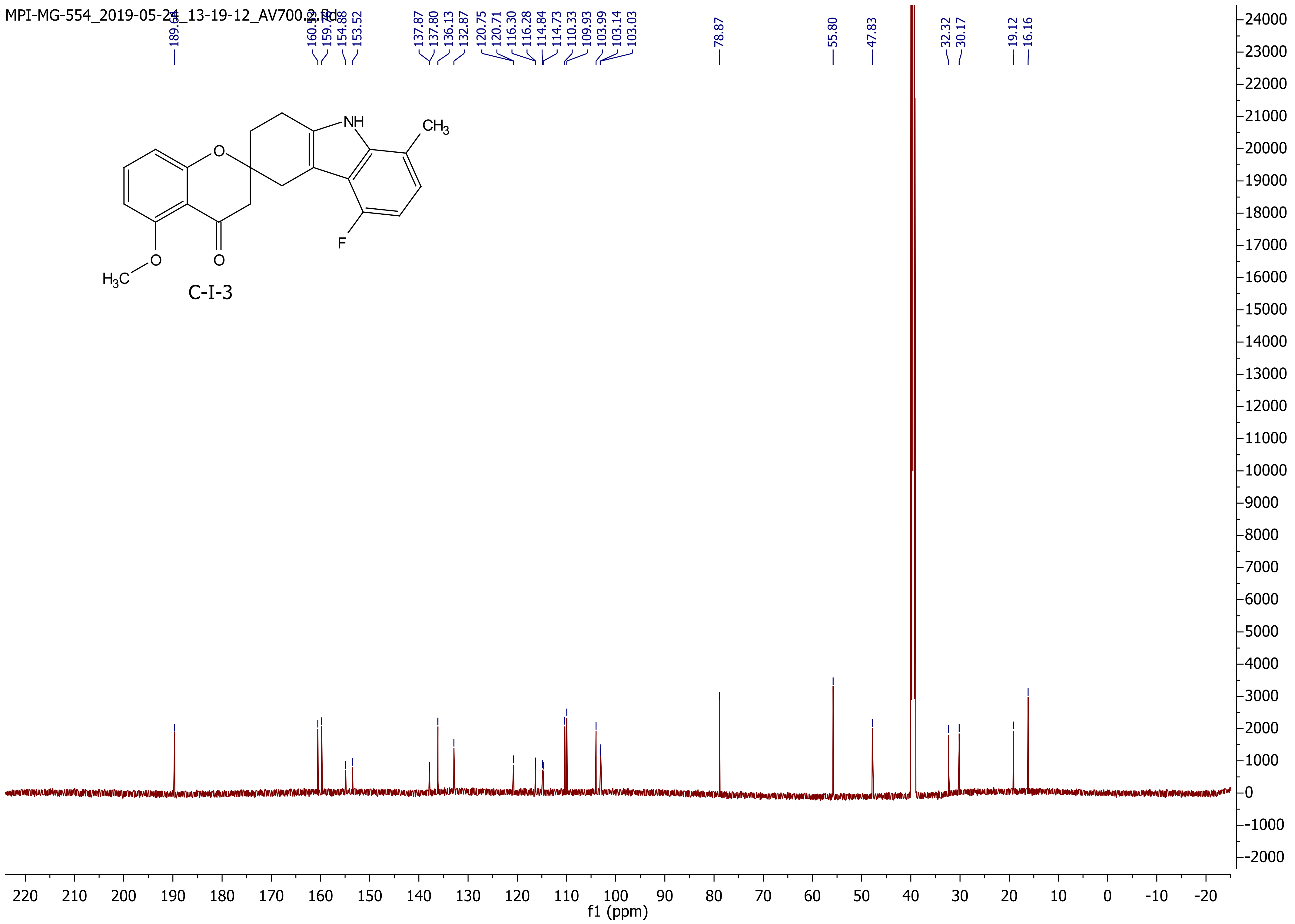
MPI-MG-554_2019-05-24_13-19-12_AV700.2.fid
154.88
153.52
137.87
137.80
136.13
132.87
120.75
120.71
116.30
116.28
114.84
114.73
110.33
109.93
103.99
103.14
103.03
78.87
55.80
47.83
32.32
30.17
19.12
16.16
NH
CH3
O
O
O
F
H3C
f1 (ppm)

C-I-3

MPI-MG-554_2019-05-27_15-36-53_AV500.10.fid

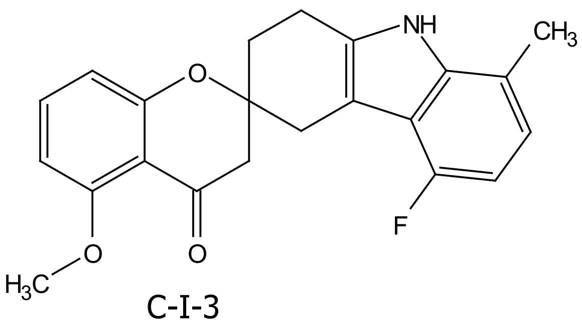

C-I-3

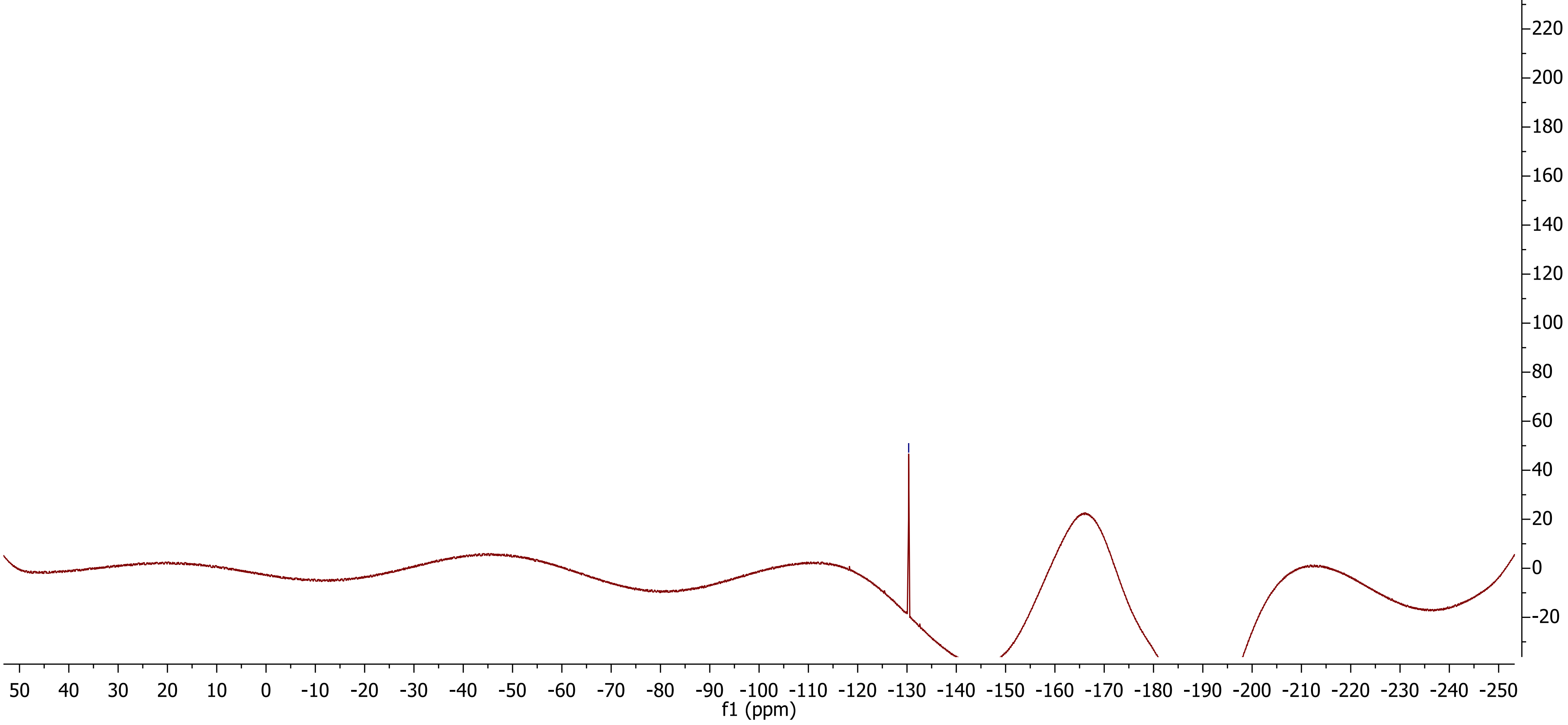

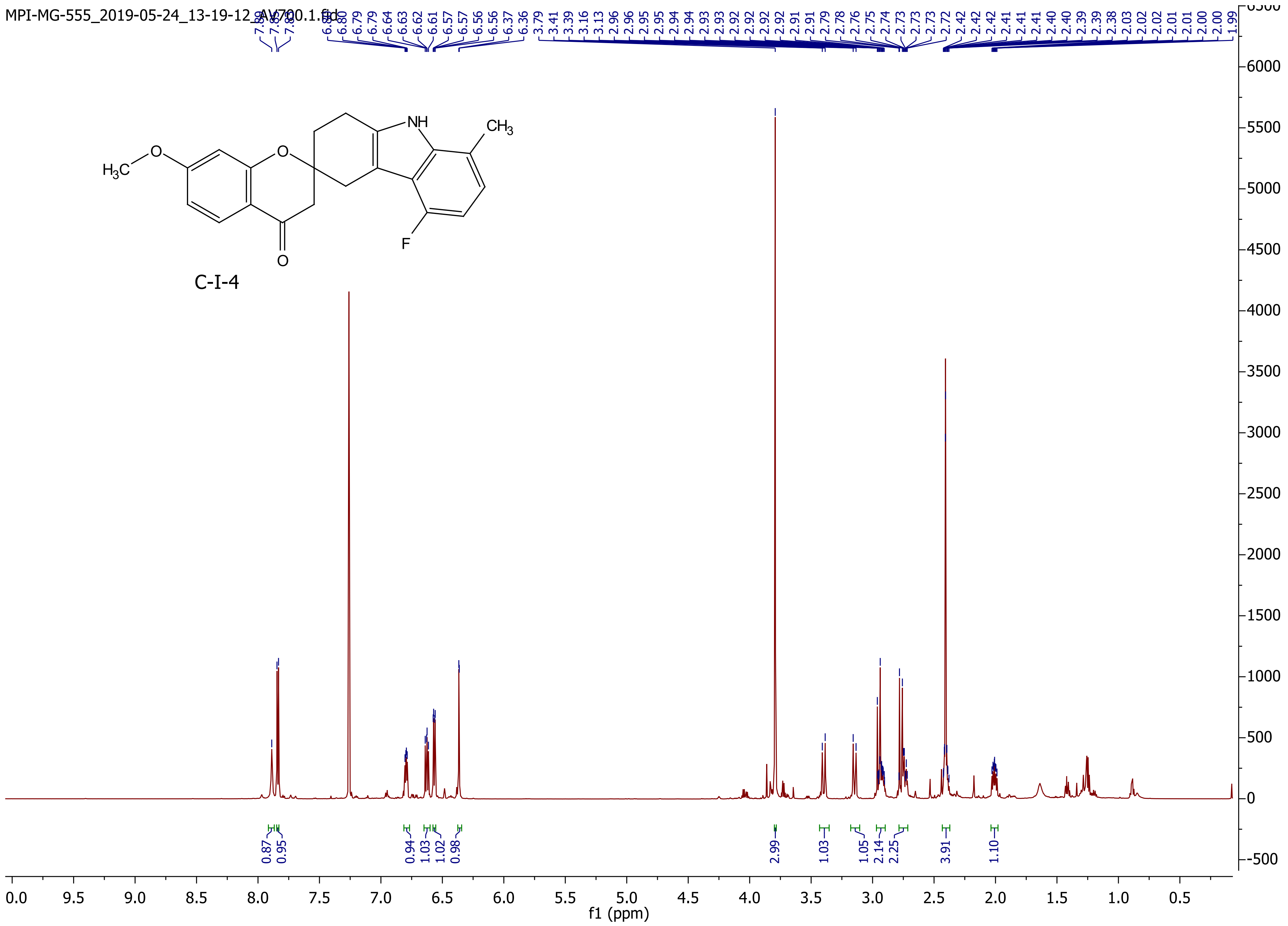
MPI-MG-555_2019-05-24_13-19-12_AV700.1.fid
6.80
6.79
6.79
6.64
6.63
6.62
6.61
6.57
6.57
6.56
6.56
6.37
6.36
3.79
3.41
3.39
3.16
3.13
2.96
2.96
2.95
2.95
2.94
2.94
2.93
2.93
2.92
2.92
2.92
2.92
2.91
2.91
2.79
2.78
2.76
2.75
2.74
2.73
2.73
2.73
2.72
2.42
2.42
2.42
2.41
2.41
2.41
2.40
2.40
2.39
2.39
2.38
2.03
2.02
2.02
2.01
2.01
2.00
2.00
1.99
NH
CH3
O
H3C
O
O
F
C-I-4
0.87
0.95
0.94
1.03
1.02
0.98
2.99
1.03
1.05
2.14
2.25
3.91
1.10
f1 (ppm)

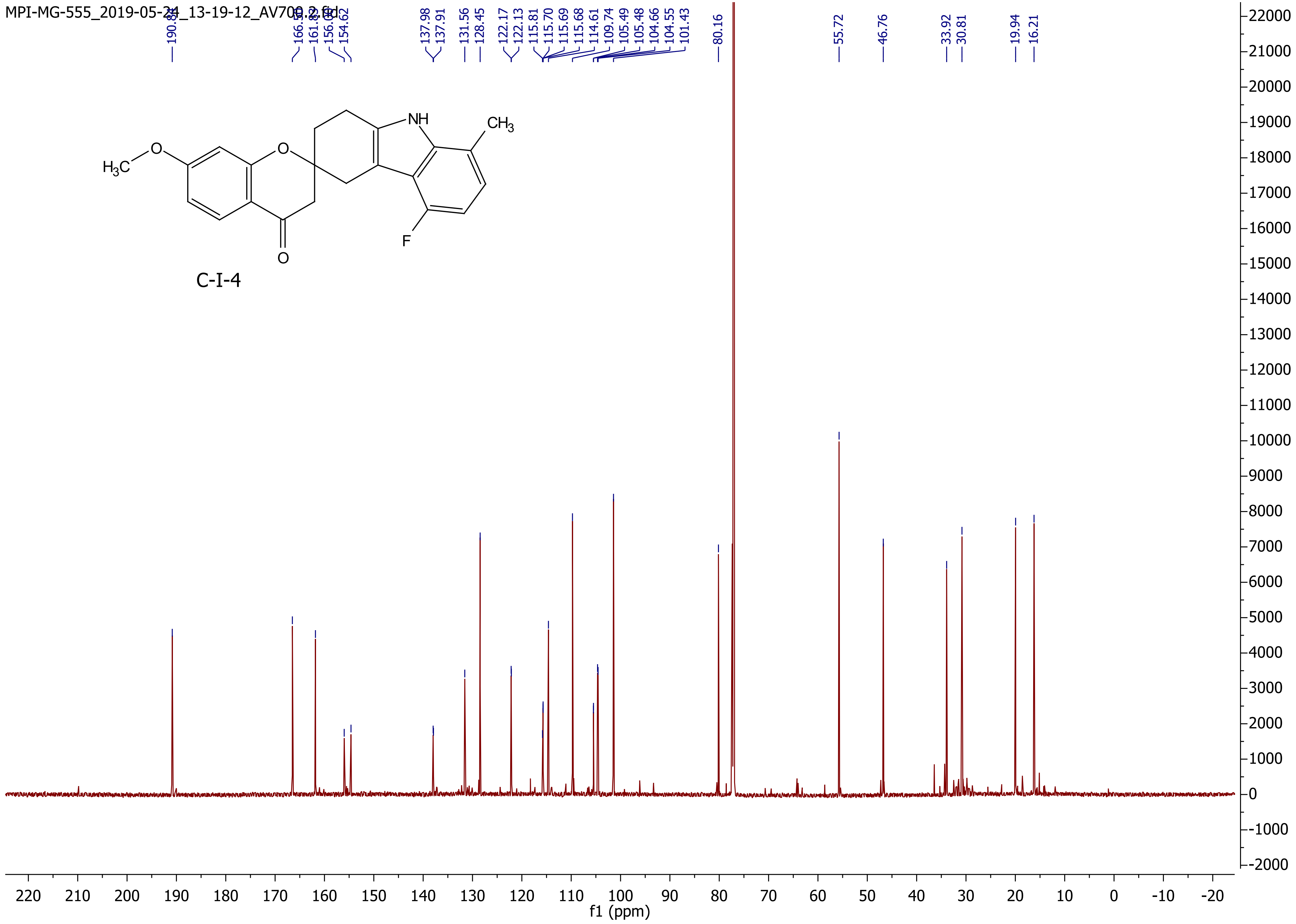

MPI-MG-555_2019-05-24_13-19-12_AV700.2.fid
137.98
137.91
131.56
128.45
122.17
122.13
115.81
115.70
115.69
115.68
114.61
109.74
105.49
105.48
104.66
104.55
101.43
80.16
55.72
46.76
33.92
30.81
19.94
16.21
154.62
161.8
NH
CH3
O
H3C
O
O
F
C-I-4
f1 (ppm)

MPI-MG-555_2019-05-27_15-36-53_AV500.10.fid

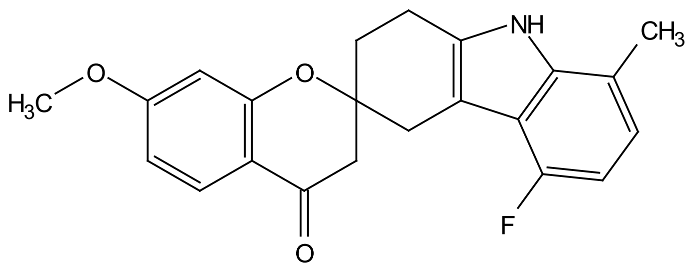

C-I-4

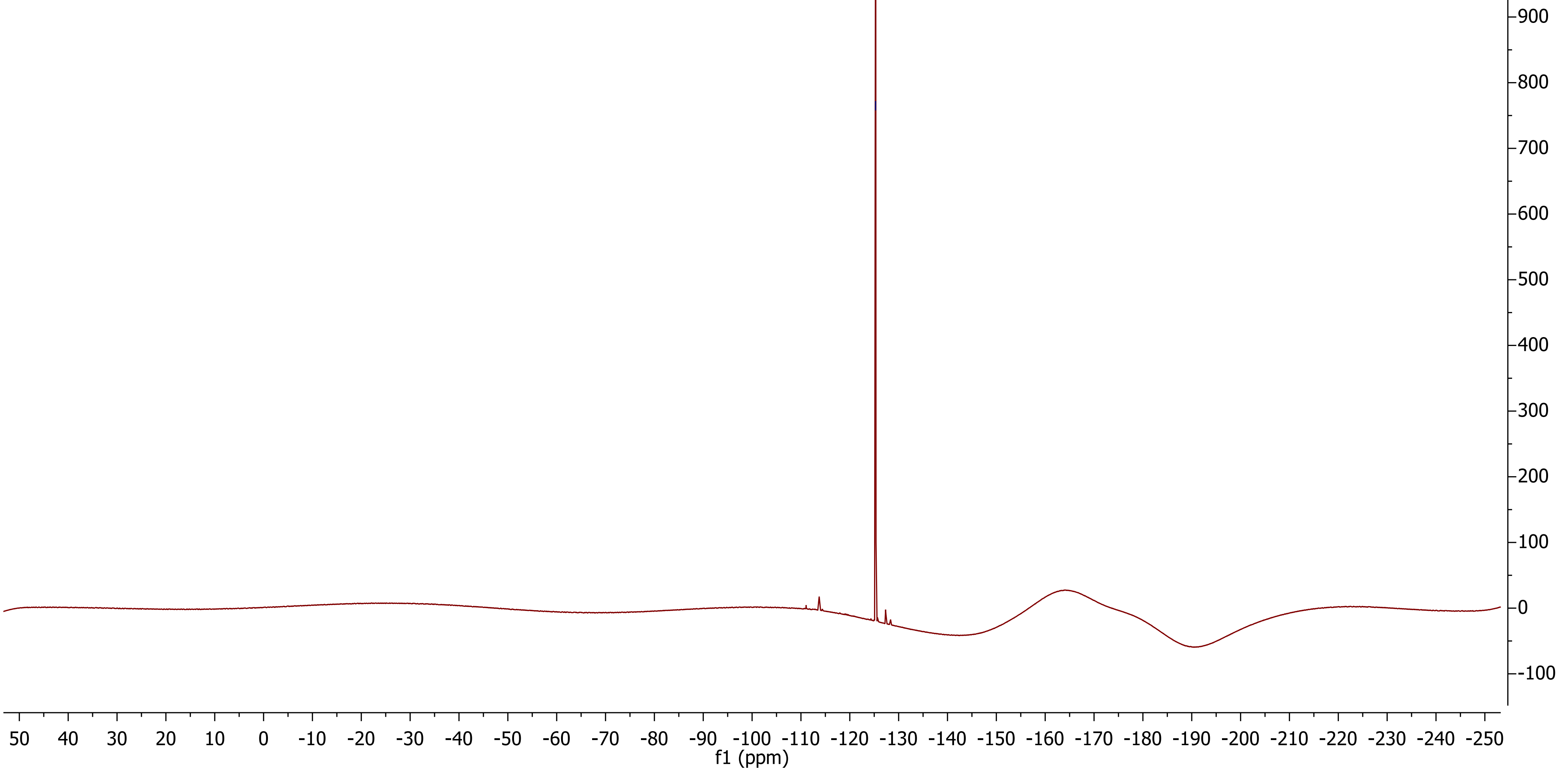

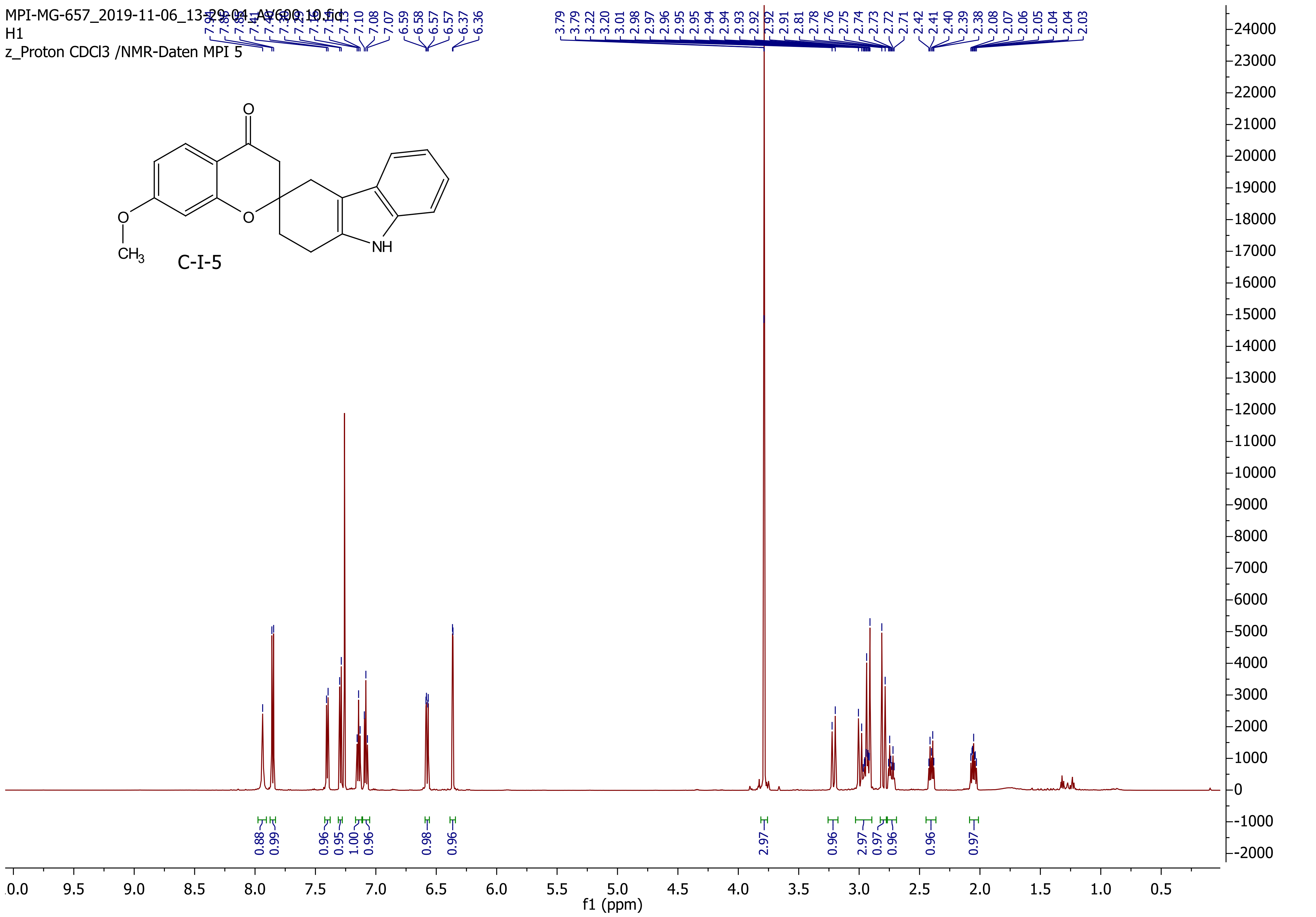

MPI-MG-657_2019-11-06_13-29-04_AV600.10.fid
H1
z_Proton CDCl3 /NMR-Daten MPI 5
C-I-5
f1 (ppm)

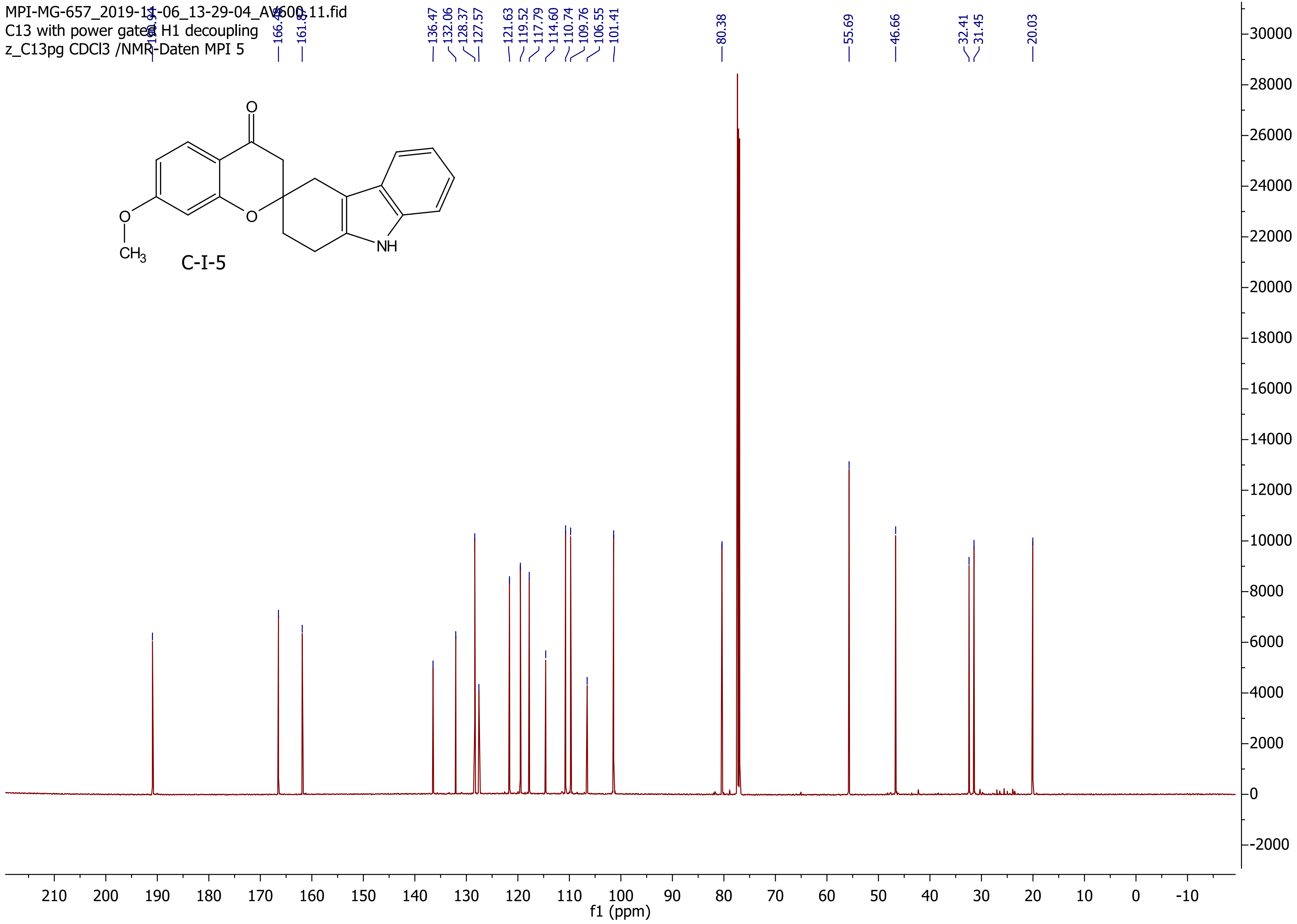
MPI-MG-657_2019-11-06_13-29-04_AV600.11.fid
C13 with power gated H1 decoupling
z_C13pg CDCl3 /NMR-Daten MPI 5
190.94
166.48
161.87
136.47
132.06
128.37
127.57
121.63
119.52
117.79
114.60
110.74
109.76
106.55
101.41
80.38
55.69
46.66
32.41
31.45
20.03
O
O
O
CH3
NH
C-I-5
f1 (ppm)

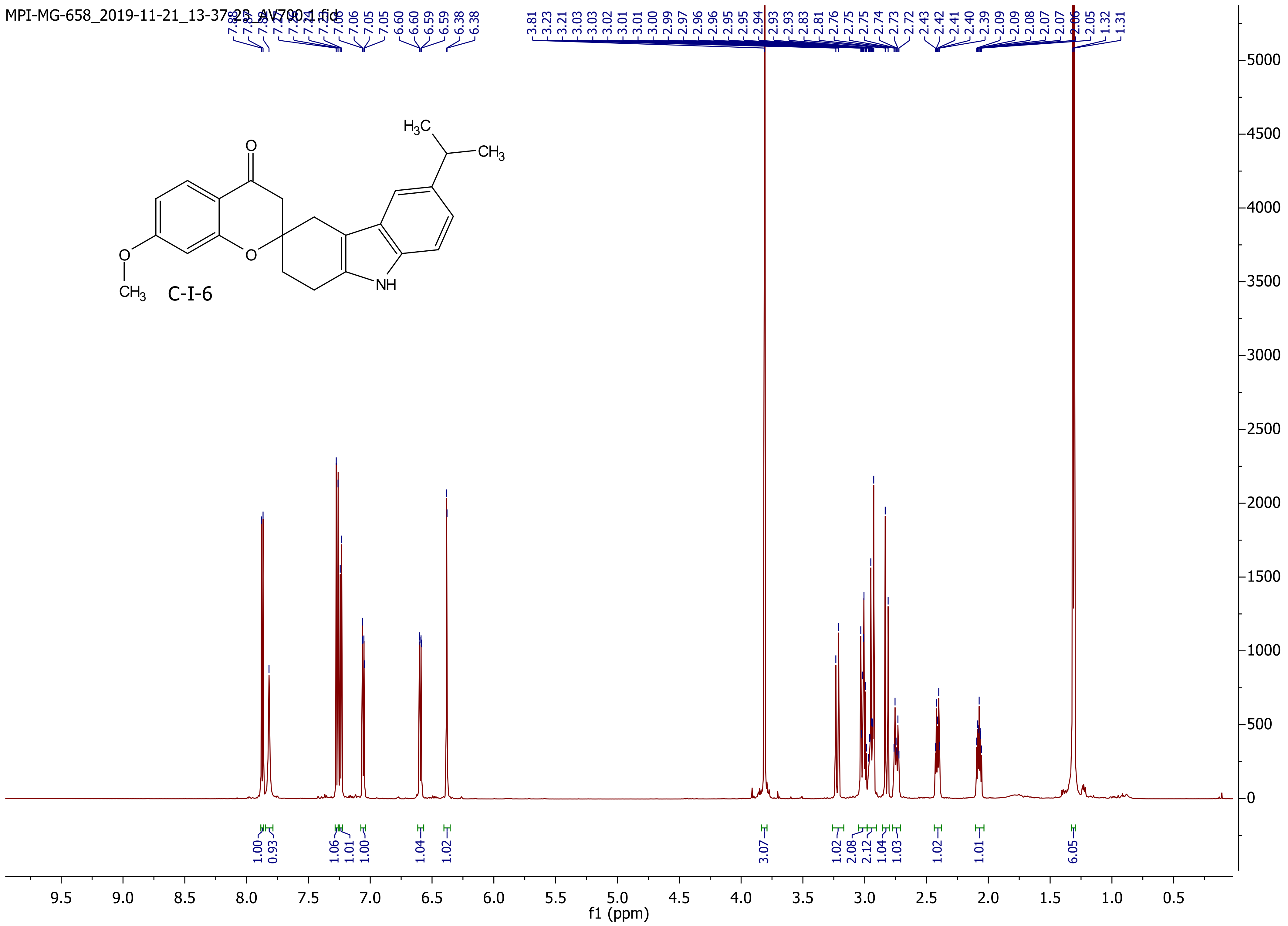
MPI-MG-658_2019-11-21_13-37-23_AV700-1.fid
C-I-6
H3C
CH3
O
O
O
CH3
NH
1.00
0.93
1.06
1.01
1.00
1.04
1.02
3.07
1.02
2.08
2.12
1.04
1.03
1.02
1.01
6.05
f1 (ppm)

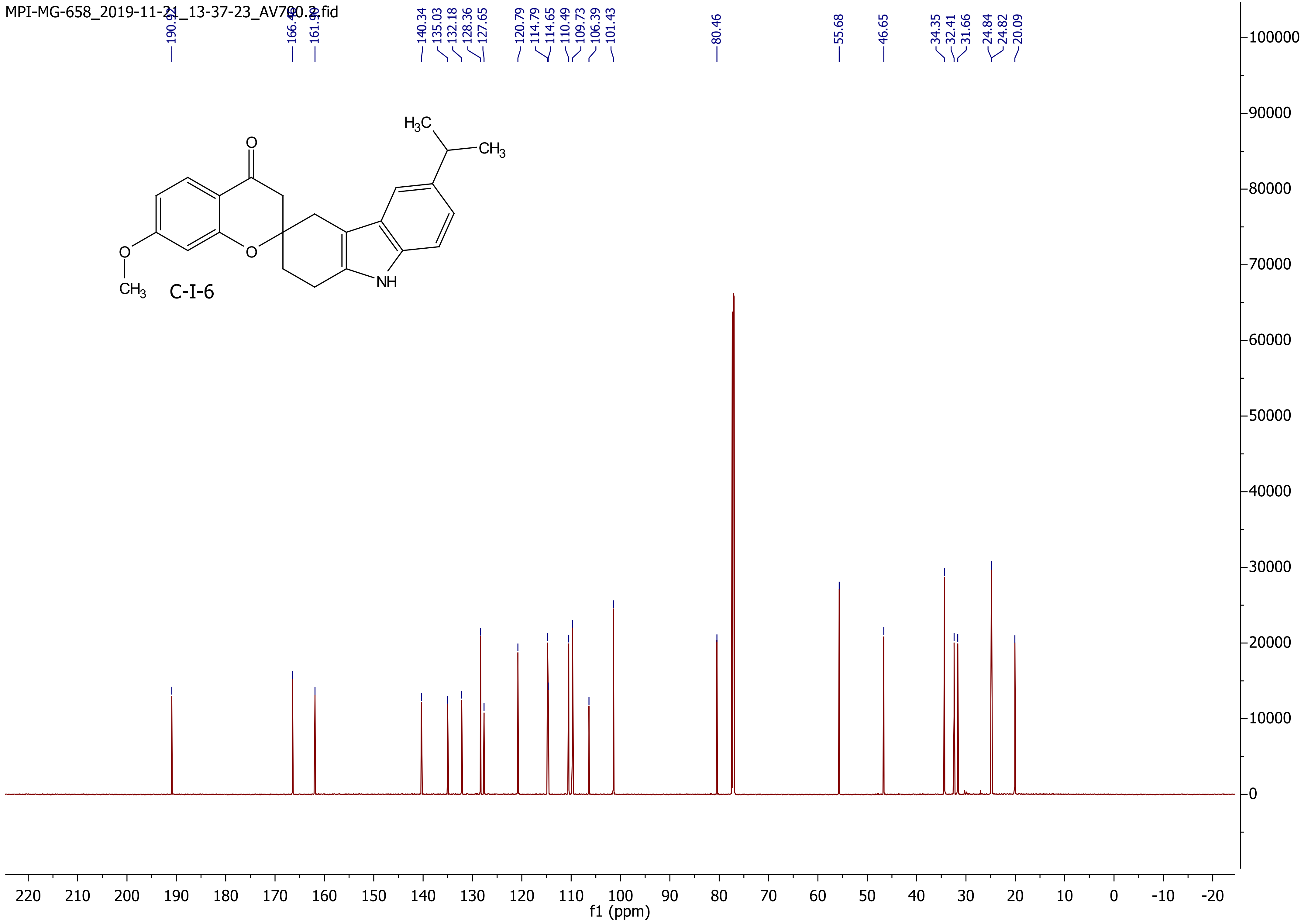

MPI-MG-658_2019-11-21_13-37-23_AV700.2.fid
140.34
135.03
132.18
128.36
127.65
120.79
114.79
114.65
110.49
109.73
106.39
101.43
80.46
55.68
46.65
34.35
32.41
31.66
24.84
24.82
20.09
H_3C
CH_3
O
O
O
NH
CH_3
C-I-6
f1 (ppm)

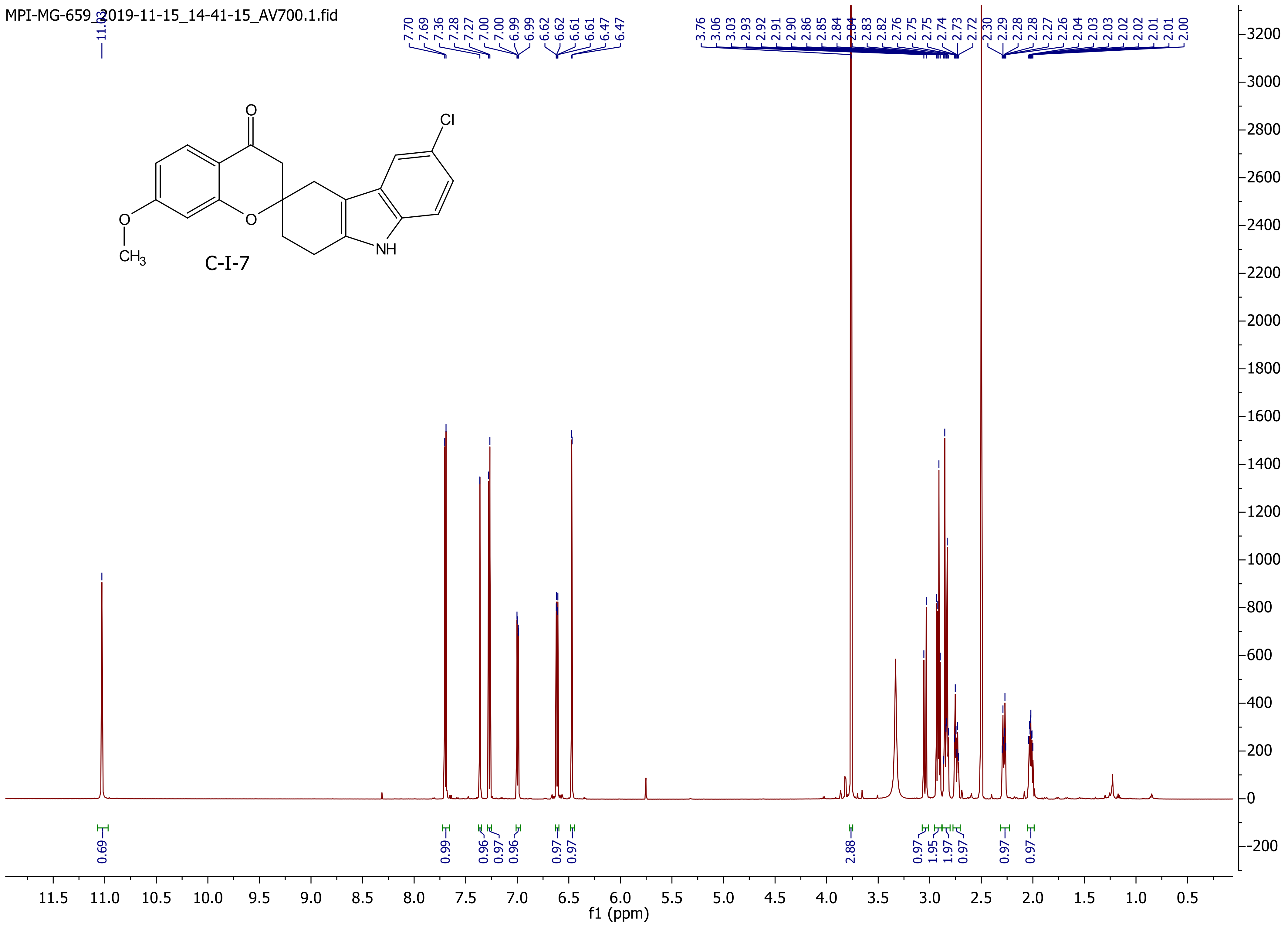

MPI-MG-659_2019-11-15_14-41-15_AV700.1.fid
7.70 7.69 7.36 7.28 7.27 7.00 7.00 6.99 6.99 6.62 6.62 6.61 6.61 6.47 6.47
3.76 3.06 3.03 2.93 2.92 2.91 2.90 2.86 2.85 2.84 2.84 2.83 2.82 2.76 2.75 2.74 2.74 2.73 2.73 2.72
2.30 2.29 2.28 2.28 2.27 2.26 2.04 2.03 2.03 2.02 2.02 2.01 2.01 2.00
O
Cl
O
O
NH
CH3
C-I-7
0.69
0.99 0.96 0.97 0.96 0.97 0.97
2.88
0.97 1.95 1.97 0.97
0.97 0.97
11.5 11.0 10.5 10.0 9.5 9.0 8.5 8.0 7.5 7.0 6.5 6.0 5.5 5.0 4.5 4.0 3.5 3.0 2.5 2.0 1.5 1.0 0.5
f1 (ppm)
3200 3000 2800 2600 2400 2200 2000 1800 1600 1400 1200 1000 800 600 400 200 0 -200

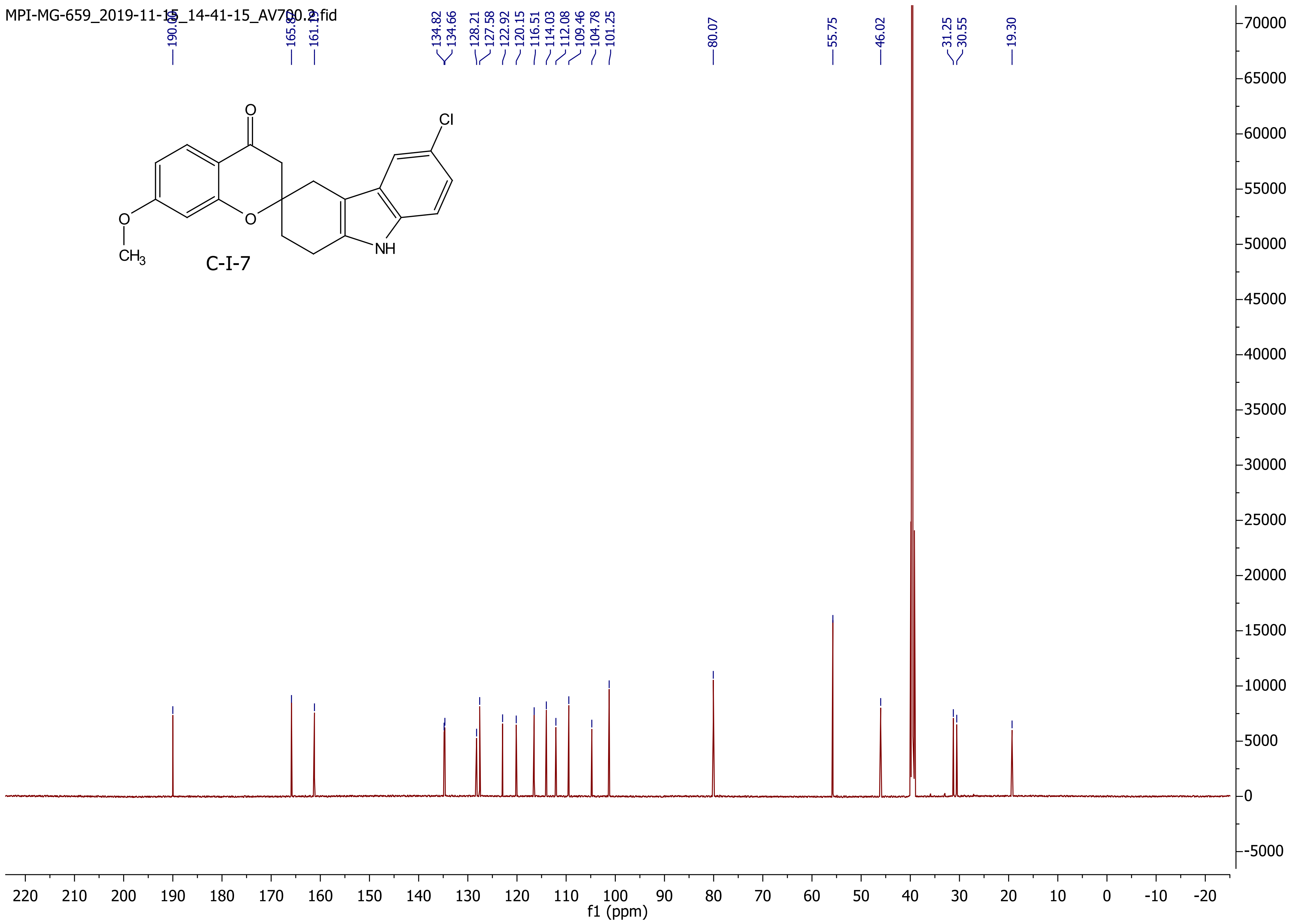
MPI-MG-659_2019-11-15_14-41-15_AV700.2.fid
134.82
134.66
128.21
127.58
122.92
120.15
116.51
114.03
112.08
109.46
104.78
101.25
80.07
55.75
46.02
31.25
30.55
19.30
O
Cl
O
O
CH3
NH
C-I-7
f1 (ppm)

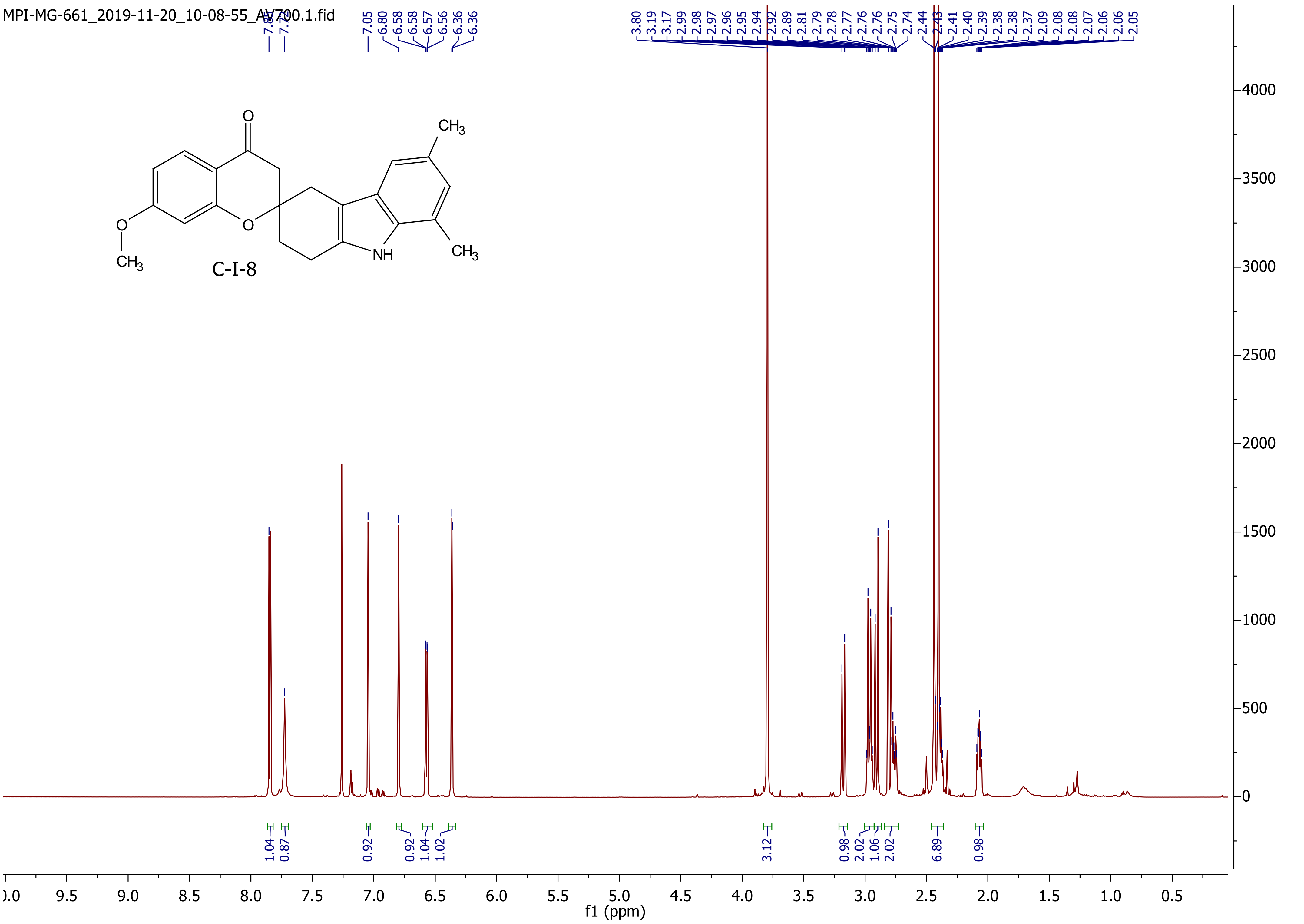

MPI-MG-661_2019-11-20_10-08-55_AV700.1.fid
7.05
6.80
6.58
6.58
6.57
6.56
6.36
6.36
3.80
3.19
3.17
2.99
2.98
2.97
2.96
2.95
2.94
2.92
2.89
2.81
2.79
2.78
2.77
2.76
2.76
2.75
2.74
2.44
2.43
2.41
2.40
2.39
2.38
2.38
2.37
2.09
2.08
2.08
2.07
2.06
2.06
2.05
O
CH_3
O
O
NH
CH_3
O
CH_3
C-I-8
1.04
0.87
0.92
0.92
1.04
1.02
3.12
0.98
2.02
1.06
2.02
6.89
0.98
4000
3500
3000
2500
2000
1500
1000
500
0
9.5
9.0
8.5
8.0
7.5
7.0
6.5
6.0
5.5
5.0
4.5
4.0
3.5
3.0
2.5
2.0
1.5
1.0
0.5
f1 (ppm)

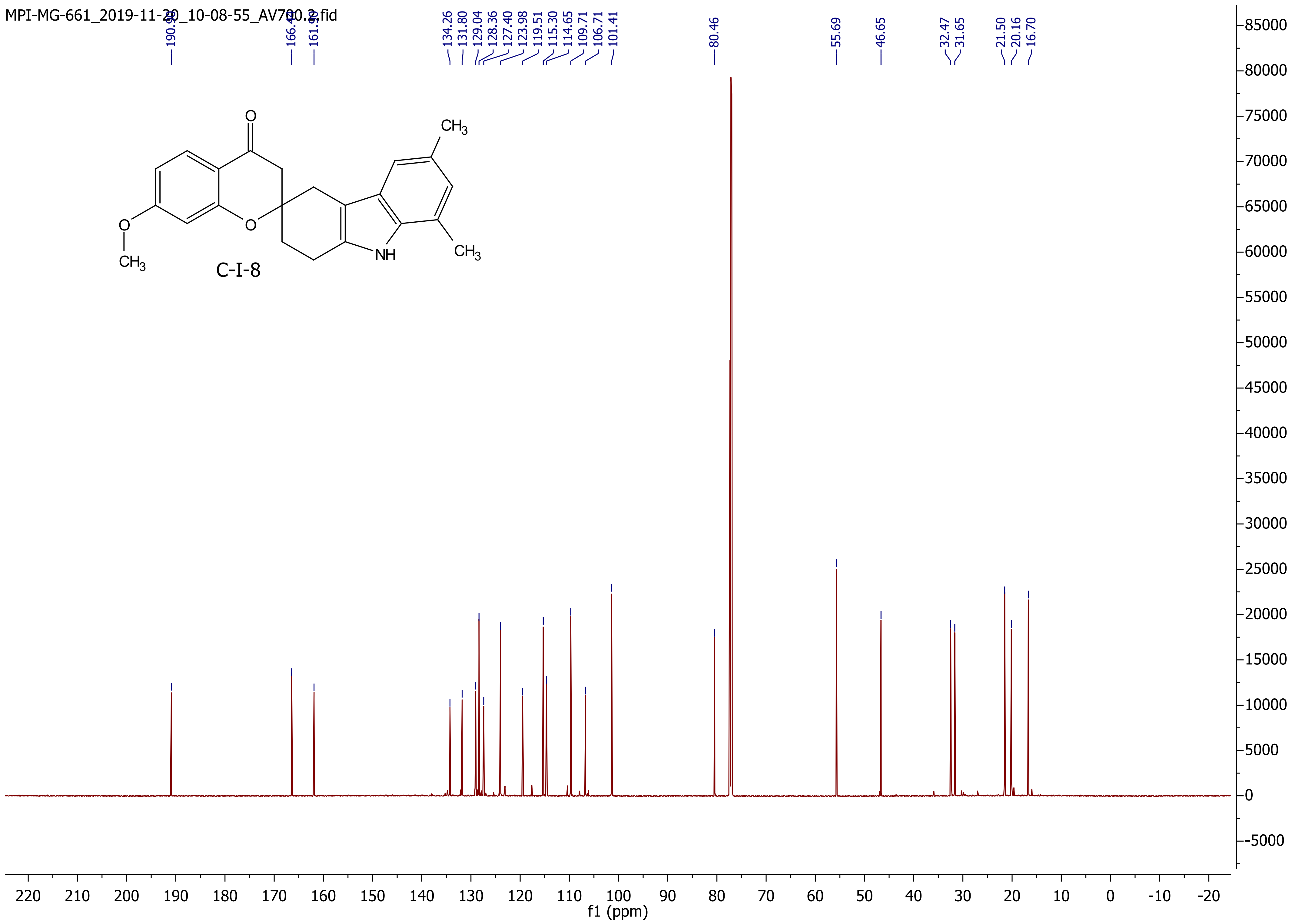
MPI-MG-661_2019-11-20_10-08-55_AV700.2.fid
134.26
131.80
129.04
128.36
127.40
123.98
119.51
115.30
114.65
109.71
106.71
101.41
80.46
55.69
46.65
32.47
31.65
21.50
20.16
16.70
O
CH3
O
O
CH3
NH
CH3
C-I-8
f1 (ppm)

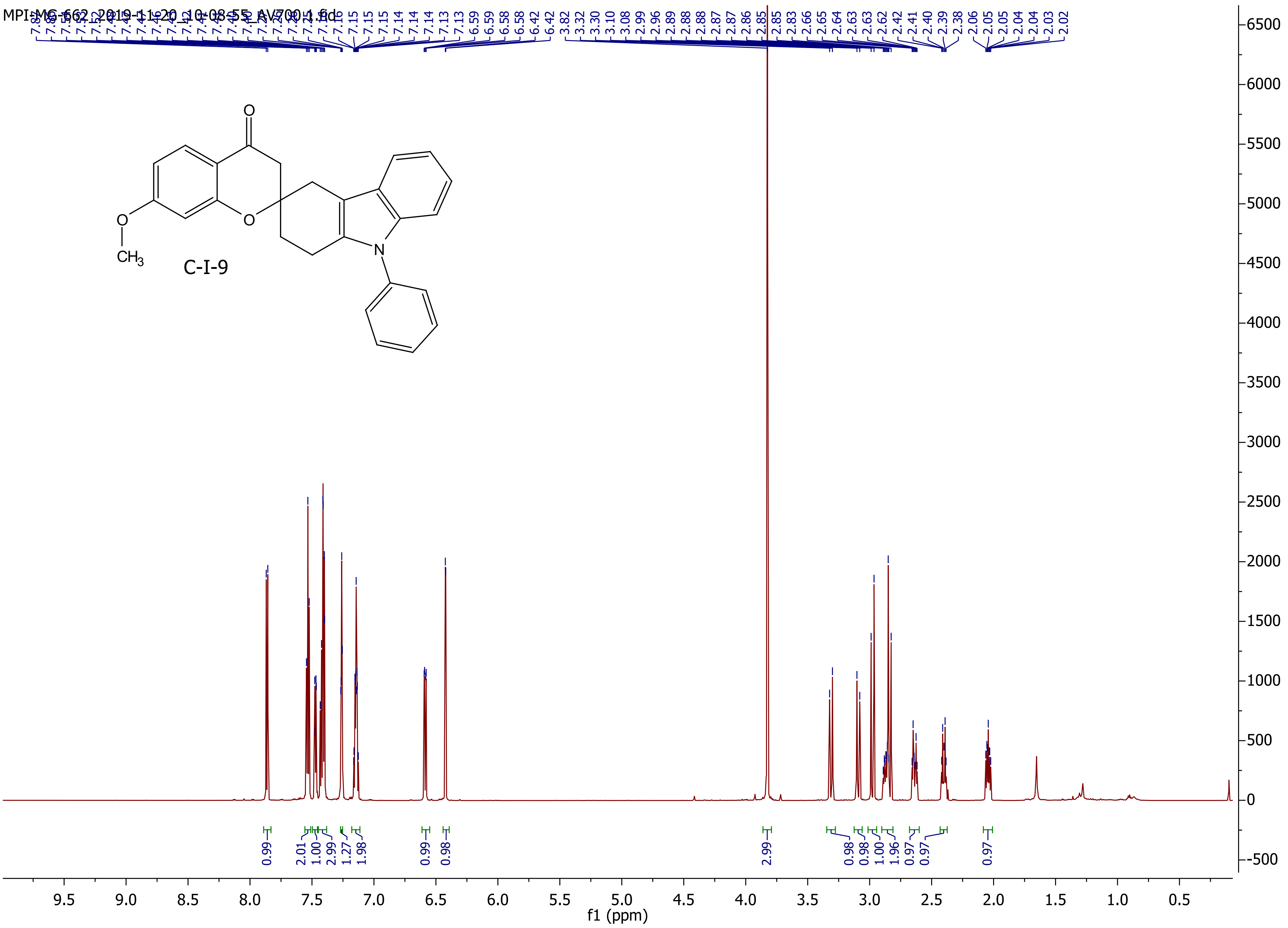
MPI-MG-662_2019-11-20_10-08-55_AV700-1.fid
7.15
7.15
7.15
7.14
7.14
7.14
7.13
6.59
6.59
6.58
6.58
6.42
3.82
3.32
3.30
3.10
3.08
2.99
2.96
2.89
2.88
2.88
2.87
2.87
2.86
2.85
2.85
2.83
2.66
2.65
2.64
2.63
2.63
2.62
2.42
2.41
2.40
2.39
2.38
2.06
2.05
2.05
2.04
2.04
2.03
2.02
O
O
O
N
CH3
C-I-9
0.99
2.01
1.00
2.99
1.27
1.98
0.99
0.98
2.99
0.98
0.98
1.00
1.96
0.97
0.97
0.97
f1 (ppm)

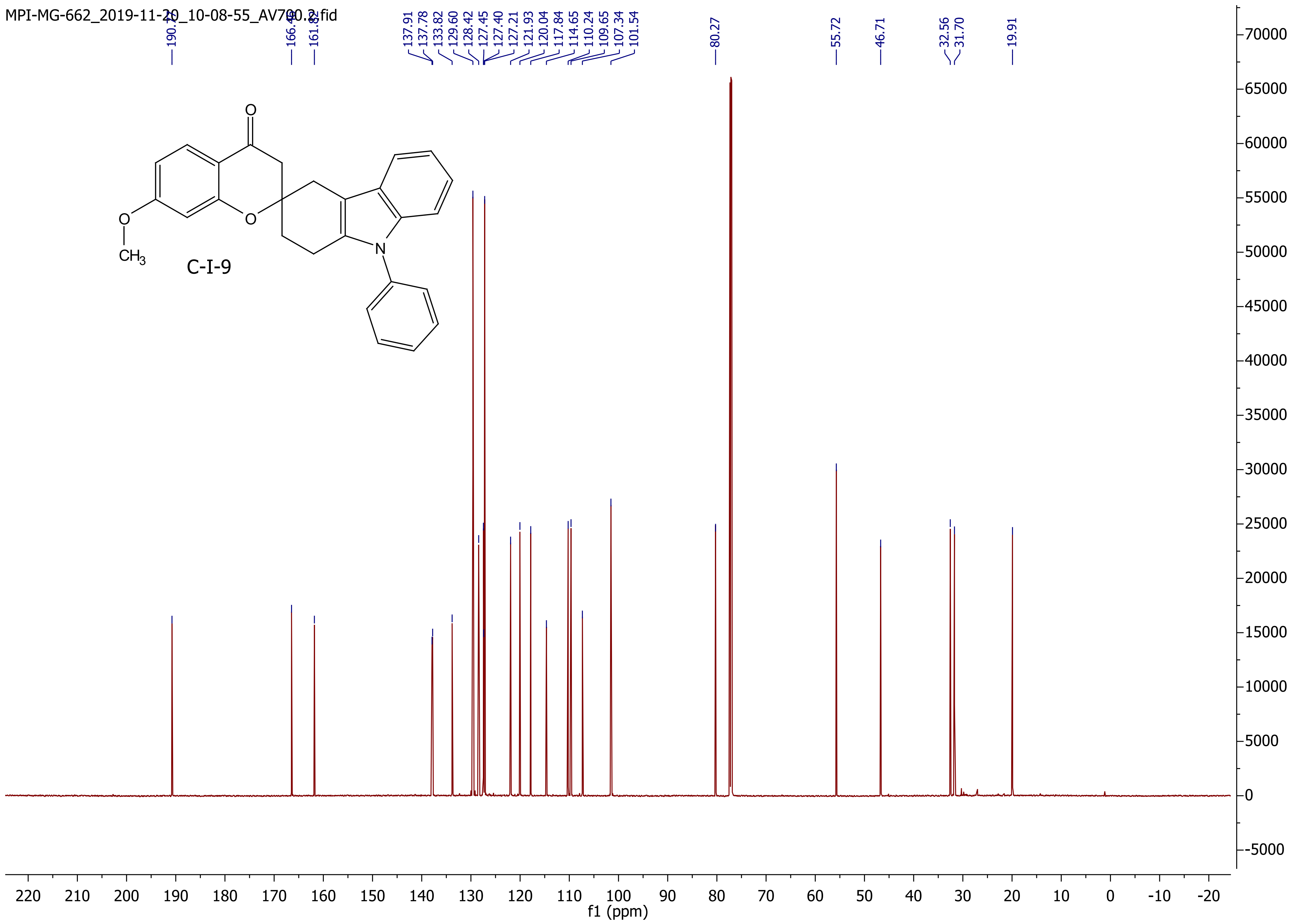
MPI-MG-662_2019-11-20_10-08-55_AV700.2.fid
190.7
166.4
161.8
137.91
137.78
133.82
129.60
128.42
127.45
127.40
127.21
121.93
120.04
117.84
114.65
110.24
109.65
107.34
101.54
80.27
55.72
46.71
32.56
31.70
19.91
O
O
O
CH3
N
C-I-9
f1 (ppm)

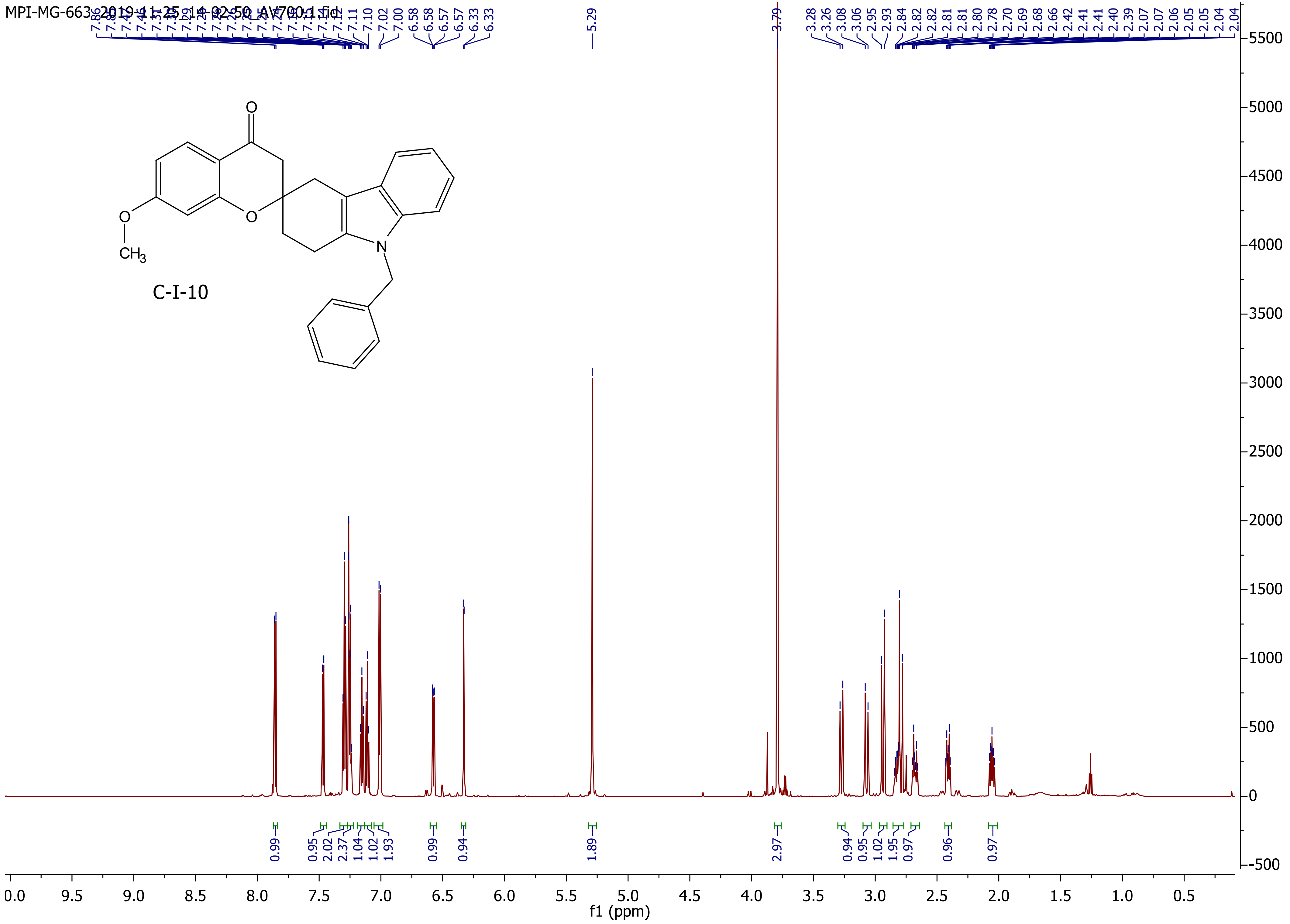
MPI-MG-663_2019-11-25_14-02-50_AV700-1.fid
7.86
7.11
7.10
7.02
7.00
6.58
6.58
6.57
6.57
6.33
6.33
5.29
3.79
3.28
3.26
3.08
3.06
2.95
2.93
2.84
2.82
2.82
2.81
2.81
2.80
2.78
2.70
2.69
2.68
2.66
2.42
2.41
2.41
2.40
2.39
2.07
2.07
2.06
2.05
2.05
2.04
2.04
O
O
O
CH3
N
C-I-10
0.99
0.95
2.02
2.37
1.04
1.02
1.93
0.99
0.94
1.89
2.97
0.94
0.95
1.02
1.95
0.97
0.96
0.97
f1 (ppm)

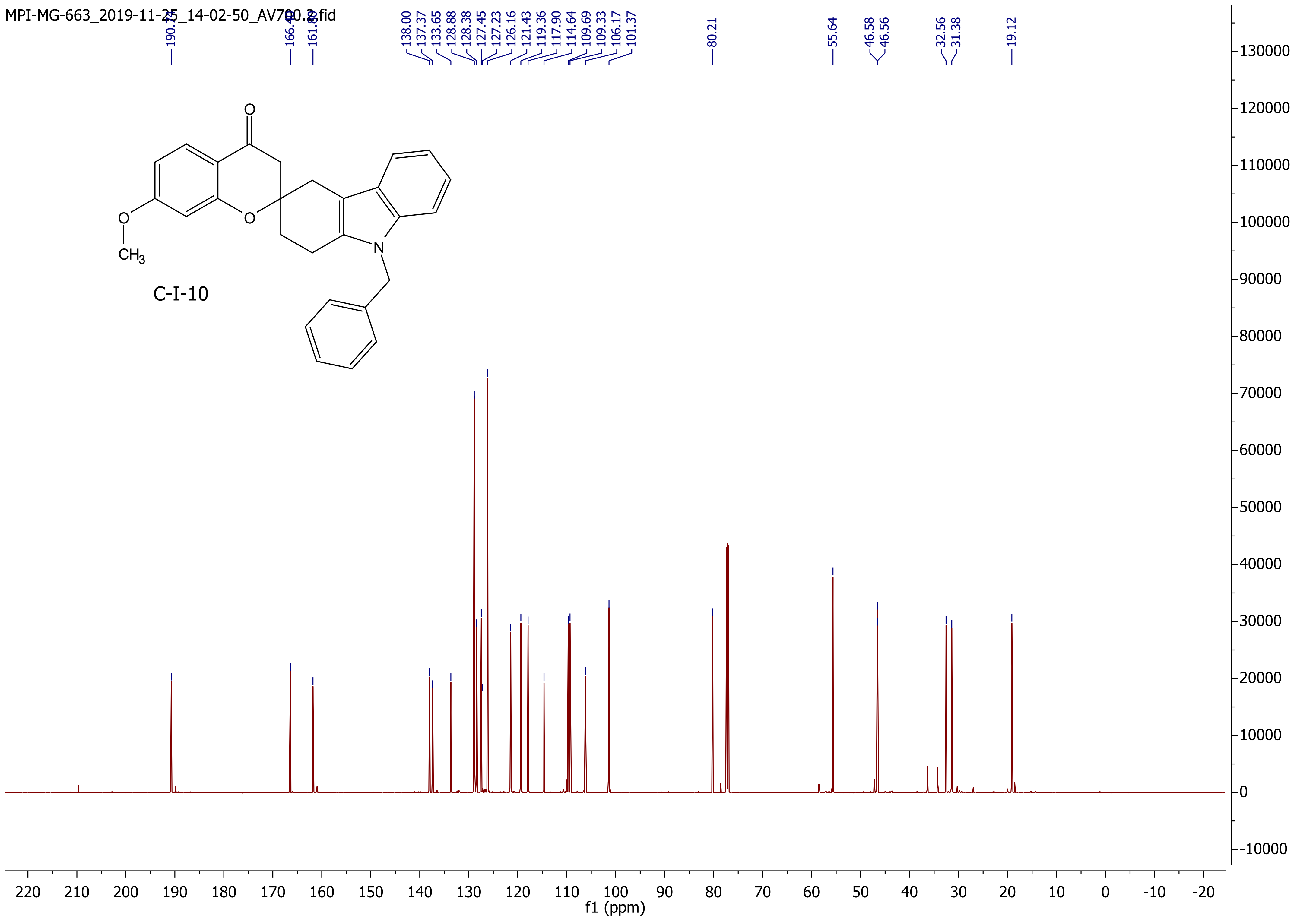

MPI-MG-663_2019-11-25_14-02-50_AV700.2.fid
138.00
137.37
133.65
128.88
128.38
127.45
127.23
126.16
121.43
119.36
117.90
114.64
109.69
109.33
106.17
101.37
80.21
55.64
46.58
46.56
32.56
31.38
19.12
O
O
O
CH3
N
C-I-10
f1 (ppm)

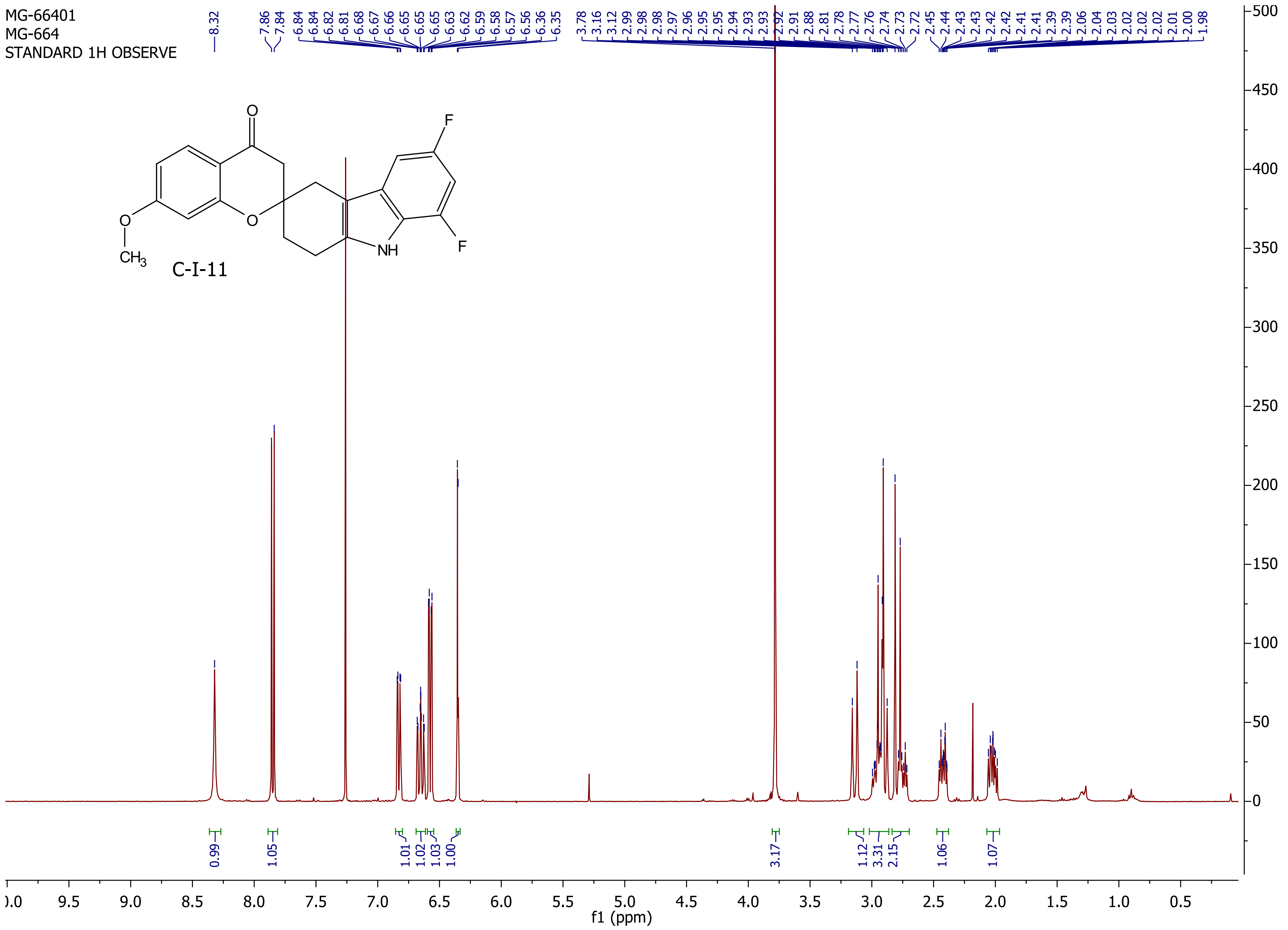
MG-66401
MG-664
STANDARD 1H OBSERVE
C-I-11
O
O
O
CH3
F
F
NH
8.32
7.86 7.84
6.84 6.84 6.82 6.81 6.68 6.67 6.66 6.65 6.65 6.65 6.63 6.62 6.59 6.58 6.57 6.56 6.36 6.35
3.78 3.16 3.12 2.99 2.98 2.98 2.97 2.96 2.95 2.95 2.94 2.93 2.93 2.92 2.91 2.88 2.81 2.78 2.77 2.76 2.74 2.73 2.72
2.45 2.44 2.43 2.43 2.42 2.42 2.41 2.41 2.39 2.06 2.04 2.03 2.02 2.02 2.02 2.01 2.00 1.98
0.99 1.05 1.01 1.02 1.03 1.00 3.17 1.12 3.31 2.15 1.06 1.07
f1 (ppm)
10.0 9.5 9.0 8.5 8.0 7.5 7.0 6.5 6.0 5.5 5.0 4.5 4.0 3.5 3.0 2.5 2.0 1.5 1.0 0.5
500 450 400 350 300 250 200 150 100 50 0

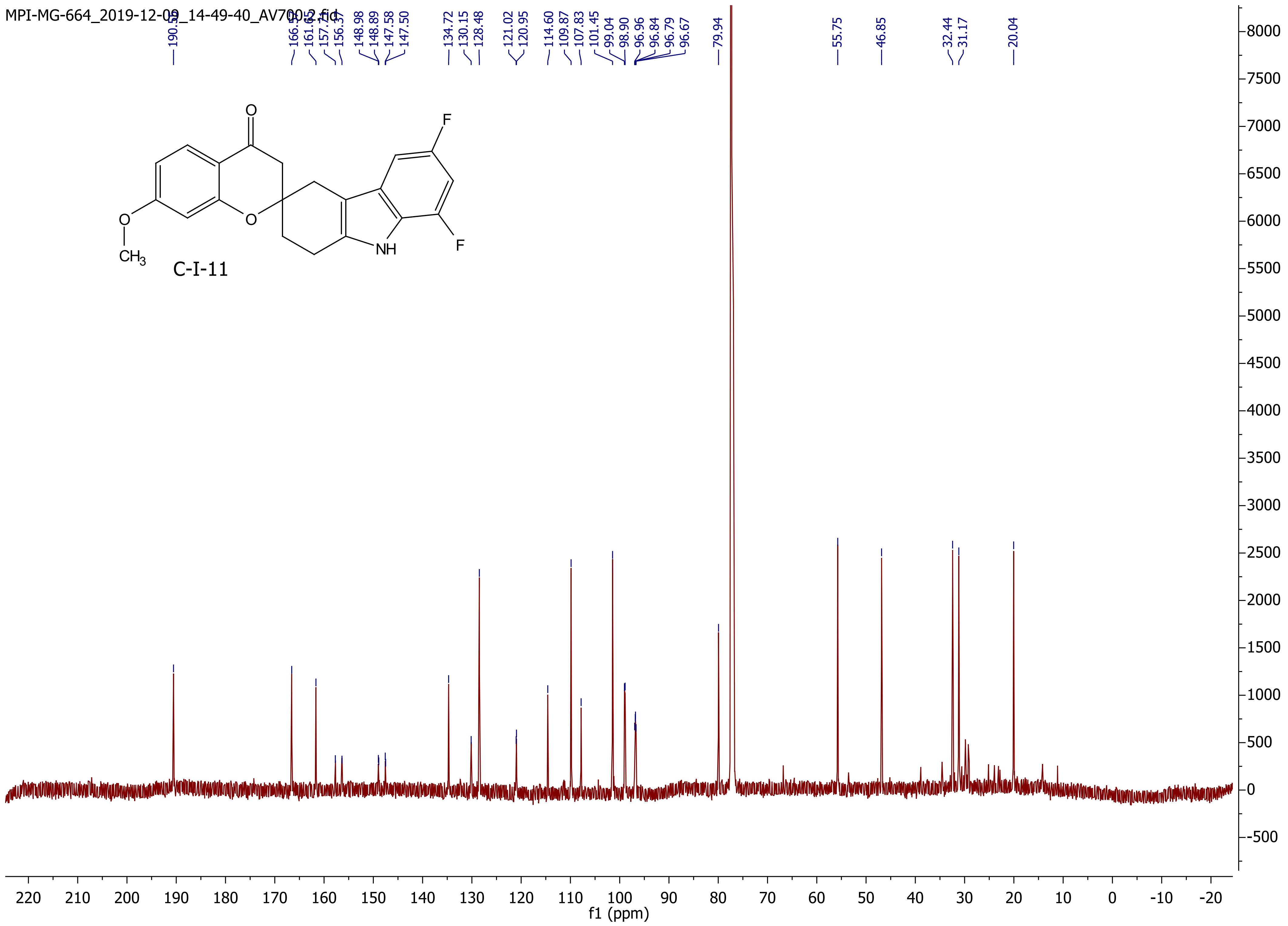
MPI-MG-664_2019-12-09_14-49-40_AV700.2.fid
190.55
166.57
161.65
157.71
156.37
148.98
148.89
147.58
147.50
134.72
130.15
128.48
121.02
120.95
114.60
109.87
107.83
101.45
99.04
98.90
96.96
96.84
96.79
96.67
79.94
55.75
46.85
32.44
31.17
20.04
O
O
O
CH3
F
F
NH
C-I-11
8000
7500
7000
6500
6000
5500
5000
4500
4000
3500
3000
2500
2000
1500
1000
500
0
-500
220
210
200
190
180
170
160
150
140
130
120
110
100
90
80
70
60
50
40
30
20
10
0
-10
-20
f1 (ppm)

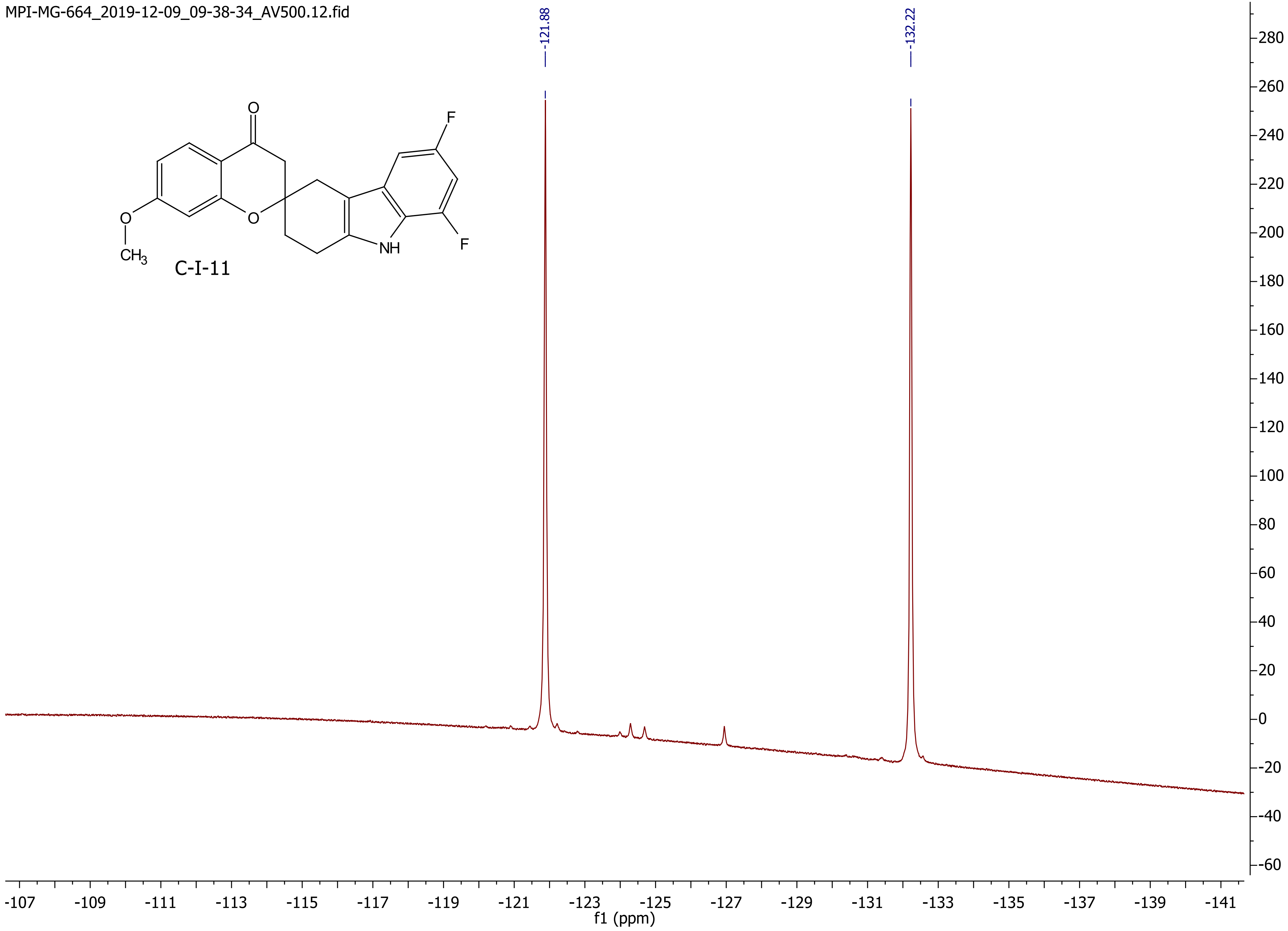
MPI-MG-664_2019-12-09_09-38-34_AV500.12.fid
-121.88
-132.22
O
F
O
O
NH
F
CH3
C-I-11
280
260
240
220
200
180
160
140
120
100
80
60
40
20
0
-20
-40
-60
-107
-109
-111
-113
-115
-117
-119
-121
-123
-125
-127
-129
-131
-133
-135
-137
-139
-141
f1 (ppm)

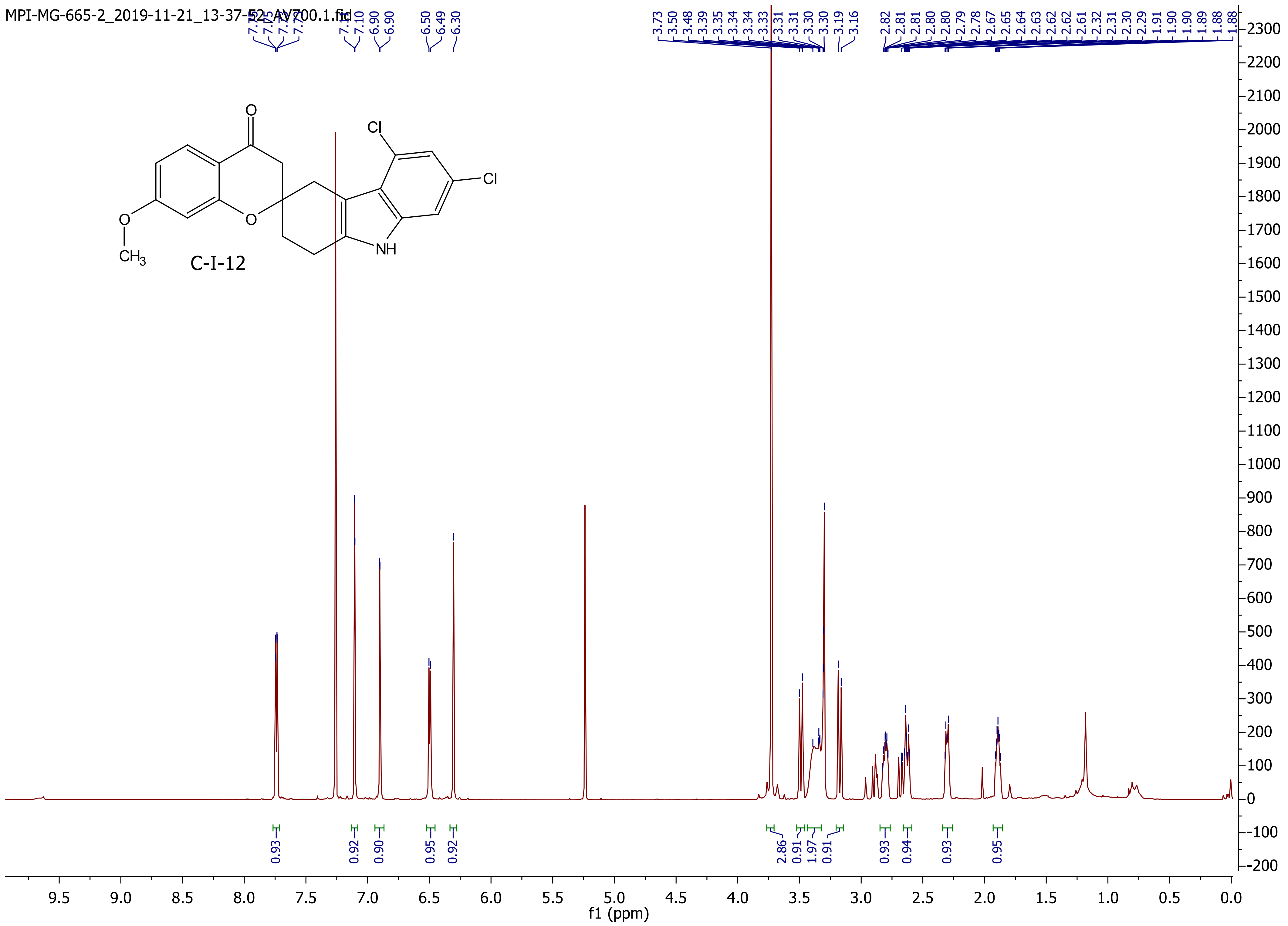
MPI-MG-665-2_2019-11-21_13-37-52_AV700.1.fid
C-I-12
f1 (ppm)

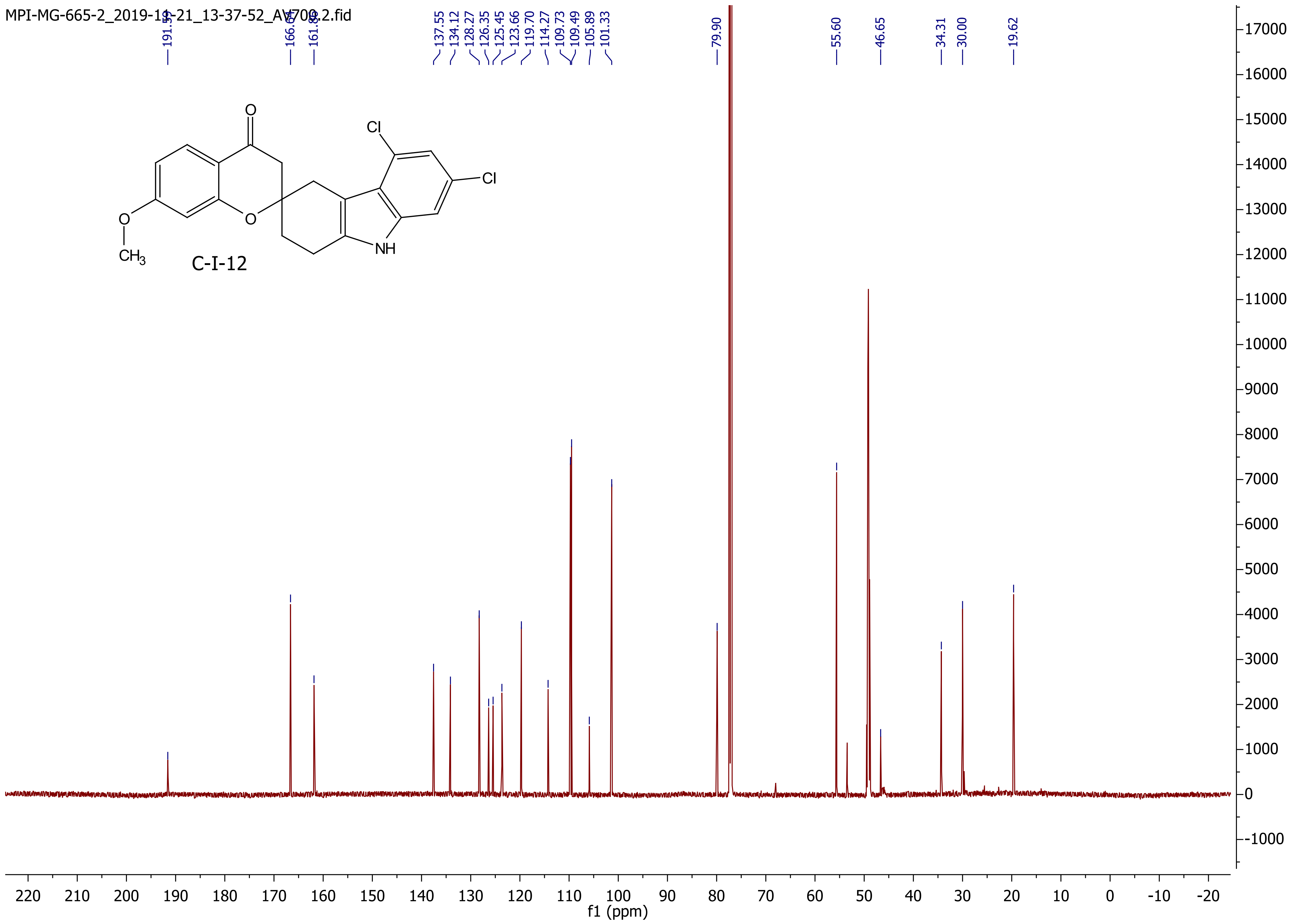

MPI-MG-665-2_2019-11-21_13-37-52_AV700.2.fid
166.6
161.8
137.55
134.12
128.27
126.35
125.45
123.66
119.70
114.27
109.73
109.49
105.89
101.33
79.90
55.60
46.65
34.31
30.00
19.62
O
O
Cl
Cl
NH
O
CH3
C-I-12
f1 (ppm)

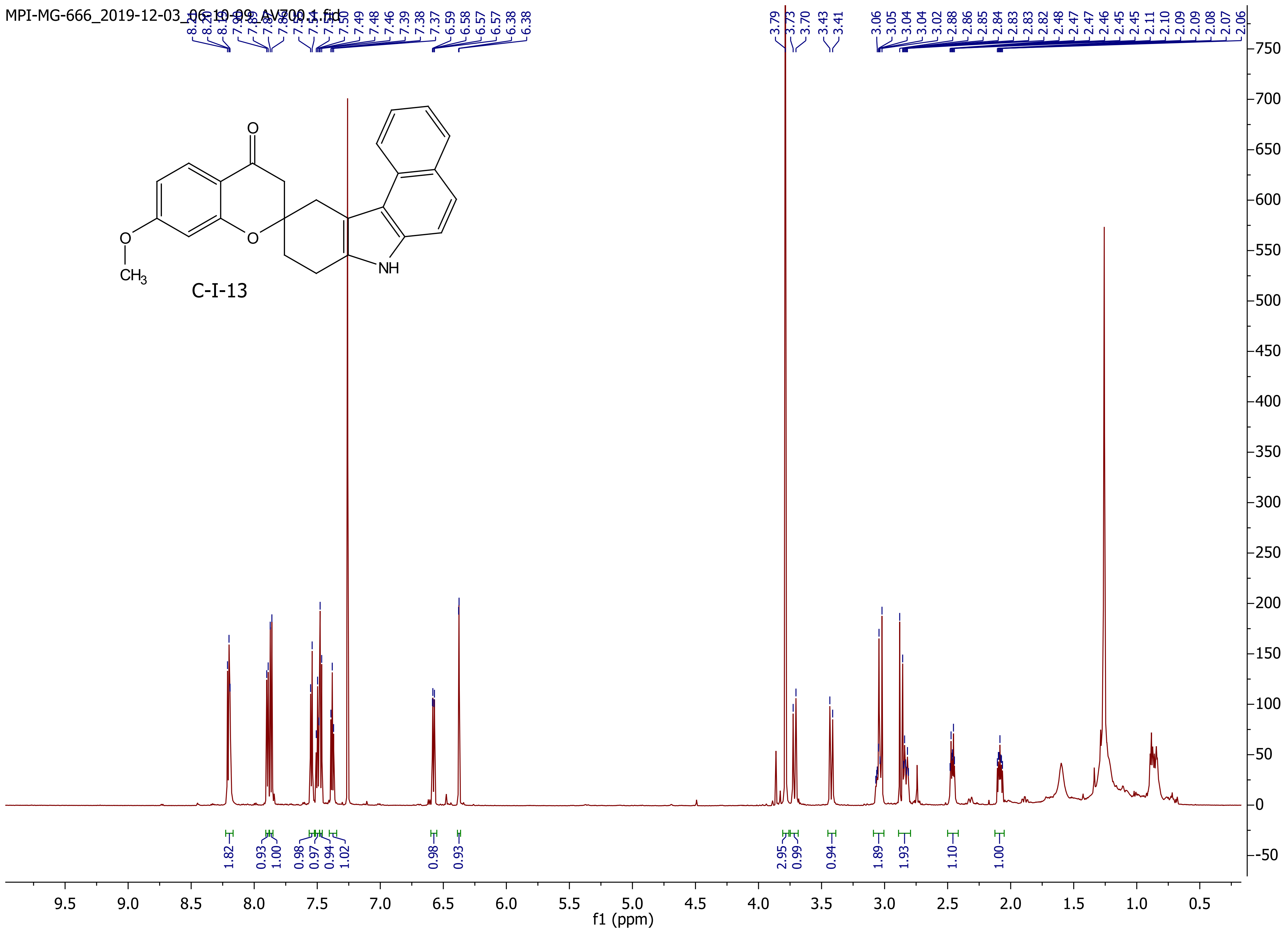

MPI-MG-666_2019-12-03_06-10-09_AV700.1.fid
7.50
7.49
7.48
7.46
7.39
7.38
7.37
6.59
6.58
6.57
6.57
6.38
6.38
3.79
3.73
3.70
3.43
3.41
3.06
3.05
3.04
3.04
3.02
2.88
2.86
2.85
2.84
2.83
2.83
2.82
2.48
2.47
2.47
2.46
2.45
2.45
2.11
2.10
2.09
2.09
2.08
2.07
2.06
O
O
O
CH3
NH
C-I-13
1.82
0.93
1.00
0.98
0.97
0.94
1.02
0.98
0.93
2.95
0.99
0.94
1.89
1.93
1.10
1.00
f1 (ppm)

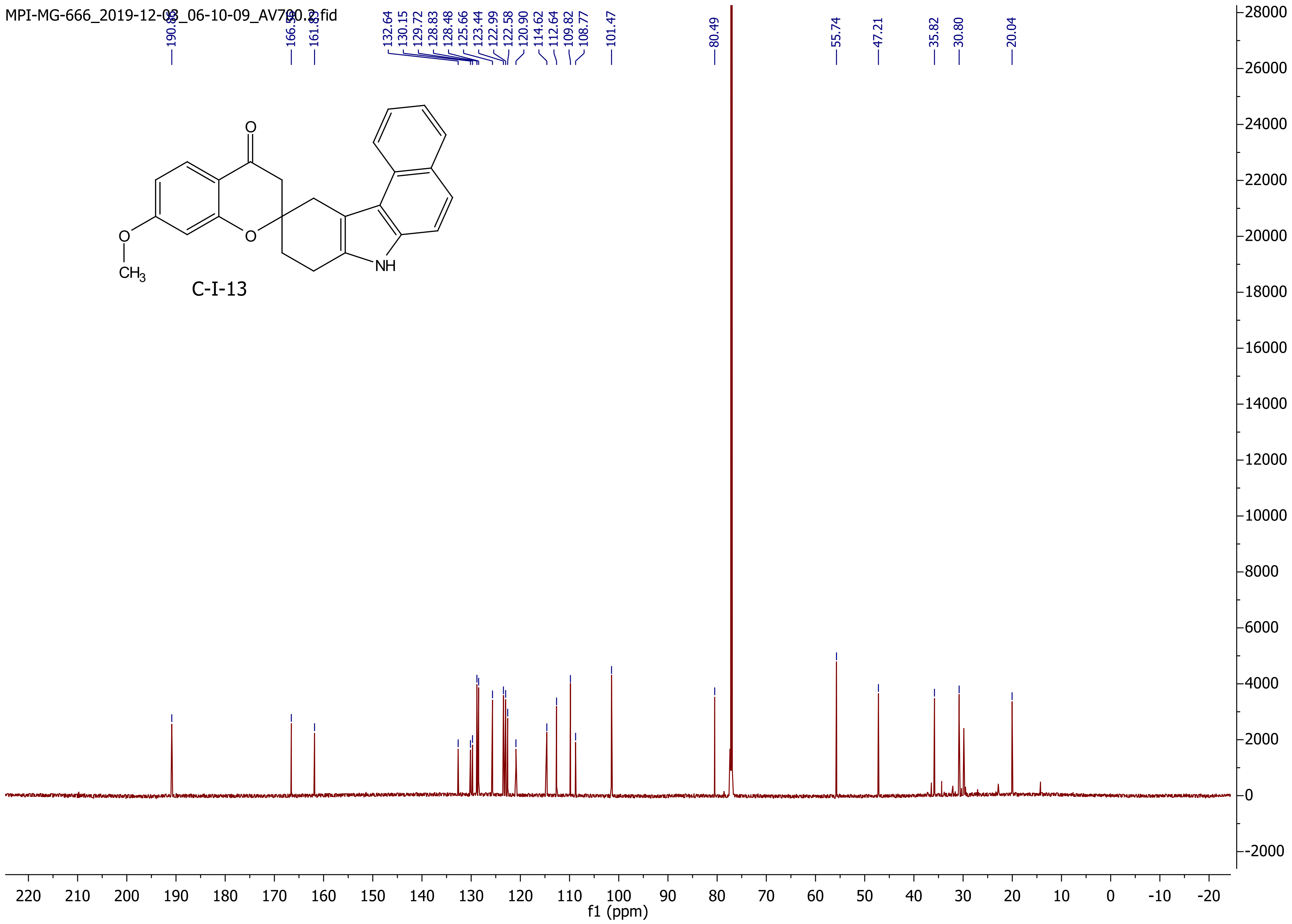

132.64
130.15
129.72
128.83
128.48
125.66
123.44
122.99
122.58
120.90
114.62
112.64
109.82
108.77
101.47
80.49
55.74
47.21
35.82
30.80
20.04
O
O
O
CH3
NH
C-I-13
f1 (ppm)

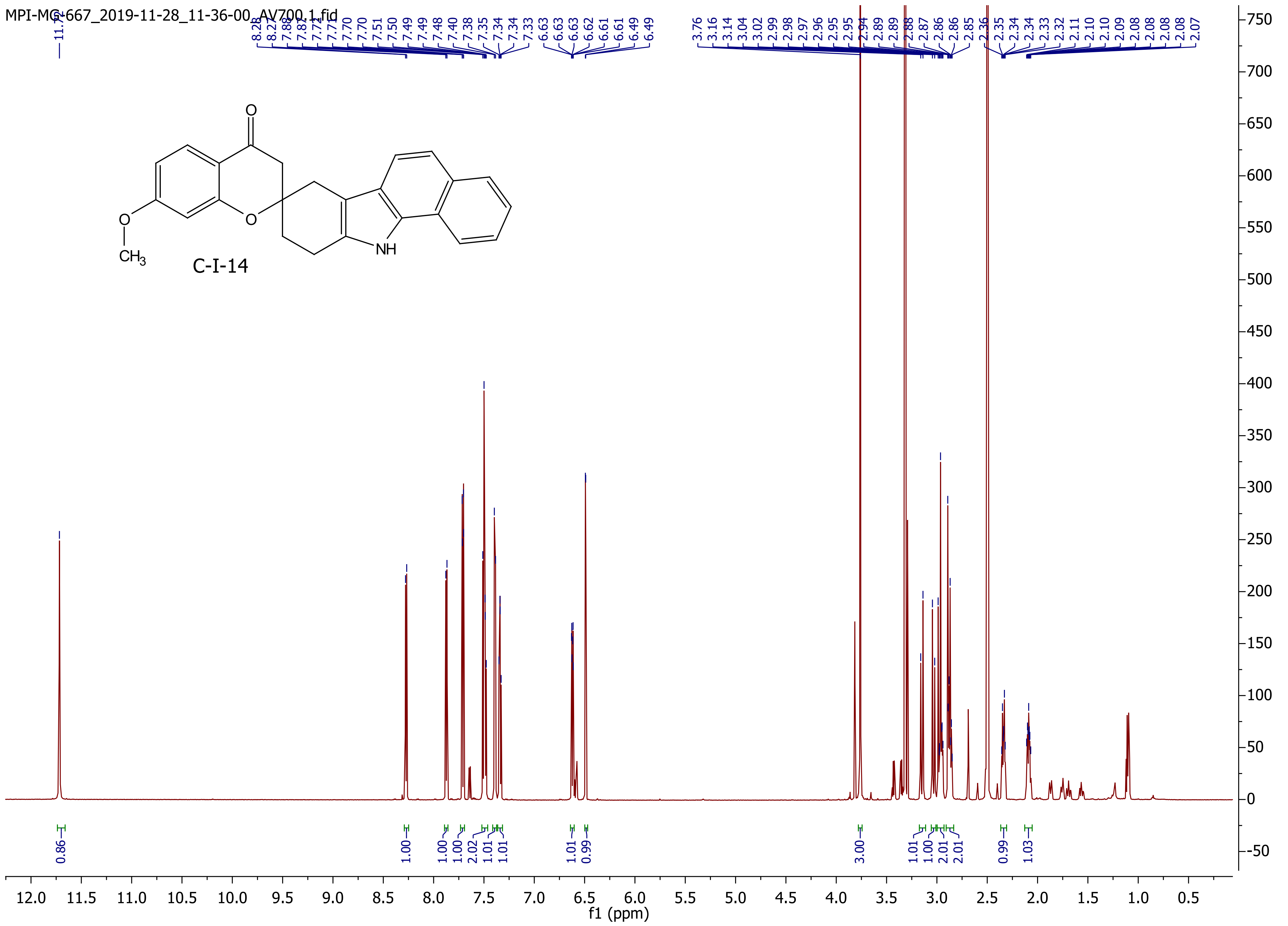
MPI-MG-667_2019-11-28_11-36-00_AV700.1.fid
O
O
O
CH3
NH
C-I-14
0.86
1.00
1.00
1.00
2.02
1.01
1.01
1.01
0.99
3.00
1.01
1.00
2.01
2.01
0.99
1.03
f1 (ppm)

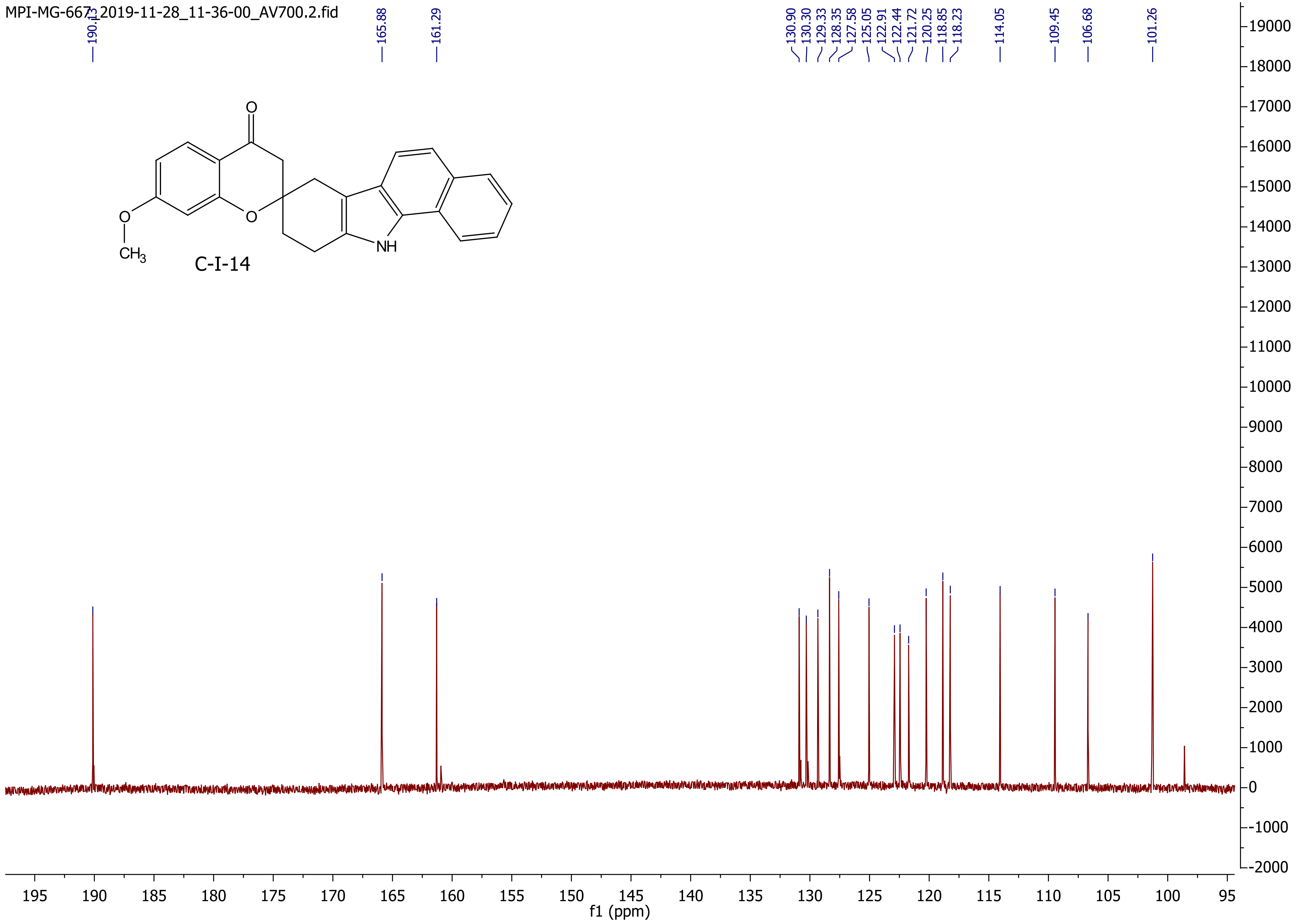
MPI-MG-667_2019-11-28_11-36-00_AV700.2.fid
190.13
165.88
161.29
130.90
130.30
129.33
128.35
127.58
125.05
122.91
122.44
121.72
120.25
118.85
118.23
114.05
109.45
106.68
101.26
O
O
O
CH3
NH
f1 (ppm)
195 190 185 180 175 170 165 160 155 150 145 140 135 130 125 120 115 110 105 100 95
19000 18000 17000 16000 15000 14000 13000 12000 11000 10000 9000 8000 7000 6000 5000 4000 3000 2000 1000 0 -1000 -2000

C-I-14

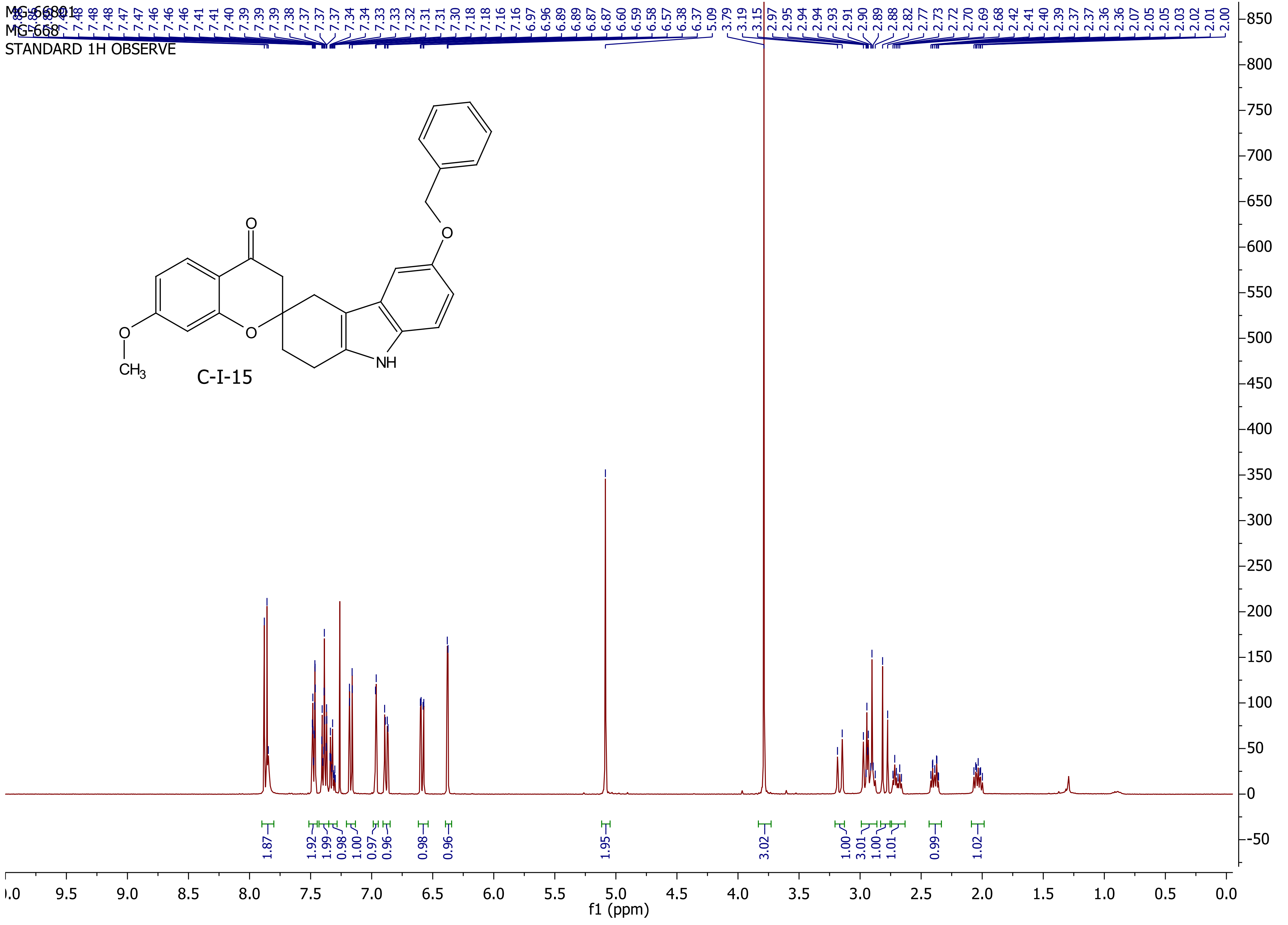
MG-66801
MG-668
STANDARD 1H OBSERVE
C-I-15
f1 (ppm)

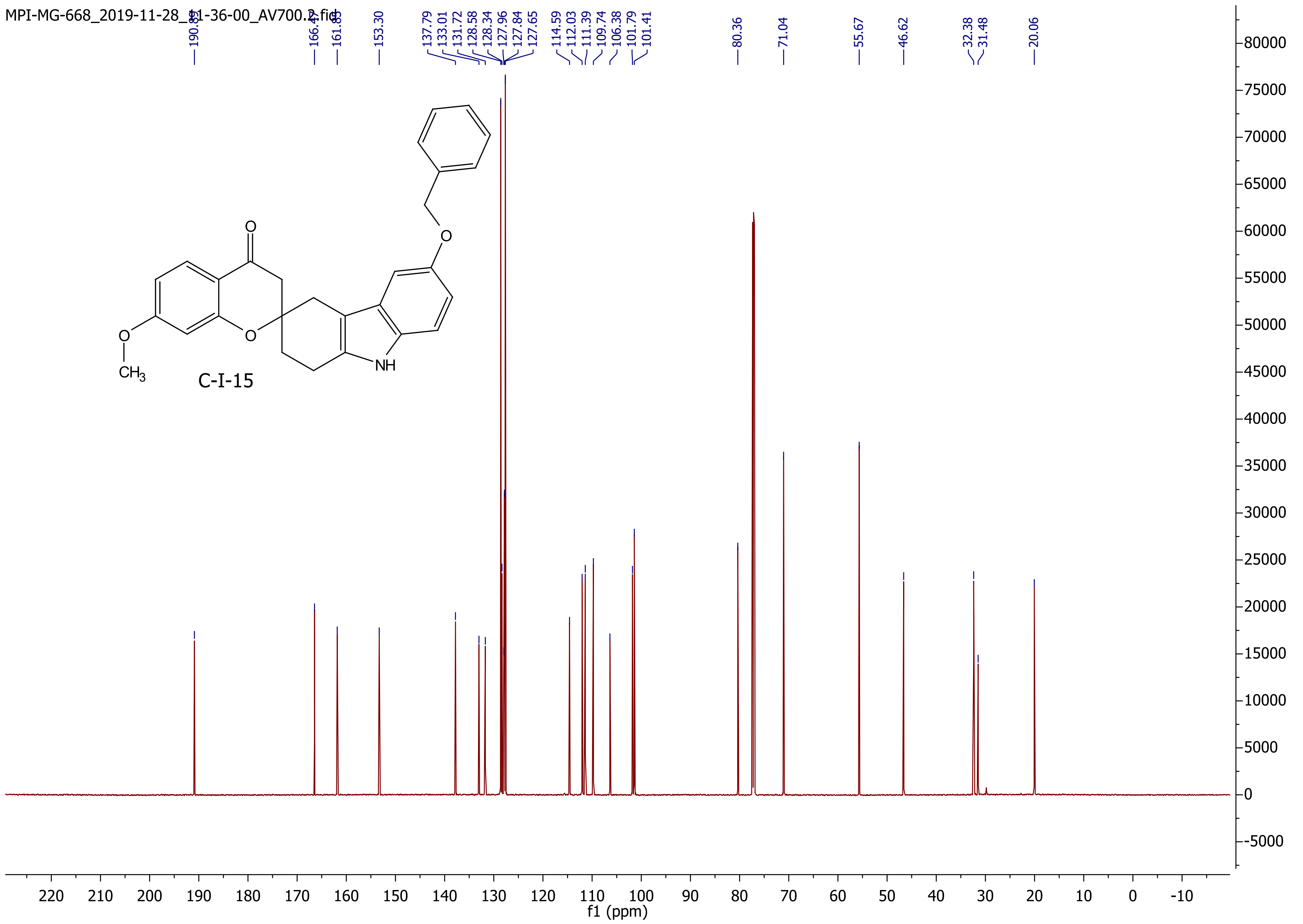

MPI-MG-668_2019-11-28_11-36-00_AV700.2.fid
190.89
166.47
161.85
153.30
137.79
133.01
131.72
128.58
128.34
127.96
127.84
127.65
114.59
112.03
111.39
109.74
106.38
101.79
101.41
80.36
71.04
55.67
46.62
32.38
31.48
20.06
O
O
O
O
CH3
NH
C-I-15
f1 (ppm)

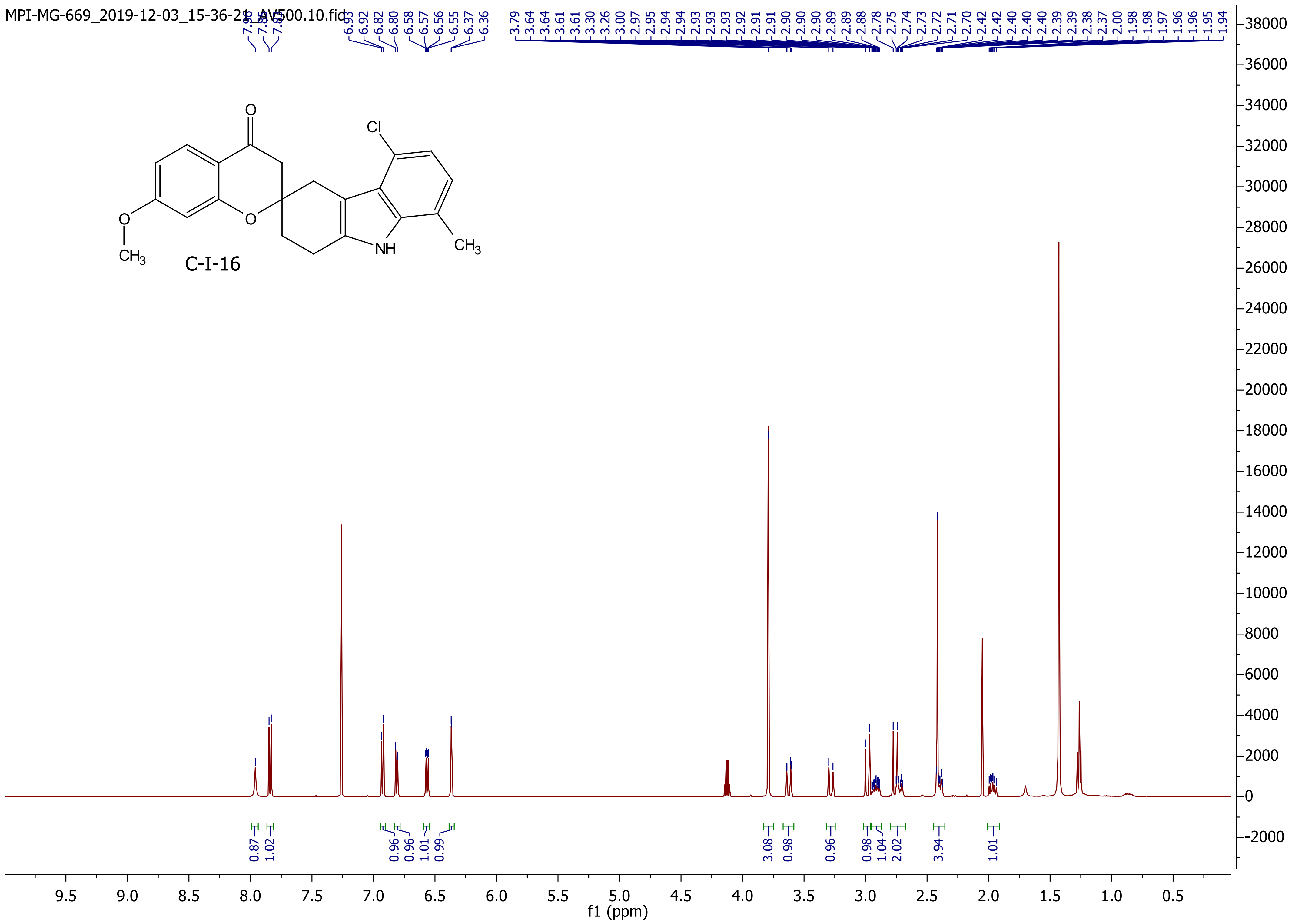
MPI-MG-669_2019-12-03_15-36-21_AV500.10.fid
6.93
6.92
6.82
6.80
6.58
6.57
6.56
6.55
6.37
6.36
3.79
3.64
3.64
3.61
3.61
3.30
3.26
3.00
2.97
2.95
2.94
2.94
2.93
2.93
2.93
2.92
2.91
2.91
2.90
2.90
2.90
2.89
2.89
2.88
2.78
2.75
2.74
2.73
2.72
2.71
2.70
2.42
2.42
2.40
2.40
2.40
2.39
2.39
2.38
2.37
2.00
1.98
1.98
1.97
1.96
1.96
1.95
1.94
O
Cl
O
O
NH
CH3
CH3
C-I-16
0.87
1.02
0.96
0.96
1.01
0.99
3.08
0.98
0.96
0.98
1.04
2.02
3.94
1.01
f1 (ppm)

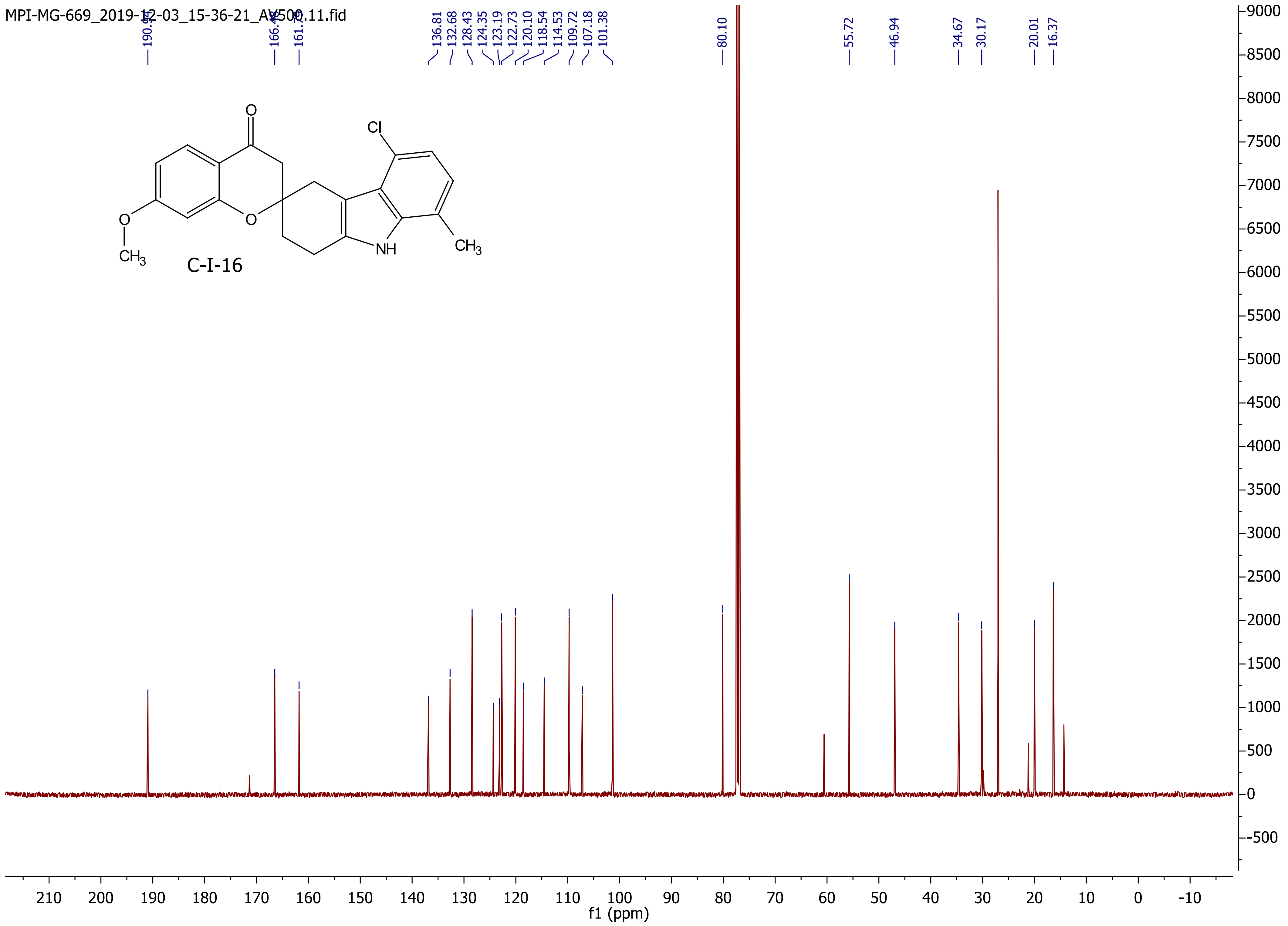

MPI-MG-669_2019-12-03_15-36-21_AV500.11.fid
136.81
132.68
128.43
124.35
123.19
122.73
120.10
118.54
114.53
109.72
107.18
101.38
80.10
55.72
46.94
34.67
30.17
20.01
16.37
O
Cl
O
O
CH3
NH
CH3
C-I-16
f1 (ppm)

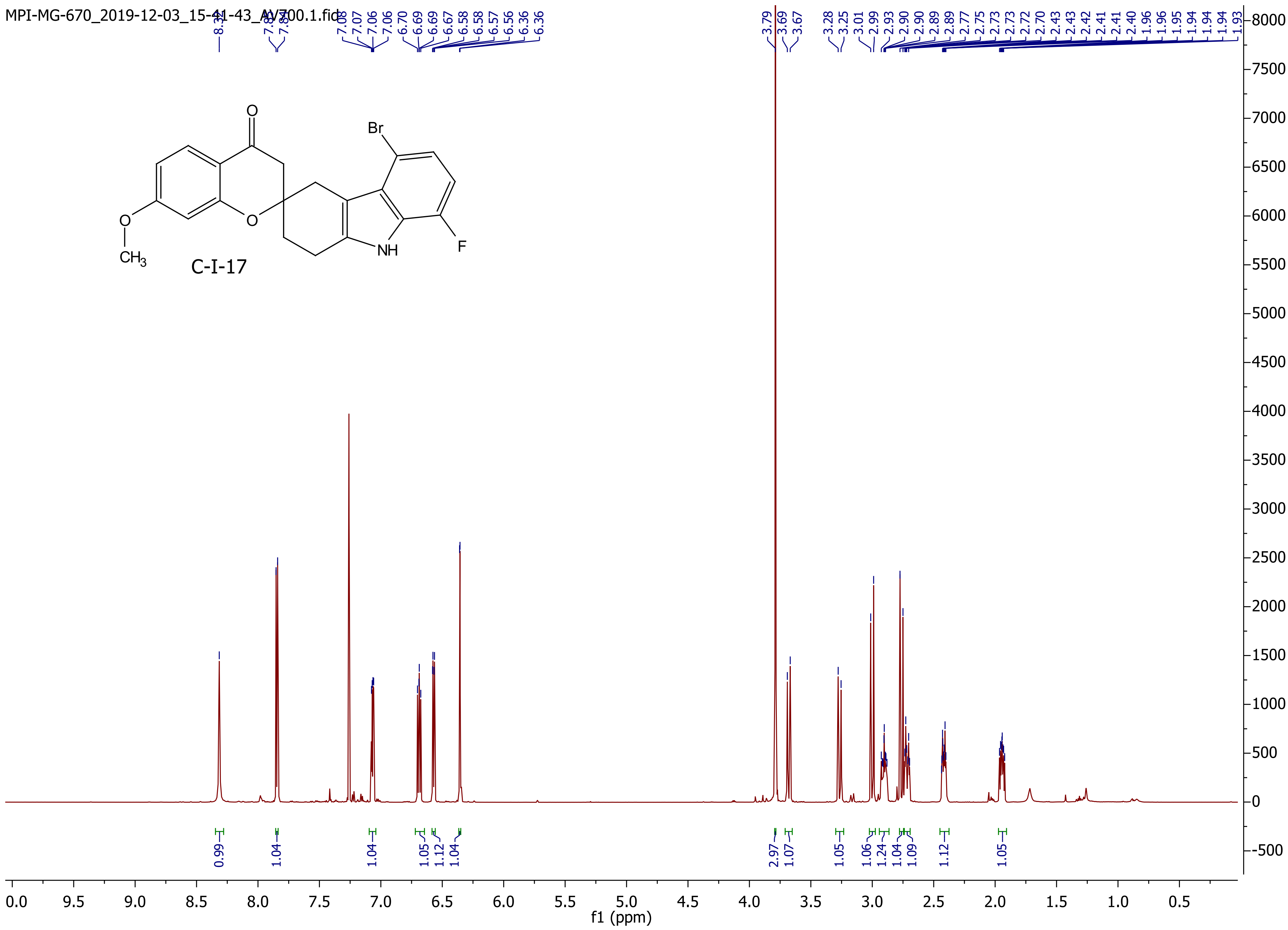

MPI-MG-670_2019-12-03_15-41-43_AV700.1.fid
7.08 7.07 7.06 7.06 6.70 6.69 6.69 6.67 6.58 6.58 6.57 6.56 6.36 6.36
3.79 3.69 3.67 3.28 3.25 3.01 2.99 2.93 2.90 2.90 2.89 2.89 2.77 2.75 2.73 2.73 2.72 2.70 2.43 2.43 2.42 2.41 2.41 2.40 1.96 1.96 1.95 1.94 1.94 1.94 1.93
O
Br
O
O
NH
F
CH3
C-I-17
0.99 1.04 1.04 1.05 1.12 1.04 2.97 1.07 1.05 1.06 1.24 1.04 1.09 1.12 1.05
f1 (ppm)

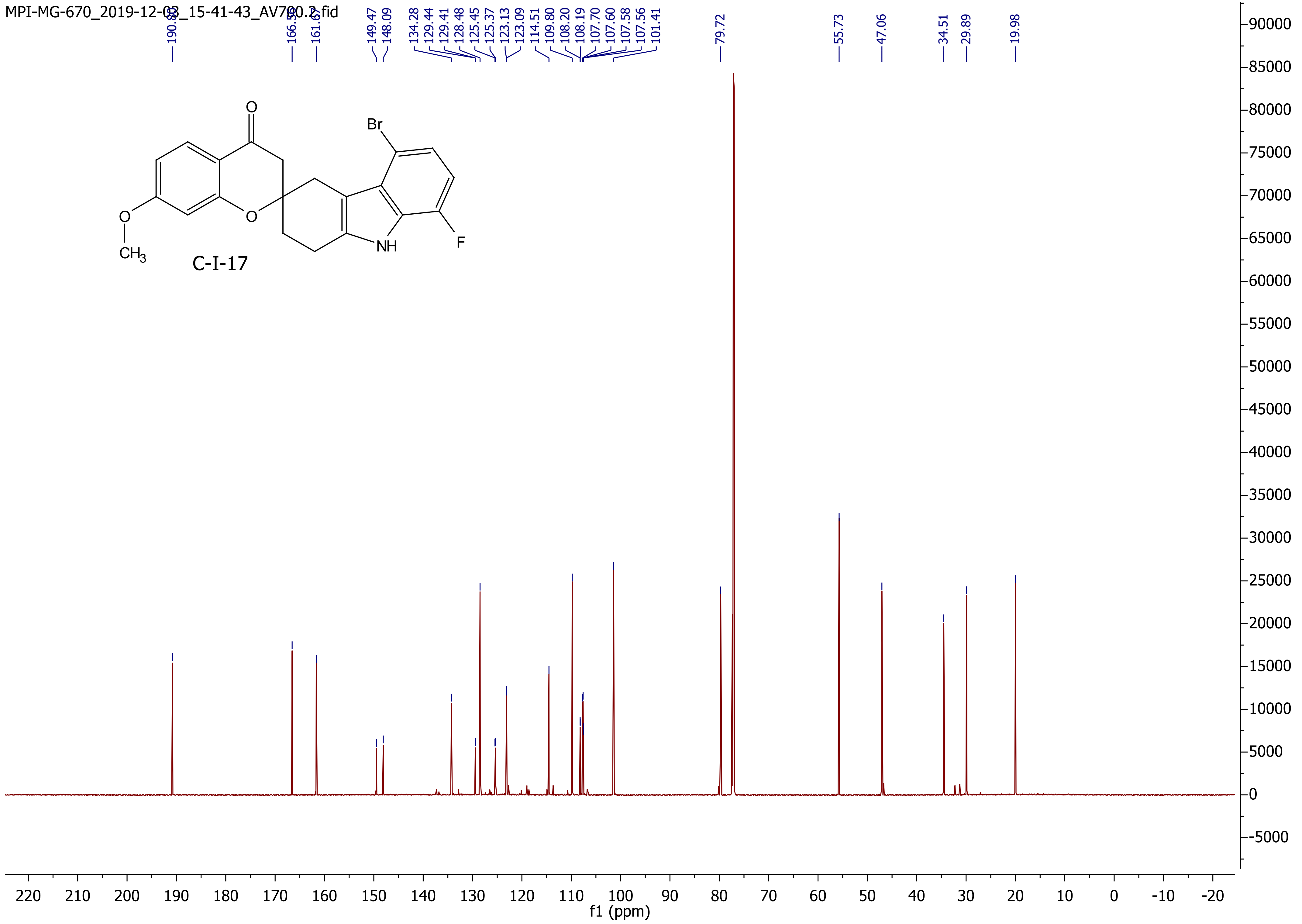
MPI-MG-670_2019-12-03_15-41-43_AV700.2.fid
149.47
148.09
134.28
129.44
129.41
128.48
125.45
125.37
123.13
123.09
114.51
109.80
108.20
108.19
107.70
107.60
107.58
107.56
101.41
79.72
55.73
47.06
34.51
29.89
19.98
Br
O
O
O
CH3
NH
F
C-I-17
f1 (ppm)

MPI-MG-670_2019-12-06_09-50-27_AV500.11.fid

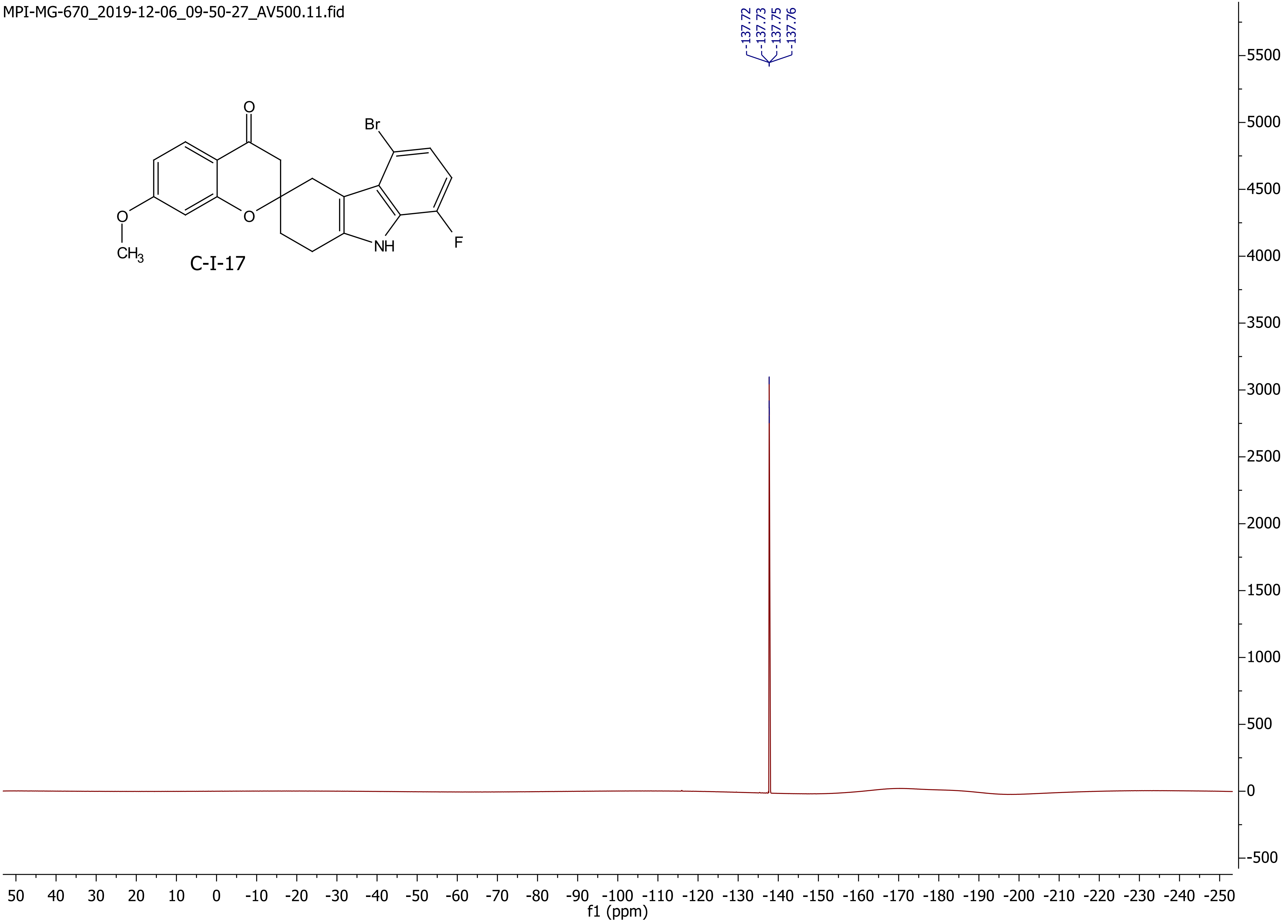

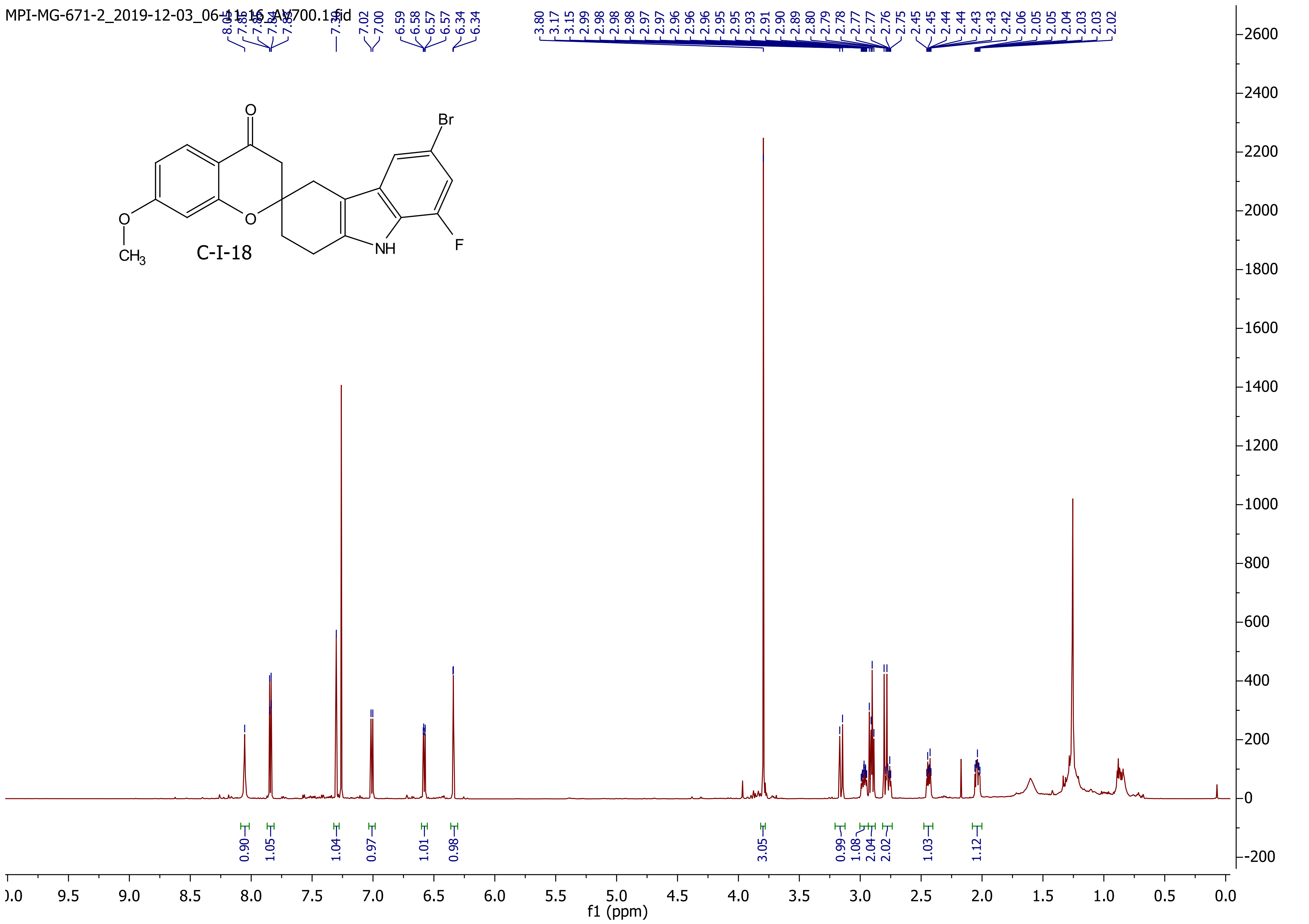

MPI-MG-671-2_2019-12-03_06-11-16_AV700.1.fid
7.02
7.00
6.59
6.58
6.57
6.57
6.34
6.34
3.80
3.17
3.15
2.99
2.98
2.98
2.98
2.97
2.97
2.96
2.96
2.96
2.95
2.95
2.93
2.91
2.90
2.89
2.80
2.79
2.78
2.77
2.77
2.76
2.75
2.45
2.45
2.44
2.44
2.43
2.43
2.42
2.06
2.05
2.05
2.04
2.03
2.03
2.02
O
Br
O
O
CH3
NH
F
C-I-18
0.90
1.05
1.04
0.97
1.01
0.98
3.05
0.99
1.08
2.04
2.02
1.03
1.12
f1 (ppm)

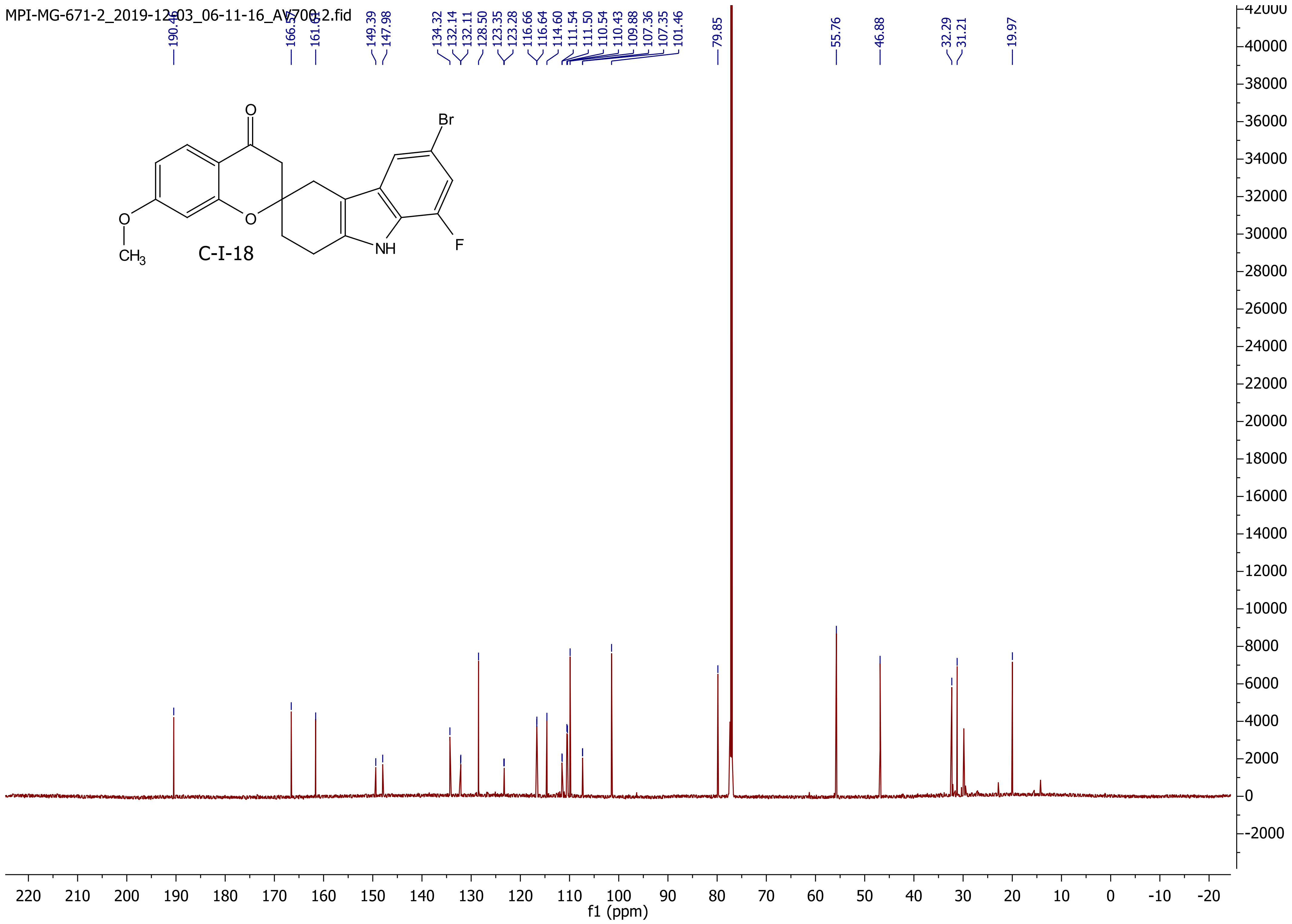
MPI-MG-671-2_2019-12-03_06-11-16_AV700-2.fid
134.32
132.14
132.11
128.50
123.35
123.28
116.66
116.64
114.60
111.54
111.50
110.54
110.43
109.88
107.36
107.35
101.46
149.39
147.98
79.85
55.76
46.88
32.29
31.21
19.97
O
Br
O
O
CH3
NH
F
C-I-18
f1 (ppm)

MPI-MG-671-2_2019-12-06_09-50-42_AV500.11.fid

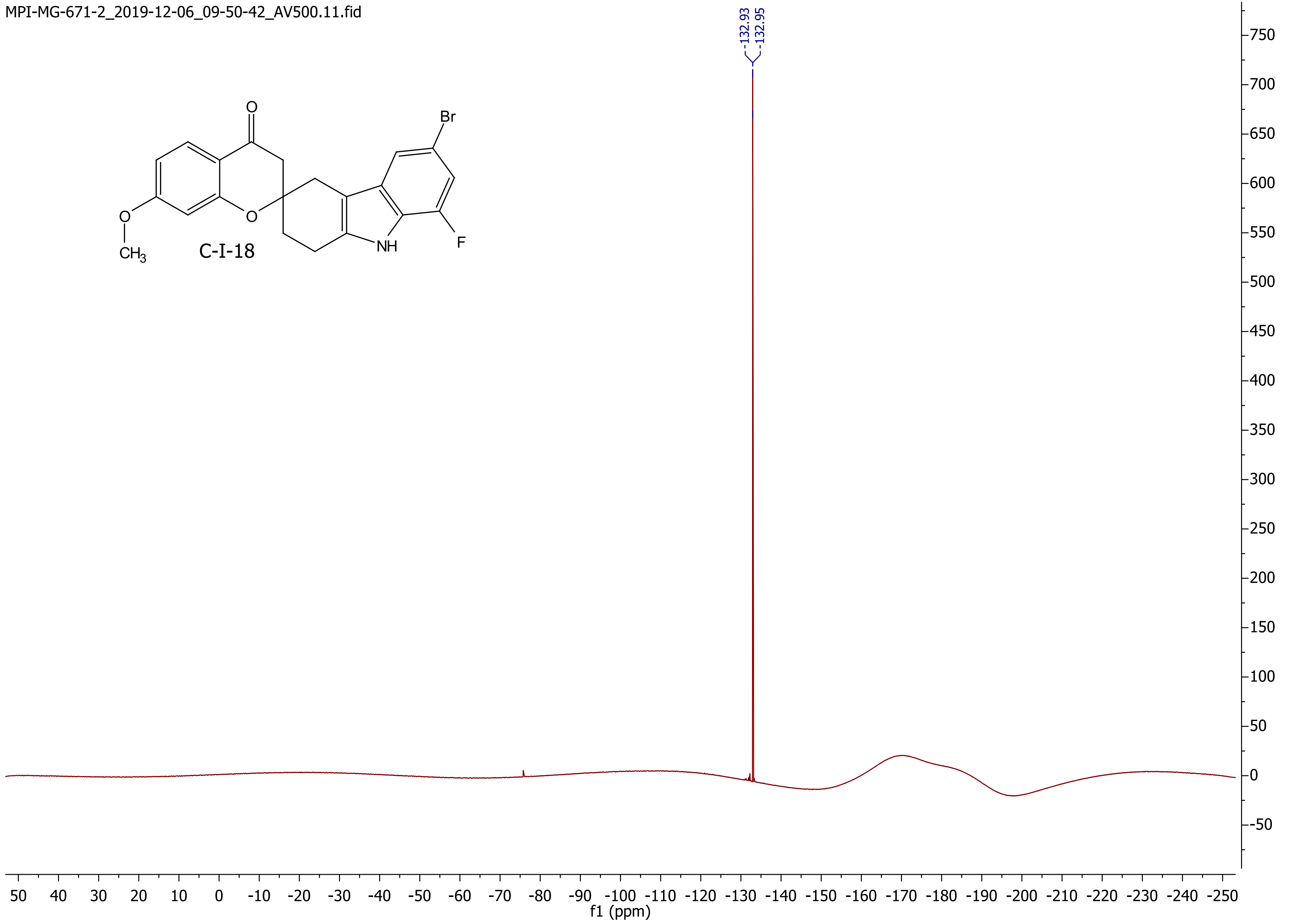

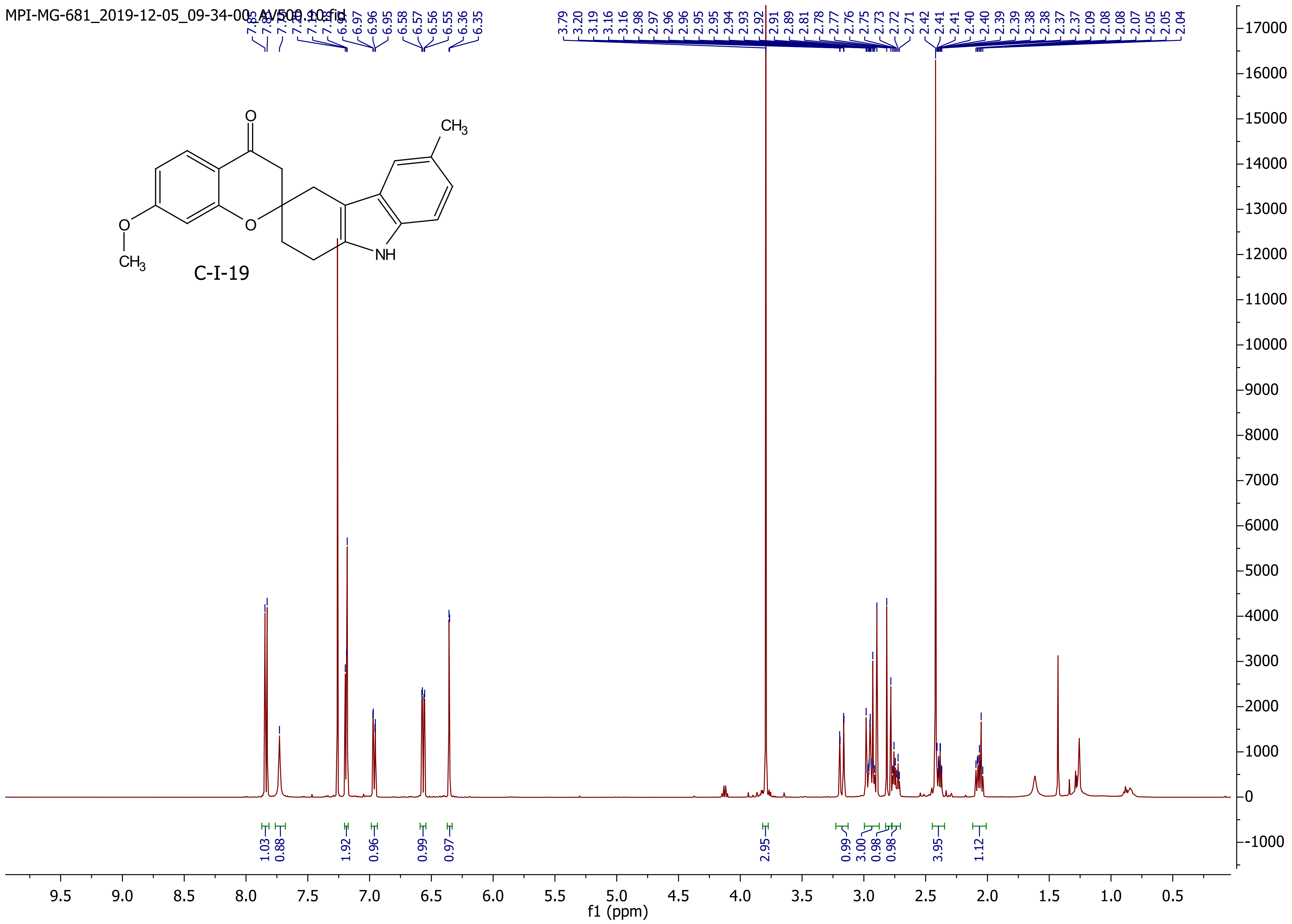
MPI-MG-681_2019-12-05_09-34-00 AV500.10.fid
6.97
6.96
6.95
6.58
6.57
6.56
6.55
6.36
6.35
3.79
3.20
3.19
3.16
3.16
2.98
2.97
2.96
2.96
2.95
2.95
2.94
2.93
2.92
2.91
2.89
2.81
2.78
2.77
2.76
2.75
2.73
2.72
2.71
2.42
2.41
2.41
2.40
2.40
2.39
2.38
2.38
2.37
2.37
2.09
2.08
2.08
2.07
2.05
2.05
2.04
O
CH_3
O
O
NH
CH_3
C-I-19
1.03
0.88
1.92
0.96
0.99
0.97
2.95
0.99
3.00
0.98
0.98
3.95
1.12
f1 (ppm)

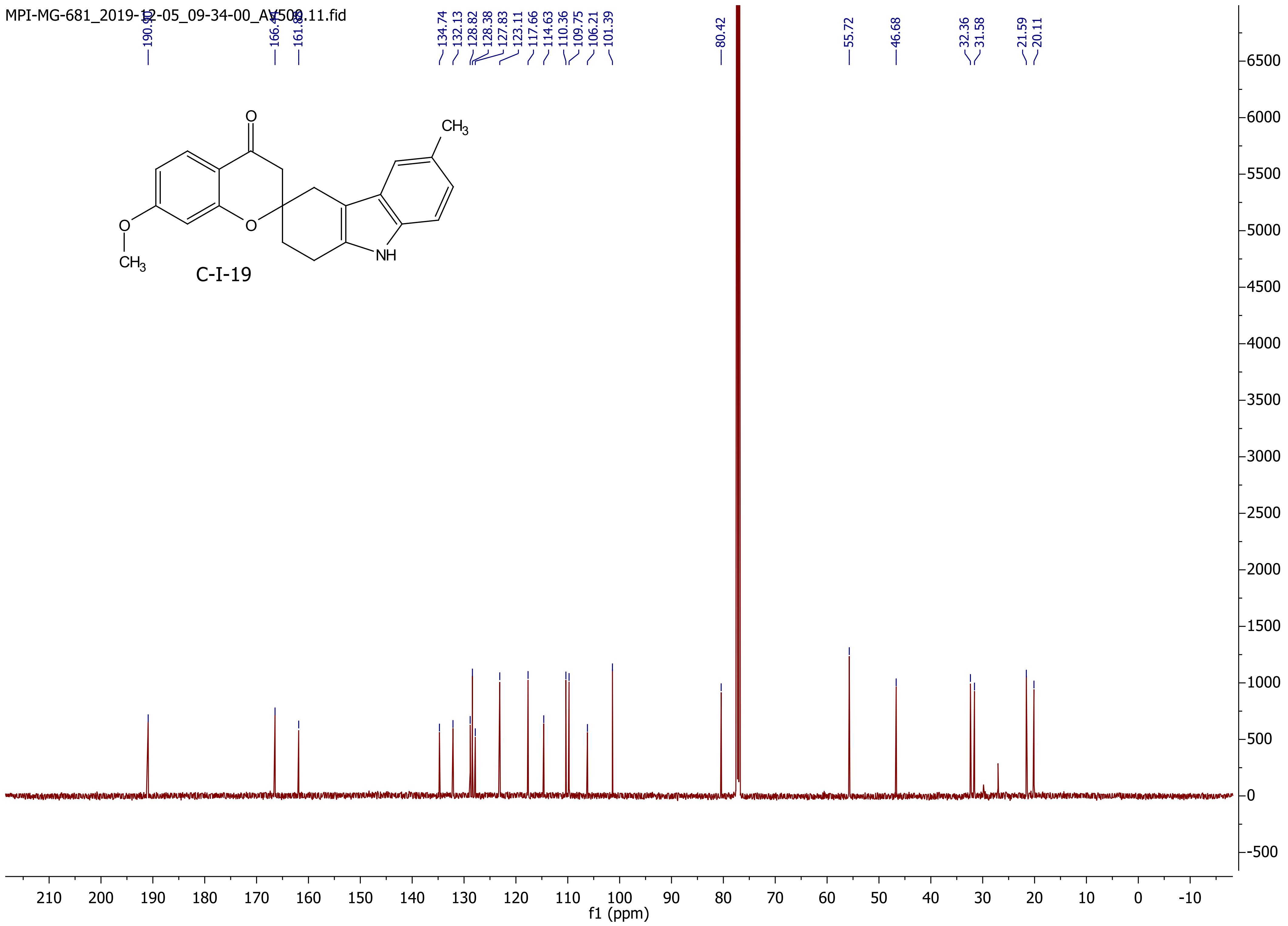
MPI-MG-681_2019-12-05_09-34-00_AV500.11.fid
190.91
166.43
161.88
134.74
132.13
128.82
128.38
127.83
123.11
117.66
114.63
110.36
109.75
106.21
101.39
80.42
55.72
46.68
32.36
31.58
21.59
20.11
C-I-19
f1 (ppm)

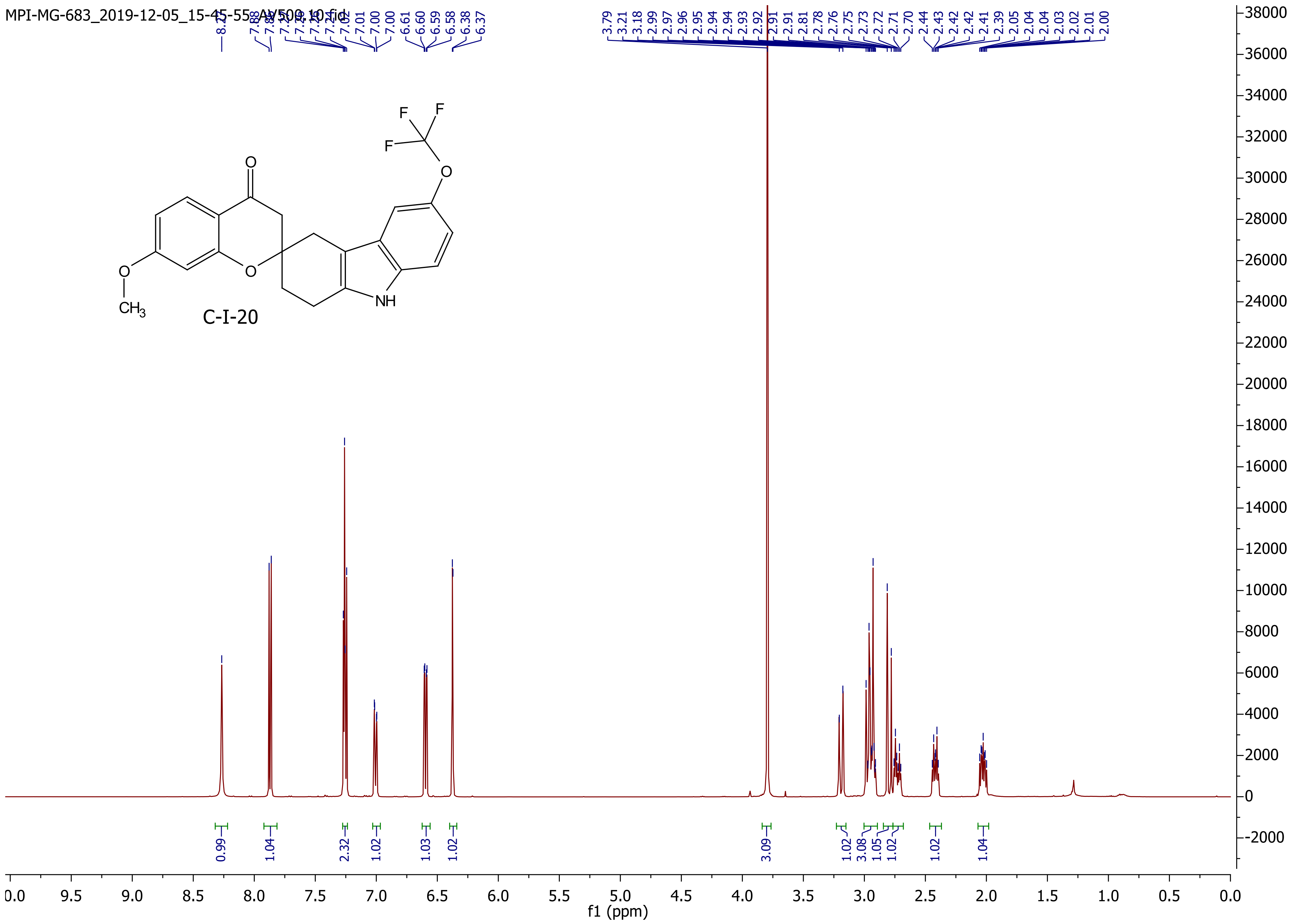

MPI-MG-683_2019-12-05_15-45-55_AV500 10 fid
3.79
3.21
3.18
2.99
2.97
2.96
2.95
2.94
2.94
2.93
2.92
2.91
2.91
2.81
2.78
2.76
2.75
2.73
2.72
2.71
2.70
2.44
2.43
2.42
2.42
2.41
2.39
2.05
2.04
2.04
2.03
2.02
2.01
2.00
7.02
7.01
7.00
7.00
6.61
6.60
6.59
6.58
6.38
6.37
F
F
F
O
O
O
O
NH
CH3
C-I-20
0.99
1.04
2.32
1.02
1.03
1.02
3.09
1.02
3.08
1.05
1.02
1.02
1.04
f1 (ppm)

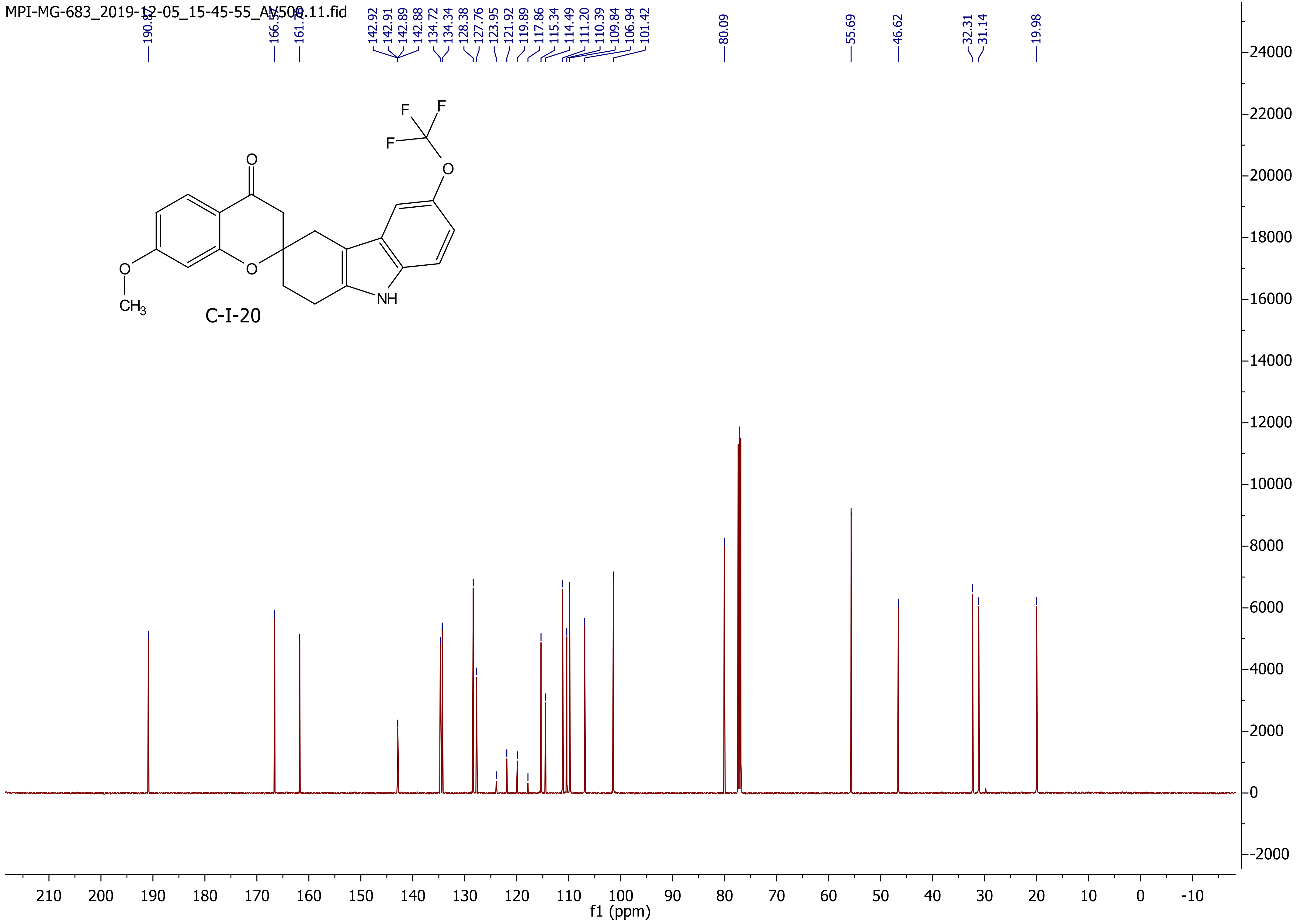

MPI-MG-683_2019-12-05_15-45-55_AV500.11.fid
142.92
142.91
142.89
142.88
134.72
134.34
128.38
127.76
123.95
121.92
119.89
117.86
115.34
114.49
111.20
110.39
109.84
106.94
101.42
80.09
55.69
46.62
32.31
31.14
19.98
F
F
F
O
O
O
O
NH
CH3
C-I-20
24000
22000
20000
18000
16000
14000
12000
10000
8000
6000
4000
2000
0
-2000
210
200
190
180
170
160
150
140
130
120
110
100
90
80
70
60
50
40
30
20
10
0
-10
f1 (ppm)

MPI-MG-683_2019-12-05_15-45-55_AV500.12.fid

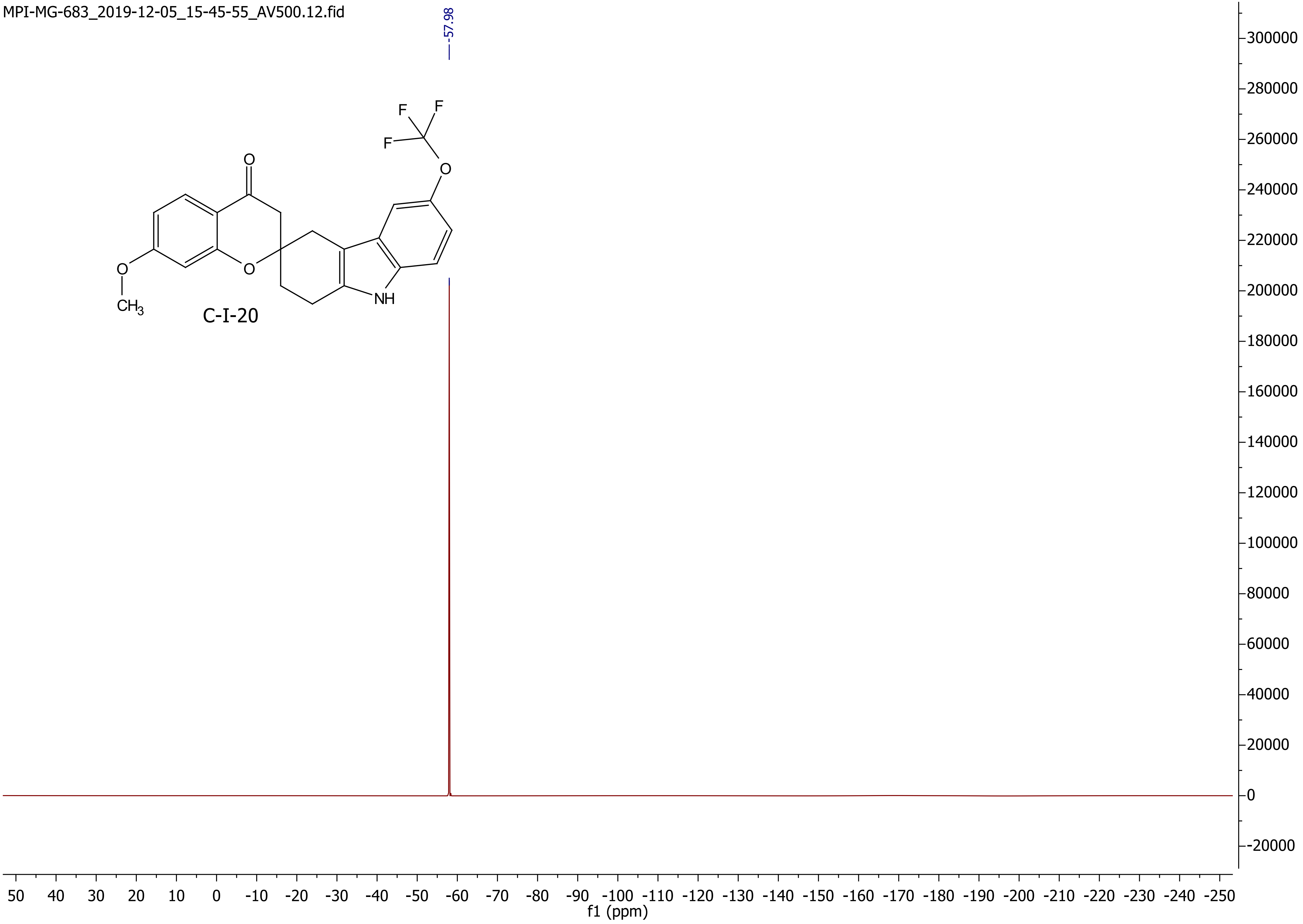

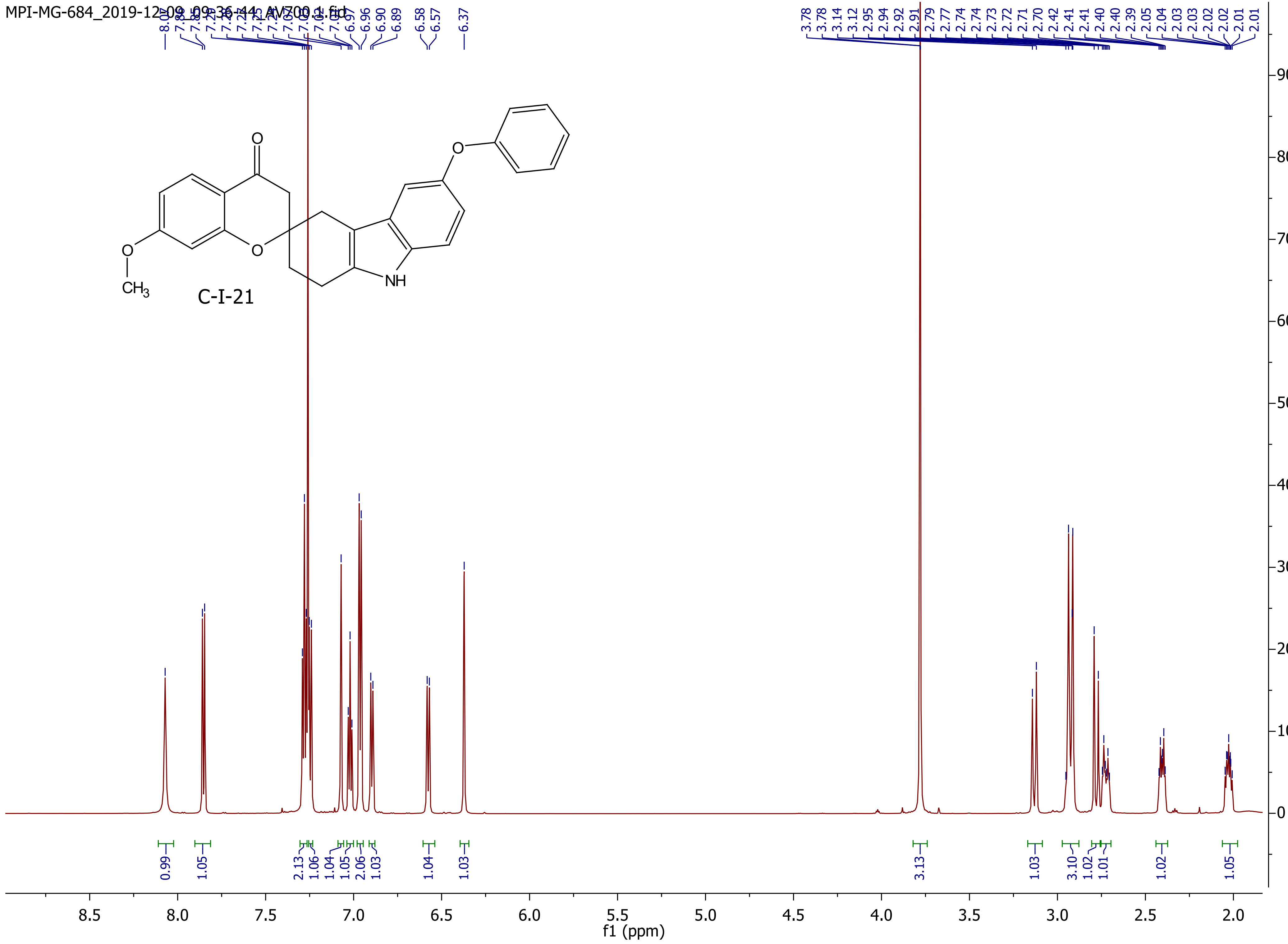
MPI-MG-684_2019-12-09_09-36-44_AV700.1.fid
6.97
6.96
6.90
6.89
6.58
6.57
6.37
3.78
3.78
3.14
3.12
2.95
2.94
2.92
2.91
2.79
2.77
2.74
2.74
2.73
2.72
2.71
2.70
2.42
2.41
2.41
2.40
2.40
2.39
2.05
2.04
2.03
2.03
2.02
2.02
2.01
2.01
O
O
O
O
CH3
NH
C-I-21
9000
8000
7000
6000
5000
4000
3000
2000
1000
0
0.99
1.05
2.13
1.06
1.04
1.05
2.06
1.03
1.04
1.03
3.13
1.03
3.10
1.02
1.01
1.02
1.05
8.5
8.0
7.5
7.0
6.5
6.0
5.5
5.0
4.5
4.0
3.5
3.0
2.5
2.0
f1 (ppm)

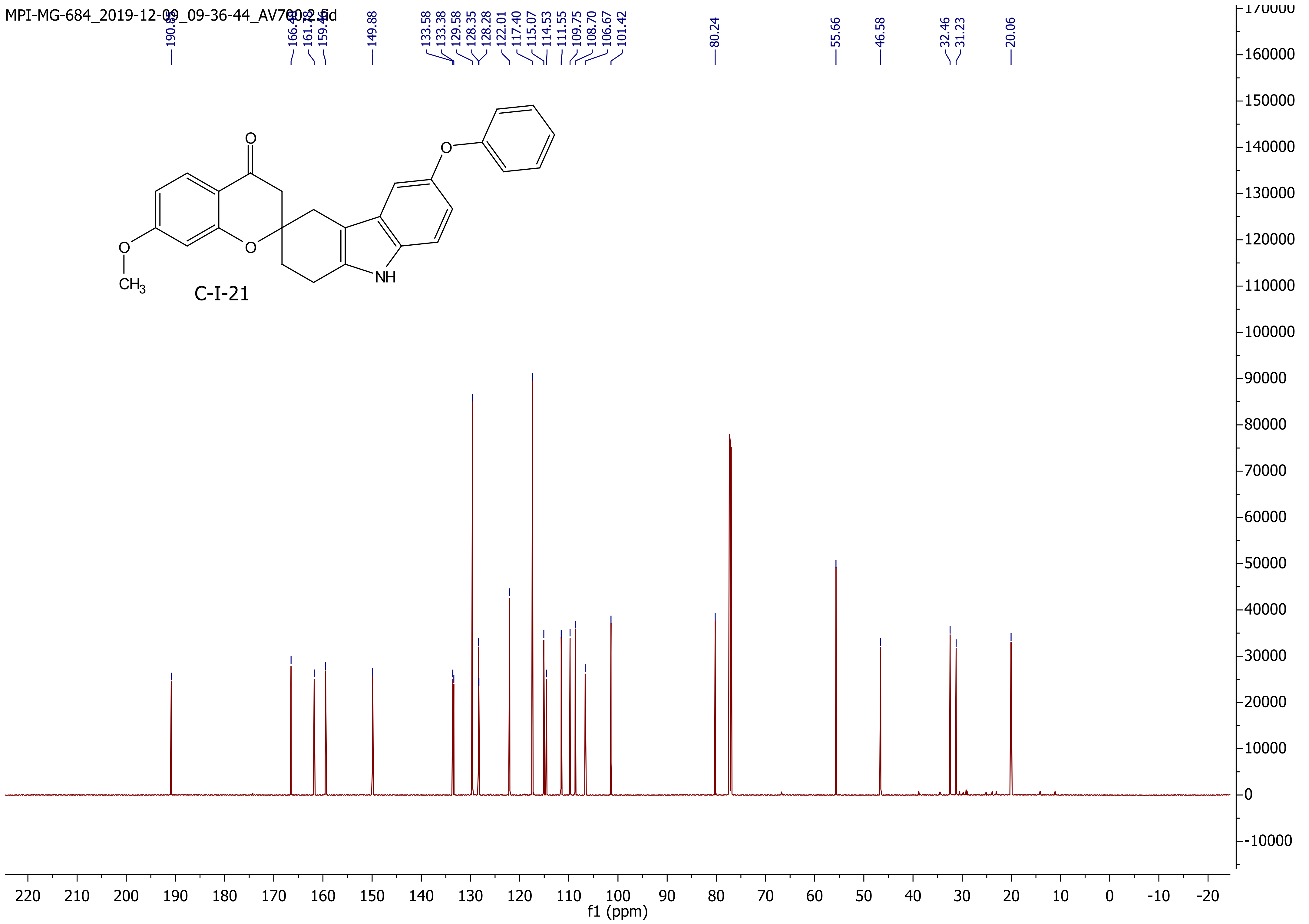
MPI-MG-684_2019-12-09_09-36-44_AV700.2.fid
149.88
133.58
133.38
129.58
128.35
128.28
122.01
117.40
115.07
114.53
111.55
109.75
108.70
106.67
101.42
80.24
55.66
46.58
32.46
31.23
20.06
O
O
O
NH
CH3
C-I-21
f1 (ppm)

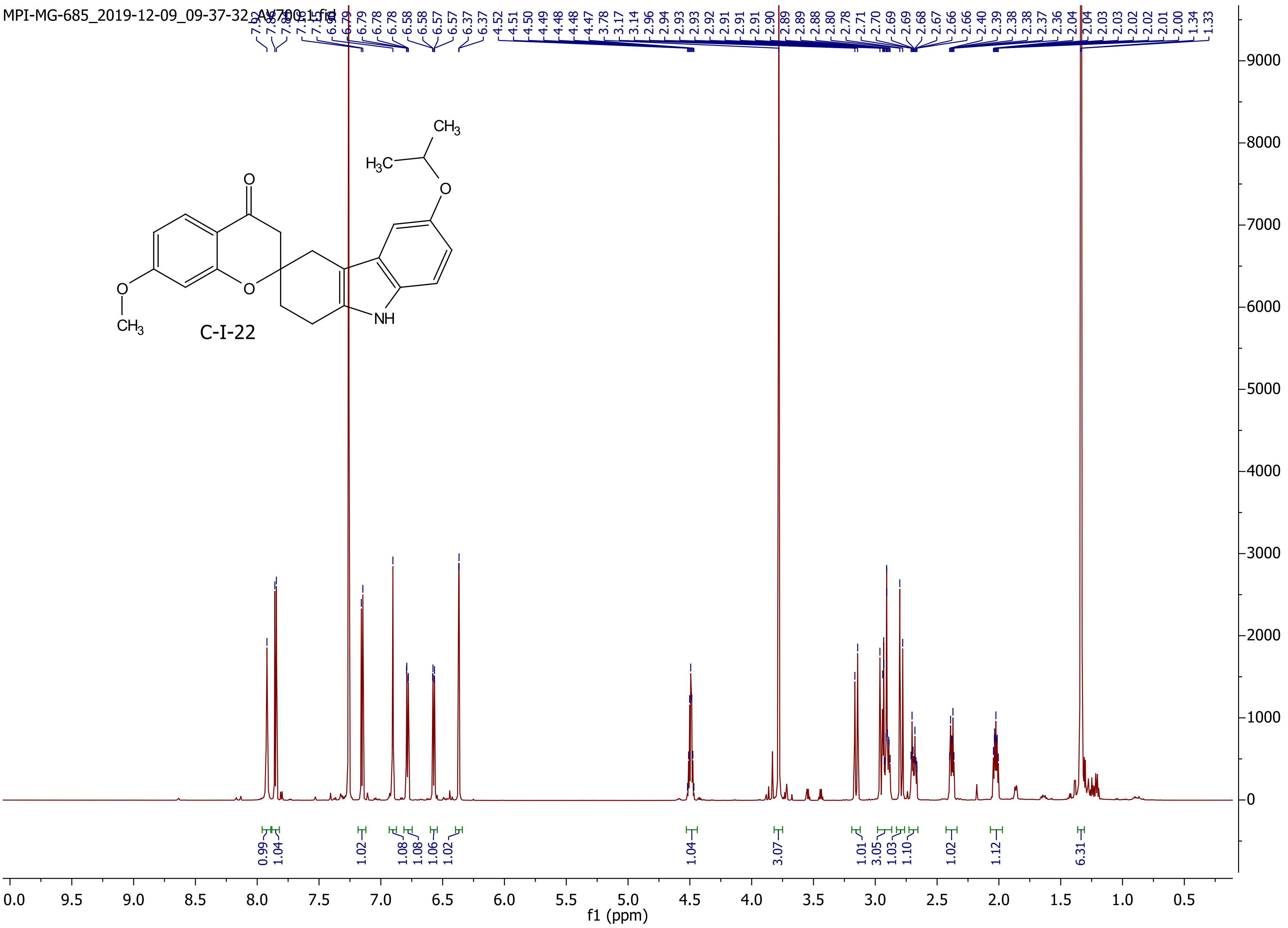
C-I-22
f1 (ppm)

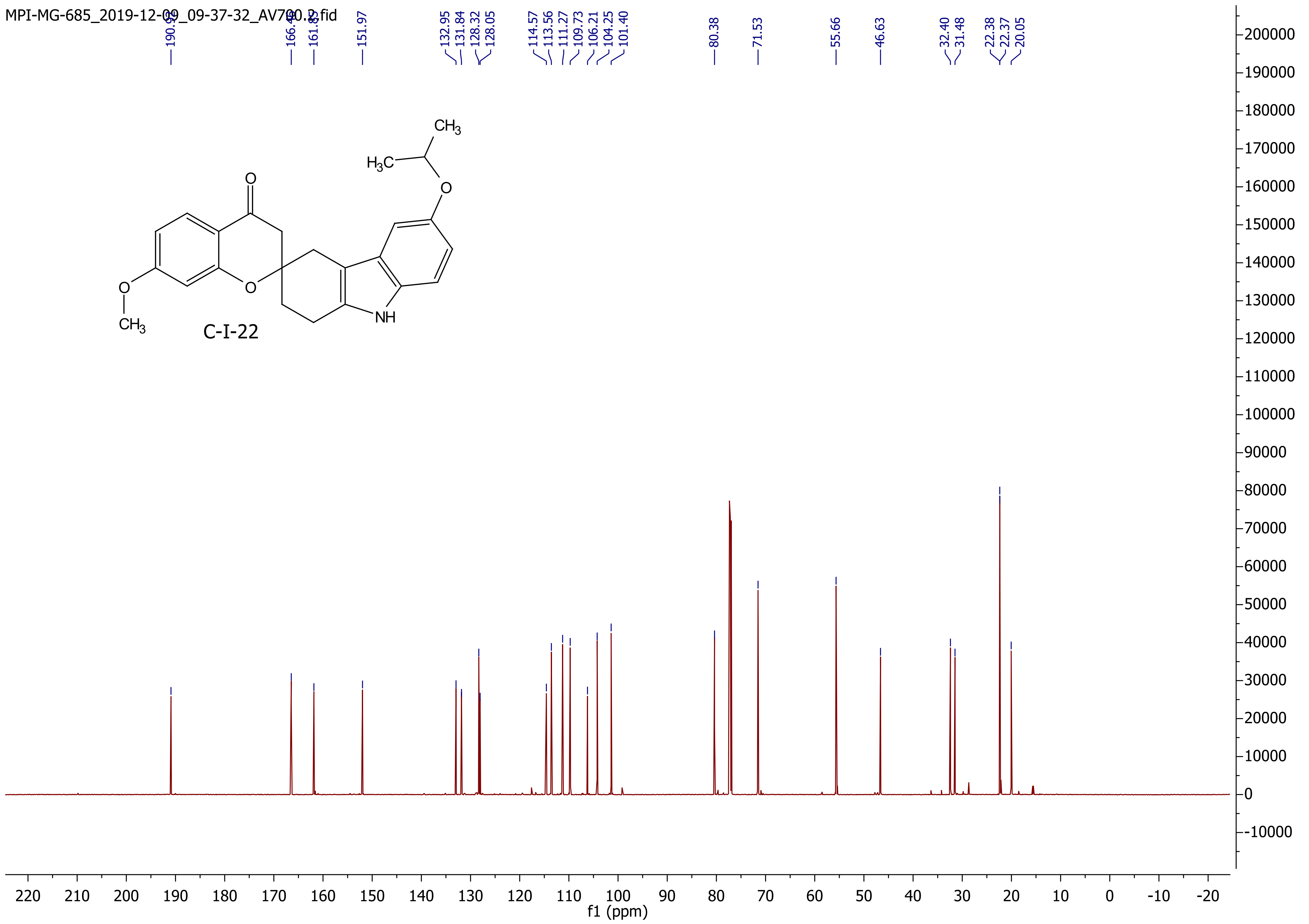

MPI-MG-685_2019-12-09_09-37-32_AV700.2.fid
151.97
132.95
131.84
128.32
128.05
114.57
113.56
111.27
109.73
106.21
104.25
101.40
80.38
71.53
55.66
46.63
32.40
31.48
22.38
22.37
20.05
CH_3
H_3C
O
O
O
O
NH
CH_3
C-I-22
f1 (ppm)

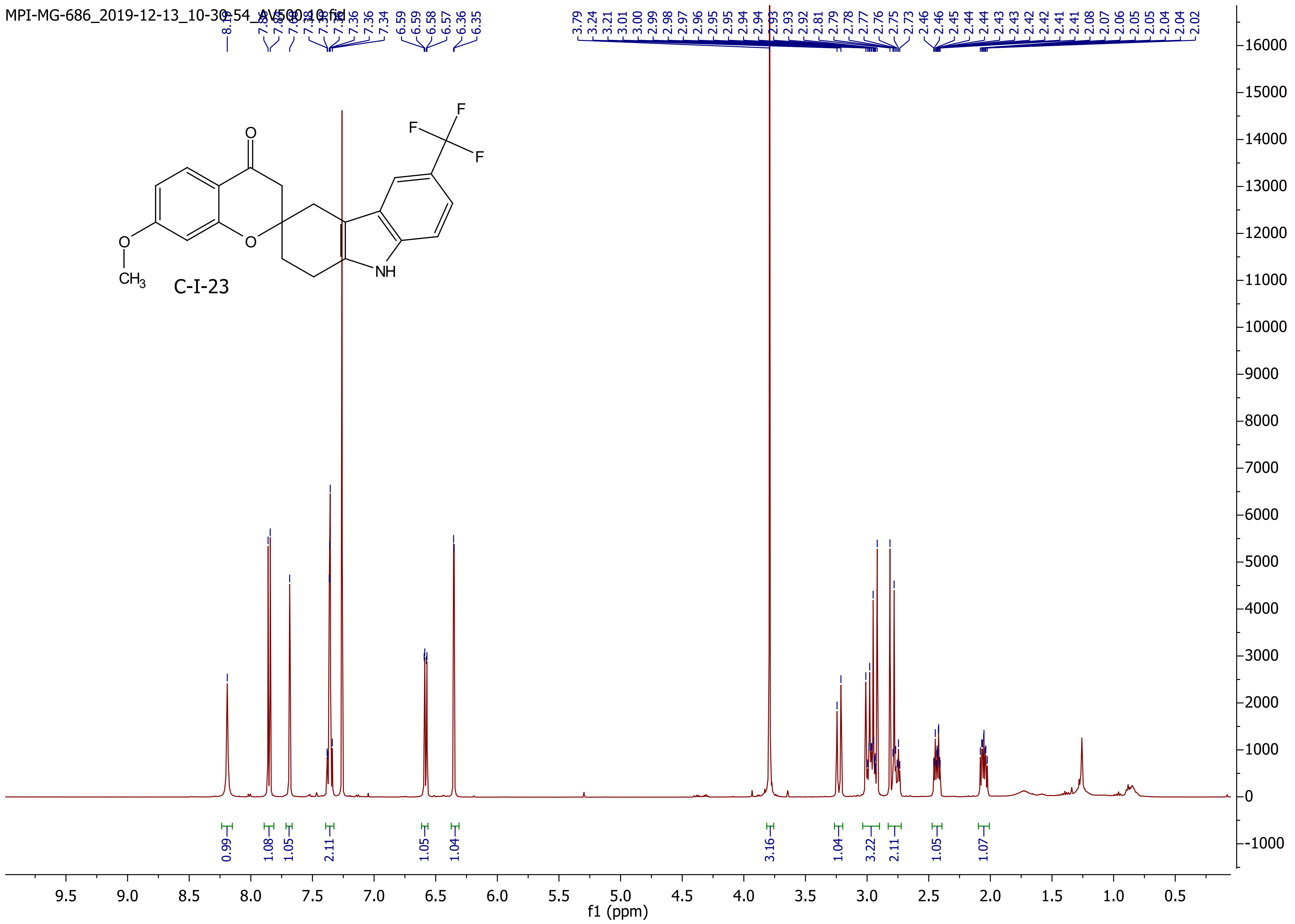

MPI-MG-686_2019-12-13_10-30-54_AV500.10.fid
7.36 7.36 7.34 6.59 6.59 6.58 6.57 6.36 6.35
3.79 3.24 3.21 3.01 3.00 2.99 2.98 2.97 2.96 2.95 2.95 2.94 2.94 2.93 2.93 2.92 2.81 2.79 2.78 2.77 2.76 2.75 2.73 2.46 2.46 2.45 2.44 2.44 2.43 2.43 2.42 2.42 2.41 2.41 2.08 2.07 2.06 2.05 2.05 2.04 2.04 2.02
O
F
F
F
O
O
NH
CH3
C-I-23
0.99 1.08 1.05 2.11 1.05 1.04 3.16 1.04 3.22 2.11 1.05 1.07
9.5 9.0 8.5 8.0 7.5 7.0 6.5 6.0 5.5 5.0 4.5 4.0 3.5 3.0 2.5 2.0 1.5 1.0 0.5
f1 (ppm)
16000 15000 14000 13000 12000 11000 10000 9000 8000 7000 6000 5000 4000 3000 2000 1000 0 -1000

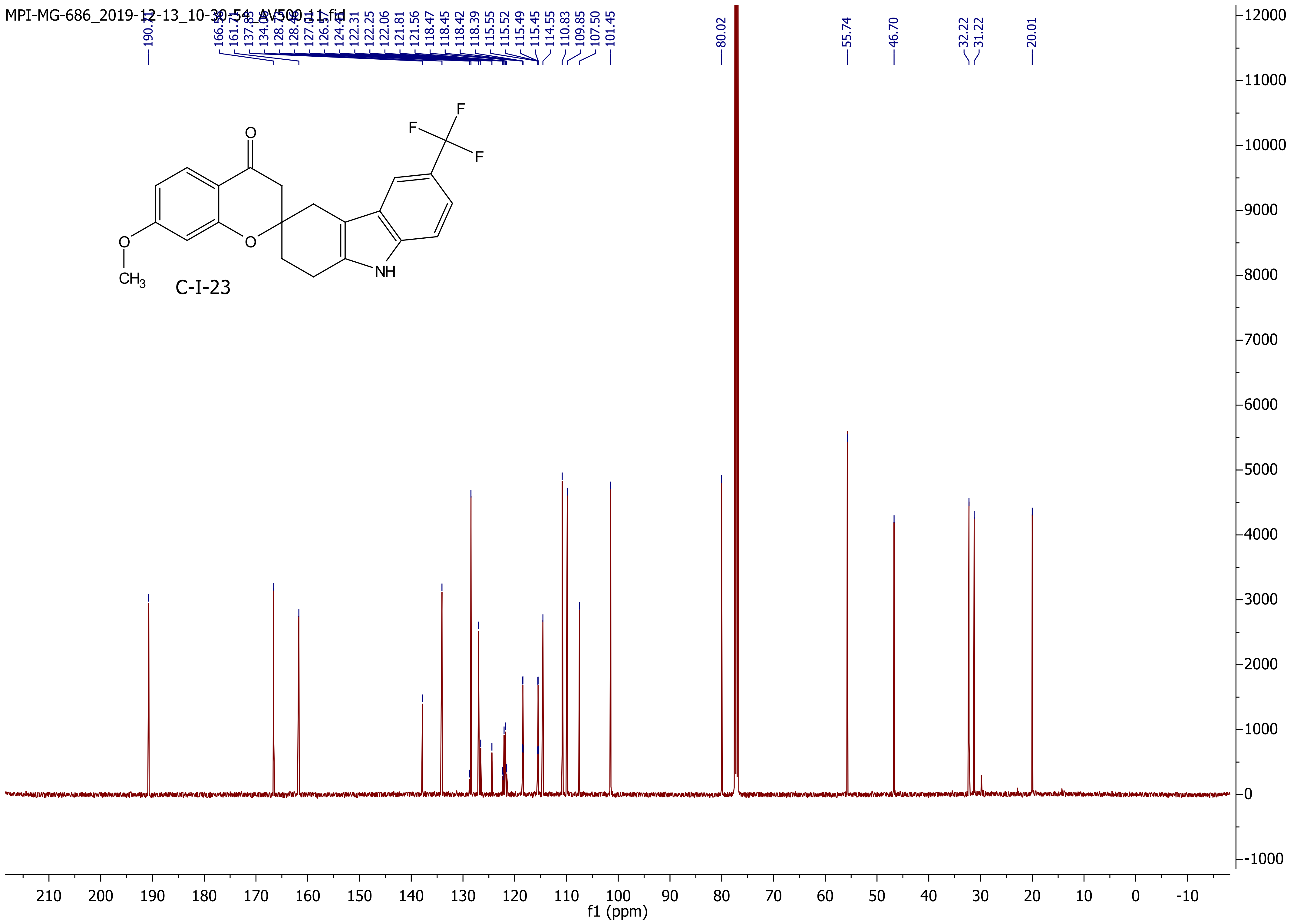

MPI-MG-686_2019-12-13_10-30-54_AV500-11.fid
122.31
122.25
122.06
121.81
121.56
118.47
118.45
118.42
118.39
115.55
115.52
115.49
115.45
114.55
110.83
109.85
107.50
101.45
80.02
55.74
46.70
32.22
31.22
20.01
O
O
O
F
F
F
NH
CH_3
C-I-23
f1 (ppm)

MPI-MG-686_2019-12-13_10-30-54_AV500.12.fid

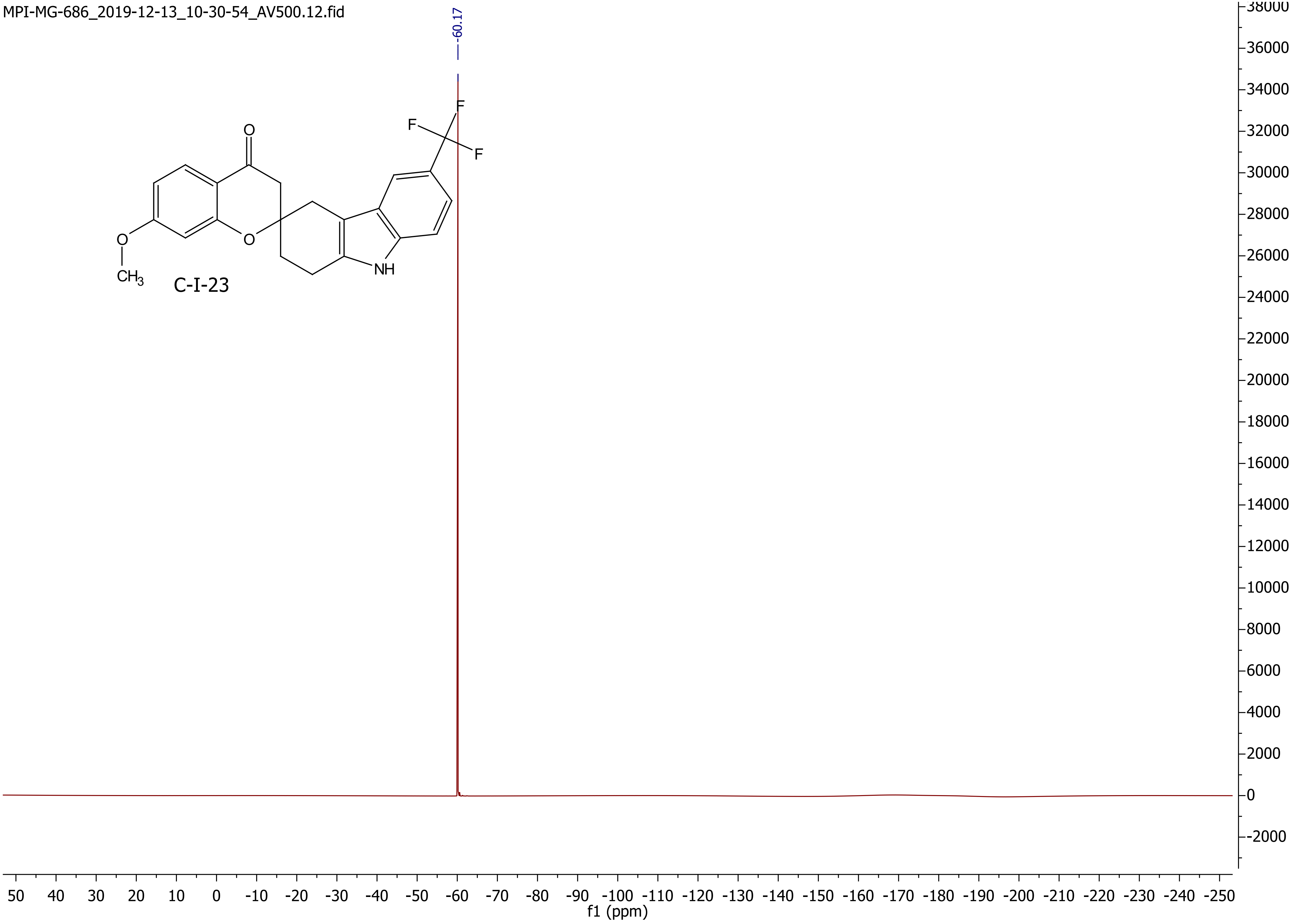

MPI-MG-687_2019-12-13_10-30-09_AV500.10.fid

C-I-24

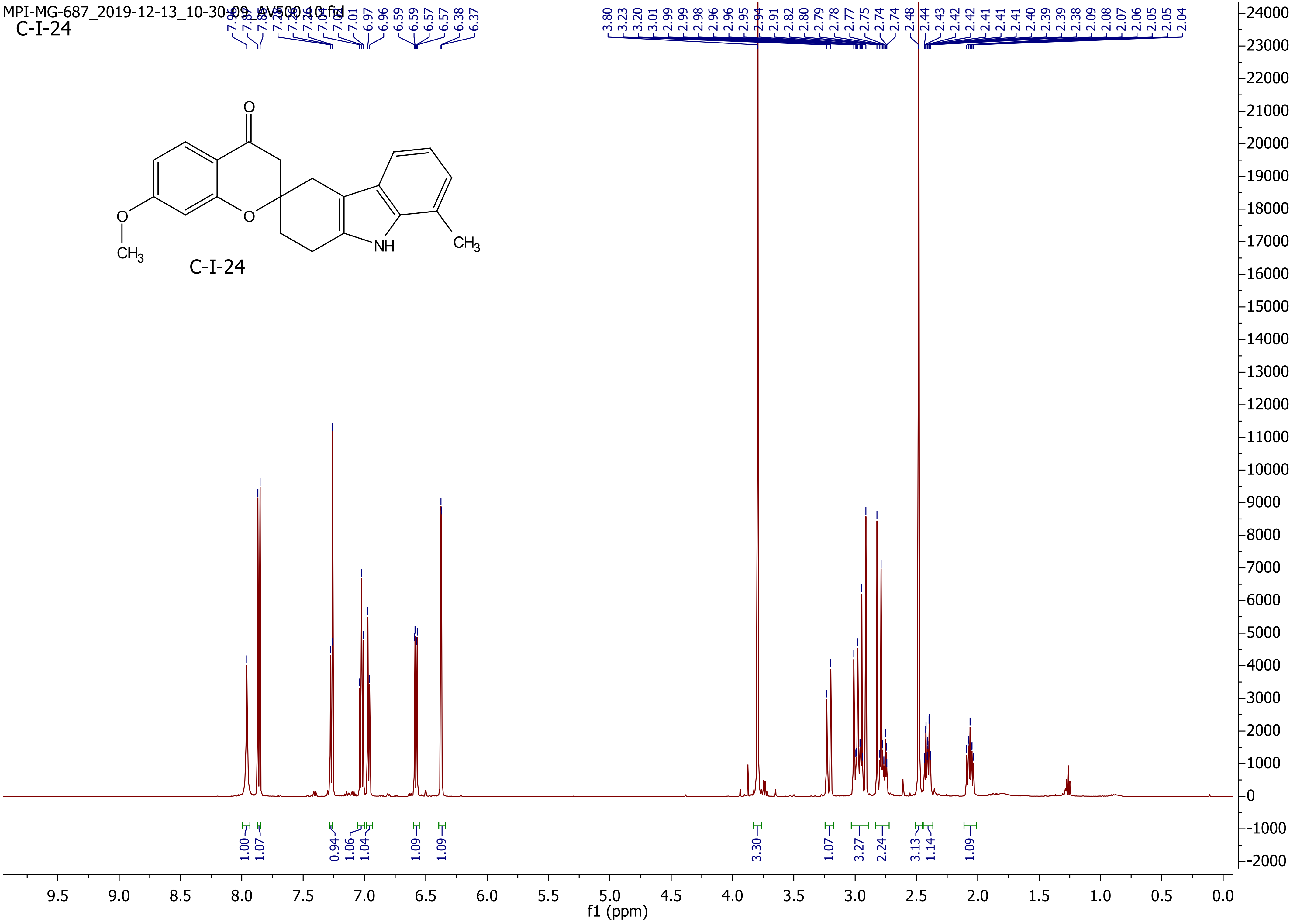

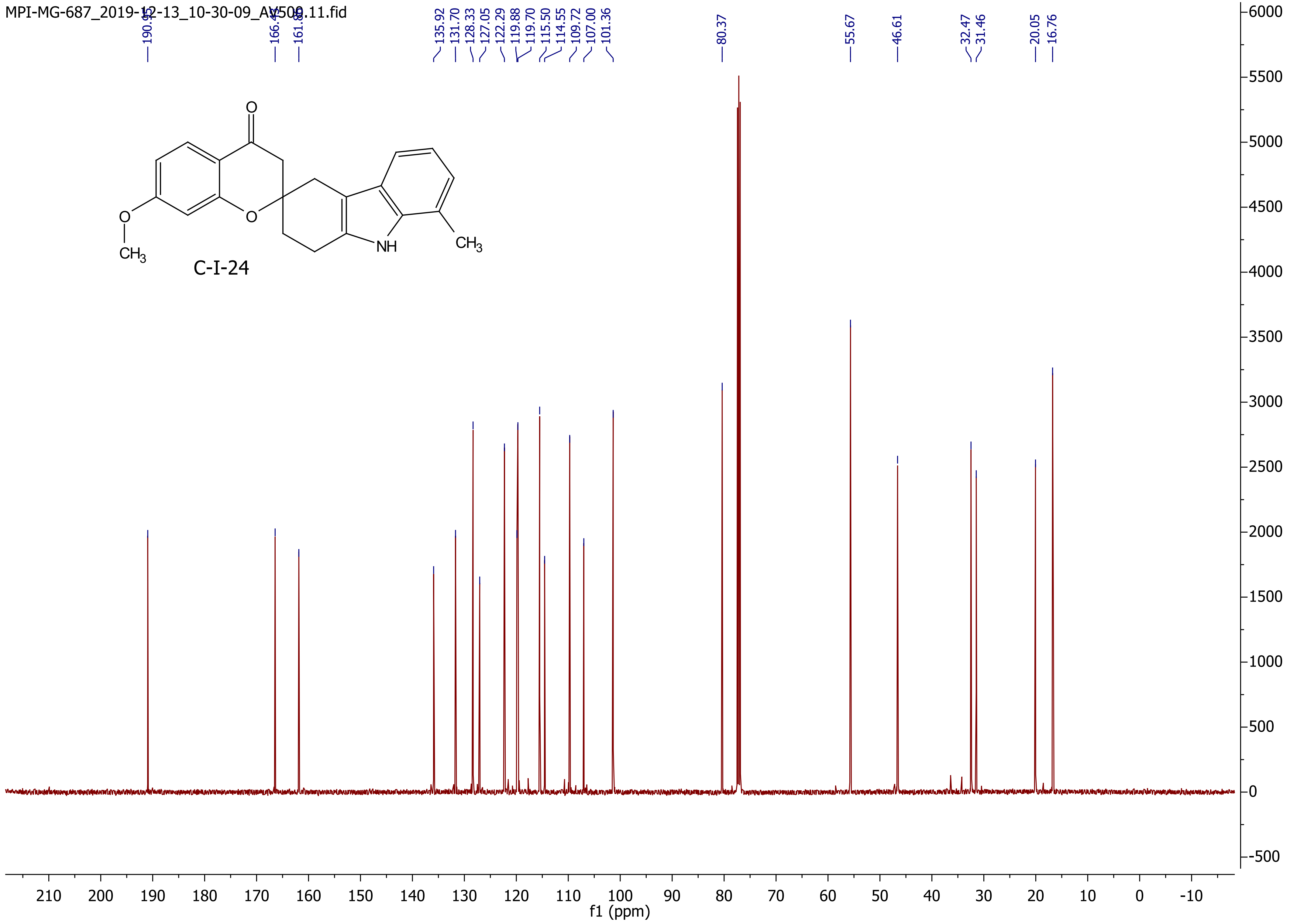

MPI-MG-687_2019-12-13_10-30-09_AV500.11.fid
135.92
131.70
128.33
127.05
122.29
119.88
119.70
115.50
114.55
109.72
107.00
101.36
80.37
55.67
46.61
32.47
31.46
20.05
16.76
O
O
O
CH3
NH
CH3
C-I-24
f1 (ppm)

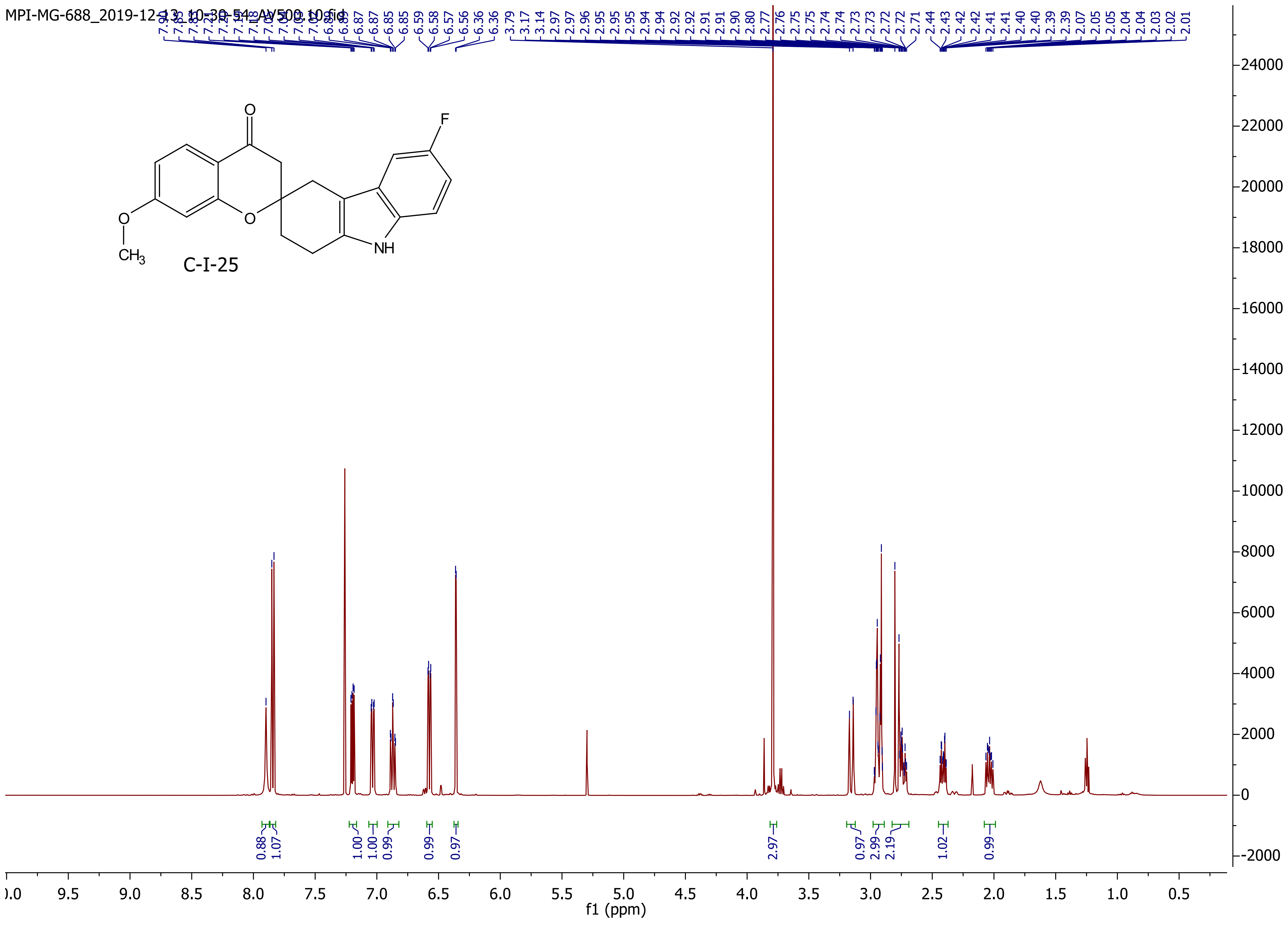

MPI-MG-688_2019-12-13
C-I-25
O
F
O
O
CH3
NH
3.79 3.17 3.14 2.97 2.97 2.96 2.95 2.95 2.95 2.94 2.94 2.92 2.92 2.91 2.91 2.90 2.80 2.77 2.76 2.75 2.75 2.74 2.74 2.73 2.73 2.72 2.72 2.71 2.44 2.43 2.42 2.42 2.41 2.41 2.40 2.40 2.39 2.39 2.07 2.05 2.05 2.04 2.04 2.03 2.02 2.01
6.87 6.87 6.85 6.85 6.59 6.58 6.57 6.56 6.36 6.36
0.88 1.07 1.00 1.00 0.99 0.99 0.97 2.97 0.97 2.99 2.19 1.02 0.99
f1 (ppm)

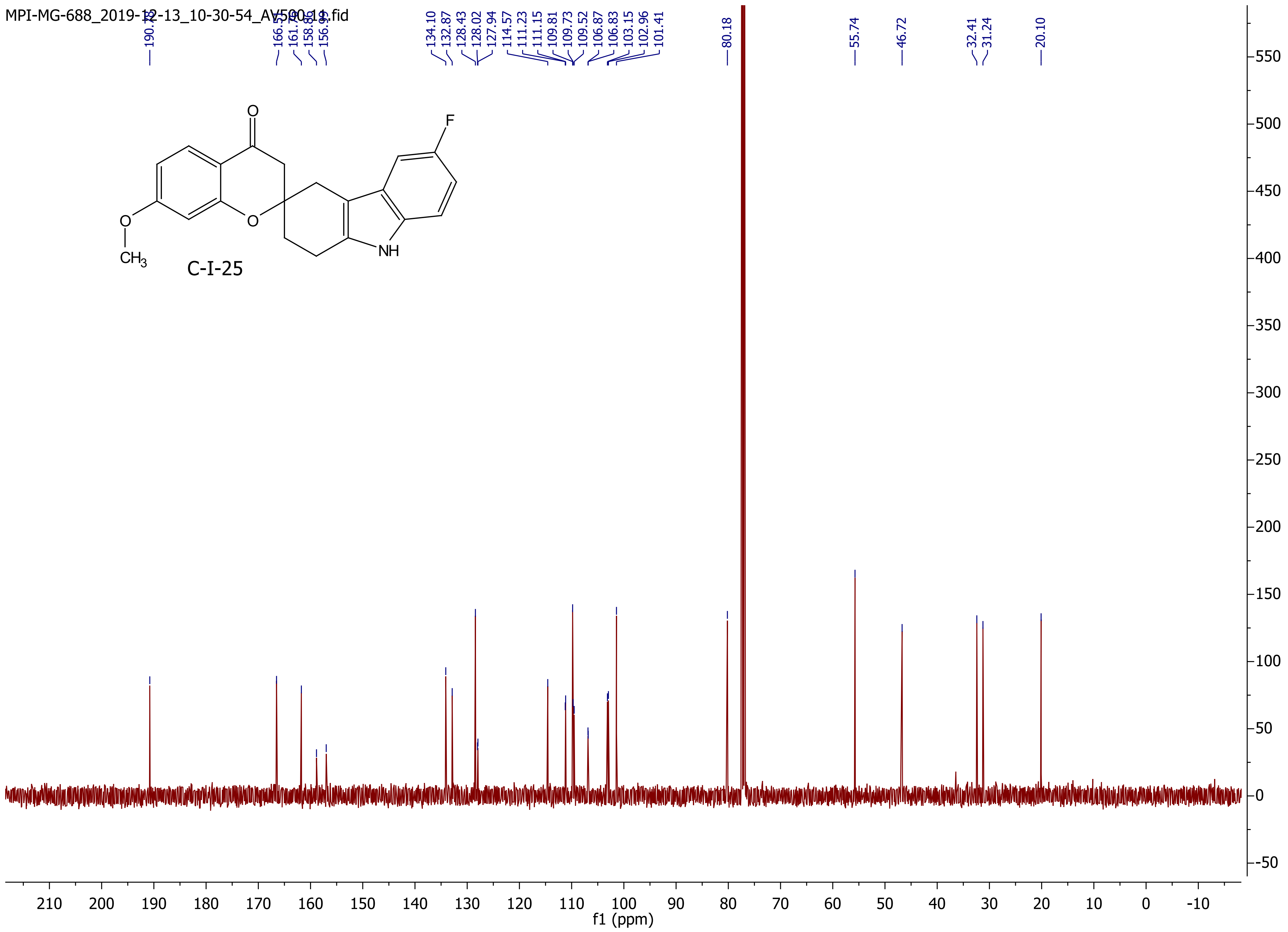
134.10
132.87
128.43
128.02
127.94
114.57
111.23
111.15
109.81
109.73
109.52
106.87
106.83
103.15
102.96
101.41
80.18
55.74
46.72
32.41
31.24
20.10
O
F
O
O
NH
CH3
C-I-25
f1 (ppm)

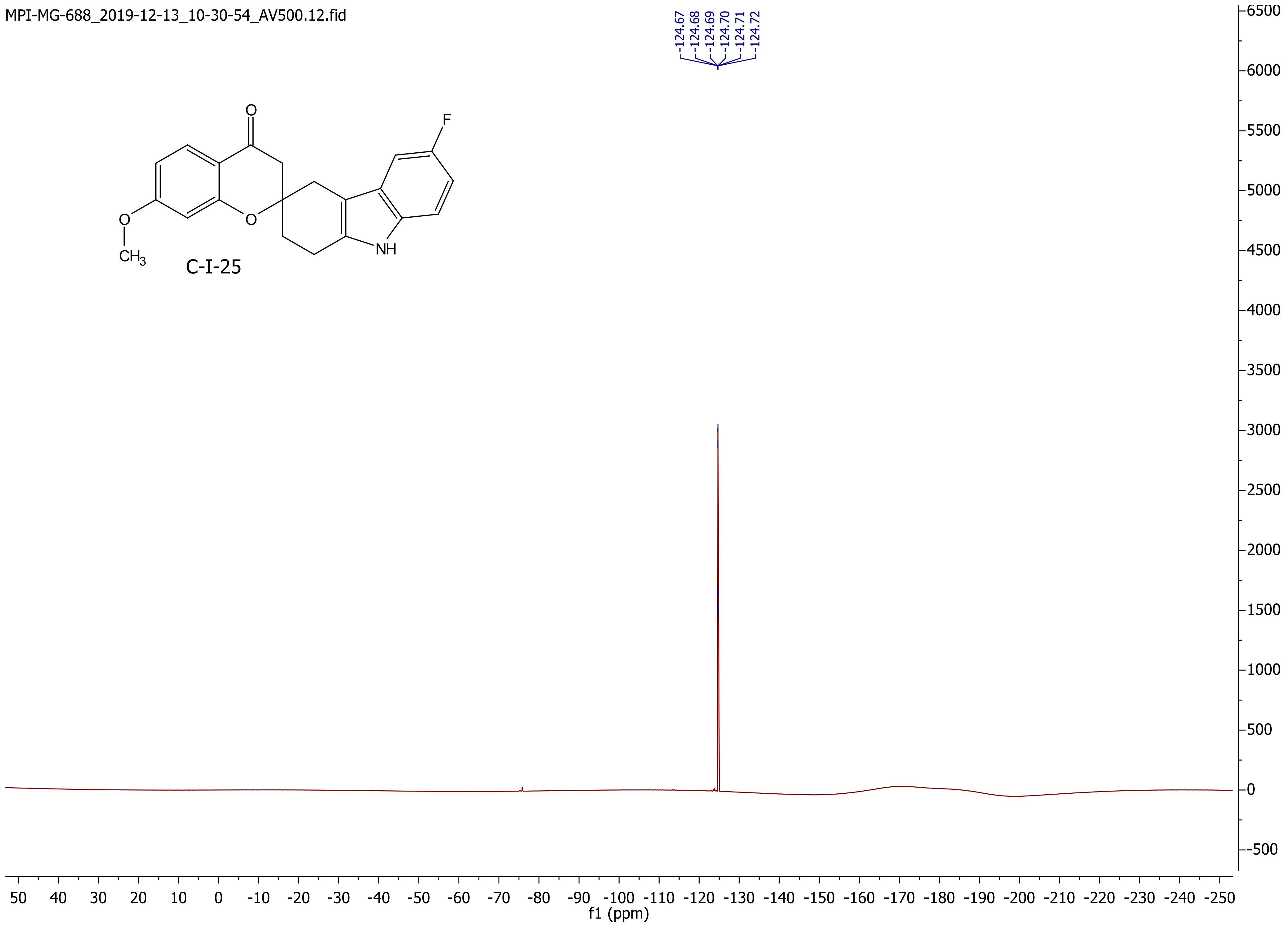
MPI-MG-688_2019-12-13_10-30-54_AV500.12.fid
-124.67
-124.68
-124.69
-124.70
-124.71
-124.72
O
F
O
O
NH
CH3
C-I-25
6500
6000
5500
5000
4500
4000
3500
3000
2500
2000
1500
1000
500
0
-500
50 40 30 20 10 0 -10 -20 -30 -40 -50 -60 -70 -80 -90 -100 -110 -120 -130 -140 -150 -160 -170 -180 -190 -200 -210 -220 -230 -240 -250
f1 (ppm)

MG-68901
MG-689
STANDARD 1H OBSERVE

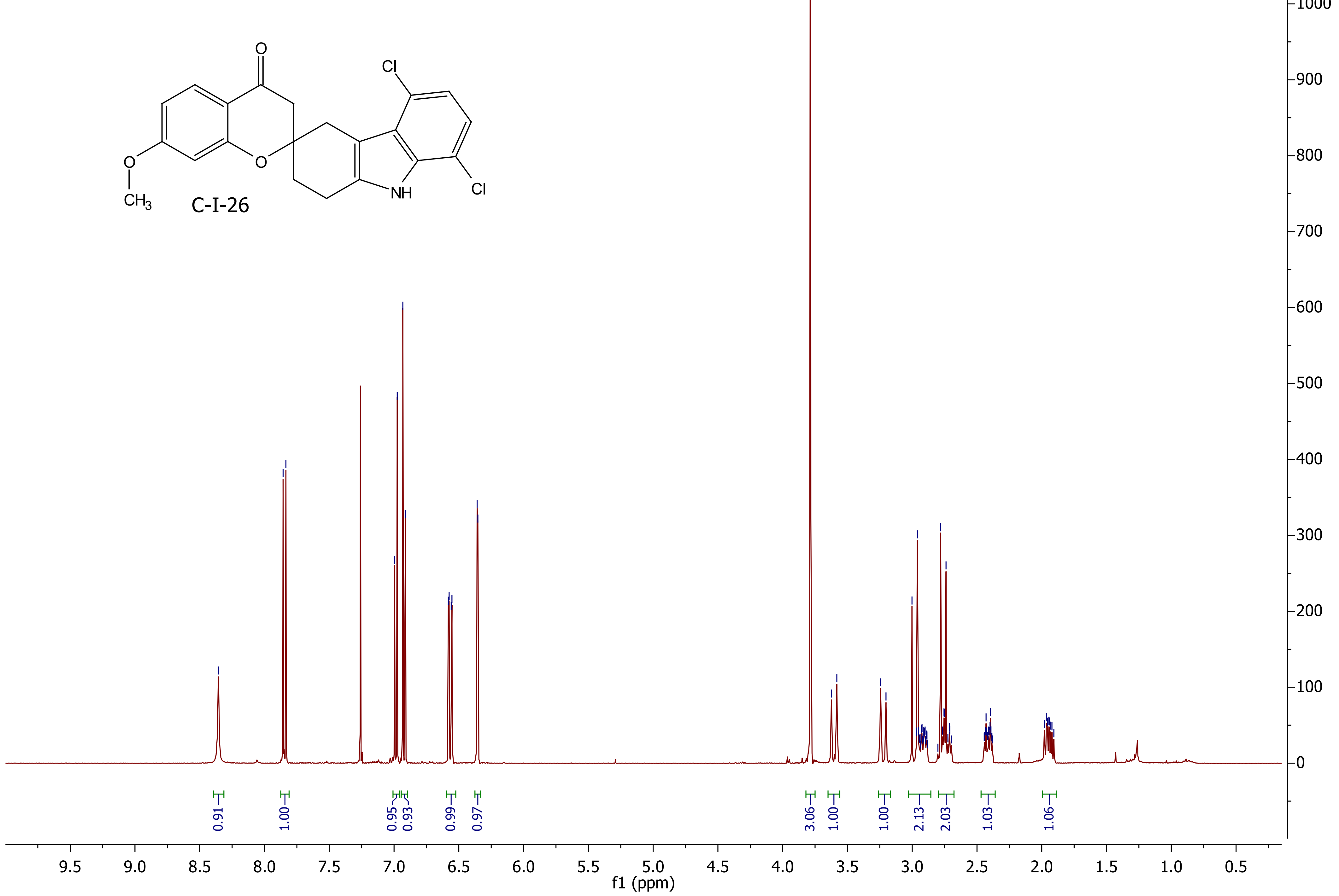

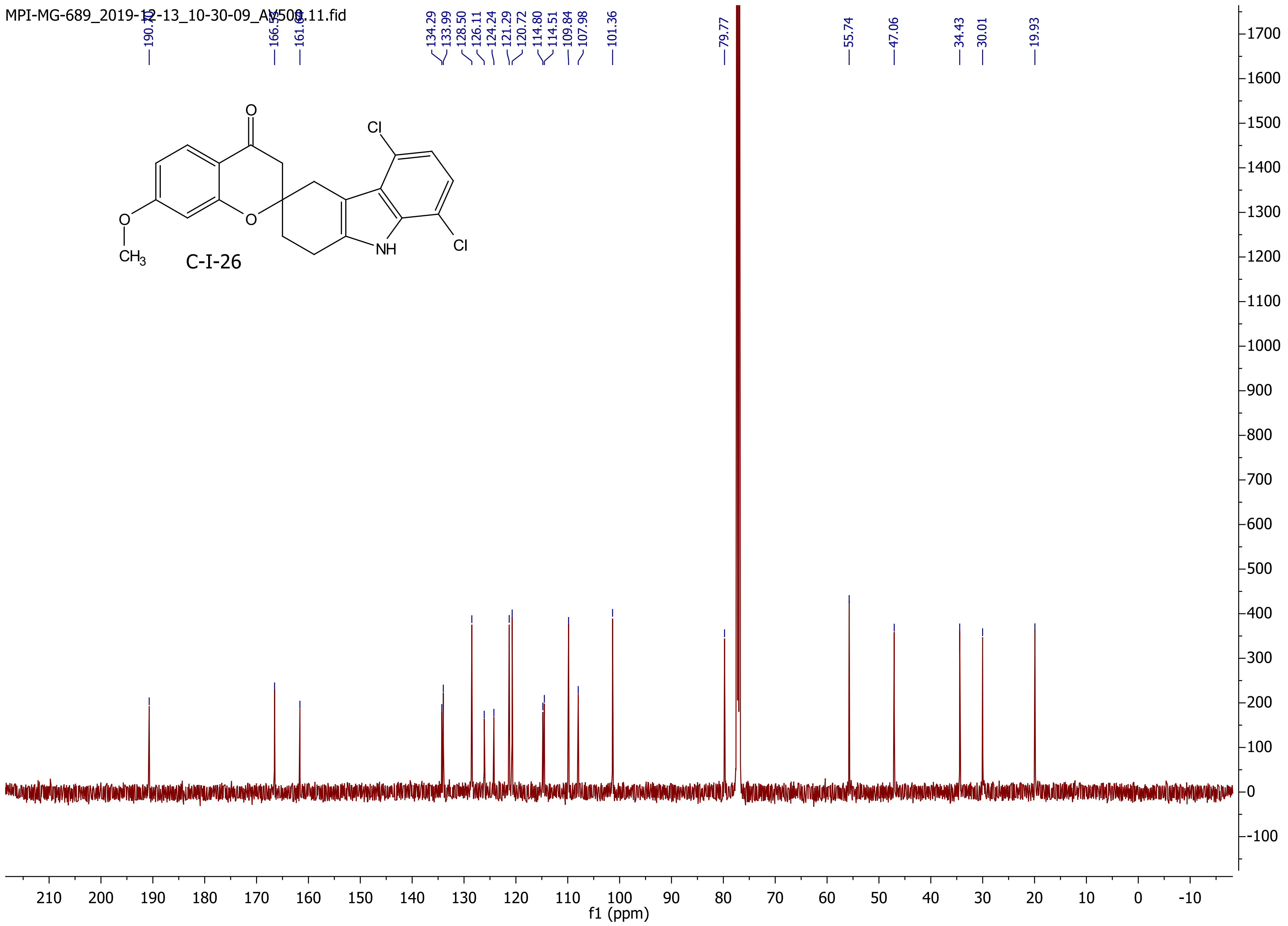

MPI-MG-689_2019-12-13_10-30-09_AV500.11.fid
134.29
133.99
128.50
126.11
124.24
121.29
120.72
114.80
114.51
109.84
107.98
101.36
79.77
55.74
47.06
34.43
30.01
19.93
O
Cl
O
O
NH
Cl
CH3
C-I-26
f1 (ppm)

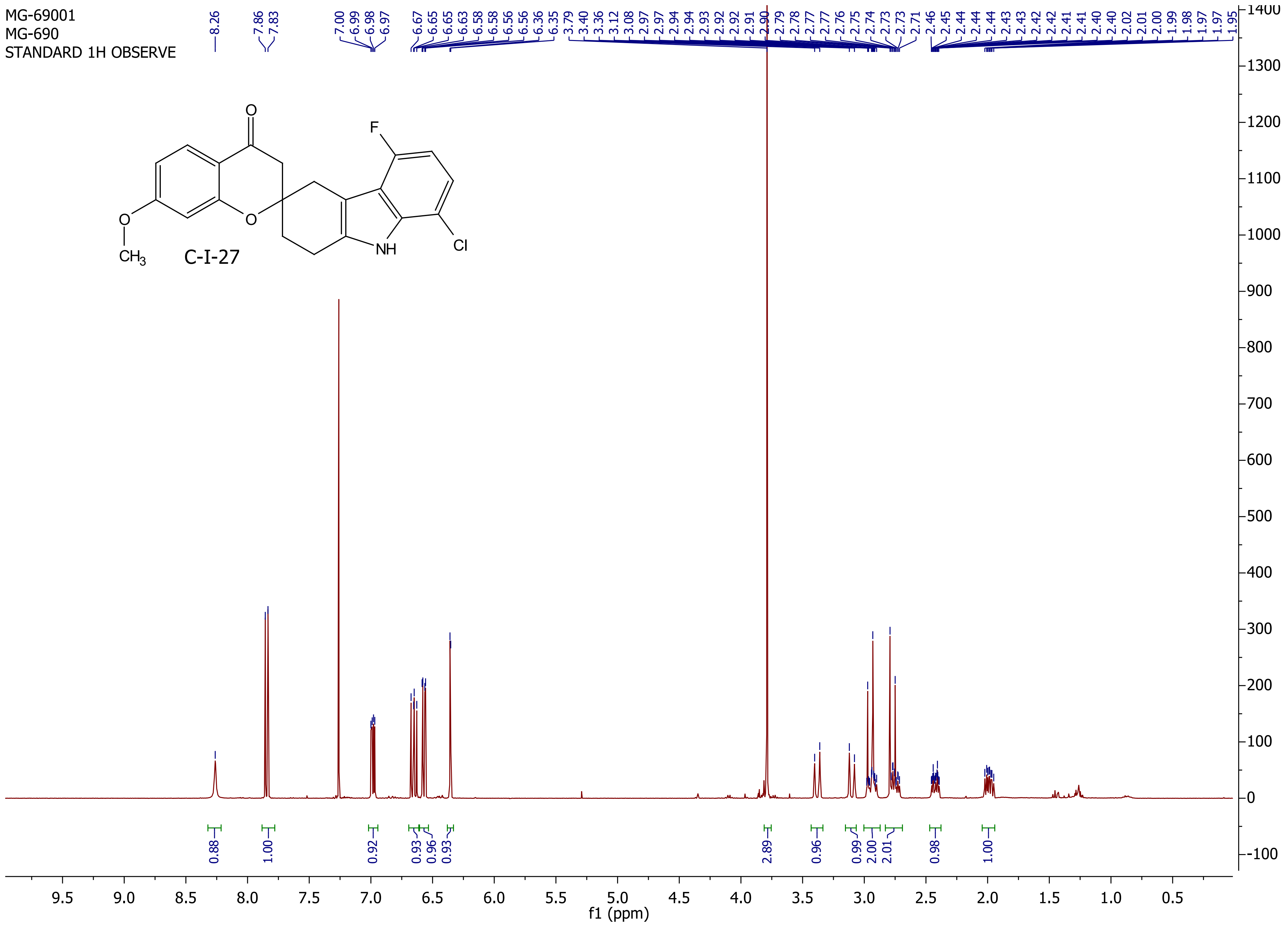
MG-69001
MG-690
STANDARD 1H OBSERVE
8.26
7.86 7.83
7.00 6.99 6.98 6.97
6.67 6.65 6.65 6.63 6.58 6.58 6.56 6.56 6.36 6.35
3.79 3.40 3.36 3.12 3.08 2.97 2.97 2.94 2.94 2.93 2.92 2.92 2.91 2.90 2.79 2.78 2.77 2.77 2.76 2.75 2.74 2.73 2.73 2.71
2.46 2.45 2.44 2.44 2.44 2.43 2.43 2.42 2.42 2.41 2.41 2.40 2.40 2.02 2.01 2.00 1.99 1.98 1.97 1.97 1.95
O
F
O
NH
Cl
O
CH3
C-I-27
0.88 1.00 0.92 0.93 0.96 0.93 2.89 0.96 0.99 2.00 2.01 0.98 1.00
f1 (ppm)

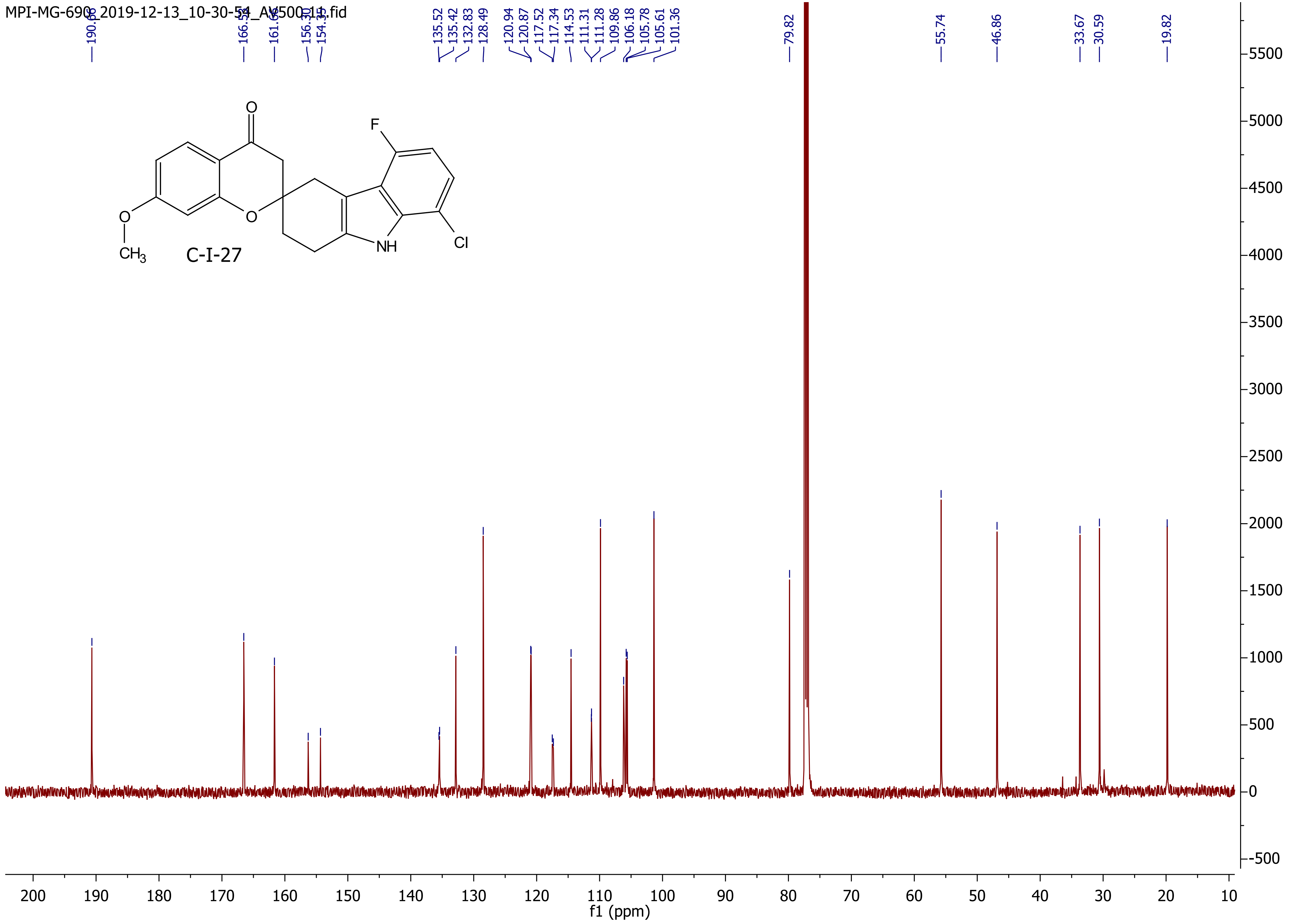
MPI-MG-690_2019-12-13_10-30-54_AV500_11.fid
135.52
135.42
132.83
128.49
120.94
120.87
117.52
117.34
114.53
111.31
111.28
109.86
106.18
105.78
105.61
101.36
79.82
55.74
46.86
33.67
30.59
19.82
F
O
O
O
CH3
NH
Cl
C-I-27
f1 (ppm)

MPI-MG-690_2019-12-13_10-30-54_AV500.12.fid

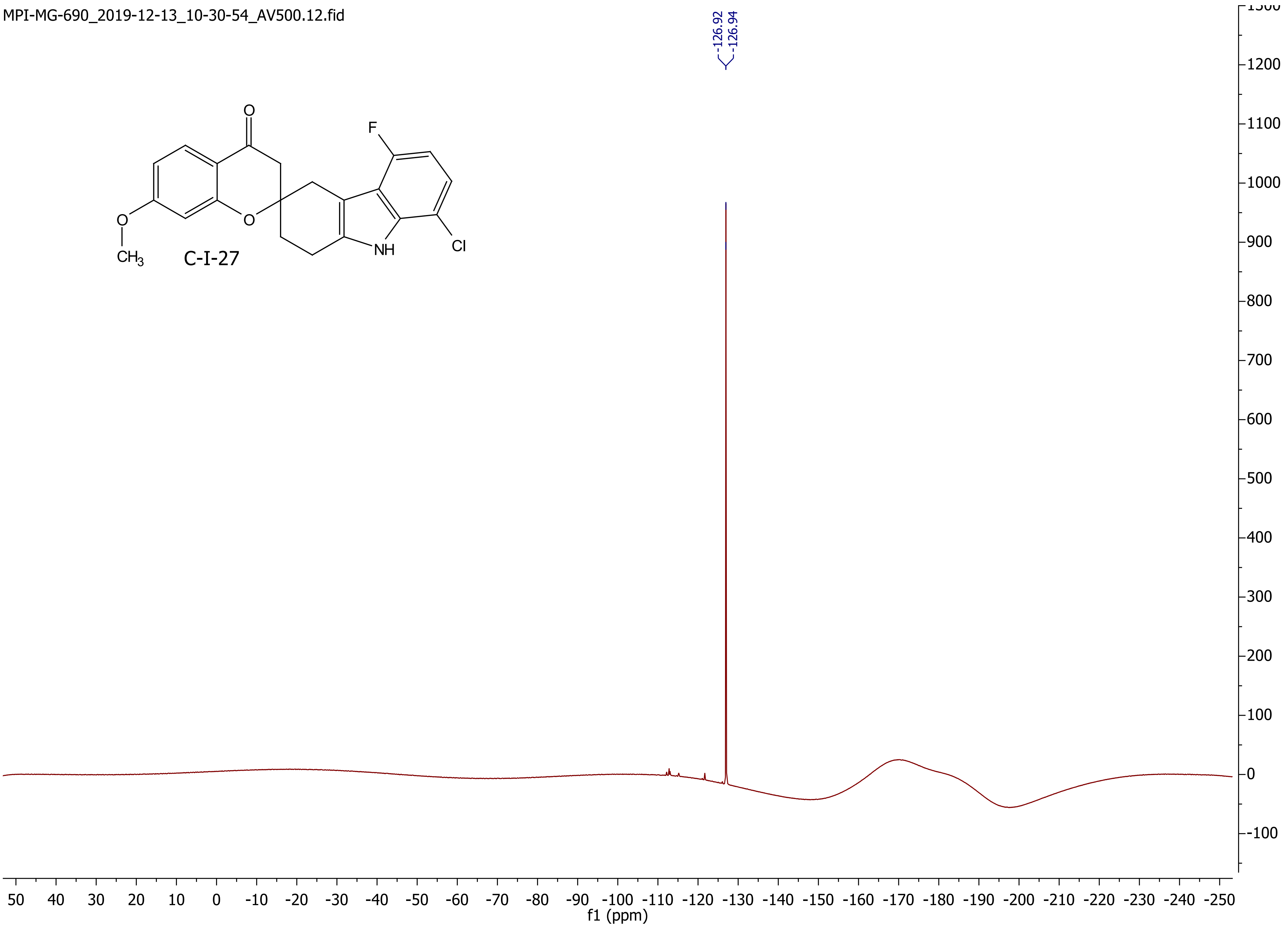

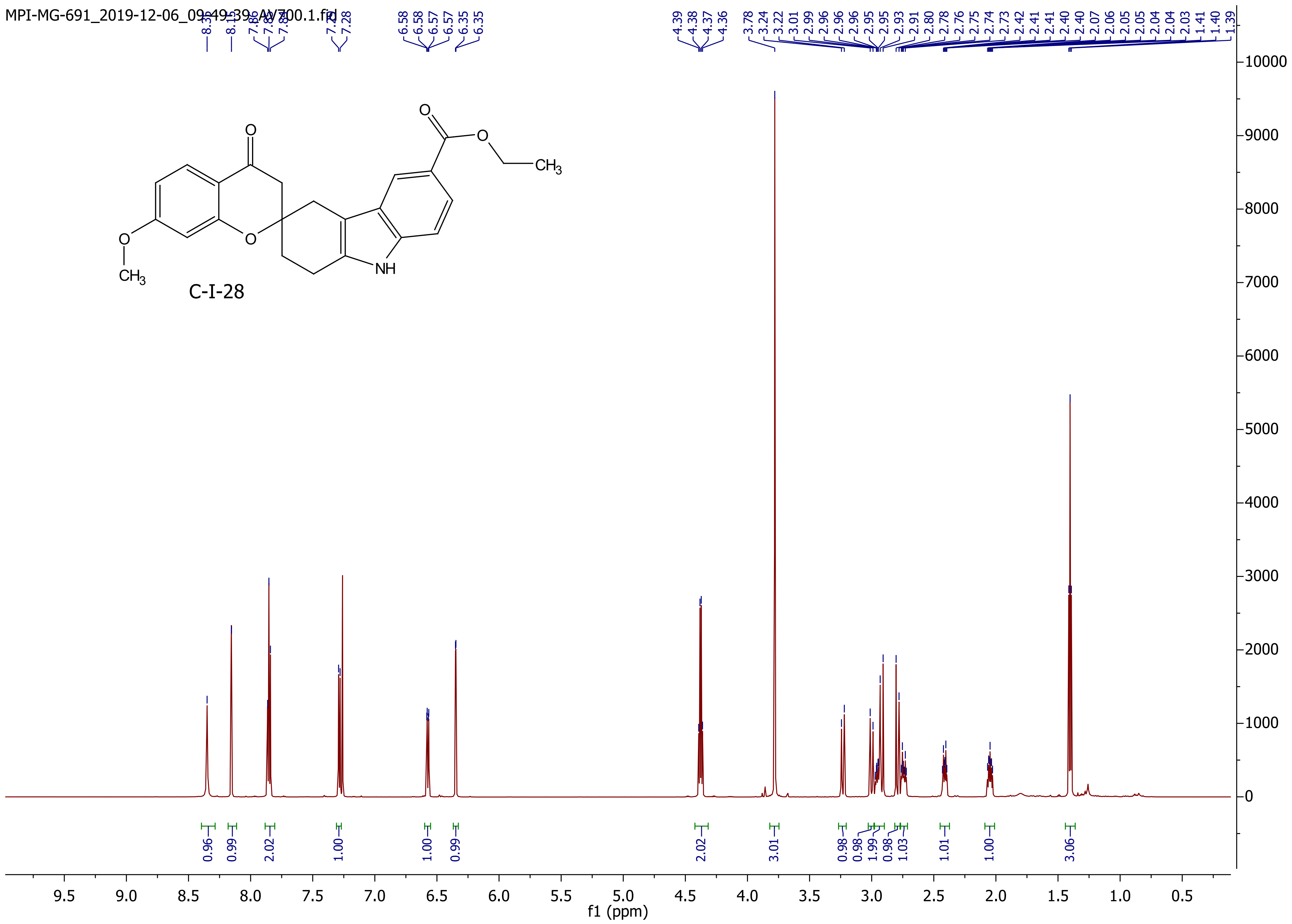

MPI-MG-691_2019-12-06_09-49-39_AV700.1.fid
7.28
6.58
6.58
6.57
6.57
6.35
6.35
4.39
4.38
4.37
4.36
3.78
3.24
3.22
3.01
2.99
2.96
2.96
2.95
2.95
2.93
2.91
2.80
2.78
2.76
2.75
2.74
2.73
2.42
2.41
2.41
2.40
2.40
2.07
2.06
2.05
2.05
2.04
2.04
2.03
1.41
1.40
1.39
O
O
O
O
O
CH3
CH3
NH
C-I-28
0.96
0.99
2.02
1.00
1.00
0.99
2.02
3.01
0.98
0.98
1.99
0.98
1.03
1.01
1.00
3.06
f1 (ppm)

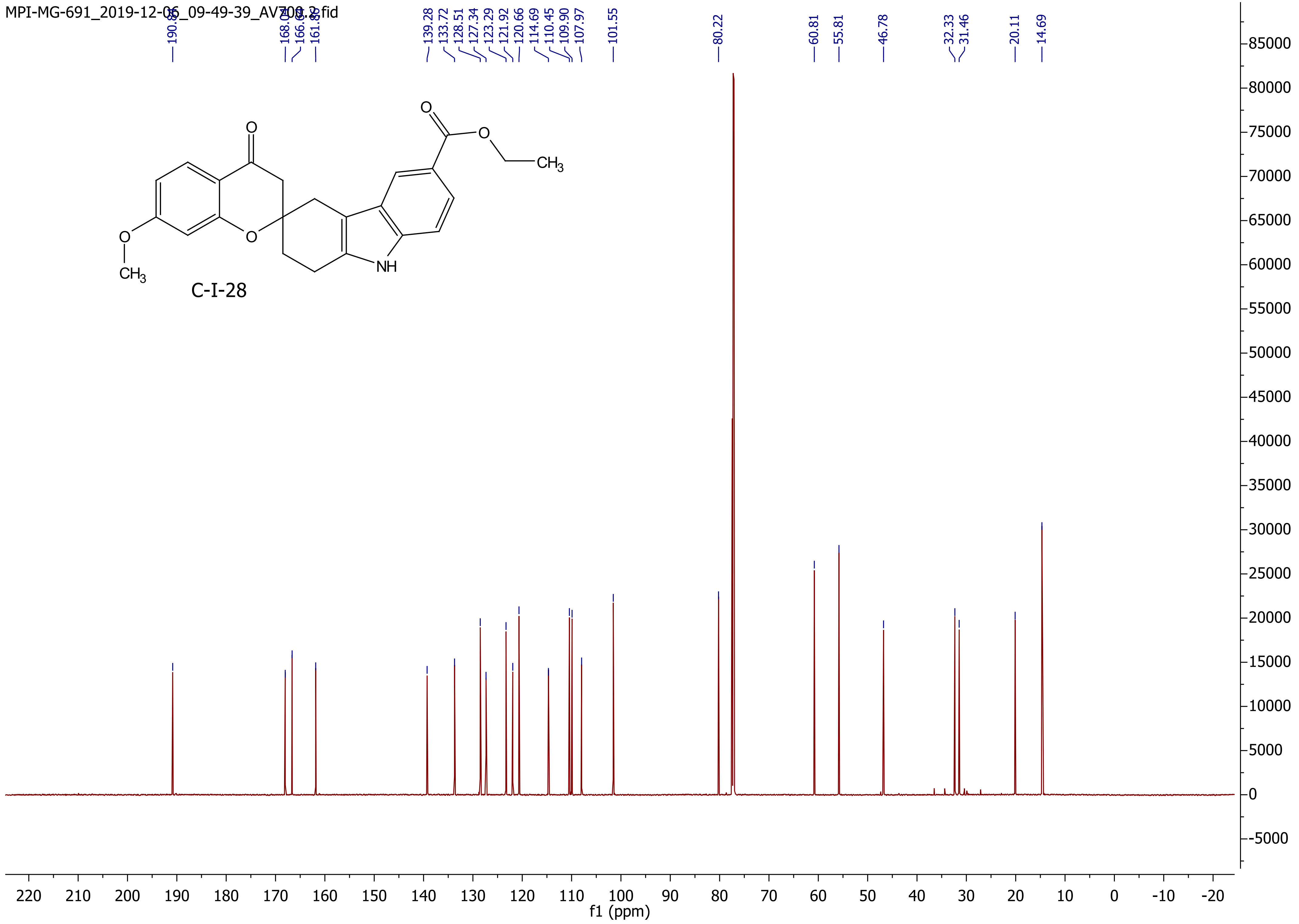

MPI-MG-691_2019-12-06_09-49-39
139.28
133.72
128.51
127.34
123.29
121.92
120.66
114.69
110.45
109.90
107.97
101.55
80.22
60.81
55.81
46.78
32.33
31.46
20.11
14.69
O
O
CH3
O
O
O
CH3
NH
C-I-28
f1 (ppm)

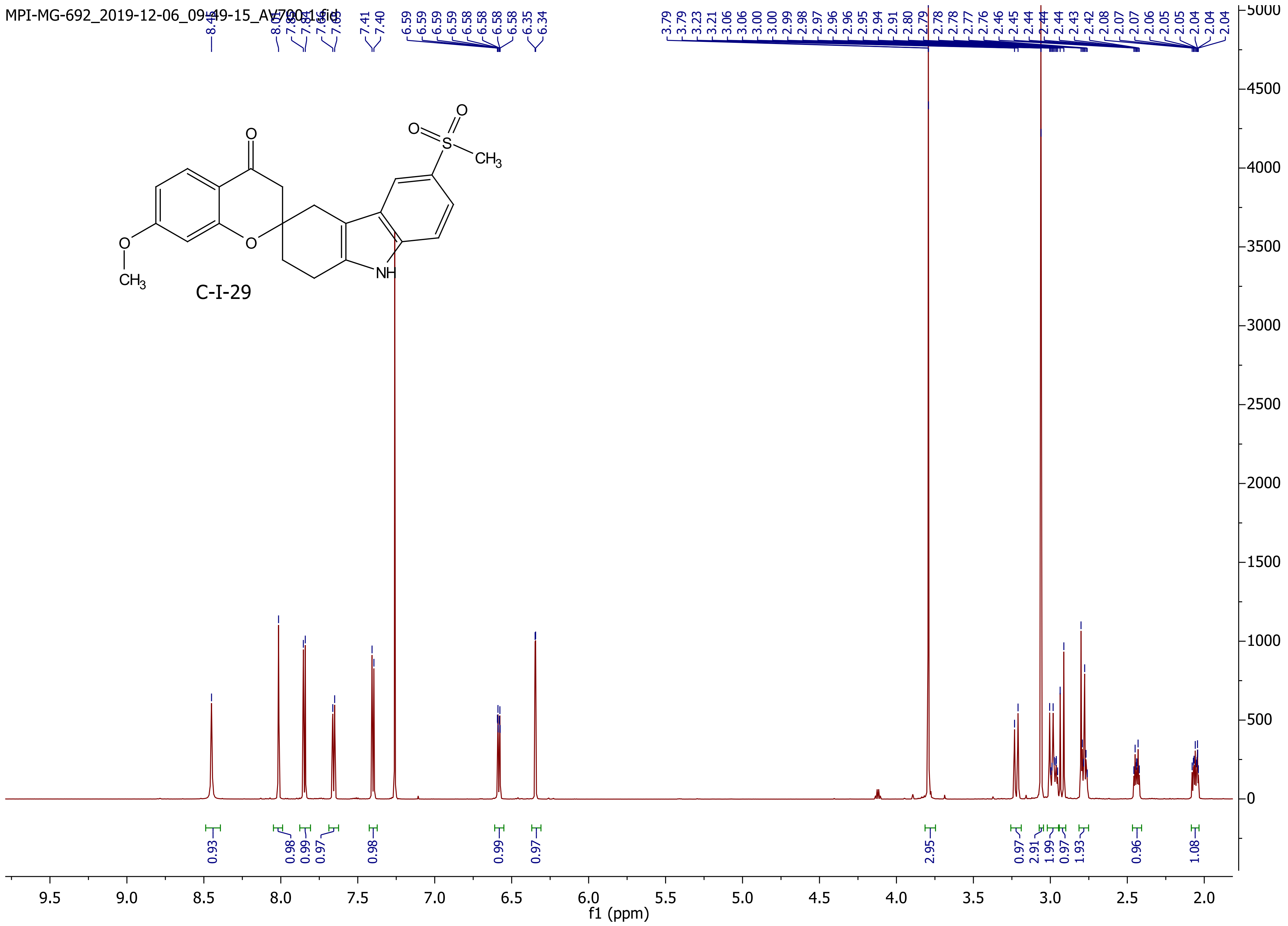
MPI-MG-692_2019-12-06_09-49-15_AV700-1.fid
C-I-29
f1 (ppm)

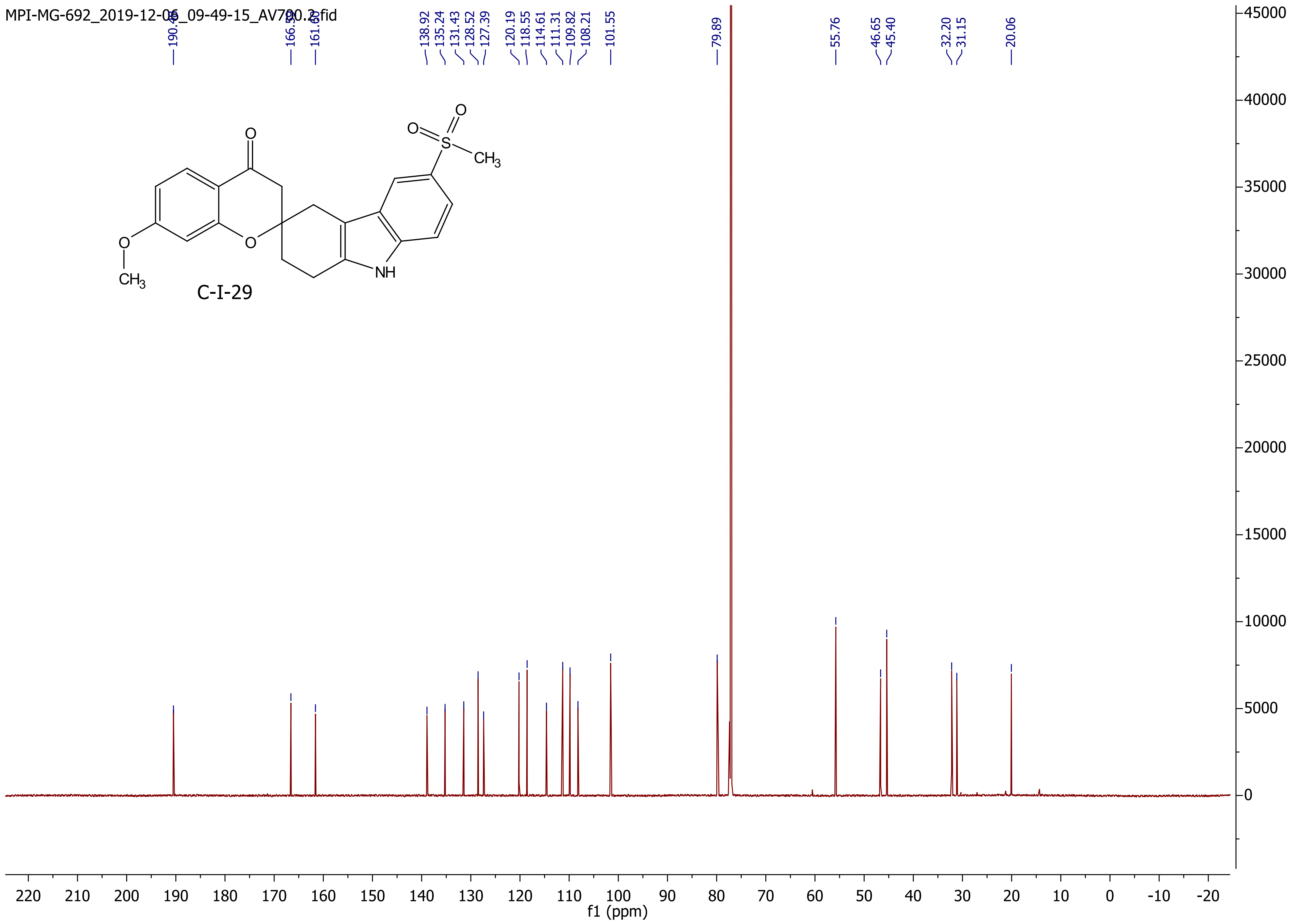
MPI-MG-692_2019-12-06_09-49-15_AV700.2.fid
138.92
135.24
131.43
128.52
127.39
120.19
118.55
114.61
111.31
109.82
108.21
101.55
79.89
55.76
46.65
45.40
32.20
31.15
20.06
C-I-29
f1 (ppm)

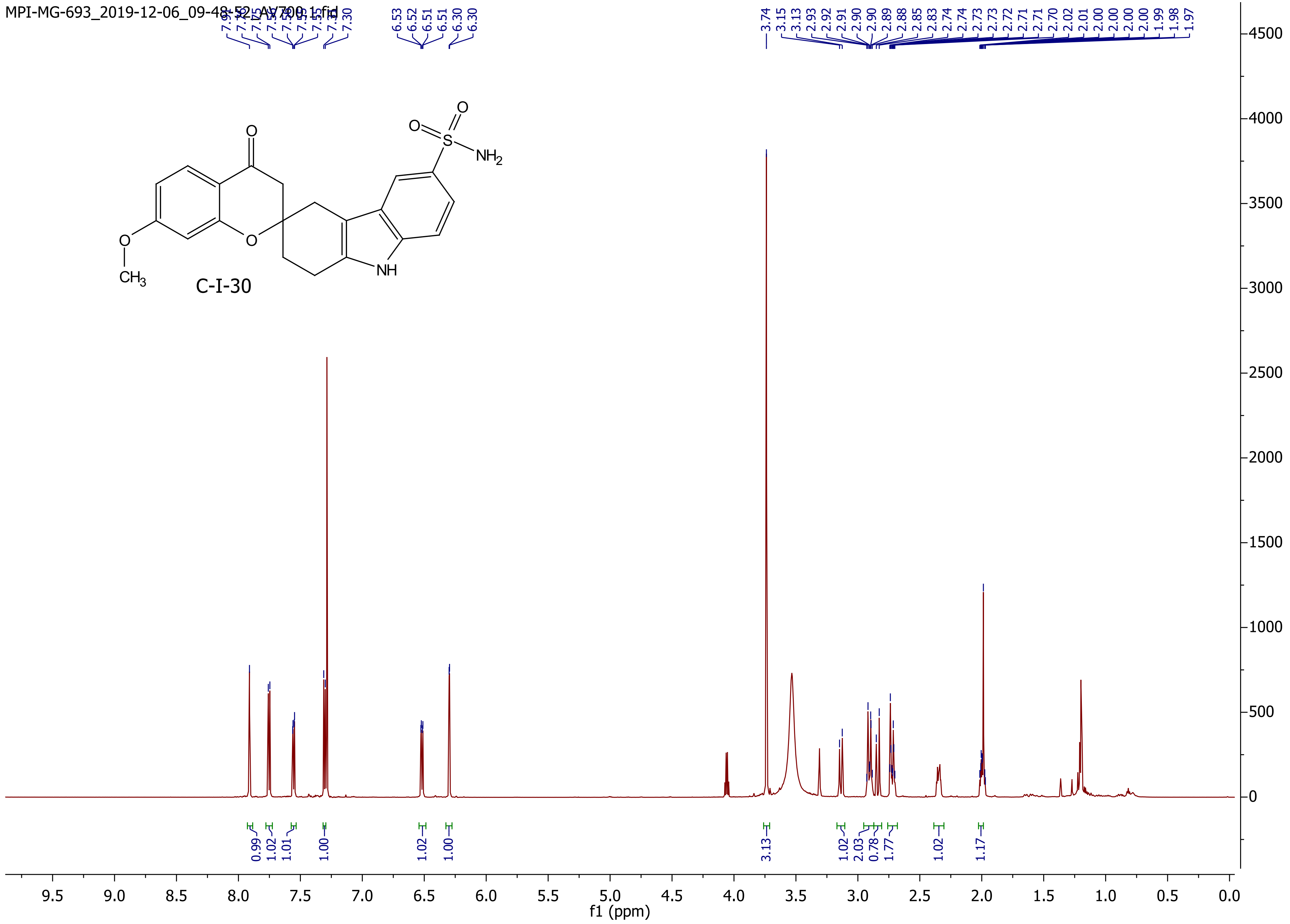
MPI-MG-693_2019-12-06_09-48-52_AV700 1.fid
C-I-30
f1 (ppm)

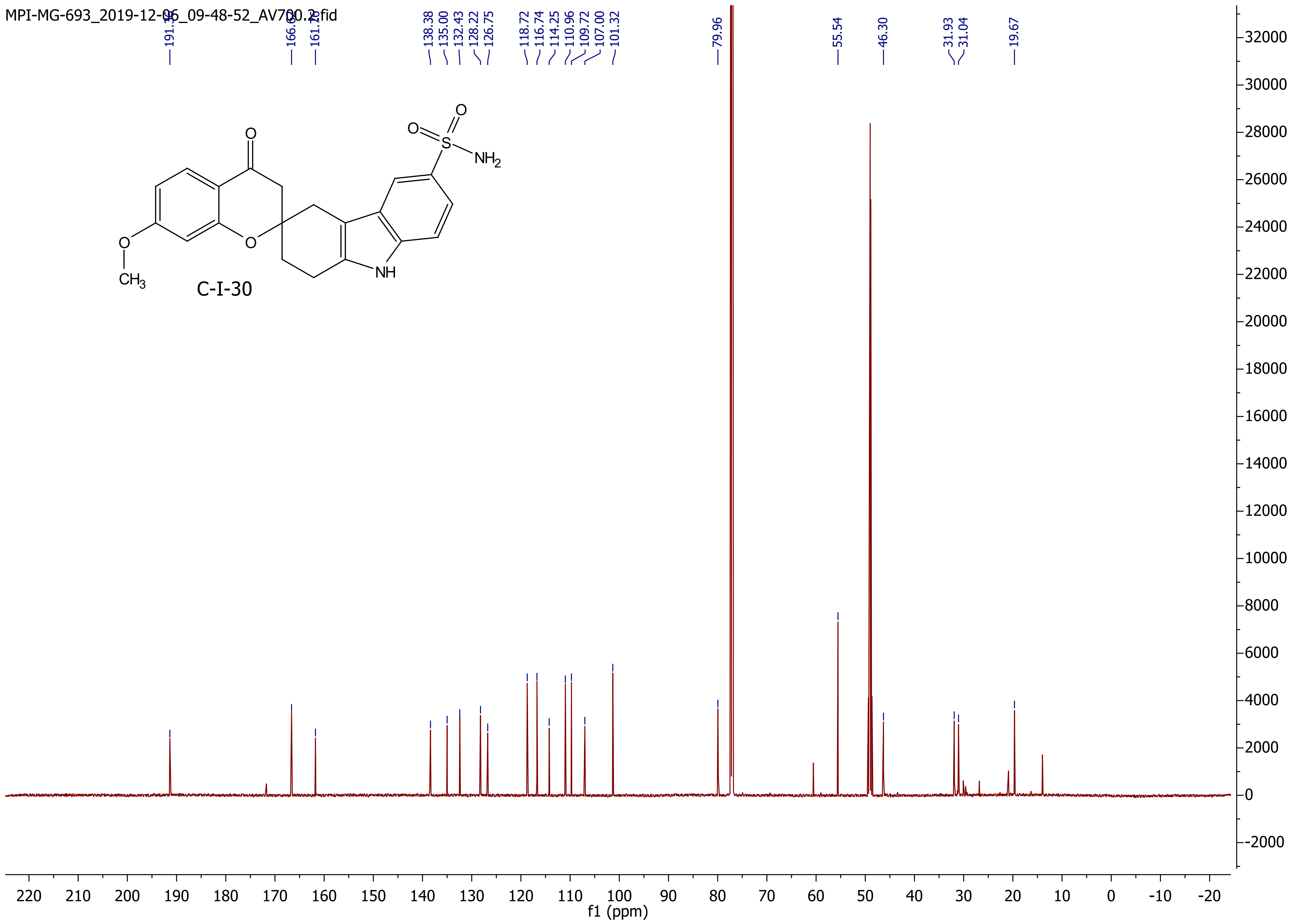

MPI-MG-693_2019-12-06_09-48-52_AV700.2.fid
191.36
166.62
161.76
138.38
135.00
132.43
128.22
126.75
118.72
116.74
114.25
110.96
109.72
107.00
101.32
79.96
55.54
46.30
31.93
31.04
19.67
O
O
S
O
NH2
O
O
NH
CH3
C-I-30
220 210 200 190 180 170 160 150 140 130 120 110 100 90 80 70 60 50 40 30 20 10 0 -10 -20
f1 (ppm)
32000 30000 28000 26000 24000 22000 20000 18000 16000 14000 12000 10000 8000 6000 4000 2000 0 -2000

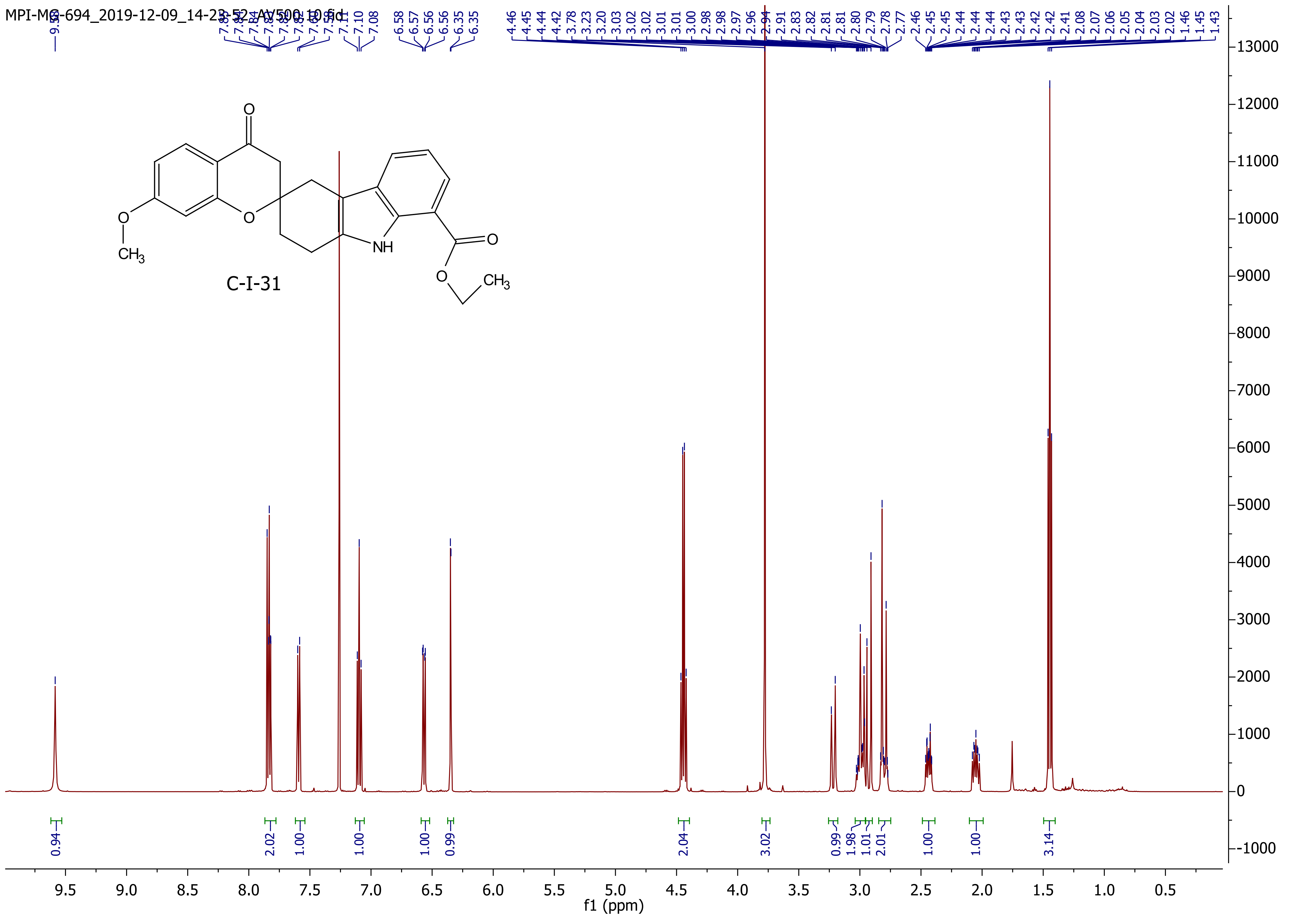

MPI-MG-694_2019-12-09_14-23-52_AV500.10.fid
C-I-31
O
NH
CH3
f1 (ppm)
0.94
2.02
1.00
1.00
1.00
0.99
2.04
3.02
0.99
1.98
1.01
2.01
1.00
1.00
3.14

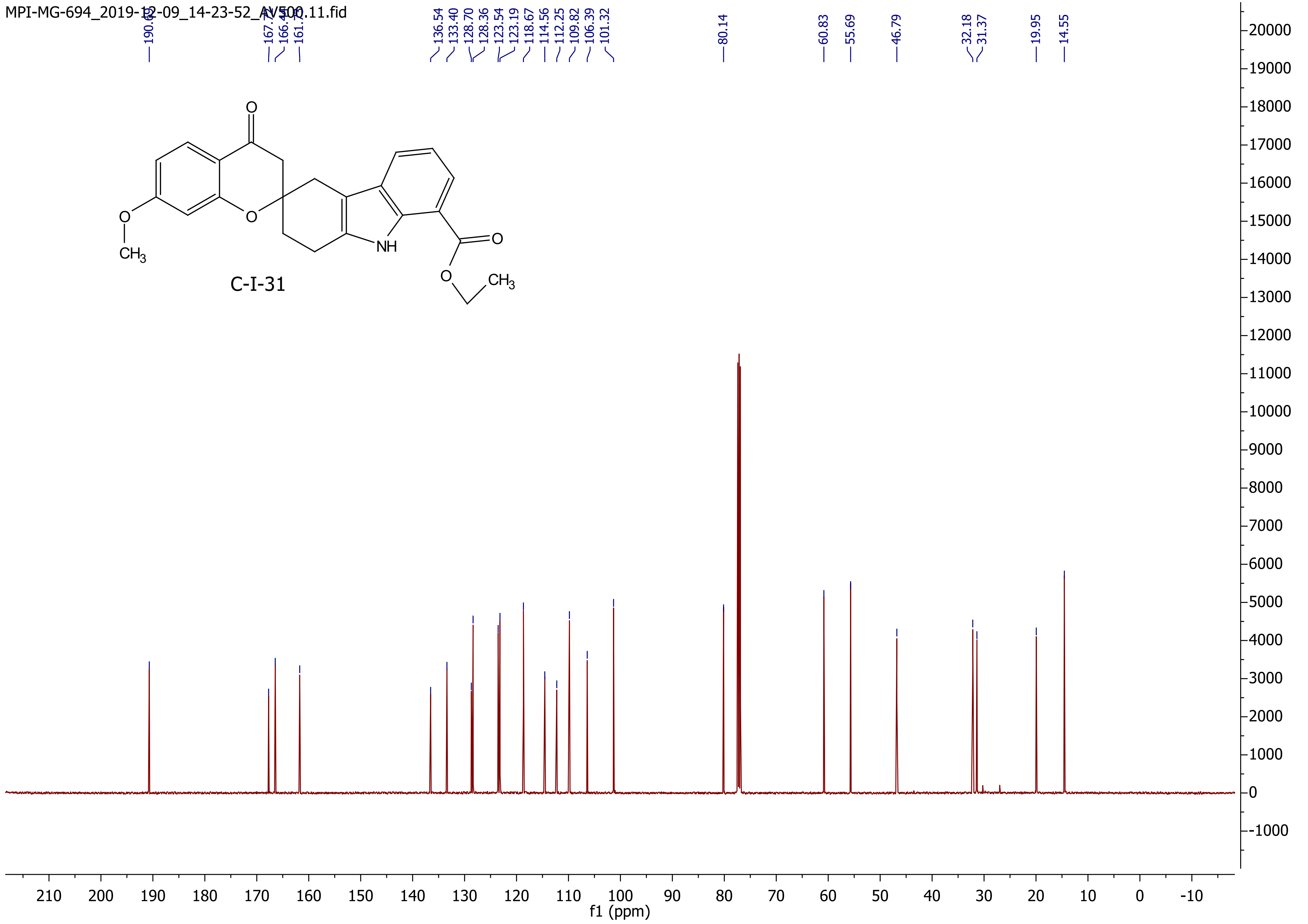

MPI-MG-694_2019-12-09_14-23-52_AV500.11.fid
136.54
133.40
128.70
128.36
123.54
123.19
118.67
114.56
112.25
109.82
106.39
101.32
80.14
60.83
55.69
46.79
32.18
31.37
19.95
14.55
O
O
O
NH
O
O
CH_3
CH_3
C-I-31
f1 (ppm)

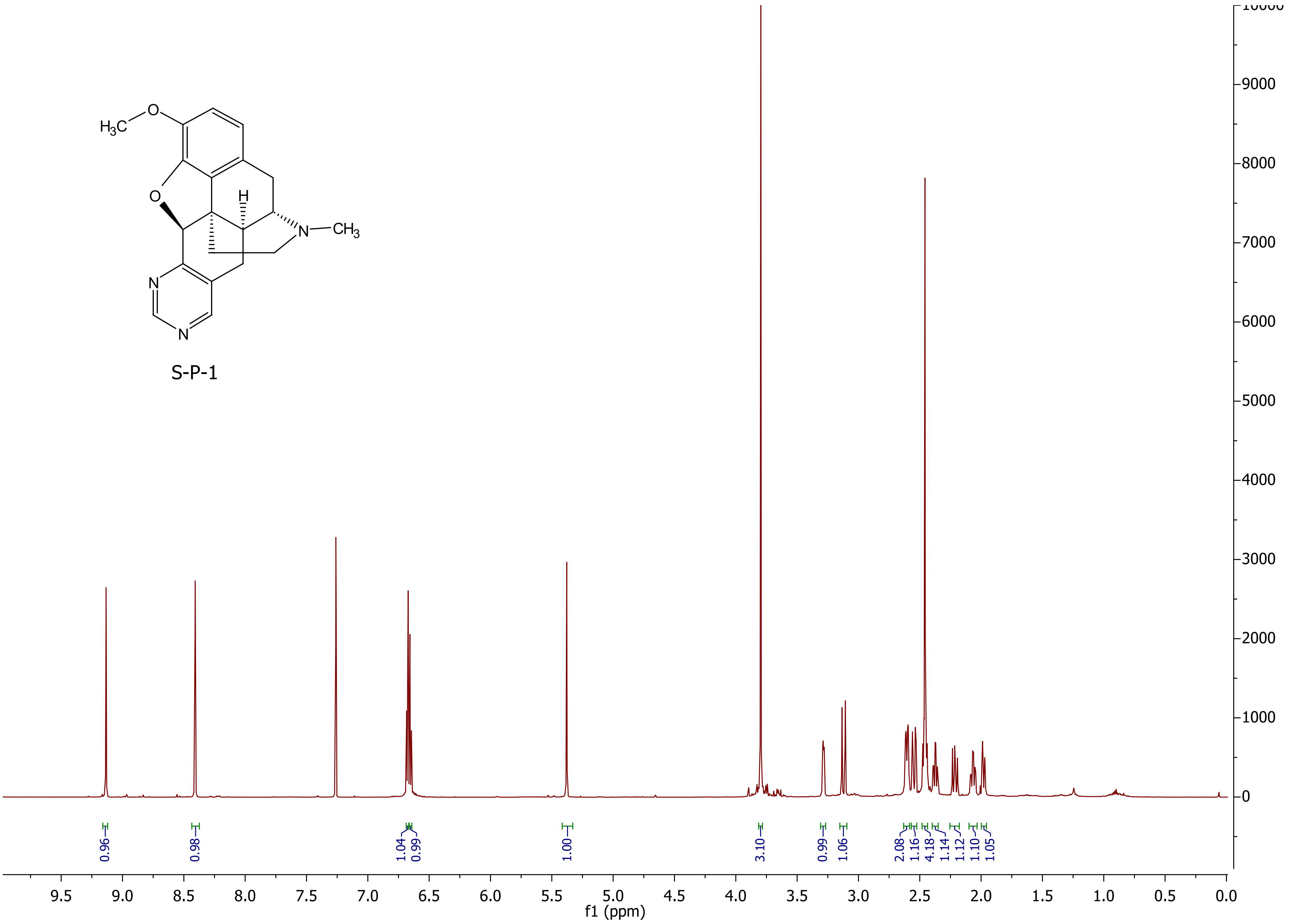
H3C
O
O
H
N
CH3
N
N
S-P-1
10000
9000
8000
7000
6000
5000
4000
3000
2000
1000
0
0.96
0.98
1.04
0.99
1.00
3.10
0.99
1.06
2.08
1.16
4.18
1.14
1.12
1.10
1.05
9.5
9.0
8.5
8.0
7.5
7.0
6.5
6.0
5.5
5.0
4.5
4.0
3.5
3.0
2.5
2.0
1.5
1.0
0.5
0.0
f1 (ppm)

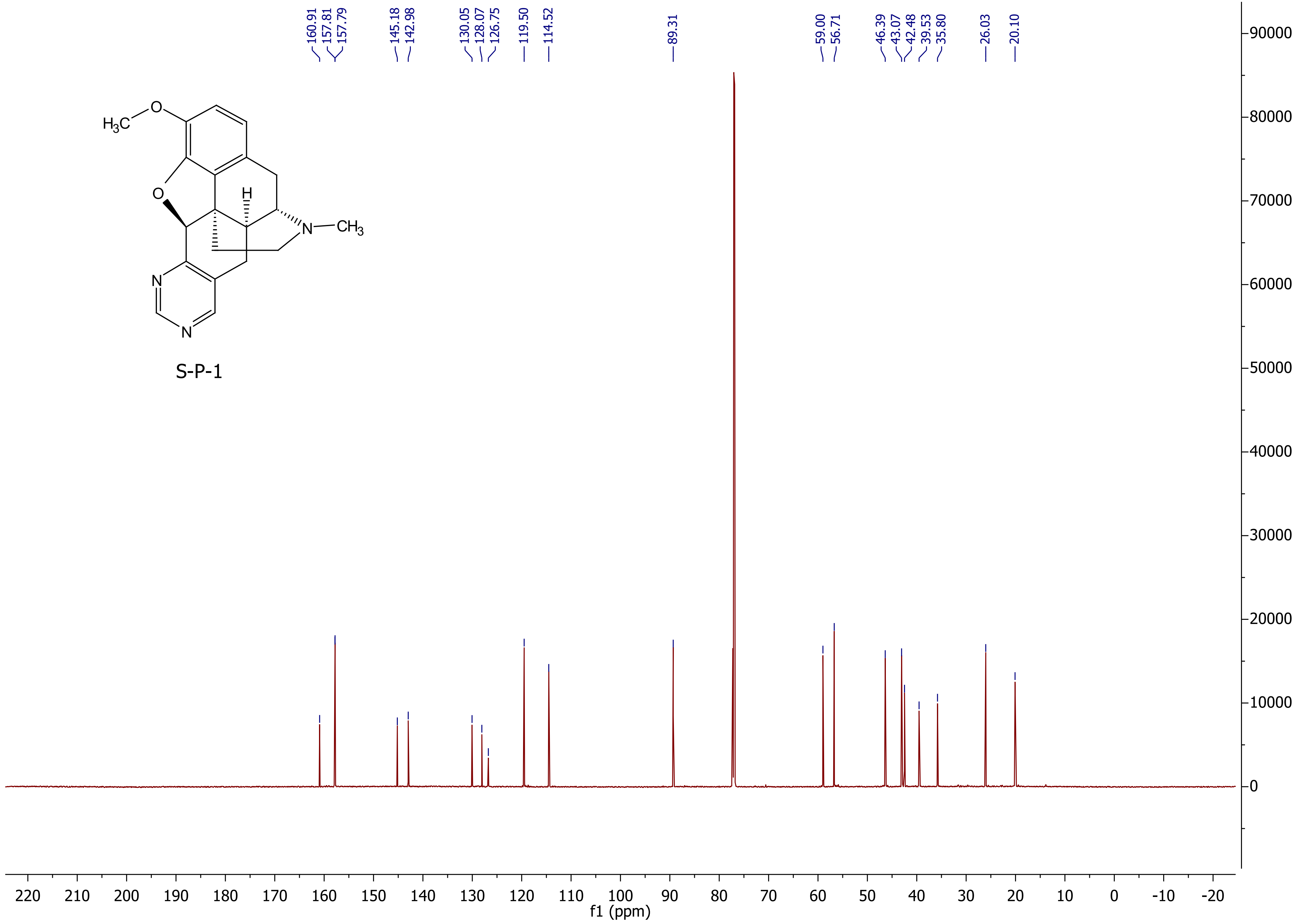

160.91
157.81
157.79
145.18
142.98
130.05
128.07
126.75
119.50
114.52
89.31
59.00
56.71
46.39
43.07
42.48
39.53
35.80
26.03
20.10
H3C
O
O
H
N
CH3
N
N
S-P-1
90000
80000
70000
60000
50000
40000
30000
20000
10000
0
220 210 200 190 180 170 160 150 140 130 120 110 100 90 80 70 60 50 40 30 20 10 0 -10 -20
f1 (ppm)

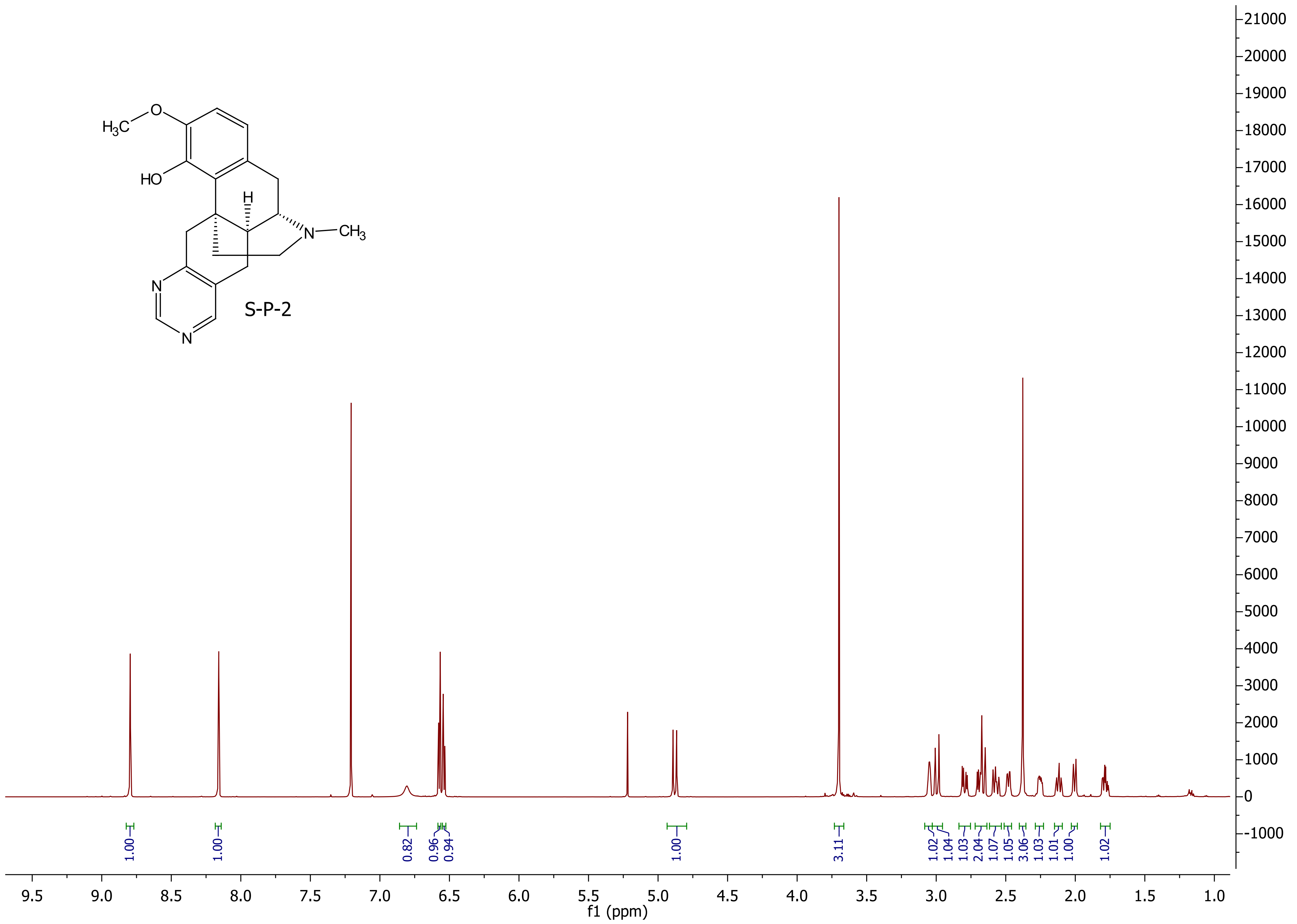

H3C
O
HO
H
N
CH3
N
N
S-P-2
1.00
1.00
0.82
0.96
0.94
1.00
3.11
1.02
1.04
1.03
2.04
1.07
1.05
3.06
1.03
1.01
1.00
1.02
f1 (ppm)

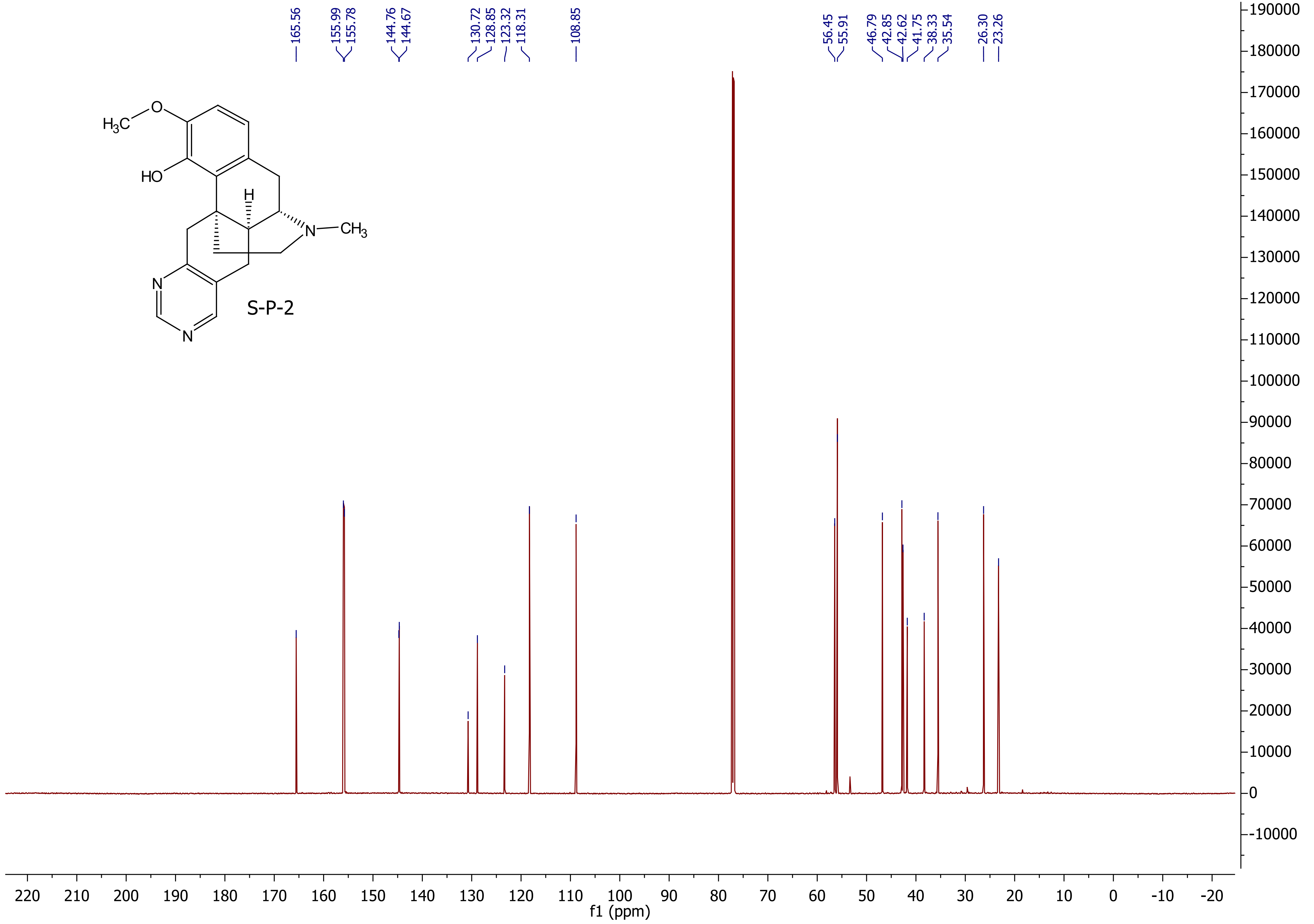

165.56
155.99
155.78
144.76
144.67
130.72
128.85
123.32
118.31
108.85
56.45
55.91
46.79
42.85
42.62
41.75
38.33
35.54
26.30
23.26
H3C
O
HO
H
N
CH3
N
N
S-P-2
f1 (ppm)

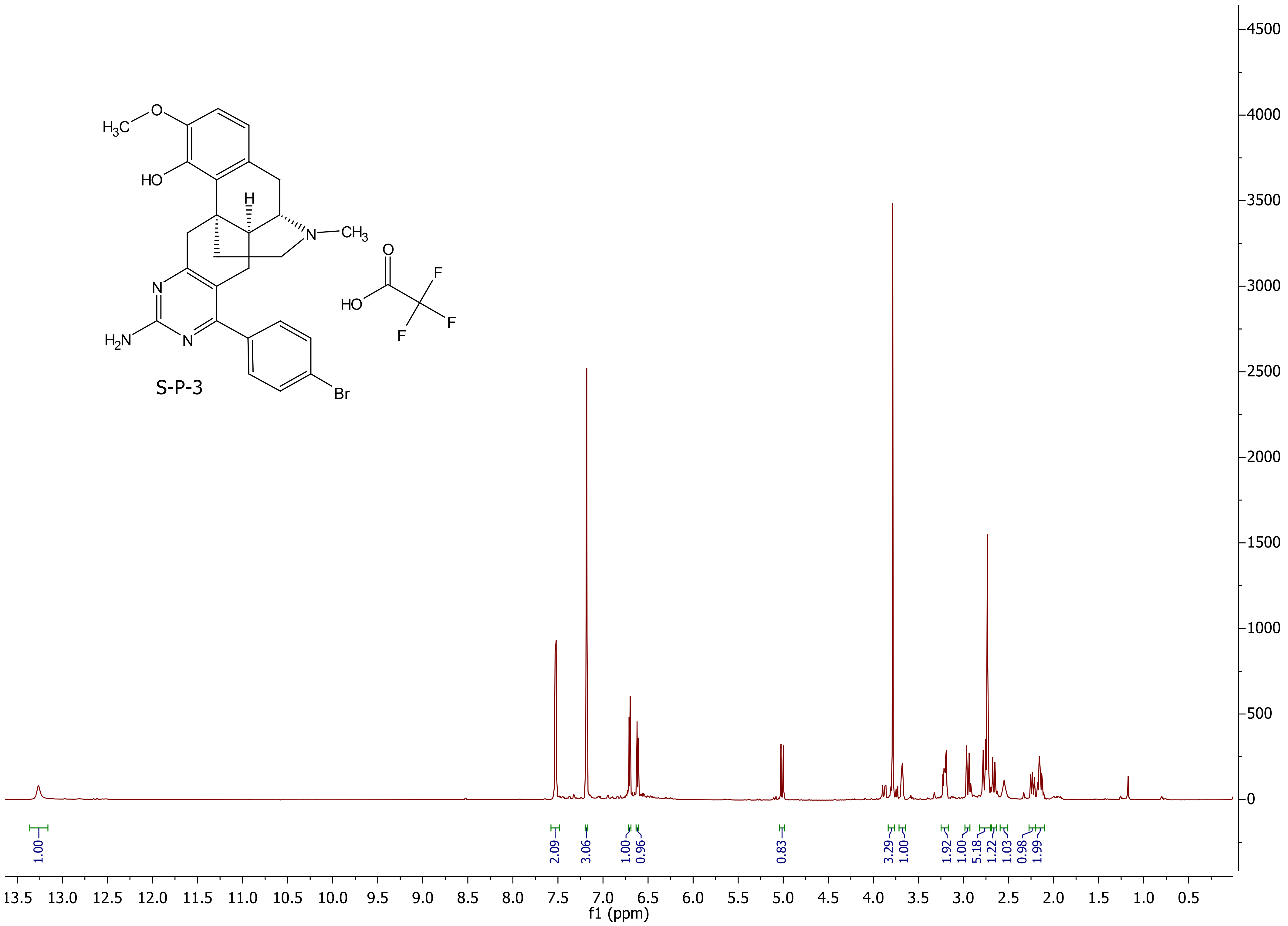

H3C
O
HO
H
N
CH3
O
F
HO
F
F
N
H2N
N
Br
S-P-3
4500
4000
3500
3000
2500
2000
1500
1000
500
0
1.00
2.09
3.06
1.00
0.96
0.83
3.29
1.00
1.92
1.00
5.18
1.22
1.03
0.98
1.99
13.5 13.0 12.5 12.0 11.5 11.0 10.5 10.0 9.5 9.0 8.5 8.0 7.5 7.0 6.5 6.0 5.5 5.0 4.5 4.0 3.5 3.0 2.5 2.0 1.5 1.0 0.5
f1 (ppm)

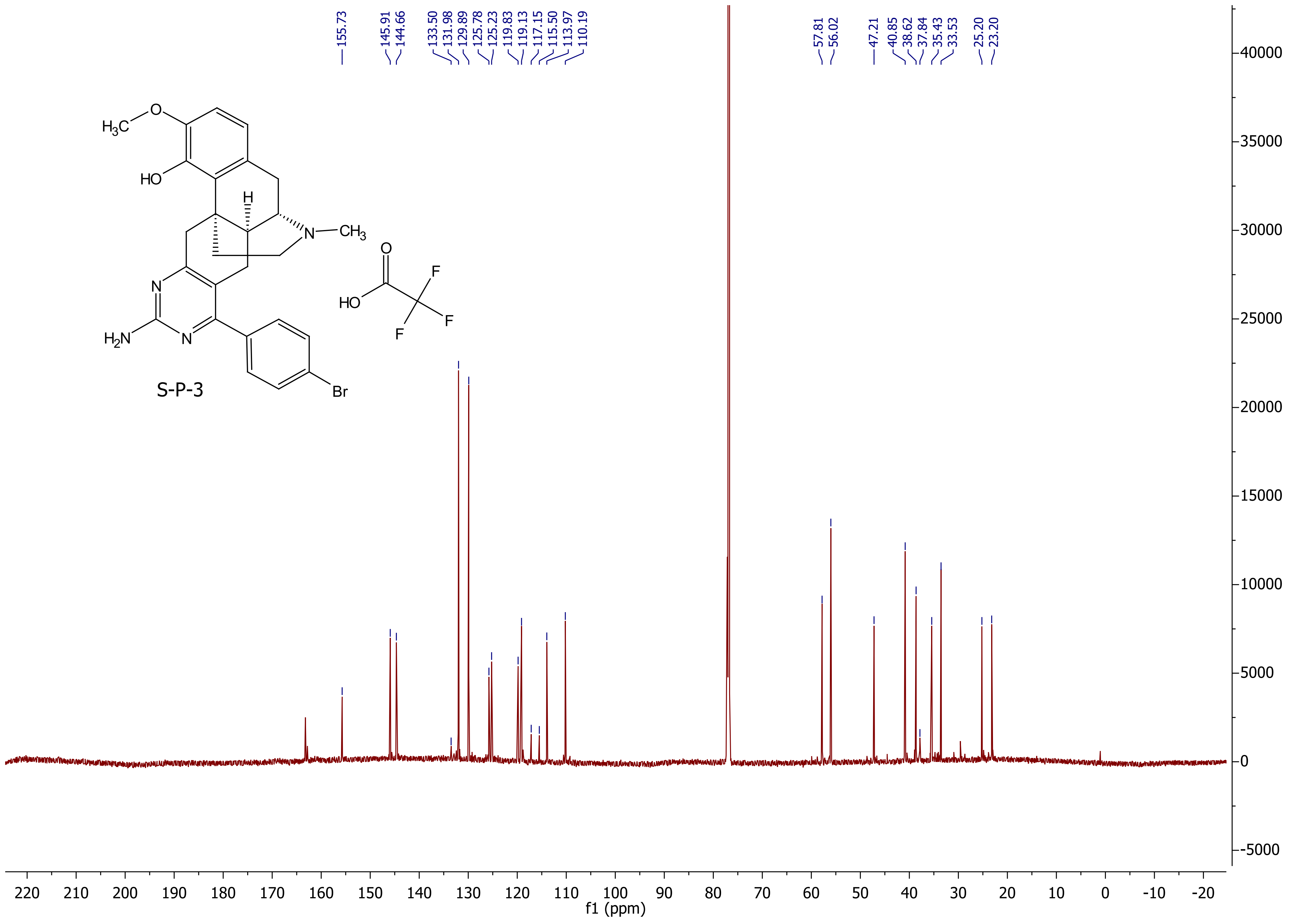
155.73
145.91
144.66
133.50
131.98
129.89
125.78
125.23
119.83
119.13
117.15
115.50
113.97
110.19
57.81
56.02
47.21
40.85
38.62
37.84
35.43
33.53
25.20
23.20
S-P-3
f1 (ppm)
220 210 200 190 180 170 160 150 140 130 120 110 100 90 80 70 60 50 40 30 20 10 0 -10 -20
40000 35000 30000 25000 20000 15000 10000 5000 0 -5000

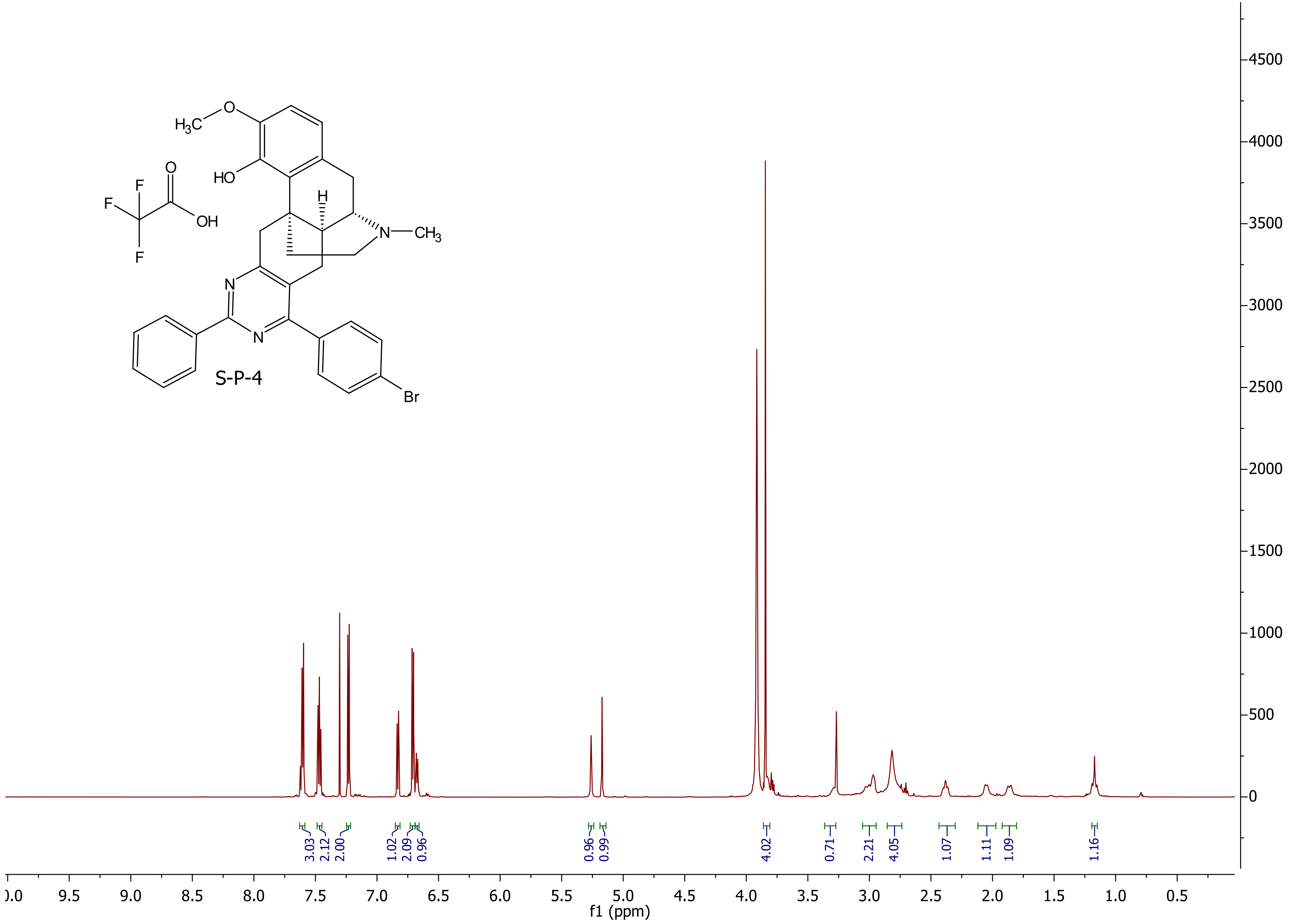
S-P-4
H3C
O
HO
F
OH
N
CH3
Br
H
3.03
2.12
2.00
1.02
2.09
0.96
0.96
0.99
4.02
0.71
2.21
4.05
1.07
1.11
1.09
1.16
f1 (ppm)
4500
4000
3500
3000
2500
2000
1500
1000
500
0

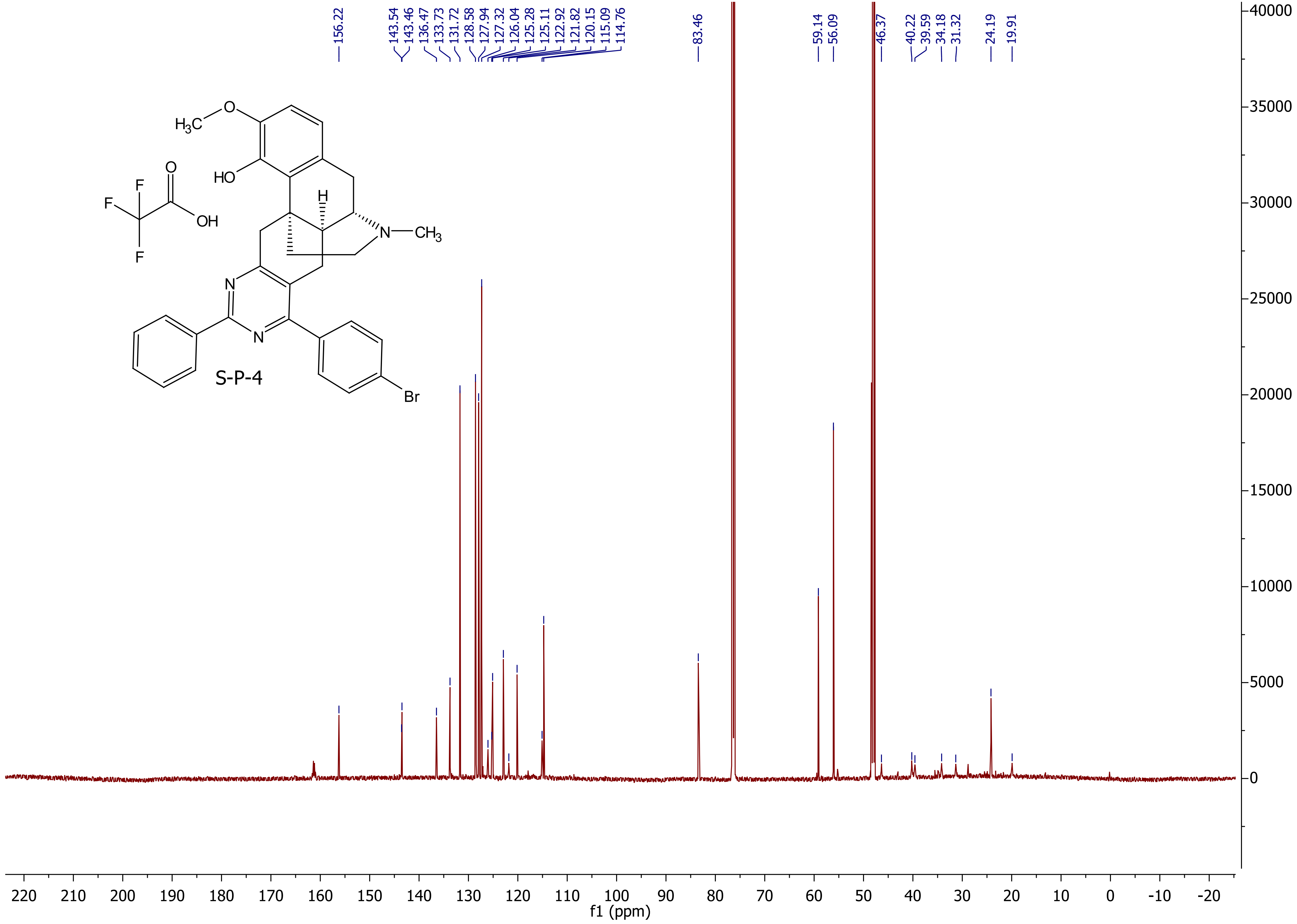

156.22
143.54
143.46
136.47
133.73
131.72
128.58
127.94
127.32
126.04
125.28
125.11
122.92
121.82
120.15
115.09
114.76
83.46
59.14
56.09
46.37
40.22
39.59
34.18
31.32
24.19
19.91
H3C
O
HO
H
N
CH3
F
F
F
O
OH
N
N
Br
S-P-4
f1 (ppm)

References

1. Foley, D. J. *et al.* Phenotyping reveals targets of a pseudo-natural-product autophagy inhibitor. *Angew. Chem. Int. Ed.* **59**, 12470–12476 (2020).

2. Tukey, J. W. *Explanatory data analysis*. (Addison-Wesley, 1977).

3. Daina, A., Michielin, O. & Zoete, V. SwissADME: A free web tool to evaluate pharmacokinetics, drug-likeness and medicinal chemistry friendliness of small molecules. *Sci. Rep.* **7**, 1–13 (2017).

4. Patlewicz, G., Jeliazkova, N., Safford, R. J., Worth, A. P. & Aleksiev, B. An evaluation of the implementation of the Cramer classification scheme in the Toxtree Software. *SAR QSAR Environ. Res.* **19**, 495 (2008).

5. Pires, D. E. V., Blundell, T. L. & Ascher, D. B. pkCSM: Predicting small-molecule pharmacokinetic and toxicity properties using graph-based signatures. *J. Med. Chem.* **58**, 4066–4072 (2015).

6. Chen, Y., De Bruyn Kops, C. & Kirchmair, J. Data resources for the computer-guided discovery of bioactive natural products. *J. Chem. Info. Model.* **57**, 2099–2111 (2017).

7. Sorokina, M. & Steinbeck, C. Review on natural products databases: Where to find data in 2020. *J. Cheminform* **12**, 1–51 (2020).

8. Sorokina, M. & Steinbeck, C. COCONUT: the COlleCtion of Open NatUral producTs. (2020) doi:10.5281/zenodo.3778405.

9. Gu, J. *et al.* Use of natural products as chemical library for drug discovery and network pharmacology. *PLoS One* **8**, 1–10 (2013).

10. Chen, Y. & Kirchmair, J. Cheminformatics in natural product-based drug discovery. *Mol. Inf.* **39**, 1–16 (2020).

11. Ertl, P., Roggo, S. & Schuffenhauer, A. Natural product-likeness score and its application for prioritization of compound libraries. *J. Chem. Inf. Model.* **48**, 68–74 (2008).

12. Chen, Y., Stork, C., Hirte, S. & Kirchmair, J. NP-scout: Machine learning approach for the quantification and visualization of the natural product-likeness of small molecules. *Biomolecules* **9**, 43 (2019).

13. Bickerton, G. R., Paolini, G. V., Besnard, J., Muresan, S. & Hopkins, A. L. Quantifying the chemical beauty of drugs. *Nat. Chem.* **4**, 90–98 (2012).

14. Lipinski, C. A., Lombardo, F., Dominy, B. W. & Feeney, P. J. Experimental and computational approaches to estimate solubility and permeability in drug discovery and development settings. *Adv. Drug Deliv. Rev.* **46**, 3–26 (2001).

15. Veber, D. F. *et al.* Molecular properties that influence the oral bioavailability of drug candidates. *J. Med. Chem.* **45**, 2615–2623 (2002).

16. Stratton, C. F., Newman, D. J. & Tan, D. S. Cheminformatic comparison of approved drugs from natural product versus synthetic origins. *Bioorg. Med. Chem. Lett.* **25**, 4802–

4807 (2015).

17. Hair, J. F., Black, W. C., Babin, B. J. & Anderson, R. E. *Multivariate Data Analysis*. (2012).

18. Riniker, S. & Landrum, G. A. Open-source platform to benchmark fingerprints for ligand-based virtual screening. *J. Cheminf.* **5**, (2013).

19. Bray, M. A. *et al.* Cell Painting, a high-content image-based assay for morphological profiling using multiplexed fluorescent dyes. *Nat. Protoc.* **11**, 1757–1774 (2016).

20. Woehrmann, M. H. *et al.* Large-scale cytological profiling for functional analysis of bioactive compounds. *Mol. BioSyst.* **9**, 2604–2617 (2013).

21. Fulmer, G. R. *et al.* NMR Chemical Shifts of Trace Impurities: Common Laboratory Solvents, Organics, and Gases in Deuterated Solvents Relevant to the Organometallic Chemist. *Organometallics* **29**, 2176–2179 (2010).

22. Gore, S., Baskaran, S. & König, B. Fischer indole synthesis in low melting mixtures. *Org. Lett.* **14**, 4568–4571 (2012).

23. Baran, P. S., Shenvi, R. A. & Nguyen, S. A. One-step synthesis of 4,5-disubstituted pyrimidines using commercially available and inexpensive reagents. *Heterocycles* **70**, 581–592 (2006).

24. Saikia, P., Gogoi, S., Gogoi, S. & Boruah, R. C. An efficient route for annulation of pyrimidines to steroids and non-steroids via a base catalyzed one-pot three component reaction. *Steroids* **88**, 1–6 (2014).

25. Dolomanov, O. V., Bourhis, L. J., Gildea, R. J., Howard, J. A. K. & Puschmann, H. OLEX2: A complete structure solution, refinement and analysis program. *J. Appl. Cryst.* **42**, 339–341 (2009).

26. Sheldrick, G. M. No Title. *Acta Cryst.* **A71**, 3–8 (2015).

27. Sheldrick, G. M. No Title. *Acta Cryst.* **C71**, 3–8 (2015).

28. Farrugia, L. J. ORTEP-3 for windows - A version of ORTEP-III with a graphical user interface (GUI). *J. Appl. Cryst.* **30**, 565 (1997).
